# A Comprehensive Analysis of the Structure-Function Relationship in Proteins Based on Local Structure Similarity

**DOI:** 10.1371/journal.pone.0006266

**Published:** 2009-07-15

**Authors:** Torgeir R. Hvidsten, Astrid Lægreid, Andriy Kryshtafovych, Gunnar Andersson, Krzysztof Fidelis, Jan Komorowski

**Affiliations:** 1 The Linnaeus Centre for Bioinformatics, Uppsala University and The Swedish University for Agricultural Sciences, Uppsala, Sweden; 2 Department of Cancer Research and Molecular Medicine, Norwegian University of Science and Technology, St. Olavs Hospital HF, Trondheim, Norway; 3 UC Davis Genome Center, Davis, California, United States of America; 4 Umeå Plant Science Centre, Department of Plant Physiology, Umeå University, Umeå, Sweden; 5 Department of Chemistry, Environment and Feed Hygiene, National Veterinary Institute, Uppsala, Sweden; 6 Interdisciplinary Centre for Mathematical and Computational Modelling, University of Warsaw, Warszawa, Poland; Weizmann Institute of Science, Israel

## Abstract

**Background:**

Sequence similarity to characterized proteins provides testable functional hypotheses for less than 50% of the proteins identified by genome sequencing projects. With structural genomics it is believed that structural similarities may give functional hypotheses for many of the remaining proteins.

**Methodology/Principal Findings:**

We provide a systematic analysis of the structure-function relationship in proteins using the novel concept of local descriptors of protein structure. A local descriptor is a small substructure of a protein which includes both short- and long-range interactions. We employ a library of commonly reoccurring local descriptors general enough to assemble most existing protein structures. We then model the relationship between these local shapes and Gene Ontology using rule-based learning. Our IF-THEN rule model offers legible, high resolution descriptions that combine local substructures and is able to discriminate functions even for functionally versatile folds such as the frequently occurring TIM barrel and Rossmann fold. By evaluating the predictive performance of the model, we provide a comprehensive quantification of the structure-function relationship based only on local structure similarity. Our findings are, among others, that conserved structure is a stronger prerequisite for enzymatic activity than for binding specificity, and that structure-based predictions complement sequence-based predictions. The model is capable of generating correct hypotheses, as confirmed by a literature study, even when no significant sequence similarity to characterized proteins exists.

**Conclusions/Significance:**

Our approach offers a new and complete description and quantification of the structure-function relationship in proteins. By demonstrating how our predictions offer higher sensitivity than using global structure, and complement the use of sequence, we show that the presented ideas could advance the development of meta-servers in function prediction.

## Introduction

Revealing functions of proteins is one of the major challenges of molecular biology. Sequence similarity search tools such as BLAST [1] revolutionized biological research by providing functional hypotheses that could be tested experimentally. However, identifying functionally characterized homologues using sequence similarity is only possible for less than 50% of the proteins predicted from genome sequencing projects. Since structure is evolutionarily more conserved than sequence, it is believed that structural information provides a solution for many of the remaining proteins [2], [3]. Indeed, the extended goal of structural genomics is to systematically solve protein structures for new protein families [4], use these structures as templates for *in silico* structure prediction methods [5], [6], and then use the solved and predicted structures to infer function [7], [8]. However, this requires new computational methods that utilize structure for function prediction. Thus understanding and predicting structure-function relationships in proteins is considered by many to be the holy grail of computational biology.

Approaches to the analysis of the structure-function relationships in proteins either rely on global similarities (fold) or local similarities (motifs) [9]–[12]. Fold similarities have been shown to associate with function [13], [14], and have also been used to infer function-specific sequence patterns [15]. However, many folds such as the TIM barrel and the Rossmann fold are found in proteins with several different functions [2], and this has led to various local structure-motif methods based on, for example, known functional sites or function-specific sequence patterns [16]–[21]. Recently, meta-servers have obtained functional predictions by allowing a large number of different evidence (including global and local properties) to independently vote for a particular function [22]–[24].

Here, we provide a comprehensive analysis of the structure-function relationship in proteins, in which a library of recurring multi-fragment structural motifs called *local descriptors of protein structure* [25], [26] are used to learn IF-THEN rules [27], [28] that associate combinations of local substructures with specific protein functions. Unlike previous studies, we investigate *all* recurring motifs and *all* annotated proteins using no prior knowledge of functional sites or any sequence information. Thus, we induce a rule-model that constitutes a complete representation of the structure-function relationship in proteins based only on structure similarity. By a computational evaluation of the model's ability generalize and predict the function of unseen proteins, we offer a full quantification of the structure-function relationship. This enables us to make critical observations about the importance of structure in various aspects of protein function. Our findings can be summarized as follows: (a) nearly two-thirds of all molecular functions are predicted with a statistically significant accuracy, (b) biological processes and cellular components are considerably harder to predict from structure than molecular function, (c) combining local similarities results in better predictive power than using global similarity, in particular for functionally versatile folds, and also allows prediction of the function of new folds, (d) catalytic activities are better predicted than most functions involving binding and this is related to protein dynamics and disorder, and (e) structure-based predictions complement sequence-based predictions and are shown through literature-validation to provide many correct predictions even when no significant sequence similarities exist.

## Results

### Library of annotated local substructures of proteins

**Figure 1:**
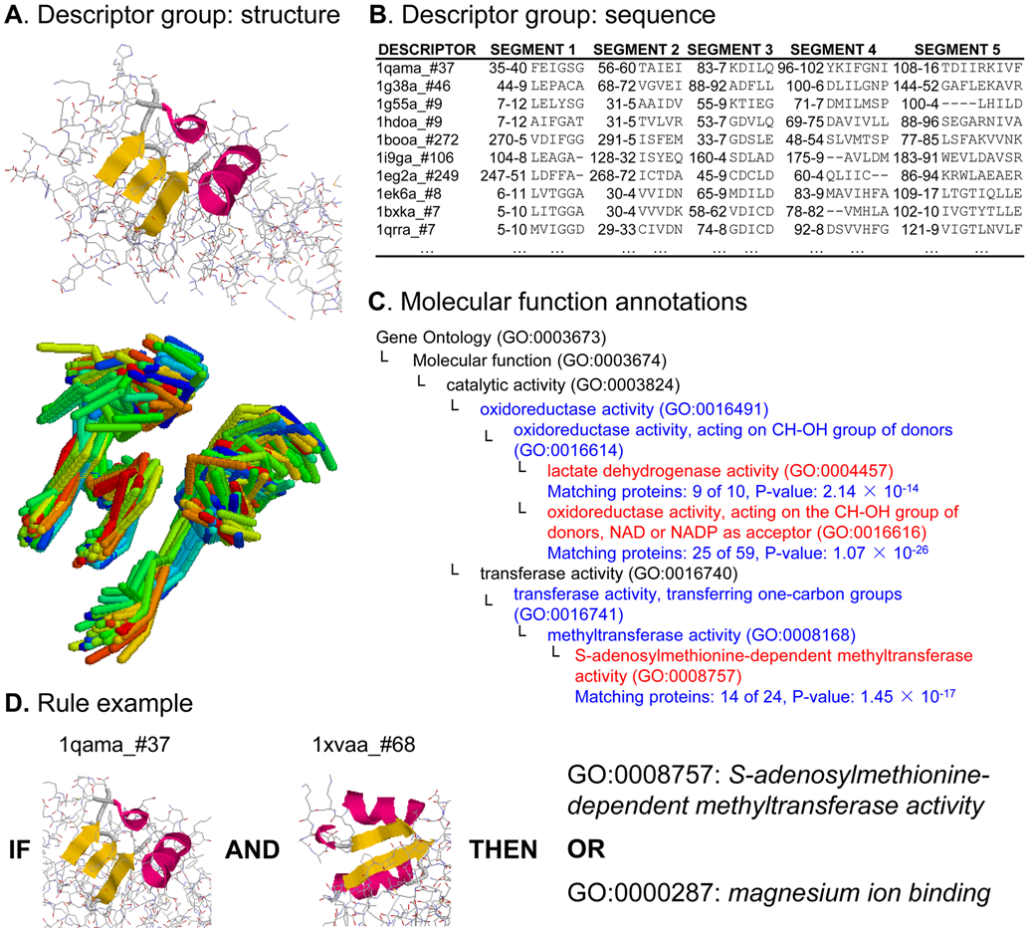
Local substructure group with central descriptor 1qama_#37. Descriptors are named: ‘PDB protein domain name’#‘central amino acid’. A) Cartoon of the secondary structure of the central descriptor and its structural alignment with the ten closest descriptors in the group. B) The sequence alignment resulting from the structural alignment in A. C) Location in Gene Ontology of the significantly overrepresented (FDR controlled at 0.05 [39]) molecular functions annotated to the 68 proteins matching the local substructure in A (marked in red). In total, 28 molecular functions were annotated to the 68 proteins. D) The rule IF (1qama_#37 AND 1xvaa_#68) THEN (GO:0008757 OR GO:0000287) combining the substructure 1qama_#37 in A with the substructure 1xvaa_#68 to uniquely describe 12 of the proteins annotated with GO:0008757: *S-adenosylmethionine-dependent methyltransferase activity*. Two of these proteins are additionally annotated with GO:0000287: *magnesium ion binding*. The rule thus effectively combines local substructures to address only one of the three statistically significant GO classes related to 1qama_#37.

A local descriptor of protein structure is a set of short continuous backbone fragments (segments) centered in three dimensions around a particular amino acid (Figure 1A, B). We built a library of 4197 such recurring local substructures [25] from a representative set of all experimentally determined protein structure domains in the Protein Data Bank (PDB) with less than 40% sequence identity to each other [29], [30]. The library was used to automatically represent all protein structures in terms of matching or not matching each of the local substructures. We then organized the Gene Ontology (GO) annotations [31], [32] of all characterized proteins into 113 classes of molecular functions, 139 classes of biological processes, and 30 classes of cellular components (see Table 1 and Materials and Methods for details).

**Table 1:** Gene Ontology annotations for molecular function, biological process and cellular component.

Gene Ontology	Number of proteins/annotations	Number of classes/proteins/annotations
Molecular function	2549/4963	113/1747/2815
Biological Process	2477/5082	139/1533/2573
Cellular Component	1379/1978	30/561/688

The second column gives the number of annotated proteins and the number of annotations for these proteins. The third column gives the number of GO classes selected and the related numbers of proteins and annotations.

### Model induction

The relationship between structure and function was modeled using IF-THEN rules [27], [28] where the IF-part of each rule specifies a minimal combination of local substructures discerning a particular protein structure from structures annotated to other GO classes (Figure 1C, D). The rule model was induced using only substructures observed in protein structures statistically overrepresented in at least one GO class (Table S1). The GO classes are not mutually exclusive. For example, the catalytic activity of a metalloendopeptidase involving a zinc ion will give rise to the GO molecular function annotations GO:0004222: *metalloendopeptidase activity* and GO:0008270: *zinc ion binding*. In addition, some functions are not completely discernible in terms of structure because, e.g., the functionally discriminating properties are too rare to be singled out by general rules. Consequently, the THEN-part of the rules often contains several GO-classes with different probabilities (Figure 1D). Our model for GO molecular function encompasses ∼20,000 rules describing various overlapping structure-function relationships at different levels of specificity (Table S2). As a point of reference, we also induced rules based on domain-specific global structural similarity in terms of orientations and connectivity of the main secondary structure elements (CATH fold, see Materials and Methods) [33].

### Quantification of the structure-function relationship

**Figure 2:**
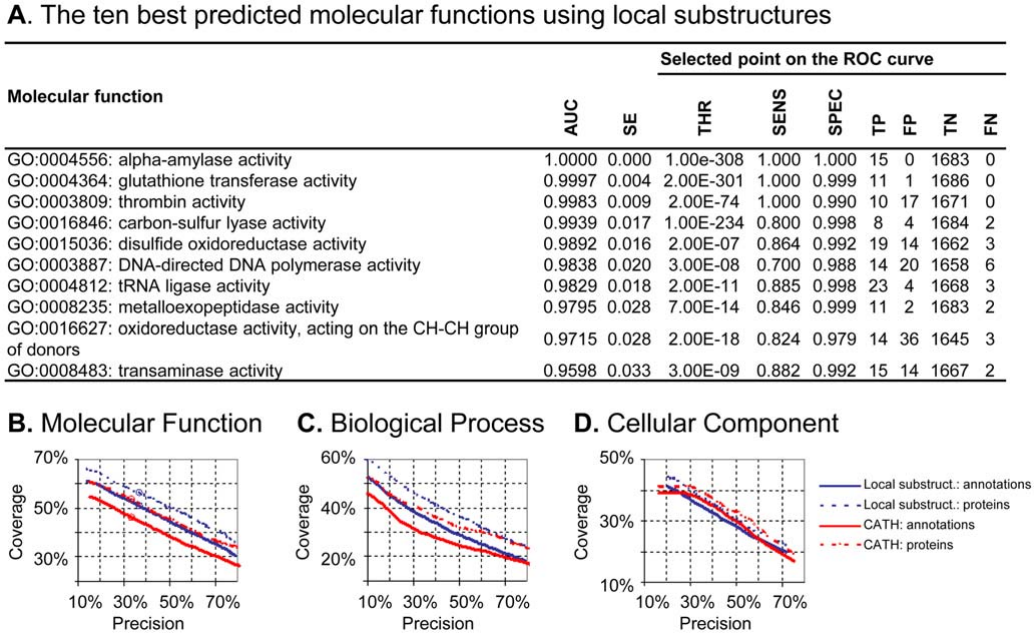
Model prediction performance using cross-validation and ROC analysis. A) List of the ten best predicted GO molecular function classes as measured by the AUC and its standard error [34]. We also report sensitivity (SENS), specificity (SPEC), and the number of true positives (TP), false positives (FP), true negatives (TN), and false negatives (FN) at one specific decision threshold (THR). See Materials and Methods for details. (B, C and D) Performance for all GO classes and all three GO subontologies using local substructures or CATH folds at different decision thresholds (resulting from varying the costs on false positives, see Materials and Methods for details). *Coverage* is the percentage of *proteins* with at least one correct prediction or the percentage of *annotations* correctly predicted, and *precision* is the percentage of predictions that are correct. Numbers corresponding to the decision thresholds in A are circled.

We argue that a rigorous evaluation of the ability of structure-based models to predict function for unseen proteins is the best way to quantify the degree to which function depends on structure. To this end, we estimated the predictive performance of the models using cross-validation and Receiver Operating Characteristic (ROC) analysis, and report the Area Under the ROC Curve (AUC) [34] for each class of molecular function, biological process and cellular component (Figure 2, Table S3).

Both the local and the global structure-based methods are better at predicting molecular function than at predicting biological process and cellular component (Figure 2). This is not unexpected since proteins sharing a cellular location or being part of a broad biological process need not be structurally related. This adds complementary evidence to other studies that have shown that gene-expression time profiles are needed to explain biological processes [35]. Consequently, we will focus our detailed analysis on molecular function.

For a selected set of decision thresholds, the local substructure approach correctly predicts 51% of the annotations, and at least one annotation for 56% of the proteins, with 37% of the predictions being correct (i.e., precision). The local approach consistently outperforms the global approach (Figure 2B) due to the flexibility associated with combining several local substructures to obtain function-specific rules. In particular, we see a pronounced difference for proteins with the same fold, but different function. For example, 69% of 169 proteins with the Rossmann fold had one function correctly predicted by the local substructure method (precision = 27%), compared to only 17% for CATH (precision = 9%), while corresponding numbers for the 50 TIM barrel proteins were 66% (precision = 21%) for local substructures and 50% (precision = 12%) for CATH. Clearly, the use of local substructures increases the resolution and allows us to functionally discriminate proteins with the same fold.

### Catalytic activities rely on conserved structure

Using local substructures, we obtain significant AUC values (i.e. AUC>0.7) for 82 of the 113 GO molecular function classes. However, not all aspects of molecular function are equally dependent on structure. When the predictive quality of GO classes was investigated in relation to groups of wider functional categories given by the hierarchical nature of GO, we found that 53 of the 63 GO molecular function classes located under GO:0003824: *catalytic activity* were significantly predicted (P<0.0020). On the other hand, 15 of 37 classes under GO:0005488: *binding* (P<0.027) and all four classes located under GO:0030528: *transcription regulator activity* (P<0.0049), three of which also were located under *binding*, were *not* significantly predicted. The same tendency was observed in the CATH-based predictions. Our results thus indicate that properties related to binding are difficult to model from the employed representations of structure while catalytic mechanisms seem to associate well with conserved structural similarity (Table S4). This may be related to the fact that the catalytic action of enzymes is not restricted to the catalytic site, but is connected to inner protein dynamics [36]. CATH folds and, to some degree, local substructures primarily describe protein cores. Thus they may be well suited for modeling *catalytic activity*. *Binding*, on the other hand, mainly requires that the protein has a surface with appropriate properties as defined by electrostatic-, hydrophobic- and van der Waals-interactions, and such a surface may be generated by alternative structures. Exceptions from the observation that *binding* is hard to predict include some of the interactions with metal ions (AUCs of 0.95, 0.92, 0.80, 0.75), which are often involved in the catalytic mechanism, and GTP and ATP binding (AUCs of 0.89 and 0.77), which play very important roles in the enzymatic activity.

**Figure 3:**
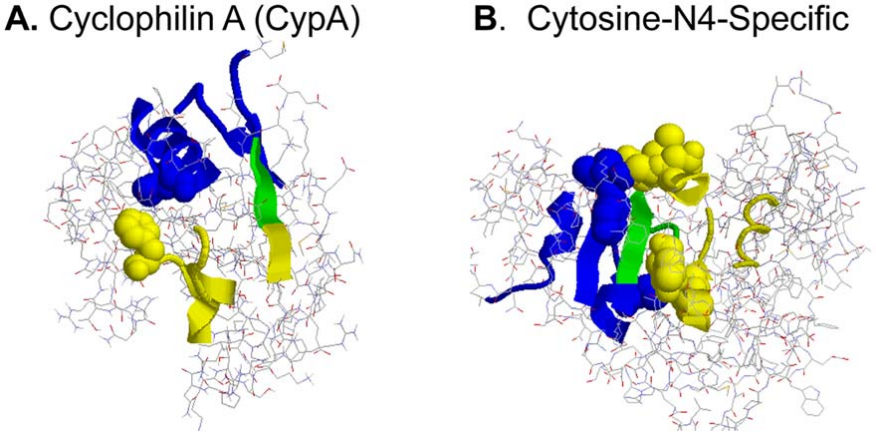
Rules combining local substructures into connected complexes. A) Structure of CypA (PDB id: 1aka). The loop region represented by Phe 67 is correlated with the dynamics of the core represented by the hydrophobic cluster including Leu 39, Phe 46, Phe 48 and Ile 78 [36]. The rule combining local substructures 1elva1#604 and 1bif_2#398 describes exactly this mechanism. 1elva1#604 (yellow) matches the loop region including Phe 67 (space filled yellow), while 1bif_2#398 (blue) matches parts of the core including Leu 39 (space filled blue). The overlap between the two local substructures is in green. Although all the residues in the hydrophobic cluster are described in our local substructure library, the minimal IF-THEN rule only needs one residue in the cluster to discriminate the function. B) Two local substructures in the rule in Figure 1D matching the enzyme Cytosine-N4-Specific (PDB id: 1boo): 1qama_#37 in yellow, 1xvaa_#68 in blue, the overlap in green and the residues in contact as space filled. The combined local substructures have a very similar number of residues in contact in the 12 matching proteins (the average contact surface included 25% of the non-overlapping residues in the two local substructures with standard deviation 6.4).

Local descriptors that co-occur in rules in the model are selected because they are function-specific. Hence, it is intriguing to observe that such co-occurring substructures, significantly more often than randomly selected substructures (P<2.2×10^−16^), form connected complexes in which one or more residues from each substructure are within 5 Å of each other (see Materials and Methods for details). The recently published contact between a loop region and a hydrophobic cluster associated with the inner dynamics of the enzyme cyclophilin A (CypA) is exactly described by one of our rules (Figure 3A) [36]. We expect that rules that combine local substructures representing stable contact surfaces found in many proteins may turn out to describe general mechanisms behind protein functions (Figure 3B). The fact that local substructure complexes emerge from rules that can predict protein function indicates that the approach chosen here is capable of generalizing and describing protein function beyond approaches based on global similarity. This also demonstrates the advantage of modeling structure-function relationships using explicit and legible IF-THEN rules.

### Protein disorder

It has become increasingly more clear that some protein functions require intrinsic disorder [37]. By using gaps of three or more residues in X-ray characterized proteins in PDB as an indication of disorder (http://www.disprot.org/), we found a significant correlation between the AUC value of each molecular function class and the degree of disorder in proteins from these classes (correlation coefficient of −0.36, which is different from no correlation at P<9.9×10^−5^). Furthermore, we found that for wider functional categories in GO such as *catalytic activity* and *binding*, GO classes that are not significantly predicted display a consistently higher degree of disorder compared to proteins in GO classes that are well predicted (Table S5). The same tendency was observed in the CATH-based prediction. This indicates that some aspects of protein function violate the assumption that sequence determines a specific structure as a prerequisite for function, and is in line with other results reported recently [38]. Examples include GO:0046983: *dimerization activity* (AUC = 0.69, disorder = 9.8%), GO:0005261: *cation channel activity* (AUC = 0.42, disorder = 8.1%) and GO:0003713: *transcription coactivator activity* (AUC = 0.58, disorder = 8.1%). Thus, such functions may only be predicted correctly by incorporating information in the rules on disorder.

### Complementarities of sequence and structure in function prediction

The ultimate validation of predictions is done experimentally. However, *in silico* validation offers advantages in that a much larger number of hypotheses may be tested and statistically sound conclusions may be drawn. We applied our model to functionally characterized proteins that were not structurally solved at the time of model induction. We divided this test set into proteins with a weak but statistically significant sequence similarity to the training set and proteins with no statistically significant similarity.

We predicted the molecular function of 429 protein structures (with 634 annotations) with a weak but statistically significant sequence similarity (less than 40% sequence identity and E-score less than 0.05) to the training set. For these proteins we were able to predict 45% of all the annotations, and at least one correct annotation for 53% of the proteins, with a precision of 29%. Since this performance is comparable to the cross-validation estimates obtained from the training set (Figure 2), we may conclude that rules based on the library of local substructures generalize well to unseen structures across the whole continuum of sequence similarity. By combining the predictions from the local descriptor approach with predictions derived from the annotations of the closest sequence-neighbor in the training set (detected by PSI-BLAST [1], see Materials and Methods for details), we could correctly predict 70% of all the annotations, and at least one correct annotation for 76% of the proteins, with a precision of 30%. Of all 444 correct predictions, 398 were made by PSI-BLAST (62%) while the remaining 46 were made exclusively by the descriptor-based method. Thus the approach that combines PSI-BLAST and our structure-based method predicts correctly more annotations than using PSI-BLAST alone, even when sequence similarity exists.

We finally challenged the system to predict function for 167 unseen proteins (with 224 annotations) with no significant sequence similarity to the training set (E-score greater than 0.05). For these rather demanding targets, the local descriptor method obtained coverage and precision of only around 10%, showing that the model is not independent from sequence even though it is based purely on structure. However, automatic annotations constitute 92.4% of the database and these annotations are generally known to be incomplete. Hence, we manually validated all predictions made by the descriptor approach of these 167 targets. This analysis revealed that out of 190 predictions made for 93 proteins, 91 predictions made for 57 proteins found some support in the scientific literature (Table S6). One example is the protein alanyl-tRNA synthetase (PDB id. 1riq) with four predictions: GO:0000049: *tRNA binding*, GO:0000287: *magnesium ion binding*, GO:0005524: *ATP binding* and GO:0004812: *tRNA ligase activity*. Only the last two predictions were annotated. However, all of them were verified as correct by literature search. Furthermore, the fold of this protein was not represented in the training set and thus this protein could not have been correctly predicted using global structural similarity. The fact that the local substructure method is fully automatic is also an advantage over methods that rely on manual assignments since predictions can be made for newly solved structural genomics targets. Only 58 of the 167 recently solved structures discussed here have so far been assigned a fold in CATH. Finally, although structural similarity in the absence of sequence similarity may be a result of convergent evolution, our results clearly show that local substructures can provide useful functional hypotheses even for these proteins.

## Discussion

Since protein function in nature depends on the global architecture, the inner dynamics of folds, and the subtle surface properties that give binding specificity, we expect that computational methods that incorporate information on all levels will be superior to exclusively sequence derived methods. Here we present a general approach for representing protein function in terms of local and global structural similarity and for quantifying the structure-function relationship. This greatly differs from any previously published work in terms of completeness, the use of multi-fragment local substructures, and the fact that the relationship to function was explored using no information about functional sites or sequence patterns. Rules are easy to interpret and allow for different types of data to be included in the model. In the future we would like to in-cooperate this approach into a meta-method where global structure similarity and sequence information is also included.

Concerns have been raised whether predicted structures will help in function prediction since these *in silico* methods mainly predict correctly the protein core while function often depends on surface-properties [3]. However, our results show that local substructures, mainly related to the core, associate strongly with some aspects of molecular function and in particular *catalytic activity*. Our evaluations show that *purely* structure-based predictions serve as a complement to predictions derived from sequence, and that correct prediction also can be provided when no sequence similarity exists. Hence, we have provided substantial support for the viability of the goal of structural genomics, i.e., reducing the number of functionally uncharacterized proteins through structure determination and function prediction.

## Materials and Methods

**Figure 4:**
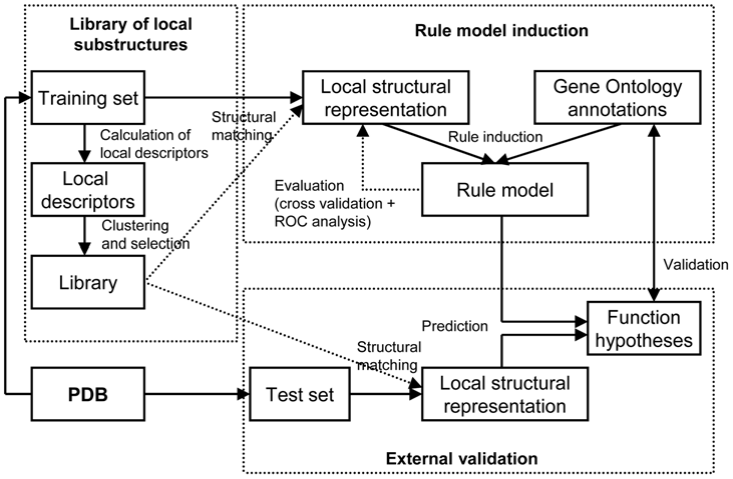
Overview of the function prediction method. A library of local descriptors of protein structure is built from a representative subset of PDB (i.e. training set). The library is used to represent protein structures, and a model that discriminates classes of Gene Ontology annotations is induced using combinations of local substructures. The model is evaluated both internally and on an external test set.

Figure 4 gives a schematic overview of our method for function prediction. Details are given in this section.

### Library of local substructures of proteins

A local descriptor of protein structure is a set of short backbone fragments centered in three dimensions around a particular amino acid [25], [26]. A local descriptor is built by a) identifying all close amino acids within a radius of 6.5 Å (an amino acid is represented as the point on the vector [C_α_,C_β_] that lies 2.5 Å away from C_α_), b) for each close amino acid, adding four sequence neighbors, two from each side, to obtain continuous backbone fragments of five amino acids, and c) merging any overlapping fragments into segments. We computed local descriptors from all amino acids in a representative set of protein domains from PDB with less than 40% sequence identity to each other (ASTRAL version 1.57 [30]). This resulted in 374,558 descriptors from 4006 domains. We then constructed a library of commonly reoccurring local descriptors by a) for each local descriptor identifying a group of structurally similar local descriptors and b) selecting a set of 4197 representative, partially overlapping *descriptor groups*. We only considered groups with at least seven descriptors with at least three non-overlapping sequence fragments.

Proteins in this study were represented as strings of 0's and 1's indicating whether the protein structure matched the corresponding local substructures or not. This was done for proteins with domains in ASTRAL 1.57 (i.e., training set) as well as for proteins in ASTRAL 1.67 with less than 40% sequence identity to the training set (i.e., external test set).

### Gene Ontology annotations

GO is an organism-independent controlled vocabulary for describing the cellular role of genes and gene products in terms of molecular functions (i.e., tasks performed by individual gene products), biological processes (i.e., broad biological goals accomplished by an ordered assembly of molecular functions), and cellular components (i.e., locations where gene products are active).

We obtained annotations from the GO homepage (http://www.geneontology.org) [32] for the proteins used to build the local substructure library described earlier (2878 proteins with 4006 protein domains in ASTRAL). We distributed these annotations (upwards) in the GO graph (version 1.419), and discarded all GO terms (nodes) used to annotate less than ten proteins. We then selected, among the remaining terms, the most specific terms as our training classes (Table 1, Table S3). By only considering GO terms used to annotate at least ten proteins, some annotations were lost. However, a majority of the proteins kept at least one annotation, indicating that there is a set of large classes providing at least one annotation for almost every protein, and that the additional annotations often are from less populated GO terms. Furthermore, selecting specific classes as training classes resulted in the loss of some general annotations.

### Significant GO classes in descriptor groups

We used the hypergeometric distribution to calculate p-values reflecting to which degree proteins annotated to a particular GO class were over-represented in the descriptor group. We then used *false discovery rate* (FDR) [39] controlled at 0.05 to define statistically significant local descriptors. FDR is a method for correcting for multiple hypotheses in statistical hypothesis testing.

In the library, 84% of the descriptor groups had a significant overrepresentation of proteins annotated to at least one of the 113 molecular function classes (FDR controlled at 0.05). Corresponding numbers for the 139 biological processes and 30 cellular components were 77% and 29%, respectively. All GO classes for all three parts of GO were significantly overrepresented in at least one descriptor group (with the exception of the cellular component GO:0005938: *cell cortex*). See Table S1 for details.

It is a fundamental principle in machine learning that a higher ratio of examples to features produces models that perform better on unseen cases (given the same class separability). To cope with the large number of structural features (i.e., 4197 local substructures) compared to the number of proteins (2815 for molecular function) in this study, we only used FDR significant descriptor groups to induce rule models.

### CATH

CATH [33] (version 2.6.0) is a classification tree that classifies domain structures, in increasing specificity, according to class (C), architecture (A), topology (T) and homologous superfamily (H). Class is assigned according to the secondary structure composition and packing of the structure domain. This is done automatically in 90% of the cases. Architecture refers to the overall shape of a domain structure in terms of the relative orientations of the secondary structure elements. Architecture is assigned manually. Topology refers to the connectivity of the secondary structure elements in otherwise similar architectures, and the assignment is done automatically. Finally, homologous superfamily refers to the proteins that are homologues as determined by sequence similarity. These assignments are also done manually.

Local descriptors are classified into groups according to the relative positioning and orientation of their segments. Hence this corresponds to architecture in CATH. However, CATH architecture is too general for function prediction (results not shown). Moreover, CATH homologous superfamily would introduce sequence similarity into the analysis and would therefore obscure the pure structure-function signal. Hence we opt for using CATH topology (i.e. fold) in this paper rather than CATH homologous superfamily or other, more manually inferred databases [11].

### Sequence-based predictions

The sequence comparison program PSI-BLAST [1] was used to obtain sequence-based predictions. Each domain in the external test set was blasted against the training set with a sequence profile obtained using the non-redundant sequence database of NCBI (ftp://ftp.ncbi.nlm.nih.gov/blast/db/nr) (PSI-BLAST was run with three iterations and an E-value threshold of 0.005 for including a sequences in the model). The annotations for the closest match in the training set (determined by E-value) were used as predictions, and predictions for a protein were taken to be the predictions for all its domains.

### Contacts between local substructures

For each rule and each matching protein, we computed the average fraction of residues in pairs of local substructure that were in contact (residues common to both of the local substructures were not considered). We defined two residues to be in contact if the shortest distance between atoms in these amino acids was less than 5 Å. This threshold is based on the hydrophobic contact distance between ligands and proteins [40]. Hence, for each rule we obtained the average number of contacts between pairs of substructures in matching proteins and the standard deviation indicating the stability of these contact surfaces over different proteins. The average contact surface of a pair of local substructures in rules encompassed 11% of the non-overlapping residues in this pair with an average standard deviation of 9.3. For comparison, we randomly sampled 1000 pairs of local substructures matching at least two proteins and where the substructures occurred in at least one of the rules. The contact surfaces for function-specific rules were significantly greater than for these randomly sampled pairs (at P<2.2×10^−16^ using the Kolmogorov-Smirnov test), while the standard deviations were significantly smaller (also at P<2.2×10^−16^). Some large contact conformations were particularly stable; 8.5% of the rules were associated with an average contact surface that included more than 20% of the residues *and* where the standard deviation was less than 5%. This was only true for 2.8% of the randomly sampled local substructure pairs.

### Rule-learning

The rough set theory [27], [28] constitutes a mathematical framework for inducing rules from examples. We used this framework, as implemented in the ROSETTA rough set system [28] (http://rosetta.lcb.uu.se), for learning IF-THEN rules associating combinations of local substructures of proteins with particular GO classes. The framework has previously been used to learn GO biological process from gene-expression time profiles [35], [41] (see Hvidsten et al. (2003) for a more theoretical/mathematical treatment of the rule-learning method).

In principle, the method finds the minimal sets of local substructures that discern a particular protein from all other proteins annotated to a different GO class. One rule is then constructed from each such set, so that the IF-part is the combination of these local substructures and the THEN-part is all GO classes used to annotate proteins matching the IF-part. If the rule includes several GO classes, it means that the corresponding protein is annotated with a GO class that cannot be uniquely defined from the local substructure data (i.e., the class is said to be *rough*). In this study, we used a genetic algorithm to find approximate minimal sets that discern each protein from a sufficiently large fraction (at least 90%) of the proteins from other GO classes. Rules from such approximate solutions are less likely to overfit the data and handle noise better than exact solutions.

We compared the approach using rules based on minimal, discerning subsets of local substructures, with the approach of using all rules based on one single local substructure. Such very simple decision rules, called 1R rules, were proposed by Holte [42]. Using this approach we found that combinations were important for the local descriptor approach, but did not help when using CATH folds.

### Prediction and evaluation

We tested the generalizing capability of our rule approach using ten-fold cross-validation. The set of proteins was randomly divided into ten equally sized subsets. A rule classifier was induced from nine subsets (the training set) and used to classify the proteins in the remaining subset (the test set). This procedure was repeated ten times, so that each protein was in the test set once and in the training set nine times.

A protein was classified by letting each matching rule cast votes to the GO classes specified by the rule. The number of votes cast by each rule to each class corresponded to the number of proteins in the training set from that class that matched that rule (i.e., the rule support). A p-value was then calculated for each class based on the votes using the hypergeometric distribution. These p-values were obtained during cross-validation and a ROC curve was computed for each class plotting sensitivity against specificity for all possible p-value thresholds. Sensitivity is TP/(TP+FN) and specificity is TN/(TN+FP) where TP is True Positives, FP is False Positives, TN is True Negatives and FN is False Negatives. The ROC curve evaluates the threshold-independent performance of the classifier. We reported the area under the ROC curve (AUC) as a measure of performance. This value is between 0 and 1, where 1 signifies perfect discrimination while 0.5 signifies no discriminatory power at all. When doing actual function predictions we used p-value thresholds from the ROC curves corresponding to the points maximizing sensitivity plus specificity (specificity was always greater than 0.90 to control the number of false positives due to the large number of classes).

By randomly shuffling the molecular function annotations we showed that cross-validation AUC values equal to or greater than 0.7 are unlikely to be obtained by chance (P<0.01). Thus AUC≥0.7 was denoted statistically significant in this study.

## Supporting Information

Table S1 All FDR significant local descriptor-GO class pairs. (a) Molecular function: All FDR significant local descriptor-GO class pairs. (b) Biological process: All FDR significant local descriptor-GO class pairs. (c) Cellular component: All FDR significant local descriptor-GO class pairs. PARAMETERS refer to the parameters in the hypergeometric distribution used to compute the p-values: N,n,k,x, where N is the number of protein-GO class pairs in the data set, n is the number of proteins matched by the local descriptor, k is the number of proteins in the GO class and x is the number of proteins matched by the local descriptor and in the GO class.
(1.28 MB PDF)

Table S2 All induced rules for molecular function. For each Gene Ontology molecular function class in the THEN-part, the p-value is given together with the parameters for the hypergeometric distribution used to compute the p-values: N,n,k,x, where N = 2725 is the number of protein-GO class pairs in the data set, n is the number of proteins matched the IF-part of the rule, k is the number of proteins in the GO class and x is the number of proteins matched by the rule and in the GO class.
(1.16 MB PDF)

Table S3 Prediction performance. (a) Prediction performance. 10-fold cross-validation AUC estimates for all molecular function classes (b) Prediction performance. 10-fold cross-validation AUC estimates for all biological process classes (c) Prediction performance. 10-fold cross-validation AUC estimates for all cellular component classes
(0.04 MB PDF)

Table S4 The overrepresentation of GO classes with significant AUC values. (a) Local substructures. The overrepresentation of GO classes with significant AUC values (AUC> = 0.7) and not significant values (AUC<0.7). P-values are calculated based on the number of proteins and the number of GO classes in each of the more general GO terms. (b) CATH folds. The overrepresentation of GO classes with significant AUC values (AUC> = 0.7) and not significant values (AUC<0.7). P-values are calculated based on the number of proteins and the number of GO classes in each of the more general GO terms.
(0.01 MB PDF)

Table S5 Protein disorder. (a) Local substructures. Protein disorder. Average disorder in the top level of Gene Ontology and correlation between predictive performance in terms of AUC cross validation and protein disorder. (b) CATH folds. Protein disorder. Average disorder in the top level of Gene Ontology and correlation between predictive performance in terms of AUC cross validation and protein disorder.
(0.03 MB PDF)

Table S6 Literature evaluation. Predictions and literature evaluation of the 167 proteins with no homology to the training set.
(0.06 MB PDF)
