# Supplementary material for: A Comprehensive Analysis of the Structure-Function Relationship in Proteins Based on Local Structure Similarity
Source: PLoS One. 2009 Jul 15;4(7):e6266. doi: 10.1371/journal.pone.0006266 (PMC2705683; doi:10.1371/journal.pone.0006266)
Supplement: Table S1 — All FDR significant local descriptor-GO class pairs. (a) Molecular function: All FDR significant local descriptor-GO class pairs. (b) Biological process: All FDR significant local descriptor-GO class pairs. (c) Cellular component: All FDR significant local descriptor-GO class pairs. PARAMETERS refer to the parameters in the hypergeometric distribution used to compute the p-values: N,n,k,x, where N is the number of protein-GO class pairs in the data set, n is the number of proteins matched by the local descriptor, k is the number of proteins in the GO class and x is the number of proteins matched by the local descriptor and in the GO class. (1.28 MB PDF) [file pone.0006266.s001.pdf]

Tab S1 (a) Molecular function: All FDR significant local descriptor-GO class pairs.

*PARAMETERS refer to the parameters in the hypergeometric distribution used to compute the p-values: N,n,k,x, where N=2725 is the number of protein-GO class pairs in the data set, n is the number of proteins matched by the local descriptor, k is the number of proteins in the GO class and x is the number of proteins matched by the local descriptor and in the GO class

LOCAL DESCRIPTOR	GENE ONTOLOGY CLASS	P-VALUE	PARAMETERS*
1danh_#197	GO:0004295: trypsin activity	4.15E-85	46,48,43
1ton__#196	GO:0004295: trypsin activity	4.86E-84	47,48,43
1dy9.1#A44	GO:0004295: trypsin activity	1.16E-83	44,48,42
1arb__#55	GO:0004295: trypsin activity	4.66E-83	48,48,43
1gg6.1#C229	GO:0004295: trypsin activity	1.97E-82	42,48,41
1arb__#208	GO:0004295: trypsin activity	3.80E-82	49,48,43
1qq4a_#143	GO:0004295: trypsin activity	1.99E-81	46,48,42
1qq4a_#63	GO:0004295: trypsin activity	1.99E-81	46,48,42
1qnja_#138	GO:0004295: trypsin activity	6.37E-78	46,48,41
1arb__#193	GO:0004295: trypsin activity	6.48E-78	50,48,42
1bqya_#103	GO:0004295: trypsin activity	1.88E-77	40,48,39
1ddja_#759	GO:0004295: trypsin activity	1.88E-77	40,48,39
1autc_#209	GO:0004295: trypsin activity	1.88E-77	40,48,39
1gdna_#123	GO:0004295: trypsin activity	1.27E-76	38,48,38
1dlea_#198	GO:0004295: trypsin activity	3.84E-76	41,48,39
1gdna_#121	GO:0004295: trypsin activity	4.92E-75	39,48,38
1arb__#24	GO:0004295: trypsin activity	5.36E-75	42,48,39
1dlea_#53	GO:0004295: trypsin activity	5.36E-75	42,48,39
1danh_#140	GO:0004295: trypsin activity	3.10E-74	37,48,37
1danh_#190	GO:0004295: trypsin activity	3.10E-74	37,48,37
1gdna_#123	GO:0004263: chymotrypsin activity	5.23E-74	38,41,36
1gdna_#121	GO:0004263: chymotrypsin activity	6.78E-73	39,41,36
1qnja_#118	GO:0004263: chymotrypsin activity	1.20E-72	36,41,35
1ton__#91	GO:0004263: chymotrypsin activity	1.20E-72	36,41,35
1ekbb_#141	GO:0004263: chymotrypsin activity	1.20E-72	36,41,35
1ton__#231	GO:0004263: chymotrypsin activity	1.20E-72	36,41,35
1gdna_#30	GO:0004295: trypsin activity	3.77E-72	45,48,39
1autc_#45	GO:0004295: trypsin activity	3.77E-72	45,48,39
2hlca_#27	GO:0004263: chymotrypsin activity	6.77E-72	40,41,36
1bqya_#103	GO:0004263: chymotrypsin activity	6.77E-72	40,41,36
1autc_#209	GO:0004263: chymotrypsin activity	6.77E-72	40,41,36
1qnja_#118	GO:0004295: trypsin activity	6.94E-72	36,48,36
1ton__#91	GO:0004295: trypsin activity	6.94E-72	36,48,36
1ekbb_#141	GO:0004295: trypsin activity	6.94E-72	36,48,36
1ton__#231	GO:0004295: trypsin activity	6.94E-72	36,48,36
1danh_#140	GO:0004263: chymotrypsin activity	2.22E-71	37,41,35
1danh_#190	GO:0004263: chymotrypsin activity	2.22E-71	37,41,35
1gdna_#155	GO:0004263: chymotrypsin activity	2.22E-71	37,41,35
1dlea_#198	GO:0004263: chymotrypsin activity	5.54E-71	41,41,36
1gdna_#155	GO:0004295: trypsin activity	2.56E-70	37,48,36
1gdna_#56	GO:0004263: chymotrypsin activity	2.80E-70	38,41,35
2hlca_#27	GO:0004295: trypsin activity	3.02E-70	40,48,37
1arb__#24	GO:0004263: chymotrypsin activity	3.87E-70	42,41,36
1dlea_#238	GO:0004263: chymotrypsin activity	3.87E-70	42,41,36
1dlea_#53	GO:0004263: chymotrypsin activity	3.87E-70	42,41,36
1ekbb_#117	GO:0004263: chymotrypsin activity	4.49E-70	35,41,34
1ton__#112	GO:0004263: chymotrypsin activity	4.49E-70	35,41,34
1ekbb_#117	GO:0004295: trypsin activity	1.44E-69	35,48,35
1ton__#112	GO:0004295: trypsin activity	1.44E-69	35,48,35
1qnja_#71	GO:0004263: chymotrypsin activity	4.33E-69	33,41,33
1gdna_#56	GO:0004295: trypsin activity	4.84E-69	38,48,36
1c5y.1#B234	GO:0004263: chymotrypsin activity	8.06E-69	36,41,34
1dy9.1#A44	GO:0004263: chymotrypsin activity	1.30E-68	44,41,36
1ddja_#759	GO:0004263: chymotrypsin activity	2.18E-68	40,41,35
1dlea_#238	GO:0004295: trypsin activity	2.58E-68	42,48,37
1azza_#51	GO:0004295: trypsin activity	5.14E-68	36,48,35
1c5y.1#B234	GO:0004295: trypsin activity	5.14E-68	36,48,35
1gdna_#30	GO:0004263: chymotrypsin activity	6.51E-68	45,41,36
1autc_#45	GO:0004263: chymotrypsin activity	6.51E-68	45,41,36

1danh_#142	GO:0004263: chymotrypsin activity	1.47E-67	34,41,33
2hlca_#68	GO:0004295: trypsin activity	1.67E-67	47,48,38
1danh_#142	GO:0004295: trypsin activity	2.76E-67	34,48,34
1danh_#197	GO:0004263: chymotrypsin activity	2.99E-67	46,41,36
1qnja_#138	GO:0004263: chymotrypsin activity	2.99E-67	46,41,36
1ton__#196	GO:0004263: chymotrypsin activity	1.27E-66	47,41,36
2hlca_#68	GO:0004263: chymotrypsin activity	1.27E-66	47,41,36
1arb__#55	GO:0004263: chymotrypsin activity	5.09E-66	48,41,36
1arb__#208	GO:0004263: chymotrypsin activity	1.91E-65	49,41,36
1ekbb_#228	GO:0004295: trypsin activity	2.99E-65	68,48,41
1azza_#51	GO:0004263: chymotrypsin activity	3.06E-65	36,41,33
1qnja_#71	GO:0004295: trypsin activity	4.95E-65	33,48,33
1qq4a_#143	GO:0004263: chymotrypsin activity	4.36E-64	46,41,35
1qq4a_#63	GO:0004263: chymotrypsin activity	4.36E-64	46,41,35
1ton__#181	GO:0004295: trypsin activity	5.24E-64	79,48,42
1gg6.1#C229	GO:0004263: chymotrypsin activity	1.49E-63	42,41,34
1fjsa_#158	GO:0004263: chymotrypsin activity	1.70E-63	47,41,35
1c5y.1#B94	GO:0004295: trypsin activity	8.34E-63	32,48,32
1c5y.1#B94	GO:0004263: chymotrypsin activity	1.11E-62	32,41,31
1elva1#604	GO:0004263: chymotrypsin activity	2.19E-62	49,41,35
1arb__#193	GO:0004263: chymotrypsin activity	7.29E-62	50,41,35
1danh_#70	GO:0004263: chymotrypsin activity	8.55E-62	30,41,30
1fjsa_#158	GO:0004295: trypsin activity	1.15E-61	47,48,36
1c5y.1#B241	GO:0004295: trypsin activity	1.55E-61	39,48,34
1a0la_#193	GO:0004295: trypsin activity	1.32E-60	31,48,31
1gg6.1#C156	GO:0004295: trypsin activity	1.32E-60	31,48,31
1ton__#179	GO:0004295: trypsin activity	1.32E-60	31,48,31
1elva1#513	GO:0004295: trypsin activity	1.32E-60	31,48,31
1elva1#604	GO:0004295: trypsin activity	1.72E-60	49,48,36
1fjsa_#163	GO:0004263: chymotrypsin activity	2.07E-60	34,41,31
1qnja_#46	GO:0004263: chymotrypsin activity	2.58E-60	60,41,36
1a0la_#193	GO:0004263: chymotrypsin activity	2.64E-60	31,41,30
1gg6.1#C156	GO:0004263: chymotrypsin activity	2.64E-60	31,41,30
1bio__#81	GO:0004263: chymotrypsin activity	2.64E-60	31,41,30
1ton__#179	GO:0004263: chymotrypsin activity	2.64E-60	31,41,30
1elva1#513	GO:0004263: chymotrypsin activity	2.64E-60	31,41,30
1fjsa_#163	GO:0004295: trypsin activity	4.62E-60	34,48,32
1ekbb_#79	GO:0004263: chymotrypsin activity	1.92E-59	29,41,29
1c5y.1#B241	GO:0004263: chymotrypsin activity	1.95E-59	39,41,32
1elva1#601	GO:0004295: trypsin activity	4.20E-59	32,48,31
1elva1#601	GO:0004263: chymotrypsin activity	4.21E-59	32,41,30
1svpa_#127	GO:0004295: trypsin activity	5.36E-59	35,48,32
2hlca_#54	GO:0004295: trypsin activity	1.07E-58	109,48,43
1elva1#566	GO:0004295: trypsin activity	1.98E-58	30,48,30
1c5y.1#B220	GO:0004295: trypsin activity	1.98E-58	30,48,30
1danh_#70	GO:0004295: trypsin activity	1.98E-58	30,48,30
1elva1#566	GO:0004263: chymotrypsin activity	5.74E-58	30,41,29
1c5y.1#B220	GO:0004263: chymotrypsin activity	5.74E-58	30,41,29
1qnja_#46	GO:0004295: trypsin activity	6.60E-58	60,48,37
1bio__#81	GO:0004295: trypsin activity	6.09E-57	31,48,30
1svpa_#127	GO:0004263: chymotrypsin activity	2.72E-56	35,41,30
1c5y.1#B191	GO:0004295: trypsin activity	2.81E-56	29,48,29
1ekbb_#79	GO:0004295: trypsin activity	2.81E-56	29,48,29
1ton__#120	GO:0004295: trypsin activity	2.81E-56	29,48,29
1c5y.1#B191	GO:0004263: chymotrypsin activity	1.15E-55	29,41,28
1g6ga_#133	GO:0004263: chymotrypsin activity	1.15E-55	29,41,28
1ton__#120	GO:0004263: chymotrypsin activity	1.15E-55	29,41,28
1qf5a_#15	GO:0005525: GTP binding	2.74E-55	43,49,33
1gg6.1#B19	GO:0004263: chymotrypsin activity	3.66E-55	59,41,34
1danh_#24	GO:0004263: chymotrypsin activity	7.68E-55	27,41,27
1bio__#108	GO:0004263: chymotrypsin activity	8.26E-55	53,41,33
1ekbb_#228	GO:0004263: chymotrypsin activity	8.60E-55	68,41,35
1ton__#211	GO:0004295: trypsin activity	1.85E-54	86,48,39
1ton__#181	GO:0004263: chymotrypsin activity	2.75E-54	79,41,36
1bqya_#216	GO:0004295: trypsin activity	3.78E-54	28,48,28
1bqya_#216	GO:0004263: chymotrypsin activity	2.14E-53	28,41,27

1gg6.1#B19	GO:0004295: trypsin activity	2.77E-53	59,48,35
1bio__#108	GO:0004295: trypsin activity	2.98E-53	53,48,34
1g6ga_#133	GO:0004295: trypsin activity	1.09E-52	29,48,28
1ton__#211	GO:0004263: chymotrypsin activity	1.46E-52	86,41,36
1fzqa_#28	GO:0005525: GTP binding	4.63E-52	49,49,33
1danh_#24	GO:0004295: trypsin activity	4.86E-52	27,48,27
1eq9a_#143	GO:0004295: trypsin activity	4.86E-52	27,48,27
2hlca_#99	GO:0004295: trypsin activity	4.86E-52	27,48,27
1eq9a_#143	GO:0004263: chymotrypsin activity	3.71E-51	27,41,26
2hlca_#99	GO:0004263: chymotrypsin activity	3.71E-51	27,41,26
1ctqa_#116	GO:0005525: GTP binding	1.43E-50	42,49,31
1c5y.1#B192	GO:0004263: chymotrypsin activity	2.33E-50	25,41,25
1h8d.1#H167	GO:0004263: chymotrypsin activity	2.33E-50	25,41,25
1danh_#242	GO:0004263: chymotrypsin activity	5.17E-50	28,41,26
1befa_#51	GO:0004295: trypsin activity	1.94E-49	29,48,27
2hlca_#54	GO:0004263: chymotrypsin activity	5.67E-48	109,41,36
1c5y.1#B192	GO:0004295: trypsin activity	7.00E-48	25,48,25
1h8d.1#H167	GO:0004295: trypsin activity	7.00E-48	25,48,25
2hlca_#226	GO:0004263: chymotrypsin activity	1.34E-47	98,41,35
1ton__#47	GO:0004263: chymotrypsin activity	1.83E-47	112,41,36
2hlca_#139	GO:0004263: chymotrypsin activity	1.83E-47	112,41,36
1danh_#242	GO:0004295: trypsin activity	2.22E-47	28,48,26
1bio__#210	GO:0004295: trypsin activity	3.06E-47	149,48,41
2hlca_#226	GO:0004295: trypsin activity	3.27E-47	98,48,37
1ton__#47	GO:0004295: trypsin activity	1.10E-46	112,48,38
2hlca_#139	GO:0004295: trypsin activity	1.10E-46	112,48,38
1h8d.1#H184	GO:0004263: chymotrypsin activity	5.56E-46	23,41,23
1gdna_#26	GO:0004295: trypsin activity	7.88E-46	24,48,24
1cipa2#326	GO:0005525: GTP binding	1.54E-45	24,49,24
1eq9a_#59	GO:0004295: trypsin activity	1.58E-45	30,48,26
1egaa1#97	GO:0005525: GTP binding	4.93E-45	27,49,25
1a7s__#148	GO:0004295: trypsin activity	7.54E-45	71,48,33
1gdna_#26	GO:0004263: chymotrypsin activity	1.33E-44	24,41,23
1ddja_#771	GO:0004263: chymotrypsin activity	1.66E-44	31,41,25
1g71a_#77	GO:0004295: trypsin activity	1.97E-44	51,48,30
1g71a_#88	GO:0004295: trypsin activity	4.64E-44	52,48,30
1arb__#210	GO:0004295: trypsin activity	5.19E-44	93,48,35
1danh_#152	GO:0004295: trypsin activity	8.52E-44	23,48,23
1h8d.1#H184	GO:0004295: trypsin activity	8.52E-44	23,48,23
1eq9a_#58	GO:0004295: trypsin activity	1.13E-43	47,48,29
1befa_#51	GO:0004263: chymotrypsin activity	4.27E-43	29,41,24
1g71a_#77	GO:0004263: chymotrypsin activity	7.04E-43	51,41,28
1g71a_#88	GO:0004263: chymotrypsin activity	1.52E-42	52,41,28
1danh_#152	GO:0004263: chymotrypsin activity	1.81E-42	23,41,22
1qf9a_#117	GO:0005525: GTP binding	2.94E-42	89,49,34
1cgha_#227	GO:0004263: chymotrypsin activity	4.03E-42	70,41,30
1ddja_#771	GO:0004295: trypsin activity	4.91E-42	31,48,25
1arb__#210	GO:0004263: chymotrypsin activity	9.18E-42	93,41,32
1autc_#188	GO:0004263: chymotrypsin activity	1.07E-41	21,41,21
1cgha_#227	GO:0004295: trypsin activity	7.41E-41	70,48,31
1cgha_#130	GO:0004263: chymotrypsin activity	2.54E-40	29,41,23
1bio__#210	GO:0004263: chymotrypsin activity	3.77E-40	149,41,35
7taa_2#119	GO:0004556: alpha-amylase activity	4.01E-40	15,15,15
1qhoa4#75	GO:0004556: alpha-amylase activity	4.01E-40	15,15,15
1eq9a_#32	GO:0004263: chymotrypsin activity	4.02E-40	132,41,34
1ekbb_#85	GO:0004263: chymotrypsin activity	4.90E-40	150,41,35
1autc_#188	GO:0004295: trypsin activity	8.87E-40	21,48,21
1eq9a_#32	GO:0004295: trypsin activity	1.37E-39	132,48,36
1bqya_#115	GO:0004263: chymotrypsin activity	1.38E-39	20,41,20
1danh_#48	GO:0004263: chymotrypsin activity	1.38E-39	20,41,20
1ekbb_#73	GO:0004295: trypsin activity	1.44E-39	27,48,23
1eq9a_#40	GO:0004295: trypsin activity	2.43E-39	86,48,32
1b3ra1#223	GO:0016616: oxidoreductase activity, acting on the CH-OH group of donors, NAD or NADP as acceptor	3.59E-39	39,59,27
1ekbb_#85	GO:0004295: trypsin activity	3.63E-39	150,48,37
1ekbb_#73	GO:0004263: chymotrypsin activity	6.17E-39	27,41,22

1gjwa2#141	GO:0004556: alpha-amylase activity	6.41E-39	16,15,15
7taa_2#295	GO:0004556: alpha-amylase activity	6.41E-39	16,15,15
1eq9a_#40	GO:0004263: chymotrypsin activity	8.78E-39	86,41,30
1cgha_#130	GO:0004295: trypsin activity	3.83E-38	29,48,23
1eh9a3#252	GO:0004556: alpha-amylase activity	5.45E-38	17,15,15
1nhp_1#282	GO:0015036: disulfide oxidoreductase activity	6.66E-38	19,22,17
1ddja_#726	GO:0004295: trypsin activity	6.78E-38	105,48,33
1bqya_#115	GO:0004295: trypsin activity	8.57E-38	20,48,20
1danh_#48	GO:0004295: trypsin activity	8.57E-38	20,48,20
1h7wa4#481	GO:0015036: disulfide oxidoreductase activity	9.63E-38	24,22,18
1a7s__#148	GO:0004263: chymotrypsin activity	1.50E-37	71,41,28
1elva1#623	GO:0004295: trypsin activity	1.92E-37	52,48,27
1fjsa_#83	GO:0004295: trypsin activity	2.88E-37	109,48,33
1eq9a_#58	GO:0004263: chymotrypsin activity	3.05E-37	47,41,25
1trb_1#285	GO:0015036: disulfide oxidoreductase activity	3.44E-37	25,22,18
1trb_1#298	GO:0015036: disulfide oxidoreductase activity	3.44E-37	25,22,18
1fjsa_#83	GO:0004263: chymotrypsin activity	4.07E-37	109,41,31
1eq9a_#59	GO:0004263: chymotrypsin activity	4.39E-37	30,41,22
1danh_#104	GO:0004263: chymotrypsin activity	1.10E-36	183,41,35
1gdna_#119	GO:0004295: trypsin activity	7.99E-36	19,48,19
1qlaa2#13	GO:0015036: disulfide oxidoreductase activity	9.34E-36	28,22,18
1pbe_1#155	GO:0015036: disulfide oxidoreductase activity	9.34E-36	28,22,18
1fl2a1#320	GO:0015036: disulfide oxidoreductase activity	9.34E-36	28,22,18
1trb_1#112	GO:0015036: disulfide oxidoreductase activity	1.02E-35	22,22,17
1ddja_#726	GO:0004263: chymotrypsin activity	1.04E-35	105,41,30
1qf6a4#362	GO:0004812: tRNA ligase activity	1.26E-35	16,26,16
1danh_#104	GO:0004295: trypsin activity	1.27E-35	183,48,37
1dar_2#264	GO:0005525: GTP binding	1.31E-35	19,49,19
1cgha_#168	GO:0004263: chymotrypsin activity	1.42E-35	24,41,20
1avaa2#204	GO:0004556: alpha-amylase activity	1.63E-35	15,15,14
1c5y.1#B18	GO:0004263: chymotrypsin activity	1.99E-35	18,41,18
1trb_1#14	GO:0015036: disulfide oxidoreductase activity	2.46E-35	29,22,18
1ddja_#746	GO:0004295: trypsin activity	3.74E-35	170,48,36
1qlaa2#35	GO:0015036: disulfide oxidoreductase activity	6.14E-35	30,22,18
1h7wa4#478	GO:0015036: disulfide oxidoreductase activity	1.70E-34	19,22,16
1bvza3#416	GO:0004556: alpha-amylase activity	1.96E-34	23,15,15
1gdna_#119	GO:0004263: chymotrypsin activity	3.76E-34	19,41,18
1ddja_#746	GO:0004263: chymotrypsin activity	4.81E-34	170,41,33
1c5y.1#B18	GO:0004295: trypsin activity	7.21E-34	18,48,18
1cgha_#168	GO:0004295: trypsin activity	8.75E-34	24,48,20
1chua2#234	GO:0015036: disulfide oxidoreductase activity	1.09E-33	16,22,15
1e39a2#278	GO:0015036: disulfide oxidoreductase activity	1.20E-33	26,22,17
1elva1#623	GO:0004263: chymotrypsin activity	1.33E-33	52,41,24
1fmja_#76	GO:0005525: GTP binding	2.05E-33	85,49,29
1danh_#93	GO:0004263: chymotrypsin activity	2.25E-33	17,41,17
1qf6a4#295	GO:0004812: tRNA ligase activity	3.10E-33	15,26,15
1atia2#67	GO:0004812: tRNA ligase activity	3.10E-33	15,26,15
1hc7a2#111	GO:0004812: tRNA ligase activity	3.10E-33	15,26,15
1hc7a2#109	GO:0004812: tRNA ligase activity	3.10E-33	15,26,15
1h6va2#219	GO:0015036: disulfide oxidoreductase activity	6.39E-33	36,22,18
1xvaa_#68	GO:0008757: S-adenosylmethionine-dependent methyltransferase activity	8.36E-33	16,24,15
1qlaa2#383	GO:0015036: disulfide oxidoreductase activity	9.25E-33	17,22,15
1eq9a_#162	GO:0004295: trypsin activity	1.16E-32	35,48,22
1fmja_#76	GO:0005524: ATP binding	1.53E-32	85,243,50
1ihua2#343	GO:0005525: GTP binding	1.80E-32	233,49,38
1ddja_#754	GO:0004295: trypsin activity	3.57E-32	31,48,21
1qnja_#124	GO:0004263: chymotrypsin activity	4.02E-32	18,41,17
1h6va1#23	GO:0015036: disulfide oxidoreductase activity	4.37E-32	39,22,18
1qf6a4#318	GO:0004812: tRNA ligase activity	4.93E-32	16,26,15
1ebda2#182	GO:0015036: disulfide oxidoreductase activity	5.54E-32	18,22,15
1danh_#93	GO:0004295: trypsin activity	6.30E-32	17,48,17
1azza_#114	GO:0004295: trypsin activity	6.30E-32	17,48,17
1gdna_#151	GO:0004295: trypsin activity	6.30E-32	17,48,17
1gdna_#230	GO:0004295: trypsin activity	6.79E-32	23,48,19
1ddja_#754	GO:0004263: chymotrypsin activity	1.07E-31	31,41,20
1e43a2#351	GO:0004556: alpha-amylase activity	1.26E-31	21,15,14

1d5ta1#12	GO:0015036: disulfide oxidoreductase activity	1.28E-31	24,22,16
1ddja_#681	GO:0004263: chymotrypsin activity	2.16E-31	253,41,35
1qnja_#77	GO:0004263: chymotrypsin activity	2.44E-31	16,41,16
1h7wa4#476	GO:0015036: disulfide oxidoreductase activity	3.46E-31	15,22,14
1nhp_1#7	GO:0015036: disulfide oxidoreductase activity	4.44E-31	33,22,17
1cipa2#34	GO:0005525: GTP binding	4.84E-31	78,49,27
1cqqa_#163	GO:0004295: trypsin activity	7.85E-31	29,48,20
1trb_1#15	GO:0015036: disulfide oxidoreductase activity	1.05E-30	20,22,15
1qnja_#124	GO:0004295: trypsin activity	1.12E-30	18,48,17
1e30a_#87	GO:0005507: copper ion binding	1.64E-30	52,38,22
1jbaa_#157	GO:0005509: calcium ion binding	1.64E-30	31,160,27
1d7ya1#255	GO:0015036: disulfide oxidoreductase activity	2.76E-30	16,22,14
1azza_#114	GO:0004263: chymotrypsin activity	4.11E-30	17,41,16
1gdna_#151	GO:0004263: chymotrypsin activity	4.11E-30	17,41,16
1qnja_#77	GO:0004295: trypsin activity	5.33E-30	16,48,16
1gnwa1#160	GO:0004364: glutathione transferase activity	7.94E-30	12,11,11
1fzqa_#165	GO:0005525: GTP binding	7.98E-30	22,49,18
1hx0a2#294	GO:0004556: alpha-amylase activity	1.26E-29	18,15,13
1e43a2#236	GO:0004556: alpha-amylase activity	1.73E-29	13,15,12
1e2fa_#14	GO:0019201: nucleotide kinase activity	1.73E-29	15,13,12
1fuma2#370	GO:0015036: disulfide oxidoreductase activity	9.74E-29	14,22,13
1a0la_#160	GO:0004295: trypsin activity	1.09E-28	73,48,25
1nat__#50	GO:0016616: oxidoreductase activity, acting on the CH-OH group of donors, NAD or NADP as acceptor	1.28E-28	297,59,42
1h4vb2#108	GO:0004812: tRNA ligase activity	1.46E-28	13,26,13
1auib_#136	GO:0005509: calcium ion binding	1.96E-28	22,160,22
1fnf_1#1146	GO:0004896: hematopoietin/interferon-class (D200-domain) cytokine receptor activity	2.12E-28	16,19,13
1foha5#218	GO:0015036: disulfide oxidoreductase activity	2.12E-28	57,22,18
1pbe_1#121	GO:0015036: disulfide oxidoreductase activity	2.18E-28	25,22,15
1trb_1#41	GO:0015036: disulfide oxidoreductase activity	2.66E-28	19,22,14
1c7wa_#105	GO:0005509: calcium ion binding	4.98E-28	29,160,25
1e79d2#36	GO:0004295: trypsin activity	6.22E-28	524,48,44
1cjca2#103	GO:0015036: disulfide oxidoreductase activity	7.28E-28	15,22,13
1bvza3#298	GO:0004556: alpha-amylase activity	9.04E-28	11,15,11
1ihua2#525	GO:0005525: GTP binding	1.09E-27	335,49,39
1hq8a_#150	GO:0005529: sugar binding	1.35E-27	17,39,15
1e87a_#121	GO:0005529: sugar binding	1.35E-27	17,39,15
1aq0a_#4	GO:0004556: alpha-amylase activity	1.67E-27	23,15,13
1qfea_#49	GO:0004556: alpha-amylase activity	1.80E-27	52,15,15
1a0la_#160	GO:0004263: chymotrypsin activity	2.29E-27	73,41,23
1h8d.1#H207	GO:0004263: chymotrypsin activity	3.16E-27	63,41,22
1e3ja2#270	GO:0016616: oxidoreductase activity, acting on the CH-OH group of donors, NAD or NADP as acceptor	3.21E-27	74,59,26
1b4va1#253	GO:0015036: disulfide oxidoreductase activity	3.87E-27	16,22,13
1ddja_#681	GO:0004295: trypsin activity	5.21E-27	253,48,35
1dr9a2#139	GO:0004896: hematopoietin/interferon-class (D200-domain) cytokine receptor activity	8.26E-27	67,19,17
1qama_#37	GO:0016616: oxidoreductase activity, acting on the CH-OH group of donors, NAD or NADP as acceptor	1.07E-26	68,59,25
2msba_#134	GO:0005529: sugar binding	1.62E-26	15,39,14
1hq8a_#223	GO:0005529: sugar binding	1.62E-26	15,39,14
1tn3__#113	GO:0005529: sugar binding	1.62E-26	15,39,14
1fyhb1#99	GO:0004896: hematopoietin/interferon-class (D200-domain) cytokine receptor activity	1.65E-26	27,19,14
1fwxa1#526	GO:0005507: copper ion binding	2.33E-26	19,38,15
1trb_1#42	GO:0015036: disulfide oxidoreductase activity	2.45E-26	13,22,12
1oaa__#14	GO:0016616: oxidoreductase activity, acting on the CH-OH group of donors, NAD or NADP as acceptor	2.89E-26	54,59,23
1a6o__#217	GO:0004674: protein serine/threonine kinase activity	3.14E-26	18,42,15
1phk__#165	GO:0004674: protein serine/threonine kinase activity	3.14E-26	18,42,15
1ldna1#94	GO:0016616: oxidoreductase activity, acting on the CH-OH group of donors, NAD or NADP as acceptor	4.29E-26	199,59,35
1cjca2#365	GO:0015036: disulfide oxidoreductase activity	5.09E-26	57,22,17
1fcda1#6	GO:0015036: disulfide oxidoreductase activity	6.37E-26	75,22,18
7taa_2#331	GO:0004556: alpha-amylase activity	7.03E-26	13,15,11
1bf2_3#575	GO:0004556: alpha-amylase activity	7.03E-26	13,15,11
1eq9a_#162	GO:0004263: chymotrypsin activity	7.88E-26	35,41,18
1pbe_1#150	GO:0015036: disulfide oxidoreductase activity	8.33E-26	76,22,18

2cuaa_#83	GO:0005507: copper ion binding	8.48E-26	46,38,19
2msba_#128	GO:0005529: sugar binding	1.29E-25	16,39,14
1fmk_3#377	GO:0004674: protein serine/threonine kinase activity	1.48E-25	19,42,15
1qlaa2#179	GO:0015036: disulfide oxidoreductase activity	1.64E-25	46,22,16
1c7na_#281	GO:0008483: transaminase activity	1.71E-25	25,17,13
1fl2a1#472	GO:0015036: disulfide oxidoreductase activity	1.71E-25	14,22,12
1qama_#39	GO:0008757: S-adenosylmethionine-dependent methyltransferase activity	2.69E-25	24,24,14
1h8d.1#H207	GO:0004295: trypsin activity	3.19E-25	63,48,22
1e2fa_#14	GO:0016776: phosphotransferase activity, phosphate group as acceptor	3.28E-25	15,14,11
1hrna_#38	GO:0004190: aspartic-type endopeptidase activity	3.57E-25	14,23,12
1fjsa_#158	GO:0004867: serine-type endopeptidase inhibitor activity	3.77E-25	47,47,20
1koba_#175	GO:0004674: protein serine/threonine kinase activity	5.85E-25	20,42,15
2dkb__#46	GO:0008483: transaminase activity	6.37E-25	13,17,11
1i2ma_#90	GO:0005525: GTP binding	7.25E-25	15,49,14
3grs_1#24	GO:0015036: disulfide oxidoreductase activity	9.02E-25	66,22,17
1aqua_#126	GO:0016616: oxidoreductase activity, acting on the CH-OH group of donors, NAD or NADP as acceptor	1.17E-24	576,59,49
1e39a2#516	GO:0015036: disulfide oxidoreductase activity	1.39E-24	21,22,13
1euva_#580	GO:0004197: cysteine-type endopeptidase activity	1.65E-24	11,24,11
1a65a2#187	GO:0005507: copper ion binding	1.69E-24	43,38,18
1tkia_#80	GO:0004674: protein serine/threonine kinase activity	2.53E-24	17,42,14
1howa_#588	GO:0004674: protein serine/threonine kinase activity	2.53E-24	17,42,14
1koba_#230	GO:0004674: protein serine/threonine kinase activity	2.53E-24	17,42,14
1tkia_#266	GO:0004674: protein serine/threonine kinase activity	2.53E-24	17,42,14
1e39a2#556	GO:0015036: disulfide oxidoreductase activity	2.59E-24	40,22,15
1bpv__#22	GO:0004896: hematopoietin/interferon-class (D200-domain) cytokine receptor activity	2.70E-24	18,19,12
1fjsa_#28	GO:0004295: trypsin activity	2.71E-24	13,48,13
1qnja_#46	GO:0004867: serine-type endopeptidase inhibitor activity	2.77E-24	60,47,21
1dik_1#848	GO:0004556: alpha-amylase activity	3.26E-24	81,15,15
1ojt_2#336	GO:0015036: disulfide oxidoreductase activity	3.40E-24	16,22,12
1qhoa4#399	GO:0004556: alpha-amylase activity	3.56E-24	24,15,12
1i2ma_#157	GO:0005525: GTP binding	3.68E-24	13,49,13
1g5ha2#319	GO:0004812: tRNA ligase activity	3.85E-24	19,26,13
1glqa2#53	GO:0004364: glutathione transferase activity	5.12E-24	26,11,11
1bd3a_#110	GO:0016763: transferase activity, transferring pentosyl groups	5.43E-24	24,28,14
1fgka_#610	GO:0004674: protein serine/threonine kinase activity	6.31E-24	22,42,15
1f3mc_#403	GO:0004674: protein serine/threonine kinase activity	6.31E-24	22,42,15
1feca1#31	GO:0015036: disulfide oxidoreductase activity	1.05E-23	75,22,17
2ae2a_#17	GO:0016616: oxidoreductase activity, acting on the CH-OH group of donors, NAD or NADP as acceptor	1.09E-23	31,59,18
1qsta_#154	GO:0004812: tRNA ligase activity	1.10E-23	20,26,13
1hq8a_#185	GO:0005529: sugar binding	1.14E-23	12,39,12
1cjca2#76	GO:0015036: disulfide oxidoreductase activity	1.15E-23	17,22,12
1gdna_#230	GO:0004263: chymotrypsin activity	1.16E-23	23,41,15
1f00i3#856	GO:0005529: sugar binding	1.18E-23	15,39,13
1a0fa1#94	GO:0004364: glutathione transferase activity	2.44E-23	10,11,9
1nsj__#178	GO:0004556: alpha-amylase activity	3.13E-23	93,15,15
1eq2a_#11	GO:0016616: oxidoreductase activity, acting on the CH-OH group of donors, NAD or NADP as acceptor	3.20E-23	45,59,20
1bvza3#373	GO:0004556: alpha-amylase activity	3.23E-23	12,15,10
1qs0a1#119	GO:0016616: oxidoreductase activity, acting on the CH-OH group of donors, NAD or NADP as acceptor	3.27E-23	454,59,44
1d2fa_#348	GO:0008483: transaminase activity	3.49E-23	11,17,10
1ek0a_#151	GO:0005525: GTP binding	3.75E-23	26,49,16
1e79d2#36	GO:0004263: chymotrypsin activity	5.20E-23	524,41,37
1qfea_#68	GO:0016616: oxidoreductase activity, acting on the CH-OH group of donors, NAD or NADP as acceptor	9.51E-23	560,59,47
1ldm_2#292	GO:0004457: lactate dehydrogenase activity	9.75E-23	12,10,9
1feca1#309	GO:0015036: disulfide oxidoreductase activity	1.06E-22	10,22,10
2msba_#195	GO:0005529: sugar binding	1.47E-22	13,39,12
1esl_1#29	GO:0005529: sugar binding	1.47E-22	13,39,12
1ljra1#165	GO:0004364: glutathione transferase activity	1.89E-22	34,11,11
1pme__#302	GO:0004674: protein serine/threonine kinase activity	1.95E-22	16,42,13
1qnja_#221	GO:0004295: trypsin activity	2.04E-22	12,48,12
1hx0a2#32	GO:0004556: alpha-amylase activity	2.06E-22	70,15,14
1gjwa2#160	GO:0004556: alpha-amylase activity	2.22E-22	9,15,9

1fjsa_#28	GO:0004263: chymotrypsin activity	2.98E-22	13,41,12
1e0ta2#275	GO:0004556: alpha-amylase activity	3.02E-22	107,15,15
7mdha2#355	GO:0004457: lactate dehydrogenase activity	3.17E-22	13,10,9
1llc_2#271	GO:0004457: lactate dehydrogenase activity	3.17E-22	13,10,9
2hlpa2#195	GO:0004457: lactate dehydrogenase activity	3.17E-22	13,10,9
1cs8a_#188	GO:0004197: cysteine-type endopeptidase activity	3.20E-22	10,24,10
1ebda2#182	GO:0016668: oxidoreductase activity, acting on sulfur group of donors, NAD or NADP as acceptor	4.69E-22	18,12,10
2pvba_#51	GO:0005509: calcium ion binding	5.10E-22	17,160,17
1bio__#88	GO:0004263: chymotrypsin activity	7.57E-22	22,41,14
1llc_2#291	GO:0004457: lactate dehydrogenase activity	8.87E-22	14,10,9
2cmd_2#251	GO:0004457: lactate dehydrogenase activity	8.87E-22	14,10,9
1ejda_#213	GO:0004263: chymotrypsin activity	9.14E-22	284,41,30
1h7wa4#478	GO:0016668: oxidoreductase activity, acting on sulfur group of donors, NAD or NADP as acceptor	9.90E-22	19,12,10
1nhp_1#282	GO:0016668: oxidoreductase activity, acting on sulfur group of donors, NAD or NADP as acceptor	9.90E-22	19,12,10
1trb_1#41	GO:0016668: oxidoreductase activity, acting on sulfur group of donors, NAD or NADP as acceptor	9.90E-22	19,12,10
1a06__#147	GO:0004674: protein serine/threonine kinase activity	1.31E-21	28,42,15
1cqqa_#163	GO:0004263: chymotrypsin activity	1.74E-21	29,41,15
1e2fa_#140	GO:0019201: nucleotide kinase activity	1.74E-21	11,13,9
1h6va2#245	GO:0015036: disulfide oxidoreductase activity	1.87E-21	99,22,17
1trb_1#15	GO:0016668: oxidoreductase activity, acting on sulfur group of donors, NAD or NADP as acceptor	1.98E-21	20,12,10
1fjsa_#84	GO:0004263: chymotrypsin activity	2.06E-21	14,41,12
1bf2_3#579	GO:0004556: alpha-amylase activity	2.22E-21	10,15,9
1llda2#177	GO:0004457: lactate dehydrogenase activity	2.22E-21	15,10,9
1gjwa2#142	GO:0004556: alpha-amylase activity	2.22E-21	10,15,9
1apme_#101	GO:0004674: protein serine/threonine kinase activity	2.89E-21	14,42,12
1danh_#242	GO:0005509: calcium ion binding	3.17E-21	28,160,21
1qlsa_#81	GO:0005509: calcium ion binding	3.22E-21	55,160,28
1ton__#120	GO:0003809: thrombin activity	3.27E-21	29,10,10
1e6ua_#10	GO:0016616: oxidoreductase activity, acting on the CH-OH group of donors, NAD or NADP as acceptor	3.79E-21	33,59,17
1e2fa_#140	GO:0016776: phosphotransferase activity, phosphate group as acceptor	4.87E-21	11,14,9
1danh_#70	GO:0003809: thrombin activity	4.91E-21	30,10,10
1eq9a_#59	GO:0003809: thrombin activity	4.91E-21	30,10,10
1fvua_#120	GO:0005529: sugar binding	5.07E-21	15,39,12
1aqua_#126	GO:0005525: GTP binding	5.33E-21	576,49,41
2cmd_2#172	GO:0004457: lactate dehydrogenase activity	5.42E-21	9,10,8
1cyx__#138	GO:0005507: copper ion binding	6.12E-21	26,38,14
1trb_1#112	GO:0016668: oxidoreductase activity, acting on sulfur group of donors, NAD or NADP as acceptor	6.92E-21	22,12,10
1bio__#81	GO:0003809: thrombin activity	7.25E-21	31,10,10
1ddja_#771	GO:0003809: thrombin activity	7.25E-21	31,10,10
1pysa_#127	GO:0004812: tRNA ligase activity	7.96E-21	21,26,12
1pme__#166	GO:0005524: ATP binding	8.60E-21	23,243,21
1atia2#189	GO:0004812: tRNA ligase activity	9.50E-21	11,26,10
1bio__#88	GO:0004295: trypsin activity	1.02E-20	22,48,14
1ir3a_#1192	GO:0004674: protein serine/threonine kinase activity	1.02E-20	31,42,15
2oata_#81	GO:0008483: transaminase activity	1.08E-20	10,17,9
1clxa_#13	GO:0004556: alpha-amylase activity	1.22E-20	11,15,9
2uaga1#54	GO:0015036: disulfide oxidoreductase activity	1.45E-20	18,22,11
2cb5a_#373	GO:0004197: cysteine-type endopeptidase activity	1.47E-20	44,24,14
1gg6.1#C232	GO:0004295: trypsin activity	1.50E-20	11,48,11
1qnja_#71	GO:0003809: thrombin activity	1.51E-20	33,10,10
1a06__#147	GO:0005524: ATP binding	1.76E-20	28,243,23
1fjsa_#84	GO:0004295: trypsin activity	1.81E-20	14,48,12
1pd211#147	GO:0004364: glutathione transferase activity	1.99E-20	9,11,8
1h7wa4#481	GO:0016668: oxidoreductase activity, acting on sulfur group of donors, NAD or NADP as acceptor	2.10E-20	24,12,10
1fjsa_#163	GO:0003809: thrombin activity	2.14E-20	34,10,10
1ceqa1#139	GO:0004457: lactate dehydrogenase activity	2.15E-20	18,10,9
1qnja_#221	GO:0004263: chymotrypsin activity	2.48E-20	12,41,11
1ekbb_#117	GO:0003809: thrombin activity	3.00E-20	35,10,10

1svpa_#127	GO:0003809: thrombin activity	3.00E-20	35,10,10
1ton__#112	GO:0003809: thrombin activity	3.00E-20	35,10,10
1gdna_#155	GO:0005509: calcium ion binding	3.04E-20	37,160,23
1howa_#551	GO:0004674: protein serine/threonine kinase activity	3.37E-20	12,42,11
1danh_#70	GO:0005509: calcium ion binding	3.47E-20	30,160,21
1trb_1#285	GO:0016668: oxidoreductase activity, acting on sulfur group of donors, NAD or NADP as acceptor	3.49E-20	25,12,10
1trb_1#298	GO:0016668: oxidoreductase activity, acting on sulfur group of donors, NAD or NADP as acceptor	3.49E-20	25,12,10
1j71a_#32	GO:0004190: aspartic-type endopeptidase activity	3.63E-20	9,23,9
1mpp__#216	GO:0004190: aspartic-type endopeptidase activity	3.63E-20	9,23,9
1pfza_#193	GO:0004190: aspartic-type endopeptidase activity	3.63E-20	9,23,9
2hlpa2#315	GO:0004457: lactate dehydrogenase activity	4.09E-20	19,10,9
1trb_1#41	GO:0050660: FAD binding	4.09E-20	19,10,9
1qnja_#118	GO:0003809: thrombin activity	4.15E-20	36,10,10
1ton__#91	GO:0003809: thrombin activity	4.15E-20	36,10,10
1ekbb_#141	GO:0003809: thrombin activity	4.15E-20	36,10,10
1ton__#231	GO:0003809: thrombin activity	4.15E-20	36,10,10
2hlpa1#98	GO:0016616: oxidoreductase activity, acting on the CH-OH group of donors, NAD or NADP as acceptor	4.19E-20	13,59,12
2hlpa2#195	GO:0016616: oxidoreductase activity, acting on the CH-OH group of donors, NAD or NADP as acceptor	4.19E-20	13,59,12
1qhoa4#230	GO:0004556: alpha-amylase activity	4.47E-20	19,15,10
1h7wa4#476	GO:0016668: oxidoreductase activity, acting on sulfur group of donors, NAD or NADP as acceptor	4.86E-20	15,12,9
1eona_#74	GO:0009036: type II site-specific deoxyribonuclease activity	4.86E-20	15,12,9
1dkia_#54	GO:0004197: cysteine-type endopeptidase activity	5.10E-20	18,24,11
1qnja_#138	GO:0004867: serine-type endopeptidase inhibitor activity	5.24E-20	46,47,17
1e39a2#278	GO:0016668: oxidoreductase activity, acting on sulfur group of donors, NAD or NADP as acceptor	5.67E-20	26,12,10
1danh_#140	GO:0003809: thrombin activity	5.69E-20	37,10,10
1gdna_#155	GO:0003809: thrombin activity	5.69E-20	37,10,10
1qs0a1#119	GO:0005525: GTP binding	5.82E-20	454,49,37
1fgka_#689	GO:0004674: protein serine/threonine kinase activity	6.72E-20	21,42,13
1qq4a_#46	GO:0004295: trypsin activity	6.83E-20	19,48,13
3lada1#116	GO:0015036: disulfide oxidoreductase activity	6.93E-20	153,22,18
1a6o__#217	GO:0005524: ATP binding	7.05E-20	18,243,18
1phk__#165	GO:0005524: ATP binding	7.05E-20	18,243,18
1bqya_#115	GO:0003809: thrombin activity	7.43E-20	20,10,9
1gdna_#123	GO:0003809: thrombin activity	7.72E-20	38,10,10
1ypta_#409	GO:0004725: protein tyrosine phosphatase activity	8.62E-20	8,15,8
1ypta_#406	GO:0004725: protein tyrosine phosphatase activity	8.62E-20	8,15,8
1f3mc_#403	GO:0005524: ATP binding	9.52E-20	22,243,20
1ceqa2#199	GO:0004457: lactate dehydrogenase activity	9.93E-20	11,10,8
1e87a_#155	GO:0005529: sugar binding	1.04E-19	10,39,10
1c5y.1#B241	GO:0003809: thrombin activity	1.04E-19	39,10,10
1gdna_#121	GO:0003809: thrombin activity	1.04E-19	39,10,10
1koba_#175	GO:0005524: ATP binding	1.08E-19	20,243,19
1chua2#234	GO:0016668: oxidoreductase activity, acting on sulfur group of donors, NAD or NADP as acceptor	1.11E-19	16,12,9
1b8pa2#197	GO:0004457: lactate dehydrogenase activity	1.30E-19	21,10,9
1e39a2#516	GO:0050660: FAD binding	1.30E-19	21,10,9
1a06__#103	GO:0004674: protein serine/threonine kinase activity	1.34E-19	28,42,14
2hlca_#27	GO:0003809: thrombin activity	1.39E-19	40,10,10
1bqya_#103	GO:0003809: thrombin activity	1.39E-19	40,10,10
1ddja_#759	GO:0003809: thrombin activity	1.39E-19	40,10,10
1autc_#209	GO:0003809: thrombin activity	1.39E-19	40,10,10
1cpt__#358	GO:0004497: monooxygenase activity	1.39E-19	9,26,9
1dz4a_#357	GO:0004497: monooxygenase activity	1.39E-19	9,26,9
1pbe_1#155	GO:0016668: oxidoreductase activity, acting on sulfur group of donors, NAD or NADP as acceptor	1.40E-19	28,12,10
1fl2a1#320	GO:0016668: oxidoreductase activity, acting on sulfur group of donors, NAD or NADP as acceptor	1.40E-19	28,12,10
1elva1#601	GO:0004867: serine-type endopeptidase inhibitor activity	1.41E-19	32,47,15
1bqya_#216	GO:0005509: calcium ion binding	1.53E-19	28,160,20
1b4va1#11	GO:0015036: disulfide oxidoreductase activity	1.54E-19	30,22,12

1qsta_#137	GO:0008080: N-acetyltransferase activity	1.55E-19	9,13,8
1hwxa1#256	GO:0015036: disulfide oxidoreductase activity	1.58E-19	57,22,14
1ddja_#759	GO:0004867: serine-type endopeptidase inhibitor activity	1.72E-19	40,47,16
1e6ca_#153	GO:0016776: phosphotransferase activity, phosphate group as acceptor	1.76E-19	14,14,9
1dlea_#198	GO:0003809: thrombin activity	1.83E-19	41,10,10
1fnf_3#1409	GO:0004896: hematopoietin/interferon-class (D200-domain) cytokine receptor activity	1.84E-19	71,19,14
2ae2a_#196	GO:0016616: oxidoreductase activity, acting on the CH-OH group of donors, NAD or NADP as acceptor	1.85E-19	11,59,11
1gcya2#331	GO:0004556: alpha-amylase activity	2.03E-19	51,15,12
1trb_1#14	GO:0016668: oxidoreductase activity, acting on sulfur group of donors, NAD or NADP as acceptor	2.13E-19	29,12,10
1bkca_#351	GO:0004222: metalloendopeptidase activity	2.24E-19	11,19,9
1h5qa_#70	GO:0016616: oxidoreductase activity, acting on the CH-OH group of donors, NAD or NADP as acceptor	2.31E-19	27,59,15
1qlaa2#383	GO:0016668: oxidoreductase activity, acting on sulfur group of donors, NAD or NADP as acceptor	2.35E-19	17,12,9
1cjca2#76	GO:0016668: oxidoreductase activity, acting on sulfur group of donors, NAD or NADP as acceptor	2.35E-19	17,12,9
1hcl__#267	GO:0004674: protein serine/threonine kinase activity	2.40E-19	10,42,10
1arb__#24	GO:0003809: thrombin activity	2.40E-19	42,10,10
1dlea_#238	GO:0003809: thrombin activity	2.40E-19	42,10,10
1dlea_#53	GO:0003809: thrombin activity	2.40E-19	42,10,10
2ebn__#77	GO:0004556: alpha-amylase activity	2.64E-19	52,15,12
1elva1#601	GO:0005509: calcium ion binding	2.83E-19	32,160,21
2cmd_2#251	GO:0016616: oxidoreductase activity, acting on the CH-OH group of donors, NAD or NADP as acceptor	2.89E-19	14,59,12
1feca1#309	GO:0016668: oxidoreductase activity, acting on sulfur group of donors, NAD or NADP as acceptor	2.98E-19	10,12,8
1tkia_#142	GO:0004674: protein serine/threonine kinase activity	3.01E-19	37,42,15
1fkna_#99	GO:0004190: aspartic-type endopeptidase activity	3.03E-19	21,23,11
1lvl_2#222	GO:0015036: disulfide oxidoreductase activity	3.15E-19	79,22,15
1qlaa2#35	GO:0016668: oxidoreductase activity, acting on sulfur group of donors, NAD or NADP as acceptor	3.20E-19	30,12,10
1bxoa_#141	GO:0004190: aspartic-type endopeptidase activity	3.61E-19	10,23,9
1h8d.1#H184	GO:0003809: thrombin activity	3.61E-19	23,10,9
1j71a_#303	GO:0004190: aspartic-type endopeptidase activity	3.61E-19	10,23,9
1pfza_#313	GO:0004190: aspartic-type endopeptidase activity	3.61E-19	10,23,9
1ton__#91	GO:0005509: calcium ion binding	3.69E-19	36,160,22
1ekbb_#141	GO:0005509: calcium ion binding	3.69E-19	36,160,22
1ton__#231	GO:0005509: calcium ion binding	3.69E-19	36,160,22
1pme__#166	GO:0004674: protein serine/threonine kinase activity	3.71E-19	23,42,13
2hlca_#27	GO:0005509: calcium ion binding	3.80E-19	40,160,23
1f5aa2#167	GO:0005525: GTP binding	3.83E-19	124,49,23
1dy9.1#A44	GO:0003809: thrombin activity	4.05E-19	44,10,10
1eh9a3#102	GO:0004556: alpha-amylase activity	4.40E-19	14,15,9
1gg6.1#C229	GO:0004867: serine-type endopeptidase inhibitor activity	4.46E-19	42,47,16
1ceqa1#139	GO:0016616: oxidoreductase activity, acting on the CH-OH group of donors, NAD or NADP as acceptor	4.49E-19	18,59,13
1g5ha2#162	GO:0004812: tRNA ligase activity	4.57E-19	27,26,12
1ekbb_#79	GO:0005509: calcium ion binding	4.68E-19	29,160,20
1gdna_#30	GO:0003809: thrombin activity	5.21E-19	45,10,10
1autc_#45	GO:0003809: thrombin activity	5.21E-19	45,10,10
1howa_#697	GO:0004674: protein serine/threonine kinase activity	5.65E-19	18,42,12
1gdna_#26	GO:0003809: thrombin activity	5.77E-19	24,10,9
1h7wa4#481	GO:0050660: FAD binding	5.77E-19	24,10,9
1danh_#197	GO:0003809: thrombin activity	6.66E-19	46,10,10
1qq4a_#63	GO:0003809: thrombin activity	6.66E-19	46,10,10
1qnja_#138	GO:0003809: thrombin activity	6.66E-19	46,10,10
1eno__#137	GO:0016616: oxidoreductase activity, acting on the CH-OH group of donors, NAD or NADP as acceptor	6.84E-19	23,59,14
1aoza1#61	GO:0005507: copper ion binding	7.06E-19	26,38,13
1qnja_#71	GO:0005509: calcium ion binding	7.39E-19	33,160,21
2hlpa1#98	GO:0004457: lactate dehydrogenase activity	7.73E-19	13,10,8
2hlpa2#272	GO:0004457: lactate dehydrogenase activity	7.73E-19	13,10,8
1fg7a_#96	GO:0016846: carbon-sulfur lyase activity	7.73E-19	13,10,8
1d6ja_#36	GO:0005525: GTP binding	8.38E-19	27,49,14

1tkia_#80	GO:0005524: ATP binding	8.45E-19	17,243,17
1howa_#588	GO:0005524: ATP binding	8.45E-19	17,243,17
1koba_#230	GO:0005524: ATP binding	8.45E-19	17,243,17
1tkia_#266	GO:0005524: ATP binding	8.45E-19	17,243,17
1bqya_#216	GO:0004867: serine-type endopeptidase inhibitor activity	8.45E-19	28,47,14
1ton__#196	GO:0003809: thrombin activity	8.46E-19	47,10,10
1eq9a_#58	GO:0003809: thrombin activity	8.46E-19	47,10,10
2hlca_#68	GO:0003809: thrombin activity	8.46E-19	47,10,10
1danh_#140	GO:0005509: calcium ion binding	8.65E-19	37,160,22
1c5y.1#B192	GO:0003809: thrombin activity	9.02E-19	25,10,9
1h8d.1#H167	GO:0003809: thrombin activity	9.02E-19	25,10,9
1hyea2#182	GO:0004457: lactate dehydrogenase activity	9.02E-19	25,10,9
1trb_1#285	GO:0050660: FAD binding	9.02E-19	25,10,9
1trb_1#298	GO:0050660: FAD binding	9.02E-19	25,10,9
1nhp_1#7	GO:0016668: oxidoreductase activity, acting on sulfur group of donors, NAD or NADP as acceptor	9.83E-19	33,12,10
1kdj__#27	GO:0005507: copper ion binding	9.94E-19	67,38,17
1phk__#221	GO:0004674: protein serine/threonine kinase activity	1.00E-18	14,42,11
1hbza_#96	GO:0004295: trypsin activity	1.05E-18	370,48,33
1arb__#55	GO:0003809: thrombin activity	1.07E-18	48,10,10
1ejda_#213	GO:0004295: trypsin activity	1.08E-18	284,48,30
3grs_2#187	GO:0016668: oxidoreductase activity, acting on sulfur group of donors, NAD or NADP as acceptor	1.09E-18	11,12,8
1dy9.1#A44	GO:0004867: serine-type endopeptidase inhibitor activity	1.09E-18	44,47,16
1g51a3#525	GO:0004263: chymotrypsin activity	1.12E-18	263,41,27
1fmk_3#377	GO:0005524: ATP binding	1.23E-18	19,243,18
1pme__#25	GO:0005524: ATP binding	1.24E-18	37,243,25
1c5y.1#B220	GO:0005509: calcium ion binding	1.33E-18	30,160,20
1arb__#208	GO:0003809: thrombin activity	1.34E-18	49,10,10
1elva1#604	GO:0003809: thrombin activity	1.34E-18	49,10,10
1e39a2#278	GO:0050660: FAD binding	1.38E-18	26,10,9
1oaa__#235	GO:0016616: oxidoreductase activity, acting on the CH-OH group of donors, NAD or NADP as acceptor	1.42E-18	15,59,12
1llda2#177	GO:0016616: oxidoreductase activity, acting on the CH-OH group of donors, NAD or NADP as acceptor	1.42E-18	15,59,12
1h5qa_#153	GO:0016616: oxidoreductase activity, acting on the CH-OH group of donors, NAD or NADP as acceptor	1.42E-18	15,59,12
1e8ca3#111	GO:0016616: oxidoreductase activity, acting on the CH-OH group of donors, NAD or NADP as acceptor	1.45E-18	280,59,33
1ia8a_#59	GO:0004674: protein serine/threonine kinase activity	1.52E-18	19,42,12
1egja_#343	GO:0004896: hematopoietin/interferon-class (D200-domain) cytokine receptor activity	1.52E-18	42,19,12
1cf9a1#736	GO:0005525: GTP binding	1.54E-18	43,49,16
1evqa_#305	GO:0016616: oxidoreductase activity, acting on the CH-OH group of donors, NAD or NADP as acceptor	1.76E-18	490,59,41
1ac6a_#34	GO:0004896: hematopoietin/interferon-class (D200-domain) cytokine receptor activity	1.78E-18	60,19,13
1qgna_#207	GO:0016846: carbon-sulfur lyase activity	1.80E-18	14,10,8
1ajsa_#139	GO:0016846: carbon-sulfur lyase activity	1.80E-18	14,10,8
1fjsa_#163	GO:0005509: calcium ion binding	1.84E-18	34,160,21
2hlca_#134	GO:0004295: trypsin activity	1.91E-18	36,48,15
1gdna_#123	GO:0005509: calcium ion binding	1.95E-18	38,160,22
1feca1#122	GO:0015036: disulfide oxidoreductase activity	1.98E-18	25,22,11
1pfza_#306	GO:0004190: aspartic-type endopeptidase activity	1.98E-18	11,23,9
1pfza_#173	GO:0004190: aspartic-type endopeptidase activity	1.98E-18	11,23,9
1j71a_#197	GO:0004190: aspartic-type endopeptidase activity	1.98E-18	11,23,9
1hrna_#136	GO:0004190: aspartic-type endopeptidase activity	1.98E-18	11,23,9
1gg6.1#C232	GO:0004263: chymotrypsin activity	1.99E-18	11,41,10
1ojt_3#488	GO:0015036: disulfide oxidoreductase activity	2.00E-18	17,22,10
1eq9a_#143	GO:0003809: thrombin activity	2.07E-18	27,10,9
1eq9a_#143	GO:0005509: calcium ion binding	2.09E-18	27,160,19
1ldm_2#292	GO:0016616: oxidoreductase activity, acting on the CH-OH group of donors, NAD or NADP as acceptor	2.19E-18	12,59,11
1hdca_#187	GO:0016616: oxidoreductase activity, acting on the CH-OH group of donors, NAD or NADP as acceptor	2.19E-18	12,59,11
1j9qa2#295	GO:0005507: copper ion binding	2.21E-18	36,38,14
1hqva_#48	GO:0005509: calcium ion binding	2.38E-18	96,160,33
1danh_#197	GO:0004867: serine-type endopeptidase inhibitor activity	2.54E-18	46,47,16

1qq4a_#143	GO:0004867: serine-type endopeptidase inhibitor activity	2.54E-18	46,47,16
1qq4a_#63	GO:0004867: serine-type endopeptidase inhibitor activity	2.54E-18	46,47,16
1hbza_#96	GO:0004263: chymotrypsin activity	2.55E-18	370,41,30
1qqga1#95	GO:0004263: chymotrypsin activity	2.56E-18	127,41,21
1elva1#623	GO:0003809: thrombin activity	2.59E-18	52,10,10
1a6o__#233	GO:0004674: protein serine/threonine kinase activity	2.62E-18	11,42,10
1h6va2#219	GO:0016668: oxidoreductase activity, acting on sulfur group of donors, NAD or NADP as acceptor	2.69E-18	36,12,10
1e7wa_#36	GO:0016616: oxidoreductase activity, acting on the CH-OH group of donors, NAD or NADP as acceptor	2.79E-18	137,59,25
1e2ka_#59	GO:0019201: nucleotide kinase activity	2.83E-18	11,13,8
1nksa_#13	GO:0019201: nucleotide kinase activity	2.89E-18	19,13,9
1danh_#70	GO:0004867: serine-type endopeptidase inhibitor activity	3.00E-18	30,47,14
1danh_#242	GO:0003809: thrombin activity	3.05E-18	28,10,9
1bqya_#216	GO:0003809: thrombin activity	3.05E-18	28,10,9
1bd3a_#195	GO:0016763: transferase activity, transferring pentosyl groups	3.05E-18	10,28,9
1fl2a1#320	GO:0050660: FAD binding	3.05E-18	28,10,9
1qtn.1#A270	GO:0016616: oxidoreductase activity, acting on the CH-OH group of donors, NAD or NADP as acceptor	3.31E-18	439,59,39
1f3ya_#6	GO:0004263: chymotrypsin activity	3.39E-18	94,41,19
1bio__#81	GO:0005509: calcium ion binding	3.58E-18	31,160,20
1ton__#196	GO:0004867: serine-type endopeptidase inhibitor activity	3.82E-18	47,47,16
1bvza3#425	GO:0004556: alpha-amylase activity	3.86E-18	10,15,8
1elua_#199	GO:0016846: carbon-sulfur lyase activity	3.86E-18	15,10,8
1el0a_#62	GO:0008009: chemokine activity	3.86E-18	15,10,8
1b16a_#184	GO:0016616: oxidoreductase activity, acting on the CH-OH group of donors, NAD or NADP as acceptor	3.94E-18	20,59,13
1gdna_#121	GO:0005509: calcium ion binding	4.26E-18	39,160,22
1ekbb_#117	GO:0005509: calcium ion binding	4.37E-18	35,160,21
1svpa_#127	GO:0005509: calcium ion binding	4.37E-18	35,160,21
1ton__#112	GO:0005509: calcium ion binding	4.37E-18	35,160,21
1g6ga_#133	GO:0003809: thrombin activity	4.42E-18	29,10,9
1ekbb_#79	GO:0003809: thrombin activity	4.42E-18	29,10,9
1trb_1#14	GO:0050660: FAD binding	4.42E-18	29,10,9
1dfoa_#344	GO:0008483: transaminase activity	4.68E-18	36,17,11
1fuma2#221	GO:0015036: disulfide oxidoreductase activity	4.79E-18	12,22,9
1e6pa2#46	GO:0004556: alpha-amylase activity	4.86E-18	95,15,13
1a0fa1#178	GO:0004364: glutathione transferase activity	4.93E-18	25,11,9
1f3ba2#7	GO:0004364: glutathione transferase activity	4.93E-18	25,11,9
1elua_#199	GO:0008483: transaminase activity	5.31E-18	15,17,9
1arb__#55	GO:0004867: serine-type endopeptidase inhibitor activity	5.66E-18	48,47,16
1elva1#566	GO:0003809: thrombin activity	6.31E-18	30,10,9
1c5y.1#B220	GO:0003809: thrombin activity	6.31E-18	30,10,9
1j71a_#220	GO:0004190: aspartic-type endopeptidase activity	6.57E-18	8,23,8
1mpp__#218	GO:0004190: aspartic-type endopeptidase activity	6.57E-18	8,23,8
1mpp__#221	GO:0004190: aspartic-type endopeptidase activity	6.57E-18	8,23,8
1htr.1#B30	GO:0004190: aspartic-type endopeptidase activity	6.57E-18	8,23,8
1e2ka_#59	GO:0016776: phosphotransferase activity, phosphate group as acceptor	6.60E-18	11,14,8
1h6va1#23	GO:0016668: oxidoreductase activity, acting on sulfur group of donors, NAD or NADP as acceptor	6.72E-18	39,12,10
1jsg__#92	GO:0004263: chymotrypsin activity	7.66E-18	607,41,35
1qsma_#95	GO:0008080: N-acetyltransferase activity	7.81E-18	7,13,7
1fkna_#324	GO:0004190: aspartic-type endopeptidase activity	7.87E-18	12,23,9
1nksa_#13	GO:0016776: phosphotransferase activity, phosphate group as acceptor	8.07E-18	19,14,9
1qqga1#95	GO:0004295: trypsin activity	8.17E-18	127,48,22
1arb__#208	GO:0004867: serine-type endopeptidase inhibitor activity	8.31E-18	49,47,16
1trb_1#42	GO:0016668: oxidoreductase activity, acting on sulfur group of donors, NAD or NADP as acceptor	8.48E-18	13,12,8
1qf9a_#117	GO:0019201: nucleotide kinase activity	8.80E-18	89,13,12
1a0la_#193	GO:0003809: thrombin activity	8.88E-18	31,10,9
1hyea2#272	GO:0004457: lactate dehydrogenase activity	8.88E-18	31,10,9
1h8d.1#H167	GO:0004867: serine-type endopeptidase inhibitor activity	8.91E-18	25,47,13
1bqya_#103	GO:0005509: calcium ion binding	9.01E-18	40,160,22
1ddja_#759	GO:0005509: calcium ion binding	9.01E-18	40,160,22
1autc_#209	GO:0005509: calcium ion binding	9.01E-18	40,160,22
1qo3c_#212	GO:0005529: sugar binding	9.41E-18	9,39,9

2msba_#156	GO:0005529: sugar binding	9.41E-18	9,39,9
1c5y.1#B94	GO:0004867: serine-type endopeptidase inhibitor activity	9.49E-18	32,47,14
1deua_#210	GO:0004197: cysteine-type endopeptidase activity	9.85E-18	8,24,8
1g38a_#47	GO:0008757: S-adenosylmethionine-dependent methyltransferase activity	9.85E-18	8,24,8
1qnja_#118	GO:0005509: calcium ion binding	9.97E-18	36,160,21
1pme__#302	GO:0005524: ATP binding	1.01E-17	16,243,16
1qf6a4#362	GO:0005524: ATP binding	1.01E-17	16,243,16
3sil__#98	GO:0004263: chymotrypsin activity	1.02E-17	228,41,25
1hwxa1#256	GO:0016616: oxidoreductase activity, acting on the CH-OH group of donors, NAD or NADP as acceptor	1.12E-17	57,59,18
1gcoa_#119	GO:0016616: oxidoreductase activity, acting on the CH-OH group of donors, NAD or NADP as acceptor	1.14E-17	40,59,16
1b8aa2#251	GO:0004812: tRNA ligase activity	1.20E-17	24,26,11
1arb__#193	GO:0004867: serine-type endopeptidase inhibitor activity	1.21E-17	50,47,16
1qnja_#46	GO:0003809: thrombin activity	1.23E-17	60,10,10
1elva1#601	GO:0003809: thrombin activity	1.24E-17	32,10,9
7mdha2#355	GO:0016616: oxidoreductase activity, acting on the CH-OH group of donors, NAD or NADP as acceptor	1.40E-17	13,59,11
1bag_2#207	GO:0004556: alpha-amylase activity	1.43E-17	30,15,10
1qama_#37	GO:0008757: S-adenosylmethionine-dependent methyltransferase activity	1.45E-17	68,24,14
1pme__#145	GO:0004674: protein serine/threonine kinase activity	1.55E-17	12,42,10
1bqya_#178	GO:0004263: chymotrypsin activity	1.55E-17	9,41,9
1hdr__#149	GO:0016616: oxidoreductase activity, acting on the CH-OH group of donors, NAD or NADP as acceptor	1.58E-17	27,59,14
1qnja_#71	GO:0004867: serine-type endopeptidase inhibitor activity	1.63E-17	33,47,14
1ecfa1#367	GO:0016763: transferase activity, transferring pentosyl groups	1.66E-17	11,28,9
1nhp_1#7	GO:0050660: FAD binding	1.70E-17	33,10,9
1c5y.1#B191	GO:0005509: calcium ion binding	1.70E-17	29,160,19
1g6ga_#133	GO:0005509: calcium ion binding	1.70E-17	29,160,19
1dlea_#198	GO:0005509: calcium ion binding	1.85E-17	41,160,22
1bjt__#890	GO:0004263: chymotrypsin activity	1.86E-17	292,41,27
1ddja_#640	GO:0004295: trypsin activity	1.94E-17	15,48,11
2hlpa2#298	GO:0004457: lactate dehydrogenase activity	1.96E-17	9,10,7
1fuma2#370	GO:0016668: oxidoreductase activity, acting on sulfur group of donors, NAD or NADP as acceptor	1.98E-17	14,12,8
1h8d.1#H207	GO:0003809: thrombin activity	2.09E-17	63,10,10
1eupa_#320	GO:0004497: monooxygenase activity	2.09E-17	8,26,8
1ile_3#170	GO:0004812: tRNA ligase activity	2.09E-17	8,26,8
1bu7a_#360	GO:0004497: monooxygenase activity	2.09E-17	8,26,8
1h6va1#27	GO:0016616: oxidoreductase activity, acting on the CH-OH group of donors, NAD or NADP as acceptor	2.12E-17	260,59,31
1danh_#190	GO:0005509: calcium ion binding	2.19E-17	37,160,21
4tmka_#141	GO:0019201: nucleotide kinase activity	2.20E-17	13,13,8
1danh_#142	GO:0003809: thrombin activity	2.31E-17	34,10,9
1ekbb_#208	GO:0004263: chymotrypsin activity	2.44E-17	17,41,11
1cpy__#93	GO:0004812: tRNA ligase activity	2.46E-17	275,26,21
1trka2#429	GO:0015036: disulfide oxidoreductase activity	2.61E-17	43,22,12
1qf9a_#117	GO:0005524: ATP binding	2.62E-17	89,243,37
1ldm_2#272	GO:0004457: lactate dehydrogenase activity	2.62E-17	18,10,8
1fjsa_#163	GO:0004867: serine-type endopeptidase inhibitor activity	2.74E-17	34,47,14
1g51a3#525	GO:0004295: trypsin activity	2.76E-17	263,48,28
1fvua_#72	GO:0005529: sugar binding	2.89E-17	13,39,10
1gdea_#243	GO:0008483: transaminase activity	2.90E-17	61,17,12
1hcl__#82	GO:0004674: protein serine/threonine kinase activity	3.17E-17	48,42,15
1eq9a_#143	GO:0004867: serine-type endopeptidase inhibitor activity	3.36E-17	27,47,13
1arb__#24	GO:0005509: calcium ion binding	3.69E-17	42,160,22
1dlea_#238	GO:0005509: calcium ion binding	3.69E-17	42,160,22
1dlea_#53	GO:0005509: calcium ion binding	3.69E-17	42,160,22
1danh_#48	GO:0005509: calcium ion binding	3.79E-17	20,160,16
1h8d.1#H184	GO:0005509: calcium ion binding	3.79E-17	23,160,17
1eny__#94	GO:0016616: oxidoreductase activity, acting on the CH-OH group of donors, NAD or NADP as acceptor	3.82E-17	35,59,15
1dpsa_#36	GO:0005126: hematopoietin/interferon-class (D200-domain) cytokine receptor binding	3.91E-17	205,20,17
1g8fa3#419	GO:0005525: GTP binding	4.13E-17	151,49,23
1h6va2#219	GO:0050660: FAD binding	4.14E-17	36,10,9

1cjca2#103	GO:0016668: oxidoreductase activity, acting on sulfur group of donors, NAD or NADP as acceptor	4.23E-17	15,12,8
1jsg__#92	GO:0004295: trypsin activity	4.24E-17	607,48,38
1elva1#566	GO:0005509: calcium ion binding	4.42E-17	30,160,19
3dfr__#63	GO:0016646: oxidoreductase activity, acting on the CH-NH group of donors, NAD or NADP as acceptor	4.52E-17	10,19,8
1edza1#206	GO:0005524: ATP binding	4.54E-17	399,243,85
1qr4a2#166	GO:0004896: hematopoietin/interferon-class (D200-domain) cytokine receptor activity	4.58E-17	16,19,9
1gdna_#56	GO:0005509: calcium ion binding	4.66E-17	38,160,21
1hcl__#82	GO:0005524: ATP binding	4.73E-17	48,243,27
1f97a1#62	GO:0005524: ATP binding	4.75E-17	707,243,121
1ekbb_#228	GO:0003809: thrombin activity	4.75E-17	68,10,10
1feca1#28	GO:0015036: disulfide oxidoreductase activity	4.98E-17	62,22,13
1danh_#142	GO:0005509: calcium ion binding	5.05E-17	34,160,20
1e6ca_#153	GO:0019201: nucleotide kinase activity	5.13E-17	14,13,8
1jlna_#383	GO:0004725: protein tyrosine phosphatase activity	5.16E-17	52,15,11
1dv8a_#213	GO:0005529: sugar binding	5.28E-17	25,39,12
1danh_#190	GO:0003809: thrombin activity	5.47E-17	37,10,9
1i50b_#966	GO:0004896: hematopoietin/interferon-class (D200-domain) cytokine receptor activity	5.72E-17	299,19,18
1b3ra1#218	GO:0016616: oxidoreductase activity, acting on the CH-OH group of donors, NAD or NADP as acceptor	5.91E-17	29,59,14
1qf9a_#117	GO:0016776: phosphotransferase activity, phosphate group as acceptor	6.00E-17	89,14,12
1qlaa2#13	GO:0016668: oxidoreductase activity, acting on sulfur group of donors, NAD or NADP as acceptor	6.62E-17	28,12,9
1b6cb_#399	GO:0004674: protein serine/threonine kinase activity	6.66E-17	13,42,10
1fgka_#668	GO:0004674: protein serine/threonine kinase activity	6.66E-17	13,42,10
1pfza_#177	GO:0004190: aspartic-type endopeptidase activity	7.09E-17	14,23,9
1bxoa_#212	GO:0004190: aspartic-type endopeptidase activity	7.09E-17	14,23,9
1gdna_#56	GO:0003809: thrombin activity	7.17E-17	38,10,9
1cyx__#133	GO:0005507: copper ion binding	7.17E-17	10,38,9
1ton__#91	GO:0004867: serine-type endopeptidase inhibitor activity	7.30E-17	36,47,14
1ekbb_#141	GO:0004867: serine-type endopeptidase inhibitor activity	7.30E-17	36,47,14
1c5y.1#B234	GO:0004867: serine-type endopeptidase inhibitor activity	7.30E-17	36,47,14
1ton__#231	GO:0004867: serine-type endopeptidase inhibitor activity	7.30E-17	36,47,14
1bqya_#178	GO:0004295: trypsin activity	7.44E-17	9,48,9
1ha6a_#20	GO:0008009: chemokine activity	7.54E-17	20,10,8
1ypta_#418	GO:0004457: lactate dehydrogenase activity	7.54E-17	20,10,8
1e79d2#36	GO:0005524: ATP binding	7.59E-17	524,243,100
1e7wa_#8	GO:0016616: oxidoreductase activity, acting on the CH-OH group of donors, NAD or NADP as acceptor	8.37E-17	111,59,22
1b4va1#253	GO:0016668: oxidoreductase activity, acting on sulfur group of donors, NAD or NADP as acceptor	8.45E-17	16,12,8
1d7ya1#255	GO:0016668: oxidoreductase activity, acting on sulfur group of donors, NAD or NADP as acceptor	8.45E-17	16,12,8
1ojt_2#336	GO:0016668: oxidoreductase activity, acting on sulfur group of donors, NAD or NADP as acceptor	8.45E-17	16,12,8
2gsaa_#246	GO:0008483: transaminase activity	8.85E-17	7,17,7
1h6va1#23	GO:0050660: FAD binding	9.31E-17	39,10,9
1nsca_#236	GO:0004896: hematopoietin/interferon-class (D200-domain) cytokine receptor activity	9.90E-17	143,19,15
1a06__#205	GO:0004674: protein serine/threonine kinase activity	1.03E-16	32,42,13
1pysa_#221	GO:0004812: tRNA ligase activity	1.06E-16	90,26,15
1quqa_#75	GO:0005525: GTP binding	1.08E-16	54,49,16
1a0la_#193	GO:0005509: calcium ion binding	1.08E-16	31,160,19
1ddja_#771	GO:0005509: calcium ion binding	1.08E-16	31,160,19
1c5y.1#B191	GO:0004867: serine-type endopeptidase inhibitor activity	1.11E-16	29,47,13
1ekbb_#79	GO:0004867: serine-type endopeptidase inhibitor activity	1.11E-16	29,47,13
1danh_#140	GO:0004867: serine-type endopeptidase inhibitor activity	1.16E-16	37,47,14
1danh_#190	GO:0004867: serine-type endopeptidase inhibitor activity	1.16E-16	37,47,14
1gdna_#155	GO:0004867: serine-type endopeptidase inhibitor activity	1.16E-16	37,47,14
1gdea_#379	GO:0008483: transaminase activity	1.20E-16	68,17,12
1qf6a4#295	GO:0005524: ATP binding	1.20E-16	15,243,15
1atia2#67	GO:0005524: ATP binding	1.20E-16	15,243,15
1hc7a2#111	GO:0005524: ATP binding	1.20E-16	15,243,15
1hc7a2#109	GO:0005524: ATP binding	1.20E-16	15,243,15
1e2fa_#14	GO:0005524: ATP binding	1.20E-16	15,243,15
1qb7a_#86	GO:0004457: lactate dehydrogenase activity	1.20E-16	40,10,9

1hyha2#179	GO:0004457: lactate dehydrogenase activity	1.22E-16	21,10,8
1fc4a_#111	GO:0016846: carbon-sulfur lyase activity	1.22E-16	21,10,8
1nksa_#13	GO:0005524: ATP binding	1.23E-16	19,243,17
3sil__#98	GO:0004295: trypsin activity	1.31E-16	228,48,26
1f3ya_#6	GO:0004295: trypsin activity	1.32E-16	94,48,19
1dy9.1#A44	GO:0005509: calcium ion binding	1.37E-16	44,160,22
2hlpa2#315	GO:0016616: oxidoreductase activity, acting on the CH-OH group of donors, NAD or NADP as acceptor	1.47E-16	19,59,12
1elva1#604	GO:0005509: calcium ion binding	1.59E-16	49,160,23
1qisa_#107	GO:0008483: transaminase activity	1.59E-16	12,17,8
1ojt_3#488	GO:0016668: oxidoreductase activity, acting on sulfur group of donors, NAD or NADP as acceptor	1.59E-16	17,12,8
1df7a_#14	GO:0016646: oxidoreductase activity, acting on the CH-NH group of donors, NAD or NADP as acceptor	1.65E-16	11,19,8
1ekbb_#208	GO:0004295: trypsin activity	1.72E-16	17,48,11
1b6e__#76	GO:0005529: sugar binding	1.73E-16	27,39,12
1gpc__#184	GO:0004896: hematopoietin/interferon-class (D200-domain) cytokine receptor activity	1.74E-16	83,19,13
3grs_2#187	GO:0050660: FAD binding	1.80E-16	11,10,7
1gdna_#123	GO:0004867: serine-type endopeptidase inhibitor activity	1.82E-16	38,47,14
1ihua2#343	GO:0005524: ATP binding	1.84E-16	233,243,61
1trb_1#112	GO:0050660: FAD binding	1.91E-16	22,10,8
1c5y.1#B220	GO:0004867: serine-type endopeptidase inhibitor activity	1.94E-16	30,47,13
1tkia_#98	GO:0004674: protein serine/threonine kinase activity	1.96E-16	10,42,9
1h4vb2#307	GO:0004812: tRNA ligase activity	2.02E-16	73,26,14
1a3c__#108	GO:0016763: transferase activity, transferring pentosyl groups	2.14E-16	13,28,9
2occb1#189	GO:0005507: copper ion binding	2.21E-16	15,38,10
1c4zd_#99	GO:0004842: ubiquitin-protein ligase activity	2.29E-16	7,19,7
1csn__#134	GO:0004674: protein serine/threonine kinase activity	2.31E-16	14,42,10
1b6cb_#337	GO:0004674: protein serine/threonine kinase activity	2.31E-16	14,42,10
1ton__#181	GO:0003809: thrombin activity	2.35E-16	79,10,10
1azza_#51	GO:0005509: calcium ion binding	2.39E-16	36,160,20
1c5y.1#B234	GO:0005509: calcium ion binding	2.39E-16	36,160,20
1gdna_#30	GO:0005509: calcium ion binding	2.54E-16	45,160,22
1autc_#45	GO:0005509: calcium ion binding	2.54E-16	45,160,22
1gdna_#121	GO:0004867: serine-type endopeptidase inhibitor activity	2.80E-16	39,47,14
1jf9a_#380	GO:0008483: transaminase activity	2.83E-16	50,17,11
1qgna_#149	GO:0016846: carbon-sulfur lyase activity	2.93E-16	23,10,8
1dbxa_#75	GO:0005525: GTP binding	3.20E-16	69,49,17
1dn2a1#261	GO:0004896: hematopoietin/interferon-class (D200-domain) cytokine receptor activity	3.29E-16	425,19,19
1gg6.1#C156	GO:0004867: serine-type endopeptidase inhibitor activity	3.29E-16	31,47,13
1bio__#81	GO:0004867: serine-type endopeptidase inhibitor activity	3.29E-16	31,47,13
5ruba1#365	GO:0016616: oxidoreductase activity, acting on the CH-OH group of donors, NAD or NADP as acceptor	3.37E-16	118,59,22
1e30a_#112	GO:0005507: copper ion binding	3.53E-16	49,38,14
1qo3c_#252	GO:0005529: sugar binding	3.56E-16	21,39,11
1oaa__#161	GO:0016616: oxidoreductase activity, acting on the CH-OH group of donors, NAD or NADP as acceptor	3.63E-16	20,59,12
1cyda_#106	GO:0016616: oxidoreductase activity, acting on the CH-OH group of donors, NAD or NADP as acceptor	3.63E-16	20,59,12
1c5y.1#B192	GO:0005509: calcium ion binding	3.67E-16	25,160,17
3grs_1#131	GO:0050660: FAD binding	3.71E-16	6,10,6
1fgka_#610	GO:0005524: ATP binding	3.72E-16	22,243,18
3grx__#55	GO:0004364: glutathione transferase activity	3.85E-16	113,11,11
1d6ja_#36	GO:0016776: phosphotransferase activity, phosphate group as acceptor	4.04E-16	27,14,9
2hlca_#27	GO:0004867: serine-type endopeptidase inhibitor activity	4.26E-16	40,47,14
1bqya_#103	GO:0004867: serine-type endopeptidase inhibitor activity	4.26E-16	40,47,14
1autc_#209	GO:0004867: serine-type endopeptidase inhibitor activity	4.26E-16	40,47,14
1cpy__#93	GO:0000287: magnesium ion binding	4.27E-16	275,128,46
1ibja_#217	GO:0016846: carbon-sulfur lyase activity	4.31E-16	12,10,7
1qisa_#107	GO:0016846: carbon-sulfur lyase activity	4.31E-16	12,10,7
3prn__#143	GO:0004896: hematopoietin/interferon-class (D200-domain) cytokine receptor activity	4.37E-16	334,19,18
1cgha_#168	GO:0003809: thrombin activity	4.39E-16	24,10,8
1g55a_#57	GO:0008757: S-adenosylmethionine-dependent methyltransferase activity	4.39E-16	10,24,8
1foha5#218	GO:0016668: oxidoreductase activity, acting on sulfur group of donors, NAD or NADP as acceptor	4.51E-16	57,12,10

1cjca2#365	GO:0016668: oxidoreductase activity, acting on sulfur group of donors, NAD or NADP as acceptor	4.51E-16	57,12,10
1danh_#197	GO:0005509: calcium ion binding	4.63E-16	46,160,22
1qq4a_#63	GO:0005509: calcium ion binding	4.63E-16	46,160,22
1qnja_#138	GO:0005509: calcium ion binding	4.63E-16	46,160,22
1bjt__#890	GO:0004295: trypsin activity	4.78E-16	292,48,28
1oaa__#175	GO:0016616: oxidoreductase activity, acting on the CH-OH group of donors, NAD or NADP as acceptor	4.80E-16	26,59,13
1ir3a_#1139	GO:0004674: protein serine/threonine kinase activity	4.82E-16	27,42,12
1qq4a_#143	GO:0003809: thrombin activity	4.83E-16	46,10,9
1jb9a2#173	GO:0016646: oxidoreductase activity, acting on the CH-NH group of donors, NAD or NADP as acceptor	4.94E-16	12,19,8
1fzqa_#165	GO:0003924: GTPase activity	5.18E-16	22,17,9
1ddja_#640	GO:0004263: chymotrypsin activity	5.22E-16	15,41,10
1tkia_#142	GO:0005524: ATP binding	5.25E-16	37,243,23
1bg6_2#24	GO:0015036: disulfide oxidoreductase activity	5.26E-16	26,22,10
1ton__#120	GO:0005509: calcium ion binding	5.32E-16	29,160,18
1b16a_#16	GO:0016616: oxidoreductase activity, acting on the CH-OH group of donors, NAD or NADP as acceptor	5.58E-16	9,59,9
2cmd_2#172	GO:0016616: oxidoreductase activity, acting on the CH-OH group of donors, NAD or NADP as acceptor	5.58E-16	9,59,9
1eq9a_#40	GO:0003809: thrombin activity	5.79E-16	86,10,10
1ton__#211	GO:0003809: thrombin activity	5.79E-16	86,10,10
3grs_1#23	GO:0005524: ATP binding	5.81E-16	168,243,50
1hcl__#280	GO:0004674: protein serine/threonine kinase activity	5.96E-16	70,42,16
1fjsa_#158	GO:0003809: thrombin activity	5.97E-16	47,10,9
1dlea_#198	GO:0004867: serine-type endopeptidase inhibitor activity	6.40E-16	41,47,14
1e8ca3#111	GO:0005524: ATP binding	6.52E-16	280,243,67
2cuaa_#121	GO:0005507: copper ion binding	6.55E-16	8,38,8
1pysb5#584	GO:0004812: tRNA ligase activity	6.63E-16	79,26,14
1gox__#124	GO:0000287: magnesium ion binding	6.67E-16	366,128,53
1mpp__#29	GO:0004190: aspartic-type endopeptidase activity	6.88E-16	37,23,11
3grs_2#187	GO:0015036: disulfide oxidoreductase activity	6.97E-16	11,22,8
1dm0a_#110	GO:0016799: hydrolase activity, hydrolyzing N-glycosyl compounds	7.06E-16	8,17,7
1qi7a_#244	GO:0016799: hydrolase activity, hydrolyzing N-glycosyl compounds	7.06E-16	8,17,7
1d6aa_#179	GO:0016799: hydrolase activity, hydrolyzing N-glycosyl compounds	7.06E-16	8,17,7
1qi7a_#208	GO:0016799: hydrolase activity, hydrolyzing N-glycosyl compounds	7.06E-16	8,17,7
2viua_#252	GO:0004896: hematopoietin/interferon-class (D200-domain) cytokine receptor activity	7.12E-16	343,19,18
1fcda2#239	GO:0015036: disulfide oxidoreductase activity	7.76E-16	7,22,7
1ton__#196	GO:0005509: calcium ion binding	8.29E-16	47,160,22
2hlca_#68	GO:0005509: calcium ion binding	8.29E-16	47,160,22
3chbd_#85	GO:0005524: ATP binding	9.02E-16	378,243,80
1dp0a4#756	GO:0004896: hematopoietin/interferon-class (D200-domain) cytokine receptor activity	9.11E-16	165,19,15
1e6wa_#24	GO:0016616: oxidoreductase activity, acting on the CH-OH group of donors, NAD or NADP as acceptor	9.11E-16	27,59,13
1arb__#24	GO:0004867: serine-type endopeptidase inhibitor activity	9.48E-16	42,47,14
1dlea_#238	GO:0004867: serine-type endopeptidase inhibitor activity	9.48E-16	42,47,14
1dlea_#53	GO:0004867: serine-type endopeptidase inhibitor activity	9.48E-16	42,47,14
1ajsa_#139	GO:0008483: transaminase activity	9.61E-16	14,17,8
1fc4a_#241	GO:0008483: transaminase activity	9.61E-16	14,17,8
1fp1d2#214	GO:0016616: oxidoreductase activity, acting on the CH-OH group of donors, NAD or NADP as acceptor	1.00E-15	173,59,25
3grs_2#189	GO:0015036: disulfide oxidoreductase activity	1.03E-15	18,22,9
1eh9a3#409	GO:0004556: alpha-amylase activity	1.05E-15	9,15,7
1koba_#293	GO:0004674: protein serine/threonine kinase activity	1.06E-15	11,42,9
1koba_#105	GO:0004674: protein serine/threonine kinase activity	1.06E-15	11,42,9
1pfza_#91	GO:0004190: aspartic-type endopeptidase activity	1.07E-15	11,23,8
1arb__#193	GO:0003809: thrombin activity	1.10E-15	50,10,9
1pfza_#125	GO:0004190: aspartic-type endopeptidase activity	1.12E-15	7,23,7
1feca1#28	GO:0016668: oxidoreductase activity, acting on sulfur group of donors, NAD or NADP as acceptor	1.12E-15	62,12,10
3grs_1#23	GO:0015036: disulfide oxidoreductase activity	1.17E-15	168,22,16
1f42a2#123	GO:0004263: chymotrypsin activity	1.21E-15	378,41,28
1h6va1#27	GO:0015036: disulfide oxidoreductase activity	1.28E-15	260,22,18
1c5y.1#B128	GO:0004263: chymotrypsin activity	1.28E-15	8,41,8
1qfea_#68	GO:0000287: magnesium ion binding	1.29E-15	560,128,66

1d7ya3#331	GO:0015036: disulfide oxidoreductase activity	1.29E-15	28,22,10
1danh_#24	GO:0003809: thrombin activity	1.32E-15	27,10,8
1llda2#161	GO:0004457: lactate dehydrogenase activity	1.32E-15	27,10,8
2hlca_#99	GO:0003809: thrombin activity	1.32E-15	27,10,8
1e39a2#516	GO:0016668: oxidoreductase activity, acting on sulfur group of donors, NAD or NADP as acceptor	1.33E-15	21,12,8
1iow_2#112	GO:0004674: protein serine/threonine kinase activity	1.41E-15	29,42,12
1apme_#101	GO:0005524: ATP binding	1.42E-15	14,243,14
1csn__#134	GO:0005524: ATP binding	1.42E-15	14,243,14
1danh_#142	GO:0004867: serine-type endopeptidase inhibitor activity	1.43E-15	34,47,13
1arb__#55	GO:0005509: calcium ion binding	1.45E-15	48,160,22
1f3ya_#6	GO:0003809: thrombin activity	1.48E-15	94,10,10
2hlca_#54	GO:0004867: serine-type endopeptidase inhibitor activity	1.56E-15	109,47,19
1f3mc_#434	GO:0004674: protein serine/threonine kinase activity	1.58E-15	8,42,8
3grs_1#131	GO:0016668: oxidoreductase activity, acting on sulfur group of donors, NAD or NADP as acceptor	1.63E-15	6,12,6
1fo4a5#1020	GO:0004896: hematopoietin/interferon-class (D200-domain) cytokine receptor activity	1.64E-15	359,19,18
1hrna_#320	GO:0004190: aspartic-type endopeptidase activity	1.69E-15	18,23,9
1qf6a4#318	GO:0005524: ATP binding	1.77E-15	16,243,15
1qlaa2#13	GO:0050660: FAD binding	1.85E-15	28,10,8
1pbe_1#155	GO:0050660: FAD binding	1.85E-15	28,10,8
1ev7a_#218	GO:0003700: transcription factor activity	1.85E-15	89,124,26
1bio__#108	GO:0003809: thrombin activity	1.94E-15	53,10,9
1c5y.1#B241	GO:0005509: calcium ion binding	1.94E-15	39,160,20
1gdha1#235	GO:0016616: oxidoreductase activity, acting on the CH-OH group of donors, NAD or NADP as acceptor	2.05E-15	113,59,21
3grs_1#24	GO:0016668: oxidoreductase activity, acting on sulfur group of donors, NAD or NADP as acceptor	2.19E-15	66,12,10
1danh_#24	GO:0004867: serine-type endopeptidase inhibitor activity	2.22E-15	27,47,12
2hlca_#99	GO:0004867: serine-type endopeptidase inhibitor activity	2.22E-15	27,47,12
1ekbb_#117	GO:0004867: serine-type endopeptidase inhibitor activity	2.25E-15	35,47,13
1svpa_#127	GO:0004867: serine-type endopeptidase inhibitor activity	2.25E-15	35,47,13
1ton__#112	GO:0004867: serine-type endopeptidase inhibitor activity	2.25E-15	35,47,13
1e42a1#711	GO:0004896: hematopoietin/interferon-class (D200-domain) cytokine receptor activity	2.26E-15	175,19,15
1iow_2#112	GO:0005524: ATP binding	2.33E-15	29,243,20
1evqa_#305	GO:0000287: magnesium ion binding	2.44E-15	490,128,61
1arb__#208	GO:0005509: calcium ion binding	2.51E-15	49,160,22
1seta2#231	GO:0000287: magnesium ion binding	2.52E-15	103,128,28
1c5y.1#B191	GO:0003809: thrombin activity	2.55E-15	29,10,8
1danh_#24	GO:0005509: calcium ion binding	2.58E-15	27,160,17
2hlca_#99	GO:0005509: calcium ion binding	2.58E-15	27,160,17
1e39a2#556	GO:0016668: oxidoreductase activity, acting on sulfur group of donors, NAD or NADP as acceptor	2.59E-15	40,12,9
1b3aa_#15	GO:0008009: chemokine activity	2.60E-15	7,10,6
1nfdb1#34	GO:0004896: hematopoietin/interferon-class (D200-domain) cytokine receptor activity	2.69E-15	177,19,15
1aqua_#126	GO:0005524: ATP binding	2.78E-15	576,243,103
2hlpa2#272	GO:0016616: oxidoreductase activity, acting on the CH-OH group of donors, NAD or NADP as acceptor	2.79E-15	13,59,10
1llc_2#271	GO:0016616: oxidoreductase activity, acting on the CH-OH group of donors, NAD or NADP as acceptor	2.79E-15	13,59,10
1gg6.1#C156	GO:0005509: calcium ion binding	2.86E-15	31,160,18
1ksia1#555	GO:0005525: GTP binding	2.88E-15	20,49,11
1gdna_#30	GO:0004867: serine-type endopeptidase inhibitor activity	2.89E-15	45,47,14
1autc_#45	GO:0004867: serine-type endopeptidase inhibitor activity	2.89E-15	45,47,14
1ekbb_#228	GO:0004867: serine-type endopeptidase inhibitor activity	2.96E-15	68,47,16
1cpt__#325	GO:0004497: monooxygenase activity	2.99E-15	7,26,7
1qnja_#46	GO:0005509: calcium ion binding	3.05E-15	60,160,24
1gox__#246	GO:0000287: magnesium ion binding	3.09E-15	177,128,36
1e5ka_#113	GO:0004263: chymotrypsin activity	3.34E-15	65,41,15
1qlaa2#35	GO:0050660: FAD binding	3.48E-15	30,10,8
1b4va1#11	GO:0050660: FAD binding	3.48E-15	30,10,8
1azza_#51	GO:0004867: serine-type endopeptidase inhibitor activity	3.48E-15	36,47,13
1qnja_#118	GO:0004867: serine-type endopeptidase inhibitor activity	3.48E-15	36,47,13
1cjca2#103	GO:0050660: FAD binding	3.49E-15	15,10,7
1h7wa4#476	GO:0050660: FAD binding	3.49E-15	15,10,7
1hyha2#171	GO:0004457: lactate dehydrogenase activity	3.49E-15	15,10,7

1fgka_#689	GO:0005524: ATP binding	3.64E-15	21,243,17
1qp8a1#153	GO:0016616: oxidoreductase activity, acting on the CH-OH group of donors, NAD or NADP as acceptor	3.71E-15	23,59,12
1ir3a_#1015	GO:0005524: ATP binding	3.74E-15	43,243,24
1danh_#242	GO:0004867: serine-type endopeptidase inhibitor activity	3.84E-15	28,47,12
1cjca2#365	GO:0050660: FAD binding	3.93E-15	57,10,9
1e8ca3#111	GO:0005525: GTP binding	3.99E-15	280,49,27
1ihua2#525	GO:0016616: oxidoreductase activity, acting on the CH-OH group of donors, NAD or NADP as acceptor	4.08E-15	335,59,32
1sek__#301	GO:0004867: serine-type endopeptidase inhibitor activity	4.21E-15	8,47,8
1ovaa_#340	GO:0004867: serine-type endopeptidase inhibitor activity	4.21E-15	8,47,8
1c8za_#386	GO:0004896: hematopoietin/interferon-class (D200-domain) cytokine receptor activity	4.43E-15	235,19,16
1gdna_#26	GO:0005509: calcium ion binding	4.68E-15	24,160,16
1ddja_#754	GO:0003809: thrombin activity	4.69E-15	31,10,8
1gg6.1#C156	GO:0003809: thrombin activity	4.69E-15	31,10,8
1ton__#179	GO:0003809: thrombin activity	4.69E-15	31,10,8
1elva1#513	GO:0003809: thrombin activity	4.69E-15	31,10,8
1ddja_#726	GO:0003809: thrombin activity	4.71E-15	105,10,10
1ho1a_#211	GO:0004556: alpha-amylase activity	4.74E-15	111,15,12
1d5ta1#12	GO:0016668: oxidoreductase activity, acting on sulfur group of donors, NAD or NADP as acceptor	4.78E-15	24,12,8
1dik_3#240	GO:0005524: ATP binding	4.93E-15	262,243,63
1d6aa_#52	GO:0004263: chymotrypsin activity	4.93E-15	293,41,25
1hhsa_#328	GO:0003887: DNA-directed DNA polymerase activity	4.95E-15	14,20,8
1c5y.1#B128	GO:0004295: trypsin activity	5.06E-15	8,48,8
1ldm_2#272	GO:0016616: oxidoreductase activity, acting on the CH-OH group of donors, NAD or NADP as acceptor	5.26E-15	18,59,11
1qp8a1#172	GO:0016616: oxidoreductase activity, acting on the CH-OH group of donors, NAD or NADP as acceptor	5.26E-15	18,59,11
1h7wa4#208	GO:0015036: disulfide oxidoreductase activity	5.39E-15	13,22,8
1qb7a_#148	GO:0016763: transferase activity, transferring pentosyl groups	5.39E-15	7,28,7
1gg6.1#B19	GO:0003809: thrombin activity	5.48E-15	59,10,9
1qf5a_#15	GO:0003924: GTPase activity	5.64E-15	43,17,10
1ec7a1#216	GO:0016616: oxidoreductase activity, acting on the CH-OH group of donors, NAD or NADP as acceptor	5.66E-15	78,59,18
2gsq_2#50	GO:0004364: glutathione transferase activity	5.71E-15	7,11,6
2hlca_#68	GO:0004867: serine-type endopeptidase inhibitor activity	5.79E-15	47,47,14
1plc__#74	GO:0005507: copper ion binding	5.84E-15	9,38,8
1aqua_#126	GO:0000287: magnesium ion binding	5.89E-15	576,128,66
1mh1__#37	GO:0005525: GTP binding	6.04E-15	8,49,8
1fl2a1#490	GO:0015036: disulfide oxidoreductase activity	6.18E-15	8,22,7
1ojt_2#336	GO:0050660: FAD binding	6.19E-15	16,10,7
1c5y.1#B94	GO:0005509: calcium ion binding	6.22E-15	32,160,18
1eq9a_#40	GO:0005509: calcium ion binding	6.22E-15	86,160,28
1c5y.1#B94	GO:0003809: thrombin activity	6.24E-15	32,10,8
1g6ga_#133	GO:0004867: serine-type endopeptidase inhibitor activity	6.47E-15	29,47,12
1gox__#124	GO:0016616: oxidoreductase activity, acting on the CH-OH group of donors, NAD or NADP as acceptor	6.54E-15	366,59,33
1fp5a2#537	GO:0004896: hematopoietin/interferon-class (D200-domain) cytokine receptor activity	6.56E-15	38,19,10
2hlca_#54	GO:0003809: thrombin activity	6.97E-15	109,10,10
1feca1#122	GO:0016668: oxidoreductase activity, acting on sulfur group of donors, NAD or NADP as acceptor	7.01E-15	25,12,8
1qbea_#92	GO:0004263: chymotrypsin activity	7.37E-15	18,41,10
1erza_#183	GO:0004556: alpha-amylase activity	7.44E-15	79,15,11
1qq4a_#143	GO:0005509: calcium ion binding	7.50E-15	46,160,21
1opy__#68	GO:0004263: chymotrypsin activity	7.58E-15	367,41,27
1gdna_#56	GO:0004867: serine-type endopeptidase inhibitor activity	7.97E-15	38,47,13
1feca1#31	GO:0016668: oxidoreductase activity, acting on sulfur group of donors, NAD or NADP as acceptor	8.56E-15	75,12,10
1fcda1#6	GO:0016668: oxidoreductase activity, acting on sulfur group of donors, NAD or NADP as acceptor	8.56E-15	75,12,10
1eh9a3#376	GO:0004556: alpha-amylase activity	8.85E-15	6,15,6
1g4us2#516	GO:0004725: protein tyrosine phosphatase activity	8.85E-15	6,15,6
1uok_2#325	GO:0004556: alpha-amylase activity	8.85E-15	6,15,6
1hx0a2#258	GO:0004556: alpha-amylase activity	8.85E-15	6,15,6
1eh9a3#170	GO:0004556: alpha-amylase activity	8.85E-15	6,15,6

1ton__#47	GO:0003809: thrombin activity	9.25E-15	112,10,10
2hlca_#139	GO:0003809: thrombin activity	9.25E-15	112,10,10
1llc_2#291	GO:0016616: oxidoreductase activity, acting on the CH-OH group of donors, NAD or NADP as acceptor	9.62E-15	14,59,10
1pbe_1#150	GO:0016668: oxidoreductase activity, acting on sulfur group of donors, NAD or NADP as acceptor	9.84E-15	76,12,10
1htr.1#B39	GO:0004190: aspartic-type endopeptidase activity	1.01E-14	21,23,9
1b8pa2#201	GO:0004457: lactate dehydrogenase activity	1.04E-14	8,10,6
1ojt_3#488	GO:0050660: FAD binding	1.05E-14	17,10,7
1elva1#566	GO:0004867: serine-type endopeptidase inhibitor activity	1.07E-14	30,47,12
1elva1#604	GO:0004867: serine-type endopeptidase inhibitor activity	1.12E-14	49,47,14
1c5y.1#B241	GO:0004867: serine-type endopeptidase inhibitor activity	1.18E-14	39,47,13
1esma_#314	GO:0004896: hematopoietin/interferon-class (D200-domain) cytokine receptor activity	1.19E-14	113,19,13
1kit_2#398	GO:0004896: hematopoietin/interferon-class (D200-domain) cytokine receptor activity	1.20E-14	83,19,12
1dz4a_#150	GO:0004497: monooxygenase activity	1.21E-14	19,26,9
1fl2a1#472	GO:0016668: oxidoreductase activity, acting on sulfur group of donors, NAD or NADP as acceptor	1.22E-14	14,12,7
1qrra_#11	GO:0016616: oxidoreductase activity, acting on the CH-OH group of donors, NAD or NADP as acceptor	1.23E-14	19,59,11
1h8d.1#H167	GO:0005509: calcium ion binding	1.24E-14	25,160,16
1gg6.1#C229	GO:0005509: calcium ion binding	1.24E-14	42,160,20
1el5a1#183	GO:0015036: disulfide oxidoreductase activity	1.25E-14	14,22,8
1fjsa_#158	GO:0005509: calcium ion binding	1.29E-14	47,160,21
1ir3a_#1028	GO:0005524: ATP binding	1.32E-14	70,243,30
1qfea_#68	GO:0005525: GTP binding	1.32E-14	560,49,35
1h8d.1#H184	GO:0004867: serine-type endopeptidase inhibitor activity	1.35E-14	23,47,11
1hyea2#182	GO:0016616: oxidoreductase activity, acting on the CH-OH group of donors, NAD or NADP as acceptor	1.38E-14	25,59,12
1mrj__#161	GO:0016799: hydrolase activity, hydrolyzing N-glycosyl compounds	1.38E-14	18,17,8
1qqka_#19	GO:0008083: growth factor activity	1.41E-14	9,42,8
1b6cb_#355	GO:0004674: protein serine/threonine kinase activity	1.41E-14	9,42,8
1leha1#177	GO:0016616: oxidoreductase activity, acting on the CH-OH group of donors, NAD or NADP as acceptor	1.49E-14	49,59,15
3grs_1#24	GO:0050660: FAD binding	1.61E-14	66,10,9
1ir3a_#1192	GO:0005524: ATP binding	1.67E-14	31,243,20
1h4vb2#108	GO:0005524: ATP binding	1.67E-14	13,243,13
4tmka_#141	GO:0005524: ATP binding	1.67E-14	13,243,13
1fgka_#668	GO:0005524: ATP binding	1.67E-14	13,243,13
1bev1_#86	GO:0004896: hematopoietin/interferon-class (D200-domain) cytokine receptor activity	1.68E-14	323,19,17
1ir3a_#1028	GO:0004674: protein serine/threonine kinase activity	1.69E-14	70,42,15
2napa1#665	GO:0030151: molybdenum ion binding	1.70E-14	21,15,8
1a0la_#193	GO:0004867: serine-type endopeptidase inhibitor activity	1.72E-14	31,47,12
1ddja_#771	GO:0004867: serine-type endopeptidase inhibitor activity	1.72E-14	31,47,12
1ton__#179	GO:0004867: serine-type endopeptidase inhibitor activity	1.72E-14	31,47,12
1elva1#513	GO:0004867: serine-type endopeptidase inhibitor activity	1.72E-14	31,47,12
3grs_2#189	GO:0050660: FAD binding	1.72E-14	18,10,7
1a6da3#190	GO:0003809: thrombin activity	1.72E-14	18,10,7
1ebda2#182	GO:0050660: FAD binding	1.72E-14	18,10,7
1cgha_#227	GO:0005509: calcium ion binding	1.77E-14	70,160,25
1mrp__#172	GO:0016616: oxidoreductase activity, acting on the CH-OH group of donors, NAD or NADP as acceptor	1.78E-14	110,59,20
1azza_#51	GO:0003809: thrombin activity	1.79E-14	36,10,8
1c5y.1#B234	GO:0003809: thrombin activity	1.79E-14	36,10,8
1nat__#50	GO:0005525: GTP binding	1.82E-14	297,49,27
1bio__#108	GO:0005509: calcium ion binding	1.91E-14	53,160,22
1d7ya3#331	GO:0016668: oxidoreductase activity, acting on sulfur group of donors, NAD or NADP as acceptor	2.01E-14	28,12,8
1aym3_#131	GO:0004896: hematopoietin/interferon-class (D200-domain) cytokine receptor activity	2.03E-14	412,19,18
1fnf_1#1146	GO:0005126: hematopoietin/interferon-class (D200-domain) cytokine receptor binding	2.10E-14	16,20,8
2ak3a1#122	GO:0019201: nucleotide kinase activity	2.12E-14	7,13,6
1qsta_#72	GO:0008080: N-acetyltransferase activity	2.12E-14	7,13,6
1qama_#37	GO:0004457: lactate dehydrogenase activity	2.14E-14	68,10,9
1gdea_#271	GO:0008483: transaminase activity	2.19E-14	6,17,6
1j71a_#176	GO:0004190: aspartic-type endopeptidase activity	2.21E-14	34,23,10
2hlca_#134	GO:0004263: chymotrypsin activity	2.28E-14	36,41,12
1ypta_#445	GO:0004725: protein tyrosine phosphatase activity	2.29E-14	12,15,7

1fzqa_#28	GO:0003924: GTPase activity	2.38E-14	49,17,10
3grs_2#211	GO:0015036: disulfide oxidoreductase activity	2.42E-14	52,22,11
1h4vb2#282	GO:0004812: tRNA ligase activity	2.49E-14	101,26,14
1jb0d_#26	GO:0004295: trypsin activity	2.55E-14	229,48,24
1bywa_#30	GO:0004896: hematopoietin/interferon-class (D200-domain) cytokine receptor activity	2.56E-14	331,19,17
1qs0a1#119	GO:0000287: magnesium ion binding	2.60E-14	454,128,57
4tmka_#141	GO:0016776: phosphotransferase activity, phosphate group as acceptor	2.64E-14	13,14,7
1d5ra2#133	GO:0004725: protein tyrosine phosphatase activity	2.67E-14	22,15,8
1h7wa4#478	GO:0050660: FAD binding	2.72E-14	19,10,7
1nhp_1#282	GO:0050660: FAD binding	2.72E-14	19,10,7
1cgha_#227	GO:0003809: thrombin activity	2.83E-14	70,10,9
1hyha2#171	GO:0016616: oxidoreductase activity, acting on the CH-OH group of donors, NAD or NADP as acceptor	2.84E-14	15,59,10
1qg6a_#93	GO:0016616: oxidoreductase activity, acting on the CH-OH group of donors, NAD or NADP as acceptor	2.84E-14	15,59,10
1cyda_#180	GO:0016616: oxidoreductase activity, acting on the CH-OH group of donors, NAD or NADP as acceptor	2.84E-14	15,59,10
1ceqa2#199	GO:0016616: oxidoreductase activity, acting on the CH-OH group of donors, NAD or NADP as acceptor	2.97E-14	11,59,9
1b8pa2#201	GO:0016616: oxidoreductase activity, acting on the CH-OH group of donors, NAD or NADP as acceptor	2.97E-14	8,59,8
1hvba_#63	GO:0008800: beta-lactamase activity	3.11E-14	9,10,6
1fu6a_#50	GO:0005524: ATP binding	3.19E-14	308,243,68
1a7s__#148	GO:0003809: thrombin activity	3.24E-14	71,10,9
1cs1a_#82	GO:0008483: transaminase activity	3.32E-14	195,17,14
1efpa1#32	GO:0016616: oxidoreductase activity, acting on the CH-OH group of donors, NAD or NADP as acceptor	3.43E-14	129,59,21
1b3ra1#223	GO:0004457: lactate dehydrogenase activity	3.63E-14	39,10,8
2ak3a1#122	GO:0016776: phosphotransferase activity, phosphate group as acceptor	3.71E-14	7,14,6
1d6ja_#36	GO:0019201: nucleotide kinase activity	3.71E-14	27,13,8
1b4va1#11	GO:0016668: oxidoreductase activity, acting on sulfur group of donors, NAD or NADP as acceptor	3.77E-14	30,12,8
1h6va1#116	GO:0016668: oxidoreductase activity, acting on sulfur group of donors, NAD or NADP as acceptor	3.77E-14	30,12,8
1hc7a2#113	GO:0004812: tRNA ligase activity	3.81E-14	21,26,9
1hxma2#150	GO:0004896: hematopoietin/interferon-class (D200-domain) cytokine receptor activity	3.84E-14	29,19,9
1ton__#181	GO:0004867: serine-type endopeptidase inhibitor activity	3.85E-14	79,47,16
1ac6a_#43	GO:0004896: hematopoietin/interferon-class (D200-domain) cytokine receptor activity	3.91E-14	163,19,14
1f5aa1#441	GO:0004896: hematopoietin/interferon-class (D200-domain) cytokine receptor activity	3.91E-14	163,19,14
1danh_#48	GO:0003809: thrombin activity	4.18E-14	20,10,7
1trb_1#15	GO:0050660: FAD binding	4.18E-14	20,10,7
1gdea_#92	GO:0016846: carbon-sulfur lyase activity	4.18E-14	20,10,7
1qbea_#92	GO:0004295: trypsin activity	4.22E-14	18,48,10
1tc1a_#158	GO:0016763: transferase activity, transferring pentosyl groups	4.28E-14	8,28,7
1c5y.1#B192	GO:0004867: serine-type endopeptidase inhibitor activity	4.33E-14	25,47,11
1oaa__#199	GO:0016616: oxidoreductase activity, acting on the CH-OH group of donors, NAD or NADP as acceptor	4.47E-14	27,59,12
1jsg__#92	GO:0005524: ATP binding	4.49E-14	607,243,104
1e39a2#556	GO:0050660: FAD binding	4.54E-14	40,10,8
1f42a2#123	GO:0004295: trypsin activity	4.62E-14	378,48,29
1pme__#25	GO:0004674: protein serine/threonine kinase activity	4.64E-14	37,42,12
8dfr__#10	GO:0016646: oxidoreductase activity, acting on the CH-NH group of donors, NAD or NADP as acceptor	4.80E-14	6,19,6
3dfr__#103	GO:0016646: oxidoreductase activity, acting on the CH-NH group of donors, NAD or NADP as acceptor	4.80E-14	6,19,6
1jb0d_#26	GO:0004263: chymotrypsin activity	4.81E-14	229,41,22
1e5ka_#113	GO:0004295: trypsin activity	5.09E-14	65,48,15
1eq9a_#32	GO:0003809: thrombin activity	5.10E-14	132,10,10
1f2la_#40	GO:0004295: trypsin activity	5.12E-14	236,48,24
1tiid_#39	GO:0005524: ATP binding	5.25E-14	239,243,58
1ad3a_#181	GO:0015036: disulfide oxidoreductase activity	5.32E-14	16,22,8
1feca1#31	GO:0050660: FAD binding	5.45E-14	75,10,9
1fcda1#6	GO:0050660: FAD binding	5.45E-14	75,10,9
1b8pa2#197	GO:0016616: oxidoreductase activity, acting on the CH-OH group of donors, NAD or NADP as acceptor	5.55E-14	21,59,11

1hyha2#179	GO:0016616: oxidoreductase activity, acting on the CH-OH group of donors, NAD or NADP as acceptor	5.55E-14	21,59,11
1cipa2#34	GO:0003924: GTPase activity	5.56E-14	78,17,11
2dpma_#194	GO:0008757: S-adenosylmethionine-dependent methyltransferase activity	5.61E-14	9,24,7
1icia_#175	GO:0004556: alpha-amylase activity	5.73E-14	63,15,10
1bqk__#78	GO:0005507: copper ion binding	5.74E-14	7,38,7
1arb__#193	GO:0005509: calcium ion binding	5.94E-14	50,160,21
1uok_2#170	GO:0004556: alpha-amylase activity	6.18E-14	7,15,6
1pbe_1#150	GO:0050660: FAD binding	6.18E-14	76,10,9
1fc4a_#111	GO:0008483: transaminase activity	6.38E-14	21,17,8
1boub_#7	GO:0005524: ATP binding	6.42E-14	240,243,58
1ton__#179	GO:0005509: calcium ion binding	6.61E-14	31,160,17
1elva1#513	GO:0005509: calcium ion binding	6.61E-14	31,160,17
1ilr1_#145	GO:0008083: growth factor activity	6.96E-14	10,42,8
1pme__#24	GO:0004674: protein serine/threonine kinase activity	6.96E-14	10,42,8
1gg6.1#C229	GO:0003809: thrombin activity	6.96E-14	42,10,8
2cpl__#53	GO:0004197: cysteine-type endopeptidase activity	7.04E-14	24,24,9
1d6aa_#52	GO:0004295: trypsin activity	7.14E-14	293,48,26
1nfdb1#21	GO:0004896: hematopoietin/interferon-class (D200-domain) cytokine receptor activity	7.52E-14	96,19,12
1howa_#697	GO:0005524: ATP binding	7.65E-14	18,243,15
1hyha2#205	GO:0004457: lactate dehydrogenase activity	7.76E-14	10,10,6
1otha2#300	GO:0016846: carbon-sulfur lyase activity	7.76E-14	10,10,6
1lgr_2#433	GO:0008235: metalloexopeptidase activity	8.46E-14	8,13,6
1e4ea2#215	GO:0005524: ATP binding	8.87E-14	490,243,90
1evqa_#305	GO:0005524: ATP binding	8.87E-14	490,243,90
1sgpe_#42	GO:0004295: trypsin activity	9.57E-14	26,48,11
1g71a_#77	GO:0005509: calcium ion binding	9.60E-14	51,160,21
3sil__#317	GO:0004896: hematopoietin/interferon-class (D200-domain) cytokine receptor activity	9.82E-14	358,19,17
1qfea_#68	GO:0005524: ATP binding	9.86E-14	560,243,98
2hlca_#54	GO:0005509: calcium ion binding	9.89E-14	109,160,30
1hbza_#214	GO:0004295: trypsin activity	9.96E-14	297,48,26
1hdr__#156	GO:0016616: oxidoreductase activity, acting on the CH-OH group of donors, NAD or NADP as acceptor	1.03E-13	55,59,15
1pma1_#17	GO:0003809: thrombin activity	1.04E-13	44,10,8
1hyha2#204	GO:0004457: lactate dehydrogenase activity	1.04E-13	44,10,8
1cpt__#401	GO:0004497: monooxygenase activity	1.05E-13	23,26,9
1gln_2#63	GO:0004812: tRNA ligase activity	1.06E-13	9,26,7
1c9la2#61	GO:0004263: chymotrypsin activity	1.10E-13	238,41,22
3grs_2#189	GO:0016668: oxidoreductase activity, acting on sulfur group of donors, NAD or NADP as acceptor	1.13E-13	18,12,7
1fmca_#248	GO:0016616: oxidoreductase activity, acting on the CH-OH group of donors, NAD or NADP as acceptor	1.17E-13	12,59,9
1cgha_#227	GO:0004867: serine-type endopeptidase inhibitor activity	1.17E-13	70,47,15
1hcl__#180	GO:0004674: protein serine/threonine kinase activity	1.23E-13	7,42,7
1exma3#99	GO:0005525: GTP binding	1.25E-13	81,49,16
1iira_#242	GO:0005525: GTP binding	1.27E-13	170,49,21
1f4la2#25	GO:0004812: tRNA ligase activity	1.29E-13	15,26,8
1danh_#152	GO:0003809: thrombin activity	1.32E-13	23,10,7
1hrna_#105	GO:0004190: aspartic-type endopeptidase activity	1.32E-13	10,23,7
3grs_1#131	GO:0015036: disulfide oxidoreductase activity	1.32E-13	6,22,6
1a7s__#148	GO:0004867: serine-type endopeptidase inhibitor activity	1.47E-13	71,47,15
1i2ma_#157	GO:0003924: GTPase activity	1.49E-13	13,17,7
1a81a1#51	GO:0004896: hematopoietin/interferon-class (D200-domain) cytokine receptor activity	1.50E-13	73,19,11
2rspa_#113	GO:0004190: aspartic-type endopeptidase activity	1.53E-13	17,23,8
1qaxa2#307	GO:0003809: thrombin activity	1.53E-13	46,10,8
1h6va2#245	GO:0016668: oxidoreductase activity, acting on sulfur group of donors, NAD or NADP as acceptor	1.58E-13	99,12,10
1ton__#211	GO:0004867: serine-type endopeptidase inhibitor activity	1.60E-13	86,47,16
1ceqa2#286	GO:0004457: lactate dehydrogenase activity	1.70E-13	11,10,6
1c4ka2#223	GO:0016846: carbon-sulfur lyase activity	1.70E-13	11,10,6
2dnja_#97	GO:0004263: chymotrypsin activity	1.76E-13	141,41,18
1bio__#210	GO:0003809: thrombin activity	1.78E-13	149,10,10
1j71a_#155	GO:0004190: aspartic-type endopeptidase activity	1.78E-13	6,23,6
1mpp__#220	GO:0004190: aspartic-type endopeptidase activity	1.78E-13	6,23,6
1bxoa_#180	GO:0004190: aspartic-type endopeptidase activity	1.78E-13	6,23,6
1ekbb_#85	GO:0003809: thrombin activity	1.91E-13	150,10,10

1gg6.1#B19	GO:0004867: serine-type endopeptidase inhibitor activity	1.96E-13	59,47,14
1howa_#551	GO:0005524: ATP binding	1.97E-13	12,243,12
1pme__#145	GO:0005524: ATP binding	1.97E-13	12,243,12
2hft_1#34	GO:0004896: hematopoietin/interferon-class (D200-domain) cytokine receptor activity	1.98E-13	34,19,9
1e3ua_#67	GO:0008800: beta-lactamase activity	2.02E-13	5,10,5
1opy__#68	GO:0004295: trypsin activity	2.09E-13	367,48,28
1b6cb_#337	GO:0005524: ATP binding	2.16E-13	14,243,13
1qs0a1#235	GO:0015036: disulfide oxidoreductase activity	2.20E-13	114,22,13
1cipa2#326	GO:0003924: GTPase activity	2.28E-13	24,17,8
1f3mc_#369	GO:0004674: protein serine/threonine kinase activity	2.45E-13	100,42,16
1lgr_2#433	GO:0004180: carboxypeptidase activity	2.46E-13	8,15,6
1apme_#168	GO:0004674: protein serine/threonine kinase activity	2.52E-13	11,42,8
1ir3a_#1056	GO:0004674: protein serine/threonine kinase activity	2.52E-13	11,42,8
1fmk_3#428	GO:0004674: protein serine/threonine kinase activity	2.52E-13	11,42,8
1hoe__#31	GO:0004556: alpha-amylase activity	2.56E-13	28,15,8
1hx0a2#234	GO:0004556: alpha-amylase activity	2.56E-13	28,15,8
1ovaa_#284	GO:0004457: lactate dehydrogenase activity	2.58E-13	25,10,7
1c7na_#281	GO:0016846: carbon-sulfur lyase activity	2.58E-13	25,10,7
1quba4#235	GO:0004896: hematopoietin/interferon-class (D200-domain) cytokine receptor activity	2.59E-13	106,19,12
1autc_#188	GO:0004867: serine-type endopeptidase inhibitor activity	2.60E-13	21,47,10
2hlpa2#298	GO:0016616: oxidoreductase activity, acting on the CH-OH group of donors, NAD or NADP as acceptor	2.63E-13	9,59,8
1a7ca_#276	GO:0004867: serine-type endopeptidase inhibitor activity	2.81E-13	15,47,9
1f0c.1#A317	GO:0004867: serine-type endopeptidase inhibitor activity	2.86E-13	7,47,7
1sek__#63	GO:0004867: serine-type endopeptidase inhibitor activity	2.86E-13	7,47,7
1a3qa2#190	GO:0004896: hematopoietin/interferon-class (D200-domain) cytokine receptor activity	3.04E-13	144,19,13
1e1aa_#178	GO:0004896: hematopoietin/interferon-class (D200-domain) cytokine receptor activity	3.16E-13	54,19,10
1ton__#120	GO:0004867: serine-type endopeptidase inhibitor activity	3.20E-13	29,47,11
1qfha1#721	GO:0005507: copper ion binding	3.22E-13	18,38,9
2cpl__#20	GO:0004896: hematopoietin/interferon-class (D200-domain) cytokine receptor activity	3.26E-13	108,19,12
1qg3a1#1179	GO:0004896: hematopoietin/interferon-class (D200-domain) cytokine receptor activity	3.34E-13	7,19,6
1jatb_#120	GO:0004842: ubiquitin-protein ligase activity	3.34E-13	7,19,6
1ia8a_#59	GO:0005524: ATP binding	3.35E-13	19,243,15
1hyea2#272	GO:0016616: oxidoreductase activity, acting on the CH-OH group of donors, NAD or NADP as acceptor	3.40E-13	31,59,12
1fuma2#221	GO:0050660: FAD binding	3.41E-13	12,10,6
1tyfa_#31	GO:0016616: oxidoreductase activity, acting on the CH-OH group of donors, NAD or NADP as acceptor	3.56E-13	162,59,22
1ir3a_#1015	GO:0004674: protein serine/threonine kinase activity	3.61E-13	43,42,12
2dkb__#114	GO:0008483: transaminase activity	3.62E-13	174,17,13
1glqa1#148	GO:0004364: glutathione transferase activity	3.70E-13	5,11,5
1hdr__#136	GO:0016616: oxidoreductase activity, acting on the CH-OH group of donors, NAD or NADP as acceptor	3.73E-13	13,59,9
1eno__#264	GO:0016616: oxidoreductase activity, acting on the CH-OH group of donors, NAD or NADP as acceptor	3.73E-13	13,59,9
1g71a_#77	GO:0003809: thrombin activity	3.73E-13	51,10,8
1bdb__#228	GO:0016616: oxidoreductase activity, acting on the CH-OH group of donors, NAD or NADP as acceptor	3.94E-13	18,59,10
1gox__#124	GO:0005525: GTP binding	3.99E-13	366,49,28
1dt6a_#376	GO:0004497: monooxygenase activity	4.07E-13	6,26,6
1dt6a_#374	GO:0004497: monooxygenase activity	4.07E-13	6,26,6
1cex__#23	GO:0016668: oxidoreductase activity, acting on sulfur group of donors, NAD or NADP as acceptor	4.10E-13	21,12,7
1arb__#210	GO:0003809: thrombin activity	4.15E-13	93,10,9
1i76a_#138	GO:0005525: GTP binding	4.17E-13	103,49,17
1edza1#206	GO:0005525: GTP binding	4.24E-13	399,49,29
1ctqa_#116	GO:0003924: GTPase activity	4.41E-13	42,17,9
1g71a_#88	GO:0003809: thrombin activity	4.41E-13	52,10,8
1hdoa_#189	GO:0016616: oxidoreductase activity, acting on the CH-OH group of donors, NAD or NADP as acceptor	4.63E-13	164,59,22
1ra9__#62	GO:0016646: oxidoreductase activity, acting on the CH-NH group of donors, NAD or NADP as acceptor	4.70E-13	23,19,8
1e9xa_#343	GO:0004497: monooxygenase activity	4.72E-13	39,26,10
1e4ea2#215	GO:0004896: hematopoietin/interferon-class (D200-domain) cytokine receptor activity	4.73E-13	490,19,18
1ekbb_#73	GO:0003809: thrombin activity	4.76E-13	27,10,7
1ek0a_#151	GO:0003924: GTPase activity	4.82E-13	26,17,8

1d6ja_#95	GO:0015036: disulfide oxidoreductase activity	4.86E-13	67,22,11
1eq9a_#59	GO:0004867: serine-type endopeptidase inhibitor activity	4.99E-13	30,47,11
1dpga2#334	GO:0004263: chymotrypsin activity	5.02E-13	287,41,23
1deua_#186	GO:0004197: cysteine-type endopeptidase activity	5.09E-13	11,24,7
1bg6_2#73	GO:0016616: oxidoreductase activity, acting on the CH-OH group of donors, NAD or NADP as acceptor	5.36E-13	32,59,12
1ab4__#89	GO:0003700: transcription factor activity	5.37E-13	159,124,31
1i2ma_#90	GO:0003924: GTPase activity	5.55E-13	15,17,7
1ddja_#681	GO:0004896: hematopoietin/interferon-class (D200-domain) cytokine receptor activity	6.14E-13	253,19,15
1d7ya3#331	GO:0050660: FAD binding	6.34E-13	28,10,7
2mpra_#190	GO:0004896: hematopoietin/interferon-class (D200-domain) cytokine receptor activity	6.52E-13	400,19,17
1hrna_#182	GO:0004190: aspartic-type endopeptidase activity	6.60E-13	31,23,9
1sek__#383	GO:0004867: serine-type endopeptidase inhibitor activity	6.69E-13	11,47,8
1sek__#296	GO:0004867: serine-type endopeptidase inhibitor activity	6.69E-13	11,47,8
2hlca_#226	GO:0003809: thrombin activity	6.78E-13	98,10,9
1egaa1#97	GO:0003924: GTPase activity	6.84E-13	27,17,8
1ddja_#746	GO:0003809: thrombin activity	6.93E-13	170,10,10
2msba_#218	GO:0005529: sugar binding	7.49E-13	49,39,12
1qtn.1#A270	GO:0005524: ATP binding	7.71E-13	439,243,82
1mpp__#103	GO:0004190: aspartic-type endopeptidase activity	7.80E-13	20,23,8
1ihua2#525	GO:0005524: ATP binding	7.98E-13	335,243,69
1cex__#23	GO:0015036: disulfide oxidoreductase activity	8.21E-13	21,22,8
1danh_#152	GO:0004867: serine-type endopeptidase inhibitor activity	8.23E-13	23,47,10
1dpga2#334	GO:0005525: GTP binding	8.33E-13	287,49,25
1g4ia_#11	GO:0003809: thrombin activity	8.35E-13	29,10,7
1ekbb_#228	GO:0005509: calcium ion binding	8.42E-13	68,160,23
1ihua2#343	GO:0000287: magnesium ion binding	8.50E-13	233,128,38
1hcl__#280	GO:0005524: ATP binding	8.76E-13	70,243,28
1a7ca_#202	GO:0004896: hematopoietin/interferon-class (D200-domain) cytokine receptor activity	8.76E-13	117,19,12
1ekbb_#85	GO:0005509: calcium ion binding	8.95E-13	150,160,34
1trka2#429	GO:0016668: oxidoreductase activity, acting on sulfur group of donors, NAD or NADP as acceptor	9.18E-13	43,12,8
1hwxa1#256	GO:0050660: FAD binding	9.65E-13	57,10,8
1foha5#218	GO:0050660: FAD binding	9.65E-13	57,10,8
1ce7a_#151	GO:0016799: hydrolase activity, hydrolyzing N-glycosyl compounds	9.84E-13	16,17,7
1hq8a_#206	GO:0004896: hematopoietin/interferon-class (D200-domain) cytokine receptor activity	1.01E-12	329,19,16
1cyda_#231	GO:0016616: oxidoreductase activity, acting on the CH-OH group of donors, NAD or NADP as acceptor	1.03E-12	14,59,9
1chma2#240	GO:0008235: metalloexopeptidase activity	1.03E-12	5,13,5
1c22a_#32	GO:0008235: metalloexopeptidase activity	1.03E-12	5,13,5
1bio__#108	GO:0004867: serine-type endopeptidase inhibitor activity	1.04E-12	53,47,13
1hbza_#214	GO:0004263: chymotrypsin activity	1.06E-12	297,41,23
1by5a_#238	GO:0004556: alpha-amylase activity	1.07E-12	237,15,13
1aky_2#133	GO:0004263: chymotrypsin activity	1.07E-12	156,41,18
1in4a1#264	GO:0003700: transcription factor activity	1.09E-12	50,124,18
1h6va1#116	GO:0050660: FAD binding	1.09E-12	30,10,7
1fl2a1#472	GO:0050660: FAD binding	1.10E-12	14,10,6
1fc4a_#241	GO:0016846: carbon-sulfur lyase activity	1.10E-12	14,10,6
1ha6a_#59	GO:0008009: chemokine activity	1.10E-12	14,10,6
1esl_1#38	GO:0005529: sugar binding	1.14E-12	28,39,10
1deua_#57	GO:0004197: cysteine-type endopeptidase activity	1.17E-12	20,24,8
1f2la_#40	GO:0004263: chymotrypsin activity	1.17E-12	236,41,21
3lada2#167	GO:0050660: FAD binding	1.21E-12	6,10,5
1fc4a_#277	GO:0016846: carbon-sulfur lyase activity	1.21E-12	6,10,5
1fg7a_#157	GO:0016846: carbon-sulfur lyase activity	1.21E-12	6,10,5
3grs_1#23	GO:0005525: GTP binding	1.24E-12	168,49,20
1hwxa1#223	GO:0016616: oxidoreductase activity, acting on the CH-OH group of donors, NAD or NADP as acceptor	1.28E-12	233,59,25
1h4ua1#404	GO:0004812: tRNA ligase activity	1.29E-12	202,26,16
1ja9a_#249	GO:0016616: oxidoreductase activity, acting on the CH-OH group of donors, NAD or NADP as acceptor	1.29E-12	10,59,8
1mdah_#329	GO:0004896: hematopoietin/interferon-class (D200-domain) cytokine receptor activity	1.35E-12	335,19,16
1f2aa_#27	GO:0004197: cysteine-type endopeptidase activity	1.37E-12	89,24,12
1mpp__#151	GO:0004190: aspartic-type endopeptidase activity	1.38E-12	94,23,12
2ctc__#196	GO:0008235: metalloexopeptidase activity	1.39E-12	11,13,6
1gdna_#26	GO:0004867: serine-type endopeptidase inhibitor activity	1.39E-12	24,47,10

1cgha_#168	GO:0004867: serine-type endopeptidase inhibitor activity	1.39E-12	24,47,10
1qo3c_#195	GO:0005529: sugar binding	1.42E-12	39,39,11
1d5ra2#25	GO:0004725: protein tyrosine phosphatase activity	1.43E-12	19,15,7
1danh_#104	GO:0003809: thrombin activity	1.48E-12	183,10,10
1qhda2#323	GO:0004896: hematopoietin/interferon-class (D200-domain) cytokine receptor activity	1.48E-12	337,19,16
1oaca1#495	GO:0003809: thrombin activity	1.49E-12	60,10,8
1fuma2#221	GO:0016668: oxidoreductase activity, acting on sulfur group of donors, NAD or NADP as acceptor	1.49E-12	12,12,6
1fc4a_#205	GO:0005525: GTP binding	1.54E-12	267,49,24
2sqca2#268	GO:0005126: hematopoietin/interferon-class (D200-domain) cytokine receptor binding	1.61E-12	59,20,10
1qlaa2#179	GO:0016668: oxidoreductase activity, acting on sulfur group of donors, NAD or NADP as acceptor	1.65E-12	46,12,8
1ekbb_#73	GO:0005509: calcium ion binding	1.68E-12	27,160,15
1pbe_1#121	GO:0016668: oxidoreductase activity, acting on sulfur group of donors, NAD or NADP as acceptor	1.68E-12	25,12,7
1bvza3#416	GO:0005509: calcium ion binding	1.71E-12	23,160,14
1dofa_#15	GO:0005126: hematopoietin/interferon-class (D200-domain) cytokine receptor binding	1.80E-12	197,20,14
1smaa1#95	GO:0004896: hematopoietin/interferon-class (D200-domain) cytokine receptor activity	1.82E-12	272,19,15
1g71a_#88	GO:0005509: calcium ion binding	1.83E-12	52,160,20
1elva1#623	GO:0005509: calcium ion binding	1.83E-12	52,160,20
1ghpa_#236	GO:0008800: beta-lactamase activity	1.84E-12	15,10,6
1feca1#28	GO:0050660: FAD binding	1.97E-12	62,10,8
1sek__#288	GO:0004867: serine-type endopeptidase inhibitor activity	1.98E-12	12,47,8
1c9la2#12	GO:0005524: ATP binding	1.98E-12	295,243,63
1pfza_#124	GO:0004190: aspartic-type endopeptidase activity	2.00E-12	51,23,10
1bu7a_#353	GO:0004497: monooxygenase activity	2.00E-12	62,26,11
1f2na_#138	GO:0005525: GTP binding	2.06E-12	96,49,16
8dfr__#115	GO:0016646: oxidoreductase activity, acting on the CH-NH group of donors, NAD or NADP as acceptor	2.10E-12	27,19,8
1eny__#147	GO:0016616: oxidoreductase activity, acting on the CH-OH group of donors, NAD or NADP as acceptor	2.10E-12	123,59,19
1ovaa_#284	GO:0004867: serine-type endopeptidase inhibitor activity	2.29E-12	25,47,10
1apme_#168	GO:0005524: ATP binding	2.30E-12	11,243,11
1ir3a_#1056	GO:0005524: ATP binding	2.30E-12	11,243,11
1e2ka_#59	GO:0005524: ATP binding	2.30E-12	11,243,11
1e2fa_#140	GO:0005524: ATP binding	2.30E-12	11,243,11
1fmk_3#428	GO:0005524: ATP binding	2.30E-12	11,243,11
1koba_#293	GO:0005524: ATP binding	2.30E-12	11,243,11
1koba_#105	GO:0005524: ATP binding	2.30E-12	11,243,11
1bg6_2#24	GO:0016668: oxidoreductase activity, acting on sulfur group of donors, NAD or NADP as acceptor	2.30E-12	26,12,7
1qu9a_#64	GO:0005529: sugar binding	2.33E-12	14,39,8
1aky_2#133	GO:0004295: trypsin activity	2.33E-12	156,48,19
1b6cb_#399	GO:0005524: ATP binding	2.35E-12	13,243,12
1lara1#1407	GO:0004725: protein tyrosine phosphatase activity	2.41E-12	5,15,5
1hqoa2#159	GO:0004364: glutathione transferase activity	2.42E-12	14,11,6
1c4zd_#101	GO:0004842: ubiquitin-protein ligase activity	2.53E-12	16,19,7
1egia_#738	GO:0005529: sugar binding	2.57E-12	30,39,10
1gg6.1#B19	GO:0005509: calcium ion binding	2.90E-12	59,160,21
1cpy__#93	GO:0005524: ATP binding	2.92E-12	275,243,60
1chua2#234	GO:0050660: FAD binding	2.94E-12	16,10,6
1ad3a_#181	GO:0050660: FAD binding	2.94E-12	16,10,6
1b4va1#253	GO:0050660: FAD binding	2.94E-12	16,10,6
1d7ya1#255	GO:0050660: FAD binding	2.94E-12	16,10,6
1cs1a_#160	GO:0004556: alpha-amylase activity	2.95E-12	187,15,12
1eq9a_#40	GO:0004867: serine-type endopeptidase inhibitor activity	2.95E-12	86,47,15
1fgga_#134	GO:0005524: ATP binding	3.07E-12	218,243,52
1h9da_#91	GO:0004896: hematopoietin/interferon-class (D200-domain) cytokine receptor activity	3.10E-12	222,19,14
1e44b_#75	GO:0003809: thrombin activity	3.31E-12	198,10,10
1bqya_#115	GO:0005509: calcium ion binding	3.32E-12	20,160,13
1bwvs_#117	GO:0005524: ATP binding	3.45E-12	329,243,67
1erv__#80	GO:0005524: ATP binding	3.47E-12	442,243,81
1bu7a_#405	GO:0004497: monooxygenase activity	3.47E-12	65,26,11
1ldna1#94	GO:0004457: lactate dehydrogenase activity	3.49E-12	199,10,10
1ef1a3#45	GO:0004263: chymotrypsin activity	3.60E-12	14,41,8
1arb__#210	GO:0005509: calcium ion binding	3.65E-12	93,160,26

1f8ra1#260	GO:0015036: disulfide oxidoreductase activity	3.66E-12	8,22,6
1as4.1#A290	GO:0004867: serine-type endopeptidase inhibitor activity	3.68E-12	26,47,10
1t7pa2#663	GO:0004867: serine-type endopeptidase inhibitor activity	3.68E-12	26,47,10
1hu4a_#267	GO:0016616: oxidoreductase activity, acting on the CH-OH group of donors, NAD or NADP as acceptor	3.77E-12	57,59,14
1ton__#211	GO:0005509: calcium ion binding	3.79E-12	86,160,25
3lada2#167	GO:0016668: oxidoreductase activity, acting on sulfur group of donors, NAD or NADP as acceptor	3.80E-12	6,12,5
1bvza3#298	GO:0005509: calcium ion binding	3.88E-12	11,160,10
1azsa_#397	GO:0003887: DNA-directed DNA polymerase activity	3.89E-12	16,20,7
1bu7a_#277	GO:0004497: monooxygenase activity	3.94E-12	21,26,8
1eq9a_#32	GO:0005509: calcium ion binding	3.96E-12	132,160,31
2ctc__#196	GO:0004180: carboxypeptidase activity	4.03E-12	11,15,6
1a65a2#247	GO:0005507: copper ion binding	4.05E-12	32,38,10
1pii_2#326	GO:0004556: alpha-amylase activity	4.07E-12	192,15,12
1hyha2#211	GO:0004457: lactate dehydrogenase activity	4.23E-12	7,10,5
1gdea_#379	GO:0016846: carbon-sulfur lyase activity	4.28E-12	68,10,8
2dkb__#133	GO:0008483: transaminase activity	4.29E-12	19,17,7
1b6cb_#346	GO:0004674: protein serine/threonine kinase activity	4.32E-12	9,42,7
1dpga2#334	GO:0004295: trypsin activity	4.35E-12	287,48,24
1gpea1#273	GO:0015036: disulfide oxidoreductase activity	4.35E-12	81,22,11
1e4ea2#210	GO:0004674: protein serine/threonine kinase activity	4.44E-12	14,42,8
1ppn__#132	GO:0005524: ATP binding	4.47E-12	234,243,54
3sil__#98	GO:0004896: hematopoietin/interferon-class (D200-domain) cytokine receptor activity	4.50E-12	228,19,14
2hlpa2#289	GO:0004457: lactate dehydrogenase activity	4.53E-12	17,10,6
1fg7a_#158	GO:0008483: transaminase activity	4.53E-12	10,17,6
1qlaa2#383	GO:0050660: FAD binding	4.53E-12	17,10,6
1cjca2#76	GO:0050660: FAD binding	4.53E-12	17,10,6
1b65a_#257	GO:0000287: magnesium ion binding	4.60E-12	210,128,35
1ceqa2#286	GO:0016616: oxidoreductase activity, acting on the CH-OH group of donors, NAD or NADP as acceptor	4.66E-12	11,59,8
2dnja_#97	GO:0004295: trypsin activity	4.68E-12	141,48,18
1fp1d2#214	GO:0005524: ATP binding	4.71E-12	173,243,45
1d2ka1#164	GO:0004556: alpha-amylase activity	4.72E-12	63,15,9
1el5a1#166	GO:0015036: disulfide oxidoreductase activity	4.81E-12	15,22,7
1el5a1#183	GO:0016668: oxidoreductase activity, acting on sulfur group of donors, NAD or NADP as acceptor	4.83E-12	14,12,6
1aoza3#351	GO:0005507: copper ion binding	4.88E-12	6,38,6
1ouna_#34	GO:0004190: aspartic-type endopeptidase activity	4.94E-12	8,23,6
1mpp__#214	GO:0004190: aspartic-type endopeptidase activity	4.94E-12	8,23,6
1f4la2#20	GO:0004812: tRNA ligase activity	4.95E-12	13,26,7
1ovaa_#364	GO:0004867: serine-type endopeptidase inhibitor activity	5.09E-12	13,47,8
1g6sa_#64	GO:0000287: magnesium ion binding	5.32E-12	108,128,25
1czan1#92	GO:0005524: ATP binding	5.62E-12	631,243,102
1gega_#58	GO:0016616: oxidoreductase activity, acting on the CH-OH group of donors, NAD or NADP as acceptor	5.83E-12	38,59,12
1gtra2#113	GO:0004812: tRNA ligase activity	6.16E-12	22,26,8
1ir3a_#1063	GO:0004674: protein serine/threonine kinase activity	6.46E-12	68,42,13
1qqsa_#124	GO:0004896: hematopoietin/interferon-class (D200-domain) cytokine receptor activity	6.50E-12	458,19,17
1a6o__#297	GO:0004674: protein serine/threonine kinase activity	6.52E-12	41,42,11
1dpja_#199	GO:0004190: aspartic-type endopeptidase activity	6.89E-12	15,23,7
1c9la2#61	GO:0004295: trypsin activity	7.02E-12	238,48,22
1fzqa_#28	GO:0019201: nucleotide kinase activity	7.27E-12	49,13,8
1a06__#103	GO:0005524: ATP binding	7.36E-12	28,243,17
1hwxa1#223	GO:0005525: GTP binding	7.59E-12	233,49,22
1a0la_#160	GO:0003809: thrombin activity	7.76E-12	73,10,8
1qtn.1#A270	GO:0000287: magnesium ion binding	7.97E-12	439,128,52
1fjfl_#67	GO:0004263: chymotrypsin activity	8.12E-12	175,41,18
1c9la2#61	GO:0004896: hematopoietin/interferon-class (D200-domain) cytokine receptor activity	8.19E-12	238,19,14
1cpt__#52	GO:0004497: monooxygenase activity	8.45E-12	35,26,9
1dt6a_#77	GO:0004497: monooxygenase activity	8.45E-12	35,26,9
1bkca_#351	GO:0008270: zinc ion binding	8.93E-12	11,108,9
2aak__#77	GO:0004842: ubiquitin-protein ligase activity	9.32E-12	5,19,5
1aym2_#185	GO:0003968: RNA-directed RNA polymerase activity	9.60E-12	6,14,5
1arb__#210	GO:0004867: serine-type endopeptidase inhibitor activity	9.77E-12	93,47,15
1dt6a_#449	GO:0004497: monooxygenase activity	9.84E-12	14,26,7

1c4ka2#223	GO:0008483: transaminase activity	9.94E-12	11,17,6
2knt__#34	GO:0004867: serine-type endopeptidase inhibitor activity	1.00E-11	9,47,7
1hle.1#B381	GO:0004867: serine-type endopeptidase inhibitor activity	1.00E-11	9,47,7
1fx7a1#44	GO:0003700: transcription factor activity	1.02E-11	56,124,18
1a4ya_#104	GO:0015036: disulfide oxidoreductase activity	1.02E-11	241,22,15
1iira_#242	GO:0005524: ATP binding	1.03E-11	170,243,44
1a06__#205	GO:0005524: ATP binding	1.05E-11	32,243,18
1bpv__#22	GO:0005126: hematopoietin/interferon-class (D200-domain) cytokine receptor binding	1.07E-11	18,20,7
1g5ha2#319	GO:0005524: ATP binding	1.09E-11	19,243,14
1pysb5#672	GO:0004812: tRNA ligase activity	1.12E-11	36,26,9
1h6la_#119	GO:0004896: hematopoietin/interferon-class (D200-domain) cytokine receptor activity	1.17E-11	308,19,15
1fmca_#86	GO:0016616: oxidoreductase activity, acting on the CH-OH group of donors, NAD or NADP as acceptor	1.19E-11	17,59,9
1ax4a_#430	GO:0008483: transaminase activity	1.19E-11	59,17,9
1fi2a_#109	GO:0004190: aspartic-type endopeptidase activity	1.19E-11	228,23,15
1qlaa3#364	GO:0004263: chymotrypsin activity	1.19E-11	73,41,13
1fmca_#88	GO:0016616: oxidoreductase activity, acting on the CH-OH group of donors, NAD or NADP as acceptor	1.22E-11	8,59,7
1cg2a1#43	GO:0003700: transcription factor activity	1.23E-11	189,124,32
1i2oa_#34	GO:0016616: oxidoreductase activity, acting on the CH-OH group of donors, NAD or NADP as acceptor	1.26E-11	192,59,22
1evqa_#305	GO:0005525: GTP binding	1.27E-11	490,49,30
1ad3a_#181	GO:0016668: oxidoreductase activity, acting on sulfur group of donors, NAD or NADP as acceptor	1.28E-11	16,12,6
1eq9a_#59	GO:0005509: calcium ion binding	1.29E-11	30,160,15
2duba_#212	GO:0005524: ATP binding	1.30E-11	62,243,25
1gpea1#271	GO:0015036: disulfide oxidoreductase activity	1.30E-11	197,22,14
1imva_#255	GO:0004896: hematopoietin/interferon-class (D200-domain) cytokine receptor activity	1.31E-11	589,19,18
1qf9a_#117	GO:0003924: GTPase activity	1.34E-11	89,17,10
7odca2#67	GO:0016831: carboxy-lyase activity	1.34E-11	15,25,7
2cmd_1#113	GO:0016616: oxidoreductase activity, acting on the CH-OH group of donors, NAD or NADP as acceptor	1.37E-11	12,59,8
1qg6a_#251	GO:0016616: oxidoreductase activity, acting on the CH-OH group of donors, NAD or NADP as acceptor	1.37E-11	12,59,8
3lada1#116	GO:0016668: oxidoreductase activity, acting on sulfur group of donors, NAD or NADP as acceptor	1.40E-11	153,12,10
1ef1a3#45	GO:0004295: trypsin activity	1.40E-11	14,48,8
1qfma2#576	GO:0016616: oxidoreductase activity, acting on the CH-OH group of donors, NAD or NADP as acceptor	1.42E-11	214,59,23
1czya1#466	GO:0004896: hematopoietin/interferon-class (D200-domain) cytokine receptor activity	1.47E-11	109,19,11
1dt6a_#181	GO:0004497: monooxygenase activity	1.47E-11	37,26,9
1bpv__#22	GO:0019955: cytokine binding	1.49E-11	18,11,6
1e43a2#257	GO:0004556: alpha-amylase activity	1.49E-11	13,15,6
1g71a_#77	GO:0004867: serine-type endopeptidase inhibitor activity	1.52E-11	51,47,12
1phk__#221	GO:0005524: ATP binding	1.52E-11	14,243,12
1e6ca_#153	GO:0005524: ATP binding	1.52E-11	14,243,12
1e8ga2#201	GO:0015036: disulfide oxidoreductase activity	1.60E-11	304,22,16
1pii_2#326	GO:0005525: GTP binding	1.61E-11	192,49,20
1e5ea_#71	GO:0008483: transaminase activity	1.63E-11	61,17,9
1ihua2#343	GO:0003924: GTPase activity	1.66E-11	233,17,13
1trka2#429	GO:0050660: FAD binding	1.70E-11	43,10,7
1fsu__#434	GO:0005524: ATP binding	1.80E-11	356,243,69
1ek6a_#33	GO:0016616: oxidoreductase activity, acting on the CH-OH group of donors, NAD or NADP as acceptor	1.94E-11	52,59,13
1cex__#23	GO:0050660: FAD binding	1.98E-11	21,10,6
1au1a_#58	GO:0005126: hematopoietin/interferon-class (D200-domain) cytokine receptor binding	2.07E-11	105,20,11
1lvl_2#262	GO:0015036: disulfide oxidoreductase activity	2.11E-11	5,22,5
1dj0a2#183	GO:0003809: thrombin activity	2.12E-11	142,10,9
1qnja_#221	GO:0005509: calcium ion binding	2.21E-11	12,160,10
1pfza_#156	GO:0004190: aspartic-type endopeptidase activity	2.23E-11	64,23,10
1nox__#88	GO:0004896: hematopoietin/interferon-class (D200-domain) cytokine receptor activity	2.24E-11	55,19,9
1h6va1#116	GO:0015036: disulfide oxidoreductase activity	2.27E-11	30,22,8
1elua_#216	GO:0008483: transaminase activity	2.28E-11	40,17,8
1avaa2#204	GO:0005509: calcium ion binding	2.28E-11	15,160,11
7taa_2#119	GO:0005509: calcium ion binding	2.28E-11	15,160,11
1qhoa4#75	GO:0005509: calcium ion binding	2.28E-11	15,160,11

1el5a1#10	GO:0016616: oxidoreductase activity, acting on the CH-OH group of donors, NAD or NADP as acceptor	2.33E-11	18,59,9
1e05i_#79	GO:0004867: serine-type endopeptidase inhibitor activity	2.48E-11	15,47,8
1e5ea_#145	GO:0016846: carbon-sulfur lyase activity	2.53E-11	9,10,5
1e3ua_#205	GO:0008800: beta-lactamase activity	2.53E-11	9,10,5
1f9va_#627	GO:0005525: GTP binding	2.61E-11	51,49,12
1b12a_#221	GO:0004896: hematopoietin/interferon-class (D200-domain) cytokine receptor activity	2.67E-11	115,19,11
1tkia_#98	GO:0005524: ATP binding	2.68E-11	10,243,10
1hcl__#267	GO:0005524: ATP binding	2.68E-11	10,243,10
1bfd_2#146	GO:0005524: ATP binding	2.69E-11	112,243,34
1e4ea2#215	GO:0004674: protein serine/threonine kinase activity	2.72E-11	490,42,27
1dlfl_#97	GO:0004896: hematopoietin/interferon-class (D200-domain) cytokine receptor activity	2.77E-11	36,19,8
1eq9a_#58	GO:0005509: calcium ion binding	2.80E-11	47,160,18
1qlaa2#179	GO:0050660: FAD binding	2.82E-11	46,10,7
1e1aa_#178	GO:0019838: growth factor binding	2.82E-11	54,9,7
1h8ua_#113	GO:0005529: sugar binding	2.82E-11	65,39,12
1dih_2#222	GO:0000287: magnesium ion binding	2.87E-11	352,128,45
1ton__#181	GO:0005509: calcium ion binding	2.93E-11	79,160,23
1eg9a2#199	GO:0005524: ATP binding	3.03E-11	74,243,27
1tyfa_#31	GO:0005524: ATP binding	3.10E-11	162,243,42
1cpza_#6	GO:0004896: hematopoietin/interferon-class (D200-domain) cytokine receptor activity	3.15E-11	157,19,12
2cuaa_#107	GO:0005507: copper ion binding	3.27E-11	128,38,15
1f0c.1#A291	GO:0004867: serine-type endopeptidase inhibitor activity	3.30E-11	10,47,7
1fa0a1#393	GO:0000287: magnesium ion binding	3.35E-11	261,128,38
3grs_1#23	GO:0016668: oxidoreductase activity, acting on sulfur group of donors, NAD or NADP as acceptor	3.63E-11	168,12,10
1dfoa_#86	GO:0008483: transaminase activity	3.66E-11	13,17,6
1eg5a_#199	GO:0008483: transaminase activity	3.66E-11	13,17,6
1cbf__#208	GO:0004896: hematopoietin/interferon-class (D200-domain) cytokine receptor activity	3.71E-11	333,19,15
1e9xa_#343	GO:0005507: copper ion binding	3.73E-11	39,38,10
1ho1a_#211	GO:0016831: carboxy-lyase activity	3.84E-11	111,25,12
1gox__#124	GO:0016763: transferase activity, transferring pentosyl groups	3.86E-11	366,28,19
2afpa_#115	GO:0005529: sugar binding	4.00E-11	7,39,6
1d6ja_#95	GO:0016668: oxidoreductase activity, acting on sulfur group of donors, NAD or NADP as acceptor	4.00E-11	67,12,8
3grx__#56	GO:0004896: hematopoietin/interferon-class (D200-domain) cytokine receptor activity	4.06E-11	335,19,15
1hyha2#204	GO:0016616: oxidoreductase activity, acting on the CH-OH group of donors, NAD or NADP as acceptor	4.12E-11	44,59,12
1jlna_#437	GO:0005507: copper ion binding	4.12E-11	53,38,11
1danh_#152	GO:0005509: calcium ion binding	4.20E-11	23,160,13
1ecfa1#367	GO:0000287: magnesium ion binding	4.29E-11	11,128,9
1d9ta_#137	GO:0016646: oxidoreductase activity, acting on the CH-NH group of donors, NAD or NADP as acceptor	4.32E-11	12,19,6
1rmg__#279	GO:0004222: metalloendopeptidase activity	4.32E-11	12,19,6
1fc4a_#205	GO:0015036: disulfide oxidoreductase activity	4.62E-11	267,22,15
1ja1a2#201	GO:0010181: FMN binding	4.63E-11	9,11,5
2napa2#292	GO:0005524: ATP binding	4.74E-11	120,243,35
1qj8a_#64	GO:0004295: trypsin activity	4.82E-11	84,48,14
1pii_2#326	GO:0000287: magnesium ion binding	4.83E-11	192,128,32
1d5ta1#12	GO:0050660: FAD binding	4.89E-11	24,10,6
1h6la_#119	GO:0005524: ATP binding	5.00E-11	308,243,62
1eu1a1#679	GO:0030151: molybdenum ion binding	5.02E-11	7,15,5
1feca1#309	GO:0050660: FAD binding	5.05E-11	10,10,5
3lada3#380	GO:0050660: FAD binding	5.05E-11	10,10,5
1ihna_#64	GO:0003809: thrombin activity	5.05E-11	10,10,5
1fg7a_#158	GO:0016846: carbon-sulfur lyase activity	5.05E-11	10,10,5
1hc7a1#288	GO:0004812: tRNA ligase activity	5.27E-11	5,26,5
1cpt__#159	GO:0004497: monooxygenase activity	5.27E-11	5,26,5
1ejda_#213	GO:0005525: GTP binding	5.28E-11	284,49,23
1dpja_#320	GO:0004190: aspartic-type endopeptidase activity	5.28E-11	19,23,7
1ir3a_#1139	GO:0005524: ATP binding	5.33E-11	27,243,16
1cpza_#6	GO:0003809: thrombin activity	5.34E-11	157,10,9
1h6va1#27	GO:0016668: oxidoreductase activity, acting on sulfur group of donors, NAD or NADP as acceptor	5.40E-11	260,12,11
1eno__#290	GO:0016616: oxidoreductase activity, acting on the CH-OH group of donors, NAD or NADP as acceptor	5.41E-11	9,59,7

1h5qa_#201	GO:0016616: oxidoreductase activity, acting on the CH-OH group of donors, NAD or NADP as acceptor	5.41E-11	9,59,7
1gtra1#468	GO:0005525: GTP binding	5.43E-11	54,49,12
1jatb_#72	GO:0004842: ubiquitin-protein ligase activity	5.57E-11	6,19,5
1fcda2#189	GO:0015036: disulfide oxidoreductase activity	5.60E-11	51,22,9
1exma3#99	GO:0005524: ATP binding	5.71E-11	81,243,28
2fnba_#67	GO:0005518: collagen binding	5.73E-11	8,13,5
1g51a3#525	GO:0003809: thrombin activity	6.00E-11	263,10,10
1ton__#47	GO:0005509: calcium ion binding	6.18E-11	112,160,27
1cbf__#208	GO:0005524: ATP binding	6.22E-11	333,243,65
1nsj__#124	GO:0016831: carboxy-lyase activity	6.31E-11	148,25,13
1a4ya_#104	GO:0004556: alpha-amylase activity	6.34E-11	241,15,12
1qgna_#207	GO:0008483: transaminase activity	6.39E-11	14,17,6
1apme_#255	GO:0004674: protein serine/threonine kinase activity	6.42E-11	7,42,6
1howa_#552	GO:0004674: protein serine/threonine kinase activity	6.42E-11	7,42,6
1pbe_1#121	GO:0050660: FAD binding	6.42E-11	25,10,6
1gjwa2#141	GO:0005509: calcium ion binding	6.93E-11	16,160,11
7taa_2#295	GO:0005509: calcium ion binding	6.93E-11	16,160,11
1gox__#246	GO:0004556: alpha-amylase activity	6.99E-11	177,15,11
3grs_2#211	GO:0050660: FAD binding	6.99E-11	52,10,7
1fkna_#18	GO:0004190: aspartic-type endopeptidase activity	7.27E-11	130,23,12
1ceza_#812	GO:0003887: DNA-directed DNA polymerase activity	7.42E-11	6,20,5
1danh_#104	GO:0005509: calcium ion binding	7.46E-11	183,160,35
1a6o__#45	GO:0005524: ATP binding	7.51E-11	303,243,61
1csn__#84	GO:0005524: ATP binding	7.55E-11	207,243,48
1qj8a_#64	GO:0004263: chymotrypsin activity	7.75E-11	84,41,13
1ihua1#119	GO:0005525: GTP binding	7.79E-11	85,49,14
1ass__#33	GO:0016668: oxidoreductase activity, acting on sulfur group of donors, NAD or NADP as acceptor	7.93E-11	9,12,5
1dyna_#42	GO:0050660: FAD binding	7.96E-11	164,10,9
1fmca_#247	GO:0016616: oxidoreductase activity, acting on the CH-OH group of donors, NAD or NADP as acceptor	7.97E-11	6,59,6
1h5qa_#21	GO:0016616: oxidoreductase activity, acting on the CH-OH group of donors, NAD or NADP as acceptor	7.97E-11	6,59,6
1thg__#223	GO:0005524: ATP binding	8.00E-11	82,243,28
1dz4a_#264	GO:0004497: monooxygenase activity	8.06E-11	44,26,9
1mpp__#100	GO:0004190: aspartic-type endopeptidase activity	8.06E-11	33,23,8
1tgoa2#409	GO:0003887: DNA-directed DNA polymerase activity	8.09E-11	23,20,7
1bg6_2#24	GO:0050660: FAD binding	8.34E-11	26,10,6
1a0la_#160	GO:0004867: serine-type endopeptidase inhibitor activity	8.38E-11	73,47,13
1qq4a_#46	GO:0004263: chymotrypsin activity	8.59E-11	19,41,8
1cgha_#168	GO:0005509: calcium ion binding	8.69E-11	24,160,13
1iira_#242	GO:0016616: oxidoreductase activity, acting on the CH-OH group of donors, NAD or NADP as acceptor	8.92E-11	170,59,20
1cewi_#14	GO:0008009: chemokine activity	8.94E-11	98,10,8
1e43a2#236	GO:0005509: calcium ion binding	9.11E-11	13,160,10
1g8fa3#419	GO:0016616: oxidoreductase activity, acting on the CH-OH group of donors, NAD or NADP as acceptor	9.12E-11	151,59,19
1f2la_#12	GO:0008009: chemokine activity	9.16E-11	4,10,4
2hlca_#226	GO:0005509: calcium ion binding	9.18E-11	98,160,25
1oaa__#14	GO:0004457: lactate dehydrogenase activity	9.24E-11	54,10,7
1pysa_#127	GO:0005524: ATP binding	9.29E-11	21,243,14
1fuia2#273	GO:0016616: oxidoreductase activity, acting on the CH-OH group of donors, NAD or NADP as acceptor	9.34E-11	133,59,18
1hw5a2#64	GO:0003809: thrombin activity	9.40E-11	167,10,9
3prn__#143	GO:0004556: alpha-amylase activity	9.52E-11	334,15,13
1b4ka_#108	GO:0016616: oxidoreductase activity, acting on the CH-OH group of donors, NAD or NADP as acceptor	9.56E-11	116,59,17
1h6va2#245	GO:0050660: FAD binding	9.72E-11	99,10,8
1mkp__#216	GO:0004725: protein tyrosine phosphatase activity	9.85E-11	129,15,10
3grs_1#23	GO:0050660: FAD binding	9.93E-11	168,10,9
1fsu__#434	GO:0004896: hematopoietin/interferon-class (D200-domain) cytokine receptor activity	9.97E-11	356,19,15
1e0ta2#275	GO:0016861: intramolecular oxidoreductase activity, interconverting aldoses and ketoses	9.97E-11	107,13,9
1bqk__#77	GO:0005507: copper ion binding	9.98E-11	74,38,12
1e9xa_#62	GO:0004497: monooxygenase activity	1.00E-10	45,26,9
1qisa_#258	GO:0008483: transaminase activity	1.03E-10	7,17,5

1bw0a_#252	GO:0008483: transaminase activity	1.03E-10	7,17,5
1b3ra2#47	GO:0016616: oxidoreductase activity, acting on the CH-OH group of donors, NAD or NADP as acceptor	1.03E-10	37,59,11
1eg5a_#197	GO:0008483: transaminase activity	1.06E-10	15,17,6
1bu7a_#278	GO:0004497: monooxygenase activity	1.08E-10	30,26,8
1xnb__#149	GO:0005525: GTP binding	1.08E-10	165,49,18
1hc7a2#258	GO:0004896: hematopoietin/interferon-class (D200-domain) cytokine receptor activity	1.10E-10	226,19,13
1qlaa3#364	GO:0004295: trypsin activity	1.13E-10	73,48,13
1ihua2#525	GO:0019201: nucleotide kinase activity	1.16E-10	335,13,12
1bwda_#117	GO:0004812: tRNA ligase activity	1.17E-10	116,26,12
1hbza_#96	GO:0005524: ATP binding	1.21E-10	370,243,69
1nsj__#178	GO:0016831: carboxy-lyase activity	1.21E-10	93,25,11
1fi2a_#109	GO:0004896: hematopoietin/interferon-class (D200-domain) cytokine receptor activity	1.23E-10	228,19,13
1boub_#7	GO:0005525: GTP binding	1.24E-10	240,49,21
1fc3a_#172	GO:0005524: ATP binding	1.25E-10	182,243,44
1qs0a1#119	GO:0005524: ATP binding	1.25E-10	454,243,79
3lada2#167	GO:0015036: disulfide oxidoreductase activity	1.26E-10	6,22,5
1jmta_#93	GO:0003729: mRNA binding	1.29E-10	9,13,5
1dih_2#235	GO:0004263: chymotrypsin activity	1.29E-10	54,41,11
1eq9a_#58	GO:0004867: serine-type endopeptidase inhibitor activity	1.29E-10	47,47,11
1jb0d_#26	GO:0004896: hematopoietin/interferon-class (D200-domain) cytokine receptor activity	1.30E-10	229,19,13
1jj2v_#118	GO:0003809: thrombin activity	1.35E-10	103,10,8
1fjfj_#68	GO:0004896: hematopoietin/interferon-class (D200-domain) cytokine receptor activity	1.37E-10	230,19,13
1b3qa2#556	GO:0005524: ATP binding	1.38E-10	40,243,19
1dt6a_#385	GO:0005507: copper ion binding	1.39E-10	76,38,12
1ytba1#125	GO:0016251: general RNA polymerase II transcription factor activity	1.39E-10	19,14,6
1bjt__#890	GO:0004896: hematopoietin/interferon-class (D200-domain) cytokine receptor activity	1.39E-10	292,19,14
1apme_#68	GO:0004674: protein serine/threonine kinase activity	1.44E-10	105,42,14
1d2na_#666	GO:0005524: ATP binding	1.52E-10	13,243,11
1el0a_#42	GO:0004896: hematopoietin/interferon-class (D200-domain) cytokine receptor activity	1.53E-10	294,19,14
1e6pa2#46	GO:0016831: carboxy-lyase activity	1.54E-10	95,25,11
1qqsa_#124	GO:0004556: alpha-amylase activity	1.54E-10	458,15,14
1qs1a2#437	GO:0004896: hematopoietin/interferon-class (D200-domain) cytokine receptor activity	1.55E-10	452,19,16
1eq9a_#32	GO:0004867: serine-type endopeptidase inhibitor activity	1.56E-10	132,47,16
1opy__#68	GO:0004896: hematopoietin/interferon-class (D200-domain) cytokine receptor activity	1.56E-10	367,19,15
1lvl_2#222	GO:0016668: oxidoreductase activity, acting on sulfur group of donors, NAD or NADP as acceptor	1.58E-10	79,12,8
1dofa_#15	GO:0005524: ATP binding	1.58E-10	197,243,46
2hlca_#139	GO:0004867: serine-type endopeptidase inhibitor activity	1.58E-10	112,47,15
2cmd_1#113	GO:0004457: lactate dehydrogenase activity	1.58E-10	12,10,5
3lada3#380	GO:0016668: oxidoreductase activity, acting on sulfur group of donors, NAD or NADP as acceptor	1.58E-10	10,12,5
1h4vb2#282	GO:0005524: ATP binding	1.62E-10	101,243,31
1ejda_#379	GO:0016616: oxidoreductase activity, acting on the CH-OH group of donors, NAD or NADP as acceptor	1.66E-10	49,59,12
1dik_3#240	GO:0004674: protein serine/threonine kinase activity	1.68E-10	262,42,20
1ghpa_#245	GO:0008800: beta-lactamase activity	1.71E-10	29,10,6
1p35a_#101	GO:0004896: hematopoietin/interferon-class (D200-domain) cytokine receptor activity	1.73E-10	136,19,11
1fgga_#134	GO:0005525: GTP binding	1.74E-10	218,49,20
1wba__#169	GO:0008083: growth factor activity	1.74E-10	20,42,8
1ihka_#164	GO:0008083: growth factor activity	1.74E-10	20,42,8
1hyha2#205	GO:0016616: oxidoreductase activity, acting on the CH-OH group of donors, NAD or NADP as acceptor	1.77E-10	10,59,7
1gdha1#237	GO:0016616: oxidoreductase activity, acting on the CH-OH group of donors, NAD or NADP as acceptor	1.77E-10	10,59,7
1cex__#109	GO:0005525: GTP binding	1.77E-10	35,49,10
1mpp__#154	GO:0004896: hematopoietin/interferon-class (D200-domain) cytokine receptor activity	1.79E-10	99,19,10
1iow_1#37	GO:0005524: ATP binding	1.80E-10	309,243,61
1b35b_#97	GO:0005524: ATP binding	1.80E-10	309,243,61
1danh_#93	GO:0005509: calcium ion binding	1.86E-10	17,160,11
1eh9a3#252	GO:0005509: calcium ion binding	1.86E-10	17,160,11
1ib2a_#974	GO:0004364: glutathione transferase activity	1.90E-10	52,11,7
1i9ga_#67	GO:0004295: trypsin activity	1.93E-10	61,48,12
1qvba_#9	GO:0004556: alpha-amylase activity	1.96E-10	60,15,8
1ja9a_#202	GO:0016616: oxidoreductase activity, acting on the CH-OH group of donors, NAD or NADP as acceptor	1.96E-10	39,59,11

1fgka_#572	GO:0004674: protein serine/threonine kinase activity	1.97E-10	13,42,7
1ilr1_#32	GO:0008083: growth factor activity	1.97E-10	13,42,7
1fjfl_#67	GO:0004295: trypsin activity	1.97E-10	175,48,18
1g6oa_#116	GO:0005524: ATP binding	2.12E-10	358,243,67
1qnja_#221	GO:0004867: serine-type endopeptidase inhibitor activity	2.13E-10	12,47,7
1seta2#306	GO:0003809: thrombin activity	2.14E-10	30,10,6
1fjsa_#83	GO:0003809: thrombin activity	2.14E-10	109,10,8
1qora2#201	GO:0016616: oxidoreductase activity, acting on the CH-OH group of donors, NAD or NADP as acceptor	2.15E-10	199,59,21
1cqka_#52	GO:0004896: hematopoietin/interferon-class (D200-domain) cytokine receptor activity	2.17E-10	70,19,9
1fsu__#434	GO:0004556: alpha-amylase activity	2.18E-10	356,15,13
1nat__#50	GO:0015036: disulfide oxidoreductase activity	2.19E-10	297,22,15
1gdea_#243	GO:0016846: carbon-sulfur lyase activity	2.26E-10	61,10,7
1e39a3#442	GO:0004263: chymotrypsin activity	2.26E-10	21,41,8
1avgi_#69	GO:0005507: copper ion binding	2.26E-10	340,38,21
1feca3#467	GO:0000287: magnesium ion binding	2.27E-10	89,128,21
1csn__#84	GO:0004674: protein serine/threonine kinase activity	2.39E-10	207,42,18
1f42a2#123	GO:0004896: hematopoietin/interferon-class (D200-domain) cytokine receptor activity	2.41E-10	378,19,15
1c8ba_#514	GO:0005525: GTP binding	2.42E-10	36,49,10
2cb5a_#312	GO:0003809: thrombin activity	2.49E-10	111,10,8
1t7pa2#513	GO:0004812: tRNA ligase activity	2.51E-10	33,26,8
1qfea_#68	GO:0016831: carboxy-lyase activity	2.54E-10	560,25,20
1je5a_#10	GO:0004457: lactate dehydrogenase activity	2.55E-10	62,10,7
1ezia_#198	GO:0016846: carbon-sulfur lyase activity	2.57E-10	13,10,5
1ei5a3#190	GO:0008800: beta-lactamase activity	2.57E-10	13,10,5
1trb_1#42	GO:0050660: FAD binding	2.57E-10	13,10,5
1gox__#246	GO:0016763: transferase activity, transferring pentosyl groups	2.59E-10	177,28,14
1qb7a_#86	GO:0016616: oxidoreductase activity, acting on the CH-OH group of donors, NAD or NADP as acceptor	2.66E-10	40,59,11
1e7wa_#8	GO:0005525: GTP binding	2.71E-10	111,49,15
1a6o__#233	GO:0005524: ATP binding	2.72E-10	11,243,10
1atia2#189	GO:0005524: ATP binding	2.72E-10	11,243,10
1p35a_#101	GO:0019838: growth factor binding	2.72E-10	136,9,8
1iow_1#37	GO:0016776: phosphotransferase activity, phosphate group as acceptor	2.74E-10	309,14,12
1qi7a_#224	GO:0016799: hydrolase activity, hydrolyzing N-glycosyl compounds	2.75E-10	8,17,5
1gtra2#173	GO:0004812: tRNA ligase activity	2.75E-10	50,26,9
1hle.1#A228	GO:0004867: serine-type endopeptidase inhibitor activity	2.76E-10	19,47,8
1qq4a_#46	GO:0004867: serine-type endopeptidase inhibitor activity	2.76E-10	19,47,8
1dlfl_#97	GO:0019838: growth factor binding	2.81E-10	36,9,6
1iq4a_#31	GO:0003887: DNA-directed DNA polymerase activity	2.88E-10	27,20,7
1fyhb1#99	GO:0005126: hematopoietin/interferon-class (D200-domain) cytokine receptor binding	2.88E-10	27,20,7
2sli_2#326	GO:0005524: ATP binding	2.88E-10	251,243,53
1dpja_#322	GO:0004896: hematopoietin/interferon-class (D200-domain) cytokine receptor activity	2.90E-10	308,19,14
1qtn.1#A270	GO:0005525: GTP binding	2.92E-10	439,49,27
1dih_2#222	GO:0003887: DNA-directed DNA polymerase activity	2.98E-10	352,20,15
1e8ca3#111	GO:0000287: magnesium ion binding	2.99E-10	280,128,38
1phm_2#253	GO:0004896: hematopoietin/interferon-class (D200-domain) cytokine receptor activity	3.03E-10	309,19,14
2hlca_#226	GO:0004867: serine-type endopeptidase inhibitor activity	3.05E-10	98,47,14
1b6cb_#346	GO:0005524: ATP binding	3.11E-10	9,243,9
1pme__#207	GO:0005524: ATP binding	3.11E-10	9,243,9
1b6cb_#355	GO:0005524: ATP binding	3.11E-10	9,243,9
1jmta_#133	GO:0003729: mRNA binding	3.12E-10	4,13,4
1h6kx_#111	GO:0003729: mRNA binding	3.12E-10	4,13,4
1b3ra2#47	GO:0005525: GTP binding	3.28E-10	37,49,10
1d0na3#305	GO:0004896: hematopoietin/interferon-class (D200-domain) cytokine receptor activity	3.31E-10	311,19,14
1fbna_#144	GO:0008757: S-adenosylmethionine-dependent methyltransferase activity	3.33E-10	79,24,10
1dkia_#185	GO:0004197: cysteine-type endopeptidase activity	3.33E-10	79,24,10
1dxea_#71	GO:0004556: alpha-amylase activity	3.41E-10	38,15,7
1im4a_#144	GO:0000287: magnesium ion binding	3.41E-10	129,128,25
1f97a1#62	GO:0004896: hematopoietin/interferon-class (D200-domain) cytokine receptor activity	3.48E-10	707,19,18
3grs_2#211	GO:0016616: oxidoreductase activity, acting on the CH-OH group of donors, NAD or NADP as acceptor	3.54E-10	52,59,12
1e5ka_#113	GO:0003809: thrombin activity	3.59E-10	65,10,7
1fmja_#76	GO:0003924: GTPase activity	3.62E-10	85,17,9
1qksa2#236	GO:0005507: copper ion binding	3.63E-10	64,38,11
1pwt__#8	GO:0005524: ATP binding	3.65E-10	314,243,61

1ddja_#754	GO:0005509: calcium ion binding	3.68E-10	31,160,14
1e2o__#346	GO:0003809: thrombin activity	3.70E-10	194,10,9
1pwt__#8	GO:0004896: hematopoietin/interferon-class (D200-domain) cytokine receptor activity	3.78E-10	314,19,14
1brwa2#100	GO:0015036: disulfide oxidoreductase activity	3.80E-10	14,22,6
1cs1a_#82	GO:0016846: carbon-sulfur lyase activity	3.88E-10	195,10,9
1j79a_#187	GO:0015036: disulfide oxidoreductase activity	3.89E-10	89,22,10
1imva_#255	GO:0005524: ATP binding	3.94E-10	589,243,93
1nsj__#124	GO:0004556: alpha-amylase activity	3.94E-10	148,15,10
1a65a3#464	GO:0005507: copper ion binding	3.98E-10	9,38,6
1fuma2#370	GO:0050660: FAD binding	3.99E-10	14,10,5
1el5a1#183	GO:0050660: FAD binding	3.99E-10	14,10,5
1kit_3#743	GO:0004896: hematopoietin/interferon-class (D200-domain) cytokine receptor activity	3.99E-10	250,19,13
1aoza3#507	GO:0005507: copper ion binding	4.02E-10	5,38,5
1kit_3#743	GO:0019838: growth factor binding	4.03E-10	250,9,9
1axca1#70	GO:0003809: thrombin activity	4.10E-10	118,10,8
1ewna_#109	GO:0004896: hematopoietin/interferon-class (D200-domain) cytokine receptor activity	4.13E-10	75,19,9
2sli_2#326	GO:0004896: hematopoietin/interferon-class (D200-domain) cytokine receptor activity	4.20E-10	251,19,13
1feca3#467	GO:0016668: oxidoreductase activity, acting on sulfur group of donors, NAD or NADP as acceptor	4.21E-10	89,12,8
1g71a_#88	GO:0004867: serine-type endopeptidase inhibitor activity	4.21E-10	52,47,11
1elva1#623	GO:0004867: serine-type endopeptidase inhibitor activity	4.21E-10	52,47,11
1gpea1#271	GO:0050660: FAD binding	4.26E-10	197,10,9
1i1b__#10	GO:0008083: growth factor activity	4.32E-10	22,42,8
1bio__#88	GO:0005509: calcium ion binding	4.35E-10	22,160,12
1aym2_#253	GO:0003968: RNA-directed RNA polymerase activity	4.37E-10	4,14,4
2mev1_#246	GO:0003968: RNA-directed RNA polymerase activity	4.37E-10	4,14,4
2mev1_#120	GO:0003968: RNA-directed RNA polymerase activity	4.37E-10	4,14,4
1b35b_#123	GO:0003968: RNA-directed RNA polymerase activity	4.37E-10	4,14,4
1f97a1#62	GO:0004295: trypsin activity	4.40E-10	707,48,33
1dgsa1#523	GO:0005509: calcium ion binding	4.42E-10	48,160,17
1d6ja_#95	GO:0050660: FAD binding	4.48E-10	67,10,7
3grs_2#211	GO:0016668: oxidoreductase activity, acting on sulfur group of donors, NAD or NADP as acceptor	4.48E-10	52,12,7
1ovaa_#297	GO:0004867: serine-type endopeptidase inhibitor activity	4.55E-10	13,47,7
1danh_#48	GO:0004867: serine-type endopeptidase inhibitor activity	4.55E-10	20,47,8
1hx0a2#294	GO:0005509: calcium ion binding	4.55E-10	18,160,11
1hd2a_#35	GO:0000287: magnesium ion binding	4.56E-10	185,128,30
1phm_2#347	GO:0008800: beta-lactamase activity	4.57E-10	5,10,4
1fwxa1#535	GO:0015078: hydrogen ion transporter activity	4.60E-10	15,21,6
1b6e__#82	GO:0005529: sugar binding	4.62E-10	5,39,5
1hwxa1#256	GO:0016638: oxidoreductase activity, acting on the CH-NH2 group of donors	4.65E-10	57,17,8
1dfoa_#362	GO:0008483: transaminase activity	4.65E-10	57,17,8
1befa_#51	GO:0004867: serine-type endopeptidase inhibitor activity	4.70E-10	29,47,9
1dpja_#322	GO:0005524: ATP binding	4.74E-10	308,243,60
1cyda_#204	GO:0016616: oxidoreductase activity, acting on the CH-OH group of donors, NAD or NADP as acceptor	4.79E-10	11,59,7
1bgva1#326	GO:0016616: oxidoreductase activity, acting on the CH-OH group of donors, NAD or NADP as acceptor	4.79E-10	11,59,7
1i6vd_#1283	GO:0004263: chymotrypsin activity	4.87E-10	141,41,15
1qdla_#211	GO:0004896: hematopoietin/interferon-class (D200-domain) cytokine receptor activity	4.90E-10	320,19,14
1fmta2#151	GO:0015036: disulfide oxidoreductase activity	4.93E-10	314,22,15
1dc1a_#241	GO:0009036: type II site-specific deoxyribonuclease activity	4.95E-10	12,12,5
1qh4a2#350	GO:0000287: magnesium ion binding	5.06E-10	67,128,18
1t7pa2#513	GO:0003700: transcription factor activity	5.08E-10	33,124,13
1vdra_#37	GO:0016646: oxidoreductase activity, acting on the CH-NH group of donors, NAD or NADP as acceptor	5.15E-10	8,19,5
2ltn.1#A63	GO:0004556: alpha-amylase activity	5.16E-10	152,15,10
1g25a_#22	GO:0005524: ATP binding	5.24E-10	415,243,73
1i9ga_#67	GO:0004263: chymotrypsin activity	5.24E-10	61,41,11
1jlxa2#166	GO:0004896: hematopoietin/interferon-class (D200-domain) cytokine receptor activity	5.27E-10	77,19,9
1aoea_#130	GO:0016646: oxidoreductase activity, acting on the CH-NH group of donors, NAD or NADP as acceptor	5.27E-10	77,19,9
1qfma1#186	GO:0050660: FAD binding	5.31E-10	326,10,10
1qj8a_#64	GO:0004867: serine-type endopeptidase inhibitor activity	5.33E-10	84,47,13
1ac6a_#34	GO:0019955: cytokine binding	5.42E-10	60,11,7
1a65a3#383	GO:0005507: copper ion binding	5.43E-10	50,38,10

1hyha2#211	GO:0016616: oxidoreductase activity, acting on the CH-OH group of donors, NAD or NADP as acceptor	5.48E-10	7,59,6
1bdb__#144	GO:0016616: oxidoreductase activity, acting on the CH-OH group of donors, NAD or NADP as acceptor	5.48E-10	7,59,6
1doka_#52	GO:0004896: hematopoietin/interferon-class (D200-domain) cytokine receptor activity	5.52E-10	51,19,8
1dpja_#265	GO:0004190: aspartic-type endopeptidase activity	5.60E-10	7,23,5
1dar_2#264	GO:0003924: GTPase activity	5.67E-10	19,17,6
8dfr__#9	GO:0016646: oxidoreductase activity, acting on the CH-NH group of donors, NAD or NADP as acceptor	5.71E-10	111,19,10
1a9xa3#78	GO:0016616: oxidoreductase activity, acting on the CH-OH group of donors, NAD or NADP as acceptor	5.72E-10	305,59,25
1e0ta2#275	GO:0016831: carboxy-lyase activity	5.76E-10	107,25,11
1eu1a2#185	GO:0030151: molybdenum ion binding	5.95E-10	4,15,4
1uok_2#97	GO:0004556: alpha-amylase activity	5.95E-10	4,15,4
1ypta_#408	GO:0004725: protein tyrosine phosphatase activity	5.95E-10	4,15,4
1p35a_#49	GO:0003809: thrombin activity	6.14E-10	124,10,8
2hlpa2#289	GO:0016616: oxidoreductase activity, acting on the CH-OH group of donors, NAD or NADP as acceptor	6.21E-10	17,59,8
1a8i__#688	GO:0005524: ATP binding	6.26E-10	241,243,51
1a4ya_#47	GO:0004556: alpha-amylase activity	6.26E-10	216,15,11
1f97a1#62	GO:0004674: protein serine/threonine kinase activity	6.49E-10	707,42,30
1e4ea2#210	GO:0005524: ATP binding	6.55E-10	14,243,11
1fs7a_#235	GO:0008083: growth factor activity	6.55E-10	23,42,8
1xnb__#149	GO:0000287: magnesium ion binding	6.61E-10	165,128,28
1dik_1#765	GO:0000287: magnesium ion binding	6.61E-10	165,128,28
1qfma1#186	GO:0016668: oxidoreductase activity, acting on sulfur group of donors, NAD or NADP as acceptor	6.63E-10	326,12,11
1dpsa_#36	GO:0005524: ATP binding	6.70E-10	205,243,46
1j9la_#82	GO:0016616: oxidoreductase activity, acting on the CH-OH group of donors, NAD or NADP as acceptor	6.77E-10	169,59,19
1cjca2#37	GO:0008757: S-adenosylmethionine-dependent methyltransferase activity	6.86E-10	25,24,7
1fo4a5#1020	GO:0005524: ATP binding	6.91E-10	359,243,66
1c4ra_#258	GO:0004263: chymotrypsin activity	7.05E-10	80,41,12
1iba__#39	GO:0005524: ATP binding	7.06E-10	351,243,65
3sil__#98	GO:0005509: calcium ion binding	7.17E-10	228,160,38
1ihua2#525	GO:0016776: phosphotransferase activity, phosphate group as acceptor	7.21E-10	335,14,12
1qsta_#154	GO:0005524: ATP binding	7.31E-10	20,243,13
1ppn__#132	GO:0004197: cysteine-type endopeptidase activity	7.36E-10	234,24,14
1smaa2#581	GO:0004896: hematopoietin/interferon-class (D200-domain) cytokine receptor activity	7.49E-10	80,19,9
1jg8a_#172	GO:0008483: transaminase activity	7.50E-10	92,17,9
1pme__#207	GO:0004674: protein serine/threonine kinase activity	7.53E-10	9,42,6
1c7na_#231	GO:0016646: oxidoreductase activity, acting on the CH-NH group of donors, NAD or NADP as acceptor	7.61E-10	263,19,13
1g5ha2#162	GO:0005524: ATP binding	7.78E-10	27,243,15
1h7wa4#208	GO:0016668: oxidoreductase activity, acting on sulfur group of donors, NAD or NADP as acceptor	8.03E-10	13,12,5
1dih_2#235	GO:0004295: trypsin activity	8.31E-10	54,48,11
1bpv__#22	GO:0019838: growth factor binding	8.52E-10	18,9,5
1aq0a_#4	GO:0005509: calcium ion binding	8.63E-10	23,160,12
1e39a3#442	GO:0004295: trypsin activity	8.67E-10	21,48,8
1aba__#17	GO:0004364: glutathione transferase activity	8.67E-10	33,11,6
1lkka_#127	GO:0005066: transmembrane receptor protein tyrosine kinase signaling protein activity	8.68E-10	16,10,5
1hwxa1#256	GO:0016668: oxidoreductase activity, acting on sulfur group of donors, NAD or NADP as acceptor	8.79E-10	57,12,7
1erv__#57	GO:0019201: nucleotide kinase activity	8.84E-10	87,13,8
1as4.1#A79	GO:0004867: serine-type endopeptidase inhibitor activity	8.97E-10	14,47,7
2cuaa_#121	GO:0015078: hydrogen ion transporter activity	9.00E-10	8,21,5
1e3ja2#270	GO:0004457: lactate dehydrogenase activity	9.21E-10	74,10,7
1i50a_#856	GO:0015036: disulfide oxidoreductase activity	9.38E-10	269,22,14
1qhda2#323	GO:0005524: ATP binding	9.41E-10	337,243,63
1p35a_#49	GO:0004263: chymotrypsin activity	9.77E-10	124,41,14
1aym3_#131	GO:0005524: ATP binding	9.97E-10	412,243,72
1bio__#210	GO:0004867: serine-type endopeptidase inhibitor activity	1.00E-09	149,47,16
1c4zd_#51	GO:0004896: hematopoietin/interferon-class (D200-domain) cytokine receptor activity	1.03E-09	160,19,11
1bxoa_#320	GO:0004190: aspartic-type endopeptidase activity	1.03E-09	66,23,9
1oaca1#438	GO:0016638: oxidoreductase activity, acting on the CH-NH2 group of donors	1.04E-09	4,17,4

1g51a3#524	GO:0005524: ATP binding	1.06E-09	102,243,30
1lvl_2#262	GO:0016668: oxidoreductase activity, acting on sulfur group of donors, NAD or NADP as acceptor	1.08E-09	5,12,4
1cpt__#325	GO:0016705: oxidoreductase activity, acting on paired donors, with incorporation or reduction of molecular oxygen	1.09E-09	7,26,5
1f7ua2#293	GO:0004812: tRNA ligase activity	1.09E-09	7,26,5
1qf6a4#304	GO:0004812: tRNA ligase activity	1.09E-09	7,26,5
1fgxa_#242	GO:0016616: oxidoreductase activity, acting on the CH-OH group of donors, NAD or NADP as acceptor	1.10E-09	18,59,8
1bfd_1#230	GO:0015036: disulfide oxidoreductase activity	1.13E-09	47,22,8
1cyda_#230	GO:0016616: oxidoreductase activity, acting on the CH-OH group of donors, NAD or NADP as acceptor	1.13E-09	12,59,7
1jeyb_#231	GO:0004725: protein tyrosine phosphatase activity	1.13E-09	24,15,6
2bb2_2#134	GO:0015036: disulfide oxidoreductase activity	1.14E-09	220,22,13
1cnv__#181	GO:0005524: ATP binding	1.16E-09	74,243,25
1d4xg_#60	GO:0004364: glutathione transferase activity	1.18E-09	115,11,8
1bqca_#24	GO:0004556: alpha-amylase activity	1.20E-09	45,15,7
1dr9a2#139	GO:0019955: cytokine binding	1.21E-09	67,11,7
1a4ya_#104	GO:0016616: oxidoreductase activity, acting on the CH-OH group of donors, NAD or NADP as acceptor	1.22E-09	241,59,22
1fzqa_#28	GO:0016776: phosphotransferase activity, phosphate group as acceptor	1.22E-09	49,14,7
1b8aa2#251	GO:0005524: ATP binding	1.22E-09	24,243,14
1elua_#95	GO:0016846: carbon-sulfur lyase activity	1.23E-09	77,10,7
1sek__#99	GO:0004867: serine-type endopeptidase inhibitor activity	1.23E-09	5,47,5
1a7ca_#92	GO:0004867: serine-type endopeptidase inhibitor activity	1.23E-09	5,47,5
1sek__#271	GO:0004867: serine-type endopeptidase inhibitor activity	1.23E-09	5,47,5
1afj__#28	GO:0015082: di-, tri-valent inorganic cation transporter activity	1.25E-09	12,14,5
1afj__#28	GO:0046915: transition metal ion transporter activity	1.25E-09	12,14,5
1epwa3#138	GO:0004896: hematopoietin/interferon-class (D200-domain) cytokine receptor activity	1.29E-09	424,19,15
1ce7a_#9	GO:0019201: nucleotide kinase activity	1.30E-09	13,13,5
1qqga2#253	GO:0004812: tRNA ligase activity	1.31E-09	110,26,11
1leha1#226	GO:0016616: oxidoreductase activity, acting on the CH-OH group of donors, NAD or NADP as acceptor	1.31E-09	26,59,9
1qq5a_#7	GO:0005524: ATP binding	1.36E-09	167,243,40
1a4sa_#364	GO:0016620: oxidoreductase activity, acting on the aldehyde or oxo group of donors, NAD or NADP as acceptor	1.37E-09	6,10,4
1d2fa_#234	GO:0016846: carbon-sulfur lyase activity	1.37E-09	6,10,4
1czan1#92	GO:0004896: hematopoietin/interferon-class (D200-domain) cytokine receptor activity	1.41E-09	631,19,17
1lvl_2#222	GO:0050660: FAD binding	1.48E-09	79,10,7
1epwa3#138	GO:0005524: ATP binding	1.49E-09	424,243,73
1f0c.1#A72	GO:0004867: serine-type endopeptidase inhibitor activity	1.53E-09	9,47,6
1egja_#346	GO:0019838: growth factor binding	1.54E-09	20,9,5
1chma2#240	GO:0004177: aminopeptidase activity	1.56E-09	5,13,4
1c22a_#32	GO:0004177: aminopeptidase activity	1.56E-09	5,13,4
1qj5a_#268	GO:0008483: transaminase activity	1.57E-09	66,17,8
1qr4a2#166	GO:0019955: cytokine binding	1.59E-09	16,11,5
1fnf_1#1146	GO:0019955: cytokine binding	1.59E-09	16,11,5
1ihna_#64	GO:0004263: chymotrypsin activity	1.60E-09	10,41,6
1mkp__#283	GO:0015036: disulfide oxidoreductase activity	1.60E-09	49,22,8
1bio__#210	GO:0004674: protein serine/threonine kinase activity	1.60E-09	149,42,15
3sil__#98	GO:0003809: thrombin activity	1.61E-09	228,10,9
1i8aa_#2	GO:0005507: copper ion binding	1.64E-09	18,38,7
1bjt__#890	GO:0019838: growth factor binding	1.67E-09	292,9,9
8dfr__#151	GO:0016646: oxidoreductase activity, acting on the CH-NH group of donors, NAD or NADP as acceptor	1.69E-09	4,19,4
1egja_#346	GO:0004896: hematopoietin/interferon-class (D200-domain) cytokine receptor activity	1.76E-09	20,19,6
1gpea1#273	GO:0050660: FAD binding	1.77E-09	81,10,7
1ihua2#525	GO:0003924: GTPase activity	1.78E-09	335,17,13
1f21a_#68	GO:0004523: ribonuclease H activity	1.79E-09	44,16,7
1qqp1_#51	GO:0015036: disulfide oxidoreductase activity	1.82E-09	31,22,7
1befa_#51	GO:0005509: calcium ion binding	1.83E-09	29,160,13
1f97a1#62	GO:0004263: chymotrypsin activity	1.84E-09	707,41,29
1dj0a2#183	GO:0004457: lactate dehydrogenase activity	1.85E-09	142,10,8
1hwxa1#223	GO:0005524: ATP binding	1.85E-09	233,243,49
1dqga_#51	GO:0008083: growth factor activity	1.86E-09	10,42,6
1ppn__#132	GO:0004674: protein serine/threonine kinase activity	1.87E-09	234,42,18

1el5a1#166	GO:0016668: oxidoreductase activity, acting on sulfur group of donors, NAD or NADP as acceptor	1.87E-09	15,12,5
1im4a_#41	GO:0004896: hematopoietin/interferon-class (D200-domain) cytokine receptor activity	1.87E-09	59,19,8
1dpja_#322	GO:0004263: chymotrypsin activity	1.88E-09	308,41,20
1lvl_2#222	GO:0004180: carboxypeptidase activity	1.91E-09	79,15,8
1ton__#47	GO:0004867: serine-type endopeptidase inhibitor activity	1.91E-09	112,47,14
1hbza_#96	GO:0003809: thrombin activity	1.92E-09	370,10,10
1llda2#161	GO:0016616: oxidoreductase activity, acting on the CH-OH group of donors, NAD or NADP as acceptor	1.93E-09	27,59,9
1je5a_#106	GO:0030151: molybdenum ion binding	1.94E-09	48,15,7
1ds1a_#172	GO:0004896: hematopoietin/interferon-class (D200-domain) cytokine receptor activity	1.94E-09	222,19,12
1mpp__#154	GO:0004190: aspartic-type endopeptidase activity	1.97E-09	99,23,10
1foha5#9	GO:0005524: ATP binding	1.98E-09	65,243,23
1prea1#50	GO:0005529: sugar binding	1.99E-09	18,39,7
1erv__#57	GO:0016776: phosphotransferase activity, phosphate group as acceptor	2.01E-09	87,14,8
1ce7a_#9	GO:0016776: phosphotransferase activity, phosphate group as acceptor	2.02E-09	13,14,5
2dpma_#237	GO:0005507: copper ion binding	2.05E-09	95,38,12
1qgna_#149	GO:0008483: transaminase activity	2.08E-09	23,17,6
1eh9a1#33	GO:0004896: hematopoietin/interferon-class (D200-domain) cytokine receptor activity	2.10E-09	37,19,7
1epwa3#248	GO:0004896: hematopoietin/interferon-class (D200-domain) cytokine receptor activity	2.10E-09	37,19,7
1hzia_#51	GO:0005126: hematopoietin/interferon-class (D200-domain) cytokine receptor binding	2.11E-09	4,20,4
1az9_2#257	GO:0008235: metalloexopeptidase activity	2.11E-09	31,13,6
1dn2a1#261	GO:0004556: alpha-amylase activity	2.14E-09	425,15,13
1iow_1#37	GO:0019201: nucleotide kinase activity	2.14E-09	309,13,11
1fp5a2#537	GO:0019955: cytokine binding	2.14E-09	38,11,6
1aym2_#185	GO:0003724: RNA helicase activity	2.15E-09	6,11,4
1bev1_#245	GO:0003968: RNA-directed RNA polymerase activity	2.18E-09	5,14,4
1b35b_#140	GO:0003968: RNA-directed RNA polymerase activity	2.18E-09	5,14,4
1qnja_#77	GO:0005509: calcium ion binding	2.18E-09	16,160,10
1ctqa_#163	GO:0005525: GTP binding	2.26E-09	15,49,7
1jb3a_#64	GO:0019838: growth factor binding	2.27E-09	302,9,9
1dbxa_#75	GO:0003924: GTPase activity	2.27E-09	69,17,8
1cpza_#6	GO:0004263: chymotrypsin activity	2.28E-09	157,41,15
1fmca_#89	GO:0016616: oxidoreductase activity, acting on the CH-OH group of donors, NAD or NADP as acceptor	2.28E-09	48,59,11
1b9ha_#276	GO:0016846: carbon-sulfur lyase activity	2.30E-09	19,10,5
1f0ia1#50	GO:0003809: thrombin activity	2.32E-09	146,10,8
1dqga_#81	GO:0008083: growth factor activity	2.34E-09	38,42,9
1f24a_#144	GO:0004497: monooxygenase activity	2.35E-09	27,26,7
1ad1a_#186	GO:0000287: magnesium ion binding	2.38E-09	24,128,11
1aoza1#115	GO:0005507: copper ion binding	2.39E-09	6,38,5
1fwxa1#526	GO:0015078: hydrogen ion transporter activity	2.45E-09	19,21,6
1bfg__#22	GO:0004896: hematopoietin/interferon-class (D200-domain) cytokine receptor activity	2.46E-09	61,19,8
1bjt__#890	GO:0005509: calcium ion binding	2.47E-09	292,160,43
1ovaa_#161	GO:0004867: serine-type endopeptidase inhibitor activity	2.52E-09	24,47,8
1qora2#201	GO:0005525: GTP binding	2.53E-09	199,49,18
1cf9a1#736	GO:0003924: GTPase activity	2.54E-09	43,17,7
1danh_#104	GO:0004867: serine-type endopeptidase inhibitor activity	2.56E-09	183,47,17
1qfea_#68	GO:0004556: alpha-amylase activity	2.56E-09	560,15,14
1qb7a_#86	GO:0016763: transferase activity, transferring pentosyl groups	2.59E-09	40,28,8
1ass__#33	GO:0015036: disulfide oxidoreductase activity	2.60E-09	9,22,5
1xgsa2#82	GO:0019838: growth factor binding	2.63E-09	307,9,9
1ddja_#726	GO:0005509: calcium ion binding	2.64E-09	105,160,24
1tiid_#39	GO:0004674: protein serine/threonine kinase activity	2.65E-09	239,42,18
2mev3_#108	GO:0004896: hematopoietin/interferon-class (D200-domain) cytokine receptor activity	2.65E-09	228,19,12
1tuba1#8	GO:0016616: oxidoreductase activity, acting on the CH-OH group of donors, NAD or NADP as acceptor	2.70E-09	124,59,16
1dpja_#322	GO:0019838: growth factor binding	2.71E-09	308,9,9
1fu6a_#50	GO:0019838: growth factor binding	2.71E-09	308,9,9
1h8ua_#110	GO:0005529: sugar binding	2.74E-09	6,39,5
1bio__#210	GO:0005509: calcium ion binding	2.76E-09	149,160,29
1a7ca_#370	GO:0004867: serine-type endopeptidase inhibitor activity	2.76E-09	47,47,10
1hdca_#126	GO:0016616: oxidoreductase activity, acting on the CH-OH group of donors, NAD or NADP as acceptor	2.80E-09	28,59,9
1ddwa_#77	GO:0004896: hematopoietin/interferon-class (D200-domain) cytokine receptor activity	2.83E-09	364,19,14
1i2oa_#34	GO:0005524: ATP binding	2.87E-09	192,243,43

1eq2a_#11	GO:0004457: lactate dehydrogenase activity	2.88E-09	45,10,6
1a6da3#190	GO:0004263: chymotrypsin activity	2.89E-09	18,41,7
1eupa_#320	GO:0016705: oxidoreductase activity, acting on paired donors, with incorporation or reduction of molecular oxygen	2.90E-09	8,26,5
1b8aa1#41	GO:0004812: tRNA ligase activity	2.90E-09	8,26,5
1qhqa_#45	GO:0005507: copper ion binding	2.90E-09	121,38,13
1ckea_#35	GO:0005524: ATP binding	2.91E-09	33,243,16
1ihua2#343	GO:0004812: tRNA ligase activity	2.92E-09	233,26,14
1el5a2#302	GO:0004180: carboxypeptidase activity	2.97E-09	5,15,4
1cnv__#181	GO:0000287: magnesium ion binding	2.97E-09	74,128,18
1nsj__#83	GO:0016861: intramolecular oxidoreductase activity, interconverting aldoses and ketoses	3.02E-09	15,13,5
1bura1#328	GO:0016616: oxidoreductase activity, acting on the CH-OH group of donors, NAD or NADP as acceptor	3.05E-09	91,59,14
1ypta_#418	GO:0016616: oxidoreductase activity, acting on the CH-OH group of donors, NAD or NADP as acceptor	3.06E-09	20,59,8
1b16a_#62	GO:0016616: oxidoreductase activity, acting on the CH-OH group of donors, NAD or NADP as acceptor	3.06E-09	20,59,8
2foka2#180	GO:0003700: transcription factor activity	3.07E-09	147,124,25
1eq9a_#162	GO:0004867: serine-type endopeptidase inhibitor activity	3.07E-09	35,47,9
1f00i2#805	GO:0004263: chymotrypsin activity	3.08E-09	135,41,14
1ej8a_#189	GO:0004896: hematopoietin/interferon-class (D200-domain) cytokine receptor activity	3.12E-09	294,19,13
1a65a1#108	GO:0004896: hematopoietin/interferon-class (D200-domain) cytokine receptor activity	3.12E-09	294,19,13
1qs0a1#235	GO:0016668: oxidoreductase activity, acting on sulfur group of donors, NAD or NADP as acceptor	3.17E-09	114,12,8
1qisa_#258	GO:0016846: carbon-sulfur lyase activity	3.19E-09	7,10,4
1bw0a_#252	GO:0016846: carbon-sulfur lyase activity	3.19E-09	7,10,4
1gox__#246	GO:0005525: GTP binding	3.19E-09	177,49,17
1flga_#161	GO:0003809: thrombin activity	3.21E-09	152,10,8
1bxoa_#307	GO:0004190: aspartic-type endopeptidase activity	3.22E-09	104,23,10
1b37a1#209	GO:0015036: disulfide oxidoreductase activity	3.35E-09	19,22,6
1kapp2#149	GO:0004222: metalloendopeptidase activity	3.35E-09	22,19,6
3lada1#116	GO:0050660: FAD binding	3.38E-09	153,10,8
2duba_#212	GO:0019201: nucleotide kinase activity	3.45E-09	62,13,7
1feca3#467	GO:0050660: FAD binding	3.47E-09	89,10,7
1d3bb_#31	GO:0030151: molybdenum ion binding	3.49E-09	52,15,7
1bywa_#30	GO:0005524: ATP binding	3.49E-09	331,243,61
1hbza_#214	GO:0004896: hematopoietin/interferon-class (D200-domain) cytokine receptor activity	3.55E-09	297,19,13
1f60a2#373	GO:0005525: GTP binding	3.58E-09	24,49,8
1ile_3#170	GO:0005524: ATP binding	3.60E-09	8,243,8
1f3mc_#434	GO:0005524: ATP binding	3.60E-09	8,243,8
1qqsa_#124	GO:0005524: ATP binding	3.62E-09	458,243,76
1a53__#236	GO:0016831: carboxy-lyase activity	3.63E-09	17,25,6
3sil__#284	GO:0004896: hematopoietin/interferon-class (D200-domain) cytokine receptor activity	3.66E-09	64,19,8
1auk__#375	GO:0015036: disulfide oxidoreductase activity	3.66E-09	34,22,7
1j79a_#187	GO:0005524: ATP binding	3.67E-09	89,243,27
2sli_2#326	GO:0004263: chymotrypsin activity	3.68E-09	251,41,18
1kid__#330	GO:0050660: FAD binding	3.76E-09	155,10,8
1cmia_#86	GO:0004896: hematopoietin/interferon-class (D200-domain) cytokine receptor activity	3.76E-09	40,19,7
1epwa3#138	GO:0004674: protein serine/threonine kinase activity	3.77E-09	424,42,23
1qfxa_#380	GO:0003809: thrombin activity	3.78E-09	47,10,6
1b3qa2#556	GO:0004674: protein serine/threonine kinase activity	3.84E-09	40,42,9
1fc4a_#205	GO:0016668: oxidoreductase activity, acting on sulfur group of donors, NAD or NADP as acceptor	3.85E-09	267,12,10
1pfza_#138	GO:0004190: aspartic-type endopeptidase activity	3.86E-09	4,23,4
1ddja_#746	GO:0005509: calcium ion binding	3.86E-09	170,160,31
1a9xa3#78	GO:0000287: magnesium ion binding	3.89E-09	305,128,38
2dri__#115	GO:0005525: GTP binding	3.96E-09	16,49,7
1f2la_#40	GO:0004896: hematopoietin/interferon-class (D200-domain) cytokine receptor activity	3.97E-09	236,19,12
1ga0a_#236	GO:0008800: beta-lactamase activity	4.01E-09	21,10,5
1cyx__#133	GO:0015078: hydrogen ion transporter activity	4.01E-09	10,21,5
1im4a_#153	GO:0000287: magnesium ion binding	4.03E-09	37,128,13
1qqka_#25	GO:0008083: growth factor activity	4.04E-09	6,42,5
1egja_#343	GO:0019955: cytokine binding	4.05E-09	42,11,6
1edza1#206	GO:0003809: thrombin activity	4.11E-09	399,10,10
1ddja_#681	GO:0003809: thrombin activity	4.13E-09	253,10,9
1fgga_#134	GO:0016763: transferase activity, transferring pentosyl groups	4.30E-09	218,28,14

1ihna_#64	GO:0004295: trypsin activity	4.32E-09	10,48,6
1autc_#188	GO:0005509: calcium ion binding	4.32E-09	21,160,11
1e43a2#351	GO:0005509: calcium ion binding	4.32E-09	21,160,11
1ihua2#521	GO:0005525: GTP binding	4.33E-09	77,49,12
1jb3a_#64	GO:0004896: hematopoietin/interferon-class (D200-domain) cytokine receptor activity	4.38E-09	302,19,13
1seta2#403	GO:0004896: hematopoietin/interferon-class (D200-domain) cytokine receptor activity	4.52E-09	41,19,7
1aym3_#131	GO:0051082: unfolded protein binding	4.55E-09	412,34,20
1htp__#16	GO:0004896: hematopoietin/interferon-class (D200-domain) cytokine receptor activity	4.56E-09	303,19,13
1qtn.1#B412	GO:0004197: cysteine-type endopeptidase activity	4.64E-09	4,24,4
1be9a_#352	GO:0005516: calmodulin binding	4.64E-09	4,24,4
1fc6a1#188	GO:0005516: calmodulin binding	4.64E-09	4,24,4
1qava_#149	GO:0005516: calmodulin binding	4.64E-09	4,24,4
2u2fa_#9	GO:0003729: mRNA binding	4.65E-09	6,13,4
2mpra_#190	GO:0005524: ATP binding	4.70E-09	400,243,69
3chbd_#85	GO:0004896: hematopoietin/interferon-class (D200-domain) cytokine receptor activity	4.71E-09	378,19,14
1qrra_#69	GO:0015036: disulfide oxidoreductase activity	4.72E-09	196,22,12
1ihua2#525	GO:0000287: magnesium ion binding	4.74E-09	335,128,40
1danh_#93	GO:0004867: serine-type endopeptidase inhibitor activity	4.89E-09	17,47,7
2hlpa2#289	GO:0004867: serine-type endopeptidase inhibitor activity	4.89E-09	17,47,7
1aa6_1#652	GO:0030151: molybdenum ion binding	4.91E-09	30,15,6
1kte__#24	GO:0004364: glutathione transferase activity	5.01E-09	7,11,4
1azza_#114	GO:0005509: calcium ion binding	5.04E-09	17,160,10
3prn__#143	GO:0005524: ATP binding	5.08E-09	334,243,61
1el0a_#28	GO:0008009: chemokine activity	5.11E-09	161,10,8
1jf9a_#386	GO:0008483: transaminase activity	5.17E-09	5,17,4
1oaca2#150	GO:0016638: oxidoreductase activity, acting on the CH-NH2 group of donors	5.17E-09	5,17,4
1ekma2#98	GO:0016638: oxidoreductase activity, acting on the CH-NH2 group of donors	5.17E-09	5,17,4
1av4_3#167	GO:0016638: oxidoreductase activity, acting on the CH-NH2 group of donors	5.17E-09	5,17,4
3lada3#380	GO:0015036: disulfide oxidoreductase activity	5.18E-09	10,22,5
1bio__#88	GO:0003809: thrombin activity	5.18E-09	22,10,5
1a7ca_#204	GO:0004867: serine-type endopeptidase inhibitor activity	5.22E-09	26,47,8
1c4ra_#258	GO:0004295: trypsin activity	5.27E-09	80,48,12
2uaga1#54	GO:0016668: oxidoreductase activity, acting on sulfur group of donors, NAD or NADP as acceptor	5.29E-09	18,12,5
1h6va1#27	GO:0050660: FAD binding	5.29E-09	260,10,9
2reb_1#138	GO:0016646: oxidoreductase activity, acting on the CH-NH group of donors, NAD or NADP as acceptor	5.33E-09	67,19,8
1by5a_#347	GO:0004556: alpha-amylase activity	5.34E-09	263,15,11
1nfdb1#34	GO:0005529: sugar binding	5.49E-09	177,39,15
1h9oa_#83	GO:0005524: ATP binding	5.51E-09	43,243,18
1qfma2#576	GO:0016776: phosphotransferase activity, phosphate group as acceptor	5.53E-09	214,14,10
1iba__#39	GO:0004556: alpha-amylase activity	5.57E-09	351,15,12
1cm9a_#57	GO:0008009: chemokine activity	5.58E-09	50,10,6
1elua_#95	GO:0008483: transaminase activity	5.58E-09	77,17,8
1egja_#346	GO:0019955: cytokine binding	5.58E-09	20,11,5
1fu6a_#50	GO:0004896: hematopoietin/interferon-class (D200-domain) cytokine receptor activity	5.60E-09	308,19,13
1leha1#255	GO:0016616: oxidoreductase activity, acting on the CH-OH group of donors, NAD or NADP as acceptor	5.61E-09	30,59,9
1ct9a1#233	GO:0016616: oxidoreductase activity, acting on the CH-OH group of donors, NAD or NADP as acceptor	5.71E-09	52,59,11
1ev2e2#286	GO:0004896: hematopoietin/interferon-class (D200-domain) cytokine receptor activity	5.74E-09	100,19,9
1cg2a1#43	GO:0005524: ATP binding	5.84E-09	189,243,42
1qmva_#40	GO:0005525: GTP binding	5.87E-09	79,49,12
1fjsa_#83	GO:0005509: calcium ion binding	5.92E-09	109,160,24
1ppn__#191	GO:0004197: cysteine-type endopeptidase activity	6.00E-09	19,24,6
1fgka_#572	GO:0005524: ATP binding	6.01E-09	13,243,10
1i6vd_#1283	GO:0004295: trypsin activity	6.13E-09	141,48,15
1axca1#70	GO:0004263: chymotrypsin activity	6.16E-09	118,41,13
1mrp__#213	GO:0008235: metalloexopeptidase activity	6.20E-09	17,13,5
1mrj__#240	GO:0016799: hydrolase activity, hydrolyzing N-glycosyl compounds	6.20E-09	13,17,5
1ce7a_#9	GO:0016799: hydrolase activity, hydrolyzing N-glycosyl compounds	6.20E-09	13,17,5
1qg6a_#252	GO:0016616: oxidoreductase activity, acting on the CH-OH group of donors, NAD or NADP as acceptor	6.36E-09	9,59,6
1i9ga_#185	GO:0016616: oxidoreductase activity, acting on the CH-OH group of donors, NAD or NADP as acceptor	6.36E-09	9,59,6
1f8ra1#260	GO:0050660: FAD binding	6.37E-09	8,10,4

1ds1a_#172	GO:0019955: cytokine binding	6.46E-09	222,11,9
1cpt__#358	GO:0016705: oxidoreductase activity, acting on paired donors, with incorporation or reduction of molecular oxygen	6.47E-09	9,26,5
1dz4a_#357	GO:0016705: oxidoreductase activity, acting on paired donors, with incorporation or reduction of molecular oxygen	6.47E-09	9,26,5
1io7a_#38	GO:0004497: monooxygenase activity	6.52E-09	4,26,4
1dz4a_#323	GO:0004497: monooxygenase activity	6.52E-09	4,26,4
1ile_1#695	GO:0004812: tRNA ligase activity	6.52E-09	4,26,4
1htr.1#B172	GO:0004190: aspartic-type endopeptidase activity	6.61E-09	10,23,5
1egza_#254	GO:0008810: cellulase activity	6.65E-09	5,18,4
2duba_#212	GO:0016776: phosphotransferase activity, phosphate group as acceptor	6.78E-09	62,14,7
1aqua_#126	GO:0016646: oxidoreductase activity, acting on the CH-NH group of donors, NAD or NADP as acceptor	6.81E-09	576,19,16
1quna1#113	GO:0005507: copper ion binding	6.85E-09	286,38,18
1nsca_#236	GO:0019955: cytokine binding	6.86E-09	143,11,8
1dmla2#313	GO:0003887: DNA-directed DNA polymerase activity	6.87E-09	41,20,7
1fjfc2#153	GO:0050660: FAD binding	6.90E-09	420,10,10
1fxla2#169	GO:0019838: growth factor binding	7.02E-09	203,9,8
1qrea_#132	GO:0004222: metalloendopeptidase activity	7.16E-09	12,19,5
2viua_#252	GO:0019838: growth factor binding	7.23E-09	343,9,9
1bik_1#76	GO:0004867: serine-type endopeptidase inhibitor activity	7.28E-09	6,47,5
1boub_#7	GO:0016616: oxidoreductase activity, acting on the CH-OH group of donors, NAD or NADP as acceptor	7.36E-09	240,59,21
1g8ka2#570	GO:0004725: protein tyrosine phosphatase activity	7.45E-09	32,15,6
1cs1a_#160	GO:0016763: transferase activity, transferring pentosyl groups	7.49E-09	187,28,13
1d8db_#78	GO:0005126: hematopoietin/interferon-class (D200-domain) cytokine receptor binding	7.70E-09	66,20,8
2sqca2#97	GO:0015078: hydrogen ion transporter activity	7.77E-09	127,21,10
1hg3a_#207	GO:0016831: carboxy-lyase activity	7.86E-09	19,25,6
1fyhb1#99	GO:0019838: growth factor binding	7.94E-09	27,9,5
1a8i__#688	GO:0016616: oxidoreductase activity, acting on the CH-OH group of donors, NAD or NADP as acceptor	7.95E-09	241,59,21
1dr9a1#39	GO:0005524: ATP binding	8.03E-09	258,243,51
1f3ya_#6	GO:0005509: calcium ion binding	8.15E-09	94,160,22
1egja_#343	GO:0005126: hematopoietin/interferon-class (D200-domain) cytokine receptor binding	8.22E-09	42,20,7
1mspa_#17	GO:0004364: glutathione transferase activity	8.22E-09	47,11,6
1gpea1#271	GO:0016668: oxidoreductase activity, acting on sulfur group of donors, NAD or NADP as acceptor	8.24E-09	197,12,9
1eova1#139	GO:0004812: tRNA ligase activity	8.33E-09	99,26,10
1gdea_#90	GO:0016846: carbon-sulfur lyase activity	8.34E-09	24,10,5
1c3pa_#6	GO:0016616: oxidoreductase activity, acting on the CH-OH group of donors, NAD or NADP as acceptor	8.39E-09	82,59,13
2sli_2#438	GO:0004842: ubiquitin-protein ligase activity	8.42E-09	5,19,4
1exma3#99	GO:0003924: GTPase activity	8.43E-09	81,17,8
1hdoa_#189	GO:0005525: GTP binding	8.49E-09	164,49,16
1dgw.1#Y353	GO:0004896: hematopoietin/interferon-class (D200-domain) cytokine receptor activity	8.70E-09	319,19,13
1dr9a2#139	GO:0005126: hematopoietin/interferon-class (D200-domain) cytokine receptor binding	8.71E-09	67,20,8
1g4us2#394	GO:0004725: protein tyrosine phosphatase activity	8.87E-09	6,15,4
1ek1a2#525	GO:0004180: carboxypeptidase activity	8.87E-09	6,15,4
1c4xa_#110	GO:0004180: carboxypeptidase activity	8.87E-09	6,15,4
1fbna_#145	GO:0008757: S-adenosylmethionine-dependent methyltransferase activity	9.08E-09	35,24,7
1gox__#124	GO:0004556: alpha-amylase activity	9.11E-09	366,15,12
1hxxa_#232	GO:0019838: growth factor binding	9.15E-09	352,9,9
1a6da3#190	GO:0004295: trypsin activity	9.22E-09	18,48,7
1ddja_#726	GO:0004867: serine-type endopeptidase inhibitor activity	9.25E-09	105,47,13
1bif_2#398	GO:0005524: ATP binding	9.26E-09	117,243,31
1phm_2#253	GO:0004295: trypsin activity	9.29E-09	309,48,21
1dfca4#1451	GO:0004295: trypsin activity	9.46E-09	67,48,11
1imva_#255	GO:0004190: aspartic-type endopeptidase activity	9.53E-09	589,23,18
2dkb__#114	GO:0016846: carbon-sulfur lyase activity	9.54E-09	174,10,8
1c9la2#61	GO:0005509: calcium ion binding	9.63E-09	238,160,37
1jbwa2#198	GO:0016763: transferase activity, transferring pentosyl groups	9.65E-09	9,28,5
1b3qa2#623	GO:0004263: chymotrypsin activity	9.73E-09	301,41,19
1fkna_#63	GO:0004190: aspartic-type endopeptidase activity	9.84E-09	37,23,7
1feca3#467	GO:0015036: disulfide oxidoreductase activity	9.89E-09	89,22,9
1a65a1#108	GO:0016668: oxidoreductase activity, acting on sulfur group of donors, NAD or NADP as acceptor	1.01E-08	294,12,10

1gdha2#47	GO:0005525: GTP binding	1.01E-08	38,49,9
1e69a_#26	GO:0016620: oxidoreductase activity, acting on the aldehyde or oxo group of donors, NAD or NADP as acceptor	1.01E-08	55,10,6
1p35a_#49	GO:0004295: trypsin activity	1.02E-08	124,48,14
2cb5a_#312	GO:0004197: cysteine-type endopeptidase activity	1.02E-08	111,24,10
1fjsa_#84	GO:0005509: calcium ion binding	1.04E-08	14,160,9
1feca1#122	GO:0050660: FAD binding	1.04E-08	25,10,5
1ekbb_#85	GO:0004867: serine-type endopeptidase inhibitor activity	1.05E-08	150,47,15
3sil__#317	GO:0019838: growth factor binding	1.07E-08	358,9,9
1c5y.1#B18	GO:0005509: calcium ion binding	1.08E-08	18,160,10
1gpea1#273	GO:0016668: oxidoreductase activity, acting on sulfur group of donors, NAD or NADP as acceptor	1.11E-08	81,12,7
1f5aa2#167	GO:0003924: GTPase activity	1.12E-08	124,17,9
1i5ga_#115	GO:0015036: disulfide oxidoreductase activity	1.14E-08	324,22,14
1ass__#33	GO:0050660: FAD binding	1.14E-08	9,10,4
1ejda_#213	GO:0003809: thrombin activity	1.18E-08	284,10,9
1htr.1#B237	GO:0004190: aspartic-type endopeptidase activity	1.21E-08	11,23,5
1hcl__#280	GO:0005126: hematopoietin/interferon-class (D200-domain) cytokine receptor binding	1.25E-08	70,20,8
1ft9a1#201	GO:0004896: hematopoietin/interferon-class (D200-domain) cytokine receptor activity	1.25E-08	109,19,9
1quna1#113	GO:0003809: thrombin activity	1.25E-08	286,10,9
1f0xa1#278	GO:0003809: thrombin activity	1.25E-08	180,10,8
1dfca4#1478	GO:0008083: growth factor activity	1.27E-08	32,42,8
2viua_#252	GO:0004263: chymotrypsin activity	1.29E-08	343,41,20
1evqa_#305	GO:0004556: alpha-amylase activity	1.32E-08	490,15,13
1aqua_#126	GO:0016763: transferase activity, transferring pentosyl groups	1.33E-08	576,28,20
2pia_1#54	GO:0019838: growth factor binding	1.34E-08	220,9,8
1nuka_#80	GO:0019838: growth factor binding	1.34E-08	220,9,8
1fxla2#169	GO:0004896: hematopoietin/interferon-class (D200-domain) cytokine receptor activity	1.34E-08	203,19,11
1e2o__#346	GO:0005524: ATP binding	1.35E-08	194,243,42
1f3mc_#369	GO:0005524: ATP binding	1.37E-08	100,243,28
1e05i_#422	GO:0004867: serine-type endopeptidase inhibitor activity	1.38E-08	29,47,8
1cqqa_#163	GO:0004867: serine-type endopeptidase inhibitor activity	1.38E-08	29,47,8
1cgha_#130	GO:0004867: serine-type endopeptidase inhibitor activity	1.38E-08	29,47,8
1dr9a2#139	GO:0019838: growth factor binding	1.40E-08	67,9,6
1fnf_3#1409	GO:0005126: hematopoietin/interferon-class (D200-domain) cytokine receptor binding	1.40E-08	71,20,8
1fl7b_#51	GO:0008083: growth factor activity	1.40E-08	7,42,5
2tgi__#80	GO:0008083: growth factor activity	1.40E-08	7,42,5
1e8ga2#201	GO:0016668: oxidoreductase activity, acting on sulfur group of donors, NAD or NADP as acceptor	1.40E-08	304,12,10
1c7na_#95	GO:0003809: thrombin activity	1.43E-08	183,10,8
1c7na_#95	GO:0016846: carbon-sulfur lyase activity	1.43E-08	183,10,8
2napa2#444	GO:0030151: molybdenum ion binding	1.44E-08	17,15,5
1hbza_#96	GO:0019838: growth factor binding	1.44E-08	370,9,9
1ds1a_#172	GO:0019838: growth factor binding	1.44E-08	222,9,8
3grx__#56	GO:0004674: protein serine/threonine kinase activity	1.44E-08	335,42,20
1qfma1#139	GO:0005524: ATP binding	1.45E-08	318,243,58
1iow_1#37	GO:0005525: GTP binding	1.46E-08	309,49,21
1d0ba_#191	GO:0016616: oxidoreductase activity, acting on the CH-OH group of donors, NAD or NADP as acceptor	1.47E-08	120,59,15
1fjsa_#83	GO:0004867: serine-type endopeptidase inhibitor activity	1.48E-08	109,47,13
1f8ra1#260	GO:0016668: oxidoreductase activity, acting on sulfur group of donors, NAD or NADP as acceptor	1.50E-08	8,12,4
1bjt__#890	GO:0003809: thrombin activity	1.51E-08	292,10,9
1qupa2#50	GO:0015082: di-, tri-valent inorganic cation transporter activity	1.51E-08	7,14,4
1qupa2#50	GO:0046915: transition metal ion transporter activity	1.51E-08	7,14,4
1jsg__#92	GO:0004896: hematopoietin/interferon-class (D200-domain) cytokine receptor activity	1.53E-08	607,19,16
1chma2#262	GO:0008235: metalloexopeptidase activity	1.54E-08	20,13,5
2oata_#120	GO:0008483: transaminase activity	1.55E-08	6,17,4
1ddja_#681	GO:0005509: calcium ion binding	1.56E-08	253,160,38
1alo_4#634	GO:0000287: magnesium ion binding	1.59E-08	64,128,16
1nls__#77	GO:0005529: sugar binding	1.59E-08	14,39,6
1d7ya1#243	GO:0015036: disulfide oxidoreductase activity	1.61E-08	12,22,5
1chua2#21	GO:0015036: disulfide oxidoreductase activity	1.61E-08	12,22,5
1a7j__#87	GO:0016776: phosphotransferase activity, phosphate group as acceptor	1.62E-08	70,14,7
1rtu__#97	GO:0004190: aspartic-type endopeptidase activity	1.64E-08	23,23,6
1g4ia_#11	GO:0004295: trypsin activity	1.64E-08	29,48,8

1c4ka1#33	GO:0005525: GTP binding	1.65E-08	40,49,9
1aky_2#133	GO:0004896: hematopoietin/interferon-class (D200-domain) cytokine receptor activity	1.69E-08	156,19,10
1clxa_#13	GO:0005509: calcium ion binding	1.69E-08	11,160,8
1bkca_#351	GO:0005509: calcium ion binding	1.69E-08	11,160,8
3lada1#116	GO:0005524: ATP binding	1.70E-08	153,243,36
1dpsa_#36	GO:0004674: protein serine/threonine kinase activity	1.71E-08	205,42,16
1f42a2#123	GO:0019838: growth factor binding	1.75E-08	378,9,9
1hbza_#214	GO:0003809: thrombin activity	1.76E-08	297,10,9
1qrra_#208	GO:0016616: oxidoreductase activity, acting on the CH-OH group of donors, NAD or NADP as acceptor	1.77E-08	87,59,13
1hbza_#96	GO:0005509: calcium ion binding	1.78E-08	370,160,48
1vola2#239	GO:0005524: ATP binding	1.79E-08	120,243,31
2e2c__#67	GO:0016251: general RNA polymerase II transcription factor activity	1.80E-08	19,14,5
1cs1a_#160	GO:0016831: carboxy-lyase activity	1.82E-08	187,25,12
1bqya_#115	GO:0004867: serine-type endopeptidase inhibitor activity	1.87E-08	20,47,7
1kit_3#743	GO:0019955: cytokine binding	1.88E-08	250,11,9
3chbd_#85	GO:0004674: protein serine/threonine kinase activity	1.88E-08	378,42,21
1amf__#196	GO:0015036: disulfide oxidoreductase activity	1.89E-08	173,22,11
1a4sa_#382	GO:0016620: oxidoreductase activity, acting on the aldehyde or oxo group of donors, NAD or NADP as acceptor	1.90E-08	10,10,4
1ayaa_#98	GO:0005066: transmembrane receptor protein tyrosine kinase signaling protein activity	1.90E-08	10,10,4
1fc4a_#205	GO:0000287: magnesium ion binding	1.91E-08	267,128,34
1e1aa_#178	GO:0019955: cytokine binding	1.95E-08	54,11,6
1fnf_3#1409	GO:0019838: growth factor binding	2.00E-08	71,9,6
1ile_2#304	GO:0004812: tRNA ligase activity	2.01E-08	21,26,6
1aoza1#61	GO:0015078: hydrogen ion transporter activity	2.01E-08	26,21,6
1qs0a1#235	GO:0050660: FAD binding	2.02E-08	114,10,7
1jb3a_#64	GO:0003809: thrombin activity	2.05E-08	302,10,9
1aa6_2#175	GO:0030151: molybdenum ion binding	2.06E-08	7,15,4
1h9ra2#238	GO:0030151: molybdenum ion binding	2.06E-08	7,15,4
1hrka_#205	GO:0005525: GTP binding	2.08E-08	41,49,9
1dgw.1#Y353	GO:0004190: aspartic-type endopeptidase activity	2.12E-08	319,23,14
1pme__#88	GO:0004674: protein serine/threonine kinase activity	2.15E-08	34,42,8
1qhoa4#230	GO:0005509: calcium ion binding	2.16E-08	19,160,10
1czan1#92	GO:0016638: oxidoreductase activity, acting on the CH-NH2 group of donors	2.18E-08	631,17,15
2bb2_2#134	GO:0016668: oxidoreductase activity, acting on sulfur group of donors, NAD or NADP as acceptor	2.21E-08	220,12,9
1f8ra2#325	GO:0004556: alpha-amylase activity	2.22E-08	222,15,10
1fwxa1#535	GO:0005507: copper ion binding	2.23E-08	15,38,6
2occb1#209	GO:0005507: copper ion binding	2.27E-08	93,38,11
1qaua_#64	GO:0005516: calmodulin binding	2.30E-08	5,24,4
3lada3#463	GO:0050660: FAD binding	2.31E-08	29,10,5
1befa_#51	GO:0003809: thrombin activity	2.31E-08	29,10,5
1fo4a5#1002	GO:0000287: magnesium ion binding	2.37E-08	93,128,19
1f0xa1#278	GO:0004295: trypsin activity	2.38E-08	180,48,16
1qs1a2#437	GO:0051082: unfolded protein binding	2.41E-08	452,34,20
1fmta2#151	GO:0005524: ATP binding	2.41E-08	314,243,57
1ovaa_#284	GO:0016616: oxidoreductase activity, acting on the CH-OH group of donors, NAD or NADP as acceptor	2.41E-08	25,59,8
1dpja_#322	GO:0003809: thrombin activity	2.44E-08	308,10,9
1tbga_#63	GO:0005509: calcium ion binding	2.46E-08	15,160,9
1ddja_#640	GO:0005509: calcium ion binding	2.46E-08	15,160,9
1ej8a_#189	GO:0004295: trypsin activity	2.46E-08	294,48,20
1ddwa_#77	GO:0005524: ATP binding	2.49E-08	364,243,63
1cfb_2#763	GO:0004896: hematopoietin/interferon-class (D200-domain) cytokine receptor activity	2.51E-08	6,19,4
1a7ca_#326	GO:0004867: serine-type endopeptidase inhibitor activity	2.52E-08	7,47,5
1c9la2#61	GO:0019838: growth factor binding	2.53E-08	238,9,8
1c3pa_#6	GO:0019201: nucleotide kinase activity	2.56E-08	82,13,7
2mpra_#190	GO:0004556: alpha-amylase activity	2.58E-08	400,15,12
1g38a_#155	GO:0008757: S-adenosylmethionine-dependent methyltransferase activity	2.59E-08	12,24,5
1iow_1#37	GO:0016616: oxidoreductase activity, acting on the CH-OH group of donors, NAD or NADP as acceptor	2.59E-08	309,59,23
1cpza_#6	GO:0004295: trypsin activity	2.76E-08	157,48,15
1e79d2#36	GO:0004896: hematopoietin/interferon-class (D200-domain) cytokine receptor activity	2.76E-08	524,19,15
1seta2#306	GO:0050660: FAD binding	2.77E-08	30,10,5
1dn2a1#261	GO:0005524: ATP binding	2.78E-08	425,243,70

1nsj__#21	GO:0000287: magnesium ion binding	2.80E-08	114,128,21
1pysb5#584	GO:0005524: ATP binding	2.81E-08	79,243,24
1fyhb1#99	GO:0019955: cytokine binding	2.87E-08	27,11,5
1fn9a_#271	GO:0004896: hematopoietin/interferon-class (D200-domain) cytokine receptor activity	2.87E-08	218,19,11
1ac5__#72	GO:0016616: oxidoreductase activity, acting on the CH-OH group of donors, NAD or NADP as acceptor	2.88E-08	47,59,10
2mpra_#190	GO:0019838: growth factor binding	2.93E-08	400,9,9
1jsg__#92	GO:0004674: protein serine/threonine kinase activity	2.93E-08	607,42,26
1qs1a2#437	GO:0005524: ATP binding	2.98E-08	452,243,73
1d2fa_#348	GO:0016846: carbon-sulfur lyase activity	2.99E-08	11,10,4
1hvba_#102	GO:0008800: beta-lactamase activity	2.99E-08	11,10,4
1phm_2#253	GO:0008201: heparin binding	3.00E-08	309,24,14
1amp__#83	GO:0016251: general RNA polymerase II transcription factor activity	3.02E-08	8,14,4
1c8na_#216	GO:0003968: RNA-directed RNA polymerase activity	3.02E-08	8,14,4
1bwda_#117	GO:0005524: ATP binding	3.04E-08	116,243,30
1ir3a_#1063	GO:0005524: ATP binding	3.06E-08	68,243,22
1qq4a_#178	GO:0030151: molybdenum ion binding	3.08E-08	40,15,6
1ddma_#130	GO:0004674: protein serine/threonine kinase activity	3.09E-08	313,42,19
1qf6a1#606	GO:0004812: tRNA ligase activity	3.12E-08	38,26,7
1f00i2#805	GO:0004295: trypsin activity	3.12E-08	135,48,14
1nsca_#236	GO:0019838: growth factor binding	3.13E-08	143,9,7
2pola2#194	GO:0003887: DNA-directed DNA polymerase activity	3.14E-08	6,20,4
2pia_1#54	GO:0004896: hematopoietin/interferon-class (D200-domain) cytokine receptor activity	3.16E-08	220,19,11
1e1oa2#423	GO:0004812: tRNA ligase activity	3.21E-08	148,26,11
1oaca1#438	GO:0005507: copper ion binding	3.22E-08	4,38,4
1a65a3#342	GO:0005507: copper ion binding	3.22E-08	4,38,4
1aoza3#362	GO:0005507: copper ion binding	3.22E-08	4,38,4
1mrj__#156	GO:0016799: hydrolase activity, hydrolyzing N-glycosyl compounds	3.24E-08	61,17,7
1qfma1#139	GO:0050660: FAD binding	3.26E-08	318,10,9
1dzfa2#211	GO:0004896: hematopoietin/interferon-class (D200-domain) cytokine receptor activity	3.28E-08	167,19,10
1fxla2#169	GO:0050660: FAD binding	3.29E-08	203,10,8
1fmja_#76	GO:0019201: nucleotide kinase activity	3.31E-08	85,13,7
1f2na_#138	GO:0003924: GTPase activity	3.32E-08	96,17,8
1hwxa1#324	GO:0016616: oxidoreductase activity, acting on the CH-OH group of donors, NAD or NADP as acceptor	3.38E-08	11,59,6
2sqca2#268	GO:0004364: glutathione transferase activity	3.38E-08	59,11,6
3grs_1#23	GO:0016616: oxidoreductase activity, acting on the CH-OH group of donors, NAD or NADP as acceptor	3.47E-08	168,59,17
1qtn.1#A270	GO:0016646: oxidoreductase activity, acting on the CH-NH group of donors, NAD or NADP as acceptor	3.51E-08	439,19,14
1ayl__#237	GO:0004896: hematopoietin/interferon-class (D200-domain) cytokine receptor activity	3.54E-08	357,19,13
1a4sa_#488	GO:0016620: oxidoreductase activity, acting on the aldehyde or oxo group of donors, NAD or NADP as acceptor	3.56E-08	3,10,3
1c7na_#36	GO:0016846: carbon-sulfur lyase activity	3.56E-08	3,10,3
3frua1#249	GO:0003887: DNA-directed DNA polymerase activity	3.56E-08	15,20,5
1jf9a_#119	GO:0008483: transaminase activity	3.59E-08	7,17,4
1elua_#388	GO:0008483: transaminase activity	3.59E-08	7,17,4
1jkma_#317	GO:0016616: oxidoreductase activity, acting on the CH-OH group of donors, NAD or NADP as acceptor	3.59E-08	76,59,12
1aiha_#226	GO:0004263: chymotrypsin activity	3.60E-08	15,41,6
1ig8a_#215	GO:0000287: magnesium ion binding	3.63E-08	59,128,15
1hdfa_#16	GO:0004896: hematopoietin/interferon-class (D200-domain) cytokine receptor activity	3.65E-08	223,19,11
1f42a2#123	GO:0005509: calcium ion binding	3.66E-08	378,160,48
1tuba1#8	GO:0004457: lactate dehydrogenase activity	3.66E-08	124,10,7
1es7a_#111	GO:0008083: growth factor activity	3.69E-08	8,42,5
1i4fb1#70	GO:0005529: sugar binding	3.69E-08	39,39,8
1b3qa2#623	GO:0004295: trypsin activity	3.71E-08	301,48,20
1nsj__#124	GO:0016616: oxidoreductase activity, acting on the CH-OH group of donors, NAD or NADP as acceptor	3.74E-08	148,59,16
1kid__#330	GO:0016668: oxidoreductase activity, acting on sulfur group of donors, NAD or NADP as acceptor	3.75E-08	155,12,8
1e87a_#174	GO:0015036: disulfide oxidoreductase activity	3.81E-08	141,22,10
1fcya_#311	GO:0003700: transcription factor activity	3.82E-08	61,124,15
1jd0a_#176	GO:0003809: thrombin activity	3.84E-08	207,10,8
1erv__#80	GO:0004896: hematopoietin/interferon-class (D200-domain) cytokine receptor activity	3.84E-08	442,19,14
1imva_#255	GO:0003887: DNA-directed DNA polymerase activity	3.85E-08	589,20,16

1d0va_#114	GO:0016646: oxidoreductase activity, acting on the CH-NH group of donors, NAD or NADP as acceptor	3.88E-08	16,19,5
1bg6_2#73	GO:0016646: oxidoreductase activity, acting on the CH-NH group of donors, NAD or NADP as acceptor	3.91E-08	32,19,6
1czya1#466	GO:0019955: cytokine binding	3.92E-08	109,11,7
1e7wa_#36	GO:0005524: ATP binding	3.92E-08	137,243,33
2viua_#252	GO:0005524: ATP binding	3.99E-08	343,243,60
3gcb__#78	GO:0004197: cysteine-type endopeptidase activity	4.02E-08	94,24,9
1d4oa_#135	GO:0015036: disulfide oxidoreductase activity	4.03E-08	14,22,5
1e2wa1#102	GO:0004896: hematopoietin/interferon-class (D200-domain) cytokine receptor activity	4.04E-08	86,19,8
1eur__#84	GO:0004896: hematopoietin/interferon-class (D200-domain) cytokine receptor activity	4.04E-08	86,19,8
1el0a_#42	GO:0005524: ATP binding	4.10E-08	294,243,54
1ayl__#237	GO:0015036: disulfide oxidoreductase activity	4.11E-08	357,22,14
1hlga_#324	GO:0004180: carboxypeptidase activity	4.11E-08	8,15,4
1bif_2#398	GO:0003887: DNA-directed DNA polymerase activity	4.12E-08	117,20,9
1h8ca_#35	GO:0000287: magnesium ion binding	4.13E-08	77,128,17
1ddja_#681	GO:0019838: growth factor binding	4.13E-08	253,9,8
1cpy__#380	GO:0004896: hematopoietin/interferon-class (D200-domain) cytokine receptor activity	4.13E-08	171,19,10
1bd3a_#110	GO:0000287: magnesium ion binding	4.13E-08	24,128,10
1hcl__#180	GO:0005524: ATP binding	4.14E-08	7,243,7
1apme_#255	GO:0005524: ATP binding	4.14E-08	7,243,7
1howa_#552	GO:0005524: ATP binding	4.14E-08	7,243,7
1tuba1#8	GO:0005524: ATP binding	4.17E-08	124,243,31
1ak7__#152	GO:0000049: tRNA binding	4.18E-08	24,13,5
1ep3a_#247	GO:0016861: intramolecular oxidoreductase activity, interconverting aldoses and ketoses	4.18E-08	24,13,5
1smaa1#95	GO:0004295: trypsin activity	4.19E-08	272,48,19
1ex1a1#284	GO:0004556: alpha-amylase activity	4.19E-08	42,15,6
1fsu__#434	GO:0004674: protein serine/threonine kinase activity	4.20E-08	356,42,20
1kit_3#743	GO:0008083: growth factor activity	4.26E-08	250,42,17
1ycsa_#162	GO:0003809: thrombin activity	4.31E-08	210,10,8
1bif_2#398	GO:0004812: tRNA ligase activity	4.32E-08	117,26,10
1qqga1#95	GO:0003809: thrombin activity	4.34E-08	127,10,7
1pud__#44	GO:0016861: intramolecular oxidoreductase activity, interconverting aldoses and ketoses	4.37E-08	50,13,6
1aop_3#276	GO:0004896: hematopoietin/interferon-class (D200-domain) cytokine receptor activity	4.37E-08	172,19,10
1aym3_#109	GO:0004896: hematopoietin/interferon-class (D200-domain) cytokine receptor activity	4.40E-08	56,19,7
1ayl__#237	GO:0004674: protein serine/threonine kinase activity	4.41E-08	357,42,20
1danh_#100	GO:0004263: chymotrypsin activity	4.42E-08	4,41,4
1dlea_#177	GO:0004263: chymotrypsin activity	4.42E-08	4,41,4
1neb__#14	GO:0005066: transmembrane receptor protein tyrosine kinase signaling protein activity	4.47E-08	12,10,4
1chua2#21	GO:0050660: FAD binding	4.47E-08	12,10,4
1ax4a_#224	GO:0016846: carbon-sulfur lyase activity	4.47E-08	12,10,4
1jf9a_#183	GO:0016846: carbon-sulfur lyase activity	4.47E-08	12,10,4
1iow_1#37	GO:0016763: transferase activity, transferring pentosyl groups	4.51E-08	309,28,15
1thw__#199	GO:0005507: copper ion binding	4.52E-08	41,38,8
2sli_2#326	GO:0008083: growth factor activity	4.52E-08	251,42,17
1f5aa2#167	GO:0005507: copper ion binding	4.59E-08	124,38,12
1thfd_#140	GO:0005507: copper ion binding	4.59E-08	124,38,12
2occb1#189	GO:0015078: hydrogen ion transporter activity	4.66E-08	15,21,5
1pme__#88	GO:0005524: ATP binding	4.67E-08	34,243,15
2cpl__#83	GO:0003755: peptidyl-prolyl cis-trans isomerase activity	4.68E-08	11,11,4
1trb_1#106	GO:0015036: disulfide oxidoreductase activity	4.74E-08	6,22,4
1ej8a_#189	GO:0004263: chymotrypsin activity	4.76E-08	294,41,18
1nuka_#80	GO:0008083: growth factor activity	4.79E-08	220,42,16
1bvza3#373	GO:0005509: calcium ion binding	4.82E-08	12,160,8
1fp5a2#537	GO:0019838: growth factor binding	4.87E-08	38,9,5
1h8d.1#H207	GO:0005509: calcium ion binding	4.87E-08	63,160,17
1iira_#326	GO:0016616: oxidoreductase activity, acting on the CH-OH group of donors, NAD or NADP as acceptor	4.91E-08	112,59,14
1tiid_#39	GO:0005525: GTP binding	4.91E-08	239,49,18
1amf__#196	GO:0030151: molybdenum ion binding	4.92E-08	173,15,9
1c3pa_#6	GO:0016776: phosphotransferase activity, phosphate group as acceptor	5.01E-08	82,14,7
1g0sa_#74	GO:0004896: hematopoietin/interferon-class (D200-domain) cytokine receptor activity	5.05E-08	294,19,12
1dn2a1#261	GO:0019838: growth factor binding	5.08E-08	425,9,9
1dzfa2#211	GO:0004263: chymotrypsin activity	5.08E-08	167,41,14
1fjsa_#84	GO:0004867: serine-type endopeptidase inhibitor activity	5.14E-08	14,47,6
1nuka_#80	GO:0005509: calcium ion binding	5.17E-08	220,160,34

1gpc__#184	GO:0019838: growth factor binding	5.21E-08	83,9,6
1axca1#70	GO:0004295: trypsin activity	5.21E-08	118,48,13
2hlca_#139	GO:0005509: calcium ion binding	5.23E-08	112,160,23
1qfma2#576	GO:0019201: nucleotide kinase activity	5.24E-08	214,13,9
1f4la2#25	GO:0005524: ATP binding	5.36E-08	15,243,10
1qtn.1#A270	GO:0003924: GTPase activity	5.37E-08	439,17,13
1hcl__#135	GO:0005524: ATP binding	5.37E-08	12,243,9
1qfea_#68	GO:0016861: intramolecular oxidoreductase activity, interconverting aldoses and ketoses	5.46E-08	560,13,12
1dhs__#99	GO:0005524: ATP binding	5.47E-08	132,243,32
1b9ha_#276	GO:0008483: transaminase activity	5.48E-08	19,17,5
1qs0a1#119	GO:0016646: oxidoreductase activity, acting on the CH-NH group of donors, NAD or NADP as acceptor	5.49E-08	454,19,14
1efpa1#32	GO:0000287: magnesium ion binding	5.50E-08	129,128,22
1hbza_#96	GO:0004896: hematopoietin/interferon-class (D200-domain) cytokine receptor activity	5.51E-08	370,19,13
1dpja_#322	GO:0004295: trypsin activity	5.53E-08	308,48,20
1a7s__#148	GO:0005509: calcium ion binding	5.54E-08	71,160,18
1fjfc2#153	GO:0003887: DNA-directed DNA polymerase activity	5.57E-08	420,20,14
1qf6a3#78	GO:0008235: metalloexopeptidase activity	5.58E-08	52,13,6
3lada3#463	GO:0015036: disulfide oxidoreductase activity	5.58E-08	29,22,6
1ddma_#130	GO:0005524: ATP binding	5.71E-08	313,243,56
1dy9.1#C229	GO:0003755: peptidyl-prolyl cis-trans isomerase activity	5.71E-08	115,11,7
1fmb__#88	GO:0004190: aspartic-type endopeptidase activity	5.73E-08	6,23,4
1e44b_#75	GO:0004263: chymotrypsin activity	5.80E-08	198,41,15
1fn9a_#271	GO:0050660: FAD binding	5.81E-08	218,10,8
1e4ea2#215	GO:0016668: oxidoreductase activity, acting on sulfur group of donors, NAD or NADP as acceptor	5.83E-08	490,12,11
1a9xa3#78	GO:0005524: ATP binding	5.84E-08	305,243,55
1e8ua_#286	GO:0004896: hematopoietin/interferon-class (D200-domain) cytokine receptor activity	5.84E-08	7,19,4
1hc7a2#258	GO:0005524: ATP binding	5.87E-08	226,243,45
1dyna_#42	GO:0016668: oxidoreductase activity, acting on sulfur group of donors, NAD or NADP as acceptor	5.87E-08	164,12,8
1ih7a1#57	GO:0030151: molybdenum ion binding	5.87E-08	77,15,7
1e32a2#345	GO:0005524: ATP binding	5.95E-08	100,243,27
1lara2#1769	GO:0003809: thrombin activity	6.03E-08	219,10,8
1dpsa_#36	GO:0015078: hydrogen ion transporter activity	6.08E-08	205,21,11
1nsj__#178	GO:0016861: intramolecular oxidoreductase activity, interconverting aldoses and ketoses	6.25E-08	93,13,7
1cex__#109	GO:0004457: lactate dehydrogenase activity	6.26E-08	35,10,5
1bywa_#30	GO:0004556: alpha-amylase activity	6.33E-08	331,15,11
1b3ra1#223	GO:0016638: oxidoreductase activity, acting on the CH-NH2 group of donors	6.36E-08	39,17,6
1fzqa_#28	GO:0005524: ATP binding	6.39E-08	49,243,18
1nsj__#124	GO:0016861: intramolecular oxidoreductase activity, interconverting aldoses and ketoses	6.42E-08	148,13,8
2viua_#252	GO:0003809: thrombin activity	6.43E-08	343,10,9
1evqa_#305	GO:0016763: transferase activity, transferring pentosyl groups	6.44E-08	490,28,18
1e79d2#36	GO:0003809: thrombin activity	6.45E-08	524,10,10
1a7ta_#80	GO:0008800: beta-lactamase activity	6.45E-08	13,10,4
2fcba2#104	GO:0008235: metalloexopeptidase activity	6.45E-08	10,13,4
2dkb__#46	GO:0016846: carbon-sulfur lyase activity	6.45E-08	13,10,4
1i1ja_#83	GO:0005066: transmembrane receptor protein tyrosine kinase signaling protein activity	6.45E-08	13,10,4
1h7wa4#208	GO:0050660: FAD binding	6.45E-08	13,10,4
1dfoa_#86	GO:0016846: carbon-sulfur lyase activity	6.45E-08	13,10,4
1fmja_#76	GO:0016776: phosphotransferase activity, phosphate group as acceptor	6.45E-08	85,14,7
1g8fa3#419	GO:0003924: GTPase activity	6.47E-08	151,17,9
1smaa2#581	GO:0005507: copper ion binding	6.54E-08	80,38,10
1fnf_1#1146	GO:0008083: growth factor activity	6.63E-08	16,42,6
1qg6a_#237	GO:0016616: oxidoreductase activity, acting on the CH-OH group of donors, NAD or NADP as acceptor	6.65E-08	12,59,6
1qp8a1#168	GO:0016616: oxidoreductase activity, acting on the CH-OH group of donors, NAD or NADP as acceptor	6.65E-08	12,59,6
1ayl__#237	GO:0016668: oxidoreductase activity, acting on sulfur group of donors, NAD or NADP as acceptor	6.87E-08	357,12,10
1gjwa2#160	GO:0005509: calcium ion binding	6.91E-08	9,160,7
1seta2#306	GO:0015036: disulfide oxidoreductase activity	6.94E-08	30,22,6
1dfoa_#237	GO:0004867: serine-type endopeptidase inhibitor activity	7.01E-08	35,47,8
1dih_2#235	GO:0008408: 3'-5' exonuclease activity	7.04E-08	54,13,6
1a9xa3#78	GO:0005525: GTP binding	7.09E-08	305,49,20
1qfea_#68	GO:0016763: transferase activity, transferring pentosyl groups	7.14E-08	560,28,19

1keva2#266	GO:0016638: oxidoreductase activity, acting on the CH-NH2 group of donors	7.16E-08	8,17,4
1gox__#334	GO:0016627: oxidoreductase activity, acting on the CH-CH group of donors	7.16E-08	8,17,4
1b9ha_#39	GO:0008483: transaminase activity	7.16E-08	8,17,4
3lada3#463	GO:0016668: oxidoreductase activity, acting on sulfur group of donors, NAD or NADP as acceptor	7.16E-08	29,12,5
1mdah_#329	GO:0004556: alpha-amylase activity	7.20E-08	335,15,11
2bpa1_#244	GO:0004896: hematopoietin/interferon-class (D200-domain) cytokine receptor activity	7.21E-08	60,19,7
1dfoa_#344	GO:0016846: carbon-sulfur lyase activity	7.26E-08	36,10,5
1ksia1#555	GO:0016638: oxidoreductase activity, acting on the CH-NH2 group of donors	7.28E-08	20,17,5
1gdea_#92	GO:0008483: transaminase activity	7.28E-08	20,17,5
2sli_2#326	GO:0004295: trypsin activity	7.33E-08	251,48,18
1g8ka2#332	GO:0030151: molybdenum ion binding	7.38E-08	9,15,4
1ac5__#458	GO:0004180: carboxypeptidase activity	7.38E-08	9,15,4
1bu7a_#405	GO:0016705: oxidoreductase activity, acting on paired donors, with incorporation or reduction of molecular oxygen	7.53E-08	65,26,8
3thia_#59	GO:0004222: metalloendopeptidase activity	7.55E-08	18,19,5
2occb1#209	GO:0004896: hematopoietin/interferon-class (D200-domain) cytokine receptor activity	7.56E-08	93,19,8
1e5ka_#113	GO:0004867: serine-type endopeptidase inhibitor activity	7.60E-08	65,47,10
1ejda_#213	GO:0003887: DNA-directed DNA polymerase activity	7.71E-08	284,20,12
1nsj__#21	GO:0016763: transferase activity, transferring pentosyl groups	7.71E-08	114,28,10
1a7ca_#325	GO:0004867: serine-type endopeptidase inhibitor activity	7.78E-08	4,47,4
1e6pa2#46	GO:0016836: hydro-lyase activity	7.86E-08	95,33,10
1a3qa2#133	GO:0005518: collagen binding	7.89E-08	27,13,5
1mrp__#172	GO:0000287: magnesium ion binding	7.89E-08	110,128,20
2scub2#126	GO:0004867: serine-type endopeptidase inhibitor activity	7.95E-08	24,47,7
1a65a1#108	GO:0019955: cytokine binding	8.00E-08	294,11,9
1ejda_#180	GO:0004457: lactate dehydrogenase activity	8.04E-08	77,10,6
1qs0a1#119	GO:0003924: GTPase activity	8.16E-08	454,17,13
1ec7a1#230	GO:0000287: magnesium ion binding	8.20E-08	54,128,14
1egja_#343	GO:0019838: growth factor binding	8.21E-08	42,9,5
1hbza_#96	GO:0004674: protein serine/threonine kinase activity	8.21E-08	370,42,20
1xgsa2#82	GO:0004896: hematopoietin/interferon-class (D200-domain) cytokine receptor activity	8.29E-08	307,19,12
3sil__#317	GO:0005524: ATP binding	8.33E-08	358,243,61
2gsta1#169	GO:0004364: glutathione transferase activity	8.35E-08	33,11,5
1f00i2#805	GO:0004896: hematopoietin/interferon-class (D200-domain) cytokine receptor activity	8.37E-08	135,19,9
1qs1a2#437	GO:0004674: protein serine/threonine kinase activity	8.39E-08	452,42,22
1imva_#191	GO:0004867: serine-type endopeptidase inhibitor activity	8.45E-08	15,47,6
1danh_#100	GO:0004295: trypsin activity	8.49E-08	4,48,4
1dlea_#177	GO:0004295: trypsin activity	8.49E-08	4,48,4
1sgpe_#127	GO:0004295: trypsin activity	8.49E-08	4,48,4
1hv8a1#84	GO:0008026: ATP-dependent helicase activity	8.49E-08	3,13,3
1rmg__#279	GO:0008270: zinc ion binding	8.52E-08	12,108,7
1seta2#306	GO:0016668: oxidoreductase activity, acting on sulfur group of donors, NAD or NADP as acceptor	8.57E-08	30,12,5
1quqb_#87	GO:0004457: lactate dehydrogenase activity	8.70E-08	78,10,6
1atg__#144	GO:0016616: oxidoreductase activity, acting on the CH-OH group of donors, NAD or NADP as acceptor	8.70E-08	82,59,12
1as4.1#A288	GO:0004867: serine-type endopeptidase inhibitor activity	8.84E-08	66,47,10
1znca_#32	GO:0016836: hydro-lyase activity	8.85E-08	5,33,4
1hle.1#B387	GO:0004867: serine-type endopeptidase inhibitor activity	8.89E-08	36,47,8
1gof_2#118	GO:0004457: lactate dehydrogenase activity	9.01E-08	14,10,4
1awx__#27	GO:0005066: transmembrane receptor protein tyrosine kinase signaling protein activity	9.01E-08	14,10,4
1i6vd_#1283	GO:0003809: thrombin activity	9.04E-08	141,10,7
2dnja_#97	GO:0003809: thrombin activity	9.04E-08	141,10,7
1iqpa2#119	GO:0005524: ATP binding	9.05E-08	19,243,11
1qqsa_#35	GO:0004812: tRNA ligase activity	9.07E-08	94,26,9
1ddma_#130	GO:0015036: disulfide oxidoreductase activity	9.09E-08	313,22,13
3prn__#143	GO:0004674: protein serine/threonine kinase activity	9.11E-08	334,42,19
1qdlb_#170	GO:0005525: GTP binding	9.18E-08	100,49,12
2sqca1#417	GO:0008810: cellulase activity	9.19E-08	8,18,4
1eona_#74	GO:0000287: magnesium ion binding	9.31E-08	15,128,8
1e44b_#75	GO:0004295: trypsin activity	9.41E-08	198,48,16
1d6aa_#52	GO:0004867: serine-type endopeptidase inhibitor activity	9.54E-08	293,47,19
1jf9a_#379	GO:0003809: thrombin activity	9.54E-08	232,10,8
1guqa1#166	GO:0004263: chymotrypsin activity	9.61E-08	28,41,7
1aiha_#226	GO:0004295: trypsin activity	9.63E-08	15,48,6

1gdha2#47	GO:0004457: lactate dehydrogenase activity	9.64E-08	38,10,5
1bawa_#65	GO:0005507: copper ion binding	9.64E-08	10,38,5
1qf6a4#360	GO:0004812: tRNA ligase activity	9.66E-08	6,26,4
1ofga1#84	GO:0016616: oxidoreductase activity, acting on the CH-OH group of donors, NAD or NADP as acceptor	9.67E-08	20,59,7
1cqka_#52	GO:0019955: cytokine binding	9.67E-08	70,11,6
1ib2a_#1101	GO:0004364: glutathione transferase activity	9.67E-08	70,11,6
1hxxa_#278	GO:0015036: disulfide oxidoreductase activity	9.70E-08	255,22,12
1b8aa2#368	GO:0005524: ATP binding	9.72E-08	135,243,32
1dy9.1#C229	GO:0015036: disulfide oxidoreductase activity	9.75E-08	115,22,9
1d6aa_#52	GO:0005524: ATP binding	9.77E-08	293,243,53
1cf1a1#33	GO:0005507: copper ion binding	9.79E-08	45,38,8
1qr4a2#166	GO:0019838: growth factor binding	9.83E-08	16,9,4
1cs6a4#363	GO:0019838: growth factor binding	9.83E-08	16,9,4
1fnf_1#1146	GO:0019838: growth factor binding	9.83E-08	16,9,4
1fjfc2#153	GO:0005524: ATP binding	9.90E-08	420,243,68
1ytba2#217	GO:0016251: general RNA polymerase II transcription factor activity	9.96E-08	26,14,5
1dt6a_#449	GO:0016705: oxidoreductase activity, acting on paired donors, with incorporation or reduction of molecular oxygen	9.96E-08	14,26,5
1c4ra_#258	GO:0003809: thrombin activity	1.02E-07	80,10,6
1mjha_#126	GO:0008483: transaminase activity	1.02E-07	159,17,9
2e2c__#67	GO:0004842: ubiquitin-protein ligase activity	1.02E-07	19,19,5
1j9la_#82	GO:0005525: GTP binding	1.03E-07	169,49,15
1htp__#16	GO:0019955: cytokine binding	1.05E-07	303,11,9
1fgga_#134	GO:0000287: magnesium ion binding	1.05E-07	218,128,29
1phm_2#253	GO:0004263: chymotrypsin activity	1.05E-07	309,41,18
1chua2#21	GO:0016668: oxidoreductase activity, acting on sulfur group of donors, NAD or NADP as acceptor	1.05E-07	12,12,4
1mrj__#156	GO:0005524: ATP binding	1.05E-07	61,243,20
1fnf_3#1409	GO:0019955: cytokine binding	1.05E-07	71,11,6
1d6ja_#36	GO:0005524: ATP binding	1.07E-07	27,243,13
3grx__#56	GO:0005524: ATP binding	1.07E-07	335,243,58
1aqb__#134	GO:0050660: FAD binding	1.10E-07	81,10,6
1f8ra1#288	GO:0005525: GTP binding	1.10E-07	49,49,9
1a2za_#209	GO:0015036: disulfide oxidoreductase activity	1.10E-07	7,22,4
1j79a_#38	GO:0004457: lactate dehydrogenase activity	1.10E-07	39,10,5
1fxla2#169	GO:0019955: cytokine binding	1.13E-07	203,11,8
1fwxa2#396	GO:0003809: thrombin activity	1.13E-07	237,10,8
1hxmb1#117	GO:0004896: hematopoietin/interferon-class (D200-domain) cytokine receptor activity	1.14E-07	64,19,7
1jf9a_#379	GO:0003887: DNA-directed DNA polymerase activity	1.14E-07	232,20,11
1flca1#294	GO:0004896: hematopoietin/interferon-class (D200-domain) cytokine receptor activity	1.15E-07	98,19,8
1b3qa2#623	GO:0005507: copper ion binding	1.16E-07	301,38,17
2bbkh_#214	GO:0004896: hematopoietin/interferon-class (D200-domain) cytokine receptor activity	1.16E-07	38,19,6
1ceza_#227	GO:0003700: transcription factor activity	1.16E-07	27,124,10
1sfp__#84	GO:0004896: hematopoietin/interferon-class (D200-domain) cytokine receptor activity	1.16E-07	8,19,4
1pvc1_#106	GO:0005507: copper ion binding	1.17E-07	64,38,9
3grs_3#435	GO:0016668: oxidoreductase activity, acting on sulfur group of donors, NAD or NADP as acceptor	1.18E-07	179,12,8
1opy__#68	GO:0003809: thrombin activity	1.18E-07	367,10,9
1djna1#86	GO:0004556: alpha-amylase activity	1.18E-07	85,15,7
1bf2_3#575	GO:0005509: calcium ion binding	1.19E-07	13,160,8
1dpja_#322	GO:0019955: cytokine binding	1.21E-07	308,11,9
1fu6a_#50	GO:0019955: cytokine binding	1.21E-07	308,11,9
1h6la_#119	GO:0019955: cytokine binding	1.21E-07	308,11,9
1c1da1#153	GO:0016616: oxidoreductase activity, acting on the CH-OH group of donors, NAD or NADP as acceptor	1.22E-07	13,59,6
1ecpa_#58	GO:0005525: GTP binding	1.22E-07	83,49,11
1el5a1#166	GO:0050660: FAD binding	1.23E-07	15,10,4
1eg5a_#197	GO:0016846: carbon-sulfur lyase activity	1.23E-07	15,10,4
1g4ia_#11	GO:0004263: chymotrypsin activity	1.25E-07	29,41,7
1nfdb1#21	GO:0019838: growth factor binding	1.26E-07	96,9,6
1kit_3#743	GO:0005509: calcium ion binding	1.28E-07	250,160,36
1f0xa1#278	GO:0003887: DNA-directed DNA polymerase activity	1.28E-07	180,20,10
1gdea_#32	GO:0008483: transaminase activity	1.28E-07	9,17,4
1h7wa2#535	GO:0016627: oxidoreductase activity, acting on the CH-CH group of donors	1.28E-07	9,17,4
1cwva2#682	GO:0019838: growth factor binding	1.28E-07	17,9,4

1cnv__#264	GO:0005525: GTP binding	1.29E-07	103,49,12
1qdla_#211	GO:0005524: ATP binding	1.29E-07	320,243,56
2pia_1#99	GO:0005524: ATP binding	1.29E-07	179,243,38
2bc2a_#46	GO:0015078: hydrogen ion transporter activity	1.31E-07	18,21,5
1dlfl_#97	GO:0019955: cytokine binding	1.32E-07	36,11,5
1bzya_#173	GO:0016763: transferase activity, transferring pentosyl groups	1.32E-07	6,28,4
1i50a_#856	GO:0016668: oxidoreductase activity, acting on sulfur group of donors, NAD or NADP as acceptor	1.32E-07	269,12,9
1f0xa1#278	GO:0004263: chymotrypsin activity	1.33E-07	180,41,14
1qnja_#77	GO:0004867: serine-type endopeptidase inhibitor activity	1.33E-07	16,47,6
1fc4a_#205	GO:0004556: alpha-amylase activity	1.34E-07	267,15,10
1ac6a_#34	GO:0005518: collagen binding	1.35E-07	60,13,6
1eh9a3#152	GO:0004556: alpha-amylase activity	1.35E-07	3,15,3
1fm2.1#B319	GO:0003887: DNA-directed DNA polymerase activity	1.35E-07	19,20,5
1cs1a_#82	GO:0000287: magnesium ion binding	1.36E-07	195,128,27
1qfea_#68	GO:0008757: S-adenosylmethionine-dependent methyltransferase activity	1.36E-07	560,24,17
1jcfa2#206	GO:0004457: lactate dehydrogenase activity	1.37E-07	84,10,6
1ej8a_#189	GO:0019838: growth factor binding	1.37E-07	294,9,8
1a65a1#108	GO:0019838: growth factor binding	1.37E-07	294,9,8
1pme__#24	GO:0005524: ATP binding	1.38E-07	10,243,8
1flga_#161	GO:0004263: chymotrypsin activity	1.38E-07	152,41,13
1g0sa_#176	GO:0003887: DNA-directed DNA polymerase activity	1.39E-07	37,20,6
1f3la_#348	GO:0008757: S-adenosylmethionine-dependent methyltransferase activity	1.40E-07	16,24,5
1ddma_#130	GO:0003755: peptidyl-prolyl cis-trans isomerase activity	1.40E-07	313,11,9
1bxoa_#320	GO:0004896: hematopoietin/interferon-class (D200-domain) cytokine receptor activity	1.42E-07	66,19,7
1h68a_#183	GO:0001584: rhodopsin-like receptor activity	1.42E-07	4,10,3
1hvba_#76	GO:0008800: beta-lactamase activity	1.42E-07	4,10,3
1e25a_#139	GO:0008800: beta-lactamase activity	1.42E-07	4,10,3
1g79a_#85	GO:0008009: chemokine activity	1.42E-07	4,10,3
1d3ga_#118	GO:0016616: oxidoreductase activity, acting on the CH-OH group of donors, NAD or NADP as acceptor	1.43E-07	42,59,9
1mjha_#126	GO:0000287: magnesium ion binding	1.46E-07	159,128,24
1hq0a_#867	GO:0000287: magnesium ion binding	1.46E-07	171,128,25
1hq0a_#867	GO:0004812: tRNA ligase activity	1.47E-07	171,26,11
1bfta_#285	GO:0004867: serine-type endopeptidase inhibitor activity	1.47E-07	9,47,5
1sek__#45	GO:0004867: serine-type endopeptidase inhibitor activity	1.47E-07	9,47,5
1e05i_#88	GO:0004867: serine-type endopeptidase inhibitor activity	1.47E-07	9,47,5
1fxma_#153	GO:0004812: tRNA ligase activity	1.48E-07	15,26,5
1e1oa1#142	GO:0004812: tRNA ligase activity	1.48E-07	15,26,5
1hbza_#214	GO:0019838: growth factor binding	1.49E-07	297,9,8
1fo4a5#1020	GO:0004556: alpha-amylase activity	1.50E-07	359,15,11
1dik_1#765	GO:0019201: nucleotide kinase activity	1.52E-07	165,13,8
1dih_2#222	GO:0005525: GTP binding	1.53E-07	352,49,21
1hbza_#96	GO:0004867: serine-type endopeptidase inhibitor activity	1.53E-07	370,47,21
1g51a3#525	GO:0005509: calcium ion binding	1.53E-07	263,160,37
1pma1_#17	GO:0004263: chymotrypsin activity	1.53E-07	44,41,8
2cuaa_#107	GO:0015078: hydrogen ion transporter activity	1.53E-07	128,21,9
1f42a2#123	GO:0003809: thrombin activity	1.54E-07	378,10,9
1cb8a2#613	GO:0005507: copper ion binding	1.54E-07	138,38,12
1euha_#349	GO:0016799: hydrolase activity, hydrolyzing N-glycosyl compounds	1.56E-07	23,17,5
1dpja_#322	GO:0004674: protein serine/threonine kinase activity	1.57E-07	308,42,18
1h6la_#119	GO:0004674: protein serine/threonine kinase activity	1.57E-07	308,42,18
1i50b_#966	GO:0019838: growth factor binding	1.57E-07	299,9,8
1am2__#163	GO:0004896: hematopoietin/interferon-class (D200-domain) cytokine receptor activity	1.58E-07	102,19,8
1cmxa_#207	GO:0003887: DNA-directed DNA polymerase activity	1.58E-07	96,20,8
1kdj__#27	GO:0004896: hematopoietin/interferon-class (D200-domain) cytokine receptor activity	1.58E-07	67,19,7
1oaca2#150	GO:0005507: copper ion binding	1.59E-07	5,38,4
1ekma2#98	GO:0005507: copper ion binding	1.59E-07	5,38,4
1av4_3#167	GO:0005507: copper ion binding	1.59E-07	5,38,4
1i6vd_#713	GO:0004896: hematopoietin/interferon-class (D200-domain) cytokine receptor activity	1.60E-07	40,19,6
1i6vd_#713	GO:0016646: oxidoreductase activity, acting on the CH-NH group of donors, NAD or NADP as acceptor	1.60E-07	40,19,6
1apme_#74	GO:0004674: protein serine/threonine kinase activity	1.60E-07	124,42,12
1h6la_#119	GO:0051082: unfolded protein binding	1.61E-07	308,34,16
1ev2e2#286	GO:0019838: growth factor binding	1.62E-07	100,9,6
1j9qa2#295	GO:0015078: hydrogen ion transporter activity	1.62E-07	36,21,6

2tgi__#106	GO:0008083: growth factor activity	1.62E-07	10,42,5
1gpea1#271	GO:0004896: hematopoietin/interferon-class (D200-domain) cytokine receptor activity	1.62E-07	197,19,10
1awx__#17	GO:0005066: transmembrane receptor protein tyrosine kinase signaling protein activity	1.63E-07	16,10,4
1ad3a_#181	GO:0016620: oxidoreductase activity, acting on the aldehyde or oxo group of donors, NAD or NADP as acceptor	1.63E-07	16,10,4
1qfma1#186	GO:0004896: hematopoietin/interferon-class (D200-domain) cytokine receptor activity	1.64E-07	326,19,12
1kcw_3#440	GO:0005507: copper ion binding	1.64E-07	20,38,6
1az9_2#257	GO:0004177: aminopeptidase activity	1.64E-07	31,13,5
1b3qa2#623	GO:0019838: growth factor binding	1.66E-07	301,9,8
1e8ga1#436	GO:0005524: ATP binding	1.67E-07	92,243,25
1aqua_#126	GO:0004457: lactate dehydrogenase activity	1.67E-07	576,10,10
1jj2i_#30	GO:0015036: disulfide oxidoreductase activity	1.69E-07	18,22,5
1cb8a2#613	GO:0005524: ATP binding	1.69E-07	138,243,32
1i76a_#138	GO:0004222: metalloendopeptidase activity	1.70E-07	103,19,8
1el0a_#42	GO:0008201: heparin binding	1.73E-07	294,24,13
1bura1#269	GO:0016831: carboxy-lyase activity	1.74E-07	16,25,5
1rb9__#49	GO:0004867: serine-type endopeptidase inhibitor activity	1.74E-07	39,47,8
1ddwa_#77	GO:0004556: alpha-amylase activity	1.74E-07	364,15,11
1dv8a_#211	GO:0005529: sugar binding	1.74E-07	86,39,10
1htp__#16	GO:0019838: growth factor binding	1.75E-07	303,9,8
3grs_1#23	GO:0019201: nucleotide kinase activity	1.75E-07	168,13,8
1edza1#206	GO:0004295: trypsin activity	1.75E-07	399,48,22
1egia_#671	GO:0005529: sugar binding	1.78E-07	5,39,4
1i9sa_#24	GO:0004725: protein tyrosine phosphatase activity	1.78E-07	53,15,6
1cpy__#93	GO:0004556: alpha-amylase activity	1.78E-07	275,15,10
1egaa1#58	GO:0003924: GTPase activity	1.78E-07	46,17,6
1edza1#206	GO:0004263: chymotrypsin activity	1.80E-07	399,41,20
1bev1_#86	GO:0005524: ATP binding	1.81E-07	323,243,56
1e4ea2#215	GO:0019838: growth factor binding	1.85E-07	490,9,9
1p35a_#101	GO:0019955: cytokine binding	1.85E-07	136,11,7
1g51a3#524	GO:0004812: tRNA ligase activity	1.87E-07	102,26,9
1b34b_#56	GO:0004674: protein serine/threonine kinase activity	1.93E-07	30,42,7
1jj2e1#42	GO:0015036: disulfide oxidoreductase activity	1.95E-07	167,22,10
2mev3_#143	GO:0003724: RNA helicase activity	1.95E-07	4,11,3
1aym2_#253	GO:0003724: RNA helicase activity	1.95E-07	4,11,3
2mev1_#246	GO:0003724: RNA helicase activity	1.95E-07	4,11,3
2mev1_#120	GO:0003724: RNA helicase activity	1.95E-07	4,11,3
2hrva_#123	GO:0003724: RNA helicase activity	1.95E-07	4,11,3
1b35b_#123	GO:0003724: RNA helicase activity	1.95E-07	4,11,3
1ekbb_#73	GO:0004867: serine-type endopeptidase inhibitor activity	1.96E-07	27,47,7
2hhma_#262	GO:0000287: magnesium ion binding	1.97E-07	95,128,18
1fn9a_#271	GO:0019955: cytokine binding	1.98E-07	218,11,8
1ja9a_#165	GO:0016616: oxidoreductase activity, acting on the CH-OH group of donors, NAD or NADP as acceptor	1.99E-07	4,59,4
1h6la_#119	GO:0019838: growth factor binding	1.99E-07	308,9,8
1hzxa_#139	GO:0004364: glutathione transferase activity	2.00E-07	39,11,5
1efpa1#32	GO:0005525: GTP binding	2.01E-07	129,49,13
1qi7a_#68	GO:0016799: hydrolase activity, hydrolyzing N-glycosyl compounds	2.02E-07	3,17,3
1phm_2#253	GO:0019838: growth factor binding	2.05E-07	309,9,8
1f97a1#62	GO:0004190: aspartic-type endopeptidase activity	2.05E-07	707,23,18
1qnja_#124	GO:0005509: calcium ion binding	2.05E-07	18,160,9
1e8ya2#433	GO:0005509: calcium ion binding	2.05E-07	18,160,9
1ii7a_#43	GO:0004457: lactate dehydrogenase activity	2.07E-07	44,10,5
1by5a_#347	GO:0004896: hematopoietin/interferon-class (D200-domain) cytokine receptor activity	2.07E-07	263,19,11
1a3k__#122	GO:0004896: hematopoietin/interferon-class (D200-domain) cytokine receptor activity	2.08E-07	9,19,4
1mjha_#126	GO:0016846: carbon-sulfur lyase activity	2.10E-07	159,10,7
1imva_#255	GO:0050660: FAD binding	2.10E-07	589,10,10
1ga6a_#198	GO:0042802: protein self binding	2.11E-07	14,12,4
1qgxa_#299	GO:0000287: magnesium ion binding	2.12E-07	5,128,5
1c7na_#231	GO:0016616: oxidoreductase activity, acting on the CH-OH group of donors, NAD or NADP as acceptor	2.13E-07	263,59,20
1danh_#93	GO:0003809: thrombin activity	2.13E-07	17,10,4
1otha2#300	GO:0008483: transaminase activity	2.13E-07	10,17,4
1ep3a_#19	GO:0016627: oxidoreductase activity, acting on the CH-CH group of donors	2.13E-07	10,17,4
1oaca1#342	GO:0016638: oxidoreductase activity, acting on the CH-NH2 group of donors	2.13E-07	10,17,4

1h5qa_#20	GO:0016616: oxidoreductase activity, acting on the CH-OH group of donors, NAD or NADP as acceptor	2.14E-07	8,59,5
1e4ea2#215	GO:0004263: chymotrypsin activity	2.14E-07	490,41,22
1aq0a_#4	GO:0008810: cellulase activity	2.15E-07	23,18,5
1b12a_#221	GO:0005507: copper ion binding	2.16E-07	115,38,11
1ct9a1#401	GO:0004222: metalloendopeptidase activity	2.17E-07	42,19,6
1i2ma_#157	GO:0005085: guanyl-nucleotide exchange factor activity	2.18E-07	13,13,4
3aky_1#194	GO:0019201: nucleotide kinase activity	2.18E-07	13,13,4
1elva1#597	GO:0004263: chymotrypsin activity	2.18E-07	5,41,4
1fiw.1#A159	GO:0004263: chymotrypsin activity	2.18E-07	5,41,4
1f8ra1#64	GO:0015036: disulfide oxidoreductase activity	2.19E-07	8,22,4
1xnb__#130	GO:0005524: ATP binding	2.20E-07	87,243,24
1erv__#57	GO:0005524: ATP binding	2.20E-07	87,243,24
1c7na_#231	GO:0005525: GTP binding	2.20E-07	263,49,18
1qaxa2#307	GO:0004263: chymotrypsin activity	2.21E-07	46,41,8
1phm_2#253	GO:0016638: oxidoreductase activity, acting on the CH-NH2 group of donors	2.23E-07	309,17,11
1qhda2#323	GO:0015036: disulfide oxidoreductase activity	2.23E-07	337,22,13
1ile_3#408	GO:0004812: tRNA ligase activity	2.24E-07	7,26,4
1cs1a_#160	GO:0000287: magnesium ion binding	2.24E-07	187,128,26
1g25a_#22	GO:0004896: hematopoietin/interferon-class (D200-domain) cytokine receptor activity	2.25E-07	415,19,13
1dlfh_#4	GO:0004896: hematopoietin/interferon-class (D200-domain) cytokine receptor activity	2.28E-07	22,19,5
1cmia_#86	GO:0019955: cytokine binding	2.28E-07	40,11,5
1erza_#183	GO:0008270: zinc ion binding	2.30E-07	79,108,15
1gg4a3#64	GO:0004457: lactate dehydrogenase activity	2.32E-07	45,10,5
3grs_3#435	GO:0004812: tRNA ligase activity	2.36E-07	179,26,11
1h6va1#27	GO:0005524: ATP binding	2.38E-07	260,243,48
1dfca1#1076	GO:0008083: growth factor activity	2.41E-07	5,42,4
1fw9a_#152	GO:0004523: ribonuclease H activity	2.42E-07	133,16,8
1bqca_#268	GO:0008810: cellulase activity	2.42E-07	3,18,3
1bhga3#540	GO:0008810: cellulase activity	2.42E-07	3,18,3
1e0ta2#275	GO:0016836: hydro-lyase activity	2.49E-07	107,33,10
1e79d2#36	GO:0004674: protein serine/threonine kinase activity	2.49E-07	524,42,23
1dik_1#765	GO:0016763: transferase activity, transferring pentosyl groups	2.52E-07	165,28,11
1f5aa2#167	GO:0016799: hydrolase activity, hydrolyzing N-glycosyl compounds	2.53E-07	124,17,8
1qoua_#90	GO:0016251: general RNA polymerase II transcription factor activity	2.54E-07	31,14,5
2mpra_#190	GO:0003809: thrombin activity	2.55E-07	400,10,9
2mpra_#190	GO:0050660: FAD binding	2.55E-07	400,10,9
1e39a3#417	GO:0004263: chymotrypsin activity	2.60E-07	11,41,5
1b6ra3#161	GO:0004457: lactate dehydrogenase activity	2.60E-07	164,10,7
1dp0a5#499	GO:0005525: GTP binding	2.63E-07	17,49,6
1a6o__#297	GO:0005126: hematopoietin/interferon-class (D200-domain) cytokine receptor binding	2.64E-07	41,20,6
1ga6a_#198	GO:0005509: calcium ion binding	2.64E-07	14,160,8
1i6vd_#1188	GO:0005525: GTP binding	2.64E-07	27,49,7
1aym3_#131	GO:0015036: disulfide oxidoreductase activity	2.66E-07	412,22,14
1fjsa_#83	GO:0004896: hematopoietin/interferon-class (D200-domain) cytokine receptor activity	2.66E-07	109,19,8
1dm9a_#57	GO:0004896: hematopoietin/interferon-class (D200-domain) cytokine receptor activity	2.66E-07	109,19,8
1e2o__#346	GO:0019838: growth factor binding	2.66E-07	194,9,7
1ea5a_#99	GO:0019838: growth factor binding	2.66E-07	194,9,7
1gpc__#184	GO:0019955: cytokine binding	2.73E-07	83,11,6
1el5a1#10	GO:0004457: lactate dehydrogenase activity	2.73E-07	18,10,4
1et0a_#118	GO:0050660: FAD binding	2.73E-07	18,10,4
2uaga1#54	GO:0050660: FAD binding	2.73E-07	18,10,4
1qbea_#92	GO:0003809: thrombin activity	2.73E-07	18,10,4
1thg__#127	GO:0016846: carbon-sulfur lyase activity	2.73E-07	18,10,4
1qp8a1#172	GO:0004457: lactate dehydrogenase activity	2.73E-07	18,10,4
1g5qa_#80	GO:0004457: lactate dehydrogenase activity	2.73E-07	18,10,4
1rypk_#12	GO:0050660: FAD binding	2.74E-07	265,10,8
7reqa2#702	GO:0000287: magnesium ion binding	2.75E-07	59,128,14
1fc3a_#172	GO:0004497: monooxygenase activity	2.80E-07	182,26,11
1cgha_#130	GO:0005509: calcium ion binding	2.81E-07	29,160,11
1jsg__#92	GO:0003809: thrombin activity	2.84E-07	607,10,10
1f3ya_#6	GO:0004867: serine-type endopeptidase inhibitor activity	2.85E-07	94,47,11
1mrp__#172	GO:0016646: oxidoreductase activity, acting on the CH-NH group of donors, NAD or NADP as acceptor	2.86E-07	110,19,8
1eqka_#47	GO:0016638: oxidoreductase activity, acting on the CH-NH2 group of donors	2.87E-07	126,17,8
1fw8a_#257	GO:0030151: molybdenum ion binding	2.87E-07	12,15,4

1rmg__#128	GO:0004222: metalloendopeptidase activity	2.88E-07	3,19,3
1hxn__#236	GO:0004222: metalloendopeptidase activity	2.88E-07	3,19,3
1fvia2#25	GO:0004896: hematopoietin/interferon-class (D200-domain) cytokine receptor activity	2.90E-07	44,19,6
1g0sa_#74	GO:0016668: oxidoreductase activity, acting on sulfur group of donors, NAD or NADP as acceptor	2.90E-07	294,12,9
1sek__#165	GO:0004867: serine-type endopeptidase inhibitor activity	2.90E-07	10,47,5
1ac5__#72	GO:0004457: lactate dehydrogenase activity	2.90E-07	47,10,5
1bev1_#86	GO:0019838: growth factor binding	2.91E-07	323,9,8
1jbqa_#152	GO:0015036: disulfide oxidoreductase activity	2.92E-07	62,22,7
21bi__#57	GO:0008083: growth factor activity	2.94E-07	11,42,5
1dbha2#460	GO:0005524: ATP binding	2.95E-07	17,243,10
1fo4a5#1020	GO:0004674: protein serine/threonine kinase activity	2.97E-07	359,42,19
1guqa1#166	GO:0004295: trypsin activity	3.00E-07	28,48,7
1qbea_#92	GO:0004867: serine-type endopeptidase inhibitor activity	3.02E-07	18,47,6
1c5y.1#B18	GO:0004867: serine-type endopeptidase inhibitor activity	3.02E-07	18,47,6
1fa0a1#393	GO:0008235: metalloexopeptidase activity	3.02E-07	261,13,9
1dbha2#460	GO:0004812: tRNA ligase activity	3.02E-07	17,26,5
1fgga_#134	GO:0016616: oxidoreductase activity, acting on the CH-OH group of donors, NAD or NADP as acceptor	3.04E-07	218,59,18
3aky_1#194	GO:0016776: phosphotransferase activity, phosphate group as acceptor	3.04E-07	13,14,4
1hhsa_#328	GO:0008408: 3'-5' exonuclease activity	3.04E-07	14,13,4
2tbva_#214	GO:0003968: RNA-directed RNA polymerase activity	3.04E-07	13,14,4
1b35b_#226	GO:0003968: RNA-directed RNA polymerase activity	3.04E-07	13,14,4
1b8oa_#241	GO:0016763: transferase activity, transferring pentosyl groups	3.06E-07	7,28,4
1b8oa_#234	GO:0016763: transferase activity, transferring pentosyl groups	3.06E-07	7,28,4
1dik_1#848	GO:0016831: carboxy-lyase activity	3.09E-07	81,25,8
1e8ca1#83	GO:0005524: ATP binding	3.11E-07	170,243,36
1d2oa1#541	GO:0005518: collagen binding	3.11E-07	35,13,5
2sli_2#326	GO:0004674: protein serine/threonine kinase activity	3.13E-07	251,42,16
1qfma1#186	GO:0019838: growth factor binding	3.13E-07	326,9,8
2viua_#252	GO:0019955: cytokine binding	3.15E-07	343,11,9
1feca1#31	GO:0016616: oxidoreductase activity, acting on the CH-OH group of donors, NAD or NADP as acceptor	3.17E-07	75,59,11
2fnba_#67	GO:0008201: heparin binding	3.17E-07	8,24,4
1fxla2#169	GO:0016668: oxidoreductase activity, acting on sulfur group of donors, NAD or NADP as acceptor	3.17E-07	203,12,8
1b3ra2#60	GO:0004725: protein tyrosine phosphatase activity	3.18E-07	30,15,5
1pa2a_#232	GO:0004601: peroxidase activity	3.21E-07	9,21,4
1jg8a_#172	GO:0016616: oxidoreductase activity, acting on the CH-OH group of donors, NAD or NADP as acceptor	3.23E-07	92,59,12
1io7a_#243	GO:0004497: monooxygenase activity	3.23E-07	78,26,8
1xgsa2#186	GO:0008235: metalloexopeptidase activity	3.29E-07	182,13,8
1plq_1#90	GO:0004896: hematopoietin/interferon-class (D200-domain) cytokine receptor activity	3.31E-07	158,19,9
1aym3_#131	GO:0050660: FAD binding	3.32E-07	412,10,9
2cpl__#32	GO:0003755: peptidyl-prolyl cis-trans isomerase activity	3.34E-07	17,11,4
1hwxa1#324	GO:0016638: oxidoreductase activity, acting on the CH-NH2 group of donors	3.34E-07	11,17,4
2arca_#64	GO:0016638: oxidoreductase activity, acting on the CH-NH2 group of donors	3.34E-07	11,17,4
1bgva1#350	GO:0016638: oxidoreductase activity, acting on the CH-NH2 group of donors	3.34E-07	11,17,4
1by5a_#238	GO:0015082: di-, tri-valent inorganic cation transporter activity	3.35E-07	237,14,9
1by5a_#238	GO:0046915: transition metal ion transporter activity	3.35E-07	237,14,9
1gox__#124	GO:0016861: intramolecular oxidoreductase activity, interconverting aldoses and ketoses	3.35E-07	366,13,10
1i50b_#966	GO:0016668: oxidoreductase activity, acting on sulfur group of donors, NAD or NADP as acceptor	3.36E-07	299,12,9
1dik_1#765	GO:0016776: phosphotransferase activity, phosphate group as acceptor	3.36E-07	165,14,8
1f9va_#627	GO:0003924: GTPase activity	3.37E-07	51,17,6
1hq8a_#206	GO:0019838: growth factor binding	3.37E-07	329,9,8
1e2wa1#102	GO:0019955: cytokine binding	3.38E-07	86,11,6
1eur__#84	GO:0019955: cytokine binding	3.38E-07	86,11,6
1tvda_#57D	GO:0008080: N-acetyltransferase activity	3.39E-07	4,13,3
3tmka_#98	GO:0019201: nucleotide kinase activity	3.39E-07	4,13,3
1e79d2#36	GO:0019838: growth factor binding	3.40E-07	524,9,9
1ejda_#213	GO:0004867: serine-type endopeptidase inhibitor activity	3.44E-07	284,47,18
1a7j__#87	GO:0019201: nucleotide kinase activity	3.45E-07	70,13,6
1e0ta2#10	GO:0000287: magnesium ion binding	3.45E-07	29,128,10
2viua_#252	GO:0004295: trypsin activity	3.46E-07	343,48,20

1brma2#268	GO:0016620: oxidoreductase activity, acting on the aldehyde or oxo group of donors, NAD or NADP as acceptor	3.46E-07	19,10,4
1qrra_#11	GO:0004457: lactate dehydrogenase activity	3.46E-07	19,10,4
1neb__#52	GO:0005066: transmembrane receptor protein tyrosine kinase signaling protein activity	3.46E-07	19,10,4
1c7na_#95	GO:0008483: transaminase activity	3.49E-07	183,17,9
1fkna_#96	GO:0004896: hematopoietin/interferon-class (D200-domain) cytokine receptor activity	3.49E-07	75,19,7
1qrra_#69	GO:0016616: oxidoreductase activity, acting on the CH-OH group of donors, NAD or NADP as acceptor	3.49E-07	196,59,17
3frua1#236	GO:0004867: serine-type endopeptidase inhibitor activity	3.53E-07	76,47,10
1bywa_#30	GO:0019838: growth factor binding	3.54E-07	331,9,8
1elua_#200	GO:0016846: carbon-sulfur lyase activity	3.55E-07	5,10,3
1d2fa_#170	GO:0016846: carbon-sulfur lyase activity	3.55E-07	5,10,3
1jg8a_#210	GO:0016846: carbon-sulfur lyase activity	3.55E-07	5,10,3
1dfca4#1451	GO:0004263: chymotrypsin activity	3.57E-07	67,41,9
1c8za_#386	GO:0019955: cytokine binding	3.59E-07	235,11,8
1ile_2#304	GO:0005524: ATP binding	3.59E-07	21,243,11
1as4.1#A336	GO:0019838: growth factor binding	3.63E-07	332,9,8
1jjya_#329	GO:0005524: ATP binding	3.64E-07	71,243,21
1g6oa_#40	GO:0004263: chymotrypsin activity	3.65E-07	21,41,6
1h5qa_#70	GO:0016627: oxidoreductase activity, acting on the CH-CH group of donors	3.69E-07	27,17,5
1eq9a_#40	GO:0004674: protein serine/threonine kinase activity	3.73E-07	86,42,10
1jjya_#329	GO:0019201: nucleotide kinase activity	3.76E-07	71,13,6
1iba__#39	GO:0004896: hematopoietin/interferon-class (D200-domain) cytokine receptor activity	3.78E-07	351,19,12
3grs_3#435	GO:0015036: disulfide oxidoreductase activity	3.79E-07	179,22,10
1kapp2#69	GO:0016799: hydrolase activity, hydrolyzing N-glycosyl compounds	3.79E-07	52,17,6
1dn2a1#261	GO:0016668: oxidoreductase activity, acting on sulfur group of donors, NAD or NADP as acceptor	3.81E-07	425,12,10
1prea2#308	GO:0004896: hematopoietin/interferon-class (D200-domain) cytokine receptor activity	3.83E-07	76,19,7
1kve.1#B199	GO:0004896: hematopoietin/interferon-class (D200-domain) cytokine receptor activity	3.83E-07	76,19,7
3grs_1#23	GO:0016776: phosphotransferase activity, phosphate group as acceptor	3.87E-07	168,14,8
1feza_#185	GO:0005351: sugar porter activity	3.89E-07	68,21,7
1mdah_#329	GO:0019838: growth factor binding	3.89E-07	335,9,8
1hxxa_#232	GO:0004896: hematopoietin/interferon-class (D200-domain) cytokine receptor activity	3.90E-07	352,19,12
1qfma2#576	GO:0005525: GTP binding	3.91E-07	214,49,16
1elja_#274	GO:0005351: sugar porter activity	3.95E-07	3,21,3
1ecpa_#156	GO:0015036: disulfide oxidoreductase activity	3.95E-07	21,22,5
1e8ca3#111	GO:0016646: oxidoreductase activity, acting on the CH-NH group of donors, NAD or NADP as acceptor	3.98E-07	280,19,11
1in4a1#264	GO:0003916: DNA topoisomerase activity	3.99E-07	50,10,5
1nat__#50	GO:0005524: ATP binding	4.05E-07	297,243,52
1foha5#9	GO:0015036: disulfide oxidoreductase activity	4.07E-07	65,22,7
1d0na6#681	GO:0005524: ATP binding	4.08E-07	143,243,32
1fvia2#25	GO:0005126: hematopoietin/interferon-class (D200-domain) cytokine receptor binding	4.09E-07	44,20,6
1svb_2#165	GO:0003968: RNA-directed RNA polymerase activity	4.12E-07	34,14,5
1i2ma_#157	GO:0005096: GTPase activator activity	4.13E-07	13,15,4
1i2ma_#90	GO:0005085: guanyl-nucleotide exchange factor activity	4.13E-07	15,13,4
1bupa1#178	GO:0005524: ATP binding	4.16E-07	14,243,9
1elva1#597	GO:0004295: trypsin activity	4.19E-07	5,48,4
1fiw.1#A159	GO:0004295: trypsin activity	4.19E-07	5,48,4
1czan1#92	GO:0050660: FAD binding	4.19E-07	631,10,10
1jj2s_#46	GO:0003755: peptidyl-prolyl cis-trans isomerase activity	4.20E-07	45,11,5
1dyka2#2937	GO:0016251: general RNA polymerase II transcription factor activity	4.24E-07	14,14,4
1qqp3_#139	GO:0003968: RNA-directed RNA polymerase activity	4.24E-07	14,14,4
1fp3a_#350	GO:0008810: cellulase activity	4.28E-07	11,18,4
2mev3_#143	GO:0003968: RNA-directed RNA polymerase activity	4.31E-07	4,14,3
2hrva_#123	GO:0003968: RNA-directed RNA polymerase activity	4.31E-07	4,14,3
3tmka_#98	GO:0016776: phosphotransferase activity, phosphate group as acceptor	4.31E-07	4,14,3
1b35a_#49	GO:0003968: RNA-directed RNA polymerase activity	4.31E-07	4,14,3
1ofga1#84	GO:0004457: lactate dehydrogenase activity	4.31E-07	20,10,4
1sek__#179	GO:0004867: serine-type endopeptidase inhibitor activity	4.32E-07	30,47,7
1qfxa_#380	GO:0004896: hematopoietin/interferon-class (D200-domain) cytokine receptor activity	4.35E-07	47,19,6
1dpja_#322	GO:0016668: oxidoreductase activity, acting on sulfur group of donors, NAD or NADP as acceptor	4.36E-07	308,12,9
1dn2a1#261	GO:0050660: FAD binding	4.38E-07	425,10,9
1avgi_#69	GO:0019838: growth factor binding	4.38E-07	340,9,8
1h9da_#91	GO:0004556: alpha-amylase activity	4.39E-07	222,15,9

1jb3a_#64	GO:0030151: molybdenum ion binding	4.39E-07	302,15,10
1fid__#237	GO:0003809: thrombin activity	4.42E-07	51,10,5
1doka_#52	GO:0008009: chemokine activity	4.42E-07	51,10,5
3grx__#56	GO:0015082: di-, tri-valent inorganic cation transporter activity	4.42E-07	335,14,10
3grx__#56	GO:0046915: transition metal ion transporter activity	4.42E-07	335,14,10
1ajsa_#184	GO:0016616: oxidoreductase activity, acting on the CH-OH group of donors, NAD or NADP as acceptor	4.44E-07	35,59,8
1fbna_#145	GO:0016616: oxidoreductase activity, acting on the CH-OH group of donors, NAD or NADP as acceptor	4.44E-07	35,59,8
2ae2a_#89	GO:0016616: oxidoreductase activity, acting on the CH-OH group of donors, NAD or NADP as acceptor	4.44E-07	35,59,8
1bu7a_#360	GO:0016705: oxidoreductase activity, acting on paired donors, with incorporation or reduction of molecular oxygen	4.45E-07	8,26,4
1kdj__#27	GO:0008083: growth factor activity	4.45E-07	67,42,9
1dr9a2#139	GO:0008083: growth factor activity	4.45E-07	67,42,9
1dfca4#1451	GO:0008083: growth factor activity	4.45E-07	67,42,9
1as4.1#A336	GO:0003887: DNA-directed DNA polymerase activity	4.50E-07	332,20,12
1ayl__#237	GO:0005524: ATP binding	4.53E-07	357,243,59
1a6o__#45	GO:0030151: molybdenum ion binding	4.53E-07	303,15,10
1cipa2#34	GO:0004222: metalloendopeptidase activity	4.59E-07	78,19,7
3sil__#317	GO:0019955: cytokine binding	4.59E-07	358,11,9
1aqb__#134	GO:0016668: oxidoreductase activity, acting on sulfur group of donors, NAD or NADP as acceptor	4.59E-07	81,12,6
2hrva_#132	GO:0004295: trypsin activity	4.62E-07	43,48,8
1hwxa1#223	GO:0000287: magnesium ion binding	4.62E-07	233,128,29
1mjha_#119	GO:0005524: ATP binding	4.63E-07	103,243,26
1nuka_#80	GO:0004896: hematopoietin/interferon-class (D200-domain) cytokine receptor activity	4.66E-07	220,19,10
1imva_#255	GO:0004674: protein serine/threonine kinase activity	4.67E-07	589,42,24
1pma1_#17	GO:0004867: serine-type endopeptidase inhibitor activity	4.70E-07	44,47,8
1gox__#124	GO:0005524: ATP binding	4.71E-07	366,243,60
1j9qa2#306	GO:0005507: copper ion binding	4.73E-07	6,38,4
1j9qa2#317	GO:0005507: copper ion binding	4.73E-07	6,38,4
1jsg__#92	GO:0008201: heparin binding	4.75E-07	607,24,17
1nbaa_#227	GO:0004364: glutathione transferase activity	4.75E-07	91,11,6
1dzfa2#211	GO:0004295: trypsin activity	4.76E-07	167,48,14
1ib2a_#1101	GO:0015078: hydrogen ion transporter activity	4.76E-07	70,21,7
1ibja_#271	GO:0005524: ATP binding	4.76E-07	78,243,22
1at0__#285	GO:0000049: tRNA binding	4.77E-07	38,13,5
3grs_3#435	GO:0050660: FAD binding	4.78E-07	179,10,7
1gtra1#468	GO:0003924: GTPase activity	4.78E-07	54,17,6
1oaa__#14	GO:0016627: oxidoreductase activity, acting on the CH-CH group of donors	4.78E-07	54,17,6
1cb8a2#613	GO:0015036: disulfide oxidoreductase activity	4.80E-07	138,22,9
1ycsb1#412	GO:0015078: hydrogen ion transporter activity	4.83E-07	146,21,9
1hdfa_#16	GO:0004190: aspartic-type endopeptidase activity	4.86E-07	223,23,11
1e69a_#26	GO:0005524: ATP binding	4.87E-07	55,243,18
2mev2_#53	GO:0003724: RNA helicase activity	4.88E-07	5,11,3
1i4fa2#21	GO:0003755: peptidyl-prolyl cis-trans isomerase activity	4.88E-07	5,11,3
1bev1_#245	GO:0003724: RNA helicase activity	4.88E-07	5,11,3
2cpl__#138	GO:0003755: peptidyl-prolyl cis-trans isomerase activity	4.88E-07	5,11,3
1b35b_#140	GO:0003724: RNA helicase activity	4.88E-07	5,11,3
1bwda_#117	GO:0005525: GTP binding	4.89E-07	116,49,12
1cfb_2#763	GO:0019838: growth factor binding	4.96E-07	6,9,3
1e69a_#40	GO:0005524: ATP binding	4.98E-07	30,243,13
1ibja_#217	GO:0008483: transaminase activity	4.98E-07	12,17,4
1qg6a_#237	GO:0016627: oxidoreductase activity, acting on the CH-CH group of donors	4.98E-07	12,17,4
1fuma2#221	GO:0016627: oxidoreductase activity, acting on the CH-CH group of donors	4.98E-07	12,17,4
1cyda_#230	GO:0016627: oxidoreductase activity, acting on the CH-CH group of donors	4.98E-07	12,17,4
1ia8a_#127	GO:0004674: protein serine/threonine kinase activity	4.99E-07	12,42,5
1hcl__#135	GO:0004674: protein serine/threonine kinase activity	4.99E-07	12,42,5
1g51a1#21	GO:0003697: single-stranded DNA binding	4.99E-07	42,12,5
1erza_#183	GO:0004222: metalloendopeptidase activity	5.02E-07	79,19,7
1a9xa3#78	GO:0016831: carboxy-lyase activity	5.06E-07	305,25,13
1qqga2#253	GO:0005524: ATP binding	5.06E-07	110,243,27
1a0la_#160	GO:0005509: calcium ion binding	5.07E-07	73,160,17
1i50b_#966	GO:0005524: ATP binding	5.07E-07	299,243,52
1hyrc1#247	GO:0008201: heparin binding	5.10E-07	61,24,7

1psda3#344	GO:0005507: copper ion binding	5.11E-07	125,38,11
1f7ua2#324	GO:0004812: tRNA ligase activity	5.13E-07	56,26,7
1dpga2#334	GO:0003809: thrombin activity	5.15E-07	287,10,8
1fhoa_#74	GO:0019838: growth factor binding	5.15E-07	347,9,8
1ac6a_#34	GO:0019838: growth factor binding	5.15E-07	60,9,5
1fmta2#151	GO:0016668: oxidoreductase activity, acting on sulfur group of donors, NAD or NADP as acceptor	5.17E-07	314,12,9
1qdlb_#170	GO:0015036: disulfide oxidoreductase activity	5.20E-07	100,22,8
1cbf__#208	GO:0004674: protein serine/threonine kinase activity	5.25E-07	333,42,18
1mdah_#329	GO:0051082: unfolded protein binding	5.27E-07	335,34,16
1nls__#128	GO:0005529: sugar binding	5.27E-07	6,39,4
1sppb_#32	GO:0005507: copper ion binding	5.31E-07	38,38,7
1epwa3#138	GO:0004295: trypsin activity	5.31E-07	424,48,22
1e39a3#442	GO:0003809: thrombin activity	5.32E-07	21,10,4
1autc_#188	GO:0003809: thrombin activity	5.32E-07	21,10,4
1e79h2#77	GO:0003809: thrombin activity	5.32E-07	21,10,4
1aqua_#126	GO:0016831: carboxy-lyase activity	5.32E-07	576,25,17
1jj2e1#42	GO:0004896: hematopoietin/interferon-class (D200-domain) cytokine receptor activity	5.36E-07	167,19,9
1xgsa2#186	GO:0008800: beta-lactamase activity	5.36E-07	182,10,7
1eur__#302	GO:0003809: thrombin activity	5.38E-07	53,10,5
1ac5__#93	GO:0004180: carboxypeptidase activity	5.38E-07	4,15,3
1dofa_#15	GO:0015078: hydrogen ion transporter activity	5.45E-07	197,21,10
1dy5a_#46	GO:0004812: tRNA ligase activity	5.50E-07	35,26,6
1fy7a_#304	GO:0008080: N-acetyltransferase activity	5.50E-07	16,13,4
1aqua_#124	GO:0016616: oxidoreductase activity, acting on the CH-OH group of donors, NAD or NADP as acceptor	5.51E-07	25,59,7
1ew2a_#237	GO:0004556: alpha-amylase activity	5.51E-07	106,15,7
2sqca2#97	GO:0005085: guanyl-nucleotide exchange factor activity	5.51E-07	127,13,7
1nbaa_#227	GO:0005524: ATP binding	5.52E-07	91,243,24
1fn9a_#271	GO:0016668: oxidoreductase activity, acting on sulfur group of donors, NAD or NADP as acceptor	5.54E-07	218,12,8
1pma1_#17	GO:0004295: trypsin activity	5.57E-07	44,48,8
1e8ca3#111	GO:0019201: nucleotide kinase activity	5.58E-07	280,13,9
1dz4a_#150	GO:0016705: oxidoreductase activity, acting on paired donors, with incorporation or reduction of molecular oxygen	5.60E-07	19,26,5
1dz4a_#150	GO:0004812: tRNA ligase activity	5.60E-07	19,26,5
1ddwa_#77	GO:0015036: disulfide oxidoreductase activity	5.62E-07	364,22,13
3prn__#145	GO:0004896: hematopoietin/interferon-class (D200-domain) cytokine receptor activity	5.64E-07	168,19,9
1qaua_#58	GO:0005516: calmodulin binding	5.67E-07	9,24,4
1cs1a_#82	GO:0004812: tRNA ligase activity	5.70E-07	195,26,11
1c4zd_#51	GO:0019955: cytokine binding	5.72E-07	160,11,7
1rypb_#114	GO:0003887: DNA-directed DNA polymerase activity	5.73E-07	158,20,9
2shpa1#387	GO:0004725: protein tyrosine phosphatase activity	5.77E-07	14,15,4
1e19a_#281	GO:0005524: ATP binding	5.77E-07	138,243,31
1c4ra_#258	GO:0004867: serine-type endopeptidase inhibitor activity	5.78E-07	80,47,10
1imva_#69	GO:0016705: oxidoreductase activity, acting on paired donors, with incorporation or reduction of molecular oxygen	5.80E-07	84,26,8
1hu4a_#267	GO:0004812: tRNA ligase activity	5.82E-07	57,26,7
1qrra_#69	GO:0000287: magnesium ion binding	5.84E-07	196,128,26
1qtn.1#A270	GO:0004457: lactate dehydrogenase activity	5.85E-07	439,10,9
1qtn.1#A270	GO:0016620: oxidoreductase activity, acting on the aldehyde or oxo group of donors, NAD or NADP as acceptor	5.85E-07	439,10,9
1e39a3#417	GO:0004295: trypsin activity	5.86E-07	11,48,5
1dt9a1#224	GO:0004364: glutathione transferase activity	5.86E-07	48,11,5
1pud__#69	GO:0016616: oxidoreductase activity, acting on the CH-OH group of donors, NAD or NADP as acceptor	5.86E-07	49,59,9
7reqa2#702	GO:0005525: GTP binding	5.86E-07	59,49,9
1e79d2#36	GO:0019955: cytokine binding	5.87E-07	524,11,10
1qqga1#95	GO:0005509: calcium ion binding	5.94E-07	127,160,23
2dkb__#239	GO:0008483: transaminase activity	5.97E-07	56,17,6
1dyoa_#153	GO:0003755: peptidyl-prolyl cis-trans isomerase activity	5.98E-07	161,11,7
1fn9a_#271	GO:0019838: growth factor binding	6.00E-07	218,9,7
1dl5a_#59	GO:0008757: S-adenosylmethionine-dependent methyltransferase activity	6.01E-07	3,24,3
1jj2s_#46	GO:0005524: ATP binding	6.02E-07	45,243,16
2sli_2#326	GO:0019955: cytokine binding	6.02E-07	251,11,8
1qfma1#186	GO:0005529: sugar binding	6.02E-07	326,39,17

1d6aa_#52	GO:0003809: thrombin activity	6.07E-07	293,10,8
1cb0a_#98	GO:0016763: transferase activity, transferring pentosyl groups	6.08E-07	8,28,4
1flca1#294	GO:0005529: sugar binding	6.11E-07	98,39,10
8dfr__#9	GO:0005524: ATP binding	6.16E-07	111,243,27
1elua_#216	GO:0005524: ATP binding	6.19E-07	40,243,15
1xvaa_#244	GO:0005518: collagen binding	6.23E-07	40,13,5
1el0a_#42	GO:0008009: chemokine activity	6.23E-07	294,10,8
1a65a1#108	GO:0050660: FAD binding	6.23E-07	294,10,8
1e6ca_#91	GO:0005524: ATP binding	6.23E-07	182,243,37
1ejda_#180	GO:0003887: DNA-directed DNA polymerase activity	6.31E-07	77,20,7
1f97a1#62	GO:0015036: disulfide oxidoreductase activity	6.31E-07	707,22,17
1ds1a_#172	GO:0016668: oxidoreductase activity, acting on sulfur group of donors, NAD or NADP as acceptor	6.39E-07	222,12,8
1thea_#79	GO:0004197: cysteine-type endopeptidase activity	6.39E-07	93,24,8
1aym3_#131	GO:0004556: alpha-amylase activity	6.42E-07	412,15,11
1jjya_#329	GO:0016776: phosphotransferase activity, phosphate group as acceptor	6.44E-07	71,14,6
1leha1#255	GO:0016638: oxidoreductase activity, acting on the CH-NH2 group of donors	6.45E-07	30,17,5
1vmoa_#106	GO:0050660: FAD binding	6.47E-07	187,10,7
1ew0a_#174	GO:0000155: two-component sensor molecule activity	6.50E-07	5,12,3
1smaa1#95	GO:0004263: chymotrypsin activity	6.50E-07	272,41,16
1e8ga1#436	GO:0000287: magnesium ion binding	6.51E-07	92,128,17
1ac6a_#43	GO:0019955: cytokine binding	6.51E-07	163,11,7
1hxxa_#232	GO:0005524: ATP binding	6.52E-07	352,243,58
1dih_2#222	GO:0005524: ATP binding	6.52E-07	352,243,58
1a4pa_#24	GO:0005509: calcium ion binding	6.58E-07	5,160,5
1g6oa_#116	GO:0019838: growth factor binding	6.60E-07	358,9,8
1qora2#201	GO:0019201: nucleotide kinase activity	6.61E-07	199,13,8
1p35a_#101	GO:0004190: aspartic-type endopeptidase activity	6.65E-07	136,23,9
1nat__#50	GO:0004457: lactate dehydrogenase activity	6.75E-07	297,10,8
1nat__#50	GO:0016620: oxidoreductase activity, acting on the aldehyde or oxo group of donors, NAD or NADP as acceptor	6.75E-07	297,10,8
1f8ra2#325	GO:0019838: growth factor binding	6.80E-07	222,9,7
1ge8a2#134	GO:0004896: hematopoietin/interferon-class (D200-domain) cytokine receptor activity	6.82E-07	229,19,10
1dik_1#765	GO:0004556: alpha-amylase activity	6.84E-07	165,15,8
1a3qa2#133	GO:0004896: hematopoietin/interferon-class (D200-domain) cytokine receptor activity	6.84E-07	27,19,5
1hq8a_#206	GO:0005529: sugar binding	6.89E-07	329,39,17
1czan1#92	GO:0004812: tRNA ligase activity	6.89E-07	631,26,18
1e44b_#75	GO:0016638: oxidoreductase activity, acting on the CH-NH2 group of donors	6.90E-07	198,17,9
1nsj__#124	GO:0000287: magnesium ion binding	6.90E-07	148,128,22
1qs1a2#437	GO:0016668: oxidoreductase activity, acting on sulfur group of donors, NAD or NADP as acceptor	6.95E-07	452,12,10
1dp0a4#756	GO:0019955: cytokine binding	7.08E-07	165,11,7
1ovb__#207	GO:0008199: ferric iron binding	7.08E-07	6,10,3
1gdea_#271	GO:0016846: carbon-sulfur lyase activity	7.08E-07	6,10,3
1qfta_#98	GO:0004896: hematopoietin/interferon-class (D200-domain) cytokine receptor activity	7.10E-07	230,19,10
1i50b_#966	GO:0050660: FAD binding	7.12E-07	299,10,8
1fwxa2#396	GO:0005524: ATP binding	7.15E-07	237,243,44
1e8ga2#180	GO:0004674: protein serine/threonine kinase activity	7.16E-07	6,42,4
1jlxa2#288	GO:0008083: growth factor activity	7.16E-07	6,42,4
1erza_#183	GO:0016861: intramolecular oxidoreductase activity, interconverting aldoses and ketoses	7.17E-07	79,13,6
1ezia_#198	GO:0008483: transaminase activity	7.17E-07	13,17,4
1h7wa4#208	GO:0016627: oxidoreductase activity, acting on the CH-CH group of donors	7.17E-07	13,17,4
1fg7a_#96	GO:0008483: transaminase activity	7.17E-07	13,17,4
1c1da1#153	GO:0016638: oxidoreductase activity, acting on the CH-NH2 group of donors	7.17E-07	13,17,4
1fjja_#5	GO:0005518: collagen binding	7.17E-07	17,13,4
1h8d.1#H207	GO:0004867: serine-type endopeptidase inhibitor activity	7.19E-07	63,47,9
1ap0__#29	GO:0019955: cytokine binding	7.22E-07	50,11,5
1dgw.1#Y353	GO:0005524: ATP binding	7.25E-07	319,243,54
1f0ia1#50	GO:0004263: chymotrypsin activity	7.35E-07	146,41,12
1ho1a_#211	GO:0004457: lactate dehydrogenase activity	7.35E-07	111,10,6
1xyza_#753	GO:0008810: cellulase activity	7.41E-07	29,18,5
1fnf_3#1409	GO:0008083: growth factor activity	7.42E-07	71,42,9
1kcw_3#440	GO:0004497: monooxygenase activity	7.42E-07	20,26,5
2cuaa_#83	GO:0015078: hydrogen ion transporter activity	7.43E-07	46,21,6
1qdla_#211	GO:0004556: alpha-amylase activity	7.64E-07	320,15,10
1qfma2#576	GO:0005524: ATP binding	7.67E-07	214,243,41

1cs6a4#363	GO:0004714: transmembrane receptor protein tyrosine kinase activity	7.67E-07	16,14,4
1qq5a_#7	GO:0016758: transferase activity, transferring hexosyl groups	7.69E-07	167,11,7
1hc7a2#258	GO:0019838: growth factor binding	7.70E-07	226,9,7
1nsca_#236	GO:0016638: oxidoreductase activity, acting on the CH-NH2 group of donors	7.71E-07	143,17,8
1ev2e2#286	GO:0004190: aspartic-type endopeptidase activity	7.73E-07	100,23,8
1pii_2#326	GO:0004457: lactate dehydrogenase activity	7.76E-07	192,10,7
1dt6a_#181	GO:0016705: oxidoreductase activity, acting on paired donors, with incorporation or reduction of molecular oxygen	7.77E-07	37,26,6
1b3qa2#623	GO:0004812: tRNA ligase activity	7.79E-07	301,26,13
1h6kx_#97	GO:0003809: thrombin activity	7.81E-07	57,10,5
1dfoa_#362	GO:0016846: carbon-sulfur lyase activity	7.81E-07	57,10,5
1i2ma_#90	GO:0005096: GTPase activator activity	7.84E-07	15,15,4
1euha_#349	GO:0016620: oxidoreductase activity, acting on the aldehyde or oxo group of donors, NAD or NADP as acceptor	7.84E-07	23,10,4
1d4ta_#80	GO:0005066: transmembrane receptor protein tyrosine kinase signaling protein activity	7.84E-07	23,10,4
1htp__#16	GO:0005524: ATP binding	7.88E-07	303,243,52
1qs0a1#119	GO:0016620: oxidoreductase activity, acting on the aldehyde or oxo group of donors, NAD or NADP as acceptor	7.89E-07	454,10,9
1e3ja1#136	GO:0005524: ATP binding	7.90E-07	31,243,13
1htp__#16	GO:0050660: FAD binding	7.90E-07	303,10,8
1wdcb_#141	GO:0004497: monooxygenase activity	7.95E-07	9,26,4
1a8h_2#186	GO:0004812: tRNA ligase activity	7.95E-07	9,26,4
1axca1#70	GO:0003887: DNA-directed DNA polymerase activity	7.96E-07	118,20,8
1qaxa2#307	GO:0004295: trypsin activity	7.99E-07	46,48,8
1fjfl_#67	GO:0004896: hematopoietin/interferon-class (D200-domain) cytokine receptor activity	8.02E-07	175,19,9
1et0a_#161	GO:0008483: transaminase activity	8.04E-07	4,17,3
1fuma2#101	GO:0016627: oxidoreductase activity, acting on the CH-CH group of donors	8.04E-07	4,17,3
1et0a_#261	GO:0008483: transaminase activity	8.04E-07	4,17,3
1ejda_#87	GO:0016638: oxidoreductase activity, acting on the CH-NH2 group of donors	8.04E-07	4,17,3
1gtma2#118	GO:0016638: oxidoreductase activity, acting on the CH-NH2 group of donors	8.04E-07	4,17,3
1et0a_#220	GO:0008483: transaminase activity	8.04E-07	4,17,3
1fuma2#132	GO:0016627: oxidoreductase activity, acting on the CH-CH group of donors	8.04E-07	4,17,3
1eg5a_#150	GO:0008483: transaminase activity	8.04E-07	4,17,3
1opy__#68	GO:0019838: growth factor binding	8.04E-07	367,9,8
1vcaa2#49	GO:0004896: hematopoietin/interferon-class (D200-domain) cytokine receptor activity	8.08E-07	12,19,4
1qora2#201	GO:0005524: ATP binding	8.11E-07	199,243,39
1eaja_#59	GO:0016638: oxidoreductase activity, acting on the CH-NH2 group of donors	8.20E-07	202,17,9
1e79d2#25	GO:0004190: aspartic-type endopeptidase activity	8.21E-07	23,23,5
1ryph_#163	GO:0004812: tRNA ligase activity	8.24E-07	121,26,9
1io1a_#311	GO:0004896: hematopoietin/interferon-class (D200-domain) cytokine receptor activity	8.25E-07	126,19,8
1by5a_#347	GO:0015082: di-, tri-valent inorganic cation transporter activity	8.27E-07	263,14,9
1by5a_#347	GO:0046915: transition metal ion transporter activity	8.27E-07	263,14,9
1cwva2#675	GO:0004896: hematopoietin/interferon-class (D200-domain) cytokine receptor activity	8.30E-07	28,19,5
1c8za_#386	GO:0004190: aspartic-type endopeptidase activity	8.32E-07	235,23,11
1b35b_#97	GO:0015036: disulfide oxidoreductase activity	8.33E-07	309,22,12
1bccb2#274	GO:0004497: monooxygenase activity	8.34E-07	60,26,7
1nsj__#178	GO:0016836: hydro-lyase activity	8.36E-07	93,33,9
1as4.1#A336	GO:0016668: oxidoreductase activity, acting on sulfur group of donors, NAD or NADP as acceptor	8.42E-07	332,12,9
1ge8a2#134	GO:0019838: growth factor binding	8.44E-07	229,9,7
1dfca4#1469	GO:0008083: growth factor activity	8.46E-07	53,42,8
1bu8a2#124	GO:0016616: oxidoreductase activity, acting on the CH-OH group of donors, NAD or NADP as acceptor	8.47E-07	140,59,14
1e39a3#442	GO:0004867: serine-type endopeptidase inhibitor activity	8.48E-07	21,47,6
1fo4a5#1020	GO:0015082: di-, tri-valent inorganic cation transporter activity	8.60E-07	359,14,10
1fo4a5#1020	GO:0046915: transition metal ion transporter activity	8.60E-07	359,14,10
1qfea_#68	GO:0004222: metalloendopeptidase activity	8.65E-07	560,19,14
1e8ca1#83	GO:0004364: glutathione transferase activity	8.70E-07	170,11,7
1clxa_#120	GO:0016836: hydro-lyase activity	8.73E-07	47,33,7
1heta2#218	GO:0000287: magnesium ion binding	8.73E-07	39,128,11
1xgsa2#82	GO:0003809: thrombin activity	8.76E-07	307,10,8
1xgsa2#82	GO:0050660: FAD binding	8.76E-07	307,10,8
1clxa_#120	GO:0000287: magnesium ion binding	8.85E-07	47,128,12
1hu4a_#267	GO:0005524: ATP binding	8.89E-07	57,243,18
1bfd_1#230	GO:0016668: oxidoreductase activity, acting on sulfur group of donors, NAD or NADP as acceptor	8.89E-07	47,12,5

1ewna_#109	GO:0003968: RNA-directed RNA polymerase activity	8.97E-07	75,14,6
1dpja_#322	GO:0050660: FAD binding	8.99E-07	308,10,8
1g51a3#525	GO:0005524: ATP binding	9.01E-07	263,243,47
1am2__#163	GO:0004190: aspartic-type endopeptidase activity	9.02E-07	102,23,8
1f06a1#296	GO:0003924: GTPase activity	9.04E-07	32,17,5
2pola2#218	GO:0016638: oxidoreductase activity, acting on the CH-NH2 group of donors	9.06E-07	146,17,8
1dp0a1#309	GO:0004896: hematopoietin/interferon-class (D200-domain) cytokine receptor activity	9.07E-07	53,19,6
2ltn.1#A63	GO:0016668: oxidoreductase activity, acting on sulfur group of donors, NAD or NADP as acceptor	9.18E-07	152,12,7
1g5qa_#80	GO:0008026: ATP-dependent helicase activity	9.20E-07	18,13,4
1iow_1#37	GO:0016620: oxidoreductase activity, acting on the aldehyde or oxo group of donors, NAD or NADP as acceptor	9.22E-07	309,10,8
1phm_2#253	GO:0003809: thrombin activity	9.22E-07	309,10,8
1ax4a_#430	GO:0016846: carbon-sulfur lyase activity	9.30E-07	59,10,5
1ed8a_#353	GO:0016620: oxidoreductase activity, acting on the aldehyde or oxo group of donors, NAD or NADP as acceptor	9.40E-07	24,10,4
1gdea_#195	GO:0016846: carbon-sulfur lyase activity	9.40E-07	24,10,4
1egaa1#58	GO:0005525: GTP binding	9.42E-07	46,49,8
1fwxa2#396	GO:0004896: hematopoietin/interferon-class (D200-domain) cytokine receptor activity	9.43E-07	237,19,10
1by5a_#238	GO:0004896: hematopoietin/interferon-class (D200-domain) cytokine receptor activity	9.43E-07	237,19,10
1e25a_#262	GO:0008800: beta-lactamase activity	9.57E-07	116,10,6
1dpsa_#36	GO:0008083: growth factor activity	9.61E-07	205,42,14
1g6oa_#40	GO:0004295: trypsin activity	9.64E-07	21,48,6
1bhga3#589	GO:0008810: cellulase activity	9.65E-07	4,18,3
1h6va3#486	GO:0004556: alpha-amylase activity	9.66E-07	70,15,6
1cyx__#138	GO:0015078: hydrogen ion transporter activity	9.68E-07	26,21,5
1cfb_2#763	GO:0019955: cytokine binding	9.73E-07	6,11,3
1urna_#93	GO:0003755: peptidyl-prolyl cis-trans isomerase activity	9.73E-07	6,11,3
1fmb__#88	GO:0003964: RNA-directed DNA polymerase activity	9.73E-07	6,11,3
1imva_#255	GO:0019838: growth factor binding	9.81E-07	589,9,9
2cb5a_#395	GO:0004197: cysteine-type endopeptidase activity	9.83E-07	67,24,7
1qf6a3#78	GO:0016616: oxidoreductase activity, acting on the CH-OH group of donors, NAD or NADP as acceptor	9.98E-07	52,59,9
1e6wa_#136	GO:0016616: oxidoreductase activity, acting on the CH-OH group of donors, NAD or NADP as acceptor	9.98E-07	52,59,9
1c8za_#386	GO:0019838: growth factor binding	1.01E-06	235,9,7
1iba__#39	GO:0051082: unfolded protein binding	1.01E-06	351,34,16
1jd0a_#176	GO:0016638: oxidoreductase activity, acting on the CH-NH2 group of donors	1.01E-06	207,17,9
1axn__#42	GO:0015078: hydrogen ion transporter activity	1.01E-06	78,21,7
1dssg1#4	GO:0016763: transferase activity, transferring pentosyl groups	1.01E-06	57,28,7
1a8l_1#74	GO:0015036: disulfide oxidoreductase activity	1.01E-06	11,22,4
1j9qa1#154	GO:0005507: copper ion binding	1.02E-06	196,38,13
1hbza_#214	GO:0005524: ATP binding	1.02E-06	297,243,51
3chbd_#85	GO:0019838: growth factor binding	1.02E-06	378,9,8
1fm2.1#B308	GO:0050660: FAD binding	1.03E-06	200,10,7
1i50a_#856	GO:0003755: peptidyl-prolyl cis-trans isomerase activity	1.04E-06	269,11,8
1g6oa_#116	GO:0003887: DNA-directed DNA polymerase activity	1.04E-06	358,20,12
1e32a1#45	GO:0030151: molybdenum ion binding	1.04E-06	16,15,4
3gcb__#78	GO:0005524: ATP binding	1.06E-06	94,243,24
5ruba1#365	GO:0016846: carbon-sulfur lyase activity	1.06E-06	118,10,6
1e6ua_#10	GO:0016627: oxidoreductase activity, acting on the CH-CH group of donors	1.06E-06	33,17,5
1kit_2#398	GO:0005126: hematopoietin/interferon-class (D200-domain) cytokine receptor binding	1.06E-06	83,20,7
1nfdb1#21	GO:0008083: growth factor activity	1.06E-06	96,42,10
1ejda_#6	GO:0000287: magnesium ion binding	1.07E-06	270,128,31
1fwxa2#396	GO:0019838: growth factor binding	1.07E-06	237,9,7
1flga_#161	GO:0004295: trypsin activity	1.07E-06	152,48,13
2mev2_#53	GO:0003968: RNA-directed RNA polymerase activity	1.07E-06	5,14,3
1e9xa_#343	GO:0016705: oxidoreductase activity, acting on paired donors, with incorporation or reduction of molecular oxygen	1.08E-06	39,26,6
1as4.1#B385	GO:0004867: serine-type endopeptidase inhibitor activity	1.08E-06	66,47,9
1euca1#64	GO:0016616: oxidoreductase activity, acting on the CH-OH group of donors, NAD or NADP as acceptor	1.08E-06	39,59,8
1wht.1#A15	GO:0005507: copper ion binding	1.09E-06	15,38,5
1e30a_#112	GO:0015078: hydrogen ion transporter activity	1.09E-06	49,21,6
1bif_2#398	GO:0000287: magnesium ion binding	1.10E-06	117,128,19
1bfg__#22	GO:0003809: thrombin activity	1.10E-06	61,10,5

1a8i__#688	GO:0016646: oxidoreductase activity, acting on the CH-NH group of donors, NAD or NADP as acceptor	1.10E-06	241,19,10
1feca1#28	GO:0016627: oxidoreductase activity, acting on the CH-CH group of donors	1.11E-06	62,17,6
1c7na_#95	GO:0004867: serine-type endopeptidase inhibitor activity	1.11E-06	183,47,14
1djna1#86	GO:0016861: intramolecular oxidoreductase activity, interconverting aldoses and ketoses	1.11E-06	85,13,6
1ga0a_#228	GO:0008800: beta-lactamase activity	1.12E-06	25,10,4
1cbf__#208	GO:0004556: alpha-amylase activity	1.12E-06	333,15,10
1f3mc_#369	GO:0005516: calmodulin binding	1.12E-06	100,24,8
1smaa1#95	GO:0005509: calcium ion binding	1.13E-06	272,160,36
1qfea_#68	GO:0004364: glutathione transferase activity	1.13E-06	560,11,10
1p35a_#155	GO:0004896: hematopoietin/interferon-class (D200-domain) cytokine receptor activity	1.14E-06	55,19,6
1i76a_#138	GO:0016616: oxidoreductase activity, acting on the CH-OH group of donors, NAD or NADP as acceptor	1.14E-06	103,59,12
1fbl_1#368	GO:0004222: metalloendopeptidase activity	1.15E-06	4,19,3
1d6aa_#52	GO:0005525: GTP binding	1.15E-06	293,49,18
1nfdb1#34	GO:0019955: cytokine binding	1.15E-06	177,11,7
1bio__#88	GO:0004867: serine-type endopeptidase inhibitor activity	1.15E-06	22,47,6
1qj8a_#64	GO:0003887: DNA-directed DNA polymerase activity	1.15E-06	84,20,7
1a4ya_#47	GO:0000287: magnesium ion binding	1.16E-06	216,128,27
1as4.1#A336	GO:0005524: ATP binding	1.16E-06	332,243,55
1qmea4#464	GO:0008235: metalloexopeptidase activity	1.16E-06	19,13,4
1deua_#151	GO:0004197: cysteine-type endopeptidase activity	1.17E-06	285,24,12
3grx__#56	GO:0004556: alpha-amylase activity	1.18E-06	335,15,10
1ihua2#525	GO:0004725: protein tyrosine phosphatase activity	1.18E-06	335,15,10
1tig__#148	GO:0016646: oxidoreductase activity, acting on the CH-NH group of donors, NAD or NADP as acceptor	1.19E-06	30,19,5
1hdma1#123	GO:0004896: hematopoietin/interferon-class (D200-domain) cytokine receptor activity	1.19E-06	30,19,5
1ax4a_#275	GO:0008483: transaminase activity	1.20E-06	102,17,7
2pgd_1#421	GO:0015078: hydrogen ion transporter activity	1.21E-06	80,21,7
1a4ya_#104	GO:0016668: oxidoreductase activity, acting on sulfur group of donors, NAD or NADP as acceptor	1.21E-06	241,12,8
1aym3_#131	GO:0008201: heparin binding	1.22E-06	412,24,14
1czan1#92	GO:0003887: DNA-directed DNA polymerase activity	1.22E-06	631,20,15
2mpra_#190	GO:0019955: cytokine binding	1.22E-06	400,11,9
1dr9a1#39	GO:0015036: disulfide oxidoreductase activity	1.22E-06	258,22,11
1qqsa_#124	GO:0016638: oxidoreductase activity, acting on the CH-NH2 group of donors	1.23E-06	458,17,12
1cmxa_#207	GO:0000287: magnesium ion binding	1.23E-06	96,128,17
1j9la_#164	GO:0000287: magnesium ion binding	1.24E-06	57,128,13
1ovb__#116	GO:0008199: ferric iron binding	1.24E-06	7,10,3
1jf9a_#119	GO:0016846: carbon-sulfur lyase activity	1.24E-06	7,10,3
1euha_#31	GO:0016620: oxidoreductase activity, acting on the aldehyde or oxo group of donors, NAD or NADP as acceptor	1.24E-06	7,10,3
1epua_#26	GO:0008483: transaminase activity	1.24E-06	34,17,5
2dri__#11	GO:0005351: sugar porter activity	1.24E-06	12,21,4
1evqa_#305	GO:0016776: phosphotransferase activity, phosphate group as acceptor	1.24E-06	490,14,11
1psda3#344	GO:0003887: DNA-directed DNA polymerase activity	1.24E-06	125,20,8
1pwt__#8	GO:0008083: growth factor activity	1.25E-06	314,42,17
1a4ya_#47	GO:0016861: intramolecular oxidoreductase activity, interconverting aldoses and ketoses	1.25E-06	216,13,8
1ab4__#89	GO:0003899: DNA-directed RNA polymerase activity	1.25E-06	159,12,7
1qhda2#323	GO:0004556: alpha-amylase activity	1.25E-06	337,15,10
1fw9a_#152	GO:0004896: hematopoietin/interferon-class (D200-domain) cytokine receptor activity	1.25E-06	133,19,8
1dcpa_#25	GO:0004896: hematopoietin/interferon-class (D200-domain) cytokine receptor activity	1.27E-06	56,19,6
1fjfc2#153	GO:0008408: 3'-5' exonuclease activity	1.27E-06	420,13,10
1e79d2#36	GO:0008083: growth factor activity	1.27E-06	524,42,22
3lada1#116	GO:0016763: transferase activity, transferring pentosyl groups	1.27E-06	153,28,10
1gln_2#63	GO:0005524: ATP binding	1.27E-06	9,243,7
1bwda_#295	GO:0005525: GTP binding	1.28E-06	104,49,11
1dyna_#19	GO:0005507: copper ion binding	1.28E-06	109,38,10
1cnv__#264	GO:0003924: GTPase activity	1.28E-06	103,17,7
1jsg__#92	GO:0019838: growth factor binding	1.29E-06	607,9,9
2sn3__#22	GO:0005524: ATP binding	1.29E-06	12,243,8
1trb_1#106	GO:0016668: oxidoreductase activity, acting on sulfur group of donors, NAD or NADP as acceptor	1.30E-06	6,12,3
1c4zd_#22	GO:0005524: ATP binding	1.30E-06	70,243,20
1kit_3#743	GO:0004263: chymotrypsin activity	1.30E-06	250,41,15
1rmg__#279	GO:0005509: calcium ion binding	1.31E-06	12,160,7

1hr0w_#69	GO:0004812: tRNA ligase activity	1.31E-06	64,26,7
1phm_2#253	GO:0005518: collagen binding	1.31E-06	309,13,9
1f97a1#62	GO:0003809: thrombin activity	1.32E-06	707,10,10
1f97a1#62	GO:0050660: FAD binding	1.32E-06	707,10,10
1qfea_#68	GO:0019201: nucleotide kinase activity	1.32E-06	560,13,11
1fa0a1#393	GO:0004812: tRNA ligase activity	1.32E-06	261,26,12
1aoea_#130	GO:0015036: disulfide oxidoreductase activity	1.33E-06	77,22,7
1g6oa_#116	GO:0051082: unfolded protein binding	1.33E-06	358,34,16
1opy__#68	GO:0016638: oxidoreductase activity, acting on the CH-NH2 group of donors	1.33E-06	367,17,11
1qksa2#236	GO:0016638: oxidoreductase activity, acting on the CH-NH2 group of donors	1.34E-06	64,17,6
1h9ra2#212	GO:0030151: molybdenum ion binding	1.34E-06	5,15,3
1clia2#273	GO:0004725: protein tyrosine phosphatase activity	1.34E-06	5,15,3
1plq_1#22	GO:0003887: DNA-directed DNA polymerase activity	1.35E-06	4,20,3
1atza_#1070	GO:0005525: GTP binding	1.35E-06	6,49,4
1fcda2#189	GO:0016668: oxidoreductase activity, acting on sulfur group of donors, NAD or NADP as acceptor	1.35E-06	51,12,5
1f97a1#62	GO:0004556: alpha-amylase activity	1.36E-06	707,15,13
1wht.1#A15	GO:0016638: oxidoreductase activity, acting on the CH-NH2 group of donors	1.36E-06	15,17,4
1mrp__#213	GO:0004180: carboxypeptidase activity	1.36E-06	17,15,4
1aa6_2#208	GO:0030151: molybdenum ion binding	1.36E-06	17,15,4
1qg6a_#93	GO:0016627: oxidoreductase activity, acting on the CH-CH group of donors	1.36E-06	15,17,4
1cyda_#180	GO:0016627: oxidoreductase activity, acting on the CH-CH group of donors	1.36E-06	15,17,4
1h6ka2#336	GO:0004497: monooxygenase activity	1.37E-06	168,26,10
1iba__#39	GO:0016668: oxidoreductase activity, acting on sulfur group of donors, NAD or NADP as acceptor	1.37E-06	351,12,9
1ig8a_#203	GO:0005525: GTP binding	1.38E-06	65,49,9
1biha3#248	GO:0019838: growth factor binding	1.38E-06	8,9,3
1ejba_#81	GO:0005525: GTP binding	1.39E-06	84,49,10
1cl1a_#184	GO:0016846: carbon-sulfur lyase activity	1.41E-06	64,10,5
1hwxa1#256	GO:0016646: oxidoreductase activity, acting on the CH-NH group of donors, NAD or NADP as acceptor	1.41E-06	57,19,6
1ejfa_#10	GO:0004263: chymotrypsin activity	1.41E-06	155,41,12
3grs_3#435	GO:0000287: magnesium ion binding	1.41E-06	179,128,24
1clxa_#120	GO:0016861: intramolecular oxidoreductase activity, interconverting aldoses and ketoses	1.43E-06	47,13,5
1e1aa_#178	GO:0005126: hematopoietin/interferon-class (D200-domain) cytokine receptor binding	1.43E-06	54,20,6
1ovb__#195	GO:0005524: ATP binding	1.43E-06	89,243,23
1mdah_#329	GO:0005509: calcium ion binding	1.43E-06	335,160,41
1g51a3#525	GO:0004812: tRNA ligase activity	1.44E-06	263,26,12
1cex__#109	GO:0003924: GTPase activity	1.44E-06	35,17,5
1eny__#94	GO:0016627: oxidoreductase activity, acting on the CH-CH group of donors	1.44E-06	35,17,5
1qora2#201	GO:0016776: phosphotransferase activity, phosphate group as acceptor	1.45E-06	199,14,8
1gox__#246	GO:0016831: carboxy-lyase activity	1.45E-06	177,25,10
1chma2#262	GO:0004177: aminopeptidase activity	1.45E-06	20,13,4
1pwt__#8	GO:0051082: unfolded protein binding	1.45E-06	314,34,15
1xgsa2#186	GO:0004180: carboxypeptidase activity	1.46E-06	182,15,8
1f5ma_#80	GO:0016638: oxidoreductase activity, acting on the CH-NH2 group of donors	1.46E-06	105,17,7
1sgpe_#42	GO:0004263: chymotrypsin activity	1.47E-06	26,41,6
1kit_2#398	GO:0005529: sugar binding	1.47E-06	83,39,9
1ayl__#482	GO:0008201: heparin binding	1.47E-06	11,24,4
2cpl__#53	GO:0003755: peptidyl-prolyl cis-trans isomerase activity	1.47E-06	24,11,4
1qfma1#139	GO:0004896: hematopoietin/interferon-class (D200-domain) cytokine receptor activity	1.48E-06	318,19,11
1efnb_#102	GO:0019843: rRNA binding	1.48E-06	49,22,6
1ecfa1#434	GO:0000287: magnesium ion binding	1.49E-06	20,128,8
1hbza_#96	GO:0003887: DNA-directed DNA polymerase activity	1.50E-06	370,20,12
1qfma1#186	GO:0015036: disulfide oxidoreductase activity	1.50E-06	326,22,12
1jb0d_#26	GO:0005509: calcium ion binding	1.51E-06	229,160,32
1hq8a_#206	GO:0050660: FAD binding	1.51E-06	329,10,8
1dpja_#322	GO:0004190: aspartic-type endopeptidase activity	1.51E-06	308,23,12
1aqua_#126	GO:0003924: GTPase activity	1.53E-06	576,17,13
1fepa_#340	GO:0016668: oxidoreductase activity, acting on sulfur group of donors, NAD or NADP as acceptor	1.53E-06	99,12,6
1d4ta_#80	GO:0004867: serine-type endopeptidase inhibitor activity	1.54E-06	23,47,6
1i50a_#469	GO:0004364: glutathione transferase activity	1.54E-06	58,11,5
1qtn.1#A331	GO:0050660: FAD binding	1.54E-06	27,10,4
1qs1a2#437	GO:0003887: DNA-directed DNA polymerase activity	1.55E-06	452,20,13
1a6ca2#312	GO:0004896: hematopoietin/interferon-class (D200-domain) cytokine receptor activity	1.55E-06	93,19,7

1p35a_#49	GO:0005509: calcium ion binding	1.55E-06	124,160,22
1e4ea2#215	GO:0050660: FAD binding	1.55E-06	490,10,9
1ew2a_#237	GO:0016616: oxidoreductase activity, acting on the CH-OH group of donors, NAD or NADP as acceptor	1.56E-06	106,59,12
1mkp__#216	GO:0005525: GTP binding	1.57E-06	129,49,12
1e30a_#87	GO:0015078: hydrogen ion transporter activity	1.57E-06	52,21,6
1amm_2#157	GO:0015078: hydrogen ion transporter activity	1.57E-06	4,21,3
1cxp.1#A97	GO:0004601: peroxidase activity	1.57E-06	4,21,3
1cpt__#401	GO:0016705: oxidoreductase activity, acting on paired donors, with incorporation or reduction of molecular oxygen	1.58E-06	23,26,5
1aym3_#131	GO:0019955: cytokine binding	1.58E-06	412,11,9
1e4ea2#215	GO:0004295: trypsin activity	1.58E-06	490,48,23
2sli_2#326	GO:0019838: growth factor binding	1.59E-06	251,9,7
1p35a_#155	GO:0005126: hematopoietin/interferon-class (D200-domain) cytokine receptor binding	1.60E-06	55,20,6
7reqb1#320	GO:0016831: carboxy-lyase activity	1.61E-06	24,25,5
1as4.1#A336	GO:0003809: thrombin activity	1.62E-06	332,10,8
1as4.1#A336	GO:0050660: FAD binding	1.62E-06	332,10,8
1ifc__#89	GO:0004896: hematopoietin/interferon-class (D200-domain) cytokine receptor activity	1.62E-06	190,19,9
1ihua2#521	GO:0005524: ATP binding	1.62E-06	77,243,21
1dnpa2#18	GO:0005524: ATP binding	1.62E-06	77,243,21
1nuka_#80	GO:0004263: chymotrypsin activity	1.64E-06	220,41,14
1ej8a_#189	GO:0008201: heparin binding	1.64E-06	294,24,12
1hqva_#48	GO:0004497: monooxygenase activity	1.64E-06	96,26,8
1fhoa_#74	GO:0004556: alpha-amylase activity	1.65E-06	347,15,10
1hcna_#12	GO:0008083: growth factor activity	1.65E-06	7,42,4
1bwvs_#117	GO:0015036: disulfide oxidoreductase activity	1.66E-06	329,22,12
1hq8a_#206	GO:0015036: disulfide oxidoreductase activity	1.66E-06	329,22,12
1hxmb1#117	GO:0005507: copper ion binding	1.66E-06	64,38,8
1c0pa1#1147	GO:0016627: oxidoreductase activity, acting on the CH-CH group of donors	1.67E-06	36,17,5
3grs_3#435	GO:0003887: DNA-directed DNA polymerase activity	1.67E-06	179,20,9
1hlwa_#82	GO:0005529: sugar binding	1.67E-06	270,39,15
1vmoa_#106	GO:0019955: cytokine binding	1.68E-06	187,11,7
1bgva1#350	GO:0016616: oxidoreductase activity, acting on the CH-OH group of donors, NAD or NADP as acceptor	1.68E-06	11,59,5
1e3ja1#136	GO:0008270: zinc ion binding	1.68E-06	31,108,9
1qgxa_#137	GO:0003729: mRNA binding	1.68E-06	6,13,3
1iqva_#122	GO:0000049: tRNA binding	1.68E-06	6,13,3
1qs1a2#437	GO:0004556: alpha-amylase activity	1.69E-06	452,15,11
3grx__#52	GO:0004364: glutathione transferase activity	1.70E-06	7,11,3
1aym1_#181	GO:0003724: RNA helicase activity	1.70E-06	7,11,3
1f8ma_#237	GO:0004812: tRNA ligase activity	1.70E-06	42,26,6
1abrb1#71	GO:0008083: growth factor activity	1.70E-06	26,42,6
1g51a1#21	GO:0004812: tRNA ligase activity	1.70E-06	42,26,6
1i7qa_#321	GO:0019838: growth factor binding	1.71E-06	148,9,6
1prea2#308	GO:0019838: growth factor binding	1.71E-06	76,9,5
1eu3a2#205	GO:0005507: copper ion binding	1.74E-06	87,38,9
1g8kb_#11	GO:0004896: hematopoietin/interferon-class (D200-domain) cytokine receptor activity	1.74E-06	59,19,6
1ihua2#525	GO:0016620: oxidoreductase activity, acting on the aldehyde or oxo group of donors, NAD or NADP as acceptor	1.74E-06	335,10,8
1fw8a_#126	GO:0030151: molybdenum ion binding	1.74E-06	18,15,4
1eu3a1#68	GO:0030151: molybdenum ion binding	1.74E-06	18,15,4
1aqua_#126	GO:0008757: S-adenosylmethionine-dependent methyltransferase activity	1.76E-06	576,24,16
1d66a1#28	GO:0008270: zinc ion binding	1.76E-06	7,108,5
1qpxa1#117	GO:0051082: unfolded protein binding	1.78E-06	3,34,3
1aqua_#126	GO:0019201: nucleotide kinase activity	1.78E-06	576,13,11
1aqua_#126	GO:0016861: intramolecular oxidoreductase activity, interconverting aldoses and ketoses	1.78E-06	576,13,11
1pysa_#127	GO:0000049: tRNA binding	1.78E-06	21,13,4
8abp__#231	GO:0005351: sugar porter activity	1.78E-06	13,21,4
1ej8a_#189	GO:0016638: oxidoreductase activity, acting on the CH-NH2 group of donors	1.80E-06	294,17,10
1jlxa2#166	GO:0019838: growth factor binding	1.83E-06	77,9,5
2bpa1_#244	GO:0019955: cytokine binding	1.83E-06	60,11,5
1czan1#92	GO:0019838: growth factor binding	1.83E-06	631,9,9
1oaca1#495	GO:0004263: chymotrypsin activity	1.86E-06	60,41,8
1fgga_#134	GO:0004457: lactate dehydrogenase activity	1.86E-06	218,10,7
1aqb__#134	GO:0015036: disulfide oxidoreductase activity	1.88E-06	81,22,7
1dih_2#222	GO:0004180: carboxypeptidase activity	1.89E-06	352,15,10

1qs1a2#437	GO:0004190: aspartic-type endopeptidase activity	1.90E-06	452,23,14
3chbd_#85	GO:0003887: DNA-directed DNA polymerase activity	1.90E-06	378,20,12
1ejda_#377	GO:0016616: oxidoreductase activity, acting on the CH-OH group of donors, NAD or NADP as acceptor	1.91E-06	108,59,12
1c7na_#231	GO:0000287: magnesium ion binding	1.92E-06	263,128,30
1g5ha2#162	GO:0000287: magnesium ion binding	1.92E-06	27,128,9
1ejba_#81	GO:0005524: ATP binding	1.92E-06	84,243,22
1e3a.1#A30	GO:0000287: magnesium ion binding	1.96E-06	157,128,22
3prn__#143	GO:0015036: disulfide oxidoreductase activity	1.96E-06	334,22,12
1ecpa_#62	GO:0016616: oxidoreductase activity, acting on the CH-OH group of donors, NAD or NADP as acceptor	1.96E-06	72,59,10
1bjt__#890	GO:0019955: cytokine binding	1.96E-06	292,11,8
1i6vd_#1283	GO:0004896: hematopoietin/interferon-class (D200-domain) cytokine receptor activity	1.97E-06	141,19,8
1dcfa_#66	GO:0000287: magnesium ion binding	1.97E-06	42,128,11
1qj5a_#316	GO:0016846: carbon-sulfur lyase activity	1.98E-06	8,10,3
1ldna1#129	GO:0004457: lactate dehydrogenase activity	1.98E-06	8,10,3
1g6za_#57	GO:0003682: chromatin binding	1.98E-06	8,10,3
1jjya_#293	GO:0004457: lactate dehydrogenase activity	1.98E-06	8,10,3
1fl2a1#490	GO:0050660: FAD binding	1.98E-06	8,10,3
1qgua_#435	GO:0005525: GTP binding	1.98E-06	23,49,6
1nat__#50	GO:0016638: oxidoreductase activity, acting on the CH-NH2 group of donors	1.98E-06	297,17,10
2pia_1#54	GO:0050660: FAD binding	1.98E-06	220,10,7
1lvl_2#222	GO:0004556: alpha-amylase activity	2.00E-06	79,15,6
1elua_#200	GO:0008483: transaminase activity	2.00E-06	5,17,3
1dgsa1#514	GO:0016799: hydrolase activity, hydrolyzing N-glycosyl compounds	2.00E-06	5,17,3
1qj5a_#136	GO:0008483: transaminase activity	2.00E-06	5,17,3
1i1ka_#69	GO:0008483: transaminase activity	2.00E-06	5,17,3
1jg8a_#210	GO:0008483: transaminase activity	2.00E-06	5,17,3
1aym3_#131	GO:0019838: growth factor binding	2.01E-06	412,9,8
3grx__#56	GO:0015036: disulfide oxidoreductase activity	2.02E-06	335,22,12
1f3oa_#154	GO:0005524: ATP binding	2.02E-06	16,243,9
1qqsa_#35	GO:0000049: tRNA binding	2.03E-06	94,13,6
1g5va_#109	GO:0004812: tRNA ligase activity	2.06E-06	11,26,4
1fjfl_#100	GO:0004812: tRNA ligase activity	2.06E-06	11,26,4
1dn2a1#261	GO:0019955: cytokine binding	2.08E-06	425,11,9
1tuba1#8	GO:0016763: transferase activity, transferring pentosyl groups	2.08E-06	124,28,9
1dpga2#334	GO:0005524: ATP binding	2.09E-06	287,243,49
1jsg__#92	GO:0005509: calcium ion binding	2.10E-06	607,160,61
1ds1a_#172	GO:0050660: FAD binding	2.11E-06	222,10,7
1bfg__#22	GO:0004263: chymotrypsin activity	2.11E-06	61,41,8
1f8ra2#325	GO:0005524: ATP binding	2.12E-06	222,243,41
1mkp__#216	GO:0004180: carboxypeptidase activity	2.13E-06	129,15,7
1g25a_#22	GO:0019838: growth factor binding	2.13E-06	415,9,8
1a7ca_#202	GO:0019955: cytokine binding	2.14E-06	117,11,6
1fxla2#169	GO:0005529: sugar binding	2.14E-06	203,39,13
1mml__#224	GO:0003968: RNA-directed RNA polymerase activity	2.14E-06	6,14,3
1ft9a1#162	GO:0004725: protein tyrosine phosphatase activity	2.15E-06	80,15,6
1fyhb1#99	GO:0008083: growth factor activity	2.16E-06	27,42,6
2dkb__#239	GO:0016866: intramolecular transferase activity	2.17E-06	56,12,5
1bwda_#295	GO:0016836: hydro-lyase activity	2.18E-06	104,33,9
1cjxa2#239	GO:0016702: oxidoreductase activity, acting on single donors with incorporation of molecular oxygen, incorporation of two atoms of oxygen	2.19E-06	24,12,4
1qmea4#464	GO:0004180: carboxypeptidase activity	2.20E-06	19,15,4
1e79d2#36	GO:0005509: calcium ion binding	2.20E-06	524,160,55
1hcl__#280	GO:0008199: ferric iron binding	2.21E-06	70,10,5
1smaa2#581	GO:0019838: growth factor binding	2.21E-06	80,9,5
1dqza_#131	GO:0015036: disulfide oxidoreductase activity	2.22E-06	83,22,7
1egja_#343	GO:0008083: growth factor activity	2.23E-06	42,42,7
1kid__#330	GO:0019838: growth factor binding	2.25E-06	155,9,6
1ihua2#343	GO:0019201: nucleotide kinase activity	2.25E-06	233,13,8
1hwxa1#223	GO:0019201: nucleotide kinase activity	2.25E-06	233,13,8
1imva_#255	GO:0004556: alpha-amylase activity	2.25E-06	589,15,12
1ldna1#94	GO:0005524: ATP binding	2.27E-06	199,243,38
1hxmb1#117	GO:0005509: calcium ion binding	2.28E-06	64,160,15
1fhoa_#74	GO:0050660: FAD binding	2.29E-06	347,10,8
1i50a_#856	GO:0030151: molybdenum ion binding	2.30E-06	269,15,9

1h9da_#91	GO:0005509: calcium ion binding	2.31E-06	222,160,31
1ayx__#69	GO:0008810: cellulase activity	2.31E-06	16,18,4
1rypk_#12	GO:0019838: growth factor binding	2.32E-06	265,9,7
1jb3a_#64	GO:0016638: oxidoreductase activity, acting on the CH-NH2 group of donors	2.32E-06	302,17,10
1ile_2#304	GO:0000287: magnesium ion binding	2.32E-06	21,128,8
1e87a_#174	GO:0005529: sugar binding	2.32E-06	141,39,11
1d66a1#31	GO:0008270: zinc ion binding	2.34E-06	4,108,4
1fw9a_#157	GO:0004896: hematopoietin/interferon-class (D200-domain) cytokine receptor activity	2.34E-06	62,19,6
1fjfc2#153	GO:0019838: growth factor binding	2.34E-06	420,9,8
1imva_#255	GO:0051082: unfolded protein binding	2.35E-06	589,34,20
1ihka_#161	GO:0016638: oxidoreductase activity, acting on the CH-NH2 group of donors	2.35E-06	17,17,4
1i50b_#966	GO:0019955: cytokine binding	2.36E-06	299,11,8
1f00i2#805	GO:0003809: thrombin activity	2.36E-06	135,10,6
2mev3_#143	GO:0004197: cysteine-type endopeptidase activity	2.39E-06	4,24,3
1aym2_#253	GO:0004197: cysteine-type endopeptidase activity	2.39E-06	4,24,3
2mev1_#246	GO:0004197: cysteine-type endopeptidase activity	2.39E-06	4,24,3
2mev1_#120	GO:0004197: cysteine-type endopeptidase activity	2.39E-06	4,24,3
1qlca_#228	GO:0005516: calmodulin binding	2.39E-06	4,24,3
1qtn.1#B433	GO:0004197: cysteine-type endopeptidase activity	2.39E-06	4,24,3
1b35b_#123	GO:0004197: cysteine-type endopeptidase activity	2.39E-06	4,24,3
1ez0a_#150	GO:0016620: oxidoreductase activity, acting on the aldehyde or oxo group of donors, NAD or NADP as acceptor	2.40E-06	30,10,4
1hlwa_#91	GO:0003809: thrombin activity	2.40E-06	30,10,4
2sqca1#553	GO:0008810: cellulase activity	2.40E-06	5,18,3
1a3qa2#133	GO:0019955: cytokine binding	2.41E-06	27,11,4
1kit_3#743	GO:0004295: trypsin activity	2.41E-06	250,48,16
1dih_2#235	GO:0004867: serine-type endopeptidase inhibitor activity	2.42E-06	54,47,8
1bwvs_#117	GO:0004674: protein serine/threonine kinase activity	2.44E-06	329,42,17
1fhga_#59	GO:0019838: growth factor binding	2.44E-06	34,9,4
1nuka_#80	GO:0004295: trypsin activity	2.48E-06	220,48,15
1fi2a_#104	GO:0051082: unfolded protein binding	2.49E-06	34,34,6
1aoza1#63	GO:0005507: copper ion binding	2.50E-06	3,38,3
1iba__#39	GO:0050660: FAD binding	2.51E-06	351,10,8
1h6va1#23	GO:0016638: oxidoreductase activity, acting on the CH-NH2 group of donors	2.52E-06	39,17,5
1epwa3#138	GO:0019838: growth factor binding	2.53E-06	424,9,8
1hxmb1#117	GO:0019955: cytokine binding	2.53E-06	64,11,5
3sil__#284	GO:0019955: cytokine binding	2.53E-06	64,11,5
1doza_#109	GO:0016616: oxidoreductase activity, acting on the CH-OH group of donors, NAD or NADP as acceptor	2.54E-06	74,59,10
1apme_#68	GO:0005524: ATP binding	2.55E-06	105,243,25
1hxxa_#232	GO:0050660: FAD binding	2.56E-06	352,10,8
1dih_2#222	GO:0003809: thrombin activity	2.56E-06	352,10,8
1fm2.1#B308	GO:0005524: ATP binding	2.58E-06	200,243,38
1jj2s_#46	GO:0004812: tRNA ligase activity	2.59E-06	45,26,6
1e9xa_#62	GO:0016705: oxidoreductase activity, acting on paired donors, with incorporation or reduction of molecular oxygen	2.59E-06	45,26,6
1avgi_#69	GO:0004263: chymotrypsin activity	2.60E-06	340,41,17
1lst__#56	GO:0005525: GTP binding	2.60E-06	24,49,6
1e44b_#75	GO:0008201: heparin binding	2.62E-06	198,24,10
1eg5a_#347	GO:0004867: serine-type endopeptidase inhibitor activity	2.62E-06	7,47,4
2pia_1#54	GO:0015036: disulfide oxidoreductase activity	2.62E-06	220,22,10
1tgoa2#409	GO:0008408: 3'-5' exonuclease activity	2.63E-06	23,13,4
1i12a_#126	GO:0008080: N-acetyltransferase activity	2.63E-06	23,13,4
1ea5a_#205	GO:0008235: metalloexopeptidase activity	2.63E-06	23,13,4
1epwa3#138	GO:0004263: chymotrypsin activity	2.65E-06	424,41,19
1fbva4#389	GO:0004180: carboxypeptidase activity	2.67E-06	6,15,3
1cf9a1#701	GO:0004180: carboxypeptidase activity	2.67E-06	6,15,3
1ecpa_#58	GO:0004725: protein tyrosine phosphatase activity	2.68E-06	83,15,6
1e8ga2#201	GO:0003755: peptidyl-prolyl cis-trans isomerase activity	2.69E-06	304,11,8
1a7ca_#202	GO:0004867: serine-type endopeptidase inhibitor activity	2.69E-06	117,47,11
1gen__#477	GO:0005509: calcium ion binding	2.69E-06	13,160,7
7taa_2#331	GO:0005509: calcium ion binding	2.69E-06	13,160,7
1e43a2#257	GO:0005509: calcium ion binding	2.69E-06	13,160,7
1erza_#183	GO:0016836: hydro-lyase activity	2.70E-06	79,33,8
1i9ga_#67	GO:0005524: ATP binding	2.71E-06	61,243,18
1e30a_#112	GO:0003968: RNA-directed RNA polymerase activity	2.71E-06	49,14,5

1jf9a_#379	GO:0005524: ATP binding	2.71E-06	232,243,42
1c8na_#216	GO:0003724: RNA helicase activity	2.71E-06	8,11,3
1hava_#141	GO:0003724: RNA helicase activity	2.71E-06	8,11,3
1pvda2#174	GO:0016831: carboxy-lyase activity	2.71E-06	4,25,3
1jb3a_#64	GO:0004263: chymotrypsin activity	2.73E-06	302,41,16
1eg2a_#132	GO:0004457: lactate dehydrogenase activity	2.75E-06	31,10,4
1smaa1#95	GO:0019838: growth factor binding	2.77E-06	272,9,7
1qs1a2#437	GO:0005529: sugar binding	2.78E-06	452,39,19
1g4ia_#11	GO:0005509: calcium ion binding	2.80E-06	29,160,10
3chbd_#85	GO:0051082: unfolded protein binding	2.80E-06	378,34,16
1el0a_#28	GO:0019838: growth factor binding	2.81E-06	161,9,6
1e79d2#36	GO:0050660: FAD binding	2.82E-06	524,10,9
1mjha_#119	GO:0004812: tRNA ligase activity	2.82E-06	103,26,8
1seta2#231	GO:0004812: tRNA ligase activity	2.82E-06	103,26,8
1g38a_#155	GO:0016616: oxidoreductase activity, acting on the CH-OH group of donors, NAD or NADP as acceptor	2.83E-06	12,59,5
1pvc1_#106	GO:0004896: hematopoietin/interferon-class (D200-domain) cytokine receptor activity	2.83E-06	64,19,6
1edza2#120	GO:0016646: oxidoreductase activity, acting on the CH-NH group of donors, NAD or NADP as acceptor	2.85E-06	5,19,3
1a4ia2#118	GO:0016646: oxidoreductase activity, acting on the CH-NH group of donors, NAD or NADP as acceptor	2.85E-06	5,19,3
1hxn__#235	GO:0004222: metalloendopeptidase activity	2.85E-06	5,19,3
1jf9a_#379	GO:0050660: FAD binding	2.86E-06	232,10,7
1ayl__#237	GO:0050660: FAD binding	2.86E-06	357,10,8
1gcoa_#119	GO:0016627: oxidoreductase activity, acting on the CH-CH group of donors	2.87E-06	40,17,5
1mla_1#281	GO:0016616: oxidoreductase activity, acting on the CH-OH group of donors, NAD or NADP as acceptor	2.87E-06	44,59,8
1pda_1#77	GO:0005351: sugar porter activity	2.88E-06	32,21,5
1bywa_#30	GO:0051082: unfolded protein binding	2.88E-06	331,34,15
1qg6a_#65	GO:0016616: oxidoreductase activity, acting on the CH-OH group of donors, NAD or NADP as acceptor	2.88E-06	6,59,4
1ppn__#132	GO:0016638: oxidoreductase activity, acting on the CH-NH2 group of donors	2.88E-06	234,17,9
1br9__#86	GO:0003887: DNA-directed DNA polymerase activity	2.88E-06	96,20,7
1fwxa2#169	GO:0016638: oxidoreductase activity, acting on the CH-NH2 group of donors	2.89E-06	116,17,7
1c9la2#12	GO:0004674: protein serine/threonine kinase activity	2.90E-06	295,42,16
1xgsa2#82	GO:0019955: cytokine binding	2.90E-06	307,11,8
1e79d2#36	GO:0016668: oxidoreductase activity, acting on sulfur group of donors, NAD or NADP as acceptor	2.92E-06	524,12,10
1iira_#242	GO:0003924: GTPase activity	2.92E-06	170,17,8
1g6oa_#116	GO:0003809: thrombin activity	2.92E-06	358,10,8
1e32a2#345	GO:0008235: metalloexopeptidase activity	2.93E-06	100,13,6
1a1va1#269	GO:0008026: ATP-dependent helicase activity	2.94E-06	7,13,3
1xsoa_#36	GO:0005518: collagen binding	2.94E-06	7,13,3
1gdea_#32	GO:0016846: carbon-sulfur lyase activity	2.96E-06	9,10,3
1i1ja_#37	GO:0005066: transmembrane receptor protein tyrosine kinase signaling protein activity	2.96E-06	9,10,3
1wdna_#152	GO:0008199: ferric iron binding	2.96E-06	9,10,3
1qmha2#136	GO:0003809: thrombin activity	2.96E-06	9,10,3
1i9ga_#185	GO:0004457: lactate dehydrogenase activity	2.96E-06	9,10,3
1f9za_#16	GO:0003887: DNA-directed DNA polymerase activity	2.97E-06	140,20,8
1g25a_#22	GO:0004674: protein serine/threonine kinase activity	2.97E-06	415,42,19
1ddja_#681	GO:0008201: heparin binding	2.99E-06	253,24,11
1ddja_#746	GO:0004867: serine-type endopeptidase inhibitor activity	2.99E-06	170,47,13
2uaga1#54	GO:0016627: oxidoreductase activity, acting on the CH-CH group of donors	3.01E-06	18,17,4
1tml__#252	GO:0008810: cellulase activity	3.01E-06	17,18,4
1ac6a_#43	GO:0019838: growth factor binding	3.03E-06	163,9,6
1evya2#38	GO:0015036: disulfide oxidoreductase activity	3.03E-06	14,22,4
1poxa3#460	GO:0000287: magnesium ion binding	3.06E-06	81,128,15
1io7a_#256	GO:0004497: monooxygenase activity	3.06E-06	12,26,4
1ajsa_#389	GO:0004812: tRNA ligase activity	3.06E-06	12,26,4
1pysb6#384	GO:0004812: tRNA ligase activity	3.06E-06	12,26,4
1gln_2#10	GO:0004812: tRNA ligase activity	3.07E-06	4,26,3
1gaxa1#681	GO:0004812: tRNA ligase activity	3.07E-06	4,26,3
1bu7a_#422	GO:0016705: oxidoreductase activity, acting on paired donors, with incorporation or reduction of molecular oxygen	3.07E-06	4,26,3
1bu7a_#422	GO:0004497: monooxygenase activity	3.07E-06	4,26,3
1bu7a_#433	GO:0004497: monooxygenase activity	3.07E-06	4,26,3

1ek1a2#500	GO:0004812: tRNA ligase activity	3.07E-06	4,26,3
2ak3a1#122	GO:0005524: ATP binding	3.08E-06	7,243,6
1a06__#85	GO:0004674: protein serine/threonine kinase activity	3.09E-06	44,42,7
1dpsa_#36	GO:0004896: hematopoietin/interferon-class (D200-domain) cytokine receptor activity	3.09E-06	205,19,9
1f4la2#20	GO:0005524: ATP binding	3.10E-06	13,243,8
1f97a1#62	GO:0016668: oxidoreductase activity, acting on sulfur group of donors, NAD or NADP as acceptor	3.11E-06	707,12,11
1hyea2#272	GO:0019843: rRNA binding	3.13E-06	31,22,5
1dyna_#42	GO:0019838: growth factor binding	3.14E-06	164,9,6
1a6da3#190	GO:0005509: calcium ion binding	3.14E-06	18,160,8
1qbea_#92	GO:0005509: calcium ion binding	3.14E-06	18,160,8
1ilr1_#32	GO:0008201: heparin binding	3.14E-06	13,24,4
1mdah_#329	GO:0008083: growth factor activity	3.15E-06	335,42,17
1jj2e1#42	GO:0004263: chymotrypsin activity	3.15E-06	167,41,12
1gdha1#235	GO:0005524: ATP binding	3.17E-06	113,243,26
1e2wa1#102	GO:0019838: growth factor binding	3.18E-06	86,9,5
1e3ja2#270	GO:0016638: oxidoreductase activity, acting on the CH-NH2 group of donors	3.19E-06	74,17,6
1ct9a1#401	GO:0008270: zinc ion binding	3.20E-06	42,108,10
1tuba1#239	GO:0005524: ATP binding	3.21E-06	120,243,27
1fu6a_#50	GO:0004714: transmembrane receptor protein tyrosine kinase activity	3.22E-06	308,14,9
5ruba1#365	GO:0008483: transaminase activity	3.25E-06	118,17,7
1azo__#144	GO:0016638: oxidoreductase activity, acting on the CH-NH2 group of donors	3.25E-06	41,17,5
1dp0a4#756	GO:0019838: growth factor binding	3.25E-06	165,9,6
1fmb__#88	GO:0004523: ribonuclease H activity	3.29E-06	6,16,3
1ea5a_#133	GO:0000287: magnesium ion binding	3.30E-06	36,128,10
1jsg__#92	GO:0005529: sugar binding	3.31E-06	607,39,22
1dik_1#765	GO:0016861: intramolecular oxidoreductase activity, interconverting aldoses and ketoses	3.32E-06	165,13,7
1io1a_#311	GO:0019955: cytokine binding	3.32E-06	126,11,6
1c9la2#61	GO:0016638: oxidoreductase activity, acting on the CH-NH2 group of donors	3.33E-06	238,17,9
1pwt__#8	GO:0016638: oxidoreductase activity, acting on the CH-NH2 group of donors	3.34E-06	314,17,10
1h6va1#27	GO:0016763: transferase activity, transferring pentosyl groups	3.36E-06	260,28,12
1iba__#39	GO:0015036: disulfide oxidoreductase activity	3.36E-06	351,22,12
1i39a_#49	GO:0005179: hormone activity	3.37E-06	21,15,4
1aru__#57	GO:0004601: peroxidase activity	3.37E-06	15,21,4
1poxa3#439	GO:0005525: GTP binding	3.38E-06	25,49,6
1e79d2#36	GO:0004867: serine-type endopeptidase inhibitor activity	3.38E-06	524,47,23
1c9la2#61	GO:0003809: thrombin activity	3.40E-06	238,10,7
1f42a2#123	GO:0005524: ATP binding	3.42E-06	378,243,59
1qhda2#323	GO:0004674: protein serine/threonine kinase activity	3.42E-06	337,42,17
1hjra_#58	GO:0000287: magnesium ion binding	3.44E-06	16,128,7
1fmta2#151	GO:0003755: peptidyl-prolyl cis-trans isomerase activity	3.45E-06	314,11,8
1g51a3#525	GO:0004867: serine-type endopeptidase inhibitor activity	3.47E-06	263,47,16
1nsca_#236	GO:0005126: hematopoietin/interferon-class (D200-domain) cytokine receptor binding	3.49E-06	143,20,8
1i76a_#138	GO:0019201: nucleotide kinase activity	3.49E-06	103,13,6
1pya.1#B236	GO:0016831: carboxy-lyase activity	3.49E-06	77,25,7
1a8d_1#87	GO:0004556: alpha-amylase activity	3.51E-06	204,15,8
1j71a_#251	GO:0004812: tRNA ligase activity	3.51E-06	106,26,8
1d66a1#28	GO:0003700: transcription factor activity	3.52E-06	7,124,5
1awx__#13	GO:0004896: hematopoietin/interferon-class (D200-domain) cytokine receptor activity	3.54E-06	37,19,5
1e79d2#36	GO:0008201: heparin binding	3.54E-06	524,24,15
1phm_2#253	GO:0005524: ATP binding	3.56E-06	309,243,51
1nhp_1#7	GO:0004457: lactate dehydrogenase activity	3.56E-06	33,10,4
1zfpe_#67	GO:0005066: transmembrane receptor protein tyrosine kinase signaling protein activity	3.56E-06	33,10,4
1quna1#113	GO:0004812: tRNA ligase activity	3.56E-06	286,26,12
1fhoa_#74	GO:0004896: hematopoietin/interferon-class (D200-domain) cytokine receptor activity	3.59E-06	347,19,11
1dzfa2#211	GO:0000049: tRNA binding	3.60E-06	167,13,7
1fl2a1#490	GO:0016668: oxidoreductase activity, acting on sulfur group of donors, NAD or NADP as acceptor	3.61E-06	8,12,3
1seta2#306	GO:0004263: chymotrypsin activity	3.62E-06	30,41,6
3prn__#145	GO:0019838: growth factor binding	3.62E-06	168,9,6
1qr4a2#166	GO:0005126: hematopoietin/interferon-class (D200-domain) cytokine receptor binding	3.63E-06	16,20,4
1e32a1#45	GO:0003887: DNA-directed DNA polymerase activity	3.63E-06	16,20,4
1dpsa_#36	GO:0005096: GTPase activator activity	3.65E-06	205,15,8
1mdah_#329	GO:0005524: ATP binding	3.65E-06	335,243,54
1h7wa5#973	GO:0016251: general RNA polymerase II transcription factor activity	3.66E-06	23,14,4
1gp1a_#173	GO:0003779: actin binding	3.69E-06	22,32,5

1ejda_#180	GO:0016616: oxidoreductase activity, acting on the CH-OH group of donors, NAD or NADP as acceptor	3.69E-06	77,59,10
1qtra_#108	GO:0016616: oxidoreductase activity, acting on the CH-OH group of donors, NAD or NADP as acceptor	3.69E-06	77,59,10
1dssg1#4	GO:0015036: disulfide oxidoreductase activity	3.69E-06	57,22,6
1a8i__#688	GO:0016620: oxidoreductase activity, acting on the aldehyde or oxo group of donors, NAD or NADP as acceptor	3.71E-06	241,10,7
1hxmb1#117	GO:0008083: growth factor activity	3.73E-06	64,42,8
1aym1_#181	GO:0003968: RNA-directed RNA polymerase activity	3.74E-06	7,14,3
3grs_1#23	GO:0008026: ATP-dependent helicase activity	3.75E-06	168,13,7
1a7ca_#202	GO:0008201: heparin binding	3.76E-06	117,24,8
1ho1a_#211	GO:0016836: hydro-lyase activity	3.79E-06	111,33,9
1aa6_2#285	GO:0016646: oxidoreductase activity, acting on the CH-NH group of donors, NAD or NADP as acceptor	3.79E-06	106,19,7
1b3qa2#623	GO:0008083: growth factor activity	3.80E-06	301,42,16
1stfi_#116	GO:0000049: tRNA binding	3.80E-06	57,13,5
1h6kx_#97	GO:0003729: mRNA binding	3.80E-06	57,13,5
1h7wa4#478	GO:0016627: oxidoreductase activity, acting on the CH-CH group of donors	3.80E-06	19,17,4
1deua_#151	GO:0019838: growth factor binding	3.82E-06	285,9,7
1i7wa_#517	GO:0003779: actin binding	3.85E-06	60,32,7
1g72a_#42	GO:0005507: copper ion binding	3.86E-06	9,38,4
1lara2#1769	GO:0005524: ATP binding	3.87E-06	219,243,40
1ddja_#746	GO:0019838: growth factor binding	3.88E-06	170,9,6
1e6ca_#91	GO:0016616: oxidoreductase activity, acting on the CH-OH group of donors, NAD or NADP as acceptor	3.91E-06	182,59,15
1b34b_#56	GO:0005524: ATP binding	3.91E-06	30,243,12
1dofa_#15	GO:0004674: protein serine/threonine kinase activity	3.91E-06	197,42,13
1fjfj_#68	GO:0015036: disulfide oxidoreductase activity	3.96E-06	230,22,10
1pjr_2#381	GO:0016638: oxidoreductase activity, acting on the CH-NH2 group of donors	3.99E-06	6,17,3
1pyma_#224	GO:0003924: GTPase activity	3.99E-06	6,17,3
1elua_#288	GO:0008483: transaminase activity	3.99E-06	6,17,3
1jg8a_#243	GO:0008483: transaminase activity	3.99E-06	6,17,3
1io1a_#202	GO:0016638: oxidoreductase activity, acting on the CH-NH2 group of donors	3.99E-06	6,17,3
1fg7a_#157	GO:0008483: transaminase activity	3.99E-06	6,17,3
1dvpa1#65	GO:0015078: hydrogen ion transporter activity	3.99E-06	187,21,9
1qqsa_#124	GO:0019955: cytokine binding	3.99E-06	458,11,9
1ejda_#379	GO:0004725: protein tyrosine phosphatase activity	4.01E-06	49,15,5
1e30a_#112	GO:0004556: alpha-amylase activity	4.01E-06	49,15,5
1g3qa_#171	GO:0005525: GTP binding	4.01E-06	15,49,5
1hava_#41	GO:0005507: copper ion binding	4.02E-06	96,38,9
1el0a_#42	GO:0051082: unfolded protein binding	4.02E-06	294,34,14
1epua_#26	GO:0016846: carbon-sulfur lyase activity	4.03E-06	34,10,4
1a3qa2#190	GO:0004295: trypsin activity	4.04E-06	144,48,12
1dih_2#222	GO:0008080: N-acetyltransferase activity	4.04E-06	352,13,9
1fds__#59	GO:0016616: oxidoreductase activity, acting on the CH-OH group of donors, NAD or NADP as acceptor	4.04E-06	61,59,9
1fwxa2#396	GO:0004263: chymotrypsin activity	4.05E-06	237,41,14
1qhoa4#399	GO:0005509: calcium ion binding	4.05E-06	24,160,9
1ejfa_#10	GO:0004896: hematopoietin/interferon-class (D200-domain) cytokine receptor activity	4.05E-06	155,19,8
1a3k__#122	GO:0019955: cytokine binding	4.06E-06	9,11,3
1cg2a1#43	GO:0004497: monooxygenase activity	4.07E-06	189,26,10
1byka_#281	GO:0003700: transcription factor activity	4.09E-06	4,124,4
1a02n1#588	GO:0003700: transcription factor activity	4.09E-06	4,124,4
1d66a1#31	GO:0003700: transcription factor activity	4.09E-06	4,124,4
1dpga2#334	GO:0000287: magnesium ion binding	4.11E-06	287,128,31
1bio__#88	GO:0030151: molybdenum ion binding	4.11E-06	22,15,4
1fzqa_#165	GO:0005096: GTPase activator activity	4.11E-06	22,15,4
1imva_#278	GO:0004867: serine-type endopeptidase inhibitor activity	4.14E-06	41,47,7
1e44b_#75	GO:0008083: growth factor activity	4.15E-06	198,42,13
1sbp__#219	GO:0016638: oxidoreductase activity, acting on the CH-NH2 group of donors	4.15E-06	43,17,5
1dp0a1#309	GO:0008201: heparin binding	4.18E-06	53,24,6
1gsa_2#218	GO:0019955: cytokine binding	4.18E-06	131,11,6
1qs1a2#437	GO:0019838: growth factor binding	4.18E-06	452,9,8
1bu7a_#278	GO:0004674: protein serine/threonine kinase activity	4.19E-06	30,42,6
1epwa3#138	GO:0015082: di-, tri-valent inorganic cation transporter activity	4.19E-06	424,14,10
1epwa3#138	GO:0046915: transition metal ion transporter activity	4.19E-06	424,14,10

1qrra_#69	GO:0005524: ATP binding	4.20E-06	196,243,37
1qgub_#197	GO:0005525: GTP binding	4.21E-06	74,49,9
1es9a_#89	GO:0004457: lactate dehydrogenase activity	4.22E-06	10,10,3
2oata_#81	GO:0016846: carbon-sulfur lyase activity	4.22E-06	10,10,3
2mpra_#190	GO:0016668: oxidoreductase activity, acting on sulfur group of donors, NAD or NADP as acceptor	4.25E-06	400,12,9
1pfza_#124	GO:0005507: copper ion binding	4.27E-06	51,38,7
1dt6a_#385	GO:0004812: tRNA ligase activity	4.27E-06	76,26,7
1jswa_#228	GO:0004867: serine-type endopeptidase inhibitor activity	4.28E-06	27,47,6
1nhp_1#7	GO:0016616: oxidoreductase activity, acting on the CH-OH group of donors, NAD or NADP as acceptor	4.28E-06	33,59,7
1fo4a5#1020	GO:0015036: disulfide oxidoreductase activity	4.29E-06	359,22,12
1qvba_#206	GO:0005525: GTP binding	4.33E-06	26,49,6
1lci__#26	GO:0015078: hydrogen ion transporter activity	4.35E-06	139,21,8
1e3ua_#164	GO:0000287: magnesium ion binding	4.35E-06	37,128,10
1dgw.1#Y353	GO:0015082: di-, tri-valent inorganic cation transporter activity	4.35E-06	319,14,9
1dgw.1#Y353	GO:0046915: transition metal ion transporter activity	4.35E-06	319,14,9
3sil__#98	GO:0005524: ATP binding	4.36E-06	228,243,41
1ct5a_#114	GO:0016831: carboxy-lyase activity	4.36E-06	29,25,5
1jjya_#324	GO:0008483: transaminase activity	4.37E-06	78,17,6
1stfi_#116	GO:0004295: trypsin activity	4.37E-06	57,48,8
1nsj__#21	GO:0016831: carboxy-lyase activity	4.38E-06	114,25,8
1qvba_#206	GO:0008235: metalloexopeptidase activity	4.40E-06	26,13,4
1i5ga_#115	GO:0003755: peptidyl-prolyl cis-trans isomerase activity	4.40E-06	324,11,8
1mkp__#216	GO:0005524: ATP binding	4.43E-06	129,243,28
1thg__#3	GO:0019838: growth factor binding	4.46E-06	92,9,5
3pah__#303	GO:0004497: monooxygenase activity	4.47E-06	28,26,5
1e6ua_#266	GO:0004812: tRNA ligase activity	4.47E-06	28,26,5
2dri__#115	GO:0005351: sugar porter activity	4.47E-06	16,21,4
2naca1#162	GO:0016616: oxidoreductase activity, acting on the CH-OH group of donors, NAD or NADP as acceptor	4.54E-06	22,59,6
1eq9a_#162	GO:0003809: thrombin activity	4.54E-06	35,10,4
1p35a_#49	GO:0016638: oxidoreductase activity, acting on the CH-NH2 group of donors	4.54E-06	124,17,7
1fa0a1#393	GO:0005524: ATP binding	4.54E-06	261,243,45
1a65a1#108	GO:0015036: disulfide oxidoreductase activity	4.56E-06	294,22,11
1fp1d2#214	GO:0008026: ATP-dependent helicase activity	4.57E-06	173,13,7
1gpea1#273	GO:0003809: thrombin activity	4.60E-06	81,10,5
1foha5#9	GO:0016668: oxidoreductase activity, acting on sulfur group of donors, NAD or NADP as acceptor	4.61E-06	65,12,5
1qfma1#186	GO:0019955: cytokine binding	4.62E-06	326,11,8
1qqsa_#124	GO:0019838: growth factor binding	4.64E-06	458,9,8
1beba_#119	GO:0003887: DNA-directed DNA polymerase activity	4.65E-06	202,20,9
1e79d3#328	GO:0005525: GTP binding	4.65E-06	40,49,7
1pvda2#174	GO:0000287: magnesium ion binding	4.65E-06	4,128,4
1ema__#13	GO:0000287: magnesium ion binding	4.65E-06	4,128,4
1xsoa_#36	GO:0004556: alpha-amylase activity	4.67E-06	7,15,3
1c4xa_#43	GO:0004180: carboxypeptidase activity	4.67E-06	7,15,3
1f0ia1#50	GO:0004295: trypsin activity	4.67E-06	146,48,12
1czan1#92	GO:0004177: aminopeptidase activity	4.68E-06	631,13,11
1g25a_#22	GO:0016638: oxidoreductase activity, acting on the CH-NH2 group of donors	4.69E-06	415,17,11
2occb1#209	GO:0019838: growth factor binding	4.71E-06	93,9,5
1qmva_#40	GO:0003924: GTPase activity	4.71E-06	79,17,6
1c9la2#12	GO:0015036: disulfide oxidoreductase activity	4.72E-06	295,22,11
1hw1a2#131	GO:0005126: hematopoietin/interferon-class (D200-domain) cytokine receptor binding	4.73E-06	17,20,4
1oaa__#161	GO:0016627: oxidoreductase activity, acting on the CH-CH group of donors	4.73E-06	20,17,4
1cyda_#106	GO:0016627: oxidoreductase activity, acting on the CH-CH group of donors	4.73E-06	20,17,4
1trb_1#15	GO:0016627: oxidoreductase activity, acting on the CH-CH group of donors	4.73E-06	20,17,4
1el0a_#42	GO:0019838: growth factor binding	4.73E-06	294,9,7
1czan1#92	GO:0000287: magnesium ion binding	4.74E-06	631,128,52
1h6va1#27	GO:0000287: magnesium ion binding	4.75E-06	260,128,29
1cmia_#86	GO:0019838: growth factor binding	4.76E-06	40,9,4
2ltn.1#A63	GO:0050660: FAD binding	4.77E-06	152,10,6
1dceb_#170	GO:0008810: cellulase activity	4.78E-06	6,18,3
1eqca_#15	GO:0008810: cellulase activity	4.78E-06	6,18,3
1f97a1#62	GO:0008201: heparin binding	4.79E-06	707,24,17
2pth__#44	GO:0004867: serine-type endopeptidase inhibitor activity	4.81E-06	3,47,3

1ovaa_#42	GO:0004867: serine-type endopeptidase inhibitor activity	4.81E-06	3,47,3
1dih_2#222	GO:0004812: tRNA ligase activity	4.81E-06	352,26,13
1ioaa_#75	GO:0005529: sugar binding	4.82E-06	33,39,6
1h6va3#486	GO:0004896: hematopoietin/interferon-class (D200-domain) cytokine receptor activity	4.83E-06	70,19,6
1ihua2#343	GO:0016776: phosphotransferase activity, phosphate group as acceptor	4.86E-06	233,14,8
1hwxa1#223	GO:0016776: phosphotransferase activity, phosphate group as acceptor	4.86E-06	233,14,8
2viua_#252	GO:0004190: aspartic-type endopeptidase activity	4.86E-06	343,23,12
1bqca_#23	GO:0008810: cellulase activity	4.86E-06	19,18,4
1kapp1#358	GO:0003724: RNA helicase activity	4.89E-06	32,11,4
1thg__#223	GO:0004457: lactate dehydrogenase activity	4.89E-06	82,10,5
2sli_2#326	GO:0003809: thrombin activity	4.89E-06	251,10,7
1ej8a_#189	GO:0030151: molybdenum ion binding	4.90E-06	294,15,9
1mspa_#17	GO:0008083: growth factor activity	4.90E-06	47,42,7
1qfxa_#380	GO:0008083: growth factor activity	4.90E-06	47,42,7
1g6oa_#116	GO:0004896: hematopoietin/interferon-class (D200-domain) cytokine receptor activity	4.91E-06	358,19,11
1f97a2#199	GO:0004263: chymotrypsin activity	4.93E-06	144,41,11
1nfdb1#34	GO:0019838: growth factor binding	4.93E-06	177,9,6
1hq8a_#206	GO:0019955: cytokine binding	4.95E-06	329,11,8
1h8ma_#8	GO:0015036: disulfide oxidoreductase activity	4.96E-06	134,22,8
1ea5a_#205	GO:0004180: carboxypeptidase activity	4.96E-06	23,15,4
1aw8.1#B59	GO:0030151: molybdenum ion binding	4.96E-06	23,15,4
1qfea_#68	GO:0016776: phosphotransferase activity, phosphate group as acceptor	5.03E-06	560,14,11
1bpv__#22	GO:0008083: growth factor activity	5.04E-06	18,42,5
1ii7a_#43	GO:0008270: zinc ion binding	5.05E-06	44,108,10
1io1a_#311	GO:0016638: oxidoreductase activity, acting on the CH-NH2 group of donors	5.06E-06	126,17,7
1qfea_#68	GO:0004457: lactate dehydrogenase activity	5.08E-06	560,10,9
1nuka_#80	GO:0019955: cytokine binding	5.08E-06	220,11,7
1e3a.1#A30	GO:0030145: manganese ion binding	5.09E-06	157,38,11
1saca_#113	GO:0005066: transmembrane receptor protein tyrosine kinase signaling protein activity	5.10E-06	36,10,4
1f97a1#62	GO:0019838: growth factor binding	5.13E-06	707,9,9
1loua_#79	GO:0000287: magnesium ion binding	5.14E-06	106,128,17
1iq4a_#31	GO:0008408: 3'-5' exonuclease activity	5.15E-06	27,13,4
1gdna_#119	GO:0005509: calcium ion binding	5.16E-06	19,160,8
1bywa_#30	GO:0019955: cytokine binding	5.19E-06	331,11,8
1foha5#77	GO:0016251: general RNA polymerase II transcription factor activity	5.19E-06	25,14,4
1nsj__#124	GO:0016836: hydro-lyase activity	5.19E-06	148,33,10
1qfea_#49	GO:0005509: calcium ion binding	5.20E-06	52,160,13
1qrra_#69	GO:0016668: oxidoreductase activity, acting on sulfur group of donors, NAD or NADP as acceptor	5.21E-06	196,12,7
1nal1_#203	GO:0000287: magnesium ion binding	5.24E-06	46,128,11
1jjya_#329	GO:0016646: oxidoreductase activity, acting on the CH-NH group of donors, NAD or NADP as acceptor	5.25E-06	71,19,6
1gox__#124	GO:0015036: disulfide oxidoreductase activity	5.29E-06	366,22,12
1dzfa2#211	GO:0005524: ATP binding	5.29E-06	167,243,33
1fo5a_#60	GO:0005524: ATP binding	5.33E-06	246,243,43
1ejfa_#10	GO:0003809: thrombin activity	5.35E-06	155,10,6
1f13a4#438	GO:0005507: copper ion binding	5.36E-06	20,38,5
1f97a1#62	GO:0019843: rRNA binding	5.36E-06	707,22,16
1ac5__#72	GO:0016763: transferase activity, transferring pentosyl groups	5.37E-06	47,28,6
1b35b_#97	GO:0004674: protein serine/threonine kinase activity	5.39E-06	309,42,16
1apy.1#B284	GO:0016811: hydrolase activity, acting on carbon-nitrogen (but not peptide) bonds, in linear amides	5.41E-06	52,15,5
1ho1a_#211	GO:0016861: intramolecular oxidoreductase activity, interconverting aldoses and ketoses	5.43E-06	111,13,6
1hc7a1#288	GO:0005524: ATP binding	5.43E-06	5,243,5
1g6ha_#179	GO:0005524: ATP binding	5.43E-06	5,243,5
1bg2__#93	GO:0005524: ATP binding	5.43E-06	5,243,5
1aym3_#131	GO:0016668: oxidoreductase activity, acting on sulfur group of donors, NAD or NADP as acceptor	5.49E-06	412,12,9
1cs1a_#160	GO:0016616: oxidoreductase activity, acting on the CH-OH group of donors, NAD or NADP as acceptor	5.50E-06	187,59,15
1miob_#338	GO:0016620: oxidoreductase activity, acting on the aldehyde or oxo group of donors, NAD or NADP as acceptor	5.52E-06	84,10,5
1i9ga_#67	GO:0019843: rRNA binding	5.54E-06	61,22,6
1evqa_#305	GO:0019201: nucleotide kinase activity	5.54E-06	490,13,10
1evqa_#305	GO:0016861: intramolecular oxidoreductase activity, interconverting aldoses and ketoses	5.54E-06	490,13,10
3prn__#143	GO:0019955: cytokine binding	5.57E-06	334,11,8

1ewna_#109	GO:0003724: RNA helicase activity	5.61E-06	75,11,5
1ecfa2#160	GO:0000287: magnesium ion binding	5.63E-06	17,128,7
1quba4#235	GO:0005126: hematopoietin/interferon-class (D200-domain) cytokine receptor binding	5.65E-06	106,20,7
1a7j__#87	GO:0005524: ATP binding	5.65E-06	70,243,19
1e42a1#711	GO:0004190: aspartic-type endopeptidase activity	5.66E-06	175,23,9
1e19a_#281	GO:0010181: FMN binding	5.68E-06	138,11,6
1aln_1#90	GO:0004222: metalloendopeptidase activity	5.68E-06	6,19,3
5ruba1#365	GO:0016831: carboxy-lyase activity	5.69E-06	118,25,8
1mdah_#329	GO:0019955: cytokine binding	5.70E-06	335,11,8
1jd0a_#176	GO:0003887: DNA-directed DNA polymerase activity	5.71E-06	207,20,9
1fkna_#63	GO:0004457: lactate dehydrogenase activity	5.71E-06	37,10,4
1awx__#13	GO:0005066: transmembrane receptor protein tyrosine kinase signaling protein activity	5.71E-06	37,10,4
1pme__#25	GO:0005066: transmembrane receptor protein tyrosine kinase signaling protein activity	5.71E-06	37,10,4
1bfd_2#146	GO:0019201: nucleotide kinase activity	5.72E-06	112,13,6
1aym1_#151	GO:0003724: RNA helicase activity	5.79E-06	10,11,3
1euha_#220	GO:0016620: oxidoreductase activity, acting on the aldehyde or oxo group of donors, NAD or NADP as acceptor	5.79E-06	11,10,3
1ftka_#61	GO:0003684: damaged DNA binding	5.79E-06	11,10,3
1ftka_#61	GO:0016620: oxidoreductase activity, acting on the aldehyde or oxo group of donors, NAD or NADP as acceptor	5.79E-06	11,10,3
1e39a3#417	GO:0003809: thrombin activity	5.79E-06	11,10,3
1nfdb1#115	GO:0019955: cytokine binding	5.79E-06	10,11,3
1hxxa_#84	GO:0003755: peptidyl-prolyl cis-trans isomerase activity	5.79E-06	10,11,3
1sek__#52	GO:0008199: ferric iron binding	5.79E-06	11,10,3
1a0i_2#222	GO:0016638: oxidoreductase activity, acting on the CH-NH2 group of donors	5.80E-06	186,17,8
1an9a2#278	GO:0016638: oxidoreductase activity, acting on the CH-NH2 group of donors	5.82E-06	21,17,4
1epwa3#138	GO:0016638: oxidoreductase activity, acting on the CH-NH2 group of donors	5.83E-06	424,17,11
1esma_#314	GO:0004842: ubiquitin-protein ligase activity	5.84E-06	113,19,7
1e1aa_#285	GO:0015036: disulfide oxidoreductase activity	5.86E-06	137,22,8
1i76a_#138	GO:0016776: phosphotransferase activity, phosphate group as acceptor	5.92E-06	103,14,6
1dgwa_#97	GO:0008201: heparin binding	5.93E-06	15,24,4
1dfca1#1076	GO:0008201: heparin binding	5.94E-06	5,24,3
2mev2_#53	GO:0004197: cysteine-type endopeptidase activity	5.94E-06	5,24,3
1bev1_#245	GO:0004197: cysteine-type endopeptidase activity	5.94E-06	5,24,3
1bg2__#93	GO:0005516: calmodulin binding	5.94E-06	5,24,3
1fhga_#48	GO:0005516: calmodulin binding	5.94E-06	5,24,3
1b35b_#140	GO:0004197: cysteine-type endopeptidase activity	5.94E-06	5,24,3
1ycsa_#162	GO:0004263: chymotrypsin activity	5.95E-06	210,41,13
2hhma_#262	GO:0004180: carboxypeptidase activity	5.96E-06	95,15,6
1jlna_#437	GO:0004725: protein tyrosine phosphatase activity	5.96E-06	53,15,5
1ddga1#278	GO:0030151: molybdenum ion binding	5.96E-06	53,15,5
1hava_#141	GO:0003968: RNA-directed RNA polymerase activity	5.96E-06	8,14,3
1f0xa1#278	GO:0000049: tRNA binding	5.98E-06	180,13,7
1rypb_#114	GO:0016846: carbon-sulfur lyase activity	5.99E-06	158,10,6
1aw1a_#169	GO:0016861: intramolecular oxidoreductase activity, interconverting aldoses and ketoses	5.99E-06	28,13,4
1evqa_#305	GO:0004812: tRNA ligase activity	6.00E-06	490,26,15
1prea2#308	GO:0019955: cytokine binding	6.00E-06	76,11,5
3lada1#116	GO:0004812: tRNA ligase activity	6.02E-06	153,26,9
1vmoa_#106	GO:0016638: oxidoreductase activity, acting on the CH-NH2 group of donors	6.04E-06	187,17,8
1mdah_#329	GO:0046983: protein dimerization activity	6.09E-06	335,17,10
1cpy__#93	GO:0016763: transferase activity, transferring pentosyl groups	6.10E-06	275,28,12
1f2da_#264	GO:0004812: tRNA ligase activity	6.12E-06	14,26,4
1dpja_#320	GO:0004896: hematopoietin/interferon-class (D200-domain) cytokine receptor activity	6.13E-06	19,19,4
1doza_#109	GO:0016763: transferase activity, transferring pentosyl groups	6.14E-06	74,28,7
1dj0a2#183	GO:0016616: oxidoreductase activity, acting on the CH-OH group of donors, NAD or NADP as acceptor	6.16E-06	142,59,13
1rkd__#186	GO:0004812: tRNA ligase activity	6.19E-06	52,26,6
2ebn__#77	GO:0004812: tRNA ligase activity	6.19E-06	52,26,6
2pia_1#54	GO:0030151: molybdenum ion binding	6.24E-06	220,15,8
1dik_1#765	GO:0016616: oxidoreductase activity, acting on the CH-OH group of donors, NAD or NADP as acceptor	6.26E-06	165,59,14
1ejfa_#10	GO:0005509: calcium ion binding	6.27E-06	155,160,24
1cbf__#208	GO:0015082: di-, tri-valent inorganic cation transporter activity	6.27E-06	333,14,9
1cbf__#208	GO:0046915: transition metal ion transporter activity	6.27E-06	333,14,9
2hft_1#34	GO:0019955: cytokine binding	6.27E-06	34,11,4
1svb_2#165	GO:0003724: RNA helicase activity	6.27E-06	34,11,4

1qoua_#64	GO:0003755: peptidyl-prolyl cis-trans isomerase activity	6.27E-06	34,11,4
1a8i__#688	GO:0016776: phosphotransferase activity, phosphate group as acceptor	6.28E-06	241,14,8
1htp__#16	GO:0004556: alpha-amylase activity	6.33E-06	303,15,9
1quqa_#75	GO:0005507: copper ion binding	6.34E-06	54,38,7
1nsj__#21	GO:0016861: intramolecular oxidoreductase activity, interconverting aldoses and ketoses	6.35E-06	114,13,6
1jfra_#83	GO:0016846: carbon-sulfur lyase activity	6.37E-06	38,10,4
1oaca1#342	GO:0005507: copper ion binding	6.37E-06	10,38,4
1a3qa2#190	GO:0008083: growth factor activity	6.38E-06	144,42,11
1avgi_#69	GO:0019955: cytokine binding	6.39E-06	340,11,8
1bu7a_#278	GO:0016705: oxidoreductase activity, acting on paired donors, with incorporation or reduction of molecular oxygen	6.39E-06	30,26,5
2cpl__#20	GO:0005126: hematopoietin/interferon-class (D200-domain) cytokine receptor binding	6.41E-06	108,20,7
1kid__#330	GO:0003887: DNA-directed DNA polymerase activity	6.43E-06	155,20,8
3prn__#143	GO:0015082: di-, tri-valent inorganic cation transporter activity	6.43E-06	334,14,9
3prn__#143	GO:0046915: transition metal ion transporter activity	6.43E-06	334,14,9
3sil__#98	GO:0019955: cytokine binding	6.48E-06	228,11,7
1oaca1#495	GO:0004295: trypsin activity	6.50E-06	60,48,8
1cex__#109	GO:0016616: oxidoreductase activity, acting on the CH-OH group of donors, NAD or NADP as acceptor	6.51E-06	35,59,7
1gc5a_#233	GO:0016616: oxidoreductase activity, acting on the CH-OH group of donors, NAD or NADP as acceptor	6.51E-06	35,59,7
1azza_#114	GO:0004867: serine-type endopeptidase inhibitor activity	6.51E-06	17,47,5
1gdna_#151	GO:0004867: serine-type endopeptidase inhibitor activity	6.51E-06	17,47,5
1a7ca_#370	GO:0016638: oxidoreductase activity, acting on the CH-NH2 group of donors	6.51E-06	47,17,5
1acc__#206	GO:0005524: ATP binding	6.55E-06	22,243,10
1doi__#42	GO:0004190: aspartic-type endopeptidase activity	6.57E-06	16,23,4
1i9ga_#97	GO:0016616: oxidoreductase activity, acting on the CH-OH group of donors, NAD or NADP as acceptor	6.62E-06	7,59,4
1zpda1#213	GO:0016616: oxidoreductase activity, acting on the CH-OH group of donors, NAD or NADP as acceptor	6.62E-06	82,59,10
1ge8a2#134	GO:0019955: cytokine binding	6.67E-06	229,11,7
1pysa_#221	GO:0005524: ATP binding	6.68E-06	90,243,22
1g4ia_#11	GO:0004867: serine-type endopeptidase inhibitor activity	6.69E-06	29,47,6
1ds1a_#172	GO:0004556: alpha-amylase activity	6.69E-06	222,15,8
1f2da_#264	GO:0005524: ATP binding	6.69E-06	14,243,8
1a9xa3#78	GO:0004556: alpha-amylase activity	6.69E-06	305,15,9
1f8ra1#288	GO:0016616: oxidoreductase activity, acting on the CH-OH group of donors, NAD or NADP as acceptor	6.71E-06	49,59,8
1mdah_#329	GO:0008201: heparin binding	6.71E-06	335,24,12
1aqua_#126	GO:0016776: phosphotransferase activity, phosphate group as acceptor	6.73E-06	576,14,11
1epwa3#138	GO:0004190: aspartic-type endopeptidase activity	6.75E-06	424,23,13
1hr0w_#69	GO:0000049: tRNA binding	6.80E-06	64,13,5
1vmoa_#106	GO:0019838: growth factor binding	6.82E-06	187,9,6
1ejda_#6	GO:0030145: manganese ion binding	6.84E-06	270,38,14
1nuka_#80	GO:0008201: heparin binding	6.88E-06	220,24,10
1e6ca_#91	GO:0000287: magnesium ion binding	6.89E-06	182,128,23
1ckea_#138	GO:0016616: oxidoreductase activity, acting on the CH-OH group of donors, NAD or NADP as acceptor	6.91E-06	14,59,5
1ejda_#6	GO:0008235: metalloexopeptidase activity	6.96E-06	270,13,8
1jj2x_#204	GO:0016638: oxidoreductase activity, acting on the CH-NH2 group of donors	6.96E-06	7,17,3
1cex__#211	GO:0016638: oxidoreductase activity, acting on the CH-NH2 group of donors	6.96E-06	7,17,3
1rypc_#66	GO:0016638: oxidoreductase activity, acting on the CH-NH2 group of donors	6.96E-06	7,17,3
1ihoa_#238	GO:0005529: sugar binding	6.99E-06	75,39,8
1ej0a_#42	GO:0000049: tRNA binding	7.03E-06	116,13,6
1e32a1#102	GO:0030151: molybdenum ion binding	7.04E-06	25,15,4
1aky_2#133	GO:0005509: calcium ion binding	7.04E-06	156,160,24
1ab4__#344	GO:0051082: unfolded protein binding	7.04E-06	4,34,3
1d2oa1#541	GO:0004364: glutathione transferase activity	7.07E-06	35,11,4
1cpza_#6	GO:0003887: DNA-directed DNA polymerase activity	7.08E-06	157,20,8
1a9xa3#78	GO:0004812: tRNA ligase activity	7.09E-06	305,26,12
1trb_1#112	GO:0016627: oxidoreductase activity, acting on the CH-CH group of donors	7.09E-06	22,17,4
1fy7a_#388	GO:0003700: transcription factor activity	7.12E-06	68,124,13
1flca1#294	GO:0005179: hormone activity	7.15E-06	98,15,6
1a28a_#688	GO:0003700: transcription factor activity	7.15E-06	18,124,7
1dl5a_#266	GO:0003968: RNA-directed RNA polymerase activity	7.16E-06	27,14,4
1fm2.1#B627	GO:0003968: RNA-directed RNA polymerase activity	7.16E-06	27,14,4

1qs1a2#437	GO:0015036: disulfide oxidoreductase activity	7.17E-06	452,22,13
1qj5a_#268	GO:0000287: magnesium ion binding	7.18E-06	66,128,13
1e4ea2#215	GO:0019955: cytokine binding	7.19E-06	490,11,9
1dpja_#322	GO:0015036: disulfide oxidoreductase activity	7.25E-06	308,22,11
1ewna_#109	GO:0004842: ubiquitin-protein ligase activity	7.26E-06	75,19,6
1pot__#138	GO:0004842: ubiquitin-protein ligase activity	7.26E-06	75,19,6
1pot__#138	GO:0004896: hematopoietin/interferon-class (D200-domain) cytokine receptor activity	7.26E-06	75,19,6
1quna1#113	GO:0004263: chymotrypsin activity	7.27E-06	286,41,15
1qqsa_#124	GO:0051082: unfolded protein binding	7.29E-06	458,34,17
1avgi_#69	GO:0004295: trypsin activity	7.29E-06	340,48,18
1jr3a2#57	GO:0005524: ATP binding	7.33E-06	18,243,9
1fo5a_#60	GO:0015082: di-, tri-valent inorganic cation transporter activity	7.34E-06	246,14,8
1fo5a_#60	GO:0046915: transition metal ion transporter activity	7.34E-06	246,14,8
1qo2a_#222	GO:0016861: intramolecular oxidoreductase activity, interconverting aldoses and ketoses	7.35E-06	65,13,5
1ig8a_#203	GO:0019201: nucleotide kinase activity	7.35E-06	65,13,5
2mev1_#123	GO:0050660: FAD binding	7.36E-06	89,10,5
1fepa_#340	GO:0015036: disulfide oxidoreductase activity	7.37E-06	99,22,7
1pii_2#326	GO:0003924: GTPase activity	7.37E-06	192,17,8
2bpa1_#45	GO:0016638: oxidoreductase activity, acting on the CH-NH2 group of donors	7.37E-06	192,17,8
1c4zd_#85	GO:0004842: ubiquitin-protein ligase activity	7.39E-06	117,19,7
1bfg__#22	GO:0004295: trypsin activity	7.39E-06	61,48,8
1j9la_#82	GO:0016776: phosphotransferase activity, phosphate group as acceptor	7.40E-06	169,14,7
1pwt__#8	GO:0019838: growth factor binding	7.43E-06	314,9,7
1b8aa2#403	GO:0004812: tRNA ligase activity	7.43E-06	117,26,8
1plq_1#90	GO:0003887: DNA-directed DNA polymerase activity	7.43E-06	158,20,8
1hcl__#280	GO:0008083: growth factor activity	7.43E-06	70,42,8
1ie5a_#102	GO:0051082: unfolded protein binding	7.43E-06	62,34,7
1eeoa_#126	GO:0004725: protein tyrosine phosphatase activity	7.44E-06	8,15,3
1a0i_1#277	GO:0030151: molybdenum ion binding	7.44E-06	8,15,3
1qfha1#721	GO:0015078: hydrogen ion transporter activity	7.45E-06	18,21,4
1fc4a_#205	GO:0050660: FAD binding	7.45E-06	267,10,7
1phm_2#253	GO:0004556: alpha-amylase activity	7.47E-06	309,15,9
1b35b_#97	GO:0030151: molybdenum ion binding	7.47E-06	309,15,9
1fhoa_#74	GO:0019955: cytokine binding	7.47E-06	347,11,8
1bmv2_#2064	GO:0005524: ATP binding	7.52E-06	147,243,30
1hd2a_#35	GO:0005524: ATP binding	7.53E-06	185,243,35
1dnv__#354	GO:0005529: sugar binding	7.61E-06	54,39,7
1ffya3#535	GO:0004812: tRNA ligase activity	7.62E-06	5,26,3
1cby__#183	GO:0016646: oxidoreductase activity, acting on the CH-NH group of donors, NAD or NADP as acceptor	7.63E-06	20,19,4
1hc7a2#258	GO:0004556: alpha-amylase activity	7.66E-06	226,15,8
1qs1a2#437	GO:0015082: di-, tri-valent inorganic cation transporter activity	7.66E-06	452,14,10
1qs1a2#437	GO:0046915: transition metal ion transporter activity	7.66E-06	452,14,10
2bpa1_#45	GO:0005529: sugar binding	7.68E-06	192,39,12
1nat__#50	GO:0016646: oxidoreductase activity, acting on the CH-NH group of donors, NAD or NADP as acceptor	7.69E-06	297,19,10
16pk__#312	GO:0003682: chromatin binding	7.70E-06	12,10,3
1qp8a1#168	GO:0016814: hydrolase activity, acting on carbon-nitrogen (but not peptide) bonds, in cyclic amidines	7.70E-06	12,10,3
1c7na_#231	GO:0003924: GTPase activity	7.73E-06	263,17,9
1dvpa1#65	GO:0008408: 3'-5' exonuclease activity	7.74E-06	187,13,7
1smaa2#581	GO:0019955: cytokine binding	7.74E-06	80,11,5
1xnb__#130	GO:0000287: magnesium ion binding	7.76E-06	87,128,15
1eucb2#188	GO:0000287: magnesium ion binding	7.76E-06	87,128,15
1dv8a_#211	GO:0016638: oxidoreductase activity, acting on the CH-NH2 group of donors	7.77E-06	86,17,6
5ruba1#365	GO:0016861: intramolecular oxidoreductase activity, interconverting aldoses and ketoses	7.78E-06	118,13,6
1elua_#288	GO:0004601: peroxidase activity	7.78E-06	6,21,3
1xgsa2#82	GO:0016668: oxidoreductase activity, acting on sulfur group of donors, NAD or NADP as acceptor	7.85E-06	307,12,8
3frua1#236	GO:0004896: hematopoietin/interferon-class (D200-domain) cytokine receptor activity	7.85E-06	76,19,6
1avgi_#69	GO:0008201: heparin binding	7.86E-06	340,24,12
1d0na3#305	GO:0030151: molybdenum ion binding	7.88E-06	311,15,9
1d0na3#305	GO:0004556: alpha-amylase activity	7.88E-06	311,15,9
1fo4a5#1020	GO:0004190: aspartic-type endopeptidase activity	7.92E-06	359,23,12
1lcl__#128	GO:0005529: sugar binding	7.94E-06	21,39,5
1fhoa_#74	GO:0005529: sugar binding	8.03E-06	347,39,16

1h8ca_#35	GO:0016763: transferase activity, transferring pentosyl groups	8.03E-06	77,28,7
1exma3#196	GO:0005525: GTP binding	8.04E-06	17,49,5
1ejda_#6	GO:0003809: thrombin activity	8.04E-06	270,10,7
1ejda_#6	GO:0004457: lactate dehydrogenase activity	8.04E-06	270,10,7
1b8aa2#368	GO:0016638: oxidoreductase activity, acting on the CH-NH2 group of donors	8.05E-06	135,17,7
1qfma1#139	GO:0019838: growth factor binding	8.10E-06	318,9,7
1kid__#330	GO:0005524: ATP binding	8.12E-06	155,243,31
8dfr__#115	GO:0005524: ATP binding	8.15E-06	27,243,11
1iira_#242	GO:0016646: oxidoreductase activity, acting on the CH-NH group of donors, NAD or NADP as acceptor	8.15E-06	170,19,8
1hxxa_#232	GO:0003887: DNA-directed DNA polymerase activity	8.15E-06	352,20,11
1e1aa_#178	GO:0005509: calcium ion binding	8.19E-06	54,160,13
1rypk_#12	GO:0046983: protein dimerization activity	8.23E-06	265,17,9
1b35b_#97	GO:0016668: oxidoreductase activity, acting on sulfur group of donors, NAD or NADP as acceptor	8.25E-06	309,12,8
1eyea_#131	GO:0004812: tRNA ligase activity	8.29E-06	15,26,4
1beba_#21	GO:0004812: tRNA ligase activity	8.29E-06	15,26,4
1qvba_#206	GO:0004180: carboxypeptidase activity	8.29E-06	26,15,4
1ek0a_#151	GO:0005096: GTPase activator activity	8.29E-06	26,15,4
1dkia_#208	GO:0016705: oxidoreductase activity, acting on paired donors, with incorporation or reduction of molecular oxygen	8.29E-06	15,26,4
1dkia_#208	GO:0004497: monooxygenase activity	8.29E-06	15,26,4
1qtn.1#A270	GO:0016627: oxidoreductase activity, acting on the CH-CH group of donors	8.29E-06	439,17,11
1jj2e1#42	GO:0003809: thrombin activity	8.30E-06	167,10,6
1dzfa2#211	GO:0003809: thrombin activity	8.30E-06	167,10,6
2mpra_#89	GO:0016251: general RNA polymerase II transcription factor activity	8.33E-06	28,14,4
1hxxa_#232	GO:0019955: cytokine binding	8.34E-06	352,11,8
1qqsa_#124	GO:0015036: disulfide oxidoreductase activity	8.35E-06	458,22,13
1fwxa2#396	GO:0019955: cytokine binding	8.42E-06	237,11,7
1h4vb2#282	GO:0015036: disulfide oxidoreductase activity	8.43E-06	101,22,7
1i50b_#334	GO:0003700: transcription factor activity	8.43E-06	69,124,13
1smaa1#95	GO:0003809: thrombin activity	8.45E-06	272,10,7
1qdla_#211	GO:0019838: growth factor binding	8.46E-06	320,9,7
1cpy__#93	GO:0030145: manganese ion binding	8.50E-06	275,38,14
1dr9a2#139	GO:0005518: collagen binding	8.55E-06	67,13,5
3grs_1#23	GO:0016620: oxidoreductase activity, acting on the aldehyde or oxo group of donors, NAD or NADP as acceptor	8.59E-06	168,10,6
1ido__#173	GO:0000287: magnesium ion binding	8.65E-06	110,128,17
1a81a1#51	GO:0005126: hematopoietin/interferon-class (D200-domain) cytokine receptor binding	8.66E-06	73,20,6
1bupa2#199	GO:0005524: ATP binding	8.66E-06	32,243,12
1thg__#3	GO:0003809: thrombin activity	8.68E-06	92,10,5
1dt9a1#209	GO:0003887: DNA-directed DNA polymerase activity	8.68E-06	113,20,7
1esma_#314	GO:0005126: hematopoietin/interferon-class (D200-domain) cytokine receptor binding	8.68E-06	113,20,7
2pia_1#99	GO:0004674: protein serine/threonine kinase activity	8.69E-06	179,42,12
1b3qa2#623	GO:0004896: hematopoietin/interferon-class (D200-domain) cytokine receptor activity	8.70E-06	301,19,10
1f2la_#40	GO:0005509: calcium ion binding	8.70E-06	236,160,31
2dpma_#189	GO:0005524: ATP binding	8.72E-06	48,243,15
1ac6a_#34	GO:0008201: heparin binding	8.75E-06	60,24,6
1fc4a_#205	GO:0008483: transaminase activity	8.77E-06	267,17,9
1ejfa_#10	GO:0004295: trypsin activity	8.78E-06	155,48,12
1fn9a_#271	GO:0003887: DNA-directed DNA polymerase activity	8.79E-06	218,20,9
1h4ua1#404	GO:0005524: ATP binding	8.83E-06	202,243,37
1epwa3#248	GO:0019955: cytokine binding	8.88E-06	37,11,4
1iira_#326	GO:0005524: ATP binding	8.88E-06	112,243,25
1a6da3#190	GO:0004867: serine-type endopeptidase inhibitor activity	8.90E-06	18,47,5
1egja_#346	GO:0008083: growth factor activity	8.91E-06	20,42,5
1apme_#74	GO:0004263: chymotrypsin activity	8.95E-06	124,41,10
1vola2#239	GO:0004812: tRNA ligase activity	8.99E-06	120,26,8
1vola2#239	GO:0004497: monooxygenase activity	8.99E-06	120,26,8
1imva_#255	GO:0016668: oxidoreductase activity, acting on sulfur group of donors, NAD or NADP as acceptor	8.99E-06	589,12,10
1nhp_2#143	GO:0015036: disulfide oxidoreductase activity	9.00E-06	6,22,3
1iqva_#122	GO:0019843: rRNA binding	9.00E-06	6,22,3
1ryph_#163	GO:0008235: metalloexopeptidase activity	9.01E-06	121,13,6
1imva_#255	GO:0005529: sugar binding	9.04E-06	589,39,21
1div_1#141	GO:0005525: GTP binding	9.04E-06	81,49,9

1et0a_#118	GO:0015036: disulfide oxidoreductase activity	9.06E-06	18,22,4
1ddma_#130	GO:0016668: oxidoreductase activity, acting on sulfur group of donors, NAD or NADP as acceptor	9.10E-06	313,12,8
1jjya_#324	GO:0016646: oxidoreductase activity, acting on the CH-NH group of donors, NAD or NADP as acceptor	9.15E-06	78,19,6
1eo6a_#107	GO:0016646: oxidoreductase activity, acting on the CH-NH group of donors, NAD or NADP as acceptor	9.15E-06	78,19,6
1nsj__#124	GO:0016763: transferase activity, transferring pentosyl groups	9.18E-06	148,28,9
1qfxa_#380	GO:0019838: growth factor binding	9.20E-06	47,9,4
1ihua1#119	GO:0016616: oxidoreductase activity, acting on the CH-OH group of donors, NAD or NADP as acceptor	9.21E-06	85,59,10
1pii_2#326	GO:0016861: intramolecular oxidoreductase activity, interconverting aldoses and ketoses	9.24E-06	192,13,7
2bpa1_#45	GO:0005518: collagen binding	9.24E-06	192,13,7
1kit_2#398	GO:0019955: cytokine binding	9.30E-06	83,11,5
1auk__#375	GO:0016668: oxidoreductase activity, acting on sulfur group of donors, NAD or NADP as acceptor	9.33E-06	34,12,4
1seta2#306	GO:0004295: trypsin activity	9.36E-06	30,48,6
1i50a_#469	GO:0030151: molybdenum ion binding	9.37E-06	58,15,5
1i39a_#49	GO:0004896: hematopoietin/interferon-class (D200-domain) cytokine receptor activity	9.39E-06	21,19,4
1f75a_#239	GO:0004222: metalloendopeptidase activity	9.39E-06	21,19,4
1ac6a_#43	GO:0005126: hematopoietin/interferon-class (D200-domain) cytokine receptor binding	9.39E-06	163,20,8
1eur__#302	GO:0004263: chymotrypsin activity	9.49E-06	53,41,7
1hbza_#214	GO:0005509: calcium ion binding	9.51E-06	297,160,36
1qfma1#139	GO:0004556: alpha-amylase activity	9.51E-06	318,15,9
1e44b_#75	GO:0019838: growth factor binding	9.55E-06	198,9,6
1deua_#151	GO:0003887: DNA-directed DNA polymerase activity	9.57E-06	285,20,10
1d3ga_#118	GO:0004457: lactate dehydrogenase activity	9.59E-06	42,10,4
1elja_#64	GO:0015036: disulfide oxidoreductase activity	9.61E-06	103,22,7
1pwt__#8	GO:0004812: tRNA ligase activity	9.64E-06	314,26,12
1fds__#59	GO:0008757: S-adenosylmethionine-dependent methyltransferase activity	9.65E-06	61,24,6
1deua_#151	GO:0005524: ATP binding	9.65E-06	285,243,47
1heta1#167	GO:0016616: oxidoreductase activity, acting on the CH-OH group of donors, NAD or NADP as acceptor	9.65E-06	3,59,3
1ldna1#94	GO:0016627: oxidoreductase activity, acting on the CH-CH group of donors	9.65E-06	199,17,8
1qora2#201	GO:0000287: magnesium ion binding	9.67E-06	199,128,24
1qtn.1#A270	GO:0004812: tRNA ligase activity	9.68E-06	439,26,14
1bfd_2#146	GO:0016776: phosphotransferase activity, phosphate group as acceptor	9.68E-06	112,14,6
1br9__#168	GO:0019829: cation-transporting ATPase activity	9.70E-06	2,9,2
1fo4a5#1020	GO:0019955: cytokine binding	9.70E-06	359,11,8
1b12a_#221	GO:0005126: hematopoietin/interferon-class (D200-domain) cytokine receptor binding	9.77E-06	115,20,7
1a9xa3#78	GO:0016646: oxidoreductase activity, acting on the CH-NH group of donors, NAD or NADP as acceptor	9.81E-06	305,19,10
1a0i_2#222	GO:0004263: chymotrypsin activity	9.83E-06	186,41,12
1aop_3#276	GO:0003809: thrombin activity	9.86E-06	172,10,6
1g25a_#22	GO:0051082: unfolded protein binding	9.87E-06	415,34,16
1akjd_#3	GO:0004896: hematopoietin/interferon-class (D200-domain) cytokine receptor activity	9.89E-06	7,19,3
1fqva2#326	GO:0004222: metalloendopeptidase activity	9.89E-06	7,19,3
2arca_#64	GO:0005507: copper ion binding	9.90E-06	11,38,4
1eny__#147	GO:0008235: metalloexopeptidase activity	9.92E-06	123,13,6
1amm_2#157	GO:0005507: copper ion binding	9.92E-06	4,38,3
1g25a_#22	GO:0008201: heparin binding	9.92E-06	415,24,13
2fcba2#104	GO:0004177: aminopeptidase activity	9.99E-06	10,13,3
3aky_1#194	GO:0008800: beta-lactamase activity	9.99E-06	13,10,3
1prxa_#28	GO:0016651: oxidoreductase activity, acting on NADH or NADPH	9.99E-06	10,13,3
1feza_#185	GO:0000287: magnesium ion binding	0.00001	68,128,13
1f97a1#62	GO:0051082: unfolded protein binding	0.00001	707,34,21
1a6o__#297	GO:0015078: hydrogen ion transporter activity	0.00001	41,21,5
1akp__#62	GO:0005509: calcium ion binding	0.00001	55,160,13
1fjja_#5	GO:0008201: heparin binding	0.00001	17,24,4
1czya1#466	GO:0019838: growth factor binding	0.00001	109,9,5
1ddja_#754	GO:0004867: serine-type endopeptidase inhibitor activity	0.00001	31,47,6
1jb3a_#64	GO:0004190: aspartic-type endopeptidase activity	0.00001	302,23,11
1fjsa_#83	GO:0019838: growth factor binding	0.00001	109,9,5
1avgi_#69	GO:0005525: GTP binding	0.00001	340,49,18
1pvc1_#106	GO:0003968: RNA-directed RNA polymerase activity	0.00001	64,14,5
1e1oa2#423	GO:0015036: disulfide oxidoreductase activity	0.00001	148,22,8

1a81a2#182	GO:0005524: ATP binding	0.00001	120,243,26
1bd8__#82	GO:0005126: hematopoietin/interferon-class (D200-domain) cytokine receptor binding	0.00001	75,20,6
1d5ta1#12	GO:0016627: oxidoreductase activity, acting on the CH-CH group of donors	0.00001	24,17,4
1jsg__#92	GO:0050660: FAD binding	0.00001	607,10,9
2bb2_1#57	GO:0004556: alpha-amylase activity	0.00001	104,15,6
1eova1#139	GO:0005524: ATP binding	0.00001	99,243,23
1a81a1#51	GO:0008083: growth factor activity	0.00001	73,42,8
1e4ea2#215	GO:0008201: heparin binding	0.00001	490,24,14
1h7wa4#481	GO:0016627: oxidoreductase activity, acting on the CH-CH group of donors	0.00001	24,17,4
1ep3a_#247	GO:0016627: oxidoreductase activity, acting on the CH-CH group of donors	0.00001	24,17,4
1qfma1#139	GO:0016668: oxidoreductase activity, acting on sulfur group of donors, NAD or NADP as acceptor	0.00001	318,12,8
1dp0a4#756	GO:0005126: hematopoietin/interferon-class (D200-domain) cytokine receptor binding	0.00001	165,20,8
1ddwa_#77	GO:0004674: protein serine/threonine kinase activity	0.00001	364,42,17
1e8ca3#111	GO:0004457: lactate dehydrogenase activity	0.00001	280,10,7
1auib_#145	GO:0005509: calcium ion binding	0.000011	4,160,4
1lml__#216	GO:0004222: metalloendopeptidase activity	0.000011	22,19,4
1dqza_#209	GO:0005525: GTP binding	0.000011	45,49,7
1znca_#32	GO:0008270: zinc ion binding	0.000011	5,108,4
1hq8a_#224	GO:0005529: sugar binding	0.000011	4,39,3
1div_1#141	GO:0016646: oxidoreductase activity, acting on the CH-NH group of donors, NAD or NADP as acceptor	0.000011	81,19,6
1hwxa2#60	GO:0016638: oxidoreductase activity, acting on the CH-NH2 group of donors	0.000011	8,17,3
1b8wa_#12	GO:0005529: sugar binding	0.000011	4,39,3
1qs0b2#269	GO:0016646: oxidoreductase activity, acting on the CH-NH group of donors, NAD or NADP as acceptor	0.000011	46,19,5
1qj5a_#316	GO:0008483: transaminase activity	0.000011	8,17,3
1rkd__#186	GO:0016616: oxidoreductase activity, acting on the CH-OH group of donors, NAD or NADP as acceptor	0.000011	52,59,8
1phm_2#347	GO:0008270: zinc ion binding	0.000011	5,108,4
1bccb2#274	GO:0004725: protein tyrosine phosphatase activity	0.000011	60,15,5
1i6vd_#1283	GO:0016638: oxidoreductase activity, acting on the CH-NH2 group of donors	0.000011	141,17,7
1muca1#245	GO:0016836: hydro-lyase activity	0.000011	45,33,6
1thg__#3	GO:0051082: unfolded protein binding	0.000011	92,34,8
1e5ma1#159	GO:0005525: GTP binding	0.000011	9,49,4
1f3ba2#4	GO:0005525: GTP binding	0.000011	45,49,7
1dgtb3#2594	GO:0008483: transaminase activity	0.000011	8,17,3
1fjfl_#67	GO:0003809: thrombin activity	0.000011	175,10,6
1epwa3#138	GO:0004556: alpha-amylase activity	0.000011	424,15,10
1epwa3#138	GO:0050660: FAD binding	0.000011	424,10,8
1prea2#308	GO:0005126: hematopoietin/interferon-class (D200-domain) cytokine receptor binding	0.000011	76,20,6
1kve.1#B199	GO:0004263: chymotrypsin activity	0.000011	76,41,8
3frua1#236	GO:0004263: chymotrypsin activity	0.000011	76,41,8
1dlfh_#4	GO:0004222: metalloendopeptidase activity	0.000011	22,19,4
1e44b_#75	GO:0005518: collagen binding	0.000011	198,13,7
1i5ga_#115	GO:0030151: molybdenum ion binding	0.000011	324,15,9
1kid__#330	GO:0051082: unfolded protein binding	0.000011	155,34,10
1enfa1#43	GO:0003697: single-stranded DNA binding	0.000011	11,12,3
1b6ra3#161	GO:0030151: molybdenum ion binding	0.000011	164,15,7
2stv__#129	GO:0003968: RNA-directed RNA polymerase activity	0.000011	30,14,4
1ft9a1#201	GO:0005509: calcium ion binding	0.000011	109,160,19
1jlna_#383	GO:0016616: oxidoreductase activity, acting on the CH-OH group of donors, NAD or NADP as acceptor	0.000011	52,59,8
1fxqa_#1009	GO:0005525: GTP binding	0.000011	9,49,4
1ezvb1#29	GO:0003887: DNA-directed DNA polymerase activity	0.000011	44,20,5
1opy__#68	GO:0019955: cytokine binding	0.000011	367,11,8
1opy__#68	GO:0008083: growth factor activity	0.000011	367,42,17
1ecsa_#69	GO:0016702: oxidoreductase activity, acting on single donors with incorporation of molecular oxygen, incorporation of two atoms of oxygen	0.000011	11,12,3
1dbga_#38	GO:0005525: GTP binding	0.000011	30,49,6
1byka_#298	GO:0005525: GTP binding	0.000011	9,49,4
1bev1_#86	GO:0004556: alpha-amylase activity	0.000011	323,15,9
1a7ca_#202	GO:0005126: hematopoietin/interferon-class (D200-domain) cytokine receptor binding	0.000011	117,20,7
1ddja_#681	GO:0004867: serine-type endopeptidase inhibitor activity	0.000011	253,47,15
1aw8.1#B59	GO:0005524: ATP binding	0.000011	23,243,10
1dv8a_#211	GO:0005524: ATP binding	0.000011	86,243,21

1jfra_#41	GO:0030145: manganese ion binding	0.000011	108,38,9
1ig8a_#203	GO:0016776: phosphotransferase activity, phosphate group as acceptor	0.000011	65,14,5
1qdla_#211	GO:0051082: unfolded protein binding	0.000011	320,34,14
1aqua_#126	GO:0004222: metalloendopeptidase activity	0.000011	576,19,13
1e3a.1#A30	GO:0005524: ATP binding	0.000011	157,243,31
1fa0a1#393	GO:0015082: di-, tri-valent inorganic cation transporter activity	0.000011	261,14,8
1fa0a1#393	GO:0046915: transition metal ion transporter activity	0.000011	261,14,8
1qfea_#68	GO:0008483: transaminase activity	0.000011	560,17,12
1kit_1#209	GO:0005529: sugar binding	0.000011	11,39,4
1quna1#113	GO:0005524: ATP binding	0.000011	286,243,47
2bpa1_#45	GO:0051082: unfolded protein binding	0.000011	192,34,11
1cpza_#6	GO:0005524: ATP binding	0.000011	157,243,31
1h4vb2#307	GO:0005524: ATP binding	0.000011	73,243,19
1gdna_#119	GO:0004867: serine-type endopeptidase inhibitor activity	0.000012	19,47,5
1qgna_#213	GO:0016846: carbon-sulfur lyase activity	0.000012	2,10,2
1vjw__#39	GO:0004457: lactate dehydrogenase activity	0.000012	2,10,2
1ejfa_#105	GO:0003887: DNA-directed DNA polymerase activity	0.000012	7,20,3
1fvua_#13	GO:0051082: unfolded protein binding	0.000012	26,34,5
1eur__#302	GO:0016638: oxidoreductase activity, acting on the CH-NH2 group of donors	0.000012	53,17,5
1eo9b_#505	GO:0008199: ferric iron binding	0.000012	2,10,2
1ap0__#29	GO:0019838: growth factor binding	0.000012	50,9,4
1gdha2#47	GO:0016616: oxidoreductase activity, acting on the CH-OH group of donors, NAD or NADP as acceptor	0.000012	38,59,7
1cewi_#14	GO:0008083: growth factor activity	0.000012	98,42,9
1aym2_#185	GO:0004197: cysteine-type endopeptidase activity	0.000012	6,24,3
1i39a_#49	GO:0005126: hematopoietin/interferon-class (D200-domain) cytokine receptor binding	0.000012	21,20,4
1jlxa2#288	GO:0008201: heparin binding	0.000012	6,24,3
1bw0a_#134	GO:0016846: carbon-sulfur lyase activity	0.000012	2,10,2
1qqka_#25	GO:0008201: heparin binding	0.000012	6,24,3
1f8ra2#325	GO:0016668: oxidoreductase activity, acting on sulfur group of donors, NAD or NADP as acceptor	0.000012	222,12,7
1i5ga_#115	GO:0016668: oxidoreductase activity, acting on sulfur group of donors, NAD or NADP as acceptor	0.000012	324,12,8
1pbe_1#121	GO:0016627: oxidoreductase activity, acting on the CH-CH group of donors	0.000012	25,17,4
1ie5a_#23	GO:0008083: growth factor activity	0.000012	21,42,5
3prn__#145	GO:0003887: DNA-directed DNA polymerase activity	0.000012	168,20,8
1mil__#30	GO:0005524: ATP binding	0.000012	61,243,17
1qo0d_#81	GO:0005525: GTP binding	0.000012	46,49,7
1bjt__#890	GO:0008201: heparin binding	0.000012	292,24,11
1eu3a2#205	GO:0004364: glutathione transferase activity	0.000012	87,11,5
1xis__#214	GO:0010181: FMN binding	0.000012	88,11,5
1fd9a_#162	GO:0003755: peptidyl-prolyl cis-trans isomerase activity	0.000012	87,11,5
1qh4a2#350	GO:0005524: ATP binding	0.000012	67,243,18
1neb__#41	GO:0003779: actin binding	0.000012	47,32,6
1f97a2#199	GO:0016638: oxidoreductase activity, acting on the CH-NH2 group of donors	0.000012	144,17,7
1hbza_#96	GO:0019955: cytokine binding	0.000012	370,11,8
1hbza_#96	GO:0051082: unfolded protein binding	0.000012	370,34,15
1amf__#196	GO:0005524: ATP binding	0.000012	173,243,33
1ldna1#94	GO:0016854: racemase and epimerase activity	0.000012	199,13,7
1trb_1#285	GO:0016627: oxidoreductase activity, acting on the CH-CH group of donors	0.000012	25,17,4
1trb_1#298	GO:0016627: oxidoreductase activity, acting on the CH-CH group of donors	0.000012	25,17,4
1hdma1#98	GO:0008201: heparin binding	0.000012	37,24,5
1ge8a2#134	GO:0005524: ATP binding	0.000012	229,243,40
1tiid_#39	GO:0004725: protein tyrosine phosphatase activity	0.000012	239,15,8
1e7wa_#36	GO:0008757: S-adenosylmethionine-dependent methyltransferase activity	0.000012	137,24,8
1cpza_#6	GO:0019955: cytokine binding	0.000012	157,11,6
1qs0a1#119	GO:0016627: oxidoreductase activity, acting on the CH-CH group of donors	0.000012	454,17,11
1nsca_#236	GO:0046983: protein dimerization activity	0.000012	143,17,7
1ckea_#138	GO:0004457: lactate dehydrogenase activity	0.000013	14,10,3
2arca_#44	GO:0016251: general RNA polymerase II transcription factor activity	0.000013	10,14,3
1aym1_#151	GO:0003968: RNA-directed RNA polymerase activity	0.000013	10,14,3
1ak5_1#353	GO:0016861: intramolecular oxidoreductase activity, interconverting aldoses and ketoses	0.000013	73,13,5
1evya2#38	GO:0008199: ferric iron binding	0.000013	14,10,3
2mev3_#107	GO:0003968: RNA-directed RNA polymerase activity	0.000013	31,14,4
1i0ha2#127	GO:0004556: alpha-amylase activity	0.000013	29,15,4
2trcp_#201	GO:0003809: thrombin activity	0.000013	14,10,3

1a9xa2#1009	GO:0008199: ferric iron binding	0.000013	14,10,3
1g5ha2#319	GO:0000287: magnesium ion binding	0.000013	19,128,7
1hzia_#51	GO:0008083: growth factor activity	0.000013	4,42,3
1eg2a_#132	GO:0016251: general RNA polymerase II transcription factor activity	0.000013	31,14,4
1by5a_#347	GO:0015036: disulfide oxidoreductase activity	0.000013	263,22,10
1ifc__#89	GO:0004295: trypsin activity	0.000013	190,48,13
1avgi_#69	GO:0005524: ATP binding	0.000013	340,243,53
1jjya_#293	GO:0016616: oxidoreductase activity, acting on the CH-OH group of donors, NAD or NADP as acceptor	0.000013	8,59,4
1a3c__#108	GO:0000287: magnesium ion binding	0.000013	13,128,6
1eqka_#47	GO:0004812: tRNA ligase activity	0.000013	126,26,8
1bwvs_#117	GO:0016668: oxidoreductase activity, acting on sulfur group of donors, NAD or NADP as acceptor	0.000013	329,12,8
3rpba_#550	GO:0030151: molybdenum ion binding	0.000013	29,15,4
1qgub_#197	GO:0005351: sugar porter activity	0.000013	74,21,6
1ddja_#681	GO:0019955: cytokine binding	0.000013	253,11,7
1dqwa_#202	GO:0016831: carboxy-lyase activity	0.000013	6,25,3
1hq8a_#206	GO:0030151: molybdenum ion binding	0.000013	329,15,9
1hq8a_#206	GO:0016668: oxidoreductase activity, acting on sulfur group of donors, NAD or NADP as acceptor	0.000013	329,12,8
2ltn.1#A63	GO:0015036: disulfide oxidoreductase activity	0.000013	152,22,8
1lcl__#110	GO:0005529: sugar binding	0.000013	23,39,5
1p35a_#49	GO:0051082: unfolded protein binding	0.000013	124,34,9
1e1aa_#178	GO:0008083: growth factor activity	0.000013	54,42,7
3grs_1#23	GO:0004812: tRNA ligase activity	0.000013	168,26,9
1hq0a_#867	GO:0003887: DNA-directed DNA polymerase activity	0.000013	171,20,8
1jf9a_#196	GO:0016846: carbon-sulfur lyase activity	0.000013	45,10,4
1ekra_#114	GO:0030145: manganese ion binding	0.000013	60,38,7
1qqp1_#51	GO:0003968: RNA-directed RNA polymerase activity	0.000013	31,14,4
1hxmb1#117	GO:0008201: heparin binding	0.000013	64,24,6
1dfca4#1451	GO:0004867: serine-type endopeptidase inhibitor activity	0.000013	67,47,8
1ge8a2#134	GO:0003887: DNA-directed DNA polymerase activity	0.000013	229,20,9
1gpea1#271	GO:0005524: ATP binding	0.000013	197,243,36
1e79d2#36	GO:0003724: RNA helicase activity	0.000013	524,11,9
1cr5a1#45	GO:0030151: molybdenum ion binding	0.000013	29,15,4
1e8ca3#111	GO:0003924: GTPase activity	0.000013	280,17,9
1brma2#268	GO:0004190: aspartic-type endopeptidase activity	0.000014	19,23,4
1j8ra_#26	GO:0005509: calcium ion binding	0.000014	41,160,11
1atia2#210	GO:0004364: glutathione transferase activity	0.000014	13,11,3
1d4xg_#60	GO:0015078: hydrogen ion transporter activity	0.000014	115,21,7
1qf6a3#92	GO:0000287: magnesium ion binding	0.000014	91,128,15
1bg2__#93	GO:0003779: actin binding	0.000014	5,32,3
1nula_#15	GO:0004497: monooxygenase activity	0.000014	35,26,5
1ezm__#146	GO:0004222: metalloendopeptidase activity	0.000014	23,19,4
2occa1#372	GO:0015078: hydrogen ion transporter activity	0.000014	7,21,3
1i6vd_#1283	GO:0005509: calcium ion binding	0.000014	141,160,22
1jj2v_#118	GO:0004263: chymotrypsin activity	0.000014	103,41,9
1j79a_#38	GO:0016616: oxidoreductase activity, acting on the CH-OH group of donors, NAD or NADP as acceptor	0.000014	39,59,7
1ej8a_#189	GO:0003809: thrombin activity	0.000014	294,10,7
1hxxa_#278	GO:0003755: peptidyl-prolyl cis-trans isomerase activity	0.000014	255,11,7
1f42a2#123	GO:0019955: cytokine binding	0.000014	378,11,8
1cpt__#52	GO:0016705: oxidoreductase activity, acting on paired donors, with incorporation or reduction of molecular oxygen	0.000014	35,26,5
1c9la2#12	GO:0005516: calmodulin binding	0.000014	295,24,11
1ii7a_#199	GO:0016616: oxidoreductase activity, acting on the CH-OH group of donors, NAD or NADP as acceptor	0.000014	54,59,8
1am2__#163	GO:0004457: lactate dehydrogenase activity	0.000014	102,10,5
1egua2#839	GO:0005507: copper ion binding	0.000014	24,38,5
1dt6a_#77	GO:0016705: oxidoreductase activity, acting on paired donors, with incorporation or reduction of molecular oxygen	0.000014	35,26,5
1qrra_#208	GO:0005524: ATP binding	0.000014	87,243,21
1hc7a2#258	GO:0016668: oxidoreductase activity, acting on sulfur group of donors, NAD or NADP as acceptor	0.000014	226,12,7
1c9la2#61	GO:0008201: heparin binding	0.000014	238,24,10
1d0na3#305	GO:0004190: aspartic-type endopeptidase activity	0.000014	311,23,11

1bywa_#30	GO:0016668: oxidoreductase activity, acting on sulfur group of donors, NAD or NADP as acceptor	0.000014	331,12,8
1g0sa_#74	GO:0050660: FAD binding	0.000014	294,10,7
1opy__#68	GO:0005524: ATP binding	0.000014	367,243,56
1h4vb2#282	GO:0050660: FAD binding	0.000014	101,10,5
1oaa__#175	GO:0016627: oxidoreductase activity, acting on the CH-CH group of donors	0.000014	26,17,4
1ejda_#377	GO:0005525: GTP binding	0.000014	108,49,10
1bhga3#529	GO:0019201: nucleotide kinase activity	0.000014	74,13,5
1dpsa_#36	GO:0005085: guanyl-nucleotide exchange factor activity	0.000014	205,13,7
2tbva_#214	GO:0003724: RNA helicase activity	0.000014	13,11,3
1b35b_#226	GO:0003724: RNA helicase activity	0.000014	13,11,3
1el0a_#42	GO:0008083: growth factor activity	0.000014	294,42,15
1el0a_#42	GO:0050660: FAD binding	0.000014	294,10,7
1e3a.1#A30	GO:0004523: ribonuclease H activity	0.000014	157,16,7
1pma1_#79	GO:0000287: magnesium ion binding	0.000014	70,128,13
1quna1#113	GO:0004295: trypsin activity	0.000014	286,48,16
1e79d2#36	GO:0004812: tRNA ligase activity	0.000014	524,26,15
1dn2a1#261	GO:0051082: unfolded protein binding	0.000014	425,34,16
1e39a2#278	GO:0016627: oxidoreductase activity, acting on the CH-CH group of donors	0.000014	26,17,4
1ax4a_#275	GO:0016846: carbon-sulfur lyase activity	0.000014	102,10,5
1acc__#206	GO:0004674: protein serine/threonine kinase activity	0.000015	22,42,5
1dt6a_#374	GO:0016705: oxidoreductase activity, acting on paired donors, with incorporation or reduction of molecular oxygen	0.000015	6,26,3
1ah7__#55	GO:0008081: phosphoric diester hydrolase activity	0.000015	2,11,2
1mml__#97	GO:0003964: RNA-directed DNA polymerase activity	0.000015	2,11,2
1hdr__#125	GO:0016616: oxidoreductase activity, acting on the CH-OH group of donors, NAD or NADP as acceptor	0.000015	16,59,5
1qhoa3#488	GO:0004556: alpha-amylase activity	0.000015	30,15,4
2dlda2#95	GO:0016616: oxidoreductase activity, acting on the CH-OH group of donors, NAD or NADP as acceptor	0.000015	16,59,5
1ah7__#184	GO:0008081: phosphoric diester hydrolase activity	0.000015	2,11,2
1afj__#28	GO:0005507: copper ion binding	0.000015	12,38,4
1bfd_1#230	GO:0050660: FAD binding	0.000015	47,10,4
1ac6a_#43	GO:0004295: trypsin activity	0.000015	163,48,12
1ihoa_#238	GO:0008235: metalloexopeptidase activity	0.000015	75,13,5
1hbza_#214	GO:0008201: heparin binding	0.000015	297,24,11
1whi__#59	GO:0019843: rRNA binding	0.000015	72,22,6
1e44b_#75	GO:0051082: unfolded protein binding	0.000015	198,34,11
1fo5a_#60	GO:0004556: alpha-amylase activity	0.000015	246,15,8
1jlxa2#166	GO:0008083: growth factor activity	0.000015	77,42,8
1aym3_#131	GO:0005518: collagen binding	0.000015	412,13,9
1b8aa2#403	GO:0019838: growth factor binding	0.000015	117,9,5
1hlwa_#82	GO:0015082: di-, tri-valent inorganic cation transporter activity	0.000015	270,14,8
1hlwa_#82	GO:0046915: transition metal ion transporter activity	0.000015	270,14,8
1e1oa2#423	GO:0004190: aspartic-type endopeptidase activity	0.000015	148,23,8
1ih7a1#150	GO:0005524: ATP binding	0.000015	62,243,17
1czan1#92	GO:0008800: beta-lactamase activity	0.000015	631,10,9
1dz4a_#264	GO:0004601: peroxidase activity	0.000015	44,21,5
1a7ca_#202	GO:0019838: growth factor binding	0.000015	117,9,5
1smaa2#581	GO:0005126: hematopoietin/interferon-class (D200-domain) cytokine receptor binding	0.000015	80,20,6
1neb__#41	GO:0005066: transmembrane receptor protein tyrosine kinase signaling protein activity	0.000015	47,10,4
1xgsa2#82	GO:0005524: ATP binding	0.000015	307,243,49
1hbza_#96	GO:0004714: transmembrane receptor protein tyrosine kinase activity	0.000015	370,14,9
1f5aa1#441	GO:0019955: cytokine binding	0.000015	163,11,6
1mkp__#216	GO:0004812: tRNA ligase activity	0.000015	129,26,8
1a7ca_#370	GO:0003809: thrombin activity	0.000015	47,10,4
1hd2a_#35	GO:0016646: oxidoreductase activity, acting on the CH-NH group of donors, NAD or NADP as acceptor	0.000015	185,19,8
1e5ka_#113	GO:0005509: calcium ion binding	0.000015	65,160,14
1ihua2#525	GO:0016866: intramolecular transferase activity	0.000015	335,12,8
1e1aa_#285	GO:0005524: ATP binding	0.000015	137,243,28
1frb__#110	GO:0004222: metalloendopeptidase activity	0.000016	8,19,3
1e42a1#711	GO:0005126: hematopoietin/interferon-class (D200-domain) cytokine receptor binding	0.000016	175,20,8
1ed1a_#94	GO:0004556: alpha-amylase activity	0.000016	10,15,3
1e8ya2#410	GO:0004620: phospholipase activity	0.000016	8,19,3
1hrna_#105	GO:0004556: alpha-amylase activity	0.000016	10,15,3

3pmga4#493	GO:0030151: molybdenum ion binding	0.000016	10,15,3
1ds9a_#119	GO:0004222: metalloendopeptidase activity	0.000016	8,19,3
1aiha_#226	GO:0003809: thrombin activity	0.000016	15,10,3
1qhda2#323	GO:0016668: oxidoreductase activity, acting on sulfur group of donors, NAD or NADP as acceptor	0.000016	337,12,8
1ap0__#29	GO:0004896: hematopoietin/interferon-class (D200-domain) cytokine receptor activity	0.000016	50,19,5
1a99a_#84	GO:0008199: ferric iron binding	0.000016	15,10,3
1j71a_#251	GO:0004190: aspartic-type endopeptidase activity	0.000016	106,23,7
1dt9a1#224	GO:0016846: carbon-sulfur lyase activity	0.000016	48,10,4
2fnba_#67	GO:0004896: hematopoietin/interferon-class (D200-domain) cytokine receptor activity	0.000016	8,19,3
1i50b_#216	GO:0005525: GTP binding	0.000016	48,49,7
1jb3a_#64	GO:0000049: tRNA binding	0.000016	302,13,8
1a0i_2#222	GO:0008009: chemokine activity	0.000016	186,10,6
1e69a_#26	GO:0016616: oxidoreductase activity, acting on the CH-OH group of donors, NAD or NADP as acceptor	0.000016	55,59,8
1cbf__#208	GO:0015036: disulfide oxidoreductase activity	0.000016	333,22,11
1imva_#255	GO:0008201: heparin binding	0.000016	589,24,15
1qfxa_#380	GO:0005126: hematopoietin/interferon-class (D200-domain) cytokine receptor binding	0.000016	47,20,5
1hr6a2#427	GO:0004222: metalloendopeptidase activity	0.000016	8,19,3
1pud__#197	GO:0000287: magnesium ion binding	0.000016	34,128,9
3sil__#98	GO:0004867: serine-type endopeptidase inhibitor activity	0.000016	228,47,14
1aqua_#126	GO:0016627: oxidoreductase activity, acting on the CH-CH group of donors	0.000016	576,17,12
1aqua_#126	GO:0008483: transaminase activity	0.000016	576,17,12
1a65a2#187	GO:0004364: glutathione transferase activity	0.000016	43,11,4
1a65a2#187	GO:0003724: RNA helicase activity	0.000016	43,11,4
1fmca_#89	GO:0005525: GTP binding	0.000016	48,49,7
1fjfb_#67	GO:0016627: oxidoreductase activity, acting on the CH-CH group of donors	0.000017	9,17,3
1b9ra_#102	GO:0016638: oxidoreductase activity, acting on the CH-NH2 group of donors	0.000017	9,17,3
1e5ea_#145	GO:0008483: transaminase activity	0.000017	9,17,3
1qj2c2#50	GO:0016836: hydro-lyase activity	0.000017	48,33,6
1ep3a_#220	GO:0016627: oxidoreductase activity, acting on the CH-CH group of donors	0.000017	9,17,3
1az9_1#73	GO:0051082: unfolded protein binding	0.000017	5,34,3
1hwxa1#256	GO:0016627: oxidoreductase activity, acting on the CH-CH group of donors	0.000017	57,17,5
1cewi_#14	GO:0016638: oxidoreductase activity, acting on the CH-NH2 group of donors	0.000017	98,17,6
1au1a_#58	GO:0008199: ferric iron binding	0.000017	105,10,5
1i2oa_#34	GO:0016776: phosphotransferase activity, phosphate group as acceptor	0.000017	192,14,7
1nsj__#21	GO:0004556: alpha-amylase activity	0.000017	114,15,6
1ac6a_#43	GO:0004263: chymotrypsin activity	0.000017	163,41,11
1j9la_#82	GO:0016836: hydro-lyase activity	0.000017	169,33,10
1gtma2#140	GO:0016638: oxidoreductase activity, acting on the CH-NH2 group of donors	0.000017	27,17,4
1fjfl_#67	GO:0030151: molybdenum ion binding	0.000017	175,15,7
1eq9a_#32	GO:0004896: hematopoietin/interferon-class (D200-domain) cytokine receptor activity	0.000017	132,19,7
1f42a2#123	GO:0008083: growth factor activity	0.000017	378,42,17
1jb3a_#64	GO:0004457: lactate dehydrogenase activity	0.000017	302,10,7
1jb3a_#64	GO:0050660: FAD binding	0.000017	302,10,7
1a0i_2#222	GO:0005509: calcium ion binding	0.000017	186,160,26
1e8ga2#201	GO:0005518: collagen binding	0.000017	304,13,8
1ihua2#521	GO:0019201: nucleotide kinase activity	0.000017	77,13,5
1ew2a_#133	GO:0005524: ATP binding	0.000017	24,243,10
1qqp3_#139	GO:0003724: RNA helicase activity	0.000017	14,11,3
2dkb__#114	GO:0004812: tRNA ligase activity	0.000017	174,26,9
1dnpa2#18	GO:0008026: ATP-dependent helicase activity	0.000017	77,13,5
1cjca2#365	GO:0016627: oxidoreductase activity, acting on the CH-CH group of donors	0.000017	57,17,5
1cjca2#365	GO:0008483: transaminase activity	0.000017	57,17,5
1ibja_#271	GO:0005351: sugar porter activity	0.000017	78,21,6
1elua_#95	GO:0019201: nucleotide kinase activity	0.000017	77,13,5
1vmoa_#106	GO:0004896: hematopoietin/interferon-class (D200-domain) cytokine receptor activity	0.000017	187,19,8
1quna1#113	GO:0051082: unfolded protein binding	0.000017	286,34,13
1ho1a_#211	GO:0016616: oxidoreductase activity, acting on the CH-OH group of donors, NAD or NADP as acceptor	0.000017	111,59,11
1bywa_#62	GO:0000155: two-component sensor molecule activity	0.000018	2,12,2
1kpf__#42	GO:0005525: GTP binding	0.000018	10,49,4
1ldm_2#272	GO:0004812: tRNA ligase activity	0.000018	18,26,4
1f97a1#62	GO:0030151: molybdenum ion binding	0.000018	707,15,12
1eo9b_#505	GO:0016702: oxidoreductase activity, acting on single donors with incorporation of molecular oxygen, incorporation of two atoms of oxygen	0.000018	2,12,2

2duba_#212	GO:0004812: tRNA ligase activity	0.000018	62,26,6
1br9__#168	GO:0015405: P-P-bond-hydrolysis-driven transporter activity	0.000018	2,12,2
1mkp__#283	GO:0050660: FAD binding	0.000018	49,10,4
1bl0a2#102	GO:0000049: tRNA binding	0.000018	12,13,3
1byb__#327	GO:0042802: protein self binding	0.000018	13,12,3
1i50b_#334	GO:0005524: ATP binding	0.000018	69,243,18
1egja_#343	GO:0005507: copper ion binding	0.000018	42,38,6
1pud__#69	GO:0004457: lactate dehydrogenase activity	0.000018	49,10,4
1io1a_#311	GO:0003887: DNA-directed DNA polymerase activity	0.000018	126,20,7
1cmia_#86	GO:0008201: heparin binding	0.000018	40,24,5
1g51a1#47	GO:0004812: tRNA ligase activity	0.000018	18,26,4
1gpc__#184	GO:0005126: hematopoietin/interferon-class (D200-domain) cytokine receptor binding	0.000018	83,20,6
1f42a2#123	GO:0016638: oxidoreductase activity, acting on the CH-NH2 group of donors	0.000018	378,17,10
3chbd_#85	GO:0016638: oxidoreductase activity, acting on the CH-NH2 group of donors	0.000018	378,17,10
2u2fa_#33	GO:0003729: mRNA binding	0.000018	12,13,3
1bjt__#890	GO:0016638: oxidoreductase activity, acting on the CH-NH2 group of donors	0.000018	292,17,9
1f21a_#68	GO:0003964: RNA-directed DNA polymerase activity	0.000018	44,11,4
1ez0a_#133	GO:0016251: general RNA polymerase II transcription factor activity	0.000018	125,14,6
1f2da_#94	GO:0019201: nucleotide kinase activity	0.000018	12,13,3
1e8ga2#201	GO:0005516: calmodulin binding	0.000018	304,24,11
1e8ga2#201	GO:0050660: FAD binding	0.000018	304,10,7
1a9xa3#78	GO:0016861: intramolecular oxidoreductase activity, interconverting aldoses and ketoses	0.000018	305,13,8
1eu3a1#68	GO:0004812: tRNA ligase activity	0.000018	18,26,4
1e4ea2#215	GO:0015036: disulfide oxidoreductase activity	0.000018	490,22,13
1qo2a_#222	GO:0016831: carboxy-lyase activity	0.000018	65,25,6
1h6va2#245	GO:0016627: oxidoreductase activity, acting on the CH-CH group of donors	0.000018	99,17,6
1sppb_#32	GO:0008083: growth factor activity	0.000018	38,42,6
1bu7a_#353	GO:0016705: oxidoreductase activity, acting on paired donors, with incorporation or reduction of molecular oxygen	0.000018	62,26,6
1epwa1#901	GO:0005529: sugar binding	0.000019	62,39,7
1brwa3#340	GO:0016763: transferase activity, transferring pentosyl groups	0.000019	6,28,3
1ecfa1#434	GO:0005525: GTP binding	0.000019	20,49,5
1cf9a2#133	GO:0000049: tRNA binding	0.000019	37,13,4
1ap0__#29	GO:0003682: chromatin binding	0.000019	50,10,4
1e3ua_#164	GO:0000049: tRNA binding	0.000019	37,13,4
1as4.1#A266	GO:0004867: serine-type endopeptidase inhibitor activity	0.000019	4,47,3
1xis__#196	GO:0016620: oxidoreductase activity, acting on the aldehyde or oxo group of donors, NAD or NADP as acceptor	0.000019	16,10,3
1heta2#212	GO:0016836: hydro-lyase activity	0.000019	49,33,6
1j9la_#82	GO:0005351: sugar porter activity	0.000019	169,21,8
1kapp2#149	GO:0005509: calcium ion binding	0.000019	22,160,8
1aym3_#109	GO:0019838: growth factor binding	0.000019	56,9,4
1whi__#59	GO:0003968: RNA-directed RNA polymerase activity	0.000019	72,14,5
1hxxa_#278	GO:0004556: alpha-amylase activity	0.000019	255,15,8
1zpda1#213	GO:0005524: ATP binding	0.000019	82,243,20
1io1a_#233	GO:0004812: tRNA ligase activity	0.000019	95,26,7
1jj2e1#42	GO:0004295: trypsin activity	0.000019	167,48,12
1fhga_#59	GO:0004714: transmembrane receptor protein tyrosine kinase activity	0.000019	34,14,4
1avgi_#69	GO:0008083: growth factor activity	0.000019	340,42,16
1d0na2#200	GO:0015078: hydrogen ion transporter activity	0.000019	79,21,6
1fgga_#134	GO:0003924: GTPase activity	0.000019	218,17,8
1qfma2#576	GO:0015036: disulfide oxidoreductase activity	0.000019	214,22,9
1boub_#7	GO:0003887: DNA-directed DNA polymerase activity	0.000019	240,20,9
1aba__#17	GO:0005525: GTP binding	0.000019	33,49,6
1a8i__#824	GO:0005524: ATP binding	0.000019	45,243,14
1hxxa_#107	GO:0016846: carbon-sulfur lyase activity	0.000019	16,10,3
1qlaa2#13	GO:0016627: oxidoreductase activity, acting on the CH-CH group of donors	0.000019	28,17,4
1jsg__#92	GO:0051082: unfolded protein binding	0.000019	607,34,19
1gpea1#273	GO:0004263: chymotrypsin activity	0.000019	81,41,8
1eq9a_#162	GO:0005509: calcium ion binding	0.000019	35,160,10
1xgsa2#82	GO:0003887: DNA-directed DNA polymerase activity	0.000019	307,20,10
1pbe_1#155	GO:0016627: oxidoreductase activity, acting on the CH-CH group of donors	0.000019	28,17,4
1e5da2#12	GO:0016638: oxidoreductase activity, acting on the CH-NH2 group of donors	0.000019	100,17,6
1nfdb1#21	GO:0019955: cytokine binding	0.000019	96,11,5
1ej0a_#42	GO:0030145: manganese ion binding	0.000019	116,38,9
1cwva2#675	GO:0046983: protein dimerization activity	0.000019	28,17,4

1fwxa2#169	GO:0005507: copper ion binding	0.000019	116,38,9
1e7wa_#36	GO:0019201: nucleotide kinase activity	0.000019	137,13,6
1jf9a_#380	GO:0016846: carbon-sulfur lyase activity	0.000019	50,10,4
1h9da_#91	GO:0019838: growth factor binding	0.000019	222,9,6
1tlfa_#275	GO:0003700: transcription factor activity	0.00002	5,124,4
1f2na_#138	GO:0005524: ATP binding	0.00002	96,243,22
1qsta_#154	GO:0000287: magnesium ion binding	0.00002	20,128,7
1au1a_#58	GO:0008083: growth factor activity	0.00002	105,42,9
1f3ba2#4	GO:0004364: glutathione transferase activity	0.00002	45,11,4
1nuka_#80	GO:0016638: oxidoreductase activity, acting on the CH-NH2 group of donors	0.00002	220,17,8
1moq__#341	GO:0000287: magnesium ion binding	0.00002	20,128,7
1qqsa_#124	GO:0050660: FAD binding	0.00002	458,10,8
1fo5a_#60	GO:0051082: unfolded protein binding	0.00002	246,34,12
1f8v.1#A116	GO:0016638: oxidoreductase activity, acting on the CH-NH2 group of donors	0.00002	101,17,6
1ft9a1#201	GO:0003809: thrombin activity	0.00002	109,10,5
1b8aa2#403	GO:0005524: ATP binding	0.00002	117,243,25
1g0sa_#74	GO:0016638: oxidoreductase activity, acting on the CH-NH2 group of donors	0.00002	294,17,9
1imva_#255	GO:0016638: oxidoreductase activity, acting on the CH-NH2 group of donors	0.00002	589,17,12
1gcua1#36	GO:0004180: carboxypeptidase activity	0.00002	32,15,4
1a4ya_#47	GO:0008235: metalloexopeptidase activity	0.00002	216,13,7
1g6oa_#40	GO:0004867: serine-type endopeptidase inhibitor activity	0.00002	21,47,5
1mjha_#119	GO:0016836: hydro-lyase activity	0.00002	103,33,8
1g8ka2#570	GO:0030151: molybdenum ion binding	0.00002	32,15,4
1jcfa2#206	GO:0003887: DNA-directed DNA polymerase activity	0.00002	84,20,6
1gai__#22	GO:0008810: cellulase activity	0.00002	9,18,3
2mev3_#108	GO:0004295: trypsin activity	0.00002	228,48,14
3sil__#98	GO:0004674: protein serine/threonine kinase activity	0.00002	228,42,13
1fu6a_#50	GO:0050660: FAD binding	0.00002	308,10,7
1mil__#31	GO:0008757: S-adenosylmethionine-dependent methyltransferase activity	0.00002	104,24,7
1fjfc2#153	GO:0000287: magnesium ion binding	0.00002	420,128,38
1qq5a_#7	GO:0000287: magnesium ion binding	0.00002	167,128,21
1aqua_#126	GO:0004180: carboxypeptidase activity	0.00002	576,15,11
1aqua_#126	GO:0004725: protein tyrosine phosphatase activity	0.00002	576,15,11
1imva_#69	GO:0005126: hematopoietin/interferon-class (D200-domain) cytokine receptor binding	0.00002	84,20,6
1dn2a1#261	GO:0004674: protein serine/threonine kinase activity	0.00002	425,42,18
1e8ca3#111	GO:0016776: phosphotransferase activity, phosphate group as acceptor	0.00002	280,14,8
1evqa_#305	GO:0016831: carboxy-lyase activity	0.00002	490,25,14
1hv8a1#109	GO:0008026: ATP-dependent helicase activity	0.000021	2,13,2
1mrp__#172	GO:0016620: oxidoreductase activity, acting on the aldehyde or oxo group of donors, NAD or NADP as acceptor	0.000021	110,10,5
1i9ga_#97	GO:0008757: S-adenosylmethionine-dependent methyltransferase activity	0.000021	7,24,3
1apme_#255	GO:0005516: calmodulin binding	0.000021	7,24,3
1fxla1#109	GO:0003729: mRNA binding	0.000021	2,13,2
1gd7a_#85	GO:0000049: tRNA binding	0.000021	2,13,2
1pysb6#217	GO:0000049: tRNA binding	0.000021	2,13,2
1dl0a_#32	GO:0008235: metalloexopeptidase activity	0.000021	38,13,4
1ha1_2#144	GO:0003729: mRNA binding	0.000021	2,13,2
1atia2#210	GO:0005507: copper ion binding	0.000021	13,38,4
1a6o__#45	GO:0004674: protein serine/threonine kinase activity	0.000021	303,42,15
1nsj__#190	GO:0016861: intramolecular oxidoreductase activity, interconverting aldoses and ketoses	0.000021	2,13,2
1c4oa1#335	GO:0008026: ATP-dependent helicase activity	0.000021	2,13,2
1nula_#15	GO:0016763: transferase activity, transferring pentosyl groups	0.000021	35,28,5
1smaa1#95	GO:0019955: cytokine binding	0.000021	272,11,7
1fcda2#189	GO:0050660: FAD binding	0.000021	51,10,4
1fiy__#475	GO:0000287: magnesium ion binding	0.000021	14,128,6
1aym1_#181	GO:0004197: cysteine-type endopeptidase activity	0.000021	7,24,3
1hw5a2#64	GO:0004263: chymotrypsin activity	0.000021	167,41,11
1fp5a2#537	GO:0005518: collagen binding	0.000021	38,13,4
1lvk_2#652	GO:0005516: calmodulin binding	0.000021	7,24,3
1dj0a2#183	GO:0000287: magnesium ion binding	0.000021	142,128,19
1a4ya_#104	GO:0016866: intramolecular transferase activity	0.000021	241,12,7
1bhga3#529	GO:0016776: phosphotransferase activity, phosphate group as acceptor	0.000021	74,14,5
1icia_#9	GO:0016651: oxidoreductase activity, acting on NADH or NADPH	0.000021	38,13,4
1qrra_#69	GO:0050660: FAD binding	0.000021	196,10,6
1a7ca_#202	GO:0005507: copper ion binding	0.000021	117,38,9
1dr9a1#39	GO:0030151: molybdenum ion binding	0.000021	258,15,8

1fu6a_#50	GO:0008201: heparin binding	0.000021	308,24,11
1rypb_#133	GO:0003968: RNA-directed RNA polymerase activity	0.000021	35,14,4
1nat__#50	GO:0016627: oxidoreductase activity, acting on the CH-CH group of donors	0.000021	297,17,9
1f5aa1#441	GO:0004674: protein serine/threonine kinase activity	0.000021	163,42,11
1j9qa1#154	GO:0003809: thrombin activity	0.000021	196,10,6
1dfca4#1451	GO:0005509: calcium ion binding	0.000021	67,160,14
1e1aa_#285	GO:0004896: hematopoietin/interferon-class (D200-domain) cytokine receptor activity	0.000021	137,19,7
1ghpa_#65	GO:0016638: oxidoreductase activity, acting on the CH-NH2 group of donors	0.000022	29,17,4
1i1b__#85	GO:0008083: growth factor activity	0.000022	12,42,4
1ffh_2#127	GO:0005524: ATP binding	0.000022	12,243,7
1c7qa_#374	GO:0005525: GTP binding	0.000022	50,49,7
1g6ha_#180	GO:0005524: ATP binding	0.000022	12,243,7
1koba_#139	GO:0004674: protein serine/threonine kinase activity	0.000022	12,42,4
1d2na_#654	GO:0005524: ATP binding	0.000022	20,243,9
1ia8a_#127	GO:0005524: ATP binding	0.000022	12,243,7
1b0ua_#38	GO:0005524: ATP binding	0.000022	12,243,7
1ii5a_#200	GO:0005525: GTP binding	0.000022	4,49,3
2foka2#180	GO:0005524: ATP binding	0.000022	147,243,29
1dik_1#848	GO:0016861: intramolecular oxidoreductase activity, interconverting aldoses and ketoses	0.000022	81,13,5
1g8fa2#258	GO:0005524: ATP binding	0.000022	12,243,7
1dpja_#322	GO:0005509: calcium ion binding	0.000022	308,160,36
1b6ra3#161	GO:0015036: disulfide oxidoreductase activity	0.000022	164,22,8
1hxxa_#173	GO:0004556: alpha-amylase activity	0.000022	260,15,8
2mev1_#123	GO:0003697: single-stranded DNA binding	0.000022	89,12,5
1b8aa2#368	GO:0004812: tRNA ligase activity	0.000022	135,26,8
1ek6a_#269	GO:0016627: oxidoreductase activity, acting on the CH-CH group of donors	0.000022	29,17,4
1hxxa_#232	GO:0016668: oxidoreductase activity, acting on sulfur group of donors, NAD or NADP as acceptor	0.000022	352,12,8
1ksia3#114	GO:0016638: oxidoreductase activity, acting on the CH-NH2 group of donors	0.000022	29,17,4
1j79a_#187	GO:0016668: oxidoreductase activity, acting on sulfur group of donors, NAD or NADP as acceptor	0.000022	89,12,5
1pwt__#8	GO:0000049: tRNA binding	0.000022	314,13,8
1i50b_#966	GO:0046983: protein dimerization activity	0.000022	299,17,9
1kit_3#743	GO:0008201: heparin binding	0.000022	250,24,10
1cnv__#264	GO:0016799: hydrolase activity, hydrolyzing N-glycosyl compounds	0.000022	103,17,6
1qfta_#98	GO:0008083: growth factor activity	0.000022	230,42,13
1fsia_#122	GO:0004812: tRNA ligase activity	0.000022	97,26,7
1e3a.1#A30	GO:0008483: transaminase activity	0.000022	157,17,7
1aa6_2#285	GO:0000287: magnesium ion binding	0.000022	106,128,16
1trb_1#14	GO:0016627: oxidoreductase activity, acting on the CH-CH group of donors	0.000022	29,17,4
1dn2a1#261	GO:0005509: calcium ion binding	0.000022	425,160,45
1ir3a_#1028	GO:0005516: calmodulin binding	0.000022	70,24,6
1h9da_#91	GO:0046983: protein dimerization activity	0.000022	222,17,8
1e42a1#711	GO:0019955: cytokine binding	0.000023	175,11,6
1ak5_1#353	GO:0000287: magnesium ion binding	0.000023	73,128,13
2bb2_2#134	GO:0003729: mRNA binding	0.000023	220,13,7
1im4a_#41	GO:0019838: growth factor binding	0.000023	59,9,4
1hg3a_#207	GO:0004497: monooxygenase activity	0.000023	19,26,4
1qf6a3#78	GO:0004457: lactate dehydrogenase activity	0.000023	52,10,4
1qf6a3#78	GO:0008800: beta-lactamase activity	0.000023	52,10,4
1bywa_#64	GO:0000155: two-component sensor molecule activity	0.000023	14,12,3
1d4oa_#135	GO:0016668: oxidoreductase activity, acting on sulfur group of donors, NAD or NADP as acceptor	0.000023	14,12,3
1jbwa2#198	GO:0000287: magnesium ion binding	0.000023	9,128,5
1ev2e2#286	GO:0019955: cytokine binding	0.000023	100,11,5
1be9a_#357	GO:0005524: ATP binding	0.000023	83,243,20
1f2da_#94	GO:0016776: phosphotransferase activity, phosphate group as acceptor	0.000023	12,14,3
1qfxa_#380	GO:0019955: cytokine binding	0.000023	47,11,4
1dj0a2#183	GO:0004177: aminopeptidase activity	0.000023	142,13,6
2dkb__#114	GO:0004867: serine-type endopeptidase inhibitor activity	0.000023	174,47,12
1euca1#64	GO:0016861: intramolecular oxidoreductase activity, interconverting aldoses and ketoses	0.000023	39,13,4
2sqca2#268	GO:0008083: growth factor activity	0.000023	59,42,7
1fcda1#6	GO:0016616: oxidoreductase activity, acting on the CH-OH group of donors, NAD or NADP as acceptor	0.000023	75,59,9
1qfta_#98	GO:0019838: growth factor binding	0.000023	230,9,6
1erv__#80	GO:0051082: unfolded protein binding	0.000023	442,34,16

1fjfc2#153	GO:0004523: ribonuclease H activity	0.000023	420,16,10
1ac5__#72	GO:0010181: FMN binding	0.000023	47,11,4
1aoza1#61	GO:0004620: phospholipase activity	0.000023	26,19,4
3sil__#8	GO:0005524: ATP binding	0.000023	125,243,26
1ihua2#525	GO:0016646: oxidoreductase activity, acting on the CH-NH group of donors, NAD or NADP as acceptor	0.000023	335,19,10
1azza_#114	GO:0003809: thrombin activity	0.000024	17,10,3
1fhga_#42	GO:0004896: hematopoietin/interferon-class (D200-domain) cytokine receptor activity	0.000024	9,19,3
1a0ca_#187	GO:0016861: intramolecular oxidoreductase activity, interconverting aldoses and ketoses	0.000024	13,13,3
1lkka_#127	GO:0005524: ATP binding	0.000024	16,243,8
1i6vc_#689	GO:0008080: N-acetyltransferase activity	0.000024	13,13,3
1jf9a_#379	GO:0019838: growth factor binding	0.000024	232,9,6
1fqva2#201	GO:0004222: metalloendopeptidase activity	0.000024	9,19,3
1f42a2#123	GO:0008201: heparin binding	0.000024	378,24,12
1ev2e1#154	GO:0004896: hematopoietin/interferon-class (D200-domain) cytokine receptor activity	0.000024	9,19,3
2dnja_#91	GO:0004896: hematopoietin/interferon-class (D200-domain) cytokine receptor activity	0.000024	9,19,3
1f7da_#97	GO:0004295: trypsin activity	0.000024	35,48,6
1amf__#168	GO:0008199: ferric iron binding	0.000024	17,10,3
1fm2.1#B308	GO:0003809: thrombin activity	0.000024	200,10,6
1imva_#255	GO:0015036: disulfide oxidoreductase activity	0.000024	589,22,14
1euca1#64	GO:0004812: tRNA ligase activity	0.000024	39,26,5
1fqva2#301	GO:0004222: metalloendopeptidase activity	0.000024	9,19,3
1gen__#475	GO:0003887: DNA-directed DNA polymerase activity	0.000024	25,20,4
1exma2#353	GO:0003697: single-stranded DNA binding	0.000024	91,12,5
1exma2#353	GO:0005525: GTP binding	0.000024	91,49,9
1nat__#50	GO:0016831: carboxy-lyase activity	0.000024	297,25,11
1h9da_#91	GO:0005518: collagen binding	0.000024	222,13,7
1a65a2#213	GO:0005507: copper ion binding	0.000025	5,38,3
1fkna_#96	GO:0004190: aspartic-type endopeptidase activity	0.000025	75,23,6
1a6o__#45	GO:0016638: oxidoreductase activity, acting on the CH-NH2 group of donors	0.000025	303,17,9
1hyua4#168	GO:0015036: disulfide oxidoreductase activity	0.000025	8,22,3
1fuia2#273	GO:0005524: ATP binding	0.000025	133,243,27
1dk5a_#111	GO:0005543: phospholipid binding	0.000025	2,14,2
1fc3a_#172	GO:0016705: oxidoreductase activity, acting on paired donors, with incorporation or reduction of molecular oxygen	0.000025	182,26,9
1dfca1#1135	GO:0003809: thrombin activity	0.000025	53,10,4
1fw9a_#152	GO:0005524: ATP binding	0.000025	133,243,27
1eaja_#59	GO:0003916: DNA topoisomerase activity	0.000025	202,10,6
1f0ia1#50	GO:0005509: calcium ion binding	0.000025	146,160,22
1g6oa_#116	GO:0016668: oxidoreductase activity, acting on sulfur group of donors, NAD or NADP as acceptor	0.000025	358,12,8
1gdna_#151	GO:0005509: calcium ion binding	0.000025	17,160,7
1aoea_#130	GO:0005524: ATP binding	0.000025	77,243,19
1pwt__#8	GO:0008201: heparin binding	0.000025	314,24,11
2bpa1_#244	GO:0019838: growth factor binding	0.000025	60,9,4
1svb_2#165	GO:0016811: hydrolase activity, acting on carbon-nitrogen (but not peptide) bonds, in linear amides	0.000025	34,15,4
1e4ea2#215	GO:0016638: oxidoreductase activity, acting on the CH-NH2 group of donors	0.000025	490,17,11
1ldna1#94	GO:0008757: S-adenosylmethionine-dependent methyltransferase activity	0.000025	199,24,9
1qs0a1#235	GO:0016620: oxidoreductase activity, acting on the aldehyde or oxo group of donors, NAD or NADP as acceptor	0.000025	114,10,5
1utea_#11	GO:0019838: growth factor binding	0.000025	130,9,5
1kit_2#398	GO:0005518: collagen binding	0.000025	83,13,5
1mdah_#329	GO:0005529: sugar binding	0.000025	335,39,15
1e0ta2#275	GO:0000287: magnesium ion binding	0.000025	107,128,16
1fgua2#360	GO:0019838: growth factor binding	0.000026	61,9,4
1qj2c2#50	GO:0003964: RNA-directed DNA polymerase activity	0.000026	48,11,4
1a7s__#148	GO:0030151: molybdenum ion binding	0.000026	71,15,5
1nfp__#171	GO:0016616: oxidoreductase activity, acting on the CH-OH group of donors, NAD or NADP as acceptor	0.000026	29,59,6
1qlaa2#35	GO:0016638: oxidoreductase activity, acting on the CH-NH2 group of donors	0.000026	30,17,4
1ig8a_#214	GO:0005524: ATP binding	0.000026	25,243,10
2cb5a_#312	GO:0004263: chymotrypsin activity	0.000026	111,41,9
1akp__#62	GO:0004896: hematopoietin/interferon-class (D200-domain) cytokine receptor activity	0.000026	55,19,5
1epwa3#140	GO:0003887: DNA-directed DNA polymerase activity	0.000026	187,20,8
1gdea_#243	GO:0019838: growth factor binding	0.000026	61,9,4

1quba4#235	GO:0016638: oxidoreductase activity, acting on the CH-NH2 group of donors	0.000026	106,17,6
1avgi_#69	GO:0004896: hematopoietin/interferon-class (D200-domain) cytokine receptor activity	0.000026	340,19,10
1ek6a_#269	GO:0016616: oxidoreductase activity, acting on the CH-OH group of donors, NAD or NADP as acceptor	0.000026	29,59,6
1fw9a_#157	GO:0046983: protein dimerization activity	0.000026	62,17,5
1bfg__#22	GO:0019838: growth factor binding	0.000026	61,9,4
1cfr__#269	GO:0016646: oxidoreductase activity, acting on the CH-NH group of donors, NAD or NADP as acceptor	0.000026	55,19,5
1ihua2#521	GO:0016776: phosphotransferase activity, phosphate group as acceptor	0.000026	77,14,5
2ebn__#77	GO:0005524: ATP binding	0.000026	52,243,15
1dvpa1#65	GO:0003887: DNA-directed DNA polymerase activity	0.000026	187,20,8
1kit_3#297	GO:0005509: calcium ion binding	0.000026	12,160,6
1dy9.1#C229	GO:0050660: FAD binding	0.000026	115,10,5
1pme__#25	GO:0004714: transmembrane receptor protein tyrosine kinase activity	0.000026	37,14,4
1f97a2#199	GO:0004295: trypsin activity	0.000026	144,48,11
1fu6a_#50	GO:0004674: protein serine/threonine kinase activity	0.000026	308,42,15
1h8ca_#35	GO:0016251: general RNA polymerase II transcription factor activity	0.000026	77,14,5
1elua_#95	GO:0016776: phosphotransferase activity, phosphate group as acceptor	0.000026	77,14,5
1arb__#55	GO:0003724: RNA helicase activity	0.000026	48,11,4
1vmoa_#106	GO:0004556: alpha-amylase activity	0.000026	187,15,7
1h6la_#119	GO:0008083: growth factor activity	0.000026	308,42,15
1e32a1#102	GO:0005524: ATP binding	0.000026	25,243,10
1ac6a_#34	GO:0008083: growth factor activity	0.000026	60,42,7
1rypk_#12	GO:0004896: hematopoietin/interferon-class (D200-domain) cytokine receptor activity	0.000026	265,19,9
1dn2a1#261	GO:0015036: disulfide oxidoreductase activity	0.000026	425,22,12
1dn2a1#261	GO:0005529: sugar binding	0.000026	425,39,17
1cpza_#6	GO:0005509: calcium ion binding	0.000026	157,160,23
1icra_#181	GO:0005529: sugar binding	0.000027	5,39,3
1gc5a_#233	GO:0005525: GTP binding	0.000027	35,49,6
1hdma1#115	GO:0030151: molybdenum ion binding	0.000027	188,15,7
1fm2.1#B308	GO:0004896: hematopoietin/interferon-class (D200-domain) cytokine receptor activity	0.000027	200,19,8
1dih_2#235	GO:0003809: thrombin activity	0.000027	54,10,4
1e5sa_#239	GO:0005524: ATP binding	0.000027	30,243,11
1pii_2#326	GO:0016831: carboxy-lyase activity	0.000027	192,25,9
1lci__#26	GO:0004812: tRNA ligase activity	0.000027	139,26,8
1ycsb1#412	GO:0005085: guanyl-nucleotide exchange factor activity	0.000027	146,13,6
3sil__#98	GO:0016638: oxidoreductase activity, acting on the CH-NH2 group of donors	0.000027	228,17,8
1a8d_1#87	GO:0050660: FAD binding	0.000027	204,10,6
1ejda_#6	GO:0005524: ATP binding	0.000027	270,243,44
1cs6a4#363	GO:0019955: cytokine binding	0.000027	16,11,3
1hx0a1#465	GO:0004556: alpha-amylase activity	0.000028	2,15,2
2bc2a_#46	GO:0008800: beta-lactamase activity	0.000028	18,10,3
1aop_3#276	GO:0004263: chymotrypsin activity	0.000028	172,41,11
1eur__#302	GO:0004295: trypsin activity	0.000028	53,48,7
1g62a_#111	GO:0003743: translation initiation factor activity	0.000028	2,15,2
7taa_2#87	GO:0004556: alpha-amylase activity	0.000028	2,15,2
1axca1#19	GO:0003887: DNA-directed DNA polymerase activity	0.000028	9,20,3
1qgua_#30	GO:0030151: molybdenum ion binding	0.000028	2,15,2
1alo_4#584	GO:0030151: molybdenum ion binding	0.000028	2,15,2
1dfoa_#88	GO:0016846: carbon-sulfur lyase activity	0.000028	18,10,3
1e30a_#112	GO:0003724: RNA helicase activity	0.000028	49,11,4
1czya1#466	GO:0008083: growth factor activity	0.000028	109,42,9
1sgpe_#42	GO:0003887: DNA-directed DNA polymerase activity	0.000028	26,20,4
1ceo__#309	GO:0008810: cellulase activity	0.000028	10,18,3
1rypl_#47	GO:0016811: hydrolase activity, acting on carbon-nitrogen (but not peptide) bonds, in linear amides	0.000028	2,15,2
1a81a2#182	GO:0000287: magnesium ion binding	0.000028	120,128,17
1g62a_#60	GO:0003743: translation initiation factor activity	0.000028	2,15,2
1ppn__#132	GO:0004812: tRNA ligase activity	0.000028	234,26,10
1qfta_#98	GO:0016638: oxidoreductase activity, acting on the CH-NH2 group of donors	0.000028	230,17,8
1i7wa_#517	GO:0005509: calcium ion binding	0.000028	60,160,13
1arb__#208	GO:0003724: RNA helicase activity	0.000028	49,11,4
1i1ka_#255	GO:0008483: transaminase activity	0.000029	31,17,4
1i9ga_#67	GO:0004674: protein serine/threonine kinase activity	0.000029	61,42,7
1mrj__#156	GO:0004674: protein serine/threonine kinase activity	0.000029	61,42,7
1e2wa1#102	GO:0004263: chymotrypsin activity	0.000029	86,41,8

1cb8a2#613	GO:0004674: protein serine/threonine kinase activity	0.000029	138,42,10
1e30a_#107	GO:0005507: copper ion binding	0.000029	14,38,4
1eaja_#59	GO:0004896: hematopoietin/interferon-class (D200-domain) cytokine receptor activity	0.000029	202,19,8
1jb3a_#64	GO:0004295: trypsin activity	0.000029	302,48,16
1qs1a2#437	GO:0004295: trypsin activity	0.000029	452,48,20
1g25a_#22	GO:0019955: cytokine binding	0.000029	415,11,8
1ryp1_#20	GO:0030151: molybdenum ion binding	0.000029	125,15,6
1bfg__#22	GO:0008083: growth factor activity	0.000029	61,42,7
1dnpa2#18	GO:0016616: oxidoreductase activity, acting on the CH-OH group of donors, NAD or NADP as acceptor	0.000029	77,59,9
1bura1#269	GO:0016836: hydro-lyase activity	0.000029	16,33,4
1e1oa1#142	GO:0003697: single-stranded DNA binding	0.000029	15,12,3
1aqua_#126	GO:0016758: transferase activity, transferring hexosyl groups	0.000029	576,11,9
1e6ca_#91	GO:0004364: glutathione transferase activity	0.000029	182,11,6
1rypb_#114	GO:0000287: magnesium ion binding	0.000029	158,128,20
1imva_#69	GO:0008083: growth factor activity	0.000029	84,42,8
1fhoa_#74	GO:0051082: unfolded protein binding	0.000029	347,34,14
1ddwa_#77	GO:0016668: oxidoreductase activity, acting on sulfur group of donors, NAD or NADP as acceptor	0.000029	364,12,8
1dyna_#42	GO:0016638: oxidoreductase activity, acting on the CH-NH2 group of donors	0.000029	164,17,7
1fmk_3#283	GO:0005524: ATP binding	0.00003	6,243,5
1pbe_2#183	GO:0003729: mRNA binding	0.00003	14,13,3
1p35a_#101	GO:0005126: hematopoietin/interferon-class (D200-domain) cytokine receptor binding	0.00003	136,20,7
1auib_#136	GO:0005516: calmodulin binding	0.00003	22,24,4
1a6o__#297	GO:0005524: ATP binding	0.00003	41,243,13
1fy7a_#388	GO:0004497: monooxygenase activity	0.00003	68,26,6
1jf9a_#379	GO:0008483: transaminase activity	0.00003	232,17,8
1fwxa2#396	GO:0004674: protein serine/threonine kinase activity	0.00003	237,42,13
1by5a_#238	GO:0004674: protein serine/threonine kinase activity	0.00003	237,42,13
1e69a_#1106	GO:0005524: ATP binding	0.00003	6,243,5
1qksa2#236	GO:0046983: protein dimerization activity	0.00003	64,17,5
1fsu__#434	GO:0015036: disulfide oxidoreductase activity	0.00003	356,22,11
3mag__#134	GO:0005525: GTP binding	0.00003	72,49,8
1d2ka1#164	GO:0016763: transferase activity, transferring pentosyl groups	0.00003	63,28,6
1e25a_#262	GO:0004523: ribonuclease H activity	0.00003	116,16,6
1ej0a_#42	GO:0004523: ribonuclease H activity	0.00003	116,16,6
1arb__#193	GO:0003724: RNA helicase activity	0.00003	50,11,4
1e79d2#36	GO:0003968: RNA-directed RNA polymerase activity	0.00003	524,14,10
1f00i1#749	GO:0005507: copper ion binding	0.000031	28,38,5
1pce__#35	GO:0004180: carboxypeptidase activity	0.000031	74,15,5
1qq9a_#178	GO:0016763: transferase activity, transferring pentosyl groups	0.000031	19,28,4
1smaa1#95	GO:0030151: molybdenum ion binding	0.000031	272,15,8
1smaa1#95	GO:0004556: alpha-amylase activity	0.000031	272,15,8
1c1da1#235	GO:0016763: transferase activity, transferring pentosyl groups	0.000031	19,28,4
1quba4#235	GO:0019955: cytokine binding	0.000031	106,11,5
1aw1a_#169	GO:0004222: metalloendopeptidase activity	0.000031	28,19,4
1tif__#58	GO:0005524: ATP binding	0.000031	65,243,17
2mpra_#190	GO:0051082: unfolded protein binding	0.000031	400,34,15
1b8aa2#403	GO:0004190: aspartic-type endopeptidase activity	0.000031	117,23,7
1imva_#255	GO:0003729: mRNA binding	0.000031	589,13,10
1c9oa_#49	GO:0004812: tRNA ligase activity	0.000031	102,26,7
1c7na_#231	GO:0005524: ATP binding	0.000031	263,243,43
1cs1a_#82	GO:0016831: carboxy-lyase activity	0.000031	195,25,9
1rypb_#114	GO:0008270: zinc ion binding	0.000031	158,108,18
1dp0a4#756	GO:0016638: oxidoreductase activity, acting on the CH-NH2 group of donors	0.000031	165,17,7
1hwxa1#223	GO:0003924: GTPase activity	0.000031	233,17,8
1c5y.1#B128	GO:0005509: calcium ion binding	0.000032	8,160,5
1fhua1#133	GO:0016836: hydro-lyase activity	0.000032	6,33,3
1mml__#97	GO:0004523: ribonuclease H activity	0.000032	2,16,2
1aop_3#276	GO:0015036: disulfide oxidoreductase activity	0.000032	172,22,8
1cyda_#204	GO:0016627: oxidoreductase activity, acting on the CH-CH group of donors	0.000032	11,17,3
3mbp__#275	GO:0005351: sugar porter activity	0.000032	9,21,3
2rspa_#113	GO:0003964: RNA-directed DNA polymerase activity	0.000032	17,11,3
1a4ia1#208	GO:0005525: GTP binding	0.000032	22,49,5
1f2aa_#27	GO:0008270: zinc ion binding	0.000032	89,108,13
1fbl_1#398	GO:0008083: growth factor activity	0.000032	62,42,7

1fwxa2#396	GO:0004295: trypsin activity	0.000032	237,48,14
1jlxa2#214	GO:0008083: growth factor activity	0.000032	13,42,4
1e79h2#77	GO:0019829: cation-transporting ATPase activity	0.000032	21,9,3
1i5ga_#115	GO:0005524: ATP binding	0.000032	324,243,50
1ie5a_#23	GO:0019838: growth factor binding	0.000032	21,9,3
1c9la2#61	GO:0008083: growth factor activity	0.000032	238,42,13
1bywa_#30	GO:0050660: FAD binding	0.000032	331,10,7
3grs_3#435	GO:0004523: ribonuclease H activity	0.000032	179,16,7
1fmta2#151	GO:0004674: protein serine/threonine kinase activity	0.000032	314,42,15
1ryph_#163	GO:0000287: magnesium ion binding	0.000032	121,128,17
1a4ya_#47	GO:0016616: oxidoreductase activity, acting on the CH-OH group of donors, NAD or NADP as acceptor	0.000032	216,59,15
1cwva2#682	GO:0019955: cytokine binding	0.000032	17,11,3
1ii7a_#43	GO:0016616: oxidoreductase activity, acting on the CH-OH group of donors, NAD or NADP as acceptor	0.000032	44,59,7
1feca1#31	GO:0008757: S-adenosylmethionine-dependent methyltransferase activity	0.000032	75,24,6
1hbza_#96	GO:0016668: oxidoreductase activity, acting on sulfur group of donors, NAD or NADP as acceptor	0.000032	370,12,8
1ie5a_#102	GO:0008083: growth factor activity	0.000032	62,42,7
1qvba_#9	GO:0016616: oxidoreductase activity, acting on the CH-OH group of donors, NAD or NADP as acceptor	0.000032	60,59,8
1fsia_#122	GO:0004620: phospholipase activity	0.000032	97,19,6
1bgva1#326	GO:0016638: oxidoreductase activity, acting on the CH-NH2 group of donors	0.000032	11,17,3
1hxn__#248	GO:0003724: RNA helicase activity	0.000032	17,11,3
1hxmb1#117	GO:0019838: growth factor binding	0.000032	64,9,4
1br9__#86	GO:0003697: single-stranded DNA binding	0.000032	96,12,5
3sil__#284	GO:0019838: growth factor binding	0.000032	64,9,4
1pfza_#156	GO:0019838: growth factor binding	0.000032	64,9,4
1j75a_#166	GO:0003809: thrombin activity	0.000032	120,10,5
1c8na_#216	GO:0004197: cysteine-type endopeptidase activity	0.000033	8,24,3
1gdna_#230	GO:0004867: serine-type endopeptidase inhibitor activity	0.000033	23,47,5
1apj__#2062	GO:0003887: DNA-directed DNA polymerase activity	0.000033	27,20,4
2hlpa2#315	GO:0016620: oxidoreductase activity, acting on the aldehyde or oxo group of donors, NAD or NADP as acceptor	0.000033	19,10,3
1rypk_#77	GO:0004197: cysteine-type endopeptidase activity	0.000033	8,24,3
1qq9a_#178	GO:0016846: carbon-sulfur lyase activity	0.000033	19,10,3
2fmr__#10	GO:0003916: DNA topoisomerase activity	0.000033	57,10,4
1dssg1#4	GO:0050660: FAD binding	0.000033	57,10,4
1doka_#52	GO:0019955: cytokine binding	0.000033	51,11,4
1jj2e1#42	GO:0016638: oxidoreductase activity, acting on the CH-NH2 group of donors	0.000033	167,17,7
1jj2e1#42	GO:0016668: oxidoreductase activity, acting on sulfur group of donors, NAD or NADP as acceptor	0.000033	167,12,6
3tdt__#201	GO:0004222: metalloendopeptidase activity	0.000033	10,19,3
1iqpa2#112	GO:0004457: lactate dehydrogenase activity	0.000033	19,10,3
1htp__#16	GO:0051082: unfolded protein binding	0.000033	303,34,13
1cl1a_#184	GO:0016763: transferase activity, transferring pentosyl groups	0.000033	64,28,6
1thfd_#140	GO:0015036: disulfide oxidoreductase activity	0.000033	124,22,7
1j9la_#164	GO:0016814: hydrolase activity, acting on carbon-nitrogen (but not peptide) bonds, in cyclic amidines	0.000033	57,10,4
1d0na6#681	GO:0004812: tRNA ligase activity	0.000033	143,26,8
1cs1a_#160	GO:0010181: FMN binding	0.000033	187,11,6
2hhma_#262	GO:0016763: transferase activity, transferring pentosyl groups	0.000033	95,28,7
1fhga_#48	GO:0004674: protein serine/threonine kinase activity	0.000033	5,42,3
1ecpa_#62	GO:0016831: carboxy-lyase activity	0.000033	72,25,6
1dr9a1#39	GO:0016668: oxidoreductase activity, acting on sulfur group of donors, NAD or NADP as acceptor	0.000033	258,12,7
1p35a_#49	GO:0004867: serine-type endopeptidase inhibitor activity	0.000033	124,47,10
1qfta_#98	GO:0005524: ATP binding	0.000033	230,243,39
1f9va_#627	GO:0004364: glutathione transferase activity	0.000033	51,11,4
1qfea_#68	GO:0004812: tRNA ligase activity	0.000033	560,26,15
1fwxa2#189	GO:0004222: metalloendopeptidase activity	0.000033	10,19,3
5ruba1#259	GO:0005524: ATP binding	0.000033	53,243,15
1evqa_#305	GO:0016620: oxidoreductase activity, acting on the aldehyde or oxo group of donors, NAD or NADP as acceptor	0.000033	490,10,8
1daba_#310	GO:0016763: transferase activity, transferring pentosyl groups	0.000033	7,28,3
1qf6a4#295	GO:0000287: magnesium ion binding	0.000034	15,128,6

1atia2#67	GO:0000287: magnesium ion binding	0.000034	15,128,6
2cpl__#20	GO:0003755: peptidyl-prolyl cis-trans isomerase activity	0.000034	108,11,5
1hc7a2#111	GO:0000287: magnesium ion binding	0.000034	15,128,6
1hc7a2#109	GO:0000287: magnesium ion binding	0.000034	15,128,6
1be9a_#357	GO:0019843: rRNA binding	0.000034	83,22,6
2mpra_#190	GO:0005507: copper ion binding	0.000034	400,38,16
1czan1#92	GO:0051082: unfolded protein binding	0.000034	631,34,19
1fxma_#153	GO:0000287: magnesium ion binding	0.000034	15,128,6
1fp1d2#214	GO:0005525: GTP binding	0.000034	173,49,12
1bmv2_#2064	GO:0004896: hematopoietin/interferon-class (D200-domain) cytokine receptor activity	0.000034	147,19,7
1ea5a_#99	GO:0003887: DNA-directed DNA polymerase activity	0.000034	194,20,8
1ej0a_#42	GO:0008270: zinc ion binding	0.000034	116,108,15
1aa6_2#285	GO:0005524: ATP binding	0.000034	106,243,23
1ihua2#525	GO:0004457: lactate dehydrogenase activity	0.000034	335,10,7
2hlca_#226	GO:0004896: hematopoietin/interferon-class (D200-domain) cytokine receptor activity	0.000034	98,19,6
1h9da_#91	GO:0005524: ATP binding	0.000034	222,243,38
1lvl_2#222	GO:0016616: oxidoreductase activity, acting on the CH-OH group of donors, NAD or NADP as acceptor	0.000035	79,59,9
1hc7a2#113	GO:0005524: ATP binding	0.000035	21,243,9
1e2wa1#102	GO:0008083: growth factor activity	0.000035	86,42,8
1j9la_#82	GO:0009036: type II site-specific deoxyribonuclease activity	0.000035	169,12,6
1tuba1#239	GO:0016616: oxidoreductase activity, acting on the CH-OH group of donors, NAD or NADP as acceptor	0.000035	120,59,11
1awx__#13	GO:0030151: molybdenum ion binding	0.000035	37,15,4
1epwa3#138	GO:0019955: cytokine binding	0.000035	424,11,8
1flga_#161	GO:0004867: serine-type endopeptidase inhibitor activity	0.000035	152,47,11
1hxxa_#173	GO:0016668: oxidoreductase activity, acting on sulfur group of donors, NAD or NADP as acceptor	0.000035	260,12,7
1ryp1_#20	GO:0015036: disulfide oxidoreductase activity	0.000035	125,22,7
1pysb5#672	GO:0005524: ATP binding	0.000035	36,243,12
1efpa1#32	GO:0004556: alpha-amylase activity	0.000035	129,15,6
1imva_#255	GO:0019955: cytokine binding	0.000035	589,11,9
1ovb__#195	GO:0019201: nucleotide kinase activity	0.000035	89,13,5
1bu7a_#277	GO:0016705: oxidoreductase activity, acting on paired donors, with incorporation or reduction of molecular oxygen	0.000035	21,26,4
1jsg__#92	GO:0004556: alpha-amylase activity	0.000035	607,15,11
1a65a3#383	GO:0015036: disulfide oxidoreductase activity	0.000035	50,22,5
1dv8a_#211	GO:0004674: protein serine/threonine kinase activity	0.000035	86,42,8
3mag__#134	GO:0005524: ATP binding	0.000035	72,243,18
1bwda_#295	GO:0004812: tRNA ligase activity	0.000035	104,26,7
1e4ea2#215	GO:0004190: aspartic-type endopeptidase activity	0.000035	490,23,13
1fjfc2#153	GO:0004812: tRNA ligase activity	0.000035	420,26,13
1i7qa_#321	GO:0004896: hematopoietin/interferon-class (D200-domain) cytokine receptor activity	0.000035	148,19,7
1eur__#84	GO:0008083: growth factor activity	0.000035	86,42,8
1h6va1#27	GO:0016866: intramolecular transferase activity	0.000035	260,12,7
1qs0a1#119	GO:0019201: nucleotide kinase activity	0.000035	454,13,9
1gof_3#436	GO:0003682: chromatin binding	0.000036	3,10,2
1gg1a_#209	GO:0004457: lactate dehydrogenase activity	0.000036	3,10,2
1qi9a_#217	GO:0004457: lactate dehydrogenase activity	0.000036	3,10,2
1qqqa_#247	GO:0008199: ferric iron binding	0.000036	3,10,2
1i6vd_#713	GO:0003968: RNA-directed RNA polymerase activity	0.000036	40,14,4
1ewqa4#78	GO:0003684: damaged DNA binding	0.000036	3,10,2
1dik_1#848	GO:0016836: hydro-lyase activity	0.000036	81,33,7
1ab4__#164	GO:0003916: DNA topoisomerase activity	0.000036	3,10,2
1cb6a2#1660	GO:0008199: ferric iron binding	0.000036	3,10,2
1epwa3#140	GO:0005529: sugar binding	0.000036	187,39,11
1prea2#308	GO:0004556: alpha-amylase activity	0.000036	76,15,5
1ej8a_#189	GO:0019955: cytokine binding	0.000036	294,11,7
1jb3a_#64	GO:0005509: calcium ion binding	0.000036	302,160,35
2mpra_#190	GO:0004674: protein serine/threonine kinase activity	0.000036	400,42,17
1ii7a_#199	GO:0005525: GTP binding	0.000036	54,49,7
1dm9a_#57	GO:0003724: RNA helicase activity	0.000036	109,11,5
1ec7a1#230	GO:0005525: GTP binding	0.000036	54,49,7
1axca1#70	GO:0005509: calcium ion binding	0.000036	118,160,19
2bpa1_#45	GO:0005507: copper ion binding	0.000036	192,38,11
1e39a2#539	GO:0016627: oxidoreductase activity, acting on the CH-CH group of donors	0.000037	2,17,2

1amp__#173	GO:0004177: aminopeptidase activity	0.000037	15,13,3
1fytd2#143	GO:0046983: protein dimerization activity	0.000037	2,17,2
1dgwa_#97	GO:0005518: collagen binding	0.000037	15,13,3
1fs7a_#235	GO:0008201: heparin binding	0.000037	23,24,4
1xis__#231	GO:0016861: intramolecular oxidoreductase activity, interconverting aldoses and ketoses	0.000037	15,13,3
1bfd_3#446	GO:0016831: carboxy-lyase activity	0.000037	8,25,3
1jf9a_#379	GO:0015036: disulfide oxidoreductase activity	0.000037	232,22,9
1dlfh_#4	GO:0019838: growth factor binding	0.000037	22,9,3
1by5a_#347	GO:0016668: oxidoreductase activity, acting on sulfur group of donors, NAD or NADP as acceptor	0.000037	263,12,7
1hxxa_#173	GO:0003887: DNA-directed DNA polymerase activity	0.000037	260,20,9
1htp__#16	GO:0005529: sugar binding	0.000037	303,39,14
1jlna_#437	GO:0015078: hydrogen ion transporter activity	0.000037	53,21,5
1a4ya_#47	GO:0016620: oxidoreductase activity, acting on the aldehyde or oxo group of donors, NAD or NADP as acceptor	0.000037	216,10,6
1ksia3#114	GO:0030145: manganese ion binding	0.000037	29,38,5
1e4ea2#215	GO:0008083: growth factor activity	0.000037	490,42,19
1qfma1#139	GO:0046983: protein dimerization activity	0.000037	318,17,9
1kit_2#398	GO:0003968: RNA-directed RNA polymerase activity	0.000037	83,14,5
3sil__#317	GO:0004674: protein serine/threonine kinase activity	0.000037	358,42,16
1doza_#109	GO:0005525: GTP binding	0.000037	74,49,8
1pbe_2#183	GO:0016251: general RNA polymerase II transcription factor activity	0.000038	14,14,3
1hhsa_#328	GO:0003968: RNA-directed RNA polymerase activity	0.000038	14,14,3
1hbza_#214	GO:0019955: cytokine binding	0.000038	297,11,7
1kit_3#331	GO:0003809: thrombin activity	0.000038	124,10,5
1d0na2#200	GO:0005524: ATP binding	0.000038	79,243,19
1f21a_#68	GO:0008270: zinc ion binding	0.000038	44,108,9
1dfca4#1469	GO:0019955: cytokine binding	0.000038	53,11,4
1opy__#68	GO:0005509: calcium ion binding	0.000038	367,160,40
1d6ja_#95	GO:0016627: oxidoreductase activity, acting on the CH-CH group of donors	0.000038	67,17,5
2cba__#144	GO:0016627: oxidoreductase activity, acting on the CH-CH group of donors	0.000038	67,17,5
1je5a_#106	GO:0003697: single-stranded DNA binding	0.000038	48,12,4
2reb_1#138	GO:0008483: transaminase activity	0.000038	67,17,5
1gpea1#271	GO:0003887: DNA-directed DNA polymerase activity	0.000038	197,20,8
1qasa3#352	GO:0016763: transferase activity, transferring pentosyl groups	0.000039	20,28,4
1f0c.1#A291	GO:0003887: DNA-directed DNA polymerase activity	0.000039	10,20,3
1aop_3#276	GO:0016668: oxidoreductase activity, acting on sulfur group of donors, NAD or NADP as acceptor	0.000039	172,12,6
1je5a_#39	GO:0003724: RNA helicase activity	0.000039	18,11,3
1eno__#83	GO:0004180: carboxypeptidase activity	0.000039	38,15,4
1kve.1#B199	GO:0004295: trypsin activity	0.000039	76,48,8
3frua1#236	GO:0004295: trypsin activity	0.000039	76,48,8
1hava_#157	GO:0003724: RNA helicase activity	0.000039	18,11,3
1cpy__#380	GO:0046983: protein dimerization activity	0.000039	171,17,7
1boub_#7	GO:0003924: GTPase activity	0.000039	240,17,8
1cpy__#346	GO:0004180: carboxypeptidase activity	0.000039	38,15,4
1erv__#80	GO:0015036: disulfide oxidoreductase activity	0.000039	442,22,12
1qs0a1#235	GO:0005524: ATP binding	0.000039	114,243,24
2bbkh_#348	GO:0005509: calcium ion binding	0.000039	24,160,8
1rypk_#12	GO:0016668: oxidoreductase activity, acting on sulfur group of donors, NAD or NADP as acceptor	0.000039	265,12,7
1e79d2#36	GO:0019843: rRNA binding	0.000039	524,22,13
1je5a_#10	GO:0016616: oxidoreductase activity, acting on the CH-OH group of donors, NAD or NADP as acceptor	0.00004	62,59,8
1aop_3#276	GO:0016638: oxidoreductase activity, acting on the CH-NH2 group of donors	0.00004	172,17,7
1azo__#144	GO:0016251: general RNA polymerase II transcription factor activity	0.00004	41,14,4
2trcp_#201	GO:0004263: chymotrypsin activity	0.00004	14,41,4
1im4a_#144	GO:0005524: ATP binding	0.00004	129,243,26
1h9oa_#83	GO:0004812: tRNA ligase activity	0.00004	43,26,5
1ycsa_#162	GO:0004295: trypsin activity	0.00004	210,48,13
1avgi_#69	GO:0005518: collagen binding	0.00004	340,13,8
1aym3_#131	GO:0046983: protein dimerization activity	0.00004	412,17,10
1e7wa_#8	GO:0000287: magnesium ion binding	0.00004	111,128,16
1huxa_#10	GO:0008800: beta-lactamase activity	0.00004	60,10,4
1apme_#74	GO:0004295: trypsin activity	0.00004	124,48,10
1opy__#68	GO:0019843: rRNA binding	0.00004	367,22,11

1amf__#196	GO:0016668: oxidoreductase activity, acting on sulfur group of donors, NAD or NADP as acceptor	0.00004	173,12,6
1qj5a_#268	GO:0016763: transferase activity, transferring pentosyl groups	0.00004	66,28,6
1qqga1#95	GO:0004867: serine-type endopeptidase inhibitor activity	0.00004	127,47,10
1dhs__#99	GO:0030151: molybdenum ion binding	0.00004	132,15,6
1e0ta2#275	GO:0008810: cellulase activity	0.00004	107,18,6
1h8va_#127	GO:0008810: cellulase activity	0.000041	2,18,2
2bb2_2#134	GO:0008009: chemokine activity	0.000041	220,10,6
1cem__#150	GO:0008810: cellulase activity	0.000041	2,18,2
1mkp__#283	GO:0016668: oxidoreductase activity, acting on sulfur group of donors, NAD or NADP as acceptor	0.000041	49,12,4
1g25a_#22	GO:0003887: DNA-directed DNA polymerase activity	0.000041	415,20,11
1phm_2#253	GO:0004714: transmembrane receptor protein tyrosine kinase activity	0.000041	309,14,8
1h4vb2#282	GO:0016668: oxidoreductase activity, acting on sulfur group of donors, NAD or NADP as acceptor	0.000041	101,12,5
1pud__#197	GO:0016836: hydro-lyase activity	0.000041	34,33,5
1a3qa2#190	GO:0004714: transmembrane receptor protein tyrosine kinase activity	0.000041	144,14,6
1a3qa2#190	GO:0019838: growth factor binding	0.000041	144,9,5
1ea5a_#99	GO:0019955: cytokine binding	0.000041	194,11,6
1f97a2#199	GO:0019838: growth factor binding	0.000041	144,9,5
2msba_#218	GO:0003697: single-stranded DNA binding	0.000041	49,12,4
1ton__#47	GO:0003724: RNA helicase activity	0.000041	112,11,5
1ck7a3#247	GO:0004812: tRNA ligase activity	0.000042	8,26,3
1cb0a_#98	GO:0004812: tRNA ligase activity	0.000042	8,26,3
1esma_#314	GO:0019955: cytokine binding	0.000042	113,11,5
1ldna1#129	GO:0004812: tRNA ligase activity	0.000042	8,26,3
1nsj__#21	GO:0016836: hydro-lyase activity	0.000042	114,33,8
1muca1#245	GO:0016854: racemase and epimerase activity	0.000042	45,13,4
1bn8a_#241	GO:0008270: zinc ion binding	0.000042	18,108,6
1flga_#161	GO:0005524: ATP binding	0.000042	152,243,29
3thia_#59	GO:0008270: zinc ion binding	0.000042	18,108,6
1hlwa_#82	GO:0005525: GTP binding	0.000042	270,49,15
1d0na3#305	GO:0004295: trypsin activity	0.000042	311,48,16
1ezvb1#29	GO:0008083: growth factor activity	0.000042	44,42,6
1a4ya_#104	GO:0016854: racemase and epimerase activity	0.000042	241,13,7
1bev1_#86	GO:0016638: oxidoreductase activity, acting on the CH-NH2 group of donors	0.000042	323,17,9
1jsg__#92	GO:0005518: collagen binding	0.000042	607,13,10
1bec_2#216	GO:0046983: protein dimerization activity	0.000042	115,17,6
1bec_2#216	GO:0016638: oxidoreductase activity, acting on the CH-NH2 group of donors	0.000042	115,17,6
1amf__#196	GO:0016638: oxidoreductase activity, acting on the CH-NH2 group of donors	0.000042	173,17,7
1a81a1#51	GO:0005524: ATP binding	0.000042	73,243,18
1e4ea2#215	GO:0005529: sugar binding	0.000042	490,39,18
1a8i__#688	GO:0019201: nucleotide kinase activity	0.000042	241,13,7
1a9xb2#1775	GO:0005524: ATP binding	0.000042	54,243,15
1p35a_#98	GO:0016799: hydrolase activity, hydrolyzing N-glycosyl compounds	0.000043	34,17,4
1i6vc_#207	GO:0019829: cation-transporting ATPase activity	0.000043	23,9,3
1dqsa_#138	GO:0000287: magnesium ion binding	0.000043	38,128,9
1f97a1#62	GO:0005509: calcium ion binding	0.000043	707,160,64
1b0ua_#38	GO:0016799: hydrolase activity, hydrolyzing N-glycosyl compounds	0.000043	12,17,3
1qs0b2#269	GO:0016616: oxidoreductase activity, acting on the CH-OH group of donors, NAD or NADP as acceptor	0.000043	46,59,7
1ajsa_#389	GO:0008483: transaminase activity	0.000043	12,17,3
1d7ya1#243	GO:0016627: oxidoreductase activity, acting on the CH-CH group of donors	0.000043	12,17,3
1d2fa_#260	GO:0008483: transaminase activity	0.000043	12,17,3
1cg2a1#43	GO:0004601: peroxidase activity	0.000043	189,21,8
1dv8a_#213	GO:0004190: aspartic-type endopeptidase activity	0.000043	25,23,4
2pia_1#99	GO:0015036: disulfide oxidoreductase activity	0.000043	179,22,8
2viua_#252	GO:0005509: calcium ion binding	0.000043	343,160,38
1mil__#30	GO:0005066: transmembrane receptor protein tyrosine kinase signaling protein activity	0.000043	61,10,4
1ax4a_#224	GO:0008483: transaminase activity	0.000043	12,17,3
1fm2.1#B308	GO:0003887: DNA-directed DNA polymerase activity	0.000043	200,20,8
1qg6a_#251	GO:0016627: oxidoreductase activity, acting on the CH-CH group of donors	0.000043	12,17,3
1jf9a_#183	GO:0008483: transaminase activity	0.000043	12,17,3
1clc_1#299	GO:0008810: cellulase activity	0.000043	32,18,4
1exma3#99	GO:0016616: oxidoreductase activity, acting on the CH-OH group of donors, NAD or NADP as acceptor	0.000043	81,59,9

1a3qa2#190	GO:0005126: hematopoietin/interferon-class (D200-domain) cytokine receptor binding	0.000043	144,20,7
1e5ea_#71	GO:0016846: carbon-sulfur lyase activity	0.000043	61,10,4
1nsj__#178	GO:0016854: racemase and epimerase activity	0.000043	93,13,5
1c8na_#165	GO:0003724: RNA helicase activity	0.000044	3,11,2
1ed1a_#94	GO:0000287: magnesium ion binding	0.000044	10,128,5
1fmb__#60	GO:0003964: RNA-directed DNA polymerase activity	0.000044	3,11,2
1b37a1#46	GO:0019955: cytokine binding	0.000044	3,11,2
1dqsa_#138	GO:0005525: GTP binding	0.000044	38,49,6
1dfna_#16	GO:0019955: cytokine binding	0.000044	3,11,2
1hnja1#167	GO:0016646: oxidoreductase activity, acting on the CH-NH group of donors, NAD or NADP as acceptor	0.000044	61,19,5
2pola3#335	GO:0008083: growth factor activity	0.000044	14,42,4
1a6o__#45	GO:0003755: peptidyl-prolyl cis-trans isomerase activity	0.000044	303,11,7
1gdea_#243	GO:0004896: hematopoietin/interferon-class (D200-domain) cytokine receptor activity	0.000044	61,19,5
1f8ra2#325	GO:0050660: FAD binding	0.000044	222,10,6
1erxa_#138	GO:0015078: hydrogen ion transporter activity	0.000044	55,21,5
1fxla2#169	GO:0004556: alpha-amylase activity	0.000044	203,15,7
1f0ia1#50	GO:0019838: growth factor binding	0.000044	146,9,5
1qqqa_#130	GO:0010181: FMN binding	0.000044	3,11,2
1icia_#9	GO:0005525: GTP binding	0.000044	38,49,6
1dz4a_#264	GO:0016705: oxidoreductase activity, acting on paired donors, with incorporation or reduction of molecular oxygen	0.000044	44,26,5
1erv__#57	GO:0015036: disulfide oxidoreductase activity	0.000044	87,22,6
1e25a_#262	GO:0046983: protein dimerization activity	0.000044	116,17,6
1bd3a_#195	GO:0000287: magnesium ion binding	0.000044	10,128,5
1aa6_1#652	GO:0005507: copper ion binding	0.000044	30,38,5
1qs0a1#119	GO:0016763: transferase activity, transferring pentosyl groups	0.000044	454,28,14
1h9da_#91	GO:0051082: unfolded protein binding	0.000044	222,34,11
1a6o__#45	GO:0004812: tRNA ligase activity	0.000045	303,26,11
1dt6a_#385	GO:0005525: GTP binding	0.000045	76,49,8
1iba__#39	GO:0005529: sugar binding	0.000045	351,39,15
1a9xa6#760	GO:0005509: calcium ion binding	0.000045	90,160,16
1bfd_2#146	GO:0000287: magnesium ion binding	0.000045	112,128,16
1pii_2#326	GO:0005524: ATP binding	0.000045	192,243,34
1bev1_#86	GO:0004674: protein serine/threonine kinase activity	0.000045	323,42,15
1jsg__#92	GO:0004867: serine-type endopeptidase inhibitor activity	0.000045	607,47,23
1dih_2#222	GO:0004523: ribonuclease H activity	0.000045	352,16,9
1fyhb2#131	GO:0004896: hematopoietin/interferon-class (D200-domain) cytokine receptor activity	0.000046	2,19,2
1aym1_#151	GO:0015078: hydrogen ion transporter activity	0.000046	10,21,3
1ah7__#55	GO:0004620: phospholipase activity	0.000046	2,19,2
1ad3a_#181	GO:0016651: oxidoreductase activity, acting on NADH or NADPH	0.000046	16,13,3
2sli_2#341	GO:0004842: ubiquitin-protein ligase activity	0.000046	2,19,2
1b12a_#221	GO:0019955: cytokine binding	0.000046	115,11,5
1tlfa_#269	GO:0016646: oxidoreductase activity, acting on the CH-NH group of donors, NAD or NADP as acceptor	0.000046	11,19,3
1im4a_#144	GO:0050660: FAD binding	0.000046	129,10,5
1azsa_#397	GO:0008408: 3'-5' exonuclease activity	0.000046	16,13,3
1ah7__#184	GO:0004620: phospholipase activity	0.000046	2,19,2
1dz4a_#150	GO:0010181: FMN binding	0.000046	19,11,3
1dn2a1#308	GO:0015078: hydrogen ion transporter activity	0.000046	10,21,3
1smpi_#11	GO:0004222: metalloendopeptidase activity	0.000046	2,19,2
1h6va3#486	GO:0019838: growth factor binding	0.000046	70,9,4
1xis__#196	GO:0016861: intramolecular oxidoreductase activity, interconverting aldoses and ketoses	0.000046	16,13,3
5ruba1#279	GO:0016836: hydro-lyase activity	0.000046	57,33,6
1jbqa_#152	GO:0050660: FAD binding	0.000046	62,10,4
1smaa1#95	GO:0008201: heparin binding	0.000046	272,24,10
1eaja_#59	GO:0003887: DNA-directed DNA polymerase activity	0.000046	202,20,8
1jb3a_#64	GO:0015036: disulfide oxidoreductase activity	0.000046	302,22,10
1cqka_#52	GO:0019838: growth factor binding	0.000046	70,9,4
1c4zd_#85	GO:0016638: oxidoreductase activity, acting on the CH-NH2 group of donors	0.000046	117,17,6
1b8aa2#403	GO:0046983: protein dimerization activity	0.000046	117,17,6
1e8ga2#201	GO:0004497: monooxygenase activity	0.000046	304,26,11
1efpa1#32	GO:0004457: lactate dehydrogenase activity	0.000046	129,10,5
1efpa1#32	GO:0016846: carbon-sulfur lyase activity	0.000046	129,10,5
1dyr__#180	GO:0016646: oxidoreductase activity, acting on the CH-NH group of donors, NAD or NADP as acceptor	0.000046	2,19,2

1qhoa4#230	GO:0008081: phosphoric diester hydrolase activity	0.000046	19,11,3
1a7ca_#202	GO:0046983: protein dimerization activity	0.000046	117,17,6
1smaa2#581	GO:0004556: alpha-amylase activity	0.000046	80,15,5
1jsg__#92	GO:0019955: cytokine binding	0.000046	607,11,9
1a8d_1#87	GO:0030151: molybdenum ion binding	0.000046	204,15,7
1fnf_1#1146	GO:0005518: collagen binding	0.000046	16,13,3
1qfma1#186	GO:0016638: oxidoreductase activity, acting on the CH-NH2 group of donors	0.000046	326,17,9
1ps1a_#83	GO:0015078: hydrogen ion transporter activity	0.000047	28,21,4
1cf9a2#133	GO:0005524: ATP binding	0.000047	37,243,12
1f06a1#296	GO:0016616: oxidoreductase activity, acting on the CH-OH group of donors, NAD or NADP as acceptor	0.000047	32,59,6
1ezwa_#245	GO:0016616: oxidoreductase activity, acting on the CH-OH group of donors, NAD or NADP as acceptor	0.000047	32,59,6
1f2aa_#27	GO:0000287: magnesium ion binding	0.000047	89,128,14
2dnja_#97	GO:0005509: calcium ion binding	0.000047	141,160,21
1aym3_#109	GO:0019955: cytokine binding	0.000047	56,11,4
1jf9a_#379	GO:0005509: calcium ion binding	0.000047	232,160,29
1hxxa_#173	GO:0019838: growth factor binding	0.000047	260,9,6
1bak__#583	GO:0015036: disulfide oxidoreductase activity	0.000047	131,22,7
1dcpa_#25	GO:0003724: RNA helicase activity	0.000047	56,11,4
1ycsb1#412	GO:0005126: hematopoietin/interferon-class (D200-domain) cytokine receptor binding	0.000047	146,20,7
1bmv2_#2064	GO:0004714: transmembrane receptor protein tyrosine kinase activity	0.000047	147,14,6
2napa1#718	GO:0030151: molybdenum ion binding	0.000047	14,15,3
1ie5a_#102	GO:0004896: hematopoietin/interferon-class (D200-domain) cytokine receptor activity	0.000047	62,19,5
1erv__#80	GO:0019955: cytokine binding	0.000047	442,11,8
1ge8a2#134	GO:0005529: sugar binding	0.000047	229,39,12
1e79d2#36	GO:0051082: unfolded protein binding	0.000047	524,34,17
1dfoa_#237	GO:0046983: protein dimerization activity	0.000048	35,17,4
1jk7a_#235	GO:0016616: oxidoreductase activity, acting on the CH-OH group of donors, NAD or NADP as acceptor	0.000048	20,59,5
1bwvs_#38	GO:0030151: molybdenum ion binding	0.000048	40,15,4
1hc7a2#258	GO:0050660: FAD binding	0.000048	226,10,6
1fxla2#169	GO:0003887: DNA-directed DNA polymerase activity	0.000048	203,20,8
1svb_2#165	GO:0051082: unfolded protein binding	0.000048	34,34,5
1fjfc2#153	GO:0016638: oxidoreductase activity, acting on the CH-NH2 group of donors	0.000048	420,17,10
1ej0a_#42	GO:0003964: RNA-directed DNA polymerase activity	0.000048	116,11,5
1a65a2#187	GO:0003968: RNA-directed RNA polymerase activity	0.000048	43,14,4
1e6pa2#46	GO:0016854: racemase and epimerase activity	0.000048	95,13,5
1qqka_#19	GO:0008201: heparin binding	0.000049	9,24,3
1nls__#128	GO:0030145: manganese ion binding	0.000049	6,38,3
1iica1#172	GO:0004197: cysteine-type endopeptidase activity	0.000049	9,24,3
1wdcb_#141	GO:0005516: calmodulin binding	0.000049	9,24,3
1io1a_#202	GO:0005507: copper ion binding	0.000049	6,38,3
1erja_#559	GO:0005507: copper ion binding	0.000049	6,38,3
2pia_1#99	GO:0016668: oxidoreductase activity, acting on sulfur group of donors, NAD or NADP as acceptor	0.000049	179,12,6
1fo0b_#82	GO:0008201: heparin binding	0.000049	9,24,3
4kbpa2#416	GO:0019829: cation-transporting ATPase activity	0.000049	24,9,3
1icia_#175	GO:0016620: oxidoreductase activity, acting on the aldehyde or oxo group of donors, NAD or NADP as acceptor	0.000049	63,10,4
1bmv2_#2064	GO:0003887: DNA-directed DNA polymerase activity	0.000049	147,20,7
1hq8a_#206	GO:0046983: protein dimerization activity	0.000049	329,17,9
1hq8a_#206	GO:0016638: oxidoreductase activity, acting on the CH-NH2 group of donors	0.000049	329,17,9
1i7qa_#321	GO:0003968: RNA-directed RNA polymerase activity	0.000049	148,14,6
1iow_1#37	GO:0004364: glutathione transferase activity	0.00005	309,11,7
1iow_1#37	GO:0016758: transferase activity, transferring hexosyl groups	0.00005	309,11,7
1e05i_#83	GO:0016251: general RNA polymerase II transcription factor activity	0.00005	88,14,5
1clxa_#120	GO:0016854: racemase and epimerase activity	0.00005	47,13,4
1b8aa2#403	GO:0019955: cytokine binding	0.00005	117,11,5
1lvk_2#652	GO:0003779: actin binding	0.00005	7,32,3
1c7na_#95	GO:0004263: chymotrypsin activity	0.00005	183,41,11
1lml__#535	GO:0005507: copper ion binding	0.00005	50,38,6
1qfma1#139	GO:0003968: RNA-directed RNA polymerase activity	0.00005	318,14,8
1et0a_#118	GO:0016668: oxidoreductase activity, acting on sulfur group of donors, NAD or NADP as acceptor	0.000051	18,12,3
1hxxa_#278	GO:0015078: hydrogen ion transporter activity	0.000051	255,21,9

2viua_#252	GO:0003887: DNA-directed DNA polymerase activity	0.000051	343,20,10
1hlwa_#82	GO:0003887: DNA-directed DNA polymerase activity	0.000051	270,20,9
1glqa2#53	GO:0004190: aspartic-type endopeptidase activity	0.000051	26,23,4
1aw8.1#B59	GO:0004812: tRNA ligase activity	0.000051	23,26,4
1f5aa1#441	GO:0000049: tRNA binding	0.000051	163,13,6
1dih_2#222	GO:0008235: metalloexopeptidase activity	0.000051	352,13,8
1qs0a1#119	GO:0004222: metalloendopeptidase activity	0.000051	454,19,11
1cpy__#93	GO:0005525: GTP binding	0.000052	275,49,15
1qf6a4#318	GO:0000287: magnesium ion binding	0.000052	16,128,6
1jb0d_#26	GO:0003809: thrombin activity	0.000052	229,10,6
1fp2a2#346	GO:0008201: heparin binding	0.000052	25,24,4
1qf6a4#362	GO:0000287: magnesium ion binding	0.000052	16,128,6
1eqga2#69	GO:0016702: oxidoreductase activity, acting on single donors with incorporation of molecular oxygen, incorporation of two atoms of oxygen	0.000053	3,12,2
1iow_1#37	GO:0000287: magnesium ion binding	0.000053	309,128,30
1fzqa_#165	GO:0003682: chromatin binding	0.000053	22,10,3
1cipa1#88	GO:0005525: GTP binding	0.000053	5,49,3
2mpra_#190	GO:0015078: hydrogen ion transporter activity	0.000053	400,21,11
3chbd_#85	GO:0015036: disulfide oxidoreductase activity	0.000053	378,22,11
2mev1_#123	GO:0003968: RNA-directed RNA polymerase activity	0.000053	89,14,5
2naca1#162	GO:0003682: chromatin binding	0.000053	22,10,3
1ovb__#195	GO:0016776: phosphotransferase activity, phosphate group as acceptor	0.000053	89,14,5
1j79a_#187	GO:0016776: phosphotransferase activity, phosphate group as acceptor	0.000053	89,14,5
1exma2#353	GO:0008083: growth factor activity	0.000053	91,42,8
1kit_3#743	GO:0005518: collagen binding	0.000053	250,13,7
1kit_3#743	GO:0016638: oxidoreductase activity, acting on the CH-NH2 group of donors	0.000053	250,17,8
1pysa_#221	GO:0000287: magnesium ion binding	0.000053	90,128,14
1ac6a_#34	GO:0005126: hematopoietin/interferon-class (D200-domain) cytokine receptor binding	0.000053	60,20,5
1dn2a1#261	GO:0046983: protein dimerization activity	0.000053	425,17,10
1kcw_3#440	GO:0004364: glutathione transferase activity	0.000054	20,11,3
1saca_#113	GO:0016638: oxidoreductase activity, acting on the CH-NH2 group of donors	0.000054	36,17,4
1jj2v_#118	GO:0004295: trypsin activity	0.000054	103,48,9
1ak5_1#376	GO:0000287: magnesium ion binding	0.000054	39,128,9
1aym3_#131	GO:0004674: protein serine/threonine kinase activity	0.000054	412,42,17
1h61a_#176	GO:0000287: magnesium ion binding	0.000054	39,128,9
1eqka_#47	GO:0000287: magnesium ion binding	0.000054	126,128,17
1qfxa_#380	GO:0004263: chymotrypsin activity	0.000054	47,41,6
1b4ka_#108	GO:0016831: carboxy-lyase activity	0.000054	116,25,7
1ecpa_#62	GO:0008483: transaminase activity	0.000054	72,17,5
1h6va2#219	GO:0016627: oxidoreductase activity, acting on the CH-CH group of donors	0.000054	36,17,4
1a81a1#51	GO:0019838: growth factor binding	0.000054	73,9,4
1gtra2#113	GO:0005524: ATP binding	0.000054	22,243,9
2bb2_2#134	GO:0004896: hematopoietin/interferon-class (D200-domain) cytokine receptor activity	0.000055	220,19,8
1g5ca_#164	GO:0016836: hydro-lyase activity	0.000055	7,33,3
1rpxa_#216	GO:0016831: carboxy-lyase activity	0.000055	9,25,3
1eaja_#59	GO:0005524: ATP binding	0.000055	202,243,35
1hp5a1#340	GO:0016831: carboxy-lyase activity	0.000055	9,25,3
1g0sa_#74	GO:0004725: protein tyrosine phosphatase activity	0.000055	294,15,8
1bev1_#86	GO:0005507: copper ion binding	0.000055	323,38,14
1pwt__#8	GO:0019955: cytokine binding	0.000055	314,11,7
1ijqa1#499	GO:0005509: calcium ion binding	0.000055	5,160,4
1ecpa_#58	GO:0004180: carboxypeptidase activity	0.000055	83,15,5
1amp__#205	GO:0000287: magnesium ion binding	0.000055	79,128,13
1qfma1#139	GO:0051082: unfolded protein binding	0.000055	318,34,13
1i76a_#138	GO:0016763: transferase activity, transferring pentosyl groups	0.000055	103,28,7
1qg8a_#90	GO:0016638: oxidoreductase activity, acting on the CH-NH2 group of donors	0.000056	13,17,3
1imt_2#41	GO:0000049: tRNA binding	0.000056	17,13,3
1pjr_1#87	GO:0008026: ATP-dependent helicase activity	0.000056	17,13,3
1htjf_#368	GO:0005085: guanyl-nucleotide exchange factor activity	0.000056	17,13,3
1hnja1#133	GO:0016651: oxidoreductase activity, acting on NADH or NADPH	0.000056	17,13,3
1c9la2#12	GO:0030151: molybdenum ion binding	0.000056	295,15,8
1qs1a2#437	GO:0019955: cytokine binding	0.000056	452,11,8
1eno__#264	GO:0016627: oxidoreductase activity, acting on the CH-CH group of donors	0.000056	13,17,3
1a53__#236	GO:0016861: intramolecular oxidoreductase activity, interconverting aldoses and ketoses	0.000056	17,13,3
1fsu__#434	GO:0005518: collagen binding	0.000056	356,13,8
1j9qa1#154	GO:0015078: hydrogen ion transporter activity	0.000056	196,21,8

1ge8a2#134	GO:0015082: di-, tri-valent inorganic cation transporter activity	0.000056	229,14,7
1ge8a2#134	GO:0046915: transition metal ion transporter activity	0.000056	229,14,7
1fhoa_#74	GO:0003887: DNA-directed DNA polymerase activity	0.000056	347,20,10
1keva2#236	GO:0019201: nucleotide kinase activity	0.000056	17,13,3
1ax4a_#275	GO:0005507: copper ion binding	0.000056	102,38,8
1qj4a_#41	GO:0004601: peroxidase activity	0.000057	2,21,2
1b6g__#56	GO:0004601: peroxidase activity	0.000057	2,21,2
1bn7a_#38	GO:0004601: peroxidase activity	0.000057	2,21,2
1qrea_#132	GO:0008270: zinc ion binding	0.000057	12,108,5
1bn7a_#253	GO:0004601: peroxidase activity	0.000057	2,21,2
1br9__#168	GO:0015078: hydrogen ion transporter activity	0.000057	2,21,2
1im4a_#41	GO:0004295: trypsin activity	0.000057	59,48,7
1ffta1#441	GO:0015078: hydrogen ion transporter activity	0.000057	2,21,2
1qj4a_#18	GO:0004601: peroxidase activity	0.000057	2,21,2
1qhba_#323	GO:0004601: peroxidase activity	0.000057	2,21,2
1pmaa_#131	GO:0004601: peroxidase activity	0.000057	2,21,2
1qqsa_#124	GO:0019843: rRNA binding	0.000057	458,22,12
1a0i_2#222	GO:0004295: trypsin activity	0.000057	186,48,12
1ehya_#41	GO:0004601: peroxidase activity	0.000057	2,21,2
1ihua2#343	GO:0016620: oxidoreductase activity, acting on the aldehyde or oxo group of donors, NAD or NADP as acceptor	0.000057	233,10,6
1mdah_#329	GO:0016638: oxidoreductase activity, acting on the CH-NH2 group of donors	0.000057	335,17,9
1qora2#233	GO:0016616: oxidoreductase activity, acting on the CH-OH group of donors, NAD or NADP as acceptor	0.000058	11,59,4
2trcp_#201	GO:0004523: ribonuclease H activity	0.000058	14,16,3
1im4a_#41	GO:0019955: cytokine binding	0.000058	59,11,4
1f0ya2#126	GO:0016616: oxidoreductase activity, acting on the CH-OH group of donors, NAD or NADP as acceptor	0.000058	11,59,4
1lkka_#127	GO:0004714: transmembrane receptor protein tyrosine kinase activity	0.000058	16,14,3
1h4ua1#486	GO:0016251: general RNA polymerase II transcription factor activity	0.000058	45,14,4
1h4ua1#486	GO:0003968: RNA-directed RNA polymerase activity	0.000058	45,14,4
1miob_#338	GO:0016616: oxidoreductase activity, acting on the CH-OH group of donors, NAD or NADP as acceptor	0.000058	84,59,9
1hxxa_#107	GO:0016251: general RNA polymerase II transcription factor activity	0.000058	16,14,3
1ddja_#681	GO:0005518: collagen binding	0.000058	253,13,7
1qksa2#236	GO:0005509: calcium ion binding	0.000058	64,160,13
1ejda_#6	GO:0019838: growth factor binding	0.000058	270,9,6
1qq5a_#7	GO:0019201: nucleotide kinase activity	0.000058	167,13,6
1g8kb_#11	GO:0019955: cytokine binding	0.000058	59,11,4
1qs0a1#119	GO:0016758: transferase activity, transferring hexosyl groups	0.000058	454,11,8
1dyna_#19	GO:0003697: single-stranded DNA binding	0.000059	109,12,5
1a65a3#409	GO:0008235: metalloexopeptidase activity	0.000059	49,13,4
1f97a1#62	GO:0008083: growth factor activity	0.000059	707,42,23
1f4la2#20	GO:0005525: GTP binding	0.000059	13,49,4
1fw8a_#298	GO:0005525: GTP binding	0.000059	13,49,4
1hbza_#214	GO:0030151: molybdenum ion binding	0.000059	297,15,8
1f8ra2#325	GO:0004896: hematopoietin/interferon-class (D200-domain) cytokine receptor activity	0.000059	222,19,8
1e87a_#174	GO:0030151: molybdenum ion binding	0.000059	141,15,6
1phm_2#253	GO:0005509: calcium ion binding	0.000059	309,160,35
1as4.1#A288	GO:0003809: thrombin activity	0.000059	66,10,4
1hrna_#182	GO:0003887: DNA-directed DNA polymerase activity	0.000059	31,20,4
1qj5a_#268	GO:0016846: carbon-sulfur lyase activity	0.000059	66,10,4
1nat__#50	GO:0004725: protein tyrosine phosphatase activity	0.000059	297,15,8
1qupa2#50	GO:0051082: unfolded protein binding	0.00006	7,34,3
1am2__#163	GO:0005126: hematopoietin/interferon-class (D200-domain) cytokine receptor binding	0.00006	102,20,6
1d0na3#305	GO:0015036: disulfide oxidoreductase activity	0.00006	311,22,10
1czan1#92	GO:0003729: mRNA binding	0.00006	631,13,10
1b3ra2#47	GO:0016627: oxidoreductase activity, acting on the CH-CH group of donors	0.00006	37,17,4
1c8za_#386	GO:0050660: FAD binding	0.00006	235,10,6
1ejda_#213	GO:0005509: calcium ion binding	0.00006	284,160,33
1qfma1#139	GO:0019955: cytokine binding	0.00006	318,11,7
1qfma1#139	GO:0003724: RNA helicase activity	0.00006	318,11,7
1tpg_1#55	GO:0004263: chymotrypsin activity	0.000061	6,41,3
1pyia1#44	GO:0008270: zinc ion binding	0.000061	3,108,3
2cmd_1#113	GO:0004222: metalloendopeptidase activity	0.000061	12,19,3
1rmg__#128	GO:0008270: zinc ion binding	0.000061	3,108,3

1hxn__#236	GO:0008270: zinc ion binding	0.000061	3,108,3
1zbdb_#109	GO:0008270: zinc ion binding	0.000061	3,108,3
1lucb_#84	GO:0009036: type II site-specific deoxyribonuclease activity	0.000061	19,12,3
1heta1#167	GO:0008270: zinc ion binding	0.000061	3,108,3
1fid__#237	GO:0008757: S-adenosylmethionine-dependent methyltransferase activity	0.000061	51,24,5
1e3ja1#319	GO:0008270: zinc ion binding	0.000061	3,108,3
1qp8a1#153	GO:0004457: lactate dehydrogenase activity	0.000061	23,10,3
1bywa_#30	GO:0004674: protein serine/threonine kinase activity	0.000061	331,42,15
1lucb_#70	GO:0008810: cellulase activity	0.000061	35,18,4
1kit_3#297	GO:0004222: metalloendopeptidase activity	0.000061	12,19,3
1fkna_#18	GO:0005529: sugar binding	0.000061	130,39,9
2ltn.1#A63	GO:0003887: DNA-directed DNA polymerase activity	0.000061	152,20,7
1nhp_2#181	GO:0015036: disulfide oxidoreductase activity	0.000062	2,22,2
1gln_2#10	GO:0005524: ATP binding	0.000062	4,243,4
1ia8a_#89	GO:0005524: ATP binding	0.000062	4,243,4
1ab4__#344	GO:0005524: ATP binding	0.000062	4,243,4
1ir3a_#1003	GO:0005524: ATP binding	0.000062	4,243,4
1wdcb_#141	GO:0016705: oxidoreductase activity, acting on paired donors, with incorporation or reduction of molecular oxygen	0.000062	9,26,3
1ile_1#695	GO:0005524: ATP binding	0.000062	4,243,4
3tmka_#98	GO:0005524: ATP binding	0.000062	4,243,4
1qrjb1#133	GO:0016705: oxidoreductase activity, acting on paired donors, with incorporation or reduction of molecular oxygen	0.000062	9,26,3
1qrjb1#133	GO:0004497: monooxygenase activity	0.000062	9,26,3
1j9la_#82	GO:0019201: nucleotide kinase activity	0.000062	169,13,6
1f2la_#40	GO:0003809: thrombin activity	0.000062	236,10,6
1hp5a1#340	GO:0004497: monooxygenase activity	0.000062	9,26,3
1f9za_#16	GO:0000287: magnesium ion binding	0.000062	140,128,18
1gpea1#273	GO:0004295: trypsin activity	0.000062	81,48,8
1qfea_#68	GO:0016646: oxidoreductase activity, acting on the CH-NH group of donors, NAD or NADP as acceptor	0.000062	560,19,12
1apy.1#B284	GO:0005507: copper ion binding	0.000062	52,38,6
1cpza_#6	GO:0019838: growth factor binding	0.000062	157,9,5
1d3ba_#16	GO:0003729: mRNA binding	0.000063	3,13,2
1gof_3#436	GO:0005085: guanyl-nucleotide exchange factor activity	0.000063	3,13,2
1qtwa_#213	GO:0016861: intramolecular oxidoreductase activity, interconverting aldoses and ketoses	0.000063	3,13,2
1dyna_#19	GO:0004896: hematopoietin/interferon-class (D200-domain) cytokine receptor activity	0.000063	109,19,6
1heta2#212	GO:0000287: magnesium ion binding	0.000063	49,128,10
1pbv__#140	GO:0004601: peroxidase activity	0.000063	11,21,3
1ie5a_#23	GO:0019955: cytokine binding	0.000063	21,11,3
1dm9a_#57	GO:0004842: ubiquitin-protein ligase activity	0.000063	109,19,6
1bjt__#890	GO:0008083: growth factor activity	0.000063	292,42,14
1qqqa_#130	GO:0016651: oxidoreductase activity, acting on NADH or NADPH	0.000063	3,13,2
1mjha_#119	GO:0000287: magnesium ion binding	0.000063	103,128,15
1ie5a_#102	GO:0005524: ATP binding	0.000063	62,243,16
2reb_1#138	GO:0004457: lactate dehydrogenase activity	0.000063	67,10,4
2reb_1#138	GO:0016620: oxidoreductase activity, acting on the aldehyde or oxo group of donors, NAD or NADP as acceptor	0.000063	67,10,4
1f5aa1#441	GO:0005524: ATP binding	0.000063	163,243,30
1hq0a_#867	GO:0005524: ATP binding	0.000063	171,243,31
1ido__#173	GO:0005524: ATP binding	0.000063	110,243,23
1ft9a1#195	GO:0003700: transcription factor activity	0.000064	17,124,6
1thfd_#140	GO:0016638: oxidoreductase activity, acting on the CH-NH2 group of donors	0.000064	124,17,6
1e19a_#281	GO:0016814: hydrolase activity, acting on carbon-nitrogen (but not peptide) bonds, in cyclic amidines	0.000064	138,10,5
1seta2#231	GO:0003887: DNA-directed DNA polymerase activity	0.000064	103,20,6
1f97a2#199	GO:0005509: calcium ion binding	0.000064	144,160,21
1qfma1#139	GO:0005529: sugar binding	0.000064	318,39,14
1iira_#242	GO:0008026: ATP-dependent helicase activity	0.000064	170,13,6
1iira_#242	GO:0016651: oxidoreductase activity, acting on NADH or NADPH	0.000064	170,13,6
1evqa_#305	GO:0008757: S-adenosylmethionine-dependent methyltransferase activity	0.000064	490,24,13
1byka_#279	GO:0005524: ATP binding	0.000065	82,243,19
1bfd_2#115	GO:0000287: magnesium ion binding	0.000065	6,128,4
1evqa_#61	GO:0004295: trypsin activity	0.000065	159,48,11
1f2la_#40	GO:0004190: aspartic-type endopeptidase activity	0.000065	236,23,9
1flga_#161	GO:0051082: unfolded protein binding	0.000065	152,34,9

1fn9a_#271	GO:0008083: growth factor activity	0.000065	218,42,12
1db3a_#266	GO:0000287: magnesium ion binding	0.000065	59,128,11
1seta2#231	GO:0005524: ATP binding	0.000065	103,243,22
1jsg__#92	GO:0008083: growth factor activity	0.000065	607,42,21
1cnv__#264	GO:0005524: ATP binding	0.000065	103,243,22
1elja_#64	GO:0005524: ATP binding	0.000065	103,243,22
1bwda_#295	GO:0005507: copper ion binding	0.000065	104,38,8
1e3a.1#A30	GO:0004812: tRNA ligase activity	0.000065	157,26,8
1hlwa_#91	GO:0004263: chymotrypsin activity	0.000065	30,41,5
1fmk_3#283	GO:0004674: protein serine/threonine kinase activity	0.000066	6,42,3
2bc2a_#46	GO:0016651: oxidoreductase activity, acting on NADH or NADPH	0.000066	18,13,3
3grs_2#189	GO:0016651: oxidoreductase activity, acting on NADH or NADPH	0.000066	18,13,3
3lada3#463	GO:0019843: rRNA binding	0.000066	29,22,4
1hava_#157	GO:0003729: mRNA binding	0.000066	18,13,3
1a3qa2#190	GO:0004556: alpha-amylase activity	0.000066	144,15,6
1feca1#31	GO:0016638: oxidoreductase activity, acting on the CH-NH2 group of donors	0.000066	75,17,5
1dr9a1#39	GO:0016638: oxidoreductase activity, acting on the CH-NH2 group of donors	0.000066	258,17,8
1evqa_#305	GO:0016854: racemase and epimerase activity	0.000066	490,13,9
1dl0a_#32	GO:0008200: ion channel inhibitor activity	0.000067	38,17,4
1ihka_#161	GO:0005507: copper ion binding	0.000067	17,38,4
1aoza3#426	GO:0005507: copper ion binding	0.000067	17,38,4
1qmua1#309	GO:0005507: copper ion binding	0.000067	17,38,4
1gdha2#47	GO:0003924: GTPase activity	0.000067	38,17,4
1fh6a_#277	GO:0005507: copper ion binding	0.000067	17,38,4
1fjja_#5	GO:0005507: copper ion binding	0.000067	17,38,4
1fa0a1#405	GO:0016638: oxidoreductase activity, acting on the CH-NH2 group of donors	0.000067	38,17,4
1psda3#344	GO:0016638: oxidoreductase activity, acting on the CH-NH2 group of donors	0.000067	125,17,6
1apme_#74	GO:0003724: RNA helicase activity	0.000067	124,11,5
1iira_#326	GO:0016668: oxidoreductase activity, acting on sulfur group of donors, NAD or NADP as acceptor	0.000067	112,12,5
1af7_2#248	GO:0016616: oxidoreductase activity, acting on the CH-OH group of donors, NAD or NADP as acceptor	0.000067	34,59,6
1icia_#9	GO:0003924: GTPase activity	0.000067	38,17,4
1bev1_#86	GO:0019955: cytokine binding	0.000067	323,11,7
1hbza_#96	GO:0050660: FAD binding	0.000067	370,10,7
1i7qa_#321	GO:0005524: ATP binding	0.000067	148,243,28
1eh9a3#409	GO:0005509: calcium ion binding	0.000068	9,160,5
1qs1a1#140	GO:0004190: aspartic-type endopeptidase activity	0.000068	2,23,2
1bqya_#178	GO:0005509: calcium ion binding	0.000068	9,160,5
1mml__#97	GO:0004190: aspartic-type endopeptidase activity	0.000068	2,23,2
1pfza_#230	GO:0004190: aspartic-type endopeptidase activity	0.000068	2,23,2
1i50b_#216	GO:0004812: tRNA ligase activity	0.000068	48,26,5
1c9oa_#49	GO:0000049: tRNA binding	0.000068	102,13,5
1c4zd_#51	GO:0019838: growth factor binding	0.000068	160,9,5
1rypb_#114	GO:0004812: tRNA ligase activity	0.000068	158,26,8
1cpza_#6	GO:0015082: di-, tri-valent inorganic cation transporter activity	0.000068	157,14,6
1cpza_#6	GO:0046915: transition metal ion transporter activity	0.000068	157,14,6
1aym1_#151	GO:0004197: cysteine-type endopeptidase activity	0.000069	10,24,3
1ilr1_#145	GO:0008201: heparin binding	0.000069	10,24,3
1tkia_#98	GO:0005516: calmodulin binding	0.000069	10,24,3
1j71a_#176	GO:0004896: hematopoietin/interferon-class (D200-domain) cytokine receptor activity	0.000069	34,19,4
1ef1a3#45	GO:0004867: serine-type endopeptidase inhibitor activity	0.000069	14,47,4
1a06__#85	GO:0005524: ATP binding	0.000069	44,243,13
1gdea_#195	GO:0004457: lactate dehydrogenase activity	0.000069	24,10,3
2viua_#252	GO:0046983: protein dimerization activity	0.000069	343,17,9
1fhga_#59	GO:0004896: hematopoietin/interferon-class (D200-domain) cytokine receptor activity	0.000069	34,19,4
1vcbb_#19	GO:0005261: cation channel activity	0.000069	24,10,3
1mrp__#170	GO:0004457: lactate dehydrogenase activity	0.000069	24,10,3
1hvba_#247	GO:0008800: beta-lactamase activity	0.000069	24,10,3
1fp1d2#214	GO:0008757: S-adenosylmethionine-dependent methyltransferase activity	0.000069	173,24,8
1gox__#124	GO:0016854: racemase and epimerase activity	0.000069	366,13,8
1bjaa_#26	GO:0003916: DNA topoisomerase activity	0.000069	24,10,3
1imva_#69	GO:0004867: serine-type endopeptidase inhibitor activity	0.000069	84,47,8
1hdoa_#189	GO:0016646: oxidoreductase activity, acting on the CH-NH group of donors, NAD or NADP as acceptor	0.000069	164,19,7
1dypa_#143	GO:0015036: disulfide oxidoreductase activity	0.000069	94,22,6

1ddwa_#77	GO:0005529: sugar binding	0.000069	364,39,15
1cyda_#231	GO:0016627: oxidoreductase activity, acting on the CH-CH group of donors	0.00007	14,17,3
1in4a1#264	GO:0005524: ATP binding	0.00007	50,243,14
1as4.1#A336	GO:0004867: serine-type endopeptidase inhibitor activity	0.00007	332,47,16
1by5a_#347	GO:0005524: ATP binding	0.00007	263,243,42
1qqsa_#124	GO:0005529: sugar binding	0.00007	458,39,17
1exba_#188	GO:0016638: oxidoreductase activity, acting on the CH-NH2 group of donors	0.00007	14,17,3
1ifc__#89	GO:0004263: chymotrypsin activity	0.00007	190,41,11
1hxxa_#173	GO:0046983: protein dimerization activity	0.00007	260,17,8
1e8ga2#201	GO:0030151: molybdenum ion binding	0.00007	304,15,8
1a3qa2#133	GO:0019838: growth factor binding	0.00007	27,9,3
1f7ua2#324	GO:0016702: oxidoreductase activity, acting on single donors with incorporation of molecular oxygen, incorporation of two atoms of oxygen	0.00007	56,12,4
1ej0a_#42	GO:0000287: magnesium ion binding	0.00007	116,128,16
1hxn__#248	GO:0003968: RNA-directed RNA polymerase activity	0.00007	17,14,3
1dp0a1#309	GO:0005507: copper ion binding	0.00007	53,38,6
1keva2#236	GO:0016776: phosphotransferase activity, phosphate group as acceptor	0.00007	17,14,3
1h6va1#27	GO:0016627: oxidoreductase activity, acting on the CH-CH group of donors	0.00007	260,17,8
1h6va1#27	GO:0016638: oxidoreductase activity, acting on the CH-NH2 group of donors	0.00007	260,17,8
1h6va1#27	GO:0008483: transaminase activity	0.00007	260,17,8
2bb2_2#134	GO:0004674: protein serine/threonine kinase activity	0.000071	220,42,12
1tmy__#14	GO:0005524: ATP binding	0.000071	18,243,8
1e87a_#174	GO:0050660: FAD binding	0.000071	141,10,5
1hw5a2#64	GO:0005509: calcium ion binding	0.000071	167,160,23
1jj2e1#42	GO:0005509: calcium ion binding	0.000071	167,160,23
1kwaa_#513	GO:0003724: RNA helicase activity	0.000071	62,11,4
1eqka_#47	GO:0046983: protein dimerization activity	0.000071	126,17,6
1tuba2#259	GO:0003887: DNA-directed DNA polymerase activity	0.000071	12,20,3
1aqua_#79	GO:0004180: carboxypeptidase activity	0.000071	44,15,4
1e25a_#262	GO:0004812: tRNA ligase activity	0.000071	116,26,7
1qtn.1#A270	GO:0008483: transaminase activity	0.000071	439,17,10
1cfe__#7	GO:0000287: magnesium ion binding	0.000071	70,128,12
1hdoa_#189	GO:0005524: ATP binding	0.000071	164,243,30
1ibja_#414	GO:0016846: carbon-sulfur lyase activity	0.000072	4,10,2
1bmv2_#2059	GO:0015036: disulfide oxidoreductase activity	0.000072	11,22,3
1ir3a_#1003	GO:0005066: transmembrane receptor protein tyrosine kinase signaling protein activity	0.000072	4,10,2
1bs0a_#252	GO:0016846: carbon-sulfur lyase activity	0.000072	4,10,2
1dik_1#848	GO:0000287: magnesium ion binding	0.000072	81,128,13
1egua1#309	GO:0003682: chromatin binding	0.000072	4,10,2
1eg5a_#150	GO:0016846: carbon-sulfur lyase activity	0.000072	4,10,2
1smaa1#95	GO:0005524: ATP binding	0.000072	272,243,43
1g61a_#2115	GO:0003682: chromatin binding	0.000072	4,10,2
1dlfh_#4	GO:0019955: cytokine binding	0.000072	22,11,3
1cqka_#52	GO:0008083: growth factor activity	0.000072	70,42,7
1gcya2#242	GO:0004556: alpha-amylase activity	0.000072	16,15,3
1fc6a2#454	GO:0004867: serine-type endopeptidase inhibitor activity	0.000072	43,47,6
1d6ja_#95	GO:0016616: oxidoreductase activity, acting on the CH-OH group of donors, NAD or NADP as acceptor	0.000072	67,59,8
1bqca_#24	GO:0016763: transferase activity, transferring pentosyl groups	0.000072	45,28,5
1alo_4#634	GO:0003887: DNA-directed DNA polymerase activity	0.000072	64,20,5
1ioaa_#75	GO:0005507: copper ion binding	0.000072	33,38,5
1fnf_1#1146	GO:0005179: hormone activity	0.000072	16,15,3
1c8na_#165	GO:0003968: RNA-directed RNA polymerase activity	0.000073	3,14,2
1e5sa_#239	GO:0004674: protein serine/threonine kinase activity	0.000073	30,42,5
1ayaa_#98	GO:0005524: ATP binding	0.000073	10,243,6
1hdma1#123	GO:0008083: growth factor activity	0.000073	30,42,5
1f3mc_#369	GO:0015078: hydrogen ion transporter activity	0.000073	100,21,6
2cb5a_#297	GO:0004197: cysteine-type endopeptidase activity	0.000074	2,24,2
2foka3#314	GO:0005524: ATP binding	0.000074	33,243,11
1cxya_#78	GO:0005516: calmodulin binding	0.000074	2,24,2
19hca_#50	GO:0005516: calmodulin binding	0.000074	2,24,2
1dfca1#1135	GO:0008201: heparin binding	0.000074	53,24,5
1exh__#44	GO:0005518: collagen binding	0.000074	52,13,4
1fi2a_#109	GO:0008201: heparin binding	0.000074	228,24,9
1aym3_#131	GO:0005529: sugar binding	0.000074	412,39,16
1huxa_#10	GO:0051082: unfolded protein binding	0.000074	60,34,6

1mdah_#329	GO:0003968: RNA-directed RNA polymerase activity	0.000074	335,14,8
1i9ga_#97	GO:0008270: zinc ion binding	0.000075	7,108,4
1e8ua_#296	GO:0016638: oxidoreductase activity, acting on the CH-NH2 group of donors	0.000075	39,17,4
2trcp_#201	GO:0004295: trypsin activity	0.000075	14,48,4
1t7pa2#513	GO:0003887: DNA-directed DNA polymerase activity	0.000075	33,20,4
2sqca2#97	GO:0004364: glutathione transferase activity	0.000075	127,11,5
1dssg1#4	GO:0016668: oxidoreductase activity, acting on sulfur group of donors, NAD or NADP as acceptor	0.000075	57,12,4
1ak5_1#376	GO:0016627: oxidoreductase activity, acting on the CH-CH group of donors	0.000075	39,17,4
1alo_4#481	GO:0016620: oxidoreductase activity, acting on the aldehyde or oxo group of donors, NAD or NADP as acceptor	0.000075	70,10,4
1ja9a_#202	GO:0016627: oxidoreductase activity, acting on the CH-CH group of donors	0.000075	39,17,4
1eqka_#47	GO:0005524: ATP binding	0.000075	126,243,25
1jkma_#317	GO:0005524: ATP binding	0.000075	76,243,18
1xgsa2#82	GO:0004556: alpha-amylase activity	0.000075	307,15,8
1f5aa1#441	GO:0019838: growth factor binding	0.000075	163,9,5
1elua_#95	GO:0016799: hydrolase activity, hydrolyzing N-glycosyl compounds	0.000075	77,17,5
1qfea_#68	GO:0016866: intramolecular transferase activity	0.000075	560,12,9
1h6va1#23	GO:0016627: oxidoreductase activity, acting on the CH-CH group of donors	0.000075	39,17,4
1arb__#55	GO:0003968: RNA-directed RNA polymerase activity	0.000075	48,14,4
1ef1a3#45	GO:0005509: calcium ion binding	0.000076	14,160,6
1f2na_#138	GO:0003968: RNA-directed RNA polymerase activity	0.000076	96,14,5
1hbza_#214	GO:0008083: growth factor activity	0.000076	297,42,14
1jb0d_#26	GO:0008201: heparin binding	0.000076	229,24,9
1a4ya_#47	GO:0016758: transferase activity, transferring hexosyl groups	0.000076	216,11,6
1cmxa_#207	GO:0016251: general RNA polymerase II transcription factor activity	0.000076	96,14,5
1nfdb1#21	GO:0004714: transmembrane receptor protein tyrosine kinase activity	0.000076	96,14,5
1nfdb1#21	GO:0003968: RNA-directed RNA polymerase activity	0.000076	96,14,5
2msba_#218	GO:0004812: tRNA ligase activity	0.000076	49,26,5
1c7na_#231	GO:0008483: transaminase activity	0.000076	263,17,8
1ekra_#114	GO:0000287: magnesium ion binding	0.000076	60,128,11
1fhoa_#74	GO:0046983: protein dimerization activity	0.000076	347,17,9
1nsca_#236	GO:0050660: FAD binding	0.000076	143,10,5
1qhda2#323	GO:0015082: di-, tri-valent inorganic cation transporter activity	0.000077	337,14,8
1qhda2#323	GO:0046915: transition metal ion transporter activity	0.000077	337,14,8
1gtra1#468	GO:0005507: copper ion binding	0.000077	54,38,6
1bev1_#86	GO:0005529: sugar binding	0.000077	323,39,14
1qq5a_#7	GO:0016646: oxidoreductase activity, acting on the CH-NH group of donors, NAD or NADP as acceptor	0.000077	167,19,7
1bio__#210	GO:0005524: ATP binding	0.000077	149,243,28
1h72c2#269	GO:0005524: ATP binding	0.000078	83,243,19
1wdna_#152	GO:0016763: transferase activity, transferring pentosyl groups	0.000078	9,28,3
1qr4a2#166	GO:0008083: growth factor activity	0.000078	16,42,4
1e8ga2#201	GO:0004190: aspartic-type endopeptidase activity	0.000078	304,23,10
1f00i2#805	GO:0005509: calcium ion binding	0.000078	135,160,20
1f97a2#199	GO:0003809: thrombin activity	0.000078	144,10,5
1cs6a4#363	GO:0008083: growth factor activity	0.000078	16,42,4
1cwva2#675	GO:0019838: growth factor binding	0.000078	28,9,3
3nul__#107	GO:0016646: oxidoreductase activity, acting on the CH-NH group of donors, NAD or NADP as acceptor	0.000079	13,19,3
1g97a1#376	GO:0004222: metalloendopeptidase activity	0.000079	13,19,3
1hg3a_#207	GO:0016861: intramolecular oxidoreductase activity, interconverting aldoses and ketoses	0.000079	19,13,3
1qq9a_#178	GO:0004177: aminopeptidase activity	0.000079	19,13,3
1nula_#15	GO:0016616: oxidoreductase activity, acting on the CH-OH group of donors, NAD or NADP as acceptor	0.000079	35,59,6
1i5na_#30	GO:0003779: actin binding	0.000079	8,32,3
1ppn__#191	GO:0000049: tRNA binding	0.000079	19,13,3
1f39a_#190	GO:0008009: chemokine activity	0.000079	25,10,3
3rpba_#644	GO:0005518: collagen binding	0.000079	19,13,3
1fp2a2#346	GO:0005066: transmembrane receptor protein tyrosine kinase signaling protein activity	0.000079	25,10,3
1bf4a_#4	GO:0005066: transmembrane receptor protein tyrosine kinase signaling protein activity	0.000079	25,10,3
1hq8a_#206	GO:0051082: unfolded protein binding	0.000079	329,34,13
1gen__#477	GO:0004222: metalloendopeptidase activity	0.000079	13,19,3
1jjya_#329	GO:0016620: oxidoreductase activity, acting on the aldehyde or oxo group of donors, NAD or NADP as acceptor	0.000079	71,10,4

1ihua2#343	GO:0016616: oxidoreductase activity, acting on the CH-OH group of donors, NAD or NADP as acceptor	0.000079	233,59,15
1nsca_#91	GO:0005518: collagen binding	0.000079	19,13,3
1pjca1#254	GO:0016616: oxidoreductase activity, acting on the CH-OH group of donors, NAD or NADP as acceptor	0.00008	22,59,5
1dfca1#1135	GO:0005518: collagen binding	0.00008	53,13,4
1quqb_#87	GO:0016638: oxidoreductase activity, acting on the CH-NH2 group of donors	0.00008	78,17,5
1f24a_#293	GO:0003697: single-stranded DNA binding	0.00008	116,12,5
1evqa_#61	GO:0004263: chymotrypsin activity	0.00008	159,41,10
1as4.1#A336	GO:0019955: cytokine binding	0.00008	332,11,7
1c9la2#12	GO:0003697: single-stranded DNA binding	0.00008	295,12,7
1c9la2#12	GO:0016668: oxidoreductase activity, acting on sulfur group of donors, NAD or NADP as acceptor	0.00008	295,12,7
1i5ga_#115	GO:0005529: sugar binding	0.00008	324,39,14
1avgi_#69	GO:0015078: hydrogen ion transporter activity	0.00008	340,21,10
1ed5a_#294	GO:0000049: tRNA binding	0.00008	53,13,4
1xnb__#149	GO:0005524: ATP binding	0.00008	165,243,30
1ig8a_#215	GO:0019843: rRNA binding	0.00008	59,22,5
1zpda1#322	GO:0016831: carboxy-lyase activity	0.000081	2,25,2
1jeya_#104	GO:0016763: transferase activity, transferring pentosyl groups	0.000081	46,28,5
1im4a_#144	GO:0016638: oxidoreductase activity, acting on the CH-NH2 group of donors	0.000081	129,17,6
3prn__#145	GO:0005529: sugar binding	0.000081	168,39,10
1pvc1_#106	GO:0019955: cytokine binding	0.000081	64,11,4
1pvc1_#106	GO:0003724: RNA helicase activity	0.000081	64,11,4
1efpa1#32	GO:0008483: transaminase activity	0.000081	129,17,6
1ekma1#545	GO:0004556: alpha-amylase activity	0.000081	90,15,5
1gox__#246	GO:0016861: intramolecular oxidoreductase activity, interconverting aldoses and ketoses	0.000081	177,13,6
1tyfa_#31	GO:0016776: phosphotransferase activity, phosphate group as acceptor	0.000081	162,14,6
1qfea_#68	GO:0015036: disulfide oxidoreductase activity	0.000081	560,22,13
1hxmb1#117	GO:0003724: RNA helicase activity	0.000081	64,11,4
1arb__#208	GO:0003968: RNA-directed RNA polymerase activity	0.000081	49,14,4
1qdea_#218	GO:0005524: ATP binding	0.000082	23,243,9
1dl5a_#283	GO:0005524: ATP binding	0.000082	28,243,10
1h61a_#176	GO:0016836: hydro-lyase activity	0.000082	39,33,5
1fc4a_#205	GO:0019201: nucleotide kinase activity	0.000082	267,13,7
1cbf__#208	GO:0019955: cytokine binding	0.000082	333,11,7
1hdfa_#16	GO:0004556: alpha-amylase activity	0.000082	223,15,7
1a65a2#247	GO:0015078: hydrogen ion transporter activity	0.000082	32,21,4
1gpea1#273	GO:0019838: growth factor binding	0.000082	81,9,4
1d2ka1#164	GO:0016836: hydro-lyase activity	0.000082	63,33,6
3mbp__#96	GO:0005351: sugar porter activity	0.000083	12,21,3
1cpt__#401	GO:0010181: FMN binding	0.000083	23,11,3
1i8na_#74	GO:0008270: zinc ion binding	0.000083	20,108,6
1jdra_#53	GO:0004601: peroxidase activity	0.000083	12,21,3
1in4a1#264	GO:0004812: tRNA ligase activity	0.000083	50,26,5
1e79h2#77	GO:0015405: P-P-bond-hydrolysis-driven transporter activity	0.000083	21,12,3
1byka_#157	GO:0005351: sugar porter activity	0.000083	12,21,3
1ecpa_#156	GO:0016668: oxidoreductase activity, acting on sulfur group of donors, NAD or NADP as acceptor	0.000083	21,12,3
1htr.1#B39	GO:0016702: oxidoreductase activity, acting on single donors with incorporation of molecular oxygen, incorporation of two atoms of oxygen	0.000083	21,12,3
2pia_3#282	GO:0016638: oxidoreductase activity, acting on the CH-NH2 group of donors	0.000083	40,17,4
1dhs__#99	GO:0005525: GTP binding	0.000083	132,49,10
1fgua1#249	GO:0003697: single-stranded DNA binding	0.000083	21,12,3
1bxoa_#320	GO:0005126: hematopoietin/interferon-class (D200-domain) cytokine receptor binding	0.000084	66,20,5
1qupa2#50	GO:0005507: copper ion binding	0.000084	7,38,3
1je5a_#39	GO:0003968: RNA-directed RNA polymerase activity	0.000084	18,14,3
2occa1#372	GO:0005507: copper ion binding	0.000084	7,38,3
1bkds_#882	GO:0008810: cellulase activity	0.000084	14,18,3
1e30a_#112	GO:0005509: calcium ion binding	0.000084	49,160,11
1hava_#157	GO:0003968: RNA-directed RNA polymerase activity	0.000084	18,14,3
1i5ga_#115	GO:0004812: tRNA ligase activity	0.000084	324,26,11
1hw5a2#64	GO:0019838: growth factor binding	0.000084	167,9,5
1avgi_#69	GO:0004674: protein serine/threonine kinase activity	0.000084	340,42,15
1ecpa_#62	GO:0016620: oxidoreductase activity, acting on the aldehyde or oxo group of donors, NAD or NADP as acceptor	0.000084	72,10,4

1rypc_#66	GO:0005507: copper ion binding	0.000084	7,38,3
1fjfc2#153	GO:0016668: oxidoreductase activity, acting on sulfur group of donors, NAD or NADP as acceptor	0.000084	420,12,8
1qs0a1#119	GO:0016776: phosphotransferase activity, phosphate group as acceptor	0.000084	454,14,9
1fl7b_#84	GO:0005179: hormone activity	0.000085	3,15,2
1soxa3#177	GO:0030151: molybdenum ion binding	0.000085	3,15,2
2pvaa_#167	GO:0016811: hydrolase activity, acting on carbon-nitrogen (but not peptide) bonds, in linear amides	0.000085	3,15,2
1fsia_#71	GO:0004556: alpha-amylase activity	0.000085	3,15,2
1ivya_#133	GO:0004180: carboxypeptidase activity	0.000085	3,15,2
1lvl_2#222	GO:0016627: oxidoreductase activity, acting on the CH-CH group of donors	0.000085	79,17,5
1f37a_#30	GO:0004725: protein tyrosine phosphatase activity	0.000085	3,15,2
1g62a_#61	GO:0003743: translation initiation factor activity	0.000085	3,15,2
1qj2b2#632	GO:0030151: molybdenum ion binding	0.000085	3,15,2
1g4us1#189	GO:0005096: GTPase activator activity	0.000085	3,15,2
1fo4a1#128	GO:0030151: molybdenum ion binding	0.000085	3,15,2
2pia_1#54	GO:0019955: cytokine binding	0.000085	220,11,6
1e2o__#346	GO:0004263: chymotrypsin activity	0.000085	194,41,11
3grx__#56	GO:0019955: cytokine binding	0.000085	335,11,7
1gci__#175	GO:0005524: ATP binding	0.000085	70,243,17
1g62a_#59	GO:0003743: translation initiation factor activity	0.000085	3,15,2
1g62a_#16	GO:0003743: translation initiation factor activity	0.000085	3,15,2
1jr3a2#166	GO:0005524: ATP binding	0.000085	39,243,12
2hft_1#34	GO:0005126: hematopoietin/interferon-class (D200-domain) cytokine receptor binding	0.000085	34,20,4
1dih_2#222	GO:0008483: transaminase activity	0.000085	352,17,9
1hcl__#280	GO:0004896: hematopoietin/interferon-class (D200-domain) cytokine receptor activity	0.000085	70,19,5
1mdah_#329	GO:0003724: RNA helicase activity	0.000085	335,11,7
1e0ta2#275	GO:0016854: racemase and epimerase activity	0.000085	107,13,5
1gky__#34	GO:0005507: copper ion binding	0.000086	18,38,4
1saca_#113	GO:0004842: ubiquitin-protein ligase activity	0.000086	36,19,4
2foka2#180	GO:0003916: DNA topoisomerase activity	0.000086	147,10,5
1h8ua_#113	GO:0003964: RNA-directed DNA polymerase activity	0.000086	65,11,4
3grs_3#435	GO:0008408: 3'-5' exonuclease activity	0.000086	179,13,6
1jj2q_#114	GO:0003729: mRNA binding	0.000086	54,13,4
1ddma_#130	GO:0004556: alpha-amylase activity	0.000086	313,15,8
1ig8a_#203	GO:0004364: glutathione transferase activity	0.000086	65,11,4
1iira_#242	GO:0000287: magnesium ion binding	0.000086	170,128,20
1as4.1#A336	GO:0051082: unfolded protein binding	0.000087	332,34,13
1bak__#583	GO:0003755: peptidyl-prolyl cis-trans isomerase activity	0.000087	131,11,5
1plq_1#90	GO:0005509: calcium ion binding	0.000087	158,160,22
1hxma2#150	GO:0019838: growth factor binding	0.000087	29,9,3
1dt6a_#164	GO:0004497: monooxygenase activity	0.000088	2,26,2
1pysb6#217	GO:0004812: tRNA ligase activity	0.000088	2,26,2
3tdt__#238	GO:0004812: tRNA ligase activity	0.000088	2,26,2
1fid__#237	GO:0005524: ATP binding	0.000088	51,243,14
1ctqa_#163	GO:0003924: GTPase activity	0.000088	15,17,3
1jjya_#96	GO:0016627: oxidoreductase activity, acting on the CH-CH group of donors	0.000088	15,17,3
1g8ka2#523	GO:0030151: molybdenum ion binding	0.000088	17,15,3
1czya1#466	GO:0005126: hematopoietin/interferon-class (D200-domain) cytokine receptor binding	0.000088	109,20,6
1fepa_#340	GO:0015082: di-, tri-valent inorganic cation transporter activity	0.000088	99,14,5
1fepa_#340	GO:0046915: transition metal ion transporter activity	0.000088	99,14,5
1fmta2#151	GO:0030151: molybdenum ion binding	0.000088	314,15,8
1a81a2#182	GO:0004812: tRNA ligase activity	0.000088	120,26,7
1h5qa_#153	GO:0016627: oxidoreductase activity, acting on the CH-CH group of donors	0.000088	15,17,3
1ea5a_#99	GO:0016638: oxidoreductase activity, acting on the CH-NH2 group of donors	0.000088	194,17,7
1oaca1#360	GO:0016638: oxidoreductase activity, acting on the CH-NH2 group of donors	0.000088	15,17,3
1jj2n_#77	GO:0005524: ATP binding	0.000088	51,243,14
1thg__#223	GO:0004812: tRNA ligase activity	0.000088	82,26,6
1arb__#193	GO:0003968: RNA-directed RNA polymerase activity	0.000088	50,14,4
1h4vb2#307	GO:0008800: beta-lactamase activity	0.000088	73,10,4
1epwa1#901	GO:0051082: unfolded protein binding	0.000089	62,34,6
1be9a_#352	GO:0010181: FMN binding	0.000089	4,11,2
1cpt__#323	GO:0004497: monooxygenase activity	0.000089	10,26,3
1exta3#123	GO:0019955: cytokine binding	0.000089	4,11,2
1bu7a_#422	GO:0010181: FMN binding	0.000089	4,11,2
1bu7a_#433	GO:0010181: FMN binding	0.000089	4,11,2

1ytba2#217	GO:0005066: transmembrane receptor protein tyrosine kinase signaling protein activity	0.000089	26,10,3
1fc6a1#188	GO:0010181: FMN binding	0.000089	4,11,2
1cpt__#144	GO:0004497: monooxygenase activity	0.000089	10,26,3
1qava_#149	GO:0010181: FMN binding	0.000089	4,11,2
1qlca_#228	GO:0010181: FMN binding	0.000089	4,11,2
1ile_2#359	GO:0004812: tRNA ligase activity	0.000089	10,26,3
1b35a_#49	GO:0003724: RNA helicase activity	0.000089	4,11,2
1hbna1#293	GO:0016705: oxidoreductase activity, acting on paired donors, with incorporation or reduction of molecular oxygen	0.000089	10,26,3
1hbna1#293	GO:0004497: monooxygenase activity	0.000089	10,26,3
1hcc__#39	GO:0019955: cytokine binding	0.000089	4,11,2
1f8ra2#325	GO:0019955: cytokine binding	0.000089	222,11,6
1g6sa_#64	GO:0008235: metalloexopeptidase activity	0.000089	108,13,5
1jfra_#41	GO:0008080: N-acetyltransferase activity	0.000089	108,13,5
1h9da_#91	GO:0019955: cytokine binding	0.000089	222,11,6
1guqa1#166	GO:0004867: serine-type endopeptidase inhibitor activity	0.00009	28,47,5
1f24a_#293	GO:0004896: hematopoietin/interferon-class (D200-domain) cytokine receptor activity	0.00009	116,19,6
1qqsa_#124	GO:0015082: di-, tri-valent inorganic cation transporter activity	0.00009	458,14,9
1qqsa_#124	GO:0046915: transition metal ion transporter activity	0.00009	458,14,9
1e7wa_#8	GO:0016763: transferase activity, transferring pentosyl groups	0.00009	111,28,7
8dfr__#9	GO:0016763: transferase activity, transferring pentosyl groups	0.00009	111,28,7
1cbf__#208	GO:0051082: unfolded protein binding	0.00009	333,34,13
1jg8a_#172	GO:0004556: alpha-amylase activity	0.00009	92,15,5
1kit_2#398	GO:0019838: growth factor binding	0.00009	83,9,4
1jj2j_#70	GO:0004896: hematopoietin/interferon-class (D200-domain) cytokine receptor activity	0.000091	71,19,5
1dqga_#30	GO:0005529: sugar binding	0.000091	7,39,3
1f8ra2#325	GO:0003887: DNA-directed DNA polymerase activity	0.000091	222,20,8
2mpra_#190	GO:0015036: disulfide oxidoreductase activity	0.000091	400,22,11
1g0sa_#74	GO:0008201: heparin binding	0.000091	294,24,10
5ruba1#259	GO:0016831: carboxy-lyase activity	0.000091	53,25,5
1h9da_#91	GO:0005126: hematopoietin/interferon-class (D200-domain) cytokine receptor binding	0.000091	222,20,8
1bvoa_#220	GO:0003700: transcription factor activity	0.000092	3,124,3
1pyia1#44	GO:0003700: transcription factor activity	0.000092	3,124,3
1fid__#237	GO:0004812: tRNA ligase activity	0.000092	51,26,5
1qsta_#154	GO:0008080: N-acetyltransferase activity	0.000092	20,13,3
1qs1a2#437	GO:0016638: oxidoreductase activity, acting on the CH-NH2 group of donors	0.000092	452,17,10
1qqga2#166	GO:0003697: single-stranded DNA binding	0.000092	200,12,6
1opy__#68	GO:0003887: DNA-directed DNA polymerase activity	0.000092	367,20,10
1puee_#237	GO:0003700: transcription factor activity	0.000092	3,124,3
1hdfa_#16	GO:0019955: cytokine binding	0.000092	223,11,6
1neb__#41	GO:0030151: molybdenum ion binding	0.000092	47,15,4
1ac5__#72	GO:0004180: carboxypeptidase activity	0.000092	47,15,4
1d0ba_#191	GO:0005524: ATP binding	0.000093	120,243,24
1jh2a_#16	GO:0005524: ATP binding	0.000093	91,243,20
1jf9a_#379	GO:0004263: chymotrypsin activity	0.000093	232,41,12
2viua_#252	GO:0004674: protein serine/threonine kinase activity	0.000093	343,42,15
1pvc1_#106	GO:0015078: hydrogen ion transporter activity	0.000093	64,21,5
1xnb__#149	GO:0004812: tRNA ligase activity	0.000093	165,26,8
1hdr__#156	GO:0016854: racemase and epimerase activity	0.000093	55,13,4
1qksa2#236	GO:0015078: hydrogen ion transporter activity	0.000093	64,21,5
1qdlb_#170	GO:0003968: RNA-directed RNA polymerase activity	0.000093	100,14,5
1qfea_#68	GO:0016627: oxidoreductase activity, acting on the CH-CH group of donors	0.000093	560,17,11
1d0ba_#191	GO:0016866: intramolecular transferase activity	0.000094	120,12,5
1eny__#256	GO:0016616: oxidoreductase activity, acting on the CH-OH group of donors, NAD or NADP as acceptor	0.000094	5,59,3
1jd0a_#176	GO:0005524: ATP binding	0.000094	207,243,35
1be9a_#357	GO:0004812: tRNA ligase activity	0.000094	83,26,6
1fw9a_#152	GO:0003964: RNA-directed DNA polymerase activity	0.000094	133,11,5
1fi2a_#109	GO:0004556: alpha-amylase activity	0.000094	228,15,7
1avgi_#69	GO:0004364: glutathione transferase activity	0.000094	340,11,7
1i50a_#856	GO:0004497: monooxygenase activity	0.000094	269,26,10
1d0na3#305	GO:0004263: chymotrypsin activity	0.000094	311,41,14
1dj0a2#183	GO:0004523: ribonuclease H activity	0.000094	142,16,6
1fds__#59	GO:0015036: disulfide oxidoreductase activity	0.000094	61,22,5
2mev3_#108	GO:0004556: alpha-amylase activity	0.000094	228,15,7
1qs0a1#119	GO:0016836: hydro-lyase activity	0.000094	454,33,15

1ir3a_#1056	GO:0005516: calmodulin binding	0.000095	11,24,3
1a6o__#233	GO:0005516: calmodulin binding	0.000095	11,24,3
1a6o__#45	GO:0016668: oxidoreductase activity, acting on sulfur group of donors, NAD or NADP as acceptor	0.000095	303,12,7
1qfla1#44	GO:0051082: unfolded protein binding	0.000095	8,34,3
1koba_#293	GO:0005516: calmodulin binding	0.000095	11,24,3
1koba_#105	GO:0005516: calmodulin binding	0.000095	11,24,3
1e2wa1#102	GO:0004295: trypsin activity	0.000095	86,48,8
1htp__#16	GO:0008083: growth factor activity	0.000095	303,42,14
1htp__#16	GO:0016668: oxidoreductase activity, acting on sulfur group of donors, NAD or NADP as acceptor	0.000095	303,12,7
1b8aa2#403	GO:0004896: hematopoietin/interferon-class (D200-domain) cytokine receptor activity	0.000095	117,19,6
1ecpa_#62	GO:0000287: magnesium ion binding	0.000095	72,128,12
1xgsa2#186	GO:0004177: aminopeptidase activity	0.000095	182,13,6
1c7wa_#105	GO:0005516: calmodulin binding	0.000095	29,24,4
2pola2#218	GO:0015036: disulfide oxidoreductase activity	0.000095	146,22,7
1fhga_#119	GO:0019838: growth factor binding	0.000096	5,9,2
1bu7a_#130	GO:0015036: disulfide oxidoreductase activity	0.000096	12,22,3
1au1a_#58	GO:0015078: hydrogen ion transporter activity	0.000096	105,21,6
1e05i_#83	GO:0004867: serine-type endopeptidase inhibitor activity	0.000096	88,47,8
1bl0a2#102	GO:0019843: rRNA binding	0.000096	12,22,3
1cpt__#52	GO:0005507: copper ion binding	0.000096	35,38,5
1fo5a_#60	GO:0005529: sugar binding	0.000096	246,39,12
3grx__#56	GO:0051082: unfolded protein binding	0.000096	335,34,13
1fm2.1#B308	GO:0015036: disulfide oxidoreductase activity	0.000096	200,22,8
1f5aa1#441	GO:0005126: hematopoietin/interferon-class (D200-domain) cytokine receptor binding	0.000096	163,20,7
1qq5a_#7	GO:0016776: phosphotransferase activity, phosphate group as acceptor	0.000096	167,14,6
1qs0a1#119	GO:0008483: transaminase activity	0.000096	454,17,10
1fmb__#60	GO:0004523: ribonuclease H activity	0.000097	3,16,2
1jb0d_#26	GO:0004556: alpha-amylase activity	0.000097	229,15,7
1f8v.1#A116	GO:0003968: RNA-directed RNA polymerase activity	0.000097	101,14,5
1bwvs_#117	GO:0004812: tRNA ligase activity	0.000097	329,26,11
1g6oa_#116	GO:0016638: oxidoreductase activity, acting on the CH-NH2 group of donors	0.000097	358,17,9
1fp1d2#214	GO:0004222: metalloendopeptidase activity	0.000097	173,19,7
1eucb2#188	GO:0016831: carboxy-lyase activity	0.000097	87,25,6
1hdma1#123	GO:0019838: growth factor binding	0.000097	30,9,3
1f7ua2#293	GO:0005524: ATP binding	0.000098	7,243,5
1qf6a4#304	GO:0005524: ATP binding	0.000098	7,243,5
1ecpa_#62	GO:0016646: oxidoreductase activity, acting on the CH-NH group of donors, NAD or NADP as acceptor	0.000098	72,19,5
1tpg_1#55	GO:0004295: trypsin activity	0.000099	6,48,3
1ft9a1#203	GO:0005066: transmembrane receptor protein tyrosine kinase signaling protein activity	0.000099	27,10,3
2cb5a_#312	GO:0004295: trypsin activity	0.000099	111,48,9
1e6wa_#24	GO:0016620: oxidoreductase activity, acting on the aldehyde or oxo group of donors, NAD or NADP as acceptor	0.000099	27,10,3
1f24a_#144	GO:0016705: oxidoreductase activity, acting on paired donors, with incorporation or reduction of molecular oxygen	0.000099	27,26,4
2btva_#597	GO:0008199: ferric iron binding	0.000099	27,10,3
1hc7a2#258	GO:0019955: cytokine binding	0.000099	226,11,6
1ceza_#227	GO:0003916: DNA topoisomerase activity	0.000099	27,10,3
1fu6a_#35	GO:0005066: transmembrane receptor protein tyrosine kinase signaling protein activity	0.000099	27,10,3
1ei5a3#311	GO:0005066: transmembrane receptor protein tyrosine kinase signaling protein activity	0.000099	27,10,3
1ei5a3#311	GO:0008800: beta-lactamase activity	0.000099	27,10,3
1e6pa2#46	GO:0000287: magnesium ion binding	0.000099	95,128,14
1hw1a2#131	GO:0008083: growth factor activity	0.0001	17,42,4
1qj2c2#50	GO:0030151: molybdenum ion binding	0.0001	48,15,4
1f0xa1#482	GO:0016638: oxidoreductase activity, acting on the CH-NH2 group of donors	0.0001	42,17,4
1egja_#343	GO:0016638: oxidoreductase activity, acting on the CH-NH2 group of donors	0.0001	42,17,4
1aym3_#109	GO:0005518: collagen binding	0.0001	56,13,4
1danh_#104	GO:0005524: ATP binding	0.0001	183,243,32
1fjfj_#68	GO:0004725: protein tyrosine phosphatase activity	0.0001	230,15,7
1ejfa_#10	GO:0004556: alpha-amylase activity	0.0001	155,15,6
1g0sa_#74	GO:0003887: DNA-directed DNA polymerase activity	0.0001	294,20,9
1d3ga_#118	GO:0016627: oxidoreductase activity, acting on the CH-CH group of donors	0.0001	42,17,4
1ec7a1#230	GO:0016831: carboxy-lyase activity	0.0001	54,25,5
1el0a_#42	GO:0003887: DNA-directed DNA polymerase activity	0.0001	294,20,9

1a65a1#108	GO:0003887: DNA-directed DNA polymerase activity	0.0001	294,20,9
1zpda3#484	GO:0000287: magnesium ion binding	0.000101	3,128,3
1je5a_#10	GO:0015036: disulfide oxidoreductase activity	0.000101	62,22,5
1poxa3#439	GO:0000287: magnesium ion binding	0.000101	25,128,7
1rypg_#159	GO:0005524: ATP binding	0.000101	34,243,11
1jb3a_#64	GO:0005524: ATP binding	0.000101	302,243,46
1fc4a_#205	GO:0005524: ATP binding	0.000101	267,243,42
1a4ya_#47	GO:0005524: ATP binding	0.000101	216,243,36
3sil__#98	GO:0008083: growth factor activity	0.000101	228,42,12
1gph12#46	GO:0016763: transferase activity, transferring pentosyl groups	0.000102	2,28,2
1f0la3#311	GO:0016763: transferase activity, transferring pentosyl groups	0.000102	2,28,2
1dd2a_#76	GO:0016763: transferase activity, transferring pentosyl groups	0.000102	2,28,2
1hw5a2#64	GO:0004295: trypsin activity	0.000102	167,48,11
1dzfa2#211	GO:0004812: tRNA ligase activity	0.000102	167,26,8
1ct9a2#27	GO:0016763: transferase activity, transferring pentosyl groups	0.000102	2,28,2
1aqua_#126	GO:0016836: hydro-lyase activity	0.000102	576,33,17
1feza_#185	GO:0010181: FMN binding	0.000103	68,11,4
1danh_#104	GO:0008201: heparin binding	0.000103	183,24,8
1kve.1#B199	GO:0003809: thrombin activity	0.000103	76,10,4
1hxxa_#278	GO:0005507: copper ion binding	0.000103	255,38,12
1e2o__#346	GO:0005509: calcium ion binding	0.000103	194,160,25
1nfdb1#21	GO:0005509: calcium ion binding	0.000103	96,160,16
1qora2#201	GO:0016627: oxidoreductase activity, acting on the CH-CH group of donors	0.000103	199,17,7
1ekbb_#228	GO:0003755: peptidyl-prolyl cis-trans isomerase activity	0.000103	68,11,4
1eur__#84	GO:0019838: growth factor binding	0.000103	86,9,4
1evqa_#305	GO:0016758: transferase activity, transferring hexosyl groups	0.000103	490,11,8
1j79a_#38	GO:0008270: zinc ion binding	0.000104	39,108,8
1ev2e2#286	GO:0008083: growth factor activity	0.000104	100,42,8
1qqsa_#124	GO:0046983: protein dimerization activity	0.000104	458,17,10
1fi2a_#109	GO:0019955: cytokine binding	0.000104	228,11,6
1iba__#39	GO:0015082: di-, tri-valent inorganic cation transporter activity	0.000104	351,14,8
1iba__#39	GO:0046915: transition metal ion transporter activity	0.000104	351,14,8
1c4ra_#258	GO:0005529: sugar binding	0.000104	80,39,7
1fhoa_#74	GO:0005524: ATP binding	0.000104	347,243,51
1e42a1#711	GO:0019838: growth factor binding	0.000105	175,9,5
1pyma_#224	GO:0005525: GTP binding	0.000105	6,49,3
1jbqa_#152	GO:0016668: oxidoreductase activity, acting on sulfur group of donors, NAD or NADP as acceptor	0.000105	62,12,4
1fjfl_#67	GO:0019838: growth factor binding	0.000105	175,9,5
1dssg1#4	GO:0030145: manganese ion binding	0.000105	57,38,6
1atg__#161	GO:0030151: molybdenum ion binding	0.000105	18,15,3
1euca2#234	GO:0008810: cellulase activity	0.000105	15,18,3
1bpv__#22	GO:0005179: hormone activity	0.000105	18,15,3
1tul__#102	GO:0005507: copper ion binding	0.000105	57,38,6
1i6vd_#759	GO:0003899: DNA-directed RNA polymerase activity	0.000106	4,12,2
1i6vd_#766	GO:0003899: DNA-directed RNA polymerase activity	0.000106	4,12,2
1byfa_#4	GO:0003697: single-stranded DNA binding	0.000106	4,12,2
1d0ba_#191	GO:0000287: magnesium ion binding	0.000106	120,128,16
1cxp.1#A97	GO:0016702: oxidoreductase activity, acting on single donors with incorporation of molecular oxygen, incorporation of two atoms of oxygen	0.000106	4,12,2
1iira_#326	GO:0019201: nucleotide kinase activity	0.000106	112,13,5
1qaxa2#307	GO:0004867: serine-type endopeptidase inhibitor activity	0.000106	46,47,6
2hhma_#262	GO:0004725: protein tyrosine phosphatase activity	0.000106	95,15,5
2dpma_#237	GO:0004180: carboxypeptidase activity	0.000106	95,15,5
1pfza_#156	GO:0051082: unfolded protein binding	0.000106	64,34,6
1brma2#268	GO:0005507: copper ion binding	0.000107	19,38,4
1dlfl_#97	GO:0005126: hematopoietin/interferon-class (D200-domain) cytokine receptor binding	0.000107	36,20,4
1ile_2#304	GO:0000049: tRNA binding	0.000107	21,13,3
1an9a2#278	GO:0008408: 3'-5' exonuclease activity	0.000107	21,13,3
2rspa_#113	GO:0004523: ribonuclease H activity	0.000107	17,16,3
1gky__#61	GO:0004364: glutathione transferase activity	0.000107	25,11,3
1i39a_#49	GO:0000049: tRNA binding	0.000107	21,13,3
1e79h2#77	GO:0003729: mRNA binding	0.000107	21,13,3
1qqga2#166	GO:0005524: ATP binding	0.000107	200,243,34
1fm2.1#B308	GO:0016638: oxidoreductase activity, acting on the CH-NH2 group of donors	0.000107	200,17,7
1a9xa3#78	GO:0016763: transferase activity, transferring pentosyl groups	0.000107	305,28,11

1ea5a_#133	GO:0003887: DNA-directed DNA polymerase activity	0.000107	36,20,4
2dri__#115	GO:0003924: GTPase activity	0.000107	16,17,3
1c0ma1#237	GO:0004867: serine-type endopeptidase inhibitor activity	0.000107	29,47,5
1d7ya1#255	GO:0016627: oxidoreductase activity, acting on the CH-CH group of donors	0.000107	16,17,3
1elja_#64	GO:0003968: RNA-directed RNA polymerase activity	0.000107	103,14,5
1ojt_2#336	GO:0016627: oxidoreductase activity, acting on the CH-CH group of donors	0.000107	16,17,3
1xis__#285	GO:0016861: intramolecular oxidoreductase activity, interconverting aldoses and ketoses	0.000107	21,13,3
1sppb_#32	GO:0004896: hematopoietin/interferon-class (D200-domain) cytokine receptor activity	0.000107	38,19,4
1dfoa_#344	GO:0003887: DNA-directed DNA polymerase activity	0.000107	36,20,4
3grx__#55	GO:0003887: DNA-directed DNA polymerase activity	0.000107	113,20,6
1gpc__#184	GO:0016638: oxidoreductase activity, acting on the CH-NH2 group of donors	0.000108	83,17,5
1hxxa_#173	GO:0050660: FAD binding	0.000108	260,10,6
1cbf__#208	GO:0005529: sugar binding	0.000108	333,39,14
1d8db_#78	GO:0015078: hydrogen ion transporter activity	0.000108	66,21,5
1a65a1#108	GO:0005524: ATP binding	0.000108	294,243,45
1e7wa_#36	GO:0010181: FMN binding	0.000108	137,11,5
1h6va1#27	GO:0016620: oxidoreductase activity, acting on the aldehyde or oxo group of donors, NAD or NADP as acceptor	0.000108	260,10,6
1mkp__#283	GO:0004725: protein tyrosine phosphatase activity	0.000109	49,15,4
1heta2#212	GO:0004556: alpha-amylase activity	0.000109	49,15,4
1d8ca_#442	GO:0004197: cysteine-type endopeptidase activity	0.000109	30,24,4
1fjfj_#68	GO:0019955: cytokine binding	0.000109	230,11,6
1cfr__#269	GO:0016831: carboxy-lyase activity	0.000109	55,25,5
1czan1#92	GO:0005529: sugar binding	0.000109	631,39,20
1aoea_#130	GO:0050660: FAD binding	0.000109	77,10,4
1qtra_#108	GO:0004457: lactate dehydrogenase activity	0.000109	77,10,4
1e79h2#39	GO:0015405: P-P-bond-hydrolysis-driven transporter activity	0.00011	23,12,3
2bbkh_#117	GO:0016638: oxidoreductase activity, acting on the CH-NH2 group of donors	0.00011	3,17,2
1i6vc_#207	GO:0015405: P-P-bond-hydrolysis-driven transporter activity	0.00011	23,12,3
1an9a2#231	GO:0016638: oxidoreductase activity, acting on the CH-NH2 group of donors	0.00011	3,17,2
2hrva_#132	GO:0016638: oxidoreductase activity, acting on the CH-NH2 group of donors	0.00011	43,17,4
1f5aa2#167	GO:0016702: oxidoreductase activity, acting on single donors with incorporation of molecular oxygen, incorporation of two atoms of oxygen	0.00011	124,12,5
1erja_#642	GO:0016638: oxidoreductase activity, acting on the CH-NH2 group of donors	0.00011	3,17,2
1fp2a2#182	GO:0016638: oxidoreductase activity, acting on the CH-NH2 group of donors	0.00011	3,17,2
1p35a_#63	GO:0016638: oxidoreductase activity, acting on the CH-NH2 group of donors	0.00011	3,17,2
1ax4a_#331	GO:0008483: transaminase activity	0.00011	3,17,2
1daaa_#15	GO:0008483: transaminase activity	0.00011	3,17,2
1a04a2#59	GO:0003700: transcription factor activity	0.00011	12,124,5
1qba_3#729	GO:0016627: oxidoreductase activity, acting on the CH-CH group of donors	0.00011	3,17,2
1cs1a_#160	GO:0016861: intramolecular oxidoreductase activity, interconverting aldoses and ketoses	0.00011	187,13,6
1a8d_1#87	GO:0015036: disulfide oxidoreductase activity	0.00011	204,22,8
1fa0a1#393	GO:0016846: carbon-sulfur lyase activity	0.00011	261,10,6
1tmy__#14	GO:0000287: magnesium ion binding	0.000111	18,128,6
1be9a_#357	GO:0005507: copper ion binding	0.000111	83,38,7
1cqka_#52	GO:0005126: hematopoietin/interferon-class (D200-domain) cytokine receptor binding	0.000111	70,20,5
1i5ga_#115	GO:0004556: alpha-amylase activity	0.000111	324,15,8
1ed5a_#294	GO:0004497: monooxygenase activity	0.000111	53,26,5
1el0a_#28	GO:0008083: growth factor activity	0.000111	161,42,10
1ejda_#377	GO:0000287: magnesium ion binding	0.000111	108,128,15
1ppn__#132	GO:0030151: molybdenum ion binding	0.000111	234,15,7
1b3qa2#623	GO:0008201: heparin binding	0.000111	301,24,10
1nfdb1#21	GO:0005179: hormone activity	0.000111	96,15,5
1gdha1#235	GO:0008026: ATP-dependent helicase activity	0.000111	113,13,5
1gdha1#235	GO:0016651: oxidoreductase activity, acting on NADH or NADPH	0.000111	113,13,5
1ecpa_#58	GO:0005507: copper ion binding	0.000111	83,38,7
1cnv__#181	GO:0016646: oxidoreductase activity, acting on the CH-NH group of donors, NAD or NADP as acceptor	0.000111	74,19,5
2hlca_#54	GO:0005529: sugar binding	0.000111	109,39,8
1br9__#86	GO:0030151: molybdenum ion binding	0.000111	96,15,5
1bxoa_#307	GO:0003968: RNA-directed RNA polymerase activity	0.000112	104,14,5
1bd8__#82	GO:0008083: growth factor activity	0.000112	75,42,7
1dik_3#240	GO:0050660: FAD binding	0.000113	262,10,6
1neb__#52	GO:0005524: ATP binding	0.000113	19,243,8
1e7wa_#36	GO:0016627: oxidoreductase activity, acting on the CH-CH group of donors	0.000113	137,17,6
1evqa_#305	GO:0015036: disulfide oxidoreductase activity	0.000113	490,22,12

1fl7b_#78	GO:0008083: growth factor activity	0.000114	7,42,3
1qs0b2#269	GO:0005524: ATP binding	0.000114	46,243,13
3pah__#303	GO:0016705: oxidoreductase activity, acting on paired donors, with incorporation or reduction of molecular oxygen	0.000114	28,26,4
1jlxa2#243	GO:0008083: growth factor activity	0.000114	7,42,3
1aqua_#126	GO:0016620: oxidoreductase activity, acting on the aldehyde or oxo group of donors, NAD or NADP as acceptor	0.000114	576,10,8
1aqua_#126	GO:0016846: carbon-sulfur lyase activity	0.000114	576,10,8
1qhqa_#45	GO:0016646: oxidoreductase activity, acting on the CH-NH group of donors, NAD or NADP as acceptor	0.000114	121,19,6
1h6va3#486	GO:0019955: cytokine binding	0.000115	70,11,4
1c4zd_#22	GO:0003724: RNA helicase activity	0.000115	70,11,4
1c4zd_#22	GO:0003755: peptidyl-prolyl cis-trans isomerase activity	0.000115	70,11,4
1aky_2#133	GO:0003809: thrombin activity	0.000115	156,10,5
1by5a_#347	GO:0050660: FAD binding	0.000115	263,10,6
1jjya_#324	GO:0016620: oxidoreductase activity, acting on the aldehyde or oxo group of donors, NAD or NADP as acceptor	0.000115	78,10,4
1jjya_#324	GO:0016846: carbon-sulfur lyase activity	0.000115	78,10,4
1axn__#42	GO:0001584: rhodopsin-like receptor activity	0.000115	78,10,4
1fsu__#434	GO:0015082: di-, tri-valent inorganic cation transporter activity	0.000115	356,14,8
1fsu__#434	GO:0046915: transition metal ion transporter activity	0.000115	356,14,8
1elja_#64	GO:0019843: rRNA binding	0.000115	103,22,6
1c7na_#231	GO:0016846: carbon-sulfur lyase activity	0.000115	263,10,6
1bjt__#890	GO:0005529: sugar binding	0.000116	292,39,13
1kdj__#27	GO:0015078: hydrogen ion transporter activity	0.000116	67,21,5
1gox__#124	GO:0003924: GTPase activity	0.000116	366,17,9
1fxla2#169	GO:0046983: protein dimerization activity	0.000117	203,17,7
1wdcb_#141	GO:0003779: actin binding	0.000118	9,32,3
1cb8a2#613	GO:0016638: oxidoreductase activity, acting on the CH-NH2 group of donors	0.000118	138,17,6
1c9la2#61	GO:0004867: serine-type endopeptidase inhibitor activity	0.000118	238,47,13
1svy__#245	GO:0003779: actin binding	0.000118	9,32,3
1qrra_#69	GO:0005525: GTP binding	0.000118	196,49,12
1bec_2#216	GO:0003887: DNA-directed DNA polymerase activity	0.000118	115,20,6
1qfta_#98	GO:0003887: DNA-directed DNA polymerase activity	0.000118	230,20,8
1eh9a1#33	GO:0005126: hematopoietin/interferon-class (D200-domain) cytokine receptor binding	0.000119	37,20,4
1f42a2#123	GO:0003887: DNA-directed DNA polymerase activity	0.000119	378,20,10
1epwa3#248	GO:0005126: hematopoietin/interferon-class (D200-domain) cytokine receptor binding	0.000119	37,20,4
1heta2#218	GO:0004222: metalloendopeptidase activity	0.000119	39,19,4
2dkb__#114	GO:0000287: magnesium ion binding	0.000119	174,128,20
1euca1#64	GO:0016646: oxidoreductase activity, acting on the CH-NH group of donors, NAD or NADP as acceptor	0.000119	39,19,4
1a3qa2#190	GO:0004190: aspartic-type endopeptidase activity	0.000119	144,23,7
1as4.1#A288	GO:0004295: trypsin activity	0.000119	66,48,7
1e1aa_#178	GO:0004714: transmembrane receptor protein tyrosine kinase activity	0.000119	54,14,4
1erv__#80	GO:0008201: heparin binding	0.000119	442,24,12
1dnv__#354	GO:0003968: RNA-directed RNA polymerase activity	0.000119	54,14,4
1g6oa_#116	GO:0004714: transmembrane receptor protein tyrosine kinase activity	0.00012	358,14,8
1qfxa_#380	GO:0004867: serine-type endopeptidase inhibitor activity	0.00012	47,47,6
1dpta_#22	GO:0000287: magnesium ion binding	0.00012	85,128,13
1dkia_#185	GO:0050660: FAD binding	0.00012	79,10,4
1edza2#120	GO:0016814: hydrolase activity, acting on carbon-nitrogen (but not peptide) bonds, in cyclic amidines	0.000121	5,10,2
1e0ca1#88	GO:0004457: lactate dehydrogenase activity	0.000121	5,10,2
1cwva1#535	GO:0008199: ferric iron binding	0.000121	5,10,2
1pud__#44	GO:0016763: transferase activity, transferring pentosyl groups	0.000121	50,28,5
1jdpa_#168	GO:0001584: rhodopsin-like receptor activity	0.000121	5,10,2
1ew0a_#174	GO:0001584: rhodopsin-like receptor activity	0.000121	5,10,2
1a4ia2#118	GO:0016814: hydrolase activity, acting on carbon-nitrogen (but not peptide) bonds, in cyclic amidines	0.000121	5,10,2
1ebma2#39	GO:0004497: monooxygenase activity	0.000121	11,26,3
1fvza_#142	GO:0004364: glutathione transferase activity	0.000121	26,11,3
1lvl_2#262	GO:0050660: FAD binding	0.000121	5,10,2
1ii7a_#275	GO:0004364: glutathione transferase activity	0.000121	26,11,3
1pma1_#17	GO:0016638: oxidoreductase activity, acting on the CH-NH2 group of donors	0.000121	44,17,4
1dz4a_#264	GO:0046983: protein dimerization activity	0.000121	44,17,4
1qhua1#47	GO:0015036: disulfide oxidoreductase activity	0.000121	104,22,6

1e39a3#442	GO:0005509: calcium ion binding	0.000122	21,160,7
1jd0a_#176	GO:0019843: rRNA binding	0.000122	207,22,8
1g97a1#376	GO:0016616: oxidoreductase activity, acting on the CH-OH group of donors, NAD or NADP as acceptor	0.000123	13,59,4
1dfaa2#211	GO:0004497: monooxygenase activity	0.000123	87,26,6
2cuaa_#107	GO:0051082: unfolded protein binding	0.000123	128,34,8
1fn9a_#271	GO:0005524: ATP binding	0.000123	218,243,36
3rpba_#557	GO:0005529: sugar binding	0.000123	57,39,6
1xnb__#130	GO:0004812: tRNA ligase activity	0.000123	87,26,6
1qs0a1#235	GO:0016616: oxidoreductase activity, acting on the CH-OH group of donors, NAD or NADP as acceptor	0.000123	114,59,10
1ejda_#213	GO:0008408: 3'-5' exonuclease activity	0.000123	284,13,7
1ghpa_#65	GO:0008800: beta-lactamase activity	0.000124	29,10,3
2mev3_#107	GO:0004197: cysteine-type endopeptidase activity	0.000124	31,24,4
1fwxa1#535	GO:0004222: metalloendopeptidase activity	0.000124	15,19,3
1tbga_#63	GO:0004222: metalloendopeptidase activity	0.000124	15,19,3
1nfp__#171	GO:0004457: lactate dehydrogenase activity	0.000124	29,10,3
1rsy__#151	GO:0005518: collagen binding	0.000124	22,13,3
1fzqa_#165	GO:0005085: guanyl-nucleotide exchange factor activity	0.000124	22,13,3
1dfca1#1135	GO:0008083: growth factor activity	0.000124	53,42,6
1ac6a_#43	GO:0008083: growth factor activity	0.000124	163,42,10
1fo5a_#60	GO:0004896: hematopoietin/interferon-class (D200-domain) cytokine receptor activity	0.000124	246,19,8
1mrp__#170	GO:0016616: oxidoreductase activity, acting on the CH-OH group of donors, NAD or NADP as acceptor	0.000124	24,59,5
1lml__#283	GO:0004222: metalloendopeptidase activity	0.000124	15,19,3
1c9la2#61	GO:0004556: alpha-amylase activity	0.000124	238,15,7
1bwvs_#117	GO:0030151: molybdenum ion binding	0.000124	329,15,8
1ib2a_#1101	GO:0003779: actin binding	0.000124	70,32,6
1e3ja1#136	GO:0005516: calmodulin binding	0.000124	31,24,4
1ekma1#545	GO:0019838: growth factor binding	0.000124	90,9,4
1gtra2#113	GO:0000049: tRNA binding	0.000124	22,13,3
1jbaa_#157	GO:0005516: calmodulin binding	0.000124	31,24,4
1jj2q_#113	GO:0005518: collagen binding	0.000125	4,13,2
1qq9a_#78	GO:0004177: aminopeptidase activity	0.000125	4,13,2
1fnha2#152	GO:0005518: collagen binding	0.000125	4,13,2
1ed8a_#97	GO:0008080: N-acetyltransferase activity	0.000125	4,13,2
1bu7a_#422	GO:0016651: oxidoreductase activity, acting on NADH or NADPH	0.000125	4,13,2
1bu7a_#433	GO:0016651: oxidoreductase activity, acting on NADH or NADPH	0.000125	4,13,2
1f0xa1#278	GO:0004896: hematopoietin/interferon-class (D200-domain) cytokine receptor activity	0.000125	180,19,7
1g6sa_#357	GO:0008080: N-acetyltransferase activity	0.000125	4,13,2
1dofa_#15	GO:0008083: growth factor activity	0.000125	197,42,11
1bura1#328	GO:0016831: carboxy-lyase activity	0.000125	91,25,6
1g61a_#2115	GO:0005085: guanyl-nucleotide exchange factor activity	0.000125	4,13,2
1ej8a_#189	GO:0005529: sugar binding	0.000125	294,39,13
1dysa_#215	GO:0016861: intramolecular oxidoreductase activity, interconverting aldoses and ketoses	0.000125	4,13,2
4kbpa2#416	GO:0015405: P-P-bond-hydrolysis-driven transporter activity	0.000125	24,12,3
1io7a_#243	GO:0005509: calcium ion binding	0.000125	78,160,14
1el0a_#42	GO:0005529: sugar binding	0.000125	294,39,13
1doza_#109	GO:0000287: magnesium ion binding	0.000125	74,128,12
1j9la_#82	GO:0005524: ATP binding	0.000126	169,243,30
1nuka_#80	GO:0005507: copper ion binding	0.000126	220,38,11
1b4ka_#108	GO:0008080: N-acetyltransferase activity	0.000126	116,13,5
1ej0a_#42	GO:0004177: aminopeptidase activity	0.000126	116,13,5
1cpy__#380	GO:0004295: trypsin activity	0.000127	171,48,11
1hfoa_#57	GO:0030145: manganese ion binding	0.000127	37,38,5
1fsu__#434	GO:0019955: cytokine binding	0.000127	356,11,7
1fsu__#434	GO:0003755: peptidyl-prolyl cis-trans isomerase activity	0.000127	356,11,7
1ihua2#343	GO:0016831: carboxy-lyase activity	0.000127	233,25,9
1i2oa_#34	GO:0019201: nucleotide kinase activity	0.000128	192,13,6
1dik_1#848	GO:0016763: transferase activity, transferring pentosyl groups	0.000128	81,28,6
1a04a2#59	GO:0000287: magnesium ion binding	0.000128	12,128,5
1htp__#16	GO:0003887: DNA-directed DNA polymerase activity	0.000128	303,20,9
1cpy__#346	GO:0016616: oxidoreductase activity, acting on the CH-OH group of donors, NAD or NADP as acceptor	0.000128	38,59,6
1ig8a_#215	GO:0030145: manganese ion binding	0.000128	59,38,6
1g8kb_#11	GO:0005507: copper ion binding	0.000128	59,38,6

1g5ca_#62	GO:0016836: hydro-lyase activity	0.000129	9,33,3
1a6ca2#312	GO:0005524: ATP binding	0.000129	93,243,20
1whi__#59	GO:0003724: RNA helicase activity	0.000129	72,11,4
1g25a_#22	GO:0015036: disulfide oxidoreductase activity	0.000129	415,22,11
1fepa_#340	GO:0004556: alpha-amylase activity	0.000129	99,15,5
1eova1#139	GO:0030151: molybdenum ion binding	0.000129	99,15,5
1ax4a_#430	GO:0005524: ATP binding	0.000129	59,243,15
1eut_1#488	GO:0046983: protein dimerization activity	0.00013	17,17,3
1eut_1#488	GO:0016638: oxidoreductase activity, acting on the CH-NH2 group of donors	0.00013	17,17,3
1dp0a5#499	GO:0003924: GTPase activity	0.00013	17,17,3
1ihna_#64	GO:0005509: calcium ion binding	0.00013	10,160,5
1bf2_3#579	GO:0005509: calcium ion binding	0.00013	10,160,5
1gjwa2#142	GO:0005509: calcium ion binding	0.00013	10,160,5
1qdla_#211	GO:0004263: chymotrypsin activity	0.00013	320,41,14
1bt0a_#26	GO:0016638: oxidoreductase activity, acting on the CH-NH2 group of donors	0.00013	17,17,3
1fwxa2#189	GO:0005509: calcium ion binding	0.00013	10,160,5
1keva2#236	GO:0016627: oxidoreductase activity, acting on the CH-CH group of donors	0.00013	17,17,3
1d4xg_#60	GO:0005524: ATP binding	0.000131	115,243,23
1nfp__#171	GO:0005525: GTP binding	0.000131	29,49,5
1danh_#104	GO:0019838: growth factor binding	0.000131	183,9,5
1i50a_#856	GO:0008009: chemokine activity	0.000131	269,10,6
1i50a_#856	GO:0050660: FAD binding	0.000131	269,10,6
1b8aa2#403	GO:0000049: tRNA binding	0.000131	117,13,5
1d0na3#305	GO:0019838: growth factor binding	0.000131	311,9,6
1apme_#74	GO:0004842: ubiquitin-protein ligase activity	0.000131	124,19,6
1apme_#74	GO:0004896: hematopoietin/interferon-class (D200-domain) cytokine receptor activity	0.000131	124,19,6
1g6oa_#116	GO:0019955: cytokine binding	0.000131	358,11,7
1xis__#214	GO:0005525: GTP binding	0.000131	88,49,8
1a7ca_#202	GO:0005518: collagen binding	0.000131	117,13,5
1dqza_#209	GO:0003924: GTPase activity	0.000132	45,17,4
1qj2c2#50	GO:0004523: ribonuclease H activity	0.000132	48,16,4
1f3ba2#4	GO:0003924: GTPase activity	0.000132	45,17,4
1cf1a1#33	GO:0016799: hydrolase activity, hydrolyzing N-glycosyl compounds	0.000132	45,17,4
1quba4#235	GO:0004263: chymotrypsin activity	0.000132	106,41,8
1kit_3#743	GO:0051082: unfolded protein binding	0.000132	250,34,11
1jf9a_#196	GO:0008483: transaminase activity	0.000132	45,17,4
1eq2a_#11	GO:0016627: oxidoreductase activity, acting on the CH-CH group of donors	0.000132	45,17,4
1qvba_#206	GO:0000287: magnesium ion binding	0.000133	26,128,7
1ksia1#555	GO:0005507: copper ion binding	0.000133	20,38,4
1im4a_#144	GO:0016668: oxidoreductase activity, acting on sulfur group of donors, NAD or NADP as acceptor	0.000133	129,12,5
1dik_1#848	GO:0016846: carbon-sulfur lyase activity	0.000133	81,10,4
1gsa_2#208	GO:0005507: copper ion binding	0.000133	20,38,4
1fp5a2#537	GO:0005126: hematopoietin/interferon-class (D200-domain) cytokine receptor binding	0.000133	38,20,4
1c9la2#61	GO:0019955: cytokine binding	0.000133	238,11,6
1dik_3#240	GO:0004714: transmembrane receptor protein tyrosine kinase activity	0.000133	262,14,7
1vrta1#439	GO:0005507: copper ion binding	0.000134	8,38,3
1aop_3#276	GO:0004295: trypsin activity	0.000134	172,48,11
1ftra2#187	GO:0005525: GTP binding	0.000134	46,49,6
1cxya_#78	GO:0003779: actin binding	0.000134	2,32,2
1mwpa_#36	GO:0008270: zinc ion binding	0.000134	14,108,5
1hx0a2#234	GO:0005509: calcium ion binding	0.000134	28,160,8
1erja_#521	GO:0005507: copper ion binding	0.000134	8,38,3
1erv__#80	GO:0004674: protein serine/threonine kinase activity	0.000134	442,42,17
1j71a_#251	GO:0015036: disulfide oxidoreductase activity	0.000135	106,22,6
1ih7a1#57	GO:0004896: hematopoietin/interferon-class (D200-domain) cytokine receptor activity	0.000135	77,19,5
1qfxa_#380	GO:0004295: trypsin activity	0.000135	47,48,6
1e32a2#345	GO:0004180: carboxypeptidase activity	0.000135	100,15,5
1bdfa2#60	GO:0015082: di-, tri-valent inorganic cation transporter activity	0.000136	21,14,3
1bdfa2#60	GO:0046915: transition metal ion transporter activity	0.000136	21,14,3
1dl5a_#266	GO:0003724: RNA helicase activity	0.000136	27,11,3
2ae2a_#89	GO:0005524: ATP binding	0.000136	35,243,11
1fm2.1#B627	GO:0003724: RNA helicase activity	0.000136	27,11,3
1deua_#151	GO:0016638: oxidoreductase activity, acting on the CH-NH2 group of donors	0.000136	285,17,8
2hft_1#34	GO:0008083: growth factor activity	0.000136	34,42,5
1a81a1#51	GO:0019955: cytokine binding	0.000136	73,11,4

1c8za_#386	GO:0008083: growth factor activity	0.000136	235,42,12
1ddwa_#77	GO:0015082: di-, tri-valent inorganic cation transporter activity	0.000136	364,14,8
1ddwa_#77	GO:0046915: transition metal ion transporter activity	0.000136	364,14,8
1d8ca_#442	GO:0004457: lactate dehydrogenase activity	0.000137	30,10,3
1f8ra2#325	GO:0015078: hydrogen ion transporter activity	0.000137	222,21,8
1c8za_#386	GO:0005126: hematopoietin/interferon-class (D200-domain) cytokine receptor binding	0.000137	235,20,8
1qqga2#253	GO:0000287: magnesium ion binding	0.000137	110,128,15
1e69a_#40	GO:0003684: damaged DNA binding	0.000137	30,10,3
1h9da_#91	GO:0005507: copper ion binding	0.000137	222,38,11
1ewfa2#313	GO:0004896: hematopoietin/interferon-class (D200-domain) cytokine receptor activity	0.000138	3,19,2
1hr6b2#255	GO:0004222: metalloendopeptidase activity	0.000138	3,19,2
1bccb1#130	GO:0004222: metalloendopeptidase activity	0.000138	3,19,2
1aym3_#109	GO:0003968: RNA-directed RNA polymerase activity	0.000138	56,14,4
1a0i_2#222	GO:0005524: ATP binding	0.000138	186,243,32
1qba_3#729	GO:0016646: oxidoreductase activity, acting on the CH-NH group of donors, NAD or NADP as acceptor	0.000138	3,19,2
1dcpa_#25	GO:0003968: RNA-directed RNA polymerase activity	0.000138	56,14,4
2pola2#218	GO:0005524: ATP binding	0.000138	146,243,27
1f24a_#293	GO:0004295: trypsin activity	0.000139	116,48,9
1kid__#330	GO:0015036: disulfide oxidoreductase activity	0.000139	155,22,7
1atg__#144	GO:0016620: oxidoreductase activity, acting on the aldehyde or oxo group of donors, NAD or NADP as acceptor	0.000139	82,10,4
1g6sa_#64	GO:0005524: ATP binding	0.000139	108,243,22
1boub_#7	GO:0010181: FMN binding	0.000139	240,11,6
1j79a_#187	GO:0004812: tRNA ligase activity	0.000139	89,26,6
1quna1#113	GO:0046983: protein dimerization activity	0.000139	286,17,8
1h6va1#27	GO:0016836: hydro-lyase activity	0.000139	260,33,11
1f97a1#62	GO:0016638: oxidoreductase activity, acting on the CH-NH2 group of donors	0.00014	707,17,12
1mrj__#156	GO:0019201: nucleotide kinase activity	0.00014	61,13,4
1czya1#466	GO:0003968: RNA-directed RNA polymerase activity	0.00014	109,14,5
1dm9a_#57	GO:0003968: RNA-directed RNA polymerase activity	0.00014	109,14,5
1edza1#206	GO:0051082: unfolded protein binding	0.00014	399,34,14
1hlwa_#91	GO:0004295: trypsin activity	0.00014	30,48,5
1cs1a_#82	GO:0008235: metalloexopeptidase activity	0.00014	195,13,6
1xo1a2#159	GO:0016616: oxidoreductase activity, acting on the CH-OH group of donors, NAD or NADP as acceptor	0.00014	55,59,7
2hlca_#54	GO:0003968: RNA-directed RNA polymerase activity	0.00014	109,14,5
1a0i_2#222	GO:0019838: growth factor binding	0.000141	186,9,5
1pwt__#8	GO:0004674: protein serine/threonine kinase activity	0.000141	314,42,14
1dfca4#1478	GO:0008201: heparin binding	0.000141	32,24,4
1foha5#77	GO:0009036: type II site-specific deoxyribonuclease activity	0.000142	25,12,3
1gjwa2#458	GO:0016854: racemase and epimerase activity	0.000142	23,13,3
1ibja_#217	GO:0016831: carboxy-lyase activity	0.000142	12,25,3
1tdj_1#35	GO:0016836: hydro-lyase activity	0.000142	2,33,2
1e8ca3#257	GO:0016831: carboxy-lyase activity	0.000142	12,25,3
1ac6a_#43	GO:0008009: chemokine activity	0.000142	163,10,5
1byka_#157	GO:0016831: carboxy-lyase activity	0.000142	12,25,3
1h4vb2#282	GO:0030151: molybdenum ion binding	0.000142	101,15,5
1ea5a_#205	GO:0008080: N-acetyltransferase activity	0.000142	23,13,3
1fxma_#153	GO:0005524: ATP binding	0.000142	15,243,7
2hft_1#34	GO:0019838: growth factor binding	0.000142	34,9,3
1mpp__#151	GO:0004523: ribonuclease H activity	0.000142	94,16,5
1e32a1#102	GO:0003899: DNA-directed RNA polymerase activity	0.000142	25,12,3
1eg9a2#199	GO:0010181: FMN binding	0.000143	74,11,4
1fi2a_#109	GO:0005524: ATP binding	0.000143	228,243,37
1fm2.1#B308	GO:0004674: protein serine/threonine kinase activity	0.000143	200,42,11
1ib2a_#1101	GO:0004601: peroxidase activity	0.000143	70,21,5
1eo6a_#107	GO:0004222: metalloendopeptidase activity	0.000143	78,19,5
1qfma1#186	GO:0004190: aspartic-type endopeptidase activity	0.000143	326,23,10
1fa0a1#405	GO:0005507: copper ion binding	0.000144	38,38,5
1qlaa2#179	GO:0016627: oxidoreductase activity, acting on the CH-CH group of donors	0.000144	46,17,4
1flga_#161	GO:0005509: calcium ion binding	0.000144	152,160,21
1qrra_#69	GO:0016861: intramolecular oxidoreductase activity, interconverting aldoses and ketoses	0.000144	196,13,6
1fjfc2#153	GO:0019843: rRNA binding	0.000144	420,22,11
1frb__#110	GO:0008270: zinc ion binding	0.000145	8,108,4
1b9ha_#276	GO:0004263: chymotrypsin activity	0.000145	19,41,4

1dm0a_#110	GO:0005529: sugar binding	0.000145	8,39,3
1qi7a_#244	GO:0005529: sugar binding	0.000145	8,39,3
1hcd__#91	GO:0005529: sugar binding	0.000145	8,39,3
1qi7a_#224	GO:0005529: sugar binding	0.000145	8,39,3
1d6aa_#179	GO:0005529: sugar binding	0.000145	8,39,3
1dpja_#322	GO:0003887: DNA-directed DNA polymerase activity	0.000145	308,20,9
1qi7a_#208	GO:0005529: sugar binding	0.000145	8,39,3
1fu6a_#50	GO:0003887: DNA-directed DNA polymerase activity	0.000145	308,20,9
1h6la_#119	GO:0003887: DNA-directed DNA polymerase activity	0.000145	308,20,9
1tmy__#34	GO:0000287: magnesium ion binding	0.000146	7,128,4
1ecfa1#434	GO:0005096: GTPase activator activity	0.000146	20,15,3
1hxxa_#112	GO:0015082: di-, tri-valent inorganic cation transporter activity	0.000146	4,14,2
1hxxa_#112	GO:0046915: transition metal ion transporter activity	0.000146	4,14,2
1fwxa2#396	GO:0003887: DNA-directed DNA polymerase activity	0.000146	237,20,8
1by5a_#238	GO:0003887: DNA-directed DNA polymerase activity	0.000146	237,20,8
1b6ra3#161	GO:0050660: FAD binding	0.000146	164,10,5
1qb7a_#148	GO:0000287: magnesium ion binding	0.000146	7,128,4
1neb__#41	GO:0005524: ATP binding	0.000146	47,243,13
1bvyf_#519	GO:0004364: glutathione transferase activity	0.000147	5,11,2
1dhn__#88	GO:0003964: RNA-directed DNA polymerase activity	0.000147	5,11,2
1cdy_2#140	GO:0003724: RNA helicase activity	0.000147	5,11,2
1ghqb1#32	GO:0019955: cytokine binding	0.000147	5,11,2
1fhga_#119	GO:0019955: cytokine binding	0.000147	5,11,2
1pvc1_#182	GO:0003724: RNA helicase activity	0.000147	5,11,2
1qfja2#201	GO:0010181: FMN binding	0.000147	5,11,2
1e32a1#26	GO:0004364: glutathione transferase activity	0.000147	5,11,2
1hxn__#235	GO:0003724: RNA helicase activity	0.000147	5,11,2
1f21a_#68	GO:0000287: magnesium ion binding	0.000147	44,128,9
1bak__#583	GO:0005524: ATP binding	0.000147	131,243,25
1qaua_#64	GO:0010181: FMN binding	0.000147	5,11,2
1utea_#11	GO:0004812: tRNA ligase activity	0.000147	130,26,7
1dofa_#15	GO:0005085: guanyl-nucleotide exchange factor activity	0.000148	197,13,6
1tuba1#239	GO:0016861: intramolecular oxidoreductase activity, interconverting aldoses and ketoses	0.000148	120,13,5
1fjfl_#67	GO:0005509: calcium ion binding	0.000148	175,160,23
2napa2#292	GO:0008026: ATP-dependent helicase activity	0.000148	120,13,5
1je5a_#10	GO:0000049: tRNA binding	0.000149	62,13,4
1gg6.1#B19	GO:0005529: sugar binding	0.000149	59,39,6
1feca1#28	GO:0008026: ATP-dependent helicase activity	0.000149	62,13,4
2pia_1#54	GO:0005524: ATP binding	0.000149	220,243,36
1cpy__#380	GO:0004263: chymotrypsin activity	0.000149	171,41,10
1phm_2#253	GO:0003887: DNA-directed DNA polymerase activity	0.000149	309,20,9
1qs0a1#119	GO:0016866: intramolecular transferase activity	0.000149	454,12,8
1kapp2#149	GO:0008270: zinc ion binding	0.00015	22,108,6
1by5a_#238	GO:0005524: ATP binding	0.00015	237,243,38
2arca_#55	GO:0016638: oxidoreductase activity, acting on the CH-NH2 group of donors	0.00015	144,17,6
1a3qa2#190	GO:0016638: oxidoreductase activity, acting on the CH-NH2 group of donors	0.00015	144,17,6
2cba__#144	GO:0005525: GTP binding	0.00015	67,49,7
1cs1a_#82	GO:0005524: ATP binding	0.00015	195,243,33
1ez0a_#150	GO:0016705: oxidoreductase activity, acting on paired donors, with incorporation or reduction of molecular oxygen	0.000151	30,26,4
1bvsa3#12	GO:0004812: tRNA ligase activity	0.000151	30,26,4
1seta2#306	GO:0004812: tRNA ligase activity	0.000151	30,26,4
1ak0__#115	GO:0004497: monooxygenase activity	0.000151	30,26,4
1ycsa_#162	GO:0051082: unfolded protein binding	0.000151	210,34,10
1a0i_2#222	GO:0005507: copper ion binding	0.000151	186,38,10
1fc4a_#205	GO:0016776: phosphotransferase activity, phosphate group as acceptor	0.000151	267,14,7
1gci__#175	GO:0004867: serine-type endopeptidase inhibitor activity	0.000151	70,47,7
1e5sa_#239	GO:0016705: oxidoreductase activity, acting on paired donors, with incorporation or reduction of molecular oxygen	0.000151	30,26,4
1dgw.1#Y353	GO:0019838: growth factor binding	0.000152	319,9,6
1e3ja1#136	GO:0005066: transmembrane receptor protein tyrosine kinase signaling protein activity	0.000152	31,10,3
1gox__#124	GO:0010181: FMN binding	0.000152	366,11,7
1cs6a4#363	GO:0004896: hematopoietin/interferon-class (D200-domain) cytokine receptor activity	0.000152	16,19,3
1t7pa2#261	GO:0003887: DNA-directed DNA polymerase activity	0.000153	3,20,2
1dzfa2#211	GO:0030151: molybdenum ion binding	0.000153	167,15,6
7reqa2#702	GO:0016831: carboxy-lyase activity	0.000153	59,25,5

1cs1a_#160	GO:0005524: ATP binding	0.000153	187,243,32
1et9a2#138	GO:0000287: magnesium ion binding	0.000153	35,128,8
1lucb_#70	GO:0000287: magnesium ion binding	0.000153	35,128,8
1ei9a_#227	GO:0003887: DNA-directed DNA polymerase activity	0.000153	3,20,2
1jd0a_#176	GO:0004263: chymotrypsin activity	0.000154	207,41,11
1by5a_#347	GO:0004190: aspartic-type endopeptidase activity	0.000154	263,23,9
1ryph_#163	GO:0000049: tRNA binding	0.000154	121,13,5
1p35a_#155	GO:0004674: protein serine/threonine kinase activity	0.000154	55,42,6
1p35a_#155	GO:0008083: growth factor activity	0.000154	55,42,6
1el5a1#10	GO:0016638: oxidoreductase activity, acting on the CH-NH2 group of donors	0.000155	18,17,3
1fw8a_#126	GO:0008483: transaminase activity	0.000155	18,17,3
1ewna_#109	GO:0005126: hematopoietin/interferon-class (D200-domain) cytokine receptor binding	0.000155	75,20,5
1bfg__#22	GO:0005507: copper ion binding	0.000155	61,38,6
1thg__#127	GO:0008483: transaminase activity	0.000155	18,17,3
1ddma_#130	GO:0005516: calmodulin binding	0.000155	313,24,10
1d2oa1#541	GO:0019838: growth factor binding	0.000155	35,9,3
1fjfc2#153	GO:0008800: beta-lactamase activity	0.000155	420,10,7
1bfg__#22	GO:0005509: calcium ion binding	0.000156	61,160,12
1qs0a1#119	GO:0008757: S-adenosylmethionine-dependent methyltransferase activity	0.000156	454,24,12
1e7ka_#53	GO:0019843: rRNA binding	0.000157	14,22,3
1eh9a3#376	GO:0005509: calcium ion binding	0.000157	6,160,4
3btaa3#194	GO:0016638: oxidoreductase activity, acting on the CH-NH2 group of donors	0.000157	47,17,4
2sli_2#326	GO:0008201: heparin binding	0.000157	251,24,9
1hx0a2#258	GO:0005509: calcium ion binding	0.000157	6,160,4
1fiy__#80	GO:0000287: magnesium ion binding	0.000157	19,128,6
1hdfa_#16	GO:0005509: calcium ion binding	0.000157	223,160,27
1ejba_#81	GO:0016763: transferase activity, transferring pentosyl groups	0.000157	84,28,6
1i7qa_#321	GO:0003724: RNA helicase activity	0.000157	148,11,5
1epwa1#901	GO:0008201: heparin binding	0.000158	62,24,5
1fbl_1#398	GO:0008201: heparin binding	0.000158	62,24,5
2pia_1#54	GO:0016668: oxidoreductase activity, acting on sulfur group of donors, NAD or NADP as acceptor	0.000158	220,12,6
3rpba_#557	GO:0004812: tRNA ligase activity	0.000158	57,26,5
1vmoa_#106	GO:0005507: copper ion binding	0.000158	187,38,10
1d0na3#305	GO:0005509: calcium ion binding	0.000159	311,160,34
1c0pa1#1147	GO:0015036: disulfide oxidoreductase activity	0.000159	36,22,4
1pii_2#326	GO:0016616: oxidoreductase activity, acting on the CH-OH group of donors, NAD or NADP as acceptor	0.000159	192,59,13
1jeta_#314	GO:0004812: tRNA ligase activity	0.00016	12,26,3
1i8da1#85	GO:0004497: monooxygenase activity	0.00016	12,26,3
1bu7a_#130	GO:0016705: oxidoreductase activity, acting on paired donors, with incorporation or reduction of molecular oxygen	0.00016	12,26,3
1bu7a_#130	GO:0004497: monooxygenase activity	0.00016	12,26,3
1a59__#341	GO:0004497: monooxygenase activity	0.00016	12,26,3
1czya1#466	GO:0005524: ATP binding	0.00016	109,243,22
1g8fa2#258	GO:0004812: tRNA ligase activity	0.00016	12,26,3
1jj2e1#42	GO:0050660: FAD binding	0.00016	167,10,5
1thfd_#140	GO:0005524: ATP binding	0.00016	124,243,24
1apme_#74	GO:0005524: ATP binding	0.00016	124,243,24
1iira_#326	GO:0016776: phosphotransferase activity, phosphate group as acceptor	0.00016	112,14,5
1dbga_#38	GO:0005524: ATP binding	0.00016	30,243,10
1bmv2_#2064	GO:0005529: sugar binding	0.00016	147,39,9
1abrb2#252	GO:0008083: growth factor activity	0.00016	19,42,4
1ton__#47	GO:0003968: RNA-directed RNA polymerase activity	0.00016	112,14,5
1a9xb2#1775	GO:0004180: carboxypeptidase activity	0.00016	54,15,4
2mpra_#267	GO:0004263: chymotrypsin activity	0.000161	36,41,5
1danh_#104	GO:0004714: transmembrane receptor protein tyrosine kinase activity	0.000161	183,14,6
1as4.1#A336	GO:0004896: hematopoietin/interferon-class (D200-domain) cytokine receptor activity	0.000161	332,19,9
1hxxa_#232	GO:0051082: unfolded protein binding	0.000161	352,34,13
1ihua1#119	GO:0003682: chromatin binding	0.000161	85,10,4
1a3qa2#190	GO:0008201: heparin binding	0.000161	144,24,7
1ad1a_#186	GO:0016861: intramolecular oxidoreductase activity, interconverting aldoses and ketoses	0.000162	24,13,3
1fgka_#668	GO:0005516: calmodulin binding	0.000162	13,24,3
1ie5a_#23	GO:0005507: copper ion binding	0.000162	21,38,4
1csn__#84	GO:0004295: trypsin activity	0.000162	207,48,12
1bwvs_#117	GO:0003697: single-stranded DNA binding	0.000162	329,12,7

1qkia1#158	GO:0008235: metalloexopeptidase activity	0.000162	24,13,3
2tbva_#214	GO:0004197: cysteine-type endopeptidase activity	0.000162	13,24,3
1b35b_#226	GO:0004197: cysteine-type endopeptidase activity	0.000162	13,24,3
1cmia_#86	GO:0005126: hematopoietin/interferon-class (D200-domain) cytokine receptor binding	0.000163	40,20,4
1rypk_#12	GO:0004190: aspartic-type endopeptidase activity	0.000163	265,23,9
1e8ua_#296	GO:0005507: copper ion binding	0.000164	39,38,5
1hbza_#214	GO:0005518: collagen binding	0.000164	297,13,7
1nat__#50	GO:0019201: nucleotide kinase activity	0.000164	297,13,7
3grs_1#23	GO:0004457: lactate dehydrogenase activity	0.000164	168,10,5
1ac6a_#34	GO:0005529: sugar binding	0.000164	60,39,6
1hdma1#115	GO:0004896: hematopoietin/interferon-class (D200-domain) cytokine receptor activity	0.000165	188,19,7
1ev2e2#286	GO:0008201: heparin binding	0.000165	100,24,6
1hxxa_#173	GO:0005529: sugar binding	0.000165	260,39,12
1d0na3#305	GO:0005507: copper ion binding	0.000165	311,38,13
1a4ya_#47	GO:0015036: disulfide oxidoreductase activity	0.000165	216,22,8
1czan1#92	GO:0003968: RNA-directed RNA polymerase activity	0.000166	631,14,10
1h6ka2#336	GO:0004601: peroxidase activity	0.000166	168,21,7
1gox__#246	GO:0016836: hydro-lyase activity	0.000166	177,33,9
1qqga2#253	GO:0019843: rRNA binding	0.000166	110,22,6
1j9qa1#154	GO:0005524: ATP binding	0.000166	196,243,33
1c4ra_#258	GO:0005509: calcium ion binding	0.000166	80,160,14
1esma_#314	GO:0003968: RNA-directed RNA polymerase activity	0.000167	113,14,5
1f97a1#62	GO:0019955: cytokine binding	0.000167	707,11,9
1bg6_2#73	GO:0016620: oxidoreductase activity, acting on the aldehyde or oxo group of donors, NAD or NADP as acceptor	0.000167	32,10,3
1jlxa2#166	GO:0019955: cytokine binding	0.000167	77,11,4
1g8ka2#570	GO:0016831: carboxy-lyase activity	0.000167	32,25,4
1eqga2#47	GO:0004263: chymotrypsin activity	0.000168	8,41,3
1bg6_1#294	GO:0008810: cellulase activity	0.000168	45,18,4
2ltn.1#A63	GO:0004190: aspartic-type endopeptidase activity	0.000168	152,23,7
1bqca_#24	GO:0008810: cellulase activity	0.000168	45,18,4
1eqga2#69	GO:0004601: peroxidase activity	0.000169	3,21,2
1sbp__#124	GO:0030151: molybdenum ion binding	0.000169	4,15,2
1bn7a_#252	GO:0004180: carboxypeptidase activity	0.000169	4,15,2
1gof_2#118	GO:0016616: oxidoreductase activity, acting on the CH-OH group of donors, NAD or NADP as acceptor	0.000169	14,59,4
1e43a1#481	GO:0004556: alpha-amylase activity	0.000169	4,15,2
1alo_3#222	GO:0030151: molybdenum ion binding	0.000169	4,15,2
1hcnb_#32	GO:0005179: hormone activity	0.000169	4,15,2
1hr6b2#255	GO:0015078: hydrogen ion transporter activity	0.000169	3,21,2
1g62a_#69	GO:0003743: translation initiation factor activity	0.000169	4,15,2
1bccb1#130	GO:0015078: hydrogen ion transporter activity	0.000169	3,21,2
1ezvd1#101	GO:0015078: hydrogen ion transporter activity	0.000169	3,21,2
1a99a_#84	GO:0005351: sugar porter activity	0.000169	15,21,3
2pia_3#270	GO:0030151: molybdenum ion binding	0.000169	4,15,2
2pia_3#280	GO:0030151: molybdenum ion binding	0.000169	4,15,2
1c4xa_#69	GO:0004601: peroxidase activity	0.000169	3,21,2
1qo7a_#149	GO:0004601: peroxidase activity	0.000169	3,21,2
1g61a_#2115	GO:0003743: translation initiation factor activity	0.000169	4,15,2
1gci__#175	GO:0042802: protein self binding	0.000169	70,12,4
1ga0a_#236	GO:0004180: carboxypeptidase activity	0.000169	21,15,3
2liv__#81	GO:0005351: sugar porter activity	0.000169	15,21,3
1fj2a_#24	GO:0004601: peroxidase activity	0.000169	3,21,2
1c9oa_#49	GO:0005524: ATP binding	0.000169	102,243,21
1cqqa_#163	GO:0003724: RNA helicase activity	0.000169	29,11,3
1gox__#124	GO:0016831: carboxy-lyase activity	0.000169	366,25,11
1qlwa_#231	GO:0004601: peroxidase activity	0.000169	3,21,2
1qlab2#76	GO:0030151: molybdenum ion binding	0.000169	4,15,2
1hxma2#150	GO:0019955: cytokine binding	0.000169	29,11,3
1iira_#242	GO:0004180: carboxypeptidase activity	0.000169	170,15,6
1iira_#242	GO:0004556: alpha-amylase activity	0.000169	170,15,6
1ax4a_#275	GO:0005524: ATP binding	0.000169	102,243,21
1f97a1#62	GO:0005518: collagen binding	0.00017	707,13,10
1fw9a_#152	GO:0004812: tRNA ligase activity	0.00017	133,26,7
1d0na6#681	GO:0003779: actin binding	0.00017	143,32,8
1smaa2#581	GO:0004674: protein serine/threonine kinase activity	0.00017	80,42,7

1jsg__#92	GO:0003916: DNA topoisomerase activity	0.00017	607,10,8
1el0a_#42	GO:0046983: protein dimerization activity	0.00017	294,17,8
1fjfc2#153	GO:0004896: hematopoietin/interferon-class (D200-domain) cytokine receptor activity	0.00017	420,19,10
1a65a1#108	GO:0046983: protein dimerization activity	0.00017	294,17,8
1qj2c2#50	GO:0008483: transaminase activity	0.000171	48,17,4
1hcl__#82	GO:0016638: oxidoreductase activity, acting on the CH-NH2 group of donors	0.000171	48,17,4
1iba__#39	GO:0019843: rRNA binding	0.000171	351,22,10
1bwda_#295	GO:0008270: zinc ion binding	0.000171	104,108,13
1f97a1#62	GO:0005529: sugar binding	0.000172	707,39,21
1vns__#487	GO:0004497: monooxygenase activity	0.000172	31,26,4
1fw9a_#152	GO:0008270: zinc ion binding	0.000172	133,108,15
1exma3#99	GO:0016646: oxidoreductase activity, acting on the CH-NH group of donors, NAD or NADP as acceptor	0.000172	81,19,5
1hrka_#343	GO:0016616: oxidoreductase activity, acting on the CH-OH group of donors, NAD or NADP as acceptor	0.000173	40,59,6
1yrga_#232	GO:0016616: oxidoreductase activity, acting on the CH-OH group of donors, NAD or NADP as acceptor	0.000173	40,59,6
1i50a_#856	GO:0005507: copper ion binding	0.000173	269,38,12
1feca3#467	GO:0030145: manganese ion binding	0.000173	89,38,7
1e79d3#328	GO:0016616: oxidoreductase activity, acting on the CH-OH group of donors, NAD or NADP as acceptor	0.000173	40,59,6
1p35a_#49	GO:0000049: tRNA binding	0.000173	124,13,5
1c9la2#12	GO:0016638: oxidoreductase activity, acting on the CH-NH2 group of donors	0.000174	295,17,8
1cpy__#380	GO:0004180: carboxypeptidase activity	0.000174	171,15,6
1qs0a1#235	GO:0016776: phosphotransferase activity, phosphate group as acceptor	0.000174	114,14,5
1gpea1#271	GO:0008201: heparin binding	0.000174	197,24,8
1iira_#242	GO:0004457: lactate dehydrogenase activity	0.000174	170,10,5
1iira_#242	GO:0016620: oxidoreductase activity, acting on the aldehyde or oxo group of donors, NAD or NADP as acceptor	0.000174	170,10,5
1f8v.1#A116	GO:0004197: cysteine-type endopeptidase activity	0.000175	101,24,6
1fxla2#169	GO:0003729: mRNA binding	0.000175	203,13,6
1e1oa2#423	GO:0005524: ATP binding	0.000175	148,243,27
5ruba1#279	GO:0016616: oxidoreductase activity, acting on the CH-OH group of donors, NAD or NADP as acceptor	0.000176	57,59,7
1dssg1#4	GO:0016616: oxidoreductase activity, acting on the CH-OH group of donors, NAD or NADP as acceptor	0.000176	57,59,7
1jlxa2#166	GO:0005126: hematopoietin/interferon-class (D200-domain) cytokine receptor binding	0.000176	77,20,5
1fn9a_#271	GO:0019843: rRNA binding	0.000176	218,22,8
3prn__#143	GO:0004190: aspartic-type endopeptidase activity	0.000176	334,23,10
1axn__#42	GO:0004364: glutathione transferase activity	0.000176	78,11,4
1bf4a_#4	GO:0005524: ATP binding	0.000176	25,243,9
1cqqa_#163	GO:0005509: calcium ion binding	0.000176	29,160,8
1eo6a_#107	GO:0004364: glutathione transferase activity	0.000176	78,11,4
1cwva1#535	GO:0016702: oxidoreductase activity, acting on single donors with incorporation of molecular oxygen, incorporation of two atoms of oxygen	0.000177	5,12,2
1ih7a1#260	GO:0000155: two-component sensor molecule activity	0.000177	5,12,2
1dfma_#147	GO:0009036: type II site-specific deoxyribonuclease activity	0.000177	5,12,2
1muca1#245	GO:0000287: magnesium ion binding	0.000177	45,128,9
1e0ca2#193	GO:0016668: oxidoreductase activity, acting on sulfur group of donors, NAD or NADP as acceptor	0.000177	5,12,2
1quba4#235	GO:0004190: aspartic-type endopeptidase activity	0.000177	106,23,6
1dmla2#297	GO:0003887: DNA-directed DNA polymerase activity	0.000178	16,20,3
1a6ca2#312	GO:0004812: tRNA ligase activity	0.000178	93,26,6
1gsa_2#218	GO:0004896: hematopoietin/interferon-class (D200-domain) cytokine receptor activity	0.000178	131,19,6
1uch__#50	GO:0016616: oxidoreductase activity, acting on the CH-OH group of donors, NAD or NADP as acceptor	0.000178	76,59,8
1f46a_#59	GO:0004725: protein tyrosine phosphatase activity	0.000178	106,15,5
1fo4a5#1002	GO:0004812: tRNA ligase activity	0.000178	93,26,6
1tif__#58	GO:0000049: tRNA binding	0.000179	65,13,4
3chbd_#85	GO:0015082: di-, tri-valent inorganic cation transporter activity	0.000179	378,14,8
3chbd_#85	GO:0004714: transmembrane receptor protein tyrosine kinase activity	0.000179	378,14,8
3chbd_#85	GO:0046915: transition metal ion transporter activity	0.000179	378,14,8
1psda3#344	GO:0008408: 3'-5' exonuclease activity	0.000179	125,13,5
1ryp1_#20	GO:0000049: tRNA binding	0.000179	125,13,5
1h8ua_#113	GO:0004177: aminopeptidase activity	0.000179	65,13,4
1foha5#9	GO:0019201: nucleotide kinase activity	0.000179	65,13,4

1ig8a_#203	GO:0016861: intramolecular oxidoreductase activity, interconverting aldoses and ketoses	0.000179	65,13,4
1hq0a_#867	GO:0008800: beta-lactamase activity	0.000179	171,10,5
1qs0a1#119	GO:0004725: protein tyrosine phosphatase activity	0.000179	454,15,9
1dqua_#456	GO:0004457: lactate dehydrogenase activity	0.00018	6,10,2
1fmk_3#283	GO:0005066: transmembrane receptor protein tyrosine kinase signaling protein activity	0.00018	6,10,2
1gsoa3#193	GO:0003682: chromatin binding	0.00018	6,10,2
2mpra_#222	GO:0016251: general RNA polymerase II transcription factor activity	0.00018	23,14,3
1qtn.1#A331	GO:0016668: oxidoreductase activity, acting on sulfur group of donors, NAD or NADP as acceptor	0.00018	27,12,3
2cpl__#20	GO:0004674: protein serine/threonine kinase activity	0.00018	108,42,8
2cpl__#20	GO:0008083: growth factor activity	0.00018	108,42,8
1iq4a_#31	GO:0016702: oxidoreductase activity, acting on single donors with incorporation of molecular oxygen, incorporation of two atoms of oxygen	0.00018	27,12,3
1dxy_1#170	GO:0003684: damaged DNA binding	0.00018	6,10,2
1qhla_#28	GO:0005516: calmodulin binding	0.00018	34,24,4
1e3ua_#187	GO:0008800: beta-lactamase activity	0.00018	6,10,2
1nhp_2#143	GO:0050660: FAD binding	0.00018	6,10,2
1hzxa_#139	GO:0015078: hydrogen ion transporter activity	0.00018	39,21,4
2oata_#120	GO:0016846: carbon-sulfur lyase activity	0.00018	6,10,2
1mpp__#154	GO:0019838: growth factor binding	0.00018	99,9,4
1fhga_#59	GO:0008201: heparin binding	0.00018	34,24,4
3grx__#56	GO:0004190: aspartic-type endopeptidase activity	0.00018	335,23,10
1apme_#74	GO:0003887: DNA-directed DNA polymerase activity	0.00018	124,20,6
1bw0a_#199	GO:0016846: carbon-sulfur lyase activity	0.00018	6,10,2
1sfp__#84	GO:0008083: growth factor activity	0.000181	8,42,3
1pmc__#10	GO:0005524: ATP binding	0.000181	36,243,11
1hcd__#91	GO:0008083: growth factor activity	0.000181	8,42,3
1jlxa1#147	GO:0008083: growth factor activity	0.000181	8,42,3
1jj2j_#88	GO:0005524: ATP binding	0.000181	36,243,11
1fsu__#434	GO:0051082: unfolded protein binding	0.000181	356,34,13
1dmla1#134	GO:0004896: hematopoietin/interferon-class (D200-domain) cytokine receptor activity	0.000182	82,19,5
1zpda1#213	GO:0016646: oxidoreductase activity, acting on the CH-NH group of donors, NAD or NADP as acceptor	0.000182	82,19,5
1psda3#344	GO:0005524: ATP binding	0.000182	125,243,24
3grx__#56	GO:0016668: oxidoreductase activity, acting on sulfur group of donors, NAD or NADP as acceptor	0.000182	335,12,7
1pma1_#79	GO:0019843: rRNA binding	0.000182	70,22,5
1nal1_#203	GO:0016836: hydro-lyase activity	0.000183	46,33,5
1epwa3#248	GO:0019838: growth factor binding	0.000183	37,9,3
1fc4a_#205	GO:0016763: transferase activity, transferring pentosyl groups	0.000183	267,28,10
1znca_#167	GO:0016836: hydro-lyase activity	0.000183	10,33,3
1jwoa_#196	GO:0005066: transmembrane receptor protein tyrosine kinase signaling protein activity	0.000183	33,10,3
1nat__#50	GO:0003924: GTPase activity	0.000183	297,17,8
1e6ua_#10	GO:0004457: lactate dehydrogenase activity	0.000183	33,10,3
2cuaa_#83	GO:0016836: hydro-lyase activity	0.000183	46,33,5
1kapp1#389	GO:0016799: hydrolase activity, hydrolyzing N-glycosyl compounds	0.000184	19,17,3
1ajsa_#326	GO:0008483: transaminase activity	0.000184	19,17,3
1ig8a_#214	GO:0008026: ATP-dependent helicase activity	0.000184	25,13,3
1oaa__#87	GO:0016616: oxidoreductase activity, acting on the CH-OH group of donors, NAD or NADP as acceptor	0.000184	6,59,3
5ruba2#41	GO:0016831: carboxy-lyase activity	0.000184	13,25,3
2foka2#180	GO:0004197: cysteine-type endopeptidase activity	0.000184	147,24,7
1qo2a_#223	GO:0016831: carboxy-lyase activity	0.000184	13,25,3
1c1da1#235	GO:0016638: oxidoreductase activity, acting on the CH-NH2 group of donors	0.000184	19,17,3
1fbl_1#398	GO:0005509: calcium ion binding	0.000184	62,160,12
1iira_#326	GO:0015036: disulfide oxidoreductase activity	0.000184	112,22,6
1dj0a2#183	GO:0004263: chymotrypsin activity	0.000184	142,41,9
1fiy__#80	GO:0008483: transaminase activity	0.000184	19,17,3
1ddja_#681	GO:0030151: molybdenum ion binding	0.000184	253,15,7
1bmv2_#2064	GO:0008201: heparin binding	0.000184	147,24,7
1nhp_1#282	GO:0016627: oxidoreductase activity, acting on the CH-CH group of donors	0.000184	19,17,3
1qtn.1#A270	GO:0016831: carboxy-lyase activity	0.000184	439,25,12
1trb_1#41	GO:0016627: oxidoreductase activity, acting on the CH-CH group of donors	0.000184	19,17,3
1hxn__#248	GO:0004222: metalloendopeptidase activity	0.000184	17,19,3
1leha1#177	GO:0016638: oxidoreductase activity, acting on the CH-NH2 group of donors	0.000185	49,17,4
1f8ra1#288	GO:0003924: GTPase activity	0.000185	49,17,4

1f8ra1#288	GO:0016638: oxidoreductase activity, acting on the CH-NH2 group of donors	0.000185	49,17,4
1h7wa2#636	GO:0016627: oxidoreductase activity, acting on the CH-CH group of donors	0.000185	49,17,4
2pia_3#282	GO:0005507: copper ion binding	0.000185	40,38,5
1rl2a1#187	GO:0019843: rRNA binding	0.000186	3,22,2
1im4a_#41	GO:0004812: tRNA ligase activity	0.000186	59,26,5
1io1a_#311	GO:0000049: tRNA binding	0.000186	126,13,5
1fo4a5#1002	GO:0046983: protein dimerization activity	0.000186	93,17,5
1db3a_#266	GO:0004812: tRNA ligase activity	0.000186	59,26,5
1xgsa2#82	GO:0051082: unfolded protein binding	0.000186	307,34,12
1arb__#210	GO:0016638: oxidoreductase activity, acting on the CH-NH2 group of donors	0.000186	93,17,5
1gpea1#271	GO:0019838: growth factor binding	0.000186	197,9,5
1qhoa3#488	GO:0019955: cytokine binding	0.000187	30,11,3
1fc3a_#172	GO:0004812: tRNA ligase activity	0.000187	182,26,8
2stv__#129	GO:0003724: RNA helicase activity	0.000187	30,11,3
3chbd_#85	GO:0019955: cytokine binding	0.000187	378,11,7
1bag_2#207	GO:0004364: glutathione transferase activity	0.000187	30,11,3
1pwt__#8	GO:0004295: trypsin activity	0.000187	314,48,15
1e4ea2#215	GO:0005509: calcium ion binding	0.000187	490,160,47
1hdma1#123	GO:0019955: cytokine binding	0.000187	30,11,3
1qfma1#139	GO:0003887: DNA-directed DNA polymerase activity	0.000187	318,20,9
1hxxa_#173	GO:0051082: unfolded protein binding	0.000188	260,34,11
1evqa_#305	GO:0003924: GTPase activity	0.000188	490,17,10
1evqa_#305	GO:0008483: transaminase activity	0.000188	490,17,10
1ffta1#441	GO:0005507: copper ion binding	0.000189	2,38,2
1fjfj_#68	GO:0005507: copper ion binding	0.000189	230,38,11
1fp1d2#214	GO:0004457: lactate dehydrogenase activity	0.000189	173,10,5
1amf__#196	GO:0004457: lactate dehydrogenase activity	0.000189	173,10,5
1amf__#196	GO:0050660: FAD binding	0.000189	173,10,5
1ej0a_#42	GO:0016251: general RNA polymerase II transcription factor activity	0.000189	116,14,5
1fo5a_#60	GO:0003887: DNA-directed DNA polymerase activity	0.00019	246,20,8
1ble__#34	GO:0000287: magnesium ion binding	0.00019	36,128,8
1eo6a_#107	GO:0008270: zinc ion binding	0.00019	78,108,11
1mrj__#156	GO:0016776: phosphotransferase activity, phosphate group as acceptor	0.000192	61,14,4
1czya1#466	GO:0004674: protein serine/threonine kinase activity	0.000192	109,42,8
1ezvb1#29	GO:0004896: hematopoietin/interferon-class (D200-domain) cytokine receptor activity	0.000192	44,19,4
1bfg__#22	GO:0004714: transmembrane receptor protein tyrosine kinase activity	0.000192	61,14,4
1b3qa2#623	GO:0005524: ATP binding	0.000192	301,243,45
1kit_2#398	GO:0004620: phospholipase activity	0.000193	83,19,5
1jj2j_#70	GO:0019843: rRNA binding	0.000194	71,22,5
1jj2v_#118	GO:0008201: heparin binding	0.000195	103,24,6
1ejfa_#10	GO:0019955: cytokine binding	0.000195	155,11,5
1mjha_#119	GO:0008757: S-adenosylmethionine-dependent methyltransferase activity	0.000195	103,24,6
1xgsa2#82	GO:0005529: sugar binding	0.000195	307,39,13
1xgsa2#186	GO:0003887: DNA-directed DNA polymerase activity	0.000195	182,20,7
1i76a_#138	GO:0005524: ATP binding	0.000195	103,243,21
1eula_#518	GO:0004812: tRNA ligase activity	0.000196	32,26,4
3prn__#143	GO:0019838: growth factor binding	0.000197	334,9,6
1bu8a2#124	GO:0016866: intramolecular transferase activity	0.000197	140,12,5
1b9ra_#102	GO:0005507: copper ion binding	0.000198	9,38,3
1e42a1#711	GO:0030151: molybdenum ion binding	0.000198	175,15,6
1qlaa3#364	GO:0004867: serine-type endopeptidase inhibitor activity	0.000198	73,47,7
1dyma_#213	GO:0019838: growth factor binding	0.000198	38,9,3
1dik_1#765	GO:0005524: ATP binding	0.000198	165,243,29
1fo3a_#593	GO:0005509: calcium ion binding	0.000199	3,160,3
1rmg__#128	GO:0005509: calcium ion binding	0.000199	3,160,3
1hxn__#236	GO:0005509: calcium ion binding	0.000199	3,160,3
1i50b_#378	GO:0003887: DNA-directed DNA polymerase activity	0.000199	79,20,5
1qfea_#68	GO:0016854: racemase and epimerase activity	0.000199	560,13,9
1dyna_#42	GO:0015036: disulfide oxidoreductase activity	0.000199	164,22,7
1lml__#535	GO:0016638: oxidoreductase activity, acting on the CH-NH2 group of donors	0.0002	50,17,4
1e4ea2#215	GO:0003887: DNA-directed DNA polymerase activity	0.0002	490,20,11
1vmoa_#106	GO:0005529: sugar binding	0.0002	187,39,10
1h4vb2#108	GO:0000287: magnesium ion binding	0.000201	13,128,5
1jc4a_#12	GO:0016702: oxidoreductase activity, acting on single donors with incorporation of molecular oxygen, incorporation of two atoms of oxygen	0.000201	28,12,3
1qo2a_#223	GO:0000287: magnesium ion binding	0.000201	13,128,5

1danh_#104	GO:0003887: DNA-directed DNA polymerase activity	0.000201	183,20,7
1f8ra2#325	GO:0015036: disulfide oxidoreductase activity	0.000201	222,22,8
1e2o__#346	GO:0004896: hematopoietin/interferon-class (D200-domain) cytokine receptor activity	0.000201	194,19,7
1avgi_#69	GO:0016702: oxidoreductase activity, acting on single donors with incorporation of molecular oxygen, incorporation of two atoms of oxygen	0.000201	340,12,7
1a9xa6#760	GO:0003809: thrombin activity	0.000201	90,10,4
1bev1_#238	GO:0003682: chromatin binding	0.000201	34,10,3
1ea5a_#99	GO:0004896: hematopoietin/interferon-class (D200-domain) cytokine receptor activity	0.000201	194,19,7
1pme__#88	GO:0005066: transmembrane receptor protein tyrosine kinase signaling protein activity	0.000201	34,10,3
1bccb2#274	GO:0016705: oxidoreductase activity, acting on paired donors, with incorporation or reduction of molecular oxygen	0.000202	60,26,5
1e8ua_#286	GO:0019838: growth factor binding	0.000202	7,9,2
1am2__#163	GO:0019838: growth factor binding	0.000202	102,9,4
1hxxa_#232	GO:0004556: alpha-amylase activity	0.000202	352,15,8
1czan1#92	GO:0016668: oxidoreductase activity, acting on sulfur group of donors, NAD or NADP as acceptor	0.000202	631,12,9
1jsg__#92	GO:0016638: oxidoreductase activity, acting on the CH-NH2 group of donors	0.000202	607,17,11
3sil__#317	GO:0019843: rRNA binding	0.000202	358,22,10
1fmb__#60	GO:0004190: aspartic-type endopeptidase activity	0.000203	3,23,2
1j71a_#264	GO:0004190: aspartic-type endopeptidase activity	0.000203	3,23,2
2bbva_#74	GO:0004190: aspartic-type endopeptidase activity	0.000203	3,23,2
1xgsa2#82	GO:0008235: metalloexopeptidase activity	0.000203	307,13,7
1e87a_#174	GO:0016668: oxidoreductase activity, acting on sulfur group of donors, NAD or NADP as acceptor	0.000204	141,12,5
1hava_#41	GO:0005524: ATP binding	0.000204	96,243,20
1leha1#255	GO:0016763: transferase activity, transferring pentosyl groups	0.000204	30,28,4
1ejda_#213	GO:0005524: ATP binding	0.000204	284,243,43
1f0ja_#414	GO:0008757: S-adenosylmethionine-dependent methyltransferase activity	0.000205	14,24,3
1abrb1#25	GO:0008201: heparin binding	0.000205	14,24,3
1kwaa_#513	GO:0003968: RNA-directed RNA polymerase activity	0.000205	62,14,4
1fw9a_#157	GO:0003968: RNA-directed RNA polymerase activity	0.000205	62,14,4
1qqp3_#139	GO:0004197: cysteine-type endopeptidase activity	0.000205	14,24,3
1a3qa2#190	GO:0004263: chymotrypsin activity	0.000205	144,41,9
1ie5a_#102	GO:0004714: transmembrane receptor protein tyrosine kinase activity	0.000205	62,14,4
1ew2a_#237	GO:0016836: hydro-lyase activity	0.000205	106,33,7
1io1a_#233	GO:0016638: oxidoreductase activity, acting on the CH-NH2 group of donors	0.000206	95,17,5
1dpga2#334	GO:0004867: serine-type endopeptidase inhibitor activity	0.000206	287,47,14
2ltn.1#A63	GO:0005529: sugar binding	0.000206	152,39,9
1e8ca1#83	GO:0016763: transferase activity, transferring pentosyl groups	0.000206	170,28,8
2dpma_#237	GO:0016799: hydrolase activity, hydrolyzing N-glycosyl compounds	0.000206	95,17,5
2mev3_#107	GO:0003724: RNA helicase activity	0.000207	31,11,3
1ek0a_#151	GO:0005085: guanyl-nucleotide exchange factor activity	0.000207	26,13,3
5ruba2#41	GO:0004497: monooxygenase activity	0.000207	13,26,3
1sgpe_#42	GO:0008408: 3'-5' exonuclease activity	0.000207	26,13,3
1io1a_#311	GO:0005524: ATP binding	0.000207	126,243,24
1c9la2#12	GO:0004812: tRNA ligase activity	0.000207	295,26,10
1mek__#17	GO:0019201: nucleotide kinase activity	0.000207	26,13,3
1fu6a_#50	GO:0000049: tRNA binding	0.000207	308,13,7
1fu6a_#50	GO:0008408: 3'-5' exonuclease activity	0.000207	308,13,7
1qtn.1#A270	GO:0016846: carbon-sulfur lyase activity	0.000207	439,10,7
1qqp1_#51	GO:0003724: RNA helicase activity	0.000207	31,11,3
1c4oa1#56	GO:0008026: ATP-dependent helicase activity	0.000208	5,13,2
1bvyf_#519	GO:0016651: oxidoreductase activity, acting on NADH or NADPH	0.000208	5,13,2
1fuia1#550	GO:0016861: intramolecular oxidoreductase activity, interconverting aldoses and ketoses	0.000208	5,13,2
1evya2#70	GO:0008408: 3'-5' exonuclease activity	0.000208	5,13,2
2pola1#113	GO:0008408: 3'-5' exonuclease activity	0.000208	5,13,2
1e44b_#15	GO:0000049: tRNA binding	0.000208	5,13,2
1jj2v_#118	GO:0051082: unfolded protein binding	0.000208	103,34,7
1f8ra2#325	GO:0046983: protein dimerization activity	0.000208	222,17,7
2mpra_#190	GO:0005529: sugar binding	0.000208	400,39,15
1aqza_#135	GO:0008408: 3'-5' exonuclease activity	0.000208	5,13,2
1f46a_#59	GO:0008270: zinc ion binding	0.000208	106,108,13
1ds1a_#172	GO:0046983: protein dimerization activity	0.000208	222,17,7
1mkp__#216	GO:0008235: metalloexopeptidase activity	0.000208	129,13,5
1e3a.1#A30	GO:0003964: RNA-directed DNA polymerase activity	0.000208	157,11,5
1h9da_#91	GO:0016638: oxidoreductase activity, acting on the CH-NH2 group of donors	0.000208	222,17,7

1dl0a_#32	GO:0004263: chymotrypsin activity	0.000209	38,41,5
2bbkh_#214	GO:0004263: chymotrypsin activity	0.000209	38,41,5
1aym3_#131	GO:0008083: growth factor activity	0.000209	412,42,16
1dr9a1#39	GO:0003755: peptidyl-prolyl cis-trans isomerase activity	0.000209	258,11,6
1d2ka1#164	GO:0016831: carboxy-lyase activity	0.000209	63,25,5
1eaja_#59	GO:0019838: growth factor binding	0.00021	202,9,5
1b8aa2#368	GO:0004896: hematopoietin/interferon-class (D200-domain) cytokine receptor activity	0.00021	135,19,6
1ojt_2#356	GO:0016646: oxidoreductase activity, acting on the CH-NH group of donors, NAD or NADP as acceptor	0.00021	45,19,4
1a8i__#824	GO:0016646: oxidoreductase activity, acting on the CH-NH group of donors, NAD or NADP as acceptor	0.00021	45,19,4
1hd2a_#35	GO:0004812: tRNA ligase activity	0.00021	185,26,8
1jeya_#104	GO:0000287: magnesium ion binding	0.000211	46,128,9
1iow_1#37	GO:0008026: ATP-dependent helicase activity	0.000211	309,13,7
1iow_1#37	GO:0016861: intramolecular oxidoreductase activity, interconverting aldoses and ketoses	0.000211	309,13,7
1htp__#16	GO:0046983: protein dimerization activity	0.000211	303,17,8
1bev1_#86	GO:0003887: DNA-directed DNA polymerase activity	0.000211	323,20,9
2bpa1_#45	GO:0003968: RNA-directed RNA polymerase activity	0.000211	192,14,6
1fo5a_#60	GO:0004674: protein serine/threonine kinase activity	0.000212	246,42,12
1uch__#50	GO:0015078: hydrogen ion transporter activity	0.000212	76,21,5
1pud__#197	GO:0016831: carboxy-lyase activity	0.000212	34,25,4
1f06a1#296	GO:0005525: GTP binding	0.000213	32,49,5
1jf9a_#379	GO:0016668: oxidoreductase activity, acting on sulfur group of donors, NAD or NADP as acceptor	0.000213	232,12,6
1kit_3#743	GO:0005126: hematopoietin/interferon-class (D200-domain) cytokine receptor binding	0.000213	250,20,8
1br9__#86	GO:0004812: tRNA ligase activity	0.000213	96,26,6
1d0ba_#191	GO:0005525: GTP binding	0.000214	120,49,9
1quqb_#87	GO:0016616: oxidoreductase activity, acting on the CH-OH group of donors, NAD or NADP as acceptor	0.000214	78,59,8
1fxla2#169	GO:0008201: heparin binding	0.000214	203,24,8
1jjya_#324	GO:0016616: oxidoreductase activity, acting on the CH-OH group of donors, NAD or NADP as acceptor	0.000214	78,59,8
1io7a_#243	GO:0016616: oxidoreductase activity, acting on the CH-OH group of donors, NAD or NADP as acceptor	0.000214	78,59,8
1thg__#223	GO:0010181: FMN binding	0.000214	82,11,4
1rypb_#114	GO:0003964: RNA-directed DNA polymerase activity	0.000214	158,11,5
1j75a_#166	GO:0005525: GTP binding	0.000214	120,49,9
1qasa3#352	GO:0016638: oxidoreductase activity, acting on the CH-NH2 group of donors	0.000215	20,17,3
1ksia1#555	GO:0003924: GTPase activity	0.000215	20,17,3
1qe3a_#7	GO:0016638: oxidoreductase activity, acting on the CH-NH2 group of donors	0.000215	20,17,3
1e6ca_#68	GO:0008483: transaminase activity	0.000215	20,17,3
1be9a_#357	GO:0004674: protein serine/threonine kinase activity	0.000215	83,42,7
1b16a_#184	GO:0016627: oxidoreductase activity, acting on the CH-CH group of donors	0.000215	20,17,3
1ea5a_#99	GO:0005507: copper ion binding	0.000215	194,38,10
1c3pa_#6	GO:0005524: ATP binding	0.000215	82,243,18
1b16a_#62	GO:0016627: oxidoreductase activity, acting on the CH-CH group of donors	0.000215	20,17,3
1xo1a2#159	GO:0005524: ATP binding	0.000215	55,243,14
1f2na_#138	GO:0016799: hydrolase activity, hydrolyzing N-glycosyl compounds	0.000216	96,17,5
1hava_#41	GO:0016638: oxidoreductase activity, acting on the CH-NH2 group of donors	0.000216	96,17,5
1d6ja_#95	GO:0000287: magnesium ion binding	0.000216	67,128,11
1nfdb1#21	GO:0046983: protein dimerization activity	0.000216	96,17,5
1a65a1#108	GO:0003809: thrombin activity	0.000216	294,10,6
1hava_#157	GO:0004295: trypsin activity	0.000217	18,48,4
1fc6a2#454	GO:0003887: DNA-directed DNA polymerase activity	0.000217	43,20,4
1erv__#80	GO:0003809: thrombin activity	0.000217	442,10,7
1erv__#80	GO:0050660: FAD binding	0.000217	442,10,7
1bf6a_#183	GO:0008810: cellulase activity	0.000217	48,18,4
1i6vd_#759	GO:0046983: protein dimerization activity	0.000218	4,17,2
1jr1a1#68	GO:0016627: oxidoreductase activity, acting on the CH-CH group of donors	0.000218	4,17,2
1i6vd_#766	GO:0046983: protein dimerization activity	0.000218	4,17,2
1hcnb_#32	GO:0046983: protein dimerization activity	0.000218	4,17,2
1hd7a_#203	GO:0016799: hydrolase activity, hydrolyzing N-glycosyl compounds	0.000218	4,17,2
2pia_3#270	GO:0016627: oxidoreductase activity, acting on the CH-CH group of donors	0.000218	4,17,2
2pia_3#280	GO:0016627: oxidoreductase activity, acting on the CH-CH group of donors	0.000218	4,17,2
1eur__#68	GO:0016638: oxidoreductase activity, acting on the CH-NH2 group of donors	0.000218	4,17,2
2dkb__#114	GO:0005524: ATP binding	0.000218	174,243,30

1fcya_#311	GO:0016705: oxidoreductase activity, acting on paired donors, with incorporation or reduction of molecular oxygen	0.000218	61,26,5
1fcya_#311	GO:0004497: monooxygenase activity	0.000218	61,26,5
2bb2_1#57	GO:0019838: growth factor binding	0.000218	104,9,4
1tiid_#39	GO:0004812: tRNA ligase activity	0.000218	239,26,9
1tiid_#39	GO:0004497: monooxygenase activity	0.000218	239,26,9
1nsca_#236	GO:0016668: oxidoreductase activity, acting on sulfur group of donors, NAD or NADP as acceptor	0.000218	143,12,5
1cex__#109	GO:0003682: chromatin binding	0.000219	35,10,3
1c8ua2#206	GO:0016814: hydrolase activity, acting on carbon-nitrogen (but not peptide) bonds, in cyclic amidines	0.000219	35,10,3
1fbna_#145	GO:0016814: hydrolase activity, acting on carbon-nitrogen (but not peptide) bonds, in cyclic amidines	0.000219	35,10,3
2ae2a_#89	GO:0016814: hydrolase activity, acting on carbon-nitrogen (but not peptide) bonds, in cyclic amidines	0.000219	35,10,3
1jg8a_#172	GO:0016846: carbon-sulfur lyase activity	0.000219	92,10,4
1eny__#94	GO:0016620: oxidoreductase activity, acting on the aldehyde or oxo group of donors, NAD or NADP as acceptor	0.000219	35,10,3
1el5a1#10	GO:0016646: oxidoreductase activity, acting on the CH-NH group of donors, NAD or NADP as acceptor	0.00022	18,19,3
1j8ra_#26	GO:0015078: hydrogen ion transporter activity	0.00022	41,21,4
1tns__#50	GO:0003755: peptidyl-prolyl cis-trans isomerase activity	0.00022	6,11,2
1d5ya3#126	GO:0003724: RNA helicase activity	0.00022	6,11,2
1wdna_#79	GO:0016646: oxidoreductase activity, acting on the CH-NH group of donors, NAD or NADP as acceptor	0.00022	18,19,3
1bn8a_#241	GO:0004222: metalloendopeptidase activity	0.00022	18,19,3
1e79h2#77	GO:0004263: chymotrypsin activity	0.00022	21,41,4
1qp8a1#172	GO:0016646: oxidoreductase activity, acting on the CH-NH group of donors, NAD or NADP as acceptor	0.00022	18,19,3
2pia_2#123	GO:0010181: FMN binding	0.00022	6,11,2
1a8p_1#55	GO:0010181: FMN binding	0.00022	6,11,2
1eqca_#15	GO:0008081: phosphoric diester hydrolase activity	0.00022	6,11,2
1e8ya2#433	GO:0004896: hematopoietin/interferon-class (D200-domain) cytokine receptor activity	0.00022	18,19,3
1hrna_#320	GO:0004896: hematopoietin/interferon-class (D200-domain) cytokine receptor activity	0.00022	18,19,3
1a0la_#176	GO:0004263: chymotrypsin activity	0.000221	2,41,2
1bio__#189	GO:0004263: chymotrypsin activity	0.000221	2,41,2
1g57a_#122	GO:0004263: chymotrypsin activity	0.000221	2,41,2
1dofa_#15	GO:0004896: hematopoietin/interferon-class (D200-domain) cytokine receptor activity	0.000221	197,19,7
1b0ua_#174	GO:0016836: hydro-lyase activity	0.000221	26,33,4
1a9xa3#78	GO:0008483: transaminase activity	0.000221	305,17,8
1bvqa_#70	GO:0008757: S-adenosylmethionine-dependent methyltransferase activity	0.000222	3,24,2
1c8na_#165	GO:0004197: cysteine-type endopeptidase activity	0.000222	3,24,2
1ghqb1#4	GO:0008201: heparin binding	0.000222	3,24,2
3grs_3#435	GO:0008800: beta-lactamase activity	0.000222	179,10,5
1jj2a1#126	GO:0019843: rRNA binding	0.000222	73,22,5
1be9a_#357	GO:0010181: FMN binding	0.000224	83,11,4
1ej8a_#189	GO:0005524: ATP binding	0.000224	294,243,44
1bak__#583	GO:0000049: tRNA binding	0.000224	131,13,5
1fa0a1#393	GO:0004180: carboxypeptidase activity	0.000224	261,15,7
1kit_2#398	GO:0003724: RNA helicase activity	0.000224	83,11,4
1im4a_#144	GO:0003887: DNA-directed DNA polymerase activity	0.000225	129,20,6
1fi2a_#109	GO:0005529: sugar binding	0.000225	228,39,11
1lci__#26	GO:0004497: monooxygenase activity	0.000225	139,26,7
1fsu__#434	GO:0005529: sugar binding	0.000225	356,39,14
1mil__#31	GO:0005524: ATP binding	0.000225	104,243,21
1mkp__#216	GO:0003887: DNA-directed DNA polymerase activity	0.000225	129,20,6
1h6va2#219	GO:0008757: S-adenosylmethionine-dependent methyltransferase activity	0.000226	36,24,4
1e39a3#417	GO:0005509: calcium ion binding	0.000227	11,160,5
1opy__#68	GO:0005507: copper ion binding	0.000227	367,38,14
1dbxa_#75	GO:0005085: guanyl-nucleotide exchange factor activity	0.000227	69,13,4
1p35a_#49	GO:0005507: copper ion binding	0.000227	124,38,8
1f0xa1#278	GO:0008009: chemokine activity	0.000228	180,10,5
1f0xa1#278	GO:0016620: oxidoreductase activity, acting on the aldehyde or oxo group of donors, NAD or NADP as acceptor	0.000228	180,10,5
1ezwa_#245	GO:0010181: FMN binding	0.000228	32,11,3
1ebma2#47	GO:0003724: RNA helicase activity	0.000228	32,11,3

1hc7a2#258	GO:0015036: disulfide oxidoreductase activity	0.000228	226,22,8
1g6oa_#116	GO:0004725: protein tyrosine phosphatase activity	0.000228	358,15,8
2sqca1#25	GO:0003779: actin binding	0.000228	11,32,3
1ddwa_#77	GO:0051082: unfolded protein binding	0.000228	364,34,13
1e7wa_#36	GO:0016646: oxidoreductase activity, acting on the CH-NH group of donors, NAD or NADP as acceptor	0.000228	137,19,6
1bag_2#207	GO:0005509: calcium ion binding	0.000229	30,160,8
1deua_#151	GO:0004714: transmembrane receptor protein tyrosine kinase activity	0.000229	285,14,7
2cb5a_#395	GO:0005516: calmodulin binding	0.000229	67,24,5
1fhoa_#74	GO:0016668: oxidoreductase activity, acting on sulfur group of donors, NAD or NADP as acceptor	0.000229	347,12,7
1edza1#206	GO:0003924: GTPase activity	0.000231	399,17,9
1b6cb_#484	GO:0004674: protein serine/threonine kinase activity	0.000232	2,42,2
1f24a_#144	GO:0019201: nucleotide kinase activity	0.000232	27,13,3
2btva_#597	GO:0008408: 3'-5' exonuclease activity	0.000232	27,13,3
1fyhb1#99	GO:0005518: collagen binding	0.000232	27,13,3
1i6vd_#1188	GO:0005085: guanyl-nucleotide exchange factor activity	0.000232	27,13,3
1ayl__#237	GO:0005529: sugar binding	0.000232	357,39,14
1tbrr1#15	GO:0016251: general RNA polymerase II transcription factor activity	0.000232	64,14,4
1bjt__#890	GO:0019843: rRNA binding	0.000232	292,22,9
1qdla_#211	GO:0004295: trypsin activity	0.000232	320,48,15
1hxmb1#117	GO:0003968: RNA-directed RNA polymerase activity	0.000232	64,14,4
1doi__#42	GO:0005524: ATP binding	0.000233	16,243,7
1hc7a2#258	GO:0016638: oxidoreductase activity, acting on the CH-NH2 group of donors	0.000233	226,17,7
1efpa1#32	GO:0016763: transferase activity, transferring pentosyl groups	0.000233	129,28,7
1fp1d2#214	GO:0016763: transferase activity, transferring pentosyl groups	0.000233	173,28,8
1cs6a4#363	GO:0005524: ATP binding	0.000233	16,243,7
1exh__#44	GO:0016638: oxidoreductase activity, acting on the CH-NH2 group of donors	0.000234	52,17,4
1aky_2#133	GO:0016638: oxidoreductase activity, acting on the CH-NH2 group of donors	0.000234	156,17,6
1b8aa2#403	GO:0019843: rRNA binding	0.000234	117,22,6
1el0a_#28	GO:0019955: cytokine binding	0.000234	161,11,5
1jlna_#383	GO:0016627: oxidoreductase activity, acting on the CH-CH group of donors	0.000234	52,17,4
1c7na_#95	GO:0004295: trypsin activity	0.000234	183,48,11
1fmta2#151	GO:0008235: metalloexopeptidase activity	0.000234	314,13,7
1qfea_#49	GO:0016627: oxidoreductase activity, acting on the CH-CH group of donors	0.000234	52,17,4
1pwt__#8	GO:0003729: mRNA binding	0.000234	314,13,7
1f0xa1#482	GO:0005507: copper ion binding	0.000235	42,38,5
1i9za_#666	GO:0051082: unfolded protein binding	0.000235	47,34,5
1quba4#235	GO:0019838: growth factor binding	0.000235	106,9,4
1fbl_1#368	GO:0008270: zinc ion binding	0.000235	4,108,3
1c7na_#231	GO:0004180: carboxypeptidase activity	0.000235	263,15,7
3btaa3#90	GO:0008270: zinc ion binding	0.000235	4,108,3
1eur__#302	GO:0004867: serine-type endopeptidase inhibitor activity	0.000236	53,47,6
1i12a_#126	GO:0030145: manganese ion binding	0.000236	23,38,4
2mpra_#190	GO:0046983: protein dimerization activity	0.000236	400,17,9
1apme_#74	GO:0005509: calcium ion binding	0.000237	124,160,18
1bwvs_#117	GO:0005516: calmodulin binding	0.000237	329,24,10
1hq8a_#206	GO:0008201: heparin binding	0.000237	329,24,10
1fu6a_#50	GO:0016638: oxidoreductase activity, acting on the CH-NH2 group of donors	0.000237	308,17,8
1fsia_#122	GO:0005524: ATP binding	0.000237	97,243,20
1h6la_#119	GO:0046983: protein dimerization activity	0.000237	308,17,8
1h6la_#119	GO:0016638: oxidoreductase activity, acting on the CH-NH2 group of donors	0.000237	308,17,8
1fbna_#144	GO:0000287: magnesium ion binding	0.000238	79,128,12
1lucb_#70	GO:0016831: carboxy-lyase activity	0.000238	35,25,4
1qlaa3#364	GO:0005509: calcium ion binding	0.000239	73,160,13
1pmc__#10	GO:0005066: transmembrane receptor protein tyrosine kinase signaling protein activity	0.000239	36,10,3
1jj2j_#88	GO:0008009: chemokine activity	0.000239	36,10,3
1h6va2#219	GO:0008800: beta-lactamase activity	0.000239	36,10,3
3sil__#317	GO:0005529: sugar binding	0.000239	358,39,14
1hx0a2#32	GO:0016861: intramolecular oxidoreductase activity, interconverting aldoses and ketoses	0.00024	70,13,4
1ryp1_#20	GO:0005507: copper ion binding	0.00024	125,38,8
1gci__#175	GO:0008026: ATP-dependent helicase activity	0.00024	70,13,4
1ib2a_#1101	GO:0005085: guanyl-nucleotide exchange factor activity	0.00024	70,13,4
1pma1_#79	GO:0000049: tRNA binding	0.00024	70,13,4
1hcl__#280	GO:0005085: guanyl-nucleotide exchange factor activity	0.00024	70,13,4
1zpda3#484	GO:0016831: carboxy-lyase activity	0.000241	3,25,2

1pii_1#48	GO:0016831: carboxy-lyase activity	0.000241	3,25,2
2hrva_#132	GO:0005524: ATP binding	0.000241	43,243,12
1thw__#66	GO:0016831: carboxy-lyase activity	0.000241	3,25,2
1f0xa1#482	GO:0015078: hydrogen ion transporter activity	0.000241	42,21,4
1yrga_#232	GO:0015036: disulfide oxidoreductase activity	0.000241	40,22,4
1fuia2#273	GO:0019201: nucleotide kinase activity	0.000241	133,13,5
1fuia2#273	GO:0016854: racemase and epimerase activity	0.000241	133,13,5
1qrra_#208	GO:0016646: oxidoreductase activity, acting on the CH-NH group of donors, NAD or NADP as acceptor	0.000241	87,19,5
1b8aa1#41	GO:0005524: ATP binding	0.000242	8,243,5
1auva2#386	GO:0005524: ATP binding	0.000242	8,243,5
1iow_1#37	GO:0003924: GTPase activity	0.000242	309,17,8
1iow_1#37	GO:0008483: transaminase activity	0.000242	309,17,8
1qfla1#44	GO:0005524: ATP binding	0.000242	8,243,5
1awx__#25	GO:0005524: ATP binding	0.000242	8,243,5
2bpa1_#244	GO:0004556: alpha-amylase activity	0.000242	60,15,4
1b35b_#97	GO:0016638: oxidoreductase activity, acting on the CH-NH2 group of donors	0.000242	309,17,8
1cdy_2#140	GO:0003968: RNA-directed RNA polymerase activity	0.000243	5,14,2
1pvc1_#182	GO:0003968: RNA-directed RNA polymerase activity	0.000243	5,14,2
1hxn__#235	GO:0003968: RNA-directed RNA polymerase activity	0.000243	5,14,2
1qqga2#166	GO:0016646: oxidoreductase activity, acting on the CH-NH group of donors, NAD or NADP as acceptor	0.000243	200,19,7
1p35a_#49	GO:0015078: hydrogen ion transporter activity	0.000243	124,21,6
1dkia_#185	GO:0003779: actin binding	0.000243	79,32,6
1gpea1#271	GO:0004714: transmembrane receptor protein tyrosine kinase activity	0.000243	197,14,6
1rypk_#12	GO:0019955: cytokine binding	0.000243	265,11,6
1cg2a1#43	GO:0004812: tRNA ligase activity	0.000244	189,26,8
1hq8a_#206	GO:0003887: DNA-directed DNA polymerase activity	0.000244	329,20,9
1cem__#132	GO:0008810: cellulase activity	0.000245	4,18,2
1cem__#281	GO:0008810: cellulase activity	0.000245	4,18,2
1fc3a_#172	GO:0004725: protein tyrosine phosphatase activity	0.000246	182,15,6
1aoea_#130	GO:0016668: oxidoreductase activity, acting on sulfur group of donors, NAD or NADP as acceptor	0.000246	77,12,4
2mev3_#108	GO:0016638: oxidoreductase activity, acting on the CH-NH2 group of donors	0.000246	228,17,7
1e6ca_#91	GO:0004180: carboxypeptidase activity	0.000246	182,15,6
1esma_#314	GO:0008083: growth factor activity	0.000247	113,42,8
1c7na_#95	GO:0005066: transmembrane receptor protein tyrosine kinase signaling protein activity	0.000247	183,10,5
1foha5#9	GO:0016776: phosphotransferase activity, phosphate group as acceptor	0.000247	65,14,4
1qfma1#139	GO:0005509: calcium ion binding	0.000247	318,160,34
1rypk_#12	GO:0004556: alpha-amylase activity	0.000247	265,15,7
1rypk_#12	GO:0004725: protein tyrosine phosphatase activity	0.000247	265,15,7
1b0ua_#174	GO:0005524: ATP binding	0.000248	26,243,9
1bjt__#890	GO:0004867: serine-type endopeptidase inhibitor activity	0.000248	292,47,14
1h7wa2#636	GO:0016836: hydro-lyase activity	0.000248	49,33,5
1egia_#738	GO:0003697: single-stranded DNA binding	0.000248	30,12,3
1bfd_1#230	GO:0016646: oxidoreductase activity, acting on the CH-NH group of donors, NAD or NADP as acceptor	0.000249	47,19,4
1f24a_#293	GO:0004263: chymotrypsin activity	0.000249	116,41,8
1mspa_#17	GO:0004896: hematopoietin/interferon-class (D200-domain) cytokine receptor activity	0.000249	47,19,4
1bywa_#30	GO:0008201: heparin binding	0.000249	331,24,10
1kit_2#398	GO:0005509: calcium ion binding	0.000249	83,160,14
1qmha2#136	GO:0004263: chymotrypsin activity	0.00025	9,41,3
1b9ma1#85	GO:0015078: hydrogen ion transporter activity	0.00025	17,21,3
1czan1#92	GO:0030145: manganese ion binding	0.00025	631,38,19
2dnja_#158	GO:0003964: RNA-directed DNA polymerase activity	0.00025	33,11,3
1e39a2#516	GO:0016627: oxidoreductase activity, acting on the CH-CH group of donors	0.00025	21,17,3
1h6va2#245	GO:0016638: oxidoreductase activity, acting on the CH-NH2 group of donors	0.00025	99,17,5
1aqua_#126	GO:0008235: metalloexopeptidase activity	0.00025	576,13,9
1aqua_#126	GO:0016651: oxidoreductase activity, acting on NADH or NADPH	0.00025	576,13,9
1aqua_#126	GO:0016854: racemase and epimerase activity	0.00025	576,13,9
1ijqa1#564	GO:0019955: cytokine binding	0.00025	33,11,3
1nsj__#178	GO:0008810: cellulase activity	0.00025	93,18,5
1e31a_#77	GO:0000287: magnesium ion binding	0.000251	47,128,9
1i5ga_#115	GO:0005507: copper ion binding	0.000251	324,38,13
1qs1a2#437	GO:0050660: FAD binding	0.000251	452,10,7
1bywa_#30	GO:0008083: growth factor activity	0.000251	331,42,14

1rypb_#114	GO:0008483: transaminase activity	0.000251	158,17,6
2gsaa_#246	GO:0016846: carbon-sulfur lyase activity	0.000252	7,10,2
1d3ya_#197	GO:0003916: DNA topoisomerase activity	0.000252	7,10,2
1dmha_#184	GO:0008199: ferric iron binding	0.000252	7,10,2
3kvt__#53	GO:0005261: cation channel activity	0.000252	7,10,2
3kvt__#53	GO:0050660: FAD binding	0.000252	7,10,2
1dpja_#265	GO:0008800: beta-lactamase activity	0.000252	7,10,2
1jj2q_#118	GO:0003682: chromatin binding	0.000252	7,10,2
1b4va1#292	GO:0050660: FAD binding	0.000252	7,10,2
1elua_#388	GO:0016846: carbon-sulfur lyase activity	0.000252	7,10,2
1b4ka_#108	GO:0000287: magnesium ion binding	0.000252	116,128,15
1fqva2#301	GO:0008270: zinc ion binding	0.000252	9,108,4
1mrj__#73	GO:0016799: hydrolase activity, hydrolyzing N-glycosyl compounds	0.000252	53,17,4
3sil__#317	GO:0005509: calcium ion binding	0.000252	358,160,37
1tiid_#39	GO:0016702: oxidoreductase activity, acting on single donors with incorporation of molecular oxygen, incorporation of two atoms of oxygen	0.000252	239,12,6
1jj2j_#70	GO:0000049: tRNA binding	0.000253	71,13,4
1jj2j_#70	GO:0005518: collagen binding	0.000253	71,13,4
1jj22_#78	GO:0005518: collagen binding	0.000253	71,13,4
1ycsa_#162	GO:0019838: growth factor binding	0.000253	210,9,5
1fnf_3#1409	GO:0005518: collagen binding	0.000253	71,13,4
1fc4a_#205	GO:0010181: FMN binding	0.000253	267,11,6
1ge8a2#134	GO:0016638: oxidoreductase activity, acting on the CH-NH2 group of donors	0.000253	229,17,7
1ihua2#525	GO:0016763: transferase activity, transferring pentosyl groups	0.000253	335,28,11
1g79a_#69	GO:0004867: serine-type endopeptidase inhibitor activity	0.000254	8,47,3
1cs1a_#160	GO:0016836: hydro-lyase activity	0.000254	187,33,9
1a3k__#199	GO:0005516: calmodulin binding	0.000255	15,24,3
1qkia1#158	GO:0004180: carboxypeptidase activity	0.000255	24,15,3
1ejfa_#10	GO:0008201: heparin binding	0.000256	155,24,7
1lara2#1769	GO:0004263: chymotrypsin activity	0.000256	219,41,11
1j75a_#166	GO:0005524: ATP binding	0.000256	120,243,23
1qs0a1#119	GO:0016831: carboxy-lyase activity	0.000256	454,25,12
1xwl_2#591	GO:0003887: DNA-directed DNA polymerase activity	0.000257	18,20,3
1qora2#201	GO:0016831: carboxy-lyase activity	0.000257	199,25,8
1hnja1#167	GO:0030151: molybdenum ion binding	0.000258	61,15,4
1quqb_#87	GO:0003697: single-stranded DNA binding	0.000258	78,12,4
1cmxa_#207	GO:0003809: thrombin activity	0.000258	96,10,4
1qs0a1#119	GO:0004457: lactate dehydrogenase activity	0.000258	454,10,7
1buoa_#44	GO:0003700: transcription factor activity	0.000259	14,124,5
1cf9a2#133	GO:0001584: rhodopsin-like receptor activity	0.000259	37,10,3
1bg6_1#294	GO:0005126: hematopoietin/interferon-class (D200-domain) cytokine receptor binding	0.000259	45,20,4
1pot__#138	GO:0051082: unfolded protein binding	0.000259	75,34,6
1apme_#74	GO:0003968: RNA-directed RNA polymerase activity	0.000259	124,14,5
1b3ra2#47	GO:0003682: chromatin binding	0.000259	37,10,3
1kapp1#389	GO:0004222: metalloendopeptidase activity	0.00026	19,19,3
2foka2#180	GO:0005509: calcium ion binding	0.00026	147,160,20
1fdr_1#26	GO:0004295: trypsin activity	0.00026	34,48,5
1nsca_#306	GO:0005524: ATP binding	0.00026	21,243,8
1e1aa_#261	GO:0008408: 3'-5' exonuclease activity	0.00026	28,13,3
1fjfj_#68	GO:0016638: oxidoreductase activity, acting on the CH-NH2 group of donors	0.00026	230,17,7
3rpba_#644	GO:0004620: phospholipase activity	0.00026	19,19,3
1qhoa4#230	GO:0004620: phospholipase activity	0.00026	19,19,3
1fp5a2#478	GO:0004896: hematopoietin/interferon-class (D200-domain) cytokine receptor activity	0.00026	19,19,3
1qtn.1#A270	GO:0016651: oxidoreductase activity, acting on NADH or NADPH	0.00026	439,13,8
1cwva2#675	GO:0005518: collagen binding	0.00026	28,13,3
1evqa_#305	GO:0016866: intramolecular transferase activity	0.00026	490,12,8
1eqga2#69	GO:0016705: oxidoreductase activity, acting on paired donors, with incorporation or reduction of molecular oxygen	0.000261	3,26,2
1thw__#66	GO:0004497: monooxygenase activity	0.000261	3,26,2
1gaxa3#88	GO:0004812: tRNA ligase activity	0.000261	3,26,2
1ac6a_#43	GO:0004190: aspartic-type endopeptidase activity	0.000261	163,23,7
1fn9a_#271	GO:0000049: tRNA binding	0.000261	218,13,6
1e8ga2#201	GO:0008009: chemokine activity	0.000261	304,10,6
1qqqa_#130	GO:0016705: oxidoreductase activity, acting on paired donors, with incorporation or reduction of molecular oxygen	0.000261	3,26,2
1qqqa_#130	GO:0004497: monooxygenase activity	0.000261	3,26,2

2hhma_#262	GO:0030145: manganese ion binding	0.000261	95,38,7
1trka2#429	GO:0016616: oxidoreductase activity, acting on the CH-OH group of donors, NAD or NADP as acceptor	0.000261	43,59,6
1fvua_#13	GO:0015082: di-, tri-valent inorganic cation transporter activity	0.000262	26,14,3
1fvua_#13	GO:0046915: transition metal ion transporter activity	0.000262	26,14,3
1pbe_2#183	GO:0016705: oxidoreductase activity, acting on paired donors, with incorporation or reduction of molecular oxygen	0.000262	14,26,3
1mek__#17	GO:0016776: phosphotransferase activity, phosphate group as acceptor	0.000262	26,14,3
1cnza_#19	GO:0016705: oxidoreductase activity, acting on paired donors, with incorporation or reduction of molecular oxygen	0.000262	14,26,3
1cnza_#19	GO:0004497: monooxygenase activity	0.000262	14,26,3
1qdlb_#170	GO:0003924: GTPase activity	0.000262	100,17,5
1dy9.1#C229	GO:0030151: molybdenum ion binding	0.000263	115,15,5
1dr9a1#39	GO:0000287: magnesium ion binding	0.000263	258,128,25
1tpg_1#55	GO:0016702: oxidoreductase activity, acting on single donors with incorporation of molecular oxygen, incorporation of two atoms of oxygen	0.000264	6,12,2
1nhp_2#143	GO:0016668: oxidoreductase activity, acting on sulfur group of donors, NAD or NADP as acceptor	0.000264	6,12,2
1elua_#288	GO:0016702: oxidoreductase activity, acting on single donors with incorporation of molecular oxygen, incorporation of two atoms of oxygen	0.000264	6,12,2
1hd2a_#47	GO:0005524: ATP binding	0.000264	56,243,14
1hbza_#214	GO:0019843: rRNA binding	0.000264	297,22,9
1opy__#68	GO:0004812: tRNA ligase activity	0.000265	367,26,11
1a65a2#187	GO:0015078: hydrogen ion transporter activity	0.000265	43,21,4
1e8ua_#296	GO:0004674: protein serine/threonine kinase activity	0.000266	39,42,5
1fw9a_#152	GO:0003887: DNA-directed DNA polymerase activity	0.000266	133,20,6
1vmoa_#84	GO:0005529: sugar binding	0.000266	42,39,5
1aky_2#133	GO:0008201: heparin binding	0.000267	156,24,7
1bjt__#890	GO:0004714: transmembrane receptor protein tyrosine kinase activity	0.000267	292,14,7
1ryp1_#18	GO:0019838: growth factor binding	0.000268	42,9,3
1whi__#59	GO:0000049: tRNA binding	0.000268	72,13,4
1whi__#59	GO:0005518: collagen binding	0.000268	72,13,4
1fo0b_#82	GO:0008083: growth factor activity	0.000268	9,42,3
2dnja_#91	GO:0004674: protein serine/threonine kinase activity	0.000268	9,42,3
1sfp__#84	GO:0019838: growth factor binding	0.000269	8,9,2
1ekbb_#208	GO:0005509: calcium ion binding	0.000269	17,160,6
2fnba_#67	GO:0019838: growth factor binding	0.000269	8,9,2
1d3bb_#31	GO:0005525: GTP binding	0.000269	52,49,6
1dceb_#134	GO:0019829: cation-transporting ATPase activity	0.000269	8,9,2
1psda3#344	GO:0015082: di-, tri-valent inorganic cation transporter activity	0.000269	125,14,5
1psda3#344	GO:0046915: transition metal ion transporter activity	0.000269	125,14,5
1c4zd_#51	GO:0016638: oxidoreductase activity, acting on the CH-NH2 group of donors	0.000269	160,17,6
1eqga2#47	GO:0004295: trypsin activity	0.00027	8,48,3
1dgw.1#Y353	GO:0051082: unfolded protein binding	0.00027	319,34,12
1qrra_#208	GO:0016758: transferase activity, transferring hexosyl groups	0.00027	87,11,4
1fsu__#434	GO:0016668: oxidoreductase activity, acting on sulfur group of donors, NAD or NADP as acceptor	0.00027	356,12,7
1erv__#80	GO:0004295: trypsin activity	0.00027	442,48,18
1b9ha_#276	GO:0004295: trypsin activity	0.000271	19,48,4
1c8ua2#206	GO:0004867: serine-type endopeptidase inhibitor activity	0.000271	35,47,5
1i05a_#132	GO:0046983: protein dimerization activity	0.000271	54,17,4
1i50b_#216	GO:0004222: metalloendopeptidase activity	0.000271	48,19,4
1bvoa_#206	GO:0004896: hematopoietin/interferon-class (D200-domain) cytokine receptor activity	0.000271	48,19,4
1ii7a_#199	GO:0016638: oxidoreductase activity, acting on the CH-NH2 group of donors	0.000271	54,17,4
1quqa_#75	GO:0003924: GTPase activity	0.000271	54,17,4
1fmta2#151	GO:0016638: oxidoreductase activity, acting on the CH-NH2 group of donors	0.000271	314,17,8
1ec7a1#230	GO:0003924: GTPase activity	0.000271	54,17,4
1pwt__#8	GO:0046983: protein dimerization activity	0.000271	314,17,8
1eo6a_#107	GO:0016836: hydro-lyase activity	0.000271	78,33,6
1pysb5#584	GO:0003899: DNA-directed RNA polymerase activity	0.000272	79,12,4
1pii_2#326	GO:0004812: tRNA ligase activity	0.000272	192,26,8
1dkia_#185	GO:0016668: oxidoreductase activity, acting on sulfur group of donors, NAD or NADP as acceptor	0.000272	79,12,4
1bwda_#117	GO:0004725: protein tyrosine phosphatase activity	0.000273	116,15,5
1erv__#80	GO:0003729: mRNA binding	0.000273	442,13,8
1erv__#80	GO:0005518: collagen binding	0.000273	442,13,8

1ldna1#94	GO:0000287: magnesium ion binding	0.000273	199,128,21
1e8ca3#111	GO:0016763: transferase activity, transferring pentosyl groups	0.000273	280,28,10
1dn0b2#214	GO:0004896: hematopoietin/interferon-class (D200-domain) cytokine receptor activity	0.000274	4,19,2
3ebx__#43	GO:0004222: metalloendopeptidase activity	0.000274	4,19,2
1ct9a2#168	GO:0004190: aspartic-type endopeptidase activity	0.000274	16,23,3
1bccb2#423	GO:0004222: metalloendopeptidase activity	0.000274	4,19,2
1qmea4#568	GO:0004222: metalloendopeptidase activity	0.000274	4,19,2
1iray1#30	GO:0004896: hematopoietin/interferon-class (D200-domain) cytokine receptor activity	0.000274	4,19,2
2pia_1#54	GO:0000049: tRNA binding	0.000274	220,13,6
1jf9a_#379	GO:0046983: protein dimerization activity	0.000274	232,17,7
1qoua_#90	GO:0016668: oxidoreductase activity, acting on sulfur group of donors, NAD or NADP as acceptor	0.000274	31,12,3
1qqsa_#124	GO:0016620: oxidoreductase activity, acting on the aldehyde or oxo group of donors, NAD or NADP as acceptor	0.000274	458,10,7
1fhga_#59	GO:0019955: cytokine binding	0.000274	34,11,3
1bev1_#238	GO:0003724: RNA helicase activity	0.000274	34,11,3
1dvpa1#65	GO:0008199: ferric iron binding	0.000274	187,10,5
1jbqa_#152	GO:0004556: alpha-amylase activity	0.000275	62,15,4
1f2da_#94	GO:0005524: ATP binding	0.000276	12,243,6
1c7na_#95	GO:0051082: unfolded protein binding	0.000276	183,34,9
1ab4__#89	GO:0005509: calcium ion binding	0.000276	159,160,21
2arca_#55	GO:0005524: ATP binding	0.000277	144,243,26
1lara2#1769	GO:0004295: trypsin activity	0.000277	219,48,12
1qfea_#68	GO:0010181: FMN binding	0.000278	560,11,8
1prea2#308	GO:0051082: unfolded protein binding	0.000279	76,34,6
1ej8a_#189	GO:0004714: transmembrane receptor protein tyrosine kinase activity	0.000279	294,14,7
2ae2a_#89	GO:0004812: tRNA ligase activity	0.000279	35,26,4
1bec_2#216	GO:0008083: growth factor activity	0.000279	115,42,8
1i50b_#966	GO:0015036: disulfide oxidoreductase activity	0.000279	299,22,9
1e0ca1#88	GO:0004725: protein tyrosine phosphatase activity	0.00028	5,15,2
1fo4a5#1150	GO:0030151: molybdenum ion binding	0.00028	5,15,2
1cewi_#14	GO:0016620: oxidoreductase activity, acting on the aldehyde or oxo group of donors, NAD or NADP as acceptor	0.00028	98,10,4
1gcoa_#5	GO:0030151: molybdenum ion binding	0.00028	5,15,2
2btva_#621	GO:0005096: GTPase activator activity	0.00028	5,15,2
1flca1#294	GO:0050660: FAD binding	0.00028	98,10,4
1fa0a1#405	GO:0008201: heparin binding	0.00028	38,24,4
1hdmb1#157	GO:0005179: hormone activity	0.00028	5,15,2
1g61a_#2202	GO:0003743: translation initiation factor activity	0.00028	5,15,2
1fiqa2#27	GO:0030151: molybdenum ion binding	0.00028	5,15,2
1hlga_#353	GO:0004180: carboxypeptidase activity	0.00028	5,15,2
1dqga_#81	GO:0008201: heparin binding	0.00028	38,24,4
1qdla_#211	GO:0005509: calcium ion binding	0.00028	320,160,34
1fumb2#36	GO:0030151: molybdenum ion binding	0.00028	5,15,2
1mdah_#329	GO:0003887: DNA-directed DNA polymerase activity	0.00028	335,20,9
1b8aa1#41	GO:0000287: magnesium ion binding	0.000281	8,128,4
1tc1a_#158	GO:0000287: magnesium ion binding	0.000281	8,128,4
1dfma_#53	GO:0000287: magnesium ion binding	0.000281	8,128,4
3lada3#463	GO:0000287: magnesium ion binding	0.000281	29,128,7
1bfd_3#446	GO:0000287: magnesium ion binding	0.000281	8,128,4
1ej8a_#189	GO:0005509: calcium ion binding	0.000281	294,160,32
1erja_#525	GO:0005507: copper ion binding	0.000281	10,38,3
1ct5a_#114	GO:0000287: magnesium ion binding	0.000281	29,128,7
1cpy__#346	GO:0004457: lactate dehydrogenase activity	0.000281	38,10,3
1qf6a1#606	GO:0003809: thrombin activity	0.000281	38,10,3
1fwxa2#189	GO:0005507: copper ion binding	0.000281	10,38,3
1h6la_#119	GO:0050660: FAD binding	0.000281	308,10,6
1g8fa3#419	GO:0009036: type II site-specific deoxyribonuclease activity	0.000282	151,12,5
2arca_#55	GO:0051082: unfolded protein binding	0.000282	144,34,8
1jj2a1#126	GO:0000049: tRNA binding	0.000282	73,13,4
1h4vb2#307	GO:0000049: tRNA binding	0.000282	73,13,4
1dxea_#71	GO:0000287: magnesium ion binding	0.000283	38,128,8
1pysa_#221	GO:0016646: oxidoreductase activity, acting on the CH-NH group of donors, NAD or NADP as acceptor	0.000283	90,19,5
1ppn__#132	GO:0005529: sugar binding	0.000284	234,39,11

1fo4a5#1020	GO:0016668: oxidoreductase activity, acting on sulfur group of donors, NAD or NADP as acceptor	0.000285	359,12,7
1jsg__#92	GO:0003887: DNA-directed DNA polymerase activity	0.000285	607,20,12
1f8ra2#325	GO:0004190: aspartic-type endopeptidase activity	0.000286	222,23,8
3prn__#145	GO:0019955: cytokine binding	0.000286	168,11,5
1ig8a_#418	GO:0005524: ATP binding	0.000287	5,243,4
1cb8a2#613	GO:0005518: collagen binding	0.000287	138,13,5
1ffya3#535	GO:0005524: ATP binding	0.000287	5,243,4
1gtra2#173	GO:0005524: ATP binding	0.000288	50,243,13
1h9oa_#83	GO:0019829: cation-transporting ATPase activity	0.000288	43,9,3
1dgw.1#Y353	GO:0005529: sugar binding	0.000288	319,39,13
1g51a3#524	GO:0046983: protein dimerization activity	0.000288	102,17,5
1g51a3#524	GO:0016638: oxidoreductase activity, acting on the CH-NH2 group of donors	0.000288	102,17,5
1fa0a1#393	GO:0003887: DNA-directed DNA polymerase activity	0.000288	261,20,8
1euwa_#71	GO:0016638: oxidoreductase activity, acting on the CH-NH2 group of donors	0.000289	22,17,3
1i9sa_#19	GO:0019843: rRNA binding	0.000289	17,22,3
1dhs__#149	GO:0000049: tRNA binding	0.000289	29,13,3
1g5va_#142	GO:0030151: molybdenum ion binding	0.000289	25,15,3
1bjt__#1066	GO:0019843: rRNA binding	0.000289	17,22,3
1nfp__#171	GO:0008026: ATP-dependent helicase activity	0.000289	29,13,3
1a4ia1#208	GO:0003924: GTPase activity	0.000289	22,17,3
1bxka_#262	GO:0016854: racemase and epimerase activity	0.000289	29,13,3
1eu3a2#174	GO:0004180: carboxypeptidase activity	0.000289	25,15,3
1e0ta2#10	GO:0016861: intramolecular oxidoreductase activity, interconverting aldoses and ketoses	0.000289	29,13,3
1epwa3#138	GO:0005507: copper ion binding	0.000289	424,38,15
1f39a_#190	GO:0030151: molybdenum ion binding	0.000289	25,15,3
3rpba_#550	GO:0005518: collagen binding	0.000289	29,13,3
1ct5a_#114	GO:0016854: racemase and epimerase activity	0.000289	29,13,3
2naca1#162	GO:0003924: GTPase activity	0.000289	22,17,3
1bf4a_#4	GO:0030151: molybdenum ion binding	0.000289	25,15,3
1b3ra1#218	GO:0019201: nucleotide kinase activity	0.000289	29,13,3
1hxma2#150	GO:0005518: collagen binding	0.000289	29,13,3
1qgxa_#53	GO:0000287: magnesium ion binding	0.00029	21,128,6
1g0sa_#74	GO:0008083: growth factor activity	0.00029	294,42,13
1a65a1#108	GO:0008083: growth factor activity	0.00029	294,42,13
2btva_#700	GO:0004867: serine-type endopeptidase inhibitor activity	0.000291	2,47,2
1hle.1#A236	GO:0004867: serine-type endopeptidase inhibitor activity	0.000291	2,47,2
1fepa_#340	GO:0050660: FAD binding	0.000291	99,10,4
1hdr__#156	GO:0016627: oxidoreductase activity, acting on the CH-CH group of donors	0.000291	55,17,4
1iqpa2#119	GO:0005525: GTP binding	0.000293	19,49,4
1f24a_#144	GO:0016776: phosphotransferase activity, phosphate group as acceptor	0.000294	27,14,3
1f2aa_#27	GO:0003964: RNA-directed DNA polymerase activity	0.000295	89,11,4
1hdma1#115	GO:0004556: alpha-amylase activity	0.000295	188,15,6
2mev1_#123	GO:0003724: RNA helicase activity	0.000295	89,11,4
1j79a_#187	GO:0010181: FMN binding	0.000295	89,11,4
1dik_3#240	GO:0003887: DNA-directed DNA polymerase activity	0.000296	262,20,8
1h6va1#27	GO:0016831: carboxy-lyase activity	0.000296	260,25,9
1czya1#466	GO:0051082: unfolded protein binding	0.000297	109,34,7
1e2wa1#102	GO:0005126: hematopoietin/interferon-class (D200-domain) cytokine receptor binding	0.000297	86,20,5
1fo4a5#1020	GO:0019838: growth factor binding	0.000297	359,9,6
1d0na3#305	GO:0050660: FAD binding	0.000297	311,10,6
1lci__#26	GO:0005085: guanyl-nucleotide exchange factor activity	0.000297	139,13,5
1c8za_#386	GO:0016638: oxidoreductase activity, acting on the CH-NH2 group of donors	0.000297	235,17,7
1cfe__#7	GO:0005524: ATP binding	0.000297	70,243,16
1eur__#84	GO:0005126: hematopoietin/interferon-class (D200-domain) cytokine receptor binding	0.000297	86,20,5
1qfma1#139	GO:0016638: oxidoreductase activity, acting on the CH-NH2 group of donors	0.000297	318,17,8
1qfma1#186	GO:0000049: tRNA binding	0.000297	326,13,7
1bhga3#529	GO:0016861: intramolecular oxidoreductase activity, interconverting aldoses and ketoses	0.000298	74,13,4
1gky__#34	GO:0015078: hydrogen ion transporter activity	0.000299	18,21,3
1gpma1#289	GO:0004364: glutathione transferase activity	0.000299	35,11,3
1qe3a_#301	GO:0010181: FMN binding	0.000299	35,11,3
1cpy__#93	GO:0016758: transferase activity, transferring hexosyl groups	0.000299	275,11,6
3thia_#59	GO:0005351: sugar porter activity	0.000299	18,21,3
1fu6a_#50	GO:0005509: calcium ion binding	0.000299	308,160,33
1rypb_#133	GO:0003724: RNA helicase activity	0.000299	35,11,3
1imva_#255	GO:0008408: 3'-5' exonuclease activity	0.0003	589,13,9

1jj2j_#70	GO:0008201: heparin binding	0.000301	71,24,5
1esma_#314	GO:0019838: growth factor binding	0.000301	113,9,4
1mla_1#95	GO:0003779: actin binding	0.000301	12,32,3
1jj22_#78	GO:0008201: heparin binding	0.000301	71,24,5
1eaja_#59	GO:0005507: copper ion binding	0.000301	202,38,10
1fnf_3#1409	GO:0008201: heparin binding	0.000301	71,24,5
1dqsa_#171	GO:0042802: protein self binding	0.000301	32,12,3
1bupa2#199	GO:0000155: two-component sensor molecule activity	0.000301	32,12,3
1deua_#151	GO:0030145: manganese ion binding	0.000301	285,38,12
1f97a2#199	GO:0004896: hematopoietin/interferon-class (D200-domain) cytokine receptor activity	0.000301	144,19,6
1jbqa_#152	GO:0016616: oxidoreductase activity, acting on the CH-OH group of donors, NAD or NADP as acceptor	0.000302	62,59,7
1csn__#84	GO:0004896: hematopoietin/interferon-class (D200-domain) cytokine receptor activity	0.000302	207,19,7
1qdlb_#170	GO:0050660: FAD binding	0.000303	100,10,4
1a0la_#176	GO:0004295: trypsin activity	0.000304	2,48,2
1bio__#189	GO:0004295: trypsin activity	0.000304	2,48,2
1dqga_#51	GO:0005529: sugar binding	0.000304	10,39,3
1g57a_#122	GO:0004295: trypsin activity	0.000304	2,48,2
1by5a_#347	GO:0003887: DNA-directed DNA polymerase activity	0.000304	263,20,8
1plq_2#197	GO:0003887: DNA-directed DNA polymerase activity	0.000304	4,20,2
1ryp1_#44	GO:0016763: transferase activity, transferring pentosyl groups	0.000304	3,28,2
1b35b_#97	GO:0004812: tRNA ligase activity	0.000306	309,26,10
1f9za_#16	GO:0008408: 3'-5' exonuclease activity	0.000307	140,13,5
1cpt__#325	GO:0010181: FMN binding	0.000308	7,11,2
1qg3a1#1179	GO:0019955: cytokine binding	0.000308	7,11,2
1e8ua_#286	GO:0019955: cytokine binding	0.000308	7,11,2
1qfxa_#380	GO:0003887: DNA-directed DNA polymerase activity	0.000308	47,20,4
1ddma_#130	GO:0003682: chromatin binding	0.000308	313,10,6
1ddma_#130	GO:0050660: FAD binding	0.000308	313,10,6
1a6o__#45	GO:0015036: disulfide oxidoreductase activity	0.000309	303,22,9
1prea2#119	GO:0005524: ATP binding	0.000309	38,243,11
1dyoa_#153	GO:0005524: ATP binding	0.000309	161,243,28
1lara2#1769	GO:0019838: growth factor binding	0.000309	219,9,5
1rb9__#49	GO:0004197: cysteine-type endopeptidase activity	0.00031	39,24,4
2mpra_#267	GO:0004867: serine-type endopeptidase inhibitor activity	0.00031	36,47,5
1i2oa_#34	GO:0016836: hydro-lyase activity	0.00031	192,33,9
1cpy__#380	GO:0019955: cytokine binding	0.000311	171,11,5
1ge8a2#134	GO:0051082: unfolded protein binding	0.000311	229,34,10
1wdcb_#134	GO:0004812: tRNA ligase activity	0.000312	36,26,4
1e6ua_#282	GO:0016854: racemase and epimerase activity	0.000312	6,13,2
2pola1#111	GO:0008408: 3'-5' exonuclease activity	0.000312	6,13,2
2pola2#194	GO:0008408: 3'-5' exonuclease activity	0.000312	6,13,2
1a77_2#183	GO:0008080: N-acetyltransferase activity	0.000312	6,13,2
1jb7a1#174	GO:0005509: calcium ion binding	0.000312	24,160,7
1gsoa3#193	GO:0005085: guanyl-nucleotide exchange factor activity	0.000312	6,13,2
1ha1_2#104	GO:0003729: mRNA binding	0.000312	6,13,2
1ha1_2#104	GO:0008026: ATP-dependent helicase activity	0.000312	6,13,2
1cfb_2#763	GO:0005518: collagen binding	0.000312	6,13,2
1hfwa_#195	GO:0000049: tRNA binding	0.000312	6,13,2
1a8h_2#133	GO:0000049: tRNA binding	0.000312	6,13,2
1dy5a_#104	GO:0000049: tRNA binding	0.000312	6,13,2
1ceza_#812	GO:0008408: 3'-5' exonuclease activity	0.000312	6,13,2
1bob__#56	GO:0000049: tRNA binding	0.000312	6,13,2
1ihoa_#75	GO:0005085: guanyl-nucleotide exchange factor activity	0.000312	6,13,2
1pyma_#224	GO:0005085: guanyl-nucleotide exchange factor activity	0.000312	6,13,2
1nhp_2#143	GO:0019201: nucleotide kinase activity	0.000312	6,13,2
1i7qa_#278	GO:0004812: tRNA ligase activity	0.000312	36,26,4
1vns__#158	GO:0008026: ATP-dependent helicase activity	0.000312	6,13,2
1htjf_#369	GO:0005085: guanyl-nucleotide exchange factor activity	0.000312	6,13,2
1pme__#302	GO:0005516: calmodulin binding	0.000312	16,24,3
1io1a_#202	GO:0016854: racemase and epimerase activity	0.000312	6,13,2
1qrra_#99	GO:0016854: racemase and epimerase activity	0.000312	6,13,2
1egua2#839	GO:0005509: calcium ion binding	0.000312	24,160,7
1e69a_#1106	GO:0019201: nucleotide kinase activity	0.000312	6,13,2
1cs6a4#363	GO:0008201: heparin binding	0.000312	16,24,3
1fnf_1#1146	GO:0008201: heparin binding	0.000312	16,24,3

1atg__#144	GO:0016866: intramolecular transferase activity	0.000314	82,12,4
1fmta2#151	GO:0050660: FAD binding	0.000314	314,10,6
1pwt__#8	GO:0005066: transmembrane receptor protein tyrosine kinase signaling protein activity	0.000314	314,10,6
1pwt__#8	GO:0050660: FAD binding	0.000314	314,10,6
1a7ca_#202	GO:0008083: growth factor activity	0.000315	117,42,8
1bxoa_#307	GO:0046983: protein dimerization activity	0.000316	104,17,5
3prn__#145	GO:0004190: aspartic-type endopeptidase activity	0.000316	168,23,7
3sil__#317	GO:0004190: aspartic-type endopeptidase activity	0.000316	358,23,10
1emsa2#165	GO:0005351: sugar porter activity	0.000317	45,21,4
1egaa1#90	GO:0005525: GTP binding	0.000317	2,49,2
1cewi_#14	GO:0005507: copper ion binding	0.000317	98,38,7
1zfja1#448	GO:0016616: oxidoreductase activity, acting on the CH-OH group of donors, NAD or NADP as acceptor	0.000317	7,59,3
1by5a_#238	GO:0015036: disulfide oxidoreductase activity	0.000317	237,22,8
1c9la2#12	GO:0003779: actin binding	0.000317	295,32,11
1jlxa2#166	GO:0004295: trypsin activity	0.000317	77,48,7
1g25a_#22	GO:0005529: sugar binding	0.000317	415,39,15
1e8ga2#176	GO:0016616: oxidoreductase activity, acting on the CH-OH group of donors, NAD or NADP as acceptor	0.000317	7,59,3
1ekra_#114	GO:0004523: ribonuclease H activity	0.000317	60,16,4
1cm9a_#57	GO:0004896: hematopoietin/interferon-class (D200-domain) cytokine receptor activity	0.000318	50,19,4
1pdc__#27	GO:0016646: oxidoreductase activity, acting on the CH-NH group of donors, NAD or NADP as acceptor	0.000318	50,19,4
1e87a_#174	GO:0000049: tRNA binding	0.000318	141,13,5
1by5a_#238	GO:0005529: sugar binding	0.000318	237,39,11
1xnb__#149	GO:0003924: GTPase activity	0.000319	165,17,6
1dhn__#88	GO:0004523: ribonuclease H activity	0.00032	5,16,2
1bvsa3#12	GO:0000049: tRNA binding	0.00032	30,13,3
1b34b_#56	GO:0003729: mRNA binding	0.00032	30,13,3
1h6va1#116	GO:0016651: oxidoreductase activity, acting on NADH or NADPH	0.00032	30,13,3
1d0ba_#191	GO:0004180: carboxypeptidase activity	0.000321	120,15,5
2napa2#292	GO:0030151: molybdenum ion binding	0.000321	120,15,5
1nfdb1#34	GO:0015036: disulfide oxidoreductase activity	0.000321	177,22,7
1b12a_#221	GO:0019838: growth factor binding	0.000322	115,9,4
1fjfj_#68	GO:0051082: unfolded protein binding	0.000322	230,34,10
1as4.1#A336	GO:0030145: manganese ion binding	0.000322	332,38,13
1j9la_#164	GO:0005524: ATP binding	0.000322	57,243,14
1gox__#124	GO:0016866: intramolecular transferase activity	0.000322	366,12,7
1qmva_#40	GO:0015036: disulfide oxidoreductase activity	0.000323	79,22,5
1dyoa_#25	GO:0016705: oxidoreductase activity, acting on paired donors, with incorporation or reduction of molecular oxygen	0.000325	15,26,3
1dyoa_#25	GO:0004497: monooxygenase activity	0.000325	15,26,3
1qvba_#206	GO:0005096: GTPase activator activity	0.000325	26,15,3
1el0a_#28	GO:0008201: heparin binding	0.000325	161,24,7
1e2o__#346	GO:0016620: oxidoreductase activity, acting on the aldehyde or oxo group of donors, NAD or NADP as acceptor	0.000326	194,10,5
1bd0a1#276	GO:0004263: chymotrypsin activity	0.000327	91,41,7
1h4ua1#486	GO:0005507: copper ion binding	0.000327	45,38,5
1ew2a_#237	GO:0000287: magnesium ion binding	0.000327	106,128,14
1ax4a_#275	GO:0005066: transmembrane receptor protein tyrosine kinase signaling protein activity	0.000327	102,10,4
1bwvs_#38	GO:0003809: thrombin activity	0.000328	40,10,3
1c4ka1#33	GO:0016620: oxidoreductase activity, acting on the aldehyde or oxo group of donors, NAD or NADP as acceptor	0.000328	40,10,3
1bywa_#30	GO:0005518: collagen binding	0.000328	331,13,7
1dj0a2#183	GO:0008235: metalloexopeptidase activity	0.000328	142,13,5
1e4ea2#215	GO:0004556: alpha-amylase activity	0.000328	490,15,9
1a06__#147	GO:0004714: transmembrane receptor protein tyrosine kinase activity	0.000328	28,14,3
1h6va3#486	GO:0003968: RNA-directed RNA polymerase activity	0.000329	70,14,4
2sqca2#97	GO:0005525: GTP binding	0.000329	127,49,9
1c4zd_#22	GO:0003968: RNA-directed RNA polymerase activity	0.000329	70,14,4
1cqka_#52	GO:0003968: RNA-directed RNA polymerase activity	0.000329	70,14,4
1dqza_#131	GO:0016668: oxidoreductase activity, acting on sulfur group of donors, NAD or NADP as acceptor	0.000329	83,12,4
1hdfa_#16	GO:0004295: trypsin activity	0.000329	223,48,12
1kve.1#B199	GO:0005518: collagen binding	0.00033	76,13,4
2sli_2#326	GO:0003697: single-stranded DNA binding	0.00033	251,12,6

3frua1#236	GO:0000049: tRNA binding	0.00033	76,13,4
1icia_#9	GO:0016831: carboxy-lyase activity	0.00033	38,25,4
1fp1d2#214	GO:0000287: magnesium ion binding	0.00033	173,128,19
1dxea_#71	GO:0016831: carboxy-lyase activity	0.00033	38,25,4
1nfdb1#21	GO:0005529: sugar binding	0.00033	96,39,7
1e8ca3#111	GO:0016758: transferase activity, transferring hexosyl groups	0.00033	280,11,6
1qgua_#435	GO:0003924: GTPase activity	0.000331	23,17,3
1tgoa2#409	GO:0016638: oxidoreductase activity, acting on the CH-NH2 group of donors	0.000331	23,17,3
1bev1_#86	GO:0046983: protein dimerization activity	0.000331	323,17,8
1mil__#31	GO:0004812: tRNA ligase activity	0.000331	104,26,6
1b65a_#257	GO:0016646: oxidoreductase activity, acting on the CH-NH group of donors, NAD or NADP as acceptor	0.000331	210,19,7
1eno__#137	GO:0016627: oxidoreductase activity, acting on the CH-CH group of donors	0.000331	23,17,3
1tiid_#39	GO:0003924: GTPase activity	0.000331	239,17,7
1qs0a1#119	GO:0016854: racemase and epimerase activity	0.000331	454,13,8
1qfma1#186	GO:0051082: unfolded protein binding	0.000332	326,34,12
1dr9a1#39	GO:0004674: protein serine/threonine kinase activity	0.000333	258,42,12
1faoa_#250	GO:0005525: GTP binding	0.000333	76,49,7
1ryph_#163	GO:0004180: carboxypeptidase activity	0.000334	121,15,5
2fmr__#10	GO:0016638: oxidoreductase activity, acting on the CH-NH2 group of donors	0.000335	57,17,4
1foha5#218	GO:0016627: oxidoreductase activity, acting on the CH-CH group of donors	0.000335	57,17,4
1foha5#218	GO:0016638: oxidoreductase activity, acting on the CH-NH2 group of donors	0.000335	57,17,4
1hu4a_#267	GO:0016627: oxidoreductase activity, acting on the CH-CH group of donors	0.000335	57,17,4
1cjca2#365	GO:0016638: oxidoreductase activity, acting on the CH-NH2 group of donors	0.000335	57,17,4
1f8ra1#64	GO:0050660: FAD binding	0.000336	8,10,2
1c5y.1#B128	GO:0003809: thrombin activity	0.000336	8,10,2
1qlwa_#110	GO:0004601: peroxidase activity	0.000336	4,21,2
1h68a_#183	GO:0015078: hydrogen ion transporter activity	0.000336	4,21,2
1bccb2#423	GO:0015078: hydrogen ion transporter activity	0.000336	4,21,2
1bn7a_#252	GO:0004601: peroxidase activity	0.000336	4,21,2
1qq9a_#78	GO:0004601: peroxidase activity	0.000336	4,21,2
1hbza_#254	GO:0004601: peroxidase activity	0.000336	4,21,2
1g6za_#57	GO:0008009: chemokine activity	0.000336	8,10,2
1ezvd1#97	GO:0015078: hydrogen ion transporter activity	0.000336	4,21,2
1eu8a_#35	GO:0005351: sugar porter activity	0.000336	4,21,2
1awx__#25	GO:0005066: transmembrane receptor protein tyrosine kinase signaling protein activity	0.000336	8,10,2
1dgtb3#2594	GO:0016846: carbon-sulfur lyase activity	0.000336	8,10,2
1tca__#106	GO:0004601: peroxidase activity	0.000336	4,21,2
1tca__#189	GO:0004601: peroxidase activity	0.000336	4,21,2
1e79d2#36	GO:0016638: oxidoreductase activity, acting on the CH-NH2 group of donors	0.000336	524,17,10
1hdma1#115	GO:0015078: hydrogen ion transporter activity	0.000337	188,21,7
1gg4a3#64	GO:0016616: oxidoreductase activity, acting on the CH-OH group of donors, NAD or NADP as acceptor	0.000337	45,59,6
1f42a2#123	GO:0030151: molybdenum ion binding	0.000337	378,15,8
1hdfa_#16	GO:0019838: growth factor binding	0.000337	223,9,5
1mil__#30	GO:0004523: ribonuclease H activity	0.000338	61,16,4
1j8ra_#26	GO:0008083: growth factor activity	0.000339	41,42,5
1a6o__#297	GO:0008083: growth factor activity	0.000339	41,42,5
1jj2v_#118	GO:0003916: DNA topoisomerase activity	0.000339	103,10,4
1jj2v_#118	GO:0016620: oxidoreductase activity, acting on the aldehyde or oxo group of donors, NAD or NADP as acceptor	0.000339	103,10,4
1i5ga_#115	GO:0015078: hydrogen ion transporter activity	0.000339	324,21,9
1elja_#64	GO:0050660: FAD binding	0.000339	103,10,4
1pma1_#79	GO:0008270: zinc ion binding	0.000339	70,108,10
1nsca_#236	GO:0005518: collagen binding	0.000339	143,13,5
1quba4#235	GO:0005509: calcium ion binding	0.00034	106,160,16
1dzfa2#211	GO:0016638: oxidoreductase activity, acting on the CH-NH2 group of donors	0.00034	167,17,6
1ddma_#130	GO:0004497: monooxygenase activity	0.00034	313,26,10
1ihua2#343	GO:0016763: transferase activity, transferring pentosyl groups	0.00034	233,28,9
1hwxa1#223	GO:0016763: transferase activity, transferring pentosyl groups	0.00034	233,28,9
1xgsa2#82	GO:0015036: disulfide oxidoreductase activity	0.000341	307,22,9
1imva_#69	GO:0015078: hydrogen ion transporter activity	0.000341	84,21,5
1aqua_#126	GO:0010181: FMN binding	0.000342	576,11,8
1j9qa1#154	GO:0016620: oxidoreductase activity, acting on the aldehyde or oxo group of donors, NAD or NADP as acceptor	0.000342	196,10,5
1hr0w_#69	GO:0005524: ATP binding	0.000343	64,243,15

2mpra_#267	GO:0004295: trypsin activity	0.000343	36,48,5
1tbrr1#15	GO:0005524: ATP binding	0.000343	64,243,15
1a81a1#51	GO:0008201: heparin binding	0.000343	73,24,5
1e8ga2#201	GO:0015082: di-, tri-valent inorganic cation transporter activity	0.000344	304,14,7
1e8ga2#201	GO:0046915: transition metal ion transporter activity	0.000344	304,14,7
1i50b_#966	GO:0004674: protein serine/threonine kinase activity	0.000344	299,42,13
1i50b_#966	GO:0008083: growth factor activity	0.000344	299,42,13
1fhga_#42	GO:0019838: growth factor binding	0.000345	9,9,2
1a3k__#122	GO:0019838: growth factor binding	0.000345	9,9,2
1ev2e1#154	GO:0019838: growth factor binding	0.000345	9,9,2
1mkp__#216	GO:0016616: oxidoreductase activity, acting on the CH-OH group of donors, NAD or NADP as acceptor	0.000345	129,59,10
1bjt__#890	GO:0004190: aspartic-type endopeptidase activity	0.000346	292,23,9
1ejda_#180	GO:0008408: 3'-5' exonuclease activity	0.000347	77,13,4
1pya.1#B236	GO:0000049: tRNA binding	0.000347	77,13,4
1qtra_#108	GO:0016651: oxidoreductase activity, acting on NADH or NADPH	0.000347	77,13,4
1e4ea2#215	GO:0051082: unfolded protein binding	0.000347	490,34,15
1jj2j_#70	GO:0003968: RNA-directed RNA polymerase activity	0.000348	71,14,4
1a4ya_#104	GO:0016627: oxidoreductase activity, acting on the CH-CH group of donors	0.000348	241,17,7
3sil__#237	GO:0005509: calcium ion binding	0.000349	7,160,4
1ryp1_#18	GO:0004190: aspartic-type endopeptidase activity	0.000349	42,23,4
1uok_2#170	GO:0005509: calcium ion binding	0.000349	7,160,4
1avaa2#235	GO:0005509: calcium ion binding	0.000349	7,160,4
1jp3a_#22	GO:0000287: magnesium ion binding	0.000349	49,128,9
1g25a_#22	GO:0015082: di-, tri-valent inorganic cation transporter activity	0.000349	415,14,8
1g25a_#22	GO:0046915: transition metal ion transporter activity	0.000349	415,14,8
1fmta2#151	GO:0004812: tRNA ligase activity	0.000349	314,26,10
2occb1#209	GO:0019955: cytokine binding	0.000349	93,11,4
1ldna1#94	GO:0004812: tRNA ligase activity	0.000349	199,26,8
1qdla_#211	GO:0003809: thrombin activity	0.000349	320,10,6
1qdla_#211	GO:0050660: FAD binding	0.000349	320,10,6
1thg__#3	GO:0004263: chymotrypsin activity	0.00035	92,41,7
1deua_#151	GO:0004896: hematopoietin/interferon-class (D200-domain) cytokine receptor activity	0.00035	285,19,8
1dofa_#15	GO:0008199: ferric iron binding	0.000351	197,10,5
3prn__#145	GO:0016638: oxidoreductase activity, acting on the CH-NH2 group of donors	0.000352	168,17,6
1mil__#31	GO:0005066: transmembrane receptor protein tyrosine kinase signaling protein activity	0.000352	104,10,4
1qhua1#47	GO:0050660: FAD binding	0.000352	104,10,4
1hdr__#125	GO:0016831: carboxy-lyase activity	0.000353	16,25,3
1bvsa3#12	GO:0000287: magnesium ion binding	0.000353	30,128,7
1lcl__#128	GO:0004222: metalloendopeptidase activity	0.000353	21,19,3
1az9_2#257	GO:0000049: tRNA binding	0.000353	31,13,3
1azo__#144	GO:0008800: beta-lactamase activity	0.000353	41,10,3
1a6o__#297	GO:0001584: rhodopsin-like receptor activity	0.000353	41,10,3
1eh9a1#33	GO:0019955: cytokine binding	0.000353	37,11,3
1ie5a_#23	GO:0004896: hematopoietin/interferon-class (D200-domain) cytokine receptor activity	0.000353	21,19,3
3grx__#56	GO:0005507: copper ion binding	0.000353	335,38,13
1qhva_#438	GO:0005351: sugar porter activity	0.000353	19,21,3
1hrna_#182	GO:0008408: 3'-5' exonuclease activity	0.000353	31,13,3
1qqp1_#51	GO:0005518: collagen binding	0.000353	31,13,3
1yrga_#194	GO:0016651: oxidoreductase activity, acting on NADH or NADPH	0.000353	31,13,3
1hdma1#98	GO:0019955: cytokine binding	0.000353	37,11,3
1qfma1#186	GO:0046983: protein dimerization activity	0.000353	326,17,8
1mdah_#329	GO:0005507: copper ion binding	0.000353	335,38,13
3grx__#56	GO:0005518: collagen binding	0.000354	335,13,7
1ihua2#525	GO:0008026: ATP-dependent helicase activity	0.000354	335,13,7
1ihua2#525	GO:0016651: oxidoreductase activity, acting on NADH or NADPH	0.000354	335,13,7
1ihua2#525	GO:0016854: racemase and epimerase activity	0.000354	335,13,7
1mdah_#329	GO:0005518: collagen binding	0.000354	335,13,7
1epwa3#140	GO:0005524: ATP binding	0.000355	187,243,31
1egja_#346	GO:0005126: hematopoietin/interferon-class (D200-domain) cytokine receptor binding	0.000356	20,20,3
1qe3a_#7	GO:0003887: DNA-directed DNA polymerase activity	0.000356	20,20,3
1a8i__#688	GO:0015036: disulfide oxidoreductase activity	0.000356	241,22,8
1pvc1_#106	GO:0008083: growth factor activity	0.000358	64,42,6
1tbrr1#15	GO:0004674: protein serine/threonine kinase activity	0.000358	64,42,6
1jsg__#92	GO:0004190: aspartic-type endopeptidase activity	0.000358	607,23,13
3sil__#284	GO:0008083: growth factor activity	0.000358	64,42,6

1jcfa2#206	GO:0016616: oxidoreductase activity, acting on the CH-OH group of donors, NAD or NADP as acceptor	0.000359	84,59,8
1qdlb_#170	GO:0005507: copper ion binding	0.000359	100,38,7
1fuia2#273	GO:0016776: phosphotransferase activity, phosphate group as acceptor	0.00036	133,14,5
1hdma1#115	GO:0005525: GTP binding	0.00036	188,49,11
1eny__#147	GO:0004180: carboxypeptidase activity	0.00036	123,15,5
1hxxa_#278	GO:0016668: oxidoreductase activity, acting on sulfur group of donors, NAD or NADP as acceptor	0.000361	255,12,6
1dceb_#82	GO:0016627: oxidoreductase activity, acting on the CH-CH group of donors	0.000362	5,17,2
1aoca_#123	GO:0003924: GTPase activity	0.000362	5,17,2
1ezvd1#213	GO:0016638: oxidoreductase activity, acting on the CH-NH2 group of donors	0.000362	5,17,2
1d5ta1#224	GO:0016799: hydrolase activity, hydrolyzing N-glycosyl compounds	0.000362	5,17,2
1qlwa_#103	GO:0016638: oxidoreductase activity, acting on the CH-NH2 group of donors	0.000362	5,17,2
1d2fa_#170	GO:0008483: transaminase activity	0.000362	5,17,2
1pii_1#40	GO:0016799: hydrolase activity, hydrolyzing N-glycosyl compounds	0.000362	5,17,2
1ajsa_#190	GO:0008483: transaminase activity	0.000362	5,17,2
1kve.1#B199	GO:0005509: calcium ion binding	0.000362	76,160,13
1elua_#95	GO:0005525: GTP binding	0.000362	77,49,7
1d5ya3#126	GO:0003968: RNA-directed RNA polymerase activity	0.000363	6,14,2
1nhp_2#143	GO:0016776: phosphotransferase activity, phosphate group as acceptor	0.000363	6,14,2
1iqva_#122	GO:0005543: phospholipid binding	0.000363	6,14,2
1ijaa_#59	GO:0005543: phospholipid binding	0.000363	6,14,2
1fc4a_#205	GO:0016831: carboxy-lyase activity	0.000363	267,25,9
1e69a_#1106	GO:0016776: phosphotransferase activity, phosphate group as acceptor	0.000363	6,14,2
1aoza1#115	GO:0015082: di-, tri-valent inorganic cation transporter activity	0.000363	6,14,2
1aoza1#115	GO:0046915: transition metal ion transporter activity	0.000363	6,14,2
1dypa_#143	GO:0003755: peptidyl-prolyl cis-trans isomerase activity	0.000364	94,11,4
1egaa1#97	GO:0005096: GTPase activator activity	0.000365	27,15,3
1bj7__#112	GO:0015082: di-, tri-valent inorganic cation transporter activity	0.000365	29,14,3
1bj7__#112	GO:0046915: transition metal ion transporter activity	0.000365	29,14,3
1ec7a1#216	GO:0016854: racemase and epimerase activity	0.000365	78,13,4
1fyhb1#99	GO:0005179: hormone activity	0.000365	27,15,3
1deua_#151	GO:0019955: cytokine binding	0.000365	285,11,6
1axn__#42	GO:0005085: guanyl-nucleotide exchange factor activity	0.000365	78,13,4
2mev3_#108	GO:0004263: chymotrypsin activity	0.000365	228,41,11
1b3ra1#218	GO:0016776: phosphotransferase activity, phosphate group as acceptor	0.000365	29,14,3
1cqqa_#163	GO:0003968: RNA-directed RNA polymerase activity	0.000365	29,14,3
1pjr_1#87	GO:0005524: ATP binding	0.000367	17,243,7
1loua_#79	GO:0004812: tRNA ligase activity	0.000367	106,26,6
1bev1_#86	GO:0016620: oxidoreductase activity, acting on the aldehyde or oxo group of donors, NAD or NADP as acceptor	0.000367	323,10,6
1b6ra3#161	GO:0005529: sugar binding	0.000368	164,39,9
2napa2#292	GO:0000287: magnesium ion binding	0.000368	120,128,15
1dmha_#184	GO:0016702: oxidoreductase activity, acting on single donors with incorporation of molecular oxygen, incorporation of two atoms of oxygen	0.000369	7,12,2
1pmi__#144	GO:0003697: single-stranded DNA binding	0.000369	7,12,2
3kvt__#53	GO:0016668: oxidoreductase activity, acting on sulfur group of donors, NAD or NADP as acceptor	0.000369	7,12,2
1fcda2#239	GO:0016668: oxidoreductase activity, acting on sulfur group of donors, NAD or NADP as acceptor	0.000369	7,12,2
1feca3#410	GO:0016668: oxidoreductase activity, acting on sulfur group of donors, NAD or NADP as acceptor	0.000369	7,12,2
1a2za_#209	GO:0016668: oxidoreductase activity, acting on sulfur group of donors, NAD or NADP as acceptor	0.000369	7,12,2
1rypi_#124	GO:0016702: oxidoreductase activity, acting on single donors with incorporation of molecular oxygen, incorporation of two atoms of oxygen	0.000369	7,12,2
1lgr_2#115	GO:0003899: DNA-directed RNA polymerase activity	0.000369	7,12,2
1rypc_#66	GO:0042802: protein self binding	0.000369	7,12,2
3grx__#8	GO:0015036: disulfide oxidoreductase activity	0.00037	4,22,2
1koba_#365	GO:0015036: disulfide oxidoreductase activity	0.00037	4,22,2
1eg7a_#1515	GO:0015036: disulfide oxidoreductase activity	0.00037	4,22,2
1bwda_#261	GO:0015036: disulfide oxidoreductase activity	0.00037	4,22,2
1ek6a_#33	GO:0016646: oxidoreductase activity, acting on the CH-NH group of donors, NAD or NADP as acceptor	0.00037	52,19,4
1rkd__#186	GO:0016646: oxidoreductase activity, acting on the CH-NH group of donors, NAD or NADP as acceptor	0.00037	52,19,4

1a0i_2#222	GO:0008083: growth factor activity	0.00037	186,42,10
1ct9a1#233	GO:0016646: oxidoreductase activity, acting on the CH-NH group of donors, NAD or NADP as acceptor	0.00037	52,19,4
1smaa2#581	GO:0051082: unfolded protein binding	0.00037	80,34,6
1fjfc2#153	GO:0019955: cytokine binding	0.00037	420,11,7
1qrea_#132	GO:0005509: calcium ion binding	0.000371	12,160,5
1as4.1#A288	GO:0004263: chymotrypsin activity	0.000371	66,41,6
1qh4a2#350	GO:0016811: hydrolase activity, acting on carbon-nitrogen (but not peptide) bonds, in linear amides	0.000372	67,15,4
1fc4a_#205	GO:0016616: oxidoreductase activity, acting on the CH-OH group of donors, NAD or NADP as acceptor	0.000373	267,59,15
1ezvb2#271	GO:0003700: transcription factor activity	0.000374	15,124,5
1fi2a_#109	GO:0019838: growth factor binding	0.000374	228,9,5
1i5ga_#115	GO:0003682: chromatin binding	0.000374	324,10,6
1i5ga_#115	GO:0008009: chemokine activity	0.000374	324,10,6
1i5ga_#115	GO:0004457: lactate dehydrogenase activity	0.000374	324,10,6
1i5ga_#115	GO:0050660: FAD binding	0.000374	324,10,6
1rypd_#93	GO:0003700: transcription factor activity	0.000374	15,124,5
2mev3_#108	GO:0019838: growth factor binding	0.000374	228,9,5
3sil__#98	GO:0019838: growth factor binding	0.000374	228,9,5
1eny__#147	GO:0005524: ATP binding	0.000374	123,243,23
1bwvs_#38	GO:0005509: calcium ion binding	0.000375	40,160,9
1fjfj_#68	GO:0005524: ATP binding	0.000375	230,243,36
1mspa_#17	GO:0019838: growth factor binding	0.000376	47,9,3
1ekbb_#85	GO:0004620: phospholipase activity	0.000376	150,19,6
1j8ra_#26	GO:0008201: heparin binding	0.000377	41,24,4
1seta2#403	GO:0008201: heparin binding	0.000377	41,24,4
1tkia_#80	GO:0005516: calmodulin binding	0.000377	17,24,3
1gdea_#90	GO:0008483: transaminase activity	0.000377	24,17,3
2cpl__#20	GO:0016638: oxidoreductase activity, acting on the CH-NH2 group of donors	0.000377	108,17,5
1howa_#588	GO:0005516: calmodulin binding	0.000377	17,24,3
1koba_#230	GO:0005516: calmodulin binding	0.000377	17,24,3
2pvba_#51	GO:0005516: calmodulin binding	0.000377	17,24,3
1tkia_#266	GO:0005516: calmodulin binding	0.000377	17,24,3
1gdea_#195	GO:0008483: transaminase activity	0.000377	24,17,3
1qqga2#166	GO:0005066: transmembrane receptor protein tyrosine kinase signaling protein activity	0.000377	200,10,5
1bwvs_#117	GO:0016638: oxidoreductase activity, acting on the CH-NH2 group of donors	0.000377	329,17,8
1ihua2#343	GO:0008026: ATP-dependent helicase activity	0.000377	233,13,6
1ihua2#343	GO:0016854: racemase and epimerase activity	0.000377	233,13,6
1hxn__#248	GO:0004197: cysteine-type endopeptidase activity	0.000377	17,24,3
1keva2#236	GO:0008757: S-adenosylmethionine-dependent methyltransferase activity	0.000377	17,24,3
1keva2#236	GO:0008270: zinc ion binding	0.000377	17,108,5
1hwxa1#223	GO:0016854: racemase and epimerase activity	0.000377	233,13,6
1hdfa_#16	GO:0004674: protein serine/threonine kinase activity	0.000378	223,42,11
2fcba2#104	GO:0008083: growth factor activity	0.000379	10,42,3
2hrva_#132	GO:0004263: chymotrypsin activity	0.000379	43,41,5
1f0xa1#482	GO:0003809: thrombin activity	0.000379	42,10,3
1dn2a1#308	GO:0008083: growth factor activity	0.000379	10,42,3
1cb8a1#326	GO:0008083: growth factor activity	0.000379	10,42,3
1io1a_#233	GO:0019955: cytokine binding	0.000379	95,11,4
1eaja_#59	GO:0005529: sugar binding	0.000379	202,39,10
1fc6a2#454	GO:0004263: chymotrypsin activity	0.000379	43,41,5
1loua_#79	GO:0008800: beta-lactamase activity	0.000379	106,10,4
1dn2a1#261	GO:0016638: oxidoreductase activity, acting on the CH-NH2 group of donors	0.000379	425,17,9
1dofa_#15	GO:0005096: GTPase activator activity	0.000381	197,15,6
1dj0a2#183	GO:0003887: DNA-directed DNA polymerase activity	0.000381	142,20,6
1psda1#233	GO:0016616: oxidoreductase activity, acting on the CH-OH group of donors, NAD or NADP as acceptor	0.000382	17,59,4
2bbkh_#214	GO:0019955: cytokine binding	0.000382	38,11,3
1eno__#83	GO:0010181: FMN binding	0.000382	38,11,3
1jb0d_#26	GO:0019838: growth factor binding	0.000382	229,9,5
1dqga_#81	GO:0019955: cytokine binding	0.000382	38,11,3
1sppb_#32	GO:0019955: cytokine binding	0.000382	38,11,3
1kit_1#209	GO:0030145: manganese ion binding	0.000382	11,38,3
1g8kb_#11	GO:0016638: oxidoreductase activity, acting on the CH-NH2 group of donors	0.000382	59,17,4

1keva2#236	GO:0016616: oxidoreductase activity, acting on the CH-OH group of donors, NAD or NADP as acceptor	0.000382	17,59,4
1fo8a_#202	GO:0000287: magnesium ion binding	0.000383	22,128,6
1nat__#62	GO:0000287: magnesium ion binding	0.000383	22,128,6
1erza_#183	GO:0019201: nucleotide kinase activity	0.000384	79,13,4
1lvl_2#222	GO:0008235: metalloexopeptidase activity	0.000384	79,13,4
1i50b_#378	GO:0008408: 3'-5' exonuclease activity	0.000384	79,13,4
1i8aa_#2	GO:0005509: calcium ion binding	0.000384	18,160,6
1dr9a1#39	GO:0003697: single-stranded DNA binding	0.000385	258,12,6
1quna1#113	GO:0005509: calcium ion binding	0.000385	286,160,31
1fvua_#13	GO:0005507: copper ion binding	0.000386	26,38,4
2foka2#180	GO:0004177: aminopeptidase activity	0.000386	147,13,5
1fxla2#169	GO:0005126: hematopoietin/interferon-class (D200-domain) cytokine receptor binding	0.000386	203,20,7
1t7pa2#513	GO:0005524: ATP binding	0.000387	33,243,10
1jwoa_#196	GO:0005524: ATP binding	0.000387	33,243,10
1gks__#70	GO:0005524: ATP binding	0.000387	33,243,10
1a81a1#51	GO:0004714: transmembrane receptor protein tyrosine kinase activity	0.000387	73,14,4
1nbaa_#227	GO:0003887: DNA-directed DNA polymerase activity	0.000388	91,20,5
1ezwa_#245	GO:0008235: metalloexopeptidase activity	0.000389	32,13,3
1d4xg_#7	GO:0003779: actin binding	0.000389	13,32,3
1gcua1#36	GO:0008235: metalloexopeptidase activity	0.000389	32,13,3
1dpta_#22	GO:0016616: oxidoreductase activity, acting on the CH-OH group of donors, NAD or NADP as acceptor	0.000389	85,59,8
2cpl__#20	GO:0005524: ATP binding	0.00039	108,243,21
1fjfj_#68	GO:0019838: growth factor binding	0.00039	230,9,5
1e3ua_#160	GO:0000287: magnesium ion binding	0.000391	4,128,3
1e5ka_#49	GO:0000287: magnesium ion binding	0.000391	4,128,3
1g0sa_#105	GO:0000287: magnesium ion binding	0.000391	4,128,3
1i50b_#334	GO:0016705: oxidoreductase activity, acting on paired donors, with incorporation or reduction of molecular oxygen	0.000391	69,26,5
1e2o__#346	GO:0004295: trypsin activity	0.000391	194,48,11
1b65a_#257	GO:0016616: oxidoreductase activity, acting on the CH-OH group of donors, NAD or NADP as acceptor	0.000391	210,59,13
1d8ca_#442	GO:0016836: hydro-lyase activity	0.000392	30,33,4
1e44b_#75	GO:0030151: molybdenum ion binding	0.000392	198,15,6
1htp__#16	GO:0004674: protein serine/threonine kinase activity	0.000392	303,42,13
1dr9a1#39	GO:0004812: tRNA ligase activity	0.000392	258,26,9
1utea_#11	GO:0005525: GTP binding	0.000392	130,49,9
1dm9a_#57	GO:0016638: oxidoreductase activity, acting on the CH-NH2 group of donors	0.000393	109,17,5
1bywa_#30	GO:0046983: protein dimerization activity	0.000393	331,17,8
1ir3a_#1063	GO:0030151: molybdenum ion binding	0.000394	68,15,4
1ec7a1#230	GO:0016836: hydro-lyase activity	0.000394	54,33,5
1cpt__#52	GO:0016702: oxidoreductase activity, acting on single donors with incorporation of molecular oxygen, incorporation of two atoms of oxygen	0.000395	35,12,3
1eaja_#59	GO:0050660: FAD binding	0.000395	202,10,5
1h8ma_#8	GO:0000287: magnesium ion binding	0.000395	134,128,16
1eu3a2#205	GO:0009036: type II site-specific deoxyribonuclease activity	0.000395	87,12,4
1beba_#119	GO:0008800: beta-lactamase activity	0.000395	202,10,5
1e3ja1#136	GO:0003779: actin binding	0.000395	31,32,4
1eq9a_#162	GO:0003697: single-stranded DNA binding	0.000395	35,12,3
1nfdb1#21	GO:0003724: RNA helicase activity	0.000395	96,11,4
1h4ua1#404	GO:0016620: oxidoreductase activity, acting on the aldehyde or oxo group of donors, NAD or NADP as acceptor	0.000395	202,10,5
1c8za_#386	GO:0000049: tRNA binding	0.000395	235,13,6
3chbd_#85	GO:0016668: oxidoreductase activity, acting on sulfur group of donors, NAD or NADP as acceptor	0.000396	378,12,7
1f0xa1#278	GO:0019955: cytokine binding	0.000397	180,11,5
1doi__#42	GO:0004812: tRNA ligase activity	0.000398	16,26,3
1svy__#161	GO:0003779: actin binding	0.000398	3,32,2
1ed5a_#294	GO:0016646: oxidoreductase activity, acting on the CH-NH group of donors, NAD or NADP as acceptor	0.000398	53,19,4
1dfca4#1469	GO:0004896: hematopoietin/interferon-class (D200-domain) cytokine receptor activity	0.000398	53,19,4
1by5a_#347	GO:0004674: protein serine/threonine kinase activity	0.000399	263,42,12
1fepa_#340	GO:0005529: sugar binding	0.000399	99,39,7
1e1oa2#423	GO:0004177: aminopeptidase activity	0.000399	148,13,5
1i7qa_#321	GO:0008235: metalloexopeptidase activity	0.000399	148,13,5

1im4a_#144	GO:0015036: disulfide oxidoreductase activity	0.0004	129,22,6
1qmha2#136	GO:0004295: trypsin activity	0.0004	9,48,3
1h9da_#91	GO:0008201: heparin binding	0.0004	222,24,8
1as4.1#A336	GO:0016638: oxidoreductase activity, acting on the CH-NH2 group of donors	0.000402	332,17,8
1bqk__#77	GO:0003700: transcription factor activity	0.000403	74,124,11
1ldna1#94	GO:0004725: protein tyrosine phosphatase activity	0.000403	199,15,6
1hdma1#115	GO:0008083: growth factor activity	0.000404	188,42,10
1ej8a_#189	GO:0005507: copper ion binding	0.000404	294,38,12
1dbga_#38	GO:0016776: phosphotransferase activity, phosphate group as acceptor	0.000404	30,14,3
1ac6a_#43	GO:0016702: oxidoreductase activity, acting on single donors with incorporation of molecular oxygen, incorporation of two atoms of oxygen	0.000405	163,12,5
1g25a_#22	GO:0004295: trypsin activity	0.000405	415,48,17
1g6sa_#64	GO:0004812: tRNA ligase activity	0.000406	108,26,6
1pwt__#8	GO:0015036: disulfide oxidoreductase activity	0.000406	314,22,9
1fvia2#134	GO:0030151: molybdenum ion binding	0.000407	28,15,3
1h9oa_#83	GO:0005066: transmembrane receptor protein tyrosine kinase signaling protein activity	0.000407	43,10,3
1lucb_#32	GO:0016646: oxidoreductase activity, acting on the CH-NH group of donors, NAD or NADP as acceptor	0.000407	22,19,3
1qf5a_#15	GO:0003682: chromatin binding	0.000407	43,10,3
1a4ia1#208	GO:0016646: oxidoreductase activity, acting on the CH-NH group of donors, NAD or NADP as acceptor	0.000407	22,19,3
1eeoa_#288	GO:0008810: cellulase activity	0.000407	5,18,2
1ppn__#191	GO:0019843: rRNA binding	0.000407	19,22,3
1fjfb_#102	GO:0015036: disulfide oxidoreductase activity	0.000407	19,22,3
1e79h2#77	GO:0004295: trypsin activity	0.000407	21,48,4
1iba__#39	GO:0008201: heparin binding	0.000407	351,24,10
1dzfa2#211	GO:0008201: heparin binding	0.000407	167,24,7
1fc6a2#454	GO:0003809: thrombin activity	0.000407	43,10,3
1ir3a_#1015	GO:0005066: transmembrane receptor protein tyrosine kinase signaling protein activity	0.000407	43,10,3
1ulo__#144	GO:0008810: cellulase activity	0.000407	5,18,2
1bccb2#322	GO:0004725: protein tyrosine phosphatase activity	0.000407	28,15,3
1cf9a1#736	GO:0003682: chromatin binding	0.000407	43,10,3
1cwva2#675	GO:0005179: hormone activity	0.000407	28,15,3
1huxa_#10	GO:0016627: oxidoreductase activity, acting on the CH-CH group of donors	0.000408	60,17,4
1air__#233	GO:0008270: zinc ion binding	0.000408	10,108,4
1g6sa_#64	GO:0005066: transmembrane receptor protein tyrosine kinase signaling protein activity	0.000408	108,10,4
1qnja_#46	GO:0016638: oxidoreductase activity, acting on the CH-NH2 group of donors	0.000408	60,17,4
1sfp__#84	GO:0019955: cytokine binding	0.000409	8,11,2
1eupa_#320	GO:0010181: FMN binding	0.000409	8,11,2
1bu7a_#360	GO:0010181: FMN binding	0.000409	8,11,2
1hv8a1#188	GO:0016758: transferase activity, transferring hexosyl groups	0.000409	8,11,2
1dosa_#144	GO:0008081: phosphoric diester hydrolase activity	0.000409	8,11,2
2fnba_#67	GO:0019955: cytokine binding	0.000409	8,11,2
1e25a_#262	GO:0005524: ATP binding	0.000409	116,243,22
1cbf__#208	GO:0016638: oxidoreductase activity, acting on the CH-NH2 group of donors	0.00041	333,17,8
2viua_#252	GO:0003729: mRNA binding	0.000411	343,13,7
2arca_#55	GO:0003887: DNA-directed DNA polymerase activity	0.000411	144,20,6
1i4ua_#154	GO:0000287: magnesium ion binding	0.000411	40,128,8
1f97a2#199	GO:0003887: DNA-directed DNA polymerase activity	0.000411	144,20,6
1by5a_#238	GO:0051082: unfolded protein binding	0.000412	237,34,10
1rb9__#49	GO:0003724: RNA helicase activity	0.000413	39,11,3
1c3ga2#272	GO:0004364: glutathione transferase activity	0.000413	39,11,3
1ejda_#213	GO:0051082: unfolded protein binding	0.000413	284,34,11
1ypta_#418	GO:0015078: hydrogen ion transporter activity	0.000414	20,21,3
1hxxa_#228	GO:0005351: sugar porter activity	0.000414	20,21,3
1ep3b1#71	GO:0003887: DNA-directed DNA polymerase activity	0.000414	21,20,3
1dn2a1#261	GO:0004714: transmembrane receptor protein tyrosine kinase activity	0.000414	425,14,8
1e7wa_#36	GO:0016776: phosphotransferase activity, phosphate group as acceptor	0.000414	137,14,5
1qlca_#210	GO:0008083: growth factor activity	0.000415	24,42,4
1quba4#235	GO:0004295: trypsin activity	0.000415	106,48,8
2bbkh_#348	GO:0008083: growth factor activity	0.000415	24,42,4
1tyfa_#31	GO:0000287: magnesium ion binding	0.000416	162,128,18
1c9la2#12	GO:0005507: copper ion binding	0.000417	295,38,12
1fgga_#134	GO:0004222: metalloendopeptidase activity	0.000417	218,19,7
1dbxa_#75	GO:0005096: GTPase activator activity	0.000417	69,15,4
1bywa_#30	GO:0005529: sugar binding	0.000418	331,39,13

1aoca_#119	GO:0005179: hormone activity	0.000419	6,15,2
1qunb1#111	GO:0004556: alpha-amylase activity	0.000419	6,15,2
1i7oa1#63	GO:0004190: aspartic-type endopeptidase activity	0.000419	44,23,4
1ema__#26	GO:0004556: alpha-amylase activity	0.000419	6,15,2
1fvia2#25	GO:0004190: aspartic-type endopeptidase activity	0.000419	44,23,4
1aa6_1#681	GO:0030151: molybdenum ion binding	0.000419	6,15,2
1atza_#1070	GO:0005096: GTPase activator activity	0.000419	6,15,2
1htjf_#369	GO:0005096: GTPase activator activity	0.000419	6,15,2
1g62a_#177	GO:0003743: translation initiation factor activity	0.000419	6,15,2
1acz__#563	GO:0004556: alpha-amylase activity	0.000419	6,15,2
1dik_3#240	GO:0016668: oxidoreductase activity, acting on sulfur group of donors, NAD or NADP as acceptor	0.000419	262,12,6
1fc3a_#172	GO:0003700: transcription factor activity	0.00042	182,124,19
1div_1#141	GO:0008408: 3'-5' exonuclease activity	0.000423	81,13,4
1doka_#52	GO:0005126: hematopoietin/interferon-class (D200-domain) cytokine receptor binding	0.000423	51,20,4
1i58a_#519	GO:0008235: metalloexopeptidase activity	0.000423	81,13,4
1dpsa_#36	GO:0008199: ferric iron binding	0.000423	205,10,5
1poxa3#460	GO:0016854: racemase and epimerase activity	0.000423	81,13,4
1bxoa_#320	GO:0008083: growth factor activity	0.000424	66,42,6
1fbna_#144	GO:0005525: GTP binding	0.000424	79,49,7
1jd0a_#176	GO:0003779: actin binding	0.000425	207,32,9
1hc7a2#258	GO:0008083: growth factor activity	0.000425	226,42,11
1e4ea2#215	GO:0003809: thrombin activity	0.000425	490,10,7
1d6aa_#52	GO:0004364: glutathione transferase activity	0.000425	293,11,6
1evqa_#305	GO:0004457: lactate dehydrogenase activity	0.000425	490,10,7
1e5ka_#168	GO:0016799: hydrolase activity, hydrolyzing N-glycosyl compounds	0.000426	25,17,3
1ckea_#35	GO:0019201: nucleotide kinase activity	0.000426	33,13,3
2foka3#314	GO:0019201: nucleotide kinase activity	0.000426	33,13,3
1poxa3#439	GO:0003924: GTPase activity	0.000426	25,17,3
1g8ka2#523	GO:0016831: carboxy-lyase activity	0.000426	17,25,3
3gcb__#176	GO:0016627: oxidoreductase activity, acting on the CH-CH group of donors	0.000426	25,17,3
1qo2a_#223	GO:0016836: hydro-lyase activity	0.000426	13,33,3
1hg3a_#43	GO:0016861: intramolecular oxidoreductase activity, interconverting aldoses and ketoses	0.000426	33,13,3
1boub_#7	GO:0016763: transferase activity, transferring pentosyl groups	0.000426	240,28,9
1bf4a_#4	GO:0016627: oxidoreductase activity, acting on the CH-CH group of donors	0.000426	25,17,3
1feca1#122	GO:0016627: oxidoreductase activity, acting on the CH-CH group of donors	0.000426	25,17,3
1nal1_#204	GO:0016831: carboxy-lyase activity	0.000426	17,25,3
1ft9a1#201	GO:0004497: monooxygenase activity	0.000427	109,26,6
3grx__#56	GO:0016638: oxidoreductase activity, acting on the CH-NH2 group of donors	0.000428	335,17,8
1ihua2#525	GO:0016799: hydrolase activity, hydrolyzing N-glycosyl compounds	0.000428	335,17,8
1bfd_1#230	GO:0016616: oxidoreductase activity, acting on the CH-OH group of donors, NAD or NADP as acceptor	0.000429	47,59,6
1e2o__#346	GO:0051082: unfolded protein binding	0.000429	194,34,9
1fo4a5#1002	GO:0003887: DNA-directed DNA polymerase activity	0.000429	93,20,5
1dik_1#765	GO:0016866: intramolecular transferase activity	0.000429	165,12,5
1c8ba_#514	GO:0042802: protein self binding	0.00043	36,12,3
1dfaa1#25	GO:0003697: single-stranded DNA binding	0.00043	36,12,3
1pot__#138	GO:0003968: RNA-directed RNA polymerase activity	0.00043	75,14,4
1c0pa1#1147	GO:0016668: oxidoreductase activity, acting on sulfur group of donors, NAD or NADP as acceptor	0.00043	36,12,3
1pwt__#8	GO:0004263: chymotrypsin activity	0.00043	314,41,13
2dpma_#194	GO:0004457: lactate dehydrogenase activity	0.000431	9,10,2
2fcba2#104	GO:0019838: growth factor binding	0.000431	10,9,2
1ep3a_#220	GO:0003684: damaged DNA binding	0.000431	9,10,2
1nfdb1#115	GO:0019838: growth factor binding	0.000431	10,9,2
1scfa_#118	GO:0008199: ferric iron binding	0.000431	9,10,2
1qq8a_#137	GO:0008199: ferric iron binding	0.000431	9,10,2
1pme__#207	GO:0005066: transmembrane receptor protein tyrosine kinase signaling protein activity	0.000431	9,10,2
1as4.1#A336	GO:0005529: sugar binding	0.000431	332,39,13
2mev1_#123	GO:0016668: oxidoreductase activity, acting on sulfur group of donors, NAD or NADP as acceptor	0.000431	89,12,4
1b8aa2#368	GO:0000287: magnesium ion binding	0.000431	135,128,16
1qfma1#238	GO:0003682: chromatin binding	0.000431	9,10,2
1ds1a_#300	GO:0005525: GTP binding	0.000432	37,49,5
1seta2#231	GO:0030145: manganese ion binding	0.000432	103,38,7
1pwt__#8	GO:0005509: calcium ion binding	0.000432	314,160,33

1h75a_#53	GO:0005525: GTP binding	0.000432	37,49,5
1elja_#64	GO:0030145: manganese ion binding	0.000432	103,38,7
1h8ua_#113	GO:0004523: ribonuclease H activity	0.000433	65,16,4
1g0sa_#74	GO:0019955: cytokine binding	0.000433	294,11,6
1el0a_#42	GO:0019955: cytokine binding	0.000433	294,11,6
1e5ka_#113	GO:0004523: ribonuclease H activity	0.000433	65,16,4
1jj2m_#38	GO:0000287: magnesium ion binding	0.000434	15,128,5
1a0ca_#273	GO:0000287: magnesium ion binding	0.000434	15,128,5
1e1oa1#142	GO:0000287: magnesium ion binding	0.000434	15,128,5
7fd1a_#81	GO:0003729: mRNA binding	0.000435	7,13,2
1qdea_#201	GO:0008408: 3'-5' exonuclease activity	0.000435	7,13,2
1cpt__#325	GO:0016651: oxidoreductase activity, acting on NADH or NADPH	0.000435	7,13,2
3sil__#237	GO:0008026: ATP-dependent helicase activity	0.000435	7,13,2
1f7ua2#293	GO:0000049: tRNA binding	0.000435	7,13,2
1b8oa_#234	GO:0004177: aminopeptidase activity	0.000435	7,13,2
1jj2q_#118	GO:0005085: guanyl-nucleotide exchange factor activity	0.000435	7,13,2
1hnua_#190	GO:0019201: nucleotide kinase activity	0.000435	7,13,2
1b8fa_#500	GO:0008408: 3'-5' exonuclease activity	0.000435	7,13,2
1e30a_#55	GO:0005518: collagen binding	0.000435	7,13,2
1mil__#30	GO:0046983: protein dimerization activity	0.000435	61,17,4
1bfg__#22	GO:0016638: oxidoreductase activity, acting on the CH-NH2 group of donors	0.000435	61,17,4
1hv8a1#134	GO:0008026: ATP-dependent helicase activity	0.000435	7,13,2
1f9za_#16	GO:0005524: ATP binding	0.000436	140,243,25
1lara2#1769	GO:0004714: transmembrane receptor protein tyrosine kinase activity	0.000437	219,14,6
16pk__#95	GO:0000287: magnesium ion binding	0.000438	31,128,7
1ib2a_#1101	GO:0005096: GTPase activator activity	0.00044	70,15,4
1pma1_#79	GO:0004725: protein tyrosine phosphatase activity	0.00044	70,15,4
1lvk_2#275	GO:0005516: calmodulin binding	0.000441	4,24,2
2hrva_#123	GO:0004197: cysteine-type endopeptidase activity	0.000441	4,24,2
1fnha2#152	GO:0008201: heparin binding	0.000441	4,24,2
1e0ga_#3	GO:0008201: heparin binding	0.000441	4,24,2
1b35a_#49	GO:0004197: cysteine-type endopeptidase activity	0.000441	4,24,2
1f2la_#40	GO:0019838: growth factor binding	0.000441	236,9,5
1c9la2#12	GO:0003755: peptidyl-prolyl cis-trans isomerase activity	0.000441	295,11,6
1jlxa2#166	GO:0008201: heparin binding	0.000441	77,24,5
1rypf_#35	GO:0005525: GTP binding	0.000441	21,49,4
1fhoa_#74	GO:0008408: 3'-5' exonuclease activity	0.000442	347,13,7
1byka_#279	GO:0016861: intramolecular oxidoreductase activity, interconverting aldoses and ketoses	0.000443	82,13,4
1d7ba_#87	GO:0004896: hematopoietin/interferon-class (D200-domain) cytokine receptor activity	0.000443	99,19,5
1zpda1#213	GO:0008026: ATP-dependent helicase activity	0.000443	82,13,4
1atg__#144	GO:0008026: ATP-dependent helicase activity	0.000443	82,13,4
1c3pa_#6	GO:0016861: intramolecular oxidoreductase activity, interconverting aldoses and ketoses	0.000443	82,13,4
2pola2#218	GO:0003887: DNA-directed DNA polymerase activity	0.000443	146,20,6
1dl0a_#32	GO:0004295: trypsin activity	0.000445	38,48,5
2bbkh_#214	GO:0004295: trypsin activity	0.000445	38,48,5
1d7ba_#87	GO:0019955: cytokine binding	0.000445	99,11,4
1i50b_#216	GO:0005507: copper ion binding	0.000445	48,38,5
1b6e__#76	GO:0004523: ribonuclease H activity	0.000446	27,16,3
1qhda2#323	GO:0046983: protein dimerization activity	0.000446	337,17,8
1qhda2#323	GO:0016638: oxidoreductase activity, acting on the CH-NH2 group of donors	0.000446	337,17,8
1i6vd_#713	GO:0019955: cytokine binding	0.000446	40,11,3
1i6vd_#713	GO:0003724: RNA helicase activity	0.000446	40,11,3
16pk__#95	GO:0016836: hydro-lyase activity	0.000446	31,33,4
1rypk_#12	GO:0042802: protein self binding	0.000446	265,12,6
1iira_#326	GO:0003924: GTPase activity	0.000447	112,17,5
1ovb__#195	GO:0005351: sugar porter activity	0.000447	89,21,5
2bb2_2#134	GO:0016251: general RNA polymerase II transcription factor activity	0.000448	220,14,6
1cnv__#181	GO:0016831: carboxy-lyase activity	0.000448	74,25,5
1dfaa1#35	GO:0005507: copper ion binding	0.000449	27,38,4
1je5a_#39	GO:0004197: cysteine-type endopeptidase activity	0.000449	18,24,3
1iq4a_#31	GO:0005507: copper ion binding	0.000449	27,38,4
1a6o__#217	GO:0005516: calmodulin binding	0.000449	18,24,3
1howa_#697	GO:0005516: calmodulin binding	0.000449	18,24,3
2sli_2#326	GO:0016638: oxidoreductase activity, acting on the CH-NH2 group of donors	0.000449	251,17,7
1phk__#165	GO:0005516: calmodulin binding	0.000449	18,24,3
1d3va_#176	GO:0008810: cellulase activity	0.000449	24,18,3

1gsoa3#172	GO:0051082: unfolded protein binding	0.00045	3,34,2
1kid__#330	GO:0004896: hematopoietin/interferon-class (D200-domain) cytokine receptor activity	0.00045	155,19,6
1a81a2#182	GO:0004523: ribonuclease H activity	0.00045	120,16,5
1f5ma_#80	GO:0005525: GTP binding	0.00045	105,49,8
1mdah_#329	GO:0050660: FAD binding	0.00045	335,10,6
1hd2a_#35	GO:0003964: RNA-directed DNA polymerase activity	0.000451	185,11,5
1vmoa_#106	GO:0019843: rRNA binding	0.000451	187,22,7
1cm9a_#57	GO:0019838: growth factor binding	0.000452	50,9,3
1h72c2#269	GO:0051082: unfolded protein binding	0.000452	83,34,6
1nfp__#171	GO:0005096: GTPase activator activity	0.000453	29,15,3
1qo7a_#109	GO:0004180: carboxypeptidase activity	0.000453	29,15,3
1qhda2#323	GO:0051082: unfolded protein binding	0.000454	337,34,12
1qs1a2#437	GO:0004263: chymotrypsin activity	0.000454	452,41,16
1a4ya_#47	GO:0016831: carboxy-lyase activity	0.000454	216,25,8
1a8i__#688	GO:0016861: intramolecular oxidoreductase activity, interconverting aldoses and ketoses	0.000454	241,13,6
1a65a2#187	GO:0004197: cysteine-type endopeptidase activity	0.000454	43,24,4
1fo8a_#367	GO:0016646: oxidoreductase activity, acting on the CH-NH group of donors, NAD or NADP as acceptor	0.000455	5,19,2
1aoca_#123	GO:0004896: hematopoietin/interferon-class (D200-domain) cytokine receptor activity	0.000455	5,19,2
1ecra_#101	GO:0004896: hematopoietin/interferon-class (D200-domain) cytokine receptor activity	0.000455	5,19,2
1fhga_#119	GO:0004896: hematopoietin/interferon-class (D200-domain) cytokine receptor activity	0.000455	5,19,2
1gen__#505	GO:0004222: metalloendopeptidase activity	0.000455	5,19,2
1ycqa_#24	GO:0004842: ubiquitin-protein ligase activity	0.000455	5,19,2
1fbl_1#418	GO:0004222: metalloendopeptidase activity	0.000455	5,19,2
1f8na1#553	GO:0004222: metalloendopeptidase activity	0.000455	5,19,2
1bjt__#890	GO:0030151: molybdenum ion binding	0.000455	292,15,7
1fhga_#48	GO:0004896: hematopoietin/interferon-class (D200-domain) cytokine receptor activity	0.000455	5,19,2
1ijqa1#499	GO:0004222: metalloendopeptidase activity	0.000455	5,19,2
1tyfa_#31	GO:0005525: GTP binding	0.000455	162,49,10
1ddja_#746	GO:0008201: heparin binding	0.000455	170,24,7
1dnv__#354	GO:0051082: unfolded protein binding	0.000455	54,34,5
1dgw.1#Y353	GO:0015036: disulfide oxidoreductase activity	0.000457	319,22,9
1aky_2#133	GO:0004674: protein serine/threonine kinase activity	0.000457	156,42,9
3prn__#143	GO:0005529: sugar binding	0.000457	334,39,13
1ea5a_#99	GO:0008270: zinc ion binding	0.000457	194,108,18
1f97a1#62	GO:0015082: di-, tri-valent inorganic cation transporter activity	0.000458	707,14,10
1f97a1#62	GO:0004714: transmembrane receptor protein tyrosine kinase activity	0.000458	707,14,10
1f97a1#62	GO:0046915: transition metal ion transporter activity	0.000458	707,14,10
1ayl__#237	GO:0003887: DNA-directed DNA polymerase activity	0.000458	357,20,9
1io1a_#311	GO:0019838: growth factor binding	0.000459	126,9,4
1fi2a_#109	GO:0008083: growth factor activity	0.000459	228,42,11
1nox__#88	GO:0004842: ubiquitin-protein ligase activity	0.00046	55,19,4
1erxa_#138	GO:0016646: oxidoreductase activity, acting on the CH-NH group of donors, NAD or NADP as acceptor	0.00046	55,19,4
1vjw__#39	GO:0016616: oxidoreductase activity, acting on the CH-OH group of donors, NAD or NADP as acceptor	0.000461	2,59,2
2naca1#329	GO:0016616: oxidoreductase activity, acting on the CH-OH group of donors, NAD or NADP as acceptor	0.000461	2,59,2
2liv__#11	GO:0016616: oxidoreductase activity, acting on the CH-OH group of donors, NAD or NADP as acceptor	0.000461	2,59,2
1e3ja1#320	GO:0016616: oxidoreductase activity, acting on the CH-OH group of donors, NAD or NADP as acceptor	0.000461	2,59,2
1dr9a2#139	GO:0004674: protein serine/threonine kinase activity	0.000461	67,42,6
1qdlb_#170	GO:0003724: RNA helicase activity	0.000462	100,11,4
1i2oa_#34	GO:0000287: magnesium ion binding	0.000463	192,128,20
1jjya_#329	GO:0030151: molybdenum ion binding	0.000465	71,15,4
1fhga_#59	GO:0005518: collagen binding	0.000466	34,13,3
1dfma_#128	GO:0009036: type II site-specific deoxyribonuclease activity	0.000466	37,12,3
1af7_2#248	GO:0019201: nucleotide kinase activity	0.000466	34,13,3
1tkia_#142	GO:0000155: two-component sensor molecule activity	0.000466	37,12,3
1bvza3#416	GO:0004222: metalloendopeptidase activity	0.000466	23,19,3
1eq2a_#11	GO:0016620: oxidoreductase activity, acting on the aldehyde or oxo group of donors, NAD or NADP as acceptor	0.000466	45,10,3
1crua_#29	GO:0004222: metalloendopeptidase activity	0.000466	23,19,3
1fvia2#134	GO:0005524: ATP binding	0.000467	28,243,9
1iba__#39	GO:0004674: protein serine/threonine kinase activity	0.000468	351,42,14

1fsia_#122	GO:0000287: magnesium ion binding	0.000468	97,128,13
1eucb2#188	GO:0005524: ATP binding	0.000468	87,243,18
3sil__#317	GO:0003887: DNA-directed DNA polymerase activity	0.000468	358,20,9
1thfd_#140	GO:0004674: protein serine/threonine kinase activity	0.000469	124,42,8
1iira_#326	GO:0050660: FAD binding	0.000469	112,10,4
1bfd_2#146	GO:0016620: oxidoreductase activity, acting on the aldehyde or oxo group of donors, NAD or NADP as acceptor	0.000469	112,10,4
1qdla_#211	GO:0015036: disulfide oxidoreductase activity	0.000469	320,22,9
1fwxa2#396	GO:0004867: serine-type endopeptidase inhibitor activity	0.00047	237,47,12
1h9da_#91	GO:0003968: RNA-directed RNA polymerase activity	0.00047	222,14,6
1oaca1#495	GO:0004867: serine-type endopeptidase inhibitor activity	0.000471	60,47,6
1qj2c2#50	GO:0008270: zinc ion binding	0.000472	48,108,8
1ddja_#681	GO:0016638: oxidoreductase activity, acting on the CH-NH2 group of donors	0.000472	253,17,7
2dnja_#97	GO:0016251: general RNA polymerase II transcription factor activity	0.000474	141,14,5
1epwa3#140	GO:0019955: cytokine binding	0.000474	187,11,5
1dpsa_#36	GO:0005179: hormone activity	0.000474	205,15,6
1i7oa1#63	GO:0008083: growth factor activity	0.000475	44,42,5
1i1ja_#83	GO:0005524: ATP binding	0.000475	13,243,6
1apme_#88	GO:0005524: ATP binding	0.000475	13,243,6
1a65a1#108	GO:0004556: alpha-amylase activity	0.000475	294,15,7
1a65a1#108	GO:0004725: protein tyrosine phosphatase activity	0.000475	294,15,7
1jf9a_#379	GO:0004295: trypsin activity	0.000476	232,48,12
1jlxa2#166	GO:0004714: transmembrane receptor protein tyrosine kinase activity	0.000476	77,14,4
1fid__#237	GO:0000287: magnesium ion binding	0.000477	51,128,9
1jb0d_#26	GO:0004674: protein serine/threonine kinase activity	0.000477	229,42,11
1a65a3#383	GO:0015078: hydrogen ion transporter activity	0.000477	50,21,4
1el0a_#28	GO:0004263: chymotrypsin activity	0.000478	161,41,9
1pii_2#326	GO:0016763: transferase activity, transferring pentosyl groups	0.000478	192,28,8
1doka_#52	GO:0019838: growth factor binding	0.000479	51,9,3
1b0pa1#28	GO:0016831: carboxy-lyase activity	0.00048	4,25,2
1a7ca_#204	GO:0046983: protein dimerization activity	0.00048	26,17,3
2cpl__#83	GO:0004263: chymotrypsin activity	0.00048	11,41,3
1t7pa2#663	GO:0046983: protein dimerization activity	0.00048	26,17,3
1apme_#85	GO:0016831: carboxy-lyase activity	0.00048	4,25,2
1e79h2#77	GO:0015078: hydrogen ion transporter activity	0.00048	21,21,3
1ie5a_#23	GO:0015078: hydrogen ion transporter activity	0.00048	21,21,3
1f8v.1#A116	GO:0003724: RNA helicase activity	0.00048	101,11,4
1djna1#86	GO:0000287: magnesium ion binding	0.00048	85,128,12
3sil__#317	GO:0008201: heparin binding	0.00048	358,24,10
1e39a2#278	GO:0016638: oxidoreductase activity, acting on the CH-NH2 group of donors	0.00048	26,17,3
1qj2c2#50	GO:0016616: oxidoreductase activity, acting on the CH-OH group of donors, NAD or NADP as acceptor	0.000482	48,59,6
2dpma_#189	GO:0016616: oxidoreductase activity, acting on the CH-OH group of donors, NAD or NADP as acceptor	0.000482	48,59,6
1mjha_#119	GO:0016763: transferase activity, transferring pentosyl groups	0.000482	103,28,6
1ddja_#746	GO:0005529: sugar binding	0.000482	170,39,9
1qj8a_#64	GO:0051082: unfolded protein binding	0.000482	84,34,6
1jj2i_#30	GO:0016616: oxidoreductase activity, acting on the CH-OH group of donors, NAD or NADP as acceptor	0.000484	18,59,4
1qnf_2#27	GO:0016616: oxidoreductase activity, acting on the CH-OH group of donors, NAD or NADP as acceptor	0.000484	18,59,4
1bn8a_#241	GO:0016616: oxidoreductase activity, acting on the CH-OH group of donors, NAD or NADP as acceptor	0.000484	18,59,4
2sqca2#268	GO:0008810: cellulase activity	0.000484	59,18,4
1dih_2#222	GO:0008408: 3'-5' exonuclease activity	0.000484	352,13,7
1nsj__#21	GO:0016627: oxidoreductase activity, acting on the CH-CH group of donors	0.000485	114,17,5
1smaa2#581	GO:0005524: ATP binding	0.000485	80,243,17
1a0la_#160	GO:0005529: sugar binding	0.000486	73,39,6
1rlr_1#66	GO:0003713: transcription coactivator activity	0.000487	67,16,4
1f8v.1#A116	GO:0004896: hematopoietin/interferon-class (D200-domain) cytokine receptor activity	0.000487	101,19,5
1dfma_#128	GO:0008270: zinc ion binding	0.000487	37,108,7
1ejda_#213	GO:0000287: magnesium ion binding	0.000487	284,128,26
1ex1a1#284	GO:0016831: carboxy-lyase activity	0.000488	42,25,4
1vmoa_#84	GO:0016831: carboxy-lyase activity	0.000488	42,25,4
2reb_1#138	GO:0016616: oxidoreductase activity, acting on the CH-OH group of donors, NAD or NADP as acceptor	0.00049	67,59,7

1eqga2#47	GO:0016702: oxidoreductase activity, acting on single donors with incorporation of molecular oxygen, incorporation of two atoms of oxygen	0.000491	8,12,2
1dk0a_#115	GO:0016866: intramolecular transferase activity	0.000491	8,12,2
1b8aa1#41	GO:0003697: single-stranded DNA binding	0.000491	8,12,2
1dlja2#177	GO:0000287: magnesium ion binding	0.000491	41,128,8
1dfma_#53	GO:0009036: type II site-specific deoxyribonuclease activity	0.000491	8,12,2
1ebma2#47	GO:0016251: general RNA polymerase II transcription factor activity	0.000491	32,14,3
1ebma2#47	GO:0003968: RNA-directed RNA polymerase activity	0.000491	32,14,3
2viua_#123	GO:0042802: protein self binding	0.000491	8,12,2
1dceb_#134	GO:0015405: P-P-bond-hydrolysis-driven transporter activity	0.000491	8,12,2
1kapp1#358	GO:0003968: RNA-directed RNA polymerase activity	0.000491	32,14,3
1qtn.1#A270	GO:0016758: transferase activity, transferring hexosyl groups	0.000491	439,11,7
1ak5_1#353	GO:0005524: ATP binding	0.000493	73,243,16
1b3qa2#623	GO:0019955: cytokine binding	0.000494	301,11,6
1qfta_#98	GO:0004674: protein serine/threonine kinase activity	0.000495	230,42,11
1ejda_#6	GO:0016702: oxidoreductase activity, acting on single donors with incorporation of molecular oxygen, incorporation of two atoms of oxygen	0.000495	270,12,6
3sil__#8	GO:0004674: protein serine/threonine kinase activity	0.000496	125,42,8
1f42a2#123	GO:0004190: aspartic-type endopeptidase activity	0.000497	378,23,10
3chbd_#85	GO:0004190: aspartic-type endopeptidase activity	0.000497	378,23,10
1jlja_#104	GO:0016763: transferase activity, transferring pentosyl groups	0.000498	16,28,3
1io1a_#233	GO:0008083: growth factor activity	0.000498	95,42,7
1hava_#41	GO:0003887: DNA-directed DNA polymerase activity	0.000498	96,20,5
1am2__#163	GO:0019955: cytokine binding	0.000499	102,11,4
1ax4a_#275	GO:0003724: RNA helicase activity	0.000499	102,11,4
1keva2#266	GO:0016616: oxidoreductase activity, acting on the CH-OH group of donors, NAD or NADP as acceptor	0.0005	8,59,3
1ldna1#129	GO:0016616: oxidoreductase activity, acting on the CH-OH group of donors, NAD or NADP as acceptor	0.0005	8,59,3
1rhs_1#10	GO:0016616: oxidoreductase activity, acting on the CH-OH group of donors, NAD or NADP as acceptor	0.0005	8,59,3
1bg6_1#316	GO:0016616: oxidoreductase activity, acting on the CH-OH group of donors, NAD or NADP as acceptor	0.0005	8,59,3
1cvl__#105	GO:0016616: oxidoreductase activity, acting on the CH-OH group of donors, NAD or NADP as acceptor	0.0005	8,59,3
1bvsa3#12	GO:0030151: molybdenum ion binding	0.000501	30,15,3
1dyna_#19	GO:0004295: trypsin activity	0.000503	109,48,8
1jb3a_#64	GO:0019955: cytokine binding	0.000503	302,11,6
1ft9a1#201	GO:0004295: trypsin activity	0.000503	109,48,8
1bev1_#86	GO:0015036: disulfide oxidoreductase activity	0.000503	323,22,9
1bev1_#86	GO:0019843: rRNA binding	0.000503	323,22,9
2viua_#252	GO:0016638: oxidoreductase activity, acting on the CH-NH2 group of donors	0.000504	343,17,8
1g31a_#15	GO:0005507: copper ion binding	0.000505	12,38,3
1ecra_#101	GO:0005126: hematopoietin/interferon-class (D200-domain) cytokine receptor binding	0.000505	5,20,2
1eno__#83	GO:0042802: protein self binding	0.000505	38,12,3
1evya2#70	GO:0003887: DNA-directed DNA polymerase activity	0.000505	5,20,2
2pola1#113	GO:0003887: DNA-directed DNA polymerase activity	0.000505	5,20,2
1aqza_#135	GO:0003887: DNA-directed DNA polymerase activity	0.000505	5,20,2
1dkra2#210	GO:0016836: hydro-lyase activity	0.000505	32,33,4
1i4ua_#154	GO:0005524: ATP binding	0.000505	40,243,11
1c7qa_#181	GO:0030145: manganese ion binding	0.000505	12,38,3
1kit_3#297	GO:0005507: copper ion binding	0.000505	12,38,3
1dxea_#71	GO:0016866: intramolecular transferase activity	0.000505	38,12,3
2scub2#19	GO:0005524: ATP binding	0.000506	9,243,5
1feua_#6	GO:0016776: phosphotransferase activity, phosphate group as acceptor	0.000507	7,14,2
1hnua_#190	GO:0016776: phosphotransferase activity, phosphate group as acceptor	0.000507	7,14,2
1cex__#109	GO:0008235: metalloexopeptidase activity	0.000509	35,13,3
1ajsa_#184	GO:0008026: ATP-dependent helicase activity	0.000509	35,13,3
1gc5a_#233	GO:0005085: guanyl-nucleotide exchange factor activity	0.000509	35,13,3
1f7da_#97	GO:0000049: tRNA binding	0.000509	35,13,3
1f0xa2#118	GO:0016651: oxidoreductase activity, acting on NADH or NADPH	0.000509	35,13,3
1thg__#127	GO:0016831: carboxy-lyase activity	0.000509	18,25,3
1fmja_#76	GO:0008026: ATP-dependent helicase activity	0.000509	85,13,4
1lucb_#70	GO:0016861: intramolecular oxidoreductase activity, interconverting aldoses and ketoses	0.000509	35,13,3
1qfta_#98	GO:0008201: heparin binding	0.00051	230,24,8
1f00i2#805	GO:0015036: disulfide oxidoreductase activity	0.000512	135,22,6

1hxxa_#173	GO:0005524: ATP binding	0.000514	260,243,39
1erv__#80	GO:0016638: oxidoreductase activity, acting on the CH-NH2 group of donors	0.000514	442,17,9
1d6aa_#52	GO:0008757: S-adenosylmethionine-dependent methyltransferase activity	0.000515	293,24,9
1hxxa_#39	GO:0003755: peptidyl-prolyl cis-trans isomerase activity	0.000516	42,11,3
1howa_#618	GO:0004674: protein serine/threonine kinase activity	0.000516	11,42,3
1vmoa_#84	GO:0019955: cytokine binding	0.000516	42,11,3
1cewi_#14	GO:0004263: chymotrypsin activity	0.000517	98,41,7
1ab4__#89	GO:0004497: monooxygenase activity	0.000517	159,26,7
1elja_#64	GO:0003724: RNA helicase activity	0.000518	103,11,4
1g5ha2#118	GO:0004812: tRNA ligase activity	0.000519	4,26,2
1dt9a1#209	GO:0004812: tRNA ligase activity	0.000519	113,26,6
1be9a_#352	GO:0016705: oxidoreductase activity, acting on paired donors, with incorporation or reduction of molecular oxygen	0.000519	4,26,2
1be9a_#352	GO:0004497: monooxygenase activity	0.000519	4,26,2
1io7a_#38	GO:0016705: oxidoreductase activity, acting on paired donors, with incorporation or reduction of molecular oxygen	0.000519	4,26,2
1seta2#403	GO:0004812: tRNA ligase activity	0.000519	41,26,4
1e53a_#367	GO:0004263: chymotrypsin activity	0.000519	26,41,4
1d4xg_#60	GO:0016620: oxidoreductase activity, acting on the aldehyde or oxo group of donors, NAD or NADP as acceptor	0.000519	115,10,4
1bu7a_#433	GO:0016705: oxidoreductase activity, acting on paired donors, with incorporation or reduction of molecular oxygen	0.000519	4,26,2
1beba_#48	GO:0004812: tRNA ligase activity	0.000519	4,26,2
1fc6a1#188	GO:0016705: oxidoreductase activity, acting on paired donors, with incorporation or reduction of molecular oxygen	0.000519	4,26,2
1fc6a1#188	GO:0004497: monooxygenase activity	0.000519	4,26,2
1dz4a_#323	GO:0016705: oxidoreductase activity, acting on paired donors, with incorporation or reduction of molecular oxygen	0.000519	4,26,2
1hbza_#254	GO:0016705: oxidoreductase activity, acting on paired donors, with incorporation or reduction of molecular oxygen	0.000519	4,26,2
1hbza_#254	GO:0004497: monooxygenase activity	0.000519	4,26,2
1qava_#149	GO:0016705: oxidoreductase activity, acting on paired donors, with incorporation or reduction of molecular oxygen	0.000519	4,26,2
1qava_#149	GO:0004497: monooxygenase activity	0.000519	4,26,2
1qlca_#228	GO:0016705: oxidoreductase activity, acting on paired donors, with incorporation or reduction of molecular oxygen	0.000519	4,26,2
1qlca_#228	GO:0004497: monooxygenase activity	0.000519	4,26,2
1cxp.1#A97	GO:0016705: oxidoreductase activity, acting on paired donors, with incorporation or reduction of molecular oxygen	0.000519	4,26,2
2dnja_#97	GO:0004867: serine-type endopeptidase inhibitor activity	0.00052	141,47,9
1czan1#92	GO:0008080: N-acetyltransferase activity	0.000522	631,13,9
1prea2#308	GO:0005507: copper ion binding	0.000524	76,38,6
1cl1a_#184	GO:0008483: transaminase activity	0.000524	64,17,4
3sil__#284	GO:0016638: oxidoreductase activity, acting on the CH-NH2 group of donors	0.000524	64,17,4
1cpt__#358	GO:0010181: FMN binding	0.000525	9,11,2
1fhga_#42	GO:0019955: cytokine binding	0.000525	9,11,2
1e3ua_#205	GO:0004364: glutathione transferase activity	0.000525	9,11,2
1erza_#183	GO:0016776: phosphotransferase activity, phosphate group as acceptor	0.000525	79,14,4
1wdcb_#141	GO:0010181: FMN binding	0.000525	9,11,2
1qrjb1#133	GO:0010181: FMN binding	0.000525	9,11,2
1fo0b_#82	GO:0019955: cytokine binding	0.000525	9,11,2
1ev2e1#154	GO:0019955: cytokine binding	0.000525	9,11,2
1dz4a_#357	GO:0010181: FMN binding	0.000525	9,11,2
1qaua_#58	GO:0010181: FMN binding	0.000525	9,11,2
1bwda_#117	GO:0003924: GTPase activity	0.000526	116,17,5
1ej0a_#42	GO:0016638: oxidoreductase activity, acting on the CH-NH2 group of donors	0.000526	116,17,5
1tuba1#8	GO:0000287: magnesium ion binding	0.000527	124,128,15
1i50b_#966	GO:0004556: alpha-amylase activity	0.000527	299,15,7
1dih_2#235	GO:0003887: DNA-directed DNA polymerase activity	0.000528	54,20,4
1a4ya_#104	GO:0008810: cellulase activity	0.000528	241,18,7
1a65a1#108	GO:0008201: heparin binding	0.000528	294,24,9
1dik_1#765	GO:0005525: GTP binding	0.000528	165,49,10
1ypci_#69	GO:0004867: serine-type endopeptidase inhibitor activity	0.00053	10,47,3
1ihna_#64	GO:0004867: serine-type endopeptidase inhibitor activity	0.00053	10,47,3
1d0na6#681	GO:0015078: hydrogen ion transporter activity	0.00053	143,21,6
1f2na_#138	GO:0004674: protein serine/threonine kinase activity	0.000531	96,42,7

1ia8a_#59	GO:0005516: calmodulin binding	0.000531	19,24,3
1nsca_#91	GO:0008201: heparin binding	0.000531	19,24,3
1fmk_3#377	GO:0005516: calmodulin binding	0.000531	19,24,3
8dfr__#9	GO:0000287: magnesium ion binding	0.000532	111,128,14
1ho1a_#211	GO:0000287: magnesium ion binding	0.000532	111,128,14
1gsa_2#218	GO:0019838: growth factor binding	0.000533	131,9,4
1f97a1#62	GO:0003916: DNA topoisomerase activity	0.000534	707,10,8
1ovb__#195	GO:0016616: oxidoreductase activity, acting on the CH-OH group of donors, NAD or NADP as acceptor	0.000534	89,59,8
1c4zd_#51	GO:0004842: ubiquitin-protein ligase activity	0.000534	160,19,6
1gjwa2#458	GO:0005524: ATP binding	0.000535	23,243,8
1cpt__#323	GO:0005066: transmembrane receptor protein tyrosine kinase signaling protein activity	0.000537	10,10,2
1tgoa1#217	GO:0016814: hydrolase activity, acting on carbon-nitrogen (but not peptide) bonds, in cyclic amidines	0.000537	10,10,2
1cb8a1#326	GO:0008199: ferric iron binding	0.000537	10,10,2
1f24a_#293	GO:0005066: transmembrane receptor protein tyrosine kinase signaling protein activity	0.000537	116,10,4
1bxoa_#307	GO:0003724: RNA helicase activity	0.000537	104,11,4
2tgi__#106	GO:0003682: chromatin binding	0.000537	10,10,2
3tdt__#201	GO:0004457: lactate dehydrogenase activity	0.000537	10,10,2
1b4ka_#108	GO:0016620: oxidoreductase activity, acting on the aldehyde or oxo group of donors, NAD or NADP as acceptor	0.000537	116,10,4
1fwxa2#189	GO:0003682: chromatin binding	0.000537	10,10,2
1qhua1#47	GO:0003755: peptidyl-prolyl cis-trans isomerase activity	0.000537	104,11,4
2bpa1_#45	GO:0003724: RNA helicase activity	0.000537	192,11,5
1dfaa1#35	GO:0016638: oxidoreductase activity, acting on the CH-NH2 group of donors	0.000538	27,17,3
1iray2#107	GO:0016836: hydro-lyase activity	0.000538	14,33,3
1ft9a1#203	GO:0016638: oxidoreductase activity, acting on the CH-NH2 group of donors	0.000538	27,17,3
1hr6a1#41	GO:0046983: protein dimerization activity	0.000538	27,17,3
1f97a1#62	GO:0004867: serine-type endopeptidase inhibitor activity	0.000538	707,47,23
1iq4a_#31	GO:0016638: oxidoreductase activity, acting on the CH-NH2 group of donors	0.000538	27,17,3
2foka3#314	GO:0016776: phosphotransferase activity, phosphate group as acceptor	0.000538	33,14,3
1d6ja_#36	GO:0003924: GTPase activity	0.000538	27,17,3
1gtxa_#150	GO:0008483: transaminase activity	0.000538	27,17,3
1dfca1#1135	GO:0019838: growth factor binding	0.000538	53,9,3
1gtma2#140	GO:0008483: transaminase activity	0.000538	27,17,3
1i6vd_#1188	GO:0003924: GTPase activity	0.000538	27,17,3
1dfca4#1469	GO:0019838: growth factor binding	0.000538	53,9,3
1zfpe_#67	GO:0015082: di-, tri-valent inorganic cation transporter activity	0.000538	33,14,3
1zfpe_#67	GO:0046915: transition metal ion transporter activity	0.000538	33,14,3
1ewqa3#159	GO:0000287: magnesium ion binding	0.000539	32,128,7
1mkp__#283	GO:0016616: oxidoreductase activity, acting on the CH-OH group of donors, NAD or NADP as acceptor	0.00054	49,59,6
1ycsa_#162	GO:0030151: molybdenum ion binding	0.000541	210,15,6
1qg6a_#65	GO:0016627: oxidoreductase activity, acting on the CH-CH group of donors	0.000542	6,17,2
1utea_#71	GO:0016799: hydrolase activity, hydrolyzing N-glycosyl compounds	0.000542	6,17,2
1ihoa_#75	GO:0003924: GTPase activity	0.000542	6,17,2
1atza_#1070	GO:0003924: GTPase activity	0.000542	6,17,2
1d2fa_#234	GO:0008483: transaminase activity	0.000542	6,17,2
1erja_#559	GO:0016638: oxidoreductase activity, acting on the CH-NH2 group of donors	0.000542	6,17,2
1bw0a_#199	GO:0008483: transaminase activity	0.000542	6,17,2
1plq_1#90	GO:0008408: 3'-5' exonuclease activity	0.000542	158,13,5
1rypd_#137	GO:0016638: oxidoreductase activity, acting on the CH-NH2 group of donors	0.000542	6,17,2
1rypb_#114	GO:0008235: metalloexopeptidase activity	0.000542	158,13,5
1cs1a_#82	GO:0004523: ribonuclease H activity	0.000543	195,16,6
1fi2a_#109	GO:0003968: RNA-directed RNA polymerase activity	0.000544	228,14,6
1h8ma_#8	GO:0005524: ATP binding	0.000544	134,243,24
1xgsa2#186	GO:0016638: oxidoreductase activity, acting on the CH-NH2 group of donors	0.000544	182,17,6
1e6ca_#91	GO:0008483: transaminase activity	0.000544	182,17,6
1qmea4#290	GO:0004556: alpha-amylase activity	0.000545	74,15,4
1dj0a2#183	GO:0005524: ATP binding	0.000545	142,243,25
1e3ja2#270	GO:0004556: alpha-amylase activity	0.000545	74,15,4
1bhga3#529	GO:0004180: carboxypeptidase activity	0.000545	74,15,4
1avgi_#69	GO:0005529: sugar binding	0.000546	340,39,13
1qo3c_#195	GO:0003697: single-stranded DNA binding	0.000546	39,12,3
1c3ga2#272	GO:0016702: oxidoreductase activity, acting on single donors with incorporation of molecular oxygen, incorporation of two atoms of oxygen	0.000546	39,12,3

1erv__#80	GO:0015082: di-, tri-valent inorganic cation transporter activity	0.000547	442,14,8
1erv__#80	GO:0046915: transition metal ion transporter activity	0.000547	442,14,8
1fhoa_#74	GO:0016638: oxidoreductase activity, acting on the CH-NH2 group of donors	0.000547	347,17,8
1fhoa_#74	GO:0003916: DNA topoisomerase activity	0.000548	347,10,6
2dnja_#97	GO:0030145: manganese ion binding	0.000549	141,38,8
1amf__#196	GO:0005529: sugar binding	0.000549	173,39,9
1b3qa2#623	GO:0005525: GTP binding	0.00055	301,49,14
1qrra_#69	GO:0016763: transferase activity, transferring pentosyl groups	0.000551	196,28,8
1el0a_#28	GO:0004896: hematopoietin/interferon-class (D200-domain) cytokine receptor activity	0.000552	161,19,6
1c4zd_#51	GO:0008083: growth factor activity	0.000552	160,42,9
1wdcb_#134	GO:0005518: collagen binding	0.000553	36,13,3
2hrva_#132	GO:0003724: RNA helicase activity	0.000553	43,11,3
1dlfl_#97	GO:0005518: collagen binding	0.000553	36,13,3
1gp1a_#173	GO:0004601: peroxidase activity	0.000553	22,21,3
1awcb_#111	GO:0005096: GTPase activator activity	0.000553	31,15,3
1hrna_#182	GO:0030151: molybdenum ion binding	0.000553	31,15,3
1fu6a_#50	GO:0004295: trypsin activity	0.000554	308,48,14
1g2912#10	GO:0005524: ATP binding	0.000555	18,243,7
1hava_#157	GO:0005524: ATP binding	0.000555	18,243,7
1heta2#218	GO:0005525: GTP binding	0.000555	39,49,5
1eu3a1#68	GO:0005524: ATP binding	0.000555	18,243,7
1xnb__#130	GO:0000049: tRNA binding	0.000556	87,13,4
1foha5#9	GO:0016627: oxidoreductase activity, acting on the CH-CH group of donors	0.000556	65,17,4
1e5ka_#113	GO:0016638: oxidoreductase activity, acting on the CH-NH2 group of donors	0.000556	65,17,4
1f0ia1#50	GO:0004714: transmembrane receptor protein tyrosine kinase activity	0.000557	146,14,5
1ge8a2#134	GO:0004714: transmembrane receptor protein tyrosine kinase activity	0.000557	229,14,6
1e79d2#36	GO:0030151: molybdenum ion binding	0.000557	524,15,9
1e79d2#36	GO:0004556: alpha-amylase activity	0.000557	524,15,9
1ek1a2#324	GO:0004601: peroxidase activity	0.000558	5,21,2
1ezvd1#213	GO:0015078: hydrogen ion transporter activity	0.000558	5,21,2
1qlwa_#103	GO:0004601: peroxidase activity	0.000558	5,21,2
1ixh__#7	GO:0015078: hydrogen ion transporter activity	0.000558	5,21,2
1ibja_#201	GO:0004601: peroxidase activity	0.000558	5,21,2
1ddja_#726	GO:0019955: cytokine binding	0.000558	105,11,4
1evqa_#61	GO:0000049: tRNA binding	0.000558	159,13,5
1hlga_#353	GO:0004601: peroxidase activity	0.000558	5,21,2
1a3qa2#190	GO:0005509: calcium ion binding	0.000558	144,160,19
1qhua1#47	GO:0004222: metalloendopeptidase activity	0.000558	104,19,5
1hxxa_#173	GO:0016638: oxidoreductase activity, acting on the CH-NH2 group of donors	0.000559	260,17,7
1ej8a_#189	GO:0051082: unfolded protein binding	0.00056	294,34,11
1g0sa_#74	GO:0051082: unfolded protein binding	0.00056	294,34,11
1fu6a_#50	GO:0003724: RNA helicase activity	0.00056	308,11,6
1c7na_#95	GO:0046983: protein dimerization activity	0.000561	183,17,6
1icia_#175	GO:0000287: magnesium ion binding	0.000561	63,128,10
1iba__#39	GO:0005507: copper ion binding	0.000562	351,38,13
1hlwa_#82	GO:0005524: ATP binding	0.000562	270,243,40
1ev7a_#265	GO:0005507: copper ion binding	0.000563	3,38,2
2bbkh_#117	GO:0005507: copper ion binding	0.000563	3,38,2
1aac__#17	GO:0005507: copper ion binding	0.000563	3,38,2
1bia_3#138	GO:0005507: copper ion binding	0.000563	3,38,2
1frpa_#140	GO:0005507: copper ion binding	0.000563	3,38,2
1erja_#642	GO:0005507: copper ion binding	0.000563	3,38,2
1fp2a2#182	GO:0005507: copper ion binding	0.000563	3,38,2
1oaca1#495	GO:0005509: calcium ion binding	0.000563	60,160,11
1ezia_#93	GO:0030145: manganese ion binding	0.000563	3,38,2
1e42a1#711	GO:0016702: oxidoreductase activity, acting on single donors with incorporation of molecular oxygen, incorporation of two atoms of oxygen	0.000565	175,12,5
2dpma_#189	GO:0003684: damaged DNA binding	0.000565	48,10,3
1opy__#68	GO:0005126: hematopoietin/interferon-class (D200-domain) cytokine receptor binding	0.000565	367,20,9
1fmca_#89	GO:0016620: oxidoreductase activity, acting on the aldehyde or oxo group of donors, NAD or NADP as acceptor	0.000565	48,10,3
1nox__#88	GO:0005126: hematopoietin/interferon-class (D200-domain) cytokine receptor binding	0.000566	55,20,4
1qhqa_#45	GO:0051082: unfolded protein binding	0.000566	121,34,7
1oaca1#495	GO:0005524: ATP binding	0.000569	60,243,14
1nat__#50	GO:0008757: S-adenosylmethionine-dependent methyltransferase activity	0.00057	297,24,9
1axca1#70	GO:0016638: oxidoreductase activity, acting on the CH-NH2 group of donors	0.00057	118,17,5

1jj2s_#91	GO:0008270: zinc ion binding	0.000571	5,108,3
1gen__#505	GO:0008270: zinc ion binding	0.000571	5,108,3
1fjfj_#68	GO:0015082: di-, tri-valent inorganic cation transporter activity	0.000571	230,14,6
1fjfj_#68	GO:0046915: transition metal ion transporter activity	0.000571	230,14,6
1hxn__#235	GO:0008270: zinc ion binding	0.000571	5,108,3
1phm_2#253	GO:0019955: cytokine binding	0.000571	309,11,6
1b35b_#97	GO:0003755: peptidyl-prolyl cis-trans isomerase activity	0.000571	309,11,6
1epwa2#1091	GO:0008270: zinc ion binding	0.000571	5,108,3
1eova1#139	GO:0000287: magnesium ion binding	0.000572	99,128,13
1qvba_#206	GO:0008810: cellulase activity	0.000573	26,18,3
1mrj__#161	GO:0016705: oxidoreductase activity, acting on paired donors, with incorporation or reduction of molecular oxygen	0.000573	18,26,3
1mrj__#161	GO:0004497: monooxygenase activity	0.000573	18,26,3
1i8aa_#2	GO:0004812: tRNA ligase activity	0.000573	18,26,3
1htp__#16	GO:0030151: molybdenum ion binding	0.000573	303,15,7
1euca1#64	GO:0016763: transferase activity, transferring pentosyl groups	0.000573	39,28,4
1fjsa_#28	GO:0005509: calcium ion binding	0.000574	13,160,5
1mpp__#154	GO:0005126: hematopoietin/interferon-class (D200-domain) cytokine receptor binding	0.000574	99,20,5
1fepa_#340	GO:0003887: DNA-directed DNA polymerase activity	0.000574	99,20,5
1ppn__#132	GO:0008201: heparin binding	0.000574	234,24,8
2bpa1_#45	GO:0005524: ATP binding	0.000574	192,243,31
2cb5a_#312	GO:0005524: ATP binding	0.000575	111,243,21
1be9a_#357	GO:0005525: GTP binding	0.000575	83,49,7
1g6oa_#116	GO:0008083: growth factor activity	0.000576	358,42,14
1dj0a2#183	GO:0030145: manganese ion binding	0.000576	142,38,8
1e19a_#281	GO:0019843: rRNA binding	0.000576	138,22,6
1cs1a_#82	GO:0003964: RNA-directed DNA polymerase activity	0.000577	195,11,5
1fa0a1#393	GO:0030145: manganese ion binding	0.000577	261,38,11
1hxxa_#232	GO:0030145: manganese ion binding	0.000578	352,38,13
1el0a_#28	GO:0004674: protein serine/threonine kinase activity	0.000578	161,42,9
1i58a_#519	GO:0003968: RNA-directed RNA polymerase activity	0.000578	81,14,4
1aqb__#134	GO:0015082: di-, tri-valent inorganic cation transporter activity	0.000578	81,14,4
1aqb__#134	GO:0046915: transition metal ion transporter activity	0.000578	81,14,4
1eupa_#320	GO:0016651: oxidoreductase activity, acting on NADH or NADPH	0.000579	8,13,2
1ile_3#170	GO:0000049: tRNA binding	0.000579	8,13,2
1e4ia_#314	GO:0008080: N-acetyltransferase activity	0.000579	8,13,2
1hava_#141	GO:0003729: mRNA binding	0.000579	8,13,2
1h70a_#211	GO:0008026: ATP-dependent helicase activity	0.000579	8,13,2
1bu7a_#360	GO:0016651: oxidoreductase activity, acting on NADH or NADPH	0.000579	8,13,2
2viua_#123	GO:0005518: collagen binding	0.000579	8,13,2
1cnv__#139	GO:0016861: intramolecular oxidoreductase activity, interconverting aldoses and ketoses	0.000579	8,13,2
1vdra_#37	GO:0019201: nucleotide kinase activity	0.000579	8,13,2
1qhla_#28	GO:0005509: calcium ion binding	0.00058	34,160,8
1beba_#119	GO:0051082: unfolded protein binding	0.00058	202,34,9
1c4zd_#51	GO:0051082: unfolded protein binding	0.00058	160,34,8
2btva_#749	GO:0000287: magnesium ion binding	0.000582	42,128,8
1ex1a1#284	GO:0000287: magnesium ion binding	0.000582	42,128,8
2cba__#144	GO:0005524: ATP binding	0.000582	67,243,15
2reb_1#138	GO:0005524: ATP binding	0.000582	67,243,15
1nfdb1#34	GO:0008201: heparin binding	0.000583	177,24,7
1fl7b_#51	GO:0005179: hormone activity	0.000585	7,15,2
1qj2b2#221	GO:0030151: molybdenum ion binding	0.000585	7,15,2
1qj2b2#221	GO:0004556: alpha-amylase activity	0.000585	7,15,2
1dmha_#184	GO:0004556: alpha-amylase activity	0.000585	7,15,2
1qg3a1#1179	GO:0005179: hormone activity	0.000585	7,15,2
2tgi__#80	GO:0005179: hormone activity	0.000585	7,15,2
1hcna_#12	GO:0005179: hormone activity	0.000585	7,15,2
1fl7b_#78	GO:0005179: hormone activity	0.000585	7,15,2
1h6va3#486	GO:0008083: growth factor activity	0.000585	70,42,6
1avaa2#235	GO:0004556: alpha-amylase activity	0.000585	7,15,2
1vns__#25	GO:0005179: hormone activity	0.000585	7,15,2
1ja1a1#497	GO:0030151: molybdenum ion binding	0.000585	7,15,2
1c4xa_#35	GO:0004180: carboxypeptidase activity	0.000585	7,15,2
1e8ga2#201	GO:0004556: alpha-amylase activity	0.000585	304,15,7
1aqua_#79	GO:0016831: carboxy-lyase activity	0.000585	44,25,4
1hv8a1#134	GO:0030151: molybdenum ion binding	0.000585	7,15,2

1booa_#254	GO:0016831: carboxy-lyase activity	0.000585	44,25,4
1g61a_#2155	GO:0003743: translation initiation factor activity	0.000585	7,15,2
1ciy_3#243	GO:0008810: cellulase activity	0.000586	62,18,4
1stfi_#116	GO:0051082: unfolded protein binding	0.000587	57,34,5
1bupa1#178	GO:0051082: unfolded protein binding	0.000588	14,34,3
1bwvs_#38	GO:0016702: oxidoreductase activity, acting on single donors with incorporation of molecular oxygen, incorporation of two atoms of oxygen	0.000588	40,12,3
1bywa_#30	GO:0015082: di-, tri-valent inorganic cation transporter activity	0.000588	331,14,7
1bywa_#30	GO:0004714: transmembrane receptor protein tyrosine kinase activity	0.000588	331,14,7
1bywa_#30	GO:0046915: transition metal ion transporter activity	0.000588	331,14,7
1bev1_#238	GO:0003968: RNA-directed RNA polymerase activity	0.000588	34,14,3
1opy__#68	GO:0008201: heparin binding	0.000589	367,24,10
2bb2_2#134	GO:0050660: FAD binding	0.00059	220,10,5
1ftra2#187	GO:0008757: S-adenosylmethionine-dependent methyltransferase activity	0.00059	46,24,4
1as4.1#B385	GO:0016638: oxidoreductase activity, acting on the CH-NH2 group of donors	0.00059	66,17,4
1d0na3#305	GO:0019955: cytokine binding	0.000591	311,11,6
1d0na3#305	GO:0003755: peptidyl-prolyl cis-trans isomerase activity	0.000591	311,11,6
1fvia2#25	GO:0019955: cytokine binding	0.000592	44,11,3
2cb5a_#373	GO:0003724: RNA helicase activity	0.000592	44,11,3
1eula_#213	GO:0005525: GTP binding	0.000592	60,49,6
1iba__#39	GO:0046983: protein dimerization activity	0.000592	351,17,8
1mjha_#126	GO:0005524: ATP binding	0.000592	159,243,27
1qs1a2#437	GO:0005507: copper ion binding	0.000593	452,38,15
1ycsb1#412	GO:0004601: peroxidase activity	0.000593	146,21,6
1e0ta2#275	GO:0016763: transferase activity, transferring pentosyl groups	0.000593	107,28,6
1lcl__#42	GO:0005529: sugar binding	0.000594	3,39,2
1dfca4#1397	GO:0005529: sugar binding	0.000594	3,39,2
1i0ha2#127	GO:0005507: copper ion binding	0.000595	29,38,4
3lada3#463	GO:0030145: manganese ion binding	0.000595	29,38,4
1ksia3#114	GO:0005507: copper ion binding	0.000595	29,38,4
1cr5a1#45	GO:0005507: copper ion binding	0.000595	29,38,4
1ddja_#681	GO:0005524: ATP binding	0.000596	253,243,38
1fd9a_#162	GO:0000287: magnesium ion binding	0.000597	87,128,12
1eoka_#127	GO:0016616: oxidoreductase activity, acting on the CH-OH group of donors, NAD or NADP as acceptor	0.000598	33,59,5
1b8aa2#368	GO:0019838: growth factor binding	0.000598	135,9,4
1fn9a_#271	GO:0005126: hematopoietin/interferon-class (D200-domain) cytokine receptor binding	0.000599	218,20,7
1dv8a_#211	GO:0004190: aspartic-type endopeptidase activity	0.000599	86,23,5
1g0sa_#176	GO:0008408: 3'-5' exonuclease activity	0.0006	37,13,3
1poxa3#439	GO:0016646: oxidoreductase activity, acting on the CH-NH group of donors, NAD or NADP as acceptor	0.0006	25,19,3
1im4a_#153	GO:0000049: tRNA binding	0.0006	37,13,3
1e6ua_#266	GO:0008483: transaminase activity	0.0006	28,17,3
1xo1a1#246	GO:0008235: metalloexopeptidase activity	0.0006	37,13,3
1eu3a2#174	GO:0004842: ubiquitin-protein ligase activity	0.0006	25,19,3
1fzqa_#28	GO:0003682: chromatin binding	0.0006	49,10,3
1hx0a2#32	GO:0005509: calcium ion binding	0.0006	70,160,12
1leha1#177	GO:0004457: lactate dehydrogenase activity	0.0006	49,10,3
1jp3a_#22	GO:0003684: damaged DNA binding	0.0006	49,10,3
1jp3a_#22	GO:0004457: lactate dehydrogenase activity	0.0006	49,10,3
1hfoa_#57	GO:0008408: 3'-5' exonuclease activity	0.0006	37,13,3
1dfma_#128	GO:0016651: oxidoreductase activity, acting on NADH or NADPH	0.0006	37,13,3
1b3ra2#47	GO:0016651: oxidoreductase activity, acting on NADH or NADPH	0.0006	37,13,3
1qlaa2#13	GO:0016638: oxidoreductase activity, acting on the CH-NH2 group of donors	0.0006	28,17,3
1ib2a_#1101	GO:0005509: calcium ion binding	0.0006	70,160,12
1gen__#475	GO:0004222: metalloendopeptidase activity	0.0006	25,19,3
1h75a_#53	GO:0003729: mRNA binding	0.0006	37,13,3
1g51a3#525	GO:0016638: oxidoreductase activity, acting on the CH-NH2 group of donors	0.0006	263,17,7
1bcca2#437	GO:0008408: 3'-5' exonuclease activity	0.0006	37,13,3
1fl2a1#320	GO:0016627: oxidoreductase activity, acting on the CH-CH group of donors	0.0006	28,17,3
1fl2a1#320	GO:0016638: oxidoreductase activity, acting on the CH-NH2 group of donors	0.0006	28,17,3
1hdma1#98	GO:0005518: collagen binding	0.0006	37,13,3
1hxxa_#173	GO:0019843: rRNA binding	0.000601	260,22,8
1ev2e2#286	GO:0005126: hematopoietin/interferon-class (D200-domain) cytokine receptor binding	0.000602	100,20,5
1e5da2#12	GO:0003887: DNA-directed DNA polymerase activity	0.000602	100,20,5
1e5ka_#49	GO:0016763: transferase activity, transferring pentosyl groups	0.000603	4,28,2

1g2oa_#52	GO:0016763: transferase activity, transferring pentosyl groups	0.000603	4,28,2
1de4c2#250	GO:0016763: transferase activity, transferring pentosyl groups	0.000603	4,28,2
1iqpa2#112	GO:0016616: oxidoreductase activity, acting on the CH-OH group of donors, NAD or NADP as acceptor	0.000603	19,59,4
2sqca2#268	GO:0004896: hematopoietin/interferon-class (D200-domain) cytokine receptor activity	0.000603	59,19,4
1a8i__#688	GO:0000287: magnesium ion binding	0.000603	241,128,23
1pud__#44	GO:0016616: oxidoreductase activity, acting on the CH-OH group of donors, NAD or NADP as acceptor	0.000604	50,59,6
1bak__#647	GO:0004725: protein tyrosine phosphatase activity	0.000604	76,15,4
1dih_2#222	GO:0016638: oxidoreductase activity, acting on the CH-NH2 group of donors	0.000604	352,17,8
1nsca_#236	GO:0005507: copper ion binding	0.000604	143,38,8
1bywa_#30	GO:0015036: disulfide oxidoreductase activity	0.000605	331,22,9
1gpea1#271	GO:0019955: cytokine binding	0.000606	197,11,5
1gpea1#271	GO:0003724: RNA helicase activity	0.000606	197,11,5
1f2aa_#27	GO:0004177: aminopeptidase activity	0.000607	89,13,4
1f2aa_#27	GO:0000049: tRNA binding	0.000607	89,13,4
1f2aa_#27	GO:0008235: metalloexopeptidase activity	0.000607	89,13,4
1aym3_#109	GO:0005126: hematopoietin/interferon-class (D200-domain) cytokine receptor binding	0.000607	56,20,4
1j79a_#187	GO:0019201: nucleotide kinase activity	0.000607	89,13,4
1feca3#467	GO:0000049: tRNA binding	0.000607	89,13,4
1fx7a1#44	GO:0003887: DNA-directed DNA polymerase activity	0.000607	56,20,4
1qqsa_#124	GO:0003887: DNA-directed DNA polymerase activity	0.000608	458,20,10
1bura1#269	GO:0000287: magnesium ion binding	0.000608	16,128,5
1edg__#81	GO:0008810: cellulase activity	0.000609	6,18,2
2hgsa2#471	GO:0003779: actin binding	0.000609	15,32,3
1ezwa_#245	GO:0004180: carboxypeptidase activity	0.000609	32,15,3
1nat__#77	GO:0030151: molybdenum ion binding	0.000609	32,15,3
1g8ka2#570	GO:0005096: GTPase activator activity	0.000609	32,15,3
1tyfa_#31	GO:0019201: nucleotide kinase activity	0.000609	162,13,5
1qs0a1#119	GO:0010181: FMN binding	0.000609	454,11,7
1dyna_#42	GO:0004896: hematopoietin/interferon-class (D200-domain) cytokine receptor activity	0.00061	164,19,6
1d0ba_#191	GO:0004457: lactate dehydrogenase activity	0.000611	120,10,4
1qs1a2#437	GO:0046983: protein dimerization activity	0.000611	452,17,9
1e0ca2#193	GO:0015036: disulfide oxidoreductase activity	0.000613	5,22,2
1jf9a_#379	GO:0004896: hematopoietin/interferon-class (D200-domain) cytokine receptor activity	0.000613	232,19,7
1e32a1#26	GO:0015036: disulfide oxidoreductase activity	0.000613	5,22,2
1fw9a_#152	GO:0004190: aspartic-type endopeptidase activity	0.000613	133,23,6
1qfea_#68	GO:0030145: manganese ion binding	0.000614	560,38,17
1dt6a_#385	GO:0004497: monooxygenase activity	0.000615	76,26,5
1bak__#647	GO:0004812: tRNA ligase activity	0.000615	76,26,5
2napa2#292	GO:0008483: transaminase activity	0.000616	120,17,5
1nsj__#21	GO:0016616: oxidoreductase activity, acting on the CH-OH group of donors, NAD or NADP as acceptor	0.000617	114,59,9
1cb8a2#613	GO:0004556: alpha-amylase activity	0.000617	138,15,5
1e19a_#281	GO:0004180: carboxypeptidase activity	0.000617	138,15,5
1hxxa_#278	GO:0000049: tRNA binding	0.000618	255,13,6
1i50b_#966	GO:0005529: sugar binding	0.000618	299,39,12
1gox__#124	GO:0016651: oxidoreductase activity, acting on NADH or NADPH	0.000618	366,13,7
1as4.1#A336	GO:0015036: disulfide oxidoreductase activity	0.000619	332,22,9
1egja_#346	GO:0008201: heparin binding	0.000621	20,24,3
2cpl__#20	GO:0019955: cytokine binding	0.000621	108,11,4
1koba_#175	GO:0005516: calmodulin binding	0.000621	20,24,3
1wba__#169	GO:0008201: heparin binding	0.000621	20,24,3
1ihka_#164	GO:0008201: heparin binding	0.000621	20,24,3
1flca1#294	GO:0016668: oxidoreductase activity, acting on sulfur group of donors, NAD or NADP as acceptor	0.000624	98,12,4
1h6la_#119	GO:0005507: copper ion binding	0.000624	308,38,12
1iba__#39	GO:0015078: hydrogen ion transporter activity	0.000625	351,21,9
1e5pa_#91	GO:0016705: oxidoreductase activity, acting on paired donors, with incorporation or reduction of molecular oxygen	0.000625	43,26,4
1e5pa_#91	GO:0004497: monooxygenase activity	0.000625	43,26,4
1kdj__#27	GO:0016638: oxidoreductase activity, acting on the CH-NH2 group of donors	0.000625	67,17,4
1nfp__#171	GO:0005524: ATP binding	0.000626	29,243,9
1be9a_#357	GO:0005516: calmodulin binding	0.000626	83,24,5
1fwxa2#396	GO:0008201: heparin binding	0.000626	237,24,8
1c0ma1#237	GO:0005524: ATP binding	0.000626	29,243,9

1b65a_#257	GO:0005524: ATP binding	0.000626	210,243,33
1kit_2#398	GO:0008201: heparin binding	0.000626	83,24,5
1bjt__#890	GO:0003887: DNA-directed DNA polymerase activity	0.000628	292,20,8
1bjt__#890	GO:0005126: hematopoietin/interferon-class (D200-domain) cytokine receptor binding	0.000628	292,20,8
1vcaa2#49	GO:0019838: growth factor binding	0.000629	12,9,2
1g93a_#229	GO:0019829: cation-transporting ATPase activity	0.000629	12,9,2
1bura1#328	GO:0016836: hydro-lyase activity	0.000629	91,33,6
1hbza_#96	GO:0008201: heparin binding	0.000629	370,24,10
1hwxa1#223	GO:0016646: oxidoreductase activity, acting on the CH-NH group of donors, NAD or NADP as acceptor	0.000629	233,19,7
1ryph_#163	GO:0008800: beta-lactamase activity	0.000631	121,10,4
1b3qa2#623	GO:0004197: cysteine-type endopeptidase activity	0.000631	301,24,9
1dik_1#765	GO:0016646: oxidoreductase activity, acting on the CH-NH group of donors, NAD or NADP as acceptor	0.000631	165,19,6
1evqa_#305	GO:0004222: metalloendopeptidase activity	0.000631	490,19,10
1fc3a_#172	GO:0000287: magnesium ion binding	0.000633	182,128,19
2pia_1#54	GO:0005126: hematopoietin/interferon-class (D200-domain) cytokine receptor binding	0.000633	220,20,7
1nuka_#80	GO:0005126: hematopoietin/interferon-class (D200-domain) cytokine receptor binding	0.000633	220,20,7
1h4ua1#486	GO:0003724: RNA helicase activity	0.000633	45,11,3
1xgsa2#186	GO:0000287: magnesium ion binding	0.000633	182,128,19
1mdah_#329	GO:0004714: transmembrane receptor protein tyrosine kinase activity	0.000633	335,14,7
1autc_#45	GO:0003724: RNA helicase activity	0.000633	45,11,3
1dpja_#322	GO:0004556: alpha-amylase activity	0.000634	308,15,7
1fu6a_#50	GO:0004725: protein tyrosine phosphatase activity	0.000634	308,15,7
1rtu__#97	GO:0005525: GTP binding	0.000636	23,49,4
1euwa_#71	GO:0019843: rRNA binding	0.000637	22,22,3
1a65a3#383	GO:0050660: FAD binding	0.000637	50,10,3
1muca1#245	GO:0016831: carboxy-lyase activity	0.000638	45,25,4
1ryph_#163	GO:0008483: transaminase activity	0.00064	121,17,5
1neb__#41	GO:0005516: calmodulin binding	0.000641	47,24,4
1c8za_#386	GO:0003968: RNA-directed RNA polymerase activity	0.000641	235,14,6
1ed5a_#294	GO:0000287: magnesium ion binding	0.000642	53,128,9
1cfe__#7	GO:0016616: oxidoreductase activity, acting on the CH-OH group of donors, NAD or NADP as acceptor	0.000642	70,59,7
1eula_#213	GO:0004896: hematopoietin/interferon-class (D200-domain) cytokine receptor activity	0.000643	60,19,4
1czya1#466	GO:0003724: RNA helicase activity	0.000643	109,11,4
1fjsa_#83	GO:0019955: cytokine binding	0.000643	109,11,4
1ft9a1#201	GO:0019955: cytokine binding	0.000643	109,11,4
2hlca_#54	GO:0003724: RNA helicase activity	0.000643	109,11,4
1hdoa_#189	GO:0016854: racemase and epimerase activity	0.000644	164,13,5
1dj0a2#183	GO:0004295: trypsin activity	0.000645	142,48,9
1fsu__#434	GO:0005507: copper ion binding	0.000646	356,38,13
1i6vd_#1283	GO:0019843: rRNA binding	0.000647	141,22,6
1phm_2#253	GO:0030151: molybdenum ion binding	0.000647	309,15,7
1fgua2#360	GO:0005525: GTP binding	0.000648	61,49,6
1i50b_#966	GO:0051082: unfolded protein binding	0.000648	299,34,11
1eova1#139	GO:0003697: single-stranded DNA binding	0.000648	99,12,4
1hdma1#115	GO:0046983: protein dimerization activity	0.000649	188,17,6
1dl0a_#32	GO:0004177: aminopeptidase activity	0.00065	38,13,3
2bbkh_#214	GO:0005518: collagen binding	0.00065	38,13,3
1eno__#83	GO:0016651: oxidoreductase activity, acting on NADH or NADPH	0.00065	38,13,3
1ii7a_#199	GO:0005524: ATP binding	0.00065	54,243,13
1g25a_#22	GO:0030151: molybdenum ion binding	0.00065	415,15,8
1dfoa_#362	GO:0003887: DNA-directed DNA polymerase activity	0.00065	57,20,4
1ycsb1#396	GO:0005507: copper ion binding	0.00065	13,38,3
1g6oa_#116	GO:0050660: FAD binding	0.000651	358,10,6
3sil__#317	GO:0003809: thrombin activity	0.000651	358,10,6
1dgw.1#Y353	GO:0004674: protein serine/threonine kinase activity	0.000652	319,42,13
1gpma1#289	GO:0005524: ATP binding	0.000653	35,243,10
1dnpa2#18	GO:0004812: tRNA ligase activity	0.000653	77,26,5
1svb_1#358	GO:0003724: RNA helicase activity	0.000655	10,11,2
1apme_#168	GO:0005066: transmembrane receptor protein tyrosine kinase signaling protein activity	0.000655	11,10,2
1ir3a_#1056	GO:0005066: transmembrane receptor protein tyrosine kinase signaling protein activity	0.000655	11,10,2
2fcba2#104	GO:0019955: cytokine binding	0.000655	10,11,2
1h6oa_#135	GO:0010181: FMN binding	0.000655	10,11,2
2cpl__#83	GO:0003809: thrombin activity	0.000655	11,10,2

1idsa2#157	GO:0003684: damaged DNA binding	0.000655	11,10,2
1tlfa_#269	GO:0016814: hydrolase activity, acting on carbon-nitrogen (but not peptide) bonds, in cyclic amidines	0.000655	11,10,2
1cpt__#144	GO:0010181: FMN binding	0.000655	10,11,2
1ha1_1#13	GO:0016620: oxidoreductase activity, acting on the aldehyde or oxo group of donors, NAD or NADP as acceptor	0.000655	11,10,2
1fmk_3#428	GO:0005066: transmembrane receptor protein tyrosine kinase signaling protein activity	0.000655	11,10,2
1hbna1#293	GO:0010181: FMN binding	0.000655	10,11,2
1koba_#105	GO:0005066: transmembrane receptor protein tyrosine kinase signaling protein activity	0.000655	11,10,2
1prxa_#28	GO:0010181: FMN binding	0.000655	10,11,2
1ecsa_#69	GO:0016846: carbon-sulfur lyase activity	0.000655	11,10,2
1e79d2#36	GO:0003916: DNA topoisomerase activity	0.000655	524,10,7
1hava_#65	GO:0004263: chymotrypsin activity	0.000656	3,41,2
1cpza_#6	GO:0005126: hematopoietin/interferon-class (D200-domain) cytokine receptor binding	0.000656	157,20,6
1thg__#3	GO:0019843: rRNA binding	0.000657	92,22,5
1zfpe_#67	GO:0000287: magnesium ion binding	0.000657	33,128,7
1e8ga1#436	GO:0019843: rRNA binding	0.000657	92,22,5
1h4ua1#404	GO:0004523: ribonuclease H activity	0.000657	202,16,6
1ej8a_#189	GO:0003887: DNA-directed DNA polymerase activity	0.000658	294,20,8
1fc4a_#205	GO:0003924: GTPase activity	0.000658	267,17,7
1a65a1#108	GO:0005126: hematopoietin/interferon-class (D200-domain) cytokine receptor binding	0.000658	294,20,8
1qf6a3#92	GO:0000049: tRNA binding	0.000661	91,13,4
1qf6a3#92	GO:0003729: mRNA binding	0.000661	91,13,4
2dnja_#97	GO:0005529: sugar binding	0.000661	141,39,8
1bura1#328	GO:0019201: nucleotide kinase activity	0.000661	91,13,4
1nbaa_#227	GO:0008408: 3'-5' exonuclease activity	0.000661	91,13,4
1c39a_#49	GO:0016638: oxidoreductase activity, acting on the CH-NH2 group of donors	0.000661	68,17,4
1qama_#37	GO:0016627: oxidoreductase activity, acting on the CH-CH group of donors	0.000661	68,17,4
1a6o__#45	GO:0005516: calmodulin binding	0.000663	303,24,9
1fgga_#134	GO:0004180: carboxypeptidase activity	0.000663	218,15,6
1dik_1#765	GO:0016651: oxidoreductase activity, acting on NADH or NADPH	0.000663	165,13,5
1xis__#214	GO:0000287: magnesium ion binding	0.000664	88,128,12
1qj8a_#64	GO:0015082: di-, tri-valent inorganic cation transporter activity	0.000665	84,14,4
1qj8a_#64	GO:0046915: transition metal ion transporter activity	0.000665	84,14,4
1e8ya2#410	GO:0005509: calcium ion binding	0.000666	8,160,4
1ayl__#237	GO:0046983: protein dimerization activity	0.000666	357,17,8
1ayl__#237	GO:0016638: oxidoreductase activity, acting on the CH-NH2 group of donors	0.000666	357,17,8
1i0ha2#127	GO:0016638: oxidoreductase activity, acting on the CH-NH2 group of donors	0.000667	29,17,3
2fmr__#10	GO:0019838: growth factor binding	0.000667	57,9,3
1fuia1#506	GO:0030151: molybdenum ion binding	0.000667	33,15,3
1cipa2#34	GO:0005096: GTPase activator activity	0.000667	78,15,4
1jjya_#324	GO:0004180: carboxypeptidase activity	0.000667	78,15,4
1czan1#92	GO:0019955: cytokine binding	0.000667	631,11,8
1czan1#92	GO:0003724: RNA helicase activity	0.000667	631,11,8
1czan1#92	GO:0003964: RNA-directed DNA polymerase activity	0.000667	631,11,8
1e6pa2#274	GO:0003924: GTPase activity	0.000667	29,17,3
2dnja_#158	GO:0030151: molybdenum ion binding	0.000667	33,15,3
1jwoa_#196	GO:0004725: protein tyrosine phosphatase activity	0.000667	33,15,3
1dr9a1#39	GO:0005529: sugar binding	0.000667	258,39,11
1trb_1#14	GO:0016638: oxidoreductase activity, acting on the CH-NH2 group of donors	0.000667	29,17,3
1hc7a2#258	GO:0003809: thrombin activity	0.000669	226,10,5
1ds1a_#172	GO:0003887: DNA-directed DNA polymerase activity	0.000669	222,20,7
1ds1a_#172	GO:0005126: hematopoietin/interferon-class (D200-domain) cytokine receptor binding	0.000669	222,20,7
2ltn.1#A63	GO:0005524: ATP binding	0.000669	152,243,26
1dhn__#88	GO:0004190: aspartic-type endopeptidase activity	0.000671	5,23,2
1flga_#161	GO:0004714: transmembrane receptor protein tyrosine kinase activity	0.000672	152,14,5
1dj0a2#183	GO:0019843: rRNA binding	0.000672	142,22,6
1qdlb_#170	GO:0016668: oxidoreductase activity, acting on sulfur group of donors, NAD or NADP as acceptor	0.000674	100,12,4
1e8ya2#410	GO:0005543: phospholipid binding	0.000675	8,14,2
1biha3#248	GO:0004714: transmembrane receptor protein tyrosine kinase activity	0.000675	8,14,2
1vdra_#37	GO:0016776: phosphotransferase activity, phosphate group as acceptor	0.000675	8,14,2
1esl_1#48	GO:0004812: tRNA ligase activity	0.000676	19,26,3
1a7ca_#204	GO:0004896: hematopoietin/interferon-class (D200-domain) cytokine receptor activity	0.000676	26,19,3
1g71a_#77	GO:0016620: oxidoreductase activity, acting on the aldehyde or oxo group of donors, NAD or NADP as acceptor	0.000676	51,10,3

1ppn__#191	GO:0004812: tRNA ligase activity	0.000676	19,26,3
1ii7a_#275	GO:0004222: metalloendopeptidase activity	0.000676	26,19,3
1qq4a_#143	GO:0003724: RNA helicase activity	0.000676	46,11,3
1qq4a_#63	GO:0003724: RNA helicase activity	0.000676	46,11,3
1egaa1#58	GO:0004364: glutathione transferase activity	0.000676	46,11,3
1fc3a_#172	GO:0009036: type II site-specific deoxyribonuclease activity	0.000678	182,12,5
1fc3a_#172	GO:0003899: DNA-directed RNA polymerase activity	0.000678	182,12,5
1jb3a_#64	GO:0005529: sugar binding	0.000678	302,39,12
3sil__#317	GO:0046983: protein dimerization activity	0.000679	358,17,8
3sil__#317	GO:0016638: oxidoreductase activity, acting on the CH-NH2 group of donors	0.000679	358,17,8
1rlw__#114	GO:0004620: phospholipase activity	0.00068	6,19,2
1vdra_#76	GO:0016646: oxidoreductase activity, acting on the CH-NH group of donors, NAD or NADP as acceptor	0.00068	6,19,2
1ema__#26	GO:0004842: ubiquitin-protein ligase activity	0.00068	6,19,2
1hcd__#114	GO:0008083: growth factor activity	0.00068	12,42,3
1vcaa2#49	GO:0008083: growth factor activity	0.00068	12,42,3
1agja_#91	GO:0004842: ubiquitin-protein ligase activity	0.00068	6,19,2
1ezvb1#185	GO:0004222: metalloendopeptidase activity	0.00068	6,19,2
1cjxa1#126	GO:0016702: oxidoreductase activity, acting on single donors with incorporation of molecular oxygen, incorporation of two atoms of oxygen	0.00068	42,12,3
2sn3__#22	GO:0004674: protein serine/threonine kinase activity	0.00068	12,42,3
1dgw.1#Y353	GO:0019955: cytokine binding	0.00068	319,11,6
1dgw.1#Y353	GO:0003964: RNA-directed DNA polymerase activity	0.00068	319,11,6
1f8ma_#237	GO:0003697: single-stranded DNA binding	0.00068	42,12,3
1fmja_#50	GO:0004842: ubiquitin-protein ligase activity	0.00068	6,19,2
1eqca_#15	GO:0004620: phospholipase activity	0.00068	6,19,2
1i6vd_#1283	GO:0004556: alpha-amylase activity	0.000681	141,15,5
1heta2#218	GO:0008270: zinc ion binding	0.000681	39,108,7
1gox__#246	GO:0005524: ATP binding	0.000682	177,243,29
1poxa3#460	GO:0016831: carboxy-lyase activity	0.000682	81,25,5
1ezvb1#29	GO:0004812: tRNA ligase activity	0.000683	44,26,4
1rypk_#12	GO:0015036: disulfide oxidoreductase activity	0.000684	265,22,8
1hyrc1#247	GO:0004896: hematopoietin/interferon-class (D200-domain) cytokine receptor activity	0.000685	61,19,4
1jd0a_#176	GO:0004295: trypsin activity	0.000686	207,48,11
1dik_1#848	GO:0005509: calcium ion binding	0.000687	81,160,13
1hxxa_#173	GO:0000049: tRNA binding	0.000687	260,13,6
1c9la2#61	GO:0004714: transmembrane receptor protein tyrosine kinase activity	0.000687	238,14,6
1h6va1#27	GO:0008026: ATP-dependent helicase activity	0.000687	260,13,6
1h6va1#27	GO:0016651: oxidoreductase activity, acting on NADH or NADPH	0.000687	260,13,6
1h6va1#27	GO:0016861: intramolecular oxidoreductase activity, interconverting aldoses and ketoses	0.000687	260,13,6
1fy7a_#388	GO:0005524: ATP binding	0.000688	68,243,15
1ido__#173	GO:0016763: transferase activity, transferring pentosyl groups	0.000688	110,28,6
1ilr1_#14	GO:0008083: growth factor activity	0.000689	3,42,2
1e58a_#223	GO:0004674: protein serine/threonine kinase activity	0.000689	3,42,2
1thg__#3	GO:0000049: tRNA binding	0.000689	92,13,4
1i50a_#856	GO:0016638: oxidoreductase activity, acting on the CH-NH2 group of donors	0.000689	269,17,7
1jl0a_#33	GO:0004674: protein serine/threonine kinase activity	0.000689	3,42,2
1e8ga1#436	GO:0000049: tRNA binding	0.000689	92,13,4
8dfr__#9	GO:0004364: glutathione transferase activity	0.00069	111,11,4
3sil__#8	GO:0051082: unfolded protein binding	0.00069	125,34,7
1eny__#147	GO:0016627: oxidoreductase activity, acting on the CH-CH group of donors	0.00069	123,17,5
1qdla_#211	GO:0019955: cytokine binding	0.000692	320,11,6
1dp0a4#756	GO:0008083: growth factor activity	0.000692	165,42,9
1csh__#95	GO:0003700: transcription factor activity	0.000693	10,124,4
1thfd_#140	GO:0005066: transmembrane receptor protein tyrosine kinase signaling protein activity	0.000693	124,10,4
1io7a_#243	GO:0016705: oxidoreductase activity, acting on paired donors, with incorporation or reduction of molecular oxygen	0.000693	78,26,5
1eo6a_#107	GO:0004812: tRNA ligase activity	0.000693	78,26,5
1ibja_#271	GO:0004812: tRNA ligase activity	0.000693	78,26,5
1jd0a_#176	GO:0051082: unfolded protein binding	0.000694	207,34,9
1dkia_#76P	GO:0003887: DNA-directed DNA polymerase activity	0.000695	58,20,4
1h6ka2#336	GO:0004620: phospholipase activity	0.000695	168,19,6
1hcl__#82	GO:0005516: calmodulin binding	0.000696	48,24,4
1nuka_#80	GO:0004556: alpha-amylase activity	0.000696	220,15,6
1g2oa_#227	GO:0016763: transferase activity, transferring pentosyl groups	0.000696	41,28,4
1hrka_#205	GO:0016763: transferase activity, transferring pentosyl groups	0.000696	41,28,4

1fi2a_#109	GO:0008800: beta-lactamase activity	0.000698	228,10,5
1hxxa_#278	GO:0005524: ATP binding	0.000699	255,243,38
1qfma2#576	GO:0016836: hydro-lyase activity	0.000699	214,33,9
1dbxa_#75	GO:0016638: oxidoreductase activity, acting on the CH-NH2 group of donors	0.000699	69,17,4
1gpc__#150	GO:0005524: ATP binding	0.000701	3,243,3
1gsoa3#172	GO:0005524: ATP binding	0.000701	3,243,3
1hv8a1#84	GO:0005524: ATP binding	0.000701	3,243,3
1ewqa4#78	GO:0005524: ATP binding	0.000701	3,243,3
1e32a2#203	GO:0005524: ATP binding	0.000701	3,243,3
1ab4__#164	GO:0005524: ATP binding	0.000701	3,243,3
1qmva_#40	GO:0004725: protein tyrosine phosphatase activity	0.000701	79,15,4
1imva_#255	GO:0019843: rRNA binding	0.000701	589,22,12
1ddma_#130	GO:0030151: molybdenum ion binding	0.000701	313,15,7
1mrj__#240	GO:0005529: sugar binding	0.000702	13,39,3
1i2oa_#173	GO:0016831: carboxy-lyase activity	0.000702	20,25,3
1dkia_#76P	GO:0019838: growth factor binding	0.000702	58,9,3
1jlxa2#214	GO:0005529: sugar binding	0.000702	13,39,3
1h61a_#176	GO:0016861: intramolecular oxidoreductase activity, interconverting aldoses and ketoses	0.000702	39,13,3
1hbza_#214	GO:0003887: DNA-directed DNA polymerase activity	0.000705	297,20,8
1ejda_#6	GO:0016638: oxidoreductase activity, acting on the CH-NH2 group of donors	0.000705	270,17,7
1i6vd_#1283	GO:0019838: growth factor binding	0.000706	141,9,4
2pia_1#99	GO:0005529: sugar binding	0.000706	179,39,9
1fjfc2#153	GO:0004556: alpha-amylase activity	0.000707	420,15,8
1epwa3#175	GO:0005507: copper ion binding	0.000709	53,38,5
1eur__#302	GO:0005507: copper ion binding	0.000709	53,38,5
1dfca1#1135	GO:0005507: copper ion binding	0.000709	53,38,5
1iira_#326	GO:0016758: transferase activity, transferring hexosyl groups	0.000714	112,11,4
1iira_#326	GO:0010181: FMN binding	0.000714	112,11,4
1bfd_2#146	GO:0010181: FMN binding	0.000714	112,11,4
1ddwa_#77	GO:0050660: FAD binding	0.000714	364,10,6
1g25a_#22	GO:0003697: single-stranded DNA binding	0.000715	415,12,7
1ryp1_#20	GO:0050660: FAD binding	0.000715	125,10,4
1fmta2#151	GO:0004180: carboxypeptidase activity	0.000715	314,15,7
1g71a_#88	GO:0016620: oxidoreductase activity, acting on the aldehyde or oxo group of donors, NAD or NADP as acceptor	0.000716	52,10,3
1exh__#44	GO:0003916: DNA topoisomerase activity	0.000716	52,10,3
1jlna_#383	GO:0004457: lactate dehydrogenase activity	0.000716	52,10,3
3grs_2#211	GO:0016620: oxidoreductase activity, acting on the aldehyde or oxo group of donors, NAD or NADP as acceptor	0.000716	52,10,3
1ihka_#161	GO:0003700: transcription factor activity	0.000717	17,124,5
1j9la_#82	GO:0016646: oxidoreductase activity, acting on the CH-NH group of donors, NAD or NADP as acceptor	0.000717	169,19,6
1nsca_#379	GO:0016638: oxidoreductase activity, acting on the CH-NH2 group of donors	0.000717	124,17,5
1apme_#74	GO:0016638: oxidoreductase activity, acting on the CH-NH2 group of donors	0.000717	124,17,5
1gox__#124	GO:0016836: hydro-lyase activity	0.000717	366,33,12
1jeyb_#231	GO:0004601: peroxidase activity	0.00072	24,21,3
1g5va_#109	GO:0004867: serine-type endopeptidase inhibitor activity	0.00072	11,47,3
1gg6.1#C232	GO:0004867: serine-type endopeptidase inhibitor activity	0.00072	11,47,3
4kbpa2#416	GO:0015078: hydrogen ion transporter activity	0.00072	24,21,3
1ie5a_#23	GO:0008201: heparin binding	0.00072	21,24,3
1mspa_#17	GO:0019955: cytokine binding	0.00072	47,11,3
1h6ka2#336	GO:0008026: ATP-dependent helicase activity	0.00072	168,13,5
1h6ka2#336	GO:0008408: 3'-5' exonuclease activity	0.00072	168,13,5
1ton__#196	GO:0003724: RNA helicase activity	0.00072	47,11,3
1eq9a_#58	GO:0003755: peptidyl-prolyl cis-trans isomerase activity	0.00072	47,11,3
1fgka_#689	GO:0005516: calmodulin binding	0.00072	21,24,3
1nsj__#178	GO:0016616: oxidoreductase activity, acting on the CH-OH group of donors, NAD or NADP as acceptor	0.00072	93,59,8
1mil__#31	GO:0003887: DNA-directed DNA polymerase activity	0.000721	104,20,5
1dik_3#240	GO:0019838: growth factor binding	0.000721	262,9,5
1h6ka2#336	GO:0016705: oxidoreductase activity, acting on paired donors, with incorporation or reduction of molecular oxygen	0.000723	168,26,7
1qqsa_#124	GO:0004674: protein serine/threonine kinase activity	0.000726	458,42,16
1el0a_#28	GO:0005524: ATP binding	0.000726	161,243,27
1c4zd_#51	GO:0005126: hematopoietin/interferon-class (D200-domain) cytokine receptor binding	0.000726	160,20,6
2bpa1_#45	GO:0046983: protein dimerization activity	0.000726	192,17,6

1d0na6#681	GO:0030151: molybdenum ion binding	0.000727	143,15,5
1eur__#84	GO:0004714: transmembrane receptor protein tyrosine kinase activity	0.000727	86,14,4
2ayh__#184	GO:0004190: aspartic-type endopeptidase activity	0.000729	22,23,3
1fbl_1#398	GO:0004222: metalloendopeptidase activity	0.000729	62,19,4
1kwaa_#513	GO:0004620: phospholipase activity	0.000729	62,19,4
1kwaa_#513	GO:0004896: hematopoietin/interferon-class (D200-domain) cytokine receptor activity	0.000729	62,19,4
1svb_2#165	GO:0004556: alpha-amylase activity	0.000729	34,15,3
1ie5a_#102	GO:0004222: metalloendopeptidase activity	0.000729	62,19,4
1h9oa_#83	GO:0015405: P-P-bond-hydrolysis-driven transporter activity	0.00073	43,12,3
1c7na_#231	GO:0008235: metalloexopeptidase activity	0.000731	263,13,6
1pvc1_#182	GO:0004197: cysteine-type endopeptidase activity	0.000732	5,24,2
1hxn__#235	GO:0004197: cysteine-type endopeptidase activity	0.000732	5,24,2
2viua_#252	GO:0004714: transmembrane receptor protein tyrosine kinase activity	0.000734	343,14,7
2viua_#252	GO:0003968: RNA-directed RNA polymerase activity	0.000734	343,14,7
1jj2a1#126	GO:0008083: growth factor activity	0.000734	73,42,6
1g51a3#525	GO:0019838: growth factor binding	0.000734	263,9,5
1hd2a_#35	GO:0030145: manganese ion binding	0.000734	185,38,9
1hd2a_#35	GO:0005507: copper ion binding	0.000734	185,38,9
1ejfa_#10	GO:0004714: transmembrane receptor protein tyrosine kinase activity	0.000735	155,14,5
1gox__#124	GO:0016846: carbon-sulfur lyase activity	0.000736	366,10,6
1bev1_#86	GO:0008083: growth factor activity	0.000737	323,42,13
1eur__#84	GO:0008201: heparin binding	0.000737	86,24,5
1dt9a1#209	GO:0005524: ATP binding	0.000738	113,243,21
1esma_#314	GO:0003724: RNA helicase activity	0.000738	113,11,4
1qlaa2#35	GO:0016627: oxidoreductase activity, acting on the CH-CH group of donors	0.000738	30,17,3
1h6va3#486	GO:0046983: protein dimerization activity	0.000738	70,17,4
1smaa1#95	GO:0016638: oxidoreductase activity, acting on the CH-NH2 group of donors	0.000738	272,17,7
1b4va1#11	GO:0016627: oxidoreductase activity, acting on the CH-CH group of donors	0.000738	30,17,3
1b4va1#11	GO:0016638: oxidoreductase activity, acting on the CH-NH2 group of donors	0.000738	30,17,3
1cmxa_#59	GO:0016638: oxidoreductase activity, acting on the CH-NH2 group of donors	0.000738	30,17,3
1fepa_#49	GO:0046983: protein dimerization activity	0.000738	70,17,4
1i50b_#966	GO:0005126: hematopoietin/interferon-class (D200-domain) cytokine receptor binding	0.000738	299,20,8
1hdma1#123	GO:0046983: protein dimerization activity	0.000738	30,17,3
1hdma1#123	GO:0016638: oxidoreductase activity, acting on the CH-NH2 group of donors	0.000738	30,17,3
1h7wa2#535	GO:0016616: oxidoreductase activity, acting on the CH-OH group of donors, NAD or NADP as acceptor	0.000739	9,59,3
1uxy_1#87	GO:0016616: oxidoreductase activity, acting on the CH-OH group of donors, NAD or NADP as acceptor	0.000739	9,59,3
1g8kb_#11	GO:0019838: growth factor binding	0.000739	59,9,3
1ii7a_#199	GO:0000287: magnesium ion binding	0.00074	54,128,9
1dt9a1#209	GO:0005525: GTP binding	0.000741	113,49,8
1cpt__#358	GO:0016651: oxidoreductase activity, acting on NADH or NADPH	0.000742	9,13,2
1deua_#127	GO:0004177: aminopeptidase activity	0.000742	9,13,2
1ja1a2#201	GO:0016651: oxidoreductase activity, acting on NADH or NADPH	0.000742	9,13,2
1iica1#172	GO:0008080: N-acetyltransferase activity	0.000742	9,13,2
1jl0a_#264	GO:0000049: tRNA binding	0.000742	9,13,2
1wdcb_#141	GO:0016651: oxidoreductase activity, acting on NADH or NADPH	0.000742	9,13,2
1a8h_2#186	GO:0000049: tRNA binding	0.000742	9,13,2
1qrjb1#133	GO:0016651: oxidoreductase activity, acting on NADH or NADPH	0.000742	9,13,2
1gln_2#63	GO:0000049: tRNA binding	0.000742	9,13,2
1dz4a_#357	GO:0016651: oxidoreductase activity, acting on NADH or NADPH	0.000742	9,13,2
1axca1#70	GO:0004867: serine-type endopeptidase inhibitor activity	0.000742	118,47,8
1qfma1#238	GO:0005085: guanyl-nucleotide exchange factor activity	0.000742	9,13,2
1e4ea2#215	GO:0004867: serine-type endopeptidase inhibitor activity	0.000743	490,47,18
1d8db_#78	GO:0008810: cellulase activity	0.000744	66,18,4
1oaca1#495	GO:0051082: unfolded protein binding	0.000745	60,34,5
1e8ya1#648	GO:0016705: oxidoreductase activity, acting on paired donors, with incorporation or reduction of molecular oxygen	0.000745	45,26,4
1e8ya1#648	GO:0004497: monooxygenase activity	0.000745	45,26,4
1hc7a2#258	GO:0003887: DNA-directed DNA polymerase activity	0.000746	226,20,7
1hc7a2#258	GO:0005126: hematopoietin/interferon-class (D200-domain) cytokine receptor binding	0.000746	226,20,7
1fxla2#169	GO:0015036: disulfide oxidoreductase activity	0.000746	203,22,7
1bd0a1#276	GO:0008270: zinc ion binding	0.000747	91,108,11
1opy__#68	GO:0003916: DNA topoisomerase activity	0.000747	367,10,6
1fxla2#169	GO:0008083: growth factor activity	0.000749	203,42,10
1hdfa_#16	GO:0030151: molybdenum ion binding	0.000749	223,15,6

1h6la_#119	GO:0008201: heparin binding	0.000749	308,24,9
1qhqa_#45	GO:0004812: tRNA ligase activity	0.00075	121,26,6
2arca_#55	GO:0004556: alpha-amylase activity	0.000751	144,15,5
1f97a2#199	GO:0004556: alpha-amylase activity	0.000751	144,15,5
1e30a_#112	GO:0004197: cysteine-type endopeptidase activity	0.000753	49,24,4
1gdea_#195	GO:0005525: GTP binding	0.000753	24,49,4
1et9a1#39	GO:0008201: heparin binding	0.000753	49,24,4
2pola1#111	GO:0003887: DNA-directed DNA polymerase activity	0.000754	6,20,2
1cfb_2#763	GO:0005126: hematopoietin/interferon-class (D200-domain) cytokine receptor binding	0.000754	6,20,2
1t7pa2#603	GO:0003887: DNA-directed DNA polymerase activity	0.000754	6,20,2
1nuka_#80	GO:0005529: sugar binding	0.000754	220,39,10
1clxa_#120	GO:0016831: carboxy-lyase activity	0.000754	47,25,4
1mjha_#119	GO:0042802: protein self binding	0.000754	103,12,4
1elja_#64	GO:0016668: oxidoreductase activity, acting on sulfur group of donors, NAD or NADP as acceptor	0.000754	103,12,4
1dhs__#99	GO:0008757: S-adenosylmethionine-dependent methyltransferase activity	0.000754	132,24,6
1i50l_#38	GO:0046983: protein dimerization activity	0.000755	7,17,2
1fl7b_#51	GO:0046983: protein dimerization activity	0.000755	7,17,2
2tgi__#80	GO:0046983: protein dimerization activity	0.000755	7,17,2
1hcna_#12	GO:0046983: protein dimerization activity	0.000755	7,17,2
1fl7b_#78	GO:0046983: protein dimerization activity	0.000755	7,17,2
1fuka_#239	GO:0016799: hydrolase activity, hydrolyzing N-glycosyl compounds	0.000755	7,17,2
1dqga_#30	GO:0016799: hydrolase activity, hydrolyzing N-glycosyl compounds	0.000755	7,17,2
1epwa3#138	GO:0030151: molybdenum ion binding	0.000755	424,15,8
1jlxa2#243	GO:0016799: hydrolase activity, hydrolyzing N-glycosyl compounds	0.000755	7,17,2
1g61a_#2155	GO:0016638: oxidoreductase activity, acting on the CH-NH2 group of donors	0.000755	7,17,2
1cpy__#380	GO:0005509: calcium ion binding	0.000756	171,160,21
1ad2__#40	GO:0005085: guanyl-nucleotide exchange factor activity	0.000757	40,13,3
1gtma2#140	GO:0004222: metalloendopeptidase activity	0.000757	27,19,3
1cmia_#86	GO:0005518: collagen binding	0.000757	40,13,3
6cel__#423	GO:0004222: metalloendopeptidase activity	0.000757	27,19,3
1e39a2#556	GO:0016651: oxidoreductase activity, acting on NADH or NADPH	0.000757	40,13,3
2sqca2#97	GO:0001584: rhodopsin-like receptor activity	0.00076	127,10,4
1fd9a_#162	GO:0016251: general RNA polymerase II transcription factor activity	0.00076	87,14,4
1qqga1#95	GO:0005066: transmembrane receptor protein tyrosine kinase signaling protein activity	0.00076	127,10,4
3ebx__#43	GO:0005509: calcium ion binding	0.000761	4,160,3
1fbl_1#368	GO:0005509: calcium ion binding	0.000761	4,160,3
1ddwa_#77	GO:0046983: protein dimerization activity	0.000761	364,17,8
1ddwa_#77	GO:0016638: oxidoreductase activity, acting on the CH-NH2 group of donors	0.000761	364,17,8
1dik_3#240	GO:0005529: sugar binding	0.000762	262,39,11
1rypk_#12	GO:0000049: tRNA binding	0.000762	265,13,6
1jd0a_#176	GO:0003964: RNA-directed DNA polymerase activity	0.000763	207,11,5
1csn__#84	GO:0003755: peptidyl-prolyl cis-trans isomerase activity	0.000763	207,11,5
2btva_#749	GO:0016763: transferase activity, transferring pentosyl groups	0.000764	42,28,4
1ex1a1#284	GO:0016763: transferase activity, transferring pentosyl groups	0.000764	42,28,4
1cwva2#675	GO:0008083: growth factor activity	0.000764	28,42,4
2arca_#55	GO:0019838: growth factor binding	0.000766	144,9,4
2cpl__#83	GO:0004295: trypsin activity	0.000767	11,48,3
1hxxa_#232	GO:0005529: sugar binding	0.000767	352,39,13
1b35b_#97	GO:0005516: calmodulin binding	0.000767	309,24,9
1e2o__#346	GO:0016638: oxidoreductase activity, acting on the CH-NH2 group of donors	0.000768	194,17,6
1fjfc2#153	GO:0015036: disulfide oxidoreductase activity	0.000768	420,22,10
1vmoa_#106	GO:0016668: oxidoreductase activity, acting on sulfur group of donors, NAD or NADP as acceptor	0.000768	187,12,5
1eyza3#196	GO:0005524: ATP binding	0.000769	14,243,6
1awx__#27	GO:0005524: ATP binding	0.000769	14,243,6
1i58a_#519	GO:0004180: carboxypeptidase activity	0.000771	81,15,4
1exma3#99	GO:0030151: molybdenum ion binding	0.000771	81,15,4
1hlwa_#82	GO:0005507: copper ion binding	0.000773	270,38,11
1dnv__#354	GO:0030145: manganese ion binding	0.000773	54,38,5
1qfma1#139	GO:0030151: molybdenum ion binding	0.000773	318,15,7
1jj2q_#114	GO:0019843: rRNA binding	0.000774	54,22,4
1eula_#213	GO:0019838: growth factor binding	0.000776	60,9,3
2viua_#123	GO:0005179: hormone activity	0.000777	8,15,2
1es7a_#111	GO:0005179: hormone activity	0.000777	8,15,2
1icia_#8	GO:0030151: molybdenum ion binding	0.000777	8,15,2

1e8ua_#180	GO:0016811: hydrolase activity, acting on carbon-nitrogen (but not peptide) bonds, in linear amides	0.000777	8,15,2
1g61a_#2111	GO:0003743: translation initiation factor activity	0.000777	8,15,2
1xnb__#130	GO:0008757: S-adenosylmethionine-dependent methyltransferase activity	0.000778	87,24,5
2hhma_#262	GO:0008235: metalloexopeptidase activity	0.000779	95,13,4
2dpma_#237	GO:0008235: metalloexopeptidase activity	0.000779	95,13,4
1jj2j_#70	GO:0046983: protein dimerization activity	0.00078	71,17,4
1jj22_#78	GO:0016638: oxidoreductase activity, acting on the CH-NH2 group of donors	0.00078	71,17,4
1e3a.1#A30	GO:0016251: general RNA polymerase II transcription factor activity	0.00078	157,14,5
1cpza_#6	GO:0003968: RNA-directed RNA polymerase activity	0.00078	157,14,5
1i7oa1#63	GO:0003697: single-stranded DNA binding	0.000781	44,12,3
1qnja_#221	GO:0003809: thrombin activity	0.000785	12,10,2
16pk__#312	GO:0003684: damaged DNA binding	0.000785	12,10,2
1koba_#139	GO:0008199: ferric iron binding	0.000785	12,10,2
1ypci_#69	GO:0042802: protein self binding	0.000785	10,12,2
1e4ia_#215	GO:0004457: lactate dehydrogenase activity	0.000785	12,10,2
1ga0a_#304	GO:0008199: ferric iron binding	0.000785	12,10,2
1ajsa_#389	GO:0016846: carbon-sulfur lyase activity	0.000785	12,10,2
1dp4a_#135	GO:0001584: rhodopsin-like receptor activity	0.000785	12,10,2
1d7ya1#243	GO:0050660: FAD binding	0.000785	12,10,2
1pysb6#384	GO:0008800: beta-lactamase activity	0.000785	12,10,2
1d2fa_#260	GO:0016846: carbon-sulfur lyase activity	0.000785	12,10,2
1bdfa2#121	GO:0003899: DNA-directed RNA polymerase activity	0.000785	10,12,2
1fw8a_#257	GO:0016620: oxidoreductase activity, acting on the aldehyde or oxo group of donors, NAD or NADP as acceptor	0.000785	12,10,2
2ts1__#109	GO:0001584: rhodopsin-like receptor activity	0.000785	12,10,2
1hcl__#135	GO:0005066: transmembrane receptor protein tyrosine kinase signaling protein activity	0.000785	12,10,2
1dc1a_#241	GO:0004457: lactate dehydrogenase activity	0.000785	12,10,2
1ffgb_#172	GO:0003899: DNA-directed RNA polymerase activity	0.000785	10,12,2
1by5a_#347	GO:0005529: sugar binding	0.000787	263,39,11
1fi2a_#109	GO:0003887: DNA-directed DNA polymerase activity	0.000787	228,20,7
1hq8a_#206	GO:0005524: ATP binding	0.000787	329,243,46
1dgw.1#Y353	GO:0004725: protein tyrosine phosphatase activity	0.000788	319,15,7
1f46a_#59	GO:0003887: DNA-directed DNA polymerase activity	0.000788	106,20,5
1fhoa_#74	GO:0015082: di-, tri-valent inorganic cation transporter activity	0.000788	347,14,7
1fhoa_#74	GO:0004714: transmembrane receptor protein tyrosine kinase activity	0.000788	347,14,7
1fhoa_#74	GO:0046915: transition metal ion transporter activity	0.000788	347,14,7
1el0a_#42	GO:0016668: oxidoreductase activity, acting on sulfur group of donors, NAD or NADP as acceptor	0.000789	294,12,6
1lvk_2#275	GO:0003779: actin binding	0.00079	4,32,2
1dxxa1#37	GO:0003779: actin binding	0.00079	4,32,2
1kcw_3#440	GO:0016705: oxidoreductase activity, acting on paired donors, with incorporation or reduction of molecular oxygen	0.00079	20,26,3
1i2oa_#173	GO:0004497: monooxygenase activity	0.00079	20,26,3
1dssg1#4	GO:0005351: sugar porter activity	0.00079	57,21,4
1mpp__#103	GO:0004812: tRNA ligase activity	0.00079	20,26,3
3rpba_#557	GO:0015078: hydrogen ion transporter activity	0.00079	57,21,4
2bpa1_#244	GO:0005126: hematopoietin/interferon-class (D200-domain) cytokine receptor binding	0.000791	60,20,4
2mpra_#190	GO:0004190: aspartic-type endopeptidase activity	0.000792	400,23,10
1pma1_#17	GO:0005509: calcium ion binding	0.000792	44,160,9
1d6aa_#52	GO:0000287: magnesium ion binding	0.000794	293,128,26
1f8ga2#36	GO:0016831: carboxy-lyase activity	0.000795	5,25,2
1pii_1#40	GO:0016831: carboxy-lyase activity	0.000795	5,25,2
1cex__#109	GO:0004180: carboxypeptidase activity	0.000795	35,15,3
1cex__#109	GO:0005096: GTPase activator activity	0.000795	35,15,3
1fjfr_#43	GO:0016831: carboxy-lyase activity	0.000795	5,25,2
1qe3a_#301	GO:0003743: translation initiation factor activity	0.000795	35,15,3
1f8ma_#237	GO:0005524: ATP binding	0.000795	42,243,11
1d0na6#681	GO:0005525: GTP binding	0.000795	143,49,9
1dfca4#1469	GO:0005509: calcium ion binding	0.000796	53,160,10
3sil__#317	GO:0051082: unfolded protein binding	0.000797	358,34,12
1ddga1#442	GO:0010181: FMN binding	0.000799	11,11,2
1doi__#76	GO:0003755: peptidyl-prolyl cis-trans isomerase activity	0.000799	11,11,2
1e2ka_#361	GO:0004364: glutathione transferase activity	0.000799	11,11,2
1fc6a2#454	GO:0004295: trypsin activity	0.000799	43,48,5
1eur__#118	GO:0003724: RNA helicase activity	0.000799	11,11,2

2sqca2#97	GO:0016627: oxidoreductase activity, acting on the CH-CH group of donors	0.0008	127,17,5
1gtra1#468	GO:0003682: chromatin binding	0.0008	54,10,3
1ii7a_#199	GO:0016620: oxidoreductase activity, acting on the aldehyde or oxo group of donors, NAD or NADP as acceptor	0.0008	54,10,3
1g25a_#22	GO:0008083: growth factor activity	0.0008	415,42,15
1ycsb1#412	GO:0005096: GTPase activator activity	0.0008	146,15,5
1quna1#113	GO:0015078: hydrogen ion transporter activity	0.0008	286,21,8
1dfca4#1469	GO:0005529: sugar binding	0.000801	53,39,5
1gpc__#58	GO:0005524: ATP binding	0.000802	6,243,4
1qf6a4#360	GO:0005524: ATP binding	0.000802	6,243,4
1e8ga2#180	GO:0005524: ATP binding	0.000802	6,243,4
1aym2_#185	GO:0005524: ATP binding	0.000802	6,243,4
1qfma1#382	GO:0005524: ATP binding	0.000802	6,243,4
1c8za_#386	GO:0003809: thrombin activity	0.000803	235,10,5
1ddwa_#77	GO:0005507: copper ion binding	0.000803	364,38,13
1bwvs_#117	GO:0003755: peptidyl-prolyl cis-trans isomerase activity	0.000805	329,11,6
1a6o__#45	GO:0003887: DNA-directed DNA polymerase activity	0.000808	303,20,8
1dmla1#134	GO:0030151: molybdenum ion binding	0.000808	82,15,4
1dmla1#134	GO:0004556: alpha-amylase activity	0.000808	82,15,4
1gsoa3#142	GO:0004556: alpha-amylase activity	0.000808	82,15,4
1hbza_#96	GO:0008083: growth factor activity	0.000809	370,42,14
1f8ra2#325	GO:0005529: sugar binding	0.00081	222,39,10
1qaxa2#307	GO:0004812: tRNA ligase activity	0.00081	46,26,4
1cmxa_#207	GO:0008408: 3'-5' exonuclease activity	0.00081	96,13,4
1nfdb1#21	GO:0005518: collagen binding	0.00081	96,13,4
1br9__#86	GO:0008408: 3'-5' exonuclease activity	0.00081	96,13,4
1h9da_#91	GO:0005529: sugar binding	0.00081	222,39,10
1dpja_#322	GO:0005529: sugar binding	0.000811	308,39,12
1fl1a_#149	GO:0005524: ATP binding	0.000813	19,243,7
1i1ka_#255	GO:0016638: oxidoreductase activity, acting on the CH-NH2 group of donors	0.000814	31,17,3
1ap0__#29	GO:0004197: cysteine-type endopeptidase activity	0.000814	50,24,4
1gky__#61	GO:0005351: sugar porter activity	0.000814	25,21,3
2dnja_#158	GO:0004523: ribonuclease H activity	0.000814	33,16,3
1awcb_#111	GO:0016627: oxidoreductase activity, acting on the CH-CH group of donors	0.000814	31,17,3
1j8ra_#26	GO:0005518: collagen binding	0.000815	41,13,3
1seta2#403	GO:0005518: collagen binding	0.000815	41,13,3
1a65a3#409	GO:0003724: RNA helicase activity	0.000815	49,11,3
1a65a3#409	GO:0003755: peptidyl-prolyl cis-trans isomerase activity	0.000815	49,11,3
1dlja2#177	GO:0000049: tRNA binding	0.000815	41,13,3
1f0ya2#126	GO:0005525: GTP binding	0.000815	11,49,3
1dmla2#313	GO:0008408: 3'-5' exonuclease activity	0.000815	41,13,3
1fds__#59	GO:0019829: cation-transporting ATPase activity	0.000815	61,9,3
1aop_3#276	GO:0005509: calcium ion binding	0.000817	172,160,21
1ovaa_#284	GO:0004295: trypsin activity	0.000817	25,48,4
1bf6a_#183	GO:0016831: carboxy-lyase activity	0.000817	48,25,4
1epwa3#138	GO:0016668: oxidoreductase activity, acting on sulfur group of donors, NAD or NADP as acceptor	0.000818	424,12,7
1esma_#314	GO:0016646: oxidoreductase activity, acting on the CH-NH group of donors, NAD or NADP as acceptor	0.000819	113,19,5
1dpe__#419	GO:0030145: manganese ion binding	0.00082	14,38,3
1j71a_#251	GO:0003700: transcription factor activity	0.00082	106,124,13
1dyma_#213	GO:0004714: transmembrane receptor protein tyrosine kinase activity	0.00082	38,14,3
1nls__#77	GO:0030145: manganese ion binding	0.00082	14,38,3
1de4c3#165	GO:0030145: manganese ion binding	0.00082	14,38,3
1fo5a_#60	GO:0016251: general RNA polymerase II transcription factor activity	0.000822	246,14,6
1ddma_#130	GO:0005525: GTP binding	0.000822	313,49,14
3mag__#134	GO:0003924: GTPase activity	0.000822	72,17,4
1tbrr1#15	GO:0004896: hematopoietin/interferon-class (D200-domain) cytokine receptor activity	0.000823	64,19,4
1cipa2#34	GO:0008270: zinc ion binding	0.000823	78,108,10
1qksa2#236	GO:0004896: hematopoietin/interferon-class (D200-domain) cytokine receptor activity	0.000823	64,19,4
1hxmb1#117	GO:0004620: phospholipase activity	0.000823	64,19,4
3sil__#284	GO:0004222: metalloendopeptidase activity	0.000823	64,19,4
1qqsa_#124	GO:0008201: heparin binding	0.000824	458,24,11
3grs_3#435	GO:0005524: ATP binding	0.000825	179,243,29
1poxa3#460	GO:0004812: tRNA ligase activity	0.000825	81,26,5
1i50a_#856	GO:0005518: collagen binding	0.000826	269,13,6

1a4ya_#104	GO:0005525: GTP binding	0.000826	241,49,12
1fc4a_#205	GO:0016836: hydro-lyase activity	0.000827	267,33,10
1bd0a1#276	GO:0005524: ATP binding	0.000828	91,243,18
1bmv2_#2064	GO:0019838: growth factor binding	0.000828	147,9,4
1acc__#206	GO:0005516: calmodulin binding	0.000829	22,24,3
1fo8a_#202	GO:0008757: S-adenosylmethionine-dependent methyltransferase activity	0.000829	22,24,3
1cjxa2#239	GO:0019843: rRNA binding	0.000829	24,22,3
1rsy__#151	GO:0008201: heparin binding	0.000829	22,24,3
1gp1a_#173	GO:0005516: calmodulin binding	0.000829	22,24,3
1fgka_#610	GO:0005516: calmodulin binding	0.000829	22,24,3
1ak7__#152	GO:0019843: rRNA binding	0.000829	24,22,3
1pkp_1#139	GO:0000287: magnesium ion binding	0.000829	17,128,5
1bqsa2#103	GO:0008201: heparin binding	0.000829	22,24,3
1f3mc_#403	GO:0005516: calmodulin binding	0.000829	22,24,3
1dyna_#42	GO:0003887: DNA-directed DNA polymerase activity	0.000829	164,20,6
1i1b__#10	GO:0008201: heparin binding	0.000829	22,24,3
1epwa3#138	GO:0015036: disulfide oxidoreductase activity	0.00083	424,22,10
1bwza2#162	GO:0016616: oxidoreductase activity, acting on the CH-OH group of donors, NAD or NADP as acceptor	0.00083	95,59,8
1utea_#11	GO:0016620: oxidoreductase activity, acting on the aldehyde or oxo group of donors, NAD or NADP as acceptor	0.00083	130,10,4
1tpg_1#55	GO:0004601: peroxidase activity	0.000833	6,21,2
1eqja2#37	GO:0005351: sugar porter activity	0.000833	6,21,2
1ek1a2#525	GO:0004601: peroxidase activity	0.000833	6,21,2
1wdcb_#134	GO:0005524: ATP binding	0.000834	36,243,10
1aop_3#276	GO:0004812: tRNA ligase activity	0.000834	172,26,7
1fwxa2#396	GO:0050660: FAD binding	0.000836	237,10,5
1g25a_#22	GO:0005507: copper ion binding	0.000836	415,38,14
1elja_#8	GO:0016763: transferase activity, transferring pentosyl groups	0.000836	43,28,4
1dpja_#322	GO:0051082: unfolded protein binding	0.000837	308,34,11
1aqua_#126	GO:0016866: intramolecular transferase activity	0.000837	576,12,8
1e87a_#174	GO:0004190: aspartic-type endopeptidase activity	0.000838	141,23,6
1ycsb1#412	GO:0005524: ATP binding	0.000838	146,243,25
1csn__#84	GO:0015036: disulfide oxidoreductase activity	0.000839	207,22,7
1rypk_#12	GO:0005529: sugar binding	0.000839	265,39,11
1gc5a_#45	GO:0016836: hydro-lyase activity	0.000841	4,33,2
1f46a_#59	GO:0009036: type II site-specific deoxyribonuclease activity	0.000841	106,12,4
1nox__#88	GO:0005507: copper ion binding	0.000842	55,38,5
1gdea_#243	GO:0005126: hematopoietin/interferon-class (D200-domain) cytokine receptor binding	0.000842	61,20,4
1hyrc1#247	GO:0005126: hematopoietin/interferon-class (D200-domain) cytokine receptor binding	0.000842	61,20,4
1gpc__#184	GO:0005507: copper ion binding	0.000842	83,38,6
1erxa_#138	GO:0005507: copper ion binding	0.000842	55,38,5
1mil__#30	GO:0003887: DNA-directed DNA polymerase activity	0.000842	61,20,4
1bfg__#22	GO:0005126: hematopoietin/interferon-class (D200-domain) cytokine receptor binding	0.000842	61,20,4
1kit_2#398	GO:0005507: copper ion binding	0.000842	83,38,6
1b8aa2#403	GO:0003964: RNA-directed DNA polymerase activity	0.000843	117,11,4
1hlwa_#82	GO:0008408: 3'-5' exonuclease activity	0.000843	270,13,6
1cnv__#264	GO:0000287: magnesium ion binding	0.000843	103,128,13
1efpa1#32	GO:0016831: carboxy-lyase activity	0.000844	129,25,6
2mev3_#108	GO:0030151: molybdenum ion binding	0.000844	228,15,6
1enfa1#77	GO:0003682: chromatin binding	0.000845	55,10,3
1dqza_#131	GO:0004180: carboxypeptidase activity	0.000846	83,15,4
1kit_2#398	GO:0005179: hormone activity	0.000846	83,15,4
1fid__#237	GO:0004263: chymotrypsin activity	0.000847	51,41,5
1doka_#52	GO:0004263: chymotrypsin activity	0.000847	51,41,5
1fkna_#96	GO:0008083: growth factor activity	0.000848	75,42,6
1pot__#138	GO:0008083: growth factor activity	0.000848	75,42,6
2hlca_#139	GO:0005529: sugar binding	0.000848	112,39,7
1fmta2#151	GO:0005525: GTP binding	0.000849	314,49,14
1cfr__#269	GO:0000287: magnesium ion binding	0.00085	55,128,9
1f00i2#805	GO:0008201: heparin binding	0.00085	135,24,6
1hq8a_#206	GO:0004190: aspartic-type endopeptidase activity	0.00085	329,23,9
1eh9a3#102	GO:0005509: calcium ion binding	0.000851	14,160,5
1i7qa_#321	GO:0004180: carboxypeptidase activity	0.000852	148,15,5
1nsca_#379	GO:0004812: tRNA ligase activity	0.000855	124,26,6
1gsa_2#218	GO:0003809: thrombin activity	0.000855	131,10,4

1kwaa_#513	GO:0019838: growth factor binding	0.000855	62,9,3
1bak__#583	GO:0050660: FAD binding	0.000855	131,10,4
1ie5a_#102	GO:0019838: growth factor binding	0.000855	62,9,3
1gox__#124	GO:0005351: sugar porter activity	0.000855	366,21,9
1vola2#239	GO:0003700: transcription factor activity	0.000856	120,124,14
1jlxa2#166	GO:0004263: chymotrypsin activity	0.000856	77,41,6
1dp0a4#756	GO:0003887: DNA-directed DNA polymerase activity	0.000856	165,20,6
1j75a_#166	GO:0003700: transcription factor activity	0.000856	120,124,14
1iknd_#183	GO:0003700: transcription factor activity	0.00086	5,124,3
1bvoa_#66	GO:0003700: transcription factor activity	0.00086	5,124,3
1efpa1#32	GO:0003924: GTPase activity	0.00086	129,17,5
1qaua_#64	GO:0016705: oxidoreductase activity, acting on paired donors, with incorporation or reduction of molecular oxygen	0.00086	5,26,2
1qaua_#64	GO:0004497: monooxygenase activity	0.00086	5,26,2
1dgw.1#Y353	GO:0005507: copper ion binding	0.000861	319,38,12
1fhoa_#74	GO:0015036: disulfide oxidoreductase activity	0.000862	347,22,9
1c8ba_#514	GO:0005096: GTPase activator activity	0.000864	36,15,3
1bea__#80	GO:0004867: serine-type endopeptidase inhibitor activity	0.000864	3,47,2
1feua_#98	GO:0004867: serine-type endopeptidase inhibitor activity	0.000864	3,47,2
1hava_#65	GO:0004867: serine-type endopeptidase inhibitor activity	0.000864	3,47,2
1c4za_#569	GO:0004867: serine-type endopeptidase inhibitor activity	0.000864	3,47,2
1jb0d_#26	GO:0030151: molybdenum ion binding	0.000864	229,15,6
1fmta2#151	GO:0005516: calmodulin binding	0.000864	314,24,9
1ea5a_#133	GO:0016811: hydrolase activity, acting on carbon-nitrogen (but not peptide) bonds, in linear amides	0.000864	36,15,3
1fp1d2#214	GO:0004812: tRNA ligase activity	0.000864	173,26,7
1i50b_#966	GO:0042802: protein self binding	0.000864	299,12,6
1ekma1#545	GO:0004714: transmembrane receptor protein tyrosine kinase activity	0.000864	90,14,4
1amf__#196	GO:0004812: tRNA ligase activity	0.000864	173,26,7
1hle.1#B387	GO:0030151: molybdenum ion binding	0.000864	36,15,3
1cm9a_#57	GO:0019955: cytokine binding	0.000865	50,11,3
1b6cb_#346	GO:0004714: transmembrane receptor protein tyrosine kinase activity	0.000865	9,14,2
2fbjh2#141	GO:0003968: RNA-directed RNA polymerase activity	0.000865	9,14,2
1fhga_#42	GO:0004714: transmembrane receptor protein tyrosine kinase activity	0.000865	9,14,2
1bmv2_#2102	GO:0019838: growth factor binding	0.000865	14,9,2
1pme__#207	GO:0004714: transmembrane receptor protein tyrosine kinase activity	0.000865	9,14,2
1g3nc2#184	GO:0005543: phospholipid binding	0.000865	9,14,2
1fo0b_#82	GO:0004714: transmembrane receptor protein tyrosine kinase activity	0.000865	9,14,2
1fxqa_#1009	GO:0005543: phospholipid binding	0.000865	9,14,2
1jj2a1#126	GO:0016638: oxidoreductase activity, acting on the CH-NH2 group of donors	0.000867	73,17,4
1i2oa_#34	GO:0009036: type II site-specific deoxyribonuclease activity	0.000868	192,12,5
1i2oa_#34	GO:0016866: intramolecular transferase activity	0.000868	192,12,5
1tiid_#39	GO:0016846: carbon-sulfur lyase activity	0.000869	239,10,5
1bu7a_#405	GO:0004620: phospholipase activity	0.000873	65,19,4
1utea_#11	GO:0051082: unfolded protein binding	0.000874	130,34,7
1ffya2#226	GO:0005518: collagen binding	0.000875	42,13,3
1dcfa_#66	GO:0016861: intramolecular oxidoreductase activity, interconverting aldoses and ketoses	0.000875	42,13,3
1p35a_#101	GO:0008083: growth factor activity	0.000875	136,42,8
1g51a1#21	GO:0000049: tRNA binding	0.000875	42,13,3
1g51a1#21	GO:0003729: mRNA binding	0.000875	42,13,3
1flca1#294	GO:0005518: collagen binding	0.000876	98,13,4
1el0a_#28	GO:0003968: RNA-directed RNA polymerase activity	0.000876	161,14,5
1hxma2#150	GO:0008083: growth factor activity	0.000876	29,42,4
1f3mc_#369	GO:0003779: actin binding	0.000877	100,32,6
1xnb__#131	GO:0004197: cysteine-type endopeptidase activity	0.000878	51,24,4
1mpp__#154	GO:0005524: ATP binding	0.000879	99,243,19
1f42a2#123	GO:0016620: oxidoreductase activity, acting on the aldehyde or oxo group of donors, NAD or NADP as acceptor	0.000879	378,10,6
3chbd_#85	GO:0050660: FAD binding	0.000879	378,10,6
1cnv__#264	GO:0005351: sugar porter activity	0.000879	103,21,5
1sgpe_#42	GO:0004867: serine-type endopeptidase inhibitor activity	0.00088	26,47,4
1dhs__#99	GO:0016620: oxidoreductase activity, acting on the aldehyde or oxo group of donors, NAD or NADP as acceptor	0.00088	132,10,4
2btva_#597	GO:0003887: DNA-directed DNA polymerase activity	0.000884	27,20,3
1a3qa2#133	GO:0005126: hematopoietin/interferon-class (D200-domain) cytokine receptor binding	0.000884	27,20,3
1evqa_#305	GO:0016836: hydro-lyase activity	0.000884	490,33,14

1rb9__#49	GO:0003968: RNA-directed RNA polymerase activity	0.000885	39,14,3
1bd0a1#276	GO:0004295: trypsin activity	0.000885	91,48,7
1e5ka_#168	GO:0005525: GTP binding	0.000885	25,49,4
1bmv2_#2102	GO:0005529: sugar binding	0.000885	14,39,3
7fabh1#71	GO:0005529: sugar binding	0.000885	14,39,3
1svb_1#318	GO:0005529: sugar binding	0.000885	14,39,3
1h7wa2#636	GO:0016831: carboxy-lyase activity	0.000885	49,25,4
1avgi_#69	GO:0005509: calcium ion binding	0.000886	340,160,34
1qdla_#211	GO:0030145: manganese ion binding	0.000886	320,38,12
1a06__#85	GO:0004295: trypsin activity	0.000889	44,48,5
3grx__#56	GO:0003755: peptidyl-prolyl cis-trans isomerase activity	0.000889	335,11,6
1ygs__#435	GO:0051082: unfolded protein binding	0.00089	16,34,3
1efnb_#102	GO:0005524: ATP binding	0.00089	49,243,12
1f24a_#293	GO:0005507: copper ion binding	0.00089	116,38,7
1qtra_#107	GO:0051082: unfolded protein binding	0.00089	16,34,3
1qo0d_#81	GO:0016866: intramolecular transferase activity	0.000891	46,12,3
1ass__#98	GO:0051082: unfolded protein binding	0.000893	4,34,2
1qfla1#42	GO:0051082: unfolded protein binding	0.000893	4,34,2
3pyp__#41	GO:0016638: oxidoreductase activity, acting on the CH-NH2 group of donors	0.000895	32,17,3
1ewqa3#159	GO:0046983: protein dimerization activity	0.000895	32,17,3
1fjsa_#83	GO:0005126: hematopoietin/interferon-class (D200-domain) cytokine receptor binding	0.000895	109,20,5
1bt3a_#49	GO:0003779: actin binding	0.000895	17,32,3
1f8na1#450	GO:0016638: oxidoreductase activity, acting on the CH-NH2 group of donors	0.000895	32,17,3
1clc_1#299	GO:0016627: oxidoreductase activity, acting on the CH-CH group of donors	0.000895	32,17,3
2hlca_#54	GO:0003887: DNA-directed DNA polymerase activity	0.000895	109,20,5
1fw9a_#157	GO:0005126: hematopoietin/interferon-class (D200-domain) cytokine receptor binding	0.000896	62,20,4
1b3qa2#623	GO:0016702: oxidoreductase activity, acting on single donors with incorporation of molecular oxygen, incorporation of two atoms of oxygen	0.000896	301,12,6
1f97a1#62	GO:0008270: zinc ion binding	0.000897	707,108,43
1dfoa_#344	GO:0051082: unfolded protein binding	0.000898	36,34,4
1beba_#119	GO:0000287: magnesium ion binding	0.000899	202,128,20
1qfma1#186	GO:0004556: alpha-amylase activity	0.0009	326,15,7
1qfma1#186	GO:0004725: protein tyrosine phosphatase activity	0.0009	326,15,7
1bura1#328	GO:0016776: phosphotransferase activity, phosphate group as acceptor	0.000901	91,14,4
1hava_#65	GO:0004295: trypsin activity	0.000902	3,48,2
1c4za_#569	GO:0004295: trypsin activity	0.000902	3,48,2
1g6sa_#64	GO:0009036: type II site-specific deoxyribonuclease activity	0.000903	108,12,4
1a4ya_#104	GO:0050660: FAD binding	0.000903	241,10,5
1ejda_#377	GO:0000155: two-component sensor molecule activity	0.000903	108,12,4
1a8i__#688	GO:0016814: hydrolase activity, acting on carbon-nitrogen (but not peptide) bonds, in cyclic amidines	0.000903	241,10,5
1a8i__#688	GO:0016846: carbon-sulfur lyase activity	0.000903	241,10,5
1h6la_#119	GO:0005126: hematopoietin/interferon-class (D200-domain) cytokine receptor binding	0.000903	308,20,8
1rypb_#114	GO:0005351: sugar porter activity	0.000905	158,21,6
1fw9a_#152	GO:0001584: rhodopsin-like receptor activity	0.000906	133,10,4
1fw9a_#152	GO:0008800: beta-lactamase activity	0.000906	133,10,4
1kve.1#B199	GO:0004674: protein serine/threonine kinase activity	0.00091	76,42,6
1imva_#255	GO:0046983: protein dimerization activity	0.00091	589,17,10
1d7ba_#87	GO:0005518: collagen binding	0.000911	99,13,4
1e2o__#346	GO:0000155: two-component sensor molecule activity	0.000911	194,12,5
1eova1#139	GO:0000049: tRNA binding	0.000911	99,13,4
1jb3a_#64	GO:0016668: oxidoreductase activity, acting on sulfur group of donors, NAD or NADP as acceptor	0.000912	302,12,6
1e3ja2#270	GO:0016627: oxidoreductase activity, acting on the CH-CH group of donors	0.000912	74,17,4
1qgub_#197	GO:0003924: GTPase activity	0.000912	74,17,4
1dzfa2#211	GO:0005126: hematopoietin/interferon-class (D200-domain) cytokine receptor binding	0.000913	167,20,6
1foha5#9	GO:0005525: GTP binding	0.000913	65,49,6
1hfwa_#195	GO:0019843: rRNA binding	0.000915	6,22,2
1a8i__#202	GO:0019843: rRNA binding	0.000915	6,22,2
1fvka2#181	GO:0015036: disulfide oxidoreductase activity	0.000915	6,22,2
1aym3_#109	GO:0005507: copper ion binding	0.000915	56,38,5
1qkma_#330	GO:0003700: transcription factor activity	0.000915	69,124,10
2sli_2#326	GO:0003968: RNA-directed RNA polymerase activity	0.000916	251,14,6
1fkna_#99	GO:0004812: tRNA ligase activity	0.000916	21,26,3
1ep3b1#71	GO:0004812: tRNA ligase activity	0.000916	21,26,3
1fgua1#249	GO:0004812: tRNA ligase activity	0.000916	21,26,3

1gox__#246	GO:0016651: oxidoreductase activity, acting on NADH or NADPH	0.000917	177,13,5
1nfdb1#34	GO:0005518: collagen binding	0.000917	177,13,5
1qhda2#323	GO:0019955: cytokine binding	0.000918	337,11,6
1e79d2#36	GO:0000049: tRNA binding	0.000918	524,13,8
1e1aa_#285	GO:0004674: protein serine/threonine kinase activity	0.000919	137,42,8
1ejda_#180	GO:0005524: ATP binding	0.000922	77,243,16
1ppn__#132	GO:0003887: DNA-directed DNA polymerase activity	0.000922	234,20,7
1qtra_#108	GO:0005524: ATP binding	0.000922	77,243,16
1be9a_#357	GO:0004497: monooxygenase activity	0.000923	83,26,5
1gpc__#184	GO:0004812: tRNA ligase activity	0.000923	83,26,5
1e25a_#262	GO:0004896: hematopoietin/interferon-class (D200-domain) cytokine receptor activity	0.000923	116,19,5
1e42a1#711	GO:0004812: tRNA ligase activity	0.000925	175,26,7
1f0ia1#50	GO:0005525: GTP binding	0.000925	146,49,9
1i2ma_#157	GO:0003682: chromatin binding	0.000926	13,10,2
1svb_1#358	GO:0005518: collagen binding	0.000926	10,13,2
1h6oa_#135	GO:0016651: oxidoreductase activity, acting on NADH or NADPH	0.000926	10,13,2
1ed1a_#94	GO:0000049: tRNA binding	0.000926	10,13,2
1mkna_#50	GO:0005518: collagen binding	0.000926	10,13,2
1g97a1#376	GO:0004457: lactate dehydrogenase activity	0.000926	13,10,2
1nfdb1#115	GO:0005518: collagen binding	0.000926	10,13,2
1mrj__#240	GO:0003684: damaged DNA binding	0.000926	13,10,2
1mwpa_#42	GO:0001584: rhodopsin-like receptor activity	0.000926	13,10,2
1dj0a1#96	GO:0003684: damaged DNA binding	0.000926	13,10,2
1cpt__#144	GO:0016651: oxidoreductase activity, acting on NADH or NADPH	0.000926	10,13,2
1e2fa_#121	GO:0019201: nucleotide kinase activity	0.000926	10,13,2
1d4xg_#7	GO:0003682: chromatin binding	0.000926	13,10,2
1fgka_#668	GO:0005066: transmembrane receptor protein tyrosine kinase signaling protein activity	0.000926	13,10,2
1hbna1#293	GO:0016651: oxidoreductase activity, acting on NADH or NADPH	0.000926	10,13,2
1fjsa_#28	GO:0003809: thrombin activity	0.000926	13,10,2
1qfja2#226	GO:0003684: damaged DNA binding	0.000926	13,10,2
2tgi__#106	GO:0005085: guanyl-nucleotide exchange factor activity	0.000926	10,13,2
3grs_1#24	GO:0004222: metalloendopeptidase activity	0.000926	66,19,4
1eg5a_#199	GO:0016846: carbon-sulfur lyase activity	0.000926	13,10,2
8abp__#231	GO:0016620: oxidoreductase activity, acting on the aldehyde or oxo group of donors, NAD or NADP as acceptor	0.000926	13,10,2
1ixh__#180	GO:0008199: ferric iron binding	0.000926	13,10,2
1fwxa2#189	GO:0005085: guanyl-nucleotide exchange factor activity	0.000926	10,13,2
1a81a2#182	GO:0003964: RNA-directed DNA polymerase activity	0.000928	120,11,4
1a6o__#45	GO:0003697: single-stranded DNA binding	0.000929	303,12,6
1fo4a5#1020	GO:0005529: sugar binding	0.000929	359,39,13
1htp__#16	GO:0042802: protein self binding	0.000929	303,12,6
1czan1#92	GO:0008201: heparin binding	0.000929	631,24,13
1dih_2#235	GO:0005509: calcium ion binding	0.000929	54,160,10
1h8ma_#8	GO:0050660: FAD binding	0.000932	134,10,4
1g8fa3#419	GO:0015036: disulfide oxidoreductase activity	0.000933	151,22,6
1dnpa2#18	GO:0016763: transferase activity, transferring pentosyl groups	0.000934	77,28,5
1ab4__#89	GO:0004601: peroxidase activity	0.000936	159,21,6
1ghpa_#65	GO:0004896: hematopoietin/interferon-class (D200-domain) cytokine receptor activity	0.000937	29,19,3
1bj7__#112	GO:0004896: hematopoietin/interferon-class (D200-domain) cytokine receptor activity	0.000937	29,19,3
1pjca1#254	GO:0016831: carboxy-lyase activity	0.000937	22,25,3
1lucb_#32	GO:0016831: carboxy-lyase activity	0.000937	22,25,3
1eu3a2#174	GO:0019843: rRNA binding	0.000937	25,22,3
1b8aa2#403	GO:0030145: manganese ion binding	0.000937	117,38,7
1jsg__#92	GO:0019843: rRNA binding	0.000937	607,22,12
1g0sa_#176	GO:0005179: hormone activity	0.000938	37,15,3
1qf5a_#15	GO:0005085: guanyl-nucleotide exchange factor activity	0.000938	43,13,3
1xo1a1#246	GO:0004180: carboxypeptidase activity	0.000938	37,15,3
1eh9a1#33	GO:0004556: alpha-amylase activity	0.000938	37,15,3
1eh9a1#33	GO:0005179: hormone activity	0.000938	37,15,3
1awx__#13	GO:0005179: hormone activity	0.000938	37,15,3
1e5pa_#91	GO:0008408: 3'-5' exonuclease activity	0.000938	43,13,3
1fc6a2#454	GO:0008408: 3'-5' exonuclease activity	0.000938	43,13,3
1cf9a1#736	GO:0005085: guanyl-nucleotide exchange factor activity	0.000938	43,13,3
1trka2#429	GO:0016854: racemase and epimerase activity	0.000938	43,13,3
1hwxa1#256	GO:0004457: lactate dehydrogenase activity	0.000939	57,10,3

1hwxa1#256	GO:0016620: oxidoreductase activity, acting on the aldehyde or oxo group of donors, NAD or NADP as acceptor	0.000939	57,10,3
1e8ga2#201	GO:0005524: ATP binding	0.000939	304,243,43
1ffgb_#172	GO:0005524: ATP binding	0.000939	10,243,5
1dar_1#395	GO:0005525: GTP binding	0.00094	3,49,2
1g4us1#189	GO:0005525: GTP binding	0.00094	3,49,2
1thg__#3	GO:0004714: transmembrane receptor protein tyrosine kinase activity	0.00094	92,14,4
1ayl__#237	GO:0015082: di-, tri-valent inorganic cation transporter activity	0.00094	357,14,7
1ayl__#237	GO:0046915: transition metal ion transporter activity	0.00094	357,14,7
1flga_#161	GO:0019838: growth factor binding	0.000941	152,9,4
2ltn.1#A63	GO:0019838: growth factor binding	0.000941	152,9,4
1f7ua2#324	GO:0005524: ATP binding	0.000942	56,243,13
1emsa2#165	GO:0000287: magnesium ion binding	0.000944	45,128,8
1qovm1#94	GO:0000287: magnesium ion binding	0.000945	5,128,3
1thg__#3	GO:0004295: trypsin activity	0.000945	92,48,7
1fjfc2#153	GO:0030145: manganese ion binding	0.000945	420,38,14
1qdlb_#170	GO:0000049: tRNA binding	0.000946	100,13,4
1e32a2#345	GO:0008026: ATP-dependent helicase activity	0.000946	100,13,4
1ghqb2#94	GO:0004842: ubiquitin-protein ligase activity	0.000948	7,19,2
1d3ya_#261	GO:0004222: metalloendopeptidase activity	0.000948	7,19,2
1hxn__#256	GO:0004222: metalloendopeptidase activity	0.000948	7,19,2
1doka_#52	GO:0008083: growth factor activity	0.000948	51,42,5
1e31a_#77	GO:0016866: intramolecular transferase activity	0.000949	47,12,3
1bfd_1#230	GO:0042802: protein self binding	0.000949	47,12,3
1neb__#41	GO:0003697: single-stranded DNA binding	0.000949	47,12,3
1b3qa2#623	GO:0005509: calcium ion binding	0.000949	301,160,31
1qfma1#139	GO:0008201: heparin binding	0.000949	318,24,9
2dnja_#97	GO:0004523: ribonuclease H activity	0.00095	141,16,5
1stfi_#116	GO:0019843: rRNA binding	0.000951	57,22,4
1alo_4#481	GO:0005524: ATP binding	0.000951	70,243,15
1by5a_#347	GO:0051082: unfolded protein binding	0.000952	263,34,10
1a8i__#688	GO:0016831: carboxy-lyase activity	0.000953	241,25,8
1e53a_#367	GO:0004295: trypsin activity	0.000954	26,48,4
1b6ra3#161	GO:0003968: RNA-directed RNA polymerase activity	0.000954	164,14,5
1pii_2#326	GO:0008757: S-adenosylmethionine-dependent methyltransferase activity	0.000954	192,24,7
2bpa1_#45	GO:0008201: heparin binding	0.000954	192,24,7
1erv__#80	GO:0019838: growth factor binding	0.000955	442,9,6
1pud__#44	GO:0016831: carboxy-lyase activity	0.000956	50,25,4
1qf6a3#92	GO:0008757: S-adenosylmethionine-dependent methyltransferase activity	0.000956	91,24,5
1tmy__#14	GO:0003700: transcription factor activity	0.000956	18,124,5
1ddja_#681	GO:0004714: transmembrane receptor protein tyrosine kinase activity	0.000956	253,14,6
1jf9a_#380	GO:0016831: carboxy-lyase activity	0.000956	50,25,4
1jb9a2#173	GO:0010181: FMN binding	0.000957	12,11,2
1bmv2_#2059	GO:0016668: oxidoreductase activity, acting on sulfur group of donors, NAD or NADP as acceptor	0.000957	11,12,2
1i8da1#85	GO:0010181: FMN binding	0.000957	12,11,2
1vcaa2#49	GO:0019955: cytokine binding	0.000957	12,11,2
1g93a_#229	GO:0016758: transferase activity, transferring hexosyl groups	0.000957	12,11,2
1bn8a_#41	GO:0003899: DNA-directed RNA polymerase activity	0.000957	11,12,2
1bu7a_#130	GO:0010181: FMN binding	0.000957	12,11,2
1ak0__#212	GO:0008081: phosphoric diester hydrolase activity	0.000957	12,11,2
1vin_2#335	GO:0000287: magnesium ion binding	0.000957	35,128,7
1i6vc_#869	GO:0003899: DNA-directed RNA polymerase activity	0.000957	11,12,2
2dnja_#5	GO:0000155: two-component sensor molecule activity	0.000957	11,12,2
1b8aa2#368	GO:0016620: oxidoreductase activity, acting on the aldehyde or oxo group of donors, NAD or NADP as acceptor	0.000958	135,10,4
1hxxa_#232	GO:0015036: disulfide oxidoreductase activity	0.000959	352,22,9
1fkna_#96	GO:0016638: oxidoreductase activity, acting on the CH-NH2 group of donors	0.00096	75,17,4
1bd8__#82	GO:0046983: protein dimerization activity	0.00096	75,17,4
1feca1#31	GO:0016627: oxidoreductase activity, acting on the CH-CH group of donors	0.00096	75,17,4
1fcda1#6	GO:0016627: oxidoreductase activity, acting on the CH-CH group of donors	0.00096	75,17,4
1i7wa_#517	GO:0015078: hydrogen ion transporter activity	0.000961	60,21,4
1mai__#108	GO:0030151: molybdenum ion binding	0.000963	152,15,5
1qdlb_#170	GO:0019843: rRNA binding	0.000964	100,22,5
1hzxa_#139	GO:0003779: actin binding	0.000966	39,32,4
1dfca4#1478	GO:0005529: sugar binding	0.000966	32,39,4

1e79h2#77	GO:0005509: calcium ion binding	0.000968	21,160,6
1f75a_#239	GO:0005509: calcium ion binding	0.000968	21,160,6
1gof_3#399	GO:0005509: calcium ion binding	0.000968	21,160,6
1mrp__#172	GO:0016866: intramolecular transferase activity	0.000969	110,12,4
1fgga_#134	GO:0010181: FMN binding	0.00097	218,11,5
1qf6a3#78	GO:0003964: RNA-directed DNA polymerase activity	0.000971	52,11,3
2sqca2#97	GO:0016705: oxidoreductase activity, acting on paired donors, with incorporation or reduction of molecular oxygen	0.000971	127,26,6
1hd2a_#47	GO:0000287: magnesium ion binding	0.000973	56,128,9
1ejba_#81	GO:0004812: tRNA ligase activity	0.000975	84,26,5
1b6ra3#161	GO:0005524: ATP binding	0.000976	164,243,27
1nat__#50	GO:0000287: magnesium ion binding	0.000978	297,128,26
1mla_1#281	GO:0005525: GTP binding	0.000979	44,49,5
1ycsa_#162	GO:0008083: growth factor activity	0.000979	210,42,10
2cba__#144	GO:0016646: oxidoreductase activity, acting on the CH-NH group of donors, NAD or NADP as acceptor	0.00098	67,19,4
1dfca4#1451	GO:0004896: hematopoietin/interferon-class (D200-domain) cytokine receptor activity	0.00098	67,19,4
1nhp_1#7	GO:0016627: oxidoreductase activity, acting on the CH-CH group of donors	0.000981	33,17,3
1nhp_1#7	GO:0016638: oxidoreductase activity, acting on the CH-NH2 group of donors	0.000981	33,17,3
1eoka_#127	GO:0016627: oxidoreductase activity, acting on the CH-CH group of donors	0.000981	33,17,3
1kit_3#282	GO:0008483: transaminase activity	0.000981	33,17,3
1erja_#457	GO:0016638: oxidoreductase activity, acting on the CH-NH2 group of donors	0.000981	33,17,3
1dpe__#402	GO:0046983: protein dimerization activity	0.000981	33,17,3
1ioaa_#75	GO:0016638: oxidoreductase activity, acting on the CH-NH2 group of donors	0.000981	33,17,3
1dp0a4#756	GO:0003968: RNA-directed RNA polymerase activity	0.000981	165,14,5
1ijqa1#564	GO:0046983: protein dimerization activity	0.000981	33,17,3
1ijqa1#564	GO:0016638: oxidoreductase activity, acting on the CH-NH2 group of donors	0.000981	33,17,3
1ppn__#132	GO:0005507: copper ion binding	0.000984	234,38,10
1ejda_#6	GO:0005529: sugar binding	0.000984	270,39,11
1dyna_#19	GO:0004263: chymotrypsin activity	0.000985	109,41,7
1erja_#457	GO:0005507: copper ion binding	0.000985	33,38,4
1ft9a1#201	GO:0004263: chymotrypsin activity	0.000985	109,41,7
1qf5a_#15	GO:0005524: ATP binding	0.000986	43,243,11
1f42a2#123	GO:0046983: protein dimerization activity	0.000986	378,17,8
3chbd_#85	GO:0046983: protein dimerization activity	0.000986	378,17,8
1i50a_#469	GO:0016620: oxidoreductase activity, acting on the aldehyde or oxo group of donors, NAD or NADP as acceptor	0.000988	58,10,3
1evqa_#305	GO:0004364: glutathione transferase activity	0.000988	490,11,7
1fw9a_#152	GO:0046983: protein dimerization activity	0.000989	133,17,5
1f0xa1#278	GO:0008408: 3'-5' exonuclease activity	0.00099	180,13,5
1fo5a_#60	GO:0016620: oxidoreductase activity, acting on the aldehyde or oxo group of donors, NAD or NADP as acceptor	0.000994	246,10,5
5ruba1#365	GO:0000287: magnesium ion binding	0.000994	118,128,14
1ayl__#237	GO:0004812: tRNA ligase activity	0.000995	357,26,10
1sfp__#66	GO:0016811: hydrolase activity, acting on carbon-nitrogen (but not peptide) bonds, in linear amides	0.000996	9,15,2
19hca_#162	GO:0003743: translation initiation factor activity	0.000996	9,15,2
1jl0a_#264	GO:0030151: molybdenum ion binding	0.000996	9,15,2
1b3ra2#358	GO:0005096: GTPase activator activity	0.000996	9,15,2
1qmha2#136	GO:0030151: molybdenum ion binding	0.000996	9,15,2
1pvc1_#106	GO:0005509: calcium ion binding	0.000996	64,160,11
1uxy_1#87	GO:0030151: molybdenum ion binding	0.000996	9,15,2
1ltda1#242	GO:0004556: alpha-amylase activity	0.000996	9,15,2
3sil__#284	GO:0005509: calcium ion binding	0.000996	64,160,11
1hvba_#63	GO:0004180: carboxypeptidase activity	0.000996	9,15,2
1hxxa_#278	GO:0015082: di-, tri-valent inorganic cation transporter activity	0.000997	255,14,6
1hxxa_#278	GO:0046915: transition metal ion transporter activity	0.000997	255,14,6
1mpp__#29	GO:0051082: unfolded protein binding	0.000998	37,34,4
1epwa3#248	GO:0051082: unfolded protein binding	0.000998	37,34,4
1iyu__#32	GO:0016763: transferase activity, transferring pentosyl groups	0.000999	5,28,2
2mev3_#108	GO:0005529: sugar binding	0.001	228,39,10
1e44b_#75	GO:0003899: DNA-directed RNA polymerase activity	0.001001	198,12,5
3btaa3#414	GO:0004190: aspartic-type endopeptidase activity	0.001002	6,23,2
1kcw_3#541	GO:0004190: aspartic-type endopeptidase activity	0.001002	6,23,2
1quna1#113	GO:0016638: oxidoreductase activity, acting on the CH-NH2 group of donors	0.001002	286,17,7

1qfea_#68	GO:0016620: oxidoreductase activity, acting on the aldehyde or oxo group of donors, NAD or NADP as acceptor	0.001003	560,10,7
1hcd__#91	GO:0016799: hydrolase activity, hydrolyzing N-glycosyl compounds	0.001004	8,17,2
1jd0a_#176	GO:0008270: zinc ion binding	0.001004	207,108,18
1hv8a1#188	GO:0003924: GTPase activity	0.001004	8,17,2
1jlxa1#147	GO:0016799: hydrolase activity, hydrolyzing N-glycosyl compounds	0.001004	8,17,2
2viua_#123	GO:0046983: protein dimerization activity	0.001004	8,17,2
1erja_#521	GO:0016638: oxidoreductase activity, acting on the CH-NH2 group of donors	0.001004	8,17,2
1es7a_#111	GO:0046983: protein dimerization activity	0.001004	8,17,2
1pvc1_#106	GO:0051082: unfolded protein binding	0.001004	64,34,5
1dceb_#134	GO:0003924: GTPase activity	0.001004	8,17,2
1ezvb1#29	GO:0008408: 3'-5' exonuclease activity	0.001004	44,13,3
1g79a_#69	GO:0016638: oxidoreductase activity, acting on the CH-NH2 group of donors	0.001004	8,17,2
1aqua_#79	GO:0008235: metalloexopeptidase activity	0.001004	44,13,3
1qksa2#236	GO:0051082: unfolded protein binding	0.001004	64,34,5
1e8ua_#180	GO:0016638: oxidoreductase activity, acting on the CH-NH2 group of donors	0.001004	8,17,2
3chbd_#85	GO:0008083: growth factor activity	0.001007	378,42,14
1cjca2#37	GO:0005524: ATP binding	0.001007	25,243,8
1erv__#80	GO:0004556: alpha-amylase activity	0.001007	442,15,8
1erv__#80	GO:0004725: protein tyrosine phosphatase activity	0.001007	442,15,8
2pia_1#99	GO:0005525: GTP binding	0.001008	179,49,10
1i50b_#216	GO:0003697: single-stranded DNA binding	0.00101	48,12,3
1i50b_#216	GO:0003899: DNA-directed RNA polymerase activity	0.00101	48,12,3
1prea2#308	GO:0016638: oxidoreductase activity, acting on the CH-NH2 group of donors	0.00101	76,17,4
1dt6a_#385	GO:0016799: hydrolase activity, hydrolyzing N-glycosyl compounds	0.00101	76,17,4
3frua1#236	GO:0046983: protein dimerization activity	0.00101	76,17,4
1uch__#50	GO:0016638: oxidoreductase activity, acting on the CH-NH2 group of donors	0.00101	76,17,4
1pbe_1#150	GO:0016627: oxidoreductase activity, acting on the CH-CH group of donors	0.00101	76,17,4
1faoa_#250	GO:0003924: GTPase activity	0.00101	76,17,4
1dfaa2#211	GO:0005096: GTPase activator activity	0.001011	87,15,4
2viua_#252	GO:0003724: RNA helicase activity	0.001011	343,11,6
1pvc1_#106	GO:0005126: hematopoietin/interferon-class (D200-domain) cytokine receptor binding	0.001011	64,20,4
1tbrr1#15	GO:0005126: hematopoietin/interferon-class (D200-domain) cytokine receptor binding	0.001011	64,20,4
1fd9a_#162	GO:0004180: carboxypeptidase activity	0.001011	87,15,4
3sil__#284	GO:0005126: hematopoietin/interferon-class (D200-domain) cytokine receptor binding	0.001011	64,20,4
1rypk_#12	GO:0051082: unfolded protein binding	0.001011	265,34,10
1d6aa_#52	GO:0004812: tRNA ligase activity	0.001013	293,26,9
1ejfa_#10	GO:0019838: growth factor binding	0.001014	155,9,4
1e1aa_#285	GO:0050660: FAD binding	0.001014	137,10,4
1a3k__#199	GO:0005507: copper ion binding	0.001015	15,38,3
1tbga_#63	GO:0005507: copper ion binding	0.001015	15,38,3
1a8q__#75	GO:0030145: manganese ion binding	0.001015	15,38,3
1at0__#285	GO:0004556: alpha-amylase activity	0.001015	38,15,3
1icia_#9	GO:0030151: molybdenum ion binding	0.001015	38,15,3
1ea5a_#99	GO:0008201: heparin binding	0.001015	194,24,7
1oaca1#360	GO:0005507: copper ion binding	0.001015	15,38,3
1h6la_#119	GO:0016668: oxidoreductase activity, acting on sulfur group of donors, NAD or NADP as acceptor	0.001015	308,12,6
1qhda2#323	GO:0004190: aspartic-type endopeptidase activity	0.001016	337,23,9
1f2la_#40	GO:0030151: molybdenum ion binding	0.001016	236,15,6
1f2la_#40	GO:0004556: alpha-amylase activity	0.001016	236,15,6
1fwxa2#396	GO:0005509: calcium ion binding	0.001016	237,160,26
1by5a_#238	GO:0005509: calcium ion binding	0.001016	237,160,26
1dfca4#1469	GO:0008201: heparin binding	0.001016	53,24,4
1mrj__#73	GO:0004197: cysteine-type endopeptidase activity	0.001016	53,24,4
1dv8a_#211	GO:0005507: copper ion binding	0.001017	86,38,6
1qtn.1#A270	GO:0016866: intramolecular transferase activity	0.001017	439,12,7
1am2__#163	GO:0000049: tRNA binding	0.00102	102,13,4
1c9la2#61	GO:0003887: DNA-directed DNA polymerase activity	0.001021	238,20,7
1c9la2#61	GO:0005126: hematopoietin/interferon-class (D200-domain) cytokine receptor binding	0.001021	238,20,7
1dpga2#334	GO:0003924: GTPase activity	0.001024	287,17,7
1dpga2#334	GO:0016638: oxidoreductase activity, acting on the CH-NH2 group of donors	0.001024	287,17,7
1fkna_#63	GO:0016616: oxidoreductase activity, acting on the CH-OH group of donors, NAD or NADP as acceptor	0.001027	37,59,5
1dfca1#1135	GO:0019955: cytokine binding	0.001027	53,11,3
1pud__#69	GO:0004812: tRNA ligase activity	0.001032	49,26,4

1by5a_#347	GO:0005509: calcium ion binding	0.001032	263,160,28
1b35b_#97	GO:0003697: single-stranded DNA binding	0.001033	309,12,6
1a8d_1#87	GO:0004190: aspartic-type endopeptidase activity	0.001035	204,23,7
1i7oa1#119	GO:0004620: phospholipase activity	0.001037	30,19,3
1hw5a2#64	GO:0004714: transmembrane receptor protein tyrosine kinase activity	0.001037	167,14,5
1jj2e1#42	GO:0004714: transmembrane receptor protein tyrosine kinase activity	0.001037	167,14,5
1dzfa2#211	GO:0004714: transmembrane receptor protein tyrosine kinase activity	0.001037	167,14,5
2hlca_#139	GO:0016702: oxidoreductase activity, acting on single donors with incorporation of molecular oxygen, incorporation of two atoms of oxygen	0.001037	112,12,4
1el0a_#42	GO:0004812: tRNA ligase activity	0.001038	294,26,9
1es9a_#89	GO:0016616: oxidoreductase activity, acting on the CH-OH group of donors, NAD or NADP as acceptor	0.001039	10,59,3
3tdt__#201	GO:0016616: oxidoreductase activity, acting on the CH-OH group of donors, NAD or NADP as acceptor	0.001039	10,59,3
1db3a_#266	GO:0003809: thrombin activity	0.001039	59,10,3
1ep3a_#19	GO:0016616: oxidoreductase activity, acting on the CH-OH group of donors, NAD or NADP as acceptor	0.001039	10,59,3
1fc3a_#172	GO:0016861: intramolecular oxidoreductase activity, interconverting aldoses and ketoses	0.001042	182,13,5
1e6ca_#91	GO:0016651: oxidoreductase activity, acting on NADH or NADPH	0.001042	182,13,5
1ds9a_#105	GO:0000287: magnesium ion binding	0.001044	68,128,10
1fm2.1#B308	GO:0016668: oxidoreductase activity, acting on sulfur group of donors, NAD or NADP as acceptor	0.001048	200,12,5
1lara2#1769	GO:0051082: unfolded protein binding	0.001049	219,34,9
1qfea_#68	GO:0016836: hydro-lyase activity	0.001049	560,33,15
1fbna_#144	GO:0016763: transferase activity, transferring pentosyl groups	0.00105	79,28,5
1amp__#205	GO:0016763: transferase activity, transferring pentosyl groups	0.00105	79,28,5
1tlfa_#269	GO:0003700: transcription factor activity	0.001051	11,124,4
1qdea_#201	GO:0003887: DNA-directed DNA polymerase activity	0.001052	7,20,2
1qg3a1#1179	GO:0005126: hematopoietin/interferon-class (D200-domain) cytokine receptor binding	0.001052	7,20,2
1b8fa_#500	GO:0003887: DNA-directed DNA polymerase activity	0.001052	7,20,2
1e8ua_#286	GO:0005126: hematopoietin/interferon-class (D200-domain) cytokine receptor binding	0.001052	7,20,2
1akjd_#3	GO:0005126: hematopoietin/interferon-class (D200-domain) cytokine receptor binding	0.001052	7,20,2
1mtyd_#390	GO:0016705: oxidoreductase activity, acting on paired donors, with incorporation or reduction of molecular oxygen	0.001053	22,26,3
1bqsa2#103	GO:0004812: tRNA ligase activity	0.001053	22,26,3
1h9da_#91	GO:0003724: RNA helicase activity	0.001054	222,11,5
1f97a1#62	GO:0004180: carboxypeptidase activity	0.001055	707,15,10
1f24a_#293	GO:0005524: ATP binding	0.001055	116,243,21
1g51a3#524	GO:0019843: rRNA binding	0.001055	102,22,5
1c9oa_#49	GO:0019843: rRNA binding	0.001055	102,22,5
1e3ua_#164	GO:0005524: ATP binding	0.001056	37,243,10
1jj2v_#118	GO:0005518: collagen binding	0.001058	103,13,4
1a4ya_#47	GO:0016763: transferase activity, transferring pentosyl groups	0.001058	216,28,8
1mjha_#119	GO:0008235: metalloexopeptidase activity	0.001058	103,13,4
1mjha_#119	GO:0016854: racemase and epimerase activity	0.001058	103,13,4
1elja_#64	GO:0005085: guanyl-nucleotide exchange factor activity	0.001058	103,13,4
1elja_#64	GO:0000049: tRNA binding	0.001058	103,13,4
1llda2#161	GO:0003700: transcription factor activity	0.001059	27,124,6
1dik_1#670	GO:0003700: transcription factor activity	0.001059	27,124,6
1f00i2#805	GO:0016638: oxidoreductase activity, acting on the CH-NH2 group of donors	0.001059	135,17,5
1a3qa2#133	GO:0003700: transcription factor activity	0.001059	27,124,6
1qtn.1#A270	GO:0016836: hydro-lyase activity	0.001059	439,33,13
1aym3_#109	GO:0004190: aspartic-type endopeptidase activity	0.00106	56,23,4
2pia_1#99	GO:0004812: tRNA ligase activity	0.00106	179,26,7
1epwa3#248	GO:0005509: calcium ion binding	0.00106	37,160,8
1io1a_#233	GO:0004714: transmembrane receptor protein tyrosine kinase activity	0.001061	95,14,4
1bwza2#162	GO:0016776: phosphotransferase activity, phosphate group as acceptor	0.001061	95,14,4
1qtra_#108	GO:0003924: GTPase activity	0.001061	77,17,4
1erv__#80	GO:0016668: oxidoreductase activity, acting on sulfur group of donors, NAD or NADP as acceptor	0.001061	442,12,7
1h8ca_#35	GO:0016638: oxidoreductase activity, acting on the CH-NH2 group of donors	0.001061	77,17,4
1e42a1#711	GO:0008083: growth factor activity	0.001063	175,42,9
1fjfl_#67	GO:0008083: growth factor activity	0.001063	175,42,9
1c9la2#61	GO:0030151: molybdenum ion binding	0.001063	238,15,6
1p35a_#49	GO:0005524: ATP binding	0.001064	124,243,22
1i8aa_#2	GO:0003779: actin binding	0.001065	18,32,3

1imt_2#41	GO:0051082: unfolded protein binding	0.001071	17,34,3
1gky__#61	GO:0004190: aspartic-type endopeptidase activity	0.001071	25,23,3
1i6vd_#1283	GO:0008201: heparin binding	0.001071	141,24,6
1rypg_#159	GO:0016638: oxidoreductase activity, acting on the CH-NH2 group of donors	0.001071	34,17,3
1lci__#26	GO:0001584: rhodopsin-like receptor activity	0.001071	139,10,4
1lci__#26	GO:0008800: beta-lactamase activity	0.001071	139,10,4
1dt9a1#209	GO:0016668: oxidoreductase activity, acting on sulfur group of donors, NAD or NADP as acceptor	0.001072	113,12,4
1emsa2#165	GO:0016861: intramolecular oxidoreductase activity, interconverting aldoses and ketoses	0.001073	45,13,3
1jfua_#53	GO:0003899: DNA-directed RNA polymerase activity	0.001073	49,12,3
2bce__#178	GO:0005525: GTP binding	0.001073	12,49,3
1f3ba2#4	GO:0005085: guanyl-nucleotide exchange factor activity	0.001073	45,13,3
1c5y.1#B85	GO:0003729: mRNA binding	0.001073	45,13,3
1f8ra1#288	GO:0042802: protein self binding	0.001073	49,12,3
1kdj__#27	GO:0019838: growth factor binding	0.001074	67,9,3
1dfca4#1451	GO:0019838: growth factor binding	0.001074	67,9,3
1dp0a4#756	GO:0005524: ATP binding	0.001074	165,243,27
1ciy_2#298	GO:0005524: ATP binding	0.001075	31,243,9
2mpra_#190	GO:0003729: mRNA binding	0.001075	400,13,7
1cs1a_#82	GO:0030145: manganese ion binding	0.001077	195,38,9
1svb_1#358	GO:0003968: RNA-directed RNA polymerase activity	0.001078	10,14,2
1iray2#107	GO:0003682: chromatin binding	0.001078	14,10,2
1bx4a_#58	GO:0016814: hydrolase activity, acting on carbon-nitrogen (but not peptide) bonds, in cyclic amidines	0.001078	14,10,2
1evya2#38	GO:0004457: lactate dehydrogenase activity	0.001078	14,10,2
2shpa1#387	GO:0003916: DNA topoisomerase activity	0.001078	14,10,2
1fjsa_#84	GO:0003809: thrombin activity	0.001078	14,10,2
1d0ba_#191	GO:0016646: oxidoreductase activity, acting on the CH-NH group of donors, NAD or NADP as acceptor	0.001078	120,19,5
1brwa2#100	GO:0050660: FAD binding	0.001078	14,10,2
1e2fa_#121	GO:0016776: phosphotransferase activity, phosphate group as acceptor	0.001078	10,14,2
1vola2#239	GO:0004222: metalloendopeptidase activity	0.001078	120,19,5
1csn__#134	GO:0005066: transmembrane receptor protein tyrosine kinase signaling protein activity	0.001078	14,10,2
1i7da_#458	GO:0003809: thrombin activity	0.001078	14,10,2
1b6cb_#337	GO:0005066: transmembrane receptor protein tyrosine kinase signaling protein activity	0.001078	14,10,2
1znca_#167	GO:0005543: phospholipid binding	0.001078	10,14,2
1de4c3#165	GO:0016620: oxidoreductase activity, acting on the aldehyde or oxo group of donors, NAD or NADP as acceptor	0.001078	14,10,2
1jbwa2#189	GO:0016846: carbon-sulfur lyase activity	0.001078	14,10,2
1ayaa_#98	GO:0004714: transmembrane receptor protein tyrosine kinase activity	0.001078	10,14,2
1ayaa_#98	GO:0005543: phospholipid binding	0.001078	10,14,2
2napa2#292	GO:0016646: oxidoreductase activity, acting on the CH-NH group of donors, NAD or NADP as acceptor	0.001078	120,19,5
1j75a_#166	GO:0004896: hematopoietin/interferon-class (D200-domain) cytokine receptor activity	0.001078	120,19,5
2mpra_#190	GO:0003887: DNA-directed DNA polymerase activity	0.00108	400,20,9
1fo4a5#1002	GO:0005524: ATP binding	0.001083	93,243,18
2pia_1#54	GO:0051082: unfolded protein binding	0.001084	220,34,9
1aky_2#133	GO:0004556: alpha-amylase activity	0.001084	156,15,5
1ryp1_#20	GO:0003755: peptidyl-prolyl cis-trans isomerase activity	0.001084	125,11,4
1qj2b2#536	GO:0016616: oxidoreductase activity, acting on the CH-OH group of donors, NAD or NADP as acceptor	0.001084	22,59,4
1dnv__#354	GO:0003724: RNA helicase activity	0.001085	54,11,3
1e9xa_#62	GO:0005525: GTP binding	0.001086	45,49,5
1e8ya1#648	GO:0005525: GTP binding	0.001086	45,49,5
1g6oa_#116	GO:0015036: disulfide oxidoreductase activity	0.001087	358,22,9
1ak5_1#376	GO:0016836: hydro-lyase activity	0.001088	39,33,4
1ciy_3#243	GO:0015078: hydrogen ion transporter activity	0.001088	62,21,4
1bu7a_#353	GO:0015078: hydrogen ion transporter activity	0.001088	62,21,4
1a81a2#182	GO:0030145: manganese ion binding	0.00109	120,38,7
1huxa_#10	GO:0016814: hydrolase activity, acting on carbon-nitrogen (but not peptide) bonds, in cyclic amidines	0.001091	60,10,3
1e1aa_#178	GO:0008201: heparin binding	0.001091	54,24,4
1ekra_#114	GO:0004457: lactate dehydrogenase activity	0.001091	60,10,3
1ekra_#114	GO:0016814: hydrolase activity, acting on carbon-nitrogen (but not peptide) bonds, in cyclic amidines	0.001091	60,10,3
1a9xb2#1775	GO:0008757: S-adenosylmethionine-dependent methyltransferase activity	0.001091	54,24,4

1oaa__#14	GO:0008757: S-adenosylmethionine-dependent methyltransferase activity	0.001091	54,24,4
1xis__#285	GO:0008270: zinc ion binding	0.001091	21,108,5
1tns__#50	GO:0004197: cysteine-type endopeptidase activity	0.001092	6,24,2
1d5ya3#126	GO:0004197: cysteine-type endopeptidase activity	0.001092	6,24,2
1cfb_2#763	GO:0008201: heparin binding	0.001092	6,24,2
1f8ea_#129	GO:0008201: heparin binding	0.001092	6,24,2
1a3k__#199	GO:0005529: sugar binding	0.001095	15,39,3
1h61a_#176	GO:0004556: alpha-amylase activity	0.001095	39,15,3
1danh_#104	GO:0004896: hematopoietin/interferon-class (D200-domain) cytokine receptor activity	0.001097	183,19,6
1ftra2#187	GO:0000287: magnesium ion binding	0.001098	46,128,8
1hxxa_#39	GO:0016251: general RNA polymerase II transcription factor activity	0.001102	42,14,3
1abrb1#25	GO:0008083: growth factor activity	0.001102	14,42,3
1ovb__#195	GO:0030151: molybdenum ion binding	0.001102	89,15,4
1p35a_#98	GO:0005507: copper ion binding	0.001105	34,38,4
1rypg_#159	GO:0005507: copper ion binding	0.001105	34,38,4
1tuba1#239	GO:0005525: GTP binding	0.001106	120,49,8
1dr9a1#39	GO:0005516: calmodulin binding	0.001108	258,24,8
1aln_1#90	GO:0008270: zinc ion binding	0.00111	6,108,3
1epwa2#1282	GO:0008270: zinc ion binding	0.00111	6,108,3
1fmb__#88	GO:0008270: zinc ion binding	0.00111	6,108,3
1dssg1#4	GO:0000287: magnesium ion binding	0.00111	57,128,9
1f46a_#59	GO:0000287: magnesium ion binding	0.00111	106,128,13
1jj2j_#70	GO:0005524: ATP binding	0.001111	71,243,15
1dj0a2#183	GO:0004197: cysteine-type endopeptidase activity	0.001111	142,24,6
1boub_#7	GO:0004180: carboxypeptidase activity	0.001111	240,15,6
1qaxa2#307	GO:0005509: calcium ion binding	0.001112	46,160,9
1opy__#68	GO:0015082: di-, tri-valent inorganic cation transporter activity	0.001114	367,14,7
1opy__#68	GO:0046915: transition metal ion transporter activity	0.001114	367,14,7
1axn__#42	GO:0046983: protein dimerization activity	0.001114	78,17,4
1ibja_#271	GO:0008483: transaminase activity	0.001114	78,17,4
1hd2a_#47	GO:0016616: oxidoreductase activity, acting on the CH-OH group of donors, NAD or NADP as acceptor	0.001115	56,59,6
1f42a2#123	GO:0004867: serine-type endopeptidase inhibitor activity	0.001116	378,47,15
3pmga1#89	GO:0005507: copper ion binding	0.001117	4,38,2
1g0sa_#105	GO:0030145: manganese ion binding	0.001117	4,38,2
1eur__#68	GO:0005507: copper ion binding	0.001117	4,38,2
1g6oa_#116	GO:0005509: calcium ion binding	0.001117	358,160,35
1ekbb_#85	GO:0005525: GTP binding	0.001125	150,49,9
1iira_#242	GO:0016776: phosphotransferase activity, phosphate group as acceptor	0.001125	170,14,5
1stfi_#116	GO:0005524: ATP binding	0.001126	57,243,13
1ceo__#295	GO:0008810: cellulase activity	0.001127	8,18,2
1fmta2#151	GO:0003697: single-stranded DNA binding	0.001127	314,12,6
1euha_#220	GO:0016861: intramolecular oxidoreductase activity, interconverting aldoses and ketoses	0.001128	11,13,2
1ayoa_#55	GO:0019955: cytokine binding	0.001128	13,11,2
1ayoa_#55	GO:0003724: RNA helicase activity	0.001128	13,11,2
1qora2#233	GO:0016651: oxidoreductase activity, acting on NADH or NADPH	0.001128	11,13,2
1atia2#210	GO:0010181: FMN binding	0.001128	13,11,2
1g5va_#109	GO:0000049: tRNA binding	0.001128	11,13,2
1csn__#52	GO:0000049: tRNA binding	0.001128	11,13,2
1vns__#306	GO:0019201: nucleotide kinase activity	0.001128	11,13,2
1pbv__#140	GO:0019201: nucleotide kinase activity	0.001128	11,13,2
2ctc__#196	GO:0019201: nucleotide kinase activity	0.001128	11,13,2
1deua_#151	GO:0008408: 3'-5' exonuclease activity	0.001128	285,13,6
1ecsa_#69	GO:0016854: racemase and epimerase activity	0.001128	11,13,2
1atia2#189	GO:0000049: tRNA binding	0.001128	11,13,2
2tbva_#214	GO:0019955: cytokine binding	0.001128	13,11,2
2dnja_#5	GO:0005518: collagen binding	0.001128	11,13,2
1b3qa2#623	GO:0015078: hydrogen ion transporter activity	0.001129	301,21,8
2dnja_#97	GO:0008800: beta-lactamase activity	0.00113	141,10,4
1e87a_#174	GO:0004457: lactate dehydrogenase activity	0.00113	141,10,4
1ddja_#681	GO:0003916: DNA topoisomerase activity	0.001131	253,10,5
1e4ea2#215	GO:0046983: protein dimerization activity	0.001131	490,17,9
1evqa_#305	GO:0016799: hydrolase activity, hydrolyzing N-glycosyl compounds	0.001131	490,17,9
1cl1a_#184	GO:0005524: ATP binding	0.001133	64,243,14
1dp0a1#309	GO:0008083: growth factor activity	0.001133	53,42,5
1ciy_2#298	GO:0004674: protein serine/threonine kinase activity	0.001135	31,42,4

2dkb__#114	GO:0003887: DNA-directed DNA polymerase activity	0.001135	174,20,6
2dri__#115	GO:0019829: cation-transporting ATPase activity	0.001136	16,9,2
1a4ya_#104	GO:0030151: molybdenum ion binding	0.001136	241,15,6
1a4ya_#104	GO:0004180: carboxypeptidase activity	0.001136	241,15,6
1au1a_#58	GO:0005085: guanyl-nucleotide exchange factor activity	0.001138	105,13,4
1apme_#68	GO:0000049: tRNA binding	0.001138	105,13,4
1f5ma_#80	GO:0000049: tRNA binding	0.001138	105,13,4
1f5ma_#80	GO:0008080: N-acetyltransferase activity	0.001138	105,13,4
1a65a3#383	GO:0016668: oxidoreductase activity, acting on sulfur group of donors, NAD or NADP as acceptor	0.001139	50,12,3
1hq8a_#206	GO:0005507: copper ion binding	0.001139	329,38,12
3pah__#303	GO:0015078: hydrogen ion transporter activity	0.001142	28,21,3
3pah__#303	GO:0004601: peroxidase activity	0.001142	28,21,3
1ecpa_#156	GO:0016763: transferase activity, transferring pentosyl groups	0.001142	21,28,3
1pysb5#672	GO:0000287: magnesium ion binding	0.001142	36,128,7
1qo3c_#252	GO:0016763: transferase activity, transferring pentosyl groups	0.001142	21,28,3
1rlw__#43	GO:0004620: phospholipase activity	0.001143	31,19,3
1dhn__#115	GO:0004620: phospholipase activity	0.001143	31,19,3
1eova1#139	GO:0004190: aspartic-type endopeptidase activity	0.001143	99,23,5
1gox__#246	GO:0016616: oxidoreductase activity, acting on the CH-OH group of donors, NAD or NADP as acceptor	0.001143	177,59,11
1yrga_#194	GO:0016646: oxidoreductase activity, acting on the CH-NH group of donors, NAD or NADP as acceptor	0.001143	31,19,3
1nal1_#203	GO:0016861: intramolecular oxidoreductase activity, interconverting aldoses and ketoses	0.001144	46,13,3
1ftra2#187	GO:0000049: tRNA binding	0.001144	46,13,3
1wdcb_#141	GO:0005509: calcium ion binding	0.001144	9,160,4
1fqva2#201	GO:0005509: calcium ion binding	0.001144	9,160,4
1gsa_2#218	GO:0004812: tRNA ligase activity	0.001144	131,26,6
1qo0d_#81	GO:0016861: intramolecular oxidoreductase activity, interconverting aldoses and ketoses	0.001144	46,13,3
1nox__#88	GO:0019955: cytokine binding	0.001145	55,11,3
1hcd__#114	GO:0016702: oxidoreductase activity, acting on single donors with incorporation of molecular oxygen, incorporation of two atoms of oxygen	0.001145	12,12,2
1g93a_#229	GO:0015405: P-P-bond-hydrolysis-driven transporter activity	0.001145	12,12,2
1afj__#28	GO:0015405: P-P-bond-hydrolysis-driven transporter activity	0.001145	12,12,2
1akp__#62	GO:0003724: RNA helicase activity	0.001145	55,11,3
1a04a2#59	GO:0000155: two-component sensor molecule activity	0.001145	12,12,2
1hcl__#135	GO:0003899: DNA-directed RNA polymerase activity	0.001145	12,12,2
1jf9a_#379	GO:0005529: sugar binding	0.001146	232,39,10
1fn9a_#271	GO:0015036: disulfide oxidoreductase activity	0.001146	218,22,7
1dy9.1#C229	GO:0016668: oxidoreductase activity, acting on sulfur group of donors, NAD or NADP as acceptor	0.001146	115,12,4
1a9xa6#760	GO:0004725: protein tyrosine phosphatase activity	0.001149	90,15,4
1quna1#113	GO:0000049: tRNA binding	0.00115	286,13,6
3chbd_#85	GO:0005507: copper ion binding	0.001156	378,38,13
1c4zd_#22	GO:0004842: ubiquitin-protein ligase activity	0.001157	70,19,4
1ds1a_#172	GO:0051082: unfolded protein binding	0.001157	222,34,9
1zyma2#177	GO:0005351: sugar porter activity	0.001161	7,21,2
1b4va1#292	GO:0004601: peroxidase activity	0.001161	7,21,2
1c4xa_#35	GO:0004601: peroxidase activity	0.001161	7,21,2
1i7wa_#518	GO:0015078: hydrogen ion transporter activity	0.001161	7,21,2
1h6la_#120	GO:0015078: hydrogen ion transporter activity	0.001161	7,21,2
1c4xa_#19	GO:0004601: peroxidase activity	0.001161	7,21,2
1c4xa_#43	GO:0004601: peroxidase activity	0.001161	7,21,2
1dqsa_#138	GO:0016616: oxidoreductase activity, acting on the CH-OH group of donors, NAD or NADP as acceptor	0.001163	38,59,5
1eno__#83	GO:0016616: oxidoreductase activity, acting on the CH-OH group of donors, NAD or NADP as acceptor	0.001163	38,59,5
1bwza2#162	GO:0008757: S-adenosylmethionine-dependent methyltransferase activity	0.001163	95,24,5
2dpma_#237	GO:0008757: S-adenosylmethionine-dependent methyltransferase activity	0.001163	95,24,5
1eq9a_#40	GO:0005524: ATP binding	0.001163	86,243,17
1nbaa_#227	GO:0016831: carboxy-lyase activity	0.001164	91,25,5
1dih_2#222	GO:0003964: RNA-directed DNA polymerase activity	0.001164	352,11,6
1dfoa_#237	GO:0008483: transaminase activity	0.001167	35,17,3
3pah__#286	GO:0016836: hydro-lyase activity	0.001167	18,33,3
2gsta1#169	GO:0008810: cellulase activity	0.001167	33,18,3
1gc5a_#233	GO:0003924: GTPase activity	0.001167	35,17,3

1hxxa_#173	GO:0008201: heparin binding	0.001167	260,24,8
1et9a2#138	GO:0046983: protein dimerization activity	0.001167	35,17,3
1edha2#106	GO:0003924: GTPase activity	0.001167	35,17,3
1qdla_#211	GO:0003887: DNA-directed DNA polymerase activity	0.001168	320,20,8
1qhqa_#45	GO:0005525: GTP binding	0.001168	121,49,8
1pysb5#584	GO:0046983: protein dimerization activity	0.001169	79,17,4
1fbna_#144	GO:0016627: oxidoreductase activity, acting on the CH-CH group of donors	0.001169	79,17,4
1dkia_#185	GO:0046983: protein dimerization activity	0.001169	79,17,4
1jsg__#92	GO:0046983: protein dimerization activity	0.00117	607,17,10
1hxxa_#278	GO:0050660: FAD binding	0.001173	255,10,5
1ycsa_#162	GO:0016638: oxidoreductase activity, acting on the CH-NH2 group of donors	0.001173	210,17,6
1dbxa_#75	GO:0000287: magnesium ion binding	0.001173	69,128,10
1c8za_#386	GO:0005524: ATP binding	0.001174	235,243,35
1clxa_#120	GO:0016763: transferase activity, transferring pentosyl groups	0.001175	47,28,4
1qs1a2#437	GO:0030151: molybdenum ion binding	0.001175	452,15,8
1dfca1#1045	GO:0005529: sugar binding	0.001176	4,39,2
1byfa_#4	GO:0005529: sugar binding	0.001176	4,39,2
1g51a3#525	GO:0003968: RNA-directed RNA polymerase activity	0.001177	263,14,6
1lara2#1769	GO:0015036: disulfide oxidoreductase activity	0.001178	219,22,7
1qtn.1#A331	GO:0015036: disulfide oxidoreductase activity	0.001179	27,22,3
1seia_#50	GO:0019843: rRNA binding	0.001179	27,22,3
1f46a_#59	GO:0008080: N-acetyltransferase activity	0.001179	106,13,4
1aa6_2#285	GO:0019201: nucleotide kinase activity	0.001179	106,13,4
1ew2a_#237	GO:0008026: ATP-dependent helicase activity	0.001179	106,13,4
1yrga_#232	GO:0004556: alpha-amylase activity	0.00118	40,15,3
1cmia_#86	GO:0005179: hormone activity	0.00118	40,15,3
1c4ka1#33	GO:0005096: GTPase activator activity	0.00118	40,15,3
1cs1a_#160	GO:0019201: nucleotide kinase activity	0.00118	187,13,5
1cs1a_#160	GO:0016651: oxidoreductase activity, acting on NADH or NADPH	0.00118	187,13,5
1dvpa1#65	GO:0016861: intramolecular oxidoreductase activity, interconverting aldoses and ketoses	0.00118	187,13,5
1erv__#80	GO:0004263: chymotrypsin activity	0.00118	442,41,15
1qb7a_#86	GO:0004180: carboxypeptidase activity	0.00118	40,15,3
1wdna_#187	GO:0030151: molybdenum ion binding	0.00118	40,15,3
2hrva_#132	GO:0003968: RNA-directed RNA polymerase activity	0.001181	43,14,3
1evqa_#61	GO:0004556: alpha-amylase activity	0.001183	159,15,5
1bwda_#117	GO:0016702: oxidoreductase activity, acting on single donors with incorporation of molecular oxygen, incorporation of two atoms of oxygen	0.001184	116,12,4
1e25a_#262	GO:0042802: protein self binding	0.001184	116,12,4
1b5ta_#59	GO:0016831: carboxy-lyase activity	0.001185	6,25,2
1qmha2#181	GO:0016831: carboxy-lyase activity	0.001185	6,25,2
1rblm_#68	GO:0016831: carboxy-lyase activity	0.001185	6,25,2
1bfd_2#115	GO:0016831: carboxy-lyase activity	0.001185	6,25,2
1ej8a_#189	GO:0046983: protein dimerization activity	0.001185	294,17,7
1c4zd_#85	GO:0005524: ATP binding	0.001185	117,243,21
1g0sa_#74	GO:0046983: protein dimerization activity	0.001185	294,17,7
1el0a_#42	GO:0016638: oxidoreductase activity, acting on the CH-NH2 group of donors	0.001185	294,17,7
1i7qa_#321	GO:0004523: ribonuclease H activity	0.001185	148,16,5
1ido__#173	GO:0005351: sugar porter activity	0.001186	110,21,5
1j7la_#86	GO:0005524: ATP binding	0.001187	15,243,6
1e1oa1#142	GO:0005524: ATP binding	0.001187	15,243,6
1e25a_#262	GO:0003887: DNA-directed DNA polymerase activity	0.001189	116,20,5
1fi2a_#109	GO:0003724: RNA helicase activity	0.001192	228,11,5
1fi2a_#109	GO:0003964: RNA-directed DNA polymerase activity	0.001192	228,11,5
1d0na6#681	GO:0003684: damaged DNA binding	0.001192	143,10,4
2mev3_#108	GO:0019955: cytokine binding	0.001192	228,11,5
1flca1#294	GO:0003968: RNA-directed RNA polymerase activity	0.001193	98,14,4
1i9sa_#24	GO:0016831: carboxy-lyase activity	0.001193	53,25,4
6cel__#423	GO:0005525: GTP binding	0.001195	27,49,4
1smaa2#581	GO:0008083: growth factor activity	0.001195	80,42,6
1a0i_2#222	GO:0004896: hematopoietin/interferon-class (D200-domain) cytokine receptor activity	0.001196	186,19,6
2arca_#55	GO:0008201: heparin binding	0.001197	144,24,6
1jh2a_#16	GO:0030151: molybdenum ion binding	0.001198	91,15,4
1hrka_#343	GO:0016836: hydro-lyase activity	0.001199	40,33,4
2mpra_#190	GO:0003916: DNA topoisomerase activity	0.0012	400,10,6
2mpra_#190	GO:0016620: oxidoreductase activity, acting on the aldehyde or oxo group of donors, NAD or NADP as acceptor	0.0012	400,10,6

1avgi_#69	GO:0004867: serine-type endopeptidase inhibitor activity	0.0012	340,47,14
1pfza_#124	GO:0004812: tRNA ligase activity	0.001201	51,26,4
1je5a_#10	GO:0050660: FAD binding	0.001202	62,10,3
1e44b_#75	GO:0005507: copper ion binding	0.001202	198,38,9
1ciy_3#243	GO:0001584: rhodopsin-like receptor activity	0.001202	62,10,3
1kdj__#27	GO:0005126: hematopoietin/interferon-class (D200-domain) cytokine receptor binding	0.001202	67,20,4
1ie5a_#102	GO:0016620: oxidoreductase activity, acting on the aldehyde or oxo group of donors, NAD or NADP as acceptor	0.001202	62,10,3
1apme_#68	GO:0019843: rRNA binding	0.001204	105,22,5
1tiid_#39	GO:0016705: oxidoreductase activity, acting on paired donors, with incorporation or reduction of molecular oxygen	0.001205	239,26,8
1qfma1#139	GO:0042802: protein self binding	0.001206	318,12,6
1eny__#147	GO:0004222: metalloendopeptidase activity	0.001206	123,19,5
1eny__#147	GO:0016646: oxidoreductase activity, acting on the CH-NH group of donors, NAD or NADP as acceptor	0.001206	123,19,5
1xnb__#131	GO:0003697: single-stranded DNA binding	0.001207	51,12,3
1aym3_#109	GO:0003724: RNA helicase activity	0.001207	56,11,3
1h6va1#116	GO:0003887: DNA-directed DNA polymerase activity	0.00121	30,20,3
1qs0a1#119	GO:0030151: molybdenum ion binding	0.001211	454,15,8
1booa_#254	GO:0005524: ATP binding	0.001212	44,243,11
1ddwa_#77	GO:0008810: cellulase activity	0.001212	364,18,8
1jsg__#92	GO:0016702: oxidoreductase activity, acting on single donors with incorporation of molecular oxygen, incorporation of two atoms of oxygen	0.001215	607,12,8
1jsg__#92	GO:0016668: oxidoreductase activity, acting on sulfur group of donors, NAD or NADP as acceptor	0.001215	607,12,8
1e31a_#55	GO:0008757: S-adenosylmethionine-dependent methyltransferase activity	0.001216	25,24,3
1dbta_#89	GO:0016831: carboxy-lyase activity	0.001216	24,25,3
1bf4a_#4	GO:0005516: calmodulin binding	0.001216	25,24,3
1ep3a_#247	GO:0016831: carboxy-lyase activity	0.001216	24,25,3
1fwxa1#535	GO:0005509: calcium ion binding	0.001217	15,160,5
1aiha_#226	GO:0005509: calcium ion binding	0.001217	15,160,5
1aru__#57	GO:0005509: calcium ion binding	0.001217	15,160,5
1jb0d_#26	GO:0019955: cytokine binding	0.001217	229,11,5
1jsg__#92	GO:0008270: zinc ion binding	0.001217	607,108,38
1qqga2#166	GO:0004197: cysteine-type endopeptidase activity	0.001218	200,24,7
1fp1d2#214	GO:0016776: phosphotransferase activity, phosphate group as acceptor	0.001218	173,14,5
1kp6a_#77	GO:0003729: mRNA binding	0.001219	47,13,3
1ldtl_#34l	GO:0004867: serine-type endopeptidase inhibitor activity	0.001219	13,47,3
1b8ba_#466	GO:0005085: guanyl-nucleotide exchange factor activity	0.001219	47,13,3
1e31a_#77	GO:0016854: racemase and epimerase activity	0.001219	47,13,3
1f2aa_#27	GO:0030145: manganese ion binding	0.001219	89,38,6
1fjsa_#28	GO:0004867: serine-type endopeptidase inhibitor activity	0.001219	13,47,3
1a7ca_#370	GO:0008408: 3'-5' exonuclease activity	0.001219	47,13,3
1eq9a_#58	GO:0008026: ATP-dependent helicase activity	0.001219	47,13,3
1nsca_#236	GO:0008083: growth factor activity	0.001219	143,42,8
1nfdb1#21	GO:0008201: heparin binding	0.00122	96,24,5
1jjya_#329	GO:0004222: metalloendopeptidase activity	0.00122	71,19,4
2hft_1#34	GO:0005529: sugar binding	0.001221	34,39,4
1deua_#151	GO:0005507: copper ion binding	0.001222	285,38,11
1bif_2#398	GO:0009036: type II site-specific deoxyribonuclease activity	0.001223	117,12,4
1hxxa_#173	GO:0004896: hematopoietin/interferon-class (D200-domain) cytokine receptor activity	0.001224	260,19,7
1h6va1#27	GO:0016646: oxidoreductase activity, acting on the CH-NH group of donors, NAD or NADP as acceptor	0.001224	260,19,7
1smaa2#581	GO:0016638: oxidoreductase activity, acting on the CH-NH2 group of donors	0.001225	80,17,4
1c4ra_#258	GO:0016638: oxidoreductase activity, acting on the CH-NH2 group of donors	0.001225	80,17,4
1hxmb1#117	GO:0015078: hydrogen ion transporter activity	0.001227	64,21,4
2viua_#252	GO:0004556: alpha-amylase activity	0.001229	343,15,7
1epwa3#140	GO:0004896: hematopoietin/interferon-class (D200-domain) cytokine receptor activity	0.00123	187,19,6
1qqsa_#35	GO:0005524: ATP binding	0.001234	94,243,18
1g0sa_#74	GO:0019838: growth factor binding	0.001235	294,9,5
1imva_#255	GO:0004812: tRNA ligase activity	0.001236	589,26,13
1edha2#106	GO:0005507: copper ion binding	0.001236	35,38,4
2cba__#144	GO:0051082: unfolded protein binding	0.001238	67,34,5
1cg2a1#43	GO:0004177: aminopeptidase activity	0.001239	189,13,5
1aiha_#226	GO:0016814: hydrolase activity, acting on carbon-nitrogen (but not peptide) bonds, in cyclic amidines	0.001241	15,10,2

1ddja_#640	GO:0003809: thrombin activity	0.001241	15,10,2
1g55a_#68	GO:0030151: molybdenum ion binding	0.001241	10,15,2
1rypf_#50	GO:0016620: oxidoreductase activity, acting on the aldehyde or oxo group of donors, NAD or NADP as acceptor	0.001241	15,10,2
1bdfa2#121	GO:0004556: alpha-amylase activity	0.001241	10,15,2
1fjfj_#68	GO:0003755: peptidyl-prolyl cis-trans isomerase activity	0.001241	230,11,5
1dmua_#199	GO:0004725: protein tyrosine phosphatase activity	0.001241	10,15,2
1dmua_#199	GO:0005179: hormone activity	0.001241	10,15,2
1qhoa1#531	GO:0004556: alpha-amylase activity	0.001241	10,15,2
2tgi__#106	GO:0005179: hormone activity	0.001241	10,15,2
1i2ma_#90	GO:0003682: chromatin binding	0.001241	15,10,2
2liv__#81	GO:0001584: rhodopsin-like receptor activity	0.001241	15,10,2
1ayaa_#98	GO:0004725: protein tyrosine phosphatase activity	0.001241	10,15,2
1qfta_#98	GO:0019955: cytokine binding	0.001241	230,11,5
1nfdb1#34	GO:0005126: hematopoietin/interferon-class (D200-domain) cytokine receptor binding	0.001243	177,20,6
1eny__#147	GO:0016763: transferase activity, transferring pentosyl groups	0.001244	123,28,6
1f0xa1#523	GO:0005529: sugar binding	0.001245	87,39,6
1qdla_#211	GO:0016668: oxidoreductase activity, acting on sulfur group of donors, NAD or NADP as acceptor	0.001248	320,12,6
1thfd_#140	GO:0004896: hematopoietin/interferon-class (D200-domain) cytokine receptor activity	0.001251	124,19,5
1dl5a_#266	GO:0000287: magnesium ion binding	0.001252	27,128,6
1gtxa_#150	GO:0000287: magnesium ion binding	0.001252	27,128,6
1aym3_#109	GO:0004197: cysteine-type endopeptidase activity	0.001252	56,24,4
1dyoa_#153	GO:0030151: molybdenum ion binding	0.001252	161,15,5
1kapp1#358	GO:0004222: metalloendopeptidase activity	0.001255	32,19,3
1kapp1#358	GO:0004896: hematopoietin/interferon-class (D200-domain) cytokine receptor activity	0.001255	32,19,3
1neb__#52	GO:0003779: actin binding	0.001255	19,32,3
1ayl__#237	GO:0019955: cytokine binding	0.001256	357,11,6
1ayl__#237	GO:0003755: peptidyl-prolyl cis-trans isomerase activity	0.001256	357,11,6
1biha3#248	GO:0004896: hematopoietin/interferon-class (D200-domain) cytokine receptor activity	0.001258	8,19,2
1hr6a1#59	GO:0004222: metalloendopeptidase activity	0.001258	8,19,2
1dosa_#144	GO:0004620: phospholipase activity	0.001258	8,19,2
2viua_#123	GO:0004896: hematopoietin/interferon-class (D200-domain) cytokine receptor activity	0.001258	8,19,2
1rypk_#76	GO:0004222: metalloendopeptidase activity	0.001258	8,19,2
1hbza_#214	GO:0046983: protein dimerization activity	0.001259	297,17,7
1hbza_#214	GO:0016638: oxidoreductase activity, acting on the CH-NH2 group of donors	0.001259	297,17,7
1nat__#50	GO:0008483: transaminase activity	0.001259	297,17,7
1fhoa_#74	GO:0004190: aspartic-type endopeptidase activity	0.00126	347,23,9
2cb5a_#373	GO:0003968: RNA-directed RNA polymerase activity	0.001263	44,14,3
1axca1#70	GO:0003899: DNA-directed RNA polymerase activity	0.001263	118,12,4
1a7ta_#80	GO:0008270: zinc ion binding	0.001265	13,108,4
1ejda_#377	GO:0008235: metalloexopeptidase activity	0.001265	108,13,4
1ea5a_#99	GO:0005529: sugar binding	0.001267	194,39,9
1wht.1#A15	GO:0004263: chymotrypsin activity	0.001269	15,41,3
1c8ba_#514	GO:0003924: GTPase activity	0.001269	36,17,3
1g2oa_#227	GO:0030151: molybdenum ion binding	0.001269	41,15,3
1ifc__#89	GO:0000049: tRNA binding	0.001269	190,13,5
1d0va_#46	GO:0016627: oxidoreductase activity, acting on the CH-CH group of donors	0.001269	36,17,3
1cbf__#208	GO:0005507: copper ion binding	0.00127	333,38,12
2fmr__#10	GO:0019955: cytokine binding	0.001272	57,11,3
1bfd_1#230	GO:0000287: magnesium ion binding	0.001272	47,128,8
1h6kx_#97	GO:0004364: glutathione transferase activity	0.001272	57,11,3
1dt9a1#224	GO:0016763: transferase activity, transferring pentosyl groups	0.001273	48,28,4
3grx__#52	GO:0015036: disulfide oxidoreductase activity	0.001275	7,22,2
1i50a_#1115	GO:0051082: unfolded protein binding	0.001275	18,34,3
3kvt__#53	GO:0015036: disulfide oxidoreductase activity	0.001275	7,22,2
1feca3#410	GO:0015036: disulfide oxidoreductase activity	0.001275	7,22,2
1jj2q_#118	GO:0019843: rRNA binding	0.001275	7,22,2
1feua_#6	GO:0019843: rRNA binding	0.001275	7,22,2
1ljra1#165	GO:0008810: cellulase activity	0.001275	34,18,3
1h75a_#33	GO:0015036: disulfide oxidoreductase activity	0.001275	7,22,2
1d66a1#28	GO:0019843: rRNA binding	0.001275	7,22,2
1g6oa_#116	GO:0003724: RNA helicase activity	0.001276	358,11,6
1exh__#44	GO:0003899: DNA-directed RNA polymerase activity	0.001277	52,12,3
1jlna_#383	GO:0016866: intramolecular transferase activity	0.001277	52,12,3

1ib2a_#974	GO:0016702: oxidoreductase activity, acting on single donors with incorporation of molecular oxygen, incorporation of two atoms of oxygen	0.001277	52,12,3
1ds1a_#172	GO:0015036: disulfide oxidoreductase activity	0.001278	222,22,7
1hdfa_#16	GO:0004263: chymotrypsin activity	0.00128	223,41,10
1f97a1#62	GO:0003887: DNA-directed DNA polymerase activity	0.001281	707,20,12
1cmxa_#188	GO:0004812: tRNA ligase activity	0.001283	6,26,2
1dt6a_#376	GO:0016705: oxidoreductase activity, acting on paired donors, with incorporation or reduction of molecular oxygen	0.001283	6,26,2
1tpg_1#55	GO:0016705: oxidoreductase activity, acting on paired donors, with incorporation or reduction of molecular oxygen	0.001283	6,26,2
1gpc__#58	GO:0004812: tRNA ligase activity	0.001283	6,26,2
1rblm_#68	GO:0004497: monooxygenase activity	0.001283	6,26,2
1a8h_2#133	GO:0004812: tRNA ligase activity	0.001283	6,26,2
1bob__#56	GO:0004812: tRNA ligase activity	0.001283	6,26,2
1dz4a_#93	GO:0016705: oxidoreductase activity, acting on paired donors, with incorporation or reduction of molecular oxygen	0.001283	6,26,2
1elua_#288	GO:0016705: oxidoreductase activity, acting on paired donors, with incorporation or reduction of molecular oxygen	0.001283	6,26,2
1h6va1#27	GO:0004457: lactate dehydrogenase activity	0.001283	260,10,5
1fjfl_#67	GO:0004714: transmembrane receptor protein tyrosine kinase activity	0.001284	175,14,5
1aqb__#134	GO:0046983: protein dimerization activity	0.001284	81,17,4
1aqb__#134	GO:0016638: oxidoreductase activity, acting on the CH-NH2 group of donors	0.001284	81,17,4
1f5na1#427	GO:0016627: oxidoreductase activity, acting on the CH-CH group of donors	0.001286	9,17,2
2knt__#34	GO:0008200: ion channel inhibitor activity	0.001286	9,17,2
1g72a_#42	GO:0016638: oxidoreductase activity, acting on the CH-NH2 group of donors	0.001286	9,17,2
1eno__#290	GO:0016627: oxidoreductase activity, acting on the CH-CH group of donors	0.001286	9,17,2
1qg6a_#252	GO:0016627: oxidoreductase activity, acting on the CH-CH group of donors	0.001286	9,17,2
1e5ma1#159	GO:0003924: GTPase activity	0.001286	9,17,2
1c3d__#72	GO:0016627: oxidoreductase activity, acting on the CH-CH group of donors	0.001286	9,17,2
1qqsa_#124	GO:0030151: molybdenum ion binding	0.001286	458,15,8
1ev2e1#154	GO:0016638: oxidoreductase activity, acting on the CH-NH2 group of donors	0.001286	9,17,2
1d3ga_#109	GO:0016627: oxidoreductase activity, acting on the CH-CH group of donors	0.001286	9,17,2
1f97a1#62	GO:0008235: metalloexopeptidase activity	0.001287	707,13,9
1h8ma_#8	GO:0004812: tRNA ligase activity	0.001288	134,26,6
1atg__#144	GO:0000287: magnesium ion binding	0.001288	82,128,11
1aym3_#131	GO:0003729: mRNA binding	0.001289	412,13,7
1i6vd_#1283	GO:0046983: protein dimerization activity	0.00129	141,17,5
2dnja_#97	GO:0016638: oxidoreductase activity, acting on the CH-NH2 group of donors	0.00129	141,17,5
1f0ia1#50	GO:0016620: oxidoreductase activity, acting on the aldehyde or oxo group of donors, NAD or NADP as acceptor	0.00129	146,10,4
1ycsb1#412	GO:0001584: rhodopsin-like receptor activity	0.00129	146,10,4
2pola2#218	GO:0050660: FAD binding	0.00129	146,10,4
1jf9a_#379	GO:0019955: cytokine binding	0.001291	232,11,5
1xgsa2#82	GO:0004263: chymotrypsin activity	0.001291	307,41,12
1eaja_#59	GO:0008201: heparin binding	0.001292	202,24,7
1fm2.1#B308	GO:0005507: copper ion binding	0.001292	200,38,9
1kapp2#69	GO:0004812: tRNA ligase activity	0.001293	52,26,4
1d3bb_#31	GO:0004812: tRNA ligase activity	0.001293	52,26,4
1ekma1#545	GO:0005507: copper ion binding	0.001293	90,38,6
2mev3_#108	GO:0005509: calcium ion binding	0.001294	228,160,25
1ldtl_#34I	GO:0004295: trypsin activity	0.001297	13,48,3
1qj2c2#50	GO:0008026: ATP-dependent helicase activity	0.001297	48,13,3
1dgsa1#523	GO:0000049: tRNA binding	0.001297	48,13,3
1dt9a1#224	GO:0019201: nucleotide kinase activity	0.001297	48,13,3
1bvoa_#206	GO:0005518: collagen binding	0.001297	48,13,3
1eu8a_#119	GO:0000049: tRNA binding	0.001297	48,13,3
1eu8a_#119	GO:0003729: mRNA binding	0.001297	48,13,3
1eu8a_#119	GO:0008408: 3'-5' exonuclease activity	0.001297	48,13,3
1je5a_#106	GO:0003729: mRNA binding	0.001297	48,13,3
1io1a_#233	GO:0005525: GTP binding	0.001298	95,49,7
1psda3#344	GO:0004842: ubiquitin-protein ligase activity	0.001298	125,19,5
1ez0a_#133	GO:0004896: hematopoietin/interferon-class (D200-domain) cytokine receptor activity	0.001298	125,19,5
1d8db_#78	GO:0005509: calcium ion binding	0.001298	66,160,11
2dpma_#237	GO:0005525: GTP binding	0.001298	95,49,7
1hwxa1#223	GO:0016836: hydro-lyase activity	0.001299	233,33,9
1bd2d1#62	GO:0004263: chymotrypsin activity	0.0013	4,41,2

1xnb__#131	GO:0005524: ATP binding	0.001302	51,243,12
3prn__#143	GO:0005507: copper ion binding	0.001304	334,38,12
1qfxa_#380	GO:0005509: calcium ion binding	0.001308	47,160,9
1czya1#466	GO:0000049: tRNA binding	0.00131	109,13,4
2hlca_#54	GO:0008026: ATP-dependent helicase activity	0.00131	109,13,4
1ycsa_#162	GO:0003899: DNA-directed RNA polymerase activity	0.001311	210,12,5
1i50b_#966	GO:0016638: oxidoreductase activity, acting on the CH-NH2 group of donors	0.001311	299,17,7
1g51a3#525	GO:0004896: hematopoietin/interferon-class (D200-domain) cytokine receptor activity	0.001311	263,19,7
1bev1_#86	GO:0000155: two-component sensor molecule activity	0.001312	323,12,6
1bev1_#86	GO:0016668: oxidoreductase activity, acting on sulfur group of donors, NAD or NADP as acceptor	0.001312	323,12,6
1h8ma_#70	GO:0003755: peptidyl-prolyl cis-trans isomerase activity	0.001313	14,11,2
1apme_#168	GO:0004714: transmembrane receptor protein tyrosine kinase activity	0.001313	11,14,2
1ir3a_#1056	GO:0004714: transmembrane receptor protein tyrosine kinase activity	0.001313	11,14,2
1fh6a_#341	GO:0016251: general RNA polymerase II transcription factor activity	0.001313	11,14,2
1ayl__#482	GO:0004714: transmembrane receptor protein tyrosine kinase activity	0.001313	11,14,2
1hhsa_#328	GO:0003964: RNA-directed DNA polymerase activity	0.001313	14,11,2
1fmk_3#428	GO:0004714: transmembrane receptor protein tyrosine kinase activity	0.001313	11,14,2
1qq9a_#100	GO:0010181: FMN binding	0.001313	14,11,2
1vns__#306	GO:0016776: phosphotransferase activity, phosphate group as acceptor	0.001313	11,14,2
1sek__#52	GO:0016251: general RNA polymerase II transcription factor activity	0.001313	11,14,2
1pbv__#140	GO:0016776: phosphotransferase activity, phosphate group as acceptor	0.001313	11,14,2
1dt6a_#449	GO:0010181: FMN binding	0.001313	14,11,2
1howa_#618	GO:0005543: phospholipid binding	0.001313	11,14,2
2ctc__#196	GO:0016776: phosphotransferase activity, phosphate group as acceptor	0.001313	11,14,2
1de4c3#165	GO:0010181: FMN binding	0.001313	14,11,2
1cnza_#19	GO:0010181: FMN binding	0.001313	14,11,2
1eur__#118	GO:0003968: RNA-directed RNA polymerase activity	0.001313	11,14,2
1hrna_#38	GO:0003964: RNA-directed DNA polymerase activity	0.001313	14,11,2
1lucb_#32	GO:0016763: transferase activity, transferring pentosyl groups	0.001314	22,28,3
1fepa_#49	GO:0000287: magnesium ion binding	0.001315	70,128,10
1fd9a_#162	GO:0004523: ribonuclease H activity	0.001315	87,16,4
1dkra2#210	GO:0008270: zinc ion binding	0.001317	32,108,6
1fhoa_#74	GO:0004725: protein tyrosine phosphatase activity	0.001318	347,15,7
1gdha2#47	GO:0005524: ATP binding	0.001322	38,243,10
1jfra_#83	GO:0005524: ATP binding	0.001322	38,243,10
1qqsa_#124	GO:0003697: single-stranded DNA binding	0.001323	458,12,7
1qqsa_#124	GO:0016668: oxidoreductase activity, acting on sulfur group of donors, NAD or NADP as acceptor	0.001323	458,12,7
1qfma1#186	GO:0005126: hematopoietin/interferon-class (D200-domain) cytokine receptor binding	0.001323	326,20,8
1c39a_#49	GO:0051082: unfolded protein binding	0.001324	68,34,5
1xgsa2#82	GO:0005509: calcium ion binding	0.001327	307,160,31
1fu6a_#50	GO:0004263: chymotrypsin activity	0.001329	308,41,12
1eq9a_#32	GO:0003755: peptidyl-prolyl cis-trans isomerase activity	0.001331	132,11,4
1cbf__#208	GO:0008201: heparin binding	0.001331	333,24,9
1ig8a_#203	GO:0005524: ATP binding	0.001331	65,243,14
1g0sa_#74	GO:0000049: tRNA binding	0.001333	294,13,6
1g0sa_#74	GO:0008408: 3'-5' exonuclease activity	0.001333	294,13,6
1el0a_#42	GO:0000049: tRNA binding	0.001333	294,13,6
1a65a1#108	GO:0000049: tRNA binding	0.001333	294,13,6
1f42a2#123	GO:0004714: transmembrane receptor protein tyrosine kinase activity	0.001334	378,14,7
1f42a2#123	GO:0003968: RNA-directed RNA polymerase activity	0.001334	378,14,7
1ce7a_#151	GO:0005529: sugar binding	0.001335	16,39,3
1gsa_2#218	GO:0005509: calcium ion binding	0.001336	131,160,17
1dkia_#76P	GO:0019955: cytokine binding	0.001338	58,11,3
1i50a_#469	GO:0005524: ATP binding	0.001338	58,243,13
1ifc__#89	GO:0004842: ubiquitin-protein ligase activity	0.001338	190,19,6
5ruba1#279	GO:0008757: S-adenosylmethionine-dependent methyltransferase activity	0.001339	57,24,4
3lada1#116	GO:0016616: oxidoreductase activity, acting on the CH-OH group of donors, NAD or NADP as acceptor	0.001342	153,59,10
1vola2#239	GO:0003899: DNA-directed RNA polymerase activity	0.001345	120,12,4
1jj2e1#42	GO:0019838: growth factor binding	0.001345	167,9,4
1dzfa2#211	GO:0019838: growth factor binding	0.001345	167,9,4
1mek__#17	GO:0005524: ATP binding	0.001345	26,243,8
1thg__#223	GO:0003924: GTPase activity	0.001345	82,17,4
1j75a_#166	GO:0003899: DNA-directed RNA polymerase activity	0.001345	120,12,4

1autc_#45	GO:0003968: RNA-directed RNA polymerase activity	0.001349	45,14,3
16pk__#312	GO:0005085: guanyl-nucleotide exchange factor activity	0.00135	12,13,2
1jeta_#314	GO:0000049: tRNA binding	0.00135	12,13,2
1g6ha_#180	GO:0008235: metalloexopeptidase activity	0.00135	12,13,2
1i8da1#85	GO:0016651: oxidoreductase activity, acting on NADH or NADPH	0.00135	12,13,2
1e0na_#28	GO:0003697: single-stranded DNA binding	0.00135	13,12,2
1e4ia_#215	GO:0016854: racemase and epimerase activity	0.00135	12,13,2
1bu7a_#130	GO:0016651: oxidoreductase activity, acting on NADH or NADPH	0.00135	12,13,2
1ajsa_#389	GO:0000049: tRNA binding	0.00135	12,13,2
1d8wa_#325	GO:0016861: intramolecular oxidoreductase activity, interconverting aldoses and ketoses	0.00135	12,13,2
1pysb5#523	GO:0000049: tRNA binding	0.00135	12,13,2
1pysb6#384	GO:0000049: tRNA binding	0.00135	12,13,2
1i6vc_#689	GO:0003899: DNA-directed RNA polymerase activity	0.00135	13,12,2
1f2da_#94	GO:0016651: oxidoreductase activity, acting on NADH or NADPH	0.00135	12,13,2
1jf9a_#183	GO:0016861: intramolecular oxidoreductase activity, interconverting aldoses and ketoses	0.00135	12,13,2
1c7qa_#181	GO:0016861: intramolecular oxidoreductase activity, interconverting aldoses and ketoses	0.00135	12,13,2
1a7j__#87	GO:0005525: GTP binding	0.001353	70,49,6
1g51a3#525	GO:0008800: beta-lactamase activity	0.001353	263,10,5
1dfma_#128	GO:0000287: magnesium ion binding	0.001354	37,128,7
1czan1#92	GO:0015036: disulfide oxidoreductase activity	0.001354	631,22,12
1mpp__#151	GO:0004725: protein tyrosine phosphatase activity	0.001354	94,15,4
1jj2a1#126	GO:0004896: hematopoietin/interferon-class (D200-domain) cytokine receptor activity	0.001355	73,19,4
1a65a1#108	GO:0005509: calcium ion binding	0.001355	294,160,30
1mrp__#172	GO:0016854: racemase and epimerase activity	0.001356	110,13,4
1qqga2#253	GO:0000049: tRNA binding	0.001356	110,13,4
3prn__#143	GO:0008201: heparin binding	0.00136	334,24,9
1g51a3#524	GO:0051082: unfolded protein binding	0.00136	102,34,6
1gsoa3#142	GO:0008083: growth factor activity	0.001361	82,42,6
1hxxa_#39	GO:0004556: alpha-amylase activity	0.001362	42,15,3
1ctqa_#116	GO:0005096: GTPase activator activity	0.001362	42,15,3
2mpra_#190	GO:0004263: chymotrypsin activity	0.001362	400,41,14
1g51a1#21	GO:0030151: molybdenum ion binding	0.001362	42,15,3
1dyna_#42	GO:0004725: protein tyrosine phosphatase activity	0.001362	164,15,5
1b8pa1#30	GO:0016616: oxidoreductase activity, acting on the CH-OH group of donors, NAD or NADP as acceptor	0.001364	3,59,2
1fmca_#70	GO:0016616: oxidoreductase activity, acting on the CH-OH group of donors, NAD or NADP as acceptor	0.001364	3,59,2
1ji6a2#497	GO:0016616: oxidoreductase activity, acting on the CH-OH group of donors, NAD or NADP as acceptor	0.001364	3,59,2
1gg1a_#209	GO:0016616: oxidoreductase activity, acting on the CH-OH group of donors, NAD or NADP as acceptor	0.001364	3,59,2
1qi9a_#217	GO:0016616: oxidoreductase activity, acting on the CH-OH group of donors, NAD or NADP as acceptor	0.001364	3,59,2
1gega_#93	GO:0016616: oxidoreductase activity, acting on the CH-OH group of donors, NAD or NADP as acceptor	0.001364	3,59,2
1e0ca1#49	GO:0016616: oxidoreductase activity, acting on the CH-OH group of donors, NAD or NADP as acceptor	0.001364	3,59,2
1e3ja1#319	GO:0016616: oxidoreductase activity, acting on the CH-OH group of donors, NAD or NADP as acceptor	0.001364	3,59,2
1dn0b2#214	GO:0008083: growth factor activity	0.001365	4,42,2
1kwaa_#569	GO:0008083: growth factor activity	0.001365	4,42,2
1ia8a_#89	GO:0004674: protein serine/threonine kinase activity	0.001365	4,42,2
1hcnb_#32	GO:0008083: growth factor activity	0.001365	4,42,2
1ir3a_#1003	GO:0004674: protein serine/threonine kinase activity	0.001365	4,42,2
1iray1#30	GO:0008083: growth factor activity	0.001365	4,42,2
1f45b_#63	GO:0008083: growth factor activity	0.001365	4,42,2
1b3qa2#623	GO:0003924: GTPase activity	0.001365	301,17,7
1b3qa2#623	GO:0016638: oxidoreductase activity, acting on the CH-NH2 group of donors	0.001365	301,17,7
1dbta_#89	GO:0004812: tRNA ligase activity	0.001367	24,26,3
1abrb1#71	GO:0008201: heparin binding	0.001367	26,24,3
1lml__#216	GO:0008270: zinc ion binding	0.001368	22,108,5
1bd0a1#276	GO:0005507: copper ion binding	0.00137	91,38,6
1jh2a_#16	GO:0005507: copper ion binding	0.00137	91,38,6
1c8za_#386	GO:0003724: RNA helicase activity	0.00137	235,11,5
2dkb__#133	GO:0016836: hydro-lyase activity	0.001375	19,33,3
1fkna_#63	GO:0016638: oxidoreductase activity, acting on the CH-NH2 group of donors	0.001375	37,17,3

1im4a_#153	GO:0016638: oxidoreductase activity, acting on the CH-NH2 group of donors	0.001375	37,17,3
1b37a1#209	GO:0016836: hydro-lyase activity	0.001375	19,33,3
1imva_#255	GO:0030151: molybdenum ion binding	0.001375	589,15,9
1b3ra2#47	GO:0003924: GTPase activity	0.001375	37,17,3
1dvpa1#65	GO:0016705: oxidoreductase activity, acting on paired donors, with incorporation or reduction of molecular oxygen	0.001375	187,26,7
1e6ua_#10	GO:0016646: oxidoreductase activity, acting on the CH-NH group of donors, NAD or NADP as acceptor	0.001375	33,19,3
1hdma1#98	GO:0016638: oxidoreductase activity, acting on the CH-NH2 group of donors	0.001375	37,17,3
1a65a1#108	GO:0019843: rRNA binding	0.001375	294,22,8
1ijqa1#564	GO:0004896: hematopoietin/interferon-class (D200-domain) cytokine receptor activity	0.001375	33,19,3
1heta2#212	GO:0016763: transferase activity, transferring pentosyl groups	0.001376	49,28,4
1pysb5#672	GO:0030145: manganese ion binding	0.001376	36,38,4
1h7wa2#636	GO:0016763: transferase activity, transferring pentosyl groups	0.001376	49,28,4
1cewi_#14	GO:0004295: trypsin activity	0.001377	98,48,7
1heta2#212	GO:0016854: racemase and epimerase activity	0.001377	49,13,3
1fzqa_#28	GO:0005085: guanyl-nucleotide exchange factor activity	0.001377	49,13,3
1h7wa2#636	GO:0016854: racemase and epimerase activity	0.001377	49,13,3
1qfma1#186	GO:0003697: single-stranded DNA binding	0.001379	326,12,6
1h8ua_#113	GO:0016814: hydrolase activity, acting on carbon-nitrogen (but not peptide) bonds, in cyclic amidines	0.00138	65,10,3
1jj2a1#126	GO:0019838: growth factor binding	0.00138	73,9,3
1qo2a_#222	GO:0004457: lactate dehydrogenase activity	0.00138	65,10,3
1foha5#9	GO:0050660: FAD binding	0.00138	65,10,3
1ewqa3#159	GO:0005524: ATP binding	0.001381	32,243,9
1eula_#518	GO:0005524: ATP binding	0.001381	32,243,9
1kit_3#743	GO:0030151: molybdenum ion binding	0.001381	250,15,6
1e1oa2#423	GO:0004197: cysteine-type endopeptidase activity	0.001382	148,24,6
1imva_#255	GO:0003809: thrombin activity	0.001383	589,10,7
1imva_#255	GO:0003916: DNA topoisomerase activity	0.001383	589,10,7
1imva_#255	GO:0016620: oxidoreductase activity, acting on the aldehyde or oxo group of donors, NAD or NADP as acceptor	0.001383	589,10,7
1hbza_#96	GO:0015036: disulfide oxidoreductase activity	0.001385	370,22,9
1a81a2#182	GO:0003887: DNA-directed DNA polymerase activity	0.001387	120,20,5
1ax4a_#275	GO:0003968: RNA-directed RNA polymerase activity	0.001387	102,14,4
2pia_1#99	GO:0000287: magnesium ion binding	0.001388	179,128,18
1ryph_#163	GO:0003899: DNA-directed RNA polymerase activity	0.001388	121,12,4
1qhqa_#45	GO:0009036: type II site-specific deoxyribonuclease activity	0.001388	121,12,4
1dfca4#1469	GO:0004812: tRNA ligase activity	0.001389	53,26,4
1beba_#119	GO:0005524: ATP binding	0.001389	202,243,31
5ruba1#259	GO:0004497: monooxygenase activity	0.001389	53,26,4
1qopb_#89	GO:0016836: hydro-lyase activity	0.00139	5,33,2
1b0xa_#961	GO:0016836: hydro-lyase activity	0.00139	5,33,2
1hf2a2#97	GO:0016836: hydro-lyase activity	0.00139	5,33,2
3grx__#56	GO:0008201: heparin binding	0.00139	335,24,9
1e8ga2#201	GO:0000287: magnesium ion binding	0.001391	304,128,26
1boub_#7	GO:0000287: magnesium ion binding	0.001394	240,128,22
1e2o__#346	GO:0005518: collagen binding	0.001395	194,13,5
1ea5a_#99	GO:0008408: 3'-5' exonuclease activity	0.001395	194,13,5
1sfp__#84	GO:0005126: hematopoietin/interferon-class (D200-domain) cytokine receptor binding	0.001396	8,20,2
1f2la_#40	GO:0019955: cytokine binding	0.001397	236,11,5
1epwa3#138	GO:0005529: sugar binding	0.001399	424,39,14
1dzfa2#211	GO:0005509: calcium ion binding	0.0014	167,160,20
1dp0a4#756	GO:0004556: alpha-amylase activity	0.0014	165,15,5
1bu7a_#278	GO:0004601: peroxidase activity	0.001402	30,21,3
1io1a_#233	GO:0005524: ATP binding	0.001402	95,243,18
1h6va1#116	GO:0015078: hydrogen ion transporter activity	0.001402	30,21,3
1bwza2#162	GO:0005524: ATP binding	0.001402	95,243,18
2cb5a_#312	GO:0004177: aminopeptidase activity	0.001403	111,13,4
2cb5a_#312	GO:0008080: N-acetyltransferase activity	0.001403	111,13,4
1ho1a_#211	GO:0016854: racemase and epimerase activity	0.001403	111,13,4
1bura1#328	GO:0004497: monooxygenase activity	0.001404	91,26,5
2sli_2#326	GO:0005126: hematopoietin/interferon-class (D200-domain) cytokine receptor binding	0.001405	251,20,7
1zfja1#379	GO:0016616: oxidoreductase activity, acting on the CH-OH group of donors, NAD or NADP as acceptor	0.001406	11,59,3
1bwvs_#117	GO:0003887: DNA-directed DNA polymerase activity	0.001406	329,20,8

1hq8a_#206	GO:0005126: hematopoietin/interferon-class (D200-domain) cytokine receptor binding	0.001406	329,20,8
1g8kb_#11	GO:0003724: RNA helicase activity	0.001406	59,11,3
2pia_1#54	GO:0008083: growth factor activity	0.001407	220,42,10
1a8d_1#87	GO:0005509: calcium ion binding	0.001407	204,160,23
1ecpa_#58	GO:0003924: GTPase activity	0.001407	83,17,4
1nat__#50	GO:0016651: oxidoreductase activity, acting on NADH or NADPH	0.001407	297,13,6
1kit_2#398	GO:0046983: protein dimerization activity	0.001407	83,17,4
1kit_2#398	GO:0016638: oxidoreductase activity, acting on the CH-NH2 group of donors	0.001407	83,17,4
1h8ma_#8	GO:0003755: peptidyl-prolyl cis-trans isomerase activity	0.001409	134,11,4
1qrra_#69	GO:0016831: carboxy-lyase activity	0.001409	196,25,7
1bwza2#162	GO:0004725: protein tyrosine phosphatase activity	0.001409	95,15,4
1aym3_#131	GO:0003809: thrombin activity	0.00141	412,10,6
1stfi_#116	GO:0004263: chymotrypsin activity	0.001413	57,41,5
1qhda2#323	GO:0005507: copper ion binding	0.001413	337,38,12
1iba__#39	GO:0004725: protein tyrosine phosphatase activity	0.001413	351,15,7
1h6kx_#97	GO:0004263: chymotrypsin activity	0.001413	57,41,5
2bpa1_#45	GO:0004620: phospholipase activity	0.001413	192,19,6
1bdfa2#171	GO:0050660: FAD binding	0.001416	16,10,2
1hx6a1#163	GO:0050660: FAD binding	0.001416	16,10,2
1ush_1#462	GO:0003684: damaged DNA binding	0.001416	16,10,2
1ush_1#462	GO:0050660: FAD binding	0.001416	16,10,2
1ush_1#462	GO:0016814: hydrolase activity, acting on carbon-nitrogen (but not peptide) bonds, in cyclic amidines	0.001416	16,10,2
1ce7a_#151	GO:0008800: beta-lactamase activity	0.001416	16,10,2
1pme__#302	GO:0005066: transmembrane receptor protein tyrosine kinase signaling protein activity	0.001416	16,10,2
1qnja_#77	GO:0003809: thrombin activity	0.001416	16,10,2
1dcea3#526	GO:0016846: carbon-sulfur lyase activity	0.001416	16,10,2
1jj7a_#528	GO:0003684: damaged DNA binding	0.001416	16,10,2
1f3la_#348	GO:0016846: carbon-sulfur lyase activity	0.001416	16,10,2
1hxxa_#107	GO:0003809: thrombin activity	0.001416	16,10,2
1cnv__#264	GO:0016616: oxidoreductase activity, acting on the CH-OH group of donors, NAD or NADP as acceptor	0.001418	103,59,8
3grs_3#435	GO:0016251: general RNA polymerase II transcription factor activity	0.001423	179,14,5
1edza1#206	GO:0016638: oxidoreductase activity, acting on the CH-NH2 group of donors	0.001423	399,17,8
1fwxa2#396	GO:0003724: RNA helicase activity	0.001424	237,11,5
1qmea4#290	GO:0004620: phospholipase activity	0.001426	74,19,4
1e3ja2#270	GO:0016646: oxidoreductase activity, acting on the CH-NH group of donors, NAD or NADP as acceptor	0.001426	74,19,4
1i05a_#132	GO:0000155: two-component sensor molecule activity	0.001427	54,12,3
1cs1a_#82	GO:0004177: aminopeptidase activity	0.001428	195,13,5
1fn9a_#271	GO:0046983: protein dimerization activity	0.001429	218,17,6
1fn9a_#271	GO:0016638: oxidoreductase activity, acting on the CH-NH2 group of donors	0.001429	218,17,6
1fgga_#134	GO:0008483: transaminase activity	0.001429	218,17,6
1qfma2#576	GO:0016668: oxidoreductase activity, acting on sulfur group of donors, NAD or NADP as acceptor	0.001429	214,12,5
1aky_2#133	GO:0004190: aspartic-type endopeptidase activity	0.00143	156,23,6
1dkia_#76P	GO:0008201: heparin binding	0.00143	58,24,4
1ryp1_#112	GO:0004197: cysteine-type endopeptidase activity	0.00143	58,24,4
1ryp1_#112	GO:0008201: heparin binding	0.00143	58,24,4
1f2aa_#27	GO:0004523: ribonuclease H activity	0.001433	89,16,4
1dih_2#222	GO:0004725: protein tyrosine phosphatase activity	0.001438	352,15,7
1qaxa2#307	GO:0016251: general RNA polymerase II transcription factor activity	0.001439	46,14,3
1qq4a_#143	GO:0003968: RNA-directed RNA polymerase activity	0.001439	46,14,3
1qq4a_#63	GO:0003968: RNA-directed RNA polymerase activity	0.001439	46,14,3
1cnv__#264	GO:0016776: phosphotransferase activity, phosphate group as acceptor	0.001439	103,14,4
1qs0a1#119	GO:0015036: disulfide oxidoreductase activity	0.001442	454,22,10
1dcfa_#66	GO:0016836: hydro-lyase activity	0.001444	42,33,4
1c3d__#72	GO:0008810: cellulase activity	0.001444	9,18,2
1b65a_#324	GO:0016836: hydro-lyase activity	0.001444	42,33,4
1e8ya2#433	GO:0019838: growth factor binding	0.001444	18,9,2
1ppn__#191	GO:0000287: magnesium ion binding	0.001445	19,128,5
1hq8a_#206	GO:0016702: oxidoreductase activity, acting on single donors with incorporation of molecular oxygen, incorporation of two atoms of oxygen	0.001448	329,12,6
1fc4a_#205	GO:0016620: oxidoreductase activity, acting on the aldehyde or oxo group of donors, NAD or NADP as acceptor	0.00145	267,10,5
1fc4a_#205	GO:0016846: carbon-sulfur lyase activity	0.00145	267,10,5

1pce__#35	GO:0004867: serine-type endopeptidase inhibitor activity	0.001451	74,47,6
1iira_#326	GO:0016651: oxidoreductase activity, acting on NADH or NADPH	0.001451	112,13,4
1ton__#47	GO:0000049: tRNA binding	0.001451	112,13,4
1fjfc2#153	GO:0003729: mRNA binding	0.001451	420,13,7
1kit_2#398	GO:0008083: growth factor activity	0.001451	83,42,6
1d7ba_#87	GO:0003700: transcription factor activity	0.001454	99,124,12
1fo5a_#60	GO:0005507: copper ion binding	0.001454	246,38,10
1aym3_#109	GO:0008083: growth factor activity	0.001457	56,42,5
1je5a_#10	GO:0030145: manganese ion binding	0.001459	62,38,5
1qf5a_#15	GO:0005096: GTPase activator activity	0.00146	43,15,3
1sbp__#219	GO:0030151: molybdenum ion binding	0.00146	43,15,3
1gtra2#173	GO:0016651: oxidoreductase activity, acting on NADH or NADPH	0.001461	50,13,3
1ap0__#29	GO:0008080: N-acetyltransferase activity	0.001461	50,13,3
1a65a3#383	GO:0016651: oxidoreductase activity, acting on NADH or NADPH	0.001461	50,13,3
1qfea_#68	GO:0016651: oxidoreductase activity, acting on NADH or NADPH	0.001461	560,13,8
1qrra_#69	GO:0008026: ATP-dependent helicase activity	0.001462	196,13,5
1cg2a1#43	GO:0016705: oxidoreductase activity, acting on paired donors, with incorporation or reduction of molecular oxygen	0.001464	189,26,7
1bywa_#30	GO:0003887: DNA-directed DNA polymerase activity	0.001464	331,20,8
1bywa_#30	GO:0005126: hematopoietin/interferon-class (D200-domain) cytokine receptor binding	0.001464	331,20,8
1ihua1#119	GO:0016763: transferase activity, transferring pentosyl groups	0.001464	85,28,5
1djna1#86	GO:0016763: transferase activity, transferring pentosyl groups	0.001464	85,28,5
1lara2#1769	GO:0016638: oxidoreductase activity, acting on the CH-NH2 group of donors	0.001464	219,17,6
1ewqa3#159	GO:0003887: DNA-directed DNA polymerase activity	0.001465	32,20,3
1cmxa_#207	GO:0004725: protein tyrosine phosphatase activity	0.001465	96,15,4
1ddma_#130	GO:0015078: hydrogen ion transporter activity	0.001466	313,21,8
1dih_2#222	GO:0003779: actin binding	0.001466	352,32,11
1eu8a_#119	GO:0000287: magnesium ion binding	0.001467	48,128,8
1hd2a_#47	GO:0016831: carboxy-lyase activity	0.001468	56,25,4
1qdlb_#170	GO:0004197: cysteine-type endopeptidase activity	0.001468	100,24,5
1erza_#183	GO:0008810: cellulase activity	0.00147	79,18,4
1cpy__#380	GO:0019838: growth factor binding	0.001471	171,9,4
1ddja_#681	GO:0003887: DNA-directed DNA polymerase activity	0.001473	253,20,7
1ddja_#681	GO:0005126: hematopoietin/interferon-class (D200-domain) cytokine receptor binding	0.001473	253,20,7
1alo_4#634	GO:0019843: rRNA binding	0.001474	64,22,4
1ig8a_#418	GO:0051082: unfolded protein binding	0.001476	5,34,2
2cpl__#138	GO:0051082: unfolded protein binding	0.001476	5,34,2
1c4ka1#33	GO:0016616: oxidoreductase activity, acting on the CH-OH group of donors, NAD or NADP as acceptor	0.001476	40,59,5
1eula_#213	GO:0019955: cytokine binding	0.001477	60,11,3
1huxa_#10	GO:0003964: RNA-directed DNA polymerase activity	0.001477	60,11,3
1hw5a2#64	GO:0030151: molybdenum ion binding	0.001479	167,15,5
1jj2e1#42	GO:0004556: alpha-amylase activity	0.001479	167,15,5
1qq5a_#7	GO:0004180: carboxypeptidase activity	0.001479	167,15,5
1c7na_#95	GO:0003887: DNA-directed DNA polymerase activity	0.001481	183,20,6
1eut_1#488	GO:0005507: copper ion binding	0.001487	17,38,3
1dqsa_#138	GO:0003924: GTPase activity	0.001487	38,17,3
1eexb_#119	GO:0008483: transaminase activity	0.001487	38,17,3
1jfra_#83	GO:0016799: hydrolase activity, hydrolyzing N-glycosyl compounds	0.001487	38,17,3
1kit_3#769	GO:0005507: copper ion binding	0.001487	17,38,3
1edg__#81	GO:0016763: transferase activity, transferring pentosyl groups	0.001489	6,28,2
1f8ea_#177	GO:0016763: transferase activity, transferring pentosyl groups	0.001489	6,28,2
1xis__#177	GO:0016763: transferase activity, transferring pentosyl groups	0.001489	6,28,2
1a3c__#13	GO:0016763: transferase activity, transferring pentosyl groups	0.001489	6,28,2
1gdoa_#24	GO:0016763: transferase activity, transferring pentosyl groups	0.001489	6,28,2
1rypk_#12	GO:0005524: ATP binding	0.001489	265,243,38
1iow_1#37	GO:0004812: tRNA ligase activity	0.001491	309,26,9
1gtra1#468	GO:0004812: tRNA ligase activity	0.001491	54,26,4
1fkna_#96	GO:0019838: growth factor binding	0.001493	75,9,3
1pysa_#221	GO:0004523: ribonuclease H activity	0.001494	90,16,4
1bywa_#30	GO:0000155: two-component sensor molecule activity	0.001496	331,12,6
1efpa1#32	GO:0016646: oxidoreductase activity, acting on the CH-NH group of donors, NAD or NADP as acceptor	0.001496	129,19,5
1gpea1#271	GO:0000049: tRNA binding	0.001496	197,13,5
2mpra_#89	GO:0004190: aspartic-type endopeptidase activity	0.0015	28,23,3
2pia_1#54	GO:0016638: oxidoreductase activity, acting on the CH-NH2 group of donors	0.0015	220,17,6

1d7ya3#331	GO:0004190: aspartic-type endopeptidase activity	0.0015	28,23,3
1c8na_#239	GO:0016763: transferase activity, transferring pentosyl groups	0.0015	23,28,3
1fcda1#6	GO:0004222: metalloendopeptidase activity	0.0015	75,19,4
1i50a_#856	GO:0003682: chromatin binding	0.001501	269,10,5
1qoua_#64	GO:0004896: hematopoietin/interferon-class (D200-domain) cytokine receptor activity	0.001501	34,19,3
1e19a_#281	GO:0004812: tRNA ligase activity	0.001503	138,26,6
1aop_3#276	GO:0019838: growth factor binding	0.001504	172,9,4
1xo1a2#159	GO:0003899: DNA-directed RNA polymerase activity	0.001505	55,12,3
1bxoa_#307	GO:0051082: unfolded protein binding	0.001506	104,34,6
1rlr_1#66	GO:0008199: ferric iron binding	0.001507	67,10,3
2reb_1#138	GO:0016846: carbon-sulfur lyase activity	0.001507	67,10,3
1f8ra2#325	GO:0008083: growth factor activity	0.001509	222,42,10
3prn__#145	GO:0005509: calcium ion binding	0.001509	168,160,20
1ds1a_#172	GO:0008083: growth factor activity	0.001509	222,42,10
1f0ia1#50	GO:0016638: oxidoreductase activity, acting on the CH-NH2 group of donors	0.001511	146,17,5
1b3qa2#623	GO:0000049: tRNA binding	0.001511	301,13,6
1iiba_#79	GO:0016758: transferase activity, transferring hexosyl groups	0.001512	15,11,2
1dyoa_#25	GO:0010181: FMN binding	0.001512	15,11,2
1h8va_#139	GO:0003724: RNA helicase activity	0.001512	15,11,2
1dpja_#199	GO:0004364: glutathione transferase activity	0.001512	15,11,2
1sek__#52	GO:0005179: hormone activity	0.001512	11,15,2
1rypf_#50	GO:0003724: RNA helicase activity	0.001512	15,11,2
1dkia_#208	GO:0010181: FMN binding	0.001512	15,11,2
1rypk_#41	GO:0004725: protein tyrosine phosphatase activity	0.001512	11,15,2
1hvba_#102	GO:0004180: carboxypeptidase activity	0.001512	11,15,2
1f97a1#62	GO:0003755: peptidyl-prolyl cis-trans isomerase activity	0.001514	707,11,8
1f97a1#62	GO:0003964: RNA-directed DNA polymerase activity	0.001514	707,11,8
3prn__#145	GO:0004556: alpha-amylase activity	0.001519	168,15,5
3prn__#145	GO:0004725: protein tyrosine phosphatase activity	0.001519	168,15,5
1hcl__#180	GO:0005516: calmodulin binding	0.00152	7,24,2
1cs6a2#164	GO:0008201: heparin binding	0.00152	7,24,2
3sil__#181	GO:0008201: heparin binding	0.00152	7,24,2
1f42a2#123	GO:0005529: sugar binding	0.001521	378,39,13
1bl0a2#102	GO:0003700: transcription factor activity	0.001522	12,124,4
1byka_#157	GO:0003700: transcription factor activity	0.001522	12,124,4
1cbf__#208	GO:0003887: DNA-directed DNA polymerase activity	0.001524	333,20,8
1cbf__#208	GO:0005126: hematopoietin/interferon-class (D200-domain) cytokine receptor binding	0.001524	333,20,8
1gdea_#195	GO:0016616: oxidoreductase activity, acting on the CH-OH group of donors, NAD or NADP as acceptor	0.001525	24,59,4
1bxka_#128	GO:0016616: oxidoreductase activity, acting on the CH-OH group of donors, NAD or NADP as acceptor	0.001525	24,59,4
1hzxa_#139	GO:0005509: calcium ion binding	0.001527	39,160,8
1hlwa_#82	GO:0003809: thrombin activity	0.001527	270,10,5
1g0sa_#176	GO:0030145: manganese ion binding	0.001528	37,38,4
1e3ua_#164	GO:0030145: manganese ion binding	0.001528	37,38,4
1epwa3#248	GO:0005507: copper ion binding	0.001528	37,38,4
1dl5a_#266	GO:0004197: cysteine-type endopeptidase activity	0.001529	27,24,3
1qtn.1#A331	GO:0004197: cysteine-type endopeptidase activity	0.001529	27,24,3
1fm2.1#B627	GO:0004197: cysteine-type endopeptidase activity	0.001529	27,24,3
1a3qa2#133	GO:0008201: heparin binding	0.001529	27,24,3
1ejda_#6	GO:0004867: serine-type endopeptidase inhibitor activity	0.001529	270,47,12
1h5qa_#70	GO:0008757: S-adenosylmethionine-dependent methyltransferase activity	0.001529	27,24,3
1e1aa_#285	GO:0003755: peptidyl-prolyl cis-trans isomerase activity	0.001531	137,11,4
1seta2#306	GO:0004867: serine-type endopeptidase inhibitor activity	0.001533	30,47,4
1dyka2#2937	GO:0004867: serine-type endopeptidase inhibitor activity	0.001533	14,47,3
1hxxa_#278	GO:0030151: molybdenum ion binding	0.001533	255,15,6
1ton__#196	GO:0003968: RNA-directed RNA polymerase activity	0.001533	47,14,3
1eq9a_#58	GO:0003968: RNA-directed RNA polymerase activity	0.001533	47,14,3
1ejda_#6	GO:0004842: ubiquitin-protein ligase activity	0.001534	270,19,7
1xgsa2#82	GO:0016638: oxidoreductase activity, acting on the CH-NH2 group of donors	0.001537	307,17,7
1a8i__#688	GO:0010181: FMN binding	0.001537	241,11,5
1ihua1#119	GO:0003924: GTPase activity	0.001539	85,17,4
1fsu__#434	GO:0004725: protein tyrosine phosphatase activity	0.001539	356,15,7
1nsca_#236	GO:0051082: unfolded protein binding	0.001539	143,34,7
1eqga2#47	GO:0004601: peroxidase activity	0.001541	8,21,2
1qo7a_#199	GO:0004601: peroxidase activity	0.001541	8,21,2

1hr6a1#59	GO:0015078: hydrogen ion transporter activity	0.001541	8,21,2
1qhba_#161	GO:0004601: peroxidase activity	0.001541	8,21,2
1hlga_#324	GO:0004601: peroxidase activity	0.001541	8,21,2
1dceb_#134	GO:0015078: hydrogen ion transporter activity	0.001541	8,21,2
1hr6a2#427	GO:0015078: hydrogen ion transporter activity	0.001541	8,21,2
1rypk_#76	GO:0015078: hydrogen ion transporter activity	0.001541	8,21,2
1foha5#77	GO:0004497: monooxygenase activity	0.001544	25,26,3
1vns__#487	GO:0004601: peroxidase activity	0.001544	31,21,3
1awcb_#111	GO:0015078: hydrogen ion transporter activity	0.001544	31,21,3
1cbf__#208	GO:0016668: oxidoreductase activity, acting on sulfur group of donors, NAD or NADP as acceptor	0.001545	333,12,6
1f5ma_#80	GO:0016251: general RNA polymerase II transcription factor activity	0.001546	105,14,4
1xnb__#131	GO:0008080: N-acetyltransferase activity	0.001548	51,13,3
1fid__#237	GO:0000049: tRNA binding	0.001548	51,13,3
1arb__#210	GO:0004812: tRNA ligase activity	0.001548	93,26,5
1f9va_#627	GO:0016651: oxidoreductase activity, acting on NADH or NADPH	0.001548	51,13,3
1gdea_#243	GO:0019955: cytokine binding	0.00155	61,11,3
1hyrc1#247	GO:0019955: cytokine binding	0.00155	61,11,3
1hyrc1#247	GO:0003755: peptidyl-prolyl cis-trans isomerase activity	0.00155	61,11,3
1ayl__#237	GO:0004190: aspartic-type endopeptidase activity	0.00155	357,23,9
1bfg__#22	GO:0019955: cytokine binding	0.00155	61,11,3
1bfg__#22	GO:0003724: RNA helicase activity	0.00155	61,11,3
1bfg__#22	GO:0003755: peptidyl-prolyl cis-trans isomerase activity	0.00155	61,11,3
1qs0a1#235	GO:0019201: nucleotide kinase activity	0.00155	114,13,4
1qs0a1#235	GO:0008026: ATP-dependent helicase activity	0.00155	114,13,4
1phm_2#253	GO:0004867: serine-type endopeptidase inhibitor activity	0.001551	309,47,13
1aqt_2#45	GO:0019829: cation-transporting ATPase activity	0.001552	76,9,3
1aqt_2#45	GO:0019838: growth factor binding	0.001552	76,9,3
1nsca_#379	GO:0005529: sugar binding	0.001553	124,39,7
3prn__#143	GO:0003887: DNA-directed DNA polymerase activity	0.001555	334,20,8
3prn__#143	GO:0005126: hematopoietin/interferon-class (D200-domain) cytokine receptor binding	0.001555	334,20,8
1fgga_#134	GO:0000155: two-component sensor molecule activity	0.001555	218,12,5
1fbl_1#398	GO:0004190: aspartic-type endopeptidase activity	0.001556	62,23,4
1ah7__#55	GO:0008270: zinc ion binding	0.001557	2,108,2
2sli_2#341	GO:0008270: zinc ion binding	0.001557	2,108,2
1e3ja1#320	GO:0008270: zinc ion binding	0.001557	2,108,2
1ah7__#184	GO:0008270: zinc ion binding	0.001557	2,108,2
1smpi_#11	GO:0008270: zinc ion binding	0.001557	2,108,2
1qj5a_#268	GO:0005524: ATP binding	0.001557	66,243,14
1bmv2_#2064	GO:0016638: oxidoreductase activity, acting on the CH-NH2 group of donors	0.001558	147,17,5
1e3a.1#A30	GO:0005525: GTP binding	0.001558	157,49,9
1xnb__#149	GO:0030145: manganese ion binding	0.00156	165,38,8
1lci__#26	GO:0016705: oxidoreductase activity, acting on paired donors, with incorporation or reduction of molecular oxygen	0.00156	139,26,6
1dp0a4#756	GO:0005507: copper ion binding	0.00156	165,38,8
1dn2a1#261	GO:0005518: collagen binding	0.00156	425,13,7
1rkd__#186	GO:0005524: ATP binding	0.001562	52,243,12
1d3bb_#31	GO:0005524: ATP binding	0.001562	52,243,12
1a6o__#45	GO:0000049: tRNA binding	0.001566	303,13,6
1ayl__#237	GO:0004556: alpha-amylase activity	0.001566	357,15,7
3chbd_#85	GO:0004812: tRNA ligase activity	0.001566	378,26,10
1qora2#201	GO:0016861: intramolecular oxidoreductase activity, interconverting aldoses and ketoses	0.001566	199,13,5
1dpja_#322	GO:0046983: protein dimerization activity	0.001567	308,17,7
1dpja_#322	GO:0016638: oxidoreductase activity, acting on the CH-NH2 group of donors	0.001567	308,17,7
1fu6a_#50	GO:0046983: protein dimerization activity	0.001567	308,17,7
1psda3#344	GO:0015405: P-P-bond-hydrolysis-driven transporter activity	0.001568	125,12,4
1ryp1_#20	GO:0003697: single-stranded DNA binding	0.001568	125,12,4
1bif_2#398	GO:0005351: sugar porter activity	0.001568	117,21,5
1b12a_#221	GO:0004674: protein serine/threonine kinase activity	0.001569	115,42,7
5ruba1#279	GO:0016831: carboxy-lyase activity	0.001569	57,25,4
3prn__#143	GO:0016668: oxidoreductase activity, acting on sulfur group of donors, NAD or NADP as acceptor	0.00157	334,12,6
1evya2#38	GO:0016668: oxidoreductase activity, acting on sulfur group of donors, NAD or NADP as acceptor	0.001571	14,12,2
1a9xa2#1009	GO:0016866: intramolecular transferase activity	0.001571	14,12,2

1brwa2#100	GO:0016668: oxidoreductase activity, acting on sulfur group of donors, NAD or NADP as acceptor	0.001571	14,12,2
1dt6a_#449	GO:0016866: intramolecular transferase activity	0.001571	14,12,2
1bl0a2#102	GO:0005543: phospholipid binding	0.001571	12,14,2
1de4c3#165	GO:0016668: oxidoreductase activity, acting on sulfur group of donors, NAD or NADP as acceptor	0.001571	14,12,2
2napa1#718	GO:0003899: DNA-directed RNA polymerase activity	0.001571	14,12,2
3chbd_#96	GO:0003697: single-stranded DNA binding	0.001571	14,12,2
1pii_2#326	GO:0016836: hydro-lyase activity	0.001573	192,33,8
1ds1a_#172	GO:0016638: oxidoreductase activity, acting on the CH-NH2 group of donors	0.001573	222,17,6
1dqaa1#596	GO:0003684: damaged DNA binding	0.001574	68,10,3
1f97a2#199	GO:0005524: ATP binding	0.001579	144,243,24
7reqa2#702	GO:0005524: ATP binding	0.001582	59,243,13
1ig8a_#215	GO:0005524: ATP binding	0.001582	59,243,13
1hw5a2#64	GO:0015036: disulfide oxidoreductase activity	0.001586	167,22,6
1dzfa2#211	GO:0019843: rRNA binding	0.001586	167,22,6
3grx__#56	GO:0003887: DNA-directed DNA polymerase activity	0.001586	335,20,8
1mdah_#329	GO:0005126: hematopoietin/interferon-class (D200-domain) cytokine receptor binding	0.001586	335,20,8
1jf9a_#379	GO:0051082: unfolded protein binding	0.001588	232,34,9
1ak5_1#353	GO:0016836: hydro-lyase activity	0.001589	73,33,5
1h4vb2#108	GO:0000049: tRNA binding	0.001591	13,13,2
1ezia_#198	GO:0019201: nucleotide kinase activity	0.001591	13,13,2
1g97a1#376	GO:0008408: 3'-5' exonuclease activity	0.001591	13,13,2
1ayoa_#55	GO:0000049: tRNA binding	0.001591	13,13,2
1atia2#210	GO:0016651: oxidoreductase activity, acting on NADH or NADPH	0.001591	13,13,2
1e9xa_#428	GO:0004177: aminopeptidase activity	0.001591	13,13,2
1b93a_#48	GO:0016861: intramolecular oxidoreductase activity, interconverting aldoses and ketoses	0.001591	13,13,2
1f4la2#20	GO:0000049: tRNA binding	0.001591	13,13,2
1d4xg_#7	GO:0005085: guanyl-nucleotide exchange factor activity	0.001591	13,13,2
1d4xg_#7	GO:0008235: metalloexopeptidase activity	0.001591	13,13,2
1b6a_1#415	GO:0004177: aminopeptidase activity	0.001591	13,13,2
1b6a_1#415	GO:0008235: metalloexopeptidase activity	0.001591	13,13,2
1elka_#21	GO:0008408: 3'-5' exonuclease activity	0.001591	13,13,2
1ilr1_#32	GO:0005518: collagen binding	0.001591	13,13,2
1fvua_#72	GO:0000049: tRNA binding	0.001591	13,13,2
2tbva_#214	GO:0005518: collagen binding	0.001591	13,13,2
1eg5a_#199	GO:0004177: aminopeptidase activity	0.001591	13,13,2
3sil__#317	GO:0004556: alpha-amylase activity	0.001592	358,15,7
1e8ga2#201	GO:0003729: mRNA binding	0.001593	304,13,6
1rypb_#114	GO:0004523: ribonuclease H activity	0.001594	158,16,5
1qlsa_#81	GO:0004497: monooxygenase activity	0.001597	55,26,4
1phm_2#253	GO:0046983: protein dimerization activity	0.001598	309,17,7
1el0a_#28	GO:0004295: trypsin activity	0.001599	161,48,9
1i9sa_#19	GO:0003682: chromatin binding	0.001601	17,10,2
1ja9a_#249	GO:0016627: oxidoreductase activity, acting on the CH-CH group of donors	0.001601	10,17,2
1mkna_#50	GO:0016627: oxidoreductase activity, acting on the CH-CH group of donors	0.001601	10,17,2
1qmua1#309	GO:0008199: ferric iron binding	0.001601	17,10,2
1qmua1#309	GO:0016846: carbon-sulfur lyase activity	0.001601	17,10,2
1tkia_#80	GO:0005066: transmembrane receptor protein tyrosine kinase signaling protein activity	0.001601	17,10,2
1fh6a_#277	GO:0016846: carbon-sulfur lyase activity	0.001601	17,10,2
1howa_#588	GO:0005066: transmembrane receptor protein tyrosine kinase signaling protein activity	0.001601	17,10,2
1koba_#230	GO:0005066: transmembrane receptor protein tyrosine kinase signaling protein activity	0.001601	17,10,2
1bdfa2#121	GO:0046983: protein dimerization activity	0.001601	10,17,2
1tkia_#266	GO:0005066: transmembrane receptor protein tyrosine kinase signaling protein activity	0.001601	17,10,2
2tgi__#106	GO:0046983: protein dimerization activity	0.001601	10,17,2
1jbwa2#252	GO:0016814: hydrolase activity, acting on carbon-nitrogen (but not peptide) bonds, in cyclic amidines	0.001601	17,10,2
1ffgb_#172	GO:0046983: protein dimerization activity	0.001601	10,17,2
1fwxa2#189	GO:0016638: oxidoreductase activity, acting on the CH-NH2 group of donors	0.001601	10,17,2
1qqga2#166	GO:0000049: tRNA binding	0.001602	200,13,5
1fm2.1#B308	GO:0000049: tRNA binding	0.001602	200,13,5
1aa6_2#285	GO:0016776: phosphotransferase activity, phosphate group as acceptor	0.001602	106,14,4
1hzxa_#139	GO:0046983: protein dimerization activity	0.001605	39,17,3
1hzxa_#139	GO:0003924: GTPase activity	0.001605	39,17,3
1ja9a_#202	GO:0003924: GTPase activity	0.001605	39,17,3
1bkpa_#41	GO:0005524: ATP binding	0.001606	21,243,7

1ecpa_#156	GO:0005524: ATP binding	0.001606	21,243,7
1e1oa2#423	GO:0016638: oxidoreductase activity, acting on the CH-NH2 group of donors	0.001606	148,17,5
1nsca_#379	GO:0003887: DNA-directed DNA polymerase activity	0.001608	124,20,5
1eur__#84	GO:0016638: oxidoreductase activity, acting on the CH-NH2 group of donors	0.001608	86,17,4
1hdfa_#16	GO:0016638: oxidoreductase activity, acting on the CH-NH2 group of donors	0.00161	223,17,6
1bvyf_#527	GO:0016646: oxidoreductase activity, acting on the CH-NH group of donors, NAD or NADP as acceptor	0.001611	9,19,2
1g72a_#42	GO:0004896: hematopoietin/interferon-class (D200-domain) cytokine receptor activity	0.001611	9,19,2
1i7oa1#119	GO:0015036: disulfide oxidoreductase activity	0.001611	30,22,3
1gai__#22	GO:0004842: ubiquitin-protein ligase activity	0.001611	9,19,2
1nsca_#91	GO:0019838: growth factor binding	0.001611	19,9,2
1bfd_2#146	GO:0015036: disulfide oxidoreductase activity	0.001612	112,22,5
1czan1#92	GO:0046983: protein dimerization activity	0.001613	631,17,10
1eqka_#47	GO:0003899: DNA-directed RNA polymerase activity	0.001616	126,12,4
1rypg_#159	GO:0008083: growth factor activity	0.001618	34,42,4
1fhga_#59	GO:0004674: protein serine/threonine kinase activity	0.001618	34,42,4
1fhga_#59	GO:0008083: growth factor activity	0.001618	34,42,4
1f42a2#123	GO:0019843: rRNA binding	0.001619	378,22,9
3chbd_#85	GO:0019843: rRNA binding	0.001619	378,22,9
1f9za_#16	GO:0004812: tRNA ligase activity	0.00162	140,26,6
1axn__#42	GO:0005509: calcium ion binding	0.001624	78,160,12
1kwaa_#513	GO:0019955: cytokine binding	0.001625	62,11,3
1kwaa_#513	GO:0008081: phosphoric diester hydrolase activity	0.001625	62,11,3
1fw9a_#157	GO:0019955: cytokine binding	0.001625	62,11,3
1fw9a_#157	GO:0003724: RNA helicase activity	0.001625	62,11,3
1ih7a1#150	GO:0016758: transferase activity, transferring hexosyl groups	0.001625	62,11,3
1ie5a_#102	GO:0019955: cytokine binding	0.001625	62,11,3
1i50b_#334	GO:0015078: hydrogen ion transporter activity	0.001628	69,21,4
1ez0a_#133	GO:0005529: sugar binding	0.001628	125,39,7
1xgsa2#82	GO:0008083: growth factor activity	0.001629	307,42,12
1epwa3#140	GO:0051082: unfolded protein binding	0.001631	187,34,8
1gdna_#230	GO:0005509: calcium ion binding	0.001632	23,160,6
1crua_#29	GO:0005509: calcium ion binding	0.001632	23,160,6
1poxa3#460	GO:0005524: ATP binding	0.001633	81,243,16
2ae2a_#89	GO:0016646: oxidoreductase activity, acting on the CH-NH group of donors, NAD or NADP as acceptor	0.001634	35,19,3
1d2oa1#541	GO:0004896: hematopoietin/interferon-class (D200-domain) cytokine receptor activity	0.001634	35,19,3
1eny__#94	GO:0016646: oxidoreductase activity, acting on the CH-NH group of donors, NAD or NADP as acceptor	0.001634	35,19,3
1ek6a_#33	GO:0016854: racemase and epimerase activity	0.001638	52,13,3
1kapp2#69	GO:0000049: tRNA binding	0.001638	52,13,3
1qf6a3#78	GO:0004177: aminopeptidase activity	0.001638	52,13,3
1d3bb_#31	GO:0003729: mRNA binding	0.001638	52,13,3
3grs_2#211	GO:0016651: oxidoreductase activity, acting on NADH or NADPH	0.001638	52,13,3
1ib2a_#974	GO:0005085: guanyl-nucleotide exchange factor activity	0.001638	52,13,3
1apy.1#B284	GO:0004177: aminopeptidase activity	0.001638	52,13,3
1e5xa_#379	GO:0005524: ATP binding	0.001641	39,243,10
1dbxa_#75	GO:0003684: damaged DNA binding	0.001642	69,10,3
1je5a_#10	GO:0005529: sugar binding	0.001644	62,39,5
1mpp__#154	GO:0004556: alpha-amylase activity	0.001644	99,15,4
1cpy__#380	GO:0030151: molybdenum ion binding	0.001646	171,15,5
1kit_3#743	GO:0005507: copper ion binding	0.001647	250,38,10
1f7da_#97	GO:0004263: chymotrypsin activity	0.001649	35,41,4
1aa6_2#175	GO:0016831: carboxy-lyase activity	0.00165	7,25,2
1dpe__#190	GO:0016831: carboxy-lyase activity	0.00165	7,25,2
2viua_#252	GO:0008201: heparin binding	0.00165	343,24,9
1h4ua1#486	GO:0008270: zinc ion binding	0.00165	45,108,7
1lvk_2#652	GO:0016831: carboxy-lyase activity	0.00165	7,25,2
1c4oa2#536	GO:0016831: carboxy-lyase activity	0.00165	7,25,2
1prea2#308	GO:0003779: actin binding	0.001651	76,32,5
1bwda_#117	GO:0004674: protein serine/threonine kinase activity	0.001651	116,42,7
1im4a_#41	GO:0004263: chymotrypsin activity	0.001653	59,41,5
1a4ya_#104	GO:0016836: hydro-lyase activity	0.001653	241,33,9
1bwda_#117	GO:0008408: 3'-5' exonuclease activity	0.001655	116,13,4
1b4ka_#108	GO:0016861: intramolecular oxidoreductase activity, interconverting aldoses and ketoses	0.001655	116,13,4
1ton__#47	GO:0005524: ATP binding	0.001655	112,243,20

1e25a_#262	GO:0000049: tRNA binding	0.001655	116,13,4
1e25a_#262	GO:0003729: mRNA binding	0.001655	116,13,4
1fwxa2#169	GO:0008408: 3'-5' exonuclease activity	0.001655	116,13,4
1bio__#210	GO:0003924: GTPase activity	0.001656	149,17,5
1quqa_#59	GO:0004674: protein serine/threonine kinase activity	0.001659	16,42,3
3prn__#143	GO:0051082: unfolded protein binding	0.001659	334,34,11
1cs6a4#363	GO:0004674: protein serine/threonine kinase activity	0.001659	16,42,3
1jj2a1#126	GO:0005126: hematopoietin/interferon-class (D200-domain) cytokine receptor binding	0.00166	73,20,4
1epwa3#138	GO:0003887: DNA-directed DNA polymerase activity	0.001661	424,20,9
1d0na3#305	GO:0016638: oxidoreductase activity, acting on the CH-NH2 group of donors	0.001661	311,17,7
1thg__#223	GO:0016616: oxidoreductase activity, acting on the CH-OH group of donors, NAD or NADP as acceptor	0.001662	82,59,7
1ycsb1#418	GO:0003700: transcription factor activity	0.001663	6,124,3
1sw6a_#352	GO:0003700: transcription factor activity	0.001663	6,124,3
1dcqa1#473	GO:0003700: transcription factor activity	0.001663	6,124,3
2sli_2#326	GO:0004812: tRNA ligase activity	0.001664	251,26,8
1e8ya1#648	GO:0003779: actin binding	0.001666	45,32,4
1dqza_#209	GO:0004180: carboxypeptidase activity	0.001667	45,15,3
1bg6_1#294	GO:0005179: hormone activity	0.001667	45,15,3
1enfa1#92	GO:0030151: molybdenum ion binding	0.001667	45,15,3
1ojt_2#356	GO:0016811: hydrolase activity, acting on carbon-nitrogen (but not peptide) bonds, in linear amides	0.001667	45,15,3
1ez0a_#133	GO:0003887: DNA-directed DNA polymerase activity	0.001667	125,20,5
3grx__#55	GO:0003779: actin binding	0.001668	113,32,6
1dn2a1#261	GO:0003809: thrombin activity	0.001669	425,10,6
1dn2a1#261	GO:0003916: DNA topoisomerase activity	0.001669	425,10,6
1im4a_#144	GO:0030145: manganese ion binding	0.00167	129,38,7
1dssg1#4	GO:0016866: intramolecular transferase activity	0.00167	57,12,3
1g0sa_#74	GO:0005524: ATP binding	0.00167	294,243,41
1dhn__#115	GO:0005509: calcium ion binding	0.001674	31,160,7
1hxxa_#232	GO:0008083: growth factor activity	0.001674	352,42,13
1jjya_#324	GO:0019829: cation-transporting ATPase activity	0.001674	78,9,3
1elja_#64	GO:0005516: calmodulin binding	0.001677	103,24,5
1xgsa2#82	GO:0004177: aminopeptidase activity	0.001679	307,13,6
1erv__#57	GO:0008483: transaminase activity	0.001679	87,17,4
1eucb2#188	GO:0046983: protein dimerization activity	0.001679	87,17,4
1eucb2#188	GO:0008483: transaminase activity	0.001679	87,17,4
1alo_4#634	GO:0030145: manganese ion binding	0.001684	64,38,5
3sil__#284	GO:0005507: copper ion binding	0.001684	64,38,5
2cuaa_#107	GO:0005525: GTP binding	0.001686	128,49,8
2msba_#218	GO:0000287: magnesium ion binding	0.001686	49,128,8
1gcya2#242	GO:0005509: calcium ion binding	0.001687	16,160,5
1ppn__#132	GO:0051082: unfolded protein binding	0.001688	234,34,9
1doza_#109	GO:0016836: hydro-lyase activity	0.001689	74,33,5
1h9da_#91	GO:0016702: oxidoreductase activity, acting on single donors with incorporation of molecular oxygen, incorporation of two atoms of oxygen	0.001689	222,12,5
1fp5a2#537	GO:0005507: copper ion binding	0.00169	38,38,4
1beba_#119	GO:0005529: sugar binding	0.00169	202,39,9
1dn2a1#261	GO:0003887: DNA-directed DNA polymerase activity	0.00169	425,20,9
1ezm__#146	GO:0008270: zinc ion binding	0.001694	23,108,5
1ezwa_#245	GO:0005351: sugar porter activity	0.001695	32,21,3
1i39a_#49	GO:0003779: actin binding	0.001695	21,32,3
1e3a.1#A30	GO:0016846: carbon-sulfur lyase activity	0.001695	157,10,4
2mpra_#89	GO:0008201: heparin binding	0.001703	28,24,3
1fvia2#134	GO:0005516: calmodulin binding	0.001703	28,24,3
3pah__#303	GO:0005516: calmodulin binding	0.001703	28,24,3
1a06__#147	GO:0005516: calmodulin binding	0.001703	28,24,3
1ihua2#343	GO:0015036: disulfide oxidoreductase activity	0.001703	233,22,7
1a06__#103	GO:0005516: calmodulin binding	0.001703	28,24,3
1ep3a_#247	GO:0016763: transferase activity, transferring pentosyl groups	0.001703	24,28,3
1cwva2#675	GO:0008201: heparin binding	0.001703	28,24,3
1mjha_#126	GO:0005525: GTP binding	0.001704	159,49,9
1i6vd_#1283	GO:0019955: cytokine binding	0.001705	141,11,4
1ldna1#94	GO:0016646: oxidoreductase activity, acting on the CH-NH group of donors, NAD or NADP as acceptor	0.001705	199,19,6
1io1a_#311	GO:0005529: sugar binding	0.001706	126,39,7

1e2o__#346	GO:0004812: tRNA ligase activity	0.001707	194,26,7
1qdlb_#170	GO:0030151: molybdenum ion binding	0.001707	100,15,4
1qdlb_#170	GO:0004725: protein tyrosine phosphatase activity	0.001707	100,15,4
1b8aa2#403	GO:0003729: mRNA binding	0.001708	117,13,4
1sgpe_#115	GO:0004867: serine-type endopeptidase inhibitor activity	0.001709	4,47,2
1epwa2#1100	GO:0004867: serine-type endopeptidase inhibitor activity	0.001709	4,47,2
1sgpe_#127	GO:0004867: serine-type endopeptidase inhibitor activity	0.001709	4,47,2
1bd2d1#62	GO:0004867: serine-type endopeptidase inhibitor activity	0.001709	4,47,2
1f42a2#123	GO:0003724: RNA helicase activity	0.00171	378,11,6
3chbd_#85	GO:0003755: peptidyl-prolyl cis-trans isomerase activity	0.00171	378,11,6
1a6o__#45	GO:0003779: actin binding	0.001711	303,32,10
1e05i_#83	GO:0016763: transferase activity, transferring pentosyl groups	0.001712	88,28,5
1hnja1#8	GO:0000287: magnesium ion binding	0.001714	12,128,4
1qs0a1#119	GO:0004812: tRNA ligase activity	0.001717	454,26,11
1alo_4#481	GO:0015078: hydrogen ion transporter activity	0.001718	70,21,4
1i0da_#297	GO:0008270: zinc ion binding	0.001718	14,108,4
1hcl__#280	GO:0015078: hydrogen ion transporter activity	0.001718	70,21,4
1ygs__#435	GO:0003724: RNA helicase activity	0.001724	16,11,2
1fy7a_#304	GO:0010181: FMN binding	0.001724	16,11,2
1azsa_#397	GO:0003964: RNA-directed DNA polymerase activity	0.001724	16,11,2
1cz4a1#76	GO:0004523: ribonuclease H activity	0.001724	11,16,2
3grx__#56	GO:0005529: sugar binding	0.001725	335,39,12
1phm_2#253	GO:0008083: growth factor activity	0.001725	309,42,12
1i6vd_#1188	GO:0016831: carboxy-lyase activity	0.001726	27,25,3
1ad2__#40	GO:0046983: protein dimerization activity	0.001728	40,17,3
1yrga_#232	GO:0003924: GTPase activity	0.001728	40,17,3
1io1a_#311	GO:0005126: hematopoietin/interferon-class (D200-domain) cytokine receptor binding	0.001728	126,20,5
1c4ka1#33	GO:0003924: GTPase activity	0.001728	40,17,3
1c4ka1#33	GO:0016638: oxidoreductase activity, acting on the CH-NH2 group of donors	0.001728	40,17,3
1bfg__#22	GO:0008201: heparin binding	0.001728	61,24,4
1e39a2#556	GO:0016627: oxidoreductase activity, acting on the CH-CH group of donors	0.001728	40,17,3
1e79d3#328	GO:0003924: GTPase activity	0.001728	40,17,3
1jsg__#92	GO:0030151: molybdenum ion binding	0.001729	607,15,9
1ckea_#138	GO:0005525: GTP binding	0.001731	14,49,3
1a65a3#409	GO:0003968: RNA-directed RNA polymerase activity	0.001731	49,14,3
1pfza_#177	GO:0005525: GTP binding	0.001731	14,49,3
1i9sa_#24	GO:0016854: racemase and epimerase activity	0.001732	53,13,3
1mrj__#73	GO:0008080: N-acetyltransferase activity	0.001732	53,13,3
5ruba1#259	GO:0008026: ATP-dependent helicase activity	0.001732	53,13,3
5ruba1#259	GO:0008408: 3'-5' exonuclease activity	0.001732	53,13,3
1dp0a1#309	GO:0005518: collagen binding	0.001732	53,13,3
1i2oa_#34	GO:0005525: GTP binding	0.001733	192,49,10
1epwa3#140	GO:0015082: di-, tri-valent inorganic cation transporter activity	0.001734	187,14,5
1epwa3#140	GO:0046915: transition metal ion transporter activity	0.001734	187,14,5
1epwa3#140	GO:0016251: general RNA polymerase II transcription factor activity	0.001734	187,14,5
1cs1a_#160	GO:0016776: phosphotransferase activity, phosphate group as acceptor	0.001734	187,14,5
1b8aa2#403	GO:0008083: growth factor activity	0.001736	117,42,7
1rypb_#114	GO:0004457: lactate dehydrogenase activity	0.001736	158,10,4
1rypb_#114	GO:0008800: beta-lactamase activity	0.001736	158,10,4
1ibja_#271	GO:0016646: oxidoreductase activity, acting on the CH-NH group of donors, NAD or NADP as acceptor	0.001737	78,19,4
1phm_2#253	GO:0000049: tRNA binding	0.001738	309,13,6
1ycsb1#412	GO:0051082: unfolded protein binding	0.001739	146,34,7
1j75a_#166	GO:0004263: chymotrypsin activity	0.001739	120,41,7
1qdea_#201	GO:0005524: ATP binding	0.00174	7,243,4
1gg4a3#24	GO:0005524: ATP binding	0.00174	7,243,4
1hnua_#190	GO:0005524: ATP binding	0.00174	7,243,4
1ile_3#408	GO:0005524: ATP binding	0.00174	7,243,4
1xvaa_#181	GO:0005524: ATP binding	0.00174	7,243,4
1dik_3#29	GO:0005524: ATP binding	0.00174	7,243,4
1d5ta1#198	GO:0005524: ATP binding	0.00174	7,243,4
1lvk_2#652	GO:0005524: ATP binding	0.00174	7,243,4
1ihua1#119	GO:0000287: magnesium ion binding	0.00174	85,128,11
1qqga2#166	GO:0004620: phospholipase activity	0.00175	200,19,6
1bxoa_#307	GO:0008201: heparin binding	0.001751	104,24,5
1xis__#214	GO:0003924: GTPase activity	0.001752	88,17,4

1a9xa3#78	GO:0015036: disulfide oxidoreductase activity	0.001753	305,22,8
1eoka_#127	GO:0005524: ATP binding	0.001754	33,243,9
1fid__#237	GO:0004295: trypsin activity	0.001754	51,48,5
1doka_#52	GO:0004295: trypsin activity	0.001754	51,48,5
1dpe__#402	GO:0005524: ATP binding	0.001754	33,243,9
1zfpe_#67	GO:0005524: ATP binding	0.001754	33,243,9
1e8ga2#201	GO:0003779: actin binding	0.001755	304,32,10
1e32a2#345	GO:0005525: GTP binding	0.001756	100,49,7
1awx__#17	GO:0005524: ATP binding	0.001759	16,243,6
1aisb2#1274	GO:0005524: ATP binding	0.001759	16,243,6
1ddwa_#77	GO:0030151: molybdenum ion binding	0.00176	364,15,7
1ddwa_#77	GO:0004725: protein tyrosine phosphatase activity	0.00176	364,15,7
1erv__#80	GO:0004190: aspartic-type endopeptidase activity	0.001764	442,23,10
1a6ca2#312	GO:0005529: sugar binding	0.001765	93,39,6
1aym3_#131	GO:0016638: oxidoreductase activity, acting on the CH-NH2 group of donors	0.001765	412,17,8
1fm2.1#B627	GO:0005524: ATP binding	0.001766	27,243,8
1fu6a_#35	GO:0005524: ATP binding	0.001766	27,243,8
1ejda_#213	GO:0016251: general RNA polymerase II transcription factor activity	0.00177	284,14,6
1nal1_#203	GO:0004556: alpha-amylase activity	0.001778	46,15,3
1qs0b2#269	GO:0030151: molybdenum ion binding	0.001778	46,15,3
1egaa1#58	GO:0005096: GTPase activator activity	0.001778	46,15,3
1sgpe_#115	GO:0004295: trypsin activity	0.001783	4,48,2
1bd2d1#62	GO:0004295: trypsin activity	0.001783	4,48,2
1qksa2#236	GO:0019955: cytokine binding	0.001783	64,11,3
1qksa2#236	GO:0003724: RNA helicase activity	0.001783	64,11,3
1pfza_#156	GO:0019955: cytokine binding	0.001783	64,11,3
1f0xa1#278	GO:0019838: growth factor binding	0.001784	180,9,4
1jj22_#78	GO:0003916: DNA topoisomerase activity	0.001784	71,10,3
1e79d2#36	GO:0004714: transmembrane receptor protein tyrosine kinase activity	0.001785	524,14,8
1dpe__#190	GO:0004497: monooxygenase activity	0.001786	7,26,2
1dcia_#233	GO:0004812: tRNA ligase activity	0.001786	7,26,2
1f2na_#138	GO:0004812: tRNA ligase activity	0.001786	96,26,5
1poxa3#439	GO:0016616: oxidoreductase activity, acting on the CH-OH group of donors, NAD or NADP as acceptor	0.001786	25,59,4
1db3a_#266	GO:0016831: carboxy-lyase activity	0.001786	59,25,4
1hqva_#48	GO:0016705: oxidoreductase activity, acting on paired donors, with incorporation or reduction of molecular oxygen	0.001786	96,26,5
1atia2#249	GO:0003887: DNA-directed DNA polymerase activity	0.001787	9,20,2
1a3k__#122	GO:0005126: hematopoietin/interferon-class (D200-domain) cytokine receptor binding	0.001787	9,20,2
1hxxa_#228	GO:0019838: growth factor binding	0.001787	20,9,2
1dpsa_#36	GO:0008408: 3'-5' exonuclease activity	0.001792	205,13,5
1bvsa3#12	GO:0005525: GTP binding	0.001794	30,49,4
1el5a1#10	GO:0016814: hydrolase activity, acting on carbon-nitrogen (but not peptide) bonds, in cyclic amidines	0.001798	18,10,2
1c0pa1#1133	GO:0050660: FAD binding	0.001798	18,10,2
1fw8a_#126	GO:0003684: damaged DNA binding	0.001798	18,10,2
1df0a3#208	GO:0016846: carbon-sulfur lyase activity	0.001798	18,10,2
1a6o__#217	GO:0005066: transmembrane receptor protein tyrosine kinase signaling protein activity	0.001798	18,10,2
1howa_#697	GO:0005066: transmembrane receptor protein tyrosine kinase signaling protein activity	0.001798	18,10,2
1qnja_#124	GO:0003809: thrombin activity	0.001798	18,10,2
1a4ya_#428	GO:0004457: lactate dehydrogenase activity	0.001798	18,10,2
1bn8a_#241	GO:0004457: lactate dehydrogenase activity	0.001798	18,10,2
1g51a1#47	GO:0003682: chromatin binding	0.001798	18,10,2
1phk__#165	GO:0005066: transmembrane receptor protein tyrosine kinase signaling protein activity	0.001798	18,10,2
3thia_#59	GO:0016846: carbon-sulfur lyase activity	0.001798	18,10,2
1d0na6#681	GO:0004364: glutathione transferase activity	0.001798	143,11,4
1qp8a1#172	GO:0016620: oxidoreductase activity, acting on the aldehyde or oxo group of donors, NAD or NADP as acceptor	0.001798	18,10,2
1xgsa2#82	GO:0004295: trypsin activity	0.001803	307,48,13
1c7na_#231	GO:0030151: molybdenum ion binding	0.001804	263,15,6
1e8ca3#111	GO:0016620: oxidoreductase activity, acting on the aldehyde or oxo group of donors, NAD or NADP as acceptor	0.001804	280,10,5
1tif__#58	GO:0005507: copper ion binding	0.001806	65,38,5
1e5ka_#113	GO:0030145: manganese ion binding	0.001806	65,38,5
1i6vd_#886	GO:0003899: DNA-directed RNA polymerase activity	0.001809	15,12,2
1qgub_#292	GO:0030151: molybdenum ion binding	0.001809	12,15,2

1g6ha_#180	GO:0004180: carboxypeptidase activity	0.001809	12,15,2
1eu3a1#51	GO:0003743: translation initiation factor activity	0.001809	12,15,2
1dyoa_#25	GO:0003697: single-stranded DNA binding	0.001809	15,12,2
1hnja1#8	GO:0030151: molybdenum ion binding	0.001809	12,15,2
1afj__#28	GO:0030151: molybdenum ion binding	0.001809	12,15,2
1e6pa3#340	GO:0003899: DNA-directed RNA polymerase activity	0.001809	15,12,2
1dzfa2#164	GO:0003899: DNA-directed RNA polymerase activity	0.001809	15,12,2
1ejda_#94	GO:0005096: GTPase activator activity	0.001809	12,15,2
1rypf_#50	GO:0003697: single-stranded DNA binding	0.001809	15,12,2
1shsa_#68	GO:0004556: alpha-amylase activity	0.001809	12,15,2
1lml__#283	GO:0009036: type II site-specific deoxyribonuclease activity	0.001809	15,12,2
1bmv2_#2064	GO:0051082: unfolded protein binding	0.001809	147,34,7
2viua_#252	GO:0042802: protein self binding	0.00181	343,12,6
1bhga3#529	GO:0005525: GTP binding	0.00181	74,49,6
1flga_#161	GO:0016638: oxidoreductase activity, acting on the CH-NH2 group of donors	0.001811	152,17,5
1mai__#108	GO:0016638: oxidoreductase activity, acting on the CH-NH2 group of donors	0.001811	152,17,5
2ltn.1#A63	GO:0008083: growth factor activity	0.001814	152,42,8
1rlr_1#66	GO:0005524: ATP binding	0.001815	67,243,14
1utea_#11	GO:0003697: single-stranded DNA binding	0.001816	130,12,4
1qhda2#323	GO:0005529: sugar binding	0.001818	337,39,12
1erza_#183	GO:0005509: calcium ion binding	0.001819	79,160,12
1ile_2#359	GO:0005509: calcium ion binding	0.00182	10,160,4
1g7sa4#55	GO:0005509: calcium ion binding	0.00182	10,160,4
1erja_#525	GO:0005509: calcium ion binding	0.00182	10,160,4
1bvza3#425	GO:0005509: calcium ion binding	0.00182	10,160,4
1f13a3#645	GO:0005509: calcium ion binding	0.001821	5,160,3
1hxn__#235	GO:0005509: calcium ion binding	0.001821	5,160,3
1qmva_#40	GO:0016646: oxidoreductase activity, acting on the CH-NH group of donors, NAD or NADP as acceptor	0.001821	79,19,4
1qaua_#64	GO:0005509: calcium ion binding	0.001821	5,160,3
1i19a1#396	GO:0000287: magnesium ion binding	0.001825	6,128,3
1gdoa_#24	GO:0000287: magnesium ion binding	0.001825	6,128,3
1axca1#70	GO:0008083: growth factor activity	0.001825	118,42,7
1stfi_#116	GO:0004812: tRNA ligase activity	0.001826	57,26,4
1dssg1#4	GO:0004812: tRNA ligase activity	0.001826	57,26,4
1h4ua1#404	GO:0004867: serine-type endopeptidase inhibitor activity	0.001826	202,47,10
2hhma_#262	GO:0004523: ribonuclease H activity	0.001828	95,16,4
1b65a_#257	GO:0030145: manganese ion binding	0.001828	210,38,9
1b65a_#257	GO:0005507: copper ion binding	0.001828	210,38,9
1gtra1#468	GO:0005085: guanyl-nucleotide exchange factor activity	0.001829	54,13,3
1quqa_#75	GO:0005085: guanyl-nucleotide exchange factor activity	0.001829	54,13,3
1e1aa_#178	GO:0005518: collagen binding	0.001829	54,13,3
2cpl__#20	GO:0051082: unfolded protein binding	0.001832	108,34,6
1fkna_#96	GO:0005126: hematopoietin/interferon-class (D200-domain) cytokine receptor binding	0.001836	75,20,4
1je5a_#10	GO:0005516: calmodulin binding	0.001836	62,24,4
1ihoa_#238	GO:0003887: DNA-directed DNA polymerase activity	0.001836	75,20,4
1pdc__#27	GO:0003968: RNA-directed RNA polymerase activity	0.001836	50,14,3
1fw9a_#157	GO:0004197: cysteine-type endopeptidase activity	0.001836	62,24,4
1ie5a_#102	GO:0008201: heparin binding	0.001836	62,24,4
1c9oa_#49	GO:0003743: translation initiation factor activity	0.001837	102,15,4
1hxxa_#278	GO:0004497: monooxygenase activity	0.001844	255,26,8
1fuia1#550	GO:0030145: manganese ion binding	0.001845	5,38,2
1ezvd1#213	GO:0005507: copper ion binding	0.001845	5,38,2
1ixh__#7	GO:0005507: copper ion binding	0.001845	5,38,2
19hca_#16	GO:0030145: manganese ion binding	0.001845	5,38,2
1ycqa_#24	GO:0005507: copper ion binding	0.001845	5,38,2
1h7ea_#106	GO:0030145: manganese ion binding	0.001845	5,38,2
1a3qa2#190	GO:0019955: cytokine binding	0.001845	144,11,4
1f97a2#199	GO:0019955: cytokine binding	0.001845	144,11,4
1db3a_#266	GO:0009036: type II site-specific deoxyribonuclease activity	0.001847	59,12,3
2sli_2#326	GO:0003724: RNA helicase activity	0.00185	251,11,5
1fi2a_#109	GO:0004674: protein serine/threonine kinase activity	0.00185	228,42,10
1jb0d_#26	GO:0046983: protein dimerization activity	0.001851	229,17,6
1jb0d_#26	GO:0016638: oxidoreductase activity, acting on the CH-NH2 group of donors	0.001851	229,17,6
1ge8a2#134	GO:0046983: protein dimerization activity	0.001851	229,17,6
1qgva_#6	GO:0008235: metalloexopeptidase activity	0.001852	14,13,2

1clia2#265	GO:0004714: transmembrane receptor protein tyrosine kinase activity	0.001852	13,14,2
1ezia_#198	GO:0016776: phosphotransferase activity, phosphate group as acceptor	0.001852	13,14,2
1ayoa_#55	GO:0003968: RNA-directed RNA polymerase activity	0.001852	13,14,2
2pola3#335	GO:0008408: 3'-5' exonuclease activity	0.001852	14,13,2
1hqoa2#159	GO:0008080: N-acetyltransferase activity	0.001852	14,13,2
1qq9a_#100	GO:0004177: aminopeptidase activity	0.001852	14,13,2
1qq9a_#100	GO:0016651: oxidoreductase activity, acting on NADH or NADPH	0.001852	14,13,2
1qq9a_#100	GO:0016861: intramolecular oxidoreductase activity, interconverting aldoses and ketoses	0.001852	14,13,2
1dt6a_#449	GO:0016651: oxidoreductase activity, acting on NADH or NADPH	0.001852	14,13,2
1fgka_#668	GO:0004714: transmembrane receptor protein tyrosine kinase activity	0.001852	13,14,2
1d4oa_#135	GO:0016651: oxidoreductase activity, acting on NADH or NADPH	0.001852	14,13,2
1ycsa_#230	GO:0005518: collagen binding	0.001852	14,13,2
1e30a_#107	GO:0000049: tRNA binding	0.001852	14,13,2
1nls__#77	GO:0005518: collagen binding	0.001852	14,13,2
1ef1a2#245	GO:0005543: phospholipid binding	0.001852	13,14,2
1i0da_#297	GO:0016854: racemase and epimerase activity	0.001852	14,13,2
1i0da_#297	GO:0016861: intramolecular oxidoreductase activity, interconverting aldoses and ketoses	0.001852	14,13,2
1cnza_#19	GO:0016651: oxidoreductase activity, acting on NADH or NADPH	0.001852	14,13,2
1acc__#79	GO:0005518: collagen binding	0.001852	14,13,2
1c7na_#231	GO:0003887: DNA-directed DNA polymerase activity	0.001853	263,20,7
2viua_#252	GO:0005126: hematopoietin/interferon-class (D200-domain) cytokine receptor binding	0.001854	343,20,8
1azo__#144	GO:0046983: protein dimerization activity	0.001858	41,17,3
1dlja2#177	GO:0016638: oxidoreductase activity, acting on the CH-NH2 group of donors	0.001858	41,17,3
1dmla2#313	GO:0046983: protein dimerization activity	0.001858	41,17,3
1imva_#278	GO:0016799: hydrolase activity, hydrolyzing N-glycosyl compounds	0.001858	41,17,3
1thw__#199	GO:0016799: hydrolase activity, hydrolyzing N-glycosyl compounds	0.001858	41,17,3
1thw__#199	GO:0016638: oxidoreductase activity, acting on the CH-NH2 group of donors	0.001858	41,17,3
1qjva_#173	GO:0005525: GTP binding	0.001858	4,49,2
1ddma_#130	GO:0005518: collagen binding	0.00186	313,13,6
1el0a_#28	GO:0003809: thrombin activity	0.001863	161,10,4
1dp0a4#756	GO:0005529: sugar binding	0.001863	165,39,8
1f3ya_#6	GO:0005529: sugar binding	0.001865	94,39,6
3lada1#116	GO:0016627: oxidoreductase activity, acting on the CH-CH group of donors	0.001865	153,17,5
1bu7a_#405	GO:0008081: phosphoric diester hydrolase activity	0.001865	65,11,3
1e5ka_#113	GO:0003964: RNA-directed DNA polymerase activity	0.001865	65,11,3
1dypa_#143	GO:0005529: sugar binding	0.001865	94,39,6
1cpy__#380	GO:0005524: ATP binding	0.001868	171,243,27
1bak__#583	GO:0016668: oxidoreductase activity, acting on sulfur group of donors, NAD or NADP as acceptor	0.001869	131,12,4
1e79d2#36	GO:0046983: protein dimerization activity	0.00187	524,17,9
1dqza_#209	GO:0016836: hydro-lyase activity	0.001873	45,33,4
1tuba1#239	GO:0019201: nucleotide kinase activity	0.001878	120,13,4
1a81a2#182	GO:0008408: 3'-5' exonuclease activity	0.001878	120,13,4
2napa2#292	GO:0019201: nucleotide kinase activity	0.001878	120,13,4
1g5ca_#164	GO:0008270: zinc ion binding	0.001887	7,108,3
1d3ya_#261	GO:0008270: zinc ion binding	0.001887	7,108,3
1f24a_#293	GO:0015036: disulfide oxidoreductase activity	0.001887	116,22,5
1fqva2#326	GO:0008270: zinc ion binding	0.001887	7,108,3
1e25a_#262	GO:0019843: rRNA binding	0.001887	116,22,5
1g4ia_#11	GO:0004197: cysteine-type endopeptidase activity	0.001888	29,24,3
3rpba_#550	GO:0008201: heparin binding	0.001888	29,24,3
1b3ra1#218	GO:0004197: cysteine-type endopeptidase activity	0.001888	29,24,3
1hxma2#150	GO:0008201: heparin binding	0.001888	29,24,3
1g8kb_#11	GO:0005509: calcium ion binding	0.001889	59,160,10
1ekma1#545	GO:0005509: calcium ion binding	0.001891	90,160,13
1bu8a2#124	GO:0000287: magnesium ion binding	0.001892	140,128,15
1wht.1#A15	GO:0004867: serine-type endopeptidase inhibitor activity	0.001893	15,47,3
1aiha_#226	GO:0004867: serine-type endopeptidase inhibitor activity	0.001893	15,47,3
1e31a_#77	GO:0030151: molybdenum ion binding	0.001893	47,15,3
1qgna_#373	GO:0004725: protein tyrosine phosphatase activity	0.001893	47,15,3
1c7na_#95	GO:0019838: growth factor binding	0.001899	183,9,4
1qtn.1#A270	GO:0019201: nucleotide kinase activity	0.001901	439,13,7
1qtn.1#A270	GO:0016861: intramolecular oxidoreductase activity, interconverting aldoses and ketoses	0.001901	439,13,7
1evqa_#61	GO:0005509: calcium ion binding	0.001902	159,160,19
1fc3a_#172	GO:0015078: hydrogen ion transporter activity	0.001904	182,21,6
1mjha_#119	GO:0004725: protein tyrosine phosphatase activity	0.001905	103,15,4

1ekma1#545	GO:0046983: protein dimerization activity	0.001905	90,17,4
1ekma1#545	GO:0016638: oxidoreductase activity, acting on the CH-NH2 group of donors	0.001905	90,17,4
1dfoa_#237	GO:0003887: DNA-directed DNA polymerase activity	0.001906	35,20,3
1mrj__#161	GO:0005529: sugar binding	0.001907	18,39,3
1dfoa_#88	GO:0005529: sugar binding	0.001907	18,39,3
1f46a_#59	GO:0008201: heparin binding	0.001907	106,24,5
1feca1#28	GO:0016616: oxidoreductase activity, acting on the CH-OH group of donors, NAD or NADP as acceptor	0.001909	62,59,6
1ih7a1#150	GO:0016616: oxidoreductase activity, acting on the CH-OH group of donors, NAD or NADP as acceptor	0.001909	62,59,6
1jb0d_#26	GO:0008083: growth factor activity	0.001913	229,42,10
1ej8a_#189	GO:0004190: aspartic-type endopeptidase activity	0.001915	294,23,8
1a4ya_#47	GO:0008757: S-adenosylmethionine-dependent methyltransferase activity	0.001919	216,24,7
1f0ia1#50	GO:0005524: ATP binding	0.001921	146,243,24
1hdma1#98	GO:0004896: hematopoietin/interferon-class (D200-domain) cytokine receptor activity	0.001923	37,19,3
1fwxa2#396	GO:0008270: zinc ion binding	0.001925	237,108,19
1dpga2#334	GO:0005509: calcium ion binding	0.001925	287,160,29
1fhoa_#74	GO:0042802: protein self binding	0.001925	347,12,6
1akp__#62	GO:0005518: collagen binding	0.001929	55,13,3
1enfa1#77	GO:0005085: guanyl-nucleotide exchange factor activity	0.001929	55,13,3
1e69a_#26	GO:0016651: oxidoreductase activity, acting on NADH or NADPH	0.001929	55,13,3
1hdr__#156	GO:0016651: oxidoreductase activity, acting on NADH or NADPH	0.001929	55,13,3
1qlaa3#364	GO:0003809: thrombin activity	0.001934	73,10,3
1a81a1#51	GO:0005066: transmembrane receptor protein tyrosine kinase signaling protein activity	0.001934	73,10,3
1rypk_#12	GO:0003887: DNA-directed DNA polymerase activity	0.001938	265,20,7
1seia_#50	GO:0004812: tRNA ligase activity	0.001939	27,26,3
1jswa_#228	GO:0004812: tRNA ligase activity	0.001939	27,26,3
1f2ri_#61	GO:0004812: tRNA ligase activity	0.001939	27,26,3
1qvba_#9	GO:0016866: intramolecular transferase activity	0.001939	60,12,3
1a8d_1#87	GO:0004896: hematopoietin/interferon-class (D200-domain) cytokine receptor activity	0.001941	204,19,6
1ihua2#525	GO:0016831: carboxy-lyase activity	0.001941	335,25,9
1i4fa1#233	GO:0005529: sugar binding	0.001943	5,39,2
2pola2#218	GO:0003755: peptidyl-prolyl cis-trans isomerase activity	0.001943	146,11,4
1doka_#52	GO:0003968: RNA-directed RNA polymerase activity	0.001945	51,14,3
1d0va_#124	GO:0003779: actin binding	0.001946	6,32,2
1jlxa2#288	GO:0003779: actin binding	0.001946	6,32,2
1ax4a_#275	GO:0016831: carboxy-lyase activity	0.001947	102,25,5
1euwa_#71	GO:0003779: actin binding	0.001948	22,32,3
1dkra2#210	GO:0019843: rRNA binding	0.001948	32,22,3
1gcua1#36	GO:0015036: disulfide oxidoreductase activity	0.001948	32,22,3
2bce__#204	GO:0010181: FMN binding	0.001949	66,11,3
1e39a3#417	GO:0016627: oxidoreductase activity, acting on the CH-CH group of donors	0.00195	11,17,2
1qora2#233	GO:0016627: oxidoreductase activity, acting on the CH-CH group of donors	0.00195	11,17,2
1bjt__#1066	GO:0003724: RNA helicase activity	0.00195	17,11,2
2napa2#444	GO:0004364: glutathione transferase activity	0.00195	17,11,2
1bn8a_#41	GO:0046983: protein dimerization activity	0.00195	11,17,2
1g4da_#18	GO:0046983: protein dimerization activity	0.00195	11,17,2
1i3ja_#233	GO:0016638: oxidoreductase activity, acting on the CH-NH2 group of donors	0.00195	11,17,2
1i6vc_#869	GO:0046983: protein dimerization activity	0.00195	11,17,2
1eur__#118	GO:0046983: protein dimerization activity	0.00195	11,17,2
1nal1_#204	GO:0003964: RNA-directed DNA polymerase activity	0.00195	17,11,2
1ac6a_#43	GO:0003809: thrombin activity	0.001951	163,10,4
1g6oa_#116	GO:0004674: protein serine/threonine kinase activity	0.001959	358,42,13
1cg2a1#43	GO:0016616: oxidoreductase activity, acting on the CH-OH group of donors, NAD or NADP as acceptor	0.00196	189,59,11
1edza1#206	GO:0004867: serine-type endopeptidase inhibitor activity	0.001963	399,47,15
1qdla_#211	GO:0046983: protein dimerization activity	0.001969	320,17,7
1qdla_#211	GO:0016638: oxidoreductase activity, acting on the CH-NH2 group of donors	0.001969	320,17,7
2napa1#665	GO:0019829: cation-transporting ATPase activity	0.001972	21,9,2
1pa2a_#232	GO:0015078: hydrogen ion transporter activity	0.001972	9,21,2
1nsca_#306	GO:0019838: growth factor binding	0.001972	21,9,2
1plc__#74	GO:0015078: hydrogen ion transporter activity	0.001972	9,21,2
1bxoa_#307	GO:0004725: protein tyrosine phosphatase activity	0.001975	104,15,4
1qora2#201	GO:0004812: tRNA ligase activity	0.001981	199,26,7
1fjfj_#68	GO:0016668: oxidoreductase activity, acting on sulfur group of donors, NAD or NADP as acceptor	0.001983	230,12,5

1ldna1#94	GO:0016836: hydro-lyase activity	0.001984	199,33,8
1jh2a_#16	GO:0016638: oxidoreductase activity, acting on the CH-NH2 group of donors	0.001985	91,17,4
1dih_2#222	GO:0008757: S-adenosylmethionine-dependent methyltransferase activity	0.001988	352,24,9
1ffya2#226	GO:0016799: hydrolase activity, hydrolyzing N-glycosyl compounds	0.001993	42,17,3
2btva_#749	GO:0016638: oxidoreductase activity, acting on the CH-NH2 group of donors	0.001993	42,17,3
1bmv2_#2064	GO:0003755: peptidyl-prolyl cis-trans isomerase activity	0.001993	147,11,4
1c4zd_#85	GO:0003779: actin binding	0.001996	117,32,6
1g0sa_#74	GO:0005529: sugar binding	0.001999	294,39,11
1gpea1#273	GO:0004896: hematopoietin/interferon-class (D200-domain) cytokine receptor activity	0.001999	81,19,4
2mev3_#108	GO:0004190: aspartic-type endopeptidase activity	0.002002	228,23,7
1qrra_#11	GO:0016620: oxidoreductase activity, acting on the aldehyde or oxo group of donors, NAD or NADP as acceptor	0.002005	19,10,2
2fcba2#104	GO:0004896: hematopoietin/interferon-class (D200-domain) cytokine receptor activity	0.002005	10,19,2
2rsla_#55	GO:0003684: damaged DNA binding	0.002005	19,10,2
1nfdb1#115	GO:0004896: hematopoietin/interferon-class (D200-domain) cytokine receptor activity	0.002005	10,19,2
2dkb__#133	GO:0016846: carbon-sulfur lyase activity	0.002005	19,10,2
1eu8a_#126	GO:0008199: ferric iron binding	0.002005	19,10,2
1dar_2#264	GO:0003682: chromatin binding	0.002005	19,10,2
1dz4a_#150	GO:0005261: cation channel activity	0.002005	19,10,2
1ajsa_#326	GO:0016846: carbon-sulfur lyase activity	0.002005	19,10,2
1tgoa1#217	GO:0016646: oxidoreductase activity, acting on the CH-NH group of donors, NAD or NADP as acceptor	0.002005	10,19,2
1dmua_#199	GO:0004896: hematopoietin/interferon-class (D200-domain) cytokine receptor activity	0.002005	10,19,2
1b87a_#47	GO:0004842: ubiquitin-protein ligase activity	0.002005	10,19,2
1air__#233	GO:0004222: metalloendopeptidase activity	0.002005	10,19,2
1dpja_#320	GO:0003809: thrombin activity	0.002005	19,10,2
1fmk_3#377	GO:0005066: transmembrane receptor protein tyrosine kinase signaling protein activity	0.002005	19,10,2
1evqa_#305	GO:0016668: oxidoreductase activity, acting on sulfur group of donors, NAD or NADP as acceptor	0.002006	490,12,7
1fjfc2#153	GO:0046983: protein dimerization activity	0.002007	420,17,8
1h4vb2#307	GO:0005351: sugar porter activity	0.002009	73,21,4
1i7oa2#399	GO:0004674: protein serine/threonine kinase activity	0.00201	36,42,4
1dlfl_#97	GO:0008083: growth factor activity	0.00201	36,42,4
1wht.1#A15	GO:0004295: trypsin activity	0.002012	15,48,3
1pce__#35	GO:0005066: transmembrane receptor protein tyrosine kinase signaling protein activity	0.002012	74,10,3
1e05i_#79	GO:0004295: trypsin activity	0.002012	15,48,3
1cnv__#181	GO:0016620: oxidoreductase activity, acting on the aldehyde or oxo group of donors, NAD or NADP as acceptor	0.002012	74,10,3
1flca1#294	GO:0030145: manganese ion binding	0.002014	98,38,6
1ecfa1#388	GO:0008757: S-adenosylmethionine-dependent methyltransferase activity	0.002016	8,24,2
1fmta2#81	GO:0008757: S-adenosylmethionine-dependent methyltransferase activity	0.002016	8,24,2
1biha3#248	GO:0005516: calmodulin binding	0.002016	8,24,2
1hava_#141	GO:0004197: cysteine-type endopeptidase activity	0.002016	8,24,2
1e4ft2#287	GO:0004197: cysteine-type endopeptidase activity	0.002016	8,24,2
1e4ft2#287	GO:0008201: heparin binding	0.002016	8,24,2
1i5na_#30	GO:0005516: calmodulin binding	0.002016	8,24,2
1jlxa1#147	GO:0008201: heparin binding	0.002016	8,24,2
3grx__#55	GO:0000287: magnesium ion binding	0.002018	113,128,13
1dpga2#334	GO:0008800: beta-lactamase activity	0.002019	287,10,5
1e32a2#328	GO:0005524: ATP binding	0.00202	40,243,10
2pia_1#99	GO:0030151: molybdenum ion binding	0.002022	179,15,5
1e8ca1#83	GO:0000287: magnesium ion binding	0.002022	170,128,17
1utea_#11	GO:0005524: ATP binding	0.002023	130,243,22
1qfma1#139	GO:0000049: tRNA binding	0.002023	318,13,6
1c9la2#61	GO:0008270: zinc ion binding	0.002024	238,108,19
1fn9a_#271	GO:0008201: heparin binding	0.002025	218,24,7
1e19a_#281	GO:0016646: oxidoreductase activity, acting on the CH-NH group of donors, NAD or NADP as acceptor	0.002025	138,19,5
1boub_#7	GO:0015036: disulfide oxidoreductase activity	0.002026	240,22,7
1hwxa1#223	GO:0016638: oxidoreductase activity, acting on the CH-NH2 group of donors	0.002026	233,17,6
1qgub_#197	GO:0000287: magnesium ion binding	0.002028	74,128,10
1a6o__#45	GO:0005507: copper ion binding	0.002031	303,38,11
1i50a_#856	GO:0004556: alpha-amylase activity	0.002031	269,15,6
1hyea2#272	GO:0005525: GTP binding	0.002032	31,49,4
1dcpa_#25	GO:0000049: tRNA binding	0.002032	56,13,3
1f7ua2#324	GO:0000049: tRNA binding	0.002032	56,13,3

1h8ma_#8	GO:0016668: oxidoreductase activity, acting on sulfur group of donors, NAD or NADP as acceptor	0.002033	134,12,4
1fgua2#360	GO:0003697: single-stranded DNA binding	0.002034	61,12,3
1i9ga_#67	GO:0003899: DNA-directed RNA polymerase activity	0.002034	61,12,3
1fds__#59	GO:0015405: P-P-bond-hydrolysis-driven transporter activity	0.002034	61,12,3
1aky_2#133	GO:0046983: protein dimerization activity	0.002035	156,17,5
1evqa_#305	GO:0004180: carboxypeptidase activity	0.002035	490,15,8
1kdj__#27	GO:0019955: cytokine binding	0.002036	67,11,3
2reb_1#138	GO:0016758: transferase activity, transferring hexosyl groups	0.002036	67,11,3
1hw5a2#64	GO:0004190: aspartic-type endopeptidase activity	0.002038	167,23,6
1g51a3#525	GO:0000287: magnesium ion binding	0.002038	263,128,23
1amp__#205	GO:0004867: serine-type endopeptidase inhibitor activity	0.00204	79,47,6
1aop_3#276	GO:0005524: ATP binding	0.002041	172,243,27
1i7qa_#321	GO:0019955: cytokine binding	0.002044	148,11,4
1au1a_#58	GO:0005096: GTPase activator activity	0.002046	105,15,4
1e2o__#346	GO:0004714: transmembrane receptor protein tyrosine kinase activity	0.002046	194,14,5
1xvaa_#244	GO:0005507: copper ion binding	0.002051	40,38,4
1djna1#86	GO:0016616: oxidoreductase activity, acting on the CH-OH group of donors, NAD or NADP as acceptor	0.002051	85,59,7
1jb0d_#26	GO:0004190: aspartic-type endopeptidase activity	0.002054	229,23,7
1nsj__#124	GO:0005509: calcium ion binding	0.002055	148,160,18
2bpa1_#45	GO:0004674: protein serine/threonine kinase activity	0.002056	192,42,9
1gsa_2#218	GO:0003887: DNA-directed DNA polymerase activity	0.002057	131,20,5
1e30a_#87	GO:0015082: di-, tri-valent inorganic cation transporter activity	0.002057	52,14,3
1e30a_#87	GO:0046915: transition metal ion transporter activity	0.002057	52,14,3
1e30a_#87	GO:0003968: RNA-directed RNA polymerase activity	0.002057	52,14,3
1dvpa1#65	GO:0019829: cation-transporting ATPase activity	0.002059	187,9,4
1doi__#42	GO:0003697: single-stranded DNA binding	0.002062	16,12,2
1bdfa2#171	GO:0003899: DNA-directed RNA polymerase activity	0.002062	16,12,2
1bdfa2#171	GO:0016668: oxidoreductase activity, acting on sulfur group of donors, NAD or NADP as acceptor	0.002062	16,12,2
1hx6a1#163	GO:0016668: oxidoreductase activity, acting on sulfur group of donors, NAD or NADP as acceptor	0.002062	16,12,2
1ush_1#462	GO:0016668: oxidoreductase activity, acting on sulfur group of donors, NAD or NADP as acceptor	0.002062	16,12,2
1j71a_#251	GO:0005524: ATP binding	0.002062	106,243,19
1e32a1#45	GO:0003899: DNA-directed RNA polymerase activity	0.002062	16,12,2
2dri__#115	GO:0015405: P-P-bond-hydrolysis-driven transporter activity	0.002062	16,12,2
1cl1a_#184	GO:0004197: cysteine-type endopeptidase activity	0.002066	64,24,4
1hxmb1#117	GO:0004197: cysteine-type endopeptidase activity	0.002066	64,24,4
1pfza_#156	GO:0008201: heparin binding	0.002066	64,24,4
1thg__#3	GO:0016638: oxidoreductase activity, acting on the CH-NH2 group of donors	0.002067	92,17,4
2viua_#252	GO:0051082: unfolded protein binding	0.002067	343,34,11
1i7qa_#278	GO:0005126: hematopoietin/interferon-class (D200-domain) cytokine receptor binding	0.00207	36,20,3
1jj2j_#88	GO:0003887: DNA-directed DNA polymerase activity	0.00207	36,20,3
1tc1a_#163	GO:0016763: transferase activity, transferring pentosyl groups	0.002072	7,28,2
1hnua_#190	GO:0016763: transferase activity, transferring pentosyl groups	0.002072	7,28,2
1qc7a_#261	GO:0016763: transferase activity, transferring pentosyl groups	0.002072	7,28,2
1imva_#255	GO:0000049: tRNA binding	0.002072	589,13,8
1imva_#255	GO:0008235: metalloexopeptidase activity	0.002072	589,13,8
1f0xa1#278	GO:0030151: molybdenum ion binding	0.002073	180,15,5
1g6sa_#64	GO:0008757: S-adenosylmethionine-dependent methyltransferase activity	0.002073	108,24,5
1icia_#175	GO:0016616: oxidoreductase activity, acting on the CH-OH group of donors, NAD or NADP as acceptor	0.002075	63,59,6
1bwvs_#117	GO:0000287: magnesium ion binding	0.002076	329,128,27
1f8ra1#233	GO:0005507: copper ion binding	0.002079	19,38,3
1hava_#41	GO:0005529: sugar binding	0.002079	96,39,6
1jkma_#317	GO:0005525: GTP binding	0.002079	76,49,6
1dqga_#81	GO:0004896: hematopoietin/interferon-class (D200-domain) cytokine receptor activity	0.002079	38,19,3
1sppb_#32	GO:0004620: phospholipase activity	0.002079	38,19,3
1br9__#86	GO:0005529: sugar binding	0.002079	96,39,6
1lara2#1769	GO:0008201: heparin binding	0.00208	219,24,7
1h8ma_#8	GO:0005507: copper ion binding	0.002084	134,38,7
1ejda_#213	GO:0008757: S-adenosylmethionine-dependent methyltransferase activity	0.002085	284,24,8
1i7oa1#119	GO:0005516: calmodulin binding	0.002086	30,24,3
2stv__#129	GO:0004197: cysteine-type endopeptidase activity	0.002086	30,24,3

1b34b_#56	GO:0005516: calmodulin binding	0.002086	30,24,3
1elja_#64	GO:0005525: GTP binding	0.002086	103,49,7
1ycsa_#162	GO:0005509: calcium ion binding	0.002087	210,160,23
1ihoa_#238	GO:0008800: beta-lactamase activity	0.002091	75,10,3
1gsoa3#142	GO:0004896: hematopoietin/interferon-class (D200-domain) cytokine receptor activity	0.002092	82,19,4
1i5ga_#115	GO:0004497: monooxygenase activity	0.002095	324,26,9
1bio__#210	GO:0003755: peptidyl-prolyl cis-trans isomerase activity	0.002096	149,11,4
3sil__#84	GO:0051082: unfolded protein binding	0.002097	45,34,4
1hdma1#115	GO:0019838: growth factor binding	0.0021	188,9,4
1ihua2#343	GO:0009036: type II site-specific deoxyribonuclease activity	0.002102	233,12,5
1b65a_#257	GO:0016831: carboxy-lyase activity	0.002114	210,25,7
1amf__#196	GO:0005507: copper ion binding	0.002118	173,38,8
1i5ga_#115	GO:0016638: oxidoreductase activity, acting on the CH-NH2 group of donors	0.002119	324,17,7
1quba4#235	GO:0030151: molybdenum ion binding	0.00212	106,15,4
1loua_#79	GO:0004725: protein tyrosine phosphatase activity	0.00212	106,15,4
1aa6_2#285	GO:0030151: molybdenum ion binding	0.00212	106,15,4
1f5aa2#167	GO:0005085: guanyl-nucleotide exchange factor activity	0.002121	124,13,4
1nsca_#379	GO:0000049: tRNA binding	0.002121	124,13,4
1kit_3#331	GO:0004177: aminopeptidase activity	0.002121	124,13,4
1kit_3#331	GO:0005518: collagen binding	0.002121	124,13,4
1pwt__#8	GO:0019843: rRNA binding	0.002121	314,22,8
8abp__#231	GO:0003700: transcription factor activity	0.002121	13,124,4
1i50a_#430	GO:0004295: trypsin activity	0.002122	32,48,4
1io7a_#243	GO:0003887: DNA-directed DNA polymerase activity	0.002124	78,20,4
1a65a1#108	GO:0004714: transmembrane receptor protein tyrosine kinase activity	0.002124	294,14,6
1dqaa1#596	GO:0003755: peptidyl-prolyl cis-trans isomerase activity	0.002125	68,11,3
1nsj__#124	GO:0008810: cellulase activity	0.002128	148,18,5
1bxka_#262	GO:0016831: carboxy-lyase activity	0.00213	29,25,3
1qo7a_#109	GO:0016831: carboxy-lyase activity	0.00213	29,25,3
1e6pa2#274	GO:0016831: carboxy-lyase activity	0.00213	29,25,3
1eyea_#131	GO:0000049: tRNA binding	0.002131	15,13,2
1qf6a4#295	GO:0000049: tRNA binding	0.002131	15,13,2
1atia2#67	GO:0000049: tRNA binding	0.002131	15,13,2
1dyoa_#25	GO:0016651: oxidoreductase activity, acting on NADH or NADPH	0.002131	15,13,2
1dgwa_#97	GO:0004177: aminopeptidase activity	0.002131	15,13,2
1dgwa_#97	GO:0008235: metalloexopeptidase activity	0.002131	15,13,2
1ekma1#386	GO:0030151: molybdenum ion binding	0.002131	13,15,2
1tbga_#63	GO:0005518: collagen binding	0.002131	15,13,2
1a0ca_#273	GO:0016861: intramolecular oxidoreductase activity, interconverting aldoses and ketoses	0.002131	15,13,2
1ctqa_#163	GO:0005085: guanyl-nucleotide exchange factor activity	0.002131	15,13,2
1dzfa2#164	GO:0000049: tRNA binding	0.002131	15,13,2
1dzfa2#164	GO:0008408: 3'-5' exonuclease activity	0.002131	15,13,2
1a0ca_#187	GO:0030151: molybdenum ion binding	0.002131	13,15,2
1hc7a2#111	GO:0000049: tRNA binding	0.002131	15,13,2
1ei5a3#190	GO:0004180: carboxypeptidase activity	0.002131	13,15,2
1hc7a2#109	GO:0000049: tRNA binding	0.002131	15,13,2
1fvua_#120	GO:0000049: tRNA binding	0.002131	15,13,2
1jlxa2#214	GO:0030151: molybdenum ion binding	0.002131	13,15,2
1dkia_#208	GO:0016651: oxidoreductase activity, acting on NADH or NADPH	0.002131	15,13,2
3frua1#249	GO:0008408: 3'-5' exonuclease activity	0.002131	15,13,2
1fxma_#153	GO:0000049: tRNA binding	0.002131	15,13,2
1f4la2#25	GO:0000049: tRNA binding	0.002131	15,13,2
1je5a_#10	GO:0016668: oxidoreductase activity, acting on sulfur group of donors, NAD or NADP as acceptor	0.002132	62,12,3
1jbqa_#152	GO:0016702: oxidoreductase activity, acting on single donors with incorporation of molecular oxygen, incorporation of two atoms of oxygen	0.002132	62,12,3
1ospo_#262	GO:0016638: oxidoreductase activity, acting on the CH-NH2 group of donors	0.002134	43,17,3
1elja_#8	GO:0003924: GTPase activity	0.002134	43,17,3
1trka2#429	GO:0016627: oxidoreductase activity, acting on the CH-CH group of donors	0.002134	43,17,3
1hw5a2#64	GO:0008009: chemokine activity	0.002135	167,10,4
1fzqa_#28	GO:0005096: GTPase activator activity	0.002136	49,15,3
1leha1#177	GO:0005096: GTPase activator activity	0.002136	49,15,3
1czan1#92	GO:0016620: oxidoreductase activity, acting on the aldehyde or oxo group of donors, NAD or NADP as acceptor	0.002137	631,10,7
1ec7a1#216	GO:0016836: hydro-lyase activity	0.002139	78,33,5
2fmr__#10	GO:0003729: mRNA binding	0.002139	57,13,3

1j9la_#164	GO:0000049: tRNA binding	0.002139	57,13,3
1tul__#102	GO:0000049: tRNA binding	0.002139	57,13,3
1f97a1#62	GO:0004812: tRNA ligase activity	0.00214	707,26,14
1el0a_#28	GO:0004197: cysteine-type endopeptidase activity	0.00214	161,24,6
1edha2#106	GO:0008270: zinc ion binding	0.002141	35,108,6
1aky_2#133	GO:0008083: growth factor activity	0.002143	156,42,8
1j9qa1#154	GO:0004714: transmembrane receptor protein tyrosine kinase activity	0.002143	196,14,5
1gdea_#243	GO:0008083: growth factor activity	0.002144	61,42,5
1hyrc1#247	GO:0008083: growth factor activity	0.002144	61,42,5
1ltda2#34	GO:0004263: chymotrypsin activity	0.002147	5,41,2
1nsj__#178	GO:0016627: oxidoreductase activity, acting on the CH-CH group of donors	0.002151	93,17,4
1apme_#101	GO:0004714: transmembrane receptor protein tyrosine kinase activity	0.002154	14,14,2
1qgva_#6	GO:0016776: phosphotransferase activity, phosphate group as acceptor	0.002154	14,14,2
2pola3#335	GO:0004714: transmembrane receptor protein tyrosine kinase activity	0.002154	14,14,2
1bmv2_#2102	GO:0004714: transmembrane receptor protein tyrosine kinase activity	0.002154	14,14,2
1csn__#134	GO:0004714: transmembrane receptor protein tyrosine kinase activity	0.002154	14,14,2
1bkds_#882	GO:0005543: phospholipid binding	0.002154	14,14,2
1plq_1#90	GO:0046983: protein dimerization activity	0.002154	158,17,5
1plq_1#90	GO:0016638: oxidoreductase activity, acting on the CH-NH2 group of donors	0.002154	158,17,5
1fsu__#434	GO:0008201: heparin binding	0.002155	356,24,9
1quna1#113	GO:0004896: hematopoietin/interferon-class (D200-domain) cytokine receptor activity	0.002157	286,19,7
1iba__#39	GO:0003887: DNA-directed DNA polymerase activity	0.002158	351,20,8
1i7qa_#321	GO:0004812: tRNA ligase activity	0.002159	148,26,6
1czya1#466	GO:0008201: heparin binding	0.00216	109,24,5
1dm9a_#57	GO:0004197: cysteine-type endopeptidase activity	0.00216	109,24,5
1bu8a2#124	GO:0004620: phospholipase activity	0.00216	140,19,5
1beba_#119	GO:0004812: tRNA ligase activity	0.002162	202,26,7
1ejda_#6	GO:0003887: DNA-directed DNA polymerase activity	0.002163	270,20,7
1h7wa2#535	GO:0015036: disulfide oxidoreductase activity	0.002165	9,22,2
1at3a_#194	GO:0019843: rRNA binding	0.002165	9,22,2
1f2la_#40	GO:0016638: oxidoreductase activity, acting on the CH-NH2 group of donors	0.002165	236,17,6
1d3ga_#109	GO:0015036: disulfide oxidoreductase activity	0.002165	9,22,2
1bec_2#216	GO:0004714: transmembrane receptor protein tyrosine kinase activity	0.00217	115,14,4
1hxxa_#173	GO:0019955: cytokine binding	0.002171	260,11,5
1hbza_#214	GO:0005529: sugar binding	0.002172	297,39,11
1jkma_#317	GO:0004457: lactate dehydrogenase activity	0.002173	76,10,3
1pbe_1#150	GO:0003916: DNA topoisomerase activity	0.002173	76,10,3
1faoa_#250	GO:0003682: chromatin binding	0.002173	76,10,3
1qfma2#576	GO:0008026: ATP-dependent helicase activity	0.002175	214,13,5
1e6ca_#91	GO:0004556: alpha-amylase activity	0.002178	182,15,5
1e44b_#75	GO:0005509: calcium ion binding	0.002179	198,160,22
1bqsa2#103	GO:0005524: ATP binding	0.002179	22,243,7
1bfg__#22	GO:0005524: ATP binding	0.002182	61,243,13
1bjt__#890	GO:0008009: chemokine activity	0.002184	292,10,5
1bjt__#890	GO:0003916: DNA topoisomerase activity	0.002184	292,10,5
1aqt_2#45	GO:0051082: unfolded protein binding	0.002185	76,34,5
1zpda3#398	GO:0016831: carboxy-lyase activity	0.002188	8,25,2
1wdna_#79	GO:0003724: RNA helicase activity	0.002189	18,11,2
1wdna_#79	GO:0010181: FMN binding	0.002189	18,11,2
1c0pa1#1133	GO:0003724: RNA helicase activity	0.002189	18,11,2
1c0pa1#1133	GO:0008081: phosphoric diester hydrolase activity	0.002189	18,11,2
1fw8a_#126	GO:0010181: FMN binding	0.002189	18,11,2
1eur__#118	GO:0008810: cellulase activity	0.002189	11,18,2
1ig8a_#203	GO:0008757: S-adenosylmethionine-dependent methyltransferase activity	0.002189	65,24,4
1e8ya2#433	GO:0019955: cytokine binding	0.002189	18,11,2
1zpda1#322	GO:0000287: magnesium ion binding	0.00219	2,128,2
1pysb6#217	GO:0000287: magnesium ion binding	0.00219	2,128,2
1obwa_#39	GO:0000287: magnesium ion binding	0.00219	2,128,2
1f3ub_#28	GO:0000287: magnesium ion binding	0.00219	2,128,2
1gph12#46	GO:0000287: magnesium ion binding	0.00219	2,128,2
1xis__#17	GO:0000287: magnesium ion binding	0.00219	2,128,2
1zpda3#392	GO:0000287: magnesium ion binding	0.00219	2,128,2
1ewka_#349	GO:0000287: magnesium ion binding	0.00219	2,128,2
1ct9a2#27	GO:0000287: magnesium ion binding	0.00219	2,128,2
1dvpa1#65	GO:0004601: peroxidase activity	0.002191	187,21,6
1gpea1#271	GO:0003968: RNA-directed RNA polymerase activity	0.002192	197,14,5

1nsj__#178	GO:0016763: transferase activity, transferring pentosyl groups	0.002192	93,28,5
1cbf__#208	GO:0019838: growth factor binding	0.002193	333,9,5
1imva_#255	GO:0005509: calcium ion binding	0.002195	589,160,50
1bev1_#86	GO:0005518: collagen binding	0.002196	323,13,6
1j6za1#84	GO:0051082: unfolded protein binding	0.002197	6,34,2
1urna_#93	GO:0051082: unfolded protein binding	0.002197	6,34,2
2ltn.1#A63	GO:0051082: unfolded protein binding	0.002197	152,34,7
1jj2n_#77	GO:0000287: magnesium ion binding	0.002199	51,128,8
1fuia2#273	GO:0003887: DNA-directed DNA polymerase activity	0.0022	133,20,5
1fhga_#59	GO:0005524: ATP binding	0.002203	34,243,9
1af7_2#248	GO:0005524: ATP binding	0.002203	34,243,9
1vin_2#335	GO:0004601: peroxidase activity	0.002204	35,21,3
1cpt__#52	GO:0015078: hydrogen ion transporter activity	0.002204	35,21,3
1f0xa2#118	GO:0015078: hydrogen ion transporter activity	0.002204	35,21,3
3chbd_#85	GO:0004725: protein tyrosine phosphatase activity	0.002207	378,15,7
1htp__#16	GO:0005509: calcium ion binding	0.002207	303,160,30
1e1aa_#285	GO:0016668: oxidoreductase activity, acting on sulfur group of donors, NAD or NADP as acceptor	0.002208	137,12,4
1j9qa1#154	GO:0051082: unfolded protein binding	0.00221	196,34,8
1fwxa2#396	GO:0016638: oxidoreductase activity, acting on the CH-NH2 group of donors	0.002213	237,17,6
1evqa_#61	GO:0046983: protein dimerization activity	0.002216	159,17,5
1evqa_#61	GO:0016638: oxidoreductase activity, acting on the CH-NH2 group of donors	0.002216	159,17,5
1mjha_#126	GO:0003924: GTPase activity	0.002216	159,17,5
1qfma1#139	GO:0004674: protein serine/threonine kinase activity	0.002216	318,42,12
1qfma1#139	GO:0008083: growth factor activity	0.002216	318,42,12
1pot__#138	GO:0005351: sugar porter activity	0.002221	75,21,4
1cpt__#144	GO:0003887: DNA-directed DNA polymerase activity	0.002224	10,20,2
1i8na_#74	GO:0008800: beta-lactamase activity	0.002224	20,10,2
1cb8a1#326	GO:0005126: hematopoietin/interferon-class (D200-domain) cytokine receptor binding	0.002224	10,20,2
3btaa2#1258	GO:0005525: GTP binding	0.002224	77,49,6
1znca_#167	GO:0003887: DNA-directed DNA polymerase activity	0.002224	10,20,2
1koba_#175	GO:0005066: transmembrane receptor protein tyrosine kinase signaling protein activity	0.002224	20,10,2
1qtra_#108	GO:0005525: GTP binding	0.002224	77,49,6
1exma2#353	GO:0005524: ATP binding	0.002229	91,243,17
1danh_#104	GO:0030151: molybdenum ion binding	0.002232	183,15,5
1i5ga_#115	GO:0000049: tRNA binding	0.002232	324,13,6
1ddja_#681	GO:0005529: sugar binding	0.002233	253,39,10
1d2ka1#164	GO:0016866: intramolecular transferase activity	0.002233	63,12,3
1f3ya_#6	GO:0046983: protein dimerization activity	0.002238	94,17,4
1ayl__#237	GO:0003697: single-stranded DNA binding	0.002238	357,12,6
1hbza_#214	GO:0004714: transmembrane receptor protein tyrosine kinase activity	0.00224	297,14,6
1hbza_#214	GO:0003968: RNA-directed RNA polymerase activity	0.00224	297,14,6
1nat__#50	GO:0016776: phosphotransferase activity, phosphate group as acceptor	0.00224	297,14,6
1fmta2#151	GO:0000287: magnesium ion binding	0.002241	314,128,26
1im4a_#153	GO:0003887: DNA-directed DNA polymerase activity	0.002242	37,20,3
1awx__#13	GO:0005126: hematopoietin/interferon-class (D200-domain) cytokine receptor binding	0.002242	37,20,3
1j79a_#38	GO:0004222: metalloendopeptidase activity	0.002242	39,19,3
1hfoa_#57	GO:0003887: DNA-directed DNA polymerase activity	0.002242	37,20,3
1fp5a2#478	GO:0005529: sugar binding	0.002242	19,39,3
1abrb2#252	GO:0005529: sugar binding	0.002242	19,39,3
1e44b_#75	GO:0004714: transmembrane receptor protein tyrosine kinase activity	0.002243	198,14,5
1g6oa_#116	GO:0004197: cysteine-type endopeptidase activity	0.002243	358,24,9
1aym3_#131	GO:0015082: di-, tri-valent inorganic cation transporter activity	0.002247	412,14,7
1aym3_#131	GO:0004714: transmembrane receptor protein tyrosine kinase activity	0.002247	412,14,7
1aym3_#131	GO:0046915: transition metal ion transporter activity	0.002247	412,14,7
1qqga2#253	GO:0008757: S-adenosylmethionine-dependent methyltransferase activity	0.002249	110,24,5
1dkia_#76P	GO:0003729: mRNA binding	0.00225	58,13,3
1fmta2#151	GO:0003779: actin binding	0.00225	314,32,10
1eqka_#47	GO:0003729: mRNA binding	0.002251	126,13,4
1aoca_#123	GO:0008083: growth factor activity	0.002252	5,42,2
1epwa2#1205	GO:0008083: growth factor activity	0.002252	5,42,2
1ecra_#101	GO:0008083: growth factor activity	0.002252	5,42,2
1fhga_#119	GO:0008083: growth factor activity	0.002252	5,42,2
1g24a_#202	GO:0004674: protein serine/threonine kinase activity	0.002252	5,42,2
1qqqa_#38	GO:0004674: protein serine/threonine kinase activity	0.002252	5,42,2
1vin_1#302	GO:0004674: protein serine/threonine kinase activity	0.002252	5,42,2

1el0a_#42	GO:0003809: thrombin activity	0.002253	294,10,5
3grx__#56	GO:0019838: growth factor binding	0.002254	335,9,5
1b4ka_#108	GO:0016836: hydro-lyase activity	0.002254	116,33,6
1ycsa_#162	GO:0004896: hematopoietin/interferon-class (D200-domain) cytokine receptor activity	0.002255	210,19,6
1b65a_#257	GO:0004222: metalloendopeptidase activity	0.002255	210,19,6
1ih7a1#57	GO:0003682: chromatin binding	0.002257	77,10,3
1jlxa2#166	GO:0003809: thrombin activity	0.002257	77,10,3
2ltn.1#A63	GO:0019955: cytokine binding	0.002257	152,11,4
1mai__#108	GO:0003755: peptidyl-prolyl cis-trans isomerase activity	0.002257	152,11,4
1smaa1#95	GO:0005126: hematopoietin/interferon-class (D200-domain) cytokine receptor binding	0.002259	272,20,7
1xgsa2#82	GO:0030145: manganese ion binding	0.002261	307,38,11
1pdc__#27	GO:0030151: molybdenum ion binding	0.002265	50,15,3
1cb8a2#613	GO:0003697: single-stranded DNA binding	0.002268	138,12,4
1cb8a2#613	GO:0016668: oxidoreductase activity, acting on sulfur group of donors, NAD or NADP as acceptor	0.002268	138,12,4
1a4ya_#47	GO:0004177: aminopeptidase activity	0.002268	216,13,5
1a4ya_#47	GO:0016651: oxidoreductase activity, acting on NADH or NADPH	0.002268	216,13,5
1fwxa2#396	GO:0016668: oxidoreductase activity, acting on sulfur group of donors, NAD or NADP as acceptor	0.00227	237,12,5
3sil__#317	GO:0016668: oxidoreductase activity, acting on sulfur group of donors, NAD or NADP as acceptor	0.002271	358,12,6
1fwxa1#535	GO:0008270: zinc ion binding	0.002272	15,108,4
1g6sa_#64	GO:0004180: carboxypeptidase activity	0.002272	108,15,4
1jfra_#41	GO:0016811: hydrolase activity, acting on carbon-nitrogen (but not peptide) bonds, in linear amides	0.002272	108,15,4
2bpa1_#45	GO:0019838: growth factor binding	0.002272	192,9,4
1qfea_#68	GO:0016758: transferase activity, transferring hexosyl groups	0.002273	560,11,7
1ee4a_#266	GO:0051082: unfolded protein binding	0.002277	46,34,4
1dgw.1#Y353	GO:0008083: growth factor activity	0.002277	319,42,12
2cuaa_#83	GO:0051082: unfolded protein binding	0.002277	46,34,4
1ac6a_#43	GO:0008201: heparin binding	0.00228	163,24,6
1f5aa1#441	GO:0008757: S-adenosylmethionine-dependent methyltransferase activity	0.00228	163,24,6
1mla_1#281	GO:0003924: GTPase activity	0.002281	44,17,3
1ezvb1#29	GO:0046983: protein dimerization activity	0.002281	44,17,3
1iira_#242	GO:0003684: damaged DNA binding	0.002282	170,10,4
1gpea1#271	GO:0051082: unfolded protein binding	0.002283	197,34,8
1edza1#206	GO:0003724: RNA helicase activity	0.002284	399,11,6
1fepa_#340	GO:0005524: ATP binding	0.002285	99,243,18
1jc4a_#12	GO:0005524: ATP binding	0.002286	28,243,8
3pah__#303	GO:0005524: ATP binding	0.002286	28,243,8
1e1aa_#261	GO:0005524: ATP binding	0.002286	28,243,8
1by5a_#347	GO:0019955: cytokine binding	0.002287	263,11,5
1by5a_#347	GO:0003964: RNA-directed DNA polymerase activity	0.002287	263,11,5
1fo4a5#1020	GO:0008201: heparin binding	0.002288	359,24,9
1c9la2#12	GO:0005066: transmembrane receptor protein tyrosine kinase signaling protein activity	0.002288	295,10,5
1fm2.1#B308	GO:0004263: chymotrypsin activity	0.002289	200,41,9
1epwa3#175	GO:0005525: GTP binding	0.00229	53,49,5
1jfua_#53	GO:0003779: actin binding	0.002292	49,32,4
1efnb_#102	GO:0003779: actin binding	0.002292	49,32,4
1dkra2#210	GO:0005525: GTP binding	0.002292	32,49,4
1hdr__#241	GO:0005525: GTP binding	0.002292	32,49,4
1eula_#518	GO:0005525: GTP binding	0.002292	32,49,4
1eg2a_#132	GO:0008757: S-adenosylmethionine-dependent methyltransferase activity	0.002296	31,24,3
1ir3a_#1192	GO:0005516: calmodulin binding	0.002296	31,24,3
1qqp1_#51	GO:0004197: cysteine-type endopeptidase activity	0.002296	31,24,3
1qqp1_#51	GO:0008201: heparin binding	0.002296	31,24,3
1yrga_#194	GO:0004197: cysteine-type endopeptidase activity	0.002296	31,24,3
1f0xa1#523	GO:0019838: growth factor binding	0.002298	87,9,3
1dfaa2#211	GO:0019829: cation-transporting ATPase activity	0.002298	87,9,3
1d2ka1#164	GO:0000287: magnesium ion binding	0.0023	63,128,9
1bu7a_#405	GO:0003700: transcription factor activity	0.002303	65,124,9
1qfma1#139	GO:0015036: disulfide oxidoreductase activity	0.002304	318,22,8
1jj2v_#118	GO:0005509: calcium ion binding	0.002305	103,160,14
1h6va3#486	GO:0003724: RNA helicase activity	0.002311	70,11,3
1cqka_#52	GO:0003724: RNA helicase activity	0.002311	70,11,3
1pma1_#79	GO:0003964: RNA-directed DNA polymerase activity	0.002311	70,11,3

1tiid_#39	GO:0008483: transaminase activity	0.002312	239,17,6
3lada1#116	GO:0004364: glutathione transferase activity	0.002313	153,11,4
1a7ca_#202	GO:0015082: di-, tri-valent inorganic cation transporter activity	0.002313	117,14,4
1a7ca_#202	GO:0046915: transition metal ion transporter activity	0.002313	117,14,4
1jlxa2#166	GO:0051082: unfolded protein binding	0.002316	77,34,5
1as4.1#A288	GO:0008201: heparin binding	0.002316	66,24,4
1czan1#92	GO:0004556: alpha-amylase activity	0.002317	631,15,9
1gpea1#273	GO:0004867: serine-type endopeptidase inhibitor activity	0.002321	81,47,6
1bqsa2#103	GO:0051082: unfolded protein binding	0.002325	22,34,3
2hhma_#262	GO:0016638: oxidoreductase activity, acting on the CH-NH2 group of donors	0.002328	95,17,4
1e6pa2#46	GO:0016627: oxidoreductase activity, acting on the CH-CH group of donors	0.002328	95,17,4
1dr9a2#139	GO:0005529: sugar binding	0.002329	67,39,5
1a6o__#45	GO:0004190: aspartic-type endopeptidase activity	0.00233	303,23,8
1qs0a1#119	GO:0016861: intramolecular oxidoreductase activity, interconverting aldoses and ketoses	0.00233	454,13,7
16pk__#312	GO:0046983: protein dimerization activity	0.002331	12,17,2
16pk__#312	GO:0003924: GTPase activity	0.002331	12,17,2
1i1b__#85	GO:0016638: oxidoreductase activity, acting on the CH-NH2 group of donors	0.002331	12,17,2
1eut_1#488	GO:0042802: protein self binding	0.002331	17,12,2
1g31a_#15	GO:0016638: oxidoreductase activity, acting on the CH-NH2 group of donors	0.002331	12,17,2
1qmua1#309	GO:0016702: oxidoreductase activity, acting on single donors with incorporation of molecular oxygen, incorporation of two atoms of oxygen	0.002331	17,12,2
1g38a_#155	GO:0016627: oxidoreductase activity, acting on the CH-CH group of donors	0.002331	12,17,2
1qrea_#132	GO:0016627: oxidoreductase activity, acting on the CH-CH group of donors	0.002331	12,17,2
1pjr_1#87	GO:0016866: intramolecular transferase activity	0.002331	17,12,2
1d7ya1#243	GO:0016638: oxidoreductase activity, acting on the CH-NH2 group of donors	0.002331	12,17,2
1bl0a2#102	GO:0016627: oxidoreductase activity, acting on the CH-CH group of donors	0.002331	12,17,2
1fmca_#248	GO:0016627: oxidoreductase activity, acting on the CH-CH group of donors	0.002331	12,17,2
1shsa_#68	GO:0016638: oxidoreductase activity, acting on the CH-NH2 group of donors	0.002331	12,17,2
2u2fa_#33	GO:0046983: protein dimerization activity	0.002331	12,17,2
1hcl__#135	GO:0046983: protein dimerization activity	0.002331	12,17,2
1kit_3#297	GO:0016638: oxidoreductase activity, acting on the CH-NH2 group of donors	0.002331	12,17,2
1aqt_2#45	GO:0015078: hydrogen ion transporter activity	0.002332	76,21,4
1dt6a_#385	GO:0015078: hydrogen ion transporter activity	0.002332	76,21,4
1qqsa_#124	GO:0004190: aspartic-type endopeptidase activity	0.002332	458,23,10
1cpy__#380	GO:0003809: thrombin activity	0.002332	171,10,4
1a9xa3#78	GO:0016836: hydro-lyase activity	0.002332	305,33,10
1bak__#647	GO:0005351: sugar porter activity	0.002332	76,21,4
1hq8a_#206	GO:0004497: monooxygenase activity	0.002336	329,26,9
1qksa2#236	GO:0042802: protein self binding	0.002337	64,12,3
1f0xa1#278	GO:0019843: rRNA binding	0.002339	180,22,6
1el0a_#28	GO:0016638: oxidoreductase activity, acting on the CH-NH2 group of donors	0.002342	161,17,5
1cipa2#34	GO:0003682: chromatin binding	0.002343	78,10,3
1jjya_#324	GO:0004457: lactate dehydrogenase activity	0.002343	78,10,3
1io7a_#243	GO:0005261: cation channel activity	0.002343	78,10,3
1fgua1#249	GO:0000287: magnesium ion binding	0.002343	21,128,5
1mil__#30	GO:0004812: tRNA ligase activity	0.00235	61,26,4
1dgw.1#Y353	GO:0019843: rRNA binding	0.002351	319,22,8
1f8v.1#A116	GO:0005507: copper ion binding	0.002351	101,38,6
1h4vb2#282	GO:0005507: copper ion binding	0.002351	101,38,6
1f00i2#805	GO:0003887: DNA-directed DNA polymerase activity	0.002351	135,20,5
1b3ra2#60	GO:0016831: carboxy-lyase activity	0.002353	30,25,3
1i7oa1#119	GO:0016831: carboxy-lyase activity	0.002353	30,25,3
1dik_3#240	GO:0005507: copper ion binding	0.002354	262,38,10
1dpsa_#36	GO:0004812: tRNA ligase activity	0.002355	205,26,7
1hbza_#214	GO:0008009: chemokine activity	0.00236	297,10,5
1qqga2#166	GO:0005525: GTP binding	0.00236	200,49,10
1nat__#50	GO:0050660: FAD binding	0.00236	297,10,5
1fn9a_#271	GO:0008408: 3'-5' exonuclease activity	0.002364	218,13,5
1fgga_#134	GO:0008235: metalloexopeptidase activity	0.002364	218,13,5
7reqa2#702	GO:0016854: racemase and epimerase activity	0.002364	59,13,3
1ig8a_#215	GO:0008408: 3'-5' exonuclease activity	0.002364	59,13,3
1ixh__#180	GO:0016616: oxidoreductase activity, acting on the CH-OH group of donors, NAD or NADP as acceptor	0.002364	13,59,3
1e8ya2#433	GO:0008083: growth factor activity	0.002366	18,42,3
1e79h2#39	GO:0019829: cation-transporting ATPase activity	0.002367	23,9,2

1eqga2#47	GO:0016705: oxidoreductase activity, acting on paired donors, with incorporation or reduction of molecular oxygen	0.002367	8,26,2
1ytba2#217	GO:0003714: transcription corepressor activity	0.002367	26,8,2
1hr6a1#59	GO:0004497: monooxygenase activity	0.002367	8,26,2
1fsu__#434	GO:0003887: DNA-directed DNA polymerase activity	0.002367	356,20,8
1fsu__#434	GO:0005126: hematopoietin/interferon-class (D200-domain) cytokine receptor binding	0.002367	356,20,8
1ec7a1#216	GO:0005525: GTP binding	0.002376	78,49,6
1f42a2#123	GO:0004620: phospholipase activity	0.002377	378,19,8
1iow_1#37	GO:0030145: manganese ion binding	0.002383	309,38,11
1b35b_#97	GO:0005507: copper ion binding	0.002383	309,38,11
1fuia1#506	GO:0004295: trypsin activity	0.002384	33,48,4
2dnja_#158	GO:0004295: trypsin activity	0.002384	33,48,4
1gcya2#331	GO:0005509: calcium ion binding	0.002384	51,160,9
1bn8a_#55	GO:0000287: magnesium ion binding	0.002386	13,128,4
1edza1#206	GO:0004812: tRNA ligase activity	0.002386	399,26,10
1ixh__#180	GO:0000287: magnesium ion binding	0.002386	13,128,4
1axn__#42	GO:0003700: transcription factor activity	0.00239	78,124,10
1ejda_#374	GO:0016705: oxidoreductase activity, acting on paired donors, with incorporation or reduction of molecular oxygen	0.002391	29,26,3
1agi__#46	GO:0004812: tRNA ligase activity	0.002391	29,26,3
1xnb__#131	GO:0004725: protein tyrosine phosphatase activity	0.002399	51,15,3
1cvra2#24	GO:0016616: oxidoreductase activity, acting on the CH-OH group of donors, NAD or NADP as acceptor	0.002399	27,59,4
1mil__#30	GO:0008270: zinc ion binding	0.002399	61,108,8
1jswa_#228	GO:0016616: oxidoreductase activity, acting on the CH-OH group of donors, NAD or NADP as acceptor	0.002399	27,59,4
1f2la_#40	GO:0004674: protein serine/threonine kinase activity	0.002403	236,42,10
1jj2j_#70	GO:0019955: cytokine binding	0.002408	71,11,3
1jj2j_#70	GO:0003724: RNA helicase activity	0.002408	71,11,3
1tyfa_#31	GO:0016799: hydrolase activity, hydrolyzing N-glycosyl compounds	0.002408	162,17,5
1e6pa2#46	GO:0016763: transferase activity, transferring pentosyl groups	0.002409	95,28,5
1c4ka1#33	GO:0016646: oxidoreductase activity, acting on the CH-NH group of donors, NAD or NADP as acceptor	0.002413	40,19,3
1e79d3#328	GO:0016646: oxidoreductase activity, acting on the CH-NH group of donors, NAD or NADP as acceptor	0.002413	40,19,3
2pia_3#282	GO:0004896: hematopoietin/interferon-class (D200-domain) cytokine receptor activity	0.002413	40,19,3
1lara2#1769	GO:0008080: N-acetyltransferase activity	0.002413	219,13,5
1wdna_#187	GO:0016646: oxidoreductase activity, acting on the CH-NH group of donors, NAD or NADP as acceptor	0.002413	40,19,3
1a4ya_#104	GO:0016638: oxidoreductase activity, acting on the CH-NH2 group of donors	0.002414	241,17,6
1hava_#41	GO:0046983: protein dimerization activity	0.002419	96,17,4
1hq8a_#206	GO:0008235: metalloexopeptidase activity	0.002419	329,13,6
1akp__#62	GO:0003968: RNA-directed RNA polymerase activity	0.00242	55,14,3
2bbkh_#214	GO:0005126: hematopoietin/interferon-class (D200-domain) cytokine receptor binding	0.002422	38,20,3
1sppb_#32	GO:0005126: hematopoietin/interferon-class (D200-domain) cytokine receptor binding	0.002422	38,20,3
1c7na_#231	GO:0030145: manganese ion binding	0.002423	263,38,10
1foha5#218	GO:0016763: transferase activity, transferring pentosyl groups	0.002424	57,28,4
1im4a_#144	GO:0015078: hydrogen ion transporter activity	0.002426	129,21,5
1kid__#330	GO:0019955: cytokine binding	0.002427	155,11,4
1dp0a4#756	GO:0008201: heparin binding	0.002428	165,24,6
1ct9a2#168	GO:0000049: tRNA binding	0.002429	16,13,2
1hf8a_#156	GO:0003713: transcription coactivator activity	0.002429	13,16,2
1quqa_#59	GO:0005518: collagen binding	0.002429	16,13,2
1e8ga2#268	GO:0005518: collagen binding	0.002429	16,13,2
1d0va_#114	GO:0019201: nucleotide kinase activity	0.002429	16,13,2
2ts1__#59	GO:0019201: nucleotide kinase activity	0.002429	16,13,2
1jlja_#104	GO:0000049: tRNA binding	0.002429	16,13,2
1hjra_#58	GO:0008408: 3'-5' exonuclease activity	0.002429	16,13,2
1b6a_1#415	GO:0004523: ribonuclease H activity	0.002429	13,16,2
1oela1#426	GO:0000049: tRNA binding	0.002429	16,13,2
1qr4a2#166	GO:0005518: collagen binding	0.002429	16,13,2
1qf6a4#318	GO:0000049: tRNA binding	0.002429	16,13,2
1jj7a_#528	GO:0005518: collagen binding	0.002429	16,13,2
1e32a1#45	GO:0008408: 3'-5' exonuclease activity	0.002429	16,13,2
2dri__#115	GO:0005085: guanyl-nucleotide exchange factor activity	0.002429	16,13,2
1qf6a4#362	GO:0000049: tRNA binding	0.002429	16,13,2

1jcfa2#206	GO:0005524: ATP binding	0.00243	84,243,16
1c8na_#239	GO:0016836: hydro-lyase activity	0.002431	23,33,3
1qqga2#253	GO:0004180: carboxypeptidase activity	0.002431	110,15,4
1qqga2#253	GO:0004725: protein tyrosine phosphatase activity	0.002431	110,15,4
1nsca_#379	GO:0008083: growth factor activity	0.002432	124,42,7
1dqza_#209	GO:0016799: hydrolase activity, hydrolyzing N-glycosyl compounds	0.002435	45,17,3
1jj2s_#46	GO:0046983: protein dimerization activity	0.002435	45,17,3
2mpra_#190	GO:0019843: rRNA binding	0.002435	400,22,9
1ton__#47	GO:0004197: cysteine-type endopeptidase activity	0.002436	112,24,5
1dbxa_#75	GO:0005524: ATP binding	0.002437	69,243,14
1euha_#220	GO:0016646: oxidoreductase activity, acting on the CH-NH group of donors, NAD or NADP as acceptor	0.002441	11,19,2
1fid__#195	GO:0004222: metalloendopeptidase activity	0.002441	11,19,2
1hava_#66	GO:0004222: metalloendopeptidase activity	0.002441	11,19,2
1ed5a_#180	GO:0010181: FMN binding	0.002441	19,11,2
1neb__#52	GO:0003964: RNA-directed DNA polymerase activity	0.002441	19,11,2
1nsca_#91	GO:0019955: cytokine binding	0.002441	19,11,2
1div_1#141	GO:0003887: DNA-directed DNA polymerase activity	0.002442	81,20,4
1cnv__#264	GO:0004812: tRNA ligase activity	0.002443	103,26,5
1elja_#64	GO:0004812: tRNA ligase activity	0.002443	103,26,5
1foha5#9	GO:0042802: protein self binding	0.002444	65,12,3
1fa0a1#393	GO:0005351: sugar porter activity	0.002444	261,21,7
1as4.1#A336	GO:0046983: protein dimerization activity	0.002448	332,17,7
1fc4a_#205	GO:0016758: transferase activity, transferring hexosyl groups	0.002448	267,11,5
1i9ga_#67	GO:0005509: calcium ion binding	0.002449	61,160,10
1dr9a2#139	GO:0005516: calmodulin binding	0.002449	67,24,4
1dr9a2#139	GO:0008201: heparin binding	0.002449	67,24,4
1eaja_#59	GO:0004714: transmembrane receptor protein tyrosine kinase activity	0.002452	202,14,5
1h6oa_#135	GO:0015078: hydrogen ion transporter activity	0.002453	10,21,2
1hxxa_#84	GO:0005351: sugar porter activity	0.002453	10,21,2
1e9ga_#78	GO:0004601: peroxidase activity	0.002453	10,21,2
1i39a_#49	GO:0016620: oxidoreductase activity, acting on the aldehyde or oxo group of donors, NAD or NADP as acceptor	0.002453	21,10,2
1nsca_#306	GO:0005066: transmembrane receptor protein tyrosine kinase signaling protein activity	0.002453	21,10,2
1ecpa_#156	GO:0003684: damaged DNA binding	0.002453	21,10,2
1ecpa_#156	GO:0050660: FAD binding	0.002453	21,10,2
1fgka_#689	GO:0005066: transmembrane receptor protein tyrosine kinase signaling protein activity	0.002453	21,10,2
1eur__#99	GO:0003682: chromatin binding	0.002453	21,10,2
1j9qa1#154	GO:0019838: growth factor binding	0.002454	196,9,4
3sil__#317	GO:0005126: hematopoietin/interferon-class (D200-domain) cytokine receptor binding	0.002455	358,20,8
1qqsa_#124	GO:0003729: mRNA binding	0.002457	458,13,7
1qqsa_#124	GO:0005518: collagen binding	0.002457	458,13,7
1epwa3#140	GO:0004556: alpha-amylase activity	0.002458	187,15,5
1epwa3#140	GO:0004725: protein tyrosine phosphatase activity	0.002458	187,15,5
1cs1a_#160	GO:0004180: carboxypeptidase activity	0.002458	187,15,5
1vmoa_#106	GO:0004725: protein tyrosine phosphatase activity	0.002458	187,15,5
1qtn.1#A270	GO:0008757: S-adenosylmethionine-dependent methyltransferase activity	0.002461	439,24,10
1nuka_#80	GO:0005518: collagen binding	0.002462	220,13,5
1fp5a2#537	GO:0008083: growth factor activity	0.002463	38,42,4
1vmoa_#84	GO:0005507: copper ion binding	0.002463	42,38,4
1fo4a5#1020	GO:0005507: copper ion binding	0.002464	359,38,12
1f5ma_#80	GO:0008270: zinc ion binding	0.002464	105,108,11
1g2oa_#227	GO:0005524: ATP binding	0.002467	41,243,10
1ac6a_#43	GO:0016627: oxidoreductase activity, acting on the CH-CH group of donors	0.002475	163,17,5
1ac6a_#43	GO:0016638: oxidoreductase activity, acting on the CH-NH2 group of donors	0.002475	163,17,5
1ckea_#138	GO:0030151: molybdenum ion binding	0.002478	14,15,2
1qgva_#6	GO:0004180: carboxypeptidase activity	0.002478	14,15,2
1cjca1#219	GO:0030151: molybdenum ion binding	0.002478	14,15,2
1dyoa_#25	GO:0003968: RNA-directed RNA polymerase activity	0.002478	15,14,2
1h8va_#139	GO:0003968: RNA-directed RNA polymerase activity	0.002478	15,14,2
1dt6a_#449	GO:0004725: protein tyrosine phosphatase activity	0.002478	14,15,2
1rypf_#50	GO:0003968: RNA-directed RNA polymerase activity	0.002478	15,14,2
1i2ma_#90	GO:0005543: phospholipid binding	0.002478	15,14,2
1pfza_#177	GO:0005096: GTPase activator activity	0.002478	14,15,2
1acc__#360	GO:0030151: molybdenum ion binding	0.002478	14,15,2
1fwxa2#396	GO:0008083: growth factor activity	0.00248	237,42,10

1ddwa_#77	GO:0003697: single-stranded DNA binding	0.00248	364,12,6
1bccb2#274	GO:0016861: intramolecular oxidoreductase activity, interconverting aldoses and ketoses	0.002481	60,13,3
1oaca1#495	GO:0000049: tRNA binding	0.002481	60,13,3
1i4fb1#70	GO:0004263: chymotrypsin activity	0.002481	39,41,4
1ekra_#114	GO:0008235: metalloexopeptidase activity	0.002481	60,13,3
1i7wa_#517	GO:0008408: 3'-5' exonuclease activity	0.002481	60,13,3
1g51a3#524	GO:0000287: magnesium ion binding	0.002485	102,128,12
1d6aa_#52	GO:0016646: oxidoreductase activity, acting on the CH-NH group of donors, NAD or NADP as acceptor	0.002486	293,19,7
1htp__#16	GO:0004714: transmembrane receptor protein tyrosine kinase activity	0.002487	303,14,6
1bak__#647	GO:0000287: magnesium ion binding	0.002488	76,128,10
1c39a_#49	GO:0005529: sugar binding	0.002488	68,39,5
1a9xb2#1775	GO:0005525: GTP binding	0.00249	54,49,5
1cbf__#208	GO:0046983: protein dimerization activity	0.002491	333,17,7
1jbqa_#152	GO:0016705: oxidoreductase activity, acting on paired donors, with incorporation or reduction of molecular oxygen	0.002496	62,26,4
1jbqa_#152	GO:0004497: monooxygenase activity	0.002496	62,26,4
1kapp2#69	GO:0000287: magnesium ion binding	0.002498	52,128,8
1rkd__#186	GO:0000287: magnesium ion binding	0.002498	52,128,8
1qqsa_#124	GO:0003809: thrombin activity	0.002498	458,10,6
1ct9a1#233	GO:0000287: magnesium ion binding	0.002498	52,128,8
1fo4a5#1020	GO:0003887: DNA-directed DNA polymerase activity	0.002501	359,20,8
1fo4a5#1020	GO:0005126: hematopoietin/interferon-class (D200-domain) cytokine receptor binding	0.002501	359,20,8
1c7na_#95	GO:0005524: ATP binding	0.002501	183,243,28
1fxla2#169	GO:0016251: general RNA polymerase II transcription factor activity	0.002507	203,14,5
1e05i_#83	GO:0016616: oxidoreductase activity, acting on the CH-OH group of donors, NAD or NADP as acceptor	0.002508	88,59,7
1by5a_#238	GO:0004190: aspartic-type endopeptidase activity	0.002508	237,23,7
2cb5a_#312	GO:0004867: serine-type endopeptidase inhibitor activity	0.002509	111,47,7
1i2oa_#34	GO:0005351: sugar porter activity	0.00251	192,21,6
1ejda_#377	GO:0016831: carboxy-lyase activity	0.00251	108,25,5
1h6va3#486	GO:0005507: copper ion binding	0.002515	70,38,5
1hx0a2#32	GO:0030145: manganese ion binding	0.002515	70,38,5
1fepa_#49	GO:0030145: manganese ion binding	0.002515	70,38,5
1mjha_#126	GO:0016831: carboxy-lyase activity	0.002515	159,25,6
1cfe__#7	GO:0030145: manganese ion binding	0.002515	70,38,5
1ir3a_#1028	GO:0005507: copper ion binding	0.002515	70,38,5
1hw1a2#131	GO:0005524: ATP binding	0.002519	17,243,6
1je5a_#129	GO:0005524: ATP binding	0.002519	17,243,6
1a06__#205	GO:0005516: calmodulin binding	0.002519	32,24,3
1ebma2#47	GO:0004197: cysteine-type endopeptidase activity	0.002519	32,24,3
1kapp1#358	GO:0004197: cysteine-type endopeptidase activity	0.002519	32,24,3
2pgd_1#421	GO:0001584: rhodopsin-like receptor activity	0.00252	80,10,3
1fjfc2#153	GO:0004714: transmembrane receptor protein tyrosine kinase activity	0.002521	420,14,7
1ojt_2#356	GO:0016616: oxidoreductase activity, acting on the CH-OH group of donors, NAD or NADP as acceptor	0.002522	45,59,5
1d4xg_#60	GO:0051082: unfolded protein binding	0.00253	115,34,6
1qmea4#290	GO:0019843: rRNA binding	0.00253	74,22,4
1bqk__#77	GO:0015036: disulfide oxidoreductase activity	0.00253	74,22,4
1e8ga2#201	GO:0016251: general RNA polymerase II transcription factor activity	0.00253	304,14,6
1f0xa2#118	GO:0019843: rRNA binding	0.00253	35,22,3
1nsca_#379	GO:0005524: ATP binding	0.002531	124,243,21
3grx__#55	GO:0004197: cysteine-type endopeptidase activity	0.002534	113,24,5
3prn__#143	GO:0016638: oxidoreductase activity, acting on the CH-NH2 group of donors	0.002536	334,17,7
1ct9a1#233	GO:0030151: molybdenum ion binding	0.002537	52,15,3
1e6wa_#136	GO:0004556: alpha-amylase activity	0.002537	52,15,3
1neb__#14	GO:0005524: ATP binding	0.00254	12,243,5
1e8ca3#257	GO:0005524: ATP binding	0.00254	12,243,5
1ajsa_#389	GO:0005524: ATP binding	0.00254	12,243,5
1pysb6#384	GO:0005524: ATP binding	0.00254	12,243,5
1tuba1#239	GO:0016776: phosphotransferase activity, phosphate group as acceptor	0.00254	120,14,4
1a04a2#59	GO:0005524: ATP binding	0.00254	12,243,5
1gsoa3#284	GO:0005524: ATP binding	0.00254	12,243,5
2napa2#292	GO:0016776: phosphotransferase activity, phosphate group as acceptor	0.00254	120,14,4
1opy__#68	GO:0004867: serine-type endopeptidase inhibitor activity	0.002541	367,47,14
1e42a1#711	GO:0003682: chromatin binding	0.002542	175,10,4

1hdoa_#189	GO:0003924: GTPase activity	0.002543	164,17,5
1hdoa_#189	GO:0016627: oxidoreductase activity, acting on the CH-CH group of donors	0.002543	164,17,5
1hdoa_#189	GO:0016638: oxidoreductase activity, acting on the CH-NH2 group of donors	0.002543	164,17,5
1dyna_#42	GO:0046983: protein dimerization activity	0.002543	164,17,5
1jb3a_#64	GO:0008009: chemokine activity	0.002545	302,10,5
1jb3a_#64	GO:0016620: oxidoreductase activity, acting on the aldehyde or oxo group of donors, NAD or NADP as acceptor	0.002545	302,10,5
2mpra_#190	GO:0004295: trypsin activity	0.002545	400,48,15
1e3a.1#A30	GO:0010181: FMN binding	0.002545	157,11,4
1cpza_#6	GO:0003724: RNA helicase activity	0.002545	157,11,4
1qtn.1#A331	GO:0003714: transcription corepressor activity	0.002552	27,8,2
1dik_1#670	GO:0003714: transcription corepressor activity	0.002552	27,8,2
1qj5a_#268	GO:0016866: intramolecular transferase activity	0.002554	66,12,3
3sil__#317	GO:0008270: zinc ion binding	0.002559	358,108,25
1nsj__#178	GO:0005509: calcium ion binding	0.00256	93,160,13
1e25a_#262	GO:0000287: magnesium ion binding	0.002562	116,128,13
1a8d_1#87	GO:0003968: RNA-directed RNA polymerase activity	0.002563	204,14,5
1f42a2#123	GO:0008270: zinc ion binding	0.002564	378,108,26
1ds1a_#172	GO:0000049: tRNA binding	0.002564	222,13,5
1ds1a_#172	GO:0008408: 3'-5' exonuclease activity	0.002564	222,13,5
1as4.1#A336	GO:0004263: chymotrypsin activity	0.002566	332,41,12
1qdlb_#170	GO:0005524: ATP binding	0.002568	100,243,18
1kit_3#743	GO:0019843: rRNA binding	0.002571	250,22,7
2foka3#314	GO:0005525: GTP binding	0.002574	33,49,4
1b6g__#63	GO:0005525: GTP binding	0.002574	33,49,4
1ejda_#6	GO:0019955: cytokine binding	0.002575	270,11,5
1fhga_#42	GO:0005516: calmodulin binding	0.002578	9,24,2
1deua_#127	GO:0004197: cysteine-type endopeptidase activity	0.002578	9,24,2
1b6cb_#355	GO:0005516: calmodulin binding	0.002578	9,24,2
1ev2e1#154	GO:0005516: calmodulin binding	0.002578	9,24,2
2dnja_#91	GO:0005516: calmodulin binding	0.002578	9,24,2
2dnja_#91	GO:0008201: heparin binding	0.002578	9,24,2
1cbf__#208	GO:0005518: collagen binding	0.002578	333,13,6
1i9ga_#185	GO:0008757: S-adenosylmethionine-dependent methyltransferase activity	0.002578	9,24,2
2bbkh_#348	GO:0019838: growth factor binding	0.002578	24,9,2
1lci__#26	GO:0005509: calcium ion binding	0.00258	139,160,17
1jj2e1#42	GO:0008201: heparin binding	0.002582	167,24,6
1ds9a_#105	GO:0004197: cysteine-type endopeptidase activity	0.002587	68,24,4
1gdea_#379	GO:0004197: cysteine-type endopeptidase activity	0.002587	68,24,4
1qj2c2#50	GO:0005524: ATP binding	0.00259	48,243,11
1dt9a1#224	GO:0005524: ATP binding	0.00259	48,243,11
1enfa2#162	GO:0004842: ubiquitin-protein ligase activity	0.002592	41,19,3
1enfa2#162	GO:0004896: hematopoietin/interferon-class (D200-domain) cytokine receptor activity	0.002592	41,19,3
1dmla2#313	GO:0016646: oxidoreductase activity, acting on the CH-NH group of donors, NAD or NADP as acceptor	0.002592	41,19,3
1dpja_#320	GO:0004263: chymotrypsin activity	0.002592	19,41,3
1ee4a_#266	GO:0003924: GTPase activity	0.002595	46,17,3
1ee4a_#266	GO:0016627: oxidoreductase activity, acting on the CH-CH group of donors	0.002595	46,17,3
1jeya_#104	GO:0016638: oxidoreductase activity, acting on the CH-NH2 group of donors	0.002595	46,17,3
1gen__#475	GO:0005509: calcium ion binding	0.002596	25,160,6
1aym3_#131	GO:0005507: copper ion binding	0.002598	412,38,13
1gsa_2#218	GO:0000049: tRNA binding	0.002599	131,13,4
1hnja1#167	GO:0005518: collagen binding	0.002602	61,13,3
1mrj__#156	GO:0005085: guanyl-nucleotide exchange factor activity	0.002602	61,13,3
1hyrc1#247	GO:0005518: collagen binding	0.002602	61,13,3
1f0ia1#50	GO:0004896: hematopoietin/interferon-class (D200-domain) cytokine receptor activity	0.002602	146,19,5
1fcya_#311	GO:0016651: oxidoreductase activity, acting on NADH or NADPH	0.002602	61,13,3
1f0xa1#523	GO:0004896: hematopoietin/interferon-class (D200-domain) cytokine receptor activity	0.002603	87,19,4
1dfaa2#211	GO:0004620: phospholipase activity	0.002603	87,19,4
1cg2a1#43	GO:0008270: zinc ion binding	0.002605	189,108,16
1i50a_#856	GO:0016705: oxidoreductase activity, acting on paired donors, with incorporation or reduction of molecular oxygen	0.002605	269,26,8
1i50a_#856	GO:0004812: tRNA ligase activity	0.002605	269,26,8
1plq_1#90	GO:0019955: cytokine binding	0.002606	158,11,4
1xnb__#149	GO:0016627: oxidoreductase activity, acting on the CH-CH group of donors	0.002612	165,17,5
1dik_1#765	GO:0016627: oxidoreductase activity, acting on the CH-CH group of donors	0.002612	165,17,5

1ihka_#164	GO:0005529: sugar binding	0.002612	20,39,3
1a6ca2#312	GO:0008083: growth factor activity	0.002613	93,42,6
1wdna_#79	GO:0000155: two-component sensor molecule activity	0.002616	18,12,2
1tmy__#14	GO:0000155: two-component sensor molecule activity	0.002616	18,12,2
1jj2i_#30	GO:0016668: oxidoreductase activity, acting on sulfur group of donors, NAD or NADP as acceptor	0.002616	18,12,2
1hg3a_#61	GO:0042802: protein self binding	0.002616	18,12,2
1aq0a_#121	GO:0008810: cellulase activity	0.002616	12,18,2
1thg__#127	GO:0016866: intramolecular transferase activity	0.002616	18,12,2
1exma2#353	GO:0019838: growth factor binding	0.002616	91,9,3
1eu3a1#68	GO:0003697: single-stranded DNA binding	0.002616	18,12,2
1e8ua_#411	GO:0008810: cellulase activity	0.002616	12,18,2
1g5ha2#118	GO:0005524: ATP binding	0.002619	4,243,3
2mev3_#143	GO:0005524: ATP binding	0.002619	4,243,3
1gaxa1#681	GO:0005524: ATP binding	0.002619	4,243,3
1aym2_#253	GO:0005524: ATP binding	0.002619	4,243,3
1cwva3#755	GO:0005524: ATP binding	0.002619	4,243,3
1ass__#98	GO:0005524: ATP binding	0.002619	4,243,3
2mev1_#246	GO:0005524: ATP binding	0.002619	4,243,3
2mev1_#120	GO:0005524: ATP binding	0.002619	4,243,3
2hrva_#123	GO:0005524: ATP binding	0.002619	4,243,3
1ek1a2#500	GO:0005524: ATP binding	0.002619	4,243,3
1qfla1#42	GO:0005524: ATP binding	0.002619	4,243,3
7odca1#391	GO:0005524: ATP binding	0.002619	4,243,3
1b35b_#123	GO:0005524: ATP binding	0.002619	4,243,3
1e8ga2#201	GO:0003682: chromatin binding	0.002622	304,10,5
1stfi_#116	GO:0004867: serine-type endopeptidase inhibitor activity	0.002629	57,47,5
3sil__#98	GO:0008201: heparin binding	0.002629	228,24,7
1e4ea2#215	GO:0019843: rRNA binding	0.002634	490,22,10
1qtn.1#A270	GO:0016763: transferase activity, transferring pentosyl groups	0.002637	439,28,11
1ifc__#89	GO:0004556: alpha-amylase activity	0.002639	190,15,5
1ak0__#115	GO:0016705: oxidoreductase activity, acting on paired donors, with incorporation or reduction of molecular oxygen	0.00264	30,26,3
1ak0__#115	GO:0004812: tRNA ligase activity	0.00264	30,26,3
1ycsa_#162	GO:0005524: ATP binding	0.00264	210,243,31
1egia_#738	GO:0004812: tRNA ligase activity	0.00264	30,26,3
1phm_2#253	GO:0004190: aspartic-type endopeptidase activity	0.002645	309,23,8
1e87a_#174	GO:0005524: ATP binding	0.002646	141,243,23
1nfdb1#34	GO:0050660: FAD binding	0.002651	177,10,4
1p35a_#101	GO:0005529: sugar binding	0.002654	136,39,7
1ihua2#525	GO:0004812: tRNA ligase activity	0.002654	335,26,9
1tbrr1#15	GO:0008083: growth factor activity	0.002655	64,42,5
1qksa2#236	GO:0008083: growth factor activity	0.002655	64,42,5
1j71a_#251	GO:0008270: zinc ion binding	0.002659	106,108,11
1quba4#235	GO:0008270: zinc ion binding	0.002659	106,108,11
1aqua_#126	GO:0030145: manganese ion binding	0.002659	576,38,16
3prn__#143	GO:0005509: calcium ion binding	0.00266	334,160,32
1ihua2#525	GO:0016861: intramolecular oxidoreductase activity, interconverting aldoses and ketoses	0.00266	335,13,6
1mdah_#329	GO:0000049: tRNA binding	0.00266	335,13,6
1mdah_#329	GO:0008408: 3'-5' exonuclease activity	0.00266	335,13,6
2cba__#144	GO:0003697: single-stranded DNA binding	0.002667	67,12,3
2cb5a_#395	GO:0016702: oxidoreductase activity, acting on single donors with incorporation of molecular oxygen, incorporation of two atoms of oxygen	0.002667	67,12,3
2reb_1#138	GO:0016866: intramolecular transferase activity	0.002667	67,12,3
1ab4__#89	GO:0010181: FMN binding	0.002667	159,11,4
1yrga_#194	GO:0000287: magnesium ion binding	0.002667	31,128,6
1h72c2#269	GO:0003887: DNA-directed DNA polymerase activity	0.002672	83,20,4
1dhs__#99	GO:0019201: nucleotide kinase activity	0.002673	132,13,4
1f2aa_#27	GO:0016616: oxidoreductase activity, acting on the CH-OH group of donors, NAD or NADP as acceptor	0.002677	89,59,7
1mrj__#73	GO:0004180: carboxypeptidase activity	0.00268	53,15,3
1dp0a1#309	GO:0004556: alpha-amylase activity	0.00268	53,15,3
1bmv2_#2064	GO:0004620: phospholipase activity	0.002682	147,19,5
3rpba_#557	GO:0005543: phospholipid binding	0.002683	57,14,3
1fo5a_#60	GO:0016638: oxidoreductase activity, acting on the CH-NH2 group of donors	0.002684	246,17,6

1f9za_#16	GO:0016616: oxidoreductase activity, acting on the CH-OH group of donors, NAD or NADP as acceptor	0.002684	140,59,9
1esma_#314	GO:0004725: protein tyrosine phosphatase activity	0.002685	113,15,4
1ospo_#262	GO:0005507: copper ion binding	0.002688	43,38,4
1fc6a2#454	GO:0030145: manganese ion binding	0.002688	43,38,4
1jr1a1#68	GO:0016616: oxidoreductase activity, acting on the CH-OH group of donors, NAD or NADP as acceptor	0.002689	4,59,2
1fjha_#118	GO:0016616: oxidoreductase activity, acting on the CH-OH group of donors, NAD or NADP as acceptor	0.002689	4,59,2
1qmva_#40	GO:0004601: peroxidase activity	0.002691	79,21,4
1amp__#205	GO:0005351: sugar porter activity	0.002691	79,21,4
1fgka_#610	GO:0005066: transmembrane receptor protein tyrosine kinase signaling protein activity	0.002693	22,10,2
1kapp2#149	GO:0003684: damaged DNA binding	0.002693	22,10,2
1ep3a_#19	GO:0015036: disulfide oxidoreductase activity	0.002693	10,22,2
1f3mc_#403	GO:0005066: transmembrane receptor protein tyrosine kinase signaling protein activity	0.002693	22,10,2
1ejda_#213	GO:0004180: carboxypeptidase activity	0.002695	284,15,6
1ge8a2#134	GO:0008201: heparin binding	0.002696	229,24,7
1enfa1#77	GO:0005525: GTP binding	0.002702	55,49,5
1e69a_#26	GO:0005525: GTP binding	0.002702	55,49,5
1qasa3#352	GO:0003964: RNA-directed DNA polymerase activity	0.002706	20,11,2
1kcw_3#440	GO:0010181: FMN binding	0.002706	20,11,2
1idsa2#157	GO:0003887: DNA-directed DNA polymerase activity	0.002706	11,20,2
1byka_#279	GO:0003684: damaged DNA binding	0.002706	82,10,3
1howa_#618	GO:0005126: hematopoietin/interferon-class (D200-domain) cytokine receptor binding	0.002706	11,20,2
1dmla1#134	GO:0003809: thrombin activity	0.002706	82,10,3
1qfma1#186	GO:0019843: rRNA binding	0.002706	326,22,8
1dpja_#322	GO:0015082: di-, tri-valent inorganic cation transporter activity	0.002708	308,14,6
1dpja_#322	GO:0004714: transmembrane receptor protein tyrosine kinase activity	0.002708	308,14,6
1dpja_#322	GO:0046915: transition metal ion transporter activity	0.002708	308,14,6
1fu6a_#50	GO:0003968: RNA-directed RNA polymerase activity	0.002708	308,14,6
1qj5a_#268	GO:0016831: carboxy-lyase activity	0.002708	66,25,4
1h6la_#119	GO:0004714: transmembrane receptor protein tyrosine kinase activity	0.002708	308,14,6
1fmta2#151	GO:0005507: copper ion binding	0.002714	314,38,11
1c3ga2#272	GO:0008083: growth factor activity	0.002714	39,42,4
1f97a2#199	GO:0004867: serine-type endopeptidase inhibitor activity	0.002716	144,47,8
1bwvs_#38	GO:0004263: chymotrypsin activity	0.002726	40,41,4
1gg6.1#C232	GO:0005509: calcium ion binding	0.00273	11,160,4
1imva_#255	GO:0008270: zinc ion binding	0.00273	589,108,36
2bb2_1#57	GO:0005507: copper ion binding	0.00273	104,38,6
2sqca1#25	GO:0005509: calcium ion binding	0.00273	11,160,4
1ddwa_#77	GO:0003887: DNA-directed DNA polymerase activity	0.002736	364,20,8
1b12a_#221	GO:0008201: heparin binding	0.002738	115,24,5
1dy9.1#C229	GO:0008201: heparin binding	0.002738	115,24,5
1dy5a_#46	GO:0005524: ATP binding	0.002739	35,243,9
1b0pa5#739	GO:0005524: ATP binding	0.002739	35,243,9
1qe3a_#301	GO:0005524: ATP binding	0.002739	35,243,9
1qunb1#111	GO:0005507: copper ion binding	0.002742	6,38,2
1io1a_#202	GO:0030145: manganese ion binding	0.002742	6,38,2
1b3qa2#623	GO:0008270: zinc ion binding	0.002742	301,108,22
1rypd_#137	GO:0005507: copper ion binding	0.002742	6,38,2
1ecfa1#388	GO:0016763: transferase activity, transferring pentosyl groups	0.002745	8,28,2
1ed5a_#236	GO:0016638: oxidoreductase activity, acting on the CH-NH2 group of donors	0.002745	13,17,2
1clia2#265	GO:0008200: ion channel inhibitor activity	0.002745	13,17,2
1psda1#233	GO:0008026: ATP-dependent helicase activity	0.002745	17,13,2
1e79d3#334	GO:0016763: transferase activity, transferring pentosyl groups	0.002745	8,28,2
1ft9a1#195	GO:0008235: metalloexopeptidase activity	0.002745	17,13,2
1bjt__#1066	GO:0000049: tRNA binding	0.002745	17,13,2
1tml__#252	GO:0016854: racemase and epimerase activity	0.002745	17,13,2
1dqwa_#169	GO:0008483: transaminase activity	0.002745	13,17,2
1ecfa2#160	GO:0000049: tRNA binding	0.002745	17,13,2
5ruba2#41	GO:0016799: hydrolase activity, hydrolyzing N-glycosyl compounds	0.002745	13,17,2
1pkp_1#139	GO:0000049: tRNA binding	0.002745	17,13,2
1jj2j_#88	GO:0015036: disulfide oxidoreductase activity	0.002745	36,22,3
1i6vc_#689	GO:0046983: protein dimerization activity	0.002745	13,17,2
1e69a_#20	GO:0016638: oxidoreductase activity, acting on the CH-NH2 group of donors	0.002745	13,17,2
1dxea_#88	GO:0016627: oxidoreductase activity, acting on the CH-CH group of donors	0.002745	13,17,2

1cwva2#682	GO:0005518: collagen binding	0.002745	17,13,2
1qfma1#186	GO:0008083: growth factor activity	0.002745	326,42,12
1ihua2#521	GO:0000287: magnesium ion binding	0.002747	77,128,10
1lst__#56	GO:0016836: hydro-lyase activity	0.002755	24,33,3
1ad1a_#186	GO:0016836: hydro-lyase activity	0.002755	24,33,3
1bxka_#128	GO:0016836: hydro-lyase activity	0.002755	24,33,3
1qkia1#158	GO:0016836: hydro-lyase activity	0.002755	24,33,3
1hw5a2#64	GO:0016638: oxidoreductase activity, acting on the CH-NH2 group of donors	0.002756	167,17,5
1kp6a_#77	GO:0016638: oxidoreductase activity, acting on the CH-NH2 group of donors	0.002761	47,17,3
1b8ba_#466	GO:0003924: GTPase activity	0.002761	47,17,3
1b8ba_#466	GO:0016627: oxidoreductase activity, acting on the CH-CH group of donors	0.002761	47,17,3
1e31a_#77	GO:0016627: oxidoreductase activity, acting on the CH-CH group of donors	0.002761	47,17,3
2sli_2#326	GO:0051082: unfolded protein binding	0.002761	251,34,9
1b8aa2#403	GO:0051082: unfolded protein binding	0.002762	117,34,6
1utea_#11	GO:0004263: chymotrypsin activity	0.002762	130,41,7
2pia_1#99	GO:0050660: FAD binding	0.002764	179,10,4
1pii_2#326	GO:0030151: molybdenum ion binding	0.002764	192,15,5
1b8aa2#403	GO:0000287: magnesium ion binding	0.002768	117,128,13
1c7qa_#374	GO:0016836: hydro-lyase activity	0.002774	50,33,4
1xgsa1#226	GO:0016836: hydro-lyase activity	0.002774	50,33,4
1ryp1_#18	GO:0004896: hematopoietin/interferon-class (D200-domain) cytokine receptor activity	0.00278	42,19,3
1f0xa1#482	GO:0004222: metalloendopeptidase activity	0.00278	42,19,3
3rpba_#644	GO:0004674: protein serine/threonine kinase activity	0.00278	19,42,3
1fp5a2#478	GO:0008083: growth factor activity	0.00278	19,42,3
1epwa2#1220	GO:0008083: growth factor activity	0.00278	19,42,3
1vmoa_#84	GO:0004620: phospholipase activity	0.00278	42,19,3
1vmoa_#84	GO:0004896: hematopoietin/interferon-class (D200-domain) cytokine receptor activity	0.00278	42,19,3
1fu6a_#50	GO:0005066: transmembrane receptor protein tyrosine kinase signaling protein activity	0.002782	308,10,5
1fu6a_#50	GO:0003809: thrombin activity	0.002782	308,10,5
1h6la_#119	GO:0003809: thrombin activity	0.002782	308,10,5
1qo0d_#81	GO:0016616: oxidoreductase activity, acting on the CH-OH group of donors, NAD or NADP as acceptor	0.002783	46,59,5
1gdea_#379	GO:0016668: oxidoreductase activity, acting on sulfur group of donors, NAD or NADP as acceptor	0.002783	68,12,3
1kve.1#B199	GO:0019843: rRNA binding	0.002791	76,22,4
1el0a_#28	GO:0003724: RNA helicase activity	0.002793	161,11,4
1dyoa_#153	GO:0003724: RNA helicase activity	0.002793	161,11,4
1f0ia1#50	GO:0016702: oxidoreductase activity, acting on single donors with incorporation of molecular oxygen, incorporation of two atoms of oxygen	0.002794	146,12,4
1ejda_#213	GO:0016831: carboxy-lyase activity	0.002794	284,25,8
2pola2#218	GO:0016668: oxidoreductase activity, acting on sulfur group of donors, NAD or NADP as acceptor	0.002794	146,12,4
1jl0a_#264	GO:0016831: carboxy-lyase activity	0.002797	9,25,2
1fiy__#232	GO:0016831: carboxy-lyase activity	0.002797	9,25,2
1dceb_#290	GO:0016831: carboxy-lyase activity	0.002797	9,25,2
1bdfa2#60	GO:0005507: copper ion binding	0.002799	21,38,3
1ile_2#304	GO:0030145: manganese ion binding	0.002799	21,38,3
1nsca_#306	GO:0030145: manganese ion binding	0.002799	21,38,3
1bkpa_#41	GO:0005507: copper ion binding	0.002799	21,38,3
1eur__#99	GO:0005507: copper ion binding	0.002799	21,38,3
1be9a_#357	GO:0005509: calcium ion binding	0.002801	83,160,12
1dypa_#143	GO:0008810: cellulase activity	0.002802	94,18,4
2sli_2#326	GO:0004867: serine-type endopeptidase inhibitor activity	0.002807	251,47,11
1hx0a2#32	GO:0005524: ATP binding	0.002808	70,243,14
1i6vd_#713	GO:0005126: hematopoietin/interferon-class (D200-domain) cytokine receptor binding	0.002811	40,20,3
2pia_3#282	GO:0005126: hematopoietin/interferon-class (D200-domain) cytokine receptor binding	0.002811	40,20,3
1ev2e2#286	GO:0016638: oxidoreductase activity, acting on the CH-NH2 group of donors	0.002812	100,17,4
1ep0a_#80	GO:0003700: transcription factor activity	0.002815	7,124,3
1avgi_#69	GO:0003924: GTPase activity	0.002815	340,17,7
1dcqa1#451	GO:0003700: transcription factor activity	0.002815	7,124,3
2pgd_1#421	GO:0004601: peroxidase activity	0.002818	80,21,4
1epwa2#1091	GO:0004867: serine-type endopeptidase inhibitor activity	0.002818	5,47,2
1fkna_#96	GO:0019955: cytokine binding	0.002819	75,11,3
1pot__#138	GO:0003724: RNA helicase activity	0.002819	75,11,3
1e8ga2#268	GO:0015082: di-, tri-valent inorganic cation transporter activity	0.002824	16,14,2
1e8ga2#268	GO:0046915: transition metal ion transporter activity	0.002824	16,14,2

1hx6a1#163	GO:0015082: di-, tri-valent inorganic cation transporter activity	0.002824	16,14,2
1hx6a1#163	GO:0046915: transition metal ion transporter activity	0.002824	16,14,2
1ygs__#435	GO:0003968: RNA-directed RNA polymerase activity	0.002824	16,14,2
1azsa_#397	GO:0003968: RNA-directed RNA polymerase activity	0.002824	16,14,2
1hhsa_#328	GO:0004523: ribonuclease H activity	0.002824	14,16,2
2ts1__#59	GO:0016776: phosphotransferase activity, phosphate group as acceptor	0.002824	16,14,2
1pme__#302	GO:0004714: transmembrane receptor protein tyrosine kinase activity	0.002824	16,14,2
1lkka_#127	GO:0005543: phospholipid binding	0.002824	16,14,2
1aisb2#1274	GO:0016251: general RNA polymerase II transcription factor activity	0.002824	16,14,2
1hrna_#38	GO:0004523: ribonuclease H activity	0.002824	14,16,2
1gtra1#468	GO:0030151: molybdenum ion binding	0.002829	54,15,3
1ii7a_#199	GO:0030151: molybdenum ion binding	0.002829	54,15,3
1ii7a_#199	GO:0005096: GTPase activator activity	0.002829	54,15,3
1quqa_#75	GO:0005096: GTPase activator activity	0.002829	54,15,3
1ec7a1#230	GO:0030151: molybdenum ion binding	0.002829	54,15,3
1dnv__#354	GO:0004556: alpha-amylase activity	0.002829	54,15,3
2mev1_#123	GO:0004896: hematopoietin/interferon-class (D200-domain) cytokine receptor activity	0.00283	89,19,4
1j79a_#187	GO:0016646: oxidoreductase activity, acting on the CH-NH group of donors, NAD or NADP as acceptor	0.00283	89,19,4
1hq0a_#867	GO:0005525: GTP binding	0.00283	171,49,9
1i50a_#856	GO:0003779: actin binding	0.002832	269,32,9
1gcua1#36	GO:0016831: carboxy-lyase activity	0.00284	32,25,3
1bf4a_#4	GO:0003779: actin binding	0.00284	25,32,3
1vmoa_#106	GO:0015036: disulfide oxidoreductase activity	0.002843	187,22,6
1ej0a_#42	GO:0008757: S-adenosylmethionine-dependent methyltransferase activity	0.002844	116,24,5
1d0na3#305	GO:0003968: RNA-directed RNA polymerase activity	0.002848	311,14,6
1wht.1#A15	GO:0004180: carboxypeptidase activity	0.00285	15,15,2
1fmta2#30	GO:0030151: molybdenum ion binding	0.00285	15,15,2
1h8va_#139	GO:0004725: protein tyrosine phosphatase activity	0.00285	15,15,2
1aiha_#226	GO:0030151: molybdenum ion binding	0.00285	15,15,2
1a8q__#75	GO:0004180: carboxypeptidase activity	0.00285	15,15,2
1e6pa3#340	GO:0030151: molybdenum ion binding	0.00285	15,15,2
1ctqa_#163	GO:0005096: GTPase activator activity	0.00285	15,15,2
7odca2#67	GO:0030151: molybdenum ion binding	0.00285	15,15,2
1eg5a_#197	GO:0004725: protein tyrosine phosphatase activity	0.00285	15,15,2
1h6la_#119	GO:0005509: calcium ion binding	0.002856	308,160,30
1tyfa_#31	GO:0016758: transferase activity, transferring hexosyl groups	0.002858	162,11,4
1d4xg_#60	GO:0030151: molybdenum ion binding	0.002864	115,15,4
1nuka_#80	GO:0004620: phospholipase activity	0.002865	220,19,6
1thfd_#140	GO:0015082: di-, tri-valent inorganic cation transporter activity	0.002866	124,14,4
1thfd_#140	GO:0046915: transition metal ion transporter activity	0.002866	124,14,4
1f5ma_#80	GO:0030145: manganese ion binding	0.002866	105,38,6
1f5ma_#80	GO:0005507: copper ion binding	0.002866	105,38,6
1e5ka_#113	GO:0000287: magnesium ion binding	0.002868	65,128,9
1fc4a_#205	GO:0004523: ribonuclease H activity	0.002872	267,16,6
1mpp__#151	GO:0019838: growth factor binding	0.002872	94,9,3
1avgi_#69	GO:0000049: tRNA binding	0.002874	340,13,6
1c9la2#61	GO:0005524: ATP binding	0.002874	238,243,34
1znca_#70	GO:0016836: hydro-lyase activity	0.002876	7,33,2
1b6ra2#45	GO:0016836: hydro-lyase activity	0.002876	7,33,2
1dcia_#233	GO:0016836: hydro-lyase activity	0.002876	7,33,2
1jb0d_#26	GO:0005524: ATP binding	0.002876	229,243,33
1lgr_2#115	GO:0016836: hydro-lyase activity	0.002876	7,33,2
1daba_#310	GO:0016836: hydro-lyase activity	0.002876	7,33,2
1h6va3#486	GO:0004197: cysteine-type endopeptidase activity	0.002878	70,24,4
1h6va3#486	GO:0008201: heparin binding	0.002878	70,24,4
1c4zd_#22	GO:0004197: cysteine-type endopeptidase activity	0.002878	70,24,4
1ib2a_#1101	GO:0005516: calmodulin binding	0.002878	70,24,4
1cgha_#227	GO:0008201: heparin binding	0.002878	70,24,4
1e30a_#112	GO:0051082: unfolded protein binding	0.002879	49,34,4
1fn9a_#271	GO:0005529: sugar binding	0.002879	218,39,9
1f7da_#97	GO:0004190: aspartic-type endopeptidase activity	0.002884	35,23,3
1hlwa_#82	GO:0000287: magnesium ion binding	0.002887	270,128,23
1h6kx_#97	GO:0004295: trypsin activity	0.002888	57,48,5
2mev3_#108	GO:0000049: tRNA binding	0.002888	228,13,5
3sil__#98	GO:0000049: tRNA binding	0.002888	228,13,5

1e2o__#346	GO:0030151: molybdenum ion binding	0.002894	194,15,5
1d4ta_#80	GO:0005524: ATP binding	0.002899	23,243,7
1miob_#338	GO:0008800: beta-lactamase activity	0.0029	84,10,3
1imva_#69	GO:0008199: ferric iron binding	0.0029	84,10,3
1j9la_#82	GO:0016627: oxidoreductase activity, acting on the CH-CH group of donors	0.002904	169,17,5
1i50b_#966	GO:0008201: heparin binding	0.002906	299,24,8
1d0na3#305	GO:0003809: thrombin activity	0.002907	311,10,5
1i50a_#856	GO:0015078: hydrogen ion transporter activity	0.002912	269,21,7
1ycsa_#162	GO:0004714: transmembrane receptor protein tyrosine kinase activity	0.002915	210,14,5
1esl_1#48	GO:0003899: DNA-directed RNA polymerase activity	0.002917	19,12,2
1jdra_#53	GO:0004896: hematopoietin/interferon-class (D200-domain) cytokine receptor activity	0.002917	12,19,2
1b37a1#209	GO:0016668: oxidoreductase activity, acting on sulfur group of donors, NAD or NADP as acceptor	0.002917	19,12,2
1ak0__#212	GO:0004620: phospholipase activity	0.002917	12,19,2
1qp8a1#168	GO:0016646: oxidoreductase activity, acting on the CH-NH group of donors, NAD or NADP as acceptor	0.002917	12,19,2
1shsa_#68	GO:0004896: hematopoietin/interferon-class (D200-domain) cytokine receptor activity	0.002917	12,19,2
1dy9.1#A71	GO:0004896: hematopoietin/interferon-class (D200-domain) cytokine receptor activity	0.002917	12,19,2
1d5ra2#25	GO:0016702: oxidoreductase activity, acting on single donors with incorporation of molecular oxygen, incorporation of two atoms of oxygen	0.002917	19,12,2
1dc1a_#241	GO:0004222: metalloendopeptidase activity	0.002917	12,19,2
1e8ua_#411	GO:0004222: metalloendopeptidase activity	0.002917	12,19,2
7reqb1#163	GO:0004222: metalloendopeptidase activity	0.002917	12,19,2
1dhs__#149	GO:0005524: ATP binding	0.002918	29,243,8
1d5ra2#133	GO:0000287: magnesium ion binding	0.00292	22,128,5
1qj2b2#536	GO:0000287: magnesium ion binding	0.00292	22,128,5
1ab4__#89	GO:0005524: ATP binding	0.002926	159,243,25
2dkb__#239	GO:0005525: GTP binding	0.002927	56,49,5
1f21a_#68	GO:0030145: manganese ion binding	0.002928	44,38,4
1ii7a_#43	GO:0030145: manganese ion binding	0.002928	44,38,4
1kve.1#B199	GO:0019955: cytokine binding	0.002929	76,11,3
1ds9a_#119	GO:0008270: zinc ion binding	0.002932	8,108,3
1sfe_2#15	GO:0004295: trypsin activity	0.002934	17,48,3
1dt9a1#224	GO:0008483: transaminase activity	0.002934	48,17,3
1i50b_#216	GO:0003924: GTPase activity	0.002934	48,17,3
1nsj__#124	GO:0016866: intramolecular transferase activity	0.002937	148,12,4
1ltda2#34	GO:0004295: trypsin activity	0.002938	5,48,2
1e6ca_#91	GO:0016846: carbon-sulfur lyase activity	0.002939	182,10,4
1dpsa_#36	GO:0051082: unfolded protein binding	0.00294	205,34,8
1dyma_#11	GO:0003809: thrombin activity	0.002944	23,10,2
1qp8a1#153	GO:0016620: oxidoreductase activity, acting on the aldehyde or oxo group of donors, NAD or NADP as acceptor	0.002944	23,10,2
1ea5a_#205	GO:0004457: lactate dehydrogenase activity	0.002944	23,10,2
1pme__#166	GO:0005066: transmembrane receptor protein tyrosine kinase signaling protein activity	0.002944	23,10,2
1avgi_#69	GO:0004812: tRNA ligase activity	0.002945	340,26,9
1ge8a2#134	GO:0005518: collagen binding	0.002945	229,13,5
1iira_#326	GO:0016831: carboxy-lyase activity	0.002947	112,25,5
1ekma1#545	GO:0004620: phospholipase activity	0.002949	90,19,4
1ljra1#165	GO:0005509: calcium ion binding	0.00295	34,160,7
1psda3#344	GO:0016251: general RNA polymerase II transcription factor activity	0.002952	125,14,4
1ez0a_#133	GO:0015082: di-, tri-valent inorganic cation transporter activity	0.002952	125,14,4
1ez0a_#133	GO:0046915: transition metal ion transporter activity	0.002952	125,14,4
1fwxa2#169	GO:0004556: alpha-amylase activity	0.002956	116,15,4
1ddwa_#77	GO:0005509: calcium ion binding	0.002956	364,160,34
1io1a_#233	GO:0019838: growth factor binding	0.00296	95,9,3
1evya2#38	GO:0016616: oxidoreductase activity, acting on the CH-OH group of donors, NAD or NADP as acceptor	0.002962	14,59,3
1exba_#188	GO:0016616: oxidoreductase activity, acting on the CH-OH group of donors, NAD or NADP as acceptor	0.002962	14,59,3
1cnza_#19	GO:0016616: oxidoreductase activity, acting on the CH-OH group of donors, NAD or NADP as acceptor	0.002962	14,59,3
1dhs__#99	GO:0000287: magnesium ion binding	0.002962	132,128,14
1g8kb_#11	GO:0003968: RNA-directed RNA polymerase activity	0.002962	59,14,3
1f0ia1#50	GO:0004867: serine-type endopeptidase inhibitor activity	0.002963	146,47,8
1jb3a_#64	GO:0004620: phospholipase activity	0.002965	302,19,7
1foha5#9	GO:0004812: tRNA ligase activity	0.002971	65,26,4

1ds1a_#300	GO:0015036: disulfide oxidoreductase activity	0.002972	37,22,3
1e3ua_#164	GO:0019843: rRNA binding	0.002972	37,22,3
1h75a_#53	GO:0015036: disulfide oxidoreductase activity	0.002972	37,22,3
1fsu__#434	GO:0019838: growth factor binding	0.002975	356,9,5
2sli_2#326	GO:0046983: protein dimerization activity	0.002977	251,17,6
1elja_#64	GO:0005529: sugar binding	0.002979	103,39,6
1ddja_#746	GO:0016638: oxidoreductase activity, acting on the CH-NH2 group of donors	0.002981	170,17,5
1nox__#88	GO:0005179: hormone activity	0.002982	55,15,3
1i6vd_#713	GO:0008083: growth factor activity	0.002982	40,42,4
1akp__#62	GO:0005179: hormone activity	0.002982	55,15,3
1cfr__#269	GO:0030151: molybdenum ion binding	0.002982	55,15,3
1hdr__#156	GO:0004180: carboxypeptidase activity	0.002982	55,15,3
1dysa_#131	GO:0010181: FMN binding	0.002984	21,11,2
1fid__#195	GO:0015078: hydrogen ion transporter activity	0.002984	11,21,2
1azsb_#1075	GO:0015078: hydrogen ion transporter activity	0.002984	11,21,2
1a8l_1#74	GO:0004601: peroxidase activity	0.002984	11,21,2
1bu7a_#277	GO:0010181: FMN binding	0.002984	21,11,2
1auoa_#121	GO:0010181: FMN binding	0.002984	21,11,2
1fkna_#99	GO:0003964: RNA-directed DNA polymerase activity	0.002984	21,11,2
1ep3b1#71	GO:0010181: FMN binding	0.002984	21,11,2
1gof_3#399	GO:0019955: cytokine binding	0.002984	21,11,2
1hr0w_#69	GO:0003729: mRNA binding	0.002988	64,13,3
1pvc1_#106	GO:0005518: collagen binding	0.002988	64,13,3
1alo_4#634	GO:0004177: aminopeptidase activity	0.002988	64,13,3
1alo_4#634	GO:0008408: 3'-5' exonuclease activity	0.002988	64,13,3
1hxmb1#117	GO:0005518: collagen binding	0.002988	64,13,3
1ryp1_#18	GO:0005524: ATP binding	0.00299	42,243,10
1b6ra3#161	GO:0003724: RNA helicase activity	0.00299	164,11,4
1b6ra3#161	GO:0003964: RNA-directed DNA polymerase activity	0.00299	164,11,4
1d3ga_#118	GO:0005524: ATP binding	0.00299	42,243,10
1dyna_#42	GO:0019955: cytokine binding	0.00299	164,11,4
1phm_2#253	GO:0005529: sugar binding	0.002991	309,39,11
1ddma_#130	GO:0008009: chemokine activity	0.002992	313,10,5
1pwt__#8	GO:0004714: transmembrane receptor protein tyrosine kinase activity	0.002994	314,14,6
1fo4a5#1020	GO:0051082: unfolded protein binding	0.002998	359,34,11
1xgsa2#186	GO:0004523: ribonuclease H activity	0.002998	182,16,5
1fjfc2#153	GO:0003964: RNA-directed DNA polymerase activity	0.002999	420,11,6
1dpta_#22	GO:0004457: lactate dehydrogenase activity	0.003	85,10,3
1fjfj_#68	GO:0000049: tRNA binding	0.003003	230,13,5
1qfta_#98	GO:0000049: tRNA binding	0.003003	230,13,5
1j71a_#176	GO:0005516: calmodulin binding	0.003004	34,24,3
1fhga_#59	GO:0005516: calmodulin binding	0.003004	34,24,3
1bev1_#238	GO:0004197: cysteine-type endopeptidase activity	0.003004	34,24,3
1af7_2#248	GO:0008757: S-adenosylmethionine-dependent methyltransferase activity	0.003004	34,24,3
1ea5a_#99	GO:0005524: ATP binding	0.003004	194,243,29
1svb_2#165	GO:0004197: cysteine-type endopeptidase activity	0.003004	34,24,3
2cpl__#53	GO:0051082: unfolded protein binding	0.003004	24,34,3
1fi2a_#104	GO:0008201: heparin binding	0.003004	34,24,3
1aym3_#131	GO:0019843: rRNA binding	0.003006	412,22,9
1jd0a_#176	GO:0019838: growth factor binding	0.003007	207,9,4
3thia_#59	GO:0005509: calcium ion binding	0.003007	18,160,5
1e87a_#174	GO:0005509: calcium ion binding	0.00301	141,160,17
1e53a_#367	GO:0008270: zinc ion binding	0.003011	26,108,5
1lst__#157	GO:0008270: zinc ion binding	0.003011	26,108,5
1ayl__#237	GO:0019838: growth factor binding	0.003013	357,9,5
1e8ua_#296	GO:0015078: hydrogen ion transporter activity	0.003017	39,21,3
1e9xa_#343	GO:0015078: hydrogen ion transporter activity	0.003017	39,21,3
1jr3a2#166	GO:0015078: hydrogen ion transporter activity	0.003017	39,21,3
1seta2#403	GO:0003887: DNA-directed DNA polymerase activity	0.003018	41,20,3
1seta2#403	GO:0005126: hematopoietin/interferon-class (D200-domain) cytokine receptor binding	0.003018	41,20,3
1imva_#278	GO:0003887: DNA-directed DNA polymerase activity	0.003018	41,20,3
1g8fa3#419	GO:0016646: oxidoreductase activity, acting on the CH-NH group of donors, NAD or NADP as acceptor	0.003019	151,19,5
1im4a_#144	GO:0019843: rRNA binding	0.003022	129,22,5
1ax4a_#275	GO:0016638: oxidoreductase activity, acting on the CH-NH2 group of donors	0.003024	102,17,4

1bvyf_#527	GO:0016705: oxidoreductase activity, acting on paired donors, with incorporation or reduction of molecular oxygen	0.003025	9,26,2
1atia2#249	GO:0004812: tRNA ligase activity	0.003025	9,26,2
1jl0a_#264	GO:0004812: tRNA ligase activity	0.003025	9,26,2
1g5ca_#62	GO:0004812: tRNA ligase activity	0.003025	9,26,2
1jb0a_#619	GO:0004812: tRNA ligase activity	0.003025	9,26,2
1qq8a_#137	GO:0016705: oxidoreductase activity, acting on paired donors, with incorporation or reduction of molecular oxygen	0.003025	9,26,2
1a7j__#87	GO:0042802: protein self binding	0.003025	70,12,3
1dceb_#290	GO:0004497: monooxygenase activity	0.003025	9,26,2
1ib2a_#1101	GO:0016702: oxidoreductase activity, acting on single donors with incorporation of molecular oxygen, incorporation of two atoms of oxygen	0.003025	70,12,3
1qaua_#58	GO:0016705: oxidoreductase activity, acting on paired donors, with incorporation or reduction of molecular oxygen	0.003025	9,26,2
1qaua_#58	GO:0004497: monooxygenase activity	0.003025	9,26,2
1cipa2#34	GO:0000287: magnesium ion binding	0.003027	78,128,10
1j9qa1#154	GO:0030151: molybdenum ion binding	0.003029	196,15,5
3prn__#145	GO:0005524: ATP binding	0.003034	168,243,26
1h6ka2#336	GO:0005524: ATP binding	0.003034	168,243,26
1fmta2#151	GO:0005066: transmembrane receptor protein tyrosine kinase signaling protein activity	0.003035	314,10,5
1pwt__#8	GO:0003809: thrombin activity	0.003035	314,10,5
1dik_1#765	GO:0016831: carboxy-lyase activity	0.003037	165,25,6
1jlxa2#166	GO:0003724: RNA helicase activity	0.00304	77,11,3
1aoea_#130	GO:0004364: glutathione transferase activity	0.00304	77,11,3
1qtra_#108	GO:0010181: FMN binding	0.00304	77,11,3
1h8ca_#35	GO:0003964: RNA-directed DNA polymerase activity	0.00304	77,11,3
1bev1_#86	GO:0005509: calcium ion binding	0.003048	323,160,31
1qfea_#68	GO:0003924: GTPase activity	0.003048	560,17,9
1jd0a_#176	GO:0005525: GTP binding	0.003049	207,49,10
1a7ca_#202	GO:0005179: hormone activity	0.003051	117,15,4
1e8ua_#286	GO:0051082: unfolded protein binding	0.003052	7,34,2
1evqa_#305	GO:0016646: oxidoreductase activity, acting on the CH-NH group of donors, NAD or NADP as acceptor	0.003056	490,19,9
1el0a_#28	GO:0051082: unfolded protein binding	0.003057	161,34,7
1dp0a4#756	GO:0003724: RNA helicase activity	0.003058	165,11,4
1aoca_#123	GO:0005525: GTP binding	0.003061	5,49,2
1bvoa_#66	GO:0005525: GTP binding	0.003061	5,49,2
2btva_#621	GO:0005525: GTP binding	0.003061	5,49,2
1dfca4#1469	GO:0003779: actin binding	0.003065	53,32,4
2pia_1#54	GO:0005529: sugar binding	0.003066	220,39,9
1axca1#70	GO:0008201: heparin binding	0.003066	118,24,5
1bd0a1#276	GO:0004896: hematopoietin/interferon-class (D200-domain) cytokine receptor activity	0.003071	91,19,4
1g6oa_#116	GO:0005529: sugar binding	0.003072	358,39,12
1fgxa_#242	GO:0016854: racemase and epimerase activity	0.00308	18,13,2
1wdna_#79	GO:0016651: oxidoreductase activity, acting on NADH or NADPH	0.00308	18,13,2
1et0a_#118	GO:0016651: oxidoreductase activity, acting on NADH or NADPH	0.00308	18,13,2
1g2912#10	GO:0000049: tRNA binding	0.00308	18,13,2
1g2912#10	GO:0008408: 3'-5' exonuclease activity	0.00308	18,13,2
1df0a3#208	GO:0000049: tRNA binding	0.00308	18,13,2
1xwl_2#591	GO:0008408: 3'-5' exonuclease activity	0.00308	18,13,2
1bn8a_#241	GO:0008408: 3'-5' exonuclease activity	0.00308	18,13,2
1g51a1#47	GO:0005085: guanyl-nucleotide exchange factor activity	0.00308	18,13,2
1bdb__#228	GO:0016854: racemase and epimerase activity	0.00308	18,13,2
1qf6a3#78	GO:0004523: ribonuclease H activity	0.003081	52,16,3
1f8ra1#288	GO:0005524: ATP binding	0.003082	49,243,11
1i6vd_#1151	GO:0000287: magnesium ion binding	0.003084	7,128,3
1tc1a_#163	GO:0000287: magnesium ion binding	0.003084	7,128,3
1h2rs_#136	GO:0000287: magnesium ion binding	0.003084	7,128,3
1h99a1#148	GO:0000287: magnesium ion binding	0.003084	7,128,3
1amk__#61	GO:0000287: magnesium ion binding	0.003084	7,128,3
1c4oa2#536	GO:0000287: magnesium ion binding	0.003084	7,128,3
1dbga_#283	GO:0000287: magnesium ion binding	0.003084	7,128,3
1daba_#310	GO:0000287: magnesium ion binding	0.003084	7,128,3
1gtra2#173	GO:0008270: zinc ion binding	0.003085	50,108,7
1byka_#279	GO:0005351: sugar porter activity	0.003085	82,21,4
1dpga2#334	GO:0003887: DNA-directed DNA polymerase activity	0.003087	287,20,7

1imva_#255	GO:0004263: chymotrypsin activity	0.003093	589,41,17
1dofa_#15	GO:0005179: hormone activity	0.003098	197,15,5
1gpea1#271	GO:0004556: alpha-amylase activity	0.003098	197,15,5
1gpea1#271	GO:0004725: protein tyrosine phosphatase activity	0.003098	197,15,5
1imva_#255	GO:0003964: RNA-directed DNA polymerase activity	0.003099	589,11,7
1ddja_#681	GO:0046983: protein dimerization activity	0.0031	253,17,6
1eq9a_#40	GO:0005066: transmembrane receptor protein tyrosine kinase signaling protein activity	0.003102	86,10,3
1qj8a_#64	GO:0005509: calcium ion binding	0.003104	84,160,12
2ltn.1#A63	GO:0004896: hematopoietin/interferon-class (D200-domain) cytokine receptor activity	0.003108	152,19,5
1huxa_#10	GO:0015082: di-, tri-valent inorganic cation transporter activity	0.003109	60,14,3
1huxa_#10	GO:0046915: transition metal ion transporter activity	0.003109	60,14,3
1fo5a_#60	GO:0004190: aspartic-type endopeptidase activity	0.00311	246,23,7
1bjt__#890	GO:0004556: alpha-amylase activity	0.003112	292,15,6
1i9sa_#19	GO:0005525: GTP binding	0.003113	17,49,3
1jfua_#53	GO:0046983: protein dimerization activity	0.003113	49,17,3
1a65a3#409	GO:0046983: protein dimerization activity	0.003113	49,17,3
1cjxa2#307	GO:0005525: GTP binding	0.003113	17,49,3
1efnb_#102	GO:0016638: oxidoreductase activity, acting on the CH-NH2 group of donors	0.003113	49,17,3
1e30a_#112	GO:0016638: oxidoreductase activity, acting on the CH-NH2 group of donors	0.003113	49,17,3
1jbwa2#252	GO:0005525: GTP binding	0.003113	17,49,3
1et9a1#39	GO:0016638: oxidoreductase activity, acting on the CH-NH2 group of donors	0.003113	49,17,3
1ab4__#89	GO:0016705: oxidoreductase activity, acting on paired donors, with incorporation or reduction of molecular oxygen	0.003114	159,26,6
1jf9a_#379	GO:0008408: 3'-5' exonuclease activity	0.00312	232,13,5
1hd2a_#35	GO:0016846: carbon-sulfur lyase activity	0.003122	185,10,4
1qgua_#435	GO:0003700: transcription factor activity	0.003127	23,124,5
1c8za_#386	GO:0008201: heparin binding	0.003128	235,24,7
1seta2#231	GO:0046983: protein dimerization activity	0.003134	103,17,4
1icia_#175	GO:0005509: calcium ion binding	0.003135	63,160,10
2dkb__#239	GO:0030151: molybdenum ion binding	0.00314	56,15,3
2dkb__#239	GO:0016811: hydrolase activity, acting on carbon-nitrogen (but not peptide) bonds, in linear amides	0.00314	56,15,3
1cb8a2#613	GO:0000049: tRNA binding	0.003148	138,13,4
1e19a_#281	GO:0019201: nucleotide kinase activity	0.003148	138,13,4
1e19a_#281	GO:0000049: tRNA binding	0.003148	138,13,4
1e19a_#281	GO:0008235: metalloexopeptidase activity	0.003148	138,13,4
1a7s__#148	GO:0003697: single-stranded DNA binding	0.00315	71,12,3
2arca_#55	GO:0030145: manganese ion binding	0.00315	144,38,7
1f97a2#199	GO:0005507: copper ion binding	0.00315	144,38,7
1jjya_#329	GO:0016866: intramolecular transferase activity	0.00315	71,12,3
1jjya_#324	GO:0016758: transferase activity, transferring hexosyl groups	0.003155	78,11,3
1pda_1#77	GO:0000287: magnesium ion binding	0.003158	32,128,6
1g8fa3#419	GO:0016668: oxidoreductase activity, acting on sulfur group of donors, NAD or NADP as acceptor	0.003162	151,12,4
1g8fa3#419	GO:0016866: intramolecular transferase activity	0.003162	151,12,4
1g25a_#22	GO:0019843: rRNA binding	0.003164	415,22,9
1htp__#16	GO:0008201: heparin binding	0.003164	303,24,8
1d7ba_#87	GO:0004263: chymotrypsin activity	0.003165	99,41,6
1fjfl_#67	GO:0008270: zinc ion binding	0.003166	175,108,15
1f21a_#68	GO:0004222: metalloendopeptidase activity	0.003178	44,19,3
1ezvb1#29	GO:0004222: metalloendopeptidase activity	0.003178	44,19,3
3sil__#84	GO:0005507: copper ion binding	0.003181	45,38,4
1ihua2#343	GO:0000049: tRNA binding	0.003181	233,13,5
2dkb__#114	GO:0004197: cysteine-type endopeptidase activity	0.003182	174,24,6
1a0i_2#222	GO:0003809: thrombin activity	0.003185	186,10,4
1a0i_2#222	GO:0050660: FAD binding	0.003185	186,10,4
1clc_1#299	GO:0016705: oxidoreductase activity, acting on paired donors, with incorporation or reduction of molecular oxygen	0.003185	32,26,3
1e79d2#36	GO:0004725: protein tyrosine phosphatase activity	0.003189	524,15,8
1e79d2#36	GO:0003743: translation initiation factor activity	0.003189	524,15,8
1fuma2#370	GO:0016627: oxidoreductase activity, acting on the CH-CH group of donors	0.003191	14,17,2
1iray2#107	GO:0003924: GTPase activity	0.003191	14,17,2
1f2da_#264	GO:0008483: transaminase activity	0.003191	14,17,2
2pola3#335	GO:0016799: hydrolase activity, hydrolyzing N-glycosyl compounds	0.003191	14,17,2
1bjt__#1066	GO:0003968: RNA-directed RNA polymerase activity	0.003191	17,14,2
1tkia_#80	GO:0004714: transmembrane receptor protein tyrosine kinase activity	0.003191	17,14,2

1howa_#588	GO:0004714: transmembrane receptor protein tyrosine kinase activity	0.003191	17,14,2
1koba_#230	GO:0004714: transmembrane receptor protein tyrosine kinase activity	0.003191	17,14,2
1fl2a1#472	GO:0016627: oxidoreductase activity, acting on the CH-CH group of donors	0.003191	14,17,2
1tkia_#266	GO:0004714: transmembrane receptor protein tyrosine kinase activity	0.003191	17,14,2
1abrb1#25	GO:0016799: hydrolase activity, hydrolyzing N-glycosyl compounds	0.003191	14,17,2
1jbwa2#189	GO:0008483: transaminase activity	0.003191	14,17,2
1cwva2#682	GO:0004714: transmembrane receptor protein tyrosine kinase activity	0.003191	17,14,2
2napa1#718	GO:0046983: protein dimerization activity	0.003191	14,17,2
1fhoa_#74	GO:0000049: tRNA binding	0.003196	347,13,6
1jj2e1#42	GO:0019955: cytokine binding	0.003197	167,11,4
1dn2a1#261	GO:0008083: growth factor activity	0.003201	425,42,14
1i7oa1#63	GO:0003700: transcription factor activity	0.003202	44,124,7
1bwvs_#117	GO:0003779: actin binding	0.003202	329,32,10
1svb_1#358	GO:0004197: cysteine-type endopeptidase activity	0.003205	10,24,2
3tdt__#213	GO:0003682: chromatin binding	0.003205	24,10,2
1htr.1#B172	GO:0008201: heparin binding	0.003205	10,24,2
1eucb2#84	GO:0004197: cysteine-type endopeptidase activity	0.003205	10,24,2
1pme__#24	GO:0005516: calmodulin binding	0.003205	10,24,2
1nfdb1#115	GO:0008201: heparin binding	0.003205	10,24,2
1dqga_#51	GO:0008201: heparin binding	0.003205	10,24,2
1g55a_#68	GO:0008757: S-adenosylmethionine-dependent methyltransferase activity	0.003205	10,24,2
1lst__#56	GO:0016620: oxidoreductase activity, acting on the aldehyde or oxo group of donors, NAD or NADP as acceptor	0.003205	24,10,2
1qlca_#210	GO:0003684: damaged DNA binding	0.003205	24,10,2
1cipa2#326	GO:0003682: chromatin binding	0.003205	24,10,2
1vcbb_#19	GO:0008800: beta-lactamase activity	0.003205	24,10,2
1mrp__#170	GO:0008199: ferric iron binding	0.003205	24,10,2
1ovaa_#161	GO:0016814: hydrolase activity, acting on carbon-nitrogen (but not peptide) bonds, in cyclic amidines	0.003205	24,10,2
1bjaa_#26	GO:0016814: hydrolase activity, acting on carbon-nitrogen (but not peptide) bonds, in cyclic amidines	0.003205	24,10,2
1ct9a1#233	GO:0016836: hydro-lyase activity	0.003206	52,33,4
1eu3a2#205	GO:0016814: hydrolase activity, acting on carbon-nitrogen (but not peptide) bonds, in cyclic amidines	0.003207	87,10,3
1fd9a_#162	GO:0008800: beta-lactamase activity	0.003207	87,10,3
1dy5a_#46	GO:0005525: GTP binding	0.003208	35,49,4
1qe3a_#301	GO:0005525: GTP binding	0.003208	35,49,4
1as4.1#A336	GO:0004674: protein serine/threonine kinase activity	0.003208	332,42,12
1f0xa2#118	GO:0005525: GTP binding	0.003208	35,49,4
1edha2#106	GO:0005525: GTP binding	0.003208	35,49,4
1euwa_#71	GO:0005507: copper ion binding	0.00321	22,38,3
1mut__#10	GO:0005507: copper ion binding	0.00321	22,38,3
1at0__#285	GO:0019843: rRNA binding	0.00321	38,22,3
1aoza1#61	GO:0005509: calcium ion binding	0.003214	26,160,6
1dkia_#185	GO:0015036: disulfide oxidoreductase activity	0.003217	79,22,4
1a9xa2#1009	GO:0000287: magnesium ion binding	0.00322	14,128,4
1bk7a_#135	GO:0000287: magnesium ion binding	0.00322	14,128,4
1am2__#163	GO:0005524: ATP binding	0.003221	102,243,18
1qtn.1#A270	GO:0005351: sugar porter activity	0.003222	439,21,9
1ej8a_#189	GO:0004725: protein tyrosine phosphatase activity	0.003223	294,15,6
1g0sa_#74	GO:0004180: carboxypeptidase activity	0.003223	294,15,6
1el0a_#42	GO:0004725: protein tyrosine phosphatase activity	0.003223	294,15,6
1hd2a_#35	GO:0004523: ribonuclease H activity	0.003223	185,16,5
1hxxa_#173	GO:0015036: disulfide oxidoreductase activity	0.003224	260,22,7
1hxxa_#278	GO:0016638: oxidoreductase activity, acting on the CH-NH2 group of donors	0.003228	255,17,6
2ayh__#33	GO:0016702: oxidoreductase activity, acting on single donors with incorporation of molecular oxygen, incorporation of two atoms of oxygen	0.003233	20,12,2
1cby__#183	GO:0000155: two-component sensor molecule activity	0.003233	20,12,2
1i2oa_#173	GO:0016866: intramolecular transferase activity	0.003233	20,12,2
1jdra_#53	GO:0005126: hematopoietin/interferon-class (D200-domain) cytokine receptor binding	0.003233	12,20,2
1dy9.1#A71	GO:0005126: hematopoietin/interferon-class (D200-domain) cytokine receptor binding	0.003233	12,20,2
1ryp1_#18	GO:0005126: hematopoietin/interferon-class (D200-domain) cytokine receptor binding	0.003235	42,20,3
1gsa_2#208	GO:0008083: growth factor activity	0.003235	20,42,3
1crza1#240	GO:0003887: DNA-directed DNA polymerase activity	0.003235	42,20,3
1vmoa_#84	GO:0005126: hematopoietin/interferon-class (D200-domain) cytokine receptor binding	0.003235	42,20,3
1jbwa1#405	GO:0005524: ATP binding	0.003238	8,243,4

1eucb2#17	GO:0005524: ATP binding	0.003238	8,243,4
1biha3#248	GO:0005524: ATP binding	0.003238	8,243,4
1ldna1#94	GO:0004556: alpha-amylase activity	0.003239	199,15,5
1e39a2#556	GO:0015078: hydrogen ion transporter activity	0.003246	40,21,3
1mil__#31	GO:0046983: protein dimerization activity	0.003246	104,17,4
1bwda_#295	GO:0003924: GTPase activity	0.003246	104,17,4
1e8ga2#268	GO:0030151: molybdenum ion binding	0.003247	16,15,2
2dlda2#95	GO:0004725: protein tyrosine phosphatase activity	0.003247	16,15,2
1lkka_#127	GO:0004725: protein tyrosine phosphatase activity	0.003247	16,15,2
1epwa3#140	GO:0005066: transmembrane receptor protein tyrosine kinase signaling protein activity	0.003249	187,10,4
1cs1a_#160	GO:0016846: carbon-sulfur lyase activity	0.003249	187,10,4
1dvpa1#65	GO:0016620: oxidoreductase activity, acting on the aldehyde or oxo group of donors, NAD or NADP as acceptor	0.003249	187,10,4
1qh4a2#350	GO:0004674: protein serine/threonine kinase activity	0.00325	67,42,5
2cb5a_#395	GO:0004674: protein serine/threonine kinase activity	0.00325	67,42,5
1dgw.1#Y353	GO:0008800: beta-lactamase activity	0.003259	319,10,5
1bfg__#22	GO:0003968: RNA-directed RNA polymerase activity	0.00326	61,14,3
1dfaa1#35	GO:0019838: growth factor binding	0.003262	27,9,2
1i6vd_#1188	GO:0019829: cation-transporting ATPase activity	0.003262	27,9,2
1ds1a_#172	GO:0005529: sugar binding	0.003262	222,39,9
2bce__#204	GO:0019201: nucleotide kinase activity	0.003263	66,13,3
2bce__#204	GO:0016651: oxidoreductase activity, acting on NADH or NADPH	0.003263	66,13,3
1e19a_#281	GO:0005351: sugar porter activity	0.003265	138,21,5
1ejda_#6	GO:0005509: calcium ion binding	0.003266	270,160,27
1b0pa5#739	GO:0008757: S-adenosylmethionine-dependent methyltransferase activity	0.003267	35,24,3
1cpt__#52	GO:0004197: cysteine-type endopeptidase activity	0.003267	35,24,3
1d2oa1#541	GO:0008201: heparin binding	0.003267	35,24,3
1rypb_#133	GO:0004197: cysteine-type endopeptidase activity	0.003267	35,24,3
1fbna_#144	GO:0016758: transferase activity, transferring hexosyl groups	0.003272	79,11,3
1b8ba_#466	GO:0008810: cellulase activity	0.003273	47,18,3
1ldm_2#272	GO:0004867: serine-type endopeptidase inhibitor activity	0.003273	18,47,3
1qnja_#124	GO:0004867: serine-type endopeptidase inhibitor activity	0.003273	18,47,3
1kapp2#149	GO:0004364: glutathione transferase activity	0.003275	22,11,2
1fjfl_#100	GO:0019843: rRNA binding	0.003275	11,22,2
1qj2b2#536	GO:0016758: transferase activity, transferring hexosyl groups	0.003275	22,11,2
1gtra2#113	GO:0010181: FMN binding	0.003275	22,11,2
1e42a1#711	GO:0008201: heparin binding	0.003276	175,24,6
3chbd_#85	GO:0008201: heparin binding	0.003294	378,24,9
1dzfa2#211	GO:0008083: growth factor activity	0.003299	167,42,8
1d0ba_#191	GO:0008757: S-adenosylmethionine-dependent methyltransferase activity	0.003301	120,24,5
1qdla_#211	GO:0015082: di-, tri-valent inorganic cation transporter activity	0.003302	320,14,6
1qdla_#211	GO:0046915: transition metal ion transporter activity	0.003302	320,14,6
1dfaa2#226	GO:0019829: cation-transporting ATPase activity	0.003303	1,9,1
1aoca_#70	GO:0019838: growth factor binding	0.003303	1,9,1
1ihua1#291	GO:0019829: cation-transporting ATPase activity	0.003303	1,9,1
1ihua1#176	GO:0019829: cation-transporting ATPase activity	0.003303	1,9,1
2duba_#212	GO:0016763: transferase activity, transferring pentosyl groups	0.003304	62,28,4
1j9la_#164	GO:0016811: hydrolase activity, acting on carbon-nitrogen (but not peptide) bonds, in linear amides	0.003304	57,15,3
2cpl__#20	GO:0030145: manganese ion binding	0.003305	108,38,6
1qdla_#211	GO:0016620: oxidoreductase activity, acting on the aldehyde or oxo group of donors, NAD or NADP as acceptor	0.003305	320,10,5
1e05i_#83	GO:0003887: DNA-directed DNA polymerase activity	0.003311	88,20,4
1fm2.1#B308	GO:0030151: molybdenum ion binding	0.003311	200,15,5
1hdma1#115	GO:0003916: DNA topoisomerase activity	0.003313	188,10,4
1czan1#92	GO:0008408: 3'-5' exonuclease activity	0.003314	631,13,8
1xis__#214	GO:0016620: oxidoreductase activity, acting on the aldehyde or oxo group of donors, NAD or NADP as acceptor	0.003314	88,10,3
1f9za_#16	GO:0016854: racemase and epimerase activity	0.003319	140,13,4
1ycsb1#412	GO:0003887: DNA-directed DNA polymerase activity	0.003322	146,20,5
1thea_#79	GO:0004620: phospholipase activity	0.003325	93,19,4
1i50b_#378	GO:0000287: magnesium ion binding	0.00333	79,128,10
1d7ba_#87	GO:0019838: growth factor binding	0.003332	99,9,3
1a4ya_#47	GO:0016836: hydro-lyase activity	0.003342	216,33,8
1cfb_2#763	GO:0008083: growth factor activity	0.003346	6,42,2
1atza_#1070	GO:0008083: growth factor activity	0.003346	6,42,2

1d0ba_#191	GO:0016811: hydrolase activity, acting on carbon-nitrogen (but not peptide) bonds, in linear amides	0.003347	120,15,4
1vola2#239	GO:0005096: GTPase activator activity	0.003347	120,15,4
1tuba1#239	GO:0030151: molybdenum ion binding	0.003347	120,15,4
2bpa1_#45	GO:0005509: calcium ion binding	0.003349	192,160,21
1c4zd_#22	GO:0016831: carboxy-lyase activity	0.00336	70,25,4
1i1ka_#255	GO:0003714: transcription corepressor activity	0.003361	31,8,2
1au1a_#58	GO:0046983: protein dimerization activity	0.003362	105,17,4
1f5ma_#80	GO:0046983: protein dimerization activity	0.003362	105,17,4
1hdfa_#16	GO:0005529: sugar binding	0.003364	223,39,9
1bf6a_#183	GO:0016616: oxidoreductase activity, acting on the CH-OH group of donors, NAD or NADP as acceptor	0.003364	48,59,5
1imva_#69	GO:0004601: peroxidase activity	0.003369	84,21,4
1iira_#326	GO:0005525: GTP binding	0.003371	112,49,7
1i7oa2#399	GO:0005524: ATP binding	0.003374	36,243,9
1d0va_#46	GO:0005524: ATP binding	0.003374	36,243,9
3prn__#143	GO:0008083: growth factor activity	0.003375	334,42,12
1iow_1#37	GO:0016646: oxidoreductase activity, acting on the CH-NH group of donors, NAD or NADP as acceptor	0.003386	309,19,7
1ejfa_#10	GO:0004620: phospholipase activity	0.003386	155,19,5
1e42a1#711	GO:0046983: protein dimerization activity	0.003387	175,17,5
1e42a1#711	GO:0016638: oxidoreductase activity, acting on the CH-NH2 group of donors	0.003387	175,17,5
1fjfl_#67	GO:0046983: protein dimerization activity	0.003387	175,17,5
1fjfl_#67	GO:0016638: oxidoreductase activity, acting on the CH-NH2 group of donors	0.003387	175,17,5
1emsa2#165	GO:0004620: phospholipase activity	0.00339	45,19,3
3sil__#84	GO:0004620: phospholipase activity	0.00339	45,19,3
1bg6_1#294	GO:0004896: hematopoietin/interferon-class (D200-domain) cytokine receptor activity	0.00339	45,19,3
1gg4a3#64	GO:0016646: oxidoreductase activity, acting on the CH-NH group of donors, NAD or NADP as acceptor	0.00339	45,19,3
1b65a_#257	GO:0005525: GTP binding	0.00339	210,49,10
1eq2a_#11	GO:0016646: oxidoreductase activity, acting on the CH-NH group of donors, NAD or NADP as acceptor	0.00339	45,19,3
1iba__#39	GO:0005518: collagen binding	0.003392	351,13,6
1c4ra_#258	GO:0019955: cytokine binding	0.003392	80,11,3
1hbza_#214	GO:0004556: alpha-amylase activity	0.003396	297,15,6
1fsu__#434	GO:0015078: hydrogen ion transporter activity	0.003396	356,21,8
1f97a1#62	GO:0005507: copper ion binding	0.003397	707,38,18
1jcfa2#206	GO:0051082: unfolded protein binding	0.003398	84,34,5
2pola2#218	GO:0005507: copper ion binding	0.003406	146,38,7
1ihoa_#238	GO:0030145: manganese ion binding	0.003409	75,38,5
1dkia_#185	GO:0005524: ATP binding	0.003411	79,243,15
1ddja_#746	GO:0019955: cytokine binding	0.003413	170,11,4
1ddja_#746	GO:0003724: RNA helicase activity	0.003413	170,11,4
1epwa1#901	GO:0004714: transmembrane receptor protein tyrosine kinase activity	0.003415	62,14,3
1epwa3#138	GO:0005509: calcium ion binding	0.003421	424,160,38
2foka2#180	GO:0003887: DNA-directed DNA polymerase activity	0.003423	147,20,5
1ovb__#195	GO:0008199: ferric iron binding	0.003423	89,10,3
1ovb__#195	GO:0016814: hydrolase activity, acting on carbon-nitrogen (but not peptide) bonds, in cyclic amidines	0.003423	89,10,3
1j79a_#187	GO:0050660: FAD binding	0.003423	89,10,3
1ev7a_#218	GO:0016814: hydrolase activity, acting on carbon-nitrogen (but not peptide) bonds, in cyclic amidines	0.003423	89,10,3
1qf9a_#117	GO:0003682: chromatin binding	0.003423	89,10,3
1qg3a2#1264	GO:0005509: calcium ion binding	0.003427	2,160,2
1hx0a1#465	GO:0005509: calcium ion binding	0.003427	2,160,2
7taa_2#87	GO:0005509: calcium ion binding	0.003427	2,160,2
1smpi_#11	GO:0005509: calcium ion binding	0.003427	2,160,2
1cxya_#78	GO:0005509: calcium ion binding	0.003427	2,160,2
1dk5a_#111	GO:0005509: calcium ion binding	0.003427	2,160,2
1fwxa2#100	GO:0005509: calcium ion binding	0.003427	2,160,2
1fwxa2#396	GO:0008080: N-acetyltransferase activity	0.00343	237,13,5
1qrra_#11	GO:0016854: racemase and epimerase activity	0.003433	19,13,2
1qg8a_#90	GO:0004222: metalloendopeptidase activity	0.003433	13,19,2
1esl_1#48	GO:0000049: tRNA binding	0.003433	19,13,2
2rsla_#55	GO:0016651: oxidoreductase activity, acting on NADH or NADPH	0.003433	19,13,2
1dar_2#264	GO:0005085: guanyl-nucleotide exchange factor activity	0.003433	19,13,2

1dz4a_#150	GO:0016651: oxidoreductase activity, acting on NADH or NADPH	0.003433	19,13,2
1hdr__#136	GO:0016646: oxidoreductase activity, acting on the CH-NH group of donors, NAD or NADP as acceptor	0.003433	13,19,2
1g5ha2#319	GO:0000049: tRNA binding	0.003433	19,13,2
1hs6a3#389	GO:0004177: aminopeptidase activity	0.003433	19,13,2
1hs6a3#389	GO:0008026: ATP-dependent helicase activity	0.003433	19,13,2
1hs6a3#389	GO:0008235: metalloexopeptidase activity	0.003433	19,13,2
1a0ca_#187	GO:0004222: metalloendopeptidase activity	0.003433	13,19,2
1fw8a_#298	GO:0016646: oxidoreductase activity, acting on the CH-NH group of donors, NAD or NADP as acceptor	0.003433	13,19,2
1fiy__#80	GO:0000049: tRNA binding	0.003433	19,13,2
1qhva_#438	GO:0003729: mRNA binding	0.003433	19,13,2
1abrb2#252	GO:0004177: aminopeptidase activity	0.003433	19,13,2
1gen__#477	GO:0004896: hematopoietin/interferon-class (D200-domain) cytokine receptor activity	0.003433	13,19,2
1iqpa2#119	GO:0008408: 3'-5' exonuclease activity	0.003433	19,13,2
1fwxa2#169	GO:0016831: carboxy-lyase activity	0.003436	116,25,5
1qfta_#98	GO:0030145: manganese ion binding	0.003439	230,38,9
1fgga_#134	GO:0016776: phosphotransferase activity, phosphate group as acceptor	0.00344	218,14,5
1hxxa_#232	GO:0008408: 3'-5' exonuclease activity	0.003442	352,13,6
1dih_2#222	GO:0004177: aminopeptidase activity	0.003442	352,13,6
1g51a3#525	GO:0019843: rRNA binding	0.003444	263,22,7
1tiid_#39	GO:0004197: cysteine-type endopeptidase activity	0.003445	239,24,7
1tiid_#39	GO:0005516: calmodulin binding	0.003445	239,24,7
1hxxa_#232	GO:0046983: protein dimerization activity	0.003447	352,17,7
1hxxa_#232	GO:0016638: oxidoreductase activity, acting on the CH-NH2 group of donors	0.003447	352,17,7
1bev1_#86	GO:0003809: thrombin activity	0.003447	323,10,5
1bev1_#86	GO:0003916: DNA topoisomerase activity	0.003447	323,10,5
1dih_2#222	GO:0003924: GTPase activity	0.003447	352,17,7
1jeya_#104	GO:0030145: manganese ion binding	0.00345	46,38,4
1ev7a_#218	GO:0003887: DNA-directed DNA polymerase activity	0.00345	89,20,4
1qhqa_#45	GO:0004725: protein tyrosine phosphatase activity	0.00345	121,15,4
1dn2a1#261	GO:0005507: copper ion binding	0.00345	425,38,13
1jd0a_#176	GO:0008083: growth factor activity	0.003458	207,42,9
1ihma_#1068	GO:0005529: sugar binding	0.003459	22,39,3
1i1b__#10	GO:0005529: sugar binding	0.003459	22,39,3
1dvpa1#65	GO:0005524: ATP binding	0.00346	187,243,28
1dm9a_#57	GO:0005507: copper ion binding	0.003462	109,38,6
1e5pa_#91	GO:0003887: DNA-directed DNA polymerase activity	0.003462	43,20,3
2hlca_#54	GO:0030145: manganese ion binding	0.003462	109,38,6
1qrra_#208	GO:0016836: hydro-lyase activity	0.003463	87,33,5
1miob_#338	GO:0005525: GTP binding	0.003465	84,49,6
1bev1_#86	GO:0004714: transmembrane receptor protein tyrosine kinase activity	0.003465	323,14,6
1bev1_#86	GO:0003968: RNA-directed RNA polymerase activity	0.003465	323,14,6
1eq9a_#32	GO:0004674: protein serine/threonine kinase activity	0.003473	132,42,7
1eq9a_#32	GO:0008083: growth factor activity	0.003473	132,42,7
1g5va_#142	GO:0016620: oxidoreductase activity, acting on the aldehyde or oxo group of donors, NAD or NADP as acceptor	0.003477	25,10,2
1e5ka_#168	GO:0003682: chromatin binding	0.003477	25,10,2
1poxa3#439	GO:0004457: lactate dehydrogenase activity	0.003477	25,10,2
1ltda1#203	GO:0008800: beta-lactamase activity	0.003477	25,10,2
1f3ba2#7	GO:0016620: oxidoreductase activity, acting on the aldehyde or oxo group of donors, NAD or NADP as acceptor	0.003477	25,10,2
1cjca2#37	GO:0003684: damaged DNA binding	0.003477	25,10,2
1j71a_#251	GO:0016638: oxidoreductase activity, acting on the CH-NH2 group of donors	0.00348	106,17,4
1loua_#79	GO:0008483: transaminase activity	0.00348	106,17,4
1ckea_#35	GO:0004812: tRNA ligase activity	0.003481	33,26,3
1fuia1#506	GO:0004812: tRNA ligase activity	0.003481	33,26,3
1dpe__#402	GO:0004812: tRNA ligase activity	0.003481	33,26,3
1g0da1#62	GO:0005509: calcium ion binding	0.003484	6,160,3
1erja_#559	GO:0005509: calcium ion binding	0.003484	6,160,3
1uok_2#325	GO:0005509: calcium ion binding	0.003484	6,160,3
1eh9a3#170	GO:0005509: calcium ion binding	0.003484	6,160,3
1cpy__#380	GO:0003755: peptidyl-prolyl cis-trans isomerase activity	0.003487	171,11,4
1g71a_#77	GO:0016638: oxidoreductase activity, acting on the CH-NH2 group of donors	0.003491	51,17,3
1f42a2#123	GO:0005126: hematopoietin/interferon-class (D200-domain) cytokine receptor binding	0.003491	378,20,8
1fa0a1#393	GO:0015405: P-P-bond-hydrolysis-driven transporter activity	0.003494	261,12,5

1dj0a2#183	GO:0000049: tRNA binding	0.003496	142,13,4
1wdna_#79	GO:0005524: ATP binding	0.003501	18,243,6
3pah__#286	GO:0005524: ATP binding	0.003501	18,243,6
1g5qa_#80	GO:0005524: ATP binding	0.003501	18,243,6
3sil__#84	GO:0005529: sugar binding	0.003503	45,39,4
1fy7a_#388	GO:0016705: oxidoreductase activity, acting on paired donors, with incorporation or reduction of molecular oxygen	0.003505	68,26,4
1gdea_#379	GO:0004497: monooxygenase activity	0.003505	68,26,4
1rypb_#133	GO:0005509: calcium ion binding	0.003509	35,160,7
1f97a1#62	GO:0003697: single-stranded DNA binding	0.003515	707,12,8
1i58a_#519	GO:0003724: RNA helicase activity	0.003515	81,11,3
1exma3#99	GO:0016758: transferase activity, transferring hexosyl groups	0.003515	81,11,3
1gpea1#273	GO:0019955: cytokine binding	0.003515	81,11,3
1i50b_#966	GO:0004725: protein tyrosine phosphatase activity	0.003515	299,15,6
1ge8a2#134	GO:0004620: phospholipase activity	0.003515	229,19,6
1i5ga_#115	GO:0016251: general RNA polymerase II transcription factor activity	0.003521	324,14,6
1kit_3#743	GO:0005524: ATP binding	0.003522	250,243,35
1div_1#141	GO:0015036: disulfide oxidoreductase activity	0.003524	81,22,4
1e1oa2#423	GO:0003887: DNA-directed DNA polymerase activity	0.003526	148,20,5
1i7qa_#321	GO:0005126: hematopoietin/interferon-class (D200-domain) cytokine receptor binding	0.003526	148,20,5
1eg9a2#199	GO:0004197: cysteine-type endopeptidase activity	0.003527	74,24,4
1fd9a_#162	GO:0005524: ATP binding	0.003528	87,243,16
2dkb__#114	GO:0016616: oxidoreductase activity, acting on the CH-OH group of donors, NAD or NADP as acceptor	0.003529	174,59,10
1ac6a_#43	GO:0004812: tRNA ligase activity	0.00353	163,26,6
1bfd_2#146	GO:0004812: tRNA ligase activity	0.003531	112,26,5
1ton__#47	GO:0004812: tRNA ligase activity	0.003531	112,26,5
1nsca_#236	GO:0005529: sugar binding	0.003531	143,39,7
2pia_1#54	GO:0004812: tRNA ligase activity	0.003532	220,26,7
1bmv2_#2064	GO:0030145: manganese ion binding	0.00354	147,38,7
1wdcb_#134	GO:0008201: heparin binding	0.003543	36,24,3
1dlfl_#97	GO:0008201: heparin binding	0.003543	36,24,3
2mpra_#267	GO:0008201: heparin binding	0.003543	36,24,3
1esma_#314	GO:0005525: GTP binding	0.003545	113,49,7
1gdha1#235	GO:0005525: GTP binding	0.003545	113,49,7
1eg9a2#199	GO:0016702: oxidoreductase activity, acting on single donors with incorporation of molecular oxygen, incorporation of two atoms of oxygen	0.003546	74,12,3
1qmea4#290	GO:0003899: DNA-directed RNA polymerase activity	0.003546	74,12,3
1el0a_#42	GO:0005126: hematopoietin/interferon-class (D200-domain) cytokine receptor binding	0.003547	294,20,7
1dqaa1#596	GO:0003729: mRNA binding	0.003554	68,13,3
1fy7a_#388	GO:0016651: oxidoreductase activity, acting on NADH or NADPH	0.003554	68,13,3
1c39a_#49	GO:0005518: collagen binding	0.003554	68,13,3
1gdea_#379	GO:0016651: oxidoreductase activity, acting on NADH or NADPH	0.003554	68,13,3
1czan1#92	GO:0008757: S-adenosylmethionine-dependent methyltransferase activity	0.003559	631,24,12
1gci__#175	GO:0016616: oxidoreductase activity, acting on the CH-OH group of donors, NAD or NADP as acceptor	0.003561	70,59,6
1dfaa1#25	GO:0005525: GTP binding	0.003562	36,49,4
1bdfa2#60	GO:0015405: P-P-bond-hydrolysis-driven transporter activity	0.003565	21,12,2
2napa1#665	GO:0015405: P-P-bond-hydrolysis-driven transporter activity	0.003565	21,12,2
1g93a_#229	GO:0015078: hydrogen ion transporter activity	0.003565	12,21,2
1dp4a_#135	GO:0005351: sugar porter activity	0.003565	12,21,2
1thg__#5	GO:0003899: DNA-directed RNA polymerase activity	0.003565	21,12,2
1htr.1#B39	GO:0003697: single-stranded DNA binding	0.003565	21,12,2
1brt__#116	GO:0004601: peroxidase activity	0.003565	12,21,2
1eur__#99	GO:0003899: DNA-directed RNA polymerase activity	0.003565	21,12,2
1b3ra2#60	GO:0016616: oxidoreductase activity, acting on the CH-OH group of donors, NAD or NADP as acceptor	0.003569	30,59,4
1d8ca_#442	GO:0016616: oxidoreductase activity, acting on the CH-OH group of donors, NAD or NADP as acceptor	0.003569	30,59,4
1cqka_#49	GO:0016616: oxidoreductase activity, acting on the CH-OH group of donors, NAD or NADP as acceptor	0.003569	30,59,4
1hdfa_#16	GO:0005524: ATP binding	0.003573	223,243,32
3rpba_#557	GO:0005524: ATP binding	0.003575	57,243,12
1i50a_#1115	GO:0004714: transmembrane receptor protein tyrosine kinase activity	0.003579	18,14,2
1qdea_#218	GO:0010181: FMN binding	0.003579	23,11,2
1wdna_#79	GO:0003968: RNA-directed RNA polymerase activity	0.003579	18,14,2

1bmv2_#2059	GO:0004190: aspartic-type endopeptidase activity	0.003579	11,23,2
1c0pa1#1133	GO:0003968: RNA-directed RNA polymerase activity	0.003579	18,14,2
1cpt__#401	GO:0004364: glutathione transferase activity	0.003579	23,11,2
1i3ja_#233	GO:0004190: aspartic-type endopeptidase activity	0.003579	11,23,2
1a6o__#217	GO:0004714: transmembrane receptor protein tyrosine kinase activity	0.003579	18,14,2
1howa_#697	GO:0004714: transmembrane receptor protein tyrosine kinase activity	0.003579	18,14,2
1eq2a_#299	GO:0016758: transferase activity, transferring hexosyl groups	0.003579	23,11,2
1hava_#66	GO:0004190: aspartic-type endopeptidase activity	0.003579	11,23,2
1phk__#165	GO:0004714: transmembrane receptor protein tyrosine kinase activity	0.003579	18,14,2
1e8ya2#433	GO:0005543: phospholipid binding	0.003579	18,14,2
1d0na2#187	GO:0003779: actin binding	0.00358	8,32,2
1i2oa_#34	GO:0016620: oxidoreductase activity, acting on the aldehyde or oxo group of donors, NAD or NADP as acceptor	0.00358	192,10,4
1dnv__#354	GO:0005509: calcium ion binding	0.00358	54,160,9
2pia_1#54	GO:0004714: transmembrane receptor protein tyrosine kinase activity	0.003582	220,14,5
1nuka_#80	GO:0004714: transmembrane receptor protein tyrosine kinase activity	0.003582	220,14,5
1hdma1#115	GO:0005507: copper ion binding	0.003583	188,38,8
1i5ga_#115	GO:0004190: aspartic-type endopeptidase activity	0.003583	324,23,8
1e4ea2#215	GO:0003916: DNA topoisomerase activity	0.003583	490,10,6
1e69a_#26	GO:0000287: magnesium ion binding	0.00359	55,128,8
1xo1a2#159	GO:0000287: magnesium ion binding	0.00359	55,128,8
1epwa3#138	GO:0051082: unfolded protein binding	0.003591	424,34,12
1qgua_#435	GO:0000287: magnesium ion binding	0.003593	23,128,5
1i12a_#126	GO:0000287: magnesium ion binding	0.003593	23,128,5
1bwza2#162	GO:0016646: oxidoreductase activity, acting on the CH-NH group of donors, NAD or NADP as acceptor	0.003593	95,19,4
1qfma1#186	GO:0003916: DNA topoisomerase activity	0.003593	326,10,5
1qfma1#186	GO:0016620: oxidoreductase activity, acting on the aldehyde or oxo group of donors, NAD or NADP as acceptor	0.003593	326,10,5
1pysa_#221	GO:0003887: DNA-directed DNA polymerase activity	0.003594	90,20,4
1ospo_#262	GO:0005524: ATP binding	0.003597	43,243,10
1a65a2#187	GO:0005524: ATP binding	0.003597	43,243,10
1e0ta2#275	GO:0016627: oxidoreductase activity, acting on the CH-CH group of donors	0.003601	107,17,4
1dhs__#99	GO:0016776: phosphotransferase activity, phosphate group as acceptor	0.003604	132,14,4
1nsj__#178	GO:0000287: magnesium ion binding	0.003604	93,128,11
1i50b_#966	GO:0005509: calcium ion binding	0.00361	299,160,29
1ee4a_#266	GO:0004842: ubiquitin-protein ligase activity	0.003611	46,19,3
1nal1_#203	GO:0016646: oxidoreductase activity, acting on the CH-NH group of donors, NAD or NADP as acceptor	0.003611	46,19,3
1g51a3#525	GO:0016702: oxidoreductase activity, acting on single donors with incorporation of molecular oxygen, incorporation of two atoms of oxygen	0.003615	263,12,5
1erxa_#138	GO:0003713: transcription coactivator activity	0.003618	55,16,3
1ddma_#130	GO:0051082: unfolded protein binding	0.003618	313,34,10
1boub_#7	GO:0008026: ATP-dependent helicase activity	0.003626	240,13,5
1boub_#7	GO:0008408: 3'-5' exonuclease activity	0.003626	240,13,5
1boub_#7	GO:0016651: oxidoreductase activity, acting on NADH or NADPH	0.003626	240,13,5
1g51a3#524	GO:0019838: growth factor binding	0.003629	102,9,3
1qfma1#186	GO:0015082: di-, tri-valent inorganic cation transporter activity	0.003634	326,14,6
1qfma1#186	GO:0046915: transition metal ion transporter activity	0.003634	326,14,6
1p35a_#101	GO:0005524: ATP binding	0.003639	136,243,22
1amf__#196	GO:0003755: peptidyl-prolyl cis-trans isomerase activity	0.003639	173,11,4
1amf__#196	GO:0003964: RNA-directed DNA polymerase activity	0.003639	173,11,4
1byka_#279	GO:0010181: FMN binding	0.00364	82,11,3
1atg__#144	GO:0016758: transferase activity, transferring hexosyl groups	0.00364	82,11,3
1xis__#214	GO:0016836: hydro-lyase activity	0.00364	88,33,5
1cm9a_#57	GO:0005524: ATP binding	0.003646	50,243,11
1im4a_#41	GO:0030151: molybdenum ion binding	0.003646	59,15,3
1im4a_#41	GO:0005179: hormone activity	0.003646	59,15,3
7reqa2#702	GO:0005096: GTPase activator activity	0.003646	59,15,3
2sqca2#268	GO:0005096: GTPase activator activity	0.003646	59,15,3
1jh2a_#16	GO:0016620: oxidoreductase activity, acting on the aldehyde or oxo group of donors, NAD or NADP as acceptor	0.003647	91,10,3
1exma2#353	GO:0003916: DNA topoisomerase activity	0.003647	91,10,3
1aqua_#126	GO:0004812: tRNA ligase activity	0.003651	576,26,12
1lcl__#110	GO:0005507: copper ion binding	0.003656	23,38,3
1crua_#29	GO:0005507: copper ion binding	0.003656	23,38,3

1hska1#54	GO:0030151: molybdenum ion binding	0.003668	17,15,2
1i9sa_#19	GO:0004725: protein tyrosine phosphatase activity	0.003668	17,15,2
1jl0a_#279	GO:0030151: molybdenum ion binding	0.003668	17,15,2
1tbga_#63	GO:0016638: oxidoreductase activity, acting on the CH-NH2 group of donors	0.003668	15,17,2
2napa2#444	GO:0003743: translation initiation factor activity	0.003668	17,15,2
1pjr_1#87	GO:0030151: molybdenum ion binding	0.003668	17,15,2
1a0ca_#273	GO:0008483: transaminase activity	0.003668	15,17,2
1aa6_2#208	GO:0016811: hydrolase activity, acting on carbon-nitrogen (but not peptide) bonds, in linear amides	0.003668	17,15,2
1pkp_1#139	GO:0030151: molybdenum ion binding	0.003668	17,15,2
1htjf_#368	GO:0005096: GTPase activator activity	0.003668	17,15,2
1ebfa1#39	GO:0046983: protein dimerization activity	0.003668	15,17,2
1imva_#191	GO:0008200: ion channel inhibitor activity	0.003668	15,17,2
1keva2#236	GO:0004556: alpha-amylase activity	0.003668	17,15,2
1as4.1#A336	GO:0004295: trypsin activity	0.003669	332,48,13
1es6a_#102	GO:0004457: lactate dehydrogenase activity	0.00367	1,10,1
1xnaa_#123	GO:0003684: damaged DNA binding	0.00367	1,10,1
1gcqc_#595	GO:0005066: transmembrane receptor protein tyrosine kinase signaling protein activity	0.00367	1,10,1
1smla_#21	GO:0008800: beta-lactamase activity	0.00367	1,10,1
1gpea2#494	GO:0050660: FAD binding	0.00367	1,10,1
1d2fa_#229	GO:0016846: carbon-sulfur lyase activity	0.00367	1,10,1
1dssg1#127	GO:0016620: oxidoreductase activity, acting on the aldehyde or oxo group of donors, NAD or NADP as acceptor	0.00367	1,10,1
1nar__#50	GO:0008009: chemokine activity	0.00367	1,10,1
1ad3a_#302	GO:0016620: oxidoreductase activity, acting on the aldehyde or oxo group of donors, NAD or NADP as acceptor	0.00367	1,10,1
1erxa_#53	GO:0050660: FAD binding	0.00367	1,10,1
1gpea2#331	GO:0050660: FAD binding	0.00367	1,10,1
1cyo__#24	GO:0003684: damaged DNA binding	0.00367	1,10,1
1eo9b_#456	GO:0008199: ferric iron binding	0.00367	1,10,1
1bjt__#985	GO:0003916: DNA topoisomerase activity	0.00367	1,10,1
1a31a1#529	GO:0003916: DNA topoisomerase activity	0.00367	1,10,1
1gpc__#66	GO:0003684: damaged DNA binding	0.00367	1,10,1
1rlr_2#709	GO:0008800: beta-lactamase activity	0.00367	1,10,1
1bjt__#589	GO:0003916: DNA topoisomerase activity	0.00367	1,10,1
1cf2o1#22	GO:0016620: oxidoreductase activity, acting on the aldehyde or oxo group of donors, NAD or NADP as acceptor	0.00367	1,10,1
2pia_1#99	GO:0005516: calmodulin binding	0.003672	179,24,6
1f97a2#199	GO:0005529: sugar binding	0.003672	144,39,7
1quna1#113	GO:0005525: GTP binding	0.003673	286,49,12
1e1oa2#423	GO:0005507: copper ion binding	0.003678	148,38,7
1bu7a_#278	GO:0005524: ATP binding	0.003679	30,243,8
1ak0__#115	GO:0005524: ATP binding	0.003679	30,243,8
2arca_#55	GO:0005518: collagen binding	0.003679	144,13,4
2stv__#129	GO:0005524: ATP binding	0.003679	30,243,8
1tig__#148	GO:0005524: ATP binding	0.003679	30,243,8
1ejda_#213	GO:0004812: tRNA ligase activity	0.003679	284,26,8
1ejda_#213	GO:0004497: monooxygenase activity	0.003679	284,26,8
1e4ea2#215	GO:0000049: tRNA binding	0.00368	490,13,7
1e4ea2#215	GO:0003729: mRNA binding	0.00368	490,13,7
1evqa_#305	GO:0008026: ATP-dependent helicase activity	0.00368	490,13,7
1evqa_#305	GO:0016651: oxidoreductase activity, acting on NADH or NADPH	0.00368	490,13,7
1fsu__#434	GO:0046983: protein dimerization activity	0.003681	356,17,7
1fsu__#434	GO:0016638: oxidoreductase activity, acting on the CH-NH2 group of donors	0.003681	356,17,7
1jj2q_#114	GO:0016836: hydro-lyase activity	0.003682	54,33,4
1heta2#212	GO:0016616: oxidoreductase activity, acting on the CH-OH group of donors, NAD or NADP as acceptor	0.003685	49,59,5
1ihoa_#238	GO:0009036: type II site-specific deoxyribonuclease activity	0.003685	75,12,3
1ihoa_#238	GO:0000155: two-component sensor molecule activity	0.003685	75,12,3
1jp3a_#22	GO:0016616: oxidoreductase activity, acting on the CH-OH group of donors, NAD or NADP as acceptor	0.003685	49,59,5
1hc7a2#258	GO:0005529: sugar binding	0.003685	226,39,9
1f00i2#805	GO:0019843: rRNA binding	0.003687	135,22,5
1g51a1#47	GO:0005525: GTP binding	0.003688	18,49,3
1g71a_#88	GO:0008483: transaminase activity	0.003691	52,17,3
1kapp2#69	GO:0046983: protein dimerization activity	0.003691	52,17,3

1exh__#44	GO:0046983: protein dimerization activity	0.003691	52,17,3
1whi__#59	GO:0005524: ATP binding	0.003691	72,243,14
2ebn__#77	GO:0008483: transaminase activity	0.003691	52,17,3
1e30a_#87	GO:0016638: oxidoreductase activity, acting on the CH-NH2 group of donors	0.003691	52,17,3
1a4ya_#104	GO:0008235: metalloexopeptidase activity	0.003693	241,13,5
1a4ya_#104	GO:0016651: oxidoreductase activity, acting on NADH or NADPH	0.003693	241,13,5
1a4ya_#104	GO:0016861: intramolecular oxidoreductase activity, interconverting aldoses and ketoses	0.003693	241,13,5
1i50b_#334	GO:0004812: tRNA ligase activity	0.003697	69,26,4
1i50b_#334	GO:0004497: monooxygenase activity	0.003697	69,26,4
1ezvb1#29	GO:0005126: hematopoietin/interferon-class (D200-domain) cytokine receptor binding	0.003697	44,20,3
1dbxa_#75	GO:0004812: tRNA ligase activity	0.003697	69,26,4
1qkma_#330	GO:0016705: oxidoreductase activity, acting on paired donors, with incorporation or reduction of molecular oxygen	0.003697	69,26,4
1qkma_#330	GO:0004497: monooxygenase activity	0.003697	69,26,4
1azsa_#397	GO:0004523: ribonuclease H activity	0.003698	16,16,2
1e0la_#20	GO:0003700: transcription factor activity	0.003703	34,124,6
1ayl__#237	GO:0008235: metalloexopeptidase activity	0.003703	357,13,6
1ewna_#109	GO:0004197: cysteine-type endopeptidase activity	0.003704	75,24,4
1pot__#138	GO:0004197: cysteine-type endopeptidase activity	0.003704	75,24,4
1dik_3#240	GO:0016638: oxidoreductase activity, acting on the CH-NH2 group of donors	0.003706	262,17,6
1dik_3#240	GO:0051082: unfolded protein binding	0.00371	262,34,9
1hg3a_#43	GO:0000287: magnesium ion binding	0.003714	33,128,6
1fmta2#151	GO:0004620: phospholipase activity	0.003714	314,19,7
1pwt__#8	GO:0016646: oxidoreductase activity, acting on the CH-NH group of donors, NAD or NADP as acceptor	0.003714	314,19,7
1f8ra2#325	GO:0016705: oxidoreductase activity, acting on paired donors, with incorporation or reduction of molecular oxygen	0.003718	222,26,7
1e2o__#346	GO:0008009: chemokine activity	0.003719	194,10,4
1qq4a_#178	GO:0019843: rRNA binding	0.00372	40,22,3
1ad2__#40	GO:0019843: rRNA binding	0.00372	40,22,3
1jsg__#92	GO:0003724: RNA helicase activity	0.003723	607,11,7
2cpl__#20	GO:0046983: protein dimerization activity	0.003725	108,17,4
2rspa_#113	GO:0008270: zinc ion binding	0.003725	17,108,4
1nal1_#204	GO:0008270: zinc ion binding	0.003725	17,108,4
1kp6a_#77	GO:0005507: copper ion binding	0.003733	47,38,4
1a7ca_#370	GO:0005507: copper ion binding	0.003733	47,38,4
1fxoa_#136	GO:0004674: protein serine/threonine kinase activity	0.003734	21,42,3
1rypb_#114	GO:0003899: DNA-directed RNA polymerase activity	0.003734	158,12,4
1tbrr1#15	GO:0004714: transmembrane receptor protein tyrosine kinase activity	0.003739	64,14,3
1qksa2#236	GO:0003968: RNA-directed RNA polymerase activity	0.003739	64,14,3
2pia_1#99	GO:0016638: oxidoreductase activity, acting on the CH-NH2 group of donors	0.00374	179,17,5
1qf6a3#92	GO:0003887: DNA-directed DNA polymerase activity	0.003741	91,20,4
1dn2a1#261	GO:0019843: rRNA binding	0.003741	425,22,9
2viua_#252	GO:0019843: rRNA binding	0.003748	343,22,8
1g6oa_#116	GO:0003729: mRNA binding	0.003757	358,13,6
1g6oa_#116	GO:0008235: metalloexopeptidase activity	0.003757	358,13,6
1g6oa_#116	GO:0008408: 3'-5' exonuclease activity	0.003757	358,13,6
1lam_2#439	GO:0004497: monooxygenase activity	0.003759	10,26,2
1eucb2#84	GO:0016705: oxidoreductase activity, acting on paired donors, with incorporation or reduction of molecular oxygen	0.003759	10,26,2
1eucb2#84	GO:0004497: monooxygenase activity	0.003759	10,26,2
1h6oa_#135	GO:0016705: oxidoreductase activity, acting on paired donors, with incorporation or reduction of molecular oxygen	0.003759	10,26,2
1h6oa_#135	GO:0004497: monooxygenase activity	0.003759	10,26,2
1ed1a_#94	GO:0004812: tRNA ligase activity	0.003759	10,26,2
1ek0a_#151	GO:0003682: chromatin binding	0.003759	26,10,2
1cpt__#144	GO:0016705: oxidoreductase activity, acting on paired donors, with incorporation or reduction of molecular oxygen	0.003759	10,26,2
1t7pa2#663	GO:0003809: thrombin activity	0.003759	26,10,2
1feza_#121	GO:0008800: beta-lactamase activity	0.003759	26,10,2
1lst__#157	GO:0003916: DNA topoisomerase activity	0.003759	26,10,2
1ii7a_#275	GO:0004457: lactate dehydrogenase activity	0.003759	26,10,2
1prxa_#28	GO:0016705: oxidoreductase activity, acting on paired donors, with incorporation or reduction of molecular oxygen	0.003759	10,26,2
1prxa_#28	GO:0004497: monooxygenase activity	0.003759	10,26,2
1cx1a_#52	GO:0019838: growth factor binding	0.00376	29,9,2

1iow_2#112	GO:0019838: growth factor binding	0.00376	29,9,2
1thg__#3	GO:0003916: DNA topoisomerase activity	0.003762	92,10,3
1a6o__#45	GO:0004556: alpha-amylase activity	0.003763	303,15,6
1h72c2#269	GO:0003755: peptidyl-prolyl cis-trans isomerase activity	0.003768	83,11,3
1be9a_#357	GO:0003755: peptidyl-prolyl cis-trans isomerase activity	0.003768	83,11,3
1f5aa2#167	GO:0005096: GTPase activator activity	0.003772	124,15,4
1thfd_#140	GO:0030151: molybdenum ion binding	0.003772	124,15,4
2liv__#81	GO:0003700: transcription factor activity	0.003772	15,124,4
1by5a_#347	GO:0046983: protein dimerization activity	0.003779	263,17,6
1by5a_#347	GO:0016638: oxidoreductase activity, acting on the CH-NH2 group of donors	0.003779	263,17,6
1evqa_#61	GO:0004842: ubiquitin-protein ligase activity	0.003785	159,19,5
1evqa_#61	GO:0004896: hematopoietin/interferon-class (D200-domain) cytokine receptor activity	0.003785	159,19,5
1qtn.1#A270	GO:0010181: FMN binding	0.003786	439,11,6
1qlca_#210	GO:0005524: ATP binding	0.003787	24,243,7
1feua_#71	GO:0005524: ATP binding	0.003787	24,243,7
1bjaa_#26	GO:0005524: ATP binding	0.003787	24,243,7
1opy__#68	GO:0005529: sugar binding	0.003794	367,39,12
1fjfl_#67	GO:0019955: cytokine binding	0.003796	175,11,4
1el0a_#42	GO:0004674: protein serine/threonine kinase activity	0.003797	294,42,11
1jsg__#92	GO:0015036: disulfide oxidoreductase activity	0.003799	607,22,11
1g97a1#376	GO:0003887: DNA-directed DNA polymerase activity	0.003804	13,20,2
1kcw_3#440	GO:0016651: oxidoreductase activity, acting on NADH or NADPH	0.003804	20,13,2
1ekma1#386	GO:0003887: DNA-directed DNA polymerase activity	0.003804	13,20,2
1d2na_#654	GO:0008026: ATP-dependent helicase activity	0.003804	20,13,2
1d2na_#654	GO:0008408: 3'-5' exonuclease activity	0.003804	20,13,2
1ksia1#555	GO:0005085: guanyl-nucleotide exchange factor activity	0.003804	20,13,2
1i2oa_#173	GO:0000049: tRNA binding	0.003804	20,13,2
1i2oa_#173	GO:0016861: intramolecular oxidoreductase activity, interconverting aldoses and ketoses	0.003804	20,13,2
1qe3a_#7	GO:0008408: 3'-5' exonuclease activity	0.003804	20,13,2
1qsta_#154	GO:0000049: tRNA binding	0.003804	20,13,2
1elka_#21	GO:0003887: DNA-directed DNA polymerase activity	0.003804	13,20,2
1cyda_#106	GO:0016854: racemase and epimerase activity	0.003804	20,13,2
1znca_#70	GO:0005507: copper ion binding	0.003806	7,38,2
1toaa_#273	GO:0030145: manganese ion binding	0.003806	7,38,2
1ile_3#408	GO:0005507: copper ion binding	0.003806	7,38,2
1jk7a_#119	GO:0005507: copper ion binding	0.003806	7,38,2
1g61a_#2155	GO:0005507: copper ion binding	0.003806	7,38,2
1dp0a5#534	GO:0016836: hydro-lyase activity	0.003806	8,33,2
1tuba1#8	GO:0008757: S-adenosylmethionine-dependent methyltransferase activity	0.003808	124,24,5
1kit_3#331	GO:0008201: heparin binding	0.003808	124,24,5
1thfd_#140	GO:0005516: calmodulin binding	0.003808	124,24,5
1apme_#74	GO:0005516: calmodulin binding	0.003808	124,24,5
1bjaa_#26	GO:0003700: transcription factor activity	0.003808	24,124,5
1bwvs_#117	GO:0003968: RNA-directed RNA polymerase activity	0.00381	329,14,6
1fo4a5#1020	GO:0005518: collagen binding	0.003812	359,13,6
2bb2_2#134	GO:0003887: DNA-directed DNA polymerase activity	0.003822	220,20,6
3btaa2#1258	GO:0005507: copper ion binding	0.003825	77,38,5
1pya.1#B236	GO:0005507: copper ion binding	0.003825	77,38,5
1jcfa1#26	GO:0004180: carboxypeptidase activity	0.003825	60,15,3
1h8ca_#35	GO:0005507: copper ion binding	0.003825	77,38,5
1ekra_#114	GO:0016811: hydrolase activity, acting on carbon-nitrogen (but not peptide) bonds, in linear amides	0.003825	60,15,3
1ac6a_#34	GO:0004556: alpha-amylase activity	0.003825	60,15,3
1aqt_2#45	GO:0015405: P-P-bond-hydrolysis-driven transporter activity	0.003827	76,12,3
1dt6a_#385	GO:0003697: single-stranded DNA binding	0.003827	76,12,3
1bak__#647	GO:0016702: oxidoreductase activity, acting on single donors with incorporation of molecular oxygen, incorporation of two atoms of oxygen	0.003827	76,12,3
1fd9a_#162	GO:0015078: hydrogen ion transporter activity	0.003828	87,21,4
3aky_1#194	GO:0005524: ATP binding	0.003831	13,243,5
1ce7a_#9	GO:0005524: ATP binding	0.003831	13,243,5
1smaa1#95	GO:0005529: sugar binding	0.003831	272,39,10
1aqua_#79	GO:0000287: magnesium ion binding	0.003831	44,128,7
1f42a2#123	GO:0005507: copper ion binding	0.003832	378,38,12
1g25a_#22	GO:0004556: alpha-amylase activity	0.003832	415,15,7
1ds1a_#300	GO:0008201: heparin binding	0.003834	37,24,3
1dfma_#128	GO:0004197: cysteine-type endopeptidase activity	0.003834	37,24,3

1tkia_#142	GO:0005516: calmodulin binding	0.003834	37,24,3
1pme__#25	GO:0005516: calmodulin binding	0.003834	37,24,3
1bxoa_#307	GO:0019838: growth factor binding	0.003836	104,9,3
1clxa_#120	GO:0016646: oxidoreductase activity, acting on the CH-NH group of donors, NAD or NADP as acceptor	0.00384	47,19,3
1dpja_#320	GO:0004867: serine-type endopeptidase inhibitor activity	0.00384	19,47,3
1epwa3#175	GO:0051082: unfolded protein binding	0.003841	53,34,4
1rrpb_#65	GO:0051082: unfolded protein binding	0.003841	53,34,4
1dp0a1#309	GO:0051082: unfolded protein binding	0.003841	53,34,4
1bywa_#30	GO:0003916: DNA topoisomerase activity	0.003846	331,10,5
1czya1#466	GO:0016638: oxidoreductase activity, acting on the CH-NH2 group of donors	0.003852	109,17,4
1h6va3#486	GO:0005518: collagen binding	0.00386	70,13,3
1fepa_#49	GO:0000049: tRNA binding	0.00386	70,13,3
1fepa_#49	GO:0008235: metalloexopeptidase activity	0.00386	70,13,3
1cgha_#227	GO:0005518: collagen binding	0.00386	70,13,3
1cfe__#7	GO:0008026: ATP-dependent helicase activity	0.00386	70,13,3
1j9qa1#154	GO:0003916: DNA topoisomerase activity	0.003862	196,10,4
1fo4a5#1020	GO:0016638: oxidoreductase activity, acting on the CH-NH2 group of donors	0.003864	359,17,7
1e79d2#36	GO:0005529: sugar binding	0.003864	524,39,15
1ycsb1#412	GO:0008408: 3'-5' exonuclease activity	0.003869	146,13,4
2pola2#218	GO:0008408: 3'-5' exonuclease activity	0.003869	146,13,4
1a06__#85	GO:0004263: chymotrypsin activity	0.003878	44,41,4
2occb1#209	GO:0003682: chromatin binding	0.00388	93,10,3
1ryp1_#20	GO:0004725: protein tyrosine phosphatase activity	0.003884	125,15,4
1ez0a_#133	GO:0004725: protein tyrosine phosphatase activity	0.003884	125,15,4
1b3qa2#623	GO:0004867: serine-type endopeptidase inhibitor activity	0.003885	301,47,12
1dt6a_#385	GO:0004197: cysteine-type endopeptidase activity	0.003887	76,24,4
3prn__#145	GO:0051082: unfolded protein binding	0.003889	168,34,7
1kapp1#389	GO:0005509: calcium ion binding	0.00389	19,160,5
1qq4a_#46	GO:0005509: calcium ion binding	0.00389	19,160,5
1qmea4#464	GO:0005509: calcium ion binding	0.00389	19,160,5
3rpba_#644	GO:0005509: calcium ion binding	0.00389	19,160,5
1c4zd_#51	GO:0016646: oxidoreductase activity, acting on the CH-NH group of donors, NAD or NADP as acceptor	0.00389	160,19,5
1jg8a_#172	GO:0003887: DNA-directed DNA polymerase activity	0.003893	92,20,4
1f46a_#59	GO:0016763: transferase activity, transferring pentosyl groups	0.003895	106,28,5
1cfe__#7	GO:0004812: tRNA ligase activity	0.003895	70,26,4
1aa6_2#285	GO:0016763: transferase activity, transferring pentosyl groups	0.003895	106,28,5
1pma1_#79	GO:0004812: tRNA ligase activity	0.003895	70,26,4
2scub2#126	GO:0003755: peptidyl-prolyl cis-trans isomerase activity	0.003896	24,11,2
1ej0a_#70	GO:0008757: S-adenosylmethionine-dependent methyltransferase activity	0.003896	11,24,2
1fmk_3#428	GO:0005516: calmodulin binding	0.003896	11,24,2
1dbta_#89	GO:0016758: transferase activity, transferring hexosyl groups	0.003896	24,11,2
1eur__#118	GO:0004197: cysteine-type endopeptidase activity	0.003896	11,24,2
2bbkh_#348	GO:0019955: cytokine binding	0.003896	24,11,2
1dfca1#1135	GO:0016638: oxidoreductase activity, acting on the CH-NH2 group of donors	0.003897	53,17,3
1ddga1#278	GO:0016638: oxidoreductase activity, acting on the CH-NH2 group of donors	0.003897	53,17,3
1ddga1#278	GO:0008483: transaminase activity	0.003897	53,17,3
1imva_#69	GO:0010181: FMN binding	0.003899	84,11,3
1i50b_#966	GO:0003887: DNA-directed DNA polymerase activity	0.003907	299,20,7
1h8ua_#113	GO:0016251: general RNA polymerase II transcription factor activity	0.003908	65,14,3
1c4zd_#51	GO:0003697: single-stranded DNA binding	0.003909	160,12,4
1hnja1#8	GO:0015036: disulfide oxidoreductase activity	0.003911	12,22,2
1aq0a_#121	GO:0015036: disulfide oxidoreductase activity	0.003911	12,22,2
1b8aa2#368	GO:0004714: transmembrane receptor protein tyrosine kinase activity	0.003911	135,14,4
2naca1#162	GO:0000155: two-component sensor molecule activity	0.003911	22,12,2
1tuba2#259	GO:0015036: disulfide oxidoreductase activity	0.003911	12,22,2
1f00i2#805	GO:0004714: transmembrane receptor protein tyrosine kinase activity	0.003911	135,14,4
1dypa_#143	GO:0000287: magnesium ion binding	0.003921	94,128,11
1erv__#80	GO:0003755: peptidyl-prolyl cis-trans isomerase activity	0.003923	442,11,6
1imva_#255	GO:0015082: di-, tri-valent inorganic cation transporter activity	0.003928	589,14,8
1imva_#255	GO:0004714: transmembrane receptor protein tyrosine kinase activity	0.003928	589,14,8
1imva_#255	GO:0046915: transition metal ion transporter activity	0.003928	589,14,8
1b8aa2#403	GO:0004523: ribonuclease H activity	0.003934	117,16,4
2btva_#597	GO:0005509: calcium ion binding	0.003935	27,160,6
1a3qa2#133	GO:0005509: calcium ion binding	0.003935	27,160,6

1bwvs_#117	GO:0005507: copper ion binding	0.003938	329,38,11
1euha_#349	GO:0005529: sugar binding	0.003939	23,39,3
1fs7a_#235	GO:0005529: sugar binding	0.003939	23,39,3
1crua_#29	GO:0005529: sugar binding	0.003939	23,39,3
1awx__#13	GO:0005525: GTP binding	0.003941	37,49,4
1apme_#68	GO:0019838: growth factor binding	0.003942	105,9,3
1h4ua1#486	GO:0003887: DNA-directed DNA polymerase activity	0.003943	45,20,3
3sil__#8	GO:0008757: S-adenosylmethionine-dependent methyltransferase activity	0.003943	125,24,5
1hbza_#214	GO:0003724: RNA helicase activity	0.003944	297,11,5
1nat__#50	GO:0016758: transferase activity, transferring hexosyl groups	0.003944	297,11,5
1fvia2#134	GO:0003779: actin binding	0.003949	28,32,3
3pah__#303	GO:0003779: actin binding	0.003949	28,32,3
1cbf__#208	GO:0050660: FAD binding	0.003951	333,10,5
1cf1a1#33	GO:0005509: calcium ion binding	0.003957	45,160,8
1nfdb1#34	GO:0003755: peptidyl-prolyl cis-trans isomerase activity	0.003957	177,11,4
1a9xa6#760	GO:0004867: serine-type endopeptidase inhibitor activity	0.00396	90,47,6
1dy9.1#C229	GO:0004497: monooxygenase activity	0.003961	115,26,5
1h4vb2#282	GO:0004674: protein serine/threonine kinase activity	0.003963	101,42,6
1bmv2_#2064	GO:0000049: tRNA binding	0.003966	147,13,4
1bmv2_#2064	GO:0005518: collagen binding	0.003966	147,13,4
1bfd_2#146	GO:0030145: manganese ion binding	0.003967	112,38,6
2dkb__#114	GO:0016831: carboxy-lyase activity	0.003969	174,25,6
1ih7a1#57	GO:0003697: single-stranded DNA binding	0.003972	77,12,3
1pya.1#B236	GO:0016702: oxidoreductase activity, acting on single donors with incorporation of molecular oxygen, incorporation of two atoms of oxygen	0.003972	77,12,3
1dnpa2#18	GO:0042802: protein self binding	0.003972	77,12,3
1h8ca_#35	GO:0009036: type II site-specific deoxyribonuclease activity	0.003972	77,12,3
1xis__#214	GO:0005524: ATP binding	0.003974	88,243,16
1ejda_#213	GO:0005351: sugar porter activity	0.003976	284,21,7
1c8za_#386	GO:0005507: copper ion binding	0.003981	235,38,9
2napa2#292	GO:0016831: carboxy-lyase activity	0.003983	120,25,5
1f8ra1#233	GO:0015082: di-, tri-valent inorganic cation transporter activity	0.003988	19,14,2
1f8ra1#233	GO:0046915: transition metal ion transporter activity	0.003988	19,14,2
1gof_2#118	GO:0004842: ubiquitin-protein ligase activity	0.003988	14,19,2
2e2c__#67	GO:0015082: di-, tri-valent inorganic cation transporter activity	0.003988	19,14,2
2e2c__#67	GO:0046915: transition metal ion transporter activity	0.003988	19,14,2
2pola3#335	GO:0004896: hematopoietin/interferon-class (D200-domain) cytokine receptor activity	0.003988	14,19,2
2aak__#28	GO:0004842: ubiquitin-protein ligase activity	0.003988	14,19,2
2aak__#28	GO:0004896: hematopoietin/interferon-class (D200-domain) cytokine receptor activity	0.003988	14,19,2
1qtra_#56	GO:0016251: general RNA polymerase II transcription factor activity	0.003988	19,14,2
1dar_2#264	GO:0005543: phospholipid binding	0.003988	19,14,2
1ia8a_#59	GO:0004714: transmembrane receptor protein tyrosine kinase activity	0.003988	19,14,2
1mwpa_#36	GO:0004842: ubiquitin-protein ligase activity	0.003988	14,19,2
3rpba_#644	GO:0005543: phospholipid binding	0.003988	19,14,2
1cnza_#19	GO:0004222: metalloendopeptidase activity	0.003988	14,19,2
1el5a1#183	GO:0004222: metalloendopeptidase activity	0.003988	14,19,2
1fmk_3#377	GO:0004714: transmembrane receptor protein tyrosine kinase activity	0.003988	19,14,2
1ble__#34	GO:0016831: carboxy-lyase activity	0.00399	36,25,3
1dfoa_#344	GO:0016831: carboxy-lyase activity	0.00399	36,25,3
1as4.1#A336	GO:0015082: di-, tri-valent inorganic cation transporter activity	0.003991	332,14,6
1as4.1#A336	GO:0004714: transmembrane receptor protein tyrosine kinase activity	0.003991	332,14,6
1as4.1#A336	GO:0046915: transition metal ion transporter activity	0.003991	332,14,6
1dlja2#177	GO:0019843: rRNA binding	0.003993	41,22,3
1elja_#8	GO:0005351: sugar porter activity	0.003994	43,21,3
1cf9a1#736	GO:0005351: sugar porter activity	0.003994	43,21,3
1edza1#206	GO:0005509: calcium ion binding	0.003995	399,160,36
3gcb__#78	GO:0016846: carbon-sulfur lyase activity	0.004	94,10,3
1mpp__#151	GO:0050660: FAD binding	0.004	94,10,3
1dypa_#143	GO:0050660: FAD binding	0.004	94,10,3
1h9da_#91	GO:0003887: DNA-directed DNA polymerase activity	0.004	222,20,6
3prn__#143	GO:0050660: FAD binding	0.004005	334,10,5
3prn__#143	GO:0008800: beta-lactamase activity	0.004005	334,10,5
2bb2_1#57	GO:0005524: ATP binding	0.004006	104,243,18
1ioaa_#215	GO:0005529: sugar binding	0.004007	7,39,2
1jlxa2#243	GO:0005529: sugar binding	0.004007	7,39,2
1e44b_#75	GO:0003916: DNA topoisomerase activity	0.004009	198,10,4

1hyrc1#247	GO:0030151: molybdenum ion binding	0.00401	61,15,3
1mil__#30	GO:0030151: molybdenum ion binding	0.00401	61,15,3
1mil__#30	GO:0004725: protein tyrosine phosphatase activity	0.00401	61,15,3
1bfg__#22	GO:0005179: hormone activity	0.00401	61,15,3
1f0xa1#278	GO:0005525: GTP binding	0.004012	180,49,9
1jj22_#78	GO:0000049: tRNA binding	0.00402	71,13,3
1jjya_#329	GO:0008408: 3'-5' exonuclease activity	0.00402	71,13,3
1cewi_#14	GO:0004896: hematopoietin/interferon-class (D200-domain) cytokine receptor activity	0.004022	98,19,4
1miob_#338	GO:0015036: disulfide oxidoreductase activity	0.004022	84,22,4
1ddja_#681	GO:0008083: growth factor activity	0.004024	253,42,10
1dcpa_#25	GO:0000287: magnesium ion binding	0.004026	56,128,8
1jb0d_#26	GO:0005529: sugar binding	0.004029	229,39,9
1hcl__#82	GO:0005507: copper ion binding	0.004032	48,38,4
1eg2a_#132	GO:0016616: oxidoreductase activity, acting on the CH-OH group of donors, NAD or NADP as acceptor	0.004032	31,59,4
1eu8a_#119	GO:0005507: copper ion binding	0.004032	48,38,4
1e3ja1#136	GO:0016616: oxidoreductase activity, acting on the CH-OH group of donors, NAD or NADP as acceptor	0.004032	31,59,4
1qf6a1#606	GO:0004295: trypsin activity	0.004032	38,48,4
1bf6a_#183	GO:0030145: manganese ion binding	0.004032	48,38,4
1dpta_#22	GO:0003964: RNA-directed DNA polymerase activity	0.004033	85,11,3
1rdr__#322	GO:0003724: RNA helicase activity	0.004037	1,11,1
1jb9a1#157	GO:0003724: RNA helicase activity	0.004037	1,11,1
1iira_#293	GO:0016758: transferase activity, transferring hexosyl groups	0.004037	1,11,1
1aqza_#146	GO:0003724: RNA helicase activity	0.004037	1,11,1
1h54a1#733	GO:0003724: RNA helicase activity	0.004037	1,11,1
1ct9a1#298	GO:0016758: transferase activity, transferring hexosyl groups	0.004037	1,11,1
1fgxa_#318	GO:0016758: transferase activity, transferring hexosyl groups	0.004037	1,11,1
1aoca_#70	GO:0019955: cytokine binding	0.004037	1,11,1
1i4ua_#142	GO:0003724: RNA helicase activity	0.004037	1,11,1
1a8i__#800	GO:0016758: transferase activity, transferring hexosyl groups	0.004037	1,11,1
1hc2_2#343	GO:0008081: phosphoric diester hydrolase activity	0.004037	1,11,1
4fiv__#38	GO:0003964: RNA-directed DNA polymerase activity	0.004037	1,11,1
2trcp_#221	GO:0051082: unfolded protein binding	0.004038	8,34,2
1fo5a_#60	GO:0000049: tRNA binding	0.004043	246,13,5
1quqb_#87	GO:0005507: copper ion binding	0.004045	78,38,5
1axn__#42	GO:0005507: copper ion binding	0.004045	78,38,5
1a6ca2#312	GO:0005126: hematopoietin/interferon-class (D200-domain) cytokine receptor binding	0.004048	93,20,4
1f97a1#62	GO:0046983: protein dimerization activity	0.004051	707,17,10
1b6e__#76	GO:0003809: thrombin activity	0.004052	27,10,2
1apj__#2062	GO:0008009: chemokine activity	0.004052	27,10,2
1d6ja_#36	GO:0003682: chromatin binding	0.004052	27,10,2
1cvra2#24	GO:0003682: chromatin binding	0.004052	27,10,2
1cvra2#24	GO:0016846: carbon-sulfur lyase activity	0.004052	27,10,2
1gtxa_#150	GO:0008199: ferric iron binding	0.004052	27,10,2
1tgoa1#65	GO:0003916: DNA topoisomerase activity	0.004052	27,10,2
1ceza_#227	GO:0016814: hydrolase activity, acting on carbon-nitrogen (but not peptide) bonds, in cyclic amidines	0.004052	27,10,2
1llda2#161	GO:0005261: cation channel activity	0.004052	27,10,2
1dik_1#670	GO:0016814: hydrolase activity, acting on carbon-nitrogen (but not peptide) bonds, in cyclic amidines	0.004052	27,10,2
1ir3a_#1139	GO:0005066: transmembrane receptor protein tyrosine kinase signaling protein activity	0.004052	27,10,2
1ei5a3#311	GO:0050660: FAD binding	0.004052	27,10,2
3grx__#56	GO:0008800: beta-lactamase activity	0.004059	335,10,5
1hlwa_#82	GO:0015405: P-P-bond-hydrolysis-driven transporter activity	0.00406	270,12,5
1hlwa_#82	GO:0016702: oxidoreductase activity, acting on single donors with incorporation of molecular oxygen, incorporation of two atoms of oxygen	0.00406	270,12,5
2mpra_#190	GO:0016702: oxidoreductase activity, acting on single donors with incorporation of molecular oxygen, incorporation of two atoms of oxygen	0.004063	400,12,6
1hbza_#96	GO:0005529: sugar binding	0.004063	370,39,12
1e1oa2#423	GO:0000049: tRNA binding	0.004065	148,13,4
1nsj__#124	GO:0016854: racemase and epimerase activity	0.004065	148,13,4
1dgw.1#Y353	GO:0004620: phospholipase activity	0.004067	319,19,7
2dpma_#189	GO:0016646: oxidoreductase activity, acting on the CH-NH group of donors, NAD or NADP as acceptor	0.004078	48,19,3
1epwa2#1220	GO:0004295: trypsin activity	0.004078	19,48,3

1dpja_#320	GO:0004295: trypsin activity	0.004078	19,48,3
1fc4a_#205	GO:0016627: oxidoreductase activity, acting on the CH-CH group of donors	0.00408	267,17,6
1fc4a_#205	GO:0016638: oxidoreductase activity, acting on the CH-NH2 group of donors	0.00408	267,17,6
2bce__#204	GO:0016776: phosphotransferase activity, phosphate group as acceptor	0.004082	66,14,3
1d8db_#78	GO:0005543: phospholipid binding	0.004082	66,14,3
1dr9a1#39	GO:0004190: aspartic-type endopeptidase activity	0.004082	258,23,7
1pii_2#326	GO:0030145: manganese ion binding	0.004084	192,38,8
1tyfa_#31	GO:0016866: intramolecular transferase activity	0.00409	162,12,4
1h6la_#119	GO:0004725: protein tyrosine phosphatase activity	0.004091	308,15,6
1b65a_#257	GO:0004180: carboxypeptidase activity	0.004103	210,15,5
1f24a_#293	GO:0005525: GTP binding	0.004108	116,49,7
1fjfc2#153	GO:0004180: carboxypeptidase activity	0.004109	420,15,7
1jj2q_#114	GO:0016638: oxidoreductase activity, acting on the CH-NH2 group of donors	0.004111	54,17,3
1a9xb2#1775	GO:0003924: GTPase activity	0.004111	54,17,3
1gtra1#468	GO:0051082: unfolded protein binding	0.004112	54,34,4
1gky__#34	GO:0030151: molybdenum ion binding	0.004113	18,15,2
1h8va_#139	GO:0008810: cellulase activity	0.004113	15,18,2
1prea1#50	GO:0004725: protein tyrosine phosphatase activity	0.004113	18,15,2
1a28a_#688	GO:0005179: hormone activity	0.004113	18,15,2
1qnf_2#27	GO:0016811: hydrolase activity, acting on carbon-nitrogen (but not peptide) bonds, in linear amides	0.004113	18,15,2
1a4ya_#428	GO:0004180: carboxypeptidase activity	0.004113	18,15,2
1hbza_#214	GO:0004674: protein serine/threonine kinase activity	0.004113	297,42,11
3thia_#59	GO:0030151: molybdenum ion binding	0.004113	18,15,2
1hc7a2#258	GO:0004812: tRNA ligase activity	0.004113	226,26,7
1qfha1#721	GO:0005179: hormone activity	0.004113	18,15,2
1hrna_#320	GO:0005179: hormone activity	0.004113	18,15,2
2sqca2#97	GO:0030151: molybdenum ion binding	0.004115	127,15,4
2sqca2#97	GO:0005096: GTPase activator activity	0.004115	127,15,4
1e7wa_#8	GO:0016627: oxidoreductase activity, acting on the CH-CH group of donors	0.004115	111,17,4
1ho1a_#211	GO:0016627: oxidoreductase activity, acting on the CH-CH group of donors	0.004115	111,17,4
1danh_#104	GO:0046983: protein dimerization activity	0.004118	183,17,5
1danh_#104	GO:0016638: oxidoreductase activity, acting on the CH-NH2 group of donors	0.004118	183,17,5
1quqb_#87	GO:0000155: two-component sensor molecule activity	0.004121	78,12,3
1jjya_#324	GO:0015405: P-P-bond-hydrolysis-driven transporter activity	0.004121	78,12,3
1gpma1#289	GO:0004812: tRNA ligase activity	0.004123	35,26,3
1b0pa5#739	GO:0004812: tRNA ligase activity	0.004123	35,26,3
3grs_3#435	GO:0003964: RNA-directed DNA polymerase activity	0.004123	179,11,4
1lucb_#70	GO:0004497: monooxygenase activity	0.004123	35,26,3
1bwza2#162	GO:0016814: hydrolase activity, acting on carbon-nitrogen (but not peptide) bonds, in cyclic amidines	0.004123	95,10,3
1e1aa_#285	GO:0004714: transmembrane receptor protein tyrosine kinase activity	0.004126	137,14,4
1gega_#58	GO:0008757: S-adenosylmethionine-dependent methyltransferase activity	0.004138	38,24,3
1prea2#119	GO:0008757: S-adenosylmethionine-dependent methyltransferase activity	0.004138	38,24,3
1dyma_#213	GO:0008201: heparin binding	0.004138	38,24,3
1fp5a2#537	GO:0008201: heparin binding	0.004138	38,24,3
2bbkh_#348	GO:0005507: copper ion binding	0.004138	24,38,3
1j9qa2#295	GO:0005509: calcium ion binding	0.004145	36,160,7
2hlca_#134	GO:0005509: calcium ion binding	0.004145	36,160,7
1bwvs_#117	GO:0016763: transferase activity, transferring pentosyl groups	0.004145	329,28,9
3frua1#236	GO:0005509: calcium ion binding	0.004148	76,160,11
1fjfj_#68	GO:0005529: sugar binding	0.00415	230,39,9
3grs_1#24	GO:0016763: transferase activity, transferring pentosyl groups	0.00415	66,28,4
1jcfa2#206	GO:0003700: transcription factor activity	0.004158	84,124,10
1iow_1#37	GO:0030151: molybdenum ion binding	0.004159	309,15,6
1fm2.1#B308	GO:0003916: DNA topoisomerase activity	0.004159	200,10,4
1b35b_#97	GO:0004556: alpha-amylase activity	0.004159	309,15,6
1am2__#163	GO:0004674: protein serine/threonine kinase activity	0.004163	102,42,6
1am2__#163	GO:0008083: growth factor activity	0.004163	102,42,6
1bio__#210	GO:0008408: 3'-5' exonuclease activity	0.004166	149,13,4
1qhda2#323	GO:0050660: FAD binding	0.004168	337,10,5
1qhda2#323	GO:0008800: beta-lactamase activity	0.004168	337,10,5
2mpra_#190	GO:0005509: calcium ion binding	0.00417	400,160,36
1mpp__#154	GO:0004620: phospholipase activity	0.004173	99,19,4
1chua2#234	GO:0016627: oxidoreductase activity, acting on the CH-CH group of donors	0.004177	16,17,2
1bdfa2#171	GO:0046983: protein dimerization activity	0.004177	16,17,2

1je5a_#129	GO:0003713: transcription coactivator activity	0.004177	17,16,2
1fy7a_#304	GO:0016638: oxidoreductase activity, acting on the CH-NH2 group of donors	0.004177	16,17,2
1jlja_#104	GO:0008483: transaminase activity	0.004177	16,17,2
2ayh__#160	GO:0046983: protein dimerization activity	0.004177	16,17,2
1qtra_#107	GO:0003924: GTPase activity	0.004177	16,17,2
1e32a1#45	GO:0046983: protein dimerization activity	0.004177	16,17,2
2btva_#866	GO:0046983: protein dimerization activity	0.004177	16,17,2
1g7da_#245	GO:0046983: protein dimerization activity	0.004177	16,17,2
1nal1_#204	GO:0004523: ribonuclease H activity	0.004177	17,16,2
1e5pa_#140	GO:0004867: serine-type endopeptidase inhibitor activity	0.00418	6,47,2
1as4.1#A336	GO:0004190: aspartic-type endopeptidase activity	0.004181	332,23,8
1ecpa_#62	GO:0016861: intramolecular oxidoreductase activity, interconverting aldoses and ketoses	0.004183	72,13,3
3mag__#134	GO:0008026: ATP-dependent helicase activity	0.004183	72,13,3
1fi2a_#109	GO:0015082: di-, tri-valent inorganic cation transporter activity	0.00419	228,14,5
1fi2a_#109	GO:0046915: transition metal ion transporter activity	0.00419	228,14,5
2mev3_#108	GO:0003968: RNA-directed RNA polymerase activity	0.00419	228,14,5
1dysa_#131	GO:0016651: oxidoreductase activity, acting on NADH or NADPH	0.004193	21,13,2
1e9xa_#428	GO:0015078: hydrogen ion transporter activity	0.004193	13,21,2
1an9a2#278	GO:0016651: oxidoreductase activity, acting on NADH or NADPH	0.004193	21,13,2
1dqwa_#169	GO:0005351: sugar porter activity	0.004193	13,21,2
1hyha2#179	GO:0019201: nucleotide kinase activity	0.004193	21,13,2
1thg__#5	GO:0008235: metalloexopeptidase activity	0.004193	21,13,2
1bu7a_#277	GO:0016651: oxidoreductase activity, acting on NADH or NADPH	0.004193	21,13,2
1auoa_#121	GO:0016651: oxidoreductase activity, acting on NADH or NADPH	0.004193	21,13,2
1ycsb1#396	GO:0015078: hydrogen ion transporter activity	0.004193	13,21,2
1xis__#285	GO:0016854: racemase and epimerase activity	0.004193	21,13,2
1eur__#99	GO:0005085: guanyl-nucleotide exchange factor activity	0.004193	21,13,2
1au1a_#58	GO:0008810: cellulase activity	0.004196	105,18,4
1egaa1#58	GO:0003887: DNA-directed DNA polymerase activity	0.004198	46,20,3
1eaja_#59	GO:0019843: rRNA binding	0.004201	202,22,6
1c5y.1#B85	GO:0004263: chymotrypsin activity	0.00421	45,41,4
1gox__#124	GO:0019201: nucleotide kinase activity	0.004211	366,13,6
1htp__#16	GO:0005126: hematopoietin/interferon-class (D200-domain) cytokine receptor binding	0.004216	303,20,7
1qqsa_#124	GO:0005509: calcium ion binding	0.00422	458,160,40
2viua_#252	GO:0008083: growth factor activity	0.004221	343,42,12
1czan1#92	GO:0004523: ribonuclease H activity	0.004221	631,16,9
1g5va_#142	GO:0003964: RNA-directed DNA polymerase activity	0.004226	25,11,2
1f0ya2#126	GO:0016831: carboxy-lyase activity	0.004226	11,25,2
1eu3a2#174	GO:0003724: RNA helicase activity	0.004226	25,11,2
1gen__#475	GO:0003724: RNA helicase activity	0.004226	25,11,2
1amp__#173	GO:0000287: magnesium ion binding	0.004233	15,128,4
1ghpa_#236	GO:0000287: magnesium ion binding	0.004233	15,128,4
1xis__#231	GO:0000287: magnesium ion binding	0.004233	15,128,4
1dusa_#177	GO:0000287: magnesium ion binding	0.004233	15,128,4
7odca2#67	GO:0000287: magnesium ion binding	0.004233	15,128,4
2cuaa_#107	GO:0004556: alpha-amylase activity	0.004233	128,15,4
2cuaa_#107	GO:0004725: protein tyrosine phosphatase activity	0.004233	128,15,4
2liv__#81	GO:0000287: magnesium ion binding	0.004233	15,128,4
1nsj__#83	GO:0000287: magnesium ion binding	0.004233	15,128,4
1fvia2#25	GO:0004674: protein serine/threonine kinase activity	0.004236	44,42,4
1fvia2#25	GO:0008083: growth factor activity	0.004236	44,42,4
1e19a_#281	GO:0016776: phosphotransferase activity, phosphate group as acceptor	0.004236	138,14,4
1c7na_#95	GO:0005509: calcium ion binding	0.004237	183,160,20
1jb3a_#64	GO:0003964: RNA-directed DNA polymerase activity	0.004247	302,11,5
1cmxa_#207	GO:0003916: DNA topoisomerase activity	0.004247	96,10,3
1bfd_2#146	GO:0016627: oxidoreductase activity, acting on the CH-CH group of donors	0.004251	112,17,4
1mrj__#156	GO:0005525: GTP binding	0.004262	61,49,5
1ekbb_#85	GO:0005518: collagen binding	0.004269	150,13,4
1mai__#108	GO:0005507: copper ion binding	0.004271	152,38,7
2cpl__#20	GO:0019838: growth factor binding	0.004272	108,9,3
1at3a_#194	GO:0008270: zinc ion binding	0.004272	9,108,3
1g5ca_#62	GO:0008270: zinc ion binding	0.004272	9,108,3
1jb0d_#26	GO:0004714: transmembrane receptor protein tyrosine kinase activity	0.004272	229,14,5
1pysb5#584	GO:0003697: single-stranded DNA binding	0.004272	79,12,3
1fqva2#201	GO:0008270: zinc ion binding	0.004272	9,108,3
1fbna_#144	GO:0016866: intramolecular transferase activity	0.004272	79,12,3

1d0na2#200	GO:0000155: two-component sensor molecule activity	0.004272	79,12,3
1dkia_#185	GO:0003899: DNA-directed RNA polymerase activity	0.004272	79,12,3
1jeta_#314	GO:0004190: aspartic-type endopeptidase activity	0.004273	12,23,2
1qq1a_#637	GO:0016668: oxidoreductase activity, acting on sulfur group of donors, NAD or NADP as acceptor	0.004273	23,12,2
1eq2a_#299	GO:0016866: intramolecular transferase activity	0.004273	23,12,2
1e79d2#25	GO:0016668: oxidoreductase activity, acting on sulfur group of donors, NAD or NADP as acceptor	0.004273	23,12,2
1aw8.1#B59	GO:0003899: DNA-directed RNA polymerase activity	0.004273	23,12,2
1c8za_#386	GO:0005509: calcium ion binding	0.004273	235,160,24
1d4ta_#80	GO:0042802: protein self binding	0.004273	23,12,2
1b6ra3#161	GO:0016668: oxidoreductase activity, acting on sulfur group of donors, NAD or NADP as acceptor	0.004277	164,12,4
1dlfh_#4	GO:0008083: growth factor activity	0.004278	22,42,3
1ac6a_#43	GO:0008270: zinc ion binding	0.004288	163,108,14
1ciy_2#298	GO:0019838: growth factor binding	0.004291	31,9,2
1qoua_#90	GO:0019838: growth factor binding	0.004291	31,9,2
1jlxa2#166	GO:0005529: sugar binding	0.004291	77,39,5
1aym3_#131	GO:0004190: aspartic-type endopeptidase activity	0.004291	412,23,9
1g25a_#22	GO:0005509: calcium ion binding	0.004291	415,160,37
1awcb_#111	GO:0019829: cation-transporting ATPase activity	0.004291	31,9,2
1e8ga2#201	GO:0003887: DNA-directed DNA polymerase activity	0.004295	304,20,7
1hq0a_#867	GO:0051082: unfolded protein binding	0.004295	171,34,7
1ezvb1#29	GO:0005524: ATP binding	0.004298	44,243,10
2foka2#180	GO:0004601: peroxidase activity	0.0043	147,21,5
1fxla2#169	GO:0019843: rRNA binding	0.004306	203,22,6
1f0xa1#523	GO:0019955: cytokine binding	0.004308	87,11,3
1dfaa2#211	GO:0004364: glutathione transferase activity	0.004308	87,11,3
1dfaa2#211	GO:0008081: phosphoric diester hydrolase activity	0.004308	87,11,3
1qrra_#208	GO:0010181: FMN binding	0.004308	87,11,3
1fd9a_#162	GO:0003964: RNA-directed DNA polymerase activity	0.004308	87,11,3
1erv__#57	GO:0016758: transferase activity, transferring hexosyl groups	0.004308	87,11,3
1bif_2#398	GO:0005525: GTP binding	0.004309	117,49,7
1e32a2#328	GO:0008270: zinc ion binding	0.004309	40,108,6
1aw1a_#169	GO:0016836: hydro-lyase activity	0.004314	28,33,3
1d8ca_#218	GO:0016763: transferase activity, transferring pentosyl groups	0.004314	33,28,3
1hlwa_#82	GO:0016638: oxidoreductase activity, acting on the CH-NH2 group of donors	0.004317	270,17,6
1hlwa_#82	GO:0008483: transaminase activity	0.004317	270,17,6
1dfca4#1469	GO:0008270: zinc ion binding	0.004317	53,108,7
1f9za_#16	GO:0019843: rRNA binding	0.004317	140,22,5
1ejda_#6	GO:0046983: protein dimerization activity	0.004317	270,17,6
3sil__#98	GO:0004812: tRNA ligase activity	0.004322	228,26,7
1pud__#69	GO:0004222: metalloendopeptidase activity	0.004325	49,19,3
1c1da1#235	GO:0005525: GTP binding	0.004325	19,49,3
1leha1#177	GO:0016646: oxidoreductase activity, acting on the CH-NH group of donors, NAD or NADP as acceptor	0.004325	49,19,3
1jp3a_#22	GO:0016646: oxidoreductase activity, acting on the CH-NH group of donors, NAD or NADP as acceptor	0.004325	49,19,3
1d5ra2#25	GO:0005525: GTP binding	0.004325	19,49,3
1e32a2#345	GO:0004222: metalloendopeptidase activity	0.004328	100,19,4
1e32a2#345	GO:0016646: oxidoreductase activity, acting on the CH-NH group of donors, NAD or NADP as acceptor	0.004328	100,19,4
1f3mc_#369	GO:0004896: hematopoietin/interferon-class (D200-domain) cytokine receptor activity	0.004328	100,19,4
1nox__#88	GO:0046983: protein dimerization activity	0.004331	55,17,3
1nox__#88	GO:0016638: oxidoreductase activity, acting on the CH-NH2 group of donors	0.004331	55,17,3
1akp__#62	GO:0046983: protein dimerization activity	0.004331	55,17,3
1enfa1#77	GO:0003924: GTPase activity	0.004331	55,17,3
1b6ra3#161	GO:0004620: phospholipase activity	0.004331	164,19,5
1qlsa_#81	GO:0046983: protein dimerization activity	0.004331	55,17,3
1f97a1#62	GO:0016620: oxidoreductase activity, acting on the aldehyde or oxo group of donors, NAD or NADP as acceptor	0.004334	707,10,7
1avgi_#69	GO:0016620: oxidoreductase activity, acting on the aldehyde or oxo group of donors, NAD or NADP as acceptor	0.004336	340,10,5
1epua_#26	GO:0000287: magnesium ion binding	0.004339	34,128,6
1epwa3#138	GO:0004725: protein tyrosine phosphatase activity	0.004342	424,15,7
1e3a.1#A30	GO:0016763: transferase activity, transferring pentosyl groups	0.004342	157,28,6

1a65a3#409	GO:0005507: copper ion binding	0.004347	49,38,4
1efnb_#102	GO:0005507: copper ion binding	0.004347	49,38,4
1cpy__#346	GO:0005525: GTP binding	0.004347	38,49,4
1et9a1#39	GO:0005507: copper ion binding	0.004347	49,38,4
1ak5_1#353	GO:0016854: racemase and epimerase activity	0.00435	73,13,3
1jj2a1#126	GO:0005518: collagen binding	0.00435	73,13,3
1a81a1#51	GO:0000049: tRNA binding	0.00435	73,13,3
1h4vb2#307	GO:0004177: aminopeptidase activity	0.00435	73,13,3
1imva_#33	GO:0003700: transcription factor activity	0.004354	8,124,3
1mkp__#216	GO:0030151: molybdenum ion binding	0.004354	129,15,4
1ed1a_#94	GO:0016763: transferase activity, transferring pentosyl groups	0.004355	10,28,2
1jc4a_#12	GO:0016846: carbon-sulfur lyase activity	0.004355	28,10,2
1fvia2#134	GO:0016814: hydrolase activity, acting on carbon-nitrogen (but not peptide) bonds, in cyclic amidines	0.004355	28,10,2
1qhoa1#531	GO:0016763: transferase activity, transferring pentosyl groups	0.004355	10,28,2
1a06__#147	GO:0005066: transmembrane receptor protein tyrosine kinase signaling protein activity	0.004355	28,10,2
1dqza_#209	GO:0000287: magnesium ion binding	0.004362	45,128,7
1jj2s_#46	GO:0000287: magnesium ion binding	0.004362	45,128,7
1ea5a_#99	GO:0004190: aspartic-type endopeptidase activity	0.004367	194,23,6
1b8aa2#251	GO:0000287: magnesium ion binding	0.00437	24,128,5
2cuaa_#107	GO:0004197: cysteine-type endopeptidase activity	0.00437	128,24,5
1c7wa_#105	GO:0003779: actin binding	0.00437	29,32,3
1g8fa3#419	GO:0005085: guanyl-nucleotide exchange factor activity	0.004373	151,13,4
1tbrr1#15	GO:0004867: serine-type endopeptidase inhibitor activity	0.004375	64,47,5
1xgsa2#186	GO:0003964: RNA-directed DNA polymerase activity	0.00438	182,11,4
1dyna_#19	GO:0019838: growth factor binding	0.004385	109,9,3
1zpda1#213	GO:0000287: magnesium ion binding	0.004385	82,128,10
1ft9a1#201	GO:0019838: growth factor binding	0.004385	109,9,3
1dn2a1#261	GO:0004725: protein tyrosine phosphatase activity	0.004402	425,15,7
1i50a_#148	GO:0003899: DNA-directed RNA polymerase activity	0.004404	1,12,1
1i50a_#110	GO:0003899: DNA-directed RNA polymerase activity	0.004404	1,12,1
1boub_#124	GO:0016702: oxidoreductase activity, acting on single donors with incorporation of molecular oxygen, incorporation of two atoms of oxygen	0.004404	1,12,1
1iyu__#47	GO:0003899: DNA-directed RNA polymerase activity	0.004404	1,12,1
1byra_#148	GO:0003899: DNA-directed RNA polymerase activity	0.004404	1,12,1
1i50f_#153	GO:0003899: DNA-directed RNA polymerase activity	0.004404	1,12,1
1dfaa2#226	GO:0015405: P-P-bond-hydrolysis-driven transporter activity	0.004404	1,12,1
2sqca2#103	GO:0016866: intramolecular transferase activity	0.004404	1,12,1
1hyoa2#174	GO:0000155: two-component sensor molecule activity	0.004404	1,12,1
1i6vc_#411	GO:0003899: DNA-directed RNA polymerase activity	0.004404	1,12,1
1mpya2#175	GO:0016702: oxidoreductase activity, acting on single donors with incorporation of molecular oxygen, incorporation of two atoms of oxygen	0.004404	1,12,1
1dpe__#399	GO:0003899: DNA-directed RNA polymerase activity	0.004404	1,12,1
1eqja2#478	GO:0016866: intramolecular transferase activity	0.004404	1,12,1
1ev7a_#291	GO:0009036: type II site-specific deoxyribonuclease activity	0.004404	1,12,1
1erxa_#53	GO:0016668: oxidoreductase activity, acting on sulfur group of donors, NAD or NADP as acceptor	0.004404	1,12,1
1eo9b_#456	GO:0016702: oxidoreductase activity, acting on single donors with incorporation of molecular oxygen, incorporation of two atoms of oxygen	0.004404	1,12,1
1i50b_#639	GO:0003899: DNA-directed RNA polymerase activity	0.004404	1,12,1
1ceza_#762	GO:0003899: DNA-directed RNA polymerase activity	0.004404	1,12,1
1eqja1#200	GO:0016866: intramolecular transferase activity	0.004404	1,12,1
1i50b_#117	GO:0003899: DNA-directed RNA polymerase activity	0.004404	1,12,1
7reqb2#633	GO:0016866: intramolecular transferase activity	0.004404	1,12,1
1ihua1#291	GO:0015405: P-P-bond-hydrolysis-driven transporter activity	0.004404	1,12,1
1ejba_#136	GO:0016866: intramolecular transferase activity	0.004404	1,12,1
1ihua1#176	GO:0015405: P-P-bond-hydrolysis-driven transporter activity	0.004404	1,12,1
1a9xa6#760	GO:0004295: trypsin activity	0.004407	90,48,6
1j75a_#166	GO:0004295: trypsin activity	0.00441	120,48,7
2sli_2#326	GO:0000049: tRNA binding	0.004417	251,13,5
2sli_2#326	GO:0005518: collagen binding	0.004417	251,13,5
1e79d2#36	GO:0015036: disulfide oxidoreductase activity	0.004417	524,22,10
2pola3#335	GO:0003887: DNA-directed DNA polymerase activity	0.004418	14,20,2
2pola3#335	GO:0005126: hematopoietin/interferon-class (D200-domain) cytokine receptor binding	0.004418	14,20,2
1i8na_#74	GO:0016251: general RNA polymerase II transcription factor activity	0.004418	20,14,2
1thg__#3	GO:0004867: serine-type endopeptidase inhibitor activity	0.004418	92,47,6

1chma2#262	GO:0015082: di-, tri-valent inorganic cation transporter activity	0.004418	20,14,2
1chma2#262	GO:0046915: transition metal ion transporter activity	0.004418	20,14,2
1ofga1#84	GO:0004714: transmembrane receptor protein tyrosine kinase activity	0.004418	20,14,2
1g3nc2#162	GO:0003887: DNA-directed DNA polymerase activity	0.004418	14,20,2
1koba_#175	GO:0004714: transmembrane receptor protein tyrosine kinase activity	0.004418	20,14,2
1ig8a_#215	GO:0004523: ribonuclease H activity	0.004419	59,16,3
1e0ca1#88	GO:0016616: oxidoreductase activity, acting on the CH-OH group of donors, NAD or NADP as acceptor	0.00442	5,59,2
1dhn__#88	GO:0016616: oxidoreductase activity, acting on the CH-OH group of donors, NAD or NADP as acceptor	0.00442	5,59,2
1j79a_#271	GO:0016616: oxidoreductase activity, acting on the CH-OH group of donors, NAD or NADP as acceptor	0.00442	5,59,2
1f0xa2#270	GO:0016616: oxidoreductase activity, acting on the CH-OH group of donors, NAD or NADP as acceptor	0.00442	5,59,2
1jj2s_#91	GO:0016616: oxidoreductase activity, acting on the CH-OH group of donors, NAD or NADP as acceptor	0.00442	5,59,2
1f0ka_#57	GO:0016616: oxidoreductase activity, acting on the CH-OH group of donors, NAD or NADP as acceptor	0.00442	5,59,2
1gpma1#326	GO:0016616: oxidoreductase activity, acting on the CH-OH group of donors, NAD or NADP as acceptor	0.00442	5,59,2
1jj2q_#118	GO:0004263: chymotrypsin activity	0.004423	7,41,2
1ft9a1#162	GO:0003899: DNA-directed RNA polymerase activity	0.004428	80,12,3
1i4fb1#70	GO:0004295: trypsin activity	0.004435	39,48,4
1a9xa3#78	GO:0016758: transferase activity, transferring hexosyl groups	0.004437	305,11,5
1a9xa3#78	GO:0010181: FMN binding	0.004437	305,11,5
1fy7a_#388	GO:0016251: general RNA polymerase II transcription factor activity	0.004443	68,14,3
1dp0a4#756	GO:0004620: phospholipase activity	0.004447	165,19,5
1i6vd_#1283	GO:0015036: disulfide oxidoreductase activity	0.004452	141,22,5
1hbza_#96	GO:0008408: 3'-5' exonuclease activity	0.004453	370,13,6
1qfma2#576	GO:0004180: carboxypeptidase activity	0.004455	214,15,5
1i9za_#666	GO:0003887: DNA-directed DNA polymerase activity	0.004464	47,20,3
1danh_#104	GO:0003724: RNA helicase activity	0.004469	183,11,4
1jj2j_#88	GO:0004812: tRNA ligase activity	0.00447	36,26,3
1pysb5#584	GO:0008201: heparin binding	0.004472	79,24,4
1dkia_#185	GO:0005516: calmodulin binding	0.004472	79,24,4
1aky_2#133	GO:0004867: serine-type endopeptidase inhibitor activity	0.004473	156,47,8
1flga_#161	GO:0000049: tRNA binding	0.004479	152,13,4
1smaa1#95	GO:0046983: protein dimerization activity	0.004481	272,17,6
1edza1#206	GO:0016763: transferase activity, transferring pentosyl groups	0.004484	399,28,10
1nbaa_#227	GO:0015078: hydrogen ion transporter activity	0.004505	91,21,4
1hrka_#343	GO:0004867: serine-type endopeptidase inhibitor activity	0.004507	40,47,4
1im4a_#144	GO:0051082: unfolded protein binding	0.00451	129,34,6
1g25a_#22	GO:0004190: aspartic-type endopeptidase activity	0.004512	415,23,9
1fmta2#151	GO:0003743: translation initiation factor activity	0.004513	314,15,6
1ft9a1#162	GO:0030145: manganese ion binding	0.004514	80,38,5
1eg9a2#199	GO:0008026: ATP-dependent helicase activity	0.004521	74,13,3
1e3ja2#270	GO:0008026: ATP-dependent helicase activity	0.004521	74,13,3
1bhga3#529	GO:0008235: metalloexopeptidase activity	0.004521	74,13,3
1f42a2#123	GO:0051082: unfolded protein binding	0.004524	378,34,11
1dvpa1#65	GO:0016799: hydrolase activity, hydrolyzing N-glycosyl compounds	0.004524	187,17,5
1gcua1#36	GO:0016616: oxidoreductase activity, acting on the CH-OH group of donors, NAD or NADP as acceptor	0.004534	32,59,4
3lada1#116	GO:0000287: magnesium ion binding	0.004535	153,128,15
1jj2a1#126	GO:0004497: monooxygenase activity	0.004535	73,26,4
1a81a1#51	GO:0004812: tRNA ligase activity	0.004535	73,26,4
1fh6a_#308	GO:0005525: GTP binding	0.004538	6,49,2
1i19a1#396	GO:0005525: GTP binding	0.004538	6,49,2
1ihoa_#75	GO:0005525: GTP binding	0.004538	6,49,2
1e69a_#1106	GO:0005525: GTP binding	0.004538	6,49,2
1ihua2#525	GO:0030145: manganese ion binding	0.00454	335,38,11
1nfdb1#21	GO:0005126: hematopoietin/interferon-class (D200-domain) cytokine receptor binding	0.004541	96,20,4
1enfa1#92	GO:0015078: hydrogen ion transporter activity	0.004547	45,21,3
1fmb__#60	GO:0008270: zinc ion binding	0.004549	3,108,2
3btaa3#262	GO:0008270: zinc ion binding	0.004549	3,108,2
1epwa2#1111	GO:0008270: zinc ion binding	0.004549	3,108,2
1hms__#42	GO:0008270: zinc ion binding	0.004549	3,108,2

1hlwa_#82	GO:0051082: unfolded protein binding	0.004551	270,34,9
1dpsa_#36	GO:0001584: rhodopsin-like receptor activity	0.004552	205,10,4
1gpc__#184	GO:0004190: aspartic-type endopeptidase activity	0.004555	83,23,4
1dcpa_#25	GO:0016638: oxidoreductase activity, acting on the CH-NH2 group of donors	0.004558	56,17,3
1bev1_#86	GO:0051082: unfolded protein binding	0.004562	323,34,10
1b3qa2#623	GO:0004674: protein serine/threonine kinase activity	0.004565	301,42,11
1a7ca_#204	GO:0019955: cytokine binding	0.004568	26,11,2
1ae9a_#239	GO:0004497: monooxygenase activity	0.004568	11,26,2
1zfja1#379	GO:0004812: tRNA ligase activity	0.004568	11,26,2
1qvba_#206	GO:0010181: FMN binding	0.004568	26,11,2
1csn__#52	GO:0004812: tRNA ligase activity	0.004568	11,26,2
1sgpe_#42	GO:0003724: RNA helicase activity	0.004568	26,11,2
1hw5a2#64	GO:0016668: oxidoreductase activity, acting on sulfur group of donors, NAD or NADP as acceptor	0.004568	167,12,4
1b9ra_#102	GO:0003779: actin binding	0.00457	9,32,2
1i1ja_#37	GO:0003779: actin binding	0.00457	9,32,2
1ryph_#52	GO:0019829: cation-transporting ATPase activity	0.00457	32,9,2
1f8na1#450	GO:0019838: growth factor binding	0.00457	32,9,2
1g0sa_#74	GO:0004812: tRNA ligase activity	0.00457	294,26,8
1uxy_1#87	GO:0003779: actin binding	0.00457	9,32,2
1eula_#518	GO:0019838: growth factor binding	0.00457	32,9,2
1kapp1#358	GO:0019838: growth factor binding	0.00457	32,9,2
1el0a_#42	GO:0004497: monooxygenase activity	0.00457	294,26,8
1ddja_#681	GO:0000049: tRNA binding	0.004573	253,13,5
1je5a_#10	GO:0005525: GTP binding	0.004574	62,49,5
1fi2a_#109	GO:0005126: hematopoietin/interferon-class (D200-domain) cytokine receptor binding	0.004574	228,20,6
1sbp__#219	GO:0019843: rRNA binding	0.004574	43,22,3
3sil__#98	GO:0005126: hematopoietin/interferon-class (D200-domain) cytokine receptor binding	0.004574	228,20,6
1i6vd_#1283	GO:0004714: transmembrane receptor protein tyrosine kinase activity	0.00458	141,14,4
2dnja_#97	GO:0004714: transmembrane receptor protein tyrosine kinase activity	0.00458	141,14,4
1pdc__#27	GO:0004896: hematopoietin/interferon-class (D200-domain) cytokine receptor activity	0.004581	50,19,3
1wht.1#A15	GO:0004842: ubiquitin-protein ligase activity	0.004583	15,19,2
1bvqa_#11	GO:0004180: carboxypeptidase activity	0.004583	19,15,2
2hgsa2#471	GO:0004896: hematopoietin/interferon-class (D200-domain) cytokine receptor activity	0.004583	15,19,2
1a3k__#199	GO:0004620: phospholipase activity	0.004583	15,19,2
1a3k__#199	GO:0004896: hematopoietin/interferon-class (D200-domain) cytokine receptor activity	0.004583	15,19,2
1eu8a_#126	GO:0030151: molybdenum ion binding	0.004583	19,15,2
1fl1a_#149	GO:0030151: molybdenum ion binding	0.004583	19,15,2
1dar_2#264	GO:0005096: GTPase activator activity	0.004583	19,15,2
1ezva1#147	GO:0004222: metalloendopeptidase activity	0.004583	15,19,2
1fm2.1#B319	GO:0004180: carboxypeptidase activity	0.004583	19,15,2
1rypf_#50	GO:0004896: hematopoietin/interferon-class (D200-domain) cytokine receptor activity	0.004583	15,19,2
1fjfb_#102	GO:0004556: alpha-amylase activity	0.004583	19,15,2
1c1da1#235	GO:0004556: alpha-amylase activity	0.004583	19,15,2
1dy9.1#A82	GO:0004556: alpha-amylase activity	0.004583	19,15,2
1iqpa2#112	GO:0003743: translation initiation factor activity	0.004583	19,15,2
1qhva_#438	GO:0004556: alpha-amylase activity	0.004583	19,15,2
1dpja_#320	GO:0005179: hormone activity	0.004583	19,15,2
1iqpa2#119	GO:0004556: alpha-amylase activity	0.004583	19,15,2
1dik_1#848	GO:0016866: intramolecular transferase activity	0.004587	81,12,3
1aqb__#134	GO:0000155: two-component sensor molecule activity	0.004587	81,12,3
1foha5#77	GO:0003700: transcription factor activity	0.004589	25,124,5
1tuba1#8	GO:0016831: carboxy-lyase activity	0.004589	124,25,5
1hr0w_#69	GO:0030151: molybdenum ion binding	0.004596	64,15,3
2mev1_#123	GO:0019955: cytokine binding	0.004596	89,11,3
1cl1a_#184	GO:0004556: alpha-amylase activity	0.004596	64,15,3
1ovb__#195	GO:0010181: FMN binding	0.004596	89,11,3
1j79a_#187	GO:0016758: transferase activity, transferring hexosyl groups	0.004596	89,11,3
1qksa2#236	GO:0005179: hormone activity	0.004596	64,15,3
3sil__#84	GO:0004674: protein serine/threonine kinase activity	0.004597	45,42,4
1gdna_#30	GO:0008083: growth factor activity	0.004597	45,42,4
1euwa_#71	GO:0000049: tRNA binding	0.0046	22,13,2
1qg8a_#90	GO:0015036: disulfide oxidoreductase activity	0.0046	13,22,2
1ayoa_#55	GO:0019843: rRNA binding	0.0046	13,22,2
2ayh__#184	GO:0005518: collagen binding	0.0046	22,13,2
1mrj__#240	GO:0015036: disulfide oxidoreductase activity	0.0046	13,22,2

1mwpa_#42	GO:0019843: rRNA binding	0.0046	13,22,2
1dj0a1#96	GO:0019843: rRNA binding	0.0046	13,22,2
1lucb_#32	GO:0016854: racemase and epimerase activity	0.0046	22,13,2
1lucb_#32	GO:0016861: intramolecular oxidoreductase activity, interconverting aldoses and ketoses	0.0046	22,13,2
1a4ia1#208	GO:0005085: guanyl-nucleotide exchange factor activity	0.0046	22,13,2
1auib_#136	GO:0005518: collagen binding	0.0046	22,13,2
1kapp2#149	GO:0005518: collagen binding	0.0046	22,13,2
1dlfh_#4	GO:0005518: collagen binding	0.0046	22,13,2
1xgsa2#186	GO:0008270: zinc ion binding	0.00462	182,108,15
1dpja_#322	GO:0005126: hematopoietin/interferon-class (D200-domain) cytokine receptor binding	0.004627	308,20,7
1hdma1#115	GO:0016638: oxidoreductase activity, acting on the CH-NH2 group of donors	0.00463	188,17,5
1jb0d_#26	GO:0004867: serine-type endopeptidase inhibitor activity	0.004631	229,47,10
1dpja_#322	GO:0003724: RNA helicase activity	0.004634	308,11,5
1d7ba_#87	GO:0005066: transmembrane receptor protein tyrosine kinase signaling protein activity	0.004635	99,10,3
1huxa_#10	GO:0004523: ribonuclease H activity	0.004635	60,16,3
1c9la2#73	GO:0008083: growth factor activity	0.004638	7,42,2
1fsia_#160	GO:0008083: growth factor activity	0.004638	7,42,2
1cs6a2#164	GO:0008083: growth factor activity	0.004638	7,42,2
1e8ua_#286	GO:0008083: growth factor activity	0.004638	7,42,2
1akjd_#3	GO:0008083: growth factor activity	0.004638	7,42,2
1a4ya_#104	GO:0016831: carboxy-lyase activity	0.004647	241,25,7
1hd2a_#35	GO:0016758: transferase activity, transferring hexosyl groups	0.004649	185,11,4
3tdt__#213	GO:0003697: single-stranded DNA binding	0.00465	24,12,2
1koba_#139	GO:0005516: calmodulin binding	0.00465	12,24,2
1hcd__#114	GO:0008201: heparin binding	0.00465	12,24,2
1vcaa2#49	GO:0005516: calmodulin binding	0.00465	12,24,2
1vcaa2#49	GO:0008201: heparin binding	0.00465	12,24,2
1howa_#551	GO:0005516: calmodulin binding	0.00465	12,24,2
1pme__#145	GO:0005516: calmodulin binding	0.00465	12,24,2
1feua_#71	GO:0003697: single-stranded DNA binding	0.00465	24,12,2
1ew2a_#133	GO:0009036: type II site-specific deoxyribonuclease activity	0.00465	24,12,2
1dbta_#89	GO:0003899: DNA-directed RNA polymerase activity	0.00465	24,12,2
1dfca1#1119	GO:0008201: heparin binding	0.00465	12,24,2
1qama_#39	GO:0009036: type II site-specific deoxyribonuclease activity	0.00465	24,12,2
1cpza_#6	GO:0004867: serine-type endopeptidase inhibitor activity	0.004652	157,47,8
1dqsa_#138	GO:0016831: carboxy-lyase activity	0.004658	38,25,3
1ltda1#203	GO:0030145: manganese ion binding	0.004658	25,38,3
1i5ga_#115	GO:0051082: unfolded protein binding	0.004666	324,34,10
3grs_1#23	GO:0016866: intramolecular transferase activity	0.004668	168,12,4
1cx1a_#52	GO:0003916: DNA topoisomerase activity	0.004669	29,10,2
1ejda_#374	GO:0016620: oxidoreductase activity, acting on the aldehyde or oxo group of donors, NAD or NADP as acceptor	0.004669	29,10,2
1e0ta2#10	GO:0016620: oxidoreductase activity, acting on the aldehyde or oxo group of donors, NAD or NADP as acceptor	0.004669	29,10,2
1agi__#46	GO:0016620: oxidoreductase activity, acting on the aldehyde or oxo group of donors, NAD or NADP as acceptor	0.004669	29,10,2
1iow_2#112	GO:0005066: transmembrane receptor protein tyrosine kinase signaling protein activity	0.004669	29,10,2
1b3ra1#218	GO:0004457: lactate dehydrogenase activity	0.004669	29,10,2
1b3ra1#218	GO:0016620: oxidoreductase activity, acting on the aldehyde or oxo group of donors, NAD or NADP as acceptor	0.004669	29,10,2
1g62a_#25	GO:0016620: oxidoreductase activity, acting on the aldehyde or oxo group of donors, NAD or NADP as acceptor	0.004669	29,10,2
1cqqa_#163	GO:0003809: thrombin activity	0.004669	29,10,2
1cgha_#130	GO:0003809: thrombin activity	0.004669	29,10,2
1h9da_#91	GO:0004263: chymotrypsin activity	0.004671	222,41,9
1jb0d_#26	GO:0005126: hematopoietin/interferon-class (D200-domain) cytokine receptor binding	0.004675	229,20,6
1b12a_#221	GO:0016638: oxidoreductase activity, acting on the CH-NH2 group of donors	0.004677	115,17,4
1a28a_#688	GO:0003713: transcription coactivator activity	0.004683	18,16,2
1hw5a2#64	GO:0004896: hematopoietin/interferon-class (D200-domain) cytokine receptor activity	0.004685	167,19,5
1jj2e1#42	GO:0004620: phospholipase activity	0.004685	167,19,5
1neb__#41	GO:0003700: transcription factor activity	0.004692	47,124,7
1ppn__#132	GO:0004714: transmembrane receptor protein tyrosine kinase activity	0.004694	234,14,5
1ppn__#132	GO:0003968: RNA-directed RNA polymerase activity	0.004694	234,14,5
1ihoa_#238	GO:0004177: aminopeptidase activity	0.004696	75,13,3
1feca1#31	GO:0016651: oxidoreductase activity, acting on NADH or NADPH	0.004696	75,13,3
1hoe__#31	GO:0051082: unfolded protein binding	0.0047	28,34,3

1iow_1#37	GO:0010181: FMN binding	0.0047	309,11,5
1af7_2#248	GO:0016763: transferase activity, transferring pentosyl groups	0.0047	34,28,3
1pud__#197	GO:0016763: transferase activity, transferring pentosyl groups	0.0047	34,28,3
1b35b_#97	GO:0003964: RNA-directed DNA polymerase activity	0.0047	309,11,5
1dpja_#322	GO:0004867: serine-type endopeptidase inhibitor activity	0.004702	308,47,12
1qgub_#197	GO:0016616: oxidoreductase activity, acting on the CH-OH group of donors, NAD or NADP as acceptor	0.004706	74,59,6
1b35b_#97	GO:0003887: DNA-directed DNA polymerase activity	0.004713	309,20,7
1i9sa_#19	GO:0003924: GTPase activity	0.004716	17,17,2
1byra_#151	GO:0003924: GTPase activity	0.004716	17,17,2
1qksa2#326	GO:0016638: oxidoreductase activity, acting on the CH-NH2 group of donors	0.004716	17,17,2
1imt_2#41	GO:0008200: ion channel inhibitor activity	0.004716	17,17,2
1eyqa_#8	GO:0016638: oxidoreductase activity, acting on the CH-NH2 group of donors	0.004716	17,17,2
1jd0a_#176	GO:0008009: chemokine activity	0.004716	207,10,4
1ecfa2#160	GO:0046983: protein dimerization activity	0.004716	17,17,2
1fmca_#86	GO:0016627: oxidoreductase activity, acting on the CH-CH group of donors	0.004716	17,17,2
1fjja_#5	GO:0016638: oxidoreductase activity, acting on the CH-NH2 group of donors	0.004716	17,17,2
1qlaa2#383	GO:0016627: oxidoreductase activity, acting on the CH-CH group of donors	0.004716	17,17,2
1hnja1#133	GO:0046983: protein dimerization activity	0.004716	17,17,2
1csn__#84	GO:0050660: FAD binding	0.004716	207,10,4
1a53__#236	GO:0016627: oxidoreductase activity, acting on the CH-CH group of donors	0.004716	17,17,2
1cjca2#76	GO:0016638: oxidoreductase activity, acting on the CH-NH2 group of donors	0.004716	17,17,2
1e25a_#262	GO:0030145: manganese ion binding	0.004723	116,38,6
1nsca_#236	GO:0015036: disulfide oxidoreductase activity	0.004731	143,22,5
1dhs__#99	GO:0004556: alpha-amylase activity	0.004732	132,15,4
1aky_2#133	GO:0005524: ATP binding	0.004733	156,243,24
1aym3_#131	GO:0016702: oxidoreductase activity, acting on single donors with incorporation of molecular oxygen, incorporation of two atoms of oxygen	0.004734	412,12,6
1aym3_#131	GO:0000155: two-component sensor molecule activity	0.004734	412,12,6
1cpy__#93	GO:0016638: oxidoreductase activity, acting on the CH-NH2 group of donors	0.004736	275,17,6
1bev1_#86	GO:0008201: heparin binding	0.004737	323,24,8
1esl_1#48	GO:0005524: ATP binding	0.004741	19,243,6
1dz4a_#150	GO:0005524: ATP binding	0.004741	19,243,6
1fsu__#434	GO:0019843: rRNA binding	0.004742	356,22,8
1a9xa6#760	GO:0003755: peptidyl-prolyl cis-trans isomerase activity	0.004743	90,11,3
1ekma1#545	GO:0019955: cytokine binding	0.004743	90,11,3
1pysa_#221	GO:0003964: RNA-directed DNA polymerase activity	0.004743	90,11,3
1d6aa_#52	GO:0015078: hydrogen ion transporter activity	0.004747	293,21,7
1fhoa_#74	GO:0016620: oxidoreductase activity, acting on the aldehyde or oxo group of donors, NAD or NADP as acceptor	0.004748	347,10,5
1byka_#279	GO:0016866: intramolecular transferase activity	0.004749	82,12,3
1dmla1#134	GO:0000155: two-component sensor molecule activity	0.004749	82,12,3
1vola2#239	GO:0016705: oxidoreductase activity, acting on paired donors, with incorporation or reduction of molecular oxygen	0.00476	120,26,5
2napa2#292	GO:0004812: tRNA ligase activity	0.00476	120,26,5
1i58a_#519	GO:0005507: copper ion binding	0.004762	81,38,5
1eg9a2#199	GO:0004812: tRNA ligase activity	0.004763	74,26,4
1bqk__#77	GO:0016705: oxidoreductase activity, acting on paired donors, with incorporation or reduction of molecular oxygen	0.004763	74,26,4
1ecfa1#388	GO:0000287: magnesium ion binding	0.004766	8,128,3
1e4ia_#314	GO:0000287: magnesium ion binding	0.004766	8,128,3
1dik_3#215	GO:0000287: magnesium ion binding	0.004766	8,128,3
1e8ca3#111	GO:0016866: intramolecular transferase activity	0.004766	280,12,5
1e5da2#12	GO:0050660: FAD binding	0.004769	100,10,3
1qdlb_#170	GO:0003682: chromatin binding	0.004769	100,10,3
1hoe__#31	GO:0005509: calcium ion binding	0.00477	28,160,6
1aw1a_#169	GO:0005509: calcium ion binding	0.00477	28,160,6
1b8ba_#507	GO:0000049: tRNA binding	0.004771	1,13,1
1qgoa_#132	GO:0008026: ATP-dependent helicase activity	0.004771	1,13,1
1fnha1#25	GO:0005518: collagen binding	0.004771	1,13,1
1xgsa2#274	GO:0004177: aminopeptidase activity	0.004771	1,13,1
1xgsa2#274	GO:0008235: metalloexopeptidase activity	0.004771	1,13,1
1a1va2#400	GO:0008026: ATP-dependent helicase activity	0.004771	1,13,1
1jb9a1#157	GO:0008026: ATP-dependent helicase activity	0.004771	1,13,1
1gcqc_#595	GO:0005085: guanyl-nucleotide exchange factor activity	0.004771	1,13,1
1cjaa_#122	GO:0016854: racemase and epimerase activity	0.004771	1,13,1

1ezia_#109	GO:0008080: N-acetyltransferase activity	0.004771	1,13,1
1dbga_#93	GO:0003729: mRNA binding	0.004771	1,13,1
1jj2s_#26	GO:0000049: tRNA binding	0.004771	1,13,1
1pmi__#341	GO:0016861: intramolecular oxidoreductase activity, interconverting aldoses and ketoses	0.004771	1,13,1
1qq9a_#194	GO:0004177: aminopeptidase activity	0.004771	1,13,1
1i05a_#113	GO:0004177: aminopeptidase activity	0.004771	1,13,1
1pbga_#334	GO:0008080: N-acetyltransferase activity	0.004771	1,13,1
1pjr_2#624	GO:0008026: ATP-dependent helicase activity	0.004771	1,13,1
1rypf_#167	GO:0008026: ATP-dependent helicase activity	0.004771	1,13,1
1a1va2#619	GO:0008026: ATP-dependent helicase activity	0.004771	1,13,1
1bkds_#974	GO:0005085: guanyl-nucleotide exchange factor activity	0.004771	1,13,1
1b6a_2#186	GO:0004177: aminopeptidase activity	0.004771	1,13,1
1b6a_2#186	GO:0008235: metalloexopeptidase activity	0.004771	1,13,1
1fjfm_#45	GO:0000049: tRNA binding	0.004771	1,13,1
1b25a1#248	GO:0000049: tRNA binding	0.004771	1,13,1
1dp0a3#129	GO:0005518: collagen binding	0.004771	1,13,1
1d4aa_#17	GO:0016651: oxidoreductase activity, acting on NADH or NADPH	0.004771	1,13,1
1hlga_#104	GO:0004177: aminopeptidase activity	0.004771	1,13,1
1hfoa_#57	GO:0003714: transcription corepressor activity	0.004772	37,8,2
3lada3#463	GO:0016836: hydro-lyase activity	0.004773	29,33,3
1e05i_#422	GO:0016836: hydro-lyase activity	0.004773	29,33,3
1i50a_#469	GO:0016836: hydro-lyase activity	0.004774	58,33,4
1fjfj_#68	GO:0003887: DNA-directed DNA polymerase activity	0.004778	230,20,6
1fjfj_#68	GO:0005126: hematopoietin/interferon-class (D200-domain) cytokine receptor binding	0.004778	230,20,6
1ja9a_#202	GO:0005525: GTP binding	0.00478	39,49,4
1fy7a_#388	GO:0008270: zinc ion binding	0.004782	68,108,8
1c8za_#386	GO:0004714: transmembrane receptor protein tyrosine kinase activity	0.004782	235,14,5
1lci__#26	GO:0005524: ATP binding	0.004787	139,243,22
1e3ja2#270	GO:0005524: ATP binding	0.004788	74,243,14
1doza_#109	GO:0005524: ATP binding	0.004788	74,243,14
1dqza_#131	GO:0000287: magnesium ion binding	0.00479	83,128,10
1ecpa_#58	GO:0000287: magnesium ion binding	0.00479	83,128,10
1i6vd_#713	GO:0004197: cysteine-type endopeptidase activity	0.004791	40,24,3
1xvaa_#244	GO:0008201: heparin binding	0.004791	40,24,3
1stfi_#116	GO:0046983: protein dimerization activity	0.004793	57,17,3
1j9la_#82	GO:0000287: magnesium ion binding	0.004793	169,128,16
1dssg1#4	GO:0016627: oxidoreductase activity, acting on the CH-CH group of donors	0.004793	57,17,3
1jsg__#92	GO:0004714: transmembrane receptor protein tyrosine kinase activity	0.004794	607,14,8
1jsg__#92	GO:0003968: RNA-directed RNA polymerase activity	0.004794	607,14,8
1tbrr1#15	GO:0004295: trypsin activity	0.004796	64,48,5
1foha5#9	GO:0030151: molybdenum ion binding	0.004803	65,15,3
1ejfa_#10	GO:0005518: collagen binding	0.004807	155,13,4
1ejfa_#10	GO:0008235: metalloexopeptidase activity	0.004807	155,13,4
1kid__#330	GO:0008408: 3'-5' exonuclease activity	0.004807	155,13,4
1h6ka2#336	GO:0004222: metalloendopeptidase activity	0.004807	168,19,5
1nbaa_#227	GO:0051082: unfolded protein binding	0.004809	91,34,5
1elja_#64	GO:0004620: phospholipase activity	0.004815	103,19,4
1i76a_#138	GO:0016646: oxidoreductase activity, acting on the CH-NH group of donors, NAD or NADP as acceptor	0.004815	103,19,4
1cqka_#49	GO:0003779: actin binding	0.004817	30,32,3
1db3a_#266	GO:0005524: ATP binding	0.004817	59,243,12
1huxa_#10	GO:0003779: actin binding	0.004818	60,32,4
1alo_4#481	GO:0004714: transmembrane receptor protein tyrosine kinase activity	0.004823	70,14,3
1fepa_#49	GO:0016251: general RNA polymerase II transcription factor activity	0.004823	70,14,3
1b4ka_#108	GO:0016627: oxidoreductase activity, acting on the CH-CH group of donors	0.004825	116,17,4
1e25a_#262	GO:0016638: oxidoreductase activity, acting on the CH-NH2 group of donors	0.004825	116,17,4
1ej0a_#42	GO:0046983: protein dimerization activity	0.004825	116,17,4
1ayl__#237	GO:0019843: rRNA binding	0.004826	357,22,8
1g8fa3#419	GO:0005351: sugar porter activity	0.004829	151,21,5
1fn9a_#271	GO:0004556: alpha-amylase activity	0.004829	218,15,5
1fn9a_#271	GO:0004725: protein tyrosine phosphatase activity	0.004829	218,15,5
1f8ra2#325	GO:0051082: unfolded protein binding	0.004831	222,34,8
1e3ua_#164	GO:0004812: tRNA ligase activity	0.004834	37,26,3
1mpp__#29	GO:0004812: tRNA ligase activity	0.004834	37,26,3
1dvpa1#65	GO:0003755: peptidyl-prolyl cis-trans isomerase activity	0.004834	187,11,4

1bcca2#437	GO:0016705: oxidoreductase activity, acting on paired donors, with incorporation or reduction of molecular oxygen	0.004834	37,26,3
1d0na3#305	GO:0003724: RNA helicase activity	0.004836	311,11,5
1ee4a_#266	GO:0015078: hydrogen ion transporter activity	0.004841	46,21,3
1qo0d_#81	GO:0005351: sugar porter activity	0.004841	46,21,3
1bjt__#890	GO:0005524: ATP binding	0.004843	292,243,39
1fcda2#189	GO:0016646: oxidoreductase activity, acting on the CH-NH group of donors, NAD or NADP as acceptor	0.004846	51,19,3
1jj2n_#77	GO:0016646: oxidoreductase activity, acting on the CH-NH group of donors, NAD or NADP as acceptor	0.004846	51,19,3
1ifc__#89	GO:0046983: protein dimerization activity	0.004847	190,17,5
1ifc__#89	GO:0016638: oxidoreductase activity, acting on the CH-NH2 group of donors	0.004847	190,17,5
1bwvs_#117	GO:0004896: hematopoietin/interferon-class (D200-domain) cytokine receptor activity	0.00485	329,19,7
3sil__#317	GO:0004263: chymotrypsin activity	0.004854	358,41,12
2mnr_1#271	GO:0016836: hydro-lyase activity	0.004856	9,33,2
1avgi_#69	GO:0004190: aspartic-type endopeptidase activity	0.004856	340,23,8
1qasa3#602	GO:0016836: hydro-lyase activity	0.004856	9,33,2
1eoka_#274	GO:0016836: hydro-lyase activity	0.004856	9,33,2
1ijqa1#564	GO:0019838: growth factor binding	0.004856	33,9,2
1fcya_#311	GO:0003713: transcription coactivator activity	0.004857	61,16,3
3chbd_#85	GO:0005529: sugar binding	0.004859	378,39,12
1dnv__#354	GO:0008810: cellulase activity	0.004864	54,18,3
1bwvs_#38	GO:0004295: trypsin activity	0.004865	40,48,4
2pia_3#282	GO:0004295: trypsin activity	0.004865	40,48,4
1bu6o1#45	GO:0008083: growth factor activity	0.004867	23,42,3
1hbza_#214	GO:0004812: tRNA ligase activity	0.004868	297,26,8
1hcc__#31	GO:0005524: ATP binding	0.004868	25,243,7
1dysa_#131	GO:0016776: phosphotransferase activity, phosphate group as acceptor	0.004869	21,14,2
1an9a2#278	GO:0016251: general RNA polymerase II transcription factor activity	0.004869	21,14,2
1hqoa2#159	GO:0015078: hydrogen ion transporter activity	0.004869	14,21,2
1as4.1#A79	GO:0015078: hydrogen ion transporter activity	0.004869	14,21,2
1cf9a2#554	GO:0003968: RNA-directed RNA polymerase activity	0.004869	21,14,2
1bkds_#882	GO:0004601: peroxidase activity	0.004869	14,21,2
1nsca_#306	GO:0004714: transmembrane receptor protein tyrosine kinase activity	0.004869	21,14,2
1e79h2#77	GO:0005543: phospholipid binding	0.004869	21,14,2
1fgua1#249	GO:0016251: general RNA polymerase II transcription factor activity	0.004869	21,14,2
1fgka_#689	GO:0004714: transmembrane receptor protein tyrosine kinase activity	0.004869	21,14,2
1rypf_#35	GO:0015082: di-, tri-valent inorganic cation transporter activity	0.004869	21,14,2
1rypf_#35	GO:0046915: transition metal ion transporter activity	0.004869	21,14,2
1ddja_#746	GO:0016702: oxidoreductase activity, acting on single donors with incorporation of molecular oxygen, incorporation of two atoms of oxygen	0.004873	170,12,4
1aqt_2#45	GO:0003729: mRNA binding	0.004876	76,13,3
1uch__#50	GO:0005518: collagen binding	0.004876	76,13,3
1pbe_1#150	GO:0008080: N-acetyltransferase activity	0.004876	76,13,3
1faoa_#250	GO:0005085: guanyl-nucleotide exchange factor activity	0.004876	76,13,3
2mpra_#190	GO:0008201: heparin binding	0.004877	400,24,9
2cb5a_#373	GO:0015036: disulfide oxidoreductase activity	0.004884	44,22,3
2cb5a_#373	GO:0019843: rRNA binding	0.004884	44,22,3
1d0na3#305	GO:0005524: ATP binding	0.004893	311,243,41
1bd0a1#276	GO:0019955: cytokine binding	0.004894	91,11,3
1bd0a1#276	GO:0003964: RNA-directed DNA polymerase activity	0.004894	91,11,3
1hxxa_#173	GO:0008083: growth factor activity	0.004906	260,42,10
1bwvs_#117	GO:0005529: sugar binding	0.004908	329,39,11
3sil__#317	GO:0015036: disulfide oxidoreductase activity	0.004911	358,22,8
1g25a_#22	GO:0016668: oxidoreductase activity, acting on sulfur group of donors, NAD or NADP as acceptor	0.004914	415,12,6
1e8ca3#111	GO:0015036: disulfide oxidoreductase activity	0.004915	280,22,7
1eqka_#47	GO:0016831: carboxy-lyase activity	0.004916	126,25,5
1erv__#80	GO:0019843: rRNA binding	0.004916	442,22,9
1aky_2#133	GO:0019201: nucleotide kinase activity	0.00492	156,13,4
1dr9a1#39	GO:0005525: GTP binding	0.004921	258,49,11
1b6e__#76	GO:0003964: RNA-directed DNA polymerase activity	0.004922	27,11,2
1g5ha2#162	GO:0003964: RNA-directed DNA polymerase activity	0.004922	27,11,2
2cnd_1#101	GO:0010181: FMN binding	0.004922	27,11,2
1edza1#206	GO:0003887: DNA-directed DNA polymerase activity	0.004927	399,20,8
1hdma1#115	GO:0019955: cytokine binding	0.004929	188,11,4

1e8ga2#201	GO:0004674: protein serine/threonine kinase activity	0.004931	304,42,11
1a7ca_#370	GO:0004263: chymotrypsin activity	0.004932	47,41,4
1f2la_#40	GO:0005529: sugar binding	0.004934	236,39,9
1czan1#92	GO:0008270: zinc ion binding	0.004934	631,108,37
2arca_#55	GO:0004714: transmembrane receptor protein tyrosine kinase activity	0.004942	144,14,4
1f97a2#199	GO:0015082: di-, tri-valent inorganic cation transporter activity	0.004942	144,14,4
1f97a2#199	GO:0046915: transition metal ion transporter activity	0.004942	144,14,4
1xyza_#753	GO:0008270: zinc ion binding	0.004945	29,108,5
2mev1_#123	GO:0015036: disulfide oxidoreductase activity	0.004956	89,22,4
1fwxa2#396	GO:0003968: RNA-directed RNA polymerase activity	0.004962	237,14,5
1e6ca_#91	GO:0016831: carboxy-lyase activity	0.004966	182,25,6
1flga_#161	GO:0005529: sugar binding	0.004967	152,39,7
1flga_#161	GO:0015078: hydrogen ion transporter activity	0.004968	152,21,5
1mai__#108	GO:0005351: sugar porter activity	0.004968	152,21,5
3chbd_#85	GO:0008408: 3'-5' exonuclease activity	0.004969	378,13,6
1ycsa_#162	GO:0008009: chemokine activity	0.00497	210,10,4
1ycsa_#162	GO:0003916: DNA topoisomerase activity	0.00497	210,10,4
1ycsa_#162	GO:0016620: oxidoreductase activity, acting on the aldehyde or oxo group of donors, NAD or NADP as acceptor	0.00497	210,10,4
1hxxa_#278	GO:0005516: calmodulin binding	0.004971	255,24,7
1b8aa2#403	GO:0016638: oxidoreductase activity, acting on the CH-NH2 group of donors	0.004976	117,17,4
1bif_2#398	GO:0003924: GTPase activity	0.004976	117,17,4
1a7ca_#202	GO:0016638: oxidoreductase activity, acting on the CH-NH2 group of donors	0.004976	117,17,4
1edza1#206	GO:0019838: growth factor binding	0.004976	399,9,5
1ftra2#187	GO:0004674: protein serine/threonine kinase activity	0.00498	46,42,4
1dr9a1#39	GO:0000049: tRNA binding	0.004982	258,13,5
1dqsa_#138	GO:0005524: ATP binding	0.004988	38,243,9
1cpy__#346	GO:0005524: ATP binding	0.004988	38,243,9
1h6ka1#134	GO:0003916: DNA topoisomerase activity	0.004992	30,10,2
1cqka_#49	GO:0004457: lactate dehydrogenase activity	0.004992	30,10,2
1cmxa_#59	GO:0005261: cation channel activity	0.004992	30,10,2
1cmxa_#59	GO:0003682: chromatin binding	0.004992	30,10,2
1e5sa_#239	GO:0003684: damaged DNA binding	0.004992	30,10,2
1h6va1#116	GO:0016620: oxidoreductase activity, acting on the aldehyde or oxo group of donors, NAD or NADP as acceptor	0.004992	30,10,2
1hlwa_#91	GO:0005261: cation channel activity	0.004992	30,10,2
1aa6_1#652	GO:0016620: oxidoreductase activity, acting on the aldehyde or oxo group of donors, NAD or NADP as acceptor	0.004992	30,10,2
1iba__#39	GO:0003916: DNA topoisomerase activity	0.004995	351,10,5
1h8ma_#8	GO:0030151: molybdenum ion binding	0.004996	134,15,4
1j9la_#164	GO:0051082: unfolded protein binding	0.005002	57,34,4
3rpba_#557	GO:0051082: unfolded protein binding	0.005002	57,34,4
1a9xa6#760	GO:0005524: ATP binding	0.005006	90,243,16
1as4.1#B385	GO:0004556: alpha-amylase activity	0.005014	66,15,3
1qj5a_#268	GO:0030151: molybdenum ion binding	0.005014	66,15,3
1ak5_1#376	GO:0016831: carboxy-lyase activity	0.005015	39,25,3
1h61a_#176	GO:0016831: carboxy-lyase activity	0.005015	39,25,3
1bywa_#30	GO:0016646: oxidoreductase activity, acting on the CH-NH group of donors, NAD or NADP as acceptor	0.005019	331,19,7
1qdea_#218	GO:0008026: ATP-dependent helicase activity	0.005024	23,13,2
1qgua_#435	GO:0005085: guanyl-nucleotide exchange factor activity	0.005024	23,13,2
1i6vc_#207	GO:0008408: 3'-5' exonuclease activity	0.005024	23,13,2
1rtu__#97	GO:0004177: aminopeptidase activity	0.005024	23,13,2
2bb2_2#134	GO:0004556: alpha-amylase activity	0.005024	220,15,5
1a65a3#409	GO:0003887: DNA-directed DNA polymerase activity	0.005024	49,20,3
1cpt__#401	GO:0016651: oxidoreductase activity, acting on NADH or NADPH	0.005024	23,13,2
1b6a_1#415	GO:0004190: aspartic-type endopeptidase activity	0.005024	13,23,2
1eq2a_#299	GO:0016854: racemase and epimerase activity	0.005024	23,13,2
1nuka_#80	GO:0030151: molybdenum ion binding	0.005024	220,15,5
1lcl__#110	GO:0005518: collagen binding	0.005024	23,13,2
1eno__#137	GO:0016854: racemase and epimerase activity	0.005024	23,13,2
1agi__#31	GO:0005507: copper ion binding	0.00503	8,38,2
1e8ua_#180	GO:0005507: copper ion binding	0.00503	8,38,2
1cpza_#6	GO:0000049: tRNA binding	0.005035	157,13,4
1cpza_#6	GO:0008408: 3'-5' exonuclease activity	0.005035	157,13,4
1ig8a_#214	GO:0003899: DNA-directed RNA polymerase activity	0.005042	25,12,2

1gky__#61	GO:0016702: oxidoreductase activity, acting on single donors with incorporation of molecular oxygen, incorporation of two atoms of oxygen	0.005042	25,12,2
1a0fa1#178	GO:0003899: DNA-directed RNA polymerase activity	0.005042	25,12,2
2ts1__#109	GO:0016831: carboxy-lyase activity	0.005042	12,25,2
1rmg__#279	GO:0016831: carboxy-lyase activity	0.005042	12,25,2
1e31a_#55	GO:0003899: DNA-directed RNA polymerase activity	0.005042	25,12,2
1brt__#116	GO:0016831: carboxy-lyase activity	0.005042	12,25,2
1g51a3#524	GO:0016620: oxidoreductase activity, acting on the aldehyde or oxo group of donors, NAD or NADP as acceptor	0.005044	102,10,3
2mev1_#123	GO:0005509: calcium ion binding	0.005045	89,160,12
1thg__#3	GO:0019955: cytokine binding	0.005048	92,11,3
1jg8a_#172	GO:0016758: transferase activity, transferring hexosyl groups	0.005048	92,11,3
1qqsa_#124	GO:0004263: chymotrypsin activity	0.005054	458,41,14
1lara2#1769	GO:0008083: growth factor activity	0.005056	219,42,9
3btaa2#1258	GO:0005518: collagen binding	0.005059	77,13,3
1ejda_#180	GO:0004177: aminopeptidase activity	0.005059	77,13,3
1ih7a1#57	GO:0000049: tRNA binding	0.005059	77,13,3
1ih7a1#57	GO:0005518: collagen binding	0.005059	77,13,3
1ih7a1#57	GO:0008408: 3'-5' exonuclease activity	0.005059	77,13,3
1pya.1#B236	GO:0003729: mRNA binding	0.005059	77,13,3
1hxxa_#232	GO:0003809: thrombin activity	0.005059	352,10,5
1hxxa_#232	GO:0003916: DNA topoisomerase activity	0.005059	352,10,5
1ihua2#521	GO:0000049: tRNA binding	0.005059	77,13,3
1dnpa2#18	GO:0000049: tRNA binding	0.005059	77,13,3
1dnpa2#18	GO:0016861: intramolecular oxidoreductase activity, interconverting aldoses and ketoses	0.005059	77,13,3
1e8ca1#83	GO:0004222: metalloendopeptidase activity	0.005059	170,19,5
1dih_2#222	GO:0008800: beta-lactamase activity	0.005059	352,10,5
1h8ca_#35	GO:0003729: mRNA binding	0.005059	77,13,3
1elua_#95	GO:0000049: tRNA binding	0.005059	77,13,3
1ddja_#746	GO:0004620: phospholipase activity	0.005059	170,19,5
1ddja_#746	GO:0004896: hematopoietin/interferon-class (D200-domain) cytokine receptor activity	0.005059	170,19,5
3gcb__#78	GO:0004601: peroxidase activity	0.005063	94,21,4
1i2oa_#34	GO:0016799: hydrolase activity, hydrolyzing N-glycosyl compounds	0.005072	192,17,5
1ejda_#213	GO:0003899: DNA-directed RNA polymerase activity	0.005072	284,12,5
1tyfa_#31	GO:0016763: transferase activity, transferring pentosyl groups	0.005072	162,28,6
1i7qa_#186	GO:0004725: protein tyrosine phosphatase activity	0.005076	20,15,2
1qe3a_#7	GO:0004556: alpha-amylase activity	0.005076	20,15,2
1dzfa2#164	GO:0003887: DNA-directed DNA polymerase activity	0.005076	15,20,2
1mpp__#103	GO:0030151: molybdenum ion binding	0.005076	20,15,2
7reqa2#702	GO:0016836: hydro-lyase activity	0.005077	59,33,4
1db3a_#266	GO:0016836: hydro-lyase activity	0.005077	59,33,4
1dyoa_#153	GO:0003887: DNA-directed DNA polymerase activity	0.005079	161,20,5
1ejba_#81	GO:0016866: intramolecular transferase activity	0.005084	84,12,3
1ycsa_#162	GO:0015036: disulfide oxidoreductase activity	0.005099	210,22,6
1eaja_#59	GO:0008270: zinc ion binding	0.005099	202,108,16
1c5y.1#B85	GO:0005524: ATP binding	0.005103	45,243,10
1vin_2#335	GO:0016763: transferase activity, transferring pentosyl groups	0.005105	35,28,3
1bec_2#216	GO:0019838: growth factor binding	0.005106	115,9,3
3lada1#116	GO:0005351: sugar porter activity	0.00511	153,21,5
1cpza_#6	GO:0005507: copper ion binding	0.005113	157,38,7
1czya1#466	GO:0004263: chymotrypsin activity	0.005116	109,41,6
1g71a_#88	GO:0004896: hematopoietin/interferon-class (D200-domain) cytokine receptor activity	0.00512	52,19,3
1ek6a_#33	GO:0004620: phospholipase activity	0.00512	52,19,3
3grs_2#211	GO:0016646: oxidoreductase activity, acting on the CH-NH group of donors, NAD or NADP as acceptor	0.00512	52,19,3
1ifc__#89	GO:0019955: cytokine binding	0.005121	190,11,4
1ifc__#89	GO:0003755: peptidyl-prolyl cis-trans isomerase activity	0.005121	190,11,4
1axca1#70	GO:0046983: protein dimerization activity	0.005131	118,17,4
5ruba1#365	GO:0016627: oxidoreductase activity, acting on the CH-CH group of donors	0.005131	118,17,4
1pma1_#79	GO:0016763: transferase activity, transferring pentosyl groups	0.005132	70,28,4
1jlxa2#199	GO:0004714: transmembrane receptor protein tyrosine kinase activity	0.005138	1,14,1
1rdr__#322	GO:0003968: RNA-directed RNA polymerase activity	0.005138	1,14,1
1a1va2#400	GO:0003968: RNA-directed RNA polymerase activity	0.005138	1,14,1
1b35c_#178	GO:0003968: RNA-directed RNA polymerase activity	0.005138	1,14,1
1jb9a1#157	GO:0003968: RNA-directed RNA polymerase activity	0.005138	1,14,1
1ev2e1#207	GO:0004714: transmembrane receptor protein tyrosine kinase activity	0.005138	1,14,1

1hhsa_#316	GO:0003968: RNA-directed RNA polymerase activity	0.005138	1,14,1
1czfa_#282	GO:0004714: transmembrane receptor protein tyrosine kinase activity	0.005138	1,14,1
1e4mm_#382	GO:0015082: di-, tri-valent inorganic cation transporter activity	0.005138	1,14,1
1e4mm_#382	GO:0046915: transition metal ion transporter activity	0.005138	1,14,1
1igra2#327	GO:0004714: transmembrane receptor protein tyrosine kinase activity	0.005138	1,14,1
1aqza_#146	GO:0003968: RNA-directed RNA polymerase activity	0.005138	1,14,1
1h54a1#733	GO:0003968: RNA-directed RNA polymerase activity	0.005138	1,14,1
1hlwa_#43	GO:0016776: phosphotransferase activity, phosphate group as acceptor	0.005138	1,14,1
1i4ua_#142	GO:0003968: RNA-directed RNA polymerase activity	0.005138	1,14,1
1hlwa_#92	GO:0016776: phosphotransferase activity, phosphate group as acceptor	0.005138	1,14,1
1rlr_2#627	GO:0003968: RNA-directed RNA polymerase activity	0.005138	1,14,1
1a1va2#619	GO:0003968: RNA-directed RNA polymerase activity	0.005138	1,14,1
1qlaa3#364	GO:0003700: transcription factor activity	0.005141	73,124,9
1gtra2#173	GO:0005529: sugar binding	0.005143	50,39,4
1e79h2#77	GO:0004867: serine-type endopeptidase inhibitor activity	0.005145	21,47,3
1e43a2#351	GO:0004867: serine-type endopeptidase inhibitor activity	0.005145	21,47,3
1cpy__#93	GO:0051082: unfolded protein binding	0.005148	275,34,9
1rypg_#159	GO:0019838: growth factor binding	0.005151	34,9,2
1qfma1#238	GO:0051082: unfolded protein binding	0.005151	9,34,2
1rypb_#114	GO:0004177: aminopeptidase activity	0.005152	158,13,4
1rypb_#114	GO:0000049: tRNA binding	0.005152	158,13,4
1rypb_#114	GO:0008408: 3'-5' exonuclease activity	0.005152	158,13,4
1fw9a_#152	GO:0008201: heparin binding	0.005153	133,24,5
1hxxa_#173	GO:0003729: mRNA binding	0.005153	260,13,5
1hxxa_#173	GO:0008408: 3'-5' exonuclease activity	0.005153	260,13,5
1apme_#68	GO:0004896: hematopoietin/interferon-class (D200-domain) cytokine receptor activity	0.005159	105,19,4
1fmta2#151	GO:0003887: DNA-directed DNA polymerase activity	0.005161	314,20,7
1bec_2#216	GO:0005529: sugar binding	0.005167	115,39,6
1cnv__#264	GO:0003682: chromatin binding	0.005186	103,10,3
1i76a_#138	GO:0016620: oxidoreductase activity, acting on the aldehyde or oxo group of donors, NAD or NADP as acceptor	0.005186	103,10,3
1amf__#196	GO:0003697: single-stranded DNA binding	0.005191	173,12,4
1amf__#196	GO:0003899: DNA-directed RNA polymerase activity	0.005191	173,12,4
1ycsb1#412	GO:0005543: phospholipid binding	0.005194	146,14,4
1dhs__#149	GO:0051082: unfolded protein binding	0.005197	29,34,3
1el0a_#42	GO:0003779: actin binding	0.005197	294,32,9
1hxma2#150	GO:0051082: unfolded protein binding	0.005197	29,34,3
2occb1#209	GO:0004364: glutathione transferase activity	0.005204	93,11,3
1nsj__#178	GO:0008081: phosphoric diester hydrolase activity	0.005204	93,11,3
1f3ba2#4	GO:0019843: rRNA binding	0.005205	45,22,3
1dcea3#526	GO:0016646: oxidoreductase activity, acting on the CH-NH group of donors, NAD or NADP as acceptor	0.005216	16,19,2
2ayh__#160	GO:0004896: hematopoietin/interferon-class (D200-domain) cytokine receptor activity	0.005216	16,19,2
1tyfa_#31	GO:0003887: DNA-directed DNA polymerase activity	0.005216	162,20,5
1neb__#52	GO:0004523: ribonuclease H activity	0.005216	19,16,2
1c9la2#61	GO:0005529: sugar binding	0.00522	238,39,9
1mspa_#17	GO:0005509: calcium ion binding	0.005222	47,160,8
1whi__#59	GO:0004714: transmembrane receptor protein tyrosine kinase activity	0.005223	72,14,3
1f8ra2#325	GO:0030151: molybdenum ion binding	0.005225	222,15,5
1ds1a_#172	GO:0004725: protein tyrosine phosphatase activity	0.005225	222,15,5
1fjfc2#153	GO:0003899: DNA-directed RNA polymerase activity	0.005227	420,12,6
1f24a_#293	GO:0019838: growth factor binding	0.005232	116,9,3
1d6ja_#95	GO:0030151: molybdenum ion binding	0.005232	67,15,3
2cb5a_#395	GO:0030151: molybdenum ion binding	0.005232	67,15,3
1czan1#92	GO:0019843: rRNA binding	0.005235	631,22,11
1fa0a1#393	GO:0003729: mRNA binding	0.00524	261,13,5
1fa0a1#393	GO:0008408: 3'-5' exonuclease activity	0.00524	261,13,5
1ad2__#40	GO:0005525: GTP binding	0.005242	40,49,4
1yrga_#232	GO:0005525: GTP binding	0.005242	40,49,4
1dt6a_#385	GO:0016705: oxidoreductase activity, acting on paired donors, with incorporation or reduction of molecular oxygen	0.005242	76,26,4
1pbe_1#150	GO:0004812: tRNA ligase activity	0.005242	76,26,4
1tbrr1#15	GO:0005525: GTP binding	0.005246	64,49,5
1ec7a1#216	GO:0019201: nucleotide kinase activity	0.005247	78,13,3
1cipa2#34	GO:0005085: guanyl-nucleotide exchange factor activity	0.005247	78,13,3
1jjya_#324	GO:0008235: metalloexopeptidase activity	0.005247	78,13,3

1io7a_#243	GO:0016651: oxidoreductase activity, acting on NADH or NADPH	0.005247	78,13,3
1dpta_#22	GO:0003899: DNA-directed RNA polymerase activity	0.005257	85,12,3
1djna1#86	GO:0016866: intramolecular transferase activity	0.005257	85,12,3
1f3mc_#369	GO:0005126: hematopoietin/interferon-class (D200-domain) cytokine receptor binding	0.005259	100,20,4
1ig8a_#214	GO:0000287: magnesium ion binding	0.00526	25,128,5
1jd0a_#176	GO:0004523: ribonuclease H activity	0.005262	207,16,5
1hc7a2#258	GO:0004263: chymotrypsin activity	0.005262	226,41,9
1p35a_#101	GO:0004725: protein tyrosine phosphatase activity	0.005269	136,15,4
1miob_#338	GO:0008270: zinc ion binding	0.005271	84,108,9
1mjha_#126	GO:0008026: ATP-dependent helicase activity	0.005271	159,13,4
1ab4__#89	GO:0004177: aminopeptidase activity	0.005271	159,13,4
1ab4__#89	GO:0008235: metalloexopeptidase activity	0.005271	159,13,4
2napa2#444	GO:0016616: oxidoreductase activity, acting on the CH-OH group of donors, NAD or NADP as acceptor	0.005284	17,59,3
1hnja1#133	GO:0016616: oxidoreductase activity, acting on the CH-OH group of donors, NAD or NADP as acceptor	0.005284	17,59,3
1i50a_#1115	GO:0046983: protein dimerization activity	0.005286	18,17,2
1fgxa_#242	GO:0016627: oxidoreductase activity, acting on the CH-CH group of donors	0.005286	18,17,2
1et0a_#118	GO:0008483: transaminase activity	0.005286	18,17,2
2tbva_#110	GO:0046983: protein dimerization activity	0.005286	18,17,2
1c0pa1#1133	GO:0016638: oxidoreductase activity, acting on the CH-NH2 group of donors	0.005286	18,17,2
1prea1#50	GO:0003924: GTPase activity	0.005286	18,17,2
1exma3#196	GO:0008810: cellulase activity	0.005286	17,18,2
1fnna2#226	GO:0046983: protein dimerization activity	0.005286	18,17,2
1a4ya_#428	GO:0016627: oxidoreductase activity, acting on the CH-CH group of donors	0.005286	18,17,2
1g51a1#47	GO:0003924: GTPase activity	0.005286	18,17,2
1atg__#161	GO:0016627: oxidoreductase activity, acting on the CH-CH group of donors	0.005286	18,17,2
1atg__#161	GO:0008483: transaminase activity	0.005286	18,17,2
1bdb__#228	GO:0016627: oxidoreductase activity, acting on the CH-CH group of donors	0.005286	18,17,2
1g5qa_#80	GO:0016627: oxidoreductase activity, acting on the CH-CH group of donors	0.005286	18,17,2
1kit_2#398	GO:0030145: manganese ion binding	0.005288	83,38,5
2mpra_#89	GO:0003964: RNA-directed DNA polymerase activity	0.005289	28,11,2
1e8ya4#800	GO:0016763: transferase activity, transferring pentosyl groups	0.005289	11,28,2
3pah__#303	GO:0010181: FMN binding	0.005289	28,11,2
1hx0a2#234	GO:0008081: phosphoric diester hydrolase activity	0.005289	28,11,2
1hdca_#126	GO:0008081: phosphoric diester hydrolase activity	0.005289	28,11,2
1cwva2#675	GO:0019955: cytokine binding	0.005289	28,11,2
1a6q__#16	GO:0005529: sugar binding	0.005294	8,39,2
1jlxa1#147	GO:0005529: sugar binding	0.005294	8,39,2
1iba__#39	GO:0016251: general RNA polymerase II transcription factor activity	0.005301	351,14,6
1ewna_#109	GO:0008083: growth factor activity	0.005304	75,42,5
1e2o__#346	GO:0046983: protein dimerization activity	0.005304	194,17,5
1ea5a_#99	GO:0046983: protein dimerization activity	0.005304	194,17,5
1dpga2#334	GO:0000155: two-component sensor molecule activity	0.00531	287,12,5
1qtn.1#A270	GO:0004180: carboxypeptidase activity	0.005311	439,15,7
1aqua_#126	GO:0009036: type II site-specific deoxyribonuclease activity	0.005315	576,12,7
1i2oa_#34	GO:0010181: FMN binding	0.005319	192,11,4
1fsu__#434	GO:0050660: FAD binding	0.005319	356,10,5
1c8za_#386	GO:0003887: DNA-directed DNA polymerase activity	0.005319	235,20,6
2bpa1_#45	GO:0019955: cytokine binding	0.005319	192,11,4
1in4a1#264	GO:0003887: DNA-directed DNA polymerase activity	0.00532	50,20,3
1xgsa2#82	GO:0004674: protein serine/threonine kinase activity	0.00532	307,42,11
1bvoa_#206	GO:0004263: chymotrypsin activity	0.005322	48,41,4
1qfma2#576	GO:0016620: oxidoreductase activity, acting on the aldehyde or oxo group of donors, NAD or NADP as acceptor	0.005323	214,10,4
1ryp1_#112	GO:0051082: unfolded protein binding	0.005325	58,34,4
1ciy_2#298	GO:0008199: ferric iron binding	0.005326	31,10,2
1i1ka_#255	GO:0003809: thrombin activity	0.005326	31,10,2
1i1ka_#255	GO:0003916: DNA topoisomerase activity	0.005326	31,10,2
1awcb_#111	GO:0008199: ferric iron binding	0.005326	31,10,2
1awcb_#111	GO:0016846: carbon-sulfur lyase activity	0.005326	31,10,2
2ae2a_#17	GO:0004457: lactate dehydrogenase activity	0.005326	31,10,2
1ir3a_#1192	GO:0005066: transmembrane receptor protein tyrosine kinase signaling protein activity	0.005326	31,10,2
1ir3a_#1192	GO:0008199: ferric iron binding	0.005326	31,10,2
1yrga_#194	GO:0016814: hydrolase activity, acting on carbon-nitrogen (but not peptide) bonds, in cyclic amidines	0.005326	31,10,2

1hdfa_#16	GO:0004180: carboxypeptidase activity	0.005328	223,15,5
1dik_3#240	GO:0008408: 3'-5' exonuclease activity	0.005328	262,13,5
1bxoa_#307	GO:0016620: oxidoreductase activity, acting on the aldehyde or oxo group of donors, NAD or NADP as acceptor	0.005329	104,10,3
2mpra_#190	GO:0008083: growth factor activity	0.005329	400,42,13
1quba4#235	GO:0004620: phospholipase activity	0.005338	106,19,4
1fgka_#610	GO:0004714: transmembrane receptor protein tyrosine kinase activity	0.00534	22,14,2
1bywa_#64	GO:0019843: rRNA binding	0.00534	14,22,2
1jj2e1#42	GO:0008270: zinc ion binding	0.00534	167,108,14
1ihma_#1068	GO:0003968: RNA-directed RNA polymerase activity	0.00534	22,14,2
1f3mc_#403	GO:0004714: transmembrane receptor protein tyrosine kinase activity	0.00534	22,14,2
1h72c2#269	GO:0008757: S-adenosylmethionine-dependent methyltransferase activity	0.005341	83,24,4
1kit_2#398	GO:0004197: cysteine-type endopeptidase activity	0.005341	83,24,4
3btaa2#1258	GO:0004263: chymotrypsin activity	0.005345	77,41,5
1ih7a1#57	GO:0004263: chymotrypsin activity	0.005345	77,41,5
1hlwa_#82	GO:0004263: chymotrypsin activity	0.005353	270,41,10
1f5aa1#441	GO:0003887: DNA-directed DNA polymerase activity	0.005356	163,20,5
1f97a1#62	GO:0004725: protein tyrosine phosphatase activity	0.005366	707,15,9
1exma2#353	GO:0019843: rRNA binding	0.005367	91,22,4
1e42a1#711	GO:0005524: ATP binding	0.005368	175,243,26
1mdah_#329	GO:0004620: phospholipase activity	0.005372	335,19,7
1dih_2#222	GO:0016251: general RNA polymerase II transcription factor activity	0.005378	352,14,6
1b8ba_#466	GO:0004674: protein serine/threonine kinase activity	0.005382	47,42,4
1f8ma_#237	GO:0004867: serine-type endopeptidase inhibitor activity	0.005382	42,47,4
1e79d3#328	GO:0016831: carboxy-lyase activity	0.005389	40,25,3
1hdoa_#189	GO:0016763: transferase activity, transferring pentosyl groups	0.005389	164,28,6
1hf8a_#156	GO:0005509: calcium ion binding	0.005391	13,160,4
1fwxa2#169	GO:0005529: sugar binding	0.005392	116,39,6
1qvba_#9	GO:0016836: hydro-lyase activity	0.005394	60,33,4
1jb3a_#64	GO:0004812: tRNA ligase activity	0.005398	302,26,8
1hc7a2#258	GO:0005509: calcium ion binding	0.005398	226,160,23
1epwa3#175	GO:0004896: hematopoietin/interferon-class (D200-domain) cytokine receptor activity	0.005403	53,19,3
1g51a3#525	GO:0008080: N-acetyltransferase activity	0.005417	263,13,5
1i1ja_#37	GO:0005524: ATP binding	0.005423	9,243,4
1fhga_#42	GO:0005524: ATP binding	0.005423	9,243,4
1g5ba_#193	GO:0005524: ATP binding	0.005423	9,243,4
1a8h_2#186	GO:0005524: ATP binding	0.005423	9,243,4
1diqa1#289	GO:0005524: ATP binding	0.005423	9,243,4
1g3nc2#184	GO:0005524: ATP binding	0.005423	9,243,4
1dq3a1#432	GO:0005524: ATP binding	0.005423	9,243,4
2dnja_#91	GO:0005524: ATP binding	0.005423	9,243,4
5nul__#43	GO:0005524: ATP binding	0.005423	9,243,4
1deua_#151	GO:0019843: rRNA binding	0.005428	285,22,7
1a81a1#51	GO:0015082: di-, tri-valent inorganic cation transporter activity	0.005431	73,14,3
1a81a1#51	GO:0046915: transition metal ion transporter activity	0.005431	73,14,3
1e2wa1#102	GO:0016702: oxidoreductase activity, acting on single donors with incorporation of molecular oxygen, incorporation of two atoms of oxygen	0.005433	86,12,3
1epwa3#140	GO:0005509: calcium ion binding	0.005433	187,160,20
1pysb5#584	GO:0000049: tRNA binding	0.005439	79,13,3
1dkia_#185	GO:0004177: aminopeptidase activity	0.005439	79,13,3
1amp__#205	GO:0004177: aminopeptidase activity	0.005439	79,13,3
1ct9a2#168	GO:0000287: magnesium ion binding	0.005442	16,128,4
1ush_1#462	GO:0000287: magnesium ion binding	0.005442	16,128,4
1jlja_#104	GO:0000287: magnesium ion binding	0.005442	16,128,4
1dcea3#526	GO:0000287: magnesium ion binding	0.005442	16,128,4
2dri__#115	GO:0000287: magnesium ion binding	0.005442	16,128,4
1thta_#71	GO:0016705: oxidoreductase activity, acting on paired donors, with incorporation or reduction of molecular oxygen	0.005449	12,26,2
1i8da1#85	GO:0016705: oxidoreductase activity, acting on paired donors, with incorporation or reduction of molecular oxygen	0.005449	12,26,2
1io7a_#256	GO:0016705: oxidoreductase activity, acting on paired donors, with incorporation or reduction of molecular oxygen	0.005449	12,26,2
1mpp__#209	GO:0004812: tRNA ligase activity	0.005449	12,26,2
1t7pa2#663	GO:0042802: protein self binding	0.005449	26,12,2
1pysb5#523	GO:0004812: tRNA ligase activity	0.005449	12,26,2
1d2fa_#260	GO:0004812: tRNA ligase activity	0.005449	12,26,2

1a59__#341	GO:0016705: oxidoreductase activity, acting on paired donors, with incorporation or reduction of molecular oxygen	0.005449	12,26,2
2ts1__#109	GO:0004497: monooxygenase activity	0.005449	12,26,2
1feza_#121	GO:0009036: type II site-specific deoxyribonuclease activity	0.005449	26,12,2
1lst__#157	GO:0016702: oxidoreductase activity, acting on single donors with incorporation of molecular oxygen, incorporation of two atoms of oxygen	0.005449	26,12,2
1qqra_#185	GO:0016702: oxidoreductase activity, acting on single donors with incorporation of molecular oxygen, incorporation of two atoms of oxygen	0.005449	26,12,2
1h4vb2#282	GO:0003887: DNA-directed DNA polymerase activity	0.00545	101,20,4
2napa2#292	GO:0003924: GTPase activity	0.00545	120,17,4
1j75a_#166	GO:0003924: GTPase activity	0.00545	120,17,4
3sil__#317	GO:0016620: oxidoreductase activity, acting on the aldehyde or oxo group of donors, NAD or NADP as acceptor	0.005452	358,10,5
3sil__#317	GO:0050660: FAD binding	0.005452	358,10,5
1gc5a_#233	GO:0019829: cation-transporting ATPase activity	0.005453	35,9,2
1fp1d2#214	GO:0016646: oxidoreductase activity, acting on the CH-NH group of donors, NAD or NADP as acceptor	0.005454	173,19,5
1ds9a_#105	GO:0030151: molybdenum ion binding	0.005455	68,15,3
1feza_#185	GO:0030151: molybdenum ion binding	0.005455	68,15,3
2viua_#252	GO:0005507: copper ion binding	0.005455	343,38,11
1dpja_#322	GO:0008083: growth factor activity	0.005455	308,42,11
1c39a_#49	GO:0004556: alpha-amylase activity	0.005455	68,15,3
1qfma1#186	GO:0030151: molybdenum ion binding	0.005457	326,15,6
1nfdb1#21	GO:0015078: hydrogen ion transporter activity	0.00546	96,21,4
1eu8a_#119	GO:0005351: sugar porter activity	0.005461	48,21,3
1jeyb_#231	GO:0008408: 3'-5' exonuclease activity	0.005467	24,13,2
1clia2#265	GO:0008201: heparin binding	0.005467	13,24,2
1ayoa_#55	GO:0004197: cysteine-type endopeptidase activity	0.005467	13,24,2
1e0na_#28	GO:0008757: S-adenosylmethionine-dependent methyltransferase activity	0.005467	13,24,2
1fg7a_#96	GO:0008757: S-adenosylmethionine-dependent methyltransferase activity	0.005467	13,24,2
1fgka_#572	GO:0005516: calmodulin binding	0.005467	13,24,2
1b6cb_#399	GO:0005516: calmodulin binding	0.005467	13,24,2
7reqb1#320	GO:0016861: intramolecular oxidoreductase activity, interconverting aldoses and ketoses	0.005467	24,13,2
4kbpa2#416	GO:0008408: 3'-5' exonuclease activity	0.005467	24,13,2
1dbta_#89	GO:0008026: ATP-dependent helicase activity	0.005467	24,13,2
1bjaa_#26	GO:0019201: nucleotide kinase activity	0.005467	24,13,2
1eq9a_#32	GO:0005524: ATP binding	0.005468	132,243,21
2bb2_2#134	GO:0005524: ATP binding	0.005471	220,243,31
1au1a_#58	GO:0016620: oxidoreductase activity, acting on the aldehyde or oxo group of donors, NAD or NADP as acceptor	0.005475	105,10,3
1apme_#68	GO:0016620: oxidoreductase activity, acting on the aldehyde or oxo group of donors, NAD or NADP as acceptor	0.005475	105,10,3
1apme_#68	GO:0050660: FAD binding	0.005475	105,10,3
1fvua_#13	GO:0003700: transcription factor activity	0.005478	26,124,5
1qvba_#206	GO:0003700: transcription factor activity	0.005478	26,124,5
1tuba1#8	GO:0004812: tRNA ligase activity	0.005478	124,26,5
1p35a_#49	GO:0004812: tRNA ligase activity	0.005478	124,26,5
1mjha_#126	GO:0030145: manganese ion binding	0.005483	159,38,7
1epwa3#138	GO:0015405: P-P-bond-hydrolysis-driven transporter activity	0.005487	424,12,6
1epwa3#138	GO:0000155: two-component sensor molecule activity	0.005487	424,12,6
1aym3_#131	GO:0004263: chymotrypsin activity	0.005489	412,41,13
1evqa_#305	GO:0016627: oxidoreductase activity, acting on the CH-CH group of donors	0.00549	490,17,8
1hd2a_#35	GO:0016616: oxidoreductase activity, acting on the CH-OH group of donors, NAD or NADP as acceptor	0.005492	185,59,10
1pya.1#B236	GO:0004497: monooxygenase activity	0.005494	77,26,4
1ffya2#226	GO:0008201: heparin binding	0.005503	42,24,3
1jb7a1#174	GO:0004674: protein serine/threonine kinase activity	0.005503	24,42,3
1feua_#71	GO:0004674: protein serine/threonine kinase activity	0.005503	24,42,3
1qba_3#397	GO:0003743: translation initiation factor activity	0.005505	1,15,1
1ruxa1#260	GO:0004556: alpha-amylase activity	0.005505	1,15,1
1g8ka1#796	GO:0030151: molybdenum ion binding	0.005505	1,15,1
2hpaa_#1057	GO:0004725: protein tyrosine phosphatase activity	0.005505	1,15,1
1fxxa_#58	GO:0004556: alpha-amylase activity	0.005505	1,15,1
1f00i1#691	GO:0004725: protein tyrosine phosphatase activity	0.005505	1,15,1
1soxa3#180	GO:0030151: molybdenum ion binding	0.005505	1,15,1
1bf2_2#695	GO:0004556: alpha-amylase activity	0.005505	1,15,1

1daba_#473	GO:0005096: GTPase activator activity	0.005505	1,15,1
1soxa2#82	GO:0030151: molybdenum ion binding	0.005505	1,15,1
1e3a.1#B363	GO:0016811: hydrolase activity, acting on carbon-nitrogen (but not peptide) bonds, in linear amides	0.005505	1,15,1
1hfwa_#167	GO:0016811: hydrolase activity, acting on carbon-nitrogen (but not peptide) bonds, in linear amides	0.005505	1,15,1
1mioa_#148	GO:0030151: molybdenum ion binding	0.005505	1,15,1
1emsa2#66	GO:0004556: alpha-amylase activity	0.005505	1,15,1
1d0ba_#138	GO:0005096: GTPase activator activity	0.005505	1,15,1
1thg__#144	GO:0016811: hydrolase activity, acting on carbon-nitrogen (but not peptide) bonds, in linear amides	0.005505	1,15,1
1bkds_#974	GO:0005096: GTPase activator activity	0.005505	1,15,1
1a4ya_#268	GO:0005096: GTPase activator activity	0.005505	1,15,1
1e3a.1#B480	GO:0016811: hydrolase activity, acting on carbon-nitrogen (but not peptide) bonds, in linear amides	0.005505	1,15,1
1rlr_2#319	GO:0004180: carboxypeptidase activity	0.005505	1,15,1
1a4ya_#47	GO:0004457: lactate dehydrogenase activity	0.005505	216,10,4
2sli_2#334	GO:0030151: molybdenum ion binding	0.005505	1,15,1
1rypi_#16	GO:0016811: hydrolase activity, acting on carbon-nitrogen (but not peptide) bonds, in linear amides	0.005505	1,15,1
1g0sa_#74	GO:0005507: copper ion binding	0.005508	294,38,10
1a65a1#108	GO:0005507: copper ion binding	0.005508	294,38,10
1htp__#16	GO:0004812: tRNA ligase activity	0.00551	303,26,8
1fo4a5#1020	GO:0050660: FAD binding	0.00552	359,10,5
1fo4a5#1020	GO:0008800: beta-lactamase activity	0.00552	359,10,5
1e0ta2#275	GO:0016646: oxidoreductase activity, acting on the CH-NH group of donors, NAD or NADP as acceptor	0.00552	107,19,4
1e2o__#346	GO:0019955: cytokine binding	0.005523	194,11,4
1f3ya_#6	GO:0051082: unfolded protein binding	0.005525	94,34,5
3gcb__#78	GO:0051082: unfolded protein binding	0.005525	94,34,5
2hhma_#262	GO:0003964: RNA-directed DNA polymerase activity	0.005527	95,11,3
1e6pa2#46	GO:0008081: phosphoric diester hydrolase activity	0.005527	95,11,3
1bk7a_#135	GO:0005524: ATP binding	0.005531	14,243,5
1bywa_#64	GO:0005524: ATP binding	0.005531	14,243,5
1qqp3_#139	GO:0005524: ATP binding	0.005531	14,243,5
1jbwa2#189	GO:0005524: ATP binding	0.005531	14,243,5
1g3nc2#162	GO:0005524: ATP binding	0.005531	14,243,5
3chbd_#96	GO:0005524: ATP binding	0.005531	14,243,5
1h9da_#91	GO:0008083: growth factor activity	0.005534	222,42,9
1quna1#113	GO:0019843: rRNA binding	0.005536	286,22,7
1eula_#213	GO:0016638: oxidoreductase activity, acting on the CH-NH2 group of donors	0.005541	60,17,3
1oaca1#495	GO:0016638: oxidoreductase activity, acting on the CH-NH2 group of donors	0.005541	60,17,3
1jcfa1#26	GO:0046983: protein dimerization activity	0.005541	60,17,3
2bpa1_#244	GO:0046983: protein dimerization activity	0.005541	60,17,3
1qrra_#69	GO:0008483: transaminase activity	0.005543	196,17,5
1dvpa1#65	GO:0000287: magnesium ion binding	0.005546	187,128,17
1by5a_#238	GO:0005126: hematopoietin/interferon-class (D200-domain) cytokine receptor binding	0.005547	237,20,6
1f21a_#68	GO:0004190: aspartic-type endopeptidase activity	0.005553	44,23,3
1dn2a1#261	GO:0016702: oxidoreductase activity, acting on single donors with incorporation of molecular oxygen, incorporation of two atoms of oxygen	0.005554	425,12,6
1dn2a1#261	GO:0042802: protein self binding	0.005554	425,12,6
1ekra_#114	GO:0005524: ATP binding	0.005555	60,243,12
1qhda2#323	GO:0004620: phospholipase activity	0.005556	337,19,7
1gpc__#184	GO:0005524: ATP binding	0.005558	83,243,15
1alo_4#634	GO:0004523: ribonuclease H activity	0.005563	64,16,3
1ejda_#213	GO:0003924: GTPase activity	0.005566	284,17,6
1ejda_#213	GO:0008483: transaminase activity	0.005566	284,17,6
1qj8a_#64	GO:0005507: copper ion binding	0.005566	84,38,5
1ac5__#72	GO:0000287: magnesium ion binding	0.005591	47,128,7
1i6vd_#886	GO:0015078: hydrogen ion transporter activity	0.005592	15,21,2
1amp__#173	GO:0004601: peroxidase activity	0.005592	15,21,2
1a8q__#75	GO:0004601: peroxidase activity	0.005592	15,21,2
1qgxa_#53	GO:0030151: molybdenum ion binding	0.005592	21,15,2
1ezva1#147	GO:0015078: hydrogen ion transporter activity	0.005592	15,21,2
1thg__#5	GO:0030151: molybdenum ion binding	0.005592	21,15,2
1thg__#5	GO:0004180: carboxypeptidase activity	0.005592	21,15,2

1phm_2#253	GO:0004674: protein serine/threonine kinase activity	0.005592	309,42,11
1fgua1#249	GO:0030151: molybdenum ion binding	0.005592	21,15,2
1rypf_#35	GO:0016811: hydrolase activity, acting on carbon-nitrogen (but not peptide) bonds, in linear amides	0.005592	21,15,2
1im4a_#144	GO:0004523: ribonuclease H activity	0.005596	129,16,4
1qf6a3#92	GO:0005524: ATP binding	0.005598	91,243,16
1qo2a_#222	GO:0005525: GTP binding	0.005607	65,49,5
1f0xa1#523	GO:0016702: oxidoreductase activity, acting on single donors with incorporation of molecular oxygen, incorporation of two atoms of oxygen	0.005613	87,12,3
1dfaa2#211	GO:0015405: P-P-bond-hydrolysis-driven transporter activity	0.005613	87,12,3
1eu3a2#205	GO:0016702: oxidoreductase activity, acting on single donors with incorporation of molecular oxygen, incorporation of two atoms of oxygen	0.005613	87,12,3
1xnb__#130	GO:0000155: two-component sensor molecule activity	0.005613	87,12,3
1erv__#57	GO:0016866: intramolecular transferase activity	0.005613	87,12,3
1eucb2#188	GO:0000155: two-component sensor molecule activity	0.005613	87,12,3
1rb9__#49	GO:0004812: tRNA ligase activity	0.005614	39,26,3
1fvua_#13	GO:0005529: sugar binding	0.005614	26,39,3
1hzxa_#139	GO:0004812: tRNA ligase activity	0.005614	39,26,3
1prea2#308	GO:0004674: protein serine/threonine kinase activity	0.005614	76,42,5
1prea2#308	GO:0008083: growth factor activity	0.005614	76,42,5
3frua1#236	GO:0008083: growth factor activity	0.005614	76,42,5
1qo3c_#195	GO:0004812: tRNA ligase activity	0.005614	39,26,3
1qo3c_#195	GO:0004497: monooxygenase activity	0.005614	39,26,3
1c3ga2#272	GO:0004812: tRNA ligase activity	0.005614	39,26,3
1abrb1#71	GO:0005529: sugar binding	0.005614	26,39,3
1ryph_#163	GO:0016799: hydrolase activity, hydrolyzing N-glycosyl compounds	0.005615	121,17,4
1ryph_#163	GO:0046983: protein dimerization activity	0.005615	121,17,4
1qhqa_#45	GO:0046983: protein dimerization activity	0.005615	121,17,4
1qhqa_#45	GO:0003924: GTPase activity	0.005615	121,17,4
1e8ga2#201	GO:0016705: oxidoreductase activity, acting on paired donors, with incorporation or reduction of molecular oxygen	0.005623	304,26,8
1e8ga2#201	GO:0004812: tRNA ligase activity	0.005623	304,26,8
1j71a_#251	GO:0005066: transmembrane receptor protein tyrosine kinase signaling protein activity	0.005624	106,10,3
1quba4#235	GO:0003809: thrombin activity	0.005624	106,10,3
1loua_#79	GO:0003809: thrombin activity	0.005624	106,10,3
1aa6_2#285	GO:0016620: oxidoreductase activity, acting on the aldehyde or oxo group of donors, NAD or NADP as acceptor	0.005624	106,10,3
1aa6_2#285	GO:0016846: carbon-sulfur lyase activity	0.005624	106,10,3
1rypk_#12	GO:0008083: growth factor activity	0.005626	265,42,10
1gox__#246	GO:0016866: intramolecular transferase activity	0.005638	177,12,4
1tyfa_#31	GO:0008408: 3'-5' exonuclease activity	0.005638	162,13,4
1nfdb1#34	GO:0016668: oxidoreductase activity, acting on sulfur group of donors, NAD or NADP as acceptor	0.005638	177,12,4
1a3qa2#190	GO:0004674: protein serine/threonine kinase activity	0.005639	144,42,7
1f97a2#199	GO:0004674: protein serine/threonine kinase activity	0.005639	144,42,7
1f97a2#199	GO:0008083: growth factor activity	0.005639	144,42,7
3pyp__#41	GO:0005524: ATP binding	0.005653	32,243,8
1ezwa_#245	GO:0005524: ATP binding	0.005653	32,243,8
1f8na1#450	GO:0005524: ATP binding	0.005653	32,243,8
1dqsa_#171	GO:0005524: ATP binding	0.005653	32,243,8
1fa0a1#393	GO:0008757: S-adenosylmethionine-dependent methyltransferase activity	0.00566	261,24,7
1deua_#151	GO:0046983: protein dimerization activity	0.005665	285,17,6
1fsia_#122	GO:0004601: peroxidase activity	0.005666	97,21,4
1gpea1#271	GO:0046983: protein dimerization activity	0.005666	197,17,5
1e44b_#75	GO:0004867: serine-type endopeptidase inhibitor activity	0.005668	198,47,9
1p35a_#101	GO:0008201: heparin binding	0.005669	136,24,5
1bj7__#112	GO:0019955: cytokine binding	0.005669	29,11,2
1bj7__#112	GO:0004364: glutathione transferase activity	0.005669	29,11,2
1bxka_#262	GO:0016758: transferase activity, transferring hexosyl groups	0.005669	29,11,2
1e6pa2#274	GO:0004364: glutathione transferase activity	0.005669	29,11,2
1c0ma1#237	GO:0003964: RNA-directed DNA polymerase activity	0.005669	29,11,2
1iow_2#112	GO:0019955: cytokine binding	0.005669	29,11,2
1xyza_#753	GO:0010181: FMN binding	0.005669	29,11,2
1ewqa3#159	GO:0003684: damaged DNA binding	0.00567	32,10,2
1bg6_2#73	GO:0004457: lactate dehydrogenase activity	0.00567	32,10,2

1bg6_2#73	GO:0016814: hydrolase activity, acting on carbon-nitrogen (but not peptide) bonds, in cyclic amidines	0.00567	32,10,2
1a06__#205	GO:0005066: transmembrane receptor protein tyrosine kinase signaling protein activity	0.00567	32,10,2
1a06__#205	GO:0008199: ferric iron binding	0.00567	32,10,2
1g7sa4#55	GO:0003779: actin binding	0.00567	10,32,2
1ryp1_#20	GO:0004812: tRNA ligase activity	0.00567	125,26,5
1dkra2#210	GO:0003682: chromatin binding	0.00567	32,10,2
1dkra2#210	GO:0016620: oxidoreductase activity, acting on the aldehyde or oxo group of donors, NAD or NADP as acceptor	0.00567	32,10,2
1hdr__#241	GO:0003682: chromatin binding	0.00567	32,10,2
1pda_1#77	GO:0008199: ferric iron binding	0.00567	32,10,2
1pda_1#77	GO:0016846: carbon-sulfur lyase activity	0.00567	32,10,2
1nat__#77	GO:0016620: oxidoreductase activity, acting on the aldehyde or oxo group of donors, NAD or NADP as acceptor	0.00567	32,10,2
1gcua1#36	GO:0016620: oxidoreductase activity, acting on the aldehyde or oxo group of donors, NAD or NADP as acceptor	0.00567	32,10,2
1g8ka2#570	GO:0016620: oxidoreductase activity, acting on the aldehyde or oxo group of donors, NAD or NADP as acceptor	0.00567	32,10,2
3sil__#8	GO:0004812: tRNA ligase activity	0.00567	125,26,5
1sppb_#32	GO:0005509: calcium ion binding	0.005676	38,160,7
1dqaa1#596	GO:0004867: serine-type endopeptidase inhibitor activity	0.005681	68,47,5
1qkma_#330	GO:0004180: carboxypeptidase activity	0.005684	69,15,3
1fi2a_#109	GO:0051082: unfolded protein binding	0.005688	228,34,8
1f2na_#138	GO:0003724: RNA helicase activity	0.005692	96,11,3
1hava_#41	GO:0003724: RNA helicase activity	0.005692	96,11,3
1fgga_#134	GO:0016620: oxidoreductase activity, acting on the aldehyde or oxo group of donors, NAD or NADP as acceptor	0.005692	218,10,4
1fgga_#134	GO:0016846: carbon-sulfur lyase activity	0.005692	218,10,4
1fsu__#434	GO:0003968: RNA-directed RNA polymerase activity	0.005695	356,14,6
1ii7a_#199	GO:0016646: oxidoreductase activity, acting on the CH-NH group of donors, NAD or NADP as acceptor	0.005696	54,19,3
1e1aa_#178	GO:0004222: metalloendopeptidase activity	0.005696	54,19,3
1oaa__#14	GO:0016646: oxidoreductase activity, acting on the CH-NH group of donors, NAD or NADP as acceptor	0.005696	54,19,3
1lci__#26	GO:0005096: GTPase activator activity	0.005698	139,15,4
1ytba1#125	GO:0008270: zinc ion binding	0.005707	19,108,4
1hw5a2#64	GO:0005524: ATP binding	0.00571	167,243,25
1qs1a2#437	GO:0008083: growth factor activity	0.00571	452,42,14
1bev1_#86	GO:0003724: RNA helicase activity	0.005712	323,11,5
1cqka_#49	GO:0051082: unfolded protein binding	0.005726	30,34,3
2stv__#129	GO:0051082: unfolded protein binding	0.005726	30,34,3
1elua_#95	GO:0016616: oxidoreductase activity, acting on the CH-OH group of donors, NAD or NADP as acceptor	0.005728	77,59,6
1qrra_#69	GO:0010181: FMN binding	0.005731	196,11,4
1qs1a2#437	GO:0019843: rRNA binding	0.005735	452,22,9
1opy__#68	GO:0015036: disulfide oxidoreductase activity	0.005735	367,22,8
1deua_#151	GO:0000287: magnesium ion binding	0.005755	285,128,23
1boub_#7	GO:0004812: tRNA ligase activity	0.005756	240,26,7
1tuba1#239	GO:0019829: cation-transporting ATPase activity	0.005758	120,9,3
1jsg__#92	GO:0004812: tRNA ligase activity	0.005759	607,26,12
1jsg__#92	GO:0004497: monooxygenase activity	0.005759	607,26,12
1am2__#163	GO:0016616: oxidoreductase activity, acting on the CH-OH group of donors, NAD or NADP as acceptor	0.00576	102,59,7
1qasa3#352	GO:0004523: ribonuclease H activity	0.005775	20,16,2
1hx6a1#163	GO:0003887: DNA-directed DNA polymerase activity	0.005775	16,20,2
1hjra_#58	GO:0003887: DNA-directed DNA polymerase activity	0.005775	16,20,2
1dyna_#19	GO:0008083: growth factor activity	0.005778	109,42,6
1fjsa_#83	GO:0008083: growth factor activity	0.005778	109,42,6
1dm9a_#57	GO:0008083: growth factor activity	0.005778	109,42,6
3mag__#134	GO:0000287: magnesium ion binding	0.00578	72,128,9
1dik_3#240	GO:0005516: calmodulin binding	0.005782	262,24,7
1dik_3#240	GO:0008201: heparin binding	0.005782	262,24,7
1f0xa1#278	GO:0004812: tRNA ligase activity	0.005783	180,26,6
1fc4a_#205	GO:0016651: oxidoreductase activity, acting on NADH or NADPH	0.005784	267,13,5
1fc4a_#205	GO:0016854: racemase and epimerase activity	0.005784	267,13,5
1fc4a_#205	GO:0016861: intramolecular oxidoreductase activity, interconverting aldoses and ketoses	0.005784	267,13,5

1jj2q_#118	GO:0004867: serine-type endopeptidase inhibitor activity	0.005788	7,47,2
1qgxa_#53	GO:0005525: GTP binding	0.005788	21,49,3
1f8ra1#288	GO:0005351: sugar porter activity	0.005788	49,21,3
1f75a_#239	GO:0005525: GTP binding	0.005788	21,49,3
1xsoa_#36	GO:0004867: serine-type endopeptidase inhibitor activity	0.005788	7,47,2
1lara2#1769	GO:0008009: chemokine activity	0.005788	219,10,4
1lara2#1769	GO:0003916: DNA topoisomerase activity	0.005788	219,10,4
1lara2#1769	GO:0050660: FAD binding	0.005788	219,10,4
1bupa2#199	GO:0003779: actin binding	0.005792	32,32,3
1fo4a5#1002	GO:0019843: rRNA binding	0.005801	93,22,4
1i9ga_#67	GO:0016638: oxidoreductase activity, acting on the CH-NH2 group of donors	0.005805	61,17,3
1hyrc1#247	GO:0046983: protein dimerization activity	0.005805	61,17,3
1e5ea_#71	GO:0016638: oxidoreductase activity, acting on the CH-NH2 group of donors	0.005805	61,17,3
1ft9a1#203	GO:0005507: copper ion binding	0.005811	27,38,3
1g5ha2#162	GO:0030145: manganese ion binding	0.005811	27,38,3
1i7qa_#278	GO:0000287: magnesium ion binding	0.005819	36,128,6
1e79h2#39	GO:0015082: di-, tri-valent inorganic cation transporter activity	0.005832	23,14,2
1e79h2#39	GO:0046915: transition metal ion transporter activity	0.005832	23,14,2
1qdea_#218	GO:0016776: phosphotransferase activity, phosphate group as acceptor	0.005832	23,14,2
2mpra_#222	GO:0015082: di-, tri-valent inorganic cation transporter activity	0.005832	23,14,2
2mpra_#222	GO:0046915: transition metal ion transporter activity	0.005832	23,14,2
1hhsa_#328	GO:0004190: aspartic-type endopeptidase activity	0.005832	14,23,2
1mwpa_#36	GO:0004190: aspartic-type endopeptidase activity	0.005832	14,23,2
1by5a_#585	GO:0015082: di-, tri-valent inorganic cation transporter activity	0.005832	23,14,2
1by5a_#585	GO:0046915: transition metal ion transporter activity	0.005832	23,14,2
1pme__#166	GO:0004714: transmembrane receptor protein tyrosine kinase activity	0.005832	23,14,2
1lcl__#110	GO:0003968: RNA-directed RNA polymerase activity	0.005832	23,14,2
1d4ta_#80	GO:0004714: transmembrane receptor protein tyrosine kinase activity	0.005832	23,14,2
1d4ta_#80	GO:0005543: phospholipid binding	0.005832	23,14,2
1cs6a2#164	GO:0005509: calcium ion binding	0.005836	7,160,3
1h75a_#33	GO:0005509: calcium ion binding	0.005836	7,160,3
1h6la_#120	GO:0005509: calcium ion binding	0.005836	7,160,3
1fqva2#326	GO:0005509: calcium ion binding	0.005836	7,160,3
1ac5__#203	GO:0005509: calcium ion binding	0.005836	7,160,3
1exma3#99	GO:0005085: guanyl-nucleotide exchange factor activity	0.005836	81,13,3
1xsoa_#36	GO:0005509: calcium ion binding	0.005836	7,160,3
1poxa3#460	GO:0016651: oxidoreductase activity, acting on NADH or NADPH	0.005836	81,13,3
1poxa3#460	GO:0016861: intramolecular oxidoreductase activity, interconverting aldoses and ketoses	0.005836	81,13,3
1a6q__#16	GO:0004263: chymotrypsin activity	0.005841	8,41,2
1f8v.1#A116	GO:0008270: zinc ion binding	0.005841	101,108,10
1g25a_#22	GO:0004263: chymotrypsin activity	0.005848	415,41,13
1e1aa_#285	GO:0005516: calmodulin binding	0.005849	137,24,5
1p35a_#101	GO:0051082: unfolded protein binding	0.005851	136,34,6
1rrpb_#65	GO:0005524: ATP binding	0.005851	53,243,11
1g6oa_#116	GO:0015082: di-, tri-valent inorganic cation transporter activity	0.005858	358,14,6
1g6oa_#116	GO:0046915: transition metal ion transporter activity	0.005858	358,14,6
1g6oa_#116	GO:0003968: RNA-directed RNA polymerase activity	0.005858	358,14,6
3sil__#317	GO:0004714: transmembrane receptor protein tyrosine kinase activity	0.005858	358,14,6
1ihoa_#238	GO:0015082: di-, tri-valent inorganic cation transporter activity	0.00586	75,14,3
1ihoa_#238	GO:0046915: transition metal ion transporter activity	0.00586	75,14,3
1ihoa_#238	GO:0016251: general RNA polymerase II transcription factor activity	0.00586	75,14,3
1ewna_#109	GO:0015082: di-, tri-valent inorganic cation transporter activity	0.00586	75,14,3
1ewna_#109	GO:0046915: transition metal ion transporter activity	0.00586	75,14,3
1pot__#138	GO:0015082: di-, tri-valent inorganic cation transporter activity	0.00586	75,14,3
1pot__#138	GO:0046915: transition metal ion transporter activity	0.00586	75,14,3
2mev3_#108	GO:0004180: carboxypeptidase activity	0.005864	228,15,5
3sil__#98	GO:0030151: molybdenum ion binding	0.005864	228,15,5
1apj__#2062	GO:0016668: oxidoreductase activity, acting on sulfur group of donors, NAD or NADP as acceptor	0.005871	27,12,2
1apj__#2062	GO:0016866: intramolecular transferase activity	0.005871	27,12,2
1hr6a1#41	GO:0003899: DNA-directed RNA polymerase activity	0.005871	27,12,2
1qtn.1#A331	GO:0003899: DNA-directed RNA polymerase activity	0.005871	27,12,2
1gtxa_#150	GO:0016866: intramolecular transferase activity	0.005871	27,12,2
2btva_#597	GO:0016702: oxidoreductase activity, acting on single donors with incorporation of molecular oxygen, incorporation of two atoms of oxygen	0.005871	27,12,2
1i6vd_#1188	GO:0015405: P-P-bond-hydrolysis-driven transporter activity	0.005871	27,12,2

1tgoa1#65	GO:0003899: DNA-directed RNA polymerase activity	0.005871	27,12,2
1llda2#161	GO:0003899: DNA-directed RNA polymerase activity	0.005871	27,12,2
1ei5a3#311	GO:0016668: oxidoreductase activity, acting on sulfur group of donors, NAD or NADP as acceptor	0.005871	27,12,2
2tnfa_#75	GO:0003713: transcription coactivator activity	0.005872	1,16,1
1ciy_2#414	GO:0004523: ribonuclease H activity	0.005872	1,16,1
4fiv__#38	GO:0004523: ribonuclease H activity	0.005872	1,16,1
1dyoa_#153	GO:0005507: copper ion binding	0.005873	161,38,7
1avgi_#69	GO:0008270: zinc ion binding	0.005875	340,108,23
1d0na3#305	GO:0004674: protein serine/threonine kinase activity	0.005876	311,42,11
2pia_1#54	GO:0008009: chemokine activity	0.005884	220,10,4
2pia_1#54	GO:0016620: oxidoreductase activity, acting on the aldehyde or oxo group of donors, NAD or NADP as acceptor	0.005884	220,10,4
1nuka_#80	GO:0003809: thrombin activity	0.005884	220,10,4
1nuka_#80	GO:0008009: chemokine activity	0.005884	220,10,4
1nuka_#80	GO:0016620: oxidoreductase activity, acting on the aldehyde or oxo group of donors, NAD or NADP as acceptor	0.005884	220,10,4
3btaa3#194	GO:0019843: rRNA binding	0.005886	47,22,3
2rsla_#55	GO:0008483: transaminase activity	0.005887	19,17,2
1qksa2#326	GO:0004896: hematopoietin/interferon-class (D200-domain) cytokine receptor activity	0.005887	17,19,2
1f8ra1#233	GO:0016638: oxidoreductase activity, acting on the CH-NH2 group of donors	0.005887	19,17,2
1je5a_#129	GO:0016646: oxidoreductase activity, acting on the CH-NH group of donors, NAD or NADP as acceptor	0.005887	17,19,2
1c8ua2#272	GO:0004222: metalloendopeptidase activity	0.005887	17,19,2
1kapp1#389	GO:0046983: protein dimerization activity	0.005887	19,17,2
1eyqa_#8	GO:0004896: hematopoietin/interferon-class (D200-domain) cytokine receptor activity	0.005887	17,19,2
1ecpa_#46	GO:0004222: metalloendopeptidase activity	0.005887	17,19,2
1ecpa_#46	GO:0004620: phospholipase activity	0.005887	17,19,2
1cjxa2#307	GO:0004222: metalloendopeptidase activity	0.005887	17,19,2
1b37a1#209	GO:0016627: oxidoreductase activity, acting on the CH-CH group of donors	0.005887	19,17,2
1b37a1#209	GO:0016638: oxidoreductase activity, acting on the CH-NH2 group of donors	0.005887	19,17,2
1qq9a_#178	GO:0008483: transaminase activity	0.005887	19,17,2
1bt3a_#49	GO:0016646: oxidoreductase activity, acting on the CH-NH group of donors, NAD or NADP as acceptor	0.005887	17,19,2
1ed5a_#180	GO:0046983: protein dimerization activity	0.005887	19,17,2
1ed5a_#180	GO:0003924: GTPase activity	0.005887	19,17,2
1bco_2#314	GO:0004222: metalloendopeptidase activity	0.005887	17,19,2
1fp5a2#478	GO:0046983: protein dimerization activity	0.005887	19,17,2
1qhva_#438	GO:0046983: protein dimerization activity	0.005887	19,17,2
1cwva2#682	GO:0004896: hematopoietin/interferon-class (D200-domain) cytokine receptor activity	0.005887	17,19,2
1epwa2#1220	GO:0016799: hydrolase activity, hydrolyzing N-glycosyl compounds	0.005887	19,17,2
1abrb2#252	GO:0016799: hydrolase activity, hydrolyzing N-glycosyl compounds	0.005887	19,17,2
1keva2#236	GO:0016646: oxidoreductase activity, acting on the CH-NH group of donors, NAD or NADP as acceptor	0.005887	17,19,2
1bywa_#30	GO:0004725: protein tyrosine phosphatase activity	0.005891	331,15,6
1qq5a_#7	GO:0016763: transferase activity, transferring pentosyl groups	0.005891	167,28,6
1b6ra3#161	GO:0000049: tRNA binding	0.005892	164,13,4
1b6ra3#161	GO:0008408: 3'-5' exonuclease activity	0.005892	164,13,4
1hdoa_#189	GO:0019201: nucleotide kinase activity	0.005892	164,13,4
1hdoa_#189	GO:0016861: intramolecular oxidoreductase activity, interconverting aldoses and ketoses	0.005892	164,13,4
1dyna_#42	GO:0008408: 3'-5' exonuclease activity	0.005892	164,13,4
1dyna_#19	GO:0004620: phospholipase activity	0.005898	109,19,4
1a65a1#108	GO:0042802: protein self binding	0.005899	294,12,5
1hlwa_#82	GO:0004295: trypsin activity	0.0059	270,48,11
1a6o__#45	GO:0005525: GTP binding	0.00591	303,49,12
1feza_#185	GO:0005524: ATP binding	0.005917	68,243,13
1hcl__#280	GO:0005096: GTPase activator activity	0.005919	70,15,3
1a8d_1#87	GO:0005507: copper ion binding	0.005921	204,38,8
1je5a_#39	GO:0008810: cellulase activity	0.005924	18,18,2
1poxa3#439	GO:0005085: guanyl-nucleotide exchange factor activity	0.005926	25,13,2
3gcb__#176	GO:0005085: guanyl-nucleotide exchange factor activity	0.005926	25,13,2
1eu3a2#174	GO:0008235: metalloexopeptidase activity	0.005926	25,13,2
1dv8a_#213	GO:0003729: mRNA binding	0.005926	25,13,2
1dxea_#88	GO:0016831: carboxy-lyase activity	0.005926	13,25,2
1gen__#475	GO:0008408: 3'-5' exonuclease activity	0.005926	25,13,2
1e2fa_#121	GO:0008270: zinc ion binding	0.005928	10,108,3

3tdt__#201	GO:0008270: zinc ion binding	0.005928	10,108,3
1znca_#167	GO:0008270: zinc ion binding	0.005928	10,108,3
1ejda_#377	GO:0008199: ferric iron binding	0.005928	108,10,3
1aqua_#126	GO:0016811: hydrolase activity, acting on carbon-nitrogen (but not peptide) bonds, in linear amides	0.005928	576,15,8
1fwxa2#189	GO:0008270: zinc ion binding	0.005928	10,108,3
1e44b_#75	GO:0019955: cytokine binding	0.005945	198,11,4
1zpda1#213	GO:0016831: carboxy-lyase activity	0.00595	82,25,4
1i50a_#856	GO:0000049: tRNA binding	0.005974	269,13,5
1ge8a2#134	GO:0004556: alpha-amylase activity	0.005975	229,15,5
1ge8a2#134	GO:0004725: protein tyrosine phosphatase activity	0.005975	229,15,5
1ds1a_#300	GO:0016763: transferase activity, transferring pentosyl groups	0.005978	37,28,3
1bd8__#103	GO:0003700: transcription factor activity	0.00598	3,124,2
1f1za1#202	GO:0003700: transcription factor activity	0.00598	3,124,2
1as4.1#A336	GO:0004556: alpha-amylase activity	0.005981	332,15,6
1f2aa_#27	GO:0003899: DNA-directed RNA polymerase activity	0.005984	89,12,3
2mev1_#123	GO:0016702: oxidoreductase activity, acting on single donors with incorporation of molecular oxygen, incorporation of two atoms of oxygen	0.005984	89,12,3
1ev7a_#218	GO:0009036: type II site-specific deoxyribonuclease activity	0.005984	89,12,3
1heta2#218	GO:0005524: ATP binding	0.005992	39,243,9
1i6vd_#1283	GO:0030151: molybdenum ion binding	0.005997	141,15,4
1akp__#62	GO:0004620: phospholipase activity	0.005998	55,19,3
1hdr__#156	GO:0016646: oxidoreductase activity, acting on the CH-NH group of donors, NAD or NADP as acceptor	0.005998	55,19,3
1mai__#108	GO:0004714: transmembrane receptor protein tyrosine kinase activity	0.006002	152,14,4
1gox__#124	GO:0016620: oxidoreductase activity, acting on the aldehyde or oxo group of donors, NAD or NADP as acceptor	0.006011	366,10,5
3sil__#317	GO:0008083: growth factor activity	0.006011	358,42,12
1eula_#213	GO:0051082: unfolded protein binding	0.006014	60,34,4
1d0na2#200	GO:0004812: tRNA ligase activity	0.006021	79,26,4
1qaxa2#307	GO:0005524: ATP binding	0.006022	46,243,10
1xnb__#149	GO:0000049: tRNA binding	0.006022	165,13,4
1xnb__#149	GO:0008080: N-acetyltransferase activity	0.006022	165,13,4
1dik_1#765	GO:0016854: racemase and epimerase activity	0.006022	165,13,4
1jd0a_#176	GO:0004190: aspartic-type endopeptidase activity	0.006023	207,23,6
1ckea_#35	GO:0003684: damaged DNA binding	0.006024	33,10,2
1kit_3#282	GO:0003684: damaged DNA binding	0.006024	33,10,2
1kit_3#282	GO:0016814: hydrolase activity, acting on carbon-nitrogen (but not peptide) bonds, in cyclic amidines	0.006024	33,10,2
1kit_3#282	GO:0008800: beta-lactamase activity	0.006024	33,10,2
3euga_#124	GO:0016836: hydro-lyase activity	0.006024	10,33,2
1erja_#457	GO:0001584: rhodopsin-like receptor activity	0.006024	33,10,2
2dnja_#158	GO:0003809: thrombin activity	0.006024	33,10,2
2dnja_#158	GO:0003684: damaged DNA binding	0.006024	33,10,2
1ijqa1#564	GO:0003682: chromatin binding	0.006024	33,10,2
1mjha_#126	GO:0005351: sugar porter activity	0.006025	159,21,5
1aym3_#131	GO:0003887: DNA-directed DNA polymerase activity	0.006027	412,20,8
1aym3_#131	GO:0005126: hematopoietin/interferon-class (D200-domain) cytokine receptor binding	0.006027	412,20,8
1qqsa_#35	GO:0019843: rRNA binding	0.006027	94,22,4
1b3qa2#556	GO:0004812: tRNA ligase activity	0.006031	40,26,3
1i4ua_#154	GO:0004812: tRNA ligase activity	0.006031	40,26,3
1jj2q_#118	GO:0004295: trypsin activity	0.006032	7,48,2
1cewi_#14	GO:0010181: FMN binding	0.006032	98,11,3
1flca1#294	GO:0003724: RNA helicase activity	0.006032	98,11,3
1zpda1#213	GO:0016651: oxidoreductase activity, acting on NADH or NADPH	0.006041	82,13,3
1atg__#144	GO:0016854: racemase and epimerase activity	0.006041	82,13,3
1qqga2#253	GO:0004674: protein serine/threonine kinase activity	0.006041	110,42,6
1thg__#223	GO:0019201: nucleotide kinase activity	0.006041	82,13,3
1thg__#223	GO:0016651: oxidoreductase activity, acting on NADH or NADPH	0.006041	82,13,3
1h6va2#245	GO:0016836: hydro-lyase activity	0.006043	99,33,5
1nfdb1#21	GO:0051082: unfolded protein binding	0.006044	96,34,5
1qqga2#166	GO:0046983: protein dimerization activity	0.006046	200,17,5
1bev1_#86	GO:0005126: hematopoietin/interferon-class (D200-domain) cytokine receptor binding	0.006049	323,20,7
2bb2_1#57	GO:0003887: DNA-directed DNA polymerase activity	0.006051	104,20,4
1qhua1#47	GO:0003887: DNA-directed DNA polymerase activity	0.006051	104,20,4
1qhoa3#488	GO:0003724: RNA helicase activity	0.00606	30,11,2

1bu7a_#278	GO:0010181: FMN binding	0.00606	30,11,2
2stv__#129	GO:0019955: cytokine binding	0.00606	30,11,2
1tig__#148	GO:0003724: RNA helicase activity	0.00606	30,11,2
1dbga_#38	GO:0016758: transferase activity, transferring hexosyl groups	0.00606	30,11,2
1hlwa_#91	GO:0003964: RNA-directed DNA polymerase activity	0.00606	30,11,2
1imva_#255	GO:0003697: single-stranded DNA binding	0.006064	589,12,7
1imva_#255	GO:0000155: two-component sensor molecule activity	0.006064	589,12,7
1eq9a_#40	GO:0005516: calmodulin binding	0.006064	86,24,4
1jbqa_#152	GO:0016836: hydro-lyase activity	0.006066	62,33,4
1hlwa_#82	GO:0003729: mRNA binding	0.006071	270,13,5
1ejda_#6	GO:0004177: aminopeptidase activity	0.006071	270,13,5
1ejda_#6	GO:0000049: tRNA binding	0.006071	270,13,5
1cbf__#208	GO:0030151: molybdenum ion binding	0.006072	333,15,6
1epwa1#901	GO:0016638: oxidoreductase activity, acting on the CH-NH2 group of donors	0.006076	62,17,3
1jbqa_#152	GO:0016627: oxidoreductase activity, acting on the CH-CH group of donors	0.006076	62,17,3
1feca1#28	GO:0016638: oxidoreductase activity, acting on the CH-NH2 group of donors	0.006076	62,17,3
1ih7a1#150	GO:0016799: hydrolase activity, hydrolyzing N-glycosyl compounds	0.006076	62,17,3
1f8ra2#325	GO:0003916: DNA topoisomerase activity	0.00608	222,10,4
1h9da_#91	GO:0003809: thrombin activity	0.00608	222,10,4
1prea2#308	GO:0004714: transmembrane receptor protein tyrosine kinase activity	0.006082	76,14,3
1kve.1#B199	GO:0003968: RNA-directed RNA polymerase activity	0.006082	76,14,3
1eh9a1#33	GO:0019838: growth factor binding	0.006083	37,9,2
1hdma1#98	GO:0019838: growth factor binding	0.006083	37,9,2
1dm9a_#57	GO:0003916: DNA topoisomerase activity	0.006084	109,10,3
1opy__#68	GO:0008009: chemokine activity	0.006084	367,10,5
1opy__#68	GO:0016846: carbon-sulfur lyase activity	0.006084	367,10,5
1fkna_#18	GO:0004867: serine-type endopeptidase inhibitor activity	0.006087	130,47,7
1qfta_#98	GO:0004556: alpha-amylase activity	0.006088	230,15,5
1fu6a_#50	GO:0004812: tRNA ligase activity	0.006093	308,26,8
1qfma1#139	GO:0004867: serine-type endopeptidase inhibitor activity	0.006106	318,47,12
1c4oa1#56	GO:0005524: ATP binding	0.006117	5,243,3
1iow_1#41	GO:0005524: ATP binding	0.006117	5,243,3
2mev2_#53	GO:0005524: ATP binding	0.006117	5,243,3
1az9_1#73	GO:0005524: ATP binding	0.006117	5,243,3
1qs1a2#407	GO:0005524: ATP binding	0.006117	5,243,3
1jixa_#283	GO:0005524: ATP binding	0.006117	5,243,3
1bev1_#245	GO:0005524: ATP binding	0.006117	5,243,3
1vin_1#302	GO:0005524: ATP binding	0.006117	5,243,3
1fhga_#48	GO:0005524: ATP binding	0.006117	5,243,3
1b35b_#140	GO:0005524: ATP binding	0.006117	5,243,3
1biha3#248	GO:0004674: protein serine/threonine kinase activity	0.006124	8,42,2
2viua_#123	GO:0008083: growth factor activity	0.006124	8,42,2
1c7qa_#374	GO:0005351: sugar porter activity	0.006127	50,21,3
1j9qa1#154	GO:0004295: trypsin activity	0.006127	196,48,9
1nsca_#379	GO:0046983: protein dimerization activity	0.006128	124,17,4
1kit_3#331	GO:0016638: oxidoreductase activity, acting on the CH-NH2 group of donors	0.006128	124,17,4
1fo8a_#202	GO:0030151: molybdenum ion binding	0.006132	22,15,2
1lml__#216	GO:0003743: translation initiation factor activity	0.006132	22,15,2
1pvda3#533	GO:0015036: disulfide oxidoreductase activity	0.006132	15,22,2
1fgka_#610	GO:0016811: hydrolase activity, acting on carbon-nitrogen (but not peptide) bonds, in linear amides	0.006132	22,15,2
1dzfa2#164	GO:0019843: rRNA binding	0.006132	15,22,2
2naca1#162	GO:0030151: molybdenum ion binding	0.006132	22,15,2
3frua1#249	GO:0019843: rRNA binding	0.006132	15,22,2
1e9ma_#99	GO:0030151: molybdenum ion binding	0.006132	22,15,2
1qj2b2#536	GO:0030151: molybdenum ion binding	0.006132	22,15,2
1aqt_2#45	GO:0005524: ATP binding	0.006137	76,243,14
1pbe_1#150	GO:0005524: ATP binding	0.006137	76,243,14
2mpra_#190	GO:0030145: manganese ion binding	0.00614	400,38,12
1plq_1#90	GO:0005529: sugar binding	0.006147	158,39,7
1i5ga_#115	GO:0005509: calcium ion binding	0.006149	324,160,30
1fo4a5#1020	GO:0008083: growth factor activity	0.00615	359,42,12
1e2wa1#102	GO:0005507: copper ion binding	0.006153	86,38,5
1jj22_#78	GO:0030151: molybdenum ion binding	0.00616	71,15,3
1fnf_3#1409	GO:0005179: hormone activity	0.00616	71,15,3
1d0na6#681	GO:0000287: magnesium ion binding	0.006163	143,128,14

1qhda2#323	GO:0008201: heparin binding	0.006164	337,24,8
1qqga2#166	GO:0019955: cytokine binding	0.006164	200,11,4
1lml__#535	GO:0004263: chymotrypsin activity	0.006164	50,41,4
1qqra_#185	GO:0005524: ATP binding	0.006166	26,243,7
1nat__#50	GO:0016668: oxidoreductase activity, acting on sulfur group of donors, NAD or NADP as acceptor	0.006166	297,12,5
1i05a_#132	GO:0005507: copper ion binding	0.006172	54,38,4
1e1aa_#178	GO:0005507: copper ion binding	0.006172	54,38,4
1dnv__#354	GO:0005507: copper ion binding	0.006172	54,38,4
1ekma1#545	GO:0000155: two-component sensor molecule activity	0.006175	90,12,3
1pysa_#221	GO:0003697: single-stranded DNA binding	0.006175	90,12,3
1pysa_#221	GO:0003899: DNA-directed RNA polymerase activity	0.006175	90,12,3
1hdfa_#16	GO:0016620: oxidoreductase activity, acting on the aldehyde or oxo group of donors, NAD or NADP as acceptor	0.006179	223,10,4
1smaa2#581	GO:0005509: calcium ion binding	0.006186	80,160,11
1dcfa_#66	GO:0016831: carboxy-lyase activity	0.006187	42,25,3
1cjxa1#126	GO:0016831: carboxy-lyase activity	0.006187	42,25,3
1fp2a2#346	GO:0008083: growth factor activity	0.006187	25,42,3
1lcl__#128	GO:0005509: calcium ion binding	0.006188	21,160,5
1g6oa_#40	GO:0005509: calcium ion binding	0.006188	21,160,5
1bccb2#274	GO:0000287: magnesium ion binding	0.006192	60,128,8
1bwvs_#117	GO:0003724: RNA helicase activity	0.006192	329,11,5
1qqga2#166	GO:0005529: sugar binding	0.006197	200,39,8
1dj0a2#183	GO:0016836: hydro-lyase activity	0.006205	142,33,6
1mpp__#154	GO:0019955: cytokine binding	0.006207	99,11,3
1sbp__#219	GO:0008270: zinc ion binding	0.006215	43,108,6
1bjt__#890	GO:0015036: disulfide oxidoreductase activity	0.006215	292,22,7
1e6ca_#91	GO:0003899: DNA-directed RNA polymerase activity	0.006232	182,12,4
1qj8a_#64	GO:0005529: sugar binding	0.006232	84,39,5
1e4ea2#215	GO:0004714: transmembrane receptor protein tyrosine kinase activity	0.006236	490,14,7
1i50a_#148	GO:0046983: protein dimerization activity	0.006239	1,17,1
1i50a_#110	GO:0046983: protein dimerization activity	0.006239	1,17,1
1fxra_#11	GO:0016627: oxidoreductase activity, acting on the CH-CH group of donors	0.006239	1,17,1
1bg1a2#370	GO:0046983: protein dimerization activity	0.006239	1,17,1
1iyu__#47	GO:0046983: protein dimerization activity	0.006239	1,17,1
1g79a_#22	GO:0016638: oxidoreductase activity, acting on the CH-NH2 group of donors	0.006239	1,17,1
1dceb_#280	GO:0003924: GTPase activity	0.006239	1,17,1
1i50f_#153	GO:0046983: protein dimerization activity	0.006239	1,17,1
1chua2#100	GO:0016638: oxidoreductase activity, acting on the CH-NH2 group of donors	0.006239	1,17,1
1mun__#41	GO:0016799: hydrolase activity, hydrolyzing N-glycosyl compounds	0.006239	1,17,1
2masa_#261	GO:0016799: hydrolase activity, hydrolyzing N-glycosyl compounds	0.006239	1,17,1
1qtoa_#93	GO:0016627: oxidoreductase activity, acting on the CH-CH group of donors	0.006239	1,17,1
1i6vc_#411	GO:0046983: protein dimerization activity	0.006239	1,17,1
2viua_#35	GO:0008200: ion channel inhibitor activity	0.006239	1,17,1
1ep3a_#15	GO:0016627: oxidoreductase activity, acting on the CH-CH group of donors	0.006239	1,17,1
1dpe__#399	GO:0046983: protein dimerization activity	0.006239	1,17,1
1fnga1#123	GO:0046983: protein dimerization activity	0.006239	1,17,1
1g99a1#91	GO:0016627: oxidoreductase activity, acting on the CH-CH group of donors	0.006239	1,17,1
1i50b_#639	GO:0046983: protein dimerization activity	0.006239	1,17,1
1ee8a_#254	GO:0016799: hydrolase activity, hydrolyzing N-glycosyl compounds	0.006239	1,17,1
1tyfa_#173	GO:0016799: hydrolase activity, hydrolyzing N-glycosyl compounds	0.006239	1,17,1
1gpc__#66	GO:0016799: hydrolase activity, hydrolyzing N-glycosyl compounds	0.006239	1,17,1
1dih_1#264	GO:0016627: oxidoreductase activity, acting on the CH-CH group of donors	0.006239	1,17,1
1i50b_#117	GO:0046983: protein dimerization activity	0.006239	1,17,1
1eny__#237	GO:0016627: oxidoreductase activity, acting on the CH-CH group of donors	0.006239	1,17,1
1bkds_#974	GO:0003924: GTPase activity	0.006239	1,17,1
1h7wa5#996	GO:0016627: oxidoreductase activity, acting on the CH-CH group of donors	0.006239	1,17,1
1dceb_#66	GO:0003924: GTPase activity	0.006239	1,17,1
1prea2#373	GO:0046983: protein dimerization activity	0.006239	1,17,1
1b3ua_#549	GO:0046983: protein dimerization activity	0.006239	1,17,1
1aj8a_#349	GO:0016627: oxidoreductase activity, acting on the CH-CH group of donors	0.006239	1,17,1
1hg3a_#61	GO:0016616: oxidoreductase activity, acting on the CH-OH group of donors, NAD or NADP as acceptor	0.006244	18,59,3
1a4ya_#428	GO:0016616: oxidoreductase activity, acting on the CH-OH group of donors, NAD or NADP as acceptor	0.006244	18,59,3

1g5qa_#80	GO:0016616: oxidoreductase activity, acting on the CH-OH group of donors, NAD or NADP as acceptor	0.006244	18,59,3
1hcl__#82	GO:0019843: rRNA binding	0.006246	48,22,3
1thg__#3	GO:0005524: ATP binding	0.006246	92,243,16
1be9a_#357	GO:0005085: guanyl-nucleotide exchange factor activity	0.00625	83,13,3
1be9a_#357	GO:0000049: tRNA binding	0.00625	83,13,3
1dqza_#131	GO:0016854: racemase and epimerase activity	0.00625	83,13,3
1ecpa_#58	GO:0008235: metalloexopeptidase activity	0.00625	83,13,3
1efnb_#102	GO:0004674: protein serine/threonine kinase activity	0.006253	49,42,4
1et9a1#39	GO:0008083: growth factor activity	0.006253	49,42,4
1kit_3#743	GO:0003968: RNA-directed RNA polymerase activity	0.006254	250,14,5
3grx__#56	GO:0030151: molybdenum ion binding	0.006257	335,15,6
1ihua2#525	GO:0030151: molybdenum ion binding	0.006257	335,15,6
1mdah_#329	GO:0030151: molybdenum ion binding	0.006257	335,15,6
1bhga3#529	GO:0016763: transferase activity, transferring pentosyl groups	0.006261	74,28,4
1am2__#163	GO:0008270: zinc ion binding	0.006264	102,108,10
1smaa1#95	GO:0000049: tRNA binding	0.006268	272,13,5
1epwa3#175	GO:0003887: DNA-directed DNA polymerase activity	0.00627	53,20,3
1dfca4#1469	GO:0005126: hematopoietin/interferon-class (D200-domain) cytokine receptor binding	0.00627	53,20,3
1ddga1#278	GO:0003887: DNA-directed DNA polymerase activity	0.00627	53,20,3
5ruba1#259	GO:0003887: DNA-directed DNA polymerase activity	0.00627	53,20,3
1dp0a1#309	GO:0005126: hematopoietin/interferon-class (D200-domain) cytokine receptor binding	0.00627	53,20,3
1ii7a_#275	GO:0000287: magnesium ion binding	0.006271	26,128,5
1qqra_#185	GO:0000287: magnesium ion binding	0.006271	26,128,5
1a06__#85	GO:0005516: calmodulin binding	0.006276	44,24,3
1ezvb1#29	GO:0008201: heparin binding	0.006276	44,24,3
1dz4a_#264	GO:0004197: cysteine-type endopeptidase activity	0.006276	44,24,3
1booa_#254	GO:0008757: S-adenosylmethionine-dependent methyltransferase activity	0.006276	44,24,3
1gsa_2#208	GO:0005524: ATP binding	0.006278	20,243,6
1f6da_#200	GO:0005525: GTP binding	0.006281	7,49,2
1nfdb1#38	GO:0005525: GTP binding	0.006281	7,49,2
1h9ra2#238	GO:0005525: GTP binding	0.006281	7,49,2
1e8ga2#176	GO:0005525: GTP binding	0.006281	7,49,2
1jb3a_#64	GO:0008270: zinc ion binding	0.006286	302,108,21
1hw5a2#64	GO:0000049: tRNA binding	0.006287	167,13,4
1jj2e1#42	GO:0008235: metalloexopeptidase activity	0.006287	167,13,4
1qq5a_#7	GO:0008026: ATP-dependent helicase activity	0.006287	167,13,4
1qq5a_#7	GO:0008235: metalloexopeptidase activity	0.006287	167,13,4
1qe3a_#301	GO:0016616: oxidoreductase activity, acting on the CH-OH group of donors, NAD or NADP as acceptor	0.006291	35,59,4
1et9a2#138	GO:0016616: oxidoreductase activity, acting on the CH-OH group of donors, NAD or NADP as acceptor	0.006291	35,59,4
1lucb_#70	GO:0016616: oxidoreductase activity, acting on the CH-OH group of donors, NAD or NADP as acceptor	0.006291	35,59,4
1qj2c2#50	GO:0000287: magnesium ion binding	0.006295	48,128,7
1i50b_#216	GO:0000287: magnesium ion binding	0.006295	48,128,7
2dpma_#189	GO:0000287: magnesium ion binding	0.006295	48,128,7
2pgd_1#421	GO:0016705: oxidoreductase activity, acting on paired donors, with incorporation or reduction of molecular oxygen	0.006296	80,26,4
2pgd_1#421	GO:0004497: monooxygenase activity	0.006296	80,26,4
1erv__#80	GO:0005529: sugar binding	0.006297	442,39,13
1ez0a_#133	GO:0046983: protein dimerization activity	0.006306	125,17,4
3sil__#8	GO:0046983: protein dimerization activity	0.006306	125,17,4
1g31a_#15	GO:0016763: transferase activity, transferring pentosyl groups	0.006307	12,28,2
1g8fa2#258	GO:0016763: transferase activity, transferring pentosyl groups	0.006307	12,28,2
1qp8a1#168	GO:0016763: transferase activity, transferring pentosyl groups	0.006307	12,28,2
1ypta_#445	GO:0016763: transferase activity, transferring pentosyl groups	0.006307	12,28,2
1cwva2#675	GO:0003899: DNA-directed RNA polymerase activity	0.006307	28,12,2
1ab4__#89	GO:0016616: oxidoreductase activity, acting on the CH-OH group of donors, NAD or NADP as acceptor	0.006308	159,59,9
1ih7a1#57	GO:0003968: RNA-directed RNA polymerase activity	0.006309	77,14,3
1jlxa2#166	GO:0003968: RNA-directed RNA polymerase activity	0.006309	77,14,3
1pya.1#B236	GO:0015082: di-, tri-valent inorganic cation transporter activity	0.006309	77,14,3
1pya.1#B236	GO:0046915: transition metal ion transporter activity	0.006309	77,14,3
1beba_#119	GO:0046983: protein dimerization activity	0.006309	202,17,5
1h4ua1#404	GO:0016638: oxidoreductase activity, acting on the CH-NH2 group of donors	0.006309	202,17,5

1bmv2_#2064	GO:0008083: growth factor activity	0.006313	147,42,7
1bfta_#285	GO:0003700: transcription factor activity	0.006316	9,124,3
1iknd_#221	GO:0003700: transcription factor activity	0.006316	9,124,3
1f3mc_#369	GO:0004601: peroxidase activity	0.006316	100,21,4
1jf9a_#379	GO:0004556: alpha-amylase activity	0.006319	232,15,5
1ir3a_#1015	GO:0004295: trypsin activity	0.00632	43,48,4
1nat__#77	GO:0016836: hydro-lyase activity	0.006321	32,33,3
1h4vb2#307	GO:0000287: magnesium ion binding	0.006336	73,128,9
1mrj__#73	GO:0005529: sugar binding	0.006341	53,39,4
1apme_#101	GO:0005516: calmodulin binding	0.006343	14,24,2
2pola3#335	GO:0008201: heparin binding	0.006343	14,24,2
2aak__#28	GO:0004197: cysteine-type endopeptidase activity	0.006343	14,24,2
1phk__#221	GO:0005516: calmodulin binding	0.006343	14,24,2
1csn__#134	GO:0005516: calmodulin binding	0.006343	14,24,2
1ga6a_#198	GO:0005516: calmodulin binding	0.006343	14,24,2
7fabh1#71	GO:0008201: heparin binding	0.006343	14,24,2
1nls__#77	GO:0008201: heparin binding	0.006343	14,24,2
1b6cb_#337	GO:0005516: calmodulin binding	0.006343	14,24,2
1qqp3_#139	GO:0008757: S-adenosylmethionine-dependent methyltransferase activity	0.006343	14,24,2
1e4ea2#210	GO:0005516: calmodulin binding	0.006343	14,24,2
1bjaa_#26	GO:0016776: phosphotransferase activity, phosphate group as acceptor	0.006343	24,14,2
1dyoa_#153	GO:0015078: hydrogen ion transporter activity	0.006354	161,21,5
1icia_#175	GO:0003924: GTPase activity	0.006356	63,17,3
1d2ka1#164	GO:0016627: oxidoreductase activity, acting on the CH-CH group of donors	0.006356	63,17,3
1bywa_#30	GO:0003755: peptidyl-prolyl cis-trans isomerase activity	0.006358	331,11,5
1i39a_#49	GO:0004523: ribonuclease H activity	0.006362	21,16,2
1qtra_#107	GO:0004601: peroxidase activity	0.006362	16,21,2
2dri__#115	GO:0015078: hydrogen ion transporter activity	0.006362	16,21,2
1fkna_#99	GO:0004523: ribonuclease H activity	0.006362	21,16,2
8dfr__#165	GO:0000287: magnesium ion binding	0.006367	3,128,2
1qtwa_#213	GO:0000287: magnesium ion binding	0.006367	3,128,2
1jb0a_#17	GO:0000287: magnesium ion binding	0.006367	3,128,2
1hpwa_#51	GO:0000287: magnesium ion binding	0.006367	3,128,2
1poxa2#127	GO:0000287: magnesium ion binding	0.006367	3,128,2
1ryp1_#44	GO:0000287: magnesium ion binding	0.006367	3,128,2
1qf6a3#92	GO:0009036: type II site-specific deoxyribonuclease activity	0.006369	91,12,3
1jh2a_#16	GO:0003697: single-stranded DNA binding	0.006369	91,12,3
1qqga2#166	GO:0005516: calmodulin binding	0.00637	200,24,6
1qfma1#186	GO:0003887: DNA-directed DNA polymerase activity	0.00637	326,20,7
1bfg__#22	GO:0051082: unfolded protein binding	0.006379	61,34,4
1fgua2#360	GO:0005524: ATP binding	0.006381	61,243,12
1e32a2#345	GO:0016758: transferase activity, transferring hexosyl groups	0.006384	100,11,3
1dr9a1#39	GO:0004896: hematopoietin/interferon-class (D200-domain) cytokine receptor activity	0.006387	258,19,6
1p35a_#98	GO:0016846: carbon-sulfur lyase activity	0.006388	34,10,2
1e0la_#20	GO:0003916: DNA topoisomerase activity	0.006388	34,10,2
1kid__#251	GO:0051082: unfolded protein binding	0.006388	10,34,2
1qhla_#28	GO:0008800: beta-lactamase activity	0.006388	34,10,2
1fdr_1#26	GO:0016814: hydrolase activity, acting on carbon-nitrogen (but not peptide) bonds, in cyclic amidines	0.006388	34,10,2
1bev1_#238	GO:0016620: oxidoreductase activity, acting on the aldehyde or oxo group of donors, NAD or NADP as acceptor	0.006388	34,10,2
1af7_2#248	GO:0001584: rhodopsin-like receptor activity	0.006388	34,10,2
1qoua_#64	GO:0003684: damaged DNA binding	0.006388	34,10,2
1eaja_#59	GO:0019955: cytokine binding	0.006389	202,11,4
1bjt__#890	GO:0046983: protein dimerization activity	0.006393	292,17,6
1iow_1#37	GO:0005351: sugar porter activity	0.006396	309,21,7
3aky_1#194	GO:0016705: oxidoreductase activity, acting on paired donors, with incorporation or reduction of molecular oxygen	0.006402	13,26,2
3aky_1#194	GO:0004497: monooxygenase activity	0.006402	13,26,2
1atia2#210	GO:0016705: oxidoreductase activity, acting on paired donors, with incorporation or reduction of molecular oxygen	0.006402	13,26,2
1atia2#210	GO:0004497: monooxygenase activity	0.006402	13,26,2
1e9xa_#428	GO:0016705: oxidoreductase activity, acting on paired donors, with incorporation or reduction of molecular oxygen	0.006402	13,26,2
1e9xa_#428	GO:0004812: tRNA ligase activity	0.006402	13,26,2
1e9xa_#428	GO:0004497: monooxygenase activity	0.006402	13,26,2

1qaxa2#103	GO:0004812: tRNA ligase activity	0.006402	13,26,2
1ek0a_#151	GO:0008026: ATP-dependent helicase activity	0.006402	26,13,2
1bg6_2#24	GO:0016651: oxidoreductase activity, acting on NADH or NADPH	0.006402	26,13,2
1leha1#226	GO:0016861: intramolecular oxidoreductase activity, interconverting aldoses and ketoses	0.006402	26,13,2
1llc_2#271	GO:0004812: tRNA ligase activity	0.006402	13,26,2
1fvza_#142	GO:0005518: collagen binding	0.006402	26,13,2
1sgpe_#42	GO:0008026: ATP-dependent helicase activity	0.006402	26,13,2
1lst__#157	GO:0016651: oxidoreductase activity, acting on NADH or NADPH	0.006402	26,13,2
1ii7a_#275	GO:0008408: 3'-5' exonuclease activity	0.006402	26,13,2
1fvua_#72	GO:0004812: tRNA ligase activity	0.006402	13,26,2
1boub_#7	GO:0016836: hydro-lyase activity	0.006402	240,33,8
1oaa__#175	GO:0016854: racemase and epimerase activity	0.006402	26,13,2
1ovaa_#364	GO:0004812: tRNA ligase activity	0.006402	13,26,2
1abrb1#71	GO:0000049: tRNA binding	0.006402	26,13,2
1e7wa_#8	GO:0016620: oxidoreductase activity, acting on the aldehyde or oxo group of donors, NAD or NADP as acceptor	0.006403	111,10,3
2bbkh_#214	GO:0019838: growth factor binding	0.00641	38,9,2
1fa0a1#405	GO:0019838: growth factor binding	0.00641	38,9,2
1dqga_#81	GO:0019838: growth factor binding	0.00641	38,9,2
1ddja_#746	GO:0003887: DNA-directed DNA polymerase activity	0.00641	170,20,5
1sppb_#32	GO:0019838: growth factor binding	0.00641	38,9,2
2pia_1#54	GO:0019843: rRNA binding	0.006416	220,22,6
1iira_#242	GO:0016763: transferase activity, transferring pentosyl groups	0.006426	170,28,6
1kid__#330	GO:0004714: transmembrane receptor protein tyrosine kinase activity	0.006436	155,14,4
1ihua2#343	GO:0016811: hydrolase activity, acting on carbon-nitrogen (but not peptide) bonds, in linear amides	0.006437	233,15,5
1hwxa1#223	GO:0004180: carboxypeptidase activity	0.006437	233,15,5
1as4.1#A336	GO:0003964: RNA-directed DNA polymerase activity	0.006442	332,11,5
1fxla2#169	GO:0016638: oxidoreductase activity, acting on the CH-NH2 group of donors	0.006443	203,17,5
1eno__#83	GO:0016763: transferase activity, transferring pentosyl groups	0.006446	38,28,3
1qhda2#323	GO:0030151: molybdenum ion binding	0.006446	337,15,6
1ej8a_#189	GO:0019843: rRNA binding	0.006455	294,22,7
1g0sa_#74	GO:0015036: disulfide oxidoreductase activity	0.006455	294,22,7
1el0a_#42	GO:0015036: disulfide oxidoreductase activity	0.006455	294,22,7
1el0a_#42	GO:0019843: rRNA binding	0.006455	294,22,7
1fsu__#434	GO:0004190: aspartic-type endopeptidase activity	0.00646	356,23,8
1eu3a2#205	GO:0030145: manganese ion binding	0.006462	87,38,5
1fd9a_#162	GO:0030145: manganese ion binding	0.006462	87,38,5
1miob_#338	GO:0019201: nucleotide kinase activity	0.006463	84,13,3
1jcfa2#206	GO:0008408: 3'-5' exonuclease activity	0.006463	84,13,3
1ejba_#81	GO:0000049: tRNA binding	0.006463	84,13,3
1ejba_#81	GO:0016854: racemase and epimerase activity	0.006463	84,13,3
1imva_#69	GO:0005085: guanyl-nucleotide exchange factor activity	0.006463	84,13,3
1imva_#69	GO:0016651: oxidoreductase activity, acting on NADH or NADPH	0.006463	84,13,3
1qj8a_#64	GO:0004177: aminopeptidase activity	0.006463	84,13,3
1qj8a_#64	GO:0008235: metalloexopeptidase activity	0.006463	84,13,3
1qj8a_#64	GO:0008408: 3'-5' exonuclease activity	0.006463	84,13,3
1rlw__#43	GO:0003724: RNA helicase activity	0.006464	31,11,2
1qoua_#90	GO:0019955: cytokine binding	0.006464	31,11,2
1e3ja1#136	GO:0003964: RNA-directed DNA polymerase activity	0.006464	31,11,2
1g2oa_#227	GO:0004812: tRNA ligase activity	0.006466	41,26,3
1d0na3#305	GO:0016705: oxidoreductase activity, acting on paired donors, with incorporation or reduction of molecular oxygen	0.006466	311,26,8
1hrka_#205	GO:0004812: tRNA ligase activity	0.006466	41,26,3
1ft9a1#203	GO:0003700: transcription factor activity	0.006483	27,124,5
1io1a_#311	GO:0046983: protein dimerization activity	0.006488	126,17,4
1eqka_#47	GO:0008483: transaminase activity	0.006488	126,17,4
1br9__#86	GO:0019843: rRNA binding	0.006495	96,22,4
1bfd_2#146	GO:0016646: oxidoreductase activity, acting on the CH-NH group of donors, NAD or NADP as acceptor	0.006497	112,19,4
1ton__#47	GO:0004620: phospholipase activity	0.006497	112,19,4
1d6aa_#52	GO:0016799: hydrolase activity, hydrolyzing N-glycosyl compounds	0.006502	293,17,6
1d6aa_#52	GO:0008483: transaminase activity	0.006502	293,17,6
1ifc__#89	GO:0005509: calcium ion binding	0.006503	190,160,20
1i7qa_#186	GO:0046983: protein dimerization activity	0.006517	20,17,2
1psda3#376	GO:0016638: oxidoreductase activity, acting on the CH-NH2 group of donors	0.006517	20,17,2

1ecfa1#434	GO:0003924: GTPase activity	0.006517	20,17,2
1htjf_#368	GO:0005126: hematopoietin/interferon-class (D200-domain) cytokine receptor binding	0.006517	17,20,2
1gsa_2#208	GO:0016638: oxidoreductase activity, acting on the CH-NH2 group of donors	0.006517	20,17,2
1wba__#169	GO:0016799: hydrolase activity, hydrolyzing N-glycosyl compounds	0.006517	20,17,2
1ihka_#164	GO:0016799: hydrolase activity, hydrolyzing N-glycosyl compounds	0.006517	20,17,2
1deua_#151	GO:0051082: unfolded protein binding	0.006529	285,34,9
1b3qa2#623	GO:0003697: single-stranded DNA binding	0.006535	301,12,5
1dqua_#456	GO:0016616: oxidoreductase activity, acting on the CH-OH group of donors, NAD or NADP as acceptor	0.006538	6,59,2
1g93a_#108	GO:0016616: oxidoreductase activity, acting on the CH-OH group of donors, NAD or NADP as acceptor	0.006538	6,59,2
1dxy_1#170	GO:0016616: oxidoreductase activity, acting on the CH-OH group of donors, NAD or NADP as acceptor	0.006538	6,59,2
1nfp__#134	GO:0016616: oxidoreductase activity, acting on the CH-OH group of donors, NAD or NADP as acceptor	0.006538	6,59,2
1qrra_#99	GO:0016616: oxidoreductase activity, acting on the CH-OH group of donors, NAD or NADP as acceptor	0.006538	6,59,2
1axn__#42	GO:0005543: phospholipid binding	0.006542	78,14,3
1a4ya_#104	GO:0005524: ATP binding	0.006546	241,243,33
1dih_2#222	GO:0016763: transferase activity, transferring pentosyl groups	0.006546	352,28,9
1gox__#124	GO:0016776: phosphotransferase activity, phosphate group as acceptor	0.006548	366,14,6
1apme_#74	GO:0005507: copper ion binding	0.00655	124,38,6
1qtn.1#A270	GO:0009036: type II site-specific deoxyribonuclease activity	0.006554	439,12,6
1qtn.1#A270	GO:0016668: oxidoreductase activity, acting on sulfur group of donors, NAD or NADP as acceptor	0.006554	439,12,6
1f8v.1#A116	GO:0019955: cytokine binding	0.006565	101,11,3
1a8i__#688	GO:0016836: hydro-lyase activity	0.006565	241,33,8
1cpy__#93	GO:0000049: tRNA binding	0.006573	275,13,5
1cpy__#93	GO:0008080: N-acetyltransferase activity	0.006573	275,13,5
1a8d_1#87	GO:0046983: protein dimerization activity	0.00658	204,17,5
1div_1#141	GO:0004497: monooxygenase activity	0.006581	81,26,4
1dik_1#848	GO:0004497: monooxygenase activity	0.006581	81,26,4
1e8ua_#296	GO:0005509: calcium ion binding	0.006583	39,160,7
1aky_2#133	GO:0004714: transmembrane receptor protein tyrosine kinase activity	0.006586	156,14,4
1aky_2#133	GO:0016776: phosphotransferase activity, phosphate group as acceptor	0.006586	156,14,4
1p35a_#155	GO:0005507: copper ion binding	0.006591	55,38,4
1dofa_#15	GO:0003779: actin binding	0.006592	197,32,7
1c0pa1#1133	GO:0004620: phospholipase activity	0.006595	18,19,2
1am2__#161	GO:0019829: cation-transporting ATPase activity	0.006596	2,9,1
1ihua1#236	GO:0019829: cation-transporting ATPase activity	0.006596	2,9,1
1c5cl2#150	GO:0019838: growth factor binding	0.006596	2,9,1
1jzta_#157	GO:0019829: cation-transporting ATPase activity	0.006596	2,9,1
1ho8a_#207	GO:0019829: cation-transporting ATPase activity	0.006596	2,9,1
1dfaa3#318	GO:0019829: cation-transporting ATPase activity	0.006596	2,9,1
1c39a_#49	GO:0003713: transcription coactivator activity	0.006596	68,16,3
1fo5a_#60	GO:0004812: tRNA ligase activity	0.006597	246,26,7
1vmoa_#106	GO:0008083: growth factor activity	0.006602	187,42,8
1ek9a_#418	GO:0008810: cellulase activity	0.006606	1,18,1
1jj2q_#114	GO:0003887: DNA-directed DNA polymerase activity	0.006608	54,20,3
1trka2#429	GO:0016831: carboxy-lyase activity	0.006611	43,25,3
1a65a1#108	GO:0016638: oxidoreductase activity, acting on the CH-NH2 group of donors	0.006613	294,17,6
2naca1#162	GO:0005525: GTP binding	0.006618	22,49,3
1a8d_1#87	GO:0019955: cytokine binding	0.006619	204,11,4
1a8d_1#87	GO:0003724: RNA helicase activity	0.006619	204,11,4
2mpra_#190	GO:0005518: collagen binding	0.006624	400,13,6
1qhqa_#45	GO:0005529: sugar binding	0.006625	121,39,6
2fmr__#10	GO:0004896: hematopoietin/interferon-class (D200-domain) cytokine receptor activity	0.00663	57,19,3
3rpba_#557	GO:0004896: hematopoietin/interferon-class (D200-domain) cytokine receptor activity	0.00663	57,19,3
1tul__#102	GO:0004896: hematopoietin/interferon-class (D200-domain) cytokine receptor activity	0.00663	57,19,3
1hwxa1#223	GO:0005351: sugar porter activity	0.006636	233,21,6
1hr0w_#69	GO:0016638: oxidoreductase activity, acting on the CH-NH2 group of donors	0.006642	64,17,3
3sil__#284	GO:0046983: protein dimerization activity	0.006642	64,17,3
1f0xa1#523	GO:0008270: zinc ion binding	0.00665	87,108,9
1xgsa1#226	GO:0003700: transcription factor activity	0.006654	50,124,7
1a81a1#51	GO:0005179: hormone activity	0.006659	73,15,3
1hbza_#96	GO:0008810: cellulase activity	0.006662	370,18,7

1qqga1#95	GO:0016638: oxidoreductase activity, acting on the CH-NH2 group of donors	0.006673	127,17,4
1ejfa_#10	GO:0019843: rRNA binding	0.006677	155,22,5
1kid__#330	GO:0019843: rRNA binding	0.006677	155,22,5
1fiy__#769	GO:0000287: magnesium ion binding	0.006683	37,128,6
1hfoa_#57	GO:0000287: magnesium ion binding	0.006683	37,128,6
1dyna_#19	GO:0005524: ATP binding	0.006685	109,243,18
1gg4a3#64	GO:0008757: S-adenosylmethionine-dependent methyltransferase activity	0.006685	45,24,3
1h4ua1#486	GO:0004197: cysteine-type endopeptidase activity	0.006685	45,24,3
1e8ya1#648	GO:0005516: calmodulin binding	0.006685	45,24,3
1kp6a_#77	GO:0004190: aspartic-type endopeptidase activity	0.006688	47,23,3
1h4ua1#404	GO:0008757: S-adenosylmethionine-dependent methyltransferase activity	0.006688	202,24,6
1i50a_#856	GO:0005516: calmodulin binding	0.00669	269,24,7
2mev3_#108	GO:0003809: thrombin activity	0.006694	228,10,4
2mev3_#108	GO:0016620: oxidoreductase activity, acting on the aldehyde or oxo group of donors, NAD or NADP as acceptor	0.006694	228,10,4
1qgua_#435	GO:0030151: molybdenum ion binding	0.006695	23,15,2
1ea5a_#205	GO:0030151: molybdenum ion binding	0.006695	23,15,2
1lcl__#110	GO:0016811: hydrolase activity, acting on carbon-nitrogen (but not peptide) bonds, in linear amides	0.006695	23,15,2
1aw8.1#B59	GO:0016811: hydrolase activity, acting on carbon-nitrogen (but not peptide) bonds, in linear amides	0.006695	23,15,2
1d4ta_#80	GO:0004725: protein tyrosine phosphatase activity	0.006695	23,15,2
1evqa_#305	GO:0010181: FMN binding	0.006698	490,11,6
1qqsa_#124	GO:0005507: copper ion binding	0.006699	458,38,13
1e8ca1#83	GO:0008408: 3'-5' exonuclease activity	0.0067	170,13,4
1e8ca1#83	GO:0016854: racemase and epimerase activity	0.0067	170,13,4
1iira_#242	GO:0019201: nucleotide kinase activity	0.0067	170,13,4
1iira_#242	GO:0008235: metalloexopeptidase activity	0.0067	170,13,4
1ihua2#525	GO:0010181: FMN binding	0.0067	335,11,5
1gdha1#235	GO:0016646: oxidoreductase activity, acting on the CH-NH group of donors, NAD or NADP as acceptor	0.006705	113,19,4
1i7qa_#321	GO:0004190: aspartic-type endopeptidase activity	0.006706	148,23,5
1ds1a_#172	GO:0019843: rRNA binding	0.006707	222,22,6
1h9da_#91	GO:0019843: rRNA binding	0.006707	222,22,6
1a06__#85	GO:0003714: transcription corepressor activity	0.006709	44,8,2
1i76a_#138	GO:0008270: zinc ion binding	0.006711	103,108,10
1eu3a2#205	GO:0000287: magnesium ion binding	0.006715	87,128,10
1cm9a_#57	GO:0008083: growth factor activity	0.006721	50,42,4
1htp__#16	GO:0003697: single-stranded DNA binding	0.006725	303,12,5
1dt9a1#209	GO:0003916: DNA topoisomerase activity	0.006732	113,10,3
1gdha1#235	GO:0016620: oxidoreductase activity, acting on the aldehyde or oxo group of donors, NAD or NADP as acceptor	0.006732	113,10,3
1a0i_2#222	GO:0016668: oxidoreductase activity, acting on sulfur group of donors, NAD or NADP as acceptor	0.006736	186,12,4
1dpsa_#36	GO:0003755: peptidyl-prolyl cis-trans isomerase activity	0.006736	205,11,4
1aop_3#276	GO:0003887: DNA-directed DNA polymerase activity	0.006737	172,20,5
1hzxa_#139	GO:0019829: cation-transporting ATPase activity	0.006745	39,9,2
1a3k__#122	GO:0005529: sugar binding	0.006745	9,39,2
1c3ga2#272	GO:0019838: growth factor binding	0.006745	39,9,2
1g51a3#524	GO:0019955: cytokine binding	0.006749	102,11,3
1ax4a_#275	GO:0003755: peptidyl-prolyl cis-trans isomerase activity	0.006749	102,11,3
1qs1a2#437	GO:0004867: serine-type endopeptidase inhibitor activity	0.006755	452,47,15
1agi__#46	GO:0016702: oxidoreductase activity, acting on single donors with incorporation of molecular oxygen, incorporation of two atoms of oxygen	0.006758	29,12,2
1e05i_#422	GO:0016702: oxidoreductase activity, acting on single donors with incorporation of molecular oxygen, incorporation of two atoms of oxygen	0.006758	29,12,2
1cr5a1#45	GO:0003899: DNA-directed RNA polymerase activity	0.006758	29,12,2
1cgha_#130	GO:0003697: single-stranded DNA binding	0.006758	29,12,2
1djna1#86	GO:0016831: carboxy-lyase activity	0.006761	85,25,4
1dfoa_#237	GO:0005066: transmembrane receptor protein tyrosine kinase signaling protein activity	0.006762	35,10,2
1dfoa_#237	GO:0016846: carbon-sulfur lyase activity	0.006762	35,10,2
1gpma1#289	GO:0016814: hydrolase activity, acting on carbon-nitrogen (but not peptide) bonds, in cyclic amidines	0.006762	35,10,2
1hw5a2#64	GO:0004867: serine-type endopeptidase inhibitor activity	0.006762	167,47,8
1jj2e1#42	GO:0004867: serine-type endopeptidase inhibitor activity	0.006762	167,47,8
1f7da_#97	GO:0003809: thrombin activity	0.006762	35,10,2

1et9a2#138	GO:0008800: beta-lactamase activity	0.006762	35,10,2
1edha2#106	GO:0003682: chromatin binding	0.006762	35,10,2
1lucb_#70	GO:0004457: lactate dehydrogenase activity	0.006762	35,10,2
2viua_#252	GO:0005529: sugar binding	0.006764	343,39,11
1i50a_#856	GO:0005525: GTP binding	0.006764	269,49,11
1fo4a5#1002	GO:0003899: DNA-directed RNA polymerase activity	0.006769	93,12,3
1nsj__#178	GO:0016866: intramolecular transferase activity	0.006769	93,12,3
1qqsa_#124	GO:0004725: protein tyrosine phosphatase activity	0.00677	458,15,7
1smaa1#95	GO:0008083: growth factor activity	0.006772	272,42,10
1erv__#80	GO:0015405: P-P-bond-hydrolysis-driven transporter activity	0.006785	442,12,6
1ge8a2#134	GO:0004674: protein serine/threonine kinase activity	0.006788	229,42,9
1f0ia1#50	GO:0030151: molybdenum ion binding	0.006791	146,15,4
2pola2#218	GO:0030151: molybdenum ion binding	0.006791	146,15,4
1fjfc2#153	GO:0008757: S-adenosylmethionine-dependent methyltransferase activity	0.006797	420,24,9
1f2la_#40	GO:0004180: carboxypeptidase activity	0.0068	236,15,5
1ge8a2#134	GO:0050660: FAD binding	0.0068	229,10,4
1gpea1#273	GO:0005509: calcium ion binding	0.006802	81,160,11
1fc6a2#454	GO:0005525: GTP binding	0.006804	43,49,4
1elja_#8	GO:0005525: GTP binding	0.006804	43,49,4
1hxxa_#278	GO:0016251: general RNA polymerase II transcription factor activity	0.00681	255,14,5
1hxxa_#278	GO:0003968: RNA-directed RNA polymerase activity	0.00681	255,14,5
1h4vb2#282	GO:0000287: magnesium ion binding	0.006816	101,128,11
1qhoa3#488	GO:0005509: calcium ion binding	0.006819	30,160,6
1seta2#306	GO:0005509: calcium ion binding	0.006819	30,160,6
1hlwa_#82	GO:0008757: S-adenosylmethionine-dependent methyltransferase activity	0.006828	270,24,7
1htp__#16	GO:0005507: copper ion binding	0.006832	303,38,10
1ek6a_#33	GO:0005351: sugar porter activity	0.006841	52,21,3
1ib2a_#974	GO:0015078: hydrogen ion transporter activity	0.006841	52,21,3
1ib2a_#974	GO:0004601: peroxidase activity	0.006841	52,21,3
1hq0a_#867	GO:0004177: aminopeptidase activity	0.006842	171,13,4
1hq0a_#867	GO:0008080: N-acetyltransferase activity	0.006842	171,13,4
1hq0a_#867	GO:0008408: 3'-5' exonuclease activity	0.006842	171,13,4
1aym3_#131	GO:0005509: calcium ion binding	0.006844	412,160,36
1imva_#255	GO:0004725: protein tyrosine phosphatase activity	0.006845	589,15,8
1ev7a_#218	GO:0005516: calmodulin binding	0.006849	89,24,4
2bb2_1#57	GO:0005509: calcium ion binding	0.006849	104,160,13
1qhua1#47	GO:0005509: calcium ion binding	0.006849	104,160,13
1utea_#11	GO:0004295: trypsin activity	0.00685	130,48,7
1fds__#59	GO:0000287: magnesium ion binding	0.006852	61,128,8
1fds__#59	GO:0008810: cellulase activity	0.006856	61,18,3
1byra_#151	GO:0000287: magnesium ion binding	0.006861	17,128,4
1g8ka2#523	GO:0000287: magnesium ion binding	0.006861	17,128,4
2cuaa_#107	GO:0046983: protein dimerization activity	0.006861	128,17,4
1cs1a_#160	GO:0016866: intramolecular transferase activity	0.006867	187,12,4
1dvpa1#65	GO:0015405: P-P-bond-hydrolysis-driven transporter activity	0.006867	187,12,4
1hdoa_#189	GO:0005351: sugar porter activity	0.006872	164,21,5
1eu3a2#174	GO:0003968: RNA-directed RNA polymerase activity	0.006875	25,14,2
1hcc__#31	GO:0004714: transmembrane receptor protein tyrosine kinase activity	0.006875	25,14,2
1gen__#475	GO:0003968: RNA-directed RNA polymerase activity	0.006875	25,14,2
1qhda2#323	GO:0003755: peptidyl-prolyl cis-trans isomerase activity	0.006876	337,11,5
1ljra1#165	GO:0003779: actin binding	0.006877	34,32,3
1bupa2#199	GO:0051082: unfolded protein binding	0.006877	32,34,3
1i50a_#430	GO:0010181: FMN binding	0.00688	32,11,2
3pyp__#41	GO:0010181: FMN binding	0.00688	32,11,2
1f8na1#450	GO:0019955: cytokine binding	0.00688	32,11,2
1dqsa_#171	GO:0010181: FMN binding	0.00688	32,11,2
1pda_1#77	GO:0010181: FMN binding	0.00688	32,11,2
1nat__#77	GO:0010181: FMN binding	0.00688	32,11,2
1gcua1#36	GO:0016758: transferase activity, transferring hexosyl groups	0.00688	32,11,2
1eula_#518	GO:0019955: cytokine binding	0.00688	32,11,2
1kapp1#358	GO:0019955: cytokine binding	0.00688	32,11,2
1dfca4#1478	GO:0019955: cytokine binding	0.00688	32,11,2
1c9la2#12	GO:0005529: sugar binding	0.006882	295,39,10
1fepa_#340	GO:0051082: unfolded protein binding	0.006885	99,34,5
1eq9a_#40	GO:0005529: sugar binding	0.006885	86,39,5
1f42a2#123	GO:0015036: disulfide oxidoreductase activity	0.006886	378,22,8

1dik_3#240	GO:0004896: hematopoietin/interferon-class (D200-domain) cytokine receptor activity	0.00689	262,19,6
1fw9a_#152	GO:0004867: serine-type endopeptidase inhibitor activity	0.006893	133,47,7
1egaa1#97	GO:0005085: guanyl-nucleotide exchange factor activity	0.006896	27,13,2
1apj__#2062	GO:0008408: 3'-5' exonuclease activity	0.006896	27,13,2
1dl5a_#266	GO:0000049: tRNA binding	0.006896	27,13,2
1iq4a_#31	GO:0000049: tRNA binding	0.006896	27,13,2
1seia_#50	GO:0000049: tRNA binding	0.006896	27,13,2
1eoka_#127	GO:0016836: hydro-lyase activity	0.006896	33,33,3
1g5ha2#162	GO:0000049: tRNA binding	0.006896	27,13,2
6cel__#423	GO:0005085: guanyl-nucleotide exchange factor activity	0.006896	27,13,2
6cel__#423	GO:0005518: collagen binding	0.006896	27,13,2
8dfr__#115	GO:0008026: ATP-dependent helicase activity	0.006896	27,13,2
1jswa_#228	GO:0000049: tRNA binding	0.006896	27,13,2
1ceza_#227	GO:0004177: aminopeptidase activity	0.006896	27,13,2
1ceza_#227	GO:0008235: metalloexopeptidase activity	0.006896	27,13,2
1hdr__#149	GO:0016854: racemase and epimerase activity	0.006896	27,13,2
1f2ri_#61	GO:0000049: tRNA binding	0.006896	27,13,2
2cnd_1#101	GO:0008080: N-acetyltransferase activity	0.006896	27,13,2
1nsj__#21	GO:0016846: carbon-sulfur lyase activity	0.0069	114,10,3
1qs0a1#235	GO:0004457: lactate dehydrogenase activity	0.0069	114,10,3
1dv8a_#211	GO:0000049: tRNA binding	0.006903	86,13,3
1eq9a_#40	GO:0000049: tRNA binding	0.006903	86,13,3
1frpa_#286	GO:0000287: magnesium ion binding	0.006906	9,128,3
1qfta_#98	GO:0050660: FAD binding	0.006908	230,10,4
1hbza_#96	GO:0015082: di-, tri-valent inorganic cation transporter activity	0.006915	370,14,6
1hbza_#96	GO:0046915: transition metal ion transporter activity	0.006915	370,14,6
1bak__#583	GO:0004812: tRNA ligase activity	0.006917	131,26,5
1qgub_#197	GO:0005096: GTPase activator activity	0.006917	74,15,3
1bhga3#529	GO:0016811: hydrolase activity, acting on carbon-nitrogen (but not peptide) bonds, in linear amides	0.006917	74,15,3
1cnv__#181	GO:0030151: molybdenum ion binding	0.006917	74,15,3
1cnv__#181	GO:0016811: hydrolase activity, acting on carbon-nitrogen (but not peptide) bonds, in linear amides	0.006917	74,15,3
1elua_#95	GO:0005524: ATP binding	0.006917	77,243,14
1doza_#109	GO:0030151: molybdenum ion binding	0.006917	74,15,3
1cjxa1#126	GO:0004497: monooxygenase activity	0.006919	42,26,3
1g6sa_#64	GO:0003887: DNA-directed DNA polymerase activity	0.006919	108,20,4
1a9xa3#78	GO:0016866: intramolecular transferase activity	0.006919	305,12,5
1b35b_#97	GO:0005525: GTP binding	0.006922	309,49,12
1fwxa2#396	GO:0004556: alpha-amylase activity	0.006924	237,15,5
1by5a_#238	GO:0030151: molybdenum ion binding	0.006924	237,15,5
1f42a2#123	GO:0003682: chromatin binding	0.006927	378,10,5
1f42a2#123	GO:0008009: chemokine activity	0.006927	378,10,5
1f42a2#123	GO:0050660: FAD binding	0.006927	378,10,5
3chbd_#85	GO:0003809: thrombin activity	0.006927	378,10,5
3chbd_#85	GO:0016620: oxidoreductase activity, acting on the aldehyde or oxo group of donors, NAD or NADP as acceptor	0.006927	378,10,5
1bhga3#529	GO:0000287: magnesium ion binding	0.006933	74,128,9
1heta2#218	GO:0016763: transferase activity, transferring pentosyl groups	0.006935	39,28,3
1a06__#103	GO:0005529: sugar binding	0.006935	28,39,3
1elja_#64	GO:0003964: RNA-directed DNA polymerase activity	0.006936	103,11,3
1tif__#58	GO:0046983: protein dimerization activity	0.006937	65,17,3
1qo2a_#222	GO:0003924: GTPase activity	0.006937	65,17,3
1ig8a_#203	GO:0016638: oxidoreductase activity, acting on the CH-NH2 group of donors	0.006937	65,17,3
1xo1a2#159	GO:0003887: DNA-directed DNA polymerase activity	0.006956	55,20,3
1bmv2_#2064	GO:0004556: alpha-amylase activity	0.006958	147,15,4
1vola2#239	GO:0008270: zinc ion binding	0.006959	120,108,11
1dkia_#76P	GO:0004896: hematopoietin/interferon-class (D200-domain) cytokine receptor activity	0.006961	58,19,3
1dvpa1#65	GO:0004497: monooxygenase activity	0.006968	187,26,6
1lml__#190	GO:0004222: metalloendopeptidase activity	0.006972	1,19,1
1pbga_#274	GO:0004842: ubiquitin-protein ligase activity	0.006972	1,19,1
1ff9a2#183	GO:0016646: oxidoreductase activity, acting on the CH-NH group of donors, NAD or NADP as acceptor	0.006972	1,19,1
1dnv__#66	GO:0004620: phospholipase activity	0.006972	1,19,1
1f3va_#60	GO:0004842: ubiquitin-protein ligase activity	0.006972	1,19,1
1b12a_#205	GO:0004842: ubiquitin-protein ligase activity	0.006972	1,19,1

1ewfa2#338	GO:0004620: phospholipase activity	0.006972	1,19,1
1cs6a3#218	GO:0004896: hematopoietin/interferon-class (D200-domain) cytokine receptor activity	0.006972	1,19,1
8tlne_#54	GO:0004222: metalloendopeptidase activity	0.006972	1,19,1
1aoca_#70	GO:0004896: hematopoietin/interferon-class (D200-domain) cytokine receptor activity	0.006972	1,19,1
1h6ka1#178	GO:0004222: metalloendopeptidase activity	0.006972	1,19,1
1hc2_2#343	GO:0004620: phospholipase activity	0.006972	1,19,1
1bdfa2#171	GO:0015036: disulfide oxidoreductase activity	0.006974	16,22,2
1hx6a1#163	GO:0015036: disulfide oxidoreductase activity	0.006974	16,22,2
1ush_1#462	GO:0015036: disulfide oxidoreductase activity	0.006974	16,22,2
1mtyd_#390	GO:0003713: transcription coactivator activity	0.006974	22,16,2
1csn__#84	GO:0003724: RNA helicase activity	0.006975	207,11,4
1mpp__#151	GO:0003697: single-stranded DNA binding	0.006975	94,12,3
1mpp__#151	GO:0016668: oxidoreductase activity, acting on sulfur group of donors, NAD or NADP as acceptor	0.006975	94,12,3
1dypa_#143	GO:0016668: oxidoreductase activity, acting on sulfur group of donors, NAD or NADP as acceptor	0.006975	94,12,3
1a8d_1#87	GO:0005529: sugar binding	0.006981	204,39,8
1fjfj_#68	GO:0004674: protein serine/threonine kinase activity	0.006984	230,42,9
1fjfj_#68	GO:0008083: growth factor activity	0.006984	230,42,9
1aop_3#276	GO:0004177: aminopeptidase activity	0.006986	172,13,4
1aop_3#276	GO:0008235: metalloexopeptidase activity	0.006986	172,13,4
1flca1#294	GO:0015036: disulfide oxidoreductase activity	0.006988	98,22,4
2hlca_#226	GO:0019843: rRNA binding	0.006988	98,22,4
1fm2.1#B308	GO:0004295: trypsin activity	0.006991	200,48,9
1e8ga2#201	GO:0005507: copper ion binding	0.006993	304,38,10
1jd0a_#176	GO:0046983: protein dimerization activity	0.007002	207,17,5
1csn__#84	GO:0016638: oxidoreductase activity, acting on the CH-NH2 group of donors	0.007002	207,17,5
1lml__#535	GO:0019843: rRNA binding	0.007004	50,22,3
1by5a_#347	GO:0004620: phospholipase activity	0.00702	263,19,6
1smaa2#581	GO:0004714: transmembrane receptor protein tyrosine kinase activity	0.007022	80,14,3
1d7ba_#87	GO:0004295: trypsin activity	0.007035	99,48,6
2viua_#252	GO:0004725: protein tyrosine phosphatase activity	0.007039	343,15,6
1cpza_#6	GO:0019843: rRNA binding	0.007049	157,22,5
1a06__#85	GO:0016831: carboxy-lyase activity	0.007052	44,25,3
1fsia_#122	GO:0005525: GTP binding	0.007056	97,49,6
1h7wa2#636	GO:0000287: magnesium ion binding	0.007064	49,128,7
1mspa_#17	GO:0005524: ATP binding	0.007065	47,243,10
1b12a_#221	GO:0003916: DNA topoisomerase activity	0.007071	115,10,3
1dy9.1#C229	GO:0003682: chromatin binding	0.007071	115,10,3
1eqka_#47	GO:0005507: copper ion binding	0.007078	126,38,6
1f97a1#62	GO:0000049: tRNA binding	0.007079	707,13,8
1f97a1#62	GO:0003729: mRNA binding	0.007079	707,13,8
1i6vd_#713	GO:0019838: growth factor binding	0.007088	40,9,2
1dih_2#222	GO:0016646: oxidoreductase activity, acting on the CH-NH group of donors, NAD or NADP as acceptor	0.007092	352,19,7
1e8ca3#111	GO:0016854: racemase and epimerase activity	0.007103	280,13,5
1ds1a_#172	GO:0003713: transcription coactivator activity	0.007105	222,16,5
1aa6_2#285	GO:0016616: oxidoreductase activity, acting on the CH-OH group of donors, NAD or NADP as acceptor	0.007105	106,59,7
1j79a_#187	GO:0030145: manganese ion binding	0.007113	89,38,5
1ghpa_#65	GO:0005507: copper ion binding	0.007121	29,38,3
1i0ha2#127	GO:0030145: manganese ion binding	0.007121	29,38,3
1xyza_#753	GO:0030145: manganese ion binding	0.007121	29,38,3
1jf9a_#379	GO:0016846: carbon-sulfur lyase activity	0.007126	232,10,4
1bxoa_#307	GO:0019955: cytokine binding	0.007126	104,11,3
1f00i2#805	GO:0005524: ATP binding	0.007128	135,243,21
1f0xa1#523	GO:0008080: N-acetyltransferase activity	0.00713	87,13,3
1dfaa2#211	GO:0008026: ATP-dependent helicase activity	0.00713	87,13,3
1qrra_#208	GO:0019201: nucleotide kinase activity	0.00713	87,13,3
1qrra_#208	GO:0016651: oxidoreductase activity, acting on NADH or NADPH	0.00713	87,13,3
1qrra_#208	GO:0016854: racemase and epimerase activity	0.00713	87,13,3
1erv__#57	GO:0008026: ATP-dependent helicase activity	0.00713	87,13,3
1eucb2#188	GO:0008408: 3'-5' exonuclease activity	0.00713	87,13,3
1fp1d2#214	GO:0019201: nucleotide kinase activity	0.007132	173,13,4
1b12a_#221	GO:0004620: phospholipase activity	0.007135	115,19,4
1gsa_2#218	GO:0004295: trypsin activity	0.00714	131,48,7

1wdcb_#134	GO:0005066: transmembrane receptor protein tyrosine kinase signaling protein activity	0.007146	36,10,2
1pmc__#10	GO:0003684: damaged DNA binding	0.007146	36,10,2
1saca_#113	GO:0050660: FAD binding	0.007146	36,10,2
1i7qa_#278	GO:0003809: thrombin activity	0.007146	36,10,2
2hlca_#134	GO:0003809: thrombin activity	0.007146	36,10,2
1c0pa1#1147	GO:0050660: FAD binding	0.007146	36,10,2
1hle.1#B387	GO:0008800: beta-lactamase activity	0.007146	36,10,2
1dyna_#19	GO:0003887: DNA-directed DNA polymerase activity	0.007148	109,20,4
1dyna_#19	GO:0005126: hematopoietin/interferon-class (D200-domain) cytokine receptor binding	0.007148	109,20,4
1ft9a1#201	GO:0005126: hematopoietin/interferon-class (D200-domain) cytokine receptor binding	0.007148	109,20,4
1pma1_#79	GO:0004523: ribonuclease H activity	0.007152	70,16,3
1czan1#92	GO:0008483: transaminase activity	0.007155	631,17,9
2mpra_#190	GO:0016638: oxidoreductase activity, acting on the CH-NH2 group of donors	0.007158	400,17,7
1be9a_#357	GO:0016705: oxidoreductase activity, acting on paired donors, with incorporation or reduction of molecular oxygen	0.007175	83,26,4
1hw1a2#131	GO:0015078: hydrogen ion transporter activity	0.007176	17,21,2
1bdfa2#60	GO:0046983: protein dimerization activity	0.007176	21,17,2
1eyqa_#8	GO:0004601: peroxidase activity	0.007176	17,21,2
1cjxa2#307	GO:0015078: hydrogen ion transporter activity	0.007176	17,21,2
1cf9a2#554	GO:0016627: oxidoreductase activity, acting on the CH-CH group of donors	0.007176	21,17,2
1exma3#196	GO:0015078: hydrogen ion transporter activity	0.007176	17,21,2
1htjf_#368	GO:0015078: hydrogen ion transporter activity	0.007176	17,21,2
1amf__#168	GO:0005351: sugar porter activity	0.007176	17,21,2
1eur__#99	GO:0046983: protein dimerization activity	0.007176	21,17,2
1eur__#99	GO:0016638: oxidoreductase activity, acting on the CH-NH2 group of donors	0.007176	21,17,2
1tiid_#39	GO:0004180: carboxypeptidase activity	0.007177	239,15,5
1fkna_#96	GO:0030151: molybdenum ion binding	0.007181	75,15,3
1ihoa_#238	GO:0004180: carboxypeptidase activity	0.007181	75,15,3
1ewna_#109	GO:0005179: hormone activity	0.007181	75,15,3
2dpma_#237	GO:0016866: intramolecular transferase activity	0.007184	95,12,3
1e6pa2#46	GO:0016866: intramolecular transferase activity	0.007184	95,12,3
1dn2a1#261	GO:0004263: chymotrypsin activity	0.007185	425,41,13
1fwxa2#396	GO:0051082: unfolded protein binding	0.007188	237,34,8
2dkb__#114	GO:0016763: transferase activity, transferring pentosyl groups	0.007196	174,28,6
1b6e__#76	GO:0004263: chymotrypsin activity	0.007199	27,41,3
1epwa3#138	GO:0005126: hematopoietin/interferon-class (D200-domain) cytokine receptor binding	0.007206	424,20,8
1bmv2_#2102	GO:0005509: calcium ion binding	0.007207	14,160,4
2trcp_#201	GO:0005509: calcium ion binding	0.007207	14,160,4
1c4zd_#51	GO:0003968: RNA-directed RNA polymerase activity	0.007207	160,14,4
1dpja_#322	GO:0042802: protein self binding	0.007218	308,12,5
1fu6a_#50	GO:0016668: oxidoreductase activity, acting on sulfur group of donors, NAD or NADP as acceptor	0.007218	308,12,5
1h6la_#119	GO:0042802: protein self binding	0.007218	308,12,5
1kit_3#743	GO:0005529: sugar binding	0.00722	250,39,9
1bvsa3#12	GO:0003697: single-stranded DNA binding	0.007223	30,12,2
1b3ra2#60	GO:0016866: intramolecular transferase activity	0.007223	30,12,2
1bu6o1#237	GO:0000155: two-component sensor molecule activity	0.007223	30,12,2
2stv__#129	GO:0016702: oxidoreductase activity, acting on single donors with incorporation of molecular oxygen, incorporation of two atoms of oxygen	0.007223	30,12,2
1b34b_#56	GO:0003697: single-stranded DNA binding	0.007223	30,12,2
1tig__#148	GO:0000155: two-component sensor molecule activity	0.007223	30,12,2
1ehya_#85	GO:0016866: intramolecular transferase activity	0.007223	30,12,2
1hdma1#123	GO:0016702: oxidoreductase activity, acting on single donors with incorporation of molecular oxygen, incorporation of two atoms of oxygen	0.007223	30,12,2
1f97a2#199	GO:0008201: heparin binding	0.007225	144,24,5
1i50b_#334	GO:0005525: GTP binding	0.007229	69,49,5
1fa0a1#393	GO:0016831: carboxy-lyase activity	0.007232	261,25,7
1b35b_#97	GO:0003779: actin binding	0.007234	309,32,9
1jsg__#92	GO:0003697: single-stranded DNA binding	0.007236	607,12,7
3grs_1#24	GO:0016627: oxidoreductase activity, acting on the CH-CH group of donors	0.007239	66,17,3
3grs_1#24	GO:0016638: oxidoreductase activity, acting on the CH-NH2 group of donors	0.007239	66,17,3
1d8db_#78	GO:0016627: oxidoreductase activity, acting on the CH-CH group of donors	0.007239	66,17,3
1as4.1#A288	GO:0016638: oxidoreductase activity, acting on the CH-NH2 group of donors	0.007239	66,17,3
1e19a_#281	GO:0016831: carboxy-lyase activity	0.00724	138,25,5
1hdma1#115	GO:0051082: unfolded protein binding	0.007241	188,34,7
3mag__#134	GO:0004867: serine-type endopeptidase inhibitor activity	0.007243	72,47,5

1eova1#139	GO:0019843: rRNA binding	0.007244	99,22,4
1e25a_#262	GO:0016814: hydrolase activity, acting on carbon-nitrogen (but not peptide) bonds, in cyclic amidines	0.007245	116,10,3
1utea_#11	GO:0016799: hydrolase activity, hydrolyzing N-glycosyl compounds	0.007249	130,17,4
1fsu__#434	GO:0005509: calcium ion binding	0.007257	356,160,32
1qhua1#47	GO:0015078: hydrogen ion transporter activity	0.007258	104,21,4
2hgsa2#471	GO:0005516: calmodulin binding	0.00728	15,24,2
1jb7a1#174	GO:0030151: molybdenum ion binding	0.00728	24,15,2
1jb7a1#174	GO:0004556: alpha-amylase activity	0.00728	24,15,2
1fmta2#30	GO:0008757: S-adenosylmethionine-dependent methyltransferase activity	0.00728	15,24,2
1cjxa2#239	GO:0003743: translation initiation factor activity	0.00728	24,15,2
1h8va_#139	GO:0004197: cysteine-type endopeptidase activity	0.00728	15,24,2
1e05i_#83	GO:0000287: magnesium ion binding	0.00728	88,128,10
1dusa_#177	GO:0008757: S-adenosylmethionine-dependent methyltransferase activity	0.00728	15,24,2
1rypf_#50	GO:0004197: cysteine-type endopeptidase activity	0.00728	15,24,2
1cipa2#326	GO:0005096: GTPase activator activity	0.00728	24,15,2
1f60a2#373	GO:0003743: translation initiation factor activity	0.00728	24,15,2
1thg__#123	GO:0008757: S-adenosylmethionine-dependent methyltransferase activity	0.00728	15,24,2
1j7la_#86	GO:0004197: cysteine-type endopeptidase activity	0.00728	15,24,2
1dkia_#208	GO:0004197: cysteine-type endopeptidase activity	0.00728	15,24,2
1feua_#71	GO:0030151: molybdenum ion binding	0.00728	24,15,2
1bxka_#128	GO:0030151: molybdenum ion binding	0.00728	24,15,2
1hvba_#247	GO:0004180: carboxypeptidase activity	0.00728	24,15,2
2dkb__#114	GO:0008026: ATP-dependent helicase activity	0.00728	174,13,4
2dkb__#114	GO:0008408: 3'-5' exonuclease activity	0.00728	174,13,4
1rypd_#93	GO:0005516: calmodulin binding	0.00728	15,24,2
1d3va_#176	GO:0004556: alpha-amylase activity	0.00728	24,15,2
1dofa_#15	GO:0005525: GTP binding	0.007286	197,49,9
1g2912#10	GO:0003887: DNA-directed DNA polymerase activity	0.007299	18,20,2
1bn8a_#241	GO:0003887: DNA-directed DNA polymerase activity	0.007299	18,20,2
1eu3a1#68	GO:0003887: DNA-directed DNA polymerase activity	0.007299	18,20,2
1qdla_#211	GO:0004674: protein serine/threonine kinase activity	0.007299	320,42,11
1hrna_#320	GO:0003887: DNA-directed DNA polymerase activity	0.007299	18,20,2
1bio__#210	GO:0030151: molybdenum ion binding	0.0073	149,15,4
1feca1#28	GO:0005524: ATP binding	0.007301	62,243,12
1kwaa_#513	GO:0005524: ATP binding	0.007301	62,243,12
2rsla_#55	GO:0016616: oxidoreductase activity, acting on the CH-OH group of donors, NAD or NADP as acceptor	0.007302	19,59,3
1lucb_#84	GO:0016616: oxidoreductase activity, acting on the CH-OH group of donors, NAD or NADP as acceptor	0.007302	19,59,3
1fm2.1#B319	GO:0016616: oxidoreductase activity, acting on the CH-OH group of donors, NAD or NADP as acceptor	0.007302	19,59,3
1ed5a_#180	GO:0016616: oxidoreductase activity, acting on the CH-OH group of donors, NAD or NADP as acceptor	0.007302	19,59,3
1ax4a_#430	GO:0016646: oxidoreductase activity, acting on the CH-NH group of donors, NAD or NADP as acceptor	0.007302	59,19,3
1boub_#7	GO:0030151: molybdenum ion binding	0.007306	240,15,5
1cz4a1#76	GO:0016836: hydro-lyase activity	0.007307	11,33,2
1dpe__#402	GO:0003964: RNA-directed DNA polymerase activity	0.007307	33,11,2
1b6g__#63	GO:0010181: FMN binding	0.007307	33,11,2
1ioaa_#75	GO:0003724: RNA helicase activity	0.007307	33,11,2
1dn2a1#261	GO:0005126: hematopoietin/interferon-class (D200-domain) cytokine receptor binding	0.007312	425,20,8
1dcpa_#25	GO:0005126: hematopoietin/interferon-class (D200-domain) cytokine receptor binding	0.007316	56,20,3
1b35b_#97	GO:0016702: oxidoreductase activity, acting on single donors with incorporation of molecular oxygen, incorporation of two atoms of oxygen	0.00732	309,12,5
1c9oa_#49	GO:0004867: serine-type endopeptidase inhibitor activity	0.007326	102,47,6
1nhp_2#181	GO:0050660: FAD binding	0.007327	2,10,1
1fmk_3#386	GO:0005066: transmembrane receptor protein tyrosine kinase signaling protein activity	0.007327	2,10,1
1f86a_#53	GO:0008199: ferric iron binding	0.007327	2,10,1
1erxa_#139	GO:0003809: thrombin activity	0.007327	2,10,1
1qg3a2#1264	GO:0003809: thrombin activity	0.007327	2,10,1
1wdna_#98	GO:0005261: cation channel activity	0.007327	2,10,1
2naca1#329	GO:0016620: oxidoreductase activity, acting on the aldehyde or oxo group of donors, NAD or NADP as acceptor	0.007327	2,10,1
1bjt__#1058	GO:0003916: DNA topoisomerase activity	0.007327	2,10,1
1a0la_#176	GO:0003809: thrombin activity	0.007327	2,10,1

1bywa_#62	GO:0005261: cation channel activity	0.007327	2,10,1
7reqa1#156	GO:0003916: DNA topoisomerase activity	0.007327	2,10,1
1fsha_#47	GO:0016814: hydrolase activity, acting on carbon-nitrogen (but not peptide) bonds, in cyclic amidines	0.007327	2,10,1
1dmha_#309	GO:0008199: ferric iron binding	0.007327	2,10,1
1ltda1#186	GO:0004457: lactate dehydrogenase activity	0.007327	2,10,1
1am2__#161	GO:0003916: DNA topoisomerase activity	0.007327	2,10,1
1bnb__#34	GO:0016846: carbon-sulfur lyase activity	0.007327	2,10,1
1ig3a1#202	GO:0016814: hydrolase activity, acting on carbon-nitrogen (but not peptide) bonds, in cyclic amidines	0.007327	2,10,1
1cs1a_#69	GO:0016846: carbon-sulfur lyase activity	0.007327	2,10,1
1gtra2#313	GO:0016846: carbon-sulfur lyase activity	0.007327	2,10,1
1g57a_#122	GO:0003809: thrombin activity	0.007327	2,10,1
1f5na2#56	GO:0016846: carbon-sulfur lyase activity	0.007327	2,10,1
1a31a1#512	GO:0003916: DNA topoisomerase activity	0.007327	2,10,1
1a4ma_#243	GO:0016814: hydrolase activity, acting on carbon-nitrogen (but not peptide) bonds, in cyclic amidines	0.007327	2,10,1
1rlr_1#180	GO:0001584: rhodopsin-like receptor activity	0.007327	2,10,1
1pmaa_#131	GO:0003682: chromatin binding	0.007327	2,10,1
1ei1a2#182	GO:0003916: DNA topoisomerase activity	0.007327	2,10,1
1poxa3#460	GO:0016616: oxidoreductase activity, acting on the CH-OH group of donors, NAD or NADP as acceptor	0.007333	81,59,6
1h97a_#81	GO:0005126: hematopoietin/interferon-class (D200-domain) cytokine receptor binding	0.007339	1,20,1
1a8l_2#207	GO:0003887: DNA-directed DNA polymerase activity	0.007339	1,20,1
1plq_1#67	GO:0003887: DNA-directed DNA polymerase activity	0.007339	1,20,1
1bgxt2#51	GO:0003887: DNA-directed DNA polymerase activity	0.007339	1,20,1
1hxmb2#144	GO:0003887: DNA-directed DNA polymerase activity	0.007339	1,20,1
1repc1#136	GO:0003887: DNA-directed DNA polymerase activity	0.007339	1,20,1
1f8ra1#233	GO:0004896: hematopoietin/interferon-class (D200-domain) cytokine receptor activity	0.00734	19,19,2
1dy9.1#A82	GO:0004620: phospholipase activity	0.00734	19,19,2
1nsca_#91	GO:0004896: hematopoietin/interferon-class (D200-domain) cytokine receptor activity	0.00734	19,19,2
1b65a_#257	GO:0016758: transferase activity, transferring hexosyl groups	0.007344	210,11,4
1gpc__#184	GO:0004263: chymotrypsin activity	0.007351	83,41,5
2bpa1_#244	GO:0005509: calcium ion binding	0.007351	60,160,9
1ac6a_#34	GO:0005509: calcium ion binding	0.007351	60,160,9
1opy__#68	GO:0004674: protein serine/threonine kinase activity	0.007352	367,42,12
1b3qa2#623	GO:0019843: rRNA binding	0.007352	301,22,7
1bwda_#117	GO:0016646: oxidoreductase activity, acting on the CH-NH group of donors, NAD or NADP as acceptor	0.007357	116,19,4
1ej0a_#42	GO:0004222: metalloendopeptidase activity	0.007357	116,19,4
1dn2a1#261	GO:0005516: calmodulin binding	0.00736	425,24,9
1dn2a1#261	GO:0008201: heparin binding	0.00736	425,24,9
1e05i_#83	GO:0008408: 3'-5' exonuclease activity	0.007361	88,13,3
1xis__#214	GO:0016854: racemase and epimerase activity	0.007361	88,13,3
1xis__#214	GO:0016861: intramolecular oxidoreductase activity, interconverting aldoses and ketoses	0.007361	88,13,3
1im4a_#144	GO:0005525: GTP binding	0.007367	129,49,7
1el0a_#28	GO:0004714: transmembrane receptor protein tyrosine kinase activity	0.007368	161,14,4
1dyoa_#153	GO:0003968: RNA-directed RNA polymerase activity	0.007368	161,14,4
1fuia2#273	GO:0004812: tRNA ligase activity	0.007374	133,26,5
1gsa_2#218	GO:0000287: magnesium ion binding	0.007379	131,128,13
1jf9a_#379	GO:0008083: growth factor activity	0.007389	232,42,9
2hrva_#132	GO:0004812: tRNA ligase activity	0.007391	43,26,3
1cmxa_#207	GO:0009036: type II site-specific deoxyribonuclease activity	0.007397	96,12,3
1cmxa_#207	GO:0003899: DNA-directed RNA polymerase activity	0.007397	96,12,3
1nfdb1#21	GO:0042802: protein self binding	0.007397	96,12,3
1br9__#86	GO:0016702: oxidoreductase activity, acting on single donors with incorporation of molecular oxygen, incorporation of two atoms of oxygen	0.007397	96,12,3
3prn__#145	GO:0030145: manganese ion binding	0.007406	168,38,7
1ldtl_#34I	GO:0016763: transferase activity, transferring pentosyl groups	0.007407	13,28,2
1ps1a_#83	GO:0008080: N-acetyltransferase activity	0.007407	28,13,2
1ayoa_#55	GO:0016763: transferase activity, transferring pentosyl groups	0.007407	13,28,2
2mpra_#89	GO:0003729: mRNA binding	0.007407	28,13,2
1fvia2#134	GO:0005518: collagen binding	0.007407	28,13,2
3pah__#303	GO:0016651: oxidoreductase activity, acting on NADH or NADPH	0.007407	28,13,2
1e6ua_#266	GO:0000049: tRNA binding	0.007407	28,13,2
1e1aa_#261	GO:0016854: racemase and epimerase activity	0.007407	28,13,2

1aw1a_#169	GO:0008026: ATP-dependent helicase activity	0.007407	28,13,2
1dr9a1#39	GO:0005507: copper ion binding	0.007408	258,38,9
1exma2#353	GO:0008201: heparin binding	0.007409	91,24,4
2sli_2#326	GO:0005529: sugar binding	0.00741	251,39,9
1ayl__#237	GO:0005507: copper ion binding	0.007411	357,38,11
3grx__#56	GO:0005126: hematopoietin/interferon-class (D200-domain) cytokine receptor binding	0.007411	335,20,7
1tgoa1#65	GO:0000287: magnesium ion binding	0.007412	27,128,5
1bif_2#398	GO:0016846: carbon-sulfur lyase activity	0.007421	117,10,3
1gof_2#118	GO:0004812: tRNA ligase activity	0.007426	14,26,2
1llc_2#291	GO:0004812: tRNA ligase activity	0.007426	14,26,2
1pbe_2#183	GO:0004497: monooxygenase activity	0.007426	14,26,2
1ek0a_#151	GO:0005543: phospholipid binding	0.007426	26,14,2
1qq9a_#100	GO:0016705: oxidoreductase activity, acting on paired donors, with incorporation or reduction of molecular oxygen	0.007426	14,26,2
1qq9a_#100	GO:0004497: monooxygenase activity	0.007426	14,26,2
1bywa_#64	GO:0004812: tRNA ligase activity	0.007426	14,26,2
1sgpe_#42	GO:0003968: RNA-directed RNA polymerase activity	0.007426	26,14,2
1e30a_#107	GO:0004812: tRNA ligase activity	0.007426	14,26,2
3chbd_#96	GO:0004812: tRNA ligase activity	0.007426	14,26,2
1b3qa2#623	GO:0016799: hydrolase activity, hydrolyzing N-glycosyl compounds	0.007428	301,17,6
1b3qa2#623	GO:0016627: oxidoreductase activity, acting on the CH-CH group of donors	0.007428	301,17,6
1e42a1#711	GO:0004177: aminopeptidase activity	0.00743	175,13,4
1e42a1#711	GO:0000049: tRNA binding	0.00743	175,13,4
1e42a1#711	GO:0008235: metalloexopeptidase activity	0.00743	175,13,4
1fjfl_#67	GO:0000049: tRNA binding	0.00743	175,13,4
1evqa_#61	GO:0019843: rRNA binding	0.007436	159,22,5
1enfa1#92	GO:0004295: trypsin activity	0.007437	45,48,4
1c5y.1#B85	GO:0004295: trypsin activity	0.007437	45,48,4
1j8ra_#26	GO:0019838: growth factor binding	0.007439	41,9,2
1seta2#403	GO:0019838: growth factor binding	0.007439	41,9,2
1enfa2#162	GO:0019838: growth factor binding	0.007439	41,9,2
1ycsa_#162	GO:0046983: protein dimerization activity	0.007444	210,17,5
1i4ua_#154	GO:0016763: transferase activity, transferring pentosyl groups	0.007446	40,28,3
1a4ya_#104	GO:0000287: magnesium ion binding	0.007449	241,128,20
1uch__#50	GO:0004556: alpha-amylase activity	0.007452	76,15,3
1pbe_1#150	GO:0030151: molybdenum ion binding	0.007452	76,15,3
1e79d2#36	GO:0003887: DNA-directed DNA polymerase activity	0.007453	524,20,9
1a9xa6#760	GO:0030145: manganese ion binding	0.007456	90,38,5
1p35a_#49	GO:0005529: sugar binding	0.007458	124,39,6
1qlaa2#179	GO:0004867: serine-type endopeptidase inhibitor activity	0.007462	46,47,4
1d0na6#681	GO:0051082: unfolded protein binding	0.007467	143,34,6
2fmr__#10	GO:0005507: copper ion binding	0.007483	57,38,4
1jb3a_#64	GO:0019843: rRNA binding	0.007488	302,22,7
1b12a_#221	GO:0008083: growth factor activity	0.007495	115,42,6
1zfpe_#67	GO:0051082: unfolded protein binding	0.007501	33,34,3
1mai__#108	GO:0004190: aspartic-type endopeptidase activity	0.007505	152,23,5
3sil__#84	GO:0016831: carboxy-lyase activity	0.00751	45,25,3
1ojt_2#356	GO:0016831: carboxy-lyase activity	0.00751	45,25,3
1quba4#235	GO:0003964: RNA-directed DNA polymerase activity	0.007516	106,11,3
1j9qa1#154	GO:0004263: chymotrypsin activity	0.00752	196,41,8
1zpda1#213	GO:0016776: phosphotransferase activity, phosphate group as acceptor	0.007523	82,14,3
1thg__#223	GO:0016776: phosphotransferase activity, phosphate group as acceptor	0.007523	82,14,3
1d0na3#305	GO:0016668: oxidoreductase activity, acting on sulfur group of donors, NAD or NADP as acceptor	0.007526	311,12,5
1jd0a_#176	GO:0008201: heparin binding	0.007533	207,24,6
1csn__#84	GO:0005516: calmodulin binding	0.007533	207,24,6
1ds1a_#300	GO:0003684: damaged DNA binding	0.007539	37,10,2
1g0sa_#176	GO:0008199: ferric iron binding	0.007539	37,10,2
1e3ua_#164	GO:0008800: beta-lactamase activity	0.007539	37,10,2
1awx__#13	GO:0003809: thrombin activity	0.007539	37,10,2
1hfoa_#57	GO:0016814: hydrolase activity, acting on carbon-nitrogen (but not peptide) bonds, in cyclic amidines	0.007539	37,10,2
1tkia_#142	GO:0005066: transmembrane receptor protein tyrosine kinase signaling protein activity	0.007539	37,10,2
1hdma1#98	GO:0016620: oxidoreductase activity, acting on the aldehyde or oxo group of donors, NAD or NADP as acceptor	0.007539	37,10,2
1pii_2#326	GO:0016866: intramolecular transferase activity	0.007545	192,12,4

2bpa1_#45	GO:0016702: oxidoreductase activity, acting on single donors with incorporation of molecular oxygen, incorporation of two atoms of oxygen	0.007545	192,12,4
1d6ja_#95	GO:0016638: oxidoreductase activity, acting on the CH-NH2 group of donors	0.007549	67,17,3
1ejda_#213	GO:0008235: metalloexopeptidase activity	0.007549	284,13,5
1kp6a_#77	GO:0008201: heparin binding	0.007551	47,24,3
1fjsa_#158	GO:0008201: heparin binding	0.007551	47,24,3
1mspa_#17	GO:0008201: heparin binding	0.007551	47,24,3
1fsu__#434	GO:0004620: phospholipase activity	0.007552	356,19,7
1je5a_#10	GO:0000287: magnesium ion binding	0.007563	62,128,8
1ciy_3#243	GO:0000287: magnesium ion binding	0.007563	62,128,8
1hxmb1#117	GO:0051082: unfolded protein binding	0.007564	64,34,4
3sil__#284	GO:0051082: unfolded protein binding	0.007564	64,34,4
1g6oa_#116	GO:0005507: copper ion binding	0.00757	358,38,11
1mai__#108	GO:0004674: protein serine/threonine kinase activity	0.00757	152,42,7
1ihoa_#238	GO:0000287: magnesium ion binding	0.007572	75,128,9
1dfca1#1135	GO:0004263: chymotrypsin activity	0.007586	53,41,4
2mev1_#123	GO:0004177: aminopeptidase activity	0.007597	89,13,3
1j79a_#187	GO:0016861: intramolecular oxidoreductase activity, interconverting aldoses and ketoses	0.007597	89,13,3
1feca3#467	GO:0016651: oxidoreductase activity, acting on NADH or NADPH	0.007597	89,13,3
1qf9a_#117	GO:0005085: guanyl-nucleotide exchange factor activity	0.007597	89,13,3
1axca1#70	GO:0003684: damaged DNA binding	0.007599	118,10,3
1axca1#70	GO:0003916: DNA topoisomerase activity	0.007599	118,10,3
1axca1#70	GO:0016620: oxidoreductase activity, acting on the aldehyde or oxo group of donors, NAD or NADP as acceptor	0.007599	118,10,3
1jj2q_#114	GO:0015078: hydrogen ion transporter activity	0.007602	54,21,3
1thfd_#140	GO:0008810: cellulase activity	0.007606	124,18,4
1azsa_#397	GO:0004190: aspartic-type endopeptidase activity	0.007612	16,23,2
1fepa_#49	GO:0005524: ATP binding	0.007619	70,243,13
2cb5a_#312	GO:0003887: DNA-directed DNA polymerase activity	0.007623	111,20,4
1a6o__#45	GO:0019843: rRNA binding	0.007625	303,22,7
1c5y.1#B128	GO:0004867: serine-type endopeptidase inhibitor activity	0.007632	8,47,2
1imva_#33	GO:0004867: serine-type endopeptidase inhibitor activity	0.007632	8,47,2
1i50a_#469	GO:0016616: oxidoreductase activity, acting on the CH-OH group of donors, NAD or NADP as acceptor	0.007632	58,59,5
1lml__#216	GO:0005509: calcium ion binding	0.007635	22,160,5
1eno__#83	GO:0000287: magnesium ion binding	0.007636	38,128,6
1cpy__#346	GO:0000287: magnesium ion binding	0.007636	38,128,6
3sil__#98	GO:0019843: rRNA binding	0.007639	228,22,6
1eq9a_#32	GO:0016638: oxidoreductase activity, acting on the CH-NH2 group of donors	0.007651	132,17,4
1dhs__#99	GO:0016627: oxidoreductase activity, acting on the CH-CH group of donors	0.007651	132,17,4
1dhs__#99	GO:0008483: transaminase activity	0.007651	132,17,4
1bccb2#274	GO:0004222: metalloendopeptidase activity	0.007652	60,19,3
1i7wa_#517	GO:0004222: metalloendopeptidase activity	0.007652	60,19,3
1qnja_#46	GO:0004896: hematopoietin/interferon-class (D200-domain) cytokine receptor activity	0.007652	60,19,3
1g8fa3#419	GO:0030151: molybdenum ion binding	0.007653	151,15,4
1qhda2#323	GO:0003887: DNA-directed DNA polymerase activity	0.007659	337,20,7
1qhda2#323	GO:0005126: hematopoietin/interferon-class (D200-domain) cytokine receptor binding	0.007659	337,20,7
1deua_#151	GO:0000049: tRNA binding	0.007664	285,13,5
1deua_#151	GO:0003729: mRNA binding	0.007664	285,13,5
1dpja_#322	GO:0030145: manganese ion binding	0.007669	308,38,10
1fu6a_#50	GO:0030145: manganese ion binding	0.007669	308,38,10
1htp__#16	GO:0016638: oxidoreductase activity, acting on the CH-NH2 group of donors	0.007674	303,17,6
1jj2v_#118	GO:0004867: serine-type endopeptidase inhibitor activity	0.007678	103,47,6
1gci__#175	GO:0005525: GTP binding	0.007681	70,49,5
1fepa_#49	GO:0005525: GTP binding	0.007681	70,49,5
1aym3_#131	GO:0000049: tRNA binding	0.007687	412,13,6
1fw9a_#152	GO:0019838: growth factor binding	0.007689	133,9,3
1ldna1#94	GO:0016831: carboxy-lyase activity	0.007691	199,25,6
1fwxa2#396	GO:0016620: oxidoreductase activity, acting on the aldehyde or oxo group of donors, NAD or NADP as acceptor	0.007693	237,10,4
1by5a_#238	GO:0008800: beta-lactamase activity	0.007693	237,10,4
1f5aa1#441	GO:0003968: RNA-directed RNA polymerase activity	0.007698	163,14,4
1awcb_#111	GO:0015405: P-P-bond-hydrolysis-driven transporter activity	0.007702	31,12,2
1qqp1_#51	GO:0000155: two-component sensor molecule activity	0.007702	31,12,2
1qqp1_#51	GO:0016668: oxidoreductase activity, acting on sulfur group of donors, NAD or NADP as acceptor	0.007702	31,12,2

2hgsa2#471	GO:0005524: ATP binding	0.007704	15,243,5
1eyea_#131	GO:0005524: ATP binding	0.007704	15,243,5
1beba_#21	GO:0005524: ATP binding	0.007704	15,243,5
1ebfa1#39	GO:0005524: ATP binding	0.007704	15,243,5
1rypd_#93	GO:0005524: ATP binding	0.007704	15,243,5
1ei5a3#311	GO:0005524: ATP binding	0.007705	27,243,7
3prn__#192	GO:0005351: sugar porter activity	0.007706	1,21,1
1c75a_#48	GO:0004601: peroxidase activity	0.007706	1,21,1
1dfaa2#226	GO:0015078: hydrogen ion transporter activity	0.007706	1,21,1
1vns__#538	GO:0004601: peroxidase activity	0.007706	1,21,1
8abp__#240	GO:0005351: sugar porter activity	0.007706	1,21,1
1cf9a1#747	GO:0004601: peroxidase activity	0.007706	1,21,1
1doza_#47	GO:0004601: peroxidase activity	0.007706	1,21,1
1ew6a_#35	GO:0004601: peroxidase activity	0.007706	1,21,1
1bn7a_#286	GO:0004601: peroxidase activity	0.007706	1,21,1
1b6ra3#161	GO:0016616: oxidoreductase activity, acting on the CH-OH group of donors, NAD or NADP as acceptor	0.007711	164,59,9
1e0ta2#275	GO:0008081: phosphoric diester hydrolase activity	0.007715	107,11,3
1f97a2#199	GO:0051082: unfolded protein binding	0.007721	144,34,6
1dypa_#143	GO:0005524: ATP binding	0.007725	94,243,16
1jlxa2#166	GO:0004556: alpha-amylase activity	0.007728	77,15,3
1ihua2#521	GO:0005096: GTPase activator activity	0.007728	77,15,3
1gox__#246	GO:0016854: racemase and epimerase activity	0.007736	177,13,4
1b35b_#97	GO:0000287: magnesium ion binding	0.007738	309,128,24
1dmla1#134	GO:0004674: protein serine/threonine kinase activity	0.007745	82,42,5
1iica1#165	GO:0051082: unfolded protein binding	0.007747	11,34,2
2cpl__#83	GO:0051082: unfolded protein binding	0.007747	11,34,2
1qhla_#28	GO:0016758: transferase activity, transferring hexosyl groups	0.007747	34,11,2
1rypg_#159	GO:0019955: cytokine binding	0.007747	34,11,2
1rypg_#159	GO:0003724: RNA helicase activity	0.007747	34,11,2
1fi2a_#104	GO:0019955: cytokine binding	0.007747	34,11,2
3sil__#8	GO:0005529: sugar binding	0.007752	125,39,6
1fn9a_#271	GO:0004190: aspartic-type endopeptidase activity	0.007756	218,23,6
1loua_#79	GO:0015078: hydrogen ion transporter activity	0.007763	106,21,4
1loua_#79	GO:0005351: sugar porter activity	0.007763	106,21,4
1qdla_#211	GO:0015078: hydrogen ion transporter activity	0.007763	320,21,7
1qs0a1#119	GO:0009036: type II site-specific deoxyribonuclease activity	0.007772	454,12,6
1f8v.1#A116	GO:0019843: rRNA binding	0.007773	101,22,4
1ec7a1#216	GO:0005524: ATP binding	0.007774	78,243,14
1eo6a_#107	GO:0005524: ATP binding	0.007774	78,243,14
1quna1#113	GO:0008080: N-acetyltransferase activity	0.00778	286,13,5
1gpc__#184	GO:0004714: transmembrane receptor protein tyrosine kinase activity	0.007781	83,14,3
1opy__#68	GO:0004190: aspartic-type endopeptidase activity	0.007785	367,23,8
1ffya2#226	GO:0019838: growth factor binding	0.007797	42,9,2
1sfp__#66	GO:0008083: growth factor activity	0.007797	9,42,2
1hxxa_#39	GO:0019838: growth factor binding	0.007797	42,9,2
1fhga_#42	GO:0004674: protein serine/threonine kinase activity	0.007797	9,42,2
1fhga_#42	GO:0008083: growth factor activity	0.007797	9,42,2
1scfa_#118	GO:0008083: growth factor activity	0.007797	9,42,2
1f5qb2#149	GO:0004674: protein serine/threonine kinase activity	0.007797	9,42,2
1g3nc2#184	GO:0004674: protein serine/threonine kinase activity	0.007797	9,42,2
1a3k__#122	GO:0008083: growth factor activity	0.007797	9,42,2
1ctqa_#116	GO:0019829: cation-transporting ATPase activity	0.007797	42,9,2
1ev2e1#154	GO:0004674: protein serine/threonine kinase activity	0.007797	9,42,2
1ev2e1#154	GO:0008083: growth factor activity	0.007797	9,42,2
1svy__#245	GO:0004674: protein serine/threonine kinase activity	0.007797	9,42,2
1eur__#84	GO:0005524: ATP binding	0.0078	86,243,15
1dpta_#22	GO:0004812: tRNA ligase activity	0.007803	85,26,4
1ihua1#119	GO:0004812: tRNA ligase activity	0.007803	85,26,4
1c9oa_#49	GO:0051082: unfolded protein binding	0.007805	102,34,5
1qf6a3#92	GO:0030145: manganese ion binding	0.007809	91,38,5
1c9la2#61	GO:0008009: chemokine activity	0.00781	238,10,4
1c9la2#61	GO:0003916: DNA topoisomerase activity	0.00781	238,10,4
1hxxa_#173	GO:0005509: calcium ion binding	0.007814	260,160,25
1exh__#44	GO:0019843: rRNA binding	0.007815	52,22,3

1e2o__#346	GO:0016702: oxidoreductase activity, acting on single donors with incorporation of molecular oxygen, incorporation of two atoms of oxygen	0.007829	194,12,4
1e2o__#346	GO:0016668: oxidoreductase activity, acting on sulfur group of donors, NAD or NADP as acceptor	0.007829	194,12,4
1mai__#108	GO:0004556: alpha-amylase activity	0.007834	152,15,4
1mai__#108	GO:0003743: translation initiation factor activity	0.007834	152,15,4
1cewi_#14	GO:0016702: oxidoreductase activity, acting on single donors with incorporation of molecular oxygen, incorporation of two atoms of oxygen	0.007835	98,12,3
1i7oa1#119	GO:0005507: copper ion binding	0.007836	30,38,3
1cqka_#49	GO:0005507: copper ion binding	0.007836	30,38,3
1cmxa_#59	GO:0005507: copper ion binding	0.007836	30,38,3
1pysa_#221	GO:0000049: tRNA binding	0.007837	90,13,3
1pysa_#221	GO:0008408: 3'-5' exonuclease activity	0.007837	90,13,3
1dyoa_#153	GO:0015036: disulfide oxidoreductase activity	0.007838	161,22,5
3sil__#284	GO:0008810: cellulase activity	0.007839	64,18,3
1pwt__#8	GO:0042802: protein self binding	0.007844	314,12,5
1qfma2#576	GO:0010181: FMN binding	0.007856	214,11,4
1acc__#206	GO:0016638: oxidoreductase activity, acting on the CH-NH2 group of donors	0.007865	22,17,2
1lucb_#32	GO:0016627: oxidoreductase activity, acting on the CH-CH group of donors	0.007865	22,17,2
1pkp_1#139	GO:0019843: rRNA binding	0.007865	17,22,2
1trb_1#112	GO:0016638: oxidoreductase activity, acting on the CH-NH2 group of donors	0.007865	22,17,2
1i1b__#10	GO:0016799: hydrolase activity, hydrolyzing N-glycosyl compounds	0.007865	22,17,2
1c39a_#49	GO:0046983: protein dimerization activity	0.007867	68,17,3
1hdoa_#189	GO:0016776: phosphotransferase activity, phosphate group as acceptor	0.007867	164,14,4
1dyna_#42	GO:0004714: transmembrane receptor protein tyrosine kinase activity	0.007867	164,14,4
2foka2#180	GO:0005516: calmodulin binding	0.00788	147,24,5
1a06__#85	GO:0004812: tRNA ligase activity	0.007882	44,26,3
1a06__#85	GO:0004497: monooxygenase activity	0.007882	44,26,3
1booa_#254	GO:0004497: monooxygenase activity	0.007882	44,26,3
1poxa3#439	GO:0030151: molybdenum ion binding	0.007888	25,15,2
1poxa3#439	GO:0005096: GTPase activator activity	0.007888	25,15,2
1f3ba2#7	GO:0030151: molybdenum ion binding	0.007888	25,15,2
1gen__#475	GO:0004725: protein tyrosine phosphatase activity	0.007888	25,15,2
1feca1#122	GO:0004725: protein tyrosine phosphatase activity	0.007888	25,15,2
1nsj__#83	GO:0016831: carboxy-lyase activity	0.007888	15,25,2
1qqga2#166	GO:0003700: transcription factor activity	0.007889	200,124,17
1i76a_#138	GO:0000287: magnesium ion binding	0.007894	103,128,11
1dpga2#334	GO:0008080: N-acetyltransferase activity	0.007897	287,13,5
1pud__#44	GO:0000287: magnesium ion binding	0.0079	50,128,7
1in4a1#264	GO:0000287: magnesium ion binding	0.0079	50,128,7
1iba__#39	GO:0005516: calmodulin binding	0.007906	351,24,8
1qora2#233	GO:0008270: zinc ion binding	0.007918	11,108,3
1cz4a1#76	GO:0008270: zinc ion binding	0.007918	11,108,3
1i2oa_#34	GO:0004812: tRNA ligase activity	0.007918	192,26,6
1fmk_3#386	GO:0005524: ATP binding	0.007922	2,243,2
1fgka_#641	GO:0005524: ATP binding	0.007922	2,243,2
1hv8a1#109	GO:0005524: ATP binding	0.007922	2,243,2
1am2__#161	GO:0005524: ATP binding	0.007922	2,243,2
1pysb6#217	GO:0005524: ATP binding	0.007922	2,243,2
3tdt__#238	GO:0005524: ATP binding	0.007922	2,243,2
1f3ub_#28	GO:0005524: ATP binding	0.007922	2,243,2
1f2t.1#B880	GO:0005524: ATP binding	0.007922	2,243,2
1e2ka_#156	GO:0005524: ATP binding	0.007922	2,243,2
1qj4a_#144	GO:0005524: ATP binding	0.007922	2,243,2
1c4oa1#335	GO:0005524: ATP binding	0.007922	2,243,2
1cxya_#78	GO:0005524: ATP binding	0.007922	2,243,2
1g2912#224	GO:0005524: ATP binding	0.007922	2,243,2
1b6cb_#484	GO:0005524: ATP binding	0.007922	2,243,2
19hca_#50	GO:0005524: ATP binding	0.007922	2,243,2
1dfaa3#318	GO:0005524: ATP binding	0.007922	2,243,2
1ei1a2#182	GO:0005524: ATP binding	0.007922	2,243,2
1ghpa_#65	GO:0003729: mRNA binding	0.007934	29,13,2
1i0ha2#127	GO:0003729: mRNA binding	0.007934	29,13,2
3lada3#463	GO:0000049: tRNA binding	0.007934	29,13,2
1e0ta2#10	GO:0016854: racemase and epimerase activity	0.007934	29,13,2
1agi__#46	GO:0008080: N-acetyltransferase activity	0.007934	29,13,2

1ek6a_#269	GO:0016854: racemase and epimerase activity	0.007934	29,13,2
1qo7a_#109	GO:0019201: nucleotide kinase activity	0.007934	29,13,2
1befa_#51	GO:0008026: ATP-dependent helicase activity	0.007934	29,13,2
1e6pa2#274	GO:0000049: tRNA binding	0.007934	29,13,2
1cqqa_#163	GO:0016854: racemase and epimerase activity	0.007934	29,13,2
1ghpa_#245	GO:0004177: aminopeptidase activity	0.007934	29,13,2
1c7wa_#105	GO:0005518: collagen binding	0.007934	29,13,2
1trb_1#14	GO:0016651: oxidoreductase activity, acting on NADH or NADPH	0.007934	29,13,2
1xyza_#753	GO:0016651: oxidoreductase activity, acting on NADH or NADPH	0.007934	29,13,2
1xyza_#753	GO:0016854: racemase and epimerase activity	0.007934	29,13,2
1lam_2#439	GO:0030145: manganese ion binding	0.007943	10,38,2
1dl0a_#32	GO:0003809: thrombin activity	0.007943	38,10,2
1dl0a_#32	GO:0003684: damaged DNA binding	0.007943	38,10,2
1dl0a_#32	GO:0050660: FAD binding	0.007943	38,10,2
1eno__#83	GO:0008800: beta-lactamase activity	0.007943	38,10,2
1gega_#58	GO:0008199: ferric iron binding	0.007943	38,10,2
1gdha2#47	GO:0003682: chromatin binding	0.007943	38,10,2
1prea2#119	GO:0003684: damaged DNA binding	0.007943	38,10,2
1fa0a1#405	GO:0016620: oxidoreductase activity, acting on the aldehyde or oxo group of donors, NAD or NADP as acceptor	0.007943	38,10,2
3euga_#124	GO:0030145: manganese ion binding	0.007943	10,38,2
1jfra_#83	GO:0008199: ferric iron binding	0.007943	38,10,2
1jfra_#83	GO:0001584: rhodopsin-like receptor activity	0.007943	38,10,2
1icia_#9	GO:0016620: oxidoreductase activity, acting on the aldehyde or oxo group of donors, NAD or NADP as acceptor	0.007943	38,10,2
1oaca1#342	GO:0030145: manganese ion binding	0.007943	10,38,2
1qf6a1#606	GO:0008199: ferric iron binding	0.007943	38,10,2
1qf6a1#606	GO:0016620: oxidoreductase activity, acting on the aldehyde or oxo group of donors, NAD or NADP as acceptor	0.007943	38,10,2
1a6q__#16	GO:0004295: trypsin activity	0.007952	8,48,2
1fi2a_#109	GO:0004523: ribonuclease H activity	0.007958	228,16,5
1fkna_#18	GO:0005509: calcium ion binding	0.00796	130,160,15
1a81a2#182	GO:0005066: transmembrane receptor protein tyrosine kinase signaling protein activity	0.007964	120,10,3
1a81a2#182	GO:0003809: thrombin activity	0.007964	120,10,3
2napa2#292	GO:0016814: hydrolase activity, acting on carbon-nitrogen (but not peptide) bonds, in cyclic amidines	0.007964	120,10,3
1j75a_#166	GO:0003684: damaged DNA binding	0.007964	120,10,3
1j75a_#166	GO:0003916: DNA topoisomerase activity	0.007964	120,10,3
1qfta_#98	GO:0019843: rRNA binding	0.00797	230,22,6
1g25a_#22	GO:0000049: tRNA binding	0.007971	415,13,6
1g25a_#22	GO:0005518: collagen binding	0.007971	415,13,6
1czan1#92	GO:0004190: aspartic-type endopeptidase activity	0.007976	631,23,11
1nat__#50	GO:0016763: transferase activity, transferring pentosyl groups	0.007977	297,28,8
1nal1_#203	GO:0016831: carboxy-lyase activity	0.007985	46,25,3
1tif__#58	GO:0051082: unfolded protein binding	0.00799	65,34,4
1dfaa1#35	GO:0004714: transmembrane receptor protein tyrosine kinase activity	0.007996	27,14,2
1egaa1#97	GO:0005543: phospholipid binding	0.007996	27,14,2
1hr6a1#41	GO:0016251: general RNA polymerase II transcription factor activity	0.007996	27,14,2
1d6ja_#36	GO:0005543: phospholipid binding	0.007996	27,14,2
1fm2.1#B627	GO:0016251: general RNA polymerase II transcription factor activity	0.007996	27,14,2
1ceza_#227	GO:0016251: general RNA polymerase II transcription factor activity	0.007996	27,14,2
1fu6a_#35	GO:0004714: transmembrane receptor protein tyrosine kinase activity	0.007996	27,14,2
1fu6a_#35	GO:0005543: phospholipid binding	0.007996	27,14,2
1ir3a_#1139	GO:0004714: transmembrane receptor protein tyrosine kinase activity	0.007996	27,14,2
1e8ca1#83	GO:0015078: hydrogen ion transporter activity	0.007997	170,21,5
2occb1#209	GO:0008201: heparin binding	0.007999	93,24,4
1cf1a1#33	GO:0005525: GTP binding	0.008001	45,49,4
1enfa1#92	GO:0005525: GTP binding	0.008001	45,49,4
1bak__#583	GO:0005525: GTP binding	0.008004	131,49,7
1a65a1#108	GO:0051082: unfolded protein binding	0.008006	294,34,9
1jjya_#324	GO:0030151: molybdenum ion binding	0.00801	78,15,3
1axn__#42	GO:0005096: GTPase activator activity	0.00801	78,15,3
1hnja1#167	GO:0004896: hematopoietin/interferon-class (D200-domain) cytokine receptor activity	0.008013	61,19,3
1i9ga_#67	GO:0004896: hematopoietin/interferon-class (D200-domain) cytokine receptor activity	0.008013	61,19,3
1hyrc1#247	GO:0016646: oxidoreductase activity, acting on the CH-NH group of donors, NAD or NADP as acceptor	0.008013	61,19,3

1i5ga_#115	GO:0004674: protein serine/threonine kinase activity	0.008014	324,42,11
1f00i2#805	GO:0019838: growth factor binding	0.008016	135,9,3
1rypk_#12	GO:0015082: di-, tri-valent inorganic cation transporter activity	0.008028	265,14,5
1rypk_#12	GO:0004714: transmembrane receptor protein tyrosine kinase activity	0.008028	265,14,5
1rypk_#12	GO:0046915: transition metal ion transporter activity	0.008028	265,14,5
1hxxa_#278	GO:0016705: oxidoreductase activity, acting on paired donors, with incorporation or reduction of molecular oxygen	0.008029	255,26,7
1hxxa_#278	GO:0004812: tRNA ligase activity	0.008029	255,26,7
1a6da3#190	GO:0015078: hydrogen ion transporter activity	0.008036	18,21,2
1xwl_2#591	GO:0015078: hydrogen ion transporter activity	0.008036	18,21,2
1jj2a1#126	GO:0003713: transcription coactivator activity	0.008039	73,16,3
1avgi_#69	GO:0005126: hematopoietin/interferon-class (D200-domain) cytokine receptor binding	0.008043	340,20,7
1hxxa_#232	GO:0008757: S-adenosylmethionine-dependent methyltransferase activity	0.008044	352,24,8
1miob_#338	GO:0016776: phosphotransferase activity, phosphate group as acceptor	0.008045	84,14,3
1boub_#7	GO:0016620: oxidoreductase activity, acting on the aldehyde or oxo group of donors, NAD or NADP as acceptor	0.008047	240,10,4
1h6ka2#336	GO:0003700: transcription factor activity	0.008049	168,124,15
2pia_1#99	GO:0000049: tRNA binding	0.00805	179,13,4
3grs_3#435	GO:0000049: tRNA binding	0.00805	179,13,4
1dbga_#197	GO:0010181: FMN binding	0.008059	2,11,1
1vrta2#286	GO:0003964: RNA-directed DNA polymerase activity	0.008059	2,11,1
1fyhb2#131	GO:0019955: cytokine binding	0.008059	2,11,1
1el5a2#277	GO:0003755: peptidyl-prolyl cis-trans isomerase activity	0.008059	2,11,1
1alo_4#836	GO:0016758: transferase activity, transferring hexosyl groups	0.008059	2,11,1
1a8i__#342	GO:0016758: transferase activity, transferring hexosyl groups	0.008059	2,11,1
1i1ka_#7	GO:0003964: RNA-directed DNA polymerase activity	0.008059	2,11,1
1rcf__#29	GO:0010181: FMN binding	0.008059	2,11,1
1esfa2#224	GO:0010181: FMN binding	0.008059	2,11,1
1klo_3#148	GO:0019955: cytokine binding	0.008059	2,11,1
1ush_1#490	GO:0003724: RNA helicase activity	0.008059	2,11,1
1c5cl2#150	GO:0019955: cytokine binding	0.008059	2,11,1
1qdla_#103	GO:0004364: glutathione transferase activity	0.008059	2,11,1
1ih7a2#812	GO:0016758: transferase activity, transferring hexosyl groups	0.008059	2,11,1
1am7a_#94	GO:0016758: transferase activity, transferring hexosyl groups	0.008059	2,11,1
1f35a_#128	GO:0004364: glutathione transferase activity	0.008059	2,11,1
1g93a_#140	GO:0016758: transferase activity, transferring hexosyl groups	0.008059	2,11,1
1tuba2#296	GO:0008081: phosphoric diester hydrolase activity	0.008059	2,11,1
1b6cb_#484	GO:0003755: peptidyl-prolyl cis-trans isomerase activity	0.008059	2,11,1
1rlr_1#180	GO:0016758: transferase activity, transferring hexosyl groups	0.008059	2,11,1
1fm2.1#B199	GO:0004364: glutathione transferase activity	0.008059	2,11,1
1alla_#124	GO:0016758: transferase activity, transferring hexosyl groups	0.008059	2,11,1
1qfma2#482	GO:0003724: RNA helicase activity	0.008059	2,11,1
1d7ba_#87	GO:0003697: single-stranded DNA binding	0.00806	99,12,3
1d7ba_#87	GO:0016668: oxidoreductase activity, acting on sulfur group of donors, NAD or NADP as acceptor	0.00806	99,12,3
1mpp__#154	GO:0000155: two-component sensor molecule activity	0.00806	99,12,3
1eova1#139	GO:0016702: oxidoreductase activity, acting on single donors with incorporation of molecular oxygen, incorporation of two atoms of oxygen	0.00806	99,12,3
1qfma2#576	GO:0003924: GTPase activity	0.008063	214,17,5
1b8ba_#507	GO:0019843: rRNA binding	0.008073	1,22,1
1fxra_#11	GO:0015036: disulfide oxidoreductase activity	0.008073	1,22,1
1hyua3#58	GO:0015036: disulfide oxidoreductase activity	0.008073	1,22,1
1chua2#100	GO:0015036: disulfide oxidoreductase activity	0.008073	1,22,1
1jj2s_#26	GO:0019843: rRNA binding	0.008073	1,22,1
1erxa_#53	GO:0015036: disulfide oxidoreductase activity	0.008073	1,22,1
1fjfm_#45	GO:0019843: rRNA binding	0.008073	1,22,1
1h7wa5#996	GO:0015036: disulfide oxidoreductase activity	0.008073	1,22,1
1b25a1#248	GO:0019843: rRNA binding	0.008073	1,22,1
1hyua4#119	GO:0015036: disulfide oxidoreductase activity	0.008073	1,22,1
1ycsa_#162	GO:0008201: heparin binding	0.008077	210,24,6
1b65a_#257	GO:0008757: S-adenosylmethionine-dependent methyltransferase activity	0.008077	210,24,6
1qf6a3#92	GO:0004177: aminopeptidase activity	0.008082	91,13,3
1qf6a3#92	GO:0008408: 3'-5' exonuclease activity	0.008082	91,13,3
1nbaa_#227	GO:0016854: racemase and epimerase activity	0.008082	91,13,3
1exma2#353	GO:0000049: tRNA binding	0.008082	91,13,3
1exma2#353	GO:0003729: mRNA binding	0.008082	91,13,3

1e79d2#36	GO:0005507: copper ion binding	0.008085	524,38,14
2pia_1#54	GO:0004190: aspartic-type endopeptidase activity	0.008107	220,23,6
1nuka_#80	GO:0004190: aspartic-type endopeptidase activity	0.008107	220,23,6
1fo5a_#60	GO:0004725: protein tyrosine phosphatase activity	0.008116	246,15,5
1dr9a1#39	GO:0003779: actin binding	0.008116	258,32,8
1cyda_#106	GO:0016646: oxidoreductase activity, acting on the CH-NH group of donors, NAD or NADP as acceptor	0.008122	20,19,2
1fp5a2#478	GO:0005126: hematopoietin/interferon-class (D200-domain) cytokine receptor binding	0.008122	19,20,2
1dpja_#320	GO:0003887: DNA-directed DNA polymerase activity	0.008122	19,20,2
1dpja_#320	GO:0005126: hematopoietin/interferon-class (D200-domain) cytokine receptor binding	0.008122	19,20,2
1iqpa2#119	GO:0003887: DNA-directed DNA polymerase activity	0.008122	19,20,2
1dyna_#19	GO:0019955: cytokine binding	0.008124	109,11,3
1qqsa_#124	GO:0015405: P-P-bond-hydrolysis-driven transporter activity	0.008124	458,12,6
1qqsa_#124	GO:0042802: protein self binding	0.008124	458,12,6
1qqsa_#124	GO:0000155: two-component sensor molecule activity	0.008124	458,12,6
1lucb_#70	GO:0016836: hydro-lyase activity	0.008137	35,33,3
1bdfa2#60	GO:0005524: ATP binding	0.008146	21,243,6
2napa1#665	GO:0005524: ATP binding	0.008146	21,243,6
1ryph_#163	GO:0016846: carbon-sulfur lyase activity	0.008151	121,10,3
1qoua_#64	GO:0051082: unfolded protein binding	0.008157	34,34,3
1a4ya_#104	GO:0016620: oxidoreductase activity, acting on the aldehyde or oxo group of donors, NAD or NADP as acceptor	0.008168	241,10,4
1a4ya_#104	GO:0016814: hydrolase activity, acting on carbon-nitrogen (but not peptide) bonds, in cyclic amidines	0.008168	241,10,4
1c9la2#12	GO:0051082: unfolded protein binding	0.008185	295,34,9
1xgsa2#82	GO:0046983: protein dimerization activity	0.008185	307,17,6
1dik_3#240	GO:0030145: manganese ion binding	0.008186	262,38,9
1i50b_#334	GO:0046983: protein dimerization activity	0.008193	69,17,3
1neb__#14	GO:0003779: actin binding	0.008195	12,32,2
1i1b__#85	GO:0003779: actin binding	0.008195	12,32,2
1hcd__#115	GO:0003779: actin binding	0.008195	12,32,2
1hcd__#114	GO:0003779: actin binding	0.008195	12,32,2
1ryph_#52	GO:0015405: P-P-bond-hydrolysis-driven transporter activity	0.008195	32,12,2
1ezwa_#245	GO:0016668: oxidoreductase activity, acting on sulfur group of donors, NAD or NADP as acceptor	0.008195	32,12,2
1f8na1#450	GO:0016702: oxidoreductase activity, acting on single donors with incorporation of molecular oxygen, incorporation of two atoms of oxygen	0.008195	32,12,2
1hdr__#241	GO:0003697: single-stranded DNA binding	0.008195	32,12,2
1clc_1#299	GO:0016702: oxidoreductase activity, acting on single donors with incorporation of molecular oxygen, incorporation of two atoms of oxygen	0.008195	32,12,2
1hq0a_#867	GO:0005351: sugar porter activity	0.008196	171,21,5
1gc5a_#233	GO:0016758: transferase activity, transferring hexosyl groups	0.008198	35,11,2
1c8ua2#206	GO:0003964: RNA-directed DNA polymerase activity	0.008198	35,11,2
1cpt__#52	GO:0010181: FMN binding	0.008198	35,11,2
1dt6a_#77	GO:0010181: FMN binding	0.008198	35,11,2
1f7da_#97	GO:0003964: RNA-directed DNA polymerase activity	0.008198	35,11,2
1f7da_#97	GO:0010181: FMN binding	0.008198	35,11,2
1d2oa1#541	GO:0019955: cytokine binding	0.008198	35,11,2
1iba__#39	GO:0003755: peptidyl-prolyl cis-trans isomerase activity	0.008203	351,11,5
1f0xa1#278	GO:0005085: guanyl-nucleotide exchange factor activity	0.008211	180,13,4
1hxxa_#278	GO:0005529: sugar binding	0.008211	255,39,9
1dgsa1#523	GO:0005524: ATP binding	0.008242	48,243,10
1f5aa1#441	GO:0019843: rRNA binding	0.008254	163,22,5
2sqca2#97	GO:0008810: cellulase activity	0.008274	127,18,4
1e8ga2#268	GO:0008201: heparin binding	0.008276	16,24,2
1ygs__#435	GO:0004197: cysteine-type endopeptidase activity	0.008276	16,24,2
1auia_#105	GO:0005516: calmodulin binding	0.008276	16,24,2
1qr4a2#166	GO:0008201: heparin binding	0.008276	16,24,2
1aisb2#1274	GO:0008757: S-adenosylmethionine-dependent methyltransferase activity	0.008276	16,24,2
1cs6a4#363	GO:0005516: calmodulin binding	0.008276	16,24,2
1e5ka_#67	GO:0005525: GTP binding	0.008279	8,49,2
1dk0a_#115	GO:0005525: GTP binding	0.008279	8,49,2
1qo7a_#199	GO:0005525: GTP binding	0.008279	8,49,2
1dfma_#53	GO:0005525: GTP binding	0.008279	8,49,2
1rhs_1#10	GO:0005525: GTP binding	0.008279	8,49,2
1hv8a1#188	GO:0005525: GTP binding	0.008279	8,49,2

1dceb_#134	GO:0005525: GTP binding	0.008279	8,49,2
1b8aa2#368	GO:0046983: protein dimerization activity	0.008282	135,17,4
1f00i2#805	GO:0046983: protein dimerization activity	0.008282	135,17,4
1ev2e2#286	GO:0016702: oxidoreductase activity, acting on single donors with incorporation of molecular oxygen, incorporation of two atoms of oxygen	0.008288	100,12,3
1e5da2#12	GO:0003697: single-stranded DNA binding	0.008288	100,12,3
1e5da2#12	GO:0016668: oxidoreductase activity, acting on sulfur group of donors, NAD or NADP as acceptor	0.008288	100,12,3
1lcl__#128	GO:0008270: zinc ion binding	0.00829	21,108,4
1f75a_#239	GO:0008270: zinc ion binding	0.00829	21,108,4
1tuba1#239	GO:0016646: oxidoreductase activity, acting on the CH-NH group of donors, NAD or NADP as acceptor	0.008291	120,19,4
1jsg__#92	GO:0004725: protein tyrosine phosphatase activity	0.008297	607,15,8
1fbna_#144	GO:0005096: GTPase activator activity	0.008299	79,15,3
1fbna_#144	GO:0004556: alpha-amylase activity	0.008299	79,15,3
1d0na2#200	GO:0004725: protein tyrosine phosphatase activity	0.008299	79,15,3
1dkia_#185	GO:0004180: carboxypeptidase activity	0.008299	79,15,3
1amp__#205	GO:0004725: protein tyrosine phosphatase activity	0.008299	79,15,3
1f3ya_#6	GO:0008201: heparin binding	0.008305	94,24,4
1nsca_#236	GO:0005509: calcium ion binding	0.008305	143,160,16
1qora2#201	GO:0016836: hydro-lyase activity	0.008306	199,33,7
1a6o__#45	GO:0005529: sugar binding	0.008307	303,39,10
1ihua1#119	GO:0016776: phosphotransferase activity, phosphate group as acceptor	0.008314	85,14,3
1icia_#175	GO:0005524: ATP binding	0.008323	63,243,12
1jg8a_#172	GO:0008235: metalloexopeptidase activity	0.008331	92,13,3
1jg8a_#172	GO:0008408: 3'-5' exonuclease activity	0.008331	92,13,3
1ekma1#545	GO:0005529: sugar binding	0.008333	90,39,5
1ido__#173	GO:0004364: glutathione transferase activity	0.008333	110,11,3
1ido__#173	GO:0010181: FMN binding	0.008333	110,11,3
1i7qa_#321	GO:0000287: magnesium ion binding	0.008336	148,128,14
1fu6a_#50	GO:0019843: rRNA binding	0.008341	308,22,7
1h6la_#119	GO:0015036: disulfide oxidoreductase activity	0.008341	308,22,7
1ir3a_#1028	GO:0003779: actin binding	0.008344	70,32,4
1j71a_#176	GO:0005524: ATP binding	0.008346	34,243,8
1bev1_#238	GO:0005524: ATP binding	0.008346	34,243,8
1qoua_#64	GO:0005524: ATP binding	0.008346	34,243,8
1g8fa3#419	GO:0016836: hydro-lyase activity	0.008351	151,33,6
1e7wa_#36	GO:0019829: cation-transporting ATPase activity	0.008352	137,9,3
1e1aa_#285	GO:0019838: growth factor binding	0.008352	137,9,3
1e9xa_#343	GO:0005261: cation channel activity	0.008355	39,10,2
1ak5_1#376	GO:0004457: lactate dehydrogenase activity	0.008355	39,10,2
1jr3a2#166	GO:0003684: damaged DNA binding	0.008355	39,10,2
1b3ra1#223	GO:0016620: oxidoreductase activity, acting on the aldehyde or oxo group of donors, NAD or NADP as acceptor	0.008355	39,10,2
1euca1#64	GO:0004457: lactate dehydrogenase activity	0.008355	39,10,2
1i4fb1#70	GO:0003809: thrombin activity	0.008355	39,10,2
1c3ga2#272	GO:0008199: ferric iron binding	0.008355	39,10,2
1h6va1#23	GO:0016620: oxidoreductase activity, acting on the aldehyde or oxo group of donors, NAD or NADP as acceptor	0.008355	39,10,2
1je5a_#10	GO:0004620: phospholipase activity	0.008383	62,19,3
1fbl_1#398	GO:0016646: oxidoreductase activity, acting on the CH-NH group of donors, NAD or NADP as acceptor	0.008383	62,19,3
1by5a_#347	GO:0005507: copper ion binding	0.00839	263,38,9
1c7na_#231	GO:0005507: copper ion binding	0.00839	263,38,9
1fn9a_#271	GO:0003964: RNA-directed DNA polymerase activity	0.008391	218,11,4
1fgga_#134	GO:0004364: glutathione transferase activity	0.008391	218,11,4
1c5y.1#B85	GO:0004812: tRNA ligase activity	0.008392	45,26,3
1a8i__#824	GO:0004812: tRNA ligase activity	0.008392	45,26,3
1ejfa_#10	GO:0030151: molybdenum ion binding	0.008394	155,15,4
1kid__#330	GO:0004725: protein tyrosine phosphatase activity	0.008394	155,15,4
1aop_3#276	GO:0005507: copper ion binding	0.008406	172,38,7
1ejfa_#10	GO:0008083: growth factor activity	0.008408	155,42,7
1kid__#330	GO:0004674: protein serine/threonine kinase activity	0.008408	155,42,7
1aym1_#151	GO:0005524: ATP binding	0.008413	10,243,4
1eucb2#84	GO:0005524: ATP binding	0.008413	10,243,4
1cpt__#144	GO:0005524: ATP binding	0.008413	10,243,4

1dr9a1#39	GO:0003887: DNA-directed DNA polymerase activity	0.008415	258,20,6
1ifc__#89	GO:0005524: ATP binding	0.008425	190,243,27
1i7oa1#119	GO:0005529: sugar binding	0.008427	30,39,3
1f8v.1#A265	GO:0004190: aspartic-type endopeptidase activity	0.00844	1,23,1
1htr.1#P26	GO:0004190: aspartic-type endopeptidase activity	0.00844	1,23,1
4fiv__#38	GO:0004190: aspartic-type endopeptidase activity	0.00844	1,23,1
1g0sa_#74	GO:0004190: aspartic-type endopeptidase activity	0.008442	294,23,7
1psda3#376	GO:0016616: oxidoreductase activity, acting on the CH-OH group of donors, NAD or NADP as acceptor	0.00846	20,59,3
1azo__#144	GO:0005524: ATP binding	0.00846	41,243,9
1im4a_#41	GO:0005126: hematopoietin/interferon-class (D200-domain) cytokine receptor binding	0.00846	59,20,3
1ig8a_#215	GO:0003887: DNA-directed DNA polymerase activity	0.00846	59,20,3
1imva_#278	GO:0005524: ATP binding	0.00846	41,243,9
1hrka_#205	GO:0005524: ATP binding	0.00846	41,243,9
1g8kb_#11	GO:0005126: hematopoietin/interferon-class (D200-domain) cytokine receptor binding	0.00846	59,20,3
1fjfc2#153	GO:0000049: tRNA binding	0.008463	420,13,6
1fjfc2#153	GO:0008235: metalloexopeptidase activity	0.008463	420,13,6
1dfaa2#211	GO:0016705: oxidoreductase activity, acting on paired donors, with incorporation or reduction of molecular oxygen	0.008468	87,26,4
1b6ra3#161	GO:0019843: rRNA binding	0.008469	164,22,5
1dyna_#42	GO:0019843: rRNA binding	0.008469	164,22,5
1ez0a_#150	GO:0004177: aminopeptidase activity	0.008478	30,13,2
1sek__#179	GO:0000049: tRNA binding	0.008478	30,13,2
1qlaa2#35	GO:0019201: nucleotide kinase activity	0.008478	30,13,2
1e31a_#77	GO:0016831: carboxy-lyase activity	0.008478	47,25,3
1bu7a_#278	GO:0016651: oxidoreductase activity, acting on NADH or NADPH	0.008478	30,13,2
1ak0__#115	GO:0016861: intramolecular oxidoreductase activity, interconverting aldoses and ketoses	0.008478	30,13,2
1d8ca_#442	GO:0008235: metalloexopeptidase activity	0.008478	30,13,2
2stv__#129	GO:0000049: tRNA binding	0.008478	30,13,2
1aym3_#131	GO:0015078: hydrogen ion transporter activity	0.008478	412,21,8
1bag_2#207	GO:0008080: N-acetyltransferase activity	0.008478	30,13,2
1dbga_#38	GO:0019201: nucleotide kinase activity	0.008478	30,13,2
1dbga_#38	GO:0008408: 3'-5' exonuclease activity	0.008478	30,13,2
1ehya_#85	GO:0016854: racemase and epimerase activity	0.008478	30,13,2
1h6va1#116	GO:0008408: 3'-5' exonuclease activity	0.008478	30,13,2
1e69a_#40	GO:0019201: nucleotide kinase activity	0.008478	30,13,2
1e69a_#40	GO:0008026: ATP-dependent helicase activity	0.008478	30,13,2
1mil__#31	GO:0000287: magnesium ion binding	0.008481	104,128,11
1jeyb_#231	GO:0005525: GTP binding	0.008482	24,49,3
1a65a3#409	GO:0004197: cysteine-type endopeptidase activity	0.008482	49,24,3
1vcbb_#19	GO:0005525: GTP binding	0.008482	24,49,3
1mrp__#170	GO:0005525: GTP binding	0.008482	24,49,3
1dbta_#89	GO:0005525: GTP binding	0.008482	24,49,3
1d3va_#176	GO:0005525: GTP binding	0.008482	24,49,3
1hwxa1#223	GO:0015036: disulfide oxidoreductase activity	0.008486	233,22,6
1iow_1#37	GO:0015036: disulfide oxidoreductase activity	0.00849	309,22,7
1b35b_#97	GO:0019843: rRNA binding	0.00849	309,22,7
2bpa1_#45	GO:0004896: hematopoietin/interferon-class (D200-domain) cytokine receptor activity	0.00849	192,19,5
1g2912#10	GO:0000287: magnesium ion binding	0.008505	18,128,4
1g51a1#47	GO:0000287: magnesium ion binding	0.008505	18,128,4
1qdla_#211	GO:0000155: two-component sensor molecule activity	0.008509	320,12,5
1iiba_#79	GO:0004812: tRNA ligase activity	0.008518	15,26,2
1ytba2#217	GO:0004725: protein tyrosine phosphatase activity	0.008518	26,15,2
1a0ca_#273	GO:0004812: tRNA ligase activity	0.008518	15,26,2
1fvua_#120	GO:0004812: tRNA ligase activity	0.008518	15,26,2
1a7ca_#276	GO:0004812: tRNA ligase activity	0.008518	15,26,2
1b0ua_#174	GO:0030151: molybdenum ion binding	0.008518	26,15,2
1qqra_#185	GO:0003743: translation initiation factor activity	0.008518	26,15,2
1c4zd_#22	GO:0046983: protein dimerization activity	0.008527	70,17,3
1cqka_#52	GO:0046983: protein dimerization activity	0.008527	70,17,3
1gci__#175	GO:0003924: GTPase activity	0.008527	70,17,3
1cgha_#227	GO:0016638: oxidoreductase activity, acting on the CH-NH2 group of donors	0.008527	70,17,3
1ir3a_#1028	GO:0046983: protein dimerization activity	0.008527	70,17,3
1ct9a1#401	GO:0016763: transferase activity, transferring pentosyl groups	0.008534	42,28,3
1f00i1#749	GO:0008083: growth factor activity	0.008534	28,42,3
1dl5a_#283	GO:0004674: protein serine/threonine kinase activity	0.008534	28,42,3

1cwva2#675	GO:0004674: protein serine/threonine kinase activity	0.008534	28,42,3
1i7oa1#63	GO:0019838: growth factor binding	0.008537	44,9,2
1fvia2#25	GO:0019838: growth factor binding	0.008537	44,9,2
1ezvb1#29	GO:0019838: growth factor binding	0.008537	44,9,2
1fc3a_#172	GO:0008026: ATP-dependent helicase activity	0.008538	182,13,4
1xgsa2#186	GO:0003729: mRNA binding	0.008538	182,13,4
1e6ca_#91	GO:0016861: intramolecular oxidoreductase activity, interconverting aldoses and ketoses	0.008538	182,13,4
1ddwa_#77	GO:0004620: phospholipase activity	0.00854	364,19,7
1e6pa2#46	GO:0005509: calcium ion binding	0.008543	95,160,12
1ho1a_#211	GO:0008081: phosphoric diester hydrolase activity	0.008545	111,11,3
3prn__#145	GO:0004714: transmembrane receptor protein tyrosine kinase activity	0.008566	168,14,4
1ldna1#94	GO:0016866: intramolecular transferase activity	0.008569	199,12,4
1ekbb_#85	GO:0008201: heparin binding	0.008575	150,24,5
1f2la_#40	GO:0005524: ATP binding	0.008576	236,243,32
1bu6o1#45	GO:0016638: oxidoreductase activity, acting on the CH-NH2 group of donors	0.008583	23,17,2
2mpra_#222	GO:0016638: oxidoreductase activity, acting on the CH-NH2 group of donors	0.008583	23,17,2
1fs7a_#235	GO:0016799: hydrolase activity, hydrolyzing N-glycosyl compounds	0.008583	23,17,2
1aw8.1#B59	GO:0046983: protein dimerization activity	0.008583	23,17,2
1crua_#29	GO:0016638: oxidoreductase activity, acting on the CH-NH2 group of donors	0.008583	23,17,2
1d4ta_#80	GO:0008200: ion channel inhibitor activity	0.008583	23,17,2
1d4ta_#80	GO:0046983: protein dimerization activity	0.008583	23,17,2
1thea_#79	GO:0016861: intramolecular oxidoreductase activity, interconverting aldoses and ketoses	0.008585	93,13,3
1a6ca2#312	GO:0000049: tRNA binding	0.008585	93,13,3
1fo4a5#1002	GO:0000049: tRNA binding	0.008585	93,13,3
1fo4a5#1002	GO:0003729: mRNA binding	0.008585	93,13,3
1h8ma_#70	GO:0016763: transferase activity, transferring pentosyl groups	0.008586	14,28,2
1jc4a_#12	GO:0016251: general RNA polymerase II transcription factor activity	0.008586	28,14,2
2mpra_#89	GO:0004714: transmembrane receptor protein tyrosine kinase activity	0.008586	28,14,2
1brwa2#100	GO:0016763: transferase activity, transferring pentosyl groups	0.008586	14,28,2
1i7da_#458	GO:0016763: transferase activity, transferring pentosyl groups	0.008586	14,28,2
1cwva2#675	GO:0004714: transmembrane receptor protein tyrosine kinase activity	0.008586	28,14,2
2pgd_1#421	GO:0005096: GTPase activator activity	0.008594	80,15,3
1qf6a3#92	GO:0016831: carboxy-lyase activity	0.008599	91,25,4
1as4.1#A336	GO:0004523: ribonuclease H activity	0.008606	332,16,6
1cb8a2#613	GO:0004812: tRNA ligase activity	0.008606	138,26,5
1fsu__#434	GO:0005516: calmodulin binding	0.008613	356,24,8
1bxoa_#307	GO:0019843: rRNA binding	0.008615	104,22,4
1exma3#99	GO:0016763: transferase activity, transferring pentosyl groups	0.008616	81,28,4
1io1a_#233	GO:0008201: heparin binding	0.008619	95,24,4
1csn__#84	GO:0003779: actin binding	0.00862	207,32,7
1hdoa_#189	GO:0000287: magnesium ion binding	0.008645	164,128,15
1qs0b2#269	GO:0005525: GTP binding	0.008649	46,49,4
1doka_#52	GO:0005509: calcium ion binding	0.008657	51,160,8
1pmc__#10	GO:0003724: RNA helicase activity	0.008661	36,11,2
2mpra_#267	GO:0003724: RNA helicase activity	0.008661	36,11,2
1jj2j_#88	GO:0003724: RNA helicase activity	0.008661	36,11,2
1pysb5#672	GO:0019955: cytokine binding	0.008661	36,11,2
1dfoa_#344	GO:0019955: cytokine binding	0.008661	36,11,2
1bd8__#82	GO:0003713: transcription coactivator activity	0.008666	75,16,3
2pia_1#54	GO:0003724: RNA helicase activity	0.008668	220,11,4
1ido__#173	GO:0016616: oxidoreductase activity, acting on the CH-OH group of donors, NAD or NADP as acceptor	0.008672	110,59,7
2cuaa_#83	GO:0008270: zinc ion binding	0.008673	46,108,6
1a7ca_#370	GO:0004295: trypsin activity	0.008677	47,48,4
1gtra1#468	GO:0019843: rRNA binding	0.00868	54,22,3
1f0xa1#523	GO:0005524: ATP binding	0.008689	87,243,15
1e6ua_#266	GO:0000287: magnesium ion binding	0.00869	28,128,5
1kit_3#743	GO:0004556: alpha-amylase activity	0.00869	250,15,5
1bccb2#322	GO:0000287: magnesium ion binding	0.00869	28,128,5
1e0ta2#275	GO:0005509: calcium ion binding	0.008693	107,160,13
1mpp__#100	GO:0003697: single-stranded DNA binding	0.008703	33,12,2
1hnja1#8	GO:0016836: hydro-lyase activity	0.008703	12,33,2
2foka3#314	GO:0016866: intramolecular transferase activity	0.008703	33,12,2
1d8wa_#325	GO:0016836: hydro-lyase activity	0.008703	12,33,2
1chua2#21	GO:0016836: hydro-lyase activity	0.008703	12,33,2
1ejda_#94	GO:0016836: hydro-lyase activity	0.008703	12,33,2

1hg3a_#43	GO:0009036: type II site-specific deoxyribonuclease activity	0.008703	33,12,2
1rmg__#279	GO:0016836: hydro-lyase activity	0.008703	12,33,2
1d8ca_#218	GO:0003899: DNA-directed RNA polymerase activity	0.008703	33,12,2
1gks__#70	GO:0003899: DNA-directed RNA polymerase activity	0.008703	33,12,2
1brt__#116	GO:0016836: hydro-lyase activity	0.008703	12,33,2
1b6g__#63	GO:0016668: oxidoreductase activity, acting on sulfur group of donors, NAD or NADP as acceptor	0.008703	33,12,2
1ijqa1#564	GO:0042802: protein self binding	0.008703	33,12,2
1c7na_#95	GO:0005518: collagen binding	0.008705	183,13,4
1amp__#205	GO:0005524: ATP binding	0.008713	79,243,14
2dnja_#158	GO:0008270: zinc ion binding	0.008714	33,108,5
1ejda_#377	GO:0016836: hydro-lyase activity	0.008714	108,33,5
1d0na3#305	GO:0046983: protein dimerization activity	0.00872	311,17,6
1fsu__#434	GO:0003724: RNA helicase activity	0.00872	356,11,5
1e1aa_#285	GO:0046983: protein dimerization activity	0.008721	137,17,4
1e1aa_#285	GO:0016638: oxidoreductase activity, acting on the CH-NH2 group of donors	0.008721	137,17,4
1f5aa2#167	GO:0003682: chromatin binding	0.008726	124,10,3
1kit_3#331	GO:0003684: damaged DNA binding	0.008726	124,10,3
1kit_3#331	GO:0003916: DNA topoisomerase activity	0.008726	124,10,3
1kit_3#331	GO:0016620: oxidoreductase activity, acting on the aldehyde or oxo group of donors, NAD or NADP as acceptor	0.008726	124,10,3
1dkia_#76P	GO:0005529: sugar binding	0.008728	58,39,4
1ryp1_#112	GO:0005529: sugar binding	0.008728	58,39,4
1czan1#92	GO:0004674: protein serine/threonine kinase activity	0.00874	631,42,17
1ej8a_#189	GO:0000049: tRNA binding	0.008753	294,13,5
1ej8a_#189	GO:0005518: collagen binding	0.008753	294,13,5
1g0sa_#74	GO:0005518: collagen binding	0.008753	294,13,5
1g0sa_#74	GO:0008235: metalloexopeptidase activity	0.008753	294,13,5
1el0a_#42	GO:0005518: collagen binding	0.008753	294,13,5
1a65a1#108	GO:0008408: 3'-5' exonuclease activity	0.008753	294,13,5
1cs1a_#82	GO:0008270: zinc ion binding	0.008755	195,108,15
1g51a3#524	GO:0003899: DNA-directed RNA polymerase activity	0.008756	102,12,3
1c9la2#12	GO:0000287: magnesium ion binding	0.008759	295,128,23
1ton__#47	GO:0003755: peptidyl-prolyl cis-trans isomerase activity	0.008761	112,11,3
1icia_#175	GO:0016646: oxidoreductase activity, acting on the CH-NH group of donors, NAD or NADP as acceptor	0.008763	63,19,3
1h8d.1#H207	GO:0004896: hematopoietin/interferon-class (D200-domain) cytokine receptor activity	0.008763	63,19,3
1edza1#206	GO:0005066: transmembrane receptor protein tyrosine kinase signaling protein activity	0.00877	399,10,5
1edza1#206	GO:0016620: oxidoreductase activity, acting on the aldehyde or oxo group of donors, NAD or NADP as acceptor	0.00877	399,10,5
1qq4a_#178	GO:0050660: FAD binding	0.008778	40,10,2
1ad2__#40	GO:0003682: chromatin binding	0.008778	40,10,2
1xvaa_#244	GO:0003916: DNA topoisomerase activity	0.008778	40,10,2
1b3qa2#556	GO:0050660: FAD binding	0.008778	40,10,2
1b3qa2#556	GO:0016846: carbon-sulfur lyase activity	0.008778	40,10,2
1c4ka1#33	GO:0003682: chromatin binding	0.008778	40,10,2
1i4ua_#154	GO:0016814: hydrolase activity, acting on carbon-nitrogen (but not peptide) bonds, in cyclic amidines	0.008778	40,10,2
1elua_#216	GO:0008800: beta-lactamase activity	0.008778	40,10,2
1elua_#216	GO:0016846: carbon-sulfur lyase activity	0.008778	40,10,2
2sqca1#520	GO:0003714: transcription corepressor activity	0.008785	3,8,1
1i2da3#510	GO:0003714: transcription corepressor activity	0.008785	3,8,1
1nhp_2#181	GO:0016668: oxidoreductase activity, acting on sulfur group of donors, NAD or NADP as acceptor	0.00879	2,12,1
1ev7a_#150	GO:0009036: type II site-specific deoxyribonuclease activity	0.00879	2,12,1
1e58a_#79	GO:0016866: intramolecular transferase activity	0.00879	2,12,1
1f86a_#53	GO:0016702: oxidoreductase activity, acting on single donors with incorporation of molecular oxygen, incorporation of two atoms of oxygen	0.00879	2,12,1
1j8fa_#223	GO:0003899: DNA-directed RNA polymerase activity	0.00879	2,12,1
7reqa1#156	GO:0016866: intramolecular transferase activity	0.00879	2,12,1
1eqja1#211	GO:0016866: intramolecular transferase activity	0.00879	2,12,1
1afra_#263	GO:0003899: DNA-directed RNA polymerase activity	0.00879	2,12,1
1ek4a1#149	GO:0003899: DNA-directed RNA polymerase activity	0.00879	2,12,1
1qh5a_#224	GO:0003899: DNA-directed RNA polymerase activity	0.00879	2,12,1
1ceza_#357	GO:0003899: DNA-directed RNA polymerase activity	0.00879	2,12,1

1dmha_#309	GO:0016702: oxidoreductase activity, acting on single donors with incorporation of molecular oxygen, incorporation of two atoms of oxygen	0.00879	2,12,1
1am2__#161	GO:0015405: P-P-bond-hydrolysis-driven transporter activity	0.00879	2,12,1
1han_1#30	GO:0016702: oxidoreductase activity, acting on single donors with incorporation of molecular oxygen, incorporation of two atoms of oxygen	0.00879	2,12,1
1ihua1#236	GO:0015405: P-P-bond-hydrolysis-driven transporter activity	0.00879	2,12,1
1fytd2#143	GO:0003899: DNA-directed RNA polymerase activity	0.00879	2,12,1
1cpy__#70	GO:0000155: two-component sensor molecule activity	0.00879	2,12,1
1uxy_1#20	GO:0009036: type II site-specific deoxyribonuclease activity	0.00879	2,12,1
1g71a_#307	GO:0003899: DNA-directed RNA polymerase activity	0.00879	2,12,1
1qfoa_#50	GO:0016866: intramolecular transferase activity	0.00879	2,12,1
1ryp1_#51	GO:0016668: oxidoreductase activity, acting on sulfur group of donors, NAD or NADP as acceptor	0.00879	2,12,1
1jzta_#157	GO:0015405: P-P-bond-hydrolysis-driven transporter activity	0.00879	2,12,1
1tgoa1#299	GO:0003899: DNA-directed RNA polymerase activity	0.00879	2,12,1
1prea2#392	GO:0000155: two-component sensor molecule activity	0.00879	2,12,1
2foka4#571	GO:0009036: type II site-specific deoxyribonuclease activity	0.00879	2,12,1
1jjya_#25	GO:0016866: intramolecular transferase activity	0.00879	2,12,1
1ev7a_#65	GO:0009036: type II site-specific deoxyribonuclease activity	0.00879	2,12,1
1ho8a_#207	GO:0015405: P-P-bond-hydrolysis-driven transporter activity	0.00879	2,12,1
1ewka_#349	GO:0016866: intramolecular transferase activity	0.00879	2,12,1
1dfaa3#318	GO:0015405: P-P-bond-hydrolysis-driven transporter activity	0.00879	2,12,1
1pmaa_#131	GO:0016702: oxidoreductase activity, acting on single donors with incorporation of molecular oxygen, incorporation of two atoms of oxygen	0.00879	2,12,1
1qj2b2#740	GO:0016702: oxidoreductase activity, acting on single donors with incorporation of molecular oxygen, incorporation of two atoms of oxygen	0.00879	2,12,1
1d8db_#148	GO:0016866: intramolecular transferase activity	0.00879	2,12,1
1g25a_#22	GO:0046983: protein dimerization activity	0.008791	415,17,7
1gox__#124	GO:0004222: metalloendopeptidase activity	0.008802	366,19,7
1i50a_#1115	GO:0019843: rRNA binding	0.008805	18,22,2
1el5a1#10	GO:0015036: disulfide oxidoreductase activity	0.008805	18,22,2
1ji6a2#458	GO:0019843: rRNA binding	0.008805	18,22,2
1df0a3#208	GO:0019843: rRNA binding	0.008805	18,22,2
1fn9a_#271	GO:0030145: manganese ion binding	0.008806	218,38,8
1xdtr_#111	GO:0008201: heparin binding	0.008807	1,24,1
1jlxa2#199	GO:0008201: heparin binding	0.008807	1,24,1
1rdr__#322	GO:0004197: cysteine-type endopeptidase activity	0.008807	1,24,1
1eg2a_#212	GO:0008757: S-adenosylmethionine-dependent methyltransferase activity	0.008807	1,24,1
1fnha1#25	GO:0008201: heparin binding	0.008807	1,24,1
1a8p_1#27	GO:0008201: heparin binding	0.008807	1,24,1
1dusa_#52	GO:0008757: S-adenosylmethionine-dependent methyltransferase activity	0.008807	1,24,1
1ev2e1#207	GO:0008201: heparin binding	0.008807	1,24,1
1vcbb_#46	GO:0004197: cysteine-type endopeptidase activity	0.008807	1,24,1
3mag__#138	GO:0008757: S-adenosylmethionine-dependent methyltransferase activity	0.008807	1,24,1
1ibc.1#A162	GO:0004197: cysteine-type endopeptidase activity	0.008807	1,24,1
1aqza_#146	GO:0004197: cysteine-type endopeptidase activity	0.008807	1,24,1
1epfa1#7	GO:0008201: heparin binding	0.008807	1,24,1
1h54a1#733	GO:0004197: cysteine-type endopeptidase activity	0.008807	1,24,1
1cs6a3#218	GO:0005516: calmodulin binding	0.008807	1,24,1
1dro__#47	GO:0005516: calmodulin binding	0.008807	1,24,1
1lvk_2#548	GO:0005516: calmodulin binding	0.008807	1,24,1
1i4ua_#142	GO:0004197: cysteine-type endopeptidase activity	0.008807	1,24,1
1fp2a2#307	GO:0008757: S-adenosylmethionine-dependent methyltransferase activity	0.008807	1,24,1
1dp0a3#129	GO:0008201: heparin binding	0.008807	1,24,1
1ckla1#4	GO:0008201: heparin binding	0.008807	1,24,1
1bqsa2#169	GO:0008201: heparin binding	0.008807	1,24,1
1e05i_#83	GO:0004497: monooxygenase activity	0.008814	88,26,4
1fo4a5#1020	GO:0030151: molybdenum ion binding	0.008823	359,15,6
1fo4a5#1020	GO:0004180: carboxypeptidase activity	0.008823	359,15,6
1qqga2#253	GO:0005351: sugar porter activity	0.008841	110,21,4
1eny__#94	GO:0051082: unfolded protein binding	0.008846	35,34,3
1bev1_#86	GO:0016702: oxidoreductase activity, acting on single donors with incorporation of molecular oxygen, incorporation of two atoms of oxygen	0.008856	323,12,5
1qkma_#330	GO:0016836: hydro-lyase activity	0.008857	69,33,4
3ebx__#43	GO:0008270: zinc ion binding	0.008863	4,108,2
1gc5a_#45	GO:0008270: zinc ion binding	0.008863	4,108,2

1qmea4#568	GO:0008270: zinc ion binding	0.008863	4,108,2
1huxa_#10	GO:0003887: DNA-directed DNA polymerase activity	0.008863	60,20,3
1dhs__#149	GO:0003700: transcription factor activity	0.008867	29,124,5
1f0xa1#523	GO:0004714: transmembrane receptor protein tyrosine kinase activity	0.008867	87,14,3
1f0xa1#523	GO:0016251: general RNA polymerase II transcription factor activity	0.008867	87,14,3
1qrra_#208	GO:0016776: phosphotransferase activity, phosphate group as acceptor	0.008867	87,14,3
1epwa3#138	GO:0003729: mRNA binding	0.008873	424,13,6
1c9la2#12	GO:0000049: tRNA binding	0.008881	295,13,5
1dr9a2#139	GO:0051082: unfolded protein binding	0.008887	67,34,4
1dfca4#1451	GO:0051082: unfolded protein binding	0.008887	67,34,4
1aqb__#134	GO:0004556: alpha-amylase activity	0.008895	81,15,3
1poxa3#460	GO:0030151: molybdenum ion binding	0.008895	81,15,3
1bwda_#117	GO:0003887: DNA-directed DNA polymerase activity	0.008899	116,20,4
1e25a_#262	GO:0005126: hematopoietin/interferon-class (D200-domain) cytokine receptor binding	0.008899	116,20,4
1g6oa_#116	GO:0008201: heparin binding	0.008909	358,24,8
1h6va1#27	GO:0004812: tRNA ligase activity	0.00892	260,26,7
1ftra2#187	GO:0004812: tRNA ligase activity	0.008921	46,26,3
1hbza_#214	GO:0004190: aspartic-type endopeptidase activity	0.008921	297,23,7
1ryp1_#20	GO:0003682: chromatin binding	0.008923	125,10,3
1ez0a_#133	GO:0003809: thrombin activity	0.008923	125,10,3
1fjfl_#67	GO:0004867: serine-type endopeptidase inhibitor activity	0.008924	175,47,8
1ddja_#746	GO:0003968: RNA-directed RNA polymerase activity	0.008931	170,14,4
3sil__#317	GO:0003755: peptidyl-prolyl cis-trans isomerase activity	0.008933	358,11,5
1jg8a_#172	GO:0016831: carboxy-lyase activity	0.008934	92,25,4
1fc3a_#172	GO:0016763: transferase activity, transferring pentosyl groups	0.008935	182,28,6
1frb__#110	GO:0005509: calcium ion binding	0.008938	8,160,3
1ds9a_#119	GO:0005509: calcium ion binding	0.008938	8,160,3
1fwxa1#467	GO:0005509: calcium ion binding	0.008938	8,160,3
1erja_#521	GO:0005509: calcium ion binding	0.008938	8,160,3
1bdfa2#60	GO:0004896: hematopoietin/interferon-class (D200-domain) cytokine receptor activity	0.00894	21,19,2
1qq4a_#46	GO:0005351: sugar porter activity	0.00894	19,21,2
1lcl__#128	GO:0004620: phospholipase activity	0.00894	21,19,2
1ie5a_#23	GO:0004222: metalloendopeptidase activity	0.00894	21,19,2
1auoa_#121	GO:0004222: metalloendopeptidase activity	0.00894	21,19,2
1xis__#285	GO:0004222: metalloendopeptidase activity	0.00894	21,19,2
1gof_3#399	GO:0004896: hematopoietin/interferon-class (D200-domain) cytokine receptor activity	0.00894	21,19,2
1f2na_#138	GO:0004197: cysteine-type endopeptidase activity	0.008941	96,24,4
1qqsa_#35	GO:0005507: copper ion binding	0.008941	94,38,5
1hqva_#48	GO:0005516: calmodulin binding	0.008941	96,24,4
1nfdb1#21	GO:0004197: cysteine-type endopeptidase activity	0.008941	96,24,4
1nfdb1#21	GO:0005516: calmodulin binding	0.008941	96,24,4
1mpp__#151	GO:0030145: manganese ion binding	0.008941	94,38,5
1br9__#86	GO:0005516: calmodulin binding	0.008941	96,24,4
1e19a_#281	GO:0008483: transaminase activity	0.008946	138,17,4
1f0xa1#523	GO:0004263: chymotrypsin activity	0.008951	87,41,5
1f8ra2#325	GO:0003724: RNA helicase activity	0.008951	222,11,4
1ds1a_#172	GO:0003724: RNA helicase activity	0.008951	222,11,4
1dyna_#42	GO:0005524: ATP binding	0.008952	164,243,24

Tab S1 (b) Biological process: All FDR significant local descriptor-GO class pairs.

*PARAMETERS refer to the parameters in the hypergeometric distribution used to compute the p-values: N,n,k,x, where N=2505 is the number of protein-GO class pairs in the data set, n is the number of proteins matched by the local descriptor, k is the number of proteins in the GO class and x is the number of proteins matched by the local descriptor and in the GO class

LOCAL DESCRIPTOR	GENE ONTOLOGY CLASS	P-VALUE	PARAMETERS*
1qf6a4#362	GO:0006418: tRNA aminoacylation for protein translation	1.10E-32	15,26,15
2hlpa2#195	GO:0006100: tricarboxylic acid cycle intermediate metabolism	7.33E-31	12,14,12
1h6va1#23	GO:0006118: electron transport	9.12E-31	40,228,34
1atia2#67	GO:0006418: tRNA aminoacylation for protein translation	2.28E-30	14,26,14
1hc7a2#111	GO:0006418: tRNA aminoacylation for protein translation	2.28E-30	14,26,14
1hc7a2#109	GO:0006418: tRNA aminoacylation for protein translation	2.28E-30	14,26,14
2cmd_2#251	GO:0006100: tricarboxylic acid cycle intermediate metabolism	9.52E-30	13,14,12
1trb_1#285	GO:0006118: electron transport	1.83E-29	27,228,27
1qf6a4#295	GO:0006418: tRNA aminoacylation for protein translation	3.40E-29	15,26,14
1qlaa2#13	GO:0006118: electron transport	3.96E-29	29,228,28
1trb_1#298	GO:0006118: electron transport	3.96E-29	29,228,28
1trb_1#14	GO:0006118: electron transport	4.73E-29	31,228,29
1h7wa4#481	GO:0006118: electron transport	2.24E-28	26,228,26
1qf6a4#318	GO:0006418: tRNA aminoacylation for protein translation	2.71E-28	16,26,14
1llda2#177	GO:0006100: tricarboxylic acid cycle intermediate metabolism	3.33E-28	15,14,12
1pbe_1#155	GO:0006118: electron transport	5.48E-28	30,228,28
1e39a2#278	GO:0006118: electron transport	5.48E-28	30,228,28
1ceqa1#139	GO:0006100: tricarboxylic acid cycle intermediate metabolism	1.33E-27	16,14,12
1i4fa1#233	GO:0030333: antigen processing	1.94E-27	18,13,12
1fl2a1#320	GO:0006118: electron transport	5.22E-27	31,228,28
2hlpa1#98	GO:0006100: tricarboxylic acid cycle intermediate metabolism	7.31E-27	12,14,11
1ldm_2#292	GO:0006100: tricarboxylic acid cycle intermediate metabolism	7.31E-27	12,14,11
1hdmb1#173	GO:0030333: antigen processing	1.02E-26	13,13,11
1hdma1#124	GO:0030333: antigen processing	1.02E-26	13,13,11
2hlpa2#315	GO:0006100: tricarboxylic acid cycle intermediate metabolism	1.35E-26	18,14,12
7mdha2#355	GO:0006100: tricarboxylic acid cycle intermediate metabolism	4.74E-26	13,14,11
1h6va2#219	GO:0006118: electron transport	5.63E-26	37,228,30
1cqka_#49	GO:0030333: antigen processing	6.20E-26	35,13,13
1hxma2#150	GO:0030333: antigen processing	6.20E-26	35,13,13
1qlaa2#35	GO:0006118: electron transport	7.19E-26	28,228,26
1h4vb2#108	GO:0006418: tRNA aminoacylation for protein translation	7.78E-26	12,26,12
1hdma1#123	GO:0030333: antigen processing	9.71E-26	36,13,13
1qoua_#90	GO:0030333: antigen processing	2.27E-25	38,13,13
2hlpa2#272	GO:0006100: tricarboxylic acid cycle intermediate metabolism	3.80E-25	10,14,10
1pbe_1#121	GO:0006118: electron transport	6.41E-25	29,228,26
1d5ta1#12	GO:0006118: electron transport	8.15E-25	27,228,25
7fabh1#71	GO:0030333: antigen processing	1.01E-24	26,13,12
1hdma1#98	GO:0030333: antigen processing	1.07E-24	42,13,13
1nhp_1#7	GO:0006118: electron transport	1.22E-24	34,228,28
1ldm_2#272	GO:0006100: tricarboxylic acid cycle intermediate metabolism	2.65E-24	16,14,11
1fp5a2#537	GO:0030333: antigen processing	3.07E-24	45,13,13
1qsta_#154	GO:0006418: tRNA aminoacylation for protein translation	3.65E-24	18,26,13
1av4_1#492	GO:0030333: antigen processing	4.13E-24	18,13,11
1trb_1#112	GO:0006118: electron transport	4.86E-24	22,228,22
1hyea2#182	GO:0006100: tricarboxylic acid cycle intermediate metabolism	7.01E-24	26,14,12
1dn0b2#124	GO:0030333: antigen processing	7.15E-24	12,13,10
1bd3a_#110	GO:0009116: nucleoside metabolism	1.64E-23	20,25,13
1g5ha2#319	GO:0006418: tRNA aminoacylation for protein translation	3.49E-23	15,26,12
1i4fb1#70	GO:0030333: antigen processing	5.70E-23	34,13,12
1h7wa4#478	GO:0006118: electron transport	5.83E-23	21,228,21

1nhp_1#282	GO:0006118: electron transport	5.83E-23	21,228,21
1trb_1#41	GO:0006118: electron transport	5.83E-23	21,228,21
1ypta_#409	GO:0006470: protein amino acid dephosphorylation	7.02E-23	10,20,10
1ypta_#406	GO:0006470: protein amino acid dephosphorylation	7.02E-23	10,20,10
1ecfa1#367	GO:0009116: nucleoside metabolism	8.91E-23	12,25,11
1b3ra1#223	GO:0006100: tricarboxylic acid cycle intermediate metabolism	1.02E-22	31,14,12
1llc_2#291	GO:0006100: tricarboxylic acid cycle intermediate metabolism	1.08E-22	13,14,10
2cmd_2#172	GO:0006100: tricarboxylic acid cycle intermediate metabolism	1.90E-22	9,14,9
1b8pa2#197	GO:0006100: tricarboxylic acid cycle intermediate metabolism	2.13E-22	21,14,11
1hyea2#272	GO:0006100: tricarboxylic acid cycle intermediate metabolism	2.13E-22	21,14,11
1gvp__#3	GO:0030333: antigen processing	3.24E-22	15,13,10
1llc_2#271	GO:0006100: tricarboxylic acid cycle intermediate metabolism	3.78E-22	14,14,10
1e79h2#39	GO:0030333: antigen processing	5.76E-22	25,13,11
1hdmb1#173	GO:0019883: antigen presentation, endogenous antigen	6.77E-22	13,10,9
1g6ga_#133	GO:0050819: negative regulation of coagulation	6.77E-22	13,10,9
1g6ga_#133	GO:0030193: regulation of blood coagulation	6.77E-22	13,10,9
1cqka_#52	GO:0030333: antigen processing	6.90E-22	65,13,13
1b4va1#253	GO:0006118: electron transport	6.96E-22	20,228,20
1c8ua2#272	GO:0030333: antigen processing	9.98E-22	26,13,11
1trb_1#15	GO:0006118: electron transport	1.18E-21	22,228,21
1hyha2#179	GO:0006100: tricarboxylic acid cycle intermediate metabolism	1.50E-21	24,14,11
1ceqa2#199	GO:0006100: tricarboxylic acid cycle intermediate metabolism	1.89E-21	10,14,9
1bio__#81	GO:0050819: negative regulation of coagulation	1.89E-21	14,10,9
1bio__#81	GO:0030193: regulation of blood coagulation	1.89E-21	14,10,9
1qnja_#71	GO:0050819: negative regulation of coagulation	1.89E-21	14,10,9
1qnja_#71	GO:0030193: regulation of blood coagulation	1.89E-21	14,10,9
1danh_#70	GO:0050819: negative regulation of coagulation	1.89E-21	14,10,9
1danh_#70	GO:0030193: regulation of blood coagulation	1.89E-21	14,10,9
1ad2__#40	GO:0030333: antigen processing	2.18E-21	44,13,12
1a3c__#108	GO:0009116: nucleoside metabolism	2.67E-21	14,25,11
1ekbb_#117	GO:0050819: negative regulation of coagulation	4.73E-21	15,10,9
1ekbb_#117	GO:0030193: regulation of blood coagulation	4.73E-21	15,10,9
1danh_#140	GO:0050819: negative regulation of coagulation	4.73E-21	15,10,9
1danh_#140	GO:0030193: regulation of blood coagulation	4.73E-21	15,10,9
1gdna_#155	GO:0050819: negative regulation of coagulation	4.73E-21	15,10,9
1gdna_#155	GO:0030193: regulation of blood coagulation	4.73E-21	15,10,9
1ton__#91	GO:0050819: negative regulation of coagulation	4.73E-21	15,10,9
1ton__#91	GO:0030193: regulation of blood coagulation	4.73E-21	15,10,9
1ekbb_#141	GO:0050819: negative regulation of coagulation	4.73E-21	15,10,9
1ekbb_#141	GO:0030193: regulation of blood coagulation	4.73E-21	15,10,9
1ddja_#759	GO:0050819: negative regulation of coagulation	4.73E-21	15,10,9
1ddja_#759	GO:0030193: regulation of blood coagulation	4.73E-21	15,10,9
1fnf_3#1409	GO:0030333: antigen processing	6.42E-21	76,13,13
1dr9a2#139	GO:0030333: antigen processing	9.26E-21	78,13,13
1a04a2#59	GO:0000160: two-component signal transduction system (phosphorelay)	9.98E-21	15,25,11
1fjsa_#163	GO:0050819: negative regulation of coagulation	1.08E-20	16,10,9
1fjsa_#163	GO:0030193: regulation of blood coagulation	1.08E-20	16,10,9
1ddja_#771	GO:0050819: negative regulation of coagulation	1.08E-20	16,10,9
1ddja_#771	GO:0030193: regulation of blood coagulation	1.08E-20	16,10,9
1gdna_#56	GO:0050819: negative regulation of coagulation	1.08E-20	16,10,9
1gdna_#56	GO:0030193: regulation of blood coagulation	1.08E-20	16,10,9
1qnja_#118	GO:0050819: negative regulation of coagulation	1.08E-20	16,10,9
1qnja_#118	GO:0030193: regulation of blood coagulation	1.08E-20	16,10,9
1gdna_#123	GO:0050819: negative regulation of coagulation	1.08E-20	16,10,9
1gdna_#123	GO:0030193: regulation of blood coagulation	1.08E-20	16,10,9
1autc_#209	GO:0050819: negative regulation of coagulation	1.08E-20	16,10,9

1autc_#209	GO:0030193: regulation of blood coagulation	1.08E-20	16,10,9
1f0xa1#523	GO:0030333: antigen processing	1.32E-20	80,13,13
1bd3a_#195	GO:0009116: nucleoside metabolism	1.36E-20	11,25,10
1hyha2#171	GO:0006100: tricarboxylic acid cycle intermediate metabolism	1.64E-20	18,14,10
2hlca_#27	GO:0050819: negative regulation of coagulation	2.30E-20	17,10,9
2hlca_#27	GO:0030193: regulation of blood coagulation	2.30E-20	17,10,9
1dy9.1#A44	GO:0050819: negative regulation of coagulation	2.30E-20	17,10,9
1dy9.1#A44	GO:0030193: regulation of blood coagulation	2.30E-20	17,10,9
1gdna_#121	GO:0050819: negative regulation of coagulation	2.30E-20	17,10,9
1gdna_#121	GO:0030193: regulation of blood coagulation	2.30E-20	17,10,9
1bqya_#103	GO:0050819: negative regulation of coagulation	2.30E-20	17,10,9
1bqya_#103	GO:0030193: regulation of blood coagulation	2.30E-20	17,10,9
1dlea_#198	GO:0050819: negative regulation of coagulation	2.30E-20	17,10,9
1dlea_#198	GO:0030193: regulation of blood coagulation	2.30E-20	17,10,9
1ton__#231	GO:0050819: negative regulation of coagulation	2.30E-20	17,10,9
1ton__#231	GO:0030193: regulation of blood coagulation	2.30E-20	17,10,9
1ekma1#545	GO:0030333: antigen processing	2.63E-20	84,13,13
1eona_#74	GO:0009307: DNA restriction	3.47E-20	14,19,10
1gdea_#243	GO:0030333: antigen processing	3.54E-20	54,13,12
1cjca2#365	GO:0006118: electron transport	3.88E-20	56,228,32
1i4fa1#233	GO:0019883: antigen presentation, endogenous antigen	4.59E-20	18,10,9
1av4_1#492	GO:0019883: antigen presentation, endogenous antigen	4.59E-20	18,10,9
1danh_#197	GO:0050819: negative regulation of coagulation	4.59E-20	18,10,9
1danh_#197	GO:0030193: regulation of blood coagulation	4.59E-20	18,10,9
1svpa_#127	GO:0050819: negative regulation of coagulation	4.59E-20	18,10,9
1svpa_#127	GO:0030193: regulation of blood coagulation	4.59E-20	18,10,9
1dlea_#53	GO:0050819: negative regulation of coagulation	4.59E-20	18,10,9
1dlea_#53	GO:0030193: regulation of blood coagulation	4.59E-20	18,10,9
2hft_1#34	GO:0030333: antigen processing	7.70E-20	36,13,11
1ton__#196	GO:0050819: negative regulation of coagulation	8.72E-20	19,10,9
1ton__#196	GO:0030193: regulation of blood coagulation	8.72E-20	19,10,9
1qq4a_#63	GO:0050819: negative regulation of coagulation	8.72E-20	19,10,9
1qq4a_#63	GO:0030193: regulation of blood coagulation	8.72E-20	19,10,9
1arb__#24	GO:0050819: negative regulation of coagulation	8.72E-20	19,10,9
1arb__#24	GO:0030193: regulation of blood coagulation	8.72E-20	19,10,9
1d7ya1#255	GO:0006118: electron transport	9.81E-20	18,228,18
1fw9a_#157	GO:0030333: antigen processing	1.44E-19	60,13,12
1arb__#55	GO:0050819: negative regulation of coagulation	1.59E-19	20,10,9
1arb__#55	GO:0030193: regulation of blood coagulation	1.59E-19	20,10,9
1gdna_#30	GO:0050819: negative regulation of coagulation	1.59E-19	20,10,9
1gdna_#30	GO:0030193: regulation of blood coagulation	1.59E-19	20,10,9
1eq9a_#143	GO:0050819: negative regulation of coagulation	1.95E-19	11,10,8
1eq9a_#143	GO:0030193: regulation of blood coagulation	1.95E-19	11,10,8
1h8d.1#H184	GO:0050819: negative regulation of coagulation	1.95E-19	11,10,8
1h8d.1#H184	GO:0030193: regulation of blood coagulation	1.95E-19	11,10,8
1c5y.1#B241	GO:0050819: negative regulation of coagulation	2.77E-19	21,10,9
1c5y.1#B241	GO:0030193: regulation of blood coagulation	2.77E-19	21,10,9
1dlea_#238	GO:0050819: negative regulation of coagulation	2.77E-19	21,10,9
1dlea_#238	GO:0030193: regulation of blood coagulation	2.77E-19	21,10,9
1arb__#208	GO:0050819: negative regulation of coagulation	2.77E-19	21,10,9
1arb__#208	GO:0030193: regulation of blood coagulation	2.77E-19	21,10,9
1autc_#45	GO:0050819: negative regulation of coagulation	2.77E-19	21,10,9
1autc_#45	GO:0030193: regulation of blood coagulation	2.77E-19	21,10,9
1qnja_#138	GO:0050819: negative regulation of coagulation	2.77E-19	21,10,9
1qnja_#138	GO:0030193: regulation of blood coagulation	2.77E-19	21,10,9
1qb7a_#86	GO:0006100: tricarboxylic acid cycle intermediate metabolism	3.56E-19	36,14,11

1dlfl_#97	GO:0030333: antigen processing	4.04E-19	41,13,11
1fp5a2#478	GO:0030333: antigen processing	5.67E-19	26,13,10
1bqya_#115	GO:0050819: negative regulation of coagulation	5.84E-19	12,10,8
1bqya_#115	GO:0030193: regulation of blood coagulation	5.84E-19	12,10,8
1h8d.1#H167	GO:0050819: negative regulation of coagulation	5.84E-19	12,10,8
1h8d.1#H167	GO:0030193: regulation of blood coagulation	5.84E-19	12,10,8
1ton__#120	GO:0050819: negative regulation of coagulation	5.84E-19	12,10,8
1ton__#120	GO:0030193: regulation of blood coagulation	5.84E-19	12,10,8
1bqya_#216	GO:0050819: negative regulation of coagulation	5.84E-19	12,10,8
1bqya_#216	GO:0030193: regulation of blood coagulation	5.84E-19	12,10,8
2cmd_1#113	GO:0006100: tricarboxylic acid cycle intermediate metabolism	7.09E-19	9,14,8
2hlpa2#298	GO:0006100: tricarboxylic acid cycle intermediate metabolism	7.09E-19	9,14,8
3dfr__#63	GO:0006544: glycine metabolism	7.14E-19	11,11,8
1kve.1#B199	GO:0030333: antigen processing	9.04E-19	69,13,12
1pysa_#127	GO:0006418: tRNA aminoacylation for protein translation	9.35E-19	19,26,11
1bec_2#216	GO:0030333: antigen processing	9.82E-19	109,13,13
1foha5#218	GO:0006118: electron transport	1.16E-18	57,228,31
1qlaa2#383	GO:0006118: electron transport	1.16E-18	17,228,17
1cjca2#103	GO:0006118: electron transport	1.16E-18	17,228,17
1tmy__#14	GO:0000160: two-component signal transduction system (phosphorelay)	1.22E-18	14,25,10
2hlca_#68	GO:0050819: negative regulation of coagulation	1.23E-18	24,10,9
2hlca_#68	GO:0030193: regulation of blood coagulation	1.23E-18	24,10,9
1e39a2#516	GO:0006118: electron transport	1.47E-18	21,228,19
1hdma1#124	GO:0019883: antigen presentation, endogenous antigen	1.52E-18	13,10,8
1ekbb_#79	GO:0050819: negative regulation of coagulation	1.52E-18	13,10,8
1ekbb_#79	GO:0030193: regulation of blood coagulation	1.52E-18	13,10,8
1a0la_#193	GO:0050819: negative regulation of coagulation	1.52E-18	13,10,8
1a0la_#193	GO:0030193: regulation of blood coagulation	1.52E-18	13,10,8
3frua1#249	GO:0019883: antigen presentation, endogenous antigen	1.52E-18	13,10,8
1e79h2#39	GO:0019883: antigen presentation, endogenous antigen	1.93E-18	25,10,9
1df7a_#14	GO:0006544: glycine metabolism	2.14E-18	12,11,8
1b4va1#11	GO:0006118: electron transport	2.53E-18	26,228,21
1c8ua2#272	GO:0019883: antigen presentation, endogenous antigen	2.94E-18	26,10,9
7fabh1#71	GO:0019883: antigen presentation, endogenous antigen	2.94E-18	26,10,9
1atia2#189	GO:0006418: tRNA aminoacylation for protein translation	2.94E-18	10,26,9
1dyoa_#25	GO:0030333: antigen processing	3.25E-18	18,13,9
1g4us2#516	GO:0006470: protein amino acid dephosphorylation	3.31E-18	8,20,8
1hyha2#204	GO:0006100: tricarboxylic acid cycle intermediate metabolism	3.38E-18	43,14,11
1ypta_#445	GO:0006470: protein amino acid dephosphorylation	3.46E-18	12,20,9
1c5y.1#B192	GO:0050819: negative regulation of coagulation	3.54E-18	14,10,8
1c5y.1#B192	GO:0030193: regulation of blood coagulation	3.54E-18	14,10,8
1c5y.1#B220	GO:0050819: negative regulation of coagulation	3.54E-18	14,10,8
1c5y.1#B220	GO:0030193: regulation of blood coagulation	3.54E-18	14,10,8
1ceqa2#286	GO:0006100: tricarboxylic acid cycle intermediate metabolism	3.54E-18	10,14,8
1b8pa2#201	GO:0006100: tricarboxylic acid cycle intermediate metabolism	3.54E-18	10,14,8
1danh_#142	GO:0050819: negative regulation of coagulation	3.54E-18	14,10,8
1danh_#142	GO:0030193: regulation of blood coagulation	3.54E-18	14,10,8
1pbe_1#150	GO:0006118: electron transport	3.67E-18	75,228,35
1i6vd_#1283	GO:0030333: antigen processing	3.68E-18	120,13,13
1elva1#604	GO:0050819: negative regulation of coagulation	4.41E-18	27,10,9
1elva1#604	GO:0030193: regulation of blood coagulation	4.41E-18	27,10,9
1e39a2#556	GO:0006118: electron transport	5.10E-18	38,228,25
1gsoa3#142	GO:0030333: antigen processing	5.23E-18	79,13,12
1eq9a_#58	GO:0050819: negative regulation of coagulation	6.50E-18	28,10,9
1eq9a_#58	GO:0030193: regulation of blood coagulation	6.50E-18	28,10,9
1gvp__#3	GO:0019883: antigen presentation, endogenous antigen	7.58E-18	15,10,8

1azza_#51	GO:0050819: negative regulation of coagulation	7.58E-18	15,10,8
1azza_#51	GO:0030193: regulation of blood coagulation	7.58E-18	15,10,8
1danh_#190	GO:0050819: negative regulation of coagulation	7.58E-18	15,10,8
1danh_#190	GO:0030193: regulation of blood coagulation	7.58E-18	15,10,8
1ton__#112	GO:0050819: negative regulation of coagulation	7.58E-18	15,10,8
1ton__#112	GO:0030193: regulation of blood coagulation	7.58E-18	15,10,8
1akp__#62	GO:0030333: antigen processing	7.65E-18	52,13,11
2ltn.1#A63	GO:0030333: antigen processing	1.22E-17	131,13,13
1h97a_#81	GO:0015671: oxygen transport	1.29E-17	8,23,8
1thg__#3	GO:0030333: antigen processing	1.34E-17	85,13,12
1chua2#234	GO:0006118: electron transport	1.36E-17	16,228,16
1h7wa4#476	GO:0006118: electron transport	1.36E-17	16,228,16
1ojt_2#336	GO:0006118: electron transport	1.36E-17	16,228,16
1dm0a_#110	GO:0017148: negative regulation of protein biosynthesis	1.41E-17	7,13,7
1d6aa_#179	GO:0017148: negative regulation of protein biosynthesis	1.41E-17	7,13,7
1qi7a_#208	GO:0017148: negative regulation of protein biosynthesis	1.41E-17	7,13,7
1feca1#31	GO:0006118: electron transport	1.44E-17	69,228,33
1i50a_#856	GO:0006118: electron transport	1.46E-17	239,228,64
1g5ha2#162	GO:0006418: tRNA aminoacylation for protein translation	1.64E-17	23,26,11
1ebda2#182	GO:0006118: electron transport	1.92E-17	18,228,17
1qnja_#46	GO:0050819: negative regulation of coagulation	2.64E-17	32,10,9
1qnja_#46	GO:0030193: regulation of blood coagulation	2.64E-17	32,10,9
3grs_1#24	GO:0006118: electron transport	2.79E-17	66,228,32
1qb7a_#148	GO:0009116: nucleoside metabolism	2.84E-17	8,25,8
1eq9a_#59	GO:0050819: negative regulation of coagulation	2.86E-17	17,10,8
1eq9a_#59	GO:0030193: regulation of blood coagulation	2.86E-17	17,10,8
1danh_#48	GO:0050819: negative regulation of coagulation	3.54E-17	9,10,7
1danh_#48	GO:0030193: regulation of blood coagulation	3.54E-17	9,10,7
1jlna_#383	GO:0006470: protein amino acid dephosphorylation	3.57E-17	46,20,12
1cpy__#93	GO:0006418: tRNA aminoacylation for protein translation	3.91E-17	218,26,20
1ile_3#170	GO:0006418: tRNA aminoacylation for protein translation	4.11E-17	8,26,8
1smaa2#581	GO:0030333: antigen processing	4.32E-17	60,13,11
3frua1#249	GO:0030333: antigen processing	4.32E-17	13,13,8
1kid__#330	GO:0030333: antigen processing	4.42E-17	144,13,13
1i4fb1#70	GO:0019883: antigen presentation, endogenous antigen	4.93E-17	34,10,9
1qq4a_#143	GO:0050819: negative regulation of coagulation	5.14E-17	18,10,8
1qq4a_#143	GO:0030193: regulation of blood coagulation	5.14E-17	18,10,8
3frua1#236	GO:0030333: antigen processing	5.26E-17	61,13,11
1llda2#161	GO:0006100: tricarboxylic acid cycle intermediate metabolism	5.46E-17	21,14,9
1fjfl_#67	GO:0030333: antigen processing	5.85E-17	147,13,13
1hw5a2#64	GO:0030333: antigen processing	6.41E-17	148,13,13
1cqka_#49	GO:0019883: antigen presentation, endogenous antigen	6.63E-17	35,10,9
1elva1#623	GO:0050819: negative regulation of coagulation	6.63E-17	35,10,9
1elva1#623	GO:0030193: regulation of blood coagulation	6.63E-17	35,10,9
1hxma2#150	GO:0019883: antigen presentation, endogenous antigen	6.63E-17	35,10,9
1hwxa1#256	GO:0006118: electron transport	7.33E-17	48,228,27
1hdma1#123	GO:0019883: antigen presentation, endogenous antigen	8.84E-17	36,10,9
1dyna_#42	GO:0030333: antigen processing	9.20E-17	152,13,13
1jb9a2#173	GO:0006544: glycine metabolism	1.04E-16	17,11,8
1qi7a_#244	GO:0017148: negative regulation of protein biosynthesis	1.12E-16	8,13,7
1e42a1#711	GO:0030333: antigen processing	1.20E-16	155,13,13
1d5ra2#133	GO:0006470: protein amino acid dephosphorylation	1.31E-16	24,20,10
1nsca_#236	GO:0030333: antigen processing	1.31E-16	156,13,13
1fjsa_#158	GO:0050819: negative regulation of coagulation	1.48E-16	20,10,8
1fjsa_#158	GO:0030193: regulation of blood coagulation	1.48E-16	20,10,8
1qoua_#90	GO:0019883: antigen presentation, endogenous antigen	1.53E-16	38,10,9

1trb_1#42	GO:0006118: electron transport	1.59E-16	15,228,15
1prea2#308	GO:0030333: antigen processing	1.61E-16	67,13,11
1e3ja2#270	GO:0006100: tricarboxylic acid cycle intermediate metabolism	1.98E-16	60,14,11
1i7qa_#186	GO:0030333: antigen processing	2.07E-16	26,13,9
1f46a_#59	GO:0030333: antigen processing	2.22E-16	106,13,12
1qfea_#68	GO:0006096: glycolysis	2.68E-16	453,38,30
1gdna_#26	GO:0050819: negative regulation of coagulation	3.24E-16	11,10,7
1gdna_#26	GO:0030193: regulation of blood coagulation	3.24E-16	11,10,7
1elva1#601	GO:0050819: negative regulation of coagulation	3.24E-16	11,10,7
1elva1#601	GO:0030193: regulation of blood coagulation	3.24E-16	11,10,7
1arb__#193	GO:0050819: negative regulation of coagulation	3.75E-16	22,10,8
1arb__#193	GO:0030193: regulation of blood coagulation	3.75E-16	22,10,8
1ezvb1#29	GO:0019883: antigen presentation, endogenous antigen	4.18E-16	42,10,9
1hdma1#98	GO:0019883: antigen presentation, endogenous antigen	4.18E-16	42,10,9
1ekbb_#228	GO:0050819: negative regulation of coagulation	5.28E-16	43,10,9
1ekbb_#228	GO:0030193: regulation of blood coagulation	5.28E-16	43,10,9
1gdna_#26	GO:0006953: acute-phase response	5.40E-16	11,20,8
1eq9a_#143	GO:0006953: acute-phase response	5.40E-16	11,20,8
1h8d.1#H184	GO:0006953: acute-phase response	5.40E-16	11,20,8
1elva1#601	GO:0006953: acute-phase response	5.40E-16	11,20,8
1i4fb1#4	GO:0019883: antigen presentation, endogenous antigen	6.16E-16	6,10,6
1ad2__#40	GO:0019883: antigen presentation, endogenous antigen	6.63E-16	44,10,9
1ycsa_#162	GO:0030333: antigen processing	7.19E-16	177,13,13
1elva1#566	GO:0050819: negative regulation of coagulation	7.76E-16	12,10,7
1elva1#566	GO:0030193: regulation of blood coagulation	7.76E-16	12,10,7
1dn0b2#124	GO:0019883: antigen presentation, endogenous antigen	7.76E-16	12,10,7
1fp5a2#537	GO:0019883: antigen presentation, endogenous antigen	8.29E-16	45,10,9
1g71a_#77	GO:0050819: negative regulation of coagulation	8.60E-16	24,10,8
1g71a_#77	GO:0030193: regulation of blood coagulation	8.60E-16	24,10,8
1ra9__#62	GO:0006544: glycine metabolism	8.71E-16	21,11,8
1aqua_#126	GO:0006096: glycolysis	8.92E-16	472,38,30
1hyha2#205	GO:0006100: tricarboxylic acid cycle intermediate metabolism	1.01E-15	9,14,7
2uaga1#54	GO:0006118: electron transport	1.03E-15	20,228,17
1fn9a_#271	GO:0030333: antigen processing	1.21E-15	184,13,13
1fxla2#169	GO:0030333: antigen processing	1.21E-15	184,13,13
8dfr__#10	GO:0006544: glycine metabolism	1.35E-15	6,11,6
3dfr__#103	GO:0006544: glycine metabolism	1.35E-15	6,11,6
1hvba_#63	GO:0046677: response to antibiotic	1.39E-15	11,40,9
1jf9a_#379	GO:0030333: antigen processing	1.40E-15	186,13,13
1hc7a2#258	GO:0030333: antigen processing	1.40E-15	186,13,13
3sil__#98	GO:0030333: antigen processing	1.40E-15	186,13,13
1fcda1#6	GO:0006118: electron transport	1.42E-15	69,228,31
1thta_#71	GO:0030333: antigen processing	1.45E-15	18,13,8
1e2o__#346	GO:0030333: antigen processing	1.51E-15	187,13,13
1f00i2#805	GO:0030333: antigen processing	1.60E-15	124,13,12
1elva1#566	GO:0006953: acute-phase response	1.61E-15	12,20,8
1h8d.1#H167	GO:0006953: acute-phase response	1.61E-15	12,20,8
1ton__#120	GO:0006953: acute-phase response	1.61E-15	12,20,8
1bqya_#216	GO:0006953: acute-phase response	1.61E-15	12,20,8
1gg6.1#C156	GO:0006953: acute-phase response	1.61E-15	12,20,8
2hlca_#99	GO:0006953: acute-phase response	1.61E-15	12,20,8
1ho1a_#211	GO:0006096: glycolysis	1.67E-15	93,38,17
1c5y.1#B191	GO:0050819: negative regulation of coagulation	1.68E-15	13,10,7
1c5y.1#B191	GO:0030193: regulation of blood coagulation	1.68E-15	13,10,7
1i4fa2#21	GO:0030333: antigen processing	1.68E-15	10,13,7
1fnga1#123	GO:0030333: antigen processing	1.68E-15	10,13,7

1c5y.1#B94	GO:0050819: negative regulation of coagulation	1.68E-15	13,10,7
1c5y.1#B94	GO:0030193: regulation of blood coagulation	1.68E-15	13,10,7
1danh_#242	GO:0050819: negative regulation of coagulation	1.68E-15	13,10,7
1danh_#242	GO:0030193: regulation of blood coagulation	1.68E-15	13,10,7
1ton__#179	GO:0050819: negative regulation of coagulation	1.68E-15	13,10,7
1ton__#179	GO:0030193: regulation of blood coagulation	1.68E-15	13,10,7
1elva1#513	GO:0050819: negative regulation of coagulation	1.68E-15	13,10,7
1elva1#513	GO:0030193: regulation of blood coagulation	1.68E-15	13,10,7
1pysb5#584	GO:0006418: tRNA aminoacylation for protein translation	1.79E-15	60,26,13
1fp5a2#478	GO:0019883: antigen presentation, endogenous antigen	1.83E-15	26,10,8
1oaa__#14	GO:0006100: tricarboxylic acid cycle intermediate metabolism	1.83E-15	29,14,9
3grx__#52	GO:0006118: electron transport	1.85E-15	14,228,14
1fuma2#370	GO:0006118: electron transport	1.85E-15	14,228,14
1ds1a_#172	GO:0030333: antigen processing	1.86E-15	190,13,13
1ton__#181	GO:0050819: negative regulation of coagulation	1.92E-15	49,10,9
1ton__#181	GO:0030193: regulation of blood coagulation	1.92E-15	49,10,9
1qlaa2#179	GO:0006118: electron transport	1.98E-15	49,228,26
1a8d_1#87	GO:0030333: antigen processing	2.00E-15	191,13,13
1ldna1#94	GO:0006100: tricarboxylic acid cycle intermediate metabolism	2.24E-15	159,14,13
1qama_#37	GO:0006100: tricarboxylic acid cycle intermediate metabolism	2.35E-15	48,14,10
1flga_#161	GO:0030333: antigen processing	2.38E-15	128,13,12
1pysa_#221	GO:0006418: tRNA aminoacylation for protein translation	2.54E-15	80,26,14
1fjsa_#158	GO:0006953: acute-phase response	2.56E-15	20,20,9
1eur__#84	GO:0030333: antigen processing	2.64E-15	85,13,11
1lara2#1769	GO:0030333: antigen processing	2.64E-15	195,13,13
1f8ra2#325	GO:0030333: antigen processing	2.83E-15	196,13,13
1eaja_#59	GO:0030333: antigen processing	2.83E-15	196,13,13
1gpea1#271	GO:0030333: antigen processing	2.83E-15	196,13,13
1io1a_#311	GO:0030333: antigen processing	2.89E-15	130,13,12
1b8aa2#368	GO:0030333: antigen processing	2.89E-15	130,13,12
1f0ia1#50	GO:0030333: antigen processing	3.18E-15	131,13,12
1ldm_2#292	GO:0006096: glycolysis	3.29E-15	12,38,9
2hlpa2#195	GO:0006096: glycolysis	3.29E-15	12,38,9
1ddja_#754	GO:0050819: negative regulation of coagulation	3.36E-15	14,10,7
1ddja_#754	GO:0030193: regulation of blood coagulation	3.36E-15	14,10,7
1ekbb_#73	GO:0050819: negative regulation of coagulation	3.36E-15	14,10,7
1ekbb_#73	GO:0030193: regulation of blood coagulation	3.36E-15	14,10,7
1ovaa_#284	GO:0006100: tricarboxylic acid cycle intermediate metabolism	3.38E-15	18,14,8
1g71a_#88	GO:0050819: negative regulation of coagulation	3.63E-15	28,10,8
1g71a_#88	GO:0030193: regulation of blood coagulation	3.63E-15	28,10,8
1nfdb1#21	GO:0030333: antigen processing	3.95E-15	88,13,11
1c5y.1#B191	GO:0006953: acute-phase response	4.17E-15	13,20,8
1c5y.1#B94	GO:0006953: acute-phase response	4.17E-15	13,20,8
1cgha_#168	GO:0006953: acute-phase response	4.17E-15	13,20,8
1a0la_#193	GO:0006953: acute-phase response	4.17E-15	13,20,8
1ton__#179	GO:0006953: acute-phase response	4.17E-15	13,20,8
1elva1#513	GO:0006953: acute-phase response	4.17E-15	13,20,8
1ejfa_#10	GO:0030333: antigen processing	4.21E-15	134,13,12
1ge8a2#134	GO:0030333: antigen processing	4.24E-15	202,13,13
1cgha_#227	GO:0006953: acute-phase response	4.34E-15	47,20,11
1ldna1#94	GO:0006096: glycolysis	4.37E-15	159,38,20
1ac6a_#34	GO:0030333: antigen processing	4.45E-15	57,13,10
1ce7a_#151	GO:0017148: negative regulation of protein biosynthesis	4.61E-15	11,13,7
1h9da_#91	GO:0030333: antigen processing	4.84E-15	204,13,13
1gdea_#243	GO:0019883: antigen presentation, endogenous antigen	4.96E-15	54,10,9
1i4fb1#4	GO:0030333: antigen processing	5.03E-15	6,13,6

1aqb__#134	GO:0030333: antigen processing	5.12E-15	90,13,11
1gln_2#63	GO:0006418: tRNA aminoacylation for protein translation	5.40E-15	7,26,7
1c9la2#61	GO:0030333: antigen processing	5.51E-15	206,13,13
2mev1_#123	GO:0030333: antigen processing	5.82E-15	91,13,11
1gg6.1#C229	GO:0050819: negative regulation of coagulation	6.29E-15	15,10,7
1gg6.1#C229	GO:0030193: regulation of blood coagulation	6.29E-15	15,10,7
3dfr__#63	GO:0009070: serine family amino acid biosynthesis	6.65E-15	11,26,8
1jb9a2#173	GO:0009070: serine family amino acid biosynthesis	6.85E-15	17,26,9
1ac6a_#34	GO:0030217: T-cell differentiation	8.38E-15	57,10,9
1axca1#70	GO:0030333: antigen processing	8.47E-15	94,13,11
1dik_3#240	GO:0030333: antigen processing	8.62E-15	213,13,13
1c5y.1#B192	GO:0006953: acute-phase response	9.70E-15	14,20,8
1c5y.1#B220	GO:0006953: acute-phase response	9.70E-15	14,20,8
1bio__#81	GO:0006953: acute-phase response	9.70E-15	14,20,8
1qnja_#71	GO:0006953: acute-phase response	9.70E-15	14,20,8
1danh_#142	GO:0006953: acute-phase response	9.70E-15	14,20,8
1danh_#70	GO:0006953: acute-phase response	9.70E-15	14,20,8
2hlca_#54	GO:0050819: negative regulation of coagulation	9.92E-15	58,10,9
2hlca_#54	GO:0030193: regulation of blood coagulation	9.92E-15	58,10,9
1ton__#211	GO:0050819: negative regulation of coagulation	9.92E-15	58,10,9
1ton__#211	GO:0030193: regulation of blood coagulation	9.92E-15	58,10,9
1vcaa2#49	GO:0030333: antigen processing	1.06E-14	22,13,8
1llc_2#291	GO:0006096: glycolysis	1.06E-14	13,38,9
7mdha2#355	GO:0006096: glycolysis	1.06E-14	13,38,9
2cmd_2#251	GO:0006096: glycolysis	1.06E-14	13,38,9
1cwva2#682	GO:0030333: antigen processing	1.10E-14	12,13,7
1c5y.1#B234	GO:0050819: negative regulation of coagulation	1.12E-14	16,10,7
1c5y.1#B234	GO:0030193: regulation of blood coagulation	1.12E-14	16,10,7
2cmd_2#172	GO:0006096: glycolysis	1.15E-14	9,38,8
1bio__#108	GO:0050819: negative regulation of coagulation	1.22E-14	32,10,8
1bio__#108	GO:0030193: regulation of blood coagulation	1.22E-14	32,10,8
1h4vb2#307	GO:0006418: tRNA aminoacylation for protein translation	1.28E-14	69,26,13
8dfr__#115	GO:0006544: glycine metabolism	1.32E-14	28,11,8
1aop_3#276	GO:0030333: antigen processing	1.33E-14	147,13,12
1ac6a_#43	GO:0030333: antigen processing	1.33E-14	147,13,12
1danh_#104	GO:0030333: antigen processing	1.33E-14	147,13,12
2bbkh_#214	GO:0030333: antigen processing	1.38E-14	39,13,9
1fw9a_#157	GO:0019883: antigen presentation, endogenous antigen	1.38E-14	60,10,9
1c7na_#95	GO:0030333: antigen processing	1.58E-14	149,13,12
1uch__#50	GO:0006118: electron transport	1.58E-14	74,228,31
1smaa1#95	GO:0030333: antigen processing	1.59E-14	223,13,13
1gg6.1#B19	GO:0050819: negative regulation of coagulation	1.61E-14	33,10,8
1gg6.1#B19	GO:0030193: regulation of blood coagulation	1.61E-14	33,10,8
1qaxa2#103	GO:0030333: antigen processing	1.62E-14	23,13,8
1hxxa_#228	GO:0030333: antigen processing	1.62E-14	23,13,8
1mrp__#172	GO:0009070: serine family amino acid biosynthesis	1.71E-14	91,26,14
1jj2e1#42	GO:0030333: antigen processing	1.71E-14	150,13,12
1dar_4#634	GO:0019883: antigen presentation, endogenous antigen	1.72E-14	8,10,6
1h6va1#27	GO:0006118: electron transport	1.79E-14	224,228,57
2btva_#866	GO:0019883: antigen presentation, endogenous antigen	1.90E-14	17,10,7
1kit_3#331	GO:0030333: antigen processing	1.94E-14	101,13,11
1df7a_#14	GO:0009070: serine family amino acid biosynthesis	1.98E-14	12,26,8
2sli_2#326	GO:0030333: antigen processing	2.02E-14	227,13,13
1ekbb_#117	GO:0006953: acute-phase response	2.07E-14	15,20,8
1danh_#140	GO:0006953: acute-phase response	2.07E-14	15,20,8
1danh_#190	GO:0006953: acute-phase response	2.07E-14	15,20,8

1gdna_#155	GO:0006953: acute-phase response	2.07E-14	15,20,8
1ton__#112	GO:0006953: acute-phase response	2.07E-14	15,20,8
1ton__#91	GO:0006953: acute-phase response	2.07E-14	15,20,8
1ekbb_#141	GO:0006953: acute-phase response	2.07E-14	15,20,8
1ddja_#759	GO:0006953: acute-phase response	2.07E-14	15,20,8
1gg6.1#C229	GO:0006953: acute-phase response	2.07E-14	15,20,8
1h7wa4#208	GO:0006118: electron transport	2.14E-14	13,228,13
2hlpa2#289	GO:0006100: tricarboxylic acid cycle intermediate metabolism	2.20E-14	12,14,7
1e9xa_#343	GO:0006118: electron transport	2.36E-14	31,228,20
1b8aa2#251	GO:0006418: tRNA aminoacylation for protein translation	2.38E-14	28,26,10
1fl2a1#472	GO:0006118: electron transport	2.55E-14	15,228,14
1eq9a_#40	GO:0050819: negative regulation of coagulation	2.56E-14	64,10,9
1eq9a_#40	GO:0030193: regulation of blood coagulation	2.56E-14	64,10,9
3prn__#145	GO:0030333: antigen processing	2.57E-14	155,13,12
1ezvb1#29	GO:0030333: antigen processing	2.88E-14	42,13,9
1llc_2#271	GO:0006096: glycolysis	2.93E-14	14,38,9
1cqka_#52	GO:0019883: antigen presentation, endogenous antigen	2.97E-14	65,10,9
1thta_#71	GO:0019883: antigen presentation, endogenous antigen	3.10E-14	18,10,7
1dyoa_#25	GO:0019883: antigen presentation, endogenous antigen	3.10E-14	18,10,7
1aqt_2#45	GO:0030333: antigen processing	3.46E-14	69,13,10
2hft_1#34	GO:0019883: antigen presentation, endogenous antigen	3.51E-14	36,10,8
1prea2#308	GO:0019883: antigen presentation, endogenous antigen	3.97E-14	67,10,9
1fjsa_#163	GO:0006953: acute-phase response	4.12E-14	16,20,8
1ddja_#771	GO:0006953: acute-phase response	4.12E-14	16,20,8
1qnja_#118	GO:0006953: acute-phase response	4.12E-14	16,20,8
1gdna_#123	GO:0006953: acute-phase response	4.12E-14	16,20,8
1c5y.1#B234	GO:0006953: acute-phase response	4.12E-14	16,20,8
1autc_#209	GO:0006953: acute-phase response	4.12E-14	16,20,8
3dfr__#63	GO:0006730: one-carbon compound metabolism	4.45E-14	11,32,8
1ac6a_#34	GO:0042088: T-helper 1 type immune response	4.53E-14	57,11,9
1aqt_2#45	GO:0019883: antigen presentation, endogenous antigen	5.26E-14	69,10,9
1ypta_#418	GO:0006100: tricarboxylic acid cycle intermediate metabolism	5.61E-14	24,14,8
1hyea2#272	GO:0006096: glycolysis	5.66E-14	21,38,10
1ceqa2#199	GO:0006096: glycolysis	5.66E-14	10,38,8
2hlpa2#272	GO:0006096: glycolysis	5.66E-14	10,38,8
1hyha2#211	GO:0006100: tricarboxylic acid cycle intermediate metabolism	6.15E-14	7,14,6
1hbza_#214	GO:0030333: antigen processing	6.94E-14	249,13,13
1llda2#177	GO:0006096: glycolysis	7.26E-14	15,38,9
1rypk_#12	GO:0030333: antigen processing	7.32E-14	250,13,13
1i50b_#966	GO:0030333: antigen processing	7.72E-14	251,13,13
2hlca_#27	GO:0006953: acute-phase response	7.75E-14	17,20,8
1dy9.1#A44	GO:0006953: acute-phase response	7.75E-14	17,20,8
1eq9a_#59	GO:0006953: acute-phase response	7.75E-14	17,20,8
1gdna_#121	GO:0006953: acute-phase response	7.75E-14	17,20,8
1bqya_#103	GO:0006953: acute-phase response	7.75E-14	17,20,8
1dlea_#198	GO:0006953: acute-phase response	7.75E-14	17,20,8
1ton__#231	GO:0006953: acute-phase response	7.75E-14	17,20,8
1feca1#28	GO:0006118: electron transport	8.06E-14	47,228,24
1a65a1#108	GO:0030333: antigen processing	8.58E-14	253,13,13
1ej8a_#189	GO:0030333: antigen processing	1.00E-13	256,13,13
1htp__#16	GO:0030333: antigen processing	1.00E-13	256,13,13
1phm_2#253	GO:0030333: antigen processing	1.06E-13	257,13,13
1bjt__#890	GO:0030333: antigen processing	1.11E-13	258,13,13
1el0a_#42	GO:0030333: antigen processing	1.11E-13	258,13,13
2hlca_#139	GO:0050819: negative regulation of coagulation	1.16E-13	75,10,9
2hlca_#139	GO:0030193: regulation of blood coagulation	1.16E-13	75,10,9

1f4la2#25	GO:0006418: tRNA aminoacylation for protein translation	1.19E-13	14,26,8
1ytba1#125	GO:0006367: transcription initiation from Pol II promoter	1.24E-13	17,12,7
1i4fa2#21	GO:0019883: antigen presentation, endogenous antigen	1.29E-13	10,10,6
1xgsa2#82	GO:0030333: antigen processing	1.30E-13	261,13,13
1dnv__#354	GO:0030333: antigen processing	1.31E-13	49,13,9
1fnf_3#1409	GO:0019883: antigen presentation, endogenous antigen	1.32E-13	76,10,9
1df7a_#14	GO:0006730: one-carbon compound metabolism	1.32E-13	12,32,8
1danh_#197	GO:0006953: acute-phase response	1.39E-13	18,20,8
1svpa_#127	GO:0006953: acute-phase response	1.39E-13	18,20,8
1qq4a_#143	GO:0006953: acute-phase response	1.39E-13	18,20,8
1dlea_#53	GO:0006953: acute-phase response	1.39E-13	18,20,8
1cwva2#675	GO:0030333: antigen processing	1.40E-13	29,13,8
1dar_4#634	GO:0030333: antigen processing	1.40E-13	8,13,6
1tc1a_#158	GO:0009116: nucleoside metabolism	1.40E-13	9,25,7
1evqa_#305	GO:0006096: glycolysis	1.43E-13	384,38,26
1h6la_#119	GO:0030333: antigen processing	1.51E-13	264,13,13
1qfma1#139	GO:0030333: antigen processing	1.51E-13	264,13,13
2bpa1_#45	GO:0030333: antigen processing	1.52E-13	179,13,12
1fu6a_#50	GO:0030333: antigen processing	1.59E-13	265,13,13
1hxmb1#117	GO:0042088: T-helper 1 type immune response	1.60E-13	65,11,9
1hxmb1#117	GO:0042108: positive regulation of cytokine biosynthesis	1.60E-13	65,11,9
1ceqa1#139	GO:0006096: glycolysis	1.64E-13	16,38,9
1f3ya_#6	GO:0030333: antigen processing	1.65E-13	80,13,10
1dr9a2#139	GO:0019883: antigen presentation, endogenous antigen	1.69E-13	78,10,9
1cjca2#76	GO:0006118: electron transport	1.82E-13	21,228,16
1gsoa3#142	GO:0019883: antigen presentation, endogenous antigen	1.90E-13	79,10,9
1dpja_#322	GO:0030333: antigen processing	1.94E-13	269,13,13
1jd0a_#176	GO:0030333: antigen processing	2.00E-13	183,13,12
1j9qa1#154	GO:0030333: antigen processing	2.00E-13	183,13,12
1danh_#152	GO:0006953: acute-phase response	2.06E-13	11,20,7
1f0xa1#523	GO:0019883: antigen presentation, endogenous antigen	2.14E-13	80,10,9
1a3qa2#190	GO:0030333: antigen processing	2.22E-13	125,13,11
1qaxa2#103	GO:0019883: antigen presentation, endogenous antigen	2.38E-13	23,10,7
1hxxa_#228	GO:0019883: antigen presentation, endogenous antigen	2.38E-13	23,10,7
1a6da3#190	GO:0006953: acute-phase response	2.39E-13	19,20,8
1ton__#196	GO:0006953: acute-phase response	2.39E-13	19,20,8
1qq4a_#63	GO:0006953: acute-phase response	2.39E-13	19,20,8
1arb__#24	GO:0006953: acute-phase response	2.39E-13	19,20,8
1a8i__#688	GO:0009070: serine family amino acid biosynthesis	2.40E-13	203,26,17
3grx__#8	GO:0006118: electron transport	2.47E-13	12,228,12
1d7ya1#243	GO:0006118: electron transport	2.47E-13	12,228,12
1fuma2#221	GO:0006118: electron transport	2.47E-13	12,228,12
2btva_#866	GO:0030333: antigen processing	2.68E-13	17,13,7
1ddja_#726	GO:0050819: negative regulation of coagulation	2.70E-13	82,10,9
1ddja_#726	GO:0030193: regulation of blood coagulation	2.70E-13	82,10,9
1ton__#47	GO:0050819: negative regulation of coagulation	2.70E-13	82,10,9
1ton__#47	GO:0030193: regulation of blood coagulation	2.70E-13	82,10,9
1as4.1#A336	GO:0030333: antigen processing	2.73E-13	276,13,13
1d0na3#305	GO:0030333: antigen processing	2.73E-13	276,13,13
1el5a1#183	GO:0006118: electron transport	2.76E-13	14,228,13
1danh_#24	GO:0050819: negative regulation of coagulation	2.82E-13	11,10,6
1danh_#24	GO:0030193: regulation of blood coagulation	2.82E-13	11,10,6
1hvba_#63	GO:0017001: antibiotic catabolism	2.82E-13	11,10,6
1jb0d_#26	GO:0030333: antigen processing	3.17E-13	190,13,12
1leha1#226	GO:0006563: L-serine metabolism	3.21E-13	19,12,7
1eq2a_#11	GO:0006100: tricarboxylic acid cycle intermediate metabolism	3.24E-13	29,14,8

1qdla_#211	GO:0030333: antigen processing	3.31E-13	280,13,13
1ekma1#545	GO:0019883: antigen presentation, endogenous antigen	3.40E-13	84,10,9
1aky_2#133	GO:0030333: antigen processing	3.47E-13	130,13,11
1hdfa_#16	GO:0030333: antigen processing	3.61E-13	192,13,12
1hxxa_#232	GO:0030333: antigen processing	3.63E-13	282,13,13
1mdah_#329	GO:0030333: antigen processing	3.63E-13	282,13,13
1thg__#3	GO:0019883: antigen presentation, endogenous antigen	3.80E-13	85,10,9
1bywa_#30	GO:0030333: antigen processing	3.81E-13	283,13,13
1arb__#55	GO:0006953: acute-phase response	3.96E-13	20,20,8
1gdna_#30	GO:0006953: acute-phase response	3.96E-13	20,20,8
1ev2e2#286	GO:0030333: antigen processing	3.99E-13	87,13,10
1qnja_#46	GO:0006953: acute-phase response	4.07E-13	32,20,9
2viua_#252	GO:0030333: antigen processing	4.18E-13	285,13,13
1fi2a_#109	GO:0030333: antigen processing	4.36E-13	195,13,12
1mrj__#161	GO:0017148: negative regulation of protein biosynthesis	4.38E-13	18,13,7
1qhoa1#531	GO:0030333: antigen processing	4.38E-13	18,13,7
1j9qa2#295	GO:0016337: cell-cell adhesion	4.48E-13	39,17,9
1bmv2_#2064	GO:0030333: antigen processing	4.50E-13	133,13,11
1e44b_#75	GO:0006953: acute-phase response	4.65E-13	165,20,14
1f0xa1#482	GO:0006118: electron transport	4.74E-13	50,228,24
1bqya_#115	GO:0006953: acute-phase response	4.93E-13	12,20,7
2mev3_#108	GO:0030333: antigen processing	4.94E-13	197,13,12
1gtra2#113	GO:0006418: tRNA aminoacylation for protein translation	5.02E-13	16,26,8
1jj2v_#118	GO:0030333: antigen processing	5.06E-13	89,13,10
1h8d.1#H207	GO:0050819: negative regulation of coagulation	5.18E-13	49,10,8
1h8d.1#H207	GO:0030193: regulation of blood coagulation	5.18E-13	49,10,8
1aqua_#126	GO:0009070: serine family amino acid biosynthesis	5.26E-13	472,26,22
1mai__#108	GO:0030333: antigen processing	5.32E-13	135,13,11
1gg6.1#C156	GO:0050819: negative regulation of coagulation	5.64E-13	12,10,6
1gg6.1#C156	GO:0030193: regulation of blood coagulation	5.64E-13	12,10,6
2hlca_#99	GO:0050819: negative regulation of coagulation	5.64E-13	12,10,6
2hlca_#99	GO:0030193: regulation of blood coagulation	5.64E-13	12,10,6
1bgva1#326	GO:0006563: L-serine metabolism	5.64E-13	10,12,6
2occb1#209	GO:0030333: antigen processing	5.69E-13	90,13,10
1mrj__#73	GO:0030333: antigen processing	5.69E-13	57,13,9
1h4vb2#282	GO:0006418: tRNA aminoacylation for protein translation	5.71E-13	91,26,13
2hlpa1#98	GO:0006096: glycolysis	6.10E-13	12,38,8
1feca1#122	GO:0006118: electron transport	6.14E-13	22,228,16
1i7qa_#186	GO:0019883: antigen presentation, endogenous antigen	6.35E-13	26,10,7
1f4la2#20	GO:0006418: tRNA aminoacylation for protein translation	6.35E-13	10,26,7
1c5y.1#B241	GO:0006953: acute-phase response	6.38E-13	21,20,8
1dlea_#238	GO:0006953: acute-phase response	6.38E-13	21,20,8
1arb__#208	GO:0006953: acute-phase response	6.38E-13	21,20,8
1autc_#45	GO:0006953: acute-phase response	6.38E-13	21,20,8
1qnja_#138	GO:0006953: acute-phase response	6.38E-13	21,20,8
1aqb__#134	GO:0019883: antigen presentation, endogenous antigen	6.50E-13	90,10,9
1qlaa3#364	GO:0030333: antigen processing	6.72E-13	58,13,9
8dfr__#10	GO:0009070: serine family amino acid biosynthesis	6.75E-13	6,26,6
3dfr__#103	GO:0009070: serine family amino acid biosynthesis	6.75E-13	6,26,6
2hlpa2#315	GO:0006096: glycolysis	6.83E-13	18,38,9
1bev1_#86	GO:0030333: antigen processing	6.91E-13	296,13,13
2bb2_1#57	GO:0030333: antigen processing	7.15E-13	92,13,10
1dzfa2#211	GO:0030333: antigen processing	7.41E-13	139,13,11
3prn__#143	GO:0030333: antigen processing	8.26E-13	300,13,13
3sil__#317	GO:0030333: antigen processing	8.26E-13	300,13,13
1f2la_#40	GO:0030333: antigen processing	8.55E-13	206,13,12

1bvqa_#11	GO:0019883: antigen presentation, endogenous antigen	8.57E-13	27,10,7
1fepa_#340	GO:0030333: antigen processing	8.95E-13	94,13,10
1g6oa_#116	GO:0030333: antigen processing	9.02E-13	302,13,13
1fgga_#134	GO:0009116: nucleoside metabolism	9.35E-13	193,25,16
1axca1#70	GO:0019883: antigen presentation, endogenous antigen	9.77E-13	94,10,9
1bwvs_#117	GO:0006118: electron transport	9.89E-13	287,228,63
1arb__#193	GO:0006953: acute-phase response	9.98E-13	22,20,8
1fwxa2#396	GO:0030333: antigen processing	1.02E-12	209,13,12
1g6ga_#133	GO:0006953: acute-phase response	1.06E-12	13,20,7
1danh_#242	GO:0006953: acute-phase response	1.06E-12	13,20,7
1ekbb_#79	GO:0006953: acute-phase response	1.06E-12	13,20,7
1qama_#37	GO:0006096: glycolysis	1.07E-12	48,38,12
1qfma1#186	GO:0030333: antigen processing	1.07E-12	306,13,13
1am2__#163	GO:0030333: antigen processing	1.11E-12	96,13,10
1fu6a_#50	GO:0007169: transmembrane receptor protein tyrosine kinase signaling pathway	1.15E-12	265,26,18
1opy__#68	GO:0030333: antigen processing	1.17E-12	308,13,13
1eq9a_#32	GO:0050819: negative regulation of coagulation	1.19E-12	96,10,9
1eq9a_#32	GO:0030193: regulation of blood coagulation	1.19E-12	96,10,9
1plq_1#90	GO:0030333: antigen processing	1.19E-12	145,13,11
1ddja_#681	GO:0030333: antigen processing	1.22E-12	212,13,12
1d9ta_#137	GO:0006544: glycine metabolism	1.24E-12	12,11,6
1gdha1#237	GO:0006563: L-serine metabolism	1.24E-12	11,12,6
1fo4a5#1020	GO:0030333: antigen processing	1.39E-12	312,13,13
1i7qa_#321	GO:0030333: antigen processing	1.39E-12	147,13,11
1qhda2#323	GO:0030333: antigen processing	1.45E-12	313,13,13
1hle.1#B387	GO:0019883: antigen presentation, endogenous antigen	1.50E-12	29,10,7
1ghpa_#245	GO:0030333: antigen processing	1.57E-12	38,13,8
1fytd2#143	GO:0030333: antigen processing	1.57E-12	5,13,5
1fhoa_#74	GO:0030333: antigen processing	1.58E-12	315,13,13
1trka2#429	GO:0006118: electron transport	1.58E-12	40,228,21
1e30a_#87	GO:0006118: electron transport	1.65E-12	44,228,22
1fjfj_#68	GO:0030333: antigen processing	1.71E-12	218,13,12
1gdha1#237	GO:0009070: serine family amino acid biosynthesis	1.74E-12	11,26,7
1ghpa_#236	GO:0017001: antibiotic catabolism	1.83E-12	14,10,6
1e3ua_#67	GO:0017001: antibiotic catabolism	1.84E-12	6,10,5
1kit_3#331	GO:0019883: antigen presentation, endogenous antigen	1.91E-12	101,10,9
1h6va3#486	GO:0030333: antigen processing	2.00E-12	65,13,9
1hbza_#96	GO:0030333: antigen processing	2.11E-12	322,13,13
1ddwa_#77	GO:0030333: antigen processing	2.11E-12	322,13,13
1leha1#255	GO:0006563: L-serine metabolism	2.18E-12	24,12,7
3chbd_#85	GO:0030333: antigen processing	2.20E-12	323,13,13
2hlca_#68	GO:0006953: acute-phase response	2.27E-12	24,20,8
1f3ya_#6	GO:0006953: acute-phase response	2.34E-12	80,20,11
1ek0a_#151	GO:0007264: small GTPase mediated signal transduction	2.45E-12	23,21,8
1fo5a_#60	GO:0030333: antigen processing	2.52E-12	225,13,12
1kit_3#743	GO:0030333: antigen processing	2.52E-12	225,13,12
8dfr__#10	GO:0006730: one-carbon compound metabolism	2.66E-12	6,32,6
3dfr__#103	GO:0006730: one-carbon compound metabolism	2.66E-12	6,32,6
1ezvd1#97	GO:0006118: electron transport	2.84E-12	11,228,11
1ezvd1#101	GO:0006118: electron transport	2.84E-12	11,228,11
1hc7a2#113	GO:0006418: tRNA aminoacylation for protein translation	2.89E-12	19,26,8
1leha1#226	GO:0009070: serine family amino acid biosynthesis	2.89E-12	19,26,8
1smaa2#581	GO:0019883: antigen presentation, endogenous antigen	2.92E-12	60,10,8
1f46a_#59	GO:0019883: antigen presentation, endogenous antigen	3.00E-12	106,10,9
1d7ba_#87	GO:0030333: antigen processing	3.06E-12	68,13,9

1danh_#104	GO:0006953: acute-phase response	3.17E-12	147,20,13
1hxxa_#173	GO:0030333: antigen processing	3.29E-12	230,13,12
1p35a_#49	GO:0006953: acute-phase response	3.31E-12	112,20,12
1mkp__#216	GO:0006470: protein amino acid dephosphorylation	3.31E-12	112,20,12
3frua1#236	GO:0019883: antigen presentation, endogenous antigen	3.35E-12	61,10,8
1utea_#11	GO:0030333: antigen processing	3.43E-12	107,13,10
1h4ua1#404	GO:0006418: tRNA aminoacylation for protein translation	3.50E-12	163,26,15
1f42a2#123	GO:0030333: antigen processing	3.57E-12	335,13,13
2hlpa2#298	GO:0006096: glycolysis	3.65E-12	9,38,7
1pysb5#672	GO:0006418: tRNA aminoacylation for protein translation	3.72E-12	30,26,9
1bec_2#216	GO:0019883: antigen presentation, endogenous antigen	3.90E-12	109,10,9
1b8pa2#197	GO:0006096: glycolysis	4.00E-12	21,38,9
1d9ta_#137	GO:0009070: serine family amino acid biosynthesis	4.14E-12	12,26,7
1oaca1#495	GO:0006953: acute-phase response	4.91E-12	41,20,9
1ie5a_#102	GO:0030217: T-cell differentiation	5.03E-12	64,10,8
1e44b_#75	GO:0030333: antigen processing	5.11E-12	165,13,11
1bio__#210	GO:0050819: negative regulation of coagulation	5.45E-12	113,10,9
1bio__#210	GO:0030193: regulation of blood coagulation	5.45E-12	113,10,9
1by5a_#347	GO:0030333: antigen processing	5.53E-12	240,13,12
1ghpa_#236	GO:0046677: response to antibiotic	5.67E-12	14,40,8
1hxmb1#117	GO:0030217: T-cell differentiation	5.73E-12	65,10,8
1amp__#83	GO:0006367: transcription initiation from Pol II promoter	5.79E-12	6,12,5
1vmoa_#106	GO:0030333: antigen processing	5.85E-12	167,13,11
1nfdb1#34	GO:0030333: antigen processing	5.85E-12	167,13,11
1feza_#185	GO:0000160: two-component signal transduction system (phosphorelay)	6.02E-12	66,25,11
2mpra_#190	GO:0030333: antigen processing	6.13E-12	349,13,13
1jb9a2#173	GO:0006730: one-carbon compound metabolism	6.22E-12	17,32,8
1am2__#163	GO:0042088: T-helper 1 type immune response	6.34E-12	96,11,9
1dhs__#99	GO:0009070: serine family amino acid biosynthesis	6.36E-12	109,26,13
1quna1#113	GO:0030333: antigen processing	6.43E-12	243,13,12
1e5sa_#239	GO:0019883: antigen presentation, endogenous antigen	6.43E-12	35,10,7
1qs0a1#119	GO:0006096: glycolysis	6.51E-12	367,38,24
1gdna_#26	GO:0006919: caspase activation	6.67E-12	11,15,6
1elva1#604	GO:0006953: acute-phase response	6.78E-12	27,20,8
1ac6a_#34	GO:0042108: positive regulation of cytokine biosynthesis	6.80E-12	57,11,8
1edza1#206	GO:0030333: antigen processing	6.87E-12	352,13,13
1dgsa1#523	GO:0015671: oxygen transport	6.90E-12	53,23,10
1d5ra2#25	GO:0006470: protein amino acid dephosphorylation	6.99E-12	16,20,7
1gdna_#56	GO:0006953: acute-phase response	6.99E-12	16,20,7
1aym3_#131	GO:0030333: antigen processing	7.13E-12	353,13,13
2foka2#180	GO:0015671: oxygen transport	7.27E-12	129,23,13
2hlca_#226	GO:0050819: negative regulation of coagulation	7.40E-12	67,10,8
2hlca_#226	GO:0030193: regulation of blood coagulation	7.40E-12	67,10,8
1ra9__#62	GO:0009070: serine family amino acid biosynthesis	7.69E-12	21,26,8
1ekbb_#228	GO:0006953: acute-phase response	7.84E-12	43,20,9
1g0sa_#74	GO:0030333: antigen processing	7.85E-12	247,13,12
1ekbb_#85	GO:0050819: negative regulation of coagulation	8.14E-12	118,10,9
1ekbb_#85	GO:0030193: regulation of blood coagulation	8.14E-12	118,10,9
1d7ba_#87	GO:0019883: antigen presentation, endogenous antigen	8.38E-12	68,10,8
1qi7a_#224	GO:0017148: negative regulation of protein biosynthesis	9.41E-12	6,13,5
1eq9a_#58	GO:0006953: acute-phase response	9.45E-12	28,20,8
1kve.1#B199	GO:0019883: antigen presentation, endogenous antigen	9.47E-12	69,10,8
1i6vd_#1283	GO:0019883: antigen presentation, endogenous antigen	9.51E-12	120,10,9
1e8ga2#201	GO:0006118: electron transport	1.00E-11	286,228,61
1ddja_#746	GO:0050819: negative regulation of coagulation	1.03E-11	121,10,9
1ddja_#746	GO:0030193: regulation of blood coagulation	1.03E-11	121,10,9

1arb__#210	GO:0050819: negative regulation of coagulation	1.07E-11	70,10,8
1arb__#210	GO:0030193: regulation of blood coagulation	1.07E-11	70,10,8
1e3ua_#67	GO:0046677: response to antibiotic	1.13E-11	6,40,6
1bjt__#890	GO:0042088: T-helper 1 type immune response	1.14E-11	258,11,11
1bjt__#890	GO:0042108: positive regulation of cytokine biosynthesis	1.14E-11	258,11,11
1vdra_#37	GO:0006544: glycine metabolism	1.18E-11	7,11,5
1g25a_#22	GO:0030333: antigen processing	1.19E-11	367,13,13
1bvqa_#11	GO:0030333: antigen processing	1.20E-11	27,13,7
1ghpa_#245	GO:0019883: antigen presentation, endogenous antigen	1.20E-11	38,10,7
1qama_#39	GO:0009307: DNA restriction	1.26E-11	18,19,7
1bqya_#115	GO:0006919: caspase activation	1.33E-11	12,15,6
1elva1#566	GO:0006919: caspase activation	1.33E-11	12,15,6
1gg6.1#C156	GO:0006919: caspase activation	1.33E-11	12,15,6
2hlca_#99	GO:0006919: caspase activation	1.33E-11	12,15,6
1epwa3#138	GO:0030333: antigen processing	1.38E-11	371,13,13
1ldm_2#272	GO:0006096: glycolysis	1.52E-11	16,38,8
1dyoa_#153	GO:0006118: electron transport	1.53E-11	144,228,40
1qb7a_#86	GO:0009116: nucleoside metabolism	1.56E-11	36,25,9
1nat__#50	GO:0006096: glycolysis	1.63E-11	242,38,20
1dn2a1#261	GO:0030333: antigen processing	1.64E-11	376,13,13
1flga_#161	GO:0019883: antigen presentation, endogenous antigen	1.73E-11	128,10,9
1ddja_#726	GO:0030333: antigen processing	1.79E-11	82,13,9
1lara1#1407	GO:0006470: protein amino acid dephosphorylation	1.89E-11	5,20,5
1ypta_#408	GO:0006470: protein amino acid dephosphorylation	1.89E-11	5,20,5
1io1a_#311	GO:0019883: antigen presentation, endogenous antigen	2.00E-11	130,10,9
1b8aa2#368	GO:0019883: antigen presentation, endogenous antigen	2.00E-11	130,10,9
1ac6a_#34	GO:0051249: regulation of lymphocyte activation	2.00E-11	57,12,8
1hwxa1#324	GO:0006563: L-serine metabolism	2.02E-11	7,12,5
1bgva1#350	GO:0006563: L-serine metabolism	2.02E-11	7,12,5
3lada1#116	GO:0006118: electron transport	2.08E-11	133,228,38
1hle.1#B387	GO:0030333: antigen processing	2.10E-11	29,13,7
1dlfl_#97	GO:0019883: antigen presentation, endogenous antigen	2.14E-11	41,10,7
1f0ia1#50	GO:0019883: antigen presentation, endogenous antigen	2.14E-11	131,10,9
2ltn.1#A63	GO:0019883: antigen presentation, endogenous antigen	2.14E-11	131,10,9
1cpt__#52	GO:0006118: electron transport	2.20E-11	29,228,17
1dt6a_#77	GO:0006118: electron transport	2.20E-11	29,228,17
1qs0a1#119	GO:0009070: serine family amino acid biosynthesis	2.29E-11	367,26,19
1qs1a2#437	GO:0030333: antigen processing	2.40E-11	387,13,13
1c5y.1#B191	GO:0006919: caspase activation	2.46E-11	13,15,6
1g6ga_#133	GO:0006919: caspase activation	2.46E-11	13,15,6
1c5y.1#B94	GO:0006919: caspase activation	2.46E-11	13,15,6
1a0la_#193	GO:0006919: caspase activation	2.46E-11	13,15,6
1hwxa1#223	GO:0009070: serine family amino acid biosynthesis	2.49E-11	186,26,15
2arca_#55	GO:0030333: antigen processing	2.53E-11	130,13,10
1lvl_2#222	GO:0006118: electron transport	2.58E-11	58,228,24
3grs_1#23	GO:0006096: glycolysis	2.69E-11	139,38,16
1h6va2#245	GO:0006118: electron transport	2.83E-11	83,228,29
1qqsa_#124	GO:0030333: antigen processing	2.85E-11	392,13,13
1ddma_#130	GO:0006118: electron transport	2.85E-11	285,228,60
3grs_2#189	GO:0006118: electron transport	3.14E-11	12,228,11
1qb7a_#148	GO:0043094: metabolic compound salvage	3.14E-11	8,11,5
1bio__#108	GO:0006953: acute-phase response	3.14E-11	32,20,8
1pma1_#17	GO:0006953: acute-phase response	3.14E-11	32,20,8
1egza_#254	GO:0030245: cellulose catabolism	3.22E-11	5,22,5
1feca1#309	GO:0006118: electron transport	3.25E-11	10,228,10
1fcda2#239	GO:0006118: electron transport	3.25E-11	10,228,10

1qfja2#201	GO:0006118: electron transport	3.25E-11	10,228,10
2pia_3#280	GO:0006118: electron transport	3.25E-11	10,228,10
1b0pa5#695	GO:0006118: electron transport	3.25E-11	10,228,10
1f3ya_#6	GO:0019883: antigen presentation, endogenous antigen	3.26E-11	80,10,8
1f3ya_#6	GO:0050819: negative regulation of coagulation	3.26E-11	80,10,8
1f3ya_#6	GO:0030193: regulation of blood coagulation	3.26E-11	80,10,8
1i2ma_#157	GO:0007264: small GTPase mediated signal transduction	3.27E-11	10,21,6
1fyhb1#99	GO:0030333: antigen processing	3.52E-11	31,13,7
8dfr__#9	GO:0009070: serine family amino acid biosynthesis	3.54E-11	97,26,12
1i5ga_#115	GO:0006118: electron transport	3.59E-11	294,228,61
1a59__#341	GO:0015671: oxygen transport	3.60E-11	28,23,8
1a7s__#148	GO:0050819: negative regulation of coagulation	3.63E-11	44,10,7
1a7s__#148	GO:0030193: regulation of blood coagulation	3.63E-11	44,10,7
1nuka_#80	GO:0030333: antigen processing	3.71E-11	197,13,11
1avgi_#69	GO:0030333: antigen processing	3.76E-11	281,13,12
1hq8a_#206	GO:0030333: antigen processing	3.76E-11	281,13,12
1erv__#80	GO:0030333: antigen processing	3.84E-11	401,13,13
1e3ua_#205	GO:0017001: antibiotic catabolism	3.85E-11	9,10,5
1qfta_#98	GO:0030333: antigen processing	3.92E-11	198,13,11
1hyea2#182	GO:0006096: glycolysis	4.04E-11	26,38,9
1gg6.1#B19	GO:0006953: acute-phase response	4.13E-11	33,20,8
2bb2_2#134	GO:0030333: antigen processing	4.15E-11	199,13,11
1tmy__#34	GO:0000160: two-component signal transduction system (phosphorelay)	4.28E-11	9,25,6
1c5y.1#B192	GO:0006919: caspase activation	4.30E-11	14,15,6
1c5y.1#B220	GO:0006919: caspase activation	4.30E-11	14,15,6
1bio__#81	GO:0006919: caspase activation	4.30E-11	14,15,6
1qnja_#71	GO:0006919: caspase activation	4.30E-11	14,15,6
1danh_#142	GO:0006919: caspase activation	4.30E-11	14,15,6
1danh_#70	GO:0006919: caspase activation	4.30E-11	14,15,6
1egaa1#97	GO:0007264: small GTPase mediated signal transduction	4.54E-11	19,21,7
2hlca_#139	GO:0006953: acute-phase response	4.58E-11	75,20,10
1jsg__#92	GO:0006953: acute-phase response	4.86E-11	519,20,18
1ra9__#62	GO:0006730: one-carbon compound metabolism	5.03E-11	21,32,8
1danh_#24	GO:0006953: acute-phase response	5.12E-11	11,20,6
1kid__#330	GO:0019883: antigen presentation, endogenous antigen	5.13E-11	144,10,9
1mpp__#151	GO:0030333: antigen processing	5.21E-11	92,13,9
1evqa_#61	GO:0030333: antigen processing	5.39E-11	140,13,10
1eur__#84	GO:0019883: antigen presentation, endogenous antigen	5.39E-11	85,10,8
1eur__#84	GO:0030217: T-cell differentiation	5.39E-11	85,10,8
1ofga1#84	GO:0006100: tricarboxylic acid cycle intermediate metabolism	5.42E-11	30,14,7
1i76a_#138	GO:0009116: nucleoside metabolism	5.45E-11	80,25,11
1jjya_#329	GO:0009070: serine family amino acid biosynthesis	5.48E-11	56,26,10
1e79d2#36	GO:0006953: acute-phase response	5.52E-11	432,20,17
1gox__#124	GO:0009116: nucleoside metabolism	5.57E-11	298,25,17
1cbf__#208	GO:0030333: antigen processing	5.74E-11	291,13,12
1qtn.1#A270	GO:0009070: serine family amino acid biosynthesis	5.76E-11	386,26,19
1lkka_#127	GO:0007169: transmembrane receptor protein tyrosine kinase signaling pathway	5.82E-11	16,26,7
1cgha_#227	GO:0050819: negative regulation of coagulation	5.94E-11	47,10,7
1cgha_#227	GO:0030193: regulation of blood coagulation	5.94E-11	47,10,7
1cg5b_#32	GO:0015671: oxygen transport	6.07E-11	10,23,6
1nat__#50	GO:0006563: L-serine metabolism	6.10E-11	242,12,11
1ac6a_#43	GO:0019883: antigen presentation, endogenous antigen	6.20E-11	147,10,9
1fjfl_#67	GO:0019883: antigen presentation, endogenous antigen	6.20E-11	147,10,9
1danh_#104	GO:0019883: antigen presentation, endogenous antigen	6.20E-11	147,10,9
1danh_#104	GO:0050819: negative regulation of coagulation	6.20E-11	147,10,9

1danh_#104	GO:0030193: regulation of blood coagulation	6.20E-11	147,10,9
1hw5a2#64	GO:0019883: antigen presentation, endogenous antigen	6.60E-11	148,10,9
1e4ea2#215	GO:0030333: antigen processing	6.86E-11	419,13,13
1elva1#623	GO:0006953: acute-phase response	6.94E-11	35,20,8
1c7na_#95	GO:0019883: antigen presentation, endogenous antigen	7.02E-11	149,10,9
1tc1a_#158	GO:0043094: metabolic compound salvage	7.05E-11	9,11,5
1ekbb_#117	GO:0006919: caspase activation	7.14E-11	15,15,6
1danh_#140	GO:0006919: caspase activation	7.14E-11	15,15,6
1danh_#190	GO:0006919: caspase activation	7.14E-11	15,15,6
1gdna_#155	GO:0006919: caspase activation	7.14E-11	15,15,6
1ton__#112	GO:0006919: caspase activation	7.14E-11	15,15,6
1ekbb_#141	GO:0006919: caspase activation	7.14E-11	15,15,6
1ddja_#759	GO:0006919: caspase activation	7.14E-11	15,15,6
1gg6.1#C229	GO:0006919: caspase activation	7.14E-11	15,15,6
2naca1#162	GO:0006563: L-serine metabolism	7.15E-11	19,12,6
1a3qa2#190	GO:0042088: T-helper 1 type immune response	7.32E-11	125,11,9
1a3qa2#190	GO:0042108: positive regulation of cytokine biosynthesis	7.32E-11	125,11,9
1qfta_#98	GO:0042088: T-helper 1 type immune response	7.88E-11	198,11,10
1jj2v_#118	GO:0019883: antigen presentation, endogenous antigen	7.88E-11	89,10,8
1c8za_#386	GO:0030333: antigen processing	7.95E-11	211,13,11
1qfea_#68	GO:0009073: aromatic amino acid family biosynthesis	7.97E-11	453,16,15
1b8aa1#41	GO:0006418: tRNA aminoacylation for protein translation	8.03E-11	5,26,5
1opy__#68	GO:0042088: T-helper 1 type immune response	8.28E-11	308,11,11
1opy__#68	GO:0042108: positive regulation of cytokine biosynthesis	8.28E-11	308,11,11
1pud__#44	GO:0006098: pentose-phosphate shunt	8.30E-11	43,11,7
1dyna_#42	GO:0019883: antigen presentation, endogenous antigen	8.43E-11	152,10,9
1g38a_#47	GO:0009307: DNA restriction	8.48E-11	6,19,5
1e5sa_#239	GO:0030333: antigen processing	8.93E-11	35,13,7
1by5a_#238	GO:0030333: antigen processing	9.30E-11	214,13,11
2mev1_#123	GO:0019883: antigen presentation, endogenous antigen	9.47E-11	91,10,8
1cyx__#138	GO:0006118: electron transport	9.78E-11	24,228,15
1ejda_#6	GO:0030333: antigen processing	9.79E-11	215,13,11
1qs0a1#235	GO:0006096: glycolysis	9.92E-11	86,38,13
1e42a1#711	GO:0019883: antigen presentation, endogenous antigen	1.01E-10	155,10,9
1e79d2#36	GO:0030333: antigen processing	1.03E-10	432,13,13
2bb2_1#57	GO:0019883: antigen presentation, endogenous antigen	1.04E-10	92,10,8
3sil__#284	GO:0030333: antigen processing	1.04E-10	62,13,8
1qp8a1#172	GO:0006100: tricarboxylic acid cycle intermediate metabolism	1.06E-10	17,14,6
1c7na_#95	GO:0006953: acute-phase response	1.06E-10	149,20,12
1nsca_#236	GO:0019883: antigen presentation, endogenous antigen	1.07E-10	156,10,9
1b35b_#97	GO:0006118: electron transport	1.09E-10	271,228,57
3grx__#56	GO:0030333: antigen processing	1.10E-10	307,13,12
8dfr__#115	GO:0009070: serine family amino acid biosynthesis	1.12E-10	28,26,8
1gdna_#56	GO:0006919: caspase activation	1.14E-10	16,15,6
1qnja_#118	GO:0006919: caspase activation	1.14E-10	16,15,6
1gdna_#123	GO:0006919: caspase activation	1.14E-10	16,15,6
1c5y.1#B234	GO:0006919: caspase activation	1.14E-10	16,15,6
1autc_#209	GO:0006919: caspase activation	1.14E-10	16,15,6
1ei5a3#190	GO:0046677: response to antibiotic	1.14E-10	12,40,7
1cpy__#93	GO:0009116: nucleoside metabolism	1.17E-10	218,25,15
1fepa_#340	GO:0019883: antigen presentation, endogenous antigen	1.24E-10	94,10,8
1akp__#62	GO:0019883: antigen presentation, endogenous antigen	1.26E-10	52,10,7
1phm_2#347	GO:0017001: antibiotic catabolism	1.28E-10	4,10,4
1dp0a4#756	GO:0030333: antigen processing	1.33E-10	153,13,10
1a9xa3#78	GO:0006568: tryptophan metabolism	1.34E-10	262,10,10

1ayaa_#98	GO:0007169: transmembrane receptor protein tyrosine kinase signaling pathway	1.38E-10	10,26,6
1ei5a3#311	GO:0019883: antigen presentation, endogenous antigen	1.38E-10	26,10,6
1bgva1#326	GO:0009070: serine family amino acid biosynthesis	1.38E-10	10,26,6
1gdna_#26	GO:0018108: peptidyl-tyrosine phosphorylation	1.41E-10	11,10,5
1danh_#152	GO:0050819: negative regulation of coagulation	1.41E-10	11,10,5
1danh_#152	GO:0030193: regulation of blood coagulation	1.41E-10	11,10,5
1idsa2#157	GO:0006801: superoxide metabolism	1.41E-10	10,11,5
1eq9a_#143	GO:0018108: peptidyl-tyrosine phosphorylation	1.41E-10	11,10,5
1h8d.1#H184	GO:0018108: peptidyl-tyrosine phosphorylation	1.41E-10	11,10,5
1elva1#601	GO:0018108: peptidyl-tyrosine phosphorylation	1.41E-10	11,10,5
1cpy__#380	GO:0030333: antigen processing	1.42E-10	154,13,10
1i2ma_#90	GO:0007264: small GTPase mediated signal transduction	1.43E-10	12,21,6
1aru__#57	GO:0015671: oxygen transport	1.45E-10	20,23,7
1hbza_#214	GO:0006953: acute-phase response	1.47E-10	249,20,14
1fsu__#434	GO:0030333: antigen processing	1.50E-10	315,13,12
1t7pa2#513	GO:0006418: tRNA aminoacylation for protein translation	1.54E-10	29,26,8
1iba__#39	GO:0030333: antigen processing	1.56E-10	316,13,12
1h4ua1#404	GO:0019883: antigen presentation, endogenous antigen	1.60E-10	163,10,9
1h8ma_#8	GO:0006118: electron transport	1.64E-10	123,228,35
1jlxa2#166	GO:0030333: antigen processing	1.75E-10	66,13,8
2hlca_#27	GO:0006919: caspase activation	1.76E-10	17,15,6
1dy9.1#A44	GO:0006919: caspase activation	1.76E-10	17,15,6
1gdna_#121	GO:0006919: caspase activation	1.76E-10	17,15,6
1bqya_#103	GO:0006919: caspase activation	1.76E-10	17,15,6
1dlea_#198	GO:0006919: caspase activation	1.76E-10	17,15,6
1ton__#231	GO:0006919: caspase activation	1.76E-10	17,15,6
1e44b_#75	GO:0019883: antigen presentation, endogenous antigen	1.79E-10	165,10,9
1ekbb_#85	GO:0006953: acute-phase response	1.87E-10	118,20,11
1qtn.1#A270	GO:0006096: glycolysis	1.90E-10	386,38,23
1eur__#84	GO:0042088: T-helper 1 type immune response	1.92E-10	85,11,8
1mrj__#240	GO:0017148: negative regulation of protein biosynthesis	1.96E-10	9,13,5
1ce7a_#9	GO:0017148: negative regulation of protein biosynthesis	1.96E-10	9,13,5
1g4ia_#11	GO:0006953: acute-phase response	2.04E-10	24,20,7
1f42a2#123	GO:0042088: T-helper 1 type immune response	2.12E-10	335,11,11
1f42a2#123	GO:0042108: positive regulation of cytokine biosynthesis	2.12E-10	335,11,11
1llda2#161	GO:0006096: glycolysis	2.28E-10	21,38,8
1jbwa2#198	GO:0009116: nucleoside metabolism	2.32E-10	11,25,6
1bqya_#115	GO:0018108: peptidyl-tyrosine phosphorylation	2.41E-10	12,10,5
1elva1#566	GO:0018108: peptidyl-tyrosine phosphorylation	2.41E-10	12,10,5
1h8d.1#H167	GO:0018108: peptidyl-tyrosine phosphorylation	2.41E-10	12,10,5
1ton__#120	GO:0018108: peptidyl-tyrosine phosphorylation	2.41E-10	12,10,5
1bqya_#216	GO:0018108: peptidyl-tyrosine phosphorylation	2.41E-10	12,10,5
1ei5a3#190	GO:0017001: antibiotic catabolism	2.41E-10	12,10,5
1gg6.1#C156	GO:0018108: peptidyl-tyrosine phosphorylation	2.41E-10	12,10,5
2hlca_#99	GO:0018108: peptidyl-tyrosine phosphorylation	2.41E-10	12,10,5
1hvba_#102	GO:0046677: response to antibiotic	2.45E-10	13,40,7
1b3ra1#223	GO:0006096: glycolysis	2.47E-10	31,38,9
1mrj__#73	GO:0019883: antigen presentation, endogenous antigen	2.47E-10	57,10,7
1ddja_#746	GO:0006953: acute-phase response	2.48E-10	121,20,11
1h4ua1#404	GO:0030333: antigen processing	2.52E-10	163,13,10
1bd3a_#195	GO:0043094: metabolic compound salvage	2.58E-10	11,11,5
1eova1#139	GO:0006418: tRNA aminoacylation for protein translation	2.61E-10	65,26,10
1danh_#197	GO:0006919: caspase activation	2.62E-10	18,15,6
1svpa_#127	GO:0006919: caspase activation	2.62E-10	18,15,6
1qq4a_#143	GO:0006919: caspase activation	2.62E-10	18,15,6

1dlea_#53	GO:0006919: caspase activation	2.62E-10	18,15,6
1cgha_#168	GO:0006606: protein-nucleus import	2.63E-10	13,21,6
1ytba2#217	GO:0006367: transcription initiation from Pol II promoter	2.64E-10	23,12,6
1ddja_#681	GO:0016477: cell migration	2.64E-10	212,23,14
1jj2v_#118	GO:0006953: acute-phase response	2.66E-10	89,20,10
1d6ja_#95	GO:0006118: electron transport	2.67E-10	54,228,22
1ifc__#89	GO:0030333: antigen processing	2.68E-10	164,13,10
1qfxa_#380	GO:0006953: acute-phase response	2.75E-10	41,20,8
1dr9a2#139	GO:0051249: regulation of lymphocyte activation	2.76E-10	78,12,8
1qlaa3#364	GO:0019883: antigen presentation, endogenous antigen	2.81E-10	58,10,7
1efpa1#32	GO:0006096: glycolysis	2.85E-10	114,38,14
1hdma1#115	GO:0030333: antigen processing	2.85E-10	165,13,10
1je5a_#10	GO:0006096: glycolysis	2.86E-10	58,38,11
1p35a_#101	GO:0030333: antigen processing	2.95E-10	111,13,9
1gox__#124	GO:0009073: aromatic amino acid family biosynthesis	3.02E-10	298,16,13
1ckla1#4	GO:0006956: complement activation	3.02E-10	4,12,4
1bg6_2#24	GO:0006118: electron transport	3.03E-10	22,228,14
1gsoa3#142	GO:0051249: regulation of lymphocyte activation	3.07E-10	79,12,8
1e1aa_#285	GO:0006118: electron transport	3.11E-10	138,228,37
2sli_2#326	GO:0042088: T-helper 1 type immune response	3.15E-10	227,11,10
2sli_2#326	GO:0042108: positive regulation of cytokine biosynthesis	3.15E-10	227,11,10
1deua_#151	GO:0030333: antigen processing	3.16E-10	239,13,11
1g51a3#525	GO:0030333: antigen processing	3.16E-10	239,13,11
1ez0a_#133	GO:0030333: antigen processing	3.20E-10	112,13,9
1ddja_#754	GO:0006953: acute-phase response	3.28E-10	14,20,6
1ekbb_#73	GO:0006953: acute-phase response	3.28E-10	14,20,6
1b0pa5#762	GO:0006118: electron transport	3.29E-10	11,228,10
1qfea_#68	GO:0009116: nucleoside metabolism	3.37E-10	453,25,19
1ycsa_#162	GO:0019883: antigen presentation, endogenous antigen	3.40E-10	177,10,9
1qp8a1#153	GO:0006563: L-serine metabolism	3.51E-10	24,12,6
1eq9a_#40	GO:0006953: acute-phase response	3.52E-10	64,20,9
1cpt__#401	GO:0006118: electron transport	3.52E-10	19,228,13
1ddja_#681	GO:0006953: acute-phase response	3.66E-10	212,20,13
1f8ra1#64	GO:0006118: electron transport	3.71E-10	9,228,9
1cpt__#358	GO:0006118: electron transport	3.71E-10	9,228,9
1kte__#24	GO:0006118: electron transport	3.71E-10	9,228,9
2pia_3#270	GO:0006118: electron transport	3.71E-10	9,228,9
1xer__#97	GO:0006118: electron transport	3.71E-10	9,228,9
1dz4a_#357	GO:0006118: electron transport	3.71E-10	9,228,9
1fxra_#6	GO:0006118: electron transport	3.71E-10	9,228,9
2bpa1_#45	GO:0019883: antigen presentation, endogenous antigen	3.77E-10	179,10,9
1ton__#196	GO:0006919: caspase activation	3.82E-10	19,15,6
1qq4a_#63	GO:0006919: caspase activation	3.82E-10	19,15,6
1arb__#24	GO:0006919: caspase activation	3.82E-10	19,15,6
1c5y.1#B191	GO:0018108: peptidyl-tyrosine phosphorylation	3.91E-10	13,10,5
1g6ga_#133	GO:0018108: peptidyl-tyrosine phosphorylation	3.91E-10	13,10,5
1c5y.1#B94	GO:0018108: peptidyl-tyrosine phosphorylation	3.91E-10	13,10,5
1cgha_#168	GO:0050819: negative regulation of coagulation	3.91E-10	13,10,5
1cgha_#168	GO:0030193: regulation of blood coagulation	3.91E-10	13,10,5
1cgha_#168	GO:0018108: peptidyl-tyrosine phosphorylation	3.91E-10	13,10,5
1a0la_#193	GO:0018108: peptidyl-tyrosine phosphorylation	3.91E-10	13,10,5
1ton__#179	GO:0018108: peptidyl-tyrosine phosphorylation	3.91E-10	13,10,5
1elva1#513	GO:0018108: peptidyl-tyrosine phosphorylation	3.91E-10	13,10,5
1ihna_#64	GO:0006953: acute-phase response	3.94E-10	7,20,5
1exma2#353	GO:0030333: antigen processing	4.05E-10	73,13,8
1e25a_#262	GO:0030333: antigen processing	4.08E-10	115,13,9

1e3ja2#270	GO:0006096: glycolysis	4.22E-10	60,38,11
1hwxa1#256	GO:0006563: L-serine metabolism	4.45E-10	48,12,7
1jd0a_#176	GO:0019883: antigen presentation, endogenous antigen	4.61E-10	183,10,9
1j9qa1#154	GO:0019883: antigen presentation, endogenous antigen	4.61E-10	183,10,9
1ct9a1#233	GO:0009070: serine family amino acid biosynthesis	4.75E-10	49,26,9
1f42a2#123	GO:0006953: acute-phase response	4.85E-10	335,20,15
1fn9a_#271	GO:0019883: antigen presentation, endogenous antigen	4.85E-10	184,10,9
1fxla2#169	GO:0019883: antigen presentation, endogenous antigen	4.85E-10	184,10,9
1f97a1#62	GO:0006953: acute-phase response	4.88E-10	591,20,18
1f97a2#199	GO:0030333: antigen processing	5.16E-10	118,13,9
1ez0a_#133	GO:0019883: antigen presentation, endogenous antigen	5.22E-10	112,10,8
1am2__#163	GO:0042108: positive regulation of cytokine biosynthesis	5.23E-10	96,11,8
1jf9a_#379	GO:0019883: antigen presentation, endogenous antigen	5.35E-10	186,10,9
1hc7a2#258	GO:0019883: antigen presentation, endogenous antigen	5.35E-10	186,10,9
3sil__#98	GO:0019883: antigen presentation, endogenous antigen	5.35E-10	186,10,9
1azza_#51	GO:0006953: acute-phase response	5.45E-10	15,20,6
1fjsa_#158	GO:0006919: caspase activation	5.45E-10	20,15,6
1arb__#55	GO:0006919: caspase activation	5.45E-10	20,15,6
1gdna_#30	GO:0006919: caspase activation	5.45E-10	20,15,6
1e2o__#346	GO:0019883: antigen presentation, endogenous antigen	5.62E-10	187,10,9
1a4ya_#104	GO:0006118: electron transport	5.63E-10	194,228,45
1mdah_#329	GO:0016337: cell-cell adhesion	5.67E-10	282,17,13
8dfr__#9	GO:0006544: glycine metabolism	5.69E-10	97,11,8
1hxxa_#278	GO:0006118: electron transport	5.80E-10	237,228,51
1el0a_#62	GO:0006935: chemotaxis	5.83E-10	14,41,7
1b3ra1#218	GO:0006563: L-serine metabolism	5.98E-10	26,12,6
1c5y.1#B192	GO:0018108: peptidyl-tyrosine phosphorylation	6.07E-10	14,10,5
1c5y.1#B220	GO:0018108: peptidyl-tyrosine phosphorylation	6.07E-10	14,10,5
1bio__#81	GO:0018108: peptidyl-tyrosine phosphorylation	6.07E-10	14,10,5
1qnja_#71	GO:0018108: peptidyl-tyrosine phosphorylation	6.07E-10	14,10,5
1danh_#142	GO:0018108: peptidyl-tyrosine phosphorylation	6.07E-10	14,10,5
1danh_#70	GO:0018108: peptidyl-tyrosine phosphorylation	6.07E-10	14,10,5
1ceqa1#139	GO:0006099: tricarboxylic acid cycle	6.09E-10	16,19,6
1fhga_#59	GO:0016337: cell-cell adhesion	6.23E-10	33,17,7
1fytd2#143	GO:0019883: antigen presentation, endogenous antigen	6.40E-10	5,10,4
1h6va3#486	GO:0019883: antigen presentation, endogenous antigen	6.45E-10	65,10,7
1cpza_#6	GO:0006953: acute-phase response	6.46E-10	132,20,11
1jb0d_#26	GO:0019883: antigen presentation, endogenous antigen	6.50E-10	190,10,9
1ds1a_#172	GO:0019883: antigen presentation, endogenous antigen	6.50E-10	190,10,9
1e9xa_#62	GO:0006118: electron transport	6.52E-10	34,228,17
1fhga_#59	GO:0030217: T-cell differentiation	6.57E-10	33,10,6
1hyha2#205	GO:0006096: glycolysis	6.58E-10	9,38,6
1e2fa_#14	GO:0006221: pyrimidine nucleotide biosynthesis	6.68E-10	7,22,5
1aq0a_#4	GO:0030245: cellulose catabolism	6.68E-10	7,22,5
1ihua2#525	GO:0009070: serine family amino acid biosynthesis	6.70E-10	278,26,16
1b12a_#221	GO:0051249: regulation of lymphocyte activation	6.79E-10	87,12,8
1a8d_1#87	GO:0019883: antigen presentation, endogenous antigen	6.82E-10	191,10,9
8dfr__#9	GO:0006730: one-carbon compound metabolism	6.83E-10	97,32,12
1g71a_#88	GO:0006953: acute-phase response	6.85E-10	28,20,7
1b3qa2#623	GO:0030333: antigen processing	7.04E-10	257,13,11
1hdfa_#16	GO:0019883: antigen presentation, endogenous antigen	7.15E-10	192,10,9
1hyrc1#247	GO:0019883: antigen presentation, endogenous antigen	7.21E-10	66,10,7
8dfr__#115	GO:0006730: one-carbon compound metabolism	7.24E-10	28,32,8
1a4ya_#47	GO:0006096: glycolysis	7.26E-10	172,38,16
1evqa_#305	GO:0009070: serine family amino acid biosynthesis	7.29E-10	384,26,18
1c5y.1#B241	GO:0006919: caspase activation	7.60E-10	21,15,6

1dlea_#238	GO:0006919: caspase activation	7.60E-10	21,15,6
1arb__#208	GO:0006919: caspase activation	7.60E-10	21,15,6
1autc_#45	GO:0006919: caspase activation	7.60E-10	21,15,6
1qnja_#138	GO:0006919: caspase activation	7.60E-10	21,15,6
1hnja1#8	GO:0006633: fatty acid biosynthesis	7.68E-10	12,27,6
1dc1a_#241	GO:0009307: DNA restriction	7.84E-10	8,19,5
1dj0a2#183	GO:0006100: tricarboxylic acid cycle intermediate metabolism	7.95E-10	111,14,9
1ypta_#418	GO:0006096: glycolysis	7.96E-10	24,38,8
1hyha2#179	GO:0006096: glycolysis	7.96E-10	24,38,8
1f97a2#199	GO:0030217: T-cell differentiation	7.99E-10	118,10,8
1fjfc2#153	GO:0030333: antigen processing	8.01E-10	362,13,12
1imva_#255	GO:0030333: antigen processing	8.23E-10	506,13,13
1fi2a_#109	GO:0019883: antigen presentation, endogenous antigen	8.24E-10	195,10,9
1lara2#1769	GO:0019883: antigen presentation, endogenous antigen	8.24E-10	195,10,9
1ycsa_#162	GO:0006953: acute-phase response	8.26E-10	177,20,12
1eu8a_#312	GO:0030333: antigen processing	8.51E-10	25,13,6
1f8ra2#325	GO:0019883: antigen presentation, endogenous antigen	8.63E-10	196,10,9
1eaja_#59	GO:0019883: antigen presentation, endogenous antigen	8.63E-10	196,10,9
1gpea1#271	GO:0019883: antigen presentation, endogenous antigen	8.63E-10	196,10,9
1qvba_#206	GO:0000160: two-component signal transduction system (phosphorelay)	8.74E-10	23,25,7
2mpra_#190	GO:0006953: acute-phase response	8.81E-10	349,20,15
1d4xg_#60	GO:0006118: electron transport	8.84E-10	94,228,29
2mev3_#108	GO:0019883: antigen presentation, endogenous antigen	9.04E-10	197,10,9
1ekbb_#117	GO:0018108: peptidyl-tyrosine phosphorylation	9.09E-10	15,10,5
1danh_#140	GO:0018108: peptidyl-tyrosine phosphorylation	9.09E-10	15,10,5
1danh_#190	GO:0018108: peptidyl-tyrosine phosphorylation	9.09E-10	15,10,5
1gdna_#155	GO:0018108: peptidyl-tyrosine phosphorylation	9.09E-10	15,10,5
1ton__#112	GO:0018108: peptidyl-tyrosine phosphorylation	9.09E-10	15,10,5
1ton__#91	GO:0018108: peptidyl-tyrosine phosphorylation	9.09E-10	15,10,5
1ekbb_#141	GO:0018108: peptidyl-tyrosine phosphorylation	9.09E-10	15,10,5
1ddja_#759	GO:0018108: peptidyl-tyrosine phosphorylation	9.09E-10	15,10,5
1gg6.1#C229	GO:0018108: peptidyl-tyrosine phosphorylation	9.09E-10	15,10,5
1b3aa_#15	GO:0006935: chemotaxis	9.15E-10	5,41,5
1e9ma_#99	GO:0006118: electron transport	9.26E-10	20,228,13
2viua_#252	GO:0006953: acute-phase response	9.31E-10	285,20,14
1mrj__#156	GO:0017148: negative regulation of protein biosynthesis	9.51E-10	48,13,7
1tca__#106	GO:0016042: lipid catabolism	9.55E-10	11,31,6
1mrp__#172	GO:0006563: L-serine metabolism	9.81E-10	91,12,8
1c4zd_#99	GO:0006511: ubiquitin-dependent protein catabolism	1.01E-09	5,11,4
8dfr__#151	GO:0006544: glycine metabolism	1.01E-09	5,11,4
1fwxa1#526	GO:0006118: electron transport	1.01E-09	17,228,12
1qf5a_#15	GO:0007264: small GTPase mediated signal transduction	1.02E-09	28,21,7
1ctqa_#116	GO:0007264: small GTPase mediated signal transduction	1.02E-09	28,21,7
1nfdb1#34	GO:0042088: T-helper 1 type immune response	1.04E-09	167,11,9
1arb__#193	GO:0006919: caspase activation	1.04E-09	22,15,6
1qfea_#68	GO:0009070: serine family amino acid biosynthesis	1.04E-09	453,26,19
1a0la_#160	GO:0006953: acute-phase response	1.05E-09	48,20,8
1qs0a1#119	GO:0006221: pyrimidine nucleotide biosynthesis	1.08E-09	367,22,16
1qb7a_#86	GO:0006096: glycolysis	1.09E-09	36,38,9
1tuba1#8	GO:0006100: tricarboxylic acid cycle intermediate metabolism	1.10E-09	115,14,9
1ei5a3#311	GO:0030333: antigen processing	1.10E-09	26,13,6
1i05a_#132	GO:0030333: antigen processing	1.11E-09	49,13,7
1d0va_#114	GO:0006544: glycine metabolism	1.11E-09	14,11,5
1a3c__#108	GO:0043094: metabolic compound salvage	1.11E-09	14,11,5
1nsj__#21	GO:0009073: aromatic amino acid family biosynthesis	1.12E-09	96,16,9
1ge8a2#134	GO:0019883: antigen presentation, endogenous antigen	1.14E-09	202,10,9

1jsg__#92	GO:0030333: antigen processing	1.15E-09	519,13,13
1dcfa_#66	GO:0042401: biogenic amine biosynthesis	1.15E-09	36,10,6
1f00i2#805	GO:0019883: antigen presentation, endogenous antigen	1.20E-09	124,10,8
2dlda2#95	GO:0006563: L-serine metabolism	1.22E-09	13,12,5
1h9da_#91	GO:0019883: antigen presentation, endogenous antigen	1.24E-09	204,10,9
1qaxa2#307	GO:0006953: acute-phase response	1.25E-09	49,20,8
1h8d.1#H207	GO:0006953: acute-phase response	1.25E-09	49,20,8
1ton__#181	GO:0006953: acute-phase response	1.25E-09	49,20,8
1fmta2#151	GO:0006118: electron transport	1.28E-09	272,228,55
1a3qa2#190	GO:0019883: antigen presentation, endogenous antigen	1.28E-09	125,10,8
1a3qa2#190	GO:0030217: T-cell differentiation	1.28E-09	125,10,8
1fjsa_#163	GO:0018108: peptidyl-tyrosine phosphorylation	1.32E-09	16,10,5
1ddja_#771	GO:0018108: peptidyl-tyrosine phosphorylation	1.32E-09	16,10,5
1gdna_#56	GO:0018108: peptidyl-tyrosine phosphorylation	1.32E-09	16,10,5
1ga0a_#236	GO:0017001: antibiotic catabolism	1.32E-09	16,10,5
1qnja_#118	GO:0018108: peptidyl-tyrosine phosphorylation	1.32E-09	16,10,5
1gdna_#123	GO:0018108: peptidyl-tyrosine phosphorylation	1.32E-09	16,10,5
1c5y.1#B234	GO:0018108: peptidyl-tyrosine phosphorylation	1.32E-09	16,10,5
1autc_#209	GO:0018108: peptidyl-tyrosine phosphorylation	1.32E-09	16,10,5
1dik_1#765	GO:0006096: glycolysis	1.33E-09	105,38,13
1f2la_#40	GO:0019883: antigen presentation, endogenous antigen	1.36E-09	206,10,9
1c9la2#61	GO:0019883: antigen presentation, endogenous antigen	1.36E-09	206,10,9
1boub_#7	GO:0006118: electron transport	1.36E-09	206,228,46
1j79a_#187	GO:0006118: electron transport	1.48E-09	79,228,26
1jf9a_#379	GO:0006953: acute-phase response	1.48E-09	186,20,12
1e5ea_#71	GO:0030333: antigen processing	1.49E-09	51,13,7
1ekbb_#228	GO:0006919: caspase activation	1.54E-09	43,15,7
1bwda_#117	GO:0006418: tRNA aminoacylation for protein translation	1.54E-09	103,26,11
1fwxa2#396	GO:0019883: antigen presentation, endogenous antigen	1.55E-09	209,10,9
1ie5a_#102	GO:0042088: T-helper 1 type immune response	1.55E-09	64,11,7
1ie5a_#102	GO:0042108: positive regulation of cytokine biosynthesis	1.55E-09	64,11,7
1io1a_#233	GO:0030333: antigen processing	1.56E-09	86,13,8
1ga0a_#236	GO:0046677: response to antibiotic	1.58E-09	16,40,7
3lada3#463	GO:0006633: fatty acid biosynthesis	1.60E-09	23,27,7
1ceqa2#286	GO:0006096: glycolysis	1.63E-09	10,38,6
1mspa_#17	GO:0030217: T-cell differentiation	1.63E-09	38,10,6
1b8pa2#201	GO:0006096: glycolysis	1.63E-09	10,38,6
1ghpa_#245	GO:0017001: antibiotic catabolism	1.63E-09	38,10,6
1danh_#152	GO:0006919: caspase activation	1.66E-09	11,15,5
1eq9a_#143	GO:0006919: caspase activation	1.66E-09	11,15,5
1h8d.1#H184	GO:0006919: caspase activation	1.66E-09	11,15,5
1elva1#601	GO:0006919: caspase activation	1.66E-09	11,15,5
1f7ua2#293	GO:0006418: tRNA aminoacylation for protein translation	1.66E-09	7,26,5
1hwxa1#324	GO:0009070: serine family amino acid biosynthesis	1.66E-09	7,26,5
1vdra_#37	GO:0009070: serine family amino acid biosynthesis	1.66E-09	7,26,5
1bgva1#350	GO:0009070: serine family amino acid biosynthesis	1.66E-09	7,26,5
1leha1#255	GO:0009070: serine family amino acid biosynthesis	1.67E-09	24,26,7
2reb_1#138	GO:0009070: serine family amino acid biosynthesis	1.68E-09	56,26,9
1c8za_#386	GO:0019883: antigen presentation, endogenous antigen	1.69E-09	211,10,9
1doka_#52	GO:0030333: antigen processing	1.71E-09	52,13,7
1b12a_#221	GO:0030333: antigen processing	1.71E-09	87,13,8
1c7wa_#105	GO:0007517: muscle development	1.74E-09	21,30,7
1aky_2#133	GO:0019883: antigen presentation, endogenous antigen	1.76E-09	130,10,8
1ddja_#681	GO:0050819: negative regulation of coagulation	1.76E-09	212,10,9
1ddja_#681	GO:0030193: regulation of blood coagulation	1.76E-09	212,10,9
1iow_1#37	GO:0009116: nucleoside metabolism	1.77E-09	263,25,15

1dp0a4#756	GO:0051249: regulation of lymphocyte activation	1.77E-09	153,12,9
1mspa_#17	GO:0016337: cell-cell adhesion	1.81E-09	38,17,7
1ad3a_#181	GO:0006118: electron transport	1.82E-09	12,228,10
1fiqa2#27	GO:0006118: electron transport	1.82E-09	12,228,10
1dik_3#240	GO:0019883: antigen presentation, endogenous antigen	1.84E-09	213,10,9
1czan1#92	GO:0030333: antigen processing	1.84E-09	538,13,13
1cipa2#326	GO:0007264: small GTPase mediated signal transduction	1.86E-09	17,21,6
2hlca_#27	GO:0018108: peptidyl-tyrosine phosphorylation	1.87E-09	17,10,5
1dy9.1#A44	GO:0018108: peptidyl-tyrosine phosphorylation	1.87E-09	17,10,5
1eq9a_#59	GO:0018108: peptidyl-tyrosine phosphorylation	1.87E-09	17,10,5
1gdna_#121	GO:0018108: peptidyl-tyrosine phosphorylation	1.87E-09	17,10,5
1bqya_#103	GO:0018108: peptidyl-tyrosine phosphorylation	1.87E-09	17,10,5
1dlea_#198	GO:0018108: peptidyl-tyrosine phosphorylation	1.87E-09	17,10,5
1ton__#231	GO:0018108: peptidyl-tyrosine phosphorylation	1.87E-09	17,10,5
2hlca_#68	GO:0006919: caspase activation	1.87E-09	24,15,6
3sil__#317	GO:0006953: acute-phase response	1.87E-09	300,20,14
1fyhb1#99	GO:0051249: regulation of lymphocyte activation	1.89E-09	31,12,6
1b3ra1#223	GO:0006563: L-serine metabolism	1.89E-09	31,12,6
1dyka2#2937	GO:0006367: transcription initiation from Pol II promoter	1.90E-09	14,12,5
2bbkh_#214	GO:0019883: antigen presentation, endogenous antigen	1.92E-09	39,10,6
1by5a_#238	GO:0019883: antigen presentation, endogenous antigen	1.92E-09	214,10,9
1e8ca3#111	GO:0006096: glycolysis	1.93E-09	244,38,18
1qfea_#68	GO:0006221: pyrimidine nucleotide biosynthesis	1.97E-09	453,22,17
1qhqa_#45	GO:0006730: one-carbon compound metabolism	1.97E-09	106,32,12
1pii_2#326	GO:0006096: glycolysis	2.06E-09	157,38,15
1pmi__#341	GO:0030333: antigen processing	2.18E-09	5,13,4
1qqsa_#35	GO:0006418: tRNA aminoacylation for protein translation	2.20E-09	80,26,10
1e6pa2#46	GO:0006207: 'de novo' pyrimidine base biosynthesis	2.20E-09	77,10,7
1nsj__#178	GO:0042401: biogenic amine biosynthesis	2.20E-09	77,10,7
1nsj__#178	GO:0006207: 'de novo' pyrimidine base biosynthesis	2.20E-09	77,10,7
1ejfa_#10	GO:0019883: antigen presentation, endogenous antigen	2.25E-09	134,10,8
2cpl__#20	GO:0030333: antigen processing	2.26E-09	90,13,8
1fjfj_#68	GO:0019883: antigen presentation, endogenous antigen	2.27E-09	218,10,9
1qs0a1#235	GO:0006118: electron transport	2.31E-09	86,228,27
1d9ta_#137	GO:0006730: one-carbon compound metabolism	2.33E-09	12,32,6
1danh_#48	GO:0006953: acute-phase response	2.34E-09	9,20,5
1kdj__#27	GO:0016337: cell-cell adhesion	2.36E-09	64,17,8
1mai__#108	GO:0019883: antigen presentation, endogenous antigen	2.39E-09	135,10,8
1dr9a2#139	GO:0030217: T-cell differentiation	2.41E-09	78,10,7
1el0a_#28	GO:0030333: antigen processing	2.45E-09	140,13,9
1doza_#109	GO:0009116: nucleoside metabolism	2.48E-09	61,25,9
1i6vd_#713	GO:0006544: glycine metabolism	2.50E-09	36,11,6
1danh_#197	GO:0018108: peptidyl-tyrosine phosphorylation	2.58E-09	18,10,5
1svpa_#127	GO:0018108: peptidyl-tyrosine phosphorylation	2.58E-09	18,10,5
1qq4a_#143	GO:0018108: peptidyl-tyrosine phosphorylation	2.58E-09	18,10,5
1dlea_#53	GO:0018108: peptidyl-tyrosine phosphorylation	2.58E-09	18,10,5
1qfxa_#380	GO:0019883: antigen presentation, endogenous antigen	2.64E-09	41,10,6
1gsoa3#142	GO:0030217: T-cell differentiation	2.65E-09	79,10,7
1hxmb1#117	GO:0016337: cell-cell adhesion	2.68E-09	65,17,8
1avgi_#69	GO:0042088: T-helper 1 type immune response	2.71E-09	281,11,10
1avgi_#69	GO:0042108: positive regulation of cytokine biosynthesis	2.71E-09	281,11,10
1fzqa_#165	GO:0007264: small GTPase mediated signal transduction	2.78E-09	18,21,6
1a8l_1#74	GO:0006118: electron transport	2.78E-09	18,228,12
1smaa1#95	GO:0019883: antigen presentation, endogenous antigen	2.79E-09	223,10,9
1c4ka1#33	GO:0006563: L-serine metabolism	2.84E-09	33,12,6
1h8d.1#H167	GO:0006919: caspase activation	2.84E-09	12,15,5

1ton__#120	GO:0006919: caspase activation	2.84E-09	12,15,5
1bqya_#216	GO:0006919: caspase activation	2.84E-09	12,15,5
1jeyb_#231	GO:0006470: protein amino acid dephosphorylation	2.90E-09	19,20,6
1bywa_#30	GO:0042088: T-helper 1 type immune response	2.91E-09	283,11,10
1ovaa_#284	GO:0006096: glycolysis	2.92E-09	18,38,7
1hyha2#171	GO:0006096: glycolysis	2.92E-09	18,38,7
1g4us2#394	GO:0006470: protein amino acid dephosphorylation	2.96E-09	4,20,4
1div_1#141	GO:0006544: glycine metabolism	2.97E-09	70,11,7
1fo5a_#60	GO:0019883: antigen presentation, endogenous antigen	3.03E-09	225,10,9
1dzfa2#211	GO:0019883: antigen presentation, endogenous antigen	3.03E-09	139,10,8
1ghpa_#245	GO:0046677: response to antibiotic	3.05E-09	38,40,9
1ihua2#343	GO:0006418: tRNA aminoacylation for protein translation	3.07E-09	176,26,13
1svb_1#318	GO:0030333: antigen processing	3.07E-09	14,13,5
2ae2a_#17	GO:0006100: tricarboxylic acid cycle intermediate metabolism	3.07E-09	13,14,5
1dgw.1#Y353	GO:0030333: antigen processing	3.08E-09	294,13,11
1j79a_#38	GO:0006100: tricarboxylic acid cycle intermediate metabolism	3.12E-09	28,14,6
1b3ra1#218	GO:0009070: serine family amino acid biosynthesis	3.13E-09	26,26,7
1evqa_#61	GO:0019883: antigen presentation, endogenous antigen	3.21E-09	140,10,8
1cs1a_#160	GO:0009064: glutamine family amino acid metabolism	3.27E-09	164,19,11
2sli_2#326	GO:0019883: antigen presentation, endogenous antigen	3.28E-09	227,10,9
2sli_2#326	GO:0030217: T-cell differentiation	3.28E-09	227,10,9
1hq0a_#867	GO:0046677: response to antibiotic	3.33E-09	153,40,15
1plc__#74	GO:0006118: electron transport	3.41E-09	10,228,9
1ddja_#726	GO:0006953: acute-phase response	3.48E-09	82,20,9
1ton__#47	GO:0006953: acute-phase response	3.48E-09	82,20,9
1dmla2#313	GO:0030333: antigen processing	3.49E-09	31,13,6
1a6da3#190	GO:0050819: negative regulation of coagulation	3.50E-09	19,10,5
1a6da3#190	GO:0030193: regulation of blood coagulation	3.50E-09	19,10,5
1a6da3#190	GO:0018108: peptidyl-tyrosine phosphorylation	3.50E-09	19,10,5
1ton__#196	GO:0018108: peptidyl-tyrosine phosphorylation	3.50E-09	19,10,5
1qq4a_#63	GO:0018108: peptidyl-tyrosine phosphorylation	3.50E-09	19,10,5
1arb__#24	GO:0018108: peptidyl-tyrosine phosphorylation	3.50E-09	19,10,5
1yaca_#112	GO:0000160: two-component signal transduction system (phosphorelay)	3.56E-09	8,25,5
1h7wa2#636	GO:0006207: 'de novo' pyrimidine base biosynthesis	3.57E-09	43,10,6
1ekbb_#228	GO:0018108: peptidyl-tyrosine phosphorylation	3.57E-09	43,10,6
1fzqa_#28	GO:0007264: small GTPase mediated signal transduction	3.59E-09	33,21,7
1a9xa3#78	GO:0009070: serine family amino acid biosynthesis	3.59E-09	262,26,15
1e79d2#36	GO:0042088: T-helper 1 type immune response	3.61E-09	432,11,11
1e79d2#36	GO:0042108: positive regulation of cytokine biosynthesis	3.61E-09	432,11,11
1hxxa_#173	GO:0019883: antigen presentation, endogenous antigen	3.70E-09	230,10,9
1aqua_#126	GO:0006100: tricarboxylic acid cycle intermediate metabolism	3.82E-09	472,14,13
1erja_#457	GO:0006118: electron transport	3.83E-09	33,228,16
1quba4#235	GO:0030333: antigen processing	3.83E-09	96,13,8
1a6da3#190	GO:0006606: protein-nucleus import	4.04E-09	19,21,6
1fjsa_#83	GO:0050819: negative regulation of coagulation	4.11E-09	84,10,7
1fjsa_#83	GO:0030193: regulation of blood coagulation	4.11E-09	84,10,7
1eupa_#320	GO:0006118: electron transport	4.21E-09	8,228,8
1f8ra1#260	GO:0006118: electron transport	4.21E-09	8,228,8
1h7wa5#996	GO:0006118: electron transport	4.21E-09	8,228,8
3grs_2#187	GO:0006118: electron transport	4.21E-09	8,228,8
3lada2#167	GO:0006118: electron transport	4.21E-09	8,228,8
1fl2a1#490	GO:0006118: electron transport	4.21E-09	8,228,8
1fi2a_#109	GO:0042088: T-helper 1 type immune response	4.23E-09	195,11,9
1plq_1#90	GO:0019883: antigen presentation, endogenous antigen	4.26E-09	145,10,8
1eq2a_#11	GO:0006096: glycolysis	4.40E-09	29,38,8
1qf6a4#304	GO:0006418: tRNA aminoacylation for protein translation	4.41E-09	8,26,5

1a4ya_#104	GO:0006096: glycolysis	4.46E-09	194,38,16
1g5ca_#164	GO:0015976: carbon utilization	4.46E-09	7,10,4
1qnja_#124	GO:0050819: negative regulation of coagulation	4.46E-09	7,10,4
1qnja_#124	GO:0030193: regulation of blood coagulation	4.46E-09	7,10,4
3tmka_#98	GO:0006221: pyrimidine nucleotide biosynthesis	4.47E-09	4,22,4
1bhga3#589	GO:0030245: cellulose catabolism	4.47E-09	4,22,4
1bqca_#268	GO:0030245: cellulose catabolism	4.47E-09	4,22,4
1bhga3#540	GO:0030245: cellulose catabolism	4.47E-09	4,22,4
1fgga_#134	GO:0006100: tricarboxylic acid cycle intermediate metabolism	4.50E-09	193,14,10
1bev1_#86	GO:0042088: T-helper 1 type immune response	4.57E-09	296,11,10
1cgha_#168	GO:0006919: caspase activation	4.60E-09	13,15,5
1danh_#242	GO:0006919: caspase activation	4.60E-09	13,15,5
1ekbb_#79	GO:0006919: caspase activation	4.60E-09	13,15,5
1ton__#179	GO:0006919: caspase activation	4.60E-09	13,15,5
1elva1#513	GO:0006919: caspase activation	4.60E-09	13,15,5
1ea5a_#205	GO:0016042: lipid catabolism	4.62E-09	23,31,7
1fjsa_#158	GO:0018108: peptidyl-tyrosine phosphorylation	4.65E-09	20,10,5
1a53__#236	GO:0006207: 'de novo' pyrimidine base biosynthesis	4.65E-09	20,10,5
1arb__#55	GO:0018108: peptidyl-tyrosine phosphorylation	4.65E-09	20,10,5
1gdna_#30	GO:0018108: peptidyl-tyrosine phosphorylation	4.65E-09	20,10,5
1aop_3#276	GO:0019883: antigen presentation, endogenous antigen	4.76E-09	147,10,8
1i7qa_#321	GO:0019883: antigen presentation, endogenous antigen	4.76E-09	147,10,8
1a0i_2#222	GO:0030333: antigen processing	4.84E-09	151,13,9
1qfta_#98	GO:0042108: positive regulation of cytokine biosynthesis	4.85E-09	198,11,9
1hbza_#96	GO:0006953: acute-phase response	4.86E-09	322,20,14
1e0ta2#275	GO:0042401: biogenic amine biosynthesis	4.87E-09	86,10,7
1e0ta2#275	GO:0006207: 'de novo' pyrimidine base biosynthesis	4.87E-09	86,10,7
1aqua_#126	GO:0009073: aromatic amino acid family biosynthesis	5.00E-09	472,16,14
1vdra_#37	GO:0006730: one-carbon compound metabolism	5.07E-09	7,32,5
2hlca_#54	GO:0006953: acute-phase response	5.12E-09	58,20,8
1ton__#211	GO:0006953: acute-phase response	5.12E-09	58,20,8
3sil__#317	GO:0042088: T-helper 1 type immune response	5.22E-09	300,11,10
1g51a3#525	GO:0019883: antigen presentation, endogenous antigen	5.23E-09	239,10,9
1fnf_3#1409	GO:0042088: T-helper 1 type immune response	5.37E-09	76,11,7
1by5a_#347	GO:0019883: antigen presentation, endogenous antigen	5.43E-09	240,10,9
1jj2e1#42	GO:0019883: antigen presentation, endogenous antigen	5.61E-09	150,10,8
1qfma1#186	GO:0006118: electron transport	5.63E-09	306,228,58
1nfdb1#21	GO:0019883: antigen presentation, endogenous antigen	5.74E-09	88,10,7
1nfdb1#21	GO:0030217: T-cell differentiation	5.74E-09	88,10,7
1c4zd_#85	GO:0030333: antigen processing	5.78E-09	101,13,8
1qhua1#47	GO:0006118: electron transport	5.79E-09	101,228,29
1nsj__#178	GO:0009073: aromatic amino acid family biosynthesis	5.84E-09	77,16,8
1aoea_#130	GO:0006544: glycine metabolism	5.90E-09	77,11,7
1nsj__#178	GO:0006098: pentose-phosphate shunt	5.90E-09	77,11,7
1quna1#113	GO:0019883: antigen presentation, endogenous antigen	6.08E-09	243,10,9
1c5y.1#B241	GO:0018108: peptidyl-tyrosine phosphorylation	6.10E-09	21,10,5
1cs6a4#363	GO:0030217: T-cell differentiation	6.10E-09	21,10,5
1dlea_#238	GO:0018108: peptidyl-tyrosine phosphorylation	6.10E-09	21,10,5
1arb__#208	GO:0018108: peptidyl-tyrosine phosphorylation	6.10E-09	21,10,5
1autc_#45	GO:0018108: peptidyl-tyrosine phosphorylation	6.10E-09	21,10,5
1qnja_#138	GO:0018108: peptidyl-tyrosine phosphorylation	6.10E-09	21,10,5
1ayl__#237	GO:0030333: antigen processing	6.11E-09	313,13,11
1gox__#246	GO:0009116: nucleoside metabolism	6.22E-09	157,25,12
2pola2#218	GO:0006118: electron transport	6.22E-09	126,228,33
1f97a1#62	GO:0030333: antigen processing	6.34E-09	591,13,13
1h9da_#91	GO:0042088: T-helper 1 type immune response	6.35E-09	204,11,9

1h9da_#91	GO:0042108: positive regulation of cytokine biosynthesis	6.35E-09	204,11,9
1qfma1#186	GO:0042088: T-helper 1 type immune response	6.37E-09	306,11,10
1dr9a2#139	GO:0042088: T-helper 1 type immune response	6.47E-09	78,11,7
1dp0a4#756	GO:0030217: T-cell differentiation	6.58E-09	153,10,8
1euha_#349	GO:0017148: negative regulation of protein biosynthesis	6.67E-09	16,13,5
1qfma2#576	GO:0016042: lipid catabolism	6.69E-09	183,31,14
1qtn.1#A270	GO:0006207: 'de novo' pyrimidine base biosynthesis	6.83E-09	386,10,10
1c9la2#61	GO:0042088: T-helper 1 type immune response	6.94E-09	206,11,9
1c9la2#61	GO:0042108: positive regulation of cytokine biosynthesis	6.94E-09	206,11,9
1b37a1#209	GO:0006118: electron transport	6.95E-09	19,228,12
1gtra2#173	GO:0006418: tRNA aminoacylation for protein translation	6.97E-09	45,26,8
2hlpa2#289	GO:0006096: glycolysis	7.00E-09	12,38,6
1qoua_#90	GO:0006367: transcription initiation from Pol II promoter	7.00E-09	38,12,6
1ekbb_#73	GO:0006919: caspase activation	7.13E-09	14,15,5
1a0la_#160	GO:0050819: negative regulation of coagulation	7.13E-09	48,10,6
1a0la_#160	GO:0030193: regulation of blood coagulation	7.13E-09	48,10,6
1hyua4#168	GO:0006118: electron transport	7.26E-09	13,228,10
1dp0a4#756	GO:0016337: cell-cell adhesion	7.29E-09	153,17,10
3prn__#145	GO:0019883: antigen presentation, endogenous antigen	7.31E-09	155,10,8
1a9xa3#78	GO:0042364: water-soluble vitamin biosynthesis	7.33E-09	262,27,15
1czya1#466	GO:0030333: antigen processing	7.33E-09	104,13,8
1je5a_#10	GO:0006100: tricarboxylic acid cycle intermediate metabolism	7.47E-09	58,14,7
1hbza_#214	GO:0019883: antigen presentation, endogenous antigen	7.58E-09	249,10,9
1ile_2#304	GO:0006418: tRNA aminoacylation for protein translation	7.74E-09	17,26,6
1rypk_#12	GO:0019883: antigen presentation, endogenous antigen	7.86E-09	250,10,9
1vcaa2#49	GO:0019883: antigen presentation, endogenous antigen	7.88E-09	22,10,5
1arb__#193	GO:0018108: peptidyl-tyrosine phosphorylation	7.88E-09	22,10,5
1mpp__#151	GO:0019883: antigen presentation, endogenous antigen	7.88E-09	92,10,7
1qqga2#253	GO:0006418: tRNA aminoacylation for protein translation	8.02E-09	91,26,10
1ha6a_#20	GO:0006935: chemotaxis	8.06E-09	19,41,7
1gox__#246	GO:0042401: biogenic amine biosynthesis	8.11E-09	157,10,8
1dih_2#222	GO:0006606: protein-nucleus import	8.11E-09	311,21,14
1csn__#84	GO:0006118: electron transport	8.14E-09	174,228,40
1i50b_#966	GO:0019883: antigen presentation, endogenous antigen	8.15E-09	251,10,9
1bd3a_#110	GO:0043094: metabolic compound salvage	8.49E-09	20,11,5
1ho1a_#211	GO:0042401: biogenic amine biosynthesis	8.52E-09	93,10,7
1ho1a_#211	GO:0006207: 'de novo' pyrimidine base biosynthesis	8.52E-09	93,10,7
1d0ba_#191	GO:0006096: glycolysis	8.59E-09	99,38,12
1c8za_#386	GO:0042088: T-helper 1 type immune response	8.61E-09	211,11,9
1e25a_#262	GO:0046677: response to antibiotic	8.66E-09	115,40,13
1a65a1#108	GO:0019883: antigen presentation, endogenous antigen	8.76E-09	253,10,9
1ddja_#681	GO:0042088: T-helper 1 type immune response	8.99E-09	212,11,9
1ddja_#681	GO:0042108: positive regulation of cytokine biosynthesis	8.99E-09	212,11,9
1h4vb2#282	GO:0006118: electron transport	9.30E-09	91,228,27
1aqua_#126	GO:0043094: metabolic compound salvage	9.66E-09	472,11,11
1hyrc1#247	GO:0030333: antigen processing	9.68E-09	66,13,7
1ej8a_#189	GO:0019883: antigen presentation, endogenous antigen	9.75E-09	256,10,9
1htp__#16	GO:0019883: antigen presentation, endogenous antigen	9.75E-09	256,10,9
1h6va1#27	GO:0042364: water-soluble vitamin biosynthesis	9.90E-09	224,27,14
1phm_2#253	GO:0019883: antigen presentation, endogenous antigen	1.01E-08	257,10,9
1phm_2#253	GO:0030217: T-cell differentiation	1.01E-08	257,10,9
1qqga2#166	GO:0030333: antigen processing	1.02E-08	164,13,9
1flga_#161	GO:0006953: acute-phase response	1.03E-08	128,20,10
1hwxa1#223	GO:0006563: L-serine metabolism	1.03E-08	186,12,9
1qrra_#11	GO:0006100: tricarboxylic acid cycle intermediate metabolism	1.03E-08	16,14,5
1e5ea_#71	GO:0019883: antigen presentation, endogenous antigen	1.04E-08	51,10,6

1bjt__#890	GO:0019883: antigen presentation, endogenous antigen	1.05E-08	258,10,9
1bjt__#890	GO:0030217: T-cell differentiation	1.05E-08	258,10,9
1el0a_#42	GO:0019883: antigen presentation, endogenous antigen	1.05E-08	258,10,9
1el0a_#42	GO:0030217: T-cell differentiation	1.05E-08	258,10,9
2arca_#44	GO:0006367: transcription initiation from Pol II promoter	1.05E-08	7,12,4
1dzfa2#211	GO:0042088: T-helper 1 type immune response	1.06E-08	139,11,8
1dzfa2#211	GO:0042108: positive regulation of cytokine biosynthesis	1.06E-08	139,11,8
1azza_#51	GO:0006919: caspase activation	1.07E-08	15,15,5
1ton__#91	GO:0006919: caspase activation	1.07E-08	15,15,5
1hxxa_#278	GO:0030333: antigen processing	1.07E-08	237,13,10
1nsj__#21	GO:0042401: biogenic amine biosynthesis	1.07E-08	96,10,7
1nsj__#21	GO:0006568: tryptophan metabolism	1.07E-08	96,10,7
1am2__#163	GO:0019883: antigen presentation, endogenous antigen	1.07E-08	96,10,7
1c4ra_#258	GO:0030333: antigen processing	1.08E-08	67,13,7
1nsj__#124	GO:0006096: glycolysis	1.08E-08	124,38,13
2e2c__#67	GO:0006367: transcription initiation from Pol II promoter	1.09E-08	19,12,5
1bpv__#22	GO:0051249: regulation of lymphocyte activation	1.09E-08	19,12,5
1cex__#109	GO:0006100: tricarboxylic acid cycle intermediate metabolism	1.10E-08	34,14,6
1qora2#201	GO:0006207: 'de novo' pyrimidine base biosynthesis	1.10E-08	163,10,8
1fjsa_#83	GO:0042108: positive regulation of cytokine biosynthesis	1.10E-08	84,11,7
1gdna_#26	GO:0006606: protein-nucleus import	1.11E-08	11,21,5
1eq9a_#143	GO:0006606: protein-nucleus import	1.11E-08	11,21,5
1h8d.1#H184	GO:0006606: protein-nucleus import	1.11E-08	11,21,5
1elva1#601	GO:0006606: protein-nucleus import	1.11E-08	11,21,5
1nsj__#124	GO:0009073: aromatic amino acid family biosynthesis	1.14E-08	124,16,9
1ecpa_#58	GO:0006470: protein amino acid dephosphorylation	1.15E-08	64,20,8
1ifc__#89	GO:0019883: antigen presentation, endogenous antigen	1.15E-08	164,10,8
1qqga2#166	GO:0019883: antigen presentation, endogenous antigen	1.15E-08	164,10,8
1cs1a_#160	GO:0006568: tryptophan metabolism	1.15E-08	164,10,8
1cs1a_#160	GO:0009167: purine ribonucleoside monophosphate metabolism	1.15E-08	164,10,8
1xgsa2#82	GO:0019883: antigen presentation, endogenous antigen	1.16E-08	261,10,9
1erv__#80	GO:0016477: cell migration	1.18E-08	401,23,16
1eur__#84	GO:0042108: positive regulation of cytokine biosynthesis	1.20E-08	85,11,7
1axca1#70	GO:0006953: acute-phase response	1.20E-08	94,20,9
1e44b_#75	GO:0050819: negative regulation of coagulation	1.21E-08	165,10,8
1e44b_#75	GO:0030193: regulation of blood coagulation	1.21E-08	165,10,8
1bio__#108	GO:0006919: caspase activation	1.23E-08	32,15,6
1qnja_#46	GO:0006919: caspase activation	1.23E-08	32,15,6
1e4ea2#215	GO:0006953: acute-phase response	1.23E-08	419,20,15
1fjfc2#153	GO:0046677: response to antibiotic	1.25E-08	362,40,21
1fnf_3#1409	GO:0051249: regulation of lymphocyte activation	1.26E-08	76,12,7
1hyha2#211	GO:0006096: glycolysis	1.26E-08	7,38,5
1epwa3#140	GO:0030333: antigen processing	1.26E-08	168,13,9
1g71a_#77	GO:0018108: peptidyl-tyrosine phosphorylation	1.27E-08	24,10,5
1lucb_#32	GO:0006207: 'de novo' pyrimidine base biosynthesis	1.27E-08	24,10,5
1g4ia_#11	GO:0050819: negative regulation of coagulation	1.27E-08	24,10,5
1g4ia_#11	GO:0030193: regulation of blood coagulation	1.27E-08	24,10,5
1g4ia_#11	GO:0018108: peptidyl-tyrosine phosphorylation	1.27E-08	24,10,5
1ie5a_#23	GO:0030217: T-cell differentiation	1.27E-08	24,10,5
1ep3a_#247	GO:0042401: biogenic amine biosynthesis	1.27E-08	24,10,5
1ep3a_#247	GO:0006568: tryptophan metabolism	1.27E-08	24,10,5
2hlca_#68	GO:0018108: peptidyl-tyrosine phosphorylation	1.27E-08	24,10,5
1bywa_#30	GO:0016337: cell-cell adhesion	1.28E-08	283,17,12
1h6la_#119	GO:0019883: antigen presentation, endogenous antigen	1.29E-08	264,10,9
1qfma1#139	GO:0019883: antigen presentation, endogenous antigen	1.29E-08	264,10,9
1a3qa2#190	GO:0051249: regulation of lymphocyte activation	1.29E-08	125,12,8

1kit_3#297	GO:0030333: antigen processing	1.30E-08	18,13,5
1e0ta2#275	GO:0006098: pentose-phosphate shunt	1.30E-08	86,11,7
1bu7a_#353	GO:0006118: electron transport	1.32E-08	54,228,20
1fu6a_#50	GO:0019883: antigen presentation, endogenous antigen	1.33E-08	265,10,9
1vmoa_#106	GO:0019883: antigen presentation, endogenous antigen	1.33E-08	167,10,8
1vmoa_#106	GO:0030217: T-cell differentiation	1.33E-08	167,10,8
1nfdb1#34	GO:0019883: antigen presentation, endogenous antigen	1.33E-08	167,10,8
1nfdb1#34	GO:0030217: T-cell differentiation	1.33E-08	167,10,8
1hq8a_#206	GO:0006118: electron transport	1.34E-08	281,228,54
1e0ta2#10	GO:0006096: glycolysis	1.36E-08	33,38,8
1ton__#211	GO:0006919: caspase activation	1.38E-08	58,15,7
1at0__#276	GO:0016485: protein processing	1.40E-08	8,11,4
1am2__#94	GO:0016485: protein processing	1.40E-08	8,11,4
1g71a_#77	GO:0006953: acute-phase response	1.40E-08	24,20,6
1smaa1#95	GO:0042088: T-helper 1 type immune response	1.42E-08	223,11,9
1e0ta2#275	GO:0009073: aromatic amino acid family biosynthesis	1.43E-08	86,16,8
1a6o__#45	GO:0006118: electron transport	1.45E-08	266,228,52
2fnba_#67	GO:0006953: acute-phase response	1.47E-08	5,20,4
3lada1#116	GO:0009116: nucleoside metabolism	1.48E-08	133,25,11
1gox__#124	GO:0009086: methionine biosynthesis	1.49E-08	298,14,11
1gg6.1#B19	GO:0006919: caspase activation	1.49E-08	33,15,6
2sli_2#326	GO:0006953: acute-phase response	1.52E-08	227,20,12
1dpja_#322	GO:0019883: antigen presentation, endogenous antigen	1.52E-08	269,10,9
1nfdb1#21	GO:0042088: T-helper 1 type immune response	1.53E-08	88,11,7
1kit_3#743	GO:0042088: T-helper 1 type immune response	1.54E-08	225,11,9
1kit_3#743	GO:0042108: positive regulation of cytokine biosynthesis	1.54E-08	225,11,9
1b8aa2#403	GO:0030333: antigen processing	1.54E-08	114,13,8
1eqka_#47	GO:0030333: antigen processing	1.54E-08	114,13,8
1fjsa_#163	GO:0006919: caspase activation	1.54E-08	16,15,5
1ddja_#771	GO:0006919: caspase activation	1.54E-08	16,15,5
1ea5a_#99	GO:0030333: antigen processing	1.56E-08	172,13,9
1imva_#69	GO:0015671: oxygen transport	1.56E-08	82,23,9
1eu8a_#312	GO:0019883: antigen presentation, endogenous antigen	1.58E-08	25,10,5
1danh_#48	GO:0018108: peptidyl-tyrosine phosphorylation	1.60E-08	9,10,4
1gox__#334	GO:0006207: 'de novo' pyrimidine base biosynthesis	1.60E-08	9,10,4
1befa_#51	GO:0050819: negative regulation of coagulation	1.60E-08	9,10,4
1befa_#51	GO:0030193: regulation of blood coagulation	1.60E-08	9,10,4
1gox__#246	GO:0006221: pyrimidine nucleotide biosynthesis	1.61E-08	157,22,11
1ihua2#525	GO:0006096: glycolysis	1.65E-08	278,38,18
1nsca_#379	GO:0030333: antigen processing	1.65E-08	115,13,8
1hxxa_#84	GO:0043123: positive regulation of I-kappaB kinase/NF-kappaB cascade	1.66E-08	6,16,4
1aym2_#185	GO:0019079: viral genome replication	1.66E-08	6,16,4
1qo2a_#222	GO:0006568: tryptophan metabolism	1.67E-08	55,10,6
2naca1#162	GO:0009070: serine family amino acid biosynthesis	1.67E-08	19,26,6
2hlca_#226	GO:0006953: acute-phase response	1.67E-08	67,20,8
1smaa1#95	GO:0016337: cell-cell adhesion	1.68E-08	223,17,11
1beba_#119	GO:0019883: antigen presentation, endogenous antigen	1.69E-08	172,10,8
1hwxa1#256	GO:0006096: glycolysis	1.72E-08	48,38,9
1h75a_#33	GO:0006118: electron transport	1.73E-08	11,228,9
2pia_2#123	GO:0006118: electron transport	1.73E-08	11,228,9
1dmla2#297	GO:0030333: antigen processing	1.76E-08	19,13,5
2mpra_#89	GO:0030333: antigen processing	1.78E-08	40,13,6
1czan1#92	GO:0046677: response to antibiotic	1.84E-08	538,40,25
1kit_3#743	GO:0016337: cell-cell adhesion	1.85E-08	225,17,11
1bqya_#115	GO:0006606: protein-nucleus import	1.90E-08	12,21,5
1elva1#566	GO:0006606: protein-nucleus import	1.90E-08	12,21,5

1h8d.1#H167	GO:0006606: protein-nucleus import	1.90E-08	12,21,5
1dar_2#264	GO:0007264: small GTPase mediated signal transduction	1.90E-08	12,21,5
1ton__#120	GO:0006606: protein-nucleus import	1.90E-08	12,21,5
1bqya_#216	GO:0006606: protein-nucleus import	1.90E-08	12,21,5
1gg6.1#C156	GO:0006606: protein-nucleus import	1.90E-08	12,21,5
2hlca_#99	GO:0006606: protein-nucleus import	1.90E-08	12,21,5
1ddga1#278	GO:0006118: electron transport	1.90E-08	50,228,19
1as4.1#A336	GO:0019883: antigen presentation, endogenous antigen	1.92E-08	276,10,9
1d0na3#305	GO:0019883: antigen presentation, endogenous antigen	1.92E-08	276,10,9
1fhga_#59	GO:0007169: transmembrane receptor protein tyrosine kinase signaling pathway	1.94E-08	33,26,7
1c4ka1#33	GO:0009070: serine family amino acid biosynthesis	1.94E-08	33,26,7
1e1oa1#142	GO:0006418: tRNA aminoacylation for protein translation	1.95E-08	10,26,5
1xyza_#753	GO:0030245: cellulose catabolism	2.01E-08	23,22,6
1a7s__#148	GO:0006953: acute-phase response	2.06E-08	44,20,7
1qfxa_#380	GO:0030333: antigen processing	2.08E-08	41,13,6
1ac6a_#34	GO:0019883: antigen presentation, endogenous antigen	2.08E-08	57,10,6
1c1da1#153	GO:0006563: L-serine metabolism	2.10E-08	8,12,4
1nsj__#21	GO:0042364: water-soluble vitamin biosynthesis	2.10E-08	96,27,10
1quqb_#87	GO:0006100: tricarboxylic acid cycle intermediate metabolism	2.11E-08	67,14,7
1e79d2#36	GO:0019883: antigen presentation, endogenous antigen	2.13E-08	432,10,10
1c0pa1#1147	GO:0006118: electron transport	2.17E-08	32,228,15
1fo0b_#82	GO:0016337: cell-cell adhesion	2.18E-08	15,17,5
1eq9a_#59	GO:0006919: caspase activation	2.18E-08	17,15,5
1aym3_#131	GO:0016477: cell migration	2.19E-08	353,23,15
1qdla_#211	GO:0019883: antigen presentation, endogenous antigen	2.19E-08	280,10,9
1znca_#32	GO:0006730: one-carbon compound metabolism	2.20E-08	4,32,4
1gox__#124	GO:0009070: serine family amino acid biosynthesis	2.21E-08	298,26,15
1jd0a_#176	GO:0006953: acute-phase response	2.22E-08	183,20,11
1e2fa_#140	GO:0006221: pyrimidine nucleotide biosynthesis	2.22E-08	5,22,4
1leha1#177	GO:0006100: tricarboxylic acid cycle intermediate metabolism	2.22E-08	38,14,6
1a8i__#688	GO:0009103: lipopolysaccharide biosynthesis	2.26E-08	203,12,9
1dp0a4#756	GO:0042088: T-helper 1 type immune response	2.29E-08	153,11,8
1utea_#11	GO:0019883: antigen presentation, endogenous antigen	2.31E-08	107,10,7
1hxxa_#232	GO:0019883: antigen presentation, endogenous antigen	2.33E-08	282,10,9
1mdah_#329	GO:0019883: antigen presentation, endogenous antigen	2.33E-08	282,10,9
1el5a1#166	GO:0006118: electron transport	2.34E-08	14,228,10
2mpra_#190	GO:0042088: T-helper 1 type immune response	2.37E-08	349,11,10
1seta2#306	GO:0006953: acute-phase response	2.38E-08	26,20,6
1b65a_#257	GO:0009070: serine family amino acid biosynthesis	2.39E-08	168,26,12
1arb__#210	GO:0006953: acute-phase response	2.39E-08	70,20,8
1elva1#604	GO:0018108: peptidyl-tyrosine phosphorylation	2.40E-08	27,10,5
1c4zd_#51	GO:0051249: regulation of lymphocyte activation	2.40E-08	135,12,8
1qora2#201	GO:0006221: pyrimidine nucleotide biosynthesis	2.40E-08	163,22,11
3grs_1#23	GO:0006118: electron transport	2.41E-08	139,228,34
1bywa_#30	GO:0019883: antigen presentation, endogenous antigen	2.41E-08	283,10,9
1bywa_#30	GO:0030217: T-cell differentiation	2.41E-08	283,10,9
2hlca_#139	GO:0030333: antigen processing	2.42E-08	75,13,7
1hu4a_#267	GO:0006418: tRNA aminoacylation for protein translation	2.42E-08	34,26,7
1dfca1#1135	GO:0006953: acute-phase response	2.43E-08	45,20,7
1a81a1#51	GO:0007169: transmembrane receptor protein tyrosine kinase signaling pathway	2.47E-08	75,26,9
1imva_#255	GO:0006118: electron transport	2.52E-08	506,228,80
1fj2a_#24	GO:0016042: lipid catabolism	2.53E-08	9,31,5
1doka_#52	GO:0042088: T-helper 1 type immune response	2.55E-08	52,11,6
1doka_#52	GO:0042108: positive regulation of cytokine biosynthesis	2.55E-08	52,11,6

1jb3a_#64	GO:0006118: electron transport	2.55E-08	247,228,49
2viua_#252	GO:0019883: antigen presentation, endogenous antigen	2.57E-08	285,10,9
1ddma_#130	GO:0019883: antigen presentation, endogenous antigen	2.57E-08	285,10,9
1fjsa_#83	GO:0051249: regulation of lymphocyte activation	2.57E-08	84,12,7
1gox__#124	GO:0006563: L-serine metabolism	2.62E-08	298,12,10
3sil__#98	GO:0006953: acute-phase response	2.64E-08	186,20,11
1aym3_#131	GO:0042088: T-helper 1 type immune response	2.66E-08	353,11,10
1fnga1#123	GO:0019883: antigen presentation, endogenous antigen	2.66E-08	10,10,4
1jbqa_#152	GO:0006118: electron transport	2.67E-08	46,228,18
2fnba_#67	GO:0016477: cell migration	2.69E-08	5,23,4
1jsg__#92	GO:0042088: T-helper 1 type immune response	2.77E-08	519,11,11
1jsg__#92	GO:0042108: positive regulation of cytokine biosynthesis	2.77E-08	519,11,11
1eur__#84	GO:0051249: regulation of lymphocyte activation	2.79E-08	85,12,7
1e2o__#346	GO:0006953: acute-phase response	2.80E-08	187,20,11
1gox__#246	GO:0043094: metabolic compound salvage	2.81E-08	157,11,8
1g71a_#88	GO:0018108: peptidyl-tyrosine phosphorylation	2.91E-08	28,10,5
1eq9a_#58	GO:0018108: peptidyl-tyrosine phosphorylation	2.91E-08	28,10,5
1nfdb1#115	GO:0030333: antigen processing	3.02E-08	8,13,4
1c5y.1#B191	GO:0006606: protein-nucleus import	3.06E-08	13,21,5
1g6ga_#133	GO:0006606: protein-nucleus import	3.06E-08	13,21,5
1c5y.1#B94	GO:0006606: protein-nucleus import	3.06E-08	13,21,5
1a0la_#193	GO:0006606: protein-nucleus import	3.06E-08	13,21,5
1ton__#179	GO:0006606: protein-nucleus import	3.06E-08	13,21,5
1elva1#513	GO:0006606: protein-nucleus import	3.06E-08	13,21,5
1cs6a4#363	GO:0030333: antigen processing	3.06E-08	21,13,5
1cbf__#208	GO:0019883: antigen presentation, endogenous antigen	3.10E-08	291,10,9
1bxka_#262	GO:0009103: lipopolysaccharide biosynthesis	3.12E-08	23,12,5
1hc7a2#258	GO:0030217: T-cell differentiation	3.17E-08	186,10,8
3sil__#98	GO:0050819: negative regulation of coagulation	3.17E-08	186,10,8
3sil__#98	GO:0030193: regulation of blood coagulation	3.17E-08	186,10,8
1f8v.1#A116	GO:0030333: antigen processing	3.20E-08	78,13,7
1cs6a4#363	GO:0007169: transmembrane receptor protein tyrosine kinase signaling pathway	3.30E-08	21,26,6
1quna1#113	GO:0006953: acute-phase response	3.33E-08	243,20,12
1dgw.1#Y353	GO:0019883: antigen presentation, endogenous antigen	3.40E-08	294,10,9
1dgw.1#Y353	GO:0030217: T-cell differentiation	3.40E-08	294,10,9
1eq2a_#11	GO:0006099: tricarboxylic acid cycle	3.41E-08	29,19,6
1qfea_#68	GO:0042401: biogenic amine biosynthesis	3.45E-08	453,10,10
1qfea_#68	GO:0006568: tryptophan metabolism	3.45E-08	453,10,10
1nat__#62	GO:0000160: two-component signal transduction system (phosphorelay)	3.49E-08	22,25,6
1bu7a_#360	GO:0006118: electron transport	3.49E-08	9,228,8
1cwva2#675	GO:0019883: antigen presentation, endogenous antigen	3.51E-08	29,10,5
1c9la2#12	GO:0006118: electron transport	3.52E-08	257,228,50
1fp5a2#478	GO:0042088: T-helper 1 type immune response	3.56E-08	26,11,5
1fp5a2#478	GO:0042108: positive regulation of cytokine biosynthesis	3.56E-08	26,11,5
5ruba1#365	GO:0006563: L-serine metabolism	3.58E-08	88,12,7
1f3mc_#369	GO:0015671: oxygen transport	3.61E-08	90,23,9
1bev1_#86	GO:0019883: antigen presentation, endogenous antigen	3.61E-08	296,10,9
1bev1_#86	GO:0030217: T-cell differentiation	3.61E-08	296,10,9
1b8aa2#403	GO:0019883: antigen presentation, endogenous antigen	3.62E-08	114,10,7
1h6va1#27	GO:0006096: glycolysis	3.71E-08	224,38,16
1b6ra3#161	GO:0006096: glycolysis	3.72E-08	137,38,13
1alo_4#634	GO:0006633: fatty acid biosynthesis	3.81E-08	53,27,8
1gox__#124	GO:0042401: biogenic amine biosynthesis	3.84E-08	298,10,9
1gox__#124	GO:0006568: tryptophan metabolism	3.84E-08	298,10,9
1e25a_#262	GO:0019883: antigen presentation, endogenous antigen	3.85E-08	115,10,7

2hlca_#226	GO:0006919: caspase activation	3.88E-08	67,15,7
1h7wa5#973	GO:0006367: transcription initiation from Pol II promoter	3.93E-08	24,12,5
1rypk_#12	GO:0042088: T-helper 1 type immune response	3.95E-08	250,11,9
1a6da3#190	GO:0006919: caspase activation	4.07E-08	19,15,5
3prn__#143	GO:0019883: antigen presentation, endogenous antigen	4.08E-08	300,10,9
3sil__#317	GO:0019883: antigen presentation, endogenous antigen	4.08E-08	300,10,9
1a7ta_#80	GO:0017001: antibiotic catabolism	4.18E-08	11,10,4
1danh_#152	GO:0018108: peptidyl-tyrosine phosphorylation	4.18E-08	11,10,4
1nat__#50	GO:0006100: tricarboxylic acid cycle intermediate metabolism	4.20E-08	242,14,10
3mbp__#275	GO:0015698: inorganic anion transport	4.23E-08	8,14,4
1dik_1#848	GO:0042401: biogenic amine biosynthesis	4.26E-08	64,10,6
1dik_1#848	GO:0006568: tryptophan metabolism	4.26E-08	64,10,6
1kdj__#27	GO:0030217: T-cell differentiation	4.26E-08	64,10,6
1eq9a_#40	GO:0018108: peptidyl-tyrosine phosphorylation	4.26E-08	64,10,6
1hw5a2#64	GO:0006953: acute-phase response	4.27E-08	148,20,10
1g6oa_#116	GO:0019883: antigen presentation, endogenous antigen	4.33E-08	302,10,9
1lara2#1769	GO:0006953: acute-phase response	4.36E-08	195,20,11
1epwa3#138	GO:0042088: T-helper 1 type immune response	4.37E-08	371,11,10
1autc_#188	GO:0006953: acute-phase response	4.39E-08	6,20,4
1a65a1#108	GO:0042088: T-helper 1 type immune response	4.40E-08	253,11,9
1i9sa_#24	GO:0006470: protein amino acid dephosphorylation	4.51E-08	49,20,7
1d4ta_#80	GO:0007169: transmembrane receptor protein tyrosine kinase signaling pathway	4.51E-08	22,26,6
1hc7a1#288	GO:0006418: tRNA aminoacylation for protein translation	4.53E-08	5,26,4
1ile_1#695	GO:0006418: tRNA aminoacylation for protein translation	4.53E-08	5,26,4
8dfr__#151	GO:0009070: serine family amino acid biosynthesis	4.53E-08	5,26,4
1e8ca3#111	GO:0006100: tricarboxylic acid cycle intermediate metabolism	4.55E-08	244,14,10
1e8ca3#111	GO:0009086: methionine biosynthesis	4.55E-08	244,14,10
1gdha2#47	GO:0006096: glycolysis	4.55E-08	38,38,8
1c5y.1#B128	GO:0050819: negative regulation of coagulation	4.59E-08	3,10,3
1c5y.1#B128	GO:0030193: regulation of blood coagulation	4.59E-08	3,10,3
1e25a_#139	GO:0017001: antibiotic catabolism	4.59E-08	3,10,3
1iba__#39	GO:0016337: cell-cell adhesion	4.60E-08	316,17,12
1vmoa_#106	GO:0042088: T-helper 1 type immune response	4.61E-08	167,11,8
1vmoa_#106	GO:0042108: positive regulation of cytokine biosynthesis	4.61E-08	167,11,8
1nfdb1#34	GO:0042108: positive regulation of cytokine biosynthesis	4.61E-08	167,11,8
1f97a2#199	GO:0019883: antigen presentation, endogenous antigen	4.61E-08	118,10,7
1fi2a_#109	GO:0030217: T-cell differentiation	4.62E-08	195,10,8
1c5y.1#B192	GO:0006606: protein-nucleus import	4.74E-08	14,21,5
1c5y.1#B220	GO:0006606: protein-nucleus import	4.74E-08	14,21,5
1bio__#81	GO:0006606: protein-nucleus import	4.74E-08	14,21,5
1qnja_#71	GO:0006606: protein-nucleus import	4.74E-08	14,21,5
1danh_#142	GO:0006606: protein-nucleus import	4.74E-08	14,21,5
1danh_#70	GO:0006606: protein-nucleus import	4.74E-08	14,21,5
1c75a_#48	GO:0006118: electron transport	4.75E-08	7,228,7
1cpt__#325	GO:0006118: electron transport	4.75E-08	7,228,7
1bqk__#78	GO:0006118: electron transport	4.75E-08	7,228,7
1qksa1#127	GO:0006118: electron transport	4.75E-08	7,228,7
1b0pa5#738	GO:0006118: electron transport	4.75E-08	7,228,7
1a65a3#383	GO:0006118: electron transport	4.80E-08	38,228,16
1f8ra2#325	GO:0030217: T-cell differentiation	4.82E-08	196,10,8
1pud__#69	GO:0006100: tricarboxylic acid cycle intermediate metabolism	4.85E-08	43,14,6
1dik_1#848	GO:0009073: aromatic amino acid family biosynthesis	4.87E-08	64,16,7
1qfma1#186	GO:0019883: antigen presentation, endogenous antigen	4.87E-08	306,10,9
1qfma1#186	GO:0030217: T-cell differentiation	4.87E-08	306,10,9
1dn2a1#261	GO:0042088: T-helper 1 type immune response	4.99E-08	376,11,10

1hrka_#343	GO:0015976: carbon utilization	5.01E-08	31,10,5
1fyhb1#99	GO:0030217: T-cell differentiation	5.01E-08	31,10,5
1dmla2#313	GO:0019883: antigen presentation, endogenous antigen	5.01E-08	31,10,5
1nuka_#80	GO:0019883: antigen presentation, endogenous antigen	5.02E-08	197,10,8
3grx__#56	GO:0019883: antigen presentation, endogenous antigen	5.02E-08	307,10,9
1gox__#124	GO:0006096: glycolysis	5.04E-08	298,38,18
1phm_2#253	GO:0042088: T-helper 1 type immune response	5.06E-08	257,11,9
1phm_2#253	GO:0042108: positive regulation of cytokine biosynthesis	5.06E-08	257,11,9
1b3qa2#623	GO:0042088: T-helper 1 type immune response	5.06E-08	257,11,9
1b3qa2#623	GO:0042108: positive regulation of cytokine biosynthesis	5.06E-08	257,11,9
1jlxa2#166	GO:0019883: antigen presentation, endogenous antigen	5.15E-08	66,10,6
1opy__#68	GO:0019883: antigen presentation, endogenous antigen	5.17E-08	308,10,9
1i6vd_#1283	GO:0050819: negative regulation of coagulation	5.19E-08	120,10,7
1i6vd_#1283	GO:0030193: regulation of blood coagulation	5.19E-08	120,10,7
1aqua_#126	GO:0015976: carbon utilization	5.22E-08	472,10,10
1aqua_#126	GO:0006568: tryptophan metabolism	5.22E-08	472,10,10
1smaa1#95	GO:0051249: regulation of lymphocyte activation	5.23E-08	223,12,9
1b3ra1#223	GO:0006099: tricarboxylic acid cycle	5.24E-08	31,19,6
1el0a_#42	GO:0042088: T-helper 1 type immune response	5.24E-08	258,11,9
1el0a_#42	GO:0042108: positive regulation of cytokine biosynthesis	5.24E-08	258,11,9
1tig__#148	GO:0006544: glycine metabolism	5.30E-08	28,11,5
1g71a_#88	GO:0006606: protein-nucleus import	5.35E-08	28,21,6
1eq9a_#58	GO:0006606: protein-nucleus import	5.35E-08	28,21,6
1fjsa_#83	GO:0030333: antigen processing	5.42E-08	84,13,7
2bb2_2#134	GO:0019883: antigen presentation, endogenous antigen	5.44E-08	199,10,8
2bb2_2#134	GO:0030217: T-cell differentiation	5.44E-08	199,10,8
1h6va1#27	GO:0006563: L-serine metabolism	5.44E-08	224,12,9
1d0na3#305	GO:0006118: electron transport	5.49E-08	276,228,52
1jsg__#92	GO:0016477: cell migration	5.50E-08	519,23,17
1leha1#177	GO:0009070: serine family amino acid biosynthesis	5.54E-08	38,26,7
1dkia_#76P	GO:0030333: antigen processing	5.58E-08	48,13,6
1phm_2#347	GO:0046677: response to antibiotic	5.58E-08	4,40,4
1hvba_#76	GO:0046677: response to antibiotic	5.58E-08	4,40,4
1qrra_#69	GO:0042364: water-soluble vitamin biosynthesis	5.66E-08	173,27,12
1kit_3#743	GO:0051249: regulation of lymphocyte activation	5.66E-08	225,12,9
1ii7a_#43	GO:0006096: glycolysis	5.67E-08	39,38,8
1fo4a5#1020	GO:0019883: antigen presentation, endogenous antigen	5.80E-08	312,10,9
1aqua_#126	GO:0042364: water-soluble vitamin biosynthesis	5.83E-08	472,27,18
1oaca1#495	GO:0006919: caspase activation	5.92E-08	41,15,6
1ak5_1#376	GO:0006207: 'de novo' pyrimidine base biosynthesis	5.92E-08	32,10,5
1bio__#108	GO:0018108: peptidyl-tyrosine phosphorylation	5.92E-08	32,10,5
1pma1_#17	GO:0050819: negative regulation of coagulation	5.92E-08	32,10,5
1pma1_#17	GO:0030193: regulation of blood coagulation	5.92E-08	32,10,5
1pma1_#17	GO:0018108: peptidyl-tyrosine phosphorylation	5.92E-08	32,10,5
1qnja_#46	GO:0018108: peptidyl-tyrosine phosphorylation	5.92E-08	32,10,5
1c4zd_#51	GO:0030333: antigen processing	5.95E-08	135,13,8
1i50b_#966	GO:0016337: cell-cell adhesion	5.95E-08	251,17,11
1qhda2#323	GO:0019883: antigen presentation, endogenous antigen	5.97E-08	313,10,9
1a9xa3#78	GO:0009396: folic acid and derivative biosynthesis	6.01E-08	262,11,9
1qo2a_#222	GO:0006096: glycolysis	6.04E-08	55,38,9
1h6va1#27	GO:0009070: serine family amino acid biosynthesis	6.07E-08	224,26,13
1ge8a2#134	GO:0030217: T-cell differentiation	6.13E-08	202,10,8
1qj5a_#268	GO:0043094: metabolic compound salvage	6.18E-08	60,11,6
1f2la_#12	GO:0006935: chemotaxis	6.19E-08	4,41,4
1bio__#210	GO:0006953: acute-phase response	6.24E-08	113,20,9
1h7wa2#535	GO:0006207: 'de novo' pyrimidine base biosynthesis	6.25E-08	12,10,4

1qbea_#92	GO:0050819: negative regulation of coagulation	6.25E-08	12,10,4
1qbea_#92	GO:0030193: regulation of blood coagulation	6.25E-08	12,10,4
1rpxa_#216	GO:0006207: 'de novo' pyrimidine base biosynthesis	6.25E-08	12,10,4
1jmta_#93	GO:0000398: nuclear mRNA splicing, via spliceosome	6.25E-08	10,12,4
1ep3a_#19	GO:0006207: 'de novo' pyrimidine base biosynthesis	6.25E-08	12,10,4
1cwva2#682	GO:0019883: antigen presentation, endogenous antigen	6.25E-08	12,10,4
1dq3a1#3	GO:0016485: protein processing	6.31E-08	3,11,3
1ryph_#131	GO:0006511: ubiquitin-dependent protein catabolism	6.31E-08	3,11,3
1rypj_#133	GO:0006511: ubiquitin-dependent protein catabolism	6.31E-08	3,11,3
1rypl_#47	GO:0006511: ubiquitin-dependent protein catabolism	6.31E-08	3,11,3
1fsu__#434	GO:0019883: antigen presentation, endogenous antigen	6.32E-08	315,10,9
1fhoa_#74	GO:0019883: antigen presentation, endogenous antigen	6.32E-08	315,10,9
1fhoa_#74	GO:0030217: T-cell differentiation	6.32E-08	315,10,9
1phm_2#253	GO:0006953: acute-phase response	6.34E-08	257,20,12
1cmia_#86	GO:0030333: antigen processing	6.34E-08	49,13,6
1erja_#521	GO:0006118: electron transport	6.37E-08	12,228,9
1a8p_1#55	GO:0006118: electron transport	6.37E-08	12,228,9
1h6la_#119	GO:0042088: T-helper 1 type immune response	6.43E-08	264,11,9
1fumb2#36	GO:0006118: electron transport	6.46E-08	15,228,10
1iba__#39	GO:0019883: antigen presentation, endogenous antigen	6.51E-08	316,10,9
1f00i2#805	GO:0050819: negative regulation of coagulation	6.54E-08	124,10,7
1f00i2#805	GO:0030193: regulation of blood coagulation	6.54E-08	124,10,7
1nsj__#124	GO:0042401: biogenic amine biosynthesis	6.54E-08	124,10,7
1nsj__#124	GO:0006568: tryptophan metabolism	6.54E-08	124,10,7
1nsj__#124	GO:0006207: 'de novo' pyrimidine base biosynthesis	6.54E-08	124,10,7
1jbwa2#198	GO:0043094: metabolic compound salvage	6.55E-08	11,11,4
1bjt__#890	GO:0006953: acute-phase response	6.62E-08	258,20,12
1el0a_#42	GO:0006953: acute-phase response	6.62E-08	258,20,12
1h9da_#91	GO:0030217: T-cell differentiation	6.63E-08	204,10,8
1qs1a2#437	GO:0042088: T-helper 1 type immune response	6.65E-08	387,11,10
1dn2a1#261	GO:0006118: electron transport	6.73E-08	376,228,64
1qfea_#68	GO:0006100: tricarboxylic acid cycle intermediate metabolism	6.91E-08	453,14,12
1gg6.1#B19	GO:0018108: peptidyl-tyrosine phosphorylation	6.97E-08	33,10,5
1h9da_#91	GO:0006953: acute-phase response	7.04E-08	204,20,11
1ekbb_#117	GO:0006606: protein-nucleus import	7.08E-08	15,21,5
1danh_#140	GO:0006606: protein-nucleus import	7.08E-08	15,21,5
1danh_#190	GO:0006606: protein-nucleus import	7.08E-08	15,21,5
1gdna_#155	GO:0006606: protein-nucleus import	7.08E-08	15,21,5
1ton__#112	GO:0006606: protein-nucleus import	7.08E-08	15,21,5
1ton__#91	GO:0006606: protein-nucleus import	7.08E-08	15,21,5
1ekbb_#141	GO:0006606: protein-nucleus import	7.08E-08	15,21,5
1ddja_#759	GO:0006606: protein-nucleus import	7.08E-08	15,21,5
1gg6.1#C229	GO:0006606: protein-nucleus import	7.08E-08	15,21,5
1c9la2#61	GO:0030217: T-cell differentiation	7.17E-08	206,10,8
1e42a1#711	GO:0051249: regulation of lymphocyte activation	7.22E-08	155,12,8
1arb__#210	GO:0018108: peptidyl-tyrosine phosphorylation	7.39E-08	70,10,6
2cmd_1#113	GO:0006096: glycolysis	7.39E-08	9,38,5
1eg2a_#132	GO:0006367: transcription initiation from Pol II promoter	7.42E-08	27,12,5
1qrra_#69	GO:0006096: glycolysis	7.50E-08	173,38,14
1hr0w_#69	GO:0006418: tRNA aminoacylation for protein translation	7.51E-08	60,26,8
1xgsa2#82	GO:0006953: acute-phase response	7.56E-08	261,20,12
1qqsa_#124	GO:0042088: T-helper 1 type immune response	7.56E-08	392,11,10
3thia_#59	GO:0015698: inorganic anion transport	7.58E-08	9,14,4
1hbza_#96	GO:0019883: antigen presentation, endogenous antigen	7.70E-08	322,10,9
1ddwa_#77	GO:0019883: antigen presentation, endogenous antigen	7.70E-08	322,10,9
1arb__#210	GO:0006118: electron transport	7.73E-08	70,228,22

1fnf_1#1146	GO:0016477: cell migration	7.79E-08	14,23,5
3chbd_#85	GO:0019883: antigen presentation, endogenous antigen	7.92E-08	323,10,9
1bjt__#890	GO:0016337: cell-cell adhesion	7.97E-08	258,17,11
2bpa1_#45	GO:0042088: T-helper 1 type immune response	8.02E-08	179,11,8
2bpa1_#45	GO:0042108: positive regulation of cytokine biosynthesis	8.02E-08	179,11,8
1qp8a1#153	GO:0009070: serine family amino acid biosynthesis	8.02E-08	24,26,6
1g51a1#21	GO:0006418: tRNA aminoacylation for protein translation	8.02E-08	24,26,6
1ie5a_#102	GO:0016337: cell-cell adhesion	8.11E-08	64,17,7
1sppb_#32	GO:0030217: T-cell differentiation	8.16E-08	34,10,5
2dpma_#194	GO:0009307: DNA restriction	8.17E-08	7,19,4
1qdlb_#98	GO:0009064: glutamine family amino acid metabolism	8.17E-08	7,19,4
1nls__#77	GO:0016337: cell-cell adhesion	8.31E-08	19,17,5
1jbaa_#157	GO:0007517: muscle development	8.34E-08	21,30,6
1danh_#100	GO:0006956: complement activation	8.41E-08	3,12,3
1ezia_#109	GO:0009103: lipopolysaccharide biosynthesis	8.41E-08	3,12,3
1jsg__#92	GO:0016337: cell-cell adhesion	8.46E-08	519,17,14
1nat__#50	GO:0000160: two-component signal transduction system (phosphorelay)	8.47E-08	242,25,13
1f5aa1#441	GO:0030217: T-cell differentiation	8.64E-08	129,10,7
1loua_#79	GO:0046677: response to antibiotic	8.64E-08	92,40,11
2hlca_#139	GO:0006919: caspase activation	8.65E-08	75,15,7
1c8za_#386	GO:0030217: T-cell differentiation	8.68E-08	211,10,8
1i5ga_#115	GO:0030333: antigen processing	8.92E-08	294,13,10
1aqua_#126	GO:0009116: nucleoside metabolism	8.92E-08	472,25,17
1fwxa2#169	GO:0006118: electron transport	8.95E-08	94,228,26
1foha5#77	GO:0006367: transcription initiation from Pol II promoter	9.01E-08	28,12,5
1hcc__#31	GO:0006956: complement activation	9.01E-08	28,12,5
1ddja_#681	GO:0019883: antigen presentation, endogenous antigen	9.01E-08	212,10,8
1ddja_#681	GO:0030217: T-cell differentiation	9.01E-08	212,10,8
1danh_#242	GO:0018108: peptidyl-tyrosine phosphorylation	9.02E-08	13,10,4
1ekbb_#79	GO:0018108: peptidyl-tyrosine phosphorylation	9.02E-08	13,10,4
1hvba_#102	GO:0017001: antibiotic catabolism	9.02E-08	13,10,4
1g8ka2#570	GO:0006470: protein amino acid dephosphorylation	9.08E-08	32,20,6
1fyhb1#99	GO:0042088: T-helper 1 type immune response	9.10E-08	31,11,5
2arca_#55	GO:0019883: antigen presentation, endogenous antigen	9.12E-08	130,10,7
1d3ga_#118	GO:0006221: pyrimidine nucleotide biosynthesis	9.15E-08	29,22,6
1e8ca3#111	GO:0009116: nucleoside metabolism	9.36E-08	244,25,13
1e8ca3#111	GO:0000160: two-component signal transduction system (phosphorelay)	9.36E-08	244,25,13
1ihua2#343	GO:0006096: glycolysis	9.36E-08	176,38,14
1qtn.1#A270	GO:0009252: peptidoglycan biosynthesis	9.42E-08	386,18,13
1erv__#80	GO:0042088: T-helper 1 type immune response	9.48E-08	401,11,10
1clxa_#120	GO:0042401: biogenic amine biosynthesis	9.50E-08	35,10,5
1clxa_#120	GO:0006568: tryptophan metabolism	9.50E-08	35,10,5
1elva1#623	GO:0018108: peptidyl-tyrosine phosphorylation	9.50E-08	35,10,5
1cvl__#111	GO:0016042: lipid catabolism	9.53E-08	5,31,4
1exma2#353	GO:0019883: antigen presentation, endogenous antigen	9.55E-08	73,10,6
1e3ua_#205	GO:0046677: response to antibiotic	9.66E-08	9,40,5
1by5a_#238	GO:0030217: T-cell differentiation	9.71E-08	214,10,8
2dlda2#95	GO:0009070: serine family amino acid biosynthesis	9.78E-08	13,26,5
1ecfa1#367	GO:0043094: metabolic compound salvage	9.80E-08	12,11,4
1hcc__#39	GO:0006956: complement activation	9.80E-08	11,12,4
1e6pa2#46	GO:0006096: glycolysis	9.84E-08	77,38,10
1cqka_#52	GO:0042088: T-helper 1 type immune response	1.01E-07	65,11,6
1cpza_#6	GO:0050819: negative regulation of coagulation	1.02E-07	132,10,7
1cpza_#6	GO:0030193: regulation of blood coagulation	1.02E-07	132,10,7
1danh_#93	GO:0006953: acute-phase response	1.02E-07	7,20,4
1fjsa_#163	GO:0006606: protein-nucleus import	1.02E-07	16,21,5

1ddja_#771	GO:0006606: protein-nucleus import	1.02E-07	16,21,5
1gdna_#56	GO:0006606: protein-nucleus import	1.02E-07	16,21,5
1qnja_#118	GO:0006606: protein-nucleus import	1.02E-07	16,21,5
1gdna_#123	GO:0006606: protein-nucleus import	1.02E-07	16,21,5
1c5y.1#B234	GO:0006606: protein-nucleus import	1.02E-07	16,21,5
1autc_#209	GO:0006606: protein-nucleus import	1.02E-07	16,21,5
1crza1#240	GO:0030333: antigen processing	1.03E-07	53,13,6
1danh_#48	GO:0006919: caspase activation	1.03E-07	9,15,4
1befa_#51	GO:0006919: caspase activation	1.03E-07	9,15,4
1nfdb1#21	GO:0007169: transmembrane receptor protein tyrosine kinase signaling pathway	1.04E-07	88,26,9
5ruba1#365	GO:0009070: serine family amino acid biosynthesis	1.04E-07	88,26,9
1smaa1#95	GO:0016477: cell migration	1.04E-07	223,23,12
1qj5a_#268	GO:0042364: water-soluble vitamin biosynthesis	1.05E-07	60,27,8
1fo8a_#202	GO:0009070: serine family amino acid biosynthesis	1.05E-07	25,26,6
1pme__#25	GO:0007169: transmembrane receptor protein tyrosine kinase signaling pathway	1.05E-07	25,26,6
1dpja_#322	GO:0006953: acute-phase response	1.07E-07	269,20,12
1bmv2_#2064	GO:0019883: antigen presentation, endogenous antigen	1.07E-07	133,10,7
1nula_#15	GO:0006563: L-serine metabolism	1.09E-07	29,12,5
1hc7a2#258	GO:0042088: T-helper 1 type immune response	1.09E-07	186,11,8
3sil__#98	GO:0042088: T-helper 1 type immune response	1.09E-07	186,11,8
3sil__#98	GO:0042108: positive regulation of cytokine biosynthesis	1.09E-07	186,11,8
8dfr__#151	GO:0006730: one-carbon compound metabolism	1.09E-07	5,32,4
1qi7a_#68	GO:0017148: negative regulation of protein biosynthesis	1.09E-07	3,13,3
1ha6a_#59	GO:0006935: chemotaxis	1.10E-07	9,41,5
1f42a2#123	GO:0019883: antigen presentation, endogenous antigen	1.10E-07	335,10,9
1dcfa_#66	GO:0006568: tryptophan metabolism	1.10E-07	36,10,5
1xo1a2#159	GO:0006568: tryptophan metabolism	1.10E-07	36,10,5
1glqa2#53	GO:0006118: electron transport	1.11E-07	19,228,11
1hq8a_#206	GO:0042088: T-helper 1 type immune response	1.12E-07	281,11,9
1fjfj_#68	GO:0006275: regulation of DNA replication	1.13E-07	218,10,8
2hlca_#139	GO:0019883: antigen presentation, endogenous antigen	1.13E-07	75,10,6
1cs1a_#160	GO:0009156: ribonucleoside monophosphate biosynthesis	1.13E-07	164,12,8
1bzya_#173	GO:0009116: nucleoside metabolism	1.14E-07	6,25,4
1mdah_#329	GO:0042088: T-helper 1 type immune response	1.16E-07	282,11,9
1au1a_#58	GO:0006879: iron ion homeostasis	1.16E-07	104,12,7
1f97a1#62	GO:0042088: T-helper 1 type immune response	1.17E-07	591,11,11
3pyp__#41	GO:0006118: electron transport	1.18E-07	31,228,14
1bywa_#30	GO:0042108: positive regulation of cytokine biosynthesis	1.19E-07	283,11,9
1smaa2#581	GO:0051249: regulation of lymphocyte activation	1.21E-07	60,12,6
1gsa_2#218	GO:0042088: T-helper 1 type immune response	1.22E-07	118,11,7
1f97a2#199	GO:0042088: T-helper 1 type immune response	1.22E-07	118,11,7
1f97a2#199	GO:0042108: positive regulation of cytokine biosynthesis	1.22E-07	118,11,7
1dqza_#131	GO:0006118: electron transport	1.23E-07	66,228,21
1ckea_#138	GO:0006100: tricarboxylic acid cycle intermediate metabolism	1.26E-07	10,14,4
1ddja_#754	GO:0018108: peptidyl-tyrosine phosphorylation	1.26E-07	14,10,4
1ekbb_#73	GO:0018108: peptidyl-tyrosine phosphorylation	1.26E-07	14,10,4
1qhva_#438	GO:0019883: antigen presentation, endogenous antigen	1.26E-07	14,10,4
1fhga_#59	GO:0042088: T-helper 1 type immune response	1.27E-07	33,11,5
1fhga_#59	GO:0042108: positive regulation of cytokine biosynthesis	1.27E-07	33,11,5
1oaa__#14	GO:0006096: glycolysis	1.27E-07	29,38,7
2viua_#252	GO:0042088: T-helper 1 type immune response	1.27E-07	285,11,9
1pud__#69	GO:0006096: glycolysis	1.28E-07	43,38,8
1hyha2#204	GO:0006096: glycolysis	1.28E-07	43,38,8
1am7a_#94	GO:0016998: cell wall catabolism	1.28E-07	4,9,3

1pa2a_#232	GO:0006979: response to oxidative stress	1.29E-07	8,18,4
1mjha_#126	GO:0043094: metabolic compound salvage	1.29E-07	119,11,7
1epwa1#901	GO:0030333: antigen processing	1.30E-07	55,13,6
1e6pa2#46	GO:0042401: biogenic amine biosynthesis	1.32E-07	77,10,6
1nsj__#178	GO:0006568: tryptophan metabolism	1.32E-07	77,10,6
1aym3_#131	GO:0006457: protein folding	1.33E-07	353,49,22
1smaa1#95	GO:0030217: T-cell differentiation	1.35E-07	223,10,8
1ile_3#408	GO:0006418: tRNA aminoacylation for protein translation	1.35E-07	6,26,4
1ei5a3#311	GO:0007169: transmembrane receptor protein tyrosine kinase signaling pathway	1.35E-07	26,26,6
1cs1a_#160	GO:0009116: nucleoside metabolism	1.36E-07	164,25,11
1el0a_#28	GO:0006935: chemotaxis	1.36E-07	140,41,13
1f3ya_#6	GO:0006919: caspase activation	1.36E-07	80,15,7
1cs1a_#160	GO:0009073: aromatic amino acid family biosynthesis	1.37E-07	164,16,9
1cgha_#227	GO:0006919: caspase activation	1.39E-07	47,15,6
1mil__#31	GO:0030333: antigen processing	1.39E-07	96,13,7
1qs1a2#437	GO:0006457: protein folding	1.42E-07	387,49,23
1as4.1#A336	GO:0006953: acute-phase response	1.43E-07	276,20,12
1jjya_#324	GO:0009070: serine family amino acid biosynthesis	1.43E-07	65,26,8
1cnv__#181	GO:0009070: serine family amino acid biosynthesis	1.43E-07	65,26,8
2hlca_#27	GO:0006606: protein-nucleus import	1.44E-07	17,21,5
1dy9.1#A44	GO:0006606: protein-nucleus import	1.44E-07	17,21,5
1eq9a_#59	GO:0006606: protein-nucleus import	1.44E-07	17,21,5
1cs6a4#363	GO:0016337: cell-cell adhesion	1.44E-07	21,17,5
1gdna_#121	GO:0006606: protein-nucleus import	1.44E-07	17,21,5
1bqya_#103	GO:0006606: protein-nucleus import	1.44E-07	17,21,5
1dlea_#198	GO:0006606: protein-nucleus import	1.44E-07	17,21,5
1ton__#231	GO:0006606: protein-nucleus import	1.44E-07	17,21,5
1mdah_#329	GO:0016477: cell migration	1.45E-07	282,23,13
1fo5a_#60	GO:0030217: T-cell differentiation	1.45E-07	225,10,8
1kit_3#743	GO:0019883: antigen presentation, endogenous antigen	1.45E-07	225,10,8
1kit_3#743	GO:0030217: T-cell differentiation	1.45E-07	225,10,8
1e79h2#77	GO:0030333: antigen processing	1.45E-07	28,13,5
1wdcb_#134	GO:0019883: antigen presentation, endogenous antigen	1.46E-07	38,10,5
1evqa_#305	GO:0009073: aromatic amino acid family biosynthesis	1.46E-07	384,16,12
1g71a_#77	GO:0006919: caspase activation	1.46E-07	24,15,5
1g4ia_#11	GO:0006919: caspase activation	1.46E-07	24,15,5
1e4ea2#215	GO:0042088: T-helper 1 type immune response	1.47E-07	419,11,10
1oaa__#175	GO:0009103: lipopolysaccharide biosynthesis	1.47E-07	12,12,4
1b6ra3#161	GO:0006100: tricarboxylic acid cycle intermediate metabolism	1.49E-07	137,14,8
1dbha2#460	GO:0006418: tRNA aminoacylation for protein translation	1.51E-07	14,26,5
1d0va_#114	GO:0009070: serine family amino acid biosynthesis	1.51E-07	14,26,5
1cs1a_#82	GO:0006418: tRNA aminoacylation for protein translation	1.51E-07	158,26,11
1eqca_#15	GO:0030245: cellulose catabolism	1.54E-07	7,22,4
1el0a_#42	GO:0006935: chemotaxis	1.54E-07	258,41,17
1nat__#50	GO:0009070: serine family amino acid biosynthesis	1.55E-07	242,26,13
1fp1d2#214	GO:0009252: peptidoglycan biosynthesis	1.57E-07	143,18,9
1fi2a_#109	GO:0042108: positive regulation of cytokine biosynthesis	1.58E-07	195,11,8
2mpra_#190	GO:0019883: antigen presentation, endogenous antigen	1.59E-07	349,10,9
2mpra_#190	GO:0030217: T-cell differentiation	1.59E-07	349,10,9
1erja_#559	GO:0006118: electron transport	1.61E-07	10,228,8
1dhs__#99	GO:0006563: L-serine metabolism	1.62E-07	109,12,7
1xvaa_#68	GO:0009307: DNA restriction	1.63E-07	8,19,4
1aym3_#131	GO:0016337: cell-cell adhesion	1.64E-07	353,17,12
1f97a1#62	GO:0006118: electron transport	1.65E-07	591,228,87
1dih_2#235	GO:0050819: negative regulation of coagulation	1.67E-07	39,10,5

1dih_2#235	GO:0030193: regulation of blood coagulation	1.67E-07	39,10,5
1dgw.1#Y353	GO:0042088: T-helper 1 type immune response	1.68E-07	294,11,9
1dgw.1#Y353	GO:0042108: positive regulation of cytokine biosynthesis	1.68E-07	294,11,9
1e8ca3#111	GO:0009070: serine family amino acid biosynthesis	1.71E-07	244,26,13
1edza1#206	GO:0019883: antigen presentation, endogenous antigen	1.71E-07	352,10,9
1azza_#51	GO:0018108: peptidyl-tyrosine phosphorylation	1.71E-07	15,10,4
1nuka_#80	GO:0042088: T-helper 1 type immune response	1.72E-07	197,11,8
1nuka_#80	GO:0042108: positive regulation of cytokine biosynthesis	1.72E-07	197,11,8
1fuia2#273	GO:0006563: L-serine metabolism	1.72E-07	110,12,7
1nsj__#124	GO:0043094: metabolic compound salvage	1.73E-07	124,11,7
1nsj__#124	GO:0006098: pentose-phosphate shunt	1.73E-07	124,11,7
1jlna_#437	GO:0006118: electron transport	1.74E-07	51,228,18
1az9_1#73	GO:0006986: response to unfolded protein	1.74E-07	3,15,3
1gof_3#399	GO:0030333: antigen processing	1.75E-07	29,13,5
1aym3_#131	GO:0019883: antigen presentation, endogenous antigen	1.76E-07	353,10,9
1aym3_#131	GO:0030217: T-cell differentiation	1.76E-07	353,10,9
1bev1_#86	GO:0042108: positive regulation of cytokine biosynthesis	1.78E-07	296,11,9
1evqa_#305	GO:0042364: water-soluble vitamin biosynthesis	1.79E-07	384,27,16
1ton__#181	GO:0006919: caspase activation	1.79E-07	49,15,6
1pfza_#156	GO:0030333: antigen processing	1.80E-07	58,13,6
1imva_#255	GO:0006953: acute-phase response	1.80E-07	506,20,15
1kit_2#398	GO:0030217: T-cell differentiation	1.80E-07	81,10,6
1ie5a_#102	GO:0051249: regulation of lymphocyte activation	1.80E-07	64,12,6
1a3qa2#190	GO:0007169: transmembrane receptor protein tyrosine kinase signaling pathway	1.80E-07	125,26,10
1iira_#242	GO:0009070: serine family amino acid biosynthesis	1.80E-07	125,26,10
1e6pa2#46	GO:0009073: aromatic amino acid family biosynthesis	1.81E-07	77,16,7
1pud__#44	GO:0042364: water-soluble vitamin biosynthesis	1.82E-07	43,27,7
1egja_#343	GO:0051249: regulation of lymphocyte activation	1.83E-07	32,12,5
1i85a_#14	GO:0030217: T-cell differentiation	1.83E-07	4,10,3
1azza_#114	GO:0050819: negative regulation of coagulation	1.83E-07	4,10,3
1azza_#114	GO:0030193: regulation of blood coagulation	1.83E-07	4,10,3
1hvba_#76	GO:0017001: antibiotic catabolism	1.83E-07	4,10,3
3tmka_#98	GO:0009219: pyrimidine deoxyribonucleotide metabolism	1.83E-07	4,10,3
1qnja_#77	GO:0050819: negative regulation of coagulation	1.83E-07	4,10,3
1qnja_#77	GO:0030193: regulation of blood coagulation	1.83E-07	4,10,3
1fmb__#88	GO:0006278: RNA-dependent DNA replication	1.83E-07	4,10,3
1dqwa_#202	GO:0006207: 'de novo' pyrimidine base biosynthesis	1.83E-07	4,10,3
1f7ua2#324	GO:0006418: tRNA aminoacylation for protein translation	1.90E-07	45,26,7
1bjt__#890	GO:0051249: regulation of lymphocyte activation	1.91E-07	258,12,9
1eur__#302	GO:0006953: acute-phase response	1.91E-07	36,20,6
1qtn.1#A270	GO:0042364: water-soluble vitamin biosynthesis	1.93E-07	386,27,16
1ddja_#726	GO:0019883: antigen presentation, endogenous antigen	1.94E-07	82,10,6
1ddja_#726	GO:0018108: peptidyl-tyrosine phosphorylation	1.94E-07	82,10,6
1dpta_#22	GO:0019883: antigen presentation, endogenous antigen	1.94E-07	82,10,6
1g97a1#376	GO:0006100: tricarboxylic acid cycle intermediate metabolism	1.97E-07	11,14,4
1hxmb1#117	GO:0051249: regulation of lymphocyte activation	1.98E-07	65,12,6
1f5aa2#167	GO:0017148: negative regulation of protein biosynthesis	1.98E-07	101,13,7
1danh_#197	GO:0006606: protein-nucleus import	1.99E-07	18,21,5
1svpa_#127	GO:0006606: protein-nucleus import	1.99E-07	18,21,5
1qq4a_#143	GO:0006606: protein-nucleus import	1.99E-07	18,21,5
1dlea_#53	GO:0006606: protein-nucleus import	1.99E-07	18,21,5
2hft_1#34	GO:0042088: T-helper 1 type immune response	2.00E-07	36,11,5
3sil__#317	GO:0042108: positive regulation of cytokine biosynthesis	2.01E-07	300,11,9
1xgsa2#186	GO:0046677: response to antibiotic	2.07E-07	149,40,13
1fp1d2#214	GO:0006100: tricarboxylic acid cycle intermediate metabolism	2.09E-07	143,14,8

1i7oa2#399	GO:0030333: antigen processing	2.10E-07	30,13,5
1auib_#136	GO:0007517: muscle development	2.10E-07	13,30,5
1e1oa2#423	GO:0006418: tRNA aminoacylation for protein translation	2.10E-07	127,26,10
2fcba2#104	GO:0030333: antigen processing	2.11E-07	12,13,4
2ayh__#160	GO:0030333: antigen processing	2.11E-07	12,13,4
1dr9a1#39	GO:0006118: electron transport	2.11E-07	224,228,44
1ep3a_#247	GO:0009073: aromatic amino acid family biosynthesis	2.11E-07	24,16,5
2mev3_#143	GO:0019079: viral genome replication	2.14E-07	3,16,3
1aym2_#253	GO:0019079: viral genome replication	2.14E-07	3,16,3
2mev1_#120	GO:0019079: viral genome replication	2.14E-07	3,16,3
1b35b_#140	GO:0019079: viral genome replication	2.14E-07	3,16,3
2cb5a_#312	GO:0006953: acute-phase response	2.14E-07	92,20,8
1aym3_#131	GO:0006953: acute-phase response	2.14E-07	353,20,13
1jcfa2#206	GO:0006100: tricarboxylic acid cycle intermediate metabolism	2.16E-07	93,14,7
1oaca1#495	GO:0050819: negative regulation of coagulation	2.17E-07	41,10,5
1oaca1#495	GO:0030193: regulation of blood coagulation	2.17E-07	41,10,5
1oaca1#495	GO:0018108: peptidyl-tyrosine phosphorylation	2.17E-07	41,10,5
1zfpe_#67	GO:0007169: transmembrane receptor protein tyrosine kinase signaling pathway	2.18E-07	28,26,6
1hxxa_#278	GO:0019883: antigen presentation, endogenous antigen	2.19E-07	237,10,8
5ruba1#279	GO:0009073: aromatic amino acid family biosynthesis	2.19E-07	47,16,6
1fjfc2#153	GO:0019883: antigen presentation, endogenous antigen	2.20E-07	362,10,9
1efpa1#32	GO:0006563: L-serine metabolism	2.21E-07	114,12,7
1elva1#623	GO:0006606: protein-nucleus import	2.22E-07	35,21,6
1f5aa1#441	GO:0042088: T-helper 1 type immune response	2.27E-07	129,11,7
1f5aa1#441	GO:0042108: positive regulation of cytokine biosynthesis	2.27E-07	129,11,7
1e39a3#442	GO:0050819: negative regulation of coagulation	2.28E-07	16,10,4
1e39a3#442	GO:0030193: regulation of blood coagulation	2.28E-07	16,10,4
1deua_#151	GO:0019883: antigen presentation, endogenous antigen	2.34E-07	239,10,8
1g51a3#525	GO:0050819: negative regulation of coagulation	2.34E-07	239,10,8
1g51a3#525	GO:0030193: regulation of blood coagulation	2.34E-07	239,10,8
1f0xa2#118	GO:0006118: electron transport	2.36E-07	37,228,15
1xgsa2#186	GO:0017001: antibiotic catabolism	2.37E-07	149,10,7
1prea2#308	GO:0051249: regulation of lymphocyte activation	2.38E-07	67,12,6
1e4ea2#215	GO:0016477: cell migration	2.38E-07	419,23,15
1qfma1#186	GO:0042108: positive regulation of cytokine biosynthesis	2.39E-07	306,11,9
1qtn.1#A270	GO:0009086: methionine biosynthesis	2.40E-07	386,14,11
1evqa_#305	GO:0009064: glutamine family amino acid metabolism	2.41E-07	384,19,13
1in4a1#264	GO:0006265: DNA topological change	2.46E-07	42,10,5
1in4a1#264	GO:0006268: DNA unwinding	2.46E-07	42,10,5
1as4.1#A288	GO:0050819: negative regulation of coagulation	2.46E-07	42,10,5
1as4.1#A288	GO:0030193: regulation of blood coagulation	2.46E-07	42,10,5
1auk__#375	GO:0006118: electron transport	2.46E-07	24,228,12
1g25a_#22	GO:0019883: antigen presentation, endogenous antigen	2.49E-07	367,10,9
1g25a_#22	GO:0030217: T-cell differentiation	2.49E-07	367,10,9
1qs0a1#119	GO:0006207: 'de novo' pyrimidine base biosynthesis	2.49E-07	367,10,9
1hr6a1#41	GO:0030333: antigen processing	2.49E-07	31,13,5
1h4ua1#486	GO:0006367: transcription initiation from Pol II promoter	2.51E-07	34,12,5
1i85a_#14	GO:0042088: T-helper 1 type immune response	2.52E-07	4,11,3
1i85a_#14	GO:0042108: positive regulation of cytokine biosynthesis	2.52E-07	4,11,3
1edza2#120	GO:0009396: folic acid and derivative biosynthesis	2.52E-07	4,11,3
1qsta_#137	GO:0016568: chromatin modification	2.52E-07	4,11,3
1ryp1_#46	GO:0006511: ubiquitin-dependent protein catabolism	2.52E-07	4,11,3
1bqca_#23	GO:0030245: cellulose catabolism	2.56E-07	18,22,5
1io1a_#233	GO:0019883: antigen presentation, endogenous antigen	2.59E-07	86,10,6
1e0ta2#275	GO:0006568: tryptophan metabolism	2.59E-07	86,10,6

1dgsa1#514	GO:0006284: base-excision repair	2.60E-07	3,17,3
1a0i_2#222	GO:0019883: antigen presentation, endogenous antigen	2.60E-07	151,10,7
1fnf_3#1409	GO:0042108: positive regulation of cytokine biosynthesis	2.62E-07	76,11,6
1poxa3#460	GO:0006098: pentose-phosphate shunt	2.62E-07	76,11,6
1mspa_#17	GO:0042088: T-helper 1 type immune response	2.65E-07	38,11,5
1mspa_#17	GO:0042108: positive regulation of cytokine biosynthesis	2.65E-07	38,11,5
1ton__#196	GO:0006606: protein-nucleus import	2.68E-07	19,21,5
1qq4a_#63	GO:0006606: protein-nucleus import	2.68E-07	19,21,5
1arb__#24	GO:0006606: protein-nucleus import	2.68E-07	19,21,5
1danh_#24	GO:0006919: caspase activation	2.69E-07	11,15,4
1c4zd_#101	GO:0006511: ubiquitin-dependent protein catabolism	2.69E-07	15,11,4
1ejda_#180	GO:0006096: glycolysis	2.73E-07	65,38,9
1epwa3#138	GO:0019883: antigen presentation, endogenous antigen	2.74E-07	371,10,9
1epwa3#138	GO:0030217: T-cell differentiation	2.74E-07	371,10,9
1epwa3#138	GO:0006275: regulation of DNA replication	2.74E-07	371,10,9
1elva1#604	GO:0006919: caspase activation	2.75E-07	27,15,5
1b12a_#221	GO:0019883: antigen presentation, endogenous antigen	2.78E-07	87,10,6
1b12a_#221	GO:0030217: T-cell differentiation	2.78E-07	87,10,6
1ev2e2#286	GO:0019883: antigen presentation, endogenous antigen	2.78E-07	87,10,6
1ryph_#163	GO:0000160: two-component signal transduction system (phosphorelay)	2.80E-07	103,25,9
1qlwa_#231	GO:0016042: lipid catabolism	2.83E-07	6,31,4
1dp0a4#756	GO:0019883: antigen presentation, endogenous antigen	2.85E-07	153,10,7
1e0ta2#275	GO:0006096: glycolysis	2.89E-07	86,38,10
2cmd_1#113	GO:0006099: tricarboxylic acid cycle	2.91E-07	9,19,4
1h6ka2#336	GO:0015671: oxygen transport	2.92E-07	152,23,10
1hdr__#156	GO:0009103: lipopolysaccharide biosynthesis	2.93E-07	35,12,5
1bio__#108	GO:0030333: antigen processing	2.94E-07	32,13,5
1pbe_2#183	GO:0006367: transcription initiation from Pol II promoter	2.95E-07	14,12,4
1qp8a1#168	GO:0006563: L-serine metabolism	2.95E-07	14,12,4
1hdr__#149	GO:0009103: lipopolysaccharide biosynthesis	2.95E-07	14,12,4
1fnf_1#1146	GO:0051249: regulation of lymphocyte activation	2.95E-07	14,12,4
1c8za_#386	GO:0042108: positive regulation of cytokine biosynthesis	2.95E-07	211,11,8
1ie5a_#23	GO:0016337: cell-cell adhesion	2.98E-07	24,17,5
1dbta_#89	GO:0006207: 'de novo' pyrimidine base biosynthesis	2.98E-07	17,10,4
1nfdb1#34	GO:0006118: electron transport	2.98E-07	167,228,36
1cpy__#380	GO:0019883: antigen presentation, endogenous antigen	2.98E-07	154,10,7
1eq9a_#32	GO:0006953: acute-phase response	3.00E-07	96,20,8
1hwxa1#256	GO:0009070: serine family amino acid biosynthesis	3.02E-07	48,26,7
1g0sa_#74	GO:0019883: antigen presentation, endogenous antigen	3.04E-07	247,10,8
1qr4a2#166	GO:0030333: antigen processing	3.04E-07	13,13,4
1hc7a2#258	GO:0051249: regulation of lymphocyte activation	3.06E-07	186,12,8
2sqca1#417	GO:0030245: cellulose catabolism	3.06E-07	8,22,4
1dr9a2#139	GO:0042108: positive regulation of cytokine biosynthesis	3.07E-07	78,11,6
1dn2a1#261	GO:0019883: antigen presentation, endogenous antigen	3.09E-07	376,10,9
1dn2a1#261	GO:0030217: T-cell differentiation	3.09E-07	376,10,9
1fhoa_#74	GO:0042088: T-helper 1 type immune response	3.09E-07	315,11,9
1fhoa_#74	GO:0001906: cell killing	3.09E-07	315,11,9
1dfna_#16	GO:0042742: defense response to bacteria	3.13E-07	7,26,4
1pysb6#384	GO:0006418: tRNA aminoacylation for protein translation	3.13E-07	7,26,4
1evqa_#305	GO:0006221: pyrimidine nucleotide biosynthesis	3.13E-07	384,22,14
1phm_2#253	GO:0007169: transmembrane receptor protein tyrosine kinase signaling pathway	3.19E-07	257,26,13
1b3qa2#623	GO:0006418: tRNA aminoacylation for protein translation	3.19E-07	257,26,13
1evqa_#305	GO:0006563: L-serine metabolism	3.19E-07	384,12,10
1nfp__#171	GO:0006100: tricarboxylic acid cycle intermediate metabolism	3.23E-07	30,14,5
1hbza_#214	GO:0050819: negative regulation of coagulation	3.24E-07	249,10,8

1hbza_#214	GO:0030193: regulation of blood coagulation	3.24E-07	249,10,8
1dr9a2#139	GO:0016337: cell-cell adhesion	3.28E-07	78,17,7
1by5a_#238	GO:0042088: T-helper 1 type immune response	3.30E-07	214,11,8
1gsoa3#142	GO:0042088: T-helper 1 type immune response	3.32E-07	79,11,6
1gsoa3#142	GO:0042108: positive regulation of cytokine biosynthesis	3.32E-07	79,11,6
1g71a_#88	GO:0006919: caspase activation	3.34E-07	28,15,5
1eq9a_#58	GO:0006919: caspase activation	3.34E-07	28,15,5
1rypk_#12	GO:0030217: T-cell differentiation	3.34E-07	250,10,8
1qtn.1#A270	GO:0006221: pyrimidine nucleotide biosynthesis	3.35E-07	386,22,14
1ovb__#207	GO:0006879: iron ion homeostasis	3.35E-07	4,12,3
1qrra_#99	GO:0009103: lipopolysaccharide biosynthesis	3.35E-07	4,12,3
1ghqb1#4	GO:0006956: complement activation	3.35E-07	4,12,3
1dn2a1#261	GO:0016337: cell-cell adhesion	3.38E-07	376,17,12
2hft_1#34	GO:0051249: regulation of lymphocyte activation	3.39E-07	36,12,5
1qf6a3#78	GO:0046677: response to antibiotic	3.41E-07	46,40,8
2occb1#209	GO:0019883: antigen presentation, endogenous antigen	3.41E-07	90,10,6
1gox__#246	GO:0006568: tryptophan metabolism	3.41E-07	157,10,7
1gox__#246	GO:0006207: 'de novo' pyrimidine base biosynthesis	3.41E-07	157,10,7
1rlr_1#66	GO:0006099: tricarboxylic acid cycle	3.43E-07	69,19,7
1i50b_#966	GO:0030217: T-cell differentiation	3.45E-07	251,10,8
1egaa1#97	GO:0045045: secretory pathway	3.45E-07	19,22,5
1ac5__#72	GO:0009116: nucleoside metabolism	3.45E-07	51,25,7
1fhga_#59	GO:0030333: antigen processing	3.46E-07	33,13,5
1fm2.1#B627	GO:0030333: antigen processing	3.46E-07	33,13,5
1iow_1#37	GO:0006221: pyrimidine nucleotide biosynthesis	3.48E-07	263,22,12
1a9xa3#78	GO:0006096: glycolysis	3.50E-07	262,38,16
1dik_3#240	GO:0007169: transmembrane receptor protein tyrosine kinase signaling pathway	3.50E-07	213,26,12
1qlab2#76	GO:0006118: electron transport	3.51E-07	8,228,7
1evqa_#305	GO:0009116: nucleoside metabolism	3.52E-07	384,25,15
1ojt_3#488	GO:0006118: electron transport	3.55E-07	17,228,10
1fjsa_#158	GO:0006606: protein-nucleus import	3.56E-07	20,21,5
1arb__#55	GO:0006606: protein-nucleus import	3.56E-07	20,21,5
1gdna_#30	GO:0006606: protein-nucleus import	3.56E-07	20,21,5
1cby__#183	GO:0006544: glycine metabolism	3.57E-07	16,11,4
1i76a_#138	GO:0043094: metabolic compound salvage	3.58E-07	80,11,6
1e79d2#36	GO:0016477: cell migration	3.63E-07	432,23,15
1befa_#51	GO:0006953: acute-phase response	3.64E-07	9,20,4
1amf__#196	GO:0006118: electron transport	3.65E-07	154,228,34
1a65a1#108	GO:0030217: T-cell differentiation	3.67E-07	253,10,8
1ct9a2#27	GO:0009064: glutamine family amino acid metabolism	3.70E-07	3,19,3
1ldna1#94	GO:0006207: 'de novo' pyrimidine base biosynthesis	3.73E-07	159,10,7
1evqa_#305	GO:0042401: biogenic amine biosynthesis	3.73E-07	384,10,9
1j9qa1#154	GO:0006118: electron transport	3.74E-07	183,228,38
1bio__#88	GO:0050819: negative regulation of coagulation	3.82E-07	18,10,4
1bio__#88	GO:0030193: regulation of blood coagulation	3.82E-07	18,10,4
1qhoa1#531	GO:0019883: antigen presentation, endogenous antigen	3.82E-07	18,10,4
1kit_3#297	GO:0019883: antigen presentation, endogenous antigen	3.82E-07	18,10,4
1eq9a_#162	GO:0050819: negative regulation of coagulation	3.82E-07	18,10,4
1eq9a_#162	GO:0030193: regulation of blood coagulation	3.82E-07	18,10,4
2cnd_1#101	GO:0006118: electron transport	3.83E-07	29,228,13
1fkna_#63	GO:0006100: tricarboxylic acid cycle intermediate metabolism	3.84E-07	31,14,5
1kit_2#398	GO:0042088: T-helper 1 type immune response	3.86E-07	81,11,6
1kit_2#398	GO:0042108: positive regulation of cytokine biosynthesis	3.86E-07	81,11,6
2cb5a_#312	GO:0018108: peptidyl-tyrosine phosphorylation	3.89E-07	92,10,6
1e69a_#26	GO:0006563: L-serine metabolism	3.91E-07	37,12,5

1g6oa_#116	GO:0006953: acute-phase response	3.94E-07	302,20,12
1qs1a2#437	GO:0019883: antigen presentation, endogenous antigen	4.00E-07	387,10,9
1qs1a2#437	GO:0030217: T-cell differentiation	4.00E-07	387,10,9
1qs1a2#437	GO:0006275: regulation of DNA replication	4.00E-07	387,10,9
1ej8a_#189	GO:0030217: T-cell differentiation	4.03E-07	256,10,8
1beba_#119	GO:0030333: antigen processing	4.04E-07	172,13,8
1p35a_#49	GO:0030333: antigen processing	4.08E-07	112,13,7
1fp1d2#214	GO:0000910: cytokinesis	4.09E-07	143,37,12
1foha5#9	GO:0006118: electron transport	4.11E-07	59,228,19
1nhp_1#7	GO:0006096: glycolysis	4.15E-07	34,38,7
1ho1a_#211	GO:0006568: tryptophan metabolism	4.16E-07	93,10,6
1b3ra1#223	GO:0009070: serine family amino acid biosynthesis	4.18E-07	31,26,6
1qf6a1#606	GO:0006418: tRNA aminoacylation for protein translation	4.18E-07	31,26,6
1qfea_#68	GO:0000160: two-component signal transduction system (phosphorelay)	4.23E-07	453,25,16
1f0xa1#278	GO:0050819: negative regulation of coagulation	4.25E-07	162,10,7
1f0xa1#278	GO:0030193: regulation of blood coagulation	4.25E-07	162,10,7
1qhva_#438	GO:0030333: antigen processing	4.25E-07	14,13,4
1bjt__#890	GO:0050819: negative regulation of coagulation	4.29E-07	258,10,8
1bjt__#890	GO:0030193: regulation of blood coagulation	4.29E-07	258,10,8
1bywa_#30	GO:0051249: regulation of lymphocyte activation	4.31E-07	283,12,9
2dpma_#189	GO:0009252: peptidoglycan biosynthesis	4.32E-07	46,18,6
1quqb_#87	GO:0030333: antigen processing	4.33E-07	67,13,6
1qhoa2#660	GO:0030333: antigen processing	4.36E-07	4,13,3
1f97a1#62	GO:0006730: one-carbon compound metabolism	4.37E-07	591,32,21
1d7ya3#331	GO:0006118: electron transport	4.37E-07	21,228,11
1fc4a_#205	GO:0006221: pyrimidine nucleotide biosynthesis	4.40E-07	215,22,11
1erza_#183	GO:0015976: carbon utilization	4.40E-07	47,10,5
1cgha_#227	GO:0018108: peptidyl-tyrosine phosphorylation	4.40E-07	47,10,5
1dp0a1#309	GO:0030217: T-cell differentiation	4.40E-07	47,10,5
1b4ka_#108	GO:0006207: 'de novo' pyrimidine base biosynthesis	4.43E-07	94,10,6
1axca1#70	GO:0018108: peptidyl-tyrosine phosphorylation	4.43E-07	94,10,6
1danh_#48	GO:0006606: protein-nucleus import	4.48E-07	9,21,4
1qqsa_#124	GO:0019883: antigen presentation, endogenous antigen	4.49E-07	392,10,9
1qqsa_#124	GO:0030217: T-cell differentiation	4.49E-07	392,10,9
1leha1#177	GO:0006563: L-serine metabolism	4.49E-07	38,12,5
1d0va_#114	GO:0006730: one-carbon compound metabolism	4.54E-07	14,32,5
2duba_#212	GO:0009116: nucleoside metabolism	4.54E-07	53,25,7
1smaa1#95	GO:0042108: positive regulation of cytokine biosynthesis	4.57E-07	223,11,8
1ejfa_#105	GO:0006275: regulation of DNA replication	4.57E-07	5,10,3
1plq_1#22	GO:0006275: regulation of DNA replication	4.57E-07	5,10,3
1h68a_#183	GO:0009582: detection of abiotic stimulus	4.57E-07	5,10,3
1ep3a_#220	GO:0006207: 'de novo' pyrimidine base biosynthesis	4.57E-07	5,10,3
1e2fa_#140	GO:0009219: pyrimidine deoxyribonucleotide metabolism	4.57E-07	5,10,3
1zfja1#448	GO:0009167: purine ribonucleoside monophosphate metabolism	4.57E-07	5,10,3
1duvg1#135	GO:0006207: 'de novo' pyrimidine base biosynthesis	4.57E-07	5,10,3
1fxma_#153	GO:0006418: tRNA aminoacylation for protein translation	4.57E-07	17,26,5
1e69a_#40	GO:0006221: pyrimidine nucleotide biosynthesis	4.58E-07	20,22,5
1dn2a1#261	GO:0006953: acute-phase response	4.61E-07	376,20,13
1cs1a_#160	GO:0042401: biogenic amine biosynthesis	4.62E-07	164,10,7
1cs1a_#160	GO:0006207: 'de novo' pyrimidine base biosynthesis	4.62E-07	164,10,7
1c5y.1#B241	GO:0006606: protein-nucleus import	4.64E-07	21,21,5
1dlea_#238	GO:0006606: protein-nucleus import	4.64E-07	21,21,5
1arb__#208	GO:0006606: protein-nucleus import	4.64E-07	21,21,5
1autc_#45	GO:0006606: protein-nucleus import	4.64E-07	21,21,5
1qnja_#138	GO:0006606: protein-nucleus import	4.64E-07	21,21,5
1ekra_#114	GO:0006633: fatty acid biosynthesis	4.66E-07	49,27,7

1d4ua1#41	GO:0030333: antigen processing	4.71E-07	35,13,5
1h6va1#27	GO:0009396: folic acid and derivative biosynthesis	4.73E-07	224,11,8
1fjsa_#83	GO:0042088: T-helper 1 type immune response	4.81E-07	84,11,6
1f97a1#62	GO:0016337: cell-cell adhesion	4.82E-07	591,17,14
1eq9a_#58	GO:0019079: viral genome replication	4.82E-07	28,16,5
3pah__#286	GO:0015976: carbon utilization	4.83E-07	19,10,4
1hg3a_#207	GO:0042401: biogenic amine biosynthesis	4.83E-07	19,10,4
1hg3a_#207	GO:0015976: carbon utilization	4.83E-07	19,10,4
1hg3a_#207	GO:0006568: tryptophan metabolism	4.83E-07	19,10,4
1a9xa3#78	GO:0042401: biogenic amine biosynthesis	4.84E-07	262,10,8
1erv__#80	GO:0051249: regulation of lymphocyte activation	4.88E-07	401,12,10
1eq9a_#32	GO:0006919: caspase activation	4.89E-07	96,15,7
1bif_2#398	GO:0006418: tRNA aminoacylation for protein translation	4.89E-07	105,26,9
1fo5a_#60	GO:0042088: T-helper 1 type immune response	4.90E-07	225,11,8
1dkia_#76P	GO:0019883: antigen presentation, endogenous antigen	4.90E-07	48,10,5
1iow_1#37	GO:0009064: glutamine family amino acid metabolism	4.91E-07	263,19,11
1opy__#68	GO:0006953: acute-phase response	4.92E-07	308,20,12
1tuba1#8	GO:0006096: glycolysis	5.00E-07	115,38,11
1hwxa1#223	GO:0000160: two-component signal transduction system (phosphorelay)	5.03E-07	186,25,11
1nsj__#21	GO:0006207: 'de novo' pyrimidine base biosynthesis	5.03E-07	96,10,6
1quba4#235	GO:0019883: antigen presentation, endogenous antigen	5.03E-07	96,10,6
1am2__#163	GO:0030217: T-cell differentiation	5.03E-07	96,10,6
2hlca_#54	GO:0006919: caspase activation	5.05E-07	58,15,6
1h8ca_#35	GO:0006367: transcription initiation from Pol II promoter	5.12E-07	76,12,6
1h6la_#119	GO:0030217: T-cell differentiation	5.14E-07	264,10,8
1hd2a_#47	GO:0006096: glycolysis	5.15E-07	51,38,8
1eq2a_#299	GO:0009103: lipopolysaccharide biosynthesis	5.34E-07	16,12,4
1e6ua_#10	GO:0009103: lipopolysaccharide biosynthesis	5.34E-07	16,12,4
1dt6a_#376	GO:0006118: electron transport	5.35E-07	6,228,6
1dt6a_#374	GO:0006118: electron transport	5.35E-07	6,228,6
3grs_1#131	GO:0006118: electron transport	5.35E-07	6,228,6
1a8l_2#207	GO:0006118: electron transport	5.35E-07	6,228,6
1etpa2#123	GO:0006118: electron transport	5.35E-07	6,228,6
1qqsa_#124	GO:0016337: cell-cell adhesion	5.42E-07	392,17,12
1chua2#21	GO:0006118: electron transport	5.44E-07	11,228,8
1cmia_#86	GO:0019883: antigen presentation, endogenous antigen	5.45E-07	49,10,5
1qaxa2#307	GO:0050819: negative regulation of coagulation	5.45E-07	49,10,5
1qaxa2#307	GO:0030193: regulation of blood coagulation	5.45E-07	49,10,5
1qaxa2#307	GO:0018108: peptidyl-tyrosine phosphorylation	5.45E-07	49,10,5
1h8d.1#H207	GO:0018108: peptidyl-tyrosine phosphorylation	5.45E-07	49,10,5
1dnv__#354	GO:0019883: antigen presentation, endogenous antigen	5.45E-07	49,10,5
1ton__#181	GO:0018108: peptidyl-tyrosine phosphorylation	5.45E-07	49,10,5
4tmka_#141	GO:0006221: pyrimidine nucleotide biosynthesis	5.47E-07	9,22,4
1e6ca_#153	GO:0006221: pyrimidine nucleotide biosynthesis	5.47E-07	9,22,4
5nul__#43	GO:0006221: pyrimidine nucleotide biosynthesis	5.47E-07	9,22,4
1erv__#80	GO:0019883: antigen presentation, endogenous antigen	5.49E-07	401,10,9
1erv__#80	GO:0030217: T-cell differentiation	5.49E-07	401,10,9
1erv__#80	GO:0006275: regulation of DNA replication	5.49E-07	401,10,9
1cbf__#208	GO:0051249: regulation of lymphocyte activation	5.50E-07	291,12,9
1hyha2#179	GO:0006099: tricarboxylic acid cycle	5.52E-07	24,19,5
1p35a_#155	GO:0008284: positive regulation of cell proliferation	5.57E-07	45,30,7
1bupa1#178	GO:0006986: response to unfolded protein	5.78E-07	13,15,4
1qora2#201	GO:0009086: methionine biosynthesis	5.83E-07	163,14,8
1gsa_2#218	GO:0030333: antigen processing	5.86E-07	118,13,7
2mpra_#89	GO:0006367: transcription initiation from Pol II promoter	5.86E-07	40,12,5
1e05i_#83	GO:0015671: oxygen transport	5.87E-07	89,23,8

1gof_2#118	GO:0006100: tricarboxylic acid cycle intermediate metabolism	5.93E-07	14,14,4
1b12a_#221	GO:0042088: T-helper 1 type immune response	5.94E-07	87,11,6
1oaca1#495	GO:0006606: protein-nucleus import	5.97E-07	41,21,6
1arb__#193	GO:0006606: protein-nucleus import	5.98E-07	22,21,5
1eur__#84	GO:0016337: cell-cell adhesion	5.98E-07	85,17,7
1a9xa3#78	GO:0009073: aromatic amino acid family biosynthesis	6.03E-07	262,16,10
2shpa1#387	GO:0006470: protein amino acid dephosphorylation	6.03E-07	10,20,4
1a53__#236	GO:0042401: biogenic amine biosynthesis	6.03E-07	20,10,4
1a53__#236	GO:0006568: tryptophan metabolism	6.03E-07	20,10,4
1d2ka1#164	GO:0006207: 'de novo' pyrimidine base biosynthesis	6.05E-07	50,10,5
1a8i__#688	GO:0006563: L-serine metabolism	6.07E-07	203,12,8
1cs1a_#160	GO:0009086: methionine biosynthesis	6.12E-07	164,14,8
1ejda_#180	GO:0006100: tricarboxylic acid cycle intermediate metabolism	6.17E-07	65,14,6
1f9za_#16	GO:0046677: response to antibiotic	6.17E-07	111,40,11
1h6va1#27	GO:0009252: peptidoglycan biosynthesis	6.21E-07	224,18,10
1c1da1#153	GO:0009070: serine family amino acid biosynthesis	6.22E-07	8,26,4
1aop_3#276	GO:0006953: acute-phase response	6.25E-07	147,20,9
1jatb_#72	GO:0006511: ubiquitin-dependent protein catabolism	6.28E-07	5,11,3
2sli_2#438	GO:0006511: ubiquitin-dependent protein catabolism	6.28E-07	5,11,3
1a4ia2#118	GO:0009396: folic acid and derivative biosynthesis	6.28E-07	5,11,3
1jatb_#120	GO:0006511: ubiquitin-dependent protein catabolism	6.28E-07	5,11,3
1rypc_#165	GO:0006511: ubiquitin-dependent protein catabolism	6.28E-07	5,11,3
1pmaa_#66	GO:0006511: ubiquitin-dependent protein catabolism	6.28E-07	5,11,3
1pma1_#128	GO:0006511: ubiquitin-dependent protein catabolism	6.28E-07	5,11,3
1fa0a1#393	GO:0009064: glutamine family amino acid metabolism	6.28E-07	209,19,10
1h9da_#91	GO:0051249: regulation of lymphocyte activation	6.31E-07	204,12,8
5ruba1#259	GO:0009396: folic acid and derivative biosynthesis	6.36E-07	45,11,5
1nfdb1#21	GO:0042108: positive regulation of cytokine biosynthesis	6.36E-07	88,11,6
1ea5a_#99	GO:0019883: antigen presentation, endogenous antigen	6.44E-07	172,10,7
1esma_#314	GO:0030217: T-cell differentiation	6.44E-07	100,10,6
1fc4a_#205	GO:0042364: water-soluble vitamin biosynthesis	6.47E-07	215,27,12
1a65a1#108	GO:0006953: acute-phase response	6.65E-07	253,20,11
1bqk__#77	GO:0030333: antigen processing	6.70E-07	72,13,6
1qrra_#69	GO:0009167: purine ribonucleoside monophosphate metabolism	6.70E-07	173,10,7
1pma1_#17	GO:0006919: caspase activation	6.75E-07	32,15,5
1ho1a_#211	GO:0009073: aromatic amino acid family biosynthesis	6.76E-07	93,16,7
1ew6a_#35	GO:0015671: oxygen transport	6.77E-07	3,23,3
1qf9a_#117	GO:0007264: small GTPase mediated signal transduction	6.84E-07	68,21,7
1ovb__#207	GO:0006826: iron ion transport	6.93E-07	4,15,3
1ab4__#344	GO:0006986: response to unfolded protein	6.93E-07	4,15,3
2bc2a_#46	GO:0006119: oxidative phosphorylation	6.96E-07	17,12,4
1lcl__#110	GO:0016337: cell-cell adhesion	6.96E-07	12,17,4
1f5aa2#167	GO:0007264: small GTPase mediated signal transduction	6.98E-07	101,21,8
1erv__#80	GO:0016337: cell-cell adhesion	7.01E-07	401,17,12
3prn__#143	GO:0051249: regulation of lymphocyte activation	7.19E-07	300,12,9
1wdcb_#134	GO:0030333: antigen processing	7.22E-07	38,13,5
1et9a1#39	GO:0030333: antigen processing	7.22E-07	38,13,5
1qfla1#42	GO:0006633: fatty acid biosynthesis	7.29E-07	8,27,4
1tuba1#8	GO:0009116: nucleoside metabolism	7.30E-07	115,25,9
1et0a_#118	GO:0006118: electron transport	7.35E-07	18,228,10
1bu7a_#277	GO:0006118: electron transport	7.35E-07	18,228,10
1qfha1#721	GO:0006118: electron transport	7.35E-07	18,228,10
1pwt__#8	GO:0006457: protein folding	7.35E-07	262,49,18
1fgga_#134	GO:0000160: two-component signal transduction system (phosphorelay)	7.35E-07	193,25,11
1e5ka_#113	GO:0019883: antigen presentation, endogenous antigen	7.39E-07	52,10,5
1e5ka_#113	GO:0050819: negative regulation of coagulation	7.39E-07	52,10,5

1e5ka_#113	GO:0030193: regulation of blood coagulation	7.39E-07	52,10,5
1beba_#21	GO:0030217: T-cell differentiation	7.43E-07	21,10,4
1ga0a_#228	GO:0017001: antibiotic catabolism	7.43E-07	21,10,4
2dri__#115	GO:0007264: small GTPase mediated signal transduction	7.43E-07	10,21,4
1dp0a4#756	GO:0042108: positive regulation of cytokine biosynthesis	7.44E-07	153,11,7
1ppn__#132	GO:0030333: antigen processing	7.44E-07	186,13,8
1i76a_#138	GO:0009070: serine family amino acid biosynthesis	7.46E-07	80,26,8
1jj2s_#46	GO:0006418: tRNA aminoacylation for protein translation	7.48E-07	34,26,6
1e4ea2#215	GO:0051249: regulation of lymphocyte activation	7.49E-07	419,12,10
3grs_2#211	GO:0006563: L-serine metabolism	7.54E-07	42,12,5
1dmla2#297	GO:0001906: cell killing	7.56E-07	19,11,4
1hg3a_#207	GO:0043094: metabolic compound salvage	7.56E-07	19,11,4
1elka_#21	GO:0006897: endocytosis	7.56E-07	11,19,4
1bpv__#22	GO:0042088: T-helper 1 type immune response	7.56E-07	19,11,4
1qs0a1#119	GO:0042364: water-soluble vitamin biosynthesis	7.60E-07	367,27,15
1dp0a1#309	GO:0008284: positive regulation of cell proliferation	7.60E-07	47,30,7
1nfdb1#21	GO:0016337: cell-cell adhesion	7.60E-07	88,17,7
1qqsa_#124	GO:0006953: acute-phase response	7.62E-07	392,20,13
2mpra_#190	GO:0042108: positive regulation of cytokine biosynthesis	7.66E-07	349,11,9
1a8i__#688	GO:0006118: electron transport	7.73E-07	203,228,40
2cuaa_#83	GO:0006118: electron transport	7.77E-07	45,228,16
1mrp__#172	GO:0006544: glycine metabolism	7.79E-07	91,11,6
1dp0a1#309	GO:0042088: T-helper 1 type immune response	7.95E-07	47,11,5
1dp0a1#309	GO:0042108: positive regulation of cytokine biosynthesis	7.95E-07	47,11,5
1ekbb_#228	GO:0006606: protein-nucleus import	8.01E-07	43,21,6
1ddja_#754	GO:0006919: caspase activation	8.06E-07	14,15,4
1fc4a_#111	GO:0009086: methionine biosynthesis	8.06E-07	15,14,4
1qama_#37	GO:0009307: DNA restriction	8.08E-07	48,19,6
1e4ea2#215	GO:0019883: antigen presentation, endogenous antigen	8.13E-07	419,10,9
1e4ea2#215	GO:0030217: T-cell differentiation	8.13E-07	419,10,9
1by5a_#347	GO:0001906: cell killing	8.13E-07	240,11,8
1e42a1#711	GO:0042088: T-helper 1 type immune response	8.14E-07	155,11,7
1czya1#466	GO:0019883: antigen presentation, endogenous antigen	8.15E-07	104,10,6
1crza1#240	GO:0019883: antigen presentation, endogenous antigen	8.15E-07	53,10,5
1qdla_#211	GO:0030217: T-cell differentiation	8.18E-07	280,10,8
1jf9a_#379	GO:0016337: cell-cell adhesion	8.21E-07	186,17,9
1tig__#148	GO:0006730: one-carbon compound metabolism	8.22E-07	28,32,6
1c8za_#386	GO:0051249: regulation of lymphocyte activation	8.22E-07	211,12,8
1flca1#294	GO:0016337: cell-cell adhesion	8.22E-07	89,17,7
1gox__#124	GO:0006779: porphyrin biosynthesis	8.36E-07	298,18,11
1zfja1#448	GO:0009156: ribonucleoside monophosphate biosynthesis	8.36E-07	5,12,3
3tdt__#160	GO:0009103: lipopolysaccharide biosynthesis	8.36E-07	5,12,3
1g97a1#354	GO:0009103: lipopolysaccharide biosynthesis	8.36E-07	5,12,3
1qrea_#80	GO:0009103: lipopolysaccharide biosynthesis	8.36E-07	5,12,3
3chbd_#85	GO:0016337: cell-cell adhesion	8.41E-07	323,17,11
1avgi_#69	GO:0019883: antigen presentation, endogenous antigen	8.41E-07	281,10,8
1hq8a_#206	GO:0019883: antigen presentation, endogenous antigen	8.41E-07	281,10,8
1hq8a_#206	GO:0030217: T-cell differentiation	8.41E-07	281,10,8
1hdoa_#189	GO:0009070: serine family amino acid biosynthesis	8.45E-07	147,26,10
1aym3_#131	GO:0042108: positive regulation of cytokine biosynthesis	8.47E-07	353,11,9
1aym3_#131	GO:0001906: cell killing	8.47E-07	353,11,9
2bpa1_#45	GO:0030217: T-cell differentiation	8.49E-07	179,10,7
1qo0d_#81	GO:0006563: L-serine metabolism	8.52E-07	43,12,5
1ddja_#681	GO:0051249: regulation of lymphocyte activation	8.53E-07	212,12,8
2mev1_#246	GO:0019079: viral genome replication	8.53E-07	4,16,3
2hrva_#123	GO:0019079: viral genome replication	8.53E-07	4,16,3

1bev1_#245	GO:0019079: viral genome replication	8.53E-07	4,16,3
2hhma_#262	GO:0009116: nucleoside metabolism	8.53E-07	85,25,8
1qfma1#186	GO:0051249: regulation of lymphocyte activation	8.55E-07	306,12,9
1hxxa_#232	GO:0030217: T-cell differentiation	8.65E-07	282,10,8
1mdah_#329	GO:0030217: T-cell differentiation	8.65E-07	282,10,8
3grx__#56	GO:0051249: regulation of lymphocyte activation	8.80E-07	307,12,9
1efpa1#32	GO:0006100: tricarboxylic acid cycle intermediate metabolism	8.88E-07	114,14,7
1efpa1#32	GO:0009086: methionine biosynthesis	8.88E-07	114,14,7
1qs0b2#269	GO:0009070: serine family amino acid biosynthesis	8.97E-07	35,26,6
1qqsa_#124	GO:0016477: cell migration	8.97E-07	392,23,14
1jj2j_#70	GO:0016477: cell migration	9.06E-07	64,23,7
1b3ra2#60	GO:0006470: protein amino acid dephosphorylation	9.10E-07	25,20,5
1i85a_#101	GO:0030217: T-cell differentiation	9.11E-07	6,10,3
1huxa_#10	GO:0046677: response to antibiotic	9.17E-07	52,40,8
1by5a_#238	GO:0051249: regulation of lymphocyte activation	9.18E-07	214,12,8
1eq9a_#40	GO:0006919: caspase activation	9.18E-07	64,15,6
1qrra_#69	GO:0009086: methionine biosynthesis	9.28E-07	173,14,8
1hvba_#247	GO:0009252: peptidoglycan biosynthesis	9.31E-07	28,18,5
1dj0a2#183	GO:0006953: acute-phase response	9.35E-07	111,20,8
1mpp__#154	GO:0051249: regulation of lymphocyte activation	9.36E-07	84,12,6
2viua_#252	GO:0050819: negative regulation of coagulation	9.40E-07	285,10,8
2viua_#252	GO:0030193: regulation of blood coagulation	9.40E-07	285,10,8
3grs_2#211	GO:0006221: pyrimidine nucleotide biosynthesis	9.40E-07	42,22,6
1bn8a_#55	GO:0000160: two-component signal transduction system (phosphorelay)	9.42E-07	9,25,4
1imva_#255	GO:0042088: T-helper 1 type immune response	9.49E-07	506,11,10
1fc4a_#205	GO:0006563: L-serine metabolism	9.52E-07	215,12,8
1g71a_#77	GO:0006606: protein-nucleus import	9.54E-07	24,21,5
1g4ia_#11	GO:0006606: protein-nucleus import	9.54E-07	24,21,5
2hlca_#68	GO:0006606: protein-nucleus import	9.54E-07	24,21,5
1cby__#183	GO:0006730: one-carbon compound metabolism	9.73E-07	16,32,5
1fp1d2#214	GO:0042364: water-soluble vitamin biosynthesis	9.84E-07	143,27,10
1epwa1#901	GO:0019883: antigen presentation, endogenous antigen	9.85E-07	55,10,5
1qo2a_#222	GO:0042401: biogenic amine biosynthesis	9.85E-07	55,10,5
1dnv__#354	GO:0001906: cell killing	9.85E-07	49,11,5
1gln_2#10	GO:0006418: tRNA aminoacylation for protein translation	9.94E-07	3,26,3
1qf6a4#360	GO:0006418: tRNA aminoacylation for protein translation	9.94E-07	3,26,3
1ffya3#535	GO:0006418: tRNA aminoacylation for protein translation	9.94E-07	3,26,3
1dvpa1#65	GO:0015671: oxygen transport	9.96E-07	173,23,10
1erv__#80	GO:0006953: acute-phase response	1.00E-06	401,20,13
1egja_#346	GO:0030333: antigen processing	1.00E-06	17,13,4
1dpga2#334	GO:0000160: two-component signal transduction system (phosphorelay)	1.01E-06	246,25,12
1fo4a5#1020	GO:0051249: regulation of lymphocyte activation	1.01E-06	312,12,9
1c9la2#61	GO:0006953: acute-phase response	1.02E-06	206,20,10
1g0sa_#74	GO:0001906: cell killing	1.02E-06	247,11,8
1opy__#68	GO:0006606: protein-nucleus import	1.02E-06	308,21,12
1gox__#124	GO:0000160: two-component signal transduction system (phosphorelay)	1.02E-06	298,25,13
1fn9a_#271	GO:0030217: T-cell differentiation	1.03E-06	184,10,7
1h6la_#119	GO:0006953: acute-phase response	1.03E-06	264,20,11
1fjfj_#68	GO:0051249: regulation of lymphocyte activation	1.06E-06	218,12,8
1e79d2#36	GO:0050819: negative regulation of coagulation	1.07E-06	432,10,9
1e79d2#36	GO:0030193: regulation of blood coagulation	1.07E-06	432,10,9
1e79d2#36	GO:0030217: T-cell differentiation	1.07E-06	432,10,9
1i6vd_#713	GO:0009070: serine family amino acid biosynthesis	1.07E-06	36,26,6
1fu6a_#50	GO:0006953: acute-phase response	1.07E-06	265,20,11
1el5a1#10	GO:0006100: tricarboxylic acid cycle intermediate metabolism	1.07E-06	16,14,4
1e6ua_#10	GO:0006100: tricarboxylic acid cycle intermediate metabolism	1.07E-06	16,14,4

1fp5a2#537	GO:0051249: regulation of lymphocyte activation	1.08E-06	45,12,5
1elva1#623	GO:0006919: caspase activation	1.08E-06	35,15,5
1cmxa_#207	GO:0006367: transcription initiation from Pol II promoter	1.08E-06	86,12,6
1ec7a1#216	GO:0009167: purine ribonucleoside monophosphate metabolism	1.08E-06	56,10,5
1bfg__#22	GO:0050819: negative regulation of coagulation	1.08E-06	56,10,5
1bfg__#22	GO:0030193: regulation of blood coagulation	1.08E-06	56,10,5
1dhs__#99	GO:0006207: 'de novo' pyrimidine base biosynthesis	1.08E-06	109,10,6
1f5aa1#441	GO:0030333: antigen processing	1.08E-06	129,13,7
1ac6a_#34	GO:0016337: cell-cell adhesion	1.08E-06	57,17,6
1dmla1#134	GO:0030333: antigen processing	1.08E-06	78,13,6
1hbza_#214	GO:0042088: T-helper 1 type immune response	1.08E-06	249,11,8
1hbza_#214	GO:0042108: positive regulation of cytokine biosynthesis	1.08E-06	249,11,8
1h7wa2#636	GO:0006221: pyrimidine nucleotide biosynthesis	1.09E-06	43,22,6
2reb_1#138	GO:0006096: glycolysis	1.09E-06	56,38,8
1a99a_#84	GO:0006826: iron ion transport	1.10E-06	15,15,4
1fsu__#434	GO:0051249: regulation of lymphocyte activation	1.10E-06	315,12,9
1fhoa_#74	GO:0051249: regulation of lymphocyte activation	1.10E-06	315,12,9
1f0xa1#278	GO:0042088: T-helper 1 type immune response	1.10E-06	162,11,7
1jf9a_#379	GO:0030217: T-cell differentiation	1.11E-06	186,10,7
3sil__#98	GO:0030217: T-cell differentiation	1.11E-06	186,10,7
1ppn__#132	GO:0019883: antigen presentation, endogenous antigen	1.11E-06	186,10,7
1aqua_#126	GO:0006779: porphyrin biosynthesis	1.11E-06	472,18,13
1cbf__#208	GO:0030217: T-cell differentiation	1.11E-06	291,10,8
1qfma2#576	GO:0006118: electron transport	1.11E-06	183,228,37
1evqa_#305	GO:0009252: peptidoglycan biosynthesis	1.11E-06	384,18,12
1evqa_#305	GO:0006779: porphyrin biosynthesis	1.11E-06	384,18,12
1g5va_#109	GO:0006418: tRNA aminoacylation for protein translation	1.11E-06	9,26,4
1gdha2#47	GO:0006100: tricarboxylic acid cycle intermediate metabolism	1.11E-06	38,14,5
1rypk_#12	GO:0042108: positive regulation of cytokine biosynthesis	1.12E-06	250,11,8
2hlpa1#98	GO:0006099: tricarboxylic acid cycle	1.13E-06	12,19,4
1ldm_2#292	GO:0006099: tricarboxylic acid cycle	1.13E-06	12,19,4
2hlpa2#195	GO:0006099: tricarboxylic acid cycle	1.13E-06	12,19,4
1g8fa3#419	GO:0009252: peptidoglycan biosynthesis	1.13E-06	129,18,8
1f42a2#123	GO:0016477: cell migration	1.14E-06	335,23,13
1el0a_#42	GO:0016337: cell-cell adhesion	1.15E-06	258,17,10
1e4ea2#215	GO:0016337: cell-cell adhesion	1.15E-06	419,17,12
1qora2#201	GO:0009396: folic acid and derivative biosynthesis	1.15E-06	163,11,7
1i50b_#966	GO:0042088: T-helper 1 type immune response	1.15E-06	251,11,8
1danh_#152	GO:0006606: protein-nucleus import	1.16E-06	11,21,4
1beba_#21	GO:0042088: T-helper 1 type immune response	1.16E-06	21,11,4
1beba_#21	GO:0042108: positive regulation of cytokine biosynthesis	1.16E-06	21,11,4
1dfaa1#25	GO:0016485: protein processing	1.16E-06	21,11,4
1cs6a4#363	GO:0042088: T-helper 1 type immune response	1.16E-06	21,11,4
1cs6a4#363	GO:0042108: positive regulation of cytokine biosynthesis	1.16E-06	21,11,4
1fwxa2#396	GO:0006953: acute-phase response	1.17E-06	209,20,10
1g25a_#22	GO:0042088: T-helper 1 type immune response	1.19E-06	367,11,9
1qs0a1#119	GO:0043094: metabolic compound salvage	1.19E-06	367,11,9
1i5ga_#115	GO:0019883: antigen presentation, endogenous antigen	1.20E-06	294,10,8
1ifc__#89	GO:0042108: positive regulation of cytokine biosynthesis	1.20E-06	164,11,7
1p35a_#101	GO:0019883: antigen presentation, endogenous antigen	1.20E-06	111,10,6
1dj0a2#183	GO:0050819: negative regulation of coagulation	1.20E-06	111,10,6
1dj0a2#183	GO:0030193: regulation of blood coagulation	1.20E-06	111,10,6
1aqua_#126	GO:0016042: lipid catabolism	1.20E-06	472,31,18
1ii7a_#199	GO:0006563: L-serine metabolism	1.20E-06	46,12,5
1kapp2#69	GO:0017148: negative regulation of protein biosynthesis	1.21E-06	42,13,5
1nsj__#178	GO:0006096: glycolysis	1.21E-06	77,38,9

1a65a1#108	GO:0042108: positive regulation of cytokine biosynthesis	1.23E-06	253,11,8
1hq0a_#867	GO:0006418: tRNA aminoacylation for protein translation	1.23E-06	153,26,10
1nfdb1#21	GO:0051249: regulation of lymphocyte activation	1.24E-06	88,12,6
1bzya_#173	GO:0043094: metabolic compound salvage	1.25E-06	6,11,3
1i85a_#101	GO:0042088: T-helper 1 type immune response	1.25E-06	6,11,3
1i85a_#101	GO:0042108: positive regulation of cytokine biosynthesis	1.25E-06	6,11,3
1dq3a1#432	GO:0016485: protein processing	1.25E-06	6,11,3
1ez0a_#133	GO:0030217: T-cell differentiation	1.27E-06	112,10,6
1ii7a_#43	GO:0006100: tricarboxylic acid cycle intermediate metabolism	1.27E-06	39,14,5
1aoza1#61	GO:0006643: membrane lipid metabolism	1.28E-06	18,13,4
1f8ra2#325	GO:0016337: cell-cell adhesion	1.29E-06	196,17,9
1gpea1#271	GO:0016337: cell-cell adhesion	1.29E-06	196,17,9
1e1aa_#178	GO:0030217: T-cell differentiation	1.29E-06	58,10,5
2hlca_#54	GO:0018108: peptidyl-tyrosine phosphorylation	1.29E-06	58,10,5
1ton__#211	GO:0018108: peptidyl-tyrosine phosphorylation	1.29E-06	58,10,5
1nat__#50	GO:0009252: peptidoglycan biosynthesis	1.29E-06	242,18,10
1dm9a_#57	GO:0006511: ubiquitin-dependent protein catabolism	1.29E-06	99,11,6
1qfla1#44	GO:0006633: fatty acid biosynthesis	1.30E-06	9,27,4
3grs_3#435	GO:0006418: tRNA aminoacylation for protein translation	1.31E-06	154,26,10
1qfea_#68	GO:0009086: methionine biosynthesis	1.31E-06	453,14,11
1epwa3#138	GO:0042108: positive regulation of cytokine biosynthesis	1.31E-06	371,11,9
1ep3a_#247	GO:0006207: 'de novo' pyrimidine base biosynthesis	1.31E-06	24,10,4
1qs0a1#235	GO:0009070: serine family amino acid biosynthesis	1.31E-06	86,26,8
1e05i_#83	GO:0006367: transcription initiation from Pol II promoter	1.32E-06	89,12,6
1cfr__#269	GO:0006544: glycine metabolism	1.33E-06	52,11,5
1gox__#124	GO:0006207: 'de novo' pyrimidine base biosynthesis	1.34E-06	298,10,8
1ddma_#130	GO:0030333: antigen processing	1.34E-06	285,13,9
1f8ra1#288	GO:0006563: L-serine metabolism	1.34E-06	47,12,5
1nuka_#80	GO:0016337: cell-cell adhesion	1.35E-06	197,17,9
1ej8a_#189	GO:0042088: T-helper 1 type immune response	1.35E-06	256,11,8
1ej8a_#189	GO:0042108: positive regulation of cytokine biosynthesis	1.35E-06	256,11,8
1htp__#16	GO:0042088: T-helper 1 type immune response	1.35E-06	256,11,8
1g6sa_#64	GO:0009307: DNA restriction	1.35E-06	84,19,7
1thg__#223	GO:0006100: tricarboxylic acid cycle intermediate metabolism	1.35E-06	74,14,6
1kit_2#398	GO:0030333: antigen processing	1.36E-06	81,13,6
1dfca4#1469	GO:0030333: antigen processing	1.37E-06	43,13,5
1esma_#314	GO:0042088: T-helper 1 type immune response	1.37E-06	100,11,6
1cgha_#227	GO:0006606: protein-nucleus import	1.38E-06	47,21,6
1ibja_#217	GO:0009086: methionine biosynthesis	1.38E-06	5,14,3
1a4ia2#118	GO:0009086: methionine biosynthesis	1.38E-06	5,14,3
1ajsa_#139	GO:0009086: methionine biosynthesis	1.38E-06	5,14,3
1gox__#124	GO:0006221: pyrimidine nucleotide biosynthesis	1.39E-06	298,22,12
1bak__#583	GO:0006118: electron transport	1.40E-06	127,228,29
1beba_#119	GO:0009064: glutamine family amino acid metabolism	1.40E-06	172,19,9
1gcoa_#119	GO:0009103: lipopolysaccharide biosynthesis	1.41E-06	20,12,4
3prn__#143	GO:0030217: T-cell differentiation	1.41E-06	300,10,8
3prn__#143	GO:0006275: regulation of DNA replication	1.41E-06	300,10,8
3sil__#317	GO:0030217: T-cell differentiation	1.41E-06	300,10,8
2cpl__#20	GO:0051249: regulation of lymphocyte activation	1.42E-06	90,12,6
1fp3a_#350	GO:0030245: cellulose catabolism	1.42E-06	11,22,4
1b65a_#257	GO:0006544: glycine metabolism	1.42E-06	168,11,7
1j71a_#251	GO:0006418: tRNA aminoacylation for protein translation	1.44E-06	87,26,8
1f0xa1#278	GO:0006953: acute-phase response	1.44E-06	162,20,9
1fc4a_#205	GO:0009073: aromatic amino acid family biosynthesis	1.44E-06	215,16,9
1gg4a3#64	GO:0006100: tricarboxylic acid cycle intermediate metabolism	1.45E-06	40,14,5
2sli_2#326	GO:0051249: regulation of lymphocyte activation	1.45E-06	227,12,8

1c4zd_#85	GO:0006511: ubiquitin-dependent protein catabolism	1.45E-06	101,11,6
2occb1#189	GO:0006118: electron transport	1.46E-06	9,228,7
1f5va_#139	GO:0006118: electron transport	1.46E-06	9,228,7
1cyx__#133	GO:0006118: electron transport	1.46E-06	9,228,7
1dpta_#22	GO:0030333: antigen processing	1.46E-06	82,13,6
1pvc1_#106	GO:0042088: T-helper 1 type immune response	1.47E-06	53,11,5
2pia_1#54	GO:0006118: electron transport	1.47E-06	185,228,37
1gdoa_#24	GO:0009064: glutamine family amino acid metabolism	1.47E-06	4,19,3
1dn2a1#261	GO:0042108: positive regulation of cytokine biosynthesis	1.48E-06	376,11,9
1a4ya_#104	GO:0006207: 'de novo' pyrimidine base biosynthesis	1.48E-06	194,10,7
1g6oa_#116	GO:0030217: T-cell differentiation	1.48E-06	302,10,8
1tlfa_#269	GO:0006730: one-carbon compound metabolism	1.48E-06	8,32,4
1nsca_#379	GO:0019883: antigen presentation, endogenous antigen	1.49E-06	115,10,6
1e25a_#262	GO:0017001: antibiotic catabolism	1.49E-06	115,10,6
1eexb_#119	GO:0009070: serine family amino acid biosynthesis	1.49E-06	38,26,6
1bio__#210	GO:0006919: caspase activation	1.51E-06	113,15,7
1smaa2#581	GO:0030217: T-cell differentiation	1.53E-06	60,10,5
1clxa_#120	GO:0009073: aromatic amino acid family biosynthesis	1.55E-06	35,16,5
1czan1#92	GO:0006418: tRNA aminoacylation for protein translation	1.55E-06	538,26,17
1g55a_#68	GO:0000160: two-component signal transduction system (phosphorelay)	1.56E-06	10,25,4
1ioaa_#75	GO:0030217: T-cell differentiation	1.56E-06	25,10,4
1f97a1#62	GO:0006457: protein folding	1.56E-06	591,49,27
1a0la_#160	GO:0006606: protein-nucleus import	1.57E-06	48,21,6
1arb__#210	GO:0006919: caspase activation	1.58E-06	70,15,6
1qs0a1#119	GO:0006099: tricarboxylic acid cycle	1.58E-06	367,19,12
1aoza1#61	GO:0016042: lipid catabolism	1.59E-06	18,31,5
1cmxa_#207	GO:0009064: glutamine family amino acid metabolism	1.59E-06	86,19,7
1danh_#93	GO:0050819: negative regulation of coagulation	1.59E-06	7,10,3
1danh_#93	GO:0030193: regulation of blood coagulation	1.59E-06	7,10,3
3aky_1#194	GO:0017001: antibiotic catabolism	1.59E-06	7,10,3
1ie5a_#104	GO:0030217: T-cell differentiation	1.59E-06	7,10,3
1ihna_#64	GO:0050819: negative regulation of coagulation	1.59E-06	7,10,3
1ihna_#64	GO:0030193: regulation of blood coagulation	1.59E-06	7,10,3
1ihna_#64	GO:0018108: peptidyl-tyrosine phosphorylation	1.59E-06	7,10,3
1e2fa_#14	GO:0009219: pyrimidine deoxyribonucleotide metabolism	1.59E-06	7,10,3
1qfea_#68	GO:0006563: L-serine metabolism	1.60E-06	453,12,10
1qfea_#68	GO:0009103: lipopolysaccharide biosynthesis	1.60E-06	453,12,10
2viua_#252	GO:0016477: cell migration	1.60E-06	285,23,12
1pwt__#8	GO:0042088: T-helper 1 type immune response	1.61E-06	262,11,8
2cmd_2#251	GO:0006099: tricarboxylic acid cycle	1.62E-06	13,19,4
2ae2a_#17	GO:0006099: tricarboxylic acid cycle	1.62E-06	13,19,4
1qfea_#68	GO:0006207: 'de novo' pyrimidine base biosynthesis	1.63E-06	453,10,9
3grs_2#211	GO:0006118: electron transport	1.64E-06	42,228,15
1a9xa3#78	GO:0009086: methionine biosynthesis	1.64E-06	262,14,9
1e79h2#77	GO:0006953: acute-phase response	1.66E-06	28,20,5
1ct9a1#233	GO:0006563: L-serine metabolism	1.66E-06	49,12,5
1fjsa_#84	GO:0006956: complement activation	1.67E-06	6,12,3
3tdt__#141	GO:0009103: lipopolysaccharide biosynthesis	1.67E-06	6,12,3
1eoka_#274	GO:0009103: lipopolysaccharide biosynthesis	1.67E-06	6,12,3
1gox__#246	GO:0009073: aromatic amino acid family biosynthesis	1.67E-06	157,16,8
3grx__#56	GO:0030217: T-cell differentiation	1.69E-06	307,10,8
3grx__#56	GO:0006275: regulation of DNA replication	1.69E-06	307,10,8
1mpp__#154	GO:0030333: antigen processing	1.69E-06	84,13,6
1iow_1#37	GO:0009086: methionine biosynthesis	1.70E-06	263,14,9
1qfta_#98	GO:0019883: antigen presentation, endogenous antigen	1.70E-06	198,10,7
1qfta_#98	GO:0030217: T-cell differentiation	1.70E-06	198,10,7

1i6vd_#1283	GO:0006953: acute-phase response	1.71E-06	120,20,8
1f0c.1#A72	GO:0015671: oxygen transport	1.71E-06	11,23,4
1ga6a_#198	GO:0030435: sporulation	1.71E-06	11,23,4
1dlfh_#4	GO:0042088: T-helper 1 type immune response	1.71E-06	23,11,4
1e8ca1#83	GO:0009116: nucleoside metabolism	1.71E-06	127,25,9
1h6la_#119	GO:0042108: positive regulation of cytokine biosynthesis	1.71E-06	264,11,8
1g51a3#524	GO:0051249: regulation of lymphocyte activation	1.72E-06	93,12,6
1xnb__#130	GO:0009156: ribonucleoside monophosphate biosynthesis	1.72E-06	93,12,6
1gtra2#173	GO:0030333: antigen processing	1.73E-06	45,13,5
1hxxa_#39	GO:0030333: antigen processing	1.73E-06	45,13,5
1dfca1#1135	GO:0030333: antigen processing	1.73E-06	45,13,5
1h68a_#183	GO:0007601: visual perception	1.73E-06	5,15,3
1opy__#68	GO:0050819: negative regulation of coagulation	1.73E-06	308,10,8
1opy__#68	GO:0030193: regulation of blood coagulation	1.73E-06	308,10,8
1opy__#68	GO:0030217: T-cell differentiation	1.73E-06	308,10,8
1czya1#466	GO:0042088: T-helper 1 type immune response	1.73E-06	104,11,6
2mpra_#222	GO:0006367: transcription initiation from Pol II promoter	1.73E-06	21,12,4
1dar_2#264	GO:0006606: protein-nucleus import	1.73E-06	12,21,4
1cs6a4#363	GO:0051249: regulation of lymphocyte activation	1.73E-06	21,12,4
1fmb__#88	GO:0015074: DNA integration	1.73E-06	4,20,3
1ekbb_#85	GO:0018108: peptidyl-tyrosine phosphorylation	1.73E-06	118,10,6
1czan1#92	GO:0042088: T-helper 1 type immune response	1.74E-06	538,11,10
1ihua2#525	GO:0006470: protein amino acid dephosphorylation	1.75E-06	278,20,11
1xnb__#130	GO:0009252: peptidoglycan biosynthesis	1.77E-06	93,18,7
1qo2a_#222	GO:0006098: pentose-phosphate shunt	1.77E-06	55,11,5
1evqa_#305	GO:0009396: folic acid and derivative biosynthesis	1.78E-06	384,11,9
1gox__#124	GO:0009064: glutamine family amino acid metabolism	1.78E-06	298,19,11
1fmja_#76	GO:0007264: small GTPase mediated signal transduction	1.78E-06	49,21,6
1qfea_#68	GO:0006099: tricarboxylic acid cycle	1.78E-06	453,19,13
1qfea_#68	GO:0009064: glutamine family amino acid metabolism	1.78E-06	453,19,13
1cfb_2#763	GO:0042113: B-cell activation	1.78E-06	8,9,3
1elva1#604	GO:0006606: protein-nucleus import	1.78E-06	27,21,5
1dcfa_#66	GO:0009073: aromatic amino acid family biosynthesis	1.79E-06	36,16,5
1dik_1#765	GO:0043094: metabolic compound salvage	1.83E-06	105,11,6
1dt6a_#385	GO:0006418: tRNA aminoacylation for protein translation	1.84E-06	62,26,7
1esma_#314	GO:0016337: cell-cell adhesion	1.84E-06	100,17,7
1f2da_#264	GO:0006418: tRNA aminoacylation for protein translation	1.84E-06	10,26,4
1a8h_2#186	GO:0006418: tRNA aminoacylation for protein translation	1.84E-06	10,26,4
1qo7a_#109	GO:0006568: tryptophan metabolism	1.84E-06	26,10,4
1fp5a2#478	GO:0030217: T-cell differentiation	1.84E-06	26,10,4
1rkd__#186	GO:0009116: nucleoside metabolism	1.86E-06	41,25,6
1dt9a1#224	GO:0009116: nucleoside metabolism	1.86E-06	41,25,6
1qtn.1#A270	GO:0009396: folic acid and derivative biosynthesis	1.86E-06	386,11,9
1qdla_#211	GO:0006953: acute-phase response	1.88E-06	280,20,11
1f42a2#123	GO:0051249: regulation of lymphocyte activation	1.88E-06	335,12,9
1qfea_#68	GO:0042364: water-soluble vitamin biosynthesis	1.88E-06	453,27,16
1qs1a2#437	GO:0042108: positive regulation of cytokine biosynthesis	1.90E-06	387,11,9
1fo4a5#1020	GO:0030217: T-cell differentiation	1.91E-06	312,10,8
1fo4a5#1020	GO:0006275: regulation of DNA replication	1.91E-06	312,10,8
1hx6a1#163	GO:0043123: positive regulation of I-kappaB kinase/NF-kappaB cascade	1.93E-06	16,16,4
1nsca_#236	GO:0051249: regulation of lymphocyte activation	1.94E-06	156,12,7
1ih7a1#57	GO:0042088: T-helper 1 type immune response	1.94E-06	56,11,5
1ih7a1#57	GO:0042108: positive regulation of cytokine biosynthesis	1.94E-06	56,11,5
2reb_1#138	GO:0006544: glycine metabolism	1.94E-06	56,11,5
1jjya_#329	GO:0006544: glycine metabolism	1.94E-06	56,11,5
1qhda2#323	GO:0030217: T-cell differentiation	1.96E-06	313,10,8

1qhda2#323	GO:0006275: regulation of DNA replication	1.96E-06	313,10,8
1ayl__#237	GO:0019883: antigen presentation, endogenous antigen	1.96E-06	313,10,8
1a4ya_#104	GO:0042364: water-soluble vitamin biosynthesis	1.97E-06	194,27,11
1dpja_#322	GO:0042088: T-helper 1 type immune response	1.98E-06	269,11,8
1e8ua_#296	GO:0006118: electron transport	1.99E-06	28,228,12
1aqua_#126	GO:0009086: methionine biosynthesis	2.02E-06	472,14,11
1mdah_#329	GO:0006953: acute-phase response	2.02E-06	282,20,11
1fjsa_#83	GO:0006953: acute-phase response	2.02E-06	84,20,7
1ekbb_#85	GO:0006919: caspase activation	2.03E-06	118,15,7
1jsg__#92	GO:0006118: electron transport	2.04E-06	519,228,76
1ie5a_#23	GO:0042088: T-helper 1 type immune response	2.05E-06	24,11,4
1ie5a_#23	GO:0042108: positive regulation of cytokine biosynthesis	2.05E-06	24,11,4
1bfd_1#230	GO:0009070: serine family amino acid biosynthesis	2.05E-06	40,26,6
1fsu__#434	GO:0030217: T-cell differentiation	2.06E-06	315,10,8
1fsu__#434	GO:0006275: regulation of DNA replication	2.06E-06	315,10,8
1ir3a_#1063	GO:0006118: electron transport	2.07E-06	59,228,18
1mjha_#126	GO:0042364: water-soluble vitamin biosynthesis	2.07E-06	119,27,9
1quba4#235	GO:0051249: regulation of lymphocyte activation	2.08E-06	96,12,6
1quba4#235	GO:0006956: complement activation	2.08E-06	96,12,6
1am2__#163	GO:0051249: regulation of lymphocyte activation	2.08E-06	96,12,6
1nat__#50	GO:0006118: electron transport	2.11E-06	242,228,44
1g8kb_#11	GO:0006118: electron transport	2.11E-06	48,228,16
1iba__#39	GO:0030217: T-cell differentiation	2.12E-06	316,10,8
1c8na_#216	GO:0019079: viral genome replication	2.12E-06	5,16,3
1b35b_#123	GO:0019079: viral genome replication	2.12E-06	5,16,3
1aym1_#181	GO:0019079: viral genome replication	2.12E-06	5,16,3
1dik_1#848	GO:0006207: 'de novo' pyrimidine base biosynthesis	2.13E-06	64,10,5
1qqsa_#124	GO:0042108: positive regulation of cytokine biosynthesis	2.13E-06	392,11,9
1smaa1#95	GO:0006953: acute-phase response	2.14E-06	223,20,10
2fmr__#10	GO:0030333: antigen processing	2.15E-06	47,13,5
1fgga_#134	GO:0009086: methionine biosynthesis	2.16E-06	193,14,8
1e8ya2#410	GO:0006690: icosanoid metabolism	2.19E-06	7,11,3
1c9la2#61	GO:0050819: negative regulation of coagulation	2.24E-06	206,10,7
1c9la2#61	GO:0030193: regulation of blood coagulation	2.24E-06	206,10,7
1by5a_#347	GO:0051249: regulation of lymphocyte activation	2.24E-06	240,12,8
1iqpa2#112	GO:0006100: tricarboxylic acid cycle intermediate metabolism	2.26E-06	19,14,4
1cqka_#52	GO:0030217: T-cell differentiation	2.30E-06	65,10,5
1jjya_#324	GO:0000051: urea cycle intermediate metabolism	2.30E-06	65,10,5
1jjya_#324	GO:0006207: 'de novo' pyrimidine base biosynthesis	2.30E-06	65,10,5
1c9la2#61	GO:0007169: transmembrane receptor protein tyrosine kinase signaling pathway	2.30E-06	206,26,11
1hlga_#353	GO:0016042: lipid catabolism	2.32E-06	9,31,4
1e1aa_#178	GO:0042088: T-helper 1 type immune response	2.32E-06	58,11,5
1e1aa_#178	GO:0042108: positive regulation of cytokine biosynthesis	2.32E-06	58,11,5
1dqwa_#202	GO:0006221: pyrimidine nucleotide biosynthesis	2.34E-06	4,22,3
1aqua_#126	GO:0042401: biogenic amine biosynthesis	2.34E-06	472,10,9
1bywa_#30	GO:0006457: protein folding	2.34E-06	283,49,18
1ej0a_#42	GO:0006367: transcription initiation from Pol II promoter	2.35E-06	98,12,6
1pii_2#326	GO:0042364: water-soluble vitamin biosynthesis	2.35E-06	157,27,10
1gox__#246	GO:0042364: water-soluble vitamin biosynthesis	2.35E-06	157,27,10
1uch__#50	GO:0006730: one-carbon compound metabolism	2.36E-06	74,32,8
1im4a_#144	GO:0006633: fatty acid biosynthesis	2.37E-06	89,27,8
1qrra_#11	GO:0006096: glycolysis	2.37E-06	16,38,5
8dfr__#9	GO:0009116: nucleoside metabolism	2.38E-06	97,25,8
1fu6a_#35	GO:0007169: transmembrane receptor protein tyrosine kinase signaling pathway	2.38E-06	23,26,5

1aqua_#126	GO:0006563: L-serine metabolism	2.38E-06	472,12,10
1dlfl_#97	GO:0007169: transmembrane receptor protein tyrosine kinase signaling pathway	2.38E-06	41,26,6
1rkd__#186	GO:0006418: tRNA aminoacylation for protein translation	2.38E-06	41,26,6
1f8ma_#237	GO:0006418: tRNA aminoacylation for protein translation	2.38E-06	41,26,6
1kcw_3#440	GO:0006118: electron transport	2.39E-06	16,228,9
1dz4a_#150	GO:0006118: electron transport	2.39E-06	16,228,9
1flca1#294	GO:0030333: antigen processing	2.39E-06	89,13,6
3rpba_#557	GO:0030333: antigen processing	2.40E-06	48,13,5
1mkp__#283	GO:0006118: electron transport	2.41E-06	38,228,14
1g51a3#524	GO:0006418: tRNA aminoacylation for protein translation	2.41E-06	93,26,8
1ypta_#418	GO:0006118: electron transport	2.41E-06	24,228,11
1c8za_#386	GO:0016337: cell-cell adhesion	2.42E-06	211,17,9
1at0__#285	GO:0016485: protein processing	2.43E-06	25,11,4
1ioaa_#75	GO:0042088: T-helper 1 type immune response	2.43E-06	25,11,4
1ioaa_#75	GO:0042108: positive regulation of cytokine biosynthesis	2.43E-06	25,11,4
1hbza_#96	GO:0050819: negative regulation of coagulation	2.45E-06	322,10,8
1hbza_#96	GO:0030193: regulation of blood coagulation	2.45E-06	322,10,8
1hbza_#96	GO:0030217: T-cell differentiation	2.45E-06	322,10,8
1ddwa_#77	GO:0030217: T-cell differentiation	2.45E-06	322,10,8
1cfr__#269	GO:0006730: one-carbon compound metabolism	2.48E-06	52,32,7
1danh_#242	GO:0006606: protein-nucleus import	2.49E-06	13,21,4
1ekbb_#79	GO:0006606: protein-nucleus import	2.49E-06	13,21,4
1ton__#47	GO:0006606: protein-nucleus import	2.50E-06	82,21,7
1e79h2#77	GO:0019883: antigen presentation, endogenous antigen	2.51E-06	28,10,4
1ddja_#640	GO:0050819: negative regulation of coagulation	2.54E-06	8,10,3
1ddja_#640	GO:0030193: regulation of blood coagulation	2.54E-06	8,10,3
1hcl__#267	GO:0000086: G2/M transition of mitotic cell cycle	2.54E-06	8,10,3
1hp5a1#340	GO:0015976: carbon utilization	2.54E-06	8,10,3
1cgha_#130	GO:0050819: negative regulation of coagulation	2.54E-06	8,10,3
1cgha_#130	GO:0030193: regulation of blood coagulation	2.54E-06	8,10,3
1jj2s_#46	GO:0006457: protein folding	2.54E-06	34,49,7
1dj0a2#183	GO:0006511: ubiquitin-dependent protein catabolism	2.55E-06	111,11,6
1e8ca3#111	GO:0042364: water-soluble vitamin biosynthesis	2.58E-06	244,27,12
1f42a2#123	GO:0006606: protein-nucleus import	2.58E-06	335,21,12
1iba__#39	GO:0006457: protein folding	2.58E-06	316,49,19
1erv__#80	GO:0042108: positive regulation of cytokine biosynthesis	2.59E-06	401,11,9
1cl1a_#184	GO:0009086: methionine biosynthesis	2.65E-06	45,14,5
1fn9a_#271	GO:0042088: T-helper 1 type immune response	2.65E-06	184,11,7
1fn9a_#271	GO:0042108: positive regulation of cytokine biosynthesis	2.65E-06	184,11,7
1fxla2#169	GO:0042088: T-helper 1 type immune response	2.65E-06	184,11,7
1fa0a1#393	GO:0006418: tRNA aminoacylation for protein translation	2.66E-06	209,26,11
1h8d.1#H207	GO:0030333: antigen processing	2.66E-06	49,13,5
1prea2#308	GO:0030217: T-cell differentiation	2.68E-06	67,10,5
1tul__#102	GO:0030217: T-cell differentiation	2.68E-06	67,10,5
1c4ra_#258	GO:0050819: negative regulation of coagulation	2.68E-06	67,10,5
1c4ra_#258	GO:0030193: regulation of blood coagulation	2.68E-06	67,10,5
2hlca_#226	GO:0018108: peptidyl-tyrosine phosphorylation	2.68E-06	67,10,5
2mpra_#190	GO:0051249: regulation of lymphocyte activation	2.69E-06	349,12,9
1ez0a_#133	GO:0042088: T-helper 1 type immune response	2.69E-06	112,11,6
1ez0a_#133	GO:0042108: positive regulation of cytokine biosynthesis	2.69E-06	112,11,6
1vin_1#302	GO:0000910: cytokinesis	2.71E-06	8,37,4
1ev7a_#218	GO:0015671: oxygen transport	2.72E-06	75,23,7
1d2ka1#164	GO:0006221: pyrimidine nucleotide biosynthesis	2.72E-06	50,22,6
1f21a_#68	GO:0006313: DNA transposition	2.73E-06	31,35,6
1ihua2#525	GO:0006100: tricarboxylic acid cycle intermediate metabolism	2.73E-06	278,14,9

1e3ua_#164	GO:0046677: response to antibiotic	2.75E-06	42,40,7
1sbp__#124	GO:0015698: inorganic anion transport	2.75E-06	6,14,3
1qj5a_#316	GO:0009086: methionine biosynthesis	2.75E-06	6,14,3
1fc4a_#277	GO:0009086: methionine biosynthesis	2.75E-06	6,14,3
1qf5a_#15	GO:0045045: secretory pathway	2.77E-06	28,22,5
1ctqa_#116	GO:0045045: secretory pathway	2.77E-06	28,22,5
1hq8a_#206	GO:0042108: positive regulation of cytokine biosynthesis	2.78E-06	281,11,8
3sil__#317	GO:0016477: cell migration	2.80E-06	300,23,12
1hc7a2#258	GO:0042108: positive regulation of cytokine biosynthesis	2.85E-06	186,11,7
1hxxa_#232	GO:0042088: T-helper 1 type immune response	2.86E-06	282,11,8
1mdah_#329	GO:0042108: positive regulation of cytokine biosynthesis	2.86E-06	282,11,8
1i0ha2#127	GO:0006801: superoxide metabolism	2.87E-06	26,11,4
1i2oa_#173	GO:0043094: metabolic compound salvage	2.87E-06	26,11,4
1aym3_#131	GO:0006118: electron transport	2.89E-06	353,228,57
1dxea_#71	GO:0006207: 'de novo' pyrimidine base biosynthesis	2.90E-06	29,10,4
1aqua_#126	GO:0009307: DNA restriction	2.91E-06	472,19,13
1f24a_#293	GO:0030333: antigen processing	2.91E-06	92,13,6
1ovb__#116	GO:0006879: iron ion homeostasis	2.91E-06	7,12,3
1sek__#52	GO:0006367: transcription initiation from Pol II promoter	2.91E-06	7,12,3
1sek__#52	GO:0006879: iron ion homeostasis	2.91E-06	7,12,3
1air__#233	GO:0009103: lipopolysaccharide biosynthesis	2.91E-06	7,12,3
1e7wa_#36	GO:0006118: electron transport	2.94E-06	104,228,25
1qf6a3#78	GO:0006100: tricarboxylic acid cycle intermediate metabolism	2.96E-06	46,14,5
1aym3_#131	GO:0051249: regulation of lymphocyte activation	2.97E-06	353,12,9
1a65a1#108	GO:0006118: electron transport	2.98E-06	253,228,45
1aiha_#226	GO:0006313: DNA transposition	2.98E-06	18,35,5
1emsa2#165	GO:0006096: glycolysis	2.98E-06	29,38,6
1dxea_#71	GO:0006096: glycolysis	2.98E-06	29,38,6
1ejda_#6	GO:0019883: antigen presentation, endogenous antigen	3.00E-06	215,10,7
1nsca_#91	GO:0030333: antigen processing	3.03E-06	22,13,4
1hqva_#48	GO:0007517: muscle development	3.05E-06	82,30,8
1ie5a_#23	GO:0051249: regulation of lymphocyte activation	3.05E-06	24,12,4
1llda2#177	GO:0006099: tricarboxylic acid cycle	3.07E-06	15,19,4
1ddja_#681	GO:0007169: transmembrane receptor protein tyrosine kinase signaling pathway	3.07E-06	212,26,11
1rypk_#12	GO:0051249: regulation of lymphocyte activation	3.07E-06	250,12,8
1cipa1#88	GO:0007186: G-protein coupled receptor protein signaling pathway	3.07E-06	4,24,3
1vmoa_#106	GO:0051249: regulation of lymphocyte activation	3.09E-06	167,12,7
1nfdb1#34	GO:0051249: regulation of lymphocyte activation	3.09E-06	167,12,7
1io1a_#311	GO:0030217: T-cell differentiation	3.09E-06	130,10,6
1apme_#68	GO:0030333: antigen processing	3.10E-06	93,13,6
2viua_#252	GO:0042108: positive regulation of cytokine biosynthesis	3.11E-06	285,11,8
1a65a2#187	GO:0006118: electron transport	3.12E-06	29,228,12
1dr9a1#39	GO:0030333: antigen processing	3.14E-06	224,13,8
1nsca_#379	GO:0042088: T-helper 1 type immune response	3.15E-06	115,11,6
1nsca_#379	GO:0042108: positive regulation of cytokine biosynthesis	3.15E-06	115,11,6
1i50b_#966	GO:0051249: regulation of lymphocyte activation	3.17E-06	251,12,8
1a8i__#688	GO:0009086: methionine biosynthesis	3.19E-06	203,14,8
1qmva_#40	GO:0006730: one-carbon compound metabolism	3.20E-06	77,32,8
1aoea_#130	GO:0006730: one-carbon compound metabolism	3.20E-06	77,32,8
1ac6a_#34	GO:0006953: acute-phase response	3.22E-06	57,20,6
2ltn.1#A63	GO:0030217: T-cell differentiation	3.23E-06	131,10,6
1a8i__#688	GO:0009252: peptidoglycan biosynthesis	3.24E-06	203,18,9
1hnja1#167	GO:0006544: glycine metabolism	3.25E-06	62,11,5
1bbpa_#119	GO:0030333: antigen processing	3.26E-06	51,13,5
1hvba_#247	GO:0046677: response to antibiotic	3.27E-06	28,40,6

1fjfj_#68	GO:0030217: T-cell differentiation	3.30E-06	218,10,7
1jb0d_#26	GO:0001906: cell killing	3.30E-06	190,11,7
1ds1a_#172	GO:0042088: T-helper 1 type immune response	3.30E-06	190,11,7
1bev1_#86	GO:0006953: acute-phase response	3.30E-06	296,20,11
2stv__#129	GO:0016337: cell-cell adhesion	3.32E-06	38,17,5
1qp8a1#172	GO:0006096: glycolysis	3.32E-06	17,38,5
1qs0b2#269	GO:0006730: one-carbon compound metabolism	3.32E-06	35,32,6
1a8d_1#87	GO:0006118: electron transport	3.33E-06	191,228,37
1f42a2#123	GO:0050819: negative regulation of coagulation	3.34E-06	335,10,8
1f42a2#123	GO:0030193: regulation of blood coagulation	3.34E-06	335,10,8
1f42a2#123	GO:0030217: T-cell differentiation	3.34E-06	335,10,8
1i7oa2#399	GO:0019883: antigen presentation, endogenous antigen	3.34E-06	30,10,4
1ayaa_#98	GO:0008284: positive regulation of cell proliferation	3.34E-06	10,30,4
1f0ia1#50	GO:0006953: acute-phase response	3.35E-06	131,20,8
1a65a1#108	GO:0051249: regulation of lymphocyte activation	3.37E-06	253,12,8
1pwt__#8	GO:0006418: tRNA aminoacylation for protein translation	3.39E-06	262,26,12
1pwt__#8	GO:0007169: transmembrane receptor protein tyrosine kinase signaling pathway	3.39E-06	262,26,12
1e79d2#36	GO:0006418: tRNA aminoacylation for protein translation	3.43E-06	432,26,15
1qq4a_#178	GO:0006118: electron transport	3.45E-06	39,228,14
1fcda2#189	GO:0006118: electron transport	3.45E-06	39,228,14
1dypa_#143	GO:0006118: electron transport	3.46E-06	79,228,21
1aa6_2#175	GO:0006118: electron transport	3.46E-06	7,228,6
1cpt__#159	GO:0006118: electron transport	3.46E-06	7,228,6
1ddja_#754	GO:0006606: protein-nucleus import	3.47E-06	14,21,4
1ekbb_#73	GO:0006606: protein-nucleus import	3.47E-06	14,21,4
1f97a1#62	GO:0016477: cell migration	3.48E-06	591,23,16
1tlfa_#269	GO:0009396: folic acid and derivative biosynthesis	3.49E-06	8,11,3
1rypi_#16	GO:0006511: ubiquitin-dependent protein catabolism	3.49E-06	8,11,3
3tmka_#98	GO:0009116: nucleoside metabolism	3.49E-06	4,25,3
1a7s__#148	GO:0006919: caspase activation	3.49E-06	44,15,5
1qfxa_#380	GO:0019735: antimicrobial humoral response (sensu Vertebrata)	3.50E-06	41,16,5
1cs1a_#160	GO:0042364: water-soluble vitamin biosynthesis	3.52E-06	164,27,10
1bu7a_#405	GO:0006118: electron transport	3.54E-06	61,228,18
1thea_#79	GO:0000910: cytokinesis	3.58E-06	67,37,8
1e5ka_#113	GO:0030333: antigen processing	3.60E-06	52,13,5
1evqa_#305	GO:0009086: methionine biosynthesis	3.61E-06	384,14,10
3chbd_#85	GO:0006457: protein folding	3.62E-06	323,49,19
1ek6a_#269	GO:0009103: lipopolysaccharide biosynthesis	3.62E-06	25,12,4
1eny__#94	GO:0009103: lipopolysaccharide biosynthesis	3.62E-06	25,12,4
1cbf__#208	GO:0042088: T-helper 1 type immune response	3.65E-06	291,11,8
1cbf__#208	GO:0001906: cell killing	3.65E-06	291,11,8
1p35a_#98	GO:0017148: negative regulation of protein biosynthesis	3.66E-06	23,13,4
1gsa_2#218	GO:0042108: positive regulation of cytokine biosynthesis	3.67E-06	118,11,6
1brt__#116	GO:0016042: lipid catabolism	3.67E-06	21,31,5
1iow_1#37	GO:0006118: electron transport	3.68E-06	263,228,46
1ofga1#84	GO:0006096: glycolysis	3.69E-06	30,38,6
1j9la_#82	GO:0015976: carbon utilization	3.69E-06	134,10,6
1ejfa_#10	GO:0050819: negative regulation of coagulation	3.69E-06	134,10,6
1ejfa_#10	GO:0030193: regulation of blood coagulation	3.69E-06	134,10,6
3lada1#116	GO:0006418: tRNA aminoacylation for protein translation	3.71E-06	133,26,9
1fgga_#134	GO:0009064: glutamine family amino acid metabolism	3.74E-06	193,19,9
1rypb_#114	GO:0015074: DNA integration	3.76E-06	133,20,8
1e25a_#139	GO:0046677: response to antibiotic	3.78E-06	3,40,3
1bd2d1#62	GO:0030333: antigen processing	3.78E-06	7,13,3
1qtn.1#A270	GO:0006100: tricarboxylic acid cycle intermediate metabolism	3.80E-06	386,14,10

1phm_2#253	GO:0051249: regulation of lymphocyte activation	3.80E-06	257,12,8
1dpga2#367	GO:0019883: antigen presentation, endogenous antigen	3.80E-06	9,10,3
1b6cb_#346	GO:0000086: G2/M transition of mitotic cell cycle	3.80E-06	9,10,3
1axca1#19	GO:0006275: regulation of DNA replication	3.80E-06	9,10,3
1fmk_3#428	GO:0000086: G2/M transition of mitotic cell cycle	3.80E-06	9,10,3
1ezvb2#271	GO:0000051: urea cycle intermediate metabolism	3.80E-06	9,10,3
1akjd_#3	GO:0019883: antigen presentation, endogenous antigen	3.80E-06	9,10,3
1befa_#51	GO:0018108: peptidyl-tyrosine phosphorylation	3.80E-06	9,10,3
1e4ea2#215	GO:0042108: positive regulation of cytokine biosynthesis	3.81E-06	419,11,9
1ihua2#525	GO:0009116: nucleoside metabolism	3.82E-06	278,25,12
1kdj__#27	GO:0042088: T-helper 1 type immune response	3.82E-06	64,11,5
1kdj__#27	GO:0042108: positive regulation of cytokine biosynthesis	3.82E-06	64,11,5
1eo6a_#107	GO:0006544: glycine metabolism	3.82E-06	64,11,5
1f21a_#68	GO:0006278: RNA-dependent DNA replication	3.83E-06	31,10,4
1bqk__#77	GO:0019883: antigen presentation, endogenous antigen	3.85E-06	72,10,5
1c4zd_#51	GO:0030217: T-cell differentiation	3.86E-06	135,10,6
1ax4a_#430	GO:0006221: pyrimidine nucleotide biosynthesis	3.87E-06	53,22,6
1el0a_#42	GO:0051249: regulation of lymphocyte activation	3.91E-06	258,12,8
1dvpa1#65	GO:0006119: oxidative phosphorylation	3.92E-06	173,12,7
1gaxa1#681	GO:0006418: tRNA aminoacylation for protein translation	3.95E-06	4,26,3
1am7a_#94	GO:0042742: defense response to bacteria	3.95E-06	4,26,3
1i6vd_#713	GO:0006730: one-carbon compound metabolism	3.95E-06	36,32,6
1dgw.1#Y353	GO:0001906: cell killing	3.96E-06	294,11,8
1aqua_#126	GO:0006118: electron transport	3.96E-06	472,228,70
1dr9a1#39	GO:0030217: T-cell differentiation	3.97E-06	224,10,7
1bqca_#24	GO:0030245: cellulose catabolism	3.98E-06	30,22,5
1ejfa_#10	GO:0006953: acute-phase response	3.98E-06	134,20,8
1a6o__#45	GO:0006418: tRNA aminoacylation for protein translation	3.99E-06	266,26,12
1iira_#326	GO:0006118: electron transport	4.02E-06	86,228,22
2mpra_#190	GO:0006606: protein-nucleus import	4.03E-06	349,21,12
1ton__#47	GO:0006919: caspase activation	4.05E-06	82,15,6
1e7wa_#36	GO:0009116: nucleoside metabolism	4.06E-06	104,25,8
1j9qa1#154	GO:0006953: acute-phase response	4.06E-06	183,20,9
1ldm_2#272	GO:0006099: tricarboxylic acid cycle	4.07E-06	16,19,4
1hg3a_#207	GO:0009073: aromatic amino acid family biosynthesis	4.07E-06	19,16,4
1e6ua_#10	GO:0006099: tricarboxylic acid cycle	4.07E-06	16,19,4
1g51a3#525	GO:0006953: acute-phase response	4.07E-06	239,20,10
1f8ra2#325	GO:0042088: T-helper 1 type immune response	4.08E-06	196,11,7
1f8ra2#325	GO:0042108: positive regulation of cytokine biosynthesis	4.08E-06	196,11,7
1cqka_#52	GO:0042108: positive regulation of cytokine biosynthesis	4.13E-06	65,11,5
1g25a_#22	GO:0051249: regulation of lymphocyte activation	4.16E-06	367,12,9
1qs0a1#119	GO:0006563: L-serine metabolism	4.16E-06	367,12,9
2reb_1#138	GO:0006730: one-carbon compound metabolism	4.16E-06	56,32,7
1jjya_#329	GO:0006730: one-carbon compound metabolism	4.16E-06	56,32,7
1bev1_#86	GO:0001906: cell killing	4.17E-06	296,11,8
1bev1_#86	GO:0016337: cell-cell adhesion	4.18E-06	296,17,10
2mev3_#108	GO:0001906: cell killing	4.22E-06	197,11,7
1ayx__#69	GO:0030245: cellulose catabolism	4.22E-06	14,22,4
1cs1a_#160	GO:0006221: pyrimidine nucleotide biosynthesis	4.23E-06	164,22,9
1mml__#224	GO:0019079: viral genome replication	4.23E-06	6,16,3
1hhsa_#328	GO:0019079: viral genome replication	4.23E-06	6,16,3
1b35b_#226	GO:0019079: viral genome replication	4.23E-06	6,16,3
1h6la_#119	GO:0006457: protein folding	4.23E-06	264,49,17
1div_1#141	GO:0009070: serine family amino acid biosynthesis	4.24E-06	70,26,7
1tbrr1#15	GO:0051249: regulation of lymphocyte activation	4.27E-06	59,12,5
1fjfl_#100	GO:0006418: tRNA aminoacylation for protein translation	4.27E-06	12,26,4

1f97a1#62	GO:0006606: protein-nucleus import	4.28E-06	591,21,15
1nsca_#236	GO:0016477: cell migration	4.31E-06	156,23,9
1aqua_#126	GO:0006221: pyrimidine nucleotide biosynthesis	4.32E-06	472,22,14
1bio__#108	GO:0006606: protein-nucleus import	4.33E-06	32,21,5
1pma1_#17	GO:0006606: protein-nucleus import	4.33E-06	32,21,5
1qnja_#46	GO:0006606: protein-nucleus import	4.33E-06	32,21,5
1imva_#255	GO:0019883: antigen presentation, endogenous antigen	4.34E-06	506,10,9
1imva_#255	GO:0030217: T-cell differentiation	4.34E-06	506,10,9
1ejda_#213	GO:0050819: negative regulation of coagulation	4.35E-06	227,10,7
1ejda_#213	GO:0030193: regulation of blood coagulation	4.35E-06	227,10,7
1f97a1#62	GO:0042108: positive regulation of cytokine biosynthesis	4.37E-06	591,11,10
1bio__#108	GO:0019883: antigen presentation, endogenous antigen	4.37E-06	32,10,4
1ie5a_#23	GO:0030333: antigen processing	4.38E-06	24,13,4
1gox__#124	GO:0009396: folic acid and derivative biosynthesis	4.39E-06	298,11,8
1edza1#206	GO:0006606: protein-nucleus import	4.42E-06	352,21,12
1cyda_#204	GO:0006633: fatty acid biosynthesis	4.44E-06	4,27,3
1qg6a_#251	GO:0006633: fatty acid biosynthesis	4.44E-06	4,27,3
1gks__#70	GO:0006118: electron transport	4.46E-06	45,228,15
1jsg__#92	GO:0019735: antimicrobial humoral response (sensu Vertebrata)	4.48E-06	519,16,12
1ax4a_#275	GO:0030333: antigen processing	4.49E-06	99,13,6
2bb2_2#134	GO:0042088: T-helper 1 type immune response	4.52E-06	199,11,7
2bb2_2#134	GO:0042108: positive regulation of cytokine biosynthesis	4.52E-06	199,11,7
1i7qa_#278	GO:0006801: superoxide metabolism	4.53E-06	29,11,4
2pia_3#282	GO:0042088: T-helper 1 type immune response	4.53E-06	29,11,4
2pia_3#282	GO:0042108: positive regulation of cytokine biosynthesis	4.53E-06	29,11,4
1cwva2#675	GO:0042088: T-helper 1 type immune response	4.53E-06	29,11,4
1cwva2#675	GO:0042108: positive regulation of cytokine biosynthesis	4.53E-06	29,11,4
1e4ia_#215	GO:0006096: glycolysis	4.55E-06	18,38,5
1e6ua_#266	GO:0006418: tRNA aminoacylation for protein translation	4.56E-06	26,26,5
1epwa3#138	GO:0051249: regulation of lymphocyte activation	4.56E-06	371,12,9
1dzfa2#211	GO:0050819: negative regulation of coagulation	4.59E-06	139,10,6
1dzfa2#211	GO:0030193: regulation of blood coagulation	4.59E-06	139,10,6
1dzfa2#211	GO:0030217: T-cell differentiation	4.59E-06	139,10,6
1i50b_#966	GO:0006730: one-carbon compound metabolism	4.61E-06	251,32,13
1h9da_#91	GO:0016477: cell migration	4.61E-06	204,23,10
1ds1a_#300	GO:0006118: electron transport	4.62E-06	21,228,10
3prn__#143	GO:0042088: T-helper 1 type immune response	4.63E-06	300,11,8
3prn__#143	GO:0001906: cell killing	4.63E-06	300,11,8
1gdna_#230	GO:0006956: complement activation	4.65E-06	8,12,3
1cfb_2#763	GO:0051249: regulation of lymphocyte activation	4.65E-06	8,12,3
1ds9a_#119	GO:0009103: lipopolysaccharide biosynthesis	4.65E-06	8,12,3
1cvl__#105	GO:0006563: L-serine metabolism	4.65E-06	8,12,3
2dnja_#97	GO:0006367: transcription initiation from Pol II promoter	4.67E-06	110,12,6
1jb9a2#173	GO:0006118: electron transport	4.67E-06	17,228,9
2bc2a_#46	GO:0006118: electron transport	4.67E-06	17,228,9
1h6la_#119	GO:0051249: regulation of lymphocyte activation	4.67E-06	264,12,8
1fjsa_#83	GO:0006919: caspase activation	4.67E-06	84,15,6
1beba_#21	GO:0016068: type I hypersensitivity	4.70E-06	21,15,4
1azza_#51	GO:0006606: protein-nucleus import	4.70E-06	15,21,4
2hlca_#139	GO:0018108: peptidyl-tyrosine phosphorylation	4.72E-06	75,10,5
3sil__#317	GO:0016337: cell-cell adhesion	4.73E-06	300,17,10
1phm_2#253	GO:0016477: cell migration	4.74E-06	257,23,11
1el0a_#28	GO:0019883: antigen presentation, endogenous antigen	4.79E-06	140,10,6
1nfdb1#34	GO:0016337: cell-cell adhesion	4.79E-06	167,17,8
1ii7a_#199	GO:0009070: serine family amino acid biosynthesis	4.79E-06	46,26,6
1qgna_#207	GO:0009086: methionine biosynthesis	4.80E-06	7,14,3

2oata_#120	GO:0009086: methionine biosynthesis	4.80E-06	7,14,3
1jjya_#293	GO:0006100: tricarboxylic acid cycle intermediate metabolism	4.80E-06	7,14,3
1i9ga_#185	GO:0006100: tricarboxylic acid cycle intermediate metabolism	4.80E-06	7,14,3
1c4ka2#223	GO:0009086: methionine biosynthesis	4.80E-06	7,14,3
1hwxa1#223	GO:0009116: nucleoside metabolism	4.91E-06	186,25,10
2hlca_#54	GO:0006606: protein-nucleus import	4.92E-06	58,21,6
1edza1#206	GO:0050819: negative regulation of coagulation	4.92E-06	352,10,8
1edza1#206	GO:0030193: regulation of blood coagulation	4.92E-06	352,10,8
1el0a_#42	GO:0016477: cell migration	4.93E-06	258,23,11
1e0ta2#275	GO:0030245: cellulose catabolism	4.94E-06	86,22,7
1e0ta2#275	GO:0006221: pyrimidine nucleotide biosynthesis	4.94E-06	86,22,7
1ejda_#6	GO:0006100: tricarboxylic acid cycle intermediate metabolism	4.96E-06	215,14,8
1fbna_#145	GO:0006207: 'de novo' pyrimidine base biosynthesis	4.96E-06	33,10,4
1fm2.1#B627	GO:0019883: antigen presentation, endogenous antigen	4.96E-06	33,10,4
1imva_#255	GO:0006457: protein folding	4.97E-06	506,49,24
1ac5__#72	GO:0006100: tricarboxylic acid cycle intermediate metabolism	5.00E-06	51,14,5
1hqva_#48	GO:0015671: oxygen transport	5.00E-06	82,23,7
1ge8a2#134	GO:0042088: T-helper 1 type immune response	5.01E-06	202,11,7
1ge8a2#134	GO:0042108: positive regulation of cytokine biosynthesis	5.01E-06	202,11,7
1hxxa_#173	GO:0016337: cell-cell adhesion	5.03E-06	230,17,9
1c8ba_#514	GO:0030435: sporulation	5.04E-06	30,23,5
1c0ma1#237	GO:0007517: muscle development	5.04E-06	23,30,5
1fnf_3#1409	GO:0030217: T-cell differentiation	5.05E-06	76,10,5
1a53__#236	GO:0009073: aromatic amino acid family biosynthesis	5.07E-06	20,16,4
1arb__#55	GO:0019079: viral genome replication	5.07E-06	20,16,4
1gg6.1#B19	GO:0006606: protein-nucleus import	5.08E-06	33,21,5
3grx__#55	GO:0009064: glutamine family amino acid metabolism	5.09E-06	102,19,7
1gox__#246	GO:0006779: porphyrin biosynthesis	5.10E-06	157,18,8
1dn2a1#261	GO:0051249: regulation of lymphocyte activation	5.12E-06	376,12,9
1nsj__#124	GO:0006221: pyrimidine nucleotide biosynthesis	5.13E-06	124,22,8
1d7ba_#87	GO:0042088: T-helper 1 type immune response	5.18E-06	68,11,5
1d7ba_#87	GO:0042108: positive regulation of cytokine biosynthesis	5.18E-06	68,11,5
1ez0a_#133	GO:0006367: transcription initiation from Pol II promoter	5.19E-06	112,12,6
2pvba_#51	GO:0007517: muscle development	5.21E-06	11,30,4
1hg3a_#43	GO:0006098: pentose-phosphate shunt	5.21E-06	30,11,4
1hg3a_#43	GO:0009396: folic acid and derivative biosynthesis	5.21E-06	30,11,4
1akjd_#3	GO:0042088: T-helper 1 type immune response	5.22E-06	9,11,3
1akjd_#3	GO:0042108: positive regulation of cytokine biosynthesis	5.22E-06	9,11,3
1rypc_#66	GO:0006511: ubiquitin-dependent protein catabolism	5.22E-06	9,11,3
1evqa_#305	GO:0006418: tRNA aminoacylation for protein translation	5.22E-06	384,26,14
1ryph_#163	GO:0006418: tRNA aminoacylation for protein translation	5.26E-06	103,26,8
1dzfa2#211	GO:0006953: acute-phase response	5.26E-06	139,20,8
1vola2#239	GO:0006118: electron transport	5.30E-06	114,228,26
1cm9a_#57	GO:0006935: chemotaxis	5.31E-06	45,41,7
1cl1a_#184	GO:0042364: water-soluble vitamin biosynthesis	5.33E-06	45,27,6
1dzfa2#211	GO:0007169: transmembrane receptor protein tyrosine kinase signaling pathway	5.37E-06	139,26,9
2dkb__#114	GO:0006418: tRNA aminoacylation for protein translation	5.37E-06	139,26,9
2dkb__#114	GO:0009070: serine family amino acid biosynthesis	5.37E-06	139,26,9
1ldna1#129	GO:0006096: glycolysis	5.38E-06	9,38,4
1fiy__#475	GO:0006096: glycolysis	5.38E-06	9,38,4
1qs1a2#437	GO:0016337: cell-cell adhesion	5.38E-06	387,17,11
1e6pa2#46	GO:0006568: tryptophan metabolism	5.39E-06	77,10,5
1nsj__#178	GO:0015976: carbon utilization	5.39E-06	77,10,5
1qfma1#186	GO:0001906: cell killing	5.39E-06	306,11,8
2pola2#218	GO:0001906: cell killing	5.41E-06	126,11,6

1apme_#168	GO:0000086: G2/M transition of mitotic cell cycle	5.42E-06	10,10,3
1fnga1#123	GO:0030217: T-cell differentiation	5.42E-06	10,10,3
1g5ca_#62	GO:0015976: carbon utilization	5.42E-06	10,10,3
1otha2#300	GO:0006207: 'de novo' pyrimidine base biosynthesis	5.42E-06	10,10,3
1jsg__#92	GO:0019883: antigen presentation, endogenous antigen	5.43E-06	519,10,9
1jsg__#92	GO:0050819: negative regulation of coagulation	5.43E-06	519,10,9
1jsg__#92	GO:0030193: regulation of blood coagulation	5.43E-06	519,10,9
1jsg__#92	GO:0030217: T-cell differentiation	5.43E-06	519,10,9
1jsg__#92	GO:0006275: regulation of DNA replication	5.43E-06	519,10,9
1a0la_#160	GO:0006919: caspase activation	5.44E-06	48,15,5
1f8ra1#288	GO:0009070: serine family amino acid biosynthesis	5.46E-06	47,26,6
1jeya_#104	GO:0009116: nucleoside metabolism	5.47E-06	28,25,5
3grx__#56	GO:0042088: T-helper 1 type immune response	5.53E-06	307,11,8
1fbl_1#398	GO:0016337: cell-cell adhesion	5.54E-06	42,17,5
1ak5_1#376	GO:0006221: pyrimidine nucleotide biosynthesis	5.55E-06	32,22,5
1kve.1#B199	GO:0001906: cell killing	5.57E-06	69,11,5
1qfea_#49	GO:0006207: 'de novo' pyrimidine base biosynthesis	5.62E-06	34,10,4
1xgsa2#186	GO:0009064: glutamine family amino acid metabolism	5.62E-06	149,19,8
1fn9a_#271	GO:0019735: antimicrobial humoral response (sensu Vertebrata)	5.64E-06	184,16,8
1f97a2#199	GO:0016337: cell-cell adhesion	5.67E-06	118,17,7
1qfma1#186	GO:0016337: cell-cell adhesion	5.69E-06	306,17,10
1f2la_#40	GO:0001906: cell killing	5.72E-06	206,11,7
1tml__#252	GO:0030245: cellulose catabolism	5.72E-06	15,22,4
1dpsa_#36	GO:0006119: oxidative phosphorylation	5.75E-06	183,12,7
2sqca2#97	GO:0007186: G-protein coupled receptor protein signaling pathway	5.76E-06	114,24,8
2sqca2#97	GO:0006119: oxidative phosphorylation	5.76E-06	114,12,6
1b8aa2#403	GO:0051249: regulation of lymphocyte activation	5.76E-06	114,12,6
1duvg1#135	GO:0006221: pyrimidine nucleotide biosynthesis	5.82E-06	5,22,3
2sqca1#553	GO:0030245: cellulose catabolism	5.82E-06	5,22,3
1qf5a_#15	GO:0007017: microtubule-based process	5.82E-06	28,12,4
1ebma2#47	GO:0006367: transcription initiation from Pol II promoter	5.82E-06	28,12,4
1kit_2#398	GO:0043123: positive regulation of I-kappaB kinase/NF-kappaB cascade	5.87E-06	81,16,6
1cpy__#93	GO:0009252: peptidoglycan biosynthesis	5.91E-06	218,18,9
1gg4a3#64	GO:0009252: peptidoglycan biosynthesis	5.91E-06	40,18,5
1cex__#109	GO:0006606: protein-nucleus import	5.92E-06	34,21,5
1jsg__#92	GO:0051249: regulation of lymphocyte activation	5.96E-06	519,12,10
1hr6a1#41	GO:0042088: T-helper 1 type immune response	5.97E-06	31,11,4
1hr6a1#41	GO:0042108: positive regulation of cytokine biosynthesis	5.97E-06	31,11,4
1fyhb1#99	GO:0042108: positive regulation of cytokine biosynthesis	5.97E-06	31,11,4
1ovb__#116	GO:0006826: iron ion transport	6.00E-06	7,15,3
1ihna_#64	GO:0006919: caspase activation	6.00E-06	7,15,3
1sek__#52	GO:0006826: iron ion transport	6.00E-06	7,15,3
1diqc_#626	GO:0006118: electron transport	6.00E-06	5,228,5
1lvl_2#262	GO:0006118: electron transport	6.00E-06	5,228,5
2cuaa_#121	GO:0006118: electron transport	6.00E-06	5,228,5
1cipa2#34	GO:0007264: small GTPase mediated signal transduction	6.02E-06	60,21,6
1hbza_#96	GO:0016477: cell migration	6.02E-06	322,23,12
1qaxa2#307	GO:0006919: caspase activation	6.03E-06	49,15,5
1h8d.1#H207	GO:0006919: caspase activation	6.03E-06	49,15,5
1fnf_3#1409	GO:0016337: cell-cell adhesion	6.07E-06	76,17,6
1jcfa2#206	GO:0006096: glycolysis	6.09E-06	93,38,9
1lucb_#32	GO:0009086: methionine biosynthesis	6.09E-06	24,14,4
1g6oa_#116	GO:0006457: protein folding	6.10E-06	302,49,18
1ftra2#187	GO:0009307: DNA restriction	6.11E-06	38,19,5
1gpea1#273	GO:0050819: negative regulation of coagulation	6.12E-06	79,10,5
1gpea1#273	GO:0030193: regulation of blood coagulation	6.12E-06	79,10,5

1f42a2#123	GO:0019735: antimicrobial humoral response (sensu Vertebrata)	6.12E-06	335,16,10
1eu3a1#68	GO:0006418: tRNA aminoacylation for protein translation	6.13E-06	13,26,4
1h6la_#119	GO:0016477: cell migration	6.20E-06	264,23,11
1bev1_#86	GO:0006606: protein-nucleus import	6.21E-06	296,21,11
1arb__#208	GO:0019079: viral genome replication	6.23E-06	21,16,4
1e1aa_#178	GO:0030333: antigen processing	6.25E-06	58,13,5
1fo4a5#1020	GO:0042088: T-helper 1 type immune response	6.26E-06	312,11,8
1fo4a5#1020	GO:0001906: cell killing	6.26E-06	312,11,8
1jsg__#92	GO:0006606: protein-nucleus import	6.27E-06	519,21,14
1qrra_#69	GO:0006118: electron transport	6.32E-06	173,228,34
1h6va1#116	GO:0006118: electron transport	6.33E-06	26,228,11
1f00i1#749	GO:0030217: T-cell differentiation	6.33E-06	35,10,4
1jb3a_#64	GO:0006096: glycolysis	6.33E-06	247,38,14
1am2__#163	GO:0006100: tricarboxylic acid cycle intermediate metabolism	6.35E-06	96,14,6
1by5a_#347	GO:0030217: T-cell differentiation	6.36E-06	240,10,7
1aop_3#276	GO:0050819: negative regulation of coagulation	6.38E-06	147,10,6
1aop_3#276	GO:0030193: regulation of blood coagulation	6.38E-06	147,10,6
1fjfl_#67	GO:0050819: negative regulation of coagulation	6.38E-06	147,10,6
1fjfl_#67	GO:0030193: regulation of blood coagulation	6.38E-06	147,10,6
3sil__#98	GO:0051249: regulation of lymphocyte activation	6.42E-06	186,12,7
1kdj__#27	GO:0051249: regulation of lymphocyte activation	6.42E-06	64,12,5
1qhda2#323	GO:0001906: cell killing	6.42E-06	313,11,8
1aky_2#133	GO:0042108: positive regulation of cytokine biosynthesis	6.50E-06	130,11,6
1aky_2#133	GO:0001906: cell killing	6.50E-06	130,11,6
1io1a_#311	GO:0042088: T-helper 1 type immune response	6.50E-06	130,11,6
1io1a_#311	GO:0042108: positive regulation of cytokine biosynthesis	6.50E-06	130,11,6
2arca_#55	GO:0001906: cell killing	6.50E-06	130,11,6
1fzqa_#28	GO:0045045: secretory pathway	6.51E-06	33,22,5
1f3ya_#6	GO:0018108: peptidyl-tyrosine phosphorylation	6.52E-06	80,10,5
1fo4a5#1002	GO:0000051: urea cycle intermediate metabolism	6.52E-06	80,10,5
1nula_#15	GO:0009116: nucleoside metabolism	6.56E-06	29,25,5
1qs1a2#437	GO:0051249: regulation of lymphocyte activation	6.57E-06	387,12,9
1jj2v_#118	GO:0006919: caspase activation	6.58E-06	89,15,6
1hw5a2#64	GO:0050819: negative regulation of coagulation	6.64E-06	148,10,6
1hw5a2#64	GO:0030193: regulation of blood coagulation	6.64E-06	148,10,6
1hw5a2#64	GO:0018108: peptidyl-tyrosine phosphorylation	6.64E-06	148,10,6
1jb3a_#64	GO:0030333: antigen processing	6.65E-06	247,13,8
2cba__#144	GO:0006730: one-carbon compound metabolism	6.68E-06	60,32,7
1qgua_#435	GO:0006935: chemotaxis	6.70E-06	18,41,5
1tig__#148	GO:0009070: serine family amino acid biosynthesis	6.72E-06	28,26,5
1nat__#50	GO:0006207: 'de novo' pyrimidine base biosynthesis	6.73E-06	242,10,7
1bg6_2#73	GO:0006563: L-serine metabolism	6.74E-06	29,12,4
1eq2a_#11	GO:0009103: lipopolysaccharide biosynthesis	6.74E-06	29,12,4
1oaa__#14	GO:0009103: lipopolysaccharide biosynthesis	6.74E-06	29,12,4
1fsu__#434	GO:0042088: T-helper 1 type immune response	6.74E-06	315,11,8
1fsu__#434	GO:0001906: cell killing	6.74E-06	315,11,8
1fhoa_#74	GO:0042108: positive regulation of cytokine biosynthesis	6.74E-06	315,11,8
2hlpa2#315	GO:0006099: tricarboxylic acid cycle	6.77E-06	18,19,4
1ovaa_#284	GO:0006099: tricarboxylic acid cycle	6.77E-06	18,19,4
1hyha2#171	GO:0006099: tricarboxylic acid cycle	6.77E-06	18,19,4
1g25a_#22	GO:0006275: regulation of DNA replication	6.81E-06	367,10,8
1qs0a1#119	GO:0015976: carbon utilization	6.81E-06	367,10,8
1eh9a1#33	GO:0006690: icosanoid metabolism	6.81E-06	32,11,4
1epwa3#138	GO:0006118: electron transport	6.85E-06	371,228,58
1f06a1#296	GO:0007264: small GTPase mediated signal transduction	6.87E-06	35,21,5
1iba__#39	GO:0001906: cell killing	6.91E-06	316,11,8

1qfea_#68	GO:0009252: peptidoglycan biosynthesis	6.92E-06	453,18,12
1cqka_#52	GO:0051249: regulation of lymphocyte activation	6.94E-06	65,12,5
16pk__#312	GO:0007017: microtubule-based process	6.95E-06	9,12,3
1qpxa1#117	GO:0006457: protein folding	7.04E-06	3,49,3
1az9_1#73	GO:0006457: protein folding	7.04E-06	3,49,3
1f97a2#199	GO:0051249: regulation of lymphocyte activation	7.06E-06	118,12,6
1f42a2#123	GO:0007169: transmembrane receptor protein tyrosine kinase signaling pathway	7.07E-06	335,26,13
1kit_3#331	GO:0006953: acute-phase response	7.09E-06	101,20,7
1bu8a2#124	GO:0015671: oxygen transport	7.10E-06	123,23,8
1dqza_#209	GO:0015976: carbon utilization	7.11E-06	36,10,4
1eur__#302	GO:0050819: negative regulation of coagulation	7.11E-06	36,10,4
1eur__#302	GO:0030193: regulation of blood coagulation	7.11E-06	36,10,4
1eur__#302	GO:0018108: peptidyl-tyrosine phosphorylation	7.11E-06	36,10,4
2hft_1#34	GO:0030217: T-cell differentiation	7.11E-06	36,10,4
1cpza_#6	GO:0042088: T-helper 1 type immune response	7.11E-06	132,11,6
1qhoa3#488	GO:0030333: antigen processing	7.17E-06	27,13,4
1az9_2#257	GO:0030333: antigen processing	7.17E-06	27,13,4
1eg9a2#199	GO:0009064: glutamine family amino acid metabolism	7.18E-06	69,19,6
1jj2e1#42	GO:0050819: negative regulation of coagulation	7.19E-06	150,10,6
1jj2e1#42	GO:0030193: regulation of blood coagulation	7.19E-06	150,10,6
1hjra_#58	GO:0000160: two-component signal transduction system (phosphorelay)	7.23E-06	14,25,4
1i7qa_#485	GO:0009064: glutamine family amino acid metabolism	7.30E-06	6,19,3
1g55a_#57	GO:0009307: DNA restriction	7.30E-06	6,19,3
1qqsa_#124	GO:0051249: regulation of lymphocyte activation	7.34E-06	392,12,9
1tmy__#34	GO:0006935: chemotaxis	7.35E-06	9,41,4
1aym1_#151	GO:0019079: viral genome replication	7.37E-06	7,16,3
2mev2_#53	GO:0019079: viral genome replication	7.37E-06	7,16,3
1dfna_#16	GO:0043123: positive regulation of I-kappaB kinase/NF-kappaB cascade	7.37E-06	7,16,3
2tbva_#214	GO:0019079: viral genome replication	7.37E-06	7,16,3
1ton__#47	GO:0018108: peptidyl-tyrosine phosphorylation	7.38E-06	82,10,5
1dssg1#4	GO:0006096: glycolysis	7.41E-06	51,38,7
1by5a_#238	GO:0042108: positive regulation of cytokine biosynthesis	7.41E-06	214,11,7
1by5a_#238	GO:0001906: cell killing	7.41E-06	214,11,7
1h6va1#27	GO:0006779: porphyrin biosynthesis	7.42E-06	224,18,9
1erv__#57	GO:0006118: electron transport	7.43E-06	70,228,19
1ayl__#482	GO:0000165: MAPKKK cascade	7.44E-06	10,11,3
1fnga1#123	GO:0042088: T-helper 1 type immune response	7.44E-06	10,11,3
1fnga1#123	GO:0042108: positive regulation of cytokine biosynthesis	7.44E-06	10,11,3
1hava_#141	GO:0006511: ubiquitin-dependent protein catabolism	7.44E-06	10,11,3
1danh_#24	GO:0018108: peptidyl-tyrosine phosphorylation	7.44E-06	11,10,3
1daba_#387	GO:0006207: 'de novo' pyrimidine base biosynthesis	7.44E-06	11,10,3
1ryp1_#44	GO:0006511: ubiquitin-dependent protein catabolism	7.44E-06	10,11,3
1qfma1#238	GO:0006511: ubiquitin-dependent protein catabolism	7.44E-06	10,11,3
1daba_#310	GO:0043094: metabolic compound salvage	7.44E-06	10,11,3
1mdah_#329	GO:0007169: transmembrane receptor protein tyrosine kinase signaling pathway	7.44E-06	282,26,12
1czan1#92	GO:0019883: antigen presentation, endogenous antigen	7.46E-06	538,10,9
1czan1#92	GO:0017001: antibiotic catabolism	7.46E-06	538,10,9
1czan1#92	GO:0030217: T-cell differentiation	7.46E-06	538,10,9
1rypb_#114	GO:0006313: DNA transposition	7.48E-06	133,35,10
1axn__#42	GO:0015992: proton transport	7.49E-06	66,12,5
1edza2#120	GO:0006730: one-carbon compound metabolism	7.52E-06	4,32,3
1qfea_#68	GO:0043094: metabolic compound salvage	7.52E-06	453,11,9
1qfea_#68	GO:0006098: pentose-phosphate shunt	7.52E-06	453,11,9
1qfea_#68	GO:0009396: folic acid and derivative biosynthesis	7.52E-06	453,11,9

1hq8a_#206	GO:0051249: regulation of lymphocyte activation	7.55E-06	281,12,8
1qqga1#95	GO:0006953: acute-phase response	7.58E-06	102,20,7
1arb__#193	GO:0019079: viral genome replication	7.59E-06	22,16,4
1hd2a_#35	GO:0009070: serine family amino acid biosynthesis	7.65E-06	145,26,9
1ejda_#6	GO:0006511: ubiquitin-dependent protein catabolism	7.65E-06	215,11,7
1ixh__#7	GO:0015698: inorganic anion transport	7.66E-06	8,14,3
1es9a_#89	GO:0006100: tricarboxylic acid cycle intermediate metabolism	7.66E-06	8,14,3
1ax4a_#224	GO:0009086: methionine biosynthesis	7.66E-06	8,14,3
1jf9a_#183	GO:0009086: methionine biosynthesis	7.66E-06	8,14,3
1elua_#199	GO:0009086: methionine biosynthesis	7.66E-06	8,14,3
1ej8a_#189	GO:0006953: acute-phase response	7.67E-06	256,20,10
1e0ta2#10	GO:0006098: pentose-phosphate shunt	7.73E-06	33,11,4
1euca1#64	GO:0006098: pentose-phosphate shunt	7.73E-06	33,11,4
1jb3a_#64	GO:0050819: negative regulation of coagulation	7.74E-06	247,10,7
1jb3a_#64	GO:0030193: regulation of blood coagulation	7.74E-06	247,10,7
1g0sa_#74	GO:0030217: T-cell differentiation	7.74E-06	247,10,7
3grx__#56	GO:0006457: protein folding	7.74E-06	307,49,18
1uxy_1#87	GO:0006118: electron transport	7.75E-06	14,228,8
1hxxa_#232	GO:0051249: regulation of lymphocyte activation	7.75E-06	282,12,8
1mdah_#329	GO:0051249: regulation of lymphocyte activation	7.75E-06	282,12,8
1dyna_#42	GO:0030217: T-cell differentiation	7.77E-06	152,10,6
1cf9a2#554	GO:0006118: electron transport	7.81E-06	22,228,10
1e5da2#12	GO:0019883: antigen presentation, endogenous antigen	7.83E-06	83,10,5
1h6va2#245	GO:0006207: 'de novo' pyrimidine base biosynthesis	7.83E-06	83,10,5
1ig8a_#215	GO:0006633: fatty acid biosynthesis	7.85E-06	48,27,6
1pii_2#326	GO:0006100: tricarboxylic acid cycle intermediate metabolism	7.87E-06	157,14,7
1eur__#302	GO:0006606: protein-nucleus import	7.94E-06	36,21,5
1g0sa_#176	GO:0006275: regulation of DNA replication	7.95E-06	37,10,4
1f5qb2#149	GO:0000910: cytokinesis	7.95E-06	10,37,4
1muca1#245	GO:0042401: biogenic amine biosynthesis	7.95E-06	37,10,4
1muca1#245	GO:0006568: tryptophan metabolism	7.95E-06	37,10,4
1e69a_#26	GO:0006207: 'de novo' pyrimidine base biosynthesis	7.95E-06	37,10,4
1ac6a_#34	GO:0016477: cell migration	7.96E-06	57,23,6
1icia_#175	GO:0006096: glycolysis	7.99E-06	34,38,6
1hbza_#96	GO:0042088: T-helper 1 type immune response	7.99E-06	322,11,8
1hbza_#96	GO:0042108: positive regulation of cytokine biosynthesis	7.99E-06	322,11,8
1ddwa_#77	GO:0042088: T-helper 1 type immune response	7.99E-06	322,11,8
1ddwa_#77	GO:0042108: positive regulation of cytokine biosynthesis	7.99E-06	322,11,8
1dbxa_#75	GO:0007264: small GTPase mediated signal transduction	8.04E-06	63,21,6
1ejda_#379	GO:0006470: protein amino acid dephosphorylation	8.05E-06	38,20,5
1j9la_#164	GO:0046677: response to antibiotic	8.06E-06	49,40,7
1bg6_2#73	GO:0009070: serine family amino acid biosynthesis	8.06E-06	29,26,5
1nula_#15	GO:0009070: serine family amino acid biosynthesis	8.06E-06	29,26,5
1tul__#102	GO:0051249: regulation of lymphocyte activation	8.07E-06	67,12,5
1aoea_#130	GO:0009070: serine family amino acid biosynthesis	8.11E-06	77,26,7
1cipa2#326	GO:0006606: protein-nucleus import	8.11E-06	17,21,4
1c4zd_#51	GO:0042088: T-helper 1 type immune response	8.12E-06	135,11,6
1c4zd_#51	GO:0001906: cell killing	8.12E-06	135,11,6
1fsia_#122	GO:0016042: lipid catabolism	8.14E-06	90,31,8
1h6la_#119	GO:0006730: one-carbon compound metabolism	8.16E-06	264,32,13
3chbd_#85	GO:0006544: glycine metabolism	8.18E-06	323,11,8
1cgha_#227	GO:0006118: electron transport	8.22E-06	47,228,15
1mpp__#154	GO:0030217: T-cell differentiation	8.32E-06	84,10,5
1fjsa_#83	GO:0019883: antigen presentation, endogenous antigen	8.32E-06	84,10,5
1fjsa_#83	GO:0018108: peptidyl-tyrosine phosphorylation	8.32E-06	84,10,5
1fjsa_#83	GO:0030217: T-cell differentiation	8.32E-06	84,10,5

1csn__#84	GO:0030333: antigen processing	8.33E-06	174,13,7
1fm2.1#B308	GO:0030333: antigen processing	8.33E-06	174,13,7
1t7pa2#663	GO:0030333: antigen processing	8.35E-06	28,13,4
2dnja_#97	GO:0030333: antigen processing	8.36E-06	110,13,6
1ho1a_#211	GO:0006221: pyrimidine nucleotide biosynthesis	8.39E-06	93,22,7
1czan1#92	GO:0051249: regulation of lymphocyte activation	8.42E-06	538,12,10
1gjwa2#458	GO:0009252: peptidoglycan biosynthesis	8.43E-06	20,18,4
1aiha_#226	GO:0015074: DNA integration	8.43E-06	18,20,4
1bio__#88	GO:0006953: acute-phase response	8.43E-06	18,20,4
1aru__#57	GO:0006979: response to oxidative stress	8.43E-06	20,18,4
1c4ra_#258	GO:0006953: acute-phase response	8.44E-06	67,20,6
1tyfa_#31	GO:0009396: folic acid and derivative biosynthesis	8.48E-06	136,11,6
1jcfa2#206	GO:0006986: response to unfolded protein	8.52E-06	93,15,6
1qp8a1#168	GO:0009070: serine family amino acid biosynthesis	8.52E-06	14,26,4
1a4ya_#104	GO:0006563: L-serine metabolism	8.53E-06	194,12,7
2cpl__#83	GO:0006457: protein folding	8.55E-06	8,49,4
1eny__#147	GO:0009116: nucleoside metabolism	8.56E-06	81,25,7
1danh_#104	GO:0007169: transmembrane receptor protein tyrosine kinase signaling pathway	8.57E-06	147,26,9
1eeoa_#126	GO:0006470: protein amino acid dephosphorylation	8.58E-06	6,20,3
1ae9a_#272	GO:0015074: DNA integration	8.58E-06	6,20,3
1clia2#273	GO:0006470: protein amino acid dephosphorylation	8.58E-06	6,20,3
1bev1_#86	GO:0006118: electron transport	8.60E-06	296,228,49
1aoza1#61	GO:0006118: electron transport	8.61E-06	18,228,9
1cr5a1#45	GO:0006118: electron transport	8.61E-06	18,228,9
1e8ga2#201	GO:0006367: transcription initiation from Pol II promoter	8.64E-06	286,12,8
1cb8a2#613	GO:0006118: electron transport	8.64E-06	131,228,28
1fuia2#273	GO:0009070: serine family amino acid biosynthesis	8.66E-06	110,26,8
1e2fa_#140	GO:0009116: nucleoside metabolism	8.67E-06	5,25,3
1gpc__#184	GO:0051249: regulation of lymphocyte activation	8.69E-06	68,12,5
1e42a1#711	GO:0030217: T-cell differentiation	8.72E-06	155,10,6
1sppb_#32	GO:0042088: T-helper 1 type immune response	8.74E-06	34,11,4
1sppb_#32	GO:0042108: positive regulation of cytokine biosynthesis	8.74E-06	34,11,4
1sppb_#32	GO:0006690: icosanoid metabolism	8.74E-06	34,11,4
1fi2a_#109	GO:0051249: regulation of lymphocyte activation	8.83E-06	195,12,7
1kit_2#398	GO:0016337: cell-cell adhesion	8.84E-06	81,17,6
1heta2#212	GO:0006207: 'de novo' pyrimidine base biosynthesis	8.87E-06	38,10,4
2ae2a_#89	GO:0006207: 'de novo' pyrimidine base biosynthesis	8.87E-06	38,10,4
1et9a1#39	GO:0019883: antigen presentation, endogenous antigen	8.87E-06	38,10,4
1mil__#30	GO:0007169: transmembrane receptor protein tyrosine kinase signaling pathway	8.90E-06	51,26,6
1ie5a_#104	GO:0016337: cell-cell adhesion	8.94E-06	7,17,3
1gox__#124	GO:0009252: peptidoglycan biosynthesis	8.96E-06	298,18,10
1bwvs_#117	GO:0006418: tRNA aminoacylation for protein translation	8.97E-06	287,26,12
1qfxa_#380	GO:0008285: negative regulation of cell proliferation	8.99E-06	41,19,5
1danh_#104	GO:0006919: caspase activation	9.00E-06	147,15,7
1b3qa2#623	GO:0017148: negative regulation of protein biosynthesis	9.00E-06	257,13,8
1dpga2#367	GO:0030333: antigen processing	9.02E-06	9,13,3
1b0ua_#38	GO:0017148: negative regulation of protein biosynthesis	9.02E-06	9,13,3
1akjd_#3	GO:0030333: antigen processing	9.02E-06	9,13,3
1g61a_#2111	GO:0017000: antibiotic biosynthesis	9.02E-06	9,13,3
1axca1#70	GO:0006919: caspase activation	9.07E-06	94,15,6
1f8ra2#325	GO:0051249: regulation of lymphocyte activation	9.14E-06	196,12,7
1g0sa_#74	GO:0016337: cell-cell adhesion	9.16E-06	247,17,9
1fc4a_#205	GO:0009064: glutamine family amino acid metabolism	9.25E-06	215,19,9
1f5ma_#80	GO:0030333: antigen processing	9.29E-06	112,13,6

1cg2a1#43	GO:0015671: oxygen transport	9.29E-06	171,23,9
1e0ta2#275	GO:0015976: carbon utilization	9.35E-06	86,10,5
1pii_2#326	GO:0015976: carbon utilization	9.40E-06	157,10,6
1e79d2#36	GO:0008285: negative regulation of cell proliferation	9.43E-06	432,19,12
1nsj__#124	GO:0009156: ribonucleoside monophosphate biosynthesis	9.44E-06	124,12,6
1nuka_#80	GO:0019735: antimicrobial humoral response (sensu Vertebrata)	9.46E-06	197,16,8
1thg__#223	GO:0006096: glycolysis	9.49E-06	74,38,8
1nal1_#203	GO:0006096: glycolysis	9.53E-06	35,38,6
1clxa_#120	GO:0006096: glycolysis	9.53E-06	35,38,6
1jsg__#92	GO:0006730: one-carbon compound metabolism	9.56E-06	519,32,18
1d2ka1#164	GO:0009073: aromatic amino acid family biosynthesis	9.57E-06	50,16,5
1qdla_#211	GO:0006457: protein folding	9.59E-06	280,49,17
1h9oa_#83	GO:0007169: transmembrane receptor protein tyrosine kinase signaling pathway	9.60E-06	30,26,5
1dik_1#670	GO:0006357: regulation of transcription from Pol II promoter	9.60E-06	26,30,5
1evqa_#305	GO:0006568: tryptophan metabolism	9.68E-06	384,10,8
1cg2a1#43	GO:0019835: cytolysis	9.73E-06	171,18,8
1smaa1#95	GO:0001906: cell killing	9.79E-06	223,11,7
1eny__#94	GO:0006986: response to unfolded protein	9.80E-06	25,15,4
1ck7a3#247	GO:0006418: tRNA aminoacylation for protein translation	9.80E-06	5,26,3
1cb0a_#98	GO:0006418: tRNA aminoacylation for protein translation	9.80E-06	5,26,3
1qfta_#98	GO:0019735: antimicrobial humoral response (sensu Vertebrata)	9.82E-06	198,16,8
1ep3b1#71	GO:0006118: electron transport	9.83E-06	27,228,11
1f00i1#749	GO:0042088: T-helper 1 type immune response	9.85E-06	35,11,4
1f00i1#749	GO:0042108: positive regulation of cytokine biosynthesis	9.85E-06	35,11,4
1saca_#113	GO:0006511: ubiquitin-dependent protein catabolism	9.85E-06	35,11,4
1clxa_#120	GO:0006098: pentose-phosphate shunt	9.85E-06	35,11,4
1j9qa2#295	GO:0030217: T-cell differentiation	9.86E-06	39,10,4
1ej8a_#189	GO:0006275: regulation of DNA replication	9.87E-06	256,10,7
1htp__#16	GO:0030217: T-cell differentiation	9.87E-06	256,10,7
1dbta_#89	GO:0006221: pyrimidine nucleotide biosynthesis	9.87E-06	17,22,4
1iira_#242	GO:0006563: L-serine metabolism	9.90E-06	125,12,6
1apme_#101	GO:0000086: G2/M transition of mitotic cell cycle	9.90E-06	12,10,3
1ir3a_#1056	GO:0000086: G2/M transition of mitotic cell cycle	9.90E-06	12,10,3
1wdna_#152	GO:0006879: iron ion homeostasis	9.90E-06	10,12,3
1h7wa2#535	GO:0009167: purine ribonucleoside monophosphate metabolism	9.90E-06	12,10,3
1zfja1#379	GO:0009167: purine ribonucleoside monophosphate metabolism	9.90E-06	12,10,3
1qrea_#132	GO:0009103: lipopolysaccharide biosynthesis	9.90E-06	10,12,3
1qbea_#92	GO:0018108: peptidyl-tyrosine phosphorylation	9.90E-06	12,10,3
5ruba2#41	GO:0015976: carbon utilization	9.90E-06	12,10,3
1qo2a_#223	GO:0006207: 'de novo' pyrimidine base biosynthesis	9.90E-06	12,10,3
1b16a_#184	GO:0009103: lipopolysaccharide biosynthesis	9.90E-06	10,12,3
1ep3a_#19	GO:0009167: purine ribonucleoside monophosphate metabolism	9.90E-06	12,10,3
1eno__#137	GO:0009103: lipopolysaccharide biosynthesis	9.90E-06	10,12,3
1b12a_#221	GO:0045595: regulation of cell differentiation	9.90E-06	87,10,5
1fmb__#88	GO:0006313: DNA transposition	9.91E-06	4,35,3
1eg2a_#132	GO:0006100: tricarboxylic acid cycle intermediate metabolism	9.97E-06	27,14,4
1fxla2#169	GO:0016337: cell-cell adhesion	9.99E-06	184,17,8
1cfr__#269	GO:0009070: serine family amino acid biosynthesis	1.00E-05	52,26,6
1exma3#99	GO:0009070: serine family amino acid biosynthesis	1.00E-05	52,26,6
1hrna_#105	GO:0006606: protein-nucleus import	0.00001	6,21,3
2bb2_2#134	GO:0051249: regulation of lymphocyte activation	0.00001	199,12,7
1fzqa_#165	GO:0006606: protein-nucleus import	0.00001	18,21,4
1dmla1#134	GO:0001906: cell killing	0.00001	78,11,5
1evqa_#61	GO:0001906: cell killing	0.00001	140,11,6
1kve.1#B199	GO:0006953: acute-phase response	0.00001	69,20,6

1bio__#108	GO:0006956: complement activation	0.00001	32,12,4
1fo5a_#60	GO:0042108: positive regulation of cytokine biosynthesis	0.00001	225,11,7
1fo5a_#60	GO:0001906: cell killing	0.00001	225,11,7
1el0a_#28	GO:0042088: T-helper 1 type immune response	0.00001	140,11,6
1el0a_#28	GO:0042108: positive regulation of cytokine biosynthesis	0.00001	140,11,6
1phm_2#253	GO:0050819: negative regulation of coagulation	0.00001	257,10,7
1phm_2#253	GO:0030193: regulation of blood coagulation	0.00001	257,10,7
1dz4a_#264	GO:0006118: electron transport	0.00001	37,228,13
1kdj__#27	GO:0030333: antigen processing	0.00001	64,13,5
1pwt__#8	GO:0030333: antigen processing	0.00001	262,13,8
1dy9.1#C229	GO:0006118: electron transport	0.00001	111,228,25
1nsj__#83	GO:0006098: pentose-phosphate shunt	0.00001	11,11,3
1b3qa2#623	GO:0019883: antigen presentation, endogenous antigen	0.00001	257,10,7
1kit_3#743	GO:0001906: cell killing	0.00001	225,11,7
1el0a_#42	GO:0006275: regulation of DNA replication	0.00001	258,10,7
1eo6a_#107	GO:0006730: one-carbon compound metabolism	0.00001	64,32,7
1g51a3#525	GO:0006418: tRNA aminoacylation for protein translation	0.00001	239,26,11
1qq5a_#7	GO:0006118: electron transport	0.00001	125,228,27
1qtn.1#A270	GO:0015976: carbon utilization	0.00001	386,10,8
2napa2#292	GO:0042364: water-soluble vitamin biosynthesis	0.00001	108,27,8
1hcl__#280	GO:0006879: iron ion homeostasis	0.00001	70,12,5
1qnja_#46	GO:0006956: complement activation	0.00001	32,12,4
1qfma1#139	GO:0006953: acute-phase response	0.00001	264,20,10
1mrp__#172	GO:0006730: one-carbon compound metabolism	0.000011	91,32,8
2mpra_#89	GO:0030217: T-cell differentiation	0.000011	40,10,4
1e3ha2#35	GO:0006633: fatty acid biosynthesis	0.000011	5,27,3
1f0xa1#278	GO:0006118: electron transport	0.000011	162,228,32
1f0xa1#278	GO:0018108: peptidyl-tyrosine phosphorylation	0.000011	162,10,6
1f0xa1#278	GO:0030217: T-cell differentiation	0.000011	162,10,6
1iow_2#289	GO:0009252: peptidoglycan biosynthesis	0.000011	7,18,3
1ldna1#129	GO:0006100: tricarboxylic acid cycle intermediate metabolism	0.000011	9,14,3
1qg6a_#237	GO:0006633: fatty acid biosynthesis	0.000011	5,27,3
1brwa2#100	GO:0006118: electron transport	0.000011	11,228,7
1dik_3#29	GO:0009252: peptidoglycan biosynthesis	0.000011	7,18,3
1f2aa_#27	GO:0006511: ubiquitin-dependent protein catabolism	0.000011	79,11,5
1vola2#239	GO:0006418: tRNA aminoacylation for protein translation	0.000011	114,26,8
1a0fa1#178	GO:0007186: G-protein coupled receptor protein signaling pathway	0.000011	16,24,4
1dgw.1#Y353	GO:0051249: regulation of lymphocyte activation	0.000011	294,12,8
1jj2v_#118	GO:0050819: negative regulation of coagulation	0.000011	89,10,5
1jj2v_#118	GO:0030193: regulation of blood coagulation	0.000011	89,10,5
1jj2v_#118	GO:0018108: peptidyl-tyrosine phosphorylation	0.000011	89,10,5
1cyda_#230	GO:0006633: fatty acid biosynthesis	0.000011	5,27,3
1fbna_#145	GO:0006563: L-serine metabolism	0.000011	33,12,4
1mpp__#154	GO:0016337: cell-cell adhesion	0.000011	84,17,6
2sli_2#326	GO:0001906: cell killing	0.000011	227,11,7
1qqsa_#124	GO:0006275: regulation of DNA replication	0.000011	392,10,8
1f42a2#123	GO:0001906: cell killing	0.000011	335,11,8
1i5ga_#115	GO:0006367: transcription initiation from Pol II promoter	0.000011	294,12,8
1fhga_#59	GO:0051249: regulation of lymphocyte activation	0.000011	33,12,4
3tdt__#201	GO:0006100: tricarboxylic acid cycle intermediate metabolism	0.000011	9,14,3
1b8aa2#403	GO:0006418: tRNA aminoacylation for protein translation	0.000011	114,26,8
1b8aa2#403	GO:0007169: transmembrane receptor protein tyrosine kinase signaling pathway	0.000011	114,26,8
1eqka_#47	GO:0006418: tRNA aminoacylation for protein translation	0.000011	114,26,8
1efpa1#32	GO:0009064: glutamine family amino acid metabolism	0.000011	114,19,7
1efpa1#32	GO:0009070: serine family amino acid biosynthesis	0.000011	114,26,8

1prxa_#28	GO:0006118: electron transport	0.000011	11,228,7
1bev1_#86	GO:0051249: regulation of lymphocyte activation	0.000011	296,12,8
1qg6a_#93	GO:0006633: fatty acid biosynthesis	0.000011	5,27,3
2hft_1#34	GO:0042108: positive regulation of cytokine biosynthesis	0.000011	36,11,4
1hq8a_#206	GO:0016477: cell migration	0.000011	281,23,11
1xgsa2#82	GO:0030217: T-cell differentiation	0.000011	261,10,7
1cyda_#180	GO:0006633: fatty acid biosynthesis	0.000011	5,27,3
1fsu__#434	GO:0006457: protein folding	0.000011	315,49,18
1gox__#246	GO:0006096: glycolysis	0.000011	157,38,11
1aqua_#126	GO:0009252: peptidoglycan biosynthesis	0.000011	472,18,12
1aqua_#126	GO:0006098: pentose-phosphate shunt	0.000011	472,11,9
1qb7a_#86	GO:0043094: metabolic compound salvage	0.000011	36,11,4
1gdna_#30	GO:0008285: negative regulation of cell proliferation	0.000011	20,19,4
1hxmb1#117	GO:0030333: antigen processing	0.000011	65,13,5
1hdma1#123	GO:0042088: T-helper 1 type immune response	0.000011	36,11,4
1ge8a2#134	GO:0051249: regulation of lymphocyte activation	0.000011	202,12,7
1qs0a1#119	GO:0009116: nucleoside metabolism	0.000011	367,25,13
1e6pa2#46	GO:0042364: water-soluble vitamin biosynthesis	0.000011	77,27,7
1eyea_#131	GO:0006418: tRNA aminoacylation for protein translation	0.000012	15,26,4
2cpl__#20	GO:0019883: antigen presentation, endogenous antigen	0.000012	90,10,5
2cpl__#20	GO:0030217: T-cell differentiation	0.000012	90,10,5
1fid__#237	GO:0006953: acute-phase response	0.000012	41,20,5
1aky_2#133	GO:0051249: regulation of lymphocyte activation	0.000012	130,12,6
2mpra_#190	GO:0006457: protein folding	0.000012	349,49,19
1bqk__#77	GO:0006118: electron transport	0.000012	72,228,19
1thfd_#140	GO:0006118: electron transport	0.000012	98,228,23
1atg__#144	GO:0006563: L-serine metabolism	0.000012	72,12,5
1bywa_#30	GO:0016477: cell migration	0.000012	283,23,11
1pda_1#77	GO:0015698: inorganic anion transport	0.000012	28,14,4
1qfxa_#380	GO:0050819: negative regulation of coagulation	0.000012	41,10,4
1qfxa_#380	GO:0030193: regulation of blood coagulation	0.000012	41,10,4
1qfxa_#380	GO:0018108: peptidyl-tyrosine phosphorylation	0.000012	41,10,4
2dri__#115	GO:0006935: chemotaxis	0.000012	10,41,4
1dceb_#170	GO:0030245: cellulose catabolism	0.000012	6,22,3
1f5aa1#441	GO:0051249: regulation of lymphocyte activation	0.000012	129,12,6
1gox__#124	GO:0046148: pigment biosynthesis	0.000012	298,12,8
1gox__#124	GO:0009156: ribonucleoside monophosphate biosynthesis	0.000012	298,12,8
1ibja_#271	GO:0015698: inorganic anion transport	0.000012	61,14,5
1hd2a_#35	GO:0006544: glycine metabolism	0.000012	145,11,6
1cf9a1#736	GO:0007264: small GTPase mediated signal transduction	0.000012	39,21,5
1qtn.1#A270	GO:0006779: porphyrin biosynthesis	0.000012	386,18,11
1fa0a1#393	GO:0006313: DNA transposition	0.000012	209,35,12
1quna1#113	GO:0006418: tRNA aminoacylation for protein translation	0.000012	243,26,11
1kit_2#398	GO:0001906: cell killing	0.000012	81,11,5
3sil__#317	GO:0051249: regulation of lymphocyte activation	0.000012	300,12,8
1cpza_#6	GO:0016477: cell migration	0.000012	132,23,8
1e3ua_#160	GO:0006096: glycolysis	0.000013	4,38,3
1b12a_#221	GO:0016337: cell-cell adhesion	0.000013	87,17,6
1ja1a2#201	GO:0006118: electron transport	0.000013	8,228,6
1fvia2#134	GO:0006118: electron transport	0.000013	23,228,10
1d6ja_#36	GO:0045045: secretory pathway	0.000013	18,22,4
1d6ja_#36	GO:0006221: pyrimidine nucleotide biosynthesis	0.000013	18,22,4
2cb5a_#312	GO:0050819: negative regulation of coagulation	0.000013	92,10,5
2cb5a_#312	GO:0015976: carbon utilization	0.000013	92,10,5
2cb5a_#312	GO:0030193: regulation of blood coagulation	0.000013	92,10,5
1ak0__#115	GO:0015671: oxygen transport	0.000013	36,23,5

1jj2i_#30	GO:0006118: electron transport	0.000013	8,228,6
1f3oa_#154	GO:0006099: tricarboxylic acid cycle	0.000013	7,19,3
1f0ya2#126	GO:0006207: 'de novo' pyrimidine base biosynthesis	0.000013	13,10,3
1ac6a_#43	GO:0042088: T-helper 1 type immune response	0.000013	147,11,6
1ac6a_#43	GO:0042108: positive regulation of cytokine biosynthesis	0.000013	147,11,6
1fjfl_#67	GO:0042088: T-helper 1 type immune response	0.000013	147,11,6
1fjfl_#67	GO:0042108: positive regulation of cytokine biosynthesis	0.000013	147,11,6
1hdma1#115	GO:0019883: antigen presentation, endogenous antigen	0.000013	165,10,6
1hyea2#272	GO:0006099: tricarboxylic acid cycle	0.000013	21,19,4
1nuka_#80	GO:0007169: transmembrane receptor protein tyrosine kinase signaling pathway	0.000013	197,26,10
1nuka_#80	GO:0007399: neurogenesis	0.000013	197,37,12
2pia_1#99	GO:0006118: electron transport	0.000013	156,228,31
1f42a2#123	GO:0016337: cell-cell adhesion	0.000013	335,17,10
1c9la2#61	GO:0051249: regulation of lymphocyte activation	0.000013	206,12,7
1c9la2#61	GO:0019735: antimicrobial humoral response (sensu Vertebrata)	0.000013	206,16,8
1ezvb1#29	GO:0030217: T-cell differentiation	0.000013	42,10,4
1phm_2#253	GO:0016337: cell-cell adhesion	0.000013	257,17,9
3grs_2#211	GO:0006207: 'de novo' pyrimidine base biosynthesis	0.000013	42,10,4
1iira_#326	GO:0009116: nucleoside metabolism	0.000013	86,25,7
2ebn__#77	GO:0006418: tRNA aminoacylation for protein translation	0.000013	32,26,5
1ehya_#85	GO:0017000: antibiotic biosynthesis	0.000013	31,13,4
1csh__#212	GO:0006099: tricarboxylic acid cycle	0.000013	7,19,3
1exma3#99	GO:0042364: water-soluble vitamin biosynthesis	0.000013	52,27,6
1ds1a_#172	GO:0016337: cell-cell adhesion	0.000013	190,17,8
1bmv2_#2064	GO:0016337: cell-cell adhesion	0.000013	133,17,7
1tul__#102	GO:0030333: antigen processing	0.000013	67,13,5
1nat__#50	GO:0006221: pyrimidine nucleotide biosynthesis	0.000013	242,22,10
1dhs__#99	GO:0009086: methionine biosynthesis	0.000013	109,14,6
1i7qa_#321	GO:0042088: T-helper 1 type immune response	0.000013	147,11,6
1hdma1#98	GO:0030217: T-cell differentiation	0.000013	42,10,4
1e79d2#36	GO:0006118: electron transport	0.000013	432,228,64
2hlca_#226	GO:0030333: antigen processing	0.000013	67,13,5
1nsca_#236	GO:0006118: electron transport	0.000013	156,228,31
3nul__#107	GO:0006730: one-carbon compound metabolism	0.000014	13,32,4
1bkpa_#275	GO:0009219: pyrimidine deoxyribonucleotide metabolism	0.000014	2,10,2
1pii_1#48	GO:0042401: biogenic amine biosynthesis	0.000014	2,10,2
1pii_1#48	GO:0006568: tryptophan metabolism	0.000014	2,10,2
1rblm_#68	GO:0015976: carbon utilization	0.000014	2,10,2
1g97a1#376	GO:0009103: lipopolysaccharide biosynthesis	0.000014	11,12,3
1nar__#85	GO:0006207: 'de novo' pyrimidine base biosynthesis	0.000014	2,10,2
1qdla_#103	GO:0042401: biogenic amine biosynthesis	0.000014	2,10,2
1qdla_#103	GO:0006568: tryptophan metabolism	0.000014	2,10,2
1qf6a3#92	GO:0009307: DNA restriction	0.000014	77,19,6
1qdla_#183	GO:0042401: biogenic amine biosynthesis	0.000014	2,10,2
1qdla_#183	GO:0006568: tryptophan metabolism	0.000014	2,10,2
1ga0a_#228	GO:0046677: response to antibiotic	0.000014	21,40,5
1qfla1#44	GO:0006986: response to unfolded protein	0.000014	9,15,3
5ruba2#41	GO:0043094: metabolic compound salvage	0.000014	12,11,3
1cewi_#14	GO:0006935: chemotaxis	0.000014	72,41,8
1epwa3#140	GO:0019883: antigen presentation, endogenous antigen	0.000014	168,10,6
1epwa3#140	GO:0030217: T-cell differentiation	0.000014	168,10,6
1apme_#68	GO:0019883: antigen presentation, endogenous antigen	0.000014	93,10,5
1qoua_#90	GO:0042088: T-helper 1 type immune response	0.000014	38,11,4
1fo5a_#60	GO:0016068: type I hypersensitivity	0.000014	225,15,8
1jb3a_#64	GO:0006100: tricarboxylic acid cycle intermediate metabolism	0.000014	247,14,8

1dpja_#322	GO:0050819: negative regulation of coagulation	0.000014	269,10,7
1dpja_#322	GO:0030193: regulation of blood coagulation	0.000014	269,10,7
1dpja_#322	GO:0030217: T-cell differentiation	0.000014	269,10,7
2mpra_#190	GO:0016477: cell migration	0.000014	349,23,12
1daba_#387	GO:0009103: lipopolysaccharide biosynthesis	0.000014	11,12,3
1avgi_#69	GO:0019735: antimicrobial humoral response (sensu Vertebrata)	0.000014	281,16,9
3chbd_#85	GO:0006730: one-carbon compound metabolism	0.000014	323,32,14
2ae2a_#89	GO:0009396: folic acid and derivative biosynthesis	0.000014	38,11,4
1j7la_#86	GO:0016485: protein processing	0.000014	12,11,3
1e1oa2#423	GO:0006118: electron transport	0.000014	127,228,27
1c7na_#95	GO:0006606: protein-nucleus import	0.000014	149,21,8
3lada1#116	GO:0006563: L-serine metabolism	0.000014	133,12,6
1mjha_#119	GO:0009116: nucleoside metabolism	0.000014	87,25,7
1plq_1#90	GO:0043123: positive regulation of I-kappaB kinase/NF-kappaB cascade	0.000014	145,16,7
1nsj__#83	GO:0006096: glycolysis	0.000014	11,38,4
1xgsa2#186	GO:0006511: ubiquitin-dependent protein catabolism	0.000014	149,11,6
1eucb2#188	GO:0043094: metabolic compound salvage	0.000014	83,11,5
1h5qa_#70	GO:0009103: lipopolysaccharide biosynthesis	0.000014	11,12,3
1ho1a_#211	GO:0015976: carbon utilization	0.000014	93,10,5
1wdna_#187	GO:0006544: glycine metabolism	0.000014	38,11,4
2bbkh_#214	GO:0042088: T-helper 1 type immune response	0.000015	39,11,4
2bbkh_#214	GO:0042108: positive regulation of cytokine biosynthesis	0.000015	39,11,4
2bbkh_#214	GO:0001906: cell killing	0.000015	39,11,4
1fvia2#25	GO:0051249: regulation of lymphocyte activation	0.000015	35,12,4
1ldm_2#272	GO:0006418: tRNA aminoacylation for protein translation	0.000015	16,26,4
1f00i1#749	GO:0051249: regulation of lymphocyte activation	0.000015	35,12,4
1cby__#183	GO:0009070: serine family amino acid biosynthesis	0.000015	16,26,4
1fy7a_#304	GO:0006118: electron transport	0.000015	19,228,9
1qs0b2#269	GO:0006563: L-serine metabolism	0.000015	35,12,4
1dz4a_#150	GO:0006418: tRNA aminoacylation for protein translation	0.000015	16,26,4
1fw8a_#126	GO:0006118: electron transport	0.000015	19,228,9
1bwvs_#38	GO:0006118: electron transport	0.000015	33,228,12
1dt6a_#449	GO:0006118: electron transport	0.000015	15,228,8
1jh2a_#16	GO:0030333: antigen processing	0.000015	69,13,5
1j9la_#82	GO:0009103: lipopolysaccharide biosynthesis	0.000015	134,12,6
1fcda2#189	GO:0009396: folic acid and derivative biosynthesis	0.000015	39,11,4
1tuba1#239	GO:0006568: tryptophan metabolism	0.000015	95,10,5
3btaa3#194	GO:0019883: antigen presentation, endogenous antigen	0.000015	43,10,4
1cmia_#86	GO:0042113: B-cell activation	0.000015	49,9,4
1fw9a_#152	GO:0046677: response to antibiotic	0.000015	124,40,10
2mpra_#190	GO:0001906: cell killing	0.000015	349,11,8
1dfca4#1469	GO:0019883: antigen presentation, endogenous antigen	0.000015	43,10,4
1opy__#68	GO:0051249: regulation of lymphocyte activation	0.000015	308,12,8
1qfxa_#380	GO:0006606: protein-nucleus import	0.000015	41,21,5
1aqua_#79	GO:0009103: lipopolysaccharide biosynthesis	0.000015	35,12,4
1pwt__#8	GO:0016337: cell-cell adhesion	0.000015	262,17,9
1axca1#70	GO:0050819: negative regulation of coagulation	0.000015	94,10,5
1axca1#70	GO:0030193: regulation of blood coagulation	0.000015	94,10,5
1e4ea2#215	GO:0007169: transmembrane receptor protein tyrosine kinase signaling pathway	0.000015	419,26,14
1qo2a_#222	GO:0009073: aromatic amino acid family biosynthesis	0.000015	55,16,5
1ddja_#746	GO:0030333: antigen processing	0.000015	121,13,6
1aa6_1#652	GO:0006118: electron transport	0.000015	28,228,11
1ddwa_#77	GO:0006457: protein folding	0.000015	322,49,18
1a7s__#148	GO:0018108: peptidyl-tyrosine phosphorylation	0.000016	44,10,4
2rspa_#113	GO:0006278: RNA-dependent DNA replication	0.000016	14,10,3

1dzfa2#164	GO:0009167: purine ribonucleoside monophosphate metabolism	0.000016	14,10,3
1i2oa_#34	GO:0042364: water-soluble vitamin biosynthesis	0.000016	152,27,9
1pme__#302	GO:0000086: G2/M transition of mitotic cell cycle	0.000016	14,10,3
1cg2a1#43	GO:0006099: tricarboxylic acid cycle	0.000016	171,19,8
1sgpe_#42	GO:0006275: regulation of DNA replication	0.000016	14,10,3
1eq9a_#32	GO:0018108: peptidyl-tyrosine phosphorylation	0.000016	96,10,5
1as4.1#A336	GO:0030217: T-cell differentiation	0.000016	276,10,7
1f42a2#123	GO:0006461: protein complex assembly	0.000016	335,24,12
1svb_1#318	GO:0019883: antigen presentation, endogenous antigen	0.000016	14,10,3
1mek__#17	GO:0009064: glutamine family amino acid metabolism	0.000016	22,19,4
1a9xa3#78	GO:0000160: two-component signal transduction system (phosphorelay)	0.000016	262,25,11
2hhma_#262	GO:0043094: metabolic compound salvage	0.000016	85,11,5
1bura1#269	GO:0042401: biogenic amine biosynthesis	0.000016	14,10,3
1euca2#234	GO:0006207: 'de novo' pyrimidine base biosynthesis	0.000016	14,10,3
1e79d3#328	GO:0006563: L-serine metabolism	0.000016	36,12,4
1hq8a_#206	GO:0006935: chemotaxis	0.000016	281,41,15
1fu6a_#50	GO:0016337: cell-cell adhesion	0.000016	265,17,9
1mil__#31	GO:0019883: antigen presentation, endogenous antigen	0.000016	96,10,5
1cqqa_#163	GO:0050819: negative regulation of coagulation	0.000016	14,10,3
1cqqa_#163	GO:0030193: regulation of blood coagulation	0.000016	14,10,3
1edza1#206	GO:0001906: cell killing	0.000016	352,11,8
1ejda_#6	GO:0006953: acute-phase response	0.000016	215,20,9
1ddja_#746	GO:0008285: negative regulation of cell proliferation	0.000016	121,19,7
1h6la_#119	GO:0016337: cell-cell adhesion	0.000016	264,17,9
1hdma1#123	GO:0051249: regulation of lymphocyte activation	0.000016	36,12,4
1eur__#84	GO:0007169: transmembrane receptor protein tyrosine kinase signaling pathway	0.000016	85,26,7
1e79d2#36	GO:0016337: cell-cell adhesion	0.000016	432,17,11
1dyna_#42	GO:0042088: T-helper 1 type immune response	0.000016	152,11,6
1dyna_#42	GO:0042108: positive regulation of cytokine biosynthesis	0.000016	152,11,6
1a9xa6#711	GO:0009252: peptidoglycan biosynthesis	0.000017	8,18,3
1a7ta_#80	GO:0046677: response to antibiotic	0.000017	11,40,4
1eucb2#17	GO:0009252: peptidoglycan biosynthesis	0.000017	8,18,3
1bxoa_#320	GO:0008284: positive regulation of cell proliferation	0.000017	49,30,6
2mpra_#89	GO:0042088: T-helper 1 type immune response	0.000017	40,11,4
2mpra_#89	GO:0042108: positive regulation of cytokine biosynthesis	0.000017	40,11,4
1f97a1#62	GO:0019883: antigen presentation, endogenous antigen	0.000017	591,10,9
1f97a1#62	GO:0050819: negative regulation of coagulation	0.000017	591,10,9
1f97a1#62	GO:0030193: regulation of blood coagulation	0.000017	591,10,9
1f97a1#62	GO:0030217: T-cell differentiation	0.000017	591,10,9
1f97a1#62	GO:0006275: regulation of DNA replication	0.000017	591,10,9
1auva2#386	GO:0009252: peptidoglycan biosynthesis	0.000017	8,18,3
1qhda2#323	GO:0051249: regulation of lymphocyte activation	0.000017	313,12,8
1qhla_#28	GO:0007517: muscle development	0.000017	29,30,5
1pyma_#224	GO:0007264: small GTPase mediated signal transduction	0.000017	7,21,3
1ihna_#64	GO:0006606: protein-nucleus import	0.000017	7,21,3
1qbkb_#684	GO:0006606: protein-nucleus import	0.000017	7,21,3
1de4c2#250	GO:0009116: nucleoside metabolism	0.000017	6,25,3
1ayl__#237	GO:0051249: regulation of lymphocyte activation	0.000017	313,12,8
1ayl__#237	GO:0016068: type I hypersensitivity	0.000017	313,15,9
1qqsa_#124	GO:0006457: protein folding	0.000017	392,49,20
1csn__#84	GO:0019883: antigen presentation, endogenous antigen	0.000017	174,10,6
1bfg__#22	GO:0019079: viral genome replication	0.000017	56,16,5
1fo4a5#1002	GO:0009064: glutamine family amino acid metabolism	0.000017	80,19,6
1tca__#189	GO:0016042: lipid catabolism	0.000017	5,31,3
1qrra_#69	GO:0009064: glutamine family amino acid metabolism	0.000017	173,19,8

1h75a_#53	GO:0006118: electron transport	0.000017	44,228,14
1quna1#113	GO:0042088: T-helper 1 type immune response	0.000017	243,11,7
1quna1#113	GO:0042108: positive regulation of cytokine biosynthesis	0.000017	243,11,7
1gpea1#271	GO:0006118: electron transport	0.000017	196,228,36
1e79d2#36	GO:0051249: regulation of lymphocyte activation	0.000017	432,12,9
1fgka_#641	GO:0000165: MAPKKK cascade	0.000018	2,11,2
1eqga2#69	GO:0006690: icosanoid metabolism	0.000018	2,11,2
3nul__#107	GO:0006544: glycine metabolism	0.000018	13,11,3
1rypa_#70	GO:0006511: ubiquitin-dependent protein catabolism	0.000018	2,11,2
1e42a1#711	GO:0042108: positive regulation of cytokine biosynthesis	0.000018	155,11,6
1rypl_#96	GO:0006511: ubiquitin-dependent protein catabolism	0.000018	2,11,2
1rypf_#148	GO:0006511: ubiquitin-dependent protein catabolism	0.000018	2,11,2
1pma1_#95	GO:0006511: ubiquitin-dependent protein catabolism	0.000018	2,11,2
1ah7__#55	GO:0001906: cell killing	0.000018	2,11,2
1evya2#38	GO:0006879: iron ion homeostasis	0.000018	12,12,3
1gtra2#173	GO:0019883: antigen presentation, endogenous antigen	0.000018	45,10,4
1rblm_#68	GO:0043094: metabolic compound salvage	0.000018	2,11,2
1am2__#161	GO:0016485: protein processing	0.000018	2,11,2
1rypl_#125	GO:0006511: ubiquitin-dependent protein catabolism	0.000018	2,11,2
1h7wa2#535	GO:0009156: ribonucleoside monophosphate biosynthesis	0.000018	12,12,3
1rypj_#39	GO:0006511: ubiquitin-dependent protein catabolism	0.000018	2,11,2
1b12a_#221	GO:0042108: positive regulation of cytokine biosynthesis	0.000018	87,11,5
1qtwa_#213	GO:0006098: pentose-phosphate shunt	0.000018	2,11,2
7ahla_#32	GO:0001906: cell killing	0.000018	2,11,2
1zfja1#379	GO:0009156: ribonucleoside monophosphate biosynthesis	0.000018	12,12,3
1dp0a5#499	GO:0007017: microtubule-based process	0.000018	12,12,3
1gp1a_#173	GO:0006979: response to oxidative stress	0.000018	24,18,4
1rypf_#167	GO:0006511: ubiquitin-dependent protein catabolism	0.000018	2,11,2
1ah7__#184	GO:0001906: cell killing	0.000018	2,11,2
1agi__#31	GO:0048514: blood vessel morphogenesis	0.000018	16,9,3
1qsta_#72	GO:0016568: chromatin modification	0.000018	2,11,2
1yrga_#232	GO:0007017: microtubule-based process	0.000018	37,12,4
1xis__#196	GO:0042364: water-soluble vitamin biosynthesis	0.000018	16,27,4
1dfca1#1135	GO:0050819: negative regulation of coagulation	0.000018	45,10,4
1dfca1#1135	GO:0030193: regulation of blood coagulation	0.000018	45,10,4
1dfca1#1135	GO:0018108: peptidyl-tyrosine phosphorylation	0.000018	45,10,4
1rypc_#153	GO:0006511: ubiquitin-dependent protein catabolism	0.000018	2,11,2
1qr4a2#166	GO:0042088: T-helper 1 type immune response	0.000018	13,11,3
1i50b_#216	GO:0006418: tRNA aminoacylation for protein translation	0.000018	34,26,5
1hnja1#133	GO:0006633: fatty acid biosynthesis	0.000018	16,27,4
1akjd_#3	GO:0019735: antimicrobial humoral response (sensu Vertebrata)	0.000018	9,16,3
1rype_#228	GO:0006511: ubiquitin-dependent protein catabolism	0.000018	2,11,2
1avgi_#69	GO:0006953: acute-phase response	0.000018	281,20,10
1avgi_#69	GO:0017148: negative regulation of protein biosynthesis	0.000018	281,13,8
1amf__#168	GO:0006879: iron ion homeostasis	0.000018	12,12,3
1czan1#92	GO:0016337: cell-cell adhesion	0.000018	538,17,12
1rype_#30	GO:0006511: ubiquitin-dependent protein catabolism	0.000018	2,11,2
1mjha_#119	GO:0006418: tRNA aminoacylation for protein translation	0.000018	87,26,7
1rypa_#28	GO:0006511: ubiquitin-dependent protein catabolism	0.000018	2,11,2
1rypj_#145	GO:0006511: ubiquitin-dependent protein catabolism	0.000018	2,11,2
1aisb2#1274	GO:0006367: transcription initiation from Pol II promoter	0.000018	12,12,3
1ep3a_#19	GO:0009156: ribonucleoside monophosphate biosynthesis	0.000018	12,12,3
1svb_2#165	GO:0019079: viral genome replication	0.000018	27,16,4
1el0a_#42	GO:0008284: positive regulation of cell proliferation	0.000018	258,30,12
3grs_1#23	GO:0006563: L-serine metabolism	0.000018	139,12,6
1rypk_#38	GO:0006511: ubiquitin-dependent protein catabolism	0.000018	2,11,2

1rypa_#35	GO:0006511: ubiquitin-dependent protein catabolism	0.000018	2,11,2
1ej0a_#42	GO:0006278: RNA-dependent DNA replication	0.000018	98,10,5
5ruba1#259	GO:0015976: carbon utilization	0.000018	45,10,4
2cuaa_#83	GO:0015976: carbon utilization	0.000018	45,10,4
1bnb__#34	GO:0042742: defense response to bacteria	0.000019	6,26,3
1dlfl_#97	GO:0042088: T-helper 1 type immune response	0.000019	41,11,4
1a4ia2#118	GO:0006730: one-carbon compound metabolism	0.000019	5,32,3
1qf6a3#78	GO:0017001: antibiotic catabolism	0.000019	46,10,4
1oela1#426	GO:0006099: tricarboxylic acid cycle	0.000019	23,19,4
1dt9a1#224	GO:0043094: metabolic compound salvage	0.000019	41,11,4
1hyrc1#247	GO:0016477: cell migration	0.000019	66,23,6
1ejfa_#10	GO:0006461: protein complex assembly	0.000019	134,24,8
1ycsa_#162	GO:0050819: negative regulation of coagulation	0.000019	177,10,6
1ycsa_#162	GO:0030193: regulation of blood coagulation	0.000019	177,10,6
1ycsa_#162	GO:0018108: peptidyl-tyrosine phosphorylation	0.000019	177,10,6
1qqsa_#124	GO:0006118: electron transport	0.000019	392,228,59
1dpja_#322	GO:0016337: cell-cell adhesion	0.000019	269,17,9
2mpra_#190	GO:0016337: cell-cell adhesion	0.000019	349,17,10
1avgi_#69	GO:0030217: T-cell differentiation	0.000019	281,10,7
1ft9a1#201	GO:0016337: cell-cell adhesion	0.000019	92,17,6
1el0a_#28	GO:0016477: cell migration	0.000019	140,23,8
1g0sa_#74	GO:0006544: glycine metabolism	0.000019	247,11,7
1dj0a2#183	GO:0006606: protein-nucleus import	0.000019	111,21,7
1boub_#7	GO:0009070: serine family amino acid biosynthesis	0.000019	206,26,10
1e1aa_#178	GO:0007169: transmembrane receptor protein tyrosine kinase signaling pathway	0.000019	58,26,6
1a81a1#51	GO:0008284: positive regulation of cell proliferation	0.000019	75,30,7
1ax4a_#275	GO:0019883: antigen presentation, endogenous antigen	0.000019	99,10,5
1wdna_#79	GO:0009070: serine family amino acid biosynthesis	0.00002	17,26,4
1wdna_#152	GO:0006826: iron ion transport	0.00002	10,15,3
3grs_2#189	GO:0006096: glycolysis	0.00002	12,38,4
1tkia_#80	GO:0000086: G2/M transition of mitotic cell cycle	0.00002	15,10,3
1azo__#144	GO:0006367: transcription initiation from Pol II promoter	0.00002	38,12,4
1eexb_#119	GO:0006563: L-serine metabolism	0.00002	38,12,4
1a6o__#217	GO:0000086: G2/M transition of mitotic cell cycle	0.00002	15,10,3
1howa_#588	GO:0000086: G2/M transition of mitotic cell cycle	0.00002	15,10,3
1koba_#230	GO:0000086: G2/M transition of mitotic cell cycle	0.00002	15,10,3
1g8ka2#523	GO:0006568: tryptophan metabolism	0.00002	15,10,3
1gc5a_#233	GO:0006563: L-serine metabolism	0.00002	38,12,4
1qo2a_#223	GO:0006096: glycolysis	0.00002	12,38,4
1j9qa2#295	GO:0006118: electron transport	0.00002	39,228,13
1j9la_#82	GO:0016042: lipid catabolism	0.00002	134,31,9
1qp8a1#168	GO:0006730: one-carbon compound metabolism	0.00002	14,32,4
1tkia_#266	GO:0000086: G2/M transition of mitotic cell cycle	0.00002	15,10,3
1dt6a_#385	GO:0006118: electron transport	0.00002	62,228,17
1pot__#138	GO:0019735: antimicrobial humoral response (sensu Vertebrata)	0.00002	58,16,5
1ej8a_#189	GO:0007169: transmembrane receptor protein tyrosine kinase signaling pathway	0.00002	256,26,11
1qoua_#90	GO:0051249: regulation of lymphocyte activation	0.00002	38,12,4
1fo0b_#82	GO:0030217: T-cell differentiation	0.00002	15,10,3
2viua_#252	GO:0030217: T-cell differentiation	0.00002	285,10,7
1c9la2#12	GO:0006418: tRNA aminoacylation for protein translation	0.00002	257,26,11
1ceo__#309	GO:0030245: cellulose catabolism	0.00002	7,22,3
1mspa_#17	GO:0051249: regulation of lymphocyte activation	0.00002	38,12,4
1phk__#165	GO:0000086: G2/M transition of mitotic cell cycle	0.00002	15,10,3
1g25a_#22	GO:0007169: transmembrane receptor protein tyrosine kinase signaling pathway	0.00002	367,26,13

1imva_#255	GO:0042108: positive regulation of cytokine biosynthesis	0.00002	506,11,9
1imva_#255	GO:0001906: cell killing	0.00002	506,11,9
1i7qa_#277	GO:0042401: biogenic amine biosynthesis	0.00002	15,10,3
1a53__#236	GO:0006221: pyrimidine nucleotide biosynthesis	0.00002	20,22,4
1eula_#518	GO:0006418: tRNA aminoacylation for protein translation	0.00002	17,26,4
1aw8.1#B59	GO:0006418: tRNA aminoacylation for protein translation	0.00002	17,26,4
2cb5a_#395	GO:0006118: electron transport	0.00002	56,228,16
1jg8a_#172	GO:0009086: methionine biosynthesis	0.00002	67,14,5
1fjfc2#153	GO:0042088: T-helper 1 type immune response	0.00002	362,11,8
1fjfc2#153	GO:0006511: ubiquitin-dependent protein catabolism	0.00002	362,11,8
1qama_#37	GO:0006099: tricarboxylic acid cycle	0.00002	48,19,5
1gox__#246	GO:0006098: pentose-phosphate shunt	0.00002	157,11,6
1e6ca_#91	GO:0006563: L-serine metabolism	0.00002	141,12,6
2hlca_#54	GO:0019079: viral genome replication	0.00002	58,16,5
1iira_#242	GO:0009064: glutamine family amino acid metabolism	0.00002	125,19,7
1i1b__#10	GO:0019883: antigen presentation, endogenous antigen	0.00002	15,10,3
1dlea_#177	GO:0006956: complement activation	0.000021	2,12,2
2naca1#329	GO:0006563: L-serine metabolism	0.000021	2,12,2
1jmta_#133	GO:0000398: nuclear mRNA splicing, via spliceosome	0.000021	2,12,2
1g40a4#229	GO:0006956: complement activation	0.000021	2,12,2
1bx4a_#163	GO:0009156: ribonucleoside monophosphate biosynthesis	0.000021	2,12,2
1qj2c2#50	GO:0015976: carbon utilization	0.000021	47,10,4
1qj2c2#50	GO:0006278: RNA-dependent DNA replication	0.000021	47,10,4
1f00i1#749	GO:0030333: antigen processing	0.000021	35,13,4
3sil__#84	GO:0042088: T-helper 1 type immune response	0.000021	42,11,4
3sil__#84	GO:0042108: positive regulation of cytokine biosynthesis	0.000021	42,11,4
1ft9a1#162	GO:0006470: protein amino acid dephosphorylation	0.000021	78,20,6
1qrra_#212	GO:0009103: lipopolysaccharide biosynthesis	0.000021	2,12,2
1saca_#113	GO:0007169: transmembrane receptor protein tyrosine kinase signaling pathway	0.000021	35,26,5
2sqca1#520	GO:0006956: complement activation	0.000021	2,12,2
5ruba1#279	GO:0042401: biogenic amine biosynthesis	0.000021	47,10,4
5ruba1#279	GO:0006568: tryptophan metabolism	0.000021	47,10,4
2fmr__#10	GO:0006265: DNA topological change	0.000021	47,10,4
2fmr__#10	GO:0006268: DNA unwinding	0.000021	47,10,4
2u2fa_#9	GO:0000398: nuclear mRNA splicing, via spliceosome	0.000021	2,12,2
1gg4a3#64	GO:0006096: glycolysis	0.000021	40,38,6
1fw9a_#152	GO:0007169: transmembrane receptor protein tyrosine kinase signaling pathway	0.000021	124,26,8
1kid__#330	GO:0016337: cell-cell adhesion	0.000021	144,17,7
1c4zd_#85	GO:0019883: antigen presentation, endogenous antigen	0.000021	101,10,5
1f8ra1#288	GO:0006207: 'de novo' pyrimidine base biosynthesis	0.000021	47,10,4
1h61a_#176	GO:0009073: aromatic amino acid family biosynthesis	0.000021	28,16,4
1bjt__#890	GO:0007169: transmembrane receptor protein tyrosine kinase signaling pathway	0.000021	258,26,11
1ezvb1#29	GO:0042088: T-helper 1 type immune response	0.000021	42,11,4
1ezvb1#29	GO:0042108: positive regulation of cytokine biosynthesis	0.000021	42,11,4
1d0na6#681	GO:0006418: tRNA aminoacylation for protein translation	0.000021	124,26,8
1imva_#255	GO:0016477: cell migration	0.000021	506,23,14
1c3ga2#272	GO:0030333: antigen processing	0.000021	35,13,4
1hbza_#96	GO:0051249: regulation of lymphocyte activation	0.000021	322,12,8
1nsj__#124	GO:0009070: serine family amino acid biosynthesis	0.000021	124,26,8
1h6kx_#111	GO:0000398: nuclear mRNA splicing, via spliceosome	0.000021	2,12,2
1gox__#124	GO:0042364: water-soluble vitamin biosynthesis	0.000021	298,27,12
1a8i__#688	GO:0006730: one-carbon compound metabolism	0.000021	203,32,11
1ldna1#94	GO:0006098: pentose-phosphate shunt	0.000021	159,11,6
1dih_2#222	GO:0006418: tRNA aminoacylation for protein translation	0.000021	311,26,12

1trka2#429	GO:0006096: glycolysis	0.000021	40,38,6
16pk__#95	GO:0006096: glycolysis	0.000021	24,38,5
1eucb2#188	GO:0009064: glutamine family amino acid metabolism	0.000021	83,19,6
1h6va1#23	GO:0006096: glycolysis	0.000021	40,38,6
1kit_2#398	GO:0051249: regulation of lymphocyte activation	0.000021	81,12,5
1rypk_#12	GO:0001906: cell killing	0.000021	250,11,7
1ddwa_#77	GO:0051249: regulation of lymphocyte activation	0.000021	322,12,8
1cyda_#231	GO:0006633: fatty acid biosynthesis	0.000022	6,27,3
1a7s__#148	GO:0006606: protein-nucleus import	0.000022	44,21,5
1bu7a_#278	GO:0006118: electron transport	0.000022	29,228,11
1fc3a_#172	GO:0006470: protein amino acid dephosphorylation	0.000022	168,20,8
1hx0a2#234	GO:0000160: two-component signal transduction system (phosphorelay)	0.000022	18,25,4
1exba_#188	GO:0042364: water-soluble vitamin biosynthesis	0.000022	6,27,3
3chbd_#85	GO:0051249: regulation of lymphocyte activation	0.000022	323,12,8
1g25a_#22	GO:0042108: positive regulation of cytokine biosynthesis	0.000022	367,11,8
1g25a_#22	GO:0001906: cell killing	0.000022	367,11,8
1exma3#99	GO:0006779: porphyrin biosynthesis	0.000022	52,18,5
1i50b_#966	GO:0042108: positive regulation of cytokine biosynthesis	0.000022	251,11,7
1i50b_#966	GO:0006544: glycine metabolism	0.000022	251,11,7
2cpl__#53	GO:0006457: protein folding	0.000022	19,49,5
1qqga1#95	GO:0050819: negative regulation of coagulation	0.000022	102,10,5
1qqga1#95	GO:0030193: regulation of blood coagulation	0.000022	102,10,5
1c7na_#231	GO:0009070: serine family amino acid biosynthesis	0.000022	210,26,10
1mjha_#126	GO:0009086: methionine biosynthesis	0.000022	119,14,6
1fnf_1#1146	GO:0042088: T-helper 1 type immune response	0.000022	14,11,3
1e79d2#36	GO:0007169: transmembrane receptor protein tyrosine kinase signaling pathway	0.000022	432,26,14
1h6va1#27	GO:0009103: lipopolysaccharide biosynthesis	0.000022	224,12,7
1qs0a1#119	GO:0009396: folic acid and derivative biosynthesis	0.000022	367,11,8
1qs0a1#119	GO:0006118: electron transport	0.000022	367,228,56
1qr4a2#166	GO:0051249: regulation of lymphocyte activation	0.000023	13,12,3
1bn8a_#241	GO:0009103: lipopolysaccharide biosynthesis	0.000023	13,12,3
1fo5a_#60	GO:0051249: regulation of lymphocyte activation	0.000023	225,12,7
1e7wa_#8	GO:0006563: L-serine metabolism	0.000023	83,12,5
1cbf__#208	GO:0006275: regulation of DNA replication	0.000023	291,10,7
1dfca4#1469	GO:0042088: T-helper 1 type immune response	0.000023	43,11,4
1ton__#179	GO:0006956: complement activation	0.000023	13,12,3
1ec7a1#230	GO:0042401: biogenic amine biosynthesis	0.000023	48,10,4
1elva1#513	GO:0006956: complement activation	0.000023	13,12,3
1ddja_#681	GO:0006606: protein-nucleus import	0.000023	212,21,9
3rpba_#557	GO:0019883: antigen presentation, endogenous antigen	0.000023	48,10,4
1ea5a_#99	GO:0016042: lipid catabolism	0.000023	172,31,10
1a0la_#160	GO:0051049: regulation of transport	0.000023	48,10,4
1a0la_#160	GO:0018108: peptidyl-tyrosine phosphorylation	0.000023	48,10,4
2bb2_1#57	GO:0001906: cell killing	0.000023	92,11,5
1feca1#31	GO:0006100: tricarboxylic acid cycle intermediate metabolism	0.000023	69,14,5
1kit_3#743	GO:0006953: acute-phase response	0.000023	225,20,9
2ae2a_#17	GO:0009103: lipopolysaccharide biosynthesis	0.000023	13,12,3
1fsia_#122	GO:0006418: tRNA aminoacylation for protein translation	0.000023	90,26,7
1fhoa_#74	GO:0006118: electron transport	0.000023	315,228,50
1hx6a1#163	GO:0006917: induction of apoptosis	0.000024	16,29,4
1dy5a_#46	GO:0006418: tRNA aminoacylation for protein translation	0.000024	36,26,5
1f0xa1#278	GO:0042108: positive regulation of cytokine biosynthesis	0.000024	162,11,6
1bg6_2#73	GO:0006730: one-carbon compound metabolism	0.000024	29,32,5
1c4zd_#22	GO:0019079: viral genome replication	0.000024	60,16,5
1eq9a_#32	GO:0016337: cell-cell adhesion	0.000024	96,17,6

1epwa3#138	GO:0001906: cell killing	0.000024	371,11,8
1ojt_2#356	GO:0009070: serine family amino acid biosynthesis	0.000024	36,26,5
1fc4a_#205	GO:0006118: electron transport	0.000024	215,228,38
1fxla2#169	GO:0030217: T-cell differentiation	0.000024	184,10,6
1fgga_#134	GO:0009252: peptidoglycan biosynthesis	0.000024	193,18,8
1a53__#236	GO:0015671: oxygen transport	0.000024	20,23,4
1smaa2#581	GO:0043123: positive regulation of I-kappaB kinase/NF-kappaB cascade	0.000024	60,16,5
1jsg__#92	GO:0001906: cell killing	0.000024	519,11,9
1erv__#80	GO:0006457: protein folding	0.000024	401,49,20
1dhs__#99	GO:0006221: pyrimidine nucleotide biosynthesis	0.000024	109,22,7
1aqua_#126	GO:0006099: tricarboxylic acid cycle	0.000024	472,19,12
1aqua_#126	GO:0009064: glutamine family amino acid metabolism	0.000024	472,19,12
1qtn.1#A270	GO:0009064: glutamine family amino acid metabolism	0.000024	386,19,11
1a65a2#187	GO:0019079: viral genome replication	0.000024	29,16,4
3sil__#317	GO:0006457: protein folding	0.000024	300,49,17
1ax4a_#275	GO:0006461: protein complex assembly	0.000024	99,24,7
1e39a3#442	GO:0018108: peptidyl-tyrosine phosphorylation	0.000025	16,10,3
1i4fa2#21	GO:0043123: positive regulation of I-kappaB kinase/NF-kappaB cascade	0.000025	10,16,3
1hava_#141	GO:0019079: viral genome replication	0.000025	10,16,3
1g72a_#42	GO:0006118: electron transport	0.000025	12,228,7
1f0xa1#278	GO:0006606: protein-nucleus import	0.000025	162,21,8
1dfca1#1135	GO:0006606: protein-nucleus import	0.000025	45,21,5
1bfd_1#230	GO:0006563: L-serine metabolism	0.000025	40,12,4
1ac6a_#43	GO:0051249: regulation of lymphocyte activation	0.000025	147,12,6
1f3ba2#4	GO:0046677: response to antibiotic	0.000025	39,40,6
1aym3_#109	GO:0016477: cell migration	0.000025	41,23,5
1i05a_#132	GO:0019883: antigen presentation, endogenous antigen	0.000025	49,10,4
1jf9a_#379	GO:0050819: negative regulation of coagulation	0.000025	186,10,6
1jf9a_#379	GO:0030193: regulation of blood coagulation	0.000025	186,10,6
1jf9a_#379	GO:0018108: peptidyl-tyrosine phosphorylation	0.000025	186,10,6
1g51a1#47	GO:0006418: tRNA aminoacylation for protein translation	0.000025	18,26,4
1hava_#157	GO:0019079: viral genome replication	0.000025	10,16,3
1qlaa2#179	GO:0019883: antigen presentation, endogenous antigen	0.000025	49,10,4
1ifc__#89	GO:0042088: T-helper 1 type immune response	0.000025	164,11,6
1ifc__#89	GO:0001906: cell killing	0.000025	164,11,6
1g25a_#22	GO:0006457: protein folding	0.000025	367,49,19
1htp__#16	GO:0042108: positive regulation of cytokine biosynthesis	0.000025	256,11,7
1phm_2#253	GO:0001906: cell killing	0.000025	257,11,7
1a9xa3#78	GO:0006418: tRNA aminoacylation for protein translation	0.000025	262,26,11
1ex1a1#284	GO:0006779: porphyrin biosynthesis	0.000025	26,18,4
3sil__#98	GO:0018108: peptidyl-tyrosine phosphorylation	0.000025	186,10,6
1p35a_#49	GO:0006919: caspase activation	0.000025	112,15,6
1nat__#50	GO:0009064: glutamine family amino acid metabolism	0.000025	242,19,9
1f5ma_#80	GO:0016068: type I hypersensitivity	0.000025	112,15,6
1qfea_#68	GO:0009156: ribonucleoside monophosphate biosynthesis	0.000025	453,12,9
1fgka_#689	GO:0000086: G2/M transition of mitotic cell cycle	0.000025	16,10,3
1hdoa_#189	GO:0009103: lipopolysaccharide biosynthesis	0.000025	147,12,6
1hxn__#248	GO:0019079: viral genome replication	0.000025	10,16,3
1fmk_3#377	GO:0000086: G2/M transition of mitotic cell cycle	0.000025	16,10,3
1mh1__#37	GO:0007186: G-protein coupled receptor protein signaling pathway	0.000026	7,24,3
1nhp_1#7	GO:0006100: tricarboxylic acid cycle intermediate metabolism	0.000026	34,14,4
1heta2#212	GO:0000160: two-component signal transduction system (phosphorelay)	0.000026	38,25,5
1e2o__#346	GO:0050819: negative regulation of coagulation	0.000026	187,10,6
1e2o__#346	GO:0030193: regulation of blood coagulation	0.000026	187,10,6
1e2o__#346	GO:0018108: peptidyl-tyrosine phosphorylation	0.000026	187,10,6
1f42a2#123	GO:0006916: anti-apoptosis	0.000026	335,18,10

1dj0a2#183	GO:0006096: glycolysis	0.000026	111,38,9
1b4ka_#108	GO:0009396: folic acid and derivative biosynthesis	0.000026	94,11,5
1kit_3#743	GO:0019735: antimicrobial humoral response (sensu Vertebrata)	0.000026	225,16,8
1el0a_#42	GO:0001906: cell killing	0.000026	258,11,7
1qdla_#211	GO:0016337: cell-cell adhesion	0.000026	280,17,9
1h6la_#119	GO:0007169: transmembrane receptor protein tyrosine kinase signaling pathway	0.000026	264,26,11
1dn2a1#261	GO:0007169: transmembrane receptor protein tyrosine kinase signaling pathway	0.000026	376,26,13
1doza_#109	GO:0009070: serine family amino acid biosynthesis	0.000026	61,26,6
1hxxa_#39	GO:0001906: cell killing	0.000027	45,11,4
1dqaa1#596	GO:0000051: urea cycle intermediate metabolism	0.000027	50,10,4
1bfd_1#230	GO:0006118: electron transport	0.000027	40,228,13
1danh_#104	GO:0016477: cell migration	0.000027	147,23,8
1e87a_#174	GO:0006118: electron transport	0.000027	124,228,26
1fp5a2#537	GO:0042088: T-helper 1 type immune response	0.000027	45,11,4
1d0na6#681	GO:0006118: electron transport	0.000027	124,228,26
1bu8a2#124	GO:0006633: fatty acid biosynthesis	0.000027	123,27,8
1axn__#42	GO:0007601: visual perception	0.000027	66,15,5
1gdha1#235	GO:0009070: serine family amino acid biosynthesis	0.000027	92,26,7
1d2ka1#164	GO:0042401: biogenic amine biosynthesis	0.000027	50,10,4
1d2ka1#164	GO:0006568: tryptophan metabolism	0.000027	50,10,4
1e3a.1#A30	GO:0046677: response to antibiotic	0.000027	133,40,10
1ffya2#226	GO:0016477: cell migration	0.000028	42,23,5
1el5a1#10	GO:0006118: electron transport	0.000028	16,228,8
1ab4__#344	GO:0006457: protein folding	0.000028	4,49,3
1dlfl_#97	GO:0051249: regulation of lymphocyte activation	0.000028	41,12,4
2mpra_#267	GO:0007399: neurogenesis	0.000028	26,37,5
1buoa_#44	GO:0042088: T-helper 1 type immune response	0.000028	15,11,3
1buoa_#44	GO:0042108: positive regulation of cytokine biosynthesis	0.000028	15,11,3
2pola3#335	GO:0042088: T-helper 1 type immune response	0.000028	15,11,3
2napa2#444	GO:0006118: electron transport	0.000028	16,228,8
2cpl__#138	GO:0006457: protein folding	0.000028	4,49,3
1ctqa_#163	GO:0006935: chemotaxis	0.000028	12,41,4
1lkka_#127	GO:0008284: positive regulation of cell proliferation	0.000028	16,30,4
1d8ca_#442	GO:0009073: aromatic amino acid family biosynthesis	0.000028	30,16,4
1aym3_#109	GO:0051249: regulation of lymphocyte activation	0.000028	41,12,4
1hbza_#214	GO:0016477: cell migration	0.000028	249,23,10
1bxoa_#307	GO:0016337: cell-cell adhesion	0.000028	99,17,6
1fo0b_#82	GO:0042088: T-helper 1 type immune response	0.000028	15,11,3
1fo0b_#82	GO:0042108: positive regulation of cytokine biosynthesis	0.000028	15,11,3
1e69a_#26	GO:0009070: serine family amino acid biosynthesis	0.000028	37,26,5
1atg__#144	GO:0015698: inorganic anion transport	0.000028	72,14,5
1fm2.1#B308	GO:0006953: acute-phase response	0.000028	174,20,8
1qfxa_#380	GO:0051249: regulation of lymphocyte activation	0.000028	41,12,4
1mjha_#119	GO:0009064: glutamine family amino acid metabolism	0.000028	87,19,6
1f00i2#805	GO:0006953: acute-phase response	0.000028	124,20,7
1xgsa2#82	GO:0042088: T-helper 1 type immune response	0.000028	261,11,7
1rypb_#133	GO:0019079: viral genome replication	0.000028	30,16,4
1cnv__#264	GO:0007017: microtubule-based process	0.000028	86,12,5
1edza1#206	GO:0006118: electron transport	0.000028	352,228,54
1qgna_#213	GO:0009086: methionine biosynthesis	0.000029	2,14,2
1elua_#200	GO:0009086: methionine biosynthesis	0.000029	2,14,2
1b8pa1#30	GO:0006100: tricarboxylic acid cycle intermediate metabolism	0.000029	2,14,2
1ekma2#98	GO:0009086: methionine biosynthesis	0.000029	2,14,2
2sli_2#341	GO:0016567: protein ubiquitination	0.000029	2,14,2

1qi9a_#217	GO:0006100: tricarboxylic acid cycle intermediate metabolism	0.000029	2,14,2
1div_1#141	GO:0006118: electron transport	0.000029	70,228,18
1ek0a_#151	GO:0006606: protein-nucleus import	0.000029	23,21,4
1nsj__#21	GO:0043094: metabolic compound salvage	0.000029	96,11,5
1nsj__#21	GO:0006098: pentose-phosphate shunt	0.000029	96,11,5
1e2wa1#102	GO:0030333: antigen processing	0.000029	79,13,5
1epwa3#140	GO:0042088: T-helper 1 type immune response	0.000029	168,11,6
1epwa3#140	GO:0042108: positive regulation of cytokine biosynthesis	0.000029	168,11,6
1bbpa_#119	GO:0019883: antigen presentation, endogenous antigen	0.000029	51,10,4
1jb0d_#26	GO:0050819: negative regulation of coagulation	0.000029	190,10,6
1jb0d_#26	GO:0030193: regulation of blood coagulation	0.000029	190,10,6
1mspa_#17	GO:0030333: antigen processing	0.000029	38,13,4
1quqa_#75	GO:0030217: T-cell differentiation	0.000029	51,10,4
1bjt__#890	GO:0006935: chemotaxis	0.000029	258,41,14
1mek__#17	GO:0006221: pyrimidine nucleotide biosynthesis	0.000029	22,22,4
1h6kx_#97	GO:0006953: acute-phase response	0.000029	49,20,5
1io7a_#243	GO:0006118: electron transport	0.000029	70,228,18
1ds1a_#172	GO:0030217: T-cell differentiation	0.000029	190,10,6
1pwt__#8	GO:0042108: positive regulation of cytokine biosynthesis	0.000029	262,11,7
1gpea1#273	GO:0030333: antigen processing	0.000029	79,13,5
1mil__#31	GO:0001906: cell killing	0.000029	96,11,5
1mkp__#216	GO:0006221: pyrimidine nucleotide biosynthesis	0.000029	112,22,7
1qs0a1#119	GO:0006100: tricarboxylic acid cycle intermediate metabolism	0.000029	367,14,9
1qs0a1#119	GO:0009086: methionine biosynthesis	0.000029	367,14,9
1dcfa_#66	GO:0042364: water-soluble vitamin biosynthesis	0.00003	36,27,5
2hlpa2#298	GO:0006099: tricarboxylic acid cycle	0.00003	9,19,3
2bc2a_#46	GO:0017001: antibiotic catabolism	0.00003	17,10,3
1seia_#50	GO:0009167: purine ribonucleoside monophosphate metabolism	0.00003	17,10,3
1a9xa2#1009	GO:0006879: iron ion homeostasis	0.00003	14,12,3
1dzfa2#164	GO:0009156: ribonucleoside monophosphate biosynthesis	0.00003	14,12,3
1ew0a_#174	GO:0000160: two-component signal transduction system (phosphorelay)	0.00003	7,25,3
1cvl__#91	GO:0006099: tricarboxylic acid cycle	0.00003	9,19,3
2cmd_2#172	GO:0006099: tricarboxylic acid cycle	0.00003	9,19,3
1e2fa_#14	GO:0009116: nucleoside metabolism	0.00003	7,25,3
1ezvb2#271	GO:0009064: glutamine family amino acid metabolism	0.00003	9,19,3
7odca2#67	GO:0006207: 'de novo' pyrimidine base biosynthesis	0.00003	17,10,3
1hbza_#214	GO:0006919: caspase activation	0.00003	249,15,8
1nuka_#80	GO:0016477: cell migration	0.00003	197,23,9
2viua_#252	GO:0016337: cell-cell adhesion	0.00003	285,17,9
1b8aa2#368	GO:0006418: tRNA aminoacylation for protein translation	0.00003	130,26,8
1amf__#168	GO:0015698: inorganic anion transport	0.00003	12,14,3
1eg5a_#197	GO:0009086: methionine biosynthesis	0.00003	12,14,3
1ceza_#227	GO:0006265: DNA topological change	0.00003	17,10,3
1fds__#59	GO:0006098: pentose-phosphate shunt	0.00003	46,11,4
1dfoa_#86	GO:0009086: methionine biosynthesis	0.00003	12,14,3
1lci__#26	GO:0006418: tRNA aminoacylation for protein translation	0.00003	130,26,8
1pme__#166	GO:0000086: G2/M transition of mitotic cell cycle	0.00003	17,10,3
1bpv__#22	GO:0042113: B-cell activation	0.00003	19,9,3
1koba_#175	GO:0000086: G2/M transition of mitotic cell cycle	0.00003	17,10,3
1jsg__#92	GO:0006457: protein folding	0.00003	519,49,23
1gpea1#273	GO:0006606: protein-nucleus import	0.00003	79,21,6
1bec_2#216	GO:0030217: T-cell differentiation	0.00003	109,10,5
1e5da2#12	GO:0006118: electron transport	0.00003	83,228,20
1hbza_#96	GO:0007169: transmembrane receptor protein tyrosine kinase signaling pathway	0.00003	322,26,12
1h6la_#119	GO:0006544: glycine metabolism	0.00003	264,11,7

1iira_#242	GO:0006100: tricarboxylic acid cycle intermediate metabolism	0.00003	125,14,6
1ds9a_#105	GO:0006118: electron transport	0.000031	64,228,17
1by5a_#238	GO:0016337: cell-cell adhesion	0.000031	214,17,8
3chbd_#85	GO:0007169: transmembrane receptor protein tyrosine kinase signaling pathway	0.000031	323,26,12
1a9xa6#760	GO:0030333: antigen processing	0.000031	80,13,5
1ezvb1#29	GO:0051249: regulation of lymphocyte activation	0.000031	42,12,4
1czan1#92	GO:0006953: acute-phase response	0.000031	538,20,13
1e8ya1#648	GO:0045045: secretory pathway	0.000031	45,22,5
1kdj__#27	GO:0006118: electron transport	0.000031	64,228,17
2occb1#209	GO:0006118: electron transport	0.000031	90,228,21
1dih_2#222	GO:0006313: DNA transposition	0.000031	311,35,14
1hdma1#98	GO:0051249: regulation of lymphocyte activation	0.000031	42,12,4
1dn2a1#261	GO:0016477: cell migration	0.000031	376,23,12
1evqa_#305	GO:0043094: metabolic compound salvage	0.000031	384,11,8
1evqa_#305	GO:0006098: pentose-phosphate shunt	0.000031	384,11,8
1prea2#119	GO:0030333: antigen processing	0.000032	39,13,4
1ad1a_#186	GO:0006096: glycolysis	0.000032	26,38,5
1fyhb1#99	GO:0019735: antimicrobial humoral response (sensu Vertebrata)	0.000032	31,16,4
1doka_#52	GO:0019883: antigen presentation, endogenous antigen	0.000032	52,10,4
1doka_#52	GO:0030217: T-cell differentiation	0.000032	52,10,4
1huxa_#10	GO:0017001: antibiotic catabolism	0.000032	52,10,4
1hyea2#182	GO:0006099: tricarboxylic acid cycle	0.000032	26,19,4
1pud__#197	GO:0009073: aromatic amino acid family biosynthesis	0.000032	31,16,4
1fp1d2#214	GO:0006779: porphyrin biosynthesis	0.000032	143,18,7
1kit_3#743	GO:0006935: chemotaxis	0.000032	225,41,13
1e5ka_#113	GO:0018108: peptidyl-tyrosine phosphorylation	0.000032	52,10,4
1qtn.1#A270	GO:0006544: glycine metabolism	0.000032	386,11,8
1sppb_#32	GO:0006917: induction of apoptosis	0.000032	34,29,5
1eqga2#69	GO:0008015: circulation	0.000033	2,15,2
1f3ya_#6	GO:0006606: protein-nucleus import	0.000033	80,21,6
1eu1a2#185	GO:0006118: electron transport	0.000033	6,228,5
1trb_1#106	GO:0006118: electron transport	0.000033	6,228,5
1erza_#183	GO:0006098: pentose-phosphate shunt	0.000033	47,11,4
1ltda2#19	GO:0006118: electron transport	0.000033	6,228,5
1hlga_#324	GO:0016042: lipid catabolism	0.000033	6,31,3
1hxxa_#278	GO:0006367: transcription initiation from Pol II promoter	0.000033	237,12,7
1cpy__#380	GO:0051249: regulation of lymphocyte activation	0.000033	154,12,6
3grs_3#435	GO:0006367: transcription initiation from Pol II promoter	0.000033	154,12,6
1czan1#92	GO:0042108: positive regulation of cytokine biosynthesis	0.000033	538,11,9
1czan1#92	GO:0001906: cell killing	0.000033	538,11,9
1dj0a2#183	GO:0018108: peptidyl-tyrosine phosphorylation	0.000033	111,10,5
1a4ya_#47	GO:0006098: pentose-phosphate shunt	0.000033	172,11,6
1qhoa4#230	GO:0016042: lipid catabolism	0.000033	6,31,3
1qrra_#69	GO:0009070: serine family amino acid biosynthesis	0.000033	173,26,9
1ea5a_#99	GO:0001906: cell killing	0.000033	172,11,6
1cgha_#227	GO:0006544: glycine metabolism	0.000033	47,11,4
1ldna1#94	GO:0006221: pyrimidine nucleotide biosynthesis	0.000033	159,22,8
1ew2a_#237	GO:0006563: L-serine metabolism	0.000033	89,12,5
1el5a1#10	GO:0009396: folic acid and derivative biosynthesis	0.000034	16,11,3
1sfp__#84	GO:0007169: transmembrane receptor protein tyrosine kinase signaling pathway	0.000034	7,26,3
1cdy_2#140	GO:0007169: transmembrane receptor protein tyrosine kinase signaling pathway	0.000034	7,26,3
1jj2j_#70	GO:0007169: transmembrane receptor protein tyrosine kinase signaling pathway	0.000034	64,26,6
1ek1a2#500	GO:0006418: tRNA aminoacylation for protein translation	0.000034	7,26,3

1ile_2#359	GO:0006418: tRNA aminoacylation for protein translation	0.000034	7,26,3
1dik_1#848	GO:0006096: glycolysis	0.000034	64,38,7
1smaa1#95	GO:0006606: protein-nucleus import	0.000034	223,21,9
1thg__#3	GO:0006953: acute-phase response	0.000034	85,20,6
1hcc__#39	GO:0043123: positive regulation of I-kappaB kinase/NF-kappaB cascade	0.000034	11,16,3
1jp3a_#22	GO:0009116: nucleoside metabolism	0.000034	40,25,5
1eaja_#59	GO:0006265: DNA topological change	0.000034	196,10,6
1dpja_#322	GO:0042108: positive regulation of cytokine biosynthesis	0.000034	269,11,7
2hhma_#262	GO:0006470: protein amino acid dephosphorylation	0.000034	85,20,6
1p35a_#49	GO:0019883: antigen presentation, endogenous antigen	0.000034	112,10,5
1p35a_#49	GO:0050819: negative regulation of coagulation	0.000034	112,10,5
1p35a_#49	GO:0030193: regulation of blood coagulation	0.000034	112,10,5
1p35a_#49	GO:0018108: peptidyl-tyrosine phosphorylation	0.000034	112,10,5
1b35b_#97	GO:0006418: tRNA aminoacylation for protein translation	0.000034	271,26,11
2msba_#218	GO:0006633: fatty acid biosynthesis	0.000034	37,27,5
1f5ma_#80	GO:0019883: antigen presentation, endogenous antigen	0.000034	112,10,5
1dfoa_#362	GO:0019883: antigen presentation, endogenous antigen	0.000034	53,10,4
1ax4a_#430	GO:0000051: urea cycle intermediate metabolism	0.000034	53,10,4
1ax4a_#430	GO:0006207: 'de novo' pyrimidine base biosynthesis	0.000034	53,10,4
1ddja_#746	GO:0006606: protein-nucleus import	0.000034	121,21,7
1quna1#113	GO:0006118: electron transport	0.000034	243,228,41
1gpea1#271	GO:0030217: T-cell differentiation	0.000034	196,10,6
1e42a1#711	GO:0016337: cell-cell adhesion	0.000035	155,17,7
1esma_#314	GO:0042108: positive regulation of cytokine biosynthesis	0.000035	100,11,5
1esma_#314	GO:0006544: glycine metabolism	0.000035	100,11,5
1qvba_#206	GO:0030245: cellulose catabolism	0.000035	23,22,4
7fd1a_#17	GO:0006118: electron transport	0.000035	9,228,6
1hqoa2#159	GO:0006118: electron transport	0.000035	9,228,6
1nsj__#21	GO:0006779: porphyrin biosynthesis	0.000035	96,18,6
1a28a_#688	GO:0015671: oxygen transport	0.000035	22,23,4
1nuka_#80	GO:0030217: T-cell differentiation	0.000035	197,10,6
1ej8a_#189	GO:0006730: one-carbon compound metabolism	0.000035	256,32,12
4kbpa2#416	GO:0009206: purine ribonucleoside triphosphate biosynthesis	0.000035	20,9,3
1hw5a2#64	GO:0006118: electron transport	0.000035	148,228,29
3prn__#145	GO:0016337: cell-cell adhesion	0.000035	155,17,7
1d8ca_#218	GO:0030435: sporulation	0.000035	22,23,4
1jlna_#437	GO:0006470: protein amino acid dephosphorylation	0.000035	51,20,5
1fmta2#151	GO:0006418: tRNA aminoacylation for protein translation	0.000035	272,26,11
1seta2#231	GO:0006418: tRNA aminoacylation for protein translation	0.000035	96,26,7
1fsu__#434	GO:0016477: cell migration	0.000035	315,23,11
1mil__#31	GO:0007169: transmembrane receptor protein tyrosine kinase signaling pathway	0.000035	96,26,7
1jg8a_#172	GO:0009116: nucleoside metabolism	0.000035	67,25,6
1bwda_#295	GO:0006418: tRNA aminoacylation for protein translation	0.000035	96,26,7
1qtn.1#A270	GO:0006418: tRNA aminoacylation for protein translation	0.000035	386,26,13
1nsca_#91	GO:0016477: cell migration	0.000035	22,23,4
1e79d2#36	GO:0008284: positive regulation of cell proliferation	0.000035	432,30,15
2bpa1_#45	GO:0006953: acute-phase response	0.000035	179,20,8
1dn2a1#261	GO:0006457: protein folding	0.000035	376,49,19
1e6pa2#46	GO:0006221: pyrimidine nucleotide biosynthesis	0.000035	77,22,6
1nsj__#178	GO:0030245: cellulose catabolism	0.000035	77,22,6
1nsj__#178	GO:0006221: pyrimidine nucleotide biosynthesis	0.000035	77,22,6
1acc__#206	GO:0009167: purine ribonucleoside monophosphate metabolism	0.000036	18,10,3
1f97a1#62	GO:0007169: transmembrane receptor protein tyrosine kinase signaling pathway	0.000036	591,26,16
1fgka_#610	GO:0000086: G2/M transition of mitotic cell cycle	0.000036	18,10,3

1d6ja_#36	GO:0009219: pyrimidine deoxyribonucleotide metabolism	0.000036	18,10,3
1fjja_#5	GO:0030217: T-cell differentiation	0.000036	18,10,3
1qqsa_#124	GO:0001906: cell killing	0.000036	392,11,8
1qhoa1#531	GO:0030217: T-cell differentiation	0.000036	18,10,3
1qs1a2#437	GO:0007169: transmembrane receptor protein tyrosine kinase signaling pathway	0.000036	387,26,13
1g6oa_#116	GO:0006730: one-carbon compound metabolism	0.000036	302,32,13
3lada1#116	GO:0009070: serine family amino acid biosynthesis	0.000036	133,26,8
1bmv2_#2064	GO:0007169: transmembrane receptor protein tyrosine kinase signaling pathway	0.000036	133,26,8
1abrb2#252	GO:0008284: positive regulation of cell proliferation	0.000036	17,30,4
1a0la_#160	GO:0042088: T-helper 1 type immune response	0.000036	48,11,4
1a0la_#160	GO:0042108: positive regulation of cytokine biosynthesis	0.000036	48,11,4
3mag__#134	GO:0042364: water-soluble vitamin biosynthesis	0.000036	62,27,6
1hd2a_#35	GO:0006096: glycolysis	0.000036	145,38,10
1e3a.1#A30	GO:0006418: tRNA aminoacylation for protein translation	0.000036	133,26,8
1rypb_#114	GO:0006418: tRNA aminoacylation for protein translation	0.000036	133,26,8
1dp0a4#756	GO:0016477: cell migration	0.000036	153,23,8
1bio__#210	GO:0018108: peptidyl-tyrosine phosphorylation	0.000036	113,10,5
1evya2#38	GO:0006826: iron ion transport	0.000037	12,15,3
1cfb_2#763	GO:0016477: cell migration	0.000037	8,23,3
1azza_#51	GO:0006956: complement activation	0.000037	15,12,3
1qbea_#92	GO:0006919: caspase activation	0.000037	12,15,3
1a99a_#84	GO:0006879: iron ion homeostasis	0.000037	15,12,3
1i6vd_#1283	GO:0006919: caspase activation	0.000037	120,15,6
1gdea_#243	GO:0030217: T-cell differentiation	0.000037	54,10,4
1kit_3#331	GO:0042088: T-helper 1 type immune response	0.000037	101,11,5
1chma2#262	GO:0006826: iron ion transport	0.000037	12,15,3
1amf__#168	GO:0006826: iron ion transport	0.000037	12,15,3
1qaxa2#307	GO:0006606: protein-nucleus import	0.000037	49,21,5
1hxxa_#107	GO:0006367: transcription initiation from Pol II promoter	0.000037	15,12,3
1qgub_#197	GO:0007264: small GTPase mediated signal transduction	0.000037	49,21,5
1d6ja_#95	GO:0009167: purine ribonucleoside monophosphate metabolism	0.000037	54,10,4
1gks__#70	GO:0000910: cytokinesis	0.000037	45,37,6
1danh_#190	GO:0006956: complement activation	0.000037	15,12,3
1h8d.1#H207	GO:0006606: protein-nucleus import	0.000037	49,21,5
1e5da2#12	GO:0030333: antigen processing	0.000037	83,13,5
1ixh__#180	GO:0006826: iron ion transport	0.000037	12,15,3
1ton__#112	GO:0006956: complement activation	0.000037	15,12,3
1ton__#91	GO:0006956: complement activation	0.000037	15,12,3
1gg6.1#C229	GO:0006956: complement activation	0.000037	15,12,3
1ton__#181	GO:0006606: protein-nucleus import	0.000037	49,21,5
1dt9a1#209	GO:0009064: glutamine family amino acid metabolism	0.000038	92,19,6
1pii_1#48	GO:0009073: aromatic amino acid family biosynthesis	0.000038	2,16,2
2fbjh2#141	GO:0030333: antigen processing	0.000038	14,13,3
1qdla_#103	GO:0009073: aromatic amino acid family biosynthesis	0.000038	2,16,2
1g62a_#111	GO:0006413: translational initiation	0.000038	2,16,2
1im4a_#144	GO:0006096: glycolysis	0.000038	89,38,8
1qdla_#183	GO:0009073: aromatic amino acid family biosynthesis	0.000038	2,16,2
1dn2a1#308	GO:0030333: antigen processing	0.000038	14,13,3
1g62a_#61	GO:0006413: translational initiation	0.000038	2,16,2
1i50b_#334	GO:0007186: G-protein coupled receptor protein signaling pathway	0.000038	71,24,6
1smaa1#95	GO:0007169: transmembrane receptor protein tyrosine kinase signaling pathway	0.000038	223,26,10
1ac6a_#43	GO:0006916: anti-apoptosis	0.000038	147,18,7
1bura1#328	GO:0006221: pyrimidine nucleotide biosynthesis	0.000038	78,22,6
1fjfl_#67	GO:0006461: protein complex assembly	0.000038	147,24,8

1ddja_#726	GO:0006606: protein-nucleus import	0.000038	82,21,6
1bn8a_#241	GO:0006100: tricarboxylic acid cycle intermediate metabolism	0.000038	13,14,3
1ayl__#237	GO:0030217: T-cell differentiation	0.000038	313,10,7
1hxn__#235	GO:0019079: viral genome replication	0.000038	2,16,2
1b8aa2#403	GO:0030217: T-cell differentiation	0.000038	114,10,5
1eqka_#47	GO:0019883: antigen presentation, endogenous antigen	0.000038	114,10,5
1g62a_#60	GO:0006413: translational initiation	0.000038	2,16,2
1ihua2#343	GO:0006098: pentose-phosphate shunt	0.000038	176,11,6
1qfea_#68	GO:0006418: tRNA aminoacylation for protein translation	0.000038	453,26,14
1nsca_#236	GO:0006917: induction of apoptosis	0.000038	156,29,9
1erza_#183	GO:0030245: cellulose catabolism	0.000039	47,22,5
1im4a_#153	GO:0046677: response to antibiotic	0.000039	42,40,6
1heta2#212	GO:0042364: water-soluble vitamin biosynthesis	0.000039	38,27,5
1bccb2#274	GO:0006470: protein amino acid dephosphorylation	0.000039	52,20,5
1dgw.1#Y353	GO:0016337: cell-cell adhesion	0.000039	294,17,9
1nsca_#379	GO:0030217: T-cell differentiation	0.000039	115,10,5
1cmia_#86	GO:0042088: T-helper 1 type immune response	0.000039	49,11,4
1ft9a1#201	GO:0051249: regulation of lymphocyte activation	0.000039	92,12,5
1dr9a1#39	GO:0006418: tRNA aminoacylation for protein translation	0.000039	224,26,10
1gdha1#235	GO:0006563: L-serine metabolism	0.000039	92,12,5
1e5ka_#113	GO:0006953: acute-phase response	0.000039	52,20,5
1a9xb2#1775	GO:0009064: glutamine family amino acid metabolism	0.000039	55,19,5
1ddja_#746	GO:0006919: caspase activation	0.000039	121,15,6
1evqa_#305	GO:0030435: sporulation	0.000039	384,23,12
1kp6a_#77	GO:0030333: antigen processing	0.00004	41,13,4
1gof_2#118	GO:0006096: glycolysis	0.00004	14,38,4
1g6za_#57	GO:0006935: chemotaxis	0.00004	5,41,3
1hjra_#58	GO:0006096: glycolysis	0.00004	14,38,4
1i12a_#126	GO:0006096: glycolysis	0.00004	14,38,4
1exh__#44	GO:0030333: antigen processing	0.00004	41,13,4
1e4ea2#215	GO:0006118: electron transport	0.00004	419,228,61
1wdna_#79	GO:0006544: glycine metabolism	0.000041	17,11,3
1hxxa_#39	GO:0006367: transcription initiation from Pol II promoter	0.000041	45,12,4
1hxxa_#39	GO:0006956: complement activation	0.000041	45,12,4
1a7ca_#204	GO:0001906: cell killing	0.000041	17,11,3
1eg9a2#199	GO:0000160: two-component signal transduction system (phosphorelay)	0.000041	69,25,6
1fyhb1#99	GO:0016337: cell-cell adhesion	0.000041	31,17,4
1gtra1#468	GO:0007264: small GTPase mediated signal transduction	0.000041	50,21,5
1jp3a_#22	GO:0009070: serine family amino acid biosynthesis	0.000041	40,26,5
1as4.1#A336	GO:0042088: T-helper 1 type immune response	0.000041	276,11,7
1jb3a_#64	GO:0006461: protein complex assembly	0.000041	247,24,10
1hlwa_#82	GO:0030333: antigen processing	0.000041	221,13,7
1heta2#218	GO:0009116: nucleoside metabolism	0.000041	21,25,4
1d0na3#305	GO:0001906: cell killing	0.000041	276,11,7
1befa_#51	GO:0006606: protein-nucleus import	0.000041	9,21,3
1d3va_#176	GO:0009116: nucleoside metabolism	0.000041	21,25,4
1hdoa_#189	GO:0006096: glycolysis	0.000041	147,38,10
1ge8a2#134	GO:0045595: regulation of cell differentiation	0.000041	202,10,6
1ak5_1#353	GO:0006096: glycolysis	0.000042	66,38,7
2cpl__#20	GO:0006457: protein folding	0.000042	90,49,9
1lucb_#32	GO:0006221: pyrimidine nucleotide biosynthesis	0.000042	24,22,4
1czya1#466	GO:0042108: positive regulation of cytokine biosynthesis	0.000042	104,11,5
1el0a_#28	GO:0008285: negative regulation of cell proliferation	0.000042	140,19,7
3lada1#116	GO:0009086: methionine biosynthesis	0.000042	133,14,6
1djna1#86	GO:0006096: glycolysis	0.000042	66,38,7
1a8i__#688	GO:0006568: tryptophan metabolism	0.000042	203,10,6

1qtn.1#A270	GO:0000910: cytokinesis	0.000042	386,37,16
2bpa1_#45	GO:0001906: cell killing	0.000042	179,11,6
1a53__#64	GO:0042401: biogenic amine biosynthesis	0.000043	3,10,2
1a53__#64	GO:0006568: tryptophan metabolism	0.000043	3,10,2
1bqya_#178	GO:0050819: negative regulation of coagulation	0.000043	3,10,2
1bqya_#178	GO:0030193: regulation of blood coagulation	0.000043	3,10,2
1a02n1#588	GO:0045893: positive regulation of transcription, DNA-dependent	0.000043	3,10,2
1mml__#97	GO:0006278: RNA-dependent DNA replication	0.000043	3,10,2
1d0ba_#191	GO:0009070: serine family amino acid biosynthesis	0.000043	99,26,7
1ev7a_#291	GO:0019059: initiation of viral infection	0.000043	3,10,2
1nsj__#190	GO:0042401: biogenic amine biosynthesis	0.000043	3,10,2
1nsj__#190	GO:0006568: tryptophan metabolism	0.000043	3,10,2
1a0ca_#273	GO:0009064: glutamine family amino acid metabolism	0.000043	10,19,3
1g57a_#122	GO:0050819: negative regulation of coagulation	0.000043	3,10,2
1g57a_#122	GO:0030193: regulation of blood coagulation	0.000043	3,10,2
3pah__#286	GO:0006559: L-phenylalanine catabolism	0.000043	19,10,3
1a4ia1#208	GO:0006207: 'de novo' pyrimidine base biosynthesis	0.000043	19,10,3
1qdlb_#66	GO:0042401: biogenic amine biosynthesis	0.000043	3,10,2
1qdlb_#66	GO:0006568: tryptophan metabolism	0.000043	3,10,2
1ec7a1#216	GO:0015976: carbon utilization	0.000043	56,10,4
1ab4__#164	GO:0006265: DNA topological change	0.000043	3,10,2
1a59__#341	GO:0006099: tricarboxylic acid cycle	0.000043	28,19,4
1fjsa_#28	GO:0050819: negative regulation of coagulation	0.000043	3,10,2
1fjsa_#28	GO:0030193: regulation of blood coagulation	0.000043	3,10,2
1fjsa_#28	GO:0018108: peptidyl-tyrosine phosphorylation	0.000043	3,10,2
1c5y.1#B18	GO:0050819: negative regulation of coagulation	0.000043	3,10,2
1c5y.1#B18	GO:0030193: regulation of blood coagulation	0.000043	3,10,2
1qlca_#210	GO:0030217: T-cell differentiation	0.000043	19,10,3
1b4aa1#50	GO:0000051: urea cycle intermediate metabolism	0.000043	3,10,2
1jf9a_#379	GO:0006919: caspase activation	0.000043	186,15,7
1f2la_#40	GO:0016477: cell migration	0.000043	206,23,9
2hlca_#134	GO:0050819: negative regulation of coagulation	0.000043	19,10,3
2hlca_#134	GO:0030193: regulation of blood coagulation	0.000043	19,10,3
1c9la2#61	GO:0016477: cell migration	0.000043	206,23,9
1g0sa_#74	GO:0051249: regulation of lymphocyte activation	0.000043	247,12,7
1efpa1#32	GO:0009073: aromatic amino acid family biosynthesis	0.000043	114,16,6
1a4ya_#47	GO:0009073: aromatic amino acid family biosynthesis	0.000043	172,16,7
1jkma_#317	GO:0006099: tricarboxylic acid cycle	0.000043	56,19,5
1b4ka_#108	GO:0009103: lipopolysaccharide biosynthesis	0.000043	94,12,5
1bpv__#22	GO:0051049: regulation of transport	0.000043	19,10,3
3sil__#98	GO:0006919: caspase activation	0.000043	186,15,7
1f3mc_#403	GO:0000086: G2/M transition of mitotic cell cycle	0.000043	19,10,3
2reb_1#138	GO:0006207: 'de novo' pyrimidine base biosynthesis	0.000043	56,10,4
1erv__#80	GO:0001906: cell killing	0.000043	401,11,8
1ryp1_#44	GO:0009064: glutamine family amino acid metabolism	0.000043	10,19,3
1eur__#302	GO:0006919: caspase activation	0.000044	36,15,4
1be9a_#357	GO:0006418: tRNA aminoacylation for protein translation	0.000044	67,26,6
1a9xa3#78	GO:0006730: one-carbon compound metabolism	0.000044	262,32,12
1xnb__#149	GO:0009252: peptidoglycan biosynthesis	0.000044	150,18,7
1fp1d2#214	GO:0009116: nucleoside metabolism	0.000044	143,25,8
1pwt__#8	GO:0016477: cell migration	0.000044	262,23,10
1hq8a_#206	GO:0006457: protein folding	0.000044	281,49,16
1c9oa_#49	GO:0030333: antigen processing	0.000044	86,13,5
1cnv__#264	GO:0017148: negative regulation of protein biosynthesis	0.000044	86,13,5
1ihua2#525	GO:0009252: peptidoglycan biosynthesis	0.000044	278,18,9
1ihua2#525	GO:0006779: porphyrin biosynthesis	0.000044	278,18,9

1i76a_#138	GO:0006221: pyrimidine nucleotide biosynthesis	0.000044	80,22,6
1qrra_#11	GO:0006563: L-serine metabolism	0.000045	16,12,3
1f0xa1#278	GO:0051249: regulation of lymphocyte activation	0.000045	162,12,6
1nsca_#379	GO:0019735: antimicrobial humoral response (sensu Vertebrata)	0.000045	115,16,6
1gdna_#56	GO:0006956: complement activation	0.000045	16,12,3
1qnja_#118	GO:0006956: complement activation	0.000045	16,12,3
1f00i2#805	GO:0006919: caspase activation	0.000045	124,15,6
1gdna_#123	GO:0006956: complement activation	0.000045	16,12,3
1ejba_#81	GO:0009116: nucleoside metabolism	0.000045	70,25,6
1ac5__#72	GO:0043094: metabolic compound salvage	0.000045	51,11,4
1qdla_#211	GO:0042088: T-helper 1 type immune response	0.000045	280,11,7
1qdla_#211	GO:0042108: positive regulation of cytokine biosynthesis	0.000045	280,11,7
1fgua1#249	GO:0006367: transcription initiation from Pol II promoter	0.000045	16,12,3
1c5y.1#B234	GO:0006956: complement activation	0.000045	16,12,3
1autc_#209	GO:0006956: complement activation	0.000045	16,12,3
1b8ba_#466	GO:0006606: protein-nucleus import	0.000046	51,21,5
1jj2s_#46	GO:0043123: positive regulation of I-kappaB kinase/NF-kappaB cascade	0.000046	34,16,4
1f2la_#40	GO:0050819: negative regulation of coagulation	0.000046	206,10,6
1f2la_#40	GO:0030193: regulation of blood coagulation	0.000046	206,10,6
1f2la_#40	GO:0006275: regulation of DNA replication	0.000046	206,10,6
2mpra_#190	GO:0006118: electron transport	0.000046	349,228,53
1quqa_#75	GO:0007264: small GTPase mediated signal transduction	0.000046	51,21,5
1hc7a2#258	GO:0006953: acute-phase response	0.000046	186,20,8
3prn__#143	GO:0016337: cell-cell adhesion	0.000046	300,17,9
1a9xa6#760	GO:0006730: one-carbon compound metabolism	0.000046	80,32,7
1fp2a2#346	GO:0008284: positive regulation of cell proliferation	0.000046	18,30,4
1hq8a_#206	GO:0001906: cell killing	0.000046	281,11,7
1qqp1_#51	GO:0006118: electron transport	0.000046	31,228,11
1ac6a_#34	GO:0045595: regulation of cell differentiation	0.000046	57,10,4
1ddwa_#77	GO:0006275: regulation of DNA replication	0.000046	322,10,7
1mdah_#329	GO:0006457: protein folding	0.000046	282,49,16
1wdna_#79	GO:0006730: one-carbon compound metabolism	0.000047	17,32,4
2mpra_#267	GO:0006935: chemotaxis	0.000047	26,41,5
1mla_1#281	GO:0006096: glycolysis	0.000047	28,38,5
1fid__#237	GO:0006418: tRNA aminoacylation for protein translation	0.000047	41,26,5
1j79a_#38	GO:0006096: glycolysis	0.000047	28,38,5
1ewna_#109	GO:0019079: viral genome replication	0.000047	69,16,5
3chbd_#85	GO:0030217: T-cell differentiation	0.000047	323,10,7
1h61a_#176	GO:0006096: glycolysis	0.000047	28,38,5
1hxxa_#232	GO:0042108: positive regulation of cytokine biosynthesis	0.000047	282,11,7
1beba_#119	GO:0046677: response to antibiotic	0.000047	172,40,11
1aqua_#126	GO:0006207: 'de novo' pyrimidine base biosynthesis	0.000047	472,10,8
1ihua2#343	GO:0007264: small GTPase mediated signal transduction	0.000047	176,21,8
1qhqa_#45	GO:0006544: glycine metabolism	0.000047	106,11,5
1fhoa_#74	GO:0006457: protein folding	0.000047	315,49,17
1mdah_#329	GO:0001906: cell killing	0.000047	282,11,7
1fvua_#13	GO:0006457: protein folding	0.000048	22,49,5
2pola3#335	GO:0017148: negative regulation of protein biosynthesis	0.000048	15,13,3
1dqwa_#169	GO:0009116: nucleoside metabolism	0.000048	8,25,3
1gox__#334	GO:0006221: pyrimidine nucleotide biosynthesis	0.000048	9,22,3
1i2oa_#34	GO:0009252: peptidoglycan biosynthesis	0.000048	152,18,7
1nsj__#21	GO:0009156: ribonucleoside monophosphate biosynthesis	0.000048	96,12,5
1i6vd_#1283	GO:0018108: peptidyl-tyrosine phosphorylation	0.000048	120,10,5
1nsca_#306	GO:0007169: transmembrane receptor protein tyrosine kinase signaling pathway	0.000048	21,26,4
3btaa3#194	GO:0030333: antigen processing	0.000048	43,13,4

2sli_2#326	GO:0016337: cell-cell adhesion	0.000048	227,17,8
1fo0b_#82	GO:0030333: antigen processing	0.000048	15,13,3
1jb3a_#64	GO:0006953: acute-phase response	0.000048	247,20,9
1bywa_#30	GO:0006544: glycine metabolism	0.000048	283,11,7
1bywa_#30	GO:0001906: cell killing	0.000048	283,11,7
1boub_#7	GO:0009086: methionine biosynthesis	0.000048	206,14,7
1gai__#22	GO:0030245: cellulose catabolism	0.000048	9,22,3
1dp0a1#309	GO:0051249: regulation of lymphocyte activation	0.000048	47,12,4
1i1b__#10	GO:0030333: antigen processing	0.000048	15,13,3
1ae9a_#272	GO:0006313: DNA transposition	0.000049	6,35,3
1ah7__#55	GO:0019835: cytolysis	0.000049	2,18,2
1qlaa3#364	GO:0051049: regulation of transport	0.000049	58,10,4
1qlaa3#364	GO:0050819: negative regulation of coagulation	0.000049	58,10,4
1qlaa3#364	GO:0030193: regulation of blood coagulation	0.000049	58,10,4
7ahla_#32	GO:0019835: cytolysis	0.000049	2,18,2
1ah7__#184	GO:0019835: cytolysis	0.000049	2,18,2
1lvl_2#222	GO:0006207: 'de novo' pyrimidine base biosynthesis	0.000049	58,10,4
1a31a1#512	GO:0006313: DNA transposition	0.000049	6,35,3
1jb0d_#26	GO:0008015: circulation	0.000049	190,15,7
1e44b_#75	GO:0006457: protein folding	0.000049	165,49,12
1fxla2#169	GO:0042108: positive regulation of cytokine biosynthesis	0.000049	184,11,6
1jlna_#383	GO:0006096: glycolysis	0.000049	46,38,6
1e3ua_#67	GO:0006313: DNA transposition	0.000049	6,35,3
1seta2#231	GO:0009064: glutamine family amino acid metabolism	0.000049	96,19,6
1hrka_#205	GO:0007186: G-protein coupled receptor protein signaling pathway	0.000049	45,24,5
1fu6a_#50	GO:0016477: cell migration	0.000049	265,23,10
3grs_1#23	GO:0006418: tRNA aminoacylation for protein translation	0.000049	139,26,8
1utea_#11	GO:0001906: cell killing	0.000049	107,11,5
1qmha2#136	GO:0006953: acute-phase response	0.00005	10,20,3
1jb9a2#222	GO:0006118: electron transport	0.00005	13,228,7
1fjja_#5	GO:0042088: T-helper 1 type immune response	0.00005	18,11,3
1fjja_#5	GO:0042108: positive regulation of cytokine biosynthesis	0.00005	18,11,3
1fwxa2#396	GO:0050819: negative regulation of coagulation	0.00005	209,10,6
1fwxa2#396	GO:0030193: regulation of blood coagulation	0.00005	209,10,6
1fwxa2#396	GO:0018108: peptidyl-tyrosine phosphorylation	0.00005	209,10,6
1fwxa2#396	GO:0030217: T-cell differentiation	0.00005	209,10,6
1qhoa1#531	GO:0042088: T-helper 1 type immune response	0.00005	18,11,3
1qhoa1#531	GO:0042108: positive regulation of cytokine biosynthesis	0.00005	18,11,3
1a8l_1#74	GO:0001906: cell killing	0.00005	18,11,3
1hlwa_#91	GO:0009064: glutamine family amino acid metabolism	0.00005	29,19,4
1iqpa2#119	GO:0006779: porphyrin biosynthesis	0.00005	11,18,3
1ddja_#746	GO:0018108: peptidyl-tyrosine phosphorylation	0.00005	121,10,5
1e69a_#40	GO:0006207: 'de novo' pyrimidine base biosynthesis	0.00005	20,10,3
1hvba_#63	GO:0009252: peptidoglycan biosynthesis	0.00005	11,18,3
1g0sa_#74	GO:0019735: antimicrobial humoral response (sensu Vertebrata)	0.000051	247,16,8
1aqua_#126	GO:0006470: protein amino acid dephosphorylation	0.000051	472,20,12
1hd2a_#35	GO:0006730: one-carbon compound metabolism	0.000051	145,32,9
1eh9a3#409	GO:0009396: folic acid and derivative biosynthesis	0.000052	3,11,2
1fo8a_#367	GO:0006544: glycine metabolism	0.000052	3,11,2
1tpg_1#55	GO:0006690: icosanoid metabolism	0.000052	3,11,2
2aak__#77	GO:0006511: ubiquitin-dependent protein catabolism	0.000052	3,11,2
1a02n1#588	GO:0042088: T-helper 1 type immune response	0.000052	3,11,2
1a02n1#588	GO:0042108: positive regulation of cytokine biosynthesis	0.000052	3,11,2
1pma1_#120	GO:0006511: ubiquitin-dependent protein catabolism	0.000052	3,11,2
1fvia2#25	GO:0019735: antimicrobial humoral response (sensu Vertebrata)	0.000052	35,16,4
1nal1_#203	GO:0009073: aromatic amino acid family biosynthesis	0.000052	35,16,4

1ryp2_#177	GO:0006511: ubiquitin-dependent protein catabolism	0.000052	3,11,2
1qsma_#95	GO:0016568: chromatin modification	0.000052	3,11,2
1rypl_#150	GO:0006511: ubiquitin-dependent protein catabolism	0.000052	3,11,2
1jf9a_#379	GO:0042088: T-helper 1 type immune response	0.000052	186,11,6
1jf9a_#379	GO:0042108: positive regulation of cytokine biosynthesis	0.000052	186,11,6
1pmaa_#141	GO:0006511: ubiquitin-dependent protein catabolism	0.000052	3,11,2
1dyr__#180	GO:0006544: glycine metabolism	0.000052	3,11,2
1a81a1#51	GO:0006461: protein complex assembly	0.000052	75,24,6
1f9va_#627	GO:0006118: electron transport	0.000052	48,228,14
1h72c2#269	GO:0000051: urea cycle intermediate metabolism	0.000053	59,10,4
1enfa2#162	GO:0030333: antigen processing	0.000053	44,13,4
1in4a1#264	GO:0006418: tRNA aminoacylation for protein translation	0.000053	42,26,5
1jj22_#78	GO:0006354: RNA elongation	0.000053	59,10,4
1pdc__#27	GO:0006544: glycine metabolism	0.000053	53,11,4
1apme_#68	GO:0006118: electron transport	0.000053	93,228,21
1pvc1_#106	GO:0042108: positive regulation of cytokine biosynthesis	0.000053	53,11,4
1ft9a1#201	GO:0006953: acute-phase response	0.000053	92,20,6
1ed5a_#294	GO:0006544: glycine metabolism	0.000053	53,11,4
1tbrr1#15	GO:0030217: T-cell differentiation	0.000053	59,10,4
1fc4a_#205	GO:0006779: porphyrin biosynthesis	0.000053	215,18,8
3grs_2#211	GO:0009070: serine family amino acid biosynthesis	0.000053	42,26,5
1bfd_2#146	GO:0006118: electron transport	0.000053	93,228,21
1ddma_#130	GO:0006457: protein folding	0.000053	285,49,16
1a0la_#160	GO:0006956: complement activation	0.000053	48,12,4
1bec_2#216	GO:0042088: T-helper 1 type immune response	0.000053	109,11,5
1bec_2#216	GO:0042108: positive regulation of cytokine biosynthesis	0.000053	109,11,5
1fu6a_#50	GO:0007399: neurogenesis	0.000053	265,37,13
1feca1#147	GO:0042742: defense response to bacteria	0.000054	8,26,3
1ej8a_#189	GO:0051249: regulation of lymphocyte activation	0.000054	256,12,7
1e2o__#346	GO:0001906: cell killing	0.000054	187,11,6
1flga_#161	GO:0006919: caspase activation	0.000054	128,15,6
1cvl__#105	GO:0009070: serine family amino acid biosynthesis	0.000054	8,26,3
1htp__#16	GO:0051249: regulation of lymphocyte activation	0.000054	256,12,7
7reqa2#702	GO:0000160: two-component signal transduction system (phosphorelay)	0.000054	44,25,5
1ddja_#681	GO:0018108: peptidyl-tyrosine phosphorylation	0.000054	212,10,6
3grs_1#23	GO:0006100: tricarboxylic acid cycle intermediate metabolism	0.000054	139,14,6
1h6va2#245	GO:0006221: pyrimidine nucleotide biosynthesis	0.000054	83,22,6
1e6ca_#91	GO:0009070: serine family amino acid biosynthesis	0.000054	141,26,8
1hdoa_#189	GO:0000160: two-component signal transduction system (phosphorelay)	0.000054	147,25,8
2fcba2#104	GO:0016337: cell-cell adhesion	0.000055	12,17,3
1qdla_#103	GO:0009064: glutamine family amino acid metabolism	0.000055	2,19,2
1gph12#46	GO:0009064: glutamine family amino acid metabolism	0.000055	2,19,2
1qi9a_#217	GO:0006099: tricarboxylic acid cycle	0.000055	2,19,2
1seia_#50	GO:0009156: ribonucleoside monophosphate biosynthesis	0.000055	17,12,3
1qdla_#183	GO:0009064: glutamine family amino acid metabolism	0.000055	2,19,2
1iira_#367	GO:0006099: tricarboxylic acid cycle	0.000055	2,19,2
1tmy__#14	GO:0006935: chemotaxis	0.000055	14,41,4
1dfma_#147	GO:0009307: DNA restriction	0.000055	2,19,2
1byb__#327	GO:0030435: sporulation	0.000055	9,23,3
2hlca_#27	GO:0006956: complement activation	0.000055	17,12,3
1fw8a_#257	GO:0006096: glycolysis	0.000055	15,38,4
1gtma2#140	GO:0006563: L-serine metabolism	0.000055	17,12,3
1epwa3#140	GO:0051249: regulation of lymphocyte activation	0.000055	168,12,6
2viua_#252	GO:0007169: transmembrane receptor protein tyrosine kinase signaling pathway	0.000055	285,26,11
1psda3#344	GO:0006367: transcription initiation from Pol II promoter	0.000055	99,12,5

1b8aa2#368	GO:0007264: small GTPase mediated signal transduction	0.000055	130,21,7
1czan1#92	GO:0006457: protein folding	0.000055	538,49,23
1c0ma1#237	GO:0048514: blood vessel morphogenesis	0.000055	23,9,3
1dy9.1#A44	GO:0006956: complement activation	0.000055	17,12,3
1i50b_#966	GO:0006953: acute-phase response	0.000055	251,20,9
1dik_3#240	GO:0030217: T-cell differentiation	0.000055	213,10,6
1gdna_#121	GO:0006956: complement activation	0.000055	17,12,3
1bqya_#103	GO:0006956: complement activation	0.000055	17,12,3
1dlea_#198	GO:0006956: complement activation	0.000055	17,12,3
1ton__#231	GO:0006956: complement activation	0.000055	17,12,3
1dpja_#322	GO:0016477: cell migration	0.000056	269,23,10
1b4ka_#108	GO:0006096: glycolysis	0.000056	94,38,8
1smaa2#581	GO:0045595: regulation of cell differentiation	0.000056	60,10,4
1qj5a_#268	GO:0006207: 'de novo' pyrimidine base biosynthesis	0.000056	60,10,4
1h8ma_#70	GO:0006457: protein folding	0.000057	12,49,4
1gdea_#243	GO:0042088: T-helper 1 type immune response	0.000057	54,11,4
1gdea_#243	GO:0042108: positive regulation of cytokine biosynthesis	0.000057	54,11,4
1fw9a_#152	GO:0006278: RNA-dependent DNA replication	0.000057	124,10,5
1by5a_#238	GO:0006275: regulation of DNA replication	0.000057	214,10,6
1f0ia1#50	GO:0006606: protein-nucleus import	0.000057	131,21,7
1opy__#68	GO:0016337: cell-cell adhesion	0.000057	308,17,9
1qaxa2#307	GO:0006367: transcription initiation from Pol II promoter	0.000057	49,12,4
1i9sa_#24	GO:0009103: lipopolysaccharide biosynthesis	0.000057	49,12,4
1i4ua_#154	GO:0009064: glutamine family amino acid metabolism	0.000057	30,19,4
1h6kx_#97	GO:0000398: nuclear mRNA splicing, via spliceosome	0.000057	49,12,4
1ecpa_#62	GO:0006098: pentose-phosphate shunt	0.000057	54,11,4
1f00i2#805	GO:0018108: peptidyl-tyrosine phosphorylation	0.000057	124,10,5
1nsj__#124	GO:0015976: carbon utilization	0.000057	124,10,5
1el0a_#42	GO:0009615: response to virus	0.000057	258,12,7
1fa0a1#393	GO:0046677: response to antibiotic	0.000057	209,40,12
1qfea_#68	GO:0006779: porphyrin biosynthesis	0.000057	453,18,11
1vmoa_#106	GO:0016337: cell-cell adhesion	0.000057	167,17,7
1qs0a1#119	GO:0009103: lipopolysaccharide biosynthesis	0.000057	367,12,8
2hlca_#226	GO:0016337: cell-cell adhesion	0.000057	67,17,5
1e8ya2#410	GO:0016042: lipid catabolism	0.000058	7,31,3
1p35a_#101	GO:0042088: T-helper 1 type immune response	0.000058	111,11,5
1esma_#314	GO:0051249: regulation of lymphocyte activation	0.000058	100,12,5
1cewi_#14	GO:0019735: antimicrobial humoral response (sensu Vertebrata)	0.000058	72,16,5
1fc4a_#205	GO:0042401: biogenic amine biosynthesis	0.000058	215,10,6
1fc4a_#205	GO:0006568: tryptophan metabolism	0.000058	215,10,6
1fc4a_#205	GO:0006207: 'de novo' pyrimidine base biosynthesis	0.000058	215,10,6
1cbf__#208	GO:0042108: positive regulation of cytokine biosynthesis	0.000058	291,11,7
2hft_1#34	GO:0019735: antimicrobial humoral response (sensu Vertebrata)	0.000058	36,16,4
1ppn__#132	GO:0006418: tRNA aminoacylation for protein translation	0.000058	186,26,9
1ppn__#132	GO:0007169: transmembrane receptor protein tyrosine kinase signaling pathway	0.000058	186,26,9
1p35a_#155	GO:0006643: membrane lipid metabolism	0.000058	45,13,4
1xo1a2#159	GO:0009073: aromatic amino acid family biosynthesis	0.000058	36,16,4
1c4xa_#110	GO:0016042: lipid catabolism	0.000058	7,31,3
1i2ma_#157	GO:0006606: protein-nucleus import	0.000059	10,21,3
1fh6a_#341	GO:0009064: glutamine family amino acid metabolism	0.000059	11,19,3
2e2c__#67	GO:0006511: ubiquitin-dependent protein catabolism	0.000059	19,11,3
1je5a_#39	GO:0019079: viral genome replication	0.000059	13,16,3
1fy7a_#304	GO:0016568: chromatin modification	0.000059	19,11,3
1hc7a2#113	GO:0042559: pteridine and derivative biosynthesis	0.000059	19,11,3
1csh__#95	GO:0006099: tricarboxylic acid cycle	0.000059	11,19,3

1hg3a_#207	GO:0006098: pentose-phosphate shunt	0.000059	19,11,3
1qlca_#210	GO:0042088: T-helper 1 type immune response	0.000059	19,11,3
1qlca_#210	GO:0042108: positive regulation of cytokine biosynthesis	0.000059	19,11,3
1aky_2#133	GO:0008015: circulation	0.000059	130,15,6
1jb0d_#26	GO:0042108: positive regulation of cytokine biosynthesis	0.000059	190,11,6
1ej8a_#189	GO:0006457: protein folding	0.000059	256,49,15
1qo0d_#81	GO:0009070: serine family amino acid biosynthesis	0.000059	43,26,5
1qqp3_#139	GO:0019079: viral genome replication	0.000059	13,16,3
1pii_2#326	GO:0006779: porphyrin biosynthesis	0.000059	157,18,7
1bpv__#22	GO:0042108: positive regulation of cytokine biosynthesis	0.000059	19,11,3
1ir3a_#1139	GO:0007169: transmembrane receptor protein tyrosine kinase signaling pathway	0.000059	22,26,4
1ds1a_#172	GO:0042108: positive regulation of cytokine biosynthesis	0.000059	190,11,6
1ds1a_#172	GO:0001906: cell killing	0.000059	190,11,6
1gox__#246	GO:0009252: peptidoglycan biosynthesis	0.000059	157,18,7
1cs6a4#363	GO:0045595: regulation of cell differentiation	0.000059	21,10,3
1cs6a4#363	GO:0019883: antigen presentation, endogenous antigen	0.000059	21,10,3
1e6ca_#91	GO:0009086: methionine biosynthesis	0.000059	141,14,6
1a06__#103	GO:0000086: G2/M transition of mitotic cell cycle	0.000059	21,10,3
1sppb_#32	GO:0016337: cell-cell adhesion	0.000059	34,17,4
1dyna_#42	GO:0006118: electron transport	0.000059	152,228,29
1iira_#242	GO:0006207: 'de novo' pyrimidine base biosynthesis	0.000059	125,10,5
1qs0a1#119	GO:0009252: peptidoglycan biosynthesis	0.000059	367,18,10
1qs0a1#119	GO:0006779: porphyrin biosynthesis	0.000059	367,18,10
1e5ma1#159	GO:0006633: fatty acid biosynthesis	0.00006	8,27,3
1jj2v_#118	GO:0006606: protein-nucleus import	0.00006	89,21,6
1bjt__#890	GO:0006461: protein complex assembly	0.00006	258,24,10
1cyda_#106	GO:0006633: fatty acid biosynthesis	0.00006	8,27,3
1cpza_#6	GO:0006606: protein-nucleus import	0.00006	132,21,7
1qnja_#221	GO:0006953: acute-phase response	0.000061	2,20,2
1a9xa2#1009	GO:0006826: iron ion transport	0.000061	14,15,3
1a99a_#84	GO:0015698: inorganic anion transport	0.000061	15,14,3
1qgna_#149	GO:0009086: methionine biosynthesis	0.000061	15,14,3
1ib2a_#1101	GO:0006690: icosanoid metabolism	0.000061	55,11,4
1cqqa_#163	GO:0006919: caspase activation	0.000061	14,15,3
1jf9a_#196	GO:0009086: methionine biosynthesis	0.000061	42,14,4
1i6vd_#1151	GO:0006096: glycolysis	0.000062	6,38,3
1j71a_#251	GO:0006118: electron transport	0.000062	87,228,20
1i5ga_#115	GO:0001906: cell killing	0.000062	294,11,7
3chbd_#85	GO:0006953: acute-phase response	0.000062	323,20,10
1xgsa2#82	GO:0051249: regulation of lymphocyte activation	0.000062	261,12,7
1hlwa_#91	GO:0006953: acute-phase response	0.000062	29,20,4
2napa2#292	GO:0006118: electron transport	0.000062	108,228,23
1gdna_#119	GO:0006956: complement activation	0.000063	3,12,2
1lxa__#255	GO:0009103: lipopolysaccharide biosynthesis	0.000063	3,12,2
1e6ua_#282	GO:0009103: lipopolysaccharide biosynthesis	0.000063	3,12,2
1klo_3#148	GO:0006956: complement activation	0.000063	3,12,2
1klo_1#46	GO:0006956: complement activation	0.000063	3,12,2
1gsoa3#193	GO:0007017: microtubule-based process	0.000063	3,12,2
1ev7a_#291	GO:0009615: response to virus	0.000063	3,12,2
1bccb1#130	GO:0006119: oxidative phosphorylation	0.000063	3,12,2
1e5xa_#282	GO:0006563: L-serine metabolism	0.000063	3,12,2
1htjf_#369	GO:0007186: G-protein coupled receptor protein signaling pathway	0.000063	9,24,3
1cb6a2#1660	GO:0006879: iron ion homeostasis	0.000063	3,12,2
1fo4a5#1020	GO:0016337: cell-cell adhesion	0.000063	312,17,9
1d8db_#148	GO:0006956: complement activation	0.000063	3,12,2

1a9xa3#78	GO:0006563: L-serine metabolism	0.000063	262,12,7
1a9xa3#78	GO:0009103: lipopolysaccharide biosynthesis	0.000063	262,12,7
1a9xa3#78	GO:0009156: ribonucleoside monophosphate biosynthesis	0.000063	262,12,7
1f24a_#144	GO:0006118: electron transport	0.000064	22,228,9
1g51a3#524	GO:0030333: antigen processing	0.000064	93,13,5
1ihua2#521	GO:0006221: pyrimidine nucleotide biosynthesis	0.000064	52,22,5
1imva_#255	GO:0051249: regulation of lymphocyte activation	0.000064	506,12,9
1znca_#167	GO:0006730: one-carbon compound metabolism	0.000064	7,32,3
1cex__#23	GO:0006118: electron transport	0.000064	22,228,9
1bd8__#82	GO:0007268: synaptic transmission	0.000064	66,28,6
3sil__#284	GO:0019883: antigen presentation, endogenous antigen	0.000064	62,10,4
1iow_1#37	GO:0009156: ribonucleoside monophosphate biosynthesis	0.000065	263,12,7
1muca1#245	GO:0009073: aromatic amino acid family biosynthesis	0.000065	37,16,4
1fgga_#134	GO:0009396: folic acid and derivative biosynthesis	0.000065	193,11,6
1dvpa1#65	GO:0006879: iron ion homeostasis	0.000065	173,12,6
1qrra_#69	GO:0009156: ribonucleoside monophosphate biosynthesis	0.000065	173,12,6
1el0a_#42	GO:0006457: protein folding	0.000065	258,49,15
1doza_#109	GO:0006099: tricarboxylic acid cycle	0.000065	61,19,5
1doza_#109	GO:0009064: glutamine family amino acid metabolism	0.000065	61,19,5
1acc__#206	GO:0009156: ribonucleoside monophosphate biosynthesis	0.000066	18,12,3
1fzqa_#165	GO:0007017: microtubule-based process	0.000066	18,12,3
1qf5a_#15	GO:0006606: protein-nucleus import	0.000066	28,21,4
1danh_#197	GO:0006956: complement activation	0.000066	18,12,3
1d8ca_#442	GO:0006096: glycolysis	0.000066	30,38,5
1ctqa_#116	GO:0006606: protein-nucleus import	0.000066	28,21,4
1hg3a_#43	GO:0006096: glycolysis	0.000066	30,38,5
1flga_#161	GO:0050819: negative regulation of coagulation	0.000066	128,10,5
1flga_#161	GO:0030193: regulation of blood coagulation	0.000066	128,10,5
1flga_#161	GO:0018108: peptidyl-tyrosine phosphorylation	0.000066	128,10,5
1b8aa2#403	GO:0042088: T-helper 1 type immune response	0.000066	114,11,5
1b8aa2#403	GO:0042108: positive regulation of cytokine biosynthesis	0.000066	114,11,5
1b8aa2#403	GO:0001906: cell killing	0.000066	114,11,5
2dkb__#114	GO:0042364: water-soluble vitamin biosynthesis	0.000066	139,27,8
1qq4a_#143	GO:0006956: complement activation	0.000066	18,12,3
1oaa__#199	GO:0009103: lipopolysaccharide biosynthesis	0.000066	18,12,3
1e4ea2#210	GO:0009252: peptidoglycan biosynthesis	0.000066	12,18,3
1c39a_#49	GO:0001906: cell killing	0.000066	56,11,4
1dlea_#53	GO:0006956: complement activation	0.000066	18,12,3
2bbkh_#117	GO:0006118: electron transport	0.000067	4,228,4
1io7a_#38	GO:0006118: electron transport	0.000067	4,228,4
1f00i1#749	GO:0016337: cell-cell adhesion	0.000067	35,17,4
1hbza_#254	GO:0006118: electron transport	0.000067	4,228,4
1atg__#144	GO:0009070: serine family amino acid biosynthesis	0.000067	72,26,6
1ayfa_#92	GO:0006118: electron transport	0.000067	4,228,4
1aqe__#76	GO:0006118: electron transport	0.000067	4,228,4
1jsg__#92	GO:0008285: negative regulation of cell proliferation	0.000067	519,19,12
1c3ga2#272	GO:0016337: cell-cell adhesion	0.000067	35,17,4
1qupa2#50	GO:0006457: protein folding	0.000068	5,49,3
1ecpa_#46	GO:0019883: antigen presentation, endogenous antigen	0.000068	22,10,3
1f24a_#144	GO:0009219: pyrimidine deoxyribonucleotide metabolism	0.000068	22,10,3
1b0ua_#174	GO:0015976: carbon utilization	0.000068	22,10,3
1otha2#300	GO:0006221: pyrimidine nucleotide biosynthesis	0.000068	10,22,3
1cbf__#208	GO:0006457: protein folding	0.000068	291,49,16
1a9xa6#760	GO:0006919: caspase activation	0.000068	80,15,5
1h8ua_#113	GO:0006278: RNA-dependent DNA replication	0.000068	63,10,4
3gcb__#78	GO:0000910: cytokinesis	0.000068	73,37,7

1c8na_#239	GO:0019883: antigen presentation, endogenous antigen	0.000068	22,10,3
1ir3a_#1139	GO:0000086: G2/M transition of mitotic cell cycle	0.000068	22,10,3
1fkna_#18	GO:0030333: antigen processing	0.000068	94,13,5
1fsu__#434	GO:0016337: cell-cell adhesion	0.000068	315,17,9
1bjaa_#26	GO:0006265: DNA topological change	0.000068	22,10,3
1bjaa_#26	GO:0006268: DNA unwinding	0.000068	22,10,3
1j75a_#166	GO:0006953: acute-phase response	0.000068	96,20,6
1dfca1#1076	GO:0048514: blood vessel morphogenesis	0.000069	4,9,2
1dgwa_#97	GO:0006953: acute-phase response	0.000069	11,20,3
2foka2#180	GO:0006265: DNA topological change	0.000069	129,10,5
2foka2#180	GO:0006268: DNA unwinding	0.000069	129,10,5
1hzia_#51	GO:0042113: B-cell activation	0.000069	4,9,2
1hg3a_#61	GO:0009396: folic acid and derivative biosynthesis	0.000069	20,11,3
1jlxa2#288	GO:0048514: blood vessel morphogenesis	0.000069	4,9,2
1tuba1#8	GO:0043094: metabolic compound salvage	0.000069	115,11,5
1cf1a1#33	GO:0017148: negative regulation of protein biosynthesis	0.000069	47,13,4
1ycsb1#412	GO:0009582: detection of abiotic stimulus	0.000069	129,10,5
1ds1a_#172	GO:0007169: transmembrane receptor protein tyrosine kinase signaling pathway	0.000069	190,26,9
1fxoa_#136	GO:0042088: T-helper 1 type immune response	0.000069	20,11,3
1fxoa_#136	GO:0042108: positive regulation of cytokine biosynthesis	0.000069	20,11,3
1f5aa1#441	GO:0019883: antigen presentation, endogenous antigen	0.000069	129,10,5
1rypk_#12	GO:0006118: electron transport	0.000069	250,228,41
1iira_#242	GO:0006096: glycolysis	0.000069	125,38,9
1au1a_#58	GO:0015992: proton transport	0.00007	104,12,5
1nuka_#80	GO:0006953: acute-phase response	0.00007	197,20,8
1ejfa_#10	GO:0006919: caspase activation	0.00007	134,15,6
2cuaa_#107	GO:0006118: electron transport	0.00007	116,228,24
1ojt_2#356	GO:0006730: one-carbon compound metabolism	0.00007	36,32,5
1deua_#151	GO:0007169: transmembrane receptor protein tyrosine kinase signaling pathway	0.00007	239,26,10
1aa6_2#285	GO:0009086: methionine biosynthesis	0.00007	87,14,5
16pk__#312	GO:0007067: mitosis	0.000071	9,25,3
1kapp1#389	GO:0017148: negative regulation of protein biosynthesis	0.000071	17,13,3
1ytba2#217	GO:0007169: transmembrane receptor protein tyrosine kinase signaling pathway	0.000071	23,26,4
1fc3a_#172	GO:0015671: oxygen transport	0.000071	168,23,8
1aky_2#133	GO:0050819: negative regulation of coagulation	0.000071	130,10,5
1aky_2#133	GO:0030193: regulation of blood coagulation	0.000071	130,10,5
1aky_2#133	GO:0030217: T-cell differentiation	0.000071	130,10,5
1fwxa2#396	GO:0006461: protein complex assembly	0.000071	209,24,9
1eaja_#59	GO:0042088: T-helper 1 type immune response	0.000071	196,11,6
1eaja_#59	GO:0042108: positive regulation of cytokine biosynthesis	0.000071	196,11,6
2arca_#55	GO:0030217: T-cell differentiation	0.000071	130,10,5
3prn__#143	GO:0042108: positive regulation of cytokine biosynthesis	0.000071	300,11,7
1feua_#71	GO:0007517: muscle development	0.000071	20,30,4
1a7ca_#202	GO:0016477: cell migration	0.000071	83,23,6
1a3qa2#190	GO:0008284: positive regulation of cell proliferation	0.000071	125,30,8
1mrj__#73	GO:0001906: cell killing	0.000071	57,11,4
3sil__#317	GO:0001906: cell killing	0.000071	300,11,7
1gpea1#271	GO:0006544: glycine metabolism	0.000071	196,11,6
1gpea1#271	GO:0001906: cell killing	0.000071	196,11,6
1el5a1#10	GO:0006096: glycolysis	0.000072	16,38,4
1smaa1#95	GO:0050819: negative regulation of coagulation	0.000072	223,10,6
1smaa1#95	GO:0030193: regulation of blood coagulation	0.000072	223,10,6
1smaa1#95	GO:0018108: peptidyl-tyrosine phosphorylation	0.000072	223,10,6
1ihoa_#238	GO:0006367: transcription initiation from Pol II promoter	0.000072	52,12,4

1doka_#52	GO:0051249: regulation of lymphocyte activation	0.000072	52,12,4
1by5a_#347	GO:0016337: cell-cell adhesion	0.000072	240,17,8
2stv__#129	GO:0043123: positive regulation of I-kappaB kinase/NF-kappaB cascade	0.000072	38,16,4
1h6ka2#336	GO:0006099: tricarboxylic acid cycle	0.000072	152,19,7
1mpp__#151	GO:0006606: protein-nucleus import	0.000072	92,21,6
1g5qa_#80	GO:0006096: glycolysis	0.000072	16,38,4
1ihua2#343	GO:0007017: microtubule-based process	0.000072	176,12,6
1faoa_#250	GO:0007017: microtubule-based process	0.000072	52,12,4
1poxa3#460	GO:0000160: two-component signal transduction system (phosphorelay)	0.000072	76,25,6
1hxmb1#117	GO:0006917: induction of apoptosis	0.000072	65,29,6
1dik_1#848	GO:0015976: carbon utilization	0.000073	64,10,4
1nuka_#80	GO:0001906: cell killing	0.000073	197,11,6
1f0ia1#50	GO:0006118: electron transport	0.000073	131,228,26
1eo6a_#107	GO:0015976: carbon utilization	0.000073	64,10,4
1h6la_#119	GO:0006461: protein complex assembly	0.000073	264,24,10
1bkpa_#275	GO:0006221: pyrimidine nucleotide biosynthesis	0.000074	2,22,2
1rkd__#224	GO:0045045: secretory pathway	0.000074	2,22,2
1qlwa_#110	GO:0017000: antibiotic biosynthesis	0.000074	3,13,2
1fbna_#183	GO:0030245: cellulose catabolism	0.000074	2,22,2
1bn7a_#252	GO:0017000: antibiotic biosynthesis	0.000074	3,13,2
1nar__#85	GO:0006221: pyrimidine nucleotide biosynthesis	0.000074	2,22,2
1qq9a_#78	GO:0017000: antibiotic biosynthesis	0.000074	3,13,2
1gsoa3#193	GO:0000082: G1/S transition of mitotic cell cycle	0.000074	3,13,2
1f97a1#62	GO:0006544: glycine metabolism	0.000074	591,11,9
1f97a1#62	GO:0001906: cell killing	0.000074	591,11,9
1cem__#132	GO:0030245: cellulose catabolism	0.000074	2,22,2
1bn7a_#253	GO:0017000: antibiotic biosynthesis	0.000074	3,13,2
1cem__#150	GO:0030245: cellulose catabolism	0.000074	2,22,2
1tuba1#239	GO:0006118: electron transport	0.000074	95,228,21
1fjfl_#67	GO:0007169: transmembrane receptor protein tyrosine kinase signaling pathway	0.000074	147,26,8
1i5ga_#115	GO:0006418: tRNA aminoacylation for protein translation	0.000074	294,26,11
1a04a2#59	GO:0006935: chemotaxis	0.000074	15,41,4
1f0ia1#50	GO:0050819: negative regulation of coagulation	0.000074	131,10,5
1f0ia1#50	GO:0030193: regulation of blood coagulation	0.000074	131,10,5
1f0ia1#50	GO:0018108: peptidyl-tyrosine phosphorylation	0.000074	131,10,5
1g6oa_#116	GO:0042088: T-helper 1 type immune response	0.000074	302,11,7
1g6oa_#116	GO:0042108: positive regulation of cytokine biosynthesis	0.000074	302,11,7
1g6oa_#116	GO:0001906: cell killing	0.000074	302,11,7
1qfxa_#380	GO:0006919: caspase activation	0.000074	41,15,4
1kit_3#743	GO:0006916: anti-apoptosis	0.000074	225,18,8
1bn7a_#286	GO:0006805: xenobiotic metabolism	0.000074	3,13,2
1h6va1#27	GO:0006207: 'de novo' pyrimidine base biosynthesis	0.000074	224,10,6
1neb__#14	GO:0006917: induction of apoptosis	0.000075	8,29,3
1i1ja_#83	GO:0006917: induction of apoptosis	0.000075	8,29,3
1dcea3#526	GO:0009086: methionine biosynthesis	0.000075	16,14,3
1as4.1#A336	GO:0006730: one-carbon compound metabolism	0.000075	276,32,12
1dpja_#322	GO:0051249: regulation of lymphocyte activation	0.000075	269,12,7
1bura1#269	GO:0009073: aromatic amino acid family biosynthesis	0.000075	14,16,3
1cqqa_#163	GO:0019079: viral genome replication	0.000075	14,16,3
1g5qa_#80	GO:0006100: tricarboxylic acid cycle intermediate metabolism	0.000075	16,14,3
1qlaa3#364	GO:0042108: positive regulation of cytokine biosynthesis	0.000076	58,11,4
1pot__#138	GO:0006511: ubiquitin-dependent protein catabolism	0.000076	58,11,4
1ej0a_#42	GO:0015074: DNA integration	0.000076	98,20,6
2napa2#292	GO:0009070: serine family amino acid biosynthesis	0.000076	108,26,7
1h6va3#486	GO:0045595: regulation of cell differentiation	0.000077	65,10,4

1ddja_#726	GO:0006919: caspase activation	0.000077	82,15,5
1apme_#74	GO:0008285: negative regulation of cell proliferation	0.000077	104,19,6
1ddja_#681	GO:0008285: negative regulation of cell proliferation	0.000077	212,19,8
1jg8a_#172	GO:0006779: porphyrin biosynthesis	0.000077	67,18,5
1cnv__#181	GO:0006568: tryptophan metabolism	0.000077	65,10,4
1hxmb1#117	GO:0045595: regulation of cell differentiation	0.000077	65,10,4
1cpza_#6	GO:0018108: peptidyl-tyrosine phosphorylation	0.000077	132,10,5
1cpza_#6	GO:0030217: T-cell differentiation	0.000077	132,10,5
1ghpa_#65	GO:0017001: antibiotic catabolism	0.000078	23,10,3
2hlpa2#289	GO:0006099: tricarboxylic acid cycle	0.000078	12,19,3
1f0xa1#278	GO:0030333: antigen processing	0.000078	162,13,6
1qlca_#210	GO:0051249: regulation of lymphocyte activation	0.000078	19,12,3
1dlfh_#4	GO:0051049: regulation of transport	0.000078	23,10,3
1jb3a_#64	GO:0006606: protein-nucleus import	0.000078	247,21,9
3grs_3#435	GO:0009064: glutamine family amino acid metabolism	0.000078	154,19,7
1imva_#255	GO:0016337: cell-cell adhesion	0.000078	506,17,11
2naca1#162	GO:0007017: microtubule-based process	0.000078	19,12,3
1cs1a_#160	GO:0006779: porphyrin biosynthesis	0.000078	164,18,7
1ton__#196	GO:0006956: complement activation	0.000078	19,12,3
1qq4a_#63	GO:0006956: complement activation	0.000078	19,12,3
1f97a2#199	GO:0001906: cell killing	0.000078	118,11,5
1iow_2#112	GO:0000086: G2/M transition of mitotic cell cycle	0.000078	23,10,3
1arb__#24	GO:0006956: complement activation	0.000078	19,12,3
1dofa_#15	GO:0006119: oxidative phosphorylation	0.000079	179,12,6
2mpra_#190	GO:0050819: negative regulation of coagulation	0.000079	349,10,7
2mpra_#190	GO:0030193: regulation of blood coagulation	0.000079	349,10,7
1beba_#21	GO:0001906: cell killing	0.00008	21,11,3
1ajsa_#389	GO:0006418: tRNA aminoacylation for protein translation	0.00008	9,26,3
1ldna1#129	GO:0006418: tRNA aminoacylation for protein translation	0.00008	9,26,3
1poxa3#439	GO:0006544: glycine metabolism	0.00008	21,11,3
1fmk_3#428	GO:0007169: transmembrane receptor protein tyrosine kinase signaling pathway	0.00008	9,26,3
1pme__#207	GO:0007169: transmembrane receptor protein tyrosine kinase signaling pathway	0.00008	9,26,3
1danh_#24	GO:0006606: protein-nucleus import	0.00008	11,21,3
1e30a_#112	GO:0019079: viral genome replication	0.00008	39,16,4
1g8fa2#258	GO:0006418: tRNA aminoacylation for protein translation	0.00008	9,26,3
1ppn__#191	GO:0006418: tRNA aminoacylation for protein translation	0.00008	9,26,3
2sli_2#326	GO:0045595: regulation of cell differentiation	0.00008	227,10,6
1rypi_#87	GO:0006511: ubiquitin-dependent protein catabolism	0.00008	21,11,3
1epwa2#1220	GO:0007264: small GTPase mediated signal transduction	0.00008	11,21,3
1bmv2_#2064	GO:0030217: T-cell differentiation	0.00008	133,10,5
1e3a.1#A30	GO:0006278: RNA-dependent DNA replication	0.00008	133,10,5
1rypb_#114	GO:0006278: RNA-dependent DNA replication	0.00008	133,10,5
1rypf_#35	GO:0006511: ubiquitin-dependent protein catabolism	0.00008	21,11,3
1evqa_#305	GO:0009103: lipopolysaccharide biosynthesis	0.00008	384,12,8
1evqa_#305	GO:0009156: ribonucleoside monophosphate biosynthesis	0.00008	384,12,8
1ciy_2#414	GO:0030435: sporulation	0.000081	2,23,2
1bupa1#178	GO:0006457: protein folding	0.000081	13,49,4
1qlaa2#179	GO:0030333: antigen processing	0.000081	49,13,4
1avgi_#69	GO:0016477: cell migration	0.000081	281,23,10
1f21a_#68	GO:0015074: DNA integration	0.000081	31,20,4
1imva_#255	GO:0006275: regulation of DNA replication	0.000081	506,10,8
1czan1#92	GO:0006313: DNA transposition	0.000081	538,35,18
1qh4a2#350	GO:0006511: ubiquitin-dependent protein catabolism	0.000081	59,11,4
1fhjb_#106	GO:0015671: oxygen transport	0.000081	2,23,2

1hbza_#96	GO:0016337: cell-cell adhesion	0.000081	322,17,9
1ie5a_#102	GO:0008284: positive regulation of cell proliferation	0.000081	64,30,6
1qqp1_#51	GO:0006953: acute-phase response	0.000081	31,20,4
1fi2a_#104	GO:0006953: acute-phase response	0.000081	31,20,4
1ge8a2#134	GO:0006457: protein folding	0.000081	202,49,13
1ddwa_#77	GO:0016337: cell-cell adhesion	0.000081	322,17,9
1aop_3#276	GO:0006118: electron transport	0.000082	147,228,28
1tbga_#63	GO:0006118: electron transport	0.000082	18,228,8
1fbna_#144	GO:0009064: glutamine family amino acid metabolism	0.000082	64,19,5
1azsb_#1075	GO:0006118: electron transport	0.000082	10,228,6
1pya.1#B236	GO:0015976: carbon utilization	0.000082	66,10,4
1e5pa_#91	GO:0016068: type I hypersensitivity	0.000082	42,15,4
1a4ya_#104	GO:0009070: serine family amino acid biosynthesis	0.000082	194,26,9
1bu8a2#124	GO:0016042: lipid catabolism	0.000082	123,31,8
1djna1#86	GO:0006568: tryptophan metabolism	0.000082	66,10,4
1djna1#86	GO:0006207: 'de novo' pyrimidine base biosynthesis	0.000082	66,10,4
1axn__#42	GO:0009582: detection of abiotic stimulus	0.000082	66,10,4
3mag__#134	GO:0016042: lipid catabolism	0.000082	62,31,6
1axca1#70	GO:0006606: protein-nucleus import	0.000082	94,21,6
1ejfa_#10	GO:0018108: peptidyl-tyrosine phosphorylation	0.000083	134,10,5
3grx__#56	GO:0042108: positive regulation of cytokine biosynthesis	0.000083	307,11,7
3grx__#56	GO:0001906: cell killing	0.000083	307,11,7
1jfra_#41	GO:0046677: response to antibiotic	0.000083	94,40,8
1ido__#173	GO:0009116: nucleoside metabolism	0.000083	78,25,6
1qtn.1#A270	GO:0006563: L-serine metabolism	0.000083	386,12,8
1h6la_#119	GO:0019735: antimicrobial humoral response (sensu Vertebrata)	0.000083	264,16,8
1qfma1#139	GO:0019079: viral genome replication	0.000083	264,16,8
1fjfl_#67	GO:0006953: acute-phase response	0.000084	147,20,7
1gdea_#243	GO:0051249: regulation of lymphocyte activation	0.000084	54,12,4
1a6ca2#312	GO:0006418: tRNA aminoacylation for protein translation	0.000084	75,26,6
1f2la_#40	GO:0008015: circulation	0.000084	206,15,7
1zpda1#213	GO:0042364: water-soluble vitamin biosynthesis	0.000084	72,27,6
1ie5a_#23	GO:0007169: transmembrane receptor protein tyrosine kinase signaling pathway	0.000084	24,26,4
1qqga2#166	GO:0006643: membrane lipid metabolism	0.000084	164,13,6
1hxxa_#232	GO:0030435: sporulation	0.000084	282,23,10
1jdra_#53	GO:0006979: response to oxidative stress	0.000085	13,18,3
1fi2a_#109	GO:0007169: transmembrane receptor protein tyrosine kinase signaling pathway	0.000085	195,26,9
1jj2e1#42	GO:0007169: transmembrane receptor protein tyrosine kinase signaling pathway	0.000085	150,26,8
1aym3_#131	GO:0006275: regulation of DNA replication	0.000085	353,10,7
1xnb__#149	GO:0006418: tRNA aminoacylation for protein translation	0.000085	150,26,8
1hvba_#102	GO:0009252: peptidoglycan biosynthesis	0.000085	13,18,3
1i50b_#1171	GO:0006354: RNA elongation	0.000086	4,10,2
1i85a_#14	GO:0045595: regulation of cell differentiation	0.000086	4,10,2
1jr1a1#68	GO:0006207: 'de novo' pyrimidine base biosynthesis	0.000086	4,10,2
1jr1a1#68	GO:0009167: purine ribonucleoside monophosphate metabolism	0.000086	4,10,2
1d3ya_#197	GO:0006265: DNA topological change	0.000086	4,10,2
1d3ya_#197	GO:0006268: DNA unwinding	0.000086	4,10,2
1gg3a3#55	GO:0030217: T-cell differentiation	0.000086	4,10,2
1jj2q_#113	GO:0045595: regulation of cell differentiation	0.000086	4,10,2
1jj2q_#113	GO:0030217: T-cell differentiation	0.000086	4,10,2
1ihka_#161	GO:0045893: positive regulation of transcription, DNA-dependent	0.000086	4,10,2
1f0c.1#A291	GO:0006275: regulation of DNA replication	0.000086	4,10,2
1hx0a2#294	GO:0000051: urea cycle intermediate metabolism	0.000086	4,10,2
7odca1#391	GO:0009167: purine ribonucleoside monophosphate metabolism	0.000086	4,10,2

1fmb__#88	GO:0009219: pyrimidine deoxyribonucleotide metabolism	0.000086	4,10,2
1e44b_#75	GO:0007399: neurogenesis	0.000086	165,37,10
1plq_2#197	GO:0006275: regulation of DNA replication	0.000086	4,10,2
1hxxa_#173	GO:0030217: T-cell differentiation	0.000086	230,10,6
1d66a1#28	GO:0045893: positive regulation of transcription, DNA-dependent	0.000086	4,10,2
1ddja_#681	GO:0006935: chemotaxis	0.000086	212,41,12
1dr9a2#139	GO:0019735: antimicrobial humoral response (sensu Vertebrata)	0.000086	78,16,5
1c4zd_#51	GO:0019883: antigen presentation, endogenous antigen	0.000086	135,10,5
1a8i__#688	GO:0009396: folic acid and derivative biosynthesis	0.000086	203,11,6
1d6aa_#52	GO:0050819: negative regulation of coagulation	0.000086	230,10,6
1d6aa_#52	GO:0030193: regulation of blood coagulation	0.000086	230,10,6
1gox__#246	GO:0000160: two-component signal transduction system (phosphorelay)	0.000086	157,25,8
1ton__#211	GO:0006606: protein-nucleus import	0.000086	58,21,5
1vjw__#39	GO:0006100: tricarboxylic acid cycle intermediate metabolism	0.000087	3,14,2
1ibja_#414	GO:0009086: methionine biosynthesis	0.000087	3,14,2
1aqua_#53	GO:0015698: inorganic anion transport	0.000087	3,14,2
1g8fa3#419	GO:0009073: aromatic amino acid family biosynthesis	0.000087	129,16,6
1eu8a_#35	GO:0015698: inorganic anion transport	0.000087	3,14,2
1c4zd_#22	GO:0006511: ubiquitin-dependent protein catabolism	0.000087	60,11,4
1bxoa_#307	GO:0030333: antigen processing	0.000087	99,13,5
1dzfa2#211	GO:0006919: caspase activation	0.000087	139,15,6
1jlna_#383	GO:0006100: tricarboxylic acid cycle intermediate metabolism	0.000087	46,14,4
1e3ja2#270	GO:0009396: folic acid and derivative biosynthesis	0.000087	60,11,4
1smaa2#581	GO:0042088: T-helper 1 type immune response	0.000087	60,11,4
1smaa2#581	GO:0042108: positive regulation of cytokine biosynthesis	0.000087	60,11,4
1c4ra_#258	GO:0019883: antigen presentation, endogenous antigen	0.000087	67,10,4
2sli_2#341	GO:0006461: protein complex assembly	0.000088	2,24,2
1g8fa3#419	GO:0006096: glycolysis	0.000088	129,38,9
2sqca1#520	GO:0007186: G-protein coupled receptor protein signaling pathway	0.000088	2,24,2
1ejda_#180	GO:0006099: tricarboxylic acid cycle	0.000088	65,19,5
1jjya_#324	GO:0009064: glutamine family amino acid metabolism	0.000088	65,19,5
1pii_2#326	GO:0009064: glutamine family amino acid metabolism	0.000088	157,19,7
1hdfa_#16	GO:0043123: positive regulation of I-kappaB kinase/NF-kappaB cascade	0.000088	192,16,7
1as4.1#A336	GO:0051249: regulation of lymphocyte activation	0.000089	276,12,7
1ie5a_#23	GO:0045595: regulation of cell differentiation	0.000089	24,10,3
1qp8a1#153	GO:0006207: 'de novo' pyrimidine base biosynthesis	0.000089	24,10,3
1d0na3#305	GO:0051249: regulation of lymphocyte activation	0.000089	276,12,7
1eu3a2#205	GO:0006118: electron transport	0.000089	69,228,17
3lada1#116	GO:0006221: pyrimidine nucleotide biosynthesis	0.000089	133,22,7
1dpsa_#36	GO:0006879: iron ion homeostasis	0.000089	183,12,6
1dpsa_#36	GO:0015992: proton transport	0.000089	183,12,6
1ddja_#746	GO:0001906: cell killing	0.000089	121,11,5
3sil__#317	GO:0007169: transmembrane receptor protein tyrosine kinase signaling pathway	0.000089	300,26,11
2bpa1_#45	GO:0016337: cell-cell adhesion	0.000089	179,17,7
1h9da_#91	GO:0001906: cell killing	0.000089	204,11,6
2dkb__#46	GO:0042364: water-soluble vitamin biosynthesis	0.00009	9,27,3
1ayl__#237	GO:0006118: electron transport	0.00009	313,228,48
1ib2a_#1101	GO:0015992: proton transport	0.00009	55,12,4
1ib2a_#1101	GO:0006119: oxidative phosphorylation	0.00009	55,12,4
1b16a_#62	GO:0006633: fatty acid biosynthesis	0.00009	9,27,3
1i2oa_#34	GO:0009086: methionine biosynthesis	0.000091	152,14,6
1e2wa1#102	GO:0019735: antimicrobial humoral response (sensu Vertebrata)	0.000091	79,16,5
1io1a_#311	GO:0019735: antimicrobial humoral response (sensu Vertebrata)	0.000091	130,16,6
1gsoa3#142	GO:0019735: antimicrobial humoral response (sensu Vertebrata)	0.000091	79,16,5
3gcb__#78	GO:0042364: water-soluble vitamin biosynthesis	0.000091	73,27,6

1e39a3#417	GO:0006953: acute-phase response	0.000092	12,20,3
1qbea_#92	GO:0006953: acute-phase response	0.000092	12,20,3
1d7ba_#87	GO:0030217: T-cell differentiation	0.000092	68,10,4
7reqb1#320	GO:0046148: pigment biosynthesis	0.000092	20,12,3
1ak5_1#376	GO:0006096: glycolysis	0.000092	32,38,5
1fjsa_#158	GO:0006956: complement activation	0.000092	20,12,3
1fo4a5#1020	GO:0042108: positive regulation of cytokine biosynthesis	0.000092	312,11,7
4kbpa2#416	GO:0015992: proton transport	0.000092	20,12,3
4kbpa2#416	GO:0006119: oxidative phosphorylation	0.000092	20,12,3
1fn9a_#271	GO:0051249: regulation of lymphocyte activation	0.000092	184,12,6
1fxla2#169	GO:0051249: regulation of lymphocyte activation	0.000092	184,12,6
1dxea_#71	GO:0006221: pyrimidine nucleotide biosynthesis	0.000092	29,22,4
1arb__#55	GO:0006956: complement activation	0.000092	20,12,3
1gdna_#30	GO:0006956: complement activation	0.000092	20,12,3
1e39a3#442	GO:0006919: caspase activation	0.000093	16,15,3
1imt_2#41	GO:0006986: response to unfolded protein	0.000093	16,15,3
1b12a_#221	GO:0016477: cell migration	0.000093	87,23,6
1gky__#34	GO:0006118: electron transport	0.000093	14,228,7
1elka_#21	GO:0045045: secretory pathway	0.000093	11,22,3
1ev2e2#286	GO:0016477: cell migration	0.000093	87,23,6
3frua1#236	GO:0042088: T-helper 1 type immune response	0.000093	61,11,4
3frua1#236	GO:0042108: positive regulation of cytokine biosynthesis	0.000093	61,11,4
1daba_#387	GO:0006221: pyrimidine nucleotide biosynthesis	0.000093	11,22,3
1b87a_#47	GO:0043123: positive regulation of I-kappaB kinase/NF-kappaB cascade	0.000093	15,16,3
1mjha_#119	GO:0030435: sporulation	0.000093	87,23,6
1ihua2#525	GO:0007017: microtubule-based process	0.000093	278,12,7
1d4ta_#80	GO:0000165: MAPKKK cascade	0.000093	22,11,3
1qhda2#323	GO:0042088: T-helper 1 type immune response	0.000094	313,11,7
1qhda2#323	GO:0042108: positive regulation of cytokine biosynthesis	0.000094	313,11,7
1i2oa_#34	GO:0009070: serine family amino acid biosynthesis	0.000094	152,26,8
1hbza_#214	GO:0006935: chemotaxis	0.000094	249,41,13
1f2la_#40	GO:0042108: positive regulation of cytokine biosynthesis	0.000094	206,11,6
1ayl__#237	GO:0042088: T-helper 1 type immune response	0.000094	313,11,7
1ayl__#237	GO:0001906: cell killing	0.000094	313,11,7
1elua_#216	GO:0000910: cytokinesis	0.000094	33,37,5
2pia_1#54	GO:0051249: regulation of lymphocyte activation	0.000095	185,12,6
1as4.1#B385	GO:0030333: antigen processing	0.000095	51,13,4
1bywa_#62	GO:0000160: two-component signal transduction system (phosphorelay)	0.000096	2,25,2
1p35a_#101	GO:0051249: regulation of lymphocyte activation	0.000096	111,12,5
1gph12#46	GO:0009116: nucleoside metabolism	0.000096	2,25,2
3lada3#463	GO:0006118: electron transport	0.000096	23,228,9
1e1aa_#261	GO:0006118: electron transport	0.000096	23,228,9
1jj2e1#42	GO:0006953: acute-phase response	0.000096	150,20,7
1hdr__#241	GO:0006118: electron transport	0.000096	23,228,9
1e19a_#281	GO:0009064: glutamine family amino acid metabolism	0.000096	108,19,6
1fjfc2#153	GO:0009064: glutamine family amino acid metabolism	0.000096	362,19,10
1ldna1#94	GO:0006099: tricarboxylic acid cycle	0.000096	159,19,7
1mkp__#216	GO:0006418: tRNA aminoacylation for protein translation	0.000096	112,26,7
1ec7a1#216	GO:0009103: lipopolysaccharide biosynthesis	0.000097	56,12,4
1ec7a1#216	GO:0009156: ribonucleoside monophosphate biosynthesis	0.000097	56,12,4
1ih7a1#57	GO:0051249: regulation of lymphocyte activation	0.000097	56,12,4
1fmta2#151	GO:0006096: glycolysis	0.000097	272,38,13
1gdha1#235	GO:0006221: pyrimidine nucleotide biosynthesis	0.000097	92,22,6
1elja_#64	GO:0019079: viral genome replication	0.000097	80,16,5
1ho1a_#211	GO:0006100: tricarboxylic acid cycle intermediate metabolism	0.000097	93,14,5
1jf9a_#379	GO:0051249: regulation of lymphocyte activation	0.000098	186,12,6

1kve.1#B199	GO:0050819: negative regulation of coagulation	0.000098	69,10,4
1kve.1#B199	GO:0030193: regulation of blood coagulation	0.000098	69,10,4
1ycsa_#162	GO:0006457: protein folding	0.000098	177,49,12
3grs_3#435	GO:0046677: response to antibiotic	0.000098	154,40,10
1jsg__#92	GO:0006265: DNA topological change	0.000098	519,10,8
1feca1#31	GO:0006207: 'de novo' pyrimidine base biosynthesis	0.000098	69,10,4
1amf__#196	GO:0015698: inorganic anion transport	0.000098	154,14,6
1fsu__#434	GO:0042108: positive regulation of cytokine biosynthesis	0.000098	315,11,7
1qdla_#211	GO:0051249: regulation of lymphocyte activation	0.000098	280,12,7
1dp0a4#756	GO:0007169: transmembrane receptor protein tyrosine kinase signaling pathway	0.000098	153,26,8
1fhoa_#74	GO:0006544: glycine metabolism	0.000098	315,11,7
1h4vb2#307	GO:0017001: antibiotic catabolism	0.000098	69,10,4
1hnja1#167	GO:0042088: T-helper 1 type immune response	0.000099	62,11,4
1hbza_#214	GO:0007169: transmembrane receptor protein tyrosine kinase signaling pathway	0.000099	249,26,10
1dzfa2#211	GO:0018108: peptidyl-tyrosine phosphorylation	0.000099	139,10,5
1cfr__#269	GO:0006096: glycolysis	0.000099	52,38,6
1nfdb1#21	GO:0016477: cell migration	0.000099	88,23,6
1fjfc2#153	GO:0006418: tRNA aminoacylation for protein translation	0.000099	362,26,12
1pma1_#79	GO:0006470: protein amino acid dephosphorylation	0.000099	63,20,5
1tpg_1#55	GO:0008015: circulation	0.0001	3,15,2
1cb6a2#1660	GO:0006826: iron ion transport	0.0001	3,15,2
1g3nc2#184	GO:0000910: cytokinesis	0.0001	7,37,3
1fjsa_#28	GO:0006919: caspase activation	0.0001	3,15,2
1avgi_#69	GO:0051249: regulation of lymphocyte activation	0.0001	281,12,7
1iba__#39	GO:0042088: T-helper 1 type immune response	0.0001	316,11,7
1iba__#39	GO:0042108: positive regulation of cytokine biosynthesis	0.0001	316,11,7
1b35b_#97	GO:0043123: positive regulation of I-kappaB kinase/NF-kappaB cascade	0.0001	271,16,8
1qora2#201	GO:0006096: glycolysis	0.0001	163,38,10
1ioaa_#75	GO:0007169: transmembrane receptor protein tyrosine kinase signaling pathway	0.0001	25,26,4
1e79h2#39	GO:0030217: T-cell differentiation	0.000101	25,10,3
1llc_2#291	GO:0006099: tricarboxylic acid cycle	0.000101	13,19,3
7mdha2#355	GO:0006099: tricarboxylic acid cycle	0.000101	13,19,3
1e6ca_#68	GO:0009064: glutamine family amino acid metabolism	0.000101	13,19,3
1pbv__#140	GO:0006099: tricarboxylic acid cycle	0.000101	13,19,3
1fc3a_#172	GO:0000910: cytokinesis	0.000101	168,37,10
1e2o__#346	GO:0051249: regulation of lymphocyte activation	0.000101	187,12,6
1e79h2#77	GO:0009206: purine ribonucleoside triphosphate biosynthesis	0.000101	28,9,3
1ez0a_#133	GO:0051249: regulation of lymphocyte activation	0.000101	112,12,5
1ct5a_#114	GO:0042401: biogenic amine biosynthesis	0.000101	25,10,3
1ddja_#681	GO:0006919: caspase activation	0.000101	212,15,7
1fjfc2#153	GO:0017001: antibiotic catabolism	0.000101	362,10,7
1fjfc2#153	GO:0030217: T-cell differentiation	0.000101	362,10,7
1a06__#147	GO:0000086: G2/M transition of mitotic cell cycle	0.000101	25,10,3
1daba_#310	GO:0009116: nucleoside metabolism	0.000101	10,25,3
1e30a_#112	GO:0006118: electron transport	0.000102	39,228,12
1fwxa2#396	GO:0042088: T-helper 1 type immune response	0.000102	209,11,6
1fwxa2#396	GO:0042108: positive regulation of cytokine biosynthesis	0.000102	209,11,6
1fwxa2#396	GO:0001906: cell killing	0.000102	209,11,6
1el0a_#28	GO:0030217: T-cell differentiation	0.000102	140,10,5
1b4ka_#108	GO:0009086: methionine biosynthesis	0.000102	94,14,5
1hbza_#96	GO:0006606: protein-nucleus import	0.000102	322,21,10
1tyfa_#31	GO:0006221: pyrimidine nucleotide biosynthesis	0.000102	136,22,7
1hwxa1#256	GO:0006100: tricarboxylic acid cycle intermediate metabolism	0.000103	48,14,4
1bmv2_#2064	GO:0019735: antimicrobial humoral response (sensu Vertebrata)	0.000103	133,16,6

2naca1#329	GO:0009070: serine family amino acid biosynthesis	0.000104	2,26,2
1ekma2#98	GO:0009070: serine family amino acid biosynthesis	0.000104	2,26,2
1pysb6#217	GO:0006418: tRNA aminoacylation for protein translation	0.000104	2,26,2
3tdt__#238	GO:0006418: tRNA aminoacylation for protein translation	0.000104	2,26,2
1a3qa2#190	GO:0001906: cell killing	0.000104	125,11,5
1erv__#80	GO:0006461: protein complex assembly	0.000104	401,24,12
1fl7b_#84	GO:0007276: gametogenesis	0.000105	4,11,2
1dfca1#1076	GO:0000165: MAPKKK cascade	0.000105	4,11,2
1ei9a_#66	GO:0009396: folic acid and derivative biosynthesis	0.000105	4,11,2
1vdra_#76	GO:0006544: glycine metabolism	0.000105	4,11,2
1gg3a3#55	GO:0042088: T-helper 1 type immune response	0.000105	4,11,2
1gg3a3#55	GO:0042108: positive regulation of cytokine biosynthesis	0.000105	4,11,2
1bmv2_#2059	GO:0006801: superoxide metabolism	0.000105	4,11,2
1jj2q_#113	GO:0042088: T-helper 1 type immune response	0.000105	4,11,2
1jj2q_#113	GO:0042108: positive regulation of cytokine biosynthesis	0.000105	4,11,2
1ihka_#161	GO:0042088: T-helper 1 type immune response	0.000105	4,11,2
1ihka_#161	GO:0042108: positive regulation of cytokine biosynthesis	0.000105	4,11,2
1hcnb_#32	GO:0007276: gametogenesis	0.000105	4,11,2
1ryp1_#51	GO:0006511: ubiquitin-dependent protein catabolism	0.000105	4,11,2
1hzia_#51	GO:0008203: cholesterol metabolism	0.000105	4,11,2
1bx4a_#75	GO:0043094: metabolic compound salvage	0.000105	4,11,2
1cxp.1#A97	GO:0006690: icosanoid metabolism	0.000105	4,11,2
1jlxa2#288	GO:0000165: MAPKKK cascade	0.000105	4,11,2
1pmaa_#131	GO:0006511: ubiquitin-dependent protein catabolism	0.000105	4,11,2
1rypi_#11	GO:0006511: ubiquitin-dependent protein catabolism	0.000105	4,11,2
1clc_1#299	GO:0030245: cellulose catabolism	0.000105	30,22,4
1xsoa_#36	GO:0006801: superoxide metabolism	0.000105	4,11,2
1f00i2#805	GO:0006461: protein complex assembly	0.000105	124,24,7
1rypd_#137	GO:0006511: ubiquitin-dependent protein catabolism	0.000105	4,11,2
1c7na_#231	GO:0006544: glycine metabolism	0.000105	210,11,6
1a65a1#108	GO:0016337: cell-cell adhesion	0.000105	253,17,8
1d4ta_#80	GO:0008284: positive regulation of cell proliferation	0.000105	22,30,4
1dgwa_#97	GO:0016477: cell migration	0.000106	11,23,3
1fvia2#134	GO:0009396: folic acid and derivative biosynthesis	0.000106	23,11,3
1whi__#59	GO:0016477: cell migration	0.000106	55,23,5
1dlfh_#4	GO:0042108: positive regulation of cytokine biosynthesis	0.000106	23,11,3
1dz4a_#264	GO:0006979: response to oxidative stress	0.000106	37,18,4
1i50b_#966	GO:0007169: transmembrane receptor protein tyrosine kinase signaling pathway	0.000106	251,26,10
1fsu__#434	GO:0006118: electron transport	0.000106	315,228,48
1a65a3#409	GO:0043123: positive regulation of I-kappaB kinase/NF-kappaB cascade	0.000107	42,16,4
1f06a1#296	GO:0009064: glutamine family amino acid metabolism	0.000107	35,19,4
1qbea_#92	GO:0006606: protein-nucleus import	0.000107	12,21,3
1ctqa_#163	GO:0007264: small GTPase mediated signal transduction	0.000107	12,21,3
1dfca1#1135	GO:0006919: caspase activation	0.000107	45,15,4
1ezvb1#29	GO:0019735: antimicrobial humoral response (sensu Vertebrata)	0.000107	42,16,4
1i2ma_#90	GO:0006606: protein-nucleus import	0.000107	12,21,3
1c5y.1#B241	GO:0006956: complement activation	0.000107	21,12,3
1c8za_#386	GO:0001906: cell killing	0.000107	211,11,6
1dlea_#238	GO:0006956: complement activation	0.000107	21,12,3
1arb__#208	GO:0006956: complement activation	0.000107	21,12,3
1autc_#45	GO:0006956: complement activation	0.000107	21,12,3
1qnja_#138	GO:0006956: complement activation	0.000107	21,12,3
1qg8a_#90	GO:0006118: electron transport	0.000108	7,228,5
1dqua_#456	GO:0006096: glycolysis	0.000108	7,38,3
1erja_#642	GO:0006118: electron transport	0.000108	7,228,5

1jjya_#293	GO:0006096: glycolysis	0.000108	7,38,3
1i9ga_#185	GO:0006096: glycolysis	0.000108	7,38,3
1mjha_#126	GO:0009116: nucleoside metabolism	0.000108	119,25,7
1c7na_#281	GO:0009086: methionine biosynthesis	0.000108	18,14,3
1zpda1#213	GO:0006779: porphyrin biosynthesis	0.000109	72,18,5
1ezvb1#29	GO:0008284: positive regulation of cell proliferation	0.000109	42,30,5
1ton__#47	GO:0019079: viral genome replication	0.000109	82,16,5
1f7ua2#324	GO:0006118: electron transport	0.000109	45,228,13
1qs0a1#119	GO:0009307: DNA restriction	0.000109	367,19,10
2sqca2#97	GO:0015992: proton transport	0.00011	114,12,5
1oaca1#495	GO:0006457: protein folding	0.00011	41,49,6
1by5a_#347	GO:0019059: initiation of viral infection	0.00011	240,10,6
2viua_#252	GO:0051249: regulation of lymphocyte activation	0.00011	285,12,7
1cbf__#208	GO:0016477: cell migration	0.00011	291,23,10
1efpa1#32	GO:0009103: lipopolysaccharide biosynthesis	0.00011	114,12,5
1ddma_#130	GO:0006367: transcription initiation from Pol II promoter	0.00011	285,12,7
1ddma_#130	GO:0006119: oxidative phosphorylation	0.00011	285,12,7
1plq_1#90	GO:0016068: type I hypersensitivity	0.00011	145,15,6
1ejda_#6	GO:0006986: response to unfolded protein	0.00011	215,15,7
1hd2a_#35	GO:0006313: DNA transposition	0.00011	145,35,9
1erza_#183	GO:0006633: fatty acid biosynthesis	0.000111	47,27,5
1jb0d_#26	GO:0051249: regulation of lymphocyte activation	0.000111	190,12,6
1ih7a1#150	GO:0042364: water-soluble vitamin biosynthesis	0.000111	47,27,5
1a81a2#182	GO:0030333: antigen processing	0.000111	104,13,5
1ds1a_#172	GO:0051249: regulation of lymphocyte activation	0.000111	190,12,6
1dfoa_#362	GO:0030333: antigen processing	0.000111	53,13,4
1qtn.1#A270	GO:0006730: one-carbon compound metabolism	0.000111	386,32,14
1eqga2#69	GO:0006633: fatty acid biosynthesis	0.000112	2,27,2
1b8ba_#466	GO:0007067: mitosis	0.000112	51,25,5
1fkna_#96	GO:0006690: icosanoid metabolism	0.000112	64,11,4
1eu8a_#126	GO:0006826: iron ion transport	0.000112	17,15,3
1im4a_#41	GO:0006418: tRNA aminoacylation for protein translation	0.000112	49,26,5
1lvl_2#222	GO:0006563: L-serine metabolism	0.000112	58,12,4
1ejba_#136	GO:0042364: water-soluble vitamin biosynthesis	0.000112	2,27,2
1dik_1#848	GO:0043094: metabolic compound salvage	0.000112	64,11,4
1dik_1#848	GO:0009396: folic acid and derivative biosynthesis	0.000112	64,11,4
1awx__#27	GO:0006917: induction of apoptosis	0.000112	9,29,3
1cmia_#86	GO:0007169: transmembrane receptor protein tyrosine kinase signaling pathway	0.000112	49,26,5
1erxa_#138	GO:0006544: glycine metabolism	0.000112	64,11,4
1e1aa_#178	GO:0051249: regulation of lymphocyte activation	0.000112	58,12,4
1e8ca1#83	GO:0043094: metabolic compound salvage	0.000112	127,11,5
1qora2#201	GO:0006099: tricarboxylic acid cycle	0.000112	163,19,7
1ge8a2#134	GO:0007169: transmembrane receptor protein tyrosine kinase signaling pathway	0.000112	202,26,9
1eq9a_#40	GO:0042108: positive regulation of cytokine biosynthesis	0.000112	64,11,4
1j79a_#187	GO:0006418: tRNA aminoacylation for protein translation	0.000113	79,26,6
1dik_3#240	GO:0042088: T-helper 1 type immune response	0.000113	213,11,6
1dik_3#240	GO:0042108: positive regulation of cytokine biosynthesis	0.000113	213,11,6
1nat__#50	GO:0042364: water-soluble vitamin biosynthesis	0.000113	242,27,10
1qfea_#68	GO:0009307: DNA restriction	0.000113	453,19,11
1ddwa_#77	GO:0006544: glycine metabolism	0.000113	322,11,7
1ddwa_#77	GO:0001906: cell killing	0.000113	322,11,7
1c8na_#165	GO:0019079: viral genome replication	0.000114	3,16,2
1esl_1#48	GO:0006418: tRNA aminoacylation for protein translation	0.000114	10,26,3
1apme_#168	GO:0007169: transmembrane receptor protein tyrosine kinase signaling pathway	0.000114	10,26,3

1d5ya3#126	GO:0019079: viral genome replication	0.000114	3,16,2
1a53__#64	GO:0009073: aromatic amino acid family biosynthesis	0.000114	3,16,2
1br9__#168	GO:0009206: purine ribonucleoside triphosphate biosynthesis	0.000114	5,9,2
1g62a_#69	GO:0006413: translational initiation	0.000114	3,16,2
1i2oa_#173	GO:0015976: carbon utilization	0.000114	26,10,3
1nsj__#190	GO:0009073: aromatic amino acid family biosynthesis	0.000114	3,16,2
1seta2#306	GO:0050819: negative regulation of coagulation	0.000114	26,10,3
1seta2#306	GO:0030193: regulation of blood coagulation	0.000114	26,10,3
1seta2#306	GO:0018108: peptidyl-tyrosine phosphorylation	0.000114	26,10,3
1b35a_#49	GO:0019079: viral genome replication	0.000114	3,16,2
1qdlb_#66	GO:0009073: aromatic amino acid family biosynthesis	0.000114	3,16,2
1eu3a2#174	GO:0019883: antigen presentation, endogenous antigen	0.000114	26,10,3
1nsca_#379	GO:0051249: regulation of lymphocyte activation	0.000114	115,12,5
1qqka_#25	GO:0048514: blood vessel morphogenesis	0.000114	5,9,2
7fabh1#71	GO:0030217: T-cell differentiation	0.000114	26,10,3
1avgi_#69	GO:0006118: electron transport	0.000114	281,228,44
1hc7a2#258	GO:0016337: cell-cell adhesion	0.000114	186,17,7
1dkra2#210	GO:0015976: carbon utilization	0.000114	26,10,3
1qo7a_#109	GO:0042401: biogenic amine biosynthesis	0.000114	26,10,3
2dnja_#158	GO:0006278: RNA-dependent DNA replication	0.000114	26,10,3
1ex1a1#284	GO:0015976: carbon utilization	0.000114	26,10,3
1b3ra1#218	GO:0009219: pyrimidine deoxyribonucleotide metabolism	0.000114	26,10,3
1ei5a3#311	GO:0017001: antibiotic catabolism	0.000114	26,10,3
1h8ca_#35	GO:0006633: fatty acid biosynthesis	0.000114	76,27,6
1i50a_#856	GO:0019835: cytolysis	0.000115	239,18,8
1iba__#39	GO:0006118: electron transport	0.000115	316,228,48
3chbd_#85	GO:0042088: T-helper 1 type immune response	0.000116	323,11,7
1qmva_#40	GO:0006118: electron transport	0.000116	77,228,18
1nat__#77	GO:0000160: two-component signal transduction system (phosphorelay)	0.000116	27,25,4
1aoea_#130	GO:0006118: electron transport	0.000116	77,228,18
1kid__#330	GO:0030217: T-cell differentiation	0.000117	144,10,5
1h6va1#116	GO:0009070: serine family amino acid biosynthesis	0.000117	26,26,4
1hdfa_#16	GO:0051249: regulation of lymphocyte activation	0.000117	192,12,6
1tiid_#39	GO:0006418: tRNA aminoacylation for protein translation	0.000117	203,26,9
1pud__#44	GO:0009073: aromatic amino acid family biosynthesis	0.000118	43,16,4
1e7wa_#8	GO:0009116: nucleoside metabolism	0.000118	83,25,6
1rypk_#41	GO:0006470: protein amino acid dephosphorylation	0.000118	13,20,3
1feca1#31	GO:0006099: tricarboxylic acid cycle	0.000118	69,19,5
1qq5a_#7	GO:0006730: one-carbon compound metabolism	0.000118	125,32,8
1quna1#113	GO:0050819: negative regulation of coagulation	0.000118	243,10,6
1quna1#113	GO:0030193: regulation of blood coagulation	0.000118	243,10,6
1quna1#113	GO:0018108: peptidyl-tyrosine phosphorylation	0.000118	243,10,6
1iira_#242	GO:0006730: one-carbon compound metabolism	0.000118	125,32,8
1cqka_#52	GO:0001906: cell killing	0.000119	65,11,4
1hava_#41	GO:0042088: T-helper 1 type immune response	0.000119	65,11,4
1tbrr1#15	GO:0006367: transcription initiation from Pol II promoter	0.000119	59,12,4
1f46a_#59	GO:0006470: protein amino acid dephosphorylation	0.000119	106,20,6
1ryp1_#112	GO:0006461: protein complex assembly	0.00012	54,24,5
1jj2q_#114	GO:0009064: glutamine family amino acid metabolism	0.00012	36,19,4
2hft_1#34	GO:0006897: endocytosis	0.00012	36,19,4
1elua_#95	GO:0017148: negative regulation of protein biosynthesis	0.00012	54,13,4
1g8fa3#419	GO:0009396: folic acid and derivative biosynthesis	0.000121	129,11,5
1icia_#9	GO:0006118: electron transport	0.000121	34,228,11
1plq_1#90	GO:0030217: T-cell differentiation	0.000121	145,10,5
1hd2a_#35	GO:0009219: pyrimidine deoxyribonucleotide metabolism	0.000121	145,10,5
1hd2a_#35	GO:0006278: RNA-dependent DNA replication	0.000121	145,10,5

1bt0a_#26	GO:0006511: ubiquitin-dependent protein catabolism	0.000121	24,11,3
1ep3a_#247	GO:0043094: metabolic compound salvage	0.000121	24,11,3
1h9da_#91	GO:0007169: transmembrane receptor protein tyrosine kinase signaling pathway	0.000121	204,26,9
1b6ra3#161	GO:0019079: viral genome replication	0.000122	137,16,6
1bmv2_#2064	GO:0016477: cell migration	0.000122	133,23,7
1h7wa2#535	GO:0006221: pyrimidine nucleotide biosynthesis	0.000123	12,22,3
1vcaa2#49	GO:0051249: regulation of lymphocyte activation	0.000123	22,12,3
1rpxa_#216	GO:0006221: pyrimidine nucleotide biosynthesis	0.000123	12,22,3
1nsj__#21	GO:0006221: pyrimidine nucleotide biosynthesis	0.000123	96,22,6
2btva_#597	GO:0006879: iron ion homeostasis	0.000123	22,12,3
1qo2a_#223	GO:0006221: pyrimidine nucleotide biosynthesis	0.000123	12,22,3
1hw5a2#64	GO:0006919: caspase activation	0.000123	148,15,6
1g3qa_#171	GO:0045045: secretory pathway	0.000123	12,22,3
1ihua2#521	GO:0000160: two-component signal transduction system (phosphorelay)	0.000123	52,25,5
1czan1#92	GO:0006118: electron transport	0.000123	538,228,72
1ep3a_#19	GO:0006221: pyrimidine nucleotide biosynthesis	0.000123	12,22,3
1ig8a_#203	GO:0006096: glycolysis	0.000123	54,38,6
1arb__#193	GO:0006956: complement activation	0.000123	22,12,3
1nsj__#178	GO:0042364: water-soluble vitamin biosynthesis	0.000123	77,27,6
1cex__#109	GO:0006096: glycolysis	0.000124	34,38,5
1hc7a2#258	GO:0006935: chemotaxis	0.000124	186,41,11
1bywa_#30	GO:0048514: blood vessel morphogenesis	0.000124	283,9,6
1aky_2#133	GO:0042088: T-helper 1 type immune response	0.000125	130,11,5
1io1a_#311	GO:0001906: cell killing	0.000125	130,11,5
2arca_#55	GO:0042088: T-helper 1 type immune response	0.000125	130,11,5
2arca_#55	GO:0042108: positive regulation of cytokine biosynthesis	0.000125	130,11,5
1b8aa2#368	GO:0042088: T-helper 1 type immune response	0.000125	130,11,5
1b8aa2#368	GO:0042108: positive regulation of cytokine biosynthesis	0.000125	130,11,5
1i85a_#14	GO:0051249: regulation of lymphocyte activation	0.000126	4,12,2
1jr1a1#68	GO:0009156: ribonucleoside monophosphate biosynthesis	0.000126	4,12,2
2gsaa_#246	GO:0046148: pigment biosynthesis	0.000126	4,12,2
3aky_1#194	GO:0046677: response to antibiotic	0.000126	7,40,3
1tdj_1#35	GO:0006563: L-serine metabolism	0.000126	4,12,2
1ezvd1#213	GO:0006119: oxidative phosphorylation	0.000126	4,12,2
1bccb2#423	GO:0006119: oxidative phosphorylation	0.000126	4,12,2
1jj2q_#113	GO:0051249: regulation of lymphocyte activation	0.000126	4,12,2
1hr6b2#255	GO:0006119: oxidative phosphorylation	0.000126	4,12,2
1tvda_#57D	GO:0051249: regulation of lymphocyte activation	0.000126	4,12,2
7odca1#391	GO:0009156: ribonucleoside monophosphate biosynthesis	0.000126	4,12,2
1hzia_#51	GO:0051249: regulation of lymphocyte activation	0.000126	4,12,2
1g61a_#2115	GO:0007017: microtubule-based process	0.000126	4,12,2
2sli_2#326	GO:0008285: negative regulation of cell proliferation	0.000126	227,19,8
1hw5a2#64	GO:0006606: protein-nucleus import	0.000126	148,21,7
1dpga2#334	GO:0050819: negative regulation of coagulation	0.000126	246,10,6
1dpga2#334	GO:0030193: regulation of blood coagulation	0.000126	246,10,6
1ds1a_#172	GO:0006118: electron transport	0.000126	190,228,33
1hq8a_#206	GO:0006461: protein complex assembly	0.000126	281,24,10
1i50b_#378	GO:0006099: tricarboxylic acid cycle	0.000127	70,19,5
1fu6a_#35	GO:0008284: positive regulation of cell proliferation	0.000127	23,30,4
1qhoa3#488	GO:0019883: antigen presentation, endogenous antigen	0.000128	27,10,3
1az9_2#257	GO:0019883: antigen presentation, endogenous antigen	0.000128	27,10,3
1a06__#205	GO:0000086: G2/M transition of mitotic cell cycle	0.000128	27,10,3
1fzqa_#28	GO:0006606: protein-nucleus import	0.000128	33,21,4
1llc_2#271	GO:0006099: tricarboxylic acid cycle	0.000128	14,19,3
1ii7a_#275	GO:0006100: tricarboxylic acid cycle intermediate metabolism	0.000128	19,14,3

1gdea_#195	GO:0006100: tricarboxylic acid cycle intermediate metabolism	0.000128	19,14,3
1flga_#161	GO:0006457: protein folding	0.000128	128,49,10
1cipa2#34	GO:0007017: microtubule-based process	0.000128	60,12,4
1cipa2#34	GO:0006221: pyrimidine nucleotide biosynthesis	0.000128	60,22,5
1c7na_#95	GO:0006919: caspase activation	0.000128	149,15,6
1bev1_#86	GO:0016477: cell migration	0.000128	296,23,10
1nfdb1#21	GO:0006461: protein complex assembly	0.000128	88,24,6
1qj5a_#268	GO:0006221: pyrimidine nucleotide biosynthesis	0.000128	60,22,5
1qj5a_#268	GO:0009156: ribonucleoside monophosphate biosynthesis	0.000128	60,12,4
1ac6a_#43	GO:0030217: T-cell differentiation	0.000129	147,10,5
1danh_#104	GO:0018108: peptidyl-tyrosine phosphorylation	0.000129	147,10,5
1fjfj_#68	GO:0042088: T-helper 1 type immune response	0.000129	218,11,6
1fjfj_#68	GO:0042108: positive regulation of cytokine biosynthesis	0.000129	218,11,6
1gsa_2#218	GO:0051249: regulation of lymphocyte activation	0.000129	118,12,5
1jjya_#324	GO:0006730: one-carbon compound metabolism	0.000129	65,32,6
1a8i__#688	GO:0006096: glycolysis	0.000129	203,38,11
1ldna1#94	GO:0006418: tRNA aminoacylation for protein translation	0.000129	159,26,8
1cnv__#181	GO:0006730: one-carbon compound metabolism	0.000129	65,32,6
1i7qa_#321	GO:0030217: T-cell differentiation	0.000129	147,10,5
1fhga_#119	GO:0016337: cell-cell adhesion	0.00013	3,17,2
2sqca2#97	GO:0006099: tricarboxylic acid cycle	0.00013	114,19,6
2ltn.1#A63	GO:0042088: T-helper 1 type immune response	0.00013	131,11,5
2ltn.1#A63	GO:0042108: positive regulation of cytokine biosynthesis	0.00013	131,11,5
2ltn.1#A63	GO:0001906: cell killing	0.00013	131,11,5
1eur__#84	GO:0019735: antimicrobial humoral response (sensu Vertebrata)	0.00013	85,16,5
1kit_2#398	GO:0007169: transmembrane receptor protein tyrosine kinase signaling pathway	0.00013	81,26,6
1dssg1#4	GO:0006100: tricarboxylic acid cycle intermediate metabolism	0.000131	51,14,4
1xgsa2#82	GO:0016337: cell-cell adhesion	0.000131	261,17,8
1e5ea_#71	GO:0009086: methionine biosynthesis	0.000131	51,14,4
1edza1#206	GO:0006953: acute-phase response	0.000132	352,20,10
1gox__#124	GO:0006099: tricarboxylic acid cycle	0.000132	298,19,9
1hf8a_#49	GO:0006313: DNA transposition	0.000133	8,35,3
1jj2e1#42	GO:0006919: caspase activation	0.000133	150,15,6
1e8ca3#111	GO:0009252: peptidoglycan biosynthesis	0.000133	244,18,8
2cb5a_#312	GO:0006919: caspase activation	0.000134	92,15,5
1quqb_#87	GO:0001906: cell killing	0.000134	67,11,4
1prea2#308	GO:0042088: T-helper 1 type immune response	0.000134	67,11,4
1prea2#308	GO:0042108: positive regulation of cytokine biosynthesis	0.000134	67,11,4
1prea2#308	GO:0001906: cell killing	0.000134	67,11,4
2arca_#55	GO:0016337: cell-cell adhesion	0.000134	130,17,6
1el0a_#28	GO:0007399: neurogenesis	0.000134	140,37,9
1tul__#102	GO:0042088: T-helper 1 type immune response	0.000134	67,11,4
1tul__#102	GO:0042108: positive regulation of cytokine biosynthesis	0.000134	67,11,4
1tul__#102	GO:0001906: cell killing	0.000134	67,11,4
1qora2#201	GO:0006100: tricarboxylic acid cycle intermediate metabolism	0.000134	163,14,6
1hcl__#280	GO:0008284: positive regulation of cell proliferation	0.000134	70,30,6
1c4ra_#258	GO:0001906: cell killing	0.000134	67,11,4
1hxxa_#84	GO:0006457: protein folding	0.000135	6,49,3
2btva_#749	GO:0009116: nucleoside metabolism	0.000135	28,25,4
1qf5a_#15	GO:0007067: mitosis	0.000135	28,25,4
1f42a2#123	GO:0006935: chemotaxis	0.000135	335,41,15
1a8d_1#87	GO:0016337: cell-cell adhesion	0.000135	191,17,7
1alo_4#634	GO:0009116: nucleoside metabolism	0.000135	53,25,5
1cpza_#6	GO:0042108: positive regulation of cytokine biosynthesis	0.000135	132,11,5
1pyma_#224	GO:0006935: chemotaxis	0.000136	7,41,3

1hbza_#214	GO:0030217: T-cell differentiation	0.000136	249,10,6
1nuka_#80	GO:0051249: regulation of lymphocyte activation	0.000136	197,12,6
1enfa1#92	GO:0006118: electron transport	0.000136	29,228,10
3frua1#236	GO:0051249: regulation of lymphocyte activation	0.000136	61,12,4
2mev3_#108	GO:0051249: regulation of lymphocyte activation	0.000136	197,12,6
1svb_2#165	GO:0006457: protein folding	0.000136	27,49,5
1ac5__#72	GO:0009070: serine family amino acid biosynthesis	0.000136	51,26,5
1quna1#113	GO:0006457: protein folding	0.000136	243,49,14
1doza_#109	GO:0006563: L-serine metabolism	0.000136	61,12,4
1qj4a_#96	GO:0016042: lipid catabolism	0.000137	9,31,3
1bccb2#274	GO:0006118: electron transport	0.000137	52,228,14
1dx4a_#180	GO:0016042: lipid catabolism	0.000137	9,31,3
1fxla2#169	GO:0016477: cell migration	0.000137	184,23,8
1e79h2#39	GO:0042088: T-helper 1 type immune response	0.000138	25,11,3
1e79h2#39	GO:0042108: positive regulation of cytokine biosynthesis	0.000138	25,11,3
1wdna_#79	GO:0019079: viral genome replication	0.000138	17,16,3
1cx1a_#52	GO:0030333: antigen processing	0.000138	21,13,3
1e5ka_#168	GO:0017148: negative regulation of protein biosynthesis	0.000138	21,13,3
1ksia1#555	GO:0007264: small GTPase mediated signal transduction	0.000138	13,21,3
1i7oa1#119	GO:0006643: membrane lipid metabolism	0.000138	21,13,3
1ihma_#1068	GO:0016337: cell-cell adhesion	0.000138	16,17,3
1c7na_#95	GO:0050819: negative regulation of coagulation	0.000138	149,10,5
1c7na_#95	GO:0030193: regulation of blood coagulation	0.000138	149,10,5
1c7na_#95	GO:0018108: peptidyl-tyrosine phosphorylation	0.000138	149,10,5
1pme__#25	GO:0000165: MAPKKK cascade	0.000138	25,11,3
1dpja_#320	GO:0006606: protein-nucleus import	0.000138	13,21,3
1a06__#147	GO:0000165: MAPKKK cascade	0.000138	25,11,3
1ioaa_#75	GO:0001906: cell killing	0.000138	25,11,3
1eq9a_#40	GO:0006606: protein-nucleus import	0.000138	64,21,5
1dkia_#76P	GO:0016068: type I hypersensitivity	0.000139	48,15,4
1a4ya_#104	GO:0030245: cellulose catabolism	0.000139	194,22,8
1a4ya_#104	GO:0006221: pyrimidine nucleotide biosynthesis	0.000139	194,22,8
1hdma1#98	GO:0016337: cell-cell adhesion	0.000139	42,17,4
1smaa1#95	GO:0016068: type I hypersensitivity	0.00014	223,15,7
1hxxa_#232	GO:0006953: acute-phase response	0.00014	282,20,9
3lada1#116	GO:0009396: folic acid and derivative biosynthesis	0.00014	133,11,5
1bmv2_#2064	GO:0042088: T-helper 1 type immune response	0.00014	133,11,5
1bmv2_#2064	GO:0042108: positive regulation of cytokine biosynthesis	0.00014	133,11,5
1bmv2_#2064	GO:0001906: cell killing	0.00014	133,11,5
1qfta_#98	GO:0051249: regulation of lymphocyte activation	0.00014	198,12,6
1vmoa_#106	GO:0006118: electron transport	0.00014	167,228,30
1rpxa_#216	GO:0015671: oxygen transport	0.000141	12,23,3
1dlfh_#4	GO:0051249: regulation of lymphocyte activation	0.000141	23,12,3
5ruba1#259	GO:0009073: aromatic amino acid family biosynthesis	0.000141	45,16,4
1ibja_#217	GO:0006559: L-phenylalanine catabolism	0.000143	5,10,2
1dhn__#88	GO:0006278: RNA-dependent DNA replication	0.000143	5,10,2
1f8ra2#324	GO:0006207: 'de novo' pyrimidine base biosynthesis	0.000143	5,10,2
1bfta_#285	GO:0045893: positive regulation of transcription, DNA-dependent	0.000143	5,10,2
1thw__#66	GO:0015976: carbon utilization	0.000143	5,10,2
1pmi__#341	GO:0019883: antigen presentation, endogenous antigen	0.000143	5,10,2
1g5ba_#193	GO:0006354: RNA elongation	0.000143	5,10,2
1qqqa_#247	GO:0009219: pyrimidine deoxyribonucleotide metabolism	0.000143	5,10,2
1hnua_#190	GO:0009167: purine ribonucleoside monophosphate metabolism	0.000143	5,10,2
1ffya2#268	GO:0019883: antigen presentation, endogenous antigen	0.000143	5,10,2
1pii_1#40	GO:0042401: biogenic amine biosynthesis	0.000143	5,10,2
1pii_1#40	GO:0006568: tryptophan metabolism	0.000143	5,10,2

1t7pa2#663	GO:0019883: antigen presentation, endogenous antigen	0.000143	28,10,3
1e3ua_#187	GO:0017001: antibiotic catabolism	0.000143	5,10,2
1duvg1#135	GO:0000051: urea cycle intermediate metabolism	0.000143	5,10,2
1vns__#158	GO:0006268: DNA unwinding	0.000143	5,10,2
1e30a_#55	GO:0015976: carbon utilization	0.000143	5,10,2
1h61a_#176	GO:0042401: biogenic amine biosynthesis	0.000143	28,10,3
1h61a_#176	GO:0006568: tryptophan metabolism	0.000143	28,10,3
1imva_#255	GO:0006313: DNA transposition	0.000143	506,35,17
1aba__#17	GO:0015976: carbon utilization	0.000143	28,10,3
1hvba_#247	GO:0017001: antibiotic catabolism	0.000143	28,10,3
1bvza3#416	GO:0006568: tryptophan metabolism	0.000143	5,10,2
1ir3a_#1192	GO:0000086: G2/M transition of mitotic cell cycle	0.000143	28,10,3
1qfma1#139	GO:0016337: cell-cell adhesion	0.000143	264,17,8
1hrna_#38	GO:0006278: RNA-dependent DNA replication	0.000143	5,10,2
2bb2_2#134	GO:0006367: transcription initiation from Pol II promoter	0.000144	199,12,6
2bb2_2#134	GO:0009615: response to virus	0.000144	199,12,6
1fyhb1#99	GO:0016477: cell migration	0.000144	31,23,4
1dr9a1#39	GO:0016068: type I hypersensitivity	0.000144	224,15,7
1fi2a_#104	GO:0016477: cell migration	0.000144	31,23,4
1f2na_#138	GO:0019079: viral genome replication	0.000145	87,16,5
1bfd_1#230	GO:0006779: porphyrin biosynthesis	0.000145	40,18,4
1j9la_#82	GO:0042559: pteridine and derivative biosynthesis	0.000145	134,11,5
1ev2e2#286	GO:0019735: antimicrobial humoral response (sensu Vertebrata)	0.000145	87,16,5
1ejfa_#10	GO:0006690: icosanoid metabolism	0.000145	134,11,5
1qrra_#208	GO:0009156: ribonucleoside monophosphate biosynthesis	0.000145	62,12,4
3mag__#134	GO:0007017: microtubule-based process	0.000145	62,12,4
3mag__#134	GO:0006563: L-serine metabolism	0.000145	62,12,4
1aqua_#126	GO:0009396: folic acid and derivative biosynthesis	0.000145	472,11,8
1ehia2#236	GO:0009252: peptidoglycan biosynthesis	0.000146	3,18,2
2dnja_#97	GO:0006953: acute-phase response	0.000147	110,20,6
1a0i_2#222	GO:0050819: negative regulation of coagulation	0.000147	151,10,5
1a0i_2#222	GO:0030193: regulation of blood coagulation	0.000147	151,10,5
1gox__#124	GO:0009103: lipopolysaccharide biosynthesis	0.000147	298,12,7
1sw6a_#464	GO:0000082: G1/S transition of mitotic cell cycle	0.000148	4,13,2
1qj4a_#41	GO:0006805: xenobiotic metabolism	0.000148	4,13,2
1qj4a_#41	GO:0017000: antibiotic biosynthesis	0.000148	4,13,2
1nox__#88	GO:0008285: negative regulation of cell proliferation	0.000148	38,19,4
1vcbb_#46	GO:0000082: G1/S transition of mitotic cell cycle	0.000148	4,13,2
1ek1a2#324	GO:0017000: antibiotic biosynthesis	0.000148	4,13,2
1bd8__#103	GO:0045786: negative regulation of cell cycle	0.000148	4,13,2
1bd8__#103	GO:0000082: G1/S transition of mitotic cell cycle	0.000148	4,13,2
1tib__#202	GO:0016042: lipid catabolism	0.000148	2,31,2
1d5ta1#224	GO:0017148: negative regulation of protein biosynthesis	0.000148	4,13,2
1i7oa1#63	GO:0008285: negative regulation of cell proliferation	0.000148	38,19,4
1bn7a_#38	GO:0006805: xenobiotic metabolism	0.000148	4,13,2
1bn7a_#38	GO:0017000: antibiotic biosynthesis	0.000148	4,13,2
1ihka_#161	GO:0045892: negative regulation of transcription, DNA-dependent	0.000148	4,13,2
1ibja_#201	GO:0017000: antibiotic biosynthesis	0.000148	4,13,2
1h72c2#235	GO:0045892: negative regulation of transcription, DNA-dependent	0.000148	4,13,2
1c4xa_#69	GO:0006805: xenobiotic metabolism	0.000148	4,13,2
1c4xa_#69	GO:0017000: antibiotic biosynthesis	0.000148	4,13,2
1g61a_#2115	GO:0000082: G1/S transition of mitotic cell cycle	0.000148	4,13,2
1leha1#177	GO:0006099: tricarboxylic acid cycle	0.000148	38,19,4
1hc7a2#258	GO:0016477: cell migration	0.000148	186,23,8
1hlga_#104	GO:0006805: xenobiotic metabolism	0.000148	4,13,2
1mrj__#73	GO:0017148: negative regulation of protein biosynthesis	0.000148	57,13,4

1qq5a_#7	GO:0009116: nucleoside metabolism	0.000148	125,25,7
1a7ca_#370	GO:0006953: acute-phase response	0.000148	36,20,4
1iira_#242	GO:0009116: nucleoside metabolism	0.000148	125,25,7
1e7wa_#8	GO:0009070: serine family amino acid biosynthesis	0.000149	83,26,6
1alo_4#634	GO:0046677: response to antibiotic	0.000149	53,40,6
1evqa_#305	GO:0006207: 'de novo' pyrimidine base biosynthesis	0.000149	384,10,7
1gjwa2#458	GO:0009086: methionine biosynthesis	0.00015	20,14,3
2rspa_#113	GO:0015074: DNA integration	0.00015	14,20,3
1ewna_#109	GO:0006511: ubiquitin-dependent protein catabolism	0.00015	69,11,4
1c4zd_#51	GO:0042108: positive regulation of cytokine biosynthesis	0.00015	135,11,5
1c4zd_#51	GO:0006544: glycine metabolism	0.00015	135,11,5
1bwda_#295	GO:0006730: one-carbon compound metabolism	0.00015	96,32,7
1d2ka1#164	GO:0042364: water-soluble vitamin biosynthesis	0.00015	50,27,5
1fnf_1#1146	GO:0006953: acute-phase response	0.00015	14,20,3
2cpl__#32	GO:0006457: protein folding	0.000151	15,49,4
1dfaa2#211	GO:0001558: regulation of cell growth	0.000151	77,10,4
1egja_#343	GO:0042113: B-cell activation	0.000151	32,9,3
1cjca2#365	GO:0006096: glycolysis	0.000151	56,38,6
1dr9a1#39	GO:0042088: T-helper 1 type immune response	0.000151	224,11,6
1dr9a1#39	GO:0042108: positive regulation of cytokine biosynthesis	0.000151	224,11,6
1e6pa2#46	GO:0015976: carbon utilization	0.000151	77,10,4
1e6pa2#46	GO:0009167: purine ribonucleoside monophosphate metabolism	0.000151	77,10,4
1i2oa_#34	GO:0015976: carbon utilization	0.000152	152,10,5
1dpja_#322	GO:0006606: protein-nucleus import	0.000153	269,21,9
1ax4a_#430	GO:0009086: methionine biosynthesis	0.000153	53,14,4
1doka_#52	GO:0006935: chemotaxis	0.000154	52,41,6
1nfdb1#21	GO:0019735: antimicrobial humoral response (sensu Vertebrata)	0.000154	88,16,5
1nat__#50	GO:0030435: sporulation	0.000154	242,23,9
1hd2a_#35	GO:0006221: pyrimidine nucleotide biosynthesis	0.000154	145,22,7
1dfoa_#237	GO:0016485: protein processing	0.000155	26,11,3
1i2oa_#173	GO:0006098: pentose-phosphate shunt	0.000155	26,11,3
1i2oa_#173	GO:0009396: folic acid and derivative biosynthesis	0.000155	26,11,3
1ad1a_#186	GO:0006098: pentose-phosphate shunt	0.000155	26,11,3
1fy7a_#388	GO:0006367: transcription initiation from Pol II promoter	0.000155	63,12,4
1fzqa_#28	GO:0006221: pyrimidine nucleotide biosynthesis	0.000155	33,22,4
1fbna_#145	GO:0006221: pyrimidine nucleotide biosynthesis	0.000155	33,22,4
1hcc__#39	GO:0042742: defense response to bacteria	0.000155	11,26,3
7fabh1#71	GO:0042088: T-helper 1 type immune response	0.000155	26,11,3
7fabh1#71	GO:0042108: positive regulation of cytokine biosynthesis	0.000155	26,11,3
7fabh1#71	GO:0001906: cell killing	0.000155	26,11,3
1g6oa_#116	GO:0006118: electron transport	0.000155	302,228,46
1dbxa_#75	GO:0007017: microtubule-based process	0.000155	63,12,4
1dnpa2#18	GO:0009064: glutamine family amino acid metabolism	0.000155	73,19,5
1fkna_#99	GO:0006418: tRNA aminoacylation for protein translation	0.000155	11,26,3
1pme__#25	GO:0006917: induction of apoptosis	0.000155	25,29,4
1qq5a_#7	GO:0009252: peptidoglycan biosynthesis	0.000155	125,18,6
1hq0a_#867	GO:0017001: antibiotic catabolism	0.000156	153,10,5
1dp0a4#756	GO:0045595: regulation of cell differentiation	0.000156	153,10,5
1hcl__#267	GO:0000910: cytokinesis	0.000158	8,37,3
1zpda1#213	GO:0006118: electron transport	0.000158	72,228,17
1aw1a_#169	GO:0009070: serine family amino acid biosynthesis	0.000158	28,26,4
1dpsa_#36	GO:0006916: anti-apoptosis	0.000158	183,18,7
1plq_1#90	GO:0006917: induction of apoptosis	0.000158	145,29,8
1c8za_#386	GO:0007169: transmembrane receptor protein tyrosine kinase signaling pathway	0.000158	211,26,9
1bdfa2#60	GO:0030333: antigen processing	0.000159	22,13,3

1ft9a1#162	GO:0009219: pyrimidine deoxyribonucleotide metabolism	0.000159	78,10,4
1ecpa_#46	GO:0030333: antigen processing	0.000159	22,13,3
1i1ka_#255	GO:0006986: response to unfolded protein	0.000159	19,15,3
1bkds_#882	GO:0030245: cellulose catabolism	0.000159	13,22,3
1f0ya2#126	GO:0006221: pyrimidine nucleotide biosynthesis	0.000159	13,22,3
1dmla1#134	GO:0019883: antigen presentation, endogenous antigen	0.000159	78,10,4
1dmla1#134	GO:0030217: T-cell differentiation	0.000159	78,10,4
1ej8a_#189	GO:0050819: negative regulation of coagulation	0.000159	256,10,6
1ej8a_#189	GO:0030193: regulation of blood coagulation	0.000159	256,10,6
1ej8a_#189	GO:0018108: peptidyl-tyrosine phosphorylation	0.000159	256,10,6
1ycsa_#230	GO:0006917: induction of apoptosis	0.000159	10,29,3
1f8v.1#A116	GO:0019883: antigen presentation, endogenous antigen	0.000159	78,10,4
1cjca2#37	GO:0009307: DNA restriction	0.000159	15,19,3
1glqa2#53	GO:0008015: circulation	0.000159	19,15,3
1a4ya_#47	GO:0009064: glutamine family amino acid metabolism	0.000159	172,19,7
1i7qa_#277	GO:0009064: glutamine family amino acid metabolism	0.000159	15,19,3
1c8na_#239	GO:0030333: antigen processing	0.000159	22,13,3
1dr9a2#139	GO:0045595: regulation of cell differentiation	0.000159	78,10,4
1erv__#57	GO:0043094: metabolic compound salvage	0.000159	70,11,4
1hlwa_#91	GO:0050819: negative regulation of coagulation	0.000159	29,10,3
1hlwa_#91	GO:0030193: regulation of blood coagulation	0.000159	29,10,3
1hlwa_#91	GO:0018108: peptidyl-tyrosine phosphorylation	0.000159	29,10,3
1cwva2#675	GO:0045595: regulation of cell differentiation	0.000159	29,10,3
1cwva2#675	GO:0030217: T-cell differentiation	0.000159	29,10,3
1br9__#86	GO:0006801: superoxide metabolism	0.000159	70,11,4
2bpa1_#45	GO:0006461: protein complex assembly	0.000159	179,24,8
1eaja_#59	GO:0016337: cell-cell adhesion	0.00016	196,17,7
1g6oa_#116	GO:0051249: regulation of lymphocyte activation	0.00016	302,12,7
1g6sa_#64	GO:0006418: tRNA aminoacylation for protein translation	0.00016	84,26,6
1g6sa_#64	GO:0007169: transmembrane receptor protein tyrosine kinase signaling pathway	0.00016	84,26,6
1ypta_#418	GO:0006119: oxidative phosphorylation	0.000161	24,12,3
3grs_3#435	GO:0017001: antibiotic catabolism	0.000161	154,10,5
1evqa_#305	GO:0006099: tricarboxylic acid cycle	0.000161	384,19,10
2hlca_#68	GO:0006956: complement activation	0.000161	24,12,3
1c9la2#12	GO:0019883: antigen presentation, endogenous antigen	0.000162	257,10,6
1phm_2#253	GO:0018108: peptidyl-tyrosine phosphorylation	0.000162	257,10,6
1phm_2#253	GO:0006275: regulation of DNA replication	0.000162	257,10,6
1dt6a_#181	GO:0006118: electron transport	0.000162	35,228,11
1b3qa2#623	GO:0030217: T-cell differentiation	0.000162	257,10,6
1qdlb_#170	GO:0019079: viral genome replication	0.000162	89,16,5
2pola2#218	GO:0019835: cytolysis	0.000162	126,18,6
1c5y.1#B128	GO:0008285: negative regulation of cell proliferation	0.000163	3,19,2
1f8ea_#177	GO:0009064: glutamine family amino acid metabolism	0.000163	3,19,2
1fiy__#300	GO:0006099: tricarboxylic acid cycle	0.000163	3,19,2
1qdlb_#66	GO:0009064: glutamine family amino acid metabolism	0.000163	3,19,2
1bd8__#151	GO:0008285: negative regulation of cell proliferation	0.000163	3,19,2
1b4aa1#50	GO:0009064: glutamine family amino acid metabolism	0.000163	3,19,2
2duba_#212	GO:0006418: tRNA aminoacylation for protein translation	0.000164	53,26,5
1db3a_#266	GO:0006418: tRNA aminoacylation for protein translation	0.000164	53,26,5
1rrpb_#65	GO:0006457: protein folding	0.000164	44,49,6
1qfma1#139	GO:0007169: transmembrane receptor protein tyrosine kinase signaling pathway	0.000164	264,26,10
1fkna_#96	GO:0051249: regulation of lymphocyte activation	0.000165	64,12,4
1eyza3#196	GO:0009252: peptidoglycan biosynthesis	0.000165	16,18,3
2pgd_1#421	GO:0015992: proton transport	0.000165	64,12,4

1fbna_#144	GO:0009103: lipopolysaccharide biosynthesis	0.000165	64,12,4
1qq4a_#143	GO:0019079: viral genome replication	0.000165	18,16,3
1ii7a_#43	GO:0009064: glutamine family amino acid metabolism	0.000165	39,19,4
1gpea1#273	GO:0006118: electron transport	0.000165	79,228,18
1qhda2#323	GO:0006457: protein folding	0.000166	313,49,16
2pia_1#99	GO:0016068: type I hypersensitivity	0.000166	156,15,6
3prn__#145	GO:0030217: T-cell differentiation	0.000166	155,10,5
1bjt__#890	GO:0045595: regulation of cell differentiation	0.000166	258,10,6
1bjt__#890	GO:0018108: peptidyl-tyrosine phosphorylation	0.000166	258,10,6
1mai__#108	GO:0016337: cell-cell adhesion	0.000166	135,17,6
1bu7a_#130	GO:0006118: electron transport	0.000167	11,228,6
1f2aa_#27	GO:0006278: RNA-dependent DNA replication	0.000167	79,10,4
1ja1a1#497	GO:0006118: electron transport	0.000167	11,228,6
1d4oa_#135	GO:0006118: electron transport	0.000167	11,228,6
1gsoa3#142	GO:0045595: regulation of cell differentiation	0.000167	79,10,4
1gpea1#273	GO:0018108: peptidyl-tyrosine phosphorylation	0.000167	79,10,4
1hbza_#96	GO:0030245: cellulose catabolism	0.000167	322,22,10
1erza_#183	GO:0009073: aromatic amino acid family biosynthesis	0.000168	47,16,4
1dcpa_#25	GO:0019079: viral genome replication	0.000168	47,16,4
1fx7a1#44	GO:0006357: regulation of transcription from Pol II promoter	0.000169	46,30,5
1qtn.1#A270	GO:0009307: DNA restriction	0.000169	386,19,10
1jj22_#78	GO:0030333: antigen processing	0.00017	59,13,4
1ekma1#545	GO:0016337: cell-cell adhesion	0.00017	84,17,5
1qqka_#19	GO:0048514: blood vessel morphogenesis	0.000171	6,9,2
3grs_2#187	GO:0006096: glycolysis	0.000171	8,38,3
1es9a_#89	GO:0006096: glycolysis	0.000171	8,38,3
3lada2#167	GO:0006096: glycolysis	0.000171	8,38,3
1rypb_#114	GO:0046677: response to antibiotic	0.000171	133,40,9
2mev3_#107	GO:0006461: protein complex assembly	0.000172	31,24,4
1jb0d_#26	GO:0016477: cell migration	0.000172	190,23,8
1ds1a_#172	GO:0016477: cell migration	0.000172	190,23,8
2hlca_#226	GO:0006606: protein-nucleus import	0.000172	67,21,5
1edza2#120	GO:0009086: methionine biosynthesis	0.000173	4,14,2
1ei9a_#66	GO:0009086: methionine biosynthesis	0.000173	4,14,2
1jf9a_#119	GO:0009086: methionine biosynthesis	0.000173	4,14,2
1gg1a_#209	GO:0006100: tricarboxylic acid cycle intermediate metabolism	0.000173	4,14,2
1ibja_#201	GO:0009086: methionine biosynthesis	0.000173	4,14,2
1ycqa_#24	GO:0016567: protein ubiquitination	0.000173	4,14,2
1bs0a_#252	GO:0009086: methionine biosynthesis	0.000173	4,14,2
1e0ca1#49	GO:0015698: inorganic anion transport	0.000173	4,14,2
1e0ca2#261	GO:0015698: inorganic anion transport	0.000173	4,14,2
1fgga_#134	GO:0006118: electron transport	0.000173	193,228,33
1fgga_#134	GO:0006935: chemotaxis	0.000173	193,41,11
1h9ra2#238	GO:0015698: inorganic anion transport	0.000173	4,14,2
1c7na_#231	GO:0006730: one-carbon compound metabolism	0.000173	210,32,10
1fl7b_#51	GO:0007276: gametogenesis	0.000174	5,11,2
1fhua1#133	GO:0009396: folic acid and derivative biosynthesis	0.000174	5,11,2
1bfta_#285	GO:0042088: T-helper 1 type immune response	0.000174	5,11,2
1bfta_#285	GO:0042108: positive regulation of cytokine biosynthesis	0.000174	5,11,2
1eqga2#47	GO:0006690: icosanoid metabolism	0.000174	5,11,2
1thw__#66	GO:0043094: metabolic compound salvage	0.000174	5,11,2
1hcna_#12	GO:0007276: gametogenesis	0.000174	5,11,2
1e9ga_#78	GO:0016485: protein processing	0.000174	5,11,2
2cb5a_#373	GO:0016485: protein processing	0.000174	27,11,3
1rypl_#97	GO:0006511: ubiquitin-dependent protein catabolism	0.000174	5,11,2
1poxa3#439	GO:0006100: tricarboxylic acid cycle intermediate metabolism	0.000174	21,14,3

1dzfa2#164	GO:0006606: protein-nucleus import	0.000174	14,21,3
1jjya_#96	GO:0006606: protein-nucleus import	0.000174	14,21,3
1oaa__#235	GO:0042559: pteridine and derivative biosynthesis	0.000174	5,11,2
1b8fa_#91	GO:0006511: ubiquitin-dependent protein catabolism	0.000174	5,11,2
1qqka_#25	GO:0000165: MAPKKK cascade	0.000174	5,11,2
1icia_#9	GO:0006221: pyrimidine nucleotide biosynthesis	0.000174	34,22,4
1dih_2#222	GO:0046677: response to antibiotic	0.000174	311,40,14
1h5qa_#70	GO:0006633: fatty acid biosynthesis	0.000174	11,27,3
1bf6a_#183	GO:0030245: cellulose catabolism	0.000174	34,22,4
1bf6a_#183	GO:0006221: pyrimidine nucleotide biosynthesis	0.000174	34,22,4
1e79d2#36	GO:0007399: neurogenesis	0.000174	432,37,16
1f3ya_#6	GO:0051049: regulation of transport	0.000175	80,10,4
1im4a_#144	GO:0009116: nucleoside metabolism	0.000175	89,25,6
1h6va3#486	GO:0051249: regulation of lymphocyte activation	0.000175	65,12,4
1f2la_#40	GO:0051249: regulation of lymphocyte activation	0.000175	206,12,6
1hxxa_#173	GO:0042088: T-helper 1 type immune response	0.000175	230,11,6
1hxxa_#173	GO:0042108: positive regulation of cytokine biosynthesis	0.000175	230,11,6
1hxxa_#173	GO:0001906: cell killing	0.000175	230,11,6
1a9xa6#760	GO:0019883: antigen presentation, endogenous antigen	0.000175	80,10,4
1a9xa6#760	GO:0050819: negative regulation of coagulation	0.000175	80,10,4
1a9xa6#760	GO:0030193: regulation of blood coagulation	0.000175	80,10,4
1a9xa6#760	GO:0018108: peptidyl-tyrosine phosphorylation	0.000175	80,10,4
1qqsa_#35	GO:0015976: carbon utilization	0.000175	80,10,4
1jjya_#324	GO:0006563: L-serine metabolism	0.000175	65,12,4
3sil__#98	GO:0006916: anti-apoptosis	0.000175	186,18,7
1cnv__#181	GO:0006563: L-serine metabolism	0.000175	65,12,4
1hwxa1#223	GO:0009252: peptidoglycan biosynthesis	0.000175	186,18,7
1e3ua_#164	GO:0009252: peptidoglycan biosynthesis	0.000176	42,18,4
1h6ka1#134	GO:0006268: DNA unwinding	0.000176	30,10,3
1d8ca_#442	GO:0042401: biogenic amine biosynthesis	0.000176	30,10,3
1d8ca_#442	GO:0006568: tryptophan metabolism	0.000176	30,10,3
1tkia_#142	GO:0000086: G2/M transition of mitotic cell cycle	0.000176	30,10,3
2hlca_#139	GO:0008285: negative regulation of cell proliferation	0.000176	75,19,5
1pii_2#326	GO:0006568: tryptophan metabolism	0.000177	157,10,5
1pii_2#326	GO:0006207: 'de novo' pyrimidine base biosynthesis	0.000177	157,10,5
1bmv2_#2064	GO:0006457: protein folding	0.000177	133,49,10
1xgsa2#82	GO:0050819: negative regulation of coagulation	0.000177	261,10,6
1xgsa2#82	GO:0030193: regulation of blood coagulation	0.000177	261,10,6
1xgsa2#82	GO:0018108: peptidyl-tyrosine phosphorylation	0.000177	261,10,6
1dp0a1#309	GO:0006118: electron transport	0.000177	47,228,13
1fjfj_#68	GO:0006457: protein folding	0.000178	218,49,13
1evqa_#61	GO:0042088: T-helper 1 type immune response	0.000179	140,11,5
1evqa_#61	GO:0042108: positive regulation of cytokine biosynthesis	0.000179	140,11,5
1e25a_#262	GO:0017000: antibiotic biosynthesis	0.000179	115,13,5
1g0sa_#74	GO:0016477: cell migration	0.00018	247,23,9
1ecpa_#62	GO:0009070: serine family amino acid biosynthesis	0.00018	54,26,5
1fjsa_#28	GO:0006953: acute-phase response	0.000181	3,20,2
1pysb5#584	GO:0030333: antigen processing	0.000181	60,13,4
2sli_2#326	GO:0008284: positive regulation of cell proliferation	0.000181	227,30,10
1a9xa3#78	GO:0009167: purine ribonucleoside monophosphate metabolism	0.000181	262,10,6
1pwt__#8	GO:0019883: antigen presentation, endogenous antigen	0.000181	262,10,6
1i7qa_#321	GO:0019079: viral genome replication	0.000181	147,16,6
1bvsa3#12	GO:0006418: tRNA aminoacylation for protein translation	0.000182	29,26,4
1dfoa_#88	GO:0030333: antigen processing	0.000182	23,13,3
1bywa_#64	GO:0000160: two-component signal transduction system (phosphorelay)	0.000182	12,25,3
1qr4a2#166	GO:0016477: cell migration	0.000182	13,23,3

1dlfh_#4	GO:0030333: antigen processing	0.000182	23,13,3
1io1a_#233	GO:0006418: tRNA aminoacylation for protein translation	0.000182	86,26,6
1h8ua_#113	GO:0006313: DNA transposition	0.000182	63,35,6
1iira_#326	GO:0009070: serine family amino acid biosynthesis	0.000182	86,26,6
1c9oa_#49	GO:0006418: tRNA aminoacylation for protein translation	0.000182	86,26,6
1abrb1#71	GO:0030333: antigen processing	0.000182	23,13,3
1cs1a_#82	GO:0019883: antigen presentation, endogenous antigen	0.000182	158,10,5
1cs1a_#82	GO:0006568: tryptophan metabolism	0.000182	158,10,5
1cs1a_#82	GO:0006278: RNA-dependent DNA replication	0.000182	158,10,5
1cwva2#675	GO:0007169: transmembrane receptor protein tyrosine kinase signaling pathway	0.000182	29,26,4
1mkp__#283	GO:0006470: protein amino acid dephosphorylation	0.000183	38,20,4
1thg__#3	GO:0006457: protein folding	0.000183	85,49,8
1kwaa_#513	GO:0019079: viral genome replication	0.000183	48,16,4
1fc4a_#205	GO:0009070: serine family amino acid biosynthesis	0.000183	215,26,9
1pii_2#326	GO:0007264: small GTPase mediated signal transduction	0.000183	157,21,7
1nfdb1#34	GO:0007169: transmembrane receptor protein tyrosine kinase signaling pathway	0.000183	167,26,8
2cuaa_#83	GO:0016337: cell-cell adhesion	0.000183	45,17,4
1fw9a_#152	GO:0006418: tRNA aminoacylation for protein translation	0.000184	124,26,7
1jsg__#92	GO:0007169: transmembrane receptor protein tyrosine kinase signaling pathway	0.000184	519,26,14
1jsg__#92	GO:0006935: chemotaxis	0.000184	519,41,19
1ihua2#343	GO:0009307: DNA restriction	0.000184	176,19,7
1eno__#83	GO:0030435: sporulation	0.000185	33,23,4
1iow_1#37	GO:0006568: tryptophan metabolism	0.000185	263,10,6
1dih_2#222	GO:0009064: glutamine family amino acid metabolism	0.000185	311,19,9
1feza_#185	GO:0006563: L-serine metabolism	0.000186	66,12,4
1dt6a_#449	GO:0006470: protein amino acid dephosphorylation	0.000186	15,20,3
1hyrc1#247	GO:0009615: response to virus	0.000186	66,12,4
1h6va2#245	GO:0042364: water-soluble vitamin biosynthesis	0.000187	83,27,6
1h6va2#245	GO:0006096: glycolysis	0.000187	83,38,7
1hnja1#167	GO:0016477: cell migration	0.000188	62,23,5
1e31a_#77	GO:0006096: glycolysis	0.000188	37,38,5
1muca1#245	GO:0006096: glycolysis	0.000188	37,38,5
1miob_#338	GO:0006099: tricarboxylic acid cycle	0.000188	76,19,5
1jr3a2#166	GO:0006118: electron transport	0.000188	30,228,10
1ie5a_#102	GO:0006457: protein folding	0.000188	64,49,7
1c3pa_#6	GO:0016568: chromatin modification	0.000188	73,11,4
1f0xa1#278	GO:0006935: chemotaxis	0.000189	162,41,10
1jjya_#324	GO:0006221: pyrimidine nucleotide biosynthesis	0.000189	65,22,5
1qfma1#139	GO:0030217: T-cell differentiation	0.000189	264,10,6
1dfaa2#226	GO:0006313: DNA transposition	0.00019	2,35,2
1f1za1#210	GO:0006313: DNA transposition	0.00019	2,35,2
1ft9a1#201	GO:0019735: antimicrobial humoral response (sensu Vertebrata)	0.00019	92,16,5
1jkma_#317	GO:0006100: tricarboxylic acid cycle intermediate metabolism	0.00019	56,14,4
1bev1_#86	GO:0043123: positive regulation of I-kappaB kinase/NF-kappaB cascade	0.00019	296,16,8
2reb_1#138	GO:0006100: tricarboxylic acid cycle intermediate metabolism	0.00019	56,14,4
1fc3a_#172	GO:0006418: tRNA aminoacylation for protein translation	0.000191	168,26,8
1bxoa_#307	GO:0016068: type I hypersensitivity	0.000191	99,15,5
1a9xa3#78	GO:0006221: pyrimidine nucleotide biosynthesis	0.000191	262,22,9
1xnb__#149	GO:0006221: pyrimidine nucleotide biosynthesis	0.000191	150,22,7
1dpja_#322	GO:0007169: transmembrane receptor protein tyrosine kinase signaling pathway	0.000192	269,26,10
2mpra_#190	GO:0016485: protein processing	0.000192	349,11,7
1foha5#218	GO:0009116: nucleoside metabolism	0.000192	57,25,5
1eny__#147	GO:0006779: porphyrin biosynthesis	0.000192	81,18,5

1ddja_#726	GO:0030217: T-cell differentiation	0.000193	82,10,4
1xis__#214	GO:0009167: purine ribonucleoside monophosphate metabolism	0.000193	82,10,4
1b3qa2#623	GO:0006916: anti-apoptosis	0.000193	257,18,8
1ge8a2#134	GO:0016337: cell-cell adhesion	0.000193	202,17,7
1apj__#2062	GO:0006275: regulation of DNA replication	0.000194	31,10,3
1hr6a1#41	GO:0051049: regulation of transport	0.000194	31,10,3
1efnb_#102	GO:0006354: RNA elongation	0.000194	31,10,3
1fyhb1#99	GO:0019883: antigen presentation, endogenous antigen	0.000194	31,10,3
1dmla2#313	GO:0006275: regulation of DNA replication	0.000194	31,10,3
1pud__#197	GO:0042401: biogenic amine biosynthesis	0.000194	31,10,3
1pud__#197	GO:0006568: tryptophan metabolism	0.000194	31,10,3
1pud__#197	GO:0006207: 'de novo' pyrimidine base biosynthesis	0.000194	31,10,3
1ejda_#213	GO:0006953: acute-phase response	0.000194	227,20,8
1qoua_#64	GO:0006457: protein folding	0.000194	29,49,5
1qrra_#11	GO:0006099: tricarboxylic acid cycle	0.000195	16,19,3
1jeya_#104	GO:0043094: metabolic compound salvage	0.000195	28,11,3
1ak7__#152	GO:0006511: ubiquitin-dependent protein catabolism	0.000195	28,11,3
1fjsa_#163	GO:0008285: negative regulation of cell proliferation	0.000195	16,19,3
1aw1a_#169	GO:0006098: pentose-phosphate shunt	0.000195	28,11,3
1nls__#77	GO:0043123: positive regulation of I-kappaB kinase/NF-kappaB cascade	0.000195	19,16,3
1epwa3#248	GO:0001906: cell killing	0.000195	28,11,3
1zfpe_#67	GO:0006511: ubiquitin-dependent protein catabolism	0.000195	28,11,3
1ton__#196	GO:0019079: viral genome replication	0.000195	19,16,3
1qq4a_#63	GO:0019079: viral genome replication	0.000195	19,16,3
2cpl__#53	GO:0019079: viral genome replication	0.000195	19,16,3
1ihua2#343	GO:0009116: nucleoside metabolism	0.000195	176,25,8
1nal1_#203	GO:0006221: pyrimidine nucleotide biosynthesis	0.000196	35,22,4
1f06a1#296	GO:0006221: pyrimidine nucleotide biosynthesis	0.000196	35,22,4
1div_1#141	GO:0006730: one-carbon compound metabolism	0.000196	70,32,6
1as4.1#A336	GO:0016337: cell-cell adhesion	0.000196	276,17,8
1quqb_#87	GO:0051249: regulation of lymphocyte activation	0.000197	67,12,4
1prea2#308	GO:0009615: response to virus	0.000197	67,12,4
1jg8a_#172	GO:0046148: pigment biosynthesis	0.000197	67,12,4
1ejda_#6	GO:0006606: protein-nucleus import	0.000197	215,21,8
1mrp__#172	GO:0000160: two-component signal transduction system (phosphorelay)	0.000198	91,25,6
1e3ua_#205	GO:0006313: DNA transposition	0.000198	9,35,3
1a6o__#297	GO:0042113: B-cell activation	0.000198	35,9,3
1uch__#50	GO:0006544: glycine metabolism	0.000198	74,11,4
1ycsb1#412	GO:0015992: proton transport	0.000198	129,12,5
1ycsb1#412	GO:0006119: oxidative phosphorylation	0.000198	129,12,5
1fp1d2#214	GO:0043094: metabolic compound salvage	0.000198	143,11,5
1dfca1#1076	GO:0045859: regulation of protein kinase activity	0.000199	4,15,2
1erxa_#139	GO:0016068: type I hypersensitivity	0.000199	4,15,2
1a7ca_#204	GO:0019835: cytolysis	0.000199	17,18,3
1seia_#50	GO:0006979: response to oxidative stress	0.000199	17,18,3
1j6za1#84	GO:0006986: response to unfolded protein	0.000199	4,15,2
1i4ua_#142	GO:0016068: type I hypersensitivity	0.000199	4,15,2
1i0da_#153	GO:0006826: iron ion transport	0.000199	4,15,2
1cxp.1#A97	GO:0008015: circulation	0.000199	4,15,2
1jlxa2#288	GO:0045859: regulation of protein kinase activity	0.000199	4,15,2
1bjt__#890	GO:0006916: anti-apoptosis	0.000199	258,18,8
1e19a_#281	GO:0009086: methionine biosynthesis	0.000199	108,14,5
1fjsa_#28	GO:0006606: protein-nucleus import	0.0002	3,21,2
1ev2e2#286	GO:0016337: cell-cell adhesion	0.0002	87,17,5
1imva_#255	GO:0006606: protein-nucleus import	0.0002	506,21,12
1qtn.1#A270	GO:0006118: electron transport	0.0002	386,228,55

1nsj__#178	GO:0006099: tricarboxylic acid cycle	0.0002	77,19,5
1euca2#234	GO:0030245: cellulose catabolism	0.000201	14,22,3
1euca2#234	GO:0006221: pyrimidine nucleotide biosynthesis	0.000201	14,22,3
1dfca4#1478	GO:0006935: chemotaxis	0.000201	19,41,4
1cs6a4#363	GO:0007399: neurogenesis	0.000202	21,37,4
1djna1#86	GO:0006221: pyrimidine nucleotide biosynthesis	0.000203	66,22,5
1edza1#206	GO:0042108: positive regulation of cytokine biosynthesis	0.000203	352,11,7
1dofa_#15	GO:0006099: tricarboxylic acid cycle	0.000204	179,19,7
1kid__#330	GO:0042088: T-helper 1 type immune response	0.000204	144,11,5
1kid__#330	GO:0042108: positive regulation of cytokine biosynthesis	0.000204	144,11,5
1crua_#29	GO:0006118: electron transport	0.000204	25,228,9
1ir3a_#1056	GO:0007169: transmembrane receptor protein tyrosine kinase signaling pathway	0.000205	12,26,3
1ps1a_#83	GO:0015992: proton transport	0.000205	26,12,3
1hzxa_#139	GO:0015992: proton transport	0.000205	26,12,3
1fgka_#668	GO:0007169: transmembrane receptor protein tyrosine kinase signaling pathway	0.000205	12,26,3
1mpp__#103	GO:0006418: tRNA aminoacylation for protein translation	0.000205	12,26,3
1gcua1#36	GO:0009103: lipopolysaccharide biosynthesis	0.000205	26,12,3
1h6va1#116	GO:0006119: oxidative phosphorylation	0.000205	26,12,3
1h6va1#116	GO:0006563: L-serine metabolism	0.000205	26,12,3
1fp5a2#478	GO:0051249: regulation of lymphocyte activation	0.000205	26,12,3
1i50b_#966	GO:0016477: cell migration	0.000205	251,23,9
1io1a_#311	GO:0051249: regulation of lymphocyte activation	0.000206	130,12,5
1dt6a_#385	GO:0017148: negative regulation of protein biosynthesis	0.000206	62,13,4
1b8aa2#368	GO:0051249: regulation of lymphocyte activation	0.000206	130,12,5
1lci__#26	GO:0015992: proton transport	0.000206	130,12,5
1c3pa_#6	GO:0016042: lipid catabolism	0.000206	73,31,6
1a6o__#297	GO:0006935: chemotaxis	0.000207	35,41,5
1hxxa_#278	GO:0001906: cell killing	0.000207	237,11,6
2scub2#126	GO:0030333: antigen processing	0.000208	24,13,3
1ksia1#555	GO:0007186: G-protein coupled receptor protein signaling pathway	0.000208	13,24,3
1h9oa_#83	GO:0006418: tRNA aminoacylation for protein translation	0.000208	30,26,4
1quba4#235	GO:0006461: protein complex assembly	0.000208	96,24,6
1nuka_#80	GO:0006935: chemotaxis	0.000208	197,41,11
1nsj__#124	GO:0006099: tricarboxylic acid cycle	0.000208	124,19,6
1j75a_#166	GO:0006461: protein complex assembly	0.000208	96,24,6
1b3aa_#15	GO:0009615: response to virus	0.000209	5,12,2
1gof_3#436	GO:0007017: microtubule-based process	0.000209	5,12,2
1h68a_#183	GO:0015992: proton transport	0.000209	5,12,2
1exta3#123	GO:0006956: complement activation	0.000209	5,12,2
1zyma2#177	GO:0009401: phosphoenolpyruvate-dependent sugar phosphotransferase system	0.000209	5,12,2
1br9__#168	GO:0015992: proton transport	0.000209	5,12,2
1br9__#168	GO:0006119: oxidative phosphorylation	0.000209	5,12,2
1jj2q_#118	GO:0007017: microtubule-based process	0.000209	5,12,2
1dxy_1#170	GO:0006563: L-serine metabolism	0.000209	5,12,2
1qqqa_#247	GO:0006879: iron ion homeostasis	0.000209	5,12,2
1hnua_#190	GO:0009156: ribonucleoside monophosphate biosynthesis	0.000209	5,12,2
1h7ea_#106	GO:0009103: lipopolysaccharide biosynthesis	0.000209	5,12,2
1jqca_#272	GO:0006367: transcription initiation from Pol II promoter	0.000209	5,12,2
3mag__#294	GO:0006367: transcription initiation from Pol II promoter	0.000209	5,12,2
1d7ba_#87	GO:0051249: regulation of lymphocyte activation	0.000209	68,12,4
1c4oa2#536	GO:0009103: lipopolysaccharide biosynthesis	0.000209	5,12,2
1feca3#467	GO:0006511: ubiquitin-dependent protein catabolism	0.000209	75,11,4
2sqca1#553	GO:0006956: complement activation	0.000209	5,12,2
2oata_#81	GO:0046148: pigment biosynthesis	0.000209	5,12,2

1qf9a_#117	GO:0007017: microtubule-based process	0.000209	68,12,4
3sil__#317	GO:0019735: antimicrobial humoral response (sensu Vertebrata)	0.000209	300,16,8
1dpja_#322	GO:0018108: peptidyl-tyrosine phosphorylation	0.00021	269,10,6
1dpja_#322	GO:0006275: regulation of DNA replication	0.00021	269,10,6
1plq_1#90	GO:0001906: cell killing	0.000211	145,11,5
1e8ca3#111	GO:0009064: glutamine family amino acid metabolism	0.000211	244,19,8
1opy__#68	GO:0006935: chemotaxis	0.000212	308,41,14
1ekma1#545	GO:0030217: T-cell differentiation	0.000212	84,10,4
1arb__#210	GO:0006606: protein-nucleus import	0.000212	70,21,5
1hcl__#180	GO:0000086: G2/M transition of mitotic cell cycle	0.000213	6,10,2
1hcl__#180	GO:0006275: regulation of DNA replication	0.000213	6,10,2
1i7qa_#485	GO:0042401: biogenic amine biosynthesis	0.000213	6,10,2
1i7qa_#485	GO:0006568: tryptophan metabolism	0.000213	6,10,2
1g6ha_#180	GO:0019883: antigen presentation, endogenous antigen	0.000213	6,10,2
1fmb__#60	GO:0006278: RNA-dependent DNA replication	0.000213	6,10,2
1i85a_#101	GO:0045595: regulation of cell differentiation	0.000213	6,10,2
1ecra_#101	GO:0030217: T-cell differentiation	0.000213	6,10,2
1dpja_#265	GO:0017001: antibiotic catabolism	0.000213	6,10,2
1hxma2#136	GO:0000051: urea cycle intermediate metabolism	0.000213	6,10,2
1d09b1#42	GO:0006207: 'de novo' pyrimidine base biosynthesis	0.000213	6,10,2
1dpe__#190	GO:0015976: carbon utilization	0.000213	6,10,2
1hhsa_#328	GO:0006278: RNA-dependent DNA replication	0.000213	6,10,2
1atia2#249	GO:0006207: 'de novo' pyrimidine base biosynthesis	0.000213	6,10,2
1qqqa_#38	GO:0009219: pyrimidine deoxyribonucleotide metabolism	0.000213	6,10,2
1mfa_1#82L	GO:0030217: T-cell differentiation	0.000213	6,10,2
1autc_#188	GO:0050819: negative regulation of coagulation	0.000213	6,10,2
1autc_#188	GO:0030193: regulation of blood coagulation	0.000213	6,10,2
1hxxa_#112	GO:0019059: initiation of viral infection	0.000213	6,10,2
1qqqa_#130	GO:0009219: pyrimidine deoxyribonucleotide metabolism	0.000213	6,10,2
1qasa3#602	GO:0015976: carbon utilization	0.000213	6,10,2
1d3ga_#109	GO:0006207: 'de novo' pyrimidine base biosynthesis	0.000213	6,10,2
2ltn.1#A63	GO:0051249: regulation of lymphocyte activation	0.000213	131,12,5
1g51a3#525	GO:0046677: response to antibiotic	0.000213	239,40,12
1ak5_1#376	GO:0042401: biogenic amine biosynthesis	0.000214	32,10,3
1ak5_1#376	GO:0006568: tryptophan metabolism	0.000214	32,10,3
1ak5_1#376	GO:0009167: purine ribonucleoside monophosphate metabolism	0.000214	32,10,3
1f7da_#97	GO:0050819: negative regulation of coagulation	0.000214	32,10,3
1f7da_#97	GO:0030193: regulation of blood coagulation	0.000214	32,10,3
1bupa2#199	GO:0019883: antigen presentation, endogenous antigen	0.000214	32,10,3
1jj2n_#77	GO:0006096: glycolysis	0.000214	38,38,5
1gpea1#271	GO:0016477: cell migration	0.000214	196,23,8
1e5ma1#159	GO:0006935: chemotaxis	0.000215	8,41,3
1iba__#39	GO:0051249: regulation of lymphocyte activation	0.000215	316,12,7
1awx__#17	GO:0006917: induction of apoptosis	0.000216	11,29,3
1bg6_2#73	GO:0009396: folic acid and derivative biosynthesis	0.000216	29,11,3
1nula_#15	GO:0043094: metabolic compound salvage	0.000216	29,11,3
1cg2a1#43	GO:0006418: tRNA aminoacylation for protein translation	0.000216	171,26,8
1h6va2#245	GO:0006779: porphyrin biosynthesis	0.000216	83,18,5
1dhs__#149	GO:0006986: response to unfolded protein	0.000217	21,15,3
1fuia2#273	GO:0009086: methionine biosynthesis	0.000217	110,14,5
1i50a_#856	GO:0001906: cell killing	0.000217	239,11,6
1gsa_2#218	GO:0006953: acute-phase response	0.000217	118,20,6
1dzfa2#211	GO:0006461: protein complex assembly	0.000217	139,24,7
1fc4a_#205	GO:0006096: glycolysis	0.000217	215,38,11
1deua_#151	GO:0001906: cell killing	0.000217	239,11,6
1f97a2#199	GO:0006953: acute-phase response	0.000217	118,20,6

1rypf_#35	GO:0006826: iron ion transport	0.000217	21,15,3
1dp0a4#756	GO:0030245: cellulose catabolism	0.000217	153,22,7
1fhoa_#74	GO:0016477: cell migration	0.000217	315,23,10
1ifc__#89	GO:0030217: T-cell differentiation	0.000218	164,10,5
1i50a_#856	GO:0008015: circulation	0.000218	239,15,7
1a65a1#108	GO:0016477: cell migration	0.000218	253,23,9
1ifc__#89	GO:0016068: type I hypersensitivity	0.000219	164,15,6
1c9la2#61	GO:0016337: cell-cell adhesion	0.000219	206,17,7
1g6oa_#116	GO:0019735: antimicrobial humoral response (sensu Vertebrata)	0.000219	302,16,8
1lucb_#70	GO:0030245: cellulose catabolism	0.000219	36,22,4
1p35a_#49	GO:0006606: protein-nucleus import	0.000219	112,21,6
1d6aa_#52	GO:0009116: nucleoside metabolism	0.000219	230,25,9
1e2ka_#59	GO:0006221: pyrimidine nucleotide biosynthesis	0.00022	3,22,2
1ceo__#63	GO:0030245: cellulose catabolism	0.00022	3,22,2
2ak3a1#122	GO:0006221: pyrimidine nucleotide biosynthesis	0.00022	3,22,2
1fnf_3#1409	GO:0001906: cell killing	0.00022	76,11,4
1e8ga2#201	GO:0030333: antigen processing	0.00022	286,13,7
1dy9.1#C229	GO:0006457: protein folding	0.00022	111,49,9
1h8ca_#35	GO:0006511: ubiquitin-dependent protein catabolism	0.00022	76,11,4
3grx__#55	GO:0006445: regulation of translation	0.00022	102,15,5
1aqt_2#45	GO:0006119: oxidative phosphorylation	0.000221	69,12,4
1fbl_1#398	GO:0008285: negative regulation of cell proliferation	0.000221	42,19,4
1by5a_#347	GO:0042088: T-helper 1 type immune response	0.000222	240,11,6
1by5a_#347	GO:0042108: positive regulation of cytokine biosynthesis	0.000222	240,11,6
1fc4a_#205	GO:0009103: lipopolysaccharide biosynthesis	0.000222	215,12,6
1eur__#84	GO:0045595: regulation of cell differentiation	0.000222	85,10,4
1hq8a_#206	GO:0016337: cell-cell adhesion	0.000223	281,17,8
1xgsa2#82	GO:0006730: one-carbon compound metabolism	0.000223	261,32,11
1f97a1#62	GO:0046677: response to antibiotic	0.000224	591,40,20
1qi9a_#217	GO:0006096: glycolysis	0.000224	2,38,2
16pk__#204	GO:0006096: glycolysis	0.000224	2,38,2
1jcfa1#26	GO:0006118: electron transport	0.000224	48,228,13
1danh_#104	GO:0006690: icosanoid metabolism	0.000225	147,11,5
1e44b_#75	GO:0018108: peptidyl-tyrosine phosphorylation	0.000225	165,10,5
1bwvs_#117	GO:0006643: membrane lipid metabolism	0.000225	287,13,7
1a9xa3#78	GO:0000910: cytokinesis	0.000225	262,37,12
1ea5a_#99	GO:0007169: transmembrane receptor protein tyrosine kinase signaling pathway	0.000225	172,26,8
1i7qa_#321	GO:0042108: positive regulation of cytokine biosynthesis	0.000225	147,11,5
1hdoa_#189	GO:0009396: folic acid and derivative biosynthesis	0.000225	147,11,5
1dp0a4#756	GO:0019735: antimicrobial humoral response (sensu Vertebrata)	0.000226	153,16,6
1e44b_#75	GO:0006919: caspase activation	0.000227	165,15,6
1qtn.1#B412	GO:0043123: positive regulation of I-kappaB kinase/NF-kappaB cascade	0.000228	4,16,2
1e39a3#442	GO:0006953: acute-phase response	0.000228	16,20,3
1b37a1#46	GO:0043123: positive regulation of I-kappaB kinase/NF-kappaB cascade	0.000228	4,16,2
1pvc1_#182	GO:0019079: viral genome replication	0.000228	4,16,2
1iow_1#37	GO:0009252: peptidoglycan biosynthesis	0.000228	263,18,8
1iow_1#37	GO:0006779: porphyrin biosynthesis	0.000228	263,18,8
1tvda_#57D	GO:0019735: antimicrobial humoral response (sensu Vertebrata)	0.000228	4,16,2
2cpl__#138	GO:0019079: viral genome replication	0.000228	4,16,2
1hzia_#51	GO:0019735: antimicrobial humoral response (sensu Vertebrata)	0.000228	4,16,2
1qtn.1#B433	GO:0043123: positive regulation of I-kappaB kinase/NF-kappaB cascade	0.000228	4,16,2
1g61a_#2115	GO:0006413: translational initiation	0.000228	4,16,2
1g61a_#2202	GO:0006413: translational initiation	0.000228	4,16,2
1g62a_#59	GO:0006413: translational initiation	0.000228	4,16,2
1g62a_#16	GO:0006413: translational initiation	0.000228	4,16,2

1g62a_#177	GO:0006413: translational initiation	0.000228	4,16,2
1cnv__#264	GO:0042364: water-soluble vitamin biosynthesis	0.000228	86,27,6
1e0ta2#275	GO:0042364: water-soluble vitamin biosynthesis	0.000228	86,27,6
1zpda1#213	GO:0006730: one-carbon compound metabolism	0.000229	72,32,6
1hxxa_#232	GO:0016337: cell-cell adhesion	0.000229	282,17,8
1e3a.1#A30	GO:0006367: transcription initiation from Pol II promoter	0.000229	133,12,5
1qj8a_#64	GO:0006457: protein folding	0.000229	66,49,7
1qhoa3#488	GO:0051249: regulation of lymphocyte activation	0.00023	27,12,3
1rypg_#159	GO:0000398: nuclear mRNA splicing, via spliceosome	0.00023	27,12,3
1oaa__#175	GO:0006633: fatty acid biosynthesis	0.00023	12,27,3
1a81a1#51	GO:0006953: acute-phase response	0.00023	75,20,5
1qfta_#98	GO:0016477: cell migration	0.00023	198,23,8
1elva1#604	GO:0006956: complement activation	0.00023	27,12,3
1bio__#210	GO:0006606: protein-nucleus import	0.00023	113,21,6
1dfaa2#211	GO:0016485: protein processing	0.000231	77,11,4
1dfoa_#88	GO:0009086: methionine biosynthesis	0.000231	23,14,3
1qmva_#40	GO:0006544: glycine metabolism	0.000231	77,11,4
1e6pa2#46	GO:0043094: metabolic compound salvage	0.000231	77,11,4
1nsj__#178	GO:0043094: metabolic compound salvage	0.000231	77,11,4
1cl1a_#184	GO:0006779: porphyrin biosynthesis	0.000232	45,18,4
1cmxa_#207	GO:0018108: peptidyl-tyrosine phosphorylation	0.000232	86,10,4
1cmxa_#207	GO:0006275: regulation of DNA replication	0.000232	86,10,4
1qfea_#68	GO:0006118: electron transport	0.000232	453,228,62
1e0ta2#275	GO:0009167: purine ribonucleoside monophosphate metabolism	0.000232	86,10,4
1euvb_#72	GO:0006457: protein folding	0.000233	7,49,3
1eq9a_#32	GO:0043123: positive regulation of I-kappaB kinase/NF-kappaB cascade	0.000233	96,16,5
1tif__#58	GO:0030333: antigen processing	0.000233	64,13,4
1hw5a2#64	GO:0001906: cell killing	0.000233	148,11,5
1ie5a_#102	GO:0030333: antigen processing	0.000233	64,13,4
1nat__#50	GO:0009396: folic acid and derivative biosynthesis	0.000233	242,11,6
1ac6a_#34	GO:0007169: transmembrane receptor protein tyrosine kinase signaling pathway	0.000233	57,26,5
1bfg__#22	GO:0006935: chemotaxis	0.000234	56,41,6
1qf9a_#117	GO:0045045: secretory pathway	0.000234	68,22,5
1qf9a_#117	GO:0006221: pyrimidine nucleotide biosynthesis	0.000234	68,22,5
1e79d2#36	GO:0006919: caspase activation	0.000234	432,15,9
1b6cb_#346	GO:0000910: cytokinesis	0.000235	9,37,3
1a6o__#233	GO:0000910: cytokinesis	0.000235	9,37,3
1fmta2#30	GO:0000160: two-component signal transduction system (phosphorelay)	0.000235	13,25,3
1f97a1#62	GO:0051249: regulation of lymphocyte activation	0.000235	591,12,9
1pjca1#254	GO:0009064: glutamine family amino acid metabolism	0.000235	17,19,3
1pme__#207	GO:0000910: cytokinesis	0.000235	9,37,3
1fhga_#59	GO:0045595: regulation of cell differentiation	0.000235	33,10,3
1fhga_#59	GO:0019883: antigen presentation, endogenous antigen	0.000235	33,10,3
1c4ka1#33	GO:0006207: 'de novo' pyrimidine base biosynthesis	0.000235	33,10,3
1euca1#64	GO:0009167: purine ribonucleoside monophosphate metabolism	0.000235	33,10,3
1dqga_#81	GO:0006643: membrane lipid metabolism	0.000235	25,13,3
2cpl__#20	GO:0016337: cell-cell adhesion	0.000236	90,17,5
1p35a_#49	GO:0006457: protein folding	0.000236	112,49,9
1f5aa1#441	GO:0007169: transmembrane receptor protein tyrosine kinase signaling pathway	0.000236	129,26,7
1h6va1#27	GO:0009073: aromatic amino acid family biosynthesis	0.000236	224,16,7
1fyhb1#99	GO:0007169: transmembrane receptor protein tyrosine kinase signaling pathway	0.000237	31,26,4
2dnja_#158	GO:0016042: lipid catabolism	0.000237	26,31,4
1ib2a_#1101	GO:0006633: fatty acid biosynthesis	0.000237	55,27,5

1ir3a_#1015	GO:0007169: transmembrane receptor protein tyrosine kinase signaling pathway	0.000237	31,26,4
2napa2#292	GO:0006221: pyrimidine nucleotide biosynthesis	0.000237	108,22,6
1av4_1#492	GO:0009252: peptidoglycan biosynthesis	0.000238	18,18,3
1as4.1#A336	GO:0007169: transmembrane receptor protein tyrosine kinase signaling pathway	0.000238	276,26,10
1a8l_1#74	GO:0019835: cytolysis	0.000238	18,18,3
1nfdb1#34	GO:0045595: regulation of cell differentiation	0.000238	167,10,5
1ilr1_#145	GO:0048514: blood vessel morphogenesis	0.000239	7,9,2
1dqga_#51	GO:0048514: blood vessel morphogenesis	0.000239	7,9,2
1jlxa1#147	GO:0048514: blood vessel morphogenesis	0.000239	7,9,2
1g25a_#22	GO:0006461: protein complex assembly	0.000239	367,24,11
1htp__#16	GO:0006457: protein folding	0.000239	256,49,14
1g6oa_#116	GO:0006470: protein amino acid dephosphorylation	0.000239	302,20,9
1e79d2#36	GO:0006606: protein-nucleus import	0.000239	432,21,11
1f3ya_#6	GO:0006897: endocytosis	0.00024	80,19,5
1cpy__#93	GO:0006563: L-serine metabolism	0.00024	218,12,6
2viua_#252	GO:0006606: protein-nucleus import	0.00024	285,21,9
1a0i_2#222	GO:0007399: neurogenesis	0.00024	151,37,9
1ofga1#84	GO:0009396: folic acid and derivative biosynthesis	0.00024	30,11,3
2sqca2#97	GO:0007264: small GTPase mediated signal transduction	0.000241	114,21,6
1b65a_#324	GO:0046677: response to antibiotic	0.000241	37,40,5
1czan1#92	GO:0016477: cell migration	0.000242	538,23,13
1quna1#113	GO:0006919: caspase activation	0.000242	243,15,7
1dmla1#134	GO:0042088: T-helper 1 type immune response	0.000243	78,11,4
1dmla1#134	GO:0042108: positive regulation of cytokine biosynthesis	0.000243	78,11,4
1bura1#328	GO:0043094: metabolic compound salvage	0.000243	78,11,4
1bura1#328	GO:0006098: pentose-phosphate shunt	0.000243	78,11,4
1as4.1#A336	GO:0050819: negative regulation of coagulation	0.000243	276,10,6
1as4.1#A336	GO:0030193: regulation of blood coagulation	0.000243	276,10,6
1as4.1#A336	GO:0018108: peptidyl-tyrosine phosphorylation	0.000243	276,10,6
1f8v.1#A116	GO:0042088: T-helper 1 type immune response	0.000243	78,11,4
1d0na3#305	GO:0019059: initiation of viral infection	0.000243	276,10,6
1d0na3#305	GO:0030217: T-cell differentiation	0.000243	276,10,6
1mjha_#119	GO:0015976: carbon utilization	0.000243	87,10,4
1bqca_#24	GO:0042364: water-soluble vitamin biosynthesis	0.000243	30,27,4
1fjfc2#153	GO:0042108: positive regulation of cytokine biosynthesis	0.000243	362,11,7
1ido__#173	GO:0009396: folic acid and derivative biosynthesis	0.000243	78,11,4
1muca1#245	GO:0006221: pyrimidine nucleotide biosynthesis	0.000244	37,22,4
1e69a_#26	GO:0006221: pyrimidine nucleotide biosynthesis	0.000244	37,22,4
1imva_#255	GO:0006544: glycine metabolism	0.000244	506,11,8
1a4ya_#47	GO:0006118: electron transport	0.000244	172,228,30
1qj5a_#268	GO:0009116: nucleoside metabolism	0.000245	60,25,5
1bwza2#162	GO:0006470: protein amino acid dephosphorylation	0.000245	76,20,5
1gof_3#436	GO:0000082: G1/S transition of mitotic cell cycle	0.000247	5,13,2
1iknd_#183	GO:0045786: negative regulation of cell cycle	0.000247	5,13,2
1iknd_#183	GO:0000082: G1/S transition of mitotic cell cycle	0.000247	5,13,2
1sw6a_#385	GO:0045786: negative regulation of cell cycle	0.000247	5,13,2
1sw6a_#385	GO:0000082: G1/S transition of mitotic cell cycle	0.000247	5,13,2
1b6g__#56	GO:0006805: xenobiotic metabolism	0.000247	5,13,2
1b6g__#56	GO:0017000: antibiotic biosynthesis	0.000247	5,13,2
1hcd__#91	GO:0017148: negative regulation of protein biosynthesis	0.000247	5,13,2
1sw6a_#383	GO:0000082: G1/S transition of mitotic cell cycle	0.000247	5,13,2
1jj2q_#118	GO:0000082: G1/S transition of mitotic cell cycle	0.000247	5,13,2
1ffya2#268	GO:0030333: antigen processing	0.000247	5,13,2
1utea_#71	GO:0017148: negative regulation of protein biosynthesis	0.000247	5,13,2

1qj4a_#18	GO:0006805: xenobiotic metabolism	0.000247	5,13,2
1qj4a_#18	GO:0017000: antibiotic biosynthesis	0.000247	5,13,2
1pii_1#40	GO:0017148: negative regulation of protein biosynthesis	0.000247	5,13,2
1dqga_#30	GO:0017148: negative regulation of protein biosynthesis	0.000247	5,13,2
1qo7a_#149	GO:0006805: xenobiotic metabolism	0.000247	5,13,2
1qo7a_#149	GO:0017000: antibiotic biosynthesis	0.000247	5,13,2
1ehya_#41	GO:0006805: xenobiotic metabolism	0.000247	5,13,2
1ehya_#41	GO:0017000: antibiotic biosynthesis	0.000247	5,13,2
1bu8a2#124	GO:0017000: antibiotic biosynthesis	0.000247	123,13,5
1tca__#189	GO:0017000: antibiotic biosynthesis	0.000247	5,13,2
1ek1a2#525	GO:0006805: xenobiotic metabolism	0.000247	5,13,2
1ek1a2#525	GO:0017000: antibiotic biosynthesis	0.000247	5,13,2
1aky_2#133	GO:0007169: transmembrane receptor protein tyrosine kinase signaling pathway	0.000248	130,26,7
2arca_#55	GO:0007169: transmembrane receptor protein tyrosine kinase signaling pathway	0.000248	130,26,7
1hava_#41	GO:0006643: membrane lipid metabolism	0.000248	65,13,4
1fm2.1#B199	GO:0046677: response to antibiotic	0.000249	2,40,2
1cipa2#34	GO:0009086: methionine biosynthesis	0.000249	60,14,4
1e3ja2#270	GO:0009086: methionine biosynthesis	0.000249	60,14,4
1kit_2#398	GO:0006917: induction of apoptosis	0.000249	81,29,6
1e79d2#36	GO:0006457: protein folding	0.000249	432,49,19
1nal1_#204	GO:0006221: pyrimidine nucleotide biosynthesis	0.00025	15,22,3
1e44b_#75	GO:0006606: protein-nucleus import	0.000251	165,21,7
1tyfa_#31	GO:0000160: two-component signal transduction system (phosphorelay)	0.000251	136,25,7
1iira_#242	GO:0042364: water-soluble vitamin biosynthesis	0.000251	125,27,7
1czya1#466	GO:0016477: cell migration	0.000252	104,23,6
1bjt__#890	GO:0016477: cell migration	0.000253	258,23,9
1pfza_#156	GO:0007169: transmembrane receptor protein tyrosine kinase signaling pathway	0.000253	58,26,5
3lada3#380	GO:0006096: glycolysis	0.000254	9,38,3
1amua_#155	GO:0006096: glycolysis	0.000254	9,38,3
2dnja_#97	GO:0046677: response to antibiotic	0.000254	110,40,8
1nfdb1#21	GO:0045595: regulation of cell differentiation	0.000254	88,10,4
1edza1#206	GO:0009116: nucleoside metabolism	0.000254	352,25,11
5ruba1#365	GO:0042401: biogenic amine biosynthesis	0.000254	88,10,4
5ruba1#365	GO:0006568: tryptophan metabolism	0.000254	88,10,4
1pii_2#326	GO:0006221: pyrimidine nucleotide biosynthesis	0.000255	157,22,7
1dnv__#354	GO:0016337: cell-cell adhesion	0.000255	49,17,4
1f97a1#62	GO:0008285: negative regulation of cell proliferation	0.000256	591,19,12
1a0i_2#222	GO:0042108: positive regulation of cytokine biosynthesis	0.000256	151,11,5
1d0na6#681	GO:0017000: antibiotic biosynthesis	0.000256	124,13,5
1dpga2#334	GO:0042559: pteridine and derivative biosynthesis	0.000256	246,11,6
1j79a_#187	GO:0009396: folic acid and derivative biosynthesis	0.000256	79,11,4
1g71a_#88	GO:0006956: complement activation	0.000257	28,12,3
1ek6a_#33	GO:0009103: lipopolysaccharide biosynthesis	0.000257	28,12,3
2sli_2#326	GO:0019735: antimicrobial humoral response (sensu Vertebrata)	0.000257	227,16,7
1e79h2#77	GO:0015992: proton transport	0.000257	28,12,3
1e79h2#77	GO:0006119: oxidative phosphorylation	0.000257	28,12,3
1h4ua1#486	GO:0006275: regulation of DNA replication	0.000257	34,10,3
1c7qa_#181	GO:0007268: synaptic transmission	0.000257	12,28,3
1f42a2#123	GO:0006919: caspase activation	0.000258	335,15,8
1hbza_#96	GO:0048514: blood vessel morphogenesis	0.000258	322,9,6
2reb_1#138	GO:0042364: water-soluble vitamin biosynthesis	0.000258	56,27,5
1g8fa3#419	GO:0009307: DNA restriction	0.000259	129,19,6
1eno__#83	GO:0009116: nucleoside metabolism	0.00026	33,25,4
1fzqa_#28	GO:0007067: mitosis	0.00026	33,25,4

1aoca_#119	GO:0007276: gametogenesis	0.000261	6,11,2
1bx4a_#31	GO:0043094: metabolic compound salvage	0.000261	6,11,2
1qqka_#19	GO:0000165: MAPKKK cascade	0.000261	6,11,2
1a77_2#183	GO:0016568: chromatin modification	0.000261	6,11,2
1tc1a_#163	GO:0043094: metabolic compound salvage	0.000261	6,11,2
1ecra_#101	GO:0042088: T-helper 1 type immune response	0.000261	6,11,2
1ecra_#101	GO:0042108: positive regulation of cytokine biosynthesis	0.000261	6,11,2
2tgi__#80	GO:0007276: gametogenesis	0.000261	6,11,2
1fl7b_#78	GO:0007276: gametogenesis	0.000261	6,11,2
1qq4a_#46	GO:0042108: positive regulation of cytokine biosynthesis	0.000261	6,11,2
1trb_1#106	GO:0006801: superoxide metabolism	0.000261	6,11,2
1dpe__#190	GO:0043094: metabolic compound salvage	0.000261	6,11,2
1mfa_1#82L	GO:0042088: T-helper 1 type immune response	0.000261	6,11,2
1mfa_1#82L	GO:0042108: positive regulation of cytokine biosynthesis	0.000261	6,11,2
1a2za_#209	GO:0006801: superoxide metabolism	0.000261	6,11,2
1rypk_#77	GO:0006511: ubiquitin-dependent protein catabolism	0.000261	6,11,2
1elua_#288	GO:0006690: icosanoid metabolism	0.000261	6,11,2
1hr6a1#59	GO:0016485: protein processing	0.000261	6,11,2
1de4c2#250	GO:0043094: metabolic compound salvage	0.000261	6,11,2
1es7a_#111	GO:0007276: gametogenesis	0.000261	6,11,2
1g0sa_#74	GO:0042088: T-helper 1 type immune response	0.000261	247,11,6
1g0sa_#74	GO:0042108: positive regulation of cytokine biosynthesis	0.000261	247,11,6
1euva_#580	GO:0006511: ubiquitin-dependent protein catabolism	0.000261	6,11,2
1g8ya_#142	GO:0006935: chemotaxis	0.000261	2,41,2
3chbd_#96	GO:0001906: cell killing	0.000261	6,11,2
1erja_#586	GO:0007186: G-protein coupled receptor protein signaling pathway	0.000262	3,24,2
1tbga_#185	GO:0007186: G-protein coupled receptor protein signaling pathway	0.000262	3,24,2
1tbga_#119	GO:0007186: G-protein coupled receptor protein signaling pathway	0.000262	3,24,2
1tbga_#204	GO:0007186: G-protein coupled receptor protein signaling pathway	0.000262	3,24,2
1f97a1#62	GO:0006265: DNA topological change	0.000262	591,10,8
1dgw.1#Y353	GO:0006643: membrane lipid metabolism	0.000262	294,13,7
1d8db_#148	GO:0007186: G-protein coupled receptor protein signaling pathway	0.000262	3,24,2
1fuia2#273	GO:0006221: pyrimidine nucleotide biosynthesis	0.000263	110,22,6
1leha1#255	GO:0006100: tricarboxylic acid cycle intermediate metabolism	0.000263	24,14,3
1qp8a1#153	GO:0006100: tricarboxylic acid cycle intermediate metabolism	0.000263	24,14,3
1ddja_#681	GO:0016337: cell-cell adhesion	0.000263	212,17,7
1qj8a_#64	GO:0030333: antigen processing	0.000263	66,13,4
1h6ka2#336	GO:0006098: pentose-phosphate shunt	0.000264	152,11,5
1e4ea2#215	GO:0006275: regulation of DNA replication	0.000264	419,10,7
3nul__#107	GO:0009070: serine family amino acid biosynthesis	0.000265	13,26,3
1e39a3#442	GO:0006606: protein-nucleus import	0.000265	16,21,3
2mev3_#107	GO:0001906: cell killing	0.000265	31,11,3
1hr6a1#41	GO:0001906: cell killing	0.000265	31,11,3
1ap0__#29	GO:0016568: chromatin modification	0.000265	31,11,3
1csn__#134	GO:0007169: transmembrane receptor protein tyrosine kinase signaling pathway	0.000265	13,26,3
1eu3a2#174	GO:0030333: antigen processing	0.000265	26,13,3
1f24a_#293	GO:0007169: transmembrane receptor protein tyrosine kinase signaling pathway	0.000265	92,26,6
6cel__#423	GO:0007264: small GTPase mediated signal transduction	0.000265	16,21,3
1heta2#218	GO:0009073: aromatic amino acid family biosynthesis	0.000265	21,16,3
1imva_#278	GO:0017148: negative regulation of protein biosynthesis	0.000265	26,13,3
1loua_#79	GO:0006418: tRNA aminoacylation for protein translation	0.000265	92,26,6
1ir3a_#1015	GO:0000165: MAPKKK cascade	0.000265	31,11,3
1ge8a2#134	GO:0016477: cell migration	0.000265	202,23,8
1autc_#45	GO:0019079: viral genome replication	0.000265	21,16,3

1nsj__#21	GO:0000160: two-component signal transduction system (phosphorelay)	0.000266	96,25,6
1jj2v_#118	GO:0006265: DNA topological change	0.000266	89,10,4
1ibja_#271	GO:0009086: methionine biosynthesis	0.000266	61,14,4
1ew2a_#237	GO:0006207: 'de novo' pyrimidine base biosynthesis	0.000266	89,10,4
1ass__#33	GO:0006118: electron transport	0.000267	8,228,5
1h6la_#120	GO:0006118: electron transport	0.000267	8,228,5
1jb3a_#64	GO:0006919: caspase activation	0.000268	247,15,7
3chbd_#85	GO:0016477: cell migration	0.000268	323,23,10
1avgi_#69	GO:0051049: regulation of transport	0.000269	281,10,6
1avgi_#69	GO:0015976: carbon utilization	0.000269	281,10,6
1pvc1_#106	GO:0019079: viral genome replication	0.000269	53,16,4
1pvc1_#106	GO:0043123: positive regulation of I-kappaB kinase/NF-kappaB cascade	0.000269	53,16,4
1dm9a_#57	GO:0019079: viral genome replication	0.000269	99,16,5
1dm9a_#57	GO:0019735: antimicrobial humoral response (sensu Vertebrata)	0.000269	99,16,5
1fo4a5#1002	GO:0006511: ubiquitin-dependent protein catabolism	0.000269	80,11,4
1pysa_#221	GO:0006544: glycine metabolism	0.000269	80,11,4
1ax4a_#275	GO:0019079: viral genome replication	0.000269	99,16,5
1flca1#294	GO:0006118: electron transport	0.00027	89,228,19
1eh9a1#33	GO:0007169: transmembrane receptor protein tyrosine kinase signaling pathway	0.00027	32,26,4
1mjha_#119	GO:0009252: peptidoglycan biosynthesis	0.00027	87,18,5
1fi2a_#104	GO:0006457: protein folding	0.00027	31,49,5
1dfoa_#344	GO:0006457: protein folding	0.00027	31,49,5
1bg6_1#294	GO:0030245: cellulose catabolism	0.000271	38,22,4
2ae2a_#89	GO:0006221: pyrimidine nucleotide biosynthesis	0.000271	38,22,4
1dik_3#240	GO:0016337: cell-cell adhesion	0.000271	213,17,7
1bcca2#437	GO:0006221: pyrimidine nucleotide biosynthesis	0.000271	38,22,4
1qfea_#68	GO:0046148: pigment biosynthesis	0.000271	453,12,8
1hc7a2#258	GO:0016042: lipid catabolism	0.000272	186,31,9
1as4.1#A288	GO:0006953: acute-phase response	0.000273	42,20,4
1dp0a4#756	GO:0001906: cell killing	0.000273	153,11,5
1cpza_#6	GO:0007169: transmembrane receptor protein tyrosine kinase signaling pathway	0.000273	132,26,7
1jj2j_#70	GO:0007399: neurogenesis	0.000274	64,37,6
1azo__#144	GO:0046677: response to antibiotic	0.000274	38,40,5
1beba_#119	GO:0017001: antibiotic catabolism	0.000274	172,10,5
1beba_#119	GO:0006275: regulation of DNA replication	0.000274	172,10,5
1a4ya_#47	GO:0042401: biogenic amine biosynthesis	0.000274	172,10,5
1qj2c2#50	GO:0006779: porphyrin biosynthesis	0.000275	47,18,4
1czan1#92	GO:0007169: transmembrane receptor protein tyrosine kinase signaling pathway	0.000276	538,26,14
1f8v.1#A116	GO:0006953: acute-phase response	0.000277	78,20,5
1xgsa2#82	GO:0016477: cell migration	0.000277	261,23,9
1aqb__#134	GO:0030217: T-cell differentiation	0.000277	90,10,4
1fsia_#122	GO:0019883: antigen presentation, endogenous antigen	0.000277	90,10,4
2sli_2#326	GO:0007169: transmembrane receptor protein tyrosine kinase signaling pathway	0.000278	227,26,9
1hxxa_#173	GO:0043123: positive regulation of I-kappaB kinase/NF-kappaB cascade	0.000279	230,16,7
1h6va1#27	GO:0009156: ribonucleoside monophosphate biosynthesis	0.000279	224,12,6
1rypk_#12	GO:0016485: protein processing	0.00028	250,11,6
1jeyb_#231	GO:0006979: response to oxidative stress	0.000281	19,18,3
1e4ia_#215	GO:0009064: glutamine family amino acid metabolism	0.000281	18,19,3
1nal1_#203	GO:0042401: biogenic amine biosynthesis	0.000281	35,10,3
1nal1_#203	GO:0006568: tryptophan metabolism	0.000281	35,10,3
1nal1_#203	GO:0006207: 'de novo' pyrimidine base biosynthesis	0.000281	35,10,3
1f06a1#296	GO:0000051: urea cycle intermediate metabolism	0.000281	35,10,3
1f06a1#296	GO:0006207: 'de novo' pyrimidine base biosynthesis	0.000281	35,10,3

1ji6a2#458	GO:0006916: anti-apoptosis	0.000281	19,18,3
1a6o__#297	GO:0009582: detection of abiotic stimulus	0.000281	35,10,3
1hbza_#214	GO:0006461: protein complex assembly	0.000281	249,24,9
1cqka_#49	GO:0030217: T-cell differentiation	0.000281	35,10,3
1cpy__#380	GO:0001906: cell killing	0.000281	154,11,5
1g51a3#524	GO:0007169: transmembrane receptor protein tyrosine kinase signaling pathway	0.000281	93,26,6
1b3ra2#47	GO:0006207: 'de novo' pyrimidine base biosynthesis	0.000281	35,10,3
1bfd_2#146	GO:0009070: serine family amino acid biosynthesis	0.000281	93,26,6
1zfpe_#67	GO:0008284: positive regulation of cell proliferation	0.000281	28,30,4
1fc6a2#454	GO:0050819: negative regulation of coagulation	0.000281	35,10,3
1fc6a2#454	GO:0030193: regulation of blood coagulation	0.000281	35,10,3
1fc6a2#454	GO:0018108: peptidyl-tyrosine phosphorylation	0.000281	35,10,3
1bpv__#22	GO:0006916: anti-apoptosis	0.000281	19,18,3
1qrra_#69	GO:0006568: tryptophan metabolism	0.000281	173,10,5
1xnb__#130	GO:0006418: tRNA aminoacylation for protein translation	0.000281	93,26,6
1hwxa1#223	GO:0009086: methionine biosynthesis	0.000281	186,14,6
1esma_#314	GO:0019735: antimicrobial humoral response (sensu Vertebrata)	0.000282	100,16,5
1cwva2#675	GO:0006917: induction of apoptosis	0.000282	29,29,4
1eny__#147	GO:0009396: folic acid and derivative biosynthesis	0.000282	81,11,4
2dkb__#114	GO:0006563: L-serine metabolism	0.000283	139,12,5
1czan1#92	GO:0017148: negative regulation of protein biosynthesis	0.000284	538,13,9
1euwa_#116	GO:0009116: nucleoside metabolism	0.000285	3,25,2
1gsoa3#193	GO:0007067: mitosis	0.000285	3,25,2
1f8ea_#177	GO:0009116: nucleoside metabolism	0.000285	3,25,2
1epwa3#138	GO:0006544: glycine metabolism	0.000285	371,11,7
1e7wa_#8	GO:0009064: glutamine family amino acid metabolism	0.000285	83,19,5
1ct9a2#27	GO:0009116: nucleoside metabolism	0.000285	3,25,2
1qs0a1#119	GO:0006730: one-carbon compound metabolism	0.000285	367,32,13
1fo5a_#60	GO:0006367: transcription initiation from Pol II promoter	0.000286	225,12,6
1qrra_#208	GO:0009116: nucleoside metabolism	0.000286	62,25,5
1cbf__#208	GO:0016337: cell-cell adhesion	0.000286	291,17,8
1e6pa2#274	GO:0006563: L-serine metabolism	0.000286	29,12,3
1ea5a_#99	GO:0016068: type I hypersensitivity	0.000286	172,15,6
1i50b_#966	GO:0001906: cell killing	0.000286	251,11,6
1qoua_#64	GO:0051249: regulation of lymphocyte activation	0.000286	29,12,3
1ghpa_#65	GO:0008015: circulation	0.000287	23,15,3
1e5ea_#145	GO:0009086: methionine biosynthesis	0.000287	5,14,2
1jatb_#72	GO:0016567: protein ubiquitination	0.000287	5,14,2
1h9ra2#212	GO:0015698: inorganic anion transport	0.000287	5,14,2
1agja_#91	GO:0016567: protein ubiquitination	0.000287	5,14,2
1qisa_#107	GO:0009086: methionine biosynthesis	0.000287	5,14,2
2sli_2#438	GO:0016567: protein ubiquitination	0.000287	5,14,2
1c4zd_#99	GO:0016567: protein ubiquitination	0.000287	5,14,2
1jg8a_#210	GO:0009086: methionine biosynthesis	0.000287	5,14,2
2sli_2#326	GO:0006606: protein-nucleus import	0.000289	227,21,8
1fm2.1#B308	GO:0019883: antigen presentation, endogenous antigen	0.000289	174,10,5
2gsaa_#246	GO:0006779: porphyrin biosynthesis	0.00029	4,18,2
1ftka_#61	GO:0009252: peptidoglycan biosynthesis	0.00029	4,18,2
1am7a_#94	GO:0019835: cytolysis	0.00029	4,18,2
1hbza_#254	GO:0006979: response to oxidative stress	0.00029	4,18,2
1qs0b2#232	GO:0009252: peptidoglycan biosynthesis	0.00029	4,18,2
1a0i_2#35	GO:0009252: peptidoglycan biosynthesis	0.00029	4,18,2
3prn__#145	GO:0042088: T-helper 1 type immune response	0.00029	155,11,5
3prn__#145	GO:0042108: positive regulation of cytokine biosynthesis	0.00029	155,11,5
1el0a_#28	GO:0006916: anti-apoptosis	0.000291	140,18,6

1qlaa3#364	GO:0006919: caspase activation	0.000292	58,15,4
1eh9a1#33	GO:0042088: T-helper 1 type immune response	0.000292	32,11,3
1egja_#343	GO:0042088: T-helper 1 type immune response	0.000292	32,11,3
1bio__#108	GO:0042108: positive regulation of cytokine biosynthesis	0.000292	32,11,3
1cqka_#52	GO:0006461: protein complex assembly	0.000292	65,24,5
1pma1_#17	GO:0006511: ubiquitin-dependent protein catabolism	0.000292	32,11,3
1gsa_2#218	GO:0006606: protein-nucleus import	0.000292	118,21,6
1ekbb_#85	GO:0006606: protein-nucleus import	0.000292	118,21,6
1pfza_#156	GO:0006986: response to unfolded protein	0.000292	58,15,4
1d7ba_#87	GO:0016477: cell migration	0.000293	68,23,5
1i5ga_#115	GO:0006457: protein folding	0.000293	294,49,15
1el0a_#28	GO:0051249: regulation of lymphocyte activation	0.000293	140,12,5
1bywa_#30	GO:0007169: transmembrane receptor protein tyrosine kinase signaling pathway	0.000294	283,26,10
1czan1#92	GO:0009252: peptidoglycan biosynthesis	0.000294	538,18,11
1gpea1#273	GO:0006953: acute-phase response	0.000294	79,20,5
2hlca_#139	GO:0006606: protein-nucleus import	0.000294	75,21,5
1cpza_#6	GO:0006897: endocytosis	0.000294	132,19,6
1f97a1#62	GO:0006313: DNA transposition	0.000295	591,35,18
1ddja_#726	GO:0042088: T-helper 1 type immune response	0.000296	82,11,4
1ddja_#726	GO:0042108: positive regulation of cytokine biosynthesis	0.000296	82,11,4
1e8ga2#201	GO:0019883: antigen presentation, endogenous antigen	0.000297	286,10,6
1e8ga2#201	GO:0030217: T-cell differentiation	0.000297	286,10,6
1e8ga2#201	GO:0006275: regulation of DNA replication	0.000297	286,10,6
1danh_#93	GO:0018108: peptidyl-tyrosine phosphorylation	0.000298	7,10,2
1fo8a_#202	GO:0009086: methionine biosynthesis	0.000298	25,14,3
1sfp__#84	GO:0030217: T-cell differentiation	0.000298	7,10,2
1ypci_#69	GO:0009167: purine ribonucleoside monophosphate metabolism	0.000298	7,10,2
1cdy_2#140	GO:0030217: T-cell differentiation	0.000298	7,10,2
1deua_#186	GO:0009219: pyrimidine deoxyribonucleotide metabolism	0.000298	7,10,2
1g5va_#142	GO:0006118: electron transport	0.000298	21,228,8
1ie5a_#104	GO:0045595: regulation of cell differentiation	0.000298	7,10,2
1qdlb_#98	GO:0042401: biogenic amine biosynthesis	0.000298	7,10,2
1qdlb_#98	GO:0006568: tryptophan metabolism	0.000298	7,10,2
1ew0a_#174	GO:0009582: detection of abiotic stimulus	0.000298	7,10,2
1pysb6#384	GO:0017001: antibiotic catabolism	0.000298	7,10,2
1bl0a2#102	GO:0006207: 'de novo' pyrimidine base biosynthesis	0.000298	7,10,2
2ts1__#109	GO:0015976: carbon utilization	0.000298	7,10,2
1hf2a2#97	GO:0015976: carbon utilization	0.000298	7,10,2
1bd2d1#62	GO:0045595: regulation of cell differentiation	0.000298	7,10,2
1bd2d1#62	GO:0019883: antigen presentation, endogenous antigen	0.000298	7,10,2
1bd2d1#62	GO:0030217: T-cell differentiation	0.000298	7,10,2
1cipa2#34	GO:0009070: serine family amino acid biosynthesis	0.000298	60,26,5
1ffgb_#172	GO:0006207: 'de novo' pyrimidine base biosynthesis	0.000298	7,10,2
1eu8a_#312	GO:0015698: inorganic anion transport	0.000298	25,14,3
1b4ka_#108	GO:0009070: serine family amino acid biosynthesis	0.000298	94,26,6
1keva2#236	GO:0009219: pyrimidine deoxyribonucleotide metabolism	0.000298	7,10,2
1a65a1#108	GO:0001906: cell killing	0.000299	253,11,6
1nsca_#236	GO:0042088: T-helper 1 type immune response	0.000299	156,11,5
1nsca_#236	GO:0001906: cell killing	0.000299	156,11,5
1nsca_#236	GO:0006690: icosanoid metabolism	0.000299	156,11,5
1ejfa_#10	GO:0007169: transmembrane receptor protein tyrosine kinase signaling pathway	0.0003	134,26,7
1eaja_#59	GO:0006461: protein complex assembly	0.0003	196,24,8
1ii7a_#43	GO:0006221: pyrimidine nucleotide biosynthesis	0.0003	39,22,4
1gdha1#235	GO:0000910: cytokinesis	0.0003	92,37,7

1dt9a1#209	GO:0000051: urea cycle intermediate metabolism	0.000302	92,10,4
1ft9a1#201	GO:0030217: T-cell differentiation	0.000302	92,10,4
1loua_#79	GO:0017001: antibiotic catabolism	0.000302	92,10,4
1qfma1#139	GO:0016477: cell migration	0.000302	264,23,9
1bwvs_#117	GO:0019883: antigen presentation, endogenous antigen	0.000303	287,10,6
1e6ca_#91	GO:0009156: ribonucleoside monophosphate biosynthesis	0.000303	141,12,5
1mpp__#154	GO:0006917: induction of apoptosis	0.000304	84,29,6
1nat__#50	GO:0006470: protein amino acid dephosphorylation	0.000304	242,20,8
1a8i__#688	GO:0006779: porphyrin biosynthesis	0.000304	203,18,7
1hcl__#82	GO:0000086: G2/M transition of mitotic cell cycle	0.000305	36,10,3
1hcl__#82	GO:0009167: purine ribonucleoside monophosphate metabolism	0.000305	36,10,3
1fbna_#145	GO:0009070: serine family amino acid biosynthesis	0.000305	33,26,4
1jwoa_#196	GO:0007169: transmembrane receptor protein tyrosine kinase signaling pathway	0.000305	33,26,4
1euca1#64	GO:0006418: tRNA aminoacylation for protein translation	0.000305	33,26,4
1e79d3#328	GO:0006568: tryptophan metabolism	0.000305	36,10,3
1e79d3#328	GO:0006207: 'de novo' pyrimidine base biosynthesis	0.000305	36,10,3
1lucb_#70	GO:0042401: biogenic amine biosynthesis	0.000305	36,10,3
1lucb_#70	GO:0006568: tryptophan metabolism	0.000305	36,10,3
1d2oa1#541	GO:0007169: transmembrane receptor protein tyrosine kinase signaling pathway	0.000305	33,26,4
1elua_#216	GO:0009070: serine family amino acid biosynthesis	0.000305	33,26,4
1xo1a2#159	GO:0042401: biogenic amine biosynthesis	0.000305	36,10,3
1hdma1#123	GO:0030217: T-cell differentiation	0.000305	36,10,3
1hxxa_#173	GO:0006457: protein folding	0.000306	230,49,13
1a81a1#51	GO:0051249: regulation of lymphocyte activation	0.000306	75,12,4
1e3a.1#A30	GO:0009064: glutamine family amino acid metabolism	0.000306	133,19,6
1hska1#54	GO:0006118: electron transport	0.000308	12,228,6
1h7wa2#535	GO:0006118: electron transport	0.000308	12,228,6
1fwxa1#535	GO:0006118: electron transport	0.000308	12,228,6
1erja_#525	GO:0006118: electron transport	0.000308	12,228,6
1ep3a_#19	GO:0006118: electron transport	0.000308	12,228,6
1d6aa_#52	GO:0006418: tRNA aminoacylation for protein translation	0.000308	230,26,9
1fwxa2#189	GO:0006118: electron transport	0.000308	12,228,6
1fo8a_#367	GO:0009070: serine family amino acid biosynthesis	0.000309	3,26,2
1aop_3#276	GO:0006461: protein complex assembly	0.000309	147,24,7
1gaxa3#88	GO:0006418: tRNA aminoacylation for protein translation	0.000309	3,26,2
1e5xa_#282	GO:0009070: serine family amino acid biosynthesis	0.000309	3,26,2
1dyr__#180	GO:0009070: serine family amino acid biosynthesis	0.000309	3,26,2
1e7wa_#8	GO:0043094: metabolic compound salvage	0.00031	83,11,4
1c9la2#61	GO:0006935: chemotaxis	0.00031	206,41,11
1a7ca_#202	GO:0042088: T-helper 1 type immune response	0.00031	83,11,4
1e8ga2#126	GO:0006118: electron transport	0.000311	5,228,4
1a8p_1#54	GO:0006118: electron transport	0.000311	5,228,4
1ft5a_#205	GO:0006118: electron transport	0.000311	5,228,4
1dz4a_#323	GO:0006118: electron transport	0.000311	5,228,4
451c__#66	GO:0006118: electron transport	0.000311	5,228,4
1alla_#124	GO:0006118: electron transport	0.000311	5,228,4
1whi__#59	GO:0019079: viral genome replication	0.000311	55,16,4
1dn2a1#261	GO:0001906: cell killing	0.000311	376,11,7
1dceb_#82	GO:0006956: complement activation	0.000312	6,12,2
1ae9a_#272	GO:0046148: pigment biosynthesis	0.000312	6,12,2
1i85a_#101	GO:0051249: regulation of lymphocyte activation	0.000312	6,12,2
1ghqb1#32	GO:0006956: complement activation	0.000312	6,12,2
1ecra_#101	GO:0051249: regulation of lymphocyte activation	0.000312	6,12,2
1qgxa_#137	GO:0000398: nuclear mRNA splicing, via spliceosome	0.000312	6,12,2

1qq4a_#46	GO:0009401: phosphoenolpyruvate-dependent sugar phosphotransferase system	0.000312	6,12,2
1d2fa_#348	GO:0046148: pigment biosynthesis	0.000312	6,12,2
1hr6a1#59	GO:0006119: oxidative phosphorylation	0.000312	6,12,2
1egua1#309	GO:0007017: microtubule-based process	0.000312	6,12,2
1egua1#309	GO:0006956: complement activation	0.000312	6,12,2
1hxxa_#112	GO:0009615: response to virus	0.000312	6,12,2
1exba_#188	GO:0009156: ribonucleoside monophosphate biosynthesis	0.000312	6,12,2
1rmg__#73	GO:0009103: lipopolysaccharide biosynthesis	0.000312	6,12,2
1a9xa6#760	GO:0006953: acute-phase response	0.000312	80,20,5
1ezia_#93	GO:0046148: pigment biosynthesis	0.000312	6,12,2
1ezia_#93	GO:0009103: lipopolysaccharide biosynthesis	0.000312	6,12,2
1dceb_#170	GO:0006956: complement activation	0.000312	6,12,2
1ciy_3#243	GO:0007601: visual perception	0.000312	59,15,4
1fjfj_#68	GO:0016337: cell-cell adhesion	0.000313	218,17,7
1ge8a2#134	GO:0048514: blood vessel morphogenesis	0.000313	202,9,5
1h9da_#91	GO:0006917: induction of apoptosis	0.000313	204,29,9
1e19a_#281	GO:0006730: one-carbon compound metabolism	0.000314	108,32,7
1i58a_#519	GO:0006461: protein complex assembly	0.000314	66,24,5
1axn__#42	GO:0007186: G-protein coupled receptor protein signaling pathway	0.000314	66,24,5
2napa2#292	GO:0006730: one-carbon compound metabolism	0.000314	108,32,7
1g51a3#524	GO:0019883: antigen presentation, endogenous antigen	0.000315	93,10,4
1j9la_#164	GO:0006730: one-carbon compound metabolism	0.000315	49,32,5
1ct9a1#233	GO:0006730: one-carbon compound metabolism	0.000315	49,32,5
1ejda_#377	GO:0015976: carbon utilization	0.000315	93,10,4
1xnb__#130	GO:0009167: purine ribonucleoside monophosphate metabolism	0.000315	93,10,4
1jr3a2#166	GO:0006119: oxidative phosphorylation	0.000317	30,12,3
1fsia_#122	GO:0009252: peptidoglycan biosynthesis	0.000317	90,18,5
1g93a_#229	GO:0009206: purine ribonucleoside triphosphate biosynthesis	0.000318	8,9,2
1cs6a2#164	GO:0016998: cell wall catabolism	0.000318	8,9,2
1jsg__#92	GO:0008284: positive regulation of cell proliferation	0.000318	519,30,15
1dyoa_#153	GO:0006730: one-carbon compound metabolism	0.000318	144,32,8
1dlfl_#97	GO:0042113: B-cell activation	0.000319	41,9,3
1j9la_#82	GO:0009307: DNA restriction	0.000319	134,19,6
1epwa3#138	GO:0006730: one-carbon compound metabolism	0.000319	371,32,13
1htp__#16	GO:0001906: cell killing	0.000319	256,11,6
1e42a1#711	GO:0016477: cell migration	0.00032	155,23,7
1xwl_2#591	GO:0006606: protein-nucleus import	0.00032	17,21,3
1dik_1#848	GO:0009086: methionine biosynthesis	0.00032	64,14,4
3prn__#145	GO:0016477: cell migration	0.00032	155,23,7
1f8na1#450	GO:0001906: cell killing	0.00032	33,11,3
1euca1#64	GO:0006544: glycine metabolism	0.00032	33,11,3
1dyna_#42	GO:0016337: cell-cell adhesion	0.00032	152,17,6
1ibja_#271	GO:0009070: serine family amino acid biosynthesis	0.000322	61,26,5
1cfe__#7	GO:0009070: serine family amino acid biosynthesis	0.000322	61,26,5
1fmja_#76	GO:0006779: porphyrin biosynthesis	0.000323	49,18,4
1fp1d2#214	GO:0006563: L-serine metabolism	0.000323	143,12,5
1e79d2#36	GO:0006265: DNA topological change	0.000323	432,10,7
1e79d2#36	GO:0006275: regulation of DNA replication	0.000323	432,10,7
1sw6a_#464	GO:0008285: negative regulation of cell proliferation	0.000324	4,19,2
1e5ka_#49	GO:0009064: glutamine family amino acid metabolism	0.000324	4,19,2
1azza_#114	GO:0008285: negative regulation of cell proliferation	0.000324	4,19,2
1e2o__#377	GO:0006099: tricarboxylic acid cycle	0.000324	4,19,2
1ezvd1#213	GO:0006099: tricarboxylic acid cycle	0.000324	4,19,2
1hx0a2#294	GO:0009064: glutamine family amino acid metabolism	0.000324	4,19,2
1qnja_#77	GO:0008285: negative regulation of cell proliferation	0.000324	4,19,2

1czya1#466	GO:0006461: protein complex assembly	0.000324	104,24,6
1lgr_2#115	GO:0009064: glutamine family amino acid metabolism	0.000324	4,19,2
1ig8a_#214	GO:0006096: glycolysis	0.000325	23,38,4
3lada3#463	GO:0006096: glycolysis	0.000325	23,38,4
1mpp__#154	GO:0042088: T-helper 1 type immune response	0.000325	84,11,4
1mpp__#154	GO:0042108: positive regulation of cytokine biosynthesis	0.000325	84,11,4
1fjsa_#83	GO:0001906: cell killing	0.000325	84,11,4
1jlxa2#166	GO:0007399: neurogenesis	0.000325	66,37,6
1e1oa2#423	GO:0006953: acute-phase response	0.000325	127,20,6
1g6sa_#64	GO:0001906: cell killing	0.000325	84,11,4
1ekma1#545	GO:0001906: cell killing	0.000325	84,11,4
1b87a_#47	GO:0006461: protein complex assembly	0.000326	15,24,3
1as4.1#B385	GO:0016042: lipid catabolism	0.000326	51,31,5
1ldna1#94	GO:0009396: folic acid and derivative biosynthesis	0.000327	159,11,5
1dj0a2#183	GO:0006919: caspase activation	0.000328	111,15,5
1fkna_#18	GO:0006354: RNA elongation	0.000328	94,10,4
1fkna_#18	GO:0019883: antigen presentation, endogenous antigen	0.000328	94,10,4
1qtn.1#A270	GO:0016042: lipid catabolism	0.000328	386,31,13
1i9sa_#19	GO:0006470: protein amino acid dephosphorylation	0.000329	18,20,3
1aiha_#226	GO:0006953: acute-phase response	0.000329	18,20,3
1fjja_#5	GO:0006953: acute-phase response	0.000329	18,20,3
7reqb1#320	GO:0006779: porphyrin biosynthesis	0.000329	20,18,3
1e31a_#55	GO:0006916: anti-apoptosis	0.000329	20,18,3
1a3qa2#133	GO:0006953: acute-phase response	0.000329	18,20,3
1eqga2#47	GO:0008015: circulation	0.000331	5,15,2
1qqqa_#247	GO:0006826: iron ion transport	0.000331	5,15,2
1qqka_#25	GO:0045859: regulation of protein kinase activity	0.000331	5,15,2
1alo_4#481	GO:0030333: antigen processing	0.000331	70,13,4
1dyna_#57	GO:0030036: actin cytoskeleton organization and biogenesis	0.000331	5,15,2
1pme__#24	GO:0000910: cytokinesis	0.000332	10,37,3
1bj7__#112	GO:0030333: antigen processing	0.000332	28,13,3
1ji6a2#458	GO:0008285: negative regulation of cell proliferation	0.000332	19,19,3
1dl5a_#283	GO:0009064: glutamine family amino acid metabolism	0.000332	19,19,3
1muca1#245	GO:0006207: 'de novo' pyrimidine base biosynthesis	0.000332	37,10,3
1cb8a2#613	GO:0006643: membrane lipid metabolism	0.000332	131,13,5
1jj2e1#42	GO:0016042: lipid catabolism	0.000332	150,31,8
1hcl__#135	GO:0000910: cytokinesis	0.000332	10,37,3
1h6va2#219	GO:0017001: antibiotic catabolism	0.000332	37,10,3
2msba_#218	GO:0042401: biogenic amine biosynthesis	0.000332	37,10,3
1qfta_#98	GO:0008284: positive regulation of cell proliferation	0.000332	198,30,9
1qfma1#139	GO:0006457: protein folding	0.000332	264,49,14
1ihua2#525	GO:0009086: methionine biosynthesis	0.000332	278,14,7
1fbna_#144	GO:0009116: nucleoside metabolism	0.000333	64,25,5
1bjt__#890	GO:0006801: superoxide metabolism	0.000333	258,11,6
1bjt__#890	GO:0001906: cell killing	0.000333	258,11,6
1tpg_1#55	GO:0006633: fatty acid biosynthesis	0.000334	3,27,2
1jf9a_#386	GO:0042364: water-soluble vitamin biosynthesis	0.000334	3,27,2
1qg6a_#65	GO:0006633: fatty acid biosynthesis	0.000334	3,27,2
1ek4a1#149	GO:0006633: fatty acid biosynthesis	0.000334	3,27,2
1ji6a2#497	GO:0042364: water-soluble vitamin biosynthesis	0.000334	3,27,2
1qg6a_#252	GO:0006633: fatty acid biosynthesis	0.000334	3,27,2
1c39a_#49	GO:0019735: antimicrobial humoral response (sensu Vertebrata)	0.000334	56,16,4
1pme__#302	GO:0007169: transmembrane receptor protein tyrosine kinase signaling pathway	0.000335	14,26,3
1ycsa_#162	GO:0006919: caspase activation	0.000335	177,15,6
1mrp__#170	GO:0006100: tricarboxylic acid cycle intermediate metabolism	0.000335	26,14,3

1mrp__#170	GO:0015698: inorganic anion transport	0.000335	26,14,3
1b3ra1#218	GO:0006100: tricarboxylic acid cycle intermediate metabolism	0.000335	26,14,3
1egia_#738	GO:0006418: tRNA aminoacylation for protein translation	0.000335	14,26,3
1ej8a_#189	GO:0006919: caspase activation	0.000336	256,15,7
1bif_2#398	GO:0006118: electron transport	0.000336	105,228,21
1io7a_#243	GO:0015671: oxygen transport	0.000336	70,23,5
1hxxa_#278	GO:0019079: viral genome replication	0.000337	237,16,7
1c9oa_#49	GO:0008285: negative regulation of cell proliferation	0.000338	86,19,5
1qmva_#40	GO:0007017: microtubule-based process	0.000339	77,12,4
1e6pa2#46	GO:0009156: ribonucleoside monophosphate biosynthesis	0.000339	77,12,4
1apme_#74	GO:0019079: viral genome replication	0.00034	104,16,5
1jjya_#324	GO:0009086: methionine biosynthesis	0.00034	65,14,4
1qtra_#108	GO:0006100: tricarboxylic acid cycle intermediate metabolism	0.00034	65,14,4
1kid__#330	GO:0006457: protein folding	0.000341	144,49,10
1dyoa_#153	GO:0006457: protein folding	0.000341	144,49,10
1hbza_#96	GO:0006935: chemotaxis	0.000341	322,41,14
1e4ea2#215	GO:0008285: negative regulation of cell proliferation	0.000341	419,19,10
1tuba1#239	GO:0042401: biogenic amine biosynthesis	0.000342	95,10,4
1g8ka2#570	GO:0006118: electron transport	0.000343	32,228,10
1as4.1#A288	GO:0048514: blood vessel morphogenesis	0.000343	42,9,3
1bf6a_#183	GO:0009070: serine family amino acid biosynthesis	0.000343	34,26,4
1cpza_#6	GO:0030333: antigen processing	0.000344	132,13,5
1plq_1#90	GO:0051249: regulation of lymphocyte activation	0.000345	145,12,5
1dgw.1#Y353	GO:0019059: initiation of viral infection	0.000346	294,10,6
1dgw.1#Y353	GO:0006275: regulation of DNA replication	0.000346	294,10,6
2mpra_#190	GO:0006986: response to unfolded protein	0.000346	349,15,8
1amf__#196	GO:0006096: glycolysis	0.000346	154,38,9
1hnja1#167	GO:0009070: serine family amino acid biosynthesis	0.000348	62,26,5
1jjya_#324	GO:0006096: glycolysis	0.000348	65,38,6
1a9xa3#78	GO:0006099: tricarboxylic acid cycle	0.000348	262,19,8
1a9xa3#78	GO:0009064: glutamine family amino acid metabolism	0.000348	262,19,8
3mag__#134	GO:0009070: serine family amino acid biosynthesis	0.000348	62,26,5
1hr6a1#41	GO:0006367: transcription initiation from Pol II promoter	0.00035	31,12,3
1ryph_#52	GO:0006511: ubiquitin-dependent protein catabolism	0.00035	34,11,3
3rpba_#644	GO:0016042: lipid catabolism	0.00035	12,31,3
2bb2_1#57	GO:0019835: cytolysis	0.000351	92,18,5
1abrb1#71	GO:0019735: antimicrobial humoral response (sensu Vertebrata)	0.000351	23,16,3
1ho1a_#211	GO:0042364: water-soluble vitamin biosynthesis	0.000351	93,27,6
1bjt__#890	GO:0006919: caspase activation	0.000353	258,15,7
1ihua2#343	GO:0042364: water-soluble vitamin biosynthesis	0.000353	176,27,8
1euca1#64	GO:0042364: water-soluble vitamin biosynthesis	0.000354	33,27,4
1edza1#206	GO:0006221: pyrimidine nucleotide biosynthesis	0.000355	352,22,10
1nsj__#21	GO:0015976: carbon utilization	0.000356	96,10,4
1nsj__#21	GO:0009167: purine ribonucleoside monophosphate metabolism	0.000356	96,10,4
1dmla1#134	GO:0051249: regulation of lymphocyte activation	0.000356	78,12,4
1bura1#328	GO:0006563: L-serine metabolism	0.000356	78,12,4
1bura1#328	GO:0009103: lipopolysaccharide biosynthesis	0.000356	78,12,4
1quba4#235	GO:0050819: negative regulation of coagulation	0.000356	96,10,4
1quba4#235	GO:0030193: regulation of blood coagulation	0.000356	96,10,4
1quba4#235	GO:0030217: T-cell differentiation	0.000356	96,10,4
1f8v.1#A116	GO:0051249: regulation of lymphocyte activation	0.000356	78,12,4
1fd9a_#162	GO:0006367: transcription initiation from Pol II promoter	0.000356	78,12,4
1fd9a_#162	GO:0006119: oxidative phosphorylation	0.000356	78,12,4
1seta2#231	GO:0000051: urea cycle intermediate metabolism	0.000356	96,10,4
1xgsa2#82	GO:0042108: positive regulation of cytokine biosynthesis	0.000356	261,11,6
1xgsa2#82	GO:0006544: glycine metabolism	0.000356	261,11,6

1xgsa2#82	GO:0001906: cell killing	0.000356	261,11,6
1bwda_#295	GO:0015976: carbon utilization	0.000356	96,10,4
1e3a.1#A30	GO:0006313: DNA transposition	0.000356	133,35,8
1e0ta2#275	GO:0043094: metabolic compound salvage	0.000356	86,11,4
1bev1_#86	GO:0008284: positive regulation of cell proliferation	0.000358	296,30,11
1qtra_#108	GO:0000160: two-component signal transduction system (phosphorelay)	0.000358	65,25,5
1e4ea2#215	GO:0019735: antimicrobial humoral response (sensu Vertebrata)	0.000358	419,16,9
1ac6a_#34	GO:0019735: antimicrobial humoral response (sensu Vertebrata)	0.000358	57,16,4
1feca1#309	GO:0006096: glycolysis	0.000359	10,38,3
1nox__#88	GO:0009582: detection of abiotic stimulus	0.000359	38,10,3
1ckea_#138	GO:0006096: glycolysis	0.000359	10,38,3
1c7qa_#374	GO:0015976: carbon utilization	0.000359	38,10,3
1azo__#144	GO:0017001: antibiotic catabolism	0.000359	38,10,3
1gc5a_#233	GO:0006207: 'de novo' pyrimidine base biosynthesis	0.000359	38,10,3
1ej8a_#189	GO:0006917: induction of apoptosis	0.000359	256,29,10
1qoua_#90	GO:0030217: T-cell differentiation	0.000359	38,10,3
1ecpa_#156	GO:0006096: glycolysis	0.000359	10,38,3
1booa_#254	GO:0015976: carbon utilization	0.000359	38,10,3
1bcca2#437	GO:0009219: pyrimidine deoxyribonucleotide metabolism	0.000359	38,10,3
1fnha2#152	GO:0006953: acute-phase response	0.00036	4,20,2
1p35a_#101	GO:0016477: cell migration	0.00036	111,23,6
1qf6a3#78	GO:0006313: DNA transposition	0.00036	46,35,5
1fxla2#169	GO:0007169: transmembrane receptor protein tyrosine kinase signaling pathway	0.00036	184,26,8
1bev1_#86	GO:0006275: regulation of DNA replication	0.00036	296,10,6
1bio__#210	GO:0006118: electron transport	0.000362	113,228,22
1avgi_#69	GO:0006916: anti-apoptosis	0.000363	281,18,8
1a9xa3#78	GO:0006544: glycine metabolism	0.000363	262,11,6
1pwt__#8	GO:0006544: glycine metabolism	0.000363	262,11,6
1ijqa1#564	GO:0006730: one-carbon compound metabolism	0.000363	28,32,4
1sfp__#84	GO:0042088: T-helper 1 type immune response	0.000364	7,11,2
1sfp__#84	GO:0042108: positive regulation of cytokine biosynthesis	0.000364	7,11,2
1rlw__#114	GO:0006690: icosanoid metabolism	0.000364	7,11,2
1cdy_2#140	GO:0042088: T-helper 1 type immune response	0.000364	7,11,2
1cdy_2#140	GO:0042108: positive regulation of cytokine biosynthesis	0.000364	7,11,2
1ilr1_#145	GO:0000165: MAPKKK cascade	0.000364	7,11,2
1ie5a_#104	GO:0042088: T-helper 1 type immune response	0.000364	7,11,2
1ie5a_#104	GO:0042108: positive regulation of cytokine biosynthesis	0.000364	7,11,2
1f7ua2#293	GO:0043094: metabolic compound salvage	0.000364	7,11,2
1dqga_#51	GO:0000165: MAPKKK cascade	0.000364	7,11,2
1iica1#172	GO:0016568: chromatin modification	0.000364	7,11,2
2sn3__#22	GO:0000165: MAPKKK cascade	0.000364	7,11,2
16vpa_#178	GO:0006511: ubiquitin-dependent protein catabolism	0.000364	7,11,2
1jlxa1#147	GO:0000165: MAPKKK cascade	0.000364	7,11,2
2ts1__#109	GO:0043094: metabolic compound salvage	0.000364	7,11,2
2tgi__#106	GO:0007276: gametogenesis	0.000364	7,11,2
1rypi_#124	GO:0006511: ubiquitin-dependent protein catabolism	0.000364	7,11,2
1i9ga_#185	GO:0006098: pentose-phosphate shunt	0.000364	7,11,2
1aqua_#126	GO:0046148: pigment biosynthesis	0.000365	472,12,8
1aqua_#126	GO:0009103: lipopolysaccharide biosynthesis	0.000365	472,12,8
1evqa_#305	GO:0006100: tricarboxylic acid cycle intermediate metabolism	0.000365	384,14,8
1jd0a_#176	GO:0050819: negative regulation of coagulation	0.000367	183,10,5
1jd0a_#176	GO:0030193: regulation of blood coagulation	0.000367	183,10,5
1jd0a_#176	GO:0018108: peptidyl-tyrosine phosphorylation	0.000367	183,10,5
1jd0a_#176	GO:0006275: regulation of DNA replication	0.000367	183,10,5
1jd0a_#176	GO:0006278: RNA-dependent DNA replication	0.000367	183,10,5

1j9qa1#154	GO:0030217: T-cell differentiation	0.000367	183,10,5
1vmoa_#106	GO:0019735: antimicrobial humoral response (sensu Vertebrata)	0.000367	167,16,6
1h6la_#119	GO:0008285: negative regulation of cell proliferation	0.000367	264,19,8
1urna_#93	GO:0006457: protein folding	0.000368	8,49,3
1fjfl_#67	GO:0051249: regulation of lymphocyte activation	0.000368	147,12,5
1epua_#26	GO:0016042: lipid catabolism	0.000368	29,31,4
1i7qa_#321	GO:0051249: regulation of lymphocyte activation	0.000368	147,12,5
1qtn.1#A270	GO:0043094: metabolic compound salvage	0.000368	386,11,7
1hdoa_#189	GO:0006563: L-serine metabolism	0.000368	147,12,5
1el5a2#277	GO:0030333: antigen processing	0.000369	6,13,2
1ycsb1#418	GO:0045786: negative regulation of cell cycle	0.000369	6,13,2
1ycsb1#418	GO:0000082: G1/S transition of mitotic cell cycle	0.000369	6,13,2
1sw6a_#352	GO:0045786: negative regulation of cell cycle	0.000369	6,13,2
1sw6a_#352	GO:0000082: G1/S transition of mitotic cell cycle	0.000369	6,13,2
1g6ha_#180	GO:0030333: antigen processing	0.000369	6,13,2
1qlwa_#103	GO:0006805: xenobiotic metabolism	0.000369	6,13,2
1qlwa_#103	GO:0017000: antibiotic biosynthesis	0.000369	6,13,2
1pjca1#254	GO:0006221: pyrimidine nucleotide biosynthesis	0.000369	17,22,3
1h9oa_#83	GO:0008284: positive regulation of cell proliferation	0.000369	30,30,4
1mfa_1#82L	GO:0030333: antigen processing	0.000369	6,13,2
1t7pa2#513	GO:0045892: negative regulation of transcription, DNA-dependent	0.000369	29,13,3
1hlga_#324	GO:0017000: antibiotic biosynthesis	0.000369	6,13,2
1c4xa_#35	GO:0006805: xenobiotic metabolism	0.000369	6,13,2
1c4xa_#35	GO:0017000: antibiotic biosynthesis	0.000369	6,13,2
7odca2#67	GO:0006221: pyrimidine nucleotide biosynthesis	0.000369	17,22,3
1qmea4#290	GO:0030333: antigen processing	0.000369	72,13,4
1dm9a_#57	GO:0016337: cell-cell adhesion	0.000369	99,17,5
1jlxa2#243	GO:0017148: negative regulation of protein biosynthesis	0.000369	6,13,2
1dcqa1#473	GO:0045786: negative regulation of cell cycle	0.000369	6,13,2
1dcqa1#473	GO:0000082: G1/S transition of mitotic cell cycle	0.000369	6,13,2
1kapp1#358	GO:0030333: antigen processing	0.000369	29,13,3
1qlwa_#231	GO:0017000: antibiotic biosynthesis	0.000369	6,13,2
2dpma_#237	GO:0017148: negative regulation of protein biosynthesis	0.000369	72,13,4
1a06__#147	GO:0045859: regulation of protein kinase activity	0.000369	25,15,3
1dcqa1#451	GO:0045786: negative regulation of cell cycle	0.000369	6,13,2
1dcqa1#451	GO:0000082: G1/S transition of mitotic cell cycle	0.000369	6,13,2
1nsca_#236	GO:0016337: cell-cell adhesion	0.000369	156,17,6
1iow_1#37	GO:0009396: folic acid and derivative biosynthesis	0.000371	263,11,6
1g6oa_#116	GO:0016337: cell-cell adhesion	0.000371	302,17,8
1a7ca_#202	GO:0006953: acute-phase response	0.000371	83,20,5
1ev2e2#286	GO:0042088: T-helper 1 type immune response	0.000372	87,11,4
1ev2e2#286	GO:0006801: superoxide metabolism	0.000372	87,11,4
1b4ka_#108	GO:0042364: water-soluble vitamin biosynthesis	0.000372	94,27,6
1aa6_2#285	GO:0009396: folic acid and derivative biosynthesis	0.000372	87,11,4
1e2wa1#102	GO:0009615: response to virus	0.000374	79,12,4
1nbaa_#227	GO:0015992: proton transport	0.000374	79,12,4
1ovb__#195	GO:0009156: ribonucleoside monophosphate biosynthesis	0.000374	79,12,4
1gox__#124	GO:0009167: purine ribonucleoside monophosphate metabolism	0.000374	298,10,6
1jj2m_#38	GO:0006633: fatty acid biosynthesis	0.000376	14,27,3
1ezwa_#245	GO:0006100: tricarboxylic acid cycle intermediate metabolism	0.000376	27,14,3
1dzfa2#211	GO:0006418: tRNA aminoacylation for protein translation	0.000376	139,26,7
3grs_1#23	GO:0009070: serine family amino acid biosynthesis	0.000376	139,26,7
1jb3a_#64	GO:0006935: chemotaxis	0.000377	247,41,12
1fxla2#169	GO:0006275: regulation of DNA replication	0.000377	184,10,5
1tns__#50	GO:0043123: positive regulation of I-kappaB kinase/NF-kappaB cascade	0.000378	5,16,2
1exta3#123	GO:0043123: positive regulation of I-kappaB kinase/NF-kappaB cascade	0.000378	5,16,2

19hca_#162	GO:0006413: translational initiation	0.000378	5,16,2
1pii_1#40	GO:0009073: aromatic amino acid family biosynthesis	0.000378	5,16,2
1a7j__#87	GO:0009252: peptidoglycan biosynthesis	0.000378	51,18,4
1p35a_#49	GO:0016477: cell migration	0.000378	112,23,6
1qqga2#166	GO:0006544: glycine metabolism	0.000379	164,11,5
1cs1a_#160	GO:0043094: metabolic compound salvage	0.000379	164,11,5
1cs1a_#160	GO:0009396: folic acid and derivative biosynthesis	0.000379	164,11,5
1qfma1#139	GO:0042088: T-helper 1 type immune response	0.000379	264,11,6
1qfma1#139	GO:0042108: positive regulation of cytokine biosynthesis	0.000379	264,11,6
1qfma1#139	GO:0001906: cell killing	0.000379	264,11,6
1hw5a2#64	GO:0051249: regulation of lymphocyte activation	0.00038	148,12,5
1qgua_#435	GO:0007264: small GTPase mediated signal transduction	0.000382	18,21,3
1fvia2#25	GO:0042088: T-helper 1 type immune response	0.000382	35,11,3
1qs0b2#269	GO:0009396: folic acid and derivative biosynthesis	0.000382	35,11,3
1a6o__#297	GO:0008203: cholesterol metabolism	0.000382	35,11,3
1hxxa_#278	GO:0006119: oxidative phosphorylation	0.000382	237,12,6
2pia_1#99	GO:0006096: glycolysis	0.000382	156,38,9
1fo5a_#60	GO:0016337: cell-cell adhesion	0.000382	225,17,7
1cqka_#49	GO:0042088: T-helper 1 type immune response	0.000382	35,11,3
1cqka_#49	GO:0042108: positive regulation of cytokine biosynthesis	0.000382	35,11,3
1clxa_#120	GO:0009396: folic acid and derivative biosynthesis	0.000382	35,11,3
1fp2a2#346	GO:0007264: small GTPase mediated signal transduction	0.000382	18,21,3
1cjca2#76	GO:0009252: peptidoglycan biosynthesis	0.000382	21,18,3
1gcya2#331	GO:0009396: folic acid and derivative biosynthesis	0.000382	35,11,3
1rypk_#12	GO:0006470: protein amino acid dephosphorylation	0.000382	250,20,8
1hvba_#63	GO:0006313: DNA transposition	0.000382	11,35,3
1hxma2#150	GO:0007169: transmembrane receptor protein tyrosine kinase signaling pathway	0.000384	35,26,4
1bg6_1#294	GO:0007186: G-protein coupled receptor protein signaling pathway	0.000385	38,24,4
3grs_1#24	GO:0009116: nucleoside metabolism	0.000385	66,25,5
1qp8a1#153	GO:0006096: glycolysis	0.000385	24,38,4
1qfta_#98	GO:0008285: negative regulation of cell proliferation	0.000385	198,19,7
1ep3a_#247	GO:0006096: glycolysis	0.000385	24,38,4
1i6vc_#689	GO:0006730: one-carbon compound metabolism	0.000386	12,32,3
1ak5_1#376	GO:0009156: ribonucleoside monophosphate biosynthesis	0.000386	32,12,3
1awcb_#111	GO:0006879: iron ion homeostasis	0.000386	32,12,3
1qdla_#211	GO:0006633: fatty acid biosynthesis	0.000386	280,27,10
1qhqa_#45	GO:0006118: electron transport	0.000386	106,228,21
1dn2a1#261	GO:0006606: protein-nucleus import	0.000386	376,21,10
1fu6a_#50	GO:0042088: T-helper 1 type immune response	0.000387	265,11,6
1prea2#119	GO:0019883: antigen presentation, endogenous antigen	0.000388	39,10,3
1j9qa2#295	GO:0045595: regulation of cell differentiation	0.000388	39,10,3
1dih_2#235	GO:0018108: peptidyl-tyrosine phosphorylation	0.000388	39,10,3
1d0va_#46	GO:0000051: urea cycle intermediate metabolism	0.000388	39,10,3
3sil__#317	GO:0019059: initiation of viral infection	0.000388	300,10,6
1ji6a2#458	GO:0006470: protein amino acid dephosphorylation	0.000389	19,20,3
1qf6a3#78	GO:0015074: DNA integration	0.000389	46,20,4
1ycsa_#162	GO:0006606: protein-nucleus import	0.000389	177,21,7
1bd3a_#110	GO:0009064: glutamine family amino acid metabolism	0.000389	20,19,3
1nfdb1#21	GO:0001906: cell killing	0.000389	88,11,4
1nfdb1#21	GO:0006690: icosanoid metabolism	0.000389	88,11,4
1hdma1#115	GO:0042088: T-helper 1 type immune response	0.00039	165,11,5
1hdma1#115	GO:0042108: positive regulation of cytokine biosynthesis	0.00039	165,11,5
1h8ua_#113	GO:0046677: response to antibiotic	0.000391	63,40,6
1fepa_#49	GO:0046677: response to antibiotic	0.000391	63,40,6
1ihua2#525	GO:0006730: one-carbon compound metabolism	0.000392	278,32,11

1f0xa1#523	GO:0006367: transcription initiation from Pol II promoter	0.000393	80,12,4
1el0a_#28	GO:0007169: transmembrane receptor protein tyrosine kinase signaling pathway	0.000393	140,26,7
1xgsa2#186	GO:0006367: transcription initiation from Pol II promoter	0.000393	149,12,5
1eur__#84	GO:0008284: positive regulation of cell proliferation	0.000395	85,30,6
1aym3_#131	GO:0007169: transmembrane receptor protein tyrosine kinase signaling pathway	0.000396	353,26,11
1gdna_#230	GO:0050819: negative regulation of coagulation	0.000397	8,10,2
1gdna_#230	GO:0030193: regulation of blood coagulation	0.000397	8,10,2
1nfdb1#115	GO:0019883: antigen presentation, endogenous antigen	0.000397	8,10,2
1c8za_#449	GO:0009167: purine ribonucleoside monophosphate metabolism	0.000397	8,10,2
1i7qa_#470	GO:0042401: biogenic amine biosynthesis	0.000397	8,10,2
1i7qa_#470	GO:0006568: tryptophan metabolism	0.000397	8,10,2
1cfb_2#763	GO:0051049: regulation of transport	0.000397	8,10,2
2cpl__#83	GO:0050819: negative regulation of coagulation	0.000397	8,10,2
2cpl__#83	GO:0030193: regulation of blood coagulation	0.000397	8,10,2
2cpl__#83	GO:0018108: peptidyl-tyrosine phosphorylation	0.000397	8,10,2
1im4a_#144	GO:0009064: glutamine family amino acid metabolism	0.000397	89,19,5
1hcl__#267	GO:0006275: regulation of DNA replication	0.000397	8,10,2
1vin_1#302	GO:0000086: G2/M transition of mitotic cell cycle	0.000397	8,10,2
1fiy__#232	GO:0015976: carbon utilization	0.000397	8,10,2
1koba_#105	GO:0000086: G2/M transition of mitotic cell cycle	0.000397	8,10,2
1cs6a2#164	GO:0019883: antigen presentation, endogenous antigen	0.000397	8,10,2
1bfd_1#230	GO:0030435: sporulation	0.000397	40,23,4
1dgtb3#2594	GO:0030217: T-cell differentiation	0.000397	8,10,2
1enfa1#43	GO:0001558: regulation of cell growth	0.000397	8,10,2
1ovaa_#161	GO:0046677: response to antibiotic	0.000397	23,40,4
1ltda1#242	GO:0006207: 'de novo' pyrimidine base biosynthesis	0.000397	8,10,2
1dfca1#1076	GO:0007264: small GTPase mediated signal transduction	0.000398	4,21,2
1ewfa2#338	GO:0006606: protein-nucleus import	0.000398	4,21,2
1atza_#1070	GO:0007264: small GTPase mediated signal transduction	0.000398	4,21,2
1jlxa2#288	GO:0007264: small GTPase mediated signal transduction	0.000398	4,21,2
1qfea_#49	GO:0042364: water-soluble vitamin biosynthesis	0.000398	34,27,4
1qq5a_#7	GO:0009086: methionine biosynthesis	0.000398	125,14,5
1a9xa6#760	GO:0006606: protein-nucleus import	0.000399	80,21,5
1elja_#64	GO:0007264: small GTPase mediated signal transduction	0.000399	80,21,5
1g51a3#525	GO:0051249: regulation of lymphocyte activation	0.0004	239,12,6
1iira_#242	GO:0007264: small GTPase mediated signal transduction	0.0004	125,21,6
1jd0a_#176	GO:0006919: caspase activation	0.000402	183,15,6
1aym3_#131	GO:0008284: positive regulation of cell proliferation	0.000402	353,30,12
1psda3#344	GO:0006275: regulation of DNA replication	0.000402	99,10,4
1ax4a_#275	GO:0006559: L-phenylalanine catabolism	0.000402	99,10,4
1g6oa_#116	GO:0050819: negative regulation of coagulation	0.000403	302,10,6
1g6oa_#116	GO:0030193: regulation of blood coagulation	0.000403	302,10,6
1g6oa_#116	GO:0018108: peptidyl-tyrosine phosphorylation	0.000403	302,10,6
1dbxa_#75	GO:0030036: actin cytoskeleton organization and biogenesis	0.000403	63,15,4
1im4a_#41	GO:0006897: endocytosis	0.000404	49,19,4
1dgw.1#Y353	GO:0007169: transmembrane receptor protein tyrosine kinase signaling pathway	0.000404	294,26,10
1ie5a_#102	GO:0007169: transmembrane receptor protein tyrosine kinase signaling pathway	0.000404	64,26,5
1eo6a_#107	GO:0006418: tRNA aminoacylation for protein translation	0.000404	64,26,5
1eo6a_#107	GO:0009070: serine family amino acid biosynthesis	0.000404	64,26,5
1ton__#181	GO:0006897: endocytosis	0.000404	49,19,4
1f8ra2#325	GO:0016042: lipid catabolism	0.000405	196,31,9
1flca1#294	GO:0042088: T-helper 1 type immune response	0.000406	89,11,4
1flca1#294	GO:0042108: positive regulation of cytokine biosynthesis	0.000406	89,11,4

1flca1#294	GO:0001906: cell killing	0.000406	89,11,4
2mpra_#190	GO:0006119: oxidative phosphorylation	0.000406	349,12,7
1fsu__#434	GO:0006643: membrane lipid metabolism	0.000407	315,13,7
1a3k__#122	GO:0042113: B-cell activation	0.000408	9,9,2
1h6la_#119	GO:0006986: response to unfolded protein	0.000408	264,15,7
1wba__#169	GO:0048514: blood vessel morphogenesis	0.000408	9,9,2
1hcd__#114	GO:0008284: positive regulation of cell proliferation	0.000409	13,30,3
1by5a_#347	GO:0009615: response to virus	0.000409	240,12,6
1pysb5#672	GO:0030333: antigen processing	0.000409	30,13,3
1fgga_#134	GO:0006096: glycolysis	0.000409	193,38,10
1quqb_#87	GO:0006096: glycolysis	0.000411	67,38,6
1dn0b2#214	GO:0008284: positive regulation of cell proliferation	0.000413	3,30,2
19hca_#50	GO:0007517: muscle development	0.000413	3,30,2
2sqca2#97	GO:0006118: electron transport	0.000413	114,228,22
1eqka_#47	GO:0006118: electron transport	0.000413	114,228,22
1fhga_#48	GO:0007517: muscle development	0.000413	3,30,2
1vmoa_#106	GO:0001906: cell killing	0.000413	167,11,5
1nfdb1#34	GO:0001906: cell killing	0.000413	167,11,5
1g51a3#525	GO:0006606: protein-nucleus import	0.000414	239,21,8
1doi__#42	GO:0006418: tRNA aminoacylation for protein translation	0.000416	15,26,3
1dcfa_#66	GO:0009396: folic acid and derivative biosynthesis	0.000416	36,11,3
2mpra_#267	GO:0030036: actin cytoskeleton organization and biogenesis	0.000416	26,15,3
2pola3#335	GO:0007169: transmembrane receptor protein tyrosine kinase signaling pathway	0.000416	15,26,3
1bmv2_#2102	GO:0007169: transmembrane receptor protein tyrosine kinase signaling pathway	0.000416	15,26,3
1tkia_#80	GO:0007169: transmembrane receptor protein tyrosine kinase signaling pathway	0.000416	15,26,3
1seta2#306	GO:0006919: caspase activation	0.000416	26,15,3
1eg9a2#199	GO:0000910: cytokinesis	0.000416	69,37,6
1a6o__#217	GO:0007169: transmembrane receptor protein tyrosine kinase signaling pathway	0.000416	15,26,3
1howa_#588	GO:0007169: transmembrane receptor protein tyrosine kinase signaling pathway	0.000416	15,26,3
1koba_#230	GO:0007169: transmembrane receptor protein tyrosine kinase signaling pathway	0.000416	15,26,3
1howa_#697	GO:0007169: transmembrane receptor protein tyrosine kinase signaling pathway	0.000416	15,26,3
1tkia_#266	GO:0007169: transmembrane receptor protein tyrosine kinase signaling pathway	0.000416	15,26,3
1fo0b_#82	GO:0007169: transmembrane receptor protein tyrosine kinase signaling pathway	0.000416	15,26,3
1phk__#165	GO:0007169: transmembrane receptor protein tyrosine kinase signaling pathway	0.000416	15,26,3
1mrp__#170	GO:0006826: iron ion transport	0.000416	26,15,3
1ojt_2#356	GO:0009396: folic acid and derivative biosynthesis	0.000416	36,11,3
1hdma1#123	GO:0042108: positive regulation of cytokine biosynthesis	0.000416	36,11,3
3grx__#56	GO:0016337: cell-cell adhesion	0.000417	307,17,8
1qhda2#323	GO:0006917: induction of apoptosis	0.000418	313,29,11
1ayl__#237	GO:0006917: induction of apoptosis	0.000418	313,29,11
1faoa_#250	GO:0006730: one-carbon compound metabolism	0.000418	52,32,5
2mpra_#89	GO:0019883: antigen presentation, endogenous antigen	0.000419	40,10,3
1esma_#314	GO:0007169: transmembrane receptor protein tyrosine kinase signaling pathway	0.000419	100,26,6
1ct9a1#401	GO:0009086: methionine biosynthesis	0.00042	28,14,3
1cpy__#346	GO:0006100: tricarboxylic acid cycle intermediate metabolism	0.00042	28,14,3
1huxa_#10	GO:0006457: protein folding	0.000421	52,49,6
1bwvs_#117	GO:0009252: peptidoglycan biosynthesis	0.000421	287,18,8
1fp5a2#537	GO:0042113: B-cell activation	0.000422	45,9,3

1e6ca_#91	GO:0009064: glutamine family amino acid metabolism	0.000422	141,19,6
1qj2c2#50	GO:0015074: DNA integration	0.000423	47,20,4
1gg6.1#B19	GO:0006956: complement activation	0.000423	33,12,3
2pgd_1#421	GO:0006118: electron transport	0.000423	64,228,15
1fzqa_#28	GO:0007017: microtubule-based process	0.000423	33,12,3
1erxa_#138	GO:0006118: electron transport	0.000423	64,228,15
1c9la2#61	GO:0006461: protein complex assembly	0.000423	206,24,8
1euca1#64	GO:0009156: ribonucleoside monophosphate biosynthesis	0.000423	33,12,3
1bec_2#216	GO:0019735: antimicrobial humoral response (sensu Vertebrata)	0.000423	109,16,5
1elua_#216	GO:0006563: L-serine metabolism	0.000423	33,12,3
1dhs__#99	GO:0009073: aromatic amino acid family biosynthesis	0.000423	109,16,5
2cpl__#20	GO:0042088: T-helper 1 type immune response	0.000424	90,11,4
2cpl__#20	GO:0042108: positive regulation of cytokine biosynthesis	0.000424	90,11,4
2occb1#209	GO:0001906: cell killing	0.000424	90,11,4
1aqb__#134	GO:0042088: T-helper 1 type immune response	0.000424	90,11,4
1aqb__#134	GO:0042108: positive regulation of cytokine biosynthesis	0.000424	90,11,4
1fsia_#122	GO:0006690: icosanoid metabolism	0.000424	90,11,4
1dpga2#334	GO:0043123: positive regulation of I-kappaB kinase/NF-kappaB cascade	0.000426	246,16,7
1bev1_#86	GO:0007169: transmembrane receptor protein tyrosine kinase signaling pathway	0.000427	296,26,10
2pgd_1#421	GO:0007601: visual perception	0.000428	64,15,4
1eg9a2#199	GO:0009086: methionine biosynthesis	0.000429	69,14,4
3prn__#145	GO:0006461: protein complex assembly	0.000429	155,24,7
1e79d3#328	GO:0009070: serine family amino acid biosynthesis	0.000429	36,26,4
1fg7a_#96	GO:0009086: methionine biosynthesis	0.00043	6,14,2
1fmja_#50	GO:0016567: protein ubiquitination	0.00043	6,14,2
1i2oa_#34	GO:0006563: L-serine metabolism	0.000431	152,12,5
1quqa_#75	GO:0006118: electron transport	0.000431	51,228,13
1ac5__#72	GO:0006118: electron transport	0.000431	51,228,13
1dyna_#42	GO:0051249: regulation of lymphocyte activation	0.000431	152,12,5
1xis__#214	GO:0009156: ribonucleoside monophosphate biosynthesis	0.000432	82,12,4
1feca3#467	GO:0030333: antigen processing	0.000432	75,13,4
1g8fa3#419	GO:0000910: cytokinesis	0.000434	129,37,8
2foka2#180	GO:0000910: cytokinesis	0.000434	129,37,8
1kit_3#331	GO:0006354: RNA elongation	0.000434	101,10,4
1kit_3#331	GO:0018108: peptidyl-tyrosine phosphorylation	0.000434	101,10,4
1h6va3#486	GO:0007169: transmembrane receptor protein tyrosine kinase signaling pathway	0.000435	65,26,5
1hxmb1#117	GO:0007169: transmembrane receptor protein tyrosine kinase signaling pathway	0.000435	65,26,5
1danh_#93	GO:0006956: complement activation	0.000436	7,12,2
1qg8a_#90	GO:0006563: L-serine metabolism	0.000436	7,12,2
1aym1_#151	GO:0006119: oxidative phosphorylation	0.000436	7,12,2
1rlw__#114	GO:0006956: complement activation	0.000436	7,12,2
1ypci_#69	GO:0009156: ribonucleoside monophosphate biosynthesis	0.000436	7,12,2
1ie5a_#104	GO:0051249: regulation of lymphocyte activation	0.000436	7,12,2
1pud__#72	GO:0006563: L-serine metabolism	0.000436	7,12,2
1qbkb_#684	GO:0007017: microtubule-based process	0.000436	7,12,2
1qopb_#89	GO:0006563: L-serine metabolism	0.000436	7,12,2
2occa1#372	GO:0015992: proton transport	0.000436	7,12,2
1c3d__#72	GO:0006956: complement activation	0.000436	7,12,2
2tgi__#106	GO:0007017: microtubule-based process	0.000436	7,12,2
1bd2d1#62	GO:0051249: regulation of lymphocyte activation	0.000436	7,12,2
1fc4a_#205	GO:0009252: peptidoglycan biosynthesis	0.000436	215,18,7
7reqb1#163	GO:0009103: lipopolysaccharide biosynthesis	0.000436	7,12,2
1cem__#281	GO:0030245: cellulose catabolism	0.000437	4,22,2
1czan1#92	GO:0019079: viral genome replication	0.000437	538,16,10

1jb0d_#26	GO:0030217: T-cell differentiation	0.000439	190,10,5
3sil__#98	GO:0008015: circulation	0.000439	186,15,6
1qfea_#68	GO:0015976: carbon utilization	0.000439	453,10,7
1qfea_#68	GO:0009167: purine ribonucleoside monophosphate metabolism	0.000439	453,10,7
1quna1#113	GO:0009156: ribonucleoside monophosphate biosynthesis	0.000439	243,12,6
1mrp__#172	GO:0006099: tricarboxylic acid cycle	0.00044	91,19,5
1pud__#44	GO:0006221: pyrimidine nucleotide biosynthesis	0.00044	43,22,4
1b0ua_#174	GO:0006779: porphyrin biosynthesis	0.000441	22,18,3
1bkpa_#41	GO:0006221: pyrimidine nucleotide biosynthesis	0.000441	18,22,3
1mrp__#172	GO:0006098: pentose-phosphate shunt	0.000442	91,11,4
1f8v.1#A116	GO:0006118: electron transport	0.000442	78,228,17
1qf9a_#117	GO:0009116: nucleoside metabolism	0.000443	68,25,5
1qrra_#69	GO:0009073: aromatic amino acid family biosynthesis	0.000445	173,16,6
1mpp__#154	GO:0016042: lipid catabolism	0.000446	84,31,6
1ekma1#545	GO:0016042: lipid catabolism	0.000446	84,31,6
1nal1_#203	GO:0042364: water-soluble vitamin biosynthesis	0.000447	35,27,4
1fp1d2#214	GO:0006418: tRNA aminoacylation for protein translation	0.000448	143,26,7
1fp1d2#214	GO:0009070: serine family amino acid biosynthesis	0.000448	143,26,7
1fjfc2#153	GO:0006445: regulation of translation	0.000448	362,15,8
1ac6a_#43	GO:0006935: chemotaxis	0.000449	147,41,9
1jb0d_#26	GO:0007169: transmembrane receptor protein tyrosine kinase signaling pathway	0.000449	190,26,8
1deua_#151	GO:0006457: protein folding	0.000449	239,49,13
1opy__#68	GO:0018108: peptidyl-tyrosine phosphorylation	0.000449	308,10,6
1opy__#68	GO:0006265: DNA topological change	0.000449	308,10,6
1e8ca3#111	GO:0006563: L-serine metabolism	0.000449	244,12,6
1i50b_#378	GO:0000910: cytokinesis	0.00045	70,37,6
1by5a_#238	GO:0006917: induction of apoptosis	0.00045	214,29,9
1a8d_1#87	GO:0019059: initiation of viral infection	0.00045	191,10,5
1a8d_1#87	GO:0030217: T-cell differentiation	0.00045	191,10,5
1i2ma_#157	GO:0006935: chemotaxis	0.000451	10,41,3
1dlfl_#97	GO:0030217: T-cell differentiation	0.000451	41,10,3
1ryp1_#18	GO:0042088: T-helper 1 type immune response	0.000451	37,11,3
1ryp1_#18	GO:0006511: ubiquitin-dependent protein catabolism	0.000451	37,11,3
1fid__#237	GO:0050819: negative regulation of coagulation	0.000451	41,10,3
1fid__#237	GO:0030193: regulation of blood coagulation	0.000451	41,10,3
1fid__#237	GO:0018108: peptidyl-tyrosine phosphorylation	0.000451	41,10,3
1phk__#221	GO:0000910: cytokinesis	0.000451	11,37,3
1dt9a1#224	GO:0009167: purine ribonucleoside monophosphate metabolism	0.000451	41,10,3
1exh__#44	GO:0019883: antigen presentation, endogenous antigen	0.000451	41,10,3
1exh__#44	GO:0006265: DNA topological change	0.000451	41,10,3
1exh__#44	GO:0006268: DNA unwinding	0.000451	41,10,3
1aym3_#109	GO:0045595: regulation of cell differentiation	0.000451	41,10,3
1aym3_#109	GO:0030217: T-cell differentiation	0.000451	41,10,3
1c7na_#95	GO:0006457: protein folding	0.000451	149,49,10
3grx__#55	GO:0000051: urea cycle intermediate metabolism	0.000451	102,10,4
1egaa1#97	GO:0006606: protein-nucleus import	0.000452	19,21,3
1b8pa2#197	GO:0006099: tricarboxylic acid cycle	0.000452	21,19,3
1ga0a_#228	GO:0008285: negative regulation of cell proliferation	0.000452	21,19,3
1a4ia1#208	GO:0007264: small GTPase mediated signal transduction	0.000452	19,21,3
1heta2#218	GO:0009064: glutamine family amino acid metabolism	0.000452	21,19,3
1fu6a_#50	GO:0006461: protein complex assembly	0.000452	265,24,9
1rypf_#35	GO:0009064: glutamine family amino acid metabolism	0.000452	21,19,3
1e2o__#346	GO:0006919: caspase activation	0.000453	187,15,6
1a7ca_#202	GO:0051249: regulation of lymphocyte activation	0.000453	83,12,4
1erv__#57	GO:0009086: methionine biosynthesis	0.000453	70,14,4

1h6va2#245	GO:0006563: L-serine metabolism	0.000453	83,12,4
1e79d2#36	GO:0019079: viral genome replication	0.000455	432,16,9
1e79d2#36	GO:0019735: antimicrobial humoral response (sensu Vertebrata)	0.000455	432,16,9
1flga_#161	GO:0006606: protein-nucleus import	0.000456	128,21,6
1fp1d2#214	GO:0006099: tricarboxylic acid cycle	0.000456	143,19,6
1jsg__#92	GO:0007399: neurogenesis	0.000458	519,37,17
1gox__#124	GO:0006118: electron transport	0.000458	298,228,44
1dpja_#322	GO:0006919: caspase activation	0.000459	269,15,7
1hbza_#214	GO:0019735: antimicrobial humoral response (sensu Vertebrata)	0.00046	249,16,7
1p35a_#101	GO:0019735: antimicrobial humoral response (sensu Vertebrata)	0.000461	111,16,5
1cg2a1#43	GO:0006098: pentose-phosphate shunt	0.000461	171,11,5
1hdfa_#16	GO:0019059: initiation of viral infection	0.000461	192,10,5
1hdfa_#16	GO:0030217: T-cell differentiation	0.000461	192,10,5
1dt9a1#209	GO:0016485: protein processing	0.000462	92,11,4
2bb2_1#57	GO:0042088: T-helper 1 type immune response	0.000462	92,11,4
2bb2_1#57	GO:0042108: positive regulation of cytokine biosynthesis	0.000462	92,11,4
2bb2_1#57	GO:0016485: protein processing	0.000462	92,11,4
1gdha1#235	GO:0009396: folic acid and derivative biosynthesis	0.000462	92,11,4
1dt9a1#209	GO:0006099: tricarboxylic acid cycle	0.000463	92,19,5
1cex__#109	GO:0007017: microtubule-based process	0.000463	34,12,3
1loua_#79	GO:0009064: glutamine family amino acid metabolism	0.000463	92,19,5
1b3qa2#623	GO:0006953: acute-phase response	0.000463	257,20,8
1bf6a_#183	GO:0009156: ribonucleoside monophosphate biosynthesis	0.000463	34,12,3
1sppb_#32	GO:0051249: regulation of lymphocyte activation	0.000463	34,12,3
1nsj__#21	GO:0006096: glycolysis	0.000464	96,38,7
1a81a1#51	GO:0016477: cell migration	0.000464	75,23,5
1quna1#113	GO:0007264: small GTPase mediated signal transduction	0.000464	243,21,8
1quna1#113	GO:0006606: protein-nucleus import	0.000464	243,21,8
1apme_#74	GO:0016337: cell-cell adhesion	0.000465	104,17,5
2dkb__#133	GO:0042364: water-soluble vitamin biosynthesis	0.000466	15,27,3
1bg6_2#73	GO:0006100: tricarboxylic acid cycle intermediate metabolism	0.000466	29,14,3
1pkp_1#139	GO:0006633: fatty acid biosynthesis	0.000466	15,27,3
1d3ga_#118	GO:0006100: tricarboxylic acid cycle intermediate metabolism	0.000466	29,14,3
1epua_#26	GO:0009086: methionine biosynthesis	0.000466	29,14,3
1neb__#52	GO:0006917: induction of apoptosis	0.000466	14,29,3
1feza_#185	GO:0009070: serine family amino acid biosynthesis	0.000467	66,26,5
1hyrc1#247	GO:0009070: serine family amino acid biosynthesis	0.000467	66,26,5
1jlxa2#166	GO:0007169: transmembrane receptor protein tyrosine kinase signaling pathway	0.000467	66,26,5
1a9xb2#1775	GO:0016042: lipid catabolism	0.000467	55,31,5
1kid__#330	GO:0007169: transmembrane receptor protein tyrosine kinase signaling pathway	0.000468	144,26,7
1ryph_#163	GO:0017001: antibiotic catabolism	0.000468	103,10,4
1dgw.1#Y353	GO:0016042: lipid catabolism	0.000469	294,31,11
1dpga2#334	GO:0009156: ribonucleoside monophosphate biosynthesis	0.000469	246,12,6
1fo8a_#367	GO:0006730: one-carbon compound metabolism	0.000471	3,32,2
1dyr__#180	GO:0006730: one-carbon compound metabolism	0.000471	3,32,2
1dssg1#4	GO:0009064: glutamine family amino acid metabolism	0.000472	51,19,4
1e44b_#75	GO:0016477: cell migration	0.000472	165,23,7
1i5ga_#115	GO:0006100: tricarboxylic acid cycle intermediate metabolism	0.000472	294,14,7
1bhga3#529	GO:0009064: glutamine family amino acid metabolism	0.000472	51,19,4
1fcya_#311	GO:0006099: tricarboxylic acid cycle	0.000472	51,19,4
3prn__#145	GO:0051249: regulation of lymphocyte activation	0.000473	155,12,5
1dih_2#222	GO:0006278: RNA-dependent DNA replication	0.000474	311,10,6
1ycsb1#412	GO:0007264: small GTPase mediated signal transduction	0.000475	129,21,6
1ekma1#545	GO:0051249: regulation of lymphocyte activation	0.000475	84,12,4

1ayl__#237	GO:0016337: cell-cell adhesion	0.000477	313,17,8
1ryp1_#18	GO:0007169: transmembrane receptor protein tyrosine kinase signaling pathway	0.000478	37,26,4
1jd0a_#176	GO:0006606: protein-nucleus import	0.000478	183,21,7
1dpsa_#36	GO:0007264: small GTPase mediated signal transduction	0.000478	183,21,7
2msba_#218	GO:0006418: tRNA aminoacylation for protein translation	0.000478	37,26,4
1pfza_#124	GO:0006418: tRNA aminoacylation for protein translation	0.000478	37,26,4
1c3ma_#58	GO:0030435: sporulation	0.000479	4,23,2
1nuka_#85	GO:0016477: cell migration	0.000479	4,23,2
1fnha2#152	GO:0016477: cell migration	0.000479	4,23,2
1hc2_2#343	GO:0015671: oxygen transport	0.000479	4,23,2
1ezvb1#29	GO:0016477: cell migration	0.00048	42,23,4
1hdma1#98	GO:0016477: cell migration	0.00048	42,23,4
1apme_#68	GO:0006801: superoxide metabolism	0.000481	93,11,4
1apme_#68	GO:0001906: cell killing	0.000481	93,11,4
1g51a3#524	GO:0042088: T-helper 1 type immune response	0.000481	93,11,4
1g51a3#524	GO:0006511: ubiquitin-dependent protein catabolism	0.000481	93,11,4
1hrka_#205	GO:0006096: glycolysis	0.000481	45,38,5
1ho1a_#211	GO:0043094: metabolic compound salvage	0.000481	93,11,4
1ho1a_#211	GO:0006098: pentose-phosphate shunt	0.000481	93,11,4
1qupa2#50	GO:0006979: response to oxidative stress	0.000482	5,18,2
1gg4a3#24	GO:0009252: peptidoglycan biosynthesis	0.000482	5,18,2
1g8pa_#210	GO:0006779: porphyrin biosynthesis	0.000482	5,18,2
1cipa2#326	GO:0007186: G-protein coupled receptor protein signaling pathway	0.000482	17,24,3
1hyrc1#247	GO:0006826: iron ion transport	0.000482	66,15,4
1jlxa2#166	GO:0006826: iron ion transport	0.000482	66,15,4
1bd8__#82	GO:0006919: caspase activation	0.000482	66,15,4
1cii_1#611	GO:0019835: cytolysis	0.000482	5,18,2
1cyx__#138	GO:0016337: cell-cell adhesion	0.000482	24,17,3
2oata_#81	GO:0006779: porphyrin biosynthesis	0.000482	5,18,2
1fo4a5#1020	GO:0019059: initiation of viral infection	0.000483	312,10,6
1feca1#31	GO:0006096: glycolysis	0.000483	69,38,6
1fcda1#6	GO:0006096: glycolysis	0.000483	69,38,6
2bpa1_#45	GO:0006457: protein folding	0.000483	179,49,11
1cmxa_#188	GO:0006511: ubiquitin-dependent protein catabolism	0.000484	8,11,2
1euha_#220	GO:0006098: pentose-phosphate shunt	0.000484	8,11,2
1euha_#220	GO:0009396: folic acid and derivative biosynthesis	0.000484	8,11,2
1hwxa2#60	GO:0016485: protein processing	0.000484	8,11,2
1dgtb3#2594	GO:0042088: T-helper 1 type immune response	0.000484	8,11,2
1dgtb3#2594	GO:0042108: positive regulation of cytokine biosynthesis	0.000484	8,11,2
1hp5a1#340	GO:0043094: metabolic compound salvage	0.000484	8,11,2
2dnja_#91	GO:0001906: cell killing	0.000484	8,11,2
1jf9a_#183	GO:0006098: pentose-phosphate shunt	0.000484	8,11,2
1cyda_#106	GO:0042559: pteridine and derivative biosynthesis	0.000484	8,11,2
1g8ka2#179	GO:0016568: chromatin modification	0.000484	8,11,2
1oaca1#360	GO:0001906: cell killing	0.000484	8,11,2
1oaca1#342	GO:0001906: cell killing	0.000484	8,11,2
1jfua_#53	GO:0006354: RNA elongation	0.000485	42,10,3
1a65a3#409	GO:0006275: regulation of DNA replication	0.000485	42,10,3
1stfi_#116	GO:0019059: initiation of viral infection	0.000485	42,10,3
1stfi_#116	GO:0009167: purine ribonucleoside monophosphate metabolism	0.000485	42,10,3
1as4.1#A336	GO:0042108: positive regulation of cytokine biosynthesis	0.000485	276,11,6
1as4.1#A336	GO:0006544: glycine metabolism	0.000485	276,11,6
1d0na3#305	GO:0042088: T-helper 1 type immune response	0.000485	276,11,6
1d0na3#305	GO:0042108: positive regulation of cytokine biosynthesis	0.000485	276,11,6
3grs_2#211	GO:0000051: urea cycle intermediate metabolism	0.000485	42,10,3

1jf9a_#196	GO:0006568: tryptophan metabolism	0.000485	42,10,3
1hdma1#98	GO:0045595: regulation of cell differentiation	0.000485	42,10,3
1dik_1#848	GO:0042364: water-soluble vitamin biosynthesis	0.000486	64,27,5
1czya1#466	GO:0030217: T-cell differentiation	0.000486	104,10,4
1a81a2#182	GO:0019883: antigen presentation, endogenous antigen	0.000486	104,10,4
1a81a2#182	GO:0006278: RNA-dependent DNA replication	0.000486	104,10,4
1qrra_#69	GO:0009396: folic acid and derivative biosynthesis	0.000487	173,11,5
1fjfc2#153	GO:0006118: electron transport	0.000487	362,228,51
1hd2a_#35	GO:0006418: tRNA aminoacylation for protein translation	0.000488	145,26,7
1i7oa1#63	GO:0042088: T-helper 1 type immune response	0.000489	38,11,3
1heta2#212	GO:0006098: pentose-phosphate shunt	0.000489	38,11,3
1qoua_#90	GO:0042108: positive regulation of cytokine biosynthesis	0.000489	38,11,3
1jj2n_#77	GO:0006544: glycine metabolism	0.000489	38,11,3
1booa_#254	GO:0043094: metabolic compound salvage	0.000489	38,11,3
1el0a_#42	GO:0006313: DNA transposition	0.00049	258,35,11
1qhda2#323	GO:0019059: initiation of viral infection	0.000492	313,10,6
1pot__#138	GO:0016337: cell-cell adhesion	0.000492	58,17,4
1ayl__#237	GO:0019059: initiation of viral infection	0.000492	313,10,6
1bwda_#117	GO:0009070: serine family amino acid biosynthesis	0.000492	103,26,6
1c9la2#61	GO:0008285: negative regulation of cell proliferation	0.000492	206,19,7
1boub_#7	GO:0009064: glutamine family amino acid metabolism	0.000492	206,19,7
1e1aa_#178	GO:0016337: cell-cell adhesion	0.000492	58,17,4
3sil__#8	GO:0006457: protein folding	0.000492	98,49,8
1jb3a_#64	GO:0016042: lipid catabolism	0.000494	247,31,10
1fnf_3#1409	GO:0016477: cell migration	0.000494	76,23,5
1g25a_#22	GO:0006826: iron ion transport	0.000494	367,15,8
1poxa3#460	GO:0030435: sporulation	0.000494	76,23,5
1qqka_#19	GO:0045859: regulation of protein kinase activity	0.000495	6,15,2
1elua_#288	GO:0008015: circulation	0.000495	6,15,2
1hxxa_#112	GO:0006826: iron ion transport	0.000495	6,15,2
1bbpa_#44	GO:0008015: circulation	0.000495	6,15,2
1ovaa_#297	GO:0006986: response to unfolded protein	0.000495	6,15,2
1howa_#552	GO:0045859: regulation of protein kinase activity	0.000495	6,15,2
1fi2a_#109	GO:0017001: antibiotic catabolism	0.000496	195,10,5
1fi2a_#109	GO:0006278: RNA-dependent DNA replication	0.000496	195,10,5
1lara2#1769	GO:0050819: negative regulation of coagulation	0.000496	195,10,5
1lara2#1769	GO:0030193: regulation of blood coagulation	0.000496	195,10,5
1lara2#1769	GO:0018108: peptidyl-tyrosine phosphorylation	0.000496	195,10,5
1lara2#1769	GO:0009167: purine ribonucleoside monophosphate metabolism	0.000496	195,10,5
1e4ea2#215	GO:0006935: chemotaxis	0.000496	419,41,16
1egja_#343	GO:0030333: antigen processing	0.000497	32,13,3
1bupa2#199	GO:0030333: antigen processing	0.000497	32,13,3
1awcb_#111	GO:0045786: negative regulation of cell cycle	0.000497	32,13,3
1ecpa_#62	GO:0006730: one-carbon compound metabolism	0.000499	54,32,5
1c8za_#386	GO:0006461: protein complex assembly	0.000499	211,24,8
1fhoa_#74	GO:0016337: cell-cell adhesion	0.000499	315,17,8
1prea2#308	GO:0007169: transmembrane receptor protein tyrosine kinase signaling pathway	0.000501	67,26,5
1fjsa_#83	GO:0006606: protein-nucleus import	0.000501	84,21,5
1axca1#70	GO:0042088: T-helper 1 type immune response	0.000501	94,11,4
1axca1#70	GO:0042108: positive regulation of cytokine biosynthesis	0.000501	94,11,4
1gsa_2#218	GO:0030435: sporulation	0.000502	118,23,6
1pii_2#326	GO:0046148: pigment biosynthesis	0.000502	157,12,5
1pii_2#326	GO:0009103: lipopolysaccharide biosynthesis	0.000502	157,12,5
1gox__#246	GO:0009156: ribonucleoside monophosphate biosynthesis	0.000502	157,12,5
1dofa_#15	GO:0006118: electron transport	0.000503	179,228,30

1qqsa_#35	GO:0030245: cellulose catabolism	0.000504	80,22,5
1g6oa_#116	GO:0007169: transmembrane receptor protein tyrosine kinase signaling pathway	0.000504	302,26,10
1dik_1#765	GO:0006207: 'de novo' pyrimidine base biosynthesis	0.000504	105,10,4
1rb9__#49	GO:0006367: transcription initiation from Pol II promoter	0.000505	35,12,3
3pah__#303	GO:0006979: response to oxidative stress	0.000505	23,18,3
1a6o__#297	GO:0015992: proton transport	0.000505	35,12,3
1hs6a3#389	GO:0015671: oxygen transport	0.000505	18,23,3
1fjja_#5	GO:0016477: cell migration	0.000505	18,23,3
1cqka_#49	GO:0051249: regulation of lymphocyte activation	0.000505	35,12,3
1clxa_#120	GO:0046148: pigment biosynthesis	0.000505	35,12,3
1b3ra2#47	GO:0007017: microtubule-based process	0.000505	35,12,3
1a3qa2#133	GO:0016477: cell migration	0.000505	18,23,3
1iow_2#112	GO:0009252: peptidoglycan biosynthesis	0.000505	23,18,3
1elja_#8	GO:0006879: iron ion homeostasis	0.000505	35,12,3
1b6g__#63	GO:0009103: lipopolysaccharide biosynthesis	0.000505	35,12,3
1ihua2#525	GO:0006098: pentose-phosphate shunt	0.000505	278,11,6
1ihua2#525	GO:0009396: folic acid and derivative biosynthesis	0.000505	278,11,6
1ihua2#525	GO:0006544: glycine metabolism	0.000505	278,11,6
1nsj__#124	GO:0030245: cellulose catabolism	0.000506	124,22,6
1ejba_#81	GO:0000160: two-component signal transduction system (phosphorelay)	0.000507	70,25,5
1erv__#57	GO:0009116: nucleoside metabolism	0.000507	70,25,5
1ge8a2#134	GO:0016042: lipid catabolism	0.000507	202,31,9
1eaja_#59	GO:0030217: T-cell differentiation	0.000508	196,10,5
1jkma_#317	GO:0016042: lipid catabolism	0.000508	56,31,5
1a8d_1#87	GO:0016068: type I hypersensitivity	0.000508	191,15,6
1a65a1#108	GO:0019735: antimicrobial humoral response (sensu Vertebrata)	0.000508	253,16,7
1qrra_#11	GO:0009070: serine family amino acid biosynthesis	0.000509	16,26,3
1b6cb_#346	GO:0006275: regulation of DNA replication	0.000509	9,10,2
1a6o__#233	GO:0000086: G2/M transition of mitotic cell cycle	0.000509	9,10,2
1a6o__#233	GO:0006275: regulation of DNA replication	0.000509	9,10,2
1ayl__#482	GO:0048514: blood vessel morphogenesis	0.000509	10,9,2
1tkia_#98	GO:0000086: G2/M transition of mitotic cell cycle	0.000509	9,10,2
1tkia_#98	GO:0006275: regulation of DNA replication	0.000509	9,10,2
1dl5a_#266	GO:0019079: viral genome replication	0.000509	26,16,3
1azsa_#397	GO:0006278: RNA-dependent DNA replication	0.000509	9,10,2
1howa_#551	GO:0000086: G2/M transition of mitotic cell cycle	0.000509	9,10,2
4tmka_#141	GO:0009219: pyrimidine deoxyribonucleotide metabolism	0.000509	9,10,2
1fmk_3#428	GO:0006275: regulation of DNA replication	0.000509	9,10,2
1e2fa_#121	GO:0009219: pyrimidine deoxyribonucleotide metabolism	0.000509	9,10,2
1e2fa_#121	GO:0045893: positive regulation of transcription, DNA-dependent	0.000509	9,10,2
1vns__#306	GO:0009219: pyrimidine deoxyribonucleotide metabolism	0.000509	9,10,2
1gox__#334	GO:0009167: purine ribonucleoside monophosphate metabolism	0.000509	9,10,2
1pme__#207	GO:0000086: G2/M transition of mitotic cell cycle	0.000509	9,10,2
1pme__#207	GO:0006275: regulation of DNA replication	0.000509	9,10,2
1a0ca_#187	GO:0042401: biogenic amine biosynthesis	0.000509	9,10,2
1a0ca_#187	GO:0006568: tryptophan metabolism	0.000509	9,10,2
1e6ca_#153	GO:0009219: pyrimidine deoxyribonucleotide metabolism	0.000509	9,10,2
1i7wa_#518	GO:0045595: regulation of cell differentiation	0.000509	9,10,2
7fabh1#71	GO:0019735: antimicrobial humoral response (sensu Vertebrata)	0.000509	26,16,3
1akjd_#3	GO:0030217: T-cell differentiation	0.000509	9,10,2
1qo7a_#109	GO:0009073: aromatic amino acid family biosynthesis	0.000509	26,16,3
5nul__#43	GO:0006568: tryptophan metabolism	0.000509	9,10,2
5nul__#43	GO:0006207: 'de novo' pyrimidine base biosynthesis	0.000509	9,10,2
2dri__#115	GO:0009206: purine ribonucleoside triphosphate biosynthesis	0.000509	10,9,2
1dceb_#290	GO:0015976: carbon utilization	0.000509	9,10,2

1dfca1#1119	GO:0009888: histogenesis	0.000509	9,10,2
1smaa2#581	GO:0006917: induction of apoptosis	0.000509	60,29,5
1i9za_#693	GO:0009219: pyrimidine deoxyribonucleotide metabolism	0.000509	9,10,2
1qvba_#9	GO:0009064: glutamine family amino acid metabolism	0.000509	52,19,4
1fgka_#689	GO:0007169: transmembrane receptor protein tyrosine kinase signaling pathway	0.000509	16,26,3
1nfdb1#34	GO:0016477: cell migration	0.000509	167,23,7
1fhoa_#74	GO:0006265: DNA topological change	0.000509	315,10,6
1fmk_3#377	GO:0007169: transmembrane receptor protein tyrosine kinase signaling pathway	0.000509	16,26,3
1ihka_#164	GO:0048514: blood vessel morphogenesis	0.000509	10,9,2
1b4ka_#108	GO:0006099: tricarboxylic acid cycle	0.000512	94,19,5
1fjfc2#153	GO:0051249: regulation of lymphocyte activation	0.000512	362,12,7
1e4ea2#215	GO:0008284: positive regulation of cell proliferation	0.000513	419,30,13
1rlw__#114	GO:0006643: membrane lipid metabolism	0.000515	7,13,2
1sw6a_#496	GO:0000082: G1/S transition of mitotic cell cycle	0.000515	7,13,2
1e8ya2#410	GO:0006643: membrane lipid metabolism	0.000515	7,13,2
1ek1a2#500	GO:0006805: xenobiotic metabolism	0.000515	7,13,2
1g4da_#18	GO:0030333: antigen processing	0.000515	7,13,2
1qo7a_#199	GO:0017000: antibiotic biosynthesis	0.000515	7,13,2
1euvb_#72	GO:0045786: negative regulation of cell cycle	0.000515	7,13,2
1jlxa1#147	GO:0017148: negative regulation of protein biosynthesis	0.000515	7,13,2
2tgi__#106	GO:0000082: G1/S transition of mitotic cell cycle	0.000515	7,13,2
1c4xa_#19	GO:0006805: xenobiotic metabolism	0.000515	7,13,2
1c4xa_#19	GO:0017000: antibiotic biosynthesis	0.000515	7,13,2
1e43a2#351	GO:0006805: xenobiotic metabolism	0.000515	7,13,2
1d8ca_#442	GO:0006100: tricarboxylic acid cycle intermediate metabolism	0.000517	30,14,3
1fnf_1#1146	GO:0008284: positive regulation of cell proliferation	0.000517	14,30,3
1hwxa1#223	GO:0042364: water-soluble vitamin biosynthesis	0.000517	186,27,8
1iba__#39	GO:0006559: L-phenylalanine catabolism	0.000518	316,10,6
1iba__#39	GO:0006275: regulation of DNA replication	0.000518	316,10,6
1a81a2#182	GO:0006418: tRNA aminoacylation for protein translation	0.000518	104,26,6
1ihua2#343	GO:0006221: pyrimidine nucleotide biosynthesis	0.000518	176,22,7
1pud__#44	GO:0042401: biogenic amine biosynthesis	0.00052	43,10,3
1pud__#44	GO:0015976: carbon utilization	0.00052	43,10,3
1pud__#44	GO:0006568: tryptophan metabolism	0.00052	43,10,3
1pud__#44	GO:0006207: 'de novo' pyrimidine base biosynthesis	0.00052	43,10,3
1pud__#44	GO:0009167: purine ribonucleoside monophosphate metabolism	0.00052	43,10,3
3btaa3#194	GO:0030217: T-cell differentiation	0.00052	43,10,3
1dfca4#1469	GO:0045595: regulation of cell differentiation	0.00052	43,10,3
1dfca4#1469	GO:0030217: T-cell differentiation	0.00052	43,10,3
1hyha2#204	GO:0019883: antigen presentation, endogenous antigen	0.00052	43,10,3
2mev3_#108	GO:0030217: T-cell differentiation	0.00052	197,10,5
1h7wa2#636	GO:0042401: biogenic amine biosynthesis	0.00052	43,10,3
1h7wa2#636	GO:0006568: tryptophan metabolism	0.00052	43,10,3
1qs0a1#235	GO:0006563: L-serine metabolism	0.00052	86,12,4
1e0ta2#275	GO:0046148: pigment biosynthesis	0.00052	86,12,4
1e0ta2#275	GO:0009156: ribonucleoside monophosphate biosynthesis	0.00052	86,12,4
1bj7__#112	GO:0016068: type I hypersensitivity	0.000521	28,15,3
1a4ia1#208	GO:0006221: pyrimidine nucleotide biosynthesis	0.000521	19,22,3
1pda_1#77	GO:0006826: iron ion transport	0.000521	28,15,3
1tbga_#76	GO:0007186: G-protein coupled receptor protein signaling pathway	0.000522	4,24,2
1agre_#169	GO:0007186: G-protein coupled receptor protein signaling pathway	0.000522	4,24,2
1tbga_#228	GO:0007186: G-protein coupled receptor protein signaling pathway	0.000522	4,24,2
1hdfa_#16	GO:0016068: type I hypersensitivity	0.000523	192,15,6
1f8ra2#325	GO:0006118: electron transport	0.000525	196,228,32

1e8ga1#436	GO:0030435: sporulation	0.000525	77,23,5
1qdla_#211	GO:0001906: cell killing	0.000525	280,11,6
1vmoa_#84	GO:0030435: sporulation	0.000526	43,23,4
1ihua2#525	GO:0009307: DNA restriction	0.000526	278,19,8
1j9qa2#295	GO:0042088: T-helper 1 type immune response	0.000528	39,11,3
1j9qa2#295	GO:0042108: positive regulation of cytokine biosynthesis	0.000528	39,11,3
1j9qa2#295	GO:0006690: icosanoid metabolism	0.000528	39,11,3
1d0va_#46	GO:0009396: folic acid and derivative biosynthesis	0.000528	39,11,3
3sil__#98	GO:0006606: protein-nucleus import	0.000528	186,21,7
1qq5a_#7	GO:0006221: pyrimidine nucleotide biosynthesis	0.000528	125,22,6
1iira_#242	GO:0006221: pyrimidine nucleotide biosynthesis	0.000528	125,22,6
1e53a_#367	GO:0006118: electron transport	0.000529	13,228,6
1g5va_#142	GO:0015074: DNA integration	0.000529	21,20,3
1hbna1#293	GO:0006118: electron transport	0.000529	13,228,6
1jb3a_#64	GO:0007169: transmembrane receptor protein tyrosine kinase signaling pathway	0.000529	247,26,9
1g0sa_#74	GO:0007169: transmembrane receptor protein tyrosine kinase signaling pathway	0.000529	247,26,9
1g0sa_#74	GO:0009070: serine family amino acid biosynthesis	0.000529	247,26,9
1lml__#535	GO:0006917: induction of apoptosis	0.000529	34,29,4
1pwt__#8	GO:0006953: acute-phase response	0.000529	262,20,8
1hdoa_#189	GO:0009064: glutamine family amino acid metabolism	0.000529	147,19,6
1wdcb_#134	GO:0006418: tRNA aminoacylation for protein translation	0.00053	38,26,4
1seta2#403	GO:0006418: tRNA aminoacylation for protein translation	0.00053	38,26,4
1dl5a_#266	GO:0006096: glycolysis	0.00053	26,38,4
1seta2#306	GO:0006096: glycolysis	0.00053	26,38,4
1e6ua_#266	GO:0006096: glycolysis	0.00053	26,38,4
1gc5a_#233	GO:0009070: serine family amino acid biosynthesis	0.00053	38,26,4
1dyma_#213	GO:0007169: transmembrane receptor protein tyrosine kinase signaling pathway	0.00053	38,26,4
1mspa_#17	GO:0007169: transmembrane receptor protein tyrosine kinase signaling pathway	0.00053	38,26,4
2ae2a_#89	GO:0006418: tRNA aminoacylation for protein translation	0.00053	38,26,4
1ex1a1#284	GO:0006096: glycolysis	0.00053	26,38,4
1jj2n_#77	GO:0009070: serine family amino acid biosynthesis	0.00053	38,26,4
1i7qa_#321	GO:0007169: transmembrane receptor protein tyrosine kinase signaling pathway	0.000531	147,26,7
1ldna1#94	GO:0006563: L-serine metabolism	0.000532	159,12,5
1rypb_#114	GO:0006100: tricarboxylic acid cycle intermediate metabolism	0.000532	133,14,5
1dnpa2#18	GO:0006100: tricarboxylic acid cycle intermediate metabolism	0.000533	73,14,4
1qfta_#98	GO:0006275: regulation of DNA replication	0.000533	198,10,5
1qf6a3#78	GO:0006096: glycolysis	0.000534	46,38,5
1ii7a_#199	GO:0006096: glycolysis	0.000534	46,38,5
1ab4__#89	GO:0000910: cytokinesis	0.000534	133,37,8
1avgi_#69	GO:0006801: superoxide metabolism	0.000536	281,11,6
1hbza_#96	GO:0006917: induction of apoptosis	0.000537	322,29,11
1qf9a_#117	GO:0009070: serine family amino acid biosynthesis	0.000537	68,26,5
1ecfa1#388	GO:0009064: glutamine family amino acid metabolism	0.000538	5,19,2
1fmta2#81	GO:0006099: tricarboxylic acid cycle	0.000538	5,19,2
1sw6a_#385	GO:0008285: negative regulation of cell proliferation	0.000538	5,19,2
1eucb2#84	GO:0006099: tricarboxylic acid cycle	0.000538	5,19,2
1ed1a_#94	GO:0009064: glutamine family amino acid metabolism	0.000538	5,19,2
1sw6a_#383	GO:0008285: negative regulation of cell proliferation	0.000538	5,19,2
1duvg1#135	GO:0009064: glutamine family amino acid metabolism	0.000538	5,19,2
1aba__#17	GO:0006118: electron transport	0.000541	28,228,9
1ycsa_#162	GO:0001906: cell killing	0.000542	177,11,5
1ddma_#130	GO:0016477: cell migration	0.000542	285,23,9

1ev2e2#286	GO:0051249: regulation of lymphocyte activation	0.000543	87,12,4
1mjha_#119	GO:0009103: lipopolysaccharide biosynthesis	0.000543	87,12,4
1jkma_#317	GO:0009252: peptidoglycan biosynthesis	0.000543	56,18,4
2reb_1#138	GO:0006779: porphyrin biosynthesis	0.000543	56,18,4
1qfla1#44	GO:0006457: protein folding	0.000544	9,49,3
1quba4#235	GO:0006690: icosanoid metabolism	0.000544	96,11,4
1am2__#163	GO:0001906: cell killing	0.000544	96,11,4
1qasa3#352	GO:0009116: nucleoside metabolism	0.000545	17,25,3
2bb2_2#134	GO:0006275: regulation of DNA replication	0.000545	199,10,5
1f8na1#450	GO:0030333: antigen processing	0.000545	33,13,3
1ej8a_#189	GO:0043123: positive regulation of I-kappaB kinase/NF-kappaB cascade	0.000546	256,16,7
1e2o__#346	GO:0006606: protein-nucleus import	0.000546	187,21,7
1htp__#16	GO:0019735: antimicrobial humoral response (sensu Vertebrata)	0.000546	256,16,7
1ryp1_#20	GO:0006418: tRNA aminoacylation for protein translation	0.000546	105,26,6
1hxxa_#232	GO:0006544: glycine metabolism	0.000546	282,11,6
1bev1_#86	GO:0006935: chemotaxis	0.000546	296,41,13
1b3qa2#623	GO:0006935: chemotaxis	0.000546	257,41,12
1mdah_#329	GO:0006690: icosanoid metabolism	0.000546	282,11,6
1ee4a_#266	GO:0006897: endocytosis	0.000547	53,19,4
2duba_#212	GO:0009064: glutamine family amino acid metabolism	0.000547	53,19,4
1a0i_2#222	GO:0006935: chemotaxis	0.000548	151,41,9
1hcl__#82	GO:0009156: ribonucleoside monophosphate biosynthesis	0.000549	36,12,3
1hbza_#214	GO:0006606: protein-nucleus import	0.000549	249,21,8
1ojt_2#356	GO:0006563: L-serine metabolism	0.000549	36,12,3
1c9la2#61	GO:0007399: neurogenesis	0.000549	206,37,10
1jj2q_#114	GO:0006119: oxidative phosphorylation	0.000549	36,12,3
1el0a_#28	GO:0006953: acute-phase response	0.000553	140,20,6
1elja_#64	GO:0006643: membrane lipid metabolism	0.000554	80,13,4
1e31a_#77	GO:0042364: water-soluble vitamin biosynthesis	0.000555	37,27,4
1cs1a_#160	GO:0006096: glycolysis	0.000555	164,38,9
1h6oa_#135	GO:0006118: electron transport	0.000556	9,228,5
19hca_#16	GO:0006118: electron transport	0.000556	9,228,5
3lada3#380	GO:0006118: electron transport	0.000556	9,228,5
1iq4a_#31	GO:0006118: electron transport	0.000556	9,228,5
1f8ra2#325	GO:0007169: transmembrane receptor protein tyrosine kinase signaling pathway	0.000556	196,26,8
1deua_#151	GO:0016337: cell-cell adhesion	0.000556	239,17,7
1e8ga2#176	GO:0006118: electron transport	0.000556	9,228,5
1gpea1#271	GO:0007169: transmembrane receptor protein tyrosine kinase signaling pathway	0.000556	196,26,8
1evqa_#305	GO:0000160: two-component signal transduction system (phosphorelay)	0.000556	384,25,11
1ad2__#40	GO:0006207: 'de novo' pyrimidine base biosynthesis	0.000557	44,10,3
7reqa2#702	GO:0042401: biogenic amine biosynthesis	0.000557	44,10,3
7reqa2#702	GO:0006568: tryptophan metabolism	0.000557	44,10,3
1h75a_#53	GO:0019059: initiation of viral infection	0.000557	44,10,3
1dr9a2#139	GO:0016477: cell migration	0.000558	78,23,5
1kit_3#743	GO:0016477: cell migration	0.000558	225,23,8
1qs0a1#119	GO:0009156: ribonucleoside monophosphate biosynthesis	0.000558	367,12,7
1iira_#326	GO:0007264: small GTPase mediated signal transduction	0.00056	86,21,5
1cmxa_#207	GO:0006606: protein-nucleus import	0.00056	86,21,5
1kdj__#27	GO:0019735: antimicrobial humoral response (sensu Vertebrata)	0.00056	64,16,4
1b3qa2#623	GO:0019735: antimicrobial humoral response (sensu Vertebrata)	0.00056	257,16,7
1ie5a_#102	GO:0019735: antimicrobial humoral response (sensu Vertebrata)	0.00056	64,16,4
1e3a.1#A30	GO:0006606: protein-nucleus import	0.000561	133,21,6
1feza_#185	GO:0042364: water-soluble vitamin biosynthesis	0.000562	66,27,5
3grs_1#24	GO:0042364: water-soluble vitamin biosynthesis	0.000562	66,27,5

1djna1#86	GO:0042364: water-soluble vitamin biosynthesis	0.000562	66,27,5
2napa2#292	GO:0009219: pyrimidine deoxyribonucleotide metabolism	0.000562	108,10,4
2napa2#292	GO:0006268: DNA unwinding	0.000562	108,10,4
1mml__#97	GO:0006313: DNA transposition	0.000564	3,35,2
1dq3a1#3	GO:0006313: DNA transposition	0.000564	3,35,2
1i1b__#85	GO:0019735: antimicrobial humoral response (sensu Vertebrata)	0.000565	6,16,2
1i7qa_#485	GO:0009073: aromatic amino acid family biosynthesis	0.000565	6,16,2
1qqka_#19	GO:0019735: antimicrobial humoral response (sensu Vertebrata)	0.000565	6,16,2
1ghqb1#32	GO:0043123: positive regulation of I-kappaB kinase/NF-kappaB cascade	0.000565	6,16,2
1ecra_#101	GO:0019735: antimicrobial humoral response (sensu Vertebrata)	0.000565	6,16,2
1nsj__#21	GO:0009064: glutamine family amino acid metabolism	0.000565	96,19,5
2viua_#123	GO:0006413: translational initiation	0.000565	6,16,2
1eq9a_#32	GO:0008285: negative regulation of cell proliferation	0.000565	96,19,5
1jlxa2#243	GO:0019735: antimicrobial humoral response (sensu Vertebrata)	0.000565	6,16,2
1g61a_#2155	GO:0006413: translational initiation	0.000565	6,16,2
1fo4a5#1020	GO:0006457: protein folding	0.000566	312,49,15
1avgi_#69	GO:0008285: negative regulation of cell proliferation	0.000566	281,19,8
8dfr__#9	GO:0043094: metabolic compound salvage	0.000566	97,11,4
8dfr__#9	GO:0009396: folic acid and derivative biosynthesis	0.000566	97,11,4
1e5ka_#49	GO:0009116: nucleoside metabolism	0.000567	4,25,2
1ek1a1#199	GO:0009116: nucleoside metabolism	0.000567	4,25,2
1chd__#270	GO:0000160: two-component signal transduction system (phosphorelay)	0.000567	4,25,2
1gdoa_#24	GO:0009116: nucleoside metabolism	0.000567	4,25,2
1chd__#330	GO:0000160: two-component signal transduction system (phosphorelay)	0.000567	4,25,2
1bx4a_#75	GO:0009116: nucleoside metabolism	0.000567	4,25,2
1fmb__#88	GO:0009116: nucleoside metabolism	0.000567	4,25,2
1g61a_#2115	GO:0007067: mitosis	0.000567	4,25,2
5ruba1#365	GO:0009103: lipopolysaccharide biosynthesis	0.000568	88,12,4
1ejda_#6	GO:0006461: protein complex assembly	0.000568	215,24,8
1ezvb1#29	GO:0006461: protein complex assembly	0.000569	42,24,4
1lara2#1769	GO:0006919: caspase activation	0.000569	195,15,6
1qhoa3#488	GO:0019735: antimicrobial humoral response (sensu Vertebrata)	0.00057	27,16,3
2cb5a_#373	GO:0019079: viral genome replication	0.00057	27,16,3
1jp3a_#22	GO:0043094: metabolic compound salvage	0.00057	40,11,3
1jp3a_#22	GO:0006544: glycine metabolism	0.00057	40,11,3
1ew2a_#133	GO:0042364: water-soluble vitamin biosynthesis	0.00057	16,27,3
1ehya_#85	GO:0009086: methionine biosynthesis	0.00057	31,14,3
1e6ua_#10	GO:0006633: fatty acid biosynthesis	0.00057	16,27,3
1elva1#604	GO:0019079: viral genome replication	0.00057	27,16,3
1ii7a_#199	GO:0006118: electron transport	0.000572	46,228,12
1fds__#59	GO:0006118: electron transport	0.000572	46,228,12
2bpa1_#45	GO:0006690: icosanoid metabolism	0.000572	179,11,5
1bjt__#890	GO:0019735: antimicrobial humoral response (sensu Vertebrata)	0.000573	258,16,7
1epwa1#901	GO:0006457: protein folding	0.000574	55,49,6
1jb7a1#174	GO:0006461: protein complex assembly	0.000574	18,24,3
1dhs__#99	GO:0006118: electron transport	0.000574	109,228,21
1qhqa_#45	GO:0006418: tRNA aminoacylation for protein translation	0.000575	106,26,6
2bpa1_#45	GO:0030245: cellulose catabolism	0.000575	179,22,7
1hbza_#96	GO:0018108: peptidyl-tyrosine phosphorylation	0.000576	322,10,6
1b87a_#47	GO:0006917: induction of apoptosis	0.000578	15,29,3
1hlwa_#91	GO:0006919: caspase activation	0.000578	29,15,3
1cwva2#675	GO:0045859: regulation of protein kinase activity	0.000578	29,15,3
1feza_#185	GO:0006935: chemotaxis	0.000579	66,41,6
2viua_#252	GO:0001906: cell killing	0.000579	285,11,6
1boub_#7	GO:0009116: nucleoside metabolism	0.000579	206,25,8
1ddma_#130	GO:0001906: cell killing	0.000579	285,11,6

1c8za_#449	GO:0009156: ribonucleoside monophosphate biosynthesis	0.00058	8,12,2
1ddja_#640	GO:0006956: complement activation	0.00058	8,12,2
1g93a_#229	GO:0015992: proton transport	0.00058	8,12,2
1g93a_#229	GO:0006119: oxidative phosphorylation	0.00058	8,12,2
1e8ya4#800	GO:0051249: regulation of lymphocyte activation	0.00058	8,12,2
1vin_1#302	GO:0006367: transcription initiation from Pol II promoter	0.00058	8,12,2
1pa2a_#232	GO:0006119: oxidative phosphorylation	0.00058	8,12,2
1qbkb_#702	GO:0007017: microtubule-based process	0.00058	8,12,2
1dgtb3#2594	GO:0051249: regulation of lymphocyte activation	0.00058	8,12,2
1dgtb3#2594	GO:0009103: lipopolysaccharide biosynthesis	0.00058	8,12,2
1fc4a_#241	GO:0006563: L-serine metabolism	0.00058	8,12,2
1oaa__#161	GO:0009103: lipopolysaccharide biosynthesis	0.00058	8,12,2
1hr6a2#427	GO:0006119: oxidative phosphorylation	0.00058	8,12,2
1bdb__#228	GO:0009103: lipopolysaccharide biosynthesis	0.00058	8,12,2
1elua_#199	GO:0006563: L-serine metabolism	0.00058	8,12,2
2sqca1#417	GO:0006956: complement activation	0.00058	8,12,2
1cgha_#130	GO:0006956: complement activation	0.00058	8,12,2
1dyna_#19	GO:0030333: antigen processing	0.000581	81,13,4
1mrj__#73	GO:0019835: cytolysis	0.000582	57,18,4
1ac6a_#34	GO:0006916: anti-apoptosis	0.000582	57,18,4
1qtn.1#A270	GO:0000160: two-component signal transduction system (phosphorelay)	0.000583	386,25,11
1cb8a2#613	GO:0006096: glycolysis	0.000584	131,38,8
1dpsa_#36	GO:0006457: protein folding	0.000584	183,49,11
1dn2a1#261	GO:0016068: type I hypersensitivity	0.000585	376,15,8
1ayl__#237	GO:0006457: protein folding	0.000586	313,49,15
1qhda2#323	GO:0008284: positive regulation of cell proliferation	0.000587	313,30,11
1aa6_2#285	GO:0006118: electron transport	0.000589	87,228,18
1f2na_#138	GO:0007264: small GTPase mediated signal transduction	0.00059	87,21,5
1xnb__#149	GO:0009064: glutamine family amino acid metabolism	0.00059	150,19,6
1tyfa_#31	GO:0006100: tricarboxylic acid cycle intermediate metabolism	0.00059	136,14,5
1erza_#183	GO:0006096: glycolysis	0.000591	47,38,5
1e8ga2#201	GO:0001906: cell killing	0.000591	286,11,6
1opy__#68	GO:0006418: tRNA aminoacylation for protein translation	0.000592	308,26,10
1fd9a_#162	GO:0006313: DNA transposition	0.000593	78,35,6
1pwt__#8	GO:0008284: positive regulation of cell proliferation	0.000593	262,30,10
1hlwa_#82	GO:0006313: DNA transposition	0.000594	221,35,10
1by5a_#238	GO:0006457: protein folding	0.000595	214,49,12
1hxmb1#117	GO:0019079: viral genome replication	0.000595	65,16,4
1hxmb1#117	GO:0043123: positive regulation of I-kappaB kinase/NF-kappaB cascade	0.000595	65,16,4
1hxmb1#117	GO:0019735: antimicrobial humoral response (sensu Vertebrata)	0.000595	65,16,4
1apme_#101	GO:0000910: cytokinesis	0.000596	12,37,3
1ryp1_#18	GO:0051249: regulation of lymphocyte activation	0.000596	37,12,3
1qtra_#56	GO:0009064: glutamine family amino acid metabolism	0.000596	23,19,3
1g0sa_#176	GO:0009103: lipopolysaccharide biosynthesis	0.000596	37,12,3
1b6cb_#399	GO:0000910: cytokinesis	0.000596	12,37,3
1dfca1#1135	GO:0019883: antigen presentation, endogenous antigen	0.000596	45,10,3
1fa0a1#405	GO:0030333: antigen processing	0.000596	34,13,3
1fp5a2#537	GO:0030217: T-cell differentiation	0.000596	45,10,3
1abrb1#25	GO:0007399: neurogenesis	0.000596	12,37,3
1f0xa2#118	GO:0006119: oxidative phosphorylation	0.000596	37,12,3
1aisb2#1274	GO:0000910: cytokinesis	0.000596	12,37,3
1bpv__#22	GO:0016477: cell migration	0.000596	19,23,3
1i50b_#966	GO:0009070: serine family amino acid biosynthesis	0.000596	251,26,9
2cuaa_#83	GO:0019883: antigen presentation, endogenous antigen	0.000596	45,10,3
1b3qa2#623	GO:0051249: regulation of lymphocyte activation	0.000597	257,12,6
1hrna_#38	GO:0015074: DNA integration	0.000597	5,20,2

1e87a_#174	GO:0016042: lipid catabolism	0.000598	124,31,7
1e7wa_#8	GO:0006221: pyrimidine nucleotide biosynthesis	0.000598	83,22,5
1a8i__#688	GO:0006207: 'de novo' pyrimidine base biosynthesis	0.000599	203,10,5
2dpma_#194	GO:0006100: tricarboxylic acid cycle intermediate metabolism	0.0006	7,14,2
1rhs_2#208	GO:0015698: inorganic anion transport	0.0006	7,14,2
1dqua_#456	GO:0006100: tricarboxylic acid cycle intermediate metabolism	0.0006	7,14,2
3pmga4#493	GO:0015698: inorganic anion transport	0.0006	7,14,2
1g38a_#155	GO:0006100: tricarboxylic acid cycle intermediate metabolism	0.0006	7,14,2
1rhs_1#10	GO:0015698: inorganic anion transport	0.0006	7,14,2
1rhs_1#84	GO:0015698: inorganic anion transport	0.0006	7,14,2
1e0ca2#193	GO:0015698: inorganic anion transport	0.0006	7,14,2
2bb2_1#57	GO:0006953: acute-phase response	0.0006	92,20,5
1f42a2#123	GO:0007399: neurogenesis	0.000601	335,37,13
2mev3_#108	GO:0008015: circulation	0.000601	197,15,6
2mev3_#108	GO:0016068: type I hypersensitivity	0.000601	197,15,6
1jg8a_#172	GO:0042364: water-soluble vitamin biosynthesis	0.000602	67,27,5
1fuia2#273	GO:0006268: DNA unwinding	0.000603	110,10,4
2dnja_#97	GO:0050819: negative regulation of coagulation	0.000603	110,10,4
2dnja_#97	GO:0030193: regulation of blood coagulation	0.000603	110,10,4
2dnja_#97	GO:0017001: antibiotic catabolism	0.000603	110,10,4
2dnja_#97	GO:0018108: peptidyl-tyrosine phosphorylation	0.000603	110,10,4
1hq8a_#206	GO:0008015: circulation	0.000603	281,15,7
1utea_#11	GO:0006418: tRNA aminoacylation for protein translation	0.000604	107,26,6
1f97a1#62	GO:0017148: negative regulation of protein biosynthesis	0.000605	591,13,9
1qqsa_#35	GO:0006118: electron transport	0.000605	80,228,17
1h6kx_#97	GO:0006606: protein-nucleus import	0.000606	49,21,4
1rsy__#151	GO:0006953: acute-phase response	0.000609	22,20,3
2mpra_#190	GO:0043123: positive regulation of I-kappaB kinase/NF-kappaB cascade	0.000609	349,16,8
1nsca_#91	GO:0006953: acute-phase response	0.000609	22,20,3
3pah__#303	GO:0006118: electron transport	0.000611	23,228,8
2bbkh_#348	GO:0006118: electron transport	0.000611	23,228,8
1d0ba_#191	GO:0009396: folic acid and derivative biosynthesis	0.000612	99,11,4
1bxoa_#307	GO:0001906: cell killing	0.000612	99,11,4
1a0i_2#222	GO:0008285: negative regulation of cell proliferation	0.000612	151,19,6
1qqga2#166	GO:0016042: lipid catabolism	0.000612	164,31,8
1pii_2#326	GO:0009116: nucleoside metabolism	0.000612	157,25,7
1dlfl_#97	GO:0042108: positive regulation of cytokine biosynthesis	0.000613	41,11,3
1rkd__#186	GO:0043094: metabolic compound salvage	0.000613	41,11,3
1qgna_#373	GO:0006801: superoxide metabolism	0.000613	41,11,3
1fid__#237	GO:0016485: protein processing	0.000613	41,11,3
1poxa3#439	GO:0007264: small GTPase mediated signal transduction	0.000613	21,21,3
1gtma2#140	GO:0009070: serine family amino acid biosynthesis	0.000613	17,26,3
1aym3_#109	GO:0042088: T-helper 1 type immune response	0.000613	41,11,3
1i8aa_#2	GO:0006418: tRNA aminoacylation for protein translation	0.000613	17,26,3
1qfxa_#380	GO:0042088: T-helper 1 type immune response	0.000613	41,11,3
1qp8a1#172	GO:0009070: serine family amino acid biosynthesis	0.000613	17,26,3
1dbta_#89	GO:0006418: tRNA aminoacylation for protein translation	0.000613	17,26,3
2sqca2#268	GO:0042113: B-cell activation	0.000613	51,9,3
1pme__#166	GO:0007169: transmembrane receptor protein tyrosine kinase signaling pathway	0.000613	17,26,3
1koba_#175	GO:0007169: transmembrane receptor protein tyrosine kinase signaling pathway	0.000613	17,26,3
1bqsa2#103	GO:0006418: tRNA aminoacylation for protein translation	0.000613	17,26,3
1f97a2#199	GO:0019735: antimicrobial humoral response (sensu Vertebrata)	0.000613	118,16,5
1ia8a_#89	GO:0007169: transmembrane receptor protein tyrosine kinase signaling pathway	0.000614	4,26,2
1vdra_#76	GO:0009070: serine family amino acid biosynthesis	0.000614	4,26,2

1tdj_1#35	GO:0009070: serine family amino acid biosynthesis	0.000614	4,26,2
1aoea_#136	GO:0009070: serine family amino acid biosynthesis	0.000614	4,26,2
1biha3#248	GO:0007169: transmembrane receptor protein tyrosine kinase signaling pathway	0.000614	4,26,2
1ir3a_#1003	GO:0007169: transmembrane receptor protein tyrosine kinase signaling pathway	0.000614	4,26,2
2foka2#180	GO:0007517: muscle development	0.000615	129,30,7
1ifc__#89	GO:0051249: regulation of lymphocyte activation	0.000615	164,12,5
1cs1a_#160	GO:0046148: pigment biosynthesis	0.000615	164,12,5
1arb__#210	GO:0006418: tRNA aminoacylation for protein translation	0.000615	70,26,5
1ejba_#81	GO:0006418: tRNA aminoacylation for protein translation	0.000615	70,26,5
1f5aa1#441	GO:0008284: positive regulation of cell proliferation	0.000615	129,30,7
1br9__#86	GO:0006418: tRNA aminoacylation for protein translation	0.000615	70,26,5
1dlja2#177	GO:0006096: glycolysis	0.000616	27,38,4
1eg2a_#132	GO:0006096: glycolysis	0.000616	27,38,4
1epwa3#138	GO:0007169: transmembrane receptor protein tyrosine kinase signaling pathway	0.000616	371,26,11
1bcca2#437	GO:0006633: fatty acid biosynthesis	0.000616	38,27,4
1quna1#113	GO:0016337: cell-cell adhesion	0.000616	243,17,7
1mdah_#329	GO:0006986: response to unfolded protein	0.000616	282,15,7
1qfta_#98	GO:0006986: response to unfolded protein	0.000618	198,15,6
1evqa_#305	GO:0006118: electron transport	0.00062	384,228,53
1f8ra1#64	GO:0008203: cholesterol metabolism	0.000621	9,11,2
16pk__#312	GO:0006801: superoxide metabolism	0.000621	9,11,2
1moq__#368	GO:0016568: chromatin modification	0.000621	9,11,2
1iq4a_#31	GO:0006690: icosanoid metabolism	0.000621	9,11,2
1fmk_3#428	GO:0000165: MAPKKK cascade	0.000621	9,11,2
1ak0__#212	GO:0001906: cell killing	0.000621	9,11,2
1koba_#293	GO:0000165: MAPKKK cascade	0.000621	9,11,2
1bg6_1#316	GO:0006098: pentose-phosphate shunt	0.000621	9,11,2
1pme__#207	GO:0000165: MAPKKK cascade	0.000621	9,11,2
1dceb_#134	GO:0009206: purine ribonucleoside triphosphate biosynthesis	0.000621	11,9,2
1dceb_#290	GO:0043094: metabolic compound salvage	0.000621	9,11,2
1dfca1#1119	GO:0007276: gametogenesis	0.000621	9,11,2
1dfca1#1119	GO:0016485: protein processing	0.000621	9,11,2
1epwa2#1220	GO:0048514: blood vessel morphogenesis	0.000621	11,9,2
1e8ua_#180	GO:0006511: ubiquitin-dependent protein catabolism	0.000621	9,11,2
1wba__#169	GO:0000165: MAPKKK cascade	0.000621	9,11,2
1poxa3#460	GO:0009086: methionine biosynthesis	0.000622	76,14,4
1e4ea2#215	GO:0001906: cell killing	0.000623	419,11,7
1e4ea2#215	GO:0016485: protein processing	0.000623	419,11,7
1p35a_#101	GO:0030217: T-cell differentiation	0.000624	111,10,4
1dfca4#1469	GO:0006461: protein complex assembly	0.000624	43,24,4
1dy9.1#C229	GO:0030217: T-cell differentiation	0.000624	111,10,4
1iray2#107	GO:0006730: one-carbon compound metabolism	0.000627	14,32,3
1qq1a_#637	GO:0006118: electron transport	0.000627	18,228,7
1kit_3#297	GO:0006118: electron transport	0.000627	18,228,7
1p35a_#155	GO:0016477: cell migration	0.000628	45,23,4
1bywa_#30	GO:0006986: response to unfolded protein	0.00063	283,15,7
1fhga_#119	GO:0007399: neurogenesis	0.000631	3,37,2
1jlxa2#166	GO:0019079: viral genome replication	0.000631	66,16,4
1bjt__#890	GO:0006118: electron transport	0.000631	258,228,39
1i58a_#519	GO:0043123: positive regulation of I-kappaB kinase/NF-kappaB cascade	0.000631	66,16,4
1el0a_#42	GO:0006118: electron transport	0.000631	258,228,39
1a9xb2#1775	GO:0009307: DNA restriction	0.000631	55,19,4
1fmja_#76	GO:0000910: cytokinesis	0.000633	49,37,5

1a65a1#108	GO:0007169: transmembrane receptor protein tyrosine kinase signaling pathway	0.000633	253,26,9
1i2oa_#34	GO:0006099: tricarboxylic acid cycle	0.000634	152,19,6
1j9qa1#154	GO:0042088: T-helper 1 type immune response	0.000634	183,11,5
1j9qa1#154	GO:0042108: positive regulation of cytokine biosynthesis	0.000634	183,11,5
1j9qa1#154	GO:0001906: cell killing	0.000634	183,11,5
1j9qa1#154	GO:0016485: protein processing	0.000634	183,11,5
1i2ma_#157	GO:0051049: regulation of transport	0.000635	10,10,2
1apme_#168	GO:0006275: regulation of DNA replication	0.000635	10,10,2
1hdr__#125	GO:0006559: L-phenylalanine catabolism	0.000635	10,10,2
1pme__#24	GO:0000086: G2/M transition of mitotic cell cycle	0.000635	10,10,2
1pme__#24	GO:0006275: regulation of DNA replication	0.000635	10,10,2
2shpa1#387	GO:0006265: DNA topological change	0.000635	10,10,2
2shpa1#387	GO:0006268: DNA unwinding	0.000635	10,10,2
1fnga1#123	GO:0045595: regulation of cell differentiation	0.000635	10,10,2
1ga0a_#304	GO:0019883: antigen presentation, endogenous antigen	0.000635	10,10,2
1sfe_2#15	GO:0015976: carbon utilization	0.000635	10,10,2
1a0ca_#273	GO:0000051: urea cycle intermediate metabolism	0.000635	10,10,2
1epwa1#1065	GO:0006207: 'de novo' pyrimidine base biosynthesis	0.000635	10,10,2
1pme__#145	GO:0000086: G2/M transition of mitotic cell cycle	0.000635	10,10,2
1dtoa_#150	GO:0045595: regulation of cell differentiation	0.000635	10,10,2
1dtoa_#150	GO:0006275: regulation of DNA replication	0.000635	10,10,2
1hcl__#135	GO:0000086: G2/M transition of mitotic cell cycle	0.000635	10,10,2
1hcl__#135	GO:0006275: regulation of DNA replication	0.000635	10,10,2
1otha2#300	GO:0000051: urea cycle intermediate metabolism	0.000635	10,10,2
1f3la_#348	GO:0000051: urea cycle intermediate metabolism	0.000635	10,10,2
1e19a_#281	GO:0006418: tRNA aminoacylation for protein translation	0.000635	108,26,6
1e19a_#281	GO:0009070: serine family amino acid biosynthesis	0.000635	108,26,6
1hxn__#248	GO:0006207: 'de novo' pyrimidine base biosynthesis	0.000635	10,10,2
1daba_#310	GO:0015976: carbon utilization	0.000635	10,10,2
1xnb__#131	GO:0009219: pyrimidine deoxyribonucleotide metabolism	0.000636	46,10,3
1esma_#314	GO:0006511: ubiquitin-dependent protein catabolism	0.000636	100,11,4
1esma_#314	GO:0001906: cell killing	0.000636	100,11,4
1qf6a3#78	GO:0006278: RNA-dependent DNA replication	0.000636	46,10,3
1ebma2#47	GO:0019079: viral genome replication	0.000636	28,16,3
1ii7a_#199	GO:0006207: 'de novo' pyrimidine base biosynthesis	0.000636	46,10,3
1jlna_#383	GO:0006207: 'de novo' pyrimidine base biosynthesis	0.000636	46,10,3
1tig__#148	GO:0019079: viral genome replication	0.000636	28,16,3
1bev1_#238	GO:0019079: viral genome replication	0.000636	28,16,3
3sil__#284	GO:0016337: cell-cell adhesion	0.000636	62,17,4
1i7oa2#399	GO:0008015: circulation	0.00064	30,15,3
2pola3#335	GO:0008284: positive regulation of cell proliferation	0.00064	15,30,3
1mfa_1#82L	GO:0016337: cell-cell adhesion	0.00064	6,17,2
1clc_1#299	GO:0008015: circulation	0.00064	30,15,3
1ejda_#6	GO:0008285: negative regulation of cell proliferation	0.00064	215,19,7
2pia_1#54	GO:0019079: viral genome replication	0.000641	185,16,6
1f2la_#40	GO:0019059: initiation of viral infection	0.000641	206,10,5
1f2la_#40	GO:0030217: T-cell differentiation	0.000641	206,10,5
1c9la2#61	GO:0018108: peptidyl-tyrosine phosphorylation	0.000641	206,10,5
1qfea_#68	GO:0006730: one-carbon compound metabolism	0.000641	453,32,14
1ir3a_#1192	GO:0000910: cytokinesis	0.000641	28,37,4
1hxxa_#173	GO:0046677: response to antibiotic	0.000643	230,40,11
1gsa_2#218	GO:0006461: protein complex assembly	0.000644	118,24,6
1f97a2#199	GO:0006461: protein complex assembly	0.000644	118,24,6
1bwda_#295	GO:0046677: response to antibiotic	0.000644	96,40,7
1tiid_#39	GO:0006470: protein amino acid dephosphorylation	0.000644	203,20,7

1byra_#151	GO:0006096: glycolysis	0.000645	12,38,3
1seta2#403	GO:0051249: regulation of lymphocyte activation	0.000645	38,12,3
1ad3a_#181	GO:0006096: glycolysis	0.000645	12,38,3
1iow_1#37	GO:0009073: aromatic amino acid family biosynthesis	0.000645	263,16,7
1ejda_#379	GO:0009103: lipopolysaccharide biosynthesis	0.000645	38,12,3
1fuma2#221	GO:0006096: glycolysis	0.000645	12,38,3
1e39a2#556	GO:0006119: oxidative phosphorylation	0.000645	38,12,3
1egaa1#58	GO:0007017: microtubule-based process	0.000645	38,12,3
1e6ca_#91	GO:0006118: electron transport	0.000646	141,228,25
1aa6_2#285	GO:0006730: one-carbon compound metabolism	0.000646	87,32,6
2mpra_#89	GO:0007169: transmembrane receptor protein tyrosine kinase signaling pathway	0.000647	40,26,4
1gg4a3#64	GO:0009070: serine family amino acid biosynthesis	0.000647	40,26,4
1ei5a3#311	GO:0046677: response to antibiotic	0.000647	26,40,4
1hxxa_#173	GO:0016477: cell migration	0.000649	230,23,8
2bce__#204	GO:0016042: lipid catabolism	0.000649	59,31,5
1evqa_#305	GO:0000910: cytokinesis	0.000649	384,37,14
1ei5a1#357	GO:0006313: DNA transposition	0.00065	13,35,3
1jd0a_#176	GO:0006313: DNA transposition	0.00065	183,35,9
1d6ja_#36	GO:0009116: nucleoside metabolism	0.00065	18,25,3
1fzqa_#165	GO:0007067: mitosis	0.00065	18,25,3
1jk7a_#235	GO:0000160: two-component signal transduction system (phosphorelay)	0.00065	18,25,3
1jfra_#83	GO:0017148: negative regulation of protein biosynthesis	0.00065	35,13,3
1fn9a_#271	GO:0001906: cell killing	0.00065	184,11,5
1fxla2#169	GO:0001906: cell killing	0.00065	184,11,5
1b6g__#63	GO:0017000: antibiotic biosynthesis	0.00065	35,13,3
1ioaa_#75	GO:0019835: cytolysis	0.00065	25,18,3
1dn2a1#261	GO:0009615: response to virus	0.000651	376,12,7
1ig8a_#215	GO:0006096: glycolysis	0.000652	48,38,5
1dyna_#42	GO:0007169: transmembrane receptor protein tyrosine kinase signaling pathway	0.000652	152,26,7
1lci__#26	GO:0045045: secretory pathway	0.000653	130,22,6
1fw9a_#152	GO:0016477: cell migration	0.000657	124,23,6
2viua_#252	GO:0006919: caspase activation	0.000658	285,15,7
1dpsa_#36	GO:0045045: secretory pathway	0.000658	183,22,7
1aoca_#123	GO:0006606: protein-nucleus import	0.000659	5,21,2
1im4a_#153	GO:0006511: ubiquitin-dependent protein catabolism	0.000659	42,11,3
1i7wa_#600	GO:0006606: protein-nucleus import	0.000659	5,21,2
1fbl_1#398	GO:0006544: glycine metabolism	0.000659	42,11,3
1fbl_1#398	GO:0006690: icosanoid metabolism	0.000659	42,11,3
1fbl_1#398	GO:0000165: MAPKKK cascade	0.000659	42,11,3
1qqka_#25	GO:0007264: small GTPase mediated signal transduction	0.000659	5,21,2
1jf9a_#196	GO:0043094: metabolic compound salvage	0.000659	42,11,3
1hdma1#98	GO:0042088: T-helper 1 type immune response	0.000659	42,11,3
1hdma1#98	GO:0042108: positive regulation of cytokine biosynthesis	0.000659	42,11,3
1jf9a_#379	GO:0019735: antimicrobial humoral response (sensu Vertebrata)	0.00066	186,16,6
1hc7a2#258	GO:0019735: antimicrobial humoral response (sensu Vertebrata)	0.00066	186,16,6
3sil__#98	GO:0019735: antimicrobial humoral response (sensu Vertebrata)	0.00066	186,16,6
1erv__#80	GO:0006606: protein-nucleus import	0.00066	401,21,10
1kit_3#331	GO:0042108: positive regulation of cytokine biosynthesis	0.000661	101,11,4
1qfma1#139	GO:0019735: antimicrobial humoral response (sensu Vertebrata)	0.000661	264,16,7
1hxxa_#232	GO:0006457: protein folding	0.000662	282,49,14
2gsaa_#246	GO:0042364: water-soluble vitamin biosynthesis	0.000663	4,27,2
1jf9a_#119	GO:0042364: water-soluble vitamin biosynthesis	0.000663	4,27,2
1xis__#177	GO:0042364: water-soluble vitamin biosynthesis	0.000663	4,27,2
1eno__#290	GO:0006633: fatty acid biosynthesis	0.000663	4,27,2

1cxp.1#A97	GO:0006633: fatty acid biosynthesis	0.000663	4,27,2
1fmca_#248	GO:0006633: fatty acid biosynthesis	0.000663	4,27,2
1eno__#264	GO:0006633: fatty acid biosynthesis	0.000663	4,27,2
1pwt__#8	GO:0000398: nuclear mRNA splicing, via spliceosome	0.000663	262,12,6
1ej8a_#189	GO:0006606: protein-nucleus import	0.000664	256,21,8
1ir3a_#1063	GO:0019835: cytolysis	0.000664	59,18,4
1kit_2#398	GO:0016477: cell migration	0.000665	81,23,5
1vjw__#39	GO:0006096: glycolysis	0.000666	3,38,2
1zpda1#322	GO:0006096: glycolysis	0.000666	3,38,2
1mpp__#154	GO:0006643: membrane lipid metabolism	0.000668	84,13,4
1g25a_#22	GO:0008285: negative regulation of cell proliferation	0.000668	367,19,9
1bec_2#216	GO:0007169: transmembrane receptor protein tyrosine kinase signaling pathway	0.000668	109,26,6
1dv8a_#211	GO:0019735: antimicrobial humoral response (sensu Vertebrata)	0.000668	67,16,4
1jf9a_#196	GO:0000160: two-component signal transduction system (phosphorelay)	0.000669	42,25,4
1hd2a_#35	GO:0015074: DNA integration	0.000669	145,20,6
1e8ga2#201	GO:0016068: type I hypersensitivity	0.000672	286,15,7
1loua_#79	GO:0006119: oxidative phosphorylation	0.000673	92,12,4
1mpp__#151	GO:0051249: regulation of lymphocyte activation	0.000673	92,12,4
1saca_#113	GO:0008284: positive regulation of cell proliferation	0.000677	35,30,4
1iow_1#37	GO:0007017: microtubule-based process	0.000677	263,12,6
1iow_1#37	GO:0009103: lipopolysaccharide biosynthesis	0.000677	263,12,6
1ec7a1#216	GO:0006099: tricarboxylic acid cycle	0.000677	56,19,4
1erza_#183	GO:0042401: biogenic amine biosynthesis	0.000678	47,10,3
1erza_#183	GO:0006568: tryptophan metabolism	0.000678	47,10,3
1lucb_#84	GO:0009307: DNA restriction	0.000678	24,19,3
5ruba1#279	GO:0009167: purine ribonucleoside monophosphate metabolism	0.000678	47,10,3
1htjf_#368	GO:0007186: G-protein coupled receptor protein signaling pathway	0.000678	19,24,3
2fmr__#10	GO:0019883: antigen presentation, endogenous antigen	0.000678	47,10,3
1cf1a1#33	GO:0045595: regulation of cell differentiation	0.000678	47,10,3
1leha1#255	GO:0006099: tricarboxylic acid cycle	0.000678	24,19,3
2bpa1_#45	GO:0006917: induction of apoptosis	0.000678	179,29,8
1dik_1#765	GO:0042364: water-soluble vitamin biosynthesis	0.000678	105,27,6
1fo4a5#1002	GO:0006313: DNA transposition	0.00068	80,35,6
1dih_2#235	GO:0006633: fatty acid biosynthesis	0.000681	39,27,4
1neb__#14	GO:0045786: negative regulation of cell cycle	0.000684	8,13,2
1cfb_2#763	GO:0030333: antigen processing	0.000684	8,13,2
1i1ja_#83	GO:0045786: negative regulation of cell cycle	0.000684	8,13,2
1cs6a2#164	GO:0030333: antigen processing	0.000684	8,13,2
1g8ka2#179	GO:0045892: negative regulation of transcription, DNA-dependent	0.000684	8,13,2
1hwxa1#223	GO:0009396: folic acid and derivative biosynthesis	0.000684	186,11,5
1opy__#68	GO:0006916: anti-apoptosis	0.000686	308,18,8
1fa0a1#393	GO:0000051: urea cycle intermediate metabolism	0.000686	209,10,5
3grx__#55	GO:0006544: glycine metabolism	0.000686	102,11,4
1fbna_#145	GO:0009086: methionine biosynthesis	0.000687	33,14,3
2arca_#55	GO:0006986: response to unfolded protein	0.000687	130,15,5
1euca1#64	GO:0006100: tricarboxylic acid cycle intermediate metabolism	0.000687	33,14,3
1fc3a_#172	GO:0006119: oxidative phosphorylation	0.000688	168,12,5
1dgw.1#Y353	GO:0006690: icosanoid metabolism	0.000688	294,11,6
1epwa3#140	GO:0006367: transcription initiation from Pol II promoter	0.000688	168,12,5
1e8ca3#111	GO:0006221: pyrimidine nucleotide biosynthesis	0.000688	244,22,8
1danh_#93	GO:0006919: caspase activation	0.000691	7,15,2
1ig8a_#418	GO:0006986: response to unfolded protein	0.000691	7,15,2
2mev2_#53	GO:0008015: circulation	0.000691	7,15,2
1ilr1_#145	GO:0045859: regulation of protein kinase activity	0.000691	7,15,2
1iica1#165	GO:0006986: response to unfolded protein	0.000691	7,15,2

1dqga_#51	GO:0045859: regulation of protein kinase activity	0.000691	7,15,2
1ew0a_#174	GO:0007601: visual perception	0.000691	7,15,2
1vola2#239	GO:0015976: carbon utilization	0.000691	114,10,4
2sqca2#97	GO:0009582: detection of abiotic stimulus	0.000691	114,10,4
1jlxa1#147	GO:0045859: regulation of protein kinase activity	0.000691	7,15,2
1b6cb_#355	GO:0045859: regulation of protein kinase activity	0.000691	7,15,2
1htp__#16	GO:0007169: transmembrane receptor protein tyrosine kinase signaling pathway	0.000691	256,26,9
1b8aa2#403	GO:0006278: RNA-dependent DNA replication	0.000691	114,10,4
1efpa1#32	GO:0015976: carbon utilization	0.000691	114,10,4
1efpa1#32	GO:0006568: tryptophan metabolism	0.000691	114,10,4
1efpa1#32	GO:0006207: 'de novo' pyrimidine base biosynthesis	0.000691	114,10,4
1efpa1#32	GO:0009167: purine ribonucleoside monophosphate metabolism	0.000691	114,10,4
1dvpa1#65	GO:0006357: regulation of transcription from Pol II promoter	0.000691	173,30,8
1ddja_#681	GO:0007399: neurogenesis	0.000691	212,37,10
1boub_#7	GO:0006096: glycolysis	0.000692	206,38,10
1euca1#64	GO:0006730: one-carbon compound metabolism	0.000693	33,32,4
2bbkh_#214	GO:0051249: regulation of lymphocyte activation	0.000697	39,12,3
1j9qa2#295	GO:0051249: regulation of lymphocyte activation	0.000697	39,12,3
1fcda2#189	GO:0006563: L-serine metabolism	0.000697	39,12,3
1cf9a1#736	GO:0007017: microtubule-based process	0.000697	39,12,3
1bjt__#890	GO:0006606: protein-nucleus import	0.0007	258,21,8
1zpda1#213	GO:0009070: serine family amino acid biosynthesis	0.000701	72,26,5
1bfd_2#146	GO:0006563: L-serine metabolism	0.000701	93,12,4
1dypa_#143	GO:0006457: protein folding	0.000701	79,49,7
1ho1a_#211	GO:0009156: ribonucleoside monophosphate biosynthesis	0.000701	93,12,4
1avgi_#69	GO:0006461: protein complex assembly	0.000703	281,24,9
1bjt__#890	GO:0016042: lipid catabolism	0.000704	258,31,10
1i6vd_#1283	GO:0006461: protein complex assembly	0.000705	120,24,6
1iira_#326	GO:0006221: pyrimidine nucleotide biosynthesis	0.000705	86,22,5
1i7qa_#321	GO:0006313: DNA transposition	0.000705	147,35,8
1edha2#106	GO:0007264: small GTPase mediated signal transduction	0.000706	22,21,3
1kapp1#358	GO:0019079: viral genome replication	0.000706	29,16,3
1fu6a_#50	GO:0051249: regulation of lymphocyte activation	0.000706	265,12,6
1amf__#196	GO:0006418: tRNA aminoacylation for protein translation	0.000706	154,26,7
1lara2#1769	GO:0006606: protein-nucleus import	0.000706	195,21,7
1d3va_#176	GO:0030245: cellulose catabolism	0.000706	21,22,3
1qoua_#64	GO:0043123: positive regulation of I-kappaB kinase/NF-kappaB cascade	0.000706	29,16,3
1cwva2#675	GO:0043123: positive regulation of I-kappaB kinase/NF-kappaB cascade	0.000706	29,16,3
1gof_3#399	GO:0043123: positive regulation of I-kappaB kinase/NF-kappaB cascade	0.000706	29,16,3
1dqza_#209	GO:0017148: negative regulation of protein biosynthesis	0.000707	36,13,3
1hx0a2#32	GO:0009396: folic acid and derivative biosynthesis	0.000707	43,11,3
3btaa3#194	GO:0042088: T-helper 1 type immune response	0.000707	43,11,3
3btaa3#194	GO:0042108: positive regulation of cytokine biosynthesis	0.000707	43,11,3
3btaa3#194	GO:0006511: ubiquitin-dependent protein catabolism	0.000707	43,11,3
1mpp__#29	GO:0006986: response to unfolded protein	0.000707	31,15,3
1dfca4#1469	GO:0042108: positive regulation of cytokine biosynthesis	0.000707	43,11,3
1dfca4#1469	GO:0001906: cell killing	0.000707	43,11,3
1a8i__#824	GO:0009396: folic acid and derivative biosynthesis	0.000707	43,11,3
2sqca2#268	GO:0007264: small GTPase mediated signal transduction	0.000707	51,21,4
1vmoa_#84	GO:0001906: cell killing	0.000707	43,11,3
1ekbb_#228	GO:0042108: positive regulation of cytokine biosynthesis	0.000707	43,11,3
1cpza_#6	GO:0008284: positive regulation of cell proliferation	0.000707	132,30,7
1e79d2#36	GO:0006935: chemotaxis	0.000708	432,41,16
1dl0a_#32	GO:0006096: glycolysis	0.000711	28,38,4
1cpy__#346	GO:0006096: glycolysis	0.000711	28,38,4

1fhoa_#74	GO:0007169: transmembrane receptor protein tyrosine kinase signaling pathway	0.000711	315,26,10
1bwda_#117	GO:0006544: glycine metabolism	0.000712	103,11,4
1f0ia1#50	GO:0006919: caspase activation	0.000712	131,15,5
1ryph_#163	GO:0042559: pteridine and derivative biosynthesis	0.000712	103,11,4
1cipa1#88	GO:0007268: synaptic transmission	0.000713	4,28,2
1tul__#102	GO:0006118: electron transport	0.000714	67,228,15
1tuba1#8	GO:0006568: tryptophan metabolism	0.000715	115,10,4
1qs1a2#437	GO:0006826: iron ion transport	0.000716	387,15,8
1qs1a2#437	GO:0006986: response to unfolded protein	0.000716	387,15,8
1f42a2#123	GO:0018108: peptidyl-tyrosine phosphorylation	0.000717	335,10,6
1qhda2#323	GO:0006118: electron transport	0.000718	313,228,45
1ae9a_#272	GO:0006779: porphyrin biosynthesis	0.000719	6,18,2
1d2fa_#348	GO:0006779: porphyrin biosynthesis	0.000719	6,18,2
1f75a_#52	GO:0009252: peptidoglycan biosynthesis	0.000719	6,18,2
1ezia_#93	GO:0006779: porphyrin biosynthesis	0.000719	6,18,2
3chbd_#96	GO:0019835: cytolysis	0.000719	6,18,2
1ac6a_#43	GO:0006953: acute-phase response	0.00072	147,20,6
1dkia_#76P	GO:0030217: T-cell differentiation	0.000721	48,10,3
1jj2e1#42	GO:0006118: electron transport	0.000721	150,228,26
1ovb__#195	GO:0015698: inorganic anion transport	0.000721	79,14,4
1j79a_#187	GO:0009086: methionine biosynthesis	0.000721	79,14,4
1ec7a1#230	GO:0006568: tryptophan metabolism	0.000721	48,10,3
1mdah_#329	GO:0006461: protein complex assembly	0.000722	282,24,9
2mpra_#190	GO:0006730: one-carbon compound metabolism	0.000724	349,32,12
1f8ra2#324	GO:0006221: pyrimidine nucleotide biosynthesis	0.000725	5,22,2
1ep3a_#220	GO:0006221: pyrimidine nucleotide biosynthesis	0.000725	5,22,2
1zyma2#177	GO:0006221: pyrimidine nucleotide biosynthesis	0.000725	5,22,2
1qqqa_#247	GO:0006221: pyrimidine nucleotide biosynthesis	0.000725	5,22,2
1f45b_#63	GO:0045045: secretory pathway	0.000725	5,22,2
1i50b_#966	GO:0006457: protein folding	0.000725	251,49,13
1im4a_#41	GO:0006221: pyrimidine nucleotide biosynthesis	0.00073	49,22,4
1fmja_#76	GO:0006221: pyrimidine nucleotide biosynthesis	0.00073	49,22,4
1gdha1#235	GO:0016042: lipid catabolism	0.00073	92,31,6
1ej0a_#42	GO:0046677: response to antibiotic	0.00073	98,40,7
1fgka_#610	GO:0007169: transmembrane receptor protein tyrosine kinase signaling pathway	0.000731	18,26,3
1fjja_#5	GO:0007169: transmembrane receptor protein tyrosine kinase signaling pathway	0.000731	18,26,3
1quba4#235	GO:0015074: DNA integration	0.000731	96,20,5
1mrp__#170	GO:0006779: porphyrin biosynthesis	0.000731	26,18,3
1b4ka_#108	GO:0006563: L-serine metabolism	0.000731	94,12,4
1el0a_#42	GO:0007169: transmembrane receptor protein tyrosine kinase signaling pathway	0.000732	258,26,9
2dkb__#239	GO:0000160: two-component signal transduction system (phosphorelay)	0.000733	43,25,4
1ddja_#681	GO:0045595: regulation of cell differentiation	0.000734	212,10,5
1ddja_#681	GO:0006265: DNA topological change	0.000734	212,10,5
1ddja_#681	GO:0006275: regulation of DNA replication	0.000734	212,10,5
1p35a_#101	GO:0007169: transmembrane receptor protein tyrosine kinase signaling pathway	0.000736	111,26,6
1f9za_#16	GO:0006418: tRNA aminoacylation for protein translation	0.000736	111,26,6
1erv__#57	GO:0042364: water-soluble vitamin biosynthesis	0.000738	70,27,5
1cpza_#6	GO:0006919: caspase activation	0.000738	132,15,5
1eh9a3#409	GO:0046677: response to antibiotic	0.000739	3,40,2
1qg6a_#65	GO:0046677: response to antibiotic	0.000739	3,40,2
2pvaa_#167	GO:0046677: response to antibiotic	0.000739	3,40,2
1hvba_#163	GO:0046677: response to antibiotic	0.000739	3,40,2

1apme_#74	GO:0006511: ubiquitin-dependent protein catabolism	0.000739	104,11,4
1gox__#124	GO:0043094: metabolic compound salvage	0.00074	298,11,6
1gox__#124	GO:0006098: pentose-phosphate shunt	0.00074	298,11,6
1cgha_#227	GO:0016477: cell migration	0.000742	47,23,4
1aa6_2#285	GO:0006221: pyrimidine nucleotide biosynthesis	0.000743	87,22,5
1evqa_#305	GO:0046148: pigment biosynthesis	0.000743	384,12,7
2occb1#189	GO:0015992: proton transport	0.000744	9,12,2
2dkb__#46	GO:0046148: pigment biosynthesis	0.000744	9,12,2
1d4xg_#7	GO:0007017: microtubule-based process	0.000744	9,12,2
1gox__#334	GO:0009156: ribonucleoside monophosphate biosynthesis	0.000744	9,12,2
1scfa_#118	GO:0006879: iron ion homeostasis	0.000744	9,12,2
1qq8a_#137	GO:0006879: iron ion homeostasis	0.000744	9,12,2
1b9ha_#39	GO:0046148: pigment biosynthesis	0.000744	9,12,2
1a3k__#122	GO:0051249: regulation of lymphocyte activation	0.000744	9,12,2
1akjd_#3	GO:0051249: regulation of lymphocyte activation	0.000744	9,12,2
3tdt__#201	GO:0009103: lipopolysaccharide biosynthesis	0.000744	9,12,2
1abrb1#25	GO:0048514: blood vessel morphogenesis	0.000744	12,9,2
1befa_#51	GO:0006956: complement activation	0.000744	9,12,2
1dceb_#290	GO:0006956: complement activation	0.000744	9,12,2
1gai__#22	GO:0006956: complement activation	0.000744	9,12,2
1qbkb_#668	GO:0006879: iron ion homeostasis	0.000744	9,12,2
1cyx__#133	GO:0015992: proton transport	0.000744	9,12,2
1h8ca_#35	GO:0009116: nucleoside metabolism	0.000744	76,25,5
1fxra_#11	GO:0006118: electron transport	0.000745	3,228,3
1fuma2#101	GO:0006118: electron transport	0.000745	3,228,3
1cqxa3#368	GO:0006118: electron transport	0.000745	3,228,3
1amm_2#157	GO:0006118: electron transport	0.000745	3,228,3
1nox__#169	GO:0006118: electron transport	0.000745	3,228,3
1qj2b2#632	GO:0006118: electron transport	0.000745	3,228,3
1dnpa2#18	GO:0006418: tRNA aminoacylation for protein translation	0.000747	73,26,5
1ewna_#109	GO:0019735: antimicrobial humoral response (sensu Vertebrata)	0.000748	69,16,4
1qqsa_#124	GO:0006917: induction of apoptosis	0.000748	392,29,12
1dik_3#240	GO:0019059: initiation of viral infection	0.00075	213,10,5
1trka2#429	GO:0006563: L-serine metabolism	0.000751	40,12,3
1hcl__#82	GO:0007517: muscle development	0.000755	36,30,4
1jb0d_#26	GO:0042088: T-helper 1 type immune response	0.000755	190,11,5
1qs1a2#437	GO:0006118: electron transport	0.000755	387,228,53
1pii_2#326	GO:0009307: DNA restriction	0.000755	157,19,6
1gox__#246	GO:0006099: tricarboxylic acid cycle	0.000755	157,19,6
1gox__#246	GO:0009064: glutamine family amino acid metabolism	0.000755	157,19,6
1a81a2#182	GO:0006096: glycolysis	0.000757	104,38,7
1h75a_#53	GO:0001906: cell killing	0.000757	44,11,3
1gox__#124	GO:0030435: sporulation	0.000757	298,23,9
1i76a_#138	GO:0006100: tricarboxylic acid cycle intermediate metabolism	0.000757	80,14,4
1exma3#99	GO:0007264: small GTPase mediated signal transduction	0.000762	52,21,4
1a8d_1#87	GO:0019079: viral genome replication	0.000762	191,16,6
1e5ka_#113	GO:0006606: protein-nucleus import	0.000762	52,21,4
1f00i1#749	GO:0006118: electron transport	0.000763	35,228,10
1qs0b2#269	GO:0006118: electron transport	0.000763	35,228,10
2pia_1#99	GO:0006418: tRNA aminoacylation for protein translation	0.000763	156,26,7
1f2na_#138	GO:0017148: negative regulation of protein biosynthesis	0.000764	87,13,4
2cb5a_#312	GO:0006606: protein-nucleus import	0.000765	92,21,5
1f24a_#293	GO:0007264: small GTPase mediated signal transduction	0.000765	92,21,5
1qtn.1#B412	GO:0006917: induction of apoptosis	0.000766	4,29,2
1f2la_#40	GO:0016068: type I hypersensitivity	0.000766	206,15,6
1pot__#138	GO:0006457: protein folding	0.000766	58,49,6

1c9la2#61	GO:0006919: caspase activation	0.000766	206,15,6
1c9la2#61	GO:0008015: circulation	0.000766	206,15,6
2naca1#162	GO:0007067: mitosis	0.000766	19,25,3
2naca1#162	GO:0000160: two-component signal transduction system (phosphorelay)	0.000766	19,25,3
1pfza_#156	GO:0006457: protein folding	0.000766	58,49,6
1dik_1#765	GO:0009396: folic acid and derivative biosynthesis	0.000766	105,11,4
1i4fa2#21	GO:0006457: protein folding	0.000767	10,49,3
1i9za_#666	GO:0019883: antigen presentation, endogenous antigen	0.000767	49,10,3
1i9za_#666	GO:0000051: urea cycle intermediate metabolism	0.000767	49,10,3
1i9za_#666	GO:0006275: regulation of DNA replication	0.000767	49,10,3
1csn__#134	GO:0000910: cytokinesis	0.000767	13,37,3
1cmia_#86	GO:0051049: regulation of transport	0.000767	49,10,3
1cmia_#86	GO:0030217: T-cell differentiation	0.000767	49,10,3
1by5a_#238	GO:0045595: regulation of cell differentiation	0.000767	214,10,5
1fmja_#76	GO:0009219: pyrimidine deoxyribonucleotide metabolism	0.000767	49,10,3
1h6kx_#97	GO:0050819: negative regulation of coagulation	0.000767	49,10,3
1h6kx_#97	GO:0030193: regulation of blood coagulation	0.000767	49,10,3
1h6kx_#97	GO:0018108: peptidyl-tyrosine phosphorylation	0.000767	49,10,3
1h6kx_#97	GO:0000051: urea cycle intermediate metabolism	0.000767	49,10,3
1qgub_#197	GO:0051049: regulation of transport	0.000767	49,10,3
1qgub_#197	GO:0015976: carbon utilization	0.000767	49,10,3
1ton__#181	GO:0001501: skeletal development	0.000767	49,10,3
1beba_#119	GO:0006367: transcription initiation from Pol II promoter	0.000768	172,12,5
1ea5a_#99	GO:0051249: regulation of lymphocyte activation	0.000768	172,12,5
1qtn.1#A270	GO:0009156: ribonucleoside monophosphate biosynthesis	0.000768	386,12,7
1xgsa2#186	GO:0006313: DNA transposition	0.000772	149,35,8
1f0xa1#523	GO:0006461: protein complex assembly	0.000773	80,24,5
1feca1#309	GO:0006801: superoxide metabolism	0.000774	10,11,2
1fh6a_#341	GO:0000051: urea cycle intermediate metabolism	0.000774	11,10,2
1hdr__#125	GO:0006098: pentose-phosphate shunt	0.000774	10,11,2
1a3k__#199	GO:0045595: regulation of cell differentiation	0.000774	11,10,2
1a3k__#199	GO:0030217: T-cell differentiation	0.000774	11,10,2
1b0ya_#73	GO:0016485: protein processing	0.000774	10,11,2
1fwxa2#231	GO:0006559: L-phenylalanine catabolism	0.000774	11,10,2
1a0ca_#273	GO:0006098: pentose-phosphate shunt	0.000774	10,11,2
1ce7a_#151	GO:0017001: antibiotic catabolism	0.000774	11,10,2
1tgoa1#217	GO:0009396: folic acid and derivative biosynthesis	0.000774	10,11,2
1phk__#221	GO:0000086: G2/M transition of mitotic cell cycle	0.000774	11,10,2
1phk__#221	GO:0006275: regulation of DNA replication	0.000774	11,10,2
1jkw_1#113	GO:0000086: G2/M transition of mitotic cell cycle	0.000774	11,10,2
1d4oa_#135	GO:0015976: carbon utilization	0.000774	11,10,2
1b16a_#184	GO:0042559: pteridine and derivative biosynthesis	0.000774	10,11,2
1nksa_#13	GO:0009219: pyrimidine deoxyribonucleotide metabolism	0.000774	11,10,2
1ayaa_#98	GO:0000165: MAPKKK cascade	0.000774	10,11,2
1fkna_#99	GO:0006278: RNA-dependent DNA replication	0.000774	11,10,2
1fkna_#99	GO:0009167: purine ribonucleoside monophosphate metabolism	0.000774	11,10,2
1nsj__#83	GO:0042401: biogenic amine biosynthesis	0.000774	11,10,2
1nsj__#83	GO:0006568: tryptophan metabolism	0.000774	11,10,2
1a8d_1#87	GO:0001906: cell killing	0.000774	191,11,5
1kit_3#743	GO:0006461: protein complex assembly	0.000774	225,24,8
1e1oa1#142	GO:0001906: cell killing	0.000774	10,11,2
1rypk_#76	GO:0006511: ubiquitin-dependent protein catabolism	0.000774	10,11,2
1eno__#137	GO:0042559: pteridine and derivative biosynthesis	0.000774	10,11,2
1acc__#360	GO:0006801: superoxide metabolism	0.000774	10,11,2
1dp0a5#534	GO:0015976: carbon utilization	0.000774	11,10,2
1ihka_#164	GO:0000165: MAPKKK cascade	0.000774	10,11,2

1g25a_#22	GO:0016477: cell migration	0.000775	367,23,10
1dn0b2#214	GO:0006935: chemotaxis	0.000776	3,41,2
1g79a_#85	GO:0006935: chemotaxis	0.000776	3,41,2
1gdna_#151	GO:0006935: chemotaxis	0.000776	3,41,2
1bupa2#199	GO:0006986: response to unfolded protein	0.000777	32,15,3
1ib2a_#1101	GO:0006470: protein amino acid dephosphorylation	0.000777	55,20,4
1awcb_#111	GO:0006826: iron ion transport	0.000777	32,15,3
1pwt__#8	GO:0007264: small GTPase mediated signal transduction	0.000777	262,21,8
1pwt__#8	GO:0006606: protein-nucleus import	0.000777	262,21,8
1fa0a1#393	GO:0006096: glycolysis	0.000777	209,38,10
1h6va1#27	GO:0009086: methionine biosynthesis	0.000778	224,14,6
1by5a_#238	GO:0016042: lipid catabolism	0.000779	214,31,9
1cs1a_#82	GO:0009064: glutamine family amino acid metabolism	0.000781	158,19,6
2hrva_#132	GO:0019079: viral genome replication	0.000782	30,16,3
1ezvb1#29	GO:0007169: transmembrane receptor protein tyrosine kinase signaling pathway	0.000782	42,26,4
1fc4a_#205	GO:0009167: purine ribonucleoside monophosphate metabolism	0.000783	215,10,5
1ejda_#6	GO:0050819: negative regulation of coagulation	0.000783	215,10,5
1ejda_#6	GO:0030193: regulation of blood coagulation	0.000783	215,10,5
1edza1#206	GO:0006730: one-carbon compound metabolism	0.000784	352,32,12
1qdlb_#170	GO:0006118: electron transport	0.000786	89,228,18
1e4ea2#215	GO:0006461: protein complex assembly	0.000786	419,24,11
1gsa_2#218	GO:0051049: regulation of transport	0.000788	118,10,4
1gsa_2#218	GO:0019883: antigen presentation, endogenous antigen	0.000788	118,10,4
1gsa_2#218	GO:0030217: T-cell differentiation	0.000788	118,10,4
1ekbb_#85	GO:0006207: 'de novo' pyrimidine base biosynthesis	0.000788	118,10,4
1f97a2#199	GO:0045595: regulation of cell differentiation	0.000788	118,10,4
1f97a2#199	GO:0006275: regulation of DNA replication	0.000788	118,10,4
1qo3c_#212	GO:0019735: antimicrobial humoral response (sensu Vertebrata)	0.000789	7,16,2
1cdy_2#140	GO:0019079: viral genome replication	0.000789	7,16,2
1ilr1_#145	GO:0019735: antimicrobial humoral response (sensu Vertebrata)	0.000789	7,16,2
1eu3a1#51	GO:0006413: translational initiation	0.000789	7,16,2
1qdlb_#98	GO:0009073: aromatic amino acid family biosynthesis	0.000789	7,16,2
1dqga_#51	GO:0019735: antimicrobial humoral response (sensu Vertebrata)	0.000789	7,16,2
1dvpa1#65	GO:0015992: proton transport	0.000789	173,12,5
2tbva_#214	GO:0043123: positive regulation of I-kappaB kinase/NF-kappaB cascade	0.000789	7,16,2
1qrra_#69	GO:0006563: L-serine metabolism	0.000789	173,12,5
1d2ka1#164	GO:0006096: glycolysis	0.00079	50,38,5
1dhn__#115	GO:0006461: protein complex assembly	0.000792	20,24,3
1nsj__#21	GO:0046148: pigment biosynthesis	0.000792	96,12,4
1fxoa_#136	GO:0006461: protein complex assembly	0.000792	20,24,3
1bwda_#295	GO:0007017: microtubule-based process	0.000792	96,12,4
1eur__#99	GO:0006461: protein complex assembly	0.000792	20,24,3
1br9__#168	GO:0015671: oxygen transport	0.000793	5,23,2
1pii_2#326	GO:0006418: tRNA aminoacylation for protein translation	0.000793	157,26,7
1hdfa_#16	GO:0042088: T-helper 1 type immune response	0.000793	192,11,5
1hdfa_#16	GO:0042108: positive regulation of cytokine biosynthesis	0.000793	192,11,5
1hdfa_#16	GO:0001906: cell killing	0.000793	192,11,5
1gox__#246	GO:0009070: serine family amino acid biosynthesis	0.000793	157,26,7
1eny__#147	GO:0009086: methionine biosynthesis	0.000794	81,14,4
1uch__#50	GO:0009070: serine family amino acid biosynthesis	0.000795	74,26,5
1f46a_#59	GO:0001906: cell killing	0.000795	106,11,4
1thg__#223	GO:0006418: tRNA aminoacylation for protein translation	0.000795	74,26,5
3prn__#143	GO:0016477: cell migration	0.000796	300,23,9
1e0ca1#88	GO:0006100: tricarboxylic acid cycle intermediate metabolism	0.000797	8,14,2
1e0ca1#88	GO:0015698: inorganic anion transport	0.000797	8,14,2

1feca1#147	GO:0009086: methionine biosynthesis	0.000797	8,14,2
1tlfa_#269	GO:0009086: methionine biosynthesis	0.000797	8,14,2
1dgtb3#2594	GO:0006100: tricarboxylic acid cycle intermediate metabolism	0.000797	8,14,2
1fc4a_#241	GO:0009086: methionine biosynthesis	0.000797	8,14,2
1dc1a_#241	GO:0006100: tricarboxylic acid cycle intermediate metabolism	0.000797	8,14,2
1g6oa_#116	GO:0006544: glycine metabolism	0.000797	302,11,6
1cs1a_#160	GO:0000160: two-component signal transduction system (phosphorelay)	0.000797	164,25,7
1hbza_#96	GO:0006457: protein folding	0.000797	322,49,15
1lci__#26	GO:0046677: response to antibiotic	0.000798	130,40,8
1xgsa2#82	GO:0007169: transmembrane receptor protein tyrosine kinase signaling pathway	0.000798	261,26,9
1hlwa_#82	GO:0006457: protein folding	0.000799	221,49,12
1i1b__#85	GO:0008285: negative regulation of cell proliferation	0.000803	6,19,2
1ycsb1#418	GO:0008285: negative regulation of cell proliferation	0.000803	6,19,2
1sw6a_#352	GO:0008285: negative regulation of cell proliferation	0.000803	6,19,2
1qqka_#19	GO:0008285: negative regulation of cell proliferation	0.000803	6,19,2
1hxma2#136	GO:0009064: glutamine family amino acid metabolism	0.000803	6,19,2
1fg7a_#96	GO:0009307: DNA restriction	0.000803	6,19,2
1jlxa2#243	GO:0008285: negative regulation of cell proliferation	0.000803	6,19,2
1dcqa1#473	GO:0008285: negative regulation of cell proliferation	0.000803	6,19,2
1dcqa1#451	GO:0008285: negative regulation of cell proliferation	0.000803	6,19,2
1kwaa_#513	GO:0016477: cell migration	0.000805	48,23,4
1qvba_#206	GO:0007264: small GTPase mediated signal transduction	0.000808	23,21,3
1bfg__#22	GO:0048514: blood vessel morphogenesis	0.000808	56,9,3
1rypi_#87	GO:0015671: oxygen transport	0.000808	21,23,3
1ldna1#94	GO:0009064: glutamine family amino acid metabolism	0.000808	159,19,6
1qgna_#373	GO:0000398: nuclear mRNA splicing, via spliceosome	0.000809	41,12,3
1dfca1#1135	GO:0001906: cell killing	0.000809	45,11,3
1fp5a2#537	GO:0042108: positive regulation of cytokine biosynthesis	0.000809	45,11,3
1i2ma_#90	GO:0006935: chemotaxis	0.000809	12,41,3
1hrka_#205	GO:0016568: chromatin modification	0.000809	45,11,3
1p35a_#155	GO:0042088: T-helper 1 type immune response	0.000809	45,11,3
1p35a_#155	GO:0006690: icosanoid metabolism	0.000809	45,11,3
2pola2#218	GO:0006730: one-carbon compound metabolism	0.000809	126,32,7
5ruba1#259	GO:0043094: metabolic compound salvage	0.000809	45,11,3
1qhda2#323	GO:0016042: lipid catabolism	0.00081	313,31,11
1fgga_#134	GO:0043094: metabolic compound salvage	0.000812	193,11,5
1f5aa1#441	GO:0016477: cell migration	0.000812	129,23,6
1f24a_#144	GO:0006221: pyrimidine nucleotide biosynthesis	0.000813	22,22,3
1f0xa1#482	GO:0045595: regulation of cell differentiation	0.000814	50,10,3
1ddga1#278	GO:0019883: antigen presentation, endogenous antigen	0.000814	50,10,3
1d2ka1#164	GO:0009167: purine ribonucleoside monophosphate metabolism	0.000814	50,10,3
1mjha_#126	GO:0006568: tryptophan metabolism	0.000814	119,10,4
1bg6_2#73	GO:0006096: glycolysis	0.000815	29,38,4
1poxa3#460	GO:0006096: glycolysis	0.000815	76,38,6
1c8za_#386	GO:0006953: acute-phase response	0.000816	211,20,7
1jb3a_#64	GO:0006643: membrane lipid metabolism	0.000818	247,13,6
1ecfa1#434	GO:0042364: water-soluble vitamin biosynthesis	0.000819	18,27,3
1saca_#113	GO:0016567: protein ubiquitination	0.000819	35,14,3
1ghpa_#236	GO:0006313: DNA transposition	0.000819	14,35,3
2rspa_#113	GO:0006313: DNA transposition	0.000819	14,35,3
1cqka_#49	GO:0006100: tricarboxylic acid cycle intermediate metabolism	0.000819	35,14,3
1elja_#8	GO:0015698: inorganic anion transport	0.000819	35,14,3
1c7na_#281	GO:0042364: water-soluble vitamin biosynthesis	0.000819	18,27,3
1neb__#52	GO:0006313: DNA transposition	0.000819	14,35,3
1sw6a_#464	GO:0006357: regulation of transcription from Pol II promoter	0.00082	4,30,2

1dfca1#1076	GO:0007517: muscle development	0.00082	4,30,2
1dfca1#1076	GO:0008284: positive regulation of cell proliferation	0.00082	4,30,2
1bd8__#103	GO:0006357: regulation of transcription from Pol II promoter	0.00082	4,30,2
1a4pa_#24	GO:0007517: muscle development	0.00082	4,30,2
1fhga_#42	GO:0007517: muscle development	0.00082	4,30,2
1bik_1#76	GO:0007517: muscle development	0.00082	4,30,2
1jlxa2#288	GO:0007517: muscle development	0.00082	4,30,2
1jlxa2#288	GO:0008284: positive regulation of cell proliferation	0.00082	4,30,2
1d66a1#28	GO:0006357: regulation of transcription from Pol II promoter	0.00082	4,30,2
1h6va1#27	GO:0006099: tricarboxylic acid cycle	0.000823	224,19,7
1h6va1#27	GO:0009064: glutamine family amino acid metabolism	0.000823	224,19,7
1h72c2#269	GO:0006099: tricarboxylic acid cycle	0.000827	59,19,4
1h72c2#269	GO:0009064: glutamine family amino acid metabolism	0.000827	59,19,4
1fjfj_#68	GO:0006118: electron transport	0.000828	218,228,34
1fwxa2#396	GO:0006919: caspase activation	0.000828	209,15,6
1a4ya_#104	GO:0009073: aromatic amino acid family biosynthesis	0.000828	194,16,6
1azo__#144	GO:0045892: negative regulation of transcription, DNA-dependent	0.00083	38,13,3
1h7wa4#208	GO:0006096: glycolysis	0.00083	13,38,3
1a4ya_#104	GO:0006098: pentose-phosphate shunt	0.000832	194,11,5
1aqua_#126	GO:0000160: two-component signal transduction system (phosphorelay)	0.000833	472,25,12
1fjfj_#68	GO:0019059: initiation of viral infection	0.000836	218,10,5
1h72c2#269	GO:0006457: protein folding	0.00084	59,49,6
1tkia_#142	GO:0000910: cytokinesis	0.00084	30,37,4
1i6vd_#1283	GO:0030217: T-cell differentiation	0.000841	120,10,4
1fw9a_#152	GO:0006461: protein complex assembly	0.000841	124,24,6
1iow_1#37	GO:0006418: tRNA aminoacylation for protein translation	0.000844	263,26,9
1qqsa_#124	GO:0006119: oxidative phosphorylation	0.000846	392,12,7
2arca_#55	GO:0016477: cell migration	0.000846	130,23,6
1kit_3#743	GO:0008285: negative regulation of cell proliferation	0.000846	225,19,7
1hdoa_#189	GO:0009086: methionine biosynthesis	0.000846	147,14,5
1ej8a_#189	GO:0016337: cell-cell adhesion	0.000848	256,17,7
1htp__#16	GO:0016337: cell-cell adhesion	0.000848	256,17,7
1b8ba_#466	GO:0045045: secretory pathway	0.000851	51,22,4
1ciy_2#298	GO:0008015: circulation	0.000851	33,15,3
1erja_#457	GO:0007601: visual perception	0.000851	33,15,3
2sqca2#268	GO:0030245: cellulose catabolism	0.000851	51,22,4
1kapp1#358	GO:0016337: cell-cell adhesion	0.000851	29,17,3
1fi2a_#109	GO:0001906: cell killing	0.000852	195,11,5
1lara2#1769	GO:0001906: cell killing	0.000852	195,11,5
1lara2#1769	GO:0016485: protein processing	0.000852	195,11,5
1ac6a_#34	GO:0042113: B-cell activation	0.000852	57,9,3
1e19a_#281	GO:0009396: folic acid and derivative biosynthesis	0.000853	108,11,4
1bif_2#398	GO:0009307: DNA restriction	0.000855	105,19,5
1quqb_#87	GO:0016337: cell-cell adhesion	0.000856	67,17,4
1pud__#69	GO:0006418: tRNA aminoacylation for protein translation	0.000856	43,26,4
1thfd_#140	GO:0000398: nuclear mRNA splicing, via spliceosome	0.000856	98,12,4
1dfca4#1469	GO:0006418: tRNA aminoacylation for protein translation	0.000856	43,26,4
1hyha2#204	GO:0007169: transmembrane receptor protein tyrosine kinase signaling pathway	0.000856	43,26,4
3sil__#8	GO:0051249: regulation of lymphocyte activation	0.000856	98,12,4
1ldna1#94	GO:0009070: serine family amino acid biosynthesis	0.000857	159,26,7
2mev3_#107	GO:0019079: viral genome replication	0.000862	31,16,3
1ewqa3#159	GO:0009064: glutamine family amino acid metabolism	0.000862	26,19,3
7fabh1#71	GO:0006897: endocytosis	0.000862	26,19,3
1g25a_#22	GO:0019735: antimicrobial humoral response (sensu Vertebrata)	0.000862	367,16,8
1thg__#127	GO:0016042: lipid catabolism	0.000862	16,31,3

1f3mc_#403	GO:0007169: transmembrane receptor protein tyrosine kinase signaling pathway	0.000862	19,26,3
1qqp1_#51	GO:0019079: viral genome replication	0.000862	31,16,3
1cg2a1#43	GO:0006979: response to oxidative stress	0.000863	171,18,6
2dpma_#189	GO:0009396: folic acid and derivative biosynthesis	0.000863	46,11,3
1quqa_#75	GO:0006559: L-phenylalanine catabolism	0.000863	51,10,3
1as4.1#B385	GO:0019059: initiation of viral infection	0.000863	51,10,3
1as4.1#B385	GO:0019883: antigen presentation, endogenous antigen	0.000863	51,10,3
1a7j__#87	GO:0015976: carbon utilization	0.000863	51,10,3
1a7j__#87	GO:0000051: urea cycle intermediate metabolism	0.000863	51,10,3
1bhga3#529	GO:0006207: 'de novo' pyrimidine base biosynthesis	0.000863	51,10,3
2sqca2#268	GO:0001558: regulation of cell growth	0.000863	51,10,3
1kit_3#743	GO:0008284: positive regulation of cell proliferation	0.000864	225,30,9
1b3aa_#15	GO:0007186: G-protein coupled receptor protein signaling pathway	0.000865	5,24,2
1c9la2#73	GO:0007186: G-protein coupled receptor protein signaling pathway	0.000865	5,24,2
1agja_#91	GO:0006461: protein complex assembly	0.000865	5,24,2
2sqca1#553	GO:0007186: G-protein coupled receptor protein signaling pathway	0.000865	5,24,2
1ayfa_#96	GO:0006118: electron transport	0.000866	6,228,4
1bx4a_#31	GO:0006118: electron transport	0.000866	6,228,4
1g8ka2#332	GO:0006118: electron transport	0.000866	6,228,4
1icra_#46	GO:0006118: electron transport	0.000866	6,228,4
1a2za_#209	GO:0006118: electron transport	0.000866	6,228,4
1f37a_#30	GO:0006118: electron transport	0.000866	6,228,4
1hr6a1#59	GO:0006118: electron transport	0.000866	6,228,4
1eu1a1#679	GO:0006118: electron transport	0.000866	6,228,4
1ac5__#72	GO:0006096: glycolysis	0.000866	51,38,5
1edza1#206	GO:0006935: chemotaxis	0.000867	352,41,14
1stfi_#116	GO:0009615: response to virus	0.000868	42,12,3
1stfi_#116	GO:0009156: ribonucleoside monophosphate biosynthesis	0.000868	42,12,3
1ddja_#746	GO:0019883: antigen presentation, endogenous antigen	0.000868	121,10,4
1h6la_#119	GO:0009070: serine family amino acid biosynthesis	0.000868	264,26,9
1cqka_#52	GO:0008284: positive regulation of cell proliferation	0.000869	65,30,5
1d0na3#305	GO:0019079: viral genome replication	0.000869	276,16,7
1fsia_#122	GO:0030333: antigen processing	0.000869	90,13,4
1fsia_#122	GO:0006643: membrane lipid metabolism	0.000869	90,13,4
1hxmb1#117	GO:0008284: positive regulation of cell proliferation	0.000869	65,30,5
1fuia2#273	GO:0042364: water-soluble vitamin biosynthesis	0.00087	110,27,6
1im4a_#41	GO:0016477: cell migration	0.000871	49,23,4
1cmia_#86	GO:0016477: cell migration	0.000871	49,23,4
1f8ra2#325	GO:0001906: cell killing	0.000872	196,11,5
1f8ra2#325	GO:0016485: protein processing	0.000872	196,11,5
1eaja_#59	GO:0001906: cell killing	0.000872	196,11,5
1gpea1#271	GO:0042088: T-helper 1 type immune response	0.000872	196,11,5
1gpea1#271	GO:0042108: positive regulation of cytokine biosynthesis	0.000872	196,11,5
1dgw.1#Y353	GO:0006096: glycolysis	0.000874	294,38,12
1f5aa2#167	GO:0046677: response to antibiotic	0.000876	101,40,7
1thta_#214	GO:0016042: lipid catabolism	0.000876	4,31,2
16pk__#312	GO:0000082: G1/S transition of mitotic cell cycle	0.000877	9,13,2
1ei5a1#357	GO:0016998: cell wall catabolism	0.000877	13,9,2
1hcd__#114	GO:0048514: blood vessel morphogenesis	0.000877	13,9,2
1moq__#368	GO:0045892: negative regulation of transcription, DNA-dependent	0.000877	9,13,2
1d4xg_#7	GO:0000082: G1/S transition of mitotic cell cycle	0.000877	9,13,2
1awx__#27	GO:0045786: negative regulation of cell cycle	0.000877	9,13,2
1qr4a2#166	GO:0042113: B-cell activation	0.000877	13,9,2
1shsa_#68	GO:0030333: antigen processing	0.000877	9,13,2
1ycsa_#162	GO:0051249: regulation of lymphocyte activation	0.000877	177,12,5

1fj2a_#24	GO:0006805: xenobiotic metabolism	0.000877	9,13,2
1fj2a_#24	GO:0017000: antibiotic biosynthesis	0.000877	9,13,2
1hlga_#353	GO:0006805: xenobiotic metabolism	0.000877	9,13,2
1hlga_#353	GO:0017000: antibiotic biosynthesis	0.000877	9,13,2
1c4xa_#43	GO:0006805: xenobiotic metabolism	0.000877	9,13,2
1c4xa_#43	GO:0017000: antibiotic biosynthesis	0.000877	9,13,2
1wba__#169	GO:0017148: negative regulation of protein biosynthesis	0.000877	9,13,2
1jf9a_#379	GO:0006917: induction of apoptosis	0.000878	186,29,8
1qmea4#290	GO:0019735: antimicrobial humoral response (sensu Vertebrata)	0.000879	72,16,4
1d0na3#305	GO:0009615: response to virus	0.000881	276,12,6
1cipa2#34	GO:0009064: glutamine family amino acid metabolism	0.000881	60,19,4
1e3ja2#270	GO:0006099: tricarboxylic acid cycle	0.000881	60,19,4
1bec_2#216	GO:0001906: cell killing	0.000884	109,11,4
1bec_2#216	GO:0016485: protein processing	0.000884	109,11,4
1opy__#68	GO:0001906: cell killing	0.000887	308,11,6
1nbaa_#227	GO:0000160: two-component signal transduction system (phosphorelay)	0.000889	79,25,5
1d0ba_#191	GO:0006563: L-serine metabolism	0.00089	99,12,4
1tuba1#8	GO:0009070: serine family amino acid biosynthesis	0.00089	115,26,6
1e25a_#262	GO:0006418: tRNA aminoacylation for protein translation	0.00089	115,26,6
1ax4a_#275	GO:0006367: transcription initiation from Pol II promoter	0.00089	99,12,4
1fmb__#60	GO:0015074: DNA integration	0.000891	6,20,2
1f37a_#30	GO:0006470: protein amino acid dephosphorylation	0.000891	6,20,2
1a31a1#512	GO:0015074: DNA integration	0.000891	6,20,2
1hlwa_#82	GO:0019883: antigen presentation, endogenous antigen	0.000891	221,10,5
1lucb_#70	GO:0006100: tricarboxylic acid cycle intermediate metabolism	0.000891	36,14,3
1ejda_#213	GO:0009307: DNA restriction	0.000892	227,19,7
1f13a3#645	GO:0016337: cell-cell adhesion	0.000893	7,17,2
1ep3b2#254	GO:0016337: cell-cell adhesion	0.000893	7,17,2
1hdmb1#157	GO:0016337: cell-cell adhesion	0.000893	7,17,2
1nuka_#80	GO:0006801: superoxide metabolism	0.000893	197,11,5
1bd2d1#62	GO:0016337: cell-cell adhesion	0.000893	7,17,2
1aym3_#131	GO:0006935: chemotaxis	0.000893	353,41,14
2mev3_#108	GO:0042088: T-helper 1 type immune response	0.000893	197,11,5
2mev3_#108	GO:0042108: positive regulation of cytokine biosynthesis	0.000893	197,11,5
1qq4a_#178	GO:0030333: antigen processing	0.000896	39,13,3
1j9qa2#295	GO:0030333: antigen processing	0.000896	39,13,3
1thw__#199	GO:0017148: negative regulation of protein biosynthesis	0.000896	39,13,3
1imva_#255	GO:0006265: DNA topological change	0.000897	506,10,7
2mpra_#190	GO:0018108: peptidyl-tyrosine phosphorylation	0.000899	349,10,6
2mpra_#190	GO:0006265: DNA topological change	0.000899	349,10,6
2mpra_#190	GO:0006275: regulation of DNA replication	0.000899	349,10,6
1fnf_3#1409	GO:0007169: transmembrane receptor protein tyrosine kinase signaling pathway	0.000899	76,26,5
1fn9a_#271	GO:0016337: cell-cell adhesion	0.000904	184,17,6
1b3qa2#623	GO:0006730: one-carbon compound metabolism	0.000904	257,32,10
1fbna_#144	GO:0009252: peptidoglycan biosynthesis	0.000906	64,18,4
1jf9a_#196	GO:0042364: water-soluble vitamin biosynthesis	0.000906	42,27,4
1b65a_#257	GO:0006730: one-carbon compound metabolism	0.000907	168,32,8
1fgga_#134	GO:0006221: pyrimidine nucleotide biosynthesis	0.000909	193,22,7
1ct9a1#401	GO:0009252: peptidoglycan biosynthesis	0.000913	28,18,3
1j79a_#38	GO:0009252: peptidoglycan biosynthesis	0.000913	28,18,3
1epwa3#248	GO:0019835: cytolysis	0.000913	28,18,3
1fgua2#360	GO:0009219: pyrimidine deoxyribonucleotide metabolism	0.000914	52,10,3
1ihoa_#238	GO:0017001: antibiotic catabolism	0.000914	52,10,3
1huxa_#10	GO:0006278: RNA-dependent DNA replication	0.000914	52,10,3
1qfta_#98	GO:0006801: superoxide metabolism	0.000914	198,11,5

1qfta_#98	GO:0001906: cell killing	0.000914	198,11,5
1faoa_#250	GO:0000051: urea cycle intermediate metabolism	0.000914	52,10,3
1e5ka_#113	GO:0006278: RNA-dependent DNA replication	0.000914	52,10,3
1ft9a1#162	GO:0006445: regulation of translation	0.000915	78,15,4
1dmla1#134	GO:0016068: type I hypersensitivity	0.000915	78,15,4
2dnja_#97	GO:0001906: cell killing	0.000915	110,11,4
1f8ra1#233	GO:0006118: electron transport	0.000916	19,228,7
1psda3#376	GO:0006118: electron transport	0.000916	19,228,7
1quba4#235	GO:0016042: lipid catabolism	0.000917	96,31,6
1ihua2#525	GO:0009156: ribonucleoside monophosphate biosynthesis	0.000917	278,12,6
1ihua2#525	GO:0000160: two-component signal transduction system (phosphorelay)	0.000917	278,25,9
1i7qa_#470	GO:0006826: iron ion transport	0.000918	8,15,2
2cpl__#83	GO:0006919: caspase activation	0.000918	8,15,2
3mbp__#275	GO:0006826: iron ion transport	0.000918	8,15,2
1qfla1#42	GO:0006986: response to unfolded protein	0.000918	8,15,2
1deua_#57	GO:0006986: response to unfolded protein	0.000918	8,15,2
1hcl__#267	GO:0045859: regulation of protein kinase activity	0.000918	8,15,2
1koba_#105	GO:0045859: regulation of protein kinase activity	0.000918	8,15,2
1fn9a_#271	GO:0016477: cell migration	0.000918	184,23,7
5ruba1#279	GO:0006098: pentose-phosphate shunt	0.00092	47,11,3
1eula_#213	GO:0001906: cell killing	0.00092	47,11,3
1eula_#213	GO:0006690: icosanoid metabolism	0.00092	47,11,3
1smaa2#581	GO:0006457: protein folding	0.00092	60,49,6
2bpa1_#244	GO:0006690: icosanoid metabolism	0.00092	47,11,3
1apy.1#B284	GO:0006511: ubiquitin-dependent protein catabolism	0.00092	47,11,3
1apy.1#B284	GO:0001906: cell killing	0.00092	47,11,3
1ddwa_#77	GO:0030245: cellulose catabolism	0.00092	322,22,9
1dofa_#15	GO:0006879: iron ion homeostasis	0.000923	179,12,5
1dofa_#15	GO:0015992: proton transport	0.000923	179,12,5
1ycsa_#162	GO:0006118: electron transport	0.000923	177,228,29
2bpa1_#45	GO:0051249: regulation of lymphocyte activation	0.000923	179,12,5
1esma_#314	GO:0009615: response to virus	0.000925	100,12,4
1f5aa1#441	GO:0019735: antimicrobial humoral response (sensu Vertebrata)	0.000925	129,16,5
1i2ma_#157	GO:0007017: microtubule-based process	0.000927	10,12,2
1apme_#101	GO:0006275: regulation of DNA replication	0.000927	12,10,2
1i6vd_#886	GO:0006119: oxidative phosphorylation	0.000927	10,12,2
1ir3a_#1056	GO:0006275: regulation of DNA replication	0.000927	12,10,2
2fcba2#104	GO:0019883: antigen presentation, endogenous antigen	0.000927	12,10,2
2fcba2#104	GO:0030217: T-cell differentiation	0.000927	12,10,2
1hf8a_#156	GO:0045595: regulation of cell differentiation	0.000927	12,10,2
1koba_#139	GO:0006879: iron ion homeostasis	0.000927	10,12,2
1e39a3#417	GO:0050819: negative regulation of coagulation	0.000927	12,10,2
1e39a3#417	GO:0030193: regulation of blood coagulation	0.000927	12,10,2
1e39a3#417	GO:0018108: peptidyl-tyrosine phosphorylation	0.000927	12,10,2
1dn0b2#124	GO:0030217: T-cell differentiation	0.000927	12,10,2
1ayoa_#55	GO:0019059: initiation of viral infection	0.000927	12,10,2
1fnga1#123	GO:0051249: regulation of lymphocyte activation	0.000927	10,12,2
1ga0a_#304	GO:0006879: iron ion homeostasis	0.000927	10,12,2
1pvda3#533	GO:0006568: tryptophan metabolism	0.000927	12,10,2
2trcp_#201	GO:0050819: negative regulation of coagulation	0.000927	12,10,2
2trcp_#201	GO:0030193: regulation of blood coagulation	0.000927	12,10,2
1dar_2#264	GO:0051049: regulation of transport	0.000927	12,10,2
1ed8a_#353	GO:0006207: 'de novo' pyrimidine base biosynthesis	0.000927	12,10,2
1fgka_#572	GO:0000086: G2/M transition of mitotic cell cycle	0.000927	12,10,2
1b6cb_#399	GO:0000086: G2/M transition of mitotic cell cycle	0.000927	12,10,2
1b6cb_#399	GO:0006275: regulation of DNA replication	0.000927	12,10,2

1f5qb2#149	GO:0006367: transcription initiation from Pol II promoter	0.000927	10,12,2
1bywa_#64	GO:0009582: detection of abiotic stimulus	0.000927	12,10,2
1qo2a_#223	GO:0042401: biogenic amine biosynthesis	0.000927	12,10,2
1qo2a_#223	GO:0006568: tryptophan metabolism	0.000927	12,10,2
1i6vc_#689	GO:0006354: RNA elongation	0.000927	12,10,2
1i6vc_#689	GO:0009219: pyrimidine deoxyribonucleotide metabolism	0.000927	12,10,2
1hava_#157	GO:0000398: nuclear mRNA splicing, via spliceosome	0.000927	10,12,2
1chma2#262	GO:0019883: antigen presentation, endogenous antigen	0.000927	12,10,2
2ayh__#160	GO:0051049: regulation of transport	0.000927	12,10,2
2ayh__#160	GO:0019883: antigen presentation, endogenous antigen	0.000927	12,10,2
1b8pa2#201	GO:0009103: lipopolysaccharide biosynthesis	0.000927	10,12,2
2u2fa_#33	GO:0051249: regulation of lymphocyte activation	0.000927	10,12,2
1ecpa_#156	GO:0006563: L-serine metabolism	0.000927	10,12,2
1i2ma_#90	GO:0051049: regulation of transport	0.000927	12,10,2
1e32a1#45	GO:0006275: regulation of DNA replication	0.000927	12,10,2
2dri__#115	GO:0015992: proton transport	0.000927	10,12,2
2dri__#115	GO:0006119: oxidative phosphorylation	0.000927	10,12,2
1lcl__#110	GO:0001501: skeletal development	0.000927	12,10,2
1rypk_#76	GO:0006119: oxidative phosphorylation	0.000927	10,12,2
1qfma1#238	GO:0007017: microtubule-based process	0.000927	10,12,2
1rsy__#151	GO:0016477: cell migration	0.00093	22,23,3
1feza_#121	GO:0006221: pyrimidine nucleotide biosynthesis	0.00093	23,22,3
1eq9a_#32	GO:0006606: protein-nucleus import	0.00093	96,21,5
1seta2#403	GO:0008284: positive regulation of cell proliferation	0.000931	38,30,4
1pud__#44	GO:0009156: ribonucleoside monophosphate biosynthesis	0.000931	43,12,3
2pia_1#54	GO:0016337: cell-cell adhesion	0.000931	185,17,6
1dfca4#1469	GO:0051249: regulation of lymphocyte activation	0.000931	43,12,3
1qlsa_#81	GO:0007517: muscle development	0.000931	38,30,4
1ekbb_#228	GO:0006956: complement activation	0.000931	43,12,3
1hyrc1#247	GO:0008284: positive regulation of cell proliferation	0.000932	66,30,5
1bjt__#890	GO:0006730: one-carbon compound metabolism	0.000932	258,32,10
1axn__#42	GO:0006357: regulation of transcription from Pol II promoter	0.000932	66,30,5
1a8i__#688	GO:0042364: water-soluble vitamin biosynthesis	0.000933	203,27,8
1vdra_#76	GO:0006730: one-carbon compound metabolism	0.000934	4,32,2
1lgr_2#115	GO:0006730: one-carbon compound metabolism	0.000934	4,32,2
1dih_2#222	GO:0006544: glycine metabolism	0.000935	311,11,6
1dih_2#222	GO:0016485: protein processing	0.000935	311,11,6
1e30a_#87	GO:0007169: transmembrane receptor protein tyrosine kinase signaling pathway	0.000935	44,26,4
1ecfa1#388	GO:0009116: nucleoside metabolism	0.000939	5,25,2
1gof_3#436	GO:0007067: mitosis	0.000939	5,25,2
1ed1a_#94	GO:0009116: nucleoside metabolism	0.000939	5,25,2
1ih7a1#260	GO:0000160: two-component signal transduction system (phosphorelay)	0.000939	5,25,2
1g5ba_#193	GO:0007067: mitosis	0.000939	5,25,2
1jj2q_#118	GO:0007067: mitosis	0.000939	5,25,2
1a3c__#13	GO:0009116: nucleoside metabolism	0.000939	5,25,2
1cex__#211	GO:0000160: two-component signal transduction system (phosphorelay)	0.000939	5,25,2
1mla_1#95	GO:0009116: nucleoside metabolism	0.000939	5,25,2
3frua1#236	GO:0006897: endocytosis	0.000939	61,19,4
1fo5a_#60	GO:0006457: protein folding	0.000939	225,49,12
1bu7a_#405	GO:0006099: tricarboxylic acid cycle	0.000939	61,19,4
1by5a_#238	GO:0016068: type I hypersensitivity	0.00094	214,15,6
1f42a2#123	GO:0006118: electron transport	0.00094	335,228,47
1rypd_#93	GO:0007517: muscle development	0.000942	17,30,3
1edza1#206	GO:0009219: pyrimidine deoxyribonucleotide metabolism	0.000942	352,10,6
1edza1#206	GO:0018108: peptidyl-tyrosine phosphorylation	0.000942	352,10,6

1bvyf_#527	GO:0006544: glycine metabolism	0.000944	11,11,2
1b93a_#48	GO:0006098: pentose-phosphate shunt	0.000944	11,11,2
1xis__#231	GO:0006098: pentose-phosphate shunt	0.000944	11,11,2
1ezva1#147	GO:0016485: protein processing	0.000944	11,11,2
1d4oa_#135	GO:0006801: superoxide metabolism	0.000944	11,11,2
1i6vc_#869	GO:0001906: cell killing	0.000944	11,11,2
1ryp1_#2	GO:0006511: ubiquitin-dependent protein catabolism	0.000944	11,11,2
1icia_#8	GO:0016568: chromatin modification	0.000944	11,11,2
1ib2a_#1101	GO:0007264: small GTPase mediated signal transduction	0.000944	55,21,4
1epwa2#1220	GO:0000165: MAPKKK cascade	0.000944	11,11,2
1ft9a1#201	GO:0030333: antigen processing	0.000945	92,13,4
3grx__#56	GO:0016477: cell migration	0.000945	307,23,9
1p35a_#101	GO:0042108: positive regulation of cytokine biosynthesis	0.000947	111,11,4
1dy9.1#C229	GO:0042088: T-helper 1 type immune response	0.000947	111,11,4
1dy9.1#C229	GO:0042108: positive regulation of cytokine biosynthesis	0.000947	111,11,4
1e4ea2#215	GO:0006606: protein-nucleus import	0.000947	419,21,10
1qvba_#9	GO:0006096: glycolysis	0.000947	52,38,5
1el5a1#10	GO:0006730: one-carbon compound metabolism	0.000948	16,32,3
1ak5_1#376	GO:0009073: aromatic amino acid family biosynthesis	0.000948	32,16,3
2ebn__#77	GO:0009073: aromatic amino acid family biosynthesis	0.000948	32,16,3
1pwt__#8	GO:0007399: neurogenesis	0.000948	262,37,11
1h6va1#27	GO:0009167: purine ribonucleoside monophosphate metabolism	0.000948	224,10,5
1hnja1#167	GO:0006730: one-carbon compound metabolism	0.00095	62,32,5
1qlaa3#364	GO:0006953: acute-phase response	0.000951	58,20,4
1fo4a5#1020	GO:0006690: icosanoid metabolism	0.000952	312,11,6
1fw9a_#152	GO:0017001: antibiotic catabolism	0.000952	124,10,4
1eqka_#47	GO:0009252: peptidoglycan biosynthesis	0.000952	114,18,5
1f00i2#805	GO:0030217: T-cell differentiation	0.000952	124,10,4
1nsj__#124	GO:0009167: purine ribonucleoside monophosphate metabolism	0.000952	124,10,4
1qqga2#166	GO:0008285: negative regulation of cell proliferation	0.000953	164,19,6
1qdla_#211	GO:0006119: oxidative phosphorylation	0.000953	280,12,6
1c4zd_#51	GO:0006457: protein folding	0.000954	135,49,9
1qmva_#40	GO:0009070: serine family amino acid biosynthesis	0.000955	77,26,5
1tiid_#39	GO:0046677: response to antibiotic	0.000955	203,40,10
1ejfa_#10	GO:0016042: lipid catabolism	0.000956	134,31,7
1aky_2#133	GO:0019735: antimicrobial humoral response (sensu Vertebrata)	0.000958	130,16,5
2arca_#55	GO:0019735: antimicrobial humoral response (sensu Vertebrata)	0.000958	130,16,5
3sil__#98	GO:0016337: cell-cell adhesion	0.000958	186,17,6
1danh_#104	GO:0006606: protein-nucleus import	0.00096	147,21,6
1kit_3#331	GO:0051249: regulation of lymphocyte activation	0.00096	101,12,4
1c4zd_#85	GO:0006367: transcription initiation from Pol II promoter	0.00096	101,12,4
1nbaa_#227	GO:0006986: response to unfolded protein	0.00096	79,15,4
1ovb__#195	GO:0006826: iron ion transport	0.00096	79,15,4
1gpea1#273	GO:0006919: caspase activation	0.00096	79,15,4
1ejda_#180	GO:0009252: peptidoglycan biosynthesis	0.000962	65,18,4
1e32a2#345	GO:0009252: peptidoglycan biosynthesis	0.000962	65,18,4
1cnv__#181	GO:0006779: porphyrin biosynthesis	0.000962	65,18,4
1dlja2#177	GO:0009064: glutamine family amino acid metabolism	0.000965	27,19,3
1eg2a_#132	GO:0009307: DNA restriction	0.000965	27,19,3
1nat__#77	GO:0009064: glutamine family amino acid metabolism	0.000965	27,19,3
1elva1#604	GO:0008285: negative regulation of cell proliferation	0.000965	27,19,3
1pme__#302	GO:0000910: cytokinesis	0.000966	14,37,3
1e5xa_#379	GO:0009086: methionine biosynthesis	0.000966	37,14,3
1rypk_#41	GO:0046677: response to antibiotic	0.000966	13,40,3
2duba_#212	GO:0009219: pyrimidine deoxyribonucleotide metabolism	0.000967	53,10,3
1pdc__#27	GO:0030217: T-cell differentiation	0.000967	53,10,3

1pvc1_#106	GO:0051049: regulation of transport	0.000967	53,10,3
1db3a_#266	GO:0042401: biogenic amine biosynthesis	0.000967	53,10,3
1ax4a_#430	GO:0006559: L-phenylalanine catabolism	0.000967	53,10,3
1fo5a_#60	GO:0019059: initiation of viral infection	0.000968	225,10,5
1fo5a_#60	GO:0006275: regulation of DNA replication	0.000968	225,10,5
1opy__#68	GO:0016477: cell migration	0.000968	308,23,9
1kit_3#743	GO:0045595: regulation of cell differentiation	0.000968	225,10,5
1ayl__#237	GO:0042108: positive regulation of cytokine biosynthesis	0.000969	313,11,6
1ayl__#237	GO:0006690: icosanoid metabolism	0.000969	313,11,6
1hq8a_#206	GO:0019735: antimicrobial humoral response (sensu Vertebrata)	0.00097	281,16,7
1e79d3#328	GO:0006730: one-carbon compound metabolism	0.000971	36,32,4
1ejda_#6	GO:0006096: glycolysis	0.000972	215,38,10
1jh2a_#16	GO:0006917: induction of apoptosis	0.000973	69,29,5
2napa2#292	GO:0009064: glutamine family amino acid metabolism	0.000973	108,19,5
1erv__#80	GO:0006367: transcription initiation from Pol II promoter	0.000975	401,12,7
1erv__#80	GO:0000398: nuclear mRNA splicing, via spliceosome	0.000975	401,12,7
1fwxa2#169	GO:0006730: one-carbon compound metabolism	0.000978	94,32,6
1hwxa1#256	GO:0006098: pentose-phosphate shunt	0.000979	48,11,3
1hwxa1#256	GO:0009396: folic acid and derivative biosynthesis	0.000979	48,11,3
1mrj__#156	GO:0000165: MAPKKK cascade	0.000979	48,11,3
1kwaa_#513	GO:0001906: cell killing	0.000979	48,11,3
1qtn.1#A270	GO:0006099: tricarboxylic acid cycle	0.000979	386,19,9
1g8kb_#11	GO:0006801: superoxide metabolism	0.000979	48,11,3
1g8kb_#11	GO:0001906: cell killing	0.000979	48,11,3
1a3qa2#190	GO:0045595: regulation of cell differentiation	0.000982	125,10,4
3sil__#284	GO:0006118: electron transport	0.000982	62,228,14
1cpt__#323	GO:0007264: small GTPase mediated signal transduction	0.000984	6,21,2
1qqka_#19	GO:0007264: small GTPase mediated signal transduction	0.000984	6,21,2
1egua1#309	GO:0006606: protein-nucleus import	0.000984	6,21,2
1e44b_#75	GO:0008285: negative regulation of cell proliferation	0.000985	165,19,6
1ryph_#163	GO:0046677: response to antibiotic	0.000985	103,40,7
2duba_#212	GO:0006221: pyrimidine nucleotide biosynthesis	0.000986	53,22,4
1aqua_#126	GO:0006730: one-carbon compound metabolism	0.000987	472,32,14
1nsca_#236	GO:0006953: acute-phase response	0.000989	156,20,6
1f5aa1#441	GO:0006118: electron transport	0.00099	129,228,23
1jsg__#92	GO:0008015: circulation	0.000991	519,15,9
1mdah_#329	GO:0019079: viral genome replication	0.000991	282,16,7
1h7wa2#636	GO:0042364: water-soluble vitamin biosynthesis	0.000992	43,27,4
1el0a_#42	GO:0048514: blood vessel morphogenesis	0.000993	258,9,5
1fjfj_#68	GO:0006470: protein amino acid dephosphorylation	0.000994	218,20,7
1qora2#201	GO:0006418: tRNA aminoacylation for protein translation	0.000995	163,26,7
1enfa2#162	GO:0006956: complement activation	0.000996	44,12,3
1h75a_#53	GO:0009615: response to virus	0.000996	44,12,3
1cnv__#264	GO:0009086: methionine biosynthesis	0.000997	86,14,4
1qs0a1#235	GO:0006100: tricarboxylic acid cycle intermediate metabolism	0.000997	86,14,4
1qs1a2#437	GO:0008285: negative regulation of cell proliferation	0.000998	387,19,9
1ek4a2#382	GO:0009252: peptidoglycan biosynthesis	0.001003	7,18,2
7reqb1#163	GO:0009252: peptidoglycan biosynthesis	0.001003	7,18,2
1ge8a2#134	GO:0001906: cell killing	0.001003	202,11,5
1fhoa_#74	GO:0016485: protein processing	0.001003	315,11,6
1feca3#467	GO:0046677: response to antibiotic	0.001006	75,40,6
2dnja_#158	GO:0015074: DNA integration	0.001007	26,20,3
1dpsa_#36	GO:0008284: positive regulation of cell proliferation	0.001007	183,30,8
2sli_2#326	GO:0050819: negative regulation of coagulation	0.001009	227,10,5
2sli_2#326	GO:0030193: regulation of blood coagulation	0.001009	227,10,5
2sli_2#326	GO:0018108: peptidyl-tyrosine phosphorylation	0.001009	227,10,5

2sli_2#326	GO:0000086: G2/M transition of mitotic cell cycle	0.001009	227,10,5
1apme_#68	GO:0030245: cellulose catabolism	0.00101	93,22,5
1bwda_#117	GO:0006470: protein amino acid dephosphorylation	0.00101	103,20,5
1bywa_#30	GO:0006119: oxidative phosphorylation	0.00101	283,12,6
1dgw.1#Y353	GO:0006457: protein folding	0.001012	294,49,14
1imva_#255	GO:0046677: response to antibiotic	0.001012	506,40,17
1et0a_#118	GO:0006917: induction of apoptosis	0.001013	18,29,3
1bu7a_#278	GO:0006979: response to oxidative stress	0.001013	29,18,3
1i7qa_#278	GO:0019835: cytolysis	0.001013	29,18,3
1bywa_#30	GO:0019735: antimicrobial humoral response (sensu Vertebrata)	0.001013	283,16,7
1epua_#26	GO:0009252: peptidoglycan biosynthesis	0.001013	29,18,3
1a6o__#297	GO:0007601: visual perception	0.001014	35,15,3
1jfra_#83	GO:0006826: iron ion transport	0.001014	35,15,3
1c9la2#12	GO:0030333: antigen processing	0.001014	257,13,6
1phm_2#253	GO:0006643: membrane lipid metabolism	0.001014	257,13,6
1fc6a2#454	GO:0006919: caspase activation	0.001014	35,15,3
1elja_#8	GO:0006826: iron ion transport	0.001014	35,15,3
1hxma2#150	GO:0045859: regulation of protein kinase activity	0.001014	35,15,3
1cbf__#208	GO:0006917: induction of apoptosis	0.001016	291,29,10
1gpc__#58	GO:0006418: tRNA aminoacylation for protein translation	0.001017	5,26,2
1ed1a_#94	GO:0006418: tRNA aminoacylation for protein translation	0.001017	5,26,2
1fmk_3#283	GO:0007169: transmembrane receptor protein tyrosine kinase signaling pathway	0.001017	5,26,2
1beba_#48	GO:0006418: tRNA aminoacylation for protein translation	0.001017	5,26,2
1dxy_1#170	GO:0009070: serine family amino acid biosynthesis	0.001017	5,26,2
1awx__#25	GO:0007169: transmembrane receptor protein tyrosine kinase signaling pathway	0.001017	5,26,2
1cii_1#611	GO:0042742: defense response to bacteria	0.001017	5,26,2
1erv__#80	GO:0000910: cytokinesis	0.001017	401,37,14
1ejda_#213	GO:0006457: protein folding	0.001017	227,49,12
3grs_1#24	GO:0006779: porphyrin biosynthesis	0.001019	66,18,4
1dn2a1#261	GO:0043123: positive regulation of I-kappaB kinase/NF-kappaB cascade	0.001019	376,16,8
1gsa_2#218	GO:0006418: tRNA aminoacylation for protein translation	0.00102	118,26,6
1gsa_2#218	GO:0007169: transmembrane receptor protein tyrosine kinase signaling pathway	0.00102	118,26,6
1f97a2#199	GO:0007169: transmembrane receptor protein tyrosine kinase signaling pathway	0.00102	118,26,6
1qfma1#186	GO:0016068: type I hypersensitivity	0.00102	306,15,7
1tmy__#34	GO:0006100: tricarboxylic acid cycle intermediate metabolism	0.001021	9,14,2
1ezia_#198	GO:0009086: methionine biosynthesis	0.001021	9,14,2
1ygs__#435	GO:0042113: B-cell activation	0.001021	14,9,2
1a0i_1#277	GO:0015698: inorganic anion transport	0.001021	9,14,2
1aop_4#433	GO:0006100: tricarboxylic acid cycle intermediate metabolism	0.001021	9,14,2
4tmka_#141	GO:0009086: methionine biosynthesis	0.001021	9,14,2
1cvl__#91	GO:0006100: tricarboxylic acid cycle intermediate metabolism	0.001021	9,14,2
1g8fa2#258	GO:0009086: methionine biosynthesis	0.001021	9,14,2
1gai__#22	GO:0016567: protein ubiquitination	0.001021	9,14,2
1elua_#95	GO:0006568: tryptophan metabolism	0.001021	54,10,3
1fnf_1#1146	GO:0042113: B-cell activation	0.001021	14,9,2
1tiid_#39	GO:0006801: superoxide metabolism	0.001026	203,11,5
1a6ca2#312	GO:0019735: antimicrobial humoral response (sensu Vertebrata)	0.001027	75,16,4
1ecpa_#156	GO:0006118: electron transport	0.001029	10,228,5
1daba_#310	GO:0006118: electron transport	0.001029	10,228,5
3prn__#143	GO:0006118: electron transport	0.00103	300,228,43
3sil__#317	GO:0006118: electron transport	0.00103	300,228,43
1c9la2#12	GO:0006096: glycolysis	0.001031	257,38,11
2cuaa_#107	GO:0006916: anti-apoptosis	0.001031	116,18,5

1qqga2#166	GO:0007169: transmembrane receptor protein tyrosine kinase signaling pathway	0.001032	164,26,7
1c4zd_#51	GO:0016477: cell migration	0.001034	135,23,6
1ejda_#6	GO:0007169: transmembrane receptor protein tyrosine kinase signaling pathway	0.001037	215,26,8
1i50a_#430	GO:0043123: positive regulation of I-kappaB kinase/NF-kappaB cascade	0.001038	33,16,3
1i6vd_#1188	GO:0007264: small GTPase mediated signal transduction	0.001038	25,21,3
1fm2.1#B627	GO:0019079: viral genome replication	0.001038	33,16,3
1dmla2#313	GO:0016337: cell-cell adhesion	0.001038	31,17,3
1ra9__#62	GO:0000160: two-component signal transduction system (phosphorelay)	0.001038	21,25,3
1dqga_#81	GO:0007264: small GTPase mediated signal transduction	0.001038	25,21,3
1ksia1#555	GO:0006935: chemotaxis	0.001039	13,41,3
1aym3_#109	GO:0030333: antigen processing	0.001039	41,13,3
1i9za_#666	GO:0001906: cell killing	0.00104	49,11,3
1im4a_#41	GO:0016485: protein processing	0.00104	49,11,3
1cmia_#86	GO:0042108: positive regulation of cytokine biosynthesis	0.00104	49,11,3
1qlaa2#179	GO:0006801: superoxide metabolism	0.00104	49,11,3
3grx__#56	GO:0016068: type I hypersensitivity	0.00104	307,15,7
1j9la_#164	GO:0009396: folic acid and derivative biosynthesis	0.00104	49,11,3
1j9la_#164	GO:0042559: pteridine and derivative biosynthesis	0.00104	49,11,3
1ct9a1#233	GO:0006544: glycine metabolism	0.00104	49,11,3
1ekra_#114	GO:0042559: pteridine and derivative biosynthesis	0.00104	49,11,3
1dnv__#354	GO:0042088: T-helper 1 type immune response	0.00104	49,11,3
1dnv__#354	GO:0042108: positive regulation of cytokine biosynthesis	0.00104	49,11,3
1ton__#181	GO:0006544: glycine metabolism	0.00104	49,11,3
1mjha_#119	GO:0009086: methionine biosynthesis	0.001041	87,14,4
1dih_2#222	GO:0006730: one-carbon compound metabolism	0.001041	311,32,11
1fn9a_#271	GO:0008284: positive regulation of cell proliferation	0.001044	184,30,8
1fuma2#370	GO:0006096: glycolysis	0.001045	14,38,3
1ejda_#379	GO:0006100: tricarboxylic acid cycle intermediate metabolism	0.001045	38,14,3
1fm2.1#B319	GO:0006096: glycolysis	0.001045	14,38,3
1bura1#269	GO:0006096: glycolysis	0.001045	14,38,3
1amf__#196	GO:0006100: tricarboxylic acid cycle intermediate metabolism	0.001047	154,14,5
1svb_1#358	GO:0019079: viral genome replication	0.001048	8,16,2
1e87a_#155	GO:0019735: antimicrobial humoral response (sensu Vertebrata)	0.001048	8,16,2
1nfdb1#115	GO:0019735: antimicrobial humoral response (sensu Vertebrata)	0.001048	8,16,2
1i7qa_#470	GO:0009073: aromatic amino acid family biosynthesis	0.001048	8,16,2
2cpl__#83	GO:0019079: viral genome replication	0.001048	8,16,2
1urna_#93	GO:0019079: viral genome replication	0.001048	8,16,2
1hq8a_#185	GO:0019735: antimicrobial humoral response (sensu Vertebrata)	0.001048	8,16,2
1efpa1#32	GO:0009396: folic acid and derivative biosynthesis	0.001048	114,11,4
1fp1d2#214	GO:0006096: glycolysis	0.00105	143,38,8
1czya1#466	GO:0006953: acute-phase response	0.001055	104,20,5
1a81a2#182	GO:0015074: DNA integration	0.001055	104,20,5
1eny__#147	GO:0006986: response to unfolded protein	0.001056	81,15,4
1fkna_#63	GO:0006096: glycolysis	0.001057	31,38,4
1qp8a1#153	GO:0006221: pyrimidine nucleotide biosynthesis	0.001057	24,22,3
1ehya_#85	GO:0006096: glycolysis	0.001057	31,38,4
1pud__#197	GO:0006096: glycolysis	0.001057	31,38,4
1ddma_#130	GO:0019079: viral genome replication	0.001057	285,16,7
1ep3a_#247	GO:0006221: pyrimidine nucleotide biosynthesis	0.001057	24,22,3
1fuia2#273	GO:0009064: glutamine family amino acid metabolism	0.001058	110,19,5
1cs1a_#82	GO:0015074: DNA integration	0.001058	158,20,6
1qs0a1#119	GO:0006470: protein amino acid dephosphorylation	0.00106	367,20,9
1fuia2#273	GO:0006096: glycolysis	0.001061	110,38,7
1opy__#68	GO:0006919: caspase activation	0.001061	308,15,7

1b4ka_#108	GO:0006221: pyrimidine nucleotide biosynthesis	0.001061	94,22,5
1nsj__#124	GO:0009116: nucleoside metabolism	0.001062	124,25,6
1hxxa_#39	GO:0051249: regulation of lymphocyte activation	0.001065	45,12,3
1fo4a5#1020	GO:0016477: cell migration	0.001065	312,23,9
1p35a_#155	GO:0051249: regulation of lymphocyte activation	0.001065	45,12,3
5ruba1#259	GO:0009156: ribonucleoside monophosphate biosynthesis	0.001065	45,12,3
1e8ga2#201	GO:0009615: response to virus	0.001069	286,12,6
1e8ga2#201	GO:0006119: oxidative phosphorylation	0.001069	286,12,6
1i76a_#138	GO:0006096: glycolysis	0.001071	80,38,6
1czya1#466	GO:0051249: regulation of lymphocyte activation	0.001073	104,12,4
1hbza_#214	GO:0006916: anti-apoptosis	0.001073	249,18,7
1fmta2#151	GO:0016042: lipid catabolism	0.001073	272,31,10
1a81a2#182	GO:0009156: ribonucleoside monophosphate biosynthesis	0.001073	104,12,4
1j79a_#187	GO:0009070: serine family amino acid biosynthesis	0.001074	79,26,5
1htp__#16	GO:0006118: electron transport	0.001075	256,228,38
1foha5#77	GO:0009307: DNA restriction	0.001076	28,19,3
1t7pa2#663	GO:0006897: endocytosis	0.001076	28,19,3
1t7pa2#663	GO:0009064: glutamine family amino acid metabolism	0.001076	28,19,3
1j79a_#38	GO:0009064: glutamine family amino acid metabolism	0.001076	28,19,3
1cmxa_#207	GO:0006461: protein complex assembly	0.001077	86,24,5
1ib2a_#1101	GO:0001558: regulation of cell growth	0.001078	55,10,3
1jj2a1#126	GO:0019883: antigen presentation, endogenous antigen	0.001078	55,10,3
1qo2a_#222	GO:0006207: 'de novo' pyrimidine base biosynthesis	0.001078	55,10,3
1a9xb2#1775	GO:0006568: tryptophan metabolism	0.001078	55,10,3
1vin_2#335	GO:0000910: cytokinesis	0.001079	32,37,4
1dkia_#185	GO:0019835: cytolysis	0.001079	67,18,4
1fnf_3#1409	GO:0019735: antimicrobial humoral response (sensu Vertebrata)	0.00108	76,16,4
1hlwa_#82	GO:0006953: acute-phase response	0.00108	221,20,7
1edg__#81	GO:0030245: cellulose catabolism	0.001081	6,22,2
1iow_1#41	GO:0006221: pyrimidine nucleotide biosynthesis	0.001081	6,22,2
1d09b1#42	GO:0006221: pyrimidine nucleotide biosynthesis	0.001081	6,22,2
1atia2#249	GO:0006221: pyrimidine nucleotide biosynthesis	0.001081	6,22,2
1qqqa_#38	GO:0006221: pyrimidine nucleotide biosynthesis	0.001081	6,22,2
1eeoa_#288	GO:0030245: cellulose catabolism	0.001081	6,22,2
1io1a_#311	GO:0006461: protein complex assembly	0.001081	130,24,6
2mpra_#190	GO:0006917: induction of apoptosis	0.001081	349,29,11
1qqqa_#130	GO:0006221: pyrimidine nucleotide biosynthesis	0.001081	6,22,2
1lci__#26	GO:0007186: G-protein coupled receptor protein signaling pathway	0.001081	130,24,6
1ulo__#144	GO:0030245: cellulose catabolism	0.001081	6,22,2
1whi__#59	GO:0007399: neurogenesis	0.001083	55,37,5
7reqa2#702	GO:0042364: water-soluble vitamin biosynthesis	0.001083	44,27,4
1bev1_#86	GO:0006457: protein folding	0.001083	296,49,14
1e25a_#262	GO:0006511: ubiquitin-dependent protein catabolism	0.001083	115,11,4
1fc3a_#172	GO:0009307: DNA restriction	0.001084	168,19,6
1b65a_#257	GO:0009307: DNA restriction	0.001084	168,19,6
1qfea_#68	GO:0000910: cytokinesis	0.001086	453,37,15
5ruba1#365	GO:0009086: methionine biosynthesis	0.001087	88,14,4
1b6ra3#161	GO:0016042: lipid catabolism	0.001092	137,31,7
1hdmb1#173	GO:0030217: T-cell differentiation	0.001093	13,10,2
1hdma1#124	GO:0030217: T-cell differentiation	0.001093	13,10,2
1ga0a_#304	GO:0030333: antigen processing	0.001093	10,13,2
1auib_#136	GO:0001501: skeletal development	0.001093	13,10,2
1pbv__#140	GO:0009219: pyrimidine deoxyribonucleotide metabolism	0.001093	13,10,2
1csn__#134	GO:0000086: G2/M transition of mitotic cell cycle	0.001093	13,10,2
1csn__#134	GO:0006275: regulation of DNA replication	0.001093	13,10,2
1qr4a2#166	GO:0051049: regulation of transport	0.001093	13,10,2

1qr4a2#166	GO:0019883: antigen presentation, endogenous antigen	0.001093	13,10,2
1doka_#52	GO:0016477: cell migration	0.001093	52,23,4
1b6cb_#337	GO:0000086: G2/M transition of mitotic cell cycle	0.001093	13,10,2
1dxea_#88	GO:0006207: 'de novo' pyrimidine base biosynthesis	0.001093	13,10,2
1eu3a1#68	GO:0006275: regulation of DNA replication	0.001093	13,10,2
1dpja_#320	GO:0050819: negative regulation of coagulation	0.001093	13,10,2
1dpja_#320	GO:0030193: regulation of blood coagulation	0.001093	13,10,2
1dpja_#320	GO:0018108: peptidyl-tyrosine phosphorylation	0.001093	13,10,2
1iknd_#221	GO:0045786: negative regulation of cell cycle	0.001093	10,13,2
1iknd_#221	GO:0000082: G1/S transition of mitotic cell cycle	0.001093	10,13,2
1qfma1#238	GO:0000082: G1/S transition of mitotic cell cycle	0.001093	10,13,2
1ihka_#164	GO:0017148: negative regulation of protein biosynthesis	0.001093	10,13,2
1ldna1#94	GO:0006470: protein amino acid dephosphorylation	0.001094	159,20,6
1ldna1#94	GO:0006633: fatty acid biosynthesis	0.001095	159,27,7
1eqga2#47	GO:0006633: fatty acid biosynthesis	0.001097	5,27,2
1mrp__#108	GO:0006633: fatty acid biosynthesis	0.001097	5,27,2
1toaa_#273	GO:0042364: water-soluble vitamin biosynthesis	0.001097	5,27,2
1h5qa_#153	GO:0006633: fatty acid biosynthesis	0.001097	5,27,2
2oata_#81	GO:0042364: water-soluble vitamin biosynthesis	0.001097	5,27,2
1f2la_#40	GO:0042088: T-helper 1 type immune response	0.001098	206,11,5
1c9la2#61	GO:0006801: superoxide metabolism	0.001098	206,11,5
1c9la2#61	GO:0001906: cell killing	0.001098	206,11,5
1fjfc2#153	GO:0006278: RNA-dependent DNA replication	0.001099	362,10,6
1dik_1#765	GO:0006470: protein amino acid dephosphorylation	0.001102	105,20,5
1hwxa1#223	GO:0009103: lipopolysaccharide biosynthesis	0.001102	186,12,5
1bwvs_#117	GO:0019079: viral genome replication	0.001103	287,16,7
1qksa2#236	GO:0001906: cell killing	0.001104	50,11,3
1d2ka1#164	GO:0006098: pentose-phosphate shunt	0.001104	50,11,3
1as4.1#A336	GO:0006606: protein-nucleus import	0.001105	276,21,8
1d0na3#305	GO:0006606: protein-nucleus import	0.001105	276,21,8
1g8fa3#419	GO:0042401: biogenic amine biosynthesis	0.001107	129,10,4
1g8fa3#419	GO:0006568: tryptophan metabolism	0.001107	129,10,4
1ycsb1#412	GO:0006268: DNA unwinding	0.001107	129,10,4
1d3bb_#31	GO:0006418: tRNA aminoacylation for protein translation	0.001108	46,26,4
1am2__#163	GO:0017148: negative regulation of protein biosynthesis	0.00111	96,13,4
1gsoa3#172	GO:0006457: protein folding	0.001111	3,49,2
1tuba1#239	GO:0006221: pyrimidine nucleotide biosynthesis	0.001113	95,22,5
1e7wa_#8	GO:0000160: two-component signal transduction system (phosphorelay)	0.001115	83,25,5
1ejda_#213	GO:0007067: mitosis	0.001115	227,25,8
3sil__#84	GO:0006643: membrane lipid metabolism	0.001116	42,13,3
1e3ua_#164	GO:0030333: antigen processing	0.001116	42,13,3
1fbl_1#398	GO:0006643: membrane lipid metabolism	0.001116	42,13,3
1f7da_#27	GO:0006313: DNA transposition	0.001118	4,35,2
1ec7a1#230	GO:0009116: nucleoside metabolism	0.001118	48,25,4
1i19a2#70	GO:0009064: glutamine family amino acid metabolism	0.001119	7,19,2
1sw6a_#496	GO:0008285: negative regulation of cell proliferation	0.001119	7,19,2
1dqua_#456	GO:0006099: tricarboxylic acid cycle	0.001119	7,19,2
1ilr1_#145	GO:0008285: negative regulation of cell proliferation	0.001119	7,19,2
1dqga_#51	GO:0008285: negative regulation of cell proliferation	0.001119	7,19,2
1dik_3#29	GO:0006099: tricarboxylic acid cycle	0.001119	7,19,2
1qnja_#124	GO:0008285: negative regulation of cell proliferation	0.001119	7,19,2
1acc__#206	GO:0007517: muscle development	0.001121	18,30,3
1fs7a_#235	GO:0007517: muscle development	0.001121	18,30,3
1jr3a2#166	GO:0006779: porphyrin biosynthesis	0.001121	30,18,3
1bqca_#24	GO:0006779: porphyrin biosynthesis	0.001121	30,18,3
1hc7a2#258	GO:0008284: positive regulation of cell proliferation	0.001122	186,30,8

1eq9a_#40	GO:0008285: negative regulation of cell proliferation	0.001126	64,19,4
3rpba_#550	GO:0006953: acute-phase response	0.001127	27,20,3
1gcoa_#119	GO:0006633: fatty acid biosynthesis	0.001127	20,27,3
1svb_2#165	GO:0006953: acute-phase response	0.001127	27,20,3
1d6ja_#95	GO:0006096: glycolysis	0.001128	54,38,5
1fcda2#189	GO:0009086: methionine biosynthesis	0.001129	39,14,3
1fgxa_#242	GO:0009103: lipopolysaccharide biosynthesis	0.00113	11,12,2
1apme_#101	GO:0000165: MAPKKK cascade	0.00113	12,11,2
2fcba2#104	GO:0042088: T-helper 1 type immune response	0.00113	12,11,2
2fcba2#104	GO:0042108: positive regulation of cytokine biosynthesis	0.00113	12,11,2
1fh6a_#341	GO:0006367: transcription initiation from Pol II promoter	0.00113	11,12,2
1evya2#38	GO:0006801: superoxide metabolism	0.00113	12,11,2
1psda1#233	GO:0006563: L-serine metabolism	0.00113	11,12,2
1dn0b2#124	GO:0042088: T-helper 1 type immune response	0.00113	12,11,2
1dn0b2#124	GO:0042108: positive regulation of cytokine biosynthesis	0.00113	12,11,2
1dn0b2#124	GO:0001906: cell killing	0.00113	12,11,2
1as4.1#A290	GO:0006879: iron ion homeostasis	0.00113	11,12,2
1dgwa_#97	GO:0006956: complement activation	0.00113	11,12,2
1a3k__#199	GO:0051249: regulation of lymphocyte activation	0.00113	11,12,2
1e9xa_#428	GO:0015992: proton transport	0.00113	11,12,2
2ts1__#59	GO:0009103: lipopolysaccharide biosynthesis	0.00113	11,12,2
1jlja_#104	GO:0042559: pteridine and derivative biosynthesis	0.00113	12,11,2
1ezvd1#97	GO:0006119: oxidative phosphorylation	0.00113	11,12,2
1ezvd1#101	GO:0006119: oxidative phosphorylation	0.00113	11,12,2
1fp3a_#350	GO:0006956: complement activation	0.00113	11,12,2
1rpxa_#216	GO:0006098: pentose-phosphate shunt	0.00113	12,11,2
1b6cb_#399	GO:0000165: MAPKKK cascade	0.00113	12,11,2
1chua2#21	GO:0009103: lipopolysaccharide biosynthesis	0.00113	11,12,2
1fgka_#668	GO:0000165: MAPKKK cascade	0.00113	12,11,2
1ezva1#147	GO:0006119: oxidative phosphorylation	0.00113	11,12,2
1aq0a_#121	GO:0006563: L-serine metabolism	0.00113	11,12,2
2ayh__#160	GO:0001906: cell killing	0.00113	12,11,2
3rpba_#644	GO:0006690: icosanoid metabolism	0.00113	12,11,2
1dceb_#134	GO:0015992: proton transport	0.00113	11,12,2
1dceb_#134	GO:0006119: oxidative phosphorylation	0.00113	11,12,2
1abrb1#25	GO:0000165: MAPKKK cascade	0.00113	12,11,2
1oaa__#175	GO:0042559: pteridine and derivative biosynthesis	0.00113	12,11,2
1fkna_#99	GO:0009156: ribonucleoside monophosphate biosynthesis	0.00113	11,12,2
1hbza_#96	GO:0001906: cell killing	0.00113	322,11,6
1h6ka1#134	GO:0046677: response to antibiotic	0.001133	30,40,4
1imva_#255	GO:0007399: neurogenesis	0.001134	506,37,16
1hdfa_#16	GO:0016337: cell-cell adhesion	0.001134	192,17,6
1h4ua1#486	GO:0019079: viral genome replication	0.001135	34,16,3
1sppb_#32	GO:0043123: positive regulation of I-kappaB kinase/NF-kappaB cascade	0.001135	34,16,3
1d3bb_#31	GO:0000398: nuclear mRNA splicing, via spliceosome	0.001136	46,12,3
2dpma_#189	GO:0009103: lipopolysaccharide biosynthesis	0.001136	46,12,3
1ih7a1#57	GO:0030217: T-cell differentiation	0.001137	56,10,3
1ih7a1#57	GO:0006207: 'de novo' pyrimidine base biosynthesis	0.001137	56,10,3
1ih7a1#57	GO:0009167: purine ribonucleoside monophosphate metabolism	0.001137	56,10,3
1bfg__#22	GO:0018108: peptidyl-tyrosine phosphorylation	0.001137	56,10,3
1c8za_#386	GO:0006606: protein-nucleus import	0.001137	211,21,7
1ac6a_#43	GO:0042113: B-cell activation	0.001138	147,9,4
1fo4a5#1002	GO:0006418: tRNA aminoacylation for protein translation	0.001138	80,26,5
1elja_#64	GO:0006418: tRNA aminoacylation for protein translation	0.001138	80,26,5
1pysa_#221	GO:0009070: serine family amino acid biosynthesis	0.001138	80,26,5
1rypk_#12	GO:0016477: cell migration	0.001138	250,23,8

1mai__#108	GO:0043123: positive regulation of I-kappaB kinase/NF-kappaB cascade	0.001139	135,16,5
1io1a_#311	GO:0006275: regulation of DNA replication	0.00114	130,10,4
1b8aa2#368	GO:0030217: T-cell differentiation	0.00114	130,10,4
1lci__#26	GO:0017001: antibiotic catabolism	0.00114	130,10,4
1lci__#26	GO:0009582: detection of abiotic stimulus	0.00114	130,10,4
1qp8a1#172	GO:0006730: one-carbon compound metabolism	0.001141	17,32,3
1qfta_#98	GO:0006457: protein folding	0.001142	198,49,11
1qf6a3#92	GO:0006633: fatty acid biosynthesis	0.001144	77,27,5
1stfi_#116	GO:0006457: protein folding	0.001148	42,49,5
1qo3c_#195	GO:0006118: electron transport	0.001148	25,228,8
3chbd_#85	GO:0006801: superoxide metabolism	0.00115	323,11,6
3chbd_#85	GO:0042108: positive regulation of cytokine biosynthesis	0.00115	323,11,6
1f46a_#59	GO:0006953: acute-phase response	0.001151	106,20,5
1a81a1#51	GO:0006935: chemotaxis	0.001151	75,41,6
1iow_1#37	GO:0042364: water-soluble vitamin biosynthesis	0.001152	263,27,9
1qlaa3#364	GO:0006606: protein-nucleus import	0.001155	58,21,4
1qf6a3#92	GO:0046677: response to antibiotic	0.001157	77,40,6
1a7ca_#202	GO:0008015: circulation	0.001158	83,15,4
1eucb2#188	GO:0006986: response to unfolded protein	0.001158	83,15,4
1g51a3#525	GO:0006461: protein complex assembly	0.00116	239,24,8
1ihua2#525	GO:0007264: small GTPase mediated signal transduction	0.00116	278,21,8
1b3qa2#623	GO:0006118: electron transport	0.001161	257,228,38
2viua_#252	GO:0008284: positive regulation of cell proliferation	0.001163	285,30,10
1bev1_#86	GO:0006917: induction of apoptosis	0.001163	296,29,10
1beba_#21	GO:0006418: tRNA aminoacylation for protein translation	0.001167	21,26,3
1beba_#21	GO:0007169: transmembrane receptor protein tyrosine kinase signaling pathway	0.001167	21,26,3
1seta2#306	GO:0006606: protein-nucleus import	0.001167	26,21,3
1poxa3#439	GO:0009070: serine family amino acid biosynthesis	0.001167	21,26,3
1ljra1#165	GO:0006606: protein-nucleus import	0.001167	26,21,3
1ja9a_#202	GO:0007264: small GTPase mediated signal transduction	0.001167	26,21,3
1qtn.1#A270	GO:0030435: sporulation	0.001167	386,23,10
1iba__#39	GO:0016477: cell migration	0.00117	316,23,9
1quqa_#75	GO:0042088: T-helper 1 type immune response	0.00117	51,11,3
1quqa_#75	GO:0042108: positive regulation of cytokine biosynthesis	0.00117	51,11,3
1f46a_#59	GO:0046677: response to antibiotic	0.00117	106,40,7
1bhga3#529	GO:0006098: pentose-phosphate shunt	0.00117	51,11,3
2sqca2#268	GO:0008203: cholesterol metabolism	0.00117	51,11,3
1smaa1#95	GO:0006919: caspase activation	0.001172	223,15,6
1h6la_#119	GO:0006643: membrane lipid metabolism	0.001172	264,13,6
1cpza_#6	GO:0006461: protein complex assembly	0.001172	132,24,6
1cb8a2#613	GO:0019059: initiation of viral infection	0.001173	131,10,4
1kit_3#331	GO:0006606: protein-nucleus import	0.001173	101,21,5
1pdc__#27	GO:0016477: cell migration	0.001175	53,23,4
1pvc1_#106	GO:0016477: cell migration	0.001175	53,23,4
1i7wa_#517	GO:0015671: oxygen transport	0.001175	53,23,4
1b6cb_#346	GO:0045859: regulation of protein kinase activity	0.001176	9,15,2
2pola3#335	GO:0048514: blood vessel morphogenesis	0.001176	15,9,2
1howa_#551	GO:0045859: regulation of protein kinase activity	0.001176	9,15,2
1g3qa_#214	GO:0006445: regulation of translation	0.001176	9,15,2
1dmua_#199	GO:0045859: regulation of protein kinase activity	0.001176	9,15,2
1fo0b_#82	GO:0048514: blood vessel morphogenesis	0.001176	15,9,2
3thia_#59	GO:0006826: iron ion transport	0.001176	9,15,2
1i1b__#10	GO:0048514: blood vessel morphogenesis	0.001176	15,9,2
1wba__#169	GO:0045859: regulation of protein kinase activity	0.001176	9,15,2
1bfg__#22	GO:0007399: neurogenesis	0.001177	56,37,5

1jkma_#317	GO:0000910: cytokinesis	0.001177	56,37,5
1cs1a_#82	GO:0009086: methionine biosynthesis	0.001178	158,14,5
1nat__#50	GO:0009086: methionine biosynthesis	0.001179	242,14,6
1ea5a_#495	GO:0007268: synaptic transmission	0.00118	5,28,2
1g25a_#22	GO:0019059: initiation of viral infection	0.001184	367,10,6
1qs0a1#119	GO:0006568: tryptophan metabolism	0.001184	367,10,6
1nfdb1#115	GO:0016337: cell-cell adhesion	0.001185	8,17,2
1cs6a2#164	GO:0016337: cell-cell adhesion	0.001185	8,17,2
1bura1#328	GO:0009073: aromatic amino acid family biosynthesis	0.001191	78,16,4
1f8v.1#A116	GO:0019079: viral genome replication	0.001191	78,16,4
1qs1a2#437	GO:0016477: cell migration	0.001191	387,23,10
1edza1#206	GO:0007264: small GTPase mediated signal transduction	0.001191	352,21,9
1epwa3#138	GO:0006457: protein folding	0.001192	371,49,16
1dmla2#297	GO:0006917: induction of apoptosis	0.001194	19,29,3
[truncated: 562,394 more chars]
