# Supplementary material for: A Comprehensive Analysis of the Structure-Function Relationship in Proteins Based on Local Structure Similarity
Source: PLoS One. 2009 Jul 15;4(7):e6266. doi: 10.1371/journal.pone.0006266 (PMC2705683; doi:10.1371/journal.pone.0006266)
Supplement: Table S2 — All induced rules for molecular function. For each Gene Ontology molecular function class in the THEN-part, the p-value is given together with the parameters for the hypergeometric distribution used to compute the p-values: N,n,k,x, where N = 2725 is the number of protein-GO class pairs in the data set, n is the number of proteins matched the IF-part of the rule, k is the number of proteins in the GO class and x is the number of proteins matched by the rule and in the GO class. (1.16 MB PDF) [file pone.0006266.s002.pdf]

Tab S2. All induced rules for molecular function.

For each Gene Ontology molecular function class in the THEN-part, the p-value is given together with the parameters for the hypergeometric distribution used to compute the p-values: N,n,k,x, where N=2725 is the number of protein-GO class pairs in the data set, n is the number of proteins matched the IF-part of the rule, k is the number of proteins in the GO class and x is the number of proteins matched by the rule and in the GO class.

17135 of 20235 rules were FDR significant at 0.05 using the best p-value from each rule
FDR significance threshold: 0.042
Only rules with p < 1e-9 are listed here.

IF 1eh9a3#252 AND 1bvza3#416
THEN
GO:0004556: alpha-amylase activity: 1.500e-32 (28,15,15) OR
GO:0005509: calcium ion binding: 1.833e-07 (28,160,11) OR
GO:0004867: serine-type endopeptidase inhibitor activity: 8.289e-02 (28,47,2)

IF 1bvza3#416 AND 7taa_2#295
THEN
GO:0004556: alpha-amylase activity: 1.500e-32 (28,15,15) OR
GO:0005509: calcium ion binding: 1.833e-07 (28,160,11) OR
GO:0004867: serine-type endopeptidase inhibitor activity: 8.289e-02 (28,47,2)

IF 1eh9a3#252 AND 1e0ta2#275
THEN
GO:0004556: alpha-amylase activity: 6.215e-32 (30,15,15) OR
GO:0005509: calcium ion binding: 4.207e-07 (30,160,11) OR
GO:0004867: serine-type endopeptidase inhibitor activity: 9.336e-02 (30,47,2) OR
GO:0016758: transferase activity, transferring hexosyl groups: 1.148e-01 (30,11,1) OR
GO:0016616: oxidoreductase activity, acting on the CH-OH group of donors, NAD or NADP as acceptor: 4.833e-01 (30,59,1)

IF 1e43a2#351 AND 1e0ta2#275
THEN
GO:0004556: alpha-amylase activity: 2.169e-29 (27,15,14) OR
GO:0005509: calcium ion binding: 1.172e-07 (27,160,11) OR
GO:0004867: serine-type endopeptidase inhibitor activity: 7.780e-02 (27,47,2)

IF 1e43a2#351 AND 1bvza3#416
THEN
GO:0004556: alpha-amylase activity: 2.169e-29 (27,15,14) OR
GO:0005509: calcium ion binding: 1.172e-07 (27,160,11) OR
GO:0004867: serine-type endopeptidase inhibitor activity: 7.780e-02 (27,47,2)

IF 1avaa2#204 AND 1e43a2#351
THEN
GO:0004556: alpha-amylase activity: 2.169e-29 (27,15,14) OR
GO:0005509: calcium ion binding: 1.172e-07 (27,160,11) OR
GO:0004867: serine-type endopeptidase inhibitor activity: 7.780e-02 (27,47,2)

IF 1qqsa_#124 AND 1bvza3#416
THEN
GO:0004556: alpha-amylase activity: 2.169e-29 (27,15,14) OR
GO:0005509: calcium ion binding: 1.307e-06 (27,160,10) OR
GO:0016854: racemase and epimerase activity: 1.217e-01 (27,13,1) OR
GO:0004867: serine-type endopeptidase inhibitor activity: 3.763e-01 (27,47,1) OR
GO:0000287: magnesium ion binding: 7.289e-01 (27,128,1)

IF 1ljra1#165 AND 1glqa2#53
THEN
GO:0004364: glutathione transferase activity: 5.164e-29 (13,11,11) OR
GO:0003714: transcription corepressor activity: 3.758e-02 (13,8,1) OR
GO:0016651: oxidoreductase activity, acting on NADH or NADPH: 6.040e-02 (13,13,1)

IF 1ljra1#165 AND 3grx__#55
THEN
GO:0004364: glutathione transferase activity: 5.164e-29 (13,11,11) OR
GO:0003714: transcription corepressor activity: 3.758e-02 (13,8,1) OR

GO:0016651: oxidoreductase activity, acting on NADH or NADPH: 6.040e-02 (13,13,1)

IF 1h8d.1#H167 AND 1e79d2#36 AND 1bio__#210
THEN
GO:0004263: chymotrypsin activity: 1.351e-28 (89,41,25) OR
GO:0004295: trypsin activity: 3.449e-26 (89,48,25) OR
GO:0003809: thrombin activity: 2.746e-13 (89,10,9) OR
GO:0004867: serine-type endopeptidase inhibitor activity: 1.129e-09 (89,47,13) OR
GO:0005509: calcium ion binding: 3.926e-05 (89,160,16) OR
GO:0005529: sugar binding: 7.286e-01 (89,39,1)

IF 1bvza3#416 AND 1qfea_#68 AND 1e0ta2#275
THEN
GO:0004556: alpha-amylase activity: 2.863e-28 (31,15,14) OR
GO:0005509: calcium ion binding: 5.593e-06 (31,160,10) OR
GO:0016831: carboxy-lyase activity: 3.194e-02 (31,25,2) OR
GO:0004867: serine-type endopeptidase inhibitor activity: 9.874e-02 (31,47,2) OR
GO:0016854: racemase and epimerase activity: 1.385e-01 (31,13,1) OR
GO:0000287: magnesium ion binding: 4.319e-01 (31,128,2)

IF 1fsu__#434 AND 7taa_2#295
THEN
GO:0004556: alpha-amylase activity: 1.674e-27 (23,15,13) OR
GO:0005509: calcium ion binding: 2.665e-06 (23,160,9) OR
GO:0004867: serine-type endopeptidase inhibitor activity: 3.309e-01 (23,47,1)

IF 1qqsa_#124 AND 1e43a2#351
THEN
GO:0004556: alpha-amylase activity: 3.649e-27 (24,15,13) OR
GO:0005509: calcium ion binding: 3.544e-07 (24,160,10) OR
GO:0004867: serine-type endopeptidase inhibitor activity: 3.425e-01 (24,47,1)

IF 1by5a_#238 AND 1bvza3#416
THEN
GO:0004556: alpha-amylase activity: 3.649e-27 (24,15,13) OR
GO:0005509: calcium ion binding: 4.051e-06 (24,160,9) OR
GO:0004867: serine-type endopeptidase inhibitor activity: 6.319e-02 (24,47,2)

IF 1avaa2#204 AND 1qfea_#68
THEN
GO:0004556: alpha-amylase activity: 7.596e-27 (25,15,13) OR
GO:0005509: calcium ion binding: 5.611e-07 (25,160,10) OR
GO:0004867: serine-type endopeptidase inhibitor activity: 6.794e-02 (25,47,2)

IF 7taa_2#295 AND 1evqa_#305
THEN
GO:0004556: alpha-amylase activity: 7.596e-27 (25,15,13) OR
GO:0005509: calcium ion binding: 5.611e-07 (25,160,10) OR
GO:0016758: transferase activity, transferring hexosyl groups: 9.658e-02 (25,11,1) OR
GO:0004867: serine-type endopeptidase inhibitor activity: 3.540e-01 (25,47,1)

IF 1gjwa2#141 AND 1evqa_#305
THEN
GO:0004556: alpha-amylase activity: 7.596e-27 (25,15,13) OR
GO:0005509: calcium ion binding: 5.611e-07 (25,160,10) OR
GO:0016758: transferase activity, transferring hexosyl groups: 9.658e-02 (25,11,1) OR
GO:0004867: serine-type endopeptidase inhibitor activity: 3.540e-01 (25,47,1)

IF 1hx0a2#294 AND 1qhoa4#75
THEN
GO:0004556: alpha-amylase activity: 1.518e-26 (26,15,13) OR
GO:0005509: calcium ion binding: 7.313e-08 (26,160,11) OR
GO:0004867: serine-type endopeptidase inhibitor activity: 7.282e-02 (26,47,2)

IF 1aq0a_#4 AND 1bvza3#416
THEN
GO:0004556: alpha-amylase activity: 1.518e-26 (26,15,13) OR
GO:0005509: calcium ion binding: 7.313e-08 (26,160,11) OR
GO:0004867: serine-type endopeptidase inhibitor activity: 7.282e-02 (26,47,2)

IF 1hx0a2#32 AND 1e43a2#351
THEN
GO:0004556: alpha-amylase activity: 1.518e-26 (26,15,13) OR
GO:0005509: calcium ion binding: 8.661e-07 (26,160,10) OR
GO:0004867: serine-type endopeptidase inhibitor activity: 7.282e-02 (26,47,2) OR
GO:0030145: manganese ion binding: 3.071e-01 (26,38,1)

IF 1gjwa2#141 AND 1aq0a_#4
THEN
GO:0004556: alpha-amylase activity: 2.926e-26 (27,15,13) OR
GO:0005509: calcium ion binding: 1.172e-07 (27,160,11) OR
GO:0004867: serine-type endopeptidase inhibitor activity: 7.780e-02 (27,47,2) OR
GO:0016758: transferase activity, transferring hexosyl groups: 1.039e-01 (27,11,1)

IF 1aq0a_#4 AND 1qfea_#49
THEN
GO:0004556: alpha-amylase activity: 9.886e-26 (29,15,13) OR
GO:0005509: calcium ion binding: 2.430e-08 (29,160,12) OR
GO:0008810: cellulase activity: 1.506e-02 (29,18,2) OR
GO:0004867: serine-type endopeptidase inhibitor activity: 8.808e-02 (29,47,2)

IF 1ton__#91 AND 1cgha_#130 AND 2hlca_#226
THEN
GO:0004263: chymotrypsin activity: 9.790e-26 (61,41,21) OR
GO:0004295: trypsin activity: 7.342e-24 (61,48,21) OR
GO:0004867: serine-type endopeptidase inhibitor activity: 6.264e-06 (61,47,8) OR
GO:0005509: calcium ion binding: 2.449e-03 (61,160,10) OR
GO:0003809: thrombin activity: 2.029e-01 (61,10,1)

IF 1hq8a_#150 AND 1e87a_#121
THEN
GO:0005529: sugar binding: 1.498e-25 (20,39,15) OR
GO:0008201: heparin binding: 1.627e-01 (20,24,1) OR
GO:0004263: chymotrypsin activity: 2.623e-01 (20,41,1) OR
GO:0004295: trypsin activity: 3.000e-01 (20,48,1) OR
GO:0005509: calcium ion binding: 3.301e-01 (20,160,2)

IF 1a7s__#148 AND 1g51a3#525 AND 1dlea_#53
THEN
GO:0004263: chymotrypsin activity: 1.748e-25 (86,41,23) OR
GO:0004295: trypsin activity: 5.716e-25 (86,48,24) OR
GO:0003809: thrombin activity: 1.989e-13 (86,10,9) OR
GO:0004867: serine-type endopeptidase inhibitor activity: 9.553e-09 (86,47,12) OR
GO:0005509: calcium ion binding: 5.836e-06 (86,160,17) OR
GO:0005529: sugar binding: 7.162e-01 (86,39,1)

IF 1iba__#39 AND 1eh9a3#252
THEN
GO:0004556: alpha-amylase activity: 3.879e-25 (21,15,12) OR
GO:0005509: calcium ion binding: 1.254e-05 (21,160,8) OR
GO:0004867: serine-type endopeptidase inhibitor activity: 3.070e-01 (21,47,1)

IF 1gjwa2#141 AND 2ebn__#77
THEN
GO:0004556: alpha-amylase activity: 3.879e-25 (21,15,12) OR
GO:0005509: calcium ion binding: 1.254e-05 (21,160,8) OR
GO:0004867: serine-type endopeptidase inhibitor activity: 3.070e-01 (21,47,1)

IF 1ebda2#182 AND 1h6va2#245
THEN
GO:0015036: disulfide oxidoreductase activity: 2.114e-25 (35,22,15) OR
GO:0016668: oxidoreductase activity, acting on sulfur group of donors, NAD or NADP as acceptor: 1.947e-18 (35,12,10) OR
GO:0050660: FAD binding: 3.575e-12 (35,10,7) OR
GO:0016651: oxidoreductase activity, acting on NADH or NADPH: 1.550e-01 (35,13,1) OR
GO:0016627: oxidoreductase activity, acting on the CH-CH group of donors: 1.978e-01 (35,17,1) OR
GO:0004601: peroxidase activity: 2.385e-01 (35,21,1)

IF 1xvaa_#68 AND 1qama_#37
THEN
GO:0008757: S-adenosylmethionine-dependent methyltransferase activity: 7.145e-25 (14,24,12) OR

GO:0000287: magnesium ion binding: 1.379e-01 (14,128,2)

IF 1eq9a_#162 AND 1arb__#55
THEN
GO:0004295: trypsin activity: 3.188e-25 (63,48,22) OR
GO:0004263: chymotrypsin activity: 3.579e-20 (63,41,18) OR
GO:0004867: serine-type endopeptidase inhibitor activity: 7.194e-07 (63,47,9) OR
GO:0003809: thrombin activity: 4.913e-05 (63,10,4) OR
GO:0005509: calcium ion binding: 3.135e-03 (63,160,10)

IF 1gjwa2#141 AND 1gcya2#331
THEN
GO:0004556: alpha-amylase activity: 8.525e-25 (22,15,12) OR
GO:0005509: calcium ion binding: 1.874e-05 (22,160,8) OR
GO:0004867: serine-type endopeptidase inhibitor activity: 5.404e-02 (22,47,2)

IF 1gg6.1#C156 AND 1gdna_#121 AND 1cgha_#130
THEN
GO:0004263: chymotrypsin activity: 8.272e-25 (56,41,20) OR
GO:0004295: trypsin activity: 4.701e-23 (56,48,20) OR
GO:0004867: serine-type endopeptidase inhibitor activity: 3.512e-05 (56,47,7) OR
GO:0005509: calcium ion binding: 1.505e-02 (56,160,8) OR
GO:0008201: heparin binding: 3.938e-01 (56,24,1)

IF 1autc_#188 AND 1ton__#179 AND 1dlea_#238
THEN
GO:0004263: chymotrypsin activity: 9.990e-25 (67,41,21) OR
GO:0004295: trypsin activity: 7.380e-23 (67,48,21) OR
GO:0004867: serine-type endopeptidase inhibitor activity: 1.026e-07 (67,47,10) OR
GO:0003809: thrombin activity: 6.277e-05 (67,10,4) OR
GO:0005509: calcium ion binding: 1.475e-03 (67,160,11)

IF 1avaa2#204 AND 3prn__#143
THEN
GO:0004556: alpha-amylase activity: 1.781e-24 (23,15,12) OR
GO:0005509: calcium ion binding: 2.665e-06 (23,160,9) OR
GO:0004867: serine-type endopeptidase inhibitor activity: 5.855e-02 (23,47,2)

IF 1gjwa2#141 AND 1a4ya_#104
THEN
GO:0004556: alpha-amylase activity: 1.781e-24 (23,15,12) OR
GO:0005509: calcium ion binding: 2.177e-07 (23,160,10) OR
GO:0004867: serine-type endopeptidase inhibitor activity: 3.309e-01 (23,47,1)

IF 1danh_#104 AND 1a0la_#160 AND 1dlea_#238
THEN
GO:0004263: chymotrypsin activity: 1.443e-24 (80,41,22) OR
GO:0004295: trypsin activity: 3.485e-24 (80,48,23) OR
GO:0003809: thrombin activity: 1.666e-11 (80,10,8) OR
GO:0004867: serine-type endopeptidase inhibitor activity: 5.157e-08 (80,47,11) OR
GO:0005509: calcium ion binding: 4.150e-05 (80,160,15) OR
GO:0005529: sugar binding: 6.898e-01 (80,39,1)

IF 1by5a_#238 AND 1qfea_#49 AND 1e6pa2#46
THEN
GO:0004556: alpha-amylase activity: 1.781e-24 (23,15,12) OR
GO:0005509: calcium ion binding: 2.731e-05 (23,160,8) OR
GO:0016836: hydro-lyase activity: 2.453e-01 (23,33,1) OR
GO:0004867: serine-type endopeptidase inhibitor activity: 3.309e-01 (23,47,1) OR
GO:0000287: magnesium ion binding: 6.708e-01 (23,128,1)

IF 1dik_1#848 AND 1qhoa4#399
THEN
GO:0004556: alpha-amylase activity: 3.558e-24 (24,15,12) OR
GO:0005509: calcium ion binding: 4.051e-06 (24,160,9) OR
GO:0004867: serine-type endopeptidase inhibitor activity: 6.319e-02 (24,47,2) OR
GO:0016836: hydro-lyase activity: 2.545e-01 (24,33,1)

IF 1gjwa2#141 AND 1ho1a_#211
THEN

GO:0004556: alpha-amylase activity: 3.558e-24 (24,15,12) OR
GO:0005509: calcium ion binding: 4.051e-06 (24,160,9) OR
GO:0004867: serine-type endopeptidase inhibitor activity: 6.319e-02 (24,47,2) OR
GO:0016758: transferase activity, transferring hexosyl groups: 9.289e-02 (24,11,1)

IF 1avaa2#204 AND 1ho1a_#211
THEN
GO:0004556: alpha-amylase activity: 3.558e-24 (24,15,12) OR
GO:0005509: calcium ion binding: 4.051e-06 (24,160,9) OR
GO:0004867: serine-type endopeptidase inhibitor activity: 6.319e-02 (24,47,2) OR
GO:0016758: transferase activity, transferring hexosyl groups: 9.289e-02 (24,11,1)

IF 1qhoa4#399 AND 1nsj__#178
THEN
GO:0004556: alpha-amylase activity: 3.558e-24 (24,15,12) OR
GO:0005509: calcium ion binding: 4.051e-06 (24,160,9) OR
GO:0004867: serine-type endopeptidase inhibitor activity: 6.319e-02 (24,47,2) OR
GO:0016836: hydro-lyase activity: 2.545e-01 (24,33,1)

IF 1h8d.1#H167 AND 1elva1#601
THEN
GO:0004263: chymotrypsin activity: 3.609e-24 (83,41,22) OR
GO:0004295: trypsin activity: 3.464e-22 (83,48,22) OR
GO:0003809: thrombin activity: 1.423e-13 (83,10,9) OR
GO:0004867: serine-type endopeptidase inhibitor activity: 4.562e-10 (83,47,13) OR
GO:0005509: calcium ion binding: 1.569e-05 (83,160,16) OR
GO:0005529: sugar binding: 7.033e-01 (83,39,1)

IF 1qhoa4#399 AND 1e0ta2#275
THEN
GO:0004556: alpha-amylase activity: 6.835e-24 (25,15,12) OR
GO:0005509: calcium ion binding: 6.015e-06 (25,160,9) OR
GO:0004867: serine-type endopeptidase inhibitor activity: 6.794e-02 (25,47,2) OR
GO:0016814: hydrolase activity, acting on carbon-nitrogen (but not peptide) bonds, in cyclic amidines: 8.819e-02 (25,10,1) OR
GO:0016836: hydro-lyase activity: 2.636e-01 (25,33,1)

IF 1aym3_#131 AND 1eh9a3#252
THEN
GO:0004556: alpha-amylase activity: 2.849e-23 (18,15,11) OR
GO:0005509: calcium ion binding: 3.881e-05 (18,160,7)

IF 1tn3__#113 AND 1e87a_#121
THEN
GO:0005529: sugar binding: 1.216e-23 (19,39,14) OR
GO:0008201: heparin binding: 1.552e-01 (19,24,1) OR
GO:0004263: chymotrypsin activity: 2.510e-01 (19,41,1) OR
GO:0004295: trypsin activity: 2.874e-01 (19,48,1) OR
GO:0005509: calcium ion binding: 3.079e-01 (19,160,2)

IF 1c7na_#281 AND 1cs1a_#82
THEN
GO:0008483: transaminase activity: 2.404e-23 (23,17,12) OR
GO:0016846: carbon-sulfur lyase activity: 1.319e-13 (23,10,7) OR
GO:0016831: carboxy-lyase activity: 1.817e-02 (23,25,2) OR
GO:0016866: intramolecular transferase activity: 9.690e-02 (23,12,1) OR
GO:0030145: manganese ion binding: 2.770e-01 (23,38,1)

IF 1danh_#24 AND 1bqya_#216
THEN
GO:0004263: chymotrypsin activity: 2.864e-23 (77,41,21) OR
GO:0004295: trypsin activity: 2.063e-21 (77,48,21) OR
GO:0003809: thrombin activity: 1.227e-09 (77,10,7) OR
GO:0004867: serine-type endopeptidase inhibitor activity: 2.563e-09 (77,47,12) OR
GO:0005509: calcium ion binding: 2.577e-05 (77,160,15) OR
GO:0004896: hematopoietin/interferon-class (D200-domain) cytokine receptor activity: 4.210e-01 (77,19,1)

IF 1trb_1#15 AND 1nhp_1#282
THEN
GO:0015036: disulfide oxidoreductase activity: 5.069e-23 (35,22,14) OR
GO:0016668: oxidoreductase activity, acting on sulfur group of donors, NAD or NADP as acceptor: 1.947e-18 (35,12,10) OR

GO:0050660: FAD binding: 3.575e-12 (35,10,7) OR
GO:0016627: oxidoreductase activity, acting on the CH-CH group of donors: 1.167e-03 (35,17,3) OR
GO:0016651: oxidoreductase activity, acting on NADH or NADPH: 1.550e-01 (35,13,1)

IF 1qama_#39 AND 1qama_#37
THEN
GO:0008757: S-adenosylmethionine-dependent methyltransferase activity: 1.278e-22 (13,24,11) OR
GO:0016620: oxidoreductase activity, acting on the aldehyde or oxo group of donors, NAD or NADP as acceptor: 4.677e-02 (13,10,1) OR
GO:0000287: magnesium ion binding: 4.657e-01 (13,128,1)

IF 1f3ba2#7 AND 1glqa2#53
THEN
GO:0004364: glutathione transferase activity: 1.341e-22 (11,11,9) OR
GO:0003714: transcription corepressor activity: 3.188e-02 (11,8,1) OR
GO:0016651: oxidoreductase activity, acting on NADH or NADPH: 5.134e-02 (11,13,1)

IF 1gjwa2#141 AND 1mdah_#329
THEN
GO:0004556: alpha-amylase activity: 1.500e-22 (20,15,11) OR
GO:0005509: calcium ion binding: 8.170e-06 (20,160,8) OR
GO:0004867: serine-type endopeptidase inhibitor activity: 2.947e-01 (20,47,1)

IF 1avaa2#204 AND 1imva_#255
THEN
GO:0004556: alpha-amylase activity: 1.500e-22 (20,15,11) OR
GO:0005509: calcium ion binding: 8.170e-06 (20,160,8) OR
GO:0004867: serine-type endopeptidase inhibitor activity: 2.947e-01 (20,47,1)

IF 1qhoa4#75 AND 1jsg__#92
THEN
GO:0004556: alpha-amylase activity: 1.500e-22 (20,15,11) OR
GO:0005509: calcium ion binding: 8.170e-06 (20,160,8) OR
GO:0004867: serine-type endopeptidase inhibitor activity: 2.947e-01 (20,47,1)

IF 1e2fa_#14 AND 1qf9a_#117
THEN
GO:0019201: nucleotide kinase activity: 1.475e-22 (39,13,12) OR
GO:0016776: phosphotransferase activity, phosphate group as acceptor: 3.926e-19 (39,14,11) OR
GO:0005524: ATP binding: 4.198e-07 (39,243,15) OR
GO:0005525: GTP binding: 5.097e-01 (39,49,1)

IF 7taa_2#119 AND 1a4ya_#47
THEN
GO:0004556: alpha-amylase activity: 3.145e-22 (21,15,11) OR
GO:0005509: calcium ion binding: 1.254e-05 (21,160,8) OR
GO:0004867: serine-type endopeptidase inhibitor activity: 4.966e-02 (21,47,2)

IF 1qhoa4#75 AND 1a4ya_#47
THEN
GO:0004556: alpha-amylase activity: 3.145e-22 (21,15,11) OR
GO:0005509: calcium ion binding: 1.254e-05 (21,160,8) OR
GO:0004867: serine-type endopeptidase inhibitor activity: 4.966e-02 (21,47,2)

IF 1avaa2#204 AND 1bf2_3#575
THEN
GO:0004556: alpha-amylase activity: 3.145e-22 (21,15,11) OR
GO:0005509: calcium ion binding: 1.254e-05 (21,160,8) OR
GO:0004867: serine-type endopeptidase inhibitor activity: 4.966e-02 (21,47,2)

IF 1c7na_#281 AND 1dfoa_#344
THEN
GO:0008483: transaminase activity: 2.571e-22 (18,17,11) OR
GO:0016846: carbon-sulfur lyase activity: 2.735e-07 (18,10,4) OR
GO:0016831: carboxy-lyase activity: 1.130e-02 (18,25,2) OR
GO:0016866: intramolecular transferase activity: 7.660e-02 (18,12,1)

IF 1c7na_#281 AND 1jf9a_#380
THEN
GO:0008483: transaminase activity: 2.571e-22 (18,17,11) OR

GO:0016846: carbon-sulfur lyase activity: 2.735e-07 (18,10,4) OR
GO:0016831: carboxy-lyase activity: 1.130e-02 (18,25,2) OR
GO:0016866: intramolecular transferase activity: 7.660e-02 (18,12,1)

IF 1cyx__#138 AND 1e30a_#87
THEN
GO:0005507: copper ion binding: 2.095e-22 (22,38,14) OR
GO:0015078: hydrogen ion transporter activity: 3.951e-07 (22,21,5) OR
GO:0015082: di-, tri-valent inorganic cation transporter activity: 1.075e-01 (22,14,1) OR
GO:0046915: transition metal ion transporter activity: 1.075e-01 (22,14,1) OR
GO:0005509: calcium ion binding: 7.372e-01 (22,160,1)

IF 1a0la_#193 AND 1gg6.1#C229 AND 1cgha_#130
THEN
GO:0004263: chymotrypsin activity: 2.776e-22 (50,41,18) OR
GO:0004295: trypsin activity: 9.273e-21 (50,48,18) OR
GO:0004867: serine-type endopeptidase inhibitor activity: 1.701e-04 (50,47,6) OR
GO:0005509: calcium ion binding: 2.488e-02 (50,160,7) OR
GO:0008201: heparin binding: 3.600e-01 (50,24,1)

IF 1gdna_#119 AND 1gg6.1#C229 AND 1ton__#211
THEN
GO:0004295: trypsin activity: 2.787e-22 (51,48,19) OR
GO:0004263: chymotrypsin activity: 4.255e-22 (51,41,18) OR
GO:0004867: serine-type endopeptidase inhibitor activity: 1.594e-03 (51,47,5) OR
GO:0005509: calcium ion binding: 8.657e-03 (51,160,8) OR
GO:0004896: hematopoietin/interferon-class (D200-domain) cytokine receptor activity: 3.024e-01 (51,19,1)

IF 1cgha_#168 AND 1ddja_#759
THEN
GO:0004263: chymotrypsin activity: 2.913e-22 (72,41,20) OR
GO:0004295: trypsin activity: 1.590e-20 (72,48,20) OR
GO:0003809: thrombin activity: 6.917e-12 (72,10,8) OR
GO:0004867: serine-type endopeptidase inhibitor activity: 2.083e-07 (72,47,10) OR
GO:0005509: calcium ion binding: 2.066e-04 (72,160,13) OR
GO:0005529: sugar binding: 6.507e-01 (72,39,1)

IF 1cgha_#168 AND 1e79d2#36 AND 1ton__#231
THEN
GO:0004263: chymotrypsin activity: 2.913e-22 (72,41,20) OR
GO:0004295: trypsin activity: 1.590e-20 (72,48,20) OR
GO:0003809: thrombin activity: 6.917e-12 (72,10,8) OR
GO:0004867: serine-type endopeptidase inhibitor activity: 2.083e-07 (72,47,10) OR
GO:0005509: calcium ion binding: 2.066e-04 (72,160,13) OR
GO:0005529: sugar binding: 6.507e-01 (72,39,1)

IF 1hx0a2#294 AND 1by5a_#238
THEN
GO:0004556: alpha-amylase activity: 6.281e-22 (22,15,11) OR
GO:0005509: calcium ion binding: 1.707e-06 (22,160,9) OR
GO:0004867: serine-type endopeptidase inhibitor activity: 5.404e-02 (22,47,2)

IF 1avaa2#204 AND 1qhoa4#399
THEN
GO:0004556: alpha-amylase activity: 6.281e-22 (22,15,11) OR
GO:0005509: calcium ion binding: 1.707e-06 (22,160,9) OR
GO:0004867: serine-type endopeptidase inhibitor activity: 5.404e-02 (22,47,2)

IF 1a7s__#148 AND 1g71a_#77 AND 2hlca_#27 AND 1ddja_#726 AND 1gdna_#123 AND 1arb__#55 AND 1elva1#604
THEN
GO:0004263: chymotrypsin activity: 3.982e-22 (73,41,20) OR
GO:0004295: trypsin activity: 5.820e-22 (73,48,21) OR
GO:0003809: thrombin activity: 8.345e-10 (73,10,7) OR
GO:0004867: serine-type endopeptidase inhibitor activity: 2.384e-07 (73,47,10) OR
GO:0005509: calcium ion binding: 5.840e-05 (73,160,14) OR
GO:0005529: sugar binding: 6.558e-01 (73,39,1)

IF 1avaa2#204 AND 1cs1a_#160
THEN
GO:0004556: alpha-amylase activity: 6.281e-22 (22,15,11) OR

GO:0005509: calcium ion binding: 1.707e-06 (22,160,9) OR
GO:0016758: transferase activity, transferring hexosyl groups: 8.546e-02 (22,11,1) OR
GO:0004867: serine-type endopeptidase inhibitor activity: 3.190e-01 (22,47,1)

IF 1avaa2#204 AND 1gox__#124
THEN
GO:0004556: alpha-amylase activity: 6.281e-22 (22,15,11) OR
GO:0005509: calcium ion binding: 1.874e-05 (22,160,8) OR
GO:0004867: serine-type endopeptidase inhibitor activity: 5.404e-02 (22,47,2) OR
GO:0016758: transferase activity, transferring hexosyl groups: 8.546e-02 (22,11,1)

IF 1a4ya_#47 AND 1qfea_#49 AND 1bvza3#416
THEN
GO:0004556: alpha-amylase activity: 6.281e-22 (22,15,11) OR
GO:0005509: calcium ion binding: 1.874e-05 (22,160,8) OR
GO:0004867: serine-type endopeptidase inhibitor activity: 5.404e-02 (22,47,2) OR
GO:0000287: magnesium ion binding: 6.545e-01 (22,128,1)

IF 1hx0a2#294 AND 1e43a2#236
THEN
GO:0004556: alpha-amylase activity: 1.202e-21 (23,15,11) OR
GO:0005509: calcium ion binding: 2.177e-07 (23,160,10) OR
GO:0004867: serine-type endopeptidase inhibitor activity: 5.855e-02 (23,47,2)

IF 1e43a2#236 AND 1e43a2#351
THEN
GO:0004556: alpha-amylase activity: 1.202e-21 (23,15,11) OR
GO:0005509: calcium ion binding: 2.177e-07 (23,160,10) OR
GO:0004867: serine-type endopeptidase inhibitor activity: 5.855e-02 (23,47,2)

IF 1e43a2#236 AND 1aq0a_#4
THEN
GO:0004556: alpha-amylase activity: 1.202e-21 (23,15,11) OR
GO:0005509: calcium ion binding: 2.177e-07 (23,160,10) OR
GO:0004867: serine-type endopeptidase inhibitor activity: 5.855e-02 (23,47,2)

IF 1e43a2#236 AND 1avaa2#204
THEN
GO:0004556: alpha-amylase activity: 1.202e-21 (23,15,11) OR
GO:0005509: calcium ion binding: 2.177e-07 (23,160,10) OR
GO:0004867: serine-type endopeptidase inhibitor activity: 5.855e-02 (23,47,2)

IF 1cjca2#103 AND 1h7wa4#478
THEN
GO:0015036: disulfide oxidoreductase activity: 7.928e-22 (30,22,13) OR
GO:0016668: oxidoreductase activity, acting on sulfur group of donors, NAD or NADP as acceptor: 3.771e-14 (30,12,8) OR
GO:0050660: FAD binding: 2.139e-10 (30,10,6) OR
GO:0016651: oxidoreductase activity, acting on NADH or NADPH: 1.343e-01 (30,13,1) OR
GO:0016627: oxidoreductase activity, acting on the CH-CH group of donors: 1.720e-01 (30,17,1) OR
GO:0004601: peroxidase activity: 2.081e-01 (30,21,1)

IF 1hx0a2#294 AND 1ho1a_#211
THEN
GO:0004556: alpha-amylase activity: 1.202e-21 (23,15,11) OR
GO:0005509: calcium ion binding: 2.665e-06 (23,160,9) OR
GO:0004867: serine-type endopeptidase inhibitor activity: 5.855e-02 (23,47,2) OR
GO:0016836: hydro-lyase activity: 2.453e-01 (23,33,1)

IF 1gdea_#243 AND 2dkb__#114 AND 1cs1a_#82
THEN
GO:0008483: transaminase activity: 1.351e-21 (20,17,11) OR
GO:0016846: carbon-sulfur lyase activity: 4.182e-14 (20,10,7) OR
GO:0016866: intramolecular transferase activity: 8.477e-02 (20,12,1) OR
GO:0016831: carboxy-lyase activity: 1.689e-01 (20,25,1)

IF 1aq0a_#4 AND 1cs1a_#160
THEN
GO:0004556: alpha-amylase activity: 1.202e-21 (23,15,11) OR
GO:0005509: calcium ion binding: 2.665e-06 (23,160,9) OR
GO:0016758: transferase activity, transferring hexosyl groups: 8.918e-02 (23,11,1) OR

GO:0005529: sugar binding: 2.832e-01 (23,39,1) OR
GO:0004867: serine-type endopeptidase inhibitor activity: 3.309e-01 (23,47,1)

IF 1cyx__#138 AND 2cuaa_#83
THEN
GO:0005507: copper ion binding: 1.264e-21 (24,38,14) OR
GO:0015078: hydrogen ion transporter activity: 6.314e-07 (24,21,5) OR
GO:0015082: di-, tri-valent inorganic cation transporter activity: 1.167e-01 (24,14,1) OR
GO:0046915: transition metal ion transporter activity: 1.167e-01 (24,14,1) OR
GO:0005509: calcium ion binding: 1.635e-01 (24,160,3)

IF 1gjwa2#141 AND 1qdla_#211
THEN
GO:0004556: alpha-amylase activity: 3.890e-21 (16,15,10) OR
GO:0005509: calcium ion binding: 1.828e-04 (16,160,6)

IF 1ayl__#237 AND 1nhp_1#282
THEN
GO:0015036: disulfide oxidoreductase activity: 1.361e-21 (31,22,13) OR
GO:0016668: oxidoreductase activity, acting on sulfur group of donors, NAD or NADP as acceptor: 1.926e-16 (31,12,9) OR
GO:0050660: FAD binding: 2.649e-10 (31,10,6) OR
GO:0016651: oxidoreductase activity, acting on NADH or NADPH: 1.385e-01 (31,13,1) OR
GO:0016638: oxidoreductase activity, acting on the CH-NH2 group of donors: 1.772e-01 (31,17,1) OR
GO:0004601: peroxidase activity: 2.143e-01 (31,21,1)

IF 1c5y.1#B18 AND 1gdna_#30
THEN
GO:0004263: chymotrypsin activity: 1.443e-21 (54,41,18) OR
GO:0004295: trypsin activity: 4.771e-20 (54,48,18) OR
GO:0004867: serine-type endopeptidase inhibitor activity: 2.625e-04 (54,47,6) OR
GO:0005509: calcium ion binding: 9.288e-04 (54,160,10) OR
GO:0003809: thrombin activity: 1.817e-01 (54,10,1) OR
GO:0004896: hematopoietin/interferon-class (D200-domain) cytokine receptor activity: 3.172e-01 (54,19,1)

IF 1c5y.1#B18 AND 1dlea_#198
THEN
GO:0004263: chymotrypsin activity: 1.443e-21 (54,41,18) OR
GO:0004295: trypsin activity: 4.771e-20 (54,48,18) OR
GO:0004867: serine-type endopeptidase inhibitor activity: 2.625e-04 (54,47,6) OR
GO:0005509: calcium ion binding: 9.288e-04 (54,160,10) OR
GO:0003809: thrombin activity: 1.817e-01 (54,10,1) OR
GO:0004896: hematopoietin/interferon-class (D200-domain) cytokine receptor activity: 3.172e-01 (54,19,1)

IF 1c5y.1#B18 AND 1arb__#193
THEN
GO:0004263: chymotrypsin activity: 1.443e-21 (54,41,18) OR
GO:0004295: trypsin activity: 4.771e-20 (54,48,18) OR
GO:0004867: serine-type endopeptidase inhibitor activity: 2.625e-04 (54,47,6) OR
GO:0005509: calcium ion binding: 9.288e-04 (54,160,10) OR
GO:0003809: thrombin activity: 1.817e-01 (54,10,1) OR
GO:0004896: hematopoietin/interferon-class (D200-domain) cytokine receptor activity: 3.172e-01 (54,19,1)

IF 1c5y.1#B18 AND 1eq9a_#32
THEN
GO:0004263: chymotrypsin activity: 1.443e-21 (54,41,18) OR
GO:0004295: trypsin activity: 4.771e-20 (54,48,18) OR
GO:0004867: serine-type endopeptidase inhibitor activity: 2.625e-04 (54,47,6) OR
GO:0005509: calcium ion binding: 9.288e-04 (54,160,10) OR
GO:0003809: thrombin activity: 1.817e-01 (54,10,1) OR
GO:0004896: hematopoietin/interferon-class (D200-domain) cytokine receptor activity: 3.172e-01 (54,19,1)

IF 1hc7a2#109 AND 1pysa_#221
THEN
GO:0004812: tRNA ligase activity: 2.423e-21 (36,26,14) OR
GO:0005524: ATP binding: 8.861e-07 (36,243,14) OR
GO:0000049: tRNA binding: 1.208e-02 (36,13,2) OR
GO:0000287: magnesium ion binding: 2.471e-02 (36,128,5) OR
GO:0003887: DNA-directed DNA polymerase activity: 2.343e-01 (36,20,1)

IF 1a4ya_#47 AND 1bvza3#416 AND 1nsj__#178

THEN
GO:0004556: alpha-amylase activity: 2.216e-21 (24,15,11) OR
GO:0005509: calcium ion binding: 3.894e-05 (24,160,8) OR
GO:0004867: serine-type endopeptidase inhibitor activity: 6.319e-02 (24,47,2) OR
GO:0008081: phosphoric diester hydrolase activity: 9.289e-02 (24,11,1) OR
GO:0004620: phospholipase activity: 1.552e-01 (24,19,1) OR
GO:0000287: magnesium ion binding: 6.864e-01 (24,128,1)

IF 1aym3_#131 AND 1e43a2#351
THEN
GO:0004556: alpha-amylase activity: 9.432e-21 (17,15,10) OR
GO:0005509: calcium ion binding: 2.494e-05 (17,160,7)

IF 1ebda2#182 AND 1trb_1#41
THEN
GO:0015036: disulfide oxidoreductase activity: 3.759e-21 (33,22,13) OR
GO:0016668: oxidoreductase activity, acting on sulfur group of donors, NAD or NADP as acceptor: 9.830e-19 (33,12,10) OR
GO:0050660: FAD binding: 2.276e-12 (33,10,7) OR
GO:0016651: oxidoreductase activity, acting on NADH or NADPH: 1.468e-01 (33,13,1) OR
GO:0016627: oxidoreductase activity, acting on the CH-CH group of donors: 1.876e-01 (33,17,1) OR
GO:0016638: oxidoreductase activity, acting on the CH-NH2 group of donors: 1.876e-01 (33,17,1)

IF 1nhp_1#282 AND 1gpea1#271
THEN
GO:0015036: disulfide oxidoreductase activity: 3.759e-21 (33,22,13) OR
GO:0016668: oxidoreductase activity, acting on sulfur group of donors, NAD or NADP as acceptor: 3.677e-16 (33,12,9) OR
GO:0050660: FAD binding: 2.276e-12 (33,10,7) OR
GO:0016627: oxidoreductase activity, acting on the CH-CH group of donors: 1.727e-02 (33,17,2) OR
GO:0016651: oxidoreductase activity, acting on NADH or NADPH: 1.468e-01 (33,13,1) OR
GO:0004601: peroxidase activity: 2.265e-01 (33,21,1)

IF 2ebn__#77 AND 1gox__#124 AND 7taa_2#295
THEN
GO:0004556: alpha-amylase activity: 9.432e-21 (17,15,10) OR
GO:0005509: calcium ion binding: 2.689e-04 (17,160,6) OR
GO:0004867: serine-type endopeptidase inhibitor activity: 2.567e-01 (17,47,1)

IF 1gdna_#119 AND 1ton__#231
THEN
GO:0004295: trypsin activity: 5.998e-21 (49,48,18) OR
GO:0004263: chymotrypsin activity: 1.116e-20 (49,41,17) OR
GO:0004867: serine-type endopeptidase inhibitor activity: 1.327e-03 (49,47,5) OR
GO:0005509: calcium ion binding: 6.776e-03 (49,160,8) OR
GO:0004896: hematopoietin/interferon-class (D200-domain) cytokine receptor activity: 2.924e-01 (49,19,1)

IF 1a7s__#148 AND 1fjsa_#163 AND 1elva1#513
THEN
GO:0004263: chymotrypsin activity: 5.301e-21 (69,41,19) OR
GO:0004295: trypsin activity: 6.072e-21 (69,48,20) OR
GO:0003809: thrombin activity: 5.548e-10 (69,10,7) OR
GO:0004867: serine-type endopeptidase inhibitor activity: 1.029e-08 (69,47,11) OR
GO:0005509: calcium ion binding: 1.890e-03 (69,160,11) OR
GO:0005529: sugar binding: 6.348e-01 (69,39,1)

IF 1j71a_#32
THEN
GO:0004190: aspartic-type endopeptidase activity: 3.627e-20 (9,23,9)

IF 1mpp__#29 AND 1pfza_#306
THEN
GO:0004190: aspartic-type endopeptidase activity: 3.627e-20 (9,23,9)

IF 1pfza_#306 AND 1hrna_#136
THEN
GO:0004190: aspartic-type endopeptidase activity: 3.627e-20 (9,23,9)

IF 1mpp__#216
THEN
GO:0004190: aspartic-type endopeptidase activity: 3.627e-20 (9,23,9)

IF 1d4xg_#60 AND 1glqa2#53
THEN
 GO:0004364: glutathione transferase activity: 1.988e-20 (9,11,8) OR
 GO:0003714: transcription corepressor activity: 2.615e-02 (9,8,1)

IF 1d4xg_#60 AND 1gnwa1#160
THEN
 GO:0004364: glutathione transferase activity: 1.988e-20 (9,11,8) OR
 GO:0003714: transcription corepressor activity: 2.615e-02 (9,8,1)

IF 1bd3a_#110 AND 1fgga_#134
THEN
 GO:0016763: transferase activity, transferring pentosyl groups: 9.801e-21 (27,28,13) OR
 GO:0000287: magnesium ion binding: 1.923e-06 (27,128,9) OR
 GO:0008757: S-adenosylmethionine-dependent methyltransferase activity: 2.282e-02 (27,24,2) OR
 GO:0016866: intramolecular transferase activity: 1.128e-01 (27,12,1) OR
 GO:0008270: zinc ion binding: 6.662e-01 (27,108,1) OR
 GO:0005524: ATP binding: 9.207e-01 (27,243,1)

IF 1avaa2#204 AND 7taa_2#331
THEN
 GO:0004556: alpha-amylase activity: 2.119e-20 (18,15,10) OR
 GO:0005509: calcium ion binding: 3.881e-05 (18,160,7) OR
 GO:0016758: transferase activity, transferring hexosyl groups: 7.043e-02 (18,11,1)

IF 7taa_2#331 AND 1evqa_#305
THEN
 GO:0004556: alpha-amylase activity: 2.119e-20 (18,15,10) OR
 GO:0005509: calcium ion binding: 3.881e-05 (18,160,7) OR
 GO:0016758: transferase activity, transferring hexosyl groups: 7.043e-02 (18,11,1)

IF 1hq8a_#150 AND 2msba_#218
THEN
 GO:0005529: sugar binding: 2.008e-20 (16,39,12) OR
 GO:0004263: chymotrypsin activity: 2.159e-01 (16,41,1) OR
 GO:0005509: calcium ion binding: 2.410e-01 (16,160,2) OR
 GO:0004295: trypsin activity: 2.481e-01 (16,48,1)

IF 1hyea2#182 AND 1qb7a_#86
THEN
 GO:0004457: lactate dehydrogenase activity: 4.086e-20 (19,10,9) OR
 GO:0016616: oxidoreductase activity, acting on the CH-OH group of donors, NAD or NADP as acceptor: 8.171e-13 (19,59,10)

IF 1llc_2#271 AND 2cmd_2#251
THEN
 GO:0004457: lactate dehydrogenase activity: 4.086e-20 (19,10,9) OR
 GO:0016616: oxidoreductase activity, acting on the CH-OH group of donors, NAD or NADP as acceptor: 8.171e-13 (19,59,10)

IF 1c5y.1#B191 AND 1gdna_#123 AND 1cgha_#130
THEN
 GO:0004263: chymotrypsin activity: 1.677e-20 (50,41,17) OR
 GO:0004295: trypsin activity: 4.326e-19 (50,48,17) OR
 GO:0004867: serine-type endopeptidase inhibitor activity: 1.636e-05 (50,47,7) OR
 GO:0005509: calcium ion binding: 7.673e-03 (50,160,8) OR
 GO:0008201: heparin binding: 3.600e-01 (50,24,1)

IF 1c5y.1#B18 AND 1gg6.1#C156
THEN
 GO:0004263: chymotrypsin activity: 1.677e-20 (50,41,17) OR
 GO:0004295: trypsin activity: 4.326e-19 (50,48,17) OR
 GO:0004867: serine-type endopeptidase inhibitor activity: 1.701e-04 (50,47,6) OR
 GO:0005509: calcium ion binding: 2.065e-03 (50,160,9) OR
 GO:0004896: hematopoietin/interferon-class (D200-domain) cytokine receptor activity: 2.975e-01 (50,19,1)

IF 1c5y.1#B18 AND 1dlea_#238 AND 1gg6.1#C229
THEN
 GO:0004263: chymotrypsin activity: 1.677e-20 (50,41,17) OR
 GO:0004295: trypsin activity: 4.326e-19 (50,48,17) OR
 GO:0004867: serine-type endopeptidase inhibitor activity: 1.701e-04 (50,47,6) OR
 GO:0005509: calcium ion binding: 2.065e-03 (50,160,9) OR

GO:0004896: hematopoietin/interferon-class (D200-domain) cytokine receptor activity: 2.975e-01 (50,19,1)

IF 1trb_1#41 AND 1fl2a1#320
THEN
GO:0015036: disulfide oxidoreductase activity: 1.501e-20 (36,22,13) OR
GO:0016668: oxidoreductase activity, acting on sulfur group of donors, NAD or NADP as acceptor: 2.694e-18 (36,12,10) OR
GO:0050660: FAD binding: 4.141e-17 (36,10,9) OR
GO:0016627: oxidoreductase activity, acting on the CH-CH group of donors: 2.038e-02 (36,17,2) OR
GO:0016651: oxidoreductase activity, acting on NADH or NADPH: 1.591e-01 (36,13,1) OR
GO:0016638: oxidoreductase activity, acting on the CH-NH2 group of donors: 2.029e-01 (36,17,1)

IF 2dkb__#46 AND 1cs1a_#82
THEN
GO:0008483: transaminase activity: 2.509e-20 (16,17,10) OR
GO:0016846: carbon-sulfur lyase activity: 1.632e-07 (16,10,4) OR
GO:0016866: intramolecular transferase activity: 6.836e-02 (16,12,1) OR
GO:0016831: carboxy-lyase activity: 1.375e-01 (16,25,1)

IF 1fvua_#120 AND 1hq8a_#150
THEN
GO:0005529: sugar binding: 2.008e-20 (16,39,12) OR
GO:0008201: heparin binding: 1.323e-01 (16,24,1) OR
GO:0004263: chymotrypsin activity: 2.159e-01 (16,41,1) OR
GO:0004295: trypsin activity: 2.481e-01 (16,48,1) OR
GO:0005509: calcium ion binding: 6.213e-01 (16,160,1)

IF 1h8ua_#113 AND 1hq8a_#150
THEN
GO:0005529: sugar binding: 2.008e-20 (16,39,12) OR
GO:0008201: heparin binding: 1.323e-01 (16,24,1) OR
GO:0004263: chymotrypsin activity: 2.159e-01 (16,41,1) OR
GO:0004295: trypsin activity: 2.481e-01 (16,48,1) OR
GO:0005509: calcium ion binding: 6.213e-01 (16,160,1)

IF 1b6e__#76 AND 1hq8a_#150
THEN
GO:0005529: sugar binding: 2.008e-20 (16,39,12) OR
GO:0008201: heparin binding: 1.323e-01 (16,24,1) OR
GO:0004263: chymotrypsin activity: 2.159e-01 (16,41,1) OR
GO:0004295: trypsin activity: 2.481e-01 (16,48,1) OR
GO:0005509: calcium ion binding: 6.213e-01 (16,160,1)

IF 1fvua_#120 AND 1e87a_#121
THEN
GO:0005529: sugar binding: 2.008e-20 (16,39,12) OR
GO:0008201: heparin binding: 1.323e-01 (16,24,1) OR
GO:0004263: chymotrypsin activity: 2.159e-01 (16,41,1) OR
GO:0004295: trypsin activity: 2.481e-01 (16,48,1) OR
GO:0005509: calcium ion binding: 6.213e-01 (16,160,1)

IF 1h8ua_#113 AND 1e87a_#121
THEN
GO:0005529: sugar binding: 2.008e-20 (16,39,12) OR
GO:0008201: heparin binding: 1.323e-01 (16,24,1) OR
GO:0004263: chymotrypsin activity: 2.159e-01 (16,41,1) OR
GO:0004295: trypsin activity: 2.481e-01 (16,48,1) OR
GO:0005509: calcium ion binding: 6.213e-01 (16,160,1)

IF 1hq8a_#185 AND 2msba_#218
THEN
GO:0005529: sugar binding: 1.039e-19 (10,39,10)

IF 1e6ua_#10 AND 2ae2a_#17
THEN
GO:0016616: oxidoreductase activity, acting on the CH-OH group of donors, NAD or NADP as acceptor: 1.820e-20 (24,59,15) OR
GO:0004457: lactate dehydrogenase activity: 3.205e-03 (24,10,2) OR
GO:0016854: racemase and epimerase activity: 5.467e-03 (24,13,2) OR
GO:0016627: oxidoreductase activity, acting on the CH-CH group of donors: 9.329e-03 (24,17,2) OR
GO:0016836: hydro-lyase activity: 3.325e-02 (24,33,2) OR
GO:0016646: oxidoreductase activity, acting on the CH-NH group of donors, NAD or NADP as acceptor: 1.552e-01 (24,19,1)

IF 1bvza3#416 AND 1fhoa_#74
THEN
GO:0004556: alpha-amylase activity: 4.465e-20 (19,15,10) OR
GO:0005509: calcium ion binding: 5.158e-06 (19,160,8) OR
GO:0004867: serine-type endopeptidase inhibitor activity: 2.823e-01 (19,47,1)

IF 1qhda2#323 AND 1bvza3#416
THEN
GO:0004556: alpha-amylase activity: 4.465e-20 (19,15,10) OR
GO:0005509: calcium ion binding: 5.158e-06 (19,160,8) OR
GO:0004867: serine-type endopeptidase inhibitor activity: 2.823e-01 (19,47,1)

IF 1qhda2#323 AND 1eh9a3#252
THEN
GO:0004556: alpha-amylase activity: 4.465e-20 (19,15,10) OR
GO:0005509: calcium ion binding: 5.158e-06 (19,160,8) OR
GO:0004867: serine-type endopeptidase inhibitor activity: 2.823e-01 (19,47,1)

IF 1gjwa2#141 AND 3grx__#56
THEN
GO:0004556: alpha-amylase activity: 4.465e-20 (19,15,10) OR
GO:0005509: calcium ion binding: 5.844e-05 (19,160,7) OR
GO:0004867: serine-type endopeptidase inhibitor activity: 4.133e-02 (19,47,2)

IF 1qhda2#323 AND 7taa_2#295
THEN
GO:0004556: alpha-amylase activity: 4.465e-20 (19,15,10) OR
GO:0005509: calcium ion binding: 5.158e-06 (19,160,8) OR
GO:0004867: serine-type endopeptidase inhibitor activity: 2.823e-01 (19,47,1)

IF 1qhda2#323 AND 1gjwa2#141
THEN
GO:0004556: alpha-amylase activity: 4.465e-20 (19,15,10) OR
GO:0005509: calcium ion binding: 5.158e-06 (19,160,8) OR
GO:0004867: serine-type endopeptidase inhibitor activity: 2.823e-01 (19,47,1)

IF 1bvza3#373 AND 1avaa2#204
THEN
GO:0004556: alpha-amylase activity: 4.465e-20 (19,15,10) OR
GO:0005509: calcium ion binding: 5.158e-06 (19,160,8) OR
GO:0004867: serine-type endopeptidase inhibitor activity: 2.823e-01 (19,47,1)

IF 1ldm_2#292 AND 2hlpa2#315
THEN
GO:0004457: lactate dehydrogenase activity: 7.427e-20 (20,10,9) OR
GO:0016616: oxidoreductase activity, acting on the CH-OH group of donors, NAD or NADP as acceptor: 2.686e-14 (20,59,11)

IF 1h7wa4#478 AND 1b4va1#253
THEN
GO:0015036: disulfide oxidoreductase activity: 3.126e-20 (27,22,12) OR
GO:0016668: oxidoreductase activity, acting on sulfur group of donors, NAD or NADP as acceptor: 1.436e-14 (27,12,8) OR
GO:0050660: FAD binding: 1.576e-08 (27,10,5) OR
GO:0016651: oxidoreductase activity, acting on NADH or NADPH: 1.217e-01 (27,13,1) OR
GO:0004601: peroxidase activity: 1.893e-01 (27,21,1)

IF 1gjwa2#141 AND 1fc4a_#205
THEN
GO:0004556: alpha-amylase activity: 4.465e-20 (19,15,10) OR
GO:0005509: calcium ion binding: 5.844e-05 (19,160,7) OR
GO:0016758: transferase activity, transferring hexosyl groups: 7.421e-02 (19,11,1) OR
GO:0004867: serine-type endopeptidase inhibitor activity: 2.823e-01 (19,47,1)

IF 1cpy__#93 AND 1bvza3#416
THEN
GO:0004556: alpha-amylase activity: 4.465e-20 (19,15,10) OR
GO:0005509: calcium ion binding: 5.341e-04 (19,160,6) OR
GO:0004867: serine-type endopeptidase inhibitor activity: 4.133e-02 (19,47,2) OR
GO:0000287: magnesium ion binding: 6.004e-01 (19,128,1)

IF 2ae2a_#196 AND 1eny__#147
THEN
GO:0016616: oxidoreductase activity, acting on the CH-OH group of donors, NAD or NADP as acceptor: 1.853e-19 (11,59,11)

IF 2ae2a_#196 AND 1h5qa_#153
THEN
GO:0016616: oxidoreductase activity, acting on the CH-OH group of donors, NAD or NADP as acceptor: 1.853e-19 (11,59,11)

IF 2ae2a_#196 AND 1b16a_#184
THEN
GO:0016616: oxidoreductase activity, acting on the CH-OH group of donors, NAD or NADP as acceptor: 1.853e-19 (11,59,11)

IF 1c5y.1#B18 AND 1fjsa_#83
THEN
GO:0004263: chymotrypsin activity: 3.674e-20 (52,41,17) OR
GO:0004295: trypsin activity: 9.432e-19 (52,48,17) OR
GO:0004867: serine-type endopeptidase inhibitor activity: 2.124e-04 (52,47,6) OR
GO:0005509: calcium ion binding: 6.800e-04 (52,160,10) OR
GO:0003809: thrombin activity: 1.755e-01 (52,10,1) OR
GO:0004896: hematopoietin/interferon-class (D200-domain) cytokine receptor activity: 3.074e-01 (52,19,1)

IF 1c5y.1#B18 AND 1svpa_#127
THEN
GO:0004263: chymotrypsin activity: 3.674e-20 (52,41,17) OR
GO:0004295: trypsin activity: 9.432e-19 (52,48,17) OR
GO:0004867: serine-type endopeptidase inhibitor activity: 2.124e-04 (52,47,6) OR
GO:0005509: calcium ion binding: 6.800e-04 (52,160,10) OR
GO:0003809: thrombin activity: 1.755e-01 (52,10,1) OR
GO:0004896: hematopoietin/interferon-class (D200-domain) cytokine receptor activity: 3.074e-01 (52,19,1)

IF 1gdea_#243 AND 1dfoa_#344
THEN
GO:0008483: transaminase activity: 6.080e-20 (17,17,10) OR
GO:0016846: carbon-sulfur lyase activity: 1.227e-09 (17,10,5) OR
GO:0016866: intramolecular transferase activity: 7.249e-02 (17,12,1) OR
GO:0016831: carboxy-lyase activity: 1.454e-01 (17,25,1)

IF 1qs1a2#437 AND 1gg6.1#C229
THEN
GO:0004295: trypsin activity: 5.119e-20 (45,48,17) OR
GO:0004263: chymotrypsin activity: 1.324e-14 (45,41,13) OR
GO:0004867: serine-type endopeptidase inhibitor activity: 7.931e-06 (45,47,7) OR
GO:0005509: calcium ion binding: 1.445e-02 (45,160,7) OR
GO:0003809: thrombin activity: 1.536e-01 (45,10,1)

IF 2cmd_2#251 AND 1hyea2#182
THEN
GO:0004457: lactate dehydrogenase activity: 1.299e-19 (21,10,9) OR
GO:0016616: oxidoreductase activity, acting on the CH-OH group of donors, NAD or NADP as acceptor: 8.330e-16 (21,59,12)

IF 2cmd_2#251 AND 1ldna1#94
THEN
GO:0004457: lactate dehydrogenase activity: 1.299e-19 (21,10,9) OR
GO:0016616: oxidoreductase activity, acting on the CH-OH group of donors, NAD or NADP as acceptor: 8.330e-16 (21,59,12)

IF 1avaa2#204 AND 1bag_2#207
THEN
GO:0004556: alpha-amylase activity: 8.915e-20 (20,15,10) OR
GO:0005509: calcium ion binding: 8.170e-06 (20,160,8) OR
GO:0004867: serine-type endopeptidase inhibitor activity: 4.542e-02 (20,47,2)

IF 1aq0a_#4 AND 1bf2_3#575
THEN
GO:0004556: alpha-amylase activity: 8.915e-20 (20,15,10) OR
GO:0005509: calcium ion binding: 8.170e-06 (20,160,8) OR
GO:0004867: serine-type endopeptidase inhibitor activity: 4.542e-02 (20,47,2)

IF 1hx0a2#294 AND 1bag_2#207
THEN
GO:0004556: alpha-amylase activity: 8.915e-20 (20,15,10) OR

GO:0005509: calcium ion binding: 8.170e-06 (20,160,8) OR
GO:0004867: serine-type endopeptidase inhibitor activity: 4.542e-02 (20,47,2)

IF 1ddja_#754 AND 1c5y.1#B241
THEN
GO:0004263: chymotrypsin activity: 4.939e-20 (64,41,18) OR
GO:0004295: trypsin activity: 1.593e-18 (64,48,18) OR
GO:0003809: thrombin activity: 2.572e-12 (64,10,8) OR
GO:0005509: calcium ion binding: 5.787e-05 (64,160,13) OR
GO:0004867: serine-type endopeptidase inhibitor activity: 6.689e-04 (64,47,6) OR
GO:0005529: sugar binding: 6.068e-01 (64,39,1)

IF 1a0fa1#178 AND 1f3ba2#7
THEN
GO:0004364: glutathione transferase activity: 9.929e-20 (10,11,8) OR
GO:0003714: transcription corepressor activity: 2.902e-02 (10,8,1) OR
GO:0016651: oxidoreductase activity, acting on NADH or NADPH: 4.677e-02 (10,13,1)

IF 2bb2_2#134 AND 1ebda2#182
THEN
GO:0015036: disulfide oxidoreductase activity: 5.452e-20 (28,22,12) OR
GO:0016668: oxidoreductase activity, acting on sulfur group of donors, NAD or NADP as acceptor: 2.008e-14 (28,12,8) OR
GO:0050660: FAD binding: 1.916e-08 (28,10,5) OR
GO:0016651: oxidoreductase activity, acting on NADH or NADPH: 1.259e-01 (28,13,1) OR
GO:0005096: GTPase activator activity: 1.439e-01 (28,15,1) OR
GO:0016627: oxidoreductase activity, acting on the CH-CH group of donors: 1.615e-01 (28,17,1)

IF 1trb_1#42 AND 1h6va1#23
THEN
GO:0015036: disulfide oxidoreductase activity: 5.452e-20 (28,22,12) OR
GO:0016668: oxidoreductase activity, acting on sulfur group of donors, NAD or NADP as acceptor: 2.008e-14 (28,12,8) OR
GO:0050660: FAD binding: 1.916e-08 (28,10,5) OR
GO:0016651: oxidoreductase activity, acting on NADH or NADPH: 1.259e-01 (28,13,1) OR
GO:0016627: oxidoreductase activity, acting on the CH-CH group of donors: 1.615e-01 (28,17,1) OR
GO:0016638: oxidoreductase activity, acting on the CH-NH2 group of donors: 1.615e-01 (28,17,1)

IF 1dv8a_#213 AND 1hq8a_#150
THEN
GO:0005529: sugar binding: 6.765e-20 (17,39,12) OR
GO:0008201: heparin binding: 1.400e-01 (17,24,1) OR
GO:0004263: chymotrypsin activity: 2.278e-01 (17,41,1) OR
GO:0004295: trypsin activity: 2.614e-01 (17,48,1) OR
GO:0005509: calcium ion binding: 2.634e-01 (17,160,2)

IF 1dv8a_#213 AND 1f00i3#856
THEN
GO:0005529: sugar binding: 6.765e-20 (17,39,12) OR
GO:0008201: heparin binding: 1.400e-01 (17,24,1) OR
GO:0004263: chymotrypsin activity: 2.278e-01 (17,41,1) OR
GO:0004295: trypsin activity: 2.614e-01 (17,48,1) OR
GO:0005509: calcium ion binding: 2.634e-01 (17,160,2)

IF 1dv8a_#213 AND 1e87a_#121
THEN
GO:0005529: sugar binding: 6.765e-20 (17,39,12) OR
GO:0008201: heparin binding: 1.400e-01 (17,24,1) OR
GO:0004263: chymotrypsin activity: 2.278e-01 (17,41,1) OR
GO:0004295: trypsin activity: 2.614e-01 (17,48,1) OR
GO:0005509: calcium ion binding: 2.634e-01 (17,160,2)

IF 1bvza3#416 AND 1gcya2#331 AND 1ho1a_#211
THEN
GO:0004556: alpha-amylase activity: 8.915e-20 (20,15,10) OR
GO:0005509: calcium ion binding: 8.551e-05 (20,160,7) OR
GO:0004867: serine-type endopeptidase inhibitor activity: 4.542e-02 (20,47,2) OR
GO:0000287: magnesium ion binding: 6.193e-01 (20,128,1)

IF 1b16a_#184 AND 1hdr__#156 AND 1qtn.1#A270
THEN
GO:0016616: oxidoreductase activity, acting on the CH-OH group of donors, NAD or NADP as acceptor: 1.268e-19 (17,59,13) OR

GO:0016627: oxidoreductase activity, acting on the CH-CH group of donors: 1.300e-04 (17,17,3) OR
GO:0016854: racemase and epimerase activity: 7.830e-02 (17,13,1)

IF 1b16a_#184 AND 1h5qa_#70
THEN
GO:0016616: oxidoreductase activity, acting on the CH-OH group of donors, NAD or NADP as acceptor: 1.268e-19 (17,59,13) OR
GO:0016627: oxidoreductase activity, acting on the CH-CH group of donors: 1.300e-04 (17,17,3) OR
GO:0016836: hydro-lyase activity: 1.876e-01 (17,33,1)

IF 1hc7a2#109 AND 1h4vb2#282
THEN
GO:0004812: tRNA ligase activity: 7.646e-20 (33,26,13) OR
GO:0005524: ATP binding: 1.864e-06 (33,243,13) OR
GO:0000287: magnesium ion binding: 1.740e-02 (33,128,5) OR
GO:0000049: tRNA binding: 1.468e-01 (33,13,1) OR
GO:0003887: DNA-directed DNA polymerase activity: 2.169e-01 (33,20,1)

IF 1cjca2#76 AND 1trb_1#285
THEN
GO:0015036: disulfide oxidoreductase activity: 9.269e-20 (29,22,12) OR
GO:0016668: oxidoreductase activity, acting on sulfur group of donors, NAD or NADP as acceptor: 9.586e-17 (29,12,9) OR
GO:0050660: FAD binding: 1.713e-10 (29,10,6) OR
GO:0016651: oxidoreductase activity, acting on NADH or NADPH: 1.301e-01 (29,13,1) OR
GO:0016638: oxidoreductase activity, acting on the CH-NH2 group of donors: 1.668e-01 (29,17,1)

IF 3grs_1#24 AND 1pbe_1#155 AND 1cjca2#76
THEN
GO:0015036: disulfide oxidoreductase activity: 9.269e-20 (29,22,12) OR
GO:0016668: oxidoreductase activity, acting on sulfur group of donors, NAD or NADP as acceptor: 9.586e-17 (29,12,9) OR
GO:0050660: FAD binding: 1.713e-10 (29,10,6) OR
GO:0016651: oxidoreductase activity, acting on NADH or NADPH: 1.301e-01 (29,13,1) OR
GO:0016638: oxidoreductase activity, acting on the CH-NH2 group of donors: 1.668e-01 (29,17,1)

IF 1chua2#234 AND 1trb_1#41
THEN
GO:0015036: disulfide oxidoreductase activity: 9.269e-20 (29,22,12) OR
GO:0016668: oxidoreductase activity, acting on sulfur group of donors, NAD or NADP as acceptor: 9.586e-17 (29,12,9) OR
GO:0050660: FAD binding: 1.713e-10 (29,10,6) OR
GO:0016651: oxidoreductase activity, acting on NADH or NADPH: 1.301e-01 (29,13,1) OR
GO:0016627: oxidoreductase activity, acting on the CH-CH group of donors: 1.668e-01 (29,17,1)

IF 1qf6a4#295 AND 1qsta_#154
THEN
GO:0004812: tRNA ligase activity: 1.232e-19 (34,26,13) OR
GO:0005524: ATP binding: 2.781e-06 (34,243,13) OR
GO:0000287: magnesium ion binding: 4.339e-03 (34,128,6) OR
GO:0000049: tRNA binding: 1.082e-02 (34,13,2)

IF 1avaa2#204 AND 1qhoa4#230
THEN
GO:0004556: alpha-amylase activity: 1.699e-19 (21,15,10) OR
GO:0005509: calcium ion binding: 1.062e-06 (21,160,9) OR
GO:0004867: serine-type endopeptidase inhibitor activity: 4.966e-02 (21,47,2)

IF 1qrra_#69 AND 1nhp_1#282
THEN
GO:0015036: disulfide oxidoreductase activity: 9.269e-20 (29,22,12) OR
GO:0016668: oxidoreductase activity, acting on sulfur group of donors, NAD or NADP as acceptor: 5.431e-12 (29,12,7) OR
GO:0050660: FAD binding: 1.713e-10 (29,10,6) OR
GO:0016627: oxidoreductase activity, acting on the CH-CH group of donors: 1.347e-02 (29,17,2) OR
GO:0016651: oxidoreductase activity, acting on NADH or NADPH: 1.301e-01 (29,13,1) OR
GO:0004601: peroxidase activity: 2.019e-01 (29,21,1)

IF 1oaa__#199 AND 1h5qa_#70
THEN
GO:0016616: oxidoreductase activity, acting on the CH-OH group of donors, NAD or NADP as acceptor: 2.888e-19 (14,59,12) OR
GO:0016836: hydro-lyase activity: 1.182e-02 (14,33,2)

IF 1icia_#175 AND 1bvza3#416
THEN

GO:0004556: alpha-amylase activity: 1.699e-19 (21,15,10) OR
GO:0005509: calcium ion binding: 1.254e-05 (21,160,8) OR
GO:0004867: serine-type endopeptidase inhibitor activity: 4.966e-02 (21,47,2) OR
GO:0000287: magnesium ion binding: 6.373e-01 (21,128,1)

IF 1icia_#175 AND 1qfea_#49
THEN
GO:0004556: alpha-amylase activity: 1.699e-19 (21,15,10) OR
GO:0005509: calcium ion binding: 1.254e-05 (21,160,8) OR
GO:0004867: serine-type endopeptidase inhibitor activity: 4.966e-02 (21,47,2) OR
GO:0000287: magnesium ion binding: 6.373e-01 (21,128,1)

IF 1avaa2#204 AND 1gox__#246
THEN
GO:0004556: alpha-amylase activity: 1.699e-19 (21,15,10) OR
GO:0005509: calcium ion binding: 1.254e-05 (21,160,8) OR
GO:0004867: serine-type endopeptidase inhibitor activity: 4.966e-02 (21,47,2) OR
GO:0016758: transferase activity, transferring hexosyl groups: 8.172e-02 (21,11,1)

IF 1avaa2#204 AND 1a4ya_#47
THEN
GO:0004556: alpha-amylase activity: 1.699e-19 (21,15,10) OR
GO:0005509: calcium ion binding: 1.254e-05 (21,160,8) OR
GO:0004867: serine-type endopeptidase inhibitor activity: 4.966e-02 (21,47,2) OR
GO:0016758: transferase activity, transferring hexosyl groups: 8.172e-02 (21,11,1)

IF 1icia_#175 AND 7taa_2#295
THEN
GO:0004556: alpha-amylase activity: 1.699e-19 (21,15,10) OR
GO:0005509: calcium ion binding: 1.254e-05 (21,160,8) OR
GO:0004867: serine-type endopeptidase inhibitor activity: 4.966e-02 (21,47,2) OR
GO:0016758: transferase activity, transferring hexosyl groups: 8.172e-02 (21,11,1)

IF 1hx0a2#294 AND 1gcya2#331
THEN
GO:0004556: alpha-amylase activity: 1.699e-19 (21,15,10) OR
GO:0005509: calcium ion binding: 1.254e-05 (21,160,8) OR
GO:0004867: serine-type endopeptidase inhibitor activity: 4.966e-02 (21,47,2) OR
GO:0003700: transcription factor activity: 6.253e-01 (21,124,1)

IF 1pfza_#313
THEN
GO:0004190: aspartic-type endopeptidase activity: 3.610e-19 (10,23,9) OR
GO:0016251: general RNA polymerase II transcription factor activity: 5.029e-02 (10,14,1)

IF 1nhp_1#7 AND 1cjca2#76
THEN
GO:0015036: disulfide oxidoreductase activity: 1.540e-19 (30,22,12) OR
GO:0016668: oxidoreductase activity, acting on sulfur group of donors, NAD or NADP as acceptor: 1.368e-16 (30,12,9) OR
GO:0050660: FAD binding: 2.139e-10 (30,10,6) OR
GO:0016638: oxidoreductase activity, acting on the CH-NH2 group of donors: 1.438e-02 (30,17,2) OR
GO:0016651: oxidoreductase activity, acting on NADH or NADPH: 1.343e-01 (30,13,1)

IF 1aq0a_#4 AND 2ebn__#77
THEN
GO:0004556: alpha-amylase activity: 1.699e-19 (21,15,10) OR
GO:0005509: calcium ion binding: 1.254e-05 (21,160,8) OR
GO:0008810: cellulase activity: 1.304e-01 (21,18,1) OR
GO:0005529: sugar binding: 2.620e-01 (21,39,1) OR
GO:0004867: serine-type endopeptidase inhibitor activity: 3.070e-01 (21,47,1)

IF 1oaa__#199 AND 1eno__#137
THEN
GO:0016616: oxidoreductase activity, acting on the CH-OH group of donors, NAD or NADP as acceptor: 2.888e-19 (14,59,12) OR
GO:0016646: oxidoreductase activity, acting on the CH-NH group of donors, NAD or NADP as acceptor: 9.353e-02 (14,19,1) OR
GO:0016836: hydro-lyase activity: 1.572e-01 (14,33,1)

IF 1eh9a3#102 AND 1bev1_#86
THEN
GO:0004556: alpha-amylase activity: 4.403e-19 (14,15,9) OR

GO:0005509: calcium ion binding: 8.514e-04 (14,160,5)

IF 1eh9a3#102 AND 7taa_2#331
THEN
GO:0004556: alpha-amylase activity: 4.403e-19 (14,15,9) OR
GO:0005509: calcium ion binding: 8.514e-04 (14,160,5)

IF 1aym3_#131 AND 7taa_2#331
THEN
GO:0004556: alpha-amylase activity: 4.403e-19 (14,15,9) OR
GO:0005509: calcium ion binding: 8.514e-04 (14,160,5)

IF 1gjwa2#141 AND 1eh9a3#102
THEN
GO:0004556: alpha-amylase activity: 4.403e-19 (14,15,9) OR
GO:0005509: calcium ion binding: 8.514e-04 (14,160,5)

IF 1erza_#183 AND 1qdla_#211 AND 1nsj__#178
THEN
GO:0004556: alpha-amylase activity: 4.403e-19 (14,15,9) OR
GO:0005509: calcium ion binding: 8.514e-04 (14,160,5)

IF 1lvl_2#222 AND 1h7wa4#476
THEN
GO:0015036: disulfide oxidoreductase activity: 1.540e-19 (30,22,12) OR
GO:0016668: oxidoreductase activity, acting on sulfur group of donors, NAD or NADP as acceptor: 3.771e-14 (30,12,8) OR
GO:0050660: FAD binding: 1.088e-12 (30,10,7) OR
GO:0016651: oxidoreductase activity, acting on NADH or NADPH: 1.343e-01 (30,13,1) OR
GO:0016627: oxidoreductase activity, acting on the CH-CH group of donors: 1.720e-01 (30,17,1) OR
GO:0004601: peroxidase activity: 2.081e-01 (30,21,1)

IF 1qrra_#69 AND 1h7wa4#481
THEN
GO:0015036: disulfide oxidoreductase activity: 1.540e-19 (30,22,12) OR
GO:0016668: oxidoreductase activity, acting on sulfur group of donors, NAD or NADP as acceptor: 7.072e-12 (30,12,7) OR
GO:0050660: FAD binding: 2.139e-10 (30,10,6) OR
GO:0016627: oxidoreductase activity, acting on the CH-CH group of donors: 7.383e-04 (30,17,3) OR
GO:0016651: oxidoreductase activity, acting on NADH or NADPH: 1.343e-01 (30,13,1) OR
GO:0004601: peroxidase activity: 2.081e-01 (30,21,1)

IF 1gdna_#119 AND 1qnja_#71 AND 1ton__#196 AND 1c5y.1#B234 AND 1ton__#211 AND 1autc_#209
THEN
GO:0004263: chymotrypsin activity: 1.859e-19 (46,41,16) OR
GO:0004295: trypsin activity: 3.776e-18 (46,48,16) OR
GO:0004867: serine-type endopeptidase inhibitor activity: 9.919e-04 (46,47,5) OR
GO:0005509: calcium ion binding: 4.556e-03 (46,160,8) OR
GO:0004896: hematopoietin/interferon-class (D200-domain) cytokine receptor activity: 2.772e-01 (46,19,1)

IF 1hx0a2#32 AND 1aq0a_#4 AND 1ho1a_#211
THEN
GO:0004556: alpha-amylase activity: 3.110e-19 (22,15,10) OR
GO:0005509: calcium ion binding: 1.707e-06 (22,160,9) OR
GO:0004867: serine-type endopeptidase inhibitor activity: 5.404e-02 (22,47,2) OR
GO:0016758: transferase activity, transferring hexosyl groups: 8.546e-02 (22,11,1)

IF 1f97a1#62 AND 1a0la_#160 AND 1dlea_#238
THEN
GO:0004295: trypsin activity: 2.207e-19 (69,48,19) OR
GO:0004263: chymotrypsin activity: 2.268e-19 (69,41,18) OR
GO:0003809: thrombin activity: 4.842e-12 (69,10,8) OR
GO:0004867: serine-type endopeptidase inhibitor activity: 1.372e-07 (69,47,10) OR
GO:0005509: calcium ion binding: 1.315e-04 (69,160,13) OR
GO:0005529: sugar binding: 6.348e-01 (69,39,1)

IF 1f3ba2#7 AND 1qfea_#68 AND 3grx__#55
THEN
GO:0004364: glutathione transferase activity: 3.637e-19 (11,11,8) OR
GO:0003714: transcription corepressor activity: 3.188e-02 (11,8,1) OR
GO:0008080: N-acetyltransferase activity: 5.134e-02 (11,13,1) OR
GO:0016651: oxidoreductase activity, acting on NADH or NADPH: 5.134e-02 (11,13,1)

IF 1fc4a_#205 AND 1ojt_2#336
THEN
GO:0015036: disulfide oxidoreductase activity: 2.503e-19 (31,22,12) OR
GO:0016668: oxidoreductase activity, acting on sulfur group of donors, NAD or NADP as acceptor: 5.076e-14 (31,12,8) OR
GO:0050660: FAD binding: 1.403e-12 (31,10,7) OR
GO:0016627: oxidoreductase activity, acting on the CH-CH group of donors: 1.532e-02 (31,17,2) OR
GO:0016651: oxidoreductase activity, acting on NADH or NADPH: 1.385e-01 (31,13,1) OR
GO:0004601: peroxidase activity: 2.143e-01 (31,21,1)

IF 1hx0a2#294 AND 1gox__#246
THEN
GO:0004556: alpha-amylase activity: 3.110e-19 (22,15,10) OR
GO:0005509: calcium ion binding: 1.874e-05 (22,160,8) OR
GO:0004867: serine-type endopeptidase inhibitor activity: 5.404e-02 (22,47,2) OR
GO:0016836: hydro-lyase activity: 2.359e-01 (22,33,1) OR
GO:0003700: transcription factor activity: 6.425e-01 (22,124,1)

IF 1qo3c_#195 AND 2msba_#128
THEN
GO:0005529: sugar binding: 3.926e-19 (14,39,11) OR
GO:0004263: chymotrypsin activity: 1.916e-01 (14,41,1) OR
GO:0004295: trypsin activity: 2.207e-01 (14,48,1) OR
GO:0005509: calcium ion binding: 5.723e-01 (14,160,1)

IF 1hq8a_#223 AND 1qo3c_#195
THEN
GO:0005529: sugar binding: 3.926e-19 (14,39,11) OR
GO:0004263: chymotrypsin activity: 1.916e-01 (14,41,1) OR
GO:0004295: trypsin activity: 2.207e-01 (14,48,1) OR
GO:0005509: calcium ion binding: 5.723e-01 (14,160,1)

IF 1qtn.1#A270 AND 1eno__#137
THEN
GO:0016616: oxidoreductase activity, acting on the CH-OH group of donors, NAD or NADP as acceptor: 2.717e-19 (22,59,14) OR
GO:0016627: oxidoreductase activity, acting on the CH-CH group of donors: 2.888e-04 (22,17,3) OR
GO:0016854: racemase and epimerase activity: 4.600e-03 (22,13,2) OR
GO:0004457: lactate dehydrogenase activity: 7.799e-02 (22,10,1) OR
GO:0016646: oxidoreductase activity, acting on the CH-NH group of donors, NAD or NADP as acceptor: 1.432e-01 (22,19,1) OR
GO:0016836: hydro-lyase activity: 2.359e-01 (22,33,1)

IF 1hdr__#149 AND 1h5qa_#70
THEN
GO:0016616: oxidoreductase activity, acting on the CH-OH group of donors, NAD or NADP as acceptor: 4.493e-19 (18,59,13) OR
GO:0016627: oxidoreductase activity, acting on the CH-CH group of donors: 5.286e-03 (18,17,2) OR
GO:0016836: hydro-lyase activity: 1.928e-02 (18,33,2) OR
GO:0016854: racemase and epimerase activity: 8.272e-02 (18,13,1)

IF 1b16a_#184 AND 1gcoa_#119
THEN
GO:0016616: oxidoreductase activity, acting on the CH-OH group of donors, NAD or NADP as acceptor: 4.493e-19 (18,59,13) OR
GO:0016627: oxidoreductase activity, acting on the CH-CH group of donors: 1.554e-04 (18,17,3) OR
GO:0016854: racemase and epimerase activity: 8.272e-02 (18,13,1) OR
GO:0016836: hydro-lyase activity: 1.975e-01 (18,33,1)

IF 1danh_#197 AND 1erv__#80
THEN
GO:0004295: trypsin activity: 3.021e-19 (59,48,18) OR
GO:0004263: chymotrypsin activity: 6.795e-16 (59,41,15) OR
GO:0003809: thrombin activity: 1.772e-10 (59,10,7) OR
GO:0004867: serine-type endopeptidase inhibitor activity: 4.969e-05 (59,47,7) OR
GO:0005509: calcium ion binding: 4.843e-04 (59,160,11) OR
GO:0004896: hematopoietin/interferon-class (D200-domain) cytokine receptor activity: 3.412e-01 (59,19,1)

IF 1ton__#179 AND 1qq4a_#143 AND 1eq9a_#162
THEN
GO:0004295: trypsin activity: 4.326e-19 (50,48,17) OR
GO:0004263: chymotrypsin activity: 8.910e-19 (50,41,16) OR
GO:0004867: serine-type endopeptidase inhibitor activity: 1.636e-05 (50,47,7) OR
GO:0005509: calcium ion binding: 7.673e-03 (50,160,8) OR

GO:0003809: thrombin activity: 1.352e-02 (50,10,2)

IF 1qq4a_#143 AND 1eq9a_#162 AND 1c5y.1#B234
THEN
GO:0004295: trypsin activity: 4.326e-19 (50,48,17) OR
GO:0004263: chymotrypsin activity: 8.910e-19 (50,41,16) OR
GO:0004867: serine-type endopeptidase inhibitor activity: 1.636e-05 (50,47,7) OR
GO:0005509: calcium ion binding: 7.673e-03 (50,160,8) OR
GO:0003809: thrombin activity: 1.352e-02 (50,10,2)

IF 2hlpa2#315 AND 1qama_#37
THEN
GO:0004457: lactate dehydrogenase activity: 3.610e-19 (23,10,9) OR
GO:0016616: oxidoreductase activity, acting on the CH-OH group of donors, NAD or NADP as acceptor: 9.471e-12 (23,59,10) OR
GO:0019829: cation-transporting ATPase activity: 7.355e-02 (23,9,1) OR
GO:0015405: P-P-bond-hydrolysis-driven transporter activity: 9.690e-02 (23,12,1) OR
GO:0015078: hydrogen ion transporter activity: 1.636e-01 (23,21,1) OR
GO:0005524: ATP binding: 8.844e-01 (23,243,1)

IF 1b3ra1#223 AND 1b3ra1#218
THEN
GO:0016616: oxidoreductase activity, acting on the CH-OH group of donors, NAD or NADP as acceptor: 4.493e-19 (18,59,13) OR
GO:0004457: lactate dehydrogenase activity: 1.798e-03 (18,10,2) OR
GO:0016620: oxidoreductase activity, acting on the aldehyde or oxo group of donors, NAD or NADP as acceptor: 6.423e-02 (18,10,1) OR
GO:0016638: oxidoreductase activity, acting on the CH-NH2 group of donors: 1.068e-01 (18,17,1) OR
GO:0015036: disulfide oxidoreductase activity: 1.362e-01 (18,22,1)

IF 1hyea2#182 AND 1ldna1#94
THEN
GO:0004457: lactate dehydrogenase activity: 5.774e-19 (24,10,9) OR
GO:0016616: oxidoreductase activity, acting on the CH-OH group of donors, NAD or NADP as acceptor: 7.301e-15 (24,59,12) OR
GO:0030145: manganese ion binding: 2.871e-01 (24,38,1) OR
GO:0005524: ATP binding: 6.452e-01 (24,243,2)

IF 1imva_#255 AND 1h7wa4#478
THEN
GO:0015036: disulfide oxidoreductase activity: 3.992e-19 (32,22,12) OR
GO:0016668: oxidoreductase activity, acting on sulfur group of donors, NAD or NADP as acceptor: 6.759e-14 (32,12,8) OR
GO:0050660: FAD binding: 1.795e-12 (32,10,7) OR
GO:0016627: oxidoreductase activity, acting on the CH-CH group of donors: 8.950e-04 (32,17,3) OR
GO:0016651: oxidoreductase activity, acting on NADH or NADPH: 1.426e-01 (32,13,1) OR
GO:0004601: peroxidase activity: 2.204e-01 (32,21,1)

IF 1nhp_1#282 AND 1ojt_2#336
THEN
GO:0015036: disulfide oxidoreductase activity: 3.992e-19 (32,22,12) OR
GO:0016668: oxidoreductase activity, acting on sulfur group of donors, NAD or NADP as acceptor: 6.759e-14 (32,12,8) OR
GO:0050660: FAD binding: 1.795e-12 (32,10,7) OR
GO:0016627: oxidoreductase activity, acting on the CH-CH group of donors: 8.950e-04 (32,17,3) OR
GO:0016651: oxidoreductase activity, acting on NADH or NADPH: 1.426e-01 (32,13,1) OR
GO:0004601: peroxidase activity: 2.204e-01 (32,21,1)

IF 1gdna_#230 AND 1gdna_#123
THEN
GO:0004263: chymotrypsin activity: 5.140e-19 (39,41,15) OR
GO:0004295: trypsin activity: 8.352e-18 (39,48,15) OR
GO:0004867: serine-type endopeptidase inhibitor activity: 2.848e-02 (39,47,3) OR
GO:0005509: calcium ion binding: 7.509e-02 (39,160,5) OR
GO:0003809: thrombin activity: 1.345e-01 (39,10,1)

IF 1gdna_#230 AND 2hlca_#27
THEN
GO:0004263: chymotrypsin activity: 5.140e-19 (39,41,15) OR
GO:0004295: trypsin activity: 8.352e-18 (39,48,15) OR
GO:0004867: serine-type endopeptidase inhibitor activity: 2.848e-02 (39,47,3) OR
GO:0005509: calcium ion binding: 7.509e-02 (39,160,5) OR
GO:0003809: thrombin activity: 1.345e-01 (39,10,1)

IF 1gdna_#230 AND 1dlea_#198

THEN
 GO:0004263: chymotrypsin activity: 5.140e-19 (39,41,15) OR
 GO:0004295: trypsin activity: 8.352e-18 (39,48,15) OR
 GO:0004867: serine-type endopeptidase inhibitor activity: 2.848e-02 (39,47,3) OR
 GO:0005509: calcium ion binding: 7.509e-02 (39,160,5) OR
 GO:0003809: thrombin activity: 1.345e-01 (39,10,1)

IF 1gdna_#230 AND 2hlca_#68
THEN
 GO:0004263: chymotrypsin activity: 5.140e-19 (39,41,15) OR
 GO:0004295: trypsin activity: 8.352e-18 (39,48,15) OR
 GO:0004867: serine-type endopeptidase inhibitor activity: 2.848e-02 (39,47,3) OR
 GO:0005509: calcium ion binding: 7.509e-02 (39,160,5) OR
 GO:0003809: thrombin activity: 1.345e-01 (39,10,1)

IF 1gcoa_#119 AND 1h5qa_#153
THEN
 GO:0016616: oxidoreductase activity, acting on the CH-OH group of donors, NAD or NADP as acceptor: 1.421e-18 (15,59,12) OR
 GO:0016627: oxidoreductase activity, acting on the CH-CH group of donors: 8.768e-05 (15,17,3)

IF 1e3ja2#270 AND 1oaa__#14
THEN
 GO:0016616: oxidoreductase activity, acting on the CH-OH group of donors, NAD or NADP as acceptor: 4.901e-19 (28,59,15) OR
 GO:0004457: lactate dehydrogenase activity: 1.360e-10 (28,10,6) OR
 GO:0016627: oxidoreductase activity, acting on the CH-CH group of donors: 1.939e-05 (28,17,4) OR
 GO:0008080: N-acetyltransferase activity: 1.259e-01 (28,13,1) OR
 GO:0016646: oxidoreductase activity, acting on the CH-NH group of donors, NAD or NADP as acceptor: 1.787e-01 (28,19,1) OR
 GO:0008270: zinc ion binding: 6.796e-01 (28,108,1)

IF 1qlaa2#383 AND 3grx__#56
THEN
 GO:0015036: disulfide oxidoreductase activity: 6.038e-19 (23,22,11) OR
 GO:0016668: oxidoreductase activity, acting on sulfur group of donors, NAD or NADP as acceptor: 1.597e-10 (23,12,6) OR
 GO:0050660: FAD binding: 7.844e-07 (23,10,4) OR
 GO:0016638: oxidoreductase activity, acting on the CH-NH2 group of donors: 1.346e-01 (23,17,1) OR
 GO:0004601: peroxidase activity: 1.636e-01 (23,21,1)

IF 1a4ya_#47 AND 1e43a2#351
THEN
 GO:0004556: alpha-amylase activity: 5.493e-19 (23,15,10) OR
 GO:0005509: calcium ion binding: 2.731e-05 (23,160,8) OR
 GO:0004867: serine-type endopeptidase inhibitor activity: 5.855e-02 (23,47,2) OR
 GO:0016702: oxidoreductase activity, acting on single donors with incorporation of molecular oxygen, incorporation of two atoms of oxygen: 9.690e-02 (23,12,1) OR
 GO:0016651: oxidoreductase activity, acting on NADH or NADPH: 1.046e-01 (23,13,1) OR
 GO:0016811: hydrolase activity, acting on carbon-nitrogen (but not peptide) bonds, in linear amides: 1.197e-01 (23,15,1)

IF 1gdna_#230 AND 1elva1#604
THEN
 GO:0004263: chymotrypsin activity: 8.149e-19 (40,41,15) OR
 GO:0004295: trypsin activity: 1.321e-17 (40,48,15) OR
 GO:0005509: calcium ion binding: 2.716e-02 (40,160,6) OR
 GO:0004867: serine-type endopeptidase inhibitor activity: 3.042e-02 (40,47,3) OR
 GO:0003809: thrombin activity: 1.377e-01 (40,10,1)

IF 1hdr__#149 AND 1oaa__#199
THEN
 GO:0016616: oxidoreductase activity, acting on the CH-OH group of donors, NAD or NADP as acceptor: 1.421e-18 (15,59,12) OR
 GO:0016836: hydro-lyase activity: 1.353e-02 (15,33,2) OR
 GO:0016646: oxidoreductase activity, acting on the CH-NH group of donors, NAD or NADP as acceptor: 9.988e-02 (15,19,1)

IF 1oaa__#199 AND 1eny__#94
THEN
 GO:0016616: oxidoreductase activity, acting on the CH-OH group of donors, NAD or NADP as acceptor: 1.421e-18 (15,59,12) OR
 GO:0016836: hydro-lyase activity: 1.353e-02 (15,33,2) OR
 GO:0016646: oxidoreductase activity, acting on the CH-NH group of donors, NAD or NADP as acceptor: 9.988e-02 (15,19,1)

IF 1hdr__#156 AND 1oaa__#199
THEN
 GO:0016616: oxidoreductase activity, acting on the CH-OH group of donors, NAD or NADP as acceptor: 1.421e-18 (15,59,12) OR

GO:0016836: hydro-lyase activity: 1.353e-02 (15,33,2) OR
GO:0016646: oxidoreductase activity, acting on the CH-NH group of donors, NAD or NADP as acceptor: 9.988e-02 (15,19,1)

IF 1hdca_#187 AND 1e6ua_#10
THEN
GO:0016616: oxidoreductase activity, acting on the CH-OH group of donors, NAD or NADP as acceptor: 2.187e-18 (12,59,11) OR
GO:0016627: oxidoreductase activity, acting on the CH-CH group of donors: 7.249e-02 (12,17,1)

IF 1oaa__#235 AND 1oaa__#199
THEN
GO:0016616: oxidoreductase activity, acting on the CH-OH group of donors, NAD or NADP as acceptor: 2.187e-18 (12,59,11) OR
GO:0016836: hydro-lyase activity: 1.363e-01 (12,33,1)

IF 1oaa__#235 AND 1e6wa_#24
THEN
GO:0016616: oxidoreductase activity, acting on the CH-OH group of donors, NAD or NADP as acceptor: 2.187e-18 (12,59,11) OR
GO:0016627: oxidoreductase activity, acting on the CH-CH group of donors: 7.249e-02 (12,17,1)

IF 1ajsa_#139 AND 1qgna_#149
THEN
GO:0016846: carbon-sulfur lyase activity: 1.803e-18 (14,10,8) OR
GO:0008483: transaminase activity: 9.606e-09 (14,17,5) OR
GO:0016831: carboxy-lyase activity: 1.213e-01 (14,25,1)

IF 1qgna_#149 AND 1fc4a_#111
THEN
GO:0016846: carbon-sulfur lyase activity: 1.803e-18 (14,10,8) OR
GO:0008483: transaminase activity: 9.606e-09 (14,17,5) OR
GO:0016831: carboxy-lyase activity: 1.213e-01 (14,25,1)

IF 1oaa__#235 AND 1nat__#50
THEN
GO:0016616: oxidoreductase activity, acting on the CH-OH group of donors, NAD or NADP as acceptor: 1.421e-18 (15,59,12) OR
GO:0016627: oxidoreductase activity, acting on the CH-CH group of donors: 8.982e-02 (15,17,1) OR
GO:0016646: oxidoreductase activity, acting on the CH-NH group of donors, NAD or NADP as acceptor: 9.988e-02 (15,19,1) OR
GO:0016836: hydro-lyase activity: 1.674e-01 (15,33,1)

IF 1e6wa_#24 AND 1e6ua_#10
THEN
GO:0016616: oxidoreductase activity, acting on the CH-OH group of donors, NAD or NADP as acceptor: 1.421e-18 (15,59,12) OR
GO:0004457: lactate dehydrogenase activity: 5.379e-02 (15,10,1) OR
GO:0016627: oxidoreductase activity, acting on the CH-CH group of donors: 8.982e-02 (15,17,1) OR
GO:0016646: oxidoreductase activity, acting on the CH-NH group of donors, NAD or NADP as acceptor: 9.988e-02 (15,19,1)

IF 1hq8a_#223 AND 2msba_#218
THEN
GO:0005529: sugar binding: 1.458e-18 (15,39,11) OR
GO:0004263: chymotrypsin activity: 2.039e-01 (15,41,1) OR
GO:0005509: calcium ion binding: 2.188e-01 (15,160,2) OR
GO:0004295: trypsin activity: 2.345e-01 (15,48,1)

IF 1f00i3#856 AND 2msba_#218
THEN
GO:0005529: sugar binding: 1.458e-18 (15,39,11) OR
GO:0004263: chymotrypsin activity: 2.039e-01 (15,41,1) OR
GO:0005509: calcium ion binding: 2.188e-01 (15,160,2) OR
GO:0004295: trypsin activity: 2.345e-01 (15,48,1)

IF 2msba_#218 AND 2msba_#128
THEN
GO:0005529: sugar binding: 1.458e-18 (15,39,11) OR
GO:0004263: chymotrypsin activity: 2.039e-01 (15,41,1) OR
GO:0005509: calcium ion binding: 2.188e-01 (15,160,2) OR
GO:0004295: trypsin activity: 2.345e-01 (15,48,1)

IF 1bxoa_#141 AND 1mpp__#221 AND 1pfza_#193
THEN
GO:0004190: aspartic-type endopeptidase activity: 6.569e-18 (8,23,8)

IF 1htr.1#B30

THEN
GO:0004190: aspartic-type endopeptidase activity: 6.569e-18 (8,23,8)

IF 1j71a_#220
THEN
GO:0004190: aspartic-type endopeptidase activity: 6.569e-18 (8,23,8)

IF 1mpp__#218
THEN
GO:0004190: aspartic-type endopeptidase activity: 6.569e-18 (8,23,8)

IF 1mpp__#154 AND 1pfza_#306
THEN
GO:0004190: aspartic-type endopeptidase activity: 6.569e-18 (8,23,8)

IF 1eq9a_#162 AND 1g51a3#525 AND 1dlea_#238
THEN
GO:0004295: trypsin activity: 1.373e-18 (53,48,17) OR
GO:0004263: chymotrypsin activity: 2.616e-18 (53,41,16) OR
GO:0003809: thrombin activity: 2.459e-05 (53,10,4) OR
GO:0004867: serine-type endopeptidase inhibitor activity: 2.364e-04 (53,47,6) OR
GO:0005509: calcium ion binding: 7.964e-04 (53,160,10)

IF 1eq9a_#32 AND 1eq9a_#162 AND 1ton__#231
THEN
GO:0004295: trypsin activity: 1.373e-18 (53,48,17) OR
GO:0004263: chymotrypsin activity: 2.616e-18 (53,41,16) OR
GO:0004867: serine-type endopeptidase inhibitor activity: 2.428e-05 (53,47,7) OR
GO:0003809: thrombin activity: 2.459e-05 (53,10,4) OR
GO:0005509: calcium ion binding: 3.138e-03 (53,160,9)

IF 1fjfc2#153 AND 1e39a2#516 AND 1trb_1#14
THEN
GO:0050660: FAD binding: 1.379e-18 (26,10,9) OR
GO:0016668: oxidoreductase activity, acting on sulfur group of donors, NAD or NADP as acceptor: 2.300e-12 (26,12,7) OR
GO:0015036: disulfide oxidoreductase activity: 6.161e-12 (26,22,8) OR
GO:0016651: oxidoreductase activity, acting on NADH or NADPH: 1.174e-01 (26,13,1) OR
GO:0016627: oxidoreductase activity, acting on the CH-CH group of donors: 1.508e-01 (26,17,1)

IF 1c7na_#95 AND 1qgna_#149
THEN
GO:0016846: carbon-sulfur lyase activity: 1.803e-18 (14,10,8) OR
GO:0008483: transaminase activity: 1.000e-06 (14,17,4) OR
GO:0004180: carboxypeptidase activity: 7.454e-02 (14,15,1) OR
GO:0016831: carboxy-lyase activity: 1.213e-01 (14,25,1)

IF 1fvua_#120 AND 1h8ua_#113
THEN
GO:0005529: sugar binding: 1.458e-18 (15,39,11) OR
GO:0008201: heparin binding: 1.246e-01 (15,24,1) OR
GO:0004263: chymotrypsin activity: 2.039e-01 (15,41,1) OR
GO:0004295: trypsin activity: 2.345e-01 (15,48,1) OR
GO:0005509: calcium ion binding: 5.975e-01 (15,160,1)

IF 1fvua_#120 AND 1dv8a_#213
THEN
GO:0005529: sugar binding: 1.458e-18 (15,39,11) OR
GO:0008201: heparin binding: 1.246e-01 (15,24,1) OR
GO:0004263: chymotrypsin activity: 2.039e-01 (15,41,1) OR
GO:0004295: trypsin activity: 2.345e-01 (15,48,1) OR
GO:0005509: calcium ion binding: 5.975e-01 (15,160,1)

IF 1cs1a_#160 AND 7taa_2#331
THEN
GO:0004556: alpha-amylase activity: 2.506e-18 (16,15,9) OR
GO:0005509: calcium ion binding: 1.828e-04 (16,160,6) OR
GO:0016758: transferase activity, transferring hexosyl groups: 6.284e-02 (16,11,1)

IF 1qnja_#77 AND 1autc_#45
THEN

GO:0004263: chymotrypsin activity: 1.287e-18 (51,41,16) OR
GO:0004295: trypsin activity: 2.582e-17 (51,48,16) OR
GO:0004867: serine-type endopeptidase inhibitor activity: 1.903e-04 (51,47,6) OR
GO:0005509: calcium ion binding: 5.781e-04 (51,160,10) OR
GO:0003809: thrombin activity: 1.404e-02 (51,10,2) OR
GO:0004896: hematopoietin/interferon-class (D200-domain) cytokine receptor activity: 3.024e-01 (51,19,1)

IF 1gdna_#119 AND 1ddja_#726
THEN
GO:0004295: trypsin activity: 1.623e-18 (44,48,16) OR
GO:0004263: chymotrypsin activity: 4.491e-18 (44,41,15) OR
GO:0005509: calcium ion binding: 3.421e-03 (44,160,8) OR
GO:0004867: serine-type endopeptidase inhibitor activity: 6.366e-03 (44,47,4) OR
GO:0004896: hematopoietin/interferon-class (D200-domain) cytokine receptor activity: 2.668e-01 (44,19,1)

IF 1hdr__#149 AND 1evqa_#305
THEN
GO:0016616: oxidoreductase activity, acting on the CH-OH group of donors, NAD or NADP as acceptor: 1.400e-18 (19,59,13) OR
GO:0016854: racemase and epimerase activity: 3.433e-03 (19,13,2) OR
GO:0016627: oxidoreductase activity, acting on the CH-CH group of donors: 1.125e-01 (19,17,1) OR
GO:0016646: oxidoreductase activity, acting on the CH-NH group of donors, NAD or NADP as acceptor: 1.249e-01 (19,19,1) OR
GO:0016836: hydro-lyase activity: 2.073e-01 (19,33,1) OR
GO:0030145: manganese ion binding: 2.349e-01 (19,38,1)

IF 1ha6a_#20 AND 1el0a_#62
THEN
GO:0008009: chemokine activity: 4.366e-18 (8,10,7) OR
GO:0008083: growth factor activity: 1.170e-01 (8,42,1)

IF 1qsta_#154 AND 1h4vb2#282
THEN
GO:0004812: tRNA ligase activity: 2.243e-18 (30,26,12) OR
GO:0005524: ATP binding: 3.906e-06 (30,243,12) OR
GO:0000287: magnesium ion binding: 1.169e-02 (30,128,5) OR
GO:0000049: tRNA binding: 1.343e-01 (30,13,1)

IF 1hq8a_#185 AND 1fvua_#72
THEN
GO:0005529: sugar binding: 9.405e-18 (9,39,9)

IF 1e87a_#155 AND 1fvua_#72
THEN
GO:0005529: sugar binding: 9.405e-18 (9,39,9)

IF 1czan1#92 AND 1xvaa_#68
THEN
GO:0008757: S-adenosylmethionine-dependent methyltransferase activity: 3.160e-18 (11,24,9) OR
GO:0008270: zinc ion binding: 3.596e-01 (11,108,1) OR
GO:0000287: magnesium ion binding: 4.115e-01 (11,128,1)

IF 1g38a_#47
THEN
GO:0008757: S-adenosylmethionine-dependent methyltransferase activity: 9.854e-18 (8,24,8)

IF 2bb2_2#134 AND 1h7wa4#476
THEN
GO:0015036: disulfide oxidoreductase activity: 1.976e-18 (25,22,11) OR
GO:0016668: oxidoreductase activity, acting on sulfur group of donors, NAD or NADP as acceptor: 1.683e-12 (25,12,7) OR
GO:0050660: FAD binding: 1.041e-08 (25,10,5) OR
GO:0016651: oxidoreductase activity, acting on NADH or NADPH: 1.132e-01 (25,13,1) OR
GO:0016627: oxidoreductase activity, acting on the CH-CH group of donors: 1.454e-01 (25,17,1)

IF 2ae2a_#196 AND 1e8ca3#111
THEN
GO:0016616: oxidoreductase activity, acting on the CH-OH group of donors, NAD or NADP as acceptor: 1.027e-17 (10,59,10)

IF 1qgna_#149 AND 1elua_#199
THEN
GO:0016846: carbon-sulfur lyase activity: 3.862e-18 (15,10,8) OR
GO:0008483: transaminase activity: 1.061e-10 (15,17,6) OR

GO:0016831: carboxy-lyase activity: 1.294e-01 (15,25,1)

IF 1ypta_#409
THEN
GO:0004725: protein tyrosine phosphatase activity: 3.862e-18 (10,15,8) OR
GO:0005096: GTPase activator activity: 5.379e-02 (10,15,1) OR
GO:0005525: GTP binding: 1.662e-01 (10,49,1)

IF 3prn__#143 AND 1h7wa4#478
THEN
GO:0015036: disulfide oxidoreductase activity: 1.976e-18 (25,22,11) OR
GO:0016668: oxidoreductase activity, acting on sulfur group of donors, NAD or NADP as acceptor: 2.791e-10 (25,12,6) OR
GO:0050660: FAD binding: 1.041e-08 (25,10,5) OR
GO:0016651: oxidoreductase activity, acting on NADH or NADPH: 1.132e-01 (25,13,1) OR
GO:0016638: oxidoreductase activity, acting on the CH-NH2 group of donors: 1.454e-01 (25,17,1) OR
GO:0004601: peroxidase activity: 1.766e-01 (25,21,1)

IF 1a6o__#217 AND 1bio__#210
THEN
GO:0004674: protein serine/threonine kinase activity: 2.606e-18 (33,42,14) OR
GO:0005524: ATP binding: 2.444e-07 (33,243,14) OR
GO:0005516: calmodulin binding: 2.755e-03 (33,24,3) OR
GO:0003755: peptidyl-prolyl cis-trans isomerase activity: 1.256e-01 (33,11,1) OR
GO:0004896: hematopoietin/interferon-class (D200-domain) cytokine receptor activity: 2.073e-01 (33,19,1)

IF 1g25a_#22 AND 1ton__#196
THEN
GO:0004295: trypsin activity: 2.490e-18 (45,48,16) OR
GO:0004263: chymotrypsin activity: 4.787e-13 (45,41,12) OR
GO:0003809: thrombin activity: 4.660e-04 (45,10,3) OR
GO:0004867: serine-type endopeptidase inhibitor activity: 8.955e-04 (45,47,5) OR
GO:0005509: calcium ion binding: 3.957e-03 (45,160,8) OR
GO:0004896: hematopoietin/interferon-class (D200-domain) cytokine receptor activity: 2.720e-01 (45,19,1)

IF 1ceqa2#199 AND 1llc_2#291
THEN
GO:0004457: lactate dehydrogenase activity: 7.719e-18 (16,10,8) OR
GO:0016616: oxidoreductase activity, acting on the CH-OH group of donors, NAD or NADP as acceptor: 3.342e-10 (16,59,8)

IF 1ceqa2#199 AND 1qb7a_#86
THEN
GO:0004457: lactate dehydrogenase activity: 7.719e-18 (16,10,8) OR
GO:0016616: oxidoreductase activity, acting on the CH-OH group of donors, NAD or NADP as acceptor: 3.342e-10 (16,59,8)

IF 1qgna_#149 AND 1gdea_#379
THEN
GO:0016846: carbon-sulfur lyase activity: 3.862e-18 (15,10,8) OR
GO:0008483: transaminase activity: 1.436e-08 (15,17,5) OR
GO:0016831: carboxy-lyase activity: 1.294e-01 (15,25,1) OR
GO:0005524: ATP binding: 7.546e-01 (15,243,1)

IF 1hdr__#156 AND 1h5qa_#70
THEN
GO:0016616: oxidoreductase activity, acting on the CH-OH group of donors, NAD or NADP as acceptor: 3.938e-18 (20,59,13) OR
GO:0016627: oxidoreductase activity, acting on the CH-CH group of donors: 4.731e-06 (20,17,4) OR
GO:0016836: hydro-lyase activity: 2.359e-02 (20,33,2) OR
GO:0016854: racemase and epimerase activity: 9.152e-02 (20,13,1)

IF 1oaa__#175 AND 1h5qa_#70
THEN
GO:0016616: oxidoreductase activity, acting on the CH-OH group of donors, NAD or NADP as acceptor: 3.938e-18 (20,59,13) OR
GO:0016627: oxidoreductase activity, acting on the CH-CH group of donors: 4.731e-06 (20,17,4) OR
GO:0016836: hydro-lyase activity: 2.359e-02 (20,33,2) OR
GO:0016854: racemase and epimerase activity: 9.152e-02 (20,13,1)

IF 1qhoa4#75 AND 1htp__#16
THEN
GO:0004556: alpha-amylase activity: 5.315e-18 (17,15,9) OR
GO:0005509: calcium ion binding: 2.494e-05 (17,160,7) OR
GO:0004867: serine-type endopeptidase inhibitor activity: 2.567e-01 (17,47,1)

IF 1avaa2#204 AND 1cpy__#93
THEN
 GO:0004556: alpha-amylase activity: 5.315e-18 (17,15,9) OR
 GO:0005509: calcium ion binding: 2.689e-04 (17,160,6) OR
 GO:0004867: serine-type endopeptidase inhibitor activity: 3.359e-02 (17,47,2)

IF 1d4xg_#60 AND 1ljra1#165
THEN
 GO:0004364: glutathione transferase activity: 2.831e-18 (13,11,8) OR
 GO:0003714: transcription corepressor activity: 3.758e-02 (13,8,1) OR
 GO:0015078: hydrogen ion transporter activity: 9.588e-02 (13,21,1) OR
 GO:0004812: tRNA ligase activity: 1.174e-01 (13,26,1) OR
 GO:0005507: copper ion binding: 1.672e-01 (13,38,1) OR
 GO:0005524: ATP binding: 7.039e-01 (13,243,1)

IF 1h8d.1#H167 AND 1autc_#188 AND 1ton__#112
THEN
 GO:0004263: chymotrypsin activity: 3.685e-18 (54,41,16) OR
 GO:0004295: trypsin activity: 7.339e-17 (54,48,16) OR
 GO:0004867: serine-type endopeptidase inhibitor activity: 1.814e-07 (54,47,9) OR
 GO:0003809: thrombin activity: 2.651e-05 (54,10,4) OR
 GO:0005509: calcium ion binding: 3.580e-03 (54,160,9)

IF 1gdna_#230 AND 1fjsa_#158
THEN
 GO:0004263: chymotrypsin activity: 4.863e-18 (35,41,14) OR
 GO:0004295: trypsin activity: 6.323e-17 (35,48,14) OR
 GO:0004867: serine-type endopeptidase inhibitor activity: 2.140e-02 (35,47,3) OR
 GO:0005509: calcium ion binding: 1.463e-01 (35,160,4)

IF 1qrra_#208 AND 1h5qa_#70
THEN
 GO:0016616: oxidoreductase activity, acting on the CH-OH group of donors, NAD or NADP as acceptor: 3.938e-18 (20,59,13) OR
 GO:0016627: oxidoreductase activity, acting on the CH-CH group of donors: 2.155e-04 (20,17,3) OR
 GO:0016836: hydro-lyase activity: 2.359e-02 (20,33,2) OR
 GO:0016854: racemase and epimerase activity: 9.152e-02 (20,13,1) OR
 GO:0016646: oxidoreductase activity, acting on the CH-NH group of donors, NAD or NADP as acceptor: 1.310e-01 (20,19,1)

IF 1qtn.1#A270 AND 1h5qa_#70 AND 1e7wa_#36
THEN
 GO:0016616: oxidoreductase activity, acting on the CH-OH group of donors, NAD or NADP as acceptor: 3.938e-18 (20,59,13) OR
 GO:0016627: oxidoreductase activity, acting on the CH-CH group of donors: 4.731e-06 (20,17,4) OR
 GO:0016854: racemase and epimerase activity: 9.152e-02 (20,13,1) OR
 GO:0016646: oxidoreductase activity, acting on the CH-NH group of donors, NAD or NADP as acceptor: 1.310e-01 (20,19,1) OR
 GO:0016836: hydro-lyase activity: 2.169e-01 (20,33,1)

IF 1hq8a_#206 AND 1tn3__#113
THEN
 GO:0005529: sugar binding: 4.622e-18 (16,39,11) OR
 GO:0008201: heparin binding: 1.323e-01 (16,24,1) OR
 GO:0004263: chymotrypsin activity: 2.159e-01 (16,41,1) OR
 GO:0005509: calcium ion binding: 2.410e-01 (16,160,2) OR
 GO:0004295: trypsin activity: 2.481e-01 (16,48,1)

IF 1gdea_#379 AND 1fc4a_#111
THEN
 GO:0016846: carbon-sulfur lyase activity: 7.719e-18 (16,10,8) OR
 GO:0008483: transaminase activity: 9.838e-13 (16,17,7) OR
 GO:0016831: carboxy-lyase activity: 1.375e-01 (16,25,1)

IF 1c7na_#95 AND 1elua_#199
THEN
 GO:0016846: carbon-sulfur lyase activity: 7.719e-18 (16,10,8) OR
 GO:0008483: transaminase activity: 9.838e-13 (16,17,7) OR
 GO:0016831: carboxy-lyase activity: 1.375e-01 (16,25,1)

IF 1atia2#67 AND 1qsta_#154
THEN
 GO:0004812: tRNA ligase activity: 5.800e-18 (32,26,12) OR

GO:0005524: ATP binding: 8.664e-06 (32,243,12) OR
GO:0000287: magnesium ion binding: 3.158e-03 (32,128,6) OR
GO:0000049: tRNA binding: 9.615e-03 (32,13,2)

IF 1h5qa_#70 AND 1e8ca3#111
THEN
GO:0016616: oxidoreductase activity, acting on the CH-OH group of donors, NAD or NADP as acceptor: 3.938e-18 (20,59,13) OR
GO:0016627: oxidoreductase activity, acting on the CH-CH group of donors: 2.155e-04 (20,17,3) OR
GO:0016846: carbon-sulfur lyase activity: 7.113e-02 (20,10,1) OR
GO:0016854: racemase and epimerase activity: 9.152e-02 (20,13,1) OR
GO:0016646: oxidoreductase activity, acting on the CH-NH group of donors, NAD or NADP as acceptor: 1.310e-01 (20,19,1) OR
GO:0008757: S-adenosylmethionine-dependent methyltransferase activity: 1.627e-01 (20,24,1)

IF 1gnwa1#160 AND 1evqa_#305
THEN
GO:0004364: glutathione transferase activity: 1.200e-17 (8,11,7) OR
GO:0003714: transcription corepressor activity: 2.328e-02 (8,8,1)

IF 1iow_1#37 AND 1gnwa1#160
THEN
GO:0004364: glutathione transferase activity: 1.200e-17 (8,11,7) OR
GO:0003714: transcription corepressor activity: 2.328e-02 (8,8,1)

IF 1ib2a_#974 AND 1gnwa1#160
THEN
GO:0004364: glutathione transferase activity: 1.200e-17 (8,11,7) OR
GO:0016651: oxidoreductase activity, acting on NADH or NADPH: 3.758e-02 (8,13,1)

IF 1glqa2#53 AND 1ib2a_#974
THEN
GO:0004364: glutathione transferase activity: 1.200e-17 (8,11,7) OR
GO:0016651: oxidoreductase activity, acting on NADH or NADPH: 3.758e-02 (8,13,1)

IF 1b16a_#184 AND 1eq2a_#11
THEN
GO:0016616: oxidoreductase activity, acting on the CH-OH group of donors, NAD or NADP as acceptor: 5.592e-18 (16,59,12) OR
GO:0016854: racemase and epimerase activity: 7.386e-02 (16,13,1) OR
GO:0016627: oxidoreductase activity, acting on the CH-CH group of donors: 9.553e-02 (16,17,1) OR
GO:0016646: oxidoreductase activity, acting on the CH-NH group of donors, NAD or NADP as acceptor: 1.062e-01 (16,19,1) OR
GO:0016836: hydro-lyase activity: 1.776e-01 (16,33,1)

IF 1qrra_#208 AND 1b16a_#184
THEN
GO:0016616: oxidoreductase activity, acting on the CH-OH group of donors, NAD or NADP as acceptor: 5.592e-18 (16,59,12) OR
GO:0016854: racemase and epimerase activity: 7.386e-02 (16,13,1) OR
GO:0016627: oxidoreductase activity, acting on the CH-CH group of donors: 9.553e-02 (16,17,1) OR
GO:0016646: oxidoreductase activity, acting on the CH-NH group of donors, NAD or NADP as acceptor: 1.062e-01 (16,19,1) OR
GO:0016836: hydro-lyase activity: 1.776e-01 (16,33,1)

IF 1oaa__#161 AND 1oaa__#199
THEN
GO:0016616: oxidoreductase activity, acting on the CH-OH group of donors, NAD or NADP as acceptor: 1.399e-17 (13,59,11) OR
GO:0016836: hydro-lyase activity: 1.021e-02 (13,33,2)

IF 1iba__#39 AND 1h7wa4#478
THEN
GO:0015036: disulfide oxidoreductase activity: 5.736e-18 (27,22,11) OR
GO:0016668: oxidoreductase activity, acting on sulfur group of donors, NAD or NADP as acceptor: 1.436e-14 (27,12,8) OR
GO:0050660: FAD binding: 1.070e-10 (27,10,6) OR
GO:0016651: oxidoreductase activity, acting on NADH or NADPH: 1.217e-01 (27,13,1) OR
GO:0004601: peroxidase activity: 1.893e-01 (27,21,1)

IF 1trb_1#41 AND 1qfma1#186
THEN
GO:0050660: FAD binding: 6.306e-18 (30,10,9) OR
GO:0016668: oxidoreductase activity, acting on sulfur group of donors, NAD or NADP as acceptor: 1.368e-16 (30,12,9) OR
GO:0015036: disulfide oxidoreductase activity: 2.928e-15 (30,22,10) OR
GO:0016651: oxidoreductase activity, acting on NADH or NADPH: 1.343e-01 (30,13,1) OR
GO:0016638: oxidoreductase activity, acting on the CH-NH2 group of donors: 1.720e-01 (30,17,1)

IF 1avaa2#204 AND 3grx__#56
THEN
GO:0004556: alpha-amylase activity: 1.061e-17 (18,15,9) OR
GO:0005509: calcium ion binding: 3.881e-05 (18,160,7) OR
GO:0004867: serine-type endopeptidase inhibitor activity: 3.738e-02 (18,47,2)

IF 1clxa_#13 AND 1e43a2#236
THEN
GO:0004556: alpha-amylase activity: 1.061e-17 (18,15,9) OR
GO:0005509: calcium ion binding: 3.142e-06 (18,160,8) OR
GO:0004867: serine-type endopeptidase inhibitor activity: 2.696e-01 (18,47,1)

IF 1cpy__#93 AND 1e43a2#351
THEN
GO:0004556: alpha-amylase activity: 1.061e-17 (18,15,9) OR
GO:0005509: calcium ion binding: 3.839e-04 (18,160,6) OR
GO:0004867: serine-type endopeptidase inhibitor activity: 3.273e-03 (18,47,3)

IF 1h7wa4#476 AND 1qrra_#69
THEN
GO:0015036: disulfide oxidoreductase activity: 5.736e-18 (27,22,11) OR
GO:0016668: oxidoreductase activity, acting on sulfur group of donors, NAD or NADP as acceptor: 3.100e-12 (27,12,7) OR
GO:0050660: FAD binding: 1.070e-10 (27,10,6) OR
GO:0016651: oxidoreductase activity, acting on NADH or NADPH: 1.217e-01 (27,13,1) OR
GO:0016627: oxidoreductase activity, acting on the CH-CH group of donors: 1.562e-01 (27,17,1) OR
GO:0004601: peroxidase activity: 1.893e-01 (27,21,1)

IF 1gcoa_#119 AND 1hdr__#156
THEN
GO:0016616: oxidoreductase activity, acting on the CH-OH group of donors, NAD or NADP as acceptor: 1.017e-17 (21,59,13) OR
GO:0016627: oxidoreductase activity, acting on the CH-CH group of donors: 5.822e-06 (21,17,4) OR
GO:0016854: racemase and epimerase activity: 4.193e-03 (21,13,2) OR
GO:0016836: hydro-lyase activity: 2.587e-02 (21,33,2)

IF 2dkb__#114 AND 1elua_#199 AND 1gdea_#379
THEN
GO:0016846: carbon-sulfur lyase activity: 1.457e-17 (17,10,8) OR
GO:0008483: transaminase activity: 7.711e-15 (17,17,8) OR
GO:0016831: carboxy-lyase activity: 1.454e-01 (17,25,1)

IF 2hlpa2#272 AND 1qama_#37
THEN
GO:0004457: lactate dehydrogenase activity: 1.457e-17 (17,10,8) OR
GO:0016616: oxidoreductase activity, acting on the CH-OH group of donors, NAD or NADP as acceptor: 6.207e-10 (17,59,8) OR
GO:0008757: S-adenosylmethionine-dependent methyltransferase activity: 1.400e-01 (17,24,1)

IF 1b4va1#11 AND 1nhp_1#282
THEN
GO:0015036: disulfide oxidoreductase activity: 9.413e-18 (28,22,11) OR
GO:0016668: oxidoreductase activity, acting on sulfur group of donors, NAD or NADP as acceptor: 2.008e-14 (28,12,8) OR
GO:0050660: FAD binding: 1.360e-10 (28,10,6) OR
GO:0016627: oxidoreductase activity, acting on the CH-CH group of donors: 1.259e-02 (28,17,2) OR
GO:0016638: oxidoreductase activity, acting on the CH-NH2 group of donors: 1.615e-01 (28,17,1)

IF 1h7wa4#476 AND 1dn2a1#261
THEN
GO:0015036: disulfide oxidoreductase activity: 9.413e-18 (28,22,11) OR
GO:0016668: oxidoreductase activity, acting on sulfur group of donors, NAD or NADP as acceptor: 2.008e-14 (28,12,8) OR
GO:0050660: FAD binding: 6.338e-13 (28,10,7) OR
GO:0016651: oxidoreductase activity, acting on NADH or NADPH: 1.259e-01 (28,13,1) OR
GO:0016627: oxidoreductase activity, acting on the CH-CH group of donors: 1.615e-01 (28,17,1)

IF 1erv__#80 AND 1autc_#209
THEN
GO:0004295: trypsin activity: 7.948e-18 (58,48,17) OR
GO:0004263: chymotrypsin activity: 5.114e-16 (58,41,15) OR
GO:0003809: thrombin activity: 1.563e-10 (58,10,7) OR
GO:0004867: serine-type endopeptidase inhibitor activity: 4.437e-05 (58,47,7) OR
GO:0005509: calcium ion binding: 4.148e-04 (58,160,11) OR
GO:0004896: hematopoietin/interferon-class (D200-domain) cytokine receptor activity: 3.364e-01 (58,19,1)

IF 1c7na_#281 AND 1e5ea_#71
THEN
GO:0008483: transaminase activity: 1.212e-17 (16,17,9) OR
GO:0016846: carbon-sulfur lyase activity: 1.632e-07 (16,10,4) OR
GO:0016831: carboxy-lyase activity: 8.964e-03 (16,25,2) OR
GO:0016866: intramolecular transferase activity: 6.836e-02 (16,12,1)

IF 1c5y.1#B18 AND 1danh_#140 AND 1c5y.1#B241
THEN
GO:0004263: chymotrypsin activity: 9.815e-18 (46,41,15) OR
GO:0004295: trypsin activity: 1.568e-16 (46,48,15) OR
GO:0004867: serine-type endopeptidase inhibitor activity: 1.057e-04 (46,47,6) OR
GO:0005509: calcium ion binding: 1.112e-03 (46,160,9) OR
GO:0003809: thrombin activity: 1.568e-01 (46,10,1)

IF 1hdr__#156 AND 1eny__#94 AND 1eno__#137
THEN
GO:0016616: oxidoreductase activity, acting on the CH-OH group of donors, NAD or NADP as acceptor: 1.017e-17 (21,59,13) OR
GO:0016627: oxidoreductase activity, acting on the CH-CH group of donors: 2.504e-04 (21,17,3) OR
GO:0016854: racemase and epimerase activity: 4.193e-03 (21,13,2) OR
GO:0016646: oxidoreductase activity, acting on the CH-NH group of donors, NAD or NADP as acceptor: 8.940e-03 (21,19,2) OR
GO:0016836: hydro-lyase activity: 2.265e-01 (21,33,1)

IF 1hdr__#149 AND 1eny__#94
THEN
GO:0016616: oxidoreductase activity, acting on the CH-OH group of donors, NAD or NADP as acceptor: 1.017e-17 (21,59,13) OR
GO:0016854: racemase and epimerase activity: 4.193e-03 (21,13,2) OR
GO:0016627: oxidoreductase activity, acting on the CH-CH group of donors: 7.176e-03 (21,17,2) OR
GO:0016646: oxidoreductase activity, acting on the CH-NH group of donors, NAD or NADP as acceptor: 8.940e-03 (21,19,2) OR
GO:0016836: hydro-lyase activity: 2.587e-02 (21,33,2)

IF 1imva_#255 AND 1trb_1#41
THEN
GO:0050660: FAD binding: 8.882e-18 (31,10,9) OR
GO:0015036: disulfide oxidoreductase activity: 4.306e-15 (31,22,10) OR
GO:0016668: oxidoreductase activity, acting on sulfur group of donors, NAD or NADP as acceptor: 5.076e-14 (31,12,8) OR
GO:0016627: oxidoreductase activity, acting on the CH-CH group of donors: 1.532e-02 (31,17,2) OR
GO:0016651: oxidoreductase activity, acting on NADH or NADPH: 1.385e-01 (31,13,1) OR
GO:0016638: oxidoreductase activity, acting on the CH-NH2 group of donors: 1.772e-01 (31,17,1)

IF 1bvza3#298 AND 1gcya2#331
THEN
GO:0004556: alpha-amylase activity: 2.012e-17 (19,15,9) OR
GO:0005509: calcium ion binding: 5.158e-06 (19,160,8) OR
GO:0004867: serine-type endopeptidase inhibitor activity: 4.133e-02 (19,47,2)

IF 1bvza3#298 AND 1qhoa4#399
THEN
GO:0004556: alpha-amylase activity: 2.012e-17 (19,15,9) OR
GO:0005509: calcium ion binding: 5.158e-06 (19,160,8) OR
GO:0004867: serine-type endopeptidase inhibitor activity: 4.133e-02 (19,47,2)

IF 1gdna_#230 AND 1ton__#112
THEN
GO:0004263: chymotrypsin activity: 1.256e-17 (37,41,14) OR
GO:0004295: trypsin activity: 1.625e-16 (37,48,14) OR
GO:0004867: serine-type endopeptidase inhibitor activity: 2.480e-02 (37,47,3) OR
GO:0005509: calcium ion binding: 6.237e-02 (37,160,5) OR
GO:0003809: thrombin activity: 1.280e-01 (37,10,1)

IF 1gdna_#119 AND 1ton__#120
THEN
GO:0004295: trypsin activity: 1.321e-17 (40,48,15) OR
GO:0004263: chymotrypsin activity: 4.641e-17 (40,41,14) OR
GO:0004867: serine-type endopeptidase inhibitor activity: 4.507e-03 (40,47,4) OR
GO:0005509: calcium ion binding: 2.716e-02 (40,160,6) OR
GO:0004896: hematopoietin/interferon-class (D200-domain) cytokine receptor activity: 2.457e-01 (40,19,1)

IF 1gdna_#119 AND 1gg6.1#C156

THEN
GO:0004295: trypsin activity: 1.321e-17 (40,48,15) OR
GO:0004263: chymotrypsin activity: 4.641e-17 (40,41,14) OR
GO:0004867: serine-type endopeptidase inhibitor activity: 4.507e-03 (40,47,4) OR
GO:0005509: calcium ion binding: 2.716e-02 (40,160,6) OR
GO:0004896: hematopoietin/interferon-class (D200-domain) cytokine receptor activity: 2.457e-01 (40,19,1)

IF 1qgna_#207 AND 2dkb__#114
THEN
GO:0016846: carbon-sulfur lyase activity: 1.457e-17 (17,10,8) OR
GO:0008483: transaminase activity: 2.607e-10 (17,17,6) OR
GO:0016831: carboxy-lyase activity: 1.454e-01 (17,25,1) OR
GO:0016763: transferase activity, transferring pentosyl groups: 1.615e-01 (17,28,1) OR
GO:0000287: magnesium ion binding: 5.597e-01 (17,128,1)

IF 1cjca2#76 AND 1fl2a1#320
THEN
GO:0015036: disulfide oxidoreductase activity: 1.511e-17 (29,22,11) OR
GO:0016668: oxidoreductase activity, acting on sulfur group of donors, NAD or NADP as acceptor: 9.586e-17 (29,12,9) OR
GO:0050660: FAD binding: 1.713e-10 (29,10,6) OR
GO:0016638: oxidoreductase activity, acting on the CH-NH2 group of donors: 1.347e-02 (29,17,2) OR
GO:0016651: oxidoreductase activity, acting on NADH or NADPH: 1.301e-01 (29,13,1)

IF 1elua_#199 AND 1c7na_#281
THEN
GO:0008483: transaminase activity: 2.568e-17 (17,17,9) OR
GO:0016846: carbon-sulfur lyase activity: 1.052e-14 (17,10,7) OR
GO:0016831: carboxy-lyase activity: 1.454e-01 (17,25,1)

IF 1a7s__#148 AND 1azza_#51 AND 1ddja_#726 AND 1ton__#179 AND 1qnja_#71 AND 1danh_#190 AND 1dy9.1#A44 AND 2hlca_#226
THEN
GO:0004263: chymotrypsin activity: 1.348e-17 (58,41,16) OR
GO:0004295: trypsin activity: 2.659e-16 (58,48,16) OR
GO:0003809: thrombin activity: 1.407e-08 (58,10,6) OR
GO:0004867: serine-type endopeptidase inhibitor activity: 3.451e-07 (58,47,9) OR
GO:0005509: calcium ion binding: 1.651e-03 (58,160,10) OR
GO:0005529: sugar binding: 5.705e-01 (58,39,1)

IF 1smaa1#95 AND 7taa_2#331
THEN
GO:0004556: alpha-amylase activity: 4.229e-17 (12,15,8) OR
GO:0005509: calcium ion binding: 3.909e-03 (12,160,4)

IF 1qnja_#77 AND 1danh_#190
THEN
GO:0004263: chymotrypsin activity: 1.429e-17 (47,41,15) OR
GO:0004295: trypsin activity: 2.276e-16 (47,48,15) OR
GO:0004867: serine-type endopeptidase inhibitor activity: 1.195e-04 (47,47,6) OR
GO:0005509: calcium ion binding: 1.308e-03 (47,160,9) OR
GO:0003809: thrombin activity: 1.599e-01 (47,10,1) OR
GO:0004896: hematopoietin/interferon-class (D200-domain) cytokine receptor activity: 2.823e-01 (47,19,1)

IF 1qnja_#77 AND 1qq4a_#143
THEN
GO:0004263: chymotrypsin activity: 1.429e-17 (47,41,15) OR
GO:0004295: trypsin activity: 2.276e-16 (47,48,15) OR
GO:0004867: serine-type endopeptidase inhibitor activity: 1.195e-04 (47,47,6) OR
GO:0005509: calcium ion binding: 1.308e-03 (47,160,9) OR
GO:0003809: thrombin activity: 1.599e-01 (47,10,1) OR
GO:0004896: hematopoietin/interferon-class (D200-domain) cytokine receptor activity: 2.823e-01 (47,19,1)

IF 2hlpa1#98 AND 2cmd_2#251
THEN
GO:0004457: lactate dehydrogenase activity: 4.524e-17 (19,10,8) OR
GO:0016616: oxidoreductase activity, acting on the CH-OH group of donors, NAD or NADP as acceptor: 1.229e-14 (19,59,11)

IF 2hlpa2#315 AND 1hyha2#204
THEN
GO:0004457: lactate dehydrogenase activity: 4.524e-17 (19,10,8) OR

GO:0016616: oxidoreductase activity, acting on the CH-OH group of donors, NAD or NADP as acceptor: 1.229e-14 (19,59,11)

IF 1pbe_1#121 AND 1trb_1#41
THEN
GO:0015036: disulfide oxidoreductase activity: 1.511e-17 (29,22,11) OR
GO:0016668: oxidoreductase activity, acting on sulfur group of donors, NAD or NADP as acceptor: 5.431e-12 (29,12,7) OR
GO:0050660: FAD binding: 1.713e-10 (29,10,6) OR
GO:0016627: oxidoreductase activity, acting on the CH-CH group of donors: 6.670e-04 (29,17,3) OR
GO:0016651: oxidoreductase activity, acting on NADH or NADPH: 1.301e-01 (29,13,1) OR
GO:0016638: oxidoreductase activity, acting on the CH-NH2 group of donors: 1.668e-01 (29,17,1)

IF 1h7wa4#476 AND 1gpea1#271
THEN
GO:0015036: disulfide oxidoreductase activity: 1.511e-17 (29,22,11) OR
GO:0016668: oxidoreductase activity, acting on sulfur group of donors, NAD or NADP as acceptor: 2.769e-14 (29,12,8) OR
GO:0050660: FAD binding: 8.346e-13 (29,10,7) OR
GO:0016651: oxidoreductase activity, acting on NADH or NADPH: 1.301e-01 (29,13,1) OR
GO:0016627: oxidoreductase activity, acting on the CH-CH group of donors: 1.668e-01 (29,17,1) OR
GO:0004601: peroxidase activity: 2.019e-01 (29,21,1)

IF 1chua2#234 AND 1gpea1#271
THEN
GO:0015036: disulfide oxidoreductase activity: 1.511e-17 (29,22,11) OR
GO:0016668: oxidoreductase activity, acting on sulfur group of donors, NAD or NADP as acceptor: 2.769e-14 (29,12,8) OR
GO:0050660: FAD binding: 1.713e-10 (29,10,6) OR
GO:0016627: oxidoreductase activity, acting on the CH-CH group of donors: 1.347e-02 (29,17,2) OR
GO:0016651: oxidoreductase activity, acting on NADH or NADPH: 1.301e-01 (29,13,1) OR
GO:0004601: peroxidase activity: 2.019e-01 (29,21,1)

IF 1feca1#122 AND 1h6va2#219
THEN
GO:0015036: disulfide oxidoreductase activity: 1.511e-17 (29,22,11) OR
GO:0016668: oxidoreductase activity, acting on sulfur group of donors, NAD or NADP as acceptor: 2.769e-14 (29,12,8) OR
GO:0050660: FAD binding: 2.312e-08 (29,10,5) OR
GO:0016627: oxidoreductase activity, acting on the CH-CH group of donors: 6.670e-04 (29,17,3) OR
GO:0016651: oxidoreductase activity, acting on NADH or NADPH: 1.301e-01 (29,13,1) OR
GO:0016638: oxidoreductase activity, acting on the CH-NH2 group of donors: 1.668e-01 (29,17,1)

IF 1h6va2#219 AND 1trb_1#41 AND 1gpea1#271
THEN
GO:0050660: FAD binding: 1.698e-17 (33,10,9) OR
GO:0015036: disulfide oxidoreductase activity: 8.326e-17 (33,22,11) OR
GO:0016668: oxidoreductase activity, acting on sulfur group of donors, NAD or NADP as acceptor: 3.677e-16 (33,12,9) OR
GO:0016627: oxidoreductase activity, acting on the CH-CH group of donors: 1.727e-02 (33,17,2) OR
GO:0016651: oxidoreductase activity, acting on NADH or NADPH: 1.468e-01 (33,13,1) OR
GO:0016638: oxidoreductase activity, acting on the CH-NH2 group of donors: 1.876e-01 (33,17,1)

IF 1qnja_#71 AND 1eq9a_#162 AND 1arb__#193
THEN
GO:0004263: chymotrypsin activity: 2.059e-17 (48,41,15) OR
GO:0004295: trypsin activity: 3.272e-16 (48,48,15) OR
GO:0004867: serine-type endopeptidase inhibitor activity: 1.238e-05 (48,47,7) OR
GO:0003809: thrombin activity: 5.647e-04 (48,10,3) OR
GO:0005509: calcium ion binding: 5.961e-03 (48,160,8)

IF 1gdna_#119 AND 1elva1#566
THEN
GO:0004295: trypsin activity: 2.059e-17 (41,48,15) OR
GO:0004263: chymotrypsin activity: 6.981e-17 (41,41,14) OR
GO:0004867: serine-type endopeptidase inhibitor activity: 4.932e-03 (41,47,4) OR
GO:0005509: calcium ion binding: 8.717e-03 (41,160,7) OR
GO:0004896: hematopoietin/interferon-class (D200-domain) cytokine receptor activity: 2.510e-01 (41,19,1)

IF 1azza_#114 AND 1ton__#231
THEN
GO:0004295: trypsin activity: 1.792e-17 (50,48,16) OR
GO:0004263: chymotrypsin activity: 4.162e-17 (50,41,15) OR
GO:0005509: calcium ion binding: 4.891e-04 (50,160,10) OR
GO:0003809: thrombin activity: 6.374e-04 (50,10,3) OR
GO:0004867: serine-type endopeptidase inhibitor activity: 1.456e-03 (50,47,5) OR

GO:0004896: hematopoietin/interferon-class (D200-domain) cytokine receptor activity: 2.975e-01 (50,19,1)

IF 2msba_#195 AND 2msba_#218
THEN
GO:0005529: sugar binding: 2.885e-17 (13,39,10) OR
GO:0004263: chymotrypsin activity: 1.792e-01 (13,41,1) OR
GO:0004295: trypsin activity: 2.067e-01 (13,48,1) OR
GO:0005509: calcium ion binding: 5.454e-01 (13,160,1)

IF 2msba_#134 AND 1qo3c_#195
THEN
GO:0005529: sugar binding: 2.885e-17 (13,39,10) OR
GO:0004263: chymotrypsin activity: 1.792e-01 (13,41,1) OR
GO:0004295: trypsin activity: 2.067e-01 (13,48,1) OR
GO:0005509: calcium ion binding: 5.454e-01 (13,160,1)

IF 1fvua_#72 AND 2msba_#128
THEN
GO:0005529: sugar binding: 2.885e-17 (13,39,10) OR
GO:0004263: chymotrypsin activity: 1.792e-01 (13,41,1) OR
GO:0004295: trypsin activity: 2.067e-01 (13,48,1) OR
GO:0005509: calcium ion binding: 5.454e-01 (13,160,1)

IF 1fvua_#72 AND 1tn3__#113
THEN
GO:0005529: sugar binding: 2.885e-17 (13,39,10) OR
GO:0004263: chymotrypsin activity: 1.792e-01 (13,41,1) OR
GO:0004295: trypsin activity: 2.067e-01 (13,48,1) OR
GO:0005509: calcium ion binding: 5.454e-01 (13,160,1)

IF 1fvua_#72 AND 1hq8a_#150
THEN
GO:0005529: sugar binding: 2.885e-17 (13,39,10) OR
GO:0004263: chymotrypsin activity: 1.792e-01 (13,41,1) OR
GO:0004295: trypsin activity: 2.067e-01 (13,48,1) OR
GO:0005509: calcium ion binding: 5.454e-01 (13,160,1)

IF 1f00i3#856 AND 1qo3c_#195
THEN
GO:0005529: sugar binding: 2.885e-17 (13,39,10) OR
GO:0004263: chymotrypsin activity: 1.792e-01 (13,41,1) OR
GO:0004295: trypsin activity: 2.067e-01 (13,48,1) OR
GO:0005509: calcium ion binding: 5.454e-01 (13,160,1)

IF 1fvua_#72 AND 1hq8a_#223
THEN
GO:0005529: sugar binding: 2.885e-17 (13,39,10) OR
GO:0004263: chymotrypsin activity: 1.792e-01 (13,41,1) OR
GO:0004295: trypsin activity: 2.067e-01 (13,48,1) OR
GO:0005509: calcium ion binding: 5.454e-01 (13,160,1)

IF 1fvua_#72 AND 1e87a_#121
THEN
GO:0005529: sugar binding: 2.885e-17 (13,39,10) OR
GO:0004263: chymotrypsin activity: 1.792e-01 (13,41,1) OR
GO:0004295: trypsin activity: 2.067e-01 (13,48,1) OR
GO:0005509: calcium ion binding: 5.454e-01 (13,160,1)

IF 3lada1#116 AND 1ojt_2#336
THEN
GO:0015036: disulfide oxidoreductase activity: 2.377e-17 (30,22,11) OR
GO:0016668: oxidoreductase activity, acting on sulfur group of donors, NAD or NADP as acceptor: 3.771e-14 (30,12,8) OR
GO:0050660: FAD binding: 1.088e-12 (30,10,7) OR
GO:0016627: oxidoreductase activity, acting on the CH-CH group of donors: 7.383e-04 (30,17,3) OR
GO:0016651: oxidoreductase activity, acting on NADH or NADPH: 1.343e-01 (30,13,1)

IF 1trb_1#15 AND 1cjca2#76
THEN
GO:0016668: oxidoreductase activity, acting on sulfur group of donors, NAD or NADP as acceptor: 3.000e-17 (26,12,9) OR
GO:0015036: disulfide oxidoreductase activity: 5.262e-16 (26,22,10) OR

GO:0050660: FAD binding: 8.335e-11 (26,10,6) OR
GO:0016651: oxidoreductase activity, acting on NADH or NADPH: 1.174e-01 (26,13,1)

IF 1eny__#94 AND 1h5qa_#70
THEN
GO:0016616: oxidoreductase activity, acting on the CH-OH group of donors, NAD or NADP as acceptor: 2.448e-17 (22,59,13) OR
GO:0016627: oxidoreductase activity, acting on the CH-CH group of donors: 1.227e-07 (22,17,5) OR
GO:0016836: hydro-lyase activity: 2.825e-02 (22,33,2) OR
GO:0016854: racemase and epimerase activity: 1.002e-01 (22,13,1) OR
GO:0016646: oxidoreductase activity, acting on the CH-NH group of donors, NAD or NADP as acceptor: 1.432e-01 (22,19,1)

IF 1qsta_#154 AND 1czan1#92
THEN
GO:0004812: tRNA ligase activity: 2.113e-17 (35,26,12) OR
GO:0005524: ATP binding: 2.508e-05 (35,243,12) OR
GO:0008080: N-acetyltransferase activity: 5.086e-04 (35,13,3) OR
GO:0000049: tRNA binding: 1.144e-02 (35,13,2) OR
GO:0000287: magnesium ion binding: 2.209e-02 (35,128,5) OR
GO:0030145: manganese ion binding: 3.902e-01 (35,38,1)

IF 1b8pa2#197 AND 1hyha2#204
THEN
GO:0004457: lactate dehydrogenase activity: 4.524e-17 (19,10,8) OR
GO:0016616: oxidoreductase activity, acting on the CH-OH group of donors, NAD or NADP as acceptor: 8.171e-13 (19,59,10) OR
GO:0019843: rRNA binding: 1.432e-01 (19,22,1)

IF 1aq0a_#4 AND 1icia_#175
THEN
GO:0004556: alpha-amylase activity: 3.650e-17 (20,15,9) OR
GO:0005509: calcium ion binding: 8.170e-06 (20,160,8) OR
GO:0004867: serine-type endopeptidase inhibitor activity: 4.542e-02 (20,47,2) OR
GO:0016758: transferase activity, transferring hexosyl groups: 7.797e-02 (20,11,1)

IF 1e3ja2#270 AND 1eq2a_#11
THEN
GO:0016616: oxidoreductase activity, acting on the CH-OH group of donors, NAD or NADP as acceptor: 2.448e-17 (22,59,13) OR
GO:0004457: lactate dehydrogenase activity: 5.182e-09 (22,10,5) OR
GO:0016620: oxidoreductase activity, acting on the aldehyde or oxo group of donors, NAD or NADP as acceptor: 7.799e-02 (22,10,1) OR
GO:0016627: oxidoreductase activity, acting on the CH-CH group of donors: 1.291e-01 (22,17,1) OR
GO:0016646: oxidoreductase activity, acting on the CH-NH group of donors, NAD or NADP as acceptor: 1.432e-01 (22,19,1) OR
GO:0005525: GTP binding: 3.302e-01 (22,49,1)

IF 1c7na_#281 AND 1qs0a1#119
THEN
GO:0008483: transaminase activity: 2.568e-17 (17,17,9) OR
GO:0016846: carbon-sulfur lyase activity: 2.131e-07 (17,10,4) OR
GO:0016866: intramolecular transferase activity: 7.249e-02 (17,12,1) OR
GO:0016831: carboxy-lyase activity: 1.454e-01 (17,25,1) OR
GO:0016616: oxidoreductase activity, acting on the CH-OH group of donors, NAD or NADP as acceptor: 3.115e-01 (17,59,1) OR
GO:0000287: magnesium ion binding: 5.597e-01 (17,128,1)

IF 1a7s__#148 AND 1befa_#51 AND 1elva1#513 AND 1qnja_#118 AND 1ekbb_#141 AND 1gdna_#30 AND 1qnja_#46 AND 1ton__#181 AND 1autc_#209
THEN
GO:0004295: trypsin activity: 2.582e-17 (51,48,16) OR
GO:0004263: chymotrypsin activity: 5.843e-17 (51,41,15) OR
GO:0004867: serine-type endopeptidase inhibitor activity: 1.871e-05 (51,47,7) OR
GO:0003809: thrombin activity: 2.106e-05 (51,10,4) OR
GO:0005509: calcium ion binding: 8.657e-03 (51,160,8) OR
GO:0005529: sugar binding: 5.239e-01 (51,39,1)

IF 1erv__#80 AND 1gg6.1#C229
THEN
GO:0004295: trypsin activity: 2.582e-17 (51,48,16) OR
GO:0004263: chymotrypsin activity: 8.146e-14 (51,41,13) OR
GO:0003809: thrombin activity: 4.420e-07 (51,10,5) OR
GO:0004867: serine-type endopeptidase inhibitor activity: 1.871e-05 (51,47,7) OR
GO:0005509: calcium ion binding: 2.384e-03 (51,160,9) OR
GO:0004896: hematopoietin/interferon-class (D200-domain) cytokine receptor activity: 3.024e-01 (51,19,1)

IF 1qgna_#149 AND 2dkb__#114
THEN
GO:0016846: carbon-sulfur lyase activity: 2.621e-17 (18,10,8) OR
GO:0008483: transaminase activity: 3.896e-10 (18,17,6) OR
GO:0004180: carboxypeptidase activity: 9.486e-02 (18,15,1) OR
GO:0016627: oxidoreductase activity, acting on the CH-CH group of donors: 1.068e-01 (18,17,1) OR
GO:0016831: carboxy-lyase activity: 1.533e-01 (18,25,1) OR
GO:0004867: serine-type endopeptidase inhibitor activity: 2.696e-01 (18,47,1)

IF 1gdna_#119 AND 1svpa_#127
THEN
GO:0004295: trypsin activity: 3.166e-17 (42,48,15) OR
GO:0004263: chymotrypsin activity: 1.037e-16 (42,41,14) OR
GO:0005509: calcium ion binding: 2.519e-03 (42,160,8) OR
GO:0004867: serine-type endopeptidase inhibitor activity: 5.382e-03 (42,47,4) OR
GO:0004896: hematopoietin/interferon-class (D200-domain) cytokine receptor activity: 2.563e-01 (42,19,1)

IF 1gnwa1#160 AND 1e8ca1#83
THEN
GO:0004364: glutathione transferase activity: 5.394e-17 (9,11,7) OR
GO:0003714: transcription corepressor activity: 2.615e-02 (9,8,1) OR
GO:0016651: oxidoreductase activity, acting on NADH or NADPH: 4.219e-02 (9,13,1)

IF 1gcoa_#119 AND 1cyda_#106
THEN
GO:0016616: oxidoreductase activity, acting on the CH-OH group of donors, NAD or NADP as acceptor: 5.522e-17 (18,59,12) OR
GO:0016627: oxidoreductase activity, acting on the CH-CH group of donors: 3.011e-06 (18,17,4) OR
GO:0016854: racemase and epimerase activity: 3.080e-03 (18,13,2)

IF 1b8pa2#197 AND 1nat__#50
THEN
GO:0004457: lactate dehydrogenase activity: 4.524e-17 (19,10,8) OR
GO:0016616: oxidoreductase activity, acting on the CH-OH group of donors, NAD or NADP as acceptor: 4.359e-11 (19,59,9) OR
GO:0005351: sugar porter activity: 1.371e-01 (19,21,1) OR
GO:0005509: calcium ion binding: 6.845e-01 (19,160,1)

IF 1a9xa3#78 AND 1eh9a3#252
THEN
GO:0004556: alpha-amylase activity: 3.650e-17 (20,15,9) OR
GO:0005509: calcium ion binding: 8.551e-05 (20,160,7) OR
GO:0004867: serine-type endopeptidase inhibitor activity: 4.542e-02 (20,47,2) OR
GO:0016758: transferase activity, transferring hexosyl groups: 7.797e-02 (20,11,1) OR
GO:0016616: oxidoreductase activity, acting on the CH-OH group of donors, NAD or NADP as acceptor: 3.555e-01 (20,59,1)

IF 1azza_#51 AND 1qnja_#124 AND 1fjsa_#158 AND 1c5y.1#B241 AND 1ton__#47 AND 1arb__#55 AND 2hlca_#139
THEN
GO:0004263: chymotrypsin activity: 3.045e-17 (39,41,14) OR
GO:0004295: trypsin activity: 3.921e-16 (39,48,14) OR
GO:0005509: calcium ion binding: 6.583e-03 (39,160,7) OR
GO:0003809: thrombin activity: 1.345e-01 (39,10,1) OR
GO:0004867: serine-type endopeptidase inhibitor activity: 1.445e-01 (39,47,2) OR
GO:0008201: heparin binding: 2.935e-01 (39,24,1)

IF 1e87a_#155 AND 1dv8a_#213
THEN
GO:0005529: sugar binding: 9.312e-17 (10,39,9) OR
GO:0008201: heparin binding: 8.480e-02 (10,24,1)

IF 2msba_#134 AND 1hlwa_#82
THEN
GO:0005529: sugar binding: 9.312e-17 (10,39,9) OR
GO:0005509: calcium ion binding: 4.545e-01 (10,160,1)

IF 1hlwa_#82 AND 1hq8a_#150
THEN
GO:0005529: sugar binding: 9.312e-17 (10,39,9) OR
GO:0005509: calcium ion binding: 4.545e-01 (10,160,1)

IF 1qo3c_#212 AND 1hq8a_#223

THEN
GO:0005529: sugar binding: 9.312e-17 (10,39,9) OR
GO:0008201: heparin binding: 8.480e-02 (10,24,1)

IF 1ha6a_#20 AND 1cewi_#14
THEN
GO:0008009: chemokine activity: 6.536e-17 (10,10,7) OR
GO:0008083: growth factor activity: 9.652e-03 (10,42,2) OR
GO:0016702: oxidoreductase activity, acting on single donors with incorporation of molecular oxygen, incorporation of two atoms of oxygen: 4.324e-02 (10,12,1)

IF 1e43a2#351 AND 1eh9a3#102
THEN
GO:0004556: alpha-amylase activity: 1.097e-16 (13,15,8) OR
GO:0005509: calcium ion binding: 5.744e-04 (13,160,5)

IF 1qrra_#208 AND 1cyda_#106
THEN
GO:0016616: oxidoreductase activity, acting on the CH-OH group of donors, NAD or NADP as acceptor: 5.522e-17 (18,59,12) OR
GO:0016854: racemase and epimerase activity: 3.080e-03 (18,13,2) OR
GO:0016627: oxidoreductase activity, acting on the CH-CH group of donors: 5.286e-03 (18,17,2) OR
GO:0016646: oxidoreductase activity, acting on the CH-NH group of donors, NAD or NADP as acceptor: 6.595e-03 (18,19,2)

IF 1qhda2#323 AND 1h7wa4#476
THEN
GO:0015036: disulfide oxidoreductase activity: 6.510e-17 (22,22,10) OR
GO:0016668: oxidoreductase activity, acting on sulfur group of donors, NAD or NADP as acceptor: 1.183e-10 (22,12,6) OR
GO:0050660: FAD binding: 5.182e-09 (22,10,5) OR
GO:0004601: peroxidase activity: 1.571e-01 (22,21,1)

IF 1hdr__#149 AND 1e7wa_#36
THEN
GO:0016616: oxidoreductase activity, acting on the CH-OH group of donors, NAD or NADP as acceptor: 5.522e-17 (18,59,12) OR
GO:0016854: racemase and epimerase activity: 3.080e-03 (18,13,2) OR
GO:0016646: oxidoreductase activity, acting on the CH-NH group of donors, NAD or NADP as acceptor: 6.595e-03 (18,19,2) OR
GO:0016627: oxidoreductase activity, acting on the CH-CH group of donors: 1.068e-01 (18,17,1) OR
GO:0016836: hydro-lyase activity: 1.975e-01 (18,33,1)

IF 1nat__#50 AND 1h5qa_#70 AND 1evqa_#305
THEN
GO:0016616: oxidoreductase activity, acting on the CH-OH group of donors, NAD or NADP as acceptor: 5.522e-17 (18,59,12) OR
GO:0016627: oxidoreductase activity, acting on the CH-CH group of donors: 1.554e-04 (18,17,3) OR
GO:0016854: racemase and epimerase activity: 8.272e-02 (18,13,1) OR
GO:0008757: S-adenosylmethionine-dependent methyltransferase activity: 1.476e-01 (18,24,1) OR
GO:0016836: hydro-lyase activity: 1.975e-01 (18,33,1)

IF 1qrra_#208 AND 1eno__#137
THEN
GO:0016616: oxidoreductase activity, acting on the CH-OH group of donors, NAD or NADP as acceptor: 5.522e-17 (18,59,12) OR
GO:0016854: racemase and epimerase activity: 3.080e-03 (18,13,2) OR
GO:0016646: oxidoreductase activity, acting on the CH-NH group of donors, NAD or NADP as acceptor: 6.595e-03 (18,19,2) OR
GO:0016627: oxidoreductase activity, acting on the CH-CH group of donors: 1.068e-01 (18,17,1) OR
GO:0016836: hydro-lyase activity: 1.975e-01 (18,33,1)

IF 1hdr__#156 AND 1e6ua_#10
THEN
GO:0016616: oxidoreductase activity, acting on the CH-OH group of donors, NAD or NADP as acceptor: 5.541e-17 (23,59,13) OR
GO:0016627: oxidoreductase activity, acting on the CH-CH group of donors: 8.547e-06 (23,17,4) OR
GO:0016854: racemase and epimerase activity: 5.024e-03 (23,13,2) OR
GO:0016646: oxidoreductase activity, acting on the CH-NH group of donors, NAD or NADP as acceptor: 1.068e-02 (23,19,2) OR
GO:0016836: hydro-lyase activity: 3.071e-02 (23,33,2)

IF 1eny__#94 AND 1e7wa_#36
THEN
GO:0016616: oxidoreductase activity, acting on the CH-OH group of donors, NAD or NADP as acceptor: 5.541e-17 (23,59,13) OR
GO:0016627: oxidoreductase activity, acting on the CH-CH group of donors: 8.547e-06 (23,17,4) OR
GO:0016646: oxidoreductase activity, acting on the CH-NH group of donors, NAD or NADP as acceptor: 4.663e-04 (23,19,3) OR
GO:0016854: racemase and epimerase activity: 5.024e-03 (23,13,2) OR
GO:0016836: hydro-lyase activity: 2.453e-01 (23,33,1)

IF 1cyx__#138 AND 1fwxa1#526
THEN
GO:0005507: copper ion binding: 5.596e-17 (19,38,11) OR
GO:0015078: hydrogen ion transporter activity: 1.770e-07 (19,21,5) OR
GO:0015082: di-, tri-valent inorganic cation transporter activity: 9.353e-02 (19,14,1) OR
GO:0046915: transition metal ion transporter activity: 9.353e-02 (19,14,1) OR
GO:0005509: calcium ion binding: 6.845e-01 (19,160,1)

IF 1jg8a_#172 AND 1c7na_#281
THEN
GO:0008483: transaminase activity: 5.122e-17 (18,17,9) OR
GO:0016846: carbon-sulfur lyase activity: 2.735e-07 (18,10,4) OR
GO:0016831: carboxy-lyase activity: 1.130e-02 (18,25,2) OR
GO:0016866: intramolecular transferase activity: 7.660e-02 (18,12,1) OR
GO:0016763: transferase activity, transferring pentosyl groups: 1.701e-01 (18,28,1) OR
GO:0000287: magnesium ion binding: 5.805e-01 (18,128,1)

IF 1b4va1#253 AND 1d7ya1#255
THEN
GO:0015036: disulfide oxidoreductase activity: 6.510e-17 (22,22,10) OR
GO:0016668: oxidoreductase activity, acting on sulfur group of donors, NAD or NADP as acceptor: 1.183e-10 (22,12,6) OR
GO:0050660: FAD binding: 6.491e-07 (22,10,4) OR
GO:0016651: oxidoreductase activity, acting on NADH or NADPH: 1.002e-01 (22,13,1) OR
GO:0004601: peroxidase activity: 1.571e-01 (22,21,1)

IF 1ayl__#237 AND 1cjca2#76
THEN
GO:0016668: oxidoreductase activity, acting on sulfur group of donors, NAD or NADP as acceptor: 6.617e-17 (28,12,9) OR
GO:0015036: disulfide oxidoreductase activity: 1.290e-15 (28,22,10) OR
GO:0050660: FAD binding: 1.360e-10 (28,10,6) OR
GO:0016638: oxidoreductase activity, acting on the CH-NH2 group of donors: 1.259e-02 (28,17,2) OR
GO:0016651: oxidoreductase activity, acting on NADH or NADPH: 1.259e-01 (28,13,1)

IF 1cgha_#168 AND 1eq9a_#40
THEN
GO:0004263: chymotrypsin activity: 5.911e-17 (63,41,16) OR
GO:0004295: trypsin activity: 1.151e-15 (63,48,16) OR
GO:0003809: thrombin activity: 2.252e-12 (63,10,8) OR
GO:0004867: serine-type endopeptidase inhibitor activity: 5.563e-08 (63,47,10) OR
GO:0005509: calcium ion binding: 2.160e-04 (63,160,12) OR
GO:0005529: sugar binding: 6.010e-01 (63,39,1)

IF 1avgi_#69 AND 1glqa2#53
THEN
GO:0004364: glutathione transferase activity: 1.796e-16 (10,11,7) OR
GO:0004190: aspartic-type endopeptidase activity: 6.069e-05 (10,23,3)

IF 1elva1#566 AND 1eq9a_#162 AND 1arb__#193
THEN
GO:0004295: trypsin activity: 7.208e-17 (44,48,15) OR
GO:0004263: chymotrypsin activity: 2.214e-16 (44,41,14) OR
GO:0004867: serine-type endopeptidase inhibitor activity: 8.172e-05 (44,47,6) OR
GO:0003809: thrombin activity: 1.056e-02 (44,10,2) OR
GO:0005509: calcium ion binding: 1.282e-02 (44,160,7)

IF 1a4ya_#47 AND 1qhoa4#399
THEN
GO:0004556: alpha-amylase activity: 6.375e-17 (21,15,9) OR
GO:0005509: calcium ion binding: 1.220e-04 (21,160,7) OR
GO:0004867: serine-type endopeptidase inhibitor activity: 4.966e-02 (21,47,2) OR
GO:0005518: collagen binding: 9.588e-02 (21,13,1) OR
GO:0008757: S-adenosylmethionine-dependent methyltransferase activity: 1.701e-01 (21,24,1) OR
GO:0000287: magnesium ion binding: 6.373e-01 (21,128,1)

IF 1a4ya_#47 AND 1bvza3#416 AND 1ho1a_#211
THEN
GO:0004556: alpha-amylase activity: 6.375e-17 (21,15,9) OR
GO:0005509: calcium ion binding: 1.220e-04 (21,160,7) OR
GO:0004867: serine-type endopeptidase inhibitor activity: 4.966e-02 (21,47,2) OR
GO:0008081: phosphoric diester hydrolase activity: 8.172e-02 (21,11,1) OR

GO:0004620: phospholipase activity: 1.371e-01 (21,19,1) OR
GO:0000287: magnesium ion binding: 6.373e-01 (21,128,1)

IF 1qnja_#124 AND 1ddja_#754
THEN
GO:0004263: chymotrypsin activity: 6.981e-17 (41,41,14) OR
GO:0004295: trypsin activity: 8.945e-16 (41,48,14) OR
GO:0005509: calcium ion binding: 2.144e-03 (41,160,8) OR
GO:0003809: thrombin activity: 9.210e-03 (41,10,2) OR
GO:0004867: serine-type endopeptidase inhibitor activity: 1.566e-01 (41,47,2) OR
GO:0004896: hematopoietin/interferon-class (D200-domain) cytokine receptor activity: 2.510e-01 (41,19,1)

IF 1fkna_#324
THEN
GO:0004190: aspartic-type endopeptidase activity: 7.094e-17 (14,23,9) OR
GO:0010181: FMN binding: 5.518e-02 (14,11,1) OR
GO:0016811: hydrolase activity, acting on carbon-nitrogen (but not peptide) bonds, in linear amides: 7.454e-02 (14,15,1) OR
GO:0016831: carboxy-lyase activity: 1.213e-01 (14,25,1) OR
GO:0000287: magnesium ion binding: 4.910e-01 (14,128,1) OR
GO:0005509: calcium ion binding: 5.723e-01 (14,160,1)

IF 1bvza3#416 AND 1h9da_#91
THEN
GO:0004556: alpha-amylase activity: 1.077e-16 (22,15,9) OR
GO:0005509: calcium ion binding: 1.707e-06 (22,160,9) OR
GO:0004222: metalloendopeptidase activity: 9.793e-03 (22,19,2) OR
GO:0008270: zinc ion binding: 2.160e-01 (22,108,2)

IF 1hu4a_#267 AND 1gcoa_#119
THEN
GO:0016616: oxidoreductase activity, acting on the CH-OH group of donors, NAD or NADP as acceptor: 2.369e-16 (15,59,11) OR
GO:0016627: oxidoreductase activity, acting on the CH-CH group of donors: 1.359e-06 (15,17,4)

IF 1hu4a_#267 AND 1aqua_#126 AND 1h5qa_#70
THEN
GO:0016616: oxidoreductase activity, acting on the CH-OH group of donors, NAD or NADP as acceptor: 2.369e-16 (15,59,11) OR
GO:0016627: oxidoreductase activity, acting on the CH-CH group of donors: 1.359e-06 (15,17,4)

IF 1hyha2#179 AND 1hyea2#182
THEN
GO:0004457: lactate dehydrogenase activity: 1.216e-16 (21,10,8) OR
GO:0016616: oxidoreductase activity, acting on the CH-OH group of donors, NAD or NADP as acceptor: 5.549e-14 (21,59,11) OR
GO:0004842: ubiquitin-protein ligase activity: 1.371e-01 (21,19,1) OR
GO:0008270: zinc ion binding: 5.736e-01 (21,108,1)

IF 1dv8a_#213 AND 1h8ua_#113
THEN
GO:0005529: sugar binding: 1.000e-16 (14,39,10) OR
GO:0008201: heparin binding: 1.167e-01 (14,24,1) OR
GO:0004263: chymotrypsin activity: 1.916e-01 (14,41,1) OR
GO:0004295: trypsin activity: 2.207e-01 (14,48,1) OR
GO:0005509: calcium ion binding: 5.723e-01 (14,160,1)

IF 1b6e__#76 AND 1f00i3#856
THEN
GO:0005529: sugar binding: 1.000e-16 (14,39,10) OR
GO:0008201: heparin binding: 1.167e-01 (14,24,1) OR
GO:0004263: chymotrypsin activity: 1.916e-01 (14,41,1) OR
GO:0004295: trypsin activity: 2.207e-01 (14,48,1) OR
GO:0005509: calcium ion binding: 5.723e-01 (14,160,1)

IF 1qf6a4#318 AND 1bwda_#117
THEN
GO:0004812: tRNA ligase activity: 1.007e-16 (28,26,11) OR
GO:0005524: ATP binding: 1.238e-05 (28,243,11) OR
GO:0000049: tRNA binding: 7.407e-03 (28,13,2) OR
GO:0000287: magnesium ion binding: 1.416e-01 (28,128,3) OR
GO:0003887: DNA-directed DNA polymerase activity: 1.872e-01 (28,20,1)

IF 1cyda_#231 AND 1hdca_#187

THEN
GO:0016616: oxidoreductase activity, acting on the CH-OH group of donors, NAD or NADP as acceptor: 5.577e-16 (9,59,9)

IF 1cyda_#231 AND 2ae2a_#196
THEN
GO:0016616: oxidoreductase activity, acting on the CH-OH group of donors, NAD or NADP as acceptor: 5.577e-16 (9,59,9)

IF 1fuma2#370 AND 1ayl__#237
THEN
GO:0015036: disulfide oxidoreductase activity: 1.147e-16 (23,22,10) OR
GO:0016668: oxidoreductase activity, acting on sulfur group of donors, NAD or NADP as acceptor: 8.613e-13 (23,12,7) OR
GO:0050660: FAD binding: 7.844e-07 (23,10,4) OR
GO:0016651: oxidoreductase activity, acting on NADH or NADPH: 1.046e-01 (23,13,1) OR
GO:0016638: oxidoreductase activity, acting on the CH-NH2 group of donors: 1.346e-01 (23,17,1)

IF 1leha1#177 AND 1b3ra1#223
THEN
GO:0016616: oxidoreductase activity, acting on the CH-OH group of donors, NAD or NADP as acceptor: 1.475e-16 (19,59,12) OR
GO:0004457: lactate dehydrogenase activity: 3.347e-05 (19,10,3) OR
GO:0016638: oxidoreductase activity, acting on the CH-NH2 group of donors: 1.839e-04 (19,17,3) OR
GO:0016620: oxidoreductase activity, acting on the aldehyde or oxo group of donors, NAD or NADP as acceptor: 6.769e-02 (19,10,1)

IF 1cyda_#106 AND 1eny__#147
THEN
GO:0016616: oxidoreductase activity, acting on the CH-OH group of donors, NAD or NADP as acceptor: 1.475e-16 (19,59,12) OR
GO:0016627: oxidoreductase activity, acting on the CH-CH group of donors: 3.799e-06 (19,17,4) OR
GO:0016854: racemase and epimerase activity: 3.433e-03 (19,13,2) OR
GO:0016646: oxidoreductase activity, acting on the CH-NH group of donors, NAD or NADP as acceptor: 1.249e-01 (19,19,1)

IF 1fwxa1#526 AND 1aoza1#61
THEN
GO:0005507: copper ion binding: 1.232e-16 (20,38,11) OR
GO:0015078: hydrogen ion transporter activity: 3.480e-09 (20,21,6) OR
GO:0015082: di-, tri-valent inorganic cation transporter activity: 9.821e-02 (20,14,1) OR
GO:0046915: transition metal ion transporter activity: 9.821e-02 (20,14,1) OR
GO:0005509: calcium ion binding: 7.032e-01 (20,160,1)

IF 1aoza1#61 AND 2cuaa_#83
THEN
GO:0005507: copper ion binding: 1.232e-16 (20,38,11) OR
GO:0015078: hydrogen ion transporter activity: 2.349e-07 (20,21,5) OR
GO:0015082: di-, tri-valent inorganic cation transporter activity: 9.821e-02 (20,14,1) OR
GO:0046915: transition metal ion transporter activity: 9.821e-02 (20,14,1) OR
GO:0005509: calcium ion binding: 3.301e-01 (20,160,2)

IF 1azza_#114 AND 1ddja_#746
THEN
GO:0004295: trypsin activity: 1.069e-16 (45,48,15) OR
GO:0004263: chymotrypsin activity: 3.184e-16 (45,41,14) OR
GO:0003809: thrombin activity: 4.660e-04 (45,10,3) OR
GO:0005509: calcium ion binding: 9.413e-04 (45,160,9) OR
GO:0004867: serine-type endopeptidase inhibitor activity: 4.117e-02 (45,47,3) OR
GO:0004896: hematopoietin/interferon-class (D200-domain) cytokine receptor activity: 2.720e-01 (45,19,1)

IF 1danh_#93 AND 1fjsa_#158 AND 1elva1#601
THEN
GO:0004263: chymotrypsin activity: 1.125e-16 (53,41,15) OR
GO:0004295: trypsin activity: 1.765e-15 (53,48,15) OR
GO:0004867: serine-type endopeptidase inhibitor activity: 2.428e-05 (53,47,7) OR
GO:0003809: thrombin activity: 2.459e-05 (53,10,4) OR
GO:0005509: calcium ion binding: 1.795e-04 (53,160,11) OR
GO:0004896: hematopoietin/interferon-class (D200-domain) cytokine receptor activity: 3.123e-01 (53,19,1)

IF 1g71a_#77 AND 2dnja_#97 AND 1ton__#91
THEN
GO:0004263: chymotrypsin activity: 1.125e-16 (53,41,15) OR
GO:0004295: trypsin activity: 1.765e-15 (53,48,15) OR
GO:0003809: thrombin activity: 5.383e-07 (53,10,5) OR
GO:0005509: calcium ion binding: 1.795e-04 (53,160,11) OR
GO:0004867: serine-type endopeptidase inhibitor activity: 2.364e-04 (53,47,6) OR

GO:0005529: sugar binding: 5.377e-01 (53,39,1)

IF 1euva_#580 AND 1ppn__#132
THEN
GO:0004197: cysteine-type endopeptidase activity: 1.133e-16 (14,24,9) OR
GO:0019955: cytokine binding: 5.518e-02 (14,11,1) OR
GO:0042802: protein self binding: 6.006e-02 (14,12,1) OR
GO:0004177: aminopeptidase activity: 6.491e-02 (14,13,1) OR
GO:0004180: carboxypeptidase activity: 7.454e-02 (14,15,1) OR
GO:0051082: unfolded protein binding: 1.616e-01 (14,34,1)

IF 2cb5a_#312 AND 1euva_#580
THEN
GO:0004197: cysteine-type endopeptidase activity: 1.133e-16 (14,24,9) OR
GO:0019955: cytokine binding: 5.518e-02 (14,11,1) OR
GO:0042802: protein self binding: 6.006e-02 (14,12,1) OR
GO:0004177: aminopeptidase activity: 6.491e-02 (14,13,1) OR
GO:0004180: carboxypeptidase activity: 7.454e-02 (14,15,1) OR
GO:0051082: unfolded protein binding: 1.616e-01 (14,34,1)

IF 1h7wa4#476 AND 1ddma_#130
THEN
GO:0015036: disulfide oxidoreductase activity: 1.147e-16 (23,22,10) OR
GO:0016668: oxidoreductase activity, acting on sulfur group of donors, NAD or NADP as acceptor: 1.597e-10 (23,12,6) OR
GO:0050660: FAD binding: 7.844e-07 (23,10,4) OR
GO:0016651: oxidoreductase activity, acting on NADH or NADPH: 1.046e-01 (23,13,1) OR
GO:0016627: oxidoreductase activity, acting on the CH-CH group of donors: 1.346e-01 (23,17,1) OR
GO:0004601: peroxidase activity: 1.636e-01 (23,21,1)

IF 1hu4a_#267 AND 1hdr__#149
THEN
GO:0016616: oxidoreductase activity, acting on the CH-OH group of donors, NAD or NADP as acceptor: 2.369e-16 (15,59,11) OR
GO:0016627: oxidoreductase activity, acting on the CH-CH group of donors: 3.668e-03 (15,17,2) OR
GO:0016646: oxidoreductase activity, acting on the CH-NH group of donors, NAD or NADP as acceptor: 4.583e-03 (15,19,2)

IF 1qfma2#576 AND 1hu4a_#267 AND 1cyda_#106
THEN
GO:0016616: oxidoreductase activity, acting on the CH-OH group of donors, NAD or NADP as acceptor: 2.369e-16 (15,59,11) OR
GO:0016627: oxidoreductase activity, acting on the CH-CH group of donors: 3.668e-03 (15,17,2) OR
GO:0016646: oxidoreductase activity, acting on the CH-NH group of donors, NAD or NADP as acceptor: 4.583e-03 (15,19,2)

IF 1gcoa_#119 AND 1e6ua_#10 AND 1qfea_#68
THEN
GO:0016616: oxidoreductase activity, acting on the CH-OH group of donors, NAD or NADP as acceptor: 1.190e-16 (24,59,13) OR
GO:0016627: oxidoreductase activity, acting on the CH-CH group of donors: 1.966e-07 (24,17,5) OR
GO:0016854: racemase and epimerase activity: 5.467e-03 (24,13,2) OR
GO:0016836: hydro-lyase activity: 3.325e-02 (24,33,2) OR
GO:0016646: oxidoreductase activity, acting on the CH-NH group of donors, NAD or NADP as acceptor: 1.552e-01 (24,19,1) OR
GO:0008757: S-adenosylmethionine-dependent methyltransferase activity: 1.920e-01 (24,24,1)

IF 1oaa__#175 AND 1eq2a_#11
THEN
GO:0016616: oxidoreductase activity, acting on the CH-OH group of donors, NAD or NADP as acceptor: 1.475e-16 (19,59,12) OR
GO:0016854: racemase and epimerase activity: 3.433e-03 (19,13,2) OR
GO:0016646: oxidoreductase activity, acting on the CH-NH group of donors, NAD or NADP as acceptor: 7.340e-03 (19,19,2) OR
GO:0016836: hydro-lyase activity: 2.139e-02 (19,33,2) OR
GO:0016627: oxidoreductase activity, acting on the CH-CH group of donors: 1.125e-01 (19,17,1)

IF 1oaa__#175 AND 2ae2a_#17
THEN
GO:0016616: oxidoreductase activity, acting on the CH-OH group of donors, NAD or NADP as acceptor: 1.475e-16 (19,59,12) OR
GO:0016854: racemase and epimerase activity: 3.433e-03 (19,13,2) OR
GO:0016627: oxidoreductase activity, acting on the CH-CH group of donors: 5.887e-03 (19,17,2) OR
GO:0016836: hydro-lyase activity: 2.139e-02 (19,33,2) OR
GO:0016646: oxidoreductase activity, acting on the CH-NH group of donors, NAD or NADP as acceptor: 1.249e-01 (19,19,1)

IF 1hdr__#156 AND 2ae2a_#17
THEN
GO:0016616: oxidoreductase activity, acting on the CH-OH group of donors, NAD or NADP as acceptor: 1.475e-16 (19,59,12) OR
GO:0016854: racemase and epimerase activity: 3.433e-03 (19,13,2) OR

GO:0016627: oxidoreductase activity, acting on the CH-CH group of donors: 5.887e-03 (19,17,2) OR
GO:0016836: hydro-lyase activity: 2.139e-02 (19,33,2) OR
GO:0016646: oxidoreductase activity, acting on the CH-NH group of donors, NAD or NADP as acceptor: 1.249e-01 (19,19,1)

IF 1gcoa_#119 AND 1eq2a_#11
THEN
GO:0016616: oxidoreductase activity, acting on the CH-OH group of donors, NAD or NADP as acceptor: 1.475e-16 (19,59,12) OR
GO:0016854: racemase and epimerase activity: 3.433e-03 (19,13,2) OR
GO:0016627: oxidoreductase activity, acting on the CH-CH group of donors: 5.887e-03 (19,17,2) OR
GO:0016836: hydro-lyase activity: 2.139e-02 (19,33,2) OR
GO:0016646: oxidoreductase activity, acting on the CH-NH group of donors, NAD or NADP as acceptor: 1.249e-01 (19,19,1)

IF 1qnja_#77 AND 1gg6.1#C156
THEN
GO:0004263: chymotrypsin activity: 1.524e-16 (43,41,14) OR
GO:0004295: trypsin activity: 1.943e-15 (43,48,14) OR
GO:0004867: serine-type endopeptidase inhibitor activity: 7.150e-05 (43,47,6) OR
GO:0005509: calcium ion binding: 2.943e-03 (43,160,8) OR
GO:0004896: hematopoietin/interferon-class (D200-domain) cytokine receptor activity: 2.616e-01 (43,19,1)

IF 1aym3_#131 AND 1qhoa4#399
THEN
GO:0004556: alpha-amylase activity: 2.554e-16 (14,15,8) OR
GO:0005509: calcium ion binding: 8.514e-04 (14,160,5) OR
GO:0016627: oxidoreductase activity, acting on the CH-CH group of donors: 8.408e-02 (14,17,1)

IF 1avaa2#204 AND 1bf2_3#579
THEN
GO:0004556: alpha-amylase activity: 2.554e-16 (14,15,8) OR
GO:0005509: calcium ion binding: 8.514e-04 (14,160,5) OR
GO:0016758: transferase activity, transferring hexosyl groups: 5.518e-02 (14,11,1)

IF 1qvba_#9 AND 7taa_2#295
THEN
GO:0004556: alpha-amylase activity: 2.554e-16 (14,15,8) OR
GO:0005509: calcium ion binding: 8.514e-04 (14,160,5) OR
GO:0004867: serine-type endopeptidase inhibitor activity: 2.166e-01 (14,47,1)

IF 1hoe__#31 AND 7taa_2#295
THEN
GO:0004556: alpha-amylase activity: 2.554e-16 (14,15,8) OR
GO:0005509: calcium ion binding: 8.514e-04 (14,160,5) OR
GO:0004867: serine-type endopeptidase inhibitor activity: 2.166e-01 (14,47,1)

IF 1bf2_3#579 AND 1ho1a_#211
THEN
GO:0004556: alpha-amylase activity: 2.554e-16 (14,15,8) OR
GO:0005509: calcium ion binding: 8.514e-04 (14,160,5) OR
GO:0016758: transferase activity, transferring hexosyl groups: 5.518e-02 (14,11,1)

IF 1qsta_#154 AND 1h4ua1#404
THEN
GO:0004812: tRNA ligase activity: 1.615e-16 (29,26,11) OR
GO:0005524: ATP binding: 1.839e-05 (29,243,11) OR
GO:0000287: magnesium ion binding: 1.011e-02 (29,128,5) OR
GO:0000049: tRNA binding: 1.301e-01 (29,13,1) OR
GO:0008080: N-acetyltransferase activity: 1.301e-01 (29,13,1)

IF 2sqca2#268 AND 3grx__#55
THEN
GO:0004364: glutathione transferase activity: 8.169e-16 (6,11,6)

IF 1ljra1#165 AND 1mspa_#17
THEN
GO:0004364: glutathione transferase activity: 8.169e-16 (6,11,6)

IF 1hq8a_#185 AND 1hlwa_#82
THEN
GO:0005529: sugar binding: 8.243e-16 (8,39,8)

IF 1qo3c_#212 AND 1fvua_#72
THEN
GO:0005529: sugar binding: 8.243e-16 (8,39,8)

IF 1f0xa1#278 AND 1arb__#24
THEN
GO:0004295: trypsin activity: 1.417e-16 (56,48,16) OR
GO:0004263: chymotrypsin activity: 9.982e-15 (56,41,14) OR
GO:0003809: thrombin activity: 8.298e-13 (56,10,8) OR
GO:0004867: serine-type endopeptidase inhibitor activity: 3.512e-05 (56,47,7) OR
GO:0005509: calcium ion binding: 1.248e-03 (56,160,10) OR
GO:0005529: sugar binding: 5.576e-01 (56,39,1)

IF 1elua_#199 AND 1qtn.1#A270
THEN
GO:0016846: carbon-sulfur lyase activity: 4.305e-16 (12,10,7) OR
GO:0008483: transaminase activity: 3.828e-09 (12,17,5)

IF 1elva1#566 AND 1bio__#81 AND 1danh_#70 AND 1cgha_#130
THEN
GO:0004263: chymotrypsin activity: 1.524e-16 (43,41,14) OR
GO:0004295: trypsin activity: 1.943e-15 (43,48,14) OR
GO:0004867: serine-type endopeptidase inhibitor activity: 7.150e-05 (43,47,6) OR
GO:0005509: calcium ion binding: 1.132e-02 (43,160,7) OR
GO:0003809: thrombin activity: 1.473e-01 (43,10,1) OR
GO:0008201: heparin binding: 3.184e-01 (43,24,1)

IF 1e2fa_#14 AND 1qs0a1#119
THEN
GO:0019201: nucleotide kinase activity: 3.095e-16 (29,13,9) OR
GO:0016776: phosphotransferase activity, phosphate group as acceptor: 1.657e-13 (29,14,8) OR
GO:0005524: ATP binding: 2.543e-06 (29,243,12)

IF 1trb_1#285 AND 1evqa_#305
THEN
GO:0015036: disulfide oxidoreductase activity: 1.959e-16 (24,22,10) OR
GO:0016668: oxidoreductase activity, acting on sulfur group of donors, NAD or NADP as acceptor: 2.125e-10 (24,12,6) OR
GO:0050660: FAD binding: 8.339e-09 (24,10,5) OR
GO:0016627: oxidoreductase activity, acting on the CH-CH group of donors: 9.329e-03 (24,17,2) OR
GO:0016651: oxidoreductase activity, acting on NADH or NADPH: 1.089e-01 (24,13,1)

IF 1qsta_#154 AND 1g5ha2#319
THEN
GO:0004812: tRNA ligase activity: 2.537e-16 (30,26,11) OR
GO:0005524: ATP binding: 2.677e-05 (30,243,11) OR
GO:0000287: magnesium ion binding: 2.236e-03 (30,128,6) OR
GO:0000049: tRNA binding: 8.478e-03 (30,13,2)

IF 1dik_1#848 AND 1jsg__#92 AND 1gox__#124 AND 1nsj__#178
THEN
GO:0004556: alpha-amylase activity: 1.765e-16 (23,15,9) OR
GO:0005509: calcium ion binding: 2.327e-04 (23,160,7) OR
GO:0016627: oxidoreductase activity, acting on the CH-CH group of donors: 8.583e-03 (23,17,2) OR
GO:0000287: magnesium ion binding: 9.038e-02 (23,128,3) OR
GO:0015036: disulfide oxidoreductase activity: 1.707e-01 (23,22,1) OR
GO:0004867: serine-type endopeptidase inhibitor activity: 3.309e-01 (23,47,1)

IF 1autc_#188 AND 1eq9a_#162 AND 1gg6.1#C229
THEN
GO:0004263: chymotrypsin activity: 2.214e-16 (44,41,14) OR
GO:0004295: trypsin activity: 2.816e-15 (44,48,14) OR
GO:0004867: serine-type endopeptidase inhibitor activity: 6.785e-06 (44,47,7) OR
GO:0003809: thrombin activity: 1.056e-02 (44,10,2) OR
GO:0005509: calcium ion binding: 1.282e-02 (44,160,7)

IF 1bxoa_#320 AND 1bxoa_#141 AND 1mpp__#221
THEN
GO:0004190: aspartic-type endopeptidase activity: 1.116e-15 (7,23,7)

IF 1pfza_#125

THEN
 GO:0004190: aspartic-type endopeptidase activity: 1.116e-15 (7,23,7)

IF 1dpja_#199 AND 1htr.1#B39
THEN
 GO:0004190: aspartic-type endopeptidase activity: 1.116e-15 (7,23,7)

IF 1erv__#80 AND 1gdna_#121
THEN
 GO:0004295: trypsin activity: 1.947e-16 (57,48,16) OR
 GO:0004263: chymotrypsin activity: 3.826e-16 (57,41,15) OR
 GO:0003809: thrombin activity: 1.376e-10 (57,10,7) OR
 GO:0004867: serine-type endopeptidase inhibitor activity: 3.952e-05 (57,47,7) OR
 GO:0005509: calcium ion binding: 3.538e-04 (57,160,11) OR
 GO:0004896: hematopoietin/interferon-class (D200-domain) cytokine receptor activity: 3.317e-01 (57,19,1)

IF 1cbf__#208 AND 1nhp_1#282
THEN
 GO:0015036: disulfide oxidoreductase activity: 1.959e-16 (24,22,10) OR
 GO:0016668: oxidoreductase activity, acting on sulfur group of donors, NAD or NADP as acceptor: 2.125e-10 (24,12,6) OR
 GO:0050660: FAD binding: 8.339e-09 (24,10,5) OR
 GO:0016651: oxidoreductase activity, acting on NADH or NADPH: 1.089e-01 (24,13,1) OR
 GO:0016638: oxidoreductase activity, acting on the CH-NH2 group of donors: 1.400e-01 (24,17,1) OR
 GO:0004601: peroxidase activity: 1.701e-01 (24,21,1)

IF 1trb_1#15 AND 1evqa_#305
THEN
 GO:0015036: disulfide oxidoreductase activity: 1.959e-16 (24,22,10) OR
 GO:0016668: oxidoreductase activity, acting on sulfur group of donors, NAD or NADP as acceptor: 2.125e-10 (24,12,6) OR
 GO:0050660: FAD binding: 9.396e-07 (24,10,4) OR
 GO:0016627: oxidoreductase activity, acting on the CH-CH group of donors: 9.329e-03 (24,17,2) OR
 GO:0016651: oxidoreductase activity, acting on NADH or NADPH: 1.089e-01 (24,13,1) OR
 GO:0016854: racemase and epimerase activity: 1.089e-01 (24,13,1)

IF 1erv__#80 AND 1trb_1#112
THEN
 GO:0015036: disulfide oxidoreductase activity: 1.959e-16 (24,22,10) OR
 GO:0016668: oxidoreductase activity, acting on sulfur group of donors, NAD or NADP as acceptor: 2.125e-10 (24,12,6) OR
 GO:0050660: FAD binding: 8.339e-09 (24,10,5) OR
 GO:0016651: oxidoreductase activity, acting on NADH or NADPH: 1.089e-01 (24,13,1) OR
 GO:0016638: oxidoreductase activity, acting on the CH-NH2 group of donors: 1.400e-01 (24,17,1) OR
 GO:0004601: peroxidase activity: 1.701e-01 (24,21,1)

IF 2pia_1#54 AND 1h7wa4#478
THEN
 GO:0015036: disulfide oxidoreductase activity: 1.959e-16 (24,22,10) OR
 GO:0016668: oxidoreductase activity, acting on sulfur group of donors, NAD or NADP as acceptor: 2.125e-10 (24,12,6) OR
 GO:0050660: FAD binding: 8.339e-09 (24,10,5) OR
 GO:0016651: oxidoreductase activity, acting on NADH or NADPH: 1.089e-01 (24,13,1) OR
 GO:0016638: oxidoreductase activity, acting on the CH-NH2 group of donors: 1.400e-01 (24,17,1) OR
 GO:0004601: peroxidase activity: 1.701e-01 (24,21,1)

IF 1bd3a_#110 AND 1bd3a_#195
THEN
 GO:0016763: transferase activity, transferring pentosyl groups: 5.946e-16 (14,28,9) OR
 GO:0000287: magnesium ion binding: 3.008e-04 (14,128,5)

IF 1d2fa_#348 AND 1ax4a_#430
THEN
 GO:0008483: transaminase activity: 4.131e-16 (13,17,8) OR
 GO:0016846: carbon-sulfur lyase activity: 6.446e-08 (13,10,4) OR
 GO:0016866: intramolecular transferase activity: 5.588e-02 (13,12,1)

IF 1d2fa_#348 AND 1c7na_#95
THEN
 GO:0008483: transaminase activity: 4.131e-16 (13,17,8) OR
 GO:0016846: carbon-sulfur lyase activity: 6.446e-08 (13,10,4) OR
 GO:0016866: intramolecular transferase activity: 5.588e-02 (13,12,1)

IF 1qgna_#207 AND 1mjha_#126

THEN
GO:0016846: carbon-sulfur lyase activity: 4.305e-16 (12,10,7) OR
GO:0008483: transaminase activity: 4.984e-07 (12,17,4) OR
GO:0005524: ATP binding: 6.748e-01 (12,243,1)

IF 1gdha1#235 AND 1h5qa_#70
THEN
GO:0016616: oxidoreductase activity, acting on the CH-OH group of donors, NAD or NADP as acceptor: 6.556e-16 (12,59,10) OR
GO:0016627: oxidoreductase activity, acting on the CH-CH group of donors: 2.331e-03 (12,17,2)

IF 1ton__#179 AND 1cqqa_#163
THEN
GO:0004263: chymotrypsin activity: 2.214e-16 (44,41,14) OR
GO:0004295: trypsin activity: 2.816e-15 (44,48,14) OR
GO:0004867: serine-type endopeptidase inhibitor activity: 8.172e-05 (44,47,6) OR
GO:0003809: thrombin activity: 1.056e-02 (44,10,2) OR
GO:0005509: calcium ion binding: 1.282e-02 (44,160,7) OR
GO:0005529: sugar binding: 4.724e-01 (44,39,1)

IF 1cyda_#106 AND 1hdr__#156
THEN
GO:0016616: oxidoreductase activity, acting on the CH-OH group of donors, NAD or NADP as acceptor: 3.628e-16 (20,59,12) OR
GO:0016627: oxidoreductase activity, acting on the CH-CH group of donors: 4.731e-06 (20,17,4) OR
GO:0016854: racemase and epimerase activity: 3.804e-03 (20,13,2) OR
GO:0016646: oxidoreductase activity, acting on the CH-NH group of donors, NAD or NADP as acceptor: 8.122e-03 (20,19,2)

IF 1cyda_#106 AND 1e6ua_#10
THEN
GO:0016616: oxidoreductase activity, acting on the CH-OH group of donors, NAD or NADP as acceptor: 3.628e-16 (20,59,12) OR
GO:0016627: oxidoreductase activity, acting on the CH-CH group of donors: 4.731e-06 (20,17,4) OR
GO:0016854: racemase and epimerase activity: 3.804e-03 (20,13,2) OR
GO:0016646: oxidoreductase activity, acting on the CH-NH group of donors, NAD or NADP as acceptor: 8.122e-03 (20,19,2)

IF 1gdna_#230 AND 1fjsa_#83
THEN
GO:0004263: chymotrypsin activity: 2.950e-16 (35,41,13) OR
GO:0004295: trypsin activity: 3.062e-15 (35,48,13) OR
GO:0004867: serine-type endopeptidase inhibitor activity: 2.140e-02 (35,47,3) OR
GO:0005509: calcium ion binding: 5.099e-02 (35,160,5) OR
GO:0003809: thrombin activity: 1.215e-01 (35,10,1)

IF 1dv8a_#213 AND 2msba_#218
THEN
GO:0005529: sugar binding: 2.971e-16 (15,39,10) OR
GO:0003729: mRNA binding: 6.939e-02 (15,13,1) OR
GO:0004263: chymotrypsin activity: 2.039e-01 (15,41,1) OR
GO:0005509: calcium ion binding: 2.188e-01 (15,160,2) OR
GO:0004295: trypsin activity: 2.345e-01 (15,48,1)

IF 1f00i3#856 AND 1egia_#738
THEN
GO:0005529: sugar binding: 2.971e-16 (15,39,10) OR
GO:0008201: heparin binding: 1.246e-01 (15,24,1) OR
GO:0004263: chymotrypsin activity: 2.039e-01 (15,41,1) OR
GO:0005509: calcium ion binding: 2.188e-01 (15,160,2) OR
GO:0004295: trypsin activity: 2.345e-01 (15,48,1)

IF 1f00i3#856 AND 1qo3c_#252
THEN
GO:0005529: sugar binding: 2.971e-16 (15,39,10) OR
GO:0008201: heparin binding: 1.246e-01 (15,24,1) OR
GO:0004263: chymotrypsin activity: 2.039e-01 (15,41,1) OR
GO:0005509: calcium ion binding: 2.188e-01 (15,160,2) OR
GO:0004295: trypsin activity: 2.345e-01 (15,48,1)

IF 1f00i3#856 AND 1hq8a_#206
THEN
GO:0005529: sugar binding: 2.971e-16 (15,39,10) OR
GO:0008201: heparin binding: 1.246e-01 (15,24,1) OR
GO:0004263: chymotrypsin activity: 2.039e-01 (15,41,1) OR

GO:0005509: calcium ion binding: 2.188e-01 (15,160,2) OR
GO:0004295: trypsin activity: 2.345e-01 (15,48,1)

IF 1dv8a_#213 AND 1qo3c_#252
THEN
GO:0005529: sugar binding: 2.971e-16 (15,39,10) OR
GO:0008201: heparin binding: 1.246e-01 (15,24,1) OR
GO:0004263: chymotrypsin activity: 2.039e-01 (15,41,1) OR
GO:0005509: calcium ion binding: 2.188e-01 (15,160,2) OR
GO:0004295: trypsin activity: 2.345e-01 (15,48,1)

IF 1ayl__#237 AND 1trb_1#42
THEN
GO:0015036: disulfide oxidoreductase activity: 3.251e-16 (25,22,10) OR
GO:0016668: oxidoreductase activity, acting on sulfur group of donors, NAD or NADP as acceptor: 7.015e-15 (25,12,8) OR
GO:0050660: FAD binding: 1.041e-08 (25,10,5) OR
GO:0016651: oxidoreductase activity, acting on NADH or NADPH: 1.132e-01 (25,13,1) OR
GO:0016638: oxidoreductase activity, acting on the CH-NH2 group of donors: 1.454e-01 (25,17,1)

IF 1cjca2#103 AND 1ayl__#237
THEN
GO:0015036: disulfide oxidoreductase activity: 3.251e-16 (25,22,10) OR
GO:0016668: oxidoreductase activity, acting on sulfur group of donors, NAD or NADP as acceptor: 1.683e-12 (25,12,7) OR
GO:0050660: FAD binding: 6.420e-11 (25,10,6) OR
GO:0016651: oxidoreductase activity, acting on NADH or NADPH: 1.132e-01 (25,13,1) OR
GO:0004601: peroxidase activity: 1.766e-01 (25,21,1)

IF 1qlaa2#383 AND 1cjca2#76
THEN
GO:0015036: disulfide oxidoreductase activity: 3.251e-16 (25,22,10) OR
GO:0016668: oxidoreductase activity, acting on sulfur group of donors, NAD or NADP as acceptor: 7.015e-15 (25,12,8) OR
GO:0050660: FAD binding: 1.041e-08 (25,10,5) OR
GO:0016651: oxidoreductase activity, acting on NADH or NADPH: 1.132e-01 (25,13,1) OR
GO:0016638: oxidoreductase activity, acting on the CH-NH2 group of donors: 1.454e-01 (25,17,1)

IF 1bg6_2#24 AND 1trb_1#285
THEN
GO:0015036: disulfide oxidoreductase activity: 3.251e-16 (25,22,10) OR
GO:0016668: oxidoreductase activity, acting on sulfur group of donors, NAD or NADP as acceptor: 1.683e-12 (25,12,7) OR
GO:0050660: FAD binding: 6.420e-11 (25,10,6) OR
GO:0016651: oxidoreductase activity, acting on NADH or NADPH: 1.132e-01 (25,13,1) OR
GO:0016627: oxidoreductase activity, acting on the CH-CH group of donors: 1.454e-01 (25,17,1)

IF 1ds1a_#172 AND 7taa_2#295
THEN
GO:0004556: alpha-amylase activity: 5.460e-16 (15,15,8) OR
GO:0005509: calcium ion binding: 1.200e-04 (15,160,6) OR
GO:0004867: serine-type endopeptidase inhibitor activity: 2.302e-01 (15,47,1)

IF 1erza_#183 AND 1aq0a_#4
THEN
GO:0004556: alpha-amylase activity: 2.819e-16 (24,15,9) OR
GO:0008810: cellulase activity: 1.306e-05 (24,18,4) OR
GO:0005509: calcium ion binding: 3.894e-05 (24,160,8) OR
GO:0016758: transferase activity, transferring hexosyl groups: 9.289e-02 (24,11,1) OR
GO:0004867: serine-type endopeptidase inhibitor activity: 3.425e-01 (24,47,1) OR
GO:0005524: ATP binding: 8.948e-01 (24,243,1)

IF 1h8ua_#113 AND 1qo3c_#195
THEN
GO:0005529: sugar binding: 2.971e-16 (15,39,10) OR
GO:0016763: transferase activity, transferring pentosyl groups: 1.439e-01 (15,28,1) OR
GO:0004263: chymotrypsin activity: 2.039e-01 (15,41,1) OR
GO:0004295: trypsin activity: 2.345e-01 (15,48,1) OR
GO:0000287: magnesium ion binding: 5.150e-01 (15,128,1) OR
GO:0005509: calcium ion binding: 5.975e-01 (15,160,1)

IF 1h8ua_#113 AND 1qo3c_#252
THEN
GO:0005529: sugar binding: 2.971e-16 (15,39,10) OR

GO:0008201: heparin binding: 1.246e-01 (15,24,1) OR
GO:0016763: transferase activity, transferring pentosyl groups: 1.439e-01 (15,28,1) OR
GO:0004263: chymotrypsin activity: 2.039e-01 (15,41,1) OR
GO:0004295: trypsin activity: 2.345e-01 (15,48,1) OR
GO:0005509: calcium ion binding: 5.975e-01 (15,160,1)

IF 1hdr__#149 AND 1oaa__#14
THEN
GO:0016616: oxidoreductase activity, acting on the CH-OH group of donors, NAD or NADP as acceptor: 3.628e-16 (20,59,12) OR
GO:0016854: racemase and epimerase activity: 3.804e-03 (20,13,2) OR
GO:0016627: oxidoreductase activity, acting on the CH-CH group of donors: 6.517e-03 (20,17,2) OR
GO:0016646: oxidoreductase activity, acting on the CH-NH group of donors, NAD or NADP as acceptor: 8.122e-03 (20,19,2) OR
GO:0016836: hydro-lyase activity: 2.359e-02 (20,33,2)

IF 1oaa__#161 AND 1e6ua_#10
THEN
GO:0016616: oxidoreductase activity, acting on the CH-OH group of donors, NAD or NADP as acceptor: 3.628e-16 (20,59,12) OR
GO:0016627: oxidoreductase activity, acting on the CH-CH group of donors: 4.731e-06 (20,17,4) OR
GO:0016836: hydro-lyase activity: 2.359e-02 (20,33,2) OR
GO:0016854: racemase and epimerase activity: 9.152e-02 (20,13,1) OR
GO:0016646: oxidoreductase activity, acting on the CH-NH group of donors, NAD or NADP as acceptor: 1.310e-01 (20,19,1)

IF 1gdna_#119 AND 1arb__#55 AND 1cgha_#130
THEN
GO:0004263: chymotrypsin activity: 4.573e-16 (36,41,13) OR
GO:0004295: trypsin activity: 4.735e-15 (36,48,13) OR
GO:0004867: serine-type endopeptidase inhibitor activity: 3.104e-04 (36,47,5) OR
GO:0005509: calcium ion binding: 5.651e-02 (36,160,5)

IF 1ekbb_#117 AND 1danh_#104 AND 1ton__#179 AND 1ekbb_#85 AND 1elva1#601 AND 1dlea_#238 AND 1arb__#193 AND 1dlea_#198 AND 1cgha_#130 AND 1autc_#209
THEN
GO:0004263: chymotrypsin activity: 4.573e-16 (36,41,13) OR
GO:0004295: trypsin activity: 4.735e-15 (36,48,13) OR
GO:0004867: serine-type endopeptidase inhibitor activity: 3.104e-04 (36,47,5) OR
GO:0005509: calcium ion binding: 5.651e-02 (36,160,5)

IF 1azza_#51 AND 1bio__#108 AND 1ton__#179 AND 1qnja_#118 AND 1ekbb_#85 AND 1dy9.1#A44 AND 1cgha_#130 AND 1ton__#211 AND 1bio__#210 AND 1autc_#45 AND 1ton__#231
THEN
GO:0004263: chymotrypsin activity: 4.573e-16 (36,41,13) OR
GO:0004295: trypsin activity: 4.735e-15 (36,48,13) OR
GO:0004867: serine-type endopeptidase inhibitor activity: 3.104e-04 (36,47,5) OR
GO:0005509: calcium ion binding: 5.651e-02 (36,160,5)

IF 2uaga1#54 AND 1cjca2#365
THEN
GO:0015036: disulfide oxidoreductase activity: 3.251e-16 (25,22,10) OR
GO:0016668: oxidoreductase activity, acting on sulfur group of donors, NAD or NADP as acceptor: 3.231e-08 (25,12,5) OR
GO:0050660: FAD binding: 1.117e-06 (25,10,4) OR
GO:0016627: oxidoreductase activity, acting on the CH-CH group of donors: 1.212e-05 (25,17,4) OR
GO:0016638: oxidoreductase activity, acting on the CH-NH2 group of donors: 1.454e-01 (25,17,1) OR
GO:0004601: peroxidase activity: 1.766e-01 (25,21,1)

IF 1ec7a1#216 AND 1h5qa_#70
THEN
GO:0016616: oxidoreductase activity, acting on the CH-OH group of donors, NAD or NADP as acceptor: 6.556e-16 (12,59,10) OR
GO:0016627: oxidoreductase activity, acting on the CH-CH group of donors: 7.249e-02 (12,17,1) OR
GO:0016836: hydro-lyase activity: 1.363e-01 (12,33,1)

IF 1h7wa4#476 AND 1dpja_#322
THEN
GO:0015036: disulfide oxidoreductase activity: 5.262e-16 (26,22,10) OR
GO:0016668: oxidoreductase activity, acting on sulfur group of donors, NAD or NADP as acceptor: 1.012e-14 (26,12,8) OR
GO:0050660: FAD binding: 3.528e-13 (26,10,7) OR
GO:0016651: oxidoreductase activity, acting on NADH or NADPH: 1.174e-01 (26,13,1)

IF 1qfma2#576 AND 1eny__#94
THEN
GO:0016616: oxidoreductase activity, acting on the CH-OH group of donors, NAD or NADP as acceptor: 3.628e-16 (20,59,12) OR

GO:0016854: racemase and epimerase activity: 3.804e-03 (20,13,2) OR
GO:0016627: oxidoreductase activity, acting on the CH-CH group of donors: 6.517e-03 (20,17,2) OR
GO:0016646: oxidoreductase activity, acting on the CH-NH group of donors, NAD or NADP as acceptor: 8.122e-03 (20,19,2) OR
GO:0016638: oxidoreductase activity, acting on the CH-NH2 group of donors: 1.180e-01 (20,17,1) OR
GO:0016836: hydro-lyase activity: 2.169e-01 (20,33,1)

IF 2ae2a_#17 AND 1eny__#147
THEN
GO:0016616: oxidoreductase activity, acting on the CH-OH group of donors, NAD or NADP as acceptor: 3.628e-16 (20,59,12) OR
GO:0016854: racemase and epimerase activity: 3.804e-03 (20,13,2) OR
GO:0016627: oxidoreductase activity, acting on the CH-CH group of donors: 6.517e-03 (20,17,2) OR
GO:0016646: oxidoreductase activity, acting on the CH-NH group of donors, NAD or NADP as acceptor: 1.310e-01 (20,19,1) OR
GO:0016836: hydro-lyase activity: 2.169e-01 (20,33,1) OR
GO:0003700: transcription factor activity: 2.302e-01 (20,124,2)

IF 1qfma2#576 AND 1e6ua_#10
THEN
GO:0016616: oxidoreductase activity, acting on the CH-OH group of donors, NAD or NADP as acceptor: 3.628e-16 (20,59,12) OR
GO:0016854: racemase and epimerase activity: 3.804e-03 (20,13,2) OR
GO:0016627: oxidoreductase activity, acting on the CH-CH group of donors: 6.517e-03 (20,17,2) OR
GO:0016646: oxidoreductase activity, acting on the CH-NH group of donors, NAD or NADP as acceptor: 8.122e-03 (20,19,2) OR
GO:0016836: hydro-lyase activity: 2.169e-01 (20,33,1) OR
GO:0000287: magnesium ion binding: 6.193e-01 (20,128,1)

IF 1glqa2#53 AND 1e8ca1#83
THEN
GO:0004364: glutathione transferase activity: 4.932e-16 (11,11,7) OR
GO:0003714: transcription corepressor activity: 3.188e-02 (11,8,1) OR
GO:0001584: rhodopsin-like receptor activity: 3.971e-02 (11,10,1) OR
GO:0016651: oxidoreductase activity, acting on NADH or NADPH: 5.134e-02 (11,13,1) OR
GO:0005179: hormone activity: 5.902e-02 (11,15,1)

IF 1ajsa_#139 AND 1dfoa_#344
THEN
GO:0008483: transaminase activity: 9.610e-16 (14,17,8) OR
GO:0016846: carbon-sulfur lyase activity: 3.988e-10 (14,10,5) OR
GO:0016831: carboxy-lyase activity: 1.213e-01 (14,25,1)

IF 1dfoa_#362 AND 1c7na_#281
THEN
GO:0008483: transaminase activity: 9.610e-16 (14,17,8) OR
GO:0016846: carbon-sulfur lyase activity: 9.009e-08 (14,10,4) OR
GO:0016831: carboxy-lyase activity: 6.875e-03 (14,25,2)

IF 1g71a_#77 AND 1gdna_#151 AND 1gdna_#123
THEN
GO:0004295: trypsin activity: 5.961e-16 (40,48,14) OR
GO:0004263: chymotrypsin activity: 2.291e-15 (40,41,13) OR
GO:0004867: serine-type endopeptidase inhibitor activity: 5.145e-04 (40,47,5) OR
GO:0005509: calcium ion binding: 7.595e-03 (40,160,7) OR
GO:0004896: hematopoietin/interferon-class (D200-domain) cytokine receptor activity: 2.457e-01 (40,19,1)

IF 1qlaa2#179 AND 1feca1#122
THEN
GO:0015036: disulfide oxidoreductase activity: 5.262e-16 (26,22,10) OR
GO:0016668: oxidoreductase activity, acting on sulfur group of donors, NAD or NADP as acceptor: 2.300e-12 (26,12,7) OR
GO:0050660: FAD binding: 1.317e-06 (26,10,4) OR
GO:0016627: oxidoreductase activity, acting on the CH-CH group of donors: 4.802e-04 (26,17,3) OR
GO:0016651: oxidoreductase activity, acting on NADH or NADPH: 1.174e-01 (26,13,1) OR
GO:0016638: oxidoreductase activity, acting on the CH-NH2 group of donors: 1.508e-01 (26,17,1)

IF 1e43a2#236 AND 2ltn.1#A63
THEN
GO:0004556: alpha-amylase activity: 1.089e-15 (16,15,8) OR
GO:0005509: calcium ion binding: 1.543e-05 (16,160,7) OR
GO:0004867: serine-type endopeptidase inhibitor activity: 2.436e-01 (16,47,1)

IF 1avaa2#204 AND 1hx0a2#234
THEN
GO:0004556: alpha-amylase activity: 1.089e-15 (16,15,8) OR

GO:0005509: calcium ion binding: 1.543e-05 (16,160,7) OR
GO:0004867: serine-type endopeptidase inhibitor activity: 2.436e-01 (16,47,1)

IF 1e5ka_#113 AND 1gdna_#121
THEN
GO:0004263: chymotrypsin activity: 6.795e-16 (59,41,15) OR
GO:0004295: trypsin activity: 1.051e-14 (59,48,15) OR
GO:0003809: thrombin activity: 1.772e-10 (59,10,7) OR
GO:0004867: serine-type endopeptidase inhibitor activity: 4.020e-07 (59,47,9) OR
GO:0005509: calcium ion binding: 2.309e-05 (59,160,13)

IF 1e5ka_#113 AND 1dlea_#198
THEN
GO:0004263: chymotrypsin activity: 6.795e-16 (59,41,15) OR
GO:0004295: trypsin activity: 1.051e-14 (59,48,15) OR
GO:0003809: thrombin activity: 1.772e-10 (59,10,7) OR
GO:0004867: serine-type endopeptidase inhibitor activity: 4.020e-07 (59,47,9) OR
GO:0005509: calcium ion binding: 2.309e-05 (59,160,13)

IF 1ceqa2#199 AND 1ypta_#418
THEN
GO:0004457: lactate dehydrogenase activity: 1.862e-15 (14,10,7) OR
GO:0016616: oxidoreductase activity, acting on the CH-OH group of donors, NAD or NADP as acceptor: 4.736e-09 (14,59,7)

IF 2cmd_2#172 AND 1qtn.1#A270
THEN
GO:0004457: lactate dehydrogenase activity: 1.862e-15 (14,10,7) OR
GO:0016616: oxidoreductase activity, acting on the CH-OH group of donors, NAD or NADP as acceptor: 4.736e-09 (14,59,7)

IF 1je5a_#10 AND 1llda2#177
THEN
GO:0004457: lactate dehydrogenase activity: 1.862e-15 (14,10,7) OR
GO:0016616: oxidoreductase activity, acting on the CH-OH group of donors, NAD or NADP as acceptor: 4.736e-09 (14,59,7)

IF 3sil__#98 AND 1cgha_#130 AND 1autc_#45
THEN
GO:0004263: chymotrypsin activity: 6.396e-16 (47,41,14) OR
GO:0004295: trypsin activity: 8.075e-15 (47,48,14) OR
GO:0004867: serine-type endopeptidase inhibitor activity: 8.017e-07 (47,47,8) OR
GO:0005509: calcium ion binding: 1.308e-03 (47,160,9) OR
GO:0003809: thrombin activity: 1.599e-01 (47,10,1) OR
GO:0008201: heparin binding: 3.425e-01 (47,24,1)

IF 1bjt__#890 AND 1gdna_#123 AND 1cgha_#130
THEN
GO:0004263: chymotrypsin activity: 6.396e-16 (47,41,14) OR
GO:0004295: trypsin activity: 8.075e-15 (47,48,14) OR
GO:0004867: serine-type endopeptidase inhibitor activity: 8.017e-07 (47,47,8) OR
GO:0005509: calcium ion binding: 1.308e-03 (47,160,9) OR
GO:0003809: thrombin activity: 1.599e-01 (47,10,1) OR
GO:0008201: heparin binding: 3.425e-01 (47,24,1)

IF 1qj5a_#268 AND 1c7na_#281
THEN
GO:0008483: transaminase activity: 9.610e-16 (14,17,8) OR
GO:0016846: carbon-sulfur lyase activity: 9.009e-08 (14,10,4) OR
GO:0016866: intramolecular transferase activity: 6.006e-02 (14,12,1) OR
GO:0016831: carboxy-lyase activity: 1.213e-01 (14,25,1)

IF 1a7s__#148 AND 1f42a2#123 AND 1elva1#513
THEN
GO:0004295: trypsin activity: 6.518e-16 (61,48,16) OR
GO:0004263: chymotrypsin activity: 1.180e-15 (61,41,15) OR
GO:0003809: thrombin activity: 2.261e-10 (61,10,7) OR
GO:0004867: serine-type endopeptidase inhibitor activity: 2.605e-09 (61,47,11) OR
GO:0005509: calcium ion binding: 6.529e-04 (61,160,11) OR
GO:0005529: sugar binding: 5.890e-01 (61,39,1)

IF 1h7wa4#476 AND 1czan1#92
THEN

GO:0015036: disulfide oxidoreductase activity: 8.323e-16 (27,22,10) OR
GO:0016668: oxidoreductase activity, acting on sulfur group of donors, NAD or NADP as acceptor: 1.436e-14 (27,12,8) OR
GO:0050660: FAD binding: 4.758e-13 (27,10,7) OR
GO:0016651: oxidoreductase activity, acting on NADH or NADPH: 1.217e-01 (27,13,1) OR
GO:0004601: peroxidase activity: 1.893e-01 (27,21,1)

IF 3grs_3#435 AND 1trb_1#14
THEN
GO:0015036: disulfide oxidoreductase activity: 8.323e-16 (27,22,10) OR
GO:0016668: oxidoreductase activity, acting on sulfur group of donors, NAD or NADP as acceptor: 1.436e-14 (27,12,8) OR
GO:0050660: FAD binding: 4.758e-13 (27,10,7) OR
GO:0016651: oxidoreductase activity, acting on NADH or NADPH: 1.217e-01 (27,13,1) OR
GO:0004601: peroxidase activity: 1.893e-01 (27,21,1)

IF 1h7wa4#476 AND 1ojt_3#488
THEN
GO:0015036: disulfide oxidoreductase activity: 8.323e-16 (27,22,10) OR
GO:0016668: oxidoreductase activity, acting on sulfur group of donors, NAD or NADP as acceptor: 1.436e-14 (27,12,8) OR
GO:0050660: FAD binding: 4.758e-13 (27,10,7) OR
GO:0016651: oxidoreductase activity, acting on NADH or NADPH: 1.217e-01 (27,13,1) OR
GO:0004601: peroxidase activity: 1.893e-01 (27,21,1)

IF 1h7wa4#476 AND 2mpra_#190
THEN
GO:0015036: disulfide oxidoreductase activity: 8.323e-16 (27,22,10) OR
GO:0016668: oxidoreductase activity, acting on sulfur group of donors, NAD or NADP as acceptor: 1.436e-14 (27,12,8) OR
GO:0050660: FAD binding: 4.758e-13 (27,10,7) OR
GO:0016651: oxidoreductase activity, acting on NADH or NADPH: 1.217e-01 (27,13,1) OR
GO:0004601: peroxidase activity: 1.893e-01 (27,21,1)

IF 1h7wa4#476 AND 1d7ya3#331
THEN
GO:0015036: disulfide oxidoreductase activity: 8.323e-16 (27,22,10) OR
GO:0016668: oxidoreductase activity, acting on sulfur group of donors, NAD or NADP as acceptor: 1.436e-14 (27,12,8) OR
GO:0050660: FAD binding: 4.758e-13 (27,10,7) OR
GO:0016651: oxidoreductase activity, acting on NADH or NADPH: 1.217e-01 (27,13,1) OR
GO:0004601: peroxidase activity: 1.893e-01 (27,21,1)

IF 1h7wa4#476 AND 1d6ja_#95
THEN
GO:0015036: disulfide oxidoreductase activity: 8.323e-16 (27,22,10) OR
GO:0016668: oxidoreductase activity, acting on sulfur group of donors, NAD or NADP as acceptor: 1.436e-14 (27,12,8) OR
GO:0050660: FAD binding: 4.758e-13 (27,10,7) OR
GO:0016651: oxidoreductase activity, acting on NADH or NADPH: 1.217e-01 (27,13,1) OR
GO:0004601: peroxidase activity: 1.893e-01 (27,21,1)

IF 1d7ya3#331 AND 1h7wa4#478
THEN
GO:0015036: disulfide oxidoreductase activity: 8.323e-16 (27,22,10) OR
GO:0016668: oxidoreductase activity, acting on sulfur group of donors, NAD or NADP as acceptor: 1.436e-14 (27,12,8) OR
GO:0050660: FAD binding: 4.758e-13 (27,10,7) OR
GO:0016651: oxidoreductase activity, acting on NADH or NADPH: 1.217e-01 (27,13,1) OR
GO:0004601: peroxidase activity: 1.893e-01 (27,21,1)

IF 1b16a_#184 AND 1e8ca3#111
THEN
GO:0016616: oxidoreductase activity, acting on the CH-OH group of donors, NAD or NADP as acceptor: 7.458e-16 (16,59,11) OR
GO:0016854: racemase and epimerase activity: 7.386e-02 (16,13,1) OR
GO:0015082: di-, tri-valent inorganic cation transporter activity: 7.932e-02 (16,14,1) OR
GO:0046915: transition metal ion transporter activity: 7.932e-02 (16,14,1) OR
GO:0016627: oxidoreductase activity, acting on the CH-CH group of donors: 9.553e-02 (16,17,1) OR
GO:0016646: oxidoreductase activity, acting on the CH-NH group of donors, NAD or NADP as acceptor: 1.062e-01 (16,19,1)

IF 1qgna_#207 AND 1qtn.1#A270
THEN
GO:0016846: carbon-sulfur lyase activity: 9.320e-16 (13,10,7) OR
GO:0008483: transaminase activity: 5.554e-05 (13,17,3) OR
GO:0016763: transferase activity, transferring pentosyl groups: 1.259e-01 (13,28,1) OR
GO:0000287: magnesium ion binding: 4.657e-01 (13,128,1) OR
GO:0005524: ATP binding: 7.039e-01 (13,243,1)

IF 1a9xa3#78 AND 1c7na_#281
THEN
GO:0008483: transaminase activity: 9.610e-16 (14,17,8) OR
GO:0016846: carbon-sulfur lyase activity: 1.269e-05 (14,10,3) OR
GO:0016866: intramolecular transferase activity: 6.006e-02 (14,12,1) OR
GO:0016831: carboxy-lyase activity: 1.213e-01 (14,25,1) OR
GO:0030145: manganese ion binding: 1.789e-01 (14,38,1)

IF 1qrra_#69 AND 1d7ya1#255
THEN
GO:0015036: disulfide oxidoreductase activity: 8.323e-16 (27,22,10) OR
GO:0050660: FAD binding: 1.070e-10 (27,10,6) OR
GO:0016668: oxidoreductase activity, acting on sulfur group of donors, NAD or NADP as acceptor: 4.647e-10 (27,12,6) OR
GO:0016627: oxidoreductase activity, acting on the CH-CH group of donors: 5.381e-04 (27,17,3) OR
GO:0016651: oxidoreductase activity, acting on NADH or NADPH: 1.217e-01 (27,13,1) OR
GO:0004601: peroxidase activity: 1.893e-01 (27,21,1)

IF 1b4va1#11 AND 1trb_1#41
THEN
GO:0015036: disulfide oxidoreductase activity: 1.290e-15 (28,22,10) OR
GO:0050660: FAD binding: 1.850e-15 (28,10,8) OR
GO:0016668: oxidoreductase activity, acting on sulfur group of donors, NAD or NADP as acceptor: 2.008e-14 (28,12,8) OR
GO:0016627: oxidoreductase activity, acting on the CH-CH group of donors: 1.259e-02 (28,17,2)

IF 1fl2a1#472 AND 3grx__#56
THEN
GO:0015036: disulfide oxidoreductase activity: 1.032e-15 (18,22,9) OR
GO:0016668: oxidoreductase activity, acting on sulfur group of donors, NAD or NADP as acceptor: 6.393e-07 (18,12,4) OR
GO:0050660: FAD binding: 2.824e-05 (18,10,3) OR
GO:0016638: oxidoreductase activity, acting on the CH-NH2 group of donors: 1.068e-01 (18,17,1) OR
GO:0004601: peroxidase activity: 1.304e-01 (18,21,1)

IF 1el0a_#28 AND 1ha6a_#59
THEN
GO:0008009: chemokine activity: 2.596e-15 (7,10,6) OR
GO:0008083: growth factor activity: 1.031e-01 (7,42,1)

IF 2hlca_#54 AND 1qj8a_#64 AND 1autc_#209
THEN
GO:0004295: trypsin activity: 8.945e-16 (41,48,14) OR
GO:0004263: chymotrypsin activity: 3.322e-15 (41,41,13) OR
GO:0004867: serine-type endopeptidase inhibitor activity: 5.784e-04 (41,47,5) OR
GO:0005509: calcium ion binding: 8.717e-03 (41,160,7) OR
GO:0003809: thrombin activity: 1.409e-01 (41,10,1) OR
GO:0004896: hematopoietin/interferon-class (D200-domain) cytokine receptor activity: 2.510e-01 (41,19,1)

IF 1arb__#193 AND 2hlca_#54 AND 1qj8a_#64
THEN
GO:0004295: trypsin activity: 8.945e-16 (41,48,14) OR
GO:0004263: chymotrypsin activity: 3.322e-15 (41,41,13) OR
GO:0004867: serine-type endopeptidase inhibitor activity: 5.784e-04 (41,47,5) OR
GO:0005509: calcium ion binding: 8.717e-03 (41,160,7) OR
GO:0003809: thrombin activity: 1.409e-01 (41,10,1) OR
GO:0004896: hematopoietin/interferon-class (D200-domain) cytokine receptor activity: 2.510e-01 (41,19,1)

IF 1ddja_#726 AND 1cqqa_#163
THEN
GO:0004295: trypsin activity: 8.945e-16 (41,48,14) OR
GO:0004263: chymotrypsin activity: 1.368e-13 (41,41,12) OR
GO:0004867: serine-type endopeptidase inhibitor activity: 5.784e-04 (41,47,5) OR
GO:0005509: calcium ion binding: 8.717e-03 (41,160,7) OR
GO:0003809: thrombin activity: 9.210e-03 (41,10,2) OR
GO:0005529: sugar binding: 4.487e-01 (41,39,1)

IF 1danh_#197 AND 1qj8a_#64
THEN
GO:0004295: trypsin activity: 8.945e-16 (41,48,14) OR
GO:0004263: chymotrypsin activity: 3.322e-15 (41,41,13) OR
GO:0004867: serine-type endopeptidase inhibitor activity: 5.784e-04 (41,47,5) OR

GO:0005509: calcium ion binding: 8.717e-03 (41,160,7) OR
GO:0003809: thrombin activity: 1.409e-01 (41,10,1) OR
GO:0004896: hematopoietin/interferon-class (D200-domain) cytokine receptor activity: 2.510e-01 (41,19,1)

IF 1arb__#55 AND 1qj8a_#64
THEN
GO:0004295: trypsin activity: 8.945e-16 (41,48,14) OR
GO:0004263: chymotrypsin activity: 3.322e-15 (41,41,13) OR
GO:0004867: serine-type endopeptidase inhibitor activity: 5.784e-04 (41,47,5) OR
GO:0005509: calcium ion binding: 8.717e-03 (41,160,7) OR
GO:0003809: thrombin activity: 1.409e-01 (41,10,1) OR
GO:0004896: hematopoietin/interferon-class (D200-domain) cytokine receptor activity: 2.510e-01 (41,19,1)

IF 2hlca_#54 AND 1qj8a_#64 AND 1gg6.1#C229
THEN
GO:0004295: trypsin activity: 8.945e-16 (41,48,14) OR
GO:0004263: chymotrypsin activity: 3.322e-15 (41,41,13) OR
GO:0004867: serine-type endopeptidase inhibitor activity: 5.784e-04 (41,47,5) OR
GO:0005509: calcium ion binding: 8.717e-03 (41,160,7) OR
GO:0003809: thrombin activity: 1.409e-01 (41,10,1) OR
GO:0004896: hematopoietin/interferon-class (D200-domain) cytokine receptor activity: 2.510e-01 (41,19,1)

IF 1trb_1#112 AND 1qfma1#139
THEN
GO:0050660: FAD binding: 9.309e-16 (26,10,8) OR
GO:0016668: oxidoreductase activity, acting on sulfur group of donors, NAD or NADP as acceptor: 2.300e-12 (26,12,7) OR
GO:0015036: disulfide oxidoreductase activity: 6.161e-12 (26,22,8) OR
GO:0016651: oxidoreductase activity, acting on NADH or NADPH: 1.174e-01 (26,13,1) OR
GO:0016627: oxidoreductase activity, acting on the CH-CH group of donors: 1.508e-01 (26,17,1) OR
GO:0016638: oxidoreductase activity, acting on the CH-NH2 group of donors: 1.508e-01 (26,17,1)

IF 1qgna_#149 AND 1c7na_#281
THEN
GO:0016846: carbon-sulfur lyase activity: 1.862e-15 (14,10,7) OR
GO:0008483: transaminase activity: 6.391e-11 (14,17,6) OR
GO:0016831: carboxy-lyase activity: 1.213e-01 (14,25,1)

IF 1gdea_#243 AND 1qgna_#149
THEN
GO:0016846: carbon-sulfur lyase activity: 1.862e-15 (14,10,7) OR
GO:0008483: transaminase activity: 6.391e-11 (14,17,6) OR
GO:0016831: carboxy-lyase activity: 1.213e-01 (14,25,1)

IF 1ja9a_#202 AND 1oaa__#161
THEN
GO:0016616: oxidoreductase activity, acting on the CH-OH group of donors, NAD or NADP as acceptor: 2.794e-15 (13,59,10) OR
GO:0016627: oxidoreductase activity, acting on the CH-CH group of donors: 5.554e-05 (13,17,3)

IF 1avaa2#204 AND 1dik_1#765
THEN
GO:0004556: alpha-amylase activity: 2.053e-15 (17,15,8) OR
GO:0005509: calcium ion binding: 2.494e-05 (17,160,7) OR
GO:0004867: serine-type endopeptidase inhibitor activity: 3.359e-02 (17,47,2)

IF 1e43a2#236 AND 1nsj__#124
THEN
GO:0004556: alpha-amylase activity: 2.053e-15 (17,15,8) OR
GO:0005509: calcium ion binding: 2.494e-05 (17,160,7) OR
GO:0004867: serine-type endopeptidase inhibitor activity: 3.359e-02 (17,47,2)

IF 1bvza3#298 AND 1bf2_3#575
THEN
GO:0004556: alpha-amylase activity: 2.053e-15 (17,15,8) OR
GO:0005509: calcium ion binding: 2.494e-05 (17,160,7) OR
GO:0004867: serine-type endopeptidase inhibitor activity: 3.359e-02 (17,47,2)

IF 1fjfj_#68 AND 1h7wa4#478
THEN
GO:0015036: disulfide oxidoreductase activity: 1.032e-15 (18,22,9) OR
GO:0016668: oxidoreductase activity, acting on sulfur group of donors, NAD or NADP as acceptor: 6.393e-07 (18,12,4) OR

GO:0050660: FAD binding: 1.798e-03 (18,10,2) OR
GO:0016651: oxidoreductase activity, acting on NADH or NADPH: 8.272e-02 (18,13,1) OR
GO:0016638: oxidoreductase activity, acting on the CH-NH2 group of donors: 1.068e-01 (18,17,1) OR
GO:0004601: peroxidase activity: 1.304e-01 (18,21,1)

IF 1hu4a_#267 AND 1oaa__#175
THEN
GO:0016616: oxidoreductase activity, acting on the CH-OH group of donors, NAD or NADP as acceptor: 2.079e-15 (17,59,11) OR
GO:0016627: oxidoreductase activity, acting on the CH-CH group of donors: 2.351e-06 (17,17,4) OR
GO:0016646: oxidoreductase activity, acting on the CH-NH group of donors, NAD or NADP as acceptor: 5.887e-03 (17,19,2)

IF 1hu4a_#267 AND 1e6ua_#10
THEN
GO:0016616: oxidoreductase activity, acting on the CH-OH group of donors, NAD or NADP as acceptor: 2.079e-15 (17,59,11) OR
GO:0016627: oxidoreductase activity, acting on the CH-CH group of donors: 2.351e-06 (17,17,4) OR
GO:0016646: oxidoreductase activity, acting on the CH-NH group of donors, NAD or NADP as acceptor: 5.887e-03 (17,19,2)

IF 1ekbb_#117 AND 1c5y.1#B241 AND 1elva1#601 AND 1gg6.1#C229 AND 1cgha_#130
THEN
GO:0004263: chymotrypsin activity: 1.561e-15 (39,41,13) OR
GO:0004295: trypsin activity: 1.605e-14 (39,48,13) OR
GO:0004867: serine-type endopeptidase inhibitor activity: 4.031e-05 (39,47,6) OR
GO:0005509: calcium ion binding: 6.583e-03 (39,160,7)

IF 1d6ja_#95 AND 1trb_1#41
THEN
GO:0015036: disulfide oxidoreductase activity: 1.290e-15 (28,22,10) OR
GO:0016668: oxidoreductase activity, acting on sulfur group of donors, NAD or NADP as acceptor: 2.008e-14 (28,12,8) OR
GO:0050660: FAD binding: 6.338e-13 (28,10,7) OR
GO:0016627: oxidoreductase activity, acting on the CH-CH group of donors: 1.259e-02 (28,17,2) OR
GO:0016651: oxidoreductase activity, acting on NADH or NADPH: 1.259e-01 (28,13,1)

IF 1iba__#39 AND 1trb_1#41
THEN
GO:0015036: disulfide oxidoreductase activity: 1.290e-15 (28,22,10) OR
GO:0050660: FAD binding: 1.850e-15 (28,10,8) OR
GO:0016668: oxidoreductase activity, acting on sulfur group of donors, NAD or NADP as acceptor: 2.008e-14 (28,12,8) OR
GO:0016651: oxidoreductase activity, acting on NADH or NADPH: 1.259e-01 (28,13,1) OR
GO:0016638: oxidoreductase activity, acting on the CH-NH2 group of donors: 1.615e-01 (28,17,1)

IF 1jb3a_#64 AND 2cmd_2#251
THEN
GO:0004457: lactate dehydrogenase activity: 3.488e-15 (15,10,7) OR
GO:0016616: oxidoreductase activity, acting on the CH-OH group of donors, NAD or NADP as acceptor: 1.699e-10 (15,59,8)

IF 2cmd_2#172 AND 1jb3a_#64
THEN
GO:0004457: lactate dehydrogenase activity: 3.488e-15 (15,10,7) OR
GO:0016616: oxidoreductase activity, acting on the CH-OH group of donors, NAD or NADP as acceptor: 1.699e-10 (15,59,8)

IF 1tuba1#8 AND 2cmd_2#251
THEN
GO:0004457: lactate dehydrogenase activity: 3.488e-15 (15,10,7) OR
GO:0016616: oxidoreductase activity, acting on the CH-OH group of donors, NAD or NADP as acceptor: 1.699e-10 (15,59,8)

IF 1hyha2#171 AND 1qb7a_#86
THEN
GO:0004457: lactate dehydrogenase activity: 3.488e-15 (15,10,7) OR
GO:0016616: oxidoreductase activity, acting on the CH-OH group of donors, NAD or NADP as acceptor: 1.699e-10 (15,59,8)

IF 2hlpa2#298 AND 2cmd_2#251
THEN
GO:0004457: lactate dehydrogenase activity: 3.488e-15 (15,10,7) OR
GO:0016616: oxidoreductase activity, acting on the CH-OH group of donors, NAD or NADP as acceptor: 1.699e-10 (15,59,8)

IF 1gjwa2#141 AND 1fi2a_#109
THEN
GO:0004556: alpha-amylase activity: 3.488e-15 (10,15,7) OR
GO:0005509: calcium ion binding: 1.756e-02 (10,160,3)

IF 1jb0d_#26 AND 1gcya2#331
THEN
GO:0004556: alpha-amylase activity: 3.488e-15 (10,15,7) OR
GO:0005509: calcium ion binding: 1.756e-02 (10,160,3)

IF 1dt6a_#385 AND 1e30a_#87
THEN
GO:0005507: copper ion binding: 1.406e-15 (17,38,10) OR
GO:0015078: hydrogen ion transporter activity: 5.822e-06 (17,21,4) OR
GO:0015082: di-, tri-valent inorganic cation transporter activity: 8.408e-02 (17,14,1) OR
GO:0046915: transition metal ion transporter activity: 8.408e-02 (17,14,1) OR
GO:0005509: calcium ion binding: 6.436e-01 (17,160,1)

IF 1qq4a_#143 AND 2hlca_#99 AND 1cgha_#130
THEN
GO:0004263: chymotrypsin activity: 1.561e-15 (39,41,13) OR
GO:0004295: trypsin activity: 1.605e-14 (39,48,13) OR
GO:0004867: serine-type endopeptidase inhibitor activity: 4.031e-05 (39,47,6) OR
GO:0005509: calcium ion binding: 2.423e-02 (39,160,6) OR
GO:0008201: heparin binding: 2.935e-01 (39,24,1)

IF 1fvua_#72 AND 2msba_#218
THEN
GO:0005529: sugar binding: 2.008e-15 (12,39,9) OR
GO:0004263: chymotrypsin activity: 1.666e-01 (12,41,1) OR
GO:0004295: trypsin activity: 1.924e-01 (12,48,1) OR
GO:0005509: calcium ion binding: 5.169e-01 (12,160,1)

IF 2msba_#195 AND 1qo3c_#195
THEN
GO:0005529: sugar binding: 2.008e-15 (12,39,9) OR
GO:0004263: chymotrypsin activity: 1.666e-01 (12,41,1) OR
GO:0004295: trypsin activity: 1.924e-01 (12,48,1) OR
GO:0005509: calcium ion binding: 5.169e-01 (12,160,1)

IF 1fvua_#120 AND 2msba_#218
THEN
GO:0005529: sugar binding: 2.008e-15 (12,39,9) OR
GO:0004263: chymotrypsin activity: 1.666e-01 (12,41,1) OR
GO:0004295: trypsin activity: 1.924e-01 (12,48,1) OR
GO:0005509: calcium ion binding: 5.169e-01 (12,160,1)

IF 1fvua_#72 AND 2msba_#195
THEN
GO:0005529: sugar binding: 2.008e-15 (12,39,9) OR
GO:0004263: chymotrypsin activity: 1.666e-01 (12,41,1) OR
GO:0004295: trypsin activity: 1.924e-01 (12,48,1) OR
GO:0005509: calcium ion binding: 5.169e-01 (12,160,1)

IF 1qhoa4#399 AND 1fhoa_#74
THEN
GO:0004556: alpha-amylase activity: 2.053e-15 (17,15,8) OR
GO:0005509: calcium ion binding: 2.494e-05 (17,160,7) OR
GO:0016627: oxidoreductase activity, acting on the CH-CH group of donors: 1.012e-01 (17,17,1) OR
GO:0004867: serine-type endopeptidase inhibitor activity: 2.567e-01 (17,47,1)

IF 1hx0a2#234 AND 1qfea_#49
THEN
GO:0004556: alpha-amylase activity: 2.053e-15 (17,15,8) OR
GO:0005509: calcium ion binding: 2.494e-05 (17,160,7) OR
GO:0008757: S-adenosylmethionine-dependent methyltransferase activity: 1.400e-01 (17,24,1) OR
GO:0004867: serine-type endopeptidase inhibitor activity: 2.567e-01 (17,47,1)

IF 1bag_2#207 AND 1gcya2#331
THEN
GO:0004556: alpha-amylase activity: 2.053e-15 (17,15,8) OR
GO:0005509: calcium ion binding: 2.689e-04 (17,160,6) OR
GO:0004867: serine-type endopeptidase inhibitor activity: 3.359e-02 (17,47,2) OR
GO:0005525: GTP binding: 2.661e-01 (17,49,1)

IF 2dkb__#114 AND 1aqua_#126 AND 1gdea_#379 AND 1dfoa_#344
THEN
GO:0008483: transaminase activity: 2.053e-15 (15,17,8) OR
GO:0016846: carbon-sulfur lyase activity: 5.974e-10 (15,10,5) OR
GO:0016866: intramolecular transferase activity: 6.422e-02 (15,12,1) OR
GO:0016831: carboxy-lyase activity: 1.294e-01 (15,25,1)

IF 1ja9a_#202 AND 2ae2a_#17
THEN
GO:0016616: oxidoreductase activity, acting on the CH-OH group of donors, NAD or NADP as acceptor: 2.794e-15 (13,59,10) OR
GO:0016627: oxidoreductase activity, acting on the CH-CH group of donors: 2.745e-03 (13,17,2) OR
GO:0016646: oxidoreductase activity, acting on the CH-NH group of donors, NAD or NADP as acceptor: 8.713e-02 (13,19,1)

IF 1qfma2#576 AND 1cyda_#180
THEN
GO:0016616: oxidoreductase activity, acting on the CH-OH group of donors, NAD or NADP as acceptor: 2.794e-15 (13,59,10) OR
GO:0016627: oxidoreductase activity, acting on the CH-CH group of donors: 2.745e-03 (13,17,2) OR
GO:0016646: oxidoreductase activity, acting on the CH-NH group of donors, NAD or NADP as acceptor: 8.713e-02 (13,19,1)

IF 1qrra_#208 AND 1cyda_#180
THEN
GO:0016616: oxidoreductase activity, acting on the CH-OH group of donors, NAD or NADP as acceptor: 2.794e-15 (13,59,10) OR
GO:0016627: oxidoreductase activity, acting on the CH-CH group of donors: 2.745e-03 (13,17,2) OR
GO:0016646: oxidoreductase activity, acting on the CH-NH group of donors, NAD or NADP as acceptor: 8.713e-02 (13,19,1)

IF 1bio__#88 AND 1arb__#55
THEN
GO:0004263: chymotrypsin activity: 1.707e-15 (50,41,14) OR
GO:0004295: trypsin activity: 2.139e-14 (50,48,14) OR
GO:0003809: thrombin activity: 3.993e-07 (50,10,5) OR
GO:0005509: calcium ion binding: 1.027e-04 (50,160,11) OR
GO:0004867: serine-type endopeptidase inhibitor activity: 1.701e-04 (50,47,6)

IF 1ypta_#406 AND 1ecpa_#58
THEN
GO:0004725: protein tyrosine phosphatase activity: 8.850e-15 (6,15,6)

IF 1ej8a_#189 AND 1ypta_#406
THEN
GO:0004725: protein tyrosine phosphatase activity: 8.850e-15 (6,15,6)

IF 1d5ra2#25 AND 1i50b_#966
THEN
GO:0004725: protein tyrosine phosphatase activity: 8.850e-15 (6,15,6)

IF 1ypta_#406 AND 1jsg__#92
THEN
GO:0004725: protein tyrosine phosphatase activity: 8.850e-15 (6,15,6)

IF 1oaa__#175 AND 1oaa__#14
THEN
GO:0016616: oxidoreductase activity, acting on the CH-OH group of donors, NAD or NADP as acceptor: 1.803e-15 (22,59,12) OR
GO:0016627: oxidoreductase activity, acting on the CH-CH group of donors: 7.088e-06 (22,17,4) OR
GO:0016854: racemase and epimerase activity: 4.600e-03 (22,13,2) OR
GO:0016646: oxidoreductase activity, acting on the CH-NH group of donors, NAD or NADP as acceptor: 9.793e-03 (22,19,2) OR
GO:0016836: hydro-lyase activity: 2.825e-02 (22,33,2)

IF 1qrra_#208 AND 1e6ua_#10
THEN
GO:0016616: oxidoreductase activity, acting on the CH-OH group of donors, NAD or NADP as acceptor: 1.803e-15 (22,59,12) OR
GO:0016627: oxidoreductase activity, acting on the CH-CH group of donors: 2.888e-04 (22,17,3) OR
GO:0016646: oxidoreductase activity, acting on the CH-NH group of donors, NAD or NADP as acceptor: 4.073e-04 (22,19,3) OR
GO:0016854: racemase and epimerase activity: 4.600e-03 (22,13,2) OR
GO:0016836: hydro-lyase activity: 2.825e-02 (22,33,2)

IF 1h8d.1#H184 AND 1a0la_#160 AND 1dlea_#238
THEN
GO:0004263: chymotrypsin activity: 1.542e-15 (62,41,15) OR
GO:0004295: trypsin activity: 2.367e-14 (62,48,15) OR
GO:0003809: thrombin activity: 2.546e-10 (62,10,7) OR

GO:0004867: serine-type endopeptidase inhibitor activity: 3.128e-09 (62,47,11) OR
GO:0005509: calcium ion binding: 4.058e-05 (62,160,13) OR
GO:0005529: sugar binding: 5.950e-01 (62,39,1)

IF 1gdna_#26 AND 1cpza_#6
THEN
GO:0003809: thrombin activity: 1.610e-15 (52,10,9) OR
GO:0004263: chymotrypsin activity: 3.202e-12 (52,41,12) OR
GO:0004295: trypsin activity: 2.564e-11 (52,48,12) OR
GO:0004867: serine-type endopeptidase inhibitor activity: 2.135e-05 (52,47,7) OR
GO:0005509: calcium ion binding: 1.498e-04 (52,160,11) OR
GO:0005529: sugar binding: 5.308e-01 (52,39,1)

IF 1gdna_#119 AND 1f97a1#62 AND 1ton__#179
THEN
GO:0004295: trypsin activity: 1.948e-15 (34,48,13) OR
GO:0004263: chymotrypsin activity: 1.019e-14 (34,41,12) OR
GO:0004867: serine-type endopeptidase inhibitor activity: 1.980e-02 (34,47,3) OR
GO:0005509: calcium ion binding: 4.579e-02 (34,160,5) OR
GO:0004896: hematopoietin/interferon-class (D200-domain) cytokine receptor activity: 2.129e-01 (34,19,1)

IF 1hwxa1#256 AND 1e39a2#516
THEN
GO:0015036: disulfide oxidoreductase activity: 1.960e-15 (29,22,10) OR
GO:0050660: FAD binding: 2.552e-15 (29,10,8) OR
GO:0016668: oxidoreductase activity, acting on sulfur group of donors, NAD or NADP as acceptor: 5.431e-12 (29,12,7) OR
GO:0016627: oxidoreductase activity, acting on the CH-CH group of donors: 6.670e-04 (29,17,3) OR
GO:0016651: oxidoreductase activity, acting on NADH or NADPH: 1.301e-01 (29,13,1)

IF 2mpra_#190 AND 1bag_2#207
THEN
GO:0004556: alpha-amylase activity: 2.053e-15 (17,15,8) OR
GO:0005509: calcium ion binding: 2.689e-04 (17,160,6) OR
GO:0004364: glutathione transferase activity: 6.664e-02 (17,11,1) OR
GO:0004867: serine-type endopeptidase inhibitor activity: 2.567e-01 (17,47,1) OR
GO:0005525: GTP binding: 2.661e-01 (17,49,1)

IF 1a4ya_#47 AND 1gcya2#331 AND 1e6pa2#46
THEN
GO:0004556: alpha-amylase activity: 2.053e-15 (17,15,8) OR
GO:0005509: calcium ion binding: 2.689e-04 (17,160,6) OR
GO:0005351: sugar porter activity: 1.236e-01 (17,21,1) OR
GO:0004867: serine-type endopeptidase inhibitor activity: 2.567e-01 (17,47,1) OR
GO:0000287: magnesium ion binding: 5.597e-01 (17,128,1)

IF 1j9la_#82 AND 1hdr__#149
THEN
GO:0016616: oxidoreductase activity, acting on the CH-OH group of donors, NAD or NADP as acceptor: 2.079e-15 (17,59,11) OR
GO:0016646: oxidoreductase activity, acting on the CH-NH group of donors, NAD or NADP as acceptor: 5.887e-03 (17,19,2) OR
GO:0016836: hydro-lyase activity: 1.727e-02 (17,33,2) OR
GO:0016854: racemase and epimerase activity: 7.830e-02 (17,13,1) OR
GO:0016627: oxidoreductase activity, acting on the CH-CH group of donors: 1.012e-01 (17,17,1)

IF 1oaa__#161 AND 1eq2a_#11
THEN
GO:0016616: oxidoreductase activity, acting on the CH-OH group of donors, NAD or NADP as acceptor: 2.079e-15 (17,59,11) OR
GO:0016627: oxidoreductase activity, acting on the CH-CH group of donors: 4.716e-03 (17,17,2) OR
GO:0016836: hydro-lyase activity: 1.727e-02 (17,33,2) OR
GO:0016854: racemase and epimerase activity: 7.830e-02 (17,13,1) OR
GO:0016646: oxidoreductase activity, acting on the CH-NH group of donors, NAD or NADP as acceptor: 1.125e-01 (17,19,1)

IF 1oaa__#235 AND 1eno__#264
THEN
GO:0016616: oxidoreductase activity, acting on the CH-OH group of donors, NAD or NADP as acceptor: 5.485e-15 (10,59,9) OR
GO:0016627: oxidoreductase activity, acting on the CH-CH group of donors: 6.076e-02 (10,17,1)

IF 1e6ua_#10 AND 1fmca_#89
THEN
GO:0016616: oxidoreductase activity, acting on the CH-OH group of donors, NAD or NADP as acceptor: 2.794e-15 (13,59,10) OR
GO:0016854: racemase and epimerase activity: 6.040e-02 (13,13,1) OR

GO:0016627: oxidoreductase activity, acting on the CH-CH group of donors: 7.830e-02 (13,17,1) OR
GO:0016646: oxidoreductase activity, acting on the CH-NH group of donors, NAD or NADP as acceptor: 8.713e-02 (13,19,1)

IF 1aba__#17 AND 1gnwa1#160
THEN
GO:0004364: glutathione transferase activity: 5.709e-15 (7,11,6) OR
GO:0003714: transcription corepressor activity: 2.039e-02 (7,8,1)

IF 1glqa2#53 AND 1aba__#17
THEN
GO:0004364: glutathione transferase activity: 5.709e-15 (7,11,6) OR
GO:0003714: transcription corepressor activity: 2.039e-02 (7,8,1)

IF 1gnwa1#160 AND 1d6aa_#52
THEN
GO:0004364: glutathione transferase activity: 5.709e-15 (7,11,6) OR
GO:0003714: transcription corepressor activity: 2.039e-02 (7,8,1)

IF 1d4xg_#60 AND 1a0fa1#94
THEN
GO:0004364: glutathione transferase activity: 5.709e-15 (7,11,6) OR
GO:0003714: transcription corepressor activity: 2.039e-02 (7,8,1)

IF 1gdna_#119 AND 1ekbb_#79 AND 1fjsa_#158 AND 1fjsa_#83 AND 1qnja_#71 AND 1dy9.1#A44 AND 1autc_#209
THEN
GO:0004263: chymotrypsin activity: 2.291e-15 (40,41,13) OR
GO:0004295: trypsin activity: 2.349e-14 (40,48,13) OR
GO:0004867: serine-type endopeptidase inhibitor activity: 5.145e-04 (40,47,5) OR
GO:0005509: calcium ion binding: 1.815e-03 (40,160,8) OR
GO:0004896: hematopoietin/interferon-class (D200-domain) cytokine receptor activity: 2.457e-01 (40,19,1)

IF 1elua_#95 AND 1c7na_#281
THEN
GO:0008483: transaminase activity: 4.094e-15 (16,17,8) OR
GO:0016846: carbon-sulfur lyase activity: 2.936e-12 (16,10,6) OR
GO:0016831: carboxy-lyase activity: 8.964e-03 (16,25,2)

IF 1hyea2#272 AND 2cmd_2#251 AND 1fgga_#134
THEN
GO:0004457: lactate dehydrogenase activity: 6.195e-15 (16,10,7) OR
GO:0016616: oxidoreductase activity, acting on the CH-OH group of donors, NAD or NADP as acceptor: 5.675e-12 (16,59,9)

IF 1j9la_#82 AND 1qfma2#576 AND 1hu4a_#267
THEN
GO:0016616: oxidoreductase activity, acting on the CH-OH group of donors, NAD or NADP as acceptor: 2.079e-15 (17,59,11) OR
GO:0016646: oxidoreductase activity, acting on the CH-NH group of donors, NAD or NADP as acceptor: 5.887e-03 (17,19,2) OR
GO:0016627: oxidoreductase activity, acting on the CH-CH group of donors: 1.012e-01 (17,17,1) OR
GO:0015036: disulfide oxidoreductase activity: 1.291e-01 (17,22,1) OR
GO:0016763: transferase activity, transferring pentosyl groups: 1.615e-01 (17,28,1) OR
GO:0003700: transcription factor activity: 5.480e-01 (17,124,1)

IF 1e7wa_#8 AND 1eq2a_#11
THEN
GO:0016616: oxidoreductase activity, acting on the CH-OH group of donors, NAD or NADP as acceptor: 2.079e-15 (17,59,11) OR
GO:0016620: oxidoreductase activity, acting on the aldehyde or oxo group of donors, NAD or NADP as acceptor: 1.601e-03 (17,10,2) OR
GO:0016854: racemase and epimerase activity: 7.830e-02 (17,13,1) OR
GO:0016627: oxidoreductase activity, acting on the CH-CH group of donors: 1.012e-01 (17,17,1) OR
GO:0016638: oxidoreductase activity, acting on the CH-NH2 group of donors: 1.012e-01 (17,17,1) OR
GO:0016646: oxidoreductase activity, acting on the CH-NH group of donors, NAD or NADP as acceptor: 1.125e-01 (17,19,1)

IF 1g8fa3#419 AND 1hdr__#149
THEN
GO:0016616: oxidoreductase activity, acting on the CH-OH group of donors, NAD or NADP as acceptor: 2.079e-15 (17,59,11) OR
GO:0016646: oxidoreductase activity, acting on the CH-NH group of donors, NAD or NADP as acceptor: 5.887e-03 (17,19,2) OR
GO:0016668: oxidoreductase activity, acting on sulfur group of donors, NAD or NADP as acceptor: 7.249e-02 (17,12,1) OR
GO:0016627: oxidoreductase activity, acting on the CH-CH group of donors: 1.012e-01 (17,17,1) OR
GO:0015036: disulfide oxidoreductase activity: 1.291e-01 (17,22,1) OR
GO:0030145: manganese ion binding: 2.129e-01 (17,38,1)

IF 1iba__#39 AND 1cjca2#76
THEN
GO:0016668: oxidoreductase activity, acting on sulfur group of donors, NAD or NADP as acceptor: 3.188e-15 (23,12,8) OR
GO:0015036: disulfide oxidoreductase activity: 1.961e-12 (23,22,8) OR
GO:0050660: FAD binding: 3.669e-11 (23,10,6) OR
GO:0016651: oxidoreductase activity, acting on NADH or NADPH: 1.046e-01 (23,13,1)

IF 1danh_#140 AND 2hlca_#54 AND 1qj8a_#64
THEN
GO:0004263: chymotrypsin activity: 2.291e-15 (40,41,13) OR
GO:0004295: trypsin activity: 2.349e-14 (40,48,13) OR
GO:0004867: serine-type endopeptidase inhibitor activity: 5.145e-04 (40,47,5) OR
GO:0005509: calcium ion binding: 7.595e-03 (40,160,7) OR
GO:0003809: thrombin activity: 1.377e-01 (40,10,1) OR
GO:0004896: hematopoietin/interferon-class (D200-domain) cytokine receptor activity: 2.457e-01 (40,19,1)

IF 1danh_#140 AND 1ton__#91 AND 1qj8a_#64
THEN
GO:0004263: chymotrypsin activity: 2.291e-15 (40,41,13) OR
GO:0004295: trypsin activity: 2.349e-14 (40,48,13) OR
GO:0004867: serine-type endopeptidase inhibitor activity: 5.145e-04 (40,47,5) OR
GO:0005509: calcium ion binding: 7.595e-03 (40,160,7) OR
GO:0003809: thrombin activity: 1.377e-01 (40,10,1) OR
GO:0004896: hematopoietin/interferon-class (D200-domain) cytokine receptor activity: 2.457e-01 (40,19,1)

IF 1feca1#28 AND 1e39a2#516
THEN
GO:0015036: disulfide oxidoreductase activity: 2.928e-15 (30,22,10) OR
GO:0050660: FAD binding: 3.478e-15 (30,10,8) OR
GO:0016668: oxidoreductase activity, acting on sulfur group of donors, NAD or NADP as acceptor: 3.771e-14 (30,12,8) OR
GO:0016627: oxidoreductase activity, acting on the CH-CH group of donors: 7.383e-04 (30,17,3) OR
GO:0016651: oxidoreductase activity, acting on NADH or NADPH: 1.343e-01 (30,13,1)

IF 1qo3c_#212 AND 1b6e__#76
THEN
GO:0005529: sugar binding: 7.344e-15 (9,39,8) OR
GO:0008201: heparin binding: 7.664e-02 (9,24,1)

IF 1h8ua_#113 AND 2msba_#156
THEN
GO:0005529: sugar binding: 7.344e-15 (9,39,8) OR
GO:0008201: heparin binding: 7.664e-02 (9,24,1)

IF 1e87a_#155 AND 1esl_1#29
THEN
GO:0005529: sugar binding: 7.344e-15 (9,39,8) OR
GO:0008201: heparin binding: 7.664e-02 (9,24,1)

IF 1qu9a_#64 AND 1e87a_#121
THEN
GO:0005529: sugar binding: 7.344e-15 (9,39,8) OR
GO:0008201: heparin binding: 7.664e-02 (9,24,1)

IF 1qu9a_#64 AND 2msba_#134
THEN
GO:0005529: sugar binding: 7.344e-15 (9,39,8) OR
GO:0008201: heparin binding: 7.664e-02 (9,24,1)

IF 1hlwa_#82 AND 1hq8a_#223
THEN
GO:0005529: sugar binding: 7.344e-15 (9,39,8) OR
GO:0005509: calcium ion binding: 4.204e-01 (9,160,1)

IF 1hlwa_#82 AND 2msba_#218 AND 1e87a_#121
THEN
GO:0005529: sugar binding: 7.344e-15 (9,39,8) OR
GO:0005509: calcium ion binding: 4.204e-01 (9,160,1)

IF 1qu9a_#64 AND 1hq8a_#150
THEN

GO:0005529: sugar binding: 7.344e-15 (9,39,8) OR
GO:0008201: heparin binding: 7.664e-02 (9,24,1)

IF 1bvza3#416 AND 1dik_1#765
THEN
GO:0004556: alpha-amylase activity: 3.687e-15 (18,15,8) OR
GO:0005509: calcium ion binding: 3.881e-05 (18,160,7) OR
GO:0004867: serine-type endopeptidase inhibitor activity: 3.738e-02 (18,47,2) OR
GO:0000287: magnesium ion binding: 5.805e-01 (18,128,1)

IF 1qq4a_#46 AND 1qq4a_#63
THEN
GO:0004295: trypsin activity: 3.062e-15 (35,48,13) OR
GO:0004263: chymotrypsin activity: 2.246e-08 (35,41,8) OR
GO:0004867: serine-type endopeptidase inhibitor activity: 7.006e-08 (35,47,8) OR
GO:0005509: calcium ion binding: 5.099e-02 (35,160,5) OR
GO:0008201: heparin binding: 2.677e-01 (35,24,1)

IF 1mjha_#126 AND 1c7na_#281
THEN
GO:0008483: transaminase activity: 4.094e-15 (16,17,8) OR
GO:0016846: carbon-sulfur lyase activity: 2.936e-12 (16,10,6) OR
GO:0016763: transferase activity, transferring pentosyl groups: 1.527e-01 (16,28,1) OR
GO:0000287: magnesium ion binding: 5.379e-01 (16,128,1)

IF 1pysa_#127 AND 1qsta_#154
THEN
GO:0004812: tRNA ligase activity: 4.230e-15 (26,26,10) OR
GO:0005524: ATP binding: 3.897e-05 (26,243,10) OR
GO:0000049: tRNA binding: 6.402e-03 (26,13,2) OR
GO:0000287: magnesium ion binding: 3.110e-02 (26,128,4)

IF 1ton__#120 AND 1danh_#104 AND 1elva1#513 AND 1danh_#142 AND 1elva1#601 AND 1dlea_#198 AND 1cgha_#130
THEN
GO:0004263: chymotrypsin activity: 4.279e-15 (32,41,12) OR
GO:0004295: trypsin activity: 3.597e-14 (32,48,12) OR
GO:0004867: serine-type endopeptidase inhibitor activity: 1.962e-03 (32,47,4) OR
GO:0005509: calcium ion binding: 1.147e-01 (32,160,4)

IF 1trb_1#41 AND 1ojt_2#336
THEN
GO:0015036: disulfide oxidoreductase activity: 2.928e-15 (30,22,10) OR
GO:0016668: oxidoreductase activity, acting on sulfur group of donors, NAD or NADP as acceptor: 3.771e-14 (30,12,8) OR
GO:0050660: FAD binding: 1.088e-12 (30,10,7) OR
GO:0016627: oxidoreductase activity, acting on the CH-CH group of donors: 7.383e-04 (30,17,3) OR
GO:0016651: oxidoreductase activity, acting on NADH or NADPH: 1.343e-01 (30,13,1) OR
GO:0016638: oxidoreductase activity, acting on the CH-NH2 group of donors: 1.720e-01 (30,17,1)

IF 1trb_1#15 AND 1ojt_2#336
THEN
GO:0015036: disulfide oxidoreductase activity: 2.928e-15 (30,22,10) OR
GO:0016668: oxidoreductase activity, acting on sulfur group of donors, NAD or NADP as acceptor: 3.771e-14 (30,12,8) OR
GO:0050660: FAD binding: 1.088e-12 (30,10,7) OR
GO:0016627: oxidoreductase activity, acting on the CH-CH group of donors: 7.383e-04 (30,17,3) OR
GO:0016651: oxidoreductase activity, acting on NADH or NADPH: 1.343e-01 (30,13,1) OR
GO:0016638: oxidoreductase activity, acting on the CH-NH2 group of donors: 1.720e-01 (30,17,1)

IF 1gdna_#230 AND 1elva1#513
THEN
GO:0004263: chymotrypsin activity: 4.573e-15 (24,41,11) OR
GO:0004295: trypsin activity: 3.170e-14 (24,48,11) OR
GO:0004867: serine-type endopeptidase inhibitor activity: 3.425e-01 (24,47,1) OR
GO:0005509: calcium ion binding: 7.674e-01 (24,160,1)

IF 1qhda2#323 AND 1bag_2#207
THEN
GO:0004556: alpha-amylase activity: 3.687e-15 (18,15,8) OR
GO:0005509: calcium ion binding: 3.839e-04 (18,160,6) OR
GO:0004867: serine-type endopeptidase inhibitor activity: 2.696e-01 (18,47,1) OR
GO:0005524: ATP binding: 4.864e-01 (18,243,2) OR

GO:0000287: magnesium ion binding: 5.805e-01 (18,128,1)

IF 1b6ra3#161 AND 1qama_#37
THEN
GO:0004457: lactate dehydrogenase activity: 6.195e-15 (16,10,7) OR
GO:0016616: oxidoreductase activity, acting on the CH-OH group of donors, NAD or NADP as acceptor: 3.342e-10 (16,59,8) OR
GO:0000287: magnesium ion binding: 5.379e-01 (16,128,1)

IF 2dnja_#97 AND 1ton__#179
THEN
GO:0004263: chymotrypsin activity: 3.159e-15 (52,41,14) OR
GO:0004295: trypsin activity: 3.940e-14 (52,48,14) OR
GO:0003809: thrombin activity: 7.130e-09 (52,10,6) OR
GO:0004867: serine-type endopeptidase inhibitor activity: 2.135e-05 (52,47,7) OR
GO:0005509: calcium ion binding: 6.800e-04 (52,160,10) OR
GO:0005529: sugar binding: 5.308e-01 (52,39,1)

IF 1dpga2#334 AND 1ton__#179 AND 1a0la_#160 AND 1dlea_#238
THEN
GO:0004263: chymotrypsin activity: 3.159e-15 (52,41,14) OR
GO:0004295: trypsin activity: 3.940e-14 (52,48,14) OR
GO:0003809: thrombin activity: 4.883e-07 (52,10,5) OR
GO:0004867: serine-type endopeptidase inhibitor activity: 1.798e-06 (52,47,8) OR
GO:0005509: calcium ion binding: 6.800e-04 (52,160,10) OR
GO:0005529: sugar binding: 5.308e-01 (52,39,1)

IF 1h7wa4#476 AND 1cjca2#76
THEN
GO:0016668: oxidoreductase activity, acting on sulfur group of donors, NAD or NADP as acceptor: 4.776e-15 (24,12,8) OR
GO:0015036: disulfide oxidoreductase activity: 2.705e-14 (24,22,9) OR
GO:0050660: FAD binding: 4.885e-11 (24,10,6) OR
GO:0016651: oxidoreductase activity, acting on NADH or NADPH: 1.089e-01 (24,13,1)

IF 1eh9a3#102 AND 1e4ea2#215
THEN
GO:0004556: alpha-amylase activity: 9.567e-15 (11,15,7) OR
GO:0005509: calcium ion binding: 2.730e-03 (11,160,4)

IF 1czan1#92 AND 1eh9a3#102
THEN
GO:0004556: alpha-amylase activity: 9.567e-15 (11,15,7) OR
GO:0005509: calcium ion binding: 2.730e-03 (11,160,4)

IF 1elva1#566 AND 1danh_#242 AND 1cgha_#130
THEN
GO:0004263: chymotrypsin activity: 3.322e-15 (41,41,13) OR
GO:0004295: trypsin activity: 3.398e-14 (41,48,13) OR
GO:0004867: serine-type endopeptidase inhibitor activity: 5.784e-04 (41,47,5) OR
GO:0005509: calcium ion binding: 2.144e-03 (41,160,8) OR
GO:0003809: thrombin activity: 1.409e-01 (41,10,1) OR
GO:0008201: heparin binding: 3.061e-01 (41,24,1)

IF 1ax4a_#430 AND 1dfoa_#344
THEN
GO:0008483: transaminase activity: 4.094e-15 (16,17,8) OR
GO:0016846: carbon-sulfur lyase activity: 8.675e-10 (16,10,5) OR
GO:0016866: intramolecular transferase activity: 6.836e-02 (16,12,1) OR
GO:0051082: unfolded protein binding: 1.825e-01 (16,34,1) OR
GO:0005524: ATP binding: 7.766e-01 (16,243,1)

IF 1fc4a_#111 AND 1dfoa_#344
THEN
GO:0008483: transaminase activity: 4.094e-15 (16,17,8) OR
GO:0016846: carbon-sulfur lyase activity: 8.675e-10 (16,10,5) OR
GO:0016831: carboxy-lyase activity: 1.375e-01 (16,25,1) OR
GO:0004812: tRNA ligase activity: 1.426e-01 (16,26,1) OR
GO:0005524: ATP binding: 7.766e-01 (16,243,1)

IF 2hlpa2#315 AND 1e3ja2#270
THEN

GO:0004457: lactate dehydrogenase activity: 1.052e-14 (17,10,7) OR
GO:0016616: oxidoreductase activity, acting on the CH-OH group of donors, NAD or NADP as acceptor: 1.778e-13 (17,59,10)

IF 1qi7a_#244 AND 1mrj__#156
THEN
GO:0016799: hydrolase activity, hydrolyzing N-glycosyl compounds: 1.052e-14 (10,17,7) OR
GO:0005529: sugar binding: 3.036e-04 (10,39,3)

IF 2cmd_2#251 AND 1e3ja2#270
THEN
GO:0004457: lactate dehydrogenase activity: 1.052e-14 (17,10,7) OR
GO:0016616: oxidoreductase activity, acting on the CH-OH group of donors, NAD or NADP as acceptor: 1.778e-13 (17,59,10)

IF 1hxxa_#278 AND 1trb_1#42
THEN
GO:0015036: disulfide oxidoreductase activity: 3.536e-15 (20,22,9) OR
GO:0016668: oxidoreductase activity, acting on sulfur group of donors, NAD or NADP as acceptor: 9.530e-09 (20,12,5) OR
GO:0050660: FAD binding: 3.930e-05 (20,10,3) OR
GO:0016651: oxidoreductase activity, acting on NADH or NADPH: 9.152e-02 (20,13,1) OR
GO:0016627: oxidoreductase activity, acting on the CH-CH group of donors: 1.180e-01 (20,17,1) OR
GO:0016638: oxidoreductase activity, acting on the CH-NH2 group of donors: 1.180e-01 (20,17,1)

IF 1by5a_#347 AND 1d2ka1#164
THEN
GO:0004556: alpha-amylase activity: 3.687e-15 (18,15,8) OR
GO:0005509: calcium ion binding: 3.007e-03 (18,160,5) OR
GO:0016763: transferase activity, transferring pentosyl groups: 1.701e-01 (18,28,1) OR
GO:0004867: serine-type endopeptidase inhibitor activity: 2.696e-01 (18,47,1) OR
GO:0005524: ATP binding: 4.864e-01 (18,243,2) OR
GO:0000287: magnesium ion binding: 5.805e-01 (18,128,1)

IF 1hdr__#156 AND 1oaa__#14
THEN
GO:0016616: oxidoreductase activity, acting on the CH-OH group of donors, NAD or NADP as acceptor: 3.710e-15 (23,59,12) OR
GO:0016627: oxidoreductase activity, acting on the CH-CH group of donors: 8.547e-06 (23,17,4) OR
GO:0016854: racemase and epimerase activity: 5.024e-03 (23,13,2) OR
GO:0016646: oxidoreductase activity, acting on the CH-NH group of donors, NAD or NADP as acceptor: 1.068e-02 (23,19,2) OR
GO:0016836: hydro-lyase activity: 3.071e-02 (23,33,2) OR
GO:0016651: oxidoreductase activity, acting on NADH or NADPH: 1.046e-01 (23,13,1)

IF 1hdr__#156 AND 1e7wa_#36 AND 1evqa_#305
THEN
GO:0016616: oxidoreductase activity, acting on the CH-OH group of donors, NAD or NADP as acceptor: 3.710e-15 (23,59,12) OR
GO:0016854: racemase and epimerase activity: 1.423e-04 (23,13,3) OR
GO:0016627: oxidoreductase activity, acting on the CH-CH group of donors: 3.309e-04 (23,17,3) OR
GO:0016646: oxidoreductase activity, acting on the CH-NH group of donors, NAD or NADP as acceptor: 1.068e-02 (23,19,2) OR
GO:0016814: hydrolase activity, acting on carbon-nitrogen (but not peptide) bonds, in cyclic amidines: 8.140e-02 (23,10,1) OR
GO:0005524: ATP binding: 6.217e-01 (23,243,2)

IF 1ypta_#418 AND 1b6ra3#161
THEN
GO:0004457: lactate dehydrogenase activity: 6.195e-15 (16,10,7) OR
GO:0016616: oxidoreductase activity, acting on the CH-OH group of donors, NAD or NADP as acceptor: 1.526e-08 (16,59,7) OR
GO:0004725: protein tyrosine phosphatase activity: 8.475e-02 (16,15,1) OR
GO:0008270: zinc ion binding: 4.774e-01 (16,108,1)

IF 1bqk__#77 AND 1kdj__#27
THEN
GO:0005507: copper ion binding: 4.977e-15 (13,38,9) OR
GO:0015082: di-, tri-valent inorganic cation transporter activity: 6.491e-02 (13,14,1) OR
GO:0046915: transition metal ion transporter activity: 6.491e-02 (13,14,1) OR
GO:0016638: oxidoreductase activity, acting on the CH-NH2 group of donors: 7.830e-02 (13,17,1) OR
GO:0008083: growth factor activity: 1.832e-01 (13,42,1)

IF 1dt6a_#77 AND 1dz4a_#357
THEN
GO:0004497: monooxygenase activity: 6.408e-15 (18,26,9) OR
GO:0016705: oxidoreductase activity, acting on paired donors, with incorporation or reduction of molecular oxygen: 4.154e-07 (18,26,5)
OR
GO:0010181: FMN binding: 2.189e-03 (18,11,2) OR

GO:0016651: oxidoreductase activity, acting on NADH or NADPH: 3.080e-03 (18,13,2)

IF 1e9xa_#343 AND 1dt6a_#77
THEN
GO:0004497: monooxygenase activity: 6.408e-15 (18,26,9) OR
GO:0016705: oxidoreductase activity, acting on paired donors, with incorporation or reduction of molecular oxygen: 4.154e-07 (18,26,5) OR
GO:0010181: FMN binding: 2.189e-03 (18,11,2) OR
GO:0016651: oxidoreductase activity, acting on NADH or NADPH: 3.080e-03 (18,13,2)

IF 1cg2a1#43 AND 1dz4a_#357
THEN
GO:0004497: monooxygenase activity: 6.408e-15 (18,26,9) OR
GO:0016705: oxidoreductase activity, acting on paired donors, with incorporation or reduction of molecular oxygen: 4.154e-07 (18,26,5) OR
GO:0010181: FMN binding: 2.189e-03 (18,11,2) OR
GO:0016651: oxidoreductase activity, acting on NADH or NADPH: 3.080e-03 (18,13,2)

IF 1cpt__#401 AND 1bu7a_#353
THEN
GO:0004497: monooxygenase activity: 6.408e-15 (18,26,9) OR
GO:0016705: oxidoreductase activity, acting on paired donors, with incorporation or reduction of molecular oxygen: 4.154e-07 (18,26,5) OR
GO:0010181: FMN binding: 2.189e-03 (18,11,2) OR
GO:0016651: oxidoreductase activity, acting on NADH or NADPH: 3.080e-03 (18,13,2)

IF 1hq8a_#150 AND 1dv8a_#211
THEN
GO:0005529: sugar binding: 6.461e-15 (13,39,9) OR
GO:0005509: calcium ion binding: 1.751e-01 (13,160,2) OR
GO:0004263: chymotrypsin activity: 1.792e-01 (13,41,1) OR
GO:0004295: trypsin activity: 2.067e-01 (13,48,1)

IF 1dv8a_#211 AND 1e87a_#121
THEN
GO:0005529: sugar binding: 6.461e-15 (13,39,9) OR
GO:0005509: calcium ion binding: 1.751e-01 (13,160,2) OR
GO:0004263: chymotrypsin activity: 1.792e-01 (13,41,1) OR
GO:0004295: trypsin activity: 2.067e-01 (13,48,1)

IF 1hq8a_#223 AND 1dv8a_#211
THEN
GO:0005529: sugar binding: 6.461e-15 (13,39,9) OR
GO:0005509: calcium ion binding: 1.751e-01 (13,160,2) OR
GO:0004263: chymotrypsin activity: 1.792e-01 (13,41,1) OR
GO:0004295: trypsin activity: 2.067e-01 (13,48,1)

IF 1h7wa4#476 AND 1i50b_#966
THEN
GO:0016668: oxidoreductase activity, acting on sulfur group of donors, NAD or NADP as acceptor: 7.015e-15 (25,12,8) OR
GO:0015036: disulfide oxidoreductase activity: 4.209e-14 (25,22,9) OR
GO:0050660: FAD binding: 2.580e-13 (25,10,7) OR
GO:0016651: oxidoreductase activity, acting on NADH or NADPH: 1.132e-01 (25,13,1)

IF 1h7wa4#476 AND 1xgsa2#82
THEN
GO:0016668: oxidoreductase activity, acting on sulfur group of donors, NAD or NADP as acceptor: 7.015e-15 (25,12,8) OR
GO:0015036: disulfide oxidoreductase activity: 4.209e-14 (25,22,9) OR
GO:0050660: FAD binding: 2.580e-13 (25,10,7) OR
GO:0016651: oxidoreductase activity, acting on NADH or NADPH: 1.132e-01 (25,13,1)

IF 1qnja_#77 AND 1c5y.1#B18
THEN
GO:0004263: chymotrypsin activity: 4.764e-15 (42,41,13) OR
GO:0004295: trypsin activity: 4.862e-14 (42,48,13) OR
GO:0005509: calcium ion binding: 5.516e-04 (42,160,9) OR
GO:0004867: serine-type endopeptidase inhibitor activity: 6.482e-04 (42,47,5) OR
GO:0003809: thrombin activity: 1.441e-01 (42,10,1) OR
GO:0004896: hematopoietin/interferon-class (D200-domain) cytokine receptor activity: 2.563e-01 (42,19,1)

IF 1bqya_#216 AND 1cqqa_#163
THEN
GO:0004263: chymotrypsin activity: 4.764e-15 (42,41,13) OR
GO:0004295: trypsin activity: 4.862e-14 (42,48,13) OR
GO:0004867: serine-type endopeptidase inhibitor activity: 6.233e-05 (42,47,6) OR
GO:0003809: thrombin activity: 9.652e-03 (42,10,2) OR
GO:0005509: calcium ion binding: 9.957e-03 (42,160,7) OR
GO:0005529: sugar binding: 4.567e-01 (42,39,1)

IF 1erza_#183 AND 1eh9a3#102
THEN
GO:0004556: alpha-amylase activity: 9.567e-15 (11,15,7) OR
GO:0005509: calcium ion binding: 2.312e-02 (11,160,3) OR
GO:0008810: cellulase activity: 7.043e-02 (11,18,1)

IF 2ae2a_#196 AND 1qora2#201
THEN
GO:0016616: oxidoreductase activity, acting on the CH-OH group of donors, NAD or NADP as acceptor: 2.971e-14 (8,59,8)

IF 1gega_#58 AND 1e6wa_#24
THEN
GO:0016616: oxidoreductase activity, acting on the CH-OH group of donors, NAD or NADP as acceptor: 2.971e-14 (8,59,8)

IF 1gega_#58 AND 2ae2a_#196
THEN
GO:0016616: oxidoreductase activity, acting on the CH-OH group of donors, NAD or NADP as acceptor: 2.971e-14 (8,59,8)

IF 2ae2a_#196 AND 1gdha1#235
THEN
GO:0016616: oxidoreductase activity, acting on the CH-OH group of donors, NAD or NADP as acceptor: 2.971e-14 (8,59,8)

IF 1ec7a1#216 AND 1fmca_#248
THEN
GO:0016616: oxidoreductase activity, acting on the CH-OH group of donors, NAD or NADP as acceptor: 2.971e-14 (8,59,8)

IF 1b16a_#184 AND 1tyfa_#31
THEN
GO:0016616: oxidoreductase activity, acting on the CH-OH group of donors, NAD or NADP as acceptor: 2.971e-14 (8,59,8)

IF 1h7wa4#476 AND 3chbd_#85
THEN
GO:0015036: disulfide oxidoreductase activity: 6.161e-15 (21,22,9) OR
GO:0016668: oxidoreductase activity, acting on sulfur group of donors, NAD or NADP as acceptor: 8.617e-11 (21,12,6) OR
GO:0050660: FAD binding: 5.320e-07 (21,10,4) OR
GO:0016651: oxidoreductase activity, acting on NADH or NADPH: 9.588e-02 (21,13,1) OR
GO:0016627: oxidoreductase activity, acting on the CH-CH group of donors: 1.236e-01 (21,17,1)

IF 1pbe_1#121 AND 1cjca2#76
THEN
GO:0015036: disulfide oxidoreductase activity: 6.161e-15 (21,22,9) OR
GO:0016668: oxidoreductase activity, acting on sulfur group of donors, NAD or NADP as acceptor: 8.617e-11 (21,12,6) OR
GO:0050660: FAD binding: 5.320e-07 (21,10,4) OR
GO:0016651: oxidoreductase activity, acting on NADH or NADPH: 9.588e-02 (21,13,1) OR
GO:0016638: oxidoreductase activity, acting on the CH-NH2 group of donors: 1.236e-01 (21,17,1)

IF 1ghpa_#236 AND 1hvba_#63
THEN
GO:0008800: beta-lactamase activity: 1.037e-14 (8,10,6) OR
GO:0004177: aminopeptidase activity: 3.758e-02 (8,13,1) OR
GO:0004180: carboxypeptidase activity: 4.325e-02 (8,15,1)

IF 1cm9a_#57 AND 1el0a_#62
THEN
GO:0008009: chemokine activity: 1.037e-14 (8,10,6) OR
GO:0030145: manganese ion binding: 1.064e-01 (8,38,1) OR
GO:0005524: ATP binding: 5.268e-01 (8,243,1)

IF 1ghpa_#236 AND 1ghpa_#245
THEN
GO:0008800: beta-lactamase activity: 1.037e-14 (8,10,6) OR

GO:0004177: aminopeptidase activity: 3.758e-02 (8,13,1) OR
GO:0004180: carboxypeptidase activity: 4.325e-02 (8,15,1)

IF 1ha6a_#59 AND 1el0a_#62
THEN
GO:0008009: chemokine activity: 1.037e-14 (8,10,6) OR
GO:0008757: S-adenosylmethionine-dependent methyltransferase activity: 6.841e-02 (8,24,1) OR
GO:0008083: growth factor activity: 1.170e-01 (8,42,1)

IF 1jb3a_#64 AND 1qama_#37
THEN
GO:0004457: lactate dehydrogenase activity: 1.052e-14 (17,10,7) OR
GO:0016616: oxidoreductase activity, acting on the CH-OH group of donors, NAD or NADP as acceptor: 1.186e-11 (17,59,9) OR
GO:0000287: magnesium ion binding: 5.597e-01 (17,128,1)

IF 1iba__#39 AND 1qhoa4#230
THEN
GO:0004556: alpha-amylase activity: 6.354e-15 (19,15,8) OR
GO:0005509: calcium ion binding: 5.158e-06 (19,160,8) OR
GO:0008081: phosphoric diester hydrolase activity: 7.421e-02 (19,11,1) OR
GO:0004620: phospholipase activity: 1.249e-01 (19,19,1) OR
GO:0004867: serine-type endopeptidase inhibitor activity: 2.823e-01 (19,47,1)

IF 1b6e__#76 AND 1dv8a_#213
THEN
GO:0005529: sugar binding: 6.461e-15 (13,39,9) OR
GO:0008201: heparin binding: 1.089e-01 (13,24,1) OR
GO:0004263: chymotrypsin activity: 1.792e-01 (13,41,1) OR
GO:0004295: trypsin activity: 2.067e-01 (13,48,1) OR
GO:0005509: calcium ion binding: 5.454e-01 (13,160,1)

IF 1eq9a_#162 AND 1ejda_#213 AND 1c5y.1#B234 AND 2hlca_#226
THEN
GO:0004263: chymotrypsin activity: 6.763e-15 (43,41,13) OR
GO:0004295: trypsin activity: 6.884e-14 (43,48,13) OR
GO:0004867: serine-type endopeptidase inhibitor activity: 5.780e-06 (43,47,7) OR
GO:0005509: calcium ion binding: 2.943e-03 (43,160,8) OR
GO:0003809: thrombin activity: 1.010e-02 (43,10,2)

IF 1danh_#48 AND 1a0la_#160 AND 1dlea_#238
THEN
GO:0004263: chymotrypsin activity: 5.687e-15 (54,41,14) OR
GO:0004295: trypsin activity: 7.058e-14 (54,48,14) OR
GO:0003809: thrombin activity: 5.923e-07 (54,10,5) OR
GO:0004867: serine-type endopeptidase inhibitor activity: 2.423e-06 (54,47,8) OR
GO:0005509: calcium ion binding: 4.416e-05 (54,160,12) OR
GO:0005529: sugar binding: 5.444e-01 (54,39,1)

IF 1gdna_#119 AND 1azza_#114
THEN
GO:0004295: trypsin activity: 7.212e-15 (37,48,13) OR
GO:0004263: chymotrypsin activity: 3.340e-14 (37,41,12) OR
GO:0004867: serine-type endopeptidase inhibitor activity: 3.383e-03 (37,47,4) OR
GO:0005509: calcium ion binding: 4.865e-03 (37,160,7) OR
GO:0004896: hematopoietin/interferon-class (D200-domain) cytokine receptor activity: 2.294e-01 (37,19,1)

IF 1fcda2#189 AND 1trb_1#285
THEN
GO:0015036: disulfide oxidoreductase activity: 6.161e-15 (21,22,9) OR
GO:0016668: oxidoreductase activity, acting on sulfur group of donors, NAD or NADP as acceptor: 1.248e-08 (21,12,5) OR
GO:0050660: FAD binding: 5.320e-07 (21,10,4) OR
GO:0016627: oxidoreductase activity, acting on the CH-CH group of donors: 1.236e-01 (21,17,1) OR
GO:0016705: oxidoreductase activity, acting on paired donors, with incorporation or reduction of molecular oxygen: 1.830e-01 (21,26,1) OR
GO:0004497: monooxygenase activity: 1.830e-01 (21,26,1)

IF 1fcda2#189 AND 1h6va1#23
THEN
GO:0015036: disulfide oxidoreductase activity: 6.161e-15 (21,22,9) OR
GO:0016668: oxidoreductase activity, acting on sulfur group of donors, NAD or NADP as acceptor: 1.248e-08 (21,12,5) OR

GO:0050660: FAD binding: 5.320e-07 (21,10,4) OR
GO:0016627: oxidoreductase activity, acting on the CH-CH group of donors: 1.236e-01 (21,17,1) OR
GO:0016705: oxidoreductase activity, acting on paired donors, with incorporation or reduction of molecular oxygen: 1.830e-01 (21,26,1) OR
GO:0004497: monooxygenase activity: 1.830e-01 (21,26,1)

IF 1fcda2#189 AND 1h6va2#219
THEN
GO:0015036: disulfide oxidoreductase activity: 6.161e-15 (21,22,9) OR
GO:0016668: oxidoreductase activity, acting on sulfur group of donors, NAD or NADP as acceptor: 1.248e-08 (21,12,5) OR
GO:0050660: FAD binding: 5.320e-07 (21,10,4) OR
GO:0016627: oxidoreductase activity, acting on the CH-CH group of donors: 1.236e-01 (21,17,1) OR
GO:0016705: oxidoreductase activity, acting on paired donors, with incorporation or reduction of molecular oxygen: 1.830e-01 (21,26,1) OR
GO:0004497: monooxygenase activity: 1.830e-01 (21,26,1)

IF 1c5y.1#B241 AND 1e5ka_#113
THEN
GO:0004263: chymotrypsin activity: 7.557e-15 (55,41,14) OR
GO:0004295: trypsin activity: 9.356e-14 (55,48,14) OR
GO:0003809: thrombin activity: 1.058e-10 (55,10,7) OR
GO:0004867: serine-type endopeptidase inhibitor activity: 2.799e-06 (55,47,8) OR
GO:0005509: calcium ion binding: 5.365e-05 (55,160,12)

IF 3chbd_#85 AND 1d5ra2#25
THEN
GO:0004725: protein tyrosine phosphatase activity: 9.567e-15 (11,15,7) OR
GO:0005525: GTP binding: 1.571e-02 (11,49,2) OR
GO:0005096: GTPase activator activity: 5.902e-02 (11,15,1) OR
GO:0016811: hydrolase activity, acting on carbon-nitrogen (but not peptide) bonds, in linear amides: 5.902e-02 (11,15,1)

IF 1hu4a_#267 AND 1eq2a_#11
THEN
GO:0016616: oxidoreductase activity, acting on the CH-OH group of donors, NAD or NADP as acceptor: 9.619e-15 (14,59,10) OR
GO:0016646: oxidoreductase activity, acting on the CH-NH group of donors, NAD or NADP as acceptor: 3.988e-03 (14,19,2) OR
GO:0016627: oxidoreductase activity, acting on the CH-CH group of donors: 8.408e-02 (14,17,1) OR
GO:0005525: GTP binding: 2.248e-01 (14,49,1)

IF 1j9qa1#154 AND 1aoza1#61
THEN
GO:0005507: copper ion binding: 6.556e-15 (19,38,10) OR
GO:0015078: hydrogen ion transporter activity: 9.386e-06 (19,21,4) OR
GO:0015082: di-, tri-valent inorganic cation transporter activity: 9.353e-02 (19,14,1) OR
GO:0046915: transition metal ion transporter activity: 9.353e-02 (19,14,1) OR
GO:0004620: phospholipase activity: 1.249e-01 (19,19,1) OR
GO:0005509: calcium ion binding: 3.079e-01 (19,160,2)

IF 1cpy__#93 AND 1bd3a_#195
THEN
GO:0016763: transferase activity, transferring pentosyl groups: 2.008e-14 (12,28,8) OR
GO:0000287: magnesium ion binding: 1.714e-03 (12,128,4)

IF 7mdha2#355 AND 1ejda_#6
THEN
GO:0004457: lactate dehydrogenase activity: 1.052e-14 (17,10,7) OR
GO:0016616: oxidoreductase activity, acting on the CH-OH group of donors, NAD or NADP as acceptor: 6.207e-10 (17,59,8) OR
GO:0016638: oxidoreductase activity, acting on the CH-NH2 group of donors: 1.012e-01 (17,17,1) OR
GO:0005525: GTP binding: 2.661e-01 (17,49,1)

IF 1hyha2#179 AND 1jb3a_#64
THEN
GO:0004457: lactate dehydrogenase activity: 1.052e-14 (17,10,7) OR
GO:0016616: oxidoreductase activity, acting on the CH-OH group of donors, NAD or NADP as acceptor: 6.207e-10 (17,59,8) OR
GO:0004842: ubiquitin-protein ligase activity: 1.125e-01 (17,19,1) OR
GO:0008270: zinc ion binding: 4.982e-01 (17,108,1)

IF 1eq9a_#162 AND 1cqqa_#163
THEN
GO:0004295: trypsin activity: 7.212e-15 (37,48,13) OR
GO:0004263: chymotrypsin activity: 4.810e-11 (37,41,10) OR

GO:0004867: serine-type endopeptidase inhibitor activity: 1.997e-06 (37,47,7) OR
GO:0005509: calcium ion binding: 6.237e-02 (37,160,5) OR
GO:0003809: thrombin activity: 1.280e-01 (37,10,1) OR
GO:0003779: actin binding: 3.560e-01 (37,32,1)

IF 1phk__#221 AND 1fmk_3#377
THEN
GO:0004674: protein serine/threonine kinase activity: 1.090e-14 (25,42,11) OR
GO:0005524: ATP binding: 3.278e-06 (25,243,11) OR
GO:0005516: calmodulin binding: 1.971e-02 (25,24,2) OR
GO:0004896: hematopoietin/interferon-class (D200-domain) cytokine receptor activity: 1.611e-01 (25,19,1)

IF 1phk__#221 AND 1tkia_#142
THEN
GO:0004674: protein serine/threonine kinase activity: 1.090e-14 (25,42,11) OR
GO:0005524: ATP binding: 3.278e-06 (25,243,11) OR
GO:0005516: calmodulin binding: 1.971e-02 (25,24,2) OR
GO:0004896: hematopoietin/interferon-class (D200-domain) cytokine receptor activity: 1.611e-01 (25,19,1)

IF 1eh9a3#409 AND 1bvza3#416
THEN
GO:0004556: alpha-amylase activity: 2.290e-14 (12,15,7) OR
GO:0005509: calcium ion binding: 3.709e-04 (12,160,5)

IF 1htp__#16 AND 1eh9a3#102
THEN
GO:0004556: alpha-amylase activity: 2.290e-14 (12,15,7) OR
GO:0005509: calcium ion binding: 3.709e-04 (12,160,5)

IF 1g71a_#77 AND 1bjt__#890 AND 1cgha_#130
THEN
GO:0004263: chymotrypsin activity: 9.506e-15 (44,41,13) OR
GO:0004295: trypsin activity: 9.653e-14 (44,48,13) OR
GO:0004867: serine-type endopeptidase inhibitor activity: 4.697e-07 (44,47,8) OR
GO:0005509: calcium ion binding: 7.923e-04 (44,160,9) OR
GO:0003809: thrombin activity: 1.505e-01 (44,10,1)

IF 1c4zd_#99 AND 1jatb_#120
THEN
GO:0004842: ubiquitin-protein ligase activity: 4.797e-14 (6,19,6)

IF 1e6wa_#24 AND 1e7wa_#8 AND 1qfma2#576
THEN
GO:0016616: oxidoreductase activity, acting on the CH-OH group of donors, NAD or NADP as acceptor: 9.619e-15 (14,59,10) OR
GO:0016627: oxidoreductase activity, acting on the CH-CH group of donors: 8.408e-02 (14,17,1) OR
GO:0016646: oxidoreductase activity, acting on the CH-NH group of donors, NAD or NADP as acceptor: 9.353e-02 (14,19,1) OR
GO:0000287: magnesium ion binding: 4.910e-01 (14,128,1) OR
GO:0005524: ATP binding: 7.304e-01 (14,243,1)

IF 1cyda_#106 AND 1oaa__#14
THEN
GO:0016616: oxidoreductase activity, acting on the CH-OH group of donors, NAD or NADP as acceptor: 1.229e-14 (19,59,11) OR
GO:0016627: oxidoreductase activity, acting on the CH-CH group of donors: 3.799e-06 (19,17,4) OR
GO:0016854: racemase and epimerase activity: 3.433e-03 (19,13,2) OR
GO:0016646: oxidoreductase activity, acting on the CH-NH group of donors, NAD or NADP as acceptor: 7.340e-03 (19,19,2)

IF 1pfza_#91
THEN
GO:0004190: aspartic-type endopeptidase activity: 8.249e-15 (13,23,8) OR
GO:0004457: lactate dehydrogenase activity: 4.677e-02 (13,10,1) OR
GO:0008080: N-acetyltransferase activity: 6.040e-02 (13,13,1) OR
GO:0004812: tRNA ligase activity: 1.174e-01 (13,26,1) OR
GO:0016616: oxidoreductase activity, acting on the CH-OH group of donors, NAD or NADP as acceptor: 2.481e-01 (13,59,1) OR
GO:0005524: ATP binding: 7.039e-01 (13,243,1)

IF 1c5y.1#B18 AND 1ekbb_#73
THEN
GO:0004263: chymotrypsin activity: 1.019e-14 (34,41,12) OR
GO:0004295: trypsin activity: 8.521e-14 (34,48,12) OR
GO:0005509: calcium ion binding: 1.272e-02 (34,160,6) OR

GO:0004867: serine-type endopeptidase inhibitor activity: 1.980e-02 (34,47,3) OR
GO:0004896: hematopoietin/interferon-class (D200-domain) cytokine receptor activity: 2.129e-01 (34,19,1)

IF 1b35b_#97 AND 1cjca2#76
THEN
GO:0015036: disulfide oxidoreductase activity: 1.038e-14 (22,22,9) OR
GO:0016668: oxidoreductase activity, acting on sulfur group of donors, NAD or NADP as acceptor: 6.001e-13 (22,12,7) OR
GO:0050660: FAD binding: 6.491e-07 (22,10,4) OR
GO:0016651: oxidoreductase activity, acting on NADH or NADPH: 1.002e-01 (22,13,1) OR
GO:0016638: oxidoreductase activity, acting on the CH-NH2 group of donors: 1.291e-01 (22,17,1)

IF 1je5a_#10 AND 1b8pa2#197
THEN
GO:0004457: lactate dehydrogenase activity: 1.052e-14 (17,10,7) OR
GO:0016616: oxidoreductase activity, acting on the CH-OH group of donors, NAD or NADP as acceptor: 2.551e-08 (17,59,7) OR
GO:0000049: tRNA binding: 7.830e-02 (17,13,1) OR
GO:0019843: rRNA binding: 1.291e-01 (17,22,1) OR
GO:0008270: zinc ion binding: 4.982e-01 (17,108,1)

IF 1gdna_#119 AND 1bqya_#216
THEN
GO:0004295: trypsin activity: 1.083e-14 (38,48,13) OR
GO:0004263: chymotrypsin activity: 4.833e-14 (38,41,12) OR
GO:0004867: serine-type endopeptidase inhibitor activity: 4.027e-04 (38,47,5) OR
GO:0005509: calcium ion binding: 5.676e-03 (38,160,7) OR
GO:0004896: hematopoietin/interferon-class (D200-domain) cytokine receptor activity: 2.349e-01 (38,19,1)

IF 2dpma_#237 AND 1e30a_#87
THEN
GO:0005507: copper ion binding: 1.380e-14 (14,38,9) OR
GO:0015082: di-, tri-valent inorganic cation transporter activity: 2.154e-03 (14,14,2) OR
GO:0046915: transition metal ion transporter activity: 2.154e-03 (14,14,2) OR
GO:0051082: unfolded protein binding: 1.616e-01 (14,34,1)

IF 1gdna_#230 AND 1arb__#210
THEN
GO:0004295: trypsin activity: 1.412e-14 (30,48,12) OR
GO:0004263: chymotrypsin activity: 9.412e-14 (30,41,11) OR
GO:0004867: serine-type endopeptidase inhibitor activity: 1.533e-03 (30,47,4) OR
GO:0005509: calcium ion binding: 2.566e-01 (30,160,3)

IF 1c5y.1#B94 AND 1ddja_#754 AND 1ton__#179
THEN
GO:0004263: chymotrypsin activity: 9.506e-15 (44,41,13) OR
GO:0004295: trypsin activity: 9.653e-14 (44,48,13) OR
GO:0003809: thrombin activity: 2.498e-09 (44,10,6) OR
GO:0004867: serine-type endopeptidase inhibitor activity: 6.366e-03 (44,47,4) OR
GO:0005509: calcium ion binding: 1.282e-02 (44,160,7) OR
GO:0005529: sugar binding: 4.724e-01 (44,39,1)

IF 2dkb__#46 AND 1elua_#199
THEN
GO:0008483: transaminase activity: 2.884e-14 (11,17,7) OR
GO:0016846: carbon-sulfur lyase activity: 2.986e-08 (11,10,4)

IF 1cpt__#401 AND 1fc3a_#172
THEN
GO:0004497: monooxygenase activity: 1.211e-14 (19,26,9) OR
GO:0016705: oxidoreductase activity, acting on paired donors, with incorporation or reduction of molecular oxygen: 5.601e-07 (19,26,5) OR
GO:0010181: FMN binding: 2.441e-03 (19,11,2) OR
GO:0016651: oxidoreductase activity, acting on NADH or NADPH: 3.433e-03 (19,13,2) OR
GO:0016814: hydrolase activity, acting on carbon-nitrogen (but not peptide) bonds, in cyclic amidines: 6.769e-02 (19,10,1)

IF 1cpt__#401 AND 1dt6a_#77
THEN
GO:0004497: monooxygenase activity: 1.211e-14 (19,26,9) OR
GO:0016705: oxidoreductase activity, acting on paired donors, with incorporation or reduction of molecular oxygen: 5.601e-07 (19,26,5) OR
GO:0010181: FMN binding: 2.441e-03 (19,11,2) OR

GO:0016651: oxidoreductase activity, acting on NADH or NADPH: 3.433e-03 (19,13,2) OR
GO:0008757: S-adenosylmethionine-dependent methyltransferase activity: 1.552e-01 (19,24,1)

IF 1qrra_#208 AND 1eq2a_#11
THEN
GO:0016616: oxidoreductase activity, acting on the CH-OH group of donors, NAD or NADP as acceptor: 1.229e-14 (19,59,11) OR
GO:0016646: oxidoreductase activity, acting on the CH-NH group of donors, NAD or NADP as acceptor: 2.597e-04 (19,19,3) OR
GO:0016854: racemase and epimerase activity: 3.433e-03 (19,13,2) OR
GO:0016836: hydro-lyase activity: 2.139e-02 (19,33,2) OR
GO:0016627: oxidoreductase activity, acting on the CH-CH group of donors: 1.125e-01 (19,17,1)

IF 3chbd_#85 AND 1ebda2#182
THEN
GO:0015036: disulfide oxidoreductase activity: 1.038e-14 (22,22,9) OR
GO:0016668: oxidoreductase activity, acting on sulfur group of donors, NAD or NADP as acceptor: 1.183e-10 (22,12,6) OR
GO:0050660: FAD binding: 6.491e-07 (22,10,4) OR
GO:0016651: oxidoreductase activity, acting on NADH or NADPH: 1.002e-01 (22,13,1) OR
GO:0005096: GTPase activator activity: 1.148e-01 (22,15,1) OR
GO:0016627: oxidoreductase activity, acting on the CH-CH group of donors: 1.291e-01 (22,17,1)

IF 1e5ka_#113 AND 1ton__#231
THEN
GO:0004263: chymotrypsin activity: 1.311e-14 (57,41,14) OR
GO:0004295: trypsin activity: 1.615e-13 (57,48,14) OR
GO:0003809: thrombin activity: 1.376e-10 (57,10,7) OR
GO:0004867: serine-type endopeptidase inhibitor activity: 2.953e-07 (57,47,9) OR
GO:0005509: calcium ion binding: 1.549e-05 (57,160,13)

IF 1hqoa2#159 AND 1ljra1#165
THEN
GO:0004364: glutathione transferase activity: 2.280e-14 (8,11,6) OR
GO:0003714: transcription corepressor activity: 2.328e-02 (8,8,1) OR
GO:0016651: oxidoreductase activity, acting on NADH or NADPH: 3.758e-02 (8,13,1)

IF 1hqoa2#159 AND 3grx__#55
THEN
GO:0004364: glutathione transferase activity: 2.280e-14 (8,11,6) OR
GO:0003714: transcription corepressor activity: 2.328e-02 (8,8,1) OR
GO:0016651: oxidoreductase activity, acting on NADH or NADPH: 3.758e-02 (8,13,1)

IF 1hqoa2#159 AND 1glqa2#53
THEN
GO:0004364: glutathione transferase activity: 2.280e-14 (8,11,6) OR
GO:0003714: transcription corepressor activity: 2.328e-02 (8,8,1) OR
GO:0016651: oxidoreductase activity, acting on NADH or NADPH: 3.758e-02 (8,13,1)

IF 1hyea2#182 AND 1ihua2#525
THEN
GO:0004457: lactate dehydrogenase activity: 1.720e-14 (18,10,7) OR
GO:0016616: oxidoreductase activity, acting on the CH-OH group of donors, NAD or NADP as acceptor: 1.099e-09 (18,59,8) OR
GO:0030145: manganese ion binding: 2.240e-01 (18,38,1) OR
GO:0005524: ATP binding: 4.864e-01 (18,243,2)

IF 1fvua_#72 AND 2msba_#156
THEN
GO:0005529: sugar binding: 7.002e-14 (7,39,7)

IF 2msba_#218 AND 2msba_#156
THEN
GO:0005529: sugar binding: 7.002e-14 (7,39,7)

IF 1e87a_#155 AND 1dv8a_#211
THEN
GO:0005529: sugar binding: 7.002e-14 (7,39,7)

IF 1qo3c_#212 AND 2msba_#218
THEN
GO:0005529: sugar binding: 7.002e-14 (7,39,7)

IF 1e2fa_#140 AND 1qfma2#576

THEN
GO:0019201: nucleotide kinase activity: 1.814e-14 (25,13,8) OR
GO:0016776: phosphotransferase activity, phosphate group as acceptor: 4.208e-14 (25,14,8) OR
GO:0005524: ATP binding: 1.007e-03 (25,243,8) OR
GO:0005525: GTP binding: 3.660e-01 (25,49,1)

IF 1e2fa_#140 AND 1qfea_#68
THEN
GO:0019201: nucleotide kinase activity: 1.814e-14 (25,13,8) OR
GO:0016776: phosphotransferase activity, phosphate group as acceptor: 4.208e-14 (25,14,8) OR
GO:0005524: ATP binding: 1.007e-03 (25,243,8) OR
GO:0005525: GTP binding: 3.660e-01 (25,49,1)

IF 1ldna1#94 AND 1h5qa_#70
THEN
GO:0016616: oxidoreductase activity, acting on the CH-OH group of donors, NAD or NADP as acceptor: 1.229e-14 (19,59,11) OR
GO:0016627: oxidoreductase activity, acting on the CH-CH group of donors: 3.799e-06 (19,17,4) OR
GO:0016854: racemase and epimerase activity: 8.713e-02 (19,13,1) OR
GO:0008757: S-adenosylmethionine-dependent methyltransferase activity: 1.552e-01 (19,24,1) OR
GO:0016836: hydro-lyase activity: 2.073e-01 (19,33,1) OR
GO:0000287: magnesium ion binding: 6.004e-01 (19,128,1)

IF 1eny__#94 AND 1qora2#201
THEN
GO:0016616: oxidoreductase activity, acting on the CH-OH group of donors, NAD or NADP as acceptor: 1.229e-14 (19,59,11) OR
GO:0016627: oxidoreductase activity, acting on the CH-CH group of donors: 1.839e-04 (19,17,3) OR
GO:0016836: hydro-lyase activity: 2.139e-02 (19,33,2) OR
GO:0004457: lactate dehydrogenase activity: 6.769e-02 (19,10,1) OR
GO:0016854: racemase and epimerase activity: 8.713e-02 (19,13,1) OR
GO:0016646: oxidoreductase activity, acting on the CH-NH group of donors, NAD or NADP as acceptor: 1.249e-01 (19,19,1)

IF 1deua_#57 AND 1dkia_#54
THEN
GO:0004197: cysteine-type endopeptidase activity: 1.235e-14 (13,24,8) OR
GO:0019955: cytokine binding: 5.134e-02 (13,11,1) OR
GO:0042802: protein self binding: 5.588e-02 (13,12,1) OR
GO:0004177: aminopeptidase activity: 6.040e-02 (13,13,1) OR
GO:0004180: carboxypeptidase activity: 6.939e-02 (13,15,1) OR
GO:0051082: unfolded protein binding: 1.509e-01 (13,34,1)

IF 2cb5a_#373 AND 1deua_#57
THEN
GO:0004197: cysteine-type endopeptidase activity: 1.235e-14 (13,24,8) OR
GO:0019955: cytokine binding: 5.134e-02 (13,11,1) OR
GO:0042802: protein self binding: 5.588e-02 (13,12,1) OR
GO:0004177: aminopeptidase activity: 6.040e-02 (13,13,1) OR
GO:0004180: carboxypeptidase activity: 6.939e-02 (13,15,1) OR
GO:0051082: unfolded protein binding: 1.509e-01 (13,34,1)

IF 1deua_#57 AND 1dkia_#185
THEN
GO:0004197: cysteine-type endopeptidase activity: 1.235e-14 (13,24,8) OR
GO:0019955: cytokine binding: 5.134e-02 (13,11,1) OR
GO:0042802: protein self binding: 5.588e-02 (13,12,1) OR
GO:0004177: aminopeptidase activity: 6.040e-02 (13,13,1) OR
GO:0004180: carboxypeptidase activity: 6.939e-02 (13,15,1) OR
GO:0051082: unfolded protein binding: 1.509e-01 (13,34,1)

IF 1ovaa_#340 AND 1a7ca_#202
THEN
GO:0004867: serine-type endopeptidase inhibitor activity: 3.744e-14 (9,47,8) OR
GO:0008201: heparin binding: 7.664e-02 (9,24,1)

IF 1sek__#301 AND 1a7ca_#202
THEN
GO:0004867: serine-type endopeptidase inhibitor activity: 3.744e-14 (9,47,8) OR
GO:0008201: heparin binding: 7.664e-02 (9,24,1)

IF 1sek__#301 AND 1ovaa_#161
THEN

GO:0004867: serine-type endopeptidase inhibitor activity: 3.744e-14 (9,47,8) OR
GO:0008201: heparin binding: 7.664e-02 (9,24,1)

IF 1hle.1#A228 AND 1e05i_#79
THEN
GO:0004867: serine-type endopeptidase inhibitor activity: 3.744e-14 (9,47,8) OR
GO:0008201: heparin binding: 7.664e-02 (9,24,1)

IF 1danh_#152 AND 1c5y.1#B18
THEN
GO:0004263: chymotrypsin activity: 1.535e-14 (35,41,12) OR
GO:0004295: trypsin activity: 1.281e-13 (35,48,12) OR
GO:0004867: serine-type endopeptidase inhibitor activity: 2.750e-03 (35,47,4) OR
GO:0005509: calcium ion binding: 1.462e-02 (35,160,6) OR
GO:0004896: hematopoietin/interferon-class (D200-domain) cytokine receptor activity: 2.184e-01 (35,19,1)

IF 2hlca_#99 AND 1danh_#142 AND 1cgha_#130
THEN
GO:0004263: chymotrypsin activity: 1.535e-14 (35,41,12) OR
GO:0004295: trypsin activity: 1.281e-13 (35,48,12) OR
GO:0004867: serine-type endopeptidase inhibitor activity: 2.708e-04 (35,47,5) OR
GO:0005509: calcium ion binding: 5.099e-02 (35,160,5) OR
GO:0008201: heparin binding: 2.677e-01 (35,24,1)

IF 1danh_#197 AND 1c5y.1#B241 AND 2hlca_#99 AND 1cgha_#130
THEN
GO:0004263: chymotrypsin activity: 1.535e-14 (35,41,12) OR
GO:0004295: trypsin activity: 1.281e-13 (35,48,12) OR
GO:0004867: serine-type endopeptidase inhibitor activity: 2.708e-04 (35,47,5) OR
GO:0005509: calcium ion binding: 5.099e-02 (35,160,5) OR
GO:0008201: heparin binding: 2.677e-01 (35,24,1)

IF 2cuaa_#107 AND 1fwxa1#526
THEN
GO:0005507: copper ion binding: 1.299e-14 (20,38,10) OR
GO:0015078: hydrogen ion transporter activity: 3.480e-09 (20,21,6) OR
GO:0015082: di-, tri-valent inorganic cation transporter activity: 9.821e-02 (20,14,1) OR
GO:0046915: transition metal ion transporter activity: 9.821e-02 (20,14,1) OR
GO:0051082: unfolded protein binding: 2.228e-01 (20,34,1) OR
GO:0005509: calcium ion binding: 7.032e-01 (20,160,1)

IF 4tmka_#141 AND 1ihua2#525
THEN
GO:0019201: nucleotide kinase activity: 2.616e-14 (26,13,8) OR
GO:0016776: phosphotransferase activity, phosphate group as acceptor: 9.844e-12 (26,14,7) OR
GO:0005524: ATP binding: 5.238e-06 (26,243,11)

IF 1gdna_#119 AND 1bjt__#890
THEN
GO:0004295: trypsin activity: 1.605e-14 (39,48,13) OR
GO:0004263: chymotrypsin activity: 6.912e-14 (39,41,12) OR
GO:0004867: serine-type endopeptidase inhibitor activity: 4.560e-04 (39,47,5) OR
GO:0005509: calcium ion binding: 1.527e-03 (39,160,8) OR
GO:0004896: hematopoietin/interferon-class (D200-domain) cytokine receptor activity: 2.403e-01 (39,19,1)

IF 1ja9a_#202 AND 1eny__#94
THEN
GO:0016616: oxidoreductase activity, acting on the CH-OH group of donors, NAD or NADP as acceptor: 2.838e-14 (15,59,10) OR
GO:0016627: oxidoreductase activity, acting on the CH-CH group of donors: 1.359e-06 (15,17,4) OR
GO:0016646: oxidoreductase activity, acting on the CH-NH group of donors, NAD or NADP as acceptor: 9.988e-02 (15,19,1)

IF 1qg6a_#93 AND 1e6ua_#10
THEN
GO:0016616: oxidoreductase activity, acting on the CH-OH group of donors, NAD or NADP as acceptor: 2.838e-14 (15,59,10) OR
GO:0016627: oxidoreductase activity, acting on the CH-CH group of donors: 1.359e-06 (15,17,4) OR
GO:0016646: oxidoreductase activity, acting on the CH-NH group of donors, NAD or NADP as acceptor: 9.988e-02 (15,19,1)

IF 1qg6a_#93 AND 1eny__#147
THEN
GO:0016616: oxidoreductase activity, acting on the CH-OH group of donors, NAD or NADP as acceptor: 2.838e-14 (15,59,10) OR

GO:0016627: oxidoreductase activity, acting on the CH-CH group of donors: 1.359e-06 (15,17,4) OR
GO:0016646: oxidoreductase activity, acting on the CH-NH group of donors, NAD or NADP as acceptor: 9.988e-02 (15,19,1)

IF 1ja9a_#202 AND 1oaa__#175
THEN
GO:0016616: oxidoreductase activity, acting on the CH-OH group of donors, NAD or NADP as acceptor: 2.838e-14 (15,59,10) OR
GO:0016627: oxidoreductase activity, acting on the CH-CH group of donors: 1.359e-06 (15,17,4) OR
GO:0016646: oxidoreductase activity, acting on the CH-NH group of donors, NAD or NADP as acceptor: 9.988e-02 (15,19,1)

IF 1fuia2#273 AND 1h5qa_#70
THEN
GO:0016616: oxidoreductase activity, acting on the CH-OH group of donors, NAD or NADP as acceptor: 2.966e-14 (11,59,9) OR
GO:0016854: racemase and epimerase activity: 5.134e-02 (11,13,1) OR
GO:0016627: oxidoreductase activity, acting on the CH-CH group of donors: 6.664e-02 (11,17,1)

IF 1hdr__#149 AND 1tyfa_#31
THEN
GO:0016616: oxidoreductase activity, acting on the CH-OH group of donors, NAD or NADP as acceptor: 2.966e-14 (11,59,9) OR
GO:0016854: racemase and epimerase activity: 5.134e-02 (11,13,1) OR
GO:0030145: manganese ion binding: 1.434e-01 (11,38,1)

IF 1e6wa_#24 AND 1ja9a_#202 AND 1cyda_#106
THEN
GO:0016616: oxidoreductase activity, acting on the CH-OH group of donors, NAD or NADP as acceptor: 2.966e-14 (11,59,9) OR
GO:0016627: oxidoreductase activity, acting on the CH-CH group of donors: 6.664e-02 (11,17,1) OR
GO:0016646: oxidoreductase activity, acting on the CH-NH group of donors, NAD or NADP as acceptor: 7.421e-02 (11,19,1)

IF 2msba_#134 AND 1esl_1#38
THEN
GO:0005529: sugar binding: 1.791e-14 (14,39,9) OR
GO:0008201: heparin binding: 1.167e-01 (14,24,1) OR
GO:0004263: chymotrypsin activity: 1.916e-01 (14,41,1) OR
GO:0005509: calcium ion binding: 1.968e-01 (14,160,2) OR
GO:0004295: trypsin activity: 2.207e-01 (14,48,1)

IF 1esl_1#29 AND 1hq8a_#206
THEN
GO:0005529: sugar binding: 1.791e-14 (14,39,9) OR
GO:0008201: heparin binding: 1.167e-01 (14,24,1) OR
GO:0004263: chymotrypsin activity: 1.916e-01 (14,41,1) OR
GO:0005509: calcium ion binding: 1.968e-01 (14,160,2) OR
GO:0004295: trypsin activity: 2.207e-01 (14,48,1)

IF 1a6o__#217 AND 1a6o__#297
THEN
GO:0004674: protein serine/threonine kinase activity: 1.869e-14 (26,42,11) OR
GO:0005524: ATP binding: 5.238e-06 (26,243,11) OR
GO:0005516: calmodulin binding: 2.124e-02 (26,24,2) OR
GO:0003755: peptidyl-prolyl cis-trans isomerase activity: 1.003e-01 (26,11,1) OR
GO:0004896: hematopoietin/interferon-class (D200-domain) cytokine receptor activity: 1.670e-01 (26,19,1)

IF 7taa_2#119 AND 1bqca_#24
THEN
GO:0004556: alpha-amylase activity: 4.949e-14 (13,15,7) OR
GO:0005509: calcium ion binding: 4.543e-05 (13,160,6)

IF 1djna1#86 AND 7taa_2#295
THEN
GO:0004556: alpha-amylase activity: 4.949e-14 (13,15,7) OR
GO:0005509: calcium ion binding: 4.543e-05 (13,160,6)

IF 1gjwa2#141 AND 1djna1#86
THEN
GO:0004556: alpha-amylase activity: 4.949e-14 (13,15,7) OR
GO:0005509: calcium ion binding: 4.543e-05 (13,160,6)

IF 1e43a2#236 AND 1h9da_#91
THEN
GO:0004556: alpha-amylase activity: 4.949e-14 (13,15,7) OR
GO:0005509: calcium ion binding: 4.543e-05 (13,160,6)

IF 1b35b_#97 AND 1trb_1#41
THEN
GO:0015036: disulfide oxidoreductase activity: 1.698e-14 (23,22,9) OR
GO:0016668: oxidoreductase activity, acting on sulfur group of donors, NAD or NADP as acceptor: 8.613e-13 (23,12,7) OR
GO:0050660: FAD binding: 7.844e-07 (23,10,4) OR
GO:0016651: oxidoreductase activity, acting on NADH or NADPH: 1.046e-01 (23,13,1) OR
GO:0016627: oxidoreductase activity, acting on the CH-CH group of donors: 1.346e-01 (23,17,1) OR
GO:0016638: oxidoreductase activity, acting on the CH-NH2 group of donors: 1.346e-01 (23,17,1)

IF 1jlna_#437 AND 1cyx__#138
THEN
GO:0005507: copper ion binding: 3.417e-14 (15,38,9) OR
GO:0015078: hydrogen ion transporter activity: 3.372e-06 (15,21,4) OR
GO:0005509: calcium ion binding: 2.188e-01 (15,160,2)

IF 1e2fa_#14 AND 1hwxa1#223
THEN
GO:0019201: nucleotide kinase activity: 2.616e-14 (26,13,8) OR
GO:0016776: phosphotransferase activity, phosphate group as acceptor: 6.067e-14 (26,14,8) OR
GO:0005524: ATP binding: 2.481e-04 (26,243,9) OR
GO:0005525: GTP binding: 3.775e-01 (26,49,1)

IF 1e2fa_#140 AND 1nksa_#13
THEN
GO:0019201: nucleotide kinase activity: 2.616e-14 (26,13,8) OR
GO:0016776: phosphotransferase activity, phosphate group as acceptor: 6.067e-14 (26,14,8) OR
GO:0005524: ATP binding: 2.481e-04 (26,243,9) OR
GO:0005525: GTP binding: 3.775e-01 (26,49,1)

IF 1fuma2#370 AND 3grx__#56
THEN
GO:0015036: disulfide oxidoreductase activity: 2.670e-14 (15,22,8) OR
GO:0016668: oxidoreductase activity, acting on sulfur group of donors, NAD or NADP as acceptor: 2.872e-07 (15,12,4) OR
GO:0050660: FAD binding: 1.241e-03 (15,10,2) OR
GO:0016638: oxidoreductase activity, acting on the CH-NH2 group of donors: 8.982e-02 (15,17,1)

IF 1c4zd_#85 AND 1c4zd_#101
THEN
GO:0004842: ubiquitin-protein ligase activity: 2.721e-14 (10,19,7) OR
GO:0008270: zinc ion binding: 3.331e-01 (10,108,1) OR
GO:0003700: transcription factor activity: 3.728e-01 (10,124,1) OR
GO:0005509: calcium ion binding: 4.545e-01 (10,160,1)

IF 2hlpa2#195 AND 1e3ja2#270
THEN
GO:0004457: lactate dehydrogenase activity: 2.721e-14 (19,10,7) OR
GO:0016616: oxidoreductase activity, acting on the CH-OH group of donors, NAD or NADP as acceptor: 8.171e-13 (19,59,10) OR
GO:0004497: monooxygenase activity: 1.670e-01 (19,26,1) OR
GO:0000287: magnesium ion binding: 6.004e-01 (19,128,1)

IF 1hdr__#149 AND 1hdoa_#189
THEN
GO:0016616: oxidoreductase activity, acting on the CH-OH group of donors, NAD or NADP as acceptor: 2.838e-14 (15,59,10) OR
GO:0016854: racemase and epimerase activity: 2.131e-03 (15,13,2) OR
GO:0016836: hydro-lyase activity: 1.353e-02 (15,33,2) OR
GO:0016627: oxidoreductase activity, acting on the CH-CH group of donors: 8.982e-02 (15,17,1)

IF 1j9la_#82 AND 1oaa__#161
THEN
GO:0016616: oxidoreductase activity, acting on the CH-OH group of donors, NAD or NADP as acceptor: 2.838e-14 (15,59,10) OR
GO:0016627: oxidoreductase activity, acting on the CH-CH group of donors: 3.668e-03 (15,17,2) OR
GO:0016836: hydro-lyase activity: 1.353e-02 (15,33,2) OR
GO:0016646: oxidoreductase activity, acting on the CH-NH group of donors, NAD or NADP as acceptor: 9.988e-02 (15,19,1)

IF 1gdna_#119 AND 1eq9a_#58 AND 1ton__#91
THEN
GO:0004295: trypsin activity: 2.276e-14 (31,48,12) OR
GO:0004263: chymotrypsin activity: 1.444e-13 (31,41,11) OR
GO:0005509: calcium ion binding: 3.218e-02 (31,160,5) OR

GO:0004867: serine-type endopeptidase inhibitor activity: 9.874e-02 (31,47,2) OR
GO:0004896: hematopoietin/interferon-class (D200-domain) cytokine receptor activity: 1.960e-01 (31,19,1)

IF 2dkb__#133 AND 2dkb__#46
THEN
GO:0008483: transaminase activity: 2.884e-14 (11,17,7) OR
GO:0016846: carbon-sulfur lyase activity: 6.552e-04 (11,10,2) OR
GO:0016866: intramolecular transferase activity: 4.747e-02 (11,12,1) OR
GO:0016831: carboxy-lyase activity: 9.658e-02 (11,25,1)

IF 2gsaa_#246 AND 2dkb__#114
THEN
GO:0008483: transaminase activity: 2.884e-14 (11,17,7) OR
GO:0016846: carbon-sulfur lyase activity: 6.552e-04 (11,10,2) OR
GO:0016866: intramolecular transferase activity: 4.747e-02 (11,12,1) OR
GO:0016831: carboxy-lyase activity: 9.658e-02 (11,25,1)

IF 2dkb__#133 AND 1c7na_#281
THEN
GO:0008483: transaminase activity: 2.884e-14 (11,17,7) OR
GO:0016846: carbon-sulfur lyase activity: 6.552e-04 (11,10,2) OR
GO:0016866: intramolecular transferase activity: 4.747e-02 (11,12,1) OR
GO:0016831: carboxy-lyase activity: 9.658e-02 (11,25,1)

IF 1ha6a_#20 AND 1ha6a_#59
THEN
GO:0008009: chemokine activity: 3.107e-14 (9,10,6) OR
GO:0004812: tRNA ligase activity: 8.278e-02 (9,26,1) OR
GO:0008083: growth factor activity: 1.306e-01 (9,42,1) OR
GO:0005524: ATP binding: 5.691e-01 (9,243,1)

IF 1cm9a_#57 AND 1cewi_#14 AND 1el0a_#28
THEN
GO:0008009: chemokine activity: 3.107e-14 (9,10,6) OR
GO:0000155: two-component sensor molecule activity: 3.900e-02 (9,12,1) OR
GO:0005525: GTP binding: 1.509e-01 (9,49,1) OR
GO:0005524: ATP binding: 5.691e-01 (9,243,1)

IF 1d6ja_#36 AND 1e6ca_#153
THEN
GO:0016776: phosphotransferase activity, phosphate group as acceptor: 4.208e-14 (25,14,8) OR
GO:0019201: nucleotide kinase activity: 3.626e-12 (25,13,7) OR
GO:0005524: ATP binding: 2.600e-05 (25,243,10)

IF 1fjsa_#158 AND 1a0la_#160 AND 1eq9a_#58
THEN
GO:0004295: trypsin activity: 2.139e-14 (50,48,14) OR
GO:0004263: chymotrypsin activity: 6.129e-14 (50,41,13) OR
GO:0003809: thrombin activity: 5.233e-11 (50,10,7) OR
GO:0004867: serine-type endopeptidase inhibitor activity: 1.701e-04 (50,47,6) OR
GO:0005509: calcium ion binding: 2.065e-03 (50,160,9) OR
GO:0005529: sugar binding: 5.168e-01 (50,39,1)

IF 1bu7a_#405 AND 1bu7a_#353
THEN
GO:0004497: monooxygenase activity: 2.189e-14 (20,26,9) OR
GO:0016705: oxidoreductase activity, acting on paired donors, with incorporation or reduction of molecular oxygen: 7.420e-07 (20,26,5) OR
GO:0010181: FMN binding: 2.706e-03 (20,11,2) OR
GO:0016651: oxidoreductase activity, acting on NADH or NADPH: 3.804e-03 (20,13,2) OR
GO:0051082: unfolded protein binding: 2.228e-01 (20,34,1) OR
GO:0005524: ATP binding: 8.466e-01 (20,243,1)

IF 1cpt__#401 AND 1dz4a_#264
THEN
GO:0004497: monooxygenase activity: 2.189e-14 (20,26,9) OR
GO:0016705: oxidoreductase activity, acting on paired donors, with incorporation or reduction of molecular oxygen: 7.420e-07 (20,26,5) OR
GO:0010181: FMN binding: 2.706e-03 (20,11,2) OR
GO:0016651: oxidoreductase activity, acting on NADH or NADPH: 3.804e-03 (20,13,2) OR

GO:0003899: DNA-directed RNA polymerase activity: 8.477e-02 (20,12,1) OR
GO:0046983: protein dimerization activity: 1.180e-01 (20,17,1)

IF 1c5y.1#B94 AND 1qnja_#77
THEN
GO:0004263: chymotrypsin activity: 3.340e-14 (37,41,12) OR
GO:0004295: trypsin activity: 2.773e-13 (37,48,12) OR
GO:0004867: serine-type endopeptidase inhibitor activity: 2.948e-05 (37,47,6) OR
GO:0005509: calcium ion binding: 4.865e-03 (37,160,7)

IF 1gcoa_#119 AND 1bu8a2#124
THEN
GO:0016616: oxidoreductase activity, acting on the CH-OH group of donors, NAD or NADP as acceptor: 2.838e-14 (15,59,10) OR
GO:0016627: oxidoreductase activity, acting on the CH-CH group of donors: 3.668e-03 (15,17,2) OR
GO:0016866: intramolecular transferase activity: 6.422e-02 (15,12,1) OR
GO:0016638: oxidoreductase activity, acting on the CH-NH2 group of donors: 8.982e-02 (15,17,1) OR
GO:0000287: magnesium ion binding: 5.150e-01 (15,128,1)

IF 1bdb__#228 AND 1eq2a_#11
THEN
GO:0016616: oxidoreductase activity, acting on the CH-OH group of donors, NAD or NADP as acceptor: 2.838e-14 (15,59,10) OR
GO:0016854: racemase and epimerase activity: 2.131e-03 (15,13,2) OR
GO:0016627: oxidoreductase activity, acting on the CH-CH group of donors: 8.982e-02 (15,17,1) OR
GO:0016646: oxidoreductase activity, acting on the CH-NH group of donors, NAD or NADP as acceptor: 9.988e-02 (15,19,1) OR
GO:0016836: hydro-lyase activity: 1.674e-01 (15,33,1)

IF 1hoe__#31 AND 1avaa2#204
THEN
GO:0004556: alpha-amylase activity: 4.949e-14 (13,15,7) OR
GO:0005509: calcium ion binding: 5.744e-04 (13,160,5) OR
GO:0004867: serine-type endopeptidase inhibitor activity: 2.028e-01 (13,47,1)

IF 1aq0a_#4 AND 1gjwa2#142
THEN
GO:0004556: alpha-amylase activity: 4.949e-14 (13,15,7) OR
GO:0005509: calcium ion binding: 5.744e-04 (13,160,5) OR
GO:0016758: transferase activity, transferring hexosyl groups: 5.134e-02 (13,11,1)

IF 1tuba1#8 AND 1a3c__#108
THEN
GO:0016763: transferase activity, transferring pentosyl groups: 5.186e-14 (13,28,8) OR
GO:0000287: magnesium ion binding: 2.386e-03 (13,128,4) OR
GO:0008483: transaminase activity: 7.830e-02 (13,17,1)

IF 1bwvs_#117 AND 1trb_1#41
THEN
GO:0015036: disulfide oxidoreductase activity: 2.705e-14 (24,22,9) OR
GO:0016668: oxidoreductase activity, acting on sulfur group of donors, NAD or NADP as acceptor: 1.214e-12 (24,12,7) OR
GO:0050660: FAD binding: 9.396e-07 (24,10,4) OR
GO:0016627: oxidoreductase activity, acting on the CH-CH group of donors: 9.329e-03 (24,17,2) OR
GO:0016651: oxidoreductase activity, acting on NADH or NADPH: 1.089e-01 (24,13,1) OR
GO:0016638: oxidoreductase activity, acting on the CH-NH2 group of donors: 1.400e-01 (24,17,1)

IF 1h7wa4#478 AND 1gpea1#273
THEN
GO:0015036: disulfide oxidoreductase activity: 2.705e-14 (24,22,9) OR
GO:0016668: oxidoreductase activity, acting on sulfur group of donors, NAD or NADP as acceptor: 1.214e-12 (24,12,7) OR
GO:0050660: FAD binding: 8.339e-09 (24,10,5) OR
GO:0016651: oxidoreductase activity, acting on NADH or NADPH: 1.089e-01 (24,13,1) OR
GO:0016627: oxidoreductase activity, acting on the CH-CH group of donors: 1.400e-01 (24,17,1) OR
GO:0004601: peroxidase activity: 1.701e-01 (24,21,1)

IF 1pbe_1#121 AND 1feca1#122
THEN
GO:0015036: disulfide oxidoreductase activity: 2.705e-14 (24,22,9) OR
GO:0016668: oxidoreductase activity, acting on sulfur group of donors, NAD or NADP as acceptor: 2.125e-10 (24,12,6) OR
GO:0050660: FAD binding: 9.396e-07 (24,10,4) OR
GO:0016627: oxidoreductase activity, acting on the CH-CH group of donors: 3.767e-04 (24,17,3) OR
GO:0016651: oxidoreductase activity, acting on NADH or NADPH: 1.089e-01 (24,13,1) OR
GO:0016638: oxidoreductase activity, acting on the CH-NH2 group of donors: 1.400e-01 (24,17,1)

IF 1gpea1#273 AND 1d7ya1#255
THEN
GO:0015036: disulfide oxidoreductase activity: 2.705e-14 (24,22,9) OR
GO:0016668: oxidoreductase activity, acting on sulfur group of donors, NAD or NADP as acceptor: 1.214e-12 (24,12,7) OR
GO:0050660: FAD binding: 8.339e-09 (24,10,5) OR
GO:0016651: oxidoreductase activity, acting on NADH or NADPH: 1.089e-01 (24,13,1) OR
GO:0016627: oxidoreductase activity, acting on the CH-CH group of donors: 1.400e-01 (24,17,1) OR
GO:0004601: peroxidase activity: 1.701e-01 (24,21,1)

IF 1feca1#309 AND 1h7wa4#476
THEN
GO:0015036: disulfide oxidoreductase activity: 2.705e-14 (24,22,9) OR
GO:0016668: oxidoreductase activity, acting on sulfur group of donors, NAD or NADP as acceptor: 1.214e-12 (24,12,7) OR
GO:0050660: FAD binding: 8.339e-09 (24,10,5) OR
GO:0016651: oxidoreductase activity, acting on NADH or NADPH: 1.089e-01 (24,13,1) OR
GO:0016627: oxidoreductase activity, acting on the CH-CH group of donors: 1.400e-01 (24,17,1) OR
GO:0004601: peroxidase activity: 1.701e-01 (24,21,1)

IF 1xvaa_#68 AND 1g55a_#57
THEN
GO:0008757: S-adenosylmethionine-dependent methyltransferase activity: 5.610e-14 (9,24,7) OR
GO:0008270: zinc ion binding: 3.055e-01 (9,108,1) OR
GO:0000287: magnesium ion binding: 3.519e-01 (9,128,1)

IF 1eny__#94 AND 1fmca_#89
THEN
GO:0016616: oxidoreductase activity, acting on the CH-OH group of donors, NAD or NADP as acceptor: 2.838e-14 (15,59,10) OR
GO:0016854: racemase and epimerase activity: 6.939e-02 (15,13,1) OR
GO:0016627: oxidoreductase activity, acting on the CH-CH group of donors: 8.982e-02 (15,17,1) OR
GO:0016646: oxidoreductase activity, acting on the CH-NH group of donors, NAD or NADP as acceptor: 9.988e-02 (15,19,1) OR
GO:0051082: unfolded protein binding: 1.721e-01 (15,34,1) OR
GO:0005524: ATP binding: 7.546e-01 (15,243,1)

IF 1hu4a_#267 AND 1aqua_#126 AND 1fmca_#89
THEN
GO:0016616: oxidoreductase activity, acting on the CH-OH group of donors, NAD or NADP as acceptor: 2.838e-14 (15,59,10) OR
GO:0016627: oxidoreductase activity, acting on the CH-CH group of donors: 8.982e-02 (15,17,1) OR
GO:0016646: oxidoreductase activity, acting on the CH-NH group of donors, NAD or NADP as acceptor: 9.988e-02 (15,19,1) OR
GO:0015036: disulfide oxidoreductase activity: 1.148e-01 (15,22,1) OR
GO:0016763: transferase activity, transferring pentosyl groups: 1.439e-01 (15,28,1) OR
GO:0003700: transcription factor activity: 5.036e-01 (15,124,1)

IF 1mrp__#172 AND 1b3ra1#218
THEN
GO:0016616: oxidoreductase activity, acting on the CH-OH group of donors, NAD or NADP as acceptor: 2.838e-14 (15,59,10) OR
GO:0016620: oxidoreductase activity, acting on the aldehyde or oxo group of donors, NAD or NADP as acceptor: 5.379e-02 (15,10,1) OR
GO:0008026: ATP-dependent helicase activity: 6.939e-02 (15,13,1) OR
GO:0016651: oxidoreductase activity, acting on NADH or NADPH: 6.939e-02 (15,13,1) OR
GO:0015036: disulfide oxidoreductase activity: 1.148e-01 (15,22,1) OR
GO:0005524: ATP binding: 7.546e-01 (15,243,1)

IF 1bxoa_#180
THEN
GO:0004190: aspartic-type endopeptidase activity: 1.785e-13 (6,23,6)

IF 1pfza_#177 AND 1mpp__#214
THEN
GO:0004190: aspartic-type endopeptidase activity: 1.785e-13 (6,23,6)

IF 1j71a_#197 AND 1mpp__#214
THEN
GO:0004190: aspartic-type endopeptidase activity: 1.785e-13 (6,23,6)

IF 1gdna_#119 AND 1gdna_#151
THEN
GO:0004295: trypsin activity: 3.597e-14 (32,48,12) OR
GO:0004263: chymotrypsin activity: 2.178e-13 (32,41,11) OR
GO:0004867: serine-type endopeptidase inhibitor activity: 1.681e-02 (32,47,3) OR

GO:0005509: calcium ion binding: 3.639e-02 (32,160,5) OR
GO:0004896: hematopoietin/interferon-class (D200-domain) cytokine receptor activity: 2.016e-01 (32,19,1)

IF 1azza_#51 AND 1g71a_#77 AND 1qnja_#124 AND 1ton__#196 AND 1arb__#55
THEN
GO:0004263: chymotrypsin activity: 3.340e-14 (37,41,12) OR
GO:0004295: trypsin activity: 2.773e-13 (37,48,12) OR
GO:0005509: calcium ion binding: 1.060e-03 (37,160,8) OR
GO:0003809: thrombin activity: 7.539e-03 (37,10,2) OR
GO:0004867: serine-type endopeptidase inhibitor activity: 1.327e-01 (37,47,2) OR
GO:0004896: hematopoietin/interferon-class (D200-domain) cytokine receptor activity: 2.294e-01 (37,19,1)

IF 1b3aa_#15 AND 1el0a_#28
THEN
GO:0008009: chemokine activity: 2.020e-13 (5,10,5)

IF 1ghpa_#236 AND 1e3ua_#205
THEN
GO:0008800: beta-lactamase activity: 2.020e-13 (5,10,5)

IF 1b3aa_#15 AND 1el0a_#62
THEN
GO:0008009: chemokine activity: 2.020e-13 (5,10,5)

IF 1cm9a_#57 AND 1b3aa_#15
THEN
GO:0008009: chemokine activity: 2.020e-13 (5,10,5)

IF 1e3ua_#67
THEN
GO:0008800: beta-lactamase activity: 2.020e-13 (5,10,5)

IF 1c5y.1#B94 AND 1i6vd_#1283 AND 1qnja_#118
THEN
GO:0004263: chymotrypsin activity: 3.398e-14 (48,41,13) OR
GO:0004295: trypsin activity: 3.417e-13 (48,48,13) OR
GO:0003809: thrombin activity: 4.320e-09 (48,10,6) OR
GO:0004867: serine-type endopeptidase inhibitor activity: 1.238e-05 (48,47,7) OR
GO:0005509: calcium ion binding: 5.961e-03 (48,160,8) OR
GO:0005529: sugar binding: 5.024e-01 (48,39,1)

IF 1aym3_#131 AND 1gdna_#123
THEN
GO:0004263: chymotrypsin activity: 3.398e-14 (48,41,13) OR
GO:0004295: trypsin activity: 3.417e-13 (48,48,13) OR
GO:0003809: thrombin activity: 4.320e-09 (48,10,6) OR
GO:0004867: serine-type endopeptidase inhibitor activity: 1.349e-04 (48,47,6) OR
GO:0005509: calcium ion binding: 1.530e-03 (48,160,9) OR
GO:0004896: hematopoietin/interferon-class (D200-domain) cytokine receptor activity: 2.874e-01 (48,19,1)

IF 1aym3_#131 AND 1dlea_#53
THEN
GO:0004263: chymotrypsin activity: 3.398e-14 (48,41,13) OR
GO:0004295: trypsin activity: 3.417e-13 (48,48,13) OR
GO:0003809: thrombin activity: 4.320e-09 (48,10,6) OR
GO:0004867: serine-type endopeptidase inhibitor activity: 1.349e-04 (48,47,6) OR
GO:0005509: calcium ion binding: 1.530e-03 (48,160,9) OR
GO:0004896: hematopoietin/interferon-class (D200-domain) cytokine receptor activity: 2.874e-01 (48,19,1)

IF 1e9xa_#343 AND 2cuaa_#83
THEN
GO:0005507: copper ion binding: 3.417e-14 (15,38,9) OR
GO:0015078: hydrogen ion transporter activity: 5.592e-03 (15,21,2) OR
GO:0015082: di-, tri-valent inorganic cation transporter activity: 7.454e-02 (15,14,1) OR
GO:0046915: transition metal ion transporter activity: 7.454e-02 (15,14,1) OR
GO:0016638: oxidoreductase activity, acting on the CH-NH2 group of donors: 8.982e-02 (15,17,1) OR
GO:0005509: calcium ion binding: 5.975e-01 (15,160,1)

IF 1nksa_#13 AND 3grs_1#23
THEN

GO:0019201: nucleotide kinase activity: 5.186e-14 (28,13,8) OR
GO:0016776: phosphotransferase activity, phosphate group as acceptor: 1.202e-13 (28,14,8) OR
GO:0005524: ATP binding: 1.238e-05 (28,243,11) OR
GO:0005525: GTP binding: 3.999e-01 (28,49,1)

IF 1gpea1#273 AND 1e39a2#516
THEN
GO:0050660: FAD binding: 4.182e-14 (20,10,7) OR
GO:0015036: disulfide oxidoreductase activity: 4.764e-09 (20,22,6) OR
GO:0016668: oxidoreductase activity, acting on sulfur group of donors, NAD or NADP as acceptor: 9.530e-09 (20,12,5) OR
GO:0016651: oxidoreductase activity, acting on NADH or NADPH: 9.152e-02 (20,13,1) OR
GO:0004601: peroxidase activity: 1.438e-01 (20,21,1)

IF 1h7wa4#476 AND 3grs_2#211
THEN
GO:0015036: disulfide oxidoreductase activity: 4.209e-14 (25,22,9) OR
GO:0050660: FAD binding: 2.580e-13 (25,10,7) OR
GO:0016668: oxidoreductase activity, acting on sulfur group of donors, NAD or NADP as acceptor: 1.683e-12 (25,12,7) OR
GO:0016651: oxidoreductase activity, acting on NADH or NADPH: 1.132e-01 (25,13,1) OR
GO:0004601: peroxidase activity: 1.766e-01 (25,21,1)

IF 1gpea1#273 AND 1trb_1#41
THEN
GO:0015036: disulfide oxidoreductase activity: 4.209e-14 (25,22,9) OR
GO:0050660: FAD binding: 2.580e-13 (25,10,7) OR
GO:0016668: oxidoreductase activity, acting on sulfur group of donors, NAD or NADP as acceptor: 1.683e-12 (25,12,7) OR
GO:0016651: oxidoreductase activity, acting on NADH or NADPH: 1.132e-01 (25,13,1) OR
GO:0016627: oxidoreductase activity, acting on the CH-CH group of donors: 1.454e-01 (25,17,1)

IF 1fjsa_#28 AND 1fjsa_#83 AND 1elva1#513
THEN
GO:0004295: trypsin activity: 3.597e-14 (32,48,12) OR
GO:0004263: chymotrypsin activity: 2.178e-13 (32,41,11) OR
GO:0003809: thrombin activity: 5.670e-03 (32,10,2) OR
GO:0004867: serine-type endopeptidase inhibitor activity: 1.042e-01 (32,47,2) OR
GO:0005509: calcium ion binding: 1.147e-01 (32,160,4) OR
GO:0005529: sugar binding: 3.712e-01 (32,39,1)

IF 1f3ya_#6 AND 1bqya_#216 AND 1qaxa2#307
THEN
GO:0003809: thrombin activity: 3.635e-14 (39,10,8) OR
GO:0004263: chymotrypsin activity: 5.620e-08 (39,41,8) OR
GO:0004295: trypsin activity: 2.066e-07 (39,48,8) OR
GO:0004867: serine-type endopeptidase inhibitor activity: 4.031e-05 (39,47,6) OR
GO:0005509: calcium ion binding: 1.527e-03 (39,160,8) OR
GO:0005529: sugar binding: 4.323e-01 (39,39,1)

IF 1e44b_#75 AND 1c7na_#95 AND 1danh_#190
THEN
GO:0003809: thrombin activity: 3.635e-14 (39,10,8) OR
GO:0004263: chymotrypsin activity: 5.620e-08 (39,41,8) OR
GO:0004295: trypsin activity: 2.066e-07 (39,48,8) OR
GO:0004867: serine-type endopeptidase inhibitor activity: 4.031e-05 (39,47,6) OR
GO:0005509: calcium ion binding: 1.527e-03 (39,160,8) OR
GO:0005529: sugar binding: 4.323e-01 (39,39,1)

IF 1hcl__#267 AND 1koba_#175
THEN
GO:0004674: protein serine/threonine kinase activity: 7.532e-14 (21,42,10) OR
GO:0005524: ATP binding: 3.875e-06 (21,243,10) OR
GO:0005516: calmodulin binding: 1.701e-01 (21,24,1)

IF 1hhsa_#328 AND 1tgoa2#409
THEN
GO:0003887: DNA-directed DNA polymerase activity: 1.145e-13 (11,20,7) OR
GO:0008408: 3'-5' exonuclease activity: 1.010e-07 (11,13,4)

IF 1b16a_#184 AND 1ihua2#525
THEN
GO:0016616: oxidoreductase activity, acting on the CH-OH group of donors, NAD or NADP as acceptor: 1.167e-13 (12,59,9) OR

GO:0016627: oxidoreductase activity, acting on the CH-CH group of donors: 4.289e-05 (12,17,3)

IF 1cyda_#231 AND 1gcoa_#119
THEN
GO:0016616: oxidoreductase activity, acting on the CH-OH group of donors, NAD or NADP as acceptor: 1.167e-13 (12,59,9) OR
GO:0016627: oxidoreductase activity, acting on the CH-CH group of donors: 4.289e-05 (12,17,3)

IF 1cyda_#231 AND 1h5qa_#70
THEN
GO:0016616: oxidoreductase activity, acting on the CH-OH group of donors, NAD or NADP as acceptor: 1.167e-13 (12,59,9) OR
GO:0016627: oxidoreductase activity, acting on the CH-CH group of donors: 4.289e-05 (12,17,3)

IF 1nuka_#80 AND 1ton__#231
THEN
GO:0004295: trypsin activity: 3.940e-14 (52,48,14) OR
GO:0004263: chymotrypsin activity: 1.076e-13 (52,41,13) OR
GO:0004867: serine-type endopeptidase inhibitor activity: 1.798e-06 (52,47,8) OR
GO:0003809: thrombin activity: 2.277e-05 (52,10,4) OR
GO:0005509: calcium ion binding: 2.946e-05 (52,160,12) OR
GO:0008201: heparin binding: 3.715e-01 (52,24,1)

IF 1fbna_#145 AND 1qama_#39
THEN
GO:0008757: S-adenosylmethionine-dependent methyltransferase activity: 2.380e-13 (6,24,6)

IF 1qnja_#77 AND 1qs1a2#437
THEN
GO:0004263: chymotrypsin activity: 4.833e-14 (38,41,12) OR
GO:0004295: trypsin activity: 4.004e-13 (38,48,12) OR
GO:0004867: serine-type endopeptidase inhibitor activity: 4.027e-04 (38,47,5) OR
GO:0005509: calcium ion binding: 5.676e-03 (38,160,7) OR
GO:0003809: thrombin activity: 7.943e-03 (38,10,2)

IF 1aqua_#126 AND 1fc4a_#111
THEN
GO:0016846: carbon-sulfur lyase activity: 4.182e-14 (20,10,7) OR
GO:0008483: transaminase activity: 6.581e-12 (20,17,7) OR
GO:0016831: carboxy-lyase activity: 1.388e-02 (20,25,2) OR
GO:0004812: tRNA ligase activity: 1.750e-01 (20,26,1) OR
GO:0004497: monooxygenase activity: 1.750e-01 (20,26,1) OR
GO:0005524: ATP binding: 5.440e-01 (20,243,2)

IF 1hyha2#204 AND 1ihua2#525
THEN
GO:0004457: lactate dehydrogenase activity: 4.182e-14 (20,10,7) OR
GO:0016616: oxidoreductase activity, acting on the CH-OH group of donors, NAD or NADP as acceptor: 3.058e-09 (20,59,8) OR
GO:0005525: GTP binding: 4.899e-02 (20,49,2) OR
GO:0004725: protein tyrosine phosphatase activity: 1.049e-01 (20,15,1) OR
GO:0030145: manganese ion binding: 2.456e-01 (20,38,1) OR
GO:0005524: ATP binding: 8.466e-01 (20,243,1)

IF 1i5ga_#115 AND 1h6va2#245 AND 1trb_1#41 AND 1evqa_#305
THEN
GO:0015036: disulfide oxidoreductase activity: 5.315e-14 (16,22,8) OR
GO:0016668: oxidoreductase activity, acting on sulfur group of donors, NAD or NADP as acceptor: 3.820e-07 (16,12,4) OR
GO:0050660: FAD binding: 1.416e-03 (16,10,2) OR
GO:0016651: oxidoreductase activity, acting on NADH or NADPH: 7.386e-02 (16,13,1) OR
GO:0016627: oxidoreductase activity, acting on the CH-CH group of donors: 9.553e-02 (16,17,1)

IF 1glqa2#53 AND 1e6ca_#91
THEN
GO:0004364: glutathione transferase activity: 6.830e-14 (9,11,6) OR
GO:0001584: rhodopsin-like receptor activity: 3.259e-02 (9,10,1) OR
GO:0016651: oxidoreductase activity, acting on NADH or NADPH: 4.219e-02 (9,13,1) OR
GO:0005179: hormone activity: 4.853e-02 (9,15,1)

IF 1iow_1#37 AND 1ljra1#165 AND 1a0fa1#178
THEN
GO:0004364: glutathione transferase activity: 6.830e-14 (9,11,6) OR
GO:0003714: transcription corepressor activity: 2.615e-02 (9,8,1) OR

GO:0004812: tRNA ligase activity: 8.278e-02 (9,26,1) OR
GO:0005524: ATP binding: 5.691e-01 (9,243,1)

IF 1danh_#93 AND 1azza_#51 AND 1ekbb_#117 AND 1cgha_#227
THEN
GO:0004263: chymotrypsin activity: 4.580e-14 (49,41,13) OR
GO:0004295: trypsin activity: 4.594e-13 (49,48,13) OR
GO:0004867: serine-type endopeptidase inhibitor activity: 1.425e-05 (49,47,7) OR
GO:0003809: thrombin activity: 1.792e-05 (49,10,4) OR
GO:0005509: calcium ion binding: 8.430e-05 (49,160,11) OR
GO:0004896: hematopoietin/interferon-class (D200-domain) cytokine receptor activity: 2.924e-01 (49,19,1)

IF 1qrra_#208 AND 1eny__#94 AND 1oaa__#14
THEN
GO:0016616: oxidoreductase activity, acting on the CH-OH group of donors, NAD or NADP as acceptor: 5.549e-14 (21,59,11) OR
GO:0016627: oxidoreductase activity, acting on the CH-CH group of donors: 2.504e-04 (21,17,3) OR
GO:0016646: oxidoreductase activity, acting on the CH-NH group of donors, NAD or NADP as acceptor: 3.533e-04 (21,19,3) OR
GO:0016854: racemase and epimerase activity: 4.193e-03 (21,13,2) OR
GO:0016836: hydro-lyase activity: 2.587e-02 (21,33,2)

IF 1fxla2#169 AND 1bvza3#416
THEN
GO:0004556: alpha-amylase activity: 9.873e-14 (14,15,7) OR
GO:0005509: calcium ion binding: 7.566e-05 (14,160,6) OR
GO:0004867: serine-type endopeptidase inhibitor activity: 2.166e-01 (14,47,1)

IF 1gjwa2#160 AND 1jsg__#92
THEN
GO:0004556: alpha-amylase activity: 9.873e-14 (14,15,7) OR
GO:0005509: calcium ion binding: 7.566e-05 (14,160,6) OR
GO:0004867: serine-type endopeptidase inhibitor activity: 2.166e-01 (14,47,1)

IF 3grx__#56 AND 1bf2_3#575
THEN
GO:0004556: alpha-amylase activity: 9.873e-14 (14,15,7) OR
GO:0005509: calcium ion binding: 8.514e-04 (14,160,5) OR
GO:0004867: serine-type endopeptidase inhibitor activity: 2.322e-02 (14,47,2)

IF 1avaa2#204 AND 1aqua_#126
THEN
GO:0004556: alpha-amylase activity: 9.873e-14 (14,15,7) OR
GO:0005509: calcium ion binding: 8.514e-04 (14,160,5) OR
GO:0004867: serine-type endopeptidase inhibitor activity: 2.322e-02 (14,47,2)

IF 1avaa2#204 AND 1ayl__#237
THEN
GO:0004556: alpha-amylase activity: 9.873e-14 (14,15,7) OR
GO:0005509: calcium ion binding: 8.514e-04 (14,160,5) OR
GO:0004867: serine-type endopeptidase inhibitor activity: 2.322e-02 (14,47,2)

IF 1hx0a2#294 AND 1ds1a_#172
THEN
GO:0004556: alpha-amylase activity: 9.873e-14 (14,15,7) OR
GO:0005509: calcium ion binding: 7.566e-05 (14,160,6) OR
GO:0004867: serine-type endopeptidase inhibitor activity: 2.166e-01 (14,47,1)

IF 1djna1#86 AND 1bvza3#416
THEN
GO:0004556: alpha-amylase activity: 9.873e-14 (14,15,7) OR
GO:0005509: calcium ion binding: 7.566e-05 (14,160,6) OR
GO:0000287: magnesium ion binding: 4.910e-01 (14,128,1)

IF 1bvza3#416 AND 1vmoa_#106
THEN
GO:0004556: alpha-amylase activity: 9.873e-14 (14,15,7) OR
GO:0005509: calcium ion binding: 7.566e-05 (14,160,6) OR
GO:0004867: serine-type endopeptidase inhibitor activity: 2.166e-01 (14,47,1)

IF 1ja9a_#202 AND 1h5qa_#70
THEN

GO:0016616: oxidoreductase activity, acting on the CH-OH group of donors, NAD or NADP as acceptor: 7.444e-14 (16,59,10) OR
GO:0016627: oxidoreductase activity, acting on the CH-CH group of donors: 1.805e-06 (16,17,4) OR
GO:0008757: S-adenosylmethionine-dependent methyltransferase activity: 1.323e-01 (16,24,1) OR
GO:0000287: magnesium ion binding: 5.379e-01 (16,128,1)

IF 1ayl__#237 AND 1f3mc_#403
THEN
GO:0004674: protein serine/threonine kinase activity: 5.087e-14 (28,42,11) OR
GO:0005524: ATP binding: 1.617e-06 (28,243,12) OR
GO:0005516: calmodulin binding: 2.444e-02 (28,24,2) OR
GO:0005066: transmembrane receptor protein tyrosine kinase signaling protein activity: 9.828e-02 (28,10,1) OR
GO:0003755: peptidyl-prolyl cis-trans isomerase activity: 1.076e-01 (28,11,1) OR
GO:0004896: hematopoietin/interferon-class (D200-domain) cytokine receptor activity: 1.787e-01 (28,19,1)

IF 1cyx__#133 AND 1e30a_#87
THEN
GO:0005507: copper ion binding: 7.735e-14 (16,38,9) OR
GO:0015078: hydrogen ion transporter activity: 6.750e-08 (16,21,5) OR
GO:0015082: di-, tri-valent inorganic cation transporter activity: 7.932e-02 (16,14,1) OR
GO:0046915: transition metal ion transporter activity: 7.932e-02 (16,14,1)

IF 1qgna_#149 AND 1rypb_#114
THEN
GO:0016846: carbon-sulfur lyase activity: 7.758e-14 (10,10,6) OR
GO:0008483: transaminase activity: 1.601e-03 (10,17,2) OR
GO:0004180: carboxypeptidase activity: 5.379e-02 (10,15,1) OR
GO:0016831: carboxy-lyase activity: 8.819e-02 (10,25,1)

IF 1hc7a2#113 AND 1hc7a2#111 AND 1hc7a2#109
THEN
GO:0004812: tRNA ligase activity: 6.409e-14 (22,26,9) OR
GO:0005524: ATP binding: 5.438e-05 (22,243,9) OR
GO:0000049: tRNA binding: 1.002e-01 (22,13,1) OR
GO:0003887: DNA-directed DNA polymerase activity: 1.501e-01 (22,20,1) OR
GO:0000287: magnesium ion binding: 2.767e-01 (22,128,2)

IF 1gdha1#235 AND 1e6ua_#10
THEN
GO:0016616: oxidoreductase activity, acting on the CH-OH group of donors, NAD or NADP as acceptor: 1.167e-13 (12,59,9) OR
GO:0016627: oxidoreductase activity, acting on the CH-CH group of donors: 2.331e-03 (12,17,2) OR
GO:0016646: oxidoreductase activity, acting on the CH-NH group of donors, NAD or NADP as acceptor: 8.069e-02 (12,19,1)

IF 1fmca_#248 AND 1e6ua_#10
THEN
GO:0016616: oxidoreductase activity, acting on the CH-OH group of donors, NAD or NADP as acceptor: 1.167e-13 (12,59,9) OR
GO:0016627: oxidoreductase activity, acting on the CH-CH group of donors: 2.331e-03 (12,17,2) OR
GO:0016646: oxidoreductase activity, acting on the CH-NH group of donors, NAD or NADP as acceptor: 8.069e-02 (12,19,1)

IF 1qrra_#208 AND 1eno__#264
THEN
GO:0016616: oxidoreductase activity, acting on the CH-OH group of donors, NAD or NADP as acceptor: 1.167e-13 (12,59,9) OR
GO:0016627: oxidoreductase activity, acting on the CH-CH group of donors: 2.331e-03 (12,17,2) OR
GO:0016646: oxidoreductase activity, acting on the CH-NH group of donors, NAD or NADP as acceptor: 8.069e-02 (12,19,1)

IF 1fmca_#248 AND 1aqua_#126
THEN
GO:0016616: oxidoreductase activity, acting on the CH-OH group of donors, NAD or NADP as acceptor: 1.167e-13 (12,59,9) OR
GO:0016627: oxidoreductase activity, acting on the CH-CH group of donors: 2.331e-03 (12,17,2) OR
GO:0016646: oxidoreductase activity, acting on the CH-NH group of donors, NAD or NADP as acceptor: 8.069e-02 (12,19,1)

IF 1hwxa1#256 AND 1ct9a1#233
THEN
GO:0016616: oxidoreductase activity, acting on the CH-OH group of donors, NAD or NADP as acceptor: 1.167e-13 (12,59,9) OR
GO:0015036: disulfide oxidoreductase activity: 3.911e-03 (12,22,2) OR
GO:0016620: oxidoreductase activity, acting on the aldehyde or oxo group of donors, NAD or NADP as acceptor: 4.324e-02 (12,10,1)

IF 1sgpe_#42 AND 1qnja_#138
THEN
GO:0004295: trypsin activity: 8.798e-14 (19,48,10) OR
GO:0004263: chymotrypsin activity: 1.867e-07 (19,41,6) OR

GO:0004867: serine-type endopeptidase inhibitor activity: 4.133e-02 (19,47,2) OR
GO:0005509: calcium ion binding: 6.845e-01 (19,160,1)

IF 1g0sa_#74 AND 1fl2a1#320
THEN
GO:0050660: FAD binding: 9.182e-14 (22,10,7) OR
GO:0016668: oxidoreductase activity, acting on sulfur group of donors, NAD or NADP as acceptor: 6.001e-13 (22,12,7) OR
GO:0015036: disulfide oxidoreductase activity: 1.231e-10 (22,22,7) OR
GO:0016638: oxidoreductase activity, acting on the CH-NH2 group of donors: 1.291e-01 (22,17,1)

IF 1h7wa4#476 AND 1dyna_#42
THEN
GO:0050660: FAD binding: 9.182e-14 (22,10,7) OR
GO:0016668: oxidoreductase activity, acting on sulfur group of donors, NAD or NADP as acceptor: 6.001e-13 (22,12,7) OR
GO:0015036: disulfide oxidoreductase activity: 1.231e-10 (22,22,7) OR
GO:0016651: oxidoreductase activity, acting on NADH or NADPH: 1.002e-01 (22,13,1)

IF 1dpga2#334 AND 1a0la_#160 AND 1arb__#210
THEN
GO:0004263: chymotrypsin activity: 6.129e-14 (50,41,13) OR
GO:0004295: trypsin activity: 6.133e-13 (50,48,13) OR
GO:0003809: thrombin activity: 3.993e-07 (50,10,5) OR
GO:0004867: serine-type endopeptidase inhibitor activity: 1.317e-06 (50,47,8) OR
GO:0005509: calcium ion binding: 4.891e-04 (50,160,10) OR
GO:0005529: sugar binding: 5.168e-01 (50,39,1)

IF 1pd211#147 AND 2sqca2#268
THEN
GO:0004364: glutathione transferase activity: 3.703e-13 (5,11,5)

IF 1d4xg_#60 AND 2gsq_2#50
THEN
GO:0004364: glutathione transferase activity: 3.703e-13 (5,11,5)

IF 1glqa1#148
THEN
GO:0004364: glutathione transferase activity: 3.703e-13 (5,11,5)

IF 1ljra1#165 AND 1i50a_#469
THEN
GO:0004364: glutathione transferase activity: 3.703e-13 (5,11,5)

IF 1qlsa_#81 AND 1hqva_#48 AND 1jbaa_#157 AND 1ab4__#89
THEN
GO:0005509: calcium ion binding: 3.716e-13 (10,160,10)

IF 1oaa__#175 AND 1bdb__#228
THEN
GO:0016616: oxidoreductase activity, acting on the CH-OH group of donors, NAD or NADP as acceptor: 7.444e-14 (16,59,10) OR
GO:0016854: racemase and epimerase activity: 2.429e-03 (16,13,2) OR
GO:0016627: oxidoreductase activity, acting on the CH-CH group of donors: 4.177e-03 (16,17,2) OR
GO:0016646: oxidoreductase activity, acting on the CH-NH group of donors, NAD or NADP as acceptor: 1.062e-01 (16,19,1) OR
GO:0016836: hydro-lyase activity: 1.776e-01 (16,33,1)

IF 1bdb__#228 AND 1e6ua_#10
THEN
GO:0016616: oxidoreductase activity, acting on the CH-OH group of donors, NAD or NADP as acceptor: 7.444e-14 (16,59,10) OR
GO:0016854: racemase and epimerase activity: 2.429e-03 (16,13,2) OR
GO:0016627: oxidoreductase activity, acting on the CH-CH group of donors: 4.177e-03 (16,17,2) OR
GO:0016646: oxidoreductase activity, acting on the CH-NH group of donors, NAD or NADP as acceptor: 1.062e-01 (16,19,1) OR
GO:0016836: hydro-lyase activity: 1.776e-01 (16,33,1)

IF 1jb3a_#64 AND 1qb7a_#86
THEN
GO:0004457: lactate dehydrogenase activity: 6.266e-14 (21,10,7) OR
GO:0016616: oxidoreductase activity, acting on the CH-OH group of donors, NAD or NADP as acceptor: 4.857e-09 (21,59,8) OR
GO:0004180: carboxypeptidase activity: 5.592e-03 (21,15,2) OR
GO:0004725: protein tyrosine phosphatase activity: 5.592e-03 (21,15,2) OR
GO:0003916: DNA topoisomerase activity: 7.456e-02 (21,10,1) OR
GO:0008080: N-acetyltransferase activity: 9.588e-02 (21,13,1)

IF 1avaa2#204 AND 1dxea_#71
THEN
GO:0004556: alpha-amylase activity: 9.873e-14 (14,15,7) OR
GO:0005509: calcium ion binding: 8.514e-04 (14,160,5) OR
GO:0016758: transferase activity, transferring hexosyl groups: 5.518e-02 (14,11,1) OR
GO:0004867: serine-type endopeptidase inhibitor activity: 2.166e-01 (14,47,1)

IF 1danh_#142 AND 1e5ka_#113
THEN
GO:0004263: chymotrypsin activity: 8.146e-14 (51,41,13) OR
GO:0004295: trypsin activity: 8.132e-13 (51,48,13) OR
GO:0003809: thrombin activity: 6.316e-09 (51,10,6) OR
GO:0004867: serine-type endopeptidase inhibitor activity: 1.542e-06 (51,47,8) OR
GO:0005509: calcium ion binding: 1.243e-04 (51,160,11)

IF 1e7wa_#8 AND 2ae2a_#17
THEN
GO:0016616: oxidoreductase activity, acting on the CH-OH group of donors, NAD or NADP as acceptor: 7.444e-14 (16,59,10) OR
GO:0016627: oxidoreductase activity, acting on the CH-CH group of donors: 4.177e-03 (16,17,2) OR
GO:0016620: oxidoreductase activity, acting on the aldehyde or oxo group of donors, NAD or NADP as acceptor: 5.728e-02 (16,10,1) OR
GO:0016854: racemase and epimerase activity: 7.386e-02 (16,13,1) OR
GO:0016646: oxidoreductase activity, acting on the CH-NH group of donors, NAD or NADP as acceptor: 1.062e-01 (16,19,1) OR
GO:0005524: ATP binding: 7.766e-01 (16,243,1)

IF 1qfma2#576 AND 1hu4a_#267 AND 1fmca_#89
THEN
GO:0016616: oxidoreductase activity, acting on the CH-OH group of donors, NAD or NADP as acceptor: 1.167e-13 (12,59,9) OR
GO:0016646: oxidoreductase activity, acting on the CH-NH group of donors, NAD or NADP as acceptor: 8.069e-02 (12,19,1) OR
GO:0016763: transferase activity, transferring pentosyl groups: 1.168e-01 (12,28,1) OR
GO:0003700: transcription factor activity: 4.288e-01 (12,124,1)

IF 1b16a_#184 AND 5ruba1#365
THEN
GO:0016616: oxidoreductase activity, acting on the CH-OH group of donors, NAD or NADP as acceptor: 1.167e-13 (12,59,9) OR
GO:0016627: oxidoreductase activity, acting on the CH-CH group of donors: 7.249e-02 (12,17,1) OR
GO:0016646: oxidoreductase activity, acting on the CH-NH group of donors, NAD or NADP as acceptor: 8.069e-02 (12,19,1) OR
GO:0016836: hydro-lyase activity: 1.363e-01 (12,33,1)

IF 1e6ua_#10 AND 1tyfa_#31
THEN
GO:0016616: oxidoreductase activity, acting on the CH-OH group of donors, NAD or NADP as acceptor: 1.167e-13 (12,59,9) OR
GO:0016854: racemase and epimerase activity: 5.588e-02 (12,13,1) OR
GO:0008757: S-adenosylmethionine-dependent methyltransferase activity: 1.009e-01 (12,24,1) OR
GO:0000287: magnesium ion binding: 4.393e-01 (12,128,1)

IF 1ia8a_#59 AND 1e4ea2#215 AND 1ir3a_#1192
THEN
GO:0004674: protein serine/threonine kinase activity: 8.109e-14 (29,42,11) OR
GO:0005524: ATP binding: 2.543e-06 (29,243,12) OR
GO:0005516: calmodulin binding: 1.888e-03 (29,24,3) OR
GO:0004714: transmembrane receptor protein tyrosine kinase activity: 1.394e-01 (29,14,1) OR
GO:0004896: hematopoietin/interferon-class (D200-domain) cytokine receptor activity: 1.845e-01 (29,19,1) OR
GO:0008201: heparin binding: 2.273e-01 (29,24,1)

IF 1danh_#242 AND 1eq9a_#162 AND 1gg6.1#C229
THEN
GO:0004263: chymotrypsin activity: 9.776e-14 (40,41,12) OR
GO:0004295: trypsin activity: 8.059e-13 (40,48,12) OR
GO:0004867: serine-type endopeptidase inhibitor activity: 4.681e-05 (40,47,6) OR
GO:0005509: calcium ion binding: 1.815e-03 (40,160,8) OR
GO:0003809: thrombin activity: 8.778e-03 (40,10,2)

IF 1eh9a3#102 AND 1a8d_1#87
THEN
GO:0004556: alpha-amylase activity: 2.464e-13 (8,15,6) OR
GO:0005509: calcium ion binding: 7.596e-02 (8,160,2)

IF 1gox__#124 AND 1trb_1#41

THEN
GO:0015036: disulfide oxidoreductase activity: 9.993e-14 (17,22,8) OR
GO:0016668: oxidoreductase activity, acting on sulfur group of donors, NAD or NADP as acceptor: 4.984e-07 (17,12,4) OR
GO:0050660: FAD binding: 2.358e-05 (17,10,3) OR
GO:0016651: oxidoreductase activity, acting on NADH or NADPH: 7.830e-02 (17,13,1) OR
GO:0016627: oxidoreductase activity, acting on the CH-CH group of donors: 1.012e-01 (17,17,1)

IF 1fjsa_#28 AND 1gdna_#155 AND 1dlea_#238
THEN
GO:0004295: trypsin activity: 8.521e-14 (34,48,12) OR
GO:0004263: chymotrypsin activity: 4.731e-13 (34,41,11) OR
GO:0003809: thrombin activity: 6.388e-03 (34,10,2) OR
GO:0004867: serine-type endopeptidase inhibitor activity: 1.980e-02 (34,47,3) OR
GO:0005509: calcium ion binding: 4.579e-02 (34,160,5) OR
GO:0005529: sugar binding: 3.893e-01 (34,39,1)

IF 1fjsa_#28 AND 1bio__#108 AND 1dlea_#238
THEN
GO:0004295: trypsin activity: 8.521e-14 (34,48,12) OR
GO:0004263: chymotrypsin activity: 4.731e-13 (34,41,11) OR
GO:0003809: thrombin activity: 6.388e-03 (34,10,2) OR
GO:0004867: serine-type endopeptidase inhibitor activity: 1.980e-02 (34,47,3) OR
GO:0005509: calcium ion binding: 4.579e-02 (34,160,5) OR
GO:0005529: sugar binding: 3.893e-01 (34,39,1)

IF 1gega_#58 AND 1b4ka_#108
THEN
GO:0016616: oxidoreductase activity, acting on the CH-OH group of donors, NAD or NADP as acceptor: 2.629e-13 (9,59,8) OR
GO:0030151: molybdenum ion binding: 4.853e-02 (9,15,1)

IF 1epwa3#138 AND 1fi2a_#109 AND 1mpp__#29 AND 1imva_#255 AND 1htr.1#B39
THEN
GO:0004190: aspartic-type endopeptidase activity: 1.319e-13 (10,23,7) OR
GO:0003964: RNA-directed DNA polymerase activity: 3.971e-02 (10,11,1) OR
GO:0004523: ribonuclease H activity: 5.728e-02 (10,16,1) OR
GO:0008270: zinc ion binding: 3.331e-01 (10,108,1)

IF 1fvua_#72 AND 1qo3c_#252
THEN
GO:0005529: sugar binding: 1.319e-13 (11,39,8) OR
GO:0004263: chymotrypsin activity: 1.539e-01 (11,41,1) OR
GO:0004295: trypsin activity: 1.779e-01 (11,48,1) OR
GO:0005509: calcium ion binding: 4.867e-01 (11,160,1)

IF 1esl_1#29 AND 1qo3c_#195
THEN
GO:0005529: sugar binding: 1.319e-13 (11,39,8) OR
GO:0004263: chymotrypsin activity: 1.539e-01 (11,41,1) OR
GO:0004295: trypsin activity: 1.779e-01 (11,48,1) OR
GO:0005509: calcium ion binding: 4.867e-01 (11,160,1)

IF 1fvua_#72 AND 1esl_1#29
THEN
GO:0005529: sugar binding: 1.319e-13 (11,39,8) OR
GO:0004263: chymotrypsin activity: 1.539e-01 (11,41,1) OR
GO:0004295: trypsin activity: 1.779e-01 (11,48,1) OR
GO:0005509: calcium ion binding: 4.867e-01 (11,160,1)

IF 1htp__#16 AND 1cjca2#365
THEN
GO:0050660: FAD binding: 9.182e-14 (22,10,7) OR
GO:0016668: oxidoreductase activity, acting on sulfur group of donors, NAD or NADP as acceptor: 1.183e-10 (22,12,6) OR
GO:0015036: disulfide oxidoreductase activity: 9.077e-09 (22,22,6) OR
GO:0016651: oxidoreductase activity, acting on NADH or NADPH: 1.002e-01 (22,13,1) OR
GO:0016638: oxidoreductase activity, acting on the CH-NH2 group of donors: 1.291e-01 (22,17,1) OR
GO:0000287: magnesium ion binding: 6.545e-01 (22,128,1)

IF 1bvza3#416 AND 1rypk_#12
THEN
GO:0004556: alpha-amylase activity: 1.846e-13 (15,15,7) OR

GO:0005509: calcium ion binding: 9.125e-06 (15,160,7) OR
GO:0004867: serine-type endopeptidase inhibitor activity: 2.302e-01 (15,47,1)

IF 1e43a2#236 AND 1a9xa3#78
THEN
GO:0004556: alpha-amylase activity: 1.846e-13 (15,15,7) OR
GO:0005509: calcium ion binding: 1.200e-04 (15,160,6) OR
GO:0004867: serine-type endopeptidase inhibitor activity: 2.651e-02 (15,47,2)

IF 1e05i_#422 AND 1a7ca_#370
THEN
GO:0004867: serine-type endopeptidase inhibitor activity: 1.848e-13 (10,47,8) OR
GO:0008201: heparin binding: 8.480e-02 (10,24,1) OR
GO:0016763: transferase activity, transferring pentosyl groups: 9.828e-02 (10,28,1)

IF 1ovaa_#161 AND 1as4.1#A288
THEN
GO:0004867: serine-type endopeptidase inhibitor activity: 1.848e-13 (10,47,8) OR
GO:0008201: heparin binding: 8.480e-02 (10,24,1) OR
GO:0005525: GTP binding: 1.662e-01 (10,49,1)

IF 1sek__#383 AND 1a7ca_#202
THEN
GO:0004867: serine-type endopeptidase inhibitor activity: 1.848e-13 (10,47,8) OR
GO:0008201: heparin binding: 8.480e-02 (10,24,1) OR
GO:0005509: calcium ion binding: 4.545e-01 (10,160,1)

IF 1a7ca_#204 AND 1ovaa_#161
THEN
GO:0004867: serine-type endopeptidase inhibitor activity: 1.848e-13 (10,47,8) OR
GO:0046983: protein dimerization activity: 6.076e-02 (10,17,1) OR
GO:0008201: heparin binding: 8.480e-02 (10,24,1)

IF 1ovaa_#161 AND 1imva_#69
THEN
GO:0004867: serine-type endopeptidase inhibitor activity: 1.848e-13 (10,47,8) OR
GO:0015078: hydrogen ion transporter activity: 7.456e-02 (10,21,1) OR
GO:0008201: heparin binding: 8.480e-02 (10,24,1)

IF 1ovaa_#161 AND 1a7ca_#370
THEN
GO:0004867: serine-type endopeptidase inhibitor activity: 1.848e-13 (10,47,8) OR
GO:0008201: heparin binding: 8.480e-02 (10,24,1) OR
GO:0051082: unfolded protein binding: 1.182e-01 (10,34,1)

IF 1d5ra2#25 AND 1fsu__#434
THEN
GO:0004725: protein tyrosine phosphatase activity: 9.873e-14 (14,15,7) OR
GO:0005525: GTP binding: 1.731e-03 (14,49,3) OR
GO:0016854: racemase and epimerase activity: 6.491e-02 (14,13,1) OR
GO:0005096: GTPase activator activity: 7.454e-02 (14,15,1) OR
GO:0003743: translation initiation factor activity: 7.454e-02 (14,15,1) OR
GO:0003924: GTPase activity: 8.408e-02 (14,17,1)

IF 1mjha_#126 AND 1dfoa_#344
THEN
GO:0008483: transaminase activity: 1.490e-13 (13,17,7) OR
GO:0016846: carbon-sulfur lyase activity: 6.446e-08 (13,10,4) OR
GO:0004812: tRNA ligase activity: 1.174e-01 (13,26,1) OR
GO:0005524: ATP binding: 7.039e-01 (13,243,1)

IF 1gdna_#119 AND 1eq9a_#162
THEN
GO:0004295: trypsin activity: 1.594e-13 (27,48,11) OR
GO:0004263: chymotrypsin activity: 1.294e-12 (27,41,10) OR
GO:0004867: serine-type endopeptidase inhibitor activity: 1.053e-02 (27,47,3) OR
GO:0005509: calcium ion binding: 2.089e-01 (27,160,3)

IF 1qf6a4#295 AND 1pysb5#672
THEN

GO:0004812: tRNA ligase activity: 1.666e-13 (24,26,9) OR
GO:0005524: ATP binding: 1.218e-04 (24,243,9) OR
GO:0000049: tRNA binding: 5.467e-03 (24,13,2) OR
GO:0000287: magnesium ion binding: 2.376e-02 (24,128,4)

IF 1pysb5#672 AND 1qf6a4#362
THEN
GO:0004812: tRNA ligase activity: 1.666e-13 (24,26,9) OR
GO:0005524: ATP binding: 1.218e-04 (24,243,9) OR
GO:0000049: tRNA binding: 5.467e-03 (24,13,2) OR
GO:0000287: magnesium ion binding: 2.376e-02 (24,128,4)

IF 1hc7a2#111 AND 1cs1a_#82
THEN
GO:0004812: tRNA ligase activity: 1.666e-13 (24,26,9) OR
GO:0005524: ATP binding: 1.218e-04 (24,243,9) OR
GO:0000049: tRNA binding: 5.467e-03 (24,13,2) OR
GO:0000287: magnesium ion binding: 2.376e-02 (24,128,4)

IF 1atia2#67 AND 1cs1a_#82
THEN
GO:0004812: tRNA ligase activity: 1.666e-13 (24,26,9) OR
GO:0005524: ATP binding: 1.218e-04 (24,243,9) OR
GO:0000049: tRNA binding: 5.467e-03 (24,13,2) OR
GO:0000287: magnesium ion binding: 2.376e-02 (24,128,4)

IF 1ceqa2#286 AND 1qama_#37
THEN
GO:0004457: lactate dehydrogenase activity: 3.405e-13 (12,10,6) OR
GO:0016616: oxidoreductase activity, acting on the CH-OH group of donors, NAD or NADP as acceptor: 6.654e-08 (12,59,6)

IF 1fjsa_#84 AND 1ekbb_#85 AND 2hlca_#68
THEN
GO:0004263: chymotrypsin activity: 1.368e-13 (41,41,12) OR
GO:0004295: trypsin activity: 1.125e-12 (41,48,12) OR
GO:0004867: serine-type endopeptidase inhibitor activity: 5.413e-05 (41,47,6) OR
GO:0005509: calcium ion binding: 4.560e-04 (41,160,9) OR
GO:0003809: thrombin activity: 9.210e-03 (41,10,2)

IF 1fcda2#189 AND 1nhp_1#282
THEN
GO:0015036: disulfide oxidoreductase activity: 1.791e-13 (18,22,8) OR
GO:0016668: oxidoreductase activity, acting on sulfur group of donors, NAD or NADP as acceptor: 5.289e-09 (18,12,5) OR
GO:0050660: FAD binding: 2.735e-07 (18,10,4) OR
GO:0016627: oxidoreductase activity, acting on the CH-CH group of donors: 1.068e-01 (18,17,1)

IF 1gdna_#230 AND 1ejda_#213
THEN
GO:0004263: chymotrypsin activity: 1.444e-13 (31,41,11) OR
GO:0004295: trypsin activity: 9.844e-13 (31,48,11) OR
GO:0004867: serine-type endopeptidase inhibitor activity: 1.542e-02 (31,47,3) OR
GO:0005509: calcium ion binding: 3.218e-02 (31,160,5) OR
GO:0003809: thrombin activity: 1.083e-01 (31,10,1)

IF 1d5ra2#25 AND 1qfma1#186
THEN
GO:0004725: protein tyrosine phosphatase activity: 2.464e-13 (8,15,6) OR
GO:0016799: hydrolase activity, hydrolyzing N-glycosyl compounds: 4.889e-02 (8,17,1) OR
GO:0005529: sugar binding: 1.091e-01 (8,39,1)

IF 1deua_#186 AND 2cb5a_#373
THEN
GO:0004197: cysteine-type endopeptidase activity: 1.860e-13 (10,24,7) OR
GO:0019955: cytokine binding: 3.971e-02 (10,11,1) OR
GO:0042802: protein self binding: 4.324e-02 (10,12,1) OR
GO:0051082: unfolded protein binding: 1.182e-01 (10,34,1)

IF 1thea_#79 AND 1cs8a_#188
THEN
GO:0004197: cysteine-type endopeptidase activity: 1.860e-13 (10,24,7) OR

GO:0019955: cytokine binding: 3.971e-02 (10,11,1) OR
GO:0042802: protein self binding: 4.324e-02 (10,12,1) OR
GO:0051082: unfolded protein binding: 1.182e-01 (10,34,1)

IF 1thea_#79 AND 1dkia_#54
THEN
GO:0004197: cysteine-type endopeptidase activity: 1.860e-13 (10,24,7) OR
GO:0019955: cytokine binding: 3.971e-02 (10,11,1) OR
GO:0042802: protein self binding: 4.324e-02 (10,12,1) OR
GO:0051082: unfolded protein binding: 1.182e-01 (10,34,1)

IF 1deua_#186 AND 3gcb__#78
THEN
GO:0004197: cysteine-type endopeptidase activity: 1.860e-13 (10,24,7) OR
GO:0019955: cytokine binding: 3.971e-02 (10,11,1) OR
GO:0042802: protein self binding: 4.324e-02 (10,12,1) OR
GO:0051082: unfolded protein binding: 1.182e-01 (10,34,1)

IF 2cpl__#53 AND 1dkia_#54
THEN
GO:0004197: cysteine-type endopeptidase activity: 1.860e-13 (10,24,7) OR
GO:0019955: cytokine binding: 3.971e-02 (10,11,1) OR
GO:0042802: protein self binding: 4.324e-02 (10,12,1) OR
GO:0051082: unfolded protein binding: 1.182e-01 (10,34,1)

IF 1deua_#57 AND 1thea_#79
THEN
GO:0004197: cysteine-type endopeptidase activity: 1.860e-13 (10,24,7) OR
GO:0019955: cytokine binding: 3.971e-02 (10,11,1) OR
GO:0042802: protein self binding: 4.324e-02 (10,12,1) OR
GO:0051082: unfolded protein binding: 1.182e-01 (10,34,1)

IF 1deua_#186 AND 1dkia_#185
THEN
GO:0004197: cysteine-type endopeptidase activity: 1.860e-13 (10,24,7) OR
GO:0019955: cytokine binding: 3.971e-02 (10,11,1) OR
GO:0042802: protein self binding: 4.324e-02 (10,12,1) OR
GO:0051082: unfolded protein binding: 1.182e-01 (10,34,1)

IF 1deua_#186 AND 1deua_#57
THEN
GO:0004197: cysteine-type endopeptidase activity: 1.860e-13 (10,24,7) OR
GO:0019955: cytokine binding: 3.971e-02 (10,11,1) OR
GO:0042802: protein self binding: 4.324e-02 (10,12,1) OR
GO:0051082: unfolded protein binding: 1.182e-01 (10,34,1)

IF 1deua_#186 AND 1cs8a_#188
THEN
GO:0004197: cysteine-type endopeptidase activity: 1.860e-13 (10,24,7) OR
GO:0019955: cytokine binding: 3.971e-02 (10,11,1) OR
GO:0042802: protein self binding: 4.324e-02 (10,12,1) OR
GO:0051082: unfolded protein binding: 1.182e-01 (10,34,1)

IF 1mrj__#156 AND 1mrj__#161
THEN
GO:0016799: hydrolase activity, hydrolyzing N-glycosyl compounds: 1.490e-13 (13,17,7) OR
GO:0005529: sugar binding: 7.024e-04 (13,39,3) OR
GO:0003899: DNA-directed RNA polymerase activity: 5.588e-02 (13,12,1) OR
GO:0046983: protein dimerization activity: 7.830e-02 (13,17,1) OR
GO:0016705: oxidoreductase activity, acting on paired donors, with incorporation or reduction of molecular oxygen: 1.174e-01 (13,26,1)

IF 2dkb__#46 AND 1qtn.1#A270
THEN
GO:0008483: transaminase activity: 1.490e-13 (13,17,7) OR
GO:0016846: carbon-sulfur lyase activity: 9.993e-06 (13,10,3) OR
GO:0016866: intramolecular transferase activity: 5.588e-02 (13,12,1) OR
GO:0004812: tRNA ligase activity: 1.174e-01 (13,26,1) OR
GO:0005524: ATP binding: 7.039e-01 (13,243,1)

IF 1ia8a_#59 AND 1a06__#103

THEN
GO:0004674: protein serine/threonine kinase activity: 1.267e-13 (30,42,11) OR
GO:0005524: ATP binding: 4.978e-07 (30,243,13) OR
GO:0005516: calmodulin binding: 2.086e-03 (30,24,3) OR
GO:0004714: transmembrane receptor protein tyrosine kinase activity: 1.439e-01 (30,14,1) OR
GO:0004896: hematopoietin/interferon-class (D200-domain) cytokine receptor activity: 1.903e-01 (30,19,1) OR
GO:0008201: heparin binding: 2.342e-01 (30,24,1)

IF 1cjca2#103 AND 1fjfc2#153
THEN
GO:0050660: FAD binding: 1.319e-13 (23,10,7) OR
GO:0016668: oxidoreductase activity, acting on sulfur group of donors, NAD or NADP as acceptor: 1.597e-10 (23,12,6) OR
GO:0015036: disulfide oxidoreductase activity: 1.761e-10 (23,22,7) OR
GO:0016651: oxidoreductase activity, acting on NADH or NADPH: 1.046e-01 (23,13,1) OR
GO:0016705: oxidoreductase activity, acting on paired donors, with incorporation or reduction of molecular oxygen: 1.986e-01 (23,26,1) OR
GO:0004497: monooxygenase activity: 1.986e-01 (23,26,1)

IF 2occb1#189 AND 1e30a_#87
THEN
GO:0005507: copper ion binding: 1.628e-13 (17,38,9) OR
GO:0015078: hydrogen ion transporter activity: 9.515e-08 (17,21,5) OR
GO:0015082: di-, tri-valent inorganic cation transporter activity: 8.408e-02 (17,14,1) OR
GO:0046915: transition metal ion transporter activity: 8.408e-02 (17,14,1) OR
GO:0005509: calcium ion binding: 6.436e-01 (17,160,1)

IF 1gg6.1#B19 AND 1dpga2#334 AND 1befa_#51 AND 1dy9.1#A44
THEN
GO:0004263: chymotrypsin activity: 1.368e-13 (41,41,12) OR
GO:0004295: trypsin activity: 1.125e-12 (41,48,12) OR
GO:0003809: thrombin activity: 3.528e-04 (41,10,3) OR
GO:0004867: serine-type endopeptidase inhibitor activity: 5.784e-04 (41,47,5) OR
GO:0005509: calcium ion binding: 2.144e-03 (41,160,8) OR
GO:0005529: sugar binding: 4.487e-01 (41,39,1)

IF 1qnja_#77 AND 1bjt__#890
THEN
GO:0004263: chymotrypsin activity: 1.368e-13 (41,41,12) OR
GO:0004295: trypsin activity: 1.125e-12 (41,48,12) OR
GO:0004867: serine-type endopeptidase inhibitor activity: 5.413e-05 (41,47,6) OR
GO:0005509: calcium ion binding: 4.560e-04 (41,160,9) OR
GO:0003809: thrombin activity: 1.409e-01 (41,10,1) OR
GO:0004896: hematopoietin/interferon-class (D200-domain) cytokine receptor activity: 2.510e-01 (41,19,1)

IF 1h7wa4#476 AND 1g0sa_#74
THEN
GO:0016668: oxidoreductase activity, acting on sulfur group of donors, NAD or NADP as acceptor: 2.737e-13 (20,12,7) OR
GO:0050660: FAD binding: 1.414e-11 (20,10,6) OR
GO:0015036: disulfide oxidoreductase activity: 5.651e-11 (20,22,7)

IF 1iq4a_#31 AND 1tgoa2#409
THEN
GO:0003887: DNA-directed DNA polymerase activity: 2.737e-13 (12,20,7) OR
GO:0008408: 3'-5' exonuclease activity: 1.511e-07 (12,13,4) OR
GO:0000287: magnesium ion binding: 4.393e-01 (12,128,1)

IF 1tgoa2#409 AND 1fjfc2#153
THEN
GO:0003887: DNA-directed DNA polymerase activity: 2.737e-13 (12,20,7) OR
GO:0008408: 3'-5' exonuclease activity: 1.511e-07 (12,13,4) OR
GO:0005524: ATP binding: 6.748e-01 (12,243,1)

IF 1ljra1#165 AND 1aba__#17
THEN
GO:0004364: glutathione transferase activity: 1.705e-13 (10,11,6) OR
GO:0003714: transcription corepressor activity: 2.902e-02 (10,8,1) OR
GO:0003682: chromatin binding: 3.616e-02 (10,10,1) OR
GO:0003924: GTPase activity: 6.076e-02 (10,17,1) OR
GO:0005525: GTP binding: 1.662e-01 (10,49,1)

IF 1ljra1#165 AND 1nbaa_#227
THEN
GO:0004364: glutathione transferase activity: 1.705e-13 (10,11,6) OR
GO:0016651: oxidoreductase activity, acting on NADH or NADPH: 4.677e-02 (10,13,1) OR
GO:0004812: tRNA ligase activity: 9.156e-02 (10,26,1) OR
GO:0005509: calcium ion binding: 4.545e-01 (10,160,1) OR
GO:0005524: ATP binding: 6.077e-01 (10,243,1)

IF 1ekbb_#117 AND 1elva1#513 AND 1ekbb_#85 AND 1elva1#601 AND 1ejda_#213 AND 1dlea_#198 AND 1cgha_#130
THEN
GO:0004263: chymotrypsin activity: 2.178e-13 (32,41,11) OR
GO:0004295: trypsin activity: 1.481e-12 (32,48,11) OR
GO:0004867: serine-type endopeptidase inhibitor activity: 1.746e-04 (32,47,5) OR
GO:0005509: calcium ion binding: 3.639e-02 (32,160,5)

IF 1gcoa_#119 AND 1hdoa_#189 AND 1h5qa_#70
THEN
GO:0016616: oxidoreductase activity, acting on the CH-OH group of donors, NAD or NADP as acceptor: 1.778e-13 (17,59,10) OR
GO:0016627: oxidoreductase activity, acting on the CH-CH group of donors: 1.300e-04 (17,17,3) OR
GO:0016836: hydro-lyase activity: 1.727e-02 (17,33,2) OR
GO:0016854: racemase and epimerase activity: 7.830e-02 (17,13,1) OR
GO:0016646: oxidoreductase activity, acting on the CH-NH group of donors, NAD or NADP as acceptor: 1.125e-01 (17,19,1)

IF 1ja9a_#202 AND 1e6ua_#10
THEN
GO:0016616: oxidoreductase activity, acting on the CH-OH group of donors, NAD or NADP as acceptor: 1.778e-13 (17,59,10) OR
GO:0016627: oxidoreductase activity, acting on the CH-CH group of donors: 2.351e-06 (17,17,4) OR
GO:0016646: oxidoreductase activity, acting on the CH-NH group of donors, NAD or NADP as acceptor: 1.125e-01 (17,19,1) OR
GO:0008757: S-adenosylmethionine-dependent methyltransferase activity: 1.400e-01 (17,24,1) OR
GO:0000287: magnesium ion binding: 5.597e-01 (17,128,1)

IF 1qj5a_#268 AND 1jf9a_#380
THEN
GO:0008483: transaminase activity: 1.490e-13 (13,17,7) OR
GO:0016846: carbon-sulfur lyase activity: 9.255e-04 (13,10,2) OR
GO:0016866: intramolecular transferase activity: 5.588e-02 (13,12,1) OR
GO:0000049: tRNA binding: 6.040e-02 (13,13,1) OR
GO:0019843: rRNA binding: 1.002e-01 (13,22,1) OR
GO:0016831: carboxy-lyase activity: 1.132e-01 (13,25,1)

IF 1hx0a2#32 AND 1c9la2#61
THEN
GO:0004556: alpha-amylase activity: 1.846e-13 (15,15,7) OR
GO:0005509: calcium ion binding: 9.385e-03 (15,160,4) OR
GO:0004812: tRNA ligase activity: 1.343e-01 (15,26,1) OR
GO:0004674: protein serine/threonine kinase activity: 2.083e-01 (15,42,1) OR
GO:0005524: ATP binding: 3.922e-01 (15,243,2)

IF 1dt6a_#385 AND 1fwxa1#526
THEN
GO:0005507: copper ion binding: 1.628e-13 (17,38,9) OR
GO:0015078: hydrogen ion transporter activity: 5.822e-06 (17,21,4) OR
GO:0015082: di-, tri-valent inorganic cation transporter activity: 8.408e-02 (17,14,1) OR
GO:0046915: transition metal ion transporter activity: 8.408e-02 (17,14,1) OR
GO:0051082: unfolded protein binding: 1.927e-01 (17,34,1) OR
GO:0005509: calcium ion binding: 6.436e-01 (17,160,1)

IF 1fcda2#239 AND 1h7wa4#478
THEN
GO:0015036: disulfide oxidoreductase activity: 2.513e-13 (11,22,7) OR
GO:0016668: oxidoreductase activity, acting on sulfur group of donors, NAD or NADP as acceptor: 9.567e-04 (11,12,2) OR
GO:0016627: oxidoreductase activity, acting on the CH-CH group of donors: 6.664e-02 (11,17,1) OR
GO:0004601: peroxidase activity: 8.172e-02 (11,21,1)

IF 2dkb__#114 AND 1elua_#199 AND 5ruba1#365
THEN
GO:0016846: carbon-sulfur lyase activity: 3.405e-13 (12,10,6) OR
GO:0008483: transaminase activity: 3.828e-09 (12,17,5) OR
GO:0016831: carboxy-lyase activity: 1.049e-01 (12,25,1)

IF 1fc4a_#241 AND 1qgna_#149
THEN
GO:0016846: carbon-sulfur lyase activity: 3.405e-13 (12,10,6) OR
GO:0008483: transaminase activity: 3.828e-09 (12,17,5) OR
GO:0016831: carboxy-lyase activity: 1.049e-01 (12,25,1)

IF 1ajsa_#139 AND 5ruba1#365
THEN
GO:0016846: carbon-sulfur lyase activity: 3.405e-13 (12,10,6) OR
GO:0008483: transaminase activity: 3.828e-09 (12,17,5) OR
GO:0016831: carboxy-lyase activity: 1.049e-01 (12,25,1)

IF 1ghpa_#236 AND 1e25a_#262
THEN
GO:0008800: beta-lactamase activity: 1.705e-13 (11,10,6) OR
GO:0004177: aminopeptidase activity: 5.134e-02 (11,13,1) OR
GO:0004180: carboxypeptidase activity: 5.902e-02 (11,15,1) OR
GO:0030145: manganese ion binding: 1.434e-01 (11,38,1) OR
GO:0000287: magnesium ion binding: 4.115e-01 (11,128,1) OR
GO:0005524: ATP binding: 6.428e-01 (11,243,1)

IF 1bu7a_#360 AND 1bu7a_#277
THEN
GO:0004497: monooxygenase activity: 2.570e-13 (16,26,8) OR
GO:0016705: oxidoreductase activity, acting on paired donors, with incorporation or reduction of molecular oxygen: 1.098e-05 (16,26,4) OR
GO:0010181: FMN binding: 1.724e-03 (16,11,2) OR
GO:0016651: oxidoreductase activity, acting on NADH or NADPH: 2.429e-03 (16,13,2)

IF 1cpt__#52 AND 1bu7a_#277
THEN
GO:0004497: monooxygenase activity: 2.570e-13 (16,26,8) OR
GO:0016705: oxidoreductase activity, acting on paired donors, with incorporation or reduction of molecular oxygen: 1.098e-05 (16,26,4) OR
GO:0010181: FMN binding: 1.724e-03 (16,11,2) OR
GO:0016651: oxidoreductase activity, acting on NADH or NADPH: 2.429e-03 (16,13,2)

IF 1dz4a_#150 AND 1bu7a_#405
THEN
GO:0004497: monooxygenase activity: 2.570e-13 (16,26,8) OR
GO:0016705: oxidoreductase activity, acting on paired donors, with incorporation or reduction of molecular oxygen: 1.098e-05 (16,26,4) OR
GO:0010181: FMN binding: 1.724e-03 (16,11,2) OR
GO:0016651: oxidoreductase activity, acting on NADH or NADPH: 2.429e-03 (16,13,2)

IF 1cpt__#358 AND 1bu7a_#277
THEN
GO:0004497: monooxygenase activity: 2.570e-13 (16,26,8) OR
GO:0016705: oxidoreductase activity, acting on paired donors, with incorporation or reduction of molecular oxygen: 1.098e-05 (16,26,4) OR
GO:0010181: FMN binding: 1.724e-03 (16,11,2) OR
GO:0016651: oxidoreductase activity, acting on NADH or NADPH: 2.429e-03 (16,13,2)

IF 1cpt__#401 AND 1bu7a_#277
THEN
GO:0004497: monooxygenase activity: 2.570e-13 (16,26,8) OR
GO:0016705: oxidoreductase activity, acting on paired donors, with incorporation or reduction of molecular oxygen: 1.098e-05 (16,26,4) OR
GO:0010181: FMN binding: 1.724e-03 (16,11,2) OR
GO:0016651: oxidoreductase activity, acting on NADH or NADPH: 2.429e-03 (16,13,2)

IF 1dz4a_#150 AND 1cpt__#52
THEN
GO:0004497: monooxygenase activity: 2.570e-13 (16,26,8) OR
GO:0016705: oxidoreductase activity, acting on paired donors, with incorporation or reduction of molecular oxygen: 1.098e-05 (16,26,4) OR
GO:0010181: FMN binding: 1.724e-03 (16,11,2) OR
GO:0016651: oxidoreductase activity, acting on NADH or NADPH: 2.429e-03 (16,13,2)

IF 1dz4a_#150 AND 1dt6a_#77

THEN
GO:0004497: monooxygenase activity: 2.570e-13 (16,26,8) OR
GO:0016705: oxidoreductase activity, acting on paired donors, with incorporation or reduction of molecular oxygen: 1.098e-05 (16,26,4) OR
GO:0010181: FMN binding: 1.724e-03 (16,11,2) OR
GO:0016651: oxidoreductase activity, acting on NADH or NADPH: 2.429e-03 (16,13,2)

IF 1dt6a_#77 AND 1bu7a_#277
THEN
GO:0004497: monooxygenase activity: 2.570e-13 (16,26,8) OR
GO:0016705: oxidoreductase activity, acting on paired donors, with incorporation or reduction of molecular oxygen: 1.098e-05 (16,26,4) OR
GO:0010181: FMN binding: 1.724e-03 (16,11,2) OR
GO:0016651: oxidoreductase activity, acting on NADH or NADPH: 2.429e-03 (16,13,2)

IF 1e9xa_#343 AND 1dz4a_#150
THEN
GO:0004497: monooxygenase activity: 2.570e-13 (16,26,8) OR
GO:0016705: oxidoreductase activity, acting on paired donors, with incorporation or reduction of molecular oxygen: 1.098e-05 (16,26,4) OR
GO:0010181: FMN binding: 1.724e-03 (16,11,2) OR
GO:0016651: oxidoreductase activity, acting on NADH or NADPH: 2.429e-03 (16,13,2)

IF 1dz4a_#150 AND 1fc3a_#172
THEN
GO:0004497: monooxygenase activity: 2.570e-13 (16,26,8) OR
GO:0016705: oxidoreductase activity, acting on paired donors, with incorporation or reduction of molecular oxygen: 1.098e-05 (16,26,4) OR
GO:0010181: FMN binding: 1.724e-03 (16,11,2) OR
GO:0016651: oxidoreductase activity, acting on NADH or NADPH: 2.429e-03 (16,13,2)

IF 1bu7a_#277 AND 1bu7a_#353
THEN
GO:0004497: monooxygenase activity: 2.570e-13 (16,26,8) OR
GO:0016705: oxidoreductase activity, acting on paired donors, with incorporation or reduction of molecular oxygen: 1.098e-05 (16,26,4) OR
GO:0010181: FMN binding: 1.724e-03 (16,11,2) OR
GO:0016651: oxidoreductase activity, acting on NADH or NADPH: 2.429e-03 (16,13,2)

IF 1f97a1#62 AND 1qsta_#154 AND 1b3qa2#623
THEN
GO:0004812: tRNA ligase activity: 2.588e-13 (25,26,9) OR
GO:0005524: ATP binding: 1.757e-04 (25,243,9) OR
GO:0000287: magnesium ion binding: 5.260e-03 (25,128,5) OR
GO:0000049: tRNA binding: 5.926e-03 (25,13,2)

IF 1gdna_#119 AND 1a0la_#160
THEN
GO:0004295: trypsin activity: 2.593e-13 (28,48,11) OR
GO:0004263: chymotrypsin activity: 1.992e-12 (28,41,10) OR
GO:0004867: serine-type endopeptidase inhibitor activity: 1.165e-02 (28,47,3) OR
GO:0005509: calcium ion binding: 7.802e-02 (28,160,4)

IF 1trb_1#42 AND 1amf__#196
THEN
GO:0015036: disulfide oxidoreductase activity: 1.791e-13 (18,22,8) OR
GO:0016668: oxidoreductase activity, acting on sulfur group of donors, NAD or NADP as acceptor: 6.393e-07 (18,12,4) OR
GO:0050660: FAD binding: 2.824e-05 (18,10,3) OR
GO:0016651: oxidoreductase activity, acting on NADH or NADPH: 8.272e-02 (18,13,1) OR
GO:0016627: oxidoreductase activity, acting on the CH-CH group of donors: 1.068e-01 (18,17,1) OR
GO:0016638: oxidoreductase activity, acting on the CH-NH2 group of donors: 1.068e-01 (18,17,1)

IF 1h7wa4#476 AND 1qfea_#68
THEN
GO:0015036: disulfide oxidoreductase activity: 1.791e-13 (18,22,8) OR
GO:0016668: oxidoreductase activity, acting on sulfur group of donors, NAD or NADP as acceptor: 6.393e-07 (18,12,4) OR
GO:0050660: FAD binding: 2.824e-05 (18,10,3) OR
GO:0016651: oxidoreductase activity, acting on NADH or NADPH: 8.272e-02 (18,13,1) OR
GO:0016627: oxidoreductase activity, acting on the CH-CH group of donors: 1.068e-01 (18,17,1) OR
GO:0004601: peroxidase activity: 1.304e-01 (18,21,1)

IF 1c5y.1#B192 AND 1fjsa_#163 AND 1a0la_#193 AND 1gdna_#56 AND 1ton__#91 AND 1cgha_#130
THEN
GO:0004263: chymotrypsin activity: 2.178e-13 (32,41,11) OR
GO:0004295: trypsin activity: 1.481e-12 (32,48,11) OR
GO:0004867: serine-type endopeptidase inhibitor activity: 1.962e-03 (32,47,4) OR
GO:0005509: calcium ion binding: 3.639e-02 (32,160,5) OR
GO:0003809: thrombin activity: 1.116e-01 (32,10,1)

IF 1qnja_#77 AND 1ton__#179
THEN
GO:0004263: chymotrypsin activity: 2.178e-13 (32,41,11) OR
GO:0004295: trypsin activity: 1.481e-12 (32,48,11) OR
GO:0004867: serine-type endopeptidase inhibitor activity: 1.962e-03 (32,47,4) OR
GO:0005509: calcium ion binding: 3.639e-02 (32,160,5) OR
GO:0004896: hematopoietin/interferon-class (D200-domain) cytokine receptor activity: 2.016e-01 (32,19,1)

IF 1a0la_#193 AND 2hlca_#27 AND 1fjsa_#158 AND 1qnja_#71 AND 1qq4a_#143 AND 1danh_#190 AND 1h8d.1#H184 AND 1cgha_#130
THEN
GO:0004263: chymotrypsin activity: 2.178e-13 (32,41,11) OR
GO:0004295: trypsin activity: 1.481e-12 (32,48,11) OR
GO:0004867: serine-type endopeptidase inhibitor activity: 1.962e-03 (32,47,4) OR
GO:0005509: calcium ion binding: 3.639e-02 (32,160,5) OR
GO:0003809: thrombin activity: 1.116e-01 (32,10,1)

IF 2bb2_2#134 AND 1cjca2#76
THEN
GO:0016668: oxidoreductase activity, acting on sulfur group of donors, NAD or NADP as acceptor: 2.737e-13 (20,12,7) OR
GO:0015036: disulfide oxidoreductase activity: 5.107e-13 (20,22,8) OR
GO:0050660: FAD binding: 4.314e-07 (20,10,4) OR
GO:0016651: oxidoreductase activity, acting on NADH or NADPH: 9.152e-02 (20,13,1)

IF 1by5a_#238 AND 1fc4a_#205 AND 1e6pa2#46
THEN
GO:0004556: alpha-amylase activity: 1.846e-13 (15,15,7) OR
GO:0005509: calcium ion binding: 9.385e-03 (15,160,4) OR
GO:0000049: tRNA binding: 6.939e-02 (15,13,1) OR
GO:0019843: rRNA binding: 1.148e-01 (15,22,1) OR
GO:0016836: hydro-lyase activity: 1.674e-01 (15,33,1) OR
GO:0000287: magnesium ion binding: 5.150e-01 (15,128,1)

IF 1esl_1#29 AND 2msba_#156
THEN
GO:0005529: sugar binding: 5.544e-13 (8,39,7) OR
GO:0008201: heparin binding: 6.841e-02 (8,24,1)

IF 1hlwa_#82 AND 2msba_#218 AND 1tn3__#113
THEN
GO:0005529: sugar binding: 5.544e-13 (8,39,7) OR
GO:0005509: calcium ion binding: 3.841e-01 (8,160,1)

IF 2msba_#156 AND 1qo3c_#252
THEN
GO:0005529: sugar binding: 5.544e-13 (8,39,7) OR
GO:0008201: heparin binding: 6.841e-02 (8,24,1)

IF 1e87a_#155 AND 2msba_#156
THEN
GO:0005529: sugar binding: 5.544e-13 (8,39,7) OR
GO:0008201: heparin binding: 6.841e-02 (8,24,1)

IF 1qu9a_#64 AND 2msba_#195
THEN
GO:0005529: sugar binding: 5.544e-13 (8,39,7) OR
GO:0008201: heparin binding: 6.841e-02 (8,24,1)

IF 1qu9a_#64 AND 1f00i3#856
THEN
GO:0005529: sugar binding: 5.544e-13 (8,39,7) OR

GO:0008201: heparin binding: 6.841e-02 (8,24,1)

IF 1qu9a_#64 AND 1h8ua_#113
THEN
GO:0005529: sugar binding: 5.544e-13 (8,39,7) OR
GO:0008201: heparin binding: 6.841e-02 (8,24,1)

IF 1qo3c_#212 AND 1esl_1#29
THEN
GO:0005529: sugar binding: 5.544e-13 (8,39,7) OR
GO:0008201: heparin binding: 6.841e-02 (8,24,1)

IF 1qg6a_#93 AND 1evqa_#305
THEN
GO:0016616: oxidoreductase activity, acting on the CH-OH group of donors, NAD or NADP as acceptor: 3.730e-13 (13,59,9) OR
GO:0016627: oxidoreductase activity, acting on the CH-CH group of donors: 5.554e-05 (13,17,3) OR
GO:0016646: oxidoreductase activity, acting on the CH-NH group of donors, NAD or NADP as acceptor: 8.713e-02 (13,19,1)

IF 1cyda_#231 AND 1oaa__#175
THEN
GO:0016616: oxidoreductase activity, acting on the CH-OH group of donors, NAD or NADP as acceptor: 3.730e-13 (13,59,9) OR
GO:0016627: oxidoreductase activity, acting on the CH-CH group of donors: 5.554e-05 (13,17,3) OR
GO:0016646: oxidoreductase activity, acting on the CH-NH group of donors, NAD or NADP as acceptor: 8.713e-02 (13,19,1)

IF 1cyda_#231 AND 1hu4a_#267
THEN
GO:0016616: oxidoreductase activity, acting on the CH-OH group of donors, NAD or NADP as acceptor: 3.730e-13 (13,59,9) OR
GO:0016627: oxidoreductase activity, acting on the CH-CH group of donors: 5.554e-05 (13,17,3) OR
GO:0016646: oxidoreductase activity, acting on the CH-NH group of donors, NAD or NADP as acceptor: 8.713e-02 (13,19,1)

IF 1c5y.1#B18 AND 1f42a2#123
THEN
GO:0004263: chymotrypsin activity: 1.897e-13 (42,41,12) OR
GO:0004295: trypsin activity: 1.556e-12 (42,48,12) OR
GO:0004867: serine-type endopeptidase inhibitor activity: 6.233e-05 (42,47,6) OR
GO:0005509: calcium ion binding: 1.055e-04 (42,160,10) OR
GO:0003809: thrombin activity: 1.441e-01 (42,10,1) OR
GO:0004896: hematopoietin/interferon-class (D200-domain) cytokine receptor activity: 2.563e-01 (42,19,1)

IF 1c5y.1#B18 AND 1opy__#68
THEN
GO:0004263: chymotrypsin activity: 1.897e-13 (42,41,12) OR
GO:0004295: trypsin activity: 1.556e-12 (42,48,12) OR
GO:0004867: serine-type endopeptidase inhibitor activity: 6.233e-05 (42,47,6) OR
GO:0005509: calcium ion binding: 1.055e-04 (42,160,10) OR
GO:0003809: thrombin activity: 1.441e-01 (42,10,1) OR
GO:0004896: hematopoietin/interferon-class (D200-domain) cytokine receptor activity: 2.563e-01 (42,19,1)

IF 1h7wa4#476 AND 1b4va1#11
THEN
GO:0016668: oxidoreductase activity, acting on sulfur group of donors, NAD or NADP as acceptor: 4.099e-13 (21,12,7) OR
GO:0015036: disulfide oxidoreductase activity: 8.212e-13 (21,22,8) OR
GO:0050660: FAD binding: 1.977e-11 (21,10,6)

IF 1h7wa4#476 AND 1f8ra2#325
THEN
GO:0016668: oxidoreductase activity, acting on sulfur group of donors, NAD or NADP as acceptor: 4.099e-13 (21,12,7) OR
GO:0015036: disulfide oxidoreductase activity: 8.212e-13 (21,22,8) OR
GO:0050660: FAD binding: 1.977e-11 (21,10,6)

IF 1h7wa4#476 AND 1epwa3#138
THEN
GO:0015036: disulfide oxidoreductase activity: 3.079e-13 (19,22,8) OR
GO:0050660: FAD binding: 2.299e-09 (19,10,5) OR
GO:0016668: oxidoreductase activity, acting on sulfur group of donors, NAD or NADP as acceptor: 7.163e-09 (19,12,5) OR
GO:0030151: molybdenum ion binding: 9.988e-02 (19,15,1)

IF 3grx__#56 AND 1cjca2#76
THEN
GO:0015036: disulfide oxidoreductase activity: 3.079e-13 (19,22,8) OR

GO:0016668: oxidoreductase activity, acting on sulfur group of donors, NAD or NADP as acceptor: 4.325e-11 (19,12,6) OR
GO:0050660: FAD binding: 3.458e-07 (19,10,4) OR
GO:0016638: oxidoreductase activity, acting on the CH-NH2 group of donors: 1.125e-01 (19,17,1)

IF 2hlpa2#289 AND 1hyha2#179
THEN
GO:0004457: lactate dehydrogenase activity: 6.316e-13 (13,10,6) OR
GO:0016616: oxidoreductase activity, acting on the CH-OH group of donors, NAD or NADP as acceptor: 2.408e-09 (13,59,7)

IF 1b6ra3#161 AND 2cmd_2#251 AND 1fgga_#134
THEN
GO:0004457: lactate dehydrogenase activity: 6.316e-13 (13,10,6) OR
GO:0016616: oxidoreductase activity, acting on the CH-OH group of donors, NAD or NADP as acceptor: 2.408e-09 (13,59,7)

IF 1ryph_#163 AND 1qf6a4#362
THEN
GO:0004812: tRNA ligase activity: 2.588e-13 (25,26,9) OR
GO:0005524: ATP binding: 1.757e-04 (25,243,9) OR
GO:0000049: tRNA binding: 5.926e-03 (25,13,2) OR
GO:0000287: magnesium ion binding: 2.728e-02 (25,128,4) OR
GO:0003887: DNA-directed DNA polymerase activity: 1.689e-01 (25,20,1)

IF 1qsta_#154 AND 1pysb5#672
THEN
GO:0004812: tRNA ligase activity: 2.588e-13 (25,26,9) OR
GO:0005524: ATP binding: 1.757e-04 (25,243,9) OR
GO:0000049: tRNA binding: 5.926e-03 (25,13,2) OR
GO:0000287: magnesium ion binding: 2.728e-02 (25,128,4) OR
GO:0030145: manganese ion binding: 2.972e-01 (25,38,1)

IF 1gdna_#119 AND 1fjsa_#163 AND 1ekbb_#73
THEN
GO:0004295: trypsin activity: 2.593e-13 (28,48,11) OR
GO:0004263: chymotrypsin activity: 1.992e-12 (28,41,10) OR
GO:0005509: calcium ion binding: 2.144e-02 (28,160,5) OR
GO:0004896: hematopoietin/interferon-class (D200-domain) cytokine receptor activity: 1.787e-01 (28,19,1) OR
GO:0004867: serine-type endopeptidase inhibitor activity: 3.871e-01 (28,47,1)

IF 1danh_#93 AND 1h8d.1#H167 AND 1fjsa_#163 AND 1ekbb_#117 AND 1fjsa_#158 AND 1ton__#91 AND 1qnja_#46 AND 1dlea_#198
THEN
GO:0004263: chymotrypsin activity: 2.605e-13 (43,41,12) OR
GO:0004295: trypsin activity: 2.132e-12 (43,48,12) OR
GO:0004867: serine-type endopeptidase inhibitor activity: 5.780e-06 (43,47,7) OR
GO:0003809: thrombin activity: 1.055e-05 (43,10,4) OR
GO:0005509: calcium ion binding: 2.943e-03 (43,160,8)

IF 1c5y.1#B18 AND 1ddja_#754
THEN
GO:0004263: chymotrypsin activity: 2.178e-13 (32,41,11) OR
GO:0004295: trypsin activity: 1.481e-12 (32,48,11) OR
GO:0005509: calcium ion binding: 9.442e-03 (32,160,6) OR
GO:0004867: serine-type endopeptidase inhibitor activity: 1.042e-01 (32,47,2) OR
GO:0003809: thrombin activity: 1.116e-01 (32,10,1) OR
GO:0004896: hematopoietin/interferon-class (D200-domain) cytokine receptor activity: 2.016e-01 (32,19,1)

IF 1cgha_#168 AND 1flga_#161
THEN
GO:0003809: thrombin activity: 2.216e-13 (48,10,8) OR
GO:0004263: chymotrypsin activity: 3.237e-11 (48,41,11) OR
GO:0004295: trypsin activity: 2.118e-10 (48,48,11) OR
GO:0004867: serine-type endopeptidase inhibitor activity: 9.497e-07 (48,47,8) OR
GO:0005509: calcium ion binding: 1.530e-03 (48,160,9) OR
GO:0005529: sugar binding: 5.024e-01 (48,39,1)

IF 1eh9a3#102 AND 1fjfc2#153
THEN
GO:0004556: alpha-amylase activity: 7.371e-13 (9,15,6) OR
GO:0005509: calcium ion binding: 1.284e-02 (9,160,3)

IF 1fi2a_#109 AND 1jsg__#92 AND 7taa_2#295
THEN
 GO:0004556: alpha-amylase activity: 7.371e-13 (9,15,6) OR
 GO:0005509: calcium ion binding: 1.284e-02 (9,160,3)

IF 1e6wa_#24 AND 1fmca_#89
THEN
 GO:0016616: oxidoreductase activity, acting on the CH-OH group of donors, NAD or NADP as acceptor: 3.730e-13 (13,59,9) OR
 GO:0016620: oxidoreductase activity, acting on the aldehyde or oxo group of donors, NAD or NADP as acceptor: 9.255e-04 (13,10,2) OR
 GO:0016646: oxidoreductase activity, acting on the CH-NH group of donors, NAD or NADP as acceptor: 8.713e-02 (13,19,1) OR
 GO:0000287: magnesium ion binding: 4.657e-01 (13,128,1)

IF 1mspa_#17 AND 3grx__#55
THEN
 GO:0004364: glutathione transferase activity: 3.744e-13 (11,11,6) OR
 GO:0004812: tRNA ligase activity: 4.568e-03 (11,26,2) OR
 GO:0008757: S-adenosylmethionine-dependent methyltransferase activity: 9.289e-02 (11,24,1) OR
 GO:0005524: ATP binding: 2.565e-01 (11,243,2)

IF 1danh_#152 AND 1qqga1#95
THEN
 GO:0004295: trypsin activity: 2.522e-13 (47,48,13) OR
 GO:0004263: chymotrypsin activity: 8.524e-13 (47,41,12) OR
 GO:0003809: thrombin activity: 2.904e-07 (47,10,5) OR
 GO:0004867: serine-type endopeptidase inhibitor activity: 1.071e-05 (47,47,7) OR
 GO:0005509: calcium ion binding: 1.308e-03 (47,160,9) OR
 GO:0005529: sugar binding: 4.951e-01 (47,39,1)

IF 1trb_1#42 AND 1feca1#122 AND 1h6va2#245
THEN
 GO:0015036: disulfide oxidoreductase activity: 3.079e-13 (19,22,8) OR
 GO:0016668: oxidoreductase activity, acting on sulfur group of donors, NAD or NADP as acceptor: 4.325e-11 (19,12,6) OR
 GO:0050660: FAD binding: 3.347e-05 (19,10,3) OR
 GO:0016651: oxidoreductase activity, acting on NADH or NADPH: 8.713e-02 (19,13,1) OR
 GO:0016627: oxidoreductase activity, acting on the CH-CH group of donors: 1.125e-01 (19,17,1)

IF 1fmca_#88 AND 1ec7a1#216
THEN
 GO:0016616: oxidoreductase activity, acting on the CH-OH group of donors, NAD or NADP as acceptor: 1.553e-12 (7,59,7)

IF 1fmca_#88 AND 1oaa__#199
THEN
 GO:0016616: oxidoreductase activity, acting on the CH-OH group of donors, NAD or NADP as acceptor: 1.553e-12 (7,59,7)

IF 1gega_#58 AND 1fmca_#248
THEN
 GO:0016616: oxidoreductase activity, acting on the CH-OH group of donors, NAD or NADP as acceptor: 1.553e-12 (7,59,7)

IF 1gega_#58 AND 1cyda_#180
THEN
 GO:0016616: oxidoreductase activity, acting on the CH-OH group of donors, NAD or NADP as acceptor: 1.553e-12 (7,59,7)

IF 1b16a_#16 AND 1fmca_#86
THEN
 GO:0016616: oxidoreductase activity, acting on the CH-OH group of donors, NAD or NADP as acceptor: 1.553e-12 (7,59,7)

IF 1fmca_#88 AND 1bu8a2#124
THEN
 GO:0016616: oxidoreductase activity, acting on the CH-OH group of donors, NAD or NADP as acceptor: 1.553e-12 (7,59,7)

IF 1cyda_#231 AND 3grs_2#211
THEN
 GO:0016616: oxidoreductase activity, acting on the CH-OH group of donors, NAD or NADP as acceptor: 1.553e-12 (7,59,7)

IF 1cyda_#231 AND 1e6wa_#136
THEN
 GO:0016616: oxidoreductase activity, acting on the CH-OH group of donors, NAD or NADP as acceptor: 1.553e-12 (7,59,7)

IF 1fmca_#88 AND 1ihua2#343

THEN
GO:0016616: oxidoreductase activity, acting on the CH-OH group of donors, NAD or NADP as acceptor: 1.553e-12 (7,59,7)

IF 1fmca_#88 AND 1qrra_#208
THEN
GO:0016616: oxidoreductase activity, acting on the CH-OH group of donors, NAD or NADP as acceptor: 1.553e-12 (7,59,7)

IF 2ae2a_#196 AND 1b16a_#62
THEN
GO:0016616: oxidoreductase activity, acting on the CH-OH group of donors, NAD or NADP as acceptor: 1.553e-12 (7,59,7)

IF 1f00i3#856 AND 1dv8a_#211
THEN
GO:0005529: sugar binding: 3.917e-13 (12,39,8) OR
GO:0005509: calcium ion binding: 1.538e-01 (12,160,2) OR
GO:0004263: chymotrypsin activity: 1.666e-01 (12,41,1) OR
GO:0004295: trypsin activity: 1.924e-01 (12,48,1)

IF 1c5y.1#B191 AND 1f0xa1#278 AND 1f42a2#123
THEN
GO:0003809: thrombin activity: 2.647e-13 (49,10,8) OR
GO:0004295: trypsin activity: 1.190e-11 (49,48,12) OR
GO:0004263: chymotrypsin activity: 4.131e-11 (49,41,11) OR
GO:0004867: serine-type endopeptidase inhibitor activity: 1.425e-05 (49,47,7) OR
GO:0005509: calcium ion binding: 4.118e-04 (49,160,10) OR
GO:0005529: sugar binding: 5.097e-01 (49,39,1)

IF 1c5y.1#B191 AND 1gg6.1#B19 AND 1c5y.1#B18 AND 1ddja_#726 AND 1befa_#51 AND 1ekbb_#228 AND 1c5y.1#B234
THEN
GO:0004263: chymotrypsin activity: 3.234e-13 (33,41,11) OR
GO:0004295: trypsin activity: 2.194e-12 (33,48,11) OR
GO:0004867: serine-type endopeptidase inhibitor activity: 2.204e-03 (33,47,4) OR
GO:0005509: calcium ion binding: 1.099e-02 (33,160,6) OR
GO:0004896: hematopoietin/interferon-class (D200-domain) cytokine receptor activity: 2.073e-01 (33,19,1)

IF 1icia_#175 AND 1ho1a_#211 AND 1e6pa2#46
THEN
GO:0004556: alpha-amylase activity: 3.274e-13 (16,15,7) OR
GO:0005509: calcium ion binding: 1.687e-03 (16,160,5) OR
GO:0016758: transferase activity, transferring hexosyl groups: 6.284e-02 (16,11,1) OR
GO:0000287: magnesium ion binding: 1.714e-01 (16,128,2) OR
GO:0004867: serine-type endopeptidase inhibitor activity: 2.436e-01 (16,47,1)

IF 1cjca2#103 AND 1cjca2#76
THEN
GO:0016668: oxidoreductase activity, acting on sulfur group of donors, NAD or NADP as acceptor: 4.099e-13 (21,12,7) OR
GO:0015036: disulfide oxidoreductase activity: 8.212e-13 (21,22,8) OR
GO:0050660: FAD binding: 4.011e-09 (21,10,5) OR
GO:0016651: oxidoreductase activity, acting on NADH or NADPH: 9.588e-02 (21,13,1)

IF 1gpea1#273 AND 1cjca2#76
THEN
GO:0016668: oxidoreductase activity, acting on sulfur group of donors, NAD or NADP as acceptor: 4.099e-13 (21,12,7) OR
GO:0015036: disulfide oxidoreductase activity: 8.212e-13 (21,22,8) OR
GO:0050660: FAD binding: 4.011e-09 (21,10,5) OR
GO:0016651: oxidoreductase activity, acting on NADH or NADPH: 9.588e-02 (21,13,1)

IF 1gdna_#119 AND 1qqga1#95
THEN
GO:0004295: trypsin activity: 4.125e-13 (29,48,11) OR
GO:0004263: chymotrypsin activity: 3.008e-12 (29,41,10) OR
GO:0004867: serine-type endopeptidase inhibitor activity: 1.284e-02 (29,47,3) OR
GO:0005509: calcium ion binding: 2.471e-02 (29,160,5)

IF 1i9ga_#67 AND 1dy9.1#A44
THEN
GO:0004295: trypsin activity: 2.773e-13 (37,48,12) OR
GO:0004263: chymotrypsin activity: 1.371e-12 (37,41,11) OR
GO:0004867: serine-type endopeptidase inhibitor activity: 3.383e-03 (37,47,4) OR
GO:0005509: calcium ion binding: 4.865e-03 (37,160,7) OR

GO:0003809: thrombin activity: 7.539e-03 (37,10,2) OR
GO:0004896: hematopoietin/interferon-class (D200-domain) cytokine receptor activity: 2.294e-01 (37,19,1)

IF 1c5y.1#B191 AND 1elva1#566 AND 1bio__#88
THEN
GO:0004263: chymotrypsin activity: 3.546e-13 (44,41,12) OR
GO:0004295: trypsin activity: 2.895e-12 (44,48,12) OR
GO:0003809: thrombin activity: 1.158e-05 (44,10,4) OR
GO:0004867: serine-type endopeptidase inhibitor activity: 8.172e-05 (44,47,6) OR
GO:0005509: calcium ion binding: 1.606e-04 (44,160,10)

IF 1danh_#93 AND 1a0la_#160 AND 1dlea_#238
THEN
GO:0004263: chymotrypsin activity: 3.546e-13 (44,41,12) OR
GO:0004295: trypsin activity: 2.895e-12 (44,48,12) OR
GO:0004867: serine-type endopeptidase inhibitor activity: 6.785e-06 (44,47,7) OR
GO:0003809: thrombin activity: 1.158e-05 (44,10,4) OR
GO:0005509: calcium ion binding: 7.923e-04 (44,160,9)

IF 1eg5a_#199 AND 1dfoa_#344
THEN
GO:0008483: transaminase activity: 6.085e-13 (8,17,6) OR
GO:0016846: carbon-sulfur lyase activity: 2.902e-02 (8,10,1) OR
GO:0016831: carboxy-lyase activity: 7.117e-02 (8,25,1)

IF 1mkp__#283 AND 1trb_1#285
THEN
GO:0015036: disulfide oxidoreductase activity: 3.079e-13 (19,22,8) OR
GO:0050660: FAD binding: 3.458e-07 (19,10,4) OR
GO:0016668: oxidoreductase activity, acting on sulfur group of donors, NAD or NADP as acceptor: 8.078e-07 (19,12,4) OR
GO:0016651: oxidoreductase activity, acting on NADH or NADPH: 8.713e-02 (19,13,1) OR
GO:0016638: oxidoreductase activity, acting on the CH-NH2 group of donors: 1.125e-01 (19,17,1) OR
GO:0004601: peroxidase activity: 1.371e-01 (19,21,1)

IF 1ejda_#379 AND 1eq2a_#11
THEN
GO:0016616: oxidoreductase activity, acting on the CH-OH group of donors, NAD or NADP as acceptor: 3.730e-13 (13,59,9) OR
GO:0004457: lactate dehydrogenase activity: 4.677e-02 (13,10,1) OR
GO:0016854: racemase and epimerase activity: 6.040e-02 (13,13,1) OR
GO:0016627: oxidoreductase activity, acting on the CH-CH group of donors: 7.830e-02 (13,17,1) OR
GO:0016836: hydro-lyase activity: 1.468e-01 (13,33,1)

IF 1ajsa_#139 AND 1c4ka2#223
THEN
GO:0016846: carbon-sulfur lyase activity: 6.316e-13 (13,10,6) OR
GO:0008483: transaminase activity: 3.665e-11 (13,17,6) OR
GO:0016831: carboxy-lyase activity: 1.132e-01 (13,25,1)

IF 1qgna_#207 AND 1c4ka2#223
THEN
GO:0016846: carbon-sulfur lyase activity: 6.316e-13 (13,10,6) OR
GO:0008483: transaminase activity: 3.665e-11 (13,17,6) OR
GO:0016831: carboxy-lyase activity: 1.132e-01 (13,25,1)

IF 1cpt__#358 AND 1bu7a_#278 AND 1dz4a_#264
THEN
GO:0004497: monooxygenase activity: 4.825e-13 (17,26,8) OR
GO:0016705: oxidoreductase activity, acting on paired donors, with incorporation or reduction of molecular oxygen: 3.019e-07 (17,26,5) OR
GO:0010181: FMN binding: 1.950e-03 (17,11,2) OR
GO:0016651: oxidoreductase activity, acting on NADH or NADPH: 2.745e-03 (17,13,2)

IF 1cpt__#358 AND 1e9xa_#62 AND 1bu7a_#405
THEN
GO:0004497: monooxygenase activity: 4.825e-13 (17,26,8) OR
GO:0016705: oxidoreductase activity, acting on paired donors, with incorporation or reduction of molecular oxygen: 3.019e-07 (17,26,5) OR
GO:0010181: FMN binding: 1.950e-03 (17,11,2) OR
GO:0016651: oxidoreductase activity, acting on NADH or NADPH: 2.745e-03 (17,13,2)

IF 1cpt__#401 AND 1e9xa_#62
THEN
GO:0004497: monooxygenase activity: 4.825e-13 (17,26,8) OR
GO:0016705: oxidoreductase activity, acting on paired donors, with incorporation or reduction of molecular oxygen: 3.019e-07 (17,26,5) OR
GO:0010181: FMN binding: 1.950e-03 (17,11,2) OR
GO:0016651: oxidoreductase activity, acting on NADH or NADPH: 2.745e-03 (17,13,2)

IF 1bu7a_#278 AND 1dt6a_#77
THEN
GO:0004497: monooxygenase activity: 4.825e-13 (17,26,8) OR
GO:0016705: oxidoreductase activity, acting on paired donors, with incorporation or reduction of molecular oxygen: 3.019e-07 (17,26,5) OR
GO:0010181: FMN binding: 1.950e-03 (17,11,2) OR
GO:0016651: oxidoreductase activity, acting on NADH or NADPH: 2.745e-03 (17,13,2)

IF 1eupa_#320 AND 1e9xa_#343
THEN
GO:0004497: monooxygenase activity: 4.825e-13 (17,26,8) OR
GO:0016705: oxidoreductase activity, acting on paired donors, with incorporation or reduction of molecular oxygen: 3.019e-07 (17,26,5) OR
GO:0010181: FMN binding: 1.950e-03 (17,11,2) OR
GO:0016651: oxidoreductase activity, acting on NADH or NADPH: 2.745e-03 (17,13,2)

IF 1e9xa_#62 AND 1dz4a_#357
THEN
GO:0004497: monooxygenase activity: 4.825e-13 (17,26,8) OR
GO:0016705: oxidoreductase activity, acting on paired donors, with incorporation or reduction of molecular oxygen: 3.019e-07 (17,26,5) OR
GO:0010181: FMN binding: 1.950e-03 (17,11,2) OR
GO:0016651: oxidoreductase activity, acting on NADH or NADPH: 2.745e-03 (17,13,2)

IF 1bu7a_#278 AND 1bu7a_#353
THEN
GO:0004497: monooxygenase activity: 4.825e-13 (17,26,8) OR
GO:0016705: oxidoreductase activity, acting on paired donors, with incorporation or reduction of molecular oxygen: 3.019e-07 (17,26,5) OR
GO:0010181: FMN binding: 1.950e-03 (17,11,2) OR
GO:0016651: oxidoreductase activity, acting on NADH or NADPH: 2.745e-03 (17,13,2)

IF 1bqya_#115 AND 1qnja_#77
THEN
GO:0004263: chymotrypsin activity: 3.234e-13 (33,41,11) OR
GO:0004295: trypsin activity: 2.194e-12 (33,48,11) OR
GO:0003809: thrombin activity: 6.024e-03 (33,10,2) OR
GO:0005509: calcium ion binding: 1.099e-02 (33,160,6) OR
GO:0004867: serine-type endopeptidase inhibitor activity: 1.097e-01 (33,47,2) OR
GO:0004896: hematopoietin/interferon-class (D200-domain) cytokine receptor activity: 2.073e-01 (33,19,1)

IF 1azza_#51 AND 1g71a_#77 AND 1qnja_#124 AND 1fjsa_#158 AND 1ton__#196 AND 1ton__#47 AND 1ekbb_#141 AND 2hlca_#139
THEN
GO:0004263: chymotrypsin activity: 3.234e-13 (33,41,11) OR
GO:0004295: trypsin activity: 2.194e-12 (33,48,11) OR
GO:0005509: calcium ion binding: 2.462e-03 (33,160,7) OR
GO:0004867: serine-type endopeptidase inhibitor activity: 1.097e-01 (33,47,2) OR
GO:0003809: thrombin activity: 1.149e-01 (33,10,1) OR
GO:0004896: hematopoietin/interferon-class (D200-domain) cytokine receptor activity: 2.073e-01 (33,19,1)

IF 2napa1#665 AND 1jb3a_#64
THEN
GO:0030151: molybdenum ion binding: 3.274e-13 (16,15,7) OR
GO:0003700: transcription factor activity: 3.336e-02 (16,124,3) OR
GO:0016620: oxidoreductase activity, acting on the aldehyde or oxo group of donors, NAD or NADP as acceptor: 5.728e-02 (16,10,1) OR
GO:0003899: DNA-directed RNA polymerase activity: 6.836e-02 (16,12,1) OR
GO:0046983: protein dimerization activity: 9.553e-02 (16,17,1) OR
GO:0005524: ATP binding: 1.656e-01 (16,243,3)

IF 1by5a_#238 AND 1fhoa_#74 AND 1e6pa2#46

THEN
GO:0004556: alpha-amylase activity: 3.274e-13 (16,15,7) OR
GO:0005509: calcium ion binding: 1.687e-03 (16,160,5) OR
GO:0000049: tRNA binding: 7.386e-02 (16,13,1) OR
GO:0019843: rRNA binding: 1.220e-01 (16,22,1) OR
GO:0016836: hydro-lyase activity: 1.776e-01 (16,33,1) OR
GO:0000287: magnesium ion binding: 5.379e-01 (16,128,1)

IF 1el5a1#183 AND 1trb_1#41
THEN
GO:0015036: disulfide oxidoreductase activity: 5.107e-13 (20,22,8) OR
GO:0016668: oxidoreductase activity, acting on sulfur group of donors, NAD or NADP as acceptor: 6.167e-11 (20,12,6) OR
GO:0050660: FAD binding: 3.060e-09 (20,10,5) OR
GO:0016638: oxidoreductase activity, acting on the CH-NH2 group of donors: 1.180e-01 (20,17,1)

IF 1c7na_#281 AND 1ax4a_#275
THEN
GO:0008483: transaminase activity: 5.552e-13 (15,17,7) OR
GO:0016846: carbon-sulfur lyase activity: 5.974e-10 (15,10,5) OR
GO:0016831: carboxy-lyase activity: 7.888e-03 (15,25,2) OR
GO:0016866: intramolecular transferase activity: 6.422e-02 (15,12,1)

IF 1gdna_#26 AND 1gg6.1#C156 AND 1f42a2#123 AND 1elva1#513
THEN
GO:0003809: thrombin activity: 3.732e-13 (51,10,8) OR
GO:0004295: trypsin activity: 1.997e-11 (51,48,12) OR
GO:0004263: chymotrypsin activity: 6.615e-11 (51,41,11) OR
GO:0004867: serine-type endopeptidase inhibitor activity: 1.542e-06 (51,47,8) OR
GO:0005509: calcium ion binding: 1.243e-04 (51,160,11) OR
GO:0005529: sugar binding: 5.239e-01 (51,39,1)

IF 1glqa2#53 AND 1d6aa_#52
THEN
GO:0004364: glutathione transferase activity: 3.744e-13 (11,11,6) OR
GO:0003714: transcription corepressor activity: 3.188e-02 (11,8,1) OR
GO:0001584: rhodopsin-like receptor activity: 3.971e-02 (11,10,1) OR
GO:0005179: hormone activity: 5.902e-02 (11,15,1) OR
GO:0003887: DNA-directed DNA polymerase activity: 7.797e-02 (11,20,1) OR
GO:0015036: disulfide oxidoreductase activity: 8.546e-02 (11,22,1)

IF 1ja9a_#202 AND 1qrra_#208
THEN
GO:0016616: oxidoreductase activity, acting on the CH-OH group of donors, NAD or NADP as acceptor: 3.935e-13 (18,59,10) OR
GO:0016758: transferase activity, transferring hexosyl groups: 2.189e-03 (18,11,2) OR
GO:0016627: oxidoreductase activity, acting on the CH-CH group of donors: 5.286e-03 (18,17,2) OR
GO:0016646: oxidoreductase activity, acting on the CH-NH group of donors, NAD or NADP as acceptor: 1.187e-01 (18,19,1) OR
GO:0030145: manganese ion binding: 2.240e-01 (18,38,1) OR
GO:0005524: ATP binding: 4.864e-01 (18,243,2)

IF 1pysb5#672 AND 1h4vb2#307
THEN
GO:0004812: tRNA ligase activity: 3.935e-13 (26,26,9) OR
GO:0005524: ATP binding: 2.481e-04 (26,243,9) OR
GO:0000049: tRNA binding: 6.402e-03 (26,13,2) OR
GO:0000287: magnesium ion binding: 3.110e-02 (26,128,4) OR
GO:0003713: transcription coactivator activity: 1.426e-01 (26,16,1) OR
GO:0003700: transcription factor activity: 7.038e-01 (26,124,1)

IF 1cex__#23 AND 1h6va1#23
THEN
GO:0016668: oxidoreductase activity, acting on sulfur group of donors, NAD or NADP as acceptor: 6.001e-13 (22,12,7) OR
GO:0015036: disulfide oxidoreductase activity: 1.285e-12 (22,22,8) OR
GO:0050660: FAD binding: 2.715e-11 (22,10,6) OR
GO:0016651: oxidoreductase activity, acting on NADH or NADPH: 1.002e-01 (22,13,1)

IF 1qlaa2#383 AND 1auk__#375
THEN
GO:0015036: disulfide oxidoreductase activity: 6.001e-13 (12,22,7) OR
GO:0016668: oxidoreductase activity, acting on sulfur group of donors, NAD or NADP as acceptor: 1.405e-05 (12,12,3) OR
GO:0016627: oxidoreductase activity, acting on the CH-CH group of donors: 7.249e-02 (12,17,1) OR

GO:0004601: peroxidase activity: 8.883e-02 (12,21,1)

IF 1fjsa_#163 AND 1fjsa_#158 AND 1g25a_#22 AND 1e79d2#36
THEN
GO:0004295: trypsin activity: 4.004e-13 (38,48,12) OR
GO:0004263: chymotrypsin activity: 1.910e-12 (38,41,11) OR
GO:0004867: serine-type endopeptidase inhibitor activity: 4.027e-04 (38,47,5) OR
GO:0005509: calcium ion binding: 5.676e-03 (38,160,7) OR
GO:0003809: thrombin activity: 7.943e-03 (38,10,2) OR
GO:0004896: hematopoietin/interferon-class (D200-domain) cytokine receptor activity: 2.349e-01 (38,19,1)

IF 2bb2_2#134 AND 1i50a_#856 AND 1el0a_#62
THEN
GO:0008009: chemokine activity: 1.210e-12 (6,10,5) OR
GO:0008083: growth factor activity: 8.906e-02 (6,42,1)

IF 1ibja_#217 AND 1gox__#124 AND 1ax4a_#275
THEN
GO:0016846: carbon-sulfur lyase activity: 1.210e-12 (6,10,5) OR
GO:0008483: transaminase activity: 3.689e-02 (6,17,1)

IF 1ha6a_#20 AND 1doka_#52
THEN
GO:0008009: chemokine activity: 1.210e-12 (6,10,5) OR
GO:0008083: growth factor activity: 8.906e-02 (6,42,1)

IF 1cewi_#14 AND 1i5ga_#115 AND 1el0a_#62
THEN
GO:0008009: chemokine activity: 1.210e-12 (6,10,5) OR
GO:0008083: growth factor activity: 8.906e-02 (6,42,1)

IF 1qgna_#149 AND 1fc4a_#277
THEN
GO:0016846: carbon-sulfur lyase activity: 1.210e-12 (6,10,5) OR
GO:0008483: transaminase activity: 3.689e-02 (6,17,1)

IF 1fzqa_#28 AND 1ejba_#81
THEN
GO:0005525: GTP binding: 4.212e-13 (15,49,9) OR
GO:0019201: nucleotide kinase activity: 6.939e-02 (15,13,1) OR
GO:0016776: phosphotransferase activity, phosphate group as acceptor: 7.454e-02 (15,14,1) OR
GO:0005096: GTPase activator activity: 7.966e-02 (15,15,1) OR
GO:0005524: ATP binding: 3.922e-01 (15,243,2) OR
GO:0000287: magnesium ion binding: 5.150e-01 (15,128,1)

IF 1f2aa_#27 AND 2cpl__#53
THEN
GO:0004197: cysteine-type endopeptidase activity: 5.086e-13 (11,24,7) OR
GO:0019955: cytokine binding: 4.360e-02 (11,11,1) OR
GO:0042802: protein self binding: 4.747e-02 (11,12,1) OR
GO:0051082: unfolded protein binding: 1.292e-01 (11,34,1) OR
GO:0005509: calcium ion binding: 4.867e-01 (11,160,1)

IF 1oaa__#161 AND 1gdha1#235
THEN
GO:0016616: oxidoreductase activity, acting on the CH-OH group of donors, NAD or NADP as acceptor: 1.293e-12 (10,59,8) OR
GO:0016627: oxidoreductase activity, acting on the CH-CH group of donors: 1.601e-03 (10,17,2)

IF 1ja9a_#249 AND 1cyda_#180
THEN
GO:0016616: oxidoreductase activity, acting on the CH-OH group of donors, NAD or NADP as acceptor: 1.293e-12 (10,59,8) OR
GO:0016627: oxidoreductase activity, acting on the CH-CH group of donors: 1.601e-03 (10,17,2)

IF 1e05i_#83 AND 1e05i_#422
THEN
GO:0004867: serine-type endopeptidase inhibitor activity: 6.689e-13 (11,47,8) OR
GO:0003887: DNA-directed DNA polymerase activity: 7.797e-02 (11,20,1) OR
GO:0008201: heparin binding: 9.289e-02 (11,24,1) OR
GO:0003700: transcription factor activity: 4.015e-01 (11,124,1)

IF 1e05i_#79 AND 1ovaa_#364
THEN
GO:0004867: serine-type endopeptidase inhibitor activity: 6.689e-13 (11,47,8) OR
GO:0008201: heparin binding: 9.289e-02 (11,24,1) OR
GO:0004812: tRNA ligase activity: 1.003e-01 (11,26,1) OR
GO:0005524: ATP binding: 6.428e-01 (11,243,1)

IF 1ovaa_#161 AND 1hle.1#B387
THEN
GO:0004867: serine-type endopeptidase inhibitor activity: 6.689e-13 (11,47,8) OR
GO:0046983: protein dimerization activity: 6.664e-02 (11,17,1) OR
GO:0015078: hydrogen ion transporter activity: 8.172e-02 (11,21,1) OR
GO:0008201: heparin binding: 9.289e-02 (11,24,1)

IF 1ovaa_#161 AND 1a7ca_#202
THEN
GO:0004867: serine-type endopeptidase inhibitor activity: 6.689e-13 (11,47,8) OR
GO:0015078: hydrogen ion transporter activity: 8.172e-02 (11,21,1) OR
GO:0008201: heparin binding: 9.289e-02 (11,24,1) OR
GO:0005509: calcium ion binding: 4.867e-01 (11,160,1)

IF 1aq0a_#4 AND 1djna1#86
THEN
GO:0004556: alpha-amylase activity: 5.552e-13 (17,15,7) OR
GO:0005509: calcium ion binding: 2.494e-05 (17,160,7) OR
GO:0008810: cellulase activity: 1.068e-01 (17,18,1) OR
GO:0016705: oxidoreductase activity, acting on paired donors, with incorporation or reduction of molecular oxygen: 1.508e-01 (17,26,1) OR
GO:0004497: monooxygenase activity: 1.508e-01 (17,26,1)

IF 1qnja_#77 AND 1ekbb_#73
THEN
GO:0004263: chymotrypsin activity: 4.731e-13 (34,41,11) OR
GO:0004295: trypsin activity: 3.202e-12 (34,48,11) OR
GO:0005509: calcium ion binding: 2.950e-03 (34,160,7) OR
GO:0004867: serine-type endopeptidase inhibitor activity: 1.980e-02 (34,47,3) OR
GO:0003809: thrombin activity: 1.182e-01 (34,10,1) OR
GO:0004896: hematopoietin/interferon-class (D200-domain) cytokine receptor activity: 2.129e-01 (34,19,1)

IF 1danh_#242 AND 1befa_#51 AND 1ton__#196 AND 1a0la_#160 AND 1elva1#601 AND 1cgha_#227 AND 1ton__#211
THEN
GO:0004263: chymotrypsin activity: 4.787e-13 (45,41,12) OR
GO:0004295: trypsin activity: 3.899e-12 (45,48,12) OR
GO:0004867: serine-type endopeptidase inhibitor activity: 7.931e-06 (45,47,7) OR
GO:0005509: calcium ion binding: 1.962e-04 (45,160,10) OR
GO:0003809: thrombin activity: 4.660e-04 (45,10,3) OR
GO:0005529: sugar binding: 4.801e-01 (45,39,1)

IF 1danh_#242 AND 1befa_#51 AND 1ton__#196 AND 1a0la_#160 AND 1elva1#601 AND 1cgha_#227 AND 1elva1#604 AND 1autc_#209 AND 1qnja_#138
THEN
GO:0004263: chymotrypsin activity: 4.787e-13 (45,41,12) OR
GO:0004295: trypsin activity: 3.899e-12 (45,48,12) OR
GO:0004867: serine-type endopeptidase inhibitor activity: 7.931e-06 (45,47,7) OR
GO:0005509: calcium ion binding: 1.962e-04 (45,160,10) OR
GO:0003809: thrombin activity: 4.660e-04 (45,10,3) OR
GO:0005529: sugar binding: 4.801e-01 (45,39,1)

IF 1cjca2#76 AND 1ddwa_#77
THEN
GO:0016668: oxidoreductase activity, acting on sulfur group of donors, NAD or NADP as acceptor: 6.001e-13 (22,12,7) OR
GO:0015036: disulfide oxidoreductase activity: 1.285e-12 (22,22,8) OR
GO:0050660: FAD binding: 5.182e-09 (22,10,5) OR
GO:0016651: oxidoreductase activity, acting on NADH or NADPH: 1.002e-01 (22,13,1) OR
GO:0016638: oxidoreductase activity, acting on the CH-NH2 group of donors: 1.291e-01 (22,17,1)

IF 1thfd_#140 AND 1h7wa4#478
THEN
GO:0015036: disulfide oxidoreductase activity: 6.001e-13 (12,22,7) OR
GO:0016668: oxidoreductase activity, acting on sulfur group of donors, NAD or NADP as acceptor: 1.145e-03 (12,12,2) OR

GO:0050660: FAD binding: 4.324e-02 (12,10,1) OR
GO:0016638: oxidoreductase activity, acting on the CH-NH2 group of donors: 7.249e-02 (12,17,1) OR
GO:0004601: peroxidase activity: 8.883e-02 (12,21,1)

IF 1c5y.1#B191 AND 1e44b_#75 AND 1f42a2#123
THEN
GO:0003809: thrombin activity: 5.188e-13 (53,10,8) OR
GO:0004295: trypsin activity: 1.402e-12 (53,48,13) OR
GO:0004263: chymotrypsin activity: 4.098e-12 (53,41,12) OR
GO:0004867: serine-type endopeptidase inhibitor activity: 2.091e-06 (53,47,8) OR
GO:0005509: calcium ion binding: 1.795e-04 (53,160,11) OR
GO:0005529: sugar binding: 5.377e-01 (53,39,1)

IF 1gdna_#119 AND 1f2la_#40
THEN
GO:0004295: trypsin activity: 6.432e-13 (30,48,11) OR
GO:0004263: chymotrypsin activity: 4.465e-12 (30,41,10) OR
GO:0004867: serine-type endopeptidase inhibitor activity: 1.409e-02 (30,47,3) OR
GO:0005509: calcium ion binding: 2.829e-02 (30,160,5) OR
GO:0004896: hematopoietin/interferon-class (D200-domain) cytokine receptor activity: 1.903e-01 (30,19,1)

IF 1bg6_2#24 AND 1h7wa4#476
THEN
GO:0015036: disulfide oxidoreductase activity: 8.212e-13 (21,22,8) OR
GO:0050660: FAD binding: 1.977e-11 (21,10,6) OR
GO:0016668: oxidoreductase activity, acting on sulfur group of donors, NAD or NADP as acceptor: 8.617e-11 (21,12,6) OR
GO:0016651: oxidoreductase activity, acting on NADH or NADPH: 9.588e-02 (21,13,1)

IF 1h7wa4#476 AND 1fhoa_#74
THEN
GO:0015036: disulfide oxidoreductase activity: 8.212e-13 (21,22,8) OR
GO:0050660: FAD binding: 1.977e-11 (21,10,6) OR
GO:0016668: oxidoreductase activity, acting on sulfur group of donors, NAD or NADP as acceptor: 8.617e-11 (21,12,6) OR
GO:0004601: peroxidase activity: 1.505e-01 (21,21,1)

IF 1dpja_#322 AND 1d7ya1#255
THEN
GO:0015036: disulfide oxidoreductase activity: 8.212e-13 (21,22,8) OR
GO:0050660: FAD binding: 1.977e-11 (21,10,6) OR
GO:0016668: oxidoreductase activity, acting on sulfur group of donors, NAD or NADP as acceptor: 8.617e-11 (21,12,6) OR
GO:0016651: oxidoreductase activity, acting on NADH or NADPH: 9.588e-02 (21,13,1)

IF 1autc_#188 AND 1eq9a_#32 AND 1fjsa_#158 AND 1gdna_#151 AND 1jsg__#92
THEN
GO:0004263: chymotrypsin activity: 8.232e-13 (26,41,10) OR
GO:0004295: trypsin activity: 4.624e-12 (26,48,10) OR
GO:0004867: serine-type endopeptidase inhibitor activity: 9.469e-03 (26,47,3) OR
GO:0005509: calcium ion binding: 1.934e-01 (26,160,3)

IF 1dxea_#71 AND 1bvza3#416
THEN
GO:0004556: alpha-amylase activity: 5.552e-13 (17,15,7) OR
GO:0005509: calcium ion binding: 2.278e-03 (17,160,5) OR
GO:0016854: racemase and epimerase activity: 7.830e-02 (17,13,1) OR
GO:0016831: carboxy-lyase activity: 1.454e-01 (17,25,1) OR
GO:0000287: magnesium ion binding: 1.885e-01 (17,128,2) OR
GO:0004867: serine-type endopeptidase inhibitor activity: 2.567e-01 (17,47,1)

IF 1apme_#74 AND 1a06__#103
THEN
GO:0004674: protein serine/threonine kinase activity: 6.685e-13 (25,42,10) OR
GO:0005524: ATP binding: 2.600e-05 (25,243,10) OR
GO:0005516: calmodulin binding: 1.216e-03 (25,24,3) OR
GO:0003755: peptidyl-prolyl cis-trans isomerase activity: 9.658e-02 (25,11,1) OR
GO:0004896: hematopoietin/interferon-class (D200-domain) cytokine receptor activity: 1.611e-01 (25,19,1)

IF 1danh_#48 AND 1cgha_#130 AND 2hlca_#226
THEN
GO:0004263: chymotrypsin activity: 6.830e-13 (35,41,11) OR
GO:0004295: trypsin activity: 4.611e-12 (35,48,11) OR

GO:0004867: serine-type endopeptidase inhibitor activity: 2.708e-04 (35,47,5) OR
GO:0005509: calcium ion binding: 3.509e-03 (35,160,7) OR
GO:0003809: thrombin activity: 1.215e-01 (35,10,1)

IF 1g71a_#77 AND 1a0la_#160 AND 1cgha_#130
THEN
GO:0004263: chymotrypsin activity: 6.830e-13 (35,41,11) OR
GO:0004295: trypsin activity: 4.611e-12 (35,48,11) OR
GO:0004867: serine-type endopeptidase inhibitor activity: 2.708e-04 (35,47,5) OR
GO:0005509: calcium ion binding: 3.509e-03 (35,160,7) OR
GO:0003809: thrombin activity: 1.215e-01 (35,10,1)

IF 1danh_#48 AND 1eq9a_#162 AND 1cgha_#130
THEN
GO:0004263: chymotrypsin activity: 6.830e-13 (35,41,11) OR
GO:0004295: trypsin activity: 4.611e-12 (35,48,11) OR
GO:0004867: serine-type endopeptidase inhibitor activity: 2.750e-03 (35,47,4) OR
GO:0005509: calcium ion binding: 3.509e-03 (35,160,7) OR
GO:0003809: thrombin activity: 6.762e-03 (35,10,2)

IF 1ayl__#237 AND 1qq4a_#63
THEN
GO:0004295: trypsin activity: 5.712e-13 (39,48,12) OR
GO:0004263: chymotrypsin activity: 5.620e-08 (39,41,8) OR
GO:0004867: serine-type endopeptidase inhibitor activity: 2.906e-06 (39,47,7) OR
GO:0003809: thrombin activity: 7.081e-06 (39,10,4) OR
GO:0005509: calcium ion binding: 6.583e-03 (39,160,7) OR
GO:0005529: sugar binding: 4.323e-01 (39,39,1)

IF 1gdea_#271 AND 1aqua_#126
THEN
GO:0008483: transaminase activity: 1.819e-12 (9,17,6) OR
GO:0016846: carbon-sulfur lyase activity: 2.958e-06 (9,10,3)

IF 1qisa_#107 AND 1qs0a1#119
THEN
GO:0008483: transaminase activity: 1.819e-12 (9,17,6) OR
GO:0016846: carbon-sulfur lyase activity: 2.958e-06 (9,10,3)

IF 1eh9a3#252 AND 1ex1a1#284
THEN
GO:0004556: alpha-amylase activity: 1.837e-12 (10,15,6) OR
GO:0005509: calcium ion binding: 1.820e-03 (10,160,4)

IF 1e43a2#351 AND 1ex1a1#284
THEN
GO:0004556: alpha-amylase activity: 1.837e-12 (10,15,6) OR
GO:0005509: calcium ion binding: 1.820e-03 (10,160,4)

IF 1gjwa2#141 AND 1ex1a1#284
THEN
GO:0004556: alpha-amylase activity: 1.837e-12 (10,15,6) OR
GO:0005509: calcium ion binding: 1.820e-03 (10,160,4)

IF 1uok_2#170 AND 1qfea_#68
THEN
GO:0004556: alpha-amylase activity: 1.837e-12 (10,15,6) OR
GO:0005509: calcium ion binding: 1.820e-03 (10,160,4)

IF 1c5y.1#B94 AND 1i6vd_#1283 AND 1arb__#210 AND 1dlea_#238 AND 1arb__#193 AND 1qnja_#138
THEN
GO:0004263: chymotrypsin activity: 6.412e-13 (46,41,12) OR
GO:0004295: trypsin activity: 5.209e-12 (46,48,12) OR
GO:0003809: thrombin activity: 3.306e-09 (46,10,6) OR
GO:0004867: serine-type endopeptidase inhibitor activity: 9.234e-06 (46,47,7) OR
GO:0005509: calcium ion binding: 4.556e-03 (46,160,8) OR
GO:0005529: sugar binding: 4.876e-01 (46,39,1)

IF 1c5y.1#B94 AND 1i6vd_#1283 AND 1arb__#210 AND 1dlea_#238 AND 1arb__#193 AND 1gg6.1#C229
THEN

GO:0004263: chymotrypsin activity: 6.412e-13 (46,41,12) OR
GO:0004295: trypsin activity: 5.209e-12 (46,48,12) OR
GO:0003809: thrombin activity: 3.306e-09 (46,10,6) OR
GO:0004867: serine-type endopeptidase inhibitor activity: 9.234e-06 (46,47,7) OR
GO:0005509: calcium ion binding: 4.556e-03 (46,160,8) OR
GO:0005529: sugar binding: 4.876e-01 (46,39,1)

IF 1fjsa_#28 AND 1befa_#51
THEN
GO:0004295: trypsin activity: 6.432e-13 (30,48,11) OR
GO:0004263: chymotrypsin activity: 4.465e-12 (30,41,10) OR
GO:0003809: thrombin activity: 4.992e-03 (30,10,2) OR
GO:0004867: serine-type endopeptidase inhibitor activity: 9.336e-02 (30,47,2) OR
GO:0005509: calcium ion binding: 9.552e-02 (30,160,4) OR
GO:0005529: sugar binding: 3.526e-01 (30,39,1)

IF 1b16a_#184 AND 1b4ka_#108
THEN
GO:0016616: oxidoreductase activity, acting on the CH-OH group of donors, NAD or NADP as acceptor: 1.293e-12 (10,59,8) OR
GO:0016854: racemase and epimerase activity: 4.677e-02 (10,13,1) OR
GO:0016646: oxidoreductase activity, acting on the CH-NH group of donors, NAD or NADP as acceptor: 6.769e-02 (10,19,1)

IF 1hdr__#136 AND 1cyda_#180
THEN
GO:0016616: oxidoreductase activity, acting on the CH-OH group of donors, NAD or NADP as acceptor: 1.293e-12 (10,59,8) OR
GO:0016627: oxidoreductase activity, acting on the CH-CH group of donors: 6.076e-02 (10,17,1) OR
GO:0016646: oxidoreductase activity, acting on the CH-NH group of donors, NAD or NADP as acceptor: 6.769e-02 (10,19,1)

IF 1cyda_#231 AND 1fmca_#89
THEN
GO:0016616: oxidoreductase activity, acting on the CH-OH group of donors, NAD or NADP as acceptor: 1.293e-12 (10,59,8) OR
GO:0016627: oxidoreductase activity, acting on the CH-CH group of donors: 6.076e-02 (10,17,1) OR
GO:0016646: oxidoreductase activity, acting on the CH-NH group of donors, NAD or NADP as acceptor: 6.769e-02 (10,19,1)

IF 1qp8a1#153 AND 1ct9a1#233
THEN
GO:0016616: oxidoreductase activity, acting on the CH-OH group of donors, NAD or NADP as acceptor: 1.293e-12 (10,59,8) OR
GO:0016620: oxidoreductase activity, acting on the aldehyde or oxo group of donors, NAD or NADP as acceptor: 3.616e-02 (10,10,1) OR
GO:0015036: disulfide oxidoreductase activity: 7.799e-02 (10,22,1)

IF 1avgi_#69 AND 1a65a3#383 AND 2cuaa_#83
THEN
GO:0005507: copper ion binding: 8.027e-13 (13,38,8) OR
GO:0015078: hydrogen ion transporter activity: 4.193e-03 (13,21,2) OR
GO:0015082: di-, tri-valent inorganic cation transporter activity: 6.491e-02 (13,14,1) OR
GO:0046915: transition metal ion transporter activity: 6.491e-02 (13,14,1) OR
GO:0004222: metalloendopeptidase activity: 8.713e-02 (13,19,1)

IF 1gg6.1#B19 AND 1azza_#51 AND 1c5y.1#B18 AND 1befa_#51 AND 1ton__#196
THEN
GO:0004263: chymotrypsin activity: 6.830e-13 (35,41,11) OR
GO:0004295: trypsin activity: 4.611e-12 (35,48,11) OR
GO:0004867: serine-type endopeptidase inhibitor activity: 2.750e-03 (35,47,4) OR
GO:0005509: calcium ion binding: 3.509e-03 (35,160,7) OR
GO:0003809: thrombin activity: 1.215e-01 (35,10,1) OR
GO:0004896: hematopoietin/interferon-class (D200-domain) cytokine receptor activity: 2.184e-01 (35,19,1)

IF 1h7wa4#478 AND 1qdla_#211
THEN
GO:0015036: disulfide oxidoreductase activity: 8.212e-13 (21,22,8) OR
GO:0016668: oxidoreductase activity, acting on sulfur group of donors, NAD or NADP as acceptor: 8.617e-11 (21,12,6) OR
GO:0050660: FAD binding: 4.011e-09 (21,10,5) OR
GO:0016651: oxidoreductase activity, acting on NADH or NADPH: 9.588e-02 (21,13,1) OR
GO:0004601: peroxidase activity: 1.505e-01 (21,21,1)

IF 3chbd_#85 AND 1trb_1#41
THEN
GO:0015036: disulfide oxidoreductase activity: 8.212e-13 (21,22,8) OR
GO:0016668: oxidoreductase activity, acting on sulfur group of donors, NAD or NADP as acceptor: 8.617e-11 (21,12,6) OR

GO:0050660: FAD binding: 4.011e-09 (21,10,5) OR
GO:0016651: oxidoreductase activity, acting on NADH or NADPH: 9.588e-02 (21,13,1) OR
GO:0016627: oxidoreductase activity, acting on the CH-CH group of donors: 1.236e-01 (21,17,1)

IF 1a0fa1#94 AND 1evqa_#305
THEN
GO:0004364: glutathione transferase activity: 2.218e-12 (6,11,5) OR
GO:0003714: transcription corepressor activity: 1.750e-02 (6,8,1)

IF 1dt9a1#224 AND 1gnwa1#160
THEN
GO:0004364: glutathione transferase activity: 2.218e-12 (6,11,5) OR
GO:0003714: transcription corepressor activity: 1.750e-02 (6,8,1)

IF 1hzxa_#139 AND 1glqa2#53
THEN
GO:0004364: glutathione transferase activity: 2.218e-12 (6,11,5) OR
GO:0003714: transcription corepressor activity: 1.750e-02 (6,8,1)

IF 1a0fa1#94 AND 1e6ca_#91
THEN
GO:0004364: glutathione transferase activity: 2.218e-12 (6,11,5) OR
GO:0016651: oxidoreductase activity, acting on NADH or NADPH: 2.831e-02 (6,13,1)

IF 1iow_1#37 AND 1a0fa1#94
THEN
GO:0004364: glutathione transferase activity: 2.218e-12 (6,11,5) OR
GO:0003714: transcription corepressor activity: 1.750e-02 (6,8,1)

IF 1a0fa1#94 AND 1d6aa_#52
THEN
GO:0004364: glutathione transferase activity: 2.218e-12 (6,11,5) OR
GO:0003714: transcription corepressor activity: 1.750e-02 (6,8,1)

IF 1f3ba2#7 AND 1e6ca_#91
THEN
GO:0004364: glutathione transferase activity: 2.218e-12 (6,11,5) OR
GO:0016651: oxidoreductase activity, acting on NADH or NADPH: 2.831e-02 (6,13,1)

IF 1hzxa_#139 AND 3grx__#55
THEN
GO:0004364: glutathione transferase activity: 2.218e-12 (6,11,5) OR
GO:0003714: transcription corepressor activity: 1.750e-02 (6,8,1)

IF 2hlpa2#289 AND 1a7ca_#202
THEN
GO:0004867: serine-type endopeptidase inhibitor activity: 2.261e-12 (8,47,7) OR
GO:0008201: heparin binding: 6.841e-02 (8,24,1)

IF 1sek__#63 AND 1a7ca_#370
THEN
GO:0004867: serine-type endopeptidase inhibitor activity: 2.261e-12 (8,47,7) OR
GO:0008201: heparin binding: 6.841e-02 (8,24,1)

IF 2hlpa2#289 AND 1a7ca_#370
THEN
GO:0004867: serine-type endopeptidase inhibitor activity: 2.261e-12 (8,47,7) OR
GO:0008201: heparin binding: 6.841e-02 (8,24,1)

IF 1ovaa_#297 AND 1ovaa_#161
THEN
GO:0004867: serine-type endopeptidase inhibitor activity: 2.261e-12 (8,47,7) OR
GO:0008201: heparin binding: 6.841e-02 (8,24,1)

IF 1ovaa_#161 AND 1qnja_#46
THEN
GO:0004867: serine-type endopeptidase inhibitor activity: 2.261e-12 (8,47,7) OR
GO:0005525: GTP binding: 1.353e-01 (8,49,1)

IF 1f0c.1#A317 AND 1e05i_#79

THEN
GO:0004867: serine-type endopeptidase inhibitor activity: 2.261e-12 (8,47,7) OR
GO:0008201: heparin binding: 6.841e-02 (8,24,1)

IF 1gdna_#26 AND 1g71a_#88 AND 1f42a2#123 AND 1elva1#513
THEN
GO:0004295: trypsin activity: 8.132e-13 (51,48,13) OR
GO:0004263: chymotrypsin activity: 2.488e-12 (51,41,12) OR
GO:0003809: thrombin activity: 6.059e-11 (51,10,7) OR
GO:0004867: serine-type endopeptidase inhibitor activity: 1.871e-05 (51,47,7) OR
GO:0005509: calcium ion binding: 1.243e-04 (51,160,11) OR
GO:0005529: sugar binding: 5.239e-01 (51,39,1)

IF 1hu4a_#267 AND 1oaa__#14
THEN
GO:0016616: oxidoreductase activity, acting on the CH-OH group of donors, NAD or NADP as acceptor: 8.171e-13 (19,59,10) OR
GO:0016627: oxidoreductase activity, acting on the CH-CH group of donors: 3.799e-06 (19,17,4) OR
GO:0016646: oxidoreductase activity, acting on the CH-NH group of donors, NAD or NADP as acceptor: 7.340e-03 (19,19,2) OR
GO:0008408: 3'-5' exonuclease activity: 8.713e-02 (19,13,1) OR
GO:0003887: DNA-directed DNA polymerase activity: 1.310e-01 (19,20,1) OR
GO:0005524: ATP binding: 8.315e-01 (19,243,1)

IF 1fm2.1#B308 AND 1h7wa4#478
THEN
GO:0015036: disulfide oxidoreductase activity: 8.212e-13 (21,22,8) OR
GO:0050660: FAD binding: 4.011e-09 (21,10,5) OR
GO:0016668: oxidoreductase activity, acting on sulfur group of donors, NAD or NADP as acceptor: 1.248e-08 (21,12,5) OR
GO:0016651: oxidoreductase activity, acting on NADH or NADPH: 9.588e-02 (21,13,1) OR
GO:0016627: oxidoreductase activity, acting on the CH-CH group of donors: 1.236e-01 (21,17,1) OR
GO:0004601: peroxidase activity: 1.505e-01 (21,21,1)

IF 2msba_#134 AND 1fxla2#169
THEN
GO:0005529: sugar binding: 1.008e-12 (13,39,8) OR
GO:0008201: heparin binding: 1.089e-01 (13,24,1) OR
GO:0005509: calcium ion binding: 1.751e-01 (13,160,2) OR
GO:0004263: chymotrypsin activity: 1.792e-01 (13,41,1) OR
GO:0004295: trypsin activity: 2.067e-01 (13,48,1)

IF 1fxla2#169 AND 1hq8a_#150
THEN
GO:0005529: sugar binding: 1.008e-12 (13,39,8) OR
GO:0008201: heparin binding: 1.089e-01 (13,24,1) OR
GO:0005509: calcium ion binding: 1.751e-01 (13,160,2) OR
GO:0004263: chymotrypsin activity: 1.792e-01 (13,41,1) OR
GO:0004295: trypsin activity: 2.067e-01 (13,48,1)

IF 1eq9a_#143 AND 1fu6a_#50
THEN
GO:0004263: chymotrypsin activity: 8.524e-13 (47,41,12) OR
GO:0004295: trypsin activity: 6.909e-12 (47,48,12) OR
GO:0003809: thrombin activity: 2.904e-07 (47,10,5) OR
GO:0004867: serine-type endopeptidase inhibitor activity: 1.071e-05 (47,47,7) OR
GO:0005509: calcium ion binding: 2.874e-04 (47,160,10) OR
GO:0004896: hematopoietin/interferon-class (D200-domain) cytokine receptor activity: 2.823e-01 (47,19,1)

IF 1ec7a1#216 AND 1hdr__#156
THEN
GO:0016616: oxidoreductase activity, acting on the CH-OH group of donors, NAD or NADP as acceptor: 1.027e-12 (14,59,9) OR
GO:0016854: racemase and epimerase activity: 1.852e-03 (14,13,2) OR
GO:0016627: oxidoreductase activity, acting on the CH-CH group of donors: 8.408e-02 (14,17,1) OR
GO:0008757: S-adenosylmethionine-dependent methyltransferase activity: 1.167e-01 (14,24,1) OR
GO:0016836: hydro-lyase activity: 1.572e-01 (14,33,1)

IF 1cyda_#231 AND 1evqa_#305
THEN
GO:0016616: oxidoreductase activity, acting on the CH-OH group of donors, NAD or NADP as acceptor: 1.027e-12 (14,59,9) OR
GO:0016627: oxidoreductase activity, acting on the CH-CH group of donors: 3.191e-03 (14,17,2) OR
GO:0016646: oxidoreductase activity, acting on the CH-NH group of donors, NAD or NADP as acceptor: 9.353e-02 (14,19,1) OR
GO:0000287: magnesium ion binding: 4.910e-01 (14,128,1) OR

GO:0005524: ATP binding: 7.304e-01 (14,243,1)

IF 1gdna_#230 AND 1gg6.1#B19 AND 1g71a_#77
THEN
GO:0004263: chymotrypsin activity: 1.294e-12 (27,41,10) OR
GO:0004295: trypsin activity: 7.250e-12 (27,48,10) OR
GO:0004867: serine-type endopeptidase inhibitor activity: 1.053e-02 (27,47,3) OR
GO:0005509: calcium ion binding: 6.994e-02 (27,160,4)

IF 1e2ka_#59 AND 1erv__#57
THEN
GO:0019201: nucleotide kinase activity: 1.294e-12 (22,13,7) OR
GO:0016776: phosphotransferase activity, phosphate group as acceptor: 2.576e-12 (22,14,7) OR
GO:0005524: ATP binding: 2.179e-03 (22,243,7) OR
GO:0005525: GTP binding: 3.302e-01 (22,49,1)

IF 1nksa_#13 AND 1c3pa_#6
THEN
GO:0019201: nucleotide kinase activity: 1.294e-12 (22,13,7) OR
GO:0016776: phosphotransferase activity, phosphate group as acceptor: 2.576e-12 (22,14,7) OR
GO:0005524: ATP binding: 2.179e-03 (22,243,7) OR
GO:0005525: GTP binding: 3.302e-01 (22,49,1)

IF 1eg5a_#199 AND 1c7na_#281
THEN
GO:0008483: transaminase activity: 1.819e-12 (9,17,6) OR
GO:0016846: carbon-sulfur lyase activity: 4.305e-04 (9,10,2) OR
GO:0016831: carboxy-lyase activity: 7.972e-02 (9,25,1)

IF 2dkb__#114 AND 1eg5a_#199
THEN
GO:0008483: transaminase activity: 1.819e-12 (9,17,6) OR
GO:0016846: carbon-sulfur lyase activity: 4.305e-04 (9,10,2) OR
GO:0016831: carboxy-lyase activity: 7.972e-02 (9,25,1)

IF 7taa_2#119 AND 1iira_#242
THEN
GO:0004556: alpha-amylase activity: 1.837e-12 (10,15,6) OR
GO:0005509: calcium ion binding: 1.756e-02 (10,160,3) OR
GO:0004867: serine-type endopeptidase inhibitor activity: 1.599e-01 (10,47,1)

IF 1jb3a_#64 AND 1b3ra1#223
THEN
GO:0004457: lactate dehydrogenase activity: 1.837e-12 (15,10,6) OR
GO:0016616: oxidoreductase activity, acting on the CH-OH group of donors, NAD or NADP as acceptor: 1.699e-10 (15,59,8) OR
GO:0016638: oxidoreductase activity, acting on the CH-NH2 group of donors: 8.982e-02 (15,17,1)

IF 1bf2_3#579 AND 1a4ya_#47
THEN
GO:0004556: alpha-amylase activity: 1.837e-12 (10,15,6) OR
GO:0005509: calcium ion binding: 1.756e-02 (10,160,3) OR
GO:0016758: transferase activity, transferring hexosyl groups: 3.971e-02 (10,11,1)

IF 1c4ra_#258 AND 1ton__#231
THEN
GO:0004263: chymotrypsin activity: 1.125e-12 (48,41,12) OR
GO:0004295: trypsin activity: 9.098e-12 (48,48,12) OR
GO:0003809: thrombin activity: 4.320e-09 (48,10,6) OR
GO:0004867: serine-type endopeptidase inhibitor activity: 6.176e-08 (48,47,9) OR
GO:0005509: calcium ion binding: 1.530e-03 (48,160,9)

IF 1eq9a_#143 AND 1eq9a_#32 AND 1eq9a_#162
THEN
GO:0004295: trypsin activity: 1.125e-12 (41,48,12) OR
GO:0004263: chymotrypsin activity: 4.863e-12 (41,41,11) OR
GO:0004867: serine-type endopeptidase inhibitor activity: 4.139e-06 (41,47,7) OR
GO:0003809: thrombin activity: 3.528e-04 (41,10,3) OR
GO:0005509: calcium ion binding: 2.144e-03 (41,160,8)

IF 1f0xa1#523 AND 1hq8a_#150

THEN
GO:0005529: sugar binding: 5.769e-12 (6,39,6)

IF 1f0xa1#523 AND 1hq8a_#223
THEN
GO:0005529: sugar binding: 5.769e-12 (6,39,6)

IF 1f97a1#62 AND 1elva1#513 AND 1gdna_#155 AND 1dy9.1#A44 AND 1ton__#47 AND 1cqqa_#163 AND 1autc_#209
THEN
GO:0004263: chymotrypsin activity: 9.735e-13 (36,41,11) OR
GO:0004295: trypsin activity: 6.557e-12 (36,48,11) OR
GO:0004867: serine-type endopeptidase inhibitor activity: 3.104e-04 (36,47,5) OR
GO:0003809: thrombin activity: 7.146e-03 (36,10,2) OR
GO:0005509: calcium ion binding: 1.672e-02 (36,160,6) OR
GO:0005529: sugar binding: 4.069e-01 (36,39,1)

IF 1i9ga_#67 AND 1ddja_#759 AND 1dlea_#53
THEN
GO:0004263: chymotrypsin activity: 9.735e-13 (36,41,11) OR
GO:0004295: trypsin activity: 6.557e-12 (36,48,11) OR
GO:0004867: serine-type endopeptidase inhibitor activity: 3.055e-03 (36,47,4) OR
GO:0005509: calcium ion binding: 4.145e-03 (36,160,7) OR
GO:0003809: thrombin activity: 7.146e-03 (36,10,2) OR
GO:0004896: hematopoietin/interferon-class (D200-domain) cytokine receptor activity: 2.239e-01 (36,19,1)

IF 1i9ga_#67 AND 1gdna_#155
THEN
GO:0004263: chymotrypsin activity: 9.735e-13 (36,41,11) OR
GO:0004295: trypsin activity: 6.557e-12 (36,48,11) OR
GO:0004867: serine-type endopeptidase inhibitor activity: 3.055e-03 (36,47,4) OR
GO:0005509: calcium ion binding: 4.145e-03 (36,160,7) OR
GO:0003809: thrombin activity: 7.146e-03 (36,10,2) OR
GO:0004896: hematopoietin/interferon-class (D200-domain) cytokine receptor activity: 2.239e-01 (36,19,1)

IF 1i9ga_#67 AND 1bio__#108
THEN
GO:0004263: chymotrypsin activity: 9.735e-13 (36,41,11) OR
GO:0004295: trypsin activity: 6.557e-12 (36,48,11) OR
GO:0004867: serine-type endopeptidase inhibitor activity: 3.055e-03 (36,47,4) OR
GO:0005509: calcium ion binding: 4.145e-03 (36,160,7) OR
GO:0003809: thrombin activity: 7.146e-03 (36,10,2) OR
GO:0004896: hematopoietin/interferon-class (D200-domain) cytokine receptor activity: 2.239e-01 (36,19,1)

IF 1b4va1#11 AND 1pbe_1#121 AND 1trb_1#112
THEN
GO:0015036: disulfide oxidoreductase activity: 1.285e-12 (22,22,8) OR
GO:0050660: FAD binding: 5.182e-09 (22,10,5) OR
GO:0016668: oxidoreductase activity, acting on sulfur group of donors, NAD or NADP as acceptor: 1.612e-08 (22,12,5) OR
GO:0016627: oxidoreductase activity, acting on the CH-CH group of donors: 2.888e-04 (22,17,3) OR
GO:0016638: oxidoreductase activity, acting on the CH-NH2 group of donors: 1.291e-01 (22,17,1)

IF 1h7wa4#208 AND 1cjca2#365
THEN
GO:0015036: disulfide oxidoreductase activity: 1.285e-12 (22,22,8) OR
GO:0016668: oxidoreductase activity, acting on sulfur group of donors, NAD or NADP as acceptor: 1.612e-08 (22,12,5) OR
GO:0050660: FAD binding: 6.491e-07 (22,10,4) OR
GO:0016627: oxidoreductase activity, acting on the CH-CH group of donors: 7.088e-06 (22,17,4) OR
GO:0016638: oxidoreductase activity, acting on the CH-NH2 group of donors: 1.291e-01 (22,17,1)

IF 1pme__#145 AND 1hcl__#82
THEN
GO:0004674: protein serine/threonine kinase activity: 1.075e-12 (26,42,10) OR
GO:0005524: ATP binding: 5.238e-06 (26,243,11) OR
GO:0005516: calmodulin binding: 2.124e-02 (26,24,2) OR
GO:0005066: transmembrane receptor protein tyrosine kinase signaling protein activity: 9.156e-02 (26,10,1) OR
GO:0004714: transmembrane receptor protein tyrosine kinase activity: 1.259e-01 (26,14,1) OR
GO:0004896: hematopoietin/interferon-class (D200-domain) cytokine receptor activity: 1.670e-01 (26,19,1)

IF 1howa_#551 AND 1pme__#145
THEN

GO:0004674: protein serine/threonine kinase activity: 1.075e-12 (26,42,10) OR
GO:0005524: ATP binding: 5.238e-06 (26,243,11) OR
GO:0005516: calmodulin binding: 2.124e-02 (26,24,2) OR
GO:0005066: transmembrane receptor protein tyrosine kinase signaling protein activity: 9.156e-02 (26,10,1) OR
GO:0004714: transmembrane receptor protein tyrosine kinase activity: 1.259e-01 (26,14,1) OR
GO:0004896: hematopoietin/interferon-class (D200-domain) cytokine receptor activity: 1.670e-01 (26,19,1)

IF 1pme__#145 AND 1a6o__#217
THEN
GO:0004674: protein serine/threonine kinase activity: 1.075e-12 (26,42,10) OR
GO:0005524: ATP binding: 5.238e-06 (26,243,11) OR
GO:0005516: calmodulin binding: 2.124e-02 (26,24,2) OR
GO:0005066: transmembrane receptor protein tyrosine kinase signaling protein activity: 9.156e-02 (26,10,1) OR
GO:0004714: transmembrane receptor protein tyrosine kinase activity: 1.259e-01 (26,14,1) OR
GO:0004896: hematopoietin/interferon-class (D200-domain) cytokine receptor activity: 1.670e-01 (26,19,1)

IF 2occb1#189 AND 1j9qa2#295
THEN
GO:0005507: copper ion binding: 1.093e-12 (20,38,9) OR
GO:0015078: hydrogen ion transporter activity: 2.349e-07 (20,21,5) OR
GO:0015082: di-, tri-valent inorganic cation transporter activity: 9.821e-02 (20,14,1) OR
GO:0046915: transition metal ion transporter activity: 9.821e-02 (20,14,1) OR
GO:0005509: calcium ion binding: 1.088e-01 (20,160,3) OR
GO:0003779: actin binding: 2.111e-01 (20,32,1)

IF 1qlsa_#81 AND 1c7wa_#105 AND 1jbaa_#157 AND 1ab4__#89
THEN
GO:0005509: calcium ion binding: 6.684e-12 (9,160,9)

IF 1bd3a_#110 AND 1h6va1#27
THEN
GO:0016763: transferase activity, transferring pentosyl groups: 1.706e-12 (18,28,8) OR
GO:0000287: magnesium ion binding: 5.618e-07 (18,128,8) OR
GO:0016866: intramolecular transferase activity: 7.660e-02 (18,12,1) OR
GO:0016651: oxidoreductase activity, acting on NADH or NADPH: 8.272e-02 (18,13,1)

IF 1fjsa_#84 AND 1ekbb_#85 AND 1ton__#112
THEN
GO:0004263: chymotrypsin activity: 1.371e-12 (37,41,11) OR
GO:0004295: trypsin activity: 9.214e-12 (37,48,11) OR
GO:0004867: serine-type endopeptidase inhibitor activity: 3.543e-04 (37,47,5) OR
GO:0005509: calcium ion binding: 1.060e-03 (37,160,8) OR
GO:0003809: thrombin activity: 7.539e-03 (37,10,2)

IF 1g71a_#77 AND 1c9la2#61 AND 1cgha_#130
THEN
GO:0004263: chymotrypsin activity: 1.371e-12 (37,41,11) OR
GO:0004295: trypsin activity: 9.214e-12 (37,48,11) OR
GO:0004867: serine-type endopeptidase inhibitor activity: 1.997e-06 (37,47,7) OR
GO:0005509: calcium ion binding: 4.865e-03 (37,160,7) OR
GO:0003809: thrombin activity: 1.280e-01 (37,10,1)

IF 1g71a_#77 AND 1autc_#188 AND 1dpga2#334
THEN
GO:0004263: chymotrypsin activity: 1.371e-12 (37,41,11) OR
GO:0004295: trypsin activity: 9.214e-12 (37,48,11) OR
GO:0004867: serine-type endopeptidase inhibitor activity: 2.948e-05 (37,47,6) OR
GO:0005509: calcium ion binding: 4.865e-03 (37,160,7) OR
GO:0003809: thrombin activity: 7.539e-03 (37,10,2)

IF 1qj5a_#268 AND 1jg8a_#172 AND 1elua_#216
THEN
GO:0008483: transaminase activity: 1.819e-12 (9,17,6) OR
GO:0016846: carbon-sulfur lyase activity: 3.259e-02 (9,10,1) OR
GO:0016866: intramolecular transferase activity: 3.900e-02 (9,12,1) OR
GO:0016831: carboxy-lyase activity: 7.972e-02 (9,25,1)

IF 2dkb__#46 AND 1qj5a_#268 AND 1e5ea_#71
THEN
GO:0008483: transaminase activity: 1.819e-12 (9,17,6) OR

GO:0016846: carbon-sulfur lyase activity: 3.259e-02 (9,10,1) OR
GO:0016866: intramolecular transferase activity: 3.900e-02 (9,12,1) OR
GO:0016831: carboxy-lyase activity: 7.972e-02 (9,25,1)

IF 2cb5a_#395 AND 1dkia_#54
THEN
GO:0004197: cysteine-type endopeptidase activity: 1.214e-12 (12,24,7) OR
GO:0019955: cytokine binding: 4.747e-02 (12,11,1) OR
GO:0042802: protein self binding: 5.168e-02 (12,12,1) OR
GO:0004177: aminopeptidase activity: 5.588e-02 (12,13,1) OR
GO:0004180: carboxypeptidase activity: 6.422e-02 (12,15,1) OR
GO:0051082: unfolded protein binding: 1.401e-01 (12,34,1)

IF 1cs8a_#188 AND 2cb5a_#395
THEN
GO:0004197: cysteine-type endopeptidase activity: 1.214e-12 (12,24,7) OR
GO:0019955: cytokine binding: 4.747e-02 (12,11,1) OR
GO:0042802: protein self binding: 5.168e-02 (12,12,1) OR
GO:0004177: aminopeptidase activity: 5.588e-02 (12,13,1) OR
GO:0004180: carboxypeptidase activity: 6.422e-02 (12,15,1) OR
GO:0051082: unfolded protein binding: 1.401e-01 (12,34,1)

IF 1pii_2#326 AND 1ceqa1#139
THEN
GO:0004457: lactate dehydrogenase activity: 1.837e-12 (15,10,6) OR
GO:0016616: oxidoreductase activity, acting on the CH-OH group of donors, NAD or NADP as acceptor: 8.731e-09 (15,59,7) OR
GO:0003700: transcription factor activity: 5.036e-01 (15,124,1) OR
GO:0005524: ATP binding: 7.546e-01 (15,243,1)

IF 1a65a3#383 AND 1fwxa1#526
THEN
GO:0005507: copper ion binding: 1.855e-12 (14,38,8) OR
GO:0015078: hydrogen ion transporter activity: 2.486e-06 (14,21,4) OR
GO:0015082: di-, tri-valent inorganic cation transporter activity: 6.973e-02 (14,14,1) OR
GO:0046915: transition metal ion transporter activity: 6.973e-02 (14,14,1)

IF 1cyx__#133 AND 1quna1#113
THEN
GO:0005507: copper ion binding: 1.855e-12 (14,38,8) OR
GO:0015078: hydrogen ion transporter activity: 2.486e-06 (14,21,4) OR
GO:0015082: di-, tri-valent inorganic cation transporter activity: 6.973e-02 (14,14,1) OR
GO:0046915: transition metal ion transporter activity: 6.973e-02 (14,14,1)

IF 1cyx__#133 AND 2cuaa_#83
THEN
GO:0005507: copper ion binding: 1.855e-12 (14,38,8) OR
GO:0015078: hydrogen ion transporter activity: 2.486e-06 (14,21,4) OR
GO:0015082: di-, tri-valent inorganic cation transporter activity: 6.973e-02 (14,14,1) OR
GO:0046915: transition metal ion transporter activity: 6.973e-02 (14,14,1)

IF 1fl2a1#472 AND 1qlaa2#13 AND 1d7ya1#255 AND 1trka2#429 AND 1h6va1#23
THEN
GO:0015036: disulfide oxidoreductase activity: 1.285e-12 (22,22,8) OR
GO:0050660: FAD binding: 5.182e-09 (22,10,5) OR
GO:0016668: oxidoreductase activity, acting on sulfur group of donors, NAD or NADP as acceptor: 1.612e-08 (22,12,5) OR
GO:0016627: oxidoreductase activity, acting on the CH-CH group of donors: 7.865e-03 (22,17,2) OR
GO:0016651: oxidoreductase activity, acting on NADH or NADPH: 1.002e-01 (22,13,1) OR
GO:0004601: peroxidase activity: 1.571e-01 (22,21,1)

IF 1i50a_#856 AND 1fc4a_#205 AND 1d6ja_#95 AND 1gpea1#271
THEN
GO:0015036: disulfide oxidoreductase activity: 1.285e-12 (22,22,8) OR
GO:0016668: oxidoreductase activity, acting on sulfur group of donors, NAD or NADP as acceptor: 1.183e-10 (22,12,6) OR
GO:0050660: FAD binding: 5.182e-09 (22,10,5) OR
GO:0016651: oxidoreductase activity, acting on NADH or NADPH: 1.002e-01 (22,13,1) OR
GO:0004725: protein tyrosine phosphatase activity: 1.148e-01 (22,15,1) OR
GO:0004601: peroxidase activity: 1.571e-01 (22,21,1)

IF 1bg6_2#24 AND 1iba__#39
THEN

GO:0015036: disulfide oxidoreductase activity: 1.285e-12 (22,22,8) OR
GO:0016668: oxidoreductase activity, acting on sulfur group of donors, NAD or NADP as acceptor: 1.183e-10 (22,12,6) OR
GO:0050660: FAD binding: 5.182e-09 (22,10,5) OR
GO:0016651: oxidoreductase activity, acting on NADH or NADPH: 1.002e-01 (22,13,1) OR
GO:0016831: carboxy-lyase activity: 1.842e-01 (22,25,1) OR
GO:0000287: magnesium ion binding: 6.545e-01 (22,128,1)

IF 1bqya_#115 AND 1a0la_#193 AND 1gdna_#151 AND 1danh_#70 AND 1gdna_#121
THEN
GO:0004263: chymotrypsin activity: 1.294e-12 (27,41,10) OR
GO:0004295: trypsin activity: 7.250e-12 (27,48,10) OR
GO:0005509: calcium ion binding: 6.994e-02 (27,160,4) OR
GO:0004896: hematopoietin/interferon-class (D200-domain) cytokine receptor activity: 1.729e-01 (27,19,1) OR
GO:0008201: heparin binding: 2.134e-01 (27,24,1) OR
GO:0004867: serine-type endopeptidase inhibitor activity: 3.763e-01 (27,47,1)

IF 1as4.1#A336 AND 1sek__#383
THEN
GO:0004867: serine-type endopeptidase inhibitor activity: 1.981e-12 (12,47,8) OR
GO:0008201: heparin binding: 1.009e-01 (12,24,1) OR
GO:0005509: calcium ion binding: 1.538e-01 (12,160,2) OR
GO:0000287: magnesium ion binding: 4.393e-01 (12,128,1)

IF 1gdna_#119 AND 1cqqa_#163
THEN
GO:0004263: chymotrypsin activity: 1.992e-12 (28,41,10) OR
GO:0004295: trypsin activity: 1.113e-11 (28,48,10) OR
GO:0004867: serine-type endopeptidase inhibitor activity: 1.175e-03 (28,47,4) OR
GO:0005509: calcium ion binding: 7.802e-02 (28,160,4)

IF 1nsj__#21 AND 7taa_2#295
THEN
GO:0004556: alpha-amylase activity: 4.031e-12 (11,15,6) OR
GO:0005509: calcium ion binding: 2.271e-04 (11,160,5)

IF 1ecfa1#367 AND 1bd3a_#195
THEN
GO:0016763: transferase activity, transferring pentosyl groups: 4.126e-12 (12,28,7) OR
GO:0000287: magnesium ion binding: 1.284e-04 (12,128,5)

IF 1ton__#120 AND 1gg6.1#C156 AND 1f42a2#123 AND 1a0la_#160
THEN
GO:0004295: trypsin activity: 1.402e-12 (53,48,13) OR
GO:0004263: chymotrypsin activity: 4.098e-12 (53,41,12) OR
GO:0003809: thrombin activity: 8.052e-11 (53,10,7) OR
GO:0004867: serine-type endopeptidase inhibitor activity: 1.531e-07 (53,47,9) OR
GO:0005509: calcium ion binding: 1.795e-04 (53,160,11) OR
GO:0005529: sugar binding: 5.377e-01 (53,39,1)

IF 1azza_#51 AND 1fjsa_#28 AND 1arb__#210
THEN
GO:0004295: trypsin activity: 1.481e-12 (32,48,11) OR
GO:0004263: chymotrypsin activity: 9.389e-12 (32,41,10) OR
GO:0003809: thrombin activity: 5.670e-03 (32,10,2) OR
GO:0004867: serine-type endopeptidase inhibitor activity: 1.681e-02 (32,47,3) OR
GO:0005509: calcium ion binding: 3.639e-02 (32,160,5) OR
GO:0005529: sugar binding: 3.712e-01 (32,39,1)

IF 1d2fa_#348 AND 1ajsa_#139
THEN
GO:0008483: transaminase activity: 4.532e-12 (10,17,6) OR
GO:0016846: carbon-sulfur lyase activity: 1.903e-08 (10,10,4)

IF 1d2fa_#348 AND 1fc4a_#111
THEN
GO:0008483: transaminase activity: 4.532e-12 (10,17,6) OR
GO:0016846: carbon-sulfur lyase activity: 1.903e-08 (10,10,4)

IF 1qisa_#107 AND 1e3a.1#A30
THEN

GO:0008483: transaminase activity: 4.532e-12 (10,17,6) OR
GO:0016846: carbon-sulfur lyase activity: 1.903e-08 (10,10,4)

IF 1gdna_#230 AND 1a7s__#148 AND 1ton__#179 AND 1gdna_#56 AND 1ekbb_#85 AND 1arb__#193 AND 1ton__#211 AND 1autc_#45
THEN
GO:0004263: chymotrypsin activity: 2.322e-12 (20,41,9) OR
GO:0004295: trypsin activity: 1.083e-11 (20,48,9) OR
GO:0004867: serine-type endopeptidase inhibitor activity: 2.947e-01 (20,47,1) OR
GO:0005509: calcium ion binding: 7.032e-01 (20,160,1)

IF 1pot__#138 AND 1jatb_#120
THEN
GO:0004842: ubiquitin-protein ligase activity: 9.321e-12 (5,19,5)

IF 1f97a1#62 AND 1vdra_#37
THEN
GO:0016646: oxidoreductase activity, acting on the CH-NH group of donors, NAD or NADP as acceptor: 9.321e-12 (5,19,5)

IF 1jb9a2#173 AND 1vdra_#37
THEN
GO:0016646: oxidoreductase activity, acting on the CH-NH group of donors, NAD or NADP as acceptor: 9.321e-12 (5,19,5)

IF 2e2c__#67 AND 1c4zd_#99
THEN
GO:0004842: ubiquitin-protein ligase activity: 9.321e-12 (5,19,5)

IF 1c4zd_#99 AND 1ifc__#89
THEN
GO:0004842: ubiquitin-protein ligase activity: 9.321e-12 (5,19,5)

IF 1gcoa_#119 AND 1b16a_#62
THEN
GO:0016616: oxidoreductase activity, acting on the CH-OH group of donors, NAD or NADP as acceptor: 4.661e-12 (11,59,8) OR
GO:0016627: oxidoreductase activity, acting on the CH-CH group of donors: 3.229e-05 (11,17,3)

IF 1h5qa_#153 AND 1ldna1#94
THEN
GO:0016616: oxidoreductase activity, acting on the CH-OH group of donors, NAD or NADP as acceptor: 4.661e-12 (11,59,8) OR
GO:0016627: oxidoreductase activity, acting on the CH-CH group of donors: 3.229e-05 (11,17,3)

IF 1b16a_#62 AND 1h5qa_#70
THEN
GO:0016616: oxidoreductase activity, acting on the CH-OH group of donors, NAD or NADP as acceptor: 4.661e-12 (11,59,8) OR
GO:0016627: oxidoreductase activity, acting on the CH-CH group of donors: 3.229e-05 (11,17,3)

IF 1g71a_#77 AND 1autc_#188 AND 1g51a3#525
THEN
GO:0004263: chymotrypsin activity: 1.910e-12 (38,41,11) OR
GO:0004295: trypsin activity: 1.280e-11 (38,48,11) OR
GO:0004867: serine-type endopeptidase inhibitor activity: 2.416e-06 (38,47,7) OR
GO:0005509: calcium ion binding: 5.676e-03 (38,160,7) OR
GO:0003809: thrombin activity: 7.943e-03 (38,10,2)

IF 1ovaa_#284 AND 1a7ca_#202
THEN
GO:0004867: serine-type endopeptidase inhibitor activity: 1.981e-12 (12,47,8) OR
GO:0003899: DNA-directed RNA polymerase activity: 5.168e-02 (12,12,1) OR
GO:0046983: protein dimerization activity: 7.249e-02 (12,17,1) OR
GO:0008201: heparin binding: 1.009e-01 (12,24,1) OR
GO:0003700: transcription factor activity: 4.288e-01 (12,124,1)

IF 1ovaa_#284 AND 1qj8a_#64
THEN
GO:0004867: serine-type endopeptidase inhibitor activity: 1.981e-12 (12,47,8) OR
GO:0003899: DNA-directed RNA polymerase activity: 5.168e-02 (12,12,1) OR
GO:0046983: protein dimerization activity: 7.249e-02 (12,17,1) OR
GO:0008201: heparin binding: 1.009e-01 (12,24,1) OR
GO:0003700: transcription factor activity: 4.288e-01 (12,124,1)

IF 1gdea_#243 AND 1elua_#95
THEN
GO:0008483: transaminase activity: 1.667e-12 (17,17,7) OR
GO:0016846: carbon-sulfur lyase activity: 4.532e-12 (17,10,6) OR
GO:0003899: DNA-directed RNA polymerase activity: 7.249e-02 (17,12,1) OR
GO:0046983: protein dimerization activity: 1.012e-01 (17,17,1) OR
GO:0016831: carboxy-lyase activity: 1.454e-01 (17,25,1) OR
GO:0030145: manganese ion binding: 2.129e-01 (17,38,1)

IF 1e3a.1#A30 AND 1gdea_#379
THEN
GO:0008483: transaminase activity: 1.667e-12 (17,17,7) OR
GO:0016846: carbon-sulfur lyase activity: 2.131e-07 (17,10,4) OR
GO:0004812: tRNA ligase activity: 1.508e-01 (17,26,1) OR
GO:0000287: magnesium ion binding: 1.885e-01 (17,128,2) OR
GO:0016616: oxidoreductase activity, acting on the CH-OH group of donors, NAD or NADP as acceptor: 3.115e-01 (17,59,1) OR
GO:0005524: ATP binding: 4.560e-01 (17,243,2)

IF 1dfoa_#362 AND 1gdea_#379
THEN
GO:0008483: transaminase activity: 1.667e-12 (17,17,7) OR
GO:0016846: carbon-sulfur lyase activity: 1.227e-09 (17,10,5) OR
GO:0016831: carboxy-lyase activity: 1.010e-02 (17,25,2) OR
GO:0016668: oxidoreductase activity, acting on sulfur group of donors, NAD or NADP as acceptor: 7.249e-02 (17,12,1) OR
GO:0016763: transferase activity, transferring pentosyl groups: 1.615e-01 (17,28,1) OR
GO:0016616: oxidoreductase activity, acting on the CH-OH group of donors, NAD or NADP as acceptor: 3.115e-01 (17,59,1)

IF 1ja9a_#202 AND 1oaa__#14
THEN
GO:0016616: oxidoreductase activity, acting on the CH-OH group of donors, NAD or NADP as acceptor: 2.525e-12 (15,59,9) OR
GO:0016627: oxidoreductase activity, acting on the CH-CH group of donors: 1.359e-06 (15,17,4) OR
GO:0016646: oxidoreductase activity, acting on the CH-NH group of donors, NAD or NADP as acceptor: 9.988e-02 (15,19,1) OR
GO:0005524: ATP binding: 7.546e-01 (15,243,1)

IF 1e3ja2#270 AND 1e6ua_#10
THEN
GO:0016616: oxidoreductase activity, acting on the CH-OH group of donors, NAD or NADP as acceptor: 2.525e-12 (15,59,9) OR
GO:0016627: oxidoreductase activity, acting on the CH-CH group of donors: 8.768e-05 (15,17,3) OR
GO:0004457: lactate dehydrogenase activity: 1.241e-03 (15,10,2) OR
GO:0016646: oxidoreductase activity, acting on the CH-NH group of donors, NAD or NADP as acceptor: 9.988e-02 (15,19,1)

IF 1fuma2#370 AND 1cb8a2#613
THEN
GO:0015036: disulfide oxidoreductase activity: 2.576e-12 (14,22,7) OR
GO:0016668: oxidoreductase activity, acting on sulfur group of donors, NAD or NADP as acceptor: 2.111e-07 (14,12,4) OR
GO:0050660: FAD binding: 1.078e-03 (14,10,2) OR
GO:0016638: oxidoreductase activity, acting on the CH-NH2 group of donors: 8.408e-02 (14,17,1)

IF 1e2ka_#59 AND 1aqua_#126
THEN
GO:0019201: nucleotide kinase activity: 2.616e-12 (24,13,7) OR
GO:0016776: phosphotransferase activity, phosphate group as acceptor: 5.203e-12 (24,14,7) OR
GO:0005524: ATP binding: 1.218e-04 (24,243,9) OR
GO:0005525: GTP binding: 3.543e-01 (24,49,1)

IF 1g71a_#77 AND 1gg6.1#C156 AND 1b3qa2#623 AND 2hlca_#54
THEN
GO:0004295: trypsin activity: 2.132e-12 (43,48,12) OR
GO:0004263: chymotrypsin activity: 8.674e-12 (43,41,11) OR
GO:0004867: serine-type endopeptidase inhibitor activity: 3.893e-07 (43,47,8) OR
GO:0005509: calcium ion binding: 1.306e-04 (43,160,10) OR
GO:0003809: thrombin activity: 1.010e-02 (43,10,2)

IF 1h8d.1#H184 AND 1cgha_#227 AND 1qqga1#95
THEN
GO:0004263: chymotrypsin activity: 1.922e-12 (50,41,12) OR
GO:0004295: trypsin activity: 1.546e-11 (50,48,12) OR
GO:0003809: thrombin activity: 5.580e-09 (50,10,6) OR
GO:0004867: serine-type endopeptidase inhibitor activity: 1.317e-06 (50,47,8) OR
GO:0005509: calcium ion binding: 1.027e-04 (50,160,11) OR

GO:0005529: sugar binding: 5.168e-01 (50,39,1)

IF 1cjca2#103 AND 1dpja_#322
THEN
GO:0015036: disulfide oxidoreductase activity: 1.961e-12 (23,22,8) OR
GO:0050660: FAD binding: 3.669e-11 (23,10,6) OR
GO:0016668: oxidoreductase activity, acting on sulfur group of donors, NAD or NADP as acceptor: 1.597e-10 (23,12,6) OR
GO:0016651: oxidoreductase activity, acting on NADH or NADPH: 1.046e-01 (23,13,1) OR
GO:0016705: oxidoreductase activity, acting on paired donors, with incorporation or reduction of molecular oxygen: 1.986e-01 (23,26,1) OR
GO:0004497: monooxygenase activity: 1.986e-01 (23,26,1)

IF 1autc_#188 AND 1eq9a_#162 AND 1cgha_#130
THEN
GO:0004263: chymotrypsin activity: 3.008e-12 (29,41,10) OR
GO:0004295: trypsin activity: 1.678e-11 (29,48,10) OR
GO:0004867: serine-type endopeptidase inhibitor activity: 1.070e-04 (29,47,5) OR
GO:0005509: calcium ion binding: 8.655e-02 (29,160,4)

IF 1hx0a2#258 AND 1bvza3#416
THEN
GO:0004556: alpha-amylase activity: 4.031e-12 (11,15,6) OR
GO:0005509: calcium ion binding: 2.730e-03 (11,160,4) OR
GO:0004867: serine-type endopeptidase inhibitor activity: 1.745e-01 (11,47,1)

IF 1nuka_#80 AND 1bvza3#416
THEN
GO:0004556: alpha-amylase activity: 4.031e-12 (11,15,6) OR
GO:0005509: calcium ion binding: 2.730e-03 (11,160,4) OR
GO:0000287: magnesium ion binding: 4.115e-01 (11,128,1)

IF 1hx0a2#294 AND 1hx0a2#258
THEN
GO:0004556: alpha-amylase activity: 4.031e-12 (11,15,6) OR
GO:0005509: calcium ion binding: 2.730e-03 (11,160,4) OR
GO:0004867: serine-type endopeptidase inhibitor activity: 1.745e-01 (11,47,1)

IF 1gcya2#331 AND 7taa_2#295 AND 1a65a1#108
THEN
GO:0004556: alpha-amylase activity: 4.031e-12 (11,15,6) OR
GO:0005509: calcium ion binding: 2.730e-03 (11,160,4) OR
GO:0004867: serine-type endopeptidase inhibitor activity: 1.745e-01 (11,47,1)

IF 1qsta_#154 AND 1evqa_#305
THEN
GO:0004812: tRNA ligase activity: 2.456e-12 (20,26,8) OR
GO:0005524: ATP binding: 1.742e-04 (20,243,8) OR
GO:0000049: tRNA binding: 9.152e-02 (20,13,1) OR
GO:0008080: N-acetyltransferase activity: 9.152e-02 (20,13,1) OR
GO:0000287: magnesium ion binding: 2.412e-01 (20,128,2)

IF 1bd3a_#195 AND 1ihua2#525
THEN
GO:0016763: transferase activity, transferring pentosyl groups: 4.126e-12 (12,28,7) OR
GO:0000287: magnesium ion binding: 1.714e-03 (12,128,4) OR
GO:0003700: transcription factor activity: 4.288e-01 (12,124,1)

IF 1cm9a_#57 AND 1ha6a_#20
THEN
GO:0008009: chemokine activity: 4.229e-12 (7,10,5) OR
GO:0004812: tRNA ligase activity: 6.498e-02 (7,26,1) OR
GO:0005524: ATP binding: 4.803e-01 (7,243,1)

IF 1e3ua_#205 AND 1czan1#92
THEN
GO:0008800: beta-lactamase activity: 4.229e-12 (7,10,5) OR
GO:0016814: hydrolase activity, acting on carbon-nitrogen (but not peptide) bonds, in cyclic amidines: 2.543e-02 (7,10,1) OR
GO:0008270: zinc ion binding: 2.468e-01 (7,108,1)

IF 1cm9a_#57 AND 1ha6a_#59

THEN
GO:0008009: chemokine activity: 4.229e-12 (7,10,5) OR
GO:0004812: tRNA ligase activity: 6.498e-02 (7,26,1) OR
GO:0005524: ATP binding: 4.803e-01 (7,243,1)

IF 1ghpa_#236 AND 1ga0a_#236
THEN
GO:0008800: beta-lactamase activity: 4.229e-12 (7,10,5) OR
GO:0004177: aminopeptidase activity: 3.296e-02 (7,13,1) OR
GO:0004180: carboxypeptidase activity: 3.794e-02 (7,15,1)

IF 1f0xa1#278 AND 1el0a_#28 AND 1el0a_#62
THEN
GO:0008009: chemokine activity: 4.229e-12 (7,10,5) OR
GO:0008408: 3'-5' exonuclease activity: 3.296e-02 (7,13,1) OR
GO:0003887: DNA-directed DNA polymerase activity: 5.031e-02 (7,20,1)

IF 1sgpe_#42 AND 1ekbb_#228 AND 1ton__#181
THEN
GO:0004295: trypsin activity: 3.219e-12 (18,48,9) OR
GO:0004263: chymotrypsin activity: 4.456e-06 (18,41,5) OR
GO:0004867: serine-type endopeptidase inhibitor activity: 3.273e-03 (18,47,3) OR
GO:0005509: calcium ion binding: 6.647e-01 (18,160,1)

IF 1gdna_#230 AND 1sgpe_#42
THEN
GO:0004295: trypsin activity: 3.219e-12 (18,48,9) OR
GO:0004263: chymotrypsin activity: 4.456e-06 (18,41,5) OR
GO:0004867: serine-type endopeptidase inhibitor activity: 3.273e-03 (18,47,3) OR
GO:0005509: calcium ion binding: 6.647e-01 (18,160,1)

IF 1qlaa2#383 AND 1qs0a1#119
THEN
GO:0015036: disulfide oxidoreductase activity: 2.576e-12 (14,22,7) OR
GO:0016668: oxidoreductase activity, acting on sulfur group of donors, NAD or NADP as acceptor: 2.111e-07 (14,12,4) OR
GO:0050660: FAD binding: 5.029e-02 (14,10,1) OR
GO:0016627: oxidoreductase activity, acting on the CH-CH group of donors: 8.408e-02 (14,17,1) OR
GO:0000287: magnesium ion binding: 4.910e-01 (14,128,1)

IF 1auk__#375 AND 1cjca2#365
THEN
GO:0015036: disulfide oxidoreductase activity: 2.576e-12 (14,22,7) OR
GO:0016668: oxidoreductase activity, acting on sulfur group of donors, NAD or NADP as acceptor: 2.313e-05 (14,12,3) OR
GO:0016627: oxidoreductase activity, acting on the CH-CH group of donors: 3.191e-03 (14,17,2) OR
GO:0050660: FAD binding: 5.029e-02 (14,10,1) OR
GO:0004601: peroxidase activity: 1.029e-01 (14,21,1)

IF 1h8d.1#H184 AND 1eq9a_#162 AND 1dlea_#53
THEN
GO:0004263: chymotrypsin activity: 2.633e-12 (39,41,11) OR
GO:0004295: trypsin activity: 1.761e-11 (39,48,11) OR
GO:0004867: serine-type endopeptidase inhibitor activity: 4.031e-05 (39,47,6) OR
GO:0003809: thrombin activity: 3.036e-04 (39,10,3) OR
GO:0005509: calcium ion binding: 1.527e-03 (39,160,8)

IF 1c4ka2#223 AND 1dfoa_#362
THEN
GO:0008483: transaminase activity: 4.532e-12 (10,17,6) OR
GO:0016846: carbon-sulfur lyase activity: 4.217e-06 (10,10,3) OR
GO:0016831: carboxy-lyase activity: 8.819e-02 (10,25,1)

IF 2oata_#81 AND 1aqua_#126
THEN
GO:0008483: transaminase activity: 4.532e-12 (10,17,6) OR
GO:0016846: carbon-sulfur lyase activity: 4.217e-06 (10,10,3) OR
GO:0016866: intramolecular transferase activity: 4.324e-02 (10,12,1)

IF 1fmca_#86 AND 1hdr__#149
THEN
GO:0016616: oxidoreductase activity, acting on the CH-OH group of donors, NAD or NADP as acceptor: 4.661e-12 (11,59,8) OR

GO:0016627: oxidoreductase activity, acting on the CH-CH group of donors: 1.950e-03 (11,17,2) OR
GO:0016646: oxidoreductase activity, acting on the CH-NH group of donors, NAD or NADP as acceptor: 7.421e-02 (11,19,1)

IF 1fmca_#248 AND 1ihua2#525
THEN
GO:0016616: oxidoreductase activity, acting on the CH-OH group of donors, NAD or NADP as acceptor: 4.661e-12 (11,59,8) OR
GO:0016627: oxidoreductase activity, acting on the CH-CH group of donors: 1.950e-03 (11,17,2) OR
GO:0016646: oxidoreductase activity, acting on the CH-NH group of donors, NAD or NADP as acceptor: 7.421e-02 (11,19,1)

IF 1b16a_#62 AND 1eno__#137
THEN
GO:0016616: oxidoreductase activity, acting on the CH-OH group of donors, NAD or NADP as acceptor: 4.661e-12 (11,59,8) OR
GO:0016627: oxidoreductase activity, acting on the CH-CH group of donors: 1.950e-03 (11,17,2) OR
GO:0016646: oxidoreductase activity, acting on the CH-NH group of donors, NAD or NADP as acceptor: 7.421e-02 (11,19,1)

IF 1b16a_#184 AND 1c3pa_#6
THEN
GO:0016616: oxidoreductase activity, acting on the CH-OH group of donors, NAD or NADP as acceptor: 4.661e-12 (11,59,8) OR
GO:0016627: oxidoreductase activity, acting on the CH-CH group of donors: 1.950e-03 (11,17,2) OR
GO:0016854: racemase and epimerase activity: 5.134e-02 (11,13,1)

IF 1gdna_#230 AND 2hlca_#134
THEN
GO:0004295: trypsin activity: 2.882e-12 (25,48,10) OR
GO:0004263: chymotrypsin activity: 1.151e-09 (25,41,8) OR
GO:0005509: calcium ion binding: 5.515e-02 (25,160,4) OR
GO:0004867: serine-type endopeptidase inhibitor activity: 6.794e-02 (25,47,2) OR
GO:0003809: thrombin activity: 8.819e-02 (25,10,1)

IF 1fuma2#221 AND 1qrra_#69
THEN
GO:0015036: disulfide oxidoreductase activity: 2.927e-12 (24,22,8) OR
GO:0050660: FAD binding: 4.885e-11 (24,10,6) OR
GO:0016668: oxidoreductase activity, acting on sulfur group of donors, NAD or NADP as acceptor: 2.125e-10 (24,12,6) OR
GO:0016627: oxidoreductase activity, acting on the CH-CH group of donors: 3.767e-04 (24,17,3) OR
GO:0016651: oxidoreductase activity, acting on NADH or NADPH: 1.089e-01 (24,13,1)

IF 1fuma2#221 AND 1ojt_2#336
THEN
GO:0015036: disulfide oxidoreductase activity: 2.927e-12 (24,22,8) OR
GO:0050660: FAD binding: 4.885e-11 (24,10,6) OR
GO:0016668: oxidoreductase activity, acting on sulfur group of donors, NAD or NADP as acceptor: 2.125e-10 (24,12,6) OR
GO:0016627: oxidoreductase activity, acting on the CH-CH group of donors: 3.767e-04 (24,17,3) OR
GO:0016651: oxidoreductase activity, acting on NADH or NADPH: 1.089e-01 (24,13,1)

IF 1fuma2#221 AND 1fc4a_#205
THEN
GO:0015036: disulfide oxidoreductase activity: 2.927e-12 (24,22,8) OR
GO:0050660: FAD binding: 4.885e-11 (24,10,6) OR
GO:0016668: oxidoreductase activity, acting on sulfur group of donors, NAD or NADP as acceptor: 2.125e-10 (24,12,6) OR
GO:0016627: oxidoreductase activity, acting on the CH-CH group of donors: 3.767e-04 (24,17,3) OR
GO:0016651: oxidoreductase activity, acting on NADH or NADPH: 1.089e-01 (24,13,1)

IF 1fuma2#221 AND 1gpea1#271
THEN
GO:0015036: disulfide oxidoreductase activity: 2.927e-12 (24,22,8) OR
GO:0050660: FAD binding: 4.885e-11 (24,10,6) OR
GO:0016668: oxidoreductase activity, acting on sulfur group of donors, NAD or NADP as acceptor: 2.125e-10 (24,12,6) OR
GO:0016627: oxidoreductase activity, acting on the CH-CH group of donors: 3.767e-04 (24,17,3) OR
GO:0016651: oxidoreductase activity, acting on NADH or NADPH: 1.089e-01 (24,13,1)

IF 1danh_#152 AND 1qnja_#77
THEN
GO:0004263: chymotrypsin activity: 3.008e-12 (29,41,10) OR
GO:0004295: trypsin activity: 1.678e-11 (29,48,10) OR
GO:0004867: serine-type endopeptidase inhibitor activity: 1.284e-02 (29,47,3) OR
GO:0005509: calcium ion binding: 2.471e-02 (29,160,5) OR
GO:0004896: hematopoietin/interferon-class (D200-domain) cytokine receptor activity: 1.845e-01 (29,19,1)

IF 1azza_#51 AND 1g71a_#77 AND 1qnja_#124 AND 1fjsa_#158 AND 1c5y.1#B241 AND 1ton__#47 AND 1arb__#55

THEN
GO:0004263: chymotrypsin activity: 3.008e-12 (29,41,10) OR
GO:0004295: trypsin activity: 1.678e-11 (29,48,10) OR
GO:0005509: calcium ion binding: 5.728e-03 (29,160,6) OR
GO:0004867: serine-type endopeptidase inhibitor activity: 8.808e-02 (29,47,2) OR
GO:0003809: thrombin activity: 1.016e-01 (29,10,1)

IF 1qp8a1#153 AND 1b3ra1#218
THEN
GO:0016616: oxidoreductase activity, acting on the CH-OH group of donors, NAD or NADP as acceptor: 2.525e-12 (15,59,9) OR
GO:0016620: oxidoreductase activity, acting on the aldehyde or oxo group of donors, NAD or NADP as acceptor: 1.241e-03 (15,10,2) OR
GO:0004457: lactate dehydrogenase activity: 5.379e-02 (15,10,1) OR
GO:0016646: oxidoreductase activity, acting on the CH-NH group of donors, NAD or NADP as acceptor: 9.988e-02 (15,19,1) OR
GO:0015036: disulfide oxidoreductase activity: 1.148e-01 (15,22,1) OR
GO:0005524: ATP binding: 7.546e-01 (15,243,1)

IF 1jlna_#437 AND 1fwxa1#526
THEN
GO:0005507: copper ion binding: 3.935e-12 (15,38,8) OR
GO:0015078: hydrogen ion transporter activity: 3.372e-06 (15,21,4) OR
GO:0051082: unfolded protein binding: 1.433e-02 (15,34,2) OR
GO:0005509: calcium ion binding: 5.975e-01 (15,160,1)

IF 1qsta_#154 AND 1atia2#189
THEN
GO:0004812: tRNA ligase activity: 3.944e-12 (21,26,8) OR
GO:0005524: ATP binding: 2.599e-04 (21,243,8) OR
GO:0000049: tRNA binding: 4.193e-03 (21,13,2) OR
GO:0000287: magnesium ion binding: 7.259e-02 (21,128,3)

IF 1h4vb2#108 AND 1pysb5#672
THEN
GO:0004812: tRNA ligase activity: 3.944e-12 (21,26,8) OR
GO:0005524: ATP binding: 2.599e-04 (21,243,8) OR
GO:0000049: tRNA binding: 4.193e-03 (21,13,2) OR
GO:0000287: magnesium ion binding: 7.259e-02 (21,128,3)

IF 1azza_#51 AND 1qqsa_#124
THEN
GO:0004263: chymotrypsin activity: 2.633e-12 (39,41,11) OR
GO:0004295: trypsin activity: 1.761e-11 (39,48,11) OR
GO:0003809: thrombin activity: 7.081e-06 (39,10,4) OR
GO:0004867: serine-type endopeptidase inhibitor activity: 4.560e-04 (39,47,5) OR
GO:0005509: calcium ion binding: 6.583e-03 (39,160,7) OR
GO:0004896: hematopoietin/interferon-class (D200-domain) cytokine receptor activity: 2.403e-01 (39,19,1)

IF 1danh_#93 AND 1danh_#48 AND 1danh_#142 AND 1qnja_#46
THEN
GO:0004263: chymotrypsin activity: 2.633e-12 (39,41,11) OR
GO:0004295: trypsin activity: 1.761e-11 (39,48,11) OR
GO:0005509: calcium ion binding: 3.054e-04 (39,160,9) OR
GO:0004867: serine-type endopeptidase inhibitor activity: 4.560e-04 (39,47,5) OR
GO:0003809: thrombin activity: 8.355e-03 (39,10,2) OR
GO:0004896: hematopoietin/interferon-class (D200-domain) cytokine receptor activity: 2.403e-01 (39,19,1)

IF 1ejfa_#10 AND 1bvza3#416
THEN
GO:0004556: alpha-amylase activity: 4.031e-12 (11,15,6) OR
GO:0005509: calcium ion binding: 2.312e-02 (11,160,3) OR
GO:0016854: racemase and epimerase activity: 5.134e-02 (11,13,1) OR
GO:0000287: magnesium ion binding: 4.115e-01 (11,128,1)

IF 1eq9a_#143 AND 1ejda_#6
THEN
GO:0003809: thrombin activity: 2.863e-12 (34,10,7) OR
GO:0004263: chymotrypsin activity: 4.088e-07 (34,41,7) OR
GO:0004295: trypsin activity: 1.259e-06 (34,48,7) OR
GO:0004867: serine-type endopeptidase inhibitor activity: 2.351e-04 (34,47,5) OR
GO:0005509: calcium ion binding: 2.950e-03 (34,160,7) OR

GO:0005529: sugar binding: 3.893e-01 (34,39,1)

IF 1eg5a_#199 AND 1qs0a1#119
THEN
GO:0008483: transaminase activity: 4.532e-12 (10,17,6) OR
GO:0016846: carbon-sulfur lyase activity: 5.371e-04 (10,10,2) OR
GO:0016831: carboxy-lyase activity: 8.819e-02 (10,25,1) OR
GO:0016616: oxidoreductase activity, acting on the CH-OH group of donors, NAD or NADP as acceptor: 1.969e-01 (10,59,1)

IF 1ec7a1#216 AND 1bdb__#228
THEN
GO:0016616: oxidoreductase activity, acting on the CH-OH group of donors, NAD or NADP as acceptor: 4.661e-12 (11,59,8) OR
GO:0016854: racemase and epimerase activity: 5.134e-02 (11,13,1) OR
GO:0016627: oxidoreductase activity, acting on the CH-CH group of donors: 6.664e-02 (11,17,1) OR
GO:0016836: hydro-lyase activity: 1.256e-01 (11,33,1)

IF 1hle.1#B381 AND 1hbza_#96
THEN
GO:0004867: serine-type endopeptidase inhibitor activity: 1.899e-11 (6,47,6)

IF 1ddja_#640 AND 1bio__#108
THEN
GO:0004295: trypsin activity: 3.202e-12 (34,48,11) OR
GO:0004263: chymotrypsin activity: 1.869e-11 (34,41,10) OR
GO:0005509: calcium ion binding: 5.796e-04 (34,160,8) OR
GO:0003809: thrombin activity: 6.388e-03 (34,10,2) OR
GO:0004867: serine-type endopeptidase inhibitor activity: 1.154e-01 (34,47,2) OR
GO:0004896: hematopoietin/interferon-class (D200-domain) cytokine receptor activity: 2.129e-01 (34,19,1)

IF 1ddja_#640 AND 2hlca_#139
THEN
GO:0004295: trypsin activity: 3.202e-12 (34,48,11) OR
GO:0004263: chymotrypsin activity: 1.869e-11 (34,41,10) OR
GO:0005509: calcium ion binding: 5.796e-04 (34,160,8) OR
GO:0003809: thrombin activity: 6.388e-03 (34,10,2) OR
GO:0004867: serine-type endopeptidase inhibitor activity: 1.154e-01 (34,47,2) OR
GO:0004896: hematopoietin/interferon-class (D200-domain) cytokine receptor activity: 2.129e-01 (34,19,1)

IF 2foka2#180 AND 1dkia_#54
THEN
GO:0004197: cysteine-type endopeptidase activity: 6.588e-12 (8,24,6) OR
GO:0004177: aminopeptidase activity: 3.758e-02 (8,13,1) OR
GO:0004180: carboxypeptidase activity: 4.325e-02 (8,15,1)

IF 1deua_#210 AND 2foka2#180
THEN
GO:0004197: cysteine-type endopeptidase activity: 6.588e-12 (8,24,6) OR
GO:0004177: aminopeptidase activity: 3.758e-02 (8,13,1) OR
GO:0004180: carboxypeptidase activity: 4.325e-02 (8,15,1)

IF 1xvaa_#68 AND 1e7wa_#36
THEN
GO:0008757: S-adenosylmethionine-dependent methyltransferase activity: 6.588e-12 (8,24,6) OR
GO:0008270: zinc ion binding: 2.767e-01 (8,108,1) OR
GO:0000287: magnesium ion binding: 3.198e-01 (8,128,1)

IF 1a6o__#233 AND 1phk__#221
THEN
GO:0004674: protein serine/threonine kinase activity: 5.097e-12 (21,42,9) OR
GO:0005524: ATP binding: 3.481e-05 (21,243,9) OR
GO:0005516: calmodulin binding: 1.410e-02 (21,24,2) OR
GO:0004896: hematopoietin/interferon-class (D200-domain) cytokine receptor activity: 1.371e-01 (21,19,1)

IF 1fbna_#145 AND 1qama_#37
THEN
GO:0008757: S-adenosylmethionine-dependent methyltransferase activity: 5.203e-12 (14,24,7) OR
GO:0016616: oxidoreductase activity, acting on the CH-OH group of donors, NAD or NADP as acceptor: 1.689e-04 (14,59,4) OR
GO:0008270: zinc ion binding: 1.039e-01 (14,108,2) OR
GO:0000287: magnesium ion binding: 4.910e-01 (14,128,1)

IF 1hwxa1#256 AND 1fuma2#221
THEN
GO:0015036: disulfide oxidoreductase activity: 4.285e-12 (25,22,8) OR
GO:0050660: FAD binding: 6.420e-11 (25,10,6) OR
GO:0016668: oxidoreductase activity, acting on sulfur group of donors, NAD or NADP as acceptor: 2.791e-10 (25,12,6) OR
GO:0016627: oxidoreductase activity, acting on the CH-CH group of donors: 1.212e-05 (25,17,4) OR
GO:0016651: oxidoreductase activity, acting on NADH or NADPH: 1.132e-01 (25,13,1)

IF 1ad3a_#181 AND 3grs_2#211
THEN
GO:0015036: disulfide oxidoreductase activity: 4.285e-12 (25,22,8) OR
GO:0050660: FAD binding: 6.420e-11 (25,10,6) OR
GO:0016668: oxidoreductase activity, acting on sulfur group of donors, NAD or NADP as acceptor: 2.791e-10 (25,12,6) OR
GO:0016620: oxidoreductase activity, acting on the aldehyde or oxo group of donors, NAD or NADP as acceptor: 7.852e-05 (25,10,3) OR
GO:0016651: oxidoreductase activity, acting on NADH or NADPH: 5.926e-03 (25,13,2)

IF 1danh_#70 AND 1eq9a_#58 AND 1cgha_#130
THEN
GO:0004263: chymotrypsin activity: 4.465e-12 (30,41,10) OR
GO:0004295: trypsin activity: 2.484e-11 (30,48,10) OR
GO:0003809: thrombin activity: 4.992e-03 (30,10,2) OR
GO:0004867: serine-type endopeptidase inhibitor activity: 1.409e-02 (30,47,3) OR
GO:0005509: calcium ion binding: 2.829e-02 (30,160,5)

IF 1e9xa_#343 AND 1f24a_#144
THEN
GO:0004497: monooxygenase activity: 1.126e-11 (8,26,6) OR
GO:0016705: oxidoreductase activity, acting on paired donors, with incorporation or reduction of molecular oxygen: 2.367e-03 (8,26,2)

IF 1f24a_#144 AND 1bu7a_#353
THEN
GO:0004497: monooxygenase activity: 1.126e-11 (8,26,6) OR
GO:0016705: oxidoreductase activity, acting on paired donors, with incorporation or reduction of molecular oxygen: 2.367e-03 (8,26,2)

IF 1cpt__#358 AND 1f24a_#144
THEN
GO:0004497: monooxygenase activity: 1.126e-11 (8,26,6) OR
GO:0016705: oxidoreductase activity, acting on paired donors, with incorporation or reduction of molecular oxygen: 2.367e-03 (8,26,2)

IF 1f24a_#144 AND 1dt6a_#77
THEN
GO:0004497: monooxygenase activity: 1.126e-11 (8,26,6) OR
GO:0016705: oxidoreductase activity, acting on paired donors, with incorporation or reduction of molecular oxygen: 2.367e-03 (8,26,2)

IF 1f24a_#144 AND 1dz4a_#357
THEN
GO:0004497: monooxygenase activity: 1.126e-11 (8,26,6) OR
GO:0016705: oxidoreductase activity, acting on paired donors, with incorporation or reduction of molecular oxygen: 2.367e-03 (8,26,2)

IF 1dt6a_#376 AND 1cpt__#401 AND 1cpt__#52
THEN
GO:0004497: monooxygenase activity: 1.126e-11 (8,26,6) OR
GO:0016705: oxidoreductase activity, acting on paired donors, with incorporation or reduction of molecular oxygen: 2.367e-03 (8,26,2)

IF 1cpt__#401 AND 1f24a_#144
THEN
GO:0004497: monooxygenase activity: 1.126e-11 (8,26,6) OR
GO:0016705: oxidoreductase activity, acting on paired donors, with incorporation or reduction of molecular oxygen: 2.367e-03 (8,26,2)

IF 1fg7a_#158 AND 1fg7a_#157
THEN
GO:0016846: carbon-sulfur lyase activity: 1.126e-11 (8,10,5) OR
GO:0008483: transaminase activity: 1.109e-05 (8,17,3)

IF 1gdea_#90 AND 1qgna_#149
THEN
GO:0016846: carbon-sulfur lyase activity: 1.126e-11 (8,10,5) OR
GO:0008483: transaminase activity: 1.109e-05 (8,17,3)

IF 2dkb__#114 AND 1fg7a_#157
THEN
GO:0016846: carbon-sulfur lyase activity: 1.126e-11 (8,10,5) OR
GO:0008483: transaminase activity: 1.109e-05 (8,17,3)

IF 1quqb_#87 AND 1hyea2#272
THEN
GO:0004457: lactate dehydrogenase activity: 4.532e-12 (17,10,6) OR
GO:0016616: oxidoreductase activity, acting on the CH-OH group of donors, NAD or NADP as acceptor: 2.551e-08 (17,59,7) OR
GO:0005525: GTP binding: 3.628e-02 (17,49,2) OR
GO:0000155: two-component sensor molecule activity: 7.249e-02 (17,12,1) OR
GO:0005524: ATP binding: 7.966e-01 (17,243,1)

IF 1hqoa2#159 AND 1f3ba2#7
THEN
GO:0004364: glutathione transferase activity: 7.748e-12 (7,11,5) OR
GO:0003714: transcription corepressor activity: 2.039e-02 (7,8,1) OR
GO:0016651: oxidoreductase activity, acting on NADH or NADPH: 3.296e-02 (7,13,1)

IF 1hqoa2#159 AND 1avgi_#69
THEN
GO:0004364: glutathione transferase activity: 7.748e-12 (7,11,5) OR
GO:0015078: hydrogen ion transporter activity: 5.277e-02 (7,21,1) OR
GO:0005507: copper ion binding: 9.372e-02 (7,38,1)

IF 1a65a3#383 AND 1a65a2#187
THEN
GO:0005507: copper ion binding: 3.935e-12 (15,38,8) OR
GO:0015078: hydrogen ion transporter activity: 1.692e-04 (15,21,3) OR
GO:0015082: di-, tri-valent inorganic cation transporter activity: 7.454e-02 (15,14,1) OR
GO:0046915: transition metal ion transporter activity: 7.454e-02 (15,14,1) OR
GO:0003779: actin binding: 1.628e-01 (15,32,1) OR
GO:0005524: ATP binding: 7.546e-01 (15,243,1)

IF 1bvza3#416 AND 1a65a1#108
THEN
GO:0004556: alpha-amylase activity: 4.806e-12 (22,15,7) OR
GO:0005509: calcium ion binding: 1.874e-05 (22,160,8) OR
GO:0004222: metalloendopeptidase activity: 4.073e-04 (22,19,3) OR
GO:0008270: zinc ion binding: 5.392e-02 (22,108,3) OR
GO:0004867: serine-type endopeptidase inhibitor activity: 3.190e-01 (22,47,1)

IF 1hoe__#31 AND 1hx0a2#294
THEN
GO:0004556: alpha-amylase activity: 8.039e-12 (12,15,6) OR
GO:0005509: calcium ion binding: 3.709e-04 (12,160,5) OR
GO:0004867: serine-type endopeptidase inhibitor activity: 1.888e-01 (12,47,1)

IF 1gega_#58 AND 1fmca_#86
THEN
GO:0016616: oxidoreductase activity, acting on the CH-OH group of donors, NAD or NADP as acceptor: 1.222e-11 (8,59,7) OR
GO:0008270: zinc ion binding: 2.767e-01 (8,108,1)

IF 1fmca_#88 AND 1qtn.1#A270
THEN
GO:0016616: oxidoreductase activity, acting on the CH-OH group of donors, NAD or NADP as acceptor: 1.222e-11 (8,59,7) OR
GO:0016627: oxidoreductase activity, acting on the CH-CH group of donors: 4.889e-02 (8,17,1)

IF 1fmca_#88 AND 1oaa__#175
THEN
GO:0016616: oxidoreductase activity, acting on the CH-OH group of donors, NAD or NADP as acceptor: 1.222e-11 (8,59,7) OR
GO:0016627: oxidoreductase activity, acting on the CH-CH group of donors: 4.889e-02 (8,17,1)

IF 1b16a_#184 AND 1oaa__#175 AND 1qrra_#69
THEN
GO:0016616: oxidoreductase activity, acting on the CH-OH group of donors, NAD or NADP as acceptor: 1.222e-11 (8,59,7) OR
GO:0016627: oxidoreductase activity, acting on the CH-CH group of donors: 4.889e-02 (8,17,1)

IF 1fmca_#88 AND 1eno__#137
THEN

GO:0016616: oxidoreductase activity, acting on the CH-OH group of donors, NAD or NADP as acceptor: 1.222e-11 (8,59,7) OR
GO:0016627: oxidoreductase activity, acting on the CH-CH group of donors: 4.889e-02 (8,17,1)

IF 1cyda_#231 AND 1gdha1#235
THEN
GO:0016616: oxidoreductase activity, acting on the CH-OH group of donors, NAD or NADP as acceptor: 1.222e-11 (8,59,7) OR
GO:0016627: oxidoreductase activity, acting on the CH-CH group of donors: 4.889e-02 (8,17,1)

IF 1fmca_#88 AND 1nat__#50
THEN
GO:0016616: oxidoreductase activity, acting on the CH-OH group of donors, NAD or NADP as acceptor: 1.222e-11 (8,59,7) OR
GO:0016627: oxidoreductase activity, acting on the CH-CH group of donors: 4.889e-02 (8,17,1)

IF 1hc7a2#111 AND 1pysb5#672
THEN
GO:0004812: tRNA ligase activity: 6.161e-12 (22,26,8) OR
GO:0005524: ATP binding: 3.774e-04 (22,243,8) OR
GO:0000049: tRNA binding: 4.600e-03 (22,13,2) OR
GO:0000287: magnesium ion binding: 1.758e-02 (22,128,4)

IF 1fuma2#221 AND 1e39a2#556
THEN
GO:0015036: disulfide oxidoreductase activity: 4.285e-12 (25,22,8) OR
GO:0050660: FAD binding: 6.420e-11 (25,10,6) OR
GO:0016668: oxidoreductase activity, acting on sulfur group of donors, NAD or NADP as acceptor: 2.791e-10 (25,12,6) OR
GO:0016627: oxidoreductase activity, acting on the CH-CH group of donors: 4.264e-04 (25,17,3) OR
GO:0016651: oxidoreductase activity, acting on NADH or NADPH: 1.132e-01 (25,13,1) OR
GO:0016638: oxidoreductase activity, acting on the CH-NH2 group of donors: 1.454e-01 (25,17,1)

IF 1fuma2#221 AND 3grs_1#24
THEN
GO:0015036: disulfide oxidoreductase activity: 4.285e-12 (25,22,8) OR
GO:0050660: FAD binding: 6.420e-11 (25,10,6) OR
GO:0016668: oxidoreductase activity, acting on sulfur group of donors, NAD or NADP as acceptor: 2.791e-10 (25,12,6) OR
GO:0016627: oxidoreductase activity, acting on the CH-CH group of donors: 4.264e-04 (25,17,3) OR
GO:0016651: oxidoreductase activity, acting on NADH or NADPH: 1.132e-01 (25,13,1) OR
GO:0016638: oxidoreductase activity, acting on the CH-NH2 group of donors: 1.454e-01 (25,17,1)

IF 1fuma2#221 AND 1e39a2#516
THEN
GO:0015036: disulfide oxidoreductase activity: 4.285e-12 (25,22,8) OR
GO:0050660: FAD binding: 6.420e-11 (25,10,6) OR
GO:0016668: oxidoreductase activity, acting on sulfur group of donors, NAD or NADP as acceptor: 2.791e-10 (25,12,6) OR
GO:0016627: oxidoreductase activity, acting on the CH-CH group of donors: 4.264e-04 (25,17,3) OR
GO:0016651: oxidoreductase activity, acting on NADH or NADPH: 1.132e-01 (25,13,1) OR
GO:0016638: oxidoreductase activity, acting on the CH-NH2 group of donors: 1.454e-01 (25,17,1)

IF 1nhp_1#7 AND 1fuma2#221
THEN
GO:0015036: disulfide oxidoreductase activity: 4.285e-12 (25,22,8) OR
GO:0050660: FAD binding: 6.420e-11 (25,10,6) OR
GO:0016668: oxidoreductase activity, acting on sulfur group of donors, NAD or NADP as acceptor: 2.791e-10 (25,12,6) OR
GO:0016627: oxidoreductase activity, acting on the CH-CH group of donors: 4.264e-04 (25,17,3) OR
GO:0016651: oxidoreductase activity, acting on NADH or NADPH: 1.132e-01 (25,13,1) OR
GO:0016638: oxidoreductase activity, acting on the CH-NH2 group of donors: 1.454e-01 (25,17,1)

IF 2dpma_#237 AND 1fwxa1#526
THEN
GO:0005507: copper ion binding: 6.690e-12 (10,38,7) OR
GO:0015082: di-, tri-valent inorganic cation transporter activity: 5.029e-02 (10,14,1) OR
GO:0046915: transition metal ion transporter activity: 5.029e-02 (10,14,1) OR
GO:0051082: unfolded protein binding: 1.182e-01 (10,34,1)

IF 1hdr__#149 AND 1ldna1#94 AND 1e6ua_#10
THEN
GO:0016616: oxidoreductase activity, acting on the CH-OH group of donors, NAD or NADP as acceptor: 5.675e-12 (16,59,9) OR
GO:0016854: racemase and epimerase activity: 2.429e-03 (16,13,2) OR
GO:0016627: oxidoreductase activity, acting on the CH-CH group of donors: 4.177e-03 (16,17,2) OR
GO:0016646: oxidoreductase activity, acting on the CH-NH group of donors, NAD or NADP as acceptor: 5.216e-03 (16,19,2) OR
GO:0016836: hydro-lyase activity: 1.776e-01 (16,33,1)

IF 1d5ra2#25 AND 1ge8a2#134
THEN
GO:0004725: protein tyrosine phosphatase activity: 1.440e-11 (6,15,5) OR
GO:0016811: hydrolase activity, acting on carbon-nitrogen (but not peptide) bonds, in linear amides: 3.261e-02 (6,15,1)

IF 1d5ra2#25 AND 1gpea1#271
THEN
GO:0004725: protein tyrosine phosphatase activity: 1.440e-11 (6,15,5) OR
GO:0016811: hydrolase activity, acting on carbon-nitrogen (but not peptide) bonds, in linear amides: 3.261e-02 (6,15,1)

IF 1qfma2#576 AND 1trb_1#15
THEN
GO:0015036: disulfide oxidoreductase activity: 4.806e-12 (15,22,7) OR
GO:0016668: oxidoreductase activity, acting on sulfur group of donors, NAD or NADP as acceptor: 2.872e-07 (15,12,4) OR
GO:0050660: FAD binding: 5.379e-02 (15,10,1) OR
GO:0016854: racemase and epimerase activity: 6.939e-02 (15,13,1) OR
GO:0016627: oxidoreductase activity, acting on the CH-CH group of donors: 8.982e-02 (15,17,1) OR
GO:0016638: oxidoreductase activity, acting on the CH-NH2 group of donors: 8.982e-02 (15,17,1)

IF 1danh_#93 AND 1ekbb_#79 AND 2hlca_#27 AND 1fjsa_#158 AND 1gdna_#56 AND 1autc_#45
THEN
GO:0004263: chymotrypsin activity: 4.863e-12 (41,41,11) OR
GO:0004295: trypsin activity: 3.237e-11 (41,48,11) OR
GO:0004867: serine-type endopeptidase inhibitor activity: 5.413e-05 (41,47,6) OR
GO:0003809: thrombin activity: 3.528e-04 (41,10,3) OR
GO:0005509: calcium ion binding: 4.560e-04 (41,160,9) OR
GO:0004896: hematopoietin/interferon-class (D200-domain) cytokine receptor activity: 2.510e-01 (41,19,1)

IF 1ddja_#754 AND 1a0la_#160 AND 1dlea_#238
THEN
GO:0004263: chymotrypsin activity: 4.863e-12 (41,41,11) OR
GO:0004295: trypsin activity: 3.237e-11 (41,48,11) OR
GO:0003809: thrombin activity: 1.597e-09 (41,10,6) OR
GO:0005509: calcium ion binding: 2.144e-03 (41,160,8) OR
GO:0004867: serine-type endopeptidase inhibitor activity: 4.932e-03 (41,47,4) OR
GO:0005529: sugar binding: 4.487e-01 (41,39,1)

IF 1egia_#738 AND 1esl_1#38
THEN
GO:0005529: sugar binding: 4.939e-12 (15,39,8) OR
GO:0005509: calcium ion binding: 5.368e-02 (15,160,3) OR
GO:0008201: heparin binding: 1.246e-01 (15,24,1) OR
GO:0016763: transferase activity, transferring pentosyl groups: 1.439e-01 (15,28,1) OR
GO:0004263: chymotrypsin activity: 2.039e-01 (15,41,1) OR
GO:0004295: trypsin activity: 2.345e-01 (15,48,1)

IF 1dv8a_#213 AND 1dv8a_#211
THEN
GO:0005529: sugar binding: 4.939e-12 (15,39,8) OR
GO:0004190: aspartic-type endopeptidase activity: 6.695e-03 (15,23,2) OR
GO:0004556: alpha-amylase activity: 7.966e-02 (15,15,1) OR
GO:0004263: chymotrypsin activity: 2.039e-01 (15,41,1) OR
GO:0005509: calcium ion binding: 2.188e-01 (15,160,2) OR
GO:0004295: trypsin activity: 2.345e-01 (15,48,1)

IF 1h7wa4#476 AND 1aqb__#134
THEN
GO:0050660: FAD binding: 9.910e-12 (19,10,6) OR
GO:0015036: disulfide oxidoreductase activity: 3.691e-11 (19,22,7) OR
GO:0016668: oxidoreductase activity, acting on sulfur group of donors, NAD or NADP as acceptor: 4.325e-11 (19,12,6)

IF 1h7wa4#476 AND 1jf9a_#379
THEN
GO:0050660: FAD binding: 9.910e-12 (19,10,6) OR
GO:0015036: disulfide oxidoreductase activity: 3.691e-11 (19,22,7) OR
GO:0016668: oxidoreductase activity, acting on sulfur group of donors, NAD or NADP as acceptor: 4.325e-11 (19,12,6)

IF 1d2fa_#348 AND 1dfoa_#362
THEN

GO:0008483: transaminase activity: 9.936e-12 (11,17,6) OR
GO:0016846: carbon-sulfur lyase activity: 2.986e-08 (11,10,4) OR
GO:0016831: carboxy-lyase activity: 9.658e-02 (11,25,1)

IF 1elua_#199 AND 1nat__#50
THEN
GO:0008483: transaminase activity: 9.936e-12 (11,17,6) OR
GO:0016846: carbon-sulfur lyase activity: 2.986e-08 (11,10,4) OR
GO:0016831: carboxy-lyase activity: 9.658e-02 (11,25,1)

IF 1ce7a_#151 AND 1b3qa2#623
THEN
GO:0016799: hydrolase activity, hydrolyzing N-glycosyl compounds: 9.936e-12 (11,17,6) OR
GO:0005529: sugar binding: 4.134e-04 (11,39,3) OR
GO:0008270: zinc ion binding: 6.778e-02 (11,108,2)

IF 1jf9a_#379 AND 1elua_#199
THEN
GO:0008483: transaminase activity: 9.936e-12 (11,17,6) OR
GO:0016846: carbon-sulfur lyase activity: 2.986e-08 (11,10,4) OR
GO:0016831: carboxy-lyase activity: 9.658e-02 (11,25,1)

IF 1as4.1#A79 AND 1ovaa_#161
THEN
GO:0004867: serine-type endopeptidase inhibitor activity: 1.004e-11 (9,47,7) OR
GO:0015078: hydrogen ion transporter activity: 6.735e-02 (9,21,1) OR
GO:0008201: heparin binding: 7.664e-02 (9,24,1)

IF 1rb9__#49 AND 1hle.1#A228
THEN
GO:0004867: serine-type endopeptidase inhibitor activity: 1.004e-11 (9,47,7) OR
GO:0008201: heparin binding: 7.664e-02 (9,24,1) OR
GO:0005507: copper ion binding: 1.189e-01 (9,38,1)

IF 1as4.1#B385 AND 1ovaa_#161
THEN
GO:0004867: serine-type endopeptidase inhibitor activity: 5.085e-12 (13,47,8) OR
GO:0016814: hydrolase activity, acting on carbon-nitrogen (but not peptide) bonds, in cyclic amidines: 4.677e-02 (13,10,1) OR
GO:0008201: heparin binding: 1.089e-01 (13,24,1) OR
GO:0051082: unfolded protein binding: 1.509e-01 (13,34,1) OR
GO:0000287: magnesium ion binding: 4.657e-01 (13,128,1) OR
GO:0005509: calcium ion binding: 5.454e-01 (13,160,1)

IF 1qf6a4#362 AND 1qqga2#253
THEN
GO:0004812: tRNA ligase activity: 6.161e-12 (22,26,8) OR
GO:0005524: ATP binding: 3.774e-04 (22,243,8) OR
GO:0000287: magnesium ion binding: 1.758e-02 (22,128,4) OR
GO:0000049: tRNA binding: 1.002e-01 (22,13,1) OR
GO:0003887: DNA-directed DNA polymerase activity: 1.501e-01 (22,20,1)

IF 1ex1a1#284 AND 1bvza3#416
THEN
GO:0004556: alpha-amylase activity: 8.039e-12 (12,15,6) OR
GO:0005509: calcium ion binding: 3.909e-03 (12,160,4) OR
GO:0016831: carboxy-lyase activity: 1.049e-01 (12,25,1) OR
GO:0000287: magnesium ion binding: 4.393e-01 (12,128,1)

IF 1erza_#183 AND 1a4ya_#47 AND 1icia_#175
THEN
GO:0004556: alpha-amylase activity: 8.039e-12 (12,15,6) OR
GO:0005509: calcium ion binding: 3.909e-03 (12,160,4) OR
GO:0016758: transferase activity, transferring hexosyl groups: 4.747e-02 (12,11,1) OR
GO:0004867: serine-type endopeptidase inhibitor activity: 1.888e-01 (12,47,1)

IF 1b6e__#76 AND 1dv8a_#211
THEN
GO:0005529: sugar binding: 8.145e-12 (10,39,7) OR
GO:0004263: chymotrypsin activity: 1.409e-01 (10,41,1) OR
GO:0004295: trypsin activity: 1.631e-01 (10,48,1) OR

GO:0005509: calcium ion binding: 4.545e-01 (10,160,1)

IF 1fvua_#72 AND 1esl_1#38
THEN
GO:0005529: sugar binding: 8.145e-12 (10,39,7) OR
GO:0004263: chymotrypsin activity: 1.409e-01 (10,41,1) OR
GO:0004295: trypsin activity: 1.631e-01 (10,48,1) OR
GO:0005509: calcium ion binding: 4.545e-01 (10,160,1)

IF 1gg6.1#C156 AND 2hlca_#99 AND 1cgha_#227 AND 1cgha_#130
THEN
GO:0004263: chymotrypsin activity: 6.523e-12 (31,41,10) OR
GO:0004295: trypsin activity: 3.620e-11 (31,48,10) OR
GO:0004867: serine-type endopeptidase inhibitor activity: 1.492e-04 (31,47,5) OR
GO:0005509: calcium ion binding: 3.218e-02 (31,160,5) OR
GO:0008201: heparin binding: 2.410e-01 (31,24,1)

IF 1ei5a3#190 AND 1hvba_#63
THEN
GO:0008800: beta-lactamase activity: 1.126e-11 (8,10,5) OR
GO:0004180: carboxypeptidase activity: 7.771e-04 (8,15,2) OR
GO:0004177: aminopeptidase activity: 3.758e-02 (8,13,1)

IF 1fg7a_#96 AND 1cl1a_#184
THEN
GO:0016846: carbon-sulfur lyase activity: 1.126e-11 (8,10,5) OR
GO:0008483: transaminase activity: 1.004e-03 (8,17,2) OR
GO:0016831: carboxy-lyase activity: 7.117e-02 (8,25,1)

IF 1ceqa1#139 AND 1evqa_#305
THEN
GO:0004457: lactate dehydrogenase activity: 6.789e-12 (18,10,6) OR
GO:0016616: oxidoreductase activity, acting on the CH-OH group of donors, NAD or NADP as acceptor: 1.099e-09 (18,59,8) OR
GO:0005525: GTP binding: 2.794e-01 (18,49,1) OR
GO:0005524: ATP binding: 4.864e-01 (18,243,2) OR
GO:0003700: transcription factor activity: 5.687e-01 (18,124,1)

IF 1mrp__#172 AND 1leha1#177
THEN
GO:0016616: oxidoreductase activity, acting on the CH-OH group of donors, NAD or NADP as acceptor: 5.675e-12 (16,59,9) OR
GO:0016620: oxidoreductase activity, acting on the aldehyde or oxo group of donors, NAD or NADP as acceptor: 1.416e-03 (16,10,2) OR
GO:0016651: oxidoreductase activity, acting on NADH or NADPH: 7.386e-02 (16,13,1) OR
GO:0016638: oxidoreductase activity, acting on the CH-NH2 group of donors: 9.553e-02 (16,17,1) OR
GO:0016646: oxidoreductase activity, acting on the CH-NH group of donors, NAD or NADP as acceptor: 1.062e-01 (16,19,1) OR
GO:0005524: ATP binding: 4.245e-01 (16,243,2)

IF 1qf6a3#92 AND 1xvaa_#68
THEN
GO:0008757: S-adenosylmethionine-dependent methyltransferase activity: 3.407e-11 (5,24,5)

IF 2dpma_#194 AND 1qama_#39
THEN
GO:0008757: S-adenosylmethionine-dependent methyltransferase activity: 3.407e-11 (5,24,5)

IF 1b3qa2#556 AND 1koba_#175
THEN
GO:0004674: protein serine/threonine kinase activity: 8.531e-12 (22,42,9) OR
GO:0005524: ATP binding: 6.552e-06 (22,243,10) OR
GO:0005516: calmodulin binding: 1.543e-02 (22,24,2) OR
GO:0005066: transmembrane receptor protein tyrosine kinase signaling protein activity: 7.799e-02 (22,10,1)

IF 1b3qa2#556 AND 1a06__#147
THEN
GO:0004674: protein serine/threonine kinase activity: 8.531e-12 (22,42,9) OR
GO:0005524: ATP binding: 6.552e-06 (22,243,10) OR
GO:0005516: calmodulin binding: 1.543e-02 (22,24,2) OR
GO:0005066: transmembrane receptor protein tyrosine kinase signaling protein activity: 7.799e-02 (22,10,1)

IF 1fgka_#610 AND 1b3qa2#556

THEN
GO:0004674: protein serine/threonine kinase activity: 8.531e-12 (22,42,9) OR
GO:0005524: ATP binding: 6.552e-06 (22,243,10) OR
GO:0005516: calmodulin binding: 1.543e-02 (22,24,2) OR
GO:0005066: transmembrane receptor protein tyrosine kinase signaling protein activity: 7.799e-02 (22,10,1)

IF 1b3qa2#556 AND 1phk__#165
THEN
GO:0004674: protein serine/threonine kinase activity: 8.531e-12 (22,42,9) OR
GO:0005524: ATP binding: 6.552e-06 (22,243,10) OR
GO:0005516: calmodulin binding: 1.543e-02 (22,24,2) OR
GO:0005066: transmembrane receptor protein tyrosine kinase signaling protein activity: 7.799e-02 (22,10,1)

IF 1b3qa2#556 AND 1a06__#103
THEN
GO:0004674: protein serine/threonine kinase activity: 8.531e-12 (22,42,9) OR
GO:0005524: ATP binding: 6.552e-06 (22,243,10) OR
GO:0005516: calmodulin binding: 1.543e-02 (22,24,2) OR
GO:0005066: transmembrane receptor protein tyrosine kinase signaling protein activity: 7.799e-02 (22,10,1)

IF 1csn__#134 AND 1f3mc_#403
THEN
GO:0004674: protein serine/threonine kinase activity: 5.821e-12 (30,42,10) OR
GO:0005524: ATP binding: 4.978e-07 (30,243,13) OR
GO:0005066: transmembrane receptor protein tyrosine kinase signaling protein activity: 4.992e-03 (30,10,2) OR
GO:0004714: transmembrane receptor protein tyrosine kinase activity: 9.823e-03 (30,14,2) OR
GO:0005516: calmodulin binding: 2.783e-02 (30,24,2) OR
GO:0008201: heparin binding: 2.342e-01 (30,24,1)

IF 1csn__#134 AND 1fmk_3#377
THEN
GO:0004674: protein serine/threonine kinase activity: 5.821e-12 (30,42,10) OR
GO:0005524: ATP binding: 4.978e-07 (30,243,13) OR
GO:0005066: transmembrane receptor protein tyrosine kinase signaling protein activity: 4.992e-03 (30,10,2) OR
GO:0004714: transmembrane receptor protein tyrosine kinase activity: 9.823e-03 (30,14,2) OR
GO:0005516: calmodulin binding: 2.783e-02 (30,24,2) OR
GO:0008201: heparin binding: 2.342e-01 (30,24,1)

IF 1bd3a_#110 AND 3lada1#116
THEN
GO:0016763: transferase activity, transferring pentosyl groups: 8.880e-12 (13,28,7) OR
GO:0000287: magnesium ion binding: 2.386e-03 (13,128,4) OR
GO:0008757: S-adenosylmethionine-dependent methyltransferase activity: 1.089e-01 (13,24,1) OR
GO:0005524: ATP binding: 7.039e-01 (13,243,1)

IF 1hcl__#267 AND 1e79d2#36
THEN
GO:0004674: protein serine/threonine kinase activity: 1.861e-11 (16,42,8) OR
GO:0005524: ATP binding: 2.445e-05 (16,243,8)

IF 1c5y.1#B191 AND 1a7s__#148 AND 1qnja_#77
THEN
GO:0004263: chymotrypsin activity: 9.389e-12 (32,41,10) OR
GO:0004295: trypsin activity: 5.199e-11 (32,48,10) OR
GO:0004867: serine-type endopeptidase inhibitor activity: 1.227e-05 (32,47,6) OR
GO:0005509: calcium ion binding: 9.442e-03 (32,160,6)

IF 1i5ga_#115 AND 1qf6a4#362
THEN
GO:0004812: tRNA ligase activity: 9.392e-12 (23,26,8) OR
GO:0005524: ATP binding: 5.347e-04 (23,243,8) OR
GO:0000287: magnesium ion binding: 3.593e-03 (23,128,5) OR
GO:0000049: tRNA binding: 5.024e-03 (23,13,2)

IF 2occb1#189 AND 1fwxa1#526
THEN
GO:0005507: copper ion binding: 7.793e-12 (16,38,8) OR
GO:0015078: hydrogen ion transporter activity: 6.750e-08 (16,21,5) OR
GO:0015082: di-, tri-valent inorganic cation transporter activity: 7.932e-02 (16,14,1) OR
GO:0046915: transition metal ion transporter activity: 7.932e-02 (16,14,1) OR

GO:0005509: calcium ion binding: 6.213e-01 (16,160,1)

IF 1f97a1#62 AND 1c9la2#61 AND 1dpga2#334 AND 1ton__#112
THEN
GO:0004263: chymotrypsin activity: 6.521e-12 (42,41,11) OR
GO:0004295: trypsin activity: 4.330e-11 (42,48,11) OR
GO:0003809: thrombin activity: 1.623e-07 (42,10,5) OR
GO:0005509: calcium ion binding: 5.516e-04 (42,160,9) OR
GO:0004867: serine-type endopeptidase inhibitor activity: 6.482e-04 (42,47,5) OR
GO:0005529: sugar binding: 4.567e-01 (42,39,1)

IF 1ds1a_#172 AND 1trb_1#41
THEN
GO:0050660: FAD binding: 9.910e-12 (19,10,6) OR
GO:0016668: oxidoreductase activity, acting on sulfur group of donors, NAD or NADP as acceptor: 4.325e-11 (19,12,6) OR
GO:0015036: disulfide oxidoreductase activity: 3.351e-09 (19,22,6) OR
GO:0016651: oxidoreductase activity, acting on NADH or NADPH: 8.713e-02 (19,13,1)

IF 2cb5a_#312 AND 1a0la_#160 AND 1dlea_#238
THEN
GO:0003809: thrombin activity: 6.690e-12 (38,10,7) OR
GO:0004263: chymotrypsin activity: 4.516e-08 (38,41,8) OR
GO:0004295: trypsin activity: 1.664e-07 (38,48,8) OR
GO:0004867: serine-type endopeptidase inhibitor activity: 3.456e-05 (38,47,6) OR
GO:0005509: calcium ion binding: 1.276e-03 (38,160,8) OR
GO:0005529: sugar binding: 4.240e-01 (38,39,1)

IF 1qnja_#71 AND 1ejda_#6
THEN
GO:0003809: thrombin activity: 6.690e-12 (38,10,7) OR
GO:0004263: chymotrypsin activity: 4.516e-08 (38,41,8) OR
GO:0004295: trypsin activity: 1.664e-07 (38,48,8) OR
GO:0004867: serine-type endopeptidase inhibitor activity: 3.456e-05 (38,47,6) OR
GO:0005509: calcium ion binding: 1.276e-03 (38,160,8) OR
GO:0005529: sugar binding: 4.240e-01 (38,39,1)

IF 2cb5a_#312 AND 1a0la_#160 AND 1eq9a_#58
THEN
GO:0003809: thrombin activity: 6.690e-12 (38,10,7) OR
GO:0004263: chymotrypsin activity: 4.516e-08 (38,41,8) OR
GO:0004295: trypsin activity: 1.664e-07 (38,48,8) OR
GO:0004867: serine-type endopeptidase inhibitor activity: 3.456e-05 (38,47,6) OR
GO:0005509: calcium ion binding: 1.276e-03 (38,160,8) OR
GO:0005529: sugar binding: 4.240e-01 (38,39,1)

IF 2cb5a_#312 AND 1danh_#190 AND 1a0la_#160
THEN
GO:0003809: thrombin activity: 6.690e-12 (38,10,7) OR
GO:0004263: chymotrypsin activity: 4.516e-08 (38,41,8) OR
GO:0004295: trypsin activity: 1.664e-07 (38,48,8) OR
GO:0004867: serine-type endopeptidase inhibitor activity: 3.456e-05 (38,47,6) OR
GO:0005509: calcium ion binding: 1.276e-03 (38,160,8) OR
GO:0005529: sugar binding: 4.240e-01 (38,39,1)

IF 1e6ua_#10 AND 1b16a_#62
THEN
GO:0016616: oxidoreductase activity, acting on the CH-OH group of donors, NAD or NADP as acceptor: 1.375e-11 (12,59,8) OR
GO:0016627: oxidoreductase activity, acting on the CH-CH group of donors: 4.289e-05 (12,17,3) OR
GO:0016646: oxidoreductase activity, acting on the CH-NH group of donors, NAD or NADP as acceptor: 8.069e-02 (12,19,1)

IF 1cyda_#231 AND 1oaa__#14
THEN
GO:0016616: oxidoreductase activity, acting on the CH-OH group of donors, NAD or NADP as acceptor: 1.375e-11 (12,59,8) OR
GO:0016627: oxidoreductase activity, acting on the CH-CH group of donors: 4.289e-05 (12,17,3) OR
GO:0016646: oxidoreductase activity, acting on the CH-NH group of donors, NAD or NADP as acceptor: 8.069e-02 (12,19,1)

IF 1eny__#94 AND 1b16a_#62
THEN
GO:0016616: oxidoreductase activity, acting on the CH-OH group of donors, NAD or NADP as acceptor: 1.375e-11 (12,59,8) OR
GO:0016627: oxidoreductase activity, acting on the CH-CH group of donors: 4.289e-05 (12,17,3) OR

GO:0016646: oxidoreductase activity, acting on the CH-NH group of donors, NAD or NADP as acceptor: 8.069e-02 (12,19,1)

IF 1qg6a_#251 AND 1qtn.1#A270
THEN
GO:0016616: oxidoreductase activity, acting on the CH-OH group of donors, NAD or NADP as acceptor: 1.375e-11 (12,59,8) OR
GO:0016627: oxidoreductase activity, acting on the CH-CH group of donors: 4.289e-05 (12,17,3) OR
GO:0016646: oxidoreductase activity, acting on the CH-NH group of donors, NAD or NADP as acceptor: 8.069e-02 (12,19,1)

IF 1phm_2#253 AND 1a0la_#160 AND 1dlea_#238
THEN
GO:0004295: trypsin activity: 6.909e-12 (47,48,12) OR
GO:0004263: chymotrypsin activity: 2.521e-11 (47,41,11) OR
GO:0003809: thrombin activity: 3.785e-09 (47,10,6) OR
GO:0004867: serine-type endopeptidase inhibitor activity: 1.071e-05 (47,47,7) OR
GO:0005509: calcium ion binding: 2.874e-04 (47,160,10) OR
GO:0005529: sugar binding: 4.951e-01 (47,39,1)

IF 1jb3a_#64 AND 3grx__#56 AND 1h7wa4#481
THEN
GO:0015036: disulfide oxidoreductase activity: 8.502e-12 (16,22,7) OR
GO:0050660: FAD binding: 1.945e-05 (16,10,3) OR
GO:0016668: oxidoreductase activity, acting on sulfur group of donors, NAD or NADP as acceptor: 3.541e-05 (16,12,3) OR
GO:0016638: oxidoreductase activity, acting on the CH-NH2 group of donors: 4.177e-03 (16,17,2) OR
GO:0004601: peroxidase activity: 1.167e-01 (16,21,1)

IF 3prn__#143 AND 1f3mc_#403
THEN
GO:0004674: protein serine/threonine kinase activity: 8.531e-12 (22,42,9) OR
GO:0005524: ATP binding: 5.438e-05 (22,243,9) OR
GO:0005516: calmodulin binding: 1.543e-02 (22,24,2) OR
GO:0003755: peptidyl-prolyl cis-trans isomerase activity: 8.546e-02 (22,11,1) OR
GO:0004896: hematopoietin/interferon-class (D200-domain) cytokine receptor activity: 1.432e-01 (22,19,1)

IF 1b6cb_#337 AND 1bio__#210
THEN
GO:0004674: protein serine/threonine kinase activity: 8.531e-12 (22,42,9) OR
GO:0005524: ATP binding: 5.438e-05 (22,243,9) OR
GO:0005516: calmodulin binding: 1.543e-02 (22,24,2) OR
GO:0003755: peptidyl-prolyl cis-trans isomerase activity: 8.546e-02 (22,11,1) OR
GO:0004896: hematopoietin/interferon-class (D200-domain) cytokine receptor activity: 1.432e-01 (22,19,1)

IF 1phk__#221 AND 1pwt__#8
THEN
GO:0004674: protein serine/threonine kinase activity: 8.531e-12 (22,42,9) OR
GO:0005524: ATP binding: 5.438e-05 (22,243,9) OR
GO:0005516: calmodulin binding: 1.543e-02 (22,24,2) OR
GO:0004896: hematopoietin/interferon-class (D200-domain) cytokine receptor activity: 1.432e-01 (22,19,1) OR
GO:0015036: disulfide oxidoreductase activity: 1.639e-01 (22,22,1)

IF 1e43a2#236 AND 1ddma_#130
THEN
GO:0004556: alpha-amylase activity: 1.489e-11 (13,15,6) OR
GO:0005509: calcium ion binding: 4.543e-05 (13,160,6) OR
GO:0004867: serine-type endopeptidase inhibitor activity: 2.028e-01 (13,47,1)

IF 1qfea_#49 AND 1e43a2#257
THEN
GO:0004556: alpha-amylase activity: 1.489e-11 (13,15,6) OR
GO:0005509: calcium ion binding: 4.543e-05 (13,160,6) OR
GO:0016836: hydro-lyase activity: 1.468e-01 (13,33,1)

IF 1bvza3#298 AND 1ddma_#130
THEN
GO:0004556: alpha-amylase activity: 1.489e-11 (13,15,6) OR
GO:0005509: calcium ion binding: 4.543e-05 (13,160,6) OR
GO:0004867: serine-type endopeptidase inhibitor activity: 2.028e-01 (13,47,1)

IF 1g6ga_#133 AND 1danh_#48 AND 1cgha_#130
THEN
GO:0004263: chymotrypsin activity: 9.389e-12 (32,41,10) OR

GO:0004295: trypsin activity: 5.199e-11 (32,48,10) OR
GO:0004867: serine-type endopeptidase inhibitor activity: 1.962e-03 (32,47,4) OR
GO:0003809: thrombin activity: 5.670e-03 (32,10,2) OR
GO:0005509: calcium ion binding: 9.442e-03 (32,160,6)

IF 1ra9__#62 AND 1h6la_#119
THEN
GO:0016646: oxidoreductase activity, acting on the CH-NH group of donors, NAD or NADP as acceptor: 9.910e-12 (10,19,6) OR
GO:0000049: tRNA binding: 4.677e-02 (10,13,1) OR
GO:0019843: rRNA binding: 7.799e-02 (10,22,1) OR
GO:0016836: hydro-lyase activity: 1.149e-01 (10,33,1) OR
GO:0008270: zinc ion binding: 3.331e-01 (10,108,1)

IF 1fg7a_#158 AND 1gdea_#92
THEN
GO:0016846: carbon-sulfur lyase activity: 2.530e-11 (9,10,5) OR
GO:0008483: transaminase activity: 1.283e-07 (9,17,4)

IF 1ezia_#198 AND 2dkb__#114
THEN
GO:0016846: carbon-sulfur lyase activity: 2.530e-11 (9,10,5) OR
GO:0008483: transaminase activity: 1.283e-07 (9,17,4)

IF 1qgna_#207 AND 1fg7a_#158
THEN
GO:0016846: carbon-sulfur lyase activity: 2.530e-11 (9,10,5) OR
GO:0008483: transaminase activity: 1.283e-07 (9,17,4)

IF 1fg7a_#158 AND 1rypb_#114
THEN
GO:0016846: carbon-sulfur lyase activity: 2.530e-11 (9,10,5) OR
GO:0008483: transaminase activity: 1.283e-07 (9,17,4)

IF 1fg7a_#158 AND 1qgna_#149
THEN
GO:0016846: carbon-sulfur lyase activity: 2.530e-11 (9,10,5) OR
GO:0008483: transaminase activity: 1.283e-07 (9,17,4)

IF 1bkca_#351 AND 1cipa2#34
THEN
GO:0004222: metalloendopeptidase activity: 1.691e-11 (20,19,7) OR
GO:0008270: zinc ion binding: 6.406e-06 (20,108,7) OR
GO:0005509: calcium ion binding: 7.263e-04 (20,160,6)

IF 1csn__#134 AND 1koba_#175
THEN
GO:0004674: protein serine/threonine kinase activity: 8.500e-12 (31,42,10) OR
GO:0005524: ATP binding: 9.326e-08 (31,243,14) OR
GO:0005066: transmembrane receptor protein tyrosine kinase signaling protein activity: 5.326e-03 (31,10,2) OR
GO:0004714: transmembrane receptor protein tyrosine kinase activity: 1.047e-02 (31,14,2) OR
GO:0005516: calmodulin binding: 2.959e-02 (31,24,2) OR
GO:0008201: heparin binding: 2.410e-01 (31,24,1)

IF 1csn__#134 AND 1tkia_#142 AND 1a06__#147
THEN
GO:0004674: protein serine/threonine kinase activity: 8.500e-12 (31,42,10) OR
GO:0005524: ATP binding: 9.326e-08 (31,243,14) OR
GO:0005066: transmembrane receptor protein tyrosine kinase signaling protein activity: 5.326e-03 (31,10,2) OR
GO:0004714: transmembrane receptor protein tyrosine kinase activity: 1.047e-02 (31,14,2) OR
GO:0005516: calmodulin binding: 2.959e-02 (31,24,2) OR
GO:0008201: heparin binding: 2.410e-01 (31,24,1)

IF 1c5y.1#B220 AND 1pwt__#8 AND 1elva1#604
THEN
GO:0004263: chymotrypsin activity: 8.674e-12 (43,41,11) OR
GO:0004295: trypsin activity: 5.745e-11 (43,48,11) OR
GO:0003809: thrombin activity: 1.834e-07 (43,10,5) OR
GO:0004867: serine-type endopeptidase inhibitor activity: 7.150e-05 (43,47,6) OR
GO:0005509: calcium ion binding: 6.631e-04 (43,160,9) OR
GO:0004896: hematopoietin/interferon-class (D200-domain) cytokine receptor activity: 2.616e-01 (43,19,1)

IF 1f97a2#199 AND 1ddja_#759
THEN
GO:0004263: chymotrypsin activity: 8.674e-12 (43,41,11) OR
GO:0004295: trypsin activity: 5.745e-11 (43,48,11) OR
GO:0003809: thrombin activity: 1.834e-07 (43,10,5) OR
GO:0004867: serine-type endopeptidase inhibitor activity: 7.150e-05 (43,47,6) OR
GO:0005509: calcium ion binding: 6.631e-04 (43,160,9) OR
GO:0004896: hematopoietin/interferon-class (D200-domain) cytokine receptor activity: 2.616e-01 (43,19,1)

IF 1danh_#93 AND 1ekbb_#79 AND 2hlca_#27 AND 1fjsa_#158 AND 1gdna_#121 AND 1bio__#210
THEN
GO:0004263: chymotrypsin activity: 8.674e-12 (43,41,11) OR
GO:0004295: trypsin activity: 5.745e-11 (43,48,11) OR
GO:0004867: serine-type endopeptidase inhibitor activity: 5.780e-06 (43,47,7) OR
GO:0003809: thrombin activity: 1.055e-05 (43,10,4) OR
GO:0005509: calcium ion binding: 6.631e-04 (43,160,9) OR
GO:0004896: hematopoietin/interferon-class (D200-domain) cytokine receptor activity: 2.616e-01 (43,19,1)

IF 1c3pa_#6 AND 1eno__#137
THEN
GO:0016616: oxidoreductase activity, acting on the CH-OH group of donors, NAD or NADP as acceptor: 1.375e-11 (12,59,8) OR
GO:0016627: oxidoreductase activity, acting on the CH-CH group of donors: 2.331e-03 (12,17,2) OR
GO:0016854: racemase and epimerase activity: 5.588e-02 (12,13,1) OR
GO:0016836: hydro-lyase activity: 1.363e-01 (12,33,1)

IF 1oaa__#161 AND 5ruba1#365
THEN
GO:0016616: oxidoreductase activity, acting on the CH-OH group of donors, NAD or NADP as acceptor: 1.375e-11 (12,59,8) OR
GO:0016627: oxidoreductase activity, acting on the CH-CH group of donors: 2.331e-03 (12,17,2) OR
GO:0016646: oxidoreductase activity, acting on the CH-NH group of donors, NAD or NADP as acceptor: 8.069e-02 (12,19,1) OR
GO:0016836: hydro-lyase activity: 1.363e-01 (12,33,1)

IF 1c7na_#231 AND 1h5qa_#70
THEN
GO:0016616: oxidoreductase activity, acting on the CH-OH group of donors, NAD or NADP as acceptor: 1.375e-11 (12,59,8) OR
GO:0016627: oxidoreductase activity, acting on the CH-CH group of donors: 2.331e-03 (12,17,2) OR
GO:0016854: racemase and epimerase activity: 5.588e-02 (12,13,1) OR
GO:0016646: oxidoreductase activity, acting on the CH-NH group of donors, NAD or NADP as acceptor: 8.069e-02 (12,19,1)

IF 1hc7a2#113 AND 1qf6a1#606 AND 1pysa_#221
THEN
GO:0004812: tRNA ligase activity: 1.835e-11 (15,26,7) OR
GO:0005524: ATP binding: 1.420e-04 (15,243,7) OR
GO:0003887: DNA-directed DNA polymerase activity: 1.049e-01 (15,20,1)

IF 1g25a_#22 AND 1g51a3#525 AND 1ddja_#759
THEN
GO:0004295: trypsin activity: 9.214e-12 (37,48,11) OR
GO:0004263: chymotrypsin activity: 4.810e-11 (37,41,10) OR
GO:0003809: thrombin activity: 2.591e-04 (37,10,3) OR
GO:0005509: calcium ion binding: 1.060e-03 (37,160,8) OR
GO:0004867: serine-type endopeptidase inhibitor activity: 3.383e-03 (37,47,4) OR
GO:0004896: hematopoietin/interferon-class (D200-domain) cytokine receptor activity: 2.294e-01 (37,19,1)

IF 1b3qa2#556 AND 1f3mc_#369
THEN
GO:0004674: protein serine/threonine kinase activity: 1.386e-11 (23,42,9) OR
GO:0005524: ATP binding: 1.170e-06 (23,243,11) OR
GO:0005516: calmodulin binding: 1.680e-02 (23,24,2) OR
GO:0005066: transmembrane receptor protein tyrosine kinase signaling protein activity: 8.140e-02 (23,10,1)

IF 1azza_#114 AND 1eq9a_#162 AND 1c5y.1#B234
THEN
GO:0004295: trypsin activity: 1.113e-11 (28,48,10) OR
GO:0004263: chymotrypsin activity: 8.767e-11 (28,41,9) OR
GO:0004867: serine-type endopeptidase inhibitor activity: 1.175e-03 (28,47,4) OR
GO:0005509: calcium ion binding: 7.802e-02 (28,160,4) OR
GO:0003809: thrombin activity: 9.828e-02 (28,10,1)

IF 1danh_#152 AND 1qs1a2#437
THEN
GO:0004295: trypsin activity: 1.113e-11 (28,48,10) OR
GO:0004263: chymotrypsin activity: 8.767e-11 (28,41,9) OR
GO:0004867: serine-type endopeptidase inhibitor activity: 1.175e-03 (28,47,4) OR
GO:0005509: calcium ion binding: 7.802e-02 (28,160,4) OR
GO:0003809: thrombin activity: 9.828e-02 (28,10,1)

IF 1qf6a4#318 AND 1dr9a1#39
THEN
GO:0004812: tRNA ligase activity: 1.400e-11 (24,26,8) OR
GO:0000287: magnesium ion binding: 6.393e-04 (24,128,6) OR
GO:0005524: ATP binding: 7.412e-04 (24,243,8) OR
GO:0000049: tRNA binding: 5.467e-03 (24,13,2)

IF 1qf6a4#362 AND 1b35b_#97
THEN
GO:0004812: tRNA ligase activity: 1.400e-11 (24,26,8) OR
GO:0000287: magnesium ion binding: 6.393e-04 (24,128,6) OR
GO:0005524: ATP binding: 7.412e-04 (24,243,8) OR
GO:0000049: tRNA binding: 5.467e-03 (24,13,2)

IF 1c5y.1#B192 AND 1qnja_#77
THEN
GO:0004263: chymotrypsin activity: 9.389e-12 (32,41,10) OR
GO:0004295: trypsin activity: 5.199e-11 (32,48,10) OR
GO:0004867: serine-type endopeptidase inhibitor activity: 1.962e-03 (32,47,4) OR
GO:0005509: calcium ion binding: 9.442e-03 (32,160,6) OR
GO:0003809: thrombin activity: 1.116e-01 (32,10,1) OR
GO:0004896: hematopoietin/interferon-class (D200-domain) cytokine receptor activity: 2.016e-01 (32,19,1)

IF 1danh_#93 AND 1ekbb_#117 AND 2hlca_#27 AND 1f2la_#40 AND 1fjsa_#158 AND 1dlea_#238
THEN
GO:0004263: chymotrypsin activity: 9.389e-12 (32,41,10) OR
GO:0004295: trypsin activity: 5.199e-11 (32,48,10) OR
GO:0003809: thrombin activity: 5.670e-03 (32,10,2) OR
GO:0005509: calcium ion binding: 9.442e-03 (32,160,6) OR
GO:0004867: serine-type endopeptidase inhibitor activity: 1.681e-02 (32,47,3) OR
GO:0004896: hematopoietin/interferon-class (D200-domain) cytokine receptor activity: 2.016e-01 (32,19,1)

IF 1qsta_#154 AND 3chbd_#85
THEN
GO:0004812: tRNA ligase activity: 9.392e-12 (23,26,8) OR
GO:0005524: ATP binding: 5.347e-04 (23,243,8) OR
GO:0000287: magnesium ion binding: 2.053e-02 (23,128,4) OR
GO:0008800: beta-lactamase activity: 8.140e-02 (23,10,1) OR
GO:0030145: manganese ion binding: 2.770e-01 (23,38,1) OR
GO:0008270: zinc ion binding: 6.070e-01 (23,108,1)

IF 1h7wa4#476 AND 1rypk_#12
THEN
GO:0050660: FAD binding: 1.414e-11 (20,10,6) OR
GO:0015036: disulfide oxidoreductase activity: 5.651e-11 (20,22,7) OR
GO:0016668: oxidoreductase activity, acting on sulfur group of donors, NAD or NADP as acceptor: 6.167e-11 (20,12,6) OR
GO:0004601: peroxidase activity: 1.438e-01 (20,21,1)

IF 1bg6_2#24 AND 1c9la2#12 AND 1h6va1#27
THEN
GO:0015036: disulfide oxidoreductase activity: 1.438e-11 (17,22,7) OR
GO:0016668: oxidoreductase activity, acting on sulfur group of donors, NAD or NADP as acceptor: 3.828e-09 (17,12,5) OR
GO:0050660: FAD binding: 2.131e-07 (17,10,4) OR
GO:0016651: oxidoreductase activity, acting on NADH or NADPH: 7.830e-02 (17,13,1)

IF 1el5a1#166 AND 1fl2a1#320
THEN
GO:0015036: disulfide oxidoreductase activity: 1.438e-11 (17,22,7) OR
GO:0016668: oxidoreductase activity, acting on sulfur group of donors, NAD or NADP as acceptor: 3.828e-09 (17,12,5) OR
GO:0050660: FAD binding: 2.131e-07 (17,10,4) OR
GO:0016638: oxidoreductase activity, acting on the CH-NH2 group of donors: 1.012e-01 (17,17,1)

IF 1el5a1#166 AND 1trb_1#112
THEN
GO:0015036: disulfide oxidoreductase activity: 1.438e-11 (17,22,7) OR
GO:0016668: oxidoreductase activity, acting on sulfur group of donors, NAD or NADP as acceptor: 3.828e-09 (17,12,5) OR
GO:0050660: FAD binding: 2.131e-07 (17,10,4) OR
GO:0016638: oxidoreductase activity, acting on the CH-NH2 group of donors: 1.012e-01 (17,17,1)

IF 1mpp__#220
THEN
GO:0004190: aspartic-type endopeptidase activity: 1.475e-11 (9,23,6) OR
GO:0003964: RNA-directed DNA polymerase activity: 3.580e-02 (9,11,1) OR
GO:0004523: ribonuclease H activity: 5.169e-02 (9,16,1) OR
GO:0008270: zinc ion binding: 3.055e-01 (9,108,1)

IF 1mpp__#154 AND 1qqsa_#124 AND 1hrna_#182
THEN
GO:0004190: aspartic-type endopeptidase activity: 1.475e-11 (9,23,6) OR
GO:0046983: protein dimerization activity: 5.484e-02 (9,17,1) OR
GO:0008483: transaminase activity: 5.484e-02 (9,17,1) OR
GO:0003887: DNA-directed DNA polymerase activity: 6.424e-02 (9,20,1)

IF 2dkb__#114 AND 1elua_#199 AND 1dfoa_#362
THEN
GO:0008483: transaminase activity: 1.980e-11 (12,17,6) OR
GO:0016846: carbon-sulfur lyase activity: 1.583e-10 (12,10,5) OR
GO:0016831: carboxy-lyase activity: 1.049e-01 (12,25,1)

IF 1qgna_#207 AND 1dfoa_#344
THEN
GO:0008483: transaminase activity: 1.980e-11 (12,17,6) OR
GO:0016846: carbon-sulfur lyase activity: 1.583e-10 (12,10,5) OR
GO:0016831: carboxy-lyase activity: 1.049e-01 (12,25,1)

IF 1qlsa_#81 AND 1hqva_#48 AND 1c7wa_#105 AND 1ab4__#89
THEN
GO:0005509: calcium ion binding: 2.212e-11 (12,160,10) OR
GO:0003779: actin binding: 1.324e-01 (12,32,1) OR
GO:0008270: zinc ion binding: 3.851e-01 (12,108,1)

IF 1danh_#48 AND 1elva1#601 AND 1cgha_#130
THEN
GO:0004263: chymotrypsin activity: 1.333e-11 (33,41,10) OR
GO:0004295: trypsin activity: 7.364e-11 (33,48,10) OR
GO:0004867: serine-type endopeptidase inhibitor activity: 2.031e-04 (33,47,5) OR
GO:0005509: calcium ion binding: 2.462e-03 (33,160,7) OR
GO:0003809: thrombin activity: 1.149e-01 (33,10,1)

IF 1e9xa_#343 AND 1hqva_#48
THEN
GO:0004497: monooxygenase activity: 3.355e-11 (9,26,6) OR
GO:0016705: oxidoreductase activity, acting on paired donors, with incorporation or reduction of molecular oxygen: 6.241e-05 (9,26,3)

IF 1cpt__#52 AND 1hqva_#48
THEN
GO:0004497: monooxygenase activity: 3.355e-11 (9,26,6) OR
GO:0016705: oxidoreductase activity, acting on paired donors, with incorporation or reduction of molecular oxygen: 6.241e-05 (9,26,3)

IF 1dz4a_#357 AND 1hqva_#48
THEN
GO:0004497: monooxygenase activity: 3.355e-11 (9,26,6) OR
GO:0016705: oxidoreductase activity, acting on paired donors, with incorporation or reduction of molecular oxygen: 6.241e-05 (9,26,3)

IF 1dt6a_#374 AND 1dz4a_#264
THEN
GO:0004497: monooxygenase activity: 3.355e-11 (9,26,6) OR
GO:0016705: oxidoreductase activity, acting on paired donors, with incorporation or reduction of molecular oxygen: 6.241e-05 (9,26,3)

IF 1bqk__#78 AND 1bqk__#77
THEN
GO:0005507: copper ion binding: 3.382e-11 (7,38,6) OR

GO:0016638: oxidoreductase activity, acting on the CH-NH2 group of donors: 4.291e-02 (7,17,1)

IF 1gg6.1#C156 AND 1fu6a_#50
THEN
GO:0004263: chymotrypsin activity: 1.145e-11 (44,41,11) OR
GO:0004295: trypsin activity: 7.563e-11 (44,48,11) OR
GO:0003809: thrombin activity: 2.066e-07 (44,10,5) OR
GO:0004867: serine-type endopeptidase inhibitor activity: 6.785e-06 (44,47,7) OR
GO:0005509: calcium ion binding: 7.923e-04 (44,160,9) OR
GO:0004896: hematopoietin/interferon-class (D200-domain) cytokine receptor activity: 2.668e-01 (44,19,1)

IF 1b4ka_#108 AND 1e6ua_#10
THEN
GO:0016616: oxidoreductase activity, acting on the CH-OH group of donors, NAD or NADP as acceptor: 1.375e-11 (12,59,8) OR
GO:0016854: racemase and epimerase activity: 5.588e-02 (12,13,1) OR
GO:0016646: oxidoreductase activity, acting on the CH-NH group of donors, NAD or NADP as acceptor: 8.069e-02 (12,19,1) OR
GO:0016836: hydro-lyase activity: 1.363e-01 (12,33,1) OR
GO:0005524: ATP binding: 6.748e-01 (12,243,1)

IF 1b4ka_#108 AND 1hdr__#149
THEN
GO:0016616: oxidoreductase activity, acting on the CH-OH group of donors, NAD or NADP as acceptor: 1.375e-11 (12,59,8) OR
GO:0016854: racemase and epimerase activity: 5.588e-02 (12,13,1) OR
GO:0016646: oxidoreductase activity, acting on the CH-NH group of donors, NAD or NADP as acceptor: 8.069e-02 (12,19,1) OR
GO:0016836: hydro-lyase activity: 1.363e-01 (12,33,1) OR
GO:0030145: manganese ion binding: 1.554e-01 (12,38,1)

IF 1fmca_#86 AND 1qrra_#208
THEN
GO:0016616: oxidoreductase activity, acting on the CH-OH group of donors, NAD or NADP as acceptor: 1.375e-11 (12,59,8) OR
GO:0016627: oxidoreductase activity, acting on the CH-CH group of donors: 7.249e-02 (12,17,1) OR
GO:0016646: oxidoreductase activity, acting on the CH-NH group of donors, NAD or NADP as acceptor: 8.069e-02 (12,19,1) OR
GO:0005525: GTP binding: 1.960e-01 (12,49,1) OR
GO:0005524: ATP binding: 6.748e-01 (12,243,1)

IF 1qf6a4#318 AND 1ppn__#132
THEN
GO:0004812: tRNA ligase activity: 1.400e-11 (24,26,8) OR
GO:0005524: ATP binding: 7.412e-04 (24,243,8) OR
GO:0000287: magnesium ion binding: 4.370e-03 (24,128,5) OR
GO:0000049: tRNA binding: 5.467e-03 (24,13,2) OR
GO:0003887: DNA-directed DNA polymerase activity: 1.627e-01 (24,20,1)

IF 2cmd_1#113 AND 1ldna1#94
THEN
GO:0016616: oxidoreductase activity, acting on the CH-OH group of donors, NAD or NADP as acceptor: 3.515e-11 (13,59,8) OR
GO:0004457: lactate dehydrogenase activity: 2.568e-10 (13,10,5)

IF 1dgw.1#Y353 AND 1h7wa4#476
THEN
GO:0015036: disulfide oxidoreductase activity: 1.438e-11 (17,22,7) OR
GO:0050660: FAD binding: 2.131e-07 (17,10,4) OR
GO:0016668: oxidoreductase activity, acting on sulfur group of donors, NAD or NADP as acceptor: 4.984e-07 (17,12,4) OR
GO:0016651: oxidoreductase activity, acting on NADH or NADPH: 7.830e-02 (17,13,1) OR
GO:0004601: peroxidase activity: 1.236e-01 (17,21,1)

IF 1fuma2#370 AND 1c9la2#12
THEN
GO:0015036: disulfide oxidoreductase activity: 1.438e-11 (17,22,7) OR
GO:0016668: oxidoreductase activity, acting on sulfur group of donors, NAD or NADP as acceptor: 3.828e-09 (17,12,5) OR
GO:0050660: FAD binding: 2.358e-05 (17,10,3) OR
GO:0016651: oxidoreductase activity, acting on NADH or NADPH: 7.830e-02 (17,13,1) OR
GO:0016638: oxidoreductase activity, acting on the CH-NH2 group of donors: 1.012e-01 (17,17,1)

IF 1hd2a_#35 AND 1fwxa1#526
THEN
GO:0005507: copper ion binding: 1.821e-11 (11,38,7) OR
GO:0051082: unfolded protein binding: 7.747e-03 (11,34,2) OR
GO:0015082: di-, tri-valent inorganic cation transporter activity: 5.518e-02 (11,14,1) OR
GO:0046915: transition metal ion transporter activity: 5.518e-02 (11,14,1)

IF 2occb1#189 AND 2cuaa_#83
THEN
GO:0005507: copper ion binding: 1.457e-11 (17,38,8) OR
GO:0015078: hydrogen ion transporter activity: 5.822e-06 (17,21,4) OR
GO:0005509: calcium ion binding: 7.367e-02 (17,160,3) OR
GO:0015082: di-, tri-valent inorganic cation transporter activity: 8.408e-02 (17,14,1) OR
GO:0046915: transition metal ion transporter activity: 8.408e-02 (17,14,1)

IF 1bu7a_#360 AND 1e9xa_#62
THEN
GO:0004497: monooxygenase activity: 1.835e-11 (15,26,7) OR
GO:0016705: oxidoreductase activity, acting on paired donors, with incorporation or reduction of molecular oxygen: 8.288e-06 (15,26,4) OR
GO:0010181: FMN binding: 1.512e-03 (15,11,2) OR
GO:0016651: oxidoreductase activity, acting on NADH or NADPH: 2.131e-03 (15,13,2)

IF 1cpt__#52 AND 1dt6a_#181
THEN
GO:0004497: monooxygenase activity: 1.835e-11 (15,26,7) OR
GO:0016705: oxidoreductase activity, acting on paired donors, with incorporation or reduction of molecular oxygen: 8.288e-06 (15,26,4) OR
GO:0010181: FMN binding: 1.512e-03 (15,11,2) OR
GO:0016651: oxidoreductase activity, acting on NADH or NADPH: 2.131e-03 (15,13,2)

IF 1dz4a_#150 AND 1io7a_#243
THEN
GO:0004497: monooxygenase activity: 1.835e-11 (15,26,7) OR
GO:0016705: oxidoreductase activity, acting on paired donors, with incorporation or reduction of molecular oxygen: 8.288e-06 (15,26,4) OR
GO:0010181: FMN binding: 1.512e-03 (15,11,2) OR
GO:0016651: oxidoreductase activity, acting on NADH or NADPH: 2.131e-03 (15,13,2)

IF 1cpt__#52 AND 1dz4a_#264 AND 1ab4__#89
THEN
GO:0004497: monooxygenase activity: 1.835e-11 (15,26,7) OR
GO:0016705: oxidoreductase activity, acting on paired donors, with incorporation or reduction of molecular oxygen: 8.288e-06 (15,26,4) OR
GO:0010181: FMN binding: 1.512e-03 (15,11,2) OR
GO:0016651: oxidoreductase activity, acting on NADH or NADPH: 2.131e-03 (15,13,2)

IF 1e9xa_#62 AND 1bu7a_#277
THEN
GO:0004497: monooxygenase activity: 1.835e-11 (15,26,7) OR
GO:0016705: oxidoreductase activity, acting on paired donors, with incorporation or reduction of molecular oxygen: 8.288e-06 (15,26,4) OR
GO:0010181: FMN binding: 1.512e-03 (15,11,2) OR
GO:0016651: oxidoreductase activity, acting on NADH or NADPH: 2.131e-03 (15,13,2)

IF 1eupa_#320 AND 1bu7a_#277
THEN
GO:0004497: monooxygenase activity: 1.835e-11 (15,26,7) OR
GO:0016705: oxidoreductase activity, acting on paired donors, with incorporation or reduction of molecular oxygen: 8.288e-06 (15,26,4) OR
GO:0010181: FMN binding: 1.512e-03 (15,11,2) OR
GO:0016651: oxidoreductase activity, acting on NADH or NADPH: 2.131e-03 (15,13,2)

IF 1fc3a_#172 AND 1cpt__#52 AND 1ab4__#89
THEN
GO:0004497: monooxygenase activity: 1.835e-11 (15,26,7) OR
GO:0016705: oxidoreductase activity, acting on paired donors, with incorporation or reduction of molecular oxygen: 8.288e-06 (15,26,4) OR
GO:0010181: FMN binding: 1.512e-03 (15,11,2) OR
GO:0016651: oxidoreductase activity, acting on NADH or NADPH: 2.131e-03 (15,13,2)

IF 1cpt__#401 AND 1dt6a_#181
THEN
GO:0004497: monooxygenase activity: 1.835e-11 (15,26,7) OR
GO:0016705: oxidoreductase activity, acting on paired donors, with incorporation or reduction of molecular oxygen: 8.288e-06 (15,26,4) OR

GO:0010181: FMN binding: 1.512e-03 (15,11,2) OR
GO:0016651: oxidoreductase activity, acting on NADH or NADPH: 2.131e-03 (15,13,2)

IF 1fc3a_#172 AND 1dt6a_#181 AND 1dz4a_#264
THEN
GO:0004497: monooxygenase activity: 1.835e-11 (15,26,7) OR
GO:0016705: oxidoreductase activity, acting on paired donors, with incorporation or reduction of molecular oxygen: 8.288e-06 (15,26,4) OR
GO:0010181: FMN binding: 1.512e-03 (15,11,2) OR
GO:0016651: oxidoreductase activity, acting on NADH or NADPH: 2.131e-03 (15,13,2)

IF 1qgna_#149 AND 1ax4a_#275
THEN
GO:0016846: carbon-sulfur lyase activity: 2.530e-11 (9,10,5) OR
GO:0008483: transaminase activity: 1.657e-05 (9,17,3) OR
GO:0016831: carboxy-lyase activity: 7.972e-02 (9,25,1)

IF 1deua_#186 AND 1ppn__#191
THEN
GO:0004197: cysteine-type endopeptidase activity: 1.965e-11 (9,24,6) OR
GO:0019955: cytokine binding: 3.580e-02 (9,11,1) OR
GO:0042802: protein self binding: 3.900e-02 (9,12,1) OR
GO:0051082: unfolded protein binding: 1.070e-01 (9,34,1)

IF 1deua_#210 AND 1deua_#186
THEN
GO:0004197: cysteine-type endopeptidase activity: 1.965e-11 (9,24,6) OR
GO:0019955: cytokine binding: 3.580e-02 (9,11,1) OR
GO:0042802: protein self binding: 3.900e-02 (9,12,1) OR
GO:0051082: unfolded protein binding: 1.070e-01 (9,34,1)

IF 2foka2#180 AND 1deua_#151 AND 3gcb__#78
THEN
GO:0004197: cysteine-type endopeptidase activity: 1.965e-11 (9,24,6) OR
GO:0004177: aminopeptidase activity: 4.219e-02 (9,13,1) OR
GO:0004180: carboxypeptidase activity: 4.853e-02 (9,15,1) OR
GO:0005524: ATP binding: 5.691e-01 (9,243,1)

IF 1deua_#186 AND 1euva_#580
THEN
GO:0004197: cysteine-type endopeptidase activity: 1.965e-11 (9,24,6) OR
GO:0019955: cytokine binding: 3.580e-02 (9,11,1) OR
GO:0042802: protein self binding: 3.900e-02 (9,12,1) OR
GO:0051082: unfolded protein binding: 1.070e-01 (9,34,1)

IF 1ppn__#191 AND 1euva_#580
THEN
GO:0004197: cysteine-type endopeptidase activity: 1.965e-11 (9,24,6) OR
GO:0019955: cytokine binding: 3.580e-02 (9,11,1) OR
GO:0042802: protein self binding: 3.900e-02 (9,12,1) OR
GO:0051082: unfolded protein binding: 1.070e-01 (9,34,1)

IF 1ppn__#191 AND 1dkia_#54
THEN
GO:0004197: cysteine-type endopeptidase activity: 1.965e-11 (9,24,6) OR
GO:0019955: cytokine binding: 3.580e-02 (9,11,1) OR
GO:0042802: protein self binding: 3.900e-02 (9,12,1) OR
GO:0051082: unfolded protein binding: 1.070e-01 (9,34,1)

IF 1thea_#79 AND 1euva_#580
THEN
GO:0004197: cysteine-type endopeptidase activity: 1.965e-11 (9,24,6) OR
GO:0019955: cytokine binding: 3.580e-02 (9,11,1) OR
GO:0042802: protein self binding: 3.900e-02 (9,12,1) OR
GO:0051082: unfolded protein binding: 1.070e-01 (9,34,1)

IF 2cb5a_#373 AND 3gcb__#78 AND 2cpl__#53
THEN
GO:0004197: cysteine-type endopeptidase activity: 1.965e-11 (9,24,6) OR
GO:0019955: cytokine binding: 3.580e-02 (9,11,1) OR

GO:0042802: protein self binding: 3.900e-02 (9,12,1) OR
GO:0051082: unfolded protein binding: 1.070e-01 (9,34,1)

IF 1fmca_#88 AND 1fmca_#248
THEN
GO:0016616: oxidoreductase activity, acting on the CH-OH group of donors, NAD or NADP as acceptor: 7.967e-11 (6,59,6)

IF 1eno__#290 AND 1ec7a1#216
THEN
GO:0016616: oxidoreductase activity, acting on the CH-OH group of donors, NAD or NADP as acceptor: 7.967e-11 (6,59,6)

IF 1ec7a1#216 AND 1h5qa_#201
THEN
GO:0016616: oxidoreductase activity, acting on the CH-OH group of donors, NAD or NADP as acceptor: 7.967e-11 (6,59,6)

IF 1h5qa_#21 AND 1e7wa_#36
THEN
GO:0016616: oxidoreductase activity, acting on the CH-OH group of donors, NAD or NADP as acceptor: 7.967e-11 (6,59,6)

IF 1fmca_#88 AND 1e8ca3#111
THEN
GO:0016616: oxidoreductase activity, acting on the CH-OH group of donors, NAD or NADP as acceptor: 7.967e-11 (6,59,6)

IF 1fmca_#88 AND 1h6va1#27
THEN
GO:0016616: oxidoreductase activity, acting on the CH-OH group of donors, NAD or NADP as acceptor: 7.967e-11 (6,59,6)

IF 1fmca_#88 AND 1hdr__#136
THEN
GO:0016616: oxidoreductase activity, acting on the CH-OH group of donors, NAD or NADP as acceptor: 7.967e-11 (6,59,6)

IF 1hdca_#187 AND 1e3ja2#270
THEN
GO:0016616: oxidoreductase activity, acting on the CH-OH group of donors, NAD or NADP as acceptor: 7.967e-11 (6,59,6)

IF 1fmca_#86 AND 1ec7a1#216
THEN
GO:0016616: oxidoreductase activity, acting on the CH-OH group of donors, NAD or NADP as acceptor: 7.967e-11 (6,59,6)

IF 1oaa__#235 AND 1e3ja2#270
THEN
GO:0016616: oxidoreductase activity, acting on the CH-OH group of donors, NAD or NADP as acceptor: 7.967e-11 (6,59,6)

IF 1cyda_#231 AND 1bdb__#144
THEN
GO:0016616: oxidoreductase activity, acting on the CH-OH group of donors, NAD or NADP as acceptor: 7.967e-11 (6,59,6)

IF 1hlwa_#82 AND 1fxla2#169 AND 2msba_#128
THEN
GO:0005529: sugar binding: 3.996e-11 (7,39,6) OR
GO:0005509: calcium ion binding: 3.456e-01 (7,160,1)

IF 1hlwa_#82 AND 1fxla2#169 AND 1tn3__#113
THEN
GO:0005529: sugar binding: 3.996e-11 (7,39,6) OR
GO:0005509: calcium ion binding: 3.456e-01 (7,160,1)

IF 1czan1#92 AND 2msba_#156
THEN
GO:0005529: sugar binding: 3.996e-11 (7,39,6) OR
GO:0008201: heparin binding: 6.011e-02 (7,24,1)

IF 1qo3c_#212 AND 2msba_#156
THEN
GO:0005529: sugar binding: 3.996e-11 (7,39,6) OR
GO:0008201: heparin binding: 6.011e-02 (7,24,1)

IF 1g6ga_#133 AND 1qj8a_#64
THEN
GO:0004263: chymotrypsin activity: 1.333e-11 (33,41,10) OR

GO:0004295: trypsin activity: 7.364e-11 (33,48,10) OR
GO:0004867: serine-type endopeptidase inhibitor activity: 2.031e-04 (33,47,5) OR
GO:0005509: calcium ion binding: 1.099e-02 (33,160,6) OR
GO:0003809: thrombin activity: 1.149e-01 (33,10,1) OR
GO:0004896: hematopoietin/interferon-class (D200-domain) cytokine receptor activity: 2.073e-01 (33,19,1)

IF 1azza_#114 AND 1danh_#48 AND 1danh_#142
THEN
GO:0004263: chymotrypsin activity: 1.333e-11 (33,41,10) OR
GO:0004295: trypsin activity: 7.364e-11 (33,48,10) OR
GO:0005509: calcium ion binding: 2.462e-03 (33,160,7) OR
GO:0003809: thrombin activity: 6.024e-03 (33,10,2) OR
GO:0004867: serine-type endopeptidase inhibitor activity: 1.827e-02 (33,47,3) OR
GO:0004896: hematopoietin/interferon-class (D200-domain) cytokine receptor activity: 2.073e-01 (33,19,1)

IF 1ib2a_#974 AND 1e8ca1#83
THEN
GO:0004364: glutathione transferase activity: 2.062e-11 (8,11,5) OR
GO:0016651: oxidoreductase activity, acting on NADH or NADPH: 3.758e-02 (8,13,1) OR
GO:0015078: hydrogen ion transporter activity: 6.009e-02 (8,21,1) OR
GO:0005507: copper ion binding: 1.064e-01 (8,38,1)

IF 1a0fa1#178 AND 1e6ca_#91
THEN
GO:0004364: glutathione transferase activity: 2.062e-11 (8,11,5) OR
GO:0003899: DNA-directed RNA polymerase activity: 3.474e-02 (8,12,1) OR
GO:0016651: oxidoreductase activity, acting on NADH or NADPH: 3.758e-02 (8,13,1) OR
GO:0008270: zinc ion binding: 2.767e-01 (8,108,1)

IF 1a0fa1#178 AND 2sqca2#268 AND 1qfea_#68
THEN
GO:0004364: glutathione transferase activity: 2.062e-11 (8,11,5) OR
GO:0004812: tRNA ligase activity: 7.392e-02 (8,26,1) OR
GO:0008270: zinc ion binding: 2.767e-01 (8,108,1) OR
GO:0005524: ATP binding: 5.268e-01 (8,243,1)

IF 1ia8a_#59 AND 1ayl__#237
THEN
GO:0004674: protein serine/threonine kinase activity: 1.386e-11 (23,42,9) OR
GO:0005524: ATP binding: 8.246e-05 (23,243,9) OR
GO:0005516: calmodulin binding: 1.680e-02 (23,24,2) OR
GO:0016799: hydrolase activity, hydrolyzing N-glycosyl compounds: 1.346e-01 (23,17,1) OR
GO:0004896: hematopoietin/interferon-class (D200-domain) cytokine receptor activity: 1.492e-01 (23,19,1) OR
GO:0016763: transferase activity, transferring pentosyl groups: 2.122e-01 (23,28,1)

IF 1cipa2#326 AND 1hxxa_#232
THEN
GO:0005525: GTP binding: 1.673e-11 (14,49,8) OR
GO:0003924: GTPase activity: 7.042e-05 (14,17,3) OR
GO:0003682: chromatin binding: 5.029e-02 (14,10,1) OR
GO:0003755: peptidyl-prolyl cis-trans isomerase activity: 5.518e-02 (14,11,1) OR
GO:0005085: guanyl-nucleotide exchange factor activity: 6.491e-02 (14,13,1)

IF 1b8aa2#251 AND 1fa0a1#393
THEN
GO:0004812: tRNA ligase activity: 1.400e-11 (24,26,8) OR
GO:0005524: ATP binding: 1.218e-04 (24,243,9) OR
GO:0000287: magnesium ion binding: 2.376e-02 (24,128,4) OR
GO:0003887: DNA-directed DNA polymerase activity: 1.627e-01 (24,20,1) OR
GO:0015078: hydrogen ion transporter activity: 1.701e-01 (24,21,1) OR
GO:0016763: transferase activity, transferring pentosyl groups: 2.204e-01 (24,28,1)

IF 1trb_1#298 AND 1vmoa_#106
THEN
GO:0050660: FAD binding: 1.414e-11 (20,10,6) OR
GO:0015036: disulfide oxidoreductase activity: 4.764e-09 (20,22,6) OR
GO:0016668: oxidoreductase activity, acting on sulfur group of donors, NAD or NADP as acceptor: 9.530e-09 (20,12,5) OR
GO:0016651: oxidoreductase activity, acting on NADH or NADPH: 9.152e-02 (20,13,1) OR
GO:0016705: oxidoreductase activity, acting on paired donors, with incorporation or reduction of molecular oxygen: 1.750e-01 (20,26,1) OR

GO:0004497: monooxygenase activity: 1.750e-01 (20,26,1)

IF 1e30a_#112 AND 1cyx__#138
THEN
GO:0005507: copper ion binding: 1.457e-11 (17,38,8) OR
GO:0015078: hydrogen ion transporter activity: 2.504e-04 (17,21,3) OR
GO:0005509: calcium ion binding: 7.367e-02 (17,160,3) OR
GO:0015082: di-, tri-valent inorganic cation transporter activity: 8.408e-02 (17,14,1) OR
GO:0046915: transition metal ion transporter activity: 8.408e-02 (17,14,1) OR
GO:0004197: cysteine-type endopeptidase activity: 1.400e-01 (17,24,1)

IF 2cuaa_#107 AND 1cyx__#133
THEN
GO:0005507: copper ion binding: 1.457e-11 (17,38,8) OR
GO:0015078: hydrogen ion transporter activity: 9.515e-08 (17,21,5) OR
GO:0009036: type II site-specific deoxyribonuclease activity: 7.249e-02 (17,12,1) OR
GO:0015082: di-, tri-valent inorganic cation transporter activity: 8.408e-02 (17,14,1) OR
GO:0046915: transition metal ion transporter activity: 8.408e-02 (17,14,1) OR
GO:0000287: magnesium ion binding: 5.597e-01 (17,128,1)

IF 1jsg__#92 AND 1tn3__#113
THEN
GO:0005529: sugar binding: 2.217e-11 (11,39,7) OR
GO:0005509: calcium ion binding: 1.331e-01 (11,160,2) OR
GO:0004263: chymotrypsin activity: 1.539e-01 (11,41,1) OR
GO:0004295: trypsin activity: 1.779e-01 (11,48,1)

IF 1esl_1#29 AND 1dv8a_#211
THEN
GO:0005529: sugar binding: 2.217e-11 (11,39,7) OR
GO:0005509: calcium ion binding: 1.331e-01 (11,160,2) OR
GO:0004263: chymotrypsin activity: 1.539e-01 (11,41,1) OR
GO:0004295: trypsin activity: 1.779e-01 (11,48,1)

IF 2msba_#134 AND 1dv8a_#211 AND 1egia_#738
THEN
GO:0005529: sugar binding: 2.217e-11 (11,39,7) OR
GO:0005509: calcium ion binding: 1.331e-01 (11,160,2) OR
GO:0004263: chymotrypsin activity: 1.539e-01 (11,41,1) OR
GO:0004295: trypsin activity: 1.779e-01 (11,48,1)

IF 2msba_#134 AND 1jsg__#92
THEN
GO:0005529: sugar binding: 2.217e-11 (11,39,7) OR
GO:0005509: calcium ion binding: 1.331e-01 (11,160,2) OR
GO:0004263: chymotrypsin activity: 1.539e-01 (11,41,1) OR
GO:0004295: trypsin activity: 1.779e-01 (11,48,1)

IF 1hq8a_#223 AND 1jsg__#92
THEN
GO:0005529: sugar binding: 2.217e-11 (11,39,7) OR
GO:0005509: calcium ion binding: 1.331e-01 (11,160,2) OR
GO:0004263: chymotrypsin activity: 1.539e-01 (11,41,1) OR
GO:0004295: trypsin activity: 1.779e-01 (11,48,1)

IF 2napa1#665 AND 1je5a_#106
THEN
GO:0030151: molybdenum ion binding: 1.489e-11 (13,15,6) OR
GO:0019829: cation-transporting ATPase activity: 4.219e-02 (13,9,1) OR
GO:0015405: P-P-bond-hydrolysis-driven transporter activity: 5.588e-02 (13,12,1) OR
GO:0015078: hydrogen ion transporter activity: 9.588e-02 (13,21,1) OR
GO:0003700: transcription factor activity: 1.155e-01 (13,124,2) OR
GO:0005524: ATP binding: 3.252e-01 (13,243,2)

IF 1by5a_#238 AND 1fjfc2#153 AND 1e6pa2#46
THEN
GO:0004556: alpha-amylase activity: 1.489e-11 (13,15,6) OR
GO:0005509: calcium ion binding: 3.676e-02 (13,160,3) OR
GO:0000049: tRNA binding: 6.040e-02 (13,13,1) OR
GO:0019843: rRNA binding: 1.002e-01 (13,22,1) OR

GO:0016836: hydro-lyase activity: 1.468e-01 (13,33,1) OR
GO:0000287: magnesium ion binding: 4.657e-01 (13,128,1)

IF 1doka_#52 AND 1el0a_#62
THEN
GO:0008009: chemokine activity: 9.161e-11 (4,10,4)

IF 1e3ua_#205 AND 1lci__#26
THEN
GO:0008800: beta-lactamase activity: 9.161e-11 (4,10,4)

IF 1doka_#52 AND 1ha6a_#59
THEN
GO:0008009: chemokine activity: 9.161e-11 (4,10,4)

IF 1f2la_#12 AND 1i5ga_#115
THEN
GO:0008009: chemokine activity: 9.161e-11 (4,10,4)

IF 1f0xa1#278 AND 1ha6a_#59
THEN
GO:0008009: chemokine activity: 9.161e-11 (4,10,4)

IF 1f2la_#12 AND 1el0a_#62
THEN
GO:0008009: chemokine activity: 9.161e-11 (4,10,4)

IF 1e3ua_#205 AND 1ei5a3#190
THEN
GO:0008800: beta-lactamase activity: 9.161e-11 (4,10,4)

IF 1danh_#197 AND 2viua_#252 AND 1eq9a_#162 AND 1ton__#112 AND 1arb__#193 AND 1autc_#209
THEN
GO:0004263: chymotrypsin activity: 1.869e-11 (34,41,10) OR
GO:0004295: trypsin activity: 1.030e-10 (34,48,10) OR
GO:0003809: thrombin activity: 2.007e-04 (34,10,3) OR
GO:0004867: serine-type endopeptidase inhibitor activity: 2.351e-04 (34,47,5) OR
GO:0005509: calcium ion binding: 1.272e-02 (34,160,6)

IF 1danh_#93 AND 1eq9a_#162 AND 1ddja_#759
THEN
GO:0004263: chymotrypsin activity: 1.869e-11 (34,41,10) OR
GO:0004295: trypsin activity: 1.030e-10 (34,48,10) OR
GO:0004867: serine-type endopeptidase inhibitor activity: 2.351e-04 (34,47,5) OR
GO:0005509: calcium ion binding: 2.950e-03 (34,160,7) OR
GO:0003809: thrombin activity: 6.388e-03 (34,10,2)

IF 1qlaa2#383 AND 1fhoa_#74
THEN
GO:0015036: disulfide oxidoreductase activity: 2.342e-11 (18,22,7) OR
GO:0050660: FAD binding: 1.697e-09 (18,10,5) OR
GO:0016668: oxidoreductase activity, acting on sulfur group of donors, NAD or NADP as acceptor: 5.289e-09 (18,12,5) OR
GO:0004601: peroxidase activity: 1.304e-01 (18,21,1)

IF 1bd3a_#110 AND 2hhma_#262
THEN
GO:0016763: transferase activity, transferring pentosyl groups: 3.285e-11 (15,28,7) OR
GO:0000287: magnesium ion binding: 2.016e-06 (15,128,7) OR
GO:0004725: protein tyrosine phosphatase activity: 7.966e-02 (15,15,1)

IF 1bf2_3#579 AND 1gjwa2#141 AND 1g25a_#22
THEN
GO:0004556: alpha-amylase activity: 5.024e-11 (7,15,5) OR
GO:0005509: calcium ion binding: 5.920e-02 (7,160,2)

IF 1evqa_#61 AND 1ton__#196 AND 2hlca_#68
THEN
GO:0004295: trypsin activity: 1.678e-11 (29,48,10) OR
GO:0004263: chymotrypsin activity: 1.258e-10 (29,41,9) OR
GO:0004867: serine-type endopeptidase inhibitor activity: 1.346e-03 (29,47,4) OR

GO:0005509: calcium ion binding: 8.655e-02 (29,160,4) OR
GO:0003809: thrombin activity: 1.016e-01 (29,10,1) OR
GO:0005529: sugar binding: 3.431e-01 (29,39,1)

IF 1evqa_#61 AND 2hlca_#68 AND 1elva1#604
THEN
GO:0004295: trypsin activity: 1.678e-11 (29,48,10) OR
GO:0004263: chymotrypsin activity: 1.258e-10 (29,41,9) OR
GO:0004867: serine-type endopeptidase inhibitor activity: 1.346e-03 (29,47,4) OR
GO:0005509: calcium ion binding: 8.655e-02 (29,160,4) OR
GO:0003809: thrombin activity: 1.016e-01 (29,10,1) OR
GO:0005529: sugar binding: 3.431e-01 (29,39,1)

IF 1ajsa_#139 AND 1fc4a_#205
THEN
GO:0016846: carbon-sulfur lyase activity: 5.051e-11 (10,10,5) OR
GO:0008483: transaminase activity: 1.227e-09 (10,17,5)

IF 1ldm_2#292 AND 1iira_#242
THEN
GO:0004457: lactate dehydrogenase activity: 5.051e-11 (10,10,5) OR
GO:0016616: oxidoreductase activity, acting on the CH-OH group of donors, NAD or NADP as acceptor: 9.304e-07 (10,59,5)

IF 1fc4a_#205 AND 1elua_#199
THEN
GO:0016846: carbon-sulfur lyase activity: 5.051e-11 (10,10,5) OR
GO:0008483: transaminase activity: 1.227e-09 (10,17,5)

IF 1hyea2#182 AND 1amf__#196
THEN
GO:0004457: lactate dehydrogenase activity: 5.051e-11 (10,10,5) OR
GO:0016616: oxidoreductase activity, acting on the CH-OH group of donors, NAD or NADP as acceptor: 9.304e-07 (10,59,5)

IF 1llc_2#291 AND 1gdha2#47
THEN
GO:0004457: lactate dehydrogenase activity: 5.051e-11 (10,10,5) OR
GO:0016616: oxidoreductase activity, acting on the CH-OH group of donors, NAD or NADP as acceptor: 9.304e-07 (10,59,5)

IF 1ceqa2#199 AND 1quqb_#87
THEN
GO:0004457: lactate dehydrogenase activity: 5.051e-11 (10,10,5) OR
GO:0016616: oxidoreductase activity, acting on the CH-OH group of donors, NAD or NADP as acceptor: 9.304e-07 (10,59,5)

IF 2cmd_2#172 AND 1b8pa2#201
THEN
GO:0004457: lactate dehydrogenase activity: 5.051e-11 (10,10,5) OR
GO:0016616: oxidoreductase activity, acting on the CH-OH group of donors, NAD or NADP as acceptor: 9.304e-07 (10,59,5)

IF 1ceqa2#199 AND 1ii7a_#43
THEN
GO:0004457: lactate dehydrogenase activity: 5.051e-11 (10,10,5) OR
GO:0016616: oxidoreductase activity, acting on the CH-OH group of donors, NAD or NADP as acceptor: 9.304e-07 (10,59,5)

IF 2bb2_2#134 AND 1ha6a_#20 AND 1ddma_#130 AND 1el0a_#42
THEN
GO:0008009: chemokine activity: 2.530e-11 (9,10,5) OR
GO:0008083: growth factor activity: 7.797e-03 (9,42,2) OR
GO:0003729: mRNA binding: 4.219e-02 (9,13,1) OR
GO:0005096: GTPase activator activity: 4.853e-02 (9,15,1)

IF 1ha6a_#20 AND 1a0i_2#222 AND 1ddma_#130
THEN
GO:0008009: chemokine activity: 2.530e-11 (9,10,5) OR
GO:0008083: growth factor activity: 7.797e-03 (9,42,2) OR
GO:0005096: GTPase activator activity: 4.853e-02 (9,15,1) OR
GO:0016763: transferase activity, transferring pentosyl groups: 8.889e-02 (9,28,1)

IF 2bb2_2#134 AND 1ha6a_#20 AND 1i5ga_#115 AND 1el0a_#42
THEN
GO:0008009: chemokine activity: 2.530e-11 (9,10,5) OR

GO:0008083: growth factor activity: 7.797e-03 (9,42,2) OR
GO:0003729: mRNA binding: 4.219e-02 (9,13,1) OR
GO:0005096: GTPase activator activity: 4.853e-02 (9,15,1)

IF 1e43a2#236 AND 1ew2a_#237
THEN
GO:0004556: alpha-amylase activity: 2.598e-11 (14,15,6) OR
GO:0005509: calcium ion binding: 7.566e-05 (14,160,6) OR
GO:0004867: serine-type endopeptidase inhibitor activity: 2.166e-01 (14,47,1) OR
GO:0005524: ATP binding: 7.304e-01 (14,243,1)

IF 1ja9a_#202 AND 1qg6a_#93
THEN
GO:0016616: oxidoreductase activity, acting on the CH-OH group of donors, NAD or NADP as acceptor: 3.515e-11 (13,59,8) OR
GO:0016627: oxidoreductase activity, acting on the CH-CH group of donors: 7.171e-07 (13,17,4) OR
GO:0016646: oxidoreductase activity, acting on the CH-NH group of donors, NAD or NADP as acceptor: 8.713e-02 (13,19,1)

IF 1a0la_#160 AND 1b3qa2#623 AND 1dlea_#238
THEN
GO:0004295: trypsin activity: 1.761e-11 (39,48,11) OR
GO:0004263: chymotrypsin activity: 8.597e-11 (39,41,10) OR
GO:0004867: serine-type endopeptidase inhibitor activity: 2.906e-06 (39,47,7) OR
GO:0005509: calcium ion binding: 1.527e-03 (39,160,8) OR
GO:0003809: thrombin activity: 8.355e-03 (39,10,2) OR
GO:0008201: heparin binding: 2.935e-01 (39,24,1)

IF 1cyda_#204 AND 1h5qa_#153
THEN
GO:0016616: oxidoreductase activity, acting on the CH-OH group of donors, NAD or NADP as acceptor: 5.405e-11 (9,59,7) OR
GO:0016627: oxidoreductase activity, acting on the CH-CH group of donors: 1.286e-03 (9,17,2)

IF 1eno__#290 AND 1qrra_#208
THEN
GO:0016616: oxidoreductase activity, acting on the CH-OH group of donors, NAD or NADP as acceptor: 5.405e-11 (9,59,7) OR
GO:0016627: oxidoreductase activity, acting on the CH-CH group of donors: 1.286e-03 (9,17,2)

IF 1h7wa4#476 AND 1e1oa2#423
THEN
GO:0015036: disulfide oxidoreductase activity: 2.715e-11 (10,22,6) OR
GO:0016668: oxidoreductase activity, acting on sulfur group of donors, NAD or NADP as acceptor: 7.847e-04 (10,12,2) OR
GO:0016627: oxidoreductase activity, acting on the CH-CH group of donors: 6.076e-02 (10,17,1) OR
GO:0004601: peroxidase activity: 7.456e-02 (10,21,1)

IF 1a3c__#108 AND 3lada1#116
THEN
GO:0016763: transferase activity, transferring pentosyl groups: 5.480e-11 (9,28,6) OR
GO:0000287: magnesium ion binding: 6.906e-03 (9,128,3)

IF 1csn__#134 AND 1e4ea2#215
THEN
GO:0004674: protein serine/threonine kinase activity: 2.194e-11 (24,42,9) OR
GO:0005524: ATP binding: 1.991e-06 (24,243,11) OR
GO:0005516: calmodulin binding: 1.823e-02 (24,24,2) OR
GO:0004714: transmembrane receptor protein tyrosine kinase activity: 1.167e-01 (24,14,1) OR
GO:0008201: heparin binding: 1.920e-01 (24,24,1)

IF 1fgka_#610 AND 1koba_#105 AND 1a06__#103
THEN
GO:0004674: protein serine/threonine kinase activity: 2.194e-11 (24,42,9) OR
GO:0005524: ATP binding: 1.691e-05 (24,243,10) OR
GO:0005516: calmodulin binding: 1.077e-03 (24,24,3) OR
GO:0005066: transmembrane receptor protein tyrosine kinase signaling protein activity: 8.480e-02 (24,10,1) OR
GO:0004896: hematopoietin/interferon-class (D200-domain) cytokine receptor activity: 1.552e-01 (24,19,1)

IF 1b3qa2#556 AND 1tkia_#142
THEN
GO:0004674: protein serine/threonine kinase activity: 2.194e-11 (24,42,9) OR
GO:0005524: ATP binding: 1.991e-06 (24,243,11) OR
GO:0005516: calmodulin binding: 1.823e-02 (24,24,2) OR
GO:0005066: transmembrane receptor protein tyrosine kinase signaling protein activity: 8.480e-02 (24,10,1) OR

GO:0000155: two-component sensor molecule activity: 1.009e-01 (24,12,1)

IF 1b3qa2#556 AND 1hcl__#280
THEN
GO:0004674: protein serine/threonine kinase activity: 2.194e-11 (24,42,9) OR
GO:0005524: ATP binding: 1.991e-06 (24,243,11) OR
GO:0005516: calmodulin binding: 1.823e-02 (24,24,2) OR
GO:0005066: transmembrane receptor protein tyrosine kinase signaling protein activity: 8.480e-02 (24,10,1) OR
GO:0003684: damaged DNA binding: 8.480e-02 (24,10,1)

IF 1qnja_#77 AND 1c9la2#61
THEN
GO:0004263: chymotrypsin activity: 1.869e-11 (34,41,10) OR
GO:0004295: trypsin activity: 1.030e-10 (34,48,10) OR
GO:0004867: serine-type endopeptidase inhibitor activity: 2.351e-04 (34,47,5) OR
GO:0005509: calcium ion binding: 2.950e-03 (34,160,7) OR
GO:0003809: thrombin activity: 1.182e-01 (34,10,1) OR
GO:0004896: hematopoietin/interferon-class (D200-domain) cytokine receptor activity: 2.129e-01 (34,19,1)

IF 1i9ga_#67 AND 1ekbb_#73
THEN
GO:0004263: chymotrypsin activity: 1.869e-11 (34,41,10) OR
GO:0004295: trypsin activity: 1.030e-10 (34,48,10) OR
GO:0004867: serine-type endopeptidase inhibitor activity: 2.467e-03 (34,47,4) OR
GO:0005509: calcium ion binding: 2.950e-03 (34,160,7) OR
GO:0003809: thrombin activity: 6.388e-03 (34,10,2) OR
GO:0004896: hematopoietin/interferon-class (D200-domain) cytokine receptor activity: 2.129e-01 (34,19,1)

IF 1jbqa_#152 AND 1pbe_1#155 AND 1h6va1#23 AND 1trb_1#14
THEN
GO:0015036: disulfide oxidoreductase activity: 2.342e-11 (18,22,7) OR
GO:0050660: FAD binding: 2.735e-07 (18,10,4) OR
GO:0016668: oxidoreductase activity, acting on sulfur group of donors, NAD or NADP as acceptor: 6.393e-07 (18,12,4) OR
GO:0016627: oxidoreductase activity, acting on the CH-CH group of donors: 5.286e-03 (18,17,2) OR
GO:0016651: oxidoreductase activity, acting on NADH or NADPH: 8.272e-02 (18,13,1)

IF 1cjca2#103 AND 1el5a1#183
THEN
GO:0015036: disulfide oxidoreductase activity: 2.342e-11 (18,22,7) OR
GO:0016668: oxidoreductase activity, acting on sulfur group of donors, NAD or NADP as acceptor: 5.289e-09 (18,12,5) OR
GO:0050660: FAD binding: 2.735e-07 (18,10,4) OR
GO:0016705: oxidoreductase activity, acting on paired donors, with incorporation or reduction of molecular oxygen: 1.590e-01 (18,26,1) OR
GO:0004497: monooxygenase activity: 1.590e-01 (18,26,1)

IF 1h7wa4#476 AND 1c9la2#12
THEN
GO:0015036: disulfide oxidoreductase activity: 2.342e-11 (18,22,7) OR
GO:0016668: oxidoreductase activity, acting on sulfur group of donors, NAD or NADP as acceptor: 5.289e-09 (18,12,5) OR
GO:0050660: FAD binding: 2.735e-07 (18,10,4) OR
GO:0016651: oxidoreductase activity, acting on NADH or NADPH: 8.272e-02 (18,13,1) OR
GO:0004601: peroxidase activity: 1.304e-01 (18,21,1)

IF 1qsta_#154 AND 1e79d2#36
THEN
GO:0004812: tRNA ligase activity: 2.047e-11 (25,26,8) OR
GO:0005524: ATP binding: 1.007e-03 (25,243,8) OR
GO:0000287: magnesium ion binding: 5.260e-03 (25,128,5) OR
GO:0000049: tRNA binding: 5.926e-03 (25,13,2) OR
GO:0030145: manganese ion binding: 2.972e-01 (25,38,1) OR
GO:0016616: oxidoreductase activity, acting on the CH-OH group of donors, NAD or NADP as acceptor: 4.229e-01 (25,59,1)

IF 1gdna_#151 AND 1eq9a_#162 AND 1e79d2#36
THEN
GO:0004295: trypsin activity: 3.126e-11 (22,48,9) OR
GO:0004263: chymotrypsin activity: 3.516e-10 (22,41,8) OR
GO:0004867: serine-type endopeptidase inhibitor activity: 5.886e-03 (22,47,3) OR
GO:0005509: calcium ion binding: 3.737e-01 (22,160,2)

IF 1autc_#188 AND 1h8d.1#H184 AND 1eq9a_#162

THEN
GO:0004263: chymotrypsin activity: 2.589e-11 (35,41,10) OR
GO:0004295: trypsin activity: 1.423e-10 (35,48,10) OR
GO:0004867: serine-type endopeptidase inhibitor activity: 2.113e-05 (35,47,6) OR
GO:0005509: calcium ion binding: 3.509e-03 (35,160,7) OR
GO:0003809: thrombin activity: 6.762e-03 (35,10,2)

IF 1ekbb_#79 AND 1elva1#513 AND 1eq9a_#162 AND 1gdna_#123 AND 1g51a3#525 AND 1ejda_#213 AND 1autc_#209
THEN
GO:0004263: chymotrypsin activity: 2.589e-11 (35,41,10) OR
GO:0004295: trypsin activity: 1.423e-10 (35,48,10) OR
GO:0004867: serine-type endopeptidase inhibitor activity: 2.113e-05 (35,47,6) OR
GO:0005509: calcium ion binding: 3.509e-03 (35,160,7) OR
GO:0003809: thrombin activity: 6.762e-03 (35,10,2)

IF 1dt6a_#449 AND 1dt6a_#77
THEN
GO:0004497: monooxygenase activity: 3.241e-11 (16,26,7) OR
GO:0016705: oxidoreductase activity, acting on paired donors, with incorporation or reduction of molecular oxygen: 2.145e-07 (16,26,5) OR
GO:0010181: FMN binding: 1.724e-03 (16,11,2) OR
GO:0016651: oxidoreductase activity, acting on NADH or NADPH: 2.429e-03 (16,13,2)

IF 1cpt__#325 AND 1bu7a_#278
THEN
GO:0004497: monooxygenase activity: 3.241e-11 (16,26,7) OR
GO:0016705: oxidoreductase activity, acting on paired donors, with incorporation or reduction of molecular oxygen: 2.145e-07 (16,26,5) OR
GO:0010181: FMN binding: 1.724e-03 (16,11,2) OR
GO:0016651: oxidoreductase activity, acting on NADH or NADPH: 2.429e-03 (16,13,2)

IF 1dt6a_#449 AND 1cpt__#52
THEN
GO:0004497: monooxygenase activity: 3.241e-11 (16,26,7) OR
GO:0016705: oxidoreductase activity, acting on paired donors, with incorporation or reduction of molecular oxygen: 2.145e-07 (16,26,5) OR
GO:0010181: FMN binding: 1.724e-03 (16,11,2) OR
GO:0016651: oxidoreductase activity, acting on NADH or NADPH: 2.429e-03 (16,13,2)

IF 1e9xa_#343 AND 1dt6a_#449
THEN
GO:0004497: monooxygenase activity: 3.241e-11 (16,26,7) OR
GO:0016705: oxidoreductase activity, acting on paired donors, with incorporation or reduction of molecular oxygen: 2.145e-07 (16,26,5) OR
GO:0010181: FMN binding: 1.724e-03 (16,11,2) OR
GO:0016651: oxidoreductase activity, acting on NADH or NADPH: 2.429e-03 (16,13,2)

IF 1ewna_#109 AND 1c4zd_#101
THEN
GO:0004842: ubiquitin-protein ligase activity: 2.171e-11 (11,19,6) OR
GO:0016854: racemase and epimerase activity: 5.134e-02 (11,13,1) OR
GO:0016638: oxidoreductase activity, acting on the CH-NH2 group of donors: 6.664e-02 (11,17,1) OR
GO:0008270: zinc ion binding: 3.596e-01 (11,108,1) OR
GO:0003700: transcription factor activity: 4.015e-01 (11,124,1) OR
GO:0005509: calcium ion binding: 4.867e-01 (11,160,1)

IF 1esma_#314 AND 1c4zd_#101
THEN
GO:0004842: ubiquitin-protein ligase activity: 2.171e-11 (11,19,6) OR
GO:0016854: racemase and epimerase activity: 5.134e-02 (11,13,1) OR
GO:0016638: oxidoreductase activity, acting on the CH-NH2 group of donors: 6.664e-02 (11,17,1) OR
GO:0008270: zinc ion binding: 3.596e-01 (11,108,1) OR
GO:0003700: transcription factor activity: 4.015e-01 (11,124,1) OR
GO:0005509: calcium ion binding: 4.867e-01 (11,160,1)

IF 1dm9a_#57 AND 1c4zd_#101
THEN
GO:0004842: ubiquitin-protein ligase activity: 2.171e-11 (11,19,6) OR
GO:0016854: racemase and epimerase activity: 5.134e-02 (11,13,1) OR
GO:0016638: oxidoreductase activity, acting on the CH-NH2 group of donors: 6.664e-02 (11,17,1) OR

GO:0008270: zinc ion binding: 3.596e-01 (11,108,1) OR
GO:0003700: transcription factor activity: 4.015e-01 (11,124,1) OR
GO:0005509: calcium ion binding: 4.867e-01 (11,160,1)

IF 1pot__#138 AND 1c4zd_#101
THEN
GO:0004842: ubiquitin-protein ligase activity: 2.171e-11 (11,19,6) OR
GO:0016854: racemase and epimerase activity: 5.134e-02 (11,13,1) OR
GO:0016638: oxidoreductase activity, acting on the CH-NH2 group of donors: 6.664e-02 (11,17,1) OR
GO:0008270: zinc ion binding: 3.596e-01 (11,108,1) OR
GO:0003700: transcription factor activity: 4.015e-01 (11,124,1) OR
GO:0005509: calcium ion binding: 4.867e-01 (11,160,1)

IF 1dpja_#322 AND 1b6cb_#337
THEN
GO:0004674: protein serine/threonine kinase activity: 2.194e-11 (24,42,9) OR
GO:0005524: ATP binding: 1.691e-05 (24,243,10) OR
GO:0005516: calmodulin binding: 1.823e-02 (24,24,2) OR
GO:0005066: transmembrane receptor protein tyrosine kinase signaling protein activity: 8.480e-02 (24,10,1) OR
GO:0003755: peptidyl-prolyl cis-trans isomerase activity: 9.289e-02 (24,11,1) OR
GO:0004896: hematopoietin/interferon-class (D200-domain) cytokine receptor activity: 1.552e-01 (24,19,1)

IF 1ia8a_#59 AND 1dpja_#322 AND 1ir3a_#1192
THEN
GO:0004674: protein serine/threonine kinase activity: 2.194e-11 (24,42,9) OR
GO:0005524: ATP binding: 1.691e-05 (24,243,10) OR
GO:0005516: calmodulin binding: 1.823e-02 (24,24,2) OR
GO:0004714: transmembrane receptor protein tyrosine kinase activity: 1.167e-01 (24,14,1) OR
GO:0004896: hematopoietin/interferon-class (D200-domain) cytokine receptor activity: 1.552e-01 (24,19,1) OR
GO:0008201: heparin binding: 1.920e-01 (24,24,1)

IF 1fo4a5#1020 AND 1koba_#175
THEN
GO:0004674: protein serine/threonine kinase activity: 2.194e-11 (24,42,9) OR
GO:0005524: ATP binding: 1.691e-05 (24,243,10) OR
GO:0005516: calmodulin binding: 1.823e-02 (24,24,2) OR
GO:0005066: transmembrane receptor protein tyrosine kinase signaling protein activity: 8.480e-02 (24,10,1) OR
GO:0003755: peptidyl-prolyl cis-trans isomerase activity: 9.289e-02 (24,11,1) OR
GO:0004896: hematopoietin/interferon-class (D200-domain) cytokine receptor activity: 1.552e-01 (24,19,1)

IF 3grx__#56 AND 1koba_#175
THEN
GO:0004674: protein serine/threonine kinase activity: 2.194e-11 (24,42,9) OR
GO:0005524: ATP binding: 1.691e-05 (24,243,10) OR
GO:0005516: calmodulin binding: 1.823e-02 (24,24,2) OR
GO:0005066: transmembrane receptor protein tyrosine kinase signaling protein activity: 8.480e-02 (24,10,1) OR
GO:0003755: peptidyl-prolyl cis-trans isomerase activity: 9.289e-02 (24,11,1) OR
GO:0004896: hematopoietin/interferon-class (D200-domain) cytokine receptor activity: 1.552e-01 (24,19,1)

IF 1ovaa_#297 AND 1a7ca_#202
THEN
GO:0004867: serine-type endopeptidase inhibitor activity: 3.304e-11 (10,47,7) OR
GO:0008201: heparin binding: 8.480e-02 (10,24,1) OR
GO:0051082: unfolded protein binding: 1.182e-01 (10,34,1) OR
GO:0005524: ATP binding: 6.077e-01 (10,243,1)

IF 1gdna_#119 AND 1ekbb_#117 AND 1qnja_#124 AND 2hlca_#27 AND 1fjsa_#158 AND 1jsg__#92 AND 1elva1#601
THEN
GO:0004263: chymotrypsin activity: 2.678e-11 (25,41,9) OR
GO:0004295: trypsin activity: 1.235e-10 (25,48,9) OR
GO:0005509: calcium ion binding: 1.336e-02 (25,160,5) OR
GO:0004896: hematopoietin/interferon-class (D200-domain) cytokine receptor activity: 1.611e-01 (25,19,1) OR
GO:0004867: serine-type endopeptidase inhibitor activity: 3.540e-01 (25,47,1)

IF 1b4va1#11 AND 1ojt_2#336
THEN
GO:0050660: FAD binding: 2.715e-11 (22,10,6) OR
GO:0016668: oxidoreductase activity, acting on sulfur group of donors, NAD or NADP as acceptor: 1.183e-10 (22,12,6) OR
GO:0015036: disulfide oxidoreductase activity: 1.231e-10 (22,22,7) OR
GO:0016627: oxidoreductase activity, acting on the CH-CH group of donors: 7.865e-03 (22,17,2) OR

GO:0005096: GTPase activator activity: 1.148e-01 (22,15,1)

IF 1qp8a1#172 AND 1ldna1#94
THEN
GO:0016616: oxidoreductase activity, acting on the CH-OH group of donors, NAD or NADP as acceptor: 2.333e-11 (18,59,9) OR
GO:0004457: lactate dehydrogenase activity: 2.735e-07 (18,10,4) OR
GO:0016646: oxidoreductase activity, acting on the CH-NH group of donors, NAD or NADP as acceptor: 6.595e-03 (18,19,2) OR
GO:0016620: oxidoreductase activity, acting on the aldehyde or oxo group of donors, NAD or NADP as acceptor: 6.423e-02 (18,10,1) OR
GO:0016814: hydrolase activity, acting on carbon-nitrogen (but not peptide) bonds, in cyclic amidines: 6.423e-02 (18,10,1) OR
GO:0016638: oxidoreductase activity, acting on the CH-NH2 group of donors: 1.068e-01 (18,17,1)

IF 1qfea_#68 AND 1trb_1#41
THEN
GO:0015036: disulfide oxidoreductase activity: 2.342e-11 (18,22,7) OR
GO:0016668: oxidoreductase activity, acting on sulfur group of donors, NAD or NADP as acceptor: 6.393e-07 (18,12,4) OR
GO:0050660: FAD binding: 2.824e-05 (18,10,3) OR
GO:0016627: oxidoreductase activity, acting on the CH-CH group of donors: 5.286e-03 (18,17,2) OR
GO:0016651: oxidoreductase activity, acting on NADH or NADPH: 8.272e-02 (18,13,1) OR
GO:0016638: oxidoreductase activity, acting on the CH-NH2 group of donors: 1.068e-01 (18,17,1)

IF 1trb_1#15 AND 1a8d_1#87 AND 1h7wa4#481
THEN
GO:0015036: disulfide oxidoreductase activity: 2.342e-11 (18,22,7) OR
GO:0050660: FAD binding: 2.735e-07 (18,10,4) OR
GO:0016668: oxidoreductase activity, acting on sulfur group of donors, NAD or NADP as acceptor: 6.393e-07 (18,12,4) OR
GO:0016651: oxidoreductase activity, acting on NADH or NADPH: 8.272e-02 (18,13,1) OR
GO:0016627: oxidoreductase activity, acting on the CH-CH group of donors: 1.068e-01 (18,17,1) OR
GO:0016638: oxidoreductase activity, acting on the CH-NH2 group of donors: 1.068e-01 (18,17,1)

IF 1cjca2#103 AND 1e87a_#174
THEN
GO:0015036: disulfide oxidoreductase activity: 2.342e-11 (18,22,7) OR
GO:0050660: FAD binding: 2.735e-07 (18,10,4) OR
GO:0016668: oxidoreductase activity, acting on sulfur group of donors, NAD or NADP as acceptor: 6.393e-07 (18,12,4) OR
GO:0016651: oxidoreductase activity, acting on NADH or NADPH: 8.272e-02 (18,13,1) OR
GO:0016627: oxidoreductase activity, acting on the CH-CH group of donors: 1.068e-01 (18,17,1) OR
GO:0004601: peroxidase activity: 1.304e-01 (18,21,1)

IF 1c3pa_#6 AND 1h5qa_#70
THEN
GO:0016616: oxidoreductase activity, acting on the CH-OH group of donors, NAD or NADP as acceptor: 3.515e-11 (13,59,8) OR
GO:0016627: oxidoreductase activity, acting on the CH-CH group of donors: 5.554e-05 (13,17,3) OR
GO:0008757: S-adenosylmethionine-dependent methyltransferase activity: 1.089e-01 (13,24,1) OR
GO:0016836: hydro-lyase activity: 1.468e-01 (13,33,1)

IF 1c3pa_#6 AND 1e6ua_#10
THEN
GO:0016616: oxidoreductase activity, acting on the CH-OH group of donors, NAD or NADP as acceptor: 3.515e-11 (13,59,8) OR
GO:0016627: oxidoreductase activity, acting on the CH-CH group of donors: 5.554e-05 (13,17,3) OR
GO:0016854: racemase and epimerase activity: 6.040e-02 (13,13,1) OR
GO:0016836: hydro-lyase activity: 1.468e-01 (13,33,1)

IF 1oaa__#175 AND 1b16a_#62
THEN
GO:0016616: oxidoreductase activity, acting on the CH-OH group of donors, NAD or NADP as acceptor: 3.515e-11 (13,59,8) OR
GO:0016627: oxidoreductase activity, acting on the CH-CH group of donors: 5.554e-05 (13,17,3) OR
GO:0016646: oxidoreductase activity, acting on the CH-NH group of donors, NAD or NADP as acceptor: 8.713e-02 (13,19,1) OR
GO:0003700: transcription factor activity: 4.549e-01 (13,124,1)

IF 1glqa2#53 AND 1d2oa1#541
THEN
GO:0004364: glutathione transferase activity: 1.440e-10 (4,11,4)

IF 1eu3a2#205 AND 1a0fa1#94
THEN
GO:0004364: glutathione transferase activity: 1.440e-10 (4,11,4)

IF 2gsq_2#50 AND 1d2oa1#541
THEN

GO:0004364: glutathione transferase activity: 1.440e-10 (4,11,4)

IF 1ljra1#165 AND 1d2oa1#541
THEN
GO:0004364: glutathione transferase activity: 1.440e-10 (4,11,4)

IF 1mspa_#17 AND 1aba__#17
THEN
GO:0004364: glutathione transferase activity: 1.440e-10 (4,11,4)

IF 1a0fa1#178 AND 1mspa_#17
THEN
GO:0004364: glutathione transferase activity: 1.440e-10 (4,11,4)

IF 1a0fa1#178 AND 1eu3a2#205
THEN
GO:0004364: glutathione transferase activity: 1.440e-10 (4,11,4)

IF 1a0fa1#94 AND 1ib2a_#1101
THEN
GO:0004364: glutathione transferase activity: 1.440e-10 (4,11,4)

IF 2gsta1#169 AND 2gsq_2#50
THEN
GO:0004364: glutathione transferase activity: 1.440e-10 (4,11,4)

IF 1gnwa1#160 AND 1ig8a_#203
THEN
GO:0004364: glutathione transferase activity: 1.440e-10 (4,11,4)

IF 1h7wa4#476 AND 1hc7a2#258
THEN
GO:0015036: disulfide oxidoreductase activity: 3.691e-11 (19,22,7) OR
GO:0016668: oxidoreductase activity, acting on sulfur group of donors, NAD or NADP as acceptor: 4.325e-11 (19,12,6) OR
GO:0050660: FAD binding: 2.299e-09 (19,10,5) OR
GO:0016651: oxidoreductase activity, acting on NADH or NADPH: 8.713e-02 (19,13,1)

IF 1h7wa4#476 AND 1hxxa_#173
THEN
GO:0015036: disulfide oxidoreductase activity: 3.691e-11 (19,22,7) OR
GO:0016668: oxidoreductase activity, acting on sulfur group of donors, NAD or NADP as acceptor: 4.325e-11 (19,12,6) OR
GO:0050660: FAD binding: 2.299e-09 (19,10,5) OR
GO:0004601: peroxidase activity: 1.371e-01 (19,21,1)

IF 1fg7a_#96 AND 1dfoa_#344
THEN
GO:0016846: carbon-sulfur lyase activity: 5.051e-11 (10,10,5) OR
GO:0008483: transaminase activity: 2.131e-07 (10,17,4) OR
GO:0016831: carboxy-lyase activity: 8.819e-02 (10,25,1)

IF 1fc4a_#241 AND 1rypb_#114
THEN
GO:0016846: carbon-sulfur lyase activity: 5.051e-11 (10,10,5) OR
GO:0008483: transaminase activity: 2.131e-07 (10,17,4) OR
GO:0016831: carboxy-lyase activity: 8.819e-02 (10,25,1)

IF 1ddja_#771 AND 1cqqa_#163
THEN
GO:0004263: chymotrypsin activity: 2.589e-11 (35,41,10) OR
GO:0004295: trypsin activity: 1.423e-10 (35,48,10) OR
GO:0004867: serine-type endopeptidase inhibitor activity: 2.708e-04 (35,47,5) OR
GO:0005509: calcium ion binding: 3.509e-03 (35,160,7) OR
GO:0003809: thrombin activity: 6.762e-03 (35,10,2) OR
GO:0005529: sugar binding: 3.981e-01 (35,39,1)

IF 1hdma1#115 AND 1fwxa1#526
THEN
GO:0005507: copper ion binding: 2.598e-11 (18,38,8) OR
GO:0015078: hydrogen ion transporter activity: 1.311e-07 (18,21,5) OR
GO:0051082: unfolded protein binding: 2.041e-02 (18,34,2) OR

GO:0015082: di-, tri-valent inorganic cation transporter activity: 8.881e-02 (18,14,1) OR
GO:0046915: transition metal ion transporter activity: 8.881e-02 (18,14,1) OR
GO:0005509: calcium ion binding: 6.647e-01 (18,160,1)

IF 1lvl_2#222 AND 1nsj__#178
THEN
GO:0004556: alpha-amylase activity: 2.598e-11 (14,15,6) OR
GO:0015036: disulfide oxidoreductase activity: 5.340e-03 (14,22,2) OR
GO:0005509: calcium ion binding: 4.482e-02 (14,160,3) OR
GO:0016627: oxidoreductase activity, acting on the CH-CH group of donors: 8.408e-02 (14,17,1) OR
GO:0016836: hydro-lyase activity: 1.572e-01 (14,33,1) OR
GO:0016616: oxidoreductase activity, acting on the CH-OH group of donors, NAD or NADP as acceptor: 2.645e-01 (14,59,1)

IF 1cg2a1#43 AND 1cpt__#52 AND 1ab4__#89
THEN
GO:0004497: monooxygenase activity: 3.241e-11 (16,26,7) OR
GO:0016705: oxidoreductase activity, acting on paired donors, with incorporation or reduction of molecular oxygen: 1.098e-05 (16,26,4) OR
GO:0010181: FMN binding: 1.724e-03 (16,11,2) OR
GO:0016651: oxidoreductase activity, acting on NADH or NADPH: 2.429e-03 (16,13,2) OR
GO:0004197: cysteine-type endopeptidase activity: 1.323e-01 (16,24,1)

IF 1dt6a_#181 AND 1bu7a_#353
THEN
GO:0004497: monooxygenase activity: 3.241e-11 (16,26,7) OR
GO:0016705: oxidoreductase activity, acting on paired donors, with incorporation or reduction of molecular oxygen: 1.098e-05 (16,26,4) OR
GO:0010181: FMN binding: 1.724e-03 (16,11,2) OR
GO:0016651: oxidoreductase activity, acting on NADH or NADPH: 2.429e-03 (16,13,2) OR
GO:0004222: metalloendopeptidase activity: 1.062e-01 (16,19,1)

IF 1iira_#326 AND 1hdr__#149
THEN
GO:0016616: oxidoreductase activity, acting on the CH-OH group of donors, NAD or NADP as acceptor: 5.405e-11 (9,59,7) OR
GO:0016627: oxidoreductase activity, acting on the CH-CH group of donors: 5.484e-02 (9,17,1) OR
GO:0016646: oxidoreductase activity, acting on the CH-NH group of donors, NAD or NADP as acceptor: 6.112e-02 (9,19,1)

IF 1fgxa_#242 AND 1ec7a1#216
THEN
GO:0016616: oxidoreductase activity, acting on the CH-OH group of donors, NAD or NADP as acceptor: 5.405e-11 (9,59,7) OR
GO:0016836: hydro-lyase activity: 1.040e-01 (9,33,1) OR
GO:0005524: ATP binding: 5.691e-01 (9,243,1)

IF 2ae2a_#89 AND 1oaa__#14
THEN
GO:0016616: oxidoreductase activity, acting on the CH-OH group of donors, NAD or NADP as acceptor: 5.405e-11 (9,59,7) OR
GO:0016627: oxidoreductase activity, acting on the CH-CH group of donors: 5.484e-02 (9,17,1) OR
GO:0016646: oxidoreductase activity, acting on the CH-NH group of donors, NAD or NADP as acceptor: 6.112e-02 (9,19,1)

IF 1oaa__#175 AND 1b4ka_#108 AND 1evqa_#305
THEN
GO:0016616: oxidoreductase activity, acting on the CH-OH group of donors, NAD or NADP as acceptor: 5.405e-11 (9,59,7) OR
GO:0016854: racemase and epimerase activity: 4.219e-02 (9,13,1) OR
GO:0008270: zinc ion binding: 3.055e-01 (9,108,1)

IF 1fmca_#248 AND 1hu4a_#267 AND 1bu8a2#124
THEN
GO:0016616: oxidoreductase activity, acting on the CH-OH group of donors, NAD or NADP as acceptor: 5.405e-11 (9,59,7) OR
GO:0016627: oxidoreductase activity, acting on the CH-CH group of donors: 5.484e-02 (9,17,1) OR
GO:0016646: oxidoreductase activity, acting on the CH-NH group of donors, NAD or NADP as acceptor: 6.112e-02 (9,19,1)

IF 1h5qa_#201 AND 1hu4a_#267
THEN
GO:0016616: oxidoreductase activity, acting on the CH-OH group of donors, NAD or NADP as acceptor: 5.405e-11 (9,59,7) OR
GO:0016627: oxidoreductase activity, acting on the CH-CH group of donors: 5.484e-02 (9,17,1) OR
GO:0016646: oxidoreductase activity, acting on the CH-NH group of donors, NAD or NADP as acceptor: 6.112e-02 (9,19,1)

IF 1qfma2#576 AND 1qg6a_#251 AND 1eno__#264
THEN
GO:0016616: oxidoreductase activity, acting on the CH-OH group of donors, NAD or NADP as acceptor: 5.405e-11 (9,59,7) OR

GO:0016627: oxidoreductase activity, acting on the CH-CH group of donors: 5.484e-02 (9,17,1) OR
GO:0016646: oxidoreductase activity, acting on the CH-NH group of donors, NAD or NADP as acceptor: 6.112e-02 (9,19,1)

IF 1fuia2#273 AND 1eno__#264
THEN
GO:0016616: oxidoreductase activity, acting on the CH-OH group of donors, NAD or NADP as acceptor: 5.405e-11 (9,59,7) OR
GO:0016627: oxidoreductase activity, acting on the CH-CH group of donors: 5.484e-02 (9,17,1) OR
GO:0016646: oxidoreductase activity, acting on the CH-NH group of donors, NAD or NADP as acceptor: 6.112e-02 (9,19,1)

IF 1bdb__#228 AND 1c3pa_#6
THEN
GO:0016616: oxidoreductase activity, acting on the CH-OH group of donors, NAD or NADP as acceptor: 5.405e-11 (9,59,7) OR
GO:0016854: racemase and epimerase activity: 4.219e-02 (9,13,1) OR
GO:0016627: oxidoreductase activity, acting on the CH-CH group of donors: 5.484e-02 (9,17,1)

IF 1cjca2#103 AND 1hxxa_#232
THEN
GO:0050660: FAD binding: 2.715e-11 (22,10,6) OR
GO:0016668: oxidoreductase activity, acting on sulfur group of donors, NAD or NADP as acceptor: 1.183e-10 (22,12,6) OR
GO:0015036: disulfide oxidoreductase activity: 1.231e-10 (22,22,7) OR
GO:0016651: oxidoreductase activity, acting on NADH or NADPH: 1.002e-01 (22,13,1) OR
GO:0016705: oxidoreductase activity, acting on paired donors, with incorporation or reduction of molecular oxygen: 1.908e-01 (22,26,1) OR
GO:0004497: monooxygenase activity: 1.908e-01 (22,26,1)

IF 1a3c__#108 AND 1qfea_#68
THEN
GO:0016763: transferase activity, transferring pentosyl groups: 3.285e-11 (15,28,7) OR
GO:0000287: magnesium ion binding: 4.344e-04 (15,128,5) OR
GO:0016758: transferase activity, transferring hexosyl groups: 5.902e-02 (15,11,1) OR
GO:0008483: transaminase activity: 8.982e-02 (15,17,1) OR
GO:0008757: S-adenosylmethionine-dependent methyltransferase activity: 1.246e-01 (15,24,1)

IF 1e2ka_#59 AND 1qora2#201
THEN
GO:0019201: nucleotide kinase activity: 5.485e-11 (18,13,6) OR
GO:0016776: phosphotransferase activity, phosphate group as acceptor: 9.563e-11 (18,14,6) OR
GO:0005524: ATP binding: 3.501e-03 (18,243,6)

IF 1c3pa_#6 AND 2ae2a_#17
THEN
GO:0016616: oxidoreductase activity, acting on the CH-OH group of donors, NAD or NADP as acceptor: 3.515e-11 (13,59,8) OR
GO:0016627: oxidoreductase activity, acting on the CH-CH group of donors: 2.745e-03 (13,17,2) OR
GO:0016620: oxidoreductase activity, acting on the aldehyde or oxo group of donors, NAD or NADP as acceptor: 4.677e-02 (13,10,1) OR
GO:0016854: racemase and epimerase activity: 6.040e-02 (13,13,1) OR
GO:0016836: hydro-lyase activity: 1.468e-01 (13,33,1)

IF 1hwxa1#256 AND 1atg__#144
THEN
GO:0016616: oxidoreductase activity, acting on the CH-OH group of donors, NAD or NADP as acceptor: 3.515e-11 (13,59,8) OR
GO:0016620: oxidoreductase activity, acting on the aldehyde or oxo group of donors, NAD or NADP as acceptor: 9.255e-04 (13,10,2) OR
GO:0016646: oxidoreductase activity, acting on the CH-NH group of donors, NAD or NADP as acceptor: 8.713e-02 (13,19,1) OR
GO:0015036: disulfide oxidoreductase activity: 1.002e-01 (13,22,1) OR
GO:0008270: zinc ion binding: 4.096e-01 (13,108,1)

IF 1b16a_#62 AND 1e7wa_#36
THEN
GO:0016616: oxidoreductase activity, acting on the CH-OH group of donors, NAD or NADP as acceptor: 3.515e-11 (13,59,8) OR
GO:0016627: oxidoreductase activity, acting on the CH-CH group of donors: 2.745e-03 (13,17,2) OR
GO:0016646: oxidoreductase activity, acting on the CH-NH group of donors, NAD or NADP as acceptor: 8.713e-02 (13,19,1) OR
GO:0005351: sugar porter activity: 9.588e-02 (13,21,1) OR
GO:0003700: transcription factor activity: 4.549e-01 (13,124,1)

IF 1bg6_2#73 AND 1qp8a1#153
THEN
GO:0016616: oxidoreductase activity, acting on the CH-OH group of donors, NAD or NADP as acceptor: 3.515e-11 (13,59,8) OR
GO:0016620: oxidoreductase activity, acting on the aldehyde or oxo group of donors, NAD or NADP as acceptor: 9.255e-04 (13,10,2) OR

GO:0004457: lactate dehydrogenase activity: 4.677e-02 (13,10,1) OR
GO:0016646: oxidoreductase activity, acting on the CH-NH group of donors, NAD or NADP as acceptor: 8.713e-02 (13,19,1) OR
GO:0015036: disulfide oxidoreductase activity: 1.002e-01 (13,22,1)

IF 1autc_#188 AND 1fjsa_#158 AND 1elva1#623 AND 1gdna_#121 AND 1bqya_#103 AND 1ddja_#746
THEN
GO:0004263: chymotrypsin activity: 3.547e-11 (36,41,10) OR
GO:0004295: trypsin activity: 1.946e-10 (36,48,10) OR
GO:0003809: thrombin activity: 5.099e-06 (36,10,4) OR
GO:0004867: serine-type endopeptidase inhibitor activity: 3.104e-04 (36,47,5) OR
GO:0005509: calcium ion binding: 4.145e-03 (36,160,7)

IF 1c5y.1#B18 AND 1b3qa2#623 AND 1dlea_#238
THEN
GO:0004263: chymotrypsin activity: 3.547e-11 (36,41,10) OR
GO:0004295: trypsin activity: 1.946e-10 (36,48,10) OR
GO:0004867: serine-type endopeptidase inhibitor activity: 2.503e-05 (36,47,6) OR
GO:0005509: calcium ion binding: 1.581e-04 (36,160,9) OR
GO:0003809: thrombin activity: 1.247e-01 (36,10,1)

IF 1jf9a_#379 AND 1fc4a_#111
THEN
GO:0008483: transaminase activity: 3.665e-11 (13,17,6) OR
GO:0016846: carbon-sulfur lyase activity: 6.446e-08 (13,10,4) OR
GO:0005066: transmembrane receptor protein tyrosine kinase signaling protein activity: 4.677e-02 (13,10,1) OR
GO:0016831: carboxy-lyase activity: 1.132e-01 (13,25,1) OR
GO:0005524: ATP binding: 7.039e-01 (13,243,1)

IF 2dkb__#114 AND 1jf9a_#380 AND 1ax4a_#275
THEN
GO:0008483: transaminase activity: 3.665e-11 (13,17,6) OR
GO:0016846: carbon-sulfur lyase activity: 9.993e-06 (13,10,3) OR
GO:0016831: carboxy-lyase activity: 5.926e-03 (13,25,2) OR
GO:0016866: intramolecular transferase activity: 5.588e-02 (13,12,1) OR
GO:0003779: actin binding: 1.426e-01 (13,32,1)

IF 1fi2a_#109 AND 1aym3_#131 AND 1htr.1#B39
THEN
GO:0004190: aspartic-type endopeptidase activity: 3.669e-11 (10,23,6) OR
GO:0008199: ferric iron binding: 3.616e-02 (10,10,1) OR
GO:0016702: oxidoreductase activity, acting on single donors with incorporation of molecular oxygen, incorporation of two atoms of oxygen: 4.324e-02 (10,12,1) OR
GO:0005529: sugar binding: 1.345e-01 (10,39,1) OR
GO:0005509: calcium ion binding: 4.545e-01 (10,160,1)

IF 1quqb_#87 AND 1i5ga_#115 AND 2cmd_2#251 AND 1fgga_#134
THEN
GO:0004457: lactate dehydrogenase activity: 9.247e-11 (11,10,5) OR
GO:0016616: oxidoreductase activity, acting on the CH-OH group of donors, NAD or NADP as acceptor: 3.383e-08 (11,59,6)

IF 1cex__#109 AND 2cmd_2#251
THEN
GO:0004457: lactate dehydrogenase activity: 9.247e-11 (11,10,5) OR
GO:0016616: oxidoreductase activity, acting on the CH-OH group of donors, NAD or NADP as acceptor: 3.383e-08 (11,59,6)

IF 1hyha2#211 AND 1ldna1#94
THEN
GO:0004457: lactate dehydrogenase activity: 9.247e-11 (11,10,5) OR
GO:0016616: oxidoreductase activity, acting on the CH-OH group of donors, NAD or NADP as acceptor: 3.383e-08 (11,59,6)

IF 1gdna_#230 AND 1befa_#51
THEN
GO:0004263: chymotrypsin activity: 5.025e-11 (18,41,8) OR
GO:0004295: trypsin activity: 1.939e-10 (18,48,8) OR
GO:0004867: serine-type endopeptidase inhibitor activity: 2.696e-01 (18,47,1) OR
GO:0005509: calcium ion binding: 6.647e-01 (18,160,1)

IF 1fc4a_#111 AND 1ax4a_#275
THEN
GO:0016846: carbon-sulfur lyase activity: 5.051e-11 (10,10,5) OR

GO:0008483: transaminase activity: 2.358e-05 (10,17,3) OR
GO:0016831: carboxy-lyase activity: 8.819e-02 (10,25,1) OR
GO:0005524: ATP binding: 6.077e-01 (10,243,1)

IF 1gdea_#90 AND 1dfoa_#362
THEN
GO:0016846: carbon-sulfur lyase activity: 5.051e-11 (10,10,5) OR
GO:0008483: transaminase activity: 2.358e-05 (10,17,3) OR
GO:0003887: DNA-directed DNA polymerase activity: 7.113e-02 (10,20,1) OR
GO:0000287: magnesium ion binding: 3.824e-01 (10,128,1)

IF 1gdea_#90 AND 1cs1a_#82
THEN
GO:0016846: carbon-sulfur lyase activity: 5.051e-11 (10,10,5) OR
GO:0008483: transaminase activity: 2.358e-05 (10,17,3) OR
GO:0003887: DNA-directed DNA polymerase activity: 7.113e-02 (10,20,1) OR
GO:0000287: magnesium ion binding: 3.824e-01 (10,128,1)

IF 1b6cb_#337 AND 1ir3a_#1139
THEN
GO:0004674: protein serine/threonine kinase activity: 3.390e-11 (25,42,9) OR
GO:0005524: ATP binding: 3.278e-06 (25,243,11) OR
GO:0005066: transmembrane receptor protein tyrosine kinase signaling protein activity: 3.477e-03 (25,10,2) OR
GO:0003755: peptidyl-prolyl cis-trans isomerase activity: 9.658e-02 (25,11,1) OR
GO:0004714: transmembrane receptor protein tyrosine kinase activity: 1.213e-01 (25,14,1) OR
GO:0005516: calmodulin binding: 1.992e-01 (25,24,1)

IF 1ce7a_#9 AND 1d6aa_#179
THEN
GO:0016799: hydrolase activity, hydrolyzing N-glycosyl compounds: 1.034e-10 (7,17,5) OR
GO:0005529: sugar binding: 4.007e-03 (7,39,2)

IF 1d6aa_#179 AND 1cnv__#264
THEN
GO:0016799: hydrolase activity, hydrolyzing N-glycosyl compounds: 1.034e-10 (7,17,5) OR
GO:0005529: sugar binding: 4.007e-03 (7,39,2)

IF 1hdca_#126 AND 1gcoa_#119
THEN
GO:0016616: oxidoreductase activity, acting on the CH-OH group of donors, NAD or NADP as acceptor: 3.515e-11 (13,59,8) OR
GO:0019829: cation-transporting ATPase activity: 4.219e-02 (13,9,1) OR
GO:0015405: P-P-bond-hydrolysis-driven transporter activity: 5.588e-02 (13,12,1) OR
GO:0016627: oxidoreductase activity, acting on the CH-CH group of donors: 7.830e-02 (13,17,1) OR
GO:0016836: hydro-lyase activity: 1.468e-01 (13,33,1) OR
GO:0005524: ATP binding: 7.039e-01 (13,243,1)

IF 1fzqa_#28 AND 1ejda_#377
THEN
GO:0005525: GTP binding: 3.537e-11 (15,49,8) OR
GO:0003924: GTPase activity: 3.668e-03 (15,17,2) OR
GO:0005085: guanyl-nucleotide exchange factor activity: 6.939e-02 (15,13,1) OR
GO:0003743: translation initiation factor activity: 7.966e-02 (15,15,1) OR
GO:0005524: ATP binding: 3.922e-01 (15,243,2) OR
GO:0000287: magnesium ion binding: 5.150e-01 (15,128,1)

IF 1danh_#48 AND 1befa_#51 AND 1c5y.1#B241 AND 2hlca_#99
THEN
GO:0004263: chymotrypsin activity: 3.547e-11 (36,41,10) OR
GO:0004295: trypsin activity: 1.946e-10 (36,48,10) OR
GO:0004867: serine-type endopeptidase inhibitor activity: 3.104e-04 (36,47,5) OR
GO:0005509: calcium ion binding: 8.734e-04 (36,160,8) OR
GO:0003809: thrombin activity: 7.146e-03 (36,10,2) OR
GO:0005529: sugar binding: 4.069e-01 (36,39,1)

IF 1h8d.1#H167 AND 1cgha_#168 AND 1f2la_#40 AND 1fjsa_#158 AND 1gdna_#121 AND 1arb__#193 AND 1elva1#604
THEN
GO:0004263: chymotrypsin activity: 3.547e-11 (36,41,10) OR
GO:0004295: trypsin activity: 1.946e-10 (36,48,10) OR
GO:0003809: thrombin activity: 5.099e-06 (36,10,4) OR
GO:0004867: serine-type endopeptidase inhibitor activity: 3.104e-04 (36,47,5) OR

GO:0005509: calcium ion binding: 1.672e-02 (36,160,6) OR
GO:0005529: sugar binding: 4.069e-01 (36,39,1)

IF 1i5ga_#115 AND 1qhoa4#399
THEN
GO:0004556: alpha-amylase activity: 4.317e-11 (15,15,6) OR
GO:0005509: calcium ion binding: 1.217e-03 (15,160,5) OR
GO:0008483: transaminase activity: 3.668e-03 (15,17,2) OR
GO:0016866: intramolecular transferase activity: 6.422e-02 (15,12,1) OR
GO:0004867: serine-type endopeptidase inhibitor activity: 2.302e-01 (15,47,1)

IF 1mrp__#172 AND 8dfr__#115
THEN
GO:0016646: oxidoreductase activity, acting on the CH-NH group of donors, NAD or NADP as acceptor: 4.325e-11 (12,19,6) OR
GO:0008026: ATP-dependent helicase activity: 1.350e-03 (12,13,2) OR
GO:0016620: oxidoreductase activity, acting on the aldehyde or oxo group of donors, NAD or NADP as acceptor: 4.324e-02 (12,10,1) OR
GO:0005524: ATP binding: 2.910e-01 (12,243,2) OR
GO:0000287: magnesium ion binding: 4.393e-01 (12,128,1)

IF 1i8aa_#2 AND 1kdj__#27 AND 2cuaa_#83
THEN
GO:0005507: copper ion binding: 4.327e-11 (12,38,7) OR
GO:0015082: di-, tri-valent inorganic cation transporter activity: 6.006e-02 (12,14,1) OR
GO:0046915: transition metal ion transporter activity: 6.006e-02 (12,14,1) OR
GO:0015078: hydrogen ion transporter activity: 8.883e-02 (12,21,1) OR
GO:0005509: calcium ion binding: 1.538e-01 (12,160,2)

IF 1ton__#196 AND 1qq4a_#63 AND 1ddwa_#77
THEN
GO:0004295: trypsin activity: 3.620e-11 (31,48,10) OR
GO:0004263: chymotrypsin activity: 7.864e-09 (31,41,8) OR
GO:0004867: serine-type endopeptidase inhibitor activity: 1.738e-03 (31,47,4) OR
GO:0003809: thrombin activity: 5.326e-03 (31,10,2) OR
GO:0005509: calcium ion binding: 8.054e-03 (31,160,6) OR
GO:0004896: hematopoietin/interferon-class (D200-domain) cytokine receptor activity: 1.960e-01 (31,19,1)

IF 1atia2#67 AND 1hu4a_#267
THEN
GO:0004812: tRNA ligase activity: 5.477e-11 (17,26,7) OR
GO:0005524: ATP binding: 3.669e-04 (17,243,7) OR
GO:0000049: tRNA binding: 7.830e-02 (17,13,1) OR
GO:0000287: magnesium ion binding: 1.885e-01 (17,128,2)

IF 1qsta_#154 AND 1ihua2#343
THEN
GO:0004812: tRNA ligase activity: 5.477e-11 (17,26,7) OR
GO:0005524: ATP binding: 3.669e-04 (17,243,7) OR
GO:0000049: tRNA binding: 7.830e-02 (17,13,1) OR
GO:0000287: magnesium ion binding: 1.885e-01 (17,128,2)

IF 1fl2a1#472 AND 1feca1#122
THEN
GO:0015036: disulfide oxidoreductase activity: 3.691e-11 (19,22,7) OR
GO:0016668: oxidoreductase activity, acting on sulfur group of donors, NAD or NADP as acceptor: 7.163e-09 (19,12,5) OR
GO:0050660: FAD binding: 3.458e-07 (19,10,4) OR
GO:0016651: oxidoreductase activity, acting on NADH or NADPH: 8.713e-02 (19,13,1) OR
GO:0016627: oxidoreductase activity, acting on the CH-CH group of donors: 1.125e-01 (19,17,1) OR
GO:0016638: oxidoreductase activity, acting on the CH-NH2 group of donors: 1.125e-01 (19,17,1)

IF 1el5a1#166 AND 1cjca2#365
THEN
GO:0015036: disulfide oxidoreductase activity: 3.691e-11 (19,22,7) OR
GO:0016668: oxidoreductase activity, acting on sulfur group of donors, NAD or NADP as acceptor: 7.163e-09 (19,12,5) OR
GO:0050660: FAD binding: 3.458e-07 (19,10,4) OR
GO:0016638: oxidoreductase activity, acting on the CH-NH2 group of donors: 1.125e-01 (19,17,1) OR
GO:0016705: oxidoreductase activity, acting on paired donors, with incorporation or reduction of molecular oxygen: 1.670e-01 (19,26,1) OR
GO:0004497: monooxygenase activity: 1.670e-01 (19,26,1)

IF 1el5a1#166 AND 1pbe_1#155
THEN
GO:0015036: disulfide oxidoreductase activity: 3.691e-11 (19,22,7) OR
GO:0016668: oxidoreductase activity, acting on sulfur group of donors, NAD or NADP as acceptor: 7.163e-09 (19,12,5) OR
GO:0050660: FAD binding: 3.458e-07 (19,10,4) OR
GO:0016638: oxidoreductase activity, acting on the CH-NH2 group of donors: 1.125e-01 (19,17,1) OR
GO:0016705: oxidoreductase activity, acting on paired donors, with incorporation or reduction of molecular oxygen: 1.670e-01 (19,26,1) OR
GO:0004497: monooxygenase activity: 1.670e-01 (19,26,1)

IF 1el5a1#166 AND 1qlaa2#35
THEN
GO:0015036: disulfide oxidoreductase activity: 3.691e-11 (19,22,7) OR
GO:0016668: oxidoreductase activity, acting on sulfur group of donors, NAD or NADP as acceptor: 7.163e-09 (19,12,5) OR
GO:0050660: FAD binding: 3.458e-07 (19,10,4) OR
GO:0016638: oxidoreductase activity, acting on the CH-NH2 group of donors: 1.125e-01 (19,17,1) OR
GO:0016705: oxidoreductase activity, acting on paired donors, with incorporation or reduction of molecular oxygen: 1.670e-01 (19,26,1) OR
GO:0004497: monooxygenase activity: 1.670e-01 (19,26,1)

IF 1el5a1#166 AND 1pbe_1#150
THEN
GO:0015036: disulfide oxidoreductase activity: 3.691e-11 (19,22,7) OR
GO:0016668: oxidoreductase activity, acting on sulfur group of donors, NAD or NADP as acceptor: 7.163e-09 (19,12,5) OR
GO:0050660: FAD binding: 3.458e-07 (19,10,4) OR
GO:0016638: oxidoreductase activity, acting on the CH-NH2 group of donors: 1.125e-01 (19,17,1) OR
GO:0016705: oxidoreductase activity, acting on paired donors, with incorporation or reduction of molecular oxygen: 1.670e-01 (19,26,1) OR
GO:0004497: monooxygenase activity: 1.670e-01 (19,26,1)

IF 1a0fa1#178 AND 1ib2a_#974
THEN
GO:0004364: glutathione transferase activity: 4.632e-11 (9,11,5) OR
GO:0016651: oxidoreductase activity, acting on NADH or NADPH: 4.219e-02 (9,13,1) OR
GO:0004812: tRNA ligase activity: 8.278e-02 (9,26,1) OR
GO:0008270: zinc ion binding: 3.055e-01 (9,108,1) OR
GO:0005524: ATP binding: 5.691e-01 (9,243,1)

IF 1auk__#375 AND 1d7ya1#255 AND 1nhp_1#282
THEN
GO:0015036: disulfide oxidoreductase activity: 5.943e-11 (11,22,6) OR
GO:0016668: oxidoreductase activity, acting on sulfur group of donors, NAD or NADP as acceptor: 9.567e-04 (11,12,2) OR
GO:0016627: oxidoreductase activity, acting on the CH-CH group of donors: 1.950e-03 (11,17,2) OR
GO:0004601: peroxidase activity: 8.172e-02 (11,21,1)

IF 1h7wa4#476 AND 1qs0a1#119
THEN
GO:0015036: disulfide oxidoreductase activity: 5.943e-11 (11,22,6) OR
GO:0016668: oxidoreductase activity, acting on sulfur group of donors, NAD or NADP as acceptor: 1.056e-05 (11,12,3) OR
GO:0050660: FAD binding: 3.971e-02 (11,10,1) OR
GO:0016627: oxidoreductase activity, acting on the CH-CH group of donors: 6.664e-02 (11,17,1)

IF 1e2fa_#140 AND 1e6ca_#153
THEN
GO:0019201: nucleotide kinase activity: 7.999e-11 (19,13,6) OR
GO:0016776: phosphotransferase activity, phosphate group as acceptor: 1.394e-10 (19,14,6) OR
GO:0005524: ATP binding: 8.131e-04 (19,243,7)

IF 1danh_#48 AND 1h8d.1#H167 AND 1eq9a_#162
THEN
GO:0004263: chymotrypsin activity: 4.810e-11 (37,41,10) OR
GO:0004295: trypsin activity: 2.632e-10 (37,48,10) OR
GO:0004867: serine-type endopeptidase inhibitor activity: 2.948e-05 (37,47,6) OR
GO:0003809: thrombin activity: 2.591e-04 (37,10,3) OR
GO:0005509: calcium ion binding: 1.060e-03 (37,160,8)

IF 1danh_#48 AND 1gg6.1#C156 AND 1cgha_#130
THEN
GO:0004263: chymotrypsin activity: 6.013e-11 (27,41,9) OR
GO:0004295: trypsin activity: 2.759e-10 (27,48,9) OR

GO:0004867: serine-type endopeptidase inhibitor activity: 1.020e-03 (27,47,4) OR
GO:0005509: calcium ion binding: 1.846e-02 (27,160,5)

IF 1danh_#48 AND 1danh_#142 AND 1gg6.1#C229 AND 1cgha_#130
THEN
GO:0004263: chymotrypsin activity: 6.013e-11 (27,41,9) OR
GO:0004295: trypsin activity: 2.759e-10 (27,48,9) OR
GO:0004867: serine-type endopeptidase inhibitor activity: 1.020e-03 (27,47,4) OR
GO:0005509: calcium ion binding: 1.846e-02 (27,160,5)

IF 1c5y.1#B18 AND 1a0la_#160 AND 1dlea_#238
THEN
GO:0004263: chymotrypsin activity: 6.013e-11 (27,41,9) OR
GO:0004295: trypsin activity: 2.759e-10 (27,48,9) OR
GO:0004867: serine-type endopeptidase inhibitor activity: 1.020e-03 (27,47,4) OR
GO:0005509: calcium ion binding: 1.846e-02 (27,160,5)

IF 2cb5a_#373 AND 1ppn__#191 AND 1dkia_#185
THEN
GO:0004197: cysteine-type endopeptidase activity: 4.885e-11 (10,24,6) OR
GO:0019955: cytokine binding: 3.971e-02 (10,11,1) OR
GO:0042802: protein self binding: 4.324e-02 (10,12,1) OR
GO:0051082: unfolded protein binding: 1.182e-01 (10,34,1) OR
GO:0000287: magnesium ion binding: 3.824e-01 (10,128,1)

IF 2foka2#180 AND 3gcb__#78 AND 1ppn__#132
THEN
GO:0004197: cysteine-type endopeptidase activity: 4.885e-11 (10,24,6) OR
GO:0004177: aminopeptidase activity: 4.677e-02 (10,13,1) OR
GO:0004180: carboxypeptidase activity: 5.379e-02 (10,15,1) OR
GO:0004674: protein serine/threonine kinase activity: 1.441e-01 (10,42,1) OR
GO:0005524: ATP binding: 6.077e-01 (10,243,1)

IF 1by5a_#238 AND 1qfma1#186 AND 1e6pa2#46
THEN
GO:0004556: alpha-amylase activity: 4.317e-11 (15,15,6) OR
GO:0005509: calcium ion binding: 9.385e-03 (15,160,4) OR
GO:0000049: tRNA binding: 6.939e-02 (15,13,1) OR
GO:0019843: rRNA binding: 1.148e-01 (15,22,1) OR
GO:0000287: magnesium ion binding: 1.545e-01 (15,128,2) OR
GO:0016836: hydro-lyase activity: 1.674e-01 (15,33,1)

IF 1bqk__#77 AND 1eu3a2#205
THEN
GO:0005507: copper ion binding: 4.327e-11 (12,38,7) OR
GO:0015082: di-, tri-valent inorganic cation transporter activity: 6.006e-02 (12,14,1) OR
GO:0046915: transition metal ion transporter activity: 6.006e-02 (12,14,1) OR
GO:0016638: oxidoreductase activity, acting on the CH-NH2 group of donors: 7.249e-02 (12,17,1) OR
GO:0030145: manganese ion binding: 1.554e-01 (12,38,1) OR
GO:0005524: ATP binding: 6.748e-01 (12,243,1)

IF 1hdoa_#189 AND 1oaa__#14
THEN
GO:0016616: oxidoreductase activity, acting on the CH-OH group of donors, NAD or NADP as acceptor: 4.359e-11 (19,59,9) OR
GO:0016627: oxidoreductase activity, acting on the CH-CH group of donors: 3.799e-06 (19,17,4) OR
GO:0016854: racemase and epimerase activity: 3.433e-03 (19,13,2) OR
GO:0016836: hydro-lyase activity: 2.139e-02 (19,33,2) OR
GO:0004457: lactate dehydrogenase activity: 6.769e-02 (19,10,1) OR
GO:0016646: oxidoreductase activity, acting on the CH-NH group of donors, NAD or NADP as acceptor: 1.249e-01 (19,19,1)

IF 1leha1#255 AND 1b3ra1#223
THEN
GO:0016616: oxidoreductase activity, acting on the CH-OH group of donors, NAD or NADP as acceptor: 4.359e-11 (19,59,9) OR
GO:0016638: oxidoreductase activity, acting on the CH-NH2 group of donors: 3.799e-06 (19,17,4) OR
GO:0005525: GTP binding: 4.459e-02 (19,49,2) OR
GO:0016620: oxidoreductase activity, acting on the aldehyde or oxo group of donors, NAD or NADP as acceptor: 6.769e-02 (19,10,1) OR
GO:0015036: disulfide oxidoreductase activity: 1.432e-01 (19,22,1) OR
GO:0005524: ATP binding: 5.158e-01 (19,243,2)

IF 1e87a_#174 AND 1tn3__#113
THEN
GO:0005529: sugar binding: 5.265e-11 (12,39,7) OR
GO:0008201: heparin binding: 1.009e-01 (12,24,1) OR
GO:0005509: calcium ion binding: 1.538e-01 (12,160,2) OR
GO:0004263: chymotrypsin activity: 1.666e-01 (12,41,1) OR
GO:0004295: trypsin activity: 1.924e-01 (12,48,1)

IF 1e87a_#174 AND 1hq8a_#223
THEN
GO:0005529: sugar binding: 5.265e-11 (12,39,7) OR
GO:0008201: heparin binding: 1.009e-01 (12,24,1) OR
GO:0005509: calcium ion binding: 1.538e-01 (12,160,2) OR
GO:0004263: chymotrypsin activity: 1.666e-01 (12,41,1) OR
GO:0004295: trypsin activity: 1.924e-01 (12,48,1)

IF 1e87a_#174 AND 1f00i3#856
THEN
GO:0005529: sugar binding: 5.265e-11 (12,39,7) OR
GO:0008201: heparin binding: 1.009e-01 (12,24,1) OR
GO:0005509: calcium ion binding: 1.538e-01 (12,160,2) OR
GO:0004263: chymotrypsin activity: 1.666e-01 (12,41,1) OR
GO:0004295: trypsin activity: 1.924e-01 (12,48,1)

IF 1e43a2#236 AND 1jb0d_#26
THEN
GO:0004556: alpha-amylase activity: 1.336e-10 (8,15,5) OR
GO:0005509: calcium ion binding: 8.938e-03 (8,160,3)

IF 1tc1a_#158 AND 1bd3a_#195
THEN
GO:0016763: transferase activity, transferring pentosyl groups: 1.360e-10 (10,28,6) OR
GO:0000287: magnesium ion binding: 7.826e-04 (10,128,4)

IF 1ecfa1#367 AND 1tuba1#8
THEN
GO:0016763: transferase activity, transferring pentosyl groups: 1.360e-10 (10,28,6) OR
GO:0000287: magnesium ion binding: 7.826e-04 (10,128,4)

IF 1ac5__#72 AND 1bd3a_#195
THEN
GO:0016763: transferase activity, transferring pentosyl groups: 1.360e-10 (10,28,6) OR
GO:0000287: magnesium ion binding: 7.826e-04 (10,128,4)

IF 2pvba_#51 AND 1qlsa_#81 AND 1c7wa_#105
THEN
GO:0005509: calcium ion binding: 9.106e-11 (13,160,10) OR
GO:0005516: calmodulin binding: 5.467e-03 (13,24,2) OR
GO:0005261: cation channel activity: 4.677e-02 (13,10,1)

IF 1bu7a_#278 AND 1e9xa_#62
THEN
GO:0004497: monooxygenase activity: 5.477e-11 (17,26,7) OR
GO:0016705: oxidoreductase activity, acting on paired donors, with incorporation or reduction of molecular oxygen: 3.019e-07 (17,26,5) OR
GO:0010181: FMN binding: 1.950e-03 (17,11,2) OR
GO:0016651: oxidoreductase activity, acting on NADH or NADPH: 2.745e-03 (17,13,2) OR
GO:0016763: transferase activity, transferring pentosyl groups: 1.615e-01 (17,28,1)

IF 1atia2#67 AND 1e79d2#36 AND 1evqa_#305
THEN
GO:0004812: tRNA ligase activity: 5.477e-11 (17,26,7) OR
GO:0005524: ATP binding: 3.669e-04 (17,243,7) OR
GO:0000049: tRNA binding: 7.830e-02 (17,13,1) OR
GO:0003887: DNA-directed DNA polymerase activity: 1.180e-01 (17,20,1) OR
GO:0000287: magnesium ion binding: 5.597e-01 (17,128,1)

IF 1hc7a2#109 AND 1hbza_#214 AND 1h4vb2#307
THEN
GO:0004812: tRNA ligase activity: 5.477e-11 (17,26,7) OR

GO:0005524: ATP binding: 3.669e-04 (17,243,7) OR
GO:0000049: tRNA binding: 7.830e-02 (17,13,1) OR
GO:0003887: DNA-directed DNA polymerase activity: 1.180e-01 (17,20,1) OR
GO:0000287: magnesium ion binding: 5.597e-01 (17,128,1)

IF 1qgna_#149 AND 1dfoa_#362
THEN
GO:0016846: carbon-sulfur lyase activity: 9.247e-11 (11,10,5) OR
GO:0008483: transaminase activity: 2.241e-09 (11,17,5) OR
GO:0016831: carboxy-lyase activity: 9.658e-02 (11,25,1)

IF 1aop_3#276 AND 1cjca2#103
THEN
GO:0015036: disulfide oxidoreductase activity: 5.943e-11 (11,22,6) OR
GO:0016668: oxidoreductase activity, acting on sulfur group of donors, NAD or NADP as acceptor: 9.567e-04 (11,12,2) OR
GO:0050660: FAD binding: 3.971e-02 (11,10,1) OR
GO:0016627: oxidoreductase activity, acting on the CH-CH group of donors: 6.664e-02 (11,17,1) OR
GO:0004601: peroxidase activity: 8.172e-02 (11,21,1)

IF 1h7wa4#478 AND 1f00i2#805
THEN
GO:0015036: disulfide oxidoreductase activity: 5.943e-11 (11,22,6) OR
GO:0016668: oxidoreductase activity, acting on sulfur group of donors, NAD or NADP as acceptor: 9.567e-04 (11,12,2) OR
GO:0050660: FAD binding: 3.971e-02 (11,10,1) OR
GO:0016627: oxidoreductase activity, acting on the CH-CH group of donors: 6.664e-02 (11,17,1) OR
GO:0004601: peroxidase activity: 8.172e-02 (11,21,1)

IF 1a8i__#688 AND 1trb_1#41
THEN
GO:0015036: disulfide oxidoreductase activity: 5.943e-11 (11,22,6) OR
GO:0016668: oxidoreductase activity, acting on sulfur group of donors, NAD or NADP as acceptor: 9.567e-04 (11,12,2) OR
GO:0050660: FAD binding: 3.971e-02 (11,10,1) OR
GO:0016651: oxidoreductase activity, acting on NADH or NADPH: 5.134e-02 (11,13,1) OR
GO:0016627: oxidoreductase activity, acting on the CH-CH group of donors: 6.664e-02 (11,17,1)

IF 1h7wa4#476 AND 1f00i2#805
THEN
GO:0015036: disulfide oxidoreductase activity: 5.943e-11 (11,22,6) OR
GO:0016668: oxidoreductase activity, acting on sulfur group of donors, NAD or NADP as acceptor: 9.567e-04 (11,12,2) OR
GO:0050660: FAD binding: 3.971e-02 (11,10,1) OR
GO:0016627: oxidoreductase activity, acting on the CH-CH group of donors: 6.664e-02 (11,17,1) OR
GO:0004601: peroxidase activity: 8.172e-02 (11,21,1)

IF 1i9ga_#67 AND 1cgha_#130
THEN
GO:0004263: chymotrypsin activity: 6.013e-11 (27,41,9) OR
GO:0004295: trypsin activity: 2.759e-10 (27,48,9) OR
GO:0004867: serine-type endopeptidase inhibitor activity: 1.053e-02 (27,47,3) OR
GO:0005509: calcium ion binding: 1.846e-02 (27,160,5) OR
GO:0003809: thrombin activity: 9.493e-02 (27,10,1)

IF 2dkb__#133 AND 1cjca2#365
THEN
GO:0008483: transaminase activity: 1.034e-10 (7,17,5) OR
GO:0016846: carbon-sulfur lyase activity: 2.543e-02 (7,10,1) OR
GO:0016866: intramolecular transferase activity: 3.045e-02 (7,12,1)

IF 1qsta_#137 AND 1hq0a_#867
THEN
GO:0008080: N-acetyltransferase activity: 3.119e-10 (4,13,4)

IF 2u2fa_#9 AND 1fxla2#169
THEN
GO:0003729: mRNA binding: 3.119e-10 (4,13,4)

IF 1h6kx_#111
THEN
GO:0003729: mRNA binding: 3.119e-10 (4,13,4)

IF 1ddja_#640 AND 1ekbb_#117

THEN
GO:0004295: trypsin activity: 5.199e-11 (32,48,10) OR
GO:0004263: chymotrypsin activity: 3.411e-10 (32,41,9) OR
GO:0005509: calcium ion binding: 3.715e-04 (32,160,8) OR
GO:0003809: thrombin activity: 5.670e-03 (32,10,2) OR
GO:0004867: serine-type endopeptidase inhibitor activity: 1.042e-01 (32,47,2) OR
GO:0004896: hematopoietin/interferon-class (D200-domain) cytokine receptor activity: 2.016e-01 (32,19,1)

IF 1nksa_#13 AND 1dik_1#765
THEN
GO:0019201: nucleotide kinase activity: 7.999e-11 (19,13,6) OR
GO:0016776: phosphotransferase activity, phosphate group as acceptor: 1.394e-10 (19,14,6) OR
GO:0005524: ATP binding: 4.741e-03 (19,243,6) OR
GO:0005525: GTP binding: 2.924e-01 (19,49,1)

IF 1iow_1#37 AND 2ak3a1#122
THEN
GO:0019201: nucleotide kinase activity: 7.999e-11 (19,13,6) OR
GO:0016776: phosphotransferase activity, phosphate group as acceptor: 1.394e-10 (19,14,6) OR
GO:0005524: ATP binding: 4.741e-03 (19,243,6) OR
GO:0005525: GTP binding: 2.924e-01 (19,49,1)

IF 1j9la_#82 AND 1nksa_#13
THEN
GO:0019201: nucleotide kinase activity: 7.999e-11 (19,13,6) OR
GO:0016776: phosphotransferase activity, phosphate group as acceptor: 1.394e-10 (19,14,6) OR
GO:0005524: ATP binding: 4.741e-03 (19,243,6) OR
GO:0005525: GTP binding: 2.924e-01 (19,49,1)

IF 1e2fa_#140 AND 1dik_1#765
THEN
GO:0019201: nucleotide kinase activity: 7.999e-11 (19,13,6) OR
GO:0016776: phosphotransferase activity, phosphate group as acceptor: 1.394e-10 (19,14,6) OR
GO:0005524: ATP binding: 4.741e-03 (19,243,6) OR
GO:0005525: GTP binding: 2.924e-01 (19,49,1)

IF 1mpp__#29 AND 2ltn.1#A63
THEN
GO:0004190: aspartic-type endopeptidase activity: 1.609e-10 (6,23,5) OR
GO:0005351: sugar porter activity: 4.540e-02 (6,21,1)

IF 1autc_#188 AND 1b3qa2#623 AND 1arb__#193
THEN
GO:0004263: chymotrypsin activity: 6.460e-11 (38,41,10) OR
GO:0004295: trypsin activity: 3.526e-10 (38,48,10) OR
GO:0004867: serine-type endopeptidase inhibitor activity: 1.400e-07 (38,47,8) OR
GO:0005509: calcium ion binding: 1.276e-03 (38,160,8) OR
GO:0003809: thrombin activity: 7.943e-03 (38,10,2)

IF 2duba_#212 AND 1nksa_#13
THEN
GO:0019201: nucleotide kinase activity: 1.140e-10 (20,13,6) OR
GO:0016776: phosphotransferase activity, phosphate group as acceptor: 1.986e-10 (20,14,6) OR
GO:0005524: ATP binding: 1.742e-04 (20,243,8)

IF 1gdna_#230 AND 1danh_#152
THEN
GO:0004263: chymotrypsin activity: 8.586e-11 (19,41,8) OR
GO:0004295: trypsin activity: 3.305e-10 (19,48,8) OR
GO:0004867: serine-type endopeptidase inhibitor activity: 2.823e-01 (19,47,1) OR
GO:0005509: calcium ion binding: 3.079e-01 (19,160,2)

IF 1nsj__#21 AND 1bvza3#416
THEN
GO:0004556: alpha-amylase activity: 6.888e-11 (16,15,6) OR
GO:0005509: calcium ion binding: 1.687e-03 (16,160,5) OR
GO:0016831: carboxy-lyase activity: 8.964e-03 (16,25,2) OR
GO:0016854: racemase and epimerase activity: 7.386e-02 (16,13,1) OR
GO:0000287: magnesium ion binding: 1.714e-01 (16,128,2)

IF 1bd3a_#110 AND 1cs1a_#160
THEN
GO:0016763: transferase activity, transferring pentosyl groups: 5.801e-11 (16,28,7) OR
GO:0000287: magnesium ion binding: 6.082e-04 (16,128,5) OR
GO:0016866: intramolecular transferase activity: 6.836e-02 (16,12,1) OR
GO:0016651: oxidoreductase activity, acting on NADH or NADPH: 7.386e-02 (16,13,1) OR
GO:0008757: S-adenosylmethionine-dependent methyltransferase activity: 1.323e-01 (16,24,1) OR
GO:0005524: ATP binding: 7.766e-01 (16,243,1)

IF 1cyda_#230 AND 1oaa__#161
THEN
GO:0016616: oxidoreductase activity, acting on the CH-OH group of donors, NAD or NADP as acceptor: 1.772e-10 (10,59,7) OR
GO:0016627: oxidoreductase activity, acting on the CH-CH group of donors: 2.358e-05 (10,17,3)

IF 1cyda_#230 AND 1h5qa_#153
THEN
GO:0016616: oxidoreductase activity, acting on the CH-OH group of donors, NAD or NADP as acceptor: 1.772e-10 (10,59,7) OR
GO:0016627: oxidoreductase activity, acting on the CH-CH group of donors: 2.358e-05 (10,17,3)

IF 2e2c__#67 AND 1cmxa_#207
THEN
GO:0016251: general RNA polymerase II transcription factor activity: 8.912e-11 (8,14,5) OR
GO:0003714: transcription corepressor activity: 2.328e-02 (8,8,1) OR
GO:0004674: protein serine/threonine kinase activity: 1.170e-01 (8,42,1) OR
GO:0005524: ATP binding: 5.268e-01 (8,243,1)

IF 1bqya_#178 AND 1danh_#104
THEN
GO:0004263: chymotrypsin activity: 6.013e-11 (27,41,9) OR
GO:0004295: trypsin activity: 2.759e-10 (27,48,9) OR
GO:0005509: calcium ion binding: 1.846e-02 (27,160,5) OR
GO:0004867: serine-type endopeptidase inhibitor activity: 7.780e-02 (27,47,2) OR
GO:0003809: thrombin activity: 9.493e-02 (27,10,1) OR
GO:0004896: hematopoietin/interferon-class (D200-domain) cytokine receptor activity: 1.729e-01 (27,19,1)

IF 1bqya_#178 AND 1dy9.1#A44
THEN
GO:0004263: chymotrypsin activity: 6.013e-11 (27,41,9) OR
GO:0004295: trypsin activity: 2.759e-10 (27,48,9) OR
GO:0005509: calcium ion binding: 1.846e-02 (27,160,5) OR
GO:0004867: serine-type endopeptidase inhibitor activity: 7.780e-02 (27,47,2) OR
GO:0003809: thrombin activity: 9.493e-02 (27,10,1) OR
GO:0004896: hematopoietin/interferon-class (D200-domain) cytokine receptor activity: 1.729e-01 (27,19,1)

IF 1bqya_#178 AND 1svpa_#127
THEN
GO:0004263: chymotrypsin activity: 6.013e-11 (27,41,9) OR
GO:0004295: trypsin activity: 2.759e-10 (27,48,9) OR
GO:0005509: calcium ion binding: 1.846e-02 (27,160,5) OR
GO:0004867: serine-type endopeptidase inhibitor activity: 7.780e-02 (27,47,2) OR
GO:0003809: thrombin activity: 9.493e-02 (27,10,1) OR
GO:0004896: hematopoietin/interferon-class (D200-domain) cytokine receptor activity: 1.729e-01 (27,19,1)

IF 1qgna_#207 AND 1fc4a_#205
THEN
GO:0016846: carbon-sulfur lyase activity: 9.247e-11 (11,10,5) OR
GO:0008483: transaminase activity: 3.335e-07 (11,17,4) OR
GO:0016763: transferase activity, transferring pentosyl groups: 1.076e-01 (11,28,1) OR
GO:0000287: magnesium ion binding: 4.115e-01 (11,128,1)

IF 1cyx__#133 AND 1a65a2#187
THEN
GO:0005507: copper ion binding: 9.282e-11 (13,38,7) OR
GO:0015078: hydrogen ion transporter activity: 1.784e-06 (13,21,4) OR
GO:0015082: di-, tri-valent inorganic cation transporter activity: 6.491e-02 (13,14,1) OR
GO:0046915: transition metal ion transporter activity: 6.491e-02 (13,14,1)

IF 1cyx__#133 AND 1cyx__#138
THEN
GO:0005507: copper ion binding: 9.282e-11 (13,38,7) OR

GO:0015078: hydrogen ion transporter activity: 1.784e-06 (13,21,4) OR
GO:0015082: di-, tri-valent inorganic cation transporter activity: 6.491e-02 (13,14,1) OR
GO:0046915: transition metal ion transporter activity: 6.491e-02 (13,14,1)

IF 1qq4a_#63 AND 1h6la_#119
THEN
GO:0004263: chymotrypsin activity: 6.460e-11 (38,41,10) OR
GO:0004295: trypsin activity: 3.526e-10 (38,48,10) OR
GO:0003809: thrombin activity: 9.637e-08 (38,10,5) OR
GO:0004867: serine-type endopeptidase inhibitor activity: 4.027e-04 (38,47,5) OR
GO:0005509: calcium ion binding: 5.676e-03 (38,160,7) OR
GO:0004896: hematopoietin/interferon-class (D200-domain) cytokine receptor activity: 2.349e-01 (38,19,1)

IF 1dy9.1#A44 AND 1h6la_#119
THEN
GO:0004263: chymotrypsin activity: 6.460e-11 (38,41,10) OR
GO:0004295: trypsin activity: 3.526e-10 (38,48,10) OR
GO:0003809: thrombin activity: 9.637e-08 (38,10,5) OR
GO:0004867: serine-type endopeptidase inhibitor activity: 4.027e-04 (38,47,5) OR
GO:0005509: calcium ion binding: 5.676e-03 (38,160,7) OR
GO:0004896: hematopoietin/interferon-class (D200-domain) cytokine receptor activity: 2.349e-01 (38,19,1)

IF 1aoza3#351
THEN
GO:0005507: copper ion binding: 1.339e-10 (8,38,6) OR
GO:0015082: di-, tri-valent inorganic cation transporter activity: 4.042e-02 (8,14,1) OR
GO:0046915: transition metal ion transporter activity: 4.042e-02 (8,14,1)

IF 1plc__#74 AND 1a65a2#187
THEN
GO:0005507: copper ion binding: 1.339e-10 (8,38,6) OR
GO:0016638: oxidoreductase activity, acting on the CH-NH2 group of donors: 4.889e-02 (8,17,1) OR
GO:0015078: hydrogen ion transporter activity: 6.009e-02 (8,21,1)

IF 1j9qa2#295 AND 2dpma_#237
THEN
GO:0005507: copper ion binding: 1.339e-10 (8,38,6) OR
GO:0015082: di-, tri-valent inorganic cation transporter activity: 4.042e-02 (8,14,1) OR
GO:0046915: transition metal ion transporter activity: 4.042e-02 (8,14,1)

IF 1e30a_#87 AND 1pfza_#124
THEN
GO:0005507: copper ion binding: 1.339e-10 (8,38,6) OR
GO:0015082: di-, tri-valent inorganic cation transporter activity: 4.042e-02 (8,14,1) OR
GO:0046915: transition metal ion transporter activity: 4.042e-02 (8,14,1)

IF 1oaa__#161 AND 1hdoa_#189
THEN
GO:0016616: oxidoreductase activity, acting on the CH-OH group of donors, NAD or NADP as acceptor: 8.065e-11 (14,59,8) OR
GO:0016627: oxidoreductase activity, acting on the CH-CH group of donors: 3.191e-03 (14,17,2) OR
GO:0016836: hydro-lyase activity: 1.182e-02 (14,33,2) OR
GO:0016854: racemase and epimerase activity: 6.491e-02 (14,13,1) OR
GO:0016646: oxidoreductase activity, acting on the CH-NH group of donors, NAD or NADP as acceptor: 9.353e-02 (14,19,1)

IF 1b16a_#62 AND 1eny__#147
THEN
GO:0016616: oxidoreductase activity, acting on the CH-OH group of donors, NAD or NADP as acceptor: 8.065e-11 (14,59,8) OR
GO:0016627: oxidoreductase activity, acting on the CH-CH group of donors: 7.042e-05 (14,17,3) OR
GO:0016646: oxidoreductase activity, acting on the CH-NH group of donors, NAD or NADP as acceptor: 9.353e-02 (14,19,1) OR
GO:0005351: sugar porter activity: 1.029e-01 (14,21,1) OR
GO:0003700: transcription factor activity: 4.798e-01 (14,124,1)

IF 3lada1#116 AND 1bd3a_#195
THEN
GO:0016763: transferase activity, transferring pentosyl groups: 1.360e-10 (10,28,6) OR
GO:0000287: magnesium ion binding: 9.530e-03 (10,128,3) OR
GO:0003700: transcription factor activity: 3.728e-01 (10,124,1)

IF 1b6cb_#399 AND 1phk__#221
THEN

GO:0004674: protein serine/threonine kinase activity: 1.057e-10 (19,42,8) OR
GO:0005524: ATP binding: 1.131e-04 (19,243,8) OR
GO:0005516: calmodulin binding: 1.160e-02 (19,24,2) OR
GO:0004896: hematopoietin/interferon-class (D200-domain) cytokine receptor activity: 1.249e-01 (19,19,1)

IF 1aq0a_#4 AND 1xyza_#753
THEN
GO:0008810: cellulase activity: 1.431e-10 (7,18,5) OR
GO:0016705: oxidoreductase activity, acting on paired donors, with incorporation or reduction of molecular oxygen: 6.498e-02 (7,26,1) OR
GO:0004497: monooxygenase activity: 6.498e-02 (7,26,1)

IF 1sgpe_#42 AND 1eq9a_#162
THEN
GO:0004295: trypsin activity: 1.092e-10 (17,48,8) OR
GO:0004263: chymotrypsin activity: 3.254e-06 (17,41,5) OR
GO:0004867: serine-type endopeptidase inhibitor activity: 2.761e-03 (17,47,3) OR
GO:0005509: calcium ion binding: 6.436e-01 (17,160,1)

IF 1a7s__#148 AND 1fjsa_#163 AND 1a0la_#193 AND 1danh_#104 AND 1gdna_#56 AND 1eq9a_#162 AND 1cgha_#130
THEN
GO:0004263: chymotrypsin activity: 8.767e-11 (28,41,9) OR
GO:0004295: trypsin activity: 4.013e-10 (28,48,9) OR
GO:0004867: serine-type endopeptidase inhibitor activity: 1.175e-03 (28,47,4) OR
GO:0005509: calcium ion binding: 2.144e-02 (28,160,5) OR
GO:0003809: thrombin activity: 9.828e-02 (28,10,1)

IF 1fjsa_#163 AND 1a0la_#193 AND 1gdna_#56 AND 1ekbb_#85 AND 1eq9a_#162 AND 1gdna_#121 AND 1cgha_#130
THEN
GO:0004263: chymotrypsin activity: 8.767e-11 (28,41,9) OR
GO:0004295: trypsin activity: 4.013e-10 (28,48,9) OR
GO:0004867: serine-type endopeptidase inhibitor activity: 1.175e-03 (28,47,4) OR
GO:0005509: calcium ion binding: 2.144e-02 (28,160,5) OR
GO:0003809: thrombin activity: 9.828e-02 (28,10,1)

IF 1c5y.1#B18 AND 1eq9a_#162 AND 1autc_#45
THEN
GO:0004263: chymotrypsin activity: 8.767e-11 (28,41,9) OR
GO:0004295: trypsin activity: 4.013e-10 (28,48,9) OR
GO:0004867: serine-type endopeptidase inhibitor activity: 1.175e-03 (28,47,4) OR
GO:0005509: calcium ion binding: 2.144e-02 (28,160,5) OR
GO:0003809: thrombin activity: 9.828e-02 (28,10,1)

IF 1h8d.1#H167 AND 1a0la_#193 AND 1h8d.1#H184 AND 1dlea_#238 AND 1cgha_#130
THEN
GO:0004263: chymotrypsin activity: 8.767e-11 (28,41,9) OR
GO:0004295: trypsin activity: 4.013e-10 (28,48,9) OR
GO:0004867: serine-type endopeptidase inhibitor activity: 1.175e-03 (28,47,4) OR
GO:0005509: calcium ion binding: 2.144e-02 (28,160,5) OR
GO:0003809: thrombin activity: 9.828e-02 (28,10,1)

IF 1c5y.1#B18 AND 1eq9a_#162 AND 1ton__#231
THEN
GO:0004263: chymotrypsin activity: 8.767e-11 (28,41,9) OR
GO:0004295: trypsin activity: 4.013e-10 (28,48,9) OR
GO:0004867: serine-type endopeptidase inhibitor activity: 1.175e-03 (28,47,4) OR
GO:0005509: calcium ion binding: 2.144e-02 (28,160,5) OR
GO:0003809: thrombin activity: 9.828e-02 (28,10,1)

IF 1fjsa_#163 AND 1a0la_#193 AND 1bio__#108 AND 1gdna_#56 AND 1eq9a_#162 AND 1g51a3#525 AND 1gdna_#121 AND 1cgha_#130 AND 1dlea_#53
THEN
GO:0004263: chymotrypsin activity: 8.767e-11 (28,41,9) OR
GO:0004295: trypsin activity: 4.013e-10 (28,48,9) OR
GO:0004867: serine-type endopeptidase inhibitor activity: 1.175e-03 (28,47,4) OR
GO:0005509: calcium ion binding: 2.144e-02 (28,160,5) OR
GO:0003809: thrombin activity: 9.828e-02 (28,10,1)

IF 1e2ka_#59 AND 1hwxa1#223
THEN

GO:0019201: nucleotide kinase activity: 1.140e-10 (20,13,6) OR
GO:0016776: phosphotransferase activity, phosphate group as acceptor: 1.986e-10 (20,14,6) OR
GO:0005524: ATP binding: 1.157e-03 (20,243,7) OR
GO:0005525: GTP binding: 3.052e-01 (20,49,1)

IF 1qu9a_#64 AND 1fvua_#72
THEN
GO:0005529: sugar binding: 4.615e-10 (5,39,5)

IF 1e87a_#155 AND 1jsg__#92
THEN
GO:0005529: sugar binding: 4.615e-10 (5,39,5)

IF 1fkna_#18 AND 2msba_#156
THEN
GO:0005529: sugar binding: 4.615e-10 (5,39,5)

IF 1hqoa2#159 AND 1qfea_#68
THEN
GO:0004364: glutathione transferase activity: 9.247e-11 (10,11,5) OR
GO:0008080: N-acetyltransferase activity: 9.255e-04 (10,13,2) OR
GO:0003714: transcription corepressor activity: 2.902e-02 (10,8,1) OR
GO:0003729: mRNA binding: 4.677e-02 (10,13,1) OR
GO:0016651: oxidoreductase activity, acting on NADH or NADPH: 4.677e-02 (10,13,1)

IF 1cl1a_#184 AND 1mjha_#126 AND 1elua_#95
THEN
GO:0016846: carbon-sulfur lyase activity: 9.247e-11 (11,10,5) OR
GO:0008483: transaminase activity: 1.950e-03 (11,17,2) OR
GO:0004222: metalloendopeptidase activity: 7.421e-02 (11,19,1) OR
GO:0000287: magnesium ion binding: 9.124e-02 (11,128,2) OR
GO:0016831: carboxy-lyase activity: 9.658e-02 (11,25,1)

IF 2sqca2#97 AND 3grx__#55
THEN
GO:0004364: glutathione transferase activity: 9.247e-11 (10,11,5) OR
GO:0015078: hydrogen ion transporter activity: 2.453e-03 (10,21,2) OR
GO:0008800: beta-lactamase activity: 3.616e-02 (10,10,1) OR
GO:0004180: carboxypeptidase activity: 5.379e-02 (10,15,1) OR
GO:0004222: metalloendopeptidase activity: 6.769e-02 (10,19,1)

IF 1ibja_#217 AND 1efpa1#32
THEN
GO:0016846: carbon-sulfur lyase activity: 9.247e-11 (11,10,5) OR
GO:0008483: transaminase activity: 3.229e-05 (11,17,3) OR
GO:0016814: hydrolase activity, acting on carbon-nitrogen (but not peptide) bonds, in cyclic amidines: 3.971e-02 (11,10,1) OR
GO:0016646: oxidoreductase activity, acting on the CH-NH group of donors, NAD or NADP as acceptor: 7.421e-02 (11,19,1) OR
GO:0005524: ATP binding: 6.428e-01 (11,243,1)

IF 1b6e__#76 AND 1f0xa1#523
THEN
GO:0005529: sugar binding: 1.582e-10 (8,39,6) OR
GO:0046983: protein dimerization activity: 4.889e-02 (8,17,1) OR
GO:0005524: ATP binding: 5.268e-01 (8,243,1)

IF 1f0xa1#523 AND 1e87a_#121
THEN
GO:0005529: sugar binding: 1.582e-10 (8,39,6) OR
GO:0030145: manganese ion binding: 1.064e-01 (8,38,1) OR
GO:0005524: ATP binding: 5.268e-01 (8,243,1)

IF 1quqb_#87 AND 1eq2a_#11
THEN
GO:0004457: lactate dehydrogenase activity: 1.583e-10 (12,10,5) OR
GO:0016616: oxidoreductase activity, acting on the CH-OH group of donors, NAD or NADP as acceptor: 6.654e-08 (12,59,6) OR
GO:0016620: oxidoreductase activity, acting on the aldehyde or oxo group of donors, NAD or NADP as acceptor: 4.324e-02 (12,10,1)

IF 1e6ca_#153 AND 1e8ca3#111
THEN
GO:0019201: nucleotide kinase activity: 1.593e-10 (21,13,6) OR

GO:0016776: phosphotransferase activity, phosphate group as acceptor: 2.774e-10 (21,14,6) OR
GO:0005524: ATP binding: 3.481e-05 (21,243,9)

IF 4tmka_#141 AND 1qora2#201
THEN
GO:0019201: nucleotide kinase activity: 1.593e-10 (21,13,6) OR
GO:0016776: phosphotransferase activity, phosphate group as acceptor: 2.774e-10 (21,14,6) OR
GO:0005524: ATP binding: 3.481e-05 (21,243,9)

IF 1j9la_#82 AND 1hu4a_#267 AND 1hwxa1#223
THEN
GO:0016616: oxidoreductase activity, acting on the CH-OH group of donors, NAD or NADP as acceptor: 8.065e-11 (14,59,8) OR
GO:0016646: oxidoreductase activity, acting on the CH-NH group of donors, NAD or NADP as acceptor: 9.353e-02 (14,19,1) OR
GO:0004812: tRNA ligase activity: 1.259e-01 (14,26,1) OR
GO:0003779: actin binding: 1.528e-01 (14,32,1) OR
GO:0005524: ATP binding: 3.590e-01 (14,243,2) OR
GO:0003700: transcription factor activity: 4.798e-01 (14,124,1)

IF 1c8ba_#514 AND 1quqa_#75
THEN
GO:0005525: GTP binding: 1.222e-10 (11,49,7) OR
GO:0005096: GTPase activator activity: 1.512e-03 (11,15,2) OR
GO:0004725: protein tyrosine phosphatase activity: 5.902e-02 (11,15,1) OR
GO:0003924: GTPase activity: 6.664e-02 (11,17,1)

IF 1danh_#48 AND 2hlca_#99 AND 1cgha_#130
THEN
GO:0004263: chymotrypsin activity: 1.258e-10 (29,41,9) OR
GO:0004295: trypsin activity: 5.744e-10 (29,48,9) OR
GO:0004867: serine-type endopeptidase inhibitor activity: 1.070e-04 (29,47,5) OR
GO:0005509: calcium ion binding: 5.728e-03 (29,160,6)

IF 1ton__#120 AND 1jb0d_#26 AND 1b3qa2#623 AND 2hlca_#54
THEN
GO:0004295: trypsin activity: 1.030e-10 (34,48,10) OR
GO:0004263: chymotrypsin activity: 6.243e-10 (34,41,9) OR
GO:0003809: thrombin activity: 2.007e-04 (34,10,3) OR
GO:0004867: serine-type endopeptidase inhibitor activity: 2.351e-04 (34,47,5) OR
GO:0005509: calcium ion binding: 2.950e-03 (34,160,7)

IF 1ton__#179 AND 1f97a2#199 AND 1arb__#208
THEN
GO:0004263: chymotrypsin activity: 8.597e-11 (39,41,10) OR
GO:0004295: trypsin activity: 4.682e-10 (39,48,10) OR
GO:0003809: thrombin activity: 7.081e-06 (39,10,4) OR
GO:0004867: serine-type endopeptidase inhibitor activity: 4.031e-05 (39,47,6) OR
GO:0005509: calcium ion binding: 1.527e-03 (39,160,8) OR
GO:0004896: hematopoietin/interferon-class (D200-domain) cytokine receptor activity: 2.403e-01 (39,19,1)

IF 1ton__#179 AND 1qq4a_#63 AND 1f97a2#199
THEN
GO:0004263: chymotrypsin activity: 8.597e-11 (39,41,10) OR
GO:0004295: trypsin activity: 4.682e-10 (39,48,10) OR
GO:0003809: thrombin activity: 7.081e-06 (39,10,4) OR
GO:0004867: serine-type endopeptidase inhibitor activity: 4.031e-05 (39,47,6) OR
GO:0005509: calcium ion binding: 1.527e-03 (39,160,8) OR
GO:0004896: hematopoietin/interferon-class (D200-domain) cytokine receptor activity: 2.403e-01 (39,19,1)

IF 1qbea_#92 AND 1dy9.1#A44 AND 1autc_#45
THEN
GO:0004263: chymotrypsin activity: 8.597e-11 (39,41,10) OR
GO:0004295: trypsin activity: 4.682e-10 (39,48,10) OR
GO:0003809: thrombin activity: 7.081e-06 (39,10,4) OR
GO:0004867: serine-type endopeptidase inhibitor activity: 4.031e-05 (39,47,6) OR
GO:0005509: calcium ion binding: 1.527e-03 (39,160,8) OR
GO:0004896: hematopoietin/interferon-class (D200-domain) cytokine receptor activity: 2.403e-01 (39,19,1)

IF 1qnja_#221 AND 1ejda_#213
THEN
GO:0004263: chymotrypsin activity: 8.597e-11 (39,41,10) OR

GO:0004295: trypsin activity: 4.682e-10 (39,48,10) OR
GO:0004867: serine-type endopeptidase inhibitor activity: 2.906e-06 (39,47,7) OR
GO:0005509: calcium ion binding: 3.054e-04 (39,160,9) OR
GO:0003809: thrombin activity: 8.355e-03 (39,10,2) OR
GO:0005529: sugar binding: 4.323e-01 (39,39,1)

IF 1tgoa2#409 AND 1hbza_#96
THEN
GO:0003887: DNA-directed DNA polymerase activity: 2.586e-10 (7,20,5) OR
GO:0008408: 3'-5' exonuclease activity: 4.354e-04 (7,13,2)

IF 1jlna_#383 AND 1i9sa_#24
THEN
GO:0004725: protein tyrosine phosphatase activity: 1.336e-10 (8,15,5) OR
GO:0010181: FMN binding: 3.188e-02 (8,11,1) OR
GO:0016763: transferase activity, transferring pentosyl groups: 7.940e-02 (8,28,1) OR
GO:0016616: oxidoreductase activity, acting on the CH-OH group of donors, NAD or NADP as acceptor: 1.608e-01 (8,59,1)

IF 1dt6a_#449 AND 1cg2a1#43
THEN
GO:0004497: monooxygenase activity: 8.907e-11 (18,26,7) OR
GO:0016705: oxidoreductase activity, acting on paired donors, with incorporation or reduction of molecular oxygen: 4.154e-07 (18,26,5) OR
GO:0010181: FMN binding: 2.189e-03 (18,11,2) OR
GO:0016651: oxidoreductase activity, acting on NADH or NADPH: 3.080e-03 (18,13,2) OR
GO:0004725: protein tyrosine phosphatase activity: 9.486e-02 (18,15,1) OR
GO:0016616: oxidoreductase activity, acting on the CH-OH group of donors, NAD or NADP as acceptor: 3.265e-01 (18,59,1)

IF 1qisa_#107 AND 1gdea_#271
THEN
GO:0008483: transaminase activity: 2.747e-10 (8,17,5) OR
GO:0016846: carbon-sulfur lyase activity: 1.976e-06 (8,10,3)

IF 1dm0a_#110 AND 1mrj__#240
THEN
GO:0016799: hydrolase activity, hydrolyzing N-glycosyl compounds: 2.747e-10 (8,17,5) OR
GO:0005529: sugar binding: 1.445e-04 (8,39,3)

IF 2dkb__#46 AND 1c4ka2#223
THEN
GO:0008483: transaminase activity: 2.747e-10 (8,17,5) OR
GO:0016846: carbon-sulfur lyase activity: 1.976e-06 (8,10,3)

IF 1d6aa_#179 AND 1dvpa1#65
THEN
GO:0016799: hydrolase activity, hydrolyzing N-glycosyl compounds: 2.747e-10 (8,17,5) OR
GO:0005529: sugar binding: 1.445e-04 (8,39,3)

IF 1gdea_#92 AND 1ax4a_#275
THEN
GO:0016846: carbon-sulfur lyase activity: 9.247e-11 (11,10,5) OR
GO:0008483: transaminase activity: 1.950e-03 (11,17,2) OR
GO:0003899: DNA-directed RNA polymerase activity: 4.747e-02 (11,12,1) OR
GO:0046983: protein dimerization activity: 6.664e-02 (11,17,1) OR
GO:0003700: transcription factor activity: 4.015e-01 (11,124,1) OR
GO:0005509: calcium ion binding: 4.867e-01 (11,160,1)

IF 1a0fa1#178 AND 1avgi_#69
THEN
GO:0004364: glutathione transferase activity: 9.247e-11 (10,11,5) OR
GO:0019829: cation-transporting ATPase activity: 3.259e-02 (10,9,1) OR
GO:0015405: P-P-bond-hydrolysis-driven transporter activity: 4.324e-02 (10,12,1) OR
GO:0015078: hydrogen ion transporter activity: 7.456e-02 (10,21,1) OR
GO:0003700: transcription factor activity: 3.728e-01 (10,124,1) OR
GO:0005524: ATP binding: 6.077e-01 (10,243,1)

IF 1gg6.1#C156 AND 1c4ra_#258 AND 1elva1#604
THEN
GO:0004263: chymotrypsin activity: 1.134e-10 (40,41,10) OR
GO:0004295: trypsin activity: 6.162e-10 (40,48,10) OR

GO:0003809: thrombin activity: 1.259e-07 (40,10,5) OR
GO:0004867: serine-type endopeptidase inhibitor activity: 2.146e-07 (40,47,8) OR
GO:0005509: calcium ion binding: 7.595e-03 (40,160,7)

IF 1ton__#179 AND 1qq4a_#63 AND 1e5ka_#113
THEN
GO:0004263: chymotrypsin activity: 1.134e-10 (40,41,10) OR
GO:0004295: trypsin activity: 6.162e-10 (40,48,10) OR
GO:0003809: thrombin activity: 1.259e-07 (40,10,5) OR
GO:0004867: serine-type endopeptidase inhibitor activity: 3.477e-06 (40,47,7) OR
GO:0005509: calcium ion binding: 1.815e-03 (40,160,8)

IF 2bb2_2#134 AND 1qhda2#323 AND 1trb_1#15 AND 1ddma_#130
THEN
GO:0015036: disulfide oxidoreductase activity: 1.183e-10 (12,22,6) OR
GO:0016668: oxidoreductase activity, acting on sulfur group of donors, NAD or NADP as acceptor: 1.405e-05 (12,12,3) OR
GO:0050660: FAD binding: 4.324e-02 (12,10,1) OR
GO:0016705: oxidoreductase activity, acting on paired donors, with incorporation or reduction of molecular oxygen: 1.089e-01 (12,26,1) OR
GO:0004497: monooxygenase activity: 1.089e-01 (12,26,1)

IF 1e1oa2#423 AND 1trb_1#41
THEN
GO:0015036: disulfide oxidoreductase activity: 1.183e-10 (12,22,6) OR
GO:0016668: oxidoreductase activity, acting on sulfur group of donors, NAD or NADP as acceptor: 1.405e-05 (12,12,3) OR
GO:0050660: FAD binding: 4.324e-02 (12,10,1) OR
GO:0016627: oxidoreductase activity, acting on the CH-CH group of donors: 7.249e-02 (12,17,1) OR
GO:0016638: oxidoreductase activity, acting on the CH-NH2 group of donors: 7.249e-02 (12,17,1)

IF 1bd3a_#110 AND 1e3a.1#A30
THEN
GO:0016763: transferase activity, transferring pentosyl groups: 2.972e-10 (11,28,6) OR
GO:0000287: magnesium ion binding: 7.785e-05 (11,128,5)

IF 1ac5__#458 AND 1cpy__#346
THEN
GO:0004180: carboxypeptidase activity: 5.954e-10 (4,15,4)

IF 1cpy__#346 AND 1c4xa_#110
THEN
GO:0004180: carboxypeptidase activity: 5.954e-10 (4,15,4)

IF 1ejda_#379 AND 1ypta_#406
THEN
GO:0004725: protein tyrosine phosphatase activity: 5.954e-10 (4,15,4)

IF 1ypta_#406 AND 1c7na_#231
THEN
GO:0004725: protein tyrosine phosphatase activity: 5.954e-10 (4,15,4)

IF 1d5ra2#25 AND 1qfta_#98
THEN
GO:0004725: protein tyrosine phosphatase activity: 5.954e-10 (4,15,4)

IF 1nsj__#21 AND 1htp__#16 AND 7taa_2#331
THEN
GO:0004556: alpha-amylase activity: 2.996e-10 (9,15,5) OR
GO:0005509: calcium ion binding: 1.144e-03 (9,160,4)

IF 1gjwa2#141 AND 2arca_#55 AND 1aym3_#131
THEN
GO:0004556: alpha-amylase activity: 2.996e-10 (9,15,5) OR
GO:0005509: calcium ion binding: 1.144e-03 (9,160,4)

IF 1aq0a_#4 AND 1ex1a1#284
THEN
GO:0004556: alpha-amylase activity: 2.996e-10 (9,15,5) OR
GO:0005509: calcium ion binding: 1.144e-03 (9,160,4)

IF 1qf5a_#15 AND 1f60a2#373

THEN
GO:0005525: GTP binding: 1.222e-10 (11,49,7) OR
GO:0003899: DNA-directed RNA polymerase activity: 4.747e-02 (11,12,1) OR
GO:0003743: translation initiation factor activity: 5.902e-02 (11,15,1) OR
GO:0003924: GTPase activity: 6.664e-02 (11,17,1) OR
GO:0005524: ATP binding: 6.428e-01 (11,243,1)

IF 1gdna_#119 AND 1aky_2#133
THEN
GO:0004295: trypsin activity: 1.235e-10 (25,48,9) OR
GO:0004263: chymotrypsin activity: 1.151e-09 (25,41,8) OR
GO:0005509: calcium ion binding: 1.336e-02 (25,160,5) OR
GO:0004867: serine-type endopeptidase inhibitor activity: 6.794e-02 (25,47,2) OR
GO:0004896: hematopoietin/interferon-class (D200-domain) cytokine receptor activity: 1.611e-01 (25,19,1)

IF 1dt6a_#376 AND 1dfaa2#211
THEN
GO:0004497: monooxygenase activity: 3.143e-10 (6,26,5) OR
GO:0016705: oxidoreductase activity, acting on paired donors, with incorporation or reduction of molecular oxygen: 5.595e-02 (6,26,1)

IF 1nula_#15 AND 1dz4a_#357
THEN
GO:0004497: monooxygenase activity: 3.143e-10 (6,26,5) OR
GO:0016705: oxidoreductase activity, acting on paired donors, with incorporation or reduction of molecular oxygen: 5.595e-02 (6,26,1)

IF 1dt6a_#376 AND 1bu7a_#277
THEN
GO:0004497: monooxygenase activity: 3.143e-10 (6,26,5) OR
GO:0016705: oxidoreductase activity, acting on paired donors, with incorporation or reduction of molecular oxygen: 5.595e-02 (6,26,1)

IF 1bu7a_#360 AND 1nula_#15
THEN
GO:0004497: monooxygenase activity: 3.143e-10 (6,26,5) OR
GO:0016705: oxidoreductase activity, acting on paired donors, with incorporation or reduction of molecular oxygen: 5.595e-02 (6,26,1)

IF 1e9xa_#343 AND 1nula_#15
THEN
GO:0004497: monooxygenase activity: 3.143e-10 (6,26,5) OR
GO:0016705: oxidoreductase activity, acting on paired donors, with incorporation or reduction of molecular oxygen: 5.595e-02 (6,26,1)

IF 1qnja_#77 AND 1eq9a_#162
THEN
GO:0004263: chymotrypsin activity: 1.258e-10 (29,41,9) OR
GO:0004295: trypsin activity: 5.744e-10 (29,48,9) OR
GO:0004867: serine-type endopeptidase inhibitor activity: 1.346e-03 (29,47,4) OR
GO:0003809: thrombin activity: 4.669e-03 (29,10,2) OR
GO:0005509: calcium ion binding: 2.471e-02 (29,160,5)

IF 1bqya_#115 AND 1autc_#188 AND 1qq4a_#143 AND 1ekbb_#228
THEN
GO:0004263: chymotrypsin activity: 1.258e-10 (29,41,9) OR
GO:0004295: trypsin activity: 5.744e-10 (29,48,9) OR
GO:0003809: thrombin activity: 1.238e-04 (29,10,3) OR
GO:0004867: serine-type endopeptidase inhibitor activity: 1.346e-03 (29,47,4) OR
GO:0005509: calcium ion binding: 8.655e-02 (29,160,4)

IF 1danh_#48 AND 1befa_#51 AND 1eq9a_#162
THEN
GO:0004263: chymotrypsin activity: 1.258e-10 (29,41,9) OR
GO:0004295: trypsin activity: 5.744e-10 (29,48,9) OR
GO:0004867: serine-type endopeptidase inhibitor activity: 1.346e-03 (29,47,4) OR
GO:0005509: calcium ion binding: 5.728e-03 (29,160,6) OR
GO:0003809: thrombin activity: 1.016e-01 (29,10,1)

IF 1b9ha_#276 AND 2dkb__#114
THEN
GO:0016846: carbon-sulfur lyase activity: 1.583e-10 (12,10,5) OR
GO:0008483: transaminase activity: 3.828e-09 (12,17,5) OR
GO:0004812: tRNA ligase activity: 1.089e-01 (12,26,1) OR
GO:0005524: ATP binding: 6.748e-01 (12,243,1)

IF 1by5a_#238 AND 1dik_1#765 AND 1e6pa2#46
THEN
GO:0004556: alpha-amylase activity: 1.061e-10 (17,15,6) OR
GO:0005509: calcium ion binding: 2.278e-03 (17,160,5) OR
GO:0000287: magnesium ion binding: 4.249e-02 (17,128,3) OR
GO:0016836: hydro-lyase activity: 1.876e-01 (17,33,1) OR
GO:0004867: serine-type endopeptidase inhibitor activity: 2.567e-01 (17,47,1) OR
GO:0005524: ATP binding: 7.966e-01 (17,243,1)

IF 2rspa_#113 AND 1mpp__#29
THEN
GO:0004190: aspartic-type endopeptidase activity: 1.597e-10 (12,23,6) OR
GO:0003964: RNA-directed DNA polymerase activity: 9.567e-04 (12,11,2) OR
GO:0004523: ribonuclease H activity: 2.062e-03 (12,16,2) OR
GO:0008270: zinc ion binding: 7.929e-02 (12,108,2)

IF 2cb5a_#312 AND 1thea_#79 AND 3gcb__#78
THEN
GO:0004197: cysteine-type endopeptidase activity: 1.069e-10 (11,24,6) OR
GO:0019955: cytokine binding: 4.360e-02 (11,11,1) OR
GO:0042802: protein self binding: 4.747e-02 (11,12,1) OR
GO:0004601: peroxidase activity: 8.172e-02 (11,21,1) OR
GO:0016831: carboxy-lyase activity: 9.658e-02 (11,25,1) OR
GO:0051082: unfolded protein binding: 1.292e-01 (11,34,1)

IF 1cjca2#37 AND 1qama_#37
THEN
GO:0008757: S-adenosylmethionine-dependent methyltransferase activity: 1.069e-10 (11,24,6) OR
GO:0016638: oxidoreductase activity, acting on the CH-NH2 group of donors: 6.664e-02 (11,17,1) OR
GO:0016646: oxidoreductase activity, acting on the CH-NH group of donors, NAD or NADP as acceptor: 7.421e-02 (11,19,1) OR
GO:0016831: carboxy-lyase activity: 9.658e-02 (11,25,1) OR
GO:0000287: magnesium ion binding: 4.115e-01 (11,128,1) OR
GO:0005524: ATP binding: 6.428e-01 (11,243,1)

IF 2cpl__#53 AND 1dkia_#185
THEN
GO:0004197: cysteine-type endopeptidase activity: 1.069e-10 (11,24,6) OR
GO:0019955: cytokine binding: 4.360e-02 (11,11,1) OR
GO:0042802: protein self binding: 4.747e-02 (11,12,1) OR
GO:0016831: carboxy-lyase activity: 9.658e-02 (11,25,1) OR
GO:0004497: monooxygenase activity: 1.003e-01 (11,26,1) OR
GO:0051082: unfolded protein binding: 1.292e-01 (11,34,1)

IF 1h7wa4#208 AND 1b4va1#253
THEN
GO:0015036: disulfide oxidoreductase activity: 2.185e-10 (13,22,6) OR
GO:0016668: oxidoreductase activity, acting on sulfur group of donors, NAD or NADP as acceptor: 1.511e-07 (13,12,4) OR
GO:0050660: FAD binding: 9.993e-06 (13,10,3)

IF 1h7wa4#208 AND 1iba__#39
THEN
GO:0015036: disulfide oxidoreductase activity: 2.185e-10 (13,22,6) OR
GO:0016668: oxidoreductase activity, acting on sulfur group of donors, NAD or NADP as acceptor: 1.511e-07 (13,12,4) OR
GO:0050660: FAD binding: 9.993e-06 (13,10,3)

IF 1koba_#230 AND 1csn__#134
THEN
GO:0004674: protein serine/threonine kinase activity: 1.109e-10 (28,42,9) OR
GO:0005524: ATP binding: 1.617e-06 (28,243,12) OR
GO:0005066: transmembrane receptor protein tyrosine kinase signaling protein activity: 4.355e-03 (28,10,2) OR
GO:0004714: transmembrane receptor protein tyrosine kinase activity: 8.586e-03 (28,14,2) OR
GO:0005516: calmodulin binding: 2.444e-02 (28,24,2) OR
GO:0008201: heparin binding: 2.204e-01 (28,24,1)

IF 1csn__#134 AND 1pwt__#8
THEN
GO:0004674: protein serine/threonine kinase activity: 1.109e-10 (28,42,9) OR
GO:0005524: ATP binding: 1.617e-06 (28,243,12) OR
GO:0005066: transmembrane receptor protein tyrosine kinase signaling protein activity: 4.355e-03 (28,10,2) OR

GO:0004714: transmembrane receptor protein tyrosine kinase activity: 8.586e-03 (28,14,2) OR
GO:0005516: calmodulin binding: 2.444e-02 (28,24,2) OR
GO:0008201: heparin binding: 2.204e-01 (28,24,1)

IF 1csn__#134 AND 1pme__#25
THEN
GO:0004674: protein serine/threonine kinase activity: 1.109e-10 (28,42,9) OR
GO:0005524: ATP binding: 1.617e-06 (28,243,12) OR
GO:0005066: transmembrane receptor protein tyrosine kinase signaling protein activity: 4.355e-03 (28,10,2) OR
GO:0004714: transmembrane receptor protein tyrosine kinase activity: 8.586e-03 (28,14,2) OR
GO:0005516: calmodulin binding: 2.444e-02 (28,24,2) OR
GO:0008201: heparin binding: 2.204e-01 (28,24,1)

IF 1howa_#588 AND 1csn__#134
THEN
GO:0004674: protein serine/threonine kinase activity: 1.109e-10 (28,42,9) OR
GO:0005524: ATP binding: 1.617e-06 (28,243,12) OR
GO:0005066: transmembrane receptor protein tyrosine kinase signaling protein activity: 4.355e-03 (28,10,2) OR
GO:0004714: transmembrane receptor protein tyrosine kinase activity: 8.586e-03 (28,14,2) OR
GO:0005516: calmodulin binding: 2.444e-02 (28,24,2) OR
GO:0008201: heparin binding: 2.204e-01 (28,24,1)

IF 1tkia_#80 AND 1csn__#134
THEN
GO:0004674: protein serine/threonine kinase activity: 1.109e-10 (28,42,9) OR
GO:0005524: ATP binding: 1.617e-06 (28,243,12) OR
GO:0005066: transmembrane receptor protein tyrosine kinase signaling protein activity: 4.355e-03 (28,10,2) OR
GO:0004714: transmembrane receptor protein tyrosine kinase activity: 8.586e-03 (28,14,2) OR
GO:0005516: calmodulin binding: 2.444e-02 (28,24,2) OR
GO:0008201: heparin binding: 2.204e-01 (28,24,1)

IF 1dv8a_#211 AND 1qo3c_#252
THEN
GO:0005529: sugar binding: 1.129e-10 (13,39,7) OR
GO:0008080: N-acetyltransferase activity: 6.040e-02 (13,13,1) OR
GO:0016763: transferase activity, transferring pentosyl groups: 1.259e-01 (13,28,1) OR
GO:0005509: calcium ion binding: 1.751e-01 (13,160,2) OR
GO:0004263: chymotrypsin activity: 1.792e-01 (13,41,1) OR
GO:0004295: trypsin activity: 2.067e-01 (13,48,1)

IF 1gg6.1#C156 AND 1pwt__#8 AND 1elva1#604
THEN
GO:0004263: chymotrypsin activity: 1.134e-10 (40,41,10) OR
GO:0004295: trypsin activity: 6.162e-10 (40,48,10) OR
GO:0003809: thrombin activity: 1.259e-07 (40,10,5) OR
GO:0004867: serine-type endopeptidase inhibitor activity: 4.681e-05 (40,47,6) OR
GO:0005509: calcium ion binding: 1.815e-03 (40,160,8) OR
GO:0004896: hematopoietin/interferon-class (D200-domain) cytokine receptor activity: 2.457e-01 (40,19,1)

IF 1g0sa_#176 AND 1rypk_#12
THEN
GO:0003887: DNA-directed DNA polymerase activity: 1.140e-10 (13,20,6) OR
GO:0008408: 3'-5' exonuclease activity: 2.362e-05 (13,13,3) OR
GO:0004725: protein tyrosine phosphatase activity: 6.939e-02 (13,15,1) OR
GO:0004601: peroxidase activity: 9.588e-02 (13,21,1) OR
GO:0030145: manganese ion binding: 1.672e-01 (13,38,1) OR
GO:0000287: magnesium ion binding: 4.657e-01 (13,128,1)

IF 1b3qa2#556 AND 1fgka_#689
THEN
GO:0004674: protein serine/threonine kinase activity: 1.742e-10 (20,42,8) OR
GO:0005524: ATP binding: 2.155e-05 (20,243,9) OR
GO:0005516: calmodulin binding: 1.282e-02 (20,24,2) OR
GO:0005066: transmembrane receptor protein tyrosine kinase signaling protein activity: 7.113e-02 (20,10,1)

IF 1koba_#105 AND 1apme_#68
THEN
GO:0004674: protein serine/threonine kinase activity: 1.742e-10 (20,42,8) OR
GO:0005524: ATP binding: 1.742e-04 (20,243,8) OR
GO:0005516: calmodulin binding: 6.206e-04 (20,24,3) OR

GO:0004896: hematopoietin/interferon-class (D200-domain) cytokine receptor activity: 1.310e-01 (20,19,1)

IF 1tkia_#98 AND 1bio__#210
THEN
GO:0004674: protein serine/threonine kinase activity: 1.742e-10 (20,42,8) OR
GO:0005524: ATP binding: 1.742e-04 (20,243,8) OR
GO:0005516: calmodulin binding: 6.206e-04 (20,24,3) OR
GO:0004896: hematopoietin/interferon-class (D200-domain) cytokine receptor activity: 1.310e-01 (20,19,1)

IF 1qrra_#69 AND 1e6ua_#10
THEN
GO:0016616: oxidoreductase activity, acting on the CH-OH group of donors, NAD or NADP as acceptor: 1.772e-10 (10,59,7) OR
GO:0016854: racemase and epimerase activity: 4.677e-02 (10,13,1) OR
GO:0016627: oxidoreductase activity, acting on the CH-CH group of donors: 6.076e-02 (10,17,1) OR
GO:0016836: hydro-lyase activity: 1.149e-01 (10,33,1)

IF 1gega_#58 AND 1ec7a1#216
THEN
GO:0016616: oxidoreductase activity, acting on the CH-OH group of donors, NAD or NADP as acceptor: 1.772e-10 (10,59,7) OR
GO:0016854: racemase and epimerase activity: 4.677e-02 (10,13,1) OR
GO:0005351: sugar porter activity: 7.456e-02 (10,21,1) OR
GO:0003700: transcription factor activity: 3.728e-01 (10,124,1)

IF 1iira_#326 AND 1oaa__#175
THEN
GO:0016616: oxidoreductase activity, acting on the CH-OH group of donors, NAD or NADP as acceptor: 1.772e-10 (10,59,7) OR
GO:0016627: oxidoreductase activity, acting on the CH-CH group of donors: 6.076e-02 (10,17,1) OR
GO:0016646: oxidoreductase activity, acting on the CH-NH group of donors, NAD or NADP as acceptor: 6.769e-02 (10,19,1) OR
GO:0003700: transcription factor activity: 3.728e-01 (10,124,1)

IF 3grs_2#211 AND 1hdr__#149
THEN
GO:0016616: oxidoreductase activity, acting on the CH-OH group of donors, NAD or NADP as acceptor: 1.772e-10 (10,59,7) OR
GO:0016627: oxidoreductase activity, acting on the CH-CH group of donors: 6.076e-02 (10,17,1) OR
GO:0016646: oxidoreductase activity, acting on the CH-NH group of donors, NAD or NADP as acceptor: 6.769e-02 (10,19,1) OR
GO:0016836: hydro-lyase activity: 1.149e-01 (10,33,1)

IF 1gega_#58 AND 1c3pa_#6
THEN
GO:0016616: oxidoreductase activity, acting on the CH-OH group of donors, NAD or NADP as acceptor: 1.772e-10 (10,59,7) OR
GO:0016854: racemase and epimerase activity: 4.677e-02 (10,13,1) OR
GO:0030145: manganese ion binding: 1.312e-01 (10,38,1) OR
GO:0005524: ATP binding: 6.077e-01 (10,243,1)

IF 1b4ka_#108 AND 1oaa__#199
THEN
GO:0016616: oxidoreductase activity, acting on the CH-OH group of donors, NAD or NADP as acceptor: 1.772e-10 (10,59,7) OR
GO:0016861: intramolecular oxidoreductase activity, interconverting aldoses and ketoses: 4.677e-02 (10,13,1) OR
GO:0016836: hydro-lyase activity: 1.149e-01 (10,33,1) OR
GO:0030145: manganese ion binding: 1.312e-01 (10,38,1)

IF 1ejda_#379 AND 2ae2a_#17
THEN
GO:0016616: oxidoreductase activity, acting on the CH-OH group of donors, NAD or NADP as acceptor: 1.772e-10 (10,59,7) OR
GO:0016854: racemase and epimerase activity: 4.677e-02 (10,13,1) OR
GO:0016627: oxidoreductase activity, acting on the CH-CH group of donors: 6.076e-02 (10,17,1) OR
GO:0016836: hydro-lyase activity: 1.149e-01 (10,33,1)

IF 1iira_#326 AND 1eny__#94
THEN
GO:0016616: oxidoreductase activity, acting on the CH-OH group of donors, NAD or NADP as acceptor: 1.772e-10 (10,59,7) OR
GO:0016627: oxidoreductase activity, acting on the CH-CH group of donors: 6.076e-02 (10,17,1) OR
GO:0016638: oxidoreductase activity, acting on the CH-NH2 group of donors: 6.076e-02 (10,17,1) OR
GO:0016646: oxidoreductase activity, acting on the CH-NH group of donors, NAD or NADP as acceptor: 6.769e-02 (10,19,1)

IF 1fuia2#273 AND 1qfma2#576 AND 1eq2a_#11
THEN
GO:0016616: oxidoreductase activity, acting on the CH-OH group of donors, NAD or NADP as acceptor: 1.772e-10 (10,59,7) OR
GO:0016854: racemase and epimerase activity: 4.677e-02 (10,13,1) OR
GO:0016627: oxidoreductase activity, acting on the CH-CH group of donors: 6.076e-02 (10,17,1) OR

GO:0016646: oxidoreductase activity, acting on the CH-NH group of donors, NAD or NADP as acceptor: 6.769e-02 (10,19,1)

IF 1bdb__#228 AND 1e6wa_#136
THEN
GO:0016616: oxidoreductase activity, acting on the CH-OH group of donors, NAD or NADP as acceptor: 1.772e-10 (10,59,7) OR
GO:0001584: rhodopsin-like receptor activity: 3.616e-02 (10,10,1) OR
GO:0005179: hormone activity: 5.379e-02 (10,15,1) OR
GO:0016627: oxidoreductase activity, acting on the CH-CH group of donors: 6.076e-02 (10,17,1)

IF 1leha1#255 AND 1b3ra1#218
THEN
GO:0016616: oxidoreductase activity, acting on the CH-OH group of donors, NAD or NADP as acceptor: 1.772e-10 (10,59,7) OR
GO:0016620: oxidoreductase activity, acting on the aldehyde or oxo group of donors, NAD or NADP as acceptor: 3.616e-02 (10,10,1) OR
GO:0016638: oxidoreductase activity, acting on the CH-NH2 group of donors: 6.076e-02 (10,17,1) OR
GO:0015036: disulfide oxidoreductase activity: 7.799e-02 (10,22,1)

IF 1cyx__#133 AND 1aoza1#61
THEN
GO:0005507: copper ion binding: 1.838e-10 (14,38,7) OR
GO:0015078: hydrogen ion transporter activity: 3.124e-08 (14,21,5) OR
GO:0015082: di-, tri-valent inorganic cation transporter activity: 6.973e-02 (14,14,1) OR
GO:0046915: transition metal ion transporter activity: 6.973e-02 (14,14,1)

IF 3lada1#116 AND 1hq8a_#206 AND 1b4va1#253
THEN
GO:0015036: disulfide oxidoreductase activity: 1.231e-10 (22,22,7) OR
GO:0050660: FAD binding: 5.182e-09 (22,10,5) OR
GO:0016668: oxidoreductase activity, acting on sulfur group of donors, NAD or NADP as acceptor: 1.612e-08 (22,12,5) OR
GO:0016705: oxidoreductase activity, acting on paired donors, with incorporation or reduction of molecular oxygen: 1.799e-02 (22,26,2) OR
GO:0004497: monooxygenase activity: 1.799e-02 (22,26,2) OR
GO:0016651: oxidoreductase activity, acting on NADH or NADPH: 1.002e-01 (22,13,1)

IF 1evqa_#61 AND 1elva1#513
THEN
GO:0004295: trypsin activity: 1.235e-10 (25,48,9) OR
GO:0004263: chymotrypsin activity: 1.151e-09 (25,41,8) OR
GO:0004867: serine-type endopeptidase inhibitor activity: 8.478e-03 (25,47,3) OR
GO:0003809: thrombin activity: 8.819e-02 (25,10,1) OR
GO:0005509: calcium ion binding: 1.783e-01 (25,160,3) OR
GO:0005529: sugar binding: 3.037e-01 (25,39,1)

IF 1evqa_#61 AND 1ton__#179
THEN
GO:0004295: trypsin activity: 1.235e-10 (25,48,9) OR
GO:0004263: chymotrypsin activity: 1.151e-09 (25,41,8) OR
GO:0004867: serine-type endopeptidase inhibitor activity: 8.478e-03 (25,47,3) OR
GO:0003809: thrombin activity: 8.819e-02 (25,10,1) OR
GO:0005509: calcium ion binding: 1.783e-01 (25,160,3) OR
GO:0005529: sugar binding: 3.037e-01 (25,39,1)

IF 1chma2#240 AND 1fa0a1#393
THEN
GO:0008235: metalloexopeptidase activity: 2.568e-10 (10,13,5) OR
GO:0004177: aminopeptidase activity: 6.446e-08 (10,13,4) OR
GO:0030145: manganese ion binding: 1.312e-01 (10,38,1)

IF 2dkb__#46 AND 1cjca2#365
THEN
GO:0008483: transaminase activity: 2.747e-10 (8,17,5) OR
GO:0016846: carbon-sulfur lyase activity: 3.355e-04 (8,10,2) OR
GO:0016866: intramolecular transferase activity: 3.474e-02 (8,12,1)

IF 1cjca2#365 AND 2oata_#81
THEN
GO:0008483: transaminase activity: 2.747e-10 (8,17,5) OR
GO:0016846: carbon-sulfur lyase activity: 3.355e-04 (8,10,2) OR
GO:0016866: intramolecular transferase activity: 3.474e-02 (8,12,1)

IF 1c7na_#95 AND 1eg5a_#199
THEN
GO:0008483: transaminase activity: 2.747e-10 (8,17,5) OR
GO:0016846: carbon-sulfur lyase activity: 3.355e-04 (8,10,2) OR
GO:0016831: carboxy-lyase activity: 7.117e-02 (8,25,1)

IF 1dfoa_#86 AND 1eg5a_#199
THEN
GO:0008483: transaminase activity: 2.747e-10 (8,17,5) OR
GO:0016846: carbon-sulfur lyase activity: 3.355e-04 (8,10,2) OR
GO:0016831: carboxy-lyase activity: 7.117e-02 (8,25,1)

IF 1cbf__#208 AND 1koba_#175
THEN
GO:0004674: protein serine/threonine kinase activity: 1.742e-10 (20,42,8) OR
GO:0005524: ATP binding: 1.742e-04 (20,243,8) OR
GO:0005516: calmodulin binding: 1.282e-02 (20,24,2) OR
GO:0003755: peptidyl-prolyl cis-trans isomerase activity: 7.797e-02 (20,11,1) OR
GO:0004896: hematopoietin/interferon-class (D200-domain) cytokine receptor activity: 1.310e-01 (20,19,1)

IF 1a6o__#297 AND 1apme_#68
THEN
GO:0004674: protein serine/threonine kinase activity: 1.742e-10 (20,42,8) OR
GO:0005524: ATP binding: 1.742e-04 (20,243,8) OR
GO:0005516: calmodulin binding: 1.282e-02 (20,24,2) OR
GO:0003755: peptidyl-prolyl cis-trans isomerase activity: 7.797e-02 (20,11,1) OR
GO:0004896: hematopoietin/interferon-class (D200-domain) cytokine receptor activity: 1.310e-01 (20,19,1)

IF 1apme_#74 AND 1b6cb_#337
THEN
GO:0004674: protein serine/threonine kinase activity: 1.742e-10 (20,42,8) OR
GO:0005524: ATP binding: 1.742e-04 (20,243,8) OR
GO:0005516: calmodulin binding: 1.282e-02 (20,24,2) OR
GO:0003755: peptidyl-prolyl cis-trans isomerase activity: 7.797e-02 (20,11,1) OR
GO:0004896: hematopoietin/interferon-class (D200-domain) cytokine receptor activity: 1.310e-01 (20,19,1)

IF 1f97a1#62 AND 1danh_#48 AND 1cgha_#130
THEN
GO:0004263: chymotrypsin activity: 1.778e-10 (30,41,9) OR
GO:0004295: trypsin activity: 8.099e-10 (30,48,9) OR
GO:0004867: serine-type endopeptidase inhibitor activity: 1.533e-03 (30,47,4) OR
GO:0003809: thrombin activity: 4.992e-03 (30,10,2) OR
GO:0005509: calcium ion binding: 6.819e-03 (30,160,6)

IF 1danh_#48 AND 1danh_#24 AND 1cgha_#130
THEN
GO:0004263: chymotrypsin activity: 1.778e-10 (30,41,9) OR
GO:0004295: trypsin activity: 8.099e-10 (30,48,9) OR
GO:0004867: serine-type endopeptidase inhibitor activity: 1.533e-03 (30,47,4) OR
GO:0003809: thrombin activity: 4.992e-03 (30,10,2) OR
GO:0005509: calcium ion binding: 6.819e-03 (30,160,6)

IF 1c5y.1#B192 AND 1pwt__#8
THEN
GO:0004263: chymotrypsin activity: 1.484e-10 (41,41,10) OR
GO:0004295: trypsin activity: 8.045e-10 (41,48,10) OR
GO:0003809: thrombin activity: 1.432e-07 (41,10,5) OR
GO:0004867: serine-type endopeptidase inhibitor activity: 5.413e-05 (41,47,6) OR
GO:0005509: calcium ion binding: 4.560e-04 (41,160,9) OR
GO:0004896: hematopoietin/interferon-class (D200-domain) cytokine receptor activity: 2.510e-01 (41,19,1)

IF 1bqya_#216 AND 1czan1#92
THEN
GO:0004263: chymotrypsin activity: 1.484e-10 (41,41,10) OR
GO:0004295: trypsin activity: 8.045e-10 (41,48,10) OR
GO:0003809: thrombin activity: 1.597e-09 (41,10,6) OR
GO:0005509: calcium ion binding: 4.560e-04 (41,160,9) OR
GO:0004867: serine-type endopeptidase inhibitor activity: 5.784e-04 (41,47,5) OR
GO:0004896: hematopoietin/interferon-class (D200-domain) cytokine receptor activity: 2.510e-01 (41,19,1)

IF 1bf2_3#579 AND 3chbd_#85
THEN
GO:0004556: alpha-amylase activity: 2.996e-10 (9,15,5) OR
GO:0005509: calcium ion binding: 1.284e-02 (9,160,3) OR
GO:0016758: transferase activity, transferring hexosyl groups: 3.580e-02 (9,11,1)

IF 1avaa2#204 AND 1iira_#242
THEN
GO:0004556: alpha-amylase activity: 2.996e-10 (9,15,5) OR
GO:0005509: calcium ion binding: 1.284e-02 (9,160,3) OR
GO:0004867: serine-type endopeptidase inhibitor activity: 1.451e-01 (9,47,1)

IF 1hx0a2#258 AND 1qfea_#68
THEN
GO:0004556: alpha-amylase activity: 2.996e-10 (9,15,5) OR
GO:0005509: calcium ion binding: 1.284e-02 (9,160,3) OR
GO:0004867: serine-type endopeptidase inhibitor activity: 1.451e-01 (9,47,1)

IF 1ac6a_#43 AND 1el0a_#62
THEN
GO:0008009: chemokine activity: 4.572e-10 (5,10,4) OR
GO:0008083: growth factor activity: 7.478e-02 (5,42,1)

IF 1e5ea_#145 AND 1gox__#124 AND 1ax4a_#275
THEN
GO:0016846: carbon-sulfur lyase activity: 4.572e-10 (5,10,4) OR
GO:0008483: transaminase activity: 3.083e-02 (5,17,1)

IF 2bb2_2#134 AND 1ha6a_#59
THEN
GO:0008009: chemokine activity: 4.572e-10 (5,10,4) OR
GO:0008083: growth factor activity: 7.478e-02 (5,42,1)

IF 1a4sa_#382 AND 2viua_#252
THEN
GO:0016620: oxidoreductase activity, acting on the aldehyde or oxo group of donors, NAD or NADP as acceptor: 4.572e-10 (5,10,4) OR
GO:0005524: ATP binding: 3.734e-01 (5,243,1)

IF 1a4sa_#382 AND 2sli_2#326
THEN
GO:0016620: oxidoreductase activity, acting on the aldehyde or oxo group of donors, NAD or NADP as acceptor: 4.572e-10 (5,10,4) OR
GO:0005524: ATP binding: 3.734e-01 (5,243,1)

IF 1gdna_#119 AND 1jb0d_#26 AND 1f42a2#123
THEN
GO:0004295: trypsin activity: 1.864e-10 (26,48,9) OR
GO:0004263: chymotrypsin activity: 1.645e-09 (26,41,8) OR
GO:0004867: serine-type endopeptidase inhibitor activity: 9.469e-03 (26,47,3) OR
GO:0005509: calcium ion binding: 1.577e-02 (26,160,5) OR
GO:0004896: hematopoietin/interferon-class (D200-domain) cytokine receptor activity: 1.670e-01 (26,19,1)

IF 1qgna_#149 AND 1a8i__#688
THEN
GO:0016846: carbon-sulfur lyase activity: 1.583e-10 (12,10,5) OR
GO:0008483: transaminase activity: 4.289e-05 (12,17,3) OR
GO:0016758: transferase activity, transferring hexosyl groups: 4.747e-02 (12,11,1) OR
GO:0005351: sugar porter activity: 8.883e-02 (12,21,1) OR
GO:0016831: carboxy-lyase activity: 1.049e-01 (12,25,1) OR
GO:0003700: transcription factor activity: 4.288e-01 (12,124,1)

IF 1bd3a_#110 AND 1qtn.1#A270
THEN
GO:0016763: transferase activity, transferring pentosyl groups: 1.592e-10 (18,28,7) OR
GO:0000287: magnesium ion binding: 8.852e-06 (18,128,7) OR
GO:0016866: intramolecular transferase activity: 7.660e-02 (18,12,1) OR
GO:0016651: oxidoreductase activity, acting on NADH or NADPH: 8.272e-02 (18,13,1) OR
GO:0008757: S-adenosylmethionine-dependent methyltransferase activity: 1.476e-01 (18,24,1) OR
GO:0005524: ATP binding: 8.149e-01 (18,243,1)

IF 1ja1a2#201 AND 1boub_#7
THEN
GO:0010181: FMN binding: 1.692e-10 (11,11,5) OR
GO:0016651: oxidoreductase activity, acting on NADH or NADPH: 1.128e-03 (11,13,2) OR
GO:0016705: oxidoreductase activity, acting on paired donors, with incorporation or reduction of molecular oxygen: 1.003e-01 (11,26,1) OR
GO:0004497: monooxygenase activity: 1.003e-01 (11,26,1) OR
GO:0030145: manganese ion binding: 1.434e-01 (11,38,1) OR
GO:0005524: ATP binding: 6.428e-01 (11,243,1)

IF 1gcoa_#119 AND 1c3pa_#6
THEN
GO:0016616: oxidoreductase activity, acting on the CH-OH group of donors, NAD or NADP as acceptor: 1.699e-10 (15,59,8) OR
GO:0016627: oxidoreductase activity, acting on the CH-CH group of donors: 8.768e-05 (15,17,3) OR
GO:0016854: racemase and epimerase activity: 6.939e-02 (15,13,1) OR
GO:0016861: intramolecular oxidoreductase activity, interconverting aldoses and ketoses: 6.939e-02 (15,13,1) OR
GO:0008810: cellulase activity: 9.486e-02 (15,18,1) OR
GO:0016836: hydro-lyase activity: 1.674e-01 (15,33,1)

IF 1gdea_#243 AND 5ruba1#365
THEN
GO:0016846: carbon-sulfur lyase activity: 2.568e-10 (13,10,5) OR
GO:0008483: transaminase activity: 6.198e-09 (13,17,5) OR
GO:0016616: oxidoreductase activity, acting on the CH-OH group of donors, NAD or NADP as acceptor: 3.084e-02 (13,59,2) OR
GO:0016831: carboxy-lyase activity: 1.132e-01 (13,25,1)

IF 1i9ga_#67 AND 1ddja_#759 AND 1ddja_#746
THEN
GO:0004263: chymotrypsin activity: 1.778e-10 (30,41,9) OR
GO:0004295: trypsin activity: 8.099e-10 (30,48,9) OR
GO:0003809: thrombin activity: 4.992e-03 (30,10,2) OR
GO:0005509: calcium ion binding: 6.819e-03 (30,160,6) OR
GO:0004867: serine-type endopeptidase inhibitor activity: 1.409e-02 (30,47,3) OR
GO:0004896: hematopoietin/interferon-class (D200-domain) cytokine receptor activity: 1.903e-01 (30,19,1)

IF 1i9ga_#67 AND 1ekbb_#79 AND 1fjsa_#158 AND 1bqya_#103 AND 1c5y.1#B234 AND 1autc_#209
THEN
GO:0004263: chymotrypsin activity: 1.778e-10 (30,41,9) OR
GO:0004295: trypsin activity: 8.099e-10 (30,48,9) OR
GO:0004867: serine-type endopeptidase inhibitor activity: 1.533e-03 (30,47,4) OR
GO:0005509: calcium ion binding: 6.819e-03 (30,160,6) OR
GO:0003809: thrombin activity: 1.050e-01 (30,10,1) OR
GO:0004896: hematopoietin/interferon-class (D200-domain) cytokine receptor activity: 1.903e-01 (30,19,1)

IF 1hdr__#136 AND 2ae2a_#89
THEN
GO:0016616: oxidoreductase activity, acting on the CH-OH group of donors, NAD or NADP as acceptor: 5.484e-10 (7,59,6) OR
GO:0016646: oxidoreductase activity, acting on the CH-NH group of donors, NAD or NADP as acceptor: 4.785e-02 (7,19,1)

IF 1nsj__#124 AND 1b3ra1#218
THEN
GO:0016616: oxidoreductase activity, acting on the CH-OH group of donors, NAD or NADP as acceptor: 5.484e-10 (7,59,6) OR
GO:0016620: oxidoreductase activity, acting on the aldehyde or oxo group of donors, NAD or NADP as acceptor: 2.543e-02 (7,10,1)

IF 1ja9a_#249 AND 5ruba1#365
THEN
GO:0016616: oxidoreductase activity, acting on the CH-OH group of donors, NAD or NADP as acceptor: 5.484e-10 (7,59,6) OR
GO:0016627: oxidoreductase activity, acting on the CH-CH group of donors: 4.291e-02 (7,17,1)

IF 1qg6a_#251 AND 1gdha1#235
THEN
GO:0016616: oxidoreductase activity, acting on the CH-OH group of donors, NAD or NADP as acceptor: 5.484e-10 (7,59,6) OR
GO:0016627: oxidoreductase activity, acting on the CH-CH group of donors: 4.291e-02 (7,17,1)

IF 1ek6a_#33 AND 1hu4a_#267 AND 1eno__#264
THEN
GO:0016616: oxidoreductase activity, acting on the CH-OH group of donors, NAD or NADP as acceptor: 5.484e-10 (7,59,6) OR
GO:0016646: oxidoreductase activity, acting on the CH-NH group of donors, NAD or NADP as acceptor: 4.785e-02 (7,19,1)

IF 1fwxa1#535 AND 1e30a_#87
THEN
GO:0015078: hydrogen ion transporter activity: 2.774e-10 (14,21,6) OR
GO:0005507: copper ion binding: 1.352e-08 (14,38,6) OR
GO:0019955: cytokine binding: 5.518e-02 (14,11,1) OR
GO:0005509: calcium ion binding: 5.723e-01 (14,160,1)

IF 1fjsa_#28 AND 1a0la_#160 AND 1ton__#231
THEN
GO:0004295: trypsin activity: 1.864e-10 (26,48,9) OR
GO:0004263: chymotrypsin activity: 1.645e-09 (26,41,8) OR
GO:0003809: thrombin activity: 3.759e-03 (26,10,2) OR
GO:0005509: calcium ion binding: 6.231e-02 (26,160,4) OR
GO:0004867: serine-type endopeptidase inhibitor activity: 7.282e-02 (26,47,2) OR
GO:0005529: sugar binding: 3.138e-01 (26,39,1)

IF 1danh_#140 AND 1a0la_#160 AND 1kit_3#743 AND 1dlea_#238
THEN
GO:0004295: trypsin activity: 1.946e-10 (36,48,10) OR
GO:0004263: chymotrypsin activity: 1.097e-09 (36,41,9) OR
GO:0004867: serine-type endopeptidase inhibitor activity: 2.503e-05 (36,47,6) OR
GO:0005509: calcium ion binding: 8.734e-04 (36,160,8) OR
GO:0003809: thrombin activity: 7.146e-03 (36,10,2) OR
GO:0008201: heparin binding: 2.742e-01 (36,24,1)

IF 1ecfa1#367 AND 2hhma_#262
THEN
GO:0016763: transferase activity, transferring pentosyl groups: 5.903e-10 (12,28,6) OR
GO:0000287: magnesium ion binding: 7.005e-06 (12,128,6)

IF 1tc1a_#158 AND 1ihua2#525
THEN
GO:0016763: transferase activity, transferring pentosyl groups: 2.972e-10 (11,28,6) OR
GO:0000287: magnesium ion binding: 1.266e-02 (11,128,3) OR
GO:0004812: tRNA ligase activity: 1.003e-01 (11,26,1) OR
GO:0005524: ATP binding: 6.428e-01 (11,243,1)

IF 1g0sa_#176 AND 1boub_#7
THEN
GO:0003887: DNA-directed DNA polymerase activity: 1.986e-10 (14,20,6) OR
GO:0008408: 3'-5' exonuclease activity: 2.998e-05 (14,13,3) OR
GO:0008199: ferric iron binding: 1.078e-03 (14,10,2) OR
GO:0008080: N-acetyltransferase activity: 6.491e-02 (14,13,1) OR
GO:0000287: magnesium ion binding: 4.910e-01 (14,128,1) OR
GO:0005524: ATP binding: 7.304e-01 (14,243,1)

IF 1plc__#74 AND 1fwxa1#526
THEN
GO:0005507: copper ion binding: 3.977e-10 (9,38,6) OR
GO:0015078: hydrogen ion transporter activity: 1.972e-03 (9,21,2) OR
GO:0005509: calcium ion binding: 4.204e-01 (9,160,1)

IF 1plc__#74 AND 1cyx__#138
THEN
GO:0005507: copper ion binding: 3.977e-10 (9,38,6) OR
GO:0015078: hydrogen ion transporter activity: 1.972e-03 (9,21,2) OR
GO:0005509: calcium ion binding: 4.204e-01 (9,160,1)

IF 1nsj__#21 AND 1e43a2#351
THEN
GO:0004556: alpha-amylase activity: 5.974e-10 (10,15,5) OR
GO:0005509: calcium ion binding: 1.300e-04 (10,160,5)

IF 1clxa_#13 AND 1bqca_#24
THEN
GO:0004556: alpha-amylase activity: 5.974e-10 (10,15,5) OR
GO:0005509: calcium ion binding: 1.300e-04 (10,160,5)

IF 1prea2#308 AND 1cs1a_#160
THEN

GO:0004556: alpha-amylase activity: 2.996e-10 (9,15,5) OR
GO:0005509: calcium ion binding: 9.399e-02 (9,160,2) OR
GO:0016836: hydro-lyase activity: 1.040e-01 (9,33,1) OR
GO:0030145: manganese ion binding: 1.189e-01 (9,38,1)

IF 1sek__#301 AND 1mjha_#126
THEN
GO:0004867: serine-type endopeptidase inhibitor activity: 1.230e-09 (5,47,5)

IF 1fg7a_#158 AND 1c7na_#231
THEN
GO:0008483: transaminase activity: 6.158e-10 (9,17,5) OR
GO:0016846: carbon-sulfur lyase activity: 1.144e-08 (9,10,4)

IF 1fg7a_#158 AND 1evqa_#305
THEN
GO:0008483: transaminase activity: 6.158e-10 (9,17,5) OR
GO:0016846: carbon-sulfur lyase activity: 1.144e-08 (9,10,4)

IF 1azza_#114 AND 1danh_#48 AND 1ekbb_#79 AND 1ejda_#213 AND 1ton__#112 AND 1ekbb_#141 AND 1elva1#604 AND 1autc_#209
THEN
GO:0004263: chymotrypsin activity: 2.478e-10 (31,41,9) OR
GO:0004295: trypsin activity: 1.126e-09 (31,48,9) OR
GO:0003809: thrombin activity: 1.517e-04 (31,10,3) OR
GO:0005509: calcium ion binding: 1.674e-03 (31,160,7) OR
GO:0004867: serine-type endopeptidase inhibitor activity: 1.542e-02 (31,47,3)

IF 1e05i_#83 AND 1imva_#278
THEN
GO:0004867: serine-type endopeptidase inhibitor activity: 2.125e-10 (12,47,7) OR
GO:0008201: heparin binding: 1.009e-01 (12,24,1) OR
GO:0003779: actin binding: 1.324e-01 (12,32,1) OR
GO:0051082: unfolded protein binding: 1.401e-01 (12,34,1) OR
GO:0016616: oxidoreductase activity, acting on the CH-OH group of donors, NAD or NADP as acceptor: 2.314e-01 (12,59,1) OR
GO:0003700: transcription factor activity: 4.288e-01 (12,124,1)

IF 1atia2#67 AND 1i50a_#856
THEN
GO:0004812: tRNA ligase activity: 3.195e-10 (21,26,7) OR
GO:0005524: ATP binding: 1.606e-03 (21,243,7) OR
GO:0000287: magnesium ion binding: 2.343e-03 (21,128,5) OR
GO:0000049: tRNA binding: 4.193e-03 (21,13,2)

IF 1hc7a2#111 AND 1qqsa_#35
THEN
GO:0004812: tRNA ligase activity: 3.195e-10 (21,26,7) OR
GO:0005524: ATP binding: 1.606e-03 (21,243,7) OR
GO:0000287: magnesium ion binding: 2.343e-03 (21,128,5) OR
GO:0000049: tRNA binding: 4.193e-03 (21,13,2)

IF 1qf6a4#318 AND 1qqsa_#35
THEN
GO:0004812: tRNA ligase activity: 3.195e-10 (21,26,7) OR
GO:0005524: ATP binding: 1.606e-03 (21,243,7) OR
GO:0000287: magnesium ion binding: 2.343e-03 (21,128,5) OR
GO:0000049: tRNA binding: 4.193e-03 (21,13,2)

IF 1qfxa_#380 AND 1danh_#70
THEN
GO:0003809: thrombin activity: 2.139e-10 (30,10,6) OR
GO:0004263: chymotrypsin activity: 3.616e-06 (30,41,6) OR
GO:0004295: trypsin activity: 9.355e-06 (30,48,6) OR
GO:0004867: serine-type endopeptidase inhibitor activity: 1.268e-04 (30,47,5) OR
GO:0005509: calcium ion binding: 6.819e-03 (30,160,6) OR
GO:0005529: sugar binding: 3.526e-01 (30,39,1)

IF 1ddja_#726 AND 1qfxa_#380 AND 1gdna_#30
THEN
GO:0003809: thrombin activity: 2.139e-10 (30,10,6) OR

GO:0004263: chymotrypsin activity: 3.616e-06 (30,41,6) OR
GO:0004295: trypsin activity: 9.355e-06 (30,48,6) OR
GO:0004867: serine-type endopeptidase inhibitor activity: 1.268e-04 (30,47,5) OR
GO:0005509: calcium ion binding: 6.819e-03 (30,160,6) OR
GO:0005529: sugar binding: 3.526e-01 (30,39,1)

IF 1qfxa_#380 AND 1h8d.1#H184
THEN
GO:0003809: thrombin activity: 2.139e-10 (30,10,6) OR
GO:0004263: chymotrypsin activity: 3.616e-06 (30,41,6) OR
GO:0004295: trypsin activity: 9.355e-06 (30,48,6) OR
GO:0004867: serine-type endopeptidase inhibitor activity: 1.268e-04 (30,47,5) OR
GO:0005509: calcium ion binding: 6.819e-03 (30,160,6) OR
GO:0005529: sugar binding: 3.526e-01 (30,39,1)

IF 1hyha2#204 AND 1pii_2#326
THEN
GO:0004457: lactate dehydrogenase activity: 2.568e-10 (13,10,5) OR
GO:0016616: oxidoreductase activity, acting on the CH-OH group of donors, NAD or NADP as acceptor: 4.520e-06 (13,59,5) OR
GO:0030145: manganese ion binding: 1.672e-01 (13,38,1) OR
GO:0005525: GTP binding: 2.105e-01 (13,49,1) OR
GO:0005524: ATP binding: 7.039e-01 (13,243,1)

IF 1brwa2#100 AND 1fl2a1#320
THEN
GO:0015036: disulfide oxidoreductase activity: 2.185e-10 (13,22,6) OR
GO:0050660: FAD binding: 9.255e-04 (13,10,2) OR
GO:0016668: oxidoreductase activity, acting on sulfur group of donors, NAD or NADP as acceptor: 1.350e-03 (13,12,2) OR
GO:0016627: oxidoreductase activity, acting on the CH-CH group of donors: 7.830e-02 (13,17,1) OR
GO:0016638: oxidoreductase activity, acting on the CH-NH2 group of donors: 7.830e-02 (13,17,1) OR
GO:0004601: peroxidase activity: 9.588e-02 (13,21,1)

IF 1b37a1#209 AND 1h6va2#219
THEN
GO:0015036: disulfide oxidoreductase activity: 2.185e-10 (13,22,6) OR
GO:0016668: oxidoreductase activity, acting on sulfur group of donors, NAD or NADP as acceptor: 1.350e-03 (13,12,2) OR
GO:0016627: oxidoreductase activity, acting on the CH-CH group of donors: 2.745e-03 (13,17,2) OR
GO:0016638: oxidoreductase activity, acting on the CH-NH2 group of donors: 7.830e-02 (13,17,1) OR
GO:0016705: oxidoreductase activity, acting on paired donors, with incorporation or reduction of molecular oxygen: 1.174e-01 (13,26,1) OR
GO:0004497: monooxygenase activity: 1.174e-01 (13,26,1)

IF 1b37a1#209 AND 1trb_1#298
THEN
GO:0015036: disulfide oxidoreductase activity: 2.185e-10 (13,22,6) OR
GO:0016668: oxidoreductase activity, acting on sulfur group of donors, NAD or NADP as acceptor: 1.350e-03 (13,12,2) OR
GO:0016627: oxidoreductase activity, acting on the CH-CH group of donors: 2.745e-03 (13,17,2) OR
GO:0016638: oxidoreductase activity, acting on the CH-NH2 group of donors: 7.830e-02 (13,17,1) OR
GO:0016705: oxidoreductase activity, acting on paired donors, with incorporation or reduction of molecular oxygen: 1.174e-01 (13,26,1) OR
GO:0004497: monooxygenase activity: 1.174e-01 (13,26,1)

IF 1cjca2#365 AND 1f00i2#805
THEN
GO:0015036: disulfide oxidoreductase activity: 2.185e-10 (13,22,6) OR
GO:0050660: FAD binding: 9.255e-04 (13,10,2) OR
GO:0016668: oxidoreductase activity, acting on sulfur group of donors, NAD or NADP as acceptor: 1.350e-03 (13,12,2) OR
GO:0016627: oxidoreductase activity, acting on the CH-CH group of donors: 7.830e-02 (13,17,1) OR
GO:0016638: oxidoreductase activity, acting on the CH-NH2 group of donors: 7.830e-02 (13,17,1) OR
GO:0004601: peroxidase activity: 9.588e-02 (13,21,1)

IF 1brwa2#100 AND 1d7ya1#255
THEN
GO:0015036: disulfide oxidoreductase activity: 2.185e-10 (13,22,6) OR
GO:0050660: FAD binding: 9.255e-04 (13,10,2) OR
GO:0016668: oxidoreductase activity, acting on sulfur group of donors, NAD or NADP as acceptor: 1.350e-03 (13,12,2) OR
GO:0016627: oxidoreductase activity, acting on the CH-CH group of donors: 7.830e-02 (13,17,1) OR
GO:0016638: oxidoreductase activity, acting on the CH-NH2 group of donors: 7.830e-02 (13,17,1) OR
GO:0004601: peroxidase activity: 9.588e-02 (13,21,1)

IF 1brwa2#100 AND 1h7wa4#478
THEN
GO:0015036: disulfide oxidoreductase activity: 2.185e-10 (13,22,6) OR
GO:0050660: FAD binding: 9.255e-04 (13,10,2) OR
GO:0016668: oxidoreductase activity, acting on sulfur group of donors, NAD or NADP as acceptor: 1.350e-03 (13,12,2) OR
GO:0016627: oxidoreductase activity, acting on the CH-CH group of donors: 7.830e-02 (13,17,1) OR
GO:0016638: oxidoreductase activity, acting on the CH-NH2 group of donors: 7.830e-02 (13,17,1) OR
GO:0004601: peroxidase activity: 9.588e-02 (13,21,1)

IF 1f42a2#123 AND 1avgi_#69 AND 1elva1#513 AND 1dy9.1#A44
THEN
GO:0004295: trypsin activity: 2.632e-10 (37,48,10) OR
GO:0004263: chymotrypsin activity: 1.434e-09 (37,41,9) OR
GO:0004867: serine-type endopeptidase inhibitor activity: 1.997e-06 (37,47,7) OR
GO:0003809: thrombin activity: 2.591e-04 (37,10,3) OR
GO:0005509: calcium ion binding: 1.060e-03 (37,160,8)

IF 1aq0a_#4 AND 1egza_#254
THEN
GO:0008810: cellulase activity: 1.335e-09 (4,18,4)

IF 1bqca_#23 AND 1egza_#254
THEN
GO:0008810: cellulase activity: 1.335e-09 (4,18,4)

IF 1egza_#254 AND 1xyza_#753
THEN
GO:0008810: cellulase activity: 1.335e-09 (4,18,4)

IF 1hdma1#115 AND 1nsj__#178
THEN
GO:0004556: alpha-amylase activity: 2.314e-10 (19,15,6) OR
GO:0005509: calcium ion binding: 5.341e-04 (19,160,6) OR
GO:0000287: magnesium ion binding: 1.039e-02 (19,128,4) OR
GO:0008235: metalloexopeptidase activity: 8.713e-02 (19,13,1) OR
GO:0004180: carboxypeptidase activity: 9.988e-02 (19,15,1) OR
GO:0016836: hydro-lyase activity: 2.073e-01 (19,33,1)

IF 1hcl__#82 AND 1b3qa2#556
THEN
GO:0004674: protein serine/threonine kinase activity: 2.782e-10 (21,42,8) OR
GO:0005524: ATP binding: 3.875e-06 (21,243,10) OR
GO:0005066: transmembrane receptor protein tyrosine kinase signaling protein activity: 7.456e-02 (21,10,1) OR
GO:0005516: calmodulin binding: 1.701e-01 (21,24,1) OR
GO:0003779: actin binding: 2.204e-01 (21,32,1)

IF 1tkia_#98 AND 1ir3a_#1139
THEN
GO:0004674: protein serine/threonine kinase activity: 2.782e-10 (21,42,8) OR
GO:0005524: ATP binding: 3.481e-05 (21,243,9) OR
GO:0005516: calmodulin binding: 1.410e-02 (21,24,2) OR
GO:0005066: transmembrane receptor protein tyrosine kinase signaling protein activity: 7.456e-02 (21,10,1) OR
GO:0004714: transmembrane receptor protein tyrosine kinase activity: 1.029e-01 (21,14,1)

IF 1tkia_#98 AND 1csn__#134
THEN
GO:0004674: protein serine/threonine kinase activity: 2.782e-10 (21,42,8) OR
GO:0005524: ATP binding: 3.481e-05 (21,243,9) OR
GO:0005516: calmodulin binding: 1.410e-02 (21,24,2) OR
GO:0005066: transmembrane receptor protein tyrosine kinase signaling protein activity: 7.456e-02 (21,10,1) OR
GO:0004714: transmembrane receptor protein tyrosine kinase activity: 1.029e-01 (21,14,1)

IF 1lgr_2#433 AND 1mkp__#216
THEN
GO:0008235: metalloexopeptidase activity: 4.696e-10 (11,13,5) OR
GO:0004180: carboxypeptidase activity: 1.092e-09 (11,15,5) OR
GO:0005509: calcium ion binding: 4.867e-01 (11,160,1)

IF 1a0fa1#178 AND 1d0na6#681
THEN

GO:0004364: glutathione transferase activity: 7.183e-10 (5,11,4) OR
GO:0003714: transcription corepressor activity: 1.460e-02 (5,8,1)

IF 1ljra1#165 AND 8dfr__#9
THEN
GO:0004364: glutathione transferase activity: 7.183e-10 (5,11,4) OR
GO:0003714: transcription corepressor activity: 1.460e-02 (5,8,1)

IF 2gsq_2#50 AND 1e6ca_#91
THEN
GO:0004364: glutathione transferase activity: 7.183e-10 (5,11,4) OR
GO:0016651: oxidoreductase activity, acting on NADH or NADPH: 2.364e-02 (5,13,1)

IF 1ja1a2#201 AND 1xis__#214
THEN
GO:0010181: FMN binding: 7.183e-10 (5,11,4) OR
GO:0016651: oxidoreductase activity, acting on NADH or NADPH: 2.364e-02 (5,13,1)

IF 1aba__#17 AND 1a0fa1#94
THEN
GO:0004364: glutathione transferase activity: 7.183e-10 (5,11,4) OR
GO:0003714: transcription corepressor activity: 1.460e-02 (5,8,1)

IF 1a0fa1#94 AND 1a4ya_#104
THEN
GO:0004364: glutathione transferase activity: 7.183e-10 (5,11,4) OR
GO:0016651: oxidoreductase activity, acting on NADH or NADPH: 2.364e-02 (5,13,1)

IF 1mspa_#17 AND 1nbaa_#227
THEN
GO:0004364: glutathione transferase activity: 7.183e-10 (5,11,4) OR
GO:0008270: zinc ion binding: 1.832e-01 (5,108,1)

IF 1d0na6#681 AND 1gnwa1#160
THEN
GO:0004364: glutathione transferase activity: 7.183e-10 (5,11,4) OR
GO:0003714: transcription corepressor activity: 1.460e-02 (5,8,1)

IF 8dfr__#9 AND 1gnwa1#160
THEN
GO:0004364: glutathione transferase activity: 7.183e-10 (5,11,4) OR
GO:0003714: transcription corepressor activity: 1.460e-02 (5,8,1)

IF 2sqca2#97 AND 1f3ba2#7
THEN
GO:0004364: glutathione transferase activity: 7.183e-10 (5,11,4) OR
GO:0004842: ubiquitin-protein ligase activity: 3.440e-02 (5,19,1)

IF 1hqoa2#159 AND 1f9va_#627
THEN
GO:0004364: glutathione transferase activity: 7.183e-10 (5,11,4) OR
GO:0016651: oxidoreductase activity, acting on NADH or NADPH: 2.364e-02 (5,13,1)

IF 2gsq_2#50 AND 1ib2a_#974
THEN
GO:0004364: glutathione transferase activity: 7.183e-10 (5,11,4) OR
GO:0016651: oxidoreductase activity, acting on NADH or NADPH: 2.364e-02 (5,13,1)

IF 1hqoa2#159 AND 1eu3a2#205
THEN
GO:0004364: glutathione transferase activity: 7.183e-10 (5,11,4) OR
GO:0008080: N-acetyltransferase activity: 2.364e-02 (5,13,1)

IF 1b16a_#62 AND 1oaa__#14
THEN
GO:0016616: oxidoreductase activity, acting on the CH-OH group of donors, NAD or NADP as acceptor: 4.790e-10 (11,59,7) OR
GO:0016627: oxidoreductase activity, acting on the CH-CH group of donors: 3.229e-05 (11,17,3) OR
GO:0016646: oxidoreductase activity, acting on the CH-NH group of donors, NAD or NADP as acceptor: 7.421e-02 (11,19,1)

IF 1cpt__#401 AND 1bccb2#274

THEN
 GO:0004497: monooxygenase activity: 3.621e-10 (12,26,6) OR
 GO:0016705: oxidoreductase activity, acting on paired donors, with incorporation or reduction of molecular oxygen: 3.065e-06 (12,26,4) OR
 GO:0010181: FMN binding: 4.747e-02 (12,11,1) OR
 GO:0016651: oxidoreductase activity, acting on NADH or NADPH: 5.588e-02 (12,13,1)

IF 1e9xa_#343 AND 1bccb2#274
THEN
 GO:0004497: monooxygenase activity: 3.621e-10 (12,26,6) OR
 GO:0016705: oxidoreductase activity, acting on paired donors, with incorporation or reduction of molecular oxygen: 3.065e-06 (12,26,4) OR
 GO:0010181: FMN binding: 4.747e-02 (12,11,1) OR
 GO:0016651: oxidoreductase activity, acting on NADH or NADPH: 5.588e-02 (12,13,1)

IF 1bccb2#274 AND 1dz4a_#357
THEN
 GO:0004497: monooxygenase activity: 3.621e-10 (12,26,6) OR
 GO:0016705: oxidoreductase activity, acting on paired donors, with incorporation or reduction of molecular oxygen: 3.065e-06 (12,26,4) OR
 GO:0010181: FMN binding: 4.747e-02 (12,11,1) OR
 GO:0016651: oxidoreductase activity, acting on NADH or NADPH: 5.588e-02 (12,13,1)

IF 1ddja_#640 AND 1ekbb_#79
THEN
 GO:0004263: chymotrypsin activity: 2.478e-10 (31,41,9) OR
 GO:0004295: trypsin activity: 1.126e-09 (31,48,9) OR
 GO:0005509: calcium ion binding: 2.930e-04 (31,160,8) OR
 GO:0003809: thrombin activity: 5.326e-03 (31,10,2) OR
 GO:0004867: serine-type endopeptidase inhibitor activity: 9.874e-02 (31,47,2) OR
 GO:0004896: hematopoietin/interferon-class (D200-domain) cytokine receptor activity: 1.960e-01 (31,19,1)

IF 1gg6.1#C156 AND 1dpga2#334 AND 1befa_#51 AND 1ton__#196
THEN
 GO:0004263: chymotrypsin activity: 2.478e-10 (31,41,9) OR
 GO:0004295: trypsin activity: 1.126e-09 (31,48,9) OR
 GO:0004867: serine-type endopeptidase inhibitor activity: 1.738e-03 (31,47,4) OR
 GO:0003809: thrombin activity: 5.326e-03 (31,10,2) OR
 GO:0005509: calcium ion binding: 8.054e-03 (31,160,6) OR
 GO:0005529: sugar binding: 3.620e-01 (31,39,1)

IF 1bqya_#216 AND 1pwt__#8 AND 3sil__#98 AND 1elva1#604
THEN
 GO:0004263: chymotrypsin activity: 2.486e-10 (43,41,10) OR
 GO:0004295: trypsin activity: 1.341e-09 (43,48,10) OR
 GO:0003809: thrombin activity: 1.834e-07 (43,10,5) OR
 GO:0004867: serine-type endopeptidase inhibitor activity: 5.780e-06 (43,47,7) OR
 GO:0005509: calcium ion binding: 1.306e-04 (43,160,10) OR
 GO:0004896: hematopoietin/interferon-class (D200-domain) cytokine receptor activity: 2.616e-01 (43,19,1)

IF 1bfd_1#230 AND 1cjca2#103
THEN
 GO:0015036: disulfide oxidoreductase activity: 3.805e-10 (14,22,6) OR
 GO:0016668: oxidoreductase activity, acting on sulfur group of donors, NAD or NADP as acceptor: 2.111e-07 (14,12,4) OR
 GO:0050660: FAD binding: 1.269e-05 (14,10,3) OR
 GO:0016627: oxidoreductase activity, acting on the CH-CH group of donors: 8.408e-02 (14,17,1)

IF 1f97a1#62 AND 1fcda2#189 AND 1cjca2#103
THEN
 GO:0015036: disulfide oxidoreductase activity: 3.805e-10 (14,22,6) OR
 GO:0016668: oxidoreductase activity, acting on sulfur group of donors, NAD or NADP as acceptor: 2.111e-07 (14,12,4) OR
 GO:0050660: FAD binding: 1.269e-05 (14,10,3) OR
 GO:0016627: oxidoreductase activity, acting on the CH-CH group of donors: 8.408e-02 (14,17,1)

IF 1jmta_#93 AND 1h6kx_#97
THEN
 GO:0003729: mRNA binding: 2.568e-10 (10,13,5) OR
 GO:0003809: thrombin activity: 3.616e-02 (10,10,1) OR
 GO:0005529: sugar binding: 1.345e-01 (10,39,1) OR
 GO:0004263: chymotrypsin activity: 1.409e-01 (10,41,1) OR

GO:0004295: trypsin activity: 1.631e-01 (10,48,1) OR
GO:0005509: calcium ion binding: 4.545e-01 (10,160,1)

IF 1e05i_#79 AND 1ejda_#6
THEN
GO:0004867: serine-type endopeptidase inhibitor activity: 5.180e-10 (8,47,6) OR
GO:0003887: DNA-directed DNA polymerase activity: 5.730e-02 (8,20,1) OR
GO:0008201: heparin binding: 6.841e-02 (8,24,1)

IF 1fc4a_#111 AND 1qfea_#68
THEN
GO:0008483: transaminase activity: 2.607e-10 (17,17,6) OR
GO:0016846: carbon-sulfur lyase activity: 1.227e-09 (17,10,5) OR
GO:0016831: carboxy-lyase activity: 1.010e-02 (17,25,2) OR
GO:0004812: tRNA ligase activity: 1.508e-01 (17,26,1) OR
GO:0004497: monooxygenase activity: 1.508e-01 (17,26,1) OR
GO:0005524: ATP binding: 4.560e-01 (17,243,2)

IF 2occb1#189 AND 1cf1a1#33
THEN
GO:0005507: copper ion binding: 3.977e-10 (9,38,6) OR
GO:0015082: di-, tri-valent inorganic cation transporter activity: 4.536e-02 (9,14,1) OR
GO:0046915: transition metal ion transporter activity: 4.536e-02 (9,14,1) OR
GO:0015078: hydrogen ion transporter activity: 6.735e-02 (9,21,1)

IF 1a65a3#464 AND 1a65a3#383
THEN
GO:0005507: copper ion binding: 3.977e-10 (9,38,6) OR
GO:0003714: transcription corepressor activity: 2.615e-02 (9,8,1) OR
GO:0015082: di-, tri-valent inorganic cation transporter activity: 4.536e-02 (9,14,1) OR
GO:0046915: transition metal ion transporter activity: 4.536e-02 (9,14,1)

IF 1cf1a1#33 AND 2cuaa_#107 AND 1e30a_#87
THEN
GO:0005507: copper ion binding: 3.977e-10 (9,38,6) OR
GO:0015082: di-, tri-valent inorganic cation transporter activity: 4.536e-02 (9,14,1) OR
GO:0046915: transition metal ion transporter activity: 4.536e-02 (9,14,1) OR
GO:0015078: hydrogen ion transporter activity: 6.735e-02 (9,21,1)

IF 1plc__#74 AND 1kdj__#27
THEN
GO:0005507: copper ion binding: 3.977e-10 (9,38,6) OR
GO:0016638: oxidoreductase activity, acting on the CH-NH2 group of donors: 5.484e-02 (9,17,1) OR
GO:0015078: hydrogen ion transporter activity: 6.735e-02 (9,21,1) OR
GO:0005509: calcium ion binding: 4.204e-01 (9,160,1)

IF 1quqa_#75 AND 1e30a_#87
THEN
GO:0005507: copper ion binding: 3.977e-10 (9,38,6) OR
GO:0015082: di-, tri-valent inorganic cation transporter activity: 4.536e-02 (9,14,1) OR
GO:0046915: transition metal ion transporter activity: 4.536e-02 (9,14,1) OR
GO:0015078: hydrogen ion transporter activity: 6.735e-02 (9,21,1)

IF 1efpa1#32 AND 1hyha2#204
THEN
GO:0004457: lactate dehydrogenase activity: 3.988e-10 (14,10,5) OR
GO:0016616: oxidoreductase activity, acting on the CH-OH group of donors, NAD or NADP as acceptor: 4.736e-09 (14,59,7) OR
GO:0030145: manganese ion binding: 1.789e-01 (14,38,1) OR
GO:0005524: ATP binding: 7.304e-01 (14,243,1)

IF 1qsta_#154 AND 1quna1#113
THEN
GO:0004812: tRNA ligase activity: 3.195e-10 (21,26,7) OR
GO:0005524: ATP binding: 1.606e-03 (21,243,7) OR
GO:0000049: tRNA binding: 4.193e-03 (21,13,2) OR
GO:0008080: N-acetyltransferase activity: 4.193e-03 (21,13,2) OR
GO:0000287: magnesium ion binding: 7.259e-02 (21,128,3)

IF 1gg6.1#C156 AND 1evqa_#61
THEN

GO:0004295: trypsin activity: 2.759e-10 (27,48,9) OR
GO:0004263: chymotrypsin activity: 2.312e-09 (27,41,8) OR
GO:0004867: serine-type endopeptidase inhibitor activity: 1.020e-03 (27,47,4) OR
GO:0005509: calcium ion binding: 6.994e-02 (27,160,4) OR
GO:0003809: thrombin activity: 9.493e-02 (27,10,1) OR
GO:0005529: sugar binding: 3.237e-01 (27,39,1)

IF 1fjsa_#163 AND 1evqa_#61 AND 2hlca_#68
THEN
GO:0004295: trypsin activity: 2.759e-10 (27,48,9) OR
GO:0004263: chymotrypsin activity: 2.312e-09 (27,41,8) OR
GO:0004867: serine-type endopeptidase inhibitor activity: 1.020e-03 (27,47,4) OR
GO:0005509: calcium ion binding: 6.994e-02 (27,160,4) OR
GO:0003809: thrombin activity: 9.493e-02 (27,10,1) OR
GO:0005529: sugar binding: 3.237e-01 (27,39,1)

IF 1leha1#177 AND 1e3ja2#270
THEN
GO:0016616: oxidoreductase activity, acting on the CH-OH group of donors, NAD or NADP as acceptor: 3.342e-10 (16,59,8) OR
GO:0004457: lactate dehydrogenase activity: 1.945e-05 (16,10,3) OR
GO:0016638: oxidoreductase activity, acting on the CH-NH2 group of donors: 1.075e-04 (16,17,3) OR
GO:0016814: hydrolase activity, acting on carbon-nitrogen (but not peptide) bonds, in cyclic amidines: 5.728e-02 (16,10,1) OR
GO:0016646: oxidoreductase activity, acting on the CH-NH group of donors, NAD or NADP as acceptor: 1.062e-01 (16,19,1)
