# Supplementary material for: A Comprehensive Analysis of the Structure-Function Relationship in Proteins Based on Local Structure Similarity
Source: PLoS One. 2009 Jul 15;4(7):e6266. doi: 10.1371/journal.pone.0006266 (PMC2705683; doi:10.1371/journal.pone.0006266)
Supplement: Table S3 — Prediction performance. (a) Prediction performance. 10-fold cross-validation AUC estimates for all molecular function classes (b) Prediction performance. 10-fold cross-validation AUC estimates for all biological process classes (c) Prediction performance. 10-fold cross-validation AUC estimates for all cellular component classes (0.04 MB PDF) [file pone.0006266.s003.pdf]

Tab S3 (a) Prediction performance. 10-fold cross-validation AUC estimates for all molecular function classes

MOLECULAR FUNCTION	AUC	SE	THR.	SENS	SPEC	TP	FP	TN	FN
GO:0004556: alpha-amylase activity	1.0000	0.00	1.00e-308	1.0000	1.0000	15	0	1683	0
GO:0004364: glutathione transferase activity	0.9997	0.00	2.00E-301	1.0000	0.9994	11	1	1686	0
GO:0003809: thrombin activity	0.9983	0.01	2.00E-74	1.0000	0.9899	10	17	1671	0
GO:0016846: carbon-sulfur lyase activity	0.9939	0.02	1.00E-234	0.8000	0.9976	8	4	1684	2
GO:0015036: disulfide oxidoreductase activity	0.9892	0.02	2.00E-07	0.8636	0.9916	19	14	1662	3
GO:0003887: DNA-directed DNA polymerase activity	0.9838	0.02	3.00E-08	0.7000	0.9881	14	20	1658	6
GO:0004812: tRNA ligase activity	0.9829	0.02	2.00E-11	0.8846	0.9976	23	4	1668	3
GO:0008235: metalloexopeptidase activity	0.9795	0.03	7.00E-14	0.8462	0.9988	11	2	1683	2
GO:0016627: oxidoreductase activity, acting on the CH-CH group of donors	0.9715	0.03	2.00E-18	0.8235	0.9786	14	36	1645	3
GO:0008483: transaminase activity	0.9598	0.03	3.00E-09	0.8824	0.9917	15	14	1667	2
GO:0019201: nucleotide kinase activity	0.9533	0.04	2.00E-25	0.9231	0.9958	12	7	1678	1
GO:0008009: chemokine activity	0.9472	0.05	2.00E-73	0.9000	0.9988	9	2	1686	1
GO:0030151: molybdenum ion binding	0.9457	0.04	2.00E-14	0.6000	0.9947	9	9	1674	6
GO:0050660: FAD binding	0.9416	0.05	5.00E-77	0.9000	0.9917	9	14	1674	1
GO:0008800: beta-lactamase activity	0.9414	0.05	4.00E-12	0.9000	0.9959	9	7	1681	1
GO:0004295: trypsin activity	0.9406	0.02	3.00E-23	0.8958	0.9994	43	1	1649	5
GO:0004457: lactate dehydrogenase activity	0.9391	0.05	1.00e-308	0.9000	0.9994	9	1	1687	1
GO:0004263: chymotrypsin activity	0.9320	0.03	2.00E-267	0.8780	0.9994	36	1	1656	5
GO:0004197: cysteine-type endopeptidase activity	0.9317	0.04	4.00E-15	0.7917	0.9994	19	1	1673	5
GO:0016763: transferase activity, transferring pentosyl groups	0.9275	0.03	2.00E-05	0.8214	0.9695	23	51	1619	5
GO:0005066: transmembrane receptor protein tyrosine kinase signaling protein activity	0.9246	0.06	9.00E-07	0.8000	0.9816	8	31	1657	2
GO:0005507: copper ion binding	0.9151	0.03	2.00E-09	0.7105	0.9813	27	31	1629	11
GO:0016776: phosphotransferase activity, phosphate group as acceptor	0.9133	0.05	2.00E-61	0.8571	0.9976	12	4	1680	2
GO:0016616: oxidoreductase activity, acting on the CH-OH group of donors, NAD or NADP as acceptor	0.9109	0.03	2.00E-07	0.8136	0.9585	48	68	1571	11
GO:0016638: oxidoreductase activity, acting on the CH-NH2 group of donors	0.9095	0.05	7.00E-12	0.5882	0.9875	10	21	1660	7
GO:0004180: carboxypeptidase activity	0.9047	0.05	7.00E-04	0.8000	0.9750	12	42	1641	3
GO:0016620: oxidoreductase activity, acting on the aldehyde or oxo group of donors, NAD or NADP as acceptor	0.9043	0.06	6.00E-59	0.4000	1.0000	4	0	1688	6
GO:0016668: oxidoreductase activity, acting on sulfur group of donors, NAD or NADP as acceptor	0.9016	0.06	3.00E-184	0.8333	0.9953	10	8	1678	2
GO:0004725: protein tyrosine phosphatase activity	0.8972	0.05	7.00E-16	0.6000	0.9988	9	2	1681	6
GO:0005525: GTP binding	0.8901	0.03	3.00E-10	0.6735	0.9879	33	20	1629	16
GO:0009036: type II site-specific deoxyribonuclease activity	0.8897	0.06	2.00E-07	0.6667	0.9941	8	10	1676	4
GO:0004177: aminopeptidase activity	0.8879	0.06	2.00E-12	0.3077	0.9941	4	10	1675	9
GO:0004896: hematopoietin/interferon-class (D200-domain) cytokine receptor activity	0.8871	0.05	2.00E-03	0.7368	0.9696	14	51	1628	5
GO:0008757: S-adenosylmethionine-dependent methyltransferase activity	0.8871	0.04	2.00E-09	0.5417	0.9916	13	14	1660	11
GO:0008199: ferric iron binding	0.8836	0.07	7.00E-09	0.7000	0.9941	7	10	1678	3
GO:0003755: peptidyl-prolyl cis-trans isomerase activity	0.8831	0.07	3.00E-08	0.6364	0.9899	7	17	1670	4
GO:0016646: oxidoreductase activity, acting on the CH-NH group of donors, NAD or NADP as acceptor	0.8796	0.05	3.00E-06	0.6316	0.9750	12	42	1637	7
GO:0003682: chromatin binding	0.8789	0.07	7.00E-05	0.6000	0.9893	6	18	1670	4
GO:0016861: intramolecular oxidoreductase activity, interconverting aldoses and ketoses	0.8765	0.06	3.00E-13	0.6154	0.9822	8	30	1655	5
GO:0019955: cytokine binding	0.8713	0.07	7.00E-06	0.3636	0.9852	4	25	1662	7
GO:0005529: sugar binding	0.8699	0.04	6.00E-06	0.6923	0.9753	27	41	1618	12

GO:0019838: growth factor binding	0.8629	0.08	9.00E-04	0.4444	0.9846	4	26	1663	5
GO:0003968: RNA-directed RNA polymerase activity	0.8604	0.06	2.00E-09	0.5000	0.9964	7	6	1678	7
GO:0004601: peroxidase activity	0.8380	0.05	6.00E-05	0.3810	0.9821	8	30	1647	13
GO:0004867: serine-type endopeptidase inhibitor activity	0.8377	0.04	6.00E-06	0.7021	0.9752	33	41	1610	14
GO:0016651: oxidoreductase activity, acting on NADH or NADPH	0.8298	0.07	6.00E-88	0.1538	0.9976	2	4	1681	11
GO:0008408: 3'-5' exonuclease activity	0.8297	0.07	4.00E-08	0.6154	0.9869	8	22	1663	5
GO:0005126: hematopoietin/interferon-class (D200-domain) cytokine receptor binding	0.8226	0.06	6.00E-04	0.5500	0.9821	11	30	1648	9
GO:0003729: mRNA binding	0.8205	0.07	2.00E-12	0.6923	0.9988	9	2	1683	4
GO:0010181: FMN binding	0.8163	0.08	7.00E-06	0.5455	0.9905	6	16	1671	5
GO:0004190: aspartic-type endopeptidase activity	0.8089	0.05	2.00E-19	0.5652	0.9988	13	2	1673	10
GO:0004222: metalloendopeptidase activity	0.8074	0.06	2.00E-10	0.5789	0.9964	11	6	1673	8
GO:0003924: GTPase activity	0.8061	0.06	3.00E-04	0.6471	0.9774	11	38	1643	6
GO:0001584: rhodopsin-like receptor activity	0.8045	0.08	4.00E-05	0.4000	0.9899	4	17	1671	6
GO:0016831: carboxy-lyase activity	0.7974	0.05	6.00E-20	0.4400	0.9845	11	26	1647	14
GO:0000287: magnesium ion binding	0.7954	0.02	2.00E-05	0.4453	0.9019	57	154	1416	71
GO:0003964: RNA-directed DNA polymerase activity	0.7951	0.08	1.18E-01	0.6364	0.9692	7	52	1635	4
GO:0003724: RNA helicase activity	0.7917	0.08	3.00E-10	0.4545	0.9964	5	6	1681	6
GO:0015082: di-, tri-valent inorganic cation transporter activity	0.7803	0.07	2.00E-09	0.2857	0.9887	4	19	1665	10
GO:0046915: transition metal ion transporter activity	0.7803	0.07	2.00E-09	0.2857	0.9887	4	19	1665	10
GO:0015078: hydrogen ion transporter activity	0.7774	0.06	2.00E-13	0.3810	0.9899	8	17	1660	13
GO:0016758: transferase activity, transferring hexosyl groups	0.7770	0.08	0.00E+00	0.0000	1.0000	0	0	1687	11
GO:0004523: ribonuclease H activity	0.7766	0.07	3.00E-04	0.4375	0.9905	7	16	1666	9
GO:0016814: hydrolase activity, acting on carbon-nitrogen (but not peptide) bonds, in cyclic amidines	0.7766	0.09	0.00E+00	0.0000	1.0000	0	0	1688	10
GO:0016836: hydro-lyase activity	0.7730	0.05	2.00E-04	0.5758	0.9441	19	93	1572	14
GO:0008080: N-acetyltransferase activity	0.7699	0.08	3.00E-12	0.5385	0.9982	7	3	1682	6
GO:0005524: ATP binding	0.7656	0.02	9.00E-04	0.5350	0.9141	130	125	1330	113
GO:0016854: racemase and epimerase activity	0.7655	0.08	7.00E-45	0.1538	0.9899	2	17	1668	11
GO:0000049: tRNA binding	0.7597	0.08	2.00E-11	0.2308	0.9923	3	13	1672	10
GO:0005518: collagen binding	0.7570	0.08	7.00E-10	0.4615	0.9923	6	13	1672	7
GO:0005509: calcium ion binding	0.7547	0.02	1.33E-01	0.6188	0.9148	99	131	1407	61
GO:0004842: ubiquitin-protein ligase activity	0.7537	0.06	6.00E-06	0.4737	0.9917	9	14	1665	10
GO:0008083: growth factor activity	0.7495	0.04	6.20E-02	0.5714	0.9390	24	101	1555	18
GO:0004497: monooxygenase activity	0.7429	0.06	4.00E-13	0.4615	0.9952	12	8	1664	14
GO:0003916: DNA topoisomerase activity	0.7406	0.09	5.00E-07	0.5000	0.9917	5	14	1674	5
GO:0008026: ATP-dependent helicase activity	0.7372	0.08	2.00E-11	0.1538	0.9976	2	4	1681	11
GO:0005516: calmodulin binding	0.7345	0.06	1.50E-02	0.5000	0.9707	12	49	1625	12
GO:0004714: transmembrane receptor protein tyrosine kinase activity	0.7237	0.08	3.00E-56	0.0714	0.9964	1	6	1678	13
GO:0005351: sugar porter activity	0.7180	0.06	5.00E-12	0.3333	0.9940	7	10	1667	14
GO:0008810: cellulase activity	0.7130	0.07	7.00E-25	0.5000	0.9958	9	7	1673	9
GO:0016799: hydrolase activity, hydrolyzing N-glycosyl compounds	0.7091	0.07	4.00E-17	0.4118	0.9976	7	4	1677	10
GO:0003697: single-stranded DNA binding	0.7022	0.08	4.00E-18	0.3333	0.9970	4	5	1681	8
GO:0019843: rRNA binding	0.6910	0.06	5.00E-37	0.0909	0.9988	2	2	1674	20
GO:0046983: protein dimerization activity	0.6856	0.07	3.00E-13	0.1765	0.9935	3	11	1670	14
GO:0003779: actin binding	0.6771	0.05	9.00E-06	0.1875	0.9838	6	27	1639	26
GO:0004674: protein serine/threonine kinase activity	0.6755	0.05	4.00E-18	0.3810	0.9976	16	4	1652	26
GO:0008270: zinc ion binding	0.6737	0.03	1.90E-02	0.4907	0.9094	53	144	1446	55
GO:0005085: guanyl-nucleotide exchange factor activity	0.6702	0.08	7.00E-11	0.1538	0.9935	2	11	1674	11
GO:0003700: transcription factor activity	0.6696	0.03	5.00E-03	0.4435	0.9104	55	141	1433	69
GO:0051082: unfolded protein binding	0.6637	0.05	7.00E-08	0.2647	0.9802	9	33	1631	25
GO:0000155: two-component sensor molecule activity	0.6522	0.09	9.00E-10	0.2500	0.9947	3	9	1677	9

GO:0016705: oxidoreductase activity, acting on paired donors, with incorporation or reduction of molecular oxygen	0.6457	0.06	2.00E-09	0.2692	0.9916	7	14	1658	19
GO:0030145: manganese ion binding	0.6413	0.05	2.00E-24	0.0789	0.9988	3	2	1658	35
GO:0004620: phospholipase activity	0.6385	0.07	3.00E-19	0.0526	1.0000	1	0	1679	18
GO:0008201: heparin binding	0.6384	0.06	2.00E-10	0.2083	0.9833	5	28	1646	19
GO:0016251: general RNA polymerase II transcription factor activity	0.6354	0.08	4.00E-17	0.2857	0.9988	4	2	1682	10
GO:0016702: oxidoreductase activity, acting on single donors with incorporation of molecular oxygen, incorporation of two atoms of oxygen	0.6194	0.09	2.00E-06	0.2500	0.9917	3	14	1672	9
GO:0042802: protein self binding	0.6192	0.09	4.00E-52	0.2500	0.9982	3	3	1683	9
GO:0005543: phospholipid binding	0.6121	0.08	2.00E-10	0.0714	0.9952	1	8	1676	13
GO:0016866: intramolecular transferase activity	0.6055	0.09	0.00E+00	0.0000	1.0000	0	0	1686	12
GO:0005096: GTPase activator activity	0.6033	0.08	3.00E-14	0.1333	0.9982	2	3	1680	13
GO:0008081: phosphoric diester hydrolase activity	0.5963	0.09	6.00E-06	0.2727	0.9947	3	9	1678	8
GO:0019829: cation-transporting ATPase activity	0.5894	0.10	2.00E-09	0.2222	0.9988	2	2	1687	7
GO:0003713: transcription coactivator activity	0.5828	0.08	5.00E-10	0.1250	0.9946	2	9	1673	14
GO:0008200: ion channel inhibitor activity	0.5754	0.07	5.00E-22	0.1176	0.9976	2	4	1677	15
GO:0003899: DNA-directed RNA polymerase activity	0.5751	0.09	2.00E-14	0.1667	0.9988	2	2	1684	10
GO:0016811: hydrolase activity, acting on carbon-nitrogen (but not peptide) bonds, in linear amides	0.5513	0.08	4.00E-08	0.1333	0.9982	2	3	1680	13
GO:0003743: translation initiation factor activity	0.5497	0.08	9.00E-08	0.1333	0.9976	2	4	1679	13
GO:0015405: P-P-bond-hydrolysis-driven transporter activity	0.5414	0.09	3.00E-09	0.1667	0.9982	2	3	1683	10
GO:0005179: hormone activity	0.5333	0.08	2.00E-07	0.1333	0.9970	2	5	1678	13
GO:0003684: damaged DNA binding	0.4776	0.09	0.00E+00	0.0000	1.0000	0	0	1688	10
GO:0003714: transcription corepressor activity	0.4509	0.10	0.00E+00	0.0000	1.0000	0	0	1690	8
GO:0005261: cation channel activity	0.4206	0.09	0.00E+00	0.0000	1.0000	0	0	1688	10
**AVERAGE/SUM**	**0.7854**	**0.06**	**0.00317**	**0.4775**	**0.9872**	**1386**	**2364**		**1339**

Tab S3 (b) Prediction performance. 10-fold cross-validation AUC estimates for all biological process classes

BIOLOGICAL PROCESS	AUC	SE	THR.	SENS	SPEC	TP	FP	TN	FN
GO:0030333: antigen processing	0.9995	0.00	2.00E-69	1.0000	0.9953	13	7	1477	0
GO:0017001: antibiotic catabolism	0.9982	0.01	4.00E-10	1.0000	0.9939	10	9	1478	0
GO:0006207: 'de novo' pyrimidine base biosynthesis	0.9970	0.01	8.00E-62	0.9000	0.9953	9	7	1480	1
GO:0042088: T-helper 1 type immune response	0.9770	0.03	5.00E-04	0.9091	0.9643	10	53	1433	1
GO:0042401: biogenic amine biosynthesis	0.9506	0.05	9.00E-31	0.6000	0.9892	6	16	1471	4
GO:0006100: tricarboxylic acid cycle intermediate metabolism	0.9477	0.04	8.00E-43	0.8571	1.0000	12	0	1483	2
GO:0043094: metabolic compound salvage	0.9474	0.05	9.00E-34	0.7273	0.9939	8	9	1477	3
GO:0006568: tryptophan metabolism	0.9467	0.05	4.00E-24	0.6000	0.9872	6	19	1468	4
GO:0006098: pentose-phosphate shunt	0.9462	0.05	2.00E-13	0.8182	0.9711	9	43	1443	2
GO:0015976: carbon utilization	0.9410	0.05	2.00E-27	0.7000	0.9980	7	3	1484	3
GO:0030193: regulation of blood coagulation	0.9349	0.05	1.00E-171	0.9000	0.9973	9	4	1483	1
GO:0050819: negative regulation of coagulation	0.9349	0.05	1.00E-171	0.9000	0.9973	9	4	1483	1
GO:0019883: antigen presentation, endogenous antigen	0.9293	0.06	5.00E-261	0.9000	1.0000	9	0	1487	1
GO:0006418: tRNA aminoacylation for protein translation	0.9283	0.04	2.00E-17	0.8077	0.9912	21	13	1458	5
GO:0030217: T-cell differentiation	0.9225	0.06	2.00E-05	0.9000	0.9711	9	43	1444	1
GO:0015671: oxygen transport	0.9208	0.04	2.00E-18	0.6087	0.9919	14	12	1462	9
GO:0042108: positive regulation of cytokine biosynthesis	0.9206	0.06	5.00E-04	0.8182	0.9637	9	54	1432	2
GO:0006563: L-serine metabolism	0.9184	0.05	3.00E-27	0.6667	0.9832	8	25	1460	4
GO:0009103: lipopolysaccharide biosynthesis	0.9174	0.06	2.00E-16	0.6667	0.9892	8	16	1469	4
GO:0006096: glycolysis	0.9134	0.03	6.00E-09	0.6579	0.9356	25	94	1365	13
GO:0009070: serine family amino acid biosynthesis	0.9105	0.04	2.00E-22	0.6923	0.9871	18	19	1452	8
GO:0006278: RNA-dependent DNA replication	0.9046	0.06	7.00E-15	0.4000	0.9966	4	5	1482	6
GO:0006979: response to oxidative stress	0.8897	0.05	1.00E-06	0.5000	0.9831	9	25	1454	9
GO:0009116: nucleoside metabolism	0.8810	0.04	6.00E-36	0.5200	0.9986	13	2	1470	12
GO:0006953: acute-phase response	0.8771	0.05	2.00E-23	0.5500	0.9932	11	10	1467	9
GO:0017148: negative regulation of protein biosynthesis	0.8740	0.06	1.00E-28	0.5385	1.0000	7	0	1484	6
GO:0001906: cell killing	0.8739	0.07	4.00E-12	0.3636	0.9899	4	15	1471	7
GO:0051249: regulation of lymphocyte activation	0.8714	0.07	3.40E-02	0.6667	0.9576	8	63	1422	4
GO:0006544: glycine metabolism	0.8680	0.07	1.00E-76	0.7273	0.9973	8	4	1482	3
GO:0006221: pyrimidine nucleotide biosynthesis	0.8663	0.05	4.00E-16	0.5000	0.9824	11	26	1449	11
GO:0009086: methionine biosynthesis	0.8560	0.06	9.00E-17	0.5714	0.9825	8	26	1457	6
GO:0006118: electron transport	0.8509	0.02	1.70E-02	0.7105	0.9046	162	121	1148	66
GO:0006606: protein-nucleus import	0.8477	0.05	8.00E-14	0.5238	0.9898	11	15	1461	10
GO:0006511: ubiquitin-dependent protein catabolism	0.8465	0.07	9.00E-22	0.6364	0.9966	7	5	1481	4
GO:0009073: aromatic amino acid family biosynthesis	0.8459	0.06	6.00E-35	0.3750	0.9905	6	14	1467	10
GO:0009307: DNA restriction	0.8449	0.06	5.00E-08	0.3684	0.9905	7	14	1464	12
GO:0000051: urea cycle intermediate metabolism	0.8382	0.08	7.00E-10	0.6000	0.9859	6	21	1466	4
GO:0006470: protein amino acid dephosphorylation	0.8376	0.06	4.00E-19	0.5500	1.0000	11	0	1477	9
GO:0009396: folic acid and derivative biosynthesis	0.8355	0.08	1.00E-26	0.5455	0.9939	6	9	1477	5
GO:0009219: pyrimidine deoxyribonucleotide metabolism	0.8339	0.08	6.00E-08	0.5000	0.9906	5	14	1473	5
GO:0006779: porphyrin biosynthesis	0.8325	0.06	0.00E+00	0.0000	1.0000	0	0	1479	18
GO:0009156: ribonucleoside monophosphate biosynthesis	0.8275	0.07	6.00E-30	0.2500	1.0000	3	0	1485	9
GO:0007264: small GTPase mediated signal transduction	0.8271	0.06	2.00E-06	0.5238	0.9797	11	30	1446	10
GO:0009167: purine ribonucleoside monophosphate metabolism	0.8252	0.08	1.00E-28	0.3000	1.0000	3	0	1487	7
GO:0006099: tricarboxylic acid cycle	0.8198	0.06	3.00E-17	0.5263	0.9878	10	18	1460	9
GO:0009064: glutamine family amino acid metabolism	0.8163	0.06	1.00E-08	0.6316	0.9709	12	43	1435	7
GO:0006633: fatty acid biosynthesis	0.8153	0.05	1.00E-05	0.5185	0.9721	14	41	1429	13
GO:0045595: regulation of cell differentiation	0.8146	0.08	0.00E+00	0.0000	1.0000	0	0	1487	10
GO:0016042: lipid catabolism	0.8105	0.05	9.00E-07	0.5161	0.9754	16	36	1430	15
GO:0042364: water-soluble vitamin biosynthesis	0.8100	0.05	9.00E-15	0.2963	0.9687	8	46	1424	19

GO:0043123: positive regulation of I-kappaB kinase/NF-kappaB cascade	0.8074	0.07	1.00E-09	0.5625	0.9905	9	14	1467	7
GO:0016337: cell-cell adhesion	0.8064	0.06	7.00E-12	0.2941	0.9804	5	29	1451	12
GO:0006956: complement activation	0.8050	0.08	2.00E-07	0.6667	0.9845	8	23	1462	4
GO:0046677: response to antibiotic	0.8033	0.04	9.00E-06	0.5000	0.9568	20	63	1394	20
GO:0009252: peptidoglycan biosynthesis	0.8017	0.06	1.00E-22	0.1111	0.9939	2	9	1470	16
GO:0006826: iron ion transport	0.7975	0.07	2.00E-07	0.4000	0.9811	6	28	1454	9
GO:0019079: viral genome replication	0.7968	0.07	5.00E-11	0.5000	0.9865	8	20	1461	8
GO:0007169: transmembrane receptor protein tyrosine kinase signaling pathway	0.7929	0.05	4.00E-07	0.4231	0.9660	11	50	1421	15
GO:0000160: two-component signal transduction system (phosphorelay)	0.7883	0.05	5.00E-20	0.4800	0.9939	12	9	1463	13
GO:0006879: iron ion homeostasis	0.7759	0.08	1.00E-07	0.5833	0.9751	7	37	1448	5
GO:0006313: DNA transposition	0.7704	0.05	3.00E-07	0.4000	0.9802	14	29	1433	21
GO:0030245: cellulose catabolism	0.7676	0.06	4.00E-44	0.4091	0.9980	9	3	1472	13
GO:0015074: DNA integration	0.7627	0.06	2.00E-09	0.3000	0.9905	6	14	1463	14
GO:0015698: inorganic anion transport	0.7624	0.07	1.00E-24	0.4286	0.9939	6	9	1474	8
GO:0006801: superoxide metabolism	0.7522	0.09	6.00E-11	0.6364	0.9812	7	28	1458	4
GO:0006275: regulation of DNA replication	0.7489	0.09	7.00E-13	0.4000	0.9946	4	8	1479	6
GO:0006730: one-carbon compound metabolism	0.7486	0.05	1.00E-09	0.4688	0.9782	15	32	1433	17
GO:0017000: antibiotic biosynthesis	0.7484	0.08	1.00E-15	0.3077	0.9933	4	10	1474	9
GO:0042559: pteridine and derivative biosynthesis	0.7457	0.09	1.00E-08	0.1818	0.9899	2	15	1471	9
GO:0007017: microtubule-based process	0.7401	0.08	2.00E-08	0.4167	0.9859	5	21	1464	7
GO:0006935: chemotaxis	0.7399	0.04	4.00E-10	0.4146	0.9863	17	20	1436	24
GO:0006919: caspase activation	0.7358	0.07	4.00E-50	0.4000	0.9946	6	8	1474	9
GO:0016477: cell migration	0.7306	0.06	1.00E-33	0.1304	0.9966	3	5	1469	20
GO:0006119: oxidative phosphorylation	0.7295	0.08	6.00E-19	0.3333	0.9980	4	3	1482	8
GO:0006265: DNA topological change	0.7247	0.09	2.00E-08	0.3000	0.9872	3	19	1468	7
GO:0016485: protein processing	0.7152	0.09	3.00E-08	0.2727	0.9872	3	19	1467	8
GO:0006643: membrane lipid metabolism	0.7063	0.08	3.00E-28	0.1538	1.0000	2	0	1484	11
GO:0006559: L-phenylalanine catabolism	0.7063	0.09	0.00E+00	0.0000	1.0000	0	0	1487	10
GO:0018108: peptidyl-tyrosine phosphorylation	0.7054	0.09	1.00E-157	0.5000	0.9966	5	5	1482	5
GO:0008285: negative regulation of cell proliferation	0.7050	0.07	0.00E+00	0.0000	1.0000	0	0	1478	19
GO:0019835: cytolysis	0.7029	0.07	3.00E-12	0.2222	0.9892	4	16	1463	14
GO:0006690: icosanoid metabolism	0.7018	0.09	2.00E-25	0.1818	1.0000	2	0	1486	9
GO:0006367: transcription initiation from Pol II promoter	0.7010	0.09	4.00E-44	0.5000	1.0000	6	0	1485	6
GO:0000398: nuclear mRNA splicing, via spliceosome	0.6966	0.09	9.00E-11	0.2500	0.9899	3	15	1470	9
GO:0009582: detection of abiotic stimulus	0.6770	0.09	7.00E-07	0.4000	0.9879	4	18	1469	6
GO:0006268: DNA unwinding	0.6763	0.09	6.00E-17	0.2000	0.9939	2	9	1478	8
GO:0009401: phosphoenolpyruvate-dependent sugar phosphotransferase system	0.6708	0.09	0.00E+00	0.0000	1.0000	0	0	1485	12
GO:0015992: proton transport	0.6696	0.09	2.00E-14	0.1667	0.9926	2	11	1474	10
GO:0045893: positive regulation of transcription, DNA-dependent	0.6684	0.09	3.00E-03	0.4000	0.9886	4	17	1470	6
GO:0051049: regulation of transport	0.6667	0.09	5.00E-17	0.2000	0.9987	2	2	1485	8
GO:0045045: secretory pathway	0.6637	0.06	1.00E-21	0.1818	0.9966	4	5	1470	18
GO:0016567: protein ubiquitination	0.6622	0.08	3.00E-09	0.2857	0.9933	4	10	1473	10
GO:0048514: blood vessel morphogenesis	0.6621	0.10	7.00E-08	0.2222	0.9966	2	5	1483	7
GO:0046148: pigment biosynthesis	0.6617	0.09	0.00E+00	0.0000	1.0000	0	0	1485	12
GO:0006457: protein folding	0.6556	0.04	2.00E-14	0.2449	0.9855	12	21	1427	37
GO:0008015: circulation	0.6553	0.08	0.00E+00	0.0000	1.0000	0	0	1482	15
GO:0008203: cholesterol metabolism	0.6544	0.09	0.00E+00	0.0000	1.0000	0	0	1486	11
GO:0000082: G1/S transition of mitotic cell cycle	0.6542	0.08	5.00E-07	0.1538	0.9879	2	18	1466	11
GO:0019735: antimicrobial humoral response (sensu Vertebrata)	0.6533	0.08	2.00E-11	0.2500	0.9899	4	15	1466	12
GO:0030435: sporulation	0.6496	0.06	7.00E-17	0.2609	0.9946	6	8	1466	17
GO:0008284: positive regulation of cell proliferation	0.6477	0.06	2.00E-05	0.2333	0.9782	7	32	1435	23

GO:0000086: G2/M transition of mitotic cell cycle	0.6349	0.10	1.00E-10	0.4000	0.9906	4	14	1473	6
GO:0016568: chromatin modification	0.6285	0.09	1.00E-04	0.4545	0.9865	5	20	1466	6
GO:0007186: G-protein coupled receptor protein signaling pathway	0.6279	0.06	4.00E-13	0.2500	0.9891	6	16	1457	18
GO:0007517: muscle development	0.6276	0.06	5.00E-06	0.3000	0.9680	9	47	1420	21
GO:0006445: regulation of translation	0.6208	0.08	3.00E-09	0.1333	0.9899	2	15	1467	13
GO:0000910: cytokinesis	0.6184	0.05	8.00E-09	0.1351	0.9822	5	26	1434	32
GO:0009615: response to virus	0.6165	0.09	7.00E-13	0.0833	0.9933	1	10	1475	11
GO:0006897: endocytosis	0.6091	0.07	7.00E-09	0.1579	0.9865	3	20	1458	16
GO:0007399: neurogenesis	0.6088	0.05	4.00E-03	0.1892	0.9568	7	63	1397	30
GO:0019059: initiation of viral infection	0.6013	0.10	4.00E-12	0.1000	0.9980	1	3	1484	9
GO:0045892: negative regulation of transcription, DNA-dependent	0.5976	0.08	0.00E+00	0.0000	1.0000	0	0	1484	13
GO:0045786: negative regulation of cell cycle	0.5915	0.08	0.00E+00	0.0000	1.0000	0	0	1484	13
GO:0006917: induction of apoptosis	0.5897	0.06	2.00E-05	0.3103	0.9768	9	34	1434	20
GO:0016068: type I hypersensitivity	0.5788	0.08	4.00E-18	0.0667	0.9980	1	3	1479	14
GO:0006986: response to unfolded protein	0.5733	0.08	1.00E-43	0.2000	1.0000	3	0	1482	12
GO:0006357: regulation of transcription from Pol II promoter	0.5707	0.05	0.00E+00	0.0000	1.0000	0	0	1467	30
GO:0006413: translational initiation	0.5685	0.07	7.00E-09	0.0625	0.9973	1	4	1477	15
GO:0000165: MAPKKK cascade	0.5683	0.09	1.00E-07	0.2727	0.9852	3	22	1464	8
GO:0016998: cell wall catabolism	0.5672	0.10	0.00E+00	0.0000	1.0000	0	0	1488	9
GO:0006461: protein complex assembly	0.5632	0.06	7.00E-41	0.0417	0.9993	1	1	1472	23
GO:0042113: B-cell activation	0.5626	0.10	0.00E+00	0.0000	1.0000	0	0	1488	9
GO:0007601: visual perception	0.5609	0.08	0.00E+00	0.0000	1.0000	0	0	1482	15
GO:0045859: regulation of protein kinase activity	0.5557	0.08	2.00E-07	0.2000	0.9838	3	24	1458	12
GO:0030036: actin cytoskeleton organization and biogenesis	0.5550	0.08	1.00E-11	0.1333	0.9960	2	6	1476	13
GO:0007067: mitosis	0.5350	0.06	6.00E-07	0.2000	0.9776	5	33	1439	20
GO:0006916: anti-apoptosis	0.5305	0.07	0.00E+00	0.0000	1.0000	0	0	1479	18
GO:0009206: purine ribonucleoside triphosphate biosynthesis	0.5303	0.10	0.00E+00	0.0000	1.0000	0	0	1488	9
GO:0009888: histogenesis	0.5167	0.09	0.00E+00	0.0000	1.0000	0	0	1487	10
GO:0007268: synaptic transmission	0.5147	0.06	7.00E-07	0.1429	0.9918	4	12	1457	24
GO:0042742: defense response to bacteria	0.5035	0.06	0.00E+00	0.0000	1.0000	0	0	1471	26
GO:0001501: skeletal development	0.5025	0.09	0.00E+00	0.0000	1.0000	0	0	1487	10
GO:0007276: gametogenesis	0.4896	0.09	0.00E+00	0.0000	1.0000	0	0	1486	11
GO:0006284: base-excision repair	0.4600	0.07	6.00E-04	0.1765	0.9818	3	27	1453	14
GO:0050832: defense response to fungi	0.4587	0.09	0.00E+00	0.0000	1.0000	0	0	1488	9
GO:0006354: RNA elongation	0.4482	0.09	0.00E+00	0.0000	1.0000	0	0	1487	10
GO:0001558: regulation of cell growth	0.4479	0.09	0.00E+00	0.0000	1.0000	0	0	1487	10
GO:0006805: xenobiotic metabolism	0.4289	0.08	1.00E-19	0.0769	0.9960	1	6	1478	12
GO:0006869: lipid transport	0.3790	0.09	0.00E+00	0.0000	1.0000	0	0	1488	9
**AVERAGE/SUM**	**0.7342**	**0.07**	**4.30E-04**	**0.3579**	**0.9892**	**962**	**2191**		**1543**

Tab S3 (c) Prediction performance. 10-fold cross-validation AUC estimates for all cellular component classes

CELLULAR COMPONENT	AUC	SE	THR.	SENS	SPEC	TP	FP	TN	FN
GO:0030288: periplasmic space (sensu Gram-negative Bacteria)	0.8101	0.05	4.00E-05	0.5185	0.9463	14	28	493	13
GO:0005746: mitochondrial electron transport chain	0.7841	0.06	1.40E-02	0.7273	0.9030	16	51	475	6
GO:0005887: integral to plasma membrane	0.7803	0.03	2.00E-03	0.5362	0.9040	37	46	433	32
GO:0005681: spliceosome complex	0.7724	0.08	1.00E-02	0.5455	0.9646	6	19	518	5
GO:0005730: nucleolus	0.7643	0.08	5.00E-05	0.4615	0.9888	6	6	529	7
GO:0019867: outer membrane	0.7611	0.06	8.00E-17	0.5000	0.9771	12	12	512	12
GO:0009579: thylakoid	0.7531	0.08	2.00E-07	0.4167	0.9851	5	8	528	7
GO:0015629: actin cytoskeleton	0.7457	0.06	3.00E-03	0.4400	0.9312	11	36	487	14
GO:0030894: replisome	0.7215	0.08	2.00E-19	0.4000	0.9981	6	1	532	9
GO:0005618: cell wall	0.7158	0.05	7.00E-05	0.4390	0.9152	18	43	464	23
GO:0009507: chloroplast	0.7122	0.06	3.00E-26	0.2800	0.9904	7	5	518	18
GO:0005777: peroxisome	0.6968	0.08	3.00E-10	0.2857	0.9906	4	5	529	10
GO:0016591: DNA-directed RNA polymerase II, holoenzyme	0.6826	0.08	4.00E-09	0.4000	1.0000	6	0	533	9
GO:0005764: lysosome	0.6799	0.07	2.00E-04	0.2778	0.9755	5	13	517	13
GO:0030136: clathrin-coated vesicle	0.6752	0.08	1.00E-10	0.1538	0.9981	2	1	534	11
GO:0012505: endomembrane system	0.6477	0.07	2.00E-05	0.2222	0.9887	4	6	524	14
GO:0005840: ribosome	0.6467	0.05	4.00E-03	0.4324	0.9041	16	49	462	21
GO:0005783: endoplasmic reticulum	0.6224	0.05	6.00E-03	0.2424	0.9534	8	24	491	25
GO:0005794: Golgi apparatus	0.6167	0.06	6.20E-02	0.3571	0.9019	10	51	469	18
GO:0005759: mitochondrial matrix	0.5971	0.10	7.00E-04	0.2000	0.9424	2	31	507	8
GO:0005667: transcription factor complex	0.5953	0.06	4.00E-04	0.3200	0.9790	8	11	512	17
GO:0005905: coated pit	0.5858	0.09	7.00E-05	0.2727	0.9907	3	5	532	8
GO:0000151: ubiquitin ligase complex	0.5856	0.09	5.00E-04	0.4167	0.9813	5	10	526	7
GO:0005829: cytosol	0.5734	0.04	5.00E-03	0.2353	0.9135	12	43	454	39
GO:0000785: chromatin	0.5712	0.08	1.00E-06	0.1875	0.9944	3	3	529	13
GO:0045202: synapse	0.5268	0.08	1.01E-01	0.2000	0.9512	3	26	507	12
GO:0005625: soluble fraction	0.5111	0.06	5.00E-27	0.1786	1.0000	5	0	520	23
GO:0005938: cell cortex	0.5043	0.08	3.00E-03	0.0833	0.9907	1	5	531	11
GO:0005624: membrane fraction	0.5035	0.07	5.00E-04	0.0526	0.9584	1	22	507	18
GO:0000228: nuclear chromosome	0.4740	0.09	1.00E-03	0.0909	0.9870	1	7	530	10
**AVERAGE/SUM**	**0.6382**	**0.07**	**1.22E-02**	**0.2803**	**0.9721**	**201**	**433**		**469**
