# Supplementary material for: A Comprehensive Analysis of the Structure-Function Relationship in Proteins Based on Local Structure Similarity
Source: PLoS One. 2009 Jul 15;4(7):e6266. doi: 10.1371/journal.pone.0006266 (PMC2705683; doi:10.1371/journal.pone.0006266)
Supplement: Table S4 — The overrepresentation of GO classes with significant AUC values. (a) Local substructures. The overrepresentation of GO classes with significant AUC values (AUC> = 0.7) and not significant values (AUC<0.7). P-values are calculated based on the number of proteins and the number of GO classes in each of the more general GO terms. (b) CATH folds. The overrepresentation of GO classes with significant AUC values (AUC> = 0.7) and not significant values (AUC<0.7). P-values are calculated based on the number of proteins and the number of GO classes in each of the more general GO terms. (0.01 MB PDF) [file pone.0006266.s004.pdf]

Tab S4 (a) Local substructures. The overrepresentation of GO classes with significant AUC values (AUC >= 0.7) and not significant values (AUC < 0.7). P-values are calculated based on the number of proteins and the number of GO classes in each of the more general GO terms.

*PARAMETERS refer to the parameters in the hypergeometric distribution used to compute the p-values: N,n,k,x, where N is the number of proteins/classes in the data set, n is the number of proteins/classes in the GO subtree, k is the number of proteins/classes that are unpredictable/predictable and x is the number of proteins/classes in the GO subtree that are unpredictable/predictable

	CALCULATIONS BASED ON PROTEINS			
**GO terms at the top level of molecular function**	**AUC<0.7**	**PARAMETERS***	**AUC>=0.7**	**PARAMETERS***
GO:0005215: transporter activity	7.884E-04	2725,118,740,48	9.996E-01	2725,118,1985,70
GO:0003824: catalytic activity	1.000E+00	2725,1119,740,170	2.461E-33	2725,1119,1985,949
GO:0016209: antioxidant activity	1.000E+00	2725,21,740,0	1.252E-03	2725,21,1985,21
GO:0005488: binding	2.261E-23	2725,1379,740,489	1.000E+00	2725,1379,1985,890
GO:0030234: enzyme regulator activity	3.297E-02	2725,75,740,28	9.816E-01	2725,75,1985,47
GO:0004871: signal transducer activity	9.980E-01	2725,152,740,27	3.734E-03	2725,152,1985,125
GO:0030528: transcription regulator activity	1.338E-98	2725,162,740,162	1.000E+00	2725,162,1985,0
GO:0045182: translation regulator activity	2.902E-09	2725,15,740,15	1.000E+00	2725,15,1985,0

	CALCULATIONS BASED ON CLASSES			
**GO terms at the top level of molecular function**	**AUC<0.7**	**PARAMETERS***	**AUC>=0.7**	**PARAMETERS***
GO:0005215: transporter activity	1.422E-01	113,8,31,4	9.654E-01	113,8,82,4
GO:0003824: catalytic activity	9.995E-01	113,63,31,10	1.962E-03	113,63,82,53
GO:0016209: antioxidant activity	1.000E+00	113,1,31,0	7.257E-01	113,1,82,1
GO:0005488: binding	2.668E-02	113,37,31,15	9.911E-01	113,37,82,22
GO:0030234: enzyme regulator activity	1.821E-01	113,3,31,2	9.808E-01	113,3,82,1
GO:0004871: signal transducer activity	7.658E-01	113,9,31,2	5.303E-01	113,9,82,7
GO:0030528: transcription regulator activity	4.887E-03	113,4,31,4	1.000E+00	113,4,82,0
GO:0045182: translation regulator activity	2.743E-01	113,1,31,1	1.000E+00	113,1,82,0

Tab S4 (b) CATH folds. The overrepresentation of GO classes with significant AUC values (AUC >= 0.7) and not significant values (AUC < 0.7). P-values are calculated based on the number of proteins and the number of GO classes in each of the more general GO terms.

*PARAMETERS refer to the parameters in the hypergeometric distribution used to compute the p-values: N,n,k,x, where N is the number of proteins/classes in the data set, n is the number of proteins/classes in the GO subtree, k is the number of proteins/classes that are unpredictable/predictable and x is the number of proteins/classes in the GO subtree that are unpredictable/predictable

	CALCULATIONS BASED ON PROTEINS			
**GO terms at the top level of molecular function**	**AUC<0.7**	**PARAMETERS***	**AUC>=0.7**	**PARAMETERS***
GO:0005215: transporter activity	2.336E-11	2641,110,521,52	1.000E+00	2641,110,2120,58
GO:0003824: catalytic activity	1.000E+00	2641,1095,521,149	9.634E-12	2641,1095,2120,946
GO:0016209: antioxidant activity	1.000E+00	2641,21,521,0	9.714E-03	2641,21,2120,21
GO:0005488: binding	1.149E-07	2641,1332,521,316	1.000E+00	2641,1332,2120,1016
GO:0030234: enzyme regulator activity	6.670E-01	2641,71,521,13	4.505E-01	2641,71,2120,58
GO:0004871: signal transducer activity	1.000E+00	2641,149,521,0	2.067E-15	2641,149,2120,149
GO:0030528: transcription regulator activity	9.664E-01	2641,152,521,22	5.436E-02	2641,152,2120,130
GO:0045182: translation regulator activity	2.265E-11	2641,15,521,15	1.000E+00	2641,15,2120,0

	CALCULATIONS BASED ON CLASSES			
**GO terms at the top level of molecular function**	**AUC<0.7**	**PARAMETERS***	**AUC>=0.7**	**PARAMETERS***
GO:0005215: transporter activity	8.045E-02	113,8,26,4	9.846E-01	113,8,87,4
GO:0003824: catalytic activity	9.877E-01	113,63,26,10	3.630E-02	113,63,87,53
GO:0016209: antioxidant activity	1.000E+00	113,1,26,0	7.699E-01	113,1,87,1
GO:0005488: binding	7.906E-02	113,37,26,12	9.694E-01	113,37,87,25
GO:0030234: enzyme regulator activity	5.473E-01	113,3,26,1	8.681E-01	113,3,87,2
GO:0004871: signal transducer activity	1.000E+00	113,9,26,0	8.591E-02	113,9,87,9
GO:0030528: transcription regulator activity	2.263E-01	113,4,26,2	9.625E-01	113,4,87,2
GO:0045182: translation regulator activity	2.301E-01	113,1,26,1	1.000E+00	113,1,87,0
