# Supplementary material for: A Comprehensive Analysis of the Structure-Function Relationship in Proteins Based on Local Structure Similarity
Source: PLoS One. 2009 Jul 15;4(7):e6266. doi: 10.1371/journal.pone.0006266 (PMC2705683; doi:10.1371/journal.pone.0006266)
Supplement: Table S5 — Protein disorder. (a) Local substructures. Protein disorder. Average disorder in the top level of Gene Ontology and correlation between predictive performance in terms of AUC cross validation and protein disorder. (b) CATH folds. Protein disorder. Average disorder in the top level of Gene Ontology and correlation between predictive performance in terms of AUC cross validation and protein disorder. (0.03 MB PDF) [file pone.0006266.s005.pdf]

Tab S5 (a) Local substructures. Protein disorder. Average disorder in the top level of Gene Ontology and correlation between predictive performance in terms of AUC cross validation and protein disorder.

DISORDER* refers to the average number of disordered amino acids in proteins from the GO class

			AUC<0.7		AUC>=0.7	
**GO terms at the top level of molecular function**		**NO. CLASSES**	**DISORDER***	**NO. CLASSES**	**DISORDER***	**NO. CLASSES**
GO:0005215	transporter activity	8	0.041	4	0.020	4
GO:0003824	catalytic activity	63	0.036	10	0.027	53
GO:0016209	antioxidant activity	1	0.000	0	0.012	1
GO:0005488	binding	37	0.050	15	0.036	22
GO:0030234	enzyme regulator activity	3	0.032	2	0.019	1
GO:0004871	signal transducer activity	9	0.065	2	0.040	7
GO:0030528	transcription regulator activity	4	0.061	4	0.000	0
GO:0045182	translation regulator activity	1	0.008	1	0.000	0

Correlation coefficient: -0.358

Significance: 9.923E-05

GO Class	GO term	AUC	NO. PROTS.	DISORDER*
GO:0003729	mRNA binding	0.821	13	0.102
GO:0046983	protein dimerization activity	0.686	17	0.098
GO:0003964	RNA-directed DNA polymerase activity	0.795	11	0.092
GO:0005261	cation channel activity	0.421	10	0.081
GO:0003713	transcription coactivator activity	0.583	16	0.081
GO:0003714	transcription corepressor activity	0.451	8	0.077
GO:0004523	ribonuclease H activity	0.777	16	0.071
GO:0019843	rRNA binding	0.691	22	0.070
GO:0003697	single-stranded DNA binding	0.702	12	0.068
GO:0005179	hormone activity	0.533	15	0.068
GO:0004725	protein tyrosine phosphatase activity	0.897	15	0.067
GO:0004714	transmembrane receptor protein tyrosine kinase activity	0.724	14	0.067
GO:0016846	carbon-sulfur lyase activity	0.994	10	0.066
GO:0008081	phosphoric diester hydrolase activity	0.596	11	0.066
GO:0000155	two-component sensor molecule activity	0.652	12	0.062
GO:0008026	ATP-dependent helicase activity	0.737	13	0.062
GO:0051082	unfolded protein binding	0.664	34	0.061
GO:0005126	hematopoietin/interferon-class (D200-domain) cytokine receptor binding	0.823	20	0.059
GO:0015078	hydrogen ion transporter activity	0.777	21	0.058
GO:0003700	transcription factor activity	0.670	124	0.057
GO:0004197	cysteine-type endopeptidase activity	0.932	24	0.056
GO:0042802	protein self binding	0.619	12	0.053
GO:0005516	calmodulin binding	0.735	24	0.052
GO:0003899	DNA-directed RNA polymerase activity	0.575	12	0.052
GO:0000049	tRNA binding	0.760	13	0.051
GO:0003724	RNA helicase activity	0.792	11	0.051
GO:0019838	growth factor binding	0.863	9	0.047
GO:0019955	cytokine binding	0.871	11	0.046
GO:0008083	growth factor activity	0.749	42	0.045
GO:0005524	ATP binding	0.766	243	0.045
GO:0008408	3'-5' exonuclease activity	0.830	13	0.044
GO:0005525	GTP binding	0.890	49	0.043

GO:0004896	hematopoietin/interferon-class (D200-domain) cytokine receptor activity	0.887	19	0.043
GO:0016758	transferase activity, transferring hexosyl groups	0.777	11	0.042
GO:0016836	hydro-lyase activity	0.773	33	0.042
GO:0005096	GTPase activator activity	0.603	15	0.040
GO:0004620	phospholipase activity	0.639	19	0.040
GO:0005507	copper ion binding	0.915	38	0.039
GO:0003916	DNA topoisomerase activity	0.741	10	0.039
GO:0030145	manganese ion binding	0.641	38	0.039
GO:0004812	tRNA ligase activity	0.983	26	0.038
GO:0005066	transmembrane receptor protein tyrosine kinase signaling protein activity	0.925	10	0.038
GO:0005543	phospholipid binding	0.612	14	0.038
GO:0003968	RNA-directed RNA polymerase activity	0.860	14	0.037
GO:0019829	cation-transporting ATPase activity	0.589	9	0.037
GO:0008757	S-adenosylmethionine-dependent methyltransferase activity	0.887	24	0.035
GO:0004177	aminopeptidase activity	0.888	13	0.034
GO:0016616	oxidoreductase activity, acting on the CH-OH group of donors, NAD or NADP as acceptor	0.911	59	0.034
GO:0016763	transferase activity, transferring pentosyl groups	0.928	28	0.034
GO:0003887	DNA-directed DNA polymerase activity	0.984	20	0.034
GO:0008080	N-acetyltransferase activity	0.770	13	0.034
GO:0016705	oxidoreductase activity, acting on paired donors, with incorporation or reduction of molecular oxygen	0.646	26	0.032
GO:0008201	heparin binding	0.638	24	0.031
GO:0000287	magnesium ion binding	0.795	128	0.031
GO:0004190	aspartic-type endopeptidase activity	0.809	23	0.031
GO:0016251	general RNA polymerase II transcription factor activity	0.635	14	0.030
GO:0008235	metalloexopeptidase activity	0.979	13	0.028
GO:0008199	ferric iron binding	0.884	10	0.028
GO:0001584	rhodopsin-like receptor activity	0.805	10	0.028
GO:0004674	protein serine/threonine kinase activity	0.675	42	0.028
GO:0004842	ubiquitin-protein ligase activity	0.754	19	0.028
GO:0016814	hydrolase activity, acting on carbon-nitrogen (but not peptide) bonds, in cyclic amidines	0.777	10	0.028
GO:0003924	GTPase activity	0.806	17	0.028
GO:0015405	P-P-bond-hydrolysis-driven transporter activity	0.541	12	0.027
GO:0009036	type II site-specific deoxyribonuclease activity	0.890	12	0.027
GO:0003684	damaged DNA binding	0.478	10	0.025
GO:0004222	metalloendopeptidase activity	0.807	19	0.025
GO:0008270	zinc ion binding	0.674	108	0.025
GO:0005085	guanyl-nucleotide exchange factor activity	0.670	13	0.024
GO:0016799	hydrolase activity, hydrolyzing N-glycosyl compounds	0.709	17	0.024
GO:0003682	chromatin binding	0.879	10	0.021
GO:0003779	actin binding	0.677	32	0.019
GO:0004867	serine-type endopeptidase inhibitor activity	0.838	47	0.019

GO:0003755	peptidyl-prolyl cis-trans isomerase activity	0.883	11	0.019
GO:0008200	ion channel inhibitor activity	0.575	17	0.019
GO:0005509	calcium ion binding	0.755	160	0.018
GO:0016702	oxidoreductase activity, acting on single donors with incorporation of molecular oxygen, incorporation of two atoms of oxygen	0.619	12	0.018
GO:0004556	alpha-amylase activity	1.000	15	0.018
GO:0016854	racemase and epimerase activity	0.766	13	0.017
GO:0004497	monooxygenase activity	0.743	26	0.016
GO:0016638	oxidoreductase activity, acting on the CH-NH2 group of donors	0.909	17	0.016
GO:0008483	transaminase activity	0.960	17	0.016
GO:0016831	carboxy-lyase activity	0.797	25	0.015
GO:0005518	collagen binding	0.757	13	0.015
GO:0016651	oxidoreductase activity, acting on NADH or NADPH	0.830	13	0.014
GO:0016811	hydrolase activity, acting on carbon-nitrogen (but not peptide) bonds, in linear amides	0.551	15	0.014
GO:0010181	FMN binding	0.816	11	0.014
GO:0003809	thrombin activity	0.998	10	0.013
GO:0016646	oxidoreductase activity, acting on the CH-NH group of donors, NAD or NADP as acceptor	0.880	19	0.013
GO:0004601	peroxidase activity	0.838	21	0.012
GO:0030151	molybdenum ion binding	0.946	15	0.012
GO:0004364	glutathione transferase activity	1.000	11	0.011
GO:0016866	intramolecular transferase activity	0.605	12	0.010
GO:0016668	oxidoreductase activity, acting on sulfur group of donors, NAD or NADP as acceptor	0.902	12	0.010
GO:0015036	disulfide oxidoreductase activity	0.989	22	0.009
GO:0016627	oxidoreductase activity, acting on the CH-CH group of donors	0.971	17	0.009
GO:0005351	sugar porter activity	0.718	21	0.009
GO:0005529	sugar binding	0.870	39	0.008
GO:0003743	translation initiation factor activity	0.550	15	0.008
GO:0016620	oxidoreductase activity, acting on the aldehyde or oxo group of donors, NAD or NADP as acceptor	0.904	10	0.008
GO:0015082	di-, tri-valent inorganic cation transporter activity	0.780	14	0.007
GO:0046915	transition metal ion transporter activity	0.780	14	0.007
GO:0004457	lactate dehydrogenase activity	0.939	10	0.007
GO:0019201	nucleotide kinase activity	0.953	13	0.006
GO:0004295	trypsin activity	0.941	48	0.006
GO:0004263	chymotrypsin activity	0.932	41	0.006
GO:0008810	cellulase activity	0.713	18	0.006
GO:0016776	phosphotransferase activity, phosphate group as acceptor	0.913	14	0.004
GO:0050660	FAD binding	0.942	10	0.004
GO:0008800	beta-lactamase activity	0.941	10	0.002
GO:0004180	carboxypeptidase activity	0.905	15	0.001
GO:0008009	chemokine activity	0.947	10	0.000
GO:0016861	intramolecular oxidoreductase activity, interconverting aldoses and ketoses	0.876	13	0.000

Tab S5 (b) CATH folds. Protein disorder. Average disorder in the top level of Gene Ontology and correlation between predictive performance in terms of AUC cross validation and protein disorder.

DISORDER* refer to the average number of disordered amino acids in proteins from the GO class

GO terms at the top level of molecular function		NO. CLASSES	AUC<0.7 DISORDER*	AUC<0.7 NO. CLASSES	AUC>=0.7 DISORDER*	AUC>=0.7 NO. CLASSES
GO:0005215	transporter activity	8	0.041	4	0.020	4
GO:0003824	catalytic activity	63	0.036	10	0.027	53
GO:0016209	antioxidant activity	1	0.000	0	0.012	1
GO:0005488	binding	37	0.050	15	0.036	22
GO:0030234	enzyme regulator activity	3	0.032	2	0.019	1
GO:0004871	signal transducer activity	9	0.065	2	0.040	7
GO:0030528	transcription regulator activity	4	0.061	4	0.000	0
GO:0045182	translation regulator activity	1	0.008	1	0.000	0

Correlation coefficient: -0.284

Significance: 2.297E-03

GO Class	GO term	AUC	NO. PROTS.	DISORDER*
GO:0003729	mRNA binding	0.818	13	0.102
GO:0046983	protein dimerization activity	0.697	17	0.098
GO:0003964	RNA-directed DNA polymerase activity	0.883	11	0.092
GO:0005179	hormone activity	0.773	12	0.085
GO:0003713	transcription coactivator activity	0.558	14	0.085
GO:0005261	cation channel activity	0.669	10	0.081
GO:0003714	transcription corepressor activity	0.468	8	0.077
GO:0004523	ribonuclease H activity	0.833	16	0.071
GO:0019843	rRNA binding	0.672	22	0.070
GO:0003697	single-stranded DNA binding	0.657	12	0.068
GO:0004714	transmembrane receptor protein tyrosine kinase activity	0.782	14	0.067
GO:0016846	carbon-sulfur lyase activity	0.994	10	0.066
GO:0008081	phosphoric diester hydrolase activity	0.560	11	0.066
GO:0000155	two-component sensor molecule activity	0.938	12	0.062
GO:0008026	ATP-dependent helicase activity	0.685	13	0.062
GO:0051082	unfolded protein binding	0.670	32	0.062
GO:0005126	hematopoietin/interferon-class (D200-domain) cytokine receptor binding	0.993	20	0.059
GO:0004197	cysteine-type endopeptidase activity	0.778	23	0.058
GO:0015078	hydrogen ion transporter activity	0.785	21	0.058
GO:0003700	transcription factor activity	0.743	117	0.056
GO:0004725	protein tyrosine phosphatase activity	0.862	14	0.054
GO:0042802	protein self binding	0.492	12	0.053
GO:0005516	calmodulin binding	0.841	24	0.052
GO:0003899	DNA-directed RNA polymerase activity	0.665	12	0.052
GO:0000049	tRNA binding	0.863	13	0.051
GO:0003724	RNA helicase activity	0.740	11	0.051
GO:0019838	growth factor binding	0.915	9	0.047
GO:0019955	cytokine binding	0.925	11	0.046
GO:0005525	GTP binding	0.851	47	0.045
GO:0008083	growth factor activity	0.900	42	0.045
GO:0005524	ATP binding	0.753	238	0.045
GO:0004896	hematopoietin/interferon-class (D200-domain) cytokine receptor activity	0.952	19	0.043
GO:0030145	manganese ion binding	0.693	35	0.042

GO:0016758	transferase activity, transferring hexosyl groups	0.815	11	0.042
GO:0016836	hydro-lyase activity	0.742	33	0.042
GO:0004620	phospholipase activity	0.603	19	0.040
GO:0005096	GTPase activator activity	0.507	13	0.039
GO:0005507	copper ion binding	0.930	38	0.039
GO:0003916	DNA topoisomerase activity	0.712	10	0.039
GO:0004812	tRNA ligase activity	0.971	26	0.038
GO:0005066	transmembrane receptor protein tyrosine kinase signaling protein activity	0.855	10	0.038
GO:0005543	phospholipid binding	0.655	14	0.038
GO:0004177	aminopeptidase activity	0.738	12	0.037
GO:0003968	RNA-directed RNA polymerase activity	0.822	14	0.037
GO:0019829	cation-transporting ATPase activity	0.610	9	0.037
GO:0008757	S-adenosylmethionine-dependent methyltransferase activity	0.838	22	0.034
GO:0016616	oxidoreductase activity, acting on the CH-OH group of donors, NAD or NADP as acceptor	0.888	59	0.034
GO:0016763	transferase activity, transferring pentosyl groups	0.810	28	0.034
GO:0008201	heparin binding	0.713	22	0.034
GO:0008080	N-acetyltransferase activity	0.866	12	0.032
GO:0016705	oxidoreductase activity, acting on paired donors, with incorporation or reduction of molecular oxygen	0.735	26	0.032
GO:0003924	GTPase activity	0.810	15	0.032
GO:0004190	aspartic-type endopeptidase activity	0.893	23	0.031
GO:0000287	magnesium ion binding	0.697	125	0.031
GO:0004842	ubiquitin-protein ligase activity	0.836	18	0.030
GO:0008200	ion channel inhibitor activity	0.816	11	0.029
GO:0008235	metalloexopeptidase activity	0.951	13	0.028
GO:0008199	ferric iron binding	0.898	10	0.028
GO:0001584	rhodopsin-like receptor activity	0.758	10	0.028
GO:0016814	hydrolase activity, acting on carbon-nitrogen (but not peptide) bonds, in cyclic amidines	0.613	10	0.028
GO:0015405	P-P-bond-hydrolysis-driven transporter activity	0.686	12	0.027
GO:0009036	type II site-specific deoxyribonuclease activity	0.791	12	0.027
GO:0003684	damaged DNA binding	0.622	10	0.025
GO:0004222	metalloendopeptidase activity	0.937	19	0.025
GO:0004674	protein serine/threonine kinase activity	0.695	40	0.024
GO:0008408	3'-5' exonuclease activity	0.632	11	0.024
GO:0016799	hydrolase activity, hydrolyzing N-glycosyl compounds	0.739	17	0.024
GO:0016251	general RNA polymerase II transcription factor activity	0.862	13	0.024
GO:0008270	zinc ion binding	0.758	100	0.023
GO:0003887	DNA-directed DNA polymerase activity	0.786	18	0.021
GO:0003682	chromatin binding	0.802	10	0.021
GO:0004867	serine-type endopeptidase inhibitor activity	0.873	46	0.020
GO:0016702	oxidoreductase activity, acting on single donors with incorporation of molecular oxygen, incorporation of two atoms of oxygen	0.857	11	0.020
GO:0003779	actin binding	0.851	32	0.019
GO:0005518	collagen binding	0.783	10	0.019

GO:0005085	guanyl-nucleotide exchange factor activity	0.753	12	0.019
GO:0005509	calcium ion binding	0.776	155	0.018
GO:0004556	alpha-amylase activity	0.994	15	0.018
GO:0016854	racemase and epimerase activity	0.710	13	0.017
GO:0004497	monooxygenase activity	0.844	26	0.016
GO:0016638	oxidoreductase activity, acting on the CH-NH2 group of donors	0.884	17	0.016
GO:0008483	transaminase activity	0.963	17	0.016
GO:0016831	carboxy-lyase activity	0.765	25	0.015
GO:0003755	peptidyl-prolyl cis-trans isomerase activity	0.939	10	0.015
GO:0016651	oxidoreductase activity, acting on NADH or NADPH	0.719	13	0.014
GO:0010181	FMN binding	0.822	11	0.014
GO:0003809	thrombin activity	0.988	10	0.013
GO:0016646	oxidoreductase activity, acting on the CH-NH group of donors, NAD or NADP as acceptor	0.853	19	0.013
GO:0004364	glutathione transferase activity	1.000	10	0.013
GO:0004601	peroxidase activity	0.819	21	0.012
GO:0030151	molybdenum ion binding	0.724	14	0.010
GO:0016668	oxidoreductase activity, acting on sulfur group of donors, NAD or NADP as acceptor	0.898	12	0.010
GO:0015036	disulfide oxidoreductase activity	0.982	21	0.009
GO:0016627	oxidoreductase activity, acting on the CH-CH group of donors	0.872	17	0.009
GO:0005529	sugar binding	0.863	38	0.009
GO:0005351	sugar porter activity	0.667	21	0.009
GO:0003743	translation initiation factor activity	0.586	15	0.008
GO:0016620	oxidoreductase activity, acting on the aldehyde or oxo group of donors, NAD or NADP as acceptor	0.901	10	0.008
GO:0016811	hydrolase activity, acting on carbon-nitrogen (but not peptide) bonds, in linear amides	0.547	13	0.008
GO:0015082	di-, tri-valent inorganic cation transporter activity	0.875	13	0.007
GO:0046915	transition metal ion transporter activity	0.875	13	0.007
GO:0016866	intramolecular transferase activity	0.589	11	0.007
GO:0004457	lactate dehydrogenase activity	0.935	10	0.007
GO:0019201	nucleotide kinase activity	0.832	13	0.006
GO:0004295	trypsin activity	0.986	47	0.006
GO:0004263	chymotrypsin activity	0.981	40	0.006
GO:0008810	cellulase activity	0.829	18	0.006
GO:0016776	phosphotransferase activity, phosphate group as acceptor	0.792	14	0.004
GO:0050660	FAD binding	0.944	10	0.004
GO:0008800	beta-lactamase activity	0.999	10	0.002
GO:0004180	carboxypeptidase activity	0.840	15	0.001
GO:0008009	chemokine activity	0.939	10	0.000
GO:0016861	intramolecular oxidoreductase activity, interconverting aldoses and ketoses	0.872	13	0.000
