# Supplementary material for: A Comprehensive Analysis of the Structure-Function Relationship in Proteins Based on Local Structure Similarity
Source: PLoS One. 2009 Jul 15;4(7):e6266. doi: 10.1371/journal.pone.0006266 (PMC2705683; doi:10.1371/journal.pone.0006266)
Supplement: Table S6 — Literature evaluation. Predictions and literature evaluation of the 167 proteins with no homology to the training set. (0.06 MB PDF) [file pone.0006266.s006.pdf]

Tab S6 Literature evaluation. Predictions and literature evaluation of the 167 proteins with no homology to the training set.

Probability key 5=explicitly true, 4= undoubtly true, 3= probably true , 2 possibly true (it would make sense), 1 = unlikely to be true (nothing pointing at it) , 0 = wrong (incompatible)

Relatedness key. 5=correct (same GO), 4 = very close (same activity on very similar substrate.e.g. different proteinases), 3= close (e.g sibling activity, related substrates etc), 2 vague similarity (e.g. Related activity on related substrate) ,1 = far sought similarity , 0 = totally unrelated

										Annotations			Predictions				
PDB id	PDB title	SWS acc	SWS id	Protein name	Synonym	Gene name	Organism	E-score	Seq. id.	GO Class	GO Term	AUC	Method	GO Class	GO Term	Probability	Relatedness
1mvh	STRUCTURE	O60016	CLR4_SCHPO	Histone-lysin	EC 2.1.1.43 Hist	clr4	Schizosacchar	10.00	0.00	GO:0008270	zinc ion bind	0.67					
1mvh	STRUCTURE	O60016	CLR4_SCHPO	Histone-lysin	EC 2.1.1.43 Hist	clr4	Schizosacchar	10.00	0.00	GO:0003682	chromatin b	0.88					
1mvh	STRUCTURE	O60016	CLR4_SCHPO	Histone-lysin	EC 2.1.1.43 Hist	clr4	Schizosacchar	10.00	0.00	GO:0008757	S-adenosyl	0.89					
1r1g	CRYSTAL ST	P83407	SC37_MESMA	Neurotoxin B	None	None	Mesobuthus m	10.00	0.00	GO:0008200	ion channel	0.58					
1ukf	CRYSTAL ST	Q52430	AVP3_PSESH	Cysteine prot	EC 3.4.22.-	avrPph3	Pseudomonas	7.90	0.01	GO:0004197	cysteine-typ	0.93					
1lmr	SOLUTION O	P58608	ADO1_AGRDO	Toxin Ado1	None	None	Agriosphodrus	6.90	0.17	GO:0008200	ion channel	0.58					
1n0z	SOLUTION S	O95218	Z265_HUMAN	Zinc finger pr	Zinc finger, splici	ZNF265	Homo sapiens	5.50	0.11	GO:0003700	transcription	0.67					
1lsl	CRYSTAL ST	P07996	TSP1_HUMAN	Thrombospor	None	THBS1	Homo sapiens	4.20	0.15	GO:0005509	calcium ion	0.75					
1l6p	N-TERMINAL	P36655	DSBD_ECOLI	Thiol:disulfide	EC 1.8.1.8 Prote	dsbD	Escherichia co	4.00	0.05	GO:0016668	oxidoreduct	0.90					
1p4d	F FACTOR T	P14565	TRI1_ECOLI	TraI protein	DNA helicase I E	traI	Escherichia co	4.00	0.04	GO:0005524	ATP binding	0.77	local descr.	GO:0005524	ATP binding	5	5
1n4k	CRYSTAL ST	P11881	IP3R_MOUSE	Inositol 1,4,5-	Type 1 inositol 1,	Itpr1	Mus musculus	3.80	0.08				local descr.	GO:0016799	hydrolase acti	1	0
1n4k	CRYSTAL ST	P11881	IP3R_MOUSE	Inositol 1,4,5-	Type 1 inositol 1,	Itpr1	Mus musculus	3.80	0.08				local descr.	GO:0005529	sugar binding	5	4
1n4k	CRYSTAL ST	P11881	IP3R_MOUSE	Inositol 1,4,5-	Type 1 inositol 1,	Itpr1	Mus musculus	3.80	0.08				local descr.	GO:0003779	actin binding	1	3
1n4k	CRYSTAL ST	P11881	IP3R_MOUSE	Inositol 1,4,5-	Type 1 inositol 1,	Itpr1	Mus musculus	3.80	0.08				local descr.	GO:0008083	growth factor	1	0
1n4k	CRYSTAL ST	P11881	IP3R_MOUSE	Inositol 1,4,5-	Type 1 inositol 1,	Itpr1	Mus musculus	3.80	0.08	GO:0005261	cation chan	0.42					
1h5o	SOLUTION S	P01475	MYXC_CRODU	Crotamine	Crt Myotoxin	None	Crotalus duriss	3.70	0.17	GO:0008200	ion channel	0.58					
1rh5	THE STRUCT	Q57817, Q	SECE_METJA	Preprotein tra	Protein transport	secE, sec	Methanococcu	3.60	0.31	GO:0015405	P-P-bond-h	0.54					
1oy5	CRYSTAL ST	O67463	TRMD_AQUAE	tRNA (Guanir	EC 2.1.1.31 M1G	trmD	Aquifex aeolicu	3.30	0.04				local descr.	GO:0008270	zinc ion bindir	2	5
1oy5	CRYSTAL ST	O67463	TRMD_AQUAE	tRNA (Guanir	EC 2.1.1.31 M1G	trmD	Aquifex aeolicu	3.30	0.04	GO:0008757	S-adenosyl	0.89					
1k8b	NMR STRUCT	Q57562	IF2B_METJA	Probable tran	eIF-2-beta	eIF2B	Methanococcu	3.10	0.12	GO:0003743	translation i	0.55					
1o63	CRYSTAL ST	Q9X0D2	HIS1_THEMA	ATP phospho	EC 2.4.2.17 ATP	hisG	Thermotoga m	3.10	0.05				local descr.	GO:0008199	ferric iron bind	1	0
1o63	CRYSTAL ST	Q9X0D2	HIS1_THEMA	ATP phospho	EC 2.4.2.17 ATP	hisG	Thermotoga m	3.10	0.05				local descr.	GO:0000287	magnesium io	3	5
1o63	CRYSTAL ST	Q9X0D2	HIS1_THEMA	ATP phospho	EC 2.4.2.17 ATP	hisG	Thermotoga m	3.10	0.05				local descr.	GO:0003700	transcription f	1	0
1o63	CRYSTAL ST	Q9X0D2	HIS1_THEMA	ATP phospho	EC 2.4.2.17 ATP	hisG	Thermotoga m	3.10	0.05				local descr.	GO:0005351	sugar porter a	1	0
1o63	CRYSTAL ST	Q9X0D2	HIS1_THEMA	ATP phospho	EC 2.4.2.17 ATP	hisG	Thermotoga m	3.10	0.05	GO:0016763	transferase	0.93					

1ovx	NMR STRUCT	P33138	CLPX_ECOLI	ATP-depende	None	clpX	Escherichia co	3.10	0.11	GO:0005524	ATP binding	0.77					
1ovx	NMR STRUCT	P33138	CLPX_ECOLI	ATP-depende	None	clpX	Escherichia co	3.10	0.11	GO:0051082	unfolded pro	0.66					
1ovx	NMR STRUCT	P33138	CLPX_ECOLI	ATP-depende	None	clpX	Escherichia co	3.10	0.11	GO:0008270	zinc ion bind	0.67					
1ovx	NMR STRUCT	P33138	CLPX_ECOLI	ATP-depende	None	clpX	Escherichia co	3.10	0.11	GO:0046983	protein dime	0.69					
1lmm	SOLUTION S	P60514	TXP1_PSACA	Psalmotoxin-	PcTx1	None	Psalmopoeus c	3.00	0.18	GO:0008200	ion channel	0.58					
1kuu	CRYSTAL ST	O27099	O27099	IMP cyclohyd	EC 3.5.4.10 Inos	purO	Methanobacter	3.00	0.03	GO:0016814	hydrolase a	0.78					
1l7l	CRYSTAL ST	Q05097	PA1L_PSEAE	PA-I galactop	PA-IL Galactose-	lecA	Pseudomonas	3.00	0.03				local descr.	GO:0003964	RNA-directed	0	0
1l7l	CRYSTAL ST	Q05097	PA1L_PSEAE	PA-I galactop	PA-IL Galactose-	lecA	Pseudomonas	3.00	0.03	GO:0005529	sugar bindin	0.87					
1owt	STRUCTURE	P05067	A4_HUMAN	Amyloid beta	APP ABPP Alzhe	APP	Homo sapiens	3.00	0.15	GO:0004867	serine-type	0.84					
1owt	STRUCTURE	P05067	A4_HUMAN	Amyloid beta	APP ABPP Alzhe	APP	Homo sapiens	3.00	0.15	GO:0008201	heparin bind	0.64					
1r4g	SOLUTION S	P04860	RRPP_SENDZ	Phosphoprote	P protein	P/V/C	Sendai virus (s	3.00	0.13	GO:0003968	RNA-directe	0.86					
1ne5	SOLUTION S	Q86QT3	SEK1_CENNO	Ergtoxin [Pre	ErgTx Ergtoxin-li	ERG1	Centruroides n	3.00	0.10	GO:0008200	ion channel	0.58					
1m9z	CRYSTAL ST	P37173	TGR2_HUMAN	TGF-beta rec	EC 2.7.1.37 TGF	TGFBR2	Homo sapiens	2.80	0.08				local descr.	GO:0003964	RNA-directed	0	0
1m9z	CRYSTAL ST	P37173	TGR2_HUMAN	TGF-beta rec	EC 2.7.1.37 TGF	TGFBR2	Homo sapiens	2.80	0.08	GO:0005524	ATP binding	0.77					
1m9z	CRYSTAL ST	P37173	TGR2_HUMAN	TGF-beta rec	EC 2.7.1.37 TGF	TGFBR2	Homo sapiens	2.80	0.08	GO:0004674	protein serin	0.68					
1pv7	CRYSTAL ST	P02920	LACY_ECOLI	Lactose perm	Lactose-proton s	lacY	Escherichia co	2.80	0.04				local descr.	GO:0016705	oxidoreductas	1	0
1pv7	CRYSTAL ST	P02920	LACY_ECOLI	Lactose perm	Lactose-proton s	lacY	Escherichia co	2.80	0.04				local descr.	GO:0000287	magnesium io	1	0
1pv7	CRYSTAL ST	P02920	LACY_ECOLI	Lactose perm	Lactose-proton s	lacY	Escherichia co	2.80	0.04				local descr.	GO:0005126	hematopoietin		
1pv7	CRYSTAL ST	P02920	LACY_ECOLI	Lactose perm	Lactose-proton s	lacY	Escherichia co	2.80	0.04				local descr.	GO:0004601	peroxidase ac	1	0
1pv7	CRYSTAL ST	P02920	LACY_ECOLI	Lactose perm	Lactose-proton s	lacY	Escherichia co	2.80	0.04				local descr.	GO:0004497	monooxygena	1	0
1pv7	CRYSTAL ST	P02920	LACY_ECOLI	Lactose perm	Lactose-proton s	lacY	Escherichia co	2.80	0.04				local descr.	GO:0008083	growth factor a	1	0
1pv7	CRYSTAL ST	P02920	LACY_ECOLI	Lactose perm	Lactose-proton s	lacY	Escherichia co	2.80	0.04	GO:0005351	sugar porter	0.72					
1s2b	STRUCTURE	P15369	PRTB_SCYLI	Scytalidopeps	EC 3.4.23.32 Aci	None	Scytalidium lign	2.60	0.04				local descr.	GO:0005509	calcium ion bi	1	0
1s2b	STRUCTURE	P15369	PRTB_SCYLI	Scytalidopeps	EC 3.4.23.32 Aci	None	Scytalidium lign	2.60	0.04				local descr.	GO:0004867	serine-type en	5	1
1s2b	STRUCTURE	P15369	PRTB_SCYLI	Scytalidopeps	EC 3.4.23.32 Aci	None	Scytalidium lign	2.60	0.04	GO:0004190	aspartic-typ	0.81					
1r6r	SOLUTION S	P12823	POLG_DEN2P	Genome poly	None	None	Dengue virus ty	2.60	0.09	GO:0003724	RNA helicas	0.79					
1r6r	SOLUTION S	P12823	POLG_DEN2P	Genome poly	None	None	Dengue virus ty	2.60	0.09	GO:0005524	ATP binding	0.77					
1r6r	SOLUTION S	P12823	POLG_DEN2P	Genome poly	None	None	Dengue virus ty	2.60	0.09	GO:0008026	ATP-depend	0.74					
1r6r	SOLUTION S	P12823	POLG_DEN2P	Genome poly	None	None	Dengue virus ty	2.60	0.09	GO:0003968	RNA-directe	0.86					
1m4z	CRYSTAL ST	P54784	ORC1_YEAST	Origin recogn	Origin recognition	ORC1	Saccharomyce	2.50	0.04				local descr.	GO:0003899	DNA-directed	5	3
1m4z	CRYSTAL ST	P54784	ORC1_YEAST	Origin recogn	Origin recognition	ORC1	Saccharomyce	2.50	0.04				local descr.	GO:0003700	transcription fa	5	3
1m4z	CRYSTAL ST	P54784	ORC1_YEAST	Origin recogn	Origin recognition	ORC1	Saccharomyce	2.50	0.04	GO:0005524	ATP binding	0.77					
1itu	HUMAN REN	P16444	MDP1_HUMAN	Microsomal d	EC 3.4.13.19 MD	DPEP1	Homo sapiens	2.30	0.03				local descr.	GO:0008270	zinc ion bindin	5	5
1itu	HUMAN REN	P16444	MDP1_HUMAN	Microsomal d	EC 3.4.13.19 MD	DPEP1	Homo sapiens	2.30	0.03	GO:0008235	metalloexop	0.98					
1k81	NMR STRUCT	Q57562	IF2B_METJA	Probable tran	eIF-2-beta	eIF2B	Methanococcus	2.30	0.17	GO:0003743	translation i	0.55					
1mby	MURINE SAK	Q64702	PLK4_MOUSE	Serine/threon	EC 2.7.1.37 Polo	Plk4	Mus musculus	1.90	0.09	GO:0005524	ATP binding	0.77	local descr.	GO:0005524	ATP binding	5	5

1mby	MURINE SAK	Q64702	PLK4_MOUSE	Serine/threon	EC 2.7.1.37 Polo	Plk4	Mus musculus	1.90	0.09	GO:0004674	protein serin	0.68					
1iw4	SOLUTION S	P16589	ITRP_HALRO	Trypsin inhibi	ATI	None	Halocynthia ro	1.80	0.20	GO:0004867	serine-type	0.84					
1jyo	STRUCTURE	P74873, Q8	P74873, Q8ZM	Effector prote	None	sptP	Salmonella typ	1.70	0.10				local descr.	GO:0008270	zinc ion bindin	2	5
1jyo	STRUCTURE	P74873, Q8	P74873, Q8ZM	Effector prote	None	sptP	Salmonella typ	1.70	0.10				local descr.	GO:0008800	beta-lactamas	5	2
1jyo	STRUCTURE	P74873, Q8	P74873, Q8ZM	Effector prote	None	sptP	Salmonella typ	1.70	0.10				local descr.	GO:0016836	hydro-lyase ac	1	1
1jyo	STRUCTURE	P74873, Q8	P74873, Q8ZM	Effector prote	None	sptP	Salmonella typ	1.70	0.10	GO:0004725	protein tyros	0.90					
1jyo	STRUCTURE	P74873, Q8	P74873, Q8ZM	Effector prote	None	sptP	Salmonella typ	1.70	0.10	GO:0005096	GTPase act	0.60					
1vju	HYPOTHETIC	P84155	P84155	LMAJ006828	None	None	Leishmania ma	1.70	0.04	GO:0016627	oxidoreduct	0.97					
1gxy	CRYSTAL ST	P20974	NRT2_RAT	T-cell ecto-AD	EC 2.4.2.31 T-ce	Art2b	Rattus norvegi	1.60	0.09	GO:0016763	transferase	0.93					
1ka8	CRYSTAL ST	P10277	PRIM_BPP4	Putative P4-s	EC 2.7.7.-	Alpha	Bacteriophage	1.60	0.09	GO:0005524	ATP binding	0.77					
1ka8	CRYSTAL ST	P10277	PRIM_BPP4	Putative P4-s	EC 2.7.7.-	Alpha	Bacteriophage	1.60	0.09	GO:0003899	DNA-directe	0.58					
1k8w	CRYSTAL ST	P60340	TRUB_ECOLI	tRNA pseudo	EC 5.4.99.- tRNA	truB	Escherichia co	1.50	0.08	GO:0016836	hydro-lyase	0.77					
1lur	CRYSTAL ST	Q966D4	Q966D4	Hypothetical	None	ORFName	Caenorhabditis	1.50	0.04				local descr.	GO:0005516	calmodulin bin	1	0
1lur	CRYSTAL ST	Q966D4	Q966D4	Hypothetical	None	ORFName	Caenorhabditis	1.50	0.04				local descr.	GO:0008201	heparin bindin	4	3
1lur	CRYSTAL ST	Q966D4	Q966D4	Hypothetical	None	ORFName	Caenorhabditis	1.50	0.04				local descr.	GO:0004896	hematopoietin	1	0
1lur	CRYSTAL ST	Q966D4	Q966D4	Hypothetical	None	ORFName	Caenorhabditis	1.50	0.04	GO:0016854	racemase a	0.77					
1odh	STRUCTURE	P70348	P70348	Chorion-spec	Glial cells missin	Gcm1	Mus musculus	1.40	0.07	GO:0008270	zinc ion bind	0.67					
1odh	STRUCTURE	P70348	P70348	Chorion-spec	Glial cells missin	Gcm1	Mus musculus	1.40	0.07	GO:0003700	transcription	0.67					
1paq	CRYSTAL ST	P32501	E2BE_YEAST	Translation in	eIF-2B GDP-GTP	GCD6	Saccharomyce	1.40	0.07	GO:0003743	translation i	0.55					
1k8t	CRYSTAL ST	P40136	CYAA_BACAN	Calmodulin-s	EC 4.6.1.1 ATP p	cya	Bacillus anthra	1.30	0.01				local descr.	GO:0008270	zinc ion bindin	5	4
1k8t	CRYSTAL ST	P40136	CYAA_BACAN	Calmodulin-s	EC 4.6.1.1 ATP p	cya	Bacillus anthra	1.30	0.01	GO:0005524	ATP binding	0.77					
1k8t	CRYSTAL ST	P40136	CYAA_BACAN	Calmodulin-s	EC 4.6.1.1 ATP p	cya	Bacillus anthra	1.30	0.01	GO:0000287	magnesium	0.80					
1k8t	CRYSTAL ST	P40136	CYAA_BACAN	Calmodulin-s	EC 4.6.1.1 ATP p	cya	Bacillus anthra	1.30	0.01	GO:0005516	calmodulin b	0.73					
1ry9	SPA15, A TYP	P35530	SPAK_SHIFL	Surface prese	Spa15 protein	spaK	Shigella flexne	1.30	0.14				local descr.	GO:0003887	DNA-directed	1	0
1ry9	SPA15, A TYP	P35530	SPAK_SHIFL	Surface prese	Spa15 protein	spaK	Shigella flexne	1.30	0.14				local descr.	GO:0005524	ATP binding	3	5
1ry9	SPA15, A TYP	P35530	SPAK_SHIFL	Surface prese	Spa15 protein	spaK	Shigella flexne	1.30	0.14	GO:0015405	P-P-bond-hy	0.54					
1ul7	SOLUTION S	Q8C6G9	Q8C6G9	Mus musculu	None	Mark3	Mus musculus	1.20	0.05	GO:0004674	protein serin	0.68					
1oxj	CRYSTAL ST	Q23972	SMG_DROME	Smaug protei	None	smg	Drosophila mel	1.20	0.10				local descr.	GO:0005509	calcium ion bin	1	0
1oxj	CRYSTAL ST	Q23972	SMG_DROME	Smaug protei	None	smg	Drosophila mel	1.20	0.10	GO:0003729	mRNA bind	0.82					
1kcf	CRYSTAL ST	Q10423	YDC2_SCHPO	Cruciform cut	EC 3.1.-.- Ydc2 p	cce1	Schizosacchar	1.20	0.03	GO:0000287	magnesium	0.80	local descr.	GO:0000287	magnesium io	5	5
1kcf	CRYSTAL ST	Q10423	YDC2_SCHPO	Cruciform cut	EC 3.1.-.- Ydc2 p	cce1	Schizosacchar	1.20	0.03				local descr.	GO:0003700	transcription fa	5	3
1jpy	CRYSTAL ST	Q96PD4	I17F_HUMAN	Interleukin-17	IL-17F Interleukin	IL17F	Homo sapiens	1.10	0.06	GO:0042802	protein self	0.62					
1jpy	CRYSTAL ST	Q96PD4	I17F_HUMAN	Interleukin-17	IL-17F Interleukin	IL17F	Homo sapiens	1.10	0.06	GO:0019955	cytokine bin	0.87					
1jpy	CRYSTAL ST	Q96PD4	I17F_HUMAN	Interleukin-17	IL-17F Interleukin	IL17F	Homo sapiens	1.10	0.06	GO:0046983	protein dime	0.69					
1oks	CRYSTAL ST	P03422	RRPP_MEASE	Phosphoprote	P protein	P/V	Measles virus (	1.10	0.11	GO:0003968	RNA-directe	0.86					
1osy	CRYSTAL ST	P80412	FVE_FLAVE	Immunomodu	None	None	Flammulina ve	1.10	0.07				local descr.	GO:0003887	DNA-directed	1	0

1osy	CRYSTAL ST	P80412	FVE_FLAVE	Immunomodu	None	None	Flammulina ve	1.10	0.07	GO:0005529	sugar bindir	0.87					
1s0p	STRUCTURE	P54654	CAP_DICDI	Adenylyl cycl	CAP	cap	Dictyostelium d	1.10	0.06				local descr.	GO:0005509	calcium ion bi	1	0
1s0p	STRUCTURE	P54654	CAP_DICDI	Adenylyl cycl	CAP	cap	Dictyostelium d	1.10	0.06	GO:0003779	actin binding	0.68					
1rso								1.00	0.23	GO:0005516	calmodulin b	0.73					
1rso								1.00	0.23	GO:0003713	transcription	0.58					
1rfm	SULFOLACTA	Q58820	COMC_METJA	L-sulfolactate	EC 1.1.1.272 (R)	comC	Methanococcus	0.99	0.03				local descr.	GO:0008270	zinc ion bindin	3	5
1rfm	SULFOLACTA	Q58820	COMC_METJA	L-sulfolactate	EC 1.1.1.272 (R)	comC	Methanococcus	0.99	0.03	GO:0016616	oxidoreduct	0.91					
1k2f	SIAH, SEVEN	P61092	SI1A_MOUSE	Ubiquitin ligas	EC 6.3.2.- Seven	Siah1a	Mus musculus	0.96	0.03				local descr.	GO:0005507	copper ion bin	5	4
1k2f	SIAH, SEVEN	P61092	SI1A_MOUSE	Ubiquitin ligas	EC 6.3.2.- Seven	Siah1a	Mus musculus	0.96	0.03	GO:0008270	zinc ion bind	0.67					
1k2f	SIAH, SEVEN	P61092	SI1A_MOUSE	Ubiquitin ligas	EC 6.3.2.- Seven	Siah1a	Mus musculus	0.96	0.03	GO:0004842	ubiquitin-pro	0.75					
1h3i	CRYSTAL ST	Q8WTS6	SET7_HUMAN	Histone-lysine	EC 2.1.1.43 Histo	SET7	Homo sapiens	0.92	0.08				local descr.	GO:0005509	calcium ion bi	0	0
1h3i	CRYSTAL ST	Q8WTS6	SET7_HUMAN	Histone-lysine	EC 2.1.1.43 Histo	SET7	Homo sapiens	0.92	0.08				local descr.	GO:0005529	sugar binding	1	0
1h3i	CRYSTAL ST	Q8WTS6	SET7_HUMAN	Histone-lysine	EC 2.1.1.43 Histo	SET7	Homo sapiens	0.92	0.08	GO:0008757	S-adenosylm	0.89					
1lva	CRYSTAL ST	Q46455	SELB_MOOTH	Selenocystein	SelB translation f	selB	Moorella therm	0.91	0.27				local descr.	GO:0004177	aminopeptidas	4	3
1lva	CRYSTAL ST	Q46455	SELB_MOOTH	Selenocystein	SelB translation f	selB	Moorella therm	0.91	0.27				local descr.	GO:0009036	type II site-spe	0	0
1lva	CRYSTAL ST	Q46455	SELB_MOOTH	Selenocystein	SelB translation f	selB	Moorella therm	0.91	0.27				local descr.	GO:0003700	transcription fa	0	3
1lva	CRYSTAL ST	Q46455	SELB_MOOTH	Selenocystein	SelB translation f	selB	Moorella therm	0.91	0.27				local descr.	GO:0003916	DNA topoisom	0	0
1lva	CRYSTAL ST	Q46455	SELB_MOOTH	Selenocystein	SelB translation f	selB	Moorella therm	0.91	0.27				local descr.	GO:0008235	metalloexopep	4	3
1lva	CRYSTAL ST	Q46455	SELB_MOOTH	Selenocystein	SelB translation f	selB	Moorella therm	0.91	0.27	GO:0005525	GTP binding	0.89					
1pzr	STRUCTURE	Not Availab	Not Available					0.88	0.18	GO:0008270	zinc ion bind	0.67					
1pzr	STRUCTURE	Not Availab	Not Available					0.88	0.18	GO:0016616	oxidoreduct	0.91					
1ml9	STRUCTURE	Q8X225	Q8X225	Histone-lysine	EC 2.1.1.43 Histo	dim-5	Neurospora cra	0.86	0.04	GO:0008270	zinc ion bind	0.67					
1ml9	STRUCTURE	Q8X225	Q8X225	Histone-lysine	EC 2.1.1.43 Histo	dim-5	Neurospora cra	0.86	0.04	GO:0008757	S-adenosylm	0.89					
1ear	CRYSTAL ST	P50049	UREE_BACPA	Urease acces	None	ureE	Bacillus pasteu	0.86	0.15	GO:0051082	unfolded pro	0.66					
1m22	X-RAY STRU	Q8RJN5	Q8RJN5	Peptide amid	None	pam	Xanthomonas m	0.82	0.02	GO:0016811	hydrolase a	0.55					
1nsz	CRYSTAL ST	Q9ZB17	Q9ZB17	Aldose 1-epim	None	galM	Lactococcus la	0.82	0.05				local descr.	GO:0051082	unfolded prote	1	0
1nsz	CRYSTAL ST	Q9ZB17	Q9ZB17	Aldose 1-epim	None	galM	Lactococcus la	0.82	0.05				local descr.	GO:0005509	calcium ion bi	3	5
1nsz	CRYSTAL ST	Q9ZB17	Q9ZB17	Aldose 1-epim	None	galM	Lactococcus la	0.82	0.05				local descr.	GO:0008201	heparin bindin	5	2
1nsz	CRYSTAL ST	Q9ZB17	Q9ZB17	Aldose 1-epim	None	galM	Lactococcus la	0.82	0.05				local descr.	GO:0004867	serine-type en	0	0
1nsz	CRYSTAL ST	Q9ZB17	Q9ZB17	Aldose 1-epim	None	galM	Lactococcus la	0.82	0.05	GO:0016854	racemase a	0.77					
1ldd	STRUCTURE	Q12440	Q12440	Anaphase pro	None	APC2	Saccharomyce	0.78	0.16				local descr.	GO:0003700	transcription fa	1	0
1ldd	STRUCTURE	Q12440	Q12440	Anaphase pro	None	APC2	Saccharomyce	0.78	0.16	GO:0004842	ubiquitin-pro	0.75					
1o8r	SOLUTION ST	Q02747	GUAN_HUMAN	Guanylin [Pre	Guanylate cyclas	GUCA2A	Homo sapiens	0.77	0.16	GO:0005179	hormone ac	0.53					
1msz	SOLUTION ST	P38935	SMB2_HUMAN	DNA-binding	Immunoglobulin m	IGHMBP2	Homo sapiens	0.74	0.11	GO:0005524	ATP binding	0.77					
1msz	SOLUTION ST	P38935	SMB2_HUMAN	DNA-binding	Immunoglobulin m	IGHMBP2	Homo sapiens	0.74	0.11	GO:0003697	single-stran	0.70					
1szq	CRYSTAL ST	P77243	PRPD_ECOLI	2-methylcitrat	EC 4.2.1.79	prpD	Escherichia co	0.72	0.02				local descr.	GO:0005524	ATP binding	1	0

1szq	CRYSTAL ST	P77243	PRPD_ECOLI	2-methylcitrat	EC 4.2.1.79	prpD	Escherichia co	0.72	0.02				local descr.	GO:0008270	zinc ion bindin	5	4
1szq	CRYSTAL ST	P77243	PRPD_ECOLI	2-methylcitrat	EC 4.2.1.79	prpD	Escherichia co	0.72	0.02	GO:0016836	hydro-lyase	0.77					
1lmz	SOLUTION S	P05100	3MG1_ECOLI	DNA-3-methy	EC 3.2.2.20 3-me	tag	Escherichia co	0.71	0.07				local descr.	GO:0005509	calcium ion bi	5	4
1lmz	SOLUTION S	P05100	3MG1_ECOLI	DNA-3-methy	EC 3.2.2.20 3-me	tag	Escherichia co	0.71	0.07	GO:0016799	hydrolase a	0.71					
1s7a	NMR STRUCT	P05455	LA_HUMAN	Lupus La pro	Sjogren syndrom	SSB	Homo sapiens	0.69	0.09	GO:0000049	tRNA bindir	0.76					
1s7a	NMR STRUCT	P05455	LA_HUMAN	Lupus La pro	Sjogren syndrom	SSB	Homo sapiens	0.69	0.09	GO:0003729	mRNA bind	0.82					
1oed								0.66	0.07				local descr.	GO:0005126	hematopoietin	4	2
1oed								0.66	0.07				local descr.	GO:0005509	calcium ion bi	3	5
1oed								0.66	0.07				local descr.	GO:0005516	calmodulin bir	1	5
1oed								0.66	0.07				local descr.	GO:0003779	actin binding	3	5
1oed								0.66	0.07				local descr.	GO:0008083	growth factor a	0	0
1oed								0.66	0.07	GO:0005261	cation chan	0.42					
1mnn	STRUCTURE	P38830	NDT80_YEAS	NDT80 protei	None	NDT80	Saccharomyce	0.62	0.08				local descr.	GO:0005509	calcium ion bi	1	1
1mnn	STRUCTURE	P38830	NDT80_YEAS	NDT80 protei	None	NDT80	Saccharomyce	0.62	0.08	GO:0003700	transcription	0.67					
1em8	CRYSTAL ST	P28632, P2	HOLC_ECOLI,	DNA polymer	EC 2.7.7.7	holD, holC	Escherichia co	0.62	0.08				local descr.	GO:0000287	magnesium io	2	5
1em8	CRYSTAL ST	P28632, P2	HOLC_ECOLI,	DNA polymer	EC 2.7.7.7	holD, holC	Escherichia co	0.62	0.08				local descr.	GO:0005524	ATP binding	2	3
1em8	CRYSTAL ST	P28632, P2	HOLC_ECOLI,	DNA polymer	EC 2.7.7.7	holD, holC	Escherichia co	0.62	0.08				local descr.	GO:0016616	oxidoreductas	1	0
1em8	CRYSTAL ST	P28632, P2	HOLC_ECOLI,	DNA polymer	EC 2.7.7.7	holD, holC	Escherichia co	0.62	0.08				local descr.	GO:0016646	oxidoreductas	1	0
1em8	CRYSTAL ST	P28632, P2	HOLC_ECOLI,	DNA polymer	EC 2.7.7.7	holD, holC	Escherichia co	0.62	0.08				local descr.	GO:0016836	hydro-lyase ac	1	0
1em8	CRYSTAL ST	P28632, P2	HOLC_ECOLI,	DNA polymer	EC 2.7.7.7	holD, holC	Escherichia co	0.62	0.08	GO:0003887	DNA-directe	0.98					
1em8	CRYSTAL ST	P28632, P2	HOLC_ECOLI,	DNA polymer	EC 2.7.7.7	holD, holC	Escherichia co	0.62	0.08	GO:0008408	3'-5' exonuc	0.83					
1v9d	CRYSTAL ST	O08808	DIA1_MOUSE	Diaphanous p	Diaphanous-relat	Diaph1	Mus musculus	0.60	0.04				local descr.	GO:0003700	transcription fa	1	0
1v9d	CRYSTAL ST	O08808	DIA1_MOUSE	Diaphanous p	Diaphanous-relat	Diaph1	Mus musculus	0.60	0.04				local descr.	GO:0005516	calmodulin bir	1	0
1v9d	CRYSTAL ST	O08808	DIA1_MOUSE	Diaphanous p	Diaphanous-relat	Diaph1	Mus musculus	0.60	0.04				local descr.	GO:0008083	growth factor a	1	0
1v9d	CRYSTAL ST	O08808	DIA1_MOUSE	Diaphanous p	Diaphanous-relat	Diaph1	Mus musculus	0.60	0.04	GO:0003779	actin binding	0.68					
1ix2	CRYSTAL ST	Q47454	PCOC_ECOLI	Copper resist	None	pcoC	Escherichia co	0.58	0.15				local descr.	GO:0005529	sugar binding	1	1
1ix2	CRYSTAL ST	Q47454	PCOC_ECOLI	Copper resist	None	pcoC	Escherichia co	0.58	0.15				local descr.	GO:0008270	zinc ion bindin	5	4
1ix2	CRYSTAL ST	Q47454	PCOC_ECOLI	Copper resist	None	pcoC	Escherichia co	0.58	0.15	GO:0005507	copper ion b	0.92					
1gmu	STRUCTURE	P18317	UREE_KLEAE	Urease acces	None	ureE	Klebsiella aero	0.58	0.16				local descr.	GO:0003964	RNA-directed	0	0
1gmu	STRUCTURE	P18317	UREE_KLEAE	Urease acces	None	ureE	Klebsiella aero	0.58	0.16	GO:0051082	unfolded pro	0.66					
1jy1	CRYSTAL ST	Q9NUW8	TYDP_HUMAN	Tyrosyl-DNA	EC 3.1.4.- Tyr-DI	TDP1	Homo sapiens	0.57	0.07	GO:0008081	phosphoric	0.60					
1oyg	CRYSTAL ST	P05655	SACB_BACSU	Levansucrase	EC 2.4.1.10 Beta	sacB	Bacillus subtilis	0.55	0.02				local descr.	GO:0003682	chromatin bind	0	0
1oyg	CRYSTAL ST	P05655	SACB_BACSU	Levansucrase	EC 2.4.1.10 Beta	sacB	Bacillus subtilis	0.55	0.02	GO:0016758	transferase	0.78					
1riq	THE CRYSTA	O67323	SYA_AQUAE	Alanyl-tRNA s	EC 6.1.1.7 Alanir	alaS	Aquifex aeolicu	0.54	0.06				local descr.	GO:0000049	tRNA binding	5	5
1riq	THE CRYSTA	O67323	SYA_AQUAE	Alanyl-tRNA s	EC 6.1.1.7 Alanir	alaS	Aquifex aeolicu	0.54	0.06				local descr.	GO:0000287	magnesium io	5	5
1riq	THE CRYSTA	O67323	SYA_AQUAE	Alanyl-tRNA s	EC 6.1.1.7 Alanir	alaS	Aquifex aeolicu	0.54	0.06	GO:0005524	ATP binding	0.77	local descr.	GO:0005524	ATP binding	5	5

1riq	THE CRYSTA	O67323	SYA_AQUAE	Alanyl-tRNA s	EC 6.1.1.7 Alanir	alaS	Aquifex aeolicu	0.54	0.06	GO:0004812	tRNA ligase	0.98	local descr.	GO:0004812	tRNA ligase a	5	5
1riq	THE CRYSTA	O67323	SYA_AQUAE	Alanyl-tRNA s	EC 6.1.1.7 Alanir	alaS	Aquifex aeolicu	0.54	0.06	GO:0008270	zinc ion bind	0.67					
1muk	REOVIRUS L	P17378	VL3_REOVD	Minor core pr	None	L1	Reovirus type 3	0.54	0.01				local descr.	GO:0016702	oxidoreductas	1	0
1muk	REOVIRUS L	P17378	VL3_REOVD	Minor core pr	None	L1	Reovirus type 3	0.54	0.01	GO:0003968	RNA-directe	0.86					
1gx1	STRUCTURE	P62617	ISPF_ECOLI	2-C-methyl-D	EC 4.6.1.12 MEC	ispF	Escherichia co	0.51	0.08				local descr.	GO:0003700	transcription fa	1	0
1gx1	STRUCTURE	P62617	ISPF_ECOLI	2-C-methyl-D	EC 4.6.1.12 MEC	ispF	Escherichia co	0.51	0.08	GO:0000287	magnesium	0.80					
1gx1	STRUCTURE	P62617	ISPF_ECOLI	2-C-methyl-D	EC 4.6.1.12 MEC	ispF	Escherichia co	0.51	0.08	GO:0030145	manganese	0.64					
1pqs	SOLUTION S	P11433	CC24_YEAST	Cell division c	Calcium regulato	CDC24	Saccharomyce	0.50	0.17	GO:0005085	guanyl-nucl	0.67			renamed as C		
1k1x	CRYSTAL ST	O32462	MALQ_THELI	4-alpha-gluca	EC 2.4.1.25 Amy	jgt	Thermococcus	0.49	0.19				local descr.	GO:0016763	transferase ac	5	4
1k1x	CRYSTAL ST	O32462	MALQ_THELI	4-alpha-gluca	EC 2.4.1.25 Amy	jgt	Thermococcus	0.49	0.19				local descr.	GO:0008270	zinc ion bindir	3	3
1k1x	CRYSTAL ST	O32462	MALQ_THELI	4-alpha-gluca	EC 2.4.1.25 Amy	jgt	Thermococcus	0.49	0.19	GO:0016758	transferase	0.78					
1j1v	CRYSTAL ST	P03004	DNAA_ECOLI	Chromosoma	None	dnaA	Escherichia co	0.48	0.05				local descr.	GO:0005509	calcium ion bi	5	4
1j1v	CRYSTAL ST	P03004	DNAA_ECOLI	Chromosoma	None	dnaA	Escherichia co	0.48	0.05	GO:0005524	ATP binding	0.77					
1k9x	STRUCTURE	Q8U3L0	Q8U3L0	Carboxypepti	None	OrderedL	Pyrococcus fur	0.48	0.03				local descr.	GO:0003700	transcription fa	1	0
1k9x	STRUCTURE	Q8U3L0	Q8U3L0	Carboxypepti	None	OrderedL	Pyrococcus fur	0.48	0.03	GO:0008235	metalloexop	0.98					
1k9x	STRUCTURE	Q8U3L0	Q8U3L0	Carboxypepti	None	OrderedL	Pyrococcus fur	0.48	0.03	GO:0004180	carboxypep	0.90					
1l2w	CRYSTAL ST	P08008	YOPE_YERPS	Outer membr	None	yopE	Yersinia pseud	0.47	0.20	GO:0005096	GTPase act	0.60					
1rhy	CRYSTAL ST	P40919	HIS7_CRYNE	Imidazoleglyc	EC 4.2.1.19 IGPI	HIS3	Cryptococcus r	0.47	0.10				local descr.	GO:0030151	molybdenum i	5	4
1rhy	CRYSTAL ST	P40919	HIS7_CRYNE	Imidazoleglyc	EC 4.2.1.19 IGPI	HIS3	Cryptococcus r	0.47	0.10				local descr.	GO:0000287	magnesium io	5	4
1rhy	CRYSTAL ST	P40919	HIS7_CRYNE	Imidazoleglyc	EC 4.2.1.19 IGPI	HIS3	Cryptococcus r	0.47	0.10				local descr.	GO:0008800	beta-lactamas	0	0
1rhy	CRYSTAL ST	P40919	HIS7_CRYNE	Imidazoleglyc	EC 4.2.1.19 IGPI	HIS3	Cryptococcus r	0.47	0.10	GO:0016836	hydro-lyase	0.77					
1o9p	CRYSTAL ST	Q9ZIV5	Q9ZIV5	Malonamidas	None	OrderedL	Bradyrhizobium	0.47	0.03				local descr.	GO:0005524	ATP binding	1	0
1o9p	CRYSTAL ST	Q9ZIV5	Q9ZIV5	Malonamidas	None	OrderedL	Bradyrhizobium	0.47	0.03				local descr.	GO:0016616	oxidoreductas	0	0
1o9p	CRYSTAL ST	Q9ZIV5	Q9ZIV5	Malonamidas	None	OrderedL	Bradyrhizobium	0.47	0.03	GO:0016811	hydrolase a	0.55					
1gxj	SMC HINGE D	Q9X0R4	Q9X0R4	Chromosome	None	OrderedL	Thermotoga ma	0.45	0.06	GO:0005524	ATP binding	0.77					
1k3e	TYPE III SEC	Q47015	CEST_ECOLI	Tir chaperone	None	cesT	Escherichia co	0.42	0.07	GO:0051082	unfolded pro	0.66					
1q92	CRYSTAL ST	Q9NPB1	Q9NPB1	5'(3')-deoxyri	EC 3.1.3.- 5',3'-n	NT5M	Homo sapiens	0.42	0.04				local descr.	GO:0016763	transferase ac	?	
1q92	CRYSTAL ST	Q9NPB1	Q9NPB1	5'(3')-deoxyri	EC 3.1.3.- 5',3'-n	NT5M	Homo sapiens	0.42	0.04	GO:0000287	magnesium	0.80					
1oz2	CRYSTAL ST	Q9Y468	LMBT_HUMAN	Lethal(3)mali	L(3)mbt-like L(3)r	L3MBTL	Homo sapiens	0.41	0.06				local descr.	GO:0008408	3'-5' exonucle		
1oz2	CRYSTAL ST	Q9Y468	LMBT_HUMAN	Lethal(3)mali	L(3)mbt-like L(3)r	L3MBTL	Homo sapiens	0.41	0.06	GO:0003700	transcription	0.67					
1px5	CRYSTAL ST	Q29599	OAS1_PIG	2'-5'-oligoade	EC 2.7.7.- (2-5')o	OAS1	Sus scrofa (Pig	0.41	0.08				local descr.	GO:0016702	oxidoreductas	?	
1q8r	STRUCTURE	P40116	RUS_ECOLI	Crossover jur	EC 3.1.22.- Hollid	rusA	Escherichia co	0.41	0.06	GO:0000287	magnesium	0.80					
1o9g	RRNA METHY	Q9F5K5	Q9F5K5	RRNA methy	None	aviRa	Streptomyces v	0.39	0.06	GO:0008757	S-adenosylm	0.89	local descr.	GO:0008757	S-adenosylme	5	5
1o9g	RRNA METHY	Q9F5K5	Q9F5K5	RRNA methy	None	aviRa	Streptomyces v	0.39	0.06				local descr.	GO:0008270	zinc ion bindir	5	5
1o9g	RRNA METHY	Q9F5K5	Q9F5K5	RRNA methy	None	aviRa	Streptomyces v	0.39	0.06				local descr.	GO:0016616	oxidoreductas	0	0
1ofz	CRYSTAL ST	P18891	LECF_ALEAU	Fucose-speci	Aleuria aurantia l	None	Aleuria aurantia	0.38	0.06				local descr.	GO:0008083	growth factor a	0	0

1ofz	CRYSTAL ST	P18891	LECF_ALEAU	Fucose-speci	Aleuria aurantia l	None	Aleuria auranti	0.38	0.06	GO:0005529	sugar bindir	0.87					
1o8b	STRUCTURE	P27252	RPIA_ECOLI					0.38	0.14	GO:0016861	intramolecu	0.88					
1m62	SOLUTION S	O95429	BAG4_HUMAN	BAG-family m	BCL2-associated	BAG4	Homo sapiens	0.36	0.07	GO:0051082	unfolded pr	0.66					
1j5s	CRYSTAL ST	Q9WXR9	UXAC_THEMA	Uronate isom	EC 5.3.1.12 Gluc	uxaC	Thermotoga m	0.35	0.03				local descr.	GO:0005509	calcium ion bi	5	4
1j5s	CRYSTAL ST	Q9WXR9	UXAC_THEMA	Uronate isom	EC 5.3.1.12 Gluc	uxaC	Thermotoga m	0.35	0.03				local descr.	GO:0008270	zinc ion bindir	5	5
1j5s	CRYSTAL ST	Q9WXR9	UXAC_THEMA	Uronate isom	EC 5.3.1.12 Gluc	uxaC	Thermotoga m	0.35	0.03	GO:0016861	intramolecu	0.88					
1hk9	CRYSTAL ST	P25521	HFQ_ECOLI					0.35	0.17				local descr.	GO:0003682	chromatin bind	0	0
1hk9	CRYSTAL ST	P25521	HFQ_ECOLI					0.35	0.17				local descr.	GO:0019838	growth factor l	5	3
1ux6	STRUCTURE	P07996	TSP1_HUMAN	Thrombospor	None	THBS1	Homo sapiens	0.33	0.07	GO:0005509	calcium ion	0.75	local descr.	GO:0005509	calcium ion bi	5	5
1ux6	STRUCTURE	P07996	TSP1_HUMAN	Thrombospor	None	THBS1	Homo sapiens	0.33	0.07	GO:0008201	heparin bind	0.64					
1mk0	CATALYTIC D	P13299	TEV1_BPT4	Intron-associa	EC 3.1.-.- I-TevI	ITEVIR	Bacteriophage	0.31	0.06	GO:0000287	magnesium	0.80					
1oi1	CRYSTAL ST	Q9UQR0	Q9UQR0	Sex comb on	None	SCML2	Homo sapiens	0.31	0.08	GO:0003700	transcription	0.67	local descr.	GO:0003700	transcription fa	5	5
1iwl	CRYSTAL ST	P61316	LOLA_ECOLI	Outer-membr	P20	lolA	Escherichia co	0.31	0.04	GO:0051082	unfolded pr	0.66					
1k75	THE L-HISTID	P06988	HISX_ECOLI	Histidinol deh	EC 1.1.1.23 HDH	hisD	Escherichia co	0.31	0.02				local descr.	GO:0000287	magnesium io	5	4
1k75	THE L-HISTID	P06988	HISX_ECOLI	Histidinol deh	EC 1.1.1.23 HDH	hisD	Escherichia co	0.31	0.02	GO:0008270	zinc ion bind	0.67					
1k75	THE L-HISTID	P06988	HISX_ECOLI	Histidinol deh	EC 1.1.1.23 HDH	hisD	Escherichia co	0.31	0.02	GO:0016616	oxidoreduct	0.91					
1omz	CRYSTAL ST	Q9ES89	EXL2_MOUSE	Exostosin-like	EC 2.4.1.223 Glu	Extl2	Mus musculus	0.31	0.07				local descr.	GO:0005509	calcium ion bi	5	4
1omz	CRYSTAL ST	Q9ES89	EXL2_MOUSE	Exostosin-like	EC 2.4.1.223 Glu	Extl2	Mus musculus	0.31	0.07	GO:0016758	transferase	0.78					
1j83	STRUCTURE	P94622	P94622	Endo-1,4-bet	EC 3.2.1.4	engF	Clostridium cel	0.30	0.06				local descr.	GO:0005509	calcium ion bi		
1j83	STRUCTURE	P94622	P94622	Endo-1,4-bet	EC 3.2.1.4	engF	Clostridium cel	0.30	0.06	GO:0008810	cellulase ac	0.71					
1v9m	CRYSTAL ST	P74902	VATC_THETH	V-type ATP s	EC 3.6.3.14 V-ty	atpC	Thermus therm	0.29	0.08				local descr.	GO:0003887	DNA-directed		
1v9m	CRYSTAL ST	P74902	VATC_THETH	V-type ATP s	EC 3.6.3.14 V-ty	atpC	Thermus therm	0.29	0.08				local descr.	GO:0003700	transcription fa		
1v9m	CRYSTAL ST	P74902	VATC_THETH	V-type ATP s	EC 3.6.3.14 V-ty	atpC	Thermus therm	0.29	0.08				local descr.	GO:0008408	3'-5' exonucle		
1v9m	CRYSTAL ST	P74902	VATC_THETH	V-type ATP s	EC 3.6.3.14 V-ty	atpC	Thermus therm	0.29	0.08	GO:0015078	hydrogen io	0.78					
1v9m	CRYSTAL ST	P74902	VATC_THETH	V-type ATP s	EC 3.6.3.14 V-ty	atpC	Thermus therm	0.29	0.08	GO:0019829	cation-trans	0.59					
1v9m	CRYSTAL ST	P74902	VATC_THETH	V-type ATP s	EC 3.6.3.14 V-ty	atpC	Thermus therm	0.29	0.08	GO:0015405	P-P-bond-h	0.54					
1p42	CRYSTAL ST	O67648	LPXC_AQUAE	UDP-3-O-[3-h	EC 3.5.1.- UDP-3	lpxC	Aquifex aeolicu	0.28	0.07	GO:0016811	hydrolase a	0.55					
1snz	CRYSTAL ST	Q8NIA2	Q8NIA2	Aldose 1-epir	EC 5.1.3.3 Galac	GALM	Homo sapiens	0.28	0.03				local descr.	GO:0005509	calcium ion bi		
1snz	CRYSTAL ST	Q8NIA2	Q8NIA2	Aldose 1-epir	EC 5.1.3.3 Galac	GALM	Homo sapiens	0.28	0.03				local descr.	GO:0004867	serine-type en		
1snz	CRYSTAL ST	Q8NIA2	Q8NIA2	Aldose 1-epir	EC 5.1.3.3 Galac	GALM	Homo sapiens	0.28	0.03	GO:0016854	racemase a	0.77					
1khy	THE CRYSTA	P63284	CLPB_ECOLI	Chaperone cl	Heat-shock prote	clpB	Escherichia co	0.26	0.04				local descr.	GO:0003700	transcription fa		
1khy	THE CRYSTA	P63284	CLPB_ECOLI	Chaperone cl	Heat-shock prote	clpB	Escherichia co	0.26	0.04				local descr.	GO:0004601	peroxidase ac		
1khy	THE CRYSTA	P63284	CLPB_ECOLI	Chaperone cl	Heat-shock prote	clpB	Escherichia co	0.26	0.04	GO:0005524	ATP binding	0.77					
1khy	THE CRYSTA	P63284	CLPB_ECOLI	Chaperone cl	Heat-shock prote	clpB	Escherichia co	0.26	0.04	GO:0051082	unfolded pr	0.66					
1nh8	ATP PHOSPH	P60759	HIS1_MYCTU	ATP phospho	EC 2.4.2.17 ATP	hisG	Mycobacterium	0.25	0.08	GO:0000287	magnesium	0.80					
1nh8	ATP PHOSPH	P60759	HIS1_MYCTU	ATP phospho	EC 2.4.2.17 ATP	hisG	Mycobacterium	0.25	0.08	GO:0016763	transferase	0.93					

1uyr	ACETYL-COA	Q00955	COAC_YEAST	Acetyl-CoA ca	EC 6.4.1.2 ACC	FAS3	Saccharomyce	0.25	0.04				local descr.	GO:0000287	magnesium io	3	5
1uyr	ACETYL-COA	Q00955	COAC_YEAST	Acetyl-CoA ca	EC 6.4.1.2 ACC	FAS3	Saccharomyce	0.25	0.04	GO:0005524	ATP binding	0.77	local descr.	GO:0005524	ATP binding	5	5
1uyr	ACETYL-COA	Q00955	COAC_YEAST	Acetyl-CoA ca	EC 6.4.1.2 ACC	FAS3	Saccharomyce	0.25	0.04				local descr.	GO:0008483	transaminase	5	2
1kc6	HINCII BOUN	P17743	T2C2_HAEIN	Type II restric	EC 3.1.21.4 Endo	hincIIR	Haemophilus in	0.25	0.04	GO:0009036	type II site-s	0.89	local descr.	GO:0009036	type II site-spe	5	5
1kc6	HINCII BOUN	P17743	T2C2_HAEIN	Type II restric	EC 3.1.21.4 Endo	hincIIR	Haemophilus in	0.25	0.04				local descr.	GO:0000287	magnesium io	5	5
1kc6	HINCII BOUN	P17743	T2C2_HAEIN	Type II restric	EC 3.1.21.4 Endo	hincIIR	Haemophilus in	0.25	0.04				local descr.	GO:0005516	calmodulin bin	5	3
1sgv	STRUCTURE	P62190	TRUB_MYCTU	tRNA pseudo	EC 5.4.99.- tRNA	truB	Mycobacterium	0.24	0.21	GO:0016836	hydro-lyase	0.77					
1q5y	NICKEL-BOU	P28910	NIKR_ECOLI					0.24	0.20				local descr.	GO:0005524	ATP binding	5	2
1q5y	NICKEL-BOU	P28910	NIKR_ECOLI					0.24	0.20				local descr.	GO:0003755	peptidyl-prolyl	0	0
1q5y	NICKEL-BOU	P28910	NIKR_ECOLI					0.24	0.20				local descr.	GO:0003964	RNA-directed	5	3
1q5y	NICKEL-BOU	P28910	NIKR_ECOLI					0.24	0.20				local descr.	GO:0008270	zinc ion bindin	5	5
1q5y	NICKEL-BOU	P28910	NIKR_ECOLI					0.24	0.20				local descr.	GO:0008800	beta-lactamas	0	0
1q5y	NICKEL-BOU	P28910	NIKR_ECOLI					0.24	0.20	GO:0003700	transcription	0.67					
1i78	CRYSTAL ST	P09169	OMPT_ECOLI	Protease VII (	EC 3.4.23.- Omp	ompT	Escherichia co	0.23	0.05				local descr.	GO:0015082	di-, tri-valent io	3	5
1i78	CRYSTAL ST	P09169	OMPT_ECOLI	Protease VII (	EC 3.4.23.- Omp	ompT	Escherichia co	0.23	0.05				local descr.	GO:0003887	DNA-directed	0	0
1i78	CRYSTAL ST	P09169	OMPT_ECOLI	Protease VII (	EC 3.4.23.- Omp	ompT	Escherichia co	0.23	0.05				local descr.	GO:0046915	transition meta	3	5
1i78	CRYSTAL ST	P09169	OMPT_ECOLI	Protease VII (	EC 3.4.23.- Omp	ompT	Escherichia co	0.23	0.05	GO:0004190	aspartic-typ	0.81					
1s4n	CRYSTAL ST	P27809	KRE2_YEAST	Glycolipid 2-a	EC 2.4.1.131 Alp	KRE2	Saccharomyce	0.23	0.04	GO:0016758	transferase	0.78					
1s4n	CRYSTAL ST	P27809	KRE2_YEAST	Glycolipid 2-a	EC 2.4.1.131 Alp	KRE2	Saccharomyce	0.23	0.04	GO:0030145	manganese	0.64					
1q5v	APO-NIKR	P28910	NIKR_ECOLI					0.22	0.19				local descr.	GO:0005524	ATP binding	5	3
1q5v	APO-NIKR	P28910	NIKR_ECOLI					0.22	0.19	GO:0003700	transcription	0.67					
1j23	CRYSTAL ST	Q8TZH8	Q8TZH8	ATP-depende	None	OrderedL	Pyrococcus fur	0.21	0.05				local descr.	GO:0009036	type II site-spe	4	5
1j23	CRYSTAL ST	Q8TZH8	Q8TZH8	ATP-depende	None	OrderedL	Pyrococcus fur	0.21	0.05				local descr.	GO:0000287	magnesium io	1	0
1j23	CRYSTAL ST	Q8TZH8	Q8TZH8	ATP-depende	None	OrderedL	Pyrococcus fur	0.21	0.05	GO:0005524	ATP binding	0.77					
1j23	CRYSTAL ST	Q8TZH8	Q8TZH8	ATP-depende	None	OrderedL	Pyrococcus fur	0.21	0.05	GO:0008026	ATP-depen	0.74					
1oxx	CRYSTAL ST	Q97UY8	Q97UY8	ABC transpor	Glucose	OrderedL	Sulfolobus solf	0.20	0.14				local descr.	GO:0015405	P-P-bond-hyd	5	5
1oxx	CRYSTAL ST	Q97UY8	Q97UY8	ABC transpor	Glucose	OrderedL	Sulfolobus solf	0.20	0.14				local descr.	GO:0019829	cation-transpo	4	5
1oxx	CRYSTAL ST	Q97UY8	Q97UY8	ABC transpor	Glucose	OrderedL	Sulfolobus solf	0.20	0.14	GO:0005524	ATP binding	0.77	local descr.	GO:0005524	ATP binding	5	5
1ni9	2.0 A STRUCT	P28860	GLPX_ECOLI	Fructose-1,6-	EC 3.1.3.11 D-fru	glpX	Escherichia co	0.19	0.03	GO:0030145	manganese	0.64					
1o8a	CRYSTAL ST	P22966	ACET_HUMAN	Angiotensin-c	EC 3.4.15.1 ACE	ACE	Homo sapiens	0.19	0.02				local descr.	GO:0003700	transcription fa	0	0
1o8a	CRYSTAL ST	P22966	ACET_HUMAN	Angiotensin-c	EC 3.4.15.1 ACE	ACE	Homo sapiens	0.19	0.02	GO:0008270	zinc ion bind	0.67					
1o8a	CRYSTAL ST	P22966	ACET_HUMAN	Angiotensin-c	EC 3.4.15.1 ACE	ACE	Homo sapiens	0.19	0.02	GO:0004180	carboxypep	0.90					
1nze	CRYSTAL ST	P12301	PSBQ_SPIOL	Oxygen-evolv	OEE3 16 kDa su	PSBQ	Spinacia olerac	0.19	0.12	GO:0005509	calcium ion	0.75					
1j7n	ANTHRAX TO	P15917	LEF_BACAN	Lethal factor (	EC 3.4.24.83 LF	lef	Bacillus anthra	0.18	0.09				local descr.	GO:0003700	transcription fa	0	0
1usu	THE STRUCT	P02829, Q1	HS82_YEAST,	ATP-depende	Heat shock prote	HSP82, A	Saccharomyce	0.18	0.04				local descr.	GO:0008270	zinc ion bindin	2	5
1usu	THE STRUCT	P02829, Q1	HS82_YEAST,	ATP-depende	Heat shock prote	HSP82, A	Saccharomyce	0.18	0.04	GO:0005524	ATP binding	0.77					

1usu	THE STRUCT	P02829, Q1	HS82_YEAST,	ATP-depende	Heat shock prote	HSP82, A	Saccharomyce	0.18	0.04	GO:0051082	unfolded pro	0.66					
1j0t	THE SOLUTIO	P55847	MIH_PENJP	Molt-inhibiting	MIH PeJ-SGP-IV	None	Penaeus japon	0.17	0.13	GO:0005179	hormone ac	0.53					
1knc	STRUCTURE	Q57353	AHPD_MYCTU	AhpD protein	None	ahpD	Mycobacterium	0.17	0.06				local descr.	GO:0008199	ferric iron bind	1	0
1knc	STRUCTURE	Q57353	AHPD_MYCTU	AhpD protein	None	ahpD	Mycobacterium	0.17	0.06	GO:0004601	peroxidase	0.84					
1itw	CRYSTAL ST	P16100	IDH_AZOVI	Isocitrate deh	EC 1.1.1.42 Oxa	icd	Azotobacter vir	0.17	0.04				local descr.	GO:0016763	transferase ac	0	0
1itw	CRYSTAL ST	P16100	IDH_AZOVI	Isocitrate deh	EC 1.1.1.42 Oxa	icd	Azotobacter vir	0.17	0.04				local descr.	GO:0000287	magnesium io	5	4
1itw	CRYSTAL ST	P16100	IDH_AZOVI	Isocitrate deh	EC 1.1.1.42 Oxa	icd	Azotobacter vir	0.17	0.04				local descr.	GO:0003700	transcription fa	0	0
1itw	CRYSTAL ST	P16100	IDH_AZOVI	Isocitrate deh	EC 1.1.1.42 Oxa	icd	Azotobacter vir	0.17	0.04				local descr.	GO:0005524	ATP binding	5	3
1itw	CRYSTAL ST	P16100	IDH_AZOVI	Isocitrate deh	EC 1.1.1.42 Oxa	icd	Azotobacter vir	0.17	0.04				local descr.	GO:0008483	transaminase	0	0
1itw	CRYSTAL ST	P16100	IDH_AZOVI	Isocitrate deh	EC 1.1.1.42 Oxa	icd	Azotobacter vir	0.17	0.04	GO:0016616	oxidoreduct	0.91					
1lj8	CRYSTAL ST	O08355	O08355	Mannitol dehy	EC 1.1.1.67	mtlD	Pseudomonas	0.17	0.06				local descr.	GO:0005507	copper ion bin	2	5
1lj8	CRYSTAL ST	O08355	O08355	Mannitol dehy	EC 1.1.1.67	mtlD	Pseudomonas	0.17	0.06	GO:0016616	oxidoreduct	0.91					
1h3d	STRUCTURE	P60757	HIS1_ECOLI	ATP phospho	EC 2.4.2.17 ATP	hisG	Escherichia co	0.17	0.12				local descr.	GO:0008199	ferric iron bind	4	4
1h3d	STRUCTURE	P60757	HIS1_ECOLI	ATP phospho	EC 2.4.2.17 ATP	hisG	Escherichia co	0.17	0.12	GO:0000287	magnesium	0.80					
1h3d	STRUCTURE	P60757	HIS1_ECOLI	ATP phospho	EC 2.4.2.17 ATP	hisG	Escherichia co	0.17	0.12	GO:0016763	transferase	0.93					
1kq1	1.55 A CRYST	Q99UG9	Q99UG9	Similar to hos	None	OrderedLo	Staphylococcus	0.16	0.13				local descr.	GO:0008083	growth factor a	0	0
1lt8	REDUCED HO	Q93088	BHMT_HUMAN	Betaine--hom	EC 2.1.1.5	BHMT	Homo sapiens	0.16	0.03				local descr.	GO:0016763	transferase ac	5	4
1lt8	REDUCED HO	Q93088	BHMT_HUMAN	Betaine--hom	EC 2.1.1.5	BHMT	Homo sapiens	0.16	0.03				local descr.	GO:0000287	magnesium io	5	4
1lt8	REDUCED HO	Q93088	BHMT_HUMAN	Betaine--hom	EC 2.1.1.5	BHMT	Homo sapiens	0.16	0.03				local descr.	GO:0005509	calcium ion bi	5	4
1lt8	REDUCED HO	Q93088	BHMT_HUMAN	Betaine--hom	EC 2.1.1.5	BHMT	Homo sapiens	0.16	0.03				local descr.	GO:0016836	hydro-lyase ac	0	0
1lt8	REDUCED HO	Q93088	BHMT_HUMAN	Betaine--hom	EC 2.1.1.5	BHMT	Homo sapiens	0.16	0.03	GO:0008757	S-adenosylm	0.89					
1suu	STRUCTURE	O51396	GYRA_BORBU	DNA gyrase s	EC 5.99.1.3	gyrA	Borrelia burgdo	0.15	0.05				local descr.	GO:0004222	metalloendope	0	0
1suu	STRUCTURE	O51396	GYRA_BORBU	DNA gyrase s	EC 5.99.1.3	gyrA	Borrelia burgdo	0.15	0.05				local descr.	GO:0005509	calcium ion bi	2	4
1suu	STRUCTURE	O51396	GYRA_BORBU	DNA gyrase s	EC 5.99.1.3	gyrA	Borrelia burgdo	0.15	0.05				local descr.	GO:0008270	zinc ion bindin	2	5
1suu	STRUCTURE	O51396	GYRA_BORBU	DNA gyrase s	EC 5.99.1.3	gyrA	Borrelia burgdo	0.15	0.05	GO:0005524	ATP binding	0.77					
1suu	STRUCTURE	O51396	GYRA_BORBU	DNA gyrase s	EC 5.99.1.3	gyrA	Borrelia burgdo	0.15	0.05	GO:0003916	DNA topoiso	0.74					
1ixd	SOLUTION ST	Q9NQC7	CYLD_HUMAN	Probable ubiq	EC 3.1.2.15 Ubiq	CYLD	Homo sapiens	0.14	0.06	GO:0004197	cysteine-typ	0.93					
1mt5	CRYSTAL ST	P97612	FAAH_RAT	Fatty-acid am	EC 3.1.-.- Oleam	Faah	Rattus norvegi	0.14	0.04				local descr.	GO:0008199	ferric iron bind	2	4
1mt5	CRYSTAL ST	P97612	FAAH_RAT	Fatty-acid am	EC 3.1.-.- Oleam	Faah	Rattus norvegi	0.14	0.04				local descr.	GO:0008270	zinc ion bindin	2	5
1mt5	CRYSTAL ST	P97612	FAAH_RAT	Fatty-acid am	EC 3.1.-.- Oleam	Faah	Rattus norvegi	0.14	0.04	GO:0016811	hydrolase a	0.55					
1umw	STRUCTURE	P53350	PLK1_HUMAN	Serine/threon	EC 2.7.1.37 Polo	PLK1	Homo sapiens	0.14	0.09				local descr.	GO:0003887	DNA-directed	0	0
1umw	STRUCTURE	P53350	PLK1_HUMAN	Serine/threon	EC 2.7.1.37 Polo	PLK1	Homo sapiens	0.14	0.09	GO:0004674	protein serin	0.68					
1mij	CRYSTAL ST	P29617	PRO_DROME	Protein prosp	None	pros	Drosophila mel	0.12	0.11				local descr.	GO:0005509	calcium ion bi	1	1
1mij	CRYSTAL ST	P29617	PRO_DROME	Protein prosp	None	pros	Drosophila mel	0.12	0.11	GO:0003700	transcription	0.67					
1k87	CRYSTAL ST	P09546	PUTA_ECOLI	Bifunctional p	None	putA	Escherichia co	0.12	0.05				local descr.	GO:0005509	calcium ion bi	1	0
1k87	CRYSTAL ST	P09546	PUTA_ECOLI	Bifunctional p	None	putA	Escherichia co	0.12	0.05				local descr.	GO:0016836	hydro-lyase ac	0	0

1k87	CRYSTAL ST	P09546	PUTA_ECOLI	Bifunctional p	None	putA	Escherichia co	0.12	0.05				local descr.	GO:0016861	intramolecular	5	4
1k87	CRYSTAL ST	P09546	PUTA_ECOLI	Bifunctional p	None	putA	Escherichia co	0.12	0.05	GO:0016646	oxidoreduct	0.88					
1q5z	CRYSTAL ST	Q56027	SIPA_SALTY	Cell invasion	Effector protein s	sipA	Salmonella typ	0.12	0.10	GO:0003779	actin binding	0.68					
1iwm	CRYSTAL ST	P61320	LOLB_ECOLI	Outer-membr	None	lolB	Escherichia co	0.11	0.07	GO:0051082	unfolded pro	0.66					
1ujx	THE FORKHE	Q9JLV6	Q9JLV6	Bifunctional p	Polynucleotide ki	Pnkp	Mus musculus	0.10	0.08	GO:0005524	ATP binding	0.77					
1r53	CRYSTAL ST	P28777	AROC_YEAST	Chorismate s	EC 4.2.3.5 5-eno	ARO2	Saccharomyce	0.10	0.03	GO:0016646	oxidoreduct	0.88					
1mki	CRYSTAL ST	O31465	GLS2_BACSU	Probable glut	EC 3.5.1.2	ybgJ	Bacillus subtilis	0.10	0.07				local descr.	GO:0003700	transcription fa	0	0
1mki	CRYSTAL ST	O31465	GLS2_BACSU	Probable glut	EC 3.5.1.2	ybgJ	Bacillus subtilis	0.10	0.07				local descr.	GO:0003713	transcription c	0	0
1mki	CRYSTAL ST	O31465	GLS2_BACSU	Probable glut	EC 3.5.1.2	ybgJ	Bacillus subtilis	0.10	0.07	GO:0016811	hydrolase a	0.55					
1nr3	SOLUTION S	O27001	TFX_METTH	DNA-binding	None	tfx	Methanobacter	0.09	0.07	GO:0003700	transcription	0.67					
1l5i	30-CONFORM	P27260	VAL1_TYLCM	AL1 protein	C1 protein	C1	Tomato yellow	0.09	0.08				local descr.	GO:0008083	growth factor a	0	0
1l5i	30-CONFORM	P27260	VAL1_TYLCM	AL1 protein	C1 protein	C1	Tomato yellow	0.09	0.08	GO:0005524	ATP binding	0.77					
1izn	CRYSTAL ST	P13127, P1	CAPB_CHICK,	F-actin cappi	CapZ 36/32 Beta	None, No	Gallus gallus (	0.09	0.08				local descr.	GO:0005524	ATP binding	2	5
1izn	CRYSTAL ST	P13127, P1	CAPB_CHICK,	F-actin cappi	CapZ 36/32 Beta	None, No	Gallus gallus (	0.09	0.08				local descr.	GO:0005529	sugar binding	1	0
1izn	CRYSTAL ST	P13127, P1	CAPB_CHICK,	F-actin cappi	CapZ 36/32 Beta	None, No	Gallus gallus (	0.09	0.08				local descr.	GO:0046983	protein dimeri	5	5
1izn	CRYSTAL ST	P13127, P1	CAPB_CHICK,	F-actin cappi	CapZ 36/32 Beta	None, No	Gallus gallus (	0.09	0.08				local descr.	GO:0003964	RNA-directed	0	0
1izn	CRYSTAL ST	P13127, P1	CAPB_CHICK,	F-actin cappi	CapZ 36/32 Beta	None, No	Gallus gallus (	0.09	0.08	GO:0003779	actin binding	0.68					
1rre	CRYSTAL ST	P08177	LON_ECOLI	ATP-depende	EC 3.4.21.53	lon	Escherichia co	0.09	0.10	GO:0005524	ATP binding	0.77	local descr.	GO:0005524	ATP binding	5	5
1s40	SOLUTION S	P32797	CC13_YEAST	Cell division c	None	CDC13	Saccharomyce	0.09	0.06	GO:0003697	single-stran	0.70					
1p9y	RIBOSOME B	P22257	TIG_ECOLI	Trigger factor	TF	tig	Escherichia co	0.09	0.14				local descr.	GO:0005529	sugar binding	3	3
1p9y	RIBOSOME B	P22257	TIG_ECOLI	Trigger factor	TF	tig	Escherichia co	0.09	0.14	GO:0051082	unfolded pro	0.66					
1p9y	RIBOSOME B	P22257	TIG_ECOLI	Trigger factor	TF	tig	Escherichia co	0.09	0.14	GO:0003755	peptidyl-pro	0.88					
1mg4	STRUCTURE	O15075	DCK1_HUMAN	Serine/threon	EC 2.7.1.37 Dou	DCAMKL1	Homo sapiens	0.08	0.16	GO:0005524	ATP binding	0.77					
1mg4	STRUCTURE	O15075	DCK1_HUMAN	Serine/threon	EC 2.7.1.37 Dou	DCAMKL1	Homo sapiens	0.08	0.16	GO:0004674	protein serin	0.68					
1v2x	TRMH	Q9FAC4	Q9FAC4	TRNA (Gm18	EC 2.1.1.34	trmH	Thermus therm	0.08	0.06				local descr.	GO:0000287	magnesium io	4	5
1v2x	TRMH	Q9FAC4	Q9FAC4	TRNA (Gm18	EC 2.1.1.34	trmH	Thermus therm	0.08	0.06				local descr.	GO:0008270	zinc ion bindin	3	5
1v2x	TRMH	Q9FAC4	Q9FAC4	TRNA (Gm18	EC 2.1.1.34	trmH	Thermus therm	0.08	0.06	GO:0008757	S-adenosylm	0.89					
1ny9	ANTIBIOTIC B	P32184	TIPA_STRCO	HTH-type tra	None	tipA	Streptomyces c	0.08	0.07	GO:0003700	transcription	0.67					
1un7	THE 3-D STR	O34450	NAGA_BACSU	N-acetylgluco	EC 3.5.1.25 GlcN	nagA	Bacillus subtilis	0.07	0.21	GO:0016811	hydrolase a	0.55					
1ojg	SENSORY DO	P39272	DCUS_ECOLI	Sensor protei	EC 2.7.3.-	dcuS	Escherichia co	0.07	0.09	GO:0005524	ATP binding	0.77					
1uqt	TREHALOSE-	P31677	OTSA_ECOLI	Alpha,alpha-t	EC 2.4.1.15 Treh	otsA	Escherichia co	0.07	0.07				local descr.	GO:0000287	magnesium io	4	5
1uqt	TREHALOSE-	P31677	OTSA_ECOLI	Alpha,alpha-t	EC 2.4.1.15 Treh	otsA	Escherichia co	0.07	0.07				local descr.	GO:0005524	ATP binding	3	4
1uqt	TREHALOSE-	P31677	OTSA_ECOLI	Alpha,alpha-t	EC 2.4.1.15 Treh	otsA	Escherichia co	0.07	0.07				local descr.	GO:0016646	oxidoreductas	0	0
1uqt	TREHALOSE-	P31677	OTSA_ECOLI	Alpha,alpha-t	EC 2.4.1.15 Treh	otsA	Escherichia co	0.07	0.07				local descr.	GO:0016836	hydro-lyase ac	0	0
1q2z	THE 3D SOLU	P13010	KU86_HUMAN	ATP-depende	Lupus Ku autoan	XRCC5	Homo sapiens	0.07	0.12	GO:0008026	ATP-depend	0.74					
1k6k	CRYSTAL ST	P15716	CLPA_ECOLI	ATP-depende	None	clpA	Escherichia co	0.07	0.13	GO:0005524	ATP binding	0.77					

1k6k	CRYSTAL ST	P15716	CLPA_ECOLI	ATP-depende	None	clpA	Escherichia co	0.07	0.13	GO:0051082	unfolded pr	0.66					
1mbx	CRYSTAL ST	P15716, P7	CLPA_ECOLI,	ATP-depende	None	clpA	Escherichia co	0.07	0.13				local descr.	GO:0003700	transcription fa	0	0
1mbx	CRYSTAL ST	P15716, P7	CLPA_ECOLI,	ATP-depende	None	clpA	Escherichia co	0.07	0.13	GO:0005524	ATP binding	0.77					
1mbx	CRYSTAL ST	P15716, P7	CLPA_ECOLI,	ATP-depende	None	clpA	Escherichia co	0.07	0.13	GO:0051082	unfolded pr	0.66					
1jr2	STRUCTURE	P10746	HEM4_HUMAN	Uroporphyrin	EC 4.2.1.75 URO	UROS	Homo sapiens	0.06	0.11				local descr.	GO:0003700	transcription fa	1	0
1jr2	STRUCTURE	P10746	HEM4_HUMAN	Uroporphyrin	EC 4.2.1.75 URO	UROS	Homo sapiens	0.06	0.11	GO:0016836	hydro-lyase	0.77					
1h2k	FACTOR INH	Q16665, Q9	HIFA_HUMAN	Hypoxia-indu	HIF-1 alpha HIF1	HIF1A, HI	Homo sapiens	0.06	0.08				local descr.	GO:0001584	rhodopsin-like	0	0
1h2k	FACTOR INH	Q16665, Q9	HIFA_HUMAN	Hypoxia-indu	HIF-1 alpha HIF1	HIF1A, HI	Homo sapiens	0.06	0.08				local descr.	GO:0016251	general RNA p	5	3
1h2k	FACTOR INH	Q16665, Q9	HIFA_HUMAN	Hypoxia-indu	HIF-1 alpha HIF1	HIF1A, HI	Homo sapiens	0.06	0.08	GO:0003700	transcription	0.67					
1h2k	FACTOR INH	Q16665, Q9	HIFA_HUMAN	Hypoxia-indu	HIF-1 alpha HIF1	HIF1A, HI	Homo sapiens	0.06	0.08	GO:0016705	oxidoreduct	0.65					
1h2k	FACTOR INH	Q16665, Q9	HIFA_HUMAN	Hypoxia-indu	HIF-1 alpha HIF1	HIF1A, HI	Homo sapiens	0.06	0.08	GO:0046983	protein dime	0.69					
1jov	CRYSTAL ST	Q9RP27	Q9RP27	HI1317 [Frag	None	None	Haemophilus in	0.06	0.02				local descr.	GO:0005509	calcium ion bi	1	1
1jov	CRYSTAL ST	Q9RP27	Q9RP27	HI1317 [Frag	None	None	Haemophilus in	0.06	0.02				local descr.	GO:0005529	sugar binding	3	4
1jov	CRYSTAL ST	Q9RP27	Q9RP27	HI1317 [Frag	None	None	Haemophilus in	0.06	0.02	GO:0016854	racemase a	0.77					
1j5w	CRYSTAL ST	Q9WY59	SYGA_THEMA	Glycyl-tRNA s	EC 6.1.1.14 Glyc	glyQ	Thermotoga ma	0.06	0.03				local descr.	GO:0000049	tRNA binding	5	5
1j5w	CRYSTAL ST	Q9WY59	SYGA_THEMA	Glycyl-tRNA s	EC 6.1.1.14 Glyc	glyQ	Thermotoga ma	0.06	0.03				local descr.	GO:0000287	magnesium io	5	5
1j5w	CRYSTAL ST	Q9WY59	SYGA_THEMA	Glycyl-tRNA s	EC 6.1.1.14 Glyc	glyQ	Thermotoga ma	0.06	0.03	GO:0005524	ATP binding	0.77	local descr.	GO:0005524	ATP binding	5	5
1j5w	CRYSTAL ST	Q9WY59	SYGA_THEMA	Glycyl-tRNA s	EC 6.1.1.14 Glyc	glyQ	Thermotoga ma	0.06	0.03	GO:0004812	tRNA ligase	0.98	local descr.	GO:0004812	tRNA ligase a	5	5
1ptm	CRYSTAL ST	P19624	PDXA_ECOLI	4-hydroxythre	EC 1.1.1.262 4-(	pdxA	Escherichia co	0.06	0.03	GO:0016616	oxidoreduct	0.91	local descr.	GO:0016616	oxidoreductas	5	5
1ltl	THE DODECA	O27798	O27798	DNA replicati	Cdc21/Cdc54	OrderedL	Methanobacter	0.05	0.04				local descr.	GO:0003700	transcription fa	4	3
1ltl	THE DODECA	O27798	O27798	DNA replicati	Cdc21/Cdc54	OrderedL	Methanobacter	0.05	0.04				local descr.	GO:0008083	growth factor a	0	0
1ltl	THE DODECA	O27798	O27798	DNA replicati	Cdc21/Cdc54	OrderedL	Methanobacter	0.05	0.04				local descr.	GO:0008408	3'-5' exonucle	4	3
1ltl	THE DODECA	O27798	O27798	DNA replicati	Cdc21/Cdc54	OrderedL	Methanobacter	0.05	0.04				local descr.	GO:0008270	zinc ion bindin	1	1
1ltl	THE DODECA	O27798	O27798	DNA replicati	Cdc21/Cdc54	OrderedL	Methanobacter	0.05	0.04	GO:0005524	ATP binding	0.77					
1nqj	CRYSTAL ST	Q9S0X0	Q9S0X0	Class1 collag	None	None	Clostridium his	0.05	0.07				local descr.	GO:0004180	carboxypeptid		
1nqj	CRYSTAL ST	Q9S0X0	Q9S0X0	Class1 collag	None	None	Clostridium his	0.05	0.07				local descr.	GO:0005507	copper ion bin		
1nqj	CRYSTAL ST	Q9S0X0	Q9S0X0	Class1 collag	None	None	Clostridium his	0.05	0.07				local descr.	GO:0005509	calcium ion bi		
